# Genome-Wide Gene Expression Analysis Implicates the Immune Response and Lymphangiogenesis in the Pathogenesis of Fetal Chylothorax

**DOI:** 10.1371/journal.pone.0034901

**Published:** 2012-04-18

**Authors:** Chen-Hsiang Yeang, Gwo-Chin Ma, Jin-Chung Shih, Yu-Shih Yang, Chih-Ping Chen, Shun-Ping Chang, Sheng-Hai Wu, Chin-San Liu, Shou-Jen Kuo, Hung-Chieh Chou, Wuh-Liang Hwu, Alan D. Cameron, Norman A. Ginsberg, Yi-Shing Lin, Ming Chen

**Affiliations:** 1 Institute of Statistical Science, Academia Sinica, Taipei, Taiwan; 2 Department of Genomic Medicine, and Center for Medical Genetics, Changhua Christian Hospital, Changhua, Taiwan; 3 Institute of Biochemistry and Biotechnology, Chung-Shan Medical University, Taichung, Taiwan; 4 Department of Obstetrics and Gynecology, National Taiwan University, Taipei, Taiwan; 5 Department of Obstetrics and Gynecology, and Department of Medical Research, Mackay Memorial Hospital, Taipei, Taiwan; 6 Department of Life Sciences, National Chung-Hsing University, Taichung, Taiwan; 7 Vascular and Genomics Center, Department of Medical Research, Changhua Christian Hospital, Changhua, Taiwan; 8 Department of Surgery, Changhua Christian Hospital, Changhua, Taiwan; 9 Department of Pediatrics, and Department of Medical Genetics, College of Medicine and Hospital, National Taiwan University, Taipei, Taiwan; 10 Ian Donald Foetal Medicine Unit, Southern General Hospital and University of Glasgow, Glasgow, United Kingdom; 11 Department of Obstetrics and Gynecology, Feinberg School of Medicine, Northwestern University, Chicago, Illinois, United States of America; 12 Welgene Biotechnology Company, NanGang Business Park, Taipei, Taiwan; 13 Department of Life Sciences, Tunghai University, Taichung, Taiwan; 14 College of Medicine, Chung-Shan Medical University, Taichung, Taiwan; University of Lausanne, Switzerland

## Abstract

Fetal chylothorax (FC) is a rare condition characterized by lymphocyte-rich pleural effusion. Although its pathogenesis remains elusive, it may involve inflammation, since there are increased concentrations of proinflammatory mediators in pleural fluids. Only a few hereditary lymphedema-associated gene loci, e.g. *VEGFR3*, *ITGA9* and *PTPN11*, were detected in human fetuses with this condition; these cases had a poorer prognosis, due to defective lymphangiogenesis. In the present study, genome-wide gene expression analysis was conducted, comparing pleural and ascitic fluids in three hydropic fetuses, one with and two without the *ITGA9* mutation. One fetus (the index case), from a dizygotic pregnancy (the cotwin was unaffected), received antenatal OK-432 pleurodesis and survived beyond the neonatal stage, despite having the *ITGA9* mutation. Genes and pathways involved in the immune response were universally up-regulated in fetal pleural fluids compared to those in ascitic fluids. Furthermore, genes involved in the lymphangiogenesis pathway were down-regulated in fetal pleural fluids (compared to ascitic fluid), but following OK-432 pleurodesis, they were up-regulated. Expression of *ITGA9* was concordant with overall trends of lymphangiogenesis. In conclusion, we inferred that both the immune response and lymphangiogenesis were implicated in the pathogenesis of fetal chylothorax. Furthermore, genome-wide gene expression microarray analysis may facilitate personalized medicine by selecting the most appropriate treatment, according to the specific circumstances of the patient, for this rare, but heterogeneous disease.

## Introduction

Congenital chylothorax (CC), or fetal chylothorax (FC), is a rare condition (estimated incidence, 1 in 12 000), characterized by accumulation of lymphocyte-rich fluid in the pleural cavity [1]. Its pathogenesis remains elusive. Although we previously detected high concentrations of proinflammatory mediators in the fetal pleural fluid [1], implying that inflammation had a role in this condition, the pathogenesis remains unclear.

Fetal therapy is available for this condition, including the gold-standard thoracoamniotic shunting and the seemingly less effective pleurodesis by OK-432, yet the prognosis is variable [2]–[4], and may be poorer for cases with a genetic component [1], [5]. Clinically, chylothorax can be regarded as the pulmonary manifestation of hereditary lymphedema, a condition likely due to aberrant lymphangiogenesis [5]–[7]. Even though genes involved in lymphangiogenesis have been extensively studied in a mouse model [8], only a few homologous genes, including *vascular endothelial growth factor receptor type 3* (*VEGFR3*), *integrinα9* (*ITGA9*), *Tyrosine-protein phosphatase non-receptor type 11* (*PTPN11*), and *Forkhead box protein C2* (*FOXC2*), were reported in humans affected with hereditary lymphedema [5], [9]. Among these causative loci, *ITGA9*, which has an important role in lymphatic valve morphogenesis (in knockout mice), is a candidate gene of autosomal-recessive primary lymphedema caused by lymphatic valve defects [7], [10]. We were the first group to identify mutations in the *ITGA9* gene in human fetuses with CC and we proposed that a specific mutant allele (p.G404S), with a reported frequency of 0.003, may be associated with a poor prognosis despite fetal therapy (specifically, OK-432 pleurodesis) [11]. Similarly, fetuses carrying mutations in the hereditary lymphedema-associated gene loci had a grave prognosis in our recent series of cases involving fetal OK-432 pleurodesis [4].

**Figure 1:**
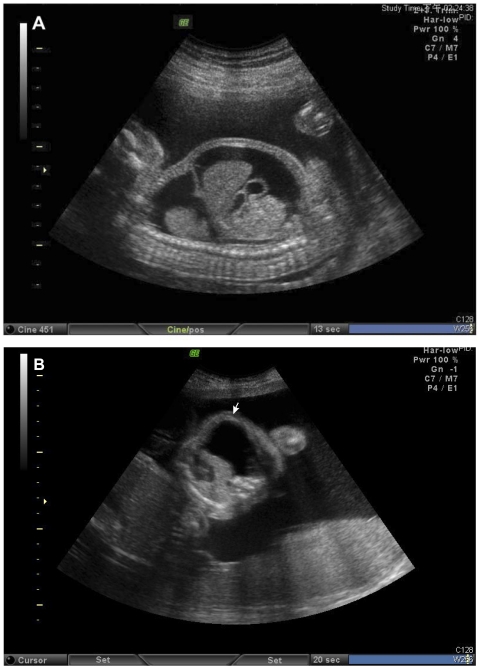
Fetal treatment of bilateral fetal pleural effusion (Ind case). The fetal chylothorax (FC) and hydrops had lessened from (A) bilateral pleural effusion and hydrops to (B) unilateral pleural effusion only (arrow) after OK-432 pleurodesis.

Here we present a dizygotic twin pregnancy complicated with one hydrops (including chylothorax, fetal lymphascites and polyhydramnios) and with a *de novo* mutation in the *ITGA9* gene (p.G404S); this index case (Ind; Figure 1) was successfully managed by antenatal OK-432 pleurodesis, combined with aggressive neonatal intensive care. A comprehensive genetic investigation was conducted to elucidate the pathogenesis of *ITGA9*-associated CC. In that regard, a genome-wide expression assay compared gene expression in pleural fluid versus ascitic fluid in the fetus, both before and after OK-432 pleurodesis. Furthermore, we also studied two additional reference cases: one was FC hydrops (FC-r) and the other was non-FC hydrops (NFC-r), with both cases lacking the *ITGA9* mutation.

The objective was to identify genes differentially expressed between pleural and ascitic fluids. Therefore, genome-wide gene expression analysis was done with gene set enrichment analysis (GSEA), a powerful bioinformatic approach to assess enrichment of relevant genes in a selected gene set [12]. All genes in the microarray were sorted by decreasing order of their expression levels. If a gene set is enriched with up-regulated genes, moving down the sorted list, the frequency of finding members of the gene set in the top portion should be higher than the frequency of finding non-members of the gene set. This is translated into a quantitative measure of gene set enrichment for up-regulated genes. Similarly, enrichment of down-regulated genes can be calculated reciprocally by reversing the order of genes in the sorted list. With this approach, we identified enriched gene sets and pathways with potential roles in the pathogenesis of FC.

## Results

### Genotyping of ITGA9, VEGFR3, FOXC2 and PTPN11 genes

A heterozygous p.G404S mutant allele was detected in the *ITGA9* gene in the Ind fetus, a female hydropic cotwin of a dizygotic twin pregnancy (see PATIENTS AND METHODS). This mutation was not detected in the healthy male cotwin or in either parent, and thus was deemed to have arisen *de novo* (Appendix S3). No other mutant allele was detected in the *VEGFR3*, *FOXC2*, or *PTPN11* genes.

### aCGH

**Figure 2:**
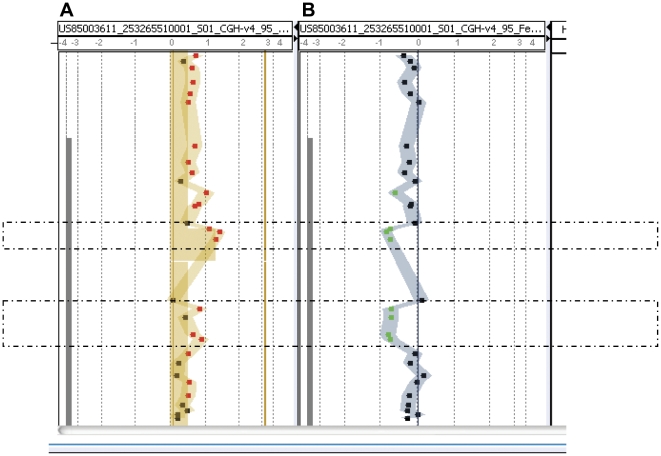
Mutation detection by aCGH. (A) Two microduplications in the 5′-region of *ITGA9*, arr 3p21.3 (37,493,992–37,494,056)×3 and arr 3p21.3 (37,494,150–37,494,255)×3, were detected. The former (64 bp) located over the exon 1-intron 1 boundary and the latter (105 bp) located within the intron 1. (B) The two microduplications were confirmed with a dye swap test. The aCGH was performed with the Agilent customer array, *ITGA9* Tiling chip (designed by Welgene Biotechnology Company and Changhua Christian Hospital, Taiwan; Appendix S1).

Two segmental microduplications were identified in the 5′-end of *ITGA9* of the Ind fetus; the first was over the exon 1-intron 1 boundary, arr 3p21.3 (37,493,992–37,494,056)×3, with a length of 64 base pairs (bp), and the second was located at the 5′end of the intron 1, arr 3p21.3 (37,494,150–37,494,255)×3, and was 100 bp (Figures 2A and 2B).

### ITGA9 cDNA analysis

In the 30 plasmids examined, there were only two sequence patterns in the Ind fetus: with or without the p.G404S mutant. No microduplications or splicing errors were detected in the cDNA.

### Real-time PCR

**Figure 3:**
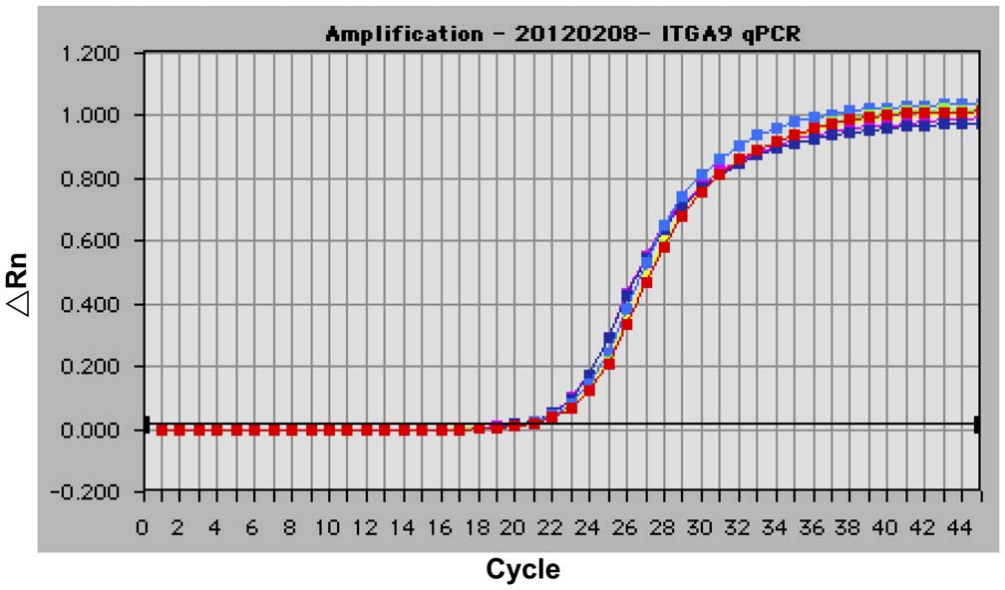
Real-time PCR analysis of *ITGA9*. Representative RT-PCR plot resulting from the amplification of the *ITGA9* gene using primer pairs of q1F/q1R (duplicated tests; red and green lines), qIVS1F/qIVS1R (duplicated tests; yellow and blue lines) and q15F/q15R (duplicated tests; violet and purple lines; PATIENTS AND METHODS). No evidence for the existence of duplicated segments in the 5′ -end of *ITGA9* indicated a false-positive result of aCGH, as shown in Figure 2.

The real-time PCR detected no *ITGA9* microduplications in the DNA (Figure 3); therefore, we concluded that the two segmental microduplications detected by aCGH at the 5′end of *ITGA9* were false positive.

### Gene Set Enrichment Analysis for differentially expressed genes

Several genes with mutations or abnormal expressions were detected in fetuses with FC [5], [9]. However, in addition to those previously reported genes, our objective was to identify functional groups or pathways that systematically exhibited differential activities in FC. Therefore, Gene Set Enrichment Analysis (GSEA) [12] was applied to the mRNA expression data from each of the four sample-pairs (Sample 1: Ind-A, fluids from the Ind fetus after fetal treatment; Sample 2: Ind-B, fluids from the Ind fetus before fetal treatment, in the index case carrying an *ITGA9* mutation (p.G404S); Sample 3: FC-r, fluids from another similar hydropic case with chylothorax, but without the *ITGA9* mutation; and Sample 4: NFC-r, fluids from an additional hydropic case without a chylothorax and no *ITGA9* mutation).

Strikingly, system-level responses were consistent with our previous findings based on individual genes; namely, chylothorax was associated with high activities of immune responses in pleural fluids. The majority of Gene Ontology (GO) categories and pathways enriched with up-regulated genes (pleural versus ascitic fluids) in multiple samples (Ind-A, Ind-B and FC-r) were related to immune or stress responses. For example, these included GO categories of MHC class II receptor activity, positive regulation of interleukin-2 biosynthesis process, and pathways of IFN-gamma signaling and canonical NF kappa B pathway. However, it was noteworthy that up-regulated genes and GO categories/pathways related to immune or stress responses decreased sharply in the Ind case after fetal OK-432 treatment was provided (Ind-A versus Ind-B; Table 1). In contrast, the GO categories and pathways enriched with down-regulated genes in multiple samples were in more diverse functional classes. Several gene sets involved in erythrocytes and blood vessels were reported, including GO categories of hemoglobin complex, regulation of angiogenesis, and pathways of heme biosynthesis and hemoglobin's chaperone.

**Table 1:** Functional Gene Ontology (GO) categories and pathways enriched with differentially expressed genes between pleural and ascitic fluids.

Direction	Type	Gene set	Ind-A	Ind-B	FC-r	NFC-r
up	GO	MHC class II receptor activity	1 (1.0000e−04)	8 (6.7000e−03)	-	2 (4.0000e−04)
up	GO	protein kinase C activity	-	7 (1.3800e−02)	12 (2.4000e−02)	-
up	GO	response to UV	13 (2.8700e−02)	13 (7.2000e−03)	-	-
up	GO	poly(A) binding	4 (1.9300e−02)	16 (1.6800e−02)	-	-
up	GO	positive regulation of interleukin-2 biosynthetic process	14 (5.7100e−02)	20 (2.8700e−02)	-	3 (7.0000e−03)
up	pathway	FasL/CD95L signaling	4 (9.3000e−03)	2 (3.3000e−03)	19 (1.1850e−01)	-
up	pathway	Canonical NF kappa B Pathway	2 (1.7000e−03)	1 (0.0000e+00)	-	-
up	pathway	cd40l signaling pathway	-	6 (1.0000e−04)	1 (3.0000e−04)	-
up	pathway	ifn gamma signaling pathway	7 (4.8500e−02)	-	3 (2.7100e−02)	-
up	pathway	MyD88 cascade	11 (5.2200e−02)	3 (1.3000e−03)	-	-
up	pathway	Effects of Botulinum Toxin	10 (1.9000e−02)	-	14 (2.6300e−02)	-
up	pathway	spliceosomal assembly	-	17 (1.8400e−02)	10 (3.2400e−02)	4 (1.5600e−02)
down	GO	hemoglobin complex	6 (1.0000e−04)	4 (0.0000e+00)	5 (1.0000e−04)	3 (0.0000e+00)
down	GO	spindle organization and biogenesis	1 (0.0000e+00)	3 (0.0000e+00)	-	4 (0.0000e+00)
down	GO	cadmium ion binding	4 (6.0000e−04)	1 (0.0000e+00)	-	6 (9.0000e−04)
down	GO	cysteine metabolic process	-	7 (4.0000e−04)	4 (1.0000e−03)	-
down	GO	cellular copper ion homeostasis	12 (1.2200e−02)	9 (3.0000e−04)	-	-
down	GO	cholesterol transport	3 (1.0000e−03)	16 (2.3000e−03)	-	-
down	GO	regulation of angiogenesis	-	19 (1.6000e−03)	12 (4.3000e−03)	16 (2.9000e−03)
down	pathway	Heme biosynthesis	-	1 (0.0000e+00)	1 (1.5000e−03)	-
down	pathway	hemoglobin's chaperone	-	4 (0.0000e+00)	2 (0.0000e+00)	-
down	pathway	lectin induced complement pathway	-	14 (0.0000e+00)	11 (1.0000e−04)	-
down	pathway	Retinoic acid receptors mediated signaling	2 (1.7000e−03)	-	15 (3.1300e−02)	-
down	pathway	Formation of ATP by chemiosmotic coupling	18 (2.1300e−02)	11 (5.0000e−04)	-	-
down	pathway	btg family proteins and cell cycle regulation	19 (8.3000e−03)	-	18 (1.9500e-02)	-
down	pathway	classical complement pathway	-	19 (0.0000e+00)	5 (0.0000e+00)	-

Functional Gene Ontology (GO) categories and pathways enriched with differentially expressed genes between pleural and ascitic fluids in the index hydropic case with an *ITGA9* p.G404S mutation before (Ind-B) and after (Ind-A) OK-432 treatment, and in hydropic cases with and without FC (FC-r and NFC-r, respectively) but without mutations in the *ITGA9*, *FOXC2*, *PTPN11*, and *VEGFR3* genes. Columns indicate the following information: direction of differential expressions (Up or Down), type of gene set (GO category or pathway), gene set name and ranks and permutation P-values (in parentheses) of the gene set enrichment scores in each sample (among the 4822 GO categories or 789 pathways).

We previously identified deleterious mutations of *ITGA9* – a key gene in lymphangiogenesis – from pleural fluids of patients with FC [11]. To examine the activities of *ITGA9* and lymphangiogenesis, *ITGA9* expressions and gene set enrichment scores of the lymphangiogenesis pathway from all four samples were evaluated. The lymphangiogenesis (VEGFR3 signaling in lymphatic endothelium) pathway was extracted from the NCI pathway interaction database [13] and did not contain *ITGA9*. In striking contrast to the homogeneously up-regulated immune response pathways, lymphangiogenesis activities exhibited heterogeneous directions among samples. In that regard, they were down-regulated in FC-r and Ind-B, but up-regulated in Ind-A and NFC-r. However, only FC-r had significant differential expression of the lymphangiogenesis pathway (rank 30 among the down-regulated pathways, P = 0.0071). Intriguingly, expression levels of *ITGA9* moved in the same direction as the lymphangiogenesis pathway; *ITGA9* was up regulated in Ind-A and NFC-r, but down regulated in FC-r and Ind-B. The expression responses of lymphangiogenesis and *ITGA9* are shown (Table 2).

**Table 2:** Expression responses of the lymphangiogenesis pathway in chylothorax fetuses.

	Ind-A	Ind-B	FC-r	NFC-r
Enrichment direction	up	down	down	up
Enrichment rank	154	165	30	212
Enrichment score	0.1937	0.2544	0.4216	0.1831
Enrichment P-value	0.3248	0.1550	0.0071	0.3766
ITGA9 log ratios	1.2466	−1.1786	−0.2068	1.2513
ITGA9 P-values	0.2573	0.0194	0.3169	<0.0001

Ind-B and Ind-A, pleural versus ascitic fluids in the index hydropic case with an *ITGA9* p.G404S mutation before (Ind-B) and after (Ind-A) OK-432 treatment, respectively. FC-r and NFC-r, pleural versus ascitic fluids in hydropic cases with and without FC, respectively; both cases lacked mutations in the *ITGA9*, *FOXC2*, *PTPN11*, and *VEGFR3* genes.

## Discussion

The pathophysiology of FC has not been definitively elucidated. In this study, we first demonstrated that immune response was implicated in the pathogenesis of FC by a genome-wide gene expression analysis. Using GSEA to compare genome-wide gene expression levels in fetal pleural fluid versus fetal ascitic fluids in the hydropic fetus yielded valuable insights regarding the pathogenesis of FC. It is noteworthy that GO categories/pathways involving immune or stress responses were universally up-regulated in the two FC samples before treatment (Ind-B and FC-r). It was unlikely that this phenomenon was caused by differential expression between pleural fluid and ascites, since a similar trend was not observed in the reference case without FC (NFC-r). Therefore, the merit of this genome-wide approach was to elucidate overall trends, instead of only a few known loci, of functional groups of genes and pathways among the tens of thousands of genes included in the microarray [12]–[14]. The present findings supported previous assertions that inflammation is one etiology of chylothorax [1], [15], [16].

Additionally, genes involved in the lymphangiogenesis pathway were down-regulated in the pleural fluid when compared to ascites in the two FC fetuses before treatment (Ind-B and FC-r), consistent with the conclusion that FC can result from defects in the lymphatic drainage system, including aberrant lymphangiogenesis [2], [5]–[7], [9]–[11]. Since genes involved in lymphangiogenesis changed their status from down-regulated to up-regulated after antenatal OK-432 pleurodesis treatment (Ind-A versus Ind-B), we inferred that OK-432 pleurodesis may regulate lymphangiogenesis. This mechanism should be further studied by assessing lymphangiogenesis before and after the OK-432 treatment, either with animal models of lymphedema [8], or postnatal human cases receiving OK-432 for cystic lymphangioma or congenital chylothorax [5], [15].

The therapeutic effect of OK-432 in sclerosing pleurodesis in the prenatal and postnatal cases (including malignant pleural effusion and chylothorax) was believed to include induction of a sclerosing adhesion between the parietal and visceral thoracic pleurae, thereby alleviating pleural effusion [16], [17]. However, the effects were also thought to be both cellular- and cytokine-mediated [15]. In that regard, the present study provided novel insight regarding a potential therapeutic mechanism of OK-432, namely up-regulation of local lymphangiogenesis.

Among the three hydropic cases we enrolled (Ind, FC-r and NFC-r), two were complicated with FC (Ind and FC-r). The Ind case had the *ITGA9* mutation. Although the FC-r case had no *ITGA9* mutation, it was included due to similar severity of FC and fetal hydrops. Both cases were subjected to diagnostic thoracocentesis, although the FC-r case died immediately after birth, despite vigorous resuscitation efforts. In contrast, the Ind case survived beyond the age of 6 months. This case was apparently the first reported to be successfully rescued, despite having a putative deleterious *ITGA9* allele (p.G404S) [4]. The *ITGA9* is a locus with autosomal recessive inheritance in mice [7], [10]; it was associated with a poor prognosis in human fetuses with a specific deleterious allele (p.G404S) [11]. In the Ind case, the expression array revealed that both *ITGA9* expression and the overall trend of genes involving the lymphangiogenesis pathway were down regulated before fetal treatment, but were subsequently up regulated after fetal treatment. Based on aCGH, the fetus probably had two microduplications at the 5′-end of the *ITGA9* gene, in addition to the confirmed p.G404S deleterious mutation. However, neither the *ITGA9* cDNA assay nor real-time PCR provided evidence to support the existence of the two proposed microduplications in *ITGA9*. Therefore, we concluded that the microduplication segments detected in the aCGH may have arisen from pseudogenes or duplicated gene segments located elsewhere in the genome [18]. Since the normal cotwin of the Ind case was healthy at birth, we inferred that these two fetuses shared a favorable maternal uterine compartment *in utero*.

Contrary to previous reports of a poor prognosis for patients with *ITGA9* mutations, the Ind case was successfully rescued by OK-432 pleurodesis, despite carrying a putatively deleterious allele (p.G404S). Since expressions of both *ITGA9* and genes in the pathway of lymphangiogenesis were up-regulated after treatment, the response of the lymphangiogenesis pathway to OK-432 pleurodesis may be a better prognostic indicator than simply the presence of the *ITGA9* mutation *per se*. Therefore, it would be of interest to determine the association between treatment-induced responses of lymphangiogenesis pathway activities and patient outcomes.

The clinical progression of fetal pleural effusion, including chylothorax, was highly variable, with an inconsistent prognosis among case series and systematic reviews [2], [19], [20]. Nevertheless, the rationale and efficacy of fetal therapy need to be carefully examined in subsets with distinct etiologies. In that regard, a better understanding of pathogenesis should guide development of more effective treatments. Currently available modalities for fetal therapy in chylothorax include thoracoamniotic shunting, repeated thoracocentesis, and OK-432 pleurodesis. Thoracoamniotic shunting is regarded as the gold standard [2], [4], despite some very rare complications [21]. However, OK-432 seemed promising in a small prenatal Danish series [3], and it is gaining acceptance for postnatal use [17], [22]. The finding that OK-432 treatment changed lymphangiogenesis from down-regulated to up-regulated in this study may offer an explanation of the therapeutic effect of this agent. Although there were safety concerns regarding prenatal use (based on studies in fetal sheep [23], [24]) and an outcome inferior to thoracoamniotic shunting in a large series [4], OK-432 treatment may be of value in selected cases, with appropriate case selection and patient counseling.

In conclusion, based on the current study, we inferred that both lymphangiogenesis and the immune response were implicated in the pathogenesis of FC. Future studies involving more cases for the three groups of fetuses (fetuses with FC and hydrops and carrying mutations in hereditary lymphedema associated loci, those with FC and hydrops but without mutations in relevant loci, and fetuses with hydrops but without FC and not-carrying mutations in relevant loci) are warranted to validate the insights obtained from this study. Genome-wide gene expression analysis may provide a more accurate and objective prognosis and facilitate personalized medicine by selecting the most appropriate treatment, according to the specific circumstances of the patient, for this rare, but heterogeneous disease.

## Patients and Methods

### Clinical Courses of Enrolled Cases

#### Case 1 (Ind): a hydropic fetus with FC and an ITGA9 mutation (p.G404S)

A 38-year-old, gravid 3, spontaneous abortion 1, para 1, Taiwanese woman without consanguineous family history was referred to Changhua Christian Hospital for further management at GA 20 weeks, due to bilateral massive pleural effusion, polyhydramnios, and fetal ascites in the female hydropic cotwin (the index case, Ind) of her dizygotic twin pregnancy. Amniocentesis at GA 17 weeks yielded 46,XY and 46,XX. Following comprehensive, non-directive genetic counseling, diagnostic thoracocentesis, palliative paracentesis, and amnioreduction were performed due to persistence of fetal hydrothorax, despite modification of the maternal diet (medium chain triglyceride oil and nutrients) for 1 week. The aspirated pleural fluid was a lymphocyte-rich (>99% of the cells) pleural effusion, a feature that is diagnostic for fetal chylothorax (FC). The pleural and aspirated fluids were sent for genetic investigation (sample Ind-B; Tables 1 and 2). Since FC persisted in the female cotwin 1 week after thoracocentesis, the couple chose fetal OK-432 pleurodesis of the affected female cotwin at GA 22 weeks. A detailed explanation of the risk and possible harm to the normal male cotwin was given. The dosage of the pleurodesis treatment (0.1 Klinische Einheit per side) followed our previous report [4]. Paracentesis was also performed. Both pleural and aspirated fluids were sent for genetic investigation (sample Ind-A; Tables 1 and 2). Two weeks later, the fetal hydrothorax had improved from severe hydrops (Figure 1A) to the much milder unilateral pleural effusion (Figure 1B), and the condition was much improved. Consequently, at GA 26 weeks, the woman was referred to the National Taiwan University Hospital for further perinatal care. Due to polyhydramnios affecting the female cotwin, 2 000 mL of amniotic fluid was drained. A scheduled cesarean section (GA 36 weeks) was uneventful. The unaffected male baby weighed 2608 gm, with Apgar scores of 8′ (1 minute) and 9′ (5 minutes). The male baby was normal and discharged with the mother.

The affected female baby weighed 2752 grams, with Apgar scores of 7′ (1 minute) and 8′ (5 minutes). At birth, this baby had neonatal respiratory distress syndrome, along with pulmonary hypertension. She received neonatal intensive care. A chest tube was placed in her left pleural cavity within a few hours after birth. Intensive respiratory supports, including high frequency oscillatory ventilation (HFOV), inhaled nitric oxide (iNO), and surfactant, were applied. The baby responded well to treatment, was healthy at discharge (42 days) and is now 6 months of age (at manuscript resubmission), with normal growth and development.

#### Case 2 (FC-r): a hydropic fetus with FC but without ITGA9 mutation

The FC-r case (please refer to Table 1, 2) is a case with hydrops fetalis (pleural effusion, ascites, and skin edema) but did not have the *ITGA9* mutation. The samples of pleural fluid and ascites from this case were obtained before OK-432 pleurodesis. This case did not survive after birth (immediate neonatal death) despite repeated OK-432 pleurodesis and thoracoamniotic shunting.

#### Case 3 (NFC-r): a hydropic fetus without FC and ITGA9 mutation

The NFC-r case (Tables 1 and 2) is a hydropic case caused by transient severe fetal anemia due to Parvovirus B19 infection. This fetus only received prenatal aspirations; the condition subsided 2 months after initial manifestation and the baby was born alive at term.

### Genotyping of ITGA9, VEGFR3, FOXC2 and PTPN11 genes

Genomic DNAs were isolated using the PUREGENE® DNA Purification Kit (Gentra Systems, Minneapolis, MN, USA), from peripheral blood lymphocytes or from cultured amniocytes of affected fetuses. Mutation screening of all coding sequences and exon-intron boundaries were performed for the four candidate genes, namely *ITGA9*, *VEGFR3*, *FOXC2*, and *PTPN11*, as previously described [6], [11].

In brief, each 20 µL PCR reaction mixture contained 5 ng DNA, 1× PCR buffer, 1.25 mmol/L MgCl_2_, 0.35 mmol/L of each dNTP, 0.5 µmol/L of each primer, 1× GC-RICH solution, and 1 U Faststart *Taq* DNA polymerase (Roche Molecular Biochemicals, Mannheim, Germany). The cycling condition were 95°C for 5 minutes, followed by 35 cycles of 95°C for 45 seconds, 55°C for 45 seconds, and 71°C for 1 minute, and a final extension cycle at 71°C for 90 seconds. Amplified products were subjected to bi-directional sequencing, using amplification primers from the Big-Dye Terminator v3.1 Cycle Sequencing Kit and an ABI Prism 3100 genetic analyzer (Applied Biosystems, Foster City, CA, USA). Sequence data were compared with the available reference sequences in the National Center for Biotechnology Information (NCBI; accession numbers: NT 022517, NT 023133, NT 010498, and NT 009775 for *ITGA9*, *PTPN11*, *VEGFR3*, *FOXC2*, and *PTPN11*, respectively) and 96 reference individuals. The DNA mutation nomenclature followed the guidelines of the Human Genome Variation Society (HGVS; http://www.HGVS.org/mutnomen), with cDNA numbering using the A of the ATG translation initiation codon as nucleotide +1. Descriptions of the mutations, at the protein level, were based on protein sequences NP 002198 for *ITGA9*, NP 002825 for *PTPN11*, NP 891555 for *VEGFR3*, and NP 005242 for *FOXC2*. The initiation codon was codon 1.

### Array Comparative Genomic Hybridization (aCGH)

#### DNA extraction

Extraction of DNA from the cultured amniotic cells was done using PUREGENE® DNA Purification Kits (Gentra Systems). Purity, quality, and concentration of DNA were assessed by ultraviolet spectrophotometry (260 and 280 nm).

#### Genomic DNA fragmentation

The DNA was intermittently sonicated using Branson digital sonifier (model 450, Branson Ultrasonics Corp., Danbury, CT, USA) for 5, 30, 90, or 120 seconds. The DNA fragments were then run on a 1.2% agarose gel to determine fragment-size distributions

#### DNA labeling and hybridization

Agilent's Genomic DNA Labeling Kit PLUS (Agilent part number 5188–5309) was used to label the sonicated DNA with either Cyanine 3 (Cy 3) or Cyanine 5 (Cy 5). As recommend by the manufacturer, 0.5 µg of genomic DNA was used as the input starting material for each labeling reaction. Samples labeled with Cy 3 and Cy5 (CyDye, PerkinElmer, Norwalk, CT, USA) were mixed and hybridized to the ITGA9 Tiling chip (Agilent Customer Array, Changhua Christian Hospital, Changhua, Taiwan) following the manufacturer's standard processing recommendations. The ITGA9 Tiling chip consisted of a probe backbone of Agilent SurePrint G3 Human CGH Microarray 8×60 K (Agilent design ID: 021924), with two customer-designed ITGA9 45-mer probe groups: 1)10,441 tiling probes with a space of 20 bp; and 2) 4,201 interval probes with space of 50 bp (Appendix S1). The customized probes spanned across chromosome 3 region of 37,483,813–37,861,279, which were designed based on the human reference genome NCBI Build 37.2 and using the tool of Agilent eArray (https://earray.chem.agilent.com/earray/). A dye swap test (modified from [25]) was also performed to confirm the results.

#### Microarray scanning and data analysis

Scanning and image analysis were conducted according to Agilent's Oligonucleotide Array-based CGH for genomic DNA analysis Protocol (Version 4.0). Microarrays were scanned using an Agilent DNA Microarray Scanner (G2565BA). Agilent's Feature Extraction software (Version 9.1.3) was used to extract data from raw microarray image files in preparation for analysis. Agilent CGH Analytics software (Version 3.4) was used to visualize, detect, and analyze aberration patterns from CGH microarray profiles. The chromosomal coordinates for fragmental rearrangements and genes included were based on NCBI Build 37.

### Analysis of Complementary DNA (cDNA)

#### RNA extraction and reverse transcription-polymerase chain reaction (RT-PCR)

Total RNA was extracted from 1.5 mL of whole blood or from 3×10^6^ cultured cells, using the QIAamp RNA Blood Mini Kit (Qiagen, Hilden, Germany). Then, cDNA was synthesized using the Quantitect Reverse Transcription kit (Qiagen), according to the manufacturer's instructions.

#### cDNA cloning of ITGA9

To confirm whether the two variants were on the same alleles, a specific oligonucleotide primer set was used to amplify (by PCR) and clone the partial sequence (from exon 1 to exon 12) of the *ITGA9* gene. The sense primer c1F (5′-CCCGCTGACTCGTTCTTC-3′) and antisense primer c12R (5′-GGATAGCCATTTCCATCCAT-3′) were designed to be complementary to the coding sequences of exons 1 and 12, respectively, of the *ITGA9* gene. The PCR reaction mixture and cycling conditions were as described above, but with an 1199-bp product. Preparation of cDNA and subcloning of plasmids were all done as described [26]. After subcloning and transformation, the host was screened by blue-white selection on a medium containing a selective antibiotic (ampicillin). Each individual colony was collected and inoculated into 2 mL of LB broth (containing the antibiotic) and incubated overnight at 37°C with shaking. Plasmid DNA was isolated with Gene-Spin MiniPrep Purification Kit (Protech Technology, Taipei, Taiwan); the presence of pure plasmids was verified by cleavage with several restriction enzymes. Finally, each pure plasmid DNA was analyzed by sequencing.

### Real-time PCR for ITGA9

Real-time PCR was performed to verify the two probably duplicated regions detected in aCGH analysis. A total of six pairs of primers were used, two for targeted sequence and four for reference exons of the same gene. Primers q1F: 5′-GGAGCATTTCCACGACAACAC-3′/q1R: 5′-CGTGGAAGAGACCGGAAAGG-3′ are specific for the 3′ -region of exon 1, and qIVS1F: 5′-GGCGATTTAAATGTCTCCGTTG-3′/qIVS1R: 5′-GGCGGAGGAGACAACTCTAGC-3′ are specific for the IVS1 region; the amplified fragments were 121 and 185 bp, respectively. The four reference primer sets, specific for exon 4, 6, 15 and 16, respectively, were designed and selected based on the closest Tm value and highest amplification efficiency (nearly 100%), which were q4F: 5′-CTGCTACATCATCCCCTCCAAC-3′/q4R: 5′-ATGTTCAGCTGGTGGTGCATAG-3′ for the exon 4 (150 bp), q6F: 5′-GCTGAACCTTACGGACAACACC-3′/q6R: 5′-TCACTCCCATTTCCCCTTTCTT-3′ for the exon 6 (121 bp), q15F: 5′-ACTTTGTGCTGCTGGGAGAGAC-3′/q15R: 5′-AAGGAGGAGAGGACTGACCTTCA-3′ for the exon 15 (116 bp), and q16F: 5′-TGTGACTGGAGAGGAGGAGAGG-3′/q16R: 5′-CCACCTTGGGTGCTGAGTATG-3′ for the exon 16 (126 bp). The qPCR was performed on the 7700 ABI Prism Sequence Detector (Applied Biosystems) in 20 µL reaction volume, which consisted of 0.5 ng DNA, 0.5 µmol/L of each primer, 1× ROX, and 1× SYBR Green PCR Master Mix (Finnzymes, Espoo, Finland). Each reaction was done in duplicate. The cycling conditions were: 95°C for 15 minutes, followed by 45 cycles at 95°C for 10 seconds, 58°C for 10 seconds, and 71°C for 10 seconds.

### Genome-wide Expression Array

#### Samples

Four pleural fluid versus ascetic fluid sample-pairs were enrolled. Two sample-pairs were from the index case (Ind-B and Ind-A for samples collected before and after fetal therapy with OK-432 pleurodesis, respectively). The remaining two sample-pairs were from two additional hydropic cases, one complicated with FC (FC-r) and one without FC (NFC-r); both cases lacked mutations in the *ITGA9*, *FOXC2*, *PTPN11*, and *VEGFR3* genes.

#### Total RNA purification

Cell lines were lyzed by Trizol® Reagent (Invitrogen, Carlsbad, CA, USA), followed by processing with an RNeasy Mini Kit (Qiagen). The isolated RNA was quantified at OD 260 nm with a ND-1000 spectrophotometer (Nanodrop Technology, Wilmington, DE, USA) and characterized with a Bioanalyzer 2100 (Agilent Technologies, Santa Clara, CA, USA) with RNA 6000 nano labchip kit (Agilent Technologies).

#### Experiments of Expression Array

The RNA from pleural and from ascitic fluids were labeled with Cy5 and Cy3, respectively. For this, 0.2 µg of total RNA was amplified by a Low Input Quick-Amp Labeling kit (Agilent Technologies) and labeled with Cy5 or Cy3 (PerkinElmer) during the *in vitro* transcription process. Then, 0.3 µg of Cy-labled cRNA was fragmented (average size of ∼50 to 100 nucleotides) by incubation with fragmentation buffer at 60°C for 30 min. Correspondingly fragmented labeled cRNA was then pooled and hybridized to a SurePrint G3 Human GE 8×60 K oligo microarray (Agilent Technologies) at 65°C for 17 h. After washing and drying (blowing with a nitrogen gun), microarrays were scanned with an Agilent microarray scanner (Agilent Technologies) at 535 and 625 nm for Cy3 and Cy5, respectively. Scanned images were analyzed by Feature extraction 10.5.1.1 software (Agilent Technologies), and image analysis and normalization software was used to quantify signal and background intensity for each feature. This software substantially normalized the background-subtracted signal by rank-consistency-filtering, using the LOWESS method. The log ratio, the log_2_ Cy5/Cy3 ratio, of a specific locus is applied to normalize the differential expression of dyes. To determine the differentially expressed genes, the P-values of the log rations are calculated by Student's *t*-test. The P-value is a measure of the confidence (viewed as a probability) that the feature is not differentially expressed. In that regard, a very small P-value (typically, <0.01), indicates to a 99% confidence level that the gene is differentially expressed.

Finally, all data were MIAME compliant and raw and processed/normalized data were deposited in the Gene Expression Omnibus (GEO) database (accession number GSE33872; Appendix S2).

### Gene Set Enrichment Analysis on differentially expressed genes

The Gene Ontology (GO) annotations of human genes were downloaded from http://www.geneontology.org/. They consisted of 4822 categories with overlapped member genes. Pathway information was extracted from three sources: Reactome [14], NCI pathway interaction database [13], and BioCarta (http://www.biocarta.com/). In total, there were 889 pathways with overlapped member genes. For each sample, Gene Set Enrichment Analysis (GSEA) was applied to identify the GO categories and pathways enriched with differentially expressed genes [12]. In brief, for each sample, log-ratios of gene expressions between pleural and ascitic fluids were sorted by a decreasing order. Denote *L* = {g_1_,…, g_N_} the sorted genes. To evaluate the enrichment of up-regulated genes in a gene set *S* (a GO category or pathway) with *N_S_* members, define a function *f* over the numbers 1,…, *N*. For each rank 1≤*i*≤*N*, *f* (*i*) evaluates the relative frequency of finding members in the gene set *S* minus the relative frequency of finding non-members of *S* among genes above or equal to rank *i*:
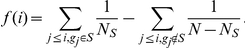



The enrichment score *ES*(*S*) is the maximum of *f* (*i*) over 1≤*i*≤*N*: *ES*(*S*) = max_1≤*i*≤N_ *f* (*i*). *f* can be viewed as a random walk along the sorted genes, with an up increment 1/*N_S_* when a gene belongs to *S* and a down increment 1/*N*−*N_S_* otherwise. Intuitively, if the majority of members of *S* have high rankings in *L*, then *f* encounters more up increments and thus yields a high maximum. The enrichment score of down-regulated genes can be evaluated reciprocally by reversing the order of genes in *L*.

To assess the significance of an enrichment score, gene orders were randomly permuted in *L*10 000 times and enrichment scores were evaluated on *S* of the permuted genes. The P-value was the fraction of random permutations that yielded higher enrichment scores than the empirical value from the unpermuted *L*.

### Ethics Statement

This study was approved by the Ethical Review Board of Changhua Christian Hospital (IRB ID: CCH-IRB-091209). All patients and/or legal guardians gave written informed consent to participate in this study and for publication of clinical pictures and details in scientific journals.

## Supporting Information

Appendix S1 **The probe list of the customer ITGA9 Tiling chip (Agilent Customer Array, Changhua Christian Hospital, Taiwan) used in this study.**
(PDF)

Appendix S2 **The results of genome-wide expression array submitted to Gene Expression Omnibus (GEO) (** http://www.ncbi.nlm.nih.gov/geo/ **).**
(RAR)

Appendix S3 **The ITGA9 genotypings for the mutation c.1210G>A, p.G404S in the familial members of the index case (Ind).**
(PDF)

## References

[1] Chen M, Hsieh CY, Shih JC, Chou CH, Ma GC (2007). Proinflammatory macrophage migratory inhibitory factor and IL-6 are concentrated in pleural effusion of human fetuses with prenatal chylothorax.. Prenat Diagn.

[2] Deurloo KL, Devlieger R, Loproire E, Klumper FJ, Oepkes D (2007). Isolated fetal hydrothorax with hydrops: a systematic review of prenatal treatment options.. Prenat Diagn.

[3] Nygaard U, Sundberg K, Nielsen HS, Hertel S, Jørgensen C (2007). New treatment of early fetal chylothorax.. Obstet Gynecol.

[4] Yang YS, Ma GC, Shih JC, Chen CP, Chou CH (2012). Experimental treatment of bilateral fetal chylothorax using in utero pleurodesis.. Ultrasound Obstet Gynecol.

[5] Chen CH, Chen TH, Kuo SJ, Chen CP, Lee DJ (2009). Genetic evaluation and management of fetal chylothorax: review and insights from a case of Noonan syndrome.. Lymphology.

[6] Tartaglia M, Kalidas K, Shaw A, Song X, Musat DL (2002). PTPN11 mutations in Noonan syndrome: molecular spectrum, genotype-phenotype correlation, and phenotypic heterogeneity.. Am J Hum Genet.

[7] Bazigou E, Xie S, Chen C, Weston A, Miura N (2009). Integrin-α9 is required for fibronectin matrix assembly during lymphatic valve morphogenesis.. Dev Cell.

[8] Saharinen P, Tammela T, Kaikkainen MJ, Alitalo K (2004). Lymphatic vasculature: development, molecular regulation, and role in tumor metastasis and inflammation.. Trends Immunol.

[9] Tammela T, Alitalo K (2010). Lymphangiogenesis: molecular mechanisms and future promise.. Cell.

[10] Huang XZ, Wu JF, Ferrando R, Lee JH, Wang YL (2000). Fetal bilateral chylothorax in mice lacking the integrin α9β1.. Mol Cell Biol.

[11] Ma GC, Liu CS, Chang SP, Yeh KT, Ke YY (2008). A recurrent ITGA9 missense mutation in human fetuses with severe chylothorax: possible correlation with poor response to fetal therapy.. Prenat Diagn.

[12] Subramanian A, Tamayo P, Mootha VK, Mukherjee S, Ebert BL (2005). Gene set enrichment analysis: A knowledge-based approach for interpreting genome-wide expression profiles.. Proc Natl Acad USA.

[13] Schaefer CF, Anthony K, Krupa S, Buchoff J, Day M (2009). PID: the Pathway Interaction Database.. Nucl Acids Res.

[14] Joshi-Tope G, Gillespie M, Vastrik I, D'Eustachio P, Schmidt E (2005). Reactome: a knowledgebase of biological pathways.. Nucl Acids Res.

[15] Samuel M, McCarthy L, Broddy SA (2000). Efficacy and safety of OK-432 sclerotherapy for giant cystic hygroma in a newborn.. Fetal Diagn Ther.

[16] Chen M, Shih JC, Wang BT, Chen CP, Yu CL (2005). Fetal OK-432 pleurodesis: complete or incomplete?. Ultrasound Obstet Gynecol.

[17] Matsukuma E, Aoki Y, Sakai M, Kawamoto N, Watanabe H (2009). Treatment of OK-432 for persistent congenital chylothorax in newborn infants resistant to octreotide.. J Pediatr Surg.

[18] Zheng D, Frankish A, Baertsch R, Kapranov P, Reymond A (2007). Pseudogenes in the ENCODE regions: Consensus annotation, analysis of transcription, and evolution.. Genome Res.

[19] Yinon Y, Kelly E, Ryan G (2008). Fetal pleural effusions.. Best Prac Res Clin Obstet Gynecol.

[20] Ruano R, Ramalho AS, Cardoso AK, Moise K, Zugaib M (2011). Prenatal diagnosis and natural history of fetuses presenting with pleural effusion.. Prenat Diagn.

[21] Chao AS, Chao A, Chang YL, Wang TH, Lien R (2010). Chest wall deformities in a newborn infant after in utero thoracoamniotic shunting for massive pleural effusion.. Eur J Obstet Gynecol Reprod Biol.

[22] Ono S, Iwai N, Chiba F, Furukawa T, Fumino S (2010). OK-432 therapy for chylous pleural effusion or ascites associated with lymphatic malformations.. J Pediatr Surg.

[23] Cowie RV, Stone PR, Parry E, Jensen EC, Gunn AJ (2009). Acute behavioral effects of intrapleural OK-432(Picibanil) administration in preterm fetal sheep.. Fetal Diagn Ther.

[24] Bennett L, Cowie RV, Stone PR, Barrett R, Naylor AS (2010). The neural and vascular effects of killed Su-Streptococcus pyogenes (OK-432) in preterm fetal sheep.. Am J Physiol Regul Integr Comp Physiol.

[25] Ma GC, Ke YY, Lee ML, Tsao LY, Yang CW (2010). De novo triple segmental aneuploid of 1p, 1q, and 4q in a girl with hypertrophic cardiomyopathy, muscle hypotonia, and multiple congenital anomalies.. Am J Med Genet A.

[26] Pemble SE, Taylor JB (1992). An evolutionary perspective on glutathione transferases inferred from class-theta glutathione transferase cDNA sequences.. Biochem J.

