# Supplementary material for: Genome-Wide Gene Expression Analysis Implicates the Immune Response and Lymphangiogenesis in the Pathogenesis of Fetal Chylothorax
Source: PLoS One. 2012 Apr 18;7(4):e34901. doi: 10.1371/journal.pone.0034901 (PMC3329545; doi:10.1371/journal.pone.0034901)

Appendix S3. The *ITGA9* genotypings for the mutation c.1210G>A, p.G404S in the familial members of the index case (Ind). The p.G404S mutation was only found in the female hydropic fetus (*i.e.* the ind, indicated by arrow) and thus was deemed to have arisen *de novo*.

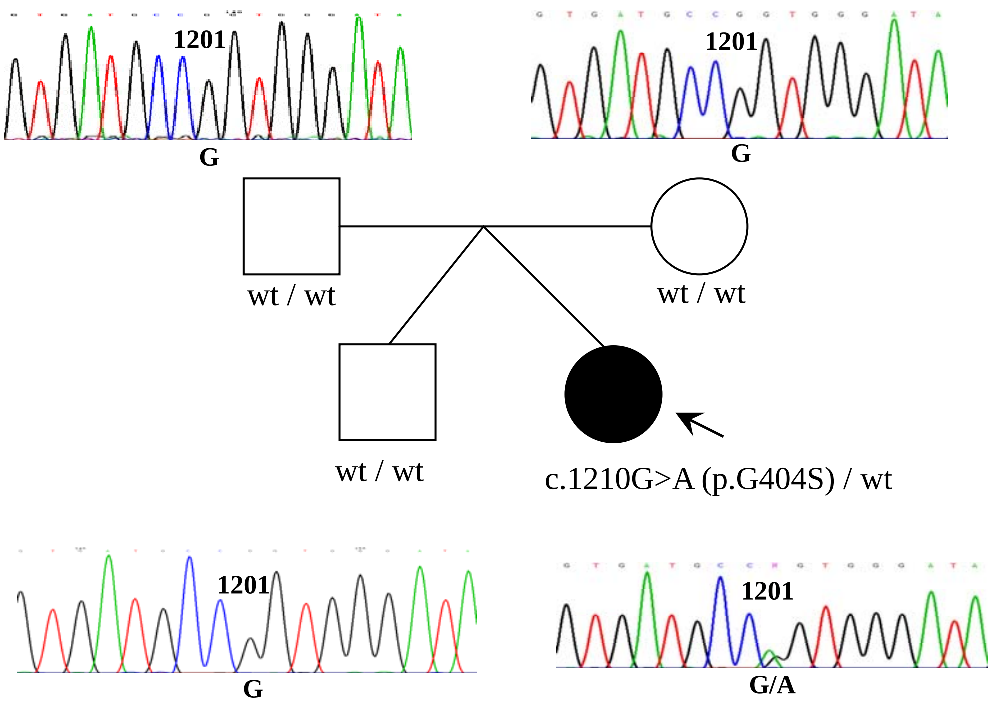

Supplement: Appendix S3 — The ITGA9 genotypings for the mutation c.1210G>A, p.G404S in the familial members of the index case (Ind). (PDF) [file pone.0034901.s003.pdf]
