# Supplementary material for: Genome-Wide Gene Expression Analysis Implicates the Immune Response and Lymphangiogenesis in the Pathogenesis of Fetal Chylothorax
Source: PLoS One. 2012 Apr 18;7(4):e34901. doi: 10.1371/journal.pone.0034901 (PMC3329545; doi:10.1371/journal.pone.0034901)
Supplement: Appendix S1 — The probe list of the customer ITGA9 Tiling chip (Agilent Customer Array, Changhua Christian Hospital, Taiwan) used in this study. (PDF) [file pone.0034901.s001.pdf]

Appendix S1: The probe list of customer ITGA9 Tiling chip (Agilent Customer Array, Changhua Christian Hospital, Taiwan).

ProbeID	Sequence
A_14_P126474	AGTATCAGTGATGCTGCCCTGTTAGACATCATTTGTAAGTGTCAGTGATTTGTATTTATA
A_14_P201291	AAGATGAAATTTCATAGAAAGTCTTCAAAGAGGAAAAGCAATGGTCTTTTCCCTCTGCTA
A_14_P129860	CTCTCGGTCCTATACAAGGAGTATGCTGAAGATGACAACATCTATCAACAGAAGATCAA
A_14_P112145	GCCAGTAAATTAGGACAGTGTTTCAACAAGCCTAGGCTATCTCGTAAGTTGAAAAATATC
A_14_P119265	CTCCTGTCACTTCTAACAGAAATAGCATAAAGTATCCAGCAGGAAAAGTGCACACAATAT
CUST_4306_PI426210822	ACAGGTTTCATGTCTTAGCCTTGGGACTACATGTTTTCTCTTTCCCTTCT
CUST_4023_PI426210822	GAAGAAGCCATGAAGGGCTCGGCCATAACCCCGGGAAAGCTCACT
CUST_2226_PI426210822	TAGTGTATTAGGACAAGGCAGAGAGTGTAGTCATTGGCTACCATGACATTCTC
CUST_1693_PI426210822	TCTTTGGAGTTTTGTCTATAGCTGAAAGATTTCCTCTAGAAATTTTGTTGATTTTAACCT
A_14_P102394	GGAGATTTGGAGCCCATTTTAGAGGCATCTGTTCTATCTTCCCATCATAAAAAAGTAAGT
CUST_3498_PI426210822	ATAATCTCTCCCTCTAGTGTTTTGGAACAATCACACACAAATGTTTATATCCACATACAG
A_14_P104870	GCCTCTATCTGCTCCTGTTGATATTTAAAAATATCTGTTATTTGATTTGGGGGTGAAGGA
A_14_P108415	ATTCACCTAAGACACATTGGCTCAAACTGGAAAGGAGTAATATTTCCTGACAAAAATTCA
A_14_P125940	AGTTATTCACATAGTGTCTACAGGGCAGAATCTCTTCCAAAGCAATTTTCTGTTCACTAA
A_18_P13888920	CACTCAGAAAACTCCACTAAATTTATGTTTCCCAATTAAAACTTCACCTATTTACCTCCA
A_16_P40463081	TCCCAACCTAGGAATATGAGAAGTATCTGTCAGGTAAATGGTTTTGTGAATAATGCTGTA
A_14_P113185	AATCATGGCTCAGGATAGAATACGCCAGTTTATTTATTCTACCACAGTCATGGCAGT
A_16_P17838182	ACAGGCCCATTAGTATATCTCTTCAGTAGATTATGCCGGATATGGATTTTACTCTGACAT
A_14_P108946	AATGAGATCACAAATGTTGGAGACAAACTGTAAGAAACTGACAAAATGACATAAAAGGCC
A_14_P126509	TTAGGATATAAGAGGGCTTTTTAAATTTGGTGCCGATGTTGAACTTTTTTTAAGGGGAGA
A_14_P113516	GACCAAGAAGCCTGGCATGAACTTGCAGAACTTTACATCAATGAACATGAGTAAGTTATT
A_16_P17716115	AACACTTCTATTGCCATCCTTACTCATTATACCACTAAACACTCTGATTTATCTCCCTGC
A_16_P18301351	AACTGATGTTGTCAACCTTTGATTATGGTCAGATTTCTACACATGCTGTAAAAACACACG
A_14_P115632	AGTGATTTGTTTAAAATGCAACATCACAGCATACTCATACTTTCAACAGGAAGCACATCC
CUST_4338_PI426210822	CTCCATAGAGCAGTGTATTTGGTTTTCTTAAGCAGGAAGAAGGGGGC

A_16_P02686289
CATGTCATTAGAAGCCTAGATTCAAGACCTTCATTTCCATCATGCATTGACATGTTCTG
A_14_P133978 TTTATAATCACCTTACAAGTAAGTTAAAGTGCATTCCCTGGTCGTTTTGGCTTTGTCTTT
A_14_P124258 TGGCTCATTTCCTCATTAAAGTTTGAATTTTCCAGTCACCAGTGTTTTGTTCTTTCTTAC
A_14_P127130 AACTTCTCTCGGCTGTTCTCAGTTCCTCCATTTATCGTTTCTTCGTCATGT
A_14_P202097 TGTATGGATTCTTCTTTCAACCTTGACGAAAGGGTATAATTAATCTGTAATTAAAGTCAT
CUST_2311_PI426210822
TTTCAACTCTTACTCAGAGTTGGAGCCATTTATTTAAGAAAGGAAAGGAAGCTTTCTCTT
A_18_P17039232 CCCAGTGACTCCTGCTATCTTCATTTTGTCCAGTTCACCTCTCAGTC
A_16_P17218405
CTTCAGTTCCCATAAAACCAAAAATCACGATTGTCTTTTCCATCATTATAGTCACTGTGG
A_14_P120490 CCCTGTTTTTCTGTTTTGTTTTGTTTCTCAAGTTTCATTTTTTACTAAGCCCCTTCTGAC
A_14_P129911 ATCTGTAAAACTTGGGGACTCCCATTTCGATCGTCAACACTAATCCCAG
A_14_P117324 CTCAAGTAATTCTTGGTTTTATTTTTGTAGGCTCAGATCACCATGAAAGTAATCCAAGGC
A_14_P200933 ACACATTAGATGTTACTGTGTGAAGAATAATGCCTTGTATGGTGTTGATACGTGTGTGAA
CUST_5490_PI426210822 GTCCAGCATCTTGCTAGCCTGGACATACTTTCCACAGTGAACCTAT
CUST_9404_PI426210822
AACTCTTTGTGAACATGTCAATATCAATATCCAAATATCTGGCTCTAACTGCTTGGAAAC
A_16_P17318754
ATTGCGCGGCATTAGAATAAACATGTATACAATGAAAAACCTCCAGATTGACACTTCTTC
A_14_P102021 GAATCAGTGAAAAGAACCTGGGAATGAAGTTTCTGAGGTTTAAGTTAGTTTCTACAGATC
A_14_P117361 CTTTAACTTTTTTTTCCTAAAACACATTGCTACTCTTCCTTTCCCATGTTCGTTTCTGGA
A_16_P21503674
AAAAAATCAAGATGGACAGTTGAAAGTCTTAGGCCAATAAAGTTCTTTACAAATATCCCT
A_14_P136493 AAGAATGATTACTTTGAGGAAGAAAGGAAGACTTTTAGGAAAGAAGCGACTTTGTTTTCG
A_16_P21184352
CTCAAAACCTGAGAGAAAAGCAAATGGCGAATGTGATTTCATAAAAATGTTTCACTGACA
A_14_P108668 CTCTTTAAAACACTGCACAGGACCAGACAACAAGTTTTTAAAAATGATGCCAGAGCATTA
A_14_P130289 CTGTCTTTTTACTTAGTCTTGCTGACACCACTTAAAAAGGAACCTCAGACAGATAGATTC
A_18_P14137986 CCTATAGAGTGAGCCTTCCTTTGGTTTTTCTTTCTCCTCTGTATCAGCATTTGATC
A_14_P104291 ACCTGTTTCTGTTTGTCTGCTTTTGCAGGAGAGTAGTTATCTTGATGGAATTAGAAGTTT
A_14_P201326 TTCCACATTTGGTTTGGTGAACAGATTCTCATTGATCACTTGTCTCTAGAAGGATTATAG
A_14_P125462 CTCCAAGTTTCTTCCAACTTGATACTTCTTTTATAACCTAGAATCCTGTGGTATGATGGT
A_16_P15076426
GTCTCTCAGATAAAGATGCCAATTCTAATGGTGTAGAACGTTCCTCTTTTTATAGTGGTG
A_16_P00836034
CCACTGCACTTTTTCAATACCAAGAAAATGGAATTACTGCCTAGTAAATTTCTCACCACT
A_18_P14114886

ACTGCAACAAAAAATACTTCTCATTGATAGGAACAGAACCAGTTATGATTCTTGAAGCCT

A_16_P02220975

GTGACTTCCTACAAAATTCCTGAAAAACTATTTGCCACTACATATGATCACACCAAGTCA

CUST_9072_PI426210822 GTGAAGTCCTTTTGTTCAAATTCTAATAGCCATGGACCTCTGCCAGCTC

A_14_P107372 GTTTTTTAATTTGGCTGTAAAGAGTAGGGTAGGAGGCTGTGCACTTGAACAGTTTGTAA

CUST_2497_PI426210822 GCCTGTGGTGGTTGTGGCTAACTGGATAAAGATACATGATTTCAAACCC

A_14_P127666 TCGACTTTATTATTAATACGGTAAAACTACAGAGAGTTTGAAACATCAGTCTGGGGCGTC

A_14_P128347 TTCGGATACTATTTGTGAAGCAAGTCAGAGGAATGAAAGACTGTCATAACAGTCTTGAAT

A_18_P14820363

GGACTAGATCTCAGAACTAATTTCCTATCCTCACAGGTATTCATCATTCACTCAATGGAA

CUST_2817_PI426210822 CATGTGTGCACACACGCCCACTCATGCAGAAATATTTACGTGAGCTA

A_14_P107007 ACAGTTGGCTTGGAATTATGTCTTTCTCTTTTAATTTGATTGAGTGGAAATCTGAGTGAA

A_14_P123051 TTGCTTTTGTATGTTGGGTCAACTTGGCCTGACGTTTCAGAGGTAGAC

A_14_P120150 TAACTCAGCAAGGCTGTGGTCTGATCTAATTTGCCTTTATAAATGCCTACTTCATGG

A_16_P02248578

TCAATACTCAAATCGTTTGCTCTAAATCTCTACCTTAGTTCTTACATCTCACCTCTCCTC

A_14_P105429 ACTTCCAATAACAGTGTATCTTTACAATATTCCAAATTCAGTCTTCAGATGCCCCCAAAT

A_18_P15651117

TACTAGCATCAGCCAATGTCTAACTCTTAATTTGTCATTTGCCACTTTTTTTCTTACCCC

A_14_P115281 AGACAAAGCCCTTAAGAGGAGATTCTCCTTGGCAACAAACTTAAAGGGTTAAAAC

A_16_P16886738

AATGACTTGGTTATCTGCATTCTTTGAGGCAATTCATGAACTCGATTTCATCTGAGGAAT

CUST_5618_PI426210822 CATATTTTGCAGTTTGGGGAGATCGTAGATGTGCCTCAGGGAGATTG

A_14_P127245 CATTGCAGTGGAATCCTGAGAAGAATGGAGAGAGAAGAATGAGGTATGAATGAAAGAT

A_14_P122141 CAAGGAAATTTTATTACAAATTCCTTATAAAGGTGGCAGTGTGGCACCATCTACCTAGAA

A_14_P106628 CCTGTGTGGTGTTGTCATGGGGATGATAATTGTTTTCTTCAAATCCAAAGGTA

A_14_P111965 CAAACAGCACTCATTTGCACCCGGAGCATTTTCTGACACTGTTTGATTTTC

A_14_P139931 CATTAGAAAAATCGAGATCTACTTACTGAAAGTTGAGGGTGTTGGAATAGAGGTGCAG

A_16_P02991999

ACTTATCTGAGTGAAGACTTCATTCACACTGAGACATGTTTGCTTATCTGAGACAAGAGT

A_14_P128154 CTATTTATCATCATATGATGTCAGCCCTGCTTTAGGTATGTAGACTTTTCCCTTCCCATA

A_14_P132564 AAAACACATCTTATATGTGGATACACAATCCTGAGGAAGAAATCCTGTCAATAACGTCTC

CUST_4054_PI426210822 ATCAAGTCACCTTGCTCATTCACAGTCATGTGTGGCTAGGACACC

A_14_P104995 CAATAATACGTCAAGCACCAAATGCTACAAAGAAATCATGAAAATAGGCCTTAAACGAGT

CUST_1673_PI426210822 CATAACACGTATTGTTTAAGGCATTGCCCTCTTGCCTGCAGGCTCTAT

A_14_P117235 CATACTAAGACTTGGGCCTTATTTTCAGGATAAAGGTGTATTTTGATTGCCAATTTCTGC

A_16_P00779908

AGGACACTTCTGCCTAAATTCTCCATTAAGATACACATTTTCCATACATACAGTGTAGCC

CUST_5729_PI426210822 GTTTTAAGGCATTTTTTTTCTCACTCCCAGTTGCCTGCCCATGTCTG

A_14_P108014 GGAAAGGAGAAAAGTAAATCACTATTTCTCATCCTCAGATTCTTACAATCTGGACAGGAA

CUST_379_PI426210822 TGCAAGGGACTCAATTTTCTTTTTTTCTAATAACACCCAGAGTGTCTGCCTG

A_14_P121839 TCTAAATACTTACCATTTTATCAGCAGACACCAAGGTCCTAAGGTAAAAGGACTTGGAAA

CUST_10095_PI426210822 ACTGAGTGTTACCCAGCTGCCACTGCTGACCGGTTGCCCACTTCA

A_14_P125204 CGAGCCCAGAAAGCCACTTTATCTGGAAGACTATTTTTTCTTTCAAGGATTATTTAGGTA

A_14_P133419 CTCTCTGCTTTTAGAGTGAATGACTAGAACACATGACTTCATCTAGAATCAGTGTAGCAT

CUST_7601_PI426210822

GAATTCAACTTGAATTCTTAGTTTGTTGACTAAGAATTTGCAGCCTCCGAGGAAATTTTT

A_16_P18783717

AAAAAAATTGTGCCACAATTTCTACTGTCTTGACAAAGATTACTTCTGATTTGCTTCCCC

CUST_143_PI426210822

TCTGAAAAGCCAATGTTGTTTTCACCCCAACATTTAATCATTCCAATATTCCTCTCTG

A_16_P18647433

AGTATTTCCTATCAGATCCCTTTCTCTACCTACAAATGTTTGATTCTTCTGTTTCGCTCA

CUST_8957_PI426210822

AGCATGAAATTAGCCATGTTCATATTAAGGAGCAATCTAAGATCATTATTGCTGTTTCTC

A_16_P18707134

AGAAACTTAGCAGTAGTTCAAACAACAGACAGTGTATTTGTAAATTCCTAACAGGCTCCA

A_16_P18325358

AAATCCAGCAAAACTTACTTTTACTCATTCATCAGTTCTATGTCACTCCTTAGTTTCCCT

A_16_P19139317

ACCCCTCCAGTTATTAACATACTCCTTCTCAATAAAACAGTAAGACACCTGATATACGTC

A_14_P115075 ATTCTGCTGCAATAAATGTTTCTACCCTCTTGCGATAGAATGGTGTCAGATGTCTAACAT

A_16_P17379648

AGAAACACAACTGAGTTTGTAAACTATCATGCTAGCAGTCCTAATTATTGAGTGCGTCTG

A_14_P138157 ACTCATGTTATTTGGGAATATTGGGATTAGACACTGAACATGTGGAAATTCATTGCTCAG

A_18_P25452176

AAACAACCTAAAACACAAGAAAGATGAGTTTTTTAAAAAATGACACGGGGAATTTAATGT

A_14_P134584 GTAATGAAATGATCATCTTGCTGCAGAAGTATATGATGACAAAGCCTCAGAGGTTATACC

A_14_P128338 CACCCTTGCCTGGAAAAAGAAGGAAGAATGAACAGAGAATAAACAGGGCAAATA

A_18_P22841637

TGCAGAAGAACAAAAAAGCCAGTTAAACTTATTCCTTTTTTTAAATAGCAATCTGGAATG

A_14_P113616 TCTGCTAAGCAAAATCTTCTAAGTTCTAAATCAGATTTGAATCATGGCAGAATTCAAGCA

CUST_9687_PI426210822 ATCCTATGCCATCAGCAGTGGGTGGTGAGTAGTTCTTTCTCTTCAC

A_14_P131050 GATCAATATGAGTTTGCCCCTGGGAGATGGATATGCTTTCAGATTACTAACAAAAATAGT

A_14_P111144 GTTTTGTAGGTGCTGACTGGGTTACTTTTTTAAACACTAGGAATGGTAATTTCTACTCTT

A_14_P123468 TCCAATAGTTTGACTTTATTAAATCAATAGAACGGGATCTCAGTGGTTAAGCCGTCTTAA

A_16_P19184575
AAAGACTTTAGCCTCTGAGATTAAAAGCATCCCTCAAGTATAATGTCACCTTCTCTTCAT
A_14_P109119 ACTGGGACATTTTCATTGATATTCTATACACCAAAGTTTTGACGGTTTACCATCATTCCA
A_16_P16847730
ATTCTTAAAAAAACACAATCTGATGAACTCTCCCCAATCTATGATTCGTTCCTCTTGGAT
A_16_P18979994 TTGGATTATCACCTCCAGTAAAGTAGACCCTACATGTGGTTAACCAGAAGACTGAT
A_14_P106137 TCCTACCTCTGGATTACCTTAAAATACTTGTCTAGGATTTCACTGTAGTTGCTGCCTTT
A_14_P137461 GGTCTAGCACTTGAAGAAATAATAGGTAGATTTGAAATGAAACAACCCAGGGATATTCCT
CUST_4865_PI426210822
TAGTTTTCTACCCTTCAAAATACTTTGTGTTAAACTCAATTACAGAGATAGATCCTTCCA
A_16_P58424117
TCTGCTACTTCCTCTTATTAACAGTGTAATCTCAGATGGGTTATTTAAGTTCTCTGTGCC
A_16_P16922957
AAATGGGAACAGAGAATACTGATGCTGTCTGTTGAATGTAGTGGAATACGAAGAAACTAA
A_14_P119687 CTTTAATCAATACATTTTGTTAAATGTGCCATAAAATGAGACTTTTTACGCCTTTATAAG
A_16_P17839268
ATACCTTCTAGGAAGCACATATCTTGATTCCCAAAGTCTGATCTTAGATGCTCCACTTT
A_16_P17733619
AAAATGAGCACCTTATTAGTTTTATCCCTGGTATGACAATTAGTTACATGACCTCCCACT
A_14_P113847 TCCGGAAAGTTTGCTCTTTAGTATTTCCGTTGTAATTTATTACCTCCTGCTTGAAATTGA
A_16_P00824169
CTACACCCTCATAGAAATGTATCTCCCTTTCTACACATATCCAGATCCTATACATCCTTC
A_16_P36230906
GTTTAAGTTTGGAATGCCTGCTGATGCCAAATACCACTTCCTTTTTCACTAGTATGAATT
A_16_P40075007
CCCAGAATTCTAAGAGAACTCATCTTCACTAAGTCTGTCTGTCATCATGAACTACCTATT
CUST_4818_PI426210822
CATTTCTTGATCATTTCAAAGTGAGGTCAGTTACAGAGTGAATGGGTTCATCCTG
A_18_P16172286
AAAAATTGGAGAGGCATTAGCAGTCTTGAGATGTCCAAACCTTTACAGAAGACGATAAAT
CUST_3360_PI426210822
TGTGATTATAAAGATCCCTTCTGCCTCAAACCTTGAACTTAGATATTCATTATAAGCATG
A_14_P202256 AATCCCATGAACAGTAACTCTCCCCAGTTCTCATTTTATTATGTTCCTTTGACATTAGAT
A_16_P58424408 CTCCTTTAAACACTAACGTCAAGTCACGCTGCTCCTCTGCTCAGAG
A_16_P15637375
GCACTTCCATGTCTAATTTTTTTCCCTTTAACACTCCTTCAGAAAACATATCGGTATCCT
A_14_P116890 TCTTTTCAGGTAATTGTGTATTTCTGCAAAGGTAGGATTAAATTAGATTGCAAGGACTCG
CUST_5689_PI426210822 GAGAAGAGTCACTCCAGACGTCAGAACCAGACAGGCTCCTGAGAC

A_14_P119852 ACTGTCACTGTGGAGAATGTTCTAAAGCAGAATGAACTACAATACAAGTAAGTTTATGTT
A_14_P108162 CAGAGGTTAGCATGATGAGCAGCAGTGCCAGTAAGAATCTGAAGTTCAAAATTAGC
A_14_P116914 GCTCTGGATATTCATTTTGTGACCTGCTACTCTTAGATCACATGATTCAACTCAAGTATG
A_14_P105682 ACTTCTTAAATTCTTAAAAGCAAGTGATACGAGAAGCCTTGACTACTTGGCTTCTCTTGA
A_16_P21661266
ATGAAATGTTATCTGAGGCTACTATCTCTCTTCTTATCCACATACCCTTTCTGTAGAACT
A_16_P18728740
TTGTCCAATTTCTTTAGTGCTGAAAAAAATGCATTGTCCATACTAATGACAACCCACTGT
A_14_P133154 CTATACACATTTCTTGTGCTGTCATAGAAATAAGACACAGTAACTGCATGCTTGCTGGTG
A_14_P101353 GTGCATGATGACGATCTTTTGTCTTCTTCTGAAGAGGAGTGCAAGGATAGG
A_16_P00766784
AGCACTGTAGCCAGATAATTTTTCATAAACAAGACTCTCATTGAATCATATTCCTCTTCA
CUST_8934_PI426210822
AGACATCTAGAGTTAAAGTTACGATCTTTTCCTTTCTGTGCTTAAGTGATACTAGGTCAT
CUST_10251_PI426210822
TTATTTTGAGTTTTTATTTTTAGAGAACTGATATTTTTTCTGTTCTTGATGAGGTAATCA
A_18_P13003465
CTGATGTTTTGATGAATCTGTGTAACTCATTCACCACATTTTCCTATGAAAATATATTCA
A_16_P02847698
GCCTCTTAGATAAAAACTCCAAACTCTTTAGAACAGTATTCACTTTCTTTAGGACCTGGC
A_14_P107160 TTCTACACAACTAAGCACTCAACCTGGACTTCTAGACAAACTTATTAATGCTTTGGGTAA
A_14_P112566 CTGGGTTAGGAGGGGCTCTTTAAAGGTCCCAAGATTCAAATGTTCTTGTA
A_16_P16315150
CTTTCAAATTTCCTCAATTTCCAAAACTTAAATGCCTCACTTTTCATTGACTTTCCAGCC
CUST_1072_PI426210822 AGATGCTGGAGAATTGGGCGCATCCTGAATGTGGTTTTAAGAGCT
A_16_P20565975
AAATCACAAATGAGTATTGGTCTAATTAGGATACTTGACAGTGACATCTTACACCGAGGC
A_14_P123557 CAGCAACTTGACTATCATATCTTGGTCCTAACATTGTCTTCAGAGGTAAGAATCTTCCTA
A_16_P01930498
ACCAACCCAATAAAATTCCTACTGAACTCTAGCATTCACTAATTCTGTCCTTACATGCAT
A_16_P40504061
AAAAACATTTTAATTCAAATAACCATAAACAAGGACATTTTTAACATGGTAAACGGGCAA
A_16_P16191152 AGATATCTGTGCTGACTTCCTTGGGTGACCAAATCATTTCTGGGAACTTAG
A_16_P20699279
TGAAGTAACATGAACAAAACTCTCATCATGACATCTAACTCTGCAAGATATCTCACTGGA
A_16_P37121696
TCAGACATATCGTGCAAACACATACTCTTCTAGTTGTGGTAAAAGATCTTACTCATCCTG
A_16_P00046732

CTGTCAGAGCCCTAAAATAACCTATGGAGATAAGCATATTATGACCCCTAGTCCAAGA

CUST_6747_PI426210822 CAGCCAGAGCACAGGGTATTATGGCCAGCAACTGGGTTTCCTCAG

A_14_P109517 GTTTGCGCAGCAGCACATCAGAAATATTAGGCATGACATCTAAGCCACTCTTT

A_14_P125822 ATTAAACAGGTGCTTGAGTTCATTTTGTAACTTTACATGTTCATTCTGCAACCTGGTTTT

CUST_2872_PI426210822

AGTAGAAAGAGAAATGTAGAAATCTGCCAGAAAATGTGCTGGCAGTGAAATGGAG

A_14_P107298 CTTTATTTGCTTATATCAGTTGTCATACTGGGCTGCTGTCAGAGAATGTTTTTTTACATG

A_14_P118621 GTAGGGATAGAAACTATTTGAATTAATAGGAAAACCTAACGTTCAAACCCAACAGCCAGA

A_14_P117506 GATGTTGAACTAGAAAGTAGATACTGACTTTGTGAGAGACCATGATGGAGTGTGAGATTA

A_16_P18591054

TGTGCATCCTTGTAGATTTCACTTCTGACTGTCTGATAATCTTTACTTCCAAAACCAAAC

A_18_P22841915

AGATCCTGCTCAATTATAAGATGATCTTTATCTGTCCGCCTTATCTATCTTCACTGACGT

A_14_P116043 GTGTAAGTTGCAGATGTGGCTTTTACTTTTTAAATGGCATATTAACAAGCCAGCAAAGTG

CUST_4515_PI426210822 TCCCCACCTCCTTTTCATCATGTCTGTGTGAAAACGAAAGCTATTGTTTATGTA

CUST_251_PI426210822 TGGGGCAGGTAGTTATCTCTTGGGAGAGCCTGAGTCTCATCTCAT

A_18_P22841389 CCATAGGGGCAGAGACCTTCTGCTAAGTCATACCGCATATCAAGAAATATATTGCTA

A_14_P110074 TGAAGTTATCCCATTAGTTGTTATGCTGCCTTTAATTTTTCCAACCAGATATCCAGTGAG

A_14_P108312 TATCTACCAACTCATATTTTGTCCAAACTTCCTAGGGGATTGCAACATGGAGAAATTATC

A_14_P110402 ATCTTTGGACTAAGAATCTGTAAAACAAGTCTTTCAAACTGCCTTCAAGTATCTCCTCTG

A_16_P00488576

CTGGTAAACTCTTTAGGAAGAATACAACTCACACCTTTTACAGTTTCCATCACATTTCCT

A_14_P125997 AGAAAAGAGAAAGAAGGGAAGTTTTCTTACTGAGTAAGATTTCAGGACCCGAGCA

CUST_1518_PI426210822 CATCCTTCTGACTCCGAGGCTTCCCAGTCCATGGAGGATTACAAT

A_14_P114816 ACAGCCCATTCGTTCATGTTCTAAAGGAGATATCTGTTTTTATTGAATGGAACTTTGCAA

A_14_P101441 GGATTTGTTTTCTTTGAAAATTCGGTTGATCTCACTACCTTGCTCATTCTTAAACTCCTC

A_14_P111901 TCTGGCCACTTAATCTTAGTGAAAACATATTTCCCCTCTTCTAACACAGGAAAGATGTAA

A_14_P113406 CCTAGAGTCTATGTGATCTCCCTAATCTCTCTTGGACAACATACACACATATTCACACTT

A_16_P15712838

AATAGGATCCGTAATACTTTAGTTTCCAGGTAAAATCCATGACATGTCAAGAGATCACAT

A_14_P122857 TTGTAGCGTTCTGTCTCATTACGAGCAAATAAATAGACTTTCATTGGAGTTCGTCACATC

A_14_P128147 ACAAAACTGTCCAAGAATAGTCTATGCCATCCTTTACTTTGACTTGCAGGAGTATTACAA

A_14_P111125 AACTCAAGCAGAAGGTTGAAAAAAATCTTCTAAAGATCATGTTTAAGAAAGGCTGCATCC

A_14_P134228 TGATCCTAAATTTGTGCAAAGACCCTGCTGTCCAAAAATTTTCAAATGAATACTGAAAGC

A_16_P58424068 AAATAACATGCTCATATCTCATTGGCCAGACACTGCACATGGCCACTCT

A_14_P138161 AAACCATGTGATGCAAAATGATTGGGTGCTGAGGGAAAAGAAATTGAATTTTCTTTTAGA

A_14_P105856 AACGAAAAAGGAGAGAAAAGTAAAACCATATGACACATAGACGACCCAGAAAACACAC

A_14_P112073 GTTGTTTTTGCCATAAGCGAACTTTGTGCCTGTCCTAGAAGTGAAAATTGTTCAGT

A_14_P121336 CCATTGAAGATTCTTGAGCAATGCATGGGAGGATAAAAACCAGTTTCAAGAAAACTAG

A_14_P127031 GTATCTTTGACTGGGGAAACATTTGCAATAAATGTTTACTATTCAGCATTGGGGCATATC

A_14_P132846 CACTCCATGATAGACTTTACAGTATCAACTTAGTAACGTATCATACAGAGCATGTCGTCG

A_14_P101544 TTTCCTAAGTAATTAGAAAAGTATTGGTCCCAATTTTGCTATCTCCCATCCCATAACAGC

A_16_P01249211

CATTTGTCCCATAAAATGTCTCTTTGATCTCCAAATTAGGCCCATCTTCAGTAATACTCA

A_14_P128705 TGACATTGTGAATTGCAAAGGCTTAGAGTAAAAACAGTATCTTCAGCACAATAGCAGAGT

A_18_P15413493

ATCTATACAGCCACTTAAAACAGTATCGTAGGTGAAGAGTATGATGGGAAAATGGTAACG

A_18_P25453096 ACTTCCTCAGGCACAAATCTTCCATCCCAGTCATTTCAGGTGAGG

A_14_P113045 CAAGAGATTCTTCTATTATTTCCAGATAGTAGGCCTGAGAAAGAAGCCTAGTGTTGACAT

A_14_P129074 CAACTTCTTTAGTTTTTAGAATATCAGGCAAATGAGCCAAGTAACAGTTGCAGGTCTTGA

A_18_P22839419 GTGCTGCTTTCTGAGCAAGTCTCTGCTTGGGGCTGCAGTCTCCTG

A_14_P102535 GAAAACTTCATATTCTGTCCACAGTGTCTTCTATTTTTACTGTCCCAAATGTGAGATTGC

CUST_3762_PI426210822 AGACAAGCAATGGGTAAGAAGACAAGGCCACGGAGGTCAAAGATA

A_14_P135709 GAAAAGTGAAATAAGCCACTTTGAAATAAGCCCAGAGTGTTCTTGCAACAATTTCTCTTA

A_14_P107936 AGGTTGTATTTGTATCTTACATCACCGGCTCTGGTCAGATTTCAATTAATGTCACAGATA

A_14_P110721 AATCTGATTTCTCTCAAAAGTTTCCATGCCTGTGATCTAGCACTTTGCAAAGATATTGTT

A_14_P114258 AAGACACAAAAACCTGTTCAAAACTAGTATCTCCACCGAAATGGGACAATATATGAATCT

A_16_P21234393

ACTCTAGGCAAGTTATAAGTAACTCTAGACTCCAAATGGATAATAGAAGACCCAGTCCTT

A_18_P14134206 ATTGTTCCTTGTCAGTGTGTAAAGTTGCATGCCAACCTTTTGTAACAGGCTG

A_18_P22843012

ACCTCTAGATTTCTTTTTTTCTAACTAACTGCAAAACAGCAATTTAATAATTTCCTCTTT

A_14_P107174 AATACGTGCTAATGGTCATATCTGTTATTTAACTGATGCAAAATTTGGGGTCAGAAGCAT

CUST_5991_PI426210822

AGTCACTAGTTTTGGTAGTTATTTTTCAAAATGCATAGTGTATCCCTTACTTAAAACTAT

A_14_P120363 TTAAACCATGTAAGTAAACTGTGCATGGTAGAACTAGAGATGGTCTATTAACCAGAGTGG

A_18_P26793737 CTCTCGGCTTCTCCACCACTGGCTTCTCTCTCTGACTCTTTCTCC

A_14_P106531 ATGCACTTTCAATAACCCTTTGGAAGGTTGTATACCCAGTTAAACTACAATTTGAGAAGT

A_14_P114972 CGTTTCAGGGGTCTAAAGGAACACAGATTTCGTTTTTGGTGGAGAAAG

A_16_P02654821

CCCACTCCTCTTTAACAATTGTGCTGTTTATCAGAAAAACTACAGAGATTAGAAACCACA

A_14_P126279 CTCATGTTTTTCCAATATCAGATGTTCCCTCCATGGTCTCTGTTCCCTC

A_16_P00685134 GTAAACTTCTCATTTTCAGATGTCACTGTTGGAGCCTTCATGTCCGACAGC

A_16_P15650726

GCACCAGTTAATGTTCACTAGTATCTCCCATTGATGTAGATTAACCATTGCCTTTCTAGA

A_14_P120810 GTCTCTCTAGGTGATGTGTTTACTTCAATTCTTTCTGTGTCTGAGAAACAAGATTGCAAA

A_14_P135831 ACTTGTCTCCAACTTTTGAGGTCTACCTTGTGGTCTTTAGAGGTAAAAACTTTGAAATAT

A_14_P120247 GAAGCAAGCCATTACAGAGAATACTATACCAAGGAAGATATTTCCACTTCCTTTCATAGC

A_18_P14137145 AATATCACTGGGAAAAACCCAGATCCCCTACAGAAATACAACACCGGTG

A_14_P132934 TACAAATGGAAGATACTTTTATTATTCCAAGCCATGGTCTTCTGTTGATACCCCAAACTG

A_16_P16747288

AAACCCACTTTGAACATTTCACTTTACTTCCCTCCATTTTACTCAGTCATTTTCAGTCTA

CUST_4344_PI426210822 TTAAAAATTGAGATTCCCATTGATCCAGATCCAGTTGGGCCTGGGAAT

A_16_P03798330

GAGCAATGTGACTGTGATTCACCTAAAAGTAGTAGAATAAATTCTAAAAAACCCAAGGGT

A_14_P124881 GGCTTCTTGGAATTCGGTGAATGTAGTATTTGGTATACTAGAAGGAAACAGATGGAATC

A_14_P132312 GAGAGAAGGAAGAGAGGAATGTCGTAGAATTATAGAAGTTATGTGTTCTACCATGTCTCT

A_14_P118436 AATTGTGTCCAAAGTATCTGTGTTGACAATTATAGGAGCAGGACTGGCCTGAAAA

CUST_9445_PI426210822 GACCTAGTAACTGCTCTTTAACTATTTACCACCCCCTTCTCCTCCATAAAGTG

A_16_P18402389

ATGGCTTTGCTGTTTTATAAAACCACAGAGGTTAACATTTATACTCTAGGCCCAGATTCT

A_14_P122083 TCAAAAGAGATTAACTGGGATTAAGTGAGATGACGCATGTAAAGATGCTGTGTAAACTGT

A_14_P200698 AGTTTCATCTGTTACCTGACAAATTAAGAAGGGAAAGGCTTTTGAGACAAAACTGTGCT

A_14_P123580 GAATGTCTGATCAAGACGTATTCATGTAAAGGCCGTAAGTATGAAATTCTGGGCAACAT

A_16_P37140731

CTAACCATTCATGCCTTTACCTACGATTTCCATATATCCCTTTCAAAAAATCCTGAGGTT

A_16_P20258822

AAAGGCCTGTTCACATTTGAATTTCTGCATTCCTCAAGAACAATTCATGGCAATAACTAA

A_14_P106627 CTAGTCTAGAAGCCTTAAATAGATAAACATTCCAAATGATTCCTGCAATGGTGATGGCTA

A_14_P108813 TGTTTTAGGTATACATGATCGTCTGAGTGGTCATAACCTTTTCTTTTATCATGTCTCGCT

A_14_P121167 AGTAAGAATTACAGTCTGTCCATAAACAGATGTGCCAAATACATCTTTAAACTGTGGAGG

A_16_P17266973

AAAACTGGAACATGGAGGATCATACTAAGAGTTACATTCAAATTGCACACTGTCATGTCT

A_14_P118213 CATTCTTGCCTCTTATGACAAAATGTTAATGACAAAATATAGGTGATCCTCCAATGGAGG

A_16_P15609434

CCTTCCACCTTCTTTTAAAACTTTGCATTTTATACTCACCGATTCCATATCAGTTTCCAC

A_14_P133540 ATGCTCTGCTTACAGCAATTGTTATCCTGTCTCCAGGTAATTCCAATGTTACATTTTAAT

A_14_P105144 ATCCACTGAGTTTTCAGTGTTCTCCATGGATACCTTTATTAACTAGAATACAAAGAAAAT

A_14_P202355 TTGTTACAAAGAACACAGGTGATTCTACAATCGTTAAAAAGGCCTGTTAAAAACAACCGT

A_14_P117896 ACAGATCATTAAAAAGAGCATGTTTGTAAAACTAATATCCTTCTGGGGGATTGCAGGAAA

A_14_P124027 AGTCTCCATCAAATGTTGTGAGTGAAAGAAGGGAAACTATTCACAGGTAAATAAAGTGTT

A_14_P122099

TATAAGATGTATAAGAAGGCATCTGAGAAAAGATGAGGAGACAAGCAACACAACAACCTT

A_18_P22442173

TGCCTAGTGCCTTATACAATTGAAGAACATTAGTCATCATCATATTCCTTTCCCCAACAA
A_14_P107903 CGATGCCAAGAAGAGCCTGAACCTCAACATCTTCCTGAAGCAATTTAAGT
A_16_P18017537
AAAAACGTGTCTAGTTTTAGAAATTACGGACTTGATTAGGAGAAAGGATTAAGAAGGGCA
A_14_P109373 GTTGGATGCTTTTGGTCCTGTGTTGCTTTTAAAAACACCTTATAAAAGAGGAGAGTATTT
A_14_P116953 AATTTGATTTGGCAAAACATACCGAACTTGATATTTTTTGAGGCTATGCTTTTGTTGGCT
A_14_P131831 CTTATCTGTTTACCTCTCTTTTTCACCTCTGTTGGTCTGTATCTTTCAGTGTCAGAAAAA
A_16_P18574990
ACTGCATCTTTCACTAACAAACGAAATGTAACCTGAACTTTGTCTTACACATAGCCTTGA
A_14_P124474 CAACTCGACACCTTTCTTAATAAAAGGCAAAGTGCAACTTAGAAATTCTAACTGCAGACA
CUST_5699_PI426210822
TAAATACTCTTTTCAAAGAGGGCACCATGGCAGAACACATGTGAAAACCATCTA
A_16_P16843119
CACTGAAGTTCGTTATCAACAGTTTTATCTGACAATATCTCACCACAAAATTCCTAAGGG
A_16_P01149916
ACCAGACTTGGAATCCATATATCTCATTGCAAACTAGATTGATTTACTAATTGGCTGCCA
A_14_P116039 CTTAGAAATCTTGGGTGGCCTTCACAGCATGGTGGTTTCTTAAAAGTATAGCATTAATAT
A_14_P135093 CTGCCATGCAAATGAACTAATGAATATTTATGAAGTCTGTTCACTGAGGTTGAAGAGACT
A_14_P105275 ATTAATTTTACGTTTGTGCGATATACAAATAAGTGAGAGCCCTTCAGTGTTCAGTGCTCA
CUST_10348_PI426210822 CTTCTTCAGGGGAGCTTATGACCATCAGGGTGGCTGTGATGCTTC
A_14_P136750 CCTCACTTAGTAGATCTTTTTTTAATCCAAAGGATTTGTCCAAGTGATGCTCACTCTATG
CUST_9535_PI426210822 TATATCATTTCCAGGGCATCTCTGGGAAGTAGTGATGCCTGTCTTCC
A_14_P126834 CTTCAAAGAGGAAGCCTTCCACAGCAATAAATAAAAATTGTGCAAATGTCACAATGTTCC
A_16_P18557013
GAAACTTCAAGAGATAAAAGTCAGCCTATACTTCATTGCTCTCCATTAATTGCTCTTCCC
A_16_P16657772
TCGGAAACTAAAACTAAATGCCCTGTAGAATTATCAATCATGTAAATCCAGAAACAGGCA
A_18_P14132352 GTTGTGTGCTTTGCTCCCAGAGGGACATGAGTATCAAAGGTCTGC
A_14_P138662 GTCTTACCAAAAGTCATTTTATCCAGCCTTCAAACAATACAGTTTACAGAAGTGTTCTCC
A_16_P00685524 CTTATATATGGGAGGTTTTTACGGGGAGCCTAATGAAGTGTAACCACTCCAAACT
A_14_P201561 CTTGTTGTTCCCTTTATATTCTAGCAGGGAATAAATAATTGTTTTAATTAGGTATTTGTT
CUST_2152_PI426210822 AAGCAGGAGACCAGTGAGAAAGCAGTCTTTTCAGGATCTGGAAGTA
A_14_P109436 TGGTCTTCCTGCAAGGACCTAATCCCTTTTCCTTGTTTAAAACTAATTATTGCATTGTTT
A_16_P19725284
TTGGGAAATAAGACTTTAAGTCCTATCTGTGCGACTTTAAATGAGTGATGCTAGTCTTGG
CUST_10227_PI426210822 ACTTTGGGTCCTGGCAGTAATGGAATAGGTAGGACTGGCTATGAAG
A_14_P104827 AGAGGATAAAAAGTAGTCATGACACACCATCCTTTCCTGTCTTCACATTTGATATGAGTA
A_14_P104796 CAAGAACTTCTCCATCTGGGATAGATGGGATACAAGGTTATGTTCTGTTTTGTTTTATTG

A_14_P117236 AACTCACAGACCTATTAACAAATTGAGTGCAGTTATTTCAGACAGAATCCTAACCAGTTC

A_14_P119386 TCTCAAACATTATACAGGGAAAAGCCTCTCCTAATTCTTTTCTCTAGGCAAAGACAAAGC

A_14_P138021 CAAAGTGAAATAAACACTATTTTGACGGCTGTCTTTTATATTTCTGAGCACACACAGAGC

CUST_6732_PI426210822 AGGCCTCCAAGGTTACCTATCTTCTCTGTCATCACACAGTCCATTAGTTAT

A_16_P38959159

TTTCTCATCTTGCCTTAGTTCATAAATGCCTTACAGATTGAGAGAATTCCCCTAACTAGC

A_14_P111002 ACACAGTTGTTTTGAAAGACATGTTTCAAAAATCCATTCAAATGTTGGGTTTTATGTGGG

A_16_P30099679

AAAAAAAAAATCACTATCTGGCAGGAAAAATTATTCCAGATTCAAAAACTATGCCTCACA

A_14_P201289 TACACCCATTGTTTTGTTTTATCACTAAAATGACAAGAATGCACTGCAGATGGAATGGAT

A_16_P20930053

AAACTTCCAGAATTAGTGATTGATATGCATTTCCTTACAAGAGAAACATAAAACCCCTCA

A_14_P132292 TAGCCACTCTTGGTCATGAGGAAGAGAACTCTAGAATAGTAGGGAAAGGACAT

A_16_P18223444

GGTTTCGAATAGTCACCTGTATCTTCATGTTAACTATCTACTAGCACTTTTGAATTCCCT

A_14_P110507 GTCCCACAAGAATTTCCAATTCCATCATGGTTCATACCAAAACTGTTTTAATGAGGAA

CUST_5581_PI426210822 AGGACAGGCAGGAGACCAAGCCTTCCCTCTATAGGGTACATAGTC

CUST_8758_PI426210822 CTGGACATCCAGAAGTCTCCGTGTGTGTTCTCATTAGCTTTCACTCT

A_14_P111763 CTTTCATTTCTGATGTCACCAGTCTTCTTAGATCTCAGGATTTACCAGAAAGTTTTGTTC

A_16_P36230105

ACCAACAAGAAGATATCAAGGCTTTTTCTTAGATCTGCCAGAAGTCTGTAATTTGCTCTA

A_14_P126224 CATTAATTCATCAGACAACACCCAGTAATTCACTTACAGAGTGTGACTTGTTCCTGTAAT

CUST_4680_PI426210822 AGACCTTGTTCCCTCTTGGGAGTTGCAGGGGAGTGTCCCAATGCT

A_16_P41758365

TTGGTTTTTCTCCTTCTACCAAATTGCTTAAAGTCTAGTTATATATCCTTGCATCCTGGC

A_14_P135132 GGCAATAACTATTAACTCCAGATTCCCCAGAGAACAGAATTTGACTTTTGTGCTTAGTTT

CUST_6604_PI426210822

ACCTATCCACTGTTTAAGATTAAAATGGCCAACAATAATAAATAATTGCCACAATAGCTT

A_14_P136375 ACTAGGGCAAATCTTTAAAAAAAGAAACATTTACAATAATAGAGAAAAAGACTGTGGCCC

A_18_P10793233

AAATGTCTTCAACACTGGTTTGTAAAAGAAGAACTGAGGAAACTCTTCTGACTCCTTTGT

CUST_5285_PI426210822

AGATTCTTGCCTTTTAGTCTGTCAGAATGACACTTTATTAGATGCTTTAGAACTGCAGAA

A_14_P115804 CTGGTTTCCACTACAGCATCTGGATGACGTAGGCCAATTTTTACTAAACAAAAATGTAAA

A_14_P102688 GTCGGAAGGGTTTTTGTTTTTGTTTCTGTTGCCATTTGTGTAAATACTAGTCTTTTTGGA

A_14_P117545 AATCTCAAACTTTGGGGAATTGGTCCAGTGTATTTTCCACAATAAAACTGGAAACCA

A_14_P132481 TCACAAATGTAGGCAAATTCATGCCTCTGAGGAGTAAATTGATGCTAGAAGGAATATGAT

A_16_P00685078

GAGGTTGCTCATTAGGTTCTTCAGTGCCCTTTGTATTATATTTGGACACTGATTACAAAT
A_16_P17940552
AGGGTCATAGAACACTAGAACAATTCTCTTAAGTTTTGAATTGGTAACATCATCGGCTCT
A_16_P17910070
AAGTTAACTACTGAAATTCACTCATCTGTGTCCTACTCATTGTCAGACAAGTTTAGAGGG
A_16_P18308587
ACCAGACTCCTTGATCATTTTACCTTCTCATAGCACGTAACATATTACATCCTAATCACC
A_14_P138547 ACTGCACTGCTTAAAAATAGACTGAGGCAAACCTTATTGAGGCTTATCGTTAAAATGACA
CUST_6930_PI426210822
AAGGCTATGTTTTGCTTTAGAAATGTCAACAGAGCTGTAGTTACGACAAGGATCAATTAA
A_14_P105651 CATTCATTCCTGAGAATGGTTCCTCCTCAGATTATAGGGTCAGAACTGAAACCTA
A_18_P14241048
ACTGGACATTTTGCAAAATTAAGAGAAACTCCATCTGTTAGAATAAGCCACAACTTCACA
CUST_1023_PI426210822 GTCATGTCCAAGCAGAAGGCAGGGGCATGTTTAGTGTGGCAGAAG
A_14_P102625 ACATATAAAATCAAAAGTTAACTGGGCTTAGTAGAGGAGCAGAAGCAGAGATGTTTGCAT
A_14_P131670 GGAAGAAAACCTGTAAGGTAGATGTTCTTCTTTGTCTCTGAAACTGTTAATGCATTACCA
A_16_P30952473 GCGCTTGCTGACTTTCATTATTGCCTGGAATAGGCAATGATTCTACAAGCACTAAT
A_14_P106373 TTATTGGTTATCAGTCTTCACACTTATTAACCATCAGCGTGGAAACACATCTTCACATTT
CUST_6175_PI426210822 CTTCCTAGGAATACTTTCATTTCATGTTTTGCCAAACACCCAACATTCTCCAGT
A_16_P03469787
GCTGTTGAAGATCATTTATTTTGGGTTCTTAGTCTCTCACAAAACCTTAGAACACTGACA
A_14_P100116 AAGGACAATATAATATATTGGTATTTCATCAACCTTATGGCCAAGGCTTCAAGTCCACAG
A_14_P109289 GGATCTTATCCAGACTATTTCTCCCCTAATTTAGAATAGGAAAGTGGTATCAGAAAGTGC
A_16_P02191036 CTCATTAGGCACCTCTCACTGATTTTCTCATGTCTAAAACTAACTGGTGAGGTAGG
A_18_P22842844
AGAAAATAATCACACTCAGAGACTCCACTTCAAATCCCACATCTCTTTTGTTGCAAACT
CUST_6348_PI426210822 CTTTTTGAGCATAAAACTGGCCCAGGTGGGTACCTATGAGGGAGG
A_16_P38392734
AGCAAAAGCACTATTGAAGAAAGACTTTGTGTAATGTCCTTTATCCAAGCCTAATGTTCA
A_14_P135473 CAAAAGAACATGCTGCAACTACACATCAGATAATATATCAAACACTGTGCTTCCTCAACT
A_18_P22842276
TAAAATAAGGACTTTTAAGGGAGATGGAAAGACACAGCAGGATATCTGTAAAGCCAGAAT
A_14_P126245 ATCACATGGTGCTGAGGATATTTCGTGCTTAATGGTTTGATTCTACACAATTTACAGAAT
A_16_P01939188
AGCACCATGTTTTATCAAAAACCTTTCAACAGAAATTCAACTATCCAACCAGGCTTACTT
A_14_P126204 ACAAGTCATCTTATGCACATTTCATTGACTTTTCTTATTGCATCCTTCAGTTGGAGAAGT
CUST_1197_PI426210822
GGGGATCTCTCCAAAATGTCTCAAGCTTTTGAATTCTTAAAAAATTGGTCTCTGGT

CUST_1858_PI426210822
TGGCTTTGATTTAATGGAGCTAGGTCCAGTCTCAGATATCTTTGAGGCTTTATTCAT
A_16_P36196912
ACAGGTCAGATGGCTTTTTGATGGTTTTCTTGGTTTCTTACACAAAATGATAAATGCTCT
A_14_P135943 AAATGATTGGTGGTCATCTCATCTCATGGTATACTCCTTACACACAAAACATTCAAACTA
A_14_P136892 CTGGTGAATTCTGAAAAGAAAGTGCCCACTTGGTATTTCATTAAGAAGAAACTTACGTTG
A_14_P103672 CCTTGACTAACCCCACTTAGTCATTTTGTGTTAATTATGTCTGCAGAAAGGAAAAGGTAT
A_18_P13695407
AAATCAAATGCCATAAGACTTGTTGGAGCATGCTACAGTTTATAAACGGGGTTTTATTGC
CUST_6284_PI426210822 AGTCCTTACTGCATAAGGGGACAGATGATGTTCAGTTACAGTCATAAGCCTTT
A_14_P108133 CTGATAGTATTTTTATGTGAATCACAAATACGAATGCCTACAATTGCCCGAAGTCTGGTT
A_16_P18680592
ATGTAGTGAACTGAGATTCCTCTAATGCCTCACAAAGATATTTTTCCAATTTCAGATGGG
A_14_P106308 TCTATTGTCTATAGATGCTGACATTAGCCCCAGAAGAGGAGTAAGAATGCTAAGAAGTG
A_16_P02959455
CCTACAGAGACCTTATAACTTTGACTACTTAGAATTGCGTTCCATTTTTAATGACCAAGC
A_14_P116276 CTGGCTTTTCGTATATTGTGTATAAAAAGATCAGATTCAGGATGATTTGGTGGGATTGCC
CUST_8884_PI426210822 CTCTCAGAAAAGCAGTGGAAGAGGTTAGATAGATGGTGATGTAGGAATCATGG
A_14_P119424 CTAAAAATAGTTACTATTTGATTGTGCATCTTCTTGTGCCAGGCACCTCTTAGGCATTTA
A_14_P104460 AGTGTGAGAGGAGAAACAAAATATGAGCGTTTAAAATACATCGCCATTCAGTTCGTTTGT
A_16_P20894443
ACACTGCACTTTGACTATAACATGCTTGTTGAAAATCCTCATCTCCGAAAATGTTTCAAA
A_16_P19182218
AAGGTAGGCAGTAATTCACTTCAGAAAGAAGCAGTATAGGTATTGAAAGTAATGGTAGGG
A_14_P122555 CAGATTCTGCTTTAGTCCCAACACCTGATTTCTACATAAGATTTTTAGAAGAACCATTGG
A_14_P202134 TTTTAATGTCTTCTACGAGAAAATGGCCTTGCTTAGGAACATACTTGTTCTGCTAGGAAA
A_14_P111774 AGGATTTTGAGATACTTATTCTTTATTTGAGCCAGTCTTGCAAGGTTAACTTCTCACTGG
A_14_P116804 CTAAAGAAGTGTTGCTTAACACGTGTGAAACGTTTGAAAATTTTGATTCAACTCAAGTCA
A_16_P02422172
TGCCCACTTTTGTTTTTAAGACCACTTAATAGACTTCATTTCTTAAACCAAAATCACTGG
A_16_P17427563
AAGTCCATACCCATAAAATGTTCTGCATGCTTAAAGATGCTCTTATACCCGGAAAAATTC
A_18_P22840691 CTTCAGGTGTTTCAAGATGGAGTGGGGTAAGGTAAGGAAACAACAGTAAGAATT
A_16_P40055451
AGCACTTCATTTTTACAACATGACTCTTGATTGACATCCCATTACTTACATGCCATTCAA
A_14_P128866 TATCTTACATGATAGGATTAAGGGCATGCTCATTAAGCAAGACCAAAACTGAATGCATCT
A_14_P116695 AAGGATAACTTTTAACAAGAGCAATAATATTATTTCCACCTTCTAGGATCTGGGGTCCTG
A_14_P135934 ACCTTAGAACCTGAGATGTCGAGTCAAAAACACTCTTCTTTAACCTTTCATATATCTGTC

A_14_P113024 ACCAGCACTGCTGAGATTTACTGTAATGTCACTAATGTGAAATTTCAATACTCCCTCTAT
A_14_P125171 TTCTCAAACATGAACGACTGGTTTGTTGAGTATGTTGAACTTGCCACTATCTAGTAAACA
A_16_P20229401
AACTACAAGCCAAAGAAACTTCAGAAAAAATGTGTATATCCCAAATCTCTAAATGCTGCT
A_16_P15048277
ATTGGCTTTTAATTTTCCTGGAAATTCTAGAAATCACCATGAATACTGCTTAGGGAGCAC
A_16_P00913328
AAATGCAAGTGCAATTTGAGTTATAAAAGAGCAAGGTTGATGTTTCACAGTTGATGGCTT
A_14_P113133 TACAAAACAATGGCAAAACCACACAATGCTTTGTTAACAAAAGAAATCACCCCCAAG
A_14_P119084 CTGGATGGCACAATAAATTATTAAGCCATGTTATGAAAATATAACTATATTTAAGTAACC
A_14_P139972 TTGCCAAAGCAAACTCGAAACTAGCCTCGATGTAATTTAAAACAGTGTCATCCAGTTATA
A_14_P137099 GCACATGCATGAAAGATAAGAAGTGAGATAGCATGATGGATTATGAGAGAGGTTGTATCA
A_16_P37254320
TCAACTTACAAAAGTTGCTTCCTAGCCAAAAGTTATGTTTGATGCTCAATTGCAACAGTT
A_16_P20722959
AACACACCTATTATTTATCACCTTTAGAAGCAGATTTCTCTGATGTATACTATGCACCCA
A_14_P136664 CCTCATCAGACACAGATCTAATTGTCATGCTTTCTGAATTCTTTTAATTGTCTCTTTCCC
CUST_3941_PI426210822
ATGTCCTGTCTTGCTTATGTTTAAGTTGGATAGGAAGAAAAAAATCCACACCAGGA
A_14_P129696 CCCCTGGTTGCGGAAAGAGCAGTTTTATGTGAGTATTCAAAATTTCTCTCAACT
A_16_P19125790
ACTGTTACAATTTGCAGTCTGCCAAATTTTCAGTTAAGTCTGACTTAAAAATCACACGTC
A_18_P22840721 CAGAGTAGATGGGCTGGCCTTACTCTGAGGTGTGGAGAAGGCTAG
A_14_P124945 AAAATTTCACATTCTGGAAAGATTTACCAGGCTGATGGTAATGATCTCATTATAGGCCAA
A_14_P117596 GTGGCCAATATCACCTTTAAAAATGTAACCCTTTTCTAGACCTCAAGGAAGCAGA
A_14_P110087 AGCATGGAAACCATAAAATGGGCACATTTTACTTGTTTATGCCAAAACACAAATTTCCAA
A_14_P127718 CTTCAAATTCCTCATGTTTTAAATGAGCACTAGAGGGAGGTTTATCACCTTTTTGGTTAC
CUST_7964_PI426210822 CATTCCCTTGGGTGCTCACAGTTTTCTGAAGAGAACTCTCAAAATTCTCAA
A_16_P02744245
CTTCAGACTTTATACCCTTGTTCATGATCGTATAAAGCCCTTTTACATGACCTTGTTCTG
A_14_P120989 GGAGAAAGGGAGCCCTAACTTCAAAGTTTGAGTTCCTCCAACTTTTTTAATAGTTAAATT
A_14_P202332 CTTGAACATGGTTTTTGTTTTGAGGGCTATGAATTTGTTGGGTATACACTGGTGTATATA
CUST_6883_PI426210822
TATGTGTTTGTATACCTTTATTTGAGGGAGTGCTTTGGCCTGTTTTTAGACAGAAGG
A_16_P19722817
AAAAATAGGACCTGCTAAGAGACATTTTCCATTCTAATTCACGATTCACTTTTCCAAGGA
A_14_P119542 CATCCAATAGCAGAGCCACTCCCTAATTATATGGCAGTGACTGAATCTTATTTTATGTGA
A_14_P202282 ATTTAGAAACTCTTTATGATTCTTTATGGTTCCCGTAAGCCCTTTAGTCTCGTAAACCCT

A_16_P03401487
AAAACAGCCACTTTGACAATTACCTCTTCTGTATCTCTACCTGAAATGCTGATGACATAA
A_16_P15562164
AAATGCTACATCCTACACCAAAAATTCATCACCTCTTTTATTTCAAATTATGCCCTGCCA
A_14_P131038 CAAGCGGTTAGTGGCTAATATAAATGGATGGGATAAAACATCCCATATTGAAAGTCAGAT
A_14_P105453 TGTGCTTAAATCTCATTGAATTATAATCACACTGAGGTGTCAGCAGGAATTTCTTTGGTC
A_14_P121931 ATTACTATTCACCATACTTACAGATCCCTTTGGGCCAGGTAATCCTATATCTCCCTAAAT
A_14_P120553 TGATTAACTGCTGTGCCATTTTTCCCCTCAAAATTCATGAAAAATGCAGAGTTACTATCT
A_16_P19551822 CCTATTTCCCCTCCTATGTTACTTATCCAGATTACAATTTCCTTCATCTAGCATTTTCTC
A_16_P19997941
TGTCTCTCATACTGAAGAAATATCCAGACTTCTCTACATACTAACCAACCCATTATGACA
A_16_P03610416
AGCCACTCAGTCTATATACTAATCTTTCATGTTCAAAGACCTATTACTGTCCTCAAATGT
A_18_P16393576 TATGCGTATTCCTAATCGTTCTTAGCATATTCTTTCCTGCACATACAGTTATTTCCACCC
A_18_P10271441
ACTGGAGGGGATGTTACTAGTTTCTGATTAAAAAAACAAAAAGACGCATATAGCAATAGA
A_16_P16939037
CAGGCTCTGATGTAATTTTCTATACCAAACTCCACACCTAATACTGTTTCTTGTGCAAAG
A_18_P16334181
GGACTCTGCAGAAAGGCTACTTTTTTTGCTTCTAGAAGTTACGTATATCAGAAAGAAACC
A_16_P16172869
ACCTTAAGTTCTGAGTCTGAATAGTGCATAGCATTTGGTGATTACAGACCAATGTGTTAG
A_16_P16644402
TGATTTCATGAAATCCAAACATTGGTTACTCAAATCCCACTTTAAATGACTCAGACTGCA
CUST_6715_PI426210822
ATTAATAAAAATAATCCCATAATCTCAGTTATACCAATAACAATTTGAGAAGAAAGTTGA
A_14_P123295 TAGGCTTTATTTGACTGCATCCCACATCTTGTTTCGTAATTTTCTTAGGATTTAAGGCAG
CUST_4973_PI426210822 AAGTCCTGCCTCTCAGATTAAGGCACCAGCATTCAGCATAAGAGG
A_14_P134445 TTAGTTCCAGCCACAATACTCCAATTATTAAAATCGAAGTTGAACAATGCTGCCTGTCTA
A_14_P108763 CACACGTCATCTTTCACACTAACTTGAAGGATCTCTGTATGGCAACATTTCCTTAATATT
A_16_P18394300
ACTTCCAGAAGTGAGAAATTCTTCAATTTACAGAATCAAGTTACCCAGAAGATTCCAACT
A_16_P16659146
CCCCATTACAGTTTCCTATACACCCTAGATACTCGATTAGCATTATTTTGTCATTTGTGG
A_14_P131407 TGCAGTATGCTTCTCACTTTCTTCTATTTGCTCATTGCAACTTTTCAAGCAATTAGCAGA
A_14_P121170 ACATTATTGTTTGTGCATGCTTTCTTGGAAGCACATGTCATGCATATGTGTGTATACACA
CUST_2791_PI426210822 ACTGGTGGAGATATAGTGCCCGGAGCCTGGCCCTTAGGGAACATT
CUST_1643_PI426210822 TTTTTCCTAGCACTTGTGAACAGCACCAGAAAGGTCACAAGCATCATC

A_14_P132966 ATTCTGTATGCCTTTGAGATGTTCCGTCTCAAGTCCTGTGTGGATTTCAAG

A_16_P30952306 GTCATTTTATTGGGCTGGAACCATCAAAGTGCTGAACCTTACGGACAAC

A_16_P16877159

CTGGATATTACCTTGCCTATAGAACTTAATTTCCTACAGCCATTTTTTTCAGACTGCAGG

A_16_P41067635

TAGGACAGTCCTTGCTATTAATGATCTCCTACCCTAATATTGTTAAATTTCCAGCCAGAC

A_14_P104953 CTTCAGCACCATTGTCACGACTTGATTCATCACATATAAATGGTTAAACCTTCAGATTTC

A_16_P00300346

AGTTGCTCACCCAGAATAAATTCCTCCTGTTCCCATTATCTAAAAATGCTGTCTCATTAG

CUST_7558_PI426210822

AAAGAGAAAGGGAGGAAGGATCATCCTAGAAGTGGATACCAGTTCTTTAAGAAG

A_16_P58424619 ATTACATCTGACAAATTTCTGGGTCAGGGATCCAGGAAGGGTTTGG

A_16_P17667044

GGACCAAAGTAAAATACCCCTAACCAAAAATTTTAGTCATGTTCAAGTATACCATGCCTG

A_14_P133478 TGATACACTTAAATACTCCCTGGCTAAATTGTTTCAGATAATCCCCTAAAAGCAGCAAGT

A_14_P109304 GACGTTTTCACTGAGTATGAATAATAACACATAGTTGGCGGTTTCACACCCATTTCTTAA

A_14_P111296 GAAAAAAAAAAAGCAAAAAACACCTCCTTCTTTGCCATGAGTTCCACCGTAAGTG

A_14_P112916 CTTTAGAATCTATAAACTTGATGTGCTTCTGCAAAACAGTTGCAGATGGTCTGAAGTGAT

CUST_2866_PI426210822

ATCAGGCTTAAAGGGGATGGTAACTGGAGAATTTTGATATCTCAATATAATAAGGCAATG

A_14_P201236 AATACACTTGCTTAAAATTCACACAAGCCAGAAACATTCTGACAGATGCACTAACAGTTT

A_16_P03809820

AAAATCGCCTAGAACCTTTCACCTTATAATAAACCACACACACCTCACTACATTTAACAG

CUST_6321_PI426210822 CTTGCCGGGTCAAGTCATACATCGCTGCACACCGGGGCTGTCTTA

A_14_P127258 TCCTTGACTATATCTCTTGTAGGTGTTTAATTAAGAGCATGTCTGTGGAAACCTTTTCCT

A_16_P02277194

AAAAGCAGCCACTTTTCTTAAGATTGAATCTCTAACTTGGACCTCAGTTGTGTTTGTAAC

A_14_P114533 CTCTAAGACAAAACTTAGTGCTTTTGCAACTTTTCTGATGATTCCTTTCATCCCAAAAGT

A_14_P139936 TGAGTGGAAACTTAGTTTGAGTAATGAAGGAATCTTCACAGAAGCAAATCAGAATATGGG

A_16_P15350855

ATGACTGGACATCTTCTTCTTTCCTACCACAAAAAAAAAAACCTGAGACACAAGTAAATCT

A_16_P01575421

AAATCCCCATCTATAGAGTAAGTCATTTAGGTAGGACTGTAATTTCAGGTTGATCCCAAA

A_16_P17026631

CCATTTCACTTCTTACCTACAGTATAGAATATCTTGCAACTAATCTATCTTCAGCCACGA

A_16_P58424444

TGCCACAATATGTTTTCTCCATTATCCTGATGATGGACATTGAAGTTGTTTCTGGTTTTA

A_16_P33503431

CAGTCAACTTCTAAACTTCCAAACACAAAATAGTTATACTCAGTCTAAGAATATCCGACC
A_14_P104046 TATTTCTTGTAGTGGGTCTGCTGATAATAAGATATTCCTACTCTTTTTGTCTGAAGCCAT
A_14_P113605 GCCCTGTTAGATCTTTAATAACTGACCATCTAATCAAGATTTCATTTCCTTCTGCATGCT
A_14_P137834 GGAATCCTACTGATGCTAACTGTAAAGTATTTAACCTTGGGAAAACCACTGAAAATTGTC
CUST_2784_PI426210822 GGTTCCTCCCCTTCTATTTCCTGCTTATCAACATGTTTCTAATGTCACACAAC
CUST_10002_PI426210822 CAGACTCTGCCCTCTGCCACAGACTGGGAGGGAGAGGGCAGTCAC
A_16_P19908455
AAAACAAAGGTATGAAACATCTTGGCTTAAACAAGAGATACTATAGGTCTAGGGCAGCAA
A_14_P126229 CTTCTGGGACAGAATTACGGTTCTCTAAGTATTTGCTTGCAGGTGGGTTTAC
A_16_P18074904
AAACAAGTGTAACAATTGCAACCCATGAACATCTTTTTCCTAGTCTTCTTGGCAAAACTC
A_14_P138016 ACATCCTCCGAACACTCTGAGTTTACTCTGAATGCTAATGGGATTAATCTGATCATTTTA
A_14_P115159 ACCTAACCTTGATACGAGGAAGTCTGGTAAAATAATTTAGCATTAGATAGGTTCAGTTGC
A_16_P00400242
AGCCTGTATTTTCATAGAACCTACAAACCTCAAATTCGAAGTTATTTTGTCCAACTCTCC
A_16_P20412081
CAGGGACAGTATGTTCACTTAAGAGTCATTCAATAGATATGTGTCCAATGCATGAAGTTC
CUST_34_PI426210822 CTAGGCTTCAACTTAAAAACTTTCTGGGGGAAGGCATAAGAAGGCTTTTCTT
A_14_P102744 CTTCTCTGGCCTGTAATAGAAAGTGCAGCAAGATCAGACGACTGACAATGTAAATC
A_14_P139402 GTCACACTCTGCAAAGGACTCTGATTCCCTACTTAAATCCTTTAAAACTGTCACATTAAA
A_14_P105129 ACCATTTGCGTATACTGAATCTGGCATGATCATATAGCGCTAGGTTCATATATTGCATGA
A_16_P00608496
AAGCAATCCACGTTAGTTTTCTGTTCTAGATTAAAGTATATCTTTCCAACCCATAGGTGG
CUST_5273_PI426210822 TTTTAAGATCACATAACCTGCAAAGTGCCCTGAGATCTGGTTCTGCATGAAT
A_18_P22842084 CTGGGTCAGAGGGCTTCCTGTTGAGTCAGAGCCTCCAGCCGATAT
A_16_P19336516
CTGCCCTCTATAAAAGGACTTGTTTTCTTGTAACTGCACCAGTTAGACTTCTCTATTGTA
A_14_P130601 TCGGAAGTAGGTATTTTCTTTGTATTTTCTTTTTTTCTCCCTTCACTCTTGCCTTAGAGG
A_16_P17504978
GGAACTCATCCCATTGACTTTAGAATTGACATGAAAATTTATGCTTATATCCCTGCCTGT
A_14_P100673 CTATCAGTTGCCTTCCAATGAATTCCAGTCTTTAAAACTAATGACCAGAAACTATGGTTG
A_14_P131981 TGGATTCTCTGGCAGAAACATTTTGGGAAACTACTAAATGGACTGATCTCTTTGACAATA
A_18_P14133989
TATCTCATGCTGTCATCTTTATGTTGTATTGGAAAAATCTGGGATTCTTTTTCCCTTGGT
A_14_P127538 TGAACACAAATCCATACGTCCCATGGCAAGGTTTTAGATATTTTCATATGGCAGAATTTT
A_14_P126915 ACACAGACTGAAGTCGATAATATACGGACTCCTCAGTTTACTTGAGAAATATGGATACAC
A_14_P123383 AAGTGCTACAAATCGCAACACCAAATGCATCAAGAGGTAGATTATTAGTATGAGTCAGTT
A_14_P111662 ACATTCTAGAGCATCATGAAAAGATAAAAACATCAAAGTAGACTTTCCAAGGTCAAGTCC

A_14_P136872 TTGTAAGACGTTCATCGCCGTGTTATCCTTGAGTAAAGGTGAGTATTAGGTGC

A_16_P18100854

AAAGCCATCAAATACCTAGAACTACCCCTATAAAATAGACTCTATTATGATTTCAGTCCA

CUST_78_PI426210822

AGCTTTAATATTTCAATTGAAGTGAGAAGTGTTTTTAAAACTTGGCATTATGTTGTACAG

A_14_P104806 TGGTTACCTACAGGAGAACAAACAATCTTTGAAGCCCATCATCTCCATATGAATTAATTC

CUST_8457_PI426210822

ATTCATGGCATAATAGGTGTCTAAAAAATGAAAGACCTCTGGACTGTTTAAAAAAAAAAA

A_14_P139867 CTGAATACCTATACACCTTGAAAATTGGCTTGGTTATTGCCCTTCTTAGAAACTTTCTTG

A_14_P136340 TTTTCCACCTCTTTCTCTTTTTTTGGACTTGTTTTTCATAGTCTGGGGCATAATTCATTT

A_16_P19493455

CCCCATTCTAATTCTTTCCTTGCTGATCATAACACAAAATCCTTTAGTTCTCACATTTCC

A_14_P202297 CATTTTCAGTAAAGGAAATGCTCACCAACACATAGTCACCAACTATTAAAGGAATCATGT

A_14_P135111 ATTTGTTCTATAGTGATGAATTATGTTTTCCATGGATGACCTGCTTAGAAGCATGGAGTC

CUST_908_PI426210822 TCAGCTCCATGGACACTGTTGCACCTCCCAGGTTTTCCCCAGAAA

A_18_P11801824

ATCTCCTGACTTGCTCAATTATTTCTGGACTTTTCTACAAAGACCCTCTGAATGTTTGTT

A_14_P109503 GCTGTGATTCACAATAAAGTCTGCCTTTATTGATGTGTATCATGTTTACTATCCCGGGT

A_16_P39354998

TCCTTCAAATCACTCCTAAAAAAACTCAACTGTTCCACAAAATTTAACTTGTCAGTGCTT

A_16_P41850814

CTAGCAAAATCCATTCTACCCAAATTTCTCATCCAATTCTGACAGAGGAAGTATATAGGC

A_14_P131380 GTTTTCAAAGAACAAAAGAGACAACTGCATCCTTGACTTGATCACATTTATTCAATATGG

A_14_P117774 GTTCCTGTAGAGTTATTTGCATTACTTCTGCAACCTTCATTAAGATGCTGTATAACCACT

A_14_P108672 TGAGATCCACAAGCAGAAGAGTAACCAGAAAGGATCAAATGTCTTTGTGTTGTTTCATTT

A_14_P119112 CATAAGCCAACTCATACATTTTACGCTTTCCTTTTACAAGAGGAAGAAGTGTTTTCGTGA

A_14_P123534 AAAAAATGTCATGAACTTGGTGTCTTAGTAAGTGAACTTAGTGGCATGAACTTAGTGTCC

A_14_P119830 TTTTCTTTTTTAAATAATCCCAAATGAAGTTTGGGAGGCATGCTTAGTATTGTGCCCTGA

A_16_P32712964

AATCTGCAACAATAAGATACTCTGGTTGATATGTAGCGAATTTATGTCCTTATGGGCTGG

A_14_P101501 ACTGAAACATTCATTTTCCAAGATTCCTTTCCAAATTCAACTCCATGGTCTTTCTTTTGT

A_18_P15521696

GGAGGTAAACTAGATTAAGTTCTCACACTGTAGAATCAGTCAAAAAACTACACGACGTTG

A_18_P13218135

AAAAAGTTCAATGCCTAAGAACAGTTTTTGGAGTGAAAAAAAATGGGAAATGCATGACACC

A_18_P14604367

AACTAAAACAAAGTTGTTCACCTTGAAAACTGTTTGTAAACTAACTGAACCTTTGCCAGG

A_16_P00392172

CGTCACTCATTACATGAAAGCCATCATCTTTATTACTTCTGAATACAATGCTAGTGCAGC

A_14_P102791 ACAAAGGTTTTTTAAAGCACATTTGTGCATGTCACTAGAGATGCTGCCGTATTTTTACTT

A_14_P129004 AATCTTTCCGGAATGTAGATAGTCCAAGGCAGAGACAATTTCTGCACCATAGAAAC

A_14_P100853 AAATGGGACAACTAATGGATACAAATCAGGATTCACTCAGACAGGTATGGGATATTTTCT

A_14_P138363 TCCAGAAAATGTAATCTCTACTTTTACCTTGTCTCCTGGGGAAGAGAAGATGAAATAAAC

A_14_P117083 TCACCTTTGTTTTTTGTTGGAGTGTTTCTAATAAACTTGGATTCTCTAACCTTTAGAAGC

A_14_P133705 AATAACTGGTTCAGGCAAGAGCAGGAAAATGGGAAATAGGTCCTGCAG

A_14_P113987 TACAGTTGCTTTGCAAAATTAAAATCTGAATGGTCTACTGCTACACAAGAGTCTGCATCT

A_16_P30952564 GAAGAAAAGTCTTGGTAAGGATTTGTTTAGTGGTTGGGGTAACACGGGTG

A_14_P201596 AAAGACATTGTTTAGTCACACAATTTTAATTGTGAATTATAGTGAATGGTAAACAGTAAA

A_16_P00685224

CTTCAAGGAAGGAAATATATTTTTACATTGAAAGAAGAGAATATGAGCCATGTGTGCCAG

A_18_P22842477 CTGCCCACAGCAGTGATCCCAAGGCTTTTGTCACCCCTTGGGAAA

A_16_P01897150

TAAGACTCTACCACATTAGGAATATGTGATTTTCGCCTCTGTAGAATTAGCAACCCTTCT

A_14_P116931 TTGGTGATCAAACAATGCTTGATCAGTATTTCCATTTGCTAGAACTTTTTCTTGGCATCT

A_14_P123909 CCATCTCCACCATTAATGCCCTTTTTCACTAAGGACCCTATTCTGAGCAG

A_14_P131084 GGAGCAATAGATTTTATCATGTGGAAAGTGATTTTTAGGCTGCCTTTCTTTATCTGGAAC

CUST_2811_PI426210822

CACCTGAAGGAAATGAATCTGGAGCTTTATGCATATTACTAGGTGCTTCATTTTTATTTA

A_18_P14132355

TGCTCACAGTTTTCTGAAGAGAACTCTCAAAATTCTCAAAACATAAATACCTGTATCCCA

A_14_P201452 TACCCTGACACTGCATTGTAGTAAATCTCTGTCTCTTTTGCTTTCATCTTGTCTAGG

A_14_P101282 TTATATGTGGACTGTAAAAAATACACTGGACTCACAGGTAATTAAGGGGTGAATCAGAGT

A_14_P105254

ACAAAAAGAAAACTGATAGGTCTGCCTCAGAGACAAAATACAGTCCAACAAGAGTTCTTA

A_14_P135868 CATGCAATCAAAGCCAGATCATAACAATTCTTTGCAAAAATGACTCAGTCTTCTTACTGG

CUST_7738_PI426210822 AAATGAGCTGCTTCTTGCAGCCCCTTCTTTGGCATCTCCCATAAG

A_14_P116593 GACAAAGCACCATGCTTGACAGACTTCAAGCATTTGAGTTATTAATTCTGTTTATTGTCC

A_16_P20672576

AAAAGCAATGATTCAGGAACTAATTTGAACAATCCAGGGATAGTCATATTGATTCCAGCC

A_16_P15294040

GAAAATGTAAGTCTTCTCTTGTTCAAGGTAACTTCCAAGGCTCTTAAACACTTACCGAAT

A_16_P16487628

ACCTCATGAGTAGCAGAATATAAGACAGGATTTTAACACACCTATACGAAAAGAGGACAT

CUST_1975_PI426210822 TGTTGCCACACAAAGAAAATCCAAACACAGGTTGCCTGATTCCTCTT

A_16_P02470056

CCCCAAGCACTATTAGACACATTACTTAAGGAAGCAGTTAATAACTTCACACAGCATTTC

A_14_P200592 ATAAAAGTGCCTTGACCGTGCTTTTTATTTCTAACATATTCTCCGATATATCGAGCACCA
A_16_P02381987
TAGGGGGTAAAACACAAATCAAAAAAGTATTAGGAAAAAAGATTTTGGCGTTGGAATGGA
A_14_P115937 TTACTGAGGTTCATCGACGAGAACTCAATGATGTCATATCAATCTGGGAAAAGAAACTTA
A_14_P201907 TGGTGCTCCTCAGTTCTTGCTATTTCTTTTGTCTTATAAAAGATCAGGAGCAGACTTATT
A_14_P121854 TTTCTGACAAAGTTTAGTAGAATATGGTGGAAATCCTTGAACCCATGTGAGTTAATGGTA
A_14_P135332 CTTCTAATGGATAATACCAGTGTCCACCAGACAAGGAGAAAAAAACTTGACATTAAGACA
A_16_P00550973
TCCTATTGTAATTATCTCACCTCTAACCTCAAATCACATGAACCTTCTAATTCTGTCCCA
CUST_940_PI426210822 AGTGCCATTACCCACCTCTAGGAGGGATGTGTTGTTTCCATACAA
A_14_P115164 CCAGTTACAGGGGCATTTAGGGAGCAGATGACTGAGAACATTAAAAAAGAACTTA
A_14_P117999 GAGAAAAAAAAATAATAATGTTCTGATTTGATATGAAGGCGAGAGATTTCCTCTGACACCC
CUST_10070_PI426210822 AGATAAAATCACAGTTGAACTCCTTTGGGGAAGAGGAGGCAAACAGAAAATG
A_14_P103074 ACACACTCAGAAGTTATGTTTGTGTTTTAAGAGAGCATTTTGTGGCAATATGGCATCAAT
A_14_P100643 GTAACCATAAAAATTCAATGACTATCACCCATACTACACCCATTTTCAATGCAAACATGC
A_14_P111043 CTTTGGGGGAAATAGTTTCCCACTATTTCACATAATATTAATCACAATCTCCCATCACAG
A_14_P113508 ATCTCAACATACTTTGAGAAGAGAAATAGGCGCCATGTAGTAATTGGCTTAACTTTGGAA
A_18_P13689450
TCACAATACTCAATGTGGTGCAATAGAAAAAGAAACCTTTGATAGGAATTCAGCCTTCAG
CUST_557_PI426210822
AGGCTTTTTCTTAGATCTGCCAGAAGTCTGTAATTTGCTCTATCCCTTGGATTTTAGT
A_18_P15909224
AATTTTCATGATGACCAAAACCTATCCACTTGAAACATACAGAAATCACCATTTTCACCG
A_14_P120389 CTAAAAACCCCCATTTCCTCAAAAATCAATTTTCTTTGTCGACTCCTATGAAAAATCTGT
A_14_P124850 AGAAAAAGTAGGTTCAGGTCTAGAAATGAAAAGTTATTTCTTGCAGACAGCCAGAGTTTG
A_16_P21512302
ATGCAGGTAAACCCTTACAGTTGACAAAATACCTCAAACTTCCCTAAACTCTACTTTCTA
A_14_P139304 CTACCCTTATTTTAAGAATTATAAATAGGTGTCAACCCACACAAAGAACAGATTGTGGAT
CUST_6696_PI426210822 CTGCAAGGTTGATAAACAGCGGATGTAAATGGCATCACCCAGCAC
A_16_P21436103
CCACCTCCAATCTAAATTCTACCAGCCATTCATACATTTCTCATAGCCCATCATTTTTTT
CUST_8256_PI426210822
ATTTTTAACTGTATCATTTATTTACTTGTCTCATCAAAGGCACAGCAAGAATATACCCAG
CUST_2940_PI426210822 GGAGAATTTGTTAGAATTTCACAGCTGAAATCTTGTTCTGACGGAGAGCAGG
A_14_P111403 TGAAATACAAATGATATTCATAATGCAGTTTTGGAGAAATTTCAGCCAAGGATGGAAGCC
A_16_P18116166
TAGAACAAATTCCTAATTCCCTACGATGAAATTCTCTTGCTATCCTAATTTTTTGCCGGC
A_14_P119190 CAGTATGTATTTATTTATACCCAACCTTTCCCTAAAAAGCCTTGGTTGCAACTTCAGATC

A_14_P134446 GGATTTTTCTCTAAAATTACCAGATGAGTTTCAGGCAGGCCATTTATCAGCAGATTACTC
A_14_P118649 ATTAAGGGAGCCAATTGAGAGATGTCTGGAACTTTGATTCTTATTTAATGATGCTCTGGA
A_16_P03403131
ACATAGGCATCCAACTAATGATTGAAAAGCAAGTTCTGAAAGTGAAATACCAGAAGGAAG
CUST_2978_PI426210822
TTTTAACATTTTAGAGAAATCTGAAAGTTTAATGTGCTGGCATGCACACACCCCG
A_18_P13101968
TATTCATCCTCTCCATCTTTTTTAGCCTACTGTGATCTAAGAGCAATGAAACTCCTAACC
A_14_P136964 TCATCATCTTTTCCTATTCACCAGATACTGATGAGTGTATATCTCTGGTGTCCCTTATTC
CUST_10245_PI426210822
TTGACTTGCATTTATTGAGAACTGTTGGGAATAATATTGTGCCTCTAGGGCATTTAGAAG
A_14_P103600 CTGAAAACCTCAGGAAATGGGATCTCTCCAAAATAAGGATGAAATGGCTTGTTTCTAAA
A_18_P12008053
AAAACTTTCCCTTTGGCTATCTCTGATACCACAAATGTCATTGAAAAGGACAGATCCTA
CUST_947_PI426210822 ATTCTAATATTTGGAGTGGGACAAGGCCCTCCTTCCCCTTTCTTATGATTA
A_14_P107324 TCCTATGAGAAGTTCCTGGAATAGGTGTAGATTCAGTAGATCTTTACAAGACCATATCTG
A_14_P136428 GGAAGGAACACCTTCAAAACTATTAGTTTTGGAAGGATAATTAAGGACACTACAGCATGA
A_14_P110473 AAAGATAGTATTCACACAGGTGATTCTAAACATGAGGCCAAATTGCCATTCTCATTTCTT
A_14_P130515 CTTTACCATCTGTTACCATGTGCATTGACTACTAGGCCAGATTTAGACATCAAGTAATGA
A_16_P18151060
TACCAAAGAACAGGGCATATTGAATCAATTTTTTTGAAGCACGTTTCTAAATGACTCAGG
A_14_P102660 ACTTGTTGCACTGGTTATAATTGCCTCTCTATTTTGTCAGTCTTTCCTACTAAGTTTTCC
A_14_P121504
AACTATCAAAGATCAAATGGCAGCAAAAGATCAGGGAAAGAAGGTAGAAAAACTATGCAG
A_14_P100899 GTTTATTCCTACAGTAGCCTCATTCTATCTTATCGTGCCAAGCATGAGGCACTTCT
A_16_P02045853
ACATACGCACATTGTTTTTTCCTCTTCCACGATTATCTGCCAGAATTCTACTCAGTTAAT
A_14_P125197 GATTTTCTATTTGGGAGACTGGTAGTGCAAATTAAAGTTCTTGCTACCCAACATTTATCC
A_16_P02299864
ACGTCTGTCACTTATAAACTGCTGTTTTTCATGCATTCACTTGTAACATACTTGCTGAGG
A_14_P122935 ACTTTGCTGTCTCCTGGATTCCATATCTACTGGATGGGTGCCATCTT
A_14_P138800 GCTGCTTTTTCAAAACGGATCCGGGCAATGCTTCGTTTTCTAAAGGATG
A_16_P01830274
AAAAAGTGGGAAAATGTATTAAAGATATGCCCTGATTTTGTCTTCTACCACGACACACTC
A_14_P200404 CTTGAGGGTGGGACGGGGTAAATAATTGGCCAGATTATTTTAATTGCTATTTGATAGTAA
CUST_2061_PI426210822
GTTGACTTTCTCCAAATAGTACACTTAAGAAATCACCAACATGTGTGCAGTTCAGATGTT
A_14_P100488 TTGGGCAGACTTGAAACCAGTTTTGTCTTTAGGTTTCTTTTCTTAATGATTGGTCTACAA

A_14_P133610 CATTTTTCTTTTTCTGAGAGTAGAGCCCAGAGGAGAAAGAATGGCTGTTTCAGTAG
A_14_P100962 AAAACAACTCTTAAGGATGCCAGATTCAGTAACCTCTAAAGAGGTGATGTGATACAAGAT
A_14_P114590 ATCTTCCATCCAGCCTTCTAAAACTCTACCTTCAAAAGAGGTCAAAAAGACTGTGTAATG
A_14_P134875 AGTGAAACTTTCTTAATTGGCATTAATGGGGCCATAGAAAATGGCCAATGCAAACTAATA
A_14_P104036 GGTGATAAGAGTAATAGCCCAACTCACTATTCATTGCTACCTAGTTTACAAATGGATGCA
A_14_P111864 GCAATATGAATCTAGTTCTTCTAGCACTCACAGTGATAGTAGCAGTGACAATGAGCAAGA
A_14_P130470 CACACCACTCAAAACTGAACAAAATTTTATAAGAGCCCTAACAAAAACCCTAACCCTAAT
A_14_P134171 AAACTTTCAGAATCCTTGTCTATACTCACTGTAATCACTTCCTTACTTCTCAACCCATTG
A_14_P127368 CTAGAGTAGGCACAGTGTTAACAAAAGGCTGATATTCTTACCTTCTCTACAAATACACGC
A_14_P130242 TGGCCCTTCTATGTCTTCTTGAGAGAGTTTTCTAAACCCAAATTATCACTTCTTATGTAT
CUST_7279_PI426210822 GTGAGTCCATGCTTTGGAAATGATGACCTGAGAGTGTGAGGAAGG
A_18_P10526046
AAGACTCACCAAAGTCATTAAACCACTCTGCTTAAATTGTCACTTACTAGAACAGACAGA
A_16_P15389042
TCTCAGTCTTCCAATTATTCCACACATACTCTCATCATGTTAATTCTCCAGAATGACCAA
A_14_P116643 TCTGCATTTCTTCTTATTTTGAAGGACAAACTCACATACGACTCTTACTCTCCTGATACC
A_14_P103163 TACCTCTCTATTCCTTGCTCAAATTATTGCAAAAGCCTCTATAGAAAGTCCTCTGTGATC
A_16_P00608361
AAATCATGAGGAGCATATGGTTTTCATTCTACCAAGACTTTCATTTCACACTGTTACCAG
A_14_P125711 AAGACTTGAAAGAACTCAGTCCTGATTTGGGAAAGTAAGTAAACAGAGTTCCTGAGAAA
A_16_P00909616
TACACACTCTACTCACATATCTTTTCCTACATCTTTGTGTCCCTTAGCAGTGATCTTAGT
A_16_P37198888
CAGTGCCATCTTTTTGCTAAAATCACTGAGTATGGAATAGGCAGTATTAGAAAGGAGAGT
A_14_P129001 GGTTAAAAATTAACATTTCTCTTTTCTTACTGTATCAGAAACTGGCCTCCAGCCATTGTT
A_14_P133377 GAGTGGATATATTTATATTTGGTGCTCTGTGTGTCTTAAAAATCTGTTTCAGTGGCTGCT
A_16_P01212250
CAGAAGAACTTTCATTTTGAAGTAACAGTTTCTACACTTTACCATTTAACAGGAAATGCC
A_16_P20456357
CCCCATTTCATTTTGATTCTGTGCTTGAATGATTTCACTGCATGCTTCTGATACTTGTAT
A_16_P03298840
ACAGACAACCTTTGTGAAATGTTTAAATATCTGCTGACCATATTAAGTTGCTGTCCCAAG
A_14_P102939 ATCTTCTTCAAACACGCTCAGTAAAGAGCGAGTCTGGAAGAAAAGAAGAAAAAAGGT
A_14_P101163 TTTGCAGATCACAACAGTATCAGGAAATGAGTTCCTTCTACAGTCAGATATTGACTTCAT
A_14_P134823 TATTTTCAGGCCGAAATAGAACTATTTGTGAACAGACTTGATTCAGTGGAATCAGTTCTT
A_14_P129855 TGTGATTTCACACATATGATTGCTTAGGATGAGACTAATGGGGGTATTAAAAAATGGTGG
A_14_P122168 CATTTGTAAATAGTTTCTTCCTTCCCCTTAACTACCTCACATTTGTCACGAGAATGAAGA
A_14_P122358 CCTTTTTTACCTGTAATGCTTTCTCCTTCTGTCTCATGCCTTTAAAAGGTAAAATTCACT

A_14_P131899 ACAAGCAGTGATTGCTATTTACCAGAGAATTTTGTCTGGTTATTTCCTGATTTCTTGTGT

A_14_P127531 TGCTTCCTAACAGACTTAGTAGAACTCAAAACTCACCTTTTCTTTTGGCAAATTTCTTTG

CUST_3717_PI426210822

TCTTCTTCAGCTTCATGTTTTAATAATGGAAGAACTTAGTGATGTTTCATATGTAGAGAT

CUST_1844_PI426210822 ATTGACCTGAACATTCTACTGGGTGGCTGGTGGGGAGGTGATGAG

A_16_P17245301

GTCTCTACAACTTACTGGTAAATTCACACAAACATTGAACATTGGATATTTCTCACCCCT

A_16_P21635109

ACTCACAGGCAGTTTTAAAACAATAACATCCCATAGGATTGGTATAACATTGTAATGGCA

A_14_P127824 CTGAAGGGGAAGATCTCAATGAATGGGTTGCAGTTAACAGTAAGTAGCCTTTTTATTTC

A_14_P104840 GACCATGTAAAACAACGACCATTAGAAGCCAAGATATGCAAATTGAGTTGATTAACTTGC

A_16_P58424224 CTGGATTGGGAATCCTTGTTTTGTCACATCATCGCTGTGTGATCCTAAG

A_18_P22840161 TTCATGGTGTTCACTCAGTCACACTGTTGTCGCTCTCATTTGCCAATGTTAAATA

A_16_P19735377

GTGAGGCATCTTTGCTAGATTCTCTTTAGGGATTATTTGACATCAGTAGTATCATGCCTT

A_14_P129670 AGCCCTTTAAAGACCTGGATTGATTGGAAGGACAAAAATTAAAAGCAATCTGATCCAG

A_18_P22842455 TCTTCTTATATGCCTTCAGAAACACCATGCTCACCTTTCCTCCTGCTGT

A_16_P36230983

GTCAGGATTAGGTTTGCCTTAAATCTTTTTCTCAACAGAAGCCAAACACTTAAAAGAAGG

A_14_P106693 AATATGAGAATGTGAATAAACAAATAGTGGCTGTGCAGAAAGAGCCCCTAAAGCATTTAG

CUST_1059_PI426210822 CTCACCTGACTTGGCTGCAGAGCCCACGGTGCTTACCAAGCATTA

A_14_P200112 AGCCAACGGGCACAAGCATTCTATATATAAGTGGCTCATTAGGTGTTTATTTTGTTCTAT

CUST_8082_PI426210822

ACATAATTTACATCACATAAAACATGACTGTGATCTAATAGAAGCCTATGCAAAGCATAG

A_16_P16158229

GTGCCCAAAATGACAAATTCTAAAATGATGATCTCTGTTGATTAATCTCATGTGCCCAAG

A_18_P12917776

AGTGTGGTAGAAAATAGATAAAGATGAGTGTAGATGGATTCTTGAGAATAGCATGGCACC

A_14_P121829 CTATTATTTTTGGGAAAGTAGGACAGAAGACGAGAGACTTAGGAGATGTTGGTGTATCTA

A_14_P112434 CGTAGAAAAATTTAAAAATACAAGCAAAGCCAGAGAAAAATGCCCGCAGTTTTACGATCT

A_14_P107510 CTCTCCAGCCACTCTAGCCTTCAATAACACAATGCTAGTGTAGAAAAGACCTTAGAT

A_16_P03800481 TGATCTGCCCTTTCTATATGTTCTCATCTTATTTCACATCACTTATCTATGGCAGTTCAT

A_14_P118472 TGCCTATAAATAAATCTTCCAAGTGCAGGTTATCATATTTGGTCCGCTTTAAAAAGGTGC

A_14_P133720 AACTTCACCTTCCCTAAATAAACCAGTTGAGAATCAGTTGAGAAGGGAAGATTCATTTTA

A_18_P15226091

AACCTGGCATTTGAAACACTATTGCTCTCTAACTCACCTTCTATTAATTGTCAAGACCTA

A_14_P115845 TCATCACCACTTTTAACTAAAGCATTCTGTCTTGCAGGAAGGTATCATCAAGATTCTAGT

A_14_P124005 CCTTCCTGTAAAATTTTTAAATGGAGAATTGGGTGCGAGATGTGGTTTCCAGC

CUST_3416_PI426210822 CTCCCGGACATACAGACTCCAGATGTGGAAAAAGCCCTTGCCATC

A_14_P103142 GGAATTTCAAAGCACTGTAAGCACTTCTGAGTTACTTGTAAGTAAAATTTGGCCAGATAA

A_16_P15949139

TAACACTCCTTTCAAAAATGCTCCATTATTTTCTAGGCTCAGTTTTTCAATTCCTTCCCT

A_16_P00847678

TCTGAGCAGTGCCTATATTACCCAGAATATCATATTTCCAATGGTGCCAGATTAATTAGG

A_14_P106052 CAAAAATCAATACAGGACTGTCCCTCAAAAGGAGTTATAATACTGAAAGAAGCGTCAACA

CUST_2804_PI426210822 AGCTGGTAACTGTCCCTGCCATTTTTCTGGCTGGGGAAAATCAAC

A_14_P112460 ATGGAAGTGTATCGGAGCCATTCTAAATTTATGAAAAATAAGGTAATGGCATGTGAGACT

A_16_P02133301

CCTATCCTTATTGTTGACAGAGATCATGCTCACTGTTGAAAAAGTTTTTAATGTTTGAGG

A_14_P129251

GCTGAAATGGTTACTGAGGTCTTAAAACAATAATAGAACAGGACACAAGAAGAAAACCTT

A_14_P139523 GGTTCATATGCAACTCTCTAGACTCTTCCATATTTCCACTCTGTGTTTGACATTTCAAAA

A_16_P16916424

GCAATGACAGAACCATAAGTATTCTTTTTCTAGGCCACAATATACAATATGTGCCCATGG

A_16_P17491813

TTTGTGGAACTAATTAGGACAGGGGGATAATTCTAAGCAACAAAGAACTGTAAGTGAATG

A_16_P17051020

TGACAGAAAACCCTGCAATTCTATAAATCTTCACCTACCCTACTACCAATATTTTGCTGT

A_14_P138179 TTCAGAATTTCATTTTGCAGATAGTCTTACCGGACTGCTTCCCTCTAGTTTTGTTTTTTT

CUST_8053_PI426210822 AGAAATGGTTACTCAGTGATAAAGCCTTGAGCCGCAAAGTGGAATTGGAT

A_14_P133028 CTTTTGCCACACAGATATCATCAAGAATTTTGCGAGTTTCTGGGACACAAATGCAAAA

A_14_P118328 CATAGGTTTTTTTGTTTTATTTTAGGTATGATGCATTGTGTATTCTCCTTCGTCACAGCA

A_18_P16437815

AAGCATTACCTAGGTTAGTATTGCCTCATGTTAATCAGTGACCATAAAGGCCATTAGGAT

A_14_P128678 CCTCTCTGAGCGCTAACTAGATTTTTAAAGAGCAAAATGTTTTGGTTTTCGGTTCCTA

A_16_P40944083

ACCAGTGCCTCATAGTAAATTCTTTCCCTAAAGACAAGTAATTATGGATCCAGAAACATT

A_14_P135538 CTTTGAACTAGAAAAACTAGAATTTCCAAAGGCCACAATGGTAAAACTCAAGCACTTGAT

A_16_P18344226

ACACCCCAACCTAATTCTTTTACCGCAGAAGTAAGTATCTCAATTATTCTACAGTGAAAA

A_14_P103136 TTTAGCCAACAATTACTTACTGGGGACATCCTTTGTAATGCTAACATGGAATTATTCCTG

A_16_P39814284

TGGATACCTTTAGTAGTTAACTCTCTTTTGTCAAACCCTCTTGTATATAACCATCGCACA

A_14_P120486 AGCAAATAACAGTAACTACTGCTTTGAATGCCAAAGTTTCTTAGAGTGGTCTGTTACGTT

CUST_458_PI426210822

ATGGAATTTTAACAACCAAAATGAATTTCGTCAGATAGTATCTACAAGAAGTCAGATGCT

A_14_P131636 CTTCTTTGCTCCAGATTTTGTCCGCTTCATGCTGCCCTTGTAAAACATTTTTCTTTAAAA

CUST_5896_PI426210822 CTCTGTCCTCAAAGCTCATAAAGCCCACCTTTCATCTTGCCTGAG

A_14_P125037 TACCTCAGCAACACAGTGGAGGCACATTTATTAAAATATGGACAGAAGTATCCAGAAAAA

A_14_P122074 CTTCCTCCTCTTGAGGCCTAAGGAAGACACAGTCTCTCTTTAGAAACAAG

A_14_P128834 CCCTTGATTTTTTCCGTTCCTAAATAATCAGTCATCAGTGGCAAAATAAGGAATCATCTT

A_14_P137300 GACTACAAGGATCAGAGTGCCTGGAAGTTTCAGATCTTGTAGAGGACG

A_14_P201087 TTTGCTGAAGATTCTACAGACTGCCTATCCCTTAATCATCTTCAGGTAAAATTTTCTGTT

A_16_P20871537

TTTTATTTGCCCACCATTTAGATTTTTACCCTCCTACAGATAGAACAATAGCCAACACAC

A_14_P128399 CCTAAACAGAAGTGTTCTTCTAAATGAAATGACCATGACCATACAGCATACTGAATGCTT

A_16_P21622568

TCCCCAAATTGTTACCTATTAACCAAAAAGCATTTATTGACTGACTAATCTTCCTTTCCC

A_14_P118983 ACCGACTCTATCAGGGTATAAATCAACTACCTAATGTTATACAGGCTCTGGAAAAACATG

A_14_P119153 ACTCTCCTCATTGTCCAGATGCTGAAGGTCTTGATACTCCTTGGTCAT

A_14_P120767 CTTCAAGGGAAAATGTCATTGCCTGTGATATTGGAACCTTCTTACAATCATTCTGTGAG

A_14_P129232 GATGTTAACATTAGCACAGGTAGATAAGTCAATACACCAGTCACATACGAAACCCTCTAT

A_14_P125314 TTGTCACATCAACTGTCTTTATGATGACTTCTCATATTTCTTGCTTGTCCCTGAGACAAG

A_16_P15215841

AGTTTGTCCATTTTAGAGTCATTCTTTCCTGTTTAATCCAAAAATCAAATTCCACCCAGC

A_18_P16469215

ATAGTGCTGACAGAATCCATTGTATCGCTTTTAAAGACCTGTAATGATTGTGATTAGCCG

A_14_P201990 TATCAAAGCAAATTACCTTTGAAGACTCTCTTATTCTTTACTGGCTGTCCAAGATCCACA

A_16_P36903553

ACCTAGAATTCACAAAAATAACAGATCTCCAAAACCATTTCATTCCATAATTTCCCCATC

A_18_P22840686 GGAGGTTTGCTTGTGTGTAGTGTATTAGGACAAGGCAGAGAGTGTAGTCATT

A_14_P133026 ATGACGTAAAGAAATCTGTCCCTTCGTGCTTATGGCCCTTAAGTTCAATTTTTATTTTCA

CUST_1792_PI426210822

AGCTAAATTTAGGAAGGCCTAATGATGACACACAATGCTCTTTCGCACTCATGATA

A_16_P03546142

GCCCCTTAGAGGTCTAATATTTGAAATCCGACATTTTACTTATCCTTCATTGCTTGCAAT

A_14_P103577 CTTCGGATTGGTGTCTATTGCCCAGTAGGACCCCTAGAGGTAAAGAAATTATATTAAC

A_16_P16282581

TTTACACATTTACATCAGAACTGTTCAGGATAATGGTCATCACTTGTGTATATCAGCCCT

A_16_P18825760

AACTATAAAGCAGACCTTACATCCATAAGACTTGAAACTGTCTTACTAGTAGTCCTGGGT

A_14_P102668 ACTAATCTTTTAAAGTTTTTAAAAGAAAAGAGTGCATAAGACCAACAGGTATAGGGGCGG

A_16_P17399935

AAAGTTTCAGGAAGAAGCATCTTAACTCAAGAAATAAAGTAGTTGCCCTAAGTTGAAGGC

A_18_P22839939
TCATGCCATGTTTAAATGTTCTTAAATTTGGGATTGTTGAAGTCTGAATTTTACTGTGCT
A_14_P112391 TGAAGTCTTGTGACAACACCCATTAGATAGAAAATGAAACACTGACAGTCTCTAATGCAA
A_14_P113108 GCTTAAGCAGGAAACACTGGGACGAGGTTCTTTAATAGGAGTAGGAAAGAGAAAAATAA
A_18_P15007500
AAAGCACGTTAACCATCATATGGAAGAACACCTTAATTTAGTTCAAACCATGAGGGAAAA
A_14_P119762 ACCAGTTGACTCATCTCAGTTATAATTTGTTTAACAGGTTTCATTTGCTTTTCCCCCTTA
A_14_P108523 AAAATTGCCCCTTTTTCTTTGGTGACTGTTAATAGAGTTGTTGATGCTGTTATTCACTTC
A_14_P102425 ACCAAAGTTTCTTTGCAAAAATAGTATCTGAGATACAAAAGGAAAGGCAGATGTGCTTGT
A_14_P100940 CTCATCAGGAAGATCATTTTCCAAGTCAAACAATGATCCAAAATCTAGAAATTAAACAGA
A_16_P02848593
AAACAAAGTCTCTTCTTTAAGAGAATTGCGATGAGTGACATTTTCTCTACCCATGAACCA
A_14_P132598 ATGGGTTAGGTAAGTGATGATGGCTGGAACTTTTGTCTCAGATCAAAACTTAAGACTATA
A_14_P118544 CTACAAGGCAAAAATGTTGATGATTCTCCGGGATTTAAAGAGTGCTTCCTCATTTTTATT
A_16_P36064590
AAGATGGCAGGGTAAATTAACAAAAATGGAAATACGTGATACTCCATATCTCATCCCTTG
A_14_P129087 TAATTCCCATGCCTTCTGGATCATGAAGTTTACCCTAAACAAAAAACAAAACTAGACTTC
A_16_P20184158
ATCGTATTGTCTTAGTTAGTGAGAGATTTCCGTTTAACACAAAATTTTTGCTCCAGAGCC
A_14_P132362 GCTCTTGGTATATTGTTCTTGGACTGATTCAGTTGTAGTTCTGTTGGATAGAGAAAAGAG
A_14_P123912 AAGTTCATAATCAGAATAGAGGACAATATGAGCAGAGTGCTGCTTCTGCTAACATAGGTA
A_14_P123992 CTGTTTGTCTTGTATTGTTTCCTGTCTAGTTCATGTCTTTCAACTTTCTTTCCCTTTGTT
A_14_P121813 ACGTTATAAACACTGTGTCAGTTTACGATGGCAGCAACGGAAGTTGTTACAAAGAAAG
A_14_P200305 GGACCTTCAATTTTTACTTCCCAGGGAAGGAAATTCATTTCCCTGAGGA
A_16_P21564471
AAACTTCAGCCAATCCAAATTTTCAAAACCCTAGTATTCTCTATTTTGGTCAGTGGGTAT
A_14_P109512 AGGAGTGCCAAATAGAGCAGGTTTTGCGCCTTTTGTTTAAAATGTATGCTAATGATTTAC
A_14_P113365 AGACATTATAACAATAACTTATGTGTTAAGACGGCAGGATTTTGGGTGCATCTTGGATGA
A_14_P128222 TATGCCTTCTTAATTGTCTTTCTCTAAGGTCTAGTATCCAAGTCTTCTGTTTTCCTAGAA
A_18_P11863590
CTGACATGACTGCTTTTGATACTCATAATCACAACCTGATGGAAAAAACTCCTGAATACA
A_14_P120549 TAGGTCAGTTAATTTTTGTGTGAACAGAGGGACATTTCTATCTCTAACCCAAGGTATTGA
A_14_P119477 ATTAGTTGTTTTTAAATAAACGTCAGCGGGCATGCTTTAATTAAGCCTTCTTCCCAGTGA
A_16_P17124285
AGTCTCTCCCAACATATACAATGTCATGGATGCTGTATTAGTCATTTGAACAAAGTGCTA
A_16_P20375863
AGCACTGGAAGTATGAATCACAGCAACAAAACACTTCTTCAGTAGTTTAGAATTCTGCAT
CUST_1588_PI426210822 GAACCGGCGGGGCAGGAGTGTGGAGGGGAAGTCCAGGTAGCTGTG

A_14_P116604 TAGATTACCAGTTAGGATACAAATTCAGACACAACCAAGATGCAAAACAACAGAGCCTTA
A_14_P132943 GTTATTTGAAGTGAACATGAAAAGGAAAAACTTGGTTGTTATTGACTGGAACTCTGGATT
A_14_P118481 CAGTCCCGGACATCTATAACTACTTTTGGGAGAAGTAATGTAAATGAGCAATTCTAATCC
A_16_P38280238
TCATTTAGCCTACTCCTCCTATTATATTTGCTGATATTGCATAACCTGGACAACTTTGCA
A_16_P20342499
CCGGCAGAAACTTTAATCTCCCCACGTTTAAAAAAGTTTTCTCAGTTATAAGACTCTCTT
A_14_P130784 TGTTGTCTATAGAAGATATAAATTGCCCCAAACTGGCCACATTGCAACTTTATGAAGTTC
A_16_P16052354
GGTGTTATACAGACCATCACAGATACATAATACTACTCCTCAAACTCTTTGATATTCACA
A_14_P124612 GAATAAAACTGGACTTACTGTATAGGCAGTAATGACCCTTCAGGGAGCAGGT
A_14_P131606 AAAACTTAGAAAAGGCTGGTTGATCTTTATCTTCTTGAGTGTTTTCTCTCAATAAGGGCT
CUST_5351_PI426210822 GGAGATTTCCAGTCTGCCCTCCAGTGTCTCTGCATGCATTCTACT
A_14_P105104 TCAATAACCATTGCATTTGGCTTTTTATCTTTAAGGGAAAAGTTCAGATGACCCAAACAG
A_14_P121195 AAAATACACAGGATAATGAATGGTTTGAAAGAGCGCATTTGAAGCAGAGAGAACCCAATT
CUST_8792_PI426210822
CATGTAGAGAAGTTGAGCTATTACCTTGACTTCCCCCTTCTCTTCCAAAAAAGATTA
A_14_P201606 TGTATGTTTTTTATTCTATCCTGGTGATGTTTTCCAGACTGAGTCCACTATTGTGGTTTC
CUST_2095_PI426210822
AATTAAGCTATAATTAAAAGTCAATATTATCTTGCTCTGTTCCCTCATCAGTTAAACAGT
A_14_P114748 CTGCAGGCCTCAATTACAATATATCATCAAAATTTAGAGACAAGATCCAGAATTGCCACT
A_14_P201375 TTTTCCAGGTGATCCTTAGGACTCAGTTGTTGATTGATACCATTTATTAACTGACAGTTC
A_16_P36059285
TCACCATCTGTCTAAATAGCATTAACCTTCTTGTAACCTCATAATATCACTGTTACGCCT
A_16_P02520660
CCACATGATAAATGTCTGTGTGTATCCTGAACAAGACTTTTAGTTAAAAGTGCTGACTGC
A_14_P136087 GAACTTACAAAATATGCAGAGGGTAGATTATGGATAACGTTTCACAGAGAAGCAGTTAGT
A_14_P110607 ACTTTTTATTGTTAGCAAGTAGAACTAACCAATTGAAGCAGCAGTCACACCTACAGAACA
CUST_1098_PI426210822 TCTCCCCTATAGAGTGAGCCTTCCTTTGGTTTTTCTTTCTCCTCTGT
A_18_P26792768 AGTGTTTGACCCATCAAAGCCAAACTCTTTCTTCCACCATCTCCTGAA
A_14_P124350 CTGGAGAGAGGAAGATAAATAATGTTTTGCTTTTCTCTCATGGCTCACACATGATTTCTT
A_16_P18676874
AAAAAAAGCAAATGGAGAAATTCCTCTTTCTGATTGGATTTTATGGTCCTATGTGGCAAC
A_16_P30710604
TGCCTAAAATCAAAAACTACAACGATGCTCTACTCTAAATATGCCCAAGATAATACTCAC
A_14_P128641 GGTTTACAATTGTGTCCAGTTTAAACTTCACTGTCATTGCTTTGTGCTTTTTGTAACAGT
A_18_P12832706
AAAAATTCAGAAATGTCTCTGATACCCCTACTTCAATACTGAATCCCTCATCATATCCCA

CUST_9202_PI426210822 TGATGCTACAACTTCTGCCTTTCAGCTTTTCAGCCATTGTTCTCCACT

A_16_P15919007

GCACAGCATGGCAAAATATTACCTCCTTCTTTCCATAACTTTCCACTTACCAACTTAAAT

A_16_P17232079

CAAGTGGCATCTAGAAAATATGAAGTCCTAGTTGGTAGAAGAAAAAATGAAGCAAAGCCA

A_14_P131808 TTGCTTATGAATATCTGTGCCACTTAGAGGAAGCCAAAAGGTAAGATCCAGACTTAGT

A_16_P02810049

TCAGACCATTTCCATACTTTCAACAATGTATCCTCTCCATACTTAAATATTCCATGGCCT

A_14_P127846 ATATACACTTAGGCAGCCAAATGCATCTTCAAGAGGCACTAAGGTAATGCAGATATTTTT

A_16_P16596169

CAGTGGCAAATAACCTTGTTTAAAATGTTTGGTAAAGTGGTAAGAGCAACTATCTCCAGT

A_14_P132653 ACCCAAATTCCTTGTGGGAACGATGATAAGGAGCAGGTTTACAGATCATAAGT

CUST_2117_PI426210822 TAGGGGGCAGCATAGCCATCCAGCCTCTGAGGGATGTTTCTTTTT

A_14_P111783 TGGTCTTTGTCAATATTTGGATACAGATACTTCAGGGGATTCTCAGGAATATTCTCAGC

A_14_P202278 TGCTCAGGCTTGAGAGAAATTTGTCTTCATAAAGACAATACTATGAAACTTCCATTCCTG

A_14_P121836 AGTGGCTTCTTTACTAGCAAAGAGAAGTGTAATACAAGTGATCATAGAAGGTGAGAATGT

A_14_P115320 CTTCATTAAGTGATACAACTTCACTCCTTATGTTGTGCATAATCCTCTAGGTTGCAAGGA

A_14_P108383 TGCCTTGGCATAAACTGCAAATCTGGTTTAATATCCTTACGGGGTAAAAATCCATTAATT

A_16_P19810582

AGCCTCTTCCTAAAATATCAAGTCTGTGAACTCAAGTAACCTTTTTACCTTCTAAGACCA

A_14_P100584 GCTAATACTTTCTTATTGGTTTTCTTAGCAAGAGAAATTGGGTGATATCTGCTTCTCCCT

A_14_P128236 TACTCATCTTTGGTCAGCAGTTTGACTATTCTTGACTGCTTGTGTAGTAGTAGAACATGA

CUST_2958_PI426210822 GCTGTGGGCAACTTGGACAGCCTGATCCACCCACCTTCCCAGGGC

A_14_P136466 CTTACAGATATTCTCTTCCTCTTCTCTCTGGGTCCTATCCTTTCTACCTAGCAC

CUST_5773_PI426210822

GAATACAAAAAAAAATTTAAATACTGTGCTAGGTGCTGGGGGTTATAAACATGAATTACA

A_16_P18007849

ATCACCCTTTACGTAACTATCAGAAAAAATAACCACTTGCTTTCATATACTCATGGCCCA

A_16_P16022614

TGGACATCCAATGATCACACATACCCAATATCTGTATTCAAGTTAAGGTAGATGAACATG

A_14_P130390 TAACATATCTATGCCCCTAAACCTACAACTTCCCCTATCCTGAATACAAAAGAGAAAACA

A_16_P16943113

AAAGTCTTTATTTCACAACACAAAATGTCTGTAATATTCCCCATACTTGAATGTCATCCA

A_18_P22839762

CTAATTATCAGGGCTGTCCTTATCTGTTCAGTGCTTCCCATCTGTCCATTTTTTTTTTTC

A_14_P103556 AATGCAGTAGGGGATCTGTCAAAAACAATAAAGATTGGTGTAAAAACAAGCCACAAGTTA

A_14_P134556 CCTCAAGATGCTGAAGTCATTGACGAAGAAGAAGAAGTCACTGGTGG

A_16_P16701045

AAAACTTGCTTACTCTAAGCCATTTCATGAACACTATCTTTCCAGGTCTCTGATTCTTCT
A_16_P00905291
AAAGTTCCCTTGTAACAAAGTGTCACTTCTTACCTTGGATTTCAAATTCATCAACCTCAT
A_16_P16766342
CTGTTAACTCCAATCATTTGAAAGGAAGCGATCTCAAACTTAAGGTTCCAGACTCTATAG
CUST_1327_PI426210822 TTTTATCCTGGTCTTTTGGAAAGGGTACAGGAAGTGACCCCACCC
A_14_P103184 TGGGTGCAGAGGCTAGAAGTCCCTTAATCTCATATTGGAAAAATTTACTGTAGAAAGAAA
A_16_P18869842
AATTCCTGTAGCACTGAAAACTTGAAACAAACATGAAAGAGGAACAAGATCACATTCTGT
A_14_P115514 ATCGATCTTTTTTTGCTGACACTACTGTATCATGCACTCACCAAATCAACTTTAGCACTA
A_14_P138825 GTTTCCCTTCATTATACCAAGGACTTAACAATACCATTAACCAGAACTCAACAAGAAAGC
A_14_P120690 CTGTGCTTTGATTGATGTGGATCTCTGAACTCCCTTAAATACAAAGACCAATTATTTAGC
A_14_P118562 ACTCATGATCTGAGATGCTTACCTGGATAGTCACCATTGCAATTTTTAGAATTTGTATGG
A_14_P137727 TGTTTTAGGACCCATTTTTTTTTAATTCTATGACCCCAATTTCTACTCTTCCCCTCTACT
A_14_P120026 GCACAGGGCAATCTACAATGGGGCCAAGTGCTAAAGCAAAAATTTATTAAAATTCATTTA
A_14_P110427
TCTCATGTCTAGAAGCAGAAAAAAAAGAAGCCAAACAAAAATACACAGATCACCTTCAGT
CUST_6632_PI426210822 TGTCTATTTTTCGTTACAGTATGGATGAGAAAACCCTGTATCTAGCTTTGGGGG
A_14_P128915 GTTAGGAGAAACTACATTTCCCATAATCCTTTGTTCCAGGGCTGGAGC
A_14_P132567 GACATTTAAGTCAGTGTGAACTAGATGAGTTTATGGACATATGATGGAGAAACCAAGGCA
A_14_P113076 AAGTAATGGCAGTGTAATTGATTCATGTCTGCTTAGGTCTTGAATCAATCATTATGCACC
A_16_P20530859
AAAACTCTGTGAATGTTCACTTCATTTTTTCCTGATATATCGAGGACTCCAGATGTAACC
A_16_P58424530
TTAAAGTTTGGTATTCAGGCAAATAGTTTTCTTCACATCTGACCTGAAATAGGACTCAGG
A_14_P127872 CACGAGACAGAATAGACTACACTCAAACTCCAGGGGAACCGTTCATTG
A_16_P02938441
GAGGAGGTATTTAAATTACAGAATTGCAAACATCTTCAAATTGAGCGTTAGGTCTCTGGT
A_16_P30952656 GCATATAAACTGTGGGTGTGGCAAGACCCCGAAGACATTTCACATCTTTATC
A_16_P15270599
ACTTGACATCTCCTCTCTTTAATCCATCATAAGTATGCACCATCACATTAATCTTCCAAA
A_18_P17037076 ACCTACTATAATCTTATATGTTTGCAATTTCCTGTCATTGAGTTTTATAACTTTCTGCCC
A_14_P120645 CTCTGAAGAGAAACTTCCTGGAACTGACCGAATTAAAATTTATACTTCGCAAAACTCAGC
A_14_P134672 GCAAGAATGATGAGTGGAATGATGTATGAAGGCTTAAGAATCTTGGATTTTGAGTTCATC
A_16_P01645175
AGAGGATGAAATTCTGGAAGGCTAAATGAAAAGAGAATGTGTCTGTTGAATTCGATAAGC
CUST_4507_PI426210822 GAGTCTGCCTCCACTGGGCAGTGCTGGCCTGGAGGAAACTTCCAT
A_14_P104118 AACAGGTAAAGGCTACATCCCAAAAGTTCCTCTCCAAAAAGAACGCCAAAATATTAATAA

CUST_7497_PI426210822
CAGCCACAAAGGCCTATTCACAGTCTTTAAGCCTGATGAAGTTCTGAATAAGTAA
A_14_P132817 GCACCATGACTTCTTCACTAAAACCACAAAGTCCTGCTTAAAACTATGGAAAACCTAAC
A_16_P20954511
TTCAGAAGGGAAGTAACAAATTCAGCATTAGGTTAGATCATTAGCATAGAGGCCATTCAT
CUST_9124_PI426210822 GTTTCTCACTCGCTTGCCCTGCCTTTCCCAGCTCCCTGCCCCTCT
A_14_P116778 AGAAATCCTCATAGAGGAGAAAAGACATAAGCCTGGTGTTTAGGCTAAGTGAAATTTGTA
A_14_P112894 TTACATTCTCTCCCCTTGTCACCCTCTAGGGAGAAAACTAAAGTTGCC
CUST_3577_PI426210822 ATTTCTGGGTGGATTGGTGCTAGGTTTCTCCTGGTCTTGGACGAG
A_14_P106696 TCCCTCTTGCTGGCCATCTTGAATGAGTAGTTTCTTTTAAACTTCTAACTAAAAGAGGAA
A_14_P118996 AACTTGATAAATGACATTATTGTCAGTTTTCCAGTAAACTTGGGCAGTTTCTCCAAGTGA
CUST_4157_PI426210822
CCTCCATCATGATTTAGTATATCATCCTTCCCAAGAGAAGTTTTGAGAACTTGTCCT
CUST_9369_PI426210822 TTTTCTGTGGAGTCCTCTACCTTCCTGTTTCTTTGAGGGTTTAACCAAAG
A_14_P129404 TTATAATTACTTTTCTAGACTATGGGCCCATCTAGTTTTCTCCGCATACAATAGCAGCCA
A_16_P39656744
TCTCCTGAAATATCTTTTCTAAACACTAAACCCTCAAAAGACGTTAACTTCTCAGACTCT
CUST_8991_PI426210822 GTCATGTTTACCCGGTTGCCGTCAGCTGCGATTGGTGTAATATGATAT
A_14_P129360 TTCCTAGCCTGGTCTTAGATATGGTTCTGAATCTACAGTTTGTCTTTCTCTTTTCTCTTT
CUST_2880_PI426210822 CTAGAATTCAGCAAGGAAGGCAGCCTCTTGGCTCTGCACAGTTTG
CUST_3205_PI426210822 GTATAGGATTTTGTGATAAGGCACATCCTCCTGAGGGGAGGGCAG
A_16_P17306357
AACATGGTCTCTATCGTCTCTTAAGTAACAACATAACACAGTGAAAAGAGCCTAGTGTCA
A_16_P19170471
GGCAAAGTGACTCCAATTACGTCCTTAATGAGTATGTTAAGCCATGTAAATCACATGTCT
A_14_P139822 ACGAAGTTAATGGGCTTTCCTGCTGTTTAACTGCTTTGAGATTTTAAAAATTCATTCAGC
A_18_P10585970
AAAGTCGGGCAGTGTATTCTATAATTACTGAGATTCACAATGAGTGCTGGAGTTTTTCTT
A_14_P102413 TTTCCTCAATGAGAACGTGGTTCATAAGTGGTTATGGGGATTACCTTAACCAATTAATTT
A_14_P120792 CGTTGCCTCCCATAAGAGCATAAAAAAAGGTTCAGGCTTTGGTGAAAGG
A_14_P202232 ACAAAGCCCGATACCAGGAAGAAATGATGAATTATGTTGGCAAGAGGAAGAAAC
CUST_642_PI426210822 GTGATTGAAGTTCACCTTTTGAGTCACCTGAGCCTGATGGGGAGG
A_14_P125517 CTGGGCATGATAAGGAGTTTTATTTTTAAAACACAAATTTCACCCTTGTTCATACTTGGG
A_16_P35286483
GCCTTCTCTAATCACATGACACCTAGAATATTACAAAATTACAAAATCATCCCTCTTGCC
CUST_9579_PI426210822
TGTACTGATTAGACATGCTGACATGTACATATAAAATATGCCTAAACCAAATTAAAAGAA
A_14_P121872 AGTTTCAAACTCTCCTTTCCTGGCTGAGATTACCTAATTCCAAGAAAGGAAACAGTTTA

A_16_P00812644
AAATTTTTGTCCTGCTTATTAGTCAATTCCCAATTGACCAAATTGTGAATTAGTCCCCTG
CUST_2070_PI426210822
TTTGTATTTCAAAACTGAGATAGGAAACTTCACATATAGAGATTTCTAATTTAACATTGA
A_16_P21101961
ACTTGAGACCCTTGTTCTTTATACATGTGGCTATTGGAAAACATTAAGACCACTATGTCT
A_18_P22842633 GAAGCCGGCTTCCTGGTGTTGTGAGGAGAGGATGAGGCCGTCATG
A_14_P115904
CCAAGACTTTAGAGAAAACATGTTAGACAACAGGGGAGAAATATCACTACTCAAAAAACC
A_14_P108856 CATTCAAACTGAGTGTTTGGACTGAGTCATTCAAGCAATTCAATAAAACAACCTGTTTGA
A_16_P00825617
AAAAAACACAGGTGACTCCTTATTCCTGAGAATTTGATGCAATTGAAACAGCGACTTTC
A_16_P15598898
CCTGACTTTCTTAACAATACAATATCAAAGATGACCAACCTAAAAATAGCCAAACTGACA
A_16_P39719647
ACCCACAGCATTTTTTTACTCCACAATATTTTTTTCCTCCTATATCTCTTCAAGGACTGT
A_14_P123778 AGGCAAAGTAAAACTTCTTGTAAGTCATAATAATCATCTCTGTCCACTTTCCCCAAGTTT
A_16_P02846621
AATTATTTGCCTGATGAGCATTATTTCATGATTCCCTTCCTAAAAGGATGAAGCATCTGG
A_16_P30952589
TGCATTTAGAAGCATTTGAATTGTTGACATCTGATTTTGGGTAAAAAAAAGGAAAAAGGA
A_14_P129188 TGATGGCTGATGACAATATAGAAGATTCTACAGCAAGGTAAGAGTTTTTGGTCGCATAAC
A_16_P19265260
AGTGATCTGCATAGAGTCAAAAATTCAAGCCATGGGTATAAAATGCAAGTAAAATCCCTG
A_14_P108043 CAGATTTTGTAGCCGATGGATGTGACTTAAATCACCTTCAAGAACAAGGTCTTGTATTAA
A_14_P129298 AGATCTTTGGGAATATTTGGGTGTTTTGTTTAGTCAAGTAAATGAGCAACCAACAATTCG
A_14_P132308 ATGGTTCTGGAAACCCCGTTATAAGTGTGTCAACCTGTCTATCAAGGACAT
CUST_6456_PI426210822
GAGGCTCATTTGCACAACATACAACTTTGGACATCAGATTTTGGTGAAACTCAAA
A_18_P22840911 AGAGACCATATCCTCCCTCACTGCTGTCAACCAAGGACCCTCTCT
A_14_P100499 CAGTTACCCTGATTCAACAGAACAAAGTTTAAAACCATTGCCATAGGGCATTAAAAAGAT
A_14_P139109 TGTCTCTTAAATAGTTCTAATACAGCATTGTAGACCAGTTCATATTGTTCCTGAAGAGCA
A_14_P116214 GTGATTCAGTTAACTCACTATCCATCTATGCAGATTTATGAGAGGAAGACCTTTCCACTA
A_14_P102208 TCCAAGTCCAAGTGCCTGGTCCCCTTTCTCCATATGTAGTAAATAATTATTATGACTAAT
A_14_P101563 CTTTTATAGAGGGTCTAAGCAGGCAGGTATTCCTAGGATACCTACACCAAGG
CUST_4268_PI426210822 TTCTTGTTTTCTGCTCTTACTCTTCCTACCTCCAACCCCATTCCAAATTTC
CUST_9392_PI426210822 ATATTTACCCCTTCCCCATCTCTCCATCAGGCATCCTCTTTCAATGTTTA
A_14_P125888 ACTTAGGATAAAGGTGAGTTGAGATCTGAGTCAAAAGAAAAGATTCAGAGTATTCAAGCC

A_14_P128648 CTTACTCTGTGCATAAGAGAATTTCACTCTTTTAACTGATTGCATTGGAATGCTGACCAA
A_16_P01165968
GCTCCTGCATTGGTTTAAAACTCTATGATCATCTTGTCCAATCTTACACAGAAGAACAG
CUST_4000_PI426210822 TCCCAGATCACTGGTTGACCTTCAGCGGGCATTTAACAGCATATT
A_14_P122161
AGAACGAGATAGCAGTCTGACAAGAGAATAAACTCTGACAAGCAGAACAAAATCCTAAAA
CUST_3838_PI426210822
TGGATGAGAATCCAAGTTATGAGAACCTATTGATGAGAGGCATGCAGAAATAAAAAGC
A_14_P121061 TTCTGTTTCTCTGAATTACTAAGTGGAAAGCAAAACTTAAAGGTGTCAACACGGTTAGC
CUST_2058_PI426210822 CATAGACAGGAGGGTTCTAACTCAGCAAACCACCGGTTCACTTCT
A_16_P00926525
ATTTGCCCATTTATAGGACGTAGAACTATCAAGATATTCTGAAAGATCACCTTCATGCCA
A_14_P119913 GTTGGCATGTAAGATGCTTATTTAGGTCTGAAATAAGAACTCATTGAGTGAGAACTGGAA
A_16_P02022553
ATTGGGTATGCACAAGTTAAATTCCTGTCCAACTCTCTCAAAATTAACTATTCCTGTGTC
A_14_P200718 CTTTCACTTTAAAGAGGCACAGATGCTGGAAACTACTACCCTTACTGAATGCAGAA
A_14_P129261 TCTGTGAATCAGGCCTCCTAAGCAGATTTATATCAGTTTGGCCTCTGTGAG
CUST_5678_PI426210822 CCCACACCCCTCAATGCATGCATATAGGAATATAGCTCATGCTATGGAAAT
CUST_5543_PI426210822
ATAGCATAAAATGCAAATGTCATTAAACTTGATTATATAAAGGTAAAAGTAGAATAAGAT
A_14_P122788 TTACCAAGCCTTTGTGAAATCTGTATAACAGGTTACTACTCCATTGAGCAAGAAAACACA
A_16_P15625132
CAAGGCAGAGGACCATTATATTAGGACAGATCATTTACAGTGGATATCAGACTCAGATTT
A_14_P117559 GACAAAAAAAAGAATCTGGGGAAAAAGAGGCGTTTCTTTTCCAAATATTCATTAAGTCCT
CUST_2112_PI426210822 AGGAATGGGGGACTTTGGGCCAGTTAGCTTTGTCTCACATTGACT
A_18_P13295287
AAAATTGTAAATTAACCCAGGATGTTACCTACAGATTGTAGGGTGATATCAGTAGGGTCG
CUST_4920_PI426210822 AGAAGGCCACATGCTCTCAGCACCAGTCAGTGGTGCTCGCTAGTC
A_14_P124894 AGAGGAGATTTCTCCATCCTGGTCAGGGAATTTGTTAAAGACTGATGAAAACATGAATA
A_14_P114990 CTTGGGGAAGTCAAAGTCTATACCAAGGCATTGCTAATAGTTGATTTTCAGGTTTTAATG
A_14_P201368 ACTGGCCTTGATGTGTCTACATGGAATTATGTTCCTTAAATAAATTCACAGCATATGAGG
A_14_P103940 TGTGGTTGGTTAACTCTTGAAGGAGCAAAATAAGACTATTAAATCCAGATCTGAAGGGAT
A_14_P107788 ACTCTTTTCGTTGTAATAAAAAGGAAAATGAGAATTTAATACCCATGCCCGTCTCAAAGG
CUST_3677_PI426210822
TTATTCCATAGCTCTCCTGAATCTGACATTTGTTCCTCTTTCATCCTATACTTCCCT
A_16_P18075120
AAAATCCTTTACTTGAGTGCAAAGACATATGTATACCACTCCACTCTTTAGCATGACTGC
A_14_P105962 AGATGGAGAGTGCTTTATTCAATTTATTTCTTGAAGGGAACTCTACAATCTGCCACTTAC

A_16_P19473827
AGAGGAAAAAGACTGTTTCAACATCTGTTATTACCCCAAATCTGATAATTATGGTGCCAG
A_16_P03718528
CTGCCTTATCTCACATTAAGTCCTTATTTCCCATATTCTCATTGCAAAGAACTGTGTAGG
A_16_P18613024
ATGGCATATTGAAAACAGCAGTATGATGTAAGAGAAAAGATCACAAATTCCTTGAGGGTG
A_14_P134123 ATGTATTGCCCCCAATTATAAATGATGTATTGTCTGAAATCCAAGGAAGGACTTCTAGGA
A_14_P125925 GGCCTATTTTTAGAATGCTCCTGATAATGCCTCAGGAGTTGCTAGATCTGAATTAAAAAC
CUST_3774_PI426210822
TGACCCTGGCTCCTATTGTCTCTGTCTCTCTATATCTATAACTTTTAATCTTGAAATAGT
CUST_434_PI426210822 TTTCTAGGGAAATGGCATTTTCAGACATTGTTCCTGGTCCTCACTCAC
A_14_P122127 ACCTCAAAACTTCTGTGTTAGATAGCATATAACCCAATATAGTCACACCCAAAAGGCAAA
A_14_P136901 CTTATGAAGATCAGACCAAGGCTAGAACACTACTCTACTTTTCTCAGACAAATTCCTGTC
A_16_P58424371 ATTTTAATCCCTTCTATGAACTACCTGGCATGGGTTCTGCTTTCCTCTCTG
A_14_P120769 GCAAGCAAATCCTGAAACATAACTAGGATTTGTATGTGACAGGAATTAGAAGAGGATCAA
A_14_P116392 TAAACATGGAGTGCTTCAGAGTATTTGACAGAAATGCAAACTAGACAATCATCAACCAAG
A_16_P16191563
CAGCATGGGATTTGGTTGTCATATCAACCCTATAAACAGTGTTGAATCCATGTCAATAAT
A_16_P58424538
CCTTTTTCATGTGCTGTCAAATTTAATCCTTATGGCACCTTCATAAGGTAAGATCAGCAT
A_14_P101305 TGGGAGGTGGGTTCATAACTTCCAAAATTAATTCATCTTTTTCAGTTTCCAGAAATTTCT
A_14_P101322 GAGGAACAATTTACTCAACGTTTCAATTTTTACCCCACTCCTGGCATATACTTTTTCAAT
A_14_P118186 CTCCTTAAGGCTCTTTTGTAAGGTTTTTGTAGTGATTTTTATGCCACCTGAATAAAGAAT
A_14_P128594 TTTTTTGGGGAGGTCTATGAAGGTGTCTACACAAATCACGTGAGTTCTAGGAT
A_14_P137744 GTGTCACAAACCAAGGTCACTAAGCCATTATTGCTGAAGAATGGGAAGACT
CUST_9927_PI426210822 AATCCTCATCTTCCTGCTGCTGGCCGTGCTGCTCTGGAAGGTGAG
A_18_P14136919
AGCATGTTTCTGTCACTTTCTCTGGCATTCATATCTCAATCTTTGCAGTAGAAAGAGAAA
A_14_P102840 AAAGTGACATTCTAAATGTTCCTCACTCTGCGAGGCTTATTTTTTAGGGACTTTGCTATA
A_16_P02975124
TATTACAGCCTCAAGTTTAAGGAAAAGTTGCATATACCTGAGTTCTTTTGCTTGGTTTCC
A_14_P129641 ATTACCTTTTGACAATCTGTTAGGAAGGTATGCAGTGTGAGAACTGAAGTATTTCTGATC
A_14_P100657 CTTTCACATTGGTGACATATAGATTTAAAAGGCCAAATACAGACCAGAAGAGTGACTGAA
A_18_P13691922
AACTCTTGGTCTTATGGGAATTACTACTTAGGAAACAGGAAAAATAGGCCTTGACTCTAA
A_16_P30952646 TTTTATATTTGTATCGATGTTTTATTATTTCTATTAAATAGTTATATAATTCACTCAAGC
A_14_P119604 GTCTTATTGCTCATTGTTCCTTTTTCTTATGAGTTCTGAGAAGTGGGCAGCAGTAGT
A_16_P00916838 CTTACCTTCCACCTAGTGAGAATCCTAACTGTTCTCATGGTAAATGTCACACTTACTG

A_16_P36714347
ACTGCCAGTTCATTTTAATCTTTCTTACCACCCTATTTTTAATTTAAGGCCCTTTAACCA
A_14_P124315 ATAAATACAATCAATTCCAAATGCCCCAACTATACATGAACCCGAAGTAGCCAATCTTTA
A_18_P22840303 TAAGTCACCTTCCTCTCTTGCCCTGCAGAGCAAGGCCTGTCATTA
A_16_P58424288 CTTTTGAGGCATGGGCTTCAGAAGTGGCATGATGTCATTTCTTACACATTCT
A_16_P18657234
AAAGGAATACTACGGGATCAACCTACTACTTCATGATCGCTGTCTAAGTAAGAAATGTCT
A_14_P132170 ATTGAAAATGCAACTGCTCCAGCATGATAAAGAGGATTATTTCTCAAGCATACCTAGCAA
A_14_P201866 CAAGAAGTAAAATTGGGGATATTTTGATTTTTCAGAGACGGTGATATGAACCTGATGGTT
A_18_P14132142
CCCATTCCAAATTTCCTCTTAAGAAAAGGAAGGAGAGTTTGTTTTTCTCAGTAGAGTAAG
A_14_P133342 TTTGACAGAATACTCTTTTCAAAATGGCATTTGCTGATTTCATAAACCTTTCACGTCTGG
A_14_P118424 CTCCAAAATCCATACAGTTTATTTAAACTGCTATGAAGCACTTCCATTTGGAAGCCGATA
A_14_P200230
GAAAAGGATAAGGGGATAAGAAAAGAGGGGATAAAGTGAGCAATGAACTGTTAATTTGTC
A_14_P117416 ACCTGATTTTGCAAATATTTTGCCAGCAGATTGTAAAGAAAAATCACCTGTATGAAAAGC
A_14_P139246 GGGGCTATACATTTGGTTACTTTTTTTAACTTTGTTTTTTCCCCGTGTCATGAAAATGTC
A_14_P100525 CCGTGGCTCAACTAAAAACAATGCAAATCATCAATCTTGTAGTCGTCACTGCAAATATTA
A_14_P137533 CAGCATGTCCACACAGATATTCTTCTTGTAGGAGTTCCTATAACTGGAAATATGAATCAG
A_14_P101471 ACAGGGAAATAAATTACTTACCTGAATAAGTTGGAAAACAAAGTGAAGCGCCGCAATAAA
A_16_P21671678
ACATCAGAACTCTCCTACGTAAAGATGGCTTAAATTTCTATTGACCCTCACCATTAAACA
A_16_P20609950 ATGCCACTGCCACAGCAAGAGAGACAGGGAAAAAATGGACATTTCT
A_14_P138921 GAATAGTTTAAATCCCCGGATTTCCAGGGTAGTGCTGATTTTTCCAAACACTATTTTAAA
CUST_9861_PI426210822 GACCAGATTAAAGCCAGCGCTCCTCCCTCTCCATCTTTCCTAACA
A_16_P15478011
AAGTCGGTCAATTTATCACTTATGTCTTATCCAGAATGAGAATAATCCGCTCCCTTTTCT
CUST_1889_PI426210822 CTCACGTTTCTGGAGCATGTGCCTCCCTCCTCCCTAACACTGATG
CUST_8628_PI426210822 TTCCGTATGCCTTCTGCAGATGTACAGCAGATAGATATTTTGGCAGAATGACA
CUST_7447_PI426210822 CAGGACCTGGAAGACCACTTGCTCAGAGTCTGAAATGTTTACACTG
CUST_2640_PI426210822 GACAAGGGAATCAGGGAGAGCAGGGAAGGCTTCAAAGACAGGCTG
A_16_P15884526
TGGGTTTTCATAGTCTCTAGGCTCTAATTCAAGCAATTTCCTATCCATACAACCAATAGA
A_14_P202534 CTTCCTGAATGTCTTGTGTATCTTTCATCTTGGCTAGAAAAGAAGTCTCTTTTCTTTCCT
A_16_P00526186
AAGGTCTTATGAGACTTTTGCACTGTAAAACTTTACACATTGTGCTATTCACTCTGCTTT
CUST_1515_PI426210822
ATTCTTACTTACTGTAAGTTTGCCCTTCGTATCAGTTCCTCCTCTCTCAAACACTT

A_14_P120106 ACCTTTGTTATCTAGGAAGTATTGGAGTCATGTTTCTCTTAGAAGGATAAACTGTTTCCC

CUST_5172_PI426210822 CTACTGATATGGATCTGCCTGGTCCACATCATCCTGGAGTGCTAG

A_14_P115297 CTTTTTGCCAAAACATTGGTGGTATAGTGGGTTTCCTTAGGAACCACTAACATTATTAAC

A_14_P104505 AGAGGCTATTGTGTATACACTCCCATAGTCTTGGTGTATATGCTGTCCCTATTAATTAGT

A_14_P102358 AACACTGTCATTGGGTGCTTACCTTGTCATGATTTTCTTTATTCCAGGCTCATTCT

A_16_P35578867

AAGTTCTCCTCCAAATATATACAGCCTAAGAAGAAATATCAGAAAAAAAAGCAAATCCCA

A_16_P19303510

AAGTTCTACTCTTCTCTTTTTGTTCTACATTCCTTCACAATTCTCAATGTTTCCTAGGGC

A_14_P138524 GAATTTTAGGCCTGCTGTTTCATTCAAAATCCTAGTCAGAATGATCTCCAAGTATCATCA

A_14_P119773 TGTGTTTAGTGCAGAACTTTAGACCTCATAATTCTGCTTCATGTGCACAACCATCTTAAA

A_16_P17858495

GACACTAATGATTCCCGAATTTGCAATATCCAGACAAAAGGTAGAAGTAAGCGTTCTTTT

A_14_P130388 CTCTCCTGTTCTCTCATCCCTAGATAGGTGACAAGTCCACATCATTGTTTTTATAAAAAA

A_14_P110026 GAAGCAGAACAGGAGTGAACATAGCATCTCTGTTACACAACTATAAATAGGAAATCAGGA

A_14_P135085 CCACAATGCTCTCCGTGGCTGATAATTTTTCGCTAACTCCATTTATTTCATTAATTAGGT

A_14_P113068 ATTGAACACACTTTTGGTTCTGAAGAAATGTTTGAGGAATGGAACTGGGTTATTGCAA

A_16_P16361284

GAGTGGACTTGGTCATAAGACAATCATAAACCATAAGATGATCCCACACTAATTTTGAGT

A_14_P119572 AAATTACTTCCATGCTATTTGCTCCAAACCATCTAGTTTGAGGTTCATTTGTCCTAAGTT

A_14_P115475 CTATTTACTATTAAAAGGTTGAATGAACAGTATGAACATGCCTCCATTCACCTGTGGGAC

CUST_9043_PI426210822 GAAACCTGTGTCCTCTCTCTTGCTCTGAGTCAACAGACAACTCTTAGAA

A_16_P20029868

AATTGATATTCAAGACCACTAGTGAGTTCTAACCATACCACAAATCCTCTTACCAGAGTT

A_14_P129556 AGTCCTGGGAAACAGAGTTATTTTTTTTTGAGAGGGGAAACTTCTAAATACAACGAGTTA

A_14_P134398 TAACAAAGGCAATCCATGCAAGAGCGAAATAAAATTGATGGAACTACCAGATTTCTTTTC

A_14_P131833 ATTTCAGCACAATCTGGACATCTTTAGCACAAAAGTGAAACAAAGTAATCAAGGACAAAG

CUST_5114_PI426210822 AAAATGCACCAGTGCCTTCCTATTGGCTGAATGTCCACTTCTGTTCTAT

A_16_P38045223

TAGATATGCATGTCATATGTCACTTGACTTATGTTCCTTGACTCAAATTGTCTGTTGGCA

A_14_P108752 CATTTGGGAGCAGCGAAGTATAAAAATTCAAAAAAAAAATATTCACAGTAGATGCCAGGG

A_16_P17431447

TTTAACCTACCTTTAAGGACAGTAGTGCAAGTAGTTAGTTCTCAAAAAGTTCCACAGTGC

A_16_P03307144

AAACTCCACTGAAGTATGGTTATAAGTTTCATCTCCCAGACAAATAAACAAGCAGCCTAT

A_14_P130128 GCATGCATCTGTATTTTTTGTCTTAGATTTCACTTGATTCTCAAATGGATCCTTGACTCC

A_14_P139689 ACTACCTCAGTCTTATGAGTATACTTGCTCACATTGATAACAACATGGTAGCATTGCTTC

CUST_5011_PI426210822

GTTAATAATTTACCCACTTAAAAGGTTAAGATCAAAGAGATCATAAACACACATGAATAC

A_14_P122683 CGAAGGGACTTCCATATGCACGAGATCAACTGTTTATTGATTTTTTTCCTCAAATACTAC

A_14_P105563 CTAAATAGGGATCAGTAACTTTATCTCTATCCTTAATGAACATTTGTTTTATTGGTGGCT

CUST_5202_PI426210822 GCCACTGCTGCTATATTCCATGCTTTTGGACAAGAGCATGTTCAGTATTGT

A_16_P20240557

AAAATGGGTTTAAAGCAGTGAGATCTGACTTAGGATCAGGCTATCTAACTAGTAAAGTCC

A_14_P138053 GGGAAGAAACCACTGACAGTATTGTAAAAGAGAAATCACTTTTACTACTGCTTTGACTTC

A_14_P129163 ATAAGTCTTGAGTCTGCACTGGTTCTCTGAGTCATATTGGATCCCTGATCATCT

A_16_P03775558

TGCTAATTTACTCTTAGAACAGGCATACATATATGGCACAGACAAAAACATAAGAATCAG

A_14_P109649 CAGTTTTGAATCTCAGGTCTAAGCCCCCAAACCAGAAATGATTTCAGGAATC

A_16_P01280187

GGAACCAAGTAAGTATTCACATAACAGAATCATAGAATCACTTTATTTAAGCCCAAACCC

A_14_P103095 ACATGAATCAAAGGTTCAATTTTATGAGAACGAATGTGTAGGGCCTGAATTTTAAACACG

CUST_9165_PI426210822

CCATGGGATTGTAAGCACAATTCTAAATTATGAATTACTAATTATTAATTTTCTGGTTTA

A_14_P108352 CAAATGATTCATAGCCTCTGGAAAACTTATCAATACCACCTTCATTTTCTCCAATGTTCT

A_16_P38429524

AAAATTCCTCACAAGGCACATACTTGAGAAAAATTTAAACTTTTTTCCCCTAGAAGCCAC

A_14_P112387 CAAAATTTCTTAATGAATCCAAACCCAGTATTAAAGTGTGGATCTAAGTGCCTTAGAGGA

A_14_P112981 CAATGACACAAAGATAATAAAAGACTCGAGCCACCTGCAACCAACAACATTCTTAAGTG

CUST_4181_PI426210822

TCAATAATGTTGGAAAAGTAATTCTAGGCATGAAAATTAGCAGTATGGTACATATGAAGT

A_14_P105353 TATGAAACCTCAGAAAGGATGTTGAGTCTTCAATAACACTAGGAAGGAAGATATGCTGTT

A_18_P22842482 CTTGATTTCAGCCCTGTTTGATTTCTGACCTTTGAAATGCCAGCCC

A_16_P00685535 AATGAGAATTTCTTTGCCCATGGAGTCCTTGCCAAGTCTATGTCTTTTCTGAGAAT

A_14_P112777 CATGGCAACTTAAACTGCAGATCTAATAGGTCTGCAACTTTTACACTAAAAATGGCACAA

A_16_P18105296

GCAGTATAGGCCACATGATATATTAACAATGAGAGACCCATAAGAAGATGAACAAGACCA

CUST_7807_PI426210822 GAGCAAATGCCTAGGACTTTCCAAGTGGTTCTCGGTGCCAGCAGC

A_16_P02027422

AGTGACACTGTGATACATTTAGCCAAGCCATATGTTTTGAATGCTATGAATCTGACCAAT

A_16_P19318732

AATGACAGGAAAGAAAATTATACCGAGGCCTAATAGCAATACATTACAAACAGACAGGGA

A_16_P16886914

AAAAAAGGCAGTCTACATTTTGATGTCTGCCAATAATTCTGGATCCATATAGCAAGACAA

A_16_P18331617

CCTTGATAACCATTTTTAGAGTTATGTTTGCACTCCAACACTAAATTAGGGCCATTCTGT

A_16_P18607669
AACAATTAGGGCACTTTCAATCCAATGATAGATTAAGTTGATTCAGACCATTTCTCCTGC
A_14_P135950 TTCGAGCAAATGTATGAAGAGATGCTTCAGACTCAACACAGGTGATTATAGATTCATACT
A_14_P128054 CTTGACAATATGATGGCAATATAAACTTACCAGCAACCATACAGACACCAAGAAGAGCC
A_14_P124694 GGAGTCATTTTTCCTTTCCAAACATCTTTAATGGCTTTTCTTTGCCCATTTAATAAGGAG
A_16_P21459221
CTACAAAACTCCTTTAGTGAGTCTTAGATGCCATCAGACAGAAATGATTTTACCTTGAGG
A_14_P107264 CCATTTATTGATAGGAAACCAAAAGGCAGAGATTATCCAAACTCACAGATCTCCTTAGTG
A_14_P117397 CTCTGGCTAAGCACTAAAAGATGGATAAAATTAACATACCCATCTCCTTACCCCAG
CUST_10093_PI426210822 ATATTCCATATGTAATTTCCTCCATTCCTTTCAAGGGGAAAGCCACTGAGTGTTA
A_14_P130648 ACAATCACACTTTGGGGTTTAAAACTAACGAGTTTCAGTTTTAAAACATGTTAAAAACCT
A_14_P115844 ATGAAATACAGGAATATTCCTAGAAAGTTCGGAGGCTCACTCTCAACGATCTGTC
A_14_P130512 CTCAGTGGTCATTTGTTCAGTCAGATGGTTATGGTGACAAGTTTGTGAACAATG
A_14_P124967 AAAAAATCCTCAAATTTATACTCACCCACTATGCTTCATATGTGCTGGTTTATCCATTCG
A_14_P124022 GAAACATTATTTTCTTTGGCAAATTCTGTTCTCCTTAAGTGGCATAGCCCATGTTAAGTC
A_14_P132994 CCTTTGAGAACACGACACAACGAACTTTATATTTTACCACTTCCTTGAATAGGTAAGACT
A_14_P111737 GAAAACGTGCTCACCTTTTACTGCCAGTAACTCCAGGCTTTGACATC
A_14_P130818 TGTTAATTGGGGATTCTGCCTTTGGAGGATCGGATCAATCACTTTTCTAGAG
A_14_P137759 TTAATGTCTTACCAAATGCCTATGTTTTCTGGCTCTACAAAGCCCATTCACACACATC
A_16_P16848563
AATACCAGATTGTGAATGTAGGAGAAAGTAGTATTCCATTAGGAAGTCAGAGACTGTTGC
A_16_P18119476
AATCAAGAAGGAGACAGTTTCTATCCAGATTTGAAGACATATATGGTGCACAGATAAGGC
A_16_P19181701
ATTTCCAGACTCAATGAAGATGAAAGGAACACTCATGATAAAAGATTCATACACTCCCTC
A_14_P111755 TAGGTCAGTGGAAGTTTCTGCTAACTCTAGAAACACTTTAACAACTTTTGTGAGGCTAAT
A_14_P101853 AAATAGTGACTTACGTGTCAACAGAAATAAGTTTCTTTTCCCCATGGTCAGAGGCATAAT
A_14_P114980 TTGTGTGAGTGTTCCCTATAGATTATTTCAAATAATCCTGAAATGAACGCTTTGAGGTGC
A_16_P17387445
TAATGTCCTTCTTCATCAGTAACATCCTAATGTCCTTTAAGTGTTAAACCCATCCCTTTC
A_14_P200482 CTGCTGCTCTACTTCTTCTATGACCACTTTGTCTATGTCACCATTGGGATCTTT
CUST_1438_PI426210822
TGGCACTGTGTTGCAATCATAGTTTCATTGTTCCTTTGTATTTTGCATTTCAAAAATAAT
A_14_P104461 GAAACAGATCTTCTGCTGTTCAGGAATGGGATGGTTGTAGTTTTACTCTAGGATCA
A_16_P03807294
CGTATTCTGTTCAGAATCCCTGAGATTACTAGAAGTTTTAGAAGGTAAAGCAACAACTTC
A_16_P19296146
AAATAGTGATAAGTTTTTGTCTAAGTCAAGTTGACGTTGAACATAATACCCGTGACGGTG

A_16_P01648431
AGCCAGGGATATTGTAAGAAAATGTCATAAAACTTGTCACATAACAATGAAACGTGAGGC
A_16_P18891519
TTTGGATCCTATACGAAAGAACAGAAACACTGAATATAGCAAGTTAGCATCACCAAAAGG
CUST_2468_PI426210822 CAAGTGTCCTAGGGAAGCTGGAATAAAGCAGTCACAGTTCTTATTTGTGACT
CUST_6960_PI426210822 CAGAGACCTGAGAGAGGTAGTTTGTGAGTGTGTATCCCTGAGATACGTA
A_18_P14135249
CCAGGAAAAACAGAGGAAATGGGAAATAAAAATGAATTTAGGACATAAACTCATGGAGCA
CUST_9177_PI426210822 TTGCCATTGCTGTCAGAAGCAAAATCAAATCACTCACCAAGAGCTCG
A_14_P118839
AAAAATAAAAAAGAAAACTTTTGCTTCTCTGTAGATAAGAGGTCCAAGATGCAGGTGGGA
A_14_P139034 AAAGTCCTATCAGTTGTTGGCAGATGGTGTAAAACTCTCTGGATTCCACATCTTTTAAAT
A_16_P17392066
AAAAGCAGTCCTTTAATCTATACCGGGAGTAGTTGGTTAATATGATGTAATTAGCAGGGT
A_14_P122578 AAGACTCGGCAGAGGTGACTTAACTATGGAATAGCACAAGGTAAGAGGTAAGAT
A_16_P20288428
ATCTCTGGATAGTGTGTTTAGTTTCATGTATCACAAATGGCAACTAATCTCAAAGCAGCA
A_14_P113113 GTGCATTTAAGTCAATGGTTGTTAAAAGAAGTTTCCCGTGTTGTAAATCATGTTTCCCTT
A_14_P104161 CCCACGACCAGATGATTTTTGCTAAGGAATCTCAAATTCAAATTCACACTAAAATGCAAA
A_16_P21360397
TTTTGTAAAGTTTTGATTGACACCAATTTGATTGCAAAGAATATAATGACAGCCCCTCGG
A_14_P131487 GATACTCACCAAATCAATTATGCCAATGTTATTCCAGCCTTGCATTATAGGGAGAAAAGA
A_14_P108111 CTTAGGACAACTACACCTTTGCCCAACCTGGGATTCAAATGAAAGTGAAAATG
A_14_P105172 TTTCCAGCACACAGACTGTCAAATCTTACTTCTAGAGAAGCCAGATAGATTAATAAGAGT
A_14_P114376 CTTTCACTCAGTTAAATACCAAGTTCACATGTGTGAAAAATTCATCCTGCCCTTAGTAAG
A_16_P58424099 AGGAAGCACAGCCTTCAAGCAGAGGTGCCATTTGGAAGCACAGAG
A_14_P123021 AAAGTTAATAGAAATGTTTGGAATTTCTGTTGTTTGGAATTATCCACACCACAGTTCGCC
A_14_P106676 CTCACAATCTAGGGAGGAAGAGATGAACAAATCCAAAGACAATTGCAATGCATTATGATA
CUST_2783_PI426210822 ACATCCTTTCCCTCTGAGGTTCCTCCCCTTCTATTTCCTGCTTAT
A_14_P106787 CTATCCCAAAATCAGCAGACAAAGTGACAGACAAAACAAAAGTGCCCTCTAG
A_14_P101714 AAGAGAGGTTTCTTCTCTCTCTGGAGATAAAGATTTGTCCCAACTTTTGTTACTTTCAGT
A_14_P111949 GCATCTGAACAAATTGAGAATTTGCCCATCACCTATTCAAAGGAAAATACTCCTTTTTCA
A_16_P19784065
AAAATCAGTCTGAAGATCAAAGGTTACCATAACATTTAACTCTTCTACCAGTGTCATCAA
CUST_303_PI426210822 ATTTGCATGGGCCACAGGTCAAAGCCACACTGCCTGGGAAAGGAAT
A_14_P126784 TTTCTCCTTTTTTAGAGATTATGCATGATGTGATAAAGAAGGTCAAGAAGAAGGGGGAAT
A_16_P16897525
GCAAACACATTCATCAATGAAACTTGTTTAAGGACAGCATTAATTCAGAGGTTTCTTGCC

A_16_P01166910
GCCCTCTTTTAGAGTTTTTTCATGTCAAAACCTAAGAACAAAAGTTCAGTAAAGTGCCTC
A_18_P11615788
ATACTTGTCAGTAACCTCATTCAAAAGCACAATGTTGAAAGTGATACTAAGGGGGAAAAG
A_14_P200423 AGAGTGCTTGGCCATACCTCAGAAGATGGAGGAGTAAAATGTCTTTTTCTATTTGAAG
A_16_P20361937
ACTTGCTATCTGGACTTCTATGTTGACTTTGCATAAAGTGATTAAATCTGATGCTCTCGC
A_14_P119543 TAGCAATGAGTTTTGATCTTTTGGGGACCATCTAGTAAAGTTTTCATAACTTACTGACCC
A_14_P113970 CTCATAAAGATGCATGCTTGACAAGTATTCTGCTTCTCTTAGGATGTGGAATATGAAGTG
A_16_P02528568
ACCCACAGTAGAGTATCGTGAAAAATTCTCCATAGAAAAGATTAAGTTGTATTCACCACA
A_14_P113753 CTATTCTCAAATACTGAACTCTGTGGGAGAAAAGCAAAAATAACCTTTACTTGCCCTCAT
A_16_P03462946
CAAATTTCAAATGAATTCCAGTTCTCCAAGATGAGCAAAAACAGGGGATTAGACTCAAAT
CUST_5571_PI426210822 CCATGTTGGTACCCAGGACTATACAGAAGGGTGGATCAAATCATGATG
CUST_7487_PI426210822 AGTGCTCTTGTAAAACTGTCTTGCCCTTGTTCTAATTGCGTATTGATCACCG
A_14_P114406 ATTAGAAGTCACTTGCTGACTGTTCTTCATATCATGTTGGCCTTTAATATTGGCTTTCTG
CUST_1835_PI426210822 CCATCCTGTTCAGGCTACGCAGTGACCGCTGGCCACTTCTCTCAC
A_14_P132971 TTCATAAACCTTGGTAGGAAATAAAACAAATTACTACAATGGACTCCAGGTCTGTGACAG
A_16_P19532140
AGGCTTTCATTACTTGAGTTTTACAACAGAATGGCATCATCTTTAGAATCAGTCCAAGGC
CUST_4274_PI426210822
TTTGGATGGTAGTGGTGATGTTACTTATTGATGGCACATTTGATGACTGTTAGTCC
CUST_6274_PI426210822 AGACTTGTGGAGTAATGTGTGGGAGACCCCTTCAGGCTGGTCCAG
A_16_P00626028
AACACGAATATTGTCATTTCCAATATCAAGGCTGTTAACTAGGTTCATTACAAAGTCGCG
CUST_7464_PI426210822 CAGACACAGAAAAGGTTGCTCAGTCAACAACTTCTGCAGGCTCAAA
A_16_P21220715
AACATCAGATAATTCAGGAAGACCTTAGGATATCTCCCAATAAATGAATTTGCAGGGTTC
A_14_P133917 ATTCATGTCAGTGATGTCAAATACTAGGACAATGCTATAGTTGGCACCTTGCATTTCTAT
CUST_8228_PI426210822 ATAATGGTCTGGAATGGGCAGGGAACCATCCCATCGGCCACACTG
CUST_5434_PI426210822 AATGAGTGAGGTGAAGAAGCAAGAATGGTAGCTTGCTGAATTGGCAGTT
A_16_P02217562
TCAGTTGTGGTCAGAAGTATGAATAGGTGTGATACTGCTAAAAATGTCAAGGTGAGTTTT
A_16_P19342669
CCTCGATAACAGGCCATATTCATCATGAAGTCTTGTTTTGAAATCACAATTAAACTCCAC
A_16_P00685289
AAACCTCAAACATTTATGTTTCCCTTTCCAATATAAAAAGTGTTAATAATTTACCCACTT

A_14_P101932 ATTTATGTGTTTTACACAGTTTTGGAAAGTGACATCACACATATTAATGCAGTTGGGGGA
A_14_P200093 TGTGTCCACACTGTGATGACTATACCTTATTTTAACTGCACACATTCATACATTCAATCT
A_16_P17032042
ATTACTCTTTTTCAACCTGAGTTAAGCAACACAAATAATCTGATCCAGATGCTACAAGCC
A_18_P11571509
AACGTTCTACAAATTGCTTTTCGACCTAATGTATGTAATGTATCATTCCATGCTAGCCAA
A_14_P118542 ATCTCATGCTTTTTATGTTCACAGAAAAGGTGGTGCTCAAACCTAATGATGTTTCAGTAT
A_16_P21659683
TCATTTACTCATGATGTAAAAATGGTTAGTCTCCACTTTTAAGGCTCTAGTTCTAGTGGC
CUST_3664_PI426210822 CCCCTCCCCGCTGCCCCGAAAGATCAGGGAAAATAAGAAGAAATT
CUST_1130_PI426210822
TTCCCAGAAGCTGTGAAAAATGTTTGTTTCTTTTTTTTCTTGGGTCTGATTACAGGTATT
A_14_P124583 GCGGTGGAGCCTTTATATCATGGGAAGCACTCTCACTTATTAAAGATCTTGGTAAATA
A_18_P22841548 AATTAGTTGCCATCTTTTCTTGGTGGCTGATTCTAAGTGGCTGAAGGAAACAC
CUST_5460_PI426210822 GTTTGGGATTTGAGATATCCATCCAGGTGTTCACGTAAGTGCTGAGATTTTTAC
A_16_P03685069
CCCTTCTAATCAGTAACCTCAGTTCCTCTAGAAATAACTGTGTCGTCTGCTTTATATAGG
A_14_P137457 GTAAACAGTCATGCGGACGGATTCTTTCTTACAGGTTGCAAGGTCAAAACTTT
A_14_P125425 TTTTTGTAATTATACTGCCTTGTTTTGGTAGGATTTAGGGACTATGCAGAAGTAGTGGGT
A_14_P108260 TCCCTTAGTGGGTTTTCTTGCTTAACAAACAACACTTTGCATAGAATTTGTTTGCAATCT
A_14_P113453 CAGGTTTAGAACAAGATTATGCCACTAGGGATGGAAAAAGAGATGTAGAGGAAGAAGAA
CUST_5530_PI426210822
AAGAAAAAAATAAAAGCATTCAAGACCTCTCAGAAAAATAGAAATAAGATAATTACCTGT
CUST_7361_PI426210822 AAGACAGTGGCGTGGGATGATGGCTCGCTGATGGGAGACAGAATTT
A_16_P02402727
ACACTCTGCTCTTTTACTTCAGAGAACGTGTAACAACTGTTATTATACGGTCATGTCATT
A_16_P19344074
TCCTTCATTTCCCCTACTATTCGATATCACAATTACAAAGATCAGTCCTTCCAAGTAGTT
CUST_6415_PI426210822 GGCTGATGTTGGCGGCATTATTCTTAGGAGACATAGAAATGTTGTGGTTTTG
A_16_P01574568
CTGTGGTCTAAAGAGTGAATCTGCTTCTATAAATGCCTCTTTAATATCATGGAGCCATGT
A_14_P111102 CAAGAGGCCCTTACCTTCAACTTCTTCCCTTTAATGTTCATAAACAAGCCATGTAA
A_14_P134032 ATTTCTATCGCAGGACGCAAAAGCCAAATGAGACTGACCAAAAAGGCAAG
A_14_P138886 CTTCTCTCTGATGATGCAGATTAATAACTTGAATGTTGAAGAGAACAGCAGTGGGGAT
A_16_P17444501
AAAATGACCTGGGATCATTAACTTTTACCTTGACAACCAAAAATGAGTCTGTTCAAATGA
A_16_P01720888
TAAATAGTCCGTAGGGTGTTAGATTGAGATTAACATCATCTATAGTGCGAGGGCACTTTA

A_16_P17178176
ATGGAAGTAACAGAACACTAACATGTGCAAGACATATTTTTCACCCTCAAAATGTAACGG
A_14_P121074 AAACACAGAAGAATCCTCTGAAAGGTTTAAACATTTCTTGGCATTTCATGTTCCTCAAAG
A_14_P119522 GTGCAAAATTTCAATCGAACAGATGATGCTTTAAATCTGTGAGCCTTCTTTTGTGGGT
A_14_P126770 GTATAAAATTTTTTGGGGGTCTATATTACTTCTTGGGATGGCTTTGCCTTAGAAGGAATC
A_14_P104614 GGAAGAAAAACGTAGGTCCTTCCAAGAGATTCTGAGGAACATAAAATGTAGGAAGTTTTT
A_14_P114012 CTGGGTGACCAAGACCACTTTCGTTTTTTTCTCTTTGGGATCCTCTT
A_14_P104107 TATATAATACAGCACTTACCCTGCTTTCTTCTAGGCTATCAAATGGACCCGGTG
A_16_P03126082
AAAAGTAAGAAAAACCTCCCACTTATTAAGTTGTTTGTGTCAAATCTTCCATCATTCCCC
A_14_P118478 TTTATGCTCTACCTAAAACTATCTGTCTCTCTACATTTCCTTACGTATTTTTTGTCTGCT
CUST_4616_PI426210822 CTGCTTTCTAGCTGATATCTCCCCTTGCTCTCAGCAAGCTCCTCT
A_14_P117072 TTTCTTACCAACTGCACTTCACCAAAAGCACCTCTTCCAATAACTTTTACAACATCATAG
A_16_P02156025
ACATTAACTCAAAGAAAGGTCTCAAATGTGTGTTAAGAATTGAGAATACTACCCTCAGGG
A_16_P39972398
AAAAAGTCTTCCAAAAGCATCAAAGCATAAAATGTATTAGGTCCTTATCCATTTGCTCAA
A_14_P126428 CCTCTGTGCATCTCCAACATGGATGCTTTTCAGGGCATTTTAAAATTCTTCCTTAAT
A_16_P18928072
CTCATCACAAGCCATTTCTCTTTTCTATCAGATAACATCCACTCAAGTATTGTCTTCCTG
A_14_P125572 TGTATTTGGCTATGTAAAACCTTTCTTTCCTCGGGACAGTTGTTTATAAGCCGG
A_16_P01187094
TTTGAGATAGCCTGCTTGATTTTACTGAAGTCCAATCTGAGTAACTAGATTTAGTGTCCC
A_14_P124196 TTAATTAAAGAAGAATATCAAATAAACAGTGCAGGGAGTGCGAATATGTGACCGAGATGA
CUST_8852_PI426210822 AGAATAAATCAAGCCAGCAGTGCTGTCTCCCTGCTGACTGAAGGC
CUST_3035_PI426210822 CTTGAATCCCCAAAACAGTTAAAAAATAAAAAGGGTTACGGAGGGGATGACC
A_14_P116245 CTGGAGTTAACATTTTTCTTTCAGATTCGTATTTCCTTAACATGAAAAGTCAACAATGAA
A_14_P133378 GCCTGGGTTTTACTTTAGCATTCTTAGAGTAACCAGGGATGATGATAATATTTCATTCCA
A_14_P102698 TAATTTTTTGGTTATTTTTGACCTTCACAGATACACCGAGGAGTTAAAGGATTTGTCCGA
A_18_P14669703
CCTGCAAAACTTAAGTGCTAGACTCTATAACGTTTCATAGTTAATAGACCCATTTGCAAC
A_16_P02161620
TCACTTTGCTAATTATCAATCCATTTAATAACCAATTCTCTAAAAATTTACCACCCTGCC
A_16_P18513312
ACTTCTGTTACAGAACTCACCTCATTACTTCATTCCGACTTGTATTATCATAAGTGGCTG
A_14_P101747 CTAGTATAACAATGCCAAAGCCATCCACAAACTAATATCAAAAATTGTTGTGTGCCTCGA
A_14_P101580 TCTAAAAGTGTTTTCAATAATAATGCATTTATGTTGATTACCTTGTTGGCTATCCTCTAC
A_14_P103534 TTGGATTCATTTGGAGGAATGGAAGAAAAACTGTTGAGAATATTGGATGCCTTTAGTTTC

A_16_P01617804 TGTAATCTTCATATATCATCCTAGCCTTTTCTTCAATTACTTTTTTGTTCTGCTCCTTCT
A_14_P136899 TCTTCTTCGCCTTTTTAACTAGATAGAGTTTCTGACAGGAGAATTTATATACAGGATGTA
A_14_P112163 GATATTCAACAAATACAAAGTATGACCCACACCAAATTAAAGCAGAAATAGCAAGTCGTC
A_14_P200793 ACAAAGAATTTTTCATTTGGAAGGAAGTTGGACTCCAGTAGTGTTTCCTAATTTCAATTG
A_16_P16848777
AAAAAAAAACATTTCCCCAGTCTATTGAGAAACAATGAGATGCCTATGGTTTTGCTTAGG
A_16_P19849781
AAAACAGGTGACCATAGGTATCTACACACAGAAAATACTTCCCTGGAAAATATCAACTCA
A_16_P00739879
TCTCTTTAACCTTCGCTCAATACTGTATCTGATGTGAATCTTCTTTATAATCGCCTCCTC
A_14_P131485 ACATAATTTTGAAAAACTGTTTGCTAATGTCTGGTGAGTAACCTTTGAAAGTGTCCCTTC
A_14_P109087 CACCATCATTAAAGGAAAAATAATAATACCTTTTTAGCCCTGCCTATCTCCAGTCTTGGA
A_16_P21578762
AATTCCTTTGGCTTCCTATGATTCAGTTCCAAGTCCATAATGTCCTTCTATCTTCATTTG
A_14_P137025 CCAGCCTAATAAAAGTTTACATAAAATGTAAAAAACAAAGCAAAGCCTCCTTTCTGCGGG
A_14_P130621 AACAGATGTGGCATCTTTTGTAAAACTGATTCTTGGAGACTAAAAAGCAGTGGACTTAAT
A_14_P136615 ATGTGCAAAATTCATATTTGTATCTGAGTATTTTTAAAGCCAGTTGGCACTGCCCATTCT
CUST_4167_PI426210822 GTTTATAATATCTAACCCTCCTATCAGATCCTAGGCCATTTGGAAGCCAGGAG
A_16_P35375856
AAAGGCATCCTATGGTAAACTGTTTTGTAAAGCAGGAATCCTTTTTTAAAATGAACCAGT
A_14_P135848 CTAGGAACTGGTAATATTGAATCAAATCTTCTTCTAGCATTCCACTGTTCATTCCTGGAT
A_16_P16191306 ACTTCACTGCCTCTTCATAATAGAGTCCTGGGACCTTCTCTGTGTTAAGCATAT
A_16_P20864281
AGGGTCTCTTCCATACAATCTTGATCCTTGTATCACAAACTGATGATCTATGAATAGCAC
A_14_P114270 CAAAAGGACACTTCAACTGTCCAGAGGGTTATTCAGGTATCTTTTTCTGATACCAATACT
CUST_732_PI426210822 GCTTCTCTCACTCCCCAGCAGGGCATCTTCCTCATGGAATCAGAT
A_16_P16191096
ATGGACTTAATGATGGTTAAATGGAACTGGTGAAGAAATTAGCAAAATGATTATGCCCCT
CUST_3632_PI426210822 ATTACCTTGTGTTGAAAGGCAGCTCCCTGGCTCCCTGGGAGGCCT
A_14_P125902 CATGGTGATCAAGCAAATGCTAATAAGAGACCCATTTCCATCCTTGTCCCTT
A_16_P01212863
TATCATTTGTCACTGACCTTTCTCTACCAATTCTCTGTTCAATGGAAGACTCAAATTTCC
A_16_P18946206
AACACAGGCTCTCTTCAAAAATAAGAATATTCCACTTTTTCTACCTCATCTGGACATTGG
A_14_P128515 GAGCAAGATTGTTCTCAGGAAGGAAACCATAAATATGGCATTTATGTAAAATCCTTGCAG
CUST_10257_PI426210822 GACACTTATTATAAGACATCTGATCCAGAGCTGCATGTTCAACTCCATCAAGCC
CUST_1331_PI426210822 CTCCATGCATTGAGTTGCGGATTGTGAACATTTGGAAACCAGTATTTTTTTTCC
A_14_P139513 GTTCTTTGAATATTGGCTTACAAACCTCATATCAGGTGTATAAGTAGAAGGATAGTGGCC

A_14_P129183 TCAAGACCTCAGTTGCTACATTATAGATTAAGGATGAAAGACCATTTCTGACTTGCTGTT

A_16_P00353026

TCCCCCCAAATCATAATCAAACAGACATAGAAACTAGAATGATGAAAGAACATTGAGGTC

A_14_P100438 ACTCCAATTATTGATCTCCACATACAGTTTACGGTAAAGGAAAATGGAATACATGCTCAG

A_14_P111732 GCAGAGACATGTAAAATTTAAGGTAGGGCTAGAAAGATAAGGAGTAAAGTGTGTTCAGAC

A_14_P127494 TCAGGCTTTTTTTCCTACTACTGTAGCAACTGATATCTAAAATGAGTCTAGAGCCCTTTC

A_14_P114279 CTTTGTAACCTACAACCCACAGTTTCGCATGTAGTGTAATACCTTCCTGTTGTATTTTTG

A_14_P201725 CTCCGAATCAATGGCTTTGATATGCTAATTAGGGAGTAATTTAATTTCAAAAGGCCGCAA

A_14_P110738 ACCTTTTTGGTTTTTCTTCTTTTTCAAGAGACTGAAAGAATTAAAGCAACGGGAATTTGC

A_16_P19251385

AAGTTTCCACATAGAACTAGGTTTTAGAATAGATCGGGCAGTTTACTTCTATGTCAAGGC

A_16_P00819444

TTGAGGATAACCCTATGTAAATTCTTTGCAAGTTGATACTTATCCCAATATCGTAGGCCA

CUST_1749_PI426210822 CTCCCCTCCTCACTTCCTTCTCTGCTTACCGTTGGATTTCTTTTT

CUST_2396_PI426210822 GTCAAGTGGAGGTTTCCAGGGCTCCTGGTTTTCATTTAGGGTTATTA

A_14_P117700 CATAGGTGAAGATTTTGCTCATGAGACCCCCCATGACATAGATGGTGTC

CUST_7784_PI426210822 GATACCAAGTGCCTGGCAGAAGTCATAGGCAACTCCCAACCAGAG

A_16_P00685195

TTTCTCATTATTATTACCTAGTTAGTCTGAGAAAGAGAAAACTCCATTCTGGATGGCCCA

A_16_P18880667

AAAAGTGGACATACGTCAAAAGTATTACTACCTCCATCATATGACCTCAGTTGCTCTCAA

A_14_P126496 TAAAACAATAACTATGTGGTCTTGCCCCAGCGCTTTCTAACAATGGCTAAAATTCTTACA

A_16_P17802273

CCCATCACTTCAATTTATCCTGTTAGCATCTCAAATTCAACGTTACATTAACATGGTTCA

A_16_P02550617

AATGTCGCCTGCAGTAAATAATTGTCTAACATTGGTATGAAATCTAATTGGCTTCCGCTA

A_14_P119641 TGCAAAGTCTTACTGTGCTCTAAAATATGCTCTTTATGACTTTGTTGGTATGGATGAACG

A_14_P126992 ATCCCAAGAACCACTTGCAACAAAATTGTCTAGATATTGTTAACCTTTGCATTTCTGTGA

A_14_P111555 AAAGATCCACCCAAGAATCAGCAACATTACTGAGGACTACAAGGAACCAAAATAAGA

A_16_P03412400 ATAAATGCGGAAACAGTTAAATGGCGATGGGAATAGGATGGGAACTCAATGG

A_14_P123325 TTTCCAGGTTAGGAGGGAAGACCTTTTTATACAGGGATATCTTGTTTTTGACCACTT

A_14_P110383 AAGATACACAATTTAGAGAGAATGAAAGTGGAAGGATCTACTTGGTTAATGGATTCAGCG

A_14_P125107 ATGGCATTCAATTAGTTTTTATTTCTTGTCATCTCTCACTAGAGAAAGAGCGCACACTTC

A_16_P02499033

AAACCAACATTTTGACATGACTCTCTTCTCTTAACAGATATATGAAACCACGTCTACTCA

A_16_P02997385

AAAACTTGTCTTTTCCATCATGCTAACTACTGTTGAGGTTCACATCTAACTTAAACTGGG

CUST_8320_PI426210822 GTCCACGTCATGAATGCCAGTTTTCCCCAAGAAGCCCTCTGTGTG

A_14_P106992 GTGATTGGAGACTGGCTCAACCTCGTCTTTAAGTGGTAAGAACCATATAGAGAG

CUST_6583_PI426210822 TGGCTGCTTCCCTTAATCACAGAGCCTTGTTTGAATCACTTGACTTTG

A_14_P123750 TTTTGCTTATCTGTCCTTTCCAGGTTGAATTTTAGATGAGAGTTTTTGATGCTTTTTGGC

CUST_5239_PI426210822

ACAGGTACAGTTTATAATAAGGATTTATTTCATAATAGGGAGCTTTTTATAGATTTTAAT

A_18_P15363762

ACACTTGATCAGTCTCATGTTCTTTCTTGTTCCTGTATATCTCATGAGCACTACTAGACT

CUST_7610_PI426210822 GCTTGCTAGTCTTTGTACTTTGGGCTTTCTGTTACGACTGGTTGACTTAGATTT

A_16_P00673326

AAGTTTATGAGAAGTGCCACTAGTTAACTTGCTTCCAATATGAAAATATGACCACAGGGA

A_14_P137813 CTGTGTCCAAATAAACGTATCAACCAATAAACTGCTAACTGGTCAATGATTAATTGCACC

A_14_P137470 GCAAAAAACTGAGAGTTTTTGTTTATCGTAATGTAAGTTCTGGGTCCTAAATCATGCTCC

A_14_P102064 AAAGGAAAGGCTTTCCAAACTTTCCTTGATAACATACCTAGGAAAACAGTCTTAAACCAT

A_16_P37084550

GTGCAGAACTATCTTGACGACACTTTTGATTTACAAACAGTAACTCTTTCCCAGAAGTTT

A_14_P127339 TAATGTTGACATTTAGTGTCCTATTTAGTGTCACTCCAACTACTGCACATGTGGTTTTCT

CUST_2238_PI426210822 ACTTTGGAGAGAGCATTGCCAGCCTGGACGATCTGGACAATGATG

CUST_6945_PI426210822

ATATATTACAAGCATATTGACAAAAGTCTTTGAACTCTTGAGGTGTTTGTATGATATTCT

A_14_P138992 GAAGAGGCACTCTGGATATAAATACAGTCCATGTCTCTACCAAATTTTCACAGATGTGTG

A_18_P14132878 GAAAATGGGAGATTTTTCTTGATTCCTGCAGTTGGCTTTGTGCCCTTG

A_14_P108995 CAAGTGCACTCGATTTTGCATTTTAAAGACTTTGTTAATGCCAGCAGCAAGTATGAAATC

A_14_P111940 ATTCATTCAGTAAAGTAAGGCACAAACAAATGAGCCTCTGTTCACTTTATTCAAAGAGTA

A_16_P15263607

TTTACGTGATTCTTTTCAGTAGTGGCATACCTTTTGCTAATTCACACACATTTGTAAGGG

CUST_10313_PI426210822

ATTGACACATTACAACATCAGCAGCCAAAGGTATGTGCTAGAGAATCCTATTTCC

A_14_P132286 CTAGATGTCAGCCTATAGTTTTTTTCTGGTTCAACACTAAAGTTTCATAAGGGCAGGATT

A_14_P200564 CTTCAAGCACACAGAAATGACTACACAGCCAGACTGTAGAAAACTGGAACTTG

A_16_P00685223 CTGGTTTTTCACTTGGGGTTCTCATTCAGATTCGTGATTCTGTTTACTGAGAGATG

A_16_P02835234

AACAGCCTAAGATTTCTCTCTATAATCATATTGTCTCCTACAGAAGATTTGTCTCTCTCT

A_14_P100547 GACAGCCTAGAAAGAGGAAGTGCGTGAGTTCCTTTCCTTTGTGCTT

CUST_9056_PI426210822 CAAGGTCTTGCCAGATCCCCTAGAGTGAACAGGGGATTCTTTATC

CUST_6784_PI426210822 CTCATAGGTTCTAAGAAATCTCCCCTCCCCACCTCATTCTGTTTTGTTTTTAG

A_16_P21545355

TTACTAGTGAGTAGGAACCAAAACTGAAAGTGTTCATGGACATCTCAGAACTAGATAGAG

A_14_P124364 ACTGTGAAGAAGGCTGGATTAGTGTTAAATTCAATAAAGTTAACAAAGCAAAGTATTGTA

A_14_P112804 TCCTGATACTTTTTTCAATTTCCCTGGTTGTAGCGCTGCGGTAAGTTTTAAATACATGT

A_14_P139857 GGAAGACTCAGAAAAATATGAAATATTCAGCCAACCAGATAGAGAAGAGTTCCTGTTTTG

CUST_5956_PI426210822 CCCTAACCTTCCTCTCCTGTCTTTCTCACAGACTGTTTTTGAAAGGAATT

CUST_866_PI426210822

TCTAATTAATTGCATGCAGGACTATTTTTTTAATGTTTTATGAAGTTATTGCATCATCCA

A_14_P134956 AACTTTTAACTTGACAATGCTGTGAGACATGTTTTGTATGTTCTGCTCTCTACTTCACTG

CUST_9061_PI426210822 GCTGACCCAGACTCTGCCACCAAGTGATGGCCACTCCTTGGGCTC

A_14_P117337

AAAGGTAGGCAACAGTTTGCAAAATTTAGACGCTTATGTGTGCACAAAGGAAAAAAAGAA

A_14_P100238 TCCTTTGTCATCCCGTTGTGAAATAAGCAGTTTAAAGAAATTTTGGTTATCTGCCTCAAA

A_14_P127427 CTTTGGAAGGATCTTTTTTTCTTTAACTAGATGGTCACCTTCTTCCCTACCACACATG

A_14_P105276 AAGTTGTTCCACTTCTAATAGGGCTTGTGAGACATGAGTGATTTCAGTCAAATAAGTAGA

CUST_9139_PI426210822 TCAGCAGAGGAATGTGCATGCCACAGGGCTGCAGAAAACAGTTTC

A_14_P139047 CTATGATAAAGTTAATTTCTTCTTCAGTCCTCCAAAAACTTCACTGCTCAAGAAACTGGG

A_14_P133256 AGATACTATATAGCAACCTTCTGTGCCCATGGATGCAAATGAATGAATTGTCTTTAGGCT

CUST_7793_PI426210822 AATTACCTTGTGGGTATGTCTGTGACTCTTGGGTGCCTGCCTGGC

A_14_P202342 CAGAAGTAACAAGGTCTACTGGGCTATGAACAAAGAACTAGAGGAAACATGTGCAAAAA

CUST_9586_PI426210822

AAAATGAGAAAATAGTCAGGATGAGGGATGAGGAGTACACATAGGAAATTTTTGTGATTT

A_16_P00685509

CAACCCATAAAACATTTTTCGAATGACAAAACAGAAAATGTATACTTTTATAATCCAGTT

A_14_P117959 AGGCACTATCTTCAGTTAAAAAATCCAGTTGCTTATCTACAAGGAATTCTACTGCAGTGA

A_16_P00368115

ACAATCTCACTCTCCCGTATCATGTGATTGAGTCTGCATATCTTATCTTTCCATCAAGTT

CUST_5858_PI426210822

GTGCTTAATTTTATCTTATCAGTACAAGCACTCATATAACCTGTAGGGATATTTGTGTAC

CUST_9023_PI426210822 GAATGGATAAACAGTGAGGTGGTGGTCAGGTGGTGTTTGGGCTTC

CUST_3117_PI426210822 GCTGGCAGGGTGGAAGACCTGGCAGTGCCTGATGCTGCAGGAATG

A_16_P38578003

CCCATACTAGAGAACTTATGGATAATTCTGGAGACTTGAATATCTACCAGCCTCAGTAAC

CUST_5034_PI426210822

TGTAAGCAGGACCAACTCTTTTATGGTGTGTTTTATACGTCTAGAGGATTTTTGTTGTTG

A_14_P132668 TCTTTACAGATTTAACAACACTTTTCTTGCCACTCATTGCCCAAACAAAATGCCAATAAC

A_14_P135601 TGTCTGTGATGAGGATTAACAAAAATGGGCAGGTGTGTATTAAGACCTCTATACAGAAAG

A_14_P121413 CAGTATAGTCCTCTGAGGACATAGTGTGTATAAAATCCAGAAGAAAATTAAGCACCAAGG

A_14_P118469 GTTTTTCTGATCTACAGAGTTCCAAAAGTGACATCCTTGGATACACTGCAAGAGACAATA

A_14_P128002 CCTTGTGGGAAGCAAAGGAAGGCAATGTCACCTTAGGTGACACTAATA

A_14_P134593 CTATTGGAGAACAGAAGTTAAATATATTACCACATTTTTGGATGCTCTTGCTCTGGACTT

A_14_P101846 GATAATTTAAATGTGTCTCTTACAGGGAGCATTTGGTGCCCTTCAGAAGATTTGTGAAG
A_16_P20192682
CAACTTTGACCCCTACAGATTAAGATACTAGGAAACGGCAAGAATTATGGACAAAAACTA
A_14_P124114 CAGCATGACTGAGTTCTTAGTCTGAGTTTCATTCTGGAGATCTGTGACCTCAT
A_14_P115816 ATCTAAACCCCAGAGCCTTCCAGAATAATAATTTCAGGGAATGGTGTTTCAAACATTC
A_16_P15012372
AAAAAGCACTAATTCATTTTCCTTGCACAAACTACTTAGAAAAGATTGTAATTGCCTCCT
CUST_7037_PI426210822 CAAAGCCCATGGCCTTTGAATAGAGAGCTCTGTTCTGTTTGCAGTG
A_14_P123145 GTTTGACTGAAACACGCTTCTACCAAATACAGTATAAATAATTAGGTAGTCTTAATGCCT
A_14_P118991 ACACATCACAGAAATGCCTGCTCGTTTGTTTTGATTCATATACAAAGTTACAAAGTATTT
A_14_P123610 ACTCATTTATGTTGAATACGTATTAAAATTAAGACAAATGGAAAATTATACTTTGAGTAT
A_14_P113897 CTTTATTTCCAGCATGATGTATTTTTTCTTTTCCTGGTCCAGTTCTTCTTCTAACCGG
A_16_P20429835
AAATCCAGAGATAGAAAAATTATAAGACGAGATGTGCTATTACAATGGTGTTCAGACTGG
A_14_P103569 ACAGTATTTATTCAAAATATCCACTGCCCTTTCCTACTGAATGCATCTCTACCAAATGTG
CUST_2829_PI426210822
CATAATTGTTTAAGATACTACAGTCTTAAGGAAAAAAGCTTTCTGATAGTTCTGGGACTC
A_14_P121627 GTGTTTATCTGTCATCCATATGGGGAATCATCTTCTTCATAATTTTGCTCAACTGGAATC
CUST_5353_PI426210822 ATTCTACTCACCTCCTCTCCCCCCGCAGCCCCGAGGAACTCTGAA
CUST_9237_PI426210822
TTGTTAGATGTAGTGATACCTAGTGATTTTATAGTTACGACTCTGTCTGGCTGAAAATTT
CUST_1948_PI426210822
TTTTCATTCCATAGGTTTATATTTTCAGAGCTGACCGAAGATCAGGCACCTTAATTAAGA
CUST_8723_PI426210822 GAAAGCAAGTCAAGATTCTTGTCTTGGGGGGACAAGTTTATGTACCAATTAGC
A_16_P02192375
AAATCGATGGTTCTGTTTGTGAAGAAGTAGGAAAATTGCCTGTTACCCTAAGAAGTCTAC
A_14_P114682 CTTCTCTAAGTATTTAATTGCCCATTCCTTCTTGAAAATTCTTTCCCCCCTTAGTATCAG
A_14_P130195 ACTAGACTGAAGCCCACAATCTTTCAATTACAGCACACTATTGAACCTTGAAGAGAAAG
A_16_P16932978
AGCCATTGACTTAGGATCAGAAAAATGACATATAACTGCGACATTTTTACTTCATCCTCA
CUST_5180_PI426210822 CTTCCCTGTCCATAGTCCCTCCCTGCCCCTCCCCCGTGATCTTGC
A_16_P00909707
CCGAGTAAAGTCATTAAAAACCTGCCTTTGTAAGTATCTGTTTACATGATTTGGACTTCC
A_16_P17972174
ATTGTAATGTCCAGTGTTCTCATACTTTGCTTCCATATCACTTCTGCTAGCATTTTCTCC
A_16_P16574305
ACCACACCTTTTGAGTCTTTATGTCTCATACACCAGAATATTAGGTTTTCCTCTTGCAAC
A_14_P132543 CTATCTTAAGGATATTACTATGCAAAGTAAGAGTTAAAACACTGGAGCAGCGATATTGAC

A_14_P114408 TCACTTGTTTGTTTTTAATGTAACCACAGTGGCATTGTCCACCTACAACGAAGTTAATG

CUST_1548_PI426210822 CCGTGTGGCGACTTCACTCTCTTCTTCCATCTCAGCAAACATTTAG

CUST_9664_PI426210822 TCAGGCAGAAACCTTTCTACTTTCTGGAGTTGCTGAGAAGGTTCAGT

CUST_1806_PI426210822 ATACACATTAGAGGCTCTTATCAGCTGCAGGGATTATCAAGGATCCGTTCA

A_14_P102378 CACCCTACACTCATCCCCCATCTCTACAGAAAATTAAAGTATACATTCATAGTTGCTGTA

A_14_P137353 GGTGGCTGTAGGTTTTTTGTTCATATGTTAAATACTTCTGTGTTTCTGTAGCACTGAGTT

A_14_P130430 TTTACCATGTTTACTATACCCAACTTTCTTTTTTTGTCAACTTTATGGAAGTGGAGCCCT

A_14_P119334 CTCAATAACCCTAGCAGTTTAGTCACAAAAGCACCATTATGGTATTTTCTGTGAGGAAAA

A_14_P136598 AAAACGAACAGGGTAGAGTTTTAGGAAATACCAGATGTTGCTGCGATTTTCTACAGTG

CUST_3502_PI426210822 AACAGAACAGAAGAAGGTTGGATGGTTCACCAAGTTGAGTGGGTC

A_14_P124984 GTCTTACTCTTTGGAATGATCTTTGACAAAGTTTAGTAGGCAGTGCCCACTTTTAATGT

A_16_P21576311

AAAAAAATGGTGAGGAAAATAACCGAGAGCAACATTATGAGAAAGATGAACAGACCCAAG

A_14_P102691 GGTTACAGCACCCAAGAAGATGAGACAATTTTGCTGTAATTAGAATAAACATCCAAGGTG

A_14_P122199 AACTTTGAAGCAGGGACTAGAGGAATAATAGATTTGGAGGAAGTATTTGAAGAGTGATGT

A_14_P130725 GTTTATTAAAAAAGTGTAAAATGGGAAGACTGTGCATCGGTCTGAGTAAAGTGGCGAACT

A_18_P22842095

CTGACTTATTTCATCATTAAGGATTTAAATGCAAAACCTTAAGATAGTCAGCCTTAGGGA

A_14_P134115 TTCAGAGGTAAAACTGGCATATATCTTTAGTGTCACCTTCTATTCCACCTGTGAGAATTT

A_14_P106979 ACCCCAGAGAATTCCATTACATAATGATTTTGCCCTTGTTATAAATGTTACCCTAGGAAT

A_14_P103689 CTCCTCTTCACCCCCGAGGATTGACTATTGACAAAAGTAGTCTACGAG

A_16_P20530658

AAAAACTGACTATAGGAGCCAAGACTAGTTTAGAGTGGAGTTTGAATATGAACATGCCTC

A_16_P00563603

GATGTTGCTACTGCTATGAGAAGATATGTCAAGCCAGAGTATACTGAATTTTGCTAGAGG

A_14_P122114 CAAGATACTGGGAAAGATCCCTGTTCTAGCATCACATTTTAATCAGATTTGTCAAAATCA

A_16_P18540712

AAAAACTATGCAATTAGTCTCAGACCAAATCCATTAATGAGCATATGGAGTTACCTAACA

A_14_P202551 TAACAATCTAAAGTTGGGGCAGGATTTAAGACACTTAGAGTCATGATTTTAAGAGTTGGG

CUST_6581_PI426210822 TTGAGACTCCTCTAAAAAAAATCTGAGTTCACATTCTGCCCTGGAAGTTGC

CUST_8467_PI426210822 GTTTTATAGAACCAAATGCTGAGACAGAAGAGGGGTACAGTTGTGCAAGTGAT

A_16_P01700930

GATCGTCTAAATATCTCGACATCCACTATTTACTTCACTCTGTATGTAGTTTCCCCTACC

A_16_P41843636

TCAGTTCACCAAAATACACAATTTACTCCCAAACTTAATCAGCAAATAACATGTCTCTGA

A_16_P58424292

ATGTTATATGCAAATTCTACACCATTTTACATCAAGGACTTGTGTGTTCTTGGGGTTTGG

A_14_P132521 CTTAGAATGATGAAAAGTTCTCTTATATGGTGTTGGCTTTTTCTCCAAAGGATGTGAGAC

A_14_P138029 CTGACATTTATTGAAAATCTGCCAGGTGAGTGGAAGGTTACAAGAATTCTAATCTGATTG

A_14_P136358

GAGGAGAGAGGCAAAAATGAAGATTTGGGACAGAACACATTATATTAATGATGAAGAAGG

CUST_2281_PI426210822

GAAGTGAATGCCCTTCTGTATATTTCCTTTATCCAGGAATGGATTTATTATCAAATAATA

A_14_P128316 ACCAATTGTTGAAATAGATGACTCTCACACCAGTTCTATTACACAAGATTTTCTGTGGTT

A_14_P115558 CATAGAGGACTGTTGTTTATAGAGAAAAGATTTGGGAAGGACTGTTTACAATGTGGGTCA

A_14_P134035 GAAAAATAAATTACTGGATATCAGATCTTAACTGAAACCTCAGGTGCTAAGCCCTTTCCA

A_14_P109847 AAAGAGTATAAAGCCTTTGCAAATGGCACATAAAAGGCAGAAAAACCTGGGTTACCTAA

A_14_P139387 TGACTGAAGATTTGTTTTCATGAAGGAACAAGTGCTATTCCAATCTAAAAGTCTCTGAGG

A_14_P125307 CTTCTTGTCTGTTCACATTCAAAAATGAATTGAAACTCACACTATTTGCCAAAAAGTGGC

A_14_P114166 AACTTTGACTAAGTCAGTGTAGTCTGATTGGTAGAAAGAAGTATTTGAAAACTGGGGGTT

A_14_P113820 CTCTGACATTTTAACTGAGAGCGTTTCTCTTTCGGGGAACCCCTAG

CUST_7661_PI426210822 GCAAAGAGGGTGGCACCCTGCTATGAGCTCTTGGAACAATTTATTTA

A_14_P115682 AGGTCTCTCTGTGGCATTCTATTTTGTTTTTATGCCTATACAGAAGCATTCTTCCATAGA

A_14_P111701 CTATGTAGGTGATGTGCAGATTGATGTGGAAGTGAAGAAATATTTTTGCAAAGCAGGAGT

A_16_P16497688

AAATAAAGCCATCACCACTACTCTAAGACCAATTTTGTGTTCCTTATCAATCCTAGAACC

A_14_P108573 GATAATGAAGTGACAATGTGTATGAAATGTAAAGAACCTTTCAATGCACTGACACGAAGG

CUST_7422_PI426210822 GGTTAAGGGTCTCATTCTGAGAGTGGCTTTGGACAGCTTGAGAATC

A_14_P109020 GAATCTATGAATGAATGCAGAAATTGGCAAGATAATACTGCACCGATGGGAAATGATACT

A_14_P127512 AGAAGAAGGTTATCATTGAGAAGGGGTGGTCTAATATTTAAAAAGGAGACAATCAATGGT

A_14_P132264 GAGAGTTCTTTTACAAAGAGATCAAAACAGAGACACTGACTTTGGGCTAAAGGACTTTTT

A_16_P16396968

AGACCAACAGAAAGATCATCTCAGATATCCATGAGTTCATACATTACAACCTTCTGGAAA

A_16_P02320542

GGGAAGATCCAAATGGAAATTATACATTATACCTTTCAACCTTCTTCATTGGGAAATGCA

A_14_P122400 TAATGGAAGAACAGAGACTTGTAACCCGTTTCTTTAAGTGTTAGTAATTCTGATCCACGA

CUST_6420_PI426210822 ATGAAGGAGAGGCCCCCAGACTGGCACTTTGAATGCAAATTCTTC

A_16_P21509904

TCACAATTCCTAAATAGTCACAACTCCTACCTAGAAAAAAAAAAATTCATTTGTCAGGCCA

A_16_P16148902

CATTATGAGATGCGGACATACATACTGTTGACTACATTTGAAACTGAGATGATGCACATC

A_16_P00766507

AGTTCCCAGAATTACCAGATAACCAGTAGATCATCGTTTTTTCATACCCAGTAGACCAT

CUST_3773_PI426210822 GAGGGCAGTGACCCTGGCTCCTATTGTCTCTGTCTCTCTATATCTATA

A_14_P106589 TGAAACCAAAGGTCAATCACATCTCTAAAAAATATCTTACCCCTGCAGAAACTGTCTACA

A_14_P116079 ATGAGGGATGTTGTTATCTGTGAGAATTTCCTTCCCTTTCTGAGGAGAGAAGA

A_14_P136603 CTGTTCTAAACTTAAAAATCTCATTAAAATCTCTCTCCTTCCAGGCTTTCCTTTCAGCAG

A_14_P127452 CTTAAGTTATCTGCACCAAACCATTCTGTGCATAATTTTTAAAGCAAATATATAGCGCCT

A_14_P124051 CTGTGAAGACTGCATCAACTCCCTTGGAACTTATACTCTAGTTTAAGGAGAAGAAATATG

A_14_P123839 CCAGTATGAACTCTGTTCTAGGGATTTCTTTGGATCTAAATACTCATTTGGCTATACCAG

A_14_P132672 TCTGACTTCAGAAATCCATACCCTTTGCTTTTTGTTAACACAAATGTTTCCTTAACCTGA

A_16_P38183846

AAAATGATTTTCGGTTCATTCTCAAACACCTTTCAGACACTCATGATCCCATTTAACTGA

CUST_1201_PI426210822 TTCCTGTTTCTGGTTGGATGGGAAAAGCTACCAGAATATCCTGTTGG

CUST_7861_PI426210822 CATCTCTCAGATATGTTCTCTCAGTGCTGAAATGTGGTGTGTGATACGACAAC

A_16_P15547674

AAGTTGTAGCCTTCTAGAACAAGACTAGTTTAGACAATGTTGAGATTAATGCTGCTGCAC

CUST_380_PI426210822 CTTTTTTTCTAATAACACCCAGAGTGTCTGCCTGCCCAGAGCCAG

A_16_P15096266

AAATCCTTATTGAAGCAAACGAAAAAGTCTCTCTGTGATTTAAGATGACCTCTGTCCTAC

A_16_P19811060

AAGATAGTCACTGATCAAAAAGAACCTATCAACTGAAACTTGCCATGAACTGACAATGTG

CUST_3467_PI426210822 GGGGAGTTTCAACTCTGGGGAAGGTGATCATTTGGGTCATTAGTA

A_16_P16750340

TCGAATAGTAGTAACAACGTGGATAGTATTCCTATGCCTTACCTCTATTTTCAGTGAAAC

A_14_P103486 TAAGAAGAAACATCAGCAACAGCATACCATTATTCGTGGCTTAGACACTGGGAG

A_14_P128693 AAATTTGTATGAACTTGGAGTATCTGTTGGCCATTACTATACATGTGCAAATAAATGTGG

A_16_P00212172

AAGGACTTGTAAGCAGTAATTTCAGGTGATCATTTAGAGAAGGACTAACCAATGACACTT

CUST_5557_PI426210822

GCAGTTCTTTCTTATTGTCCTTTTCCTGATTATGGGGAAAAAAAAAAAAAAAAAAAGATA

A_14_P121615 TTACCCCTGGAAGTGTTCATTCAAGTCTGAGAGCAGATTAAATAATGAATAGGAATGTCA

A_14_P103862 CAGAAAGTAATTCCCAGCAGGAAGATAAGAAGTTTTACACTTTTTCTTTATCCGGGAAGA

A_14_P125183 CTCTGTGTTTCTCTTTATGGGGTTCAAAACCTTTCAAGGACCTCTCTCCAT

A_14_P115659 TTTAGATTTCCCTCAATGATTATCTACAATTAACCTACACTTCTGGACCACCTGGCTAG

A_14_P102811 GTCCCTCTGTGACATTTCCTTTGTTCTTTCCCTGGAAAAAATCTCTTTTTAATTCTCATA

A_18_P12987039

ATCATACTTAAATCCAAACTCCTTACTGATCAAGGACCTATAAGACGTCACCTCCTCAAA

A_16_P39637838

CCAGAGTAGTTCAAAATAGTCTTCAATATTCAAAATTGCCTCTAATATGCACTGTCTAGG

A_16_P36096590

ACCTAGATTTCCATTTCCACATCTCATTATACCACTCTTCCTGTTTATATAGTCTTCACA

A_16_P00573324

TGTGGTTACAGACATCAACTGATTTTCTAAGTATATCCTAGTAACCCTTTACTCACTGCT

A_16_P17467495
AAATGCAAGTGAGGATATGTCTGGGATAAAGTGTTAGTAAACCATTGAATGACTTTTGGC
A_14_P138337 GAAGTAGGAAATTTTTATGTTTGAACTTGTCAGGGAGAAGCCAGAAGTAAAAGGACATTT
A_14_P201578 ATTTTCTTTCTTTTCCCACATGATTGTGGTTACAAAATTTGTTCAGGGTCTGTTCTTTCA
A_16_P03228857
TTCTCCCACGCATTCTTAAATAGAATATTATGAACTGTTACGATAAACACCCTTTACAGT
A_14_P111990 ACTCTATCACAGTATGAAAGAAAAGTCACTTTTCACATTGGACTCAAGCCGTTTTTGCAA
CUST_9441_PI426210822 TATCTTTCTTAGACCTTTCCTAAGACAGACCACATCTGCCTTACCCGTC
A_14_P129190 GAAAATGAGTGGGACTTGTTTTGTGATGAAGAATTTTATGTCTTAGATCTATAGAAACCT
A_16_P36159717
GAGCCTCTTTGTCTAAAATAGCCTTCCCTATTTTTACTTCCATTTAACTAAAAGCAGCCA
A_14_P200122 CTTTCAGTGACAGTTTTTGTGAAGCATTTTTACTGTCATTGCCTCTATTCTCTGCAATTT
A_16_P17891481
AAGGCACAGATGAATTAAGAAACACTTCCTGCTATGTATGATCTTTGTCCTTCTCATTCT
A_14_P122524 ACACTCTGCTAAATGGAAGTTAATGACTGAAAGTGAATCATGTGCTTGCTGTAAGGAAAT
A_16_P15451256
ACCAAGAGTGGACCTATTACAAAGATAAATGGGATTGATTAATTTAAGGCTGAGTTTGCG
CUST_6641_PI426210822 CTGTAAAAAGAGCAGTTGTTCCATCTGGTGCGCTTGCTGACTTTCATT
A_16_P38576851
GACCTTCCTTCAAAGAATCATACATGTTCACTGTCACATTACACTCATATATCCATAACC
A_16_P19811276
AGTTACTCCTGTGATATCTAAAAGTCTGATAATGCCCAAAAGGATGATTACATGCTGGAA
A_16_P20210394
AAAAGATAGTGCCATTTCCTACCATAAAAAGGCCACTACCTTTAAACTATTTGTCCAAGG
A_14_P124786 CTTAGAATGACTTACAAACTACTCAACAGCCACACCTTGTTTACCTGGCTATTCTGAT
CUST_10099_PI426210822 ACAGTGTTGAAGTCCTCTTCCCTTTACAGAACCGTGGCTCTTGAAT
A_14_P118560 CTAACTCATCCGAACTCTCAGATGCTTTTTCTTTGGATACAACACAAGACAGTGAC
A_14_P125941 CTGATATTTGTATTCATCTCATATAAAATCTTTCAAGCACACCTCATGTCTTCCTTCTCC
A_14_P134616 GTATCTTCTGGTTGGAGGATATCAAGATGTTTGTTAAACAGAAAATTGATGTCCAAGAGC
A_14_P124031 ATCTGCCATCTTTTGCCAATACTGATCAGTCTTAATTCATGTGATATGTGTTTCCTCCTA
A_14_P118400 CTGAAAGATGACAGCCTAGTTCTCTTTGCCTTCATTATGATACTAACAAAAACCAGAAGT
A_16_P00277410
CTGGTGACTAGTGCAATTTCTTATGAATACAACTGACTCTAAATGCCTAGTGTAAGCCAA
CUST_5913_PI426210822 TTCGTTGGCCAGCACGTTTGCTGGCGCCCACACTGACACTCTGAG
A_14_P117445 AAATTTTTATTGATGACTGTTGGGTTGATGGTCTCTATCAAAACCAGTAAGGTCTTTGAC
A_14_P136365 ACAGATAATGTGCTGCTTCCTATACGCATGGCTTAATTTTTTAAAGGTTCAGACAATGAG
A_14_P103325 TTTAGATACTCTTATAAAAAGCAGATAAACTTCAAGCAAGTTGGGATGGAGGGGTCTGAA
A_14_P119295 ATTCTATAACAGTGCATATGTGTGCATACACGAGAAATTGAACAAACACATTTAGGGGTC

A_14_P128931 CTACCAGAAGAAATTTAGGCCAGTTAAGACAGGGATGTTCTTACTCAATTGGTCATTAAA
A_16_P19921233
ATCACAATAGAACAATGTTTCCAAAGTCATGGTATCACAACATACTTTCTCCAGTTACCT
A_14_P121420 ACTCAGCATACTTTCATTAAAAATTTGGCACAATATGATAATTGTTAATATTCTGTTCTT
A_14_P117752 CAGACTATCCAGATCCTGTTTGGAATTATGACCTTTTCTTTTGGAGTTATCTTCCTTTTC
A_16_P19925519
TGTTGAGAATAGCATCACCATACTGAGTTAGAACCTCATTTCATCTTAAACTAAGTGGCA
A_16_P03513289
AGGTAATTTGAGAATTCGATACAGCATGTCTTATCTCCATTATTTGTTCATGCCATGGCT
A_14_P134694 CTTTTTAGTGCAGACCAAAAAATACCTTACCAGATAAGGAATAGCCAGACCCTTAAAATT
A_16_P00684998
CCCAGTATTCTTTGTCTTAGACCTTGAATGTATTTTTTGCTGTATGTATGCTTGAATGTG
A_16_P15858652
AACTCAAGTTTTGGCATTAAAAAGTGTATCCTCAGAGGTTTGTATCCAGTTGTAAGGCAT
A_14_P111109 CTACAAAGAAAATGTCCTTTCTTACATTTGGAAGTGGAGGGCAGTTCTTCCTAGATGTA
A_16_P17286487
AAAGATAACACCTCCATAAATCAGTAGAAAGTTGGGTCAATAAGTAACGATTCTGGTTCC
A_16_P18065777
GCCAAGTGTCACAGATAATACTCTACATCATAAACAAAAGTGCACAGTAATTGTCCTTGG
A_16_P15499772
AAACCTAAGAGTATCCACAAAACAGAATAGGGCAGAGGTTTTTATTCACAGCAGTCATTT
A_14_P102437 CTTTTAAATATAAGGAAATTCGGCTTACCTACTTGACAGTGTTTGCTGTCTCTCTCTCTC
A_14_P117467 ATTACTGCCACTACTTGGAAATTCTGCTTTTCTGTCATTGACCTCTACTGTTTTTTCTTC
A_14_P108910 GTTGTTCTTTTAAGGTGTGAATCCTCCTACACAGTTTTGCTACTTGGATTATCGATTTTT
CUST_8242_PI426210822 CCTTGACCCAAATGCAAGCTGAACTTTCTGGAGGTTCACAGTTTCTAATTT
CUST_280_PI426210822 ACTCTGAGACCAGCCCACTAAGAATCATGCTGTTCAGGAGATCAG
A_18_P14903952
AAATTCAGAAATGCAACATAGAGAACTATATTTGCAAGTCAGGATAGATAGATGGCCTCA
A_14_P129352
GAGGAAAAAAAAAAGAAACATGTTTCAGGTCTGTGTCTGTGTAATACAAAGATGACTGCA
A_14_P122157 CACAAGATGTCTTCTCTTAACTTTTTTATGGAAAGTAGTTAAAGACAAGCAGACTGACCC
A_16_P20154283
TCCCTCATCACTATTCAGAAACATTGCCTTACATCCAGTTTATTAAGTCCATACTGCATA
A_14_P133930 TCATTTCATTCTGAGAAAAAGATAATTCCCAAATCCCTTCCCATGTTGCCTTTAAATCTT
A_14_P112865 AAACCTCATGTCATTTCGGACCAGATTTTGAAATTAGATACCTCCTTAGCCCCATTTTTA
A_14_P104262 AAGGTTTAGCAAGTCATGCTTGACCCTGTTAGTGCTTTTTTATTCCTACATCATATTGAG
A_16_P01163208
AAAGCATAAGTTTTGTTCTCTAGAAAATGACATCAACTTTAGACGTCAGATTGCCTGTCC

A_18_P22840334 CATCCCTCCTTCCCTCTGTCCATCACAGCCTCCCCTTAGGGTCAT

A_14_P114606 CTCACAACCGTGAGTGAAAAAGGATCCATGTAGTGTAACTGAAGTCATATAAACTTTAAT

A_14_P113632 TGCTAGACAGAACAAATCTTTAAACAGAAAAAAAAAGCACGTAATCCTTCAGGCAAAGGA

A_14_P103585 CTTGAGTTTAGTCGTATTTGGGCATGCAGGGACATAAAAATACTTAAGAGATAACCACTT

A_14_P117939 CTGTTACCTACCTTGCTTCAGTCAATCCCATTTCTATGAGAAAATGATGGAGGG

A_18_P14134553 TATAAAATGTAGATCAGATGCCTTCTGTGGAATGCCAGGCCTAGATTTGCTCTACA

A_14_P106019 GTGAAAAAGAAAAATAGAATATGGCCAGACTGGCATTAGCATAATTCACTCCGAAAACTG

A_14_P126172 AATTCCACCCCAAGTTCTCACTAATATCATTCCTATAGCCTCTAATGGGATCAACATAAT

A_14_P115015 TGACAGGGGAAACTGAAAACATTCCTCTTTTCTTTTCTATGGAAAAGGATTATGAAGAGA

CUST_849_PI426210822 GTAAATCAAAGGGTAGCCCAAGGTCCTGATTCAGAACAAAAAGGAGGT

A_14_P116017 CCTCAATACTATAAAACAGACTAAGTAGAAGAAACATGCTCCTTGTCTTCAAAACTCCCC

CUST_69_PI426210822 AATAGCTATTTTAAAAGAGTGGAATAAATGACATAATAATAATGGCACTAAATTGCTAAC

A_14_P103376 GATGACTGGCCTTTCTCTCTGTTTTCAGAGAAGTTACAGATTAGACTATTCAGTTTAGAC

A_14_P137945 AAATTTTCATGTCTATGTATGGTTTACTTGACTTGGAGAAGTAGAGAGAAGGAATCTCCC

A_14_P201459 CCTTTCTGTGGTGATTTCTTGAGTTACGTTTCTGAAGCAGTTTGGAGTTTCTAATATTTG

A_14_P126060 TGCAAACATATAGGAGTCAATATCCATAAGGAGGTTGAACATTGGGTAATTGTGAACTTG

A_16_P19862939 AGAACCACATATAACTGCTATAATCTTTTCAACATTAGGCTGACATAATTTGGATGCTCA

A_14_P123210 TCAGAGTGAAACATCTTTCAACCTAATATCAGAAAAATGTGACATTCTATCCATTCTTCG

A_14_P126247 TACCTTTTAAACTGACAAGGGCTTTTGCTGAAGAGCCTGCAATTAAAAATAAAACAAATC

A_16_P21531701 TCAGTATTACATGTGAAGCAAACCAATTCTAGTCTCAGAAAAGTGATAGCCCTTGATGTT

A_18_P14135710 CTCAGATGCTTCTCAGTGGGGCTGGGCTGTGTCTTCTGCCAGGAC

A_16_P20328479 CCGTTGTGAGAGTGTTTATTCCGCTGTATTGTTTTATTTTTCTGTTTCCACAATTGAGAC

A_14_P134043 TACTCTTTTTGTCCCATTAGTTCTAAAACAGTATCCACAATGCTGTGACTGCAAAGAAAC

A_14_P111331 GTTGTGATCGGCAGAAGAAACGAGATTGAGAATCAGATTAAATTGAAATTAGGCAATTTT

A_14_P116798 AACGTTATGAGCATTTGCTTATAATGAACAAGAAGAGAGGAAGGACCTGCAATTTCAAAT

A_14_P132898 ACTCCTTCTTAGCACTTCCTGTGCCAAGAAATCTGGAAGTATAGAAGTATTAGAAGATTG

A_14_P122270 ATTTCATAAATATCATCATCTTCTAGTGATTGATGTTTACCAAGCCTGGTGTGGACCTTG

A_14_P118876 ATATTAAAAAGAAAAATACAGTAGAGCACCAAATAGAATATTCTCAACAAATGTCTCAAA

A_14_P200271 ACTTATTGAGACCTGAGTCTGTGCCAGTCTCCATTATATTTTCTTCCTCAAATATACCTC

A_14_P126289 CTTTGTTGTCGTGTTTCTGGATTCTCTTCCCCATTTTGCGTAAATATTGGTCTTATATAT

A_16_P21579737 AAAATCCAACTTCCAACTTACATCCTAATGATCCCATGCATTCTTAAACACAACTCTTC

A_16_P21457818

AAAGTCCCTTTTGATTACATGTCCTCCTAAGTTCTAATCCGATTTGATCTAACTTTCTCC

A_14_P102213 CAGAAACCTCAATCACAGCGCCAAAACCATTCCATACCAGGGATG

CUST_4151_PI426210822 TGAGAAAGGGGAGCAAGCAGAGAGGGAGAAGAGGAAGAAGCCAAA

A_16_P36230857 GAATATCATGTTTCCTCCAGGGAGGGCTCAGTGACATGGAGCAGT

A_14_P133799 ACATGCTGAATATACACTGTCACAGGAATTTGCAAATCTGACTCCATTAAGAAGTCCAG

A_14_P131634 ACTTTTCATCTTCAAAGTGATTAGACAGATTATGTGCCCAAGTTTTATAAGGCTCAGCC

CUST_4339_PI426210822 TTTTCTTAAGCAGGAAGAAGGGGGCCCCCCCAATTTTAATTTACTGAAT

CUST_4298_PI426210822

CAGAGTAGCTCACCTGTGGGTCATGATGAGAAATAGCCACTGTATTTTATTTGAG

A_14_P113779 TCTGAAATAACTTAGTGTTTTCCCCTTTTGAGGATTAGTAGCAGAACTCTCTGATTCTCT

A_16_P58424402

GAACCTTCTCAACCCAGTCTTTCCCATCTCAATAAATGACAACTTTTCTCTTCTAGTTAT

A_14_P124576 ACCATGTGATATCCACTCTCATTTTGCAAAACAGGAAATTGAGAAAGAGAAATGGAGTTA

A_16_P19424154

AACCAGTATCTTCCAGAAACCTTTGAAAAACAACCTATTTAAAACCAATGTCCCCTAGAT

A_16_P18378578

AAAGATGAGTGCCAAGTTTAGTCAATTTACCCTACCTGGAATACTATATACAACTCTGGG

A_14_P131193 TATGTGGGGCAACATTTCTCAAGCATTTTGTTCTCTAGGATCTTTACACACTCTTTTTTT

A_16_P38377248

CCCCAAACTCCATAGATTTGAATTCCGGTCAATCTAAATCCTAAAAACATACCCTAAATG

A_14_P126011 ATGAAGAAAAGGACTAATGAGGTGTAGATTCCATGCAACACGCAAATGAAAAATTTACGC

A_18_P22840880

AAATTTAAATACTGTGCTAGGTGCTGGGGGTTATAAACATGAATTACATGATTAGGGCCT

A_14_P103277 TTTGTTTCGTGTGCTTTTAAATTCAGGTTAAATGTTGCAATAATCTGATGCAGAAGACTC

A_14_P107738 CTAAGTGACGAAGTAAAGTTCTTATCAAAATAATTTTGATGTCCCATTGGCATGTCTGGG

A_14_P111463 CTATTTCTTGAGCAGTGGTGACAAGATTCGATTCTTCTTTGAGAAAGGCGTTTTTGATG

A_16_P03356466

AAAAAGGCTGTTTCTCTATTGATGTGCTATAACTCTCAAGTATTCCAAAAAGTCACTGAA

CUST_7092_PI426210822 AGCTGCGATTTGTCTAAACAAGGCTTTTCCTTCAGTACCCAAGGATTCT

CUST_1267_PI426210822 AGAGGTCTCCAGCAGGGCCTCTCTGGGGCTTTCCAGGTTTCCTTG

A_14_P125163 CTGGATTTTGGAGAGACTATGGATATGGCATCACCTGTATTTACAAAAGTGATCTTCTAG

CUST_8102_PI426210822 GTGCAACAGCTTGGCCCCAGAGCATGAGGTTGATGAATTTGATAA

CUST_1542_PI426210822

CATGCACATTTGGCCAAATCTATCAGTCTTTTTTTTTTTTTCCCTCTAGATTCTAAACTT

A_18_P22840318 GATGTAGTTGCCAAAATGCTCGCCTTCCTGGGTTCTGCTTTCATTAGTTT

A_14_P105787 ACACAGATAGAAACATTTGCTTGCATGAAGAAAACTTTTCCACACTCCTATTTTCTTTCC

A_16_P41585292

CCCTCCATATCTAAGCATACCAAGAAACAATTTATAGCCTAAGAATCTATGCCATTAAGC

A_14_P137401 AGCAATAGATAATACACTACCTAATTGTCCCAGGGATCAAAGACTCACTTGACTGATTC

A_14_P139543 ATCTATGCTGATGTGTTGCTGTATAAGTATAGAGTTACCATACACACTGGCTTTAAAGGC

A_14_P125328 TTCCAAATATTTTATCTTCACCTTTGCCTCTTGAATACCGCATGTCTTGTAGTGACAATC

A_16_P03417617

CGGGTCACAGAATTTAATCAATTCCCACTTGTTTATCCCATTATGTCCCTTATAAACAAC

CUST_3133_PI426210822 CAGCTGCCTGAGGGCTTGGGATGCTGGCCATTTATAAAGCTGCTC

A_16_P16660703

AAAAAGTGGGCAAAAGTTTTTAGATGAAACACAAGATGTCAAAAGGCACATTTCTCCG

A_14_P115006 ACTGATGAATAAATCTAAGCAATGTCCCCAGAAAATTAATCATTTTGTTCTCTGGGCAGA

A_14_P116638 GTCTTTGGGTAGGGGAAGGGTATTGAGACCTTTTCTAGTATGAATATTTTTTAAGTTTGG

A_14_P116916 TCCAACACTAGGATATAGCCCATGTGGGAGTCTCTGAAAATAGAGTCTGTCT

A_14_P107135 GCACACAATTCAGATGTGAATATTAGAAAGGTTTCTGAACTGTCATTTGGTAATAGCCCA

A_14_P132333 ATTGCTAAATAGTGCAGAAAAAGACTCCAGACATCTAATCTGGTTATATTCAGCCTCCTT

A_14_P202088 TTGTTCCTTAATTTCAGGAGGTTTACTTTTAGGACTGTGGACATTCTCTTTAAGAGTTCT

A_14_P106598 GGCACTACATTTCCTGAATTATTGAGTCTGACTAGTTAGATGGTGATCACTTAATCTGGT

A_16_P16191869

CTTAGAGGATGTCTAGTTCACCAAGAGAACAGGTGAAGAAGAGAACATTCCAGATAG

A_14_P136970 TTTGTTCTTGATTCTTGTGTGTTGCTTTCTCTTGTAGCAAAACAGTGCAGCAGACTT

CUST_5821_PI426210822 ATAACTAGACAGGGCTTTGGCCAACTAGCAGGTGAAGTCTGGAGG

CUST_7373_PI426210822 CACACAAAGTTGGCACTCCCTCCTCCACCAACCCTCAGCTAAAAG

A_16_P16144211

AACTGTCCATGTGAGAAATAACTTCATTGAAAGTCAGTTTTTGAAATGTGAGCATCTGCA

CUST_5249_PI426210822

ATTTTAACTGTGATCTTATTTATTATGAGTATAGAGATAAAGGAAAAGGCATCCCACTTC

A_14_P101973 AGAACACACAAGAAGCAGCGAAGTTTGGGAATTGGTAATATGAATTGTTAGAAGATCAAT

A_18_P12433868

AAAGAAAAGGATTAATTTTTACTGTCTTTTTTTTGTTTTTAGTTTTTTTTAGACGTTGGG

A_16_P15862211

GGGCACACTAACAGAGATCAAACTAAGTTTTTCCACTAACATCAGACCTTTGCAATTTAA

A_14_P136041 CTTCAATTTTTCTTTTTTCAGTGGCATTTGGAAGTTTCACTTCTCCTCTATCTCCTTTTG

A_14_P134799 GAACATGTGTCCTGTCTGCATGGAAACACATACCGACTTAATTTGGGTTAAATATACATT

CUST_4739_PI426210822

CAGTAGGGGAGATCATCACCTCTGTAATGGTATAATATGAACTCAGGAGTTGAGATT

A_18_P22841712 AATGCCTTTTTCTCTTGCTGCAGGGTCCTTGTGGGCGCACCAAAG

A_14_P125273 TCCTAATGGACTTATAGAAGACCGAGGTTCAGTGCTTTGCATTACATAAGAACAAAATCT

A_14_P124848 CAAAAACCTAAGATGCTTATGGACTGAAAGGTTCAATGCTACAATACAAGTGGATACTGT

CUST_6782_PI426210822 CTGCTGTTACAGGCCTGTCAGGACTTTACCACTCATAGGTTCTAAGAA

A_18_P14133153

GCTTCATGCTAATTAACTACCTGTTTTACAGGTGAAAAGATGTGTTGTCTCTAGGGAAAG

A_14_P128151 CCCTTGGCTTCACTGAAACCATTCCTTCTTGTCTTAGAAAGCAATCAGAAATATAGTTAA

A_14_P120867 TTACAAGTCTTATTCTTCCTTGGTCAAGGCACTTAGGAAAACCCCATCCTTGATA

A_16_P37084875

CACTCGCCTATAACTTACTTAATTTCACGTCTCAACAAATATAGTATTATTTCACCCACA

CUST_9768_PI426210822 GAAGCCCTCCATCCAGCAGAAGCCTGGGTGAGATGTACTGCCATT

A_14_P111146 AGATCAATGCACTTGACCCCTTTTATTTGATTATTCTTCTTCTGATGTTTGGATGCCATT

A_16_P19091233 AACTGGTTATCTTCCAAGTCCTTTAGTTCTTCTTCCTTTATCCCCACTAATTGCAAGTC

A_16_P16825299

AAAAGATTCCCACAAAATCATTGGTCATTGAAGAAAAACTGTTTATGTCCCATGTAGCAG

CUST_6577_PI426210822

TAAAAATAACTTTGGAAGCAGAGATGTGCTGTTTTAGTCAAAGACTTTTTTAGCTTTGGA

A_16_P21036709

AGCCGCCAGTCAAATTAGTATTTTAGATGAAGACTGCATTTGAGACATACTTGAACAACA

A_14_P129055 CCTGTCTCATCTTGCTCTTCATCACACACGAAAGGAAAATAATCTGAGATGGTGTAA

A_14_P124087 TTTGGGGACCAACGGGATATTATTTCACAGTTATTAGTAAGACCTACTTTCTGTGTAACT

A_14_P123063 AGTGATGGCAAAGAAGAAAACAGATGCCCAGAATGAATGTGTTACAATATATTGCACAAT

A_16_P15655594

ATAGGACTCTATACCATTGCATATGTCTACTTCAGAGGTCTAATATCATGCACTGTCCTG

A_14_P112762 ATCTGTGCTAAAATTGTGTTCAGTCTCTTCGCAATCTCTGTCTGCAAAGGCAA

A_14_P200460 CACCTTTTTGATTATTTTGGAACCAATCCCTTGACCTGTTGTGGAGAGAAAGA

A_14_P130674 CTTGTTTTTAAATCTCCAAAGTTTTCAAGCACAATGTTTTCTGCCTAGAAAAACGCTTGC

A_14_P129295 CCAGTGTATCCAGAAATTCAACTGTCAGATATTTTGATAATGGTATGTGTTGAGTAGCCT

A_14_P135672 GATGTTGATGTCAGAGGCTTGAAGTTCAATTCTTAAATGCAGAACTTAGTGTTTTAAGGG

A_16_P58424323

CCAAGATTGAGGAATATTTGGTGCATGAAAGGAAAGGCTAAAATAAACCAGGAGAATAAC

A_14_P120296

CTGTGACAGTCAAGAAGCAAATGACTGTAATAGCACAACTGGAAACAGTTAAAAACATTG

A_16_P19697295

GCCCTGATATTCAAGCAGATATAGACAGTTTCCTTAGTGATTGACTCCTAATGACTTTCA

A_14_P200171 CCAGGAAACTTATGCCCCAGAATACAGAATAATTTTGCATACATATGAATAGTAGGGCAA

A_16_P01784070

GTGCTTAAATTATGTGCTTGTAAACAGAACTCTTGTGACATAGTTGCTGACTAGACCACT

A_14_P110967 ACTATTACTTTCCCCCTCTGTTTTGGGGGAAATTATAAAAAGATGTCTATTTAGGTCATG

A_14_P119802 ATCACTCTCTTTTGATGCAGCATCATGATTTTTCACAGAAAATTATTTGGCTCCAAGATG

A_18_P12359076

TGCATGAACGACAGTAGATATTTTTACTAGCAGCAACTTTGTAGTTGATAATGGGTCGTT

A_16_P15133571

TAGAAGCCACAAATATCCATCCTTATCTACCAAGTCTCTTCCTTATTAAATGTCTCTGGC
A_16_P18560564
ATCATTTCTCTTTGACCAACTGTTCATGAACTAATTCACATTTACACACAATAGCAGGCC
A_14_P130302 CAAAAAGAAAAATAAATTCCTTTGTATCTCCCAACACACTGCTCTCTCCCCTAAGATCTG
A_18_P14131903 GGGGGTTTAAACACTTTTTTCAAAGATGAGAGATTTATGGGACCAGGAAAGGAT
A_14_P124702 TAATGGTGTGTCAATTCCAGATGCCCAAATAAAATACAAAATCAAGCAAAACCACACATC
A_16_P20908875
CCCTCACTTACGAATTTTCCTAATCATGCCCCATAACTTTGTCCTATGAATTCACATATT
A_16_P20169000
AAATAGCCAGTTCCTGCTTATTAATACTCAAACACATCAATTATTCATCTGCACTTGGGT
A_14_P127793 CTAGACATCATTTAGAAGCAGACGGGTTAAAATAGACAAGAAATAGCAAAGACACATCCT
A_14_P102810 TAAAATGTGGTCCATCCTTGTGATTCTGCAAAAAAATTATGTTTCCTCCCCTTTGAATTT
A_14_P119919 GTGTTATACCACTTTACATAAAATAATCTTAGACGGGAATTTTTAAATGTAATTTATTAT
A_14_P201885 AAGGAGAATCCAGCCAAGCACAGTCTACGTGTGCAATGAAAACCAG
A_14_P104280 CACAAAGTTCATTAGGTTCAGACAGTAAAAAGGACGCACACTTTCTGGAACAATATACAA
A_16_P16479707
ATCTTAACCCATGGAATCTCTACTATAACTTGTAAGCCTCCTTCACTTGAAACTACAGTG
A_14_P121003 AACATTAATACTAACAGCAAAAACCTTGAATACCACCAGTGGTTACTAGAGCAGTTGACA
A_14_P120202 AAATTAGAGAGGTAATCATCCCACTCCAACACATCTGAGCATAAAAACGTATCATCAGAA
A_14_P126801 GTTGAGTAGGGCTTTAGGTAAGTATGTAAGTAGGTAAAACTGCTGATGGTAATTAGAGGG
A_14_P106773 TTCATTTCTAGGTGACTTTTATACTGAAAAAGGCTACAATACACACTGGAACAACTGGTT
A_16_P39068466
AACTTTCAATCCTTTTACCATGTCATAAAGCAAATGCTAACTCCTACTTCCTAAGCATCC
A_16_P21683292
AACACCTGAGAAACATTTGAGTATATTTTCAAATACTAATAATTACATGGTTTATAATTT
A_14_P137729 TCATTCTGTCAATATATTCCCTTGCTTTATCATGGCGACCAATGTGCCATAAAAATAAGC
A_14_P137262 GCATAAATATGGCACATTTAGGGCTTAAATAATCTCCTGTTACTGTCACCTGGACTTCAA
A_14_P125664
TCCGGAGTAGGAAAAAGGTTTAGGAAATAAACAAGTGGAAAAATATCTGGAAAATGGAAC
CUST_3593_PI426210822 ACTTGGCAAGTGAAGCATTCCCATGCCTTCCAGACTCCTGGCTCT
A_16_P03546270
AAAGGTTTCTTGGATATTACGCACATTGTTGATTTTGCCAGATAGCAAGTCTCCAATCTA
A_14_P135148 ACTTGTATAGAGAGGTAGGGGGAGATATAATAAGGGAATATGGTAGTGACGGAAAAAAAT
A_18_P14198788
AAGAGAACTGTGTGTAATCTTAGCATGATAATCGGATTCCACTTATCACTTGGAACTGTG
A_14_P105224 CTCAAATAGCGGATCAGATCCTTCACGCTCTCTTCAGAGACAGAACAAAACAT
CUST_1720_PI426210822 CTATTGCTGCCTTGTGGTCCCCTCTTCCAGTTTGTGTTTTTCCTTA
A_16_P17590890

CCCTCTTTTGCTACCATAATTGATACCTACTCAAAGTTTTCTGCATTTAGAAACTCTGCC
A_14_P127058 GAGACAGTATTTAGTTCAGAAAAGACTAGTGGAGACTGACCCAATAGGACAACACAGTA
A_14_P131696 AAAGTAACTTACAATAACTTCCCTAAAGGATTCCTGGTCACTGATTGTCTTGTCTTCTAC
A_14_P133362 CAACCAGAAGATGTGGCAACCACTCCTTATATTTTGCTTGTGCTTTAATTGCATGATTTA
A_16_P37509047
TCCAAAGTAGTCTTCCTACAACCAATTTCCTTCTCTAATACATTCCTTCTACGACAAACA
A_14_P107052 TAAATTGTGAATGCAAAGATAACATGAACTATTGTCCCAGGGGTCATTGCCTTTGTAAAT
A_16_P00851224
AATGGTATCTCTCAGGCAATAAACATCTATTACTGGGTCATATGTCATAGTGTGCAACAG
A_14_P121798 AGAAAATGTGGTCTTACATGAAATCAGCGGAGCCATCTGTGTTTACCAAAACAAC
A_14_P109190 CTAGACAGACGTCGACACAAACTTTCAAATTGGATTGTCAGTTTCATCAAATTTGGAATA
A_14_P102433 CATTTTAAAAAATGTATATAGAGAGAAGGAAGTTTCTCTCTCCCTGTCCTCCTTTGGCTT
CUST_7081_PI426210822 AGGCTGACTCCAGTGTGCTCTAATGCATCTTCTTTGCATAAAGAGGCTTAT
A_18_P17026939
AAAATGACATCATGGCAAAGTATTAATGTGCTTTCTCACGTAGAATGTAGAGAGGGAAGA
A_14_P115155 CTGTAGAATAGAATTCAACAGAAAGTGATCCATTTGGGCTCCATTCAAAAAAAGGCAAAA
CUST_3937_PI426210822
CTTTTAAAGATTTATTTATATTTAGCTCAGGCCTTAAAAGTTCCATGTACTATCTAAAAA
A_14_P121018 GATGCTAACTGGAAAACATTCAAAGTAGGCAATATTGTATGTCCATTTGCAATCTGGAAC
A_16_P15754037
ACCCCAAATTAAGTCAGGAAAATATGTTGTAACTAAACGCAACCTGTAATTTCAGTGTCG
A_14_P139962 ACAAGACTTAATCTGGATTTCCCAGACTTTGCATCCTTCTATAGTCCTCATTCTTTTTTT
A_18_P15564721
AACTGACCCCAAACCTATTTCAGACCATTTTTAATAACAGAACATTTAGAGGCTCTCTTC
A_14_P133145 GTTTCCAAGTCACCCGTTTTGGATGTGCATTTCATGTGACAATACAGATGACAT
A_14_P201244 GGTGTTCGCTATCTCTTGTGTCAAATCTGGTGAACGCTACGCTTACATATATTGTCTTT
CUST_7575_PI426210822 CAATGCTTGTCCCAAAGTCTGCTCAGATAAGGGCCGTGTGAATAG
A_16_P00685454
GGGGCACTTAAGTGGATTAAAATCAATATGTAGTCACATAAGTCTGCTAAGCCATCCTTA
A_16_P01070632
AGCCATAGACAAGTAGTAACTCTTTTCCACTGAATAGAGGACATTAGTAAGATGAGACGT
A_14_P200418 GAGGAGTTTCCTCTATGTCTCCAAAGTTTTCATGACTTGACATTTCCAATTCTTTCAAAA
A_16_P02304570
AAAAACAGCCTGCTAACATAACAATATACCAACTTCAGGAAAATTAGTGTGGGCTTTCTG
CUST_1323_PI426210822
CAAATGGGTAATATGAAGGGAGATTATTAGGAAGAATATATGTTATGCTGTATGTGAATC
A_16_P17100697
AAATGGGATGAGGTAAAATAACTGCCAGTTTTAACCATAGACTATTTCAAAGACTCCCTG

A_14_P138478 TGATTCTGTTGTAGGTAGACTCATTTAGAGGCAGTCCTAGATGCTCTTTCTTGTCTATTA
A_14_P138703 CAAGAAGTCAGTTCCAGGCATTTAAATGAATAACTCATGAATACCTTTGGGTCTATGTCA
A_14_P134947 CACTTCAGCCCTCTTCCAGAATTTCAATGAAAATTGTCTGAAACTCTGAAATGCC
A_16_P18386840
AAAAAAAAATCAGATTAGGTTCCAAGCATTAAGAAAGAAAAGGTTAGATCATGAAACACA
A_14_P104515 CTCTTTCTAATTATACCTCCCTTCTGTCATTTGAAAATGGGTCGCTTTTTCCTACACTTA
A_14_P129976 AAATATAACATCATACCCATGCAAGAAGCAACAGATTTCTTTTCACCGTTCCACAATATC
A_14_P111422 ATAACCACCAAAAAAAAAAAGCCCTGGTAGTTTTTTGGCACCTTATGTTTAAATCAGATT
A_16_P36230343
CTGGAGAATTTTGATATCTCAATATAATAAGGCAATGCAGAAAATTATTCATCCCCAGCA
A_16_P40915326
AGCATCCCCTTTGATGTTAAGTAATACAACAAACTCCTAAATGAAAATCCACTAAGAGAA
A_14_P129336 GTGGAATTAAAGTCATACTTGCTTAGCAAATGCATTCCTGATTGCCACAAACTCAGTAAA
A_14_P103566 CCATGATGTTAATCTTTCTTCACTGTTCTTGGTGGGTTTTCTAAAACCACTACACAAAGA
A_14_P109351 ACGTCTACTTTTTAATGGTTCACTGCTATCCAAGGCGAATGATTTGTTATATCCCTTAC
A_16_P02515896
GGCGGATACAGAATATTGTGCATCTCCTATTAGTTACTATAAAATCTCACTGGAATCCCA
A_16_P17829404
AATAGGTCCCTTGAACCAAATTTAGTATCCCAGAATATGCATTGTCCTAAGAACTTTTGC
CUST_2040_PI426210822 GAAAGGCCCAGGTATTCAACTTCTTGGCTTCGTAGCCATTTGACTTTTT
A_14_P119686 TACATCAAAGGAGAAAAGCACCTTCATCAACATGACCAGAGTGGATTCAGACAT
A_18_P10001390
CAATGAGTTGTCATTTTATCCAAATCTAAAAAAAAACATACATACAGTATCAAGTTCTGG
A_16_P20083400
TGTCCCTAATTCTCCAATTAAACTACAAATCATTGTCCTCCCTTTTCAAGTTCACATCTT
A_16_P20110242
TTAGCACCTTTCATGACATATTCCAGCATCCTGTTAAATTGATCAGAAGACCAATTGCAC
A_14_P100641 GAAAATATGGACTATGCTAACTTGGGCATATGGTCTAGTTTTTGTCCCCTAACTTTTTGA
A_16_P30952340 CATCCTTGTGTGAGAGCAGACACTGAATGCTTTGTTGCAACTCAACTTG
A_16_P30952600 GTTGTGTTGTTTCTTCCACAGGACAGTTCGTCTGTCATCCAGTTCATGT
A_14_P102565 CCATCTCGGCTTATGTCCTCTTTCACCAAAGAATGATACAAATAAAGACAACAAACAGAA
A_18_P13009105
AGACATTTTTTGTAGAGGAAAAACTCTGAAGCACTTTCACACTTTGTTCAATATCAGGGA
A_18_P15193116
CTCCAAGTAGGGTTAGGTATGTAAACAAGTGCTAACAGATTGATATAGGCTTTCTGCTTC
CUST_7720_PI426210822
GGTCATCTTCAGTTTAACAAACACATGTCAGAACTAGAAAGTTGGAAGTGAGACTTCA
CUST_2253_PI426210822 CTCAGTGTTCTTTGTTGTTTGCAGTGGAAGCAAACCTTGGGGCTG

A_14_P104346 AGGTATGTGTTACACTTTCTAACATGCCATTTAGAAAGTGTTAGACTTTCTCACCTGCCA

A_14_P126340 TCGACGCAAAATAGAACATTTTGAGTGAAATTTAAACACAGTTTTTGAACGGTGCTGTTC

A_14_P136122 CTGTCACCTTATTTATTTTAACAACGTCTTGTCCACAGATACCTGTCATGTTTGAAAACC

A_16_P20046426

TGGGATCGCATTTGAAAATAACAGTCATGCTAAGAATACTAAATAATGCTTCAACAAGGG

A_14_P105929 GTTGAAGGATTGAAGAGTTCTAAGCATAAAATAAGTGGCATTTTCTGACTTCTTCCTCCT

A_16_P18070249

AATTGACCACTTGAACACAATTCTTTCCCTCTTATTTCCAAATTCTATTCCTGTGTAGCA

CUST_7662_PI426210822

GTGGCACCCTGCTATGAGCTCTTGGAACAATTTATTTATAAGTTCCTTATAGCCTG

A_14_P133727 GTGTAACATGTTTATTCTCAGCATATGCTTGTGAATGAACTAGGAGAGGGGGCTTT

A_16_P19089855

AGGGAATTACTAACACATGACTACTAAAATCAGCCTTTTTTCATTTCTGCTGTAACTCAA

CUST_8168_PI426210822 GCAGAGAGAGGGCACAGAGCAGACAGGGACTCAGCAGATGTTCAG

A_16_P58424331 GCGGTATTAACACTCGTTGATGGCTTAGCAGGCATGGTGTTCTGTAGAA

A_14_P102797 TTGGCTTCTGTTTCTAGTTGAGGAGTTTCCTAAAAGTTCATAACAGTGCCATTGTCTTTA

A_16_P03100415

GGAAAACCCCATTAAAGTGACTTCTTTTTCCTTTCACCTAACACATTGGCAAATAAAAAC

CUST_2686_PI426210822 AATACTTGGAGTTGAAACAAAAAGGTTGAAGGGAACTGATCCGACAGCTG

A_14_P119254 ACAAGGGCCAGATATTAACACAATCACTAAATAAACATTAGATAGCAGAGCATCTGGCAA

A_16_P02658517

AAACCTGACCCCTCATATAACCTCAAAAGTTTCAAAAGTTCTTTAACCAGATAAACTCCA

A_14_P105668 TATATAAATGAATGAATGAGTGCGTTTTGTAAAGGAATCTGGGAGTCAAAATCTGGCCAG

A_14_P128365 CTCTCTTGGAGTCTTCATTGCTTAGATAGGGCAGTTCTCTTTTATTTATAGCCTAGGGA

A_14_P100276 ACACCCAGTCCAATTGTTGGGAGAAAGATATTACATCATTTAAGGAAGTTGTTCAAAGAG

A_14_P134191 ATTGGATCTGTGCCAGTAATGACAAAATTATTTTTTTGACTTGCTTGCCTGAATAAATTG

A_14_P117343 CTAAGCATCAAGTATGAGAATGCCATGACAAAATTCAGGATGTTCGAATTATCTGCTCAT

A_14_P101769 TAATACTTTAGAAGTTGGATTTATTAGGAGGGGAGGAAAGAGGGTAATGTTGCAAAATGC

A_14_P104493 AACAATTCATTCACACTCTGCAAATATCGGAAAACTGGATTTGGAAGGTCCTGATAAC

A_18_P15046629

CCTCTCAATCTCACGTAAACAATCAGAACCAGACTAATGATTTTTCTTCCAACTCTAACA

A_16_P01766547

GCTAGTCATTCAGCCCTTAAAATAAACATATTCACAGTTTTTTCTCAGTAGTAGTGGTCA

A_14_P114808 GATCTGCTCAAGGTATTCCTTGTCATAGACCACAATAGGCTCGGATTCATTGAT

A_14_P106747 GTCATTGCTTCTTGCTGATCTTGACAAAGAAGAAAAGGAAAAAGACTGGTATTACGCTC

A_14_P124235 TCATTTTGAGTATTTGTATTAGTTACATTCATCAGATGTTCTTCCTGAGGGCAATTATGT

CUST_10158_PI426210822 ATTTGACCCTTTTGCAAACCGAGATGGTTTGGAGCAAAGGGAAGT

A_16_P01720931

TGCACCTGTGAATCACATTATAGATTAGGCCTGTCTTACCAAATGAGTCAAATACTGAAT
A_18_P17185373
AAATGATGCTCCTGAGAAAATGACATTCACTTTGCTAGATCTCTCAACAAATAGGAACGT
CUST_7014_PI426210822
AAGACAAAATACAGAATACAGAGCAGCAGTTCACGGGCTATAGCATTTGAGGA
CUST_7068_PI426210822
CTTATCTGTGCAATACAACCTAATGTAAGTCTACTTTGTAAGTATTTGTTCTGAGATTAT
A_14_P102556 CTTGGCTTCTGTTCTTCCTAGAGGATATGTTTGCATGGCAAATTGATTTCAATGGTG
CUST_5654_PI426210822 GAGAAGATCTGGGGAGGACCCATATAACCAGAACTCGGGAGCCAG
A_14_P123962 ACCATCTTTCAAAAGAGCAGTCACTGAAGAGTAGAAGTGGAAAATCATAGTTAGTGGTAA
A_14_P124591 ACTTTTGTCTCTTTTTAGGTCTAGACAAACTGAAAGGCAATAAATCCTATGTGAACATGG
A_14_P111736 TACTGCCTCCTCTGTGTATGAAAAAAGTCCTCAATACCCTATATTCCCATAATGACTTTT
A_14_P137340 CTGTTTTTCTTTTCCCTTAGGTTCATTTTCCAGGGTAGAGGAAATAACAAGTTGTTAGTT
CUST_6096_PI426210822 CAAAACAGGCCATTGTCAAAACGTCCAGGAACAGGGATGAAACTT
CUST_147_PI426210822
GCAGTATAACCATCACTTCTTTTGCATTGTTACAGTAGCAGTAACAATTCAACAAAGTAT
A_14_P126748 GATATAGCGACTAGTGTCAGAAATCTTCCAGAATTAAAGTGAGTGAGAAGTAGTTCTGCC
A_14_P113929 TAACTTATTTAGGGATAGAAATGATTACCTGGCATAACCAATGATAAGGCTGCCAAAGAC
A_14_P133207 TACTGGATTTTGTGGAAAGATTCCACAATCTGAGCATGTGAGAAATGGAAGACTTAATAC
A_14_P105531 CCTTATCTACATGCCGATTAATCCTGTTTCTGCCTGAAGCCAATAAATTTAAAAATAGCA
A_14_P121153 GCTTCGGCGGCTAAATGGCATTTTGAAGCCCAGTCATTCTCTAAAAAG
A_16_P18000500
GAACTCTCCACAATATTGTCCTAATCTCCCCTTATTACCAAAAATGACCAAGGTATCATT
A_14_P126814 CTTGCCAAGGCTAAACCACTGCTTTTACATGCTTTTGCAAGTTAATATTTTTTTTTGCCA
A_16_P17166932
AAAAAAAACTACAGTGAATCCTTCTTGAACTCCTAATTTTGATTCTGTCCTCAAAAGGCT
A_16_P03605463
ATATCACACAGAGACAACTTAGATCTTTCTTCTCAGAAGTCCAATAGTCTTCAGAGGAAC
A_14_P133605 ACACTACCAACCTTTTTTTCCATATGATCCAAATCATAGGACTGAAAATGTTCTCTCAGT
A_14_P201161 GTTGTCAATCTGTGTCATTTTGAATATTGTTCGACTTTCACTTGGTTTTATAACCATTGG
A_14_P135974 ACATGATTTTTCTTTTCTGGAAAGTGAGAAGTTTTGCTGTGATTCTATCATTCTGTGGAC
A_16_P16190993
CTTGTGTTTTGTGTTCTACAACGAGAATTGATCATTTTTCACTCTCACACAACCAAAACA
A_14_P201600 CCAAGTTAAAAGATTCTTTGTGGAGCCATGTAGATAATGAGTGATTCTGAAATGCAGTTG
A_16_P16549063
CCCTGAACAGATTCTGAATCCAACTAAAGATCTAATTAAGTAACCCCAAGAAAATTTCCC
A_14_P135745 CCCCGCCTTCCTTCTTCCTTTTTATATACAATTTGTTATTGTCAAATAAAAGTAGGAAAT
A_18_P27218367

TGGGATTACTAGGCTAAGATATCCGACTCATTGCCGAGCATAAATTGATTTATTCAAAGA
A_14_P105736 GATGAAGCCACGTGACAGTGTATAAAGCAGTTTAAAGATCTTAATAAACGAGGCCCTC
A_18_P14137150 ACAAAAGACACACAGGTGGTGGCATCTGGGGGGTCAATGTGCATG
A_14_P109700 GTGGCCATGAATTTTCATGCTTCTTTATATTTTCATCCTCCTCAACCTAGCCTAGATAAT
A_16_P03171129 CACGGCCAATAAATCTGAGGTGCAACCTAATATCAAGGCTACAAACTACAGAGAAG
A_14_P124801 CAAGTGTCCCTTTCTTATTCCCTACATAACAGATGAACCTTTTCTGTCAAAAACTCACTA
A_14_P109556 TATTACAAAGATTGTAGCAAGTAGGTCTTAATCCATGTGTTAGGCATGGGAACATTTGCT
A_14_P117144 GTGGTAGGATAGGATGGTGTAATAGTTGTTTCTCTAAGGGTTTGATATCAAAGCATTGAG
A_14_P103654 AACTGGTTCAATTTCCATTTCCTCTCCATTTTCCGTTAAAACAATCAGGGCC
A_14_P104214 TAATATTTACTTTCATTGTGATTCTCAACAATCCAACCTAGCCAAAGAACTTCTCCACTT
A_16_P37917098
GGGGGAGATAGTCTAACATGCATTGATCTAACATCCATTCGTTGATTTTTCTTGAACTAG
A_16_P37873607
GGAGAACAGGTGACAATATACACATGTGCGCTAATCGTAAAATGAGCATCTTAGTCTTTA
CUST_2799_PI426210822 ATGGTGGGATTCGGATGCTGGTGTTACAGAGGCTTAGGTTTCTAAA
A_16_P17023364
AAGCCACACACTTCAATACTTCACTAGATTTCTCCTAGAATGTGACATACAGAGTAAACT
A_14_P135392 TCAGCACTTAGAAGAGACATTCAAATACATTTCATTTCCTGTTACCCAGATTGTTCGGAA
A_14_P111695 AAGAAAGAAGGTAAGTCCTAAGATGTTTGCTTCCTATTCCTTGTAAGGTTTAGCCATGTA
A_14_P200972 TGCAATGAGAAATTAGAGTTCAACTATTTGGGTTTTTATTGCCTGCTCTGCTAGTGAAAG
CUST_8043_PI426210822 TTCCTTTTTCACTAGTATGAATTCAGCGTGATCTTTGATACAAGCCACCTGTCTG
A_14_P131270 CTGCAGCGAGGTTGTGATGTTCTTAATAGATAGTCCTGTGATCAGTTTAAGGAACAAAC
CUST_9581_PI426210822
CCTAAACCAAATTAAAAGAAAACAAAATATATTCCATGGCAATCTTGAAAAGTCAGGGAG
A_14_P134121 AATGATCAAATGAGATGCATTTTGAAAATAAGAAACTACCACACAAAGTGAACAGGAGAC
A_16_P02661623
TTCTCACATTTGATCATCTCTTACCAAAACTCTTTCTTGAATTTCCACTATACCAGCACA
A_16_P21244763
AAGCATTTCATCCTTTGTATAATCAAAGGCCTTCATACAATAAAACTACTGGGTTGGTGC
A_14_P124546 TGGTTACAATTCAACTCCATTTTGCAGAATGGTAAACCAAAACCCAAAGATACTGGATAA
A_14_P112921 CTCTAATAGGTTGTTTCTTTCTTATTGTCAGTAGTAGCCATTGTTCATGGAGCACATCTG
A_14_P117019 ATAACATCTATTTAAGTCTCAGTAACATTACATTGCATTCCAGAAATCTGTCATTCCCCC
CUST_9450_PI426210822 GTGGAGGTGTGGGAGAGGCTGGAGGGAAGCTATGGCCTTGGAATT
A_14_P105250 AAAGAGCATTATAATTAACAGGCTATACCCTCAGTAAAAGGTGTTAAGTTCAGCAAAGGG
CUST_5350_PI426210822 TTCATCATGGCCGTCTCCTCTCTGGGGCTGCTGCGGGAGATTTCC
A_14_P138156 GGACCCTCTTAATAATTCTTTTAGGCCGCGAATATCGCTACTGGCTAATATTGTTAGTAA
A_16_P18781928
CTGCCTGTTTTAATTCACATGTCCCTTTCTAAGACTTACAACTGCATTTCTCATAAAGGA

A_14_P127565 TGAATGCAGCCACTTTTGGTTGGGTATATTTTGTTACTGACTTAGTCTCTTTTCTAACTG

A_14_P132649 GAAAAGAATCTCAGAGAACTCGGTGGGTTCTTGTGTTTACAGTTTATTTAATGGAAAAGA

A_14_P136854 CAGGGATGCAAATATGGTGTGATATCAGAAATGCATGTCTTTACTTGTAAAATCAGCCAA

A_14_P123583 CTTCTGTGCAATATCTAGTCCTACACTCTAAATGGAAGAAGCCCTTGAGAAATACTTGAT

A_18_P14134392

TCAAAACTTTAAAATGAAGTGCTCATCAACAGTCATTTCTTGTCTACACCCTCCTGAATT

A_14_P118390 CTGTCTTTGGAAAAGGTTGTATACTTCTTGACCGTGTGGGACTAATGAATGCTTCATA

A_16_P15753051

AAAAAAGATCCCTTACACACTGTTTCAGTCCTATTCCTATAGTCTCATTCTAGCAGTTCC

A_18_P15732212

AATAACATCCCTATCATTTATTCATTGCAAATTGCTCAGGTTGTATATCACCCTCCAAGG

A_14_P124667 CTTTTACACAGTCTGCCATGGTTTTACACAGTTTAAAACTGGAAGACAGATTCTGCTGAT

A_14_P109666 GCAACATTTTCAAGGAAAAGAAAAGGGTATTTTTTTTCTTGTTTTGTAAAATGCCCAGGC

CUST_1865_PI426210822 ACTGGAAATATGTGGGTCTGGCTTTCCCTAAACTATAGTATCTGCCAAAAAAACC

A_16_P01572054

AGTGAACCCATATCTGGAATACACAGTTCATTATCAGTCATTACGTATACCACGAACTTC

A_18_P22841086 TTTTCCTTATTGAAATTGCCAATGCCTGTTCTCTAGGTTCTAAGAGCAGCCCT

A_14_P113975 TAAGATTTCCATAGGACATTGTGCCTTTGAACTTTCTGGACTTACATTTGGGTTCACATT

A_14_P123641 GTTACTGACTAAAGAAATGACCATTAGTTTCCTCTGGATGTGTTCAGTCATTCTTGTGAA

A_14_P124783 CTCCCTGCTGCTACCTGGTTTTATCTGTGCCTTAAGATAAATTATTTATCTTCTATGTGC

A_14_P139548 ATGTAACTGCCAGTCAAAGCATTTGGTTATCATGAAGTAACTGGTGTTACTTCAGTATCA

A_16_P21116887

AAAAAGTGCAAAGGATTATATTGGAGACAGAGGGTTAAGACAATGACGCTAGGATAAAGA

CUST_4569_PI426210822 GAAGCCAGGAGTCCAAGGACCCCTTGGGTTTCTGGTTTGGAGAAC

A_14_P111169 CTAAGTTCCATCCTAAATGCTGTGAAAAGTGATTTCCTTCATGTAACTTAAAGTCCAGCA

CUST_5454_PI426210822 AAAGCTGTGGAACCACCTTTCACAGTGCTTCAGACCACTGTAAGT

A_16_P35544748

AAATGTCATGCCTTTGTATATTTGCAGGATTTTAGACCTCATCTCATACACAGATGGTCT

A_14_P132388 GGTAGAATGTTGATTTGTCCTTTTCTTTGATAATGTGTCTCCAATCATCCCTACTTCTTC

A_14_P136034 CCAATGACCAGGTCAAGAAATACTTCGTTACTTCATATTAGTAAGATCCGGGGACT

CUST_10267_PI426210822

TTGTTTCTTTCAAAAATTAACAGATTTTTAAAAATTGAAAATTGAATTTTCAAAAAATTT

A_16_P15857382

TTCCTTTCCCCATAACTGAATTCCTACTCAATAAGCACCTTAAAGATTCTAAGACAGTCT

A_14_P130410 CGTTCCAACATCCCAAACTTTTACACTTTTGTCAGACGAACTATTTTAAAAAGTGAACAT

CUST_924_PI426210822

AGCTTCTAGATTATTTTTGTGCAGAAGCAGTATTCTCTGGTTCTCTTTGTCATTTCCTTT

A_14_P119538 TCCTTTGGGATTTTGTCCAAGACTCTGTAAATTAATTTTTCTGTGCCTGTTTCCTCATAA

A_16_P16191798 GAAAGGTGCTCCTTGACAGTGAGGACAACTTAAACAGAGGTGTGAACATAAG

CUST_9622_PI426210822

GTTGTGCTTTACAGAGCATTTGGGTGAATAACTAGAAATATGCCATAGATTCTTGAGGC

A_14_P133726 TACTTATTCTTTGCAACAGGAGGTGGTATAACAGACAGAAATCTACAAAGGATCCTTGAG

CUST_8423_PI426210822 GTTTTCTAGGGAAGAGCTAGCTGGAGCTAGATCTTCATACATCCTGTCATGT

A_14_P122320 ATGGTTCTCAGAGCATTGGAACAAGACAACAAGTTTTCCACTTGGAAATACTTTTTAAAA

A_14_P136986 CTGAGGGACAACCCAAGAAATCACACTTTATGTTTTTTCTTCTGCTTATTTTTTGAAGCA

A_14_P103892 CTGCAGTCTCTCTACAGGGGCTTTAAGAATGTGAGTGTTCCCCATTC

A_14_P110536 TATTACATTCCTCATAGCACATGATGTATTGGTCCACATCCTGAAGAATTTCTGATGCAA

CUST_7052_PI426210822 CCCGGGCATCTTCATTATAAAAGCTTAGTCTTTCTAAATGCAGCCAAGCTCT

CUST_5602_PI426210822

GAGTCACAGACAGTCCAGGAATAATTTACAAAGTGAGATTTAGATTAGTGTGTAAGAATT

A_16_P15857025

AAATCCCACAAGAAACGTTATAGGATAATGGAATGTGCAAAATTAATCGCTTTTCAAGGG

A_16_P16394022

AAGCATCCCAGTGGATAAATTCTCCTTTAAATACTCGAGTATGTTTAATCAGTAAGGCCT

CUST_9086_PI426210822

CGGTTAAAACCAAGTTGTGGGAGGATGGATAGTCAGTAATTGTTTATCTGTGTGAT

A_14_P106215 ACGTGTGAGTAAAGGCAGAAGAATCACAGTCACTTATTTTTAACAAAGTGAGTCCATTTC

A_14_P113026 CCACTCCAAACTCTCAATCATCTCCCACGTTATGTTTATAGAATGTAACATCTTTTCTCT

A_16_P16790941

AAACACCATGATACCAGAATCACCATTTCTTTCACATCATCACTCTAGTAAGTTGCATCT

A_14_P132405 ACAGTTCCATATAACTTACATTTTCCATTGTGTCATTAATTTCTTCCTCTGCACCCTGAA

CUST_8976_PI426210822

ATTTATCTTTCTAGAACTAGACACCTTAGGTCAGGCTAATCAACCAGCTGGTATCATTTC

A_18_P22841426

AGAGTTGGATTTCCACTGTGGAGCCAGGACTATTTATATCTTTCTTAGACCTTTCCTAA

A_14_P112501 TGTGGATCGTGGTCGTTATAACCCTGTGCTTCACGATTTATGATGTATTAATGAATTATA

A_14_P125384 ACTTTTCAACCAATGGATTGCATGGCTTGGGACAGGCAGAAATTATTATTCTATTGTTAT

A_14_P100037 CACACAATCCTCTTTTCTTGTCCTTACAATGCACAAGGGTGGCCTC

A_14_P120506 TTCCTGTGTGCGAGTGGCAAACTATTTTGAATGTGGAGAGGCTATTTTAAAGTTTTAATT

A_16_P01210553 CCCACTATATCAAATTTTCATTCGCTTTCTACATTCTAATATACCTATTTCGCACATGGA

A_18_P22840535

TCTAAGGGCTTCTCTTGATCTTTTTTATTTAAATCTTTGTCTTCTGAAGCATCTTAAAGT

A_14_P110704 TTGTTTCTTCCCTTAGATAACTCCTCTCCTTGTATTCTTTAAGTTAGACGATGGCAACTC

A_16_P18516810

AATCCTTGTCAAGAGATTTGGAGTTGAATTAAACAGAGCAGATGAATTAACTACAGCCAG

A_14_P122856 CAGCACTAATCGCTATTTTCTTTGGAAACTTCCTTTGATTCTTTACCTCCTTCACCTTC

A_14_P114543 AAAATTGAGTGTGCATTAGTGCACTTGAGTTCTCTTTGAATGCATCTAGAATACAAGTGC

A_14_P110432 TATACGTCTGGCCAAAATACCGTGTTTAACATTCTTTAACTAGTAACAGTTCTTTCCCTT

A_14_P115590 ACTTCTAATCACCATGACTCAGATACCACAATTCCTAAAAACAGAAGTGGTCAAGAAATG

A_14_P109329 AGTAACTGGTAATACAGGTGTTTTTTAGTCTCGAAACTGTCATCCAGCAATAGTTTCTC

A_14_P121278 TAGACTGGTGATGTTACTAAGTGCATTATTTTTAGCAGAGAGGTTAAGCAGGTGGTAAC

CUST_6218_PI426210822 ATAGAAGAGGCTGGCCACGTGCTCATAGCGGCCTTCCTCCTGAGA

A_14_P112180 CTCTTTTCTTTTGTGAGAATTAATGGTGCACATTCTGAAGGTGGGAAAGAGTCATTATTT

A_16_P19045145

AAAAGTCACCTTATGCAGTGAGAAATACAAGTATAGAATGTGTTATATAGACAGGTGGTT

A_14_P112134 TCTAAAAAGAGCAGAGGATAGTCATTTTAGGACCAGAAGAAATCTATTCCTTTTCTGCCT

A_14_P111373 TTATCATATCCTGCTCATTTCATTCATTGGTTCATTCAAAATTCACTGAACTGTGTGCCA

A_14_P117669 GGGTATGAAGCGAGAAGAGTCTAAGTTTGTATCTTCCGTTTGAAAGGTAATTGTTTTTTG

A_14_P116735 ATCTGTATGTCAGAACAAGATGCATTAAGAATCCACTGAAGAACATAAGAAATGGCACTG

CUST_62_PI426210822 CACCTGAAGCACCTGGGGGTTTGAGGGCTTGAACACTTAGACAAA

A_18_P14919553

AAGTCACTGGCTATCAAGATCAGAGTGAATTTAATGAATAACATAGCCCAGGGTTATCTC

CUST_8787_PI426210822 CCATTCTGGTATCCCTCAAATCCCTTGTTATGACTATCCACAATTCTCCATG

A_14_P118666 CTTGGGGAAACCAACATAGACTTGTCACTTTACCTTTATATGTTTGTTTGCCAACTACAA

A_14_P121541 GCCTAGGCATAATAAGAAAGAAAACAGAAACAAAGCAATTGTTTTGCTCTCAGTCTGAGC

A_18_P14136015

ACGTGCCATTTAGTTGACCTTCCTAAAATAATTTTTTTCTGAGACATTCTGTGCCTCTT

A_16_P39339599

TCCCCTTGCTTTCTTATCATAACTATACTTTGCTAAATCTCCATCACTGTATCTCTGACA

A_14_P133515 AAGATGTGAAGACTAAAGATGCTTTGGGCATGGCAAAGTGTTCTACAGATATTAGAATTG

A_14_P115321 CCCTTTTCTTCCAGAACATTTCCTTCTTATTCTTGTTGCAACTACAGGATTTGCTTTATT

A_16_P16191740 GTTTGATGTCCAGTAAGCCTGAGCAGCCCTGCAAATACAGATGAGC

A_14_P115812 AACAGATCTGTAACAAGAATAGACTGAAAAGTAGTCTGCATGGAGGTATGGATTTGTGGT

A_14_P125947 ACACTCAAACACATTTTTTTGTAAAGCAGAGGTGATCCAGAGATATCAGGATAGGAGATA

CUST_8986_PI426210822

GGCAATGGGTAATTGAATTAGCAAAGTAGACATTCAAAACATTCTCTGAGTTTTCAAACT

A_16_P58424169 CTAGGACCTGAACCTCTGAGGAGGGGGCTGGTGCTGCTGATCTCT

A_14_P103772 TCTTAAAATAATCAAGTCCCCTTCCAATCTTAATCATCCATCCATTGTTGAACCTGTTGA

A_14_P109220 GAGTGATGTTCTACTACTGGGTCTGAAATACTTCATAATTCAGCAGTCCATTCTGAAAAG

A_14_P137650 CTATGTGCTTAGAATACAGGTGTGGGGAAGAAATATACAGGGCAAACCCTATAAATTATC

CUST_1044_PI426210822 CTACTATGAAGCCGACCACATCCTACCCCATGGCTTCTGCTACAT

A_16_P02758750

ACTGTTACCCAACTTTTGTCATAATGGTGTCTTAAATGACTACTCTACGAAACCACCAGT

A_14_P103370 AAAGCCCTTTCAACTAAGTTGCATGAAATGTTATTTTCAGTTATCGTATCAGGGCATCTG

A_16_P00281572
AAAAAGGGGAATGCTGTATTTTTGGTAATATCCCAATGCCCTATCTAAAGGAAAGTCAAA
A_16_P41460279
GAGAAAGAGAGAACTCTGAACAAGGCTTTCTTCACTTACAAACCTGATTCAATCCAATTT
A_16_P02023094
AAACACTGCGGCTATGTAGAATCTCACTTGATTTGCTTTAATTGTTAAAGGTTTTGGAGG
A_14_P108889 ACATCATCTTCAGGTAGAAAGCCCAAATCATCTGCAGTTTGGAATTTTTTAAAAACACC
A_14_P138062 AGAGATTTTAGATTTTAAGGGAAGACTCTGGAGAGTTATCACTGCCCTCACTGTTTATAT
A_16_P00685438
ACATTAAATCTAAAAATGTCTAAAAAGTTAGGACATTTAACCTATACTATACAAGAAGTT
CUST_312_PI426210822 GGGTAAATGGTAGGGAGTGGGTGTGGAGCAGTAATGCTGTGCATA
A_14_P123989 TAGACATCCTCAATGTTTACCATCATGAAGAGCAGTGTTGCTGTAGACGTCCTTAAT
A_14_P102280
AGCGCAAAGCCACAATAGACTTAGTCACAGAATCATTCACAAAACCTAAAAGAAAATGAG
A_14_P139119 ATCTGATACTAAATGCAATAAATAATCCCAGATACCTAACCTTGAGTGGGAAGGCTGAAA
A_14_P133590 ACCTATTACTTAGCACAGAGAAACCTTTTGCTTGAAGGGATTTTCACATTTTATCTGAAC
A_16_P00685388
CATCATTGTTAAATATAGTTGTGGTTGGTAGATACAATACCGATAATAATAATGATGGTG
A_14_P110884 ACTGTGCCGTTTTTATTTTATTCCTTTTATTTTCCCCCTTTTCACAGAGAAATAAAGGTC
A_14_P116703 GGCCTCTTCCTCTGTGAGAATTCAGCATTTTGCAACCATCCAAAAATGTT
A_14_P102376 ATGGAATTACTCAGGTAGGAAGGGACTGAAAATATACTGCTATCTGCTGTCTTGTTAAAA
A_14_P116151 AGTTTTTTTAGAAAACAGGGTCAGAAAACTTCCTCAATGTAGTAGTCAAAGCATTTTGGC
A_16_P17252355
AATGAGCCAGTCCACTTAATAAGTTCATCTGTGTGTTTACCTTTTAGCAATAGTCTGACT
A_14_P135773 TGTTTTTACTGCACATTTGGGCACACAGCCATAAACTGCCTTGTATTAATTGCTTTTTAT
A_16_P16191932
GAAGTGTCACATGACTTCTTAGCCCTCTTATTCACCTACCTAATAAATAGGCATGAAAGT
A_16_P21548870
CATGATCCTGGTTATATACATAGAGCATTTTCCTAGTTACTCTACTATCCCACAGTCACC
A_14_P100509 TGATAGCACCATTCAAAACAACATTGTAACTGTTATTAAAACAAAACCTTACTTGTCTGG
A_14_P131015 CTCCATATGAGAAATTATGGCAGAGAGCAAATATTTCAATTAAGATAGTTGACAGATTTC
A_16_P18720989
AAGTGTGACAAGGCCATAATCTGTAACAAGGAATGTCTATCATAGTCACAAAAGAGAACC
A_14_P114908 ACACTTGTGTAAATTTCAGTTACTGGTCATAGAAGGCTTTCAATGTTGAGTGGCCTTTTA
A_16_P38904648
AAAACACACAGTGCTGTTAAAACACAAATTACCTATTTCTTAAAGTGTGACATCAGCCAA
A_14_P119795 AGAGTTTTTGTGCAAACTCTGATTTGGAAAATGTTCCCTTGAGTTTTAAACCCTGAGA
A_14_P128840 CTCGATACTTACAAGTAATAGGAGTAGGAATAAGCATTTCTTCTATATATTGATGGGCAG

A_16_P02718340
ACCATACTGTTCGGATGATTTTCAAAAAAGTGACATTTAGCATTTTGATAAATGGCCGTC
A_14_P108078 CAACTCTGGACTTCAAGTAATTTGAGGGTCTTGCTATTCGAATCACAAAATATTCACTGA
A_16_P18338927
TCTTATGAGACAACGATTATCCACTATGTCCATTTGTATTATCTGGCAAGAAACCAGTAT
A_14_P114742
ACAGTAAAACAAGTTGTCAAAGATATAACCAGAGTCCAGAGAATAGGACACAACCTACAA
A_18_P16424809
ATCCACTCCTCACCTAATTTTCATTATTCAGCGCTAACATTTAAACAGTATTCTCTCCCA
A_14_P109526 TCTGGTCCTCCAAAGTGGGATTTTTTCTTAAGTTCTGTCATTGCATTTTTATATGCTTCC
A_14_P135840 CTATAACTTTGGTGAATTAAAAAATGCTTTCAGAACAGGGGACTTGTGAGAAGAAGTAAC
A_14_P122874 AGAAGAAAAAATGAGGAATGTGAAGGCACATGCTTGGAAAGTGAAAAGCCTACTG
A_14_P201758 TATTGATTTCCAGATACCATCTAACCTCCAATGAATGATAACCATTTCTCTGATAGAAAA
A_14_P118250 TAACTGTGACGTATACGGCATTCTGTAACTTTTTCAAACCAGGTAGAATGAATATGGCAT
CUST_4120_PI426210822
AAGTTTTGGATGAGCTCATTTCATAAGGATTTATGTGCCTTATAGGCCAGTGTGG
A_14_P116901 TTCCTTGGAGAGATTTCTCCTACTCTTCACCTATTCACTTTCTTAAGCAAATGTTTTCTC
A_14_P110625 TTCGTTAAACAGAAATGATATTTGCCACCTTTTCTTCTCTAAGGCCTAAAGAGATGACAG
A_14_P125049 GTCAGTGTGGGACTACCAACCAAGCCAGGTGATTTCCTAAAAATGTCATAAAAATATTC
A_14_P101115 AAAAGTTCCAAGTGACCTTCATCATTTCCCTCTCTATTTACATTTCCCCTAAATGATTTC
A_14_P138641 ATTTCTTATTCCTATTGCTCAGGCCTCATCATCCATGAAGGCTCCTCTGT
A_14_P114831 AATTTGTTGAATGAAACAGTTGGGTATGTTCGAAAGAGAGTATGCTCTAGGAGGCAAAAA
CUST_6310_PI426210822
CATTGCTTGTGACTATTTATGGCCGTATTTGGTTCCTGAGCTAGAAACAGAAAAGTT
A_18_P10997673
TTTATCCCTGGCAAAAACATTAAGACTCTCCACACAGTTAATCATTCACTTCTTATGCTC
A_16_P15141087
AACTTGGCTCTCCAGTTAAGAAGAATCATTTTTTACACTTTAACTCAGACTGTTGGTGAA
A_14_P108682 CACCTGAACTTTCCCACCCTTTCTTGTCATTTTTATTTGACTCAGGCTATATTCATTCAT
A_14_P107998 CCCTGCTTGTCATACATTAGAAAGACCAGTCTTCCTGTGGAGAGTTAAAATAATTAAACC
A_14_P128558 GAACTGCTGTCATACGTATCCAGAACCCAGTCAAAAAACACATTCAAATACTAATGACAA
A_14_P120170 GCAGACAAAGCACAGTAAAACTGGCCACTAAATTGTGTCTGAAACTTACTCATCAAAATA
A_14_P113699 ATTCAAGTAGGTGATTACCTGTGTCATTCATTGAGATAAAAGGCTGTCTCTTCAGAAGTA
A_16_P38116590
AAAAAACTGTTACTGCCACATTTGAAGTGGATTTGCGTGTTAGGACAAAGAATTTCAAGA
A_16_P00720459
ACCTGCAGCATAAATGAGATCCCAAGATATCTAGTGTCTTAGTTCAGTGCTTCTAAATGG
A_16_P02747039

ACTTTCTGTTACCTAGATCTAATCATGTGTGTCCTAACATCACATTGCTCCATAACAAGG
A_14_P135102 TTTTATTTCAGACCTAATTAGAGCCAAAACCCTGAATTCCATCTCCTATGTGTGATCATC
A_14_P108905 ACTAGCCCCTATATTTTAATGTAAGAGTTACTCTGCAATCTAAGCAAAGCACCCAACAAT
A_16_P00092670
AAAAGTGGAAGGTGTTACTAGAACTCAAATGAAATTTCACCGTATGTCCTTTCCTTCAAT
A_18_P13210552
GCATGTAAGAGACGTTATTTCCTCTATGGTATGCCTAATAGTTTATAAGTAGCCCTCTCC
A_16_P03188449
AAACACCTCTCAAATTGTGATTCCATCAAGTGTCTTCTTATTCATGTCCTCTCATGATAA
A_16_P03619080
ATCTTCTAATCATTTCCTGTGCATTACTTGATCTCTCTTCATTCGACTAAAACCCCTTCT
A_14_P119591 GTTAGGGTCCAAATCATGAGGAAAAGAGCCTCCTATACTAGAAAAGTCTCCTTGTA
A_14_P114610 ATTTAATTCATAATTGTTTCTTTACAGGTTTCTTTACCTCCAGAAAGAAGAATATTGGCC
A_16_P21616903
GTCCTTTGCCCACGATAATTTTTTAATGAGAAACGAAACAATTCCCATTCTTTTTTGCAC
A_14_P136239 CATTTCTACATCAGATCCTGTGGCCTAAGTCTTCTCCCTTCCCTTATCC
A_14_P104393 ACAACTTACAATAGGGCATTTTTTAAAAAGCAAAACACTGAAATGTTGAGGTGCCACAAT
A_16_P02840154
ACATTTGGTAGCAGGATAATTAATTAGAGCATAGCAGATGGAATGATCTTACTGACCAGT
A_16_P16957185
GGAGTCCTGGAATCATATGTAATCTCATCACCCAATCATTTGCTCTACCATACAACTTTA
A_14_P202450 ACAGCATTTCGCAGGTCTGAGCCTATTTATAAACACTGTTTATTCATGAATTTTAGCCAC
A_16_P03459507
AGAACAGAGTTGGGAGAGATTTGAAACTTCTAGAGGAGGACCTTGAATTTTTCTGAAA
A_16_P01866890
CCATCTATCCTACCTTCTGAAATCAGTGATAATCAAAAGCCAAAATAGCATAACAACCCA
A_16_P30952420 AGGCCTGAATTATGTTCTGATGGCTGACGTGGCCAAAAAGGAGAA
A_14_P139369 GCAACTCCCCAGAAAGACTGTATTTCTACAGATAAATATTGCATTGAGATGCCAAATAGA
A_14_P137325 CAGTCCCTGGTAATACAAGCAGTGGCAAACCTTGTTGATCAATTCCAACATATAAA
A_16_P17352673
AATAAATGCCATACCCCTTTGATTCTTCATTGCCAATTAAGTCTACTCCACAGAACTTGT
A_14_P115950 GATTAAAGCATCATGTTTTACAAATTCCCCCTTTTCTGGTTACCCACAGCATACCAT
A_16_P16215059
GAGTTAGGTCATATCCTGCTAACAACAGTCTTATTGATCAGTTGTAACCCCATATGTTGT
A_14_P123215 TGGCTCTTCTCAACACCCAGTTATTATTACTACTTTATGGCAAGACTTCTCCTTTATTGT
CUST_4520_PI426210822
TTTTCCATTTTCATATTCAGTTTAGACCTTGGCTGAAGTAGGGAGGTTATGTAACTGG
CUST_7915_PI426210822 CTTGTACCCTCCCCACACCCTGGGGAGACTTGCTGGTGGACAGCC

A_16_P00928470
CTTTCATCACACTATTCAACAACAGATACTCCTGTAAGCAGATTCATACCCAAGAAAAGC
A_14_P202497 CTTGACAGTGGCAGAAAATAGGTCATTCCCGTAGTAGGGATATTAGAGATGTCAC
CUST_6242_PI426210822 CTCATCAGCACCCCTCCCAAGTTTGTTCTGAGCCACCTGATATAA
A_14_P122312 TATAATCAGAGGTTTGAAAATCTATGGGATGTTTAAAGGGAGGTCACATCCACTTACACA
A_16_P17958949 ACGTTCCTTAATATACTCACATACTCTCCTACTCTATGTTCTATATGCCATTTAAGTCTT
A_14_P135086 CTTTCCTTGATGATCTAGAATTGTCCTTATGAATATCAAGTGACTATGGTGCTGTCTCTG
A_16_P36230711
CTCTCTGGAGTTAATAACCATATGGATCTGTTTTGCCTGATTAGAAGGGATTTCTAAAGC
A_14_P101676 ACACTAGCAAAAATAGAGAACCACATGTGGTTTTTCCGGAGATTACTACTCACATCTATA
A_14_P103191 GCAACTGTGGGAAATATAGTCAGTTCCACAAATCCCTGTAAAGATAGAGAATAACAACTT
A_14_P104570 ACAGATTATAGTGTTTCCAGAAGATGGCATTCATGGATTCAACTTTACAAGAACATCCAT
CUST_1598_PI426210822 CAGTGGTGAGTGGGAAGGCAGCCCCTCTGGGCGCCACTCTGTCCT
A_14_P200011 AAGAAGGCCAGCGATGTCAACATCTCCAATGATAGGGCGAAAGAAG
A_18_P16628655
ATTGGTAAGGTCTGTATTACTCCAGATTTGCGAATATTCAAAATATGCCCTGTTTGATGG
CUST_3375_PI426210822 ACCTTCTCTGTGTTAAGCATATGGCCCCCAGAGGTGACAGCTTTT
A_16_P03717971
TCTCCTGAATCTCTAAAGTCATCTCCCAATTTTTTATACCCTTAAAACTCAGACAAGCA
A_16_P15481125
AAGTCCTATACGTTTTTGTCTCCATATCAGGTATTCCACACTTTATATCTTGTTCCCTCT
A_16_P20408572
CAAGACGTGTTGTGTTCATTTAAACCACTTGTGTTCATGAATCTATAAAGCCATGTTTGG
A_14_P109251 GAAACTGCTTTGTAAACAGGGCCATGATGTGAGGAAATGGTATTCTCAGAAAG
CUST_10004_PI426210822 CAGGGACACACCGTGTGCCCAGACCAGGGCTTGCCGTAACTAACG
A_14_P106677 TATTGTCATACAGATTGATGAATACACAACAGCATTGTGTATATTCACAGGCCCCAAACA
A_14_P131783 TAGAGTTTCCGCTTGAAAAAGAGTCCATGTTATACCGGGATAAATATAAGGATCTTCACA
A_14_P100732 CATGAATACATGCTTTTTGCAGGGTGGAATACCTGAAAGACAATGGAGAAGGTAA
A_14_P100017 TTTCAATGCATGAGTAGTCATTTTGTTTTCAATTTAAACCACTAGTTTGGCCCTTGGGTA
A_14_P102586 TCAATTATTTTTTGAGGCTGAACTCCTGCTTGTCACAGCAGAAACAAACATGTTCTTAAT
A_16_P40996294
AATCCAGTTTCCAAACTGTAATTCTAACTCTGATCTTCCATGTAAAAGCATGTTCTCCAG
A_16_P16684587
AAGCATAAGCGATACAACCTATTTGCAACAAACTAAAAAGTTGTATGGGGATATCTGTCA
A_16_P00807839
CTAGGAACACCAAAATTCGAAGAAAAAGGAGTGTGATTAATTAGCCATGAAGTGACCTAT
A_14_P116601 ATTAAATTTGTCTGGCTTAATTTAATGGGGACTTCTGGGACCTGCAGAGACTGTAAA
A_16_P03810508 AAGTCTCATTCCTTTCAAAAGGGAAACTTCTGCTGTTTCCACGCCTAACA

A_14_P122414 GATAAATCTAAAGGATGGGTAGGATTTAAGTGGTAAGGGAGGATAAAGAGGCTATAACTG
CUST_1104_PI426210822
ATCTGTATTGTCCCAAACAGAAGAAGAACAAACTTCATCTTAAAAATAGAGGATGATATA
A_14_P128267 GCAGATTTATTAAAGGTCGACTGTTTAGCAGAACAACGTAGATTTTTTACAACGCTTTCC
A_18_P22840321
ATTGTTTATGTAAAAGGACCATTTGCCTAATGTCCCTGGAATTTATTTGCTGGAGCATTT
A_14_P134640 CACACAGGTGAAAAAAACAAGCCATTTGCCCTTTCCTCTCTATACCATTTCTTAC
A_14_P105210 AAATGTTCTATTTCACAAAGGCAGTTTTCCTCCAACTCACTAATGTTTGTTGATCTGAAC
A_14_P125009 GGACTGTGTGAACCCCAAATACAAGCAGAAGAGACGCAGTTATAAGAACT
A_14_P116720 TTCTAAATGAGGTTGACCATGGAAAATTAAGGAGGTTATTGATGTTAGTTGCAGGTAGAG
A_14_P129588 ACCAGAAACAAAGAGTATGATCTAAATCAGAAGTTAGGAAGCCAGTGTGGTATAAATGGA
A_18_P19751309
TTCACCTGCTTAAAAACATTCCAGAGTTTACCTTTCTACCTCATTTCCTACAATATTGCC
A_16_P01306215
AATGTTCACCACCAACTACATAGTAATGCACACTTTCAAAAAAATAAAGGCCCTGTCTAG
A_14_P132085 GACTGAAATTTGAACTGTGCTTATTAATGGAATAATTAGTCAGGTGCATGCAGATATCAT
A_14_P127958 ACCTTTAGTGGTTTCCACTTCTTCTAGTAACACCTGGGAGATATAGTGGATGCT
A_14_P124736 GTTCCTTTTTTCCAGACTGACGAGTAAAATAAGTCACTTTTCCTCTTATAGCAGAAGTCT
A_16_P02985388 TATCCTCCTTAGTCATTGTATTCATTCTCCTTAGTCATTGTATTCACTTCCCTGTAGTCC
A_16_P18256518
ACTCTTTTCAGATGTGACATATTTTCCACAGAACTTGATTCTCATCTTTGCTATGACTGC
A_14_P114133 AAGTTTACCAGATAGAGATTTCTTTGCTTTCATTTTCTGAAAGAGCAGATTTTGAGGGGA
A_14_P138094 CTACCTTTGAAAAAAGTCCTTCTCCAGCACTGGAAATAAGAGATTCAGCAACATAAACC
A_16_P16550701
AAACACACTGTTTCATCCCAATCCTTTCTTAGTATCCTTTAACTGTCACTAAACAACACC
A_14_P107049 CTCATATGGGCAAAAAGATCAAAATTCTACAGAAGAATCTGATGGCTAAATGGCTAATTT
A_14_P123788 GAAACCGCTTCTAGACAATCCTGTATTATTTAGATCCACATAGAGATACACGAAAACCCT
A_14_P200148
ACAGTCATCAATCCAAAAAAGCACCAGAAAAAAAAGTTAACACTTAGATTAAGCCTGACC
A_14_P126150 ATCCAAACTCTTAGACCATTGCATTTGCAGTAAAAGCAATCGTTTTTATCCTACTTCCTT
A_14_P103779 CTGAACATGGATGTCTGTCTACAAGTGTTTCTTTGTATTTGCAAGGATGTTTGCAAAAAT
CUST_6825_PI426210822
TAGAGACAGTGTTAAACTGAACATTTTTTAATTTAATGAGTTGCTTGTACTTAAGAAAAT
A_16_P21626151
GGTCACCACTGAATATTATGGTTCTGGTCCTAGATAATGGATAGATAATACTAGGCACAC
A_14_P102819 GTTTGTATAAGCCTAGATTTCTGGAAGGATGCACAAAACCTAAAAACAGTGTATGCTTCT
A_18_P14134772 CCTTTTGTGAGATCAAATACCTATGCATGACAGGCAAATAAGCAGGCTCTTCAG
A_14_P113157 ACTTAACCCTGGACTTGATGTGAACTTGGCTACATCAACAATTTTTACAGCAAATTGTTG

A_18_P22841420 AAATGGGTCAGGCTGGAGAATGCTGAGGTTTCCAGGCTAGTGGTTT
CUST_4027_PI426210822 TAATTCTTCCCTGGATGCTGGTGGTCTCCAGAGACACAGGGGTAA
A_18_P16260535
AAACATCAAAGTTGCTTGAGTTTAGGTTGAAAAAGCAAAACCATTCCAATTTCTTGTGCT
A_14_P128980 TGAGTCATATGCCTTTAAGATTGAGACAGACTGGGATAGAGACTCTGTGATCAATACTAT
A_14_P136111 GGTCATGTTACTTACGTTACTTAAATCTGACTCAACCTATAGAGACACACTGTGAGAGTT
A_14_P202243 AAGGAGAGATTTTTAATTCCTTAGTGCAATACTTTGGTGACAACTTGGGGCGAAAAGTTA
A_16_P16198118
CCTCATTTTGTTTTCAGTTGTTTTCTCCAAAACATTGTTTGCTAGTTTTTCACACATGCC
A_14_P127626 TAATTTTCCTTCTTTTCAAAGATGCCACTAAATAGGCCACCAGAGAGTATCAAGTGAATG
A_16_P00065903
TAAAGACAGGTTCTATTTTGCCAAGGGCAGTAAGTAAATCAGTAGTGAGACATGCCATAT
A_14_P126693 GTCCATATGTTTATGATGACAACAACCAGGACTGTTTTCTTTTTACCCTGAAAGTCAACT
A_14_P105508 ATGCAGTAGGAGGAGAGCAAAGTTGGAGATTTACTTTTGCACTCGGG
A_16_P19623915
ATAAAGCCCTAAATGTTGTGGATCTTCTGATAAATGCATAATTGAGCAAAAACCTGGACG
A_14_P139651 CTAATAGCATTCGTTATAATAGGGCTTCGTTAACAATATTAACAGTAATCGCTGACCTTG
A_14_P115330 CTGTGGGTATTATTGCAAAGACGGAAATCTGATTCTATTTGCTCTGTAACAGGGTTAAAA
CUST_7797_PI426210822 TTTTAAGGTTATGCCCTGGGCATGATCTGAAACCAGGAGAACAAAGTG
A_18_P13227435
GAGTCAGATCTTGTCCTTAGTCGTATCTTCTCCTTTACACACTAATAATTCCCCATATCA
CUST_9113_PI426210822 CACAACTGGCCAAGATTTTCATTCACCGCAGACTCAGTCCTCATTT
A_16_P02526471
AATGCTCCTCTATTAAGTCTGCAGAAAATGACATTAAAGGTATTGGTTAAAGACACGGCT
A_14_P128874 TGTGTCAGGTTTACATGCACACCATATTGCTTATTCTGTATTAGAGAAACCATTAGGCAT
A_14_P108866 GTTATGTTAAGGACTTTGTCCTCATTGAGGACCAGTGCTGTTATTTGTCTTGATTTAAAT
A_14_P116843 CTACCTTATTAAACGTGAATAAAACCTGCATCCAGAGACACTTAAAATGTTGGCCAATTC
A_14_P133447 CTTGGCCCTGCTATTTATTTTTGTATTTATGTCTTAATCTCTTCCACTGATGCATCCTCC
A_14_P201186 TATCTGAAGACATGAACAGAAGTCAAATACCAGCCATATCAGTAGAGCCTTCCATTCAA
CUST_498_PI426210822 GGATGCTTATTCATGACCCACTGGCTACTCTTGGGGGGATGCTAG
CUST_9224_PI426210822
AACTCCTAATTTTCAGTCTGAGGTTTCAATCAAAATGTTAAATTTACATTCCAAATCCTA
A_14_P101913 ACAAACTAGTAATGACACACAGTGCTTAAATAAGTCTGACAACATAGATGGAAAGGCATC
A_14_P201729 CTTGGCTTACTGTAGGCATCATCATAATTACTACCATTTTCACTGTGCTCTGAAATGCTA
A_14_P123637 GGAGGATGTGAACTTGTTCGTATTCTAAAAAAAGAAATGAAATTCCTCACCAAAGAGGC
A_14_P104489 ACTCTTCTAGTATTCCCAATTTTTTTTTTCTTTTACTGCAACCAGTCAGAAATACATCTT
A_14_P202077 ACTTTCTCTAGAAAAATTCTAGAGTCTTAGCAGAGTAGGTAAGAGAAGATTCCCTCTGTG
CUST_8906_PI426210822 TCACTGCCTCTCTCAGACTACTTCCCATCTGTATCGCTTTAACTCCAATAAA

CUST_7678_PI426210822 TCTTTCATGACCCCTGCTGGATTGACTACATGAGCCTCTTTCAGA

A_14_P130189 TATAGTTTTATATGGATTATAACAACCACCATGTTTGCCCCCTACTTTAAGGAGTAAGAA

A_14_P117826 ATTTACAAGAGGAGACACACAATTTGCCGGAATGATCAAACCTGTAATTAGTAACAAGAA

A_18_P14341350
CCCACTTCTTTCATTAACTTGTCATGTTCTCTTTTTCTCCTAGCATTCTCGATACTGAAA

A_14_P112801 TATAACTCACAGGACCTATTTCTTTGAAAGATTTGTGTCTCCTAAAGACATGGCATTTCC

CUST_5871_PI426210822 AGAAGTCTTTGCATCTCACATGGCTAAGAGACAGTACTTTGGTTAGGGG

A_14_P116831 TCCTGAAATTGGTTAGAGCGCAATAACCTAATTTGTTCCTCTTTCTTATACTCGCTGTTT

A_14_P108379 CTCTGCTAATTAGGCTGCACATTTGAAATCTTAAACATGTGTAGAATTGTGAGGGATCTT

A_16_P20150944
CCTAGGTTAAAATGTGCAGAATTCAGAAAATACCACACAGGAATTACAGAATTGCCATCT

A_16_P01774538
ATTCTTCTGCAACAGAATTCTACAAACAACTTCTCATAGGTCATATACACATCACCTGGG

A_18_P22839045
AAGAGGAGTAACTCAATTGACATTCTTTGTCATTTAGCAGTCAGTTAATGGCCACTTACT

A_14_P115891 ACATGGGTGTATACGTGAATTTTAGTGAATTGTGTGTAAATGTGAGCAGTGTGAATTTGA

CUST_2460_PI426210822
GTGGATTTTTTTCATTAACACAGAACTTAGAAAAAATTTTAAATGATGACGTTTGGCCAA

CUST_6397_PI426210822
GAATCGAAAACAATAGGCAGTCACTAACTAAAATGGGACAGCAAGACAGAAACAGT

A_14_P129147
ATTAAATGGCAATGCCCAAGGACAGAAAAACCAAAATGAAAGAGAAAGGAGATTTCTATG

A_16_P15796264
AATGCAATCTGTCTTCCTAAACATTTTTCTCTATGATATCCATGATAGTCCATCTTCCCA

A_14_P113363 ACTACAAGTAACTACAAGTCTGTCTGCAGGAATATACTAGTGCTACAAACACTGGTTTCA

A_18_P10462427
ATGGCCATCAATATTAAATACCAGATTGTTCCAAAACGTCAGTCTTAATGTGACACTGTC

A_14_P118437 ACTTGTCTAGTCAGTCCTCTACGGTGTTATCAGAGTGGTAATATATCCAAAACATTACAA

A_14_P135517 TTGTCTTGCAAACATCTGGGAGATGATTTCTTATTCTTTACATGACCCATCAAATGTCTT

A_16_P17019764
CAGAGCCTGAGACACTAATTACTTCTGCTATTGACCATATTGCTCCTTCTTTCACATAAA

A_18_P22841045
GAGTATAAAAGGAATCCTGGCTGGGCATTGAGTGAATACAATCATAAAACCCAAATGATG

A_16_P15434701
GTAAACTGTCCATTTCTACCAAACACATTGAAACATAACAGAAATACAAACCAGTGCAGC

A_14_P120508 GAAAGTAACAGTGTCTACAAAGGGCCTAGTGGTAAGGAATGAAACAGGGC

CUST_803_PI426210822 CTGTGTTTAAGTGCCGTGTTCACACCAACCCTGACCGGAGATGCA

A_14_P125488 GATGAAGTTCTTTTGGGATTTGAAGTCTTGATCCTATGTAGCAAATGACACAAAACTCAT

A_16_P03578848
CCACTGTCCTCTGAAGAACAGTATCTTTAATTTTTAGTAATAGATCATTAGAAACCTGGA
CUST_83_PI426210822
ACTAAATTTAGATCTCTGGAAAAAAATTTGTTTGAGCATGGAAACAGGTTGGAAATATTG
A_18_P14793130
AGGTGCAATTTGAGAATGAATCTTTCCTACATAAGAAGACATTGTAAATATGCAGGTGGG
A_14_P134259 TCTTTCGATTTAAATCCTCAAGTGTTACAACAGTTAGATAGTAAACGACAGCAGGTCCAA
A_16_P18027724
AAACATCTGTGTGTGTATATCTCATCCAAACCCATTAAATTTCAGCAAATCCTCAAGAGA
A_16_P03798818
AAAAACTTAATGTGAACCCTTCAAACACAATTAGGTTGATACTGAAGTTCCACACAGTCA
A_14_P134721 AGAGAGAATTGGGGAAACACTGCTTGAGAGAAAAATATTGAAATGGTTTTAAAAGGGTTT
A_14_P107202 CTTGTATTAGTCCATGCTATCAGTCTTCTTGCAGATTTTTTCACTGAACATTTTTCTTGC
CUST_3012_PI426210822 ACACAGCCTTGCCTTGTAAGTCAACTGTTTGCTGGCGTTAAACAAGT
A_16_P17990584
CCAAATCACTTCAACATGAAAAATGCACAAATTTTCCTATCCTCTCTTCTGTTCTACTGC
A_14_P201555 GACACAACTATTCAGTAATCTTTACAGTGCATTATTTATAAGACGCCACAAGTCACACAG
A_16_P17082781
AAACTTCAGTCTAACTTACCTCTTTTGCTAGGCCTTAACCAAAAGATCTACTTTCTCCAT
CUST_628_PI426210822 TACCTGCAGATTACCCTCAAAACATGAGACAGGTTCAGAGCGAGG
A_14_P136985 ACAGCCTGCTTCTTAATTTTTAAAATGAGGATGAATGGCTTCCTTAGAACGTGTTCTATT
CUST_5609_PI426210822 TAGGATTTGGGTCCTCAGGGAATGGAGGGGCTGATGGATGCTAAA
A_14_P107744 TCTTTCTCATTGTTGAGATGGCATCTCACAGTAGGTGAGGAACTTTGTAGATTTAATTCT
A_14_P115675 GATTAGGGAGAAAAGCAGTTTGAATCTAATATCTAGGAGCAATCATTTCCAGCAAAACTG
A_16_P18871056
AACTGGTGTGAATTTAGTGTGACTGATATGTGATGTTAAATGTCCTCCATTGAAAATCGG
A_16_P03160663
TATCATAGATCCAACATCAGCATTCATATTACTACCTTTGAACATACTAACCCCCAGTGG
A_16_P00426002
TAGGCACTAAAGAAGCATATGCTGAAGGGATTGGAATGAAATTGGATCATAGAGAGCATA
A_14_P124630 TCTACCAAGTGTGTGAACTTCAAGCAATACCATTGCCAGTGTAATTATTTTCTCAATCTC
A_14_P127753 CTGACCTAAATGCAAAAGCAGGAAGATGGTGTATCTGATTATCTTGTAAATGCAGCGTTC
CUST_798_PI426210822 CTTATCGTAGGTTGTGACAGAATGCCTTTTTCTCTTGCTGCAGGGTC
CUST_2895_PI426210822 AGGCACCTCTCAATGTCTGCATATCTTGGGCTGAGTTTGTTAGCAG
A_16_P01638976
TCAGCGTTGATTTTCTCTTCTCTACATGAAAACACTATGAGACTCTCATTTGACTGCAAA
A_14_P122246 GACATGACTAAAAATTACTGAATGACTGTGAATAGTTCATGCCTCCTTAACTCTGTTCCT
A_14_P120780 ACTTTTTAATTTAGTAACTCAAAACTTTTTAATTTTAGAGTGTGATGTGAAGGATTATGG

A_18_P16702351
TTTTCCTACAGAGATAACAACCAATTGCATTTCCTTTCTTAAGAGCCAGTGATGAATGTG
A_16_P00010327
AGCACTTAACACTTAACAAATTACTCCTGATCACCAATACCTAGCAGATGAAGCATAACC
A_14_P106633 TAATAGAACAATCATGCCAACAGTAGACAAATGGTGCCATTTGTGTTGGAAAACTAGTAG
A_16_P20548193
ATGTTGCTTTTCATACTTCACCCTTCAATCCTTATCTTCTCTAATTTATTGGACAGACCC
A_16_P01897561
AAGAGGTCAATCGAAGTGATGTTTACCACATTCTCTCTAAATGAAACTTACTCTGCATGT
A_16_P19401237
AAAAATGTGACAAGTGCATCTAAGAAGAGGACAGCAGTAACTGTTACATTTGGTAATCCT
A_14_P132009 GGGAGATCTGCTTTTCAACATTTACCTGAACTAAGAACACTGTAAGTTTCTATGTCTCTT
A_14_P104481 CTGCATTTTGTGTGGGAAAGAGTGAAAATTCTTCGAGATTATGCTGGCCTTATACTTTTC
A_14_P101781 ATTGGGGGAAGTTAGACTAGAAGTATTATTATACAGGGTGATTCTTACGAAAGAAGACCC
A_14_P121229 TTTAACTTGTTTCTTTTCCATTTATTAGCCTACTCGCCCTCCTAATGGTCAAGGAC
A_16_P19050931
AAAACCATCTTGGTATGACTTCATTTTCTTAGAGACACATGATTGATCTGGCTATTGGTG
A_16_P17033806
ACTTGACTCACAACTAACAGTCTGTATTATAGCACTGACACCTACAAATTTACAATGGCC
A_14_P201292 TGAAAAGAATGATTTTTTCATTACAGCCCCCAAAGAATCTTGTCTCGTTGGCTAAAAGTA
A_14_P111508 CTAAAATAATAAAACTTCATGTGAGGCAAATTCCACCTTAATTATTGCCCTTTGGGGTGG
CUST_573_PI426210822 GCTCTCCCTCCATTTACTGTAACCTAGGCTCTCCAGGATAAAAATTATTATTGCC
A_18_P14138687
AAGACTTTATAAGAGGTTAAAAGGGCAATTGAAATGTAGGATTATTTTAAAGACCTACGT
A_16_P01159675
AATTATGCTCCCTGTTTAACCATCTAACTCAGATTAACACTATCTTACTTGACAGCCACC
A_16_P17717747
GCAAATTGAAGCCTGTTAAGTCCTTTTCTAAATCAGTTACTTGTCCAACATTTGCTTTCC
CUST_6193_PI426210822 CTCAGGCGTAAGCACAACCCATGACTCTGGGATGCCCCTCAATTG
A_14_P126523 GCCTCTCGCACTTTTTAGATATTAGTGTGTTAAGATGGTAGGTTGTTCCCTTCTC
CUST_8360_PI426210822 CTTCTGACCAACTAAGCCCCTGCTGTCACACCCTGCTCTTGATTT
A_18_P15390907
AAAGTGTAGAAATTTGAGCATGGAACAGTATTGAAGTGGAAAAGGTCAATGGTTACCATC
A_14_P123721 TACTGATAAAATTCACATTGGCAAATGTGGCTTTATCATTTAGCAAATGGGTCCCTTGAT
A_14_P128349 TAAAATGGGGGGAAGGATTATTATTCATGTTATTTTCCCTGCCCAAGACTTTATTTAACC
CUST_7876_PI426210822 GAACAGGATGGGCCAAAATGGAAGCTGGGATCTTAAGATTTGAGACTT
A_14_P127612 TAGACTGGTTTGTATTAAAATGTGTCAATTGCTAAGAAATACCTGTGGCTGGTCTGTAAG
A_14_P119778 GAGCAGATTTGAAACAATGTTCCTTGTGCTTTCTTATCTGCCCTTTTCTTTTTCAATTTG

A_16_P17716866
AAAACTCCCCTATAGAAATGCTTCGAAAGAAATCCATATGCTCTGATAAAGTTTGGCAAG
A_16_P18388086
AATATCTAGTTCTGTCTCTTTGTAATGGGAACAAAAACACTGAAACTGAGATGCTGACTG
A_16_P03609266
CCTCTCAGACTTTTCCTATTCTGTAAACAGGAGATTTTAACCAGGGTAACAACACTTTAA
CUST_9677_PI426210822
ATTCCTTTGGTGTACTGCTTCTAGAGGCAAAGTTTACTGCTTTTACTGTCATGAAATAGA
A_14_P139312 ACAAAGTCTCAAAATGTTGATCCACTTGGAGACTTTGAACAGCATATTGCTTATGGATAC
A_14_P121086 TGTAGGTTGTTTGCTATTTCAGAAAAATGTTTTGCACGTGAAAGAAAGAATAGCCCTTAT
A_18_P17038253 TTCCTGTCATCGAGAACCCTGAAATAACCCCAGTCTTTCCTTGAGT
A_14_P200378 ATTTTTGATTAGTTCGTGCATGGAGATTTGTTTGAGATGAGAAACCTTAAGTTTGCACAC
A_14_P105597 GAGCACTTATTTCACTCACATCTTTACACGAAGATTTGGTGGGTGTTTACTTTGTTTTTA
A_14_P100787 GGAATCTACACTGCCTATCAGTAACACTTATCCACATACTTCTAGACAGTCATAGTGATC
A_14_P105905 CCAAACAGGTTATACATTAAGTCTTCAGTAAATATTAGTGTAATGTATGCTGGCTGGGGG
A_16_P17759242
GCCTTTCTTCCTCTTCAATATTCAATAAATGTGTCACAGAACTACAGAAACCTTAAAGGC
A_16_P20830297
ATTGTTGTGAATTACCATGTAGATAGATGGAGTTACACTAGTAATTGTAGGATCCCCTCC
A_14_P103206 ACATTGTTTATGCCCAAGTAAATTGATTTTCCATGCTACTAGCACATCCTAATCTGTGTC
A_16_P39741668
GACAGATCATCCATCTTTTCATCTGAACTCCGTATCTATAAATCCTGATTATCTGCCCAT
A_14_P126876 AAATCAGAGTTAGTGACTGCACTGCCTTCGAGAATATTTTGATAAAAAGGATTTTGGCAT
CUST_331_PI426210822
CCAGTATTCTTTGTCTTAGACCTTGAATGTATTTTTTGCTGTATGTATGCTTGAATGTGT
A_14_P133091 TTATACATTCCTGAAACATTTTCATCTGTATCTGCCCTGTATTTTTCATGTGATATATAT
A_14_P110859 TGGTTTATATAATACCAAAGTCACATACAGGTAATTTCCCACCATTGAACCTTCCTGTTG
A_16_P18051015
AATCACTCTACCACGGATAAATTATTCCATACCACTAAATAAGTTGAATCCCTTCCAGCA
A_14_P139000 GATGTTTTGGATAATAAAGCATGCATGTGTTTTCATATACCATTGCCTTGCAGATCAAGG
A_14_P130008 TAGTTAGGGTCTATTTTATATTTATTGTCTCAGACTAAATTTTATTATGGCAGTGTTTCA
A_14_P132808 AAACCAGATCAGTAAGTCATTAACTAAAAATCTAGGTTGACGGGCACTTAATTTGACCTT
A_14_P122240 ACCAGACCCAATAGGTTAATTTCAAATTTTGTTTTGGTCAGGCTCATGATGACTTGTTAA
A_14_P127888
GGATCAAAATGAAGCAGAAGTAAAGAAACTAAGATTACGAGTGGAAGAACTAAAGCAGGG
CUST_731_PI426210822 GCAGCCTGGGTACCTGCACCATTGGGAGTCTTGCTTCTCTCACTC
A_14_P110700 CTTCAAAAGCAACTGTATTAAAGGGAGTTACAGATGGATATATACAGTAAAATTGTTTAA
A_18_P22840675 TAAGTTCACAAGCAGGTGACACACTGGCAGATGGCAGTGTTGAAAG

A_16_P03200617
AAATGCCAGTTATTGCTATTCCAGTTACTTTAAGAGACTAGATCATCCTCAAACAGCAGC
A_14_P125144 ATAACCCTGCTACATTAGATGACCTGTAGAACATCTTTAATGATTCACCTTCAACTCAGT
CUST_5504_PI426210822 ATTATAAATGAGTAGATCGGAACAGGGAGGACAACACTGTGAAAGAAAGGGC
A_14_P128593 CAAATCCTCTTCTTCATTCCCTTCTTCTACATTTTCATCATTTTCCAAAACCCTCTGAAA
CUST_1141_PI426210822
CCATTTTGTGTCCTGGTAATTAATGGTAATTTCTGTTAGTTTGTTTTTTAGGGTTTCTAA
A_18_P22839950
ATAATAATAAGGAATGGTCCTTTATCCAGGACCTCAGACTTTCTCAGTTACACAATAGCC
A_16_P02022430
TCGATACTTTCCTAACTCCACTCATAACCCAATAATGCACATTCAACTCCTTAGTAGATC
A_14_P111294 CTTCACTGGAATGCATATAAATTTCCTTTGCTTTGCTGCAGATATTTTGCTTGCTAAGCA
A_14_P109653 CTTTTTTTTGTGTGCTAAACACAAAAGCCTCTTTTTGGTCATTGCACATCAAAGCATTTT
A_14_P101790 CTACCATGGTCTACAAGACTCTCTTCGCTCTTTGCATCTTAACTGCAGGTAATTG
A_14_P122346 ATATCTTCCCCTTTAATGATAAAATCAGGATCCCTGATATATTCATCCCAAAAGCAAACC
A_14_P138318 AATAACTGGTGGTGAAACTTGAGTTGTCTGTCCTGTAACTGCTTTACTATTAATTGGTTT
A_14_P138619 CAGGCAAGTGGGGCAGAAAAGGTTAAAAATCACATTTGATTACAGAAATTTAGAAGATGT
A_14_P105762 AAGAATCCTTTACCTGACCAGTTTTACTGCTTCACTTCTATTTGAATTCCCAATTTCTTC
CUST_6589_PI426210822 TAGATGTCCCTGGCCTGATCCCTTACCCTTTACATATAAGGAAAGGAAG
A_16_P20781923
TTCTATAGGAAAAACATGTTTCACCTGATAATATGCAGATTTCTATCCCCCTTCAACACC
A_14_P110707 TCAGACCATAGATGTCTTGTTTGTGACTTGTCTACTTTGCAGTTTACTGAGAAAAGTTCA
A_14_P119719 TCCTGAATCAGTTCCTTGAAACTGTTTTCCAAGCAAATTCTCTGAATTTGAAATAGCGAT
A_16_P18928713
ACTTCTCCAGAGAATGTGTATCAGGTAACCAACAATATAAGAGGTAATAGACAGCATGGG
A_16_P01352856
GGATCTTTCATCTAAGAAGAGACACAGCATACTATACCATGCATGTTAATTTCCATAGCC
A_14_P129305 AACTCTGCATTTGACCCAGAAAACATGTCAAATCCTATGATCTTGTCTAAAGTTCAACAA
CUST_8212_PI426210822 CACCCACCTGGTAGATAATCTGAGAAGTTTGATGACAAGTTTAAGCCCCATAAT
A_14_P200075 CAAGAACTGAGATATCTTGATTCCTAATTACCTGTGTTAATTCAAACTACATTTGGGGGG
A_16_P18136434
AATGAAACCTTCAACATATTCTTCCACTGAAAAAGCCAAAGTTCCATTTCAGACATGAAG
A_16_P16278195
AAAACACTAGTTGTGGATCTTCACTAACCTACCGGAAATTGATTTTCTTCTGTCAATTCA
CUST_3230_PI426210822 AGCTACTGTCCTAGGCCCTGGGGATGCCACTGTCATCTTCCTGAC
A_14_P123941 GATTACCTATAACAACTGAGAGTTGATTTTTTAGTATACCAGTCAGGCAGGTAAAAGGCG
A_16_P02394674
GCCTAAGCCTTATTACTCACATGAATCATGACGTTTAATGCATTTACTAAGACCTTTCCA

CUST_2170_PI426210822 GCAGAGGTTTAGCTATAACTAAGTGGGCAGATGCCAGAAAGGAAGGAA
A_14_P131661 CTGGTTTCTGTATCCAAAGACATGAAAATTATGATGAACACATTTCAAACTTCCACTCCA
A_16_P18192770
AATTGAATCTTCTGACAAGAGTTCCTACAGATTCTCATTTAGTCCAAATTGTTAGCCCAG
A_16_P19531818
ATTTTGCACATTTTCTCGAGTATGGGTAAGTAATCAAGTAATCCTACATACTGGTGGCTG
A_14_P127535 TTTTTCTTTCTCTCTCTGCTCTCTCTTTTCAATAACAGCAATAACAAATCCCTAGCCTTG
CUST_8502_PI426210822 ACCATTTGGTTAATGCTGTAAGAAGACCTCTTTGGAGATGGGGAAGAGTTAA
A_14_P128893 ATTCTTCCTAATTTACTGTGATTTGTTAATGGATGAATTGTATTTTGCAAAGATAGTTAG
A_14_P135954 CTATTTAATCCAAATTCATGGGATGAGGCATAAAGAAGATGGAGGGGGAGATTAGAAATA
A_14_P106323 CCAACTGTGTGTGTGTTTTTTTAAGCCATAAAAATATCCACGATGTAGGTGGTTCATTTT
A_14_P105963 ATTTTGATTTCTTTTGTAAGCCTCTTCTTTCCACAAATACTATGACTGCTTTCTGGCCAA
CUST_1748_PI426210822 TTCTCATTGCATTCCCCTCCTCCCCTCCTCACTTCCTTCTCTGCTT
A_14_P115873 CCATGTTCATCTATCCCCTTCTGCATTCATTATTTTAATAGCATCCACATAAGACCACTT
A_18_P14362604
AACCCAGTTCTATCATCTATTACCCTATGTTGTTTTTGTCAGTTTCATACAGTCCCAAAC
A_14_P130386 TCTGTAGCATGGAGGGAAAGATAGAAGATGTGGAATGCAACATGTTTTATGTGTTTAAAG
CUST_4252_PI426210822
AATAGCAGCAAACAAACACCATAGTGGTGATTTCATACTCATGAAATTATTGTCATGGAG
A_14_P122968 GAGTTTGTTAGTTCCCACCCCTGTAAGACTTGACACGTAGTCTTCATTTCTG
A_14_P104735 CTAATCCCATTCACAGTTATAGAGGTAACACTTCCTAATTCCATACAGGCTTGACTGTTA
A_14_P114459 AAATATCCAAAGCCTGATGCCAACCATAAAAGTGTGATTGATTCACCTGTTAAAACTTCT
A_14_P112603 ATATGTTTCCTCTCACCTGGGTTTTCTTAGCCCTCTACTTTTCAAGACACCAA
A_14_P101436 GGAGGGAGCCATCTTCAAACATAAATTTGGTGAGTTTAACAATCAGAGATTTGAAAAACA
A_16_P02334544
AGATTCTTTGATCTTGGCTTTCTGATCTAAAACTCTAGTTACCAACTTTAGCCTTCTCCA
A_14_P102032 GGAAACATATTCTCAGATTGCCATTATCTCATTATCCTCCTAAAACCATCTTGGCTAATG
A_16_P21190577
GGCACCAGGTAAAATAACCATAACTGCTACTTAATATAAACAAATCCATTTTCTGTCTCT
A_14_P100516 CCCCTACTAAAGAATTCAAGAAGACGTTCTCTTTCCTCAGCGCTTTGTTAAACAAAT
A_14_P116659 AGAATCCATTAAATTACAGGGAAACATTCTGTTTGCTAAGACATTTGCCTTAGCCTTTCA
CUST_9670_PI426210822 ACATGTGAGTTGCCCCTTCACACCACTCCCTAACACTGCCCTCAC
A_16_P02726077
AAAATCGCTTGTATTGCTTACTCCACAATTTCTTTGAATTTACCCATCCTAAAAATAGAT
A_14_P136821 ATAAACCTTAGCCATTTGGCCCCACTAGATAGTGTAGGGATCTAAACTAAACTAGATAAC
A_14_P132622 TTCTTTCAAACATCATGGCCTCCCATCCAATCAACATCATCAAATTACATGTGTAATCAA
A_16_P15704633
ATACTTTAAAGTGAACATGCATAGTTAGCCACAAATCCCATTACTCTCCAATTCCAGGTT

A_16_P20204985
AAAAAAACCCTCCAAATCCTAAATACCGCTTTCTCATAATGCATTAGGGGTTTGCTTTTT
A_16_P20171527
GAAGAGAAATATCAACAGTGTCAGATTTAACCGCCAAACTACTTAATATAACTTCCGCCC
A_14_P131822 ATTTTTTTCAGGCTTTCTGAAACCTACATGTGAATCATCGGAGGATATTTGCTGAATGAA
A_14_P125464 CTAATTCCTTAGTGAAAAGTCAGATGGCAGGGGTCAAAAGAAGTTAGAAACATTCATAAG
A_14_P100478 GAACAAATTAACGAAGCATCCACAGATCCCTCAAAACATTTTAAAAGGTCAGTCTTATGG
A_14_P110166 CTGAAAAAGACCCACTTTGGGAACATTCCTTACCTAACAATCTCAACAATTATAAAAGGT
A_14_P117282
GGGAAAGTCCAGGAAAGAGAAGAGTGCTTAAAAAAAAATTTAAACCTTTGCCATTAAAGC
A_14_P108069 ATTTGTTAAATAAAAATAAAGTCTTTATTCTGTTTTTCTTGAAATGGCTTTGTAGAAACA
A_16_P00685406 CAGGAATCACCCCCACCCCCCATTTTTGTTTAATGAACTACTATGTCCTTAAAT
A_16_P18420486
ACCATGGATGTCTACAAACCATTACTGACTTTCTCCATAGAAACTGCATCAAATTTTACC
CUST_6563_PI426210822 TCGTTTTACTATTTCAGCAGGACTGTCAGAGAGGTTGGTCTTTGTGGA
CUST_8641_PI426210822
TAGCTACTGACCTCCCTAGCTTACTTTTTTCCTTCTATTGGACAGTCATTCTAAAAAACT
A_14_P125194 TACCATATTTGTCCAAGAAGAGAAATACAAAAGTATCTGCAATGGTGATGAGAACGCCAC
A_14_P108393 AGAGGCAGTGTGCACACACAAAGTAGATCTGCTACTGCTAAATTAATCATGTATACTTGG
A_16_P38766360
AATTTTTACATTCCCTTCCTATGCTTCAAATTGTTGATGCCAGATCAAGAGTTCTACTGC
A_14_P133939 ACAGTTTATCCATTAAATATTTGCTTGAGGTGGGATAGTGCTTGAATCCTTTCTTTGCAT
A_14_P134582 TAAATTCTTGCCTTGACTGAAGATGGCTTGAGATTCCATGAAAAATTTTAAGCCTGAGAA
A_16_P03593313
AAAATTCACAACTGCTAAAACAGTGTTCCTTCTAGTGTAATTTTCACCCTTAGAGACCTG
A_16_P20064872
GGCTTCAGAATTTATGGCAGTGGTTGAATTTATTCCAGAAAATTTGTCTCATGATCTGTC
A_14_P127190 ATTTTTAAAAGACATGATGAAATGATGGTTGCTACGGCCATAAAGAAAAGACACAGAGGA
CUST_10433_PI426210822 CCGAAGACATTTCACATCTTTATCGCCTCGATCAAGTGTGGAGTCAC
A_14_P134036 GTAGTGCAGAATGTAACAAATCTTCTAAATGTGGTTATTCTTGGCTTTCTGCTCACATGA
A_14_P135098 CAGATCATAGAGTTTGTGAATGTAGCGTATATACATCTCCTCCTTGTTCAGTTCAGTCTT
A_14_P135454 CACACCTCACAGTGTTAAGATTTATATTGTGAAATGAGACACCCTACCTTCAATTGTTCA
A_14_P123659 CTTCAAAGAAAGATGCATCTAGTCAGTCTTCTTTACGAAAAGCAGTTCCTGTGTTTTAGA
A_14_P123394 AACTCTAAATTCTACGCAGAACTTATCAATTCCCAGTGGACAATGTTGCTTTCTTCTCTA
A_14_P123554 CTAATAGTAAGTGCATGAGCCTCCATATGATGAATGGGAATGAATGGCTTTTTTAACTGT
A_14_P131250 CTGTTTCGCTCTAGTCCTTGTAAAATCTTGCCCTGTATATACTAGAAAATGGTATTTGGC
A_14_P119069 CTCCTGCACAGATATGTGGTTGTTTCGGTAAAACACACATTTATGAAATTCATCACTTTT
A_14_P132464 TGGTTTCTAGGTCATGCAGTTTTTGAATAAGATGGCTGGTAATGAATATGTTGGATTCAG

A_16_P02019303
TGGGAATATGCATCTATGTGTTTTACTTTCAGCAGATCAGATCTTACTAATCACAACCGA
A_14_P131974
ATTTCAGATGATGAAATTGAAGGAAAAAGAACTTTTGACTTGGAAGAGAAACTGCACACC
A_16_P21047371
GCATGCCAGAAAAAAATATTTACAGTAACTATGGAACACAAAGGTTTATTACATCCATAA
A_14_P104233 AAGACCACCACAAAATCTAAAATCCATACAAACATTTATTCTGTCCCTGCCTGGAG
A_14_P123608 TGGTTTTCTGATTTCATTTATGTCCCATTCATAAGCCATTTTAGGGGACTATATTCCATA
CUST_3758_PI426210822 CTATAGTGTTGTCCTAGACTGGGGTGGTGTTAGTGGTCAGGCAAA
CUST_457_PI426210822
CTTGCTTTCAAGGGGTTGCATGGAATTTTAACAACCAAAATGAATTTCGTCAGATAGTA
A_14_P116670 AAACACTCATCTCCTCTGTAATGATCAGATGAACTCATGTCTTTCTCAGAGGACAATAAA
CUST_5288_PI426210822
GCTTCAATTTTAGAATTTTCAGTTTGTAGCGTTATTTTTTTAAGCAGTAAAATTTCAGC
A_18_P22839787
TTAATTGTTTTGTTATATTTTTGGTGGCCTCCTACACTATGGAAGACTTTCTGAAAGCCC
A_14_P107545 GGAGAAATATTTAATAAAATCAGAGTCGAGAGAAAGTCACTTGCACAATTCATCTCCTAC
A_16_P01419774
AAAACATCTGCAATTGAATATGCAAGCCTTTAGAAAAGAGTATCTCAGGTGTATTTCCCC
A_14_P200190 TTGAGGGAGTCAGTAGAAGTAGTAGATAAGTTTTTAGATCCAGTCTCCTCTTTATGCAAG
A_14_P126816 CTACAGACATGAAGATGCTTTCCTGTGTTACAAAAAAGGATAGTTTAAATAATTCCCTGC
A_14_P109117 CTCTATCTTGGTTTGTGTTTAGTGTTAACTCGTCCGTAGAGAACCACGATTTATCTCC
A_14_P103834 AGCATCAGTTTTGGAGTAAAGAGCATTGGTAAGGATGAAATTTAGTGGTTAGTTTTGTGT
A_16_P18148837
TCTATAACACTGATGGAAAGAGACAGTAAACCTGAGGAAGCACAAAAGATAGGATCATTT
A_14_P132708 TGCCTTTGCCTTTTCTGTCTCTCCACCTCTAACCATAAATACAATTAGGTAAAGATTACT
A_14_P136578 GTCTCGCTTAAAAACACACACAGATCATGGCGTTACTAATTCCTATAGAGAGTCCAT
A_14_P135912 TACCCAAACGTTTTAGCAAGTGTTTGAGAACAAAACAGCCTTTGAAACCAAAATTCTTTT
A_14_P127785 ACTGCAATCTCAGTCCCCAATCTGGAATTCATATTTAGTTCAGTAGTTAACTTCTTCCTT
A_14_P101146 CTAACTTATGTAAATCATATTTATGCTAATTGAAGAGAATATATTTCACCTTCAGGATAT
A_18_P10522842
AAAAATCTTCGTGTTATGCTAGAATCTGAGAGAGAACAGAATGTCAAGAATCAGGACTTG
A_16_P15001594
TACGCTACAGAGCGTAAATTCAGATTTAGAGATCTTATGTTCCATCATAAATTGGGCTGG
A_14_P200393 AAACCTCTCACTGCATCATCATTGTCGGAATCAAGTTTACTCAACAAAACATTGGTTATT
CUST_7187_PI426210822 AGTTCATTTTTTACACTCTGGTTCCATCTCCTTCCCCCTGCACAC
A_14_P111506 AACTTTTTAGTGAGGCAAGGGGCCCATTTCTAACGGAAATTGCTTGGTTTTATAG
A_16_P20390889

AAAAAAAAAAAAGGCTTATTGACTCTTAGGGACCTTATCTGAAAGTGTCATGACCATGTCT

A_18_P14135520 TTCATGACTATCTGGAGGAAAGAGAGAATGCGTGTTCTTAGGCCATGTTG

A_14_P115837 CCGGTTTTGTTAAAGATTTAAGTGAACTAGTGATAGAACAAAAAGATTAACTGGAGTAGG

CUST_676_PI426210822 GGGCTGCGGAGGAAGCCACGATGTTCTGTAAACACATAGATACAA

CUST_8305_PI426210822 GACGCAGCCAACTTCATTCAGCTGGATGACCTGGAGTGTCACTTT

A_16_P20232737

GGCAGTTAGAATGTGGTTATAGTTTTCTTGGCAGATAATAGTTGATGTCTCACAGAAACC

CUST_3396_PI426210822 GGGTTATTACCTCCCACAGGACTGTTTAAGCTCTGGAAGTTTTCCTAC

A_14_P106973 CAAACACACAGTTTCTGTGAGAGCAAAATTTGTCCCAGAGTTCAGATTTCAACAATTAAA

A_14_P128964 ACTTTAAAAAATAGAATGGTCTCCTTTTGAGCAAATCTGTTTAGACTTAGGATTAGTAAA

A_16_P18389300

AAGAGTCACCACAACTAAATACTACCTTTTTATCTAACTTGAACAACTAAACAACCTCTT

A_16_P01246243

AAACGTCTTCAGTATCGATTCCTACCATCTTCTACCAATAATCTCCTGATTCTCCATTAG

A_14_P115383 TGCTATATAAAATTCTTGTCAAGGTTGATGTAAGTTACTGGTTCAGATCCCATCTGTCAG

CUST_1379_PI426210822 GTGGAAGGTGTGGAATGTGAAACTGTCTAATCCAATAACACCCACTAACTTTC

A_16_P17061801

ACAGCATATTCTAAGTTAACGCCTTTCATGATCTAGTTACACCTGACCTAATATGTTCCA

CUST_8367_PI426210822

TCTGAATTGCTTCTCAGAGTTATTTTATTTTTCTCCTTTGCTTTTCGTAATTGGGTAGGA

CUST_10294_PI426210822

TAGCAAGAATTGCATACATAAAAACTTGAGCACATGAAAGCCACATGTAAGAGTGAGTTC

A_16_P18849067

AGGTATTCAACAGTAGCAAGTTACATCATTTCAGAGTAGTAGCACATCTGTTTCTTTGCC

A_16_P00898004

GCTCGTTACTAGAATGCCAAACTGAATTAGGTCATTTCTCAATCTAGAAGATGCTTTTCC

A_14_P125992 TCTGATGACAAATGGCACACTATGTCTTTTAACCTGATTGCAGACCACAAATGTTTTGT

A_16_P18194457

ACAGCCATACTGCCTACATTTTTCCATCGACCCTTAACTATATTCCAAATGAACAGTATT

A_14_P139313 ATGGTTCACCTATCGTTTGAAGAAATTACGAAAATTAGTCCTATAGAAGTAAGTTGGAAA

A_14_P102019 CTTACTCTGTCCATTTATCCACAGGAAAGTGTTATTTTTCAAGGAAGGTTCATGTAGAGA

A_14_P105633 TGAGCCCTGAAGGTAATTGTCTTAAAGCAATGGTAATATTAAGAGAAACACTCAAACTGG

A_14_P123349 ACTCTCTTCTTCTCTCTCTACCTAGGTCCTCAATTTAGTGATGTCTCCCTGTT

A_14_P101778 TGAAGATAATAATTTTATTTTTCCCACCAGTGTCTACTTTATGTGATGCTCACATCGGCC

A_14_P107349 AATACCTTCTGGTTGCTTCAAAAGAAGGCTCAGATTCATTTACACTGCAAGTATAGCAAA

A_14_P138903 CTTTACTTCAACTTTAAGAGTTATTAACTCTGAGAAGTAGGAGTTGGTGGCAACGTAGTG

A_14_P135489 ATCAGACAGTGAAGATATAACAAGATCAGCAAATCTAACTGACCAGGTATGCGCTTTTCA

A_16_P17295646

TCCCAAACTCCAATCATTTAAAACTTCACCTCCATACCTATTGAAATACCCTGAATCATT

A_14_P103054 CTACTCTTTGTTCATTTTATTTCAGCCTTTAGAAATAAGAGGGCCATATGACGTGGCAAA

CUST_3097_PI426210822 CTAGGAGACCAGTGATGGTCCCTGGCTTTGACTTGTTCTTTTGAG

CUST_1222_PI426210822

AACAAGTTGTCTTTTTATTGTGACCGAAAAACCTGATGAGTGTGGAATAGTTTGGG

CUST_6000_PI426210822

TTTACTTAGGGCAATAGTGGTAGATTAAAATAGCACACGTTTTGCCAAGCTTACAAAATT

A_18_P14133980

CTTCAAAGAGTCCTAAGACAAATGCAGGAGATAATAAGGGGGCAGACTGTTAAGTTT

A_18_P13409265

GGGCTTCCTTTCATATATCACTACAGAGTTGTCAGATGGAAAAAAAAAAATGCACCATGT

A_14_P123817 TACTGCAAATTCATTTCCGTCGGAGAACTGGATTCTGGTTCCAACTATTATGATCATAAG

A_18_P12172336

CCCACAAATCAATGTCATAGCCTCTTTTCTCTTAATGAATATGCCTCCATAGTTTTCTTC

A_16_P16191059

AAGGAGGTATTTTGTTATAATCATGACTGCAAGCATTTCTTTCTCCAAAGTCTTTTTGCA

A_14_P120884 TTACTCTACTGGAAAACCAGTATTAGATTACTCACCTATCATATCATTTCTGATGCTGAG

A_18_P11102566

AAAGAAGTTTACATCCCCATCTTAATGGTAGAAGGCAAACTAAATTGGTTCCAGATTCAT

A_14_P118095 GTCAATGGCTCTCAAAAATTACCTAATAAATGGTCTTGTAACAGCCAGAAGTGTTCTGAT

A_18_P22841632 AAGGAAAAGTGGGACTGGGGAAAAGGCCAGTTATGTGAATCCTCTTAAAATAG

A_14_P139471 AAGTGTCTGCTAGTTATGTGCATGCTTGAATGGAATCCACCTCATAAATTCAGTGTCT

A_14_P106028 CTAATGATGATGGTCAAAGAAAAGATGATCACAATTGAGGAAGCACTTGCTAGGGTAAG

A_14_P200435 CTTGAGGACTTTCCTCACTTAGGTGTATTATTCTAGGAGACTGTCCTTCCTTTTACTG

A_16_P16753917

AGTGGTTCAGATGTCATTAATACTTGTCACACAAACAAAACTTGATGAACTTCATGGCAG

A_14_P103734 GCCTTGGCATCCTCCGTGGAAATGATATTTTTTATTTTAGCACTATGGAGAGAAAGAAAC

A_16_P16763906

ATGTGGTGTATCTCGTAATGTCAATAGTTCAACAGTAACATTCAACTGAACAAGGCCTAA

A_14_P130823 CAACCTGATATTCAGCCTATAACTGGCTCAATACATAGCCTTATTTCCTAAAATCATATT

A_14_P128387 GCTGTGCAGACCAGTTTATAAAATCCAGAGTGGCTGCATGTAGAACATTTTTTTAAATCA

CUST_3300_PI426210822

TAGAAATCTGTGTGTGTACCTCCTTTTATTTTATGCAAATAGTAGTATACTGTGTGCACA

A_14_P110889 TGCCACGTTTGTTTGTTTTTTAATTTTTTTGCAAATCATGGTGGAAACTTAGGGTCTTCA

A_16_P20049786

ATCTTTCTGATCCCAAAACCTATGTTGAATCCTTGCTATTGCATGAATGCTGAAGATAGA

A_14_P105131 CTTACTGTCTCCATGGTCTTGGAACTACTAATTTTCTTGGGAATAAAGAAAAGAGATTGG

A_14_P139054 TGATTGATTGATACGTTAAAGATTATTTCCTCCTTCCTCAGAATGCTACACTTCGCTCAT

A_16_P16507975
TTCTACGATTTGGCATAGTCTATGAAGCAAGTAAACACCAAAATCAACACATTTCCTGTG
CUST_161_PI426210822
GGACAAGAGGTTCAGTTTTTCAAAAGTCTCCATTCCATAAAGCAAATCTACATTTTGCAT
A_16_P01894435
AGTTCCACTGTTAGGTTCAGAAACATCATTTCATAAGGGTATTCAGAAGATAACGTCACT
A_14_P112247 CTTTGTCATGTAAACTCACTAAACCCTCCAAAATGAAGCCATGAAAACATGAACAGAAAA
CUST_10112_PI426210822 AGCACCTTCACTAGGCAAAGAGTAGTAGGTCCCTACTAGGTGGCA
A_14_P135469 GCTGCAACTTCCAGATTTCTCAGAAGAATCATTTGCCAAGAGCAAGTAAATATTTCTTCT
A_14_P138691 ATTACCGAGTAGCATACAGAAGTAAAACCAAGTTTTAAAAAAATGCACCGCAGTTCTGAA
A_14_P116756 CCTGCGGCTTTAACAACTGTAATACAACCTTTTGAATTAGTTCCGTGTATAGATAATTAA
A_14_P111091 CCCAATCACCTAAGTTACTTGTCTCTGAATTGCCTTCAATTTGTCTTCATCTTTCTAGAA
A_16_P20459387
TAGAAACACACTTTGGTCTCTCTCTCTTAAAAATGCAAATACTTCCTGTTACCTTTGGAG
A_14_P131272 CAAAAGCCTTAAGGGGGTTTCTCTCGGCTAAAAATTTTATTGAAAACACAAGATTCATTC
A_16_P15433708
AAATTGATCAACGTGATCCAAACTATGGTTATTTGATTCTATGGAATCCTAACTCACCGC
A_14_P134933 CTCTGCACCTTATTTCCCATCTGTGAAATGCCCTTAAACACCCCTTCTAATTTTAATATT
A_14_P115630 AAAAGATTAGGAACAATCAAATCAACCAGGTATGCATCGTTCTCAGTGTCAAGTTAAACT
A_16_P17704453
CCCCATACCTTAGTCATAATTCTTATTCATTATCCATGCTAGAAACATGAGACTCATCCC
A_14_P131419 AATATGACATTGAAGCCAAATATGAAGTATTTGATGCAACAACTGACTTCAGGACCCTTG
A_14_P122155 TATAGCACACAGGCCAAACTTTAAGAGTAACAGTGTGGCAAATGAACCTATTTACTCTTT
A_16_P36305790
AACACCTACAAACATTTTTAAATCCAAATTTGAGGGCTAGTAGATATAAACGAACCCACA
A_18_P21079106
GGTCCTAGTGGTTATGATTCTGCTAATGATAATGTTTGTCTCTTGCTTATGGATAGCCTT
A_16_P00375940
AACAGAGTAGCAGGTCTAAAACTAGAACTCTTGTCTTAGGAAACAAAACGCATAGAGATG
CUST_10161_PI426210822 CTATGGCCATGTCTTCCCAGGAGGCCCAGGACCTACTTGCTCAAG
A_14_P103058 ACCATTTGATTCAGAGCAGCATAAAAACTCAAGACACACATTTTCATTCAATGCACTTTC
A_16_P17983225
ATCTATAATATGGTTTTTGGAGGATTGCTGAGGGACTCAGTAAGAAACAATAGTAGGGAG
A_14_P107588 CTGAAGGCTGTCACAGGTTGGTGGACTAAATTAATTTGAATTTTACATCTTCCTATTCTG
A_16_P19882372
AAAAAAATCCCTGGTCTACTGTAACATCCTAATTGTAATCTAAGATAAACACAACACGCT
A_16_P36131029
TCAGAGAAATGTTCTTGCATTTGAAATCAAATCAACAAGTTTAATTGGCCAGAGAGTTCC

A_14_P110077 TTTTGCACTGGTTTATGGCGATTGTTTTCTTGGACGGATAGTGTAAAATAAACTTCTCTG

A_16_P16392877

AAGTCAGACAGTATGAGGTAGACTTCAAAACGTATAATACTATTTGTCTTTCTTCCCATT

A_14_P113931 TCCTGCCAAATCTTCAGACCATAAATGTTGACATAGTCCTGTATGTATTCAAAAGAACGA

A_14_P200970 ACACCTAAAATGACATGTTGGTTCCAAATAGTAATTATATTCTAACTGGTGCTGACTTGA

A_14_P134609 GTTCTGTTCCCTGGCTGTCCATCTGAACTGCTTTTCAGGCTCATAT

A_14_P135961

ACAGATGCAGAGATGTAAGAAAAGGAGAAGAAATGAAAAGCAGAAATGGTTTAAGGGAAC

A_16_P20751697

TACGTGACTTCTAACATTTTTCTTATGCCCATTTTTTTTTCACGTAGACGTTCTCAGAGC

A_14_P125020 CAAAACCACACACTGTTCTCATTAGTGTGAAGTGCAAGATTCTCTCTTCTATGCTAATTT

A_14_P132281 GTTACTCAATAGTTGTTTTCCTATAATTAAATATCTGGACACATTGGGCTGCCATCCACT

CUST_9675_PI426210822 CATGGCAAGCAAGATTACTCCAAAAAAATGCCACTTACCATTCCTTTGGTGTA

A_14_P119482 ATGAAAAATCTTGCTCTTTCCATAGTAAAGAAGAATTAAGAGATGGACAGGTAAGCATGC

A_14_P111060 ACAGAATTAAATTGGCATTACTGCTTAGTTCATGACTGGCTTTTATTCTAACAGTGCACC

A_14_P111562 GGGGGTATCCAGAATTGGTTGTAAATACTTTGCATATTGTCTGATTAAACACAAACAGAC

CUST_4159_PI426210822 TTTTGAGAACTTGTCCTCTGGTATCCAGGAAATGGCCTCCCCAGC

A_14_P112704 AGTCCGGAAATAACATAGAAGCCTGAAAGATTGGTGCATGAATTCATTTATTCATTCTGT

A_16_P16669386

AAATGGCCTTACCTCATACACTAAGTCTCTGGTAAATGGGTCTTAAGAACAATTGAGTTA

A_16_P15134568

AATCTTTGAAGTCACTTCTAGATTTACCAATCACTGGATTACAGACTGACAAGGCAGAAT

A_14_P126479 AACAAGCCTAACACTCTTTAACATTTTTTCCACGTTGCATAAAGAACATGGCAAAATACC

A_14_P129443 ATTCTCTGACTCCTACCTTTAGGAGAACGAATAAAGGCATCTTAAGGAGCAGTGATT

A_16_P18845493

TGGAATGTCAGCAAAGATAACACAGTATTCATCTCACCATTACTAAGGAAGAAACTCTGA

A_14_P120299 GACAGATTCCAGAGGCTTGGTTTTATTGTGCAGTTTTCTTTGTCCTCAATGAGAT

CUST_852_PI426210822 AGCATAACACAAGATGCTCTTAAGGGCATCGAGCCTAGAGTCAGAGTTT

A_14_P136146 AGTATTTTCCCACTCACATCTGAAAAAAAAAGGATGTCTCCTCCTTAGGTCTGAATTTTA

A_14_P110252 GGGGAAGAGAATCAAGCCTTTCTTTCTTAACAGTTGTTTATGTCATATTTCTGGTAAGAC

A_14_P107371 CTATTAGAGTGTTATTGGTAGTGTCTAGCCTTTTCCTCCACGACATTCCTTGACTTAATC

A_16_P15526806

AGGCTATTTCATGCCTGATAACTAGAGCATTAATCATCCCGTTAAAGATATGTGTATGCA

A_14_P112173 TTAACAGACATTTCTCAAAATTTGTTCTAATTCCCAAGCATGTGTCAGCAGTCTCTGTAG

A_14_P109816 CTAATGGTTCCAAAACAAAATGATTAAGTATTTGAATCCTGGAGCATCAACACTGCCTTT

A_18_P22841396 GAGATGATACAGTTGCTGTCTCCAGCACACACATGTTCTAGAGAATTCCAGTTTTAG

A_18_P22728513

AAGCCATATTTTTTTTGGTGCTGGAATTTATACACGAAAATACAGGACGATCCCATCAAA

A_16_P00685533

CATCACACAAGTAGGAGTAAAATAATGAGTATTTGAATAATGCAACTAGAGTTGCGATCA

A_16_P15560659

AAAATGCCTTCTTAAAAAGACAAATCCCATATAGCACTGAATTACTCCAGTGACCTTGAG

A_16_P15880822

CTGCTTTGAATGAGTCCAACTTGTGTTATATGCTGCAAAATCTGACCTTAATCTCAAGG

A_16_P20375665

AAATAGTTACTGCTTTTCACACTTGGTTTTCACAGTATCCATCCTTTCAGGTAGACTTTG

A_16_P19530772

AAAATACCAAGATTGCATGATAATGAACAACATCGAAAGAAAACACTTGTCTGGGTCAGA

A_14_P123417 AATAAATGTTGATAACAGCCACTGGAAATTTGAAGCAGTCCTCATTCTTTTATTGTCCAC

A_16_P20610439

CGGTGTTTTTGGTTTCTTATTTTTCAACAGGATTTCTTTATGCGTATATGATCATTCACA

A_14_P122429 GAATTACTAAAGAAGAAATCTTCAGAATCGACCACATGCTCTATTCCACAGTTTGAAATC

A_14_P108124 CTTCAAGCACCCTATGTAGCAAAAGCCATAAACCCAATTTATTTCAGAGTAGTTTTCAGA

A_14_P129687 CATCACTATTTGGTTTTTCTCTTTGGTCTCATTTAGAGCCAAGTTGAGAAATTTCCAGTT

A_14_P107320 TTTCTTGTTCTACCTTCATCGGCCGAGGTAAAAGTGCTGAAACCATGTGAATAAAATA

A_14_P121248

CACACACAAAAAAAAAACCTGTTACGAAATGCCAAACAACAAAATGTGTTCTACAGGTAG

A_14_P107311 CCTAAAATTCTGACAGGAGAGTCAACATGATGGTTAAGGCCTATTTCATATTCAAACTTC

A_14_P129229 AGTTAAATAGGAAGGTCTAGACACAAATAAGGTGAAATGTCATTAACGCAGTCTCAAAGG

A_16_P30952525 CTCCTCTGTCCGTGGGGGCTAACGGAAGGGCACATGGGATGTCTC

A_14_P135609 CTGAACAAGTCACGTGCGTCCTTACCTTTATTATATCATCAAGTTCTGAAAGAGTTAGAC

A_14_P200526 ACCTCTCCATGGTTCATTTTCTCCCTAACTTTTCCTCTTGTTATTTTATGAAAATTTCAA

A_14_P102758 CTAAGGAGAAAATCAGCACTTAGAAACTTGGATTAGATTTCACCCAACCCTTAACAGTAT

A_14_P120121 AAATCAAAAATTCTTTGATGAACTTCGAATGAACTACATCAAGGAACTCGATCGTATCAT

A_16_P15997098

CAGAATTTCAATGACTCAGAAACTATACCACAGTTGATAATTAGGCCAGTTTCTCGAAGG

A_16_P17205046

ATCCACATCAAATGGTTAATGATCTCCAGTTATGTCTCTACAATTGGCATCATCAGTGAT

A_14_P127098 TACCCACAATGTAATGCCATTTAAACGAAACACGAAACTATGTGCAACAATTCTGTATGT

A_14_P108788 ACTTTTCTGGGCATTAAGCATTACCATGGAATAAAGGAACAAATGAAAATTGGCATACGT

A_16_P36230199

GGAAGTGGCACTGTGTTGCAATCATAGTTTCATTGTTCCTTTGTATTTTGCATTTCAAAA

A_16_P17807240

ATTTCCAAATATTTCTCACCTTTACTACCAAGTCCAACAAAAAATGGTTTATGAGCGGCA

A_16_P03045605

AAGAAGATGAACTCAGTCCTACGTAGTAACAAGAATGTGACATCTATGTTGTTGCCAAAA

A_14_P116363 TACTCTAGTATTCCAGACCTGAAGAATCACAATAATTTTCTACCTGGTCTCTCCTTGTTC

CUST_2881_PI426210822 GCTCTGCACAGTTTGGAAGGTCAGTCTTCAGATGGAGGAAAGATG

A_14_P129492 CTTTTGAAACAGGCTTCTTAACGTATTCTTGTGAAATTTCTCATGAGCAGTTCATTTCTC

A_14_P119957 CTGCTGGACATTAACTTTGATATTGCTTCCACATGTATCTTCAAAACATCCTTCTCAGAA

A_16_P30952327
AGGACTCCCAGGAGAGTGCTGATAAATATCTTTGATCATTGATTTCATCCTAACTAGATT

CUST_6998_PI426210822 GACCAAGGCCATGCCTGCATATTCCTCCCTGGACCCCTCATTCAA

A_18_P10073704
AGCATGATTATAGATGACAAGTAAGTGTGCAAGTGAGTTAAAAGACTTACCAACTCTGCC

A_14_P105020 CACTGCTTCGTGTATCCTTAAATGCGTTTTTGTTTTTGTTTTTTTAACCATATGGAGCCA

A_14_P136720 GCCTGCAAATATTATGTGTTTGCCTGCTGCAAAATTGAAGGTGTATTTTTATAGACAAGG

A_16_P37083887
ATTAAGATTGTCAGCAGAAAGTAAGGCGTTGTGACTTCAGATCTCAACAGTTGAGTATTT

A_16_P00000357
TTTGCAAACGAGTTAGAACATACGCATTTGTAAATACAGTGTAAAATGTTTGCTGGTGGG

A_18_P11803289
AAGAACATTCACAATCTTGTCTGAAACTATTTCTCAAGCCTTATCTCTACATTTTCTGCA

A_14_P138895 ACAAAAGGAAAATTGGGTCCCAATCACTGACAACATCTTAAACTACCAGATGCTTTTTTA

CUST_9415_PI426210822 TACATGGAATCATGGAATTTCAGTTCTGCCCAGTCCCTCTCAAGG

A_16_P21430585
ATTCATGTCACAGTAACTATGACCCACTAGTTCCAATTATATCACCCACTCTAACCTTTG

A_14_P100892 GAGCATCAGCACGAATAGACTTTGTAGAGGTAGTTCTCCCAAACTATATTATGAAACCAA

CUST_5708_PI426210822 TCATCAAAGGTTAGACCATGTCCTGAAGTGACCTGTTGAATTTGCAAAACTTC

A_14_P126351 ATGGGCAACACAAATTATATTGGGTGTAACTTGCTTTGCTCATAAAAATCATCATGCATG

A_14_P121370 TTTTACTTAAATCAATGACGTAGTTACCTAGGGAAGACAAAGATGTGTTCAGTCCCTGAA

A_16_P21554172
ACACGACAGTTTTTTTTTAAAGTAATACGTCATAGCCAATCAAAATTCCCAAGCCAGAAA

A_16_P00685558
AACTTGAGGATATAAACCTTCACCTTATTTATTTTCAGACATATATAGTAAATCCTGGAA

A_14_P131837 ACAGAAAGTGGAAGAGAAAAAACTACTTGGCTCTACAGTCCTATTGTAAAATGTCTTTGC

CUST_4867_PI426210822 GAGATAGATCCTTCCATTCATCAAGTGAATTTTAGAAGCCACACCTTTGGGTG

CUST_830_PI426210822 CTTCATTTGGGTCACATGCTGCAAAGCTGAGGGTTTCGTGTGTATTTT

A_16_P01916101
CTTCGGCGACTTAATCTTTATCGACCTCTTTTAAATGGCAAAAATTCTGATCAACTTCTC

CUST_5267_PI426210822 CTTTCTTCGGGTCATAGAGTGAGGGTGGGAATCAAGTCACTCAGA

A_14_P124790 AAACCTATCCTTGTTTTTATCCAGTCCCTGACAAGAATTTACAAAAATACCGACTTGTGA

A_14_P124312 GTATCCAGATGTTGATCACCACTGTAGGAGCCTTTGCCGCTTTTAGTTTAATGA

A_14_P131488 CTGCAATGAAACCTATAAACTTTAACATACCTGGCTTTTATGGGGAAGTTCTGTGAAATG

A_16_P16067575
AAACACTGTTAGAAGTCCCACTACACAGTTCTAATCCAATTGATCTATGTGCTGTCTTTT
A_14_P127263 GATAGTCCTTAAGGTTAAGAATGCATAATGAAAGCCAATCAGTAACCCATAGAGACCAGT
A_16_P17829857
AAAAATCAATCACTTGAAAGCATACGTCTCTGATTTCTATCCCTTGTCTTATAGGCAGGT
CUST_4619_PI426210822
GTAAAGCATAAGGAGAGGACTCAACAGTAAGGACTAATATTTGACAGTGAGGAGCA
A_14_P130609 CTTCATCGTCTTCTAGTGGTGAGTCTCCCGATCCTACAATAAAAAAGAACAGAAACT
A_18_P14131885 CCACACTATGTTTTCGTGAAGGCATAAGACCATGGGCCAACTTTCTG
A_16_P20362763
AAAATCCCATCTCCCTACAAGAATAAATGCTAACCTGCTACGAAAATTCTCAAGAACTCT
A_16_P02250159
ACACCATTTTGTGATTCAACTGCTTCTCTTAAGACAACCACATCAACTTTATCAACTCAA
CUST_3102_PI426210822 TTGGAAGCATGTGCCTCTGGGTCTGCAGTGATGCTGGATCACACT
A_14_P107118 AGGTAGCATTACCTTAGGCATCCAGTGACGTTGTAGAATATGTCAAAATCGAGCAAA
A_16_P35884749
GGAACATAAAATCCATCCAATATACAGTTGTATCACCTGAAAGTCTACATTCACACTTCC
A_14_P125245 GTAGTTACAACGTTTTCCCATTCTATCATCTTTCTCTCCTTACACACTTCTTTGGCATAC
A_14_P137755 TCATGACAATTGCTATTTGGGTTAAGTTACTGCTACATCTTTAATATTCCCTTGGGGTGA
A_16_P16492392
AAATAACAGTGCTAAAGAAGGCTTACAGATCCTTACTTAGAAAACTGCTCACCTAACAGG
A_14_P124124
AGAACTCAACTCACTTTAAAATGGAAACACGCAACAAAAAACAGATGCTGCTTTCTAGAA
A_16_P18996951
ACAGTTGAACTAGAAACACAATCTTATGCCTACAACTTGAAATATATGAAGAGCCGACCC
A_14_P106781 GCTCTCTCGTTCTTTTTTTGCTCCAGAGTAAATTTTAAAATGAAGAAGGGCTGGAATAAG
A_14_P113064 AAGTCAGCATGTATGAGAACACCAATCTATAAGAGTGTTCAACAAAGGAAGGCATTGTTA
A_18_P14602078
CCGCATTGAAAACTAGTAACTATCACCCCTGAAATACATAAACAAAACACTGATTTGTCC
A_16_P38036102
AACACCATTCTAGAAAATTAGAAATCCATCAGTATTACACACAAAGTTACCAACTGATCA
A_14_P124136 GGCTACATGTCTTTGTCTCAATGCTGGCTCCTGTTGAGTTATAGATTCTTTTCCTA
A_14_P100182 CTCATTTTACATTAACCATACACCCTTGTTCTCTGTTCCTGCTATATCAAATTGGACAGT
A_14_P131264 GTGCTGCTGTGTAACAGATTGCTGCTGACTTTGGAAGGCTTATATCTGTAACGATAATTT
CUST_9498_PI426210822
ATTTTGAGCACTATGGTGATATTTTCATTATCAAGAGTTCTTGGGAAGAGGCCGAG
A_16_P17367199
AATTTTTTGTTCCAGCACACATAGTTGAAATCAGAAGGATAATTGCATGCACAGTGAGAA

A_16_P18311605
ATTTGACTTTACCTTGACCCAAGAGTAAATAACTGAACACCTATCTGGCATCTCATGAAT
CUST_4723_PI426210822 GTGACCAGTTAAGCCCAGTGGGTGATGCCTTTCCTTTGTTACTTT
A_14_P137604 AAGCAAAATTGGGATACTGTTATAGAGGATAATGGAAGGTCAACAGCAGAAAAACAATGG
A_14_P201192 TCTTACTCATTATTCCAGGGATATTCTGTGCATTTACAGCCATAGACTTATGCATTCCTA
A_16_P21118317 TCATGGTGTCATCCACACTATCCTTTGTAAACAGAGGTTCACACAGATACCATAC
A_14_P127970 TTGATGAATTTCACCTATTCCATTCTTCACGTTTCAGAATTGGTCGACTTTGTTAGAAGA
A_14_P111769 GTTTTTTAATGGTGAACCTTTCTGAGGATCATAGACGTTGTGTCTTACCTACCTTAAACT
A_16_P15820551
ACATTAGGACTCAATATTGGGAAGGCAAAAAGCAAAGTTATCAAGAGATTTAACCACTTA
A_16_P20316184
AACACGTTCTTCACATTTTTCATTCAAATTCCAGGTCACTGAATCTAAACAACCCACTTT
A_14_P109352 CAAAGGGAGATATGATAAAGTTTGTTATCAGGAAGGATAAGTGTCACTTCAATGAGATCC
A_14_P135694 GACATAAGACTAAATTACACCTTAGACCTGACATCTGGCTATCACACAGACAGAATCCAA
A_16_P16398415
TGCTGTGCTCTTTATAATCCTCCCATTTTTGCTAATTATGAAAGATATTTGTGCTGAGGG
A_14_P101329 ACAACCAAAAGGTTCAATAAGAAGACCTTTGTTTTCTCTTCTGAAAGTCTAAGACAACAC
A_16_P18162366
AATTTTTGGAAGATGTCAGGAGAACTTCTCAAACTTGGAAGAAATTGCTACACTCTACTC
A_16_P19243906
GCTTAAAGCCATCAATATAGCAATTACACTTGAACACTGAAATTATGTGGTTATGCCTGG
A_14_P137848 AAAATATAAATGTTTTCCCTTTGAAGATTTGTTTTCTTTCCTGCCGTCCTGACAGATCAA
CUST_9589_PI426210822
TCTTCATTTTGATTGTATTGCTTTCTTGTCTTCAGGAGGGAAGATTTTGACTTCAAAAGT
A_14_P125849 GAGAACACAGTGCCCAGATTATTTATCATTTTTGAATTTGGGAATTGTGATTTCCTCCAA
A_16_P15802982
TGATAGATTTCACTGCATAACATACAGAGTATACACTGAGGACAAAATACCAACTGGAGG
A_14_P200056 CTGAGGTTCATTTTCCCATTCCTCAAGGTAAGGGTAGACTACCTAGGAACTTATT
A_14_P117505 AGACAGACAGAAATGTGGCCTTCCCTAGAGAGAGAGACAGACGTTTTT
CUST_1174_PI426210822 GTGTGGAGAGAGAATTTCCTGCCAGTGTGTACTGCTGGAGCCCGT
A_16_P16191537
TGATGAGCAAGTGGCAAATATTCAGTATATAGTGCAGATTGAATATGTTTCTTTTATGGG
A_16_P16191461
TTCCTTAATTTAATGAAATGTATCAAGCAGAAACCCACAATAAGCATCATACTTAAAGGA
A_14_P123290 AAATGAAACTTTCCTTTAAGGAACAAGTGCATTTTTAATAGATTCTGGAACTCGGCTTGC
A_14_P132865 GAAGCAAAATGAAAAATAAGCATTCAATCTTATCTCACCATCTCGCCATAGGTGGCTAAA
A_14_P201293 ACATGTTATGTCATGACTGTCATGATAAAGTCTTGCCTTTTAGACATGAACCTGATTCAG
A_14_P111445 GTATTCACAGGTTTTAAGTGTATCAAGGCAAGTTAAGCAGTGTTCAAGTGAGAATCATCT

A_16_P16759714
TTTTAGTAAAGTTAGTCACAAATCAAATACTCGCTCACGTTACACTAGTTTTCAGCCCGT
A_14_P135384 CTCTCCAGTGCTCTTCATAACCATTTCCATATATCTGTGGTTTACATAGTTGCCATTTTT
CUST_4853_PI426210822 TAAGACATCATCAAGGTTCCTCCAACCCCTGCTCCTCATCACACT
CUST_4890_PI426210822 CTTCCATGAAAGTTCTAAGGCTCATGGGTGAGGCCTAAGAAAGGC
A_16_P03652122
AATGCTACTTTTGTCTCATTTTTGGAGTTATAAAACCATGTGGATTAATCCCAACCAGGT
A_16_P21631065
AAAAAAGATTTTACTTTCATTGAGTCCTTCACCAATCCTGTAAGAATGACTTGGCAAGGA
A_14_P127472 AAGAAGAAATTATTCATTTTAAGAAAGAACTTCGAGAACCACAATTTCGGGATGCTGAGG
A_16_P58424324
CTCAGTGGAAGTATTAGAAGACAATGGTAAATAATTTCCCAATGGAAGTGCTAAAAGACA
A_14_P107730 GTGATGTTATAAATAAAGTTGTCTTCAGTTTAGATGAGCCTTGGTAGGTAGTCTCGTGCA
CUST_2216_PI426210822
GGATTTTCTGAACCATGACTCCCTCTATCAAGATAACTCATATATGCACTTTGCAAAACA
A_16_P35407491
TCCACTTATTCCAATCACTCATAGTTGCATAAGGAAAGAAGACTTACATTAGGCTTTGCT
A_14_P119476 GTATGAAAATACACTGGTCATCTTAGTGGTGTCTTGTGGGAGAGAAAACCTAAAGTATAT
CUST_3156_PI426210822 ATTTCTTCACTCAAGCAAAACCTGGCTGCCAAGAAGAGAGATTGAGATGAT
A_14_P102320 TGATGGAGTTACTAATGCTTTGGAGACAGTCCCTAGGAATTTTTTTAAAATGTAGTGGTC
A_14_P133479 CTTAGTGGGAGAAAAATAACAAAATCACTTTCCACTTCCTTTCATGCTCTGATGATGTTT
A_16_P03096692
ACACTCTCCTTTATAATGCCGATTCTCCAACATTTTAAACCTATGTCCCATTTTGATCAA
A_14_P120421 TTTCTTAAACTGAATTTCTTAGCAGACACGCTGGGATGTTCAACCATAACACACGTTT
A_14_P100245 TGGCTTTACTTACTAGGAAGCCTGGAATTCATTATTTTTTTCCTTTTATGTGCCACTGTG
A_14_P103709 CTCATGAGCAGGGAAAACCCTGCCTATTCGATTGTTGTCTTAAAACTCTTTATTTATTGC
A_16_P01768247
ACTCTCAGCAATTCTCATACTGTTCCTCCAAAACTAAAAAGGGTAATTTCTCCTAATTGG
A_16_P16149948
ATGGACTCACAAGATCCTATCATTCCTACAGACTTCTCAAAGCAAAACAATTCCCTATTA
CUST_9406_PI426210822 CTTGGAAACCACAGCACACATCTTGCGTGTGTTGATTACTGGCAAA
CUST_8955_PI426210822 TCCTCATTACCTTCAAATGGGGTGATGCACACACGAGTAGCATGAAATTA
A_14_P106783
AACAAAGAAAAATTCTGAATAATATGTTGTGGGACAAGAGGAAGATGCCAGTGAAGAAAG
A_16_P41327510
ACTCTACTCAATTCTAAGACACAATTATGCCTAGTTCGTTATAATCATAGAAAAACCCCA
A_16_P37544735
TGACTTGGTTATAAATGATTGCACTTTTCTACTATTTCTAACTACAGTATAGCAGTGCTG

A_14_P201392 CTCGTTTTAAATTCATGTTGATTCTTGTAAGCACTGGACTGTCTTCATCAAGTATTTCCC

CUST_611_PI426210822 GCTGCTTTCTGCCCAGTAAGGGATGCGGACAATTCCTGGGGTTTC

A_16_P55127820

ACGCAAAAATAGGAAGCAGTGAGCCAAACTCACTTTGAAAACAGATACAAAAGTTCTAAA

A_18_P15831421

AAAGAGTGAAAAATACAAACATTGGTTAGTAGTCTCCACTTTACAACTCTTGTCTCACCC

A_14_P200986 GTAAGACTATATTAAGTGCTGTTGAAAGAGAAACCCTAAGTTGTGCAGAATAGTCCCTTC

A_14_P137569 TCACTGACCTGGAAGAAAAGAGTTCCACTCATGAATTCTAAGGAAATTTATCCTCAAAAT

A_16_P35529400

ACGATCTAATGAACAGAATAACTTTGACCAAATTTTCCAGTTACTTTTTCCTCCCTTCCT

A_14_P112798 GGGGGGTGTTAATTTATGCACTTATAAGGTGTTTTCTGTGTAACCATTTTATAAAGTGCT

A_14_P109110 CCATTCAGTAGTATCATACCCCTTAGAGATTTCAACTTGGAAAACTGAATAATCAGCCAT

A_14_P101788 TGACTTGATGACTTCACAAGTTGACCATATGTTGTAGAACATAAACACGGGCAC

CUST_1214_PI426210822

AGCACTATAAAACATTAATTACTATTAACCAAGCTATGATATTTTAGGACTCCGAAATGT

A_14_P109782 CTCTGGCTTAGATCTACAGGATATTTACTGGCTATGTGGATTATCTCTAATCTCATCCTG

CUST_10385_PI426210822 GAATGTCAGGTCCCTGGTTTTTTTCCCCACCGGAATTAATGGTAATG

A_18_P18462460

CTGTTGACTTTGGCTTATGATTCTATACAGCACAACCAAACTATACTAAAACCTACACCA

A_14_P105608 GAAAATTCTTAAATGAAGATGTCTTCTTTCAGTCTGGCCGGGACCTTCAACAGTATC

A_16_P15915606

AAATGTGTTTGACATGTTTAAGTTGATGCAATTGGTATATCTGAAATAATTCCAGCCTGT

A_14_P107572 TTTTGGTGCAGTCCCTATCAGGGAAAGTTAAGAGGGACAATAATCTTCTCAAATAATGTA

A_16_P58424148 CTTATCTTCTTGGCTTTTAGCATAATGGCCTGCAGATGTCTGTGGGATAACTTAAGC

A_14_P117929 AGGCAATGCAACAATAATTGCAAGTTATTTAGTTAACATCCCAATCACTCTTCCAAGCAT

A_14_P133620 AACTCTTTGGACAGAATGTTTAAGAACCTCCTACAGCCTGATGCTTAACCCTTT

A_16_P15085755

AAAGTCTGCAGACTAATTAACCCCATTGTTCAGAATTAATCACCTCAGGAATATTTGGAG

A_14_P131204 GCGATGCCCAGTCATTCTTAGATAGAAAATGAAGATCACATGAAATTTGGAAAGAAGTTT

A_14_P138367 CTAGTTTTTCTTCCCTAGGGAAAATGTCATCCCACTTCAAATATCAACAACTTTGACATT

A_14_P139024 AGAAACATTTACAAAATGCACAGGGGAGTTGGGATCCCCGAACATAAAATTTTATAGTAA

A_14_P138914 CTTTTAATAGTTCATGGATCCTCACTCTCCTTGGTTTCCAGCACATCGTCAGTTTAT

CUST_5876_PI426210822 CATTTGGCTCCCCATTTGAGTGGTCACTCTGTCTCTCAGAGCAAT

A_14_P116339 TCTATGGTGTATTTGTAGGGATTTTGCAGATACCTGGAAAAACAATTCAGTGACTTAAAG

A_14_P125234 CAACAGTCAAAGAAGGGAACTTCCCGGGAAGATCAGGTAACTTCAAAAA

A_16_P18292471

GAGCATAACAACAATAAACACATCTAAAGACCAATTGTGAATTGTGAATCACCAACTGGG

A_14_P106296 CTCCATAATAGTGATAATGAGGCAGGAACCTTGAGATAAAAAGCAGATATTACAGGTTCC

A_14_P120346 GTCACAGAAGAAGTGGTAGCGTGAGCAAGTGTGCAAACATCCTATTTGTAAAC

A_14_P120948 GATGTGATTCAGGACACTTTCCATCCTAGGAATAAAGGGTAGTGCAATCATCAAGT

A_14_P118832 TTTCAGATGACCTTCAGCAAAATCCATTTTATTTTCTTGTCTTTCTGGAGTGTGACTTTG

CUST_4655_PI426210822 TTGCCCCATGTGTGGCAGCAGAAGGCAATCCAGCCCTCAGCCTGG

A_16_P16227360
AATTGATCAAAAGTAACTTACCCTAGCATCCATATCTTCTCTTTCCACTCTGAAAGGCTT

A_14_P119008 GTTTTTCTTTTAAATATATCAGTGTGTTAGTTACAGGTCATCCCCAACTCATGACAACCC

A_14_P125727 CTATTTAGGGAATGAAAATGTTGGTTGAGAGAGGAAATTGCAGATTATCCCATTTCCATT

A_14_P126274 AGAATCTGTGTTTCCTTGTGTTGAAAATCCCTGCACTTTTAAAACTGGAAAAAGTCTTAG

A_14_P200866 CTCACTTAGGAAGGTATTTCAGAAGGTTCAGGATATTTTGAGACTGGTATATGGCAATTT

A_16_P17374075
AAAAAAAACCTCTAGATTTCACACGAACCATTTTGAATACAAAGTTTGCTATGTTGGGGC

A_14_P117974 GAGTCAGTGGTAAATAGCAGCAAACGATATAAAATACATTTCAGAGGTGGAACTGATAGG

A_16_P18531358
TTCCCAGTCTATTTAAACAGCCTCTTTTCCTAATTTTCCTAAAGCATCACTTACTCATGG

A_14_P200651 CTTAATCCATTTCCGTATTCCAATCCTCTCCTTTCCCAAATGAGTCCCC

A_14_P110636 TTGTTGTTGGATATTTTTGTTTCCCATAAAAGCACATCATTTCAACCCTAAACCTGTGTG

A_14_P105201 CTGATGAAACTTACCAAGTGATATGTCTGGGCCTTTAAAACGTTGAGATCAATAAAGTTT

A_14_P103189 AAATGTTCTGAGGGAATCAGACACTGTAATTGCTCACACTGACACTACTACAGAAAAATA

A_14_P136690 GATGTGACATCCTCAAAATAACACTCTTCAAACAGGGAATCCTCAAAGGACACT

A_14_P137501 AAGATTTCCTTTGCTCTTCTCTCTTTTGCAATAGCATATGCTTAAGCACAAGCAAGCATA

A_16_P17615426
CGGAACCCTTAGATATCAATGTCAGAACATACTCTCCTTGAAGACATTAGACTCAGTTTT

A_14_P113038 CCATCGGCAGTGATGCTCACACAACAATGGTCTACAAATAACGTTTGCAATAAAT

A_16_P03775374
ACCACAACAACCTCAGATTACATTTTAATCTACTATCTATAGATCAATGGTAGCAGTGCA

A_14_P106139 ATGTGAGTAATTTCTTCAGGTTTTCCATGACCATGTCAGTAGGCTGATGTTGTTTATATC

A_14_P128442 AGCCAAATGAACATTAATGTCAAAAAAGTTTTTGTAAAAAAATCTGGCCTTGCACCTGCA

A_14_P136669 GTTTTCCATGTTGGTTTTCTTCATATTACCCTAGTTCTGCAGTAAGACAGATATCACCTT

A_16_P00685556
CTTTGGAGTGAAAAAGTGATGGATATTAAGAGCACAACTACACACATTCGGATTCTCAAA

A_14_P104263 AAAAGATGCCTTTTGAAAACTTCTTCTTTACCCTGAAATTAGTTTTCCTTTGCCTTAGCC

A_18_P15961033
CCAAGTTTTAAAAAGATGAATGTGGTATAATCAGCATTAAAGTTTCCTGTTCGGTCTGGG

A_14_P125487 TCAAGATCTTGACCAGCCTGAACAAATGTTAAAGACTTGTAGGATACAATCATTAGACCA

A_16_P36863139
GCACTACATAAAAAAACGATGCTAGTTTGTTAACACCACAATTTGACTTTCAGCAAATGC

A_14_P109039 ACACATTTCAAAGTTCCTCACATGAATACTAACATATCCTGTTCACTTTGCAAGTTCCTT

A_14_P100586 GAGTTTATGCATTTTGAAGGTCCTGCTGAGTTGTATCCGTTTTGCTTTTATCTCATTAAC

A_14_P108085 ATTGATGAAGCACATAGGTTAAAAAATAAAAATTGTAAACTCTTAGAGGGCCTGAAACTC

A_14_P138398 TCATCTTTCTTATAAACAACCTCATGCACATTCTGTGTTTGAGCCAAGACTAGTCACC

A_14_P126828 AATTTCACTCACCACTAATTCCTTGACTTGGAAAGATTTTTCTTTTCACAGAAACAAGGT

A_18_P11153110AATGGATTGTAGGGAAAGCAAATGAAAAACACCACTGAACATCATTGAACAAGTCCTTT

A_14_P117943 CTTCGCAATGAGAAAAGCCACTCCAAACATCTACCAGTTACTTTTCTTGATGATTAGTAA

A_14_P133432 GTAAAAGAACTGAATACAGTTGGTAATAGAGTGGAATACAATGAACAAATGGCCAAGCCA

A_14_P111474 CAAATTCCAGCAGATGCTAACTTCCACTCTGCGTCTTTCTCTGACACA

A_14_P129017 TCTATAGTAAATATGTATTCTTTCCAAAATCAGACAAATAATAAACACTATAAATTGTTT

CUST_9740_PI426210822 ACAGATGTGCAGTCAAGGGGAGAATGAAGACTCCTCGGGATTCTC

A_18_P14133161 ACATGTGAGTTGCCCCTTCACACCACTCCCTAACACTGCCCTCAC

A_14_P107276 ACATTATCAGGTTCAAGTTCTTACTCAAGTCCATTTTTCAAGGCCCTGTAGACTTCTTC

A_14_P120324 GGCTGAGATCAAAAGAGAAGTTTCCTCAATTCCAAAACAGGTTTGGACTTGATCT

A_14_P120914 GTTTTGGTAACAGAGGAATGGACCTCAAAGTATTATTTCTTTGGCATATTTCATCTGCTC

CUST_2491_PI426210822

CCCCTACTCTTGAAATAAAATGATGGAGAAAGATTTGGAGTTAAAAGAAAACCAAGTCCT

A_14_P134219 AAAAGTGGTTTAACTCAGCCACACAGTAATCAAAAAGACAGGTGTAAATTAGCAGAACTT

A_16_P03522345

CACATAGCATTTGCCTGATTTCTTGTATGTTGCATACACGCTGGATGCTGATTTAGAAG

A_16_P03808684

GCGAAATGTGGCATATCTTAATATTTGCACTTAAGGAAAGATGCTTTCAATGCCAACTGT

CUST_2733_PI426210822 TGATTTACTCTTTGTTTTTGTGGGATGTCCTTGGGGAGCCCACAG

A_16_P19652600

ACTCCTAGAAAAACAAGATGCTCTGATCAACAAATACATGTATAAAATCTGTTTGGTGCT

A_16_P16191311 CCACCTCCCGGACATACAGACTCCAGATGTGGAAAAAGCCCTTGC

CUST_4177_PI426210822

AAAAGGTTAACTTCTTCCCAGAGAGCTTTTACATAATTATCCATTTAATGTGTTTTTAAT

A_18_P22840124 TATATGGGTGCTGGGCAAAGAGGAGCATCTGACACCATCTGCTTT

A_14_P102859 AGAAGACCAGAAACAGGCCAAAGTTTATGGAAATTTAATATATGATCAAAGGGGCATTCA

A_16_P41571022

ACTAGATCCAAACGCCAGATTAACAATTCTTGCAGTAAATATCATCCACTAATAGTCCCA

A_14_P101070 CTACCTACCTAAGCGATGAAATCCAAAGGTGAATCTTTTGGTTGGTGATTTTGATGATAG

A_14_P101774 ACTGTTTTATTTTATGCTTTCAGATATCAACATGGCAGGAGAGCCCAAACCATACAGAC

A_14_P122315 CTACCTAGTGTGTGTTGCATTATTAACCCCTAGTTTGTAGTGTATTCTGGAGTCAAGTTG

A_14_P108484 ACGTGTTTTCTCTTGTTCCTATCCTTCATTCCCGATTATTGCTTCCTCTGATTCT

A_18_P14135060 AGCCCAGATGACCCCATGAGGACCCCTTTCTGTTCTCCATCATTC

A_14_P122150 AATATCAACAGAATGACCCAGTTCTCTGTCTTGTTAAATATCAGAGCAAATCTCCACATC

A_14_P130397 CTTTTTTGGTTTTTCTGTCTTATTCCTCTTGTGCTCAATCCTGTTGACCTTCTTAAACAT

A_14_P117672 GAGTGTGAAACAGATGAACAAAAACAGGGCTCTGATGAGAACATGTCAGAATG

A_14_P118307 AGGCTTTTTTTCTGGAAATTCCTAAACCAAGGCTAAGAACCACTGTTCTAAAAGAAGTAA

A_14_P201031 TTTCCTCCTGGTATGGAGGTGACAAACGTGAACTTTACATAAAGAAAAAGAAACATTTGA

A_14_P133317 GAAGCCAAAAGAGATGTCTCAACATTTCCAGAGGTATAACCAAACTGCTAACTCATTTTA

A_14_P130066 ATGGGTGTCAATGAAAATGACTATAACCCTGGCTCCATGAACATTGTGAGGTAAT

A_16_P01717246

AAACCCTACGCCATCTTTTAAAATGTTAAAATTCAAGAGTTTGGAGTGTTTTTCACAGCG

A_14_P101179 CTCACCTGGCTATACCTTGACCATAACCATATCAGCAATATTGACGAAAATGCTTTTAAT

A_14_P112097 CTATTTTTGCACCACACATTTGTATACATGCACAGTGTATCAGGGACATAAATGATCCTT

A_14_P135153 AATAGGGTGTATTTCATTAGATCTAATATTGACAGCACTGCTCTAGCATGATGGACATGC

A_14_P100198 ACATTCCAAATGACTTAGGTTTCAAGCACAGAACTTCAGACACTGTGAAAGATAACATTT

A_14_P102092 TAAAAATTTTTCTATAATCTTACCTCCCAGTCCAAATAATCACAGACATCTTCAAAGTTA

A_14_P127557 AGGATTTTCCAACAGGATGACACTCTTAGGATATTCTCACTTTCTTCTAGAAATGGTAGA

A_16_P17802024

GTCCGGTTATTTTCCATTTCAGTTACTTCCAAGACTCTTCGATATGCACTTACATACTTC

A_16_P17478739

CCGTGGAAAGAAATTAGATACCAATGCTAAAGACTTACCAGATGGATATTCAATCAGTGC

A_14_P137565 GTTGATGACAGAGTCCTTGAATTTGATATGCAGCCGGTAGTTAGAGATGGCAAT

CUST_8624_PI426210822 AGTATCTGCTGTGCTGGGAGCTACACCCGGTTCTCCATTCTGTTT

A_14_P132859 AGGACGTATAGACAACTATTTTGACCTTAGAGTTCTTCAGTCATACCTGAAGCAGTTTTT

CUST_829_PI426210822 AGTAATACTAGCTGCCGCTTATGGAGGGCTTCATTTGGGTCACATG

A_16_P36283190

AAGTGTTCCTTTAATTTAAATCAAACACCCCATCTGATAATCACAGTCACTTAGCCTGAC

A_14_P102653 AAGAACTTTGGGTCATGCATCTAATTCCCTTCTAACTTCTAAAATGGAAAGTAGTTTCCT

A_16_P16792495

ACATGGGGTGAATTTCTTAAAAGGAGTGTATAGGCTAAATACAACCATTGTTCTGAGTTC

A_14_P136040 TCCCTCTTTTGTAAAAGTCAATGCCCTCAAGGAATTTATTTCCTGAAAACTTATGGCTAA

A_18_P16730490

AAGTGACGTTGGGCTAATAATCATCCTGCATTGACATCAGTATCTGAACATGTGCTTTAT

A_14_P133055 ACGTGTTTTCCATCCCACATAAATACAGTATTCCCACTCTTATCTTACAACTCCCC

CUST_1579_PI426210822 GAGACACCTCCCTTTAGATGGGAGGAGAAGCTAGATTGATGGGAG

A_16_P41564467

TATCCAGTATGAATTGTAGGTTCCAAATGCATTGAATCATATCATCATGGTGGGTGTAGT

A_14_P111948 GCTTCAGTCAGATGGAAAATTTGTTTTTTGCTTCTAGATGTCCTGTGTTGAAGGTTATTT

A_18_P12084771 CTTATGTGCCAATCCAACTCTTATTCCCATGTATGGTCTTTCATTTAGCCAAGTGTAAG

CUST_529_PI426210822 CTCTTTTGCACCAGGATGGGTCCTAGAGGGTGGATGTTCCAGATT

CUST_9828_PI426210822 CCGGTCTCACAGAGGAGAGGCCTCTAACTGGCTAGAATGTAAATATG

A_14_P138721 TGAGCGGATAGAGAGTTTAAAATCTAATTGAGGAGATGAGCAACCAATATGTGGGTAATT

A_16_P17328859
ACCTTCTGCTTTCACTAAAACAGTTCTCAAGTATTACAGTGTAGTTGCTTTCAAGAACGT
A_14_P104540 GACACTAATACCCTTTGACTCAGACATGTTAACTATGATCTGAGTTGGAACGATATTTGG
A_14_P117828 AAAACTGTATACCCCAGCCTTGTATTAACACTTTTTTGTCTAATCTAGGGGAAATTGCAT
A_14_P201328 ACAATATTCCCTATTGCCTAAAACCTCTGAACAAGACAATGTTTCAATTGCTCTGTCAAA
A_14_P105951 TACTGTAAGCCATAAGATACCTGTTTAGGGAAGAAGTCACTGTCCTAAAAATCAGAATGC
A_16_P03312583
AAGACATAATTCAGACTTGGCTCTTATTCTCAAGAAGTGGACTCTAGACTTTTAGAAGCC
A_16_P36230478
TGGATATGCAGTTATAAATGATTTCTTGTTAAGCATTTTTAAAAGGTTAACTTCTTCCCA
CUST_6328_PI426210822
AGTGAACCTGATTTGTTCCAGATGTCCTAAGATTTAGTGATTCCGGAAACTACTC
A_16_P01150490
AAGGCTGCCTAACTAATAGACACTTAAATCCAGATGAAGATTAACAAGTCCTCAGAAGAG
A_14_P200024 GCCCTCCAAACTACTTACAGGAATAACTGCGTAGACTGTATCTTTTGCTGCAAAATATTG
A_14_P125529 CTTTCCAGTGGTAAGAGACAGAATTGATTAGGGAAATGAGGCAGATTCACTAAACTC
A_16_P17919489
AAATAGCCTTTTATCCCTGTTTACAAGAAGTTCAAATACCCTTCGTGATATCTCTCTTCC
A_16_P00334902
TGGGCCTTATACTTCTTTACTTTTCAGTTTCACTTCTACCATAATTTTCACCTTCTACCA
A_16_P19076182
AAAGCAAGAGACTGTAGATTGACTCAATAATTCAGGCTAATATAATCCGGTCATCTCCAC
A_14_P102860 TACCTGACCATAAACATGTCTGCATTTGAATTCTCTAAACCCTAAATCTGTGTCTATGAA
A_16_P21495252
AACTGGAGAACAAAGTTCTACCTATGGAAGAGAACAGAAATATTTATGAAGGCCAACACT
A_14_P110374 TTCAGGTTAAATTAAACCAAGGAGTCTGATTGCAGGAAGGGAAGAGCATGTAAGAAATAA
A_14_P138080 CTGAGTCTCCCACCCAAGATTGGTTATAAGGACCTCAGGCCATTA
A_14_P124622 CGTAAATTTAAAAGTCAAATATGTTTAGCACCCACTCAAGGAGATACTCCCACTAAACAA
CUST_7076_PI426210822
TAAAATATTGACATGCTAAGTTTTTAGTCTTCTCTTTTTTGACCTATAAAAAAGGTCAAT
A_14_P112940 TGGAAGTTAACATACTTCATTAAACCTTTCACAGTTCTTGAAGATCAAACGGACATCGG
A_14_P202258 TTGATAGTCCTTCTTATCTTTCCAAGGGTTTAGTATTCAGTGTTCTGTTCACTTGCTGAA
A_14_P116732 CAAAACTGATTTCCAATCACAGCACAGATCCTTTAATGGTAAACAAAAGTGAACACCATT
A_14_P103301 CTCCACAGTTCTGGTTGGTTTGTCAAGGACGTTTTTAATCTCCATGGTAAAC
A_14_P108210 AATGATACCTTTTCATGTTCATTTGTTTAGATATGCCACACTTCTGCCTGCTGTTGG
A_14_P112197 ACTGAATCTCTTCTTTCATTGTGATTTCTGACATTATAAGGTGGTTTGTGCAACACATTG
A_18_P22842130
TATTATTTCCATTGATATCTGAACTCAGTATAGAGATGGGACCATAAGCGATTTCCCAGG

A_14_P123098 ACTCTAAAGCAACTCATCCATACTGTCTTCAATTGATATATTAGTGAGGGAAGCAACTGA

A_14_P138229 CTGAAAGTGGTCACAGCCGCATCTTTGAAGGTTAGCATAACATTTTGATATGCTTT

A_14_P104904 TTCAACTGAGCAACCCAGAGATTGCCAGACTTGTGAGTTATGGTTTTAAAAATATTTCTT

A_14_P129235 CAGTTGCACATGCCCTACTTTTGCCCATTCTTTGCAAATCCCCTAAG

A_14_P200298 ACCTGTTTTCTGTGCCACATTTTAAATAATCGTGTCTCTAAAAACAGGGTAGAAAAGGAA

A_14_P115815 TAACCTGTAAAAGTTATTGGTTAGAGTCCTTAACCAGAACAACAGACACTATGATACCCT

A_14_P113490 GAAAGGCCCCTTCATGTTATTTTTGTTGGAAACAAATCTATGGGTTTGGAAGTTTTATAG

CUST_605_PI426210822 CTTTCAGGTCCCATGGCTCCATTGGGCAAGCATTGGTCCCTAGTG

A_14_P137237 TAAATGTGTTTGACACATCATAGTTTACAAATCACCTTCACTCATATTCTCTCACTACTC

A_16_P18113521

AATGTATCCTCATTCCAGAAAAATTAGAAAACAGACCAGCAATTTAGGAACTGTGTAGGC

A_14_P136420 TCAGTTTCCCTTCTCTTTTGTCCATAACCCGGAGTCTCCTTATTTTCGAG

A_16_P19862896

AACAATATGGGGATCTTCAATGACATATGCAAACATGAATTCATGTATAAGGGTGTTGGG

A_14_P122214 AGTTGGCTCTGTTAGGCTGATAGCAATCTGTTTAGTTGAAAAGACATCCATTTTATCAAC

CUST_7969_PI426210822

AAGAAATTAAGTCACTTTGGTATTGGTATGGAGACAGATTTTCTAAGTTCAAATAGTATA

A_14_P103470 CTTAACTGCTCCTCTAGATATTACAGAGTTGAAAACTCTGAAGGGGTTGGCACT

A_14_P134661 TCCCCCATGTCCCTTTTTGACTCAACTTATGCTTGTTTATTTTTTAAAACAAAAAGCCAT

A_14_P126838 AGTTTACCAGAGCCAAAAATTTGTAGTAATTTGTTGTGACCGTGACAGAAACCTAAAAAC

A_16_P00685058

ACCAAGGAAAATACTCCCATTTTTAGAATGGAGGAGCCATACGACTTAATGAATTAAAGC

A_16_P00901629

ACTCCAACATTTCACATTTATCTCTTGTTATATGTGGCTAGTAACCATTGATCACCTTCC

CUST_4261_PI426210822 GAACCTGTTGAGTCAAGATGGGCAGGGGATTTATTTCTCTGGCC

CUST_5635_PI426210822

TCAATCCCTGTACACAGAATAGCGCTCTGAAAATTATAACTCTTCTCAGGCTTCA

A_14_P105434 TTATTCACTGAGTCTCATGGAGAGATGTCTGTTCTAAGTCCACTTAAATAATCCTCTATG

A_14_P131205 CTGATGAGTTGAGTTCTGTCTTAATCTTCGATTTTTCAAGATCTTCTTTATATTCCTTCT

A_14_P108448

GGATACAGAACACATAAATGGAACACAAGAAAACAAACAAATGGGCATTTTCAACGCATA

A_14_P134634 AGGAGTTGAGGTATGTTGGATAGATGGGGGAAAGTAAAAGTGATCTAATATTTGCACTAA

A_14_P123086 CAATGGAATAGAGAAAACCTTACCCCTTTAAGGTGGCTCCTAAGTTCATCTGATT

A_16_P19634042

ATCCTCCCCACTTAGATCTAAACCAAGAGGCTATGTTAATGCACAAGTATCATAAATCAC

A_14_P128675 CTCTAGACCTCATTGCATATCACTAAACTCAACCCAAGAGAATATTTAGTCATGCAGTCA

A_14_P126028

GCTGGAGAGTGAAAAACTTTTGCAGAAGTAGAACACGCTAAAGCAGCAATACATTAAAGT

A_14_P127082 ACCACTTTAATGCCAGTTCTTTCTTTTAGTTTGAAAAAGAACTTCAGAGAATTTCGGAAG
CUST_416_PI426210822
AACAATGAAAACTTTTTGCTCACAATCTGACAGGTGAGAAAATTGTGGGGTTCTAG
A_14_P117873 GTGGTCCATAGATCTGATCTAGTGACTTAGTTCTGCATGTATAATTAATTGGCAGCATGT
A_18_P15419249
AAAGGTGAGCGTAATCATTTCTTCTGGAATTTCTTAGTCGTTGCAATAATAAGAATGGGC
A_16_P00685490
ACACAAATTTAATTAAAATCTTTTGAGTAACAGATAATAGGCTTAACTGGAAAACTTGCA
A_18_P14135746
GTGGAAACAGAAACACATTTTTATTTAGTCATGAGCGTCACTTGGAGTAGAAATAGAGCC
A_16_P02253466
AGTGTTACAATGCAATTTCTCAAGCCATAAACTGGAGAAAGATCAAAAGAGCCTCTTGT
CUST_5271_PI426210822 ATGCTACCCCCTTCCCAGCTCTCCCTAGCTTTTAAGATCACATAAC
A_16_P00297486
AGGTAACAATAGATGCATGACAACCATTATTCAGGAATAGAAAGAACTATTGGCAAGGGT
A_14_P139366 CTATTAATTGCAAATGTAATGAGGCTGGCATAACTGTATGGAGGTTTCCCATCTTTGTG
A_14_P201204
GGGAACAAAAAAACCATGCTATCACATAATACTATGATGAAGCAGAGAAAACAGCAAGCA
A_14_P139425 GGAAAAAAAACCAACTGCTCCAAAAGAATGTGTTTTTCTCCCATTCTGGAAATCAACA
A_14_P119799 CTGTTTGCAGTTTTTAGCCTGATTTTGGGGTCTATAGAGATTGCTTTATTGGATACTTCA
A_16_P18193492
AAAGTCAGGGGAAAAAGCATATTCACCAAATTTTAGAATGTTATTCCATCTGTCCGCATT
A_18_P22839612 AGACTCTGAGTTGAGAAACCTGTTCTCCCTATCCAGAGCATACAATTCAGAAAATG
A_16_P00695397
TGTCTTCACCGCATTTAATTTCTCATAGACATACTACCATTCCCATATTTTACTTTCGGC
A_14_P127772 CTGCCTAAGTGAACTTGACCACTTGATCATGAAGATCTTGATGTTGAATGTTATTCATGA
A_14_P114586 AAGAAAAACTCTGAGGCTCATTCACCCAGATTTGTGATTGTTAACTGGTGTTAAAATGAT
A_14_P108859 CTCATAGGTTTGTCTTTTCACTTTTTAGAAAATTCTGCATCGCCAGACAAATATCCCAAC
A_14_P101297 CGTTACCTTGATAATCATAGTAGTCTGGGTTGTCTGCAAACAAAGATGAAAGTGGAATTG
A_18_P26795865
AATTCCCGCTTTACTCTTCTAAAAAATGAAATCATCCTTACTATCACCCTAAGAAAATTT
A_16_P16140205
AAAAAATGCAGCAGTAGACTTCTTATCCAGTCCTAAACTGTTTCTTGGATGATGTTTGTG
A_14_P101371 TTCAAGTTTCCTAAAAATGCATCTAAAAATCCTCCTAAAAACCTGGACCAAACTTCACAG
A_16_P19403667
TGCAGTCCCTATAAAGTCCAAACTTAAAGTGCTCTTTCAAGTTTTGCTACATAGAGATGC
A_14_P111944 TATATCCTCCATACTGCTGACCTTGTCCCTGTGGGTAGTTAGGATACTGA
CUST_3053_PI426210822

GCACGACTCAACTTTGTTGAGATGAGTTAAAATTATCAGACGAGATGGTTCCCT
A_18_P15638920
GCCTCAATAGTTGATACCAAGATATTTGTTTCCCAAAAACTTCTTAGCATGCATTCAAGG
A_16_P16046766
TCAACATGTCATTTCGTCACATATGCATATGCTAACATTTGAGATTTTGAGGTCCATTCC
CUST_2738_PI426210822 TATTTATTGAGTTTCATTTGGTCATAAGACTGGGCAGGTTGATTTCTTCTGCCCT
A_14_P127682 GCAGGGGGAATTTTTGTTTGTTTGTTTTAGGTTCTCCCAAAGAATATTTGGAAACTTTCA
A_16_P20787382
ACAAGCCAAGGTTGTTTAAATTATCCAACTGTGACTATGATCAGAGAGCAATGACAGTAA
CUST_5877_PI426210822 AAAAGTGTGTGGAACTGTGAGAGCTCACGATGGGCCTGAACTTGC
A_18_P17134661
GAGCCAAAGAATTCCCAATATATGAAGAGAAAGAATCACAGACATAACTTATTCCCCACT
A_14_P119450 CAACTTCCCCTTGTATTACTCACTCCTATAAGATGGAATTACTGATAGTTTCCTGATGAC
A_14_P131071 GACTCACTTTATCCTGAAGTCATCCAGTGTCATGCGAGTGTTGTCAATGTC
A_18_P22843226 CAGAGACACGGGAGGGTGTGGCAAACCACGAGGCTGAGAGTGGAG
A_16_P18823975
AAGACAATTCAGCCAGAAAAACCAGTAAAGACCAAAAGACCTAAATATGCCCTTAGTAAG
A_14_P110364 GTTCTCTGTTTTAATGGTGTTAAGATTGTTCAGTGGAGCAATCTGATTCTTTAAGGTTGA
A_14_P100227 TTAGGTTTTTCCACGCAATCGATGGATTGTGTCCTAGGAAGACTTTTCTTTTCC
A_14_P113371 TTTTATTGTCCTGAGTTGTCTTAAACCTGCAAAATATACACTACCCATTTTTTTTTTCCA
A_16_P21581340
TAACCATTGTTTTCAATCTATCGTTCACCAAATGTTCTCAACTATAACATCCAGGCTCAC
A_14_P109122 TCTCTAAAAGTCTAAAGTCTTTTGTAGCCCTAAGTTTCTATGTTTCAGTGCCTTGAAGAA
CUST_9397_PI426210822 GAGTTAAAAGCAACTCATATGAAGGGTTTGAGGGTTGTGGTCAAGGCA
A_14_P119691 CCATGCACTTACCTGAAATTCAAGTCTTTGACGGAAAACTTGGTTTCAATGATGC
A_14_P111355 ATGGACACACGCAAAATCAGTGTAAAATAATCAGTGTAAGTGGAGGCCACGTT
A_14_P108420 TCACTGAACCGTAAAAAGTGGTTACAAGACAAAAATAATGGATCTGTTGGGAGGATTAAT
A_14_P139897 AATCAATCTAGGTTAGAAAACATCAATTTGCAAGAGTTCTATGGCTGAGGAAAATGGCAT
A_16_P58424225 TGATGTGACCTCCCTGCACTTCAATTTCCTCACCTCTAAAGTTGGGATAATA
A_14_P122191 ACTGACGTTTAAGAAGAAGACGGAGAACAAATTGCATGTGGATGTGAACGTGT
A_14_P202110 CTCACAACTGGCATGGGATGAACAAACTCATTAGAGAATGAATGAATGGAGTTAATCTTA
A_14_P100076 CTACCTGACTTACTCAAGAAAGCATGCCTTCATACTGAGTTCTTGTATAAAATTCCTCTG
A_14_P102100
CAGAGTCTAAAAGAAATCAGATGAGGCCCAAAAGAAATCAAGCAGAAGCCAAGATTAAAA
A_14_P117238 CTGATAGTATTTCTGAAGTTAGGCAATTAAGATTACTTCAACCACCAGATCTCCTCTGTC
A_18_P16718566
ACACTCTCAAAACTTGTGTTTACTTGAAAATAAACAGTGTCAGATTTTTCATTAGGGGCA
A_14_P137800 GTAAGGGATCACCGTTTTCGAAGCCTCTTCATGAAGCAGCAAGTGAT

A_16_P16452495
AAAAAAATTTCTCCCTGCAAATTAAAGTCAGAACACCTATGAAATTGGAAGGGTCTCTTG
A_14_P122000 TGCCCACCAGACTTAGGGCCTCTAGTAGGAAATAATCAAATCTATTTATTTACAAGTGAT
A_16_P02047762
GCTAGGTCTTATCACCATGTGCATCAGATATTGCTTTGCCTTTATCACTGATTTCAGATG
CUST_2094_PI426210822
CTTGTTCATGTCCATCAACCCAGGGGCCTTTTATTAGAAAACATTTATTTTAAAAGAATA
A_14_P119503 GTCACTTGATCTTCTTGGTCATTGGTCATGCTGGGAAATTATTATTATCATATTGTGCCA
A_14_P115544 CTGGAATCTGTGGATACATTATAGGTATGTATGACTCCTCCAGTCCCTAAGATATGTAGA
A_14_P138573 CAAACATCGGGAACCCTGTTTTCTTCTGGGTGTGTCTCACTTAGAAATC
A_16_P02553896
AATCCCACATACAGTTTACTCATTGATCATTGCTCTTTTTCACATACACATATAGGGGCT
A_16_P00785875
CATCCACACCTTATAACTTTCATGTAAGCCATTCTTTGATACATCATAGGACCTCAACAC
A_14_P109347
CAGGCCCAAGGTGAGGTGACTCACCTTAATTTGATTTAGAAAAAAACAAAAACAAAAAGA
A_14_P116270 AGCAAGCATTCAGAAGAAAAAGAAGCAACCTCAGTAATTTAGAAATCATTTTGCAATCCC
CUST_5479_PI426210822 GGTTGGCCTGGGGACACAGTAAAGCATGGTATTATGGTAACTTCTACAT
A_16_P58424086
TCTTTGAACACCTACTTTGTAGCAGGCACTATGATGGGTGTTTTATAAACATTCTTTTGC
A_14_P137409 AAATCTAGAATACGGACCTAATTAATTGACCAACCTAAAGAGCAAATTCCCTACTTCAGG
A_16_P16575874
CAAGATGGTTTCAACCTTTGTGATTTCTGCTCTATCTACACACCAACAAATACTACCTAC
A_14_P108044 GGTCCAGATATTTCCAAGGCATAAATTCTTGCCTGGAAACTTTGGAGCAATTAATTAAAT
A_14_P135750 GAAACACAGAAAACACGGGCAAGAGAGTTGAGGAACCGTTAAAGGATAAAGAAA
A_14_P115411 CATTTTTGATCAAAATCTTCCATTGCATTCACTTGTCTTCCAAATCCCATCTTGACAAAT
A_14_P121154 CTGAGATCTGCAATATATAACGGGACAGTCAAAGGGCAATGTTTTTCTGTAACATATTGG
A_16_P20368702
AAAACAACCACATGTTCAATCATCCAGAAAGAAAGTTTCTCAGTCAGAAAGTTTAGCAGT
A_14_P133780 CTCTTCATGACTGTCTCCAGCAATAGGATGATTTACTATAAATTTCATCCAACTACTTGT
A_16_P20987084
GTCACAATATGAAATTGCTTTTAATTGAGATGACCAACTTCCATTACATTGTTCAACCCC
A_18_P14288055
AAAATGGAGGAAACATAGCAACATCTCACATTTGTCTCTAATTATGGGTTCCACTGATGT
A_14_P104116 TAAACCACAAAAATGAGCAATACCCAACAATTGATTTATGGCTTCTACTCACAAGCCATT
A_16_P15059239
TGGGACCAGGTAATAAACTTCTATTTTTCCTATTTTCTTCCCCAAGAAACTTTTGTATTG
A_14_P122816 CATTAAACAAGGGATGGGAAAATATATTAAGCAGAGGAGAAATTCTAAGGGATGACAACC

A_14_P122910 ATATCCATGTGAATGAATGGATTTCACTCATCCATACTCAACGTGAATTCTTTTGTCTGT
A_16_P18090558
CTTATTGGCTTCATTCAACCATAACCATGATCCTTCAATTGCAGATAACCTAAGACCTTT
CUST_138_PI426210822
AATACTCACCTGGAGGAACTATGTGTATTTCCTGCTATAGTAATATAGCATAGTATTTGT
A_16_P16191112
CTTTGAAAGACAGAACTTTCTCAAATGGGTAATATGAAGGGAGATTATTAGGAAGAATAT
A_14_P103528 TTTCTAACCCTATCTCCCAGAATTTTCACCAGTCTCTTTTGTTATTTCCTCTCTTAAAGG
A_14_P126829 TTTCCTTCCTATTTTCAAACAGTTCTGTAGTTCAGGAACCTTGTCTGTTGTAGCAG
A_14_P131744 TTCTTAGAAAACACAACAAAACCATATTTACCATCACGTGCACTAACAAGACAGCAAGTT
A_14_P127779 ACATGCTCCTGTAATGCCGTGTGTTAATGTCAGGGAATAAATTCACAATCTGATTTGATT
A_16_P30952347 CGCAGTTGACCTGAATGGGGACGGCCTCTCTGACCTGCTGGTGGG
A_14_P117374 TAATGCTTTTTAAAAATCTACTCATCTTGAAGCATCTTACAAACAAGGATGTTCAGTGCT
CUST_5534_PI426210822
ATTCTATCTGAAGTAGAAGTTGAATATTCCTGGACTTTAACTTTGCAACTGTAGAGGAGG
A_18_P22841392 ATGTTCACTCTTCAGAGCCAGGGACAATGTGAGGGAAGATACAATCAGTTAA
CUST_7161_PI426210822 CACGTGTATTCCGTGAAATGCTCTACCTGCTTTCTGTATCGGCTTTCT
CUST_10413_PI426210822 GCCAGCACCACTTCCTTTAAAGATGAACTCTGAACTTTGGAGAGTGAG
A_14_P116248 CTAACTGTCTTGCTTCTTTCTATCCCTGCTGCACTGATTACACAAAATTAAGAAAGCAGA
A_16_P35052127
GAAATCCAACCAGCATCTTAGGTGAAAGGATCCTGATTCTAAACTTTTAGACACTATCTC
A_16_P18098022
AATGCCACTTTACTTTTAGATACTACATTCCATCAGAAAACCAGAATGACCTGAGTAACT
A_16_P02446597
GAGGTATATGTAGATGGCATATTTACCAGGATAGTGTTAGACATCTAAAGGCAATTCAGG
A_16_P20095481
AAAATGGAGCAACTAATCATGTAGCAATCCTTTCATACTCCCTACACTCGTATTCAACAT
A_16_P16294438
CCCAAAGCAATGATATTGAGTAAAATAATAGTAATCTGAAGAAGCATACATATAGTGGCC
A_14_P111633 CTTTTGTATTCTTTGAATTGGATACTCTGTGCCACATCTGACCTCTTGAAAAATAAGAGG
A_16_P38253157
AGACGTTATGAACTATCCTCGTCTCATTTCACATAAAACACAAATTTCCTCTACCTCATT
A_16_P01247271
ACGTATCTACTTGTGAAATACCTTACCTATCCCCACTAGTCTATAAATCCCTAAGAGCAA
A_14_P136797 TCTTTGACAGATCATCTTGGACTTCAGGAAATGCAATTATGATAACGTTTGTAGACAGTC
CUST_4318_PI426210822 CTTCTCTTCTAGCCTGAAGAGCCCATGGAATGTCAGCTTGGGAAT
A_16_P30179444
GTAACCCTTGGCAATTCTAATTAGAACAGAAATTCTTCTCCATTAGTATGTGCTTCCCAA

CUST_7043_PI426210822 GTTAAGCAGAGAAAACCGTCAGGCAGGAGAGGTGGGGCTGTATTT

A_14_P106970 TCTCTTTGTTTTGAATTGTTAGCCTCTTAACTAACCAGAACCGTATGTGTTTTTCCTGCA

A_14_P124784 ATCAACCATGCTTCCAGTTGGTTCTTTGATAGAAACAGTGTATGTGTTTTCAATCTTACT

A_16_P18270720

CACCATCCTACTTCTTACACCAAAAACGTATCATAGCCATTGCTTATGTCTTTAACTGTC

CUST_2786_PI426210822 TAATGTCACACAACCTCCAGCTCTAGGACAAATAATGTTGGACCCCA

A_14_P132692 GCTGGATCAAGGGCAAAAACTGGTCATTAAGTCATCTGACATTAAATCATTTAGCCA

A_16_P03059666

TTGGCCTAAATCTATAGTCTGAGGAAGCATATCACATCTTTATCATTCATGACAAGCAGG

A_16_P18739862

AAATTCAGACCCTAACACTTGACTGAAAGTCTTTTCCTATACTCTATTCTGCCCTAATGC

A_14_P200559 TATTTGTCTCTCTTACTTCAAACTCCCTTAACTCCCACTTATCTTTGCCTTTGAAAACCA

A_14_P112388 GATGCCTCTCTTCTACTAAAGGCATGGAAACGTCTGTGTCAGAACGAAGTATATTC

CUST_8366_PI426210822

GTGAACAGCACCTCTGCATTTCTGAATTGCTTCTCAGAGTTATTTTATTTTTCTCCTTTG

A_14_P138782 ATATTGTTTATTTCATGGCCACCGTTGAGGGGAAACTGAGACTTGAAGTAGCAAT

A_14_P110188 GTGTTGAAAAAAATTGTTTCCATTCTGAGGTCTGTCTTGTGAAGAGGAAAAAAAAAATCC

A_16_P17565854

AAACCTGTGGTTGTGTAAGAGTTAACATAGTCTCAAAGCAGTTATTGAAAATGTGCCATC

A_18_P11536812

GGCTATATTAATACTGTCACTAGGAAAACCATTCAGAACAGATACCTTACCAATGCCAGT

A_14_P117890 CCTGAGGGGAGAGTATACAGCAAGTTTCATTGTTTAAATTTCCTCTAACTGTGAGTATTT

A_14_P132535 ATTGGATGTAAATAATCCTATTTGCGGATATGCAATATGGTAAGGGGATGGTTGTCTGTA

CUST_9171_PI426210822

ACTTCATTTTTCTAACAGTTCCTCAATTTAAACATCAGAAAGAGTGCTGTTCTTAAGGAA

A_14_P134224 TTAGGCAAGTTAGGCAGCAGGAAAAAAGGGCCTACTGAATGCTAATAATAAACTTTAGTT

CUST_7905_PI426210822 GTCTTGATTTGCTTTCAGTGACTCTGAGAAGCCCTGTGGTTTGTGAA

A_16_P21349921

GTAGTAGGTGAACAAGAACAGTAAAATATCCAACTCTGAAAAATCTGCATCCATGTGACC

A_14_P127479 AATCCAGGATTTTAGACTCAAAGCATTTTTAGAAGTCATTCAGTTCCAAAGCCCATATTT

A_14_P118553 TTTATTGGCCCAGTGCAGGCATAATGCAATTGGCAATTTTTAATGTATGCAAATTTTCAC

A_16_P18903174

CCCCCATATTCAGTTGTTTTGCTTATCTTAGTTCTTACTGTTATAGCCTGAGTTTTCCTG

A_14_P122476 GGACATGTAGTTAGTTACCCTTGTTCCTTGTTTTTTTGTAACAAACATGGAAAACCTGAA

A_14_P121511

GAATCATTGGGATGGCAAAGGCAAGAGTTTTCCCACTTCCTTAAAAGTAAAAAAACAAAA

A_14_P117336 TGTTGCAGCAGCCACAGTCTCATAAATATCAGTATCAGATTCATAACAGGGTGC

CUST_6172_PI426210822 CTGAAATTCCAACAGGAGTATCATATCACCTTTTGACATGGAGGCTTCTTCC

CUST_9094_PI426210822 GGAGTCATTTTTTTTTGCTACAGTGGTGGAAATGGGTCAGGCTGG
A_14_P126446 AATAAGTATTTGTTGAATGAATGAAATCCCTCAGAATGGGCTAACCACCATGCTACCT
CUST_7916_PI426210822 AGACTTGCTGGTGGACAGCCCAGCCCTCACTGCTTCAGGCTTTAG
CUST_3392_PI426210822 TCTGAAGATGCACAGCAGCCTCTCTGTACTCTGAAGACGCATGAAT
CUST_4700_PI426210822 AGAGTGTGCTGAAAGGTGGGCAGAAGCCTCTCTGCCCTGGGGGAG
CUST_659_PI426210822
CTCAAGGGCAATCTCTTCTACTGGCTCCAAATGAACATAATTATTTGTAATCAGTTTAGA
CUST_3178_PI426210822 TAGCTAGCTGTCCACAGAGGAGGCAGCAGGCACCGCCCCGGCTTA
A_14_P136200 ATTTTCACTGAGTTTGAGTGGGAATGGAACTGACTATATATCTTACCCTTCCTCCTC
A_14_P119296 ATATCATGGCTATATTTTTGGTTTCTACAAGTGGCATCATGAACCTGCTGAATTCCAGG
A_14_P117567 CTGGAGTAATTTTTCCTTCAAACAAGCCTCCAAACTGACACCTTCAATCCTTAAACT
A_14_P133764 CTTTGAAGAATGAGCACAATTCCATTTTACAAAGTTTGCTGGAGACACTGAAGTGTTTGA
A_16_P15224400
AAAGAATCCTGGAATAACAATCAAGATGTCTTTCCCTCACTAGTTCCTCAAAGTCAAAAT
A_16_P36471598
AAGCCCAGTATAATGACATCAAAGAACAGTGCAGGTAAGTCAGTAAGATAGGTCTACTTA
CUST_502_PI426210822 ATCACTGAGTGAACAGGGACCCAATGATTCTGCCAGGGACATTTG
A_16_P20062934
GAAAACTGAAAATTACCTACTTTCCGCCTATACATTTCTGTTTATGCCATTACCGTGAGC
A_14_P128186 GGTCAAAGCAAATAAACACAGTTTATGTTTCAGCCAAATTATTGCATTCAAGAAGGCTGT
A_16_P41162240
TGTTTTGGGAAAAAAAGTCTTTCTGCTACTTTGTTATCCACATAAAAAAACATATCCTCT
A_16_P19751024
AAGAGAGGGTAGTATTTAAGGATTGAGATGACACTTGTATGAATGAAATAAAGGCCAGGA
A_14_P115819 TTTTTTAAATGGAAGAAGGAATCTAAGTGTTCCTTCAAGGTGATGAACCTTGCCATTCC
A_16_P19348651
AACTGAGCCAAACAATCATCTTTTCCATAAAATTGTTCTACCTAAGATCAGTGAAGACCC
A_16_P15780329
GGTTCTCTTAGCACAGTTATCTTGATATACAGCATGTGATACATAAGGTCAGAAACTGTC
A_16_P01500298
CATGAAAGGCTATTGCACTACAAAGTAAACCCTGAATTCAATATGAATCCCCCTGTAATG
A_18_P16838670
TTGAAAACAGAAATAAATTCTTGGAGAAACTTGACAGTATTCTACACACTTTGCTCTTGA
A_14_P112265 TCATGGTTATTTTGAAATCTTTCAGGGCCTAGGAAAGACTCTTCAAACAATTTCTCTTCT
A_14_P119523 GTGATCATCGATGTGGATATGCACATTTCTGTCATCATCTCTGCACTCATTGC
A_14_P105039 AAACCATGCAGAGAAATGTAATATAGTGAGCCATGTTTCTGTTTTCGTTCTAATATTTTG
A_16_P20511954
AAAAGATAATTGAGCCCTTTTTACATGCTCTTCTCATCTGTAGGCTAACCTCAAGTCAAA

A_16_P17858925
AAAATCAGATTTGGAGACTTGCTATCTACCACAATTTGGAGATTCTAGTCAAAAGCATGC
A_14_P128418 CTTCAGTCAAATCTGCAGCAGCATGATTTAAGGTAACGTATCAACATTTCTCTTGGGT
A_16_P19064296
AAAAATGGTGTCATCATTGAGATGATCACACTTGATCCTATACCAGTGGCAAAGTTCTTT
CUST_3160_PI426210822 TCGAGTGCAGATCCAGCACAGACCTTGACGTGATTATTTTGGTCATT
A_14_P121565 AAAACTGGGAATTCTAAAACAATTTGCTTCCTTTATTTTCCTCAGTCAGTCCTATTCCTC
A_14_P103245 GCACACCACAATTTACCAATTGAATTGGGTGTAGGTAAATGTCCTCATAATCTTTCTATC
A_14_P122286 GTCTTCAGTCATTACTTCAGGAAGTTTTCCCCTATTATTTTTATCCCAACTGATTTCTCC
A_14_P120812 GTCTAGTTCCAATTCAACAAACATATGGACTGACGAAGAGAAAGATAGCACAAATTACCT
CUST_3849_PI426210822
AGAAGGAACTTTCCCTTTAGGAGTGGTAATTTAGAAGAAAAGAACAAGCCCAATGAAT
A_14_P127578 CCCAAGAAAACTTAATATTACTTACCTCTGCAACCTTGAGAAATTCAGACACTCGTGTCA
A_16_P17539665
AAATTAAACTAGTCCATCCATATCCACCATTTGAACAGAATCTTAGACCAAAGAACCTCC
A_14_P104672 CCCCTGCTCTGGACCTCTCATTTCTCTTCATTGGTTTATTTTTCAATGCATCTTTAATTT
A_14_P119238 AGGTAGCAGGAAGGTTTATATTTTTGAAGGTCTTAAAGGAAGTTAGGGGAATAGGAGATA
A_14_P127572 TAGCAATCCTAATTAAGATGAGAAATGGGGTAACTTTCACATGACAGTCACACATAACCT
CUST_9902_PI426210822 AATTGCTGTGGCTCCCATGGGAAACTACAGTACAGCCTCTCGAAC
A_14_P116005 ACACCAATAGCACCAATCTGGAAGAAGTATTTAAGTTGGGAAACAAGGTGAGATAATTTT
A_16_P17102832
AAATCTAATGTTCACCCAGATACATTTCCAACCCACATATAGACACCGAATTTAACGATG
A_14_P131306 GGTTCATGAGATCTGAGAGCCATCAGGAGTATATCTTTAACATTTAGATCTATGGGTTAG
CUST_1992_PI426210822 TAATTTGGAGGTCTTGGTGGAGGCCTTAGTGGGATCTGGGAAGAA
CUST_6374_PI426210822 TTCAGCTGGAGCTGGGCCAGACTACTAGGTTGTGTATTTAGAGAAACTA
A_16_P17406688
AAAAAAAAAGCCATGTAATCATGAAGAGTGAGAACCGTAAATAGAACAAACTTTCCCTGG
A_14_P116280 TTCCAATATATCTAGGAGAAGAAAGTATGGTCCCCCACACAAAGAATCATAACTTGAACA
A_14_P137013 GGCAGCAACTCCTTTAACCTCTAAATCCATTTGAGTTCCTGTGTTTATCATGCAGTATAC
A_16_P39695760
TCTTAGTGCTGTAATTGTTGATCCTGAATGAACTTAACAAACTTCCGAAGAGATGAGACT
A_14_P121606 GAAGAACACTCATTTATTCACCAACTTCCCACTCAGGAAAAATATATGACACTGTAGGAA
A_14_P108790 TAAGGTGAACGGGCGAAAATGTCACCTATGAAATCTCTAACTTCCCTTTACACAAA
A_14_P123931 GCTTCAGATATTTTTTTGTTCTCCAACCTTTGATCTTTGTCTTGTCTGGTTTTGTCTTTG
A_16_P58424485
CTTAATAGTTACATGGATATTGCCAAGTAGCCTGAGTTCTCAGAGGCTTGATATCCTCAT
A_14_P113700 CTGTAGGCAAGAGTTATGTGTTCAGGTATGTATAATGGAAAAATTCTGTCTCAAATGAGG
CUST_10150_PI426210822 TATATAAGCCAGCCTGGTTTCTGTGGAAAGAATGAAGATGATGGGGTGTTT

A_18_P26794658 CGAAACACTCCTCTTGCCCCAGTAAAAGGATGCCCATTTCAATCTC

A_14_P112808 AGGTATGTCCAGCCAATTTGTTCTGTGCCTCAGTTTACCTTTTTGCAAAACTAG

A_14_P117687 TAATAGTAGTATCTGGAATCTCTACTTTCTCTCCACCTGGATCATTTACGCTTCAAGTCT

A_14_P111678 GCCTAATTACACTTTTTATTACTTTTTAGTGGTTGGATTTCATCCCTTACTGGTCTCAAC

A_14_P107172 ATCTATATTCATACATATTATACTCTTGTGTGTAGTGCACGTGCTATTGGTGGTTTGTCT

A_14_P107479 GCTAGTGAGTGGAGTTGGGTTACAACAGCATTATTCCAAAAATGGAATTGGAAAATTATT

A_14_P114746 ATCCATCAGTTTTAGCCTTTCATTTCTATTGCTTCCTTTTGGGCTTCTATTCATTGAACA

A_14_P104232 TTAAGTTTCTTACCTACAATGTCTATAAGGACACTGTAGATAAGTAGGCCATCTGGAGGT

CUST_4579_PI426210822 CTTCTGCCAACCCCTTGCAGCTATAGTGGAGAAAAACTGTTTGGTAG

A_14_P131738 ACATTGATAACCTTATCTACTACTGTGGGGAATCCTGACCATTTGGAGAAACTATTATCA

A_14_P125762 AGCAGAAAAGAATGTCTTACATATAAGCACCCTCAATAAAAATAAGACGTGTGAATTAAC

A_16_P19830654

ACAGATCTCTACTTGCTACACCAGATAATCTCTTGCTTAACTTATGTTCCTCCAGAATGT

A_14_P106568 AAGAGAGATAGTCTGGGAATCCTATACAATATGAGTGTCTGATGTTAGTAAGAAACCCAG

A_14_P201507 GTAAAACTGTTACCCTAAGGATAAACAGAGTTCAGGCACTCAAACTGGGTCTAAGT

A_14_P121385 TTTTTTAATGTAATGCCAGGATAATGTCTTCTCTGAAGAACTGTTTCTGACCATAGGCCC

A_16_P00056166

TCCCAAGTCAAAACATCAATAGATACCTATCATCCTTTTCAACTAATTCATATCCTCCAA

A_14_P133484 CTCTAGGCAAAATGACCTTTTCCTTCACCCTTCCTGAGTAATGATTATCAGGATTTTTAT

A_14_P126888 CCAACAAGGGGATGAAAATAGACTTAAGGGCTATAGCAATGTGATAAACATATTTGGGAA

A_16_P36028070

GGGTCCCCTACTAATTTTACAAAGATGCGTGATAATATCCAACATATGGCTTTAACTACG

A_16_P36380997

TGTCACCTTTAAAACCTCACATTCTCAAGTAATAACCTCTTACCTTATCTTCCTACTGCT

A_14_P119089 AATTCTGTTTGAAATGGAATTTCATAATTTGGGGGCATATACTTCCTAGTAGAACAAAGA

CUST_768_PI426210822 AAATGGCTCGTAGCTCAGCACAAACCGTGCCTGATTAGTGTTGTTT

A_14_P133247 ATAAAGGTGTTTTGGAATTCTCTTCACATATTCAGAATCGTAGGTCATTCAGGCTTCTAG

A_16_P58424513

GAGTTCAGCATTGCTCTGTTTTCATTGTGTTCATTTTTACAACTACCTTCTCTTTTTGGC

A_16_P21540226

AAGATTGAGTTCAAGGTCATAAAACCTAGTCAAGCAAATCCAAGACTATTCCTTTGAAGC

A_18_P14056427

CCTTCTGCACAAATACTTAGACAGTTCTTTGCAAATGAATAATCATACCACCAGAGTTTC

A_16_P35184729

TGGATCTAGCATCTTCATTATAATAGCACCTTAGCATGTAATTCAGTCTCCTCATGACAA

A_16_P16294089

AAAAAAAACTTTCAGATGATTACCATGAACTCACACTATACCTTCAGTTACAGAAAAAGT

A_18_P22843231 CTTTAACCTCTCTGTGTCCACAAAACCAGACCTCCAACGTGGCGTTG

A_14_P139253 ACTGGTAAGCACTTTATAACATACCTTTGTAGAATGCCATTTTCCTGTGGTATTACACCA
A_16_P01740736
TCCCAAGGGTATTCAAAATCTAGGCACTAATCATCATGTAACTTCCTTTTACTAGCCAAA
A_14_P104547 GCCAGCCACTCACTTCAGTAGGATCTCCTGGGTCTAGAGAAATAG
A_14_P201295 CAGGAAATTATAGCCAGTTGAACCCATATGTATGCATATGCCAACAATTGAGTTTCCATA
A_14_P133093 AAATGCAACAAGTTGAACTCCAAGTTCTGGAAACGTTTACAGTATGAAATGCCTTTTAAG
CUST_9514_PI426210822
CATAGAGCCTCGCCACCAAATTTAAATTAGGCAAAGTTTCAAAGATTTTAGATTAGGCAC
A_14_P116722 CAGTTATTTACCCCAGTCTCTCAACTGGCTTTTAATGAAGCAATTTTCAGCATCAGATTA
A_14_P112114 CATTTCCCAATCTCTGCTTACCACAAACTTAGAGACTTTTCTCTCCTCGATGC
CUST_3735_PI426210822
GCCACTGCATACGTGGGTGTATTTTTAAAAATTGTGACACATCATCAAGATGGATATACT
A_14_P103208 CTTACCTCATAACAATGTGCAGATCCAGTGAAAACTTTCCCAATATCCAGCAATTCAA
A_14_P134188 GGAGAAAACACTAGACTTAGAGTCAAGTCTTAATTCTGCCTTTGCCATTTAACTGCTCAC
A_16_P58424163 CTTGTTTTTCCCCTTACTGAGTAGCACTCCATTGACTGAATATCCTTGAGTTTGTTCAT
A_14_P110737 GACAAACTTCATATTATCCTGTGTTCTTTCCTGACTGGTAATATTGTGTGGGATTTCACA
A_18_P15208024
GGAATGCACAAAATATGAAGCCTGAAACTATAAGACCCAATGATCTGAAAAATGCTCACA
A_16_P18750316
AGCCAAAATACCTTAAATTATCCCACTATCCTAGAGAATACACATTATCTCAGGCCATGT
A_14_P121752 AAGTAAATAAGTGTTTCCTTTTATTTAGTTCTTCTCACCACTAAATCCAATACGAGTTCA
A_16_P00564304
CCACCGAATTACAATATGATTGTCATGGTATCCTATCAAACCAATCATAAAAGCCCATGT
A_14_P132200 CTTTGCACCTACCACATCCAAATTCAGCATTTTTTTACACAGGTGAAATTTACTGACAA
A_16_P39794746
GGAACTGGACTCAGAAATAAACCTATCAATTTAATAAGTTAGAACATTTCACTCATGCAC
A_14_P130643 TGTTTTTTCCTCCTTTGTTACCTTTTATAGATACTTGGAGATGAAGTTCAACTTTTTTCA
CUST_10386_PI426210822
GTTTTTTTCCCCACCGGAATTAATGGTAATGGATCCTTTACTTTCCACTGTACATT
A_16_P00685076
CCTGGGAGGTTTATTTATTTTTGTAGATGAGGCTAACAGAGGTAGTAGATACTGCTTAGC
A_18_P14547977
AGCCCATTTCTAACATATCCCAACTTAATTTCCTAAATCTGTCCTATCTACATACCACAA
A_16_P02785109
GGGAGAGAGTATGCATGATTGATCAGACTCAGCATAGATGTTTGCATTCATAACAAAAAG
A_16_P36230975
ATCAGGGTTCTCAGCATAAATGTTGACTTCTATTTTGGTGAAGCAGAATATAACTTTAAT
A_14_P107767 TTTTGTCCCCATCCCCCAAGTTATAAAACTATAATGCAATTCTTTGGATTTTTCTTTAAA

A_14_P101839 CAAACTTGCCAACATTTCTGTTTATAGATTTTAGGTGTTATGTCTGTCTTGAGTCTTCCC

A_14_P133778 AGTTTTTTAGAATCACTTCTTTCAGCAATTCTCTCAATTCAGTTCAAATAGGATGCCCCA

A_14_P110096 TTAAACGAAGTGTAAACCTGCCACACTAATACTTTGGATCTGGCAAATAGAAAGTTTCTG

CUST_2248_PI426210822 GAGATAGGACACGTTTAGAGGTGCTCTCCCTGTAGACACGCAGGG

A_14_P112676 AACTTCAATTGCTTTTTCAGTTCCGTGGTAACTGCCCTCTTTCCATTATACATAAAAAGA

A_14_P124412 ATGTAACTTCAATCAAAATTGACCCCTACATTAACATTGATGCAGGAACATTCTCTCCTT

A_16_P00806182

TGCTCCACCTAAGTTACTTTAGGCTTTTCCTACTAGGTAAAAACCAGATTACTTTGACAA

A_14_P136727 CTGTGCCTTTTAGATTTTTCAATGGCAGAAACCCATGAGTTACAGGAGGCTTTAATTAAA

A_16_P01801974

AAACAGATTCAAACCTCGTCTCAATTACTGTTGTGCTGGATAATTCTTGAATAGATCACA

A_16_P00347285

ACTCATATTCAATATGCGCCTTAAAACGACTTTAAATCTCTCTTGATTTTTCACCATTGA

A_14_P106264 CTTGCCTTCCTGACTTCAGTTTTTCAATTGCTTTCTTTTCAAATTATCTTACACACCTCT

A_14_P107108 AAGGTAGTTAGAGAAGAGAGTTGAATATGACCTTTGCATAGAAGGAGAACTTACCTTGTT

A_14_P122613 TGCGTATATGTGGGATGTATGGGTTTCTTATTAACAGATCTGTGAGTTAAACTCAGGG

A_18_P16982945

ATCTTTGCTCCCTAAAAACTCCTTTAGAACTGGTCTCATGATAAAGCACACTCCATAAG

A_14_P115083 CTAAACCACTGTTTTGTTCACCCAAAGACTCAAACTGAAATATTTATCTTTTCCTCCTGT

CUST_7078_PI426210822 TACTTAATCTCTCTCCTACAAATACCAGCTTCCTTGGTGAGTATTGGGGCT

A_14_P107514 GGCCAAGAGAATATCCGAACTTTAATTTCAGGAATTGAATGGGTTTGCTAGAATGTGATA

A_14_P102270 CTGGTCAGGTTTTAAAGAACAGTTCAGCAACCATGAATTTGTTTTCTGAACATATATAAG

A_14_P128944

GAAAAGAAATTGTTGACATGAAAAACACAGCAGTAACAACCATTCAAACTTCTGATCAGG

A_14_P123691 ACCTAACACATACTGTGGAAAAGGAGTCTGCTTACTTACTGGAATTATGGTAAGTGTCTT

CUST_9107_PI426210822 GTCTGAATCAGACTTATGTAGCACCAGGCAACCTTCAATCTCCTTTTGAAAG

CUST_5706_PI426210822 AAGGTTAACCCCAACGGGAAAGGAGAGGGGACTCATCAAAGGTTA

A_16_P16191529 CTTTCACAGTGCTTCAGACCACTGTAAGTGTAGGGGAAAACTAGCAGATTGT

A_14_P103525 GGTCAAAGAAAAACTCTTCATTTCTCCCTGATTCTTAAACGAAGGTGGTTAATAGAAACT

A_14_P129469 ACATACAGAAGTATGGCTCTGTTCTTTCTATACTGTATGTTTGGTGCTTTCCTTTTGTCT

CUST_1644_PI426210822

CACCAGAAAGGTCACAAGCATCATCCATAGGGAACAGAAAAAACAACTATGAG

A_14_P128997 AAAGGGGGGGAACAAGAGGAAGGGAAACTTTCTCTTTTATTTTAAAAGTGTATTACTAAG

A_14_P105706 GTTTAGAAATTATGGAACAGATGGCCTTACTTCAAGAAACGGCTTATGAAAGACTTTACC

A_18_P17062650

AAAGAGTAACAAGATGAAGGGGTTAGAATGATTTGTGATGGATACATTGTGCATTGGAAA

A_14_P104359 TTCATCCTCAAAAGGAGACTCTTTTGCTCAACATTAAATTGTGCTTCCTAACCCTAAATC

A_14_P131032 CTTTAAAACATGCACATAAATTGATATAAGAAGGCACTTATGAAGTCCATACAGTCAAAT

CUST_536_PI426210822
TTTATGAAACCAATGGTCTTTTTCATCCTGACTTGAGATGCTGAGTTTTCTTTCAGTATT
A_14_P133156 TTAGTCAACCTAGAAAAATTAAGTGTAAAAATTAGTCTGGTCCCTAGGAATCCAAGGTGG
A_14_P100324 ATCTCCTCCTCCATTTTGAATAGTATTATTTACTTGGTCATCTGGGAACCTTAATGTTGG
A_16_P37059914
TGCCCAAATAGTTTGCTTATACCAGATGTTTCACATCATGAAGAGAATATGGATTTGCAA
A_14_P101939 ACATTTCTCCTTTTCAAACCAACACAGAAGAAATTTCTATCCCAATTTAGTTACTCTCCC
A_14_P106208 CTAAGTGACTTAGCCCACACAGAAATTCACATGGATTACCAACTGGTAATTATGAAATCT
A_14_P102399 AAGAGCCCATTTGTATTATTTCACTGCCTAAATTCCAGAAAGCAACAAAAACCAAATCAC
A_16_P21288802
AAAAACACAATGACGATGAATGTGATGATGGTTCAAATACACAACTGAAGTTTAGTGGCA
A_14_P123390 CATTAGGTGACAATTTTAATAATCCTCTGTTTTCCGTTCACACTTAGGAGTCTGTTTCTG
A_16_P16191480
TGGGATACCAATGCAAACAAACATAGATTTAGTGGAAAATGACTCAACCAAGTAAGATTT
CUST_10166_PI426210822 CAAACCCTCTATCAGCTGGGGCTGTTCCAAGAGCCTGCTGCTTTA
A_14_P139501 CTGGTTAATGGTCATTTTACGGTCCACATACTACTCTGAAATCTATTAGAGTGTGGCTTA
A_14_P112753 CTTTTAAATTCTGTTCATCAATAGGATTCAATCAACCTGGAATCTCCTCTTCTTTGTCGA
A_16_P36351479
AAAGCCCAACTTTGTAACATAGAGCATGTTAAAATTTAAGTTAGAACTTCTACCACTGCA
A_14_P104820 TCCTTCCTCTTGAGTAGGCTGCATAACATTTTAGTCACTTTTGGTTTTCAAAAATGTCTT
A_14_P114325 TTAACAAATAAACCGAATTTTTAACTTGTGAAGAGTTGCACCTTATGGGTGAAGACACCA
A_18_P22840493 AAGGCACAAAAATTCTAATCCAAAGGCTTTGCCCGCCTTAAGTCCTTTTTTGAAAT
A_16_P17008175
TGTGTGTATTTTCCCCTTAGCAATTATACTCCTCCACTGAAATCTATATGTTCCATACCG
A_18_P26794477 CAACACTCCCCCATCTCATGCCATCCATAGGTGAGATATCCTCTG
A_14_P104736 ACAGTGGAGAAATATTTATAAACTTAATTTTGTAAAACAGAACTGCCATTCTTTCGTGCC
A_14_P121063 ATAAATGATGTGGGGTTTGTTTTGTTCCTCTTCATCTTCTTGTTCATCCATAGACTGTAG
A_14_P139956 ACTTTGTATGGACATACAGATTCTGTGAACAGCATTGAGTTTTTTCCTTTCTCCAATACT
CUST_4960_PI426210822 ATGGATGAGCTAATACATCCATCATCCTATCTCAGGAGGATGCTTGGATTTGT
A_14_P115010 TAGAAGCAAGATGGCTGAACTCAATACTCATGTGAATGTCAAGGAAAAGGTAAGATCAAG
A_14_P122909 ATGATAACCACAGAACAACAGTATGCTTGACTTCATTCACCATTCCCTAAAATTTAAGCC
A_14_P109761 CTCTGTGAGTGTAAAATTGCTAGATTAAAAGCCAGTGTGCATGGTCAAGATACTAGATCA
CUST_7374_PI426210822 CAACCCTCAGCTAAAAGGTTCAGTGGAAGGGACTGATGCTGTCTC
A_14_P134701 TGATTCCTGAGCCTTAACACTGTGCTCTTTCCTTCTGTATATACCATGGTCTTACTTT
A_14_P117477 TGAGGTAGAGGTATTTCGGAAGGAATATAGGGAACATAACTTATTATTCTCTGATTCCTA
A_16_P18823659
TTCCTTCCTCAATTACAACTTTCATCTTCCAATGTTCTGCTAATTCAAACATCACATCGT
CUST_4370_PI426210822 CAGGGGGAAGCTCTGACTAACTGATCTCTGGAGAGGAACACAGAT

A_14_P132938 CAAGTTCTTTCATTTGACAGAGGCATAAATCTCCACTCTATTACTTGAATTCACATCCCA
A_14_P119777 TTAATTTTAAGTCTACTAACTTACCCATATAGATAGGAGTCCCACATGTGGCCTGCAGC
A_14_P113734 ACCAAGCCAATTGAAATGATAAGAATCTCAATATGGGAAGACCAATTGAAATAAGAAGGG
A_16_P19262799
TCTCCAGGCTCATTTAATCATCCACAAAGTCCCATACATATACTGTAGAGTTTTCAACAA
A_16_P03458678
TTTTATCAGCAGGAACATCAAGGCTTAACACTGAAAGTATCAAGGCAGAATGTTGAG
A_14_P135183 GACTTTCTGGCTTTGGACACTCCCAATGGTATATTTCTTTGTGGTGTTTTTATATTTGGA
A_18_P13675690
GACTTTGCCTGGATGTAGTAATAACCTAACTGATTCCTAGTTAGAAAGGACAGCCTTAGT
A_16_P19741567
TTCCCTTACTTGACCTGTGATGAAAAACTTACCAAATGGATTATAATATGCTTCTCGTGA
A_14_P131359 TTGTAAGATAATTGGTAAGTTTTGGAAAATCTCACAGTGGCTGGGTAATTCGATTTCCTT
A_14_P121651 ATCCTGAAATGTCTCTTATGGAATTAGAACTGTGTCCATTACTAAGCATACACTGACCTT
A_14_P125928 ATCGTTTTAGCCATGCTGTGCAATCCACAAATGAAAGTTAGTTTAGTGCTTTGTAAAACT
A_16_P18194327
GGCCAATTTGAACATTCTGACATTTCTATTACCAACGCCAATCTAAAATGTGAAACAGAG
A_16_P00957722
AATCAGGGAAGTCTTAGATAAAATATGAACTGTGGACTATGGATTTCACCTCTGCTAGAC
A_14_P129385 TTTGTGACAAACCAAAAGAATAAGAGGATTTAGAACAGGACTGCTTTTCCCCTATGATTT
A_14_P137279 TCATCCTCTAGGGTGTTCTTGTTAGGGGAAGATTTGGCAAACATGCTAAAAATAATTACA
A_14_P124395 AAGATAAAATTCTCACATGTGGCAATTATCAGATAGCCTGTGGACACTTATTCTCATGCA
A_14_P108857 CCACCCGATGCTGAAAAGTTCACAAACCCACAATTCTAAAGGAATTTTAAAAAGATTATT
A_14_P139065 CATTTGTTGAAAAAGGAGACAAAGCCATATCCCTTAGACTTTCCTGTTGCCATG
A_14_P118470
CTCAGAAGAATTAAAAAAACTCATCGAGGAGGTGAAGGTTCTGGATGAAGCAACATTAAA
A_14_P121280 TGCCCTTATAAAACTGGGTTTTTCTGCTTAAGTCGGATTTCATTTTTGGCATTGAAGAAA
CUST_1829_PI426210822 CTGCCATTTTTTTCCTGGTAGTTTATGTTCCAAGAGTAGGGAGCATGCTTG
A_16_P00685337 GACATGAAATGTGTCCCATAAAAGGCCACCTAGTCCCAGGCAGAT
A_14_P126258 TTTAAGAGAGTTTTTAAAATACCTGGCACTGGCTATCTTAGCGATAAAGGCAAGACATGG
CUST_1926_PI426210822 GTCTACAGTACTCATCTCCACCTATCCCTTTATCTTCTGTTGTAATGCCTGGTA
A_14_P102157 ATATGAAATGAAACACTTTTTGGGTGCAAAAGCATTTTCTTCCCCTTTCTAGTCAAGGTA
CUST_6105_PI426210822
TATGGCAAGTGAGCTTTAAAAAAATTCTTTTTTTGATGCATTCCATCAGAAATCAGTGGA
A_14_P132829 AGTTGAGTGGGAAGAGAGAGGGATTGTAAAAATTCCATGTAATGGAATTTATACGGAAAT
A_16_P02511360
GGCCTCTGAATCTTCTACCTTTGTTCAATTTTCCAGTGTTTAGATCCGAAAACAGAATTA
A_14_P126072 ATGTATTGGATCATGTAGCAAAAACACTCAATTCAAAGACAGTGCGTCTAAACGCCAG

A_14_P126197 CCATATACAGAATTAGGTGCAAACCTTTCTTCTGAAGAATTAGGTAATGCCCTTTTAGAT

A_14_P105304 AGAAACAACTCAGAACCGCTATGGAGAGGTATTTTTTCCCAACATTCTATGAAAACTTAC

A_14_P100897 ATCCTGGTGAATAAATTAACCCTCAAGGTAAAAGCAGAGAAGCCAGAGGC

A_14_P113009 ATCCAAAAATTCCATACCTGTCTAAATTTCCATTCCTGTTCATGTTCTGATTCTCTTTGC

CUST_6027_PI426210822 ATCAATAATGCTTGAACAACTAGTGTGGTGGCCAGGACCCAGTCC

A_14_P113984 CTAAGTTTTGACAAATGGTGGAATTCATTGTTATCAACAATGCCATCTTCTGGTTACCAT

A_18_P14135661

TCCTCCCCTAGAATAACAACATCATAACTGACTTATTTCATCATTAAGGATTTAAATGCA

A_14_P124220 AGTCCTTTCCTTATTATCTGTTTTTGCAGGTTGCAAAAGGACTGTGATCATATGAAGATC

A_14_P107797 CTAAAACTACATCAGAGTTTCATAGTGGGTTTGACAAGTGGCATATCTGATTATAGCCGA

A_16_P17600562

AAAAAGCATGTGATACAGAGTAAGACAGCAAATACACAGTCACACAAGGTTTATGCTTGT

A_14_P132271 CTTCAATGTCAGGTTCAGGTTTTCTGGTGTCTGACTAATGAACATTTCCTGAATTAAAAT

A_14_P122589 AGAATTCTATTTGGAGAAGCCCTTCTCCATTGTCTCAGTCACTTCAGGGT

A_14_P202339 TTGCAAACTTTTTCTTGTATTTCCAATTCCTAGTTCCCTCTTTTTCTAATTAACCCCCAG

CUST_1338_PI426210822

GAGCCATACGACTTAATGAATTAAAGCATGTAATGCAGGCCAAGTAATTCAAACCCA

A_16_P03500985

TTCTCGTGTTTACCAATATCTTCATATCAGTTTGCCAAAGTAATTCTTGATACCACACCC

A_14_P107569 TAGACTTTTTTCTTAGGAGGATATTTAGAGAGGGAAGGGGAAGATTTCCAAGAAGTAAAT

A_16_P21546230

TAGGCCAAAAAGGAAAGTTAAGTTCTCAGAGAGAAAGTCAAAATCTTGTGAGCATACTTG

A_16_P00423432

CCCATTTTGAACAACAGTAACAACAAATATCTCTTAACAGTCATCACAATTCTTTGTTCC

A_14_P110110 TAGTAGAAAGAATAAAGGGCTTTCTGTTTATGAGGATCAGGCTCATCAGCAGATACAAAA

A_14_P129378 ACTTGATCATCGATCCATATGTCATCTTTTCTCTTAAGTGATAGAACTTGTTTCTCCTGC

A_14_P120224 GATTGGAAGTGGGATTAACTCAGAATCAAAATTTTGTCGATGGGTTGGAGGAAG

CUST_4398_PI426210822 ATAATATAAGCCCAAGGACTTGGAGGGATGAGAGCTGTGATGCCC

A_16_P17712020

CAAATGCTAAGAATAGTCACTCCCACTTGTATATGGTTTTCAATGTCTCTTTGCTGTCTC

A_14_P129666 CTTTCGCATATGCGATCTACCTGTTACATTTTTGTCTTCCTCAGAGTAACTTCAAATCAT

A_16_P38686856

CAGCCTGTAAACTTGACTTTTAAGACTCACAAATCCATTAACTATCCCCTTCAGAGATTA

CUST_6201_PI426210822 GGAACCTGGCCTGTCTTTAGGTGGAAACAAGTTTTGGAAATTCCTC

A_18_P17387997

GACACACTAAAGAGCATCACTTATTTCTCTAAATAACACTTTCAGTTAGTTGCCTCTTAA

A_14_P111908 ACATTTTTGTTGTTACGAAATTTATCGTGCTGTGGTATTGTCCTCTGTTCAAAAATGAGG

A_14_P125662 CCAGGACATTCTCTAAATGAGTTACAAATCAGTTTCTGGAAAGGAAGTGCTCCAT

A_16_P17696809
ACTTTTCTCTCCATGACTTTGATCCTTATAGCACAATTGACTTCAACAGTAGTTTCGAGA
A_14_P117740 GATGTTAGAAGAAATGTCCAAAAAGATACAGGTAGGTATAATATCACGCAACCACACTCA
CUST_5211_PI426210822
CAGCTACATATTGTTTTACCTTTGAAGAGAAAAAAGCATCAGTGGGGTTATGTGATTTTC
A_16_P02818890
TAGACTCAGTATCTCCTGTTTAACCTCATATAGAAAGCACATCATATTGAAGCCCTCTGT
A_14_P138243 GGTTTTTCATAACATCACTATAGTCCTCAATAGGCATTTTTAGTTTCCTATCCGGCAGAA
A_18_P13944186
CCAGAAGACATCAGGAAGAAACCTCTATTTTCTCTACAAACATCCTTTGAGAACATTTCA
A_16_P19964231
AAAAACCGCAAGTATAGCATGAAATTGACTAGAAACCCTATTTGTCAGGTAATCTTCGCA
CUST_2096_PI426210822
TCAATATTATCTTGCTCTGTTCCCTCATCAGTTAAACAGTCTTAAAAGTTGTACCTTTTT
A_16_P00483964
GGCCTATGCATAAGTATGAGCGTATTACCGTCTATTACTAACATAGTGGTCAAAATCCAG
A_14_P113353 ATTTAGAGGAAATATCACCCCGTGCCTCTCTTCCTTACTTTTCATTTCTTCAGTTTATAG
A_14_P103842 TTCTTTTTAGGTATAGTTTTTCCTAATTGGGTTTGACATTGCTTCATAGTGCCTCTGTTT
A_16_P17059412
ATTAGTGTAAAGAAAGCAAATCCTGGTATGTTGAAAGAGACTACTGAGGTTAAGGGTGTT
A_14_P129192 AAATTATGAAAAGTTTAAACAACCCTGCCATGTTGCAAGTTCTTCTACAGCCCCAG
A_16_P18035568
ATTTCAAGATGACCCAGTATTTAGTGACTTTACCTAATGCTTCCGGTTGAAATGTTTCTC
A_16_P18592375
AATAGTCTCTTGATACAAGTGCTACTCATTGTTCTGATAATTCACTGATCCATTAGGCCC
A_16_P17263376
TGACAACTCACATAGTCTACTTTTCATCATTATCCCCACATTCCAATATCATAGCCCTTT
A_18_P16878550
AAAACTACTAGCCCTACACTGATTACACATAAAGACTGGACTCTAACATTGCTCTGTGTT
A_14_P138173 CTCTGATGATATTCGACTTCTACAAGCAGAACAAAACCACCAGAGACCAGATG
A_16_P16710511
AAAACCCTTTGAACTGACAATCTTTTGTGCACTCAAATTCTCAATACTGAAACCAGTAGT
A_16_P02528913
CGAAGATTCTCCTTAGGTAAAAACTTAGCCATGAACTAACCCTACAAACAAAAGTGTCAA
CUST_154_PI426210822 AATGCTAGGTGAGCTGATAGAGCATGCTGTTCAGTTTGCAGCCACTA
CUST_7823_PI426210822 CTAGAGATGATGTCTTTGGTTTACTCTTTTGGCCTGTCCTCACCAAGATC
A_16_P01412878
AAATACTTTTAGGCCATGTTTGCATGTAGATTTTCCTCTTTAAACCTGTGCTTTTTGCCT

A_16_P00042692
TAAATCTATGTCTACTTCCATGACTTCGATTCAGTAAACACTCAGGGAGTCTTACAATGG
CUST_6850_PI426210822 AAAGAAACAGTGCTGACAGGCCAGGAAGGTCAGAGACTCAGTGTT
A_14_P124799 TTTTTGGGTAAGTGTGTGAAAAGGATTGGTAACCGTATTTGAAAATCAATGATCATGTCG
A_14_P110491 TGTTTCCAAAATTGACACTGAGATGAGGAAAAGACTATGTGGAGAATTTGAGTATTTCCG
CUST_6972_PI426210822
TAGCCACAGCACAATTCTATTTATTCTTGAGGCATTCCCTCTTTTATTTTCTTGGTCTTT
A_16_P37784068
CCTCATGAATGGTAGACTTCACAAACTTGTAAGTGAAATAACTAAGACTGTCCAGACTTC
A_14_P124923
AAGAACACAAGGAACAGAAAGATAGACATGAAACATAAACCAATAATTGCCAAGGATGGA
A_18_P18331191
ATTATCCTATAGTCTTCCCTATTTGGCCTAACTTGTGTAGAATCAGAAATACCATGTGCC
A_14_P123639 TTGTTTTCCTTCTTTGAGAATGTCGTATGGAACTGCAGACCATCCTCAGGATAAA
A_14_P128583 GATCACAACAGGAAACTGGAGAACTGTTGATTTATTACAGTTCTCAAATTAGAAACCTCC
A_14_P201148 CCAAAGGCAGATTTTAGTTTAGGATTTTGTGCTAACATGATCTTTGTGATAAGTCAGGTG
A_16_P17288386
CTGTCTCTCTAACTATCCATCATCATGTCACATTAGACTTTAGTAAGTCCTCGTAAAGGC
A_14_P132753 AAACAGCCAACTACATTAGTGCTCACACTAAAGACGTAAGGACCGTATTTTACCTCT
A_16_P36061110
TTCAGACTTCCAGTGATAAAACAAGAAATTGCTATAGGCCTTTAGACTTAAGAGTCACCC
A_16_P17731293
AAAAACGCACATACAATTTTGTAAGATTGATTAGCCCCCAAGTAAGTAGATTTCCCCTTG
A_14_P110995 AAGCCCAAAGCCCCAAATAGTCCTTTGAATTTGTTCTCCATTTTTTCATTTATTTTATTA
A_16_P16799849
CCCATCCTCTGTAAATTCTCTCAACCTTCTCTCATTTTTAATTTCATACCAAGACCTAGA
A_14_P129026 ATAGCCCTTCCACCAAAATTGTATACGTTGATTCAAATGAACATCTATTCCTCTTTGCTT
A_14_P118769 CTTTCCTTGTAGCAAAGGCTGAGTTTGCCTATTCAGGGACATTTATAACTATTTCAAACA
A_14_P128293 ATGGCAGTTTTCTTTCAAAGTGAAAATATTTGCTTCTTTGATACTCCTCAGTGCCTTGCA
A_16_P16338255
ACACCTTTGAAATCTAAGATATCCAGTCTGTCAAATAAGAACTTTCTGACCCTTCTTTCC
A_18_P16045387
AAGGGGGTCTTTGTAAATTAAAGGAAGCCAGTGTATAATTAGCAATAGCAGATACAATGA
A_16_P19224244
AGATGACTCACTCTTCTTAAAACATGTCCTATATGCTCTAACTACCAAATCATGCTGGAG
A_14_P138282 GAATATACCTAGATTGGTAAAAAGAATAGGAGGGCATGGAGATATAGAAGGGACAGAATG
CUST_1704_PI426210822 AGCCTCTGCACAATGGGAGCTTCGTCTTAATGCTTGTAAAATGTCATCTC
CUST_7802_PI426210822

AACCTTTATAAACTGCAGTCAGGACTGTTTTCTCTTCCTTAGTATGGATTTAAACAAGAA

A_14_P102114 CCTCTCTATACATGATCATAACTTCACTCTCTAGCCATTGTTTTTCACTAAAGGCAGTGA

A_14_P126851 CCTCACGTTCCTGTTATTAGATTTCTGACTTACACAAGAGCCTAGAACAGAAAGAAAGTA

A_14_P133202 CCCCAAATATTTTCCAACAGAAGTAGAAAACAGGGCATATTAAACAAACAACAAACCAAC

A_14_P105945 TAAAAGTATCAGAGATCCCGATTTCATTCATTCCTGACACCAAGTCACTTGAAAAAGCA

A_16_P55126285 ATAGAAGACTTCCTCAACTTGACAAAGACCCTTTGCCAGAAACCTATAGGAAACTT

A_16_P16307491

AAATGTGCCTTTTTGATGAGAACAAGCATTAGTTTCCTGCTATCTTGAGTATGACAGATG

A_14_P114499 CCACCCACAAGGTCTCTAATAAATAGGGAGCAGTTTTTCACTTCATCACAATCTC

A_18_P16654512

GGAACAAAGGAGGAGTATAGCAAAAAGATACTAGCAGTAAGTATAAAACCAGGATGCACT

A_14_P103357 CTACATATAGTCTAGAACAGCATGCGACATGTATCTTCTAGGAATATGTGCCAGTCTCAA

A_14_P136607 CTACAGCCTAGTAATAGAGGAGGAGACATTTCTAAAATCGCACCCAGAACTGT

A_16_P18031614

TCTAAGTTAACCTCTTACCTAATCCAATGTTACTTTCCACCAGATACTTGAATCCCTTGA

A_14_P129201 TTTCAAGCATAATGCATCAATCTGTATCCTCTAAGCCCTGCTTCCAGTCTATATAAACAC

A_14_P119250 CTGTCTCAAATGCTGCTTCTGGTGCCTGGAGAAGTTCATCAAATTCCTTAATAG

A_14_P119282 CACTTTTTTCTTTCCTATGTGTTAGGCCTATATGTTCAGTTCAGCCCATTGACTTGAACT

A_16_P19188043

TCAGTGAACGAATATGTCTCCTCTGTCTAAATAATCCTTAATTACACCATACGTAACCCC

A_16_P37923439

AGGACAATTTTTAGTATTCCTCAATGACTGTTTCTTCAACCAGTATCAACCACATTGTCA

A_14_P108341 CTTCATAGAGAAGACAATTACATTGAGCCTTGAATTTGAGTAAAATGGAAGGAAGGAAGC

A_16_P03762699

TGAGCCTCAAAGTCTTAGAACACTGATGATACATAATCCTACTTTATATACGCTATTACT

CUST_5396_PI426210822 TCAATCGACTGTTCCCTTGGGGCTTGAGATTCTTGGATTCCAAAG

A_14_P121908 CTGGACTGGGGCTATGAAACACAGTCACCAGCACTATACTCACTC

CUST_6443_PI426210822 CTTGAACCACATCAGTAGAAATTGAGGCTATGGCACCTGGGATGTC

A_14_P117171 CAGAACTCTATGCAGGTAACAGCAGAAATGATGGTGGTTTTCAGGAACAACTGAAAATAT

A_16_P37007417

GATCCATAAGTCTAAGGATGCTTTACACATATTTAGGGTGCCATTATAGATTACTGGGGA

A_14_P117864 TTTATGTTTTCCAGGAATCTGAATTTCTGTGTTTGGAATTTGATGAGGTCAAAGTCAACC

A_14_P104127 TATAATATTCTCCTTCATGTTCGAGTGAAGAAAACTGCATGAAGCGCTGCTTACGAAGA

A_14_P131841 CACATGCTCTTTAGGCATCCTCTATGACAATCCAAACAGGCTCTTTGGAAG

A_14_P126096 GTCTCAATTATGAACTTCCCACTTAGAAATAACCAGTTGCATGTTCCATTTCAAACCTTC

A_14_P107605 AGTGCTAGAGTGCAGAAAACATGTCCATCACCCTCAAAATGGTGACG

A_14_P100860 CTTTTGCTGCTTATTTTTGTTTTCAGTGCACTAATTTTGTTTTCAGTGCACTTTACGGGA

A_16_P02155842

AGTGTAGTTTAAGCCATGATAACCACAAACTTTATTCATATTCTACCATTAGCCCTCTCA
A_18_P14877191
CCAGAAAGCATAAACTTAGCCATCAAAGTGTATCCCTAATAACGTTTTACATCTTTAGTG
A_14_P125771 TGTGACCCTTGACTGATGGCTTCATTAGCATTTTGTTTTTCTTTTTGGATCCTTAATAGA
A_14_P122660 AGTTTCCTAAACCCACATATATCTAATTATGCCTCCATATACCTAATTGTCCCTAGGACC
CUST_2208_PI426210822 CCCAAGTTAGATGACTTTTCCTGCCTTTGTACACCATTAACCCCCT
A_16_P01006791
AAAGTTACTCCAGTGAAAGATCAAAGGTAGATATTGGCAAGTCTTCCTAATCTGACATGG
A_14_P130267 CCGTAGGACTGGAAGAAACCTCAGTAATGGGAATAAGACTTTGTCCAATAGG
A_14_P127302 CGAGAATGTTACAGACCAGTGGCAGCAAGAGCATCTCTTCTTTATTTTGTTATTAATGAC
A_14_P100718 CAATTTCAGTGTTATCTCTTAATTCAGAGCAAGCCCTGCTTTTCTAAGGAATAGTCAGTG
A_14_P200076 AAATGCAGCATTTCAGCAGCCTTGAAATGACAAAGCCTGGAGAGTAGG
A_14_P119623 ACATACCCACTCTTATTTCAGTGCTGAAATTTATGAAGATCTCTGAATTCAGGTCTCAAG
A_14_P112716 AACCCACAAGTTTTTCTGCAAATTCCAGTAACAGGTTATAATTTCATTGTCTTTCGACTC
A_14_P200325 GGTTACAGCAAATTACATGATGACCTAGGAGAGTTTCCATATGGTAAGTAAATAACACGA
A_16_P41538201
AGATGGTAGTATAAACACACACACTTAAATATAAATAACAAGTCACTAGAAGGTATGGGA
CUST_2198_PI426210822
GTGTTTATAGTGAAAAAGAAGACTTGGTCATGATAAAAGCCAGACACTCGTGGGAT
A_14_P124882 CTGATTTGCTTCAACATTGATTTGGTAAGATGCTAGACACAAAATAATACCACAAACCGC
CUST_771_PI426210822
GGTATTCTTTGCTATAGATCTTCTGTTGACTGCTTTCCGGAAACAATGCATAAAACTGTA
A_14_P201910 GTGAAACTTTAAACTACAGCCGGGATAATTCAGGACTGATCTCATTATCTTTGTAACCAT
CUST_1580_PI426210822 CCTTTAGATGGGAGGAGAAGCTAGATTGATGGGAGACATCAGAACTCA
CUST_3884_PI426210822 TCTGCTCTATGAATTCTGGGCTAGTGGGGATCATGGGGATTTTGTAAATT
A_16_P39955749
GCACTCTCATCAAACAAATCTCTTTGAAAATGCATTAGATAACACGGTTCCTTCAATTCC
A_16_P19421019
AACCCAGATAACTCCACAAGAATGTCTAATCCCCAAGATAATTCTATCTGTGATTTCTCT
A_14_P132937 CTGATTTGCTGCTGCTTTTTATTTTTAAAGGAAAAATTGAAAAACATTCTGAAGCCTGGG
A_16_P15247423
AAAGTGGAATAACGACAGACATCAATCTATTCTCCAAAGAGGTATAGCATTTCAGCACAA
CUST_1229_PI426210822
TGTTAGACCTAAATTGTGGAATCTGGCTTTACTACTCACTTATTGCTATGATTAAACCTA
A_14_P129063 ATCATGGTCTGCAAAATTTGGAAATTGTTTTAAAGGTGAACTTAGTGGCAAGATGCATAA
A_14_P131055
AGAGAGAAGGAGGAAAAGAAACTAGTGTGATAAAAAAAGAAATGCTACAGAAATGGGGTT
A_16_P39142289

TAAGTTGTCTTCACATACTTCTCTGAATAGTTTGGTCCCACCAATAAACCTATCACTGTT

A_14_P127292 AGATCAGTTTAACATTGGAAAGCCCTGAATGTAAAATCTTTAACTTTCCCCCTTAAACAG

A_14_P131726 CTTTTTACATTTGCAGGTTTTTCACGACTGAAAACAACATAGCAAAATAAGCCAAGATGT

A_16_P39971558

ATATCACGTTCAATATTCCTGTTGATTCAGAAAGAACTAATGTTTACTAAGCCATCCCCC

A_14_P136647 CCACCAAAACATTTCTGAAGAATGACATTAACATGTTTCTTTGTGTGCACTCATGTATGC

A_16_P17020832

AGCCACAAAGAGATGTCTAAATATATTTGAACTCCACACTACAGAATTTCACACTCCCTT

CUST_8534_PI426210822 CCCTCTTAAATATTATTAACCATGCTGACCATGCCACATTTCCCCAGCATCTA

A_16_P17766893

AGGATGTCTGCATATTTCAAAATGGCAATTCATACCTTCAAAAAAAGCAGGAAATGTTGG

A_14_P103895 TAACATATGAATTATTGAATGTATTTCAAGGGCTTTCAGCGATTCTGGTTTCAGTCCTGG

A_18_P16207443

CCTCACTCTGGATATTCAATTAGTTTGGACAAATCACAATTGATGCCAATTAACTGCACT

A_14_P118793 CTGGTTGCATAATCTGGGGAATAGTCATAATTTCATGTGTTATACAGTTTAAAAAGCCTG

A_14_P114088 GATTTAAGGAACTGTTTCTGGGATTCCTTCCAAATCCATTACCTGAAGAGAAATTCATAC

A_14_P106822 AACAAACAAGAAAAGCAACTATTCTGAGACCTTTTCTGCCCATCAGTTAGATGATTTAGG

A_16_P35881175

GCTTTCCTCAGTTAAATACTAATCATTGAACTCAATTCTCTAAGCCTACAATTAGCCAGG

A_14_P202365 CCATGTGACTAATTCGGTCTCTATGATCAAAGCCTTATAAGAAAGAATGACTGGTTGACC

A_16_P02009760

AATCCGTGAACACCATAATATCCTTAACTAGAGTTGGAGTTGATTCTATTGCCTGAAACT

A_14_P100597

AGGAAAAAAACAGCAGGCAGGCAGAAAAGCAAATTTTAAGAAGAATCTATGTGCTAAGAA

A_14_P103135 AGTGTGTAAATTCAAGGCTCACAAAAGATGTGCAGTGAGAGCAACAAATAACTGTAAATG

A_14_P107765 CTTTCACTTTTTTCCAACCTAGTTTCTAATAAGTGACCGTGACCCACAGTGCAA

A_16_P00109951

CCATCAATTTAACTCAATCTTTTCCACTTCTTACATCTATATCACAATCTTATCGCTGCA

A_14_P128471 TAAATAAAATACATCCACAGCAGTTTACAACTCTGCCATGATGTCATTTTGTCAGATGGC

A_16_P39866695

CACCTCTTCCCAATGTTATTAAAACAGTAAACTATCAGGAAAGGATTTGAGGCTTTCCAT

A_14_P137801 TTCCAAATCTCAGGCTTATGCACAAGATGGAAGAGCACTGTTAAAATTAAAAAATGGACC

A_14_P122263 CACAGTTCTCATAATCTCATTACTAGAGAGAAAAGGAGAAAGGGAAGCATCAAGGTAGTA

A_14_P107994 AACAAAATGATCCCCTCCTCCCCAAAATTGCATCATGTATTTGTTCATTTAACTTTTCAA

A_14_P120337 CAAGATCTTTCCATCTCAGACTTTAATGGTCATCCTTTCTATCTTCAGAAATGGGTATGT

A_14_P115690 ATTTTTTTTTTGCCATGTCATGGATATCTTTGTGGAATTTGTCCTAAATCTGGTGTGCTG

A_14_P102332 AGGGATTGATGCCTAACTGATGGATTCTCTTTGAGACTATTTAGATATTATGTGAGCAAT

A_14_P122796 GAAGTCTGGCAGGCTCGGAATTGTCATCTTCCTCAGGCAAATAGTTTT

A_14_P138391 AAAAGGTAATATTTCAGGTGTTGCCACCTAAAATACTAATCTACATCCTCTGGCTTCATG

A_14_P136503 CTATTCTAGGTTCTTACCTGTATATGTGACCCTCAGAATGTCCCTCTCCTCTAAGT

A_14_P119914 TTACCTTGATTCCATATAAAACGGTTACTTGGAGTGTATTGGTCATTCTGTTCCTGCTTC

A_14_P125106 GGAGAGAAGCACGGATGAGCCACATGTTTAAAATTCTAGTTTGTAACATATTCAAGAGAT

A_16_P15896330

AAGGTTGGAATTTTCTGGATTGTTAACAAGATCAAAAAGCAAAGATATAAAGCCAAGGGG

A_14_P106823 TAGATTTTTTTTTCCTTCCAAATTAATGACTATGACTTTTTGAATCCTCCCCTCCACTCC

CUST_7975_PI426210822

CAAACATAGGTAAAAAGATGTGGTGATTGGAGGCAAAACTAGAATCTAAAAGACCAAGTC

A_16_P20627273

GATGGAGCAGTCTAACAGATAAGCAGTATGATTTACTAGCCCACAGAGAATGATTACTGG

A_14_P121976 TGAAACAATCAACACATACACAGGAGGTTACTAGGAAACAGGAAAAATGTTTTCCTGAAT

CUST_9119_PI426210822 GCCACCCCAGAAACTCCCGGCGGGAGACAGAAACTAACTTCTGAG

A_14_P125347 CTGTTGGTGATGCATTTTAGCAAAATCACACAAACCTAGAAACATGGAAATAACCGCAAT

A_14_P101851 AGCACTGTTTTTCTTCAAACTGAAGATTTAACGGTATCAAACTAACACAGGGTAGTGTTT

A_14_P129754 GGCTATGGAAAAACAGTATTTTTGCTATTTTTAGAAGCAACCACCCTCTCTTTCTGAGTA

A_16_P18212022

ACTCACTGCGACTAAATTTAACGCTTAATCATGTCCACCTTCTAGTCATCCTTTTAAAGA

A_14_P117702 TTTAGAACATGGCAAAAGCAGAAAAATTAAAATAATGTCTTATACCCCTTCCCCCTGATG

A_14_P129920 TGAAAGATTTTTTTTTAAGGGTAAATGATGGTAAAAAGATGGCCTTGAAATGGCCCCAA

A_14_P123880 TCAATGTTTCAAAACATGCTCAGAGCAAAATGGTTATGTTCTCACATTGTCAAATGCACA

CUST_3630_PI426210822 TGTGTAACCTTTAAGGAAAACCACACGGAGCCATTACCTTGTGTTGAAAG

A_14_P138005 GCTGGTGTTGATAGTGTGGTAGATGAGGATGTGGTATGTAATGAAGTAGATGGACTT

A_16_P37561948

TTGTCCTTGTCTAACAAACTTGTTATACATTCTTTCAACTTCGCATACCACATTCAGACC

A_14_P133147 TGAACTTCATGAAGTGTTTTGTGGCACAATTTTACATTGGACTGAAGATGCTTTTATTCC

CUST_10212_PI426210822 ACAGTTAACAGAGCCGCTTGCCCCTCAAATGGTGAAGCAAGCACT

A_14_P122752 ACCTTTTCACTAAAGGCAGAAATAATTTTGGGAGTTCCATCTGCAGGAAAACAAATTATG

A_14_P124300 CTGGAGTATACATAGGAAATTTGGTAACTTGGATAAGGCAAAGTCTTTTCCCTTTAGGAG

A_16_P02302218

ATTGTAATAGTGAACATGAAGTTGTTGGTGCTATCTGAGAACATCTGTATGGAATTCAGG

A_16_P58424357 GAGCACTTGCTATGGGTATGCCAGACCTGGTGGAAATAGACACATAATG

A_14_P107315 AAATTACTTTTAACAAAAGGGATTTAGCAGAAGCACCTGGGAATCTATGGCATTTGAGAA

A_14_P126665 CCCTTATTACCATTTAAGTCCCCACATCTCAGTGCACTATCACTGCTTTTATCATATACT

A_14_P114048 CTTGAGCAACTGGCATGCATTCCAAGAATCCTCATAACAGCCCCAT

A_14_P104630 ATGTTGTTGTGTCAAGAATATAAAGATCTACAGAAAATGATGAAAGTGGTCCAAGTGGCC

A_16_P02714432

TGAAGTGTTCCAAGGTAAAGGAAAAAATACAGTGATACATCATGACTGCTTAGTGTTCTG

A_18_P14133598 ATCTGCAGTTGCATGCGTCTGAATGCATTTTGCTGAGTGCATTTTGCAC

A_14_P103633 TGCTGTTGAAAATCAAAATGAAGCAGAAAATACCATTCAAAATGAGGCACAGCGATAACT

A_16_P18554237 ACGTGCCTATCTTTATCCTATCCTCCAATTTTTCACATCTATATTTTGTCTTTTCTGCCT

A_16_P03733848

ATCTCTGAGTGAATTCTTAGTAGACACATTGACATGGTATAAAATACATCACACGGCTGT

A_14_P116241 ACATGTATGTTAACAAAAATAAGGAATGTGTCCGATATATGCAGACTTGGGACAATTCTT

A_14_P129624

ACACCTTGCTGAAGAAAACCATGAGAAAATAGTTTTTAAAATCAACCCGGACAAAAAAAA

A_14_P117588 TTTTGAAAAGGTCATTAACTCAGCGTCCAAACACTAGTCTGTGATCTTGTCTCAAATCTA

A_14_P134359 CATATTGAGGTTATCAGAGTAGATGGAGGAAGTCAGAATCTCCAAAGTGGAAGAATTTAG

A_14_P112995 TTTGACTTTGAGTTTGTGGTAACTCCTGTGGTGTAAATCATTGCTTCAGGTTCTG

A_16_P17394801

TTTGCCGACCAAACTTTTTATAATCTCGAGACTAGATCAACCCTAGTGATTTCATCAAAC

A_16_P00331697

AAAACATTGGCAGTAGAACACAAATCAGGACAGAGATAATAAACCTTCTCAGGCTACTTC

A_14_P123747 CTCACATCCCTTAAATTTGGTGTCAGATGTGTATTTAAAAATGTGCTTCTTTGTGGCTTT

CUST_4698_PI426210822

ATTCTGCCTATGACGCAGGTATAGAAATAAAAAAGACTATGAAAAGATTAAAAAGAGCTC

CUST_2425_PI426210822 GGGGACCATGCAGATAAAGGCAGAAGGAGTAGGTCAGACCCCAGA

A_16_P21659580

TGCAAACACTATCATTTAACACCTAGTTCACAGTCATAGATTATCCCCCTGTTTCTGAAT

A_14_P102940 TTTTATGGAGTGAAGCAATCAGAAGAAGAGAAGTAGCACCTTTGCCCTTAAAAAATTTCA

A_14_P137973 CTGTCATAGTCACTTGACTCAATCTGTTTCTCCTTGAGATCAGCCTCAAAACG

A_14_P136207 ACTTATTCTGTCTTCATTTAATGAACATACATAGCCCTTACAAAATGCTTGGCGCAGTTC

A_14_P131715 TTTTTCTCATTTTTACTTTGTGGGAAGGAGTATGGTTTTGAAAATTCCATGGATTTGGCT

A_14_P111219 AAGCCACGAATTGAACAGAAACAAATAAAAGCAAAATGTCGTTAGGCGCATGATTTAATC

A_16_P38083186

TGGGCTACTCTTGGATCTTACTTGATCTGCCTCATATCAACTTAATTTTTCTCAACTTCT

A_18_P16095954

ACTCAAGGTCTTGAAGCATTTGTTTAGGCTACAGTATATAATTGGCTAAATATTCTCCCC

A_14_P132662 CTTAGTTTCTCCTTTAGTTTGAGAAAAGACAGACTTGGGGTGGGTTTGTTTTTGTTTTTT

A_16_P39938516

AGAGACCGGATTCTAGTCTATCAGGCTTTTTTATTAACCACTATAGAAGTGCTGACCATA

A_16_P19712323 GTTTATTTTTACCGGGGGCTTTATTCTTCCATTGAGCCCCAGATACACTCT

A_16_P17977578

AAATCCTGACATAGAATCTTCTAACTCAAGGTCATCTCTTTCATGATCCATGATGCAAGA

A_16_P03302471

TCCCTTTTCTACACACAATTCAGGTCATCTATGATTATGCCATCTAAGAGATACTGTGTC

A_14_P106337 AGTATTTTAAAGAAGAAATCGACTACTTTAATGTAGGCGGAAGATCCTATGGTAGAATCT
A_16_P21231526
AAAAATAATGTGGAACATCATATGGCATGACCAATATTCAAAGCATGAGCCATTAGAACA
CUST_4849_PI426210822 AAAGCAGGCTACCTATTTTATCCAGGAAGCAGACAGAGGATACGGATG
A_14_P131057 CCTTGTTGCTTCATAACATTTAAGTCCAAGGAATATCCAGAACAGTTTTTTGTTTTTGGG
A_14_P111907 CTTCTTTTGGTTTCAGAGAAAAACATCTCCTTAAAATGTTTATTTCCCAAGGATTCTTGA
A_14_P105319 GAGTAACATGGACTTGTTTGATTTTTGGTAGAACGTGGTCATTTTTTGTTTGTATAGATA
A_16_P03016522
ACTTTGAGCATCTACAGATTCTTCACAAACAAAGGTATTCTTATGCATTACTTGACACAT
CUST_3431_PI426210822 CTTCCCTTCGTGCTGTGTGTCACAATAAGATAGGACACAGATGCAAAAG
A_14_P110986 CTTTTGAGTAGAGTATTTTACCCCGTTGGGGATCAGTTCTGTTAAATGAAAATAACCGTA
CUST_2609_PI426210822 AATAAGAGCATGGAAGGCCCAAGAAGAGTGGTCGGGCCATGACAG
A_14_P200908 GTCTGTTCAGAAAAAGAAACTTAGAAAATATTCCTCACCCACCAACTCACTAAATGTAGG
A_14_P108437 CTCAAGTCCAAAAGACCCTTTAGGATCATTTTACAAATATTTGTTTGTGGCTCTGACTTT
A_14_P200558 CATCTCACACACAATGACTCTCACAAATGACCACATGACACTAACTTAATCTTCCAG
A_14_P104830 ATGGCTGCGAGTCCATGGTCAGAACTGGACGATGTCTTCAAATAAAAC
A_14_P200187 AAGGATGCAAAAGTGGTTTTCTGCTTTCGATGATGCAATCATTCAGCGACAG
A_14_P100838 ACAAAAGGAATATAGTCTAAGTTTCCTTGTGGTCTTCGTAATCCAAGGGTAAGATAATGG
CUST_7691_PI426210822 AGTGTATCATCAGGGCCTGAGCAAGTGGCAGGAGACAAGGTATAG
A_16_P19810045
CCTGCTACATAAGGTCTCTACCATCATTGAACAGTTTAGGTCTTTATTAGAACATGCACA
A_14_P132471 ACAGACTCACCGTCCCTTGTCCAATTATCATATTCATCACCCGCAAGATAT
A_14_P103098 GCCTTTGTCATTTTCTTGCTTTAAAGGAATATATTCGACTCTGTCAGAAATATTACTCCT
A_16_P02589709
GTTTGTAAAGCATGTGACATAAGACCTGAAAAACATCAACTATTCGTTTTGAAGAAGGGC
A_14_P112299 CAAGTTTTACCTTGTCCTGAAATCCAGGAGTTCGGATGCTCTCAAGTTCT
A_14_P117877 TACAAGATCCATGCAAGGAATCCAGTTACACACAAGACACATTTAAAACCTGGTTAAAAC
A_14_P102917 CACCACTTATACTCTAACATTAACTCAACCTTTCTCAGGATGAGGTCTCTCACCTTAG
A_14_P133736 TTGCACTCAATTACAGTGATTATTGATGAAAGCGATGCATGGATATTTTAATACTTCCTA
A_14_P139023 TGGTTACCTTAATGGTAAGGTCTTCTTTTCCCAAGACAACAATAACCTCTATTGGTGTAA
A_16_P15649089
ACAGAGAAGACCACATTGTATGCAAAAAGAGTTTTAGATCAGGGCAACATTTCTAAACTG
CUST_9374_PI426210822 CAAGACACCTTGCCTGTGGAGCCCCTCCTGCCCCACAGGTAGAAT
A_14_P129609 GCCCTGCCTTAATTTGTTCTTCCTTTTCCATTTCTCCTTTGGACAACTTG
A_16_P03087690
GTTTATTCCATCTGATGAGCGGATTGTTCAGAAACTTAAAATGATTAACTCAGCCTCCGT
A_16_P00936129
AAAACCACCTTTCCATTGAAATCCCAAAGAAAATAGGACATTGCATTTTTTTAGAGGTCA

CUST_6623_PI426210822 GGGAGCATCTGTTCTCACATCGCTGCCACTTGATCACCCTTAGAT

A_14_P125982 ACCTCACATGACACATTTTTTGTCAAATGCATTAGATGAAAACTGCTGACATGATCTTCA

A_16_P00685128 CTATTATCTGTTTCAGAAACTGTCTGGGCAGAAGATAAATCCAGTGCTCCGGAT

A_16_P02525590

AAAAATCAATTCAAAGCAAAAACTGATGTCACTGTGAGTTCTTTTCACCTAGACACAGCA

A_14_P120741 AGGACCCATTCATTTTTAACCACCTTGCTTTATCTGTTCAAAAGAGAAACAAATTCAGCA

CUST_3127_PI426210822 GTATAGAGAGATGCCCCACACACCCATGGCCCAGAGCCCATGAGT

A_14_P128274 GTAGACTTACAGTAATTTCAGAATACCCAAAGTCACAAGAAGTTGTCCACCTGATTGTTC

A_14_P130851 GGTGGAGCAGTTTACATTTAATCTCAGGTATTCGGTTTTGAAAAAACACTTCAGACATTT

CUST_1282_PI426210822

ACACTATAGACTTGTTAAGCTATGTTTCAGTTTTGCAATTATAGATACACTCTGTAGACA

A_14_P138445 CAAGCATTTCTTTTCTTTTAGGGATGTCTGAAAGTCACATCCAGTTACATTACTGTGTTC

A_14_P102024 CTTAGGTATGTTTGAGTTCAAAGGAGGAAACCTGAGAGATAAAACAGTTTAGGCTTGTAT

A_14_P119963 TCTAGTTCTGTTATCTGTTGATTTCCCATCACCTGACAGTAACTTTCATGACATAGGATT

A_14_P115126 CCACAGATAAATGTAATGTGTATCATTACACTTACATTGCTTTTATCTAGCACAAAACCT

A_16_P01404438

CTCCTTTTCTTGGCTTAATTGGATTTGGATCTCTAATTATACCTTGAATGACCCTGACAT

CUST_5854_PI426210822 CTAAGACAAAATTGCTATCACTGCAAGCTCCTACCCCTGGCTAAATGCTAAT

A_14_P131019 AAAAGTTGTTACCAAATACACATTTCACATTGAATCATGTAGGATCTTGATGGGCCTTTG

A_16_P00685312 GAAGAAGGGGCTGTCCTTTTGGGGGTGTTGGCCTGTTTCTAATTG

A_14_P137055 AAACTTCCTTTTGCTGATGATCTTTATGGCTACTTTCTTACATGTTTTCCTCTCGAAAGC

A_14_P114064 TCTCATTGCCATAATAACTTCTCCGTGATTCTGTTTTGATACTTGTCTGACTTGTCTGAT

A_14_P201053 CTCACACTAAATAGCAAAAGAACATCATGATTTAACAAAACAGGGCCTGAAATAGATTTA

A_14_P108059 CTCTGTATCATATCATTGTGCTTGGTGCACAGTAATATTTGTGGAATTTTGGTGACCTAG

A_14_P113782 TTTCTGAGACTATAATCAGAGCGGAGATAGAATCTTTGACTCTAGACTGTCACCTTACTT

A_14_P130467 ATTCTGCCTCCCTTGATAAGGAGACGGTCACTCTAGAAAAGAAGGAAAACAT

A_16_P00809624

CCCAAATCATAACAGAATCACAGACAATCACATCTCTAGGTGACTTTTCATGTCATTCAT

A_18_P11852700

AAAAAAAATCAATAACACTCCTACAAGAAGATTTAGGCTACTATATATGCATAGCACAGG

A_16_P20771208

AATCTGTAGTCAGTATTCATCCATCACTCAGTTTTGGTGTATTTTGTTCTCCTTGGTTTC

A_16_P20550437

AACCTTTCTAACTGAAAACAGGATCACTACTCATTTTCTCTTTCTACCCTCATTCAACAC

A_16_P16887107

ATTCCACTAAATTCTATTCAATCCCATCCTTACCAAAATGTTTGTCTTCTTGCCTACTGT

A_16_P21545895

ACTAATTAATCTCCCTTCTACTTCAAATCAATCCTGGTTTAACCCTTGGAACAGTGGTTT

A_16_P19210036
GTGATGGACAAGAAGACTTATTTTCAACTATGATGCAAGAAACTCTAAAGGGTCATGTGT
A_14_P110192 ACTTCAGATTCCAAACCTGTGTTGGAATATACGTCTCATACATAGGACTAATCTTTCCAA
A_14_P133752 TATCCTCCGTGGTTTGAGTTGAAAATAAGTTTTAAAAATCCTGTGAGTCATGGTTTTGCA
A_16_P16109838
AAAAACGTCAAATGACATGTTAAGTCAAAATGGGGCTATTCCTTGATGTTTGCTTAGAGT
A_14_P126717 ACATCCTAGTTAGAAAAAAAAAAGACTCTGCCTAACTTCATGGGGTTAAAGAGAGGTTAA
A_14_P104140 TATGTTGCTCCCTTGAAAATCTTCCACTTATTTTCCTGTAAACTTCCAAGCCACAAATAT
A_14_P124429 CTAAAGTTCGTGTTTCTATATTGCCACTCCATATTTTTCAGTGTTCTTTAGTGCCCATCA
A_14_P202462 GACTGAAATAAGCATTTCTCTTTTCAGACTCCCAAGAAGAGTCACCAGAAATCTCTG
A_14_P134972 CTCCTTGAAAAACGCAGGAGAAATTTTGCAGAATTATCTCTCCTTTCCACTTTGCTATAA
A_16_P36230267 CTCTTACTTCGGCTCCTCCTTGTGCGCAGTTGACCTGAATGGGGA
A_16_P19501820
AATGAAGCCCTTCTATTCCTAGTAGACCTCAAAGTGAAGATGAATGGAAAGGAACAAAAT
A_16_P58424170 TGGCTCTGGGAGAATGGGGGGAAAATTGAAAACTACAATTAACTGCTGTT
CUST_3888_PI426210822 CAGGATTTCAGCGGAATCCAAACCATGTGACATGGACCATTATCATTCA
A_14_P132904 ACTCTGCCATATTCATGCCCTCTTGGAGGTTTTTCAATGGATGAATCTGATGATA
A_14_P101128 CTTGTCTTAATACCATAAGGACTGGATGACTTCTCCTGAGATAGAACCGTTTGGTT
A_14_P127749 ACCACAGGACAAGTCTGGCTATTTCTTTCTAAGATAAAATGAATCATCTACAAAGGACAG
A_14_P116591 TAGGTATTGGTGATCTCTGTATCTAGAGAACATTAGTTAGTCTCTGAGATGGGTTGAGAT
A_14_P113827 CTTTTGAAGACCGTAGTATGATATAGAAGAATGATCACATCACTGGTTGAAGAGTCAGTG
A_14_P123474 TTCCTTGGCTACTTAGTCCCGAGAGCCTAGATATTAAAGAGATCAATGGGAATAAAATCA
A_14_P108684 GAGTTTTGGTTTTGAAATACCGTTTTATTCATGATAAGTTGAGGTCACTTTTGGCACTCT
A_14_P122309 TTTCCAAGTGGTGCTATTCTTAGCACTTGATACCAAAAGGAGAAGACAAAATGAGAAGAT
A_14_P133234 ACAACAAACTTCCTCAGGAGTTCCATCTTCTCAACAAATATTTTCTAACTGAATCAGAGT
A_18_P13304079
TCATGTTTATTTGTGGATTATCACTTCACTTAAAGATTTTATACAATGCCGTTCTAGCAA
A_16_P18392332
TTCAAAGAATCTTATTCCACCAACAAACCATACAAAGTTTTAGATCATGTTCTTGCCAGT
A_14_P134830 AAAACTCCAGCAGGAAGTAGAATCCTGGTGTTTAATGATTTCTTCTCTGTCTTACTCATT
A_14_P119607 CTTACCATTTTCAGAGTTAGAAAACTGAGGTGCTCCAAATTCTCTCATTTGAGAATGTTT
A_14_P119534 GGCTGCGTCTCACCCACATACTTGAATAGATTACGGTATTCAATTTGTTTTAAAATCTCT
A_14_P127303 CACAAAGACACTGCAGCATATGCGTTCCTTTGCTGAAAATGTTCCAAAGAAAC
CUST_3750_PI426210822 TGAGGTTAAAGGGTTTGAACAAAATGGGCCTGATCTTGGGCTTCT
A_14_P138692 CTTGAAATTGTTTTAAGTGAACTATGGCTGCGGATTTTTTACTGTTTTATCTCAGGGAGC
A_14_P132544 AAATTATAAACAGGACTTTGTAGTTTGGGAAGCCAAATTGATAATATTCTATGTTCTAAA
A_16_P02342057
AAGACAGTAACGGTCAAATGAAACATAAATCCATGCTAACGACTATGAAATCTTTTGGCC

A_14_P117982 ACAATTTGTGTTCTGCTCATTTAGCAGCATCTCATGACATTCAACCTCTGTCTGTC
A_14_P138965 AGAGAAAAAAATATTTTTTGGCTTAGAACTCTACCTGAAATCCCCTTCAGAAGAACTTGG
A_16_P00093950
GTTCCAGAGTAGAACAACAAGTGATAAATTGGTTATTGAAAATACATGAAACAAGCCCCC
A_16_P19742250
AAACTTTACATCATTGATCTGCCACTAAAAAATGTTTGTGTAAAATTCCTGCCTCGAAGC
A_14_P202520 TTAGCAAGCATGGCTTGGTATTTTCATTCTTGTAAAGTCTCTTTATGAAGATTCTTGGGC
A_16_P02015472 AAAATTTAACTGTCGGGTAGATAAGATGCTCACAGTATCTGGATCAGGGATTATCTCC
A_14_P104731 CTGCGCTATATTAAAAAGGAAACAAAAAAATTTATGATATCCCATCATCTTTGCCTCTCA
A_16_P00905964
ATATTCTGCGTTTATCTTTTCTCTTCTGAGGAAGCATACAGTTGGGAGTTTCTCATTGTT
CUST_4994_PI426210822 CCGTCTTCACCATTAATGGCCATTCTTCAAAGCTACTTTCATTCCTGTGAG
CUST_7082_PI426210822
GTGTGCTCTAATGCATCTTCTTTGCATAAAGAGGCTTATTTACATGTCTTTTGGGGTATG
A_14_P128993 TTTCTTCAGGTGTTGAGAAATCCAATAGAGACCTCTGCTTGTCTCCTCCTTT
CUST_8966_PI426210822
TGGTGATACGTGAAAGTATTTGTCCTAGGATTCATTAATATTATTATATTTTAGTCTTTG
A_14_P133007 CTACCTCCACTTAACCCCCCAAAAAAGAGTTTTAAACCCTCCTGTCTATAAGTA
A_14_P122474 TATATTCACAAAAATTTCCCAACAGTTTCAATGAGTTCACACATCATTAAGGAATCCTAC
A_14_P110457 GAAAGGAAATCTGCTTTGGTGATGATCTTTCAGGCAATCTCAACTTACTTCTTTAATCAA
A_16_P16112422
AAAACTTGACAAAGATTAACCTTCAAATTGGTTAACCATGTCATATTCCTAGCCTGCCTC
A_16_P00685369 CTTGGCTTCCAAACATAGCCATCCATTGGAGGTTTTATGTGGAAGCATCTTT
A_14_P139652 CTTTGACTATGCAAATGAGCATCTCTCCCTATGAGAAATTAGAGATATAGGGAATGTGGA
A_14_P107271 CTGTTTATACCCTGGGATGGGGATAAAATAATTAGTCTAATTAAGGAAACTTCACCTTGG
A_14_P136405 TGTAAAGTCTCTCCCTTAATGCTCAGTTCTTAGAAGACCGAGGTAGGTGG
A_14_P201196 TCTTTCCCTGATAGTGGATGTGACAAAGAAAATGATATTGTTGATCTTTATGATGGCAGG
A_16_P00685125 TAGGTCAGACCCCAGAAGATGGGACTTTTCTGATTCAAGAAGTTCAGTATCTTT
A_16_P16769577
ACTCTCCTATCTGACAGATCACTTATACTTTAACCAGTTCATTGTATCTTAACAAGGCAC
CUST_3931_PI426210822 CTAGGCAGAATGAGCAGGGCACCAATCTAATGGCTCTTCTGGATAA
CUST_6178_PI426210822 CTATGATAACTATTCTCTATTGCATTCGTCTGGACCCTTTTTTCCCCTTCACTAGC
A_14_P102886 CAACCACTAGTCAGGGAAATATACAGCACAGTAATAAAGATGGGGAATAAATCTGTAAGG
A_16_P17766723
AAAAAAATGCAAATCTTGAACTAGAAAAACCAAGGTAGAAAATTCCTGGAAATCCAGCCA
A_16_P58424512 TTTTCAACAAAGATGGAGTAGACCTTAGGCTCAGAGGCTTCCAAAGGC
A_16_P17586446
AAGGCCACATATCTTCAAACACATTATGCCCTTTAAATTATTTCCTCTCAATCCTAGCAA

A_14_P135492 AGCGCTCAATAACTTGACATCAATTGCTTCCAGTTCTGACTTTATTAGTAGAAATTCTAC
A_14_P133692 GTCCTAATTTCCACTTACGCTTGCTTTTATTCATTGGCGAACTTAAATTCTAGTGACTCT
A_14_P115724 TTAAAAAACATACGTTTTACATTGCCAGTCATTAGCACTAAATAGGCCTCGGCTCTTTTC
A_16_P00198430
CTAAGGTTGACAAAACACAAACAGCATTACGTTCACACTATTGGTCAGTTCAGACTGAA
A_14_P114260 GGATTTTGTAATTAACCCAGAAGGCCAAAGTAAAAGCATATTTTACCCTGCATAACTCTG
A_18_P25836655
AATTTGGCCTCAATCTGTATCTTTGGTGTATTAATCTCCTCATGATCCAGATAACCTCTT
A_18_P22840132 AGATTGGCAAGGCCACTGAAATAGTGACTGGCAACTCGGTTTCTTTC
A_14_P106592 CTCCTAGTCAATATCCACCCCATCCAATTTATCAAGGAAGAAATGGTTCAGAAAATATTT
A_14_P100803 TAACTAGTAATTCTATACTGCCACTCAGCAGTCTTAAAATCCCAAGTCTGATTGTGTCAC
A_14_P111446 ACCAAATTGGTAATTATAATAATGGTCTTGGAAACCATGAATGGAATTCAGTTCAGACCC
A_14_P128138 GGAAAAGGTAACACAAAGAACTTTGGTCTATGAAGGACACAAATGAATTCTCATCTTCAA
A_14_P110341 CCCTTGGATTTATGAGACATATACAAACATGCTTATCATAAATGGGCTGTGACAGTCTTT
A_16_P16527183
AGAATGCTGCTTCTGCTTTTTAACATGCAAACATAAGTATGAGGCTTTTGCTATGAGTCA
A_14_P132314 ATCTGCAAGGATTCGGAGTTGTGCTAAAAGGACTTTGATTTTTTTCCCCCTTTA
A_14_P109116 TCACACACTTTTAGTGAAAATGGATTATTTTCAACAGCACCAAGAAGTCAATGGTGACTT
A_14_P135539 CATCTGGGGACTCTCTGAAATGAAATAAAATGCCTGGACAACTAACAAATGCAAAG
A_18_P22842255 GTCATGCATTCACCCCACAAATATTCCATCTATCTCTGAACTAGGCAGAATGAG
CUST_1399_PI426210822 CTGAAAACAGTGCTTCATGCTGCCTGAAACAGGGGTGCCTTTAAG
CUST_4032_PI426210822 GTAATGACAAAATGTGGGCCAGCGCCTTCTTGCAGCTGGGCACAC
A_16_P20130519
AAATGACGAATAAAACCCAGATCTCAAAACTTAACACCCAAACTAAAAGATGCTGCAACT
A_14_P122862 CTGTATTGACTGTCTTCAAAAGTGTCTGGAGAAATGGAAAGTCAGTAGCAACATTAAACA
A_14_P119571 TTACTGTATATAGAAAAGATGTCCGCAGCGTTCAGTAAACACAGACACTAATGAGACTCA
A_14_P110878 GGCAGCATTTATTACCCCATTTGTAAGTTCATTGCTATTAACCTAGGGAAAAATTGGAAA
A_14_P127155 AGTAGGGGACAGATGTCTATATTAGTGCCATTTTTTAAAAGATGCCAACACTAACAAAGG
CUST_832_PI426210822 CGTGTGTATTTTATCCCAGGGAAACAGGTTTTCCTTAAGGCACTAGGC
A_14_P117149 GTAAAGTTGAATCTAGTGAAAATAATCTTTATTTGACATTTAGAGAACAGGATTGTGGGG
A_14_P137375 TAAATTCCTGTCTTTGTCTTCTTGGAACAGTCTTCTACTTGTTACAGGATCTTCCTATCT
A_16_P17425941
AAAAACAGTTTTTTACTGGCTAGGAACTAAAAATCTCTGACCAGATAAGGGTTTAACCGG
A_14_P112037 GCTTTTTCATTTAACTGATGCCTGCTGTCCTCTTGTGAGTCAGTATAACCAAGAG
A_16_P18232920
TCTTGCCTAAAGTCTCACTACTCTTTGATTCTTACCACTATTAAAGCATTCAGACTTGGT
A_14_P138483 TCTCCAAAACCCTTTAGTAAACTATTTTCCAGCAATAAAACCCACTTTAAATTCAGGCAG
A_18_P22842641 AATCAAGGACTGCAGGAGGGGTGTGCTTTCCATCCACTTTCTATATTCTAAAAATTA

A_18_P14133135 CTGGGCTCTCTGGGGAAGGAGCACAGGCTGTGCAGTGAGATCAGG

A_14_P119258 AGGGAACATAGACATACACAAGTTTGTAGCAAGATACGCAGATACAGAAATACGGACATG

A_16_P37682025

TCCACGTTCTCTTTTGAAAGATAAACATGCACTCTATAAACTTCATCGGACTAAACAACA

A_14_P121730 TATTAACAAAATTCTCACTTTTGGCTTTGGATTGGCTTTGCAGGAGTGCAGATTGATCAT

A_14_P119417 GTGGATCATTTACAATGCAGATTTACAGATTTCTCTCAGATTCACTTCTTCCCAATGAAT

A_14_P131596 TCATCCTTAGAAAGTCTTTTGAAAGGTTTATCGTGTTTGGGTTTTTTTGCTCTACCACC

A_14_P116097 CCAGTGCCACAGCATTGTGTTCAAATAAAGGGAAAGTCTCAGAATTATCTATAAGAAACA

A_16_P36230632 GAGTCTGTGTGATACCCCCCAGAGAGTTGACTCCTAATTAATTGCTAGTTG

A_18_P17338418

AATTGCCATTCATCAATGTGCTTCTATGTCTACTTGTTCTTATACACGTCATATTGCACT

A_14_P139855 CCATTTGAAAGCCTAGGTGCAATGAGTGAAGAAATCTTTTCTTAAAAACTCCAAATAGGT

A_14_P133564 CCTACGTTCCTGTGTTTACATACACAAGTAGCAAATTCGAGTATGCATAGTGTAGATGGA

CUST_1108_PI426210822 TTGGTTTCAGAAACGCAAAGCATGTGTCAAGCCTGGGTTTCCACT

A_14_P137869 TCAGAATTTTCAATCACTGCTATTTGGACAATTGGTTTAAAAACAGAACGATCGATGGTC

A_16_P21018962

GTGTTCTCACATGGTAAAGATAAGGCATGTTCTGGATCTTCTCAAGAACCTTTGACAT

CUST_5793_PI426210822

TTAGATCAGAAGTGAAGATTTTCTGGTACAGATTTGGGTGGGAGAAATGAGCTAC

A_14_P138256 AATAAAATCTCTAGGAAACTGTTTTGCTGCTGCTTATAGGGGACCTACATTCCAATTAGA

A_14_P126255 CTCATCCATTTATCCATTCCTCATAATCCAGTGTCCAAAGAGCACCCC

A_14_P109551 ACATGGCAGTAGATGAGATTAGGGATTGAAGTCACTTTAAGTCCAAGAAAAGTATTGAAG

A_14_P138904 ACCAAACACGATTGTTGTTTCTTGGACTGCAGAAATTGGAAGAGTAAGTAAATTTTTGTT

A_14_P126310 AAATGGATTTACAGCCTTGCATTTAGCAGTTTACAAGGTAGGACACTTTAATTCCCATAA

CUST_7993_PI426210822

GAGATAAAGGAGGACGACCACAATCCATGGACACTATTGCCTATAGTAATGTATGT

A_14_P125010 CCTAAACAAAAGCCTTCCTGGTGGATTATCTGGTATTTGGATATCTTTCATCATTTTGTT

A_14_P109424 AAAACAATGATTAAGAATGGGATGCATCATCCCTTTGACTTGTAGGTAAGACTTAATGGT

A_14_P115652 ACCTATGGCAAGAGCATTGACCTAGTCAAATTTCTTCTTGATCAGAATGTCATAAACATC

A_14_P136304 TTACTAATTGGTATTCCACAGGGACCAAAGTAGTAAAAGTTTTCTTGACAGCACTCTAGT

A_16_P01246588

AAAAAAATCTTTTCTGCTGTTTCCTAGGTAAGACACAATCTTTGGACTCACTCTTGTTGA

A_18_P22841890 ACTTCCTCTGCTCTCCTTGGCCTTGGCCTCTTTGCTTTAGGATAT

A_14_P107265 TTAAATTCTGGTTTTAGAGCCCACTGTGTTTAACCTCTCCATCCATTAAAAGAGATGATT

A_14_P110524 AAAATTGGAAAGTAACACCATGAATAGTTGAATCTTTCATTTTGGGCTACAGATCCTTGC

A_18_P13529607

AGCAGAGTCATTTCCGATATCTAGTCTACATCGTGAATATAAGAAATATGTCCCTCATCT

A_14_P127812 AAAGAAAAATGAGTCCCTTCACTTACACAGATGATTTCATTTTTCCAGTGCTAGTTATCC

CUST_1767_PI426210822
ACACAAGGTAGAGAGGAAAACCAAGGAGGGAACTGAATTAAAACTGTATGCTTTAATTAA
A_14_P120908 AATTCCATGTCACATTTTCTACCCAATCATCTGGATTTCAAGATTCCTTTTAAGATCTCA
CUST_2858_PI426210822 GAAGCAACCTGGCTTATATCTGACATTAGGTGGGGCCCATTCAGC
A_14_P114379 CAACCATCATTACATAACTGTTGAAGAGTTTGCATGCTGAACACTTCACACAATCTACAA
A_16_P16564440
AGAAGCCTACTGGGATTCTGTTATTGAGATCTATCTGTATCTAAAAATGTGGGTCTCATG
A_14_P139658 CTTGTTTTAGCATGGCAAAATTTGGCGTTCACAGAATCCTTCTTCTGGCTATTTCT
A_14_P123252 CCCAGCAATTGAAGTCTTGTGTGTATTTAATGTCCACCGATTTTAACCTTGTTATTACTC
CUST_7694_PI426210822
AAGAGAATGTCTTGACAGCTGGGGTCTGTAATTCATATGCTGTTCAAATGACAG
A_16_P00888777
CTCTGTCCTAATCCTTATCATTCTCACCAACTATAGCAATACTCTCTGATACGCCTTCTT
A_14_P103693 AAACAAATTTTCGTCGTTTACCTTTTGACCACTGCAGGTAAGAGTTTTGCGAGTTTAC
A_14_P124654 TCTGATTCCTGGCTAATATTATTCCTATATGTGTCTACTTCATTATCAGTCCCACAGTGG
A_14_P124440 CGAAGGGATTTGTGTTTTTTTAGGTTGGACACAAATTTTTGAGAACATAACTGCTAAGCA
A_14_P113645 TTTAGGGATTCCATTTTACTGCACTTACTAGGTTAAAACAGGAATGGCAAAAACAGGAC
CUST_10033_PI426210822 AAAGAATAGCTGTCTCTCCTTAAGATAAGTTCAGACAAAGGATGGGTGCCC
A_14_P200205 TCCAAAATCATGTTGGGGGCATCTACATTTCAGTATAAAAATTAGGTGAATTGGCATTTT
A_16_P16339564
AAAATCTCATGTGGAATAGGGTAAGTTTGCATATCACTAAAAGGTGTTACCAGTTGACTG
A_16_P00962102
CTTAGCCCTCTCCTGATATTAACAGACTAGCCAAAATCTGATCTAATTCATGCAGACCAT
A_14_P124444 CAAGAAAAAGTAGAAAGTTCCTTTGGCCAACTGCTTAGAAGCCCAACACAAC
A_14_P110556 ATTTAATTAGAAGGAGATTCTCCAGGTCATCCTCAGACACTTTAATCTTATCTTTGCCTG
A_18_P10834703
TCACTATCTGCACATGATACCTAAGTTTTTCTCAGTATCCAGAATGAGCATTACTTCTCC
A_14_P100785
ACAAAAACACAAAAAAACGCCAAAAAAGGATAATGAAGCAACATACAACTTACTACAGGAT
A_14_P201478 ACAGGACTTTGGAAGAGGACTACTTTGCCCATATGAAATAAACTACATCTCCTTTCTATC
A_16_P20391348
AACTTAACCTTCTGAATCCTCAAGTAGCATTTTTATCACCCACCATTAGAAAAGAGATGG
A_16_P36230203 GCTGTGGTTTGCTTCTCTCTCCTTCCTTCCCCCTAGAGTATAAGAAG
A_14_P108738 CTTCCTTCGTTGTGATGCTGTGAACCTCTTGTGCATAAATTATCATGTGCATCTCTAA
A_14_P101838 CAGTGCTAAAATGCAGAGGAATCTTCATTCACTGCAATATTTCTTCTCTTTTTTGGTGAT
A_16_P17567812
AAATTGTCGGAGATCTAAACATTTGCTACCTCATTATTCATGACTGAACCTGATACCTGT
A_14_P109869 AAAAGGTGGTGATGTCTGGGTTGTAGCCCAGAACAGAATGACTTTAAAACAAC

A_14_P133060 GTAACCAATTCTGTTTTTCTCCTTGTAGAATGCTGAGATGTATAAACTGTCTTCAGAGCA
A_18_P22839792 CTGATGTGGCACAGGTGGTCAGTGGGGCAGGGAAGACAGAAAGAT
A_16_P15008404
CATGTCCCCCTTGATTGTAAATTGCTTCTGTTCTGTTTATAAGTAAACTGTGCATGACTC
A_14_P120847 TTTCTCTTCCTTTCCTTCAGCCAAATTTCAACACTAACTTTGAAGACAGAAATGCATTTG
A_14_P110517 GTGCTATCATTTATCTTGGCTTATAATATCTGGGGTTTGATTGCCTTGTGAAATGTCCAA
A_14_P106322 CCAATTGCTTGAATTCCATCATCCACTGTAGTAAGCAGTTGTAAAATGTGCATGTAAACA
A_14_P121638 CATTTTTGATCCAGGAGAGGAGTTTGGGAGACGTTACCATATAGTATATTATACTCTGCA
A_14_P126064 ACCATATCACTCGTAGTTTCCGATCACTAATCACTCAACATACTCATATCTTTTATGGCC
CUST_6646_PI426210822
TCTGTGGACTATCATTTAGAATGATTAGAACTTTTTTCTTCCTGGGCTTAGAAAACATGT
A_14_P126741 ATTACAAGATCAACAGTTTACTTACATCACTGTAGTTTATTCGGTTGAGTTTGGCTAACA
A_14_P122707 CAGACTTAAGTAAATCCATGTCGGGGAGGCAGAAACTTGAAGCACAACTAAC
A_18_P22842102 AAAATCACTTTCTGTGGAAAACCTTCACTCTGCCCACCGATGAGCA
CUST_7019_PI426210822 CATAACGAGGTCATCTCTGCTTTCAGGGAGCTCTCAGAGTCTCAG
A_16_P03701706
TCATCCCCAAACCCCTTAATTACAAACACTTAATCACTAAGAAGCATAGATTTCATAAGA
CUST_5506_PI426210822
TGAAAGAAAGGGCATCAGCTAATAATAAGAGTCCAGTAATTTTCTGGTATGAGTGAAGTA
A_14_P138603 CAGAGATTATCTTAAATAAACTGAAATTTGTGCCACACGGACCAAAGTCTTGCCACTATA
A_14_P108704 TTTCAGAAGTAACACAAGGAGGTATAATTCTTCTTACATGACAAAAAGTGGCCCCAATTT
A_16_P00075045
AACAGAACTGATATAGTCCTTAAAACAACTCCTTTGGCATTTGATCTCCATCTCTGGTAT
A_16_P00848512
TTAAGTTTTCCTCACACACATTTCTTAATGCGTTGATGCTTCTTACAACTTATTGGCCTG
A_18_P16933035
AAATCAGATGGGTAAAAATGAACCACAGTGCTTTAGAAAGAATGTAGAATGGCCACATTA
A_16_P30952418 ATTTATTGGCAGGCCTGAATTATGTTCTGATGGCTGACGTGGCCAAAAAG
A_16_P01151328
CTAGACAACAAAGGCCTTTTAGTCATTGCAATCTTGTTAGTCTTTTCTGAGTAGTCTCAC
CUST_6938_PI426210822
AAAACAGCAATTTAATAATTTCCTCTTTTAAGGGGAAAGGGGGGAGTCTTGGATTTTAAA
A_16_P18971680 ATCATGCCATGCTAATCACCTACACGAGTAAATTAGTCTTGTCAAGAGCCATTAAGGT
A_14_P115696 TAGAAGTGCATTCATACCTGTTTTAGTAAATAGTGTTTGTGGGCTTTTTGCCACTCCTTT
A_14_P107726 TTTGGTTTTCACAGGAAGCAGATTTTAAAGTTCTTGGATGCTGAAAAGGACATTTCTGTC
A_14_P121034 CCTTTTTCAAAGATCCAGCGCTTGTAGAGGTTTAGTGGTCAGTCTTTGCAG
A_14_P107549 TATCAATACAGTGATATTCACCCTCATCGATCCTTTTGAACTTAGGCAAATGTTCTGGTT
A_16_P30952438 GTGTTTGAAGCAGCCTACAGCCTCAGTGAGCATGTGACTGGAGAG

A_18_P22841787
CAGTAACCCAAAGACCTGTTAATTGATGGTCTAAACTCCAGCCTCATATTTGACTTATAC
A_14_P122836 AATCTTTTTCTGGATTTAAGAAGTTGTGGAGGTAATGGAATGGCTTTAGTTGACTTGTTC
A_14_P117123 GGACGTAATAGCCACATTGGTTGTTCACATGTCAACTTCTTTTCTTTCTGTTAAATCTCT
A_16_P19309648
GCATTCAGTTCCAATTTATCATCAGTAGCAAATAAACTCTTCCATGTGTATAACCAGTCC
A_16_P01374278
AATTAAAGACCTTTAGTTCAGGATTGCACTAACAGTTTGATAGTTACGGATGACTGAGGA
A_14_P111177 GTGCCAGTTGTTTTACTGAAGCAAACAATGACAGCAAAGGTCTACTGAATAAATGAACTA
A_14_P137151 ACTTGACTTACGTCATTCCAAGAACTCTAGTATAAAAATCCAGTGACTTCTTAGGATCCT
A_14_P103606 ACAGGTGTTCTTTATTTTACATGAGGGCTACTTTCCAACCAAATAAAGTCAATTTTTCTA
A_14_P103411 GTAAAACACATGGACACAGGTAACTATGCTGTAAAGCAAAATCCCCAAGTCCTACA
A_14_P128346 CAAACCTCATTTTCTAAAAAACAAACACAAAAGCAACCTTGTATGCATCCTTGCATGTTC
A_14_P111354 ACTTTCTATACTATCCAGACGTATGTCTTCCCTGTCACTACTTGCTTGGCACA
A_14_P104187 CAGTAGAACAAGCATGGAACTCCAATTTGCACTGCAAAAGAGAGACACAACAG
CUST_640_PI426210822 ACACCATGACAGCAGTGTCAATGCTTTGGAAAAGCAAGGTGATTGAAG
A_16_P02762555
GCTCAGTATGTCAGCACATTAACCAAAGTCAATGTATGCCTTAGAGGTAATTGGTTCTTA
A_14_P106315 GACTAATTGAAGAATAATTTGAATTTTCTTTGGCAATCCAGGAGAGGAGAGATTTGTGCC
CUST_3844_PI426210822
ATCCAGAAACAGTGGGCCTTTTATAAATTAAGCCTGCCATTCTCCAATTGCTTAA
A_14_P137480 CAGACCTTGTCTGTTAAAAATGAAAGAAAAAAGTAGAGGGCAGAGTGGTGACTACAG
A_16_P16191969
CGTCTCAGTCTGTGTGAGAAGCGTGCATGAATTCTTGCTATTTTCTTGATATTTTCAAAT
A_14_P105085 CTTCTTCTTGTGAAGGAAATCCCGTTTTTTTCTAGACAGACACTTTGGTGCAGA
A_14_P105270 CTTGGAAGCAATTAAGAAACCCAAGATAGAGCAGGGGATTATGCTCGCTC
A_16_P02035747
AACTGAATTCTGGAATCACAGATCTTCATATCATTCCAACCAAACCTTTACTTTAGCAGC
CUST_268_PI426210822 GAAGTTCCCCCGTTCAAGGTCGTGCCTCATGGGGGCCCAGGTGAC
linker ATAACCGACGCCTAAATAACCGACGCCTAA
A_14_P117674 CAACAACTGTCATGCAGAACTGAGATAAAAGTCAATCCAGAGATTCCTCCTGCTTA
A_16_P17736126
AAACAGTGAAATGCCATAAAATAACCCTCTCTCGAAGTATATTTGGCCTCACAGGAAA
CUST_6385_PI426210822
GATTTTCTCTAATATATTTAGAATTGAACCTTTTTGGGTACAATATTCTTTTTTAGTATT
A_14_P131494 TGATGGCAGTTTTTGGCAATGACAATGATTATCCAAACTTCCAGTTATTAAGCAGTCATT
A_14_P118905 ACACTTATGCCCAATAGGCCACTTACATTGAATAAAATATGTGGATTTTGCATCCTGTTT
A_14_P114718 ATTTATATGTTGGCTGAATGCATTGGAAACTGGTAATCTATTTTCTTATAACTGTTAAGA

A_14_P115211 CTTTTCAAAGAAAGACACTTGAAATAATGGAGGATCCATTCTTTTCCTCTGTCAGATTGT
A_14_P112151 CTATAATAGCACAGCATTTCAGAACGTTGGCCTGTAAGAATATGATGCAACTCTGTAACA
A_16_P40639535
AAATGACTTTTGCACAAGTTTCAACCATCTTGAATGTGTATCCATGAAAACAAACCATCC
A_14_P137778 CCAACGCTACAGATATGCCAATGAAAAGTCGTTTACAAAGGATCACAATTCTGAATAAGT
A_14_P119955 AGAAATAATCTTTGGTATGACTCCAATTGTGAGATCCTGCTGTGAATCAAAGAAAGATTG
A_14_P125076 GACCCTGCAATGTAGATATTCTTTCTTTTAAAAGTAGTAGAGTTCAGAAGGGGCAGAAAT
A_14_P125491
CTGGGCAAATAGAGAGGAAGTCTGGAAAATCATGTTAAACAAGGAAAAGACATACTTAAG
CUST_2606_PI426210822 AGAAGAACTCCAGGGAGAGCACATATCAAGAGAAGAGACCTAGGAGA
CUST_10203_PI426210822 ATATGAGAAAATTGTGTATGTCTGTTACGAGGGTTCAGGGAGCTGCTCTTTG
CUST_3873_PI426210822 AATCCGCTTTCTGCTTTCTTAGGCACTAGGAAGGCAAGGAGATACTTG
A_16_P19426111
ATGTCGGTGCTCCATATACATCCAAAAGCGTAATATTGAAAAACAGATTCTGACCTTGG
A_18_P27169945
ATCAGGAAGAGAAAAATACCATTAGAAAGTTTACTCACCTACTACTGATAAGGCCAGGAC
A_14_P137284 AACTTTCCACAGGTCTAAAAATTGCTTCCATTTTATAATTTGAGGTGTTGCATGGGAATT
A_14_P139146 CTACGAAAGACAGAATATGCCATTTGATGAACTCAGAGTTAAGTGAGATTTCTCCTGTTT
A_14_P202197 TTCAAATCTATTGATCCTTAGGTTTATCCAAAGATTCTTTAATCCTGATCACCTCTCGGG
A_14_P117288 AAAACCTTTTCTCTTAGGATTTGGTGCTATGGAGAAATTTTTGGTAGAATATAAGAGTGC
A_16_P21581155
GTGAAGTGCTTTAGTTACAGGTGATCTATGTTCCAGGATTGACATGATATCAGCATAACT
A_14_P104318 AGAATGTGTATACAGAATGAAGTAAGACATGGAGGATTTGATCCAGAAAACTCTTGAGAC
A_14_P115500 ACGTAAGTATTCACTTACCTGCTTTTGATCAACAATTATTTCGAGGTCTTCATCACTGCT
A_14_P129614 CCATCAAATCATATATGTTGACCTGTCTGAATTATAAACCAGCCTGACCTTTCCTTTAGC
A_14_P130155 ACAGTTATTTAATAGATCGACACAGACAAATATCCTCCCCCAGAAAGGATCCTCTATTTT
CUST_818_PI426210822 GAAGACCGCGATGATGAGTGGATGGGGGTGAGCCTGGCCCGACAG
A_14_P202373 AAAAGTGGCATGATCCTTTGCAGAAATGACTACATTAGGTAAGACTTTGCTGTCTTTCC
A_14_P118671 ATATTTATGCCACATGTTCTTTCTGGGTAGAGAAGCCTGAAGTTATTCTGTTTATCCTGA
CUST_7359_PI426210822 AGCTCTGGAAGTGACTGGATTAAAACCCCACCAACTGAAGACAGT
A_16_P18474058
AGTTCCTAAGGCCAGTTTTTAAGTGGATCTGTATAGAGAAAATGTTACCATGAGAATCCA
CUST_6896_PI426210822
CTTGTCAAGTTAATAGTAGCCTCAAGTAAAATTCAACCCTTAGATGTTTATAGTCATCTT
CUST_7764_PI426210822
AATTTTGACTCCTTCAACAGTTAACTGATCTAACTCTGCCAGATCCAGATTTATCAGTG
A_14_P109703 GCCAGATCTCTAAAAACTATCTTTGTTCTTTAACTTCCCTGTGTCTTTTAAGGAAGGCTT
A_16_P00685178 TAGGTAGCATAGATGGCCTGAATGTTTGGTAAGGGGTGCTGGAAACTAG

CUST_1733_PI426210822 TTGCTTACTGCTTTTCATGAGTGGTGTTCCCCAGAATCTGTGGTTG

A_14_P132836 AGAATTTAGGGATGGTGATAAGCCAGGAATTTAATAATTATTCATTCTCCTCCACCTTTA

A_14_P126805 AGGAAAAAGTGTGGATAGTTTTCAATTTCCCTGGTAAGGACTTTGAGCAGGTTTC

A_16_P18310558

ACTGAAATCACTCTACATTACACCTCTCACAAAACCTTATTACTTCCAAATTCAAACTCT

A_16_P19749610

AACTGAAACCAAAGACAAAGGAACTATGGAGGAAAAAAGATTTAAAAGACATTCACTGGC

A_14_P127861 CTAACAATACTCACTCTATGATCTTCTTCCTCCACCTCTTGTTGTCACTGGAGTG

A_14_P133539 CACACAATTGTCTCTTACAACTGAAGGGACATTTAAACTGTCAGCCTAATAATAAAGTAG

A_14_P135623 TATCTTAACTAAAAAGCAATACAAACCTAAACTGTCAGCAATTATTCTGTTTATAGTGCT

CUST_8221_PI426210822 AGAGATTATCACCAGATGTGTCTCCAGTCCTGGCTCACAGGAGGA

A_14_P115922 CTCTTGGCCTAAAGATCCAACATGTAACTTCAGGGGAGTCCTTCATTAATTAATACTACT

A_14_P129865 CTTCAAGGAAATTAAACAGCCATCAAACAGGTTTAAACAAATGCCTAATGCCTATCGTTT

A_14_P120287 ACGCCATTTCTTTCCAACTTAAATCCTTCAGTAACAACAAACACCACTCACTTCAAGAT

A_14_P137166 GAGAAAATAAGGGGTTAATAATTTATCCCTGAAGGGATTCTGTTATTTTGTGCCATTGCC

A_14_P104765 TGCCAATATTATCAATGGAAAGCCACATGCTGCAGATGTAACTCAATGAATTGGATTAAG

A_14_P128856 GACTCCTACAAACTATTATGCTCACCCTGAAAAAGTGACTAGTGACTATTCTACATGACT

A_14_P109091 GGGTCCCTTGAATTATAAAGAAATGCATGTAGACATGTATGTGCTTAAAACTCACACAGT

A_14_P120342 GCAGATTCTAAAACAGTGGGGCTTTGTAAACCTTTGTTGTTTCAAAATACTGTGGCTT

A_16_P01660657

AACACCATTCCTTTTAAGTGATCAATTGCCTTTACAAAATTCTACAATGGTTCCTCATGG

A_14_P125681 ACGGAAATAAAGTGTGGATTATTTTTATTGATGCCTAATTTCCACTGGCAAAGAAGTCAG

A_14_P116408 CAACAGTTACAGCAGAAAGTTTTCATGGAGAAATGGAATCACACTTCAAATGATTTCATT

CUST_9685_PI426210822 GATGTAGGATGAAGAGGAGGGTTGTTGAAGGGTCATCACCGCCCC

CUST_5758_PI426210822

GAAGGACAATTTGTACAGACCCTGAGATAGAAGGATTCCTCAGCTTTAATTTCATGAA

CUST_2437_PI426210822 GTTGTGAACTCTGGTTGGTTGAGGGGGCTGTTTTCTAGAGGGGAG

A_16_P38233280

ACAGCAGTTCCAATCTTGAAACTGGCCAATCTTGAAAAATGAAGAGCAGATATTGAAAG

A_14_P133169 AGTGGGCGTGAAAGGGTTTATTTGTAAGAGATATTGCTGCTGACAGAATTTTATCTTTTT

A_14_P110675 CTTAGAGGCCTCAAAACGCCATAGGCTAACAGTTCATCATAAAAACTGAAATAAATCAAT

A_14_P114033 GACCCTTAGATATGAACATATCTTGAACATCCAGAATTCCAGTTTACTTCATCTGTGAGG

A_16_P18577258

CCCACATAAATCTGACATAGGAATTACATTTGGAAACTGGAATAACATGCTTTGTCTCCT

A_14_P138534 TGAATTCTTTGTTACTTTTACTTTTGTAGTTGTGTGAATTACGTGCCTGAGCCTGAGAAC

A_14_P133594 ACTTGAGCAGATAGAATAGTCCTCTGAAAATTAATCACACCGTTGGCTACATCCATTTTA

CUST_1902_PI426210822 TGTAGAAATGGTAAAAATGCCTACGTCTAGTTCACAGTGATGCGGGTGC

A_14_P127414

CTACAAGTGGAAAGCATGCTATCAGAAATCAAAATGGAACAGGCTAAAACAGAGGATGAA

A_14_P101719 TGTATTGGTGTTTAAACTTCAATTTCTGCCTTCTCTGATTGTTGTCCTTTCAAGAGTTCT

A_14_P115858 CATCCATGGCTACTGAAGAGTTGGATAATTTCGTCTACAAAATTGAATACATGTTTGTCC

A_14_P113710 ACATTTTATATTGGAGGAAAAGGTTCACTGAACAGCCCATCACAGATTTTTGTAGTGTAA

A_16_P35353999

ACACTCATACCTTTTACACTGAGTTCCTCACATACACATTATCTCAAAGCATCACTTTGA

A_16_P00011664

ACATTTCTTCTCAAAACTTGGATACTATGAGGCCTTTGGAATCTTTTCTGGGACTGATT

A_16_P02197547

CCGACTCACTAGTGATATGTCAACAATAAAACTGGATTGTCATCTTTGTAGAGCAGTAGT

A_14_P136321 CATGTAACCTGTAAAACTTCTTTGGGTAAGCGATTTGTTGCTACTGGCTAATAAGACTTG

A_16_P21254356

CGGGTATCATTAAAATCAGAATAATGTGAAGTCGTGCATAACAATGACAGATATACAGAC

A_14_P131090 TTTTATCTTTAATTTATTCTAGGAAAGAAGTTGATGGCAGAAAGGGCACTACAATTAGCG

A_14_P127733 TCTCAATAAAATGTTTGACTGTCTTAATAGCCTTTCTAGTCATCTCTTTACGGGCTTCCA

CUST_7362_PI426210822 GATGATGGCTCGCTGATGGGAGACAGAATTTCTGTCTCTCCTGTC

A_14_P126277 GTTATTTATGGATTAGGTTAGTCCCTGATAGTTCCCTACTTTGTTGACCTAGGTATCACA

A_16_P21387807

TGTGGCTTTGCATTCAGATAAGTATTCATAATTGCCTCAGAAATCGATTACTGCATCTCA

A_16_P00685311

AGCAGGAAAAGGTAGATTCTTGCCTTTTAGTCTGTCAGAATGACACTTTATTAGATGCTT

A_16_P00852548

AAGTGTGCATAGTTCAAAGATGCTTTAATGACTCTATCACGGAATTCCAGTAAATCCACT

A_16_P36174059

TCACTACCTACATTACACATAACTTTCTGCGACTCTAGCATTGGTCTTATGATTTTGTTT

A_14_P200614 CAACAGAGATAACCACGGCTTCCAATGCAAACAGGAAAGGATTCTCTATAGAAAAAA

A_14_P135218

GCAAAGCAAACTTTGCCTTTCTCAGGACTCAGAAACTATGAAATGAACAGAAAGAAATCA

A_16_P03611973

TATGCAATCTTTACCCACAACTCTACTCACTATGTCTGATCACATTCAGCATTTTCCTAC

A_16_P02017065

ACATGGCTCTTTTTATCGTCTACTCAAGATAATACTGAAATACAATCGGGTGTTAGTGGC

A_16_P18676280

AAGCACCTAGTAACTAATCAGCAATCTGATAGACAACATGCTTATTATCCTCAAGGACCT

CUST_2744_PI426210822 ATGAATTGGTGGAGGTAAATGATGCAGGAGGTTTGACCAACTGCATG

A_14_P129323 TATTCTTAGGCTCATGATGCACATGGCTCACCCGTGAATGGTTCAGAAAAATATTATAAA

A_14_P109569 CCAAGAGGTCATTTCGCTTCAGGCATCCTTCATGTTTCAAATCCATTTTTAAATAAATTG

A_16_P16891804

AATCAATATGCCCCACATAACTTAAAGTTCCCTTTTTGTTAGGACATCATGAATTGCACA

A_14_P113758 CATTGTGACTTAATTTTAATCATTTGTTTGCTGGTCAAAATTCTACAAAAACTCGGGCCC

A_16_P39433444

ATGTAAGGTTTAACTGCCTCCATTAAACAAGATAAGCCCTTTTCTAAGATTTTGCCACTG

A_14_P111705 CTCTGCAGGTGAAACATGAACTTGAGTATTTCAAAGTTCATATTGCTGAACGTAAAATCC

A_18_P13358344

AATTTCCCATGACGTTACTTCTAAACTCTGAAGAGATTGAAAGAATTATCCCTTCCCTAC

A_14_P100543

CTAAAGAAAAAACTGAGAACATGACAGATTCAGAGTGCAGAGATTGATGGTCTTGAAAAG

A_14_P114607 TTAGGGCAGAGGCAATAAGTCAGAAGTCTTGAAGTTGAAATAGTTATATGTGTGTCATTG

A_14_P121283 GATTGATGTCTACCTCTATGAGGTAGATATTAAACCAGACAAGTGTCCTAGGAGAGTGAA

CUST_9743_PI426210822 CCTCTGCAAACTCTGACAGAGAAAGGGATTTTGGAAACATGACACCT

A_14_P109149 CTGGACTATCTGGCTGGTCCAGCCACTCTGTAAAATGACATTAGTAACAATAATATCTAC

A_14_P128272 GAGTGGCAGGTTTATTTTTTTTTATAGCATTTCTTAACATAAGCCAGGGGCATGCTATC

A_14_P131990 ATTACCTCTTCATTCTCCTCATCAGGACTCCCCTTCAGAGACTGAAGAT

A_18_P20213229

ATCTGGAAAAGCCTTTTCTTTTCACCTATGATAGGGAGTTTTGGTGATGATAGGTTGTT

A_14_P200197 AAAATAACTGGGGATGCTTTTATTTGAGGCCAAAATATTCACAGGTCATCTAGTATGGAA

A_14_P135323 ACTTAAGAGATTTGAGAACTTCATAAAATCTCGTCCTCCTTTTGATGTTGTCATTGATGG

CUST_5023_PI426210822

ACTCCAGACACTTCACTGAAACTGTGTGAATCATTTTAAACAGGTTTGTCCTTG

A_14_P101867 TGATGTAGCGATAGCGGTTGTATTTCCACAGGTTATTTGAAATGGATTCCATATCTAAGT

A_16_P38271039

ATGGCTGAGGACTTTATTTACAAAGGATCATGCAGTTGTATTATTACACGCAAAGCATCA

CUST_3585_PI426210822 CTTCTGTTTGATCTTCCCTGCCTGTCTGTGGCCTCTGCAGTTTAA

A_14_P107774 GGTATACAAGATGGGGTTATTTCTACTTCAGACACGTAGGATGAAACAAATCTAAGAGAG

A_14_P136390 TTTTGCAGGACTTTTCTGACAATTTTTTCATGTTAATTATGCATGTGATGAACTTTAGAA

CUST_7521_PI426210822

GAGAGCATGAACAAAGGAATGTAAGATGTAACAGAAAACATACGTGTCTCACACATGAG

A_14_P135749 CTGTGATAAGATCTTAACATAATAAATGCAAAATACACCCCTTCCTCAGGTTGCAAAACC

A_16_P35632671

CCTTCTACTTTGTGCATTTTTAAATCTAAAATTCTCCCAGATTACTTAGCAGCACAGCCA

A_16_P18861503

AAACACCATTCACAAACTTACATTGAGTCGACAAAGGTTAGAAAAATATTGTTTTGGCGG

A_14_P124451 CACCTGCTGACATGATGGACATTAAAAAAAAAAAAAAGCCAATAGTTGACCTTCAGTTCA

A_14_P114721 GTTTACCGTGGTGAAATTGAACTTACTCTGAAACAGATGAAAAGGGACATGCAAAATTG

A_14_P138344 ATAATTCCCTAACTTTGGCAAAAACACCACATACCCAGATACTTAAACACATATCCTACC

A_14_P202313 CAATGACTGCCCCTATTGAACATACGACATTTCAGATCCTTACCTTTATGTGTTATGATT

A_14_P138199 TAAAGGGTTTTCTGAAGACCATTTCCCAGAATTATAAGAAAAATCCTGTCCCCTAAGAAA
A_16_P19558817
GGGTAAAGTCTAGTTAGTCCTTCACATTCAAAGCATCACCGTTTAAAGTGACTTAACAAA
A_14_P127863 ATTTTGATTCCAGTGAAAGTTCTTACCTTTCTCTTTTGTCATTCTCAACCTCTTACCTGA
A_14_P129495 GGCCTTTGCTAGACTTATTTTAAGCGTAACCTCTTGTGATTCAATTTACCTTTTTGCTTG
A_16_P01898181
AAGGACTCTCTATGAATTATACGAGATTTTTACAAACTGTAGTTTCGCATCTTCACACCA
A_14_P110319 CTAAGGAACAAAAGAGTGGAGAATCCTTTTTGGATATGAGATTGTTAACTTCTCTTGAGG
CUST_2844_PI426210822 CAAGATGCAAGTTAGAGCAAGAGTGATTCAGAGCATGAGACAAGTGGTAGAG
A_14_P124097 CTTCAACTGCAATATTTGTATGCAACTACGGACCTGCGTGAGTTATTTTCTCTTAAAAAT
CUST_8576_PI426210822 CACTTTGGCCTTATGGGACTTTGAATGGAGCAGGGCTTTCATTCT
A_16_P16662976
CACCAGTATTAAAAATCCTTCATTAAACAAATGTTTGTAGTTCCCCGTAGATTCATGAGG
A_16_P16136916
ATAATTCAGGGGTGATTTATTCACATTTAACAATCCAGACATTCAGTGAAGTCTCTCACC
A_14_P123285 CTTAAAATGAAAATGTTAAAAGCCTCAGAGGTTATATCTGGCCATCAAATTCAAGCCTCT
A_16_P39861897
TGACAATATTATAGTAGGGTTAAGAAAGGTGGAAAGCAAAATTACTTCAGCACAACAGGA
A_14_P125489 TGACCAGTCTTGGGATATATCTGCAAAAATGAAAGCACAATTAAGTAAAATGAGAGAGGG
A_14_P118304 CACCGTCTCTTTAACCAGCGACATATTACTAGGGTCATTGACACTAAAGAAAGAAATGA
CUST_1243_PI426210822
AACTCATGCCATGTTTAAATGTTCTTAAATTTGGGATTGTTGAAGTCTGAATTTTACTGT
A_14_P112682 GCAAATCAAAGAAGATAGGTTCTGGACATGTGCCTATCGTTTGTATTATGCTCTAAAAGA
A_14_P121877 CTTTAAAGTCACTGAAATGTTTCAAATCAGCCCCATTCACTGAATTTGGAGAAAGTATCT
CUST_6270_PI426210822
AAAAAAAACAGTTGCTCATTGGCTTTTTATTTGCCCTCTTGGAATTGCAGAGGA
A_14_P134469
TCAGATGCCCAAAGACAAGAAAGACCTGTAAGAAAACAATGCATAAAATCCCTAAAACAA
A_18_P10704774
GACTGCACATGCTCAAATATATTGCCCCTGGTTATTTAAATGTCTATTAAAAGTGTTCCA
A_14_P113343 AGGCACTTCACAGAAATTCCAGAACTCCTGAGAGAAATGGCCATACTTATCT
CUST_5859_PI426210822
AGTACAAGCACTCATATAACCTGTAGGGATATTTGTGTACATGTGCATGTGTGCATATAC
CUST_8177_PI426210822
AGAGCCACGCATTTTCCCAAGGAATAATGATCACAATTCCTATTAGAAATTTAATAAAGT
A_14_P202527 TATGGTGTGAGGAACCAAATGGCCAACAATTTAAATATGCACAAAGGTCTCTCATTTATT
A_16_P00293299
AGACCTGGATCAAACATTACATACTTTGTTACATCTTTTTGAGAATGAGTTGGAGTGAGG

CUST_5902_PI426210822 AGCAAATATATTGAAATCTCCTGGAAATTTGGCTCTCTCCTTGACCCCAGT
CUST_9266_PI426210822 TTTTCTTCTCAGGACTGTGAAAAACCAGGAATTTCTTGCCTAACAGCACAC
A_14_P121305 TCTTAGCAGGTAACCTTCCTTTCCTCTCCAGACTGAGGAATCAGAGTT
A_16_P18640006
CACCTGTTTCTAACCACAATTACTTGTAAACCCTAACTCGAAATTAAAACGTGTCTGTGA
CUST_7795_PI426210822 CCAAGAAGACATGTGGCTCCATGATGACTCTGCTTTTTAAGGTTATGCC
A_14_P101712 CTTTTAGATGATCCAGAAAGTTCTGTTCTTCTTAGAGTCTCCTCAAAATGAAAACACATC
A_18_P14945996
CATTTCTGCCATATTCTCTGACTATTCAATCTGCTTTCTAACTTCCACGAATCAACTGAC
A_18_P18169396
AAAAACACCTTATTTCACCAGATGAAGAAAAGATGGACATATTCTGATTTCATGCCACAG
A_16_P00652699
AATCTCCAAATGCAAGTTATTTTTACCACGGCTAGATTAATTACCCACCTGTCTTTTACC
A_16_P36239990
GCCACCATGACTTAAATCATCTGTCATTTTACTACATGGGCTTGTTATATCCTAAATCCG
A_14_P101816 CCTTTGGGTTATCCACTGCCTTAAAATTATACCTATTTCATGTTTAAAAAGATATCAATC
A_14_P125894 CACCTGAAAAGATTCCTAAATTACAAAAAATGGATTTCAATTTGGCAGTGTGCACTGTTC
CUST_9490_PI426210822
AGAATCCCTTGTGTAGATGAAAAAAAAAAATCACTGGGCATATTGCCTTCCATTATAGGA
A_14_P121253 ACCTAAACACCTTCTGAATTTTCACAAAATAGTTCACCTCTCTCTAAACTCTTGAGCAAA
CUST_5798_PI426210822 AGCGGACACTGAGCTGCTATTTCTGAATCACATGTCCCCAAAGCT
A_14_P108643 ATGGAAGCATTGTTAAGCGTAGGTTGGATTTTCTCTTCATAACCAATACAGGATTCAATT
A_14_P107536 TAACAAATGACATTTGATCTGTTTCAGCAGGTGGTCCATTATGGCTGACATGCAAAAT
A_18_P22841777 GTAGAAATGTTGATCCCTTGCAGGGAGAGCCATCCCTGCACTAGC
A_14_P100038 GAATTGACTAAAAAATTGCTCCTGACCAACAGTTTTAAAATTCAGTCAACTTCACAGGGA
A_14_P121249 TTGAATTTCCATTACATCATCAGACATTTACAACAACCAACAAGCCGAAGGAAACCAATA
A_14_P115204 CCAACATTTCCCCCATCTCAATTCATTCACTAATTCAAAAAACACGAATTACCAACAACA
A_14_P105904 AAAAGAGGAAGTGAATATTCTAAATGGACTTCAGTATGAGGGAGGTTCTTCTGTCTAAAC
A_16_P00798597
AAACAGTTTGAGACAACTTTAAGTTAAATGAGGCTCATCAAAGTTAACATGTAGGGGAGG
A_16_P16569751
AAAGAACCCTCACTTACAGTTTGTAAGCCATGTGTTCTTCTCAAACGAAATGTGAAAGA
A_16_P02349703
AAAAAGGTCAGACTTACATTGAAGTGCTTAAGTGGGATAACTAATACCAGGTCCACTATG
A_14_P110946 AGAAACTGCTCCTTATGTGCAGACAACATTGAAGGAAAGACATTTGTTTCAACAGTAAAT
A_16_P38150183
TCACTTTGGACCATTCTATCAAGTCTCATACTCGAAACATAAATGAACTATCCAACAACC
A_14_P200402 CTACAGGAAAAAGATAGGCATTAAAAAAAAAGAATAAATGTGACGCTTTCCTTGGAGCAT

A_14_P103639 TCCGTGTTTAGGAAAATGCAAACAAGACCAATATCTTTCAAAGCAAAAAGTAAAAGCCC
A_16_P17693040
AAAAAGTTACACCGATAAGTTCAACAAACTGTCCATTTTTGATATTTGCATGCTCCCCTA
A_16_P19655176
AAATGGCTACAGGTAAAAGAAGTGGTGTTCACTGAATTCATAGACTCAAACAGTGGTTAT
CUST_2344_PI426210822 TGCTGAGCTCAGTGCCTCACCTGCAGGAGACAGGGCTTTGGGAGG
A_16_P15333750
CCATACCAGAAAACAGTTTAACTGCAGATCAAATTGCTAATACGATTAGTGCCTAGTGGA
A_16_P02308707
AAGAACAAGTGAAGATCTACCCAAAATTCTGCTTAAAGACCTCGAAGATACCAAAATACC
A_14_P115938 AATTTGAAAGGAATGTGGTAAGATTCAGTTGTCCTGTTGAATTTGTGAGAGATCGTTATT
CUST_8342_PI426210822 ATGAAGTGAAGGAGATGGGCCCAACTCACCCATAGGACACAACAG
A_14_P132716 CTTCTGTATGTAGGAGGTGGAATGAAGTTTTTCATATTTCTGACCTTTGGAGAAAGTTTG
A_14_P129720 GTTTTTACGTCATGGATCCTATTTCCTCACACATTAGATGGAGGTTAGTGTATTTCGTTA
A_16_P36863994
ACATACTGCTATGTCCCTATTTTCAATGCAAAAATTTTAATACCACCTAAAACTCCACCT
A_14_P106796 AATGATCAGTTAAACCTCTGTGTTTCAACAAATATTGAATGTCTCAGACTGGCCTCTTTT
A_14_P134488 AAAAGAAATGAAAACCAGATGGTCTATGCTAAGAAGTGAAGGCATTTTGTTGTCTTCAGA
A_14_P102945 TCTACTCTTCAGACAACCAACGGCCTATCAAGTAGTGTCATTTATGGAGAAAAAAAAATG
A_14_P122929 CTTCATGGTGAGGGACTGAAGTATGCTACATATCTCAATTTAAATGCTGAAGGAACATTT
A_14_P138393 ATAAGGGAAAATATAGTCAGTAGATAACGATCCAGATCTGACAGTTGGATTAACCATTTG
A_14_P126697 TCTTTAAAGTTAACCAGACTCTCCATTGCCTTCAATTTAATCCAAATTTCACACTGCTGT
A_14_P105464 TAGAGTTTCTGGCTCACCTTGTCATCATAGTGATTCTAATTCTTCTGAGAAACACATGTC
A_14_P139399
TAAATCTGTAGGTGTGTCTAACCGGCAGAATAAGAAAGTAGAAGAAGAAGAAAAGTTGCT
A_14_P124174 CAAAACCAAGTAAAATCTCCTGCTTATGGATTCAGTTACTTCTGACTTCAAAGCAAAGAC
CUST_1418_PI426210822
AAGAGTTACAATTGGGTGTTTAGTCTCTAGAGTCAGTGGGATGGATATGCTTTTTG
A_14_P130913 ACTTTTTTTTTTAATAGGGAAAATTTGTTGGCAGATTGTTTGCAGGGTTGTTTTGTGGTG
A_14_P107957 GGGAGAAGAGAATGATGAAGGAAACCGAAAAATTGGAAATTTTGATTGTGAATTTGGTTC
A_16_P16737813
AAGTAATTGCCTTGCTGCTACAAATTTGGAACTTAACATTTTGTTGGGCCTATTTTTGCT
A_16_P36546352
AACAGGGATTCCTAGAAATCTAAATCATGCCCTTTAAAAATTTCGTGTCCAAACCTGATA
A_14_P123719 TTAGGCAAGAGAAAAGGAAGTTTTTTTTTGCTAAACAGGAGTAAATGAGAGGTGGTAACT
A_16_P19311268
TAAATCATAATAGAGTCACTGCAGTATTTCACTTGGAGGAATCAGATGTATGCCTAGGTC
A_14_P107844 GTTGACAAGTCAAGTGCAGTAATGATGTCATGGTAACCAATATGTTTCCAAACTTTCCTA

A_14_P117592 CTGACCACGATGAATTTTAGCCATATTCTTCTCTTCTAAACTTTCAGCCTGAGAAAAAGA

A_14_P138664 TTTCTAGGAACAGTCTTCAGTGTTTCATCCTTTACTTGATTATCTCAGAAATGTTGGAGG

A_14_P103020 CTGTGATGAATTGGTGAGAAATCCATCTGTAGATGACAAAATCCAACACTTCAGATTAAG

A_14_P114094 TGAAATAAATGAAACAGAAAATGATGGCAAGACTGCTAAGAACATCCTTTGCTTTGCTCT

A_14_P128090 GTTGACTCAGGAGGCCATTGATGAAATTAAAAACTTCTCTTCCTTTGTCTTTAACTCTTT

A_16_P19319019

ACAAACCAAGATTAAATCCTCTGAAGCATATGTTTTATAAAGACACAAGTGGCAACTCAA

A_14_P105895 ATATGGCTGTGGGAAAACACACCTTGGAATTCATGATGAGACAGTCCAAC

A_18_P16261534

AAAAGATCAAGAACAGGCAAATACCTTCTGTTTGACCAGTATTGTGAGTAAATAGGCCTG

A_16_P15628906

AGAAATTACCCACAATCCTATCACATATGCTGAAGTGACCTTTTTTTTCCTATAGGCTCT

A_16_P37231672

ACACACGAGTATTAATGAGGCAGGTTTAACTCTTACTAACAAAATACTACCATCTTTGCA

A_16_P15519828

AAAAAACCTTTTTGTGCACAGAATATGAAAAAGGGACTACACAATAAGCGAGAGTCTTCG

A_18_P14136405 CCTTGGCTCTAATGGCCTCAGGAGCACATCATGCTCAGACTTATTT

A_14_P103908 CTCTATTTTCATCTTTTCTGATTCAGCCTTCAGTTTATTGACGACAATCTCATGTTCCCT

A_16_P03658436

ACTCCATACTTGTAATTGGACAGTGTATTTCTTTTCATTTAGAAATGGCACAGCAGATGG

A_14_P110971 GGATGATGTTGACAAACAACTTGGAACAAAGTGAAGTAAATTCCCAGGAAATTAGATGAA

A_14_P130866 CTACACCTGCTCAACCACTAATTTAATGAGAGAATGCCTTAGAAATTGCAACTGGTAAAT

A_14_P136455 TGACACGTGAATGCCATTTGGATACATATTCTAATCTTTCAAGTAGAAAGGACAGTCAGA

A_14_P123428 GGACATATAGTTGCACATATTTTGGTTTCTTCTTTCACACTGTATTGAGACACCTTGCAA

A_14_P127242

CTCAGACTCAGGTCTTTTAAAAAAAAAAGATAGAAAAAGGAAGTCCTCATAGCAACTGGT

A_16_P19597043

AAGAGCATATGCATATTTTTCCCAGGTTAATCATCTAGTTCAGAGTCTCCATAGATGTGG

A_14_P135106 TTGTAATGTCCTTACTGTAAATGTAGGGTTTGAACTCAAGACTGACCATAAGACTTCTGT

A_16_P35566606

TATGCCAGTTTTATAGGTCCCATACATTTCCTTAAATGTTGATGAAAATCCTTAGCCCAC

A_16_P21078683

GGTCAATCCGAAATATTTCAAAACCGCACACTTATTTTAGAGCCATTTGTTAACAAGAGC

A_14_P104528 CTTACAACTCTCTTTAAGTCACCCATCTTTATCTCACACCTTTCACTCTTAGTAGACAAC

A_14_P105949 CAGACTCTAGAATTACTGAGTCCTAAATTTCCCAACTAAGTATGTTCTCTAAACCACTCG

CUST_6513_PI426210822

GTAGCATTTGTAAAGAGATGACACCTTATGTTCTAAATATTCTCCCAGTATTCTAAAATA

A_14_P112172 TCATCTAAACCACTAGGAATTTCAACTAGAGGGGAAGAAAATGTTAATGTAACAGACCAC

CUST_8819_PI426210822 ATCCTCAGACACACAGTCACCCCTTGTATCTCCTGGGGATTGGCT

A_14_P127910 CTTGAAAAAACATCTTCAGGTCAAGCCAAAGCAGAAATATATGAGATGAGACCTCTCTCA

A_14_P114021 TCAGTTAAATCTATGACAAAGGATGTGATCATCACTAGGAAAGCAGTCAGGTATTAGACA

A_14_P117016 CACCAATAACTGAGGATTGCTCAGATGCTTTTTCAAAGTGAGAGAAGGTATTTTAGCAAT

A_14_P134780 AACTGGGCAGATTAAAACCAGGCAAGTCAAACAATATTTTTTATCATTACAGACCATTGG

A_14_P139854 GCGTTGGGAGGGGTTGATTTAGATTCCATCTTATATAAAAAGACCAGAACATTTTTGATT

A_16_P18158589

AATCTTACTCCATCATGAGATCAATCATCTGGAAATCTTGATAGAAAACCTACAGCCACA

A_16_P19863613

AGTAACAGTAAAGTAGTGCAGTCTATGGTCTTAAACACGAAGAACATTTCAAGACTACCC

A_14_P119227 TGAGTTTACTTTCGAATTTGGGGAACAATTAGAATGTGAATGCTCAGAACATGTCAACAG

CUST_4499_PI426210822 CTTTGTTTCAGGAGGAACTCTGCCAGTGGAAACCCCATGTCCTTA

A_16_P01362695

AAGCATAGCATTGTCTTTGACTAATGAGACCCTGATTCATTTTGACAGGAAGACAGATG

A_14_P107550 TGAAAACTGTTGTGGATTAAAATGGAAATGCCAACTGTTGTGTGTTACTCATTTTCAGTG

A_14_P134148 AATTAGCACCCATTGTCTTTATGCTCACTTACCTCTGCGTATTGTTGTTTCTTCATAAAT

A_14_P113461 TATTATCAACTGTTTCCACAGATGCATTCCTCTTGACTAATCCCTTCCACATTTTGTTAG

A_16_P02028688

GAGTGAATCCTCAACTGCATTTTCAATTACGTATAGAAATTTCCCTTTAGAACCCTCAGA

A_16_P15955123

AGCATAATCCTAATCTTTTACCACTATTCCACTCACTTATCCTCACTACTTTGTCACTCT

A_14_P103850 GCGTGTTGTATTCCAGATTCCTAACAGTTAGGTTATTTGCTACTTCTGAAAATGTGAGTT

CUST_5843_PI426210822

CAGTGCGTGACTCAGGTCTCTTGATTCCATGGTGTATTGTTTTTTTACCTATTAAACAAT

A_14_P106769 CACCTTGGAGAGTTGGGTAATGATTTATCTTTTGTGAAAAGTGTGAAATCAAGAATCTGT

A_14_P106728 CTTTGGAGACTATGGCAAAGATATCACCTTCTGTTTTGTGAGCATCAAAGAGTTCATTAA

A_18_P12056361

ATTTGACACCTTGAGATTTTGTCTACAGAAAGTAGCAACATCATCCTTATACAAAGACCC

A_14_P110521 AGCCAAACAGGTCTGTGTAATCTACTTTCAACTGTTTTTTCCCAAATCAATACTTACACT

A_14_P130146 CCTCCATCCTCCTTTTAGGAAGAAAACTTGGATACACCTTCAACAATCAGAACTTT

A_14_P108864 ACATATTCTTACTCTGGTTGCCTTCAGCGGAACTGAGAAACTTCCAAAAGGTT

A_16_P03191319

GAGGGGTTCACATTTTATGCAAGAGGAAAATGAACACTGACATGTATAGTAAATCTGGTT

A_14_P104192 GTAACCAAAAAGAAAGAAATGTGAATCACACCACCTCAAAGATTTCATGGTAAATCAAAT

A_14_P106573 GGTGATATTTCTGGGGGAGATAAATCCAAGAAAGGGGTAAAACGGAAGAAGATTT

A_18_P14132730

TTTGAGAACCACTACATGACTTCTTGATAAGATTCTTTCAACTTTACCCTATGCCTGGAT

CUST_1051_PI426210822 CTTCCCACCTCTATGCACCACCAGCTGAACATTTCCCCAGCTTTC

A_16_P31307581
CCCAATGAAATCAGATCAAATGTTACCATACAGTAGTCTTACTCAAGAATGTCGAAGAGA
A_16_P15989026
GAGCCTAATCCAATATACTCTGTCCACATATTTCCCTTAGCAACAAAAATGTTTTTCCCA
A_14_P122007 AAGCACTGGAGTGTTAGTGATTACTATGAAAGTCAAACTTCTATTGGAGAAAGAGTTGGT
A_14_P122133 CACCCTTAGCATAGGGAATTTCAAAATAGACATTTACAGTTGGCTGCTTACAACATAGTT
A_14_P127018 CTAGAGGCGATAGATATGAAGGTAACTCCAGATTTTCAAGGTTCCTCTTTTGGCA
CUST_8181_PI426210822 CTTCCATGATGTTTATAGTGGTAGGTTTGGTCAGGGCCTCTTTCCTG
A_16_P02634743
CCTTCCCCTTCAGTAATAGTAACAATTTATTCCTTTCTGACAAAATGGTAAGCGTCTTCT
A_14_P201463 CATAGATTTTGCTTTTAAATCATTATAACCCTGTCCACTGGTATCTGTGGCTCAGTGAAT
A_16_P02574975
TATTGCTTCAAAATCTGGAGTTTTCTATTGCATTGTGTTCCTCTATTTTCAGCATCTCCC
A_16_P00007895
AAACATCACTAACCAACCTCAAGAAGTAAACAGTATGGCTGGTTTTATAAGTTGGGAATG
A_16_P15967391
AACCTACCCAAAGTTAAGAAACCTTAAAGACAATGCAAAATCAAACTGTTCACATCCTGT
A_16_P39752742
GCCCAGAAAAAGAATGATTTGACTTTTCACTAATAACATCACAGAAAATGATTCCACACC
A_14_P103199 TCTGTTTTATACGTGTGTGGCTAACACCCTAATGTATTCCTGGTATTAAAAATGGGAACA
A_16_P35944905
TGTCCTTCATGACAATCTTACTATCCCAATGAGTGAATGACTATATGACAAAACAATCTT
A_14_P126573 GAAAAAGTGTCCCTCATTATTTTGAAACTTCATTCCTCAAGTAATTCTTTGCCCTTTTGC
A_14_P104909 TATTCGATATCACTTTTGTTTAACTGCTTATGTTGCCACTTTGAACCTGTCCTCTTTCAG
CUST_577_PI426210822 TGATTTAATGCTAAACTGACTGTTCTCAGGGTTAGCCATGCTACCCTCC
A_14_P107050
CAAAGAAAAGAAGGAAAAGGAGAGAATCTCTGAGAACTACTCTGACAAATCAGATATTGA
A_16_P17574778
AGCATAAGTTAGAGTTTAACCCAGAGAGAAACGATTAGTATCAACACGTATGCTTTGTGG
A_16_P03659412
AAAATGTAACAGGAAACAATCAATCACTACCCGATATCACGATCTTTCAATGACTGCTTC
A_14_P135463 AAATCATGATGAAGTGTTGGGATTATAAACCTGAAAATCGCCCTAAGTTCAGTGAACTTC
A_16_P18669801
AAAATCCATCAACAAGGACTAAAGTCAATTCTTCCAATGGTAAAATGATCCCCATTCAAG
A_14_P103868 CATGCCTTAACAGAACACCCAGATGCCTTAAAAGACTTTTACAAAATGCAAATTTCTGAT
A_14_P138195 CTGACCAAAAAGATGCTGTATGACATGGAAAATCCACCTGCTGACGAATAC
A_14_P121077 CTTAAGACACCATAATTTTAAGCAAAACTTCCCCGAGGTAGGTAAAATGTGAAGTTTAGG
A_14_P135124 ACATCTGTATTCTGTAGTGTTAAGGGATCAGATCTTGATCCTGAAGAACCAATCAAAGTT

A_16_P21645369
TCCTGTTCATGCATTACACATAAGATTAGACTTACACTCCTAAATTAAGCAAGCAGGTCA
CUST_7656_PI426210822 TCCCATCACAGTATTCCACCCAAATGAGGCAAATTCTTATCCTTTCAAGAC
A_16_P19254295
AAAAACTAAGGAAACACTGAAACTCCTTTTTCCATAAAGTCACTTCAGAATGCCCTTAGA
A_16_P19469357 GCAATTTCCTCCACATATTCACTCATTACTTTTACTCATTATTTACTCTATACCAGCCCA
A_14_P105870 CTGTCTTCCTTCTTGAAGGATTTCAGCATCTGTTTGGTCTTTATCTAAAGTATCTTCCAA
A_16_P20232489
AAAAGTCTATCCACCAACTAGTTAAGTTGTTACAGTTTATGGGGCTCTAAAGTTTTTGGG
A_18_P17041792 CAGTAATGACAGTGCAGTAAAAAACCACCTGATAAATCCCCTGTCATCCC
A_14_P136314 TGCCTTGTCCTCAAAAACTTCTAAAAGCCAGATTGTCAAAAGCAATTTAATTTTTGGAGG
A_14_P135397 CTTTGCAATTCAGACATAGCCTGCGTAAATATGGCCTTCTTTTGTGCTGTTTAATGG
A_16_P16931260
AATCTAGGTGAAGATATCTTGCTGTTATTCTCTTGCCAACACATCTTATTCGTGCTACGT
A_18_P16340422
ACGTGTTTCCCAAGAGTTAGTCATGTTTGCTCCTAAAACTTATTGTAATCGAGGAATCTA
CUST_8015_PI426210822
GTGCTCTTTAATGTTTTTAATTTAATTCAGATCTCCTCCCAGTGCTGACTCTACACCAG
A_16_P21094080
AACCTAAACGCTCATCATCTTGCTATCCAGACTTTCCTTGCTTAAGTGCTAAATGTTTAT
CUST_9643_PI426210822 CCTTCAGAAAGAAAATGAGTAGGCTCCGGAGACGTTCATCTGTCTTC
A_14_P200013 CTGTCTGCACTCTATGAGGACTCAAATATTAAGGAGATGTCTCCCAGAAAACTAGAC
A_14_P112980 ACGGATGTGGGTTCTAACTATACTTTCTCCCCAAATAATCAACTAGTTGCTTTAATGACA
CUST_3927_PI426210822 GCTTAGGTCACTGTGGACACAGTGGCAGCAGTGGACAAGGACAAG
A_14_P100713 GATGCTTTTCTTTATTTTTCTCTTTGTAGGGAAACTGAACAGCCAAGACTCCTATAACAA
CUST_7060_PI426210822 AAAAGAGCTCTGCCTCCCGGAAGGTTCCAGGTGTTGGGGTAAATA
A_14_P131667 ATTCTTTTCTCTTAAAGAATCGTCTAGAGTCGTTGAGAAAACTGAAGGCTTCCTTACAAG
A_14_P130078 TTTTGAAAGGAGGATAGGAAATAAATCATGGTTTTGTCCTCAGTCCTAAAAAGTTCGGAA
A_14_P134443 CTGAACGCTGATGGGCAGTGTCTGATTAATCGAATGAAGTAAAAATAAACAGGTTTTAAC
A_16_P19136449
ACACTACCTTTACTTTGTAGGCAGAAGATTCTCTTTGTAACAAGAATGAACAAACCATGG
A_14_P120726 ATGTGTGGGGGACATAGCAAATGTTCATTGCAGTATCTGTAATGAGGAAACCAAAAT
A_16_P19564691
AAAACTCTGACATTCTCACACTGATTATATGACCATCTGAATAAAGTGGAAATGCAAGCC
A_14_P108271 TCAGTGAAACTTATTTCACCGTGTTTTTCACTTACATATTTAGGTGTTCATAATACAGAT
A_16_P19933268
ACTCTTGCTATTCTCACATCCTAATGCAAAATCGAAACTAACCAACTTTTTGTTTGTTCT
A_14_P100558 ACATGGGAAAAGATCTGAAGGCTACTGCATAAACTGCTAACAACAATTATGATAATTAAT

A_14_P131599 AGAATTAGTAAAAACTGGCTTTTTCCTTACACCCAATTTTTGCCTTCTCATTCTCTCATG
A_16_P00628077
AATAGTCTAACCTAGAACTACCAGATCTTTGGCTACAATGATTCCTCCTTCATTGTCAGT
A_14_P124222 ACCTTATATGAGGAATAGGAAAGGGTATCCAGTGTTATATGCTCTTTTCTTCCTTCAATT
A_18_P13383358
AATGGTGCTTTTTTAATGGAACGTCAAAAGTTCTCAGATCTTTCAATTGTCTGCAAGACT
A_14_P128262 TGTGGGTTCATGACTTCTCACTGTGAAGAAGTGCATATTGGCCATTTAAGATTTTTTAAA
CUST_9045_PI426210822 TCTTAGAAGTGCCTGACTGAGCCTACAACATGGAAATGCCATTTCCT
A_16_P20175183
GAAGGCCACTCTATCAGAATATCAAAGTGTTTCTCCTTAATCACAAAGAGAAAACGAGTT
A_14_P135064 AACTATCAAGGTAAAGGAAATTTACCAATTAAATACAAAGTAGTAAAATTCAAAATCACA
A_14_P134974 AAGGTGCAAAGTTGTGTTTATTTTTCAGATCTCTAGCAGAGAATACTCTAATCCCATGAG
CUST_6234_PI426210822 TTATTCTGTGTGGCTGTGTGTGTTGGTCCCTTGCCAATAATCCTAGA
A_14_P139494 ACCTATAATCAGGCATTTCTTTACTTTAGGGTTATTTGTCATGTCAGACAGCATGGACTT
A_14_P104384 GTAGTTACCTGATCCATCAACATCCCGAGCAATCTGAAAGGCAGAATTTTAAAGAAAAAA
A_14_P123745 CAACATGTAACCTGATACTGAGTTTGAGTATCTTCTTCTCAAAGACTCAAACACTGCAAC
CUST_4774_PI426210822 CAGAAAGCACTCTTTCTCTTCTGCCTACAACATCCTCCTCCCATC
A_14_P200486 GTCATGTTTACTAAACTGGGCAAGCACTAATATTTATAAGGCTTTTCCAATATGGGTTGA
A_16_P38515598
TAATGACGAATCAAGGTTGAATGAACAGAGATAAGTTGACAAATAGCCATACGTAAGCCA
A_16_P00685210
TGGTAATGGAATTGGTTGTATATTTTAGTATGATAGTTCTTATAGATGATAAAGACCTCA
A_14_P106722 CACAGACAGTTCTACTGTGGCAACACAGTTTTCCATAATAGAAAATCGATGAACTGGAAA
CUST_681_PI426210822 CCCTTATCTCTAGCCTGATTTTCCATTGCACAGGATTTGGGAAAAACCTT
A_16_P17694582
AAACTCACAAGATCAATTGAAAGGGCAAAATAACAGTATTTTATCAATACTCCCCTGGCC
A_16_P39250272
TCCATGTGAACTTACTTTCTCTAGTTGCTGAAGACAAAAGTAGTCAACCATATTTCCTGA
A_14_P129682 GCCTATTCTGTGGCTTCAACCTAGAAATTTCTTTTCCTTTTGATGTTTAGGACTTTACTC
A_16_P00017478
AAGCATACAGGAATAAACATGAGCGTTCTAAAAAACAAGTTGATATGTGGAGTTGAGGAG
A_18_P14418358
AAAGACCTTAGTTACCCAGGAACATCTATTTCGTTCAGAGAAGAAAAATAGCGCTTATGA
A_14_P127907
ATAAATATGAAATTTTACTCGACAACAGAAAAGGAGAAACAGGAAAAAAGGTGCCTCGAG
A_16_P36913901
TTTGATCCTTGACAATTTGTCCACTATACTATCCATTTCTTGAACCATAAAGCCATCTGC
A_14_P103517

GATTAAAAACAGCAAAATTGGAAGGAAAGAGACTTCTATGGCTAGGAAACTGACATTAGG

A_14_P101649 TGCCCTCAGGTGGCACACAAACAACATTTATTTTAATAATTATGGATTTGTTTTAATGCA

CUST_10259_PI426210822 AACTCCATCAAGCCTTGCCTACTTTCCTCATCGGTAAAATTGACCTTAAAATC

A_16_P19562778

ATTCCAACTAAAGCATGTTATCGACTTGACTTAGTAACCTTTACTATGCATCTACCTGGC

A_14_P101386 GTTTTTTCCTTTTTTTTCTTTATGCTCATCTTTAATGTGACGGTAAGTTCTGCCTGCTTT

A_16_P16473861

AAAGGCCTCACTAACATGAAAATTGTTCTGAAGTGCAAAAATGTAGATCAAGACACTACG

A_16_P01086879

AGTTACCCATTAGTCAAAACATGCATCATACACTGAACACTGAATACAAAGGAGTCTGAA

A_14_P126975 CTAATTTTAGAAACATGCCACCATACACTAAGTAAGCAGGAGGGCAGAATTTTCTAACAT

A_14_P106946 AGAAATCAGTCAGGAGAAATAACTAAAGAGTCAGGATTCAGGCTTACATTTCATGTGATG

A_16_P02127282

ATTGAGTGTGTATTTCATGATTGAGGGAATCTAGTCATCTAGAACATGGTCAGAACGTGT

A_16_P35518649

GCACTACCACCATTATCAAAAAAGCAGTTATTGATCGTTTCATTAATTAAGCCCATGTGA

A_16_P31945167

ATCCATCAGGTATCTTTCTGTGATTTCATTTTGGAAATGATGTTTTAAAGGCCTCTGACT

A_14_P105933 TGATCCATTTTCCGAATACATCTTTTGCAAATACTGAAAAGGAGAGTTTGATTTTTCAGT

A_14_P134976 GCCTAACTATGCAATTCATTCACTGTTGTATTGGGTTTTTCTGTTTCTTTGTCTATTTGT

CUST_4500_PI426210822 CAGTGGAAACCCCATGTCCTTAGCGCAGCCCTGGGGGTTATCAGG

A_14_P118779 AAACTGATCCTCATGCATAAATCTCTTTGGTTATGCAAGACTTTGTGTCAAAGAAGATGC

A_14_P119088 AGGTTTCTAATTACTGAAAATCAGGGGAGGAAAATTAAATGTTGGAGAAGGTTGGTAATC

A_14_P119840 ATGCAGAAACGAAATATCGTGTCTTGAAGATTGATAGAACAGAACCAAAAGATTTGGTCA

A_16_P02020073

ATTCAGGTAACAAAGCCATTTCCTCTTCTTAACCCTCTACTGTGGAAAGTTATTGACTTA

A_14_P115466 TACTTTTCATTGTAGTCTGAATTAGGTGACTTAGATACCTGGCAGGAAAATACCAATGCA

A_16_P00685553

GGATAGTCAGTAATTGTTTATCTGTGTGATGAGATAAGGTAGTCCCCTGCTGTAATGAAA

A_14_P127296 CCAGCCTCTGGCAGAGAAACAAACTAAGAGTATCTCTTCACCTGTTTCAAAG

A_16_P18846778

AAAATGCATGTAGGTAAAGTCCTTTAGAGTCTCCTATATTTCATCTGCTTATTAGGGCCG

A_14_P129979 TAATCAGTAGCAACGATAAAACAACTGTAGAATATTAGGGCACATTCCTTCCTTTCACAT

A_14_P115214 CTGAGTAAATGTAGCCCAGTGCTGTATTTTAAACAGTCCCTTAAGGTAAAGTGATTTGTC

A_14_P134706 ACCCTCGGTGCAGAAATCAGAATATGATTTTTAGGTTTTTTTCCTATGTGGTTAGTGAAA

A_16_P01001125

AAATAAACACACAGTAAGAGCAATGTAATTCACCACTTTCAAGTTTGAGGTCATGGAGAG

A_16_P15280058

AAATGTTTCTTTAAGAGACTCATGTGAGAAAGCAATCTACACATTCTTTAACCAGCCTGG
A_14_P202253 CTACAGTCATCTCTATAACCATATAAAGGGGGATGCGTGCAGAAAATGACTGAAAAAAGA
A_14_P121708 TTCCTGGTTAGTAAACTGGGGTGTTTATCACCAAAAATTACCTGCTTATTAATCCCTAAC
A_14_P134686 ATTACTAACTTCAAATTAAAATATACTCACTTCATCAGCCACAAACTCCTTACTCAGACC
A_14_P119009 CAGATCTTCCAACTTCTCAGAGCAACCGAAGTCACTAATTTTACAGACATCCTG
A_16_P20779909
AAATGACTGACATAAACCCCATCTTAGTGAGAATAGGTTGCCAATATAACCACCTTTTTC
A_16_P01474386
ACAACTGAACATATCTGGGTCAATTTGTAAAACACTGTATGAAAAACTTCCTCCATCTGG
CUST_2949_PI426210822 GAAAGTATTACTCGGGGCTGATCAGCTTTTCCATCACCATGACATCATTC
A_14_P124446 TTTTTCAGATATTGATGAATGTTTGAATCCAAGCACTTGTCCAGATGAGCAGTGTGTGAA
CUST_2479_PI426210822
AATGAAGATAAATGCATGAAGTAGAATAATTTTCAGTCTGGTTAGCAATCATCACATCCA
A_16_P18130072
GCCTCATTGATGTATTGCTTAGACAGTGTTAACCTATTTGCAAACTTTAGATGTAAGCCC
A_14_P116101 TAAAAAGATTTTACTCATTTCAAACACTACCTCATTCAAAAAGGCGACCATAAATTCCTG
A_14_P108883 CCTGTTTTTTGGCCCCAGAAAATGCAATATTTTGACACAGAAAAAAACATTAGGATGAGT
A_14_P134506 TTATTTCAAAACATCCTTGCAACATTACTGAAGAAGACTATCAGCCACTAATGAAGTTGG
A_16_P00685014 CATAGTTGGATAATTGGTTTGGACTTAGCGGGAATTAGTGGGCGATGGAATTAC
A_14_P138032 GGTTTTCATCCTGATTCTATACTTTGTGATTGACAACTTTGTGATAAATCGCAGACCATG
A_18_P13100551
AAGAAAACAATGATCCCAATCACCTTTACTGAAGCAAATCCTATCCTCTGGTTCTATCTT
A_14_P126153 TTATGCATCTGTCATAGAATAAAGGATTGCTGGTATTGGGTTAAAAACTTGAGAACCCTG
A_14_P201704 ACTTTAAAAAATGAAACGAAAGTGACAGAGTGTTCTAAGTCAAGCAACCTTTTCAGATGG
A_14_P101797 ACAGGACCACTTCCCTAAAATGTATCAACACCTTAAGCAGTTTCAGGTTATTACAAAATT
A_16_P00837527
ACTCCCCTACTAAATCCTCACAGGCTTTACAATATCTGAACTACTCAGTCTTCAGTTTAC
CUST_3764_PI426210822
AAGATACAGAAAGATAATTGCCATATATGCTATGCAGCCAGTTCCCCTACCTCATG
A_16_P18608923
AGCCTTTCTGTAGATGTGTAACTTATTAAGCCAGTCAAATATTTGTTCCATTTTGCCTCA
A_16_P58424461 ACTTTCTTCCTTGTAGCCTCAGTGGCCTCCTTCTGTTCCTTGAAAC
A_14_P122970 TGCCTAATGAGTTACTTAGATCTCATCATTCACCTAACACACGTGAAATATCTCATCCAG
A_16_P58424276 GTGATTCTGGGACTGCAGTTGCTTTCCATTGCTGCTGCTGCAATT
A_14_P124166
GAGAACTACAAAGTTTTTGACTTGTCCTGAAGAAATGAGAGAAATCCAACAAGAGTTGTT
A_14_P105082 GCAGATGTTTTCTTTAAGTTCTATCTGAATTCAAGGTCAGGCTGTTTGACTCTGGATAAA
A_16_P21163276

AAATCAGACATTCTAGAGTCTGTCATGAAATGCTACCAAAGTTTTCTTTTGCTTAAGGGC

CUST_7564_PI426210822 AGACAGGGCTCCATGAGCTGACAGGTTGATCTGTGGAGATACACT

A_14_P124102 TAAAGAGTAGTTTTTGTGGGGGGAGGAGGATCCATTATACTAAAGACAATCCTTCAAATA

A_16_P02930363

ATGGCACTATTACTGATGTTTGTTATTGGAACCTGTAACATATTTCTGATTGACCAGCCA

A_14_P116918 TAGATCTTGATATGTATATGGAGAACCTGGAGTGTGACATGGATAACATCATCAGTGACC

A_14_P105223 GCAGAAGGCTATAAAGCAGTGTTACCTGTCCTTCATATTCAAAAGCATTTTGACATTCAA

CUST_9170_PI426210822

TATGGTGATATATAAAGAAGACTTCATTTTTCTAACAGTTCCTCAATTTAAACATCAGAA

A_16_P19361173

AACTCCAAATCAAAATCTATGAAGAACAGTGTTCACGTATAGTATAACCTGGCCCGAATT

A_16_P00200710

AACACCAATTGAGATCCTAACCCAGAAATCAAAGTCTATCCCATTTAACACAATACCTAC

A_16_P16410932

CCCGGATTAATGACAAAACACTATCTTATTCAAACCAAGAATTATCTACAACCCTCCTTC

A_14_P103491 CTACAAGATTCTTTCAACACTATGCCCTCAAAATTGACTGTTCATGTATGTGCAGACATA

A_16_P16683247

AATGTTGAGGTCTAGAGAACAAAGCAAGTAACATGTGGATTAGATGTGCATTCTGTGAAG

A_14_P127048 AGAGATGCAAAGAGTCTAGCAAATTATTTCTAGCATAATCACACATTTAACTTCAGACTC

A_18_P13150594

TGACACTCCAATATGCAAAACACTTACCTTCATTATTGACAATTATAGAAGCAGTTCCTG

A_18_P22840683 ATTCTGGTGCCGCCTACCTTTTCTCTAACATGAGAAAACGCTTATGGAGATAATC

A_18_P22842113 CCATGTTGGAGCCAGCCATGGTCTGCAGAGTTCATTTCTCTGAGT

A_18_P22841639

CCTAAATCTGGTTTTAATCAATTTGACTTACAAAGGCACAGACTGTGGTCAACTCCTAAT

A_14_P139614 GATACTGAGCATGGTAATGTGGTTTGGTATGAGGTAACTGTTGGAAAAACATGAGAATAT

A_14_P110723 GGGCTTTTTGGTAACATGGGAGTGCCTAATGTTGCTTTTGTTATGTCAAGTTGAAA

CUST_3536_PI426210822 TTTATGTGACCTAGTGCAGCTCGCCAGCCCACGCTGATGTGTGTTAA

A_16_P16881050

TCCAAGAATTACTATTTAACATGCAAAACAGGTTCTTACACTGGATCCCTTCTATGACAC

A_14_P116632 ACAGTTGCACAGTGCTTATGTCTATAATATACATGAATCTGAATGTCCATCAGCGAGATG

A_16_P03740472

AGTCATTGTTTTTCATTCACCCTTATTTGTCAGTGTATTGCATTACTATTCCCTGTTGGC

A_16_P16123291

AAGGAAATCAATGTCCTTTGTCCCTAATTCCACATTAATCATCTTAAAAGCACTGAAGCG

A_14_P101875 CTGTTAATAGGACCAAGTTTAACATTATAGGATGAACTGTTTCTTTTACCCAACTGGGCA

A_14_P103460 ACCGTGAGTTGCTATGTGGCTATGGTGATTTCATCTTTCTGATCCTTGATTTAACTATAA

A_14_P105587 TAGTAAAACCAACACAGTGAAAACCACGAAGAGAAATCGGCGATAAACCAGAATGTAA

A_16_P16191054 ATACAGAGGTTCATTCTTGCTCCTGTTTGCATGCAAACTGTTCTATTTCTGTTTTGCA
A_16_P21457373
ACAGTTTGTTTTCTCTGGAAGTATCCTTATGTCTATGTCTCAAATGTTACTCTGATGGCT
A_14_P115375 TAGGGCAGGTGAAGAAGTAACTTCATAAATGCTTTCCAATAAAATTATGCTTTACAGGGG
A_16_P17370843
TTTCCACTGACTTCATAAACTTCAGCATTAAAAGATAGTCTTTAGAATCCAACTCCCTGC
A_16_P17564450
AACAGGTGAAAAACTAGTCTGGACTTGAAGCATCTTCCATAAGGCTTGCTATTCTTTTAC
A_18_P16817341
AAAAAAAACACCCATTCAGACATTGGCATTTAATATAATTTTTGTCAAATGAGTCCGTCA
A_16_P02985313
ATTTTATCGGAAACAGAATTCTTCATGATGGCTTTGCTTATCTCTGACATGCATTCTGCT
A_14_P101265 GTAGAGGGAAAACTATTGAATGTGAGCAGTCATTGAAAGATGTCTTCTTTCTCTTGTCTT
A_16_P16191277 CTACCGTGGTCATAGTTTTGTTGAAAGGATAGGCACTACAAAATGTTTGATCACCAC
A_16_P30524775
AAAAATAAAGCCATTCTCCATTTGAAAATGTGACCCATTTATTTGAACTGAAAGGCATCA
A_14_P133010 AGAATGCATATATCACCTTCTTATGGCCACCTGATACAGACACTGCAAAAATGTAAAAGA
CUST_8739_PI426210822
AACTTTAATAATGCTCCCTTAGGATTCTGAGAGAAAGTTGTTAGATTCAGACCAAAAAGT
A_14_P139518 CTGATCTGTTTGTTCTCTCAACAGACACCTTTGTTCGTTTACTCAGAGAACAGAATAAAA
A_14_P201126 TGTCGAGAAGGGTCTTTGTTTAACTGGCATTTTAAGGTTTAATCTTAAAGATTACGCCCT
A_16_P00122210
ACCTTGCTAGACTCAACATGCTAAGACTGACTTCTACATCTGAACTATTCCATTGAATTC
CUST_6398_PI426210822
ACTAACTAAAATGGGACAGCAAGACAGAAACAGTTTCCTATCCCTGTAGGAAAG
CUST_2731_PI426210822 AGCAGATGTCTGCAGTGGGAGCAGAATGGTGTGATTTACTCTTTGTTTT
A_14_P106038 CAGGGAATATTTCCCAAAAGGTTTGATTTAGCAAGAGGAGCACAAGTCAGC
CUST_2403_PI426210822 ACGTGGCTTTTAAGAAATGTGCAGACACACGTACATGTTTGTTTAGACAGAGC
A_14_P104661 GAATACATGAATGTTATAATGTTAAATTACTAACCTTTTAAAAATATTAAGCATTGCAAT
A_14_P110982 TTTGGAGTTGCATAAAAGTAGGGTTGTGGATTTGATTGAACCATACTTAGCAAAAGTGTT
A_16_P19296678
TTGGAAAGGACCATTTTACAGTTTCTCAATTCATACATCACTACCACAACAGATGTTCTC
A_16_P00458005
CTGAGATTCCATCATTTAAAAACTCAATTAACCTGAGTCCTCAAGTTACTCCAAACTCCT
A_18_P14135735 CCACACACACAGTATCTTTTACAGAATCGCATGTGCTGTGATGCCATCTC
A_16_P16191606
AAAGAAAGGCAAAATGCTCAACCAAATGGAACTTTTTGAATAAGAGTTGACAGATTGCTC
A_14_P105078 ACTTTGAAATGCTGGAAAATATTTCAAGAACTAGTGGAGAGAACTCTAGCACAATCCATA

A_14_P138426 TCATTAGAATTTGACACTTTTGAAAAGTAACCTAAGTTGCAAAAATTTCCTGGAGGTCCT

CUST_5683_PI426210822 GGTGCCCAGCTGGCTTAACATGCCATTATTTACAGCTCTAAGGTTTATTTTCAT

A_16_P20139722

AAAATTTCACAGATTGTGCAGATCACTTTTAAACTCACATAGGTCGGTGTCTTTACAGTG

A_14_P104603 ATCTCAAACAGTTTATGCTTCCAACTGGCAAAAAAAAATAAAGTCAGGAATTTCTCAGGT

CUST_2065_PI426210822 ATGGGGAGCTCTGATCATCGGACACACACCCCAAAGCATGTCATT

A_14_P128605 ATGAAGAAGAAAAGATCTTGAAAGTTCAGGCCTCACACAAGCCTTCTGAAATTCTG

A_16_P19005976

AAAAGGAAATTGCCAATTTACTTGCTTTTTGCCTACTAAAGAGACTTCTCAACTGGTCAT

A_14_P120320 CAAAGGGTGAATGCGTATCCACCCAGAAAGGTGAACAGATTTACTTCTTAATAATATATC

A_14_P138343 GCAGTAGCAAATCCTTCATATGGGAAAAAACAGGTTGTCAGAAATAGTGATCAATATCCA

CUST_3481_PI426210822 AGGAAGAAGTGGGACATGCAAAGGGGCTCTTTATAATCTGGTCTGA

A_14_P202186 TGCTTTATGTGATGGAATAAATTGTTCCCGCAAGCAGCAAATGTAATGAGTTTAGTGCTA

A_14_P109164 TTGTTTTTAGGTTTGCAGATTTTATCAGGGGTATGCTGAAACTAATTCTTCTCCTCCTGT

A_14_P115613 ACAGAATATTGGAACTGTAAAAACCAACTGCATTAAATAGATGTGGTATGTGGCAGTGAC

CUST_1546_PI426210822 TTATGTATACTGCAAGTTGGCATGAACAGCTGGGCAGGAGAGTGC

A_14_P134714 TGTCTATAGTGTTTACTAAAAGCACATTATGGGCAGTTGTCATTTTAAGGGCAAACAGAG

A_16_P02114978

GCCACAGAGGAAATTAAACTGCTAATTATGTGTGTCTAAATCACTAAAAATGGGTCCAGT

A_16_P35650446

CCAATGACTGAAAACTTGAAAATTCCAAACTTCAACTTTCAATATTATGACAGCCTCTCC

A_16_P40327080

TCTTCATAAATGTCTTTCAATGTCATTGTGCTCAATGGTATCAAAAATCATCAAGGCCCT

CUST_1864_PI426210822 CAAAGGCTGGACTGGAAATATGTGGGTCTGGCTTTCCCTAAACTATAGTA

A_14_P132757 GGATTTTTTGCCGAGTATAGTAAAGTGCTTTGATATTCTCCAAGAGGATTCTTTTCAGGT

CUST_5025_PI426210822 TCCTTGGGCCCCAGATAGTACATTGGCATTTGTGAATCATTTTAGATGAGTT

A_16_P15802427

AAAGATCCCAAACATTAGTCTGAGTCTCTTTTTGAAACATCTTGCTACAAACAATTGGCA

A_16_P20052106

AATCAGAATACCCACAGATTTACTTGTCCCAACATGAATAGACTCACATTCTTTGGATTC

A_14_P133915 AGTAAAAGGCACAGATACCCAGTTACTATATACACTCAAGACTATGTTCCCAAGAAAACT

CUST_1890_PI426210822 CCCTCCTCCCTAACACTGATGCTGTGGAAAGAGGTTCTTCAAAAC

A_14_P121707 AATAGATGACTTGACAAGGTAGAGATCTGATATTTTGAGATTTTGGAGACCATTTCCTGT

A_14_P115569 GAAGTCTTTTAAGTTCCGAAACCAGCAGAAGATTGCGAGTCAGTAGTAAGTGATG

A_16_P15036280

AGAATGTCTTCCTTACATACTACCGGCTGCTTTTAAATATTGTTAACAGATCCCCCTTAA

A_16_P20484553

AAAGGCAAAGAAACAAGGAAAAATCACCAAACTTCCACTATATGTGTGATTTCTACCACA

A_14_P118152 TGTGGCTGGCACATTTATCCATCTGTTTAAATTACTTGAAAATGCTTTTATAGGTGAGCG
A_16_P20342329
TGTTTTTAATGCTGATCATAAACCACTCAATCCATCAGAGAATCTATCAAAGTCCGTCAG
A_14_P126716 AATTTTAAACTGAATTATGAACCCAAACCTCAAGGTTGGGGGAGATTTCAATTGAAAATG
A_14_P137631 TGTAATATCCAAAGCCACCGAGAAAGAGACTTTCACTGATAGTAACCAAAGCAAAACTAA
CUST_7686_PI426210822
ATATTTTCTTAGTTATGTATCATGTAGTCTTCCTTGATTCAAGAGTACCATGACTTGTGT
A_16_P16001834
AAACAGGATCTTAAATCTCGTCCCAAGAATTTGATAATGACAATACCAAAAGGCCATCTT
A_14_P116507 TTATAAGCAATGACATGAACATAAACTCACATCACTGGGACTTGGGCATTCAAACATGAA
A_14_P122259 TGGCCACCAAAAACTAGGAAGAATTTTTTTAAACAGATGGTAATAATGAAGGGGCAAAG
A_14_P200947 TTGAGAGTGAAATGTTTTAAATATTGCCTGGATTTAAAATTGGGTAAGGAGGTCAGCACT
A_16_P21245765
AAAAGCCATTTTTACCCATCTCTTTACATCACAAAGCAATTATTTCTCACCTGCCACTTT
A_14_P106481 AAATCTAACATTTTGTAGTAATTGCTCGCACATATAGTGCCTTGGTAATGCAGGCCTCAT
A_16_P20824084
GGGCCAGATAAAGATAAACTGCTTCATCACTGATAATGTTAAGTCCAGTAGTCTTGCAAT
A_14_P201063 AAAGAATGCTTGTGCTAGTTAATTTCCCTGCCAAAGGGTTCAAAATTAGTAGTTTTGTTC
A_14_P111882 CTAACATGCCAAAATGTTTAGTGTTACGTTCATTGTGTGTCACATGAAGTGCTATCATCT
A_14_P201921 TTTGAGTAGACCCAGATGTTATGATAATATCTAAAATTTCTTGGAAACTATTTCAATAAT
A_16_P18191382
TAAACAACGCCATTGTGAATATGGAGTGAGTCAGAGTCTTTCTACAAATGCTTGAAAATC
A_16_P38170387
GATGTATTTCAGCACAGTTTAGGAATTGGTCAACTATTGAACACCTTCCATTTTTCTGCT
A_16_P16711208
TGAGCCTAGTCAGAAGGATATAACAATGTAATGAGAGACTTAGAAATCAGAATGCAAGGC
A_16_P15708350
AAAAGTCCCTTTTATATCTCTCAGAAAGCAAATTAGTCCATAAGTAACTCCAGGCACGTT
A_14_P125005 AGACACTGACATTTATCACAGTGTCTAACCCAAAGTAGATTTCCATAAAGGTTGATAGGA
A_14_P132000 GTCAGACAGCAACTCAGAGAATAACCAGAGAACAACCAGGTATTTCAATGATTTCCAT
A_14_P106246 TAATACTCTTACTTTTGAAGGCACTTGCCAGTGCATTATTGCCATTTGGTTCTCTAGAAA
A_14_P124558 GGCTTTGCATCAAACTGTGCTTATCACATTGATCACATGATAAAAAGGGAATTTTGTCAT
A_14_P125450 CTGTCCAAATCAAATTAACCTTTAGAGGCATACAAATTCAAGATGTTTTGCAACAGGCTT
A_18_P22839221 CTCTTAAGGGCATCGAGCCTAGAGTCAGAGTTTGCTCCTGTGAAAATATTTC
A_16_P36141085
TCCCCAAATAACTCAATGTTCAAATGCTAGACTACCCATATAACATAGGATCATCTGAGA
A_14_P102463 GTGGTGTTTATTCCTAAAACCATATAGGATCATTGCAGAACATGAAGGAAAAATGGGTAG
CUST_5406_PI426210822 TCATCGAAACACTGAATGCAAAGGGTTAGCAGAGTGAGCACAGATGTT

A_16_P41658928
ACATAGAGTTTTAGTCAACATCCTATAGTCCATTTACCCAACATCATTTTGTGTTCCACC
CUST_3494_PI426210822
TTGAGGTCACATTGACTGTTTCACGTTGTACTTAGACATGCTGATTAAAACCTGAG
A_16_P19693676
AATGCATCTCCATATTCTCAAATCAGACAGCATTTGAAAAACTACACATTCAGGCCTTTT
A_16_P21424502
ACCTATCTTTCTCAATAGTTATTGGACCATGATAAGTGATACCTCAATACCACATACCCC
A_14_P110622 CTTTAGTTTTGAAACATCCCTTCTCACTCATTAGCACAATCCTTTAATAATGCACCTTGG
A_14_P103122 TGGATGACATCATGATTAATTTTCTTGTCTGTTCATCAAGGCCACTCAGTAATAACCTCC
A_16_P19603637
TATGTCTTAGAAGAACCCATTTAACTTTACAAAGCCAGTAATTTGAACATACCAGGGAGC
A_14_P128800 TGTATTGCTGTCCTTGAATGCCATAGTCAAAGAGAGTTTTTAATAGAACCATGTTGGTTG
A_16_P18104410
TCCATGTATAAACAGATCTCCCTTTACTCAACTCTAAACTTTCTTCCTTTCACTAACTGC
A_14_P135651 TTACAAGTAGGGAACACATTATCTTCATCTGTGTAACTTGTGCTGCTTTCTGGCTCAT
A_16_P21366844
GCGCTATTAATCCCATTCTAAACCTCTTCTTCATGCAATAATGTTAAAACAGTCCTTCCA
A_14_P117427 GAAGATCTAAAACTTTCCAGAATTTCAGTGCTTTGTTTCCCCCTAGCAATTAAGTTCAAT
A_14_P133435 CTAGATACCTCCACACATCCATACACTGTGATGAGACCTAGAATCATCTAGAACAC
A_14_P125350
TTCACTCATGTTGGGTCTAGAAGAAAAACATGACAAAACAGAAGTTGAGAGAAGCATTTT
CUST_8767_PI426210822 GAGGATAATTGCAACAAGAAGTCTCCCCTGCAGCCAGCTTATTGG
A_14_P108540 GCAATCCTGTTATTAATAATAGTGGCAGGGTCAGATGAATGGCACATTATAAAGTTGGTT
A_14_P124669 CTTGTTAAAAAGGAGGACTGTGTAGACAAAGTCTTTGAATGAAAATGGCCTGTTGTATTT
A_14_P129100 GTTCCGCACTTCCGTGATGTTGTTCAAACTCAGATCTAACACTTCTAAGGAAAG
A_18_P16534651
AACCTCTCTCTTACAAACAAATCTGCCTTTTCCAGATACTAAATCTAACGAGATGACACA
A_14_P136746 CTTGACACATGCTCTGTAGGTTGAACACATAATCACAAATATTCTAGCAAATGCTGCCTT
A_16_P02634036
TGAGAAAGCCTTATATCCCATTTCATAAGAAATCGGTCTAGTAGTTATGCCTCAACCAAA
A_16_P16309774
AATTTATGCCACATATCAACTTTTCTTCTAGGCTTTCTTCAGTGTATTCCAATGTTGGCC
A_16_P18931752
GGAGTATGTAATTGTGTGATAGGTGGAAATTCATAAGGAGAATGGATCAACTACAAGTGG
A_16_P17422896
ATATGCTCTACAAAAATCAGTCTGCTTCTAACATTGTTCCTCAACCACTTAAGAGTCCTT
A_16_P01703739

ATTTCTCATACCAGGTCTTTTTAACAAAAGCACATTCATAATTTTCCTCCCAAAGCACC
A_14_P137062 TCTGACTGTGACACATTGGTGAAATGAAACTTTCTGTGCGGAATTTATTGTTTAATAAAT
A_16_P16070429
AGATTGTTGTAAAAACTTGCCTTATGAGAAAGTAACACATTCGATCACTGTCCATAGGTC
A_14_P102123 AACATCTTTTAAATGTGGATAAGGGAGGTAGGTAGAGGTTTGAGAATTTTGAAAAGGTGA
A_14_P132666 CACATTGAGGTATGAAGGTCTCCAGTAACCAGACTCTCCTCTACCTTGTT
A_14_P106059 CACATCCTGATTATTCTTACTTAGCATTCAGTCCATGTGTGACTGCACTTATAATACCAC
CUST_4143_PI426210822 TCATAATCTCAGCAGGCTCCTGTGTCTGAGCTATTTCTGTATCCAGGT
A_14_P115189 CACTGCTTTATTTTCAATAGATTAAACCTGATTTCTGAGAGGTCCTGAAGTTGGGCTTAT
CUST_5878_PI426210822 CTGAACTTGCCCAGGGACTCAGAGAAGTGGTCTGGATTTGCTTGT
A_14_P127079 ATAACACTCAAAAATACAGGTGAGGGGGATTCTGTATATTCTTTCTAGGAAATCTGGATG
A_16_P36841395
GAGCAGAAACTTGAAACCATTCAACTTAAAGAAGACTTTCCCTCTCTCTTATGTGAAGTG
A_14_P103309 CTATTTTCCTCTCCCCTAATATCCTTTCTTCTCTCCTGGACTATAATCAGTCTTCAGG
A_14_P122467 ATATTGGAACTCTGTGAAGTCAGATTAATTGTATTTTGTAGCAGTCCAATCCAGGAGAGA
A_14_P130168 CAACGCTAAGTGTCCTATGAGATGTGGTATTTGTTGTGTATGCAGTAGCCCAAATAGT
A_14_P101743 CTTACAGAAAAAAGTCCTCTTCTTCATCTGAATCATCTTCCAAAAGATTTCTCTGTCAAA
A_14_P100533 GAGAAGGAAAGGAAAACTGACAAGAAGGGACTCAGCCAACAACAGTG
CUST_6141_PI426210822 ATGTAGGTTGCATTTTCTCTCCTTGGAGAGCCTCAGATTCAATGGTGA
A_14_P123012 GTATAATACACATGCATAGGCAGCACTTATGTATGATTTCATGCAGAACTGTGACAGTCA
A_16_P03137188
AGTGATCTCACTTGACAATAAACCCCAAGAGAACTTAGAACCCTAGTATCCAAAAAAAAC
A_14_P119528 CAGTCTTTCTGGGACCTGTGATCTAGAAGACAGGAGAAGATGAACACA
A_14_P200416 CTTCACGGCCTTCATTTTGTTACTAAGTTATTTTCTGGAAGAACAGCAAAAATTTCAGGT
A_14_P130628 TGTTTCCAGTTTTTTACCTCCACAATAGAAAGGAAATCTTAAGTCCACTCTACTGTAGCA
A_14_P116987 GGCTGCTTTTTACTAGAAGGTTGCTTTTATGAGCATATTTATACTGCTGAAGGATGAGTG
A_14_P129849 ATCTCAAGAGTATGTCTGCTATACTGAAGATACGATTTTACATGTCACGGCCTAAAACTG
A_14_P126482 CTGTGTAGCATAATGTTTGTTTTCCATGTTGATCTGCAAAAGAGAGAGATGACAGAAAGA
A_16_P20824381
AAAATGTTGTTCTTTAAACACTTCGGTCTCAAAATTTTTGTTCATTCACAGGTCCATTGC
CUST_5161_PI426210822
CTTTCTGAGCTTTGCCTTTTGTAAATCTCAGTGCTTCACACACATCAGACAAAGATATT
A_18_P14135854
GACTGATGTCTTCAGAGCAATGCAAAAAGACAGTGTTTCCATGAAACAGAATAAACTAGA
A_14_P137675 AATTCTAATTGATATTGTCAGAGCACTGGGCTTTCTTTGAAGAAAGCCACATTTTTTGAC
A_14_P126637 TATGTAGGTGCTTCTTGGTCGCACTCATGCATTGAAGGATTCAGAGTAAAATTTCTAAAA
A_16_P16306285
ACTCTGATAATCCAATGTAATCCACCTTCCAAAGAATTGTAAACCCAGACCAAATTACAA

A_16_P03717294
TATGGAAATTTTGGAAAATACACAGAAACAAACACGATGACCTGTATGCACTAGCACCA
A_16_P18718546
AAGGAGTTAGTGTATAGAAGGAGTCATTGATATCGGAGCATGTTAGCACAATAGACCTAA
A_18_P14363738
GCCCAAAATAGACATCATAGCAGCAGAATATAATAACAAAAACCCATATACACTAACCAC
A_16_P36946874
AAGAAAGATTCCTTTGGTCCTCATTATCCTCATTCCCTTACAATTCTTCTCACATTTCTC
A_14_P120743 GATTGATGTTCCATCAAGTATACGTGGAAAGTTTCAGTATAACCGTTAGGACACTGTAAA
A_16_P17215500
TTTTCACATTAAGTGTGTCACCTAAATTCAAACTGCAAATCCAAATCCACTTCTGGGTTA
A_16_P17505558
AATTGACTCAACCTTTGGAAGAAAGAAAAGTGGAAAGTTAGACTAACTTCATAAGTGGGG
A_14_P201922 AAGTTTCTCACATCACTCAGTCAAAACTGAAAAGACTGCTTGCTTTGGAGGTAGTTTAG
A_14_P118251 CTCACTTTCTTCTCACATGCGCAAAAGTAGTCTTAGGATTTCTCATTTTCTTTTTGAGTC
CUST_2727_PI426210822 GTGAAGGCCAGGGCTCCTTAGAGGTAAAAGGGTCTCTTTGTGAGG
A_14_P116089 TTGCGTCCATATCCAAAGTTCTGTTATTCAATAATGTCTTTGTCCATTCGTTCCTCTTTT
A_14_P102266 CATCCATGAAACAATTTATGGAAACTTTAGGGAGCATTGAGATCTAGTTTCTCTTCCCTA
A_16_P02963987
AGAAACTTTTCCAGCAATCCTCTACTACTGGTTCACATACTGATCTGATATGTTAAAGCC
A_16_P20926695
ATTTCGATGTTTCAGAATGATCTCCCTTTAATCCTAAGACTACAAAGTAAGACACCAGGA
A_14_P136387 AAATTTTTTCACCTTTGGAGGAGAATATATTAGAGTTGTGGGTAATTTTTCACAGCCACC
A_14_P128596 TCTTGCTGTGATCATGGCAGTTAACAAAGTAAATGCATCTGAATTTTCCAAACAGGATGA
CUST_3499_PI426210822
TGGAACAATCACACACAAATGTTTATATCCACATACAGGCCCCATATACAATGGG
A_14_P111878 CCATTTAAAAAAAAAAATCTTTGTCTGCATAGCATTTGGAAATCAGCCTTCAGGACTTCC
A_16_P02886463
AGTAAAGAATGCGATCTTGCTCTAAAAACGAAGTCTGTAAAACTCTGACGTATGTGGTAA
CUST_7041_PI426210822 AATTCTCTAAAGGGAAAAAAAAAATGAGGTGGCTGCTCTCTGGGGTTAAGC
A_16_P02089288
TGCCTCACAATATAATTCCATCTGCAACATACATTTAGATATCCATTAAGAGTTTGGCCA
A_14_P131248 AAGGAAAAAAACACATATATTCACCAGTGGTTTAAGGTGGTGGGAAGATGCATTGG
A_14_P115907 CTCTCTGCATTCCCAAATAGATAAGAAAGACAGGAAGGAGGAGTATTTTTGTGCCT
A_14_P121478 ACTCTCTGGAAGAAAACTCCTAAAGAGATGCCCACCCATAATCAGAAGCAAAATT
A_14_P112266 CACTGAAAGGCTTATTACATTATCTTTGTGTAGTTACTCTCCAGTATAAACCCTGTGATG
A_16_P16162589
GACTCAGTTCTCTGCACATATTTCAGTTTCCTTTCAGATCTATTTTTAGCACTATCACCC

A_14_P108892 TTCATTCTTCTCTCCTTTTTTCTTTTGTAGCCCCATCAGTAGCACCTTTAAATGTCACT

A_14_P124865 CCTTATCATATTGGCATACAACAACATTTTCTGTGTCAAAGAGTTCCTGTCCTTCCTCAT

A_16_P38429088

AATCAGCCTCCACATAATTAAAAACTGATTGACATCTGTATAGTCACAGACTCCTTGTGC

A_16_P00311136

CTATGCCAGACACAAATAATGACACTGATACATGAAGTCACTTGATTCTTTCCTGTTCTC

A_16_P00685577

CAGGCACTAATTTTAATGATAGAAACCAAATGGAATGATGCTGGGTCATTTACTACTTAG

A_14_P134000 GCACATCAAACTTTTACACGCAAATATAGCCTTGAACTGGACAAGCCAAGATAACTTTAA

A_14_P201872 CTTTTTGTATTGTCAAGTCTCTAACCCGAACCAAAACTCGATCTGTAATAACAGTAAACG

A_16_P02457477

CACACCCTTTTCAGTTTATGTTCTCTTGCTGTATTTTATGAATGCTCATTAAGCCACCTT

CUST_6126_PI426210822 TTTTATGAAGGGAAAGACACCCTTGTTTAGTGAGCACTGACCAAGCACTA

A_14_P101385 TTATATGGATTCATCTTTAGGCAATCCAATGGTGTGAAGACTTTTAGCAGTTGCAGTTTG

CUST_862_PI426210822 TTTAGCAAAGGGAGCCAAGAAGGCAGCACTAGAGCATTTGTGGATAA

A_16_P16499032

CTAGCCCAAATGACTTCTTCTATTTCAACTCCACTATCATTGCATTTAACTGGTCACAC

CUST_1385_PI426210822

GTAGAAGGATTAGAACTGGATTCTACTCTACCTTGAAGGGATTAATGACAGTCACAGA

A_18_P22842828 CTCAGCAGGCTGATTCTTCCCCTCTCCTATGAGACCATCCTCCAT

A_14_P116696 ATTATTCACTCCTTCAAGAGAACTTGCCACTTGAAAGGTAAGTGACCTTTGTTTTGAACA

A_16_P02591874

CTGATTTCTAATTACTTTCCCTGACCATGATTCTTACCTAATCCTTGCCTATCTACTGCT

A_14_P136349 ATTGTGATTATTTTTGTTCACCAACATAACCTAAATTAGAACTCTCCATGCGGGCTTTCA

A_14_P137616

AAATAAATACATCTGAAGAAGGATGATAGATAATGGAAGAGATGGTGAAGGAGAACAAGG

A_14_P200777 TCGGTTCACTCACCTTTTTCTGTTTGAGAGATGTTTGACTCATTTTTTTACCTGTTGAAA

A_18_P14134910 GGTTTGATGTCCAGTAAGCCTGAGCAGCCCTGCAAATACAGATGAG

A_14_P119137 GATTCTCTGCACAACAGGAACTTACCACATTTCCAAAGAATGGAAACTGTATACACAATA

A_14_P103318 ACATAGGTGATTTGCATTTGGGGACCTTGCAATATGAGAAATGCATGTGTTTAAACAGT

A_14_P134911 CAATGGTGGAGTAGATCCGGGATATTGAGTAACAGGTAGACAAGCCAATG

A_14_P128946 CCCTTTTGATCTCTCTTGCCATACTCCTCGAAAAACACTTTATATTTCACCTTTGTATTC

A_14_P201883 GTAGATCCTTAGTGAGTAGGAAGAGAAAATGTATACCAGAACCCTCTTGCTTATCTGAAA

A_18_P16340710

ATGGCTCATCCTAACTAAAATCAGTTCTAATGCCTACAAAAAAGCACTTTCTATTTCCTT

A_16_P15150730

ACCCCACATGCATTATCAACTCCTATCACATCATCTTTTAAATACTTGATATCTGCCCTT

A_16_P18327405

AAAAGGGGAACAAAATATGAGATTGTTAGTCACATGATTTCCTTACCGTGAACAACAGTT

A_14_P117126 CACAAAGGCATCTGGTTTAAAGCAGACTAGCAAGGATAGGAAGATTCCCTG

A_14_P135353 ACATTGGAATTAAAGTGCTTGGATGTTTTTCCCCCACTTTAAAAAAACTTTTGAGGTTTT

A_14_P137772 TTAGGTAAACATAGTTTAAGGAAAGTCTTCTTCCCATTTCTCCAATTCCCAAAGACATGA

A_14_P123082 CATGCAAACCAGGATTAATCTTCTCCTTACAAAAGAATTTTATTTATACTGCACTGATTC

A_14_P107470 TAGATGTATTCTTGGGGAACTATTCACAAAGAAGCCTATTTTTCAAGCCAATCTGGAACT

CUST_8504_PI426210822 GAGATGGGGAAGAGTTAATTACGAACCAGCTCCTGGGATGGACTT

A_16_P01012782

AACCACAGTGCTAATGATACTGCTATCCAAACAACTACATATACCAACGTGTATTTTCGG

A_14_P100477 ATCTTTTTCGTAATGTAGCCGATCCAGTGCACCACTCACTACTGTATTTACCTACAAATC

CUST_1336_PI426210822

CAGCTAAACCAAGGAAAATACTCCCATTTTTAGAATGGAGGAGCCATACGACTTA

A_16_P15104167

ACACCATAGTAATTCTTTGTCCAGCAATAAAAATACCACCATAGCACATACATTAGTTAC

A_16_P17593336

ATGGCATTTCATCCGTTTATGCACTCCATCTTGTATTTAAAGGGTAAACCTGGTATTGTG

CUST_6021_PI426210822

TTATATTAAGATGTAAAATTCAAGGCTCAGTATAGCACCTGGTGGCTAAAGCTGCCTC

A_18_P14137707

TTTAGTCAACGTATTAGTATTAATATCATGGCCTCTTTTCTTCTCATTTTGGAAGGGGAA

A_14_P121078 AGTTTCTGATCAGTATGAAGGCTACCTTCATACATGTGTGATCTTACATACATGTGTGAC

A_16_P19408775

ATTCATCTTAATCACTTTATGGATCCTGAAGCAATAGCACACTAATTTTTTTGACCCCCC

A_14_P121394 GTGGGTAGAAGTGACTTTTGCCCTTAACTCTTTTATAGTCTGAGAAGAAGGATTACTATG

A_14_P101417 TCCTTTGCTTCCCTCTCCACAATGATTTTTAAGAAAAATCCCCATGATTCTTATTAATGG

A_16_P18183897

AAAAAATACAGTGGCACTCTAATCAGCAGTAAACTCATGCTCAAAATGCTCTTTACGGAA

A_14_P116319 CTTTAGAGCAAGTTTCTGTTTTGAACAGAGTCCATTGATGTGCTTTAAGAGATTGCTGAA

A_16_P16106325

AACATCTACCATGAATTGAGGTTTGTCATTACCTACAAAGAGTATCCCAATTTTGATGGC

A_14_P120877 TCTATTGGGCAGACACCCTTATAAACTTCATGAAGGAAGAATATTAACACTGTGCATTTT

A_14_P111640 AGTCCACTCACCTTAACCCATTGAACAGTTGCTTAGATTTATCCAGAAGATAACAAAAAT

A_14_P116008 CCCTAATATAGTCCTCTGAATTTTTACCATTGTGGGTTTTGAAATTTGGTGATTTCCAGT

A_14_P115861 ACTGGATAATGAGAAGTAAATATGCCTAATTGAGTGTGTATGGGGAATGAGGTTAATGCT

A_14_P107683 TGCAAATACAACTTACTACTTCTGTTACCTTGAACTTGGAAAAAAACAGTGCTCTACCGA

A_16_P16805686

AACCAACTCAAAATGTTATGTCCTGTAGTCTTTTTTATTTATTAATGTTGCCGGTCCTCT

CUST_5465_PI426210822 TTTTGATTTAAAACACGAACCTTCTTTCACAACACAAGTGCCCCAGGAGA

A_14_P121774 GTTCTGGGGAAAAGCAGAATAATATTTGAGACTAAATAATATACTATTGGATACAATTAG

A_14_P116991 CCCTACTTTAAAAATAGTCAGAACTTCAAACATGGACTCCCAAGGAAAACAAACAGTC

CUST_3763_PI426210822

GAAGACAAGGCCACGGAGGTCAAAGATACAGAAAGATAATTGCCATATATGCTATG

A_14_P121045 AAAATGCAGCCAACTCACTTCAATCAACTGGAACCTCCCCAAAAGGTATAG

A_16_P21229743

AATCTTTTTACCACAATCTGAACCATGAAATAAGCACATTGAAAGCATACTTCTCCTCAG

CUST_9699_PI426210822 GCACTCTGTTAATAGTGGCTGTCATTGCATAAAGAGATGGCCAAGGAGAT

A_14_P112208 TACATGCTTTAAAAAATTCTCATTTTAAGGTTAGTCATGAAAGAAGATGGTGCCAACTTG

A_16_P19680444

AACAGCAAAGCATCTCATTTTTCTTTTTGCTACTTCAGATACATCAGACTTCCAGACAAG

A_14_P101864 CTGTTTATACTGTCTAAATCTAAAGATCTCGTCACTAACTAGGTCTCTACTGAGTGTGGC

A_14_P113082 CTCATATATTTTGAATTTGTTAAAAGACTCTTCCCCGTGTGTGTGGATTTTGCCTGTAGG

A_14_P127773 AAAGACCTGAGATTAAGGTTCTTTTATTTTGTGGGATAATATTCGCCCAAATTGGCCATA

A_16_P00345385

ACTCCCTGCCAAAAAATCATTATTTGCCTTGTAGACTTTTATGAAATCATAGTAGCCTGC

A_18_P13468682

TGCCACATCAGATCATCAATGCTCTTAACCCAATGTTCTCTGAATTGTATCAAATTATCC

A_14_P127541 CAAGGCTCGCAATTTTGAGAGAGTTTTGGGAAAGGTATGGGAAAGGTAG

A_16_P17469597

TGAAGTAGGTTATAACAGGTCTTTTCTAAGCCCAATGCATATGATCAGTGAAATAGGAGT

A_14_P100415 CAAAGAGGCTATAGGGGTAACACTATCACTGACACTAGGGTAGAGACTGAG

A_14_P136675 CAAATGCACTCTTCCATTCCAGTAGGCATGTATAAACTAGAACGTCAGATTTCTAAAGCA

A_14_P106583 TTCTTTGAAACAGTCTCTAGAGGTGACTCTAAGTATCATTTCTGTCAGACATAAGAGGAC

A_14_P200092 ACATGCCTGCGTTGATCTTATTGGACACAAACACCTGTGGCTTCTC

A_14_P122900 TTCAAAGCGATTCCATTTGGAGGAAGGAAAGAATGAGTAATGCATTATATACCAAGATCG

A_14_P114964 TTACTTTAAGGATCAAATCCTTAGTTTGAGAGCAGGACAGATGAGCATAGTTTGTCTTGT

A_18_P13365260

GCCAGGAAAACCTAATAAATTATTGCCGTAAATATGCTGTAAACATAACTTGACCCCTCA

A_14_P111012 CACTATCGAGTCTCATGGGGTTGAAGCAAGCATTTTTTGGCTTATATATAAACACAAATG

A_14_P106885 ACAAAGAGAAGCACCTGAACACACCTTACATAGTGGCCTGTGACC

A_16_P00881430

TCCAGTTTTAACCAGTGATCCAGATATATCTCATTCCTACCCAAAGATAAAATAGCACCA

A_14_P134659 TCTCTCCGTTCTCTCCTGGCTCCGCTTTAAAAAAATGTTTTTGTAAATATTGACCTGT

CUST_197_PI426210822 GCTAGAACCTGGAGTCTAAACTCGTCCTTCACCCGGGTTGCTCTG

A_14_P120673 CTCTTTGGATGGGAGTCTGGCAAGAGGAAATTGGAAGATAAAATAAATAATAAGTGAAAT

CUST_7085_PI426210822 GCTTTGTAGACATTACCCTCTGGAGTGCCAGGTGCTTTGTGAATT

A_18_P13932876

CTCCTTCACTTACTTTGACCTGTTACTGACTATTTGATACATGCAAGAATTACTCAGCCT
A_16_P00685393
TAATGAGGATTCCACCCCCCCAGAAATTAGTATTTCAAAGTATTTTTGCCTATGGAGATT
A_14_P134571 TGACATTTCTGTAGTAGGAATGAATGCAACTGACTAGCAGAAAGATTGTTTACTCCAAGT
A_16_P19897009
AAAAAACCCTGATACAATTGAGTTGGCTGACATATAGGCATAAAGTCTGCTGAACAGATT
A_14_P131608 TCCAAAAGCAGCGGGCTTTGTATTGTCTGAGTGCTTGAAATGTTTCACAATTAAAATTTA
A_14_P102920 GTGTGTTTTATGTGTGGAGGTGTTTTTAGTGTTGGGTTAATCCTAGAAAGTCAACACAAA
A_16_P02837304
GTGCCTATCCATTGACTTATCCAACTTTATTTCCATGTTAGATACCAGCAGTTCAGAAAC
A_16_P21564674
CCTCCATAACATCTGACCATTTTGATCTCTACACAAGCACAAAATTTCAGTAGCCTTTTT
A_16_P00011128
CATGCTTCCACAAGTTCCTTTGATTATTTAGAAAAAGGCTTACATAATTCAACCCGAAGG
A_14_P103533 GAAGGATATTCCCTCTCTGTGCCATTCTACTACTGGCTTGTTTGGGTTTAATACAAT
A_14_P119289 TCCCGTTAATTTTCTTGCAGAAAAGTTAAGTCTAATTGCCCATTGCCATAAATTTTGCCT
CUST_1585_PI426210822 AGTGTCATTATGTTTGCCGCAGCAGCTGGCAAGTCAGGCATTGGG
A_18_P14596428
AAAAAATGAAAAGCATGAACACAGTTGGTCAGTTATAAAACATTACATAGACACCAGCCA
A_16_P38144693
ATGATACATTAAGGCCTCAGTGTTACTTTAATCATCTTGGAAATTGTCACCATGTTTGCC
A_14_P135891 ATCACTCCAAAATGCAAGAACTAAATTTGAAAGAGTGCTTGGACATGCAGAACTGAAATA
A_16_P03075935
TAGTGGAGTTGTTGATGAATAGAACCAAGACTAGAACAAATGTATCTGCCACATCAATGA
A_16_P01799905
GATGAGACATTGACTTTCATTCGCTATTCTCCAGAACAATCCACTATTCAGAAAGAAAGC
A_14_P126543 ATCCATCTGCCAAGTCTCATTTCAAGGAAACTGGAAGTTGTATTTGGTTGTATAATAACT
A_14_P104724 CATCACCCCTAGCAATATGTATTAAAGTCTGAAGTGTTGCCATGGAAACCTCAGT
A_14_P124938 TGGGCTTCTTGGAAATGATTTGATATTTGATGGTTCCAAACAGCATCAAAGTAATCAATG
A_14_P133684 TGGCCTTATGTTCCCCTCATTTAGAAGAATCAAAACCAAAGGCATTAGTATGTATAATGG
A_14_P139064 CTTTTTCCCCCCGAAAGCCTCTTTCATGATTTTCATTATGGTTCAGAAAAGTAAAGAAAA
A_14_P131153 GTGAGGTTAGTTGACAATCTCCTGAAGTTAGGATGGCATAAACTGATAAGACTAAAAGTT
A_18_P18190853
ATGTCATACCCTAGGATTTTTAGTTCCAAGCCTCAGAAATTCTCTTTCAAGAATCAGATC
A_16_P02843356
CTGCCTTGCAATACCTTATATACTGATGACTCATGCAAACTAAACAGGTAGATCTACCAG
CUST_1021_PI426210822 ATCTGGATGTGCAGAGACATGCACGGGAACTGGGGCCACTGTCAT
A_14_P110947 AGAATGTGAACAAGTAAACTATGCAAAATCTCATTCTGAAGGACTTTACAGTTGACTGGG

A_16_P18159905
AACAAGGAGAGATTTTTCTGAAACAACCCATTAAATTCCCCTATGCAAAACATTAGCCTA
A_16_P03234929
AAAAACATCTTGGTCAAGCATTCAATCATGAGTTACTTTTTGAGTTCTCACCTCTTTTGG
A_14_P137144 CATTGTTGCAGTTTCTTCTTTGTGCTTTTCCTGGAGGTGAGAAGAAAAGACAAT
A_14_P134581 AGGCCATTTATGAAGTTAGTATTTGAGCCCCATAAGATCTTTAAAAAGCCTCCAATCATT
CUST_4916_PI426210822 GAGTATGATATAGTGTGCATGTGTGACATGCTGCGAGTGTGAGGAATGC
A_14_P135387 TTTACATAAACCTACCATTCGGTAACCATGTGAAAGGATGGACCAATATCAAGGTAATTT
CUST_10429_PI426210822 ACAGATGAAATAAGTTGAAGAAACCTCATGTAATGAATCCACCAGGCTGGCA
A_16_P39622637
AGTCCTCCACCTTAAATAATCTCATTTGTAACCCAAAGAATCTCATACAGGAAACCTTTT
A_16_P02168216
AGACGCCCAGTAATTCTTAACTAGTTATGCCTAACATCAAATAAGAACCCCCTCAATTTT
A_14_P124304 CTGTGCCTGCTGTTATTATCATTGTTGCTGTAATTATGCTTACAACATGAAAGAACTACC
A_16_P37935939
ACATTGACATGCACCAAAAATGATTTTATCCAATCCAATCTACATTTCCCTTCATTCAAT
A_14_P131988 CTGTTAACTCTCCTCGGGCACTTCTCTGGCTTGATATCCAATTCATAATGATAGATG
A_14_P120313 GTTTCCCCTGTCTTTGTCCACAGATACCTGATGGATCCAGACACATT
A_14_P134376 AGCGTTCCTCTTTTGCTAAAAAACCCTGATTATTTTTTCAAGGAAGCGAATACTACTATT
A_16_P01118100
GGATCTGACTTCCTAAATTATTGGCAGGCTTAATTTACGTAACAAATGAATGTTCCACCT
CUST_10376_PI426210822 GTGGTGCACAGACAGCACAAATTCCATGGGTTTGAGAGAAAGATGT
A_14_P101899 CATTTATATGTGAAAATAGCACCTTTAACAACAAAACTCTTTGTTTGGAGGTAAGAGGGT
A_14_P114390 CTTTTTATAACCTGTGAATCAGCCCAGGATCGAGTATGAGGATTGAGACGG
CUST_2087_PI426210822 ATTTCTGAGCGGCCACTACAAAGCCCTCCTGTGACCCTGTTAGCT
A_14_P138464 AACAATTAGGAATTTTTTATCGTTATAGATGTTGTTAAAGGACTCCAGTAGCAAAGATCA
A_14_P133414 CTAGAAATGCCTTGGTAATAAGAACTTTCATCAGCATCCATGGTGAGACATCTAGAACAG
CUST_5662_PI426210822
TTCACTAGCAGATTCAGCAGAGCTATAAAATTAAAAGAAATGTGTTCTGTAATCCCTCTG
CUST_345_PI426210822
GGAAGGTAAAATATAGACATGAAAGGGAAAAGCAGTATAGCAAAACTTTGAAAGGAAAAG
A_14_P139933 CCTTAAATCACAGTGCTATTATCATCTCTGGAGGACCTAATTCTGTGTATGCTGAAGATG
A_14_P125630 GAATGTTTTGATGTTTGCAGTGACATCGATTGTGAAAAAGGTTAGCCAGAAAGGAAAC
A_14_P112816 ACTGAATCTTCTGTGAAATGTTTAGACTGCCAACAACACTAAAAACTCAAAAACAACTCC
A_14_P134231 TATGTAGTTGGTTTTGGATTGATCAGTTTTGGTGAATGCTTTGCTGATATCTTGATGGC
A_14_P133417 CAGATTTTGAGTCAACCAAATTTCAGTGAAGATAGACTAGCATGCTAAACTCATCGTCTC
A_18_P17162450
AAGAGTCCAGAATGTCCATAACTATAGGCCTGTAACTAATAATCCTCACTTATTGTCTGA

A_16_P15611739
GCCTGTAGAATGAACACATGAAAGCATTAAGGACATTATGATAATTTCAGGATTTGCCCA
A_14_P119744 ACTTCTTCCACTAACTATGATACCAGCACAACTTCTACTGACTCTAGCCAAGTATATTCT
A_14_P138962 CAAAGCCACTTGTAATAAACACCCACATACAAGGAAAGGCCAAAGCAGG
A_14_P112749 GTTTCTAAGATCTCCTACACTTCTGTTTTTGCCCTGTATGAGGCATTTTAAAGTTTGTTC
A_14_P116947 TTTTGCAATCTGGAGCCTGTTCTTGTGCAGTTGATTCATGTAAGTATTTTCATTCATTAG
A_16_P02139095
ACAATCTGGATTCTTGATGTAGTATAGCATAGAAACGTATGAACTCACAGATTACTGTCC
A_18_P11415657
ACCCCAATCTTAAATGTCTCTTAAATCACCCATCACATATTCAATACTTACTCCTAACCC
A_14_P112223 CAGAAGAGGGAATGGTATTGATGAACTGTGAAGTTTCAAATATGTGTGGATCTAATTTGC
A_16_P18090976
CCTCATCAAAATTTTAACAGCATTCCAATACTTACCATCTTTCACATCTAACTCTTTCAC
A_14_P110409 CTTGTGTTCTCAGAATCTGATGCTTTCTGATCAACAAAACTGAGCAAGGAAGATCC
A_18_P22840861 GAGGTGACTTTCTATTTACAGTTATTCATTTACACTGGGTTCATCTATTAATATATACTG
A_16_P15599848
AAAAGGCAACACTACAACTCTGTCCATCAAAAATTGAACAAACCTGAATAGGAAATAGGA
A_14_P105990 ATGATTGACAGCAGGACAAAGTGTTTTCTGGGTGAAGTTTATTCTGTTTTCACATCTAG
A_16_P21615739
AAACTAATAGAAGGGTCGATATGATGTCTCTATGGTAATTATCATGCCACTTCTCCTCTC
A_16_P58424343
ACATAGTCATCACGCTATACATTCAATCTCTAAATTTTTTCATGCTACCTAACCAAAACT
A_16_P16191561
TTGCCTTTCATTGATTCTTGAGGATATGAATGTTTTAGAAAGCAACAGTGTATGCCTTGA
A_16_P19554610 GATAGGAGGTCACTGGCATTGCTTCAGTAACTCATCTCTGTCAGGATTAAACTCTAT
CUST_451_PI426210822 GGTGACATGCTGCTGTTCAGGAAATGGTCTCCTGATAACTTCTATTGTGAT
A_14_P137535 GAATGCTTGTAGATGTGGATTCTGTTAAGGATAAAGGGAATGTTGACTAAATTGCAAAGG
A_18_P13930125
AATTGGTGAGAAGGAATTAAGACACTGAAATCAGTCATGGAAACAATCACTCTGTTCACT
A_14_P106897 GAAAAATTGGCCTTCAGACTTTGAAAAACATATTATGCAGACTTTCTCATTCAAGGCTGT
A_14_P122501 AAATTTAATCTTATAAAAGTTCTAGGATGATTCATGCTACCCTTCCTTCCCCCAAATCGT
A_14_P128713 AAAACAGAACGCACCTCAACAGAATAAACACTGATATAAACATCAACACACTGATTTCTA
A_16_P16249098
GGAAAACATTCACACACATTAACAGAACTGCATCTTTTCTTCAGTAAAATCAGGTCTTCC
A_16_P20375396 GCTTTCCCTGAGTTCTAACCAAGAACTTGTCCAAACTACTTCTCTGAAGTCAG
A_14_P139562 ATCTTTGATGCCTTTTAGGAGAACACTTGATTAGCATTAGTGTCCTAAGTCACTAAAGAG
CUST_1903_PI426210822 TAAAAATGCCTACGTCTAGTTCACAGTGATGCGGGTGCCTCTATGCTAAT
A_14_P132199 ACTCATGAAAGTCAAGGATGAAGATTTCAGTCTTGTCCTGTCTTTTCTCTTTTTCTTACT

A_14_P123845 CGGAGGCATTATGACTCTGCCACTGCAAAATCTACAAGGTAATCCATAATGTC
A_14_P132154 CACTTCAGATTTGCTGACCATGAAAGAATATCACTGTTTGCTGCAATTACTGTGTCCT
A_18_P22840355
AAATGCTAGGGAAAACAAGAGAGGCAAAGTTTTATAGAACCAAATGCTGAGACAGAAGAG
A_16_P19757782
ATGTCCAGATATTTTACCAAATACTCTGCTTTCATTACTGTTCAGTGTCAAAGTTCCAGG
A_14_P105676 TTGACTATTTGGAAACCAAGCATCATTAAAATTCTCTCAAACTCCTAATTGCGAAGAATC
CUST_5304_PI426210822
TGCCCAGAGCTACTGGGACATGTAATAAGAAGCTGCTTTTCAGACTTCTTAATTA
CUST_6861_PI426210822 GTTGCATTGTCTCTGATCTCCTTGAGAGACTGAGATAGATGGATAGGCC
A_14_P109221 AAATCCAACTTCTGTTCTATATAAACGCATACAACTCACAGAGATTACTTTCCCTTTGGC
A_16_P15410017
ATTTTTCCCTTATTCTCAGGTCACTATTCAGCATTCAAAGTCCAGATGACAGAATCAGTC
A_16_P03583641
AAATCTCCAAGAACAGAAACTTCTCTTGACAGTGCATTCTGTTACCTTCAGTATGAAAAC
A_14_P135316 ACTGAGATTAGTAAGGGGTTAAGTGAGGTTTCACATGTAGAAAAAGGCCACACTATTTTT
A_18_P13166759
AGTGATCTCCAAAATACCAGATGTAAACAGTAAAACCAGATAATTCAAAGTCATCCCAGC
A_14_P111247 CACCACATAACTTCCTAATGTCTTCAAATAGCGAAAGTGCTAGCCGGAAACTG
A_14_P130357 CAGTGCTGAAAAAGGAGATGAGGAATATGATTCATGTAAACCTAATCAGTTCTTAAAACA
A_14_P111548 TTTGCCTCCCTATGCATACATATATCACACAGTCTCAAAGTGAATATACTTACTCTGTGC
A_14_P102126 ACAAATAGCACCCTGCTCCTTCACTTTAAATCATATGATGTTTTCAACGGTGTAATAGAG
A_16_P00806516 GGCTTTTGAATTCCTGCCAGTTGAAATACTTCCCATAGCAACTGACTCTCAAAAT
A_14_P135580 CTATTCCCACCAATTTTCATATTATTTCATGTGACCCTTGAGAATGTATTGCCATGTCTC
A_14_P105180 CATGTGCCACTACGTCGTGAGTCTGAAGTAAGGCCTAGTGTTTTATGAAGAAAAAA
CUST_4353_PI426210822
AAGAGCATGTCACATTTAGAAATACTGTAACTGGTTGCTGAGGTTTTACATATATTCTTT
A_14_P121049 ACTGTAGTCCTTGAAGAAGCCAAAGAGAAGTGGGATTGTGAATCTATTTGTAGTAAGTAT
A_14_P137053 ACAAAGAAGTGGCGATCAAAAAGACTGAGCCCAGAATTTGCTGAGAATGTC
CUST_6103_PI426210822
CAGTACCTAAGAAAGCATCACATATGATAAAGAAAAAGACTATGGCAAGTGAGCTTTAAA
A_14_P105894 TTCACCTCTGGTTCATTTACTTGAATTGTGTCATCTTCAGGGTAGAAGTAGATTTTATAG
CUST_3628_PI426210822
GAAAGAATGAGGGAATGACTCAAACAATTTCAGGAAATGGTTCAAGGCTGTGTA
A_14_P120448 AAGTGTCACCGGTCACACTTTAATTCCAGTCTAAAATTAAAGTCTTCAGTCTCCACATTC
A_14_P115420 GTAGCAGACTTGAGTTGCTGAAGTAAGGGATCCACAGTTATGGTTACAGG
A_14_P201466 AACCTCAAGTGTAGGTCTAATTAGTGTTTCTGGGATCCAAAGTTAGAGGAAAATTTAGAT
CUST_7157_PI426210822 TTTCTTCTTTCTTCCACGTCTCTGAAAGTCCAGCTTCGTGTGGGA

A_14_P119942 GGTATGGGCATTTTTTTATTTTCTGGAGAGTTAACAGTTATTAGCCTTTGTAAGAGGCTT
A_14_P121224 TGTTTCAGTCTGTTGGAAATAAGAAAATCGAGACAAAAATATCACAGCCGTGAAACTGGT
CUST_6853_PI426210822 AAAGACAAGTGAGGAAGAGGGGTGATTGTGAACTCAGCAGGGAGA
A_18_P22841262 AATTTGAGACATTTTCAGGGTCCCAGTTCCATCCCCTAGGAATCTGTAAAG
A_14_P110727 TTAATGTTCTACAAATACAAAGTAGGGCCAAAGTAAACCTTCTTGAAGGGAACATTGGTT
A_16_P00494880
AATCACTTTGCTTCAGTCTTTCATATTAGTCTTAGCATGTGTTATTGCGGATGTCTCCTG
A_14_P132220 ATTATCCAGGCTTTACCCACAATGGGAATAGTTTTCTCTCCATATAATCATCAACCTAAC
A_14_P103231 CCTTCCCCTAGGATGCAATCTCCAGAAAAGATAGCATTTTCCTCTTCTAAGAGTA
CUST_1772_PI426210822
TTTCTCATTTTATCAATTGTTGAAGTTCAGATGGTCAGGTAGAAATGAGTGTGATGGAGT
A_18_P15377280
AGGCTGTTTAGGTAAACTAGGAGCAATGAATATTACAACTTCTTTAAGGGCTAAGTGGAG
A_14_P119980 CGGCCCCAAGTAATAAACAACTAATTAAAAATAGAGGAAATTTTGGCAAGTGCTGTCTAC
A_14_P138480 TTTTCTTCTTTTTTTTATGGTAGATATCCCAGAGGAGGAAGCCAGATATTGGACCTACAA
A_14_P106533 AACCTTATCCTACAGACACATAGGGATATACCCGAAGTCTTAAATACACAGATGCACTTG
A_18_P13969187
AAATGTTTCTGCAATGCATAAGTGAAATAAATACCAGGAAGCCTTATGGTTCGATCACAC
A_14_P115324 GGATTTACTTGTTTTACCATGTTGTCTTGGAAGGATTTACTTCTTACAAGTCATTAGGCC
A_16_P16780808
AAATACCTCTGATGGTATATGGACTAAGGTAGCAAAAAGGGAACTATTGAACAAACTGGC
A_14_P138620 ACAGGGCAATCTGTTTAAATGACTAATTACAGAAATCATTAAAGGCACCAAGCAAATGTC
A_16_P19426393 GCTCATTACCTACTGCATTTTTATTCATCTAATTCCCCATATGAACTCCTTATCTTGGCC
A_16_P41718703
TGACAGTGCTATTAAATAAACCATGGTCCTTAGAATTACAGATACTGCATAATACTGACC
A_16_P02177606
ACTCTACAGTTCTTGGTTACAGACTTTTCTAAGGCTTGGATACACTTCCTTTGAGAAAAC
A_14_P135658 CTGAAGAAACACATACATAAGACAGTGTTTGAAAACATAATATGCATGTGCTTCAGCCCT
A_14_P108157 TCTATTGGCCCAAAGATATTGTATTCACTGGACAAGTTTATGATCTCATTCCAGGGTTAA
A_14_P123461 CTTAGTTCAATGGCTGAAACACAGTCGAAAAGAATACTGTGAATTATGCAAGCACAGATT
A_14_P117122 CTAATGAACCTGTTAGTGACCTCTCTTACTTTGACTGCATTGAGAGTGTGATGGAAAA
A_14_P104788 TGCAGGCATCATAAGAGATTCTTCAACTTTTATTTCCATGTCATTTTCTGTCGAAAAGGT
A_14_P135037 CAAGAAGAATTGAAAAGACTCTATAAGGCTCAGGTCAGTAAATAAACTGGACATGAGTGA
CUST_6885_PI426210822 GTTTTTAGACAGAAGGTTCCATACAGAAACTGCATCTGGGCAGGAGAAC
A_14_P106591 ACAATTCCCTTTAGCCAATGTTACCGAAGTCAGTGTCAAGAAAACTTAACAGAAAAAAAA
A_14_P112495 CTTTTAACAAGAAAGTAACAATGAACAAAAGGCTAGGATGCTGTCCCTTGAAGATTACAG
A_14_P108781 AGAAAATGAAATGAAATATGATACCAATAATAATGAAGAGGAAGAGGGAGAACAGTTCGA
CUST_7735_PI426210822 GCAAGGTATGTCTGGATGAGAGAAGGTCTTTGCCAAGACAAGCATC

A_16_P58424135 TTCAGACATGTTTACCAGTTGACACTCCCCTGCATTGCTTCTGTCTCTAT

A_14_P113468 CATGTTTATGTAAACCAGTGATGCTGCAAAAAGTTACAGCCCCATAAATGAAGTTGTAGA

CUST_2300_PI426210822 TAGCTGTTTTTTTACCCTCAGATGTGGCCATTGGTGCACCCAAGG

A_16_P36323208

ACTTTATCACCACCATACGTTTTCCTATATTCATTGGCTTTCAAGATGTTCAAAGACAGC

A_14_P116493 CCAGTCCAACAAAAAGTTAAAAGGATGCTCATCTCTTTCTCGGCATCACTGAT

A_14_P136294 GTATAAACATCAAAATCATAGGGACCTTTTTACAAACTACACATACCCCATATGCCCCAA

A_14_P125344 CATTACACTTGACATTTGGAAATGAAATCCACGTTTTTGTGTGACATACGGAACAAATTG

A_14_P136681 CTTTAGTTAATGGGCTGGATGGAATATTTGCATCTAGAAAGTTCCCCTGATGTTTTAAAG

A_14_P106906 GTATCGCAGAATATGGCCCTTATCCTATTGAAGGAGGACTCTTTGGATGTAAGT

A_14_P117226 TATCTTGCCAAAAGGTAACAAAGAATCTGGAATGTTTCCCAATACACCATATCTTAGGAG

A_18_P16280508

TCTGTGTCAGTTACTTGAAGAAAAATTGTGCCACTCAAATACTCTACACCTATGTCTAGC

CUST_5846_PI426210822 CTATGGAAGGTCTTACCTAAGCCAAGACTTTGGCAGGGATTGGATTG

CUST_4396_PI426210822

CTTTCTTCTGTCTTTTGCAAAATCTATGACACTGTTGAATGAATAATATAAGCCCAAGGA

A_14_P108172 GATTTGATACCCCAAAGATTTTCCATTTCCTTAGACGTATCTTTGGATCCATCTTCTTTT

A_14_P126360 TATGGCCCAATCAACAACATGAAGAATGTATTTGCAGAACCTCAGGTTTGTTTTTGAAG

A_16_P19979344

AGTTTGGAGTTCAGGATAGAAACTAGGAAAATTTTTACCCCAACTGTTTAGTTCAAAGCA

A_16_P35334114

GGGCTTATAACTAACCAAGATATGGCGTATAACAATGGATATTTTTCACAAGGAGGACCT

A_16_P00279722

CAGACTACTCACAACCAAACTATATCTTTCAGAATTCTACCTTTCAGAGCATTTTAGCGA

A_14_P101155 TAAAGTAAAGCCAGTTTGGGAAGTTCCAAGCATTTTAAACCAACTGTGGTCTGTAG

A_16_P20907399

ACAAGTAACCAAGAAAGGCTAGAAAGACCTAACACTTGACCATTTTACATGTGATGTTTC

A_14_P130259 GCAGACAATGAGACACAATCAAATCACATTCAGCACAATATGAATTAAGCATCTGAGAGT

CUST_10013_PI426210822 AGTCCTTGCTTTGCCTGGACTAGGCACTGGGATGCAGCAATAACT

A_18_P16903972

ACTATTTTTCCCCAAAGACACTATAATACAAGAAGTTAATTTTTTTGCACATGTATAGGT

A_14_P124001 TCAATTCCTGACATGACCTTTTAACAGGCAGTTAATGTTTTCATTCTATGGACTTTCCTT

A_16_P30952424 TCGTCACTATGTGGCCCATGTGAAGGTCAGTCCTCTCCTCCTTTT

A_16_P15652195

AAATTGCCCTACAATCTAACACTACGAAGACAAAGATTGAACTTAGTGGCTATTTGCTCA

A_14_P111862 AGGTCTTGTTGCCCGTGCCCTTGCAATACAGGTATGCCTAAATTATTTACT

A_14_P110121 AGAGCAACCACTCCATGGTTTCTTAGCACCAGGATCTGTAGAGAAATAAATAGTCAATTA

A_16_P02049664

ACCCACAGTCCTATATTCACATCCTTTCATCTAAATCTAGTTGACTAAAGAATATTGGCT

A_14_P201015 AACTTCCATAGCACTTGACTGTGCTGTTTAAATTCGACCTAACATCTGTGATACATGTAT

CUST_4075_PI426210822 GCTGAGAGTGGTGTTGGCACCTTCATAGCTGACATCAAATGGATC

A_14_P118275 AAGAGGATGATCAGCAGCGTCTGGATCATGATGATACCATCCTTCAG

A_14_P135271 TTTTGCAATGTTGTTACTCACTATCCATCTACTTGGCCAACAGCCTCTTCATAAACTTAA

A_18_P14135724 CTGCCTCTGCACTGTTAGTCCTCCCTGACTTCCTGCCCCAGGCTC

A_14_P126485 AGGCTACTTTTAACCTAAAATTTCACCTACTATTCTGTCATGGTATCTCCTTCTTCCTCT

A_14_P106066 CTTTCTGTCTCTATGTGAATGAATGACTGCAAACCTGAGAGGTATTTAAAACATCCAACA

CUST_9305_PI426210822 CTCCCTTTGTACCTCTCCCTTTATAAAGGACAGTGTTGGAGGAGG

A_18_P22842473 CTTAGAGGAACACAAATTGCCAGACTGACAGTCAGATATGGAGACACCAG

A_14_P125786 TTTTCCTTTTCTGACAAGTGTTTTTGGAAATCGTCAGTGAGAAGTCCAGATACTCTCT

A_16_P21367238

GTAACAATTCTCCACTTCAGAACTTTTATCTCACAAACCTTTATAGGCTCCAACAAAGCA

A_14_P128411 AAATCACTAAATACTACTCTGAGGGGCTTAGAAATTAACAGGGTAAGTTGAATATCATCT

A_14_P106967 ATTTCATGGTGACATCCTCCAGTTACAACTTAACCACAGACGACACTGACAAAT

A_14_P100600 GCATCTTACTTGATGTTTACTTATGGGAACCCCTTCCAAACTAGATATGGCTTTCAGT

A_14_P136215 ACCTTTAGTAAGCCTAAATATATGTAATCCAACGCACCTAAGCAGAAACGGTAAGGCAAG

A_14_P130015 ATAATTTCAAGTTGCGTGAACATTGATTGCCCTGGAAAATATATCAGTGAGGCTTTCCTA

A_14_P135843 CTGAAAGCCTTGAAGCCGGGAGTTATTCAAATCTTGGGAGTCAAGACAT

A_14_P124904 CACTTCAGTTCCTGAGTCCTGATATACGTCCTTTCCAAATACCACATAAAAGTAAAATGA

A_18_P26795630 CTCCTGAGGAGGGTCACCCTGCGCTTACTCCCCGTCATACCCATC

A_14_P122850 AAGTAAGTGAAAAGAAAAGAGACGTCACTCATAAGCACTCTGTCTGGAAATAGTCATTTG

A_16_P19723614

AACCACACTGTCAAAGAGTATAACCTCCTTCATTTATTATTTCCTACAATCCCAACATGA

A_16_P00685123

ATGATAAGGGCAGAGATTTAAAAGAAAAAAAATTCTTTAATGGCAGATTCTTGCAGGCCC

A_16_P02102899

AACCCTAAGTCCTTGCATAAGATAACCTCTTAGACTTTTGTCACATTTTGTTTCAACTGC

A_18_P11655211

ACCATCAGTTTTTCCATTCTCTCCTTGTTGAATTAACCCATTATCAATACGGTCATCTGT

CUST_6786_PI426210822 TGTTTTGTTTTTAGCCCATTCTGGATTTCTGCACTCATGCATGCCTCATG

A_16_P21198395

AACAGGTGTTTCTCACTAGACAACAATGTCCGTTTACTTTAAAATTCAGAATTTCCCCAT

A_14_P118493 CAACGTCTCAAATAATGTGAAATGTCACTTTTGCAGTTGACTGTATTAGCACTAAAGGGA

A_16_P19374597

AGGACTCCTTCAGAGATTAGAACTAGGCTTAAAAAATTGGTCACTAAATATGCCTGCAAT

A_16_P30952294 GGCTTCTTCACCGAGGTGGGTGTCTGCTGTCTGGGCATTTCTGTT

A_16_P20139028

TATAACATGCTCGAATCCAATGACAAGTGCTTCTAACAATTTCCAAGACTAAATACGCCT

A_16_P00523328

TTGCCCTCTGATCCATTATTATTCTTAACTTGCCTTTTATCATTCAACCAATCCCTACAG

A_16_P15005041

CCACTCTCAGAATAAGTTCCTTGCATTTATTCCAAATAATCTCGTTTACTCTCACCTGTT

A_16_P19994266

AAAAAGCAATCCCTTTTACTCCTCTATTTCTATCCATCAGACCCATTACAGACTAGACA

A_14_P202138 GGCAGAGACTAACTGGCTTCAAAATCCCTTTAAAGAAAGATGCTTCCGAGTAA

A_14_P103867 TCTGAATATCTCCAGTGTTTCTTTGTTTGTTCTTACTTTTCTTTTCAGACTTGGGTGTAA

A_18_P13535609

AAGCCCTCCTATAAATTCTGTGATGTCCACTAATACATGCTATCTCCTTCTCTGTAGATA

A_14_P131685 CTTTCTTGCTTCTCAAATTGAACATGGTCATCCATATTTTGCCTCATGATTACATTGGTT

A_18_P22841023

AATTACATTCATTAATGTAGGCTCCATGCATTGAGTTGCGGATTGTGAACATTTGGAAAC

A_14_P103985 GTTATGATCACCAGTGACAAGTGTTTTACCAACAAACACATGAGTCGGGG

CUST_3410_PI426210822 TCATTTGGAGAATTCTCTGTTCCAATAAGTGGGATCCACATGGCTCAAG

A_14_P111112 TTTGCCACACAGACTCAGAGTATGTATTACCTCAGTTACTTTTCTTTCTACTTCTGTTCC

A_14_P100475 CTGCAATAATCTTTAACATGACACATAAATTCAAGGAGAGGCATCCTCAGGCACAACTAT

A_16_P18041438

AACCAACTCAACATCTGAGAAGTCCTATGCTAATCTCAAAATAGATGATCACTAAGGCTG

A_14_P100009 TCTTCCCACAATATTACTTCCTTGGCATGTTTAGAGTGAAAATAAGTTTTCGTGGCTTTT

A_14_P101444 AAAGCCTACACTGAAGAGATTAAATCTAGATTCGTTAGCCAGCCACATAACTTGATTCAT

A_14_P123410 CTTTCAAACCAAATGAAAAAGTAGTGGGTCAGGTAGAAAGCAGTAATTTTGCTATTGGTT

A_16_P19750359

AATGTGTTGTTCTCAGATATCCCTTCACACTTAATTGCTTATTGAACAAAGTAGAACCCC

A_14_P125578

AATGAAGAGTCCATATAGTTCAATGAACAAAAGGGAAAGTTCATGAGGACAAAGATCACA

CUST_7000_PI426210822 TCATTCAAAGCCAGAATCTCTTTCCTGCCAGAGCAGTCCTTTGTC

A_16_P39361539

ACTGCCACCTTTTTACCATTCAAGATTTTACTGAAGAAATTATTTAGCCCCCATAAGGAA

A_14_P128025 AAATTCATCTGGAAAGTTCTGGAATGAGTGTCCTATAACCACATGACAACTCTATAGACA

A_14_P122431 CTTAAAACTTCGAGCGTGGACGCTGAATGGGTTTGTAAAGTTTCGGTAAGT

A_14_P113514 AGTTGCTGATCTGGTAGATCCTATGGCTGATTCACGATGACAATTGTTATTAAAAAGAGA

A_14_P122457 CATGCACATGTTAAACATGTGATAGGTTCAGTTGAATCAAACAAAGGGAGGAAAAATAGT

A_14_P116042 TTCAGGTTATCACTATAATCTACAGGATGTCTTTCACTTGATGTTCTTTCTTGATTGCCC

A_14_P124831 ACTGTGACTGTTGGACCTGCTGTCTTTCTCAAGGGTTATATTTTTGTTTACATTTTACTT

A_14_P134949 ATCAGGTAGAGGCTATTGATTAGATCATGCTCCTCTATTCTACAATGCCTTTTTTCTTGC

A_18_P15138954

AAAAACCTTGATTATTACTCATGAATGTAACTTACCAAGTATTCACTAAGGGGCCAAGGG

A_14_P123449 TCTTGTGATAATATACAACTGGATTCTTTCCTTGGGAGAATGGGATATGTTAGAATTGCT

A_14_P130646 GGAACATACCGTTTCTGCTGTTGTAGCACAGATTTATGTAATGTCAACTTTACTGAGAAT

A_14_P124279

GTAAAGGAAAGGGGAAAAAACACATTTTAGGAGAAAGAGGTAAAATCACAACACAAACTG

A_16_P00772204

CAACTTATACCAAGACATTGTCCTATCCAGAATTTTCAGCCAATATAGCATATTCCCCAC

CUST_9162_PI426210822

CAACTATATTTTACTCAAGGGCATTATAGACAGGGCAATTTAACAGATGCCTGTGGTTAG

CUST_5486_PI426210822 TCGGGGTCCTGGGAAGAATTTCAGGGTCCTCTGTGCTTATTTGTT

A_14_P108483 ACAAAGACAAAATCCCCCTCTAAACTCTTAGTTAAAAGGTATATGCCAACCTGCAGTATA

CUST_3895_PI426210822

GACAACAGAAAACCTTGTTTTTTTCCTGTGTTCTGGTGAAAGTCTTTTCTGTCTTTC

A_14_P118048

TTATGTTTGAAGTAGAAGAAAAGGAGCCCATGGAGAAAGAATGAAGATATTAGAGAGACA

A_14_P124466 GTTACTGTTAGACATGTTCTTCAATAGAATACAGTGGCCTCTATGGACTCCTAAAGAAAC

A_14_P126191 CTTCTGTATTTTCAAAGACTGCCACGTGCCTTAGGAATACTGTTTTATCTCCATACTTTG

A_18_P10632222

AAAATTCTTTTTTACCCGAATAGAGTCAAAGCAAACCATCCTAATGACCATCCCTCTGTT

A_16_P17974121

AACTCTTCCATCTTCTCTTTTTCGTGTCTTTTGACATTTTTGAGCATAGATAAACCAGGC

A_16_P16468767

AAGTAGCCACTAGTGATGTTCTACTACTCATTCTTAAATCACAGTGCCATCCTTAAAAGG

A_14_P119568 CTTATTAATGGGAGACAGTTTAAGCATATGTGTAGACATAACTGAGTTCTTCCTGGAGCA

A_14_P117180 CTGCTTCTACTGATTATGAACAAAGCACAGGAATGCAGGAGTGCAGAAAGTATTTTAAAA

A_14_P132951 TCTAAGTTCACCCTTACTTCTATCAATGTCAATGTTAACTCTCCTGGTTAAACAGGGTTA

A_16_P16200598

AAGACAAATGTCCTCACCTTATGTAACAAGTCTCAGAAAAAAAATGTCCAAATAGTCAGCA

A_14_P202396 CTTCCTCCATTTCCTGAAAGTTTATTGATCAAAGAAATGTTGTCCTGGGTGTGTTTTTTC

A_16_P17586656

ACCATTGCATACTTTCTGATTTTTACTGATTGTAATGCTTACCTTCTAAATGCTGCAGGC

A_16_P21397007

TTTCTATTTCCCACTATCTCAGATCATATGAACCATCTCCTGTTTTGTAGTTAAGCACCT

A_14_P114674 TCCCTAGGGAACAGTGGGTTTGGTAGAACTCTCTTCTAAATCTCTTTTTCATAGAATTTA

A_14_P139225 TTTTAAGTGTATTAAGCATTTTGTGTCCTTCTTCACAGAAGAGAATGTCCTGGGTAGAAC

A_16_P20793724

GTAACACAAAAACAGTTCAGATCCTACAGAATCAAGACATCTTCAACAAATAAAACCCCC

A_14_P128630 TTATCATTTCCCTAATGATTGTCTTGAAACTGCTAGACTAGAAAGAGAGTTGGAGCAACA

A_16_P03195637
AAGAGAAATTCAATGCCAGCATTCAATTCCTTTCATGTAAATCATCAAATTTCCAGGCTG
A_14_P124372 CAAAGAAATTCACCAGGATCACACAACTGCTGATCTGAAAATGAAAATGTTTCGAAATAA
A_16_P37517728
ACCGCATTTATGGCACATTACATCTTACATGGTTATTATTCTGCATTCTTCATCCAATCA
CUST_4983_PI426210822 AGGGGTTAAAATGTCTGGTGCCATCTGTCACAGTAATGAGTTGTGCA
A_16_P36230479
AAGGGTTTCACCACTAGTCAGTATCAGTAGATTGTTGGGGAGAAATCTTTATGAAAAATG
CUST_4910_PI426210822 GGCGGCTGGTTAAAAATCACATGGTGTGGGCATTTATAGGGGTAATA
CUST_5688_PI426210822 AGAGTAGAAGAGGAGAGGGAGAAGAGTCACTCCAGACGTCAGAAC
A_14_P117663 GGTAAGGCTTTTGTTTCTCTTAAGAGGGGGTTTTCCTGTTGTTCAATTTAATTTTAATGG
A_14_P122859 ACAGTTCTTAGACACTTCATATGTAAATCTGGGCAGAGTAAGTTCTGATGTGAGGAAAAA
A_14_P123516 TAATTTGAGAACATGATCTGAGAAACCAGAAAGGATAATGGTCCCTCCCCTAATGTAAAA
A_14_P107427 AGCAGGGGAGGCAAGAAATGGGAAAAAATTGTCTTTTAAAAAGATTTACCATTCTCATTT
A_14_P124345 GTTTCCATCACTCGCTAATAAATCAACAGAAACACAAATGGCTTGGCTCTCATGTT
A_16_P15468583
ACACCTTCCTACACATAATACCCCAATTGTTTTCTTGTAAGACCATAGATAGAAGTCACA
A_14_P133501 ACTTTCACCAGGTTTTCTTCCTTCAGCATAGTTGAGATGCTTTTATTTCAAATGCCTTAA
A_14_P133522 GAAAACATTTTTCTTACCTTAATGTCAGCCAGAGATGCCATTGCCCACGCTATT
A_14_P201495 ACCATGCCTAATTAAACACTTAAGTGTGAAAGAATTCTTGCAGTGGAGAGTAGGTAAAGT
A_16_P16908381
AAATGAATTGTCCCTTCATACAATGTTCCTTTCCCAAATGTTTGTATCTGTTCATTCAGC
A_16_P16191091
AAGTGCTGGACTATTTATTTCTTAATTCCCCTCCTACTTTATTTGGACAGAAGTCTCACT
A_16_P00313561
TCCTTTTACTTTTACTAGTGACCAATCTGAGTTTTTTCATTACACGGCAATGATTCACCA
CUST_6659_PI426210822
AGTAAGGGCAGATCACTGCTTGTCGTGTTAGTTATTATGTATGATGTACTTGATGTTTCT
A_16_P41618514
AGCACAAAAACCCTTTTATAGCCTCACAGTAATTTTTTGTATACTTCTCCTGACTTATGT
A_14_P103818 ATTTATCTGTAAGTGAGCCGTTGGGGAAGGATTGAATACAGAGACGCTGTC
CUST_9633_PI426210822
GGTGCCAATTACATGTTGAATTTCATCTTCAACCCAACTCTACTTACCTTTCAGG
A_14_P107704 CAATATAGAACAGTTAATCCCTGTGCCAGAGGCCTTAGATCAAAGCCCTTTCTATAC
A_14_P126804 TATAGTGAGAGCAATTTTAGCGTTGATGACCAAGACCTTTCTAGGACAGAGTTTGATTC
CUST_2387_PI426210822 TGGGTAAATTGTGAGATCAGGGCCTACACTTCTTCCACATTGCTCTTTAA
A_14_P133790 ACCAAAAAGAATGTTTTCTTCTTCATACCGTCCAAAAGAGATCACTTCCTTCATGATTGA
A_14_P104879 AACTGCTTACTTACTAATGTGGTCACATTAATGTTATAAATGCCTGATGGGGAATGTCTG

A_16_P02927237
AAGATTAGTCCAGCATTTAGGCAAGAAGAATGTTAGTCCAGTCATCTGCTACTTTTGCTA
A_16_P16189634
AATCTGTTGATCAGAAAAGAATGTCACAGGAGTAGATAAGGATCCACACAGTATATCCAC
A_16_P17920027
GTCATCCTTGAAGCCCTATATAATGCAGATGTTTAACCAAGATGCTACTAAATACTCCCA
A_14_P115133 GGGTGGTGGACTATGTTTGATGATGACTTTACAAAATTACCTTCCCAATTATGTTTTCTT
A_14_P124111 AACATAGTAAATAGATGCAGTGCTGACAGTGCTTTAGGACTGGAGCATTGTTTTGTATAA
A_14_P109535 AGAATCCATCCTTGAAAGGTGAAAGTTCAATAGAGTATCATGGTAGACATGGGTTGTATA
A_14_P118044 TGAGTTTCAGATTTGCGACTACAAAAATTAGGCATTTCTTTCTTTCTCCCTTTGCTTGAA
A_14_P107795 TAGGGTAGCCAGTCCTATAAACGTTTTTGCTTTAACTTTGATTCTTTGTTGCTTTACTGG
A_14_P125105 TCAAAATGAACCATCACAGCAATCTTGGCTTTTTGATTTGGACCCTCATTTCTCTTATAA
CUST_835_PI426210822 CCTCTCTAAGGAGCCCACTTTTGTGTTAAACTTCTTCATGGCATAAGCTGTTA
A_14_P121149 ATAATTAAAAAGGTTTTAGTTGCCTTCATTGAAAAGACCAACAACTACAGAAGAAACTGG
A_14_P120956 GGTATCTTGCTGCATTCAGTTCAACAAAGTCTTCACTGTAATTCACTGAGAAGTTGAG
A_16_P15536690
TTCTTCTCGGAAAGGTAAAAAGCATATATCATTATTCCTCTAAACAAGTGAAGTGGGTCC
A_16_P41631734
TCCTGTCTATTCACAAAATCATACTATCCTTCAATTTCACCATAATGCTTAGACCTCCAA
A_14_P115867 GAGACAAAGTCTGCTGATCTACTGTCAAATACAATTCGTTTTTCCTTTACTTTGAGGTCA
A_14_P105980 CTTTCCTTCTCAAAAACATTTGTCACCTATTTTGGAGTTAATTCAGAGGATTGCTCCAAA
A_18_P10026487
GGGGGAAAAACTTAGGATACAAACAGACGTTATTTCAGCAACTCAATCATTGTTTAATCC
A_16_P16241203
CCTGCCCAACTAAATTACTTAAATGGAACCAAAATAACCAAATGATTAGCCTTTCTCAGG
A_14_P134702 CTGACGGGGTATTATCTTTCATTGTAAATGAGTAAAATGACATCAGTCTCCTGTGAGGAT
A_16_P00455423
AATCTCACTTTAACATGTGTTTGATGCTTTATCTGAGTTTTGGGTCATCTTAAGGGAGTG
A_14_P109154 CCCATCGATGACTCATCGGAGGAGTTTGTCAATTTTGCAGCCATTTTAG
A_14_P133282 TCAGGAACCTTCTCATGGTCTAGAACCCTTTTCAACTGGATATTTACTTTTGATTTTTCT
A_14_P106326 TTAATACACACTGTCATAGTCCTAGGATAGAAGAGTCCTCAGAACACTGCTCCAC
A_14_P117903 ACCTCTGGAGTATGAAAATAAGCAACACCATTATGGTAAGTATAAGGATATCCAGTTGAA
A_14_P115821 AATGTGCCATTTTCACACAACTTCTCTCTGTATAGGCCTCTGAAATATCAATAAGGCTAA
A_14_P106551 CTTCAAATCGTGTTACATCAAAGTGCAGTAAACTGGCCAAGTCCCAACTATAATAAAGTA
A_14_P201429 TAAAATGAATGTGTCATGAGAATGGGCTTAACACATCGTTGGTTTGAAAACTTCCATTTT
A_14_P112743 ATTGAAAATGATCCTGGAAATGGATTAATGTAGAGATGGCCCTGTTTTATTTTGGTAGAA
A_14_P116201 CTGAAGACATCCTTCTGACAATCATTTGGTGATGCAAAGAATTCTTTTGTTAACAATAGT
A_18_P14120613

ACAGGTCCAATTACTAGTTCATATTGCAATGAAGCCTAAAAACACCATATTAACCCTGTA

A_14_P114246 TTAGCACATCACTGGCATCATACAAAAGTTGGTCTTAGGCTTTAAAATGCTATGACTCTT

A_16_P15454676

GTTTTAAGTTATGCAGAGTATCCAGGAATAACACTTCTCTCATATGAAAAGCAAGTGCCC

A_16_P16360920

TTGCTTTCTCAATCATGTTCCAATCAGCATTAGCAAGATCTTTGTCAAATTTAAGGGCAG

A_16_P18423561

AGATGCCATCTGGTTTAAAAAAGAAGTAACCACCCAATATACCCTATGATTATAGCCAGG

A_18_P22840876 GTGATTTTCAGCCCAGGCCCTGAACCTGTCATAAAATGTAATGGATGA

A_14_P125788 TTATATTTGAAAGAAGTGATTTACAACCACTGTATTGCCATAGCCATCCCACAATGCAAC

A_14_P121135 GTTGCTTTTCAATGTATTCTCCTTAGAGAATCATGAAAAATGGCTTTTTACTTTAAAGGG

A_14_P124416 TAAGAATAAAGCGTATTGTTTCAGGAAAGCAAATTTCCCTGGATTCAGAGATTTCAAGGA

A_14_P115096 GCTGCTATTGCTAAAACTTCAGATTCTTCCCTGAAAGATGGTCTTGTGTTTGTATTGTAT

A_16_P19460270

ATAATGTATCCCTCCCTTTCAAATAACTCAAAATAGGCCATGATATTACAGCGAATCCCT

A_16_P00304675

ATGTGATTGCATCAGTGAAGAAGAACATATACCACGAGTTAAAATAAATTTCCCGTGCTG

A_16_P17425453

AAAAAAGCCGTGGAATTTGTCAAAAGAATGAACAGCAATCATGAGAGTATGATATGCCTT

A_14_P136886 GGATCCTGCTTTGTCTTATAGCACATATCCTATAGAATAGAGATCATGGAAGATGCATGG

A_14_P120304 TAACCACTACAACAACATCACCTCATGTTAACCAACCTTAATTTCCTCAAAGATATGCAC

A_14_P100357 CTGGCCGCCTTTCCTCTCAGGACATACTTCTGGAGATCAAAAAAAAAAATTACAAA

A_14_P114654 GAAGGGTAATTTTTTTGTCAAAATTTAACTTCCTTTTGCCTGAAAGCATTTGAGCACAGT

CUST_10117_PI426210822 GACTTGGCCTGTGCCACCAGCCCCTGGCCCCACCTACGCATGCAC

A_16_P16481265

AAAAGTAGTAACTTGGGACCTTTAATTTGCCTATCTACCTGCTCTCATTGAGTATAGTGC

A_16_P15797827

AAAGATTAGTCATTAACACCATAGCAGGAAACTTTAGCACTAACACCTTAGCATCATGGG

CUST_5652_PI426210822 CAAAGATGCCTGTTAGTCTGTCGCAGAGGGAGAAGATCTGGGGAG

A_14_P126997 TGTGGCCACTTTGTTTATGACATTCTCTAAGCCAGTTTCATTATTTTCCTGTTGAAACAA

CUST_10336_PI426210822 GAACACAAGACTCTTGCCAGTTGTTCTAAGAAAGCTTCTGCTCTTTCCC

A_16_P03082640

CCTCCATCATTGGTTCATCCAAACTACTTCCTAATGTCAGAATAAGAAACAAGTCTCTTT

A_16_P35069118

TATCTTTAGCGGGATTGAATTGAATTTTTGCTGTTGAGTGTAATAGCCAGATCCTCTGTA

CUST_218_PI426210822 GCGCCGAGGGGCGCCGGGAGGCTCCGCGCGCTGCTGCTGGCGCTG

CUST_6706_PI426210822

GAGAGCTTCCTGAAAAGTAGTTATTTACTGATAAACTACTCAAGAATACAAAAGTAGTTG

A_14_P113272 CAAATATAGATTCTCTTTCCCACAGCGATATTAATACTGGGCCTGCTATCAACTTGCTAA

A_14_P200817 GATTCCCACATCTTTACTAGTTTGCAGAAATAAGATCCTAGCATATGCAGTGTGTAGGAA

A_16_P21392538

GAAGACTGTGCACTCAAAAATAATCAATTCACTGTCCAGAATACTCACTGCTTTATCTCC

A_16_P16097637

AGAGTTCCTTCTATTCTGACACACATCTCTAAATCACATTATTACCTTGAAAAGCCAGCA

A_18_P14137420

ACAGATAGTTAAGAAAGGGAGATTAAGTTGGAGTTAATAAGAACTCTGAGAAAGAGGAGA

A_14_P116443 CTCACTTTTCTTTTGCCCGCTTCAATAAAAGTTCTGAAACGATTTTCATATGTGCAATTT

A_16_P02729945

GTCTGCAGTGTATCAGAAAACAAAAATGTCTCCATATGGTTATACAAATTGTTCCTTGGA

A_16_P21504546

GGCAAACACCATATCCTATGCTTCCTTTTATATCCAGAGGTCTCACATAAAACTGTTTAC

A_14_P100374 TTGTTTTTTTTTTAGGTCTTCAATAAACTCATCAGGAGATATAAGTATTTGGAGAAGGCA

A_14_P103132 AAATACAGTAAGAAGAGAGTGTGATTACAGACACCCTCTCTCTTTAGACTCTCACAAGTA

A_16_P16191643 GCCAAGTATGTTTCATATACATTGATACTATTTAGTTGCATTTTTATATGCTTTAGGAGC

A_16_P16191424 CATGTTGCACACCCCTCTCTACCTGCAGCCGCCATCCCCTAACAG

A_16_P19487467

AAAAACATACTTCTTCCTGCAGATAACATTCTGATTGACAATTTTTGCTTCACCAGGCTT

A_16_P15306803

AATAACAAAGTCTGTTGCTATACTTTTCCTTGAGGTCGTCTGAAAACTTTCTTCCAGAAC

CUST_6_PI426210822 TCATATCTTTTAATGTCTCAGTAAAACAGCTTGAGCCCTCCCCTTATCACCTTG

A_14_P101164 TTTCCTTGTCTCAGTCAAGAATGGTATAAAGGGTTTACCATTTTGATGGTATAAAGTGGT

A_16_P18679520

ACCCAAATCTATAGCGTTTTTTAAGCCCTTTATTGATCTTTCACTGAAACTTCTTTCTCC

CUST_6101_PI426210822

GTAAAAGGATGCAACTATGTTTTGTCTTTTTGAATACTCACAGTACCTAAGAAAGCATCA

A_14_P117381 GAATCTGATTCAAGTTAACCTTCTGAGTTGCAACACCAATACCCCAGATTCCTAAA

A_14_P133776 CAAATATGCAGGAAGTAAAAGGCAGAGTTGGTAACCAAAGGTATAGAAGCCATTTTCTAT

A_14_P105059 AAATACAACAGTTGATTTCTGAAAGTCACATGTGCAGTGCATTCTTTGGAATCTTCTTTG

A_14_P132500 ATTTTTCTGTGAGTTGTTTCTCTAGAAGTGAGGAATTGAATTATAGACCCAAACCCTCAT

A_14_P104338 TATTCGTCTATGTATTTAATGAGCACCTCTTCAGATTGCCTCTCATCTTAAGATGCCATT

A_14_P137713 TAGATGAGTTTAAATAGAGACTATTGAACCTTCTCTTCTCTCTAGTCACTTTGCCTTCCC

A_14_P121348 ATCAGTATAAAACCTTTGTGAGTTTTTTTTTTTTTCCGCATGAAATCTTATGTGGAAAG

A_14_P111624 CTGAATTGTGGATGCTATACTGTGTGGCTTGAATTATTCTCTAGTGCAAATTCTGTAGTG

A_14_P131251 GCCTTAAGGTAGAAGGCACATAGAAATAACATCTCATCTTTCTGCTGACCATTTTAGTGA

A_14_P110730 AGAGGCATCCAGCAACTCTGAGATTAAATTCATCATTGATCAATTCGTTATTGTTTGGAA

CUST_5277_PI426210822 ACTTGACTTCCCACCTTTGGGAGTCAGATGCAGTCCGCTGGACAG

A_14_P139285 ATCTTCCTGTTCTACCTCCTCATTCAGACTGTAGGCTTCTTCGGCTAC

A_14_P104212 ATACCCACAACGAAACTCAGACTCACGTGATGCAGAGAACTGGTTG

A_14_P133438 GTGATTTACAAAAAGTTTCTTATTTCCAGTTTTAAGGGGGAAGGTAATAGATGCATGTGG

A_14_P101382 ACTGCTCCAGAAGGGGAGTAAAAATTTCGTTCTCCATTTTACAGAGTATGTATTTGAAAA

A_14_P103200 ACATTAGGAAACATGCTAGAATTACCTGGAACTTCATCATCATCTACTTCACAGGAATTG

A_14_P129165 AGGTGTGAAGACAGAACCAAATCAGGCAAAATGCTGATTACTTGGTATAGTTTATGTCAA

A_16_P40418210

AAAAATGACCATTTTAGACGACTCCTGACATAAACACAGAAAAATGCTACCAAATGTCCT

A_16_P18323518

AGTCATGTTATACAGGTGTGTCAAAAGGTATCTTGGTCATTAAGTATTGTGCAGTGCATT

A_14_P124476 ATGTGCTTTCAAATTGAAAAGTGATTTGAATCATTCCAATAACTACCACCTGTGTCACTG

A_16_P40423211

AACAATTTGTGACTGGAATGATTTAAAGCCAATACACATGTTATGTAATCTTCTGCCCTG

A_16_P18021049

GCAGCCTACTTTATCTATTGCACAGTTCATAGGAAATGACCATTACTCAGATGCTTGTAA

A_18_P12173146

GGATTCATCGGACTTTCAACTGTTTAAACCACAAAACTAAAGCAAGAATTCTCTATCACC

A_14_P123001 TCTTTTGGATTGTCCCGGACTCTAACTAAAGTCCTCAGTTTCCAATCTGTCT

A_14_P127707 AAGATCTTTGAAGAGCAGGGCCAGACTCAAGAGAAATGCAGCAAGGAATA

A_14_P107325 CATGGAGTTAAGACGCTCCATATGAAGCCTAGCCTGGGATATCCGT

A_18_P14132927 AAGGAGAAAGGGGTGTCCTCATCCCCATGTCTAGGATGATTTGTTT

A_14_P119612 AAAAATCCTCTGGGATTATGTAATTCTGAACTGGCGTCCACATTTTGTTGTATTCTGTAT

A_16_P03013991 TCTTTCTGTCCCAGTTACAAGGAGGAAATGGAAAGCAAATGAGAAAACTGCTAGAT

CUST_3875_PI426210822 CTGGAAGGTGGCAGGGGAGCATGGAAGGGAGGAGTCCTTGTCCAT

A_16_P19170684

TTGACTTCCTCTGTCCTATTTAGCAAAAGCAAATCCCAATAGATATAAAGGCTCTCCTTT

CUST_629_PI426210822 AAACATGAGACAGGTTCAGAGCGAGGTGGGCGGCTGGAATGGAGC

A_14_P129209 GTGCATGACCAATGTATGTCTATGAACACTGCATTGTTTCAGGTGGACATTTTATCATTT

A_16_P36230858

CCTTTTCATCCAAACACCATTCATGAGTTGTTTTTATGCCATGAGGTTAAATTCTTTCCT

A_14_P136573 CAAAACCTCTAGATTCCACAGTTTAATTAGACCGTCAGAAAATATTACCCAATGGAAAGG

A_14_P116035 ATTACAGAGATTAGTGTAACCTCTTATACTGCGGTAGGCTAAGCCGCTATGATCATAGTA

A_16_P16191373

GTTAGGATCAAGTGTGGTGTAATAAAATATCAGTCCCAAGAGTCACCTTTGCTGATTTTT

A_18_P22839934 CTTGGTCCACCAATGTCTGTTCTGACCAAAAAGGACTTCTGTGTTGAGTTTAAATTA

A_14_P129422 CCGTGAGGTGGGAATGCATGTAGTCTACTCAGCAAATGTAAAAAACTGAATATAAGATTT

A_14_P109240 ATAGGGCACTAGGATAAAAGAAAGAAGTATCTGTTGCTTCTCAATGGTCTTTTTCCACTA

A_14_P124335 AAGATCCTCAAAGTTCTTAACTGAAGTCAAATTACAGAAAACTCTGAAGTCAACATATGG

CUST_5867_PI426210822 CAGGGACAGGAAATAGTAGAGTTCAAGGAAAAGTTAGGACAAGGAAAATGAGTCTTTA
A_14_P127967 CTTACCCAGTGTTCCACAGGGCTTATTTTTATAAGTGCAGATATCATATCTATGGAGAAA
CUST_9024_PI426210822 GGTGGTCAGGTGGTGTTTGGGCTTCAGTATGTGGAATAGCTACTAT
A_16_P16191882 AAAAAAGTATCCTATTGTAAAAATTGTTATTGTCTCCCTAAAAAAATCCAAGAGAATCAA
A_14_P126809 ATAATTTCCAACCTGCACAGATCCTGAACAACAAATGAATAACGATGAATGTCTTTTTGG
CUST_5746_PI426210822 AAGAGCACTGGGCTGGGAGTCCCATGGGGCCAGGGCCTCACCAAG
A_14_P130925 TCATCTGTGGCTCTTAATACTCAACCTAATTTTACAGAAGTCTGGGATACAAAGAGACTA
A_14_P137495 CTTCAAAGAGTGTTACATGCTTATCGAAGAGTGAAAGTGGAAAGAATGCAGAAATGAAAG
A_16_P03213883 GTGCCATCATAACCATACATGAAGTTGAAGCATTCTTAAAGTGAATACCCATTCCACATT
A_14_P129825 CTAAAGGTTTTCCCCCTATGACAAAGCACTAGCACTTTCTTTCATGATAATTCTCTACTT
A_14_P123743 ATTATTTACACAAACTACTCCCTGAGATCCTACAATCCGCTTCATTTCTAACTGCTAATG
A_14_P138639 CAACAGGAGAATTCAGAAATTACAGGTTGGAGCCTGAAGATGTAGAATTTTGCCATATAA
A_14_P103342 AGATCTCACTTTCTAGGTAAGTATAATGTTCCTTTCCTTACATACCTGAAACTGCCTGTT
A_16_P15487227 ACACATAAGAAAAACCAGTAATCAGACATCTAAGGATTCCAGATATAAGCACTCTCCTGC
CUST_5462_PI426210822 AGTTCACAGTGCCCACTACACATCAAAACACACCCTCTGGGGAAT
A_14_P103518 TTATTAAAAGGAGTTACACATTGGATCATCCCAAAGCCAGTTCCTTTTCATTTATCAGGT
A_14_P201975 AGTCTTATTTCCCTTACTTCCAAGTCCTTGAGATGGGAGGAAGTAACCTCTTTT
CUST_7931_PI426210822 ACCTGTCTTCCCAGGGAACCCATGAAAAAGTGGGCAGCTGGAAGG
A_16_P19068800 AAATTCTCTTACACCTGATCAGCATACTTTATCCAATTTGAACATGGCTTTCTGATCTCA
A_14_P129790 ACCATGTCCCTTACGTCCAGTGCCAAGCAATCTGAGATCTAGACATTAC
A_14_P135027 CTTTTGTCTTTAAGAAATAAAACCATTTTTTTAAGCCCCAAAAGGTTGCAGGAAGGTGG
CUST_3164_PI426210822 CTTCAGTGAATTGGGACCTAGAGTCATTTGGATACAGTGGTTGCTTGG
A_14_P132359 CAGTATAATGCATCCAAAAAGTCCCTAGAACTTGTTATGGACATGTTCAAGAGGCTTAAA
A_14_P139809 GTAGCATCCAAGGACAAAAAGAATGTTTGCCAGCAAATCTTGGCTGTCAATTTTAAAG
A_16_P33568742 AAGGTGTAACTGCTTAGAACCACTTATCCCTTCAACCTTAAAAATTATTTACAAGCCCAG
A_14_P132254 CCTTTTAAAATTATTTGCAGGAGAGGGCTGCTCACAATTTATATGGCCAACTTGGTAAG
A_16_P17239252 ACCACATGAATACAAGACTAAGTTGCTACATCAGTCATAAAGGAACATCATGTTACCCTG
A_14_P124298 CAGTCCTGGGGAAAATGATTGTTTTTTTCCTAGAGTTAATCTTTTTCACTTGAAAACTGC
CUST_6772_PI426210822 CATTGCAGAGCTAGGGTGGGTGGAATGAGTCAGTTTATTTGGCTG
A_16_P15163737

TACCCCCTTCTCATTTTAGTAGTTTCCCATATTGCGAATCCAATGTAAACTCCAATTTTT
A_16_P20303922
ACACTTCTCCTTTGCTTTTATGTTCCTAAGGCAGTTTATTTAAATAAGTGTTCCCGCATG
A_14_P109282 GTTGGAAAAGTTTATGCTTTTGGTTTTGATCCAAGAGGCATAAATTGTCAACATGCCAAC
A_14_P109855 TCTATACATATCGAGTCAGGTAATCTACCTGGCTATTATTCTCAGAGGTCCTCAATACCT
A_14_P137893 GGGAGAACAGGAATATATTTAATATTTGGAAAGAAGCACAAGGCAGTGTAGATCCAGGT
A_14_P126484 CTTCTTTTTAAAATCAAGAGACTTCGAGCGATCCCAGTTTCCATTTCAATCTGTATTCAC
A_14_P201657 ACAAATGAAGAAAATCTGAAAATCAATGGAAGCACGAGGACATTCCTAGTCATTTTCTCA
A_16_P00685139 CTCTAGATCAGTGATTAGGCCAAGAACGTCTTTGAGCCATTTTCTGGTCTC
CUST_6928_PI426210822
TATGAAAAGCTTCATTTGTGTATTCACTGCGTTTGGAAGGAAGGCTATGTTTTGCTTT
A_14_P134942 TTAAGTGTTGATAAATGAACTCTTGCCATATCCTTTTTCCATGCATGACTGGCTATGTTC
A_16_P15226698
TGAGTAAACGATCATCAACCTCTCCTAAACATGTTAAGAAGTTCATTTCCGTCTGAATTG
A_14_P201833 TCTCTCTTAACTGTGCATTTTCTACTTTGCTTAGTGTTTTGCTCTTGGTTGACATTTCAC
A_14_P107309 GAGATCTTGGAATGGGAACTACAAATAAAACAGAACACACAGACGTTTATCTCCTTGTAA
A_14_P120275 TCTCATCAGATGGAAAACCATTTGAGTATGACAACTATAGCATGAAACATCTAAGGTAGG
A_16_P18545973
CATACTAGGTGATTCCCAAAGAAAATGGCTAACTCATGTATGAACTAAACCACTACCAAG
A_16_P21256017
GCTTGCCAAATTACTCTGAAATTACATTTTGTTGTCCATCTGTAGATGTAGACCAAGGAA
A_16_P01675517
TGACCCTACTTACCACATACAGATAACAGAGACTAGAAGAACAATTTGATCCTCTTCATG
A_14_P107176 TACAGGGGTCTTTTCTGCTCCTGTCACCGCTTAAAACTATCAGATGGTT
A_16_P58424242
GCCTGGCAAAGTGTTAACCTATACAATTGCCAATATTGGAAAACTGGGATACTTTGTTTA
A_14_P139707 TAATACTGACAACACTAATCATCTTTTTTATGGAGTGTTTCCTTGGTGCCAGGCAGTTC
A_18_P25145702
GCAGGAAAAACTATACTAGACAATACTATCCCCAAGTCTCATATGTAAGAAACAAAGCCA
A_14_P138630 CTCTTGGTAAAGTCTATCATCGTCGTCCTGCACTCCGTCATCTGTAAAAGA
A_18_P22842654
TCTGAAAATAATTCGTTTGTGACTATTTAACAGAAGGAAAAACTTCCTGAGTGAGCATCA
A_16_P01917875
AAAAGACAGGTTGATCCAAATATCACTAAGAATCAATGCAAGTCATTGAGTCAAGAGGAG
A_14_P107413
TGGAAAAAGAGAAGCATGAAAAAGGTGCTCAGAAAACAGATTGTCAAAAGTAAGTCTTAC
A_14_P100261 CTCACCTCTCTGTGTAGTGGGGAATGGAAGAAATTAACTAGTATATAATCCAGAAATGAC
A_18_P17831843

AATCTGTTTCTTACCAGCAGGAGTAGAAGTCTGAACATTTCTCTTGGTATCATTTCTCTT

A_14_P116710 CAAAAATGTATAGGGCTAGAAATCCTCAAATTGTTGGAGTCCCCTAAGATGTAACAAATG

A_14_P200636 ATTAAGGATGATAACAGTATTTGGAACTGAAGACCTGATGGGAAAAAAACCTTCAAGTTC

A_14_P113079 TAGTTGTTACGACCAATCTTTCATACTTCTTGGTTAAGAATCTGTCCGGTTCTAAAGAGT

A_16_P19971729 AAGACTCCCCAAAGTTTAATTCCTATCTGCTTTTTATTGGACTTCTACATATGAGGCTCT

CUST_6856_PI426210822 CTGATCATGCTCTAGCGCACAGAGCACTGTAAGCTCGTGAGGAAT

A_16_P01523083 AGCCCTATCTCCAACTTAAATAATGTTTGCAAGTTTCTAGTTAGGCAGTGAGACAGAAAA

CUST_6765_PI426210822 CAAAGGACTTTGGCTCTTCTTGCCTTTGTCAAACAGCCATGAATTTGTTTG

A_14_P104778 TAGATTTAAGCCTTTTCAGACCTGTTTATGCACCTAAGGATTTTCTTGAGGTAGGTTCTA

A_16_P19698908 TTTCAATACGTCAACAAAGTTAACTGACCCAATACAAGATATGAACCCTAGAGTCTCAGA

A_14_P129362 ACTAAAATTGGGAACTTATTTGCAATTTTTGACCCTGACCACTAACTAGTGATTCTTCTC

A_14_P120698 CAGTGGTCCAGGTGAGCAAGTTCACAAACCATTCAGGAAATAAAGACAG

A_18_P16629413 AACAAGCGCTAATTCTACATCTTAAGACATAGTTCAACAAGCATATGAGTTGAGGTCATT

A_16_P17464991 AAAAGAGGAGGTCGTCATATATACCAGACTAGTTAACACATAACCTATCCTAACTGAACA

A_16_P18335949 AAGACCAAGTTACAAACTACTCCTTTCATTCCTGCATACATTCTCTATACCTGTTGACAA

A_14_P139428 GTTTCTGGGCTATACAGATACTAATATTTCACACATTTGTAGTCTCCACCTTGTTGCATC

A_16_P15774142 AAAGGAAATCAGAGTTACAGTTTATTCAAGCAAGATCTCCATTACAGACTTAAGTGGCCT

A_14_P106079 ACTATACACATCCAACAAACATTTTATATTTGCCCATCATCCTCTTCAAGCATCTCAGAG

A_14_P102753 GCTTCCTCCATAAAGAAAAATCCCCAATAAAAGCAAAGGCAGAGAAGACAAAGAG

A_14_P126818 GCACACACAAGAGTATACAGAATAATGGGAAACACCCTTATCATTATCACTCAGACTACA

A_14_P118540 CTACTCCAACACAATTTTCTCCTAGAGAGTAAAGTGAACTCTCATAGAGAGTAAAGGGAA

A_14_P114749 CAAAGAGAAGAGAAAAAAAAAACTTAAGGAGGTTCATTGTTCACAGCAAATAGGTCTGCT

A_14_P125258 GCCATAAGAAATAATTGGAGAAAGAAGGAATTGGGAATTGGGAAGAGAAAAGAGAGAAAA

A_14_P108340 CCTCATCCCCAGAAATTATTTTTCTACACCAAATTGAGCAATTTGACCAAGATTTCTAGT

A_16_P16372528 GCACAGCCAAGTATAATAAATTAACTCAGTCCACTACAATCATGAATGATTTCTAACACC

A_14_P111919 CCTGGCCTAGACCTCTGGAATTGAGATTAAACAATTTGGAGTTGGATACCTG

CUST_3854_PI426210822 GATTGCATAGCAATACTGCCTACCCAGAGTTGTTGTTCCCAGGCTA

A_14_P118287 CTTTGGAAGTATGAGCCATGCTTCTCTCAAACATTTTCTGGTTTATTTTATAGGAACCTG

A_14_P200169 CAAAGGCAAATAATGTCTCCATAGAATCACTTTCCAATACAACGGCTCTTTTCAGAGC
A_14_P102845 TTTCCTAAGTTAGCCATTCCCTTGAGTTGAACCAATCTTTCTTCAGATGACTATCATTAG
A_16_P15126656
TTTGTTCCATTCAAACCTAAAATGTATGAATCCGCAGAATTTGATGTCAAAAGAAGCCTC
A_14_P200237 TTTAGGATATGAATTTTTTTTTCCTGCTGGGTAGATTGTCCAGTGACTTATGGCATGAAT
A_14_P116423 AAGACAAAAAGGATTTGCAGTGCCGTGATCTGCTAGTGCCTTAAAATACTGC
A_14_P108819 TGCAAGGATAGGTGTTTCACAATGGAATTTCATTTGCTTAGGCTTTTTATTTCTTCTGTG
A_14_P104172 TTGCCTCTATACCAGACTCAGTTCACTGCAAAAATTAAAGGTTACTACCTTTTCTGTTTA
A_14_P105640 CTCATGTCAATCTGCCAATAAATGTGGTTTTCAGAAAATGGCAGTGATACAGAAAACTAG
A_14_P104857 AGTGAAGGTGCACATACATACTTTCTTACAAATACACACAAGTGGCGTATGTGTATATAT
A_16_P00011400
CCAGGCTCCATAAGAAATCTGCTTTCAGAATCAAAGACCTGAAAATTAGAAATGCTTTTC
A_14_P122001
GAACTAGAACAAGAAGCACAGGAAGTGTATATCCTGTGGCAAAAAGTTAAGGAAAATAGT
A_14_P120390 CTTTTGTTGCAGATGGAAGAGAAGAGGCGAAAATACTCCATCAGCAGTGAC
A_16_P36230534
GTGATGCCTTTCCTTTGTTACTTTTCATGCAGACATCTGGAAAGAATTTGTCAAGAATTA
A_16_P02417113
CCTGCTAATTCTTACCTTTACAATTCAAAGAAGTTTGACATATGCAGTAATCCAACCTCC
A_14_P137078 CAACATGGGTATGAAAAATAATAACATGGGAAGATGGTATCTATCATTTGACGGGCTTTC
A_14_P128359 CAATAAAGAAGGTAATGGGCACCAACCTTCTTTGATCAGAAAATGACTTGTCCTTGT
A_14_P110685 TCTAACATGCTTGTATTTATTATATTAAATTACAAATGCCTGGAGTTACATTTGCTTGTT
A_14_P127852 CTGTAAACATGATTTGAAATTTGACAGCAGACTTTGTGGATTTCATTAGTGTTAAGGGCT
A_14_P133712 TGATATTACATTGGGATATTTGAAAATAGCCTTTAGTGACCAGCATGTGGGTCTTGTTTC
A_14_P117224 ATCTTCTTGAGGGCTTCCTTCAGTATTTTCATTCTAAAAGATAGCAAAGTTAGGGTTGGA
A_14_P128565 TAGGGAAATGGGTTATTAAGCATTGAATGGTGGTCTCTTCAAATTACACGTCAGATAATG
A_14_P128413 TGGCATCTATGAACACAACCACCATACTCACATTTGTGCCTAATGAAAATTTTACATCAG
CUST_8752_PI426210822 AGTCCTTGCCAAGTCTATGTCTTTTCTGAGAATTCTCTCCCCCTTGATT
A_14_P120590 AGTTATGTTGGATCCCTGATGTAAAGTTTGACAGTGTATGTTGTGTTCAAAATCAACAGT
A_16_P39244204
GTGTGATCTGCAAATGTCGATCGTGTTTGGTTACATTTAATAGAGGAGAGGAAAATTTCT
A_14_P200991 AGACTCCCTAACATTCATAACAATGTTTTATCCCAGTATCCATTGGGAATCTGGAGTATA
A_14_P107644 CAGGATACCAAACTGGAACGTGCTCTTCACATTCAATATATCCAGGTCTTGCAT
A_16_P16746907
AGGCCACAATAACTTGAACTGATACTGGTAAGCCATGTCTATAATCTCAAGAAAAATGTT
CUST_9881_PI426210822 GGATGGAAGCCAGGCCCTAAAAAGGTTAAATAAGCAGTGCTAGGCATTATAA
A_14_P114211 ATCATCCTCAGCACCTCATGGTTTTATAGCGCAGTTTTATAGCCAACATACACAGT
CUST_8798_PI426210822 ATAAAAATTCAAAACTGAGCAGCAAATCCTGTGCCTCTTCATCCAACCCC

A_14_P111626 ACGATGACCCCTGAAAGGTATGAGCCCTTCTTTCTCATCACAGAGATTAA

A_16_P18930135

CTTCTGACACTGTTTTTCCATATGTGCAACTTGTAAAGAATGAGATTAATCCTCACTGCA

A_14_P120794 CTAAATGATTTGGGGACTCTAGGAATATGGGCAAAAGTAAAACCTAATTTCACACAGATG

A_14_P139447 GGGACGCAGTTATTTTTGTTTGTTTTCTATTTCTAATTTTCCCCCTCCTTACAAATGCAA

A_14_P129266 TTACCAATTTATCAGTCCCATGATCTGTCTTCACCTCAGAACTAAGTGGAAGAAATAAGT

A_14_P117048 CTATCAGAGAAAAACAGAATTTAGGGCAAAATATTACTAAAAGATGCTGGGCCATCTCAT

A_16_P36959322

CAAGGAAACTCATTAGCCTAACTAGTCCTTCATCTACCTCTATTGCAAAAGGATTCTTTG

A_16_P00343228

CTGTAACTGTGCTCAAAGATTAAATAAATTACCCCAGTTTACAGAGTTACCTTACCCAGC

A_14_P106724 CTCAGAGAGCAGCACAACTCATCTCTGTGTAAGACCTGAAATTCCTTCAG

CUST_8100_PI426210822 CTGATGGCACTGAGGAGCCACTGAAAGTTTTTGAAAAGGGGAGTG

A_16_P02056673

AAGGTGTAAGTTAGTATGTCTTCAGTAGGCTAGACACTTATCTTAGTTACCCACAACAGA

CUST_8774_PI426210822 CCATCATGAACACATGCTTCTTTTTTTCCTCGCCTTCACCCTCAATTTTTT

A_14_P132304 CTCCAGATCACTGATTTTTCAAACATTTCTTCAGCAGCAGAATTCTTTTCTTCCAAAGAA

A_14_P108823 GTCTGCTGTCTAAGTGCTAGGATTTTATCCTGATGAATAGACCTGATTGTAGAAGGAATC

A_16_P16480723

ACTGAAGTAGAGAAAAATGCTGATATTGTGCTTGACATGTGAATAATGATGCTTTGCCCT

A_14_P116027 ACTGAAGTAACTTTTTTCCCCTGAGAACAGGATCATTTGATTTTTTGAGATGTAAACCTC

A_14_P104454 CTTATTCGCTCACAATGCATGCCAGTTAGATATGGGTATTTAGCATGCTGTATTTTAGGA

A_14_P115066 CATCTGCACCCTGTTGAGTTGTAGTTGGATTTGTCTGTTTATGCTTGGATTCAC

CUST_10219_PI426210822 CAGCACCTTCAGGGTGGGCTCAGCTGGCAACAAAAACAGAGATTT

A_14_P128140 CTATAAACACCAACCAGAACATCGAAAAGATTGATTTGCTTGTTTCATTGCAAAACCAAG

A_14_P117720 TGGATGAGTGTGTATATCCTTTGATACTTATTGGGGATATGGACCCTTAGTTATGTTTCT

A_14_P111165 TGTGGACATAGATACAGAAGACCCTTTGAGAGATCTCTTTCTGAAAGGATCTAAAATGAT

A_14_P112661 CAAACTCCAGTGGCATGAAGATCTAATTTTCAAAAGGGTAAAAACTGCATGGCATATATA

A_16_P00960821

GGCACACAAATTCTACCTTTATTCTAAGCATACCCTTTTTCTAAGTCTCATGAATCCCAA

A_14_P121319 TCTTTTTATTGCTTTCTCCAGCCCAAGAATCTATCATTTTGAAGCCAAAGATGGCCTTA

CUST_1764_PI426210822 GTTGTTCTAATGACTTACATCTTTAGCCAGGGCATCTTCACATCTAGCTCCAC

CUST_4652_PI426210822 AAAGAAAGCTTTGGGGCAGTTTCCTGCCACCCAGTTTTTATGAACAT

A_14_P124857 AAGGGCATGTTTCTGCTGCAACTAATTTTACTACTGTTGTTTCTATTGCATCAGTAAAGC

A_14_P138348 TTCCATGAAGACAAAATGTAGTTCTTCGGTCTTTCTTTATTGTCTCAATAAAGGCATCAT

A_14_P111991 CTCAGCCATTATATGATGAACGATCTTTGAATTTGTCAGAAAAGGAAGTATTGGATCTCT

A_14_P114430 CCTTGAGACTTTCTGAGAAGTTTATTCTTCTCCTTATTCTTAGTGCCTTCATCACTCTGT

CUST_1163_PI426210822 GTAGTGAGAGGGCTCTTGTCTGTGCTCTGTTGGCTTTAGGCATCT

CUST_10201_PI426210822
CTGTTGTTTCCCTAGGAGTTTTCTGGTTGCTTTTGGATACATGGTTATATGAGAAAATTG
CUST_3598_PI426210822 CTGACTTTCCACCACCCTGACCTGCGATTCCTCTCTGCTAACCCT
A_14_P107887 TCAAATTCAGGAAAGGAATGGGAGACAGAGACGCTTTCTGAAAAAGATTTTTTTCATTAG
A_14_P102204 GAAATGAATTTTGGTATGAACATTACCTTGACATCGACCCAAAGACATGACTTCAATTTT
CUST_5393_PI426210822 TGACGCTGTGTGCAGAAAAGCCAAGAGCTCCAGGGCTGAACAGAT
A_14_P130910 TCATAAAACAACTTACTCTGGGTTTTAAGGTCCTCGTTCCACTGATCATCCTTC
A_16_P21446775
ACCTCATTATTCACATCAGTTCCTTATTCATCTCAGGCATATCTACCTAAGCAACAACAC
A_14_P201266 TTCTAATACTTTCCATATGTTCACTATTACAGAATCCTGCAATATGTCCTTGGAAGGTCT
A_14_P117997 CAGAGGTTTGACATTTCCCATCATCGCCATTGCATTTGGGAGATTTTATTTTTTTAGTAA
A_18_P10856090
GAAGTGTTCCTGAAAGTTACTATAGGCCTGGTTATGAACAGTATGCATCTTTTGTCTACA
A_16_P19137671
ACAGTGAAGTTCCATTGACAAATTTCTCTTGCCTAACCATCTCAGTATGACAATCAACTG
A_14_P106394 CAGTAAATAAATGTCCAGGGATATTGGGGGCTGGGAAGTTGAATAAATGGTCTAATCTA
A_18_P14136126 ATAGATGCATAGGTGCAGGTGGTTTATTTGAGTAGCGCCCCAGAAAGTAAG
A_16_P19725619
AACTCTGCAGTATCAAGTGGAGATTTACGTAAGAGGAGGTTTAGATGGTTAATGATAACC
A_16_P16203325
CACAGACTATCAGGTAGTGTCCATTTATGAACCTCTCTATCATCTTCTTGCATACAAACA
A_14_P125490 GTCCATATGACATAGTTTTAAATATTTAGCCACATAATGCTAGGAAAGAAAAAAAACGGT
CUST_4627_PI426210822 CCTAGTCATCTCCCTCAAGAATTTCCCAGTTACTTCTCCTGGAAGATC
A_16_P19775043
TAATACTTTCGTAGTCTTTCACAGGTAATCATGGACATTCTCTACCTAATTCCACACTGC
CUST_3563_PI426210822 CAGGCTGAGGAAACCCACCCCTTCGAAGCAGCCATCCATAGGTAG
CUST_8345_PI426210822 CATATCTCCTCACTCTCTGGTTCTACCTCCCAGAACTCTTCTCTGC
A_14_P118194 GATTTTCTACTTGGCACTGGGGCAGTGTATTTATGAGTAAAACATTTCTTCCTAAAATGG
A_14_P114456 ACTTTGACTTAGGTGCTTTGGGATCTAAGTGTGGTTTGTGACTATCCCCATTATTT
A_14_P118938 CATCAGAGATTTTCAAAATAATGGGCCCAAAGGGCTGAAGTGATAAATACTTTATTGGAG
A_16_P02862351
AGCCTTGAGACGTTACAATTTGTTTAACTGTAGTAACTCCCTCTTTAGTGTTACCCAAAA
A_14_P200722 CATTCTAGGCAGGATGAACACTGTAGTCAGAAGTGCAAGTTCATATGACATTGTGAAAG
A_14_P134122 CTGGAAACCAGTGTAAAAAAAATTATTCCAAACCTGATCTGGGTCAGGTTTTTACATTAC
A_14_P114425 CTGGTTGCCTCTGTTGTATAAGATTCAGGGAAGCATTTCTTAGGGATAACACCATATTTT
CUST_8550_PI426210822 GACAACTGCCTGGCTATCGTTCTGGCTGAGTGGACCCTCAGATAC
CUST_2342_PI426210822 GTTGTCATTCGTGCCTCAGATCCCCTCTCTACTGGCCTCCTGCTG
A_14_P202447 CATGTTTGCTTCTTTTCAAAGTGAACTACCATGGAAAAATTGTTCTTCGTGGTCAGATAA

A_18_P16113335
GAATTATCATCCTACTAAACTAAAACCTTACCAGTTACGAACAAAACCTGAGTGATTCCA
CUST_4503_PI426210822 CTCAGATGCAGGAAGTCCTCGCCTCTGCTGTCCTGCTCTGCCCCA
A_16_P16544034
AAAGTGCAGATCCTAACTTTTTTTTTCATGCAGTAATCCTCAAAAAGGTAGACGTCTACC
A_14_P118109 CTGGCTTGAGTGCGGGAGAACACTTTTTAAAGACTCACCTTGGAA
CUST_5170_PI426210822 GAAGTGTCAAAATGAGACTCTGCATGTAACTCCCCATGCCTCTCC
CUST_8734_PI426210822
TCTTAAAAAAAAAAGTATCCTATTGTAAAAATTGTTATTGTCTCCCTAAAAAAATCCAAG
A_14_P118944 ACAAAGATTGTGGTATAGATTTTTATCTTGACTCATCTCAAACACTTTCTCCCATTCTTT
A_14_P113030 ATGTCCTTATTTTTGCTTCTTGTCTAAACAGGACAATTACTTCTATGGTCCATCTAAGCC
A_14_P132631
AGGAGAGAAACAGTTCTGTGAGTGAAACAGAAGTATGTAAAACTGCTTGCAATCTCTAAA
A_14_P100274 CCAGCATCTCTTCTGAATGGTGCCTGTATCCTACTGTTTGTTAAATATTTCACTTCTTAA
A_14_P112702 ACCATGGAAAGCAAAACTGTAAGAGAGGACTACTATAGTTTTGTTTCTTCCTCCTTCTTA
A_16_P01582561
AAAGGGCTCCATGATTTACTGTTAGTAAAAAGAACCGGTTCTAAACAGTATACAAATGCC
CUST_7375_PI426210822 CTAAAAGGTTCAGTGGAAGGGACTGATGCTGTCTCCAGATGCAGA
A_18_P14244909
AAAGCACAATACCAGCAAATTCACTACCTTACCACTTTTATCCAAATCTAACTTTTCTTA
CUST_5422_PI426210822 TGGCAGCTGCACAGAGCAGAAATAATGGCCCAAAAGTTGAGAGAG
A_18_P12208474
ACATGGAAGTCACTTTATCCTACTCCATACATCTGATTTCAACATATCCCTCATTTCTGA
A_16_P03543944
AAATGAAGTAAACTCGAGAAATCCATTAAGCCAGATTAGTATTGAACTCCAGTCAAGACC
A_16_P38629893
TCATTGACATTCCCTAATTAAGAGTAACACACATAACAACCTAAATCCTTAATTTCCTGC
A_14_P138081 ATTGTAAGTAAATTGAGCGGAGGTTGAAGAGACGTGGAAAATGAGCAAAGTTGATCTT
A_14_P133779 CTTGGTAGGTTTGATCGCTTCCTGATCCTGTTCTGCCTAGTTCTGT
A_16_P20208945
ACATGGTGTTACTCACTATCCAAACCTTCACTATTCAAACTCAAGAACCACATAGATTGA
A_14_P101376 AATTTTGTTTCCTTAAGTCTCTACCTCACTCTATGTTGTCTTGGGTAGGTAAGAATTGAC
A_14_P128385 TTTCATTCTTTTCGTCAATTTGTAGACCCCGTTGATAATCTCATGAAAGTGCTGGAGAT
A_14_P124512 ACACACAAAACTCAGGGTGGGAACTTGATTTAGTTGCTTCTTTCTTGGTTAAGTTATTTA
A_14_P100828 AACTAAGGATCATGTATTTTGAAACATTACACACCTGTGGTCAGTGTTAAGGAAATTGCT
CUST_9442_PI426210822
AAACACTGGGTGTTTTAAAAACAAGTTGTTTGAATGACTTAAAGAGATTACATCCAAATT
A_14_P110002 TAGTCAAGATCTTTCCCAATTTCTGCTGTATGATTGGACATACAACTGTCTTTCTTGTTG

CUST_4629_PI426210822 CAGCTCCACCACCAGGTCAGACAGTACCCTTGGCAACCTGTGGAC

A_14_P102798 CTAGTTATCTGTAATTAGCATACATGGTTAATCATGAGCACGCTAACTGCAGGGGGAAAA

A_18_P21455053

AATTACAAGAAACTAGCCCCATAGACATAAATATACTGGACTGAACTAAAACTGCACTCA

A_14_P102694 CTCTTGCACATTTTCTTGCAAACTTCAGGGTCTGCATCAGTCTACAGAAATGAG

A_16_P02763628

AAAATCCTCACTACCTGATTCGTATCTAAAAGAGTCACACAATTGCTTTGTTTTGGAGTC

A_16_P18329800

ATTTCCACATTCCATTCTCATTTTCTATCCATCAACCTAAAACAAGTAAGTGGTCATTGG

CUST_6256_PI426210822 TGTGAGACTTGGGTTTAACGTGACCTGTCCGTAAATCATTTTGCTGAAATTG

A_14_P101671 CTCTGTTCTTTCCCCATGTATAGGTGTCACCAAGAGTGACCTACACAC

A_16_P03115093

CTATCATGGCATCTTGTTCCTATCATAGCACAGTAACCATAATAGATCCCATCTGTTTCC

A_14_P118215 GTCTCACTCTATTCTTAGGCACTAAAGCAATCTTCAACCGAACTCCTCTTTGGA

CUST_4056_PI426210822 ACACCATGTGGTCCAGTCTGACACAGGCTGATCCTTTCCCCTCAG

A_14_P116122 TCATGTAATGACATGAATGACAGTATCATCCATTGGACTGAACACCCGAATAGTATAGAG

CUST_7141_PI426210822

ACTTTAGAAGATAAGCCAAAAAATTTGACCTTAATGAAAATAGAGGATTTTTAATGGAGA

A_14_P118827 CTTGAGTCAGTCATCTTGGCTACATCGACAAAAAGTCCAACTCAAAATGGTGATATCT

A_14_P117751 ATAATCGAAAGAACATCAGGGTGAGCATAACTAGCCATTTATTCATCCATCTAACCAACA

A_18_P14132336 GAACCTTTCAGAAAAACGTTGCTTTCCCCGATTGAAGTGATTCCCATGTAAC

CUST_9148_PI426210822

TTTCCAAATTATTAGACACTTAAATTTTAAAGGACAGTAAGTCCCAAACTGTGAGAAACT

A_14_P131669 GCATATAAATGATGATCCTGTATTATCAAGGACAAATGCTGTATAGAATCCTGTGGCACC

A_14_P128163 CACTTCACATTATGAGTATTGTTTCAAAAGAGAAGCGATTCATGGAATTAAAACATCCAC

A_14_P127685 GGATTGGCCAGGTCATTTAAAAACATAGTATTAAGTAAAAGAATGTTTGCACGAGTCAGG

A_14_P139020 TGACCAAATTGTTTTAATACGCATAGACTGGTGACATTTGCACTGGAATCCTGTAATAGA

A_14_P115534 GCTGGAACAGCAGTTCTTACCTCTCTAGAATCCAGGTTTAAAGGTATATAAGTAAGAAAG

A_14_P126594 TATTCTCTCTTTTTAATTAATAAGGTTCCCCAATATCAACTCCAAGCCCATCACCATTGC

A_14_P117494 CTCATTGCCCACAAATCAGGCTGGGAAATCTAGATATTTAAATAAGTAGTTTGTCACCAA

CUST_3855_PI426210822 AATACTGCCTACCCAGAGTTGTTGTTCCCAGGCTAGGGTCCAACT

A_14_P129585 CTGATGCTGATCATGGAGACTTTTATTATTGAGACCTCCCTATACTTTGGACACTTCAAA

A_14_P137021 TCTGTATTATAAATGGGTCTCTGTTTTTTGGTCACTTTCCCAGAATCTGATTAACTGGAG

A_14_P120021 CTCTAGTATTTCTCAGCACATACACAGAAGAGGGAATTAAATGGTCTTTGATACCCCTAA

A_14_P106791 TACCAGCCTGAGATATACTAATTGGTCAGAAGTGAAAAGAGAGGAAAAGAAATCTCAGTT

A_16_P18256320

TGCAATGAGATATGAGAAACGAAAGAAACAAAAACTGTCAAATGAAGATATGCCAGGGTT

A_16_P18209944

CGTAGTTCCAAAATTTCAGAAAACTCTACAATGACCTAACACATACTATAAAGCGTAGCA
A_14_P118755 CTTTAATCTCCTTACTTGGGAGTTCCTGCTCATTTCTGGAAATGTTTAAATAAGCAAGTC
A_16_P20781052
ACTATATGGCCAGTTACTTGACTAATCACTTAACACGAAATCTTCAGGAGTGTCATCTGT
CUST_4081_PI426210822 CCCAGCCATCTTCTGGTGCATTTCTAACATACCCTTAAAAGGCATAAAAAAGTG
A_14_P100938 TGCAACAAAAATAGAATAGACCCAAATTCCTTTCGGTGGAATTAAGAGTGATCACTGATT
A_14_P102931 AGCAGTATGATATTACTGTATCGGACCTGAATCAGCCCATGCTTGTTAGTCTGTTAAAG
A_16_P35654972
AAGAAGCATAAAGACTTGCTATCCACCAATGGTTAGGATTTAAAGAAACGCAAACACTTG
A_16_P01415626
GACACTGAAAGTTCTAAGTATCTGTAGCCATTACACATCTTTATCTTCCTCCACAAGTGT
CUST_10202_PI426210822
TCTGGTTGCTTTTGGATACATGGTTATATGAGAAAATTGTGTATGTCTGTTACGAGGGT
CUST_2694_PI426210822 CACTGTCTTCTGCAGAGCTCTTTTGTGAGAGGAGGATTCGGCCTC
A_14_P104288 TTCTTATTGCCTATCAAGATTCAAATAATGCTCAGAACACTCATGGCTACTGGGTTTTTG
CUST_349_PI426210822
TGTATCTTACGTCTCTCTTCTCGAAGAAATTATTCTTTGTTACTTAATTCAATATTTTGC
A_16_P37673158
CGAATCTACATCCTGAGTTTTATAAAAACAGTGTTCCAAATAACTCTACCAGTGAAGCCA
A_14_P104041 CTCCCAACAACCTATCTGGTTCCCCAAAATTATATAAAGAAGTGTGGTGGCTTTTT
A_16_P18767974
CTCAGGTAGTTGAAAACAGTTGCTTAAAAAATTGAATCTCTAGAATTGTAGCACGGGTGT
A_16_P17023562
ATGGTCCTAGAACCTTTATTTTGTGGAGACGTTACTTATTCATCTTTAACCCTAAGCACA
A_14_P131157 CTATATTGAAAAAAGGTTATCTTTCCAAAATACTGGCCTTTTTGAAACGTGAGGGAAGCA
A_16_P03801979
ACCTGTGCCATCAAAATAATTAGCAACGGATTTCTAAAAAACATTTGCTCAGGTTACAGA
A_16_P36230987
CAGATACAATTCCAAGGACCATGTTTTGTCTCATATGGGGGCTATTCACTCATTTATTAC
A_14_P135702 AAGCAGTATGGTAAATACCATTGCATGCATTTGGGTTTTCAACATGTTAAGGATTGAAGA
A_14_P112813 ATCTCTAATCTACATTTACATGGGAAAAAAGCAAGGTGAAGTCCAGTTTACAGACTTACC
A_16_P16505146
CCCTGATTCCAAGTTGATCTGCATCTATATGAGAAAAATCTACATAGTCCCTTAGCGATA
A_14_P108865 CTGGTCAGTCTGGTCTTTGAAAGTATTAGAATAACAGAAAGAGTCTACTCTTGACGATGA
A_14_P102486 AATCAGTGCAAACTTAAAAATGATTTTCCTTCTAGTGCATTAAAAGAAACTGAGCAGGCT
CUST_8291_PI426210822
AATCTTGTCATTTCAGAAAGGTAATCCCAGTGCATATTTCTCCATGCAGCTTATTGAC
A_14_P122676 AAGTAGTGGTCATTTTGTCAGTGGCAAGAAACTTGGGTGAAATCTCAGTTGATC

A_16_P20025303
AGACATAACTGATAGAACTCAAGCCGTCAAAAATTTCAAGGTCTCAAATCACACAATCTG
A_14_P118298 CTTTTCTTGAAATCATGACTTGTTTCTAATTCTACCCTCAGGGGCCTGTAGATGTTG
A_16_P18079633
GGCTTTTACTCCATATCACTAAATCGTTTTCTAAGAGTTTAACCAATATTTCCTGCCACC
CUST_2834_PI426210822
GTGCCAGCTCATCACGTAGTTGGACTCTGTTAAAAAACATTTTTAAAGAATGAAGATTAA
CUST_5753_PI426210822
TCTAGTTTTTCCACAGGACCATAAAGCAAAATTGGTGCCGGGTTTGTATTTAAAG
CUST_9602_PI426210822 TGATGTGTTTCAGCAGCTTTTACCTGCGCGGAAGACACAAGATCT
CUST_4090_PI426210822
TTTAATTAGGTTGTGGTACAAGAGGAGCAATTAATAAAACCTTCCATTTCCACATCTACT
A_14_P126323 ACGCAATAAATGTTTATTGATGGTGAAGATGAGAAAACCCTATTTCTGCTGATCTTTCCT
A_14_P108118 AATTAGCACCTCTTTTTTTCACCCACTCCTGAATTTATGAAATGAGATACATAAAACAAA
A_14_P113643 TTTTTCAATCTGTCAGATTCTAAAACACTAAATTCTCCTACTTCCCGACCATGGATTAGC
A_14_P119815 CTTCCTAACTGTCCTGGTTTTCTTCTTGGATTCTTCTATGTGTGTTTACAAAACATACAC
A_16_P19682232
AATATGCCGCACTTTACTATTTTGACATGGTCTGGAGATGTTTTCTACAGTTACTGACAA
A_16_P37205741
ACCTAGAAAAAACTTCAGTCATCTAGTCTACAATGCTACCATACTCTTAATACAGTTCCA
A_16_P00685179 ATTGATTTATTTCTTGGGTGCAATGAAGGGCTCTTAATCTTCTGCATTTGGACACTTG
A_14_P132442 TCTGCCAAAAACAAGAAAACAGAAGCAGCCATAGAGAATTAGGATCTTATTGCTCTAGAT
A_16_P02765891
ATTGCTTCAAAATCTTAGTGGGCTTTCTGATATCTTTGCTTCAAGTCAATTTCTGCAACT
A_16_P16191833 GTCAAGTCCACGGTCATTCAGTGCCGCTGGCATTGTTGGAAAGCA
A_14_P105481 TGCTCATAAAATAGAGAAGCAACACGCAGGGAATATTTATTTCTAAGGAAGCAGTTGCAT
CUST_542_PI426210822
ATGAGATATTCTAACATAGACACACTCTTCATTATGTGTGGCCTCTAACATTTTTGTTAT
A_14_P100685 CCAAGGATCTGGCATAATGTTTTTGTGCTTCATGTTTATTTTAGGAGAGTATTGGGGA
A_14_P105613 CTATGCTGATCATCAAACAGAAACCAATTATGAAGTATGAAGGCTCACCACTAAAACAGT
A_14_P126582 CATGATAAGACGTCGGAAAAAGTAAGTCCAGGTGATATAATCCAATGCATCTTGCTTAGA
A_16_P15264986
AAAACATACTCTTCATAGAAAGATGGACTCAAACTGCTATTTGGATTTCAAAACTCCCCA
A_14_P135216 AGGTTGAGTTTAGATGTCCAAGACAGAGTGACAGATCTGGAGTAGAGGAAAG
A_14_P126572 CTAGTTTTTCTCAGAATGGCTGATGCTGTATATAAATCAAGCCAAAATGGCATTTGAGTA
CUST_376_PI426210822 GACCAGAATATCCTGGACAATGTTCCAGATGACGTTTGTATCAGCTAGGATAC
A_16_P20775600
AGTCACTCCTAATTCACTACAGCAATAGAACGATGCAATGTCTATCCTCAAATCCTAAGA

A_16_P17692696
AAAACCCATAACATTTTGTTTTTATTCAACTCTGCCTTATTAATCCTCCCTCCCAAAAGG
A_16_P36493825
ACTTAAGGCTCATTCTTAAATTCCACATGCTCATAACAATTGAGTGGTCCATTTGTAAGG
A_14_P119627 TCATTTGTGACACTCAGTATATGTTCTGGTCTCAGGTAATAGCACCTTATAAGAAATCTA
A_14_P126184 TAATGCATAGACATTCACTCTGAAAGTTAAAGGATTCCTTTCTTCTCTCCCATGTGTTTG
A_14_P100354 GTATTTTGTTATTGTTCAATGATATTAACTGCATGCTAGACTTAGGAGTGGGGCAGGATC
A_16_P00685366
AATTTATTCTGAAATTCCAACAGGAGTATCATATCACCTTTTGACATGGAGGCTTCTTCC
A_14_P100508 AATTTTTTTTAGGAGATAAGAGAATCACACGAGGTATGACTTCTGCTGAGACAAGAATGC
A_14_P132743 AATATGACCCCTGATCTTGCTTGGATGGGAGATTTGTAAAGTATTATGAAAAACACACTT
A_16_P17358744
AAAACTCACAAGGGTTTGTATGATGTAATTCCAAGGAAACTAACTATTCCCAGGTATTCC
A_16_P16864437
AATTCATCTTACCACCAATCCACAATAACATACAGAACTTTAGAACACAAATCATTTGCT
A_14_P107735 TTAAACACTCCACATAAAAACATGTGCACCCTCTCCTTCTACATTTAATCATTAGTAGTC
A_14_P112719 GGCCATGTAAAAAGAGAATTAAACTCTTGTTGCTTTTTGTATACCTGTATGCGTTTGTGT
A_16_P01882672
TGCATTGCAAAAACATTAGCAAGTCCAGTATCTTATATCCATCACTCTTTCTTCACATGA
A_18_P22840306
CCAGAATGATTGCACTTGGTGAATTGGATAATGCGGTATGTCTGTGATTGTATGTTTTAA
A_14_P120825
ATAAGTAAAAAAATTGATGCAAAGGGAAAACGTAGGAAAAATAAGTCGAAGCCAAGCGAG
CUST_4560_PI426210822 CTCTTTCATCCCTCTATGGACTGAGACATCTCCCTTTACACCAGTTTTTTACTT
A_16_P18522496
CCACCATTGTTGGATGAAGAATATAAATCTCAAAAGCAAAACTTTACAACTTCTGTCTTG
A_14_P114842 CTGCCTCTCTCTTTGTGTCTATTTCTGCTTATCAATTAGTGATTACGTATTGAGCACCTA
CUST_4183_PI426210822
CAGTATGGTACATATGAAGTACATACCAAGTACTACCTGATTCCAGATATTAGGAAAACA
A_16_P35981508
CTGATGCTCCAATACAATTGATTATCTCAATCCAGAAACTACTCTAATAGCCCTACACGT
A_14_P114053 ACCATCACTCTTGACTCTGGTTTGCTAACCTTTTATTTTTTGCTTTTCACTTTCAGCAGA
CUST_5583_PI426210822 TAGGGTACATAGTCCTGGGAGAAGACGGGTTCCAGGATAGGGCTTT
A_16_P00304995
ACTCAAGTCTCCTCATCTATTTCAGTTCTAAAAGGTTGCATAGATTAAATACCCACACCA
A_14_P113538 GTTGTCTGCATTCAAGTTTATGCAACTAGAGTTTCTTCAATATTTTCTAGGTGTGTATAG
A_14_P126377 TCAAACCACCAAACATTAGCCAGAATAGTTTTTCAACAAGGTTTCTTTTATTTATTTAGC
A_16_P15532355

AATTTGGGCCTCAATATTAATTCATGCTCATCTGATGTATCTCTTTCTCCTACAAGCAGA
A_14_P100269 TCATGGTTATATCCAAGAAAGCATAAAATAACCAATGTCCTGATATGCAATCTGGATGTG
A_14_P113737 ATGAATTCTGCAATTATTACAAGAGTGAAGATACTCGGCAGGGCACTTTCCATGTC
A_14_P124925 ACATTTTGATGCTTTTTTTCCCTATCACAGCGCATTTTTACTCAGATACATGATCTCCTT
A_14_P122699 AAACTGGGGTTCTATCACTTGCATCATATGTTTTACTTGTGTTGGTTCACAACTATCAAT
A_14_P120168 ACCCAAGCAATTATCTGTATTCATAATCCTTCCTCCTTAGTGGGTATTCTGAAAGTGTAA
A_14_P127173 TTGTGGAAGAGTTCCAAGTTTTATCTCTGTCCTAGTTGGTTAAACATCATTTTCTCTTTT
A_14_P108142 AAGGCACCTGGGCTGTTTTCAACTTAACATTCTAAAGACAACAAATATAGAAGAACAGTT
A_14_P111045
GAGAGAGAGAGTTCTGTGTAACCTTAAATAACAAAGCACAGTCTGGTAAAACAAACCAAA
A_14_P124378 TGGATCATGATTCAGGAACATGTCAAGTCCTTTTCTTGGTAAGTTGTGTCATCTAAGAAA
A_18_P22843023 AGACACTTCCTGCCTCCACCTAATTCAGGGCTAATCATAACAGTCAGAG
A_14_P132302 GCCAAAACACTGAACTGGCTCTTATGTCTGCACAAGCCTAAAACCAAAATTAGTAAAAC
CUST_1941_PI426210822 TGACTTCTTCATGATCATCAGCTAGCATAAGCACATCTGCTGTGCACCT
A_16_P41883816
GGCTGCAGATTTTGATATTACAGAAAAAAACATGCATCATAGAGTTGGCTCCACATATCC
A_14_P105282 ACATGGATTTTGTAGCCCATGTATGATTGCTGTAATACAATGTGGAGAAGATGTATCTGT
A_14_P105233 TACCAAGTGAAGAATTCATTGTGTATTTATTATTCACAGTTAATCACTACCTACCAAATG
A_16_P19650719
AGCAGTTGGAAATTTGACATTGACTCCTATAACAGTCTTATGATACCATCACTTTCCCAG
CUST_8137_PI426210822 ATAACATTGAGACTGAGCAGTTTCTTGAACCTCTCCTCTCAGCAGCC
A_16_P18815225
AAAAAGATTTCAATCAGACTTAAGGCAAACACTTCAATTCACTCCATACTGGTGAACACT
A_18_P22842088 ATGCTTTTGTTTCTTTACTGCCATCAGTGTCTCTCTAAGGGAGGGCG
A_14_P138415 CTTGGAAAGAGAAAACACGTCTAAGGAAAGTAGAACAGTGTAAATCTGAATGATTTGTCC
A_14_P126021 AAAAAGATACATTTAGCAGAGACTCCAAGGAGATTCCAAGTTACTGAGATGTTCTACTCT
A_14_P123733 TTTGCAGGAAGTTCTGCTTTGATTCCTAGATAATTGCTTTATGAGACAAGCCTGTGTTTA
CUST_7530_PI426210822 AGAGTTCATTTCTCTGAGTCCCTGCCCCAGAGGCACATGACATAG
A_14_P132114 TGCATCTGTGAATAAAGACTAAAAGGGCACATGGAGAAATTAAGAAGTTATGTTAGCCTT
CUST_2636_PI426210822 GAATAAGGTCAGGGCGAGGTTCAGTTTGGGATGTGGATGAGGATG
A_14_P107167 CTGTAAAGGGTGCATGGCACTAAACAGCCCACAGTCTGCAAAAGAAT
A_16_P16414821
CTATAGTTTCTTGTCATGTTTCTCAGATACCACCCTAAGAACTAAGAGCCAGAAGAAACC
A_14_P121802 GTTATTTCATGTGTCTATCTTGACTTCTTTCCAAGTCCCCTCTGTTTTAAATGAAACTCC
A_16_P16191697 ATTGAAATTGTTTGCTACAGTCGGTTTACCTTCAACTGGTTTCCTTCTTGTTCTGAAG
A_14_P120816 CAGTTGATCAGAAAATAGATGAAATTAACAAGGAGATGGGCAGTCTTTTTTGATGCCAAA
A_14_P134774 CCTGCCTAACTGCCTATTTATCCCAAATCCATATATTATTTTGGAACATTCAGTATTGCA
A_14_P101752 CCAGTGTTTGGACTGATGACAATGACTTTTTAAAATCTGAACACCCAAAAATTTACACAG

A_14_P134343
AGATACTTAGTGGACAAGAGATCACAAAATAAAACACAAAATGAGACACGGATGAGTTCA
A_16_P19920573
AAAAAAACCATTGACCAATATTGATAAGATATCACAAACCCAAGAGTATATGCCTGAAGT
CUST_4758_PI426210822 CTATTCCTGTGCTACACTCGAGCCAAGGTGGCCTCTTCCCTAGCC
A_14_P115791 AACTCGAGTAAAGCACATGAAGCCTTTAGTATTGAGTCTGGAAGACAGTTTCAGTTATAT
A_14_P135506 GAGAATTAAATGGATTTTATGCTCTGCAGATTAATTCCTTGGTTGATGTAAGTCAGCCTG
A_14_P117841 GAGTCCAAATGTGAGTGATAATCGGCAGAAAATCTGGCAAAATCGGATTTCAAAG
A_14_P133327
ATAAAAAGTTTTTGAAAGAAAGAAAATAGAACATGGAGGAGCCAGCCTCAGAGATGAGAA
A_16_P17604611
TGGACATCCTCTATTCATTTACACAGGACTATAGCAATCATGTAAGCCTACTTTTTCAGA
A_16_P02533244
CGGGAGCATCTTTAACTACAAATGATAACGATCCCTTATGTTCGTATCCACTTCGTAATA
A_16_P20259267
AAAATAGTTCACCAATAATACACTCAATGATCAGGCAAAATTCACATCGGTATCAGTGGC
A_16_P17372124
AAACTCATTTTCCACTGACTATGTAACACAAGTTAAGACTTATAACAGATGCCCCAGTGA
CUST_10165_PI426210822 AATGTGTTTGCCAGCCCTCTGAAAAGGGCTGCCTCAAACCCTCTAT
A_16_P01973724
AATGACCCTGAGTAGAGTTACATATTTTTCACACCTCCTACTAAAACTCAGCAAATGCTT
CUST_2442_PI426210822 GACCATGGAGTTCTAGATTTACTGGGTAAAACTGTTCACTCCTGTCTGTGA
A_18_P13426726
GCTATAAACCAAAAAACTCTTCCAAATGCAAAACATTGATGATTTGATTTCCCCACTTGG
A_18_P22841101
TGAAATACTTGCCCAACATGAGAGGTTTAGAATGTTCTGGTAAACCACAGTGTTAATAAG
A_14_P128875 CGCTCTTGTGAAACTGAAGACAATTTCCTTTTCTGTCATAAAACAAATTATTGCTCATCA
A_18_P10619298
GCCACTGATTGTGAAATAATCTGAAGTTTGAGAGTCTCAGTTAATAACCTATAGGTGCTG
A_18_P16349792
CACCAATTATCTTGTGATCAGAGTTCAGACTAAACAGTCAGAAAACAGTGTTTCAAACCT
A_14_P139997 GTTGTTTTTAACTGCCCACCTAGCAAGTGCATTTCTGGTTACTCTTATGAAATCTAAACA
A_14_P102582 GATTGTAGAGTCAGAAATGAATTACCCTGCATTAGAGATGCTGGATGATACAATAGGTTG
A_14_P129380 ACCTTGTCATTCCAAAGTCTGAAATTTTTACTATGCATGTTGAACTGACCAAACAATTCC
A_14_P106664 AACAAGCGATATATTTCTCCTTCAAGACAGATCGGCACGGATATTTAACATATAGATGAT
A_16_P03225661
AGATGGATGTTAATATTATTTGGAAACATTTTCATTTATAGTATTGTGAGCAAACCATCA
A_14_P123766 TCAAATTCCTCTGGACTAATTTGACGGCAAAATGATGGAGCAGATAAAGTGATATCCATA

A_16_P15849644
AATTAATTTGACATGGCTAGGTTTATTGTGCATAACCAAAAGTGAGAAGGTATTCCCAGG
A_16_P20390156
ATGCCATGTTACAACTTGTAAACCGTCATATTATGTAGCCACTTAAAGGATGAAGTAGGC
A_16_P16618926
AAACACTCATAACCCTTTGTTAACTTTCCAGAAGCCATAATCCAGAAGTAAAACCTGAAT
A_16_P58424286 CAGATGTTTTAGAGTTGGCTGAGGTGACAGTGTGGCTGGGCCAAT
A_16_P20946094
ATCAGAAGTGGGATTCTCATGCATAGCATTTACTATCAGATATATTAACTCCACAGAGGC
A_14_P121474 AGGAGTAACATTAGGGTCTACCTCTACCTCAATTTAGTTAGCGATTTACTACAATTTCAG
CUST_1397_PI426210822 GCAGTCCCAGCTTGGGAAGGCGGAGGCTGGCCTGAAAACAGTGCT
A_14_P130363 AGCATGACCTTTAAACACAATTGGACTTAAGACAAATTTAGTAAATGCAGATTTCTGGGG
A_14_P200861 TCTTTAAATTTCCTCAGTGAAGCCAGGCCTGGATAATAAACTTCATGCTCAGTATATCAT
A_14_P113413 GGCAATTGCTGTTTTTGCATCAAGATATTCTTACATCACCTGTGTCTGGAATATTAAACC
CUST_1213_PI426210822
TACTTGTAAATTAAAGGGCAAGCACTATAAAACATTAATTACTATTAACCAAGCTATGAT
A_16_P17443252
TCAGTTAACGAAAAAATGTAGGTGTTTACATGAATGCAAATCACAGTGAGTTATTGGGGC
A_16_P18130478
TCAAATGATGAACTCTTAAAACGTATCCTGAAGTCAAGTAAGTTGTTGAAGCCAATGTCC
A_14_P105529 CAACAGGAGGATAAAATCTGGATAGATTAAGACAATCCATTCTCTGGCCATTTCATCTAT
A_16_P00107122
AAATCTAACAGTCATACGAAAGACACTTAAAGGAAAAGGAATTTAAACTAAGCCCCCACA
A_14_P132035 ACCATTATCTTTCTGATGATGCTCTGAGAGATTATCCTTATGGCTGAGATTTATGTGGTT
A_16_P00474049
GCAGCATGTTATTTCCTTGTTTGCTTAATCTTGCTTCCTACTTCTGTTTGTTGCTATCAA
A_14_P120901 AATATACACAGAGACAGGTGCTTTAAAAAAACAAAGCAAACCCTGGGATCAACTTTATTG
A_14_P134511 TCCTGACACCAAAGTCTATAATGATAACTGATGAAAACAGAGATTCACTCATCACATCCT
A_14_P103332 CACACGCGGCAAAGAACACCATGTTAAACAATTAGAAAGCAAAATAAGCATCATAAACTC
A_14_P112705 ACTTTAGACTTTGTGAGAAATTCATAAAGGTGGCTGAGTGGATTTGCATGCTTTAGAACT
A_14_P101500 CAATATTATTCTCAGCCACTGTAGTTTTTCATTTCTTCCCCATAATCCTCTTCCACTCAA
A_14_P104848 ATTTCGTTAGGTTTTGTCCGGTCTTAACATTTATTCCAACTGGATCATTACCAAGACCT
A_16_P20879399
AAAAACACCCTTTTGAAGATATTCCCATCAATCCTGAGTCTGATTTTCATCTCGTCTTAT
A_16_P00347177
AACCAAGGTTCATACTACATTTCTTGTTCTAATATACTCCTATAATATTTGCCTCCGACA
A_14_P100529 TGGAATGGCTAACGTGAAGCCTGAATTAAATGTGGTTTTTAGTTTTTACCATCACCAATT
A_14_P202058 GAGCATCTGTCCTTTCTACCCGTTCCTCTTTATCTTTAGTGTTCAGTAGCAGC

A_14_P139458 CAAATCACAGTGCTGTTTTTAATGCCTGAGAAATGCACTTTAGGGGTAGACTTATACCTT
A_16_P17626303
AAACTGTGCTTCAATTATCTTCCCTTAGTAACTTCAGTTATCTTCCCTGATTTCCCAAAC
A_14_P200956 CTGAATCTTGGATATCAAATCCAGTGAATATTGTATCACAATCTAGACTGGGTAGTCCTG
CUST_2219_PI426210822
GTTTTATAAACTTGTACCAAAAGGCACAGGAAAGAAAAAATAGAAAAATGTATACCCTTT
A_14_P133696 TTTAAAATATTTGTGACCATAAGTAGCATAAATTCCTAGTTCCACCCTTGTAAAGTGCCC
CUST_17_PI426210822 AGTGTGATCAGGGCTTAGTGTCCTGCTTCTGCCACCTCCGCAACT
A_14_P110846 CTCCATTCTGCCCTACATGATGGGTAACTGTGATCTTTCTTCCCTGTTAG
A_14_P114967 GATGTTCCCATCAGATTAGGCTCCTGCCTTTCAGTGTGATCCTTGAAA
A_16_P16822954
GTTCCAGCATATAAAAGCCTTTCCAAAAGTGATCTCTAGTATACTTGGACAGTCAACAAG
A_16_P17877434
GTTTAGATGAGCATGTTCTACTGTTAGAGAACCAAGGTTAGATCATTGTCTAGTTGGCAT
A_14_P130413 TTCTCTCTTTCTTTAAGGTCCAGTTGAAAAGTGAAGAATCCAAAACGTTTGCAATGAAGA
A_14_P128063 CATTCAGACATATCCTGCTCCGATGTGACTATTTATGCATAAACAGCAGACACTGAC
CUST_8642_PI426210822
TTACTTTTTTCCTTCTATTGGACAGTCATTCTAAAAAACTCACCTGATTTCCAGGAATAA
A_14_P132509 CTGCTTACCTTCAGAACTCATTAGCAAAAAGTATATCGGCTATACATGTTGTCTGCCTTT
CUST_9016_PI426210822 CATTCTCCTACTTCAAAGCTTTCCCTGGTTCCTATTACCTTCTGGGTAACTA
A_14_P135579 GTCTGAAACTTTCATCCCACTACAGAAACCAGATGGAATATCTCACAATCAAATAACAGA
A_16_P18584883
ACACATTTTTTCATTCCGTTATCACCAAGAATCATTTATTGAACCATGATATACCCCACT
A_14_P125587 AGTCTTTGGATTCACCATATCATTGTGAATCTTGGAAGAGTCCACAATCCTTAAACAAAA
A_14_P119173
AAAACAAAAAGCCCAAGTTTATCTGATTAATAATGTGCTGAAGAAAGCAGGAAGTGAGGA
CUST_9997_PI426210822 GTGTTTGTTTAAAAAAGCATGGTTGCAGGAGATGCTGTCATGGGTGG
A_16_P00924375
GACTTTTCAAAGACTTCCTCAATTGGCCTTAGGATAACATGCAAATTTCACAAGAGGATC
A_14_P105335 TAGAGTAACGATTCCAATGAGTCTGCCAATACTGGTGACATAAGCATGGTCC
A_14_P100186 ACCAGGATTAACCACATTTGCTCACATTCTTTTAAAAATAGTGATTTGCATCCTTGGATG
A_16_P03771768
TGTGGACTATTAAAGGATTAAAAATGAATTCAAACTAAGTGCGAAGAGATAACAGGTTGC
A_14_P117831 TATTACAGATCCAAAGATCTAGAAGGAGCCCAACATGGAAAATAAAACATAAAGCCCCTA
A_16_P00685225
AAGTTTATTTTACATTTATGTGAAGTTTATTTTTGGTTCTGATATTCTTCATAGGGTAGA
A_14_P119967 ACAGTTTGGTTGTTGCTGTAAATAGGGTAGCGTTTTGTTGTTGTTGTTTTTTCATGCC
A_14_P130329 CAACAACCACAAATTTAAAAAAAATTCTCCCTTTGGCATAACCTAATGTTCCCTGACACT

A_14_P131404 AAACTTTACCTCTTGAAATGATTCACCAGCACTCCAACAAGTTCTCCAATTCGAC

A_14_P122306 AACCTCCTTTAAGCACACACAGGACCATGATGTCACTATATCCTATGTCTTTCATAATAT

A_14_P103381 GTGACTAAACCTGATCATTTTATAAGTTCTGTTCAGGCTTTTTTGGCCTAGTCTGCAGT

A_14_P112370 TACTTTTATTTCTCTCACCTATAGGTGAAGTATTTGACTATTTGGTTGCACATGGCAGGA

A_16_P01731798

ATGACAACAGATGTGTATCGTAGGATTCCATATATGGGGGTAAAATGTATTTGCAGTTGA

A_14_P138763 GCTTTCAACAGATTTCCAAGGAGTCTCTGAGTAAAAAGATTCAGAGCCATTGCAAG

A_14_P135842 AAAACAGAAGTCCTCTAGAGAATCCATTCACTAATGAACTGTTCCTTTCAGTCAAAACAT

A_14_P112236 CTCAGTGATACAGAAACAGGCTTTTTTTTTTTCTAACAAGATCCCAAACTCATTCTGTGT

CUST_643_PI426210822 TCACCTGAGCCTGATGGGGAGGGGAAGGTCTTTTCTCTTCCAAAC

A_16_P19664168

AGAATGATGGGAAAATAAGCCAAAGAATTGTGAGAGTAGAACTAAAAGTGCACAAGACAG

A_16_P15503375

CCTCACTCTCCTTATCAACTATCCAAATCTTATCTACTCTTTAAAGGCCAGATTTCAACC

A_16_P03578358

ATCTGTGAGGGAGTTGTAAAATTATTGGCACCAAAAAAGAGATATTGCACCTAATTGCTG

A_16_P21486107

ACAGGCAGACCTTTCAATATTATTGACTTAACATCCTAGACCATAGTGATGAAGACTCAG

A_16_P18874589

AGAAGGGGAAGTTAATGTAAAGCCAGAGGGAATAATCTATTCATGAGTTTTAAATACCGT

A_16_P37027436

TGCTTATCTTTCTTTGTTGATTTTTACCTCTCCCTAAACTACATTCCACACCATAACACA

A_18_P16190686

AACACGATTCACGAGAGATATGTGTAGTAGGAGTGCCAATACATAAAACACAAGGTGAAG

A_14_P137029 CTTGATCTTCCTACCCTGAAAGGGAAGATTGAATGATGTTTTTAAATCCTTTCTTCTTCT

A_14_P113112 CTCATTCCAGTCCTCATCAGGAGTTAGACAAAGAAGCAACGTTCTACTTTGTTTTTTAAA

A_16_P19758410

AGACTGAAGTCATTGCCTTACACACAAATAGACATACTGAACTCAGAAGAACACATACTG

A_14_P131201 CCCCAGATTTAGATGAATCCTTCTTTCATGCTGATTCTGTTTTTTAGTCTCTTTTTCACT

A_16_P15610741

AGGGGGACTACTATTTTCTATCACCTGAAAATCTCGATGAAAGTGTTTTAGCCTAAAGAT

CUST_3305_PI426210822

TTCTTTTTCACTATTCCTTGATTTATTTAACCTGTCCAGGTGGTCTTCTATATGTCAGTT

A_14_P122557 TTTTCTCCCTTGATTTTCTCAATTGGGAGCCAGAAGTTTTCAAGCGATAACAACAA

A_16_P02444256

AGATTCTCAGTCCTCCTTATGCAAGATACCTTATGTGCTTCATCTCAAATCCCAATTTTA

A_14_P117540 TTATTGAGTATTGGCCATGATGTTACAGAGACGAACATTCAGTTTTGAGTCACTTCTACT

A_16_P15603437

AAACATTATGAATTAGTTCCTCCCATATGTCTCACCTTAACCACATCCATTCTGAAAACG

CUST_3963_PI426210822

AATCTGAAATTAAAACTTATGGTTTTAACCAATAAGCTATAAAGCTGTTGAACTCCCACT

A_14_P127620 CTATTAGACGAAATAACAAAAACTTTTGTCCAAGGTTTTAGCCCTTTTTATTGACCTTCT

A_16_P17971839

AAAAATGTGGAATAACCAGATAAAGGACCCTTAATTGGTTGTAGAGGAACAACTTTTTGG

A_14_P127880 AAAACAGAACCATTTACTTTCAGTTCTTCACACTTTGAGGGAACCAGTTTATTGTTTTCA

A_14_P132025 GAGAAGGTCACTCACTGAATTACAATAACATCAAAACTTTTACATGGGGGTATAGGGTAT

A_16_P18268437

ATTCCTAGCAAATCTTCATGTATAGTGTGTCAGCAACTAGTCTCAAGTGTCTAACGTTCT

A_16_P37913083

AACCTCTTCCATGGAATTTATAATTGCAAAAAATGAACGTAAGGATTCTACACCTCAGCA

A_16_P02623450

AACCCAACCTCTAACTAACAATTTCTCCAGCATAAAAATGAAACATCAGATTCGTTAGCC

A_16_P03550833

TCTCCCAGGAGAATTAAGAGATTTTTTTTTCTCTTTTCTAAAGGGAACAAATAAAGGCCC

A_14_P120581 GTTATTTAGGGAATAACTCCCAAAAATATCTCGCTTGGTGGAAAAACTGCGGAAAAC

CUST_5487_PI426210822 GAAGAATTTCAGGGTCCTCTGTGCTTATTTGTTTCTGAATTGACCCAGTGA

A_14_P135544 ATATTTCTGGTGGACCCATCGCTTTCAGTCAACTTTATAAGAACTAACTAGACCTACCA

CUST_7767_PI426210822

TTTTGCAGTGGTTTTATCCATTTTTCAACATCACTTTCATTGATTTCATGAAAGTCTGCA

A_14_P122981 AGGATGGCTCGGATGTCCTCTTCCAGTAATGTCGACTTGACATAATAAACAAAAG

A_14_P137412 AGATTAGCACCAGGGCTAGAATTGTGATTTGCCATTCAGTCATTTCATACACATTTACTA

A_14_P136184 GTCAGATTTGCCAGGCATTGTTTGAAGTTCTTCCCAGCCCAGAAA

A_14_P122108 CTTTTTCTTTAAACAGAGGTGAAATATCTGTGGCTAAAAAGTTTGAGATTTGTGATAACT

A_18_P22841216 CTTTCTAACTGCCTCTGCATTTGGAAGGGGTCAAGACTCGTTTCATAGTTTTTT

A_14_P103716 GTGGAAACATGTATTAAGGAAAGAGCAGTCACCTAAAGACTCATCAGTGTGTTTATTGAT

CUST_109_PI426210822

CAACCTTTTGTAACAGGCTGTGTAAGGTGCTGATTCAACCAAATAGTGGATAGTAT

A_16_P01649196

ACTTGCCTTTCTCAGAACTAAAACTTCCGATTTGAGGTATCAGTAACAAAGCCTAAGAAC

CUST_7856_PI426210822 CACTTGCCATCACACAGCTATGTAAACACCTATGTCTCTGCTGGGA

A_16_P16251972

TCTCAGTCCTTAAAAACTTTGTCAGTCTACTCAACTAGCCAGAAAGAATATCTGTTTCCA

A_14_P116515 ACCAAGTTGATATAAGATGGTCAACTAAAATCTCTTAATGTGGGTGCTTGCTCTCTTCAG

A_14_P131729 CTATTTGATAGACCAATGCACGTCAAGATGGTAAGTCAGTAGGATCTTTCTCTGTGA

A_16_P20088316

TCTTTTCTCGAATGGTCAAAGTCAACTAACACATATAATTCCTAGTATTCTGCCCTCACA

CUST_8425_PI426210822	TCCTGTCATGTGTGAAGACCTGGTAAGATTTCTGGCTTTTTGTTGGC
A_14_P128182	GAAGAGGCCATTTTTCCCCCTAATGCGAAGCAAAACCTGGTGTTT
A_14_P201315	GCCCTTTACAAGCCAGTTCTAGTTTTCTATTTTCATTTGCACTTAAACACTAATTCACCC
A_14_P124325
CTTTGAAAAGAGAGGAAAAGGAAAAGTAGTAGACCATCTCTCCTACTCTACACTAAGAGA
A_16_P15524189
AACGACTGTGGATTACCATTCTTGACCTCATTACTTTCTATCCCCAAATATTAATCACTT
CUST_8229_PI426210822	ATCGGCCACACTGTCTGTCTGGGTCAAGTCCACGGTCATTCAGTG
A_16_P36230284
ATTGGATGGGTCAGTGGTTTTTCTTACGAGCATAAGGTAGAAACTTTTTTGGAACTAAAT
A_16_P19571438
CAAGAATAATCAATGCAATGTCATAAGCCAAGTCAGAAAACGATAATTTCCCATGGTTCC
A_14_P111499	GAGGTTCTGTGCCACATGGGAGTGTTAACAAACGTAATTTTATTCCAAAAGATTCCAA
A_16_P21458768
CTTTTATACAGCATGGCCCAGATATACAAGTCAACCTCTTCCACTTCATACACTGATTT
A_14_P128945	TAAACAAATGAGTGAACTGATGAGTGGATGAGAATGGACACAGGAAGTAGATTCTTCTG
A_14_P114681	TAACAAACATTAGTCAGTGTATTCAAATGGTAACACAATCTGGTCAGCAACAGAGGAAAG
CUST_694_PI426210822	CAGGGTCCCCTCCTGCTTAGATAACCTATGAATCTGTGTCTGTGAATT
A_14_P132059	CGCTTTCCATAATACTTTCATAGGTTAATCGTTTTCAATTTCCTCAGCCAGAATCAAGAG
A_14_P121556	TTCTCAGAGAGGAAGATTTTCATCATGCCTAAAAAATTAATGAAAACCAAACGGTTCTGG
A_16_P16571591
ACGAGGGCACTCAAGAATATTCAGATGGAAACCATCTTAGATCTAATTGGCTCATTAAAT
A_14_P124640	CATTAGAATTTAGTGGTATAAAGGATTAGGTCCCTTTTATAGGCCTGAAGTGGCTGTAAG
A_18_P22840309	ATTTCTTCACTCAAGCAAAACCTGGCTGCCAAGAAGAGAGATTGAGATGATG
A_14_P121985
CTGACTGGGACGCAAGACAGATCTAACTTTAGACTGAAAGGAAAGAAGAGAAAGATATAA
CUST_419_PI426210822	CTCAAATGAAATTTAAGATCTAGGCTCTGATGGGGGAGAGGCTTGTCC
A_14_P110448	CTGCTTAATTCAGACTTTGAGGATGTTCAGGAACAGGTAATGCTTAGATTTGGTGTGATT
A_16_P16157638
ACCTGAAACTGAGTCTAAAACCACTTCTCTCTACTTCCTCTTGTCTTTTTCATTTAAACA
A_14_P113911	ACAACTAATCGACCTTACCACTTTCACAATCTGCTAGCAAAGGTTATGCAGCG
CUST_3584_PI426210822	AGGAGTCCTGGGGACTGGAGGTTCTCTGCACTTCTGTTTGATCTT
CUST_537_PI426210822
TTTCATCCTGACTTGAGATGCTGAGTTTTCTTTCAGTATTTTTGTTGATTCATTCATTTA
A_14_P134293	CAAAATGCATGTGTGCCAATTCCAAATAAGAGACTCTAAGTGGTCAGGGTTGT
A_16_P00685241
ATTTTGGATGGTAGTGGTGATGTTACTTATTGATGGCACATTTGATGACTGTTAGTCCAT
A_14_P102853	AAACCATGGATTAGTAAAATCTTCCATTTTACAAATGAGAAGAGTGAGGACTCAAGGTCT

A_16_P18032513
CAGCACTTGAGATTCATGTAGTTAACGAAGTCCATATAGTTTTGTGCTTTTACCATGGTG
A_14_P139201 GCAAATTATTTAAAAAATGCCCAGCCAAGGATTCCATACAAACTATGGTATAGGAAGAGG
A_14_P113336 GAATGTCTCTTATGCAATAAAAGCAGCAGCAGAGAGTTGGAGTCACCTTGAC
A_14_P116948 ATGGTATGGTCTGAGAAAGCCTCATTAAATTATTCCTTTCAATGGCTGCAAACAAAAACA
A_16_P16386132
AGGCCTTCATTATCACCTAATTGTGTTCCCACTAAACTCTCATTAATTAGCCACTCTAAA
A_14_P101887 AATTGATTGTGTTTGGATATGTGCCGAGAACACTACAACTAAGGTTGAGACCATATAGAG
A_16_P01878833
CTGAGCGCTCTCATACATAGTCCTCTGTTGAGACTTCTGTTGGTAAAAATTCTGTAATGA
A_18_P13125106
TGAATGTGCTTCTATCGTAAGAGACCAATAATTCAACCTTTTAATCGTAGACAGTTCCAG
A_14_P125516 CTTCAGCATAGTTCAAAGCCTTTCCAAACTTTTCCACCTGATCAACAGAGTTAATACA
A_14_P202452 ACAGTAGGAGCAGTAGGCAAATGTCAACTCTCTAAAGATACTTAATAGCATCATTGTTGC
A_14_P110579 CAAGCCTGTTTTCTCGATCTGGACTTTGTCCTTAGAAAATTAAAGAGACTATTCTTGTGA
A_14_P130409 CTATTTTCTGGCAAGCGACAATAACTGGATCCAAAAAAAAAATTAACCATGAGGAATTGG
A_14_P200978 ATCCTGACTCTGAAGTTGTGTCATAAGTCAAGAAATAACTTTCTTCAAAACCGTTTCCAA
A_14_P124569 CCTTTAATTCCAGACCACGTGATTACAACTCCCCATCGCCAATTATATTCATAATTTGAA
A_14_P133120 ACATAGGATGGGCAGTAACAACCACAAATGGGTTATGTTCATTTGAGATTACGAAGTATA
A_14_P126894 CCCGAAACACTGAAGCGTCTTTTTGTTGTTAAAGGTAAGTTGGGAATTTCTTGTGATAAA
A_14_P130841 GGTTTTGATTTCAATTGGATGAGGGTTCTGTGGCTACAACACGAATACCTGAAAA
A_14_P119194 ATTGTTTTCCTTACAGAATGTTTCATCCATTTGTGGACCAAAAGATGGAGTTGGTTTTTA
A_14_P108092 AGAAGTCCCTTTATAGGGATGACAGGAAGGAGAACTATGGGAGTATTACTTCCAT
A_16_P39754128
AGGTCGCATAAGAAGCAATAGGAACTTTACTAAGTAACAGAGTTTTAGATACCCAAAGCA
A_14_P118658 ATAATTAAGGAGAAGAAAATCAGCCAGTGGCTGTTGTTTTACTCAGCATCTTTCAGATAC
A_16_P18863292
AAAAGAACATGAAAACATTGCAGTTTCTGGTAATCTCCCAAATCATGTGCTTGTCAGTTC
A_16_P15394100
GCACCCAATTTACTCTCATCTATAGTCACTACTTAATCCCTGCTTTTTCTAATCCTGCTT
CUST_4262_PI426210822 GATTTTATTTCTCTGGCCCTGAGGTCATTTGTCTTGTTTAAGAAGGGGCA
A_18_P14132324
CCAGACATGCCAGGATGTTTCTTTTTAATTCTGTGCACTCATGTTTCACTTCAAATTAGT
A_14_P133250 ACATTTCCTTCTGCTTTTTGGTTTTAAGCCTATATTTGAAGCCTTAGATCTCTCCAGCAC
A_14_P133784 TGTGTTTGTTATGTGTGACTGAGGTTCACTCCCAGCATTCTGTTTAAATTTGGAATTTAT
CUST_5835_PI426210822 ACTGCATTGTCATCTAGTATTCCAAGGCTTTAGTCTGTCCACCCTCC
A_16_P01421028
AAATTGGTCCACTGAAATAATGACTCAAAAATTGTGGTAAAGTTTAAATATGCGCCCTGC

A_14_P117922 ATGCTGGTAGATCAAGTGGTCTGAAACTATGTAAAATATTTTTGAGTTGGCAACTCTTGG

A_14_P103809 CTTTCCTCTAATCATCAGGAAATATTCACCATCTTCACTGGCACCTTTAAGTCTTTTTAC

A_18_P15826834

TGCCGAAGGATACTGTTTACATTCATGTATAGCATGCAATAGGGATTTTGTTTATCTGGC

CUST_500_PI426210822 GGATGCTAGGGTTCTGTTTTCTGGAGATGTTTGATCACTGAGTGAACAG

CUST_1627_PI426210822 GCACTTGGGTGGGAGTTTGTTCAGCACGGACTTCTGTTGTAGATC

A_14_P201491 AAAATCTAAACCAATTGCATATTGAAGAAACCTAAGAAAGCCTGCTTAAAAGCCAACACC

A_16_P30952385 GTTGTGTATGGGCTTCAGCAAGTTTCTGAACGCTGTAAACTTCTCATTTTCAGA

A_14_P114572 TTGTAGGATAATTAGACTCTATTCTCTTCCAACATCGTAATTGGCATGAGATGCGTGATC

CUST_6684_PI426210822 ATTGAATTCCAGCAACCTTTGAAACCTTTGTCACAAGGAACCCTAGGTTT

A_14_P130949 GGCTAGAGCAGGTCTTCATCATGCCTTGTCTTTTTTAACTGAGAAAGGAGATTTTT

CUST_3980_PI426210822 GATCAGAATGACTTCACCCAGACTTCTGGACCTGGAACTCATTCTC

A_14_P127545 TTGATGCTGTGTATTGTCACTCGGTTGAACTTCGAAATATGTTTGGTGAAACACTTCATA

A_16_P15111310

TGTCCTTTATTACTAGTTAAGTTCTCTTCACCACTGAAAGAAAAAGTCCAAATGAACCCA

A_14_P103153 CTCTTTCTAGAACACTGGTTAAGGCTTCTGTTTATCACACATAGGCCCACACA

A_16_P36168102

AAAAATCCCGATGTAACAACAATATTGCAGTTTCCACATTAGTAATTTAGGACAACTACA

A_14_P121376 GGGAAGCAGTGTGACATTTATATGAAATATATGAGGTGAGAGAGATGTAATACGGGAAAC

A_14_P110999 GTGTTATACTTTATTTTAATTAGTAATTATATACAGTGTCATGGTTCTATCCAATGACTT

A_14_P138559 CTTTCCTTTTCTCAAAGGGCATGGATACACCTGGTTCTCAATGCTCTCTTTT

A_14_P102284 AGAGGGGGAGGGAAAACAAGTATTTCATAGGATAGTGCTCATTTTGTTATGATTTCATAT

A_16_P00685632

GAATTTGGATGGAAAACCTGATCCCTAGCAAGAAGTCTGCTCTGTATCACCTTTATATAG

A_16_P16191799

CTACCAAACATCATTATCCGGCAGAATCACTAATTTTATCACTCAATAATTTTATGTATT

A_18_P17042933

AAAAGCCACTCTGATAAAACAACTACAATGGTGAAGTTAATAGGATCCCACAAATGACTC

A_14_P201566 AATACAATTTCCAACATCATCTTACCTTTTTCTGCTGGGCTCAAAACAGTCACTGTGAA

A_16_P16191406

AAAACAAATCAAGCATCCATTCCTCTGAGAGGTTATATAATCACTGAGAATAGGCTTCCA

A_14_P119925 AACGATTCACGAATTATTTTGACATATATTAAGCAATGACTTCTCCAGGCCTCATACTGG

A_14_P107502 GGGGCCTCCCTTTATCTTCATGAGGTATGGTTATTTGTAATATGCTGGTTTATTTTAACT

A_14_P122632 GTAACTCTTTATTCCTTGTGTGAAAATCCAGGGCTGTTAAAGTTTAGTGGCAGGAG

A_16_P17680361

AACATGTATTTGTCTCCCTTGATAAAACCTCTGACTCATCTTTATCATCCACAGCACTAT

A_18_P14886635

AAGTGTTTCGTAGGTTTCAAATGAATTGTGCCTGTTTCATACATTTTCGTGGCAAAGTTA

A_16_P02129404
AAGTGACTTGCAAATCCACTTTCATCTGCCATAGCATGTTAAAAGTTCTCAAAAACCACA
A_14_P115450 TCATGGACAGCAATATTATAATAGGACCTTGATATTAAGTGAGGACTGCTATGAAGGCCA
A_14_P111608 ACTCAACAGGGTATACAACAAATAGCAAAATAATCCACTTTGAAATCAAAGACAGGTTCG
A_16_P16261604
CCCATTGCATAGTAAGGGTTTTTCACAAGTTCTATTTCCAATAACTCTTTTGACAAAGCC
CUST_1196_PI426210822
GCAGAGACTCTAAATATGTGGGGATCTCTCCAAAATGTCTCAAGCTTTTGAATTCTTAA
A_14_P118552 CTACACTTTGTGGAGAACAGGGATCTCTTCTAACTTTCTGAAGTTATTTTCTTGTTCACA
CUST_10268_PI426210822
CAGATTTTTAAAAATTGAAAATTGAATTTTCAAAAAATTTCTCAAAAATCTTTGAGAAAA
A_14_P201627 AATATGCACTCTTTGGAAAATTCCCTTATCGATATTATGAGAGCAGAGCATGATCCTCTT
A_14_P122941 AAAAAAAAGACAGCCCTGAAGTATATTTTTGTTGCTGTGAGGGCAATATGTGTAATGAAA
A_14_P135296 TCATTTTTGGCTATTCCAAAAAAGCATATCGTGGGGTATATCAGAATTCTTCGGACTTTC
A_14_P135079 CAAGAAAAAGTGGGTAATGGTGCCCATGATGAGTTTGCATCACCTGACTATAC
A_14_P113429 CTCTTTCTGGACTACAAGACTGAGGTAAGATAGATGCTTACTAGTTTCAGAGATAGACAC
A_16_P18114635
ATGCACAATATCATATAAAACACAATACCTTTATCACAAAACACAATACCTTTCTCACCT
A_14_P106947 GGACTCTTAACTCCTTCTTCTTTACATTTGTTGTTGTCATCATGAGGCATGTTAAACCTA
A_14_P123769 TTTTTACTCAAGATAAAAACTATGACCATTGTGTGAACATTACTGCTTCACCTCCCTCTC
A_14_P125249 GTGAGGTGAGTGTTCACCTAACTGAAAACTGGAATGTTTGTGGGAATATTTCAGAAA
A_16_P37274344
AGGGTCTTTTACGTATGTTCTTCCAGGTATTCCTCAAAATAAAACTGAATCTCATAGCGA
A_14_P135531 ACTTCAACAAGCCCAAGTTCCTATAGCCAAATTTTTTAACTTGGCAAAAAATAAAGGACC
A_16_P16481983
AAAAATGGATTCCCTGACTATGAAATGACTTTTTCCTTGCATCTACTTTCTGTCTTCCAT
CUST_2569_PI426210822 AGGTATGAGATGATAACATCCTGGGAGCTTCTAGGTAGGGGTTGG
CUST_3812_PI426210822 AATATTGAGTGTCCAAAAGATTCGTGAACTGCATCTCAGCTGGGTGG
A_16_P19064562
TATGGAAAATGTTTGATAAGCCAAAAGGAAAAAACTGAAATCTCCCACAATTCCATCAGC
CUST_5567_PI426210822
AAATTCATCAGAACTCTCTAGGTCATGTTGCCAGATAACATTAGGGTTCCACTACT
A_16_P32319853
AAGTCCACAGTAGTAAATACTCACGTAAGCCTTAAGATTAGATCTTTTCGTTCAGTGGTC
A_16_P58424564 ATAGGAATCAGAACTATGATTGCCTTTGAGGTGGGGGGAGTGGGC
A_14_P134708 ATAATCTCTCACCTAATGTAAGTTCACCTAGAAAGTCATTTACCAGGAGGTTTCATGAGA
A_14_P122744 TTCCTAACTTCCTCAGGACCTAGGTCACCTTTATTAAAAGGAAGAATTCACTCTTTTTTC
A_14_P103918 ATTTGGTCTTTTGATGATGGTATAAATACTAAGTGAGGCATTATGTGAAAAATGAATATT

A_14_P118164 AAAGAAATGTCTATGACAATTACTTGCATCTTCTTCTATTTACACTGAAGCCAGGCGTCA

CUST_1101_PI426210822 CATTTGATCAGCTCTGGCTGGGCCATGTAGGGAAATGAAACTCTG

A_16_P16599208

ATGGCTTAGAATTGCTCAAAATTGCTTGACATTTAGATTGTGCTTCATGAAAAAGGAGGA

A_16_P00654199

AAATTGACATTGGATATCTAAACAAAATGGGAACTTACGGGAAAGATATTAGAGGCTCGG

A_16_P00685102

CGTTCTGCTTTTGAAATAGGCCATGACCTTTCTTTATACAACTTATTCTACTTCTTGCCA

A_16_P15276082

TTCTGCTAACTTGGTCCTTAGAAGAAAAAACGCAACATGTTTCTTATCATGCTCCTAAAC

A_16_P16541974

AAGGCCTTTCTTATCAGTAGATTCTCCTAAGCATTTATGAGTGTAACAGGATGGAATAGG

A_16_P19483414

GGAGTGGATGACAAAGATAGGGAAGTAGTAGAAGACCTTTAAGAAATCAGAGGTTCTATT

A_14_P131592 TCTGCCAATACTTTCTATCAGTCTGTCATGTTTACTGTCTTTTCCTCCATACAGAAGTTT

A_14_P108961 TTGTTTTCAGAAATAAATTCACCTTCCGGGGCTAAAAAAATCCTAACCATTTCCTACTTC

CUST_1001_PI426210822 TCACTCGGTGACGTGGGGCATTCCATTCGACCCCAGAGTGGTTAT

A_14_P101617 GCATTGTTTGCTTCATGACCTAAACAAATACTGGCTTTGAAGTCTAGGTTCTATTTCCTA

A_14_P100251 GGGTATATATTGTCTCAATAAAGTGGACCTATCTGTGTGTCCTGGACACAGTAAATAACA

A_16_P19781107

GCCACCATCTTTAATGTTTACCTCAACAGATTATTATGATGTGTTCCCAGTAGATATGCC

A_14_P119565 CTGGGTAATCAAGATAACTTCTCCCGAGTAAGAAGTGTCTAAAATTAGAAAACGATCCTT

A_14_P111278 TGCAGGAGAATCCACTTAGAAGCCTGGGTAAAATTGTATTACATGATCTTGGGTTT

A_14_P125050 AATAGGAATCTTGCAAGGTAATTCTTATTTGCAAGTGGGTTATGTGTTCACTCTCCTCTA

A_14_P111490 CCTTTTGTCTTAAAGTAATAACCATTATGCTTACGAGTCATAACATAACAGGTTAGGGAG

A_16_P15398429

ACTGCATATGAGACTACCTACAATTAAGAGCAGTTTGACTAAATCACTTACTTGAGCCCT

A_16_P15936618

AATATCCCCAAGAGTCCTAAAATTCTTTACAGTCTTCTCTTTACAAACGCAGGAAACTTC

A_14_P201048 TCATATACCCTATCCTGGTGGAAATGACCCTATTTGATATGCTGTCCCTTAAAATAACTT

A_14_P102261 CTGAGTCACAATTGTTTCGGAGTTTTCAGTAAAGAAATGTATTTTTGCACCCATTTTTTC

A_14_P200302 TTATAATTTGATACTTTCATATTTATTGTGGGCCGATTATGGTCAGGCATGTGCTCAACT

A_14_P139584 TTAGGATGCACTTCTTGCTTATTCAATAAAGTTAATAATTTCTAATTTTTCTACTTTTCA

A_14_P112546 AAAACAAAAAAAATGACCCACCCTGACAAAACTACACCATATATGAGCATCAGTAAAACG

A_16_P17119575

AGGTTAGGATTTCACACAATGCATTTATTTTTCTCACTAGTTATGTTTGACAATGGGGCT

A_14_P129432 AATCCAAAGACAAAGTTATCTCAGATGAAGATGTCCTAGGAACATTACTGAAAGTTTTCC

A_14_P101981 CACTCCAGCCAACAGCAAATTGCATTTCTTCCTTTCCTTTATCACTATATTTCCATTTTG

A_14_P103536 TTTTTTTTTTCATATTTTAGGATGCCAGAAGCCTCAAAAATGGAAAACCCCCTGTGC
A_14_P132052 ACCTGGGTCACAAAATGACAAAGGCTAAAGAAGTTCGAAATGTTTAATATTTCATGCAGT
A_16_P02052945
CTTCTCATCCTCCAATCCATAATAGCCTCTAGATGTAACTGCTATCGTTGTCTTTTCTCT
A_14_P123744 AATTAACCCAGTTGGTGATGGAGAATTACTGGAAAGTTATAAGAAACATAGACACCTACC
CUST_6631_PI426210822 TGTAGGCCTCAGGGCATTCATTGCTGTCTATTTTTCGTTACAGTATGGATG
A_16_P16006744
TTCCTAGGACAATACGCATAACCAGATCTTACTTGATTCCTTGCATCTATCAAAAGTCAA
A_14_P128670 TTTAAGTAATTAAAATGGGAACTAGTGTTTCCAGTGTCAATGATCTCACTGCACGTATCC
A_14_P135907 GTAAATCAGTCATTAATATAACTTTATATTATCAATGCTCACACTGGAGTTCATAAAATA
A_14_P128826 GTAAAAAAAAAATGGCTTTATGCTCCCTGTTTTACATATGCAAATAAGGGGCTGTGTGAT
CUST_180_PI426210822
TTTAGTTAAATCAAACACCAATGCTATAATTTTTAACCGATATCATTTTTAGGGTTTGGT
A_14_P202160 ATGGGAAATAAAAGATCTCATGAAAGTTTTAGTAGGTAAACCTTGTGAAGTCTGTGGAGT
A_14_P139620 CGAGGAAGTCACCATCAAACCACTTTATCTACAGTTAGCATAAGATGTGAGAAGTGT
A_16_P21258057
TTTTAGGGAGGTAAGAACACAATAGGTCAGGATCTGTCATATTAACTGAGGTCATCTGAT
A_14_P133080 ATCCATCTATCTGGTAGACTAGAAGAAGACCAGTGGATATCTGAAGCATTTACTGGTG
A_14_P124365 CAGATTTGTGGATTTGTATACGTATCTGCGTGTGATTATATGTAGGAGTTAACATCAGCA
A_14_P105758 CCTATGGTTTTACTGATTCAGTATAACCTCTTTAATGGCATGCTCTTACAATGCATGGAC
A_16_P02218040
CACACCTTTTCTTCTCTAATCATCTCCCTATTTAACACATCACAGGAAAGATTGCTGAAT
A_14_P133150 CTTTGATGACAGAAGGCAAATAAATTCTACAGCACTCATTTTTAGCAAGCATGGAGTTTG
A_14_P109788 CACAACAAACGAACTCCATCTAAGCGATCAATTATCCTATCGTGGAACCCAAA
A_16_P17809311
TGATCTCCTTTTGATAACAACCACAATAATGGGTCAGATTAATTGAACACTGCAATGAGC
A_16_P17281674
GGCATTGTATACCCAAATCATATTCTGTATCAGCAATTTAAGGGGATTTCAAGAGCACTT
A_16_P17651731
CAGAATCCTTTTGAAGTAGTTTGCCCATCTAATTCAGTTTGTTAATACCTTCAGCCATCT
A_14_P132875 AGTTATTTACAATGGCTTTAAATGACAAGGCCCGGCTATGTGCCAGAAACCT
CUST_6045_PI426210822 TGAATTTGGTTGGCAAAGGCATAGTGGCTCCAAGCAGAGGTAAAC
CUST_8332_PI426210822 TTATAAGAACAGAGGAGTGTGAGCCCCTGTGAGCCCCCACGGGAG
A_16_P15660446
TCCCTAAAGATCATTCCAATTTTAGTCTAGTATACAAGTATCCCATTTAAGAACCAAGGC
A_16_P17430989
GTAGTGCACATTTCATACCTCCAAATGTTTTAAGCCAAATTATAATTGCAGACAGCCCTT
CUST_7724_PI426210822 CTGACCACAAAGATATTCTGTTCCCAAAGAGCACCATATTCGTGGTCAAG

A_14_P121073 CTTGTGGCTTTCTGTCTAAAATTCACCTTAGGCTTGGTAATATGAAGTTCTTTCACCATT
A_14_P200870 TGCTTGTGATAAACTGTCAGGAAGCAATGTAATACAGTTACTAAATAGTGCAGACCTCTG
A_16_P16814360
GGAAATTTCCTTCTCATCTGTATGCAATGAATGATAGCACTTACCAACAGATACCAAGAC
A_14_P127881 ATTCTCTATGGGAGGATGCATGGCAATACATTTAGCATATAGAAATCATCAAGATGTGGC
A_14_P129515 CCAAAATAAATTCATGGCTTTTAAAATGTCATAGAAATGTCTATTCAGGTATCTTTTTTT
A_14_P115684 ACATCATCTCATTTAACCTTGAAATAACTACACTGCCTCTGGATCTTCCATTCAAAGTCG
A_16_P00431573
ACATCACTTTTGATATCAGCATTTACTAGATCTACAAGTTAGACTCAGTATGTCCCTGCT
A_16_P40826529
CTCAGCCTGAACTTATTCAAACTTAACTTACTCGATTTCACATCGTCTTGATGCTAACAA
A_14_P100010 GGAGGGAGACAGCAGTTGATGAGGATGGTAACAAAAACCAAAAAAAGTTACCATTTA
A_14_P116563 TTAACTACTTTATACCAATGTATTACATTCTCTGGATTATCTAAGCAAGGGGCCAGTCCG
A_14_P100934 ATGGGCTTTAGAGAAAAATCTTGACTGTCTCCGCTTACAGAAGTTGTATATGCTCATTAT
A_14_P116956 GAGGTTTGATTATATTTAAAAAATTGTCAAAACAAATTTATTTTTCTGTAGGAAATTGGA
A_16_P15540528
AAAAACAACAACCATTCTTTTCTCATGGACAATCATTTATTTACAAGCCAGTGAAGTTCA
A_16_P58424424 ATTTATAAGCAGGTTACTGCCGTAGGCACCTTGGGCTTGTCTTCCT
A_14_P120451 CTAGAACAAGCATTCCTCAGTCCATTACAAATCTTACACACTTATACAGTAGCCTCTCTT
A_16_P02729255
TTGGCTCATCATCTCATAATCCATTCTCATTGATCTATGCTCCATCCTTATTGGAAAATG
CUST_5736_PI426210822
CCTCTTCTGACTAATTAACAGCAACTAAAAAAGAAAAATGAAACATAGAACACAAAAAAT
A_16_P36230668 GCCCTAACCTTCCTCTCCTGTCTTTCTCACAGACTGTTTTTGAAAGGAATT
A_16_P00372572
GATTATCACCTTAAAAATCCTGGATCATTTCTCACTTACTGGTATTACTCATGCACACCC
CUST_7200_PI426210822 TGCCCTCAGAATGCTACTGAAGATGGTACAAAGCTGAAATCATCAGAGTTG
A_16_P21270561
CCTTCCAATGTTAGTCTTTCAAGTGAACCTACTTTCCTATTAGCAGTCACCTAAAAACAA
A_14_P138134 AATGAAGCATTCTTCTTTGGAATAAGCAATCTACTGTTTCATGAACAGAAGTTGGGTTCA
A_14_P106793 ATGTGATTCAAGAACCCAAACATCTTTTGACATAAAGGGAGAAAATTGACATAAAGCGAG
A_14_P129315 TCTTGTATTCATCATCATCTTCCTCTACTGGGTCTCTTGTTTTCTCTGAAAGAGAATTTG
A_16_P01043879
GGTGTCGTATATCACTAGTGGAGTTTCTTACCTACATTTAAGTATCCTCACTAGCCTTCA
A_16_P17392721
TCGAGTCCCTTCATCATTCATAAACACATTAGGTGCAATTTACTGCTGTTATTCTTGGG
A_16_P03635894
AAGGGCCTATTCCATATTCTCTACACGTTTACAATCTCACAAACCTAAAATATTCACCGA

A_14_P120904 GAACGCTGAAAATAGCACGTCTTTTTCTTTCTTTGTGTTCAAAACTATTTTCTTTCTTCA

A_14_P102875 TTCAGAAAATAGTATCAATAGTATCAATATGTTTGACAGATCCCTTGACACAACTGGGGA

A_14_P111164 AGTTTATTAGTCTTCTCACAGAGGATGTTGATGTCATGGATCACGATTTTATAAACACCC

CUST_4612_PI426210822 ATACCAGGAGCAAGTGGAGCTGGGGAGGGGACTCACATCTCTGTC

A_16_P30952551 TTGAGAATGACTCCTCTGACAGGCCTCTTTTCATTTGCAGGTGGG

A_14_P119856 ATATTTGAAAACCTCAACAAACATGCATTTCCTCTTTCTAACGGACAGGTAAATACACCA

A_16_P21277038

CTAATCACGAACCAATCAGAATAGAGTTCCTCCAATTCAAGACACTTCATGACCTTAC

A_14_P114981 CCATTCTTTTCTATTTCTAAAGCACACTGTCTTCTACCTTTGATAAGATGGCTAACTTCA

CUST_64_PI426210822 ACACTTAGACAAAGGAAGGCTCTAAAATGGCCCACTGAAACATGCAGA

A_14_P102718 CTAAAAATCTGTTGGGGGTCGTCAGTTGTCAGTTGGGATCCCCTTTATAATTAA

A_18_P14134550

TGGTATTATAAAGGTTGCTTTCACACTTACAAAAGTTAAGAAGCATGGTTCTGACTTTCC

A_14_P132642 CTGTATCCTTCTTCAGTTTCATCTAACGTCACAAAGAACGCTGCTTTCTCCAC

CUST_6413_PI426210822 GATTTCATGAAGGCATTTACCCTGGCATTGTACCACTAAAGGCTGATGTTG

A_14_P128860 CTTCCATGTCATGTCTGAGACCTTACAAACTCAAGTTCCCTGTAGGTGTAAAA

A_14_P131989 AAATTTTAATGCATAAGGCACAGTGAGAGGCTGGAATCATTAAGCATCCTCAAACACAA

A_14_P202513 CCAGAAGAGGTAAATGAACAATACTTCTTACAAGAGGCCATTTAAGGAATCTGGTGTAAA

A_14_P102996 CAAATTACAGGAGTCAGTTTGTGTATTTGCTTGATGAGAATGGCTTCTATCACTTTGGTT

CUST_3377_PI426210822

GTCATATCACTCAAGTCATGCCTGATACACAGATGTTTGATTATCAGAGTCTTTTTTCTT

A_14_P122336 TCATCGAGGGAATAGACACGATCAATTGATTCCTCAATTCTTGAATGCAAATCTACTATG

A_16_P19164343

AAAAAGTGAGTTGCCAAATAATGTGTAAGTGTGATCCTCTTCAAGTAAGCCTTTTACCTG

A_16_P16964162

AAAATCAAATTGGCTCAAAACTAACAATCCACAAATTTGTAGACTAAATTGAGGCCTGGG

A_14_P131600 ACGTCTATCATTTCTTTGTCTTAGGAATGTCACCAAATACAAAATGCTCAACTTTCAGGT

A_14_P117624 AGTTTGTGATTCTTTATGATACATTAATGCTTTCATTTTCCTAGGGGCTTGGATGTGCAT

A_14_P139920 TTGAGTGAATGAAAGACACTATGAAAACAACAGGCTCACTATAAAGATTCGTTGTCGTTG

A_16_P15876417

GCCCAGTTACATTCTGCTAGTCAATCAAGTCATCCAATCACAAATGCTTAGGAATTTTAA

A_16_P16191758 TCATTCAGTGCTCTTGTAAAACTGTCTTGCCCTTGTTCTAATTGCGTATTGATCACC

A_16_P16909912

GTCAATTCCCCTAATAAAAACACATCTAGCGCATTTATATTACAACCTCAACCTTACACA

CUST_7705_PI426210822

GTAAGGTTCAAGCTGACTCATCATTGAGTGCTGCTAATAAAGTGTTTGCTGAGAATG

A_14_P123056 TCCCCCCAATTACTACCATAAGTGTTCGTTTTCTTACAGACATAAGTCACCTATTTAACT

A_16_P00993203

TAGCAAGCACGATTATAGATGTAAAGTCCCACATGTAAAATTATATCCTCCTGCTTCCAC
CUST_3400_PI426210822
AATTAGTACAGAGTGAACTAGCTGTGAACAATCTGGTTTTCTTGCTTTGTGCCTTG
A_14_P121182 GACATCAATTTTCTAATGCTAGGCTCTATTTCCACTCATCACCATCATGCAAAATGCTAT
A_14_P113996 TAATAGTGTTAAATTAACAATAGTCACAGTGCCTATGGTGGGTAAAGAGGGTTCTAGTGC
A_14_P201650 GATTACATTCCCCTTTTTATTTTTGATCAGATATCTGCTGTGCTTCCTTCAAACTCTGAC
A_14_P127617 CACCAAGAAAAAAAGTGCCTTGCCCATCTTTTCATACTGAGAAGTGGAGAG
CUST_6471_PI426210822
ACATTTTTTAATATCTTCACTTTGGTGTTTTATTATTGTCAAATTACTTGCAATAATGTA
A_16_P17565199
AGGGATCCATTTCATCATACTGAGCAAAAATTACCAAGAGTAAAGTCAAAATTGGAGCAG
A_16_P18718696
AGATCCATTGTTATACTTGTCACATTAAGAGCCTCATGATCAGATTTTGCCTTGTGCTTA
A_16_P21222091
CAAGTTATTAAACTACCATGGACTAGTGAAGTATGACCCATATCCATATGACCCACCTTA
A_14_P108079 AAAAACAGCATTGCTTTCTGAAATTAGGGCCCAATTAATAATCAGCAAGAATTTGATCGT
A_14_P200591 ATCCCAGAAAATGTTTTAATGATTAAAATAAAACAATTGCCCTCTCCTCCTTGGAGCCC
A_14_P122379 TTTCTGAAATAGAAAAACTGGCATTCGTATCAGATAGGTTCATTCTATCTGCGAGATGGT
CUST_4747_PI426210822
TGAAATGAGGCTTTCAGTAAACAGAAGATGGGCATTTCATCCTTTCTCATTACTGA
A_14_P126968 GGTGAACCAATGAACTGCACATGTCGGTAGTGATGAGTGAATTAATTTCTGGTATATTGA
CUST_9777_PI426210822 TTAGGTTCACCCACTGCCTCAGACTGGAAGCTGGCTTCTGGCCAC
A_16_P35296899
AGATCCTCTTAACCAGTATCAGTATAATTGTTCCCTTTAGATTTATTGCTGTCACCCCAA
A_16_P16921234
CTGTCCACCAATAATTTTTTTCCAATACAACCAGAAGTAAACAAATCACAAAAGGGGTCT
A_16_P35557422
AGCCAAAATCATGTTTCTTCAATAAAAATCTTGCAAGCATCTCTTCTAAACACAAAACCA
A_18_P16745676
ACCTGATTTCCATACAAGAACATCACAAAGTTACATTTCTTAATCTCTTAATCCCATGCA
A_14_P105839 GAAAACAATAGTCATAGGGCCTGCTTTGTCCTATTTAACTATAAATGTTACACATTTACA
A_16_P02109098 TGTTGTTAGTTGGCTGGTCTTCAAAACTCTCATGTTTAGGGTTGACAATGTTGG
A_14_P113222 CAAATTATTCAGTGTTCTTGCAGTTTCACTGAAACTCAGCCAATATCTGATCTGAGAACA
A_18_P12275458
CTCTTCTCTCCGTGCATTATATTCAAACATGTAACTCCAAACAGAACATACAAGCATAGG
A_16_P00072251
AAAAACTCACGAGTCCAATAATAGATATCTCCACCGCAAGAGATATATTTAAACTCCCCC
CUST_7273_PI426210822 TTAAGTGTCCCAAGGCCAACCCTGAACCTTGGAAGCCTTCATTTA

A_16_P19897871
AGCCTACTTCAATATCCATACAACATATTATCTTTTCACTAACCCATCCAAATTTAACCT
A_16_P37991211
AAGCACCACTGAAATTTCGTAACAGTGTTGATTTGTCACCTATATGTCTATACCCAATGT
A_14_P200831 ATGCATTAAGCCAGTTCTTTGCAACTGAAAATTACCTGTTTCTCCTTCCCTTTCAC
A_18_P25583551
AAAGAAGTTGACATGTTTTTGGAATGGTCATACTAATTGCGTTGACATAGTGTATCAGGG
A_14_P111132 ATAGACAGAACCAAAACTGAACAATGAACAATAACAAGCATCTAAACCTCACTGTTCCTT
A_14_P106095 AGGTTTTGGAGAGACTTATGATGAAAGAGAAAGTCTTTATAGGAGACAGTCCTTGGTAAG
A_14_P134414 TGACTTGAGGCAATATTCTACAAGTTTCTCTAGTTTCCACTGAAACTGGAACATCTGTAT
A_16_P58424194
CTTATCCCTAACATCAGGGATATTTCCTGAGAACAAGGACATTCTTTTACGTAACCATAG
A_14_P104497 TCTTTGTCTGTAAGAGAATCCACATCTATCATTTTGGTCTGAATTGGAACCAATGTTAGA
A_14_P102937 AAATATCGAGACATTTATCAGTGCTTCCTTTCACCAGTATGACATTGTGCTATTGTTTGC
A_14_P202200 ACTTCAGCAGGTTACCTTCAGTTATGGAGTCTGTAATACTCTGTGTAAGAATCATTTCAA
A_16_P40184418
TGCTTTCCTATTTGCTTTTGACTACAATGCTTGATTATTAACAAAACAGTCTCTGTGAGT
A_16_P15224602
AAAAAGGGCATATTAGGACCACAAAAACATGAGAAACCTAATTAAACAGACATGTAGGCC
A_16_P20946317
CTGTGCTTACTCCTTTTGAACTGTGCTTAGCAATCAGGATTTCACTTAATCAGTTCAATG
A_14_P131925 TAAAAAAGAGATCTCACCACTTCTTGTTTCTGGGCTTCATTAAGTTTTTCAGTCAATTCT
A_14_P131562 CTCAGAATATGAGTTTCAAAGACCTTTGGAAGACCAGAACTTTATGTCATATCCCTACTT
A_14_P125364 TTTTCAGGCTATCTTTGTCACGAGTGAATTGTTCTCAGTTTCTCGGTTTATGTGCT
A_14_P108693 GACCTCACTCAAATATTTACATCTCTCCTAATATTTAAATGATGAAGCAGGCTCCTGCAC
A_16_P03618825
AAAACTGGCAAGAAATGTTTTCCTGATTTAAAGCCAACTGTCTAGAGTGATGACTGTTAG
A_14_P106968 GACAGTGAAGCCAACTGTCAAAATGAATTCAGTCCTAAAAATTAAAGTATGCTTGCTGAG
A_14_P118608 TGCATATTTGGGAACAGCAAGCACTTAGTTTGAGAAAATGAGGACTTAAAACAGTTGAAT
A_18_P14129359 GTTTCCATTTTCATATCCAACAAAATGCCTTGGGACCATGGCTTCAATCACATTACAT
A_14_P101685 GTGCTTGTCATAGTGACACTCATTCATTGTGGAGAAGTGAACATCCTAGAAAGAAATTTA
A_14_P116989 GCGGTCGTTGGTATACATCATACATTTATTAGTGAATATCCCTCTGAAATCAGCAACAAT
A_16_P15857748
AACAAGAATTGGTAAAGACAATGATAGTGGCAATGATAGAATTATGAAAGTCCGGAAGGC
CUST_1182_PI426210822 ATGGTGGAAAGACCGTGGGAGGGGCTGTGGGTTGAGGACGGCTCT
A_16_P15103836
TTCCTCTTTTATAGCCCTTGTCAAATTCCAAAGCAAGTCGTAAATTCATTAACCTCGTAG
A_16_P01783538

TGGCATTGAAAGTCAAAAAATAAGATTACCCGTTTCTCTCATAAATTATAGGTCCCTCGT

A_16_P18023254

AAAAGAGAAACATGACTTTATCCATTTGCAAGATAAGAGATAGAAGCCAGATAAGAGGGA

A_14_P120286 CAGACTCATTTGGTAATGAATATGACATAGATCCTTTCCTCAAGGATGCCCTAGTCTAAT

A_14_P123822 AGTCAGTTGGATAGATCTGGATTCTATTCTTAGCAATTCTGTGATCTTGGGCAATTTACA

A_14_P109074 AAACTAGGGAAGAGAGACTGAGTTATGGAAAGGATGCTACCATTTTTCTCAAAGCATG

A_16_P19669692

TCGTAACCAAATTTTAGAACCAACAACTCATACTTCCTGCTTAGAATGAACCTTCTCATC

CUST_9817_PI426210822

TACTTGCCTTTAAAATGCTCAATTTAAAAAATAGTAAAACGTAGAAAAGAGAAATAATAT

A_16_P02508104

ACTAGCATGCTCTAATTTTTCATAGGTGGTTGTTCTTATGAATATTGATCTGCTTCGGCA

A_14_P129124 AGGACTAGAGGATTACTTTAGTGAAGATTCTTAGGCAGTTTTGTAACCCTTTTTCCTCAA

A_14_P106807 ACAGGACTGGTGAACCATCTCTACACGATATTCATATCTAAAATTGAAAAACAGACCATT

CUST_3365_PI426210822

GAAATTTCTTCCATGGTTTGTTTATTCACTTTTATCATGGTCCATTTTTACTCTAGGAGT

A_16_P16403259

CCTGCTTTGCTGTGAAACACCTCAATGTCATGACTTCCTCTGATATGTTCTTTAATTAAT

A_14_P129022 TCAGTGCATATCTATCCTTCATGTGCTAACTTGTATAAAAGGTCAGTTAACTGCTGAACC

A_14_P112572 ATCTGCTCTTGCTGGGAGTCAATGTCAAATACATAGTCAAAAGTAAAAGCCTTATCTTTC

A_16_P37425649

TCCTTCCATCTCAATTATCCATCACAAATAACACCATAAATCTTCAAACCATGTATTCCA

A_14_P138733 TTCTGTGTTATGATTTACATTTTTCTTTATTTCAGGTAAGCCAGCATGATTCTATTTTTG

A_16_P17658044

TTTAGTGCCCTTTAAATGCAAAGTGACAGAGTATAAACCGATCCCACAGTATAGTAGATG

A_16_P17966172

GTAGACTGTTATGGATAGATAAAATGCTGTTCCACCAATTTTCAGAAGGATATATTGGGT

A_16_P02553251

GGCTCATTTTCCTTTACATCACACTGACTGTCTAAATCATCTCCTTCACTTACTTGAATG

A_14_P125677 GCAAAATTACCTTTGCTTCAAATTCCAACCTTGGAAATGTTTCTTGAGTTTCTACAGGTA

A_16_P17026183

AAGAGTTCTCAAAGCATAAGGTTACCATATGGGCATCAGAAGTTTTATTCATTCCAAACC

A_14_P126659 ATGTTTTAATAAGAGTTGAACGCACAAATTGGCTTCCTTGCAATGTTTACTTCAAGCAGT

CUST_3355_PI426210822

CTATTAATATATACTGTTGATGCCCTTTTAACACTGGTATTCTTTGAAGTCACACATGAT

A_14_P129984 TTACTTTATCTTTTACCTTTTTTCTTAAACATCTGAAGCAAAGTCCCCACCTGCCAATGC

A_14_P114411 GGCAACAATTCCAAGTGTTTTTACAGTGATTATAGGATGTTCTTGTCTTCCTGTGCAG

CUST_462_PI426210822 TTGAGTCTTTGTAACATCCTGGTGAGTGGTTTCCAGTTTGCTATTTGGG

A_14_P108557 TCTTTGTCTTATGCACGTAGTCTGCCTTGTTGTTGCAAATAACATTTCTGCTATAATCAC
A_16_P03147056
ACTTTTGCTGTGCAATCATACTCTTGTAAAATCTGCTTTTCATATCCCTTGAGTTGTTGC
CUST_6955_PI426210822 CTTACAGACAGTCTGGGCTTCCACTGCCCCCATGCTCTGCCTGTG
A_16_P02119608
AAATGTGTCGTCTAATCACTAAGGTGTCATGAACTCAAAAATTTAACTGGCAGTGAAGAG
A_16_P01306371
AAAACCCTAGAACCTCATTACTGTGGATAAAAACATAAGCCAAAACTTCTTCCTAGATCC
A_14_P139766 AAGGGAATTCTTCCAAGTTACTCCTTCCAATGTGTGAAAACTTCATTTACTTCCTAAATC
A_14_P139509 AATCTAAGTGTCACCACATCTCAGAAGAATATCATGTGATGAACTAAAGCACTGCAAATT
CUST_6241_PI426210822 ATACTTTTCTTGTTGTCCTGCACACACCCTCTCATCAGCACCCCT
A_16_P17991716
AAAAACTAGAGCGAAAACTAGAGGAATGTCACTACCCAGAAATACTTTCTAGAAGGTCAG
A_14_P111869 ATTCTGGGTATCTGGCAGATGTGTTGCAACACATAACAAATATTCATCATAGAGCCATTA
A_14_P129769 ATAACACGCACAGAGGCAAATACGAATTTCACCCAGGAATTGTGCTCAAACATTA
CUST_9533_PI426210822
TACTACAAATGCCTCATTCTGTTATCACGTTGAATTCTTCAGCATCTTCTATATCATTTC
A_16_P16768771
CCTCTTTCCCTAGTTAAATCTCTAATGAAGATCAGTCCTAACATATTACAGGCCTCAGAA
A_14_P121558 AAATATCCCTAGTCATAGAGGCATGTAAGTTTGGAATTAGAGATGAACAGGATTTGTGGA
A_14_P120238 TAGAACCTCAGTTCTTTTGAGTAAAATTGATGAAGAGACCTAACAGAGGGTTATCAGAGT
A_16_P20454595
CCTGAAAGCGATCCATAGTTATGTTTCTGATTTAAAAATTGTATCTTCCACATGAACACG
CUST_9306_PI426210822 TTTATAAAGGACAGTGTTGGAGGAGGACCAGGCCTGACTTAGAGC
CUST_5378_PI426210822 AACTTCACACAGAGAGTAGTATGAAGTCCTCATCTCTGCACTTGTGGC
A_14_P106280 CTCCTCACCTCCTTAAGGTTCCGGAAGAGAAGGTCATTGTTTTTGTC
A_18_P22842114 AAATCTTTCAAAGCACTTTTTTCCTCAGGGCCAAGTGGACTCTTTGGG
A_14_P119637 CTGTTGCAAAACACTGTGCTTTCGTATCTTTTCTTTCTACCTGTTTGGAAATGTGAAAAT
A_14_P200518 CTCTGCAAACAATTTCTGGTTACTGAGGAAGATATCAGGAAACAGGAAGGGAAAG
A_14_P112963 GCCATTTGACTGGCAAACATATTCATTACAGTTTCAAAAGAAAACACCCTATTTGACTCA
A_16_P21629293
AAACATCTTATCACCCTTCAAACTGTTTATGTCTGATTGAAATGCTCCATGTGATTCTGC
A_18_P22842110 CACTAGTGTTGATGAAGGACGAGGGTCTCACCTTTCCAGTCCTTG
A_16_P15242657
AAAAACTTGGGATAAACATACAGTCAATTCCTTCAAATGACCAATTGGATTAGATGCCCT
A_16_P58424493 ATCTGTTGCTTCCCAAACGTGTGGCCTCTGGTTGGTTTCTGGAAC
A_14_P103418 TATACATGCAAAGGAGAATGGGCAAATCCAAATCAGAGTGAAGGATTTTAATCAATGTCT
A_14_P116220 ACATTCAACATTTCTGTCATTTTTTGTGTGTGATAATCCCTTCAGGTCTATCTTTTCAAC

A_18_P14132146 TTGTTCAGAGGGGCAGAAGAGAAACTCCTGTGAAGGGAAAACATTTAGAAG
A_14_P120120 CTTGATTCTATTAATACCAAGCCTGTGTGAATTGGAGTGGAAAGCAGAAGTGCT
A_14_P110504 CATTCCAAATGAAATGTTTTTAGTCCTTGCAGACAGCCCTAGATAATACTCACAGTGAAA
A_16_P37151257 TCATGATAAAATATTATATACTTCATATGTAAGCAGGCCCTCCTAATTATATTAAGTTGT
A_16_P19913867
AATCAAACACAGATTAAACACCCACATAATTACTTTTCGGATAGTTTCTTTGCCACTGCA
A_14_P137700 GGGCTCTTTGCCATGGGAGATTTTCATGATTATGAGTTCAAGTATACTTACCTTTCTATT
CUST_4423_PI426210822
TTAGAACCAGAACAACATTTCATTATTTTATGTTAAACGGACTCTCCTGCCCGGA
A_14_P137799 GACGTAAACACCTTTGATATAACTGTTTACTTGTTGCAAGGGAGAAGACTCCTACAAC
A_16_P20888592
TACGTTTCTGACTCTACCAAAAATTCACACTAATCAGTTGATGAAAAAATGCCCATTCCT
A_16_P16665049
AAACACAAACTGTAGGTATATTTCTAAGGAACACTCACAAAATTTCTCAGCAGACATGGG
CUST_9372_PI426210822 AAGTGTAAGCTCCTTGCATGACAGGCAAGCTGGGTAGATGTAGGT
A_16_P17431850
TTTACATCTTTTTGCCCACGTATACCTAATCCTACAGTTTTCTACATTAAAACACCTCGG
A_16_P00719701
ACCAATTTAGCCACATATGCAAATAGGAAAAGGTCTCTCTTTTACTCTAAGTCCCATCTT
A_16_P19624355
TTTAGTCCTGGTCAGATATCTCCAACACCTTTACAGACTCTTTACAACTTTCTCTTTCAT
A_14_P200354 GTTTTCGACACTGGACTGGAAGAACAAGTCTATAAAAATAAAAATGGACAATTAACAGTG
A_14_P136402 CCAGCCTACAAAATACTAGGACCATGGGCATTAAGAGTTCTTAAGAATATATACTTCTTC
A_14_P123883 CTTCAAGCAAATACAAACCAGATCTGAACACATGTGAATATTTGGGGGAAACGTCAA
A_14_P138998 TTTTTTGCATCCTAGTTTGAGGGAAAAAACTAGAATTTGGTTGTTGTAGTGGGGTTATTG
A_14_P122638 TCTTGGCAGCGTAACATACAAATTAGTATCTCTTGCTACTTATTCTCCAAGGAGAAACTG
A_16_P36231015
CCAACAGCAAAAAAAGTTTTTGTTCCCTACCATGAATAAAAAGTTGAAACATTCTTGAAC
CUST_4535_PI426210822 GGTCCAGACTTTCCTTGCACACAAAGCCTAGGCCTTTCCTATCAG
A_16_P21612731
TTCCAGATAGTCTCTTACATCTTATCAGCCTGAACAATATTATTGCCTGCATAGATTCCC
CUST_9783_PI426210822 CTGTTTGCCCTCCTCAAAGGCCCCCTTGAGACAGATCCTCATACT
A_14_P126254 CATCATCTATTCGAGTCGAGAGTATTCAGTTATCAGCCTCAGAAATCCAGAAGGAAC
A_16_P00685220
AGTTCCCAACAATTAGATAGGAAATGAACTGAATGGGAGTTCACATTTTATCTTCAGGAA
A_14_P121994 TGTGTCAATTATCTTTGGGGCTGCTTTTACATTAGCCATGCCTACAAAAGTAAGTAAAAG
A_14_P132547 GGGCCACTAAACCAATTTTAAAAGGCAAAAAATTTGGGCCATTTGTTGGTGATAAGAAAA
A_14_P131491 ACCCGAGAAATGAGTTTTTGTTTCTACAATTATCATCTCTAGGCAGTATAACTATGGGAG

A_14_P105047 GCAATAATTTTAGGACAAGCAAAAACCTACATACGAGAGGAAGTGGAATTGCCAATATTG

A_14_P129376 ACTGTTAATGAGGCTGGAGACTTAACAACCTAGTTTTAATTCTGACTGTATGTCTCCAAA

A_14_P100927 AAGAAGTCTAAGTTTTAAGGCTTGTTATGGAAAATTGGCATAAATCTCCCTCGCTTTCAA

A_14_P125022 CCTTTCAAATGAGATAATAAAAATTGCTTTTCAATGAAATGGAGGGAATTGTGGCCTCTC

A_16_P21663335

TTTCTGTAGCAGTCCTCTAACAAGACAAACATTTTTCAAACATGCATCTCCTTTTAGTCC

A_14_P102031 CCCAGATTTTAACACTGCTCCTAGGAAAAAAAAATATTTCCTTTTATGATGCCACTTCCT

A_14_P106667 CCGGGAATGAAAAATGGTTTTAATTCCCTCCCAGCAGCAATAATTTGATTCTTCATAA

A_14_P109711 GGCATAACATGTCCTAGAAAAATAAAGATTTAGGATACAAAGGAGACACAGCGGCAG

A_14_P127294 TTGCAAGGAAAGGAACATGTTTGAATTTCAGATGTGTTCCTCAATGAATCACGTAAGTTT

A_16_P17126418

AGGATTTTCATTCGCTAACTCAAATTGCTAGTCTTCTAGAGTTTTCATTTGCTACCAAGT

A_14_P110944 ACTTACATTCTCAGGTATGCTGGGAAGTTCTCTGGTATCAGGCACAGTAAAATAAGAAT

A_16_P20889854

AGAGGTATAATGTATGAGGCCCTGTATAGATACTGATTTTATCTTTAAGCAAGCGGTTGT

A_14_P137517 GACCTCGATAACTCTCCCCATTGCAGGTCATGCATTCAACTAATAAAATTAAAGTATGG

CUST_3173_PI426210822

ACTTTATAACCATGAGAATGTTTATTTCTGGCAGTTACTGTCTGGGCTGTAGCAGA

A_14_P104699 CATTCTTTATAGTTTTAAAACGTTTCTGAAAAACACTGCTTTCAATATGGCCAGAGGCAT

A_14_P121242 TCAAGATGGTAATCTTATTACCTGCTTAAACAAATCCCCATTTAAACCACCAAGAGGTTT

CUST_6559_PI426210822

GAATAAAATTTCTTGAAGATTAGAAACACTGTTCCTCAATAAATAAGCACCCCTGGAATG

A_14_P136591 CTCTTCTTTGTATTTGTCATTGCAGATGGCGTTTAATGATTGCTTCAGTTTGAACTACCC

A_14_P130214 AAAAGCAAAATCCTCTTATCTTCTAGATATGGGAGAAAGTGACATAGGTTCTAACCAATC

A_14_P135470

AAGGAAATAGAGAAATTGAGAATAGAACTGGATGAAAGCAAACAACACTTGGAACAGGAG

A_14_P101418 AAAGGGAATTATACAAGGATATCTTTATGAGTTGCTGCCAAATTTTGTTCTAGACCACGG

A_16_P18542061

CATGTCTTACCACTCACTATGTTTCTTGCATCACTGAATATTGAGCCTTGGATACTTTTG

A_14_P121623 CAAAGCACTTTTTTTTTGACATTTGTTTTGCCTGCTGCTCTAAGATCAGACCAGACAT

A_16_P20087750

TGTGGTCTACAGTGTCTACTCCTATCATAAAATATGCAACAGTCACAATAAACGATGTTG

A_16_P19076048

ACAATTGCCATTTAATTATGAAGTCCATCAGTATTGACAGAAGACGTTACAGTGAAGTGC

A_16_P16191986 GTCTCACAGAGGAGAGGCCTCTAACTGGCTAGAATGTAAATATGTGTCCTG

A_14_P104457 CAGTAAAGGACTGAAGGTCAGGATTCCAGGCTCAATATTCTTTATTGATAAGCATTAGTG

A_14_P119952 GGCTGTGAAAACAAATCATTAAATAAGTGAGAACTGTTGGTAAATGTTGAGTCCCACTAG

A_14_P202168 CACTCTCACATGAATGCTAGGGTTTGTTTAGTAGGAAAGGAAACTTCTTAACCAAGCATT

A_16_P58424503 TAATTATACCAGAATGACAGAAATGAACCAGGACCGTCCCCAGGAAACC

A_16_P18467017

AGACTTGCAAGCCATATACTGTTACTATACTTCATGAATTCTTCCCTCATCTATTAGCAA

A_14_P112797 TTTTCAAGCCAGACAGTCTTCAGCAAAGTGCTCCATTTAACATCGAAAATCTTTATTTCT

A_14_P124389 ACAGAGAGGGGACACATTTTCTGTGAAAATCTAGGTATTCTTTTACACATTATAACTCCG

A_14_P138407 CTCAGGCACTTTTAGGGTGAGGAACTTTAACTTTCTGACCTGACTTAAAATGTTACTAAA

A_14_P118283 TTATAACTAAGGTGTAGAGTTAGGTAAGGGTGGTCAGAAAAATTTGCAGGCTAAGGAAAT

A_16_P16258544

TAAAAAGTGACCTGGATACATACATATCTCTCTCCCTGAAATATTGCTAGAGCATACCCT

A_14_P130241 TGGATAGCAGACCTTCAAACAGAATTCAAGTGAACTCTGTAGATGGACAAGGATATTTAA

A_14_P125430 CTCCAGAATATTTTTTTGACCTTGTCCACATGAAGTGAAAGGTTTTTGAACACTTCTTCT

A_14_P124371 CTGTCTATCCCTAAATACCAATGCTGAAAAATATGTAGGCTTATTCTAGTGAAGGCAGGA

A_16_P00127260

CCTATCATCTGCACATAATCCTATTCTCTCCATTATTGTCTTAATACCAAGAACAGCACA

A_14_P110356 GAAATTTCCTAATAGCAAGACCAGCGAAGCGGTTGCACCCTTTTCAATCTT

A_16_P18351192

AAAAAGAGTATTTGATCAGTGACCAACAGTTAAAACAGACTATCACTAGGAGGCTTAGAA

A_16_P00806830

CTCAGTCCTATCTATTCTGTGATTCAAACCATCAACATTAACTTTGGATTCCAACAACCT

A_14_P131934 TGTTTATTCATCAGCAGGGCTATCTTATACTGGTCATAGTTTTCTATGGCTCCTACTCTT

A_16_P16309102

ACACCTTATGGATTTGTTAGCATAGTATCACCAACAAAATCTAGAACACACTAAGAGGCT

A_14_P117817 CTTTTTGAAACTGGCTCCTATTTCTTCTACTTGGATGGGGAAAATGTTACTCTGCAG

A_14_P136057 CTAGGGGAAAGGCCCTGCCAATGTTTAACTTTTAAAAGCATCTTATCTAAAAGAAAGGC

A_16_P19485824

AACCAAGAACAAGTGATTTGCTATACACAAGTAAAGGTGAAGGAATTTCGAAATGTGGAA

A_14_P112672 CTACAGCCATGCTCAGAAAAATACCATATACACACCATGTTTCTCACAATTTCAGAGAAT

A_16_P38153938

TAACATGTAATCTAGTCTTTGCTCTACTACTAATTGCTGGGGAATATGATACTGGCTGGG

CUST_1321_PI426210822

AAATTGATGTGTTAAAAAGCTTTGAAAGACAGAACTTTCTCAAATGGGTAATATGAAGGG

A_18_P21172437

AAAAAAAAACTGTTAATATTTGAATGACCTAGTTGAGCAACTATAACAACCGGATGAAAA

A_14_P117143 CCTTTGATCTCATGACCATTTGCCTTCTGAACATACTTTCTTTTTTTCTATTTTGGCCTA

A_14_P100232 ACAATTCACTCAACTGCACTAATTGTTTCTTCATCTATAAACCGAGTCCTTGTGCCTAGA

A_14_P104834 TGTGCATCCTTATTCTCTATATTTTTCTTCCCCTCCCCTCTAACTTCTTCCTTATAAACT

A_14_P201951 CTGGGCCCTTAATAAAGAAAACAAAGATTATTAAACATTTTTCAAAAGTATTTGGCGGAG

A_16_P19220206

AAATGCAAAGACTATCCTCATTTCTCTTAGCCTCCAAAACAAAGTCATAAATGTTCAGGC
A_16_P17919161
TGCCATTTTCTCTGAAAACTATCACTGCTACATCCAAAAGACATATGTGACATTCGAAAG
A_14_P118715 TTTTGCAGAGTTCTAAAGTTCCTGTTGCTTCAGACAATGGATGAGCAATCACAAG
A_16_P01731850
AAAGACACATCTTTCAAAGGCACATGTCTAACATCATTCTCTTCATGAAACCTTCCCT
A_16_P16170563
ACCAGCAAGACCTTCTATTCTCATGAATTCTTCGCTTTCATTTTTTAATAACAAGGCTGC
A_14_P127483 CTGCTTGACGATATCTTTCCACAATAGTTTCCAAAGTGAAGTTTCGAAACAGACCATTG
A_14_P119756 AATTGTTGCAAGGGCAAAGATAACTCTTAAAAAACCGTCGAGATTACAATGCTCTAGAAT
A_14_P128634 GCCCAGTCTGATGCAGTCAGTAAACATTTGCCAAGTGAGTGAGTAAATGAATC
A_14_P112451 ACTTTTTCTTTCCTTCTCTTTATATGTTTTATTATAGGAGTGTCACAGGGCTCACTGCAC
A_16_P03497989
AAAATTTTTAATTGTTCTTACCTATACTCTGGTAATGCCATCGAAAGAAAATTCGTTCAG
CUST_645_PI426210822 ATGCCCTTTGACATGACGGAGCTTTCACATCAGAGTTCTCACTTCA
CUST_192_PI426210822 CGTGGCTACTATTGTTATGAACAACAACAGCCGGCGTTTACTGAGCA
A_16_P16191171
TTCTCCCTAATTGCAGAAAACATGATCCAGTTTTATAGTTTTTCCTTCAGATGATTGTGA
A_14_P116472 AGGGATGGCATAAACAAACAGTTAGCATTCCTACACTCACTGGATTTAGAGAGCAT
CUST_9742_PI426210822 GAATTAGAAAGCCCAGCTCATTCCTCTGCAAACTCTGACAGAGAAAGG
A_16_P30977912 AAGGGCAATGTCATTTTCAATTATTCCAGGAGACGTAAAATATCGGTGGATAACGAC
A_14_P139536 TGTGTTGTGGTTCATGATTTTGAGTTGCTACTTTTATTTGCATTTTCTCAGGCTCTCAAT
A_16_P17387409
CTTCTCCATGTCCCTAATCTTAATCACCCAGAATTACACATTTAGAAACACTTCTTCAGT
A_18_P22841756 GTTTCTAATTGCTGCACCCTCCTCTGCCTCCACCCATGCCTCAGT
A_16_P16652385
GCCCTTCTTTTCTCTACTTGACAACTTCTGATAACCACATGCTTACAATTTTATGACGTT
A_14_P108625 GCAAGTTCCCTGACATGCTTTATCATCTTCACTGGGTTATTTTCTTCAACTTTAATTCTC
CUST_3245_PI426210822 GTTTCTGGTTTATGTGTTCTTACCTGGATGACAGCTGTGCTCATCCTCTAA
A_16_P20329259
AAAAATCTGAAAATCTGATATGCTTTGGAAATCAAAGGTAAATTTTATGTCCCCTGGGGG
A_14_P104512 TTCCCAGGCAACTTTTCAAGCCCACCGATAATCAAGAAACTTAGTTTTATTTCAAATTGT
A_14_P116932 CTTTGGCTTTGAGAAGTGTTTTTAACAAACTTTCCCGAAGCAAATTTGGTTTTAGAAAGT
A_14_P110519 ATATTCCTGTCTAGTCACAAGAGGTCTAATCTGTGTATGGCAGTGTCATTGTTTCATAAT
A_16_P16456961
ACTGCATCAATCCTACATAATCAGTCCTTCTGAATATCATAATGTTCCTGCTGCTATTCC
A_16_P16191089 AACTTTCAGCATTTCATGACATTTGAGGTCTGACTTTCCTTCCCATGTGGT
CUST_5932_PI426210822 CTCCACAGATAGGCCCAGGCCCAGGCACAGCCAGCAAGCTCTCTG

A_14_P111493 AATTGGTTGAAAAAAATATTTGATATAGGCAGATTCCATTGGAGAAATTCACCTTCCCCT
A_16_P17186205
AAACCTAGTTTGCACAATGCTAATTCACAAACATTTTGGATTTCAGTTACGGACCTCTTT
A_16_P16191220
CTACACCTGTGTAAAAACAAACAGAACCTGCATTAGGAATGGAAGTTAAACTTTATCCAT
A_14_P137548 TATACATAGGGTAAGCGCAGCCATTTTGGATTTTGCAGTTAATGTCTTATTTTGGCTGTG
A_16_P01408362
ACAGTTCATGCTCTGTAAACATATGTCGAAAATTAATTCTATATGCTACTTCCCCAAACA
A_14_P118993 TCGACCTTACTTCTGGTGACTATTTCCTCCAATCCCTGTTAAATAATCTAGTGGGTATT
A_14_P115423 CTGGAAAAAGAATCACAATGCAGGGCATTACTACTTCTGTTTCTCTGACATAATAACAGT
A_14_P112601 CAAAGAGGCATCTCCTTTTAGTAATTGTGAGACTTCAATTTTTGCACTACTAAAGTCAGT
A_14_P105760 TTTTGGAAACAGTCTTAGTCTTTTAACTTGGAAAGATGAGGTATTAATCCCCTCCATTGC
A_14_P106869 AAATTTCTTGTTTTTTACCTGATTAAGATGGCAGAGCATGGAGTTTTCTACTTAGTTCCA
A_14_P124203 CTCGAGCCCTATCTTCTGCATTGGTTCTTATCTCAGATTTATTCTTCCTCCAG
A_14_P104266 TTCACTCTCATCCCATGTCATTAAGGGAAGGATAGAGCAATTTGGAAAAATGCTTTTAAT
A_14_P121783 CTACAGTTCAATCTCGAATGTCTTTTGCAGGTCGCCAGAAAAGGGGTT
A_14_P132599 GATGTCTTTTTGCCTTATTTCCTTCTCTCTCCTTCATTCATTCTCATGGTCATCATTTAG
A_16_P01105355
TAGATCCTAACTGATTGCTTTAATTGTGCACCTTAGCAGAAATATTACTTTCCCCTCGTT
A_14_P126141 TCTAAATCCTACCCATTTTTCAAGGTCCAGCACAATCATTGCTTATTCTTCAAAACTCTT
A_14_P135863 TATTTGGAAGAGAAAACATGCCTTTCTTTGTTGAATCACATTAGTATGATGAGTGAGTCA
A_18_P18218993
GCAATCCTAGTAAGTTGTGTAGTAGTTTCTCTAACTAGAGTCAAACATGACAGAGTCACC
A_14_P100000 TTTTCATTTGGGTCCTATTATTGTCCCCAGAAATTTCAGCCTCATGACATTTTACAAATC
A_14_P119458 GCTTCAGATGATCCATCTTCAATTGAACAGTATGAGAACACCAGAACATATGAACTTCAA
A_14_P122553 GACACAGTTTGTTGTTCTGGCCCTGACATGAAACAGGTAATCAAGTAAACGTATATTTTA
A_14_P113439 AAGACTCTTTATGAAGAAAAGGTTCTTCAAAAAGAGGGCAGAGTCCCCAGTATGTAAA
A_14_P105585 CAGCAAAGATGAGATTAGGGGTTTCTAAACAAAGAGAAACAAGGGCATAATATTCTCACA
A_16_P16105221
AAAGTGCCTATAAACCATTTGCTGATAGAAAGACAACCTCTTGAATACAAATTGACTCCC
A_16_P15665387
TGCAACCGAGATCAAAATTATAGTAGACTCATTCAGACACAAAGAGACAATAGGAGGAAA
A_14_P200748 AAAGAGACTCTCCTCTCTCCACTCCTATAATTATGAGGAACTTTTATTCAACTCTGAAAT
A_14_P116868 AGAGAGGAGAAAAGGTATGAAATGAGGAAAGTGTTATTTGCCACAGGAAATATTGGATTC
A_16_P19752461
AATTATGCAGGGGATTAGACATATTATGAATAGTGAGGAATCTGACAAATCTACGTGGCC
A_14_P112791 ACACTGGAATCTGGTTTTGCTACATTCTATTCACAATCCCAAAGAAATGCTATTTCAATG
A_14_P102579 AAAGTTGGGTATTTTGCCTGCAATCTGATTGGCTGAATTTTCATCTTCATTGTCATCAG

A_16_P21521049
GGGTCTGTATTGATGTCATATAGTTCAGCCTATCACTAGTTCTTTTGTCCAGTCAAACAA
A_14_P106539 AACTTATATCCCTTCTTGAAAACATCCCCAAAGATAGTCTGTCAGAAATCCTTCGGT
A_14_P136422 AACATCTTGGTTTGAGGAACTTGGATCTCTCTCTTTTAAAGGCTCAGTAAATCAAAATCA
A_14_P118831 GGACCTTCTGGGAGCAGGTGCTAACATAGTGTTCAGAATCAATATGTGAGA
A_14_P138477 CCTTTTGTCTAGTGAATATAAAATGTGTTCCAAATATATTCCCTTCCTCCTATTGCCTGC
A_14_P117768 CCTTCATATTTTTCTTAACCCTTCCTTTCTTCTTTAATTTTTTCCTGTGGGGGCATGTT
A_14_P130577 CTTTTAAAAGTGGGAAAATAGTTACCTCTCCGCGAGGCTTCAAAACTATCAAAAAGGTTA
CUST_407_PI426210822
AGGATCAAAAGGAGAGGTAAGAAAAAGAAAAAGAAAAAAAATCAACCCATAGCATATGTG
A_16_P18405034
AAAAATAAAATGGATCACCTTACAAAAAATGGATCCTCCTTCTTCAAAGACAAATGGGGC
A_16_P03286229
TCTATAATCCATTCCCCTTCTATGTATGTCCTCTCCTAAGTAAATCCATCATTCCAAAGG
A_14_P101827 GGTTCAAGTTGATGTGACTACTTTATTGGTATCATCACATCAATTTGATGAATGGTCCTG
A_18_P13304203
ACTATAAAAAAAAATCTAACTCATCCTGATCATTTTAAAAAAGAAATTATTGCAAGTAGA
A_14_P106824 AATATTTTAGTTCTTCCGTAAAAACACGCCATCTACGTGTTTCCCATCTTCTAAGACACT
A_14_P112570 ATTTTAATGGTAGACTGTAGCAAGAACATGAAGTAGGTCAGTTGAAGAAGATAAGCCTGT
A_16_P36158302
GGTCTTGTCCAATACTCATCTAGTTGCCTCCATTATTTATAATTCTATGACCAGATGCCT
A_14_P125200 GTGTTAGACATGCAATTATCATAAAGACTCTATGCGGTGGATGACCAGATCATACAAATC
CUST_7648_PI426210822 CTTTTAGTTTATCTGATCTGTGCTCCACACCTCTTCAGACTCCATTTCCATGAAT
A_14_P105236 AGAATATGAATTGGATACTCTGTTTGCAGTTACATACTGGTAACTGTCTGAATGGCATGT
A_16_P01850541
CCAAAAGATTAAAATCACATCCCTCTACATTTCAACTGTAAGCCTAATTCAGTCTCTCCA
CUST_2960_PI426210822 GACCGCGGAGCCCGGGCAGCTGATCGGCTGCTTCCCAGACATGTC
A_14_P107916 TGCCTGGGCAGATAAAAATCAGACATGTTTAATAGGAAGTTGTTATGTTTTGTTTCCCCT
A_16_P16631074
AACTAAAGATATCAAGATGAATGGTCTACCTTAGCATCAATGTCTTCCTCTACTCGTTGC
A_18_P22841550 CAGTCAACAACCCTCACAAAGCAAGAAAGGGTGACTGAACTATCCCAAAAATA
A_14_P132916 CAAAAAACAATAACTCAAAATATCACTAACAAAATGGCACTGACAGAACCTGGGGTTTTA
CUST_7480_PI426210822
ATTAACTGAAAAATGTCAAAAACAAATATAGGAGAAAAGTAAGATAATCTTGGAAGGCAA
A_14_P134731 AATTTCAATGGGTTATTCTATACCAAATCCAAGTGTTTACATCCAAGATGTCACAGAGGT
A_14_P200771 TGGACTGTGAGGCAGTATTTATTGCCATTTTCATAGTCAAGGATTTGCTTAGCATGAATA
A_14_P128731 GTCTTCAACTCTTTGGGGAACACTCATTTGGTTACCCATTGCTCCTATAATG
A_16_P19423350

CCTCACTCTTTAGTTACATGACTCTCTTTAAATCTACTGAAATTTACGTATCTCCCTCCC
A_14_P139067 TGTCTGGACATATCCATAGAGCACGCATTTTAAATCTGAAAGAATATATGTAGTCTCCCC
A_14_P118693 CTAGCAGCCGGATGGATGGGATCTACAAACACCGAACATCTCTTAAAATG
A_16_P03215066
ACCCCATTACACATATAATATCCATTAACCCGATCTAATGACATCAGAGTGATCATGGTT
A_16_P21094261
AAAAAATGGATTGAATCCAGTATATGAAGGACCCTCTGTAATAACTATGCTTTCTCCCAG
A_14_P137474 ATTTTAGTTGCTGATTAAAATGTGGCAACTCCTATGGACAAAGAGTGTTGTTTGTTCTCA
A_16_P19026284
TCTTCCCAGAAAGCGTTTATGAAAATATCACTCAAAAAGTTCTATTCTAGACGGTGTTGG
A_14_P128876 TACAATTTATCTTCTTACGTTGTTTTAGGAGTTGGAACAAGGAAGATTCCCACCTCTTTT
A_16_P01882038
AACCTCTCCGTTTTTCTTATCTTCAGTTTTTGCAAGATACACTAGATGCACTCTTTAACA
A_14_P108450 TATTCTTTTGTCTTTTCCAGAATTTTCCAATGAGAAAAGTGCCCTGTAAGAAAGATGCTG
A_14_P100210 ACCTAGTATCTTCACACATCCTTTGACAGTGACCATTTATTACACACATCAGACTGG
A_14_P130103 CTTCCTTCTGTTGAACCATTAAGAAAAGATGGCGAAAGTCAACATAACTAGAGACCTCAT
A_18_P15536040
TTGCCAAGACCAAATAATTCCTAAACTATTGTCTGCTATGCTTCCTAAAAGTCACTTCAA
A_16_P00685529
GTTGATGTGGGAACTTCAATTAATTTATAGTGGGACTGCTCACATCCCTATGAAGAAAAT
A_14_P200545 CTGAGGAGTAGATGGGAATTTGAGGGAGAATACAGAGATACTATGAACGTATTTGACATT
A_18_P15415826
GACAGATGGCATCAATATCCTCTAAGACAGCAAAGTTCGATATTCCCAAGTCACAAAA
CUST_76_PI426210822
TCATCATTTTAATATTTTATGTTTTGCTAAGCCATAGGCTAGCTTTAATATTTCAATTGA
A_16_P37063836
AACCAACGTGGTTCTCTTTTTTTAAACATTCAAGTATGCGAGAACAAGAAGTTTAGGTGT
CUST_332_PI426210822
CCTTGAATGTATTTTTTGCTGTATGTATGCTTGAATGTGTTTTTTACCCAGGCTTGGTAA
A_14_P102391 CCATGTTGTTTATCTGAGGCTGAAGAAAATTGACATTAAACTGATGTGGTTTTTGTCTGT
A_14_P121383 ATTTCAGGAACCATGCTGACATTATTTTTTGCCTGTTTGTTTTCAATGGAGTCATCTGAA
A_14_P121884 ATAGGCTGTCTATACTCTTGACTACACAGCAATGTCTTAGTTATTAGGAACCAAATATTC
A_16_P01080034
GCCTTACTCAAAGAATTGCCATGAAAGATCTGATTATACCTCCCTTTAAACAAGTCTAGG
A_14_P107180 ACAGGTGTAAATTTGACAACGCTTTCTCAAGGCATCAAGTTTTAATTTCATCTTTGCTTT
A_14_P129265 ATCCTCACATCTTGCAAATGCATTTCACATTGTCACATGATTACTCCTGAATTTTGGTTA
A_14_P117113 TTTGATGGGTCCCTGTTTGCTGTTTTTCCTTCTCTAAATGAAGAGCAAACACT
A_16_P01770759

ATCATACCTACACCTTTGACTTCTTTAGCACCTTTTCATTATCAATTGAACCAGCCATCT

A_14_P127203 TTTGCAAGATGTTACATTTCATATTTTGGGTTTGTTGGTGGTAGAATACTGGACCATGAT

A_14_P103347 TATGCCCTGGAATTTTCAAAATCACAAATATGTAAACAACGACAAATGTGCCCCAATTCA

A_14_P121097 AGACACACTTTTGGGTATGGCTACACAATGTGTTCAAGTCAAGAATGTAATAAAAACATC

A_14_P117874 ACATATTAATAGTGATGGAACTTTAGCAACATCGTTTTTCCCCAAAGAAACATGACTCC

A_14_P136447 GGTTGAGAGGCTGCCAGTCATTTACTATGTAAGTGATTTTAAGGAAAGAACTTTTTCTCA

A_14_P119749 CTATTTTCAACAACCTTCAAATGTAAATCCAGCCAGCCTGTCATCATGAACATCCTTATT

A_14_P200157 CTATTGTCTTAGAGAATGAAATGACATAAGGTGATGAGGCTTGGTGTATCATCTGAGACA

A_14_P128450

TAGTGTGAAAGGAAGGAGAATACAATCTAGGCAAAGAGTTAATAAGCAACAGCCAAAGAT

A_14_P122552 CCTCTTTACTAAAAATATACCTGAGTCATTTACACTGCTGTTCCTCTTGGCATAAGCACT

A_14_P100651 TTATTGCATTTAACTGAAAGGGCACTGTTGGAACAGGGACAATGTAGTAAGTTAACTGTA

A_16_P21599891

GCTTTCAGTGGAATCAACAAATGTTTTATTAACTTACTAATTCCCTCTAACAACAGAGCC

A_16_P18158832

AAATGAAGGAAATGATACTATGACACAGACAACCATTTTTCAATCAAGTTATTCTGCCAT

A_16_P03506006

TCTGCCTGATGATAGTTTTGGTGCTATGCCTAACATTCACATTTACTTATATTCCTAGGG

A_16_P41867000

TACCATGAAACTAAAGCAGAGACATTAACCCAAATTTCATTATAGAGGGAAATCCAACGC

A_14_P103225 ACTTTATCGCCTTTCTCAAATTCCAAGATTCTCATCACATTTTTTTCTTCCCAAACTCT

A_16_P00473561

AAAAACCATTCAGAGATTGTTAGATCTACGTAAACTCCCTCATTACCAATTCAACTTCAA

A_14_P109482 TTTTCAATCCCTTAGATTTAAACTCAGTGTGGAGAATCTGTGATAAGCACCTTCCAAATT

A_16_P58424617 CTTAAGATAAGTTCAGACAAAGGATGGGTGCCCTTAATCAGGGTGGG

CUST_6158_PI426210822 ATGTGGGCTTTCTAGGAGGGGGCAGCAGATGCCTCTCCTCACATC

A_14_P130956 GCTACAGATCCAAAACCACATTCCCCAATTGGTTTTATTAGTAAAACCAATTACATTTGA

A_16_P02564087

AGAGATTTCCACCATCTAAATAGAACTGCATCTTTTCTTGTCTTCTTTCCTTAAAGGTCC

CUST_8689_PI426210822

TCAGTTGATGTGGGAACTTCAATTAATTTATAGTGGGACTGCTCACATCCCTATG

A_16_P00067574

TGACGAGGCAAAAAATATAATAGATAGGGTTGATCAACTTCATTAGGATGACATTGGCCT

A_14_P107677 AGAAATGATGGACATTCCCCAAGCAAGATGATAATGAGCACCATCCCTAATATGT

A_16_P03085085

AGTATGTGGATTCGACTGATTGTATTTAGGAACAAAAACAGACATTTTAACACCTGTGCG

A_16_P19569515

GTTGCTGATAACCATCTCTGTTGATTCTAAGTGAATTCTGATGTAAAGACATGCATGACC

CUST_2645_PI426210822 AGGCTGGATGTCTGCAGGGGCAGCCGTTGATAAAGTCCCAGGATT
CUST_2571_PI426210822 CCTAACTCTTAAGCCTAAGGTAGGGGTTGGGGTAGCCTAACTCTTAAG
A_14_P131410 CTATGCTTCCAGGGGGAATGATTCACAACCTGGGAAACCTAGACTTTA
A_14_P112186 ACCTTTTATCCATTTAGGATTTTAGGTGATATGTATGCATCTAACTTTCGGGGATGATGG
A_16_P01106792 TGCACTAAAGATGATCCTCCTCATACCTCTAACCAATATCTATGTCACCATAAGAATGTC
A_16_P00348512 AGCCTTTATCAGAACTGTAAGATCATCTTGTTAAGACTCTCACATTATCTAGTTGTGCAG
A_16_P18222843 GTCTTCGGCTACATTTTGACACATGTATCAGGGTAGTTTTGCATAAGTTGTTCATACTTC
A_14_P103605 TTAAGTTCCAGAGAAGAGTTAGGGCATTTTCTTTCCTGAATAGACCATCAACACACTATT
CUST_7202_PI426210822 AATCATCAGAGTTGGGCCAGAAAGAAGTGACACAGAACATCTAAGGCATATC
A_14_P117743 GGGTGCTTTGTGCATATTTGTTGAATGACAGTGAATAATTTCTAATATAAACCCCAGACC
A_16_P02094184 AGGCTAGGAAAGAATTAATGGGAGAATGCAAAAACAATCAAAAGATCTGAATCACAACCT
A_14_P134362 AGTCTACCACCTATTGACACAATGGATTCTACTTCCATAGCAGTATCCCTAATCAAAAAT
CUST_7676_PI426210822 GGATACAAAGAAAAGCACCCTCTCTGTGACTTCTCAGATCCCATCTG
A_14_P107114 CTGACGAGATTGGGAATAAAGAGGCATAAAGAATTCTCTGTGTGTATGTGTGACCAA
A_16_P02309290 CCAGAAAGCATTTAGAGTTAATCCTACCATTCTGGAATAGCAATAAAACTTCCTGAGGAA
A_14_P132334 AACTTTTAATTTTGTTGTTACAGAAAAGCAAAGCCCAAAGAAAACACGATTTACCTTAAG
A_14_P130083 AGTAACACCATTAGTAAATGTTTGCTGAATACTGGGGGTTTGACTGTAATTCCATTTTCC
A_14_P103786 CTCCAGGAACTTAAGAATTTAAAACTTGACGATAATGAATTGATTCAATTTCCTTGCAAG
A_16_P35681678 CCTATCCAATACCATGTTTACCATAAATCCCCTTAACTATGAATAATGCATCTACACTCC
CUST_4313_PI426210822 GATCACAGGCAGTGTAATTATTTGAAGACCATAAGGTTGGGCATACAGCATAGAG
CUST_7180_PI426210822 ATTCATTCTCTTCCTGGTGGGGACTTAAGGTTGGAGGCAGCAAAA
A_14_P105401 GGTATGTGTTAGTGCTGAGTAAAACCCTGGATAATAAAAACATGCTTTGACTTGCAAAGA
A_14_P126478 CTGGTTACCAAATCAACGTCCATTTCATCATCTAAAAGGAATTCCAAACTGTCTTCTGAA
A_14_P114857 AAGAACAGTAGAAGATGTAATACTTACATACAAAGGTCTCTACATGTGTGCACAAGTCGC
A_14_P112924 TTGATGCACACTGCAGATGAATGTTTACCCCTGCTGTAGATTTAAAGAACAGCATAGATA
A_14_P112367 TAGGTTTCCTCTGATCACTTTGAACTGCTTATGAGTGAGCATCATAGCCAGAAACTAA
CUST_5224_PI426210822 ACACTGATTTTCTTACCACTTATTTCCTGCTCTCATGACAGAATTTCTGGAGTTCTTTG
A_14_P138762 CTGGCTTCTTTCTTGGCAGATAAGTGCATGACGGAACAATAGGTGAGC
A_14_P135990 TATCCTCTGTAAATGATGCTGCTACTAAGTGCGACAGACCTCAAGGGAAAAAAAAAAAAG
A_14_P106307 TTGGTGAATAGAGTCTATGGCTAATACTGTTTCTGCTATATAAAACTGAGTCTCCTCTTC

A_14_P132150 CTCTTTAGTAGAGATTTTATCATTCCTGGGAGGTAAATACAGATACACCTTTAAGGAGGG

A_14_P114735 ATACTTGTAGCACCCAGCAGTATCCTGATCGAAATTTTTAAATCTCATAGTTCACAGGAA

A_14_P130188 GTATAAAAAATATCTCAACCTAGGGGAGGGCCTTGTTGACTGTTTGAACATGGAAAC

A_14_P101948 ACTTAAAAAACAGAAAAATTGCCAGTTTCACATTCACTATCTTTATTGATGGGTTCAAGT

A_14_P117698 ATATATGTTCTTGACTGTCTGCAATTCTTTCCTTTACTGATTTTGCAGGTGCTTTTTTGC

A_14_P123757 TGGACAACAGTCAAGAGCAAAGTGTAGAATATCTGTTCTTATCACAAGGAACTTACACTT

A_14_P200994 TTTAGAATGAATAATCTCCAAGCAGTGTTCTTGCAACTGTTGCAAGGACCATGTTAAATT

A_18_P11754966

AAAAAATACTGAAGCCTCTATACTGTCTGCTTCTGAACTACTATTCACAGCGGATATCCA

A_16_P38203094

CCTCTTAATGTTGCCTTAATTACTTAACTTGACAAACTCTTAGTCTTGCATTCTCTAACG

A_14_P134159 TTCTGTCACTGAATTATCTCCCAAGTGCTGGCAGACTGAATGTTGATGTCATT

A_14_P114708 TGTTTCTTTTTTGTTGTTAGAATAATAACCTGGAAAGAGCACTGGATTGGATCTTTAGCC

A_16_P03808674

CACAAAGCAGTGAAACTGTCCAGAAAATGTCCCTTTTTATTCCATACGCAAATAAGTAGA

A_14_P106587 ACAGACTGCACACTAGATAGTAATTCTTCAGGTCTTTTACATAACCACCAAGAAACAGAT

A_16_P38084613

ATTACACATAAGACTCACTCCAAACACATTTTCTACAGTAGATTCTCTAATTCCGATTGA

A_14_P120961 TTTTTATGCGCTTTTCAACTCGGTCTTTACTTAGTCTTGTGGTGGCTCTCAGTTTT

A_16_P37280941

AAGTTCTGTGGATACCCAATACCACTTTAAACACTGATTTGATGTTATTGCTTCAACCAA

A_14_P108787 GGTGACACTAGTTTTTCTTCACTTATACAGAGCCTTATAATGTAAGAACCTTTCTCCCTG

A_14_P110130 CAGTAACTTCAGAACAACATAACATGATTAGCCAAACAGAAGTGAAAAGATAGCCACTGT

A_14_P104609 CTTCTCTCAAAGCCCAAGAGTCACAAAAGTGGTTTCATTTAATAAATATTTGCTTTGCTC

A_16_P00387742

CCAATGGAGTGCATCATCATAACATTCAGAATCTTCTACTAAACAATGCCATAAGATGTC

A_14_P117704 TTATTCATTTCATTTAGATCTTGACCTTCTGTCCTAGAGCCTTTGCTTTTTCTCTCCATG

A_14_P126525 CAAGCGTTAGTTGTGAAGGTTTATTTTGTTGCAAATCCCAGTCCTGCATGAAAATG

A_16_P58424143 ATTTAGGAAGTTAAATGTCAGTGATCGTCTTTGCAGTGGCGATGCATAGTTCACATG

CUST_980_PI426210822

TAAGTGATTCAAGTTTGCGAGCCATAAGAATTGAGTCAGAACTTTAAAAATCAATTATTT

A_14_P109374 CTGTCTTGCTCTATATTGTTAAGTCTCATCATCTACTTCAGAACTCATCTGCTTTTGGCA

A_14_P107504 GATGAAATCACTTGCATCCATAGAACTTTATCAGGGCTGTGTGAGAATTATCTGAAGAGA

A_14_P202035 TTTCTGTTTTAAGTGAGATTTCCAAGTTAATTTAAGTTGTGTGGAACAGGACACTCCCTT

A_14_P128511 TCTGTTGAAATGTGGGCTTACTTCATTTATTCTTAAGGTAAAAGACAGCAGACTGCTACA

CUST_8141_PI426210822 GCTCATTAGCTTTTGGAATGACGGGAAGCCTTTTGATGTTTGACGTCTTT

A_18_P22841946

TGAGTTAAGAGTCCTAGACTTACTTAGCAAGTCCTCAATTCTGACACTTATTATAAGACA

A_14_P118994 TAGTTCATTTCATTAAGTGGCTACATCCTAACATATGCATTTGGTCAAGGTTGCAGAAGA

A_14_P123765 GAGTCATTCCTTTTCTAACTCTTCTCCTGGTTCATTGAATATTGGACAAGTCCTTTTTTC

A_16_P16951100

GGATACAACTGAATCTGAATGAATCTTTCTCCCATGATGAAACAAAAAATTCCAGCACAA

A_14_P124083

CTGGAGAAAATGGGAAATCAAAATTGTTGAAAGGCAAAGGATTAGAGTTTAAAATGGACC

A_14_P108371 AGATAGTTCACGGTCAAGAGGTTTTGATGTATAAAGAGATCCATTTCCTGAATGAATGTT

CUST_6534_PI426210822

TTAAATATAGTTGTGGTTGGTAGATACAATACCGATAATAATAATGATGGTGTAATGAGA

A_14_P117094 AGTGAGAATATAAGGTGGTATCTCCGGGCTAATCTAATTTTGGTTTTGGTCTTAAGATTG

A_16_P39201728

AACCCATAGTCCTGTAATTAAATCTGTATCTCTTCAGAAGATTATGACCCATCCAAAGAA

A_14_P111513 AGGCATTTGAGTTTTCTGCTTCACTCAAGGACACAGTAATCTTCTTTTTAATATCCTGCA

A_14_P120880 GTATAGTAACTATTCTTACCGTTCTCTGGATGTAACTCTACGTCTCTTCTCATCCTGTGT

A_14_P129903 CTGCATCCTACGTTTTTATATGGAACCGTTTTATCACGTTCAGTTCAAAGGAATAGAGG

A_16_P18599583

AGATTAACTGGTGAACTATGAGAAGTCTGTTTTGAATCCCGAGTAATACTGAAGGTGATT

A_16_P20847335

AAGTCATTTATCCAGTGCTTCAATACATGCAATCTTTCAAAAGAGTAACCAGACACAAGA

A_14_P125843 CTCGAAGAACTTCTTATTTGGCTCATGAAAATATTCGTGCAGTTTATTGAGTAGTTTGGG

CUST_5173_PI426210822 ACATCATCCTGGAGTGCTAGGGTGACCCTAGCACTGTATCCAGCT

A_16_P17815552

AAGCAACCAAGTTGAATAAAAAAGTCTTTAGTCCACAATGAAACATTTGAGCAGATGACT

A_16_P58424466 CTGCAGCAGTGCCTGGATGTGTGGTAGGATCTCAATCTTTGTTGAATAAACTAA

A_14_P100931 TAAGGTAATTTATTCCCCTACCTTTATATGATGGCTGTGGATGAATTAAAGACTCCAGAG

A_16_P00343442

AACTGAGAATGAACAACTCACAGTCAAACTAGATCAAACTTGAAGCACATTTGGAAACTC

A_16_P03488216

GCAGGCTGTGTTATGAAAAACGAATGATAACTGTCTAATTGTAATTGGTTGGTTATAGCC

A_16_P16181257

CTTAAACCTTAGTCTTGACAGTGTGGTTTGTCCTACCTTAAAATCGTTGATATATGCACC

CUST_1161_PI426210822 TTTCTAGAAGACTGGACTGGACGGGTGGGGCTGGGAGGATGCCGG

CUST_10334_PI426210822 GGAACTCAATATCTGTTTACAAAACTGGCCTTGAGGATTGAAGCTCAGAACAC

A_14_P110714 TTGGGCTGTGTTATTATTACTCTTGTTAAAGATCAATCGGCCAAGCACTTTAGGTTGAG

A_14_P123622 GATGAAAAACTCCCTACTCTAGGAACAGGGGGAAATGGAATTAGAAAAACTACTTAGATG

A_16_P41771854

TGCCAAAAAGTCTGCTACTAGTCATATTTTACTTCCGTCTAACAAGAAACTTTCCTACCA

CUST_8351_PI426210822 TTCCTTGTGGCCTCTCCTAGGTGGACTTAGGGGATGACAAATAAT

A_18_P14132683
AGAATCATCTGGTGGCAACATATAATTGTCCTATGGTTGTAACCTTGATAGTGCTGTTAC
A_16_P00376064
CTATTCTTTCAATGATCTGTCCTATCACGTTCAGTAAAATCAGTTCACTATTCCCCAAGG
A_14_P106964 CAAGGAGTAGTGGTTTTAAAGAACTGTAAAGCATTCCAGTAAAGTAAGTCCAGTTGGTAT
A_16_P02887096
ACTGAATTCCTCATTGTTTCAGACCTAGCAACTATTTGTGGATTATTCCTATCTTTCGCT
A_14_P121064 AGGGCTCAGAGTTTACAAAAAAATTTATTTGTGTCTGAGAGCCACGATTGAGAACATTAA
A_14_P122308 CAATCATTACTGATAACTGGTATCACTCAGGTATCCACTTCATTCAAGACTTCCAAAACT
A_16_P17499416
CTTTGGGTGATAATAACACATACACATAGATGAGACTATGAACAAATATTAGGAGAAGCA
A_14_P106107 GGTGAGAGGGGACCAGAATCCTGTTTACAATACATTCAATTTATATGTAACTTTTCTGGA
A_18_P22842127 GTATAAGAGCCAGACCTGGCACTTTAAACAGCCCAGCAAATAACCCAAAA
A_16_P00685442 AAAGAGATTTTTCTCTCCCAGCAAAGTAGTAAACAGGCTCTCTCAAGCATTCATCC
CUST_9710_PI426210822 GAGGTTCCTGGAACTCCCTCTTGGTGGCTTCCATACCCCCTCACT
A_14_P127442 GTGAGTGAAGTTGAAATAAAATCTTTAAAATTTACTATATTGCCAGTGGTTTCACAACAG
A_16_P00685472 TAGGAACTTTATGGCTCAAGCCCGAAGGATTCCCCTCTGGGAATA
CUST_2608_PI426210822 GGGAGCCCTGCCCTGAATAAGAGCATGGAAGGCCCAAGAAGAGTG
A_14_P113205 CCATCTTTAAGTTCAACATTTGGAATGTTCATAGGCTTATCAATATGGCCAGACGTAGAT
A_16_P03810244
AATGTGAAAGTCGAGGCATCAGGAAGTTAGTTTAATGGGTTGCAGAAAATCTAAGAGC
A_14_P135857 TCTCAGCCTTTTTTCCATCCTTAGAATTCAGCACATAATTTGGACATCAAAGATGTCAAT
A_14_P120327 GTCTGGAACACAGTGCTTAACAACAGTAATGCCAACTATCAGTGCTAACATAAAAACATT
A_16_P03072747
ATGACTCAGCCTCATATAGAAACCTCACTCCATAATCTTCTGCAAAATTAGCACCAAATT
CUST_2271_PI426210822 AGGCCAGCCATCCAGACGTCTTTGATACCTCCTACCACTGGCTAC
A_14_P120329 GTGGTATTTTAAAACGTAACTGGAGTCACAGTGAATGTATCACTCTGCAACTTGCTTTAT
A_14_P109024 CAAGGTGCGCAGATAGAGAATGTAACAGGCGCCGAAAAAGACAGC
A_16_P17511592
GAATAGCCCATTCCTTTTAGTTTCGTTCTCCTACAGTTGAACATTGATTAGAAATGCACA
A_14_P107708 AACTTACAATGGGCTTGGTAAGGTTAAGGAAATATATAAGGGATAATGCTTGCAACCATG
A_14_P102884 TCCAAAGGCTCTGGTTTTTTTTCACCCATCTTTGCTTTTTTAACATTTACAGTTTGTTAG
A_16_P36231020
GTTAAATTTACATTCCAAATCCTATTACCTGGAAAAGGGATAAATCTTTGCAACCCACCA
CUST_7489_PI426210822 TATTGATCACCGAGGTTCTTGGCATCGGGCTTTTCTTTGATGTGTG
A_16_P21570177
TATTAAAGATGACATCAGTAAGACAACTTCTCAGGACTATGCTATATGCCATCTCCTCCT
CUST_7244_PI426210822 CCCAGGGGACATAAGTACAAAGTGAAATGAGGCGATGTCAGGATC

A_14_P100660 CTTGTTGATGTATGTTTATCCATTAGGAATGAAATCATGTTGCTCTGCCTTACAGAGGAT
A_14_P135944 ACCAAAATAGGGGACTTGTTGAGACTTGGATACATATCTTAGATTTAGGGAATTCTGATG
A_14_P129780 CTACACTGGTCACTAGAATGCCATTCCACAACCTTTCTTTTAGTTTTGTAGAGCAATTAG
A_16_P00913809
AAAGTTGAAAAAGGGTAAAAGTTTTCAGGAATATTCGGGCTCTCTATTGCTAAGCATAGC
A_14_P121944
TGCTGGAAACAGTGAAAAACAATATAGAGTGGCTAAAACAACATAGAAACACCATCAGAG
CUST_824_PI426210822
TCAACACGAAAGGAATTTCTAACACTGCAGAGGTTTTGTTTTTGTCAATTTTTCCTTTAA
CUST_1317_PI426210822
AGAATAAAGACTGGCAAGCTATTTTTCCCCCAGTATTTTATTTCAGAGCTAAGCTAAGGA
A_18_P14790139
ATCTTTTTATCAGGCATACACCATAGCAACTTTTTCTTTTGTTATCCAAACCACCACAGT
A_16_P03074147
TCTTATCCTGTTTACATCACACCATCCTTTAAATCCATCTGAGATTTCACTTTTCCACAA
A_16_P36803954
AAGTAACTCTGTTATTCTGAGCATTAAACTTCAGTGGAGGTTATAGTTACCCTTCTACCC
A_14_P110142 ACAAGCGTTTTGAATTTACAGTCATGAAGGAAATGCAAAAGATGATGTCTTCATAGCGAA
A_16_P16408157
ATGCATTAAACTCAAAGTCCTGTATTTGTGAACACTTGTTTCATTACCCATTGCTGTAGC
A_14_P102662 CAGCATGTCTGGGATAGACTGGTCTAGAATAAAAATGAAGTTTCCATTGCTTTGTTTGCT
A_14_P133713 CATCTCTGTGAATATGAATTCTCCTCCTTCAAAGTCATCATTCATATATAGGAGAGCACT
A_18_P20496958
AAACTGCTTCTATTCAACTGTTTTGACCTCAAAGCATCCATTTCATAAGGCTCCATTTTA
A_14_P111353 CTTTGCTGAGAAATTGAGAAATTCTTCTTGGTTAGAGTCCTGAATTACATTAAATGGAGG
A_16_P21080779
AACTTGTCAGCAGAATAAAAACACGAAAGGAAGTATTATTAGGGAGAGGTTGCAAATCTG
A_14_P129719 AAACATTAATCCCTTCAGATGAGAATAAGACTTATTTCTTCCCCAAAGTGACCATTCTCT
A_16_P16096501
CTGGACAATACTGAGCAATCTACAGATATCAACGTAGCATAATTTGAGCCTCCTTGTTTT
A_16_P01941493
AATTACCCAGAGATATCCTATTCAATGAACTGTGTCATCATATCAGAACCATTTCACAGG
A_14_P201472
AGAAAGAGGAGACTCACAGACTTAAAGTAGAAGCAAAACAAACATGATGGCATGCATATA
A_14_P132240
AAATCAACAACCAAACGTTCACCCAAACCACCAAACAAGAAGAAGACTAAGAAAGTTATA
CUST_7013_PI426210822 TGGTCCTCACTAAGACAAAATACAGAATACAGAGCAGCAGTTCACGGG
A_14_P115448 CTACTGATTAGTTCCCATTACTTACGAAGTGAGACCTTAAAAATCCACAGAACAGAGTTG

A_18_P14135496 CGACAGCCCAAGGCTGATGGCCGTGTGTTGGTAAGTCCCTCCTGG
A_14_P132465
TTAAAAACTGGAAGTTTTTCTATAGACTCAGAAGACAAAGCCTAAAAGAAAAGGGGGGAA
A_14_P117530 TGGTGAAATATCATGGCATCTGTAAAAACCACATCTCTAAGCATAGTTAATGAAACCGGA
CUST_8374_PI426210822
CAATTAAAGATGTAGTTCAGAAAATTATTTAGAAGGGTTCAAGTAACACAGACCATGTAA
A_14_P201765 ACCGTAATTCTCCCTTTTCTTCAGCCTAAATGTCTTCTTCTCTTCAAAATGTTATCAAGT
A_16_P03506411
GAGCCAAACCCTTTAGGTAAATATTTACGTATAGATTCATTTGTTCCCCTTATTCCCAGA
A_14_P200829 ATTAAACTGTAGTTTACTGCGATTTGGAAATGCCCTAAAACCCCAGGCATTTCTATTATG
A_14_P109882 CTTGGAGACCATGGTCTTTCCAATTCAGTGAATTTTTTTTTTTTGAAATGGCATTCAAAC
A_14_P128544
AAGTATCTAAAGGAAAAAAGGGTTGAAAATCAAAGAGAAGGTTTTATCAGGGGGAGTTTG
A_14_P120579 CCAGATGAGTTGTTCTAGAGTGAATACCCTGGGAACAAATGGTCTGAAAAGATC
A_14_P126159 GTCAAATAGTAGTAATCCACCAACCCAAACATTTGTCTCTTGTAAAACTATCTCGTCTGT
A_14_P200323 CTTGCGATAAATCCAGTGGGATGAGTTAGTGGCCTAGATTCAGATCTAATTACAATTTAG
A_16_P20362646
GACCCTCTAAGTTTCTATCAATTGCTCACTCTAAATCCATCATTGTCCAATGTGTCAAAA
A_16_P00961158
ATAAGTTCTGGGTTATCATGGTTATGACTTCCTCTGTTCTCAAAGACATTCATCCATGTT
A_14_P122775 TTGGTTTTTGGTTTTCTTGCCGTGGTGCAAAACTGTAGAAAGTTGCTTATTCACT
A_16_P00684981
TTTTTTGGTGAATGTTCCATTGCATCTGAATGATATTGAAGTGAACTTCTCTTACCTGCA
A_14_P102022 AGAAATAGCACTGCTGCCATGTATTTCCTCACTAAAAGCAGTGTTTGTATGATTCATTTC
A_14_P132268 GACCAAAAACGAATCGCTGAAAAAAAAAAAAATCCAGCAACCTTATATTTTACACACAGCC
A_16_P01683005
CCTGAACCTTGCCAAATATGTTTTTTTGAACAGTTGATCAAAACTCATCAAGCACAATGT
CUST_3234_PI426210822 TGCTTTGGGGATACTTGTCAGGCAGAACCTTGTTGGAGTGACTAAT
A_14_P102328 GAGTGTGTTACAATGGCAAGTTTTAACGGTTGAGTATTTTTACCATGATATGTTTGGTGC
A_14_P136807 ATTGATGGAATAAATGCACAAGGAATGACTCATCTTGAAGCCCAGAATAAGATTAAGGGT
A_16_P00423953
GGCTTTGAATAGTTTGTGTTCCTTAATTGTGTCCATAAGTTGTAACTTGCGTGACAAGTA
A_16_P01536784
TAAGATGAAAATTCACTGACTTGTGCACTGATTGTTCAGATGGAGAAAGCCGAATAAAGT
CUST_1605_PI426210822 TCTTTGAACTCACTGCCTGCCTTGTTTCTGGAAGTTCAGCTGTTTG
A_14_P125400
CACAACTAAAAGCACCCCTCAACCAAACATAAAATACAGAAGTGAATACAAAAGATCTGG
A_14_P132369 TTAAATTCTTACGTCCCAAGTTTTCAGTTTCAAGTCTGAATTCCACACAAATTCTGCTTG

CUST_6524_PI426210822 GTATCTGTGATTTTTTTGGAAGCTGAATCCTTACTGAGGGCATGCTGCC
A_16_P38912163
ACAGGACAGAAATACATATCCCACCAATGATATATTACAACTTACCTTGATCTTCTTCCT
CUST_10239_PI426210822
AAGTCATTATCTTTACTTAAGGGGATGACAAAAACCTTGCTAAATTTAGTTTTTCACTTC
A_14_P132147 TTTTCCTAAGGAAATCAAGTTTTTATACTCTTCTGCAAGTCACCACAGTAACATGCTGTG
A_14_P104020 TTACCTCAGACTCATCTGTAGATTCTTCAAGGGACCCAGTTTCCTATAAAGCATAGAAA
A_14_P136502 TAGATGATGCAAGTTCCAGTTCAGTCACCATTTCCCCGTTTCTATACTTTGTCAATAAAA
A_14_P103412 CTCATCAAACCTTTTAGGAATCAGCAACAGCAAGAAAGAATATAGTGTGGAGTCATTTGA
A_16_P03068525
CGCTATTCAAAGACAAGATGAAATGCACTGTGAATAACCAGCAATCAAAGAAAATCATGG
A_14_P121794 CAAAAATTTCACAACGGCCATATCCTACTTTTGCAAATAGAAAACAGTGTTCTTCAGCAT
A_16_P20179195
GGACAGCCCTTTCAAAAGTGTTTTCCACTGCTTATACTGATGAAGGTAGAAAAAAATTTC
A_14_P111998 AAAAGATGAAAACTTCTGTCTAACATTGAAGGGAAGTGTATACAGCCTCAGAGCAATCTA
A_14_P111855 AATAACTTTTGTTTTTTTGAAGGTGCCAAATCAATCATTCAGGAGTTTCCAGAGATCACA
CUST_9417_PI426210822
CAAGGCCCAGCCATTATCATAGAAAAATCTTCAAGTAAGTTGTGTTGTTTCTTCCAC
A_14_P101475 CTTGGGTTAAATCATTTGCTAACTTGGATCTTTGCTATCTCGGCTAACTTTGAATAGTCT
A_14_P132797 AACCCTTAACTTGTGCCTAGAAACTACAGCACATATAAAATATGTAAACACCAGCCTGTT
A_14_P133197 GAGATCTTTTACCTGTGCTTACTCCGTCTGCGTTTCCTCCATCTACG
A_14_P111606 CTAAAGGATGATGAACATCAAAGAAGAGTCATACAGTGTTTGCAGAAATTACACGAGGAC
CUST_4077_PI426210822 ACATCAAATGGATCTCCAAGCCCAAACACTAACAGTGGAACCAGCTAT
A_16_P20528703
AGCAGACTTCAGTTAAGCATCTCAAGGTAAAAATCATTTTTCAATGTATCAGAACCCTCC
A_16_P21105833
AACTAAAAGATAAATAAACAGACAGGCGGATTTTTTGTTTTTTTGCAAGGATACGTCGGG
A_14_P108818 CTTTATGAGATTCAAATTCAGTTCCAACTGGGCTTTGTCCATTTCTCTGGCTTTAGC
A_16_P19953595
AATGTCATGGTTTGCACATACTGAAACACTTAAACAGGAACATTTTGCTATACTGCATCC
A_16_P39556458
AACATGAATTCAAAACATCTTTACTGAGGAAAGCAAAATTGGTCTTCTAGCAAATCCAGG
A_14_P103806 TGATTGATATTCTGACCTTATTTCTTCTTTAAAGAACTTCTTGCAAATAGATGTCCCGAT
A_16_P18565037
AACACCAGCCATAAAAACAATTATTGGGTCACAATTGTATACTGGTTGACTATCACGTTC
A_14_P107996 AGAACTTCTTATTTCCTACATACAGTGGGGCAAGAGGTTTTAGGTAATGTGTAATGTGAC
CUST_2613_PI426210822 GCCTGGCACAGTGTGAGCACTTCATTTGTGTTCATTCTTTGTTATTGTTTTCTTT
A_14_P114512 CTTCTACATTCCCCCTAAGGGCCAACTTTAAGTTCCTTGAGTCTCTTAAAGTC

A_14_P130981 TGAAATGTGACACTGAAGCATAAGAACACAACTGAAGACTGCAAACAACCTAATTCATTT

A_16_P41592263

ACCTATCAATCGATAATGCAAACTCTCTAACCTAAGGATTCTGCAGATTTTACCTCACTT

A_18_P14022959

GTGACATCACAGTCATTAACAATAAGAAAGATTCTCCTGGTATCTACAGGTGTTCATTGG

A_14_P201484 GCCTTTCATCTCATTTGCCGTTGTCCAAATTCTAATTTAAAACTCATGTGTTACTTGCTG

CUST_7933_PI426210822 AGACGGCCTGGTCAGGGCATCTCGTCCCACTGGTGCCTACCGTCT

A_14_P100915 CTATTAATGCAGAAAATGAAAGGATTGGCGAGTTATCTTTGAAACTGTTTCAGAAGCGAG

A_14_P117692 AGGGACATACTCCTGAACTTACTTCATTTCTAAAGAACGGAGTATTCTCTTGTTACTCAT

A_16_P41870802

AGCATTTCACTATTGCCACATTTAGTTAACCTACATTTAACCTACAGTGCCTACAACTTT

A_14_P139018 CCATATATTTGGGAGAAAACAAACCTACCTAATTCTCTAGCAGTGAGTCCTTCAGAAAGT

A_16_P17828110

AATCACGTTATCAGAAACCATCCATTACATGACAGTGAAAAATAATGCCAACGTTGGATT

A_16_P18553366

AAACTGTAATCTGTATCATCTCATGGGTAAAATGCGAATGAGTGGTCTAAGAATCAGTCA

A_18_P17039736 TACGTATCTCTTCGAACTCCAACCTGTCACCGTTTTAGATCCAAACCC

A_16_P36230928

GCTTATGATCATCTTCTTCAACCCATAAAACATTTTTCGAATGACAAAACAGAAAATGTA

CUST_2579_PI426210822 TTCATCTGTACACCCTGCATTTGTACCTCCAGTCAGTCCACAACA

A_14_P116210 CTGGAAGAACCCTTGGTTTTCAAATATATCCAATACCTAGGAAGAGACAAAGCCAAG

A_16_P58424325

CAATTTACAATCACAATAAACCAAAATTATCATAGACACATCATTTTGAAATGTCAGTGC

A_14_P119600 CAAAGTATTTGATGACTCACCCTACAAGGAAAAAAGTTTCAGCATCCACATATTTGTAGC

A_14_P132066 GCCTGTAATTCTCAAATAATGTTTAGAAATGATCTGAGGAAAGTTTGCTGGAAGGATGAG

A_14_P202422 TGAGACAATAACAACCCTAAATGTAGGGTCCAATGCAAATCAGTTATTATGCAAAATGCC

A_18_P22842098 AAGGATGAATGGACATGTGGGTGTCTTCCGTGGGAGTTACCTGGC

A_16_P18805702

AAAAACACACAATCCAGAACTCTCAATATCCACACATAGTATGTTCCCATTAAATAGCCC

CUST_3913_PI426210822

CCTGTTACTGCTTTCATCATCACCTCTCACTCTCATTTTCTAATAAGCCTGAAAAGC

A_14_P137443 GACATATTTTCACATGTTCTGTTCATTAAAGTGTCAGAGTCCAGGTATGAAACAACGAAC

A_14_P112758 ACTTAAGCAACAGAAGCACAGATAGCCAGAATTCTTTTGCAAGTTGTAAACTTACACAGA

CUST_3626_PI426210822 TCTGTTGCACGTGCCTGTCCAGCTTACTTAAAATATGTCAGAAAGAATGAGG

CUST_5853_PI426210822

AATGTTAAATAGTGATGCACAGTGCCTAAGACAAAATTGCTATCACTGCAAGCTCCT

A_14_P129189 GCGGGACCAGGGAGAATTAATAAAGTTCTGGACTTTTGCTATATGGTGCTTT

A_14_P105200 GTTGGAGTCAGGTTTTAGTTTAAGCCACTGTGTATTATTTCAAAGTGTAACTACAGTGGT

A_14_P137104 AAATAAAACAAGGCAAACACTTACCATCTGGCATCCTTATTCTGTTGATACATCTCCCAA
A_14_P118787 CTTTCTTCCCAGAGGAACAGAAGAAGTAATGTGCTAGTAAATGCACTAATTCTGTATCTG
A_14_P107300 CTTTCACCTTTGTATCATCCACGATTTCTTTCTTCAGGAAATCCTTCTCTTCTTTTACAC
A_14_P121093 CTTCCTGAACTCATCCCATTGCAGAAAAGATGGATAAAATGGTAAATTCTCCTTAATGTC
A_14_P116078 AGCATTTAAAACAAATTCTATCTTATTTGGTCTCCCCTAAGGGATAGGCACACAGGTATA
A_14_P110916 TAAATCCAACCTCTCAGAAAAAATGGAAAATTATGAACATAACCACTTGTGCACCCTAAC
A_18_P16015063
AAATTGTCTCCCAACTGTTTTGATTCCATAACACATAGACCTCCTGATAGTTAGCACATT
A_18_P22841789 GTTATCACCTTCCCTTCAAACTGGGCTGGGAGGGCATCCTTGCTT
A_14_P121503
AGGAAAAACATCTTGATCTGGAGAAAAACTGGATGTTGGTAGAAAAAGCAAGAGTTTGTT
A_14_P101073 TCCTCTCAACTCCCTTGGTAATCATGAAGATGAAAATATTTATGGACAATTTTCCTCACT
A_14_P121401 GCAGAAAAGATTCCGGGATTTACTGATCTCCCCAAAGAAGATCAGACATTACTTAT
A_16_P16738544
TGATTAATGCATGACCAAACCTAAACATAACCACTTCAGCCTTTAACTTTCTCATCTGAC
A_14_P119838 AGGTTCATCTAGGAGGACATTATCTCCAGGATTAATGATCATTTCAAACACCTAACAAAA
A_14_P107676 AACCCAAAGAGACTTCTGTCTGTGGAGTTTGTATGGCAGCGTTACTTAGAC
A_16_P00917147 CTCAGATTGCCTCTCGTGCTTGTGTTCACAGATTCTTGATGATTGGTCATTC
A_14_P101054 TTGTGGTCTTTAACTGAGAAGAGAGACTTCCCTGATGTAGTCTAGCACTGACT
A_14_P113422 CTTTTTATCTCCTAGAGACTACAAAGCAAACTTCAAGAGGTAACACATCAAGGTGAAAGT
CUST_3587_PI426210822 GCCTCTGCAGTTTAAACAATGACTGTCACTTAAACCTCTTCTGAAGAGCTTGA
CUST_2710_PI426210822 TATTGTCTGACTTCCGTTTGTCCTGTGGCCAGATTTGCTCTGGAA
CUST_6980_PI426210822 GTATACAACTAAAGAAAAAGAGAAAAGGTTCGCTGGGCCAGAGAGTGGCTA
A_14_P120971 TACTTGCACTTTGCTCCAGCCTTTTCACCAACCTTCAAAAAGTTTTCGTAATTGATC
A_18_P18603880
AGCAGCACTTTGTTTTTTTAACCTAACAGCAAGATGGTATTACAACACTTTCAGAAGGTG
A_16_P00961620
ATAAAACTTTCTCTCTCCAACATATGTGCTCATGTATCAACATCTATGTTTCTCCTCCCA
A_16_P18001603
ACCTACAGCCTAACATATTCCTCCTTTTTCAGAAATTTCCAGATACATCACAGTCATAGT
A_16_P17435961 ATCCTCTGATTGATACCTTTCTTCACTTATTCCCCATTTTCAATAATTTGCCTCATCTGG
CUST_5657_PI426210822 AAGAAAATCCGGACACATGCTAAGGCCCACGAGAGCCTGTACAGT
A_16_P18284199
AAAACAAAACCTAGAAATGGAGGATGTGTTAGTCAATTGTTATATCTGGTGTCAGCAACC
A_14_P132756 TTGTTGTTGCTCTACATCCAACTTCACTTTTCTAAGTTCCCAGATGTATGTTCTATCTCT
A_14_P124478 GTTGTTGAGAACTGAGCGCTTAGCCAGGGAAATATCTGCCGTCAC
A_14_P123312 CTATCAGTAAATACAGTGTTTGGCACCCTTGCTATTAGTTCTTTTCTTACCTTTGCCTTT
A_14_P130845 ACTGATGTTAACTCCATCTTAACAGAGGTCATCTCAGAACAAGCATGAAACCG

A_16_P19412904
AAACAGATTCTAGTGTCTTAAGGCATACTTCTCCAATACAATCATTCTGACACCATCACA
A_14_P107922 GTCAGTTGTGAGCAGATTTCTTTTATGTGTGTGGTTCTCCATCATATTTAACTTTGGATG
A_16_P01734945
TTAGCCATTACTACCAGTAATCCATCACACCATAACTGTCTAAAGAATTACCAATGGGCA
A_16_P03045468
TGGGGAATCCTTTAGAGAATTCAGTTAACTCTCAACAAACATAGGATCTAATTTCGACAG
A_14_P116759 CTCTCTCCAAATTTATCTGACCTATATTCATTCCTGAAATGTCATGCTTCCTGGGATATT
A_14_P136792 TAAATAAATAGTGAACGGATTGTTTCTGCCCCTAAGATGAAATCCCACAGATCTTATGCT
CUST_3514_PI426210822 GGAAAGATTCCCTTTCAACTCAACATGTGTGAAGCACATGAACTGAAGCA
CUST_9512_PI426210822 CCTGGCCTTCTGTCAACTATCAGTCTGAAGGGGAATTTCCATAGAG
A_16_P19146409
AGAGAAAACTAACTTTGAGATCTTCTAGACAATCACTGATTGCAAGACACTCACTCTCAA
A_14_P117470
AGAAACACTTCTAATGAAATCTAAAACTCAGAAGCCCTGTGTGTAAAACAGTCCAAAGCA
A_16_P19908615
AGAATATGTGTGTAATCATCAGAATCTGGGATTTTCAACTGATAAGCCTTTACCCTCTGT
A_18_P15581460
CCCCAAGTAAAAATACACTCTAACTAAATACATTATGTTGGCTATAATACCACACTTAGG
A_16_P16010211
AAATGACCACTTTTGAATACGATTGTAACGACACATACTAACGTCTATGACTTTCCAGCC
A_14_P106920 CTAAGGACTGGACAAAACACATTTACAGGTTATGTAGTCCAAATCTACTCCTAAAAAGAC
A_18_P14132753 CTTTTATAAGTATTGTGGTTCAGGAGCAGTGGAGGTGATGTCTGCCTCAG
A_16_P15815289
GGGAGCCATAATGCTATTCATGAACTCTAGAAAAAGTAAGATTTGCAATTAGGTGATGCA
CUST_5015_PI426210822
CAGCTATTTCTACTAACAGAAAGTGAGTTTATGGGATACCAATGCAAACAAACATAGATT
CUST_8260_PI426210822 GAGAAAACATTCTCCCCACCACCCCCAGACACTTGATCATGTAAT
A_16_P02773057
AACCAACCTCTTTGATCTTGTAATTCAGAAACTAATTCGTGTAAGTGGCAAAATAGCAGG
A_14_P131774 CTAGAGACTGTGCTCTTAACCCCTAAGTGATTCTTCTACTTATTTTTATTATTAGTTTTC
A_14_P121833 CCTTCAGTTGCTCTTGTATCTGCTCCAAGAACTATGCCATCCTATTAAAAAAAAAAGTTT
A_14_P126320 TTCCAAATTAATCCCAGACCCCCTAAAATATTGACAACCTTGACAACCCC
A_14_P124674 TTATTTACTAGCCTCAATTCTCTAATCTGCAAAATGGGCAGCACACTTCTGTATCCACT
A_16_P03654734
ACCCTGAACAAAAACCTAAATATTTGATTACTTCCATAACACTATACCTATCTTTCACCA
A_14_P128454 CCATTGTAATTAACTCTTTAAGAAAACTGAGTTCCATTTCTGCATGTTGCTCAGACAAGG
A_14_P136693 CTGAGAACGACAGAGCCCAAGACTTCAAATCCTAATGCCCATCCAAA

CUST_5541_PI426210822
TTTCAATATTTATAATTAAGCCAAAATCAGAGCTATGGTGATAGCATAAAATGCAAATGT
A_16_P39851048
TTCAGTGCGTGCTAATTTTCAACTTTATCAGATTCTCACTTCACATTATACACCAGCAGA
A_16_P00703929
CATTTGAGGATAATAGGATACAGGCCTTTGTTGTAACACTAGTCAGTTTTTTACTTCCCC
A_14_P126176 CTATTCTTTCATTGGCTCCTCTTCTCTAAAGATCCGCCCCCTGTTAGT
A_14_P133583 CATTTATATCCTGTCAGAGTTAACTATCTCCAGGGAATATTGGTAGTGTGTTCAATGGAG
A_14_P137882 AACATTGATATTCTATCGGGACTTTTGTTCTGTATTAACAGATGGGCACTTGTTCTGCTA
A_14_P200778 CTGTAGGATTTGCGTTTATGAAATGCTGACTTTATCATATGCCTCAACTTGCTAGACCTA
A_16_P03704942
CATTTTTCTTGTTTTGTCAACTTATCTATCCTTAACCATTGCTTCCTATATACACACAGG
A_18_P22842093 TGTCCTGTATGTCTGTTATGTTTATTTCTGAATTTTCCTTCCTTTTTCCTCTCCATGCTT
A_16_P01927412
AAACCATCTTAGCCTAATGCCATTACCCTTTGAAATGATATAACAAACTTCCTGGTTCTG
A_14_P108675 GAAAACAGGATCAACAGAACGAAGGGCAGGCTAATTTCCTATTACAAATGTTTTTCATTT
A_16_P02183614 CAAGTTACCCAAAGCAGGACGGAAAGGAACAAAAACAAAGAGTTAGTGAGT
A_14_P127822 CATCTACACAGATGTCTTAGAGTTTGACGATGGTAAGAGGCCTCCAGTCT
A_16_P00184926
AGGTGAATTGGATTAACCCTGACATTATTTCCCTAATTGAGTATATCACAGCAGACAGAA
A_16_P01590859
CACCATTCTTCTCAATTCCTCTATCCACAGTGTATCTTCACCATTTCCACTTTAATTAGA
A_14_P119240 CTAAGATGTGTTTCTCTGCCAGGTATTGGTCATCATTGATGTGAAGCCGAAG
CUST_9188_PI426210822 GAAAACCTCTGGCAGCAGATCATCCATCAGGCTTGGTCCCAAGTT
CUST_4527_PI426210822 CTGTATATTCAAGTCGCCTCTGTGGCTGCCAGAGTTGGGGAGCTG
A_14_P116950 ATCATCCTTTTTTTTAAATATGATTCACCCTCAAAAAGATGAGTGGCTATGCCAGATCCA
A_16_P41862864
TTTTCAGTCAAAATGCCTTGATGAAGGACATAACGAAACATGCTTAATTCCTCACCATAT
A_16_P19342549
GCAGTCAACTTTGTAGAGAACTATATGAAAACAGGACAAGATCTTAAACTGGAATGGCTG
A_16_P03012106
AAAATCTTGCTCACATACTTCACGTAAAATAAGAATGACATCTGGATACCATAAGGCGGA
A_14_P109202 GTTCCACCACAGAACTGAGGTTTTCTGTTTTTGCATTAAATGGACTTGCTTATTTTTCAT
A_14_P123929 CTGTTTTCATTGTTCTGAAGATGGTCAACTATCGACTACACAGAGCACTTGATGCT
A_16_P03455922
AAATAGCCAACAGAGATTCAAAGAGACATGATGAAAGAGTTCCTTGGAGAGACATGAAAA
A_16_P58424199 GTGACCTCACTGACGAGGTGTGATTTGAGCAAAGCATGGAAGGAG
A_16_P17838804

AATGTTATTTACGCAAACTTTTTAGGAAGTCAGTTACACAAGGTTGAGGTTCATCTGAGG
CUST_837_PI426210822 TTCATGGCATAAGCTGTTAATCTGAATTCCGTCCTCAGCACAATTAAGCATCTC
A_14_P125084 CTGTATTTCCTTTGCCTGTTGCTCAAATGGGGGTGATTATTTATAATTGTGGGTTTAAAT
CUST_516_PI426210822 TAAAGAAACCCACACAGAGTCAAACTTCTGCACCAGTGACCAACAATATGT
A_16_P00034214
AATGGTTTTATAGTAAAATCCTTATTCCTTTCAGGGTATTCAGTGCCTATTTCTGACCCC
A_14_P129044 CACTGTCACTTCATTTCTATGTCTTCGCATTGTCTAGAAAAGAAAAACAAAGATTACAGA
A_18_P14134134 CTGTGCATATTCTTGTTGCCAGTTGAGATGTGGGACTGGTATTTCAGG
A_16_P16191695 GTGTTTGTATTTCCTCACCTGGATTACCTGTGGCCCTTGCATCAG
A_16_P17918690
ACCCTATCACATTATTCCTCAATTTAAGAGAATGGAACTATGATCAAATCAACGTGTGCA
A_18_P16760089
GTCACTCAAATATTAACATCCATTCTCTCTTAAAGGTCACTTCCTACAAATAAATCCCAC
A_16_P19036137
GCATTCTTACCAAGCGTTAAACAACTAATATGAATTATCACTCATAGCAAGATCTGGGCC
A_14_P138549 TATAGAAACAGTCTTCATATAAGGTGATACCACACATAGTAACACTGGGTTTCAGTTGCC
A_14_P119661 AAAAGGCCCTTAAAACAAATTCAGTTCCGGTGACATCCAAAAGAGCATATTAAAAAGCAA
CUST_1624_PI426210822 AGATACTGCTTAGCAGCAAGAGAGGATTTGGACCAGCTGGGCTGG
CUST_8060_PI426210822 AAATTGTCTGGCTGGGCCCACTGCTGCTTTTGGACATGGTGCTGTAT
A_14_P138406 CAACAGTGCAGTGATTTATTGACCAGACTTTGCAGCAAGAACACAGCGAAG
CUST_3327_PI426210822 AAGCCGCTGCTCATGCGATATGCTCTGCTCACACAGATGGCACTA
A_14_P127391 CTTAATCCTCCTCTCTCAGCACATACTGCTTTGCAACACCACAGAAACG
A_16_P00685411 ACAGAGTGATAGAGGAGGCCATGCTAGGAGAGAAGGGAGAACAAG
A_14_P109570 CCTTCTCTCTTAATTGAGCGCCTTGCATATAAGGAATGGTAACTGATGATTCTAACAGTA
A_16_P01506080
AGCACAGATTTAAAACTTAGCAAATTTTGTCATAAACCTTGAATAGACAACCAAAAGCCA
A_14_P200229 CTATGACAGAGTCTGAAAATGATCAATTACCATAATGAATTCGATTGGAATGGGCACTGG
A_14_P136994
AATAAAGAAACACCAAATGGAGAGAAAAATATGGAAAAGTCTGGGAAATGGGAAGACAAT
A_14_P118492 TAAATTTAACCATACACACCAAGGCTTTTCTTATCTTCTTCCTCTAACTGGTGGGTAGAA
A_18_P15349240
AAATACAGACAGCAGATAGGTTTTAGTGCAATCTTACACAGAAAGCAAAGATCTAGGTGC
A_14_P116390
TGTCAGGGGAAAAGAAAGTTGAACAAGAGGAAAAAGAAATTGCACGTAAGGAATTTGTAA
A_14_P104089 TCTTCTTACAGGGCAACATTTTAAAAGTCCCACTTTTGTTCCATTCTGAAAGAATAAAAA
CUST_8178_PI426210822 TCATGTTCACCTTTCCAGAATGATCTCACAAGAATCCCCGTTCTGTG
A_18_P15302336
ATGTGAAGAATCACTTGCTAAACTTTCTGATTTGCTAGACTAGGTTTTCCTAAATTGGCC

A_16_P15261968
TATTACATGACCTAAATCTACCATCCTGTTTTGTCTTTCATTAGTCCACTTCACACACAC
A_14_P116728 CTATAAATAAAACCAAGACTTCCGTTCTGGCTCTCACTCTAGTTTCCTGGTGCATT
A_14_P111974 GATTTTGTGGATTTCTGTAAGTGAAGTAAGTGAGTCAAAGACATGTATGGTAAAGCCTCT
A_14_P103598 AAAACAGGAACCCCTCAATGCTATGGGTGGAAACTAAGAAAAAAAGGAAAAGAAAAG
A_14_P132650 CTTAACTACTGCCTCCTCATTTTTCAGTATGTGTTCTAAGTATAAAAAGTCCTTGGTTCT
A_14_P108979 ACATTAGGCACACTGTATACAGATTTCATCTTTACTTGACCTTTGCCAAAGAACTATGCA
CUST_3806_PI426210822 CTTCTCATGGATGCAGCAAAAACCCAGTAGGATGCACCTCTCAAG
A_16_P19138075
GGCCTATCCTGAATTCTCAAGCATAAACTACCCTGATTCTTAATCTTAGCCTTGCAATAT
A_18_P17388414
AGAATTAGGTTGGTTAGCAGGAAAAGAAAATGCTTACTGAGTTGACTGAGGTTTTTGATG
A_16_P01165504
CAGTCACCATAAAATACTAGTCCAAGCATTATTCCAAATACAAATATCCAATTCTAAGCA
A_14_P137408 ACTTAAGCAACAAACATGGTTCTAGGGGAAAAACAAGTAATTGACTAACTGACCTTTTGA
CUST_187_PI426210822 GGTGGGGGGCAACGGGAAGTTTTCAACCATTATCCAAACTCTGTT
A_14_P137979 GGAGTCTTATTCCATGTATTTTTGTTGACCTTTAAAAGTTGTTCAAGGACTGCCTTCATT
A_16_P15807437
TAACTTCAGACCGCTATTGTTCCTTTGTATCAACTACCCTGTTATTCAAATTAACCCAAC
A_14_P101594 AATGCTTGGGCAGGAGCATTCAGAGGATGTGTATTTGGTTTTGTTAATATTCTATTTCTT
CUST_905_PI426210822 TCTGGCAAGGACCAGATTGTCAGGCTGGTCTGGCAGGTTGACCTT
A_14_P107070 CTGCTTCCTTTTACTATGCTGCTATCTACCATATATGACCTCCAGTCAAAAATTTCCTTC
A_14_P116458 AAGAGATTTTTATGAAGCCCTTGCTGTGTGTGAAACACCACAATCTCCGTC
A_14_P126720 GTGGTTTAATTTGCCAGGGTCATAAGATACTTATAGAGCAATGGGAGAACTAACTGTAAG
A_18_P13711261
CTGGACTTGCTCTTCTCCTAATAAATGGACAGTGATCAATGAATTACACCCATGAAATAG
A_18_P21339816
GCATAGTTATGCAGTGTTTGTAGACAACCTCATTCAAACACATTCACTAGTATTTTCTAC
A_14_P129793 CTGAGGCAAAACGAAAATCAGGGCGGATGATATAATTTTCACTTTCTGATAAAAAGGAAT
A_14_P127947 AGGAAACTCCACTGCTATAAAGCAAATGTCATCTGCATTACAAAAAAAAAACTTCCTAAG
CUST_9044_PI426210822 GTCAACAGACAACTCTTAGAAGTGCCTGACTGAGCCTACAACATGGAAAT
A_14_P132440 ACTACTGATGTAACTTAGACAAAATATTCCTCTCGATCCCTAAAGAGAGGGAAAATTCCA
CUST_4675_PI426210822 AGGAGCTATTTAAAAGGGGTTGTACCTTAGGTGACCCTAGGGAGAG
A_16_P18549564
AAAAATCTTTATGAATTTCTAGGTTAGATGCTTCCGATATTCTATTTCCCTGACAACCAA
A_14_P136732 TGTTGTTTTACTTGTTGGTTGTTTTGTCTATTGATTCCACGAAAGCCTCAGCACTAACTA
A_14_P102862 CTTTACAGAGTTCCAAAATCTGCATGTTTTTATGTCTTGTTTAAAAAGGGGGTTGGTAAT
A_14_P135081 TCCTGTAAGTAGTCTAACAATTTTCCAGCCCTTTTTCTACTTGATGTTCGACAAGAAATT

A_14_P133502 TTGGATAACAACTGGTAACAAATCCTACTCTGGCTTGGATAATAACTGCTAACAAATGCT
A_14_P124939 CTCTGCTTATTTCATTGGTTGCTATGAACACCTGCCTGAAACTTTTACTTCTTACAAGAA
A_14_P123543 AAAAAGAAGGGATTCCATTTTCAATACACATTTATAACTCCTGCCAGTAACAGACACTGT
A_14_P139309 CTACACTAAGTTTTATCACTTCCTACCTGATCCTCACATCCCCATTTCTTTTTCTTGTAT
A_16_P36567113
CTATTCACTTGCTACTCTGAAACATGTTGCAATCTTGACCTTTATATTTGCCACCGTTTT
A_16_P21558489
AACATGTTTCTCTGTGGAACTATAAACATAGTCCTCATAAACTCAAGCACTAGAGGTGAC
A_14_P112209 CTGTCTAAAATGAGTTGTGTCTCATCTTACGCAGGTTTTGATCTTTCTGATCCTGAAAAA
CUST_9843_PI426210822 ACAGCATCCCTAAAAATAATAAGAGATGAAGCCCACATTCCGGGGAAGT
A_18_P26795169 CATCGTTGCCATGACCTCCCCTACCCTCACTCCCACACCTGAGAG
CUST_3698_PI426210822 TCAATGTTACAGCCATTTATGTCCCCTCAGATGTTGGGTTGGTCC
A_14_P113994 AGAGGACTAGAGGATTCTGTTCTTTTCTTTCCCTTAGGAAACTGAAATTAGCCTAATTAG
A_16_P00685627
ATGTTTGGCTCACAGTGAAGAAGCATGAGGACAAATACACAGACAACAAAAACATTTGAT
A_14_P104789 GCACTTTTAGGTAAGTGGACTTCTGTTCATTTGCAGAGAAATCTGATAAACTGCACATTT
A_14_P105503
CTTCAACAAACACAGAAATGGGAATTTAATTGCGAGTCACCAAGAAAACAACAAAATAGC
A_14_P111417
AAATAGTCAAGTTGCTACTGGAACCCCATTTGCAGAAAATAAAAGGAAACAGTGTAAGAA
A_16_P15659273
ACCTCCACAAACTACAAACAAAACTTTTGATGCTTCAAATTAGTCCATACCAGAGTGTTT
A_14_P104500 GATATGTATTGCTCCAATTTCAACTTGTCACTTGATCACATTTACAAAACTCTTCCCCAC
A_18_P25342829 AGGAATTTACTGGCAGACAACGAACATCCCTTGAAAGCATCAGAGCATTAAGAAA
CUST_2697_PI426210822 GATGATGGGGCGGGCTAGGCTTTTCTTCTGTACCATGACCAGGCG
A_14_P105150
ACAGAGAAAAATTTGGTTCAAGGAGGCTGTATAGGAGAAAAGAGATGTCAAAGTTTATGA
A_16_P03705284
ATGGTTCAAAGGTCAAAGTAAAGTCTGATAGCATGATTGATATATGCAGCCGAAAAGATT
A_14_P129840 GAAAGAAACAAGTTACGCTTTGCCACTATGTTTTGTTCCTTAAAATATAGGTGCAAGTGC
A_14_P137974 TGGAATATTACCAGACTTGACTGAATTTTCTCTGGATCTTACCCTTTTTTCTTTTCCTGA
A_18_P22841731 ATTAAACCCCAGAACAAGATAGAACCAGTGTTGATTGGAATTCAGAGCACGTGAT
A_14_P118397 TCACATGAAAGAACAGCATAACATTAATCATCCAAATGCATATCAGAGCAAACTCTAGGC
A_14_P130108 ATACGAGTCCCAATATGCCCATTACATTTGTTTATGGGAGATACTGCAAGTTTAAGGTAA
A_18_P14133536 CCAACCTCAGTATCTAAGGGTTAAGGGTGTGCAAGAGAGTGCAATCAGTATT
A_14_P116090 GTAGTCAATAATTGACTACAGATACTTGAAAGATGACTCATCAAAGCATGAGGGGGAATT
A_14_P125315 CTAAGTTAGAAAAATTTTGTGACTGCCATTTGTAAGATATGGAGACACATAAGCCAGCCT
A_14_P136255 AACGGAACTCAAATTCTTGGTGATGAAACCCATAATAGAGATAGAGACATATACCAAGCA

CUST_591_PI426210822
AATAGTTGTCAGCTGCATAGTTGGATAATTGGTTTGGACTTAGCGGGAATTAGTG
CUST_4699_PI426210822 GTGTGAGTGAAGAGGTGGGAGAGTGTGCTGAAAGGTGGGCAGAAG
A_16_P03241705
CTCACTGAGCACACCAATATCATCACATAGAATTATCACAGAGAACTGTATAAACTTAGT
A_14_P116243 AATTTTCCTAAGCCCCTCAGCCACAGATATATAATGTGTAGTTTCTGAATGAGTGAATGA
A_16_P00764858
GTCCTTCTTAGTCAACTCATATTTGAAGAGTCACATATCATCTCCTATCACATACCAACA
CUST_3150_PI426210822 GAGTGGCAATAGTGAAGTGCAGGCCAGAGGAAGGGCTGTTTCTAT
A_16_P02446001
TGGATAGACTTTAATATCCAGATAGACCATCTTTAACATATTAAAAATAGATATGAGGGA
CUST_8623_PI426210822 TCACCACTTGTGAGTGTATGATGAGTATCTGCTGTGCTGGGAGCTA
A_14_P126302 CTTATTTCTGGGAACAATTTATTTTGTCATCTGGAAGCAAGGCAGAGAAGACTACTCATT
A_14_P121885 AGTAATTATGCTATCTACAGGAGGCCAAGGTTTTTCACTTGGTAATTTAATTCAGCAAGC
A_16_P01397873
AACACGCAACACTCTATTAACGCATGAATGTATTCTCACCGAACAAGATTCAAACAGTAT
A_14_P139908 CAAGAGATGCTTGCTTGTCTATTGCTGATGGTTTCTAACAATCATCGGCTTTATTTCATT
A_14_P201516 ATCCACAGAGAAAGATGATCCTCGTATTTCAACAAGTTTCACTTTTACTATTCAAGGCTT
A_14_P200218 TTCTGGTGCTCTCTGAAGTTAGGAAGTATTTAGAAGAGGAAGATTTGTGCTTCCAG
A_16_P30952660 CATCTTTATCGCCTCGATCAAGTGTGGAGTCACATGCTAATGTGTGCTAAAGAA
A_14_P126321 CTTCCTTTTACCATGCCGGCATTAAACTTTTCTCAGTCACAAGTAGTAGAACATTAATCT
CUST_4086_PI426210822 AATTTTGGAATAACCAGCCTTCCTTCAACCCCCTTACATGCATTAGAAACTTC
A_16_P19891523
AGTTTCCAGAAGAAATTGTATGCATCGTAATGTAATGTGCAACTCCTCATAGTTTTGTGG
CUST_7765_PI426210822
TTAACTGATCTAACTCTGCCAGATCCAGATTTATCAGTGGTTTTGCAGTGGTTTTATC
A_14_P107906
AGAAAGAAGAAGGAATATTAAAGATCGGAGAGGAAGTTCAAGAGAGAAAAGAGTTTTCTC
CUST_4932_PI426210822 GTCATCCAAGAGGGCCTCAAGGTGCCCAGAGAGACCAACTGAAAA
CUST_7119_PI426210822
AATGATTGATTATATCCTGTGTCAGCTGGAATCCTGCGACATGTTCTAGTTTCCTA
A_14_P113094 AAAACATCATTACTTCTCAAAGGCAACTTGAACTGAGTCTCCTGCAATTTTAAAATGACC
A_14_P122390 AAGTTCTTTTGTTCTTTGAAACAGTAATTGGGAACCTCTCTTCTCTTGTCATTCCTTTAC
CUST_10172_PI426210822 TTAGAAAACAAATCTGTGAAATGGCTCAGAGAATGTGTAAGGTTTGGCAACCC
A_14_P139132 CTCCATGACTGTGTGTTGTGAAATAAAATGGTGAAAGATCACGATTAGCAAGTGTTTTCT
A_18_P16256109
AAACCATGCAATTACACACATGAAAGAAGTTTTCTGCGTCAAATATTTTCATTGCTCCAG
A_14_P115920 AAACTCATTGCCATTAACAAGGTGCAGCCAAAACTGACATTATTCATGTGTATAAATCCT

A_16_P38941347
ATGTTATTTTCCCCAGTTATTATGAAGCCTTCCTGAAATGAACTTTAAATATCGGCCACC
A_14_P133758 ACTGTAGGTGAACCTAAATCTAATATACCCACCTTTACACTGCTGTATCAGTGATCTTTC
A_14_P119941 AGAGATATGTGGAGAAAATCATCATTCATGAAAAATACAACTCTGCGACAGAGGGAAATG
A_14_P120377 GTGGCCCATGGATATCCATTCCTTCTCTATCAAGATCTTCATTTTATAGAAAAGAAAGTG
A_14_P139697 AAAACAGGCCCAGAAACCAGACATATATTGATTGAAGTTAAGGGAAATCCTTCCAG
A_18_P13854123
TCAATTAATTTGTTTTATTTTATTCCAATGAGGTAAAAAATGCATCAACCAAAATGCAGA
A_14_P125229 CCTTAGGAAATGATTCTGCTCCATAGGATATTGTTATCATAAACTAGCCAGAGCCATTTC
A_16_P16579532
GTGTTTCCCTTTTAGTTACATAACAGACACTCATTACGGAATTGAACAGTTTTAGACCCA
A_14_P105948 TGTGCTAACTTTTGCCTTTCAGATTTTTAATACGGAGACTGGAGAACTTTAATCTTACCC
A_14_P129760 GTGAAGAACTCATCAACCAGAGCCAGGTTGAACAGGTAGAGAAGCAG
A_14_P130029 GGCATTGAGAAAAAAAAAAATGAAGGCATTTTACAGTCTCTAAACATCTTTAAGTGCCATG
A_14_P110897 ATGTGAAAGGTTCACTACTAGTAAACTGCAGTCCTTTGAGGATTTAGCCAGTATTTCTAC
A_14_P110810 GTAGTGAAAGCCCTCTTCTGATATGCTATCATGAAGTCGCACTAGAAAAAAATAAGAGAA
CUST_4152_PI426210822 AAGCAGAGAGGGAGAAGAGGAAGAAGCCAAATGAGATGGTTCGTAATT
A_16_P16191356 CCAATTTCATATGGTCATTGCCTCTGGCTTGTGGCTGATCATCTTGTTCT
A_14_P138342 AAACTGACAATGATGTTCTACCTACTTGTTACATGCTCATGGAAGACCGTGCAGTATT
A_14_P135282 TTATCATGTTAGAAAGTTTGCCATCTGGCCTGGAAGCAACTATTTTTAAGTCTGTTTAGG
A_16_P19818139
AAAGAATGGGATGGTCTATAAGAAGAATCGCAATAACATGGCATAAGAACTCAACCTTTG
CUST_10358_PI426210822 TTAAGGGTTTTCCAGTATGACTTGGGAATTGCAAGCTGATGATACCATGGTA
A_14_P136164 ACTCATCAGTAAATCAACCTCATGTTCTAATCTTAATATGTGTCAGACACCCACAAAAGC
A_14_P107818
GAAAAGAGAGGACAAGGAAGAAAATGAAGTGTATAAGGCAGAGAAGACACTAAAGACTAA
A_18_P15129593
ATTTGAGGCTGATATTCCTGTTCACTTCTTCTACCAAAATTTGATCAATAGGACCTTTGC
A_14_P119722 CTCTGTTAATTTGTCAGTAAGGTCAATTTGTCTGCTCTACTTGGAACATATGGGTTTGAA
A_16_P15751512
AAATGCCAATGCTATAAGGAATACCATCAGTGAACTTATCCCAAAAGGTTATCTGCTACA
A_14_P115031 ATCCTTAGTTAAGAAGAGGCAAATGTTCCTTCTCCATTAGCAGCATCATTTAAACAGATG
A_16_P18023353
TTGGGATTAAGGTCTTCACACTGCATACTTTTCAATAATCACTTAGAAAGTCAAGAAGCC
A_18_P11977812
TAACCAGGAAAATACTCAATCACCTATATCTGTAATCCCCATCATAGTTTTTGTCCCACA
CUST_10068_PI426210822
TTAAAGTAGATGAAACCTCATCTCTCTCCCATAGTGTCATAGATAAAATCACAGTTGAAC

A_16_P21586826
ACCTTGACTTTGACAGAATTTACTCAGGACTATAACAATCATGATAAAGCCATAGACCCA
A_16_P41728449
CCAGGTTGATAGGACAGAATCAGTTTTAAGAACAACTTATCTTATAGTCATCCCCAACAC
A_14_P201572 CATTATAAAAGCCATGGTTCCCCTCCTTCACATAGCCCTTTTGGTATTATTTGTAATCAT
A_18_P13077067
GCCCATTTTCATTCCATACTACTAGCACAATATAAATACTATATCCACCATTGTCTTGTG
A_16_P19173919
AACATGTCAGTTCAGATGCAAAACCTTCATTCTATAGCCAAAATTTTTGTCCTCTTGCTC
A_14_P124806 TGCTATTGAAAACTTTCTAATACAAAAAAGAGAGTTCCTGCGACAGAGGTTTACAGTGAT
A_16_P15962231
AAGCCTTCAACAATATAGCAACATTCCTAGAATCCTACTAACTTCAACTCATGAATGGGT
A_16_P33580345
AAAAAAGACAGGATTATAAAGAGATATCAAAGCACGATTTTAGATAACCTAAACGGCCCA
A_18_P14138676
TGCTCAGATAACATATTTATGTATCGCTTTTTCACTAAAGAGAATCTGACGGCATCTGGA
A_14_P132811 ACTGAACAAGATTTGAGATATTCAAGGTGGACCTTAATTTAATTCAATCAAACAAGCGGG
A_14_P121548 ATTCTTCATTATGGACAGGCCATTATGAAAAACTCTTGAGGGTTGATTATATTTGTGCCT
A_14_P120104 TTCTTATTTCTGTGCGTTGCAAAACCCCGAGAGAAATCCACTAGCGTC
A_16_P00783820
AATGGAAAAACCAAAAACGACCATACCTATTCTTTTCGCAGCAACGTTATTAACGAAGTG
A_14_P129958 CTTTCTTTTCACATGATGCCTTGTTCCTACGGAAACCATTTTTGTCTTGTTTTTAGATGA
A_14_P116038 TATTTAGATATGGTGTCCTTTTCTGTTTTTGGGGGGGGAGTTTTGTTGTGTTTTTTTAAA
A_16_P18026211
AACTTCCACATTACCAGTGTAATTCATACCACAAAGCAATTCACTTTACCTTCTAGTTGG
A_14_P109382 ATGTCACTTATAAGGGGAGAAGTGTTCTTAAAAAGTCAACCAGAAAACTGTTATGCCTTT
A_16_P18357095
AATGGGAGTTAAGTTACCTAAAATTGCATGAATCACAAGACCTTTTTACACTGCAGACAG
A_14_P130781 TGCTGTCACTTCTATCTCCCCATGATTCTGTTGGTTTCTTCTGATATTTACAGCATAAAT
CUST_10360_PI426210822 GATGATACCATGGTACCACAACCTGCAGTGTGGCAAGCCTACCAG
A_14_P135339 GTCTAGTCAGGGGGAAAGCCAAGGCAACCAATATTTTGGTTTTTATAATTTTCATTTGTG
A_18_P17179246
AGCAGAACTTTTTATGATGACACTACGGGTTGAAAAAAGGCATAGTTTTGGTTTTATGAA
A_14_P131671 CTCATTGCTAGCAATGTCCACTCCTTTAAGTGATTCGAAATGGCTCAAAATTTCCAC
A_14_P111976 CTCTGTTACTTGGAGTGTAGTATCAGAAAAACTAGGACATCGGTTTGATGTAAGTAAGGA
A_14_P137916 CTGCAAAGCCTGAATTTTCTTGAATAAAGTGTTACTAATGAATTCTTCTTCCTGCTCAGC
A_14_P117100 ACAATAACAACCACTGCATATCAGCCATACTCATGTCAGTCTTTCTGACATTCATTTACA
A_14_P101919 CCATAAATTTGATGGTAATGCTTCTAAATACTTGGCCTTTGGATTCTGCCTGAACTAGAA

A_14_P117560 CTGTGATTTGTAAAAATAAAATCTTTAAATCTCTCGAGCCCCACGTCTCTTCTTTCAGAG

CUST_4232_PI426210822 TTTAATTGTCAGAACAAAGTGAAAGCGAACTCAGAGTGGGACTTGGGATG

A_16_P00907342

CTATAAAGACGTCCTTTCACTCCTAATCACAATTGATTGTCAATTAGACCCCCATTTGTG

A_16_P18889997

AAACATCCCAATCCAAATTTTAGGAAGAGGTATGTGATGACATCAAATAGCACAGGACA

A_14_P200801 GGGCTTGAATTTGAAGCATGCTGAAACGTGTGTCTTGTTAAACAAAAATGGTTGTTTATA

A_14_P201036 ATCATCTCTTTGCCCATACTGATCCACAAAAGTTTTCATTTCAGGTGATAACTCCTAAAA

A_14_P128540 GTATTTAGTGTTTCTGGTTCTAACAGCATAATTTCTTTCAGCAGTGTCATAAGTATGGCT

A_16_P03748788

ACTTCTATTTTGCTTTTTCCAATGCAAATACACAGTCTGATCCCTTAAATACCAGAAGCG

A_14_P129302 CTGGTATCAAATATTTTGCATAGATCTTAAGGTTGTTCAGCAGCCACTTCAAAGAGACTG

A_14_P122499 ACATTTCTGTCCTGTATGTATCCAGGGTTATAGGACGTGATTATAGGACACGCATATATG

A_14_P120620 ACAGTTCAGGCATCTCAAGATGAAAATTTTGGTAATACTACACCAAGAAACCAGGTTATC

A_14_P132572 CCAATGACCCATTTCTGGCTCCTCAGATGAGGAAGAAGAAGAAGAG

A_16_P15832556

ATCCTTGCCATCATTAAGAATAATCCCAGTGTAAGTCATTTCTAAACTACTTCACGGTGT

A_14_P131566 TATCACTTTTCAAGAAGGCAAAAACCCCCACACAATCCTATTGTTATCTTCTTGACTAAA

A_14_P106071 ACATTTGGCTTCTATTTTCATCTCTTTGAATGGGTAACATGTAATCTTATGGTGTCCCTT

CUST_1740_PI426210822 TGAATTCTGTCACCTTGCTCAGTCTCTTCACATCTTTAGTCCTGATAATCCC

A_16_P00685428 TTTGCAGTGACTCCCCTCAAAAGGCAGAATGCAATTCTCTAAAGGGAAAAAAAAAT

A_14_P112386 CTGATATCTCTCCACAGGACTAGGCTCTAAGCCCCTCATTTCAATGAG

A_14_P125329 AACTTTATGGAGGCTAGCCAACTACTGACAGATTCTAGTGCTAGAAAAGAAAATACCTAC

A_16_P18375686

ACTGACACCCGATAAATAACAATTACAAATGATAGGGACCAGTAGAGATTCTCACTATGT

A_14_P106988 AGAGAAGAGACTGCATTATGCTCCCCTTACCACATCCCTTACAAGTAAATGTAAAAATTC

A_18_P14965298

ATGCGAATGTTTGAAGAAGAAATAGACTACCAGCAAAATATAACTCGTTTGAATCGTGGC

A_14_P131601 ACATTACGAAGACATTCCATTCACAAGAACAGATCCAGTGGTTGCAGATTACAAGTATG

A_14_P117656 CTTGGAAGTCTCCTCAGTTCGAACAGACTGCTTATCTTTCTTGCTACAGATC

A_16_P17998154

TCAGAATGTCTGCTAAATCAACTTTTTTGACTGGTATTACTCACGTTATCTATGGAACGC

A_16_P18500997

ACTGCACTCTCATCAATTTGCTCTCCTTCTCTAAACTCCTAATGATATTTCTCATGACAG

A_14_P124764 GGATACAATAAGATGACAATTTTGATTCCAGTTCCTACTTGACAAAGACTAAAAGCACCC

CUST_5614_PI426210822 CAGTCTGAAGCCACTTGTTCACTGGCTGAGGAGTTGGATGTGATT

CUST_9613_PI426210822 TTGGAATGCCTGATCCTGCGAGTCACTGCAGCCCCCATCTCATAA

A_14_P125100 CTGTCTCTGTTTTTTGACATTCTCCACTGCATGGCTGAGAACGTGAC

A_18_P22839783 CTTGAGATGATGGAGAAGAGAATTTGGGATGCATGGTGTGTGCCAAAGAATAAAT
A_18_P11657933
CGATGCTCCCAAAACTGATAACAAGATGTATGCTTTATTGAGAACAAGATTCCAACACTT
A_16_P01748101
CCCCCATGATTTTTGTTAAACTCTACAACTCTCAACACATCAATAAGCAGTCTGGATTTA
CUST_3530_PI426210822 AGCTGGCAGTTGATGCAGGTATCAGAAGCAAGTGGGCCTCGGAGG
A_14_P117653 TTCACTCCGGAACTTCTGATAGCAAGACCAGAGAAACATGATAAATTAAACCAAAGACTA
A_14_P110464 GCACTGAAAGATTAGTTATAATCCAACGTGAACTTGAAAAGGGGAAAGCAAGCGATATCT
A_14_P113839 TTAAACATTTCAAATCCTCCTCCTCCCTATGGGTTCCTCTCTTGTTAATGTGATTATATG
A_18_P12594721
AGTTTCTATAGTTACTTCACAAGAAAAACAAGGGAGAGTGGAAGAGGCTAACAGTCAGA
A_14_P138613 TTCCAAAGATGCATATGATTATAGGATTATAGGAACTTTGAGGAAAGTGCTCTAGGCAAT
A_14_P116301 CTGCCACTATAATCATGTTTTGGCTGAACATATTTCTTGCTTGAGTTTATGTGGCTTTTG
A_16_P15704801
ATCGTATGTTAACTACATGACTGCTTACCCATTAGTCTGAATTAACAAGTTCCTCTACCC
A_16_P15274163
AAAACAGGTTCTGGTTCTACTTCTTCTTCCTGTAAATAATTCAGCATGTGAATAATGGGC
A_16_P35833441
AGTCCTCATGTTAATGATACTAACGATAATGACAAACCAATTTTCACTGAGGTCCACAGT
A_16_P16988005
AAAATGTCCCTACCTTTACCAGAAAATAATCTGTGATCACAAGTTTTGCCTTTCTTGCTT
A_14_P123714 ACTGCACTGGTCATTGATAAAGATGTATTCAAAGGCTCACTGAAAAGAGTCAAAAATATT
A_14_P119769 CTGTGAACTTGATTCCAACATGCTTCTAAAACCAAGTGGACAGCCTTGTTGT
A_14_P138984 TGAATAATCCAGTAAAGATTTAGATAAACGCGTTAGGAATTCAGGGAAAAGAACGGCCTA
A_16_P03326208
AATGTCGTTGTCCAAGAGATATGTTGAAACCCAAAGAAGAATTGACCTAAAACCATGTAT
A_14_P116903 TATATACAGTCTTAGAAACATTGAGTTCATTTATGGGGTCAGAGAAACTCCCAAGGAATA
A_18_P16460028
TACGTTATGAGTCGATATTTCTTAAAGCATGTCTACCTTCCATAATGCCAGAATTCCTCC
A_14_P130732 AGGATGAGAATTTTAAAGAGGAAGCATGGTATCTCTTGTCACTTCAGCAAAGTGTATCTT
A_18_P14136159 GATTCCCATGTTTGCTAGTTACATATGGCCAAGCCCTTCAGCCTTTCT
A_14_P123920 CATTTAAATATCCAGGATCCATCCCCCAATAGGGCGTCACCATAAACAACTATCTTAA
A_14_P127273 GCGACTGGACTGCTATTGTAAAAATTCTTTTGCAACTTTTCTTTCCCATTCTGTATTTGT
A_14_P110954 GAAATCCTTTGCTTGATTCCATTTCTTGTTTCCCAAGGGCTATATATGCCAAACTTTTAT
A_16_P21221282
TTTTAAAGGCTTGCTTAGTTGATACAAATAATGCTCTCATAATATCCTCATCTGTGCGCA
A_14_P128201 ATCTAACCCCTTCCTAAATCCCCCACCATTAAAAAAAACTGTTGTAAGTTTAGAATGCAA
CUST_8693_PI426210822

ATCACTGACCATAACAGGAGCTTAAAATTAGACATAAATGGAGGAATGAATTGTTTTCCC

A_14_P122762 TTGCTTTAGTTGCTAGAATTTTACCCGAACGATGCAGTTATAGATGAGGGACCCTTATAA

A_16_P00994845

TAATTCCAGTGTATTAATACTTCACAAACTTTTGGAACTTCTTGTTTCCAATGACAGGTG

A_18_P11512454

ACTACCAATCTACACTTAACATGACTAGTATCTCCTTGAGTTATCTCCAGTCTTTGTGGC

A_14_P134716 TTTTCAACGTCTACTTTTGTATTTGCAGAACATCATCAAGAAGGTGATCGGGCAGAA

A_14_P113189 CAAGTCCTTTTTCCGTCTTTGACCAGGGCTTTTCCTTTCCAAATCTGAT

A_18_P22842665 TTCTGACCAACTAAGCCCCTGCTGTCACACCCTGCTCTTGATTTA

A_14_P119771 GCAATTTTTTAAACGTCAGGACTGTCATCTGTGGAATCAATTGAAAAGGGAATCAACACT

A_14_P201935 TGCTCTTTACATAGGGTGGAGAATTGTCATGTCTTCTCTAATTTTTCCAAGTAAAGTGGT

A_16_P19804719

GACATCAGGACATCAGTAGACATCATCAGCATCAGTTACAGATACCCAAAGAGTAATAAG

A_14_P123262 GTTAATAGTCTGGGAGTGGACTCTCAAGAAGTCCGATTCTGAGAAAGGC

A_14_P124916 CTTCCTATGGGAAAGACCAAGTGATTTCTGGAACTCATAGTCACATATTACAATCTCTTT

A_14_P115467 ACAGAAGCAAAATCAAACCTGCTATTTCAGCACTCCTGTTTTTAACTTGGTGTCTTTAGT

A_14_P136657 AAAAGTTGTTTGGAAGAGAGAAAAGGCTAGTAAGATCAATGGTGCTTATTTCTGTGAAGG

A_14_P107437 CCCAGATCGCAGTATAATACCTTAGCACTGGTAATTGATCCAAAAGGAGAAAATTCTTTC

A_14_P107533

ACAAAGAGTTTGCAAAGAAAATCTCAAAAAAATTTTGTAAAAAGGTGATCACTGCCCACC

A_14_P129132 CTCCTCAAAACTAAGACCACTGTTATTCTTACGCTGGATTTCTTGAATTGCATTTTTCAG

A_18_P16594193 CCTACCTCAGTTTACACTCTTCTCTTTCATATCATCCATATAAATGTTGCCTCTCCTCAT

A_14_P115763 ATAGATGGGGCATTTTAACTGAAGTATAGGGATAGTTTTAATAGGTGGAACTGGCAGAAA

A_16_P15018747

AACCCTTCCTTTAAAAGTTCCTACATTCCACTGTAGGTCAATTACACTGTAACCTGAATT

A_18_P22840726

CCAGTTTCAGGGACAGGAAATAGTAGAGTTCAAGGAAAAGTTAGGACAAGGAAAAT

A_14_P116482 CACCACAGAGAGATACATGATACCATACACATTCTTTGAGTTTTCTTGTGTTGTGTTTGA

CUST_8513_PI426210822 ACGTGAAGGACATTCAGCCAAAGGGACAGTTTTTCCATAGATAGCTTGA

A_16_P20787201

TATTTATTGCCCTTAAATAGTCTCCTATCCAATCGTCTTGTATTTCAACACACCACTGGA

A_14_P131825 TGGCTTTCTTCATACTTCTAAGTATATTTGCTGGAAAGAAGTTAAAACAATGAACAAGCC

CUST_5679_PI426210822 GCATGCATATAGGAATATAGCTCATGCTATGGAAATCCACTGTTTGCACAGCCT

CUST_10160_PI426210822 CAAAGGGAAGTGAGACTGCTTGGCCAGCCCTATGGCCATGTCTTC

A_14_P122959 CTGTGGAAGGTTACACATCAAAATTTGGCCTACAGATTCCAGAATTTATGCAATAGATTC

CUST_5754_PI426210822

ATAAAGCAAAATTGGTGCCGGGTTTGTATTTAAAGGGAAACCAAGAGGGTTTTTTTAT

CUST_3979_PI426210822 GATTGGGGCTCCAGCAGGATCAGAATGACTTCACCCAGACTTCTG

A_14_P110360 TTTGTTGTTTCTGAATTTCCACATGTTCTGAAGACAAATTTTTTATAGCCGCTTATGGCC
A_16_P16192025
TTGTTTCCCTAGGAGTTTTCTGGTTGCTTTTGGATACATGGTTATATGAGAAAATTGTGT
A_14_P130381 CTGCTTTTATAACTCTCTCTTTATTCCCTTTCTGCCTCTTTGAACATCTTTTATTTGCCC
CUST_3413_PI426210822 GAATCACTCTTTGAGTAGTAGCTGGTCCCTCAGTCTACCCCAAAGA
A_14_P118034 CTCATCTTACATAATTTTATTATTTTCAATTGCTGCCCGTCAATCTCAGGTGATGGTTCC
A_18_P22841903 GATTTCAGATTGCACACTTGGAGTCAGGGCAGCAGTTTGGCTCAG
CUST_3103_PI426210822 GTGATGCTGGATCACACTGCACCTGATGCCTTTCTCAGTGGTCTA
A_14_P107117 GTAAGCCCGTAAGAAGATAAAGTCAACAGTATTTTCTTCCTTCTCAGTGACAGATTCCA
A_16_P18224592
GAAATGCAAATATTTGTCCTTGAAGTCTCTGTAGTCATAGGATTAGCAACATTATGGGGC
A_14_P113049 CTTCCCTTTCTCACAGGCTAATTTTGATAACACTACCAGCAAACATGCTTTGAAATTATC
A_14_P122813 TATTTCCTTTAGAAAAGCAGCCTCCTTATGTGTAAACTATAAACCAATGCAACCCTCACA
A_14_P106545 AGGACTGATGGACATACATTATCTGAGAAAAGAAACTACCAGGTAAGACCAGTCTTTCTT
A_14_P101480 TTCTAGTTCTGAGGAAATGAAGAATGGGATTTTGTAATTCCCGTAACATATCTCGTCTTG
A_16_P16378588
AGGGGAATTTCTTGAAATATCAGAGCCATTCATAACTGAGGACATAACTGGAATATTTGG
A_16_P38183115 TGATGACAGCAACTTACATCCATATAATATCTTTGTCCATCCTATTCTATCTTCCACCTT
A_14_P103112 ACTCTTCATAAAAATGTGAGAAAAGCACAGTGGTTATAAAAACAGAAGGGTCTTCCACAA
A_18_P25891818
ACATCGCCAAAGATATGAATCATCATAGAAAACAACGAGAGAAATACTACTACTCTAGGA
A_14_P103511 CTCTGGAGAGGTTGGATTTTGCTTTTGTAAACACATGAATCCTTATGATAAAAGTCTGTC
CUST_2852_PI426210822 TTCACAGACTGCTAAGCATAATTAGAATGAAGGTCTAGGCTTAGCCATAGCCC
A_14_P129864 ACGCCTATTTCTGTTTCCATTATTTTTCCTGCTCTTAATCAGGACTGAGACTTTTCAAAA
A_14_P111661 AATAGGCCTGAGTCTTCTCCAGTATATTTCCTATGTGCTTCTTAAATCTCTCATTTCCTT
A_16_P19564646
CCAGTGAATATCACATAATTGACTCCAACTAACCCTTTTTAACCTTACAGCATAGACCAC
A_14_P108102 AAGCAGTGAGGAAGTTTAAAGATGCATGAAATCTGTAAAGTCTCAGAACAAGAATTCTCC
A_14_P124895 CTGAAGGTGCTTCTCTTGCGATTCTTATTTTAGCCATATGTTCATAGGATTTCTGTAGGA
A_14_P118237 ACTTCCCTTATCAAGTAGTGTTGGATACATACTGTCCTGTTACTTAGCAAATACTGAGTG
A_14_P123382 GAGATTCCGATACAATTAGTGCAAACATTTAGAAGATAAATTGGCCTCCATTCCAAGGTA
A_14_P136818 GTTCTGCTAATGATTTGGGAAGATTAAGTGGTATTTCACTTAGTTGATTGTGGGACAGAT
A_14_P119746 CTTCTTTTTGATCTGCTGCTATGACATTTCCACTCACTTGCTTAAAACAGTTTCCTTTTT
A_14_P134287 TAATACCAAACTTAGCCAGTCTAACTGTAACTCAGATGATAATCTGACCACTTCCTGGTA
A_16_P03430126
CAGGTGGTGTTATGTCCAATGTAAGTATCCTAAGAGCATGTGAAATGAATTTTTGGTTGG
A_14_P106400 TATTTGGGAATTGGCCCCAGTCAATACTTGAAAAGAAAATATTGTTTGTCGAGTGTCTAT
A_14_P137557 GTGCAATCAAGCATGTAAAACTATAAACCAGTGAATGTTCCTTGCTGTTTTTTGGCAAAA

A_18_P10781005
CACACCCAAAGTTCAGTAATCCAGTTGTTGCATGATATTCAATTAAATTGCACCTTTACG
A_18_P22840868 CTGGGTTCTTGTTCTCTGACCAATTCCCAGTCATGGCTCTCCCAT
A_16_P19626025
AACATCAGGATGGCTATGTCTAAAAAATAGGATGAACAATAACTAACCTCTTCAGCCTCC
A_14_P112099 GTTATATTTGCGACAAGTAGGATACTTGTGGTTGACTTCTTGACTGATAGAATACCTTCA
A_16_P15093138
TCACCCTTACCAACACTAGATATAACTGTTAAATACATAAATAAATCAATCTCTGCTGGT
A_14_P114792 GAGCAAATGGGATGGTGCATATGATCGGAAAATATATGGTAAACTAAAACTCACCATTCA
A_14_P135298 TGGCTGATTTGGACTATAAGACCAAAAGGAGTGTGGCCAGAAGCAAGAAATAA
A_14_P135910 TTACTATGACATGAGGCGGCTGTATACAATTTTGGGGTCTTCTCTATTTTACAAGGTAAG
A_16_P15589507
TGCCCCATACTAGGCTATGAAATAGAAGATAGGATTAAAAATAGACCTGGACATTTCTGC
A_16_P16843776
GAAGTTCAAACACATCATCCATAGCAACTTGAAGTAGACTCCTATTCTGTTTAACCATGC
A_18_P22840134 TAAGAGAGGAAGCAAGGTGGGGCAGGCAAAGGGGTTGCTGAGCAT
A_14_P114907 CAGTTTTCTTGGGCCTCATGGTAATGTTCACGTGGTTGATACCAAATGA
A_18_P10526611
ACCGAGCATCAACTTTTAATTACCAGCAGTTACTTTTTTTTAAAAACCTGCCACTTGAGA
A_14_P201697 CAATCATATGGCAGCGCCAATATTTTCCACATCATATAGGTTTCAAATCACCTAGTCATT
A_18_P19003338
CTTCCCATTACTGAATCTTGCACTTTTGTTAATGCCATGACAATTCTCAAACTCACCATC
CUST_8791_PI426210822
GAAAAATAATTGCCTAGTTCCCTCATGTAGAGAAGTTGAGCTATTACCTTGACTTCCC
A_14_P137346 CAGTAATGTATTTGAAGGAAAAATACCCTGATTTGAAACCAACAGCAGATGTTGCAAACT
A_14_P116928
AAACAGAACTTGAAGAAAGGACAAAAACAAATATAAGAAACTGCAGCAAATGTCAGCAGG
A_14_P118603 ACTCCTCCTGAGACTTAGGGATTTTCAAACCATTTCAAATAGAAAATGAAAGTGTAAGGA
A_16_P20311340
ATCCAAGTATTTCCACAATTTCAGTTCAATCCTATCTTCAAATTCAACCACCTTCACTCA
CUST_5722_PI426210822
CGTTAGCCTCTCTTTTCTTATCTTATGAGATAATTTAGGAAATCAAGTACAAGGCCCTTG
A_14_P130627 TGATGTTTGGCATTTCTAGATGTTATTTCAAGGGTATAGGCATATTGTTGGCATCTAGGT
A_14_P115230 CCCACCAAAGCATAAACATTCTGAATAGAAGAAGAGATGTTCTATGAAAAGCAATGGAAG
CUST_8816_PI426210822 TTCAAGACCCAGTTCATATGTCCTCTCTGTCAAGGCTTCCCAGAT
A_14_P200223 ACAATATCTAAAAAGAAAGTGACATGAAGGAAGCAATCTACAACTTCCTTCCGCTTAGCG
A_14_P113485 GAAGGAAGGAAATACAGAATATTATGGCCATGGGAAAGAGGTGTCAGTGTGAA
A_14_P121014 TTTCTCCTGGGGAGAGAAAGTATGGGAATATCCATCTAACATTCTGACTTTTTTTTTCTC

A_14_P111819 AGAAACTGGGGCCACAGAACTTCATTGATGAGACCAGTGATATAGAAAATTTACTCAAA

A_14_P128280 AGGGGAGTATCAAAATCACCAGGGAACTCTTTGAAAATCATTCACCTTGATTATTTTTTT

CUST_9309_PI426210822 CTGATTCCATAAGGGAGGCTGGAAAACCCCACGTCTGGAGAAGTA

A_16_P18103972

AGGCTAGACTACACACATCTTTTTTGTGACTATAATGAACTGAACTGAAAAGGCTTTCCT

A_16_P18864406

AATCTTACTTCTAGAACGTAGTCACTGGTCATTTCTATGCAAAATTTACCCTCTTCCAGT

A_14_P137657 TTCCACTTCCCTTTTGTTGTTTTATATTTTCTCCATCTCTCTTCATCATAATTGGCACGA

A_16_P17731119

ATTGTGTTGTCATACAATGATGTCCTGAAGAGTAACTGATGTGTCAAATACAGTCACCTT

A_16_P58424366 TGGAGTCTATGTCCTCTTGCTGATCTGTGAACCCGGATAAGGAAATGATT

A_16_P02621035

CTCAAGGAGTTGACTCTAATAAAGATGACAACACAAAAGGAGATAGAAGACATCACATCC

A_14_P134133 TTAAATCTTTCTTGTTTAGGTTTGTTCGGACAGTGTTACCATTTTCTCAGGAATTTCAAA

A_14_P123604 CAAATGTTGGGCTCATAAATGCAGACAATGGGAGATGAAGATTCCCCAGAATATA

A_14_P100502 AGAGGAGACTCAAATCTCCCTACTGTATACTCAAAACAATGTGACTTCCTTCCATTATAT

A_16_P02865765

TGATAGACCCACCCTATAGAATTGTGATAGACACACCCTATCCATCTAACCATTAATAGA

A_16_P21523620

AGTTCAGTTCTTCAGGCTTCATTTTGATGTTTTCCTTTCATCTTAGTCTCTAACTTGTGA

CUST_142_PI426210822

AAGACTTTTTTGAGGCTTCTGAAAAGCCAATGTTGTTTTCACCCCAACATTTAATCA

A_16_P15227765

AAGAGTAAGAGAAGTGGAAAAATACCTGACTGTGATACAATCTCCAAAGACAAAAGAAGG

A_18_P22840115 GGGGCTTCAGGGCGCTCAAACATCTGTCTTGTGAAGTTCTGTTCT

A_16_P16141203

TGTGGACATCATTAGACAGTAATATCTCTGTGACCAAACATATGTATTCACTGCCAGAAA

A_16_P19971394

GATGCAGTTCTGTTATCTCATCTTCACAATTCCATCTGATAATTTAACCATACCCTCCTC

A_14_P122875 ATAGTTTTCCTCTCATACTTTCCCAACTTTGTCTCATTCTCTCCTGAACTCATATGAATC

A_16_P18881837

AACCCAAGCATCATAACTCAATTGAAATCTTCACTTTTTCTATTGTCACAGAAAGGCACA

A_14_P103725 CTGATAAACAAGAAAAGGAATCTTAAAATTATAAATTTGCTGTAGAAAAGATAAAAAACA

A_14_P117670 CTTCAGTTCCTGGAGTTACAGAAGGACTTAGTAGATGATTTAGGATACGATTAACACAA

CUST_8247_PI426210822 GTTGATCCCTTGCAGGGAGAGCCATCCCTGCACTAGCCCGCACTG

A_14_P123958 GGTAAGATAATTATTATAGATAACTCAGAGCAAGCAGACCCCATCTTTCTTTGTCTAGGG

CUST_9882_PI426210822

CTAAAAAGGTTAAATAAGCAGTGCTAGGCATTATAACATCAGTATTGTGCCCAGTCACTG

A_14_P130699 TCATGATAGTTCATTAATTTCTCGAATAGGTGAACAAACAGATGTAATGGTGGGAAAGCA
A_14_P127321 CTTGATTTCATTACTTCTACTTCCTCTCCTAACTGGATTAGATGGCAGTCTATGAAATCC
A_14_P126683 ACGAATAGGAATTCCATTTTTTTCATCTTGCATCCGTGCTATGACGTCTTCCATCTAAAC
CUST_8499_PI426210822 AGACATATCTGGAGAGGCCAGGGGACCTGTGATACCTGAATTTCT
A_16_P18527317
AAACAACTAGATGACAATTTGGTCTTCACAAATCCCTTTTAGTCATTCACCTGGATAGAC
CUST_4953_PI426210822 GCACAGATGCTTGAAAATCAATGGTTTCTTGACACATCTTGGCTTGTGTGA
A_14_P105626 AAAACAGAGAATGGCATATCCTGTCAAGCACCTAATGTTTTCTGAGTAAACTCTTGTTTT
A_16_P39207872
TTTTAATCCATTATCCTCTAACGCTATAGTCTGTGACTTTCCTTACTTGAGTCTAGGTGG
A_16_P02311484
AATGGCAATACCTTTTTACTCCTTAATTCCCTTCTGTGAGTCATAAGAACCATATCGACT
A_14_P126308 TTGATCGATAAAAATGAATAAAAAACACTTCATGAGCACATCCCATGACATGCTGCAAGT
A_16_P15975873
ATCACTGCCGAAATTAATTTCTTTCTAGCAAGACCTCAAGTTACTATCACTAAATGGGCA
A_18_P14133387 GTAAGGAATGCTTATCCTGGAGGGCTGGGAGTGATCCAGTGTCAT
A_14_P139103 ACTCTGAAGAGTTACCTAGAAGCACCTTCAACTACAGACCTGGAGCATTCT
A_14_P134529 ACTTTAATTTTTCATTTTCTTTTAGGATATTTAGACCACCCTTGTTCCACGCAAACCAGA
A_14_P124606 CATTGAGGACGTGAATGACAATGCACCCTATTTTCTGCCTGAGAATAAGACTTTTG
A_14_P112245 CCTCTCAAACAGTTTATAACCTAAGGGATGTGAAGAGGGAATAAGTAAAACTTCATCAGT
A_14_P115584 GCCAGACTGATTTCTGTAATAATTGCTTGGTGTATTAATGGGTGCTCAGTATATTGATGG
A_14_P103279 GACCCATACAAAACCAGAGTAAGGGAACTCTATTGAGACTGATTAAACATACTAAGTGTC
A_16_P16532852
AGATTGAATGGATGCCCTATTGCAATAACTATTCTCACTAATGAACCGGCTTGTTTTCAT
A_14_P114740 GATGCTTTTCTTGAGTTCTTGCCAGCAAATTTTGGCAGGTAAATTATGGTTTCTTCTAAA
A_14_P202388 AAAGGTGAAAATCTTTCTCTGATCAATGCCTCCAAATTGGGCATCTACTTTTAAATACTC
A_14_P134442 AAAGAGTTGCATTGTGAAGTCAGAGGAACATAAAATCAGAAGTTTTACCCAATGTCAAAC
A_14_P100378 AAATGGACATTCCCTTACTTGGCAGCCACAGTTCAGATGGTAGTGATGATT
CUST_5259_PI426210822
TTAGAAGATTTTCCCTAAACTAATTATCTGGCTTAATGCTAACTGCTTATTTGCTGACCT
A_16_P39092801
CAACACAGACATCTCAATTTTACTGAAGAAAATCCCTACACATTTAACATGTTGGTTGCC
A_18_P22840336
TGCCACAATCTCTAGAATCTTCTAAATCAGGAAATTTTAATGATTTTCACAGCAGTGAAA
A_16_P21084494
GACTTGCTAATAACCAAAGACCATCCAGATATTCATGAGCACTACTAAACGATCATTGCT
A_14_P101077 AAAATAAGGCTTATAAAGTTTCCAGAGAATCACTTATGCTTAGGACATAGCCAGTGGTCT
A_16_P18299663

AAGACTTGGAGGTAGAGTAATGGGATCTAGAACTTCTTTGTATTAGACTTAGCAGAGACA
A_14_P118103 TTTGGGTTGGCCCATAAAATTGACTTTGGAAATGAATTTAAAAACATATTTGGGACTCCA
A_14_P103335 ATTCATTGAAACAGGCAGGTTTTATTTTTGGTCTGTCCAGCACTAAATGATTTTCTTTTG
A_16_P00685398
GTTGCCTTGTTCTTTATGTTAAAATGGAACCAAAATCACTTTCTGTGGAAAACCTTCACT
A_18_P11798845
AAAAACACCATTTCTAAAAACGGAGACCTAGAACTTAGAGCGTTAGTAACACTGTAATAA
A_14_P129595 CCACAGGCACATGTAAGCATTCCATGTTGTATTATTGCAATACTGTGAAATTCGTCTTTT
A_16_P16691929
CCACACGACTAGACCCTTAACATTCCTCGTAGGTATTTTATTGAAGTTATTCACTCTTCT
A_14_P126686 CTAGAGTTCCACCTCAAGTCTCAGTCTCTTTTCCATTTCCAATATTAATTGCAGTAGTTT
A_18_P12168053
GCTTTCACAAAACCATGAGGTCAGTTTATCACATTTGATTGTTAGACGTTAGTGGGATTC
A_14_P102820 TTAAACAAACAGGTCCTTAGCCCAAGATCTTTCCTATCATTTGGTTTTAATTTTGAGGCA
A_16_P40506724
AAAATTACTGAAGACATCTGTTGCTTCAGAAAATTTAGGGAGTAAATCAAGCATGTCGGG
A_14_P123393 AACTAGACTTTCAAGGAACACGTAATTCCATTGATGCATATGCTATTTCTTAGTGGGTTG
A_14_P201949 CTGCATCTGAATCTTCTAAATCACCCATGGCTGGTAAGATCATTTATACTCTAGTGACAA
CUST_377_PI426210822 ACAATGTTCCAGATGACGTTTGTATCAGCTAGGATACAAACATGCAAGGGAC
A_16_P39860486
AAGAGTTACTTAAAAACTACCACAGTTTATCATCCTGTATCACAATTACTAAGCCAGTCA
A_14_P111401 TTTCAGGAACTCTTTCCAGTTAACAGACAGAGTGTGGATCATTTTGCTAAATACTTCACT
A_16_P38106599
CCAAATCTATCTGTATGTTCCTAAAACTAGCACTTGTATATAATTGTTCACACCACTCCC
A_14_P123130 CAGTGCCTCCTCCCTTTCGAAGTCCTTAAAATGGTAGGTATATTGATATCTTATGAATTG
CUST_1484_PI426210822 ATGGCTGTGGTTTGCTTCTCTCTCCTTCCTTCCCCCTAGAGTATA
A_14_P100954 CTCAGTGTCAGACGTTTACTGGGATTATTATCATTGCTCAAAAAAGCCATTTATTTCACC
A_14_P116001 AGGTAGTCATTGATACAGACATCAATATGGTAGTCACCCCTCTGGAATCTGTTAGA
A_16_P02786472
CCATTCATCTTGCTAACACTAACCTTTGCAATAACTCCAATACTGTTCATGAGTTTCCAA
A_18_P22841599
TTTTTAGATTTAAGCAGCCCAATCAAAGCCAGAGATGGAGGGACAGATTAGTATTGAAAT
A_16_P16611056
GCGTCAAAGGTTTCAGTATTGTTCACTAATGTTCAATTGTGCACTAATCACTCTACAGAG
A_14_P133073 AAAGCACAATTCTCCTAGATAACAGCCCTACGAACAGTAAGCATATCTCAAATGAGAAAA
A_14_P137839 GAGAAATGGGATAGTCTCTTGGAATTGATTGTAATGGGATATGACATCTGTCATTGTCAA
CUST_3714_PI426210822
TTCTATGAATAACTTGGCTTTATGCCTTCATATACCTTTTCTCTCTACTCATGTAGAAAC

A_14_P133541 TCCCTTGCTATTGCCCTTGAACAGCCTTTGTAAAGTCATTGAAGGCTTACATTAATAATA

A_16_P16793998

CTTTGAACCAAGTCTCCTACTCTTTCAACAGTTGCTCAATATTCCGTCATTGAAATAGAG

A_14_P137152 TATGCAAATGTGTTGTGTGTCCACACACACACAAACAATCTTATTTGATCCTCACTTTAG

A_18_P15928983

AAAAAAAGGCGCTGATATATGCTTTCTGTAATTTGACTCAATGGGTTTTTGAATCCTTCA

CUST_5245_PI426210822 TTCTGGGTCACTGGTCGTCTTTTCTGCTGTGTTCAATCTCCAGTAAAATA

A_16_P19138459

TTCAAAACAAGTTGCCTTTTGATAAATCTGATCTGTCCTCTTCATCTTACCCAATAAGGC

CUST_3703_PI426210822 GGTGTGGCTTGGTTTCTCATACCTTTTTATTTCAGTCCCTGGTGTTTTTG

A_16_P15504959

ACAGGCCATTTCATTTTTCATTTTTTAACAGCATGCTTTAACAACAATGACATTGGATCT

CUST_3098_PI426210822 GGTCCCTGGCTTTGACTTGTTCTTTTGAGAGGTTTCTCATCCTCT

A_18_P14988284

AAGCCAATTAACCTAATAAAAAACATTTCAGCCCTAGTTTAAAGTTTAGAAGCCACAGGA

A_16_P19779046

GAAAAGTGAGTTGCTTATACTACCACATGACTTTTACGTTTATTTCTGCCATTCAGAGCC

A_16_P19164052

GGTGAGACAACCCTATTATAGCCCTATTACAATAAACAGGAAAATTTTTGCAGCAGTCTC

A_14_P119712 CTGGACAGAAAGAAGTCAATGTCACGAAATGATTTTCTATTGTAGATACTTTGTCCCTTG

A_14_P121359 TTTCTCCAGTGATCAGAAACATTGATATCAATCCCTATTAAATTAGTGGGGGGAATATTA

CUST_1401_PI426210822 TTCCCCTTGCTGGCCTTCAGCCTGGGCCAGAAGTGGGGTATTCTTA

A_16_P18661271

TAGCACCTTAGACCAAAAGAGATCGGAAAATTGAGTTACCTGTATAATCTGGGCATAATT

A_14_P139587 AAGGGATTCTCATCTTGTGACCTGAAAAAAAAATGCTGGTAATAGCCACTTAAATTCATC

A_14_P110233 TACTTCTAGAATGAACCTTGTCAAAGACTCTAATGTGCACTTCCTGCTTTTATAAGAGTG

A_14_P136668 TGAAAAGCAGTCTGGAGATTGTTATTGCTATTTGGATCTTTCCTTGCTTTGCTTTCCTTT

A_14_P105022 ACAATTAAGTATTTCTGAAGTTATGTTTTTAGAAAAAATTACTACCAGTATAAAACCTCT

A_16_P19933819

CTATTTGAGAACCTGAGAAACCTAAAGACAGTCCCTTTAAAAATCCTCAAGCACACATTT

A_16_P03798871

GGAGACTGAAAACACTCAGATGGTAGGCAAGTCATAATTGTCAATCACAAACTCAAAAAT

A_14_P114362 ACATTGGTGCATTGGATCTCACTTTAGATAATCCCTTGATGGAAGTAGAAACATCAAAAT

A_14_P123589 CAAAGCAAATATGGTGGCAAAATGTTAAAATGTGAGAAAACTGGCAATAAGTATGTGGGT

A_14_P123742 ACCTACGTTCACCCCAGTTTTAGGATAAATAAAAGTTTCTCAGAGTTCTTTTCCATTTTG

A_14_P119058 AAAGAAATTTGATACATTTATTCCACTTGAGCCTCTTCCACAAATTCCCAAGTGAGTTCT

CUST_2601_PI426210822

CCATCTGGCTGAAGGTCCATCATATAGACTCTGCTTTCTCATCATTCTTGTATTTT

A_16_P16933893
AACATGGAATGCAAATGTCTTCTTAAAGTGCCTACACTGATATAGTTTACCTACGCAGTC
A_14_P104189 ATGGGGAATAATATTTGGTTAAGGCTGTTGAAGCCCTTGCTTTTGAGGTTTTACATTATT
A_14_P124617 CACATCATAGTTGCGGAATGTGCTAGTGGTTTTTGTCTTGAAGTTATCACCATCTTGAT
A_14_P119179 GGAGAATATTGAGAAGCATCTGAAATGGTAATGCTTGTGATACACAAAACCATGACAAAC
A_16_P01833969
ACACTCACATCCAATTTGGAATCATATCATTCCCAGACTGATCTATGATAATCTGCTTCT
A_18_P26792384 TATCACTGTTCTTCTGTAACCCCATAACTCCTTCACCTCCCCACC
A_14_P135466 AAAGTTCATTGTCATACATAGAGGTGTCAATATCCTCAAACAGGCCCTCAAGC
A_14_P131618 GTGATTTCATTTAAAGAGATTGTATGCATTTGTGGCTTTCCTGATTTCTGAGTCTGTGTT
CUST_8152_PI426210822 CACAGCAGTGATCCCAAGGCTTTTGTCACCCCTTGGGAAAGGAAG
A_16_P16878571
AAGGAAAACAAATCTGATTATCAAACACACGATTACTATTCTCTCATCACCGGATTACAA
A_14_P134498 TCTCCGACGCTGAATTATCAGGGCATTCTAAATCGGCTCAAGCAGTT
CUST_602_PI426210822 CCACTGCTTAAATACCAGGAGCCTCAGTTTCCCCAAATATAAGAAGAGCTT
A_14_P200005 CCTGTGGTTTTTAGTAAACTCTCTCCAGTATCTACCACCAAAATATAACACATCTCAGAG
CUST_2767_PI426210822 CTTACCACGACTAAGTCACCTTCCTCTCTTGCCCTGCAGAGCAAG
A_18_P13556858
ACCTGTCTTAAAAGAACATAGTCTGCCCATAAAAACAATATTGTATGCTCCGTTTAGAGA
A_14_P110204 CTCTGTGCAAATTTTGGAGGATTGTCATTAACATCAGTAAGAGTCACTGTAAGTGTCGT
A_16_P01555940
ACAGTTCTTGTTGCTAATCCATCCTAACCTAAAAAGAATCATAGTCAAACCCATATTGGC
A_14_P110200 GCCTACACCTGTATACTCTGCTAGACCCTAATTCAACATATCCAAAACCTCTTTATTACT
A_14_P110598 TTGTTTTACTCTGATGAGTTTGTTTCCTTTGCAAGTATATCTTCAGGGAAGGTGCTTTTT
A_16_P19473524
TCCACTATCAACATACCTCAACTTCTAAAATATGACTCCCACTATAAAGATGAAACTCCT
A_16_P30952516 ATTGAGACAAATGTTTGCTTCTCAGAATGGCAGTGACCATGGGTGCTT
CUST_136_PI426210822 GGAAAAAGTATGGCTCAATACAGGGTCCAGAAATACTCACCTGGAGG
A_14_P133460 GAAATAACAGCATCATTGCTTTCGATTATTGGTGTTGCTACCCTCCATTTTATTACATGC
A_14_P128120 AATAAAAAATTTCTTTACCGCAACATGACCTTCCTCATGGCTGAGATAGTTGTGAACATC
A_14_P130160 ACTTGATTATCACTTGCTCTGTAGAAATAGCAAGAAAAAAATGCCTTTATAAGTAACACA
A_14_P118022 CCATGACCCTGGCTAATCTCATGACCTCTTAGCCTTGGCTTATTCATC
A_18_P11577394
TGTAAGGCCTTAGAAAACACTTTCTCCATTCTCATTCTTACTACTTAATCTTGACCACCT
A_14_P103959 ATCCTCTATGTCAGAACCATTCAAATTTAGCATCAATCTTAATACGTTTCCCTAAGCCAC
A_16_P01235560
ACCTTCAGGCATACTTGACTAAATAAAATCCTCTTTTTGCTAGTTCCTTTTCTGCAAAGC
A_14_P131000 AAACAGGGGGCTTTATAGGCTGAAAAATATCTTAGACTTCAGAACAGAATACCAATCAAA

CUST_6088_PI426210822 GCCAGAGATCAAGCCATAGGAAATGCTGTCTGTGACCAGGATTCAT
A_14_P101630 GGTATCTGTAAAAACCTTAAGAATAGTTACTCTTCCCAAGAGAATTACAGCGTCTAAGGG
A_14_P138472 AATATTTAAACAGCAGCGCTTTGAAATTGAGGCAGCCAAGATATAACCTAGTGGAGATAT
CUST_8339_PI426210822 ATCCTGAAGGTCCCAGAGCTGGGAGAAGCCGGGCTGGGGCCAAGA
A_18_P22840671
AAGGCCTTTGAAATAAAGTGTTTATTTTCTTTCTCTTTTTCCTCTTTTGGGAGGAAGAGC
CUST_10193_PI426210822 CCAAGAGACCAGATGTCATGGTGTTGTCCAGCTGGTCCCTCTCAC
A_16_P16953901
AGACATGACCGTAGACAATAGAAGACTAGATTGCCTATCAATAGAGCAAAGGTATCACTC
A_14_P201709 ACAAGGCTCATATACTGGGTATATAAAGAAAATGTATTTAAGCACCTTTCCTCCCAGGAA
A_16_P00685508
AAAATGGATGAGTTGTTGTCTATTCGGACTTAGTCTCAAAAATCTGCCATTGTTGTGTTT
A_14_P115570 CAGGGAATATAATCAGATAGATATTACCCTGGGTCATAATAAAAGCAGCCTAACATGGGA
A_14_P128215 AGGGAAAAAAGACCATTGCTCAGAATGAATGACAAAAGCATTTTATTGAAGATTCACCTG
A_14_P139299 CAAGTGGTCCTCTTGTTTGTATCCTCTTACCTTGAATAGTAAGACAGTGCAAAAACTAGA
A_16_P18097616
CTCACCGTTACTCTTTGTAAATCAAACGTGTCTATCAATACCATGTTTATTAGACCCCAG
A_14_P201575 ACTGCAAGGGATACCGAAGACTCATAAATGAGTAATTAAACTGCTTTAATAACACCCAGA
A_14_P139219 TTTACCTTTGGGACAGTTAGATGTGCACAAATGCACCATGTGTTTAAAGATCACC
A_14_P118325 GCTGAGGATGTGAGATCTGAACTCAGTAAGAGATCATAGACCTGAATTGCTACAATATAT
CUST_5024_PI426210822 AATCATTTTAAACAGGTTTGTCCTTGGGCCCCAGATAGTACATTGGCATTTG
A_18_P22841645 CTCCTTCTCCCCAAATCCTCAAGTCCTGGTCAGTTTACTCTAAATCAAAG
A_18_P17402499
TGAAATGGAGGAGAAAGAAAAAACACTTTGGATTGTTTCAGGTGAATTAGCAATTCTCTG
A_14_P107424
CTTGGGAAAAAGTGTATTTATCAGCAGTTAGTGAGAGGAAGAAAAATCAGAAGAAGTTCA
A_14_P116766 AGTAGAATTCAAACACTATCTCTTCGAAGGTTCTAGCAAGTCTCAAATTATTGAACCCAG
A_14_P136651 ACTACAAACACCTTTGAGCACTTAGGCAATTTTGGTGATATCAACTCCTTGAAGTAATAC
CUST_6066_PI426210822 AAAGGGAGAGAAACCAGATTGTGGAACACTTATGAGTCTGGACTTCATCTT
CUST_687_PI426210822
TATTGTTGTTAGAACCATTGAGTACCTTGGGCTTGGAAGGAAATAATAATTCAGCAGG
A_14_P201238 CTCAATCAGAGATCAAATTCACCAACTGCAATTAAGCACTATCTTAGATTTACTCCTTCC
A_14_P100781 AATATAATCTCGAACAGTCACTGAGCCTTCAATTTCTTCCATATATAAGCAGTTGGAAGC
CUST_8655_PI426210822 ATAACACATCTGTCACAAGCTTCATGGCTTATGAAGGGAAGAGGGTAGTG
A_14_P101139 TGCCCCTCGGCCTTACTATAATCAGAAAGCAAATTTCAAAACCAATCTGATTCTTAAATT
A_14_P122566 ATCTTGCTTAACTTTCATGGGAATTAACACAGCCACCGACAAGATTTTATTTGGTTGTAA
A_14_P135165 AAATATCCTATTTTGATCCCCCAGAGGGTTTTCCTCAGTGTGACTTTGAAGAC
A_14_P113943 ATGGCCTGGCACATTATAAGTAGGCAAAAATACTTTATGCTACTTTATGCAGTATTTAGC

A_14_P115246 CTTTCTAAAACCAGTGAGAGCACACACTAAAGAGAAATTCTATAACTTTAATGGGGTAAC
CUST_3653_PI426210822 GTGCTGGTGGGTCTCCTCCTATTCTCAGAGCAGAGAGCCACGAAT
A_14_P100030 CCTGAAAATAATTTTTTAAAAAAGGCCTTCAGTCAGAGTTTAAGACAATGGTATGCTAAA
A_14_P136369 CTCCAGAGGTGAACAATATTTTCATCAAACAAGAACTTCCTACACCAGATCTTCATCTTT
A_14_P117175 GCTGAGTTAAATCCTTAAGATTGTTGATTTTGTCTTCGGTATCACTCTGTTTCTTTGAGA
A_14_P134918 AAAGCAGTGGGAACCATTTCTCCAAAGAATAATTTTATTCAAAATGGGGAAGGGTTAG
A_14_P121886 GTTCCTTTTAAATCAAATCCAGTGTTGTTCTAAAGACTGTAGACCAAATTCTACTGTGGC
A_14_P134085 ATCACTAAAGGTCATTGGTTCTCTTCCTTGTTTTCTTAGACAAATCATCTCCCTGTATTT
A_16_P00632494
ACCTCCCCCAATAAAGAGATAGATGCCAAAACTTTGAGTTATTTATGCCAGTGCTATATC
A_14_P116021 CTAATAAACACAACAAAGTATGCGAAAAGCACTTCAGTTTTTCTGCATCTTCTCCAAGAC
A_16_P20259799
AATAACTAACCAGTCCATAAAACCCTAACTACTGTTGTATACTCAGTCACGAAAGGTTGC
A_14_P115642 TAACCAACCAAAAAACCTCTGTTCTCATTTTAGTTTTCCTACCACTTATATTAAAAAAGA
CUST_1965_PI426210822 TAGTTCAGACTGTCATCAAAGTGACTGCATGCCCTGTCTCTCTGC
A_14_P127060 TGAGTAGTGATTATTTTTTCCTCTTATCAGTATGAAGAGAGGGATACACTGGCTCACAGT
CUST_2461_PI426210822
CAGAACTTAGAAAAAATTTTAAATGATGACGTTTGGCCAAGACAAGTAATAATAAAGGAG
A_16_P15097329 TTAGATTCTGGCCTTGTCCTCAAATGCCAATTATCTTCTTCCTGTCCAAACTCTTA
A_14_P116791 TTTGGGATACAATCCAGTGTTTATTACAGATGGAGGCTCAGTTTTCTGGAAAAGAGT
A_18_P22839945 ATGGGGGGTGGAGGGAGGATGAAGGGTGCTCTCTGGCTTCCAAAG
A_16_P21590714
AAAAGGAAACAACATTCCAGTTTGATTTCCTGTTGATACAATTATCACTGTGGCCATCTA
CUST_1435_PI426210822 TATATTTCCCCAGGGCCCTTTTTCCCCTTGCCTGGAGTAGACACT
A_14_P103308 CTTCTGCTTTTCAGAAGGAGGCGGTGTGATGCAAGGAAAGTTACAAACT
A_14_P113584 CTGAACAGAACTGGTCAGTAACTTTTAACAATTCATAGGGAAATAAGAAAGTTAGAAGCA
A_16_P18076412
AAGAATTGGCTTTAGGGAAAATCTGTTAACTTAGCAAATGTTGATACGCAAAGAAGCAGG
A_16_P00071399
AGAAATGCTGGCCTAAATAACCTCTAATCAGTCCTAAAAATATGATTCATGTGTGTGGCT
A_14_P118225 GGAGGAAGTTTCACTGTAATCTTCATGCCTTATACAAAGGTAACTTTGCCTTTTACTTCA
A_16_P16056445
ATGGTGAAACTTTTAGTATGAGGCCTTATGCTCTTTAGAAACCCCCTTTACTTTTCAAAA
A_16_P20190307
AACCTAAACCTTTTTAAACCTATGCTGCAGACATAATTTCTACCATCTTCCTAGATCTCA
A_14_P129642 TTTCCTTCATTTGTTTCTTATTTTAGGAAGACCAACACTGTCATCTGAAGTTGGAATCAT
A_14_P104226 TATGACTCCAGTTTCTTTTACCTGTTTCTTTAAGTTTCTTCTTGACTGGCTATTGGGAAG
A_18_P22839416

GGGAAATTGACAAATTCATCAGGTTCTTTTATTTTTAAACATATAGCAAATTTAAAGACT
A_16_P15458068
AGACAGTAACTGCAGTCAATGATTAAGCAATGTCCCTCTAAAATTATCCAAGGGCTTATT
A_14_P115761 GTGTTTAAAGGCCAAGCAAATTCTAAACACATAATTTGGGCCATACTTTTAGTCTGGTAG
CUST_9713_PI426210822 CCACTGCTATGGAAAGTTGTATTCTCAGAAGGTTGCCCAGTCCATC
A_14_P114420
TGAATGAAGAAGAACAATCATGAAATGGAAGAAGGAGGAAGAGCATGAAGGATCTTATAA
A_14_P128543 GTGCAATTTATCTTTATATAGGAATACATTTCTAGGGCTTCCTTCAAGCCCACTCTCTTC
A_14_P103086 GGAAGAAAGGAAATTATATTCTGGTTTTTATTGCCTTAGTCAGCATGGTTTATGGTGCTG
A_16_P15259323
ACATCCTACATCATGCTACATAAGAAAGATTTAACAGCCCAAACAATAATCATGAGATTT
A_18_P13075383
TTTGACATCATCTAACGCTACAAACTCTATCGACCTTTTCATAATTGCTTCCTGATGACT
A_16_P01453025
ACTATCTGGGTATGGAAAAGGTTACTAGGCAAGATTGTGATTGAAGAGAAAGGATAATGA
A_14_P102727 TTGAAAAGGCATTTGCCAAAAGATTTGGACGATCATTCCAATACATGCAATATGGATTTC
A_14_P128194 TGAAAAAAGAAGTCTTTGCTGTTTATAAGAATTGCATTGAAAAAGAGTGATCCCAAGGGC
A_18_P10550201
GCTTTTTGAACATTGTCTTCCAATTACTTTTCATCACCCCTTTTAATTACTTACAAACCC
A_14_P124519 AGAAAAGCCAGTATTGCTTGTGCTCCTAACAACCTGTCTCTTGGTAAGTTAACTCTAA
A_18_P13693108
AATTTGAGCGACTTCAGTAGTATCTCTTAGTCTACGCTTTTCATACACAAAACACTGTGG
A_18_P22841413
ATTTCCCCACCTGAAAAACAATGGCCAAAAATAATGCAGATATTACACATGAACATTTGC
A_14_P117870 CTGGACAATTCCACAAAGGATTTCGACTAAACATTAAGTCGTCAGAGACTCAATTAACAT
A_16_P36369164
CGTCGTATCTTCTAACAGTTTTTTTCCATAGCCAGAATATTGTTCATATTGCAACCCAGT
A_16_P17800693
AAGTTCATTGTAGCAGATGTGTCAACTGAAGATCACTACCCATTAAAATCAAACAACCAG
A_14_P114760 TTAATTTCTAAAACAAGCAATTCAAGAATGTGGTGGTCCTAAGTGACTGCATCTATGGAG
A_14_P112978 CTCAAGAATGTCATTTACTTTGAAACAATCCCAATCTTCATAAACCTAACAAGAGACCAC
A_16_P02368291
CTCATGCATATCTGCGGTTTTAGAAAACACTCTTAACGAACAAAAAAACCTATGGTCAGT
A_14_P139252 CTGCATTTTCCAGGCCTTAAACCTAGTAATTATATTGTCAAGATAATGGCCTCTGTATCT
A_14_P138934 ACCATATTCCTCAGAGATTTGGTTCGTCGATAATCATGGTGTTATCAAATCAGATACTCT
A_14_P106419 CTTCACTTCTAAAGTCCCAGGAACCAATTCCTGATGATCAAAAGGTGAGTATAAGAGATT
A_16_P36141916
GCTGACACAATTCAGAATCTGACTTTGTTACTGATTGGACTTAAAGAAGGTCATGAGTAG

A_14_P137915 AGAACCTGCAGCAAAAGCAGTTTTGATTTTTTTCACTTTTTAGAAAGTTTTCCCATGTGA
A_14_P113072 CCTGCAGTCACACAAAGTGTTTTTCCACTAAGACTCAGTAAGTCAGTTTGGTTTTGTAT
A_18_P13425289
AAAAGATCATTGTCCATCTAAGGAATAACATTGCACTATTATACATCAATGGCACCTTTC
CUST_7808_PI426210822 ATTCAAGCTGTTGTACTTATCTAGTAAGGGGTTCTGAGTGGAGCACCAC
A_16_P18837636
AAATGCAACTACTCCAACTCTAGCATATTTGAATTTTATGTCTGTATCCCAAATGGCTGG
A_16_P17361461
AAGCAGAAAGATTCTACGATACATCTATTACTCCCACTATTATGCACTTGAACATCCTGT
A_16_P30952624 ATATTTGGAAGAAAAAAATCTTCTCCAGATTTTTCGGAGGCCCCACTGATGCT
A_14_P117830 CTGATAGTGTCACTACTTGCTAAAAACTTTTCAAGTTTCCCAGCATCTACCCATTTAAGT
CUST_7617_PI426210822
CTTTATTCACTTTGGAAAAATGTAAAGATTAGTCCTAAGCAGGGTTGATTCACCGCACTA
A_16_P00238795
CCTGTATCTTGATCTGTTCACGAAGCATTTCTACTTGATGTCCTCAAATTAACCCTCATA
CUST_9380_PI426210822 TACCACTGGCTTGAGGTTCTGGCTTCTCCCCAGACCACAGTTTTT
A_16_P16672766
AAAGTCCTAGGTTAGTCTTACTCTCATACCACAATTTTCAGAAAAGTCCTTCACTGTCAT
A_14_P100815 CTATCTACAACCAAAATGTAAGCCACTGTCTTAGATGCGTCTATTACTTGTGGCTACTCC
A_16_P41863103
TGCTTTTGCAACATGGAATAAGTCACCTTCTAACTACCTAACTACTAATTGTAAGAAGCC
A_16_P18942670
ACATGTTTTGAAAATACCAGGTATTGAGGAGTATGCAGAGTAGTATAATAACCCAACCCT
A_16_P02447502
GAAAGTCAGAATCCTTTTTACTCAATCACATTTCGTTACTTCTGCTAGATAAGCCAGTCC
A_16_P16957032
AAAATATGTTTCCAAAGAAGAGGAATGTCTCCAGAAATTGTCTTAAATTCATCCCCTCAG
CUST_10043_PI426210822 AAGCCCCTTCTCTCTACTTGGCTACACATCCGCATCATTCTCTTC
A_14_P103104 TGGGGCAGACGAGAAGTTCTGATAATAGTTTTTAGGGAACATGATAAAATTCTGACCTTA
A_16_P01239601
AAAGCACGCTCGAGTATTTTTACTGTCTGCACACTATTTAACCTCATGTCTTATTGTGTA
A_16_P20239014
AAACAGCAGATTTAATGACCCGGAAATTAACTCAACTAACTATGCCTTGATCTTGTGAAA
A_16_P17294619
ACTGTCTGATCCTCAAACTTCTGATTGTTCACATCTTCATTTTGCTGCTACTGATAGACT
A_16_P00838246
CTTGCCTTAAACTCAACTAATCAAGATGGTAATTCCTAAGTAATTCATGGTTCCTAGCGG
A_14_P105881 CTTCCTTGTAGGTAAGGCAAAAAGCAAGCAAAGTATTTTAGGCAATTCCCATAACTAAAG

A_14_P132789 GTGTTCCTGCAGAAATCCTATCCATTATTTTTCACTGCCAGTCCTTAATGAAGTCTC
A_16_P19316692
AAACACTCCACTGAAAACTAACTTTGGGTTTTGTAAAAACAACTGTGCAGTTAGATGACT
A_14_P201668 CACCCTTATAAAGGACTTAGTCCTGAATGCACAAATTGATCAGATAGTGTATTATGCTCC
A_14_P130801 CCACTTATAAAGAGAAAAGCCCAGTGGCTGAATTAGAAATCTGAATTGAAATCAGGGATT
A_16_P16585820
TCTCTGTCCCCTGTATTATGTCTTTATTCTTTCCAAATCACCAAATATTTCCAATACCCA
CUST_10178_PI426210822 TCCAAAGAAAAAAAATGAACAGACACATGCCCATGTGCTTGCACACACA
A_14_P127179 ACCTGGTTATCTTTCAGAGTGTCATTGTTAAATAATCATTTCAGTAAGCAGTGATGGCTG
A_14_P129328 CTCAAACATCAATAAGCAATATTTTATCACATGCCAGCAAGTTTTAGATCCAATGTGCCC
A_14_P127732 CTATGTAGACGCAGTTCTTCAAACAAGTTATATTTACTGCTTCCCCTTCTTTTGTGTGAC
A_16_P37999166
GTTGACAGTTGGTAGCCAAATCTAATAGCGCAAGTTTGAAATTCATTTTTGAGGATTCTG
CUST_7787_PI426210822 GTTGCTTAGAGCTGGGGACAGCTTAGATTAGCCATATCTCCCCAAAATTT
A_14_P112018 ATGACCCATGTGTGCTTTGTAAGGCACCAGCCACATACTGGAAAC
A_16_P16097743
AAAAATTCTCAGACCTCATGTAACCACAATTCAGGAAAACCTTTGAAAAAGATGCTCAAG
A_18_P22841035 GGCACCTTGAGCAACATGTCAAAGGGCATCCCAACTGGTTGTATTATTTA
A_14_P124812 GAAGCACTGTGGTGACTGGTGTGTAGATAGAAGTTGCTGAGTTAAACATTTTTGTTTTAA
A_14_P135710 CTGTGCTACAAAGAAAATAAGATGATCTATCAGAGAGCGACCTGGAGGACTACTTTAAAA
A_14_P133847 TCTAGCAACCTCAACAAAGGGCAGGAAGATATGACATTATGATACAAGAACAAAAACTCA
A_14_P119938 GTTAGTTAGTGCATATTTTTTCCTGCATTTGGGAGGTGTGCTGTTCTTGGTTTCTG
A_16_P17792613
AAACAATGATCGGGAATAGAACAAACTGTCATACTTCTAACCCCAAGGATAAGTTTTCAG
A_14_P130636 GAAATCCTAATCAAAGGTTAGGTTATTTGGGAAAGAGAAAGAGCAGGTGTCAAAAGATTT
A_18_P22842843 CAGTTGGCTTTGGAACTCTTCCCATTGTGTCCTGGTCCTTAACTAGAT
A_14_P122043 AACATGTATGGTATATTTGGGAGGTGATAAAAAGGGAAAACTCAGTCATTTTTTGTGGCT
A_18_P15825880
AAGTCCCAAAGCCTAACTAACATTCTATAGTCCAAACAGACACAGTATTATTGGGTTCTT
CUST_9762_PI426210822 TTCCTTCTATTGAGACAAGTGTGTGATGTTGACTGCAGAGAGCATCTTGC
A_16_P20323940
AAAACCAGAGATAATGACTGAGCGATGAATACATGAAACCTGTGACATTTTCTTTCTTTT
A_18_P22839954 CATGAGGTTTAAATCAGGGAGAGCCGTGAAGTATGACACAAGGAGAGTCATTA
CUST_3912_PI426210822
TTAAGCCTCGTTTAATTTTTTACCTGTTACTGCTTTCATCATCACCTCTCACTCTCATTT
A_14_P126114 CTTAAACTCCGTGGTCCCCATTTAATTAATCGAAATGACTTTTGTCTGCTGGTAATTTAC
CUST_9336_PI426210822 AGCTGCTCCCAGAGCTTCAGTTGCCCTGAAGCCAGCTACAGCCTC
A_14_P113102 AAAAGAGGTTTATGATGATGTCCTCTTCTTCCTGGCCAAGAAGGAGAAGGTAAT

CUST_7125_PI426210822
AAGCCCTTATTTTTGTTTTGTTTTATTTTAAGTCAAAAGCAATTTCTACCAGTAAGAGAC
A_14_P111110 TGTGATGTTCTGCCTAAAATAAGCCTGTTTTCATTTCTCTCCATTTCAATTTTCTGCTCT
CUST_9152_PI426210822
TTCTAATAGTTTAACTTGAGGATATAAACCTTCACCTTATTTATTTTCAGACATATATAG
CUST_7408_PI426210822 TTAAGTTTTCTAGCCTCATTTATTTTAGAAGGGGAACTGGAGGCAGCTTGGA
A_16_P19433399
ACTGCTTTGTAGACAATTACCTTTATGCCTGTTATTTTCAGTGCTTAGATCCTTCCCATT
A_14_P110853 TTCTTTCTGTCTCACTTGACAGTATTTTAATTACCTGTATACCTACAAAATGCCTGGGGA
A_14_P123955 AATGTCTGACTATCCTGCTTGCAAATGGGGCTGACATCAACAGCAAGAAT
CUST_1461_PI426210822 CAGTCCCTTCTTCTTGCGGTCCTCTGTGGACGGCTTGTTTCTTTT
A_14_P121425 TAATCAACACACTGGCTTATTTTGTTCTCCTCACTATTAGACGCATGTCCCCAAAC
A_18_P22840362 ATTTGCAGGTGGGCCAAGAGAAGGGAAACTGCTCTTTCCAGAAAA
A_14_P201845 CCAGATACAAAGATAAGCAACACAATTCGTGGTTTTACCATTCTATAAATAAGACCAGGA
CUST_9478_PI426210822 CAGATATTTGGAGTTGGTGTTTCCCTTTTCTTCCCTGCCTTCTGG
A_14_P115576 ATTTTATTCACCTGGTTTGAACTTAAGCAAAGCCTTCTTGTTGGAACAGGTGC
A_14_P123491 ATGGCTTCAGAGCGATAATACACTATTATCTTCTTAAACCAGGAAAAAATAAAAGGGGGG
A_14_P107211 AGGTTTCACTGTTATCCACACTTTGGCAATGATTTTTCTTTTATGTCAAAGGATAGTTCC
CUST_6156_PI426210822 TAAGAGTTGACAGATTGCTCATCCTTGCCCTCCTCAGAACAAGTGTTT
A_14_P118821 TAATGTATGCACAAGTAATCACAGAATTTTAAACATCAACCTTTTACAACCCATTCAATG
A_14_P108351 TGAGCAAAATACCATCTAAAAGGAGAATGTGAATAGTTGTATTTTGAAATGATGTGTCAG
A_14_P104177 GGCACAGATCAGAATATTCTACTACAGTTAAAGTAGTCCAAATTGGGTAGGACTTGAATC
A_14_P119656 GATTAAGTGCAACACAGACATTTTCATTGGCGGAGTCCCCAATTATGATGATGT
CUST_8122_PI426210822
AGGATCTGTAGCTTCAATAATATAAAAATTTCTAGTTTCTTAAATCACAAATGTTAAGAT
A_14_P101848 TAATTTATTCCATTGTATAGAAGTAATGGGCAGAGTCATGTATTGATTAAAGTGATCCAA
CUST_6138_PI426210822 TGTTCAATAGCTTTTCCTCCTGGAGACACAAGCAATCTGTCCTGCT
A_14_P118889 ATGAGATTTACACAGGGAAACCCTAATTAAGAGGTTAGTCTCCAGTTTAATTGCCTTCAA
CUST_1604_PI426210822 CTTAAACAGGAGGTCTTTGAACTCACTGCCTGCCTTGTTTCTGGAA
A_14_P201423 CTTTTGTTGGTTGGGTTTGAAGTTTTGGTTAAAGGAAAAATGCACATTGGAACTAAATCA
A_14_P112820 GGGCCCATGCACCTGCTTTTTAAACAAGTTTTAAAAACGTAACTAACTACACACTAGTAA
A_14_P117316 TGAAAAGTGTTTTGCTTCTGATCACCAGGTTCCATTAAATTTCATAAATGAGGCTTCATC
CUST_6963_PI426210822 GGAAGGACTGCCCAGTGAGTCTACTCCCTTCAGTATTTGTCAGAT
A_14_P137952 TTTTATTGAGGCAAAAGGCATTCTAGTTGTTTAAAAGACTAACCTAGGCAGGGCATAAAG
A_14_P107877 ACATACAAAAATCTAATGGTAATACTTACCTATCAGCCCCTTCTCAAGAGTGAAGGTTTT
A_16_P16191945
TGTTTTAAGAAAAGAATGATAATTGGTGCATAACTCCCAAGTCACATATACAGACCAGGG
A_16_P30952642 AGTATTTTTATAAATATAAGCCTTTTATACTGATTATGTCTTTTATATTTGTATCGATGT

CUST_3232_PI426210822 GTCATCTTCCTGACTTCTTGGAGCTTGTTCCATGCTGCCTGACTC

A_14_P122402 TATATTTTATGTTTCACCCTCTAGGACAAGCATGACTTTCAGGACTCTGTGAGAATAACG

CUST_4161_PI426210822 ATAGTCCCAGGGAAGCCGTCTCATGAGCCCCTCCTTCTCTTGACT

A_18_P10566469

AAAACCCTCCACTTCCTTAGAATAAAATTCCCGTTCCTCATTTTTTTTGTGAAAGTTTGC

A_18_P17167856

TAAGATTAATTAGCCTGAATGAATCAAGATCGCATTCACTATTAGGGAAGGGTAAGCGAG

A_16_P19567870

GTTAGCCACTCTGAGAATCAATTTTCCCCACTTGATAAATACCACTTTCTTCAGACTAAA

A_14_P107527 TTTACGGCTGCTCTAACAAAACTGGAAGATTTGTTGTAAGTGTCCTTAAAAATAGTGCGA

A_14_P107750 TTTGTCCACAGGGGAAAAAAGTAATCAAATCATGCCAGTTAGAGACTTTTTCAAGTTAAG

A_16_P18090786

AAAAAAATGGTGACTCCATAAGGAACACAAAAGCCTTTAAAGAATTCTTGCCACATCTTC

A_14_P100479 CAATTGTGTGTTCTGGAGACAAGCACATGGTGGGTGGTGATCTTACAAATA

A_14_P133944 CATAAAGAGCAAGATTTTTACAGAAATTCTCCCTCATCCCATGGTAGATCTGATTTCTCA

A_16_P35618533

AAAGGAGAACTTTTTAATGCCTTACCTGTATTTAGAATCTGTTCTAGTGACAACACCTCC

CUST_6427_PI426210822 GGCTGAGGAGTAAACACCATACCAACATTCTGGGACAAAATAGCCTG

A_14_P125199 AAGGTTTAGATTTCACTGAACTTCAGATTTTGCTAAACTTTCCTTCCCCACTTTTAGTTC

A_14_P105887 GTAAGATGAAGAACATCCAGAAGTGAATTCCGAACACAAGAATGACCTGGAAGATG

CUST_1493_PI426210822 ATTTTGGCAACCCTTAGAGGTGGGACTTCTGGAGTCCCAGATACT

A_14_P122867 AACATTGTGATGGTCGTTGAAATTAATCTGTTTGTGTCATAAGATTTGTTGCAAACAGGC

A_14_P117087 ACTTTTAGGCCGCTCAATTTATGAATATTATCATGCTTTGGACTCTGATCATCTGACCAA

A_14_P131568 GTGCTTTCCTTTATTTGGACAGAGAGAAAATTCATGAACCCTTTCAACATGCCTAATCTG

CUST_5537_PI426210822

ATGTATAATCATGACACTTGTCTTGGCTCAACTGTGAGCAATAATCATGAACCATAATAA

CUST_4_PI426210822

TAGTCACTTTTCTGTCTTGAGGCACACAAAAAAAACAAGGTCATATCTTTTAATGTCTCA

A_14_P105857 TATTTGTAGGGAAAATTTTAAAGTGGTTCTAAGTATTTTCCTGTTTGTCTGGCACCAGGT

A_14_P200623 AAGTTGTCCTTTTCTGGTTTCGTGTTCACCATGGAACATTTTGATTATAGTTAATCCTTC

A_14_P117783 GAAAAATCAGGGGAGGAGTTAAAATGAGGATCATAATGTCTCTTAACTGACTCTCAAAGA

A_14_P200156 CAGATATGATTATCTATGTTGTGGACAATGGACAAGTGAGTTTGTAAATTTGTATGTGTT

A_14_P102094 CTGATTTACAGATTTTTAATATCCACCTTACGTCAGGGAATCCAAGCAACCGACAG

A_14_P117489 TCCTGGGAAAAGGAATATAAATGACAGCAAGACACATTTTAGTTGCTACTAAAGAACAGC

A_14_P135435 CTCCATCTATTAGACTGCACCCTAGACCTTGGAAAATCTTATGGCTTACACTAAAACTAA

A_18_P14532431

GGGAACAATCTAAAATACTTAGCATTCATCAGTTGGAATAATTAAATGTGCAGGACCAGG

A_14_P123205 TATTTGTTAAAAATACACATGTTCCTGGTATTTCCTGAGCCTGAGTCAGAATCTCCAGAA

A_14_P134485 ATTTGCCGTTTGCTATTCATGTTACAGGTATTATATGATGCTTCTGGGGTTCTTGAGAAA

A_14_P108569 CTCATCACAAAGAAAAGTCCATCTAGTTTCAGAGTTCAATGAATAAGCCCGAAGTCTTAA

A_16_P02407273 TCTCACTTTTCTTTACTCATTCTCACAGTCTTGAAATTTACAACCCTTAAGTCCACAAGG

A_16_P01499906 GAGAATTGGTCCTCTTGAATATGCTTTAAATAAACACTCTCTCTACAAACCATCCACTCC

A_14_P135447 AGAGCCTTCCCATCTATTTAGTGGGCTATATAATTTCTAATGAAAGCCGGTTATTGTCAT

A_14_P122961 CAATCAGAAGATTGGAGACATAACCTTTCAAGGTCAGTTAGAAAATCCAGAGTCAGTATC

A_14_P109126 GGGAGAACAGAGGTTAGAATGAGAAAAAAAAAACCAAAAAAACTCAGTGTCAGAATTCTC

A_14_P114772 GGACTCTACATCATGCACTTACCTTTACAAACAGAGATGTGATCTTCTCAGAGGTATTAT

A_14_P201370 CTTCTAACCTCATAAAACAAATCATGCATGAACACTAAAGGTCATTCTTTCCATCACTGC

A_18_P14131305 CTAAACATCTCTATCTCCAACCTCGGAGATGATGCCTATGATGCCAACGT

CUST_1798_PI426210822 AGGGAGTCGTTGACTTGCCTTTTCCCTTCTAACTACAAGTTTTTTGCC

A_14_P103281 GGAAGAACATCTGGAATTTATTAAATATGCCAAGGGGCTTCTGAGACTTAACTTTCTATG

A_14_P122537 CTGGTGTGTTTTTTCCATATACAAGAGAGGTGAAAAATCTATTTCCTAACTTCCATGCAG

A_14_P131146 ATCAGAACATGAAAGCATTCCAGTTAGGACCTTGTGATATTGAAACTAGAATAAGCAACC

A_14_P125003 TGAAATGATTCCACAAAGATTAAACTGGCTATCATTTGTTGATTTCCCCCTTCTTACATT

A_14_P114951 TAATAAATTTGGCTTATTTGCTTGGAAGTGATTATACCGAAGGAATACCAACTGTGGGTT

A_14_P117800 ACAACCTTTGGCTGGATGATTTTCAAGGGTTACTTATTCATTCCTTACCCTATATTGAGT

A_16_P21403624 AGGTAGGCCTGTGTCATAATTCTAATCTCGTTTCTTTTGCTTATGTAAAGACCTAGTGAC

A_14_P125041 CTTGGAGCATGCTTAAATGGTGAGCAAAAACCAGAAATTTAAGCGAATCATTCTGAAGC

A_14_P201716 AAACAGAGAAGATCTACGTGTTAGATGGGACACAGAATGACACAGCCTTTGTC

CUST_5733_PI426210822 TTCTTTTCTCTGACAGCACCAGCCCCTTTATTTGAACTTCCTAGCTGT

A_14_P129438 ATTAGGAACCTCTGCTTTCTTTCCAGATGCGTCCAGGACTAATTGAGAGATTAAG

A_16_P02340932 CACTCAACTAGAATGACATTATGTAGCCCAAACTCTAAAAATGATGCATATCCCTCAGAA

A_14_P116361 TTTGTGATGGTGACTCCAGGAAGGAAACTCACCATGACCCTGTATATACATAG

A_14_P116554 GACTAGTCTCAACACTGGATCAAGGTAATCTACCTATATTCTATCTGGTTGCCAAAAGAA

A_16_P00010205 TATTACAGTCACGGCAATAACTTTAATGATGACAACGACTTTTTCCTTCAATTAGGTCCC

A_18_P14134527 CTCCCTCTGGAAATGAAGGGTTTGGTGTGGTCAGAGGTGGCTATC

A_14_P202218 CTTCCAACATGAAGCACAAAGAAAAACATGCACTACCCTTTATAATCAGACCAAGACTAA

A_16_P21542404 AAAGGTGAGCATTGGATTCATAGAACCAACTTGTTTAAAAAAAGAGTAGAACTATGGGGC

CUST_2047_PI426210822 TGCCTTCACTTCCTTCTACTAGTGTCTCCAACTAGATTTACCCTGTCATCAT

CUST_10146_PI426210822 GAAAGTTAAATTGCTCAGAGGTGCAGGAGGGATAACAACTTTGTGTGTGAC

A_16_P03595389
ATCATCCTTGAATCCACCCACATTCCAAATTCATTCTCAAAAATGAGATTCCAAATTTCA
A_14_P112106 CTCTTTTTCAAAAAAACAAAACCACTTAATTTTTTTCTACCTAAAACCATAACAAGAAAA
A_14_P127280
GAAGTCCACTGAAAGATCCAGAAGAAAAAAGATGATGGCTAAAACTTAGGTAACTAGAGT
A_16_P36496277
AAAGCCTCATGAATTCCTTTTACATATTTGCCATTAACTCATTTTGTCATAGCAACCTCA
A_16_P16262364
ACAGCCAAATACCAACTTTTATTTCATTAGTCCATGTCCACAATTAGTCCACCAATTTGT
A_14_P132850 CTCACAGTAGCCTTAGGGGAATCTAGAGCAGGCAAATGGAAATGCAG
A_16_P00100480
AAACTTTCTCCTGCCGTAATAAATCTGTGAATGGAGCATAATCATGTCTGAAGTTGCATA
CUST_4067_PI426210822 TTACATATGGCCAAGCCCTTCAGCCTTTCTGCCCCACTTCCAGTTT
A_14_P106092 TTTTGTCAGAACGCATGATACCCATTTGCTTGTTATTATAGGAGGATTGTAGATAAGGTT
A_14_P139888 ATTCAAGGACTGACCCCTAATACAGGTTCTCTGTTTAAATATGTAGCCCAGTAAACTAAA
A_18_P22841102 CAACTATCAGTCTGAAGGGGAATTTCCATAGAGCCTCGCCACCAAATTTAAATTAG
A_14_P138676 AGGCAAATGTGACTATACCCACACAGTAGGTGCAGAGTAAATGTAGTTAAATGTTTCAAA
A_18_P15596973
GGTGAGACCCACTAGTGTAACCATCTGAAAAATCTATGGTTAAAAAGCCGCTATTAATTA
A_16_P19538118
GCTTGACATTTTCCCAGATACTGCCAAATTTCAAAAAATACTCTAGATACTTCAAAGCCA
CUST_9359_PI426210822
AAAGTGAGTTAACTGGAGTAACTCTTTTGAATGACAAGCTTCCCAAAAGCATTTAAAATG
A_14_P102050 TTAGATTACATTCCATCAAGTAGAAATTTAAATATGGTCCCAGAAGGGCAGGTGAGTTTC
A_16_P15447966
CAGCCAAACCTTTAGTTTTTCTCAGATAGTCCATTTACCTTTATCCTGAAAATCGTGACT
A_16_P18076887
AGGGAATTGTGTAACGTTAACATTGGTAACAGGTAAAAGATGTAATAGTGCTGCCTACTG
CUST_8368_PI426210822
TATTTTATTTTTCTCCTTTGCTTTTCGTAATTGGGTAGGAAATAGATAAAGACCTGATGG
A_14_P138765 CTCAAAGTTTGAATTCATGGCACTTTTATCCACTGTGACATAGTTTTGAGTGACTAGAAC
A_14_P126030 GCTTCTATTTCTCCTCTTTTAACTCAGGCACTTTTTGGCTAGAAGTGAATGCATTCTATT
A_14_P120979 ATGTTCATCAGGAAGTCGGAACTTGTTCTCCTTTCTGTTTTATAACGATTTTTAAGACTG
A_16_P03603412
GCCTCAACTACAACAATCCAAACTTTTGGTTCATATTGCATTTTTCTTTGTTATCCTCCA
A_18_P16457341
GATTTCATCCACAGAAATAAACTCTCTAGATTCACTCCAGAAATATCACTTGCATGCTCC
A_14_P200408 AAACTGACTTCCTGAATGTATCCACTGGGATAAATTTCTCAAAATGGAATTGGTAGATCA

A_14_P139643 CTTCTCTATTTGACTCAAGAATTACTCTGGGGTGTTTTTCAGTAGGTTGTTTTCACTACA
A_16_P38389677
AAAACTCTAACTCATAGAAATTCAAGAAGTGTAGCAAGTCATAATTTATTCAACCTCCCT
A_18_P10599217
CTTGCCACTCATGTCTTTATCTCATTAAAACCTATTTTCTCCTGAATATTGACCACAGCA
A_14_P119930 GATCCGAAACTATCACAGTGGAAATGATGTAGAGGCTTATGAGTATCTTAACAAGGTAAG
A_14_P120146 CTTTTTTGCTTGGCCTGTTTTGCTTTCAGAGCCTGAGATGGACGTAAG
A_14_P133059 TTTTGTGCTTCCATACATTCTGTAATCTCTATTGCTTTTTTCAGGCAACGTCTGAATAGG
A_14_P111484 AGATGTGATTACTTCCCGGCAAATGGTTGAGTCAAACCCAGTATAGCAATG
A_14_P116520 CATCAGAGAAAGAACCATCAGCATGTCTGCGGCCAAGTTCTTCAACAAT
A_16_P36838703
GCTAGGGACAAAAGTATGGTTTTTTTAAGTGTAATTCTAGTGCACATAAATATCCTGGGG
A_16_P15263658
AAAGGCAAAATCTTGGTCATATGACATTCCTGCTGAAATATCTCCAACATCTTATGTTGT
A_16_P37047943
AAAATGCACGATTCTATTTCCCTTATTTGTGCTAAGTCCATTAACAAATCCACGACTGAG
CUST_6332_PI426210822
ATTTGAATTCCTCTGTTAGCTGGGAACCAGCAGTTCTTTTCACAGTTAAACAGAT
A_14_P135981 CTTTTTGTCTTTCAATCTTGCCTGCCTAGAATCACTCAAACTTTCTGTGAGAGACTATAT
A_14_P134510 CTCAGTAATTTTTCCCAAAGCAAAATCTTAAGACCAAGAGCAAAACTGGAAGGTTTTTCT
CUST_84_PI426210822
AAAAAATTTGTTTGAGCATGGAAACAGGTTGGAAATATTGCACCCAATAATAACAGTTTA
A_14_P105940 ACTGCAGAGAATGTAAGTTTCCATATTCCTCTGGGTTCTCATTCATTTTTTTTCTGACAA
CUST_9615_PI426210822 ATCTCATAACATGATGCCCTCAGGTTTACGCCACTTGTGGGAACA
A_14_P131575 ACTAAGAAGCAAGCACTACCTTCTAACCATCAAAACATATCCTCTGATTTCAGGTATTTA
A_18_P13055982
TGGCATAGTTTAAAGCAAAGCATTTGAAATTCTGAAGTCCTCATAAGTTGCAAACAGTTG
A_14_P122801 CATGTGTGTGAGCCACTGTAACTATGTGTATGAACATGGCAGTGTCTATGCCTAT
A_14_P124231 CAAGAAACACTTTCCCTTGAATTCCCAAAAAAAGTGGGCAAAATACACATGGAGTATAA
A_16_P31233547
ATTTCCAGTTCCACAAAAGAAAGTAATCAAACAGATGTTCCTCCTTAAGTCTTAAACCAC
A_18_P10922140
AAAAATCGTTGCCTTCATTCTAAAACAAATGCTGCCAAATTTAATTTAACCGATGGCTGG
A_16_P20364403
CATCCAAACTTGGTGCTAATTCCACTTAAGAATTTGAAAAAGCCTTTTTCCTCCAACAAC
A_16_P38658267
AGGAAAACTTGTTAGATACAGCAAACTTAGATCATGACTTCTTTCATTGTTCCTTCTGGG
A_16_P35974361

GTGGCCAGTAATTACACTACAGTTGTTACTATGCATTACCGAAACTTCATTTTTTGACCA

A_14_P118532 CTCCAAATATTACCTTTCTACCGGCGCAAAAGAATAGAGAGCGATGAGCAG

A_14_P127922 ATGAGAATGAACTCTACAATCAGGCTCAGGTCCAGCGACTTGAACATCTTAC

A_18_P13126952

AAAAGTAAGTGGACCAAATAACACAGAGTTACCTTTAATGTGAACCCCATTTTCTAGACG

A_16_P21380337

AGACAGTAGGAAAAGATACAGGTTTATTGTGGACAGTTAACTTAGACAAGGAATAGGCTG

A_16_P16191599 CTAGGTCAGAAGTGAAGTTTGTCCTGTTTTATGAAGGGAAAGACACCCTTGTTTAG

A_14_P100792 AAACCAGAACTAGGACAGTAAATGGAAATGGATCTGAGATGGACCTTTAAGATGCAAA

A_14_P139946 AGTAGATGAGTCCCCCCGCTGTTTTATACAGAAACTCCTGTAGGAAAGAC

A_16_P01187971

ACACCGTTAGGCTCCATATTTCTACCTCAATGATATACATAAAGTGCATGAAATTCCACT

A_14_P120697 CGCCCCTTTGTGCACAAGATGTTTAACTCTTCTTTCCGTCTTTTCTAAAATAGTCA

A_16_P03635519

CAAATTTCTGTGATTAAAATGAATTCATCAGTTCCTCCAAGTCTTTGAGCATCTGTGGTG

A_14_P124079 GTCCCTAGATTCAGCCACATCAGAAATAAACTGTGACTACACTTGGCTGTG

A_16_P41295808

AAATATCTACCAAACCACAGAACTCTATGAACTTCTTGTTCACAGTTACTCATTCCAGCA

A_18_P14806270

TCACCCAATAAAACATCTGTTCTCTTCCAAATCATGACATTAAAATGCAATAACCCTTAC

A_14_P117630 GATATTTCTGCCTGTGATTCCAAATCTTCATGAATGTCTTCCTTGTGAAGAAACTCCTTG

A_16_P18021295

CCCTCCAGTTTTGTGTTAAATAATTCATTGCAATTGAGTCTCAGTAAGAAACCAAGGACA

A_14_P109880 CATGGCAGGACAAAAGTCACTAATGTTTGGCAAGGAGATTTTTCAAAACATCAATTAGAA

A_16_P15224484

AAAACAGCCCAATGTTTCAACTCTCTTTTTTCAAGAATCAAAAACAATTCCTGAGATGGA

A_14_P112324 AAAACTTTTAACTGCTCATTGCTTTGCTTACACAACTCAAATGCTCTTTGATTTCAAGCC

A_16_P21040942

TGAGCACATCAGGAAGTTTAGATACCTGCTTTGTCGTTTAGTAACTGCTTTTAATACGTT

A_14_P137516 CATGACTTATTTTTTTCCTCTCAAAGCCCCAGTGAAAATGCAAAGAACTCCACTT

A_16_P03708025

GGAGAACTTCATTGTTACACCATCTAAAAAGATGCATAAAAACCATGGTTGTCTCTGAGA

A_14_P101430 CATTCTAATGGATAATGAAATGGTAATGGATAATGCAACAAGTATTTCTACCACTATGGT

A_16_P02353731

TATACAGCAAGAACACTTCTAGGATCTGTGGGTCTCATTTAACAAGATGCTCTCCAAAAT

CUST_2910_PI426210822 ACAGTGCCTACTTGGTCATGGGAGTGTTCAGGACCAGCTCATTAG

A_14_P200647 ATGAATGCAGATGCAGTGTTCTCATAGAATAACTGTTCCTGCACTTTTACAGACAAATCT

A_14_P135328

CTAGGCAGAAAACTGGATAAAATGACGAAAAGAGAAACTGTCTACAAAAGCATCTATGGT

A_14_P132862 TTTTTGAGTTGTTTGTTTCTGTTTTGTAAAGAGAATCCTTATTGGACACCAGTGAAGGTG

A_18_P22841596

ACCCTTTAATAGGGAAGGGTTTCTTCTTCCTATGAATTTTTACCTCACTTTGTGTCTTTT

CUST_8725_PI426210822

TTATGTACCAATTAGCTCAAGGCTTTGGTAAAATAAATGTTTAAATATGTAAAATTTTAA

CUST_10111_PI426210822 CTTTATGCTTGCAAGATGGCCAGCACCTTCACTAGGCAAAGAGTAGT

A_18_P16741853

AACTGAGTTTTTTTGATGGCATACAGGAATACCTGAATACACAGGGATCTGGATAGTTAT

A_16_P19141485

ATTTTACCATCCCTTTGTAGATTTGTATTGGCCCTAGTTCATTGAAATCTCATCTGTGTC

A_14_P109536 TCTCTCCTACCTTTCAGAGAGTTACCCATTAAGTGCTGCGTTTGGTTCTT

A_14_P109237 ATGTCTTTGTTGTTGCAGATACAGATGTGGTGTATAAAAGCGAGAATGGACATGTCATTA

A_14_P117855 CCAACTTTGAATAAAACAGATGATGTCCTGTGACTGCCCCACAGAGATAAG

CUST_9609_PI426210822 ATCTGCTCTCACAGGAGGCTGTTGGTAGAGGGGTGCCTGGGAGCA

A_14_P134562 CTATGAAGAGAAGTTCTTCCACCAAACATACAAAATCAGGATATGCAACTTATAGAGTTC

A_14_P138995 CACTTGCCATTTAGTGTTAATAGATACACCACCCACCTTAAGTTAAGAATCGAAATTGCT

A_14_P107086 TATCCCATCATTAGGTATAACACTTTTGCCTGCAAATCTATCAGCAACCTCTATTACCCA

A_14_P112984 TCATTGTAAGGATTTTCTTTTTAAGTTTGGTTTCACCTCTGCCTCCGAATTTTGTATGCA

A_14_P112328 TCTAATTTGTGCTTATTCCTAACTAGTTAAAGAGCGAGTTTTGACTGCACTTTACCCCTT

A_16_P19015044

CCATGAGGCCTCAAAATTAGTCATCAGATTTTACCACTCTTTCCCATATCTACACAAAAT

A_14_P202123

ACTAATTAAAAGGCTTCATCAAAAAAACAGGGTAAGGAAAAACCAAGACCAACATGACAT

A_14_P130489 GTAGCGTTAATCATCCTCCTCTGTTCCTGGAGTATAATTATCATCATTCTTTGCAGTCAT

CUST_10318_PI426210822 GCCACATCTGGCCATGTTTTCTTAGCCAACCACATCTTTATCGCAAAA

A_16_P19894311

AAGTGAACAGTTTGCCTAATCATCTCCCTAATATTCCTTCCAATCTACTTATGTCCATGT

A_14_P106199 GCCTTGCAATCGTTTGCCTTGATGTTATGTCTAAAGCCCCACAATTCTCTAAAAA

A_14_P106387 GGAAAAAGTAGAAAATGTTAGTGAAAACAGGGTTTAAACCCTTAGGAAATTCATTTCTGT

A_14_P105803 TAATCATTAAGGAGCAAATGAAGAGACATGTATATGTGGCCACATTCAAGACCACGTTAT

A_14_P124245 GAATTGCACATTTATGACTGTCTTCTGTCGGACTGTCATTGTTTGTTTAACTGTTAGACA

A_14_P136167 ACACTTAGTATGGTATTCCCCAGTCTGAACATGTGTTCCCTTCCTCTG

A_14_P128419 GAAATTTCACCATCACAAAATACTTTCCTCACTGAAATGAAAGTTCTAAGCCTGTGTTGT

CUST_8871_PI426210822 ATGCTTCGGAAGCGCAAAAACAGGAAAACGCTTTTCTGACATACACC

A_16_P41001815

AAAAGAAAAAGCCCTGCTTAATAATGTAGCAACAAATATTTCAAACATACCCCAGAAACT

CUST_9001_PI426210822 TACCTTTTAGGTTTTTTTTTTTTACCCAAAAACCACACTGGGGATGACTCCAG

A_14_P120399 ACTGGAGATAAATATGCTAAAGAAATACTCTCATCACAGAAACATTGCAACATATTATGG
A_18_P10525078
AAATGAATATCCCTTCAAGACTTAGGATACCTACACTGAATGTTTCTACCTCCTTCTCTC
A_18_P11537194
AAAATGCCTGATGCTATTTAATGGACAATGTTATAAGCAGTAAAGCCTGATGATCTCCGA
A_14_P102467 ATTTTTAAATACTGTGTTTCTTGTGATCGCTACATACAGTGCTGCTCAGAGGCATAAAGT
A_14_P126491 GAACACATTGGGCGACTTCTTAATAAAATCCTTGAACTCTGGAAAGTTCATATGTCTCTA
A_16_P19759202
ACTTCTCGCTGTATTGTCAGAATAAAAACATGTAATTGAGGTTCCTTGTAGGCTAAAGGT
A_16_P16540054
AAGAACACAGAAAATTGAGTTAGTCTTTTGAAACCTTGCATCCATTGTATCTGAATGCCA
A_16_P39073325
AATAAAAGCAATGGTTTAATTAGGCCTCAGGATACGAAAAATATGGAAAAGGCGCTTCTT
A_14_P115930 TTTGTTTTCCTCACTACCTATTTAATCCATAACTCCCTAACCTTCACTGCTCCACTAAAA
A_16_P18300075
AAAGTTTTGCCAGCAATTAATCTCCTTAGCCGTTAGAATAGATTATACACCAGTCCTTGG
A_14_P124587 TGTTAGAGTGAGGGAAGGCTGAAGACACTTTATATAATTGGACCTAGTTTTGGTGTTATT
A_14_P121180 ACCTCAAGCACCAAAGGAAGTTTACTTTCATCATCCTCAGTATAGAAATTTCCAAAAAGA
CUST_533_PI426210822 CATTGAATCCCTGGTTCCACAGCAACAACCAGATCCACGATTTTTG
A_16_P38852890
AAAAAACTGACATATGAGGTATTTTTATACCGCACAGTATAAAATGATATGAGGCCTCTC
A_14_P133001 TACAAGACTGATTGCTTGTATCAATTAAGAGAGGTATGGAAATTGGGTGAAACCTCTAGT
A_16_P35990357
CCTCAAAACCTTAATCACCTCCATTTTCATTCAATTGCTAATCAATTTCATAAGTCTGCC
A_14_P114318 AACAGAGGGTATGGGTATGCGGACTACCAGCAAAAAATAGAAACTTGATTTTTTTAAAAG
A_18_P14136413 AAATTTGGTGCACTAATGAACACGGGGTCTCTTTTCACAAAGCAAAAGACCAT
A_16_P21435426
AAGCACAATAACCTGAACTATCTGAAGAAACAAGCATCATAGAGCCATAATTGCTGTGTT
A_14_P125521 TTAAATGATGAGGCAGAATGGATCACCCCTCTCACCACTATGCAGGAT
A_16_P02952966
TGGACCTCAAACTCAAGATTCTTTTTCCTCTACACTTCACTACTTTAACTCTCCTGATTA
A_14_P111495 CTACAGGAAGTTGTTCAGCATATTTCAGAGGTTCCATTAGTTTCTCATCCATCTGGT
A_18_P12784988
ATTGACCTGTTGTTAACCACAAAACTGATTGAATTCTACAGATCTTATGCCTAGGCAGTT
A_16_P01597392
GATCTTCCATGGCAAAATATATGAGGAAACTAACGAAAGTGTTTTTGACTATGCAGAGGC
A_16_P03002795
AAACAGGCCTACTCATTTACTAATTCCTCTTCTTTAGGTCATAGAATATGGAGCACTCAC

CUST_5070_PI426210822 GATGAGAGGTCCAAGGCAAGATGGATAAGTAGGAATAAGCAGCCTTGTTT
A_14_P109853 AGCATTGAGAAACTAAGATTTAGCAGAAGTTTAAGCCAGATTTCCAGTTCATCTCTCTTC
CUST_6458_PI426210822
AGATTTTGGTGAAACTCAAAATTACTTGGTGAGAAAGAAAAGCACACATATGTTACGCTT
A_14_P103898 CTCATTTTTGCTTGCTGAAAAATAAAAGTATCACAAGGAGAAAAATTGAAGTGCCCCAGT
A_18_P14270424
CCTCCTCTAATGTCTCGTGTTTTTCATTGTTTATTCCCATAGTATCAGATACACCATGA
A_16_P00805070
TATATGACATTCAATATCTCTGATGTCCTCATCACCAAATCATAAAACCTGACGGAAGGA
A_14_P108537 GTAACTAATTGTAAGTGGAGTGAAGGTGTTTCATTGAAGTCTCCTATGGAAGGTAACATT
CUST_3644_PI426210822 TATTATTCTCAGGGATTTGGAGGGTGCAGAGGTAGATGAAGTAAGCAAGC
A_14_P117821
ACACTATTACTAGTGAGCCTCCAAGAGAAAAAACTCTGTAATGGAAACACGTAGACAAAA
A_14_P128567 CTTTTCTTTTTAGATAGTCTTTTCTTCTGTCTGGCTAGCAGGTCTGACCAGCATTTTAAT
CUST_4386_PI426210822 ATTAGAGTTGCTGGGCAGCCTGGGCTGCCTTTTAACTTACGGGTC
A_14_P129755 TGAGTCTTCTCCAAGTCAACATGACTAGATCCACCTCTATGCTTACTCTGAGTAATC
A_14_P106745 CTTCAAAACCAAAAGTTTCTGCAGTGGATGTTATTTCTGTTTTCCTTTCAATACAGGAAC
A_14_P101074 TGCCTTCTAATTCTTGACACAAATATATAATGACCATTTTAGATCGGGGAACTCCCTTTC
A_14_P135243 CTATTTTGAGTCACTGAAAGTCCTTGTCAGGTAAAGGCAGGGTATAAAGAAAGAAATGAT
A_14_P137738 CAGCAGGAAGGTCAATAATGTGAGAATGAAAGTTTTTCTGGGTCATTATCAAGAACTTTT
A_14_P123138 AAAGTGGATACAGAGACAACTTTTTCTAGAAAGTTCAGAAAAGTGTATTTTGAGGACGGA
CUST_8094_PI426210822 ACCTTCTCAGAAGAAGGATGGGGAGCACACAAGCACAGTGGGAAT
A_16_P17341946
ACAGCACATGACGTAAAACAGATCCATTGATATTCTGGTCCTTACTTAGAAATCCTAAGA
CUST_8979_PI426210822
TCAGTTATTGTATGACTACTATCTTTATTTCAAACCCTGAAGTCCTAAAATTAATGTCAC
A_14_P122293 TTTCATTAAAGAGTCACTTGTCCAAAAATGTTTCATCTCCCTTTCTGGGATATTTGCAGA
A_16_P01412603
AAAGCCATATCAGACCATATAAAAGACAGGCTAGACAGTAAGTAAACTTGTAACCATGCC
A_16_P36632274
ACAGGGTGAAATAGAAGGTGTTCTGATTTTATAGAAACGGATTGCAAGATAGTCATAGCA
A_14_P115406 AAAAGTATCCCAAGTCTTGAAGATAAACTGTTAGTTTTATGGGGTCGCTTTTACTCCTAC
A_14_P104227 CAATAAAAGACAAGTCATATCAGGAAACTAGTGGTTAGAAGTGCTAAATAGATGGGCAGC
A_14_P108075 CCTGTGATATCATTAATGACATGACTACTTCTTAAAAATTCCACACCTGGATGCAAAACC
CUST_5387_PI426210822 GAGCAGCCCACACGCACCCCCGCCGAGCTCACTGTCTCAGAATGT
A_14_P117370 ATCTACTACTAGAAAATGAGACAGGACAGAAGTATTCTCACCCTAAAACACTGGATACAG
A_16_P18153551
AATAACGACCTAACTGTCCTTTAAGCCTATGTCCCAAGAATCAGTTCCTCAGATATAATC

A_16_P18157250
TATTCAATTTGTCAGCCAATCACATTAACTCTATGTCCAATTGATTTCCCATATGCCTGC
A_18_P15800771
AAAAAAAAAAAGTCATCTACCTATAAAGTCACTCCAATTTAAGGCTGATCCTATCTGAGG
A_16_P03604862
CGTGTTCATGAAGATAGTTTAGGAAATGGGTGTTTAACTGATCACAATCTCGCTGAAAAT
CUST_5564_PI426210822
TAACCTGTGTATATATACACAACAGTTTGACACAGAGCATCTCACCTCTTTCATAGGAAG
A_14_P101093 TAAAGGTGATTTTCAGGTTTTCTCTGAGACCATTTTCTTCGAAAGAGTCAGATCATCAAT
A_16_P18034658
AAATGCATTTATGTTCTTGAGCCTACTTTCTAGCCATTTCTAATCAGAGACTACCACGTG
A_16_P40018271
CGCTACAGATACCATTTAAAGACACATGAAATCATACTCACTAGTTAAAAATCCAGCCAC
A_14_P104156 TTGACAGGATAATAATCCCTTAGTTGTCTCCAAATTGCCCGGTTCCTTATCC
A_14_P113041 TTTCTGCTAGATATCTCTGAAAGTGTGAACTTCGATGAGGAGACTGATGGAATATCC
CUST_7948_PI426210822 CTCCCTGAGGTGAGGAGTCCTCCCACCCCCTGCCTCCCATAGCAG
A_14_P108263 ACCTTGTGATGGCTGAAAAAATATTTCTGTTTCCTTTTTCTCCATATCTTCCTTATGTGG
CUST_6810_PI426210822 AGATAAATTAGTCAAAGAGATGAGAGTTTGGGGCTTCAAGTGTCCGAGTGTC
A_16_P35113860
AGTTGAATGAGAAAAGACTGCAGAATCAAATGCTGAATTATGCTTTAACAATTAAGGAGA
CUST_7523_PI426210822
TACGTGTCTCACACATGAGAATTATTGTGTAGAAGCTGTTTCCCATTTGAGACAAAATAA
A_14_P131036 CTGAAGACTCTGAGATCTGGGATTCCAAACAAATATACACTTCCATTCTGAACTAATCAT
A_16_P02761878
CCTCAACTTATCAAGAAATCACTAGTCCTTCAAAAGTCAGATGTTTCAAAAAGATTGCCC
A_14_P127567 AACATAATCTGTGTCTGCATCAGCGATATTTCTGGTGACCCAGCCAGAAAT
A_14_P139612 CTGGAATAGTCACACTTAAACTCCACAGTTTCTCTGGAGCAGTTGAATTTAGAAAGAAAC
A_16_P16621365
AAGCGACATAACTTTACCAAAGACGACATGAGTAGATCCTGATCAGTTACACATCACTTC
A_14_P125879 TTCAGGATAAAGCCAAAGTGATGTTTCTGGGAGTGATATAAAGTGTGTAATGTTACCAGT
A_16_P21337914
TGTTAAGGGGTTAACCTCTGAGAGATTACATTGTATTCAGCGCTCTGTTAAAGTTTGAAT
A_14_P135418 CAAAACAAGGGAGTCGTGGTCAATTCTTCAGTGATGGTGAAATTCCTCTTTC
A_14_P115589 TGGTAGAAACACCCTGTGTCTCATTTCGTTCATTCATATAGTAGGCTACATCAGTTAAAA
A_14_P119318 TGAAGAGGGTATTATGCTCTAAATATGTGACTTTCCTGAGAGCATATAGATCCATTTCCC
CUST_433_PI426210822 CCGCTTCCTCATTTGAATTTTTCTAGGGAAATGGCATTTTCAGACATTGTTCC
A_14_P123746 GTGTAGTTTCTGATGGGTGGTGAACATCAAATAGGGCCATTGTAATTAATCTATGTTTGA
A_14_P104312 TCACATGTTAACACTGCAAGGAAGACAGTTGTTTTAGATGTTCTGTTTCAATACTCCCTA

CUST_5790_PI426210822 TCCAAGAAACTGATCCTCCTCTGAACATGATAGCTGGACTAGGCTAAGAAT

CUST_5952_PI426210822 AGGGGCTCAGGGGAGGAATTCCCACTGATTTGCAAGAAAGGAATT

CUST_1087_PI426210822 CTCCTACTTTATTTGGACAGAAGTCTCACTGGCTTATGTCCACATTGCCTT

A_14_P129193 CATGTGAATTTGTTTTCCATTTTCCTTGTTTGACTGACTGGCTATGTCTTCACTTTTTTC

A_18_P16234926

AAGTCCTACCGTCTCTTACATAGAAAAATCTGCAACACTCCTTTTATCCTGTCATTGTAA

A_14_P120016 CCTTCAGGATTTTTACAACCACTCTCTCATTGTTGGTGATATTAATGGCCTCAAATACTT

A_16_P02218844

AAATGATTGGAACAACACATTTGCATTCTCACATAGACTATATTTCGCATCACTCTTGGC

CUST_9435_PI426210822

CTTTAGGATGCCATCATTATGTGAATGTTTCCTGCTATGATTGTCCAAAGGGAAAATG

CUST_1234_PI426210822

AGGTGTCTAACATATCCTGTTTTAGTGACTAGATAATGAAGGGTAGAAAGCTGGATATTC

A_18_P17038578 ATAGAAGGCACCTCGTTCCCTATTGCCCCTCACTAACTCCCGTGA

A_14_P131385 GAGAGAGGACATGTTGACAGAAGTGAGGTAAATTAGAGAAATTTCTTCAACTTCAAATCC

A_14_P135175 AATGGAGTTAGAGTGAACCTACCAAAAAAGAGATGGAAACAAGTTATCTCTCTTCGTTTC

A_14_P120176 AGAATCATCACCGATAACATATTTCAGGAGACAAAAACAAGAGGTGCATTGATTTTTTCC

A_14_P127727 GAATGAGATTCCTTCTTCCTAAATAGTGATTGGATAGAACTTCTGGTGTTTGGATACGAC

A_14_P109278 ACACTAATTTATTTTCTTATTTAAACAGGCACTCGTCTGCGAAATGTTCCTGTTCTTTTT

A_14_P114255 CAAGAATTTCAGAAAGTGTGTTGGTTCCTAAATATTCAGAGAGTTCTACTAATGGGGTGT

A_16_P17001400

ATCCTCGACATGGATACGTTTATTCAGGTTTTTATCCAAAATGAGATAAGGGCTATGCAA

A_14_P129901 GTTTCCTCTTCAAAGCCTCTTCAGATATTTTAATTAGCCACAGAGGCGGGTTTTT

CUST_2743_PI426210822

TTCTTTTAGGAAATTACCCAATGAATTGGTGGAGGTAAATGATGCAGGAGGTTTG

CUST_3840_PI426210822

CATGCAGAAATAAAAAGCCCAAATTCAGTGATCTAAATCAGGGCTCAGATGAGG

A_16_P00639849

TCGTGTCTTACCACTGTAATCGTTTGTTTGAACAAGAGTTTTATTGCTGAAAAGACATCA

A_14_P121057 TAGGAAGATGGAATGGGTAGGGCCTTGGTTTCAGAATTTATAGGACAGATTATAAGAATT

CUST_7538_PI426210822 TAACTTCAAAAATGGCAGTGGCTACATTAGCTCTTGGGACCAGTGTTTCA

A_14_P132238 GAAGATCCTAGAACTTCTGCAACTCACTGATTCTCCTCTGTGGTGAAGTTTTTCTTAT

A_14_P124863 GGAGAGTTGTATTATTATTTGTAGATGAAGAAAACTGGTTCTGTCCAACCAATGCAGAAT

A_14_P118226 ATCATCGATGCTGTAACTTGTCTGGTAGAAATCCGTGTTTAAGTTTTCAAAGCCTGAC

A_16_P35742979

AAATCTCTCCCCAAAACAATTTTAACGTTCAAAAACAGAAGTGTCCTGATTCTCAGTTTC

A_18_P16310728

AGCCTAAAGGTTAATGTTGGTAAACAGAAAAAGATGTGGTTATGAAAGGGTCATCTTGAG

CUST_7649_PI426210822 CTCTTCAGACTCCATTTCCATGAATGTTTTGGGAGAGGTGAGGAAGAA
A_18_P15522837
AAAAACCCTCAATTCCAAACATAATCAATCCAACAATGCATTTTTCAGTCCACAAGAGAA
A_14_P100472 TAAGTAACATTTTTTCCTTCTTCACTCACTAAACCTTTACTGAACTCCAACTAAGTGCCC
A_14_P200501 GATTTTGCTGCTGGGCAACTTCTTCTGCATTAGGAAGAAGCCCAAAG
A_16_P02721335
AAAGCAGGGATTATAACTTCAATGGAGCAATGTAGACAACTTCACAGAGAACATAAGCAT
A_14_P121174 CTACAGCGACTATGATATGCCTTTGGATGAAGATGAGGATGTGACCAATTCCA
A_16_P18738075
AACTTGCAGTAATCGTTATACTTTGGAAGGTTTAGACAGGAATAGAAATGAGGGTAGTCC
A_14_P103120 TGGCAAAAAAGTTCCCTTTTCATGTTTTAACAATGCTTCCTTCTCTGCCTCAAATATCTA
CUST_4126_PI426210822 CTTTTTGGTGGGAAACGTAAAGGCCAGTCAGCTTTTGGATGAATTTTCAA
A_16_P19615564
ATCTTCTATTCTCGCAGATTGTCTCATTAACCTTGTTCGTCTCATAGTAACAACATCTCC
CUST_10040_PI426210822 GAGGCTAGGAGACAAGGCTGGGCTTGCCTCACTCCCTGTCGCCCC
A_16_P00149595
ATCAGAATGAGTAAGATTACGCCATGAAGTTCTTAGAAATACAATTTTCCACCCATTGCC
A_16_P18145042
ACTATTTCCTGGTTAGAGATCAAAGTCACTTCTGTGTAATGATAGCAAATGTGAGAACCA
A_14_P133733 ATTCACTTGTTTTATAAATGTCTGACCTGTCTTGAGTAAGTTTGTGCTGTCGTCAATCTT
CUST_5976_PI426210822 GGCAGGCTGCCCTGATCCAAGTGAGCAGATAGTCTCCTTATTGTT
A_14_P133490 TGCTAGTTACATAATTGCTTTAATATTTAGTTCTAGAACTGGGGAACAAAAGTGACCCCC
A_14_P105879 TTGGAATTTCCTACCTGCCTTTTCTCCTTAATTGTTGCCTTCTTTGAACTGTTG
CUST_75_PI426210822
TAAACTGCCTCCAACAAATATCATCATTTTAATATTTTATGTTTTGCTAAGCCATAGGCT
A_16_P21434380
AGCCTTGCATCATAAGTAAGCCCTAATTCTGCAAAAATTGAGTATTGTTTCATTTCCTGA
A_14_P109755 CTTACCTGTGCATACTCTCTTTCAATAGCAGCCTTCTTCTGACTGAATGTCCTAAAAATA
A_14_P131008 TTGTTAATTACTATGCGAAGAGTTAGACACATCTCCTCTGAAACCACGTAAATCCTCCTA
A_14_P128631 CATGGCATGTGGGTTATAAACGTTGCTGTCAAAAGATTTACCAGGTCTACACCATTAT
A_14_P123676
AGGCAACAAAAAGAAAATCAGAGGCAAAAGATTTAGACCTCGGTCTAATTCAACTGAGTA
A_14_P122804 CTTCTTCTTTGCCCCCATGGTAAGAGACTCAAATTTATTCAGATCTTCTTCAGTAAGG
A_14_P133102 TGTGGTTTTAAACAAAGAAATCATTGTAGGTGAAGTTGAAATCTCCTTTGTTCTCTTCCC
A_14_P123888 GCTTAAAGCAGGCTCTGGAGTTTGCATACACAGGACAGGTATGGTATTAAATGT
A_14_P201054 ATGCTCACAGAGACTTTGAAAACCGTGATCGGTATTATTCAAATATATGAGCACAATGAC
A_18_P14134624
TTCACACTTTTTTCCTTCTCTGAGGTATGCTGTTTTTTGTCAAAACAGAAAAAACACACT

CUST_1549_PI426210822
TTCACTCTCTTCTTCCATCTCAGCAAACATTTAGATTGTGTGACAACTATGGCTG
A_18_P22840701 AAACAGAACAGAAGAAGGTTGGATGGTTCACCAAGTTGAGTGGGTC
A_16_P21362380
AACAGGAACAGAGTGAGAAAACTATGGTTACATTTTAAGACATATAGGCAGCAATCCAAA
A_14_P112812 GGTTTATTTGTTTATATTCACAAGAGAAATGGGTTTACGGATGGGAATTTTGTGCTTTCT
CUST_4529_PI426210822 CTGGAGTGATAGAGAAGGAGTTGGCAGCATTTAGAATGGAGTCAGGA
A_14_P106263 ATTTATTTCCCACTTAGGTTTTATGGCAGGCACACAAAGAAACCCTCAGATGG
A_16_P21374699
TGACTATGTGACTACGGATTACCAGTAACCTTATTAACCTACGCATTAAGAGAAAAAGCC
A_16_P03763212
AAAAAGCACCATTGTCCCTAAATATTATGACCCTGGAATATAAGATTGATGCCTAAAGCC
A_14_P110515 TTACAGCCTTCTTCTGTGTGTTTATGTGCATATATGTAGCCATAGGGAAAAAAATCAAAA
A_14_P102642 CTGTGTGCTCCAGCCTCTTCCTATGTGTGTAACTTCAATAAAACCAAGCAAAG
A_16_P00685176
TAAGATTGTTCTTCTATGTTCCTGAAGGATTTTTCAAGGAACTTTGAAACCTAAGTGGCT
A_16_P58424423 CCAAGCATGCTGGACAAGGAGGCTCTAGGAAGTCAAAGACAATGT
A_14_P127516 AGGAGTTTTTGTTCTAGGAAACTTAGTAGGCTTCCCTGTTATGGTTGCACTCAG
A_14_P123624 ATCACATAAACAAATGACTATACAAGACTACATGTGAAACTGAGTTCAGGAATGGCCAGA
A_14_P136856 CTAAAATGTAAAATATATATTTACCTGGTTGTGTTTGTGGGATTCTTGGGCGTGGTGATG
CUST_6761_PI426210822 GAGATGTACAGCTCTATGGCCCTGGAGTCAAGGTAAAGCCTCATT
A_14_P110157 CAACATATAAATGCTTCAAGGACATGCTATACGTGTTGCTACCCCTTGTTCTCTAAAGAA
A_14_P134760 TGTTATTGGTATAGCCATGGTAAATGTGAATGTTGGCTCTCCTCTTTGCTTTTTCTGAAC
A_16_P01449825
TCAAAAGCAGCACAACTCTTAGGTTTGAACACTTTTTTTAATGGTATTTCAATAGATCCA
A_16_P21478468
TGCTTTCTATGATAATGTCTAAAGTCTTAGCATGACCTAAAAGATTCTGTATGGCATACC
A_14_P138902 CTTCCTCTTTAGAAGTTCTTGAACCAAATTTATGGTTCACTGTAAGCCAGTGTATAGGAT
A_14_P116874 TCTTATTTCTCCTCCACTCTCTCTAGTGAACATTTTCATAGATACTTCAGAGGCTACTCT
A_16_P19366604
AAATGACCTAGGCAAATTAACAACATTTCCCTCTACAACCAAATGTAAAATAGATGCCCC
A_14_P121266 TCCCTTGTTCTTGGTCAGGACATTTCAGCATCATCAAAAATCCCAATAAAAATACCTTTT
A_14_P123842 ATCAGCACCAACTTTCTTTCCTTTATGTGGGTATTCAAAGGAGTTTACATAAAAGGTTTC
A_14_P118747 AACCTTGCATCTTCAGAGTCACAAATCATATTCAATTTGGCAAGCACACATTTTTAGTGA
A_16_P34852300
ACATTGAGTTCTAGACCAACATACCTGTTTTTGAGTCTTTAACATGCTTCATATCTTGCC
A_16_P21661712
AAAAAAGCAAAGCATTTGTAATGTTGACCAACAAGAGGTATTGAGGTGCTAAGATGAGAA

A_14_P200285
TTGACTTGATTTGGTCCACAGAAGAGTAAAAAATAGAAGGAAGTAGTATAGGAGACAGCA
A_14_P100321 GTCTGCACAGTTTAAAATGTCTAACTAAAACAGTAATAACTCGTTCGACATTCGTTTGGA
CUST_4866_PI426210822
TACTTTGTGTTAAACTCAATTACAGAGATAGATCCTTCCATTCATCAAGTGAATTTTAGA
A_16_P16510297
AAAAATGGAACACTCTCCCTCAATTGCTTTAATGTAGATAACATCCCAGAACTTTCAAGA
CUST_3473_PI426210822 TTCACAGAGGTATCAGACAGGAATTCCACTCAGCTTGCAGGAATTGA
A_14_P133142 CTTTGGTCTCTTCCACTTTCTGAACCCGTCCAGACACAGTTTTGTC
A_14_P129951 CAGAAAGAAATCTCACCCTTGTAGAGACTGCAGGAGTCAGTGCTCACTTAAAA
A_14_P119328 CAGTGCGATGCCTCTGACATTAAAATTCACATCATGAATACCAAAGAGAATACTTGGAAA
A_14_P101666 ACCTTGATTGCCTTACAAAGTTATTTGTTTACAAACAGCGACCATATAAAAGCCTCCTGC
CUST_4506_PI426210822 ATGGGAGAGTCTGCCTCCACTGGGCAGTGCTGGCCTGGAGGAAAC
A_14_P108572 AGTGGATCAGTAGCCATACAAATATACTGGTAATTTATGGTCATTCCTTTTAGAGGGGAT
A_14_P130119 GCACTGACTTAAGCACCAGGAAAATGCTACTCACTTGGAATTTAGAATTATTTGATTTGG
A_16_P17208796
AAAAAAAACATGTTCAATACCATTGTTGTTTGAACAGGTAACAGAAGTTTCCAAATGGGA
A_14_P108888 CTGGAGACATTTTGTGACATGCATACAGATAGCATGTGTTATTAAAAAGAGTTGCCTATT
A_16_P19426616
AGATTTAAAACAATGCAGTTCTTAAAACACCATGTCTCAAATTGCTACCAGACTACCCTG
CUST_8697_PI426210822
TTCTTAAGTTGGGGCCATCAAATCAGTCGATATAATCCAAAGCTAAAGAGTAAAGAAATT
A_14_P114468 AATAGCAGCAAGTGCTCAGTTAATTGGTCAATAAAGGGGAGAAATTTGCTTACAAAAAAG
A_18_P11518373
TCAACTTCTAAACAATGTCTCACTTATCCCTTGATCCTTAAAATAATAAACCAGCCCTGC
A_14_P100894 TCCTCACTCTCCTCTTACTACAAAATGAAGTCCATGTGGCTCAATGACTTAATTACAAAA
A_16_P01395894
TTGCAAGCATGATTTCTTGATTTTCCAGGACAGACAATTAGAAGCAGCAGTTTAGTAAAG
A_14_P104466 AAAATGCATGCCACAGCATTAATTTGCTATGCTAAACCGATGTTGACAAACATAAACCAA
CUST_5802_PI426210822 ACTGTAAGAGAAGCACAGCCAGCCCCAGCGTCCTTACTCTGACTC
A_14_P127634 ACTAAGCAGTTATATCCAGGGCATATTTTCGTTGATTGTAATTTTGGGGAAAGTTTTCCA
A_14_P116198 GATCTCTTAGTCGAGATATTTGTGGGAAGAGACTTGGAAAAACCATTGAAAGAGTTAAGA
CUST_11_PI426210822 AAAATGTCAATCCCTGTCCTTTCCACCTTGCCTTTGCAGTCAGAA
A_16_P18329382
TTTTGAATGACCAGAAAAATAGACTAACTCATCATGACCAAAATCCTATAATGGCCACGC
A_14_P200600 ACCTTCACTCTTGCTATTACTGTTTTACTTGAAGGTGATTACCCCTCAAAAATGAAAACT
A_14_P105515 ATGAGTTGACAATCTGGAAAACATGACAGATGAATAGAAATAATGGCAGTCCTCGATATC
A_16_P01762524

GGTGCTAGTGAAAAGTTAAACAGTATGGTATTTAGATGGCTCAACTGACTTACCTATGGT
A_14_P201059 ATAATCAAGTTTTTGGCACACAAACTGTTATTGTGATTTAATGTAATTTTTTTGAGAGAG
A_14_P104590 AGTTATGGTTTGAGTTTGTTTCCCTTTTTCGTTAGTAAGGCCACATTTTTGTGAACATTT
A_16_P00396164
AAAACATCCATCCTTCAAATCATTCCACAGGTTAAAGAACAAATGTCAGGTTACACTCTT
CUST_182_PI426210822
TATCATTTTTAGGGTTTGGTTTCCAAAGTTGGAAATCAGTGTGTTTTAGATCAGTGGTTT
A_14_P200262 GCGGGGTGGAAATTTTATATAAGGACCTTGTTAAGGCCAAGAAAGTCAGTTTAATCT
A_14_P115458 ATGCTTCAAATTCTGAATCCCTAGAGTTAAATGGAATGTTGTTATGACTTAGGATGGGTT
A_14_P102784 GGCGAGTCTCTCTTTTTTATGTTGCTTTGATTTCAAGATCTCTTAGATATACTAGGTAGT
A_14_P102101 ATGAAGCAAATATGCTCTTAAATTTTTTAAATTTTTCAGCACATGAGACAGCCTTCTCCT
A_14_P201974 GCAGGAAGTGACTAGAATGACAAATCATCAGCCAGGAAAGAAAAAGATTTTGTCTTTATA
A_14_P109442 CTGCTTCAACAAGATATTTACTAGTATTAGACTATCAGGAATACACCCTTGCGAGATTAT
A_14_P101037 CTACAGTATGTTTGAAGGGAAACAAACCTTTGAAAATTGTATCAGTCCTACTGCACATTC
A_16_P03224289
ATGAGTTCTGTTTCTTAACACTGGATGTTACTGAGTATAAGCACCTCTGACGGTTCTAG
A_14_P103194 CAGTCCTGTTGGGGCAAAAAATGATCCTTAAAGCATTAAACATTGTTCATCTTAATCCAA
A_14_P130839 GAACATCCGCAAGTTACATGTTATAGCACACTCACATACGCACAAAATCCTATATGCTAA
A_14_P116797 ATAAATAGAGACCTGTAAATGATGAACAGTGAACTTGGTGGAGAAGTGAGATGGGC
A_14_P134089 CTGAGGGGCTTATCACTGGAACAGTCCTGACACAGTTTGATGTAAGTAATATCATTATAT
A_14_P106016 GCAAACTACTCAGTGAAGAAGTGCATCTTCTTACTCTTCATCAACCGTAAGTTAAAAAGA
A_14_P109844 AATGTCAGGAATTCCCAGAAGTAAGACCATCTTTGGGACAAGAGACAATGAAG
CUST_10098_PI426210822 AAGACAGCGGTTGGCGGGGACAGTGTTGAAGTCCTCTTCCCTTTA
A_14_P114899 CTTAAAATATCTCTTATTGCAGCCTGGGTAAGCACACATGAAGGGGCGTTTC
A_18_P22839615 GAGTGTGAGGAATGCCAGAGGGAGACCCACGAAACTGCCCTGCAG
A_18_P14017883
CCCTGCCACTTGTTACTCTATTACTTTATTCCACATGTCATCTGTTACTTTGGAAAAACA
A_16_P19032129
ATTCTTCTTCCAGTGTATGAAGTCACTACCCCATTTTCAGTCTATAAACCAAAGAAAGGA
A_18_P14170925
AACTCCAAGTTAGGCTGATACTCAGGAACATGCACTATTTATGCTTTTATGGTTTCATGT
A_16_P17620642
AAAATGAAAAGCAAGTAAGAAAGGGAGACAAATCTACATTCCATCATTATTTCGGAGCCA
A_14_P110939 GTTAATAAATGACAGTCATTCTCCAAAACAATGTTCTCAGTGGGGAGTTTAAGAGATTGG
CUST_4925_PI426210822
ATAAAGTTTCATAAGTCTTATATGGCTTTGAACAAAGCAAGTCTATTTTTCATCATAGTA
A_14_P124154 ACTATGCAGGTTGTTTTAGGTATTTTGTCCTAAGAATGTAAATGTGAAAGGCTGGGAAAT
A_14_P101931 ACTCAAGGCTTTGAATTACCTGAGAAGTTCAACTAGTTTTGCTTTAGAGAGCATCAGTTA

A_14_P119875 ACTCTGCAATAGTCATACGAAGTAGATGCCATCCAGATCTCCCCTTTAGAGT

A_14_P134075 ACAAAGATCTGTGTTTCTATAAAAACGAAAAACAAGACTCATCCCCAGAAGTCCTGG

A_14_P110834 AAACAAAGTTTGCGATGCTCATAATCATCTTCAGCAGTGGCAACATTTAACTTTTTGAGT

CUST_10190_PI426210822 AGAATCACTGTCTAGGAAGCAACCCAAAGGCCGCGGAGTGGGTAG

A_14_P137646 CTTTTCTAATCTGAGAGGTTTCATGTTGAAGAAAATCAGTGTTGGGGTTGCAGGA

A_18_P22842075 GATTGGTGCTAGGTTTCTCCTGGTCTTGGACGAGGTTGCACTTTT

A_16_P18328789

GACTTACCAAAACGACTTCAAATTTAACCTTTGCAATTGAGTTCAACATTCTCCCATCAT

A_16_P16582625

ATTATTGCCAAAAAAAAGTGAAATAGAAGTCCTGTCAAGCATACTACAGTTCCAAGAGGG

CUST_9074_PI426210822 TCCCAGAGGCCCTGAGAGATAAGCAGCCATGTCATGAGAAGCATT

A_16_P17800876

AGCAAACAGGATACCAAATTATAACGAGACCACAAATTCTTATCTGCAGAATCCTCATGT

A_16_P19707930

AACTACTGCTCTGATTTAGGGAATAGTCAAGTCTTTAGTTGAACACTCACCTTAAGAACC

A_16_P02924132

ATCACTGGTTCTTATTCATGGATGCCCTTGTTATACTGGTTGACCAACATTTTGAACATT

CUST_3309_PI426210822 GACTAGTTATCTTAGTTTGCTTCTCCAGAAGAGGCTCCTCTTCTCTCCC

A_14_P129016 ACTAGGAAAAGGGCTAGTCTTTCTGTGAATATACATGAATTCTTCCTATAAGATGTGGGT

A_14_P132788 TTGGCACTCTGGCCGAATTCTGCATTACAAACAGTGATCTCTATGGAGATATAGATTATA

A_14_P139704 TTGATCAAGACTCAGAATTTCAACTCCAGTAGCCCCTTTATTAGGTTTTAGAAGTCAATG

CUST_58_PI426210822 AAAAAGGAGCTGGCTAGCCGGCCGCTGATGTGAGAGTGAGGTCAT

A_18_P10844933

AGTTCTAATTCAGACTCTTTGCTTCATACCATTCCTTCTCATAATCAGAGCCTGTAGTGT

A_14_P112990 GAGTGTTTAGTCACACTACTTCATGATTATATCCCATATAATATAGACGTATCCGCGGGC

A_16_P03425624

CGGTTAGCACAGTAGATGCTCAAAAAGGTATCTGTGACAATATTTGTGTTGCAAATGATG

A_16_P20465924

AGTTCTCTGCATGTCTACATCTATTACTTAACCAAGAACAACTCATGTATTCTGCCTTCC

A_14_P102735 AAAGAGAACTTGTGATCAGACAAAACTGAGAGAGTAAGTGTCTCACCTGTATTTGCATTA

CUST_1939_PI426210822 TTGAAGGACCCTTTTTTTTGCAGGGCTGGTAAAATGACAGCTGACTT

A_14_P133767 GGATCCCGAGGGATTACTTTTTAGACCTTCTTTCACATTCAGAAAAGTAGTATAGATTCA

A_14_P101860 TCTCATCCCACCTGAAATTCGGTTTTCTTCCAAATTAAATTTGTGGTAGAAACATCCTAT

A_14_P120404 TTATTAAAAGCCATACCCATCTTTTAAGGTTCGTGTTTAAAATCATGCCAGGCTGCTATC

CUST_6587_PI426210822 ATTAAGTGACAGAGGCCCAAGGTTTGGGGATTGGCAGAAGTTTAG

A_14_P200759 CTGAGATCTTATTAACATTAGTTGTCACTTTATTGGTTTGACCTAAGTGACAATATGCTG

A_14_P101446

AGCAAAACACTGTGAAACAAAGAGGAGAAACAGCACTTTAAACTTTTGCCACTGTATTAA

A_14_P117867 GGGCACTCTTTATTTTTAACCTGTCTAGACTGTGTATAAAGTAGTAAAGGGTCACTGAAG

A_14_P128938 GACATAAATTCAGTTAGAGATAAACTTACAGTCAGTTTAATGGCCTTTTCTGGAGCAACC

CUST_8887_PI426210822

CCAGAAAATGAGTGTGATTTACCCTTTGATAAAAGGGAACTAATAAAGCAATATATGCAG

A_14_P200098 TGTGGCTTCACTCATGAAATTTACTTCAGTTAATATGAGACTGGGGGTTAAGTTGTAACT

A_14_P133899 CTGATCTCATTTGTCACCTGAACAAAAAGCAATACTTAAACTGAATTAAAACTACCCACA

A_14_P106874 CTGGAAAACCAATTAGCATTCTTTTTATATCTGGGTGTTAATACTTGGGCCTTAGAGCAA

CUST_257_PI426210822 AAGGGGTCCAGTTGTCCCCTGAGTCCGGAGGGGTGGGCCTAAGAA

A_16_P19610099

CCTTTCCTTTTTAAGACACAACCATCTTTACTGTAAGCCTTTAACAAAACACCTTGTCTG

CUST_340_PI426210822

GAGTAGTTTTGCAAAGGGCTGCTCTAGTGCACTGAAAGAGTATAGAACCTACAT

A_18_P14134939 CTTGGTCCCAAGTTCCAGACCTGGGTTCTTGACTCCATGACTGTT

A_16_P32194009

AAGAGAGATGATTACTGAGGTTTACACGTCATGTATTTTTAAAAACACACTTTGTCCCTG

A_16_P02464808

TTCAGTTGTTATTAGAGGTCGTCGTTATCTTTCTCATGAGTGACTAAAGCCTATTATCCC

A_16_P19522127

AAATGTGTTGAATCCCTACTGAACTCATTCTTTCCAATAGAAACGAAGTCATACATGAGG

A_14_P137120 CTGGGAACTTCCAGAATCAACTGTTATTGATGAAATGTCATAACAGTAGTGCTATTGCTT

A_14_P137732 ACGCTATATTGGAATGGAACCACAGTTAGTGCAATCATATTATATTTTCTCTGGTGCACT

A_14_P123930 ATATTGCTGAAAGAAGGGAACAAACAATAAGGACCTATAAGGTGTTACAGTTACTCCAAC

A_14_P137437 TCTTTTCTATGAGTCTCCATTTTCATTCGAGCAATTCTGGCAGGTATATCCAGTGAATAT

A_16_P38056960

AGTCAGACATACAACAATTGTGCATTTCACTTGAAACTAGTATCTCGTTGATTGCTACCA

A_16_P16076858

CTCCTACCAGCAAGTATTCTTCATGTCTTGGATATAGCAATAACCATCATTACTTCTGGA

A_14_P133878 CAACCTGTTGTCTTCAAAAACATCAACTCCAGTATTAAAAGGATTCTGGCATATTCCTTT

CUST_4748_PI426210822 AACAGAAGATGGGCATTTCATCCTTTCTCATTACTGAGCCTTCCTCAAGA

CUST_4587_PI426210822 GTATTGTCTCTCTAAGCTGCGGGGAAAGGGAAGGCAGCAGATTGG

A_14_P124499 ATTTGACTCCATGATACTCACCTGATTTCTTGGCCCCAGCATCAGAG

A_14_P113269 CATAGGAAAACCATTACAAGCAAAATAACAGAATGAGCATTCTTAATTTGGGATGCCCAG

CUST_313_PI426210822 GTGGGTGTGGAGCAGTAATGCTGTGCATATTCTTGTTGCCAGTTG

A_14_P131214 TAGAGTTGCTGTTAGGTCATCAACAGTAGCAATACTACGATCATCTCTTGCAAGATCAAA

CUST_10021_PI426210822 GTGGGAAAATAAGCAGGGTTTGTGGAGAGCAGCTTGTAAAGTTTGTCA

A_16_P18555883

AACCCGCTCAACTTTAGTAATTCCATCTACCTTTGAGTAAACTTTATCATGCATTGCCTA

A_14_P129511 TTTAATTGTTATAACCGAATCAATTATTGGAAAGTGAAAAACACCTCCCGGTCACACAAC

CUST_9502_PI426210822 AAAAATTAACACACATGGGAATTCCACAATGCCCCTTCTCCCTCCT
A_16_P02372495
AGCAGAGGTCCTTTTCAGTTTGCAAACAGTAGTATACAAACTTGGTCCATATCAAATACT
A_16_P36146612
TTGGCTTTTGTATTCTAACCTTATTGTCTCATCTCCCTCTAACACATGATACACTGCAAG
A_14_P109317 TTGCTGACCTCTTTAACCTCACTTTGTTGGAGAAGGCAGTGATCGATTTCTTA
A_14_P135051 AAGGGAAATCCGATATGATTGATACTTCTTAGGGTCATCTGCATATAATTCCTCAAAATA
CUST_4347_PI426210822 TATCCTGAGTCATATTTGGGAAGCACTGGGCTCTGCTTATGATTCTCAC
A_18_P22841254
CATTAACTGGTTATCCGATTAAAACTGGCCACTCACATGGATTTATTTGTTCAAAGGTC
A_14_P138806 CCAAGATATTTCATTAACTAAAGCCTTCCTTAATTATCCCTACCCTCTAACCCTCGAAAG
A_14_P124906 GTATAATTACTCCTTTTTATTGCAGTCTTCAGTTCACGATCTTCTAGTTGCAGCGATGAG
A_14_P124311 TTCTTGTATCCTTCTTTCTTCTTTTCATAACTTTGGGTTATGTTTCTGCTACCAGCAGAG
A_18_P22842285 CTGCCGGGCAAGGAACCAGCAAAGCCTCACTACAGAGTGACAATT
A_16_P02021722
GTGAACTCACGAGAAATATGCTTCAGTAATCTTCTGTCTTAACAAAAAGGCTTTCTCCAA
A_14_P122717 TCTGTCTATCTCATGAACTCTCCATAAATCCAAGAACTGCCTGTAGTTTTCAAAATGACA
A_14_P120537 ACAAAATGACGATGCACTTTACTAAAGAATTCATCATGGGATTCCTATAAGTGGTGATTT
A_14_P110702 TCAATGTGCTGTCCTAAATATGGTTTGTTGCATTACTCTCAAAAGGTTAAGAAGACACTG
CUST_8790_PI426210822
GACCCTTTCTTAGCTAAAATGAAAAATAATTGCCTAGTTCCCTCATGTAGAGAAGTTGAG
CUST_5078_PI426210822
TATTTACCCCAGAGGAAGATTATTTTTTTCATTCTCTAAATTGTTTAATTAAAACTCATA
A_14_P115111 AGTATGCGTTCTTCAGCATCATCAATGTGCCTTCAAGGACAATCTGTGGT
A_14_P118072 ACAAAACTAGTTAACATATTGTCAAACCATCTTTCTCTGAAGCCTACAGAGATTTGGCTC
A_14_P109684 CTACTCGGTTATAGCACACATGCTGACTTCGTCCTTGAAATGAACACTGCAAA
A_14_P119753 GGCAGTTCTCCTGACACACCAAAGAAATATTTAATAAAGGACATAGAATAAAGCCAGTGA
CUST_10059_PI426210822 GCTAGTGGGGGAGAAAGATGCAGGATGGTGGTTTAGGGGATACTG
A_18_P22841559 AAATTAACCATAACACGTATTGTTTAAGGCATTGCCCTCTTGCCTGCAGGCT
A_16_P21561406
AATTGAGCAGAAACAGTCTATTACACTTTGAGGCAAGAATCAAGTTACAAGGAAGTAGGT
A_14_P124522 GAAAAATGTGTTCTCTAAATATTATTTTTACCTCCTAAAGGAGGAAGAGAAAGGATCTTG
A_16_P20321593
AACATATTCAGTTATTTCCCTTCTATGCCTACATATGCTATAAAAAACTTCACACACGCA
A_16_P39055416
TTGAAAGCCTTGAATGGATATAAGAAACGAAAGTAGGAAGATTTACCTTCTCATTCACGC
A_14_P118681 CTCAGGACAAATAGAGAAGATTGTTTTGAATCTGAGTCTGAAGATGAGTTCTATAAGCAG
A_16_P58424164

GTTTGTTCATCCATCCTCTTGTTGACAGAACACATGACAACTCTTAGTCTTCTTAACAAT
A_14_P123016 GAAAAATGGCACCTCCAGTTAGATTGAATTTGGTTTACACTTTTAAGAGTGAGCAGTATG
A_14_P130255 AATTAAGTAACATGACAAGGCGACCTCACATGAAAGATCAACATACCCACTTTATTACTG
A_16_P19307609
TCCAATGCCTTTAACCACTAATTTGAACCTAAACTGATACTGAAAGAAAACTACCAGGAC
CUST_4116_PI426210822
TGTGTTTCTGTTGGAAAGAAAGGTTTGATTTTGGAAGTTTTAAATGTACTGCATGATGTT
A_14_P112156 ACGTGGTATAATCTGTAACTTTACCCATATTCTAATGCTGCACCTTCTAGCAGAAGATTC
CUST_2547_PI426210822
GTGACAATTCCATGTTCTTCAGAACCTAGAGCATTTTCCAAGAGTCACTGAATTC
A_14_P117034 TTTGTTTAAACCTACTTACCTTTTCCCAACAAATACATGCAGTTTAAATGGGAACCTGTC
A_14_P116474 TATTTTTTATGTCCCAGTCCTGAGCAGAACCGGCTTTCAGAATGTGAAGAAC
A_14_P201609 CTGCAAATTATTGGGGAAAAAATAGGTATGCAGAATGAAGAGGGTATATATGTTAATCCA
A_16_P18680543
ACCCAAAAGAATCCCTTTATTATGATCTTGTCTTCTTCTACCACACAATAGTCAGTCTGA
A_14_P139270 TTCATGATTATACTTACCATAACAGTCTAGCATCAGCAGTAAGCCCCGCAATTGAGATAC
CUST_3248_PI426210822 CAATACCTTGAGAGCACAGGCCTTTTGTGAGATCAAATACCTATGCATGACA
A_16_P17475322
TTGCCTATGTTTTATAGCACCTAATAAATTGTCCAATCCCTTAAAAACACTCCATCTGCC
A_14_P103667 ATGGGTATGTGTGGTGCATTTTAAAGAGATTCTGGTGAACTAACAAGGTGTAATAATTGT
A_14_P117017 TTGCTGAACATGGATATCTACATTATACCGCAGAGTATTTGAAGTCACACTGTAACCTCA
A_14_P111003 ACTTTGAGTTCTAAGGTTATAAGAGGCAATATCACCAATTGTTGGTCAAGGTGATCTAGA
A_18_P14790801
AAAAACAGGGATCATTTTTTTACTTCAACCTGCATGAAGAATTAGTTTTACCGAGTGGG
A_14_P106342 GAGATTATTTCTTTCAGGAAAAATTATGTGTGTAGTCTGTGTCTCCAACCCAGGTAATGA
A_16_P18798654
AGTGGGAACTAACAGAATTCTTTAAAATTGGGACCAACATTAACTTAGTGCATTCCAGAA
A_14_P101663 CTAGTTCCCACTAATTTCTAGGAGTAACTTCATTCTGTTTGTTGTCTCTCTTTGGTTGCA
A_16_P18146385
ACTGATTATGACTAATTATGGCAGATCATGACCATTATCCAAACAAAGATAACCCAGGGT
A_14_P127397 ACATACTGAAATAACTTCAGGGGAGAATACTTTACAGGTAAAAAGATGGACGAGTTCTTG
A_16_P21407886
TGTATACATCGACTCACAGAAGTATTTGAGTAAGCCTAATCATTGTTGAACTGTGATGGG
A_14_P122265 CTTTAGGGAACTATTTCTATAGGACGAGAAACTCACAAGTTCTGAATCTCTGCATAAAGC
A_16_P17056312
TGCTACCACAATTCTTCTAACGTTGACTAACATTTACCACACTTTCCATAATGACAACTT
A_14_P119120 ATATCTCACATTTGCATTATCTGCATGAGAGAAAAAGGGATGGAGATGCACCTAACTATG
A_14_P124170 TGAGTATTAGGTGGGTTTGAACGTGTTGGTGTTAGTCCATAAGTGAAAATATATTCCTCT

CUST_2121_PI426210822 AGACACTGAATGCTTTGTTGCAACTCAACTTGGTTGCAGATGGGC

A_14_P133507 CACTTTGTCAGAAGGGATTGAGTCTGCTAACTTAAACTTTCCTTGATTCAGGAATACAAA

A_14_P119605 CCAAACAACTTTGGAGTATATGCCTATAGAGTTCTTTCTACCGTAATCCTCTTGGTTATG

A_16_P03425864

AGATGTCTTCTGCTAGAGTTAAAAATTTCCACGGAATTATCTCCAATCTCCTCTCACAG

CUST_6636_PI426210822 AACATCTCTATCTCCAACCTCGGAGATGATGCCTATGATGCCAACG

A_16_P21659063

GCCATATCCCTAATTCCACCTAATTCCAACATTTTAAAATTGATTTTCTGAGGCCACTAC

A_14_P120845 GGGATTTTTTTATTCCATTAAATGTTCTTCCTTGCAGGTTTAAGACTTATCCTTGGGCAA

A_14_P127070 GCATTCATACCTTTTCTTTCTGCAGAAGTAGTTCACATATGCACCTCAATGTGATCAAAA

A_16_P00685279 AGGTTATAATAGGGAAGGGATGTGTGATCTTTCCTGAGAACTGAGGCATGCTATTAA

A_14_P201050 ATTTCCAGGTCTTGATAGAAATTTCTTTAAGTGTCTCTTCTCTCTCAGAAGTAAAGCCTC

A_14_P129299 CCCAAGGACGTGGTGTCCATTTTTTGAATTTTATCATCTTCACCCATTTCCATTTT

A_14_P201158 ACCATATGCTGTTCCTGTTATAATGATTCTTATAGTCAGAGATCTGTAGACTGAGTGAGG

A_14_P125255 AAATACTAAAAGCAGACAATATTTTGTCCTTTCTCCACTATTTAGACCACCAAGGCTTGG

A_16_P16474072

GCAGACTAATGGACAGAAATAACTTCATCCAAAAACAATCTAATAACCTCCCACTCATGA

A_14_P112089 CTTTAATACGATTGGAAACATTGAAGAGATTGCATATAATGCTGTTTCCTTTACCTGGGA

A_14_P121608 ACAATATTTTCCCCAATCTACCTACTTTGCTTCCTGAAAATGTTGCAACAATACACTTCA

A_16_P30952571 TTAGTCACCAAGTTCATGGAGCCCACTACTCTGTAAATTGTTGTGGATTTGTCTTT

CUST_8491_PI426210822 ATCGACTCTCATCTGGTGGTGCAGAGATGTTTCATGTCCAGGAAAT

A_14_P113857 ATCAGGTCTGTTCCTGTTACTGTTGGGTCTTGGCTGAAAAAAAAAAATGCTTTTAAAAAA

A_14_P202060 AACACTACTAGTTTCCTTTCCCCAAAAAACTTAAACTTTTATGGTTTAGTGGTTCAACAC

A_14_P113697 TTATAGGAAATCGAACATCTTCCTTGCAACATATTATTTCCATCCAGTTTTACCTGCTGG

A_16_P40074166

ACATTACCAACCCTCTTGTTTAAATGTAAAACCCATATTGGAGGTTTACACCAATGTCAT

A_16_P00685302

TCTCTTCCTCAGATGTCTAGTGTTTATTAGACCTCCATAGTATCTTCACCAACTGAGAAG

A_14_P119029 CACATACTGGCCTATAGTGCAAAAGAGAATATGTATATGTTTGTCAATGAGAATGGCTGG

A_16_P21485080

AAGAAAGAGACTCTGGAAACTCATTATCAGGTCTATCAACTCTTGTATTTGTTCTCCCAG

CUST_9921_PI426210822 GTAGTTCTCTCCAAATGTCAAGCCCAGGCTGGGCTCCATCATGCT

A_16_P16625131

AAACAACTTCAAATACTCAACTCCTAGCAAACATATGGATCACTCTCTTTTGGAAAGGAG

A_14_P117994 AGCATTTCTTACTTTCATTTGCTTATCTCCATTGGCGAAATTATTGGTAATTGCAAGCGA

A_14_P105212 ACACACACATAGTTTTCTTTTGTGTCATTCCATTGTGGAAAAGTTTTCAAGAGGATTGTT

A_14_P127495 AGCATGGGATATGCAACTTACATGGTGAGACCAGATTTAGGTAACTAAATATTCATGCAT

A_14_P133268 CACTTCAACGTTTGGATGTCTATGTGAACAACTTATTGGTCTGTCCAAAAACTACAATAT

A_16_P01642024
AAGCATCTGGCTGTTGAATAATGGAATGGCTTAACTTAGTTACACCCCAAATATCTGAAT
A_16_P20351038
TAAACAGTAACGCATCTTAAACCATCATCTCGATCCTCTAACTTGCTCTTTTTTTCTCCT
A_18_P13672322
CACTTGTCTATCCAATAAACCAATCACTCAATTATTTTACTCATAATCAAGTTTCCTGCC
CUST_2162_PI426210822
CAGTCCTCAGAAATATGCCAAATACATAGAAAATGTGAATAACAGCTCTCTGGATAGATG
A_14_P119801 ATGTGAAAGGAAAGGAAGATCATTTCATGCCTTGTTGATAAAGGTAAGTTCATAGTAATT
A_14_P138525 CTTTCTAACACAAGACAGCATTCCTTCACTCTACTATTTGTTTTTCCCAAACAGATAAGC
A_18_P22841022 TTTTGGAAATTGTTCTAGAAGACACCAGTGAGGCAGGGGGAAGTAAGAATG
A_16_P15318362
GACCCCAATCAGAATCTTATGACTTCAAAATCCTCACTCTCTCTATTTTATCACACTACC
A_14_P139162 TCACTATTATAGTTCAGCGAAAGAGCCTCCGGGATAATTTTTTCCTTTCAGTTCATAACA
A_16_P02874497
ATCCTTATGAACCAGTTTTTCACATATTTTCAAAGTCCTCAAATATATCATCCCCCCTCA
A_14_P103240 GGCAAAAAAAAAGAACAGATTTTAAATGGCATTGATTTCCTAGGGCAGCACAAACTGATT
A_18_P22842262 CTGAGCCTCACTGCTTGAGCCTTGGCTGGATTAGGGAAATGTCTC
CUST_5191_PI426210822 TATTCTTCCCTCATTGGATTGCCACCATTCTTGTAAGGGCACCTTC
A_14_P125132 GAGACATAAATGGTGTGTCTTTCCTTACCAATGATTCCTTTAGTGTTATTCACCCCAAAA
A_14_P118244 TTGTATGCATATGAAAAACCATGGTGGGATTAAAAACAAGTGCTTCCTTTCACCAAGATT
A_14_P133659 GTCACCATTTACATTGTTCTTGGATTTCAGGTTGCATAAAATACACGTCTTCTTTTTTGC
A_14_P139245 ATTCCATGACGCACTTTCTCGGTTCCTCAGAATCCTACCTACTTAGTAGC
A_18_P10111359
TGCATCTACATCAGAGACATCTAGATTAACACAGTATGTCTAACTCTCTCTTCATGACCC
CUST_7500_PI426210822
ACCCTTAGCTACATCAGGCAGTTTATGAATGGTGTTTTTTGTTACTGTCATTTGTAATAG
A_16_P55113301 AGAAATGGAGAGCGGGCACACTCACGCAAAAATAGGAAGCAGTGA
A_14_P111179 AACAGCGTCTTCCAAAAAATTTCAAAATCGGGCTTAATGCCAATTACACGATCATAATCC
A_14_P200394 ATTTTCCTATAGCCAAGGTTTAAGCAGCAAAAAATGAGACTGCACAAGACATGCTTTCTA
CUST_8836_PI426210822
CTTCTTTCTGCACATGTATGAATGCAAGCTGAATAGTCAAGTTCCTGAAATGGGT
A_14_P130657
CAAAACAAAAACAAACAAGAATTAATGATCATTTCACCATGGAGGGCAACAGAGTAAAGC
CUST_3881_PI426210822
GTCTCAGAAATTCCAAAAGACAGCTTATGACCCTGCATTTTTGGTTAAAGAAAGGA
A_14_P117773 ACTTCTCCATTATGTTTTTGCTGAGGCTAATGTTTAAATTGGCTGGCATGTCTTGAATAT
A_14_P124330 CTTCAACAAGATCACCTTTGTCTTCATAAGTTTAGCCAAGATCAGCATTCAGTTTTCCTG

A_14_P124165 CTGCCTTCATCCATCAAGAAGTCCTAGATTCTATTTGTCTGATTCTGGTTATCATTCAAT

CUST_3905_PI426210822 GTGATATTTATGTATATGCTTTTTAAGGACTACAATCCTTAGTTTCCTACCTACTCAAGG

A_14_P114472 ATGGAAAAACTAACAAGAATGGTGACATTAGGTTTTTCTGTCTTGAACATGCTGCTTACA

A_18_P22841935 ATTGTTTTGTTTCTGATGTTGCTGTCACTCAGCACCGTTGCCATTGCT

CUST_9000_PI426210822 CAAACATAGCTTTTAGATCATACCTTTTAGGTTTTTTTTTTTTTACCCAAAAACCACACT

A_14_P119763 TTTGTTATTCTTAGAGATGAACTCAGAAGCCCAAGTAGAATAGATGTGATACCCCTTGTC

A_18_P22840282 CCATCCTTTTTTCTCTTTCCCTTTTTCCCAGTATTCTTTGTCTTAGACCTTGAATGTATT

A_16_P16509520 AAGTTCATGAAAGCCAGTTATTTTCTCCGATTTCTTTACAGTCACAATGTTGAACAACCT

A_14_P101394 ACTACTACAAACAAAAGGATGATGATGATATTTGAATAAATGAATAAGGATTATACTCAA

A_14_P114439 ACAACTAAAACATAAAAGAAAATACTGTCCCACATCCACTGTTCACAACAAGGAAGAGCA

A_14_P125608 CTTTTAAGCATTTTTATTTTTGGAGTTCCACAGTTAGGCTGGTTGTTGTAGTTTGCAACT

A_14_P125274 AGGAAGACAGGTTTCTTTTCAGAGTTTCACCTTTAGCCAGAATTTTCTTATCAGTCATAT

CUST_9196_PI426210822 CATGGAGTATAAGGCTTATTTTACTCCTTAAGTCTTCCTATTTCCTAGTCACATCTCATT

A_14_P114384 GATTCATCATTTTTAGAAGCATCAGCAGTTGAATTATCTTGGTCCGATTGGTTCAAAGTT

A_18_P10006640 AAAAGCCACAAATTTAGATGGGGATACAGTAAACTTCATGAAGTAGTCTCGCGTCTTATC

A_14_P108466 GCACACACACAAAATCTTCACCAAGAGAACAGCAAACTAATAAGACATTACCTTTGGTTA

A_18_P22841381 ATTGCTTTTGCCATCTCAGCATTTCACCGCTGTCCTCGGTATGAGATTAT

A_14_P121883 CCAGGCTGAATGTAATTGTCCTCGATGAAGCCATCATCATCCTCATCTTC

A_14_P136078 AAAGGATATGCCTGTGTGAAGAAATATCACGATACCAAAGTCCAGATAAGACCAATTTAC

A_16_P03598414 AGCCCGATATAAAGTCAGAGTAAGGATTTTCATCAAAATATTAATTCTGCAGAATGGCCT

A_14_P133359 GGGGTGAAAATTAAAATTCAGAAGAATTAGGTAAGATACCATCACGAGTGCAAAGGTAGA

A_14_P105045 GTCTGGCCTCGCTGTGATCTGATTTGGGATTAAAGGTTTGGAAATTTAACAACTTT

A_16_P01068976 CCTAGAAATCAGTTGTATAGCCCAAAATGTTACCTAGACTACTAATGAACAGCAAGGTCT

A_14_P134425 TTTTTGAGTTTCATTACCTTTATCAAGAGATGCTCCAGCCATATGGTAAAAACTCAATGC

A_14_P132690 AACATCTCGGATCCTATTCAAATGTCAACACTAAGCAAGCGCAGGAATTTAGTTGAG

A_14_P135864 TTCTCTTAATGCATCCTCTTCATTTAGTTTGGGTCTGCATTCTTAACTTTAACCAGTCAC

A_16_P36231055 ATTAGGCAAAGTTTCAAAGATTTTAGATTAGGCACCAAATTGTTATGGCCTTGGAAGGAA

A_14_P133653 AACACTTCTTTTCTTTAACCTCTTTCAGATTGAAGACATCATTACAAAGATGCAAGATGA

A_14_P118468 CATATAAGAAGGGTCAAAAGCCATCATGCTACATTAGGCCCTTTGAAAAAAGTATCAGAA

A_14_P107603 AACTGATTCGGCTACGGTAAAGTAGTAAACAGAATCTGCATGAATTTTTGGAGAAGTTAG
A_14_P134579 GAAGCGAGAAGTGTTCTAGTCTAAGCACTAATAAGGTGATTAAATCACAATGGACATGTG
A_18_P16437853
GCAAGCATTCATAGCATAGTATATCATGTAGCAGGGTTTAAAATAAGCATAGAAGAGGGT
A_14_P100072 GGAAACATTGCAGATCACCCAGTCTTAAGGTTTCAAAAACTCTTTGACATTAGATTTCAC
A_14_P130352 CGACAGCAGAATAAAGGCAATAAGATGATTCTCTTGCCCCTTCTTATTCCTCCT
A_14_P103677 CTGAAGGAAGAATTGAGGTTAGAAGAAGCAAAACTCGTGTTGTTGAAAAAGTTGCG
A_14_P124299 CTGTGCTCAAAGGTCACTGAGCGGCAGTGATTATGTAGCATTCTGCATATA
A_14_P123645 TTAAAATTTGCTTCGTCTTACAGACCATTGTGATGAATGACTGTATTATCCGAGGGGATC
A_16_P00192831
GAACGAAGTTTCCCAAATAAGACTGATTCATGAGTTCTCCTTCAATATCCCAAGATCTAA
A_18_P17118634
ATCCTTGCTCTTACCTGGTAACTCATTACTTGTCCTATACATTCCACTTGACTTGTTTCT
A_14_P112579 CCCATACTTTTACAGTTCTAATGCCACCTGTCGTCTTATCATCTGATTGCAGACAAA
A_16_P16191049 CATTTGAGTTACAGCAGACAAGGGCCAGCCAAACTGCTCTTGTGT
A_16_P00898228
AAGCACCATCCTAACATTTGGAGTTAACATACAGCACTTGTAAAAACCTGACTTAGAAGT
A_14_P127111 CTCTTCAACCTCTGCCACTGTGATAGGTTGTAGTATCAGAGGAGAAATAAAAATCATATG
A_16_P39923927
TGTCATATGCAATAGCAGAGTGTAAAATCTCTTGACTTAACTACATACCATAGAAGCCAC
A_16_P15143738
AGATTCAGCAACATTTCCTTTCCTTACAGCATTGGTTTTCAAGTTTCAAAGTAAGCACAT
A_14_P121779 CTGTTCTGAGTATGTTTAAACCCTGCTAGTATTTACTGAGTTTTTTCCTACATATGGGGC
A_14_P137518 AATTTCAGAGTAAGCAAATGGACAAGTCACAAATTTTAAAATCGCTCTCCTCTTTGCAGA
A_14_P132820 ACGATTCAGATTCGCACTGTCCTGTGTATTTTCATAGTGGTCTTAATCTACTCAGTAGC
A_14_P109182 CCGTCTGCCCAAGCAACACAGACCACAATTTCTACTTTTGATGATTCATGTATTATTT
A_16_P20316649
AGCAAAGCACAATTCACTAAAAAAACCAGTTTGATTACACAGACATTACCCATCTGAACA
CUST_6357_PI426210822
TAACCATATGGATCTGTTTTGCCTGATTAGAAGGGATTTCTAAAGCATGATGAGATGG
A_16_P00685626
TGATGTCTATGACTTTTTCTTAATTATCCAGAAAATTCTGGCCAAGCACCTTTTCTTGCA
A_14_P127124 TTAACACCTACTGGTATACACTTGTTTTTGAGAACTGTCCAAGATCATGTCCTTTCTACT
A_14_P104223 CAAGAAGTTGTGAGACAGTCACAAATGTTACTCATGCAAAAGAAGTCACAGATACTTTTC
A_14_P128987 CCAGAATTGACCATCGTTCCTCATCGTTACCTAGAGTATGAGTTTTTCAGGTTT
A_14_P138677 CATTATTTGTTTTTCTCCTGAAATCAATATTCCCTCAGTAACAAATTCAGCAGGGATGCC
A_14_P133814 CTATATTAGGCAGGAATTTACTAGCAGGGAGTATTCAGAATGCCATTAACATCAGTCTGT
A_16_P01189000

AAGCAACTTACCACAACTAACTTCCAATTTATTGTCTCAAGTTTGAATTACTGGGCAAGG
CUST_2771_PI426210822 GCTCCATCAACATCACAGCGCCTCAGTGTCACGACGGACAGCAGC
A_14_P119278 TTACTTTTTTTCCATCTGTTCCCTATATTACAGTATACCTGCTTCTACGGAATTCCCGTT
A_14_P106954 CCAAACAGGGATATGAAAATAAAATATATAGGAAAAACCTGATTTGATTAGGCTTGTGCT
A_18_P13704664
AAAATCTCCTCTGTCTCAATTAGTGGAATCCAGTTAGAGGTTTGAACTATGATTCTACCA
A_16_P01727740
AGACACGCACAAAATCATTCATTTTTATGCCTGATAAATCTCTATTTTCCCTGAGGACAC
CUST_3615_PI426210822
TTGAACTTGAAAGGAGAGATTTTTTTCCCTCTTGTAATAAATCAAACTTTTCTGAATCTG
A_16_P02276566
ACCCCTATCTAAGTCAAGTATCAAAGATTTTTCTTCATGCCATTACTACAGAAACAGGGT
A_18_P15306673
AGGATCTTCATGCATGCAAATTAATTTAGTGTTTTCCACTTTGACAATGTGACAGTAGGC
A_14_P105409 ACAAATGTCAAATTTTGGAAGTATATTCAATACCAATGCTGTATGAGTGGGCTGAATCCA
A_14_P105696 AAAGAGTTACACTCACTTTATTAATCCAGATGAGCCCCGGGATTTGGTTGGAATTAAT
A_16_P15837312
ATTTCTTTCTAAGGTAATATATGTGAGAGTTGTAGAATGGTAGCATTCAAAGTAAGTGGT
A_14_P119791 TTTATCCAGATTAATATAAGTGAAAGCCTTTAAATGCAGGGACGAAAGGTATCCCTAGAA
CUST_7883_PI426210822
CAGCAGCTACCAAACATCATTATCCGGCAGAATCACTAATTTTATCACTCAATAATTTTA
A_14_P137085 CCTAGAAAATTGTGGTGCAACACCCATGTCATTATCGTCCTTATAATTTTTCATAGGCTT
A_14_P101510 CAGGTTATCGTGTGAAAATTGAATTTATTTAAATTCGAGGACCAGCAGAGAGGTCTTCTA
A_14_P110836 CCTTGCTGGCTTATGCAACAGAATAAAACCTAATATCCTAGGATCAATGAAAAAACGACA
A_14_P118452 AGAGAAGTAAAGTGACCTGCTTAGTGCTTAAACATCAAATAACTTTGCTAATCTGCCTCT
CUST_3696_PI426210822 CAGATTCCAGCCGTGGCTTTGCTGGGTCAATGTTACAGCCATTTA
A_14_P104913 TAGTTTTTACTTCTTGCAACCATGAAGCCATACATTGGGAAGTATTTTTGTTTGTTTTGT
A_14_P104582 CGCGTAAGATAGCACTGTGTTTTAAACTGTTGCATTACACTGTCTTTGCAATGATGTAAA
A_14_P137985 TATTCATGGAAAGGACAACAAGGAGTTCAAATATTAGAAGTGGTGTCTAATGTCCACTTC
A_14_P111592 ATTTCTTCATGTTCATGGGCAAACTTCTCTTTCTCTTGTGTTAAGGAAGCACGTC
A_14_P128807 TAGCATGGATACTTTTGGAGTAGTCATTTTGCACAAAATATTCTTTTCTACCAGGCCAGA
A_14_P129929 GAACTTTGCTATTTCTGTGCTGAAAGTGTGGTGAAATTTGTAATCTTCTCTCAGGTTGTT
A_16_P20509391
ACTATGGTCATATCTTGAAGTGACTCATAAAATTAAACTTTCCAAAGACTCAAAAGGAAA
A_14_P125886 CTCTCTAAGGGATACGAAATCTGATTTTATCCAAAGAAATCACAGCAGCCTTTGAAAGAC
A_16_P02561846
GTCCATGTCATATACCAATAAAAATGGTAACGCTCCTTAGATTCATCTCTTCCTGGATTC
A_16_P00403691

AAAAACTGGTAATCCCATGAGATTTCACCAATGGCATAAAGTTGTAAAATGAATTGGGAG
A_14_P131435 CACCTCTTTTCCAGGCTGAGAAGTCAGAATAGTAATCTGAAAATAAATCCAAGAATCAAT
A_14_P111585 TTCATAAATGGTATTGCAGGATAACCCAAAGCAAGAAAAAGAATTGCTGATGGTTTGTGA
A_18_P22841096
ATGACTGCCTTAAGGGATGGGGAAAGGGTTAGAGAAAGCAAGTATTTTATTTAGAAGAC
A_14_P123667 TGGTTTTTCATTGTTTTTAGTGGTGCAAAAAGCAAACCATATCTTACCGTTGATCAGATG
A_16_P41864696
TATTTAGCAAGTTCTGAGAACAACCTATAGGCTACATTCCTCACATTTGCAAGATTTCCC
A_16_P16805560
AAGCCCTTTAATTCTGCTACTTCTTACCCTTGAGTTATAAAACAGGCTAGGATTATGGAG
A_14_P128392 CCATGTGCTTTAACGTTACGGTAATACTTTACTTTAGGCATCCCTCCTGTTGC
A_16_P15533678
GACAGGCCTTAAAAAAAGTTGTAGTATACCCAAACGCAAATTTACACCTTGATTTACACT
A_14_P135715 AAAGCCAAGTATGGTTGGAAAGGAGAAGGACTGAGAAAAGACGATGGC
A_16_P20710061
ACTCCCACTCACACAATAAAAAGACCTGGGTAACATATGTGTTCTCATTAGCCACATAT
A_18_P18695476
GATGCCTACTTTAGACTATCACAGAATGACAAACCTCTATTGCATCAAACCACTGAAATT
A_14_P110970 AAGTCAAAATCTAAATGTTGATTGGGCCTATTAGTGTTTGCACATGAACACATGGCTATT
A_14_P117715 ATGTTACTCAGAAGATTTACCATCAAAGTGGCATTGTTATGAGCGATTACCTTGAAAACG
A_14_P131691 TGGTGAATCATAGGTCTCTTGCCCATTCACAATAATTGGTTCTTTATCCAAAATGGCTA
A_14_P110508 TACCCTAAAATATGCATTCCTTTTGCCAAAAAGATTAAAGTTCCAGGTTCTTTTGTGTCC
A_14_P201232 ACTTGGTTTCATTTGCACAGGCTTTGCCAAACTTTAATTGAATTCAGAGAGTTCTACAAA
CUST_828_PI426210822 CAAGAGGGGAAAGTAATACTAGCTGCCGCTTATGGAGGGCTTCATT
A_16_P16191991 AGGTGGTCTCATTTTCTTTCCTTATCACCAGGGAAGAGCCAGTAAGTAATTCATT
A_14_P112645 CAGCACGAGATACTTTCTGTGTCTCTTTTTTCTTTATAAGAAAACCTGGAGGTGGA
A_14_P115379 AGTTTATACAGAGAAGCAAAGCCTGAATCCAAAAGAATGCTTCTACTGTGTGAATTTTCC
A_18_P16452201
GAAATCGGATATAAATCACCAACCTTTTATCTCACCTGCAGATTCATATACCATAGGACG
A_18_P17146528
ACCAGCATAATCACACCATTAGTCCAGATTGTTTCACATATTTAAAAAATTCTTAGCGCA
A_18_P16005214 CCTTCATACTCTTTCCATCCTATCCATTCATCATTAGTTCATCTACCTTATTCATCTCCC
A_14_P104841 CTCCTACTTTATAAGTAGTTCCATCATGACCTCTTCCAGGATACACTAATCTAACAAGGT
A_16_P15723094
AAAATTCAAGTCCTAGAGAATTTGGAGCAGAGTAGTATGAAAACCAAGACAGAATCATGG
A_16_P38257306
ACGTCTCCAACTATACCAAAGTTGAAAATAGTTCTTCGAAGTCGAAATTATGAGACTCAA
A_14_P115644 CTGTGTAGGTATTTTTTCTTGTGTGTCCATTATAATTAATTAAATTGATTAGGATCATTT

A_16_P16681879
TGCTGATAGAGAGGAAATTACTCATGGAAGAAATATCTCTGATGCATAACACACAGTCTG
A_16_P00685273 CTTCATGACTCACTGTGATCTACAACTCCTCAGAGCCAGCCTTAAAGTG
CUST_219_PI426210822 GGAGGCTCCGCGCGCTGCTGCTGGCGCTGGTGGTCGCGGGGATCC
A_16_P02672899
AACAACTTACTAATCTAACCAATTTCACTTTTGCTGATTCCATTCATCTCTACCTTCAGA
A_14_P124976 AAGATACATTAAAACCATCAGTGTGTTACACTTGTTCAAAACAGAACTCATAAGGCAGAC
A_14_P127931 GTTCTTGTCATTCTTATCATTCCTTGCTGTTTTCCTTATGACAAAGACACACTCCAAATG
A_14_P101604 GGCATTCTCTGGGTGTTATATTAGTCTTGACAGTAGACGTTTTCTACCCTTTGATAACTA
A_16_P18881240
AATACTCATCAGAAGTCGAAACTAGAATGAAACAGGCTTTTTACCCTAAAGGTTTCTGC
A_14_P115926 ATGACATTTGTGTAATTTCTTCTTTGGGCTACTCAATTTGCTTCTTGTCAGTTAGGATGT
A_14_P116462 CGGTGTATTTCTCTACTTACCTGTAATAATGCTTTTGTCTTAATAGGGTGGTTCTCTTCC
A_16_P15926984
AAGTTAATCTTCATGTAGTTGGCCTCATTTTTGTCATCCTAGAATACATTCTCAAACAGA
CUST_7133_PI426210822
CTCTTAGAAGTACCAAACTTACAAATGGGAAAGTGGATGCTTAGAATGGCATGAATGTT
A_14_P138627 GTTTCAGGATTGTAGGGAGCAGATTCCTACTGTTCTTAAAGGACAGTAATGCCTTTT
A_14_P107850 CAATAATTTGAAGAAGAATCACATGTGTGTTGTTAAGTGAAGCAACCAAGGTGCAAGATT
A_14_P103312 CTTTATGCCATCATTAGTGGTTAAGAAGCAACGAGCAAAAAACCTTTCTGTAGGCTGA
A_14_P105030 CTTTGGAAATCCAAATTCACTGTTAAAGGTTTTAGTTGCTCAGTTATGACCCAGGTATCA
A_16_P37295657
ACCCTTCCTTCACTTTATCACTTCTGCAATTACAATTTCCAAGAACTCTACTTATTCTAC
A_14_P131096
CTTTTGGAGAAAGTCAAAGTGAGGATGAACAATTTGAAAATGACTTAGAGACAGATCCAC
A_14_P123050 ATCTTGCCTAATGGACTAGATTGGACTGTATCAACATTGATTTACTCCACTTTTTATGCC
A_14_P131473 CTCACCTCATTCTAGGATGAGAAAACAGGGTTAAATGTTGAGTCTTACAGGGTTAAATAT
CUST_3482_PI426210822 CTTTATAATCTGGTCTGAGGCCCCTGTAAAGAGCCTCCAGTGACT
A_14_P111481 GTCAGAATAACAGCCAAATCCTCACCTATTTTCCGAAACATTCTTCTGATTATAGCGTCT
CUST_10377_PI426210822
ACAAATTCCATGGGTTTGAGAGAAAGATGTAAATGACAGCAAGGTAGAATGCAGA
A_14_P101331 ACAAACTTTGATGGAGAATTTCACACCACGCTGGAAAAATGCCGGTTATGAA
A_16_P58424157 CACTGCATACGCTGTGTGACTACCACCCCACCACAATAAAGAATACTT
A_14_P129764 TGTTTTCCTTACACTTTGACCTCTCTAGGATAAAATGTCTGTAACAGATGAAAGGCTCAA
A_14_P106889 TCTGACTTTAACACCCTAGAAACCCCCTCACAGTTTAGAGTAAGTATCTCAGATTTAG
A_14_P132655 GGACAACAGGCAACATATATGAGAATGGTTTTTGGGTCAGTGTTGTTTTTAAAGTCATTT
CUST_5731_PI426210822 CATGTCTGGTTTAGCAGCTTTTAGAAACGTGGCTGGCATCCTTTTTTCTTTT
CUST_614_PI426210822 ACACACAGTGAAGAATTTGACTCATGTAACCAAAACGTGGGTCCCTG

A_14_P110274 TAGAGCCATTTCAATATGTTAGTAAAGCCGACTAAACAACGACACCTGCACGAAAAAG

CUST_10338_PI426210822 AGCTTCTGCTCTTTCCCAGTCATTCAAACTGCACCACCTTATCGA

A_14_P111163 CTTGCCCTTGTAAATTTCCAAGTAGTGAAGGAAATTTCCTGGACCATTAATCTATTTTTG

A_14_P104804 CCTAACCAAACTTCCTGGCTTTCGGTATAAACAAATCCTTTCTAGAGAAATAAAGCATTC

CUST_7613_PI426210822

ATTTGTTTATTTAGCTTCTTAATTTCATATCCTGTGGTCCTGAAGAGGACAATCACCAGG

CUST_8540_PI426210822

GCCTTTTTTTTTCCTATGGGCAAAATCATCTATTGACCAAAACAAGACATTTAAGTCACT

A_14_P107410 AAAAATCCTCTCTCCTTTTTCAAAATATTTGTGGGCATTTACTCTTCAGAGAAACTTGAG

A_14_P116178 GAAGAGCGAATATCCTGACTTATTCGAGTGGTTTTGTGTGAAGACACTGAAAGTGT

A_14_P101308 CTTCTCCCCTGTTACCCATAATTCTTGCCTCTTTCCATAATCCGTGGTTT

A_14_P117697 CACCAATTTTCATTTGCACTTGAATGTCTACCCCGTTTTACTCTATTGTATCTCCTTAGA

A_14_P101571 GTTCTTAGGGAAAGGGGCAAGAGAAATAAATTCTCCCTCATACTTACAACATAACAATGA

A_14_P119732 GAGTTCAATTATGCTCATTAATTCAATTTGAGGCAGGAATGTGTGTAAATAAACGCTGGC

A_14_P133448 TTCTGGGCTGTCTATGACTTACGATATCACCTCTTCTTTTTAAATGTCCAAGTGGAAATA

A_18_P22841278

CCCAAAAGCATTTAAAATGTGTCTTATGGTTGTTTTAAGAAAAGAATGATAATTGGTGCA

A_14_P101052 ATTTTCTGTCAGGACTTTTACACACACTATAACCTAAGCATTGCCCAATGACATCTCATA

A_16_P17949732

AAAGTTTTCCCAGTATATCATGAGTCTCCATTGATTCAAACTGGTCTATCTGTTTTCTGG

A_14_P119096 AACGGTGGTCTTTAGATATTTTGTCACAACCTAAAAACAAGAAAGACAGTTCATTGATTA

CUST_4681_PI426210822 AGTGTCCCAATGCTCAGGAAGCTCCAGGGAACCTTCAGTGTGCCT

A_16_P17299126

CATTGAAGCAGCGTTCATAAATGATCTACAATTTGAGATAGACTAATTCAGACTGCTCCC

A_14_P105622 CTAGGAGAATTTTTTTTGTCGATGTGTTAACCATTCCTTTTTCTCCAACTGCATTCTACT

A_14_P125065 ACTGTATTGATTATTTCTGGCAACATCATTGTGATTTTTGTATTTCACTGTGCACCTTTG

A_14_P117897 GTAATTAGACTATAATGGTGACTTTTAAGATTCCCCCTTCATTTCAGCCGTTTTCTGTGA

A_18_P14041145

ATTATGTTGAGGGACTCCACTATAAATTTGAAGACACCCATAATCCATGTATCAGGCAGA

CUST_7201_PI426210822 TACAAAGCTGAAATCATCAGAGTTGGGCCAGAAAGAAGTGACACAGAACAT

A_14_P124465 ACTTTTCTCATGGGCTAGTGGATATTAATATTCCACTGACATGCTCATATTTGCCAATCA

A_14_P125921 GCTGTATATCAAAAGGCAAGTGGCCATTCATCAATAAAAGGCATTAGTTACTTAGCCTTT

CUST_3372_PI426210822

TAATAATACAGGAATTAGTATTAAAGAATGTAGTAATTAAACTCCTTTTGAAAATGATTT

A_16_P18489811

AAGTTCTTCTGACCATATATTTGCAAATATTGCACCACATATCTCTCTACCATCCCACTC

A_14_P108443 TCAGGCTTTGGCTCCAAATGTTTGGCTTCAAATATGAAAAAGTTACATGAAAGATGAAAC

CUST_6248_PI426210822 CAGTAGTCACTGGCCACGGTGAGACCTGGGTTTAACATGACCTGC

CUST_2654_PI426210822 GGAGGGCTAGGAGACCACGCATGTGCAAGCACACATAGATATATATTG

A_16_P18535025

AAAAATGTAGCCAAACATATCTCCACCTCCTAATAACTATGTGGATCAGAGGAACTGAAA

CUST_5413_PI426210822 GGTGCAGGACGTCATCAGCCCGATCGTGTTTGAAGCAGCCTACAG

A_14_P117063 ACATAGTCAGTTAATAACATCCCATCTGGAGTCAAAACCAATAAGTGTGTGTCATGCTTA

A_16_P02776336

AAACATACACCCTAATGCATGTGTATATGGGGATGAAACTCTGATAGCAAAAAGACTTGT

A_14_P111759 GGATACTTAAAGACATGTGGAATAACTATGAGAATTCCTGTTCCTGGAAAGTTTGTCCTC

A_16_P20009783

AGGAGAGTGACATTTTATCCAACCTATTGTAGTCACATTAGTTCATAGCAGATGAGATCA

CUST_4995_PI426210822 CCATTCTTCAAAGCTACTTTCATTCCTGTGAGGGATGGCCAGACA

A_16_P16191232 ATTACACTTTGGAGTGGGAGCCAATTCTTAGGTTTTGTTAATAACCTGATGGCAGAA

A_14_P109495 GGGGTGGTGGGTATTTTCATTCATAATTGTCAGATTACTTTCATAAACAATGGAAACAGT

A_16_P19551704

AATGCTTTTTACCTGATCTTTGGCTACATCACATACATGATCATCAAATCACCCTTGAAG

A_16_P02951117

ATGGGAAAAACAACTGTTTTACTGCTCTAGAATCTAACAGAAATCATAATGTCACCCTGG

A_16_P00685365 TGTAAGGGACAGTCACACTCTGGGAAAGACATCGAAGGGTGACAT

A_16_P21539731

AAGCCTTTTTAAAAGTCCAATGCCCAATATTCATCAAGACTTTGAAATCATACCGCTGAT

CUST_7864_PI426210822 TCAGGGCGCGTTCTGAATCCTGTGCCATTTGCATGTTACATACAT

CUST_7246_PI426210822 AATGAGGCGATGTCAGGATCACCTGTGCACATTCTGCTTCTCTCC

A_14_P116634 AACCGAAAACTAACGATACTGTTTCTATAGAGAAGATCCCACCACCTGACAAATATTACT

A_14_P129577 CTCTGTTGCCAAAATATACTAGAAGCACTGAGCCTCCTCATATCCAAACTAAATGTTAGA

A_14_P131579 ATGTCAGTTCCCTTTTTGTTAGCAGGCTTACACACATAAATTGCCTACTAATAAGCAAAC

A_14_P118258 GGCATGCTGCAGAATACAGTAGACATTTAATTGATATCTGCTGAAAGCATTATTTTCCCA

A_14_P117722 TAATGACATTTAAACCCAATTATTAACAGAAAAGGGATAAGAACTGTGGTTCCTTGGCCT

CUST_1427_PI426210822 GATGCAGGATTTGTTCATTGCTTTGCACTAGTCTCACTCGCAGCC

CUST_1048_PI426210822 TGATCCCTTGCTATGAAGGTGAGCATGGATTGATTTTTCCTCATCCC

A_16_P01667051

AAGATCTTGAGCCCTAGATGAAAACAAAATTTTCTATCTTGTAATTCACATGTCCTGCCT

A_14_P100523 GCCCAGTCCTAGAACAGACAATTCAATTCATAGCGTTACCATAAAATTACTGTCAAAAAA

A_16_P03743239

AAAAAAATCCTTCCAAAATGAGGATACTTTTCTTGCTATAAACCCCACCACATCATTACC

CUST_6327_PI426210822

GTCTTTAATCCAGTTGTTACAGTGAGTGAACCTGATTTGTTCCAGATGTCCTAAGATTT

A_14_P110837 CTTTTGGTTAAACTTAATGCATGGTTGGAGATGTTATGGCGACACTAAACAAAGTATTCC

A_14_P132677 CTTCCTAGTAGTATGTGCTTTCACCATCAGTTTTTAGGGGAAGTTTTCAATCATCAGTTT

A_18_P13614097
TCCAGTTAACTCACAAACATACTTCTAGAACCTAACTTGCTTTTTAATTAGAGACTGCCA
A_16_P15108396
GAATGAAGATTGAAGATTCATGGATTTGATCCTGAGATTTAGAAATGCTTTTCTGCTGGG
CUST_4813_PI426210822
CACGCCTCATCAACATGAGCAGTATTAGTCTTTATCTCTGACATCAATCAAACTTGAAC
A_16_P01184417
AAAAAACACTAGTGAGTTAACCAAGATCAGATTTATTCTCTTAAAACGTTCCCATACAGT
CUST_8238_PI426210822
AGAAAACAGAGTGCATACCTCTTTGATAGTGGAAAAAAGATTTACACAATTAGTTTAGAT
A_18_P22840301 ATCTCTAGTGGCATGCTCCTGGGTCTACTGATCCCCTTCGAAGGC
A_16_P36141825
TGCCACAAGTATCAAGTAAACTTCTTCGAGAGTATAATATAGATTGTAAAGCAGCACGTT
A_14_P136036 AAAGAGAATTCACCACAGTTCTTAGTAAGATCTCAAAGGACAGTAGGGAGAGTCTTTAAA
A_14_P136838 ATTCTCATTTGTTGTAGGAGTGAGGACCTAGAATTTTAATGTCTAATATGCGGTGTGAAT
A_16_P21347286
GCAAATTGGGCAAATTTCAATATCCCCTGTCTACAGTTTTTCAGAACCAAATCCTTAAAG
A_14_P137283 TGGCTCTGAAATATCTGACACAATGTAAACATTGCAGGCACCTGCATTTTATGTTTTTTT
A_16_P18176827
ACGAAACAAAATCACGTGTCTGTGTATAGTGTTCCATCTTCTTAAGAACAATGCTACTTT
A_14_P139841 AAGAAGCCTGTAAACTCTCTTACAAATACATTTGGTTATTCACCATGAGGTTAGCAAAGC
A_16_P41349476
CGTTTGCCAAAAAAAGAAAAGTTCCTTAAAGACGTTTAAAATGATTACATTATTGCGGCA
A_14_P119632 TGTCCTGCTTGGAGAGTGACTTGTAAGATGCTGAATTATTCATGATAATACAGTGAATGT
CUST_4988_PI426210822 CTAGACCTCAGAGTGACATCAGGGGGCGGGGACCTGATAGATGTG
A_16_P15120952
TGCAAACTTCAAATTCCTTAGTGATCCACTTGTAATGATTGCTTTCTGAAAAAGGCTACT
A_14_P138850 ACCTGTAAAGCACAGGGTGAGATATAAATACAGTGACGTGCTTTTTCAAACTATTTTCTG
A_14_P129882 AGAGCCAATGATTACTTAGGAACTGCGAAAGAAATAACGTATCTTTACGATGGAGAGATT
A_16_P16094476
AAAGTGATTCAAATAACAACGAAAACCTTCTTGATCTCAAATTCCCTTTGTCCGAATGAC
A_14_P126654 ACTAACTTGTGAAAGTTTTGTTTAAGTTCTAGTAAGAGAAACAAGTGAGTGTAGTGGGTT
A_16_P19899355
AAGCACATCATGATTTAGAGCAGAAATACTAGAACAGAGACAGAACTTATCCACCAAGTT
A_14_P102812 GTATTCTACCCACCAAACAATAGAATATAGATTCTGCCCCAACATAGAGCAGTCACAATA
A_14_P136467 TTTGAAGAAGTGTAATAACTTTAAAATCACTTCAGAAGCATCTCCTCATGTGCATGACGG
A_14_P100590 CCATCTGTGGAAAATCAATCATGGTGATATGGTCACTTTCATCCAAAATGAGATTAAATT
A_14_P132327 CTCCCAGAAGGAAGTTTAACATGTTTTCTGGAACTTGTTTCTTCAGACTTCAATAAAAAT

A_14_P107648 TCTTCAAAATTTTGGTATTTACCTTTCTAAGCCAGAATTTAAGAAGATCACAGAACTGAC
CUST_215_PI426210822 ATTCCGCCCGTGTCCAGGCGCAGAGCTCCCGCCCCGGGGAGCTTC
A_14_P200931 CTGGTCTCAGGGATTTTAGGTCTGTAGAAGTGTTATTATCCACAGTCCCAGTTTT
A_16_P17898633
ATTCCCTTCACTCCATAGTTATTTATTGCCCAGAACTAATTTCCTAGCCTTTATGAAGCA
CUST_1618_PI426210822 AGCTTAACAAAGCCTTCGGAGCTATGGATACCACCTGAATTTTAGCCTG
A_14_P104139 CTCGTTTTCTATTTTTCTTCAGATTTTCAAAAGAAACAGCCAGACGATGATTCCGCTC
A_16_P00685226 TTACCTTTTCCTAAACTTCCTGCATTGTCTCCTTTTAGCCCCACAGTTGATTTCTTATT
A_14_P130447 GAAAGGTGGGTATAGAGAGGTGGAAACTTTCCTTTATTATGACTCTACCGTTATCTTATG
A_18_P13060381
CTAGGAATGAAATGATTGCGATGAGAGACAAAAACAGGGAACTTGGTTAGATTTGTGG
A_16_P20137489
TGAGGTCATGTAATACACAATCATTCCTCTACCTGACAATAGAATACAAGTGCTTTTCGA
A_14_P136178 TGTTCATTATAAATCTCTTTCTAATCCTTTAAGGTTCTGAAATTTCCTCAACAGACCCTC
A_14_P100805 TGAGGAACTTTTAGATGGAGCAGGTGTTATTATGGACTTTCAAACATCTGAAGATGACAA
A_14_P123513 ATCCGTGATATTATGTCGATTTTTAACTGAGGGGAAATTAACTAGTCCAGCCTAAAATGC
CUST_8074_PI426210822 CAGATCCAGCATGCCTTGAAGCAGCATCTAGGTAGCACTTGGCTG
A_16_P35747584
TGACAAGCATAGATCTCTACTGTAGCACGTTTGTATTTGACTTCATTCGCTCTTTTATGG
A_14_P117471 CTCTTCATAACATGGGCTGCATGAAATCAAAGCAAACTTTCCCATTTCCTACCATATATG
CUST_10246_PI426210822
ACTGTTGGGAATAATATTGTGCCTCTAGGGCATTTAGAAGATAACATGCTTTTTTCTGG
A_14_P103705 GCAAAGAGAGATAGACTAACTCGGTCTTTACCAAACTTCAAGGATTTTCTCCTTTGCAA
A_18_P14137119
TTCCCAAAGCAGACAAAGAGAAAACACATGGGAATGGTAATTAAATGAGTCCATGTTTAA
A_18_P12362357
CATTCGATTCCAAAAATTAAGTCCTAATCACAACCTACCACTTACTAGAAAGTCACCCAA
CUST_274_PI426210822 CTGATGCTGGTGCTCTCAGCATGTAGTTATGCAGGAGAGTCACTG
A_14_P118426 CTTGTATTGTGATTTGATTTTAGTGGAAGATGTGGTTGGAAGTCACATAGAAGATGCAGT
CUST_5800_PI426210822 AAAGCTGCCCTGGCCTTGGTGACCATGGCACTGTAAGAGAAGCAC
A_18_P22841717 AAGAACATCTACTATGAAGCCGACCACATCCTACCCCATGGCTTCT
A_14_P114004 TTTTCTCCTTTACAGTTGTGTTCGATATATACAGAGACAGTAGGGTAAGTAAAAACTCTT
A_14_P106903 CACCGGGCAAGGTGTTACAATATTTTATGTTGATAGACTTGGCTACTATCCTTACATAGA
A_14_P133228 TGGGCTTAGGAACAAATCTCACAGCAATTCTTTCTATGTATTGGAGTGGGATTTTATTTT
A_14_P100214 TGTTTTGCAGACTTATTTACCAAGCATTGGAGGAATATCGTAGGTAAAAATGCCTATTGG
A_18_P13166390
AAAAATCTTATTAGCCCACATCTATTGTCCATCCCTAAATTAAATTTGAATGTAGCCCAA
A_16_P21495882

TCTTTTGAGGTGTGCACAATATGCTTCTTATTTTAGTCCTTGGTCTTTGCTAGATCTCAT

A_16_P15808943

AGGCTTCACTGGAATAAAATATTCGAGCATTAATTGCCAAACTTTCACATGATGACTTGC

CUST_10281_PI426210822

TCTTCAGACTCTAAAAAAAGGAAGACATACATCAGTGGTAGCTCTGATCTCCCAT

CUST_7858_PI426210822 GGTCCCCACCTCTTCTGAACTCTGAGCTCTACGAGGACAGCGATT

A_14_P128255 ACAGAAGTGACATGTAAGATGATACCCCAGAATCTTAAGTAGTTCGAGTTGTGTTTGATC

A_16_P18968624

ACAATTCAAAGAGTTGCTACTAGGTGAACAACAGTGATATTTAGGATTTTTCAGGGCAAC

A_14_P108635 CTCCTGAACACAATTAGGCGTGACAGTCCACAAATAAGAAAGAACTGGAGAATTAATTAA

A_14_P201082 CTTATTTCCCAGCATTCTCTTACAAGTGTTTGAAGTGCCAGGCAGACAAAGTATTTG

A_16_P15262635

ATGGGTCCATTAAGACATGATTGAGACAAACATAAAAGATATGCAGAGAGTCAAACCTTC

A_16_P30952293 AGGCTGGGATAGCGGGCTTCTTCACCGAGGTGGGTGTCTGCTGTC

A_16_P30952477 CTGCCTTTTGTGCAGCCGGCCCATCTGTTGGTGCTTTTTCTTTCA

CUST_3966_PI426210822 TAGTTACCTTTTCCTAAACTTCCTGCATTGTCTCCTTTTAGCCCCACAGTT

A_14_P101601 CTCTGCTACCGTCAGGAAAACATTTTCAGAATTGTTCAAAGAATCCATATCACATTCTTT

A_14_P114232 ACTTGCTATGTGTAAGAAAATTGAGCATTTTCTCCTTCAATTCACATATGATTGTGGGCT

A_16_P00685010

TCTCTTGTTACTTGATCTGATTTATGAAACCAATGGTCTTTTTCATCCTGACTTGAGATG

A_16_P03800710

CCATTCCAGACAGATTTGTTCTAAAATCCTAAATAATAATAAACACAACACATCTGCCCA

CUST_5263_PI426210822 CTTCCTGTTTTCCTTTAATTTACCCCGGCAGCTGCTGGTTAGCTA

A_14_P118357 ACTTTTTTCCACACTACATCTAGGTATGGTTTCTAGTGGTAATAGACGTGTCATCCAAAA

A_14_P127431 CATGGTATACTTTTCCCCAGCCTATTGTCTTGAGATATCTTCTACAGCCTAAATTTGCTA

A_16_P03108116

GGTTTGCCAACTCTAAATTGCTATACAAATAAGTCCTGATTATGTGCACTGAACAAAAGG

A_18_P15509910 ATGACCATACCCAATCTAAACACTTAAACCCATCTTTATATGGAGAATCGGTGAGGC

CUST_5968_PI426210822

ATTCTGTGGTTTTAAAAAAATGTATGTTACAAAATAATTATAACAAAAGCTTTGTTGGGA

A_14_P129307 CACAGGTTTCTGTTCATGGTAAATAATTCCTGTCCTTTATGCTTTGTTGGAAGATTTTTC

CUST_2586_PI426210822 TTGCTGAGGCCCGTGCAGTCTGCATCTCTCCTGCAAAGGCCAGTG

A_14_P202016 CTTGTCCTGTGAGTTAGTGTTGGAACTAAGGTGAATTTCTTAATCCTTGATAGAATTCCA

A_16_P38970775

GAAACAACATAATTTAGACTCGAGACAAAATAAACTCTTCTCTACTTAGACTTGACCCCA

A_14_P138304 TTTCCACAACCCTATCTTAGGAAACGTTGGAAGGATTAACAAAATCCTATTTGCAAATCA

A_16_P17885091

ATCAGTGCAATTTCATGCCTACAGATTTTCAGTTTCTAACGTTCAATGACTAAATGGTGG

A_14_P132960 GTATATAGTCAGCCATACTAGAAATCTGCCAGTGTCGGTAATGAATGTTCAGTTGACAAA
A_14_P135935 AGTAACCCAAAGGAAGGAACTGAGATAACTACTAATGGATTACTTAGAGCGTAAGTCATT
A_14_P124152 CTTTAAATGAAACTTGCCTAAAATCTTAGGTCATACACAGAAGAGACTCCAATCGACAAG
A_16_P16191674
ACAAAAGTAGTTGAATAAAAAGTGTGCCACAGCCAAATAAGCATAGAATGAAGCAAAACA
A_14_P129578
CTCTAGCACAATGAAGAGTAGAAATTGGAAGGATATCAAAAAGGGGAGAAGCAAAACAAT
A_14_P115505 CTTCAGTTACCTTTGAAAATCGGGCAGATTTTCCTCCATATTGAAATGCATCCATTTC
A_18_P14138049
TTTGACACAGAGCATCTCACCTCTTTCATAGGAAGGAATAAATTCATCAGAACTCTCTAG
A_14_P120982 CTCATAGAATTGCCACACATGGCCAATAATCCCCATTTTACATGCAAAGTTATGATAGGA
A_14_P127920 TTGTATACGTATTCCAAAGCAAAGATTATTGCTGGAAGGCCTTAATGGTAATCAACTCCA
A_14_P126385 GGAATGCCTGTTTACTATAGTGTCATTCCACCTGGTCAACAAAACAATTTAAGGTGAGTA
A_14_P131288 CTTACAGTTATAGAGGACCTCAGGATTTCAATTCTTTTGTCCTCGAGCAGCATGAATATA
A_16_P19744618
GGTATTTCTCGTTATCAAGCATAGGTGGTATAGTAGCAGAAGTCAAAAATCATACTGTCC
A_14_P120978 AGGTTCTAAATTACCTCTTTCTGGAAAGATGTTGTTTTTGGTGAGTTTGACGCTTTTGG
A_14_P135615 GTTAGACTCTGATGATATTTACTATCTGATTTGGGGATAAACCAACCCACTAGGTCAGAC
A_14_P114536 CTAAGAAATGTATGAAAATGTATAATACTGGGGAAAACTGCCTAGCAGATAGCGGAAGCA
CUST_397_PI426210822 CTTCCTGCTCCCTCTCCTGGGACACATCAAGACGGCTGTGCTTAG
A_14_P119850 ATAGTGAGTTCTGTGTGTTTAGAAACACAAGAGGCTGTGTGTCTTAGTGTCTGTTTTGTA
A_16_P18290063
AAAAAGCCTAGTAATACGTGACCTTATCAGAGAGATTTATGTTCAGTTGAAAGCCAAGTT
CUST_3384_PI426210822
TTTTATGTTTGTACATTGGTTCAGAGGTACTCTGTATTTTGACTGGATTTATTGGATAGT
A_16_P00767240
GCATCTCCTGAGTATAAATTGTATCCTTGCCCATCTATGTCATGAACTTTCCAAAAGATG
A_16_P17980427
TTGTTCTCCTCTATGAAACCACTAATAGCCTAATAAAGAAATATGCCAATCTGCCAACCA
A_14_P124699 CTGATTTGGTTTGACTGGCTGACATAAGCAGATAATTGAGACTCTTACTGATTTGCTGAA
A_14_P104991 GGAAGTTAAAAATTTGGGGATTTTTTAGATGACAAGTCCAGACTTTCAATATGACACCCA
A_14_P111488 CTGTCACTTACTGACTGTCACTCATCAATCCCTATTACCACTCTAATAATATGGGTTGAA
A_16_P18548435
AAGTCCACTGTAGATGCTTAGTCAATTCTCCTGCTTAATTTGTGTGTATTCGTTTGTCAT
A_14_P100994 AGCAAACTACAGGCCTCCTCTTTTCATGTGTCCTGCGAACTAAAAATGCTTTTAAAAT
CUST_5718_PI426210822 ACCTTGTGCAAAAGTATGTGTGGGGTGAGAGGTGTTTAAACACACC
A_14_P201402 CAAATACAGAATATGCTTGTTGTTGTAATCGAGCCAAGAAATTTGCAAGGCATGTATCTG
A_14_P117860 CCAAACCAAACACCTACTCTATGGTATTTTCTATGTCAGCATAAGACAAGGTGTGAAAAT

A_16_P00721547
AAAACACACTTTCTCTCCACGTAAACAAATGAAAGAATTTGACTATTCAGCAAACTGCCT
A_14_P130485 AAGTCACCATACCTCCTACAACATGCCTACAATCCTGTGGACTGGT
A_14_P127982 GAAGACCAGTATATCTAGATGAGCAAACAAAACTTCAGTTGATTCTGTGAACACTGGAGA
A_18_P13079225
ACACCATTTATACAGACAAACTTTCAGTCAATTCATTACAAATAGAGCAGGCTCAATTCA
A_16_P01423350
CGCGTTCATACAGTTTTAGGCAAAAGAAAACTTGACATTTTAATACACCTGTGAGTCCTA
CUST_9950_PI426210822 ACTTGAGTAACTGAGATGGGCTACAGTCCAGAGTAGCTTCATCATAACTTCTC
A_14_P121563 AACTAATTTTCTCATGGTTACAGAGCAAACTTATGGTAGAAGTATATTGGAAAATTCAAG
A_14_P134413 AGATGTTTTAGACATGGATGATATTCAAATGACAAGTAGAGAAGGTAGCCTAAGGGTGAT
A_16_P18614278
AAAGTTGCCCACATTTCTAAAAATGCTGTAATTCTGTGTTCATCTTTCCTGTTCTTCCTA
A_16_P02570724
CCTTATGTGCTTGGTCAAAAATAAAGAAGCCCATTTAAATATTTGTGACATCAGTGGAGC
A_16_P20260087
GCAGATGGTATCCATACACAGTTTGATTTGTTTAGAAACCTACATAAAGAGCAGCATGTG
A_16_P17847483
AACACAGCATACACAAAATACTCTTTCTCCTTATTCTCTCTATTGATGTTTCTCTTCCCA
A_18_P12324621
ATAGACCTTCAAAAACCAATTCAAATCTCCAAGAATCCAAGTCAGAAGAAATTGCTGCC
A_14_P108777 GCAGCAGTGCTATTGGATCCTTGATGCCAGCATGGACAATTTATTAACTATTCTTTATTT
CUST_6244_PI426210822 CTGATATAACCTCAGGTGAACAAGAAGGCTGCCCTGTGGCTCTTC
A_18_P13088645
AAAGTCATCACGCATTTATTGAAGTCTTCCTAATATGGTTATGCTCTACTGCTACTGCAT
A_16_P21224356
AGTCTTTTAAAGCCTCATTGAAGCAAATAACTACCACTGAAATTTTAAAAGTCCCTGGGA
A_14_P114744 CTTATTAGCCTCTGCATCTTGGTCAATTACCTCATGTTTTTGTTGCATTATCTATCCCTT
A_16_P15807973
ATTAGCCACCACTTTATAATGTGAGAGCCATTGAATTAGGTATCCATTAGTGTCTACTCC
A_14_P125390 TTAAGACCAAGTCCATTGGGCTGTTTATGTGCAATGATTAAAAACTGGGAAAATTTAAGG
A_16_P19480369
TCCCTTTACTCCATCTCCATTATGATGTCTCTCAAGATTACACCATATAACAAAACTGGA
A_14_P117824 ACCACCTAACAGTCTTTTAAAAGCCTAAAGACAGGAACATTCTACCACTTAATATGGAAG
A_14_P129092 AAAGTTAAATGCCTGGGTATCTATTTTCCTGGTATAGAAAGCAGCAGACACAATGCATTA
A_14_P109944 ATGTGTAAGTTTTTTAAACATCCCAGTGGCCTCCAGGATGGAAATCTCATTGTTATTC
A_14_P113899 GTCCTGTAATTTTAACGTTATTTTGAGTGGCCTGGCTAATGTTATCTCTTGGAAGAACAA
A_16_P58424511

TTTAACACTTCTCTATCACCAGCAAATCTCCCTTTTCAACAAAGATGGAGTAGACCTTAG
A_16_P18291307
ATCAGAGCCCAAACCATTAAAGGACTAACAGATTTGACCAAGAACCATAAACAAAGTAAA
A_14_P128380 GAAACCTGTATTTCTAAGTTGGCAGTAGACGTTAAATGAATTGATTTACTTGAGAGCGTA
A_14_P102793 ATGTCCAGTCACTGTTGAATTTCTCCAACTACTAATTCTAGAGAAGAATCTAGATCCCAC
A_18_P22841434 CTTTTCTTGCAGAGGGGAGAAGGTGAAGAGGTTTCAGGAGAGAAAAGTATATG
A_16_P01505638 ACCCTGGTGATAAAATTTGTGAACTCAAGAACGTGTATGAAACTGTGTGTCCACATT
A_14_P128897 TTACTCATCAAATTAGCAAAAACCAGAAACTTACATTTCACATTGGCTGGGCAAAAGACA
A_14_P123848 CTCAGGCTACAAGAAATTGATCAAACAGGGTAATGAGACTATTTCTTCACAGAGGTTATT
CUST_9313_PI426210822 TGGAGGACTATGCCTCTGCATATGTACCTGTCCTGTTTGTATTCTTCAAAAATG
CUST_7898_PI426210822
CAGTTGGACACAAAATAAAAATGTTTTTCATCCACCTGTCTAGATGCCTTGAAATGTTAG
A_18_P22843219
AATTAGCAAAGTAGACATTCAAAACATTCTCTGAGTTTTCAAACTCAAGGAGCGTGTGAC
A_14_P115216 GGTCCTTCCAAGTTTAGATAGGGGATATCCATGACCCTCATAAGAAATAACCTACAA
A_14_P125979 AGAAGTCATATTTGAGTTAGAGCCAGATGAAAACCTCAAGAATTACCAGTTATTCCACTG
A_16_P17528734
CATCTTGGGGCTCATTTCATCTAATAGCCTTAGTATCCTGTTTCAAACCTTAAAACACAA
A_14_P111035 TCTTTCATAGAATATTGAAAGGAAGCCCATTTGTAACTGATATCAACGCTCTCCAAATCC
CUST_7105_PI426210822 TATCCTGTTTTCTCATTTGCCCGTTGAGCACTGAACGACTGGAAGTA
CUST_8681_PI426210822
ACCATTAGGACATTGACTTCAATATGGCATGAATACCAACATCTGTTAGAATATTCCAAG
A_14_P100540 GATCAACTCTTATTACCAAGCATGACATGTTTCATTTTGCAAGCCACTTTCTAATTCAGC
CUST_8281_PI426210822 CGAAATCCAAGGGAAAATGCTATCAATGAACGGATTCAGCTTTCAAATGCCTA
A_16_P00685022 CAATGTGATCTGTTTGGGAAAGTGCTTGCTTTGATTTCCAAAGGGCTGTG
A_14_P137892 AAAACAAAACCAACTCAGACCAGAACTGTTTTCTCAACCTGGGACTTCCTAAA
CUST_7189_PI426210822 CACACATGAGTCAGCTCCAGGAGGACCCCGTGTCCTTGACTCTTC
CUST_7266_PI426210822 CACCATAAATCCCCAGTCATGGTGCTGACCTGGCATGTGAAGCTG
CUST_8219_PI426210822 CTGGGATTAAAATCTCAGCTGGAAGTTGCTGGGCCAGAGATTATCAC
A_16_P17087425
AAAAATCTCGACTTTACAAATCAGTCCATGAAATTCCTTCAGTCCTTTAGTCAGACAGAG
A_16_P19594509
CTCCACTTGCTAATGAAACTAGACAACTGAAAGATCTCCTACATTACCTTCTTCATCACT
CUST_7687_PI426210822 TGTAGTCTTCCTTGATTCAAGAGTACCATGACTTGTGTTTGTTCTGACTCCAG
CUST_6860_PI426210822
ACTGAATTTGGCTAATAAGATGTTGCATTGTCTCTGATCTCCTTGAGAGACTGAGA
A_14_P128284 AAACACGTCTTGACTTGCTCTTAAAACAGAGATATGTGAAGGAGTTTCAGCATCAGATTA
CUST_2864_PI426210822 TTTACACTCTAAGGGTGTGTTTCCCCGATAATCAGGCTTAAAGGGGAT

A_16_P16191762 AGTCCTCACCAGCATAAGAATCCCAGAGAAGGCATTTTTTTCTCACATACTCTAG
A_16_P38107915
ATCCAAGAAATGGCACTAACTAAAACAACCAGATCTGGATTTCAAAACATGTATTGTGCA
A_16_P21304864
ACTAGCCCTATTCATAGATTAATCTTAATGCCAGATATTTCATTGTAACAGTTTCAGCCC
A_14_P139335 ACATTTACAGATCGACTATTTCCTTTAACCTCCTAGAAGAAAGTTTTTGTGGGGAAAGAT
CUST_5597_PI426210822
GAGGAGTATTTTAAAAGACTATAAAGCTGGAAGCAATTCAATTGTGTGTCTACTTTATGT
A_14_P115141 CTGGGAAAAAGATTAGAAGACATGGGAAATGTCCAACATTTTACGTGTAGGATTTTTTG
A_16_P16684297
TGACATACATCACATTTTCTGGACATCTCTCATCTATTCCTATGACTTAAAATTCCCCCT
A_14_P116860 CAACATGTGTTCAAAGTCAAGGATATGGAAAACCTGGAAGATGTTTTCTACCAAATGATC
A_16_P03624052 GTTGCAGTTGGTCCTTATTTATTCCTCCAGCATCTTCAATATCATCCCACATATGTTCT
A_14_P128769 AATACAGGTAGGAAATATGTGAGATGTGAATCTGTCTTTTCTCCTGAAATTGGCTACAAC
A_14_P101518 GGCCTTGGACAGATATATGCTCTATTACTGAAAAAAGAGATGTGAAAACAAACTCAGGTT
A_16_P17427885
AAAGTAGAGACTACATTCAGTTGCTTTTTATAAGAACCTGATGCATTGGATGATCCTGGA
A_16_P03028841
TCAACAGAAGATACCAAGTCTATAGATCTAAGAAGAACCCATCTTGACTTAACTCCTTGC
A_14_P119344 TTTTGTTTTAATTGAGTAGAATCAGCAGAATCAGGAGCACTTCTGTATGTGAGAAAACGC
A_14_P114618 ACCTTGTATGTTTCTTTGAAACCTATCTGCCTGAAGAAGTATTACAAACATATCATAGCA
A_14_P101499 AAAGAACAGATCATTTCAGTGGGACCTATTAGGAGAAAAAGTATGTATGTTCCCTAAAAC
A_14_P114455 ACAATCTGTGGGTAAACTGTGTCCTTCGTTATGTCTGTTAATACTGCAGAAGAAGCATAT
A_14_P128624 ATGTAGAATAAGTTTTCTTACAGGGTTAACCATTTCATCTTGCCATTGGATACTGGGAAT
A_16_P00009371
CATGACATGTTAAAAGCCGTCTTGATACTATTTGATATTCAGTGCTAACAACTCACCACC
CUST_10438_PI426210822 GTACTTTTCATTGGAAGATTCCCAACAAGAATTTGGATGGAAAACCTGATCCC
A_16_P36664479
TCCATCTACTCCACTTTATAAGCAAATTTCTGTGAATACTTCTAACTCTCACCTACCTCA
CUST_10144_PI426210822
GCATCTCTTCAAGACAATCATAAAGGCCTGTTTAGGAGGAGAAAGTTAAATTGCTCA
A_14_P111388 CCTCCCGATTCAGAAAAATGTAGAACATTCAGACCTTCAAAATCATCATTGTATCCACTT
A_14_P137585 CACTTGCTGTTTGATTCTAAGTCTGCTCTATGCCAGAAATTAGTGAGAAATCCAAGG
CUST_1668_PI426210822 ATCAAAAAGCAAGCCTTTCCGGCGAGAATCTAGTGGCTTTTCTTCC
A_16_P02766760
TATTTGTTCCCATGATACTCTCTACACATACCCCATTATTACTGAAGGTCTCATTACACC
CUST_3283_PI426210822
AGAACTCTGAGAAAGAGGAGAAAGAAAAACAGCAAAAGTTTCTTCTTTATCTGGAAAATA

A_16_P00481922
CGGATGATTTCTATCATGTTTGTTCCATCATAGAGGGCTGAATAAATAGCATCTGACCTA
A_16_P15150633
AGTTACCACCATACATCAAATGATTTTCCCAAAAAGATAACCCGTATACTGTGATTCGTG
A_14_P136595 ATACAGAAAGGTGATCTTTGACCAACTCACCACACCAATGGAGAAATAGTTGTTCAT
A_14_P114149 GTATTCTGATTATAGAGAGGATTAGGCTTCTGCAGCACTGTGCTAACATTTTGCAAAGG
A_16_P15297284
AAAATTGCACTTGTATTAACCTTCCATTAAATGTCTCTTTCTTCCTCTTCCATCTCCATT
A_14_P110336 AGTGTCCTGCAACATATTAAGTTTTACATGGTGACCAGAAAGTTAAATAAAACATCCCCT
A_14_P121203 GGCTATATGGTAAAGTTGCTTACAGATTCTCGTTTGCCAGAAGAGGAACATGAATTCTT
CUST_1762_PI426210822 ATTGTTGGAAAGAGGAGTTAGTTGGCTTTGACGCCTGTTGTTCTAATGACT
A_16_P16307625
AAAAAACCCTAAAAGCACAAGATACCTAGTCCTTTATTGGCTTAACTTTGGAAATGGCTT
A_14_P200030 CACTGTATGGACACAAACTGCCTGTTATATGCATGGACATCTCTCATGTAAGTAGTTTAA
A_14_P109297 CATATTTTTTAAGCAGTAGTCTCAGTTTACATCACACGTTCCCATTCAACACAGTCCATT
A_14_P128821 AATAAAAGACACTTATTGGAGGAGGTAGCAATAAGGAGTAAGACATGTCATTTGTCCTCA
A_14_P101058 ATTACTTTCCTTTTGACATGATTTCTAGAGGCAGATCACATTGTTTTCTTCTGCATGCTT
A_14_P132107 TATAGAGAGTGAGGAGATTGCAGAAAAAAAAAGGCAGATACATGAAATGTATGCCTTTCC
A_14_P100795 GTTTCTGTCTCATGATCCTCACTCTAATTCAGCAACAATAATAGTAACAGTGTCTTGTGC
A_14_P119739 GTGTCTTACACATCTCCCAACAGCCGACTTAGATGTGATCCGTTCTC
A_16_P38122913 CACTCTCCACCATCAACGGCAAACTATGAGCCATTAATGGCAAACTATGAG
A_14_P127708 TCTTTTAAGCCCTGCTCTTTCTCTGAGAACCAAAAGATGCCTTGAATATTTATTCAGTGA
A_16_P15195108
TCAACACGATGAAATAACTCCTATTCAAACTCAGAACATTGCTTCCTTATATCCTGTGCT
A_14_P111482 CTTCTCAAAAAATGTTAATCCTTTTCTGTTCTCATCTCAGTGCATCTCAGATTTCTCTGC
A_16_P36230603
TGCAAAACCTTAAGATAGTCAGCCTTAGGGAAACACTGAAGAATTTATTTTTAAACACAT
A_14_P105113 CCAAAAGAATGCAGTTGAAATTTTAACTTTATTATTTTCCTATTACAAAAGCAACACATA
A_14_P115414 TTTCATCAATACAGTATAGCGACAAAGCCCAGAGGGAATATAAGAGATGTAGGCAATGTA
A_14_P120378 ACTCACCTTGCATATAAATCAAAGCATCATAAATTTTCACCACCTTATCAATGGTTGCCT
CUST_6444_PI426210822 ACCTGGGATGTCTCCTTGTGACAGTTACGCAAGAAGGAAAGTGAA
A_14_P114800 TCCATCTTCTGACATACAACTGAGGTCATGACAAGGAAAAAAAACACCACATTTATCTAA
A_14_P105054 CTCCTTCTCCTTATTCTGGAGTGCACATCCAGTAATTAAGAAAACATGAGTGAAGAATG
A_14_P110314 GGGGCCAAAGAGCAGTTGATTTTTTTTCAAAGTCTAGTATTTCTCTGAAGATTCTACATC
A_16_P21093267
CTTGGTTATTTGCAGGGATTTCATTTAGTGATCTGAATCCAGAGATTGTCCATCAAAGTC
A_14_P202107 TCTACTCAAGTAAGTCAATGTTTCTGTCTCAACTGGTTGATTGGAACAACATACAAACTG
A_16_P19323808

TCCAAAGTCAAGACTTACAAGATCCACAAACCTAAGTAAAGTAGACAACAAATGGTGAAC
A_14_P115160 TTTTTGAAGTGCAGATTTCTGGCTTCTTGAGGCTTTGAAGGTAAAGGCTTC
A_14_P111160 GAAGAAGATTTATCTTTTCTCTACAAGAGCAGCCCAGGAAGAAAGCATCAGGTAAG
A_16_P39547742
TGAGAATTTTGAGTGTCAGTGATCAACAATTAACCCTACGTCTCAATTCATCATAAGCAA
A_16_P01222684
GCAGGAATATAGGCAATACTTTAGTCTTACTCAGCAATCGTTTAAGAACTTGACAGGAGA
A_14_P108988 AATTTACTTTGTTCCATGTAGGAACAAAGTAGGAAGTATTTATGTCACTCGGTATGGGAC
A_14_P128117 GATGATGTCACCTTTGCTGAATTTAAGGTCTCCAGGCTCTTTTCCTTCATAGTTGTATA
A_14_P121199 TCTGGCAACATACGGATTTAAAATGAAATAAGTTCTACTTTAGCACAGTCCATTGGGATG
A_16_P18224333
AACTCTAGCCATCCTTATCACAAAGAATTGACCTTCCAAATCGAATCAGAACCTAAAAAT
A_14_P101819 GAGAGAGAAAGGACCTTCTCAGTCTCACGAGAAACTAAAAAGTTGCATAAAGTTAACTAT
A_14_P111140 CACAAAGCCCTTTAAAAAACACTTATTTGCAAAATGAAGTGTCTGAAAAGCCTAACTGGG
A_16_P17634840
GCACAGCCATACTTAAGTCATCATCAGCAGTCAAATTTTTCATTTCCAGTCACAAATTCT
A_18_P15448191
AGAATCTCTGACACCAGTATTACTTCTCTCCCGATTAGTAAATTTGTTTGCTCCCATTTT
A_14_P105230 GAAATAGGTAATCACATACAGCCAATACATATCAGAACTGATTCAAATCAGGGCTATGCC
A_14_P126893 TGTAAATAAAATGCAAATAAGCCTTTACTGTTAGCACTTGCCTTCTAGTGATGGCTCGAA
A_14_P125187 ACCTCATATATGCTCCGATAGCATCCTACATGTGTAATCACATATCCTTCTGTATGTGAT
A_16_P39069885
AAATAGTCTTAAAATAACCTCAGTTAATCCTTTGTCTCCATGAATACCATCTACCCTGTT
A_14_P117876 ACCACATAATGAAAGTGCATGGAACTATTTGAAAGGGTAAGAGGTTGTTTTTGCTTTTTT
A_16_P20664058
AAACTGAAAATTGTCAATCCCATGATACTGTTCCAAACCTATAAATCATTGCTCTAGGCC
A_14_P115689 CTTTCTTAGCAATTTCTCTTTTCTGAAACAGTGAAAATGTAAACTTTAGGGTAGCCTCTG
A_14_P101541 TATCAGTAAAAAGGCATGTGATTTTTTACATTCAACAAAGGAGGAAGCGGTCAAGAGAAA
A_14_P134804 GAAGAATAAAATGTAAACTATTAAGTTATGAGATCTTTTTTGTCCCCCCCCAGCCCATTT
A_14_P100333 CTGTGGATTCAACTCTGGCCTGAGATTGATTCCCTTGAGATAAGTTGTTTTCT
A_14_P132835 TGTATGACTTTCTAAATCACTTCTGAGACTTAGTTGGAGACAATACAGCAGAGTTGTTGT
A_14_P139627 TAACAGTTAAATTCCAAAATACCAGTCCAAACCTTCTCTTTTCTTCCCCTCAAATCTACT
A_16_P20563641
TCGTTAGTCTTTAGTAAACCAAACCCAGACATTGTATGTGCTGTTTTGCATATTGCTACA
A_18_P26181138
AAAATACTGTGGTGATGTTAAAGAAAAGTTCCTAGGTTCAAAATTTGGCACAGAGAGCAA
A_16_P17747681
ATCTTGAGTTTGCCCTATTCATGACTTTTCAGTGTCAATAACAAAAACACGAAGACCAAT

A_14_P123853 CTTGCTGGAAGATCGAGTTTTAGACGCATTTTTAAAAATCTTAAAGACTAAAACACTTCC

A_14_P136376 CTTTTCTGGTTCACAGGTTTCAAAACCCTCATATCCAAAGAAAGTCATTAGAGTTCTCTA

A_16_P02892653

ATGGACCTTAGTTTATGAACCAAATTTCTTTGCACCAATACTTAAGACAACCCAAGGAAT

A_14_P109029 TCAATGTAATGTGTGAGAGTCATCTGTCTTTACGTTGTGTTTCGTTTGTGTTTAGTCTCA

A_16_P35621551

TGAATCCCAAAACTTTTATCCGAATAACTGTGATCGATTATGCTTAACTGAACTACTCCA

A_16_P35882344

ACTTCACAGATGCAACTATGTCTACCTATTTCCTAACACAAATCCTGATGACTTGTTACA

A_14_P116597 TTTTAAAGGATTTGCTTGCTTTGATGGAGGAAAGGAAGCCTCATTCTTCCCAA

CUST_5848_PI426210822 GATTGGATTGTGCAGCCCATTTTTGTTAATAGCATTGCCCAAGTTAGGGAA

A_18_P22841634 ATCAATGTGCTCTCTTGGTCCTTCCACACCTGAGGCAGCCTGTAT

A_14_P108489 GCATCATGTCACTGCAAAGCCGTTGCAGATAAACTTTGTATTTTAATTTGCTTTGGTGAT

CUST_4406_PI426210822

ATATCTATTTTCTCATTTTGCATTACAATAGCATCTAGTAACATTCAAGCCTCAATTAAT

A_14_P118366 GTGTGTGTTTTAAGTTAGTTTGCAGAATTACTCTCCTATGATGGCACAGAAATCACAAGG

A_16_P15030174

TTGACTCCTACATTGTTTCTCACAACATCTCTTACTCACAACATCTCTTACTCTAGAAGG

A_18_P14134110

AGCAAACATTTCCAAATAGGGATTGACTTTTAAGGGGAGGAAGATGGCTAAATGTATATG

A_16_P00928508

TCCCACTCTCAGGTTAATATGCTTTCCCAAATAAAGGAATTAGTTTTCACTGTAACTCTG

A_14_P117815 TTATCTTACTCTATTCTACCCCTGACCTATGTATGCAGGCCACCTACCTCTATTTTATTT

A_16_P16191482

AATTAAGTGGTGTCATACTCTGATCCTTCTTCCACTGAACATCACATTCTTCATGAGAAT

CUST_70_PI426210822

GGAATAAATGACATAATAATAATGGCACTAAATTGCTAACATGTACCAGTTGCTTCCCTC

A_14_P101112 CCGCATATGTCAGGTGTCTTTTTCTTTCTTTTCCAAAATGTAGTTGCACAAATTCTGAAT

A_14_P202412 AAAATTGTGCGCATCTGCAAGTTAAAACCATGACATATAGCAGGCTAAAATTGAACAGAA

A_16_P16982083

AAAGTGTCTCACAATGAATACATTGCTATTTCTCACCATTAGGTATATGGTCTCAACCCA

A_18_P11126172

ACAAAACCCAGCAATTTTACCTTGATGTTTTTTAACTAAATCCAATCTTCATTCCTGCTT

A_18_P13243370

CCACTACAACATGAAGAAATCTCTTAAAAGCACCATTAGAATATAGCAGAACTGACTGGG

A_14_P132803 ATCCTGTAACATTCTGGAAGTAAGAAGGATCACTTGTTAAGTCGCCATCTAGTAGTTTAT

A_16_P00966196

TGAGCCCAAGCATTAGATAAAAAATCATTCCTTTCCTATCTTCCTAGTGAACACTTTTCA

A_14_P100569 ACTTACTATCACTCGTCCTCCATGTGACAGAAGACTCAAGTCTTTACTAAGATTTACATT

A_14_P114407 GATCTTCTGAGTTTCTTCATCAAAGCCAGATTTGTGCAGCCTGGGAAAATAGAAATAA

A_14_P127963 TCATAAAATTGCATTTACCTTCATCTCAAAACACACTTTGCCTTTTGACACACCATAGGA

A_14_P104862 TGGCATAAACTCTTTAGTTCTCAGACTGCTTACTCATAAGTTCTTCTTGAGAACTTGTCA

A_14_P104207 ATTGATCTGAAAGGACAAATGGAAGAAATACAATGCAATGTTGATGAAATTTCATCCTCT

A_14_P202428 CTGCAACTAAGGCAGTTGGGCAGCAGTAGAAACAAAAAGTTGATATTTATTTTCTTTCAG

A_18_P17039638 CCTGTTTCTCATCTGTTCTCAGAACCTTTCTCTCAGCCACTAACCTTTAGGATAAA

CUST_4584_PI426210822

GACCAATCTGTTTTCAAATACAAAGCTAGTAGTTAGAATAACCAGGGGGAAAAAAGCATA

A_18_P12841778

AACCCTTTGTGCCCTCTATTACTCAGAAAACTAAATTAATTAGAACCCTCCATTTTCCAA

A_16_P03646697

AGTAAGCCTAAAAGCAAGACATGATTTTCCTAAAATTGGCCATGTAAAATCAGAGAGGTG

A_14_P118891 CTAAACATTCTCAACAATAATTATAAGATTCTGCAGGCGGACCAAGAACTGTGACCACA

A_16_P39314902

AACAGGCTACTAAACTTCTGTGGGAATCAAAAAAAGAATGTAGTGTAAATTGTGTCAGGC

A_16_P03279363

TGGTGTTCCTGTTACATTTTACAACTCTTCATATGCCACTTAAGAAATGACCTCTCTAGA

A_14_P123715 CTATTAGAATGATCCCTTATTCATTTGAAAATTGGCATGGGATTTTGACTATCTGCCGAG

A_14_P108187 CGTGCGAAAAGATGGGATATTTGAATTGTGCCCTGGGAGATTATTAGTAACTACACAAT

CUST_9844_PI426210822 AAAAATAATAAGAGATGAAGCCCACATTCCGGGGAAGTGACAGGGC

A_14_P122840 CAGGATCTCTTTATTGCACAGACTGAATGGCTTTACATGTTTCTAATGTGAATTAGGCAT

A_18_P15041967

TCAGAAATTCGACTAATCTTTCAAGATCCACTTCAAAGTCCTGTATCTAATACTGCCACT

A_18_P10532453

GCGATACACTGTGCTTGAATCCATCACTGAAAAAAAAATAATGTTCGTGGAAGTGTTCTTG

A_14_P119106 ACCAAATGGCAATGGATTTTCAGCACAAACTTAATACTTTCCTAACACTACAGCAATAGT

A_16_P21377288

AAAACATCAGGATTGTGTTCAAACATTTTTTTCCATGTTATCTTTCCTACTGCCTCCTTG

A_14_P126725 GGAGAAATGCAACACAGAATATAAAATCAGGCAAAACAAACACTAGTCACCATGGAAGT

A_14_P115504 ATGCTTGTAACATGGAAATAAGAACCGTTTCTAACTCACAGTCTTATGCAGATTCATCTG

A_18_P14132314

GATTCTAAGTGGCTGAAGGAAACACCAGGAAACATTCTGTAAAATGTTCCTTGGAAAT

A_14_P102187 GAGAGAAATTCAGTTTTCTTCTTTTTCTCCTTCTCTGTGGAATCTTGGATGATTCTTTCT

CUST_3217_PI426210822 AACACCTCCCCTCAGCATCGGGCTCTCCATGCCCTGCCTGACCTTA

A_18_P14133155 GCATGTGCAAGCACACATAGATATATATTGCCCATCGTTCCTAACCCTCCTAAG

A_14_P110824 GTTGATATGTCAATGACTAATTAAAAGGGGTTGTCAGTGTTACACATCATTGGTTAAATT

A_14_P126023 CACAGATTGAGGAAATAATTTCTGGTGGGATGATCAGTGAAAATTTTATGGAGAAAGTGT

A_14_P101414 AGGTCTAGGACAAGACCAAATTCTTGATACAGACAAATAATGTTTGCTCAGGCTCATGTA
A_16_P16612985
ACATTCATAAATCCTACCTCTTGTCACTATGCTTCTACCTTAATCCACTGACCTTCAAAA
A_18_P22839606 CATCTTTTTCTCCCCATGGCTTGGCCATGCCACAGTGGTCCCTCG
A_16_P02350259
GGATATGGCCATACTAGTGTGTATGATGAAATAACAAAGTCCATTTATGTTCATGGAGGG
CUST_4570_PI426210822 CTTGGGTTTCTGGTTTGGAGAACAGGGTGGATGGTGCCTCTGAAG
A_16_P19850004
AATAAGACCGTTTCTGATCTCATGATTTCACAAATACCCAATCTTGTAACCACTATCACC
A_16_P19914946
AGTTTCATCCGTTTCTGGTATCAACAAAATCAAAGCGTCTAACTTTTTCAAACACAAAGG
A_14_P118781 CCTGCATCCATATTTTGATTCCTGCTAGTTCAATGTCTTATCCTCCACTGTTTTGAATAA
A_14_P136485 CTTTTTCTTAAAGCCTGTTCTGTGTAAGTGGACTCACCGTTTCAAAAAGTCACATAATTA
A_16_P30952302 CTTTGTTGTGACTGTGGTTCTCATTGCATTCCCCTCCTCCCCTCC
A_14_P138133 AATTAGGAAGGGGAAGTAGGATAGGGGACACGATTACGTTCCTAAGTGAAG
CUST_2156_PI426210822 GAACAACATGGTTCAATGAAAACATTCTGGTGCCGCCTACCTTTTCTCTAA
A_18_P15065066
TGCCACTCCTACATATTAAATGGAATGAAAACCAACTTAAGAATTACTTCAGGTGTCTGG
CUST_6306_PI426210822 TCCCATAGGGCTACCTCACCTCCCAGGAGAAAAGATTCAAAATCTA
A_14_P202114 ATGTTAACCGGCTTTCCAAAAGTAATGATCTTCTAGCACCACAGTCATTTTCTATTTAAA
A_14_P134320 ATCCCTATTCTAAATGCTTTGCTCTGAACACAGTTGATGCTAAATAAATGCTTGTGAGTG
A_14_P108385 GATGGAGCAGGAGATCTCTTCATTATTAATGAAAACACAGGCGACATACAGGC
A_18_P14135924 TTATGATTCTCACGTTCTGGTCTGTGACTGAGGAGACGTGCCATGAT
A_16_P15567948
TGATCGTCTACCCAAAATCATAAAAACGGAAAGTGCTTTTAATTATACCAGGCTCATCAC
A_14_P136131 ATTTCTTTTGTCAACCTGCATCTTTAATGGTTAATTCAAGCAGAATCAGCGAGGTGAATT
CUST_3900_PI426210822
TTAAGAGTACTTTTTAAGTTCCCAACAATTAGATAGGAAATGAACTGAATGGGAGTTCAC
A_14_P100320 CGGCCAGTTGTGTGTGATCCACTGTGCACATTCATTTTATTATTGAGAATATTATGTTTT
A_16_P20187123
ACCTAAAATTCCCACTAATTTGCTTTTCCCAAAAACTGTATCTGTCTTGAATGTTAGCAC
A_14_P116761 AATCTGTGAACAATTCCATTTTTCATCAGATAGCAGAACAACTACAACAGCAAAACCTAG
A_14_P138875 CACATGGTAGCCAAAGTTACCTGCTTTTAAGTAATAAATGTGAACACCTACTGCTATCAC
A_16_P02427121
CTTCATCTTACACTCCCTTCCTAAAAATGGTCTCATTTTGCTTGTCTCAGTATCCATGTT
A_14_P101623 CATGAGGGCTCATGGAAATGCATACATAACTGCCCACATATATAAATCAGCAATGATTTT
A_14_P123147 GAGAATAATTGACACCCAGCAAGCACAGTTCTTGAAGCAGTCTGTGCATTC
A_16_P03267089

TCCTACTCTGTGTTTATTACTTCTTTCCATCACTTAAAAACAAGTTCCAACATTGTCCCA

A_14_P104048 CTTAGCAATAGATCTCACCTAACAGTAGGATCTAAGGACAGGCCCTGCTTAC

A_16_P21035534

AAAATAGCCACAGATCCATAGTTCAGGTTGAAAGTCACAAACAGTAGCCTTTTTGAGAAA

A_16_P17900145

AGAGGGACTTGTTGGGAATTTTTTTTCTCTTATTTAGAAATAAAACTAAAAAGGGAAGCA

A_16_P01129765

AGATGATGTTTTGACGTTTTACTTGACTTCTGATCCTAGTTCAAGAGTAGCAACTAGCAC

A_14_P119436 AGATTCATGATGAGATTTCAGTGTCAAGCATGGATGCTTCTAGACAACTAATGTTGAATG

CUST_6214_PI426210822 TGAGATTTTCCAGCTTCGACTTTCTAGATGGTGAGAGGATGGGGAT

A_14_P129501 TATGATATCAAAGTTAAATTTATGATGAATGACTAACTGGACAAAACTGAGAGCAACCAC

A_18_P14688813

ATCTCAAGGACCAAGGAAATAATATGCAGCACTAGATTTTATTAAGCAAACAACAGGCCT

CUST_6538_PI426210822

GTTGGATGCATTTGATTAACACTTGTGCTAGCCTTTACTCAGGCTAGATTTATGATGG

A_16_P00685133

GTTCTTCAGAACCTAGAGCATTTTCCAAGAGTCACTGAATTCTAGGTATGTCTTTCTCTA

A_16_P18930292

AAAACGTATGCCAAATAGTAAATGCGAACTCTGAACCATTTTTGATCAAGGTCTTCACAA

A_16_P17168477

GAAAAACATCCTGGTTAACAAAATATGACACCTAACAACTTTGTATCCATTTCCACCTGC

A_14_P105329 CTTGGTAACTTCATCAATCCTCTATTTATTCAAACCTAAAATGGTGCTAATGGGTCTGCA

A_14_P134008 ATTCTGTCCCTTTTGAGATTACTTTGCATTAATATGACCAACCACTGCGATCAATTTTAT

A_18_P24671437

TACTGCGATCATCAACAGTTTGTTATTTAAAACCTTCCTACACCCATCTTTGATCTGAAG

A_14_P201941 CAGAAAGTGGGTGATAAACACTGAGTATCTCAAACAAATTTATCTACTCAAAACATGGGG

A_14_P130840 ACATCTTATAAGACTTTATAGGACTAAGGAAGTTAATCCATCCTTAGCAGGGATGCCAGA

CUST_998_PI426210822 GAGCTTAGAAGGCTGTGATCTTATCATAGAATGATGTCAGGGAGAACCAGG

A_14_P121685 CACATGAACTATTGGCCATTCTCTTTTTTGCTTCTGGGATTGTAATCTGTTAAAATCCTT

A_14_P111199 AAAAAAAACTCTTCGGAATAAAGAGGGCTGTAAATTTTGAATTCCAGTGTCAGATCCTTT

CUST_6359_PI426210822

AAAGCATGATGAGATGGGGAAGCACAATTCAAACTACATGGCATAGTAACTCAC

A_14_P201762 TCCCAAGGCTACATAATTACAGTGAGAACTTTAAGAAATCCACGTTTACGTGTGTTCTTT

A_14_P138388 TTTAAAAGGTAAAAAGGATTTATGGTGAAAAATTCCCCAAACATCCACACAGTTTCCCTT

A_14_P121682 CTGCAAGTGATTGAAAGTATAATGCGTTGCATGGCAATCTGCTAAAAAGTGACAGAATAT

A_14_P115026 GTAAGCCTTGACGTGCTTAAGAACAGGTTTCAGATTATTTTCATATCTGACACACAGATC

A_14_P125535 CTCATTCTATGCCATCTGCCCATCGCCTTTTATTTTATAATCGACATCAATAAACTCCAT

A_14_P121255 ACAGCATATAATGGTTTTTTGTCACTGCAAAGATGAGGTTGATGTTGTTCTCTGCCAATT

A_18_P15362659
TCACCACGACACATTATATATGCTACTCCCATGAATAATTGAAATTGTATCCCACAGAGT
A_14_P123975 TTTTACCCTGTGTCAGGGTGATGTAATGAGAGAGTAACAAAGCAAAATCTTCTTCTTAGA
A_14_P109118 ACACTGCTGAATTAAACTGTTTTCCATGAAAATGTCCACAATCTGAAACATATCCAAGCC
CUST_3488_PI426210822 CAGACACTGGACAGACAACTCACAGCTCCTGCCACAGTACAGCAT
A_14_P127254 CTTGAAGAGCCACAAAACCTTCCTTCCAAAATACTGAAACTTCACAGTAAACATCTTCAT
A_16_P03549788
CATCTGTCCAGATCAAGTTTAACTCACCTACTCTAAGAACTGCTCTCCACAATTATTTTG
A_16_P15422228
TCCTTTCATTTCATGAAGGTTTCAGGCTCTCATTTGCCTTATAGTCTTTGTCTACTGTAT
A_14_P126764 GAGGCGGAAAAGAAAAAACCAAAAACTCAAATAGTATGTCCTTTTCTTGAATTGAGTTGC
A_14_P113107 ATATTATTTGCAGTGGTAAAACCTGCATCATTGAGGGTTTCCCACTTCAGGATTAAATTG
A_16_P02018422
AAAGGTATCAACTGACTTGGAATAATATGATCCATCGGCTTAACTAAGAACTTTGCCACA
A_16_P19376388
ACGCACTCACAAAATACATAACAAGGTTCTAATATGAGATAAGCATCTGCATTGATCAGA
A_14_P202468 TTTCGACCATTCATTCAGATATAATCCACACACTTTGATGTTACCACTTGATACCAACAC
A_16_P15849145
TAAAAATCTAGTTCCAAATTCCATACTTCCATCCAAAGTCTATGCACATTCCCAATTCCA
A_18_P22840723
AAAAAAAAAAAAGATAATCTTCCAGATGTTGGCTCAGTTTGAGGAGGAGGAAAGATTCTG
A_14_P106550 AACTGGCTCTGGAATATAAAGAAGAGTAAAACAGGCACTTCTCACAATGATAATGATGAG
A_14_P118017 GAACATGTCCAAATCCATGTCAAAGTCTTATGACTGAAAAATGCCTTTTAATTCCTGGAA
A_14_P115640 TAAATGGTTGACAACTTGTTCAAATAGGATATTACTTAGTAGAACTTCCTCTGATGGGAA
CUST_4487_PI426210822 TCTGGTGCTGGAAGGCTCTCTTGGGTTCTTTGCCAAAGTTCAGAT
A_14_P117476 TGACAATCAGTTGTTGCCTTGCTGAGAAGCATACTAATCATAATTCAGTCTTCTCTAGAG
A_16_P41770027
TGCCCAGCATTATGGATTTTATTTTAGAAAAGCCCTTGATTATGTTATCTTTTTCTCATG
A_14_P139662 TAGATCTTAAGTGTGGAATGCATCTATCCAACATAAATGCCCATGTTGAAAGAAGGAAAG
A_14_P129439 AACTCTACTTACAATAGCATGCATTATCTGCCACAATACATGCTACTTTGCTCTTCAGAC
A_14_P127022 TTTGGGGCTGAGGATACTTTCTCTCCATAATTGAATTCTTTGCTTTAGAGATTTCTATGG
A_14_P138117 AAAAGAAGAAAAGTTAGAAGAGCAATATGTTTCCTTCTCTGTAACAGTGTCCTAACAGTG
A_14_P132012 TCTCGACCCGAAGGTTCTTCGTATTGTCCACCGACTCAGTATTTGAT
A_14_P122594 CATTTTTAAGTGTCTCTGGCTCATTTTAGAGCCAGGTGACTTAACAAAGGTTTCATTTAC
A_14_P132483 AAACACAACTTCCACAAACTTGGACTTGTCAGTTGCCAATTCATTAAGAATATTGATCAG
A_14_P127849 GCATTTATGGCTGGAGAAAGCGCTGTCTCTACTTGTTTGTTCTTCTTTTACTCATCA
A_18_P22841401 CATAACTAGTGAGCCTGTCCTGCTCTCCCAAGCAAGTCTGTGTTTAAA
A_16_P03004640

TTGGACAAGGTTAAACATACTTTATAGCAAATCCCATTCATTTAATAACGCGAGTCCTCC

A_14_P112374 CAACCCTATTGTTTCCTTTTCATTTTCAGAACAAGCAATCAAGATAATTTGGCTCCAATT

A_14_P137674 CCTGGCCAAAGACAGATAGATGTCCTTTAATGAGCAACAATTAGATCAGAGGTTACAA

A_14_P130316 TATTTATTAGTTATGCAGTCCCCATACCATTAAAAGGCCTTTATAAGTTGGTGAGGCATC

A_16_P19953452

AATTACAGATTGTTTCCCTCAAAAAACACATACATGGATGACCAACCTCTAGTGACTTTG

A_14_P200911 TTAGCAAGTCATTAAATCCTCAAAATAATTCTTGAAGACACTTGAAGTCCCACTATTCAC

A_14_P115691 ATTTGCATTTTCCCTTGAGGTTATGCATTTCACAAATCCAGAAGTTTTTCTTTGAGAATT

A_16_P21561827

ATCACCTTCAGCCACTATTAACTCATGCTTAAAGATCCAACTCTCAAATATCACCTTTGT

A_14_P135878 GGTCCTTCTCAGAAAAATTTGTTATTGTGTTATAGTATAAGTCATTTCGCTGTTCCCAGA

A_14_P136161 GTATAAGGCAAATAACACAGGGAGACTTCCCAGTGATACTATTAAAGAGTTGACATTTCG

A_18_P22839799 CTATCTCAGGAGGATGCTTGGATTTGTGTTTAGAAATTGGGTAACTCAGTTGTTCAT

A_14_P122502 AGCACATATCCATGTTCCCAAAGGTTTGCATCTCTAATAGAGTTAAAATTGCACTTATGC

A_14_P101228 CTGGCCAATATTTTCTTCAATATTCAAGACGCAAGACTTACAATGCATTCCATCTATTCT

A_16_P02992133

TCTTAATGATCATTAAATTATAATTGCGATTCAGACCTTGCTACGTCTCTGAGTCAAAAA

A_14_P112709 GAAAATACAAACAAGATCCACTCACCAATCCATTTATCTGTTTTGGACCAGGAAAGATC

A_14_P101087 GATGGAAACTCTCTATTGCCTATAACTGACACCACAATTCTTTACACTGGCATTTACAAT

A_18_P22839988

TGGTTTAGGGATATTGACTTTTTAAAACCAAGATATATAATTGTTCAAATATGTGATGCA

A_14_P109902 AATTTGCATCTGGAACCTTAGCAGTAAGACAGCCTGGAAAATGTATTTAGTGTCTGATCT

CUST_6993_PI426210822 CTTGCTGTATGGGATCAGGCCTGAAAAGTGAGTTAAGTCTGTTGGTG

A_16_P01062907

GGGATGAGAACTGACATACACGTAGACATCTATTCCATTTACATGGCTCTTAATTGAGTT

A_14_P104797 TATCATCTTTGTCTGAAAGAGCAAAGGAAGTATTCGTTATATTAATGATGCTGGCGACCA

A_14_P100206 CCTCCCTCTTTTTTAATGTAAAGGTTTTCTCAAAAATTCTCCTAAAGGTGAGGGTCTCTT

A_14_P114010 ATTTCCTCAATGTCTGTAACAGGATTAAATAAGAAGTAGTCTCTCCATTCATTCCAGTCC

A_14_P120519 GGTCACTTGGAAAATAGACGAATGACTTGATAATGCATGAGCAACATTAGTTTCAACTTT

CUST_884_PI426210822

AAACATTCTGTAAAATGTTCCTTGGAAATGGCAGGCAAAAACAAGATACAGTGAGTG

A_18_P22841091

AAGAGGGTATGAGTGTTGAAAGAAGGAAAAGAAGAACAGGGGTTGCACAGAATATA

A_16_P01131121

AACCCCAAGTATATTACAGAAAACTTCAGAAAGGATAGCATCAGATTCTACAAGATGAAA

CUST_2998_PI426210822

TGGAAATTCACTTCATTTAAATGTAATCTTTCTTACTTCAGGGCAGTTGTTCAGGGAAGT

A_14_P201525 CAGTTGGCAAACATAAAGCCTAGACTGACGGCCATGAAGAGGACTC

A_14_P114824 CTCACCACTTCTATGCTTACAGTGTTTAAATTCTCTGGCATGAAACTTTTAGGCTTGAAA
A_14_P134678 GCTCAATAAAAATTTTTGATGTTCTTAATGCTGCTTATCGGGTGCTATCTACCCTCACAC
A_14_P105107 TTTCTAAGAAGAAAATGCAACTTCCTCTCTGCCATTACTTAGCAAAAGTGTATCCCATTC
CUST_5059_PI426210822
AATGTTCTACCCTTTAATAGGGAAGGGTTTCTTCTTCCTATGAATTTTTACCTCACTTTG
A_14_P105066 CAAAGGCCTAGTCAATGGACTCAAGGCAGGACCAAGTAAGTATATCATGAAACTT
A_16_P19663737
AAAAAGCAGAATGAACTGGAAAAGAAACACAGAGAAACATTCGATAATCATTCCACCAAC
A_14_P124753 TGCATGTAATGACTAGTTGGAGGTAGCAAATTTCCCTGTGACATGTATTTTACTCTGTGT
A_16_P15286557
AAACGTGAACTCCATTTGACTATGCCAATAGAAAAATAGACCAGTTAGTTTAAGGACACT
CUST_921_PI426210822 GTCGCCCCTTTTCATCAGACTTGCCAAGGCTCAGACAACAAATAATTT
A_14_P127471 CAGCCATCTGCACAGAGAAGATAAACATATAACGATTGAGGATTTATGGAAACGATGGAA
CUST_10319_PI426210822
CCATGTTTTCTTAGCCAACCACATCTTTATCGCAAAAGAATGACATTTCAAGCAGTAG
A_16_P00685484
CAGTTGGACACAAAATAAAAATGTTTTTCATCCACCTGTCTAGATGCCTTGAAATGTTAG
A_14_P120693 AATACATCTTGAAACGTCTTTGTGAAGGCTCTTAGTTTTAATGCATGGATGCTGTTATTT
CUST_4477_PI426210822 TCTCTGACCAATTCCCAGTCATGGCTCTCCCATGGGGTTTCACAG
A_14_P105034 TTTTCCTTCCTTCTAAGTTCACGGGGCTTCATTAAAACACTATGCTTATATTTGGGATAA
A_16_P18981118
TTTTACCTGACATTTCTAATGGCATTAATCAATTAACCGATCAGTTATTTAGCCCCTCCT
CUST_2977_PI426210822
GGTATGTCTGTGATTGTATGTTTTAACATTTTAGAGAAATCTGAAAGTTTAATGTGCTGG
A_14_P115090 CTATGAACACTGGCACCTTGAAATTTATTTCCAACCCTCTAGAAACTTCAAAAGATACAA
A_14_P136631 GTGAAACATTTGAGAAATTAGCAAATTTTCCACTTCCAAGATCTTGCTGCAAAGTGATGA
A_14_P120219 GGTGCCAGTATTTCTTGTTGTATGCCCAGAAGTATTGTGGCTCTTTGTTAATTGATTAAT
A_14_P105400 ACCATGAAAAAGTTGAAACAGACTTACACAGTTACATAGGCATATTCTCCACAGAACTGC
A_14_P132697 TTTATACCCGCTTTTCCCCAAACCCCAGACTTAAATAAAAATGTTCGTTGTTAGAAATGA
A_14_P123813 AATTCGCCATCGAGATATACATGCTTCGGTTCTATTTTGCATTTCTGCACTCAATGAG
A_14_P122503 CGAAATCATCAATAATTACCTTTTCTTCCGCTAGTCAAAACTCAGTGAGAATCCACCAT
A_14_P110031 ATAAAATTAAGATTAACACATTGCCTCAGATGACACACAGAGCATGGACTTGAACTTAGG
A_16_P00685219 AATCCGCTTTCTGCTTTCTTAGGCACTAGGAAGGCAAGGAGATACTTG
A_14_P103443 GCCTCCTTTTATCTACCATACCTATCCAGGGGTCTTTTTTCTTATTTTTGGCTAGTGATA
A_14_P131128 TTGTTACTCTTTCTCATGGCTGACCTTCAGCATAATAGAACAGGCTATTACTGAGCATTA
A_14_P121674 TGGTCCTGCAGTGTTGCTGTAAATCCCCATTGGAGACTGCATTAG
A_14_P117809 AACCCCAACTTCTATTCCATTAAATACCTTAGATGCATGTTCTAAATACTGTTTTCCTGC
A_14_P122061 TTAAAATACCAGTAAATTCAACAGGAACAGACAAGTTAATGACAGGTGTCATTAGCCCTG

A_14_P128706 CTAGAAGAAACTTCAGATGTCTCTGGGACTTTCTGCAAAGTCAATAGTAATTCTCCATTT

A_16_P20117248 ATTGCAGATTACACACCTAAACTCCCTATAGCAATCCCAGAGAAAACTTAACATGATGAA

A_14_P127356 GTTGTGAACGTCCAGCCACAAAACCATAAAAAAGGTAAATAAAACAAACCCAAAAGGA

A_14_P137826 ACTGGGGAAAAACTGGTTGTGTTGGATTGCATACAGAAAGCCAGATATACTGTATATTAT

A_14_P132860 ACCCAACACAAAAACTAAAATCTTCTCAGGAAACCCTTAGACCTCAAATTATTCCTAGAA

A_16_P16190994 TTTTTCACTCTCACACAACCAAAACATGAAGAAAGTTAAAAGTCTCCTTCAAAATCTCCC

A_16_P03746128 ACCTTTCTCTTTTCTAAACCAGGCTATACAGTTTAACACGGTTATAAGATAAGGTCCCAT

A_14_P100866 TGATGTTTTCTAAGGCCCTTTTTCCTAGTGGTGTCATTTTTGAATGCCTCATAAATTAAT

A_16_P20793229 ACGGTTTAAATGGCAAGTCTCAGTTTACACAATTACTTAGTGATGCACCTGATAACAGAA

A_14_P200033 GCTTCTTTTCTCTCATTTCCCATGGAAGGACTTCGGAAATGCCTTAAGTTC

A_16_P18977293 AAAGTATTATAACAGATTTCAAAACACTGTTTCCGGTAGCATCTCTTAGTAAGGCCAAGG

A_14_P100851 CGAGATGTATTCTCAGAGCCCAAAGACAAGACCTTTGACAGATGCAGG

CUST_7766_PI426210822 AGATCCAGATTTATCAGTGGTTTTGCAGTGGTTTTATCCATTTTTCAACATCACTTTCAT

CUST_7503_PI426210822 CTGGTATGGTACATAGTATGACTATGGTAGTCCAGTATTGGATTCCATAATTTAAATAAC

A_16_P17055455 AAATTCTTAAGTCACAGTTTGTTCAAGGACAGTCTCAGACAAAATAGCGTTCACTAAAGG

A_16_P20093936 ACAAACGTTTCTGAAGCAAAAGAAAATGTATCACTGTAGAGAGTCTAGGACCTTTAGTGT

A_14_P104939 CTCCTGGCAGGGTATCAGAAACTCGTAGGTAGTTTCTATTAATAGATTGTTATGGAGTCA

A_16_P18153932 AATGTCAACTCATCACATTTCAGTTAAGCAGACATCAAATCAAATGACTCATGTCACAGA

A_14_P201378 CACTGTGTCTCCATCTCAACTTATCCTTGTTTCTGTGAAATACAATTTCATCTACTAGGC

A_16_P36231101 TCCCAGAAATAACCATGATGAGCATAGTATTGCCTCATTTTGACCTTTTTCTCTGCATAT

A_16_P19570888 TTAAGTGTGTTGCTACATCTTATTTAGGCATACCACAACATCTATTTAACCCACCAACGG

A_16_P19750471 CGCCACCCTTTAAAGAAATTTCTCCTTTATAGTGTTTTACAGTAACACCTTAACCTTTGC

A_14_P137775 GATGTTATACTTACTTTTGAGTCTATGCATGGGTTGGACAATAGTCAGAATGAAGAAGTC

A_16_P00124386 GTCATGACTTTCAGTCTGCTGTTTAGCAAAATTTACAAACACCTAGAGGTAAGAGAAGAG

A_14_P116350 CTTTTGAGGTAAACAAGTGAGAAAGAAGAGACAATAAAGTGATCTTTCTTTACCATCTAC
A_16_P19740543
CCTAACATCTCAAGGATCCTAAGTTTTACACCTTAATTTCATGTTACCAGGAACTACAGG
A_14_P135182 ATTTCCAATTAAATGTTATACAAAATCTTGACTGGGAGTTCTGCGGATTGCCAAAATCTC
A_14_P108792 CGCACCCCTCCGTTCTTTCTGCATAGACTCACTTGTTAAATAGCAGTT
A_14_P123680 TGTTGTTAGGCAGCAGAGGCCTTTCTTTATAAAACTCCATAATGACCTAAAACAGATTTG
CUST_411_PI426210822
TGCTTACATCTAACTAAAAATTATTGCCTGCTGAAGAGGTTGAATTGTCTTTCTACTCAT
A_14_P136524
GGAACCACTGCTACTCTCTAAAAAAGGCAAGCAGATAAAAGAGAACACGAAAAATATTCC
A_16_P18174193
ATACTAGGATAGACTATGGAAGATGTGCTTACTGAGTTCTTACCCTCAATGTCAAAAACC
A_18_P14746970
TATCTGTGCCAGAAACATTAAATTGATTGCGTCTTTCTGATCATCAAATAGACAGGTTGG
A_16_P19807473
AAGGTCTACATGGTATGGCATTATTTGCAGTCATGATAAATTCTTATCTGCCCTTTGGTA
A_16_P17439820
CCTGGAGTTATCTTTATCACCTCTCCTCAAGTTTTATCTTGCACCATATAAGTCACATTG
A_16_P19623555
ACGTGAAAATCTAAATAATCCTTACCTTCCTATCCTTCTATAAGTATCACTGCAGCACAT
A_14_P105944 GAGGTCATTAAAAAGCCTGTGGTGAACTACACCGACATAAACCTTTTATAGAAACAATCA
A_16_P20368816
AAGAGACTACCAAGGTGTAAATATTATAGGAGCAAAAAGGTATCACTTCTCTTACTGGGG
A_14_P104910 ACTGGACAAGTTGGTAGAGAAGATTAAGAAGTTACCCTTACTCAAGGGCCATC
CUST_5252_PI426210822
GAAGATTTCTCGTTTCTAGGAGTGTCTACAAGATTATCATAGGTGAAAGTAGGGTTGGA
A_16_P37431659
AGCACACAAAAATCTTCAGTAGTTTCATGTCCTCTTAGAAGCCATTCATAATCTACAGGT
A_16_P03649137
TCCTACCACCTATTGACAGTTACTCTGATAGCCTTTCATTACACATTGCATTTTGATGTT
A_14_P133164 AACGTTGGCAATGAAAGATATGTCTTTGAAGCAGTAATTCGATGGATAGCACATGATACA
A_16_P19308469
AAAAAAACCCACCAGGATTAAACACTCATTCAGATTCATGCTAAATGTCTGATCATTGTG
A_14_P202557 ATTGTGAAAGCAAACCTCCAAATATTCATTTGTAAGCCATAAGAGGATAAGCACAACCAT
A_14_P121162 ACTTGTTAGTATAGGTATGTAAGCAGTGCTTTAGTTCTGCCTGGTGTAGTTAAATCTGGA
A_16_P21661102
CCTTCTCACTCAGATGAATCCAAAGTTTTATGCTTATACTCACTGGTTCTCTATTGTGTG
A_16_P16514585

ATTACCTGAATTTCACCATTCTGTAAATCCAGTCATTGCTGTATACTTTTCTAGCCCTGA
A_14_P201695 TAGTCACAAACCTGTGTGGGAAAAATACCACTAGCAATAGATTATAAAAGGCCACTGAAA
A_16_P20587611
AAAAGAAGTGGCTTTTTTCTAAGGTTAATAGCAGATTGTTCGTAGTTCATACCTGAAGGC
A_16_P16632388
AAAAAAAAACAACAAAATATCTTCCACCTTTAAGACAATCCCTAACACTGCTCAAATAGA
A_14_P108236 CAAAGTGCCCAATATACAGAAGAGTTGAGTTCCTAAGTAACTCGCTCAGAGTCG
A_18_P14624068
TCAACCAAACACTGTAGGTAAGTTGTTTTTAGTCTTCATATCAATGATGCGCTCACAATT
A_16_P00018114
GGCTGACAATTTGGAGAGAGATGAAAAATACCACAATTGCTTACAATAGAACCTGACAAA
A_16_P36195989
AGTTGGGCTATTAATCATACATTTACATTCGAATCATCATCCAATTTCTCAGTTGTCAGA
A_16_P17143357
ACTTCTGATATAAAGACTGTGATACTATCCTCCCCTATCATTACCTCAACTACTCAGTGG
A_14_P118140 TTATGTTCCCATTTTATCCTATGAAATGCTAGAGGTTTCCTTTCACTATGCAAACCACTG
A_14_P111126 ACTTCTCTCCTGATTATGTCATTACTACCTTCCTTTCATGACTTTTGTTCATGTCTCTCA
A_16_P00340136
CTCCTCCTGGCTTTCTAAATAACTTTTGCATCTTTCTAGTCCTAATCCTTCCCTTTAAAA
A_14_P111178 CTACTTTCTTTTACCTCCGAAATATATACCCTTTATATCTCTTTCCACTGCCTCCAGGAA
A_14_P139771 GAGGGCATTAATTGCTGAACTCAACCAAAAAAACATTCTTTTTCTGCCTGTTCATTTATC
A_14_P116880 GACATGGGGCAAATAGGAATCATGAGGATCTATTTGCTAGATCTGTGTATACCACAC
CUST_8750_PI426210822 CCAAAGCTATCTTACGGGAGTTTGCAATGAGAATTTCTTTGCCCATGGA
A_18_P22841013
TTAGAAAAATACATTAATTTATTCAGACACTGGAGGCTTGGGAGTAGTTTTGCAAAGGGC
A_14_P105412 CTCATATGCTTGAAAACAGAAATACCCACATAAATGACCTTGAGATTCCTGATTGCATCA
A_14_P105284 TTGTGGCATTATGAGTAGTTTTCTGAATTTTTCCTTCCCTTTTTGATATGTCAGACCTGT
A_16_P19881029
CAACCATCCCTTGAAATAATCCTAATCAATCTTTTAACCAGTAATACACAACGTTCCTCA
A_14_P125981 CTTGTCAAAAGTCTGTTTACTAACCAGAATTTGGTTGGTCACATGTGTAGACTCTGTAAA
A_18_P13621937
ACCAGCAGATATATAATCCTACCCAGTATCATCCTATTCAACATTAAAGCAGAAAGTTCA
A_16_P16186162
AATTCAGCATGTGTATCAGCATTTGACTGCTTAAACCATAAAACTGAGTTAGGATCCCAG
CUST_364_PI426210822
CCCATAACTGAGCATTTCTCTCAATTATTTTGTAGACCAAAATTGTCACTAATCGGAGGA
A_16_P18342224
GGACACCATATTTGACCAAGAAGCAATATCTACATCTTTTACCCAATAACAATGAACACC

A_14_P101645 CTTACTGGTTTGTTGTTAGGCAATTAACAGAAAGATCTGGGACAATAGGCATCACAAAGT
A_14_P127059 AAGTGGTGGTGTCTTTCTCAGTTTATATAGTCACATTTAACTCTTACTGGAATGGAGGGA
CUST_3522_PI426210822
GAAAGTATGGAGCAAATAAGAAATGTGGGAAGAGAAGAGGGCAAATGGTAAGG
A_14_P106910 ATTAGACAAAATTCCAAAGCAGATTTAAAATTATGCCATTCTTGGTATTTCCTGGCTGGG
A_14_P119715 ATTTCCTGGATAACTTGATTGTCTTCTATGTCCTGTCCTATCTTCAACCAGTAAGTAAAT
CUST_6642_PI426210822 GTTGTTCCATCTGGTGCGCTTGCTGACTTTCATTATTGCCTGGAATAG
A_14_P116067 ACTGTAATAGTGTGATTACTATGCTCTAGAGAAAAGTCTACCCCTGCTAAGGAGTTCTCA
A_16_P58424563
TATATATGCCTAAATATAGGGCAAGCCCCAAATAGCACATCCTCCATTTCCCTAATTAGA
A_16_P30952618 GGACAGACTGCCTTGGGGTCACAATGGCTGATTCTTCAGCACTTC
A_14_P201785 TGGCTCACTTTCCGAAAGATGGAAACTGCTGGGAATTTATTATTTTTCCTCTCTATTTAT
A_14_P125569 CTTCCCCCAAAAGTAGAAGAAGTGCTATGATTTAGAAATGGATGGTGCTTCTCTATTTAT
A_14_P132832 GAGCACACCAGAAGATAACACCTTTAGTCTCAGTCTTACCTCTTTTGCAAAAAGCATTAT
A_14_P123396 CAGACATCCTGCACTTTGGCCAGATCCTATCAGACATCCTGTTACTG
A_14_P126188
ACAATTCTCTGAACAAAATAAAATGCAAGGATTTGCTCAAGGAATACAAGAAGGGAGAAG
A_14_P104440 ATTGAAAAATAAATCCCCTACTGTTAACTCTAGATACTGCTTACTATTGTGTGATGTGAT
A_18_P22841709 GATCCAAAAAGCATCTTCTCGGGGTTTCTGGGAAATTTCTCTGAAGATCT
A_14_P200202 ATGGGCTGGAGGAATTATCTGATGCCCTAAACTGTATAGGAGGTTGTCAG
A_14_P121818 GAGTTGCAGGCAGAGTGTTTTAAAGGAGACAGAATTGATCATTTATCTACCTTTCTTGAA
A_16_P00613071
CTGCTAACTCTAGTGACACCTTCTAATCCACTTTGATTACAGACATTTTGGTGTATCACC
A_16_P58424460 CCGTTGCTGCCTCTCTGAACATGTGTCCTGTCACTTTCTTCCTTG
A_16_P36579106
CTAAATCCAAGTAAATGTGCTAGACTGAATCTCGAAACCCACTTTTTTTCAGATGCCTAG
CUST_9956_PI426210822 AAGGAGAAAGGGGTGTCCTCATCCCCATGTCTAGGATGATTTGTT
A_14_P123699 ATTTTTCCTTATTGCCATCAGTTCAACCTATTTGTGAGGGGGTGATTTTTACTTTGATTT
A_14_P200506 ATAATTGGATCTCTGTGGATTCTGTCACATCTGAAATTAAACTTGCAAAACTTCCTGATT
A_14_P130547 TAAAGACCGTTCTGGTAAGATAAAGTGTGTGCCAACAGTTTAATTCAATTTTCACATCAC
A_14_P103730 AAATTTTCAACATGCTGTCATGGTCCTCAGGAATTAGTCTTGTTTTTTTCTCTGAAAAAC
A_16_P21659167
TAAATGATTGATTTCCGCTTCTATCTTTGTGTTCAGACAAGAGAGTTCTACCTGTTTGAC
A_18_P23423426
AAATTAACCTTTGAAATGAATGAACGTAGCACAAGAGTTATATTCTGGTCCTGAGAAGCG
A_14_P132699 AAGAAGAGGACTATATGTCTGATTCCTTCATTAATGTCCAGTAAGTAAATGTGCACACCC
A_14_P136357 TAAAACCAGGGCTCTAATTGGGACGTAGAAATATTTTCATTAGACACCGATTTCTCTTGT
A_14_P101657 TACACTTTTGATGGGAACGAAGAGGATAAAACAGAGACTATAGATTACAAGGACCCGT

A_14_P104135 AAGACATTAACCACTGCTTAATACTGCTACTGATGGCAAATTCTTAAACTGGGTTCATTT
A_14_P106325 GGATTTCCATTGTAGGTCTGCTCATTGTGTTTTTCAGGAAAAAGGAAGGGAAAG
A_16_P00685521
TCCTATGGGCAAAATCATCTATTGACCAAAACAAGACATTTAAGTCACTCAGATTCTGAA
A_16_P20235776
AAAAATCTGAGCACTTTCGAGAAGTTCCATCTGTGTTAAAAAGCCTTGTATCTCATCTTC
CUST_3622_PI426210822
CTCTTGTTAGCCTTTTGTGAGTTAATTCTTTGTTTAATAACAAAGAGCTGCCAGATGGTA
A_14_P122649 AAATCATTTTTAACTGCAGTCTCACCTTACACATCTCACCAGCACTGTAGTTCATTAGTG
A_16_P18773751
TACATCACTGTCAAAACTGCTTTCTATACGTATCTGCATTTACACATTAGTTACCCACGC
CUST_10140_PI426210822 TAAAGGAAGAAAAGTCCAAATGAGTCCCCTCTCCTTTCCTGCAGC
A_18_P16694304
AAGTGTCCAGTTTAACTCACCATAACAGAGAAAAACATTCACTAATCAAGGCAGAGACTG
A_16_P02498753
AAGGAGCAAAAGATTCCCTATGCACAGATAGTTCTTTATCTTTATACTACGTAAGAGGCC
A_14_P107351 TGCTAAACTGCAGTTTTCACAACTGGGGGTTTACAAAAAAGTCTGAAAAGATGAGTATTT
A_18_P22841643 GTTAATTTACTCTCTGACAACAGTGGAGACACCACATGTCAAAATGTCAGAATGGG
A_16_P17883710
ACCAAATGCTACCCTAAAAACTTCTCTGTAAAGTCATGACAATATCCTCTTCTTGCAACT
CUST_6100_PI426210822
CAGGACTGAAAATTCCATTTGTAAAAGGATGCAACTATGTTTTGTCTTTTTGAATACTCA
A_16_P39973432
TTCTTATTAAGGCAAAATCCCTATTAGGCTAACACAAGAAGGATGTTGATTGCAGTAGGA
A_16_P39059572
TATTTGCAAAGCCACATATTAGACTCACTTTCTCTTGAAGACAGGACTATCTCAGCATGA
CUST_9555_PI426210822 GCCTGCCTCTGAAGGCCACGGGACTTACCTGTTTGTTTGTCAAAG
A_14_P135855 TGTCTATTTTGATTATTTTTAACCTCCAGGCTCATGTGGCCTGGAAATATGCATTACATC
A_14_P113236 TTATACATGAGGCTTAATTATTCACAAGAGCAGGAACTTTACTAGGAGGACTCATTCCAC
A_14_P120057 TGTATCTAGTTTGGATCATATATGAACAGGTGATGATTGCAGCACTAGACTATGGTCGG
CUST_7332_PI426210822 GTATTGGGCACAGCCACGAGTGTCATCCCAGGGAACTCATGTGAG
A_14_P132559 TTTGTCATTTCCTAGAGTAACCTAGAGAGAGATTTCCAAGCATTTCCCCATGAACA
A_16_P16191865 CAGTGCCCTTGGTGTCTGTATTGCCTGTGATCATCATCCATAGAGCATAAT
A_16_P39066666
AGATAAGCATTGGTCTTCTTAGGATAGTATGCCATATGTTAGATTGCCACTGAAGTCTCT
A_14_P200381 GTAGAGTGGAACACAAAAATCTTTGATGTGGGCACTGAAGTCTTTATATCATATTTCCCA
A_14_P125239 CTACAAAGTGAGGCAGTCCCAAAAGATTTTAGGTTTGAATTTTATCTTACCCCATTGAAT
A_16_P00439521

TTCAACTTCCATTCCCAGATATCAAGGCAAATTAAAGACACTTAGCAGAATACTTTCAGG

A_14_P127003 ACAAGATTTATTCGAGGCAGGGAGATTTATTTAGCACATTTATGCTCAACAGAGAAGACA

A_18_P14099642

TACTCACACAGCATTTAGTGTATGCCATTCTTAGTCTTTTTAATAGCCTCGATGTCACTG

A_14_P105265 GTGGACTCCCAAATATTTGGTCCTTGGACAGCATAATGAAAAACTCCTCTTCTATATAAA

CUST_6010_PI426210822

AGTGTTTTAGGGAAAAAATAACAAGAAACAAAAATATATGGATTTGTTTTGTCTTGAGGC

A_14_P100614 TGCAAAACCTGGATAAGAAGATTGATGTGATTCGTAGAAAGGTTTCAAAAATCCAACGTT

A_18_P15516829

AAGTATTCTTACCTCCCAGGTATTTGATCTCATCATACATTTTTCATTGTCAGCAGCAGA

A_14_P101720 AGGTAGGAGAAAGAAACTACACAACTACACACAGAATGCTTGGATTATATCCTTTGGTAT

CUST_5704_PI426210822 GAAGTCTGGCCTTATCTGTCTGTCTCAGGCTTCTTCTCCAAGGTT

A_14_P116007 TGAACTTGTAAAACACTGACATATACCCCAGAGAAAGGCATTAGATCTTCATGTCTATCA

A_14_P117116 AAATAGTTACATACTTACTTTTTGTCACCTGGGACTCTGCACACTTTTGATATAGTTTGA

CUST_10414_PI426210822 CTTTAAAGATGAACTCTGAACTTTGGAGAGTGAGCTACAGAGCCGAGCAATA

A_14_P138108 CTAGCATTTGGTGGGAAGATTTCTTAGATTTTTCATGTTAGTGCAGTGTTTGCCAAATTT

A_14_P135635 CTCTGCCTTACAGAAGACTAACTCTGCCACCATCTGTGAAAAAAATACACACATG

A_16_P18824161

AAAAAGGTAACCAAAAGTCAGACCTAGAATGGGATTTAAAAAAACTCAAACCTTCATGCC

A_14_P107663

TTGACCTCTGCAACAGAGTCTAATGAATCAAGCAGCACAACTGGAAGAATATAAAAGAAA

CUST_4069_PI426210822 TTTCTTTCTTGGGTGGGAATGCCTTGAGCCTCAGTTAATTGTTTGAGTTTT

A_14_P112666 ACTTTGAAATCCTTTTTCTGAACGAAACTGAAGGAGTCCAGAGTGAATTGATAACTCTAA

A_14_P118737 CCAGGAGGTGACAAGGAAATACCAGGAAATGACGGGACAAGTTTG

A_16_P30952457 TTTCCTTCAGAGCATTGTTCTCAGGCTCCTTTTTCTTGACCCCAG

A_16_P01367104

AAGAGCCTGTCTATTTTTATTACAACCTACTGTGTCCTTTTTGAAAACTTCTCACTGGTG

A_14_P105802 GTTTGTATGCACGACTTGAAGACACGCACTTATCATTCAGTGGGTAAGTAGAACTAC

A_16_P18323102

ACCATAAGCAGCAAAATAGTCTTCAGTATCCAATAGGCAAATGTCCTATGATTTCACTCT

A_14_P129707 ATAAATAAAGCCAAACAAAACTGTTTCTACCGCTGCGGTGGATGATTTTTTAAAGGCATC

A_14_P100181 CTCATCTAGTGAGACAGATTCCAAACACAGCCATTATGATGGCCACCTATGAATT

A_18_P13104356

AACCCTACATAATGTATCTTTCCACACACTCAATTCTCAAATACATCCAAATAGTCTTCA

A_16_P01032730

CGGTTGCTTCTATCTATCAAGTTCCTTAAAAATACAGTTCACACATAGTGCTCTAGCCAT

A_14_P105183 AAGATATTCTGTCAATTTAACCAAGTCATTTCCTTCTTAAGGCCTCTGGTTACCTTTCTT

CUST_2709_PI426210822 GACTAAGGTGTTGGCCTTCTTTCAGGCACTGCTATTGTCTGACTTC

A_16_P30952384 CGGTTGTGTATGGGCTTCAGCAAGTTTCTGAACGCTGTAAACTTCTCA
A_14_P138385 CAAAGGTGATGAGAAAAAATCTCATGCAAACTCCGGGCATACAATAAAAATAACTCAAAT
A_14_P109961 ACCTGTGAAATAGAAAATAAACCCTAGACAAAAGACTCCAGAATACTGAGATGCAAATGT
CUST_7791_PI426210822
TGAATCCATTTTAAACATGGGATTTTATTTTTATTTAAAGAATTACCTTGTGGGTATGTC
A_14_P112428 GTCACTAGAAATAGACTTACTGACATACACACGCAAAAGGCTATACAGAATCCCCTAAAG
A_14_P123000 GTTACCTGATTAACACAGTTCCTTATTGGTGAGGGATTCTCTACTGTGGTCCTT
A_16_P41647032
ACCCAGAACACTCATACTCAACAAGTCCCACAATTAATTCATTGTCTTTTTCTCAAATAC
A_16_P39874098
TGCATTAGAATTGGTTTTCTAAATCTTGACACTATAGAAATTTTGACCAAGTGATTATTT
A_14_P115829 CTCGTCAACGAATTCATCAACAGGCAGAAACGCAAGGAATTGTCCAATAG
A_16_P18194649
CCTTTTAAATGTGTTTCATACCATCGAATTTCAACAATCCTCTCTTAAAAAACCCGTCCA
CUST_1055_PI426210822 CAGATGGTCTCTTGGTCCCCAAGGCCCTGCCCAGAGAAGAGGCAG
A_14_P105835 TGGGATTTAGGTATACATTCCAGAAAGGTTGAGTTAAATTCAATGCCAAAAATCACCTTT
A_14_P112286 CAAAGCAGCCATTATCTAAGAAATATCACTAGTCTTTCCCGACATCTCCCTTGAAATCAT
A_16_P17014283
ATCCTTGGTCCCACATTTTAAGAGAGAAATAGCAATAACTGTTCCAATTTTATCTCCTGC
A_18_P22840511 AATATCTAGTGAGGGAAAGGGGAGAGGCCATCTGGACTGGAGATT
A_14_P117167 TTTCTGCCTCAGATATAGAGTGGCATTAGGTTTCAATTTTCTGTTCTAACCCAAAGTAGA
CUST_432_PI426210822 GAAGGTGTAGGTCCGCTTCCTCATTTGAATTTTTCTAGGGAAATGGCATT
A_14_P131700 AGACTTACGTAATTTGTAGCCCACCAGGATTTTAACTTCAAATACCACTGTAATCTTGGT
A_14_P101928 TGCTCCAACAACCCATTTGCCTGACTTCTATGCTTTGAGTGTATTAATATTTTGAGGATA
A_18_P22841586
GGTCAAGGTTTTCATAATTGAGTTTTAGGGGGGCTAATTTTAGACTTTCTGAAAATCAGT
A_14_P118076 CTTATTTCTCCGGCATCTTATAAAATTGTTCAAACCTACAAAAAGTGAGAAGAATCATAC
A_14_P119268
TAATAGGGACAGGAGACAAGCAACAAACTCCCCCTAAGTTAAAAAAAAAAAAAGCATACA
A_16_P30952228 CTACGCAGTTCTGGAGCATTTCCACGACAACACGCGCTGGTGAGT
CUST_1787_PI426210822
AATTTAAGAACAGACATAGGCTGTCTTTGCTTGTGTAGATGACAATTACAAGACTCTTTT
A_16_P19805461
AAAGAAAACATATGAAACAACTCACCTGTGATAGCAAATCTCTTTGAGTCAGAACCTTCC
A_14_P110279 AGGCGCTCTGAAAAATCTTTGAAGACACATGAGGATATTGTAGCCCTTTTTCCT
A_14_P126352 GGGGTTAATTAATAACAGATGTAGGTGGGACCACTGGATATTCAAAGATCTTTGGAAATA
A_14_P101823 TGTGATTTAACCAAGAATGCTGATGTTGAATTCTTTGGGCCTAGAATATACTTGAGAAAG
A_16_P01333278

TGCCAACCAATTGTATTCTTATAATGCTGCTTGTTTTTTCCAAATCAATTAACACGTGCC
A_14_P138370 AATCCCTCAAGGGAAAAGCAGAGACTAAATCAGCATCACCCCACTTAC
A_14_P138023 TCATGCAGATTACGTAATCAGGATGTATGATTTTCCAAGCAGTTCATGAATCCTGAAAAC
A_18_P22840357 TACAAAGGTTCAGTGCTTCACAAAGGAACCTCTTCAAGTCAGGGCTTAG
A_16_P58424474 GATGTAGTTAAAGGGAGGGTTGGAGAGTGGGCGTTTTTGCAAACTATC
A_18_P16378618
AAAAAAAAGAATCCTGCCTAGAAAACAAAATTTACACATTCATATGGTTATCTCTGGACG
A_16_P20881116
AAAGGTGAGGCTACTAACAGTCCTATGAATATACTGTGTCTACCACTTTAGATCACATGA
A_14_P111938 TGGGTGATTTTTCTTATCTTTCCATTTGGTAATTCTAGGTGATCTGGTTTAGTCAGCATG
A_18_P15969228
CATCCATCGTTGGTATCAAATAAATAAGCGTTAGCAGTTCATAAAGACTCTGTCAGCAGA
A_14_P115846 AATAAACCTTTAAGCAGTTTTCAAAGTCAGGTTAACCCTGATATGTATCCTGGAAGGGTA
A_14_P118628 CCGATAGGTGTGGCAGACCAAATGGACATTTCTCTAAAAAACCAGTATATATAGTCTGTT
CUST_946_PI426210822 ATCCTTATACCTTGAAATGTGTCAGATTCTAATATTTGGAGTGGGACAAGGCCC
A_14_P201992 GAAGAAGTAACATTTTTGCCAAAAGATGACTGGGGTTTTCCGTTTGTTAATATTAAGCAC
A_16_P20954036
AAACGTATAATTGACATTAAGCAGGAGTTACCTCTATCAAGACAATGAATGTGGATGGCA
A_16_P58424523 GTTACCTGATTTATCTGACTTGCTCCTTACCTGTCAAAGGTGGGTGCTC
A_14_P107120
GGAGAAGACCAAAAAATTCAAACTCTGGGGAAAAAAATTACTATGAAAAAAAATCAGGGG
A_14_P120883 GATCTGGACTCGAACTCCTTTGAACTGGACTTACAGTTTTCCGAAGATGAG
A_14_P135875 ATAACAGGCATGTTCTTGTTTTCTCCTTAGGTGGCCATAAAAGTCATTGATAAGAAGAGA
A_14_P103274 CTGCTTTCTCATAATAAAGACTATTTGCATTTGACATCTGTTCCCTTTCACCGGCAAC
CUST_4360_PI426210822
TTTTAGATGTTCTAATTGATACCAGAAGCAAAGACGTAGAAACCGTCATGTTATAAACAT
A_14_P123575 CTTGTATGTTTCTTTCTAGCAGAGCCACATTTTTAAGGCAAATGAGTTTGAACCTCAGTA
A_14_P201296 TAAGAAAAGAAATTAAATCCCAGAATTTATTGCAACAATCCACAACCTCGAATTGTTTTT
A_14_P108453 ACCAACACTTCTGCTTCTTATTTGGCATATGTCCAAGTTTCTGCTGTGGTAACATTT
A_16_P00333217
ACCTTCCAAGATATGCTGTTTAAATGCTCTTTACCTCATCCAAATTTGTCTACTATGGCA
A_16_P00380366
GAAACCATGTTTTATTCCAGTTACCTCTGCTATCTTTTATCCCCAGAATCTCATATAGGC
A_14_P122393 TCCATGAGAGTGGAATATTTGGTTCTGGAAGACCTCCTATTAGTAGTGTGTTAATCTTTT
A_14_P110795 CAAGGATTCCTTGAAAGAAGACATTCAGAAAGCAGATCTTGTTATTAGTCACGCAGGTA
A_16_P19647957
ATAGTTTCATCAAGTGGATGTTAAGGTTTCATGCTCTCTGATTCAGTGAAGGACAAAATC
A_14_P111203 TGTGGTGATCTGGCTCTGGAGGTAAAATATTCAGAAAAGGTGATTTATGATATGTGTTTG

A_14_P137417 TTGCTTCTTCTGCATATGTGAACCTTGTGAATATACAGTAAGTGGTAATGGGTTTTTTCC

A_14_P131843 AGTCAATAAACAGCAAACAAATCATAATATACAGACTCTGGCATCAGACTGGCAAACAGA

A_14_P129612 TCCCATCATACCTGTGTTTCCATTTCCATCATCCTTTGTTTTATTTCTCTTATTTCTGCT

A_16_P21603386

CTCTGAAGCAGTGTTTTCTCTATCCACTTCATGCTATGATGCTAGTTCTTCTGGAATATT

CUST_6590_PI426210822

CCTGATCCCTTACCCTTTACATATAAGGAAAGGAAGCTGATCACATGAACAACTAAA

A_16_P40579846

ACAAAGGAGTTTAGAAATCCAGGCACACCAAAGATATTACGAATGTTTAAGTATAACTGA

A_14_P102306 GATGATGGATTTGTGGACATCCTAGAGAGTGACTTAAAGGTAAACAGCCTTGTC

A_14_P110644 TCTTCTTTTTTTTTAAACAAACAAAACGTATTCTCCATGAAATCGCCCAGTGCTTTGGT

A_16_P17216047

ATTTTTGTGCCCAATGTAATCATCACAGAACATACTACTTTTGACCGGTAATGAATGAGC

A_14_P114478 TCTGGCTAAGTGAGACATTATTTTGTAATTGAGACACTCCTCCTTATATGGTCAGGTTTT

A_14_P116968 ACAATTTGAGGAGTTTACTTACAGATTCAGAATAAGTTCATGGAGAAAAGTGCACATCTG

A_16_P20018145

AACTGGTCTTTACTATTCCCCAGAGAACAATATCACTACAAAGCACTAAATTACCCCAAT

CUST_2234_PI426210822 CATTTTCTCCTCCATCCTGTTTCCCAGGGAGCCCTCGAGGAGCAG

A_16_P02972996

AAAGGAAGGCAACTAGACTTTGAAGACAACAACTCTTTCAGAGAAAGTAATGCTCTACTC

A_14_P127577 CATCCAAACTGTGAAAAATGTGTAGCAGACATGGTTCCTTAGTGTTTCCTCCATTTTATA

CUST_3995_PI426210822 ACTCATCCTAGGGCATTCCTGCTTGGCAGTAGCAGATTCTAGATTGT

A_14_P135076 ACTTGGTATATGTATGTTGAGAAATCCCAAGGTGAATCAAAGGTCTGAGGTATTAGATGA

A_14_P135876 GCTTCTAGTTGAAAATGAACTACCTCCACCAGAAAATATAGAAGTCAGTGTCCAAAATCA

A_16_P20089675

AAGATGAACTAAGGTTACAAAATCATCAGTGCTGATCATGAGATTTTTCCAGGTGATGTG

A_14_P129512 CTTTAAAACCATGAATGAGGGCCATTGTTGTGTAATTCAAGTTCAGACATGTTACAGGAT

A_14_P139048 CCTTGAAATGATTTATGGCTAGAGGGAATCTGGGATCATTTAAAATCTGTCATAGAGGAA

A_16_P17810024 TATGTGGAAGGTCCTCACTTCCTCATTTCTGCACCATCCGCACTT

A_16_P01581136

AAAAGGAGAGTCAATGTATTACAAAGTAAGCATATTCCATTAAAACTGCAATTCCACCAT

A_14_P133857 CCTTTCTGTCCAGAGTGACTGTCTTCCTGCTTTTCTGTGTTTTGCAATT

A_14_P109663 TACCTCCTCCCATTTTCTAGTGCAAAAATAACTCCCTAAGGAAGAATTGTAGCAATGTAA

A_14_P122642 GACTGTCTGCTGCCATCAATCATGGAATTCTCACTGTCGTTTTTCTCACAG

A_16_P41024956

CAAATTGCATTTATTTGCTCATTTCTAATCACATCCTAGGATCTCATGCTCTCTTGATGG

A_14_P108747 TTATACATTTCTGCCACCTTCTTTGAAAATCTGGCCTTTATTCAGAGAAAAGACTCTTCT

A_14_P121715 TATAAAATGCAAACATCCTTCCACTGCTCTCAGTTCTTCTGTCTAGTGACAAATCATCTG

A_16_P20374933
CCAGCCTTTTAGCATTTGTTTTATGTCCATATAAACTTCTTCAGTGTAATGTGTTGTGTG
A_16_P01917763
AAACCCACTAGGTAAACTAGGTTAACTCAGGTTGTGCTGTTACTTAAACACAAAAGTGTA
A_14_P103935 CTTAGACAAATAAAGGCAAGTGCTGCTTCATAAGATGAGGTTCTTAAAAACTGTGGTTCA
A_18_P26731679
AAATGGTGATAAATGCAGGAATTACTCACAAGGATCTTTTCACATATTTTTCCTCAAGCC
A_16_P17108841
AAACGTTTTGCAACTCAGAAATATTTTCCTGACCTCTAAAACTGAACTTGACATTGCTGT
CUST_10215_PI426210822 ATGCAGAAAAACATTGACTCCATTGGCTTGGAAGTGAGTGTGGGG
A_16_P21541975
GTGAGCACATGTTACTCAAAATTCATTGATCAATTCAGGAAGCATTTGAAAATTGCCCAA
CUST_8626_PI426210822
CTGTTTGCCTAGCTCTTATACAAATAGGCAAGAGTAAGTGTTCCGTATGCCTTCT
A_16_P20623112
AAATCCCTCCTTTTCTAAACCATAGCCAAGTCTATTACTGTTTAACAATGACCATCTTGG
A_14_P105517 GGCTCTTGCTATATAATAGGGTCGATCACAACATCTGGGAAGTTTATACCAATTTAAGTG
A_16_P18869976
AACGCCTTCATTCCAATTAATTGTCCCTTTTAACTCTTTCAGTATTTCCTACTTAGCAGC
CUST_6776_PI426210822 TAGAAGAGAGCAAAGGAGGAGGGGCAGGAGAGAACCCGGTAGGAT
A_14_P200254 GTTCCAAATTAACTCTTAGAAGAAACCCCACAAATAAAGCAATTCAATCAAGGCTGTTCT
A_14_P136683 AAATACCAATATAGAGCCTGCTTTTCTTTTCTGATGCCTTGGTAAACGTTTTTGGAGAAA
A_14_P130871 TTCTGGTAATATTGCACAATATGGGGCTTGAATCACATGACATTCAAAAATAGGTTTGGT
CUST_4703_PI426210822 TTGTATTTTGCTGGCACTGGGGAGCCATGGAAATTTCTGTAGGAAG
A_14_P132747 TGGCAGAGAGAAAATAGTGGGAAAAAAGTATTTCCAACAACATCCTCAGAAGAGTTATAG
A_16_P16648484
AAACCATGAAATGGAACGATCAACCTTGAAATACGGAGTTTATTCATAAGCAAATGAGCC
A_14_P129313 GGGTGAAACTATTCCCCCTAAGACTGTTCAACTTGTAGATACATCAACTTTGAAATTATT
A_14_P105167 AGTCTAGAGAATCTGTAAGAACAGCCTAAGAGATGATTGCCAATATTTCTGTAGCACTTC
A_16_P35699543
ACTAACTGACAGAATCTCTACCTTTATTAGGCCAGTTTAGCCATATTGTATCCCAGAGAT
A_14_P128855 GGTGTGGCATGGTAGAAAATGTTACCATGCTATTTCTTTTGCCTTTTTCTACTTAAAGAG
A_14_P107220 CTTGACTGGAACACCCGATCAAATGCATAAGGCTTGGACTGAAAAACAAAAAAG
A_14_P105216 ATGCCTTAAAAAAAGCAACTTATTTACCTCATCTTCTGTGATGTCACCTTGTTTGTCTTT
A_16_P18813395
AACAACACCCACTTATAATCAAGCATCACTACCTTTCTTTGAAACTTGTAAGCATTCGTG
A_14_P130483 CTCCTGTAGAAAGCCCTGGTCAGTGACCAGTAGGTATAAGTGACTC
A_14_P138987 AGAACGAGTATTTCTTTGATCGGCATCCAGGAGCCTTCACTTCCATTTTAAATTTCTA

A_14_P105809 CTAGTGCCATCATAGGAATCATATTCATCAAGGAAAAGTGGAAACCGAAAGACTTTCTGA

A_14_P127680 ATAAGTATTCATGTTTCTGATTTCCCTCCATTAAAGGGAAAATCCAATGTTTTGGCTTGG

A_16_P02100628

AAACTCAGTTAGACAATAGAACGGCATTTGAATCAAGGATTCTCTCGTTGGAGTCAAATC

A_16_P00572557

ACAGATGTTTCAATAACCTAACTGCTAGCAAGTATTACCTGAAATTTAACCCGATTGTTC

A_14_P121991 AACACTTTTGCTATTCCTGAAACCTGGTAGAAAACCATTATCTCGAAGAAAGTGTTAGTT

A_14_P104283 ACAACACTTTCACCTGATTGATTTATGTTTTTTGGTTTGCCCAGATATGATCTAATGGTG

A_14_P120031 TAGTAACAATGAGGACAATAACTTCTTAGAGCACCTTCATTCAGGAGTTGAATCACAGTT

A_16_P37251673

AACCACATTAGCACGTCTACTCTTGAAATATTATAAAAACCCTTGCAAATTTTCTAACAA

A_14_P201451 TAGACCTCACTTTAAGCCTGCAAAATTGTTATGAAAATAATACTCCAGAGGCAGGAAGAA

A_14_P129771 AATATTTCTTGTATTGCACTCCAGGATGTTATCGGCTTCAATATGGCTGGTCTTGATTAT

A_16_P01649062

TGAAGGGTCTTACTTTAAATAAATACTGGCTTCTTAAGCATTAACTGTCCACGTAGAGCC

CUST_5625_PI426210822 AGTTGACTCCTAATTAATTGCTAGTTGGTCTCAGCTGCTGAAACAGAGGGTA

A_14_P200193 CTGAAGTTCAGTATCAGAACATGATGGCTATTCTTAGTAATCGCGGTGATTGATAACAAC

A_14_P109056 TCTTCAGATGCCGAAAGTTCAGTATAAATCGAACTGTAAACCATCCACATTTGCATATCC

A_14_P121724 CCAGTGGAAAATTAAAGTAGATGTAGACTTCTGTCCTACCCTTTGTTCTTTTCTCTTTGA

A_14_P137088 CAAAAACAGGTAAAAAATTTTCCTTATTTCTCCACTGACCTGTTTATATGTTCTCTCTGC

A_14_P122544 CTCTACTAGGCTCCGTAAGACCATCTTCAGATTGCTGACCTAGGGAG

A_14_P201387 GACTTAACTATTAAAAATTCCTGATACTGAAGGCAAAAGGCAAAGATCTTCCACGCATCA

CUST_6344_PI426210822

TACCTTAATTATCTGAATGTTGTTGCTAGGGAAACCACTATTTTGGCTCAGATCATCTTA

CUST_2536_PI426210822 TCTGTGAAGTAGTAGTGGGAACCCCAGGGCCAGGGATTTATATGG

A_14_P110253 AATGCTGTGACTCAGAACATTTAAGAGAACTTCGTCTGTAAGTAATTTGTCTTAAAGCCC

CUST_1455_PI426210822 GGTAGCAGTGTTGTGAGCTGAGGGGGTGGAGGAGAAAAGCTTATC

A_14_P134134 CATAGCCAAATAACCACATAAGCACGAATTACTTCAAAAGGAAGGTGATTTAGTCTGTCT

A_14_P115647 GAAGTATATAATATCAGAGGGTTATGAGGAAACTAGAGACTGAGTTCTACTCTTCCTATG

A_18_P22840902 GTAGTCCCCTGCTGTAATGAAAATGCACAGACAGTTCATGAAGTGAGGAATTAG

A_14_P125544 AGAATTTAATTTAGAGTCTTACTACCCTCTGGGTCTCCTGTGATCTTCATGTTTGTTTCT

A_14_P122279 CTGTAGTTCGTCATTTTATGCTTGAAAAAATTCTGCCCTTAAAAGTGCAAGTAACTCTGC

A_16_P01419552

TCCTGGTGTCAGAAATTACTCATTCGAAAAATAGTAGTTGTATATGTTCGCTCTAGCAGG

A_14_P114906 ACGTGGAGTCTCAAAACATTAAAATCTATTTGATAGGCTCAGGAATCCTGTGACATAACA

CUST_4883_PI426210822 AGGATGTAGTTTTTGGCAGATGTCCTTTGTAGGGTTCAGTATTTCTCACTCAC

A_16_P01113776

TTTAAAAGGCTAGTAAGGTTACCTGATGTATTTCCGGCTTTGATTCAACTTCTTGGAAAG

CUST_7419_PI426210822 CAGGAAGCTGCTTCCCTCCCAAGGGAAGGAAGCCACCAGTGTTTC
A_14_P114551 TTTTCTCATAACCAGTTTACTCACCAGATCACAGTCACCCAGATCTAATTTCTCTAATGA
A_14_P116070
AAATATTTTGGGTGAAGGGAGGTTGAAGAAGGAAGAGTAACATTTCAAAAATGTGAAGTT
A_16_P17500148
AAAGAAAACCATTTTTCTAAGGCAGTGCAATTCTTATTCTACCCTCAACTTTTGACTTCA
A_14_P129286 CTCTCCTTGCATAAAACAAGAGCCTGACTTGTGTGAAAAAGTGATAAGAAGATATTCAGT
A_16_P21626347
CCTGCCAGGTATCTGTTTATCCTACATACTTCCACAACTCAGAAATAATCAATACCTTGT
A_14_P113598 AGACTCTGCGTGGCATTAAAACAAAGAATTAAACAAAAACCTCCTCAGGAATCATTTGC
A_16_P36468276
AACTTCTAGGAATTACCAACCCTAACTGGATCATTGAACTCTCTGAACTTACTATCATCG
A_14_P115032 ATTCTTCTTTCACGGGTCCTTACGATCTTGGTGAAATTGATTAATTCCACATTTAAACTG
A_14_P130191 ACACATATGGCTGTAATGACAATTTGCCAAATTTTCAGAGATCACAATCACAAGGATCAA
A_14_P104499 TTTGGACACTGTTGATTTAAAAAATCTTATTTTCAGGATCATAATTACGGAGCCCCTCCT
A_16_P02484230
AGTATTCCCACAATACCTTCAGTTCACTTACATTCCCATAATACCTTCAGTTCGCTTAAA
CUST_5003_PI426210822 CTCTGGGTGTGGATGGGGGCATCTGTGTACTCTTTATGGCTATATTT
A_16_P16191340 CTCCTCCTATTCTCAGAGCAGAGAGCCACGAATGGGGCACTCGGG
A_14_P120710 GGAATCAGTAAAAAATAAGCAGTCTCTGCCCCTACAATGCTATGTCATATGTGGTAACTA
A_16_P21217141
AAAACACAACATGTTGCTATCTTTTTTCCTTGGATATGTGATTGTGAATCACTAAGGGGC
A_14_P112900 CCTTCTTTGAGAAGAGTAAAGACTCCTTGTTTATCCATGTTCAGATCCAAGGATAAATGA
A_16_P03747490
TCCAGTTGATCTTTTAATCTTCAGAAATAACACCAGAAATCAGTTATGCCATCACTTTCC
A_16_P21527575
AAACTCATCTTGACTCACACTGTATCTTCCTACTTGTAACCTGATGTGATCATTCTCAAA
A_14_P138633 ATTTCACAGTTACCTTCTGTAAATCTATTTTCTCATCTACTGAATAGAATCAGGCGCCCT
A_14_P138946
CAAAGGAGATCATAAGAAAACTCCTGGGAAAAAAGTAGAAACAGGTCAGATAGAAAATGG
A_14_P102147 AGGATTTACAACTATAGGATCATCCACCAAGAGTTTAATTGACGGTGATGCTAAAAGACA
A_14_P122329 AAAGGGTATTCAGAGATGAATCATGAGAATGGGAGTAGAATTACTTGTGTGTGTAACTGA
A_18_P14136130 TTTAACTAAAAAGGAAGAATTGTCTGTGGCTTCTTAGAAAGTGGTGTGTGGACAGG
A_16_P19743745
AACTATAAATGCCACGTCTATTAGCAAACTACAGGTCTATTCTTCCCAACGTTTGATCTG
A_14_P126233 AAAGATGGCTTTAAAAGTGCTACTAGAACAAGAGAAAACGTTTTTCACTCTTTTAGTATT
A_14_P135522 CTTATGTGTCTTGGAACAAGAGAGTAATGTTAAAACCATAACCACACAGAAAGGGCTTTA
A_16_P16762106

AAATCATCCTAGCCTTTTCAAACATCTTGCATTCTCCAATATCCCTCAGTGAAAAATGAA

CUST_3198_PI426210822

TCCTTTTCCCTGGAAGTTTTCTTGCCATATATGGTGATAAGTGACTTTATCATGGAAG

A_14_P119335 CATTGTGAGTTACATTGCATATAGTGTTATTCTAAGGGCATCACCCAGAGAAAAAGGACG

A_18_P14137168 AGTCCTTGCCAAGTCTATGTCTTTTCTGAGAATTCTCTCCCCCTTGATTTC

A_18_P14976532

GCATGTGAAGAAACAGATCATATCAAAGGAGGATAACTGTTTTCAGATAACTGCTGTGAC

A_14_P118595 AGCAGTGACTTCTCCTGGACTGAACTGAAGAGACTCAATGCTGGAT

A_16_P17665793

CCTACACAGATGGCATAATTATTGGGCTTGATCATTTTTATCCCTTATTCACACAAGACA

A_14_P121388 GAGTCCCTTTACTACACAAAAAAGCCAAAGAATACAAGAATACAGAGACATACTTCCTGA

A_14_P132495 GCTACAGTAAGAAAAAATATGTGTGTAAACTGCTTTTCATCTTCCTGCTTGGCCATGACA

A_16_P17545550

AACTCAGTCCCTCTATGTTCAACACTTTCCTGAACTTTCCATCTAAATAATAGACTGTGA

A_18_P15727810

AAGACATGATTGGACCAGAAAAAGTATTATCCCTCACTATCACTTTCCTGGACATAAAGA

A_16_P01644062

CCTGCAAACCCTGTGATTAAATCCCATGATTCCTTTACAACTCTAATTACACACCTAATT

A_14_P136051 TGTCTTTATTTTTATGTTTCTTGCAGAAAATGTGGGATCAAGAAAAGGACCATTTGAAAA

CUST_494_PI426210822 ACATCTGTCTTGTGAAGTTCTGTTCTGCCTGCTTTCCTGGAAAAGTTATTGAAT

A_16_P37210100

CAACCTTTCCTACCTCAAATATGCCAATTATTCCAGAAAATTCTATTCAAGCCCTCAAAA

A_16_P17696614

AGTTTTGCTAGACCATCATATTGTAGAGCCCTCTTTTATTCCAATTGTTGAGACTGATGT

CUST_538_PI426210822

CTGAGTTTTCTTTCAGTATTTTTGTTGATTCATTCATTTATTCATCAGACTTTGAGTGTT

A_18_P13405219

TTGGACAGTCTATCTTTTAGCCCTGACTAAGTATACTCCTAGCAAAACTTGAATTCAGAG

A_14_P123485 TAAAGCATAGTATTTACCAGTCTTGTCACCTAAGTCTAGAGTGTTTCCATCAAGAAACAG

A_14_P132270 GATGTTAGGAAACCTGCTGTAATTAAACATAAAATGTCATTCAGGGTTTCCTGACTGCTA

CUST_1670_PI426210822 CTAGTGGCTTTTCTTCCATCTGTGTTCCTGCCCTATCCTTTGTTAAGG

A_14_P116640 AAATCAATGATCTCTGATTCCTGAAACAAACAAGAAAATTGTAGGGAGAGCATTAAAAAA

A_14_P122807 AATGCACCATATTTGCTTCTCCCTGATGCTTGCATGTGAGACATAACTCCATTTAC

A_18_P10827329

TGCTTAGAATTACCCCTCATCTCAATCATTACATCCCCTTAAACTCCTTAAATCTAGCAA

A_14_P114089 CTTTCGAATATGAATCATGTGCTTTAGGTGGGAATTGTGAATTCTGAAGATACAGATGAC

A_14_P109559 TGAGAATAAACCCTCAAGAACTTCCTCAAAGAAAAAAGCAATTCACAAAGGTATGTCCAT

A_14_P124541 CTGTTGAGATTTCTAGGCAGCCTTGACTTAAAACAAGTAAAATCTGTTCTCTCCACCT

A_16_P03136955
CCAGTGCTCTAAAACATTCTCTATTCCAAGCCATCATCTACAAAATCCTTTACATCTCTC
A_14_P102271 CATTCTTATTATTCTTATTCTCTTGGCGTCACATGCGTTTAGTGGATCTGGAAATCAAAG
A_16_P17254314
AAACAGCCTTATACACTGATCTAACATTCAACAGGTGCATACTATTCTGGTTTATCAGCC
A_14_P109111 CTTTTTAAAAAATCATTCTTGTTTAAGGCATGCAGCAGGAGATTGTAGTGAAGTCTCTGC
A_14_P114992 ATGAACAGAGACCTGTGATTATTCCAGATTTTACAGAGAATTGCTCTCTGCAGGGTATA
A_14_P119508 CTCAGAAGCACTTACTTCTGGAACAGAAGGACCTGTAGATGTGGAGTATCTTG
A_16_P16579313
AACCATTCTTAAATAGGTCTCAGGATCTCTATGTCGCTTCCTTATGACAGAACCTATTTC
A_14_P200770 CTGGAGAGGAAGGCATTTTGAACATCTATCACTATTATATTAGGAACTTCACATGAACCA
A_14_P116532 AGTTTATATACCATTGTTTAACTGGTCCTCTGAGTGATCCTGCATGATTGATAATCCACA
A_14_P130684 TTTCTTTCTGTTATGTGCCACATTTTTTTTCTTCCAAAAGATACTCACTTGTGGTAACCC
A_14_P132306 ACAGAGTCTGGCTAGATATTTACCATCATTTTTCTTTTTAAAGGCCTATTGGAAGAAAGC
A_14_P136646 GGGCATGTGGCTTTAGACCTTCCATTTAGTTATAAATGTGTCGGAAATGTCCTTAAAATA
A_14_P125541 GATACAGGGATCAAATTTCTACAGGCTTCCAGAGATTCCTATAAAAATGGAGTGAAGGTT
A_16_P01400579
AAACACGAGATAGGAAACACTATGGTCTACGATGAATTAGACGCACACAAAAGAGAAATT
A_14_P111788 GAAGTCCATGTCTCAAGGATGTCCAGACTAAACATCCAAAAGAGATAAGAACTATTTGAT
A_14_P101078 GCATGAGCCTTGCCTAAAAAAAATACCTATATATCTCACAATGCTGTTTGGTAAAGCCTT
A_14_P122527 CTTCTGCACTTGACAAATTATTTTCATGCACGAGGCCCTTGAAAATGTTGACAGT
A_14_P123599 CATTAACGTTACGACCGTGAAATATGACAATAAAATGATAGCCGTATGGTCGCAAATTTG
A_16_P18717506
ACAGTTGAGGAGAATAGCAATTTGGGAAATACATCAATGATTGACATGGGTAAAAGAGAG
CUST_2967_PI426210822 ATTTCTTCTTGCCTCCTCAGGCAGGGTCTCTCGTTGTGACTCTTG
A_14_P100482 CTTAAGAAATGTATAAAAACTCCAGAAATCCCTTCTAAACATGTTAGGAACTGGGTCCCC
A_16_P18337896
AAACATCCTCTTCTATGAGTGAGAATCTTCTGTTTTGTAGTATGCTGTCATTCTAGGAGC
CUST_4946_PI426210822 AATGACCACTACTCCGAGCATGGGCTTTCTTCTGTTTGATCTCGTT
A_14_P102671 ACGAGTGGGCAGGTTTGTGTGAATGAGAATAGAAAATCACATCTAGAAATGTTTTACTTT
A_14_P129481 CTTTCCACTGAAATCTTTAGGATGAAACTTCCAAAGCAATGACAAATCAGTCAACAAAAG
A_14_P105624 GAAAATGATTTTGGAACTCACAGATGCAAGTATGCCGGTCATCTGCAAACTCATA
A_14_P138816 GTATTTCCTCACTTTTTATAACTTACTTTGATCCGGCCTCTCTTTGTCCTCGCTTC
A_16_P17718781
TCTGACAATTTGCTGGTTTGTATAAGTTTGAGTTAAGACACCGTAATAATGTCCTGCAGT
CUST_680_PI426210822 ATGCATGTGTGTGCATGCCCCCTTATCTCTAGCCTGATTTTCCATT
A_14_P200750 ATGTATTCTTTTGTGAATGACAAGTGCGTTTCTTTCCAGTAGGCCTATATCCAAGTTATC
A_16_P20124665

AACGTTCTTTCCAGGAAATAAGATGTGAGCACCATATGTAAGGAATCCATTGAAACCTAC
A_14_P116150 ACAGAATTGGATAGTTTTCAAAGAACAGGCCGATAGGTGTATGAGTGGTAAAAATGCAG
A_14_P130122 CAATAGGGGAATAAAAAGGCTGATTATCCCTATCATCCTTACCATTTACTGAGAACTAAA
A_14_P130337 TCTCATTTTAGTATCCGTGTAAACTTTATTGGACAAGGAGGGTGATAAAAACTGTGCATG
CUST_9807_PI426210822 GTAGTCTGGGTTCTAGCTGCCCACCTAAGAAGCTCCATTGTATACAC
A_14_P140003
AGCAGCAAGAACAAGAAAAGTTTTAAAGAAAGATGTTGCTTACTATGAGTGTCAAGGTGA
A_16_P02343335
AAATTTCAGGGGTTCTTAAGTTGTTTCTCAACCATTTCTTCTAGCAAAACAATGCCAGTC
A_14_P135404 GAAAACCTTCTTAGCCTACCTATATACTTTTCTCCCATAGCATTATATCGGCAACTCTGT
A_16_P21063771
CAATGCATAGTCCAGACTTAATTACATGACAGACAGAAAGTATAAGAGGAGGAGAGAACA
A_14_P137959 TTGAAAGTCCTTTCATGAATACATCCATGGCTAATGAATTCCTTTACACCACACTGGAAA
A_14_P120574 GGATTATTTTGGATAATGACAGTTGGAAATTGCCTGCTGCTTCTCAAGTTTAATTTTCCT
CUST_4996_PI426210822 GGATGGCCAGACACGAGAAAGCCAAGCAGGGATTTTCAGTTACAG
A_14_P119650 CTACAGAATTACTAAGACTGTCATATGGAAGGTGCTGCTCTGTGCAGCAATCTC
A_14_P134475 CTCTGAAAACACATTAGAAAAGTAGTCACCTGACAAATTCTGTCTGCTGGTTTCATTTTC
A_14_P131265 AGTTCAATTCCAATCATGACTCATTGGCTTAGCCATCAAAGTTTATCTTTCCTTTTGAAC
A_14_P139790 TTTCCTTAATCGTTCAGAGTGTTTTTCTTTGTCCCAGGTTTATATACCAAGAATACGGG
A_14_P110215 ATGGTGATGGTAGAAGAGTGCTTCTTTTCTGAATCTGTTACTCTGGCACAACAG
A_14_P131998 CTCTCAGTAGCAGTTTTGAGCCTCTGAGTGTTTAGAAAGAAAAATTGGGAAAATATGAAC
A_14_P111236 TTTGTGTGAGATCTCTTGGTTATTAAATCCACGTTGTAAGAGAGTATAACAGGACCAACT
A_14_P119806 TCCTTTTTCTGTTTTTCTGGTCTCAATTATGCTGTTAATTAATTCTCTGAGGAGGCATAC
A_14_P115746 ATATCAGTTTCTGCTTATCTCTCTCTTGAACTGTGAAATAAACCCTTGAACCTCATAGTG
A_16_P41777870
AACCCAAAGAACTGTTCAATTCAATTCTGTCTTTACACTCAATGTCTCACATTGCATCTT
A_16_P01649564
CTGTCTGATCCAAGGATCTGTCAATCAGAGAATTTTAAAAAAGGTGAAACAGTTCACACA
A_16_P17220645
ATGGAAAGTAAAACCACATCCATCTTGTTTTGAAAAATCCTGACACCAATATAAAAGCCC
A_14_P102195 CTGTATGCTGACCAGAGTTTTTCTGTAATATCTCCCGATATGCATTTTAAAAGCACAGTG
A_14_P105466 TATAGTCTATTTGCTTGGGAATATTTGCTTGGGAATAGCCAAGCAAATTCCCATCACTTA
A_14_P111563
GTGATATAAGGAGAAATGCTACATAGAACCAAAAATAAGAAAAAGGAACCATCCGGCAAC
A_16_P03772170
TGGGGGCATACAGAAATTATAATTCATGACTAACATAGGAAAAAAGCATCAACTCTAGTG
CUST_2002_PI426210822
ACTTAGTGTATAATTGTCATTATCTTTTGAATAAAAAATGCATCACCAAACTTGGTTTTG

CUST_3353_PI426210822
TGACTTTCTATTTACAGTTATTCATTTACACTGGGTTCATCTATTAATATATACTGTTGA
A_16_P00685603
CCCATTTGCTTAAAAAAGATCAGAAGGCGAACTATAGAACAATTCTTCCTTCTTTTCCTC
A_18_P22840688 AAGCATTGTCAGGATATTTGTGGATGTCTGCCTGTGGAGTGGCAC
A_16_P18320075
AAACTCAAGTTCCACTTTTTGACACAACTGCAAATCTGTTTATGGTCGTATTAATCTTCA
A_14_P114897 GGATCCCACCAAAGGAAACTGTTACATGCATTATGGTGAGTGGTTGATAGTTTTTTTT
A_16_P17010842
ACTCTTGCTATGACACCTCTACAATTGTAAAAACCACATTAGAGTTCTTCTATGAGCCTC
CUST_8544_PI426210822 CCTTTTCCTCTTTACTGATCATCGAGCCAAGGAAAAGCCACTGGAA
A_14_P202018 AGCGTGGAAGAGAAGTTTAAGAATGAACCACTGAATGTTTCGCTTTTATTTTTAAATTTA
A_14_P138526 CTGTGTGACATTAGGACTGAAAATTGGTAAGAACTTTGATTTTTGCAGCATGATTCCATT
A_14_P132360 CCAAATGGATCTCTCTTCAATAACAGTTCTCCATGAGAACCTATTCATGGTTTAACAAAC
A_14_P111955 CTGCTCATCTGAACAAATGCCTTTTGTATCAATCATTTAGCACCTCTAATTTCTAGGCTT
A_14_P121583 CTTTGAAGAAAGGTGGCGTTTTACTACCAAATGATCTCCTTGAAAAAGTGGATTCAATTA
A_14_P119496 CTATCAGAATTGCAGCGATTTTCCCCCTAAATCCTAACAATATAGGAATGTTCATTTGCA
A_14_P105610 CTCTCCAGACAGTGGTAATATTAGCAATCTGACTTCTCTTTCAAAACAGAGAATACAGAA
A_18_P13417399
ATTCAGCATGTCCAATATTCATTCCAATAACGTCATATTCTCATCATTGCACCAACTTCC
A_14_P201432 GCATTCAAACTTACCGAACAGAGACTAAATCAAATTCAAACTTACGGAAAGGGACAAAAT
A_14_P129863 GGAACCAGTTGACATTGAATAAAAAGAACATGACAAACCCACACTGGCATTGGATA
CUST_9304_PI426210822 ACTCACACTGCAGTGCTTTCTTCTCCCTTTGTACCTCTCCCTTTATAAAG
A_16_P19802055
AGTTGTCCTGAATTGATGAACGAGTCTCTTTTTAGTAAATGCTTCTTTTAACGGTCATGG
A_18_P15975171
AACGCTCTCTCTATAAACTTTCACACAATCTAAAGGTGTTTTTGTCATAGTCAGTTGCCT
A_14_P104907 TTTGTAGACACAAGGAAAACAAGTTTGGCTTCCCTGGTCTCCATTTCTTCA
A_14_P110595 TCTATGACTTTCTCTGTTTAGTTAGACTAATAGGATAGGAGCCTGCATAAATATGGGGGA
A_14_P128034 TTGAGAAACCACAGAGTTTTCTGTTTAATGGAAGAAAGAAATCCAGCCTCTCTCC
CUST_5339_PI426210822
CCCCTATCACTCTCTTGGAGAAGTCCTGTGTTTTATTTATGCTGAATGATTGAATCTATT
A_14_P124885 ATTATACCTACTTTCTTTCCACAGACTGCTTTTCTTTTAGGAGATCATTTAGTTTGTGCT
A_14_P107733 CATCTCTGTTTTTTTCCTTTCAGTGTTCAAGGAGAAACCTAAATATCCCCCCAG
A_18_P22841429 TTTTTAGAACAAACACGATCTTCCCTGCGTGATTCTTCTGAATGCCTGGC
A_16_P20366364 GCTTGGCCATATCTCTACTGCTATTTCATTCATCATTCATTATTTCACTTTACAGACCCC
A_14_P133553 CCTTCTGTTTCTCAATAAAGGCAGAATGTTGCATGTAATTGTTTTAGGAATCATGACGTT
A_16_P01550528 TTCCTTCCCTTCTAATATGCCATAATACGCTCCTAATCTTTATGATTTGATGTTTCTGCA

A_16_P30952661 TTTATCGCCTCGATCAAGTGTGGAGTCACATGCTAATGTGTGCTAAAGAACTGTAA
CUST_1757_PI426210822 AAGTGATCATGAACAGGCGGTACACCTACCTGGGTGAGTACTCAG
A_14_P110612 GAAACAAGAAAAGTAAAACTAAGAGAACTTAGCATCTGATTGACTGTTCCCTTTCTTTTA
CUST_9204_PI426210822 CAGCCATTGTTCTCCACTTCAAAATGGACAAGGAGGAAAGGTGAATTG
A_18_P14134543
CTCTCTCTGCTTTCTGAAAGTTTCCATTATACTGCCCTGTTGTCTAAAATTAAGCCAATT
A_14_P110882 AAGTTCTGGCTTGTCAATTAAAAATACAGAGTTCCTGTTTTCACATGAAAGGGAAGGATA
A_14_P111246 TAATTCTCAAAAAATGCTGCCAAGAGCCTTTCTAGAAAACATGGCTTATATTATTTGGTT
A_14_P109350 TGGCAGGAAATGGCTACAGAAAGAGGCTTGTTCGGGTTAGAAATGTAAGTA
A_18_P15078003
AAGTCAACTCTTGAGGTAGTTAGGATGAGAAAATATGCAAATTAAATTGCAGCATGGACC
A_14_P136904 ACCTGGAAATGACACTGCTACAACTCACACCACATTTCAATCAAGGTCCATAAATAAAAA
A_14_P134685 TATAGGGGCAGGTGATAAAAAAATTTCAATTTCACTAACTCTGACTACAAAATTAAACTT
A_18_P22843229 ATTTTGGAAACATGACACCTGGCAGGCATGGGGCATGTTTGACAT
A_14_P108809 GTCCATTCTGCTTGAGAGCATTGTATCTGATCTTGAAAAATCTCTTGGAACAGGTTTATC
A_16_P58424116 GAGTTCAAGTCCCGGCTCTGCTACTTCCTCTTATTAACAGTGTAATCTCAGAT
A_14_P134927 CTAACTCTATGATGTTGTCACGCTGACATGAATAGAGAGGGTTTTGTTTTTTAAGCCAAA
A_14_P132742 CTAAGTCCAGGTGTTTGTATTCGGGCTAGAAAAGGCAATGTCCCAAGT
A_14_P130667 TCCATTCTGGCCTTCATTTTTTTCAACAATCCTTTGTGGATTGAATGTGGAATGAATAAT
A_14_P102674
ACTGGCAGTTAGAACAGGAAGGAAGTCAATGAGAAAAAAAAATGTTTTAGACCTACTTGA
A_14_P124740 ATGCCTCCTAGACTGCCTTTTATTTTAATTAAGCCTGAAGCACCTTAACTTTGCATTTCT
A_14_P124652 ACTTTCAGTATCTGTTCGTTCACAAATTGTCCAATGACCAAAAATAACCATGCATTCAGC
A_14_P202205 AAACGTGTTTCAATCAAAACTGACCATGAGATAAAGGAAAGAGATGTGGCTTTTGTGATA
CUST_5549_PI426210822
TGATACAGTAGAGGAGTTAAACTAATAATTTGCCCATCAAAATTATGGAGGTGAAGGAGT
A_14_P200074 GGGGAGCAAACTCTATTGACTTAATCCACTATAGTTCATCTTTTGTCCACTCTCAGTTAT
A_16_P21219283
ACCAATTACTTCCTTGTCTCAGTGTATTTTTACCCACTAGAATCACAAGTGACATCATTC
A_16_P00011351
ACTTTTATGGGGAAATAAACGAAACCAGTTCAATGATTGATATTTTCAGGAGTAGTGGCC
A_14_P104278 CATGATCAGGACTATTCTAATTTCTCTTCATCCATGGAAAAAGAGTCTGAGGATGGTC
A_14_P108957 GCAAAGATCTAATTCTAAAGGATGAATACTTCAGGAGGATAAGATCATGGGAAGAAAGCA
A_14_P131688 CTGTTTTTGCTTTTTTTAAACTGATTTATTTAAAATAAGCCTAACTTTGAAGGCTGCCAC
A_14_P135479 AGGGAAACAACAGAGAAAGTTAAACCACCCAACTTAAATCTAATTCATGCTAACGATCTG
A_16_P02240957
AAATGCTATGGACAAAATATCCCTCTGACAGTAAATTCGGAGTATTCTTTGAGGCATACA
A_14_P130252 ACGGTCTTAACTGCTGGTGATTAATCTTGATTACATCATGCAGTCTCTTTTTTGAAAGAC

A_16_P15703157
GTCAATACAAAAGTCCTTTCTTACAGTCTGACCTAATACCAACTTCACCCTATGTTTTCA
A_18_P15948888
CCAGAGAAGAATAGCAACACAAATCCTATACGATATCTTACGGTGATATCTATAGACCCC
A_18_P16366839
ACTAGGTTCCTTCACCTTATGTTACTAACTAACCCAAGATCTTAAAATCAACTTCTGCCG
A_14_P120504 CTATGACACTCATGAAATTGAGAAGGTAACTTAAGTATTCCAAAAAAGAGGGTTTCACTC
A_18_P13269857
AAAGTGGAATACTGAGTTGAATTCCTATGTCAGAAGTTGAGTAAAGATAAGTCACACCCT
A_16_P16353557
AAATCAGTGCAATTCCATCTACTCTGCATTGCTGAATTAAGTCAGAAAAGTCTCAGGTC
A_14_P116575 ATTTTAGATACAGTCGTTGTTTCTTGAGTGTAGTGCGTTTGTTAACTTTTTCCACCTGGT
A_14_P104231 CTTCTTATGAGGAACTCCTTATCCACTAGATCACATGAGTAAACCTGGATTCCTTAAGTA
A_14_P101338 TTGGCCCCAAACAGTAATTATCAAGATGTTGAGACCCTCTATAACTTCCTAATCAAGTAT
A_16_P00004729
AAATACTATCAGAGAACTCCAGGGTAGAGATAAGTTGAACTCCTGCATCTCTTAGACATC
A_14_P114124 TCAGGAATTTCTTTGGATTAGAATCAGATTAAAAACTTGCAGTTTGCTTGGGATTTGAGG
A_14_P134243 TATGATTGCAAAATGAAAGAATTGTTTCCAAGAGAACTAAGTTAACCGCCCTAGAAAGAC
A_16_P19464763
AACATCTGCTGGACATGCTTACTCAAAATTGAGACAAAGTATCATTTTTCATCCATAGCC
A_14_P122946 ACCTATACAGAATTATGCTGATTCTGCAAATTGTGTGTATGTAGGAAAATGCTGGAAGGA
A_14_P115825 TATGTTTAAAGACACGTCACTTCCTTTAGTGTGCTGGTTTTTCTCTATGTAAACTGAGGA
A_14_P114585 ACCTTGTCTACAGGTAATAAGAAGTAGGTATATAATGGGCAGATGAAAGCATGGATAGCA
CUST_1207_PI426210822
TCAGTCAGTTTAGTTTGTGTTTGTCTGATGTGAGAACATCATTACCTACCATATGTTCTT
A_16_P03304074
TCTGGTAAACTCCCTTAATTGACAAAAATGCTGTTTTTCCAAATCATTTCAAGATGCCTG
CUST_5030_PI426210822
GACACTTCTCTCTTCCAATAAAAGGCTTTTACTAGATGTTTCAGTGACATAAAGTTGAAG
CUST_1041_PI426210822 TTTCCTGCTCTCAACTGCTCTCTATCCCTTTAGGCCTGTGCTCAT
A_16_P16191441 ATTAGAAGCCCCATTTGTGTGTCTCAGCATGGCAGGAAGGTCAAA
A_14_P136625 CTAAGACCCAAAAAAACACAAAGTCTCCAAATTTTCTTCAGCCTGCTACTTAAAAAACCA
CUST_9332_PI426210822 TTTCAGCAGAAACCAGGAGCACTTCCAGGTGCAGAGAAAAGCTTA
A_14_P130446 TAGGTTACAATCTGTGGGGATCTTTTAAGATTAGAGGGCCTTATAGGAGGGTC
A_16_P40402283
GTGCCACCTTAAAAACTTAAATCCAACTGAGACAGAATTTTATTTAGTGAAATGCTCCCC
A_18_P15277594
ACAGTTTTGGAATTACACATGTTGTTCATACTAGCGCAGAACATAAACTATTGTCCCCTA

A_14_P115801 ATTTTCACTACTAGTATAATGTCACAGGCTAACCTTGAGCCAGATGAATGGGAAGTTATT
A_14_P135741 GTGACTGATCACCTGAGAATATATCCTGTCTTCTAGTGATTTGATATCTCTTCCTTCTCT
A_14_P110792 CTAGTCAGGTTTCACAAACTCCTTCCCAATTCATCTCATTACTGCAAAAAATAAAGACTG
A_18_P14924186
CTGCTAGGATTAAACCTCCATCAAGATCACAAGTAAGGCATGACTAAGTAAAAACCTACT
A_14_P119647 GCCACATTACCTCCAAAACAAAAGCATCTTTTTTCCCATGTGAAAGGTCTAATTCTGTAA
A_14_P123103 TATCTGTGTGCCTTAGAGACAGACACATCTGCATCCTTGGAAAATCATACTTGGTTTTAT
A_14_P115465 ACAAATGAATTTGTCATAATTTGAGAGGGAAATGGAGCATTGTTTGCTATCATCTGGTGA
A_14_P114759 AGAAAAGAAGTGTGCGGAAAGTAGATAACACATGGTTACTGTCAACAGTCTATAGTTCTT
A_14_P112519 TAGGAAAAGCAGGATCTGTCACTTATCCAATTGACTATATCTAGGAGGGGATTGATATAC
A_14_P139158 ATTCCATACCTGTTACTCCTTAACCTTATCAAAACTTCCCTGTTCTTTATCCCATTCTTT
A_16_P00379822
TTTCTCCGGTATGTCTTAATTTACTCTGCAGCATGTAGTAATCACATTCTGAAACGACTG
A_14_P129222 TGCAGATGAAATAAAGTGTAATCCTTTATTAACATCTCTTTGCAGTGTCCCCAATGAGGT
A_14_P100810
GAAACATGGGAAAGTAGCAGTATGAACAAGAGTAAAACAGGTAGTAGAAAACAACAGCAT
A_16_P01936709
CAAGCCATGAAATGTTAAACCAGTATCCTTGAATAGCATAGTCCCTATTCCTTTCCAAAA
A_14_P110382 ACAGACAAGGAGCGAACTCAGAATTTTCTTTTCTTTTTGCTTCTCAATCAGTGATCTTAA
A_14_P113366 GCACAGAAAGACACTCCTTCAGTTCATTCACATCTAGAAAATAAGACAAGAAAACATCTC
CUST_5942_PI426210822 GTTGTGATTCCCTCAGCCTGATGGCCTAATTGCCTTTGCCTTTTC
A_16_P16060806
AAAAATCCCACTTTTAACACTTGACCTTCATATCCGAGAAGTATCAATAAAGAGCCAGAC
A_18_P17040694
ACCTCTAAAAAATAAAAAAGTAAAAAAGAAACACAGCCTTACAGAATTCTAGGATAAAGA
CUST_1861_PI426210822
CAAATTATTCTCGTATTTATTTCCCCAGTGTTTGAGAAGGAACATTGGGTTCCCA
CUST_372_PI426210822 CAGCTTCTCTCAGAGTACTTGCTTGAGTGGTCCCGCTGTCACTGT
A_14_P112375 TCCCTCATCTTAGCACAGTGCCATACTCCCAATAAGCATACAACTATTAAGAAGTAAAAG
A_16_P20763330
AGGCCACTAGACAAAATTATCATACATCTTCAATCATCAATGTCCAGAGTAATGAGTCAA
A_16_P16191552 AGTAAATAGTTGAAAGAGCCCTTCTGGAAGAACACAAGGCTAGTTCCTTTGG
A_16_P18142144
AATTCTTTTACGGAGTCCAAATGGTAGTTTACAACACCAATTTAAGCCTTATCGCATGAG
A_14_P131873 CTTTAGTCTAAACTTCAAATCTTCAACCCTGTTCCCTAATCCAACACATTCCCCAAAT
CUST_4431_PI426210822 AGAGGGATCAGCTCTTGTTTCTTGGCTTTGCTCTTTACAGCCTCTTAG
A_14_P106770 TAACTCAAAAAGTTACCTTTTTAAGAGCACAGGCTTTGGATTCAGATACAATTGCTCAGA
A_14_P112829 GCTATATTTATCAACAGAATTTGCTTACAAGTGCTGGGAAGAAACATTTTCCTAATGGCC

A_14_P103992 TCTTTTCTGATATAGCAATGTCCTTAGTGAGTTCTATTTAGGTGTTAAGCATACAGCCTT
A_14_P128863 TATTAACATCATCACCTAATGAACTGAGCCACCCAGAATTTGGGGATAAACTAGAGATTA
A_18_P24091069
AAAGAAGCACAAAAGTAATTCAGAACTGACAGAATCAAAGCAGAGAGTTTAAGAAGTGGG
A_16_P19044419
AAAAGGATTCTATTCAGAGACTATCCAGAATTTCCATTTACTAATCAGGCCTTGTGCTGT
A_16_P30952458 CTCCTTTTTCTTGACCCCAGGTCCCAAACCTGAATGTGCAGATTT
A_18_P14134917 AATGCCAGTTTGGGAGACACAGTGGAGGAAGAACCTGTTGTTTCTT
CUST_2937_PI426210822 TTGCATTTTGCACTCTGACCCAGTGCTCCTGTGTTTCCATGGTTA
A_14_P117731 AACTGCAAGAATAGAGACAGAATCAAGTAGCCTCTCTATGTTTCTCTGCATATGTATTCC
CUST_4078_PI426210822 AACACTAACAGTGGAACCAGCTATTGCTGCTTCTGGTGTATCATCCAAATG
A_14_P139552
ATCAAAGAAGGCAAAAAAAATTGTTCATGGCATGCAAATTAGAAAATGAGAAATTGGCGG
A_14_P138715 TAGTGATCTGAAGCCATAGTCAATTTCAGACTTTCGCAACAATGATTATTCTGCTTTTGG
A_14_P132610 CATCAGATCCCAGAAACCTTTTATCGACTTGTTCTCAGTGATGTTTAAAGAATTGCAGG
A_16_P30952576
TGTCTTTGTTCTTAATTCCATTGGAAATTTTTCTGACTTTTCTTCTCAGGACTGTGAAAA
CUST_2995_PI426210822 GCTTGTCTTCCAGCAATGTGTATCTGTAGCCATGACTTTGCTTAAGCGAA
A_14_P138704 CATCAGAGAAGGAACAGTTCTAGAAGGTGCAGGAGGTAGTAAAAGAACAGAAAAATCAT
A_16_P16191490
GGGTTGGGGATAAATATGTTAGCCAGTAAATGCCATTTATTGCTTTAGATGTCATCTCAG
A_16_P36580826
TGGACTATCTTGAACATTCCTCACATTTTACACAACGATTCTATACTTTCCCACGAAATG
A_14_P115823 CATTTGTTTTCTCTTATCTTGCTTTATTAGGTCGGAGACCATGAGAAACAGCGTCAAATC
A_16_P41689685
CCCAGCCATCTTAACTAGTCTCACTTTAATTACTTTTAGTAGGTCCTACCATACTGTGAG
A_14_P201634 TTAGAAGTTTATAAAGTAAAGACCTGTAAAGCATGTGGGTGGAATGTTTCCATGCTCTTG
A_14_P120546 AGTTTATTCCAAATATTTGGTTCATTTCTTCTCTGATCTTCTGTAGCAATCTTGTCCCCT
CUST_8493_PI426210822 AAATGGTGGTGAGTTCTCCATTTGTTTTCACTATTGCTTTCAGCTTTGAGCC
CUST_4783_PI426210822
TTCTTTTAACCTGCAATTACTGGGTGAAAAGAGTAGGATATAGTAAAATGCAAATGCTAG
A_14_P114045 CTGACCTTCTAGTTCTAATATTCTAGGAAACCAATTGCTCTTAATGTAACAATGATAAGG
A_16_P01625705
AACAGTCTCAGGTCTATAGCATGTCTAAAGCAATCAGTAATCACATTTGGATGTTGTGTT
A_16_P03277568
GGAAGCATTATGAACGATCTTAATACCCTCTCCCATGTCAATTAAACTAGTCACGAATGA
A_14_P201276 TCTTTTGCAGAAAATTTTGCAGTTAGCAAAACTTTGTAATGATGGGAAGGGAAAAACCTT
A_14_P104121 TTTACTAACTGCCAAAATCTTGAGTCTAATAACCAACCCAAACTTGAAGAGATCACTGTC

A_14_P131995 CTACTGAGGAACAATTTAAGAGTATAAATGTAGACCCACTTGATGCAGATGAACACAGTC
A_14_P124712 GGGTGACCACTCAGATTAATTTGGATTGTATCTATCTTACTGAAGCACAATTTAAGGGAT
A_14_P104105
TGATGACAAAACAGTTAGGAGCCCATCTGGAACAACTAAAAATGACCATAAAATCAAAGA
A_14_P121426 TGCCCAGAAATCTTAGGAGTCTGCAGACACAAATTTAAGAACCTTCTAAGTTTGATTTAC
A_14_P133735 CTGTCCTAGACCAGACTTGGACAACTACATGTTATTTTATGCACTAGTAACAGGTTATCA
A_16_P36827177
ACCATTCATTGTTCTTTTTACACCTTCACAATATCTCTTTCTAACAACAATATTCTGCCC
A_14_P118121 CACCTTGTTTTAAACCTGAATCTTTTTTTCTTTGGATAAAAGACTTAGATGCACCCTGGA
A_14_P109170 AGCGAACATCTTTCTTACATGAACAATGCTGCTCCCAGTAACAGACTGTTTTATATACAA
CUST_6116_PI426210822 AAGTGCATTTCCCAAAACTGTTGGCTAGCTGAGGGATGAGAGCTC
A_16_P19051544
TTCTAATTTGAGACATTTTGCATATGTGAGGTTTGAACGAAATGATATTCCCTGCTCACC
A_14_P120361 CCAGAGTATCCCTCTCAGGGAACACATCTGAATAAAGGCCTATGGAAATATAATACATTA
A_18_P12152634
AACACTTAGGTCAAAGGAAATTTTCTGGAGTCATTAAAGTCTTCAAAATGAATTGTGGGC
A_14_P129288 ACTCAAATCCAAGTGTAAAAGTAAAATGATTTGCTTTCGTTTTGTTTTCCTTGCTTAGGG
A_16_P00905332
AGAGCATTGACATCTTAGAAACTTTGAGCATTCAGATCTACCTATAATTTCCTCTGGACA
A_16_P18101626
AAGCCCAATTTGATATCTTGATGCCTTCTAAATTTTGCAAGCCCATATTCAACTGTCATT
A_14_P110006 TCTAACTCTGTTAAGACGTCAATCTGTAGGGAAGGGTCCACACCAAG
CUST_4861_PI426210822
ACATGAGGCTTAGAGAGATGCTAACTAAGTAGTCATGAGTCGAATCTCCCATTTAGT
A_14_P125917 CTCAGATACTGTGATGGACCAGTCCAAGACCAATGTTTTTTTTTCCTTCATTAAAACAAA
A_14_P127500 CTACAAAGTAGTTCCTGTTTCGCTCTCAGAGGTTTATTTACTCCAGTGCAATATGAAGTT
A_14_P103025 GTTTTGTTTCAGATCATTATGTAGAAGTTCTGGAATGCAAAATACAGTGTGAAGAGAACC
A_14_P122209 AGAATATCTACCTATTGACTTGTAGGATAAAAATCCTGCCCTAAAAAGATTTCATGGCCC
A_14_P129027 CTTGCAAAAAAAAATGAGCACCAAACCTGATATGATTCAAAAGTGTTTGTGGCTTGAGATC
CUST_1870_PI426210822 GGGCCTGAATTGTGAACTACGCTTGTTGGTTCTTTATCATGAAGGTACAG
CUST_132_PI426210822 GGAAGCTTTGTCAGGGAGAAATTAGCATTTCCCATCGGAGACCAT
A_14_P139534 GTATTCAACACAACATTGACTACCTAAAACATCTCTCAAAAGTCAAAAAAATCCCCAGTG
A_14_P118684 CAGCAGATCTCCTGAAGGTTCTAGATTTTCACAACTTGCCTGATGGAATAACAA
A_16_P58424606 ATTTGTTTGCCAAATACTCCCACCATAGCCCATTTCAAACTGCCATTGCAAT
A_14_P108505 GACTAGATTTCCTAACATAGTATACCACTGTCTTTTGGGTTTTTTTTCCAAATCTCCTGG
A_14_P104388 CTTACTGTAATACAATCAAGGCTAACAGAAGAAAGGAAGGAAGGGATTATCATGGAACTT
A_14_P129529 AGGAACTTCCGGTCTAGATGGCAGAAGATCTTCTAGTTCCTTTATTAAAACAAACAAAAA
A_18_P11123380

CTCTGCACCTCACTCTTATGTAGAAACTTGACTGACCACAAACACAAATGTTTTAACATT

A_14_P200077 ACTACTCCCAAGCACTTTAACATATTGGGCAATGCACTGTTCATTTAAAGATTTTCTACC

CUST_3430_PI426210822 GAAGTGGGGGAGCCAGCTTCCCTTCGTGCTGTGTGTCACAATAAG

A_14_P139984 GAGAGCGCTCGTGAACTGGAATCAACTGCTTCAGGGAAAAAGAAAAAAAAAAAA

A_14_P138750 CTGGGGATACTTTTGAAGTTTGTGAAGATGAAAGAGATTGTAAGTAGTCTCAAACCTTTC

A_14_P100447 CCTTTATAGGTCACCATTTGCTGCTGTGACAGACTATTCAGTTACAAGAAATAATGTCAT

A_14_P113332 ATATAGTTGCTATGCCCTGTATATTCACCAGCACTCAACAGTGACCAAAATAAAGTCATT

A_14_P120014 CCACTTCATGTCTAAACTAACTGTTCTTTGAAACATCTCAGACTCTAAAAGATGTCCCAT

CUST_2513_PI426210822 GAAATTCCTCAAGACAACAGAGCCAGGAGGAAGTAAGGGAATAGAAAGAAG

A_14_P124121 TTTCTAGACCAAGTCACAACTTCTATTCCATGCTTATTCAGGGGCCTGAAAGAT

A_14_P138545 GTAAACATAACTTTCAGGCTACTCTGGCATAAGGTTTCAATGTGCCAAACAAAACTAAGA

A_14_P138616 ACTTTAGAAACTGAACCTTCCTAATTTACCTGATTGGATCTCATTCCCAGATGAAATGAT

A_14_P123003 TGCCCATGTTCAAATTGGGTAGAGAATGTATTCGAGAGTGTCTGTTATTTTTACTACAGT

A_14_P135654 CATTTCCCTCTTCCATTTGTTCCACACTTATTATTTGTTTATTGAAATGCTCCACTTGCT

A_14_P111638 TGTTTATACACACACACAGTTTTGTATATACTTGTTCACTCGAAGAGGCTCCAACAACTT

A_14_P125210 TGTGTCTAGATTTCTGTCCATGCTAGAAGAAGAAGTATATAGTCAAAACTCTCCCATCTG

A_16_P17360774

AAAAAAAAAATGCATGGCAATATCCTTAGTGTTAAAATTGCTCATTTCACTCTCTAGCCT

A_14_P131141 GTGACAATGACAGTTAATGCCGCCTCTTCTTGTTCACTTCCCCTTTAGAAAT

A_14_P116120 TTTCTTTTAGAATGATTACGGACCTGAAGCCAAAGAACAAGATGCGCTAGTGGA

A_14_P134903 TGCTCACTCTGGATTATCTCTGGGTCTCATTTTCATATTTCCTTCCCTTTATTACCATAC

A_16_P03573169

CCCCAGAAATGTTGTTACAATTCACACTCCGTTAACATTACAAGGTTTCCATCCTTATTA

A_16_P16575815

GCCAACACCTTTCTTTAGTTAAAACAGTTTTATCTATGCAAGCATTCAAAGACACTTCGC

A_14_P118760 TGGCAAGGGCTTTCAGGGTATTTTTCTCTTCTTTATAAAAATGAAATTACTTTTCCCCTG

A_14_P102441 CATATTAATATTTCCCAACTCAGTTGACTTACATGTCGACAATAAACCTTGACAGCAGGT

CUST_3780_PI426210822 TATTTTTCAACTACCATTAGCCCCACTTTGGCAAGGCCAGGTGGG

A_14_P136747 CTCTTACCTCCCTTTAAGAAAATGTTCTCCTCCTTTTACCCATGTGGATTACAAAAGATA

A_18_P22841211

TATTTCATGCTTTAATTAAATGCTGGGTTGAAGGCCTAGGATTTATCCTGTTGGTCTAGT

A_16_P17325193

GCCTTCCAATTATTAAGACTCTTAACACTTTGTCTCATCACAAGTAGCAAATATTGTCCA

A_16_P02018254

ACTTCCTTGAGGAGAAAATATTGTGCGTTATGGAATTTTATACTCTCAGCCCAATGACTG

CUST_3030_PI426210822 CTGGGTCATCTGCCTTCTGTTGCTTAGCCATCTTGCCTTGTCATT

A_14_P123460 ACTTGCCAAAACAATATTAACAAAGTGGATTCAAAGGTAAGAGGATAGCAGTTTGCTGAT

CUST_9360_PI426210822

ACTCTTTTGAATGACAAGCTTCCCAAAAGCATTTAAAATGTGTCTTATGGTTGTTTTAAG

CUST_5186_PI426210822 ATAGTATCTTCACCAACTGAGAAGGTGCCCCATCAGCCTGAGGAA

A_14_P137298 CTCAACAGTCACACCTTAAATATGTCCAGCACTTCAGTTACACAAGGTTTTTATCTCTAA

A_16_P19310863

CACTGAACTCATTGATCCAGGTAGTAAATAGCATAAACTTCAATGTTTGGTCCTAAGTGC

A_14_P124301 ACTTTAGAACAGGGATCTGTGAAACAAGTCAATTTTTACTTGTGAACGATCAGAATCCAA

A_14_P124567 CTTTCCAGTTCTACTTGATATCGTAAGAAATACTAAGTCACCCAAAGTGTAACACCTGTT

A_16_P15172957

ATTGCCATACTCTTATTGCAATCAGTAGCCAAATTGTCTGTTTTAAACCAATGACCAGAC

A_14_P117178 ACAACAAAAACAAAACTACTACTTACATCATAAGTTAGTGAGTCTGCTTCATTGGCCACC

A_16_P00691580

TTGTGGAGCCTCTAAAAAAAGTTACATGAATACATGAGATACACCTGAGATAGAGCCC

A_14_P130617 ACTGCATGAGGTTTTTTGCAGCGTGCATGAGTTTTAGTGACCTTGTTATTTAAGAAGTTA

A_14_P138331 CATGATGAAACACTGAATTCTTTTCTTAAGGGCAATCAAAGACAATGATGTTTTTGGGAG

A_16_P03592295

TTTTAGGCATTCAGTCCAGATACAAGGATTATGAAACTTAAAAAGCAGCATCTTAAGCCC

A_18_P22839595 CTCCATCTGCCACATGGGTTCATAGATAAAGTCAAACATCCCTTTACACTCTAAG

A_14_P101812 CCAGGAAGTAACATGTTCATCATCATGGAAACGCCATTAGACTCTCAAATGTTATAAAAG

A_16_P16688625

AACGGTGGTAAAAAAACTGAACTGTATGGGGTTATCTCAAAATAGTAGTGTTTGCTTACC

A_14_P111675 GCCCCAGAACAACAGTTTCACTTTGTGGCTTTTAATTATTCTAGAATTTTAACTGCATCT

A_14_P139840 CCTGTGGTTACATGCTTATTTTATGGTTTCCTAGGTTCCCATTAGTTTTACGTTCCTTAA

A_14_P108588 CTTATTTTGGAAATACGGGTTGCGATCGAAGTAGAACTTGATTTTGTAGCCCAATCTG

A_14_P101893 CTGGATGTTCTTTAAAGTTTTCGCTCCAGCATCAGGAAAGTTCTAAATATATGTAAGTGT

A_16_P02311750

AAAGGGATAACAGAATTGACTTCTCTCAAAGCATTAAGACTGATCATTACATCAACACCC

A_16_P01651155

CTTAGTCCAAACCCCTTAATATCCCTATTCTGTGTTCCTATAGCATCTATCTATCACAGG

A_14_P200669 ACTCATATAGCATATTTCAAAAATGTTGTCATTCATTAAGGCCTCTTAAATAGACCACTA

A_16_P02158127

AGGAGCGGCAGTAATTTTTTTTCCTAATAAATCCATTTACAAAGCATCTGGTCTCATTCA

CUST_8808_PI426210822 ATTTTCTCCAATAGCCCCTCATCCCTGACCTTGTCTTCCAGCCTC

A_18_P14156998

CCCGAGGAGAGTTCATGTATACGTTACACATTATAGGGTTCACTTACATTCACAAAAAAA

A_14_P126434 CAAGTAATAGAAAATTCTGTTGCAGACTAGAACTTTGCCCCAGAATGTTGTTTTAGCAGA

A_16_P16958981

GCACAACTTGTTGAGGTATATATGAGTTTTAAACTATTTGAACTATCTGCACAACCGAGA

A_14_P129460 ATCATTATGTGGTAATATTTCCTGAACAAGAGCATAGGCGTTGTCAGGCAAAAATGTCTA

A_16_P02000024
AAAATGAGGCTAACCAAGAAACATTTGACATGGATGATGTTACAAACACACTATGCTTCA
A_14_P125427 ACCCTCGAATTTCTAAAATGAACACAATCTCTTTTGTCTCTTTGGAAGCCCATTTCTATA
A_16_P01663221
AATGAAAAGCAATCCAAAGTCATCTCTAGTCCGTAACATGCTCATAGTTGATGTTCCTAA
A_16_P17207052
CAGCCTGGAACATTATGATCAAGAGCCTTATTAACAGAAGAAATAATGAACGTAGACACA
CUST_9571_PI426210822
ACTATCACATATGAAGAATGAACTAAACACCTAACTTGGAAAGTAAAACTTAAATGAGGG
CUST_6788_PI426210822 GCATGCCTCATGTGACCAGGGATAGGCAGGGAGAAAAACTCAAGG
A_14_P131296 ACATGGTCTTTGAACTCTCGTGTCGAAAGAGTTGAACACAACTAAACTTTAATGTGAAAA
CUST_259_PI426210822 GCAGCTCTCCCCCATTAAGCACCATAAGCCGAGTGGTACAGAGCT
CUST_3077_PI426210822 CCTCTCATTTATTGGCAGGCCTGAATTATGTTCTGATGGCTGACGTG
A_14_P139028 ATTATAGTTCCAGAATTGTGGGAGTTTTTTTTTCTGAGAAAATCATTTTTAGTAATTATT
A_14_P107337 TAATTGAGGGGTCCTCTGGGAATACAGTTTACTACAATAAGTTTTGAACCAAAGAGTGTA
CUST_2090_PI426210822 ATAGATTGGAGTGTCCTCTACCTCTCTCCCTCAAACTCCCTGGGT
A_18_P22841762 TTCAGTTATATGGGATTTATCCAGACCTTTGGATGTTCACTCTTCAGAGCCAG
CUST_5384_PI426210822 GACTCGGCTGTTTGAGCCACTTTCCTTTGACACGTCTCAGACTTG
CUST_2086_PI426210822 CTTGGCATATCACTGGGGAAGGATTTCTGAGCGGCCACTACAAAG
A_14_P116905 TTCTACACTATGGTCACACTCTTGGTTACTCATAAACCATAGCAGGTGAATAATACCTCA
A_14_P111245 ACTCTAGGATATTCGAGGTAGTCAGATCCAGCCAATTCAATCAAAATATTTCTTTTGGAG
A_14_P130229 ACCAGAAAGAAGCCAATCCATTTAGGACTGTAAAAATCAGGAAATACTTAGAACAGCACT
A_14_P106012 TGATGAGCAATTACTTGGCTTATGTGGGATCTGTTTCATAGTTTAAGTTCCCTTAAAAGA
A_18_P13284971
AGGTCCAAGGTAATCATACTGTTTAGATACCACACTTGAGTATATGCATTACGGGTAGTA
A_16_P17338714
AAGTTCTTTTCATCCCAGAAGAGTGAAAAAAAAGAAAATTCTAACCACATGTTACCACCG
A_16_P00685446
GGACACTCCTCACGGACAAGTTCAGTGTTGTATTTACTTTTCTGGATTTATTTTCACAAA
A_14_P122773 CTCAGTGGTTGTCAAAGGTGTATGATGACATTGGTTCTTGCAAGTGTCATTAAATAAATG
A_16_P00486151
ACCAAGACTATACCTAACCAACCTACTTTTACAAAAGACAAAAGTTAGACCAAACAGTCA
A_14_P121500 CTCTGATTCTTATTGATGGGTTTATTGCTTTCTGCCTCTTGTCTGTGCTTTGCAG
CUST_5922_PI426210822 GCTCTTCTCTCCAAATTCCACTGAAATGAGCAAAGCCATCTAACAGTAGTGA
A_14_P125454 TATTCTTCTCTTCTGTTGTGTTTTCCTCAACACTTTCTGGGTTATTTTTCCATTCCTTCA
A_14_P132249 CTTTCTCTAGTGTTGATCTACAGCAGCAAATTATGACCATTATAGATGAACTGGGCAAGG
A_14_P127858 CCCCTTCACCTTTATTCTGCCTTTTCAGTGATTTAATTTTTTATCTTATTTTGGCCATTT
A_16_P15965374

AGTTGTCAGTAAAGGTCTCCATTGTTATCAAAGGCACTTCTCTAGAAAAACGTGACTATT

CUST_5375_PI426210822 GGCCTTTAGAACCTCTACTTCTTTGAACACAGTGCTACTCTGCTCCAAA

A_14_P108424 CTGTGATCAATCATTTCTTCTTGACGTGTGCCATGCAACCCTACAAAAATAGAGAAG

CUST_2081_PI426210822 AACTCGGTTTCTTTCCTCCTCACCCTCTGTGATTTCAACCCTTTC

A_14_P135810 CGACCTTATCGTGGGCAATGAATACTATTTCCGAGTTTACACCGAGAACATC

CUST_2075_PI426210822 CTCAGCCAGTGAGCGCAGCCAGCAGCTTGGCTCTTTGGGTCTTTG

A_14_P130763 TCAGAGCAAGCACGGGAAATGATGAAAACATTACAAATTGTGCTTAAAACCACATAAACA

CUST_2642_PI426210822 TAAGAGAGGAAGCAAGGTGGGGCAGGCAAAGGGGTTGCTGAGCAT

A_16_P35487690

TTTGTTTATGCAAGTCCCAGTAATTCAGTCTTTTTCACTATGCTTTGTAACTACCACCAC

A_16_P16453290

AAAAGTGATGCTAAGTGCTGAGATAATCTGTCACACCATTTAAGTAGGGGTATTGCAATG

A_14_P129744 ATTTAGTCTTTCCATTTTTTCCTTCCAATCTTTTGAAAGTAGTTTTTCCATGCAGGAGGA

A_18_P13309668

TGTAGACTTTAAACTTCTAAACCTCAAGATATATTCAAGAAGTGTTGCAACAAATCTGGG

A_16_P01728571

GCTTGTTAGGATTGATACCCATTTAGTGGACAATCTCATCAATGTTTGCATGGAATGTAA

CUST_4122_PI426210822 CTTATAGGCCAGTGTGGGGTAAGGGGTGGAGAAGCTTGCCTCCTT

A_16_P38318324

AAGCCCTGAAATACAAGATAAGCCCTGAAATACAAAATAAACCCTAGTCAATGAATTCTG

A_16_P21428038

ATCCTCCTTGTTGAGACATAATTTAATGCTACTGTTTTGTCACATACTCCTCCAATTCCT

A_14_P118292 CTTTTGCCAACTTTCCAATTAAAGGTTGACATTCCTGCATAAGCATTTCTCTGTGAAAAT

CUST_2423_PI426210822 CAAAGCATTTCTCTTGTCCTCCACAGTGAGGACATCAGGAAGTGAG

CUST_7254_PI426210822

TAATGTTTTCACCTATGGATGTCAGTTCTACTTCTCCTTGGGGCTTTAACTAAAAAGG

A_14_P132018 ACACTCCTAACTTTTAATCTATTGCCTTCATTTGCAGACTGTTCAATCATTGTTTTCCCC

A_16_P30952614 CTGCTGCTGGCCGTGCTGCTCTGGAAGGTGAGTCTGGTGATTGCA

CUST_3619_PI426210822 AAGAGTCCTCTGTAAATCCACTGAGCTGGAATTTCCAAGCAGGTGT

A_14_P103254 CTTACAAAATTACACCAGCCAGAAGACTTAATTTAGCCTTAAGGAATAGGGTTCTCTGTT

A_18_P22607477

TTCGTGTTAAAGAAAACATTACAATATTCTCAAAAAAATCCATATCCATCACCATTGCAT

A_16_P17442069

AAATACTCTGGTATGCCATTCAACAAACTGATAGACGTTCTCTTCTTATTTTCGGCAGTA

A_16_P16459214

AGTGCCTCTTATGATCTTCACACCTGATAAATGAATGAGTGACTGGATATACTGTAGAGT

A_14_P116167 GGGAAATACACACAGTCTTCTAAGACTTGAAACTAGATGTTCCTAATGGAGAGGTTAGTA

A_16_P16573991

AGATTCCCATTAAACTCAATGCAACTTCCATTTTAAAAGACTTCTAGGACTCTTACACCC
A_16_P02810681
AACTCAGAGAGAATAAAACTGGAAACTATAGTCATTCTCAGTTCATAATGCAAGCCCCAA
A_14_P102705 ATTCCAGGTTAAACTCTACAAAAATGTTTTTTTCCTCTTAACTTGCCATGCTCACACCTA
A_16_P01592468
TCACTTTTAAGCCCTATACAATACTCCCCATGTCATAAATTTTCTAACTCTAACCCATCT
A_16_P21531899
AGTCTACCACAATTGGCTGTTTTTAATTATTTCCCCATTAACTCATTATTCCCTTGCCAA
A_14_P105813 GTGTCTACAAGATTATCATAGGTGAAAGTAGGGTTGGATGCAGAGAAATAGTAAGAAAGG
A_16_P18086737
GCTTTGTCATGTGGTTCTACTACGGTATACCCCAAGAGTAACTTGTATATGAGACCTTAG
A_14_P101452 GCAATGTAACATTAGCAACCCACGTCAGAAGATTTGTTCATTAGCATAAAGCATTTCAGA
A_14_P116926 TAGGTGATAGTCAATAAGTGGTATGGCCCACTATCTTTCTCTTTTTCCTTTTTTTGTTGG
A_16_P00502625
TGTCTACGTTTAATGCCAACTTTAACATCTCCAAATTACCCTAATAAGGCTATGACCTGA
A_14_P101205 TCAATTAAATCTTCCTCACTCTCTCAGCCAGTTTATTTGTCCATTTGAGTCTTAAAGAGG
A_14_P134855 GGTCTTTACTCTTCAACTGACAACAGTAAATATTTTGAATTTACTGTGATGGGACGCTTA
A_14_P119020 TCTATTTCTGCGTAACCTACTATTGATTAAAGACATCACCCATGTAACCAGTCACCTAAG
A_14_P109787 CCTTTAGAGACTGTTGTGATGATTAGGATTTAACCTTTGTATTCCCAAAGGACTTGGTTG
A_14_P117518 TAAGGTGGAAGTCAGCAGAAACTATCAATCTGAGATACCATATGCAAGTAAATTACAACT
A_14_P119098 TTATAGATATGACTGGCTCTTTGGACCTGTGATGTGCAAAGTTTTTGGTTCTTTTCTTAC
A_16_P18738772
AACACCTTCTAGTAAAATACACAGAATGGAATACATGGGCTAATTGTTCACTAAGTTCCA
CUST_7834_PI426210822
TAATTAAACTGAGGTTTCCTCGAGTTCCAGAGTACATTTTTGAAGACATTTATTTCACAC
A_16_P15823848
AGGCAGGATCAGATACCTTTTTTAACCATTACGAAGAAAAACCAACACATGAACTATCTC
A_18_P11014816
ACAAAAACATCAAAGACCTTTGCTTCTATTGACTTGGCTTTTGCTTATTTTCACTAAGGT
CUST_8404_PI426210822
CACATGAACATTTGCAGAGAATCCAGTATAAAGACATTTGTTAAAAATAAAGATGGTAAT
A_14_P108222 GAATTGTTTTGTTTGTTCCGCAGATACAATGGCTCAAATCATGGCAGAACAAGAAGT
A_14_P127426 CTGGGAAAACTCAGATTCAAGCCATAAGGCTTTTTCTATAGATAGTGAACATTTTTCCTC
A_14_P200250 TCAGCCCATTTCATAATACAGATGACCGGTCTGAGTCTAATGGATGATCAAGTTTAAGAT
A_16_P01481417
TAACTGACCCAATGACTATGTCTATAATGTAGCAATCCTTTCCTTTAATAGAGCCGAGAC
CUST_7292_PI426210822 GGAAACAGCACATTCTTGTGAGAAGATAGGTTTTGGAAAGTCCGGAGTAG
A_16_P00685155

GAGCAGGTTTACAACGTATTTCATAAAGCACTTTAAAATAGGAGTTCTTCCTGTCCCTAA

A_16_P30952350 CATGTTTTCTGAGATCAGGGATGAGGGACAGGTCACTGTCTACATCAAC

A_16_P00685403

AAGCAATAAAAAAGGATGACATGAGGACTATATTGTGGAAGGTAGAAACTGGAAAACAGC

A_14_P128011 ATAAAGTATCAACGAGCCTAACATTCAGTAGCACTGTTCTACCATATCGTTAGCACTCAA

A_14_P101742 TGACTAATTGTCTTTGCAGTGCTCAAGAGCGCTGTATATAAATGTTCACGAACTGAAAAT

A_18_P10009303

CCTCCGAGTAAGTATCAGATGAGTTGCTAATGTTGGAAAAAAAGCGAGAAACATGCTT

A_16_P30952316 GATGGATGTTTTTTTTTCCCTTCCCTTCTTTTTCAACCGTGTGCTTGGTTTC

A_16_P00871245

AGAATTTTTCAAACTATGTCCTCATGTCTTATCACCTTGCTTATAATGCCCTTCATTCCC

A_18_P22840712

CAAATAAATAAGGTTTTACTTTTCTTCTTTGGGATTTCTTAGCATGCCCGTTGGGACAAT

A_16_P17215942

AAGGGGTTTTTAAAACAATTACAACATGGATTGAGCAGACCATACTACTTTCAAGAACCT

A_14_P202543 TCTCTTTGAGGATTATCTGCGGTGCCTGAAATCTAAGCACTACACATTTTAAACTCAAAT

A_16_P15351883

AGTCACCTTTTAACTCTAACATGAAGAACACAACTGATCGTAGACTGTAAATCTCATCCA

CUST_1157_PI426210822 AAAAATGGGGGACTAAATTGAAAGTTTCTGTAGTTCTTGGCCCTGTAAGAGG

CUST_9273_PI426210822

CCTAATGAACCGCACTTGTGGATGCATTTAGAAGCATTTGAATTGTTGACATCTG

A_14_P124469 AAGAAAAGGCAAATAAAGACAGGGTATTTGCAAGGTAAGCCAAATCTGTTTCATTGTTTG

A_14_P109580 CACACACATATGTTATCCACAAAGTCAGGTGAATGTTATCCACAAAGTCAGGTGAATG

A_16_P03212604

ACACACACGCTATTTCATAAGTAATATCAGGAGGTTTCTACACTACTTCAGAACCCTACT

A_14_P119320 GAATGCCTTTGAGATTACCCGGTTTATTCATTTACCTACGTTATTTATCAAGAGCCACAC

A_14_P126149 ATGTGTGCGTTTGTGTATTTACATGCACACGTATGTAAATCACAGGCTGCGTTC

A_14_P116296 ATTTCCTGTCATGTTTATATGTCATGGGTTTTTATTTCTTGCCATGGAAGTTGAAAGTGG

CUST_9917_PI426210822

ATCCATCAGTTTGCAACCAGAAAATCGTTCAGCGGAGCTTATGTATTTATTTATTAGTAA

CUST_9065_PI426210822 CCACCATTTCATGGTTTCAGTTTGCACAATGTCCTCTTTGGGGTAAAG

A_16_P58424265 GGCAGGGATTTCTGTTGTCCTGGGCACACAGAGCATATCTGCAAG

A_14_P126452 ATACTCCGTGATCTTTCTTGATGGCCAGACTGTGTAAAATTCATACAGTGTTTACTACAG

A_16_P39924975

GCACCTAAGACTATGTTATCCACCTCTATGACTATACAAACCTATGCTTGACACATAGAA

A_14_P117359 TCTTTTCTGGGAAACCACTTGCTTTTGGAGACACTGAAAACTAAATCAAAAGGTTAAGAA

A_14_P109996 GCACTAAACATCGTGGGGGATCTCTTACGGAAAGTAGGGGTAAGTTTCAATTATTTA

A_18_P14773553

AAAAAGAAGGGAACAATGTAGAAGGAACAATTGATGAGTAATTGAAGGGAACAATTCTGG
A_14_P132148 AATACTGCCTAAAAATGGTTTAAGCAGTTAATACATCCACAGTTTCTGATCTCAGAGGCA
A_14_P127554 CACCAAAAGGAAAGAAAATTTGTTTTGCTACCTTAGTTTTTATAATCTCAACCTCTAGAG
A_14_P103860 CATTGAAGATAATCGATCCCAAGATGATTAAAATAGGGTTTCTGATGGATGTTGAATGGC
A_14_P132227 TCATTTAGATAATGTTTCCAGATACCTCCAAGTGAGCGTGAATATCAGTGTTTGAGCAAT
A_14_P115365 CTCTAAGGACCTGTTTTGCTTCTGTTTAAACCAAATGGGCAGTCTGTCATTACA
A_14_P126056 TGGAGAAAGATGTGTCAAAATTGGGCATGGAATAAGGTATAAAGTAGGGAAGAGATGATA
A_14_P109403 AAATATTTTTCCAAGAAATTTTGCTGCCCCAATTGCTCCAAATCATAAATAACCCACTCT
A_14_P122614 AAGGTGAAAATTAAAACTTACCTCATAATACCACTGTGATATTGGCAAACTGTGAGTGAT
A_16_P17014191
CCATGCCATTGATAGAAACCAAAGTCCTAAAAAATTCCTCTAAAGCCATAAATGAACCAG
A_14_P123987 CTACTTTATACAAAGTTACCTGGGTATGGAAATTTTCCCATGACAGTTCAAACCTTCTTC
A_14_P128047 AGCAGCAAGTTTGTCAAGAGAAAGTGACCTGGATTAAGGATGATGAAAAGGTC
A_16_P03234806
AAAAACTCACGATCTTCCATATTGGTGAACAAGAACCTAAGGTGAACAGTTAGCATAAGA
A_16_P03294682
AAGTCAGAAGTTCAAAAAAGAGAAACAGAAAACATTAGCACCTTTATCCTACTGACCTGC
A_14_P111931 ACTTCAAAGTTTTCATACACTCATGTTCTATTGGCAGAATGGAGAAACTAACCCATTCAT
A_16_P20935501
AATTGCTCTAGAAAACAGTAATGAGGGAAAGCAAAACAGAAGTCATTTATGGTCACGAAC
A_14_P108036 ACCTCTGAAGTCTTGCTTCAGTCGCTATTAATATTACCAAGTAATAATAACAGCACAGGC
A_16_P16332351
AAATTCAAAACTGGTGAAGATAGGCTATGTTGAAATGCATATCAAAACTCCTGCCTGATC
A_14_P109131 GGGATCCCCAGTCCAGGAAGTTGTTTGAGGTGCATCATAGGAAATTAT
A_16_P02713901
GCTGGATTATATAACCAAAGATCTTTATAACCTGCTCACAATTATCTAAACTCACAGGGC
A_14_P128315 CTCTGATGGAAGGTATGGCTGAAGAAATAGGGTAAATTATTTAGATACTAAAGTGCAGGG
A_14_P123434 AGAAGTAAAATGAACTAAAATTCATACTAAGATGGTAGTCCCTTGCTGTATGACAAGCCT
A_14_P134908 CTACATTTGGTTCCTTGGGTTTTTCAGATGCTACTTCATTAATCATCCACTTGTCTGC
A_16_P00685526 CTGTTTGATACTAGAGTCTAACTACATGTCATGGACATTGTCAGCAAACCACCCTT
A_14_P123160 CTTGTGCATTTTAATCATCATCTGGAAATCGTTCCTTGGAAGATCTGTGATTTGATCTAC
A_14_P100462 GGGAGTGTTATCCTGTGCGTTTTAAATGGGAAATCCTTCATTATTTGTGTTAATCCAATT
A_14_P137620 CTTCCTGAAAAATTTATTTACTGTGACTGTGATCCGCAGGAGCCAATTGATGTTTATCTA
A_14_P121010 ACCAAGGACAAGTGCTGGTTTATGCAATTGAGCAGTAGAGGATGCTGTATC
A_14_P132499 TCATATTCAGACCAGGTAAGCCTCATTTGCACAACAGTCAAATTGTTTGTTCCTTTAAAA
A_14_P133475 ATGTGTTTTCTTTTTTTGGCACTCAGATGAGTATAGGATCAAACCAGTGGAAGAGGT
A_14_P121208 TATTCTTACAATATCCCTAGAAGAGATTCTTTGCTCCTTTCCCAGTAAGCCACTCTACTA
A_14_P123644 TATTTCTATAACCATTTTCTTACACTAAACACAGGATCAGGATCTGAAAGAGACACGGTC

A_14_P130898 GGGCCTCAAATGACAAATTTTGTCTCTAAATCTTCCTCTCATTAGAAAGATATACACCCT

CUST_1242_PI426210822

TGTAGATGACCTAAAACTTTAACTCATGCCATGTTTAAATGTTCTTAAATTTGGGATTGT

CUST_2230_PI426210822 CTATGGGAGACCATCCTGCTAAGCAGGTAAAAGGGACGCATTTTG

A_14_P108761 CAATTTCCATGTATTTAGCGTCCAAATTGGTAGAGGTAAGCGAAATTTTTGTGCAAGAAT

A_14_P104678

TTAAAGAAAACACAGAACAAGTCCTAAATCAGACAAATTGCACAAAAGAGAGAGAAGAAA

A_16_P00685109 CTCTCTGGATAGATGTTTAGTTTTATCTTTATCCAAATAGTGAGAATATAATTTCATATT

A_14_P103903 GGATGATAGTGATGCAGGAAAAAAGACATCTGTGCAAACAGCAATGTATTTTAAGTTGTC

A_14_P101857 TCTTCTTTCTGTCCGTGTCTTCTTCAAAAAGTGTCCCAGCATTGTGCAAAATTTT

A_16_P37321596

TGACCATAGATGCTACTTTTTCTCATCCATAATGCTACTGCTTAAAAATCCTTTAACCAA

A_14_P115206 CCAGAACATGTAGTCAGCAATCATGGCACTCCTAAGAACAAGGACAGATTAAAAAAA

A_14_P113234 CCTGCCTCTGTTTAAAATCTCCTGCCTGGGAGATATATGCTTATTCACTTATGTTCT

A_14_P119032 ACTCCTGAGATAAGAGAACATTATCTTGACTCTAAAAAAGACGTAGACCGTCATCTGAAA

A_14_P129948 CTTTACTTACTTGACTGGATTGGACTAAAAGCACTGATCAGAGGCCACGACATAAAA

A_14_P126559 TCAGTCTCCCTCATCCACTACAAGTTCTGTATTATCTGCTGATGGAAATTTTTATTCATT

CUST_8667_PI426210822 TGATAGCTAACAGGGAGTGAAGGGCTAGAACTTTTATTCCAGGTGAAAGTACT

A_14_P117286 AATATTCACCGTCTGGAGTTAAAAATTCATCTGTTATATCCTCAGAAGACTCTGGTGAAC

A_14_P125290 CAGGCTTGTTCCATAGAAATGGTATTTAACATTTTTTAAGACACAAGTCTGGAGGAGATG

A_18_P10385255

CCCCCTAGGATATGTCTAGATATGGATCTTTGACCATTAATCTCATGTCACCTTTTTCTT

A_14_P133869 TACACGTCTCTCTTATCTCTGCAGTGTGTAACGGAATAGGTATTGGTGAATTTAAAGACT

A_14_P132691 AAAGTGATTTGTTCAAAATTGAAATTTGAAACTTGACATTTGGTCAGTGGGCCCTATGTA

CUST_10185_PI426210822

ACTGTCGACATCTCGAGACAGAGACATGCTATGTAAATAGTCAAGTAACTGAAGAGA

A_14_P117823 TTATACCCCACACAAAAACATTCTAGATGGATAAAAAAGACCATGCATACCAAATGGATG

A_16_P16410362

ACCTAATCCTATCAGTGAGCATCTAAAGATACATTTTCCTCCTTCTACCTATGATCGTGA

A_14_P101912 CAAAGAAGTTATTGACAAATGCCAATCATTCAATCAGTTGGTGTATCACCAAAACGAGAT

A_14_P115698 CTATATTATTACTCTAGTCAAATTTGCCTGAGAAACCCAATTGCTTTCACCATCCCAACA

A_14_P105122 CAAGACTGACTATTGATTTGATTGATTGTAGTCTACGGAGATCTCCAGGAGTGAATTTTT

A_18_P16715369

CTGAATCATGTTGTAAACTGAATGCACTGATTGATACCTCTCTAAACACTAATGTTGTGA

A_14_P119119 TGCTTATTCTGAAACTCTCAATGTGTCTCTGAGAGAGGGCAAATATTGGACATTTGATAC

A_14_P110078 CCAGTGGCTTTCCTACATGCTGTCTTTCTCCACACAAGATTAGACAAATAACAAATATGA

A_16_P19881751

GGAACACTATCACTTGAATTTGCAATCTGACATTTATACTCTGCTAATCTACCTTCAGGG

A_16_P18239224
AAAAAGTAGTCACACCTTGTAGGCTTATCAGACAGTTGCAGAAGTAAATAAGAACGTGTT
A_14_P106717 TGCTGATGTTAAATTAACTGACCCGTGTATGTCATAAGGTTTCTCTCAATTCTGTTCACA
A_16_P38653898
CCTCAATGATAAGAGCAACTAATCCCTATACTTCACTACTTTCCTAGAATCCCTGTAACA
A_14_P103045 CTATGATGGCCATGAAGGCTGATCCCTTTACTAAAGTCTCTGTGGCTACT
A_16_P35863269
ACAACTTCCCAGAAATGTAACTATCAGCCTTCAGAATCAAATTCACATCTCAAAGTCTCT
CUST_8489_PI426210822 GTCATCTGTCAGCATCTCTTTCCCTAATCGACTCTCATCTGGTGGT
A_14_P113271 AGACAAAGTTCTCATATGTTGCATTACAGAGTTTTCTTACAAGGATTTATCCACCTACCC
A_14_P104518 CAGGCCAAAATCCACTACATCAAGAACCATGAGTTTATCGCCACCTTCTTT
A_14_P135766 AAAGAGCGATATGGAAAATCCCTATTTCATGATATCAGAGTAAGTTTCCGACACATGATT
A_16_P19221767
GCCAGTTAATCCTCTTAATTTCCCTAACCTCATTTACATTTTCAATGCTAATTTCCCACC
A_14_P127684 TCCTGAGAAGATCCTGTATTCTTGGGGAGAGTTGCTAGGAAAATGGTAAAATTTTATTTT
A_14_P105877 AAAAGTTTAACACGTGAATGTATGGAAAAGCCCTTAGAATTTGGATCGTATCATGATGGT
A_14_P126443 CTTTATGATTTCATTCACACCTCTTTTGCAGAAGTCGTATCAAAAGGAAAAGGGAAAAAG
A_14_P119441 CGTGAAGCGCATCTTGTATATTGCTAGTTGAATAGAGCCCAATTGGCGAGTTATA
A_14_P139710 CTTGGCAATGATGACTTACACTGCCTAACAAGAATAGCCCATTTGGAATTTCTCTGAT
A_16_P19750942
ATAGACCAAAACCACTTTCTTATAATCTCACCGACTGTGTATAATGAATGGCTGTCCTTT
A_16_P00684987
TCCTGCTATAGTAATATAGCATAGTATTTGTAATGAAAAATTGTATTCTAAACCTAAAGA
A_14_P110557 ATTTCCTGCATTTAAATGCAGTTCATTTACCTGTCTTCTTTGCCTCAATGAGATTTCAGC
A_14_P138678 AAGACTGTAAGTTAAACATATCAGGGGAAAGCCTTGTTTCAGGCTAGCGTTTCATGTAAT
CUST_4364_PI426210822
TTTAGCCCACCCTTTGAGAACCACTACATGACTTCTTGATAAGATTCTTTCAACTTTAC
A_16_P17559296
AAAAAGATTACGCATTGATTAAATAGACGTCACCAATAGGGAAAAAATGCTTGGAAGTGC
A_14_P139332 TATGTGATCTAAGAAAATTGCTAATCTTTCCCTGCCATTTTGAGAAACACAGTCCAAACA
A_16_P21081598
CCCAAATTCTCACTTATTGAATTCACTCACAGAATTCTACTTCCCACTACAGTTCTATCA
A_14_P107534
GCTAGGAATGAGGATGAAATGTGAAGTTAAAGAAATATCAAAATGGAAGCAGTAGGGAGA
A_14_P107880 CTGTCCAAGGCAAATTGAAAGGAACAGTGGAAGACAAATAAAGTTGCTTTATGATTACAC
A_16_P00421491
TCAGCCAGATGTTTAGTATCTTGTAGATGGAAGGTTAACTTGATTTAGTGCTTAAATGCA
A_14_P100541 AGGTAAATGCCTTACTTTGTAGCCAGGAGCATGTTTTTTCTTGTCACTTGCTCATT

A_16_P16733836
AAATATTGACTACCCAACTTTTTAATTATCCCCAAACTTGTTTTTTCAGAATCGGCCACC
A_14_P116589 TGCAAGCAAAGAACCCATTTGCCATGCTGCTATGAAGACTACTTTTAGATCAACAATAAA
A_14_P136433 CCATCTACACTCTGCCTCAGACTCCATTTAAGGACGCTGTTGTCATTACTATG
A_14_P130414 TATCTCTCTCAGTATGATTGTGTCACATTTCTTAATCTTCTGGAATCTCTGAGAGCAACG
A_16_P18798811
AATTTTTATACTGTTTGAGCCATCTATCCAATTGTCTCACTTTAAAAGCCACATAGCCGG
A_14_P128707 TCTAGAAATTGCACTTCTATAGACTCTCATGTTTCCTCAAATCCTGTGTCCAAATGGTAT
A_14_P110209 TCGTAAGTCTTACATTTTTTCCTGAATGGAATATGGAATTGGAGTTTAGGGGATTTTGCT
A_16_P34915638
TGACCCCTCCAATACTGAATATATAATGTATAATGAGTATATTATAGACTGGTGTAGATG
A_16_P03616570
TAGGAAAAGTATAAGCGGCTGTGGAATTTAGTAATTCAGTGCAACAAATGTTTGCTGAAC
A_14_P139195 AAGCAGAATGGGCACTTATTTATTTGCTCCAAAACCTGAATAAAAGGCAAACTTTAACAC
A_16_P21672158
AAAAAGGAGAATCAAGTTCCCAAAGATATCTGAAGCCCACATCATTAATCACATCTTTCT
CUST_9910_PI426210822
ATCAAGAACATAGTGTGTCCTCTAAGTTTTTCAGTACACTTTTGTTCACTTCATGATATT
A_14_P110779 GAATTCCTGCATGAACTGAATGTTCCATTTTTCAAAGTTGGATCTGGAGACACTAATAAT
A_14_P134748 TACAGGTTATATCAGGTCAATTCTTATCAGATAAGAAAATTGGCACCTACGTAGAGGTGG
A_14_P103077 TATTGTGTGGTTTTACTTTTGTGCTGGTATGAATTCGATTCCATTTTAATGCATCCTGTT
A_18_P16475773
CGTCATATGATTCCCCTATACTACTGTTTTAACTGTTTGGAGATCCATATGTCTGTTGCA
A_14_P130597 CAAATGTATCACAAAGAAGCAAAGGACTATCATGGACTTATCCAATGGAGAGAGTTACAT
A_14_P113270 CTGTAAATCTGTGACTTCTGCGACATGGGATTTAATTTGGCTAAAGCACCC
A_14_P110716 TTATGTAGGAGAACATTACTGACTATTATGCAGGAAGAGGGAGAGAACAATGTAGAGGAA
CUST_3889_PI426210822 GAATCCAAACCATGTGACATGGACCATTATCATTCAGCTGCCACCT
A_16_P17122354
AAAATCACCTGTCAACAATACAGTAGATCCCATAAACTGGTCTGATAAAAATGTGACACC
A_16_P41329709
TGCACTATTTTCAAATCAAAATCCATCTCCTTACATCGTATTTTCCAAATCACTTAGCCA
CUST_5469_PI426210822 TTGGAACAGGCCCTGGGGGAGTGAGGGAGGACACGTGGTGTCTAA
A_16_P31230317
GAGACTTGAGATGCTTTGGCTGATATTTACATGTTTTCGATAAGAAAGGGATCGCTGATT
A_18_P14137612 GATGGGCAAGCACTTCTATCAGATGCTGGTTCTGTTGTAAGACATCATCAAG
CUST_6611_PI426210822 CCCGTGCTTCCAAAGCAAGGCCATCATATTTCTCATTGCACATGTT
A_16_P35297373
GGTGTTCTGTCATAATTACCCCAAATGATCAGAAGTTTCTTACACAAAATGGGATGCTTG

A_14_P138449 GTTGGTTCTTTCCTTTATTGTTGAATGCTCCCATTTAATGCTTTATGGCTTTTACTGTGT

A_16_P16191341 TTTATACCTTTATTCCTCTTTAGTTGACCTTTTAGATACTTTTCTAACCATGCCCCCTCC

CUST_1671_PI426210822 GTGTTCCTGCCCTATCCTTTGTTAAGGCTCTTCCCCAAATTAACCAT

A_14_P119052 TCTCTAACTAAAAACCAAAAAAGCCAAACATATCTTTCTGCTCACTTCCGTAGTCTTCAC

A_14_P137983 TCTATACACATTTTCACACTTGAACATCTACCTATTTTGTTTTGCTTTAGGGGGATGAGG

A_14_P127933 CTCAAGGCAAAGGTGGGATATCATGGCATCTATCTGGGTAAGTAAAAATTAAGCCTT

A_16_P00500417

ATAGAGCCAATATTTTCCTCACACAGTAACTTGGACTCATGAATTACCTGCTCAAACATT

A_16_P16644246

ATCTGAATCTGATCTCAACACTATCATACGACTTTCCTTTTGCACATAAAACGCTGATCA

A_14_P104749 TCTTCCCTGGATTAAAGACCAGTTAAAATATGGATCTGTAAACTCAAGGTAAGAGCACAA

A_14_P136600 TTGCAAGAAAGGCATGGACATTTAGTGCAGTGAGAAAATGAATGGACAGAAATGTTTTAA

A_16_P01164850

TCATTGCAAATCAGTGATTTTTATCAGGCAAGTAAAAATAACAACTTCCTAATAGTGCTT

A_14_P114043 AGAAGAAATTCTTCCTCATTAGAGAAATGAAATGCAGTTGTCTCAGATGCTAGTGAGAGA

A_14_P200349 GAGGACTATAAAACATCCTTCTCTTATTCTCCATACTGTCTTCTACACCTTTAAAGCCTG

A_14_P124942 TCTTTTCTTCTCAACTCGTCTATGTTTTTGTGTCATGGAGTCTGCACTGAAAAAGAAATT

A_14_P137379 AATATTTCAAAATAACTATAAAAAGAAGTTAGAAGCAGGTGAATTGCCCCACAGAGGGGA

A_16_P16192030

AAATTTCTCAAAAATCTTTGAGAAAAATTGAGATGGGACAAAGACTCTGTGCCTTGTTCC

CUST_1413_PI426210822

CATGTCAGAAATTTATGAGGCTGAACTGGTGGTGTGTCTTGCACTATAGTTTCATT

A_14_P109229 CCTCATCAGTAGTCAAAAGCATTTCTCCTTTTCAACATAAACCCTTCCAAAACATCATTT

A_16_P02380972 CTCCATTTCCACACATCTTTTTTATACACCATAGCCCATATTTCATACCACATTGTCCTT

CUST_6457_PI426210822

AACATACAACTTTGGACATCAGATTTTGGTGAAACTCAAAATTACTTGGTGAGAAAGAAA

A_14_P117797 AATCTCCAATTTTCTCCAAAAATAGACCAGGAATCACTTCCTGTGAACTTTACAATCTCT

A_16_P16191667

TTGCCTGAGAATTTTGGCTTGTTTTTACCTTGTGACTGACTAGGCTTCATTCTCTTTATT

A_18_P22839596

TAAAATAGGAGTTCTTCCTGTCCCTAATAGTATTATTCATCACTTCATTCTTGGCCTGGG

A_14_P105458 AAGGAATCTGTTACCCAAAAGATTGAGAACTACTGTTGACAGATCTCTCAAACATATTCC

A_14_P200324 CTAGTATGTTTTATTATTTTATGGGTCTTGAGTGCATTTGAAGGTGTCCTTCAGTTCCCA

A_14_P108113 GAGGGCAGGAAATAAAACTCAGGCAGGCCTATAAGAAGGAGTTGTTTCTATC

CUST_4650_PI426210822 GTTTGGGTTTGTAGGCAGGATGGCCATGTATGCACAAAGAAAGCTTT

A_16_P15384110

TGCTGCTACTTTTGCTATTTAATCTCCTCCTTCATACAAAACATAGTTGACCTTCTCAGC

A_18_P13699283 GTCATGTCAGGGAGGCCTATTCTTTCTCTAAGTCCCATCTTCCAGAAG

A_14_P129259 GGCTGCCTGAAAAATTATATTACTTGCTGTTTACTTCCCTTGATGGAGTTTTAGGCATTA
A_16_P01730976
AGAATGAATGGGTTCTCTAACTTAACACAGAGAATAAGACGAGTCTGAGAGAGAATTCTG
A_14_P118567 CTCAACACAGGCAAATTTCTAAAAAAATTATACCCATCATCTTTGACTTGCACCTTCAGC
A_14_P105297 TCGGAAATGAGATGTTTTGTTGGTAGGAAATGGATAGTTATTTCCAGGTTTTATTGCTGT
A_14_P101079 ACCCAGACACACTACAAAAAACTCATTCATAACACAGTAAGGGCAAACATTATTCATGTA
A_16_P18962909
AAATGGTATGGAAGGTCTCAAGAAAAGTTACACACCTTATCAAGTGGAGTCACAACATAG
A_14_P122145 CTTCTTGTGCAGGGTAGCATTTTAATTCAGTCTCTAATTTTTGCCCCTTCTGTTATTGTA
A_14_P125819 CAGCCATAGCAAATAAATTTCACACAGAGAGAGACCTGAACCTGGAAATTATTGTCCTT
A_16_P00354812
AAGCCAAGCCTTATAACATCTGTCAGATGCTAAACAATCCTACCAAGAAGTAATGCCAT
A_14_P137232 ATTGCGGTTTATCATTTGCTTCACCCATTTTTCTACTTGGACGTTGAATTTCATGAAAAC
A_16_P00132500
ACTGCCATTTGTTTCCATAAACACCTGAAATCTATCCTCAGTTGCCATTTATAATTGCAG
A_14_P108696 TTGGATCCTTCATGGAGATCTTTCCATTTCTTAGAACATTTGCAGAACAACGTCTAATAC
A_14_P124198 ATACTTGAAGGGAACATTTTCATCCAGTGTAACTCATGGAAATTCAATTTCCTTCTTCTG
A_14_P202296 TACCTGAATATATTTCTTGCTATAATTGTAACCTGGCTGTAGGGATCCGAAATCTACTTT
A_14_P121395 ATCTTAGACTTCTCAAATGAGATATTGCTGGTCTCTAGAATTTTAGGATTTGGGAGGCTA
A_14_P200467 ATTCTGGGTTAGCCAGTCCCTACATTTTTTTCACTGAATTTATTGCTCCTGACATTCTAG
A_14_P111952 AGTCCCCTTTTCCTTTTATAAATGAAGAAAAACAAAGGCTCACTCCAAGAGTCAGAC
A_14_P125191 TTTTAGAATCTTATTATCACTCTAGAATCAGACTTCTCTGCTCCGTGATTCCAGAACATC
CUST_4744_PI426210822 AAATGGACTATGTGGGAAAGCGGGACTCTTATCCTTGCAGAAGTCA
A_18_P16043218
AGTAAGTCAATATAAAAGTATATAATTAAAATACGGTGATTAATAGAAAAATACAAAGAT
CUST_10293_PI426210822
AGTGCCTTCACCTTGTTGTTAGCAAGAATTGCATACATAAAAACTTGAGCACATGAAAG
A_16_P18182607
AAAAAGATTTCACTAACAAGACTGCAACCTATATTTGTGGCTATGAATTCCCCAGATTGC
A_16_P58424393 TCCAGTTAGGCTTTCCTCCCATCGTGCCACTTCAGCAGTTCTTAT
A_14_P105467 CACCTTGATATTTTTTCTCCCCCAGATACACCTATGAAGATTATAAGAACACTGCAGAAT
A_14_P135551 CCCTCTATATAGTCACTAGACCCTGAATTTGAAGTCTTAGTTTTTCGAGTTAATCTTCCC
A_14_P133877 AACTGGAAAGACACACGCAATTCTGTAAATCACAATTTATAAGCAAGCAGCAATTGAACT
A_18_P22840141 GTCCCACACTTGTCCCTCCCACCCCTTGCTTCTCTATTTTCAATT
A_14_P129777 TCATTGTAACTTTCCATCTGCTATTGGAATCAAGAACTAAAAACTTATCAGCAGGCAGCA
A_18_P14132345
TTATTTTTAGGCAACAGAACCCATTTCTAAATTGAAATATGCTTCAGTTGACTCAGGGGT
A_14_P138857 CTTTCCCCCACTTTTCTCTCTATTTGTATTGTTAGCCATCTTGAAGTGATGTTGTTTAAC

A_14_P117148 GAGCCAACACTCCTATGGCTCTTCTCAGAACTGGGAGTTATTTTTTAAATATATCATCGC

CUST_5610_PI426210822 GATGGATGCTAAAGGTCTCTCACGTGGCTGAATATCTAGTGAGGGAAA

A_14_P113925 AATCCAAAGTATATAAGATTCCGAGCCAAGTGGACCACCTTAATAAAAAAAAAAAAGCGG

A_18_P18303780

AATGACTTTAAGGTTGTTCTAAGGATTGATGACATTCACATTTGTAATAGTGCACTCCGC

A_16_P16191447 ATAGGTGACAGATATGAAACGATAAATGTGGAGGCAGGGAGACCAGGTAAG

A_16_P03128747

AAGAATTCTAATCATTTTCATTGCTCATCTCCAAACTTCTTCAACTATCAACCAAACTCA

A_14_P107350 CTCCTTTGTTTATGGTTTTATTGGCCTCTTATCAAGCAGTTACCAGTTGAAGCCCATAAT

A_14_P200225 GGGAACCATAGACAAAGGTAGATAAGGCAACTAATGTTTCATGATCAGGAGGAAAGA

A_16_P40248434

ACTCTTTCAGGTATCTCTAAGATAAACTAGCATCTGAGATGGTTCATTCAGATGGATCTC

A_14_P101268 GAAAACAGACAGGTGTCAATGAGCACCTCTGTTGAAGTGAAATATTAAACAAGATCTTCA

A_14_P112356 TGTATCAAGCCTAGATGTTTTATACAGATGCCATATAGTGTTATGAGCCAGGCTGTTGAA

A_14_P108251 AACACAATGCTAAATTTTACACTGTCTGTGCTTCTAGGCCTATTGGACATACGTTGATAT

A_16_P01208083

AAAACTTTTCCCCCAAGTTAGATAATTCTAGTTGCTAAGAATTTTGGCTCATACACCTGC

A_14_P130017 ATTTCTGCACCATGAAATTAAAATCATCTCATTCCTGAAGATGAATGTAGGACCAGACAT

A_14_P110602 GTAAAACGACCTGTGGCCTAAGTCAGAAAGTGAGTGAAAGGAACCTACAG

A_14_P112726 TCCGACATGATTTACTCAACTTTTTCTTCTACTCTTCTTTCTTCAAAACAACCAGACAAG

A_16_P40116679

TTCAGACTTCTCTCCTATGAATAACCCCTTAGTGTCAATATTGCAATCCCTATTATGGAT

A_14_P200857 AATTTGACGGCGAAGTGTTGTAGAGACCGTCTCCTTGAATCATTGCAAAG

A_14_P124200 TCTTCAATTTTGTCAGGCTTAAGTATTTCACAAAACCTCACATGCTTGCATCAAAACAGA

A_14_P119648 CTCCCATTCTCATCAGTCAAAATCTTACCCTACTCAGAAGATGATCAAAAGCAAAACTAA

A_14_P128479 ACAGAGAACATGCTAAAGGATCCCATCTCTATGAAGTTCATGCAGTTATGAAAGTGTC

A_14_P125703 GTTCTTATTTGGATATTTGTTTCTTTAGGCCTGTCAACTACTTGGTCCCAGAATTCCC

A_14_P122095 CTTGCGGAAGAGATTCTTATCATGGTTCTCATAATCATCTTCTTCCACCTCCTCATAG

A_14_P136667 AAAAGCAATCTTACCGTAGTGCAGAGTAGACGCCCAAAGACAAAAACGAATAATCAG

A_14_P117152 ATTACTTACACCAGTTTGTCAGCATTCACACCTGAATTATTCTATGTTTGGTGGTATGTC

A_16_P03192846

CACGTGTTGATTTTTGGTTCTGGAATTCTGATCACCTTATTTTTTATTGTAAGCCTGGAA

A_14_P112875 GGACAGCACAATCCAAATCTGTTTTAATTATTTTAACATGGCATACACACTATATCGGGG

CUST_10051_PI426210822 AGAATGATCAGCCACCTCTGATTCCTGGTCCCACATATCTGGGAG

A_14_P100387 TAGCCACAAGTTTTTATACCTAAAGGATAACGGGAGCACCACACCAGG

A_16_P01860902

AGAGAATACCCAACCTAATCACAATCAACAACAGAAAGTTCTAGTGAAAGAGAAATGGTG

A_16_P40061488

CCTGTATCATCTGGCCTATAAATAGAACTCCCCAGAATATCTACAAAGTGGTAATGCATT
A_16_P40212924
ACTTCCATCATATCAGTTATTTCCATACCATCATAGTCTCAAAATACTGCTCAATGGACT
A_18_P22840466 AGACGAGAGCAGATCTGACAGAGAAAAGGTGTTCAGCCTACTTGGATTTAAAAC
A_14_P125667 CACAAAAACAGTGAGAACTCCTGTGTTTTTACAATGTGTTTTGGCTTACAAAGTATCCTC
A_16_P02186332
CCTTAGAAATAGACCTTAAACTTTGCCTAAATGTAGCATTCAGTTATGAGTAGCCCAGGT
A_14_P109608 CTTCTTTGTGGCTTACACTGGAAATTATGAAAGCAGTTTTTCTCCTAAGACTTTTGGTTT
CUST_10363_PI426210822 ATGTTAGTGAGCCAAGGACGAGAGATGCCGAGTCTCTCAGAGCAT
A_14_P124952 ATGTTTCTCTTGCTACCAAACAGTCATGTATTAACTCTCTTTGGATGATGAAGTTTAAAG
A_16_P20274873 CTGCTTGCAAAACAACAGGTGAGAAGAAGCCATAGGGTGCCACTC
A_14_P131222 ATTTTCAAAAGCAAACCAAGAAATCCAAGTTTTAGCAAGGTGCTCATTCTCATGTTGAAT
A_14_P136389 TTAGGCAAAGTCTATAGTCAGGACACCTTAGTGTCTTTCAGAAGGCAGGTAAACATATAA
A_14_P113552 TTCAAAACATGAACATTACCACATGTTTGAAAATAGCGAACACAAATCTACAGATGAGAA
A_18_P14130895
ACTCACATGGATTTATTTTGTTCAAAGGTCGCATCACACAATAATCATCAGTCTCTCATG
A_14_P107938 AATTTCTTAACTGGATACAATTAGGTGGGATCACATCACAGAACCCATAATGGTAATCAC
A_14_P101299 ATTACACAATTTTAAAAAGTGGAAGAATATCAAGTGACCATACATACACAAAAAGGTGGG
A_14_P103955 CAGAAAGGGAAAGAAGAACTCACTGGCTCAGAATATGACCATTGTTTATAGTTTACCTAC
A_16_P16191947
CCTCAACTCTTTGTGAACATGTCAATATCAATATCCAAATATCTGGCTCTAACTGCTTGG
A_16_P02367847
ACATCTCTCATCAGAAATGTTACCTTGAGTCTTTATCTAGATGCAGTTGCTGTTTAGACC
A_14_P101422 GATTTCAGAAATGTCATTCTAACCTTGATCTCTTCGAGATGCCTGTTTATAACACAGCAT
A_14_P118021 ACATTTTTCAACCACTCCCTGAATATTTCTGTTGTATTATCCACATTGTGATCAGTGGCT
A_14_P126349 TTTTTTTTTTTCCTAATGGGGGTTCCAATTCCCCATGTGTCTGATATTCATTTGAAATGA
A_14_P135425 AATAAATGAAGCAAATAACCATGATTATGGCTCCCATTAACAGGATATATGGAGACAGCA
A_16_P35741597
GCTGCTAACTTGACCTTATGTCTGTAATGGTAAAAATGAATCCTGATGGACTTCAAACAA
A_14_P114098 TGTTTTGGAAACTTACATTAAGAAACACTTTAGTGCAACGTTAGCCTACACGTAAGTTCC
A_14_P131124 GTGTGAGTGCTATCACATAGCACCCATGCCAGGCTGTAATGTTATTTATTAATTTTTTGC
A_16_P19988446
AACTGTGGATGCAATATCAAACTCGTCTGTGTATGTAAAAACCTATGCTCCATTTATGCA
A_16_P18059451
GAATACTGAGCCTAAAACCAATTCTATGCAAACATCACTAGTTTTGCTTGTTATTCCGCC
A_18_P10240138
TGCTGACCACAAGACTAATCACTGTGAGATACTTTAATTTTTGAATGACAAACAAGTGCT
A_14_P119696 GACCAAAAAAACGAGAGTCAGAAACATTGCATACTGCAGGATAAGAAAGAAAGTCCAA

A_16_P37231444
AACCTGTGAAACTGAATACTCCAACTTGACAAGGATGACTTAAAAAAAGATCAGGCAAAA
A_16_P18160176
AAATGTAGTCAATCACTGGATGCTCTCAAGTCAACTCTAAATGTTCCTGAAATCAGCCTA
CUST_5079_PI426210822
TATTTTTTTCATTCTCTAAATTGTTTAATTAAAACTCATAGTTCATGTTAAATTTGTTCA
A_14_P201968 GTCACCAAAAGTAAGTGTATTGAAAATGTGACCCATCCAGGGTTATAATGAGAAGTGTTA
A_16_P01342381
ACTCTTGAATTGATCTAACCTTTGGCCAAACATATCGATATGACTGCTTAGTTCAGACTT
A_16_P18723661
AGGGGCTGACTATAGATTCTCAGTGCTAATAGTTCACATGGAATTTTTATTACCGTTTCC
A_14_P107169 GATGTGGTGATGCTCCTCCTTTATCTTGGAATTGAAGTAATTCGCCTGTTTTTTG
A_16_P02215827
TGAGATAAAATCTCCCCTGTGTGTAAGGATACCAATTGAAATTAGCAACCAAAGCCAAA
A_14_P124492 TGCATTTTTGCATAACATTAAAGGAAAGACTCGTTCATGAAGCCAGTATTTGCGATTGTT
A_16_P00403649
TGAATATGCAAAACCTGTCCTAATAAAACCATTTCTACCCATGATCATGACTTCTTCTCC
A_14_P128157 CTGAGAAGATGGAAAATGGAATTAATACAAAGTCTGCCTACCTCTTTGCTTTATTAATCT
A_14_P123682 ATCTGGGAGATACTGTCTTTCTACATCAATTCAATGCTCAAACTTTATGACAAGCATTCA
A_18_P11209850
CAATGAACTATGATTCCCCTAAAGATTTAGAGCACCATTTAGTATTTTACAGCACCAGGC
A_16_P01412332
AAATGACAGAGTTACATCTTACCCTGAAATACGTAGATCTCAACCAAACATCCCTAGATC
A_14_P133894 CTTTGCTGCTTCCTGTAAGAAGATAGAATAAAGACATGGGTTTGTCAGCACTAGATTTTC
A_16_P19375068
ACAAGGACCTGTATTTTTTTTATGCCAGTAACAGTGACTAAGACATATGAGCATCCATCA
A_14_P107506 AAGAGGGTCCTTACTGTCAAAAACATGAACTTAACACCTCTGAAATTGAGGAAGAAGA
A_16_P02566102
CCACATCAGGTTTTCTTTTCACGTAAATCTCATCCCATAATCTCTCTTATCTTCACCAAT
A_16_P40413727
CCCTTTCTGAAAATTATGACCATTAAGGATTACCCCTCTAAAATAATCAACCAACCGAAA
A_14_P112622 GTTTTGAAAAAGTCGCTGTGCATGGTCAAGTGCCACTGGAAAGAGAAG
A_14_P134020 CTATAAAAGAAGACAATAATCGACATAGAACAGCATCCAACCTGATTTCCTTGGGAAACA
A_14_P139139 CAGCATCTGTTCCTCTGTCATTTCTGATAACAGTAAAAGCCAGCATGGAAAAAACC
A_14_P121614 TGTCCCTACAAAAACTAAATGCCTTGGGTTTGTCCCTATAGAAACTCTCTTGTTATCTTA
A_14_P123727 CTTATGCAGGGTATATGGTAAGAATATCACACTTCCTTCCTTGCTCCTCATGCAAC
A_14_P124290 GCTAAGTGAAATGACTACGTTTCTCTAGACAGTGGTGGATGATATTCAACTTGCAAAAAT
A_14_P111962 CCACAATCGCTAACCATTTAATGCCATTAGATTTTCCCCACCCTTTTTATGAAAGTTAAT

A_14_P137846
AGACAGAAGAGAAACGGATAGAAGGCAGTTTAGAAACTTCTAGATCAAAGAGACTTTTTG
A_16_P41808290
GGATATTTGTAAATGATTTTGTCCACCTTCTCTGATATTTTTAGCAGTAGTGTTAGTCCA
A_16_P17519156
AAACACTGAGACTTTGTTATTCTCTGCAACAAAATCATTTATACCAAGTCAGGTCTGGAC
A_14_P123831 ACTTCCTATGTCCTATGCCACTAAGTCTTCTCTCTCTCTCATTTAAAAATCTCTGTTGTT
A_16_P15220570
AAGTCCATGTAGAAATTCAGACTCTCAAACTGTCTTATACTATTTATGTCCTGGCCTTGG
A_14_P103267 ACAGATCAGTGAGTGTGAGCAGTAGTGAACTAACACTATGCTTTGTAATTCATCATATCC
A_18_P22841760 GTGTGGGATTATTCAGTCCAATTCTTCTACTTTTTTTAGGCCTCAGCATGGGATTT
A_14_P136175 CAATTGTTCCATCATAGACAGAAATATAAATTAAGGCACAGGGTTTTGTGGTGGTTTGTT
CUST_8786_PI426210822
GCAAGTTCTCTTTGTTAAGCCATTCTGGTATCCCTCAAATCCCTTGTTATGACTAT
A_16_P03027521
CCTGAAGCCAAATAAGACAGATAAAAATTTCCTTCTAAAACTAAAGCAAGACAACTGACA
A_14_P100337 ATTCAGGACACAGGGAAATCAGGGTTACAAATCTTCTTGATCCACTTCTCTC
A_14_P118596 CAGTCATCCATTCACACACCACTTCAATGCCTCATATTTCCTGACAGTCTTATTCTAAAA
CUST_6334_PI426210822 AGTTAAACAGATGAGGCAGGAGGAGGGAGACTGAGCTGCCTTGAG
A_16_P02624541
TCCTCCCATTTTCATATCTCCATACAAAAGTTATGTCTCTAAATTGCATATTCTGCACCC
A_14_P121595 CATGAATGACTATTATGATGAAATCCACGTCTTGATGGAGTTCTGTGTGCCTTTATTCCA
A_16_P21468462
AAGAACCTCCAATAACTTGTAAATCTCCCAACATTTACTCCTCATACAAATTGCTTCAGA
A_14_P135640 TCTCTAAATGGTGACTTCTTTGCTGTAGACTCTGACTTAAGGCCTTCGCATTTTC
A_14_P129029 ATGGATAATTCTGCATCACCACACCATAAATATCATTAGGATGGACACCATGCTTTATTC
A_16_P36996914
CAGAAAACCTTTGGCCTTATATCACTACTACACCTACTTCAAATATTTTACAGTCTGTGG
A_14_P112513 ACAAAAACGCATTATTTATTTTCAAGATGAGGGCTCTCTGACCAAGAAACTTTGTGAACA
A_14_P102805 AGTTCTAATTTATCAGAAAATTGTGTTTTGGGATTGAGTTCTTTGTCTCAGCCCAGAATC
A_14_P117661 GCCTAGTAGATGACTATTGTTTCTAATGACTGTAACATCACTAATTGGCAGAAGGTTCAC
A_14_P121505 AATAAAAGTCAACTCTAAAGACACTCGAGGGAACAAATATGGATTCATAGACTCTCAGAG
A_14_P118389 TTAAGGGAAGGCTAAAACTTTGCTGATATGTGATAAAACTTGAACTCTAAATTGCCTCCT
A_14_P127310 ACGCTGCGGGAAGGAAAAAAGATATCACAATTTAAACCAATCTATTGATGACTGTCAAGT
A_18_P16734337
AAAATCTCCCAGAATGAAGTCATATTGTGTTGGCAAACTTAGTCATAATCCCCATTTCCT
CUST_2023_PI426210822 AAACTGCTTTTCCTTCCCTGCCCTACTCCTTACTGTCTGTGCCCT
CUST_6890_PI426210822 CTTAGGCAGAAGTGAGCCTCATCCCTCAGTGCTGGGACAGATTAC

A_14_P120257 GGTGAAGGTTTATACGTTATACACAAGGCCAGGTAAGCGCTCATAATTCACACATAATAA

A_14_P103614 AATTCAGTGTGCATGAAGATCTAAGCATTTGAGTGAATATAACGACTCCAGAGATCAGAT

CUST_9429_PI426210822 AGTCTCTGAGCCAAGATGTTGGGTTTGTGGCAGACCCAAGACTAT

A_16_P16191751 GTTAATGTTGAGGCAGCCCACCCAGGTTAAGGGTCTCATTCTGAG

CUST_9696_PI426210822 TATCCTGGAGGGCTGGGAGTGATCCAGTGTCATCTCGATGCAAAG

A_14_P129729 CATCATGGGAAGGAAGCAACTTCACACCTGGCCTTTTATAGAGGAGAT

A_16_P17176590

AATGCCCTAAAAACGTAATATGACATTTACCACTGAAAAAGCGTTATGATTCCAGGTGTC

A_14_P108159 ACAAACTCAATGTCTAGGTTTGTCTTGTTTCTACAGTATATGGCTATAAAAACCTTCCGG

CUST_9511_PI426210822 GTGATTCTTCTGAATGCCTGGCCTTCTGTCAACTATCAGTCTGAAGG

CUST_3960_PI426210822

TTTCCTGTTATTCCATATTCATGCAGGTCTTGCAGCCAAGGATTGTTACATTAAGATC

A_14_P129842

AAAAGAAACCAAAATCCAGAGAAAAAGAATTAACAAGATTTAGGAGCAAACGAGTTCAGG

A_18_P16316189

ACTCATGTCCAGATTTTTGAACCAACTCAACCTAAGTAAAGTCCTATCCTTATGCTGAG

A_14_P129640 CACCTAAAATTCAGTTGGACCACATATATTCATCCAAAAAGCATAGGCTTCTGTGTAGAT

A_14_P100430 ATTCATATATAACAGGCAGTATATTATAGGCATCATCTAGTTATGGGAATGGGAGGGGCA

A_14_P112739 CTATTCAACCAGTTGCACAGAGTGGAATATAAGGAAAAGCACATAGAAACTTCATTCACT

A_14_P116344 TAGGCAGAGGTAATATCCTAATTGAACAATAAGGAGAACCAAATTCTTAGTCACCAACGT

A_14_P139654 TGTTAGTTAGCACTGTTAGTCTCCTCTTTTCTCATGTTTTTTTCCTATGGGGCTTTAGAA

A_18_P14134505

ATGTAAGATTATTTTTACTTATTTTAAATCTTTCAAAGCACTTTTTTCCTCAGGGCCAAG

A_14_P139641 GAGGCCAAATGAATGTTTGAAGTAGGTATGCTTTGTTTCCAGAATATTCCAAAAGTTAGA

A_14_P114564 ATCAATAACGTTGATTTGCACAAAACAAAGGGTCTTGAAATCTGAAAACCAAAGTCATAT

A_14_P122334 AGCATACAAGTGAGCCCTTCTCTGCTATAAAGGAAACTGTCCATAAATCACTTAAAATGA

A_14_P123277 AATTAAGTAGTATGCTGACTTGGCCTGTTCAAAAGTTCATGCTAGAGTGCTTATTGGTAT

A_14_P127256 TGCAATATTTTTGCTACTACCCAATAACTATGCCACTTTGGGTAATTATTGGGAGTCTCT

A_16_P01567131

AGAGCCAGTGATTACAAAAGAAACCTCTCCTTATACCTTCATAGTAGTTTATTGTAGGCA

A_16_P02634606

ACTCTTGTTGACTCTTTACTCCAAGTTCATTCTTCATTGCTAATGTCTCCAAATAGTGCT

A_14_P139987 GGGTCAGGTGGTCTCTATTATTTGGGGTGTTATGATATTAAGTCATGAATCCAATAAACT

A_14_P111196 ACAAAACACTTGCTTTAAAAGAAATTTAAAATTATAAAAACTCCGAGCATTACTATCATG

A_14_P107192 AACAATTATTTTCTCATTTATGCTGAGCCCTTACAGGATTTTGGAAAGTCAAAGGTGAAC

A_16_P17943564

ATGACCTATCTTCCCTTTATGCCTTTTGATCTTTTGTCAATTACCCTGTAAATGTATGCC

A_14_P112929 TTACTGTTTACTGTTTCTTGGTTCTGTTCTATTTGTTGGGTGCAGTATTAGTTTGGATCC

A_16_P21598182
CAAAAAAAATACACACACTTTAGGATTGAACCTAGTTGTCTTCAATTTATAGGGCCTGCC
A_18_P22839774
ACTGCCCTGTTGTCTAAAATTAAGCCAATTAGAGAGTCCTGCTATTAATAAGCAACAGTT
A_14_P111066 ATCAAGATATAGGTGAAGATTTAGGTTTTATCATGTGAATATTGTGTGGTAGTGCACATT
CUST_10383_PI426210822
TAAGATCTCTCAAGATACTAGGTTTATCAAAAGCCAAATAAGCTTGGGGAATGTCAGGT
A_18_P26792696 AGCACTCACCACCACGTCAGAGTCCAACACACGTTCCCACAGGAC
CUST_4084_PI426210822
GCATAGATGATGGCTGGAAAGATTAAGTTCTTTCTGGTGGCTTCTTTAATTTTGGAATAA
A_16_P17617399
AAGCCCTCTTATCTGATTCTATATTTGGTCTTGTCACCATGAAAAACTATGCTCAGACTT
A_14_P106297 CTACTGAAAATAGGGAGATGTAAAGCACTAAACTAACCCACTATTAAAAAGGAGAGAACC
A_14_P110899 CCACCTAAGTTCTTTTACACAAGAAAACTTCTTGGGGTAAGTATAAATTCCATCCCATGC
CUST_8246_PI426210822 CCTTGATTAGCAGTAGAAATGTTGATCCCTTGCAGGGAGAGCCATC
A_18_P14132860 GATTAAAAGCAGAGGTGAGGTTAAAGGGTTTGAACAAAATGGGCCTGATCTTG
A_16_P38676374
AAATTCCCTCACAACAGATATCTTTGCACAATGAAAAGATTTTTTCAAGCCCTCAGTTTG
A_14_P130905 CTAGATCCTGGCCTCCAAGATTCTATTTCTCCATTATATTCTAGAACCTAAGTTTGGAAG
A_16_P15079562
AAGGAGCATATGCATTACACTTACAGTCAGTAAACAGGTATTTTACAAAAGAGGCAGAGG
CUST_9036_PI426210822 CATAAGCGATTTCCCAGGATGATGTGCTTGGAGCATGACACTTAGAAAAAAG
A_14_P121777 TTCTTAGATACTTAAAATGTGCCAATGCCTAAGTCTTGACTTGCACGTAGTCTGTCTTAG
A_14_P122800 ATGCTTCTGTTATGTCAAAATTGTGAACATTTGCCCTTTGTGGATAAAGTTCTCTTGATG
A_14_P200627 GGTGACCAAATCTGTTTACTGATCCCTGATAGAGGTCACATTCTTTGAGCAAAATATTTA
A_14_P105057 CTCATTTCACTTTGTTCCTTTTGATTTAATGCAAGGAATTACTCAAGAGCAGAAAGGGAT
A_14_P132140 GCCAGGCCTGTCTTTTTTCTACTAGTGATATGAACAATTTAGTTAGCAAGACAGATAGGA
A_16_P00685448
TTTCCTAAAAGTTCTTGCAAAAATGTCATAGAGTCTGATTGGTCCAATTGAGTTAAGCCT
CUST_10284_PI426210822 AAGAGATTACGCATAGGCTGTGTTAGGAACAGGACTGGAGACCCT
A_16_P16173790
AAAACTAGTCATTAAGATGTGCAATCTGTATCCGATGTATATCTTCAACCTAGCCCTTCC
A_16_P16905240
AGTCAATCTGCCAAAAAATAAATGTGCCTAAATTATCAAAATCACCTATGTTCCAACCGT
A_16_P30952400 CTTACCACGACTAAGTCACCTTCCTCTCTTGCCCTGCAGAGCAAG
A_16_P30952411 ATGTGATCCTGTGACAAGGAAAAGAGGTGGCCTCATCACAGTGTG
A_14_P122886 CTAAGGTAGTCAAAAGAGAATCAGTCTTGAAACACTGGATGTTTATGAATAGACCTTGGT
A_16_P37838345

CCCTAACATAAAACAAAACAATCATTTCCCTAAAAATAGTCAATATACAGTGTGCAGTGC

A_14_P139250 ATAAATTACCGACAGAAACCATAAAGTGGGTGGGGTTTGACTGCTAGTAAATGACTATTA

A_18_P12317848

TGACACGTAGTATACAGAATCCTCAGTTTACTTACACTCTTCTAATAGTGCCAAATGTCA

A_14_P128640 GTTTTGGTGAGAAAGGAAATAATGGAACTTAGAGAGGATGAAGTCTTTGAGCCAATGTG

A_16_P15923043

GCTGCAAAATGGAACGAAGTAAAAAAACTCCCTATCAAAGTCTATGGCCATAATGTGATT

A_16_P03022464

CCTGTCTTTCGGATATATGCTCCTCTCTCAAGTATACATGAACTTTTAGCAACCTTTCAT

A_16_P17734208

AGTGCCTATTTCCTTTCAATACCTAAGCCCAAGACTACAAGTAAAAAAGGATCTCCATTT

A_14_P201667 CTAAACTCTGTGGCCATTTCAGTTGAAAAATAAATGGTTATGTGGATGTTTTGAAGACAT

A_14_P101629 CTTCAGAGTGTCAGGGCCTCACTTGTATAGAATGTAATGTTCTCCTCAAACATTTATGTT

A_14_P115813 TAGTTCAGGAAGTAAGTAACAGATTATTATTTTTCTCCCAGTGGCAGGGTCACACTATTT

A_14_P110102 CTTTGTCCAAGGCAAAATTGTAATCATTAGGTCTACTACCCAAGGCTGCTAAATGAATAT

A_14_P129096 AGCAATAATGGCTACTGAAAAATTAATAGTGTCGGCTTTGAATGAAAAAATTCCCTTCCC

CUST_4606_PI426210822 TCAGCAGCAGTCATTGGAGCAGGGATCTGGGGAGGCTTTTCACGT

A_16_P15144546

ACTCTATCCACTTGTCTTCATAAAGCCTTTTCATATGCTTACATTGCTCAATCCCATTGT

A_14_P120279 AGCCACAGATTATGACACATGCTAATTATTATCACTGTAGGTTCAATAAACTGGGGGTAG

A_14_P134885 TGTATTTTTATTCCTATCGGTCAGTGAAGTGGTGGCTCTTTTGGAAAATCTAAGGTATTC

CUST_3666_PI426210822 GAAAATAAGAAGAAATTCAAACCCTTGTTTTGCTCCTCATCAGCGACAGTGGT

A_14_P120875 ACCTATCTCTGATCTTACCCTCTAACAATTCCCAACAAGAAATGACTCCATGATAGTGAT

A_14_P119059 AAAATCCATACAACTTCAGATGGAATGAGCAGTATTTCTGAAAGAGACTTAGCGTCATCA

CUST_7288_PI426210822 TCAGACCTCCTTGTATGGGAACATCATCACCTTCACGCTTTGTCT

A_14_P102246 ACACTTTGTTTCTGAATGAGTTTGTGACACATAAACTTGGAGAATGGAATTATAGCCTGG

A_14_P112521 ACAATCATTCCTTGTGCTTTTAAATTTGGAGATCCGAGAGAAAACAGCCTTTTTGTTGC

A_14_P128172 TTTGTTACCCAAGAAAGCAAAAATGCTTTGTGACACAGGTTATCTGTAGTGCTACTTAGA

A_16_P38431159

GATCCCAACTCCAAAAATTTAGACTTCCCTTGTCATTTAATCTCTAGTCCCCTATTTACA

A_18_P14136298

ATTCATCATGTGAGATGATAAGTGTCTTTCCTTGAACCAGTTCTTCTTCTTCCTCCTAG

A_14_P116108 GATTCGCATAGGTGTTACTCTTCTATATTTCTCTCTAACTGCTGACTATAATATCACTTG

A_14_P100599 GAGAAACCACCCTACATATCTGGTGCTATTTCATCCATCTACAAGCAGGAG

A_14_P119342 TGCTCCATAATCATAGGCAGAAAAAGATAGGGAGTGCAAATATTGTAAACATCCAATGGA

A_14_P201610 GTGGACCTCACACCTTTAATTACTGGATACAGAAAAAGATACCTGAAGTATTGAAAGGGA

CUST_7280_PI426210822 GAAATGATGACCTGAGAGTGTGAGGAAGGGGAGAGCCAGGAGCTG

A_14_P131437 AGAATCTCAACTTTGATTTCACTCGGAGTTACCTGGACTTGATTGTAACTTACACCTC

CUST_1880_PI426210822 TCATATCTCCCAGGGCTTGGAGAAGTGGAATCTGGATTCTGGATTA

A_14_P201851 ATCCTCAGTATCTAACTCATTGCCTGGCATGAAATCTACATAAACAACAAACGTAAAAGG

A_14_P135792 TGATTCATTTCTTCATTTCCTTCATGTCTACAGGAATAAATCACAGAAGGCTAGTTCTGG

A_16_P15628480

ATAAAATCTCGACATGGATACATAGTCATAGGGTGAATGTAAAAATCATGGACTGAAGGG

A_16_P17846649

ACTGAATAAGAAGACTGTTGACATCCTGTAACTTATACTTTATATGCAACCCCTTCACGG

CUST_8101_PI426210822 ACTGAAAGTTTTTGAAAAGGGGAGTGGCACAGTGCAACAGCTTGG

CUST_9499_PI426210822 ATTTTCATTATCAAGAGTTCTTGGGAAGAGGCCGAGAGGTTTTTTTAGCACTC

A_14_P107270 TTTACCCTCGCTATGACTACAGAGTTTCCTGGGACCTCCTCTCTTT

A_14_P135333 CACCCATTTTCTTAATTAGAAAGGGAAAAGACTGTGGCCTTGGGAGAGTTAAGAATAGTA

A_14_P115162 TAGAGGTTGTCTATGAAAATTAAGTGGCTATGATCGACCTTGCAGTAAACCTGAAAGATT

CUST_5641_PI426210822 CTGTTCCCTTCCTGGGAAGCCGGCTTCCTGGTGTTGTGAGGAGAG

A_16_P16230288

CCATTCGTATGCTCAACCATATGCAATATTGCCTGAATGTGAAATCTTCTGTAGGAAATG

A_14_P119196 GGATAGCCTGTGAAGATTTCAAGAAAAGCAAGGGACCTCAACAAATTCACCTTAAA

A_14_P109341 GGATGCTTATTTTAATGACGCTATACTTTGGTGGTAGCAGCATTTACTCAGTCAACACTG

A_14_P102728 GAATGGCAAAGTCCACTATTTTCATACATTTGTTTCTCTATAACCTTTTTCCCTCAGAGC

CUST_5146_PI426210822 AGGACCAAATTCTTAAAACCCCAGGGGCCCTGGGGTGATTTTCAG

A_14_P132280 CTTCAAATAGAGGCCGATTTCCTTGGTCAATGAAGAGAACTTGGTCATTCAAATTTCTTA

CUST_9826_PI426210822 CATTTCAGGAGGCAAGAGCAGCCTGCAGAGAGGCTGGGGACCACC

A_14_P136023 CTTTACTCACCCAATTCAATACAAGTGATGCCAAGTGACCAAATATCAACTTTCCCATC

A_14_P117281 CTCCCAGACTTCCTCTTTGTTTCTTTGTTGGGGATTTGTTGAGGAGAG

A_14_P122925 GCCCCCCCTCAAATACTTCCACATAGTTCCATAAAAATAAGTAAATAAAGGAAATATCCA

A_18_P16180029

ACTGCATGCAAATCATCCAGAACTTTTCAAAACCCTTGAACCTATTACATGTAAAATCCA

A_14_P130709 TCTTTCTCCATATTTTGACTCTTTTTGCTCTTTCCTTCACTTGCAAACATAGTCAGGTTT

A_16_P16894772

CCTCACTCCTTTAAATTGGTCCAAATGTTCTAAAGAAGGCACCATTGATTATTTAAACGC

A_14_P200209 CACCTACTCATCCAGAGAATTTAAATTGCTTTTGTTCATTTTCCATACTACTCCCCACA

A_14_P115655 ATACCCCCATTTGACATTGACTTACCATTACTGATTTCAGGGAGGTCAAACTTAGT

A_14_P129962 CAGTGGTAAGGTGGCAGAAGATGAGAGGAAAATTATTTTAAATCAATGCAGAGCACTTTA

A_14_P102741 AATAGAGGAATTTTACCATTTTTGTGATAGTAGGTGACCTCCACTTTCCTCTCTTCTGAC

A_14_P111084 CATGAAAACATTTTCTCAGGAGAAGGCAACTGTGAAGTTGAGAAGTCTGCTTTCTAAAC

A_14_P122143 ACATGACCCTAAAGGATGAAAGAAAGGAATTTAGAAATCTCTCCTCATTTCTCTCTCTTT

A_16_P58424394 ACCAGTGACCTCCAGAAGGCTGATTCCAATGGCCAGTTCTAATTATTC

A_14_P106857 ACACACTACCTCTTTCACTCTTTTAATTCTCTATCTATAGGCCATTAGTTTCTTGTGATT

A_18_P23434627

ACAAAAACAAAAACGAGTCTTTCAGAAAGTAGCCGATTCAAAATTCAAATGCACGTCTTC
CUST_7508_PI426210822 ATAACAATGAGCTGTAAAATACGACCTGTGGGGTGGGAATGGGGA
CUST_3405_PI426210822
CATTGCTTTAATCAGAAATAACATATCAAGGTGGCCCAAATTGTGTCACTATTCTTTCCT
A_14_P101892 AGTCCCAGTGATGAATCAGGCTTTGTATACTATATGAGAGAACATTACCTCCATAGAACT
A_14_P135494 AAATGTCATGGCTTTACTTTGGTCAATTAAAGGGGGGAATTTTTTAAAAAATGTGCCTT
A_14_P117068 ATAAACAGGGAATTACACAAAATATTTATGGCCCATTTGTAATAAGGGCCATGAAGGGA
A_14_P202143 CATTCTTGGATAGACTATTAGATTGGAATATACTAGGTAGACAAACCTTTGCTGTCCCAC
A_14_P106500 TGGAAAACTGAGACTAGAGGTGAACTAAAAGTTGAAAAACTAGTTCTTGATTGCCTGCTA
A_14_P135002 AGTCTGAAATCTCCATGAATCAAATTCTCTTCATTGTCATTATCGGGCAAGTTCTTCATT
A_14_P101513 CACAAAATTACTCCCTAACAAGAAAATTGAAATATCCAAGGCCAAGTTCAAGGTAAAGAC
A_14_P115265 CACTTTTCCCTTGGTGAAGTGAGTTTCACTACTGAGAAAGTAATCAAATGCTAAATCATG
A_14_P113146 GTTGCCTTGGAAAAACTCCGAGATGTGATTGCTCAGTGTATTCTCCC
A_14_P131794 GACAAAGTCAAGGAAATCAAATCTTCAATGAATGAAAAACTCAGTGCCATCTGGGG
A_14_P137555 AGTATATTTACAAATGAGACATTGCATGAACGGTCTTAGGAAGAAAGTGCCTGCTAAATC
A_14_P105598 CACGCTCTACTGCATACAGGAAAACCGAGGAAAAATGAAATTGGGAAGAAG
A_16_P37755895
TCTTCAAACCTGTCACAATCTTTTCATGTAGTCCATAACCTGTAAAATTCACAGCCTATG
A_14_P125064 TTACACAAAGGAGACTACTTCTGAGTGAAGGATCTAGTTGAATTGTGTAGCCTTTTCTAG
A_14_P114652 ACCATTTTGGAAATTCACAGTAATAGGAAGCACAGAACAGTAGGTAATTGGACCATAGG
A_14_P130662 AATATTGCCATGGACAAATCTGATTTGATCATTGAAGGGACAAGCCTCTGTTTTAGAATT
A_16_P19934091
ATGATGTTGAATAAGTATGTCCTCATTAGCCTTCAAAACATTCTCACATTGTTGGACTGG
A_14_P117132 AACATGATCAAAACCACAGCAGGAACCACAATAAGGAACAAGACTCAGGTTAAAG
A_16_P19256327
TCTGTTGCAAATGGAAGTTGTTAATGAGACTCAACAAGTTAGTGGAAAATGTTTTGAGTG
A_14_P119389 AAATGGTTATTCTTAATAAAAGGTCATTCCTAATTGGTGGATGTTGGGTGAGAGAACAGA
A_16_P01791176
AAAGTAAATACAAGTGAGCCTACTAATACATCAACCCTGATTGATTTTAATTTTCCCCAC
A_16_P21458402
AGCGGCAAGACTTTCCTAATTAACTTACATATGCTGGTTACTTTGCTATAGGGATCAAAG
A_14_P121310
GCAAGGAAATTGCAAGAATAGAAAATGAAAGAGGAAGTAGAAAGGTTTGCGGTATAAGAA
A_14_P139622 ACATTTTGCTGTGGAAATGTATCCACAGGCCATTTTAGTTGACTTGAGAAATCAAAGTTA
A_14_P111096 ACCCAGGTGGCGTCAGTCACTGAGTATATGGTGAGATAATAAATACTAGTTGTTGAGATA
CUST_8075_PI426210822 CTTGAAGCAGCATCTAGGTAGCACTTGGCTGGCAGCTCCAGTGGA
A_14_P133818 TCCTTCACAGGCAAGAGAAATATGTGTTAAATTCACTCGTGGTGTGGACTTCAAT
A_14_P131495 AACATTTATATCTTTCTTTCTGTTTCAGGTGCGTATAGCAGCAAAATTCATCATTCATGC

A_14_P121917 AAAAAGTTTTCAGTTAGAACAAAAATGGAAAACAGAACCACACAAACTCATCATTTATGT

A_16_P18682495

CAAATTTTTCCTGATAAACCCTCTCATCTATGTCAGTCATAGTTCAATCAGAAGCAGACG

A_14_P100595 AAATCTATTAATGGTAGCAAAGTGCATAAGACAGGGATTTCTTTGAGATGAAAGGAGTGC

A_14_P127456 GCTTGGCGACATAAACCTCATACTTGGATTTATTCCTGCATCTTTCCACCTC

A_16_P00799708

ACCAGCATCCTTGATAACAGTTTACCACAATGCATTAAATAAATCATAGAACACCAAGGA

A_14_P111453 CCTGCATTCTTAAGAGAATGTGAGTAAATATGTTGAGTGGCAATGGTCAAGTTCAATGAA

A_14_P134015 CCCTTCATCCAGTCTTTTAATTCTGTAGTGTTTTACAACTGCTCCATCTAAAACTGGAAC

CUST_3151_PI426210822 CAGAGGAAGGGCTGTTTCTATGGTGGCCCTGAGGGTTACTGGGAA

A_16_P20344523

GGTATATGGCACTATTGTTAAGACCTGAATTAGACCTGCTTAGATATCCCAATCTGTGAA

A_16_P20863822

TAAGAAAGATTGAATGCTTAAGTTCAGAAGGTTCAGACAAGAAGATTTTCTACCTGCCAC

A_14_P134531 CAACCAAAATGTAATTCGCCTTGATACATTCAAGTTAATTGATGCTTGTTCCTACACTAG

A_14_P112071 GAGTGTTGAGAAGTCCCTCCATATGCCTTTCTCTCCAGAAACAACTGTGTTTATATAAAA

A_18_P14644205

TCAATTGCATTCCATTGATAGAATCCCTTGATTCAGCATATTCTATAGCCACTCCTTGAT

A_16_P03487895

TACTACGATCCTGCTTTTAAATATCCTCTGCTGACGGTTACACTGTTCTGAAGAAGTAAT

A_18_P17379992

ACCAGTATCTAAAAAGATCAAAATAACTCTTACAAACAGACAACAATCTAGCCTAGGAGA

A_14_P139731 CAATTGCCTAGAGTCTTGGACATTGGATAAGCATAACAGTTCAACATAATGTTGGTGAAA

A_14_P101118 GGATGGGGCACAATTTTAAGTCTCTTTCAATCTAAAAAGGGGAAATGAGTTGAAGATTTA

A_14_P130456 GAATTACCTGGGATTACAGACTAAATCCCAGTATGAATTCCAACAAGCAGAATTCAATTG

A_14_P114008 CGACAATGTCATGCATGTATTTAGAACCTTGGTGTGTATCTTAGACTATGCCAGATTGTA

A_14_P136749 TGTCAGAGGAAGTGTGTGTCTAATCTGTCACTTTTTATGGAAAAGCCACTAGATTTTACA

A_14_P133099 CCACCTGATTTTAGAAAATAAAACAATGCTGGTTAATTTTTCAGTCTTGTTTGCTTACAT

A_14_P105151 ATTTGGAAGGATGTTTACCAATGTATTTGTGGTTTATCATCGGATAGGAGATCTCAGTTT

A_14_P129557 AACAAAAGATTCTTCAATGGACAGAATTGTCACCTGAAATATAGCAATTTGGCAGAAGCA

A_14_P128481 GGTATCTCCAGTCTATTATAAAACTACTTTACAGAGATCCTCCTCAGCCTGAGAGATTTT

A_14_P127604 CTTGAGTTCTTTTAAAGGAGGGTAAAGACAGCAGAAGGAGTGATGATGTTAGATAATGTT

A_14_P131944 TCCAGCAACAATTTATATCTGAAAAATGTTTCCAAGCCAAAAACCTGTAGCAAATCGTCA

A_16_P00907455

TTTCATTAAACAAACCTATAACATTACGTGCCTCATTATGTATTTCGAGACTTCCCCTGC

A_14_P105292 CTGTGAACTCCTTGCATGCCCTGTAAAGGCTTTAAAATACAAATATCCTAGTTCTGAAAA

A_14_P111038 AACCTTACTGAGGCTTTCGTCAGATACTCGTGATAAAGCACCTTCTTTTTGTAATTTGC

A_16_P16960288

AGTGCATATCATCGTCATTCATCTCACTTTTCCTTCTAACATCAAGGATCTATACAAGGC
A_14_P105790 TGATTTATTCATAAAAAGCCATTATTTGTGCCAAAGAAGAACTGAGCCGCTGGCTTTTTA
A_14_P101363 GACACGAAGATGTGCATTGACAGAACCATAGAGACTCTACAAATATTCATTATCCTTCAT
A_14_P117407 AGAGTATTGGGACTTTGGAGGAAGCCAGTGCTATCAATATTTACAAGCATAAAGCC
A_16_P41251123
ACAATCTCAATCATTTCAGTAAACATTTAAGTAAGTCAGTCTCACTTACTGTGGATTCCA
A_14_P139363 CAGTAACTTGCTTTTTAAAGTCCACTAAGCAATCTGTGGCTTCTGAAATGGAAACAAAAG
A_14_P116072 GAAATGTCACTAACAAAAAATTACATTATTTGTGGGAAAGATGGGAAGATGACTGCGTGT
A_14_P200272 CTGGCAGAGTTTATTGACTTTCTGGACAAGAATGATGAGGAGTATATGAAATACCTGGC
CUST_8119_PI426210822 ACTAAATTCAAGAACACCCCCTATGTACATTAACAGTGCTGCATAGCTTCAGGA
A_18_P12198319
AATGAAGAATCACTACTGTTATGGCCTACATTGTAGACATTGTTCTTTACAGTCAGACCA
A_16_P15235057
CCGGGTATACTTTGAGATATTGGGTCATATTACTCTCTCTAGTAACAATACTCATCACAC
A_14_P106186 AGAACTGATGCTCTTACCTTGTTAATAATTCCTCCGTTTTCAACAACTCCTTCAGCAC
A_14_P200481 CTATGTCTCCATTCATGAGTAGGAAAATTCCAGTATAAATGAGAATCTCAAATCATGGCG
CUST_4187_PI426210822
TAGTCAGTATCAGTAGATTGTTGGGGAGAAATCTTTATGAAAAATGCCACTTTTCACTCT
A_16_P00238229
TCCCTCCCATATTTTCATACTTCAGCCTGTTGATATTTTACCAACCTTAAAAATCCACTT
A_14_P100094 CTTCATTAGGATAAGAGATGAGCATCTAATTCTTGCTATCAAAATGTGGTGAAGCCAGTG
CUST_9544_PI426210822 TTAGGTCATAAACTACAGAACCTTAGGTCATATATTCTCACCGGGCGCTTGTTT
A_14_P132279 AACCTATTCACATATATCTTTTAAATTTCTGGTGTCTTTGAGGATGGGCAAACTGAAGGT
A_14_P112789 GATGTGAAAACCAACAAAGTGAGCAGGTTTATCACAGACACGAGAAAGGCAT
A_14_P110033 CTTTACGCTCAGCCTCCTACTGGTTTTCATTATTAGTGAAATGTCTGTTAGTGCAATATG
A_14_P201829 AGTGTTAAGTCAGAAGATTGAGTAGATGGCTACTGTTTGCTTAAACATAAGTGTAAAAAA
A_16_P00235540
CTAGGGACAAACCCGAAGTAAAACGACTATACTTACCTCAAGTTGAACTTTTCAGAGAG
A_16_P20371706
CTGAAATCCAGGGCATTTGACAAATGAAAGTGATTATCACTGTTTAATTCCAAGTTCTCC
A_14_P128433 AAGAGTCATGCCTACAATTGAAACAGGAAAATTAATCAGTTCAGGATCTCAGAAAGTGAG
A_14_P115314 AGCAGTCTATGACAGAAAAAGTGATAGGCCCTCTTTCTACAAATATTCATACAAGGACTT
A_14_P127475 GATCTGATTCCCATCATATTTTGCTCTTCTACTCTTATCCCCTAGACTTCCATTTTCTTT
CUST_1181_PI426210822 GCCTCTAAACCCACTTCTTGTGGGATGGTGGAAAGACCGTGGGAG
A_14_P113742 CCAACTTTTATTGACCATTTACTATGTATCAGAGTTCCTGCCCTGAAGAAGTTAAGTCTG
A_16_P16367305
CAGAGATTTCCATAGGCAGAATAACATTAGAGATTACGTCATTACAACAGATAGCCCTCA
A_14_P107236 TCATAAGAGGTTCTAACTTCTGTTGACAAAGGAAACACATACTCTCTCATTCAGGCTTCT

A_14_P118652 CAGCAAATTTCCATAAAAAATAAATTATGTCAATGTAAGAAGGGTGTTGATCCCATCGGT
A_14_P102781 AGACAGTGTCTTCATCTCATTAGATAAAGGAGGCTCAGAAAAGATAACAGTTTAAGACCT
A_14_P102069 AAGCATTTCTAGATGAGAAAAAGAAACTTAAGTGTTATTTCCCCCCTACAGTTTTGAAGA
A_16_P02658035 ACTGAACTTCCCCTGTTAAAATGATCCACATTCTACATGGCAATTACGTTTTTCAAATCT
A_14_P117753 AATACTACTATCTAGCCTTCTCCAAGATAAGTGCTTTGGTAGGCTAATCTTTCAATGGGT
A_18_P14375740 ACCTTGCTCCTGATTAACTTAATTATCACCAGGCTAAATATAGATCGAGTTCTACATGGA
A_14_P132536 GGGTTCTATTTTTAAGACCTGGATTGAACCTTCAGTCATTAATGTGATTTGAGGCTGTC
A_16_P03435271 CCCCATCATAATTCTAAGCAATCATTCATGTTTCCAACATCTCTTATTGTCTCATCTCCA
A_18_P11903891 ACAGAAATTGCCAACTTACTTTATGAAAAGACACCTGAAATGATGATGCATGACCATTGT
A_16_P15550756 AAATATCAGAAATCTCAGATAGTGCTTCAGACAATGTGTTTATGTCCGTTTCATCTCCGT
A_16_P16480112 CCAGCCTTAAACAAGATCTCAACATTCCTGTGAATTATAATGCAGTCTTCTAACTCATGC
CUST_4351_PI426210822 TGAGAGTGATGATGGTGATGGTGGCAGACAAGAGCATGTCACATTT
A_16_P00748742 AGGTTAGTGACTCTCCTCTATACCCTAAAACACAATCTTCTATCTTAAGATATCCCACCT
CUST_7671_PI426210822 GATGATGTATAAGTCCTTTCAGAGGGAAACTTTGAGTATGTTAAGACCCCAGGAA
A_16_P15220727 TACTCTTCAATGAACTAAACGACTCCTTTAGAAACTACAGACCAAATAGTATGAGGGTGC
A_18_P22839414 AGAAAGATTTGGAGTTAAAAGAAAACCAAGTCCTCTTGCGTGTGGACAAAGCAG
A_14_P127741 ACCTTCTAGGCATCACAGTATTAAGAACCATCCATAGTTTATTGACTGTTTGCAGAATTC
A_14_P110838 TCTAAAGACTGTTAGTGGGGTGTTAGTTGATTTTTCCTGGTATACTGTTTCTTGGCTG
A_18_P10353913 AACCCCAATTCTACATTAACTCAGGAGTATTTTTTCATTTTTGCCCTATGACACTGAACA
A_14_P112120 ATGACTTCTGATCGAGTTTTCTCTTTGGGTAACTCCGAGGTGCGTATATTATTAATTCTT
A_14_P114584 ATTCCGCCTTTTTTTTTTCTAATGGGGAGCAAAAAGAAATTCACTGTCTTTTAATAGTGA
A_16_P16191724 CCAAAGTGCTCTTTTCAGTCCATTGTTTTTCCTGAGATGTAATGACTCAAGGGTAAAT
A_18_P10892520 AAGTTGCTTATCTTGACAACTTTTCTAGTCTCAGATCCTAAGATAAAAATGGCTGCTCCC
A_14_P104054 ATCTCTGGGATGTTTTGAATGAGAGAGGCTGAAATAAATGCATTCTCTTCTGGATTTTAT
A_16_P18089354 GGCCCAGATTTAGAGCAATACCTTTTATGCCAATGATTATAAGCAAAACAACTTTGACCT
A_14_P122839 AAAACGCATAAAGTGACTTTAAAAACAGACATTCTGTAAACTCCAGACCTTTGTTCCTTC

A_14_P127335 CTAAATGGCCATGAATAGAAAAACAGAGATAATCTCCTGTCCTATCTCTGAGTCTACTTG

A_14_P137922 CCAAATAACTGCTTGGTTGTGTAGTTTCTCACTCTTCAGTTTCTTTAAACATGTAAGCAG

A_16_P21671228 CCTTCCATTACCATAACATTCATGTTCACGTTAGTGCTGTAATACGGAAACACATATACA

A_16_P15717408 TATATATGCAGGAATAAGTCAGAGAGACCTAAGACTCGAATGCTTCTTATGGCTTCTGAA

A_16_P35198280 AAATGACCTGTAACAAATGTGGAGAATAAAGAATGCCAAAACAATGCTATTTTTCTCAGT

A_16_P03200031 AAGAAAAGAAAGCGTGTAGATGATTTACCTCTTAAGTCTCAATCTCATGGTGAAACTCCT

A_14_P115522 TAAAAGGGTCCCAGTGAGAGTCATCTTAAATGTATTCACCAAGAGAATCATATGAATCCA

A_16_P02989704 TTAGACAGAAAAAAATATCACTCCTCACTATGGAAGTTGAAAAAGATGAATACCCCCAGC

A_14_P120629 CTTACAGTATGAGAGAATCGTTTGGTTCTCCTAATATAGTGTTTGCCTCGATCACTTTGA

A_16_P01019049 AAGACAGCATTTAGATTGAGAAGAGACAAGTAAGGTTTGTTAGAACTCTATCCTGGATCC

A_18_P22841607 AGTAACTGTGGCAACCATAGAAGAGGCTGGCCACGTGCTCATAGC

A_14_P138796 AGTAATTTGGAAAAATCCCAAGGTTGAAATGTAACTATCAGGGAACAACGAAAAATGCTT

CUST_8596_PI426210822 ATGGGCTTACTCGCCCTATGGGCCCTGGGCTGTCACCCTACCTAC

A_14_P130459 AATGTCTGCGCTTAGGTAAAATAAAAGGAGGAGAGTCATGAAGATTTTATACAGTGGTCA

A_14_P127480 AATATCATTTGGAGTGGGATGTGTCCTGTATGACTGGAATCGTTTTCTATTTGTAACAGA

A_14_P125153 TCAAGTCTATTGGACGTAATTCGGGAGATTAGGTTTATTGAAAATATGGCTTGAGGCTAT

A_14_P104958 ATGTTTGAATCCCAGTTTGGGTGGTAGAAGTGTTTCTTATTTTTCTAAAACATCCTCTCT

A_14_P106466 GGCATTCTGCTTTTCTCTACTAATCTTGAAGTGCTCAGTGATTTGTGTATTTGTGTCTTT

A_14_P115184 TCCTAAACATTAGCATGACACCTTAAACAGAGCAAACTTTCAGAACATGTTTGATGAGAG

A_16_P58424303 ATTAGTGACCTTGTTAAAACCAGTAGGAATGGGGTGGCAAGAAGGAGAGTA

A_16_P01657141 AAAAAGCAAAAGATTTACCTGAACTCAGTATTCAACCAAAGTTGTTTGACTCAATGAGGG

A_16_P03754683 AACTTTGACAGGCAATATTTATCTGGTCATTGGTGCAATTTCATAGACCTAATTATGGCG

CUST_2266_PI426210822 GCTTGTTTGAGCCTCTTCACCTCGCTGCATACACAGCATACAGAC

A_14_P136353 CTATGGACTCCATACTGTTGGATATATTGTCTCTTGTGTCTTCTGCTGACTGCAGATTAA

CUST_4669_PI426210822 TCTTCCAGAGTCTTGGAGAGCACAAAGATGTGTGTTGATGTACAGCTTAAAG

A_14_P111155 ACATAGAAGCACTTTATCCATCATTGCAGGCTCTTCAGAAGGACTATGAAAAATATGCTG

A_14_P105436 CCGTCGACTGAGACAGAGTTCTCACCCTGTGTAAGGAAAAAAGATG

A_14_P113162 AAACTGAAGGCACATAAAACACCACTCCAAAATAGACGGTAGGTTCCAATGCATA

A_14_P108331 ATCCATATTCTTTAATATGGAAAATGTTGGGATCCTCATAGCCCACCAAAATTCAAGTGA

CUST_9121_PI426210822 ACAGAAACTAACTTCTGAGCTGTGACTCTGCCCCTTGGGGTTGAC

A_14_P126535 CGTTGATCTTGAGTCGACTTTTGTGACCAAATTTGGAAACAATTGCAGTTTGAGA

A_14_P113803 TGATTAGCATAGTGTTTTGGTAACTTCTTGACAGTGTCAATGTCCATTTCTTCATCATCT

A_14_P132178 TTGGGGTGGTGAAAACTTTGAGATCTCATACAAGGTAACATTTTATTTCAACAGATGGAA

CUST_6933_PI426210822 CAGAGCCGCAAGACTTGAGTGAATCTGTGGGGGGAATTTAGAAAA

A_14_P127585 ACACCAAATTTTAATGCTAACATTTGGCTTAGTCTTTGGTAATCAGATGTCCAAATTGGG

A_14_P102891 AAAATTAAACTTGGGCATTTTGATCTTGGACTTTTTCAGTTTGACTTCAGACTCTTCCCA

CUST_8692_PI426210822

AGAAAATATGCACCTGATACATCACTGACCATAACAGGAGCTTAAAATTAGACATAAATG

A_16_P21081823

AAATTGATCCAGTGTGGTAACAAGAATGCTATTCTCCTGTTTGATCTCTTAGGCTTTTTC

A_16_P03153995

GCAAAGGGAAACATATGATTTATAAAAACTAAGCCTGTATTGAATAAGAGCCTGCATTGC

A_16_P02711265

AAGCCCAATGATGTCACTAAATAGAGTTCAACTAACTCAGGCTATTGATGAATGTCAAGA

A_16_P20956688

ACTTGGACAGAACACATATGAAGTCACAATCATCAAATACACCAAGTTAAGGTAGAGTCA

A_14_P113224 ACTAGGTTATTTAACCACATTGAAAGACTTCATCTGAAAGGGCAACGTGAAAAGTGTTAT

A_14_P112759 TCCTGTCTCCTTTATAAGGCCACAGATTTTATCTGTAAAACAAAGCACTACTTTCCTTGA

A_14_P103106 ACTTAATCCTCTGTCCCACTTAATCTTCCTATGCCAAATGTTTACAGCACCTAAATAGAA

CUST_2500_PI426210822 AAGTGATATCTGGCCTAGGTCAAATTGCCTGGGTGGTGGAGTAGT

A_16_P02940090

ACAAGGACATACTTCATGAACCTCTTCATTGCAACTTTACTGTATTTTTCCTAACATCCC

A_16_P21612255

AATGCATTTGAGCAGACTAAAGATGAAACAGAGATTTTTTCATGTCTTTTGACCCTGAGA

A_14_P101420 GCCATGTCGTCCTAGAAGGGTCCAGAAGATTATTTTACGTTGAGTCCATTTTTAATGT

A_14_P117338 GTTTTTGCAAATTGTAGCAAGATGTGAGATACAGAGAGACAGATGTTATCTGGGTCAAAC

A_14_P106163 AATGCCTTAATATAAACTGACTCAGTTGAATACCTAATTTGCTGAGACCTCAGCGTTTCC

A_14_P116600 CTCCTTCTGAGAAGGAAGTATTATTGAGCCTACATTGTGTTGGGAGGGAAAAC

A_14_P123986 TGCGCTGTTTTTCTTTCATCTCCATGTTTTGGTTCTTTTTTCACGTATTTTATCTTCTCC

A_16_P15245983

TCCTCTACATTCTTCTTCTACAACAGTTTATGCACATTACTCTAAAAACAACACATGGAA

A_14_P100111 TATCTTTTGTGAAAATTGAAGAGGTCATCGGAGCAGGTATGGCTCTTCCCT

A_16_P17361826

ACGTCTTTCACTTCCAATAGGCTAATAACTACCAGAGTTTTAAAATCAGCACGATCTCAA

A_18_P13683842

AAAGGATACTGAATTGTAGACAAACTCCCACAGACATCTTGAAAACATATTTTCCAGCAT

A_14_P107979 TAATTTTATGCATATTACTCCCAAGTATTTTAACACTTGTTGGAGAAGCAATATCTGGAT

A_14_P113859 AATAATGTGAGAAAAGGCCACTATGGGAGAGGATAATCTCATATGTTGAAAACGGATAAC

A_14_P106595 AGACAGGAAACAGTTTAGGGTAAGAAATGCTTTTAAAAAATCATTCTCACGCCAGATGAA
A_16_P20779379
AAAACTGGGGACTGATGATCAAAAAATTACCTGGACCACAATAGATTAAAGGATACTACC
A_16_P02857907
AGCCATGTTACTTCTAAACTGGATTGCTATAACAATTGCCTTTTAACTCATCTGTCTCCA
A_14_P121412 TACACCCTGATGTTTGGTAAAGAGAATTTACTAACCCCTTGGTTCGTTCTATATCTTTCA
CUST_3013_PI426210822 TGTAAGTCAACTGTTTGCTGGCGTTAAACAAGTTGGGGCAGATGG
A_14_P118479 GTATAAGGGTGTGATATTTTTTCAACAGGAAAGAGAGGCACACCCATTGATCATGAATAT
A_14_P108758 CCACTTTTTGTCTCATACGTCCACACCAAGAAGCCTGTCTGAGAAC
A_14_P129369 TATGGATGTCTTCCTCCTTGCCCTATTTATTTTTGAGAACTTACTCTCACGGAGC
A_14_P113314 GGTGATTGTAAAAAGTGAGGAGTTGATTTAGACATGTTCTGTTTGAGGTGATAGTAGGAT
A_16_P21476962
ACCATACAACCAAATTCATCATAACAGTGTAGTTTTCCTTCCATAGATCTGATCCACTCT
A_14_P127855 TATCTTGAGAAATGTCAATACTATCTTAACGCAATTGCCTCTCAAGGGTGGTAAAGTCTT
CUST_8365_PI426210822 CAGTAGAGTGATTGCTGTGAACAGCACCTCTGCATTTCTGAATTGCTTCT
A_14_P137246
AAGAGTGCTCCAAAATAGAAGTAAAAGAAGAAAGCCCTCAATCAAAAGCAGAAACAGAAT
A_14_P136650 AGTCTGCAAGGAAGAAATGCTTTACTAGGAAGAAAAGAGCAGCATTTTAAATTGAATCCT
A_14_P108533 TGCCCCTATTTCTCTACCTGCTAATGAAAATGCTTTTGAAAGTTTGAGTAACAGTATTGG
CUST_5778_PI426210822 AGTTCAGAAATGTCACTCCTGTCATCCAGACAGGATGCTTCACCAT
A_14_P113305 CTCACCAACTGGTGCTTTTTTTTTTGTGGAAAAAATATTCCAATAATATATTAAGCAATT
A_16_P03805858
TAGAAGACTTAACTCACCAAGAAAGTATGCCTCTAGACTGATTATGCCCCTACTTACAAA
A_16_P00685421 CTCTTGGAGCCAAACGAAGACTGAAGCCACAGTGTGCATGTAAAG
CUST_4884_PI426210822 TGTCCTTTGTAGGGTTCAGTATTTCTCACTCACCCCTTTAAGACCCTAAA
A_14_P123093 TAATTTATCAATGACCAACTGCATGTCAGACACTTCCACAAAGGTTATGATTATATGATT
A_14_P132802 ACATGTTATTATTATTATTAGTGCATACACCAGATTGCACTGTCTCCAATGGCAAATGAG
A_14_P107919 GGATACTTTGGTTTTGCACATTTTGCCACACCTTTTCTTCTCTTTTCTTTATCTTTCACT
A_16_P21170111TTCACACAACACTCTTCAAAAATCCTGCTATTTGTGTAAAATGTTGAACACCACATGACA
A_14_P108218 ATCAAACTGTGCATGCAGGTTGATGAAAATAACATAAAAAATAGGCAAGCAGCCAATCAA
A_14_P103204 TATCTTTTTTTTTTTCAAGAATAAAGGTTCTCTTCAGTTTGAAGACAAATGGGATTTTAT
A_14_P134765 ATAGGCACGATTCCACGACTCCCAGCCATAAATTTTCATAATGAAGTTGAAATTAAAGAT
A_16_P36230591
CATTCACAATCCTATAAATTAAAAAGCAAACAACTTCTGGGTCACTGGTCGTCTTTTCTG
A_14_P101443 TGAAATTGAGAGTCATAAATACTGTGGGTTAGAATAAAAACCATTTGCCAAAGCAACACT
A_14_P123091 ACTCTTGTAATTTGAAATGTATTTCTTCGAAACCATCCTGGAAATTAAGCAAAAGGAGGC
A_14_P114298 CTCAGTATTAAGCAACAGAAAATGAGACTCATCGTAGACTCAGCATAGACCCATCACA
A_16_P03303601

CCCTACAACATGTTCTCTAAGATGCACTTTCACTAACTTCAAGTCTTTTTCTAGAGCAAA
A_18_P17103290
AAAACTCTGATGCCAAAAGTAAAAGTCTAACCCTCCCATGTAATTCTATACTTTCTGGCA
A_16_P02991594
GGATGTTCACTAGGATAACTAATTGACCCATAATATGGCATTATGGAACTAAACCCAGCC
A_16_P39324410
ATGAAGAATGCTCACATGGATCTGATATGTCTGGTTTGCTATATTTGGATTTCTGAGTGA
A_14_P202220 AATTAAGAAGACAAAATGAAGATATTGAGCCAACATCCTCAAGATCAAATGTGGTCAGAG
CUST_5501_PI426210822 CAAGCACAAATGTTGATGTCTGTGCCTTCTGGTACCCCATTGCAAT
A_14_P103061 CTTAATTACCTGCTTAGTTTCAAAGTTAACAAAACAGTAGCCTCGAGGCTGTCCCT
A_14_P120592 ATAAGCACAGAAGCAAACAAAAATGGAAATAACCATTTTGAACTCCAACATGAACAACCT
A_14_P201404 ACTGTTTTTTATTGGACTCTGCTCCCTGAGGTAAAATCATGTTGAAAACCTAGTTTCAAA
A_14_P110719 ATGTGTTTGCACAAAACTGGTGTCATTACAACTAATAGCCTTAAAAAGTCTGTTGGTGAT
A_16_P30528576
TAAGGAGTGCCAGACTTATCTTTGATGGGAATACAGTATGAACCCTGCTTGATGTAAAAT
A_14_P103321 TTGGGATGGGATATCAGAGGAAGAAGCATACAAGTGGGAAAAACTGTGATTAGAAA
A_14_P119407 CTTAGTTGACATTTCAGCATGCCATTTCTGACTATATAAACCTCTATTCTCAGGAGGAGA
A_14_P137348 GATTTTCTCTTGCTTTTTTAAAAAAATAAACAACAAGGTTTGCTTGTCTCTCTGCCCTGC
A_14_P104174 CATCATCTTCCTCTAATCCGTCATAGGTAATTGCTATATTCCACATCTTAGTTGACATTC
A_14_P124423 ACTATATTGTCAGCACTCATGCTGCCTTAGCATAGAGCCTAAATACTTTAAAATTCTGCC
A_18_P14131924 ATACAATCAGTTAAGTGCTAGTGGAAACCAGGGTCTGAATTTGGTTGGCAAAG
A_14_P124846 ACACACGGCTGTGACTTGATTCAAAAGAAAATGTTATAAGATGCAGTAAACTAATAACAG
A_14_P108433 ACAGCAATTACTCTGCTAATGGCTTCCACATTCATACAGGATTTCCATCTAAGAGAGAT
A_14_P100847 CTATTCCTTTCAGAATCCATCCAGTTTTGATCCTAGTATGCTGCTGCAAAGATTTTCAGT
A_16_P00685266 CAGAGAAAAGCCAACACCCAAAGACACCAGTTCCAACTAGGTGTG
A_16_P03047270
AACAACCTAAAAACCTCAGAACATTAATTTTAAACACTGCTGCATCTAACATTTTCTTCA
A_14_P201591 AGCCTTGACTTTTAACCTGATTCTGGAATACTTAGAGGTAGAAGATGAGGTCCCTA
A_14_P127411 ATTTGAACAGGTTTTAAAAATGTAGCAAAAATAAATGATGGTAAAAAGGCAATAAGGGTG
CUST_6432_PI426210822 CTCTTACAGGAGTGCATGCCCTATAGACAGAGGCTCATGAACTTGTG
A_14_P119644 ATCTACATCCTGTCCTCTGCCAAAGCAAAGGAGCATATCTTTATTGAAGGAAATAGTTTA
A_16_P17422401
CCAATAACTGGACTTTTTCACAAATTAGTAGCAGATTTGATTAGATGCCCACTGAATGCA
A_16_P20374420
AAATTATACCTGTCTCCCTACAGAAATTCATTAAGAGACTCATGCTCAGACTGTTGTCTG
A_14_P131048 AGTGTCAAGAGGAAAGGGAAGAAATGGTTTAATATCCAGCGATGGTTGCAAAG
CUST_3359_PI426210822
TTTTGTCTTTATATTTGAGATGTGATTATAAAGATCCCTTCTGCCTCAAACCTTGAACTT

A_14_P102382 TCTCATGGCAAGAAAATTATTATGTATTCTGTGGGGACACATCTTTTTCATAGGCCACTA

A_14_P112724 TGTCAATATAAAATTAGCAGTATGGATGGGGAGAGGAGGGCTAACTCATGATACAAATAG

A_14_P107390 TTCTCTCTACTCCTGTGACGATCTGAGTCTGATTTGAATTGGAATGTAGAAGACTGAT

A_14_P124941 GATCACGGTCCAAGATGTGGAAACAGATAAGAATTAATTTAACAATCCTGGATAACGCTG

A_14_P100406 CCTCAGCACCTATCCCTTTTCCTGCAGATGATGGGAGAGGAATATAA

A_14_P202329 CTCTTTTCTGTTTGTATAGTTGATCAGATGTTTTGGGAGGTTATGCAGTTGAGAAAAGAG

A_14_P201200 TCTTATTAGTTACTGAACTTGGAGGCAATATTTTTGGTGTCTCTGAATTCCAGTTCCATC

A_18_P14132756 TTCTCATAACCTCAGTGGTGAGCATCTTTGCAGAGTTCCTCTGTCCT

A_16_P00508804

AAAAGGCAAATCACACACAAAATTGAAGTTCAGGGAGTTTCTATGGAATCTAGGGTTTTC

CUST_2136_PI426210822

TATGTGATATCTGTCTTGAATAAGCTCAATGGCCCATTAATGGAAAGTTATATACAATGT

A_14_P120628 CTTTGTCTCTGAAATCATCGGTATCTACTACCCAAGTGATGAGTCTGTCCAAGATG

A_14_P121221 TAGGGAACAAGTCTTATCCAGGAGAACATATATGTGACAAAATACAAAGCAGCAAGTGCT

A_14_P112202 AAGTGTCTGCATTTTCATTTTTGAGTAGGTGAGAATTATTTTTCAGCCTGCTCAGATCAT

A_14_P125558 TCCATTGCTAAAAACAACAATGTGTAAAGTCCTGAACTTGGATTCCCTCAGAAAAATGTA

A_16_P39600120

GCGTGCTTAATCATGATGTATGGCCTGATAGAACAAATATTTGAAATGATAACTGGCAAC

A_14_P128781 CTATAATTTTAAAACTGCGAGAGTAAGACAAGCCAAATTTTCTTTATTTATTTCCCACCT

A_14_P120010 ACATAGCAGCCCAGTATTCCAATAGAAATTCCTGCATAATCTAATGCCATCCATCTTC

A_14_P129218 TGCTGACTTCCTCTAGTGCAGAATCCTCAGGATATAACATACTACTACATGTTACCAGT

A_14_P126298 AGAACAAACTGGAAGGAACAGTATGGACCGAAATTGATGATACAAAAGTCTTCAAAATTC

A_14_P116542 TGTTAAAAGCGTAAATAACGGAAACAGTCTTTAGAGCCAAGACTCCGGTTTGAATTTGTA

A_14_P130544 ACCTGAAGAATTTTTGTTTTTGACTGATTTGGGTGTTGTTGGAGATTTAGTTGGTGATGT

A_14_P103384 ACAGAACTCAACTTGTTTACAACTAGATAGGTTGACTTAATACCCAAACTAGCACTTGCT

CUST_7719_PI426210822

GATATGGAGACACCAGGGTCATCTTCAGTTTAACAAACACATGTCAGAACTAGAAA

A_14_P101873 GAAAGAAGTCAGATTTCTCATCAAGTATGATTCACATATTTTGGCCTGAGGAGTGAAAAG

A_14_P115222 CTTTTCTGTTCCATCTTTGTTTCTCTCACTAAAAAAATGGCTAGGATGTCTTGAGACTTT

A_16_P00303168

ATGGCTTATTAGACAAAGAAGATTCTCTGGATGCTATTCTCTTCACAGTAAAGGTCACTC

A_14_P133129 TTAATTGAGCCTGTGCCTACATTTTTGCATGCAACATGGAAATTAACAGCAGAGTGATAA

A_14_P113100 CTGCTGTTCAAAGATTGCTAGTGATTCTCCATACTTACTGCTTAGGCATTTTTATGACTC

CUST_2358_PI426210822 AGGGATGCTCTTCCCAGGGTGACAAGCTAAAAGTCACCCAAGGAG

A_14_P129370 CCTGCAAGCGGGCTATGGTTTCATTGCCCTTCAGTAACTCATACAT

A_14_P107815 TTTACAGAATTGATATATAAGGAAGTTATGGACTTGGAGGAGAGAACCAAGAATGGAGTT

A_14_P125082 TTACTTACCTTTAAAGGTTGAGTTGACTGCTTATTGCTTTTGTGACTCAGATGTCTTCTT

A_16_P21545755

GGAAACATTTGATAATGACCCAGTCCTTTATAATCCTCTACAGATCACCAAAAGGTATTC
A_16_P03111739
AACCCATTCAAAATCTCCAATTTCATCCACCATCTAATCTGTTAATTCCCTTTCAAATGC
CUST_1460_PI426210822 CACACAGCAATAGTGTAACCGAATGCCAGAGCAGTCCCTTCTTCT
CUST_82_PI426210822
TTGTTGCTTAAAGCCGTTTCACTAAATTTAGATCTCTGGAAAAAAATTTGTTTGAGCATG
A_16_P58424590 GCCCTGCTCAAAATCCTCCAGTGGCTTCTCATTCCTCTTAAGGTAAA
CUST_4297_PI426210822 CTTGTCCTCCCAGAGTAGCTCACCTGTGGGTCATGATGAGAAATA
A_14_P102028 AGAACTTTCCATACTATTGGCTGCGAAACCTGCCACTCATATGCGACTC
A_14_P106939 ACCCAAGTAGCAGAAACTTTTGGGAGCGTCGTTAATAGTTTCGATTAGCATAAAGATTTT
CUST_1967_PI426210822 CCATCCTTTCTCAACGAATCTCCACCTCCAGAGAGAGCAGATCTT
A_14_P107946 ATGGCAAGTTTTTGGTAATGATGATTCAATCATTTTGGGAAATTCTTGCACTGTATCCAA
A_18_P16978317
AAAGGCTGTTTGATTAGTATTTCGTGGATGACATAACTATAGACCTCTTAATGACTGTTA
A_14_P109198 ACTGTCCCCATTAGGGAGTCTAGAAGAGGCACTCTATAAAGATCAATATGCAG
A_14_P117364 GAAATAACTTGATTCTTCTCGTCTCATTTTAGACAGTGCTAAACAGAGTTCATGCCACTT
A_18_P14192417
AAAATCCAGAAAAGCCTATAAAAGAATAGAAAACCCGAATACGTAACTTCCTCCTGTGAC
A_14_P126653 TAACCCTCAGTTTCCACTATTAAAGAGTATCTTTTGACGTCTGCTTGGAAAATGAATAGT
A_14_P201652 CTATCTCCATAGAATGGCCTAATGCCCATGTCTAAATCATGTTACTCTCATGCTCAG
A_14_P115300 TGGAAGGGCATGTTTACAGTAGATGTTTAAGCCAAGATTTTGTATTAAGGCATCTCTCAA
CUST_8176_PI426210822 GAACATGTACCCAAGAGCCACGCATTTTCCCAAGGAATAATGATCACAATT
CUST_9960_PI426210822 TTTTCCTTCAGAGCCTCTCTATTCATCAGTAATGGTATTGGAGGCTTTAGCTGAT
A_14_P109184 CCAGTATCTATGGCCATAATAACCTCTACTTATTCTACCTTAAGCCTAGGTATACCATCC
A_14_P101783 CTAAACTTACCTGCTTGTAATAAGATTCATACAACGAATTTCCAGTGGGAATCTGTAAAT
A_16_P00685361
AGAGTTTGAAACAAATTGTTCTCCTTCTCAAAAGTGGCAGAATGGTTAGAATCATCAACT
A_14_P126281 TCTACCATTGTCTCTCTGCATTCTCCTCATTCCACCAAAGAAATGGCACTTTA
A_14_P117694 AGGGAAGTCCAGAGACACCTAGGAAATGATTCAGATGGTTTGGTATATTTAAAAATTTCT
A_18_P22842678 AATAACTGTGCCCTTCCCTGCACTTGACCGCCTCACCACCTAGCAATA
A_14_P100353 GCTGTCACCTTTCTGCGACGCAAAATCCTTTCTAACTCAGTTTCACTGTTT
A_16_P01777846
CCTGTCTAGTCCTAGAAGATTGACTTTCTTAGCAGATTGACTACAACAGACCTTTGATAT
A_14_P125380 TCCCGATGTAACCAATATCTCTTTGAGATATAAGCATGAAACAGTTCTATGGCTTCCAAA
A_14_P100327 AAATCCCTGACTCCTTTGCAGTGGTGTAGACATGTCCTAGATAACCCAA
A_16_P17390012
GCCCCCATATCAGAAGTTAAAAGATAGGAAGTCAATAGAGCATCTTAAGCAATTCAATGC
A_14_P134515 GTTTGGGAAGGGTAAGAGAAATCCAGTTCTATTTAAGAGAAATCCAGTTCTATTTTTGCC

A_14_P100322 CTCTTGGCCCTTCATGTTCTCCAGATAATAGAATCCTAATATGTTCCATTGATACTCAGT
A_14_P108205 ACACCCTCGAATAATGACGCATACCTATCCTACTGTTACTGATCACCTAGTAATAATCTT
A_14_P121218 AGAAAGTAGGCAGGTTTGATACTCCTTTAACAAAGGAAGAACATAAATCCCTTCTCATTT
A_16_P20652769
TTCCAAGCAGGAGAATAAAATAGAAGGTATTTCAAAGAGCAATTATAGTAGGCATGGCTT
A_14_P121560 TAAATATTTGAGATTGGAGATGAGATTTGCAGACGATACTGTATTTCAGTGGCAGAAGT
A_14_P133332 AAAATATGATATGGAAAATGACTGTATCAGAGGCACAAGATCTTGGCCCACCCAAAA
A_16_P35742239
TTTTGGTTGTCACCCTAAATTATTCAACATGCTTACATAAGATTGGAGAAAGTGATCGCG
A_14_P115268 AAAGACTGAAAACATTGCTCTTTATAGTTTGCGGGAATTTTTAATGCCCTAAACAGAAAC
CUST_3094_PI426210822 GACAGCCCTTTTATTTATCTTACTGTGTTGATTTCAGCCTTTAAGGGGGGTC
A_16_P16697108
AATTGCAGGAAACCTTTTATCAGGATATCAAGTATGAATCTCTTACCCCCACTATTCACT
A_16_P20029242
GCCTTTTCTTCTCTTATGCCAAACATTGAAGATCATGAAAATTTGCTTGCTTCCAAAGAA
A_14_P130880 GTTTTTGTCGGTTTCTTATTGTGCTATACCCTGTGAACAAAACAGGTTCCAGCAT
A_14_P129121 TGCTTGTTCTGATAAGTTTCATCGTTACTTGGTCTTCTCATAAACTCAAATGACCTGTTC
A_16_P40012575
TAGGCAGGCTATATCAAAGAAAACTCATCTAGGACATCTAATGCTTACTTCTGAGGTAGT
A_16_P20027809
AATGTGATTGCTCTTACCATCTATTGCCTAAAACATCACAGTAAATTCCAAATTGGTGCC
A_14_P118984 AGTTTGATGACATGAATACCAGTTAAGACACATTGTCCCATTCACTGAAGATGAGAACTC
A_14_P103289 GGTCATGCTCTTAAACCTCAGGTTTCCTCTATTTTTACATCATTTTTGGACGGATTAAAA
A_16_P35705285
GCAGAAAAACAATAGTCAGAGATAGGCTTAAGATCCTCATGTTTCATGTTTCAGGTATGG
CUST_5674_PI426210822
TTTTAGTCTAATGGTAATTTTTAACTATGTTGAAGCCATACACTTTGGGTCTCCATTGTG
A_14_P105816 CTCCCTTAATCCTGGATTACATTTAAACCTGTTTGGGTGGAAAGATGTTCTGTTTATGC
A_16_P18409473
GAACCATTTCCTCTCACATGAGTTTACCCTTCACATCAGCAAATTTGCATTTTAGAGAAG
CUST_7995_PI426210822 CACAGAAGTATGAACATTGGAATGTTGGTTGTTTTCCCAGCTGGTAATGCTAC
A_14_P137112 ACATTCATCTAGGGTCTTTCCTGCAGTGTATAAACTGATTAACACAGAGAATTATCAGGT
A_14_P138356 TTCTTCTAAACTTCCTGAATAAATCATGTCTTACACTGAAACATCCAAGTTGAAGGAGGG
A_14_P114140 CTGTAACTGAGTAAGTAGAAACTTGTCATTGCATTGTGCCTGATGTCTACAGATATTTGG
A_14_P116377 GATTTTAATGCTTCTCATGACAGCATCCTTAGTTATGCATGGGCTAATTGGAAAAACAAC
A_14_P138726 CTCACCTTTTCTTGGAGTGACAAGAGAGACTTCCAGAAGGCATGTTG
A_16_P16682168
ATTCTCAGCATATTCAAGCAGTCACAAAGGTATCTGATAAAAGATCACCCTCCTTAACTT

A_14_P127834 CACAGGTATGAACACATAACATTCATTTTAGGAAAAGGGTCTCCAATCAGGAATCTAATG
A_16_P16942627
TCGTAGTGATAAGGGCAAACATATTGGAATCTGATCTTGCATGAATGCTAACTTTGTGAT
A_16_P01632197
TTAAGTATCTCATTCAGGATTCCATGAAACATTGCTTCATTTCTCAATACGTCTGTCCTC
A_14_P123197 ACCAGAGGGTGTAATTAATGTGCATTACTACTATCATTACATTCTCTCCCATAACAGTTT
A_14_P108565 TGTGATATATTGTTGTGGTAATCTTTCATGGTTTCTTTGTCGTTGACAGGTGATTTGGAT
A_14_P100937 CCCGAGTCTCACTCTCTGTTCTTTTATTTCTGTAGTAAACTCTCTGGAGGTGA
A_14_P107934 CACAAATAAAGAAAGCACCTGAGGGCTATTACTGACCACAATTCAAATGAGTATTCACAA
A_14_P111026 TGTTGAGATCTAGATATTCCACCTAGAGTAGGTGTTTGACAATCTATAATCCCAATGTCC
A_18_P13859005
TGTCTTTCATTTACTAGCAGAAATAAAATCTTAGCCTTGATTTAGTAAATTCCTCCATTT
A_18_P14134347 TTCACTGCCGCTTTCATTCAGCGCAGGGACTGATTTTCATTGTGTT
CUST_4424_PI426210822 ATTATTTTATGTTAAACGGACTCTCCTGCCCGGACCCAGGCCCAC
A_14_P115158 TACTTAGTATCTGTTTCAATAGTGTCTTCAATGCCTTTAGGGAGGGATAGATGACCTCAG
A_16_P01851954
ATCTCATCAGTAAACATAAGTTACTCCCAAGTTGGATCTATTACTGACCATCATTGCTCC
A_14_P105103 TATACATACGTTATTTTAAACCTCCCAGCCCTGTGAATTGGTGGTGGTATTATCCATAAT
A_16_P16708965
TCCTTTCTTATCCTTACTCTTGCAATAACAACTAGCATTACTGTCTTTAAAATCCACGGC
A_16_P15348075
AAAGTCCCTTCAGAAAAACATGTGACCTTGAAACTTTAGAAGTATACCCTTGAAGATCAA
A_14_P104657 ATTTTCTAGGGATGCGGTTTAATATTTTCATGTGATTCCTAAAAGAATCTGAGGCTCAAG
A_14_P115568 TCTTAAACAGAGTATGCTTTTAGATATCTTCAGTTCCTGCAAGTTTGTGGGGAACTAGAA
A_16_P16751597
ACTCTATCACCATAACTCACGATTCAAATTTCTCCCATTTTCATTCAGTCCTTGTCAAAA
A_16_P01955507
TCTTGGCTTCCACCTATAATTAATGATCTCCATCACCTTATCTTACACACAACCATTTCT
A_14_P101120 GGAATTATTTCTAGGCTCTTTTATCCTCCCTCACAGTTAAGAACTCATAAGACTCCTAAC
CUST_9422_PI426210822 ATCCTGCCCTAAGGGTGGTGGAAATAGCTCATGGGAACCCAGAAG
A_14_P127217 CTGACAAGGCTATTAATTGCTAGTTACGTCCTACATTAAGTTTGTGTTCCTCACATCTTC
A_14_P136037 GAAGAGGCTGTGGATGACAGACTATTTTGGTCCTTGTGATTAGGAACTTTTTATAGTCTC
CUST_3961_PI426210822
CATGCAGGTCTTGCAGCCAAGGATTGTTACATTAAGATCCAATCTGAAATTAAAACTTAT
A_14_P106673 CTGCTGGTGAGGAAGCAATTGCAGGATTTTTACAAAGTATAATGAAGTTAGAAATGGTTC
A_14_P100705 TATGAAGATAACGCCTTCCCTAAGTCACATATCAGAATAGGAAGATATGCCACTAACTTC
A_14_P106492 GAATTCAGACCTTTTGAACAGTAGGAATGAGTCTTGCAGTAGAGATGAGTAAGAAGG
A_18_P25322196

AAAACTGGCAATTGTTGAATAGCCAAATTGGCTCAGAATAGATTCAAAGTATCACTCCAA

A_14_P135563 TTTTACTTATTCTGGAAGATCTGGCTGAAGGATTATTCATGGAACAGGAAGAAGCGTAAA

A_14_P128409 TCTTAGCACTTACCTGTCAACACAACTTTTCCAGATACAAAGATAAGCAACACAATTCGT

A_14_P201805 TATGTTAAGTATTGATACTTACCCAGCACTCGGAGAATACTGAAGTGCTCTCTTCATCAT

A_14_P117456 AACTCTCTGTATAACTCTAGTGAATCCCCAGTAAGTGCAAATTAAAATGGTGACCTGTAT

A_14_P118327 CTTTCTTATGCAACTCCAAATCAGTCTCCCAGATCTGTCTTTCCCCTTTGT

CUST_9477_PI426210822 GTCAGTTTTTCTGGCAGGTCAGATATTTGGAGTTGGTGTTTCCCTTTTCT

A_18_P22842269 GGTTTCCCTCCTCAGCCACCATGGCTTGAATGGCTGCATTTAGAA

A_14_P101383 GAAACTATTTTTCCTCAACATAATTCCAGTTAAGACCAAAGATGCAGTTTGCAGGTGAAC

A_14_P122220 ACAACTGAGGTAATTATTTCACAATGATGGGTGGCTCAGTGAGATTCTGATTGCAT

A_14_P100916 CTGAACAATTTGTTGCTGAATACGAGGCAGGAATATCTGCATTGCTATTATAAGAGGTTC

A_16_P02929332

AAAAATCTCCCCTACCTTACCTTATCTAGAGTAAGTCCTTACTGAGATGATCCACTACAT

A_14_P133634 TCCCTTTGAAAAGTATGCTAGCAGACAAGCATTTGGTTTTATGAAAGAGGCACTCTTATA

CUST_6367_PI426210822

CAGTTATGGATTTAAAACAAAAGCAGAACTTAATTAATGTTGCTGTTTGGATTTTTTTCA

A_16_P18698002

GAGGCTTTCTAGAATTACTCTTGTTTCAGCATATTTTGTCAGCCAAAGATTAGTCAGAGG

A_14_P121367 AGAGTGAAATAAGTGACAAATAAATAGGTTTTTGCTATAACGTGGCCCTTTCCTTACTCC

A_14_P139087 GCTTGGAGCCTTTTTACCCCCTTCACACTCTAAAGTAAGAATAGTTTGAATTACAAAGAA

A_16_P20753546

CCAGTCTCTTTATCTTTCTAGACATGCCCTTTTACATAGTCATGATATTGAGTGCAGCAA

A_16_P16718877

TTCAATTCCGAGAGACTATGATAATAAGCCAACTGTTTACCTATCATCCTCACCTGTAAC

A_16_P00685497 ATTCAAACCTGAAAATTCTCATTCTCTTGCCATTTCTTGGATCCACAGAACACTCG

A_14_P106634 CTGAATGAAATCTCAGATGGCTGAAATGTTAGAGAAACAGAGCACCAAGTCCC

A_14_P135304 TGAAATCTATCTCCATGACAACATTCACACAACAGGAAATTGAATTCTTACAAAAACATG

A_14_P133264 GTATTGTCTTCTATTTCTGCATCCTGTTGCCTCCTGGTTTCGATTTCTTGGAG

CUST_4518_PI426210822

CCATTTGCCTAATGTCCCTGGAATTTATTTGCTGGAGCATTTTCCATTTTCATATTCA

A_16_P16818194

AAACACGGTAAAATGCAAATACTCGAAGATCCATGAATCCATGTCATTTTTTATTGGGAG

A_16_P18131192

AGCCTCTTGATGATATCTTCAATAAAATACCTTCTCATCCCCTTAAAACTCTAACACCCT

A_14_P127267 CTCTCCTGCTTGACACTGACTTACTACTTGAAACTTTATTTATTGCACCATGTTGGTGT

A_18_P14529129

TCCATTGACCTAAACATTTTCTGAGCAGACATATTCCCTTACCTACATAAAACCTCTTTG

A_14_P127930 GTGGAATATCATCCACTCTTTCCCCAAATGTGTGTCATGTCCTGTGTG

A_16_P20866267
CTTCTCTAGGCCCTAAGAATTATTACCTCCCCTTTCTAATTCTAGCAAACATGGAACATT
A_16_P21547937
AAATTCCTACATCCACAAAGTCATGAATGTCATATTTTCCCCAATAGTGAGCAAATGTGC
A_14_P122617 GCTGGATGTCATTCACACAGAAAATAAACTCTACCTGGTTTTTGAATTTCTGCACCAA
A_14_P123279 TCTTCCACAACTGATGACACCTGCTTCACAAACTATAAATTTTCTCTTTCCCTTTTCAAA
A_14_P108875 CAATCAGTGGTGATAGGCTTACTCATCAAATCATGGATCTCAACCTTGATACTATGTATT
A_16_P18830016
AAAACCAAGTATGACCTTTTCAGAGCATCTACCTCTTTTTGTCTTCTCATAACACAATGC
A_14_P113197 TAGACATAACAAAACACTCGGCATTTCAAATGTGCTGTCAAAACAAGTTTTTCTGTCAAG
A_14_P105903 ACTTTTGTAATGACTGTGAAATACACTTTTCCCTCACTACGACTGCTTCTTTATTTGCTG
A_16_P15802340
ACGTAATGGCTTATAATCAACTTTTAACTACTCAAACTGTCCTATGCTACATTCAATCCA
A_14_P112381 CTTTATTTCCCTGCTGTGCTGAGTGAATCTTTTAGCAGACTTGCCGAAACAAG
A_16_P01788180
TGGAGACATAATGTCATCTTTAGCATCTGTTGTTACTCTACCATGTGATGAATAGGATGG
CUST_5397_PI426210822 ACTGTTCCCTTGGGGCTTGAGATTCTTGGATTCCAAAGGAAAGAA
A_14_P116833 AACAGAACTTGAAAGTAATGGGGGATAACTGCAATGCTTTGAACTGTAACAATGTAAACC
A_14_P129349 ACCTCCCTTTTTTCAGCATTGAAGATAATGATGATGACAGCAAAATGGCAGATCTCTT
CUST_2282_PI426210822
TATTTCCTTTATCCAGGAATGGATTTATTATCAAATAATAATAAGGAATGGTCCTTTATC
A_14_P137142 CTCACCTTTAAAATGTCCGACGTCATGGTTTTTAGTGGTATCAGCCGG
A_14_P138166 CTACAGAGAATAAGTTCTACAGTCATTTTGTTTCTGCCTATTTCCTCCAGATTCAAAGGC
A_14_P124053 AGATGGACATTGTTAGCGTTACTTCCATCACTTTTCTTTAGTTTAGGAATATGCCACAGA
CUST_6544_PI426210822
AGAATATTATTAAGCACCATCCAAAAAATTATCTGGATAGAAGAGGAAATTTTTGCTTTA
A_14_P123032 CTCCCAACATAGTATTATATTTTGTTTCCTTTCAGTGACTCCTTAGGTAACTGCAAGCAC
A_16_P01843968
TGAACAGCAGATCTTTATTTTCCGTTCATTTCTTTCACTAATTCAGGTTATCGATTGCCA
A_14_P119628 TTGGTTTACATTTAGATTCGTCTGTGATGGCTGTTTAAAGAAAAGTGCACGAACTAGGAA
A_14_P133970 CTGTTTGCAAACTTCCTCTACCTTTGTTTTCTGGCTGTTCAGAATTATTTTTTATGGTAA
A_16_P41407842
AAGACACAACATTGCATAATATTAAACTTCCGTAGAAGAATTACAGATCTTTTCCACCCA
A_14_P119038 CTAATTAATTAATGAACTCATTAACATGTTCTCCTGCTGGGCTGTAGTTAGGGCCAAATT
CUST_5096_PI426210822 TGTCTGTCTCTGTTATTGCATCAGGATCTCACATTATCCAGTAAACCCAAATGC
A_14_P122349 AATAAGACAAAAAATAGTTCCAGGAGTTCTCACTCAACCATTTGCCTTTCTTCTTTTCTG
A_14_P115918 CTCTTAAACAATGTTAGCAGTCTTCAGTTCAAATACTTTTGTGTCCTTCTGGCAGTATGT
CUST_7907_PI426210822 GTTTGTGAAGAGCCTCTTGGCTTTCCTTTGACCTTGGTTGGAAGC

A_16_P18672960
AAAACAGCCACGAGTATTTGAACATGAAATGACACACAATTATTGACAGAGCAACCATTT
CUST_8273_PI426210822 TTGTGTTTAGGTGAGTGAGTGGCCTCCAGCATCTAGAAGTCGATATTTG
A_16_P03616140
ACAGGCATCAATATGTTCACAAGTTAGAATTCATGCATTGCTAGTGTTAAAGAGAAGGGT
A_16_P17122305
ACTTCAAATGCAACATAATCCAAAACAGAATTGAGAATCTTTCCTTATTCCCATTCTGCC
A_16_P16421771
GGTATAGCACCCTTATGTTACTAAAGCCTAGTCTAACACTGAACTCATGTTCTTGATGAG
A_14_P101148 CCCTGTTGTCAGTTGGAAAAGAAATTAACTTGAAGGTGGTCTAGTTCTCATTTTTAGAAA
A_16_P01863523
GCATAACCTTAAATCCAATCCTAACCCCTAAATTACTACCTTAACAAGCAGAACAGTGAC
CUST_2983_PI426210822 TTGTTTCTCTCTTCCTTGAGTTGCAAGAGATCAGTACTTCCGGATCAGTAC
A_14_P102197 ATTTTACCCTCATAAAGGCAAAATCAAAAATGATTAACACGGCTTTTCTTTCAGCAGGAT
A_16_P03807725
ACACGTAAATACTGTGATGATAAGGGATCACCCTTTCTTTTTGGCATAAAACCAAAAAGA
A_14_P111813 TGTAAATGTTAGTCACACATCTCATTGGACACATTGTTAGAGTGTGTTGTCCATGTTTTC
A_14_P132857 CTAATGGCTTCTTAAAGGTAATAAAACCCTTCCAACGTAATTGGTCAGATAAAACTTTTT
A_14_P138444 GTAGCAAACTGCTCTAAATGTTTTAGGGTAATTTCTTTGTATTTTGACGTCTCTGCCAGT
A_14_P101588 ACTACACAACTGCTTCTCATAGTAAAGCACAAGTGTGTTAATTGCCAGGAAGAATAACAA
A_14_P110071 CTTTTAATACATCACGGCTGAATTTCTGGGAAGTCTGATTGTGTTTCTGAAATAGACTTG
A_14_P130885 GCACTCAGACCTACCAGAGAGGAAAAACTTTTCTTTTTCATTATGTAATTCCTCCCTAAT
A_16_P01166993
TTCAGGCTCCCTTTAACATACTATCTGACTTAAGTATTCACAACTTTGATAACATGGCCA
A_14_P129507 CAGGATTTGGAAAAATGCCACTGATTTCATGATTCATATGCACACCATGGAAATAAACAA
A_14_P130043 GGGGTATGACAGTGAGAAAGAGAAAAAAGAAGAGAAGAAACCAATAGAAACAGGTTC
A_16_P17042269
ACTGATTTGCTGACAATACCATTTAAGAGCCCTCAAATTATTCCTCTAAGGCAAAAAACC
CUST_428_PI426210822 GGGAGCCCTGCCTTCTCATAACCTCAGTGGTGAGCATCTTTGCAG
CUST_2355_PI426210822 AAGAGAGCTTTGGGCACAGTTTGGGGGCCACATCTGAGTCACAAG
CUST_2506_PI426210822
TGGTCATGGAATTCAGGAAACATTTCTCTAAATTTTATAGTCATCTTGCTTTTGAGAAAT
A_14_P122990 GAATATTTGGAAGAAATAAGAGAATCCACAATAGAGACCTTGCTAAGTAAACGGTGGTGC
A_14_P101145 AAATCAAAGGACTTAGAAGATTCAATTTACTTATTTGTGTTTGCCAACGTTGAAAACTAT
A_16_P21694327
CCAGTTCCGAAGAATAAATTTACCCAGATACAATACAGAAAACTTCACATGTCTCTCCTT
A_16_P16581530
GAGAATTTCTTCCATCGTGTAAAAGTTAGACTAGTCAGAATGTGCATCTCAGTTTTAGGG

A_16_P21479550

AAAAACAAGGTTGTCCTTGGAGAAGTAACTCTCTTGTCAAAACTTCCACTTGCATAACTT

A_16_P37996846

CCACTGTGACTATACATGACTAAGAAACCAAATACATGCTTCATTGCTACATACCCTTGT

A_16_P00438389

ACGTCTTTAGTTAATGCATAGAGCCTTAAAATAGTTCTTGACATAAGTCTTTCCACTCCC

A_16_P01921194

ATCAACCATGCTCACTTTAAATTCACGACTTTTTTTGAACCTACTGAACAACTGAGACAA

A_14_P102494 AAAAAACTAGAAATGACAAATATCCTGCAAGTCTAGATTGTTGAACATGCCTGATCAGGT

A_14_P124608 GTCCTCCTTACACTGAATGTTGATTAAATGTATGCAAATCTGTTGCCTTTCCTAAGTTTG

A_18_P22840534 GAGCCCCATGTTGCACAGGGCAACTATGTCACTCATTGGAAATTA

A_14_P133521 TGTGTGGTTTTAAAAAGATTACAGAGAACCAGATTCTGGATTTCAGAAACTGCTTGTCTT

A_14_P107171 TGGGCCAATTTATGATTATGAATTTCTTAGATGATGGAGTGAATACGTGTAGAGGGAGAT

A_16_P16557663

ATGGCTAGGAAAGGACTACAGATACAGTGGATACTCATTAAAACACCTGTTGATTTCTAC

A_14_P118413 CATGAAGACTCTGCATGAATCTCTTTTCTTCCCATTTCCCAAATATGAAAATAACTTGGT

A_14_P111861 ACATGATACAGAGGTAAACCGTTATTTCCTCTTCTTCAATACATACCCCTTAAATCCCTT

A_14_P201643 AAGGAACATCTTTTGTTTTTGCCATCTAGTAATGATTCTCCAAACTTCCAGTAACATATT

A_14_P135913 CTACAGTAAGTTTACAAGCCACAGTTCATGGTCCCTTCTCTCTGTAGAAAATTCTAGTTA

A_16_P32383980

GCCTCCTCTTACAAATTTATCACAGACATTCAACATCTAAAACAAGATCTGAACTACCCA

A_16_P17609238

AGATGCCCAGAAAACATATAAACCCACCTCTATATCATCTTCTTCCTTTCTAACTTCTGC

A_16_P00685309

TGTTTGGTGAATCCTCTTTTAAAAACACTTAGAAGATTTTCCCTAAACTAATTATCTGGC

A_16_P02585702

CATATGAACAGACTTGATTTATTCCAACTCACAAAACACTGATGTATAAGCCAGTTGCCA

A_16_P03808740

CCTGTCTGGAAACGTGTTAATGATCTTAATATTTAGGAGAAAGTGTCTTCAGATGGAGAG

CUST_9062_PI426210822 CAAGTGATGGCCACTCCTTGGGCTCCCACAGCCAGGGTTCCTGAC

A_14_P117497 CAGTTCTTTTGCTGGGTCAGCCTTTATGCCAAGGTAGGTCTTAAGGAAATTTAC

A_14_P108395 ACCTCAATAGATTTTAACTCTCCTCCATCCTGATTTTGTTGGTTACTGTATAAACTGACC

A_18_P22841242

AGAAAATATTTTCAAAGACAACTGTATTCTCCAGGGAAACCTGAGATGAAGACCAAGGT

A_16_P39685867

GCATGAGAAACTGAACTTCTATTTCTGTGCAATTCAATGTTATGAGGAAGAGTGATGCAT

A_14_P106650 CCAATTTTGTGGAGGTCATTTTATTTTTGACGTTATGACATAGGCAAGTAGAAGCCTCTA

A_14_P110526 CTCTATGAGAAGATCTAGATTCTAACATCCAAATTTACATTCACAGTCTCTGCCAGGGTA

A_14_P114475 TCTTTATTGGCTTTCTTTTTTGCTTCACCAAAGAGCAGAAAAACATTGTGTGAGTGCAG
CUST_7565_PI426210822 TGACAGGTTGATCTGTGGAGATACACTCTGAAGGTGCTAGACTAGGAA
CUST_4316_PI426210822 GTTTTTCCTCTCCTCTGGCCTCTACATCACTAAATGGTCACTTCTCTTCT
A_14_P119261 TCAGGCTTTCTGGGCTTAAAAAAAAAGTCCTGTTTCTTGGATATTTTAATTCAGTCTGCA
A_16_P17415423
TCAAAATAGCCTAGAAAAATTTCAAAGTCATCATGACCAACTGATCTTAAAGATGCCCCA
A_16_P02067805
AGGTGTCAGAGGAATAAGATACGATGCTCTAATAGGTTTCAATGACAGAATGTGAGAAAG
A_14_P123314 CTACATACCAATATTCCATTTATATCTCTGTCACTGACCTCTTAGGAGACCTAAGGTAAG
A_14_P139236 CTTTTCCAGGCACCTTAATTTCAAAATATCCACATTCCCATTCCTTTCAATGCCAAATAT
CUST_1666_PI426210822
TTTAAAATGAAGTGCTCATCAACAGTCATTTCTTGTCTACACCCTCCTGAATTATCAAAA
A_18_P17038999 CAGCACCATTTTTACTCCCCTTTCCCAAGCAAATCGTGCATTTTTGTCTAA
A_16_P18040421
TACTTACCCGTTATGATTAGGCATCTTGAACTCTAATGAGTCGCTCGAATTACTATTGCT
A_16_P16759577
CCCCCAAAATGATCAGAACACTCAATTACACTTAATCTAATAAATTCAGGTTATGCGGCA
A_14_P128504 CATAAAATTATAAGATAAAGGGTTTAGGCAGACCCTTGGGGAATAATAACAATTGAGGGG
A_16_P17190858
TCTGCCTTCTTTGTTTATGCTCACATCCTTTTTTTCTAGCCAATCATATGTCTCAGAAAC
A_14_P118204 TGCCCCTGGGCTTCATGTATTAATGAGCATTCAACAAATAGTTCCTGGAATTAATTAGTA
A_16_P21450530
AAAATCTCCCTTTTTACAAGACTTCTAGGCTAGTCAAGAAGAAATAACAGTGCTAAGGCA
A_14_P119893 ATGTCCACACACTCTTAGGTATGAATTACATAATGGCATGAATTCATGTTTCTCCAGAAG
A_14_P122526 CTGTTCCTATTATAATTTAAGACCCGTGGACATCTAGAAGTTCAAGGAAGTTTCCGAAAA
CUST_7297_PI426210822
CAAACCAAGTTGAGTGTTGTATTTACTTTTCTGGATTTATTTTCACAAATATTCAGCTCA
A_16_P03641827
AACTGCAGAAGTGCTATCAGATTTCAGGGACTAAAATTAGAAACTCAAACCCCTTCAG
A_14_P108843 CCCCCATGGTGGTTTTTTAAACACTTCTTTTCCTTCTCTTCCTCGTTTTGATT
A_16_P01117617
AAAAAGAAACAAAGGCAAGAACATTCATACATCGGTTTCCAGAACAAATTTTGTCAACAC
A_16_P39278974
TGCGTTGTAGATCACATTATCCTAGAAAGCCAGAATAATATGAAATAATCCTTGACAGCA
A_14_P201574 TAATTTTAAAACAGAACCACAATCATGCCTATGAAGTGTTTTTAAGCCTTTGACTGCCCT
A_14_P131920 TCTCAACCTCTTTGGAATCTTCTTCATCTTGGCTGCCCATGAACATTATTCTATTGAT
A_14_P112562 TACTAAAAATGTAATGATTAGGATGAGGTCTGAAGGATGAATAGGGGCTGATGCATTGAT
A_16_P18238574

ATGCTTCTCCTAACTTTGTAAAACAGCCTCTGCATTAAACTTTGCTCAAACATTCAGTTG

A_14_P116878 TTGTATTTGCACTTCTTGTTTGAAGGAAATACATACAACACAGGTAGCGTCTCTACACTT

A_16_P00685487

TCTCAAAATTCTCAAAACATAAATACCTGTATCCCACTTCATTTTTAAGAAGAGTAAAGA

A_14_P131513 CATGTCATTGTGATTGTAGGTGTCAAGCATGTATGTAGTAAATCTAGTGAAAAGCCTTTT

A_14_P115521 ATATCTTTGACCAATTCCAGAAAGAACTGACGCTCATTATGCTTTTTCAGATGGCACTT

A_16_P16191659

TCAGGGCATTCATTGCTGTCTATTTTTCGTTACAGTATGGATGAGAAAACCCTGTATCTA

A_14_P137465 GAAAAAGTCAAAGGGCTTTTTCTTCTAAATTTGATGGTTTGCGTTCTTCGTGTCTCTAG

A_14_P105910 TGGATCAAATTTTTAGATAACCTCGCTAGTCATTTATGAGTTATTGGAAGGCAGACACCA

CUST_4795_PI426210822 TCCTAGCCTTATTGGATGGAAAAGAGGGAACAGTGGTTCCTTTCTTTC

A_16_P36230571

TTCTTTTTACTTCCTACTTGCAAAGACTCTGGAAATGTAATGATGGTTCAGAGTGTTCTG

A_16_P19800561

GGCTTACCTTATAAACTCAACCTAATCAATTCTCCTACTTCTGATCCTATCTATGCTCCC

A_14_P126847 CTTGTGCAGGACCAAAAGATCTCAGATTGCTATAACTGAAGGTATATTTGAACTTCCAAA

A_14_P128372 ACAGAACTCAGACTAAAACTCGTCTTTCCAACTCTGCGTATGTATTTGAACTTTCTACAA

A_14_P113183 CCCACTTTCGAATTGCCTTTCAAACACAGTTTTTATTAGCAGTCTTTCTTGGTTTGGAT

CUST_7140_PI426210822

TACCGTTATCATTTTTCCTTACTTTAGAAGATAAGCCAAAAAATTTGACCTTAATGAAAA

A_14_P126839 TCCTGGCCTTATCTCTTGATAGAGGATCATCACTAACTTGCTCATTCTCAAGAGTATTTA

A_16_P01306311

ACTATCGTCTTACCACTAGTAAGAACTCTGACTCCTACAAGTATATTCTGCGGTCAAAAC

A_16_P03471449

AAAAGGTGTTCAAGACATGTAGTTAGATGAAAAGGTAAGTTTCAATACAATAGGTGGGGC

A_14_P119030 CATTTCATGGCTCCTTTTAAAATCATCTGCATGACACATCCTGTCACTTAACAGTATTTG

A_14_P118182 AGGAGTTGATGGCAGTTTCAATAACAGGTCATTGCCGAGAAAAGGTAAGTTTTAACTTTA

A_14_P132719 TGCTGAAGTTGAGTCATCAAAGAATGCTTCAGAGGACAATCATTCTGAGAATACTTTGTA

CUST_317_PI426210822 ATGTGTCTGGGCTGCTGAGCTCAAAAGTGCCCTCTCTTGTTCTGAT

A_16_P16399318

CCACACTCCTAAATCTTAGGTTCAAACTAGGTAGTTGTCTCAATTTTGCTGAAATAGTCC

A_14_P112692 ATAACATCAAGGTAATGGTGGTAAGAAAAATAAAGCAAATTTAGAAGAACAACCTAGAGT

A_14_P135167 GGTGTGTTTACTATTTAGTCGTTATGGTCTGTCTGGTTTCTAACAATACAAAAACCACCA

A_18_P11560301

AAAACCTTTGATCAGAACGATCTGTGGAAGAGTAACTCCATTTCTATATGAGTGAGTGTC

A_16_P17487919

CCAGTTCTTACTCCAAACCTATTAGTCCATACTGCTCTTCTTGTTATTAAAGCCTGTAGT

A_16_P39409005

TGGAATGCACATCCTTACATAGCAGACTGCACATTTCAAAAATCAGTTCAAAATCACAAT
A_18_P16135422 TACCCAGAGTGGAATTTGTTTCTTCAGTTCACTGAAGTTCAGTTCTTCAGTTCACTA
A_16_P19356925
CACAACAGTGAAACAGTTAAGTAGTCATAAGCACTAGATTTAAGCCATCTTGCAGAATGA
A_18_P14778778
AAAAGACCTTTCCAGTAATAAACTCTTGAGATGCTTAGAATTATTGCCACCCTTTGATGC
CUST_9032_PI426210822
TCAGCATATGCCGTTCTCTAACATACTAATAGCACCTAATATTGTATTATTTCCATTGAT
A_14_P109973 CCAGAAGAAGTTCTGCGGCGACAATTCTCTAAAGCAGGGCTTAAGTC
CUST_1656_PI426210822
TACTCTTTCTAGAACTAGCGTGGTCTCTTATGCAGGACAGATGAGTGTAAAAAGAATG
A_14_P105252 GTGGCAAAAAAAAACTTCATGGTAATGGCAATATCCAAAGCATAGTTGAGTAAAAGCCAT
A_14_P125386
AACTTGAAAAGAAGAGAGATTCTGAAAAATAGTCAGAAAAGAAGGGTAAATGGAAATGAT
A_14_P132303 AATCTGTCTACTTTATTGTTTGCCTTTTCCCCTGGTTTTTGTTTGTTTTTTAATTTGGCC
CUST_6080_PI426210822 AAAAGTCGAAGCAAAGGGGTCACAGAGAAAGATTTCTCTGATGACTGTGTA
A_16_P00346036
AGCCAGTTATTCAAGCAACTATGTGCCTTTATTTGTTTATAAGATACAGTCCACAGCACA
A_16_P01809575
AAAAAGATGAATGTCCATCCAGTTCATACAAACCGTAATCTTTGTTTTGGTAGAAGGGAT
CUST_8724_PI426210822
TTGTCTTGGGGGGACAAGTTTATGTACCAATTAGCTCAAGGCTTTGGTAAAATAAAT
A_16_P30952587
CTAATGAACCGCACTTGTGGATGCATTTAGAAGCATTTGAATTGTTGACATCTGATTTTG
CUST_8811_PI426210822 GACCATGAATGCTGCTGAAGGCTCTCACTTTGTACTTCCCTGGGC
A_18_P22841376 CTTAAACCACTTTGAGAAACACTGTCCTGGAGGTAAGCAGCCCCT
A_14_P128602 GGGGAAAACTAGTTCTGTGGGATTTTAAAATGTGTTTAGTGAAAAAAGCATTTTTGCCAT
A_14_P121759 GATTCTTCTTAGTGAATTATCCAGAAGGCGTATCCGTTCTATCAACAAACTCATCCGAAT
A_16_P19003802
CCTGGCATGTTTGATTTTATGAGTCTGTGAGATTACTTAGTATATAGTGCACCCCATGTT
A_16_P02979576
ACCATCCCGAGATACTCATAGAATAATTCACGTTATTGTTGCTTAATCGACCGAACATTT
A_18_P22840139
CTGTTGATGCCCTTTTAACACTGGTATTCTTTGAAGTCACACATGATAATGTGTTCTGTT
A_16_P01278953
CCTGCTAGACACTATCCAAAGTTAAGTTGCTTTTATTCTGTTCTTATTTTGCCCATGTGC
A_14_P114359 AGCAGCACAAATTGGAACTCTTACCTTTCTTGCCCAAGTCAGAGATTTTATTGAAAAAAA
A_14_P122395 ACAACGCTCTGAAAAATAGTTTTGTCCCAAAATCCAGCGCCTAGGGT

CUST_2913_PI426210822
CAAGCTGCTGTAGCCTCTGTAAATACTGTAGGAAATACAGACTTTCTCTGTAGGAG
A_14_P110114 TGTGCTGTGGATGAAATCTTATTAATAGATGTGGGAGACACTAATTAGAACACCACAACT
CUST_10352_PI426210822 GACAAATACACAGACAACAAAAACATTTGATGTGCCCATGACAGCTGGGT
A_14_P132624 CTTACGAATCTTGTCCTGAACAAAACACAAGACCCTGCTGTAAAGTTTTATCTGCAATG
A_16_P00081182
TCCTAGTTTCCATTTCCCAATCATTACTCTCATCAATGTTGCCATCCTTTTTGTTAGTTT
A_14_P106501 CTTACAGAGTGTGGAGGATATTGTCAAGTATAGTTTTGTTAACCAACAACTCCTATGCAG
A_18_P17401005
TGCCCTTTAACCTATCTTTTTTTTAGATTACTCTCACAATTGGCATATCTGCACAAAGGA
CUST_2836_PI426210822
TTTTAAAGAATGAAGATTAATTTTGATACACATAAGAATGAGAACTATTCTAGAACTCTC
A_14_P129076 CCAAAAGTCACTTTAGAGTTATCTGCAAATGATTAATTGTTGGATTTGCTCTGAGTCCTG
A_14_P132821 CTCACTTCAATCGTCTTGGCAAGTAACTGTGTGTGAGGAAAATTATACTTGTATACTTCA
A_14_P110990 TCAAGTCCTAAGGGCATGGTTAGAGATCAGAGTATTTTAACTGAATAGGAAAATACTTTG
CUST_190_PI426210822 GTGTACGAGACCAAGTGACTAGAATATGGAGGGGTGCTCTGCTCG
A_16_P20118826
TAGGAGATAATCAATTACCACAGTTAGGGTTTTACAGTCATTTGCATACTAGACCAGCAG
A_14_P104619 CATCCCTTGCCGGAAGCGCAAAGTTCTTAATAATTTTGACTTCCAAGTTCAGAAATTTA
A_14_P139928 AACTGGTGTGGAAAAGGTTTCTATTTTATAAAAGAAAGTATCCAGAAGGCTAAAGGCAGT
A_14_P102410 TTTCATAATGAAAAATGAAGAATGAAACTGCCAAAGCATAGGACTGTGGTTTACTTTGGG
A_14_P116496 CCAGTTTTTAAGAAGAAACAACTTTTGATCCTTTTGGATATCTTTTATGTGTCTCCCCCA
A_16_P37318950
AGAGAGGTTCAGTTTGACTCAGATTTCAGGAAAAGTCAGTTATGTAAAATTCTCCTAGAA
A_14_P104690 AACAACAGGCTAATTTTCTTTTCTCCTTTTAAAAAGCATCCATGTGCTCATAAGCAATGG
A_14_P133562 ACTTGCCTGTCAGGGAAACTTCTCTGTTTTATTCTCTGCAAAAAGATAAAGATAAAATGT
A_14_P139032 CTGATGGTTACAAGGCTTTCTTTCTATGCTGATAATCCCTGTGGAAAGAAAACATTGTG
CUST_8935_PI426210822
CGATCTTTTCCTTTCTGTGCTTAAGTGATACTAGGTCATTCTTTAACTTCTTGCTCCAC
A_14_P118701 AAGTGTAATGATTTTCAAAAACAGCCTTGCTCTTTCTGTTACTCCCTCCTGATCATTTAA
A_16_P38912217
TCATAAAGAACACTTTCAGCAAAGATCAAGTCTCATCTCTATGATTATCATACTCCCCCT
A_18_P22842291
CGCTTCTTTTTACTGGTCCACAAGACTTGGTTATTTTCTATTGACACTATTTTCAAGATC
A_14_P108117 GAGATGTGCAGTTATCAGTCTCACAGAATATTCCTATAACAGGTAAATCTTCAAAGTTTA
A_16_P03771106
ACTTCCCTTCAGTCCAATTAGTTCAAAGTCACATTCCACTGTATCAGTAATAGTTGCTAG
A_14_P123023 ACTGCATTATGAGCATGAATTTGTGTTACATGATTCTATTCTAGACTCGGGAAACAGCAA

A_14_P115770 ACTAATCCAAACAGTCATGGTTTTGTTCAAGATAGTCAGTATTCAGGTATTGGCAGTATG
A_14_P127551 TCATTGTAGAACAAGTGAGGACAGCACGCAGTGAATCTTTTAAAGAAGGAAAAGAAAG
A_14_P122404 ATCATCATCGTCTATGATTTCTTCTTCAGCAACATCCAGTGAACTCTCAGTAATCATGTC
A_14_P115065 TGTTTGTGATTTAGATTTGGAACAAATTCAGTGAGGCGGTTACCTATTTAGTCAAAATGC
A_14_P131782 TTGCAATCCACTGAGTCCAATAAGATCATTAATTATTTTGCTGACCATTAAGCACTGGTC
A_14_P115782 GTAAACCTTTTGGTGTGGTTACAAACATACTGAACTTGGATACTTGATTGTTTGATGGTT
A_14_P202568 TTGAAACAAAAGAAATCCTAAGTCTGACACACAATGTCATGTTTAAATTCCCCTTTCTCC
A_14_P113438 CTTTCTAGTTCCATAGTGGAGACCAATAGTTAGATTTTGGTCTGTTGACACTGCTTTTAT
A_14_P130870 CTACAATCATACCCATTTACTGTAGGTTTCAGAGAAAGAAAAACAACCCTCACCTTTTCT
CUST_8653_PI426210822 TAGCCCCTGAGCACAGGTCTAAGATTGCACCAAGATAACACATCTG
A_14_P134587 GTTGTAGGAGATTTTTGAAGACAGCGTCTTATCGTAATACTCCAGAATATGGAAGAACTC
A_16_P19798054
AAAAACAAAGGGAAATTGACTCTGGAAAACTGTATTCTTCACAATCCAATTCATGAAGCC
A_16_P01096843
AAACTGTAGGAGATAAGACTGAAAAAGACTTAGAGGCAAAATACATATCAATGCTTGTCA
A_16_P17057342
AACGTCTTCCTCTCATATTCCTTGCATCTTATCATCATATTAACCATGCACAATCTTGCA
A_16_P00350153
ATGTTTTCCCAAAGTCTTCGTCAATGTTAATAGTTTTCTGCAATCTAGTTTATCCCCACC
A_16_P36230073 GTCTAAACTCGTCCTTCACCCGGGTTGCTCTGGATCTTACTGGTT
A_14_P105789 TGACTGTTTTTGGAGATCATGAATGTTTCTACAACACTGCTGCATTCATTTGACCATTTG
CUST_5195_PI426210822 CCTATTTTTCTCTTCTCAGCTTCCCTATGCCTCATGATGCCAGGCATA
A_14_P108762 ATAGGTATCCTGAATAAATGAGAACTCTAGAAAATCAAGGTTTCAAAATTCTACCCTTCC
A_14_P131872 TTTTGTGAATATTTGAGTCGTGTTTGAGTTCCCTGTATCAGGGTCTGTATTTGTTTCAAA
A_14_P134595 TAACATTCTGACCAAGAAGTGTCACTTCTTTCAGCCCCTAAACATGGGAAAATATATTG
A_16_P16003572
ACGTTAGCAATATTGGGATGAGTTGAAGAACTTCTAAAAAGGTGAACTAAGGACGTAAGA
A_14_P133179 CTGGTTTCATGATTTCTTTGCGTCTGTAGGCAACGCGGTAAAAATATTGCTTCG
A_18_P10144354
AATCTGTCAGTCCATAAAGAGTCCAAAAATGTCTGCTAATCCCTTGAATTTTCTTGGAAT
A_14_P123627 AAGAGTGTCAAAAATGTCCTCGGCATTTGTTTTCTCAGAAAAAGAATTTAGATATTGGAG
A_14_P122593 TGAAATGCTAGTATAAAGGTTATTTTTTTTCTTTCCTAAATAACTAAAGTGAGGTGTAGA
A_14_P124113 CTCCCATCCACTTTATACTTTGTCATCTTTATTGTTGTTGTTTCTCGTGACATTGACCTA
A_14_P127125 GGTAACACAAAAATCTTTCGAAATATTTGTAGTTTTTACAGGCGGCGTTGTCTTTGATAG
A_18_P16723308
AGGAAAAAAAGAAACACTGCCTCTAATTGCTTTTTAATCCTTTTTATCAGCCCAATTGCA
A_14_P110422 ATTATGAAAAAATAGCAATTACCCTTTATTTTCAGAATCACGAGCCACCATTACAAATGT
A_14_P102008 AAATTAGACTTCCTTGCAGGCAGTCTATCAGTGATATCCAATAAAGACTCAATAATGGCT

A_14_P116519 AATGTAATTTTGGTCTCCCAACGTAATTACACTGTATCTCACTACAGTCTCTGAAAGGAA
A_14_P123155 CTGAACCCAGAAAATTAAACCATACAGACGCACACAAAACATGAAGAAAAAATGTCCTAT
A_16_P17295788
AAAGATAACCCCATTGACAACTAATCAAAATATTTCCAGCATATTAGTCACCATTACCAT
A_14_P122314 TCTCACGCAAAACAAACTCAACAAACTCTCTTGCATCAAAAAGATTGCATTGTTCTACAT
CUST_3681_PI426210822
TGTGTTGGAGAAATGTGGCATGTGGCAGATGTATCCTTGCTTGTTTAATTAATGA
A_16_P16811398
AAGAGTTGATACCCTGAAACAATTTGACCAAAACTTGAATAGGCATAGTGATGTTGCATT
A_16_P19150651
GTTATGGCATGGATCAATTAGAAGGGCAGAAGTTTACAATAAGATCAAGAGTTACCAGTG
A_16_P15634431
GGAGAGAATAGTATAAGCAAGTGAGATGTAGACTAAGCAAAATTTAGATGGAGAAGCACA
A_14_P124562 TGTCAGAGATGTGATTCATCCATATGCTAGAAACCAACAGAGTGACTTTTACAAAATTCC
A_14_P100605 ATTTAACCTATGGGAACAATGTTCTTTAATTAAGTTCTTTAGTTTAAAGTCACTGGCAAG
A_16_P39942678
TTCAATGACCCCCAATGATATCTCTCTTGAATGTCAATCTTCCTACAACAAAAGCAAATT
A_16_P03269708
GCAACCCTAGGATTTTTTTAAATAGATGCTGCTTGCTATGTTTTCAAACCTTTTTGAGCC
A_14_P134512 TAGGACTATGTGCATAGACTACTGGACTCAAGAGAACCTAGGAAATCAGACTGCTA
A_14_P109069 CAAGGACCACAGAAAGAGCCTGATGATTATTCTAACTTTGTAATCGAGGATTCTGTAACA
A_14_P119767 ACTCTTTCCAGTAGGTGATGTAGTCTCTGAGAGTCATGTGCTCTTTGGG
CUST_1884_PI426210822 GAGGTCAGGATGATCAGGGAGCTGACGAACTTTCCAGTTGACTAG
CUST_7734_PI426210822 GCTGGGACAGGCAAGGTATGTCTGGATGAGAGAAGGTCTTTGCCA
A_14_P102316 ACAATCCTTGCACATGACTTTAGTTCTTTATTAATGTTTTTTGAAGGGATGTAGTTCCCA
A_14_P130997 GTTAATCATGAGAACTGTGGTTGTCTTTTCCATGGGGCTCTTCACCT
A_14_P136782 TCACTGAATTACAGAATAAGCAAAATACTAGGTAAACAGAATCACGCTTGGGGGCTATAT
A_14_P120343 AAGTTTCAAGAAGGTTCTTATGGAAAAAAGGCTGTGAGCATAGAAAGCAGTCATAGGAG
A_14_P116712 CTTTTAATATCAACAGGAATACTGGTCAAGAGTCCACATTATGCAGGTTGTAAATGGTAG
A_16_P36230107 GACTGATTTAATGCTAAACTGACTGTTCTCAGGGTTAGCCATGCTACCCTC
A_14_P139076 GTGCAACAGACAAAAGATGAAGAACCAGTGACCAAATCCTGTTAGTAGAAAACAGAA
A_18_P11055233
ACGGACTTTAAACTCAATGGATATTCTAAATGCCCTTAATTTATTTTCCCCTCTGTCCTT
A_16_P39014334
TGAAAAGTGGGAGAGACTCTTTATTGATCACTGGCTGATTTATAATCAAGTATTAGGGGA
A_16_P36670143
AAGGTAGGAGATAACAAACAGTTGGTAAAACAGAACTCTACGAACAATAGCATTGAGACT
A_14_P135932 TGTGAAAAAATTGCCCCTAATGTAGTGATGGTGTTTTTTAAAAAGCACCATTTGGGAAGT

A_14_P134454
ATAATGAAATAATAAAAGGGAAAAAAAAACCAGGACAACTTTGTCCCTTCAAAGTAGAGG
A_18_P10139031
AAACACTTCAAAATTGGCTTCAGACAATTACTCAGAAGAGAGAAGAATACTGTGACCTTC
A_14_P202199 GGATAAAGTTCTTCCTTTATAAAACGTAGGTGCTTCTGTCTATTGGTCACCCAAGTAACA
A_16_P17847719
AATAGATGCTATTGAGGCTCCTTATTTCCTACTTGGCTATGCTAGATAAGAATCCCTGTT
A_14_P115958 AATCACCATCCACTGGAATTCAATCAGATTTTCCTAACAAGATGTTAGAAAGTGTCTAGG
A_14_P115863 ACAGTCCTATCACAAAGCAGAATAAAAACATGAAACCTATAAATGGGAATGCCATAAAGT
A_14_P117866 GAGGTAAAGTGAAGAGGAGAATCCGAAGTTATTGTGGCTTCTTCTCTTATTTCAAATAGT
A_14_P128072
ACAAGAAAGTATATCCTAGCACATTTGTGTAGTAGAAAGGTTAGAAAAAACAGGAGGAGG
A_16_P16763192
AACACTGATGAGTCTACAAAGATGACTATGAAATGCACATCTTTAGTCCTAATCCCTCTC
A_16_P02282423
GCCTTGACCATGATAAACTTTAAAATGAAATCACACAAATCATAACTTCACCATTACCGG
A_14_P114537 CCTAGTTGAGATAGATCTACGACAGACAAGTAGATTTATCCTATGATAGATCTGTGTGGG
A_14_P105641 GGTGCACAGTCAGTTGATGAATTCATGGCATTTCATGTTTGTTTCATTTTTTTGTTTGTT
CUST_8487_PI426210822 TGTGTGTGTTCCACACCCACAGGTCTACAACACTGGCCCAAGCAC
A_14_P201736 GTATAAATATTGTGAAGGGAAAACTCTACCTGTCAAAACAAATGGACTCTGGCCTTTCTA
A_16_P37494877
GGTTGGCTTGTATGAGACTGCTTTGTTAAATCACTTCTGTAATTTTACCCAGCAAAATCT
A_16_P21482571
GCAGTGGAATTAGTCAAAGGACATGTTTTTCAGATGACCTTATCCTGAATGTGACTTATT
A_18_P22840843
CATACGACTTAATGAATTAAAGCATGTAATGCAGGCCAAGTAATTCAAACCCACCCATG
A_16_P00403120
TCACACAACGCTTATTACCTAAACCTAATTTCCAAGAAAGAAATCGTTATGATCACCACC
A_14_P138657 ACTTCCTTGTGAATACAAATTCAAAGAAAATTCCACTCAATGCATCTAAGGAGACTGCAA
A_14_P123256 AAGATACACATAAAGTAAAGGTGTGTTACCATTCTAAACCATCCATGTGCACAGATGACA
CUST_4064_PI426210822 ATGACACCACCCTGTGGGATGAATGGGGATTCCCATGTTTGCTAG
A_18_P15514999
AACTCTGCACCTATAGAGAGATTCATAATTGTTCCCTTTTCCATATTGAAGAAATCCCGT
CUST_5491_PI426210822 CCTGGACATACTTTCCACAGTGAACCTATCTCTCATTCCTGCAAGTCT
A_14_P201383 ACTTGGAGAACTAAATATTCTACCCAATACACAAAAGGTAACTTGCTTTTATCAGCCTGC
A_16_P16666067
AAAGTCAACTCAGCATCAAATACTGAGCGATATTTGGTTAATACTGGTTGGAATGCATCT
A_18_P22841750 TAAAATGTCTGGTGCCATCTGTCACAGTAATGAGTTGTGCAAAGCCCAATG

A_14_P108613 AAAAATCTGGTATCAGAAATGAACTTACAGGAAGAAATACAGTCAAGTAGCCCAAATGCC

CUST_7089_PI426210822 TTTTTGTGGAGGGGGGCAATGGGCAAGGAAGCATTGATGAATTTT

A_14_P109368 AGCCAATGAGTTGCTTTTAGACTAAAATGGTAATTGTGAAACTCCATATTCAAGAGAGGG

A_14_P137737 AGGGTGGTTATAATGTCAGGTTCTGTTTATGATGTCTGGATGCCTTGAAAATTAATCATT

A_14_P126945 GTCTTGACCAAAGTCAGGCACACAGATTGTAACATTTTTCCCATGTTCATCTAGAA

A_14_P122305 TCAGTGCTGAATTAGTGCCTACCTCATCATGGAACATCTCAAGTGAACTCAAC

A_16_P41644646

AATGTCTGTCCTGAATTAACTTCATGAAGGCTTATGATAAACATCTCCCTAACCCTTTTG

A_18_P13239397

CCCATCATCCAATCTAAGAAGCAAAACATTACTAACCTCACAAATTGTTAAGTCACTCTG

A_16_P00023631

CTTTCCAAAGAAAACTGTAAAGGTCTCAGAATGCTATTTCAGTAGATCAGGCTTTCAGAG

CUST_5089_PI426210822

AGAGTGTTCTGCTATAAATCCTAACAGCTTAAACAATCTCCTGGGATCATATTGGCAA

A_14_P121954 AAACTTCATGTCAATGGAAAATCAACCTGGACTAATCAAAACACCTATGAAGAAACAGGA

CUST_9692_PI426210822 GAGGAGACAGAGCAAGTGGACAGTATGTCTATGTCAGTGAAGGTGC

A_14_P138435 CTTTTCCCCAAACATCCCTGTGTTGTGCTTCCGATCTCAGCAAAT

CUST_566_PI426210822 CTTTCACTCCCTAACACACAAGCTGGGGACCAATGTCACTCCAAG

A_14_P111259 ACTATCTTTACCAAGATTCTTGATGTTCTAAGGTGTTTTTCAGTTTCAGAGATGTGTTGC

A_14_P114827 ATATAAGAACAGTCCCACTCTAAGGGTATTGGAAGTCTTAATGAATGACGTAAAGCCAAT

A_16_P03235563

AAAAAGATTACCTGATGCATAATGTCCTTTGTGATGTTATTAAAAGTCCTCCCAGGTAGC

A_16_P40662709

TCACAAAGAGTTGATGTGTCATATACGGTCTTTCCTAAACTACTATTCCAAAAAACGCCT

A_14_P138970 TTTGTATATATCACTCTCTCCCTCTCCTGAAAGACCAGAGATTGTGTATTTTCAGTGTC

A_14_P122718

CTCCCAGAACTTGTTAAAAGAACTTCCCCATTTGAAAACAAACAGAACAACAGAAAACTA

A_14_P126802 ACCACAACTCTCAACTCTTGTGGGGACATGCTACTATACTCCAATTATCCACAAAATA

A_16_P38184757

CTCTACCTTTAACTGAAGATCTAGAACCATCCAAAGTTAATCATCATTGTTAGCACTGGG

CUST_2372_PI426210822 AGCCTGCCTTTGTCAGCACGCAGGCTGGCTCCTTTGCTTTGACAA

A_18_P21406694 CAGGGTCTCAAAAATATGATGTGTATTTCATCATATATATAATATAATCGTATACACACA

CUST_6315_PI426210822 GCTTTCTTGAGCTGTCTGCCCCGCTAGCTTTGATGAAATGCACATA

A_14_P114730 AGACTTACCTCGTTCATCTAGAAGAATATTCTCCATCTGAAAGATCACAGGTGAGAAGTA

A_14_P101117 TTTTCTTTCCCATATCACATCTTTGAATTCTGAAAGGGAGGCTTTATTGTTGTTCCTACA

A_16_P16809999

GTGACCACAATTCTCCTGTTCTAAATGTGAAGATCTGTTCTGCAAACAATCATTTTAACC

A_16_P20823546

AAAAGGTAGAAATCCTGTGCTAAATTTCATTATGTTCTGACTGCTGGTCTAAGATGTGGT
A_14_P119310 TTCACAAACCTTGAGTATTTTCTTAGTTTTAATGCAGTGCCTCTCTCCATAGAGAGTCAT
A_14_P102907
CTGTTAAAAAAAAAAAGTTCAGTGTGAGGGAGGGAACATGGAAAAGTCAAAGGTCTATTA
A_14_P131031 ATACAAATATGGCCAAAATAAGGCAGGATGTGGTATAGGACAACGCAAAATAATCCATTT
A_14_P201655 CTATTTGCATGGGTGGATTATATCATGTTAAGGGAATTCTTTATCTCAGCAGAGGGAACA
A_14_P109228 CCTCAGAGCCTCGGTGATATCTTCTGGCTTGAAGTTATTAGATCCTTCTAGAG
A_18_P22841015 GGAAATGGAAGATGTGCTGGGCTTTTTGCTTTGTTAAAGTTGTCAGAGTGTT
CUST_5142_PI426210822 GGGAAACAGATGCTTTCCTATAACTCTGAGCACTGAAGCAGCTTTGCT
A_14_P100917 CTATAAAGCGCCAACGAAAAGGAAAAACAGCGTGAGCAGGGTATGACAAG
A_18_P12352208
AAATGAAAACAGACCTGATGAAATTTGGAGTCTAAAATCAGTCCCCATACTAGAAAGGAG
CUST_8087_PI426210822 GGAGGTAGCATTTAAACAGAGGAGTCTGTTTTCAGGCTGAAGCAACATG
A_14_P126758 TTGTTTTTAAAATAAACTTCAATTGGCAAAATGAAACCAGCATACCCAGATTCTTCTACT
A_14_P119990 AGTTGACTGATCTCTTAGGGAGAGGAAGCCCTCATTAATTTGTCTAAGGTAAATTTAACA
A_14_P124891 AAGACTATTTTTGGCCTCCAGGCCCTATAGAGCAAGAATTTAAGAACTAGATAAATTTGG
A_16_P02100962
AAGCACTTATACAGTTCACTAGATGCTATCATCACAAATGTTTGCTAGATCTTTGTGCCC
CUST_7460_PI426210822
CACCTGAAAAAATTTTCAAAGGGAGAAAAAGAATGAGCAAAACTATCCTGTAGAGGAGAG
A_14_P131674 CCTATAGATATGAAATGTTATCATTGCACATCACATGGACAGGAATGAAGAAACTGGGAT
A_14_P122673 GTGGTCAAGGACAACGGAACCTTTCGTTGACTTCTGCAAAAATAATTCAGTTTTATATTT
A_14_P124176 GTGGTTACCTTGTAGACATCATGATGATCAACAATGTGGTCAAAACTCTTATAGATGTCA
A_14_P131013 CTCAAATTTTGGGTCTTACCATGGTTTCAAGATGCCCAATAACTTCAAAGGTGTATC
A_14_P115869 ATGAGCCTCTTCTAATTTTCATGTTATTAACAGGTTTGCAGTATCAGTCAGTGCTTGTCA
A_14_P113668 CACATTAATATTGCAGAGGCAGATACATAGGATTATGTAAGAGGAGTTGAGGAAAGGTCA
A_14_P102099 ACATCCTGATGGACAAGTCCTAATTCCTTACAATAATATTCACCCACCTGAAGGAAATC
A_16_P15008538
TGTATGTATATTTACAGACTGTTACGATGCTTCTTACCCTGAAATATTCAAACGCCTGCC
A_16_P20770458
AAACGTCCTTTTGTCTTTACTGTCTCATATGGCTTTCTTCCTGACTATACAGAAACCATA
A_14_P133794 CTCTGGGAGACAAAGAAAGTATGTAAGAACTCTGCTGTAATGTTAAAGCAAGAAGTAGAT
A_14_P115378 CTATAGAATAAAGGAAGACAGACACAGAAAACACAGCAAAGGCTTAGCCATGATCAATTT
A_14_P100465 AGAACGGTAACTGTCACCAGGATAATAAAGCACAAAGATATGCTAATAACGTTAACAAAA
A_14_P200360 TAGTTTATTGGTTTCTTCTGCACTTAGGGTTCTGATATTGAAGATGAGGACATGGAAGAA
A_16_P16081279
CGGCTCACCTCTTCATATGATTTGCTCAGATTAATTCTCCAGTATTTCACATTCTCAAAG
A_14_P123709 CCACTTTTTTATTGCACTGGCTTGAATACAGTAGCAGTGTTGATAGAATCATTTTATTCA

A_14_P138092 TTTAGTAGACATTAAAAGTATGAGGTTGGACTGTGAGTTTGCAGAACTTAAAGACGTCCT
A_16_P41703025
GAAAATACCAACAAAACTGAGGGGATATTTTGATAAACTAAAAAAATTAAGCAATGAACA
A_14_P111044 TCTGCCAGTTGGTTTTATTTGGAGGTTTGTGGGCTTTTTTTAAAAAAAAAAAGTCCTCAA
CUST_7620_PI426210822
CAGAGGGACACGTTAGGAACGAGACAGCTTATTCAAGTGCTTTTGAATTTGTAT
A_14_P108445 CACTTTCTCTAGGATTCTCTTTTAAAATATAAATCTTCTCTGGAGGTGGAAGCCCATTCA
A_14_P101473 AGAGAAGCAAACAGATGTATTTGACTGTATTTCAAAGGCAGTGACTGTAAACAAACTTGG
A_14_P137385 AATGTCAGTGCAGAAAATAACCTGCACTTCTCTTTGTCCTGGTGAGATATATGCAGATT
A_16_P38500926
ATTGCCTACTCAGTCCAAATTTTAGGGTTTACAACTTAAACTAATAACTCACTGGCACCT
A_18_P22842107 TTGAGAGACTGAGATAGATGGATAGGCCCTAGGTCCTTATTTCCTCCATG
A_14_P103435 CAATAAATTCACGTGAAACAAACAAACATACTGCATGGCCAGGAGACAGATTCACAATC
A_14_P117851 CCAAAATAAGTTTTAAAAATATGTAGTTTATAGACTCCAGAGCAATGGGGATGTCCCTTC
A_14_P126034 CTGCAGACAATCTAGACAAAATTCACGACGAAAATGGAAACAATCTATTACATATTGCGG
A_16_P36372434
ACTGATACCATGAGGCCTTTACCTAAAAACTACTCATGTATTGTTTTGCAAATCTCTGGT
A_16_P00666367
GAGTAATCAACACATTCCCCCTTATTTTAAATGCCTAGAAAGACATTAGTTCCTGCATGG
A_14_P135381 GTGGTTCTTCTCCGTCTATTCCTTCCTTTTCCTGTATTTAAGGTGAGTTTCCAC
A_16_P20963078
ATACGGCTCATTATCCAAGTATTTACACCATTATGTGTTTAGGGAAGCAAAAGTCATCCT
A_14_P106154 TCTTCTAAAAGGTCAGATGGTTTCACAAGTAATGGGCTAGTGGGATTTGTATGATCTAAT
A_18_P22840543 GTCTGCTATTTTCCACTGAGACTTGCTGTGTGTGTTCCACACCCA
A_14_P117516 TCTGTCATTTCCAATTTATAATAAAAATTCAGAAAGTGGGGAAGGACAGATCTTCTGGGC
A_14_P128455 ATAAGTAGACCCCAAGCATCCTTTTCTAAAAAGTGACTTAAAATAAGCCAACAGACTCTC
A_14_P132720 GAGAAAAACAAATGAAAACCATCACTTTGCACTTGACTTTTAGTGCAGTGATTCTGGAAA
A_16_P58424114 CAGTGGCTTGTTCCTGTTCATGCAGTTTTCTGATGCCCAGAAAAGTG
A_18_P14136590
TGGTTTTGTCTTTATATTTGAGATGTGATTATAAAGATCCCTTCTGCCTCAAACCTTGAA
A_14_P120974 AGGAGTTTCACTTCATCCCTTTTCATTTTGAAGGTAGGAGTTTTTATGATGTCATTGGTA
CUST_9928_PI426210822 TGCTGCTGGCCGTGCTGCTCTGGAAGGTGAGTCTGGTGATTGCAG
A_14_P129872 GCACAAATTTCTGGCTCTTCGACACACTCATCAATATCTAAAAGAATCACATGAGTCAAA
A_14_P116508 TTTTAACCTCCATCCTTTTTTTTTTTCCTTGGTGGTCGAAGAGTTTTACTGATTTTTTAA
CUST_6358_PI426210822
CTGATTAGAAGGGATTTCTAAAGCATGATGAGATGGGGAAGCACAATTCAAACTACAT
A_14_P133387 CCTTAGATAGCACTGACCTAGTTTTTTGTGCTCATTTTAAAAACCGCAATGAAAGACCTA
A_14_P101406 TTTTTTTTAACTTCCCTTCCCAATCTAGAAGCGTGCTCAAATAATTAGCCTTGATTGTGT

A_14_P137217 CCCGTGTTGAACATGAGTCCTGTAAGAAAATACGAAACAGCATTAATTTTCCTACTGAGA

A_14_P118629 CTGTTCCCGTGTGCAGTGTCTACTATTTATCTACAAATTAGAATTCAAAGTGAGATAGCG

CUST_324_PI426210822

AAAATAATGCTTCTTTTTCCTCCCTCACCCACCATATTGACTTAATTTTTTCTTAGATTG

A_14_P109157 TGTGGGGTTCAGTGTTGCAAAGTCAAAGACCAGGTTGTCAAGGATAAAT

A_14_P104165 AGGGCAAATTAGTTTTCAAAACCTCAAATCTTTGGTTTCGGATAACCTAGAAGAGTTAGA

A_16_P15755518

AATATTGCTCTTCATAACTGGCTAACGTTTTCTCTATCCTCCATATCCCAATTTTCAGGA

A_14_P140002 ACTTGAAGTATATTTCCTCCCTGTCCCCTCCTATATATATTTGTTTTGCTGACTGTAGTT

A_14_P113052 GCATATACTGAGTATTTAGAATGTGAAGGACTAATTCCAACAAGGAATGCTGTCTGGGAG

A_14_P106021 TTGAGATAAATTAGGAATGGCTAAGTTAAAATTGTCTCTGCTCCAGACTCTCTCATCTGC

CUST_4302_PI426210822 ATTGTATGTTGAGCACCTAGAAAACATATGCATGTGCATGATGAGCACCCTGTA

CUST_1058_PI426210822 AGCAGTCCCAGGCCTTTCCAACCCTCACCTGACTTGGCTGCAGAG

A_16_P03520414

AGTCCATCTCTATAAACGTATCCATAGATTCCTCACAGAGAAAAAGATATGAATGCTGGT

A_14_P125299 GTTGTTATCACTTTTAGGATTAGGACAGGGCAGATAATAAAAGTTCAGTCAGTTGGTCAT

A_14_P116745 ATTGAGGGAGATGAAACTTCTACTGAAGCAGCAACACGCCTGTAAGTTACGTAAT

A_14_P101094 GAAAAAGAAATAGGTCAGTCTTTAAAATAATGAAACCCCCCACAGGCTCATAGATATTCG

A_16_P00723299

CTCCTAAACAAGCACAAATATGCAAAGTTGACTTTTTGATAATCCCTTTCACTTGTGTCC

A_14_P112910 CTTGCTAACTAGATAACTATTCAGGATTTCCCCCAAAGAATTTGGACATACATTTTGTGG

A_14_P118193 GTTAGTGCACAAATTGGTAAATCATACACAGACTCGAGAACACACGCCTATCCTC

A_14_P100414 AGTTTTTGAAGTTAAATAGTGAATACTCTTTTTATTCAGAAGAATGCATTTTTAATAGAA

A_16_P21527741

CCTTAGCATGCCTAGTGATTTGTAGTTGTGTGATATGTAATTCATAGCCTCCAATGTAGA

A_16_P40685886

CAACCTTGCTAAAAAGAAACTTACCGTAGAGAAACTTAACATCAGTTACCATAGTTGCTG

A_16_P03580535

CCAGATGCCACAGTTAAATTAGCAAAAGAACTCAAATAACCCTGAATAGAAGAAAGAGGA

A_16_P41618901

GCATCGCGTAACAATTATAACCTATTTAGATCCAAGATCCACTTAAGACATGTTGACTTC

A_16_P16259968

ACATCAAGCCCATCTTTATAATCCCTTGTCTCATTCTTTACTTTCTGTTTATGCCTTTGC

A_16_P21320479

TAACAGCATAATCAGAGTGAACTCCTATCTTGAATCAGTATAATTTCCAGTGGCACCTTG

A_18_P12066516

ACATCCTGTCATCAGTAAAACCAAGTGTCAGTTATCCACACAATTAATCAAGTTCTCACT

A_14_P135178 CTCTAAAACCTACCTGAGTCCCCACGCTCAATTGTAGAGCAGGCTAATTA

A_18_P16325219
GAATTCTACCCAGTTAAGAACTGCATTTGGTTCTTCCAGATGACAATTTTCACTGAATTC
A_14_P201066 TGATATAAATAAGTGTCCTAGTTAACCCCACAGATTTGGTAACATTCACGCCACAGAAAA
A_14_P105889 GAATTGCTGCCACTGAAGTTGTAATGGGGAATCAATCAGGATATATTATGCTGTTGTATT
A_16_P02128124
CCCTAATAGAGTGGATAAGAGATCAAAGACTTGAACAAGATCTATGCATTGCCTTGAAAC
A_14_P118288 CTGTTTCCAGTGATAAAACTCAAAATAAACAGAATTAAGTGAAAAATTGGGCATTGGCCC
A_14_P101018 ACAGACTTTAATGTTGCAGAGAAACAGAAATGCCTCTTGTTTCAAGAGTCTTTGTTCAAA
A_16_P02551989
AAAAACAATCCCCAACACAATTCATGTTGTCGTTAGGCATAAAAAAGGTATTTTCCAGAG
CUST_3043_PI426210822 ATTCAAGACTGTGTTACAAAAGTTCTGCCATCTGGCATACACATTCAGGTATGG
CUST_9527_PI426210822
TCTTAAAACAATTATTGTATTGAACTAAAAATAAACTAGGTAGTTTGTTCACATAACAAA
A_16_P00909981
ATCCATTCACCCATCAACCTAACTAAACTTGTTTTCTCCCATGTAACACAATACTTATCC
A_18_P14137558 GCTCACCATGGGTGGACTGAGCAGACGCCTGCCCTCACACACAAC
A_14_P134989 AAAGTGTAGACATGGTCCAATTATAGGTGAGTATCATTTTGTGTGCTTGTGTAACAGTGA
A_16_P01497240
CCAATCCCCTTCAAGTATTGTAAGGATTTAAAAATCAGTATTGCTCCATGTTACACCACT
A_16_P39620931
GACATCCAACACCCTTTTAAATGTTATCTAGTCATTCAGAAGTTAAATAACCCAGGTTGC
A_14_P126909 ATTCATGTTTTTCTCTTCTTCCCTGTGGAGTATATTTATTGACTGCAGATGGAAGCACAT
A_14_P126198 GCAGGTTCTTCTAGAGTATCATATTGCCTATCTAAAGGTTTGTATACGGTTCATACTATA
A_14_P133141 TTCAGAATCCCTGTTGTTAAAGCACTTTCCAAAATCAGTAAAGTGCTGAACAAATGCTAA
A_14_P111529 ACCACCATTTTCATTCAGATAAAAGAAATGACCTTTTGAAATCATGGGCATCAAATTTGG
A_16_P18751262
ACTTCTGTGTTGTGCAATATGAACTTCTCAGCCTAACAATCAAAGTCCTGCATCATTAGT
CUST_107_PI426210822 CAGGGATTGTTCCTTGTCAGTGTGTAAAGTTGCATGCCAACCTTTTGTAA
A_14_P137269 ACTCATCAATGTAGCGTCGACTATGATAATCTTTCTCATTTATAGACCTGCTTTCCAAAT
A_14_P133071 CAGTCCATGCCAAAGTAGTATAAAATGAAGCCAGAAGTCTTCAAAAAGAAAACATTTTCC
CUST_3678_PI426210822 CATTTGTTCCTCTTTCATCCTATACTTCCCTGGGCATGATACCCTTCTTTC
CUST_6676_PI426210822
ACTTAGTCTTACTATTTTATAAACTCACTAGCCTCCTGTATCTTGCTTTGCTCCCCC
A_16_P16191915 CACTTTTGTCTCCCATGGTCTAGGCAGGAGTCATTTTTTTTGCTACAGTG
A_14_P131099 TACTATGAAAACCTGAAAGGTTGAAATCTGGTGGTTTACCTAACCTTTGAAAACGTCAAT
CUST_1974_PI426210822 CAACTGTCATTGCCAAGGTGACTGTTGCCACACAAAGAAAATCCAAAC
A_16_P19295924
ACTTTTCTCCATGATTTGTCTATTAATACCTTTTCTCCCAAATAACTGAAACCTGCCCAA

A_16_P41477963
CATGTTGTATTACCACAATTACCCACTTTCTTTATAAAGCGGTAGTAAGTCTTTCAGCCA
A_16_P00311243
TTATGGCTGCTTTTATTTTAAAGGAATCTTACCAAGTGCTATCATTGTAGAGTCCCACCT
A_18_P17384769
TTTTTTGGAAGTTTGTGTAATTGCTCAAAATCACAGACATTGAAGTTGAGTGCTTCATGG
CUST_4407_PI426210822 TATGTATGCCCCAGGACAGAGATTAGGCTATGGTGGGCATTTGAG
A_14_P137307 TTGTTTTCCAGGTGCGATTTGAAGGTTTAACAGGAAACGTGCAGTTTAATGAGAAAG
A_14_P138461 CAACATCAAGTTCCTTTGTTTTATTTACCAGATGACCAGGAATCATAGATGAGTTTGGCT
A_14_P109438 CTAGCCTTTTATCTACTCTACATCCTCAAAGTAATCTGCGTTTGTCATTTACCTCCATGA
A_14_P132823 CTTGATTCTTATGGCAGTCACTATCAGTGGTTATTGGCCAATATTTTAGACGTGTCTTTT
A_18_P22839944
ATTCTTACTTACTGTAAGTTTGCCCTTCGTATCAGTTCCTCCTCTCTCAAACACTTTCAT
A_16_P19987526
GTGCAGATTGGACCAAATACAAACCTCTAACAGTATCAGTTTACTCATTGCCATCTTGAT
A_14_P116572 GAATGTCTAGCCACAAATTCTCAACAGAAGCGTATGCAGAGTGTATCATTAGATGGC
CUST_5658_PI426210822 GTAAATAGTTGAAAGAGCCCTTCTGGAAGAACACAAGGCTAGTTCCTTTGG
A_14_P105349 TATCTGGGATGGGATTGGGCACCAAAATAAAATCTGAGTAATTGGAAAAGGG
A_14_P111988 GATCTGTTTCCTTATACCAGATCCTCAGTCTCACTAAAGACAGCGATATGGGAG
A_14_P139779 TTGGTCTTCAGAGGTGGTTGTTTTAGAGATAGGATAAGAGGAGACTAAATATTCTTGGTT
A_14_P128845 CTGTATTACCAGTAGACAACACAGTAAATGCCTGGCACCAAAAGTGCACTAAACAAA
A_16_P16067808
AACACTAATCCTTACCTATGCCTGTAAATAAGCCACTAATAACACTTGACTTCATCTGGC
A_14_P115862 ATCATTCTACATTAGCCCGAGGGGTTCGCTCAACACAAATATTAGAATATACTTGAGATT
CUST_7519_PI426210822
CCAACAAAGGACACTGAGGCTACTCAGTAATTATTAAATGAGAGCATGAACAAAGGAAT
A_14_P108209 ACTTGTGGTTTGAAAAAAAGTCACCATGTCTTTTTCATTCTTGGGCATACCTTGATTTAT
A_16_P03070647
GGCAACTTTTAAACCTCAAGGTAATTAGCAAAACCAATCTCCTTTAAAGAGACAAGTTGC
A_14_P102737 CTTGAGTAAGGGGCTCATTTTATTTTATTTTTTCTACAGACTGCAATCAATTGAGGGATG
A_14_P136463 GTTCTTTAATCTGGAAACATCATTTTTGGTGTCAAGAATGTTCTTTTGTAGGATCCCAGT
A_16_P18307879
AAGGTTTATCAAGCAGCAGAAGATATTCTCCCACATGATTTTTATTCAAACACTCTCTCC
A_16_P02333343
TTGAGGAAGAAAAGGCTAAATCATGCATGTAGGGTCATAGGAAGAACTAAAGGCATAATT
A_14_P113361 CAGGTATTGTGTGTATGCATTTAGTTGTGCACACACAAATATGCTCCTATACTGGCATTA
A_14_P125280 CTTTTCTATGACTCTATGAAATGCCACAAAGTTAACAGGGAGGTGAATTTTACAAAACTG
A_16_P16442484

ACTCCATGGAAATATTCACCAATGTAAACTGTTCTTTCTACCTAGACTGGAACTTAACCT
A_16_P15013319
ATGTCAAATAAAAACTCAGACTAAAAGAGATGAACTTCCCCTTAATATACCGTGATCGCC
A_14_P135206 ACATCCCATCAATGCATTTATCAAGCAAGTATGGTCCTATTTCTTACAAAGAACAATTCC
A_14_P139798 ATTTTCATTTTGGGAATGAGAACGTTCTTAATTTGTCCAATTTCTGTTCTGCTCCATTCT
A_14_P115231 AAAACGGATGTGCATAACAGAAAATGTCTTCCCTTGGGATTTTTCTGATTGTTTTGATTA
A_14_P104598 TTCATGATCCCTTTCTCAGATGGATCAACTCTGCTTATCCTTGAAGACTTCAAAGAAAT
A_14_P202525 TCAACCTCCATAACAAGTTAGGTATATCAAATACTGCTGTAAAACCCATCCAGCAAAAAC
CUST_5048_PI426210822 CGCCCAAATTAAGTGGTGTCATACTCTGATCCTTCTTCCACTGAACATC
A_16_P16191048
TAAATTACAAGTGGCAATTAGTCACTGTGCTGATGTAAATTTGGAGTTGGTGGGAATTTT
A_14_P123978 CTGAAAATGCAAAGAGAAGGGAAAATGCCTTAAATTGTTCTGGCTAATTTAGAAGCAGCA
A_14_P133496 TCTACAATTAAACAATATTTCCTCTTGGCCGTCCATTATTTTCTGAAGCAGATGGTTCAT
A_14_P128249 ATTTATGATGTATACCAAACCATGAACAGTAGTAATTTCTGGAAACAGGTCTGTCTGATT
A_14_P137265 CTCAATTTAAACTGGCATCCAGTAGAGAAGCAACAACTCACCTCATATAATGTTTTCCAA
A_14_P126022 CTCCCCATTCAAAAACTCCTGGAATCTTCTTTATTTAATACTAAATTCCTACTAATGGTG
A_14_P129456 AGGAAGAACCATGTTTTTCTATCCTTTGAGACAGTGGAAAATTATTTTGGTCTCCTCTAA
A_14_P127041 AGGGAGAATCTGACTACAGTTTCATATTCTTCCCCAGAAATCATACAGATTTCTCCAC
A_14_P102287 CATTTGCCAGGATGTATCTGAATCTCCTTGGCAGCATCTATTACCGTGAAAG
CUST_1202_PI426210822 GGATGGGAAAAGCTACCAGAATATCCTGTTGGAACCAGGAAAAACAGA
CUST_1977_PI426210822 CTCTTAGAACCTTGAAGCAGAAATAGCTCAGGGTGCTGCCCTAAAGTT
A_16_P39579553
CTCTCCTAATTAAAACACTTCCAACTTCAGTAACTGTTATTTACAATACCTCCCCTCAGA
A_14_P139767 GTGATAGGACATGAGAAATAAATTTCCATCAAGTGTAATCTACCGTCTCATGATGACCAC
A_14_P100184 TCCCTCTTTCCCTTGGTTATTGTAAATGGAAACTTTTCGGCTTGTATTTCCTTAGATTTT
A_16_P17691892
AAAGAAAAAGTTCATGACCTTGCTAACCTGTTCTTAAGTCTGAAATGCTCCACTAGAATG
A_14_P133729 GAGAGGTGTGCCTAACAAGTGAGGTCATTTATGGACATTATGTTCTCTGTGC
A_14_P124171
GAGGGTGGATTGTATTGGGTCAAGGAGTCAACAGAAAAAAGTTGAAACAACAAATATAAA
A_16_P16504888
AAAGAAGGACCAAATGAAAGTAGATGAGATGTGAAATCAAGGAGTCAGACTATCTTCTGT
A_14_P111392 ATGTGACCTTTATTCCTCATGTTTCTCTGACTTGAGTGGGGGTTTTGGAG
A_16_P00911659
ATCAACGACTTCCTTTGAATGGTGCTAACAATGCATTTTCACGGTTAGATGTAGATTTTG
A_14_P135776 TTAGCCAATGAAAAAGTAGAAGAGTTCACCACATTTGTGAAGGTTTGAATTACTTGTCGT
CUST_3123_PI426210822 CAGATTAGAGATGAAGTGTCCTCCTGTTAGTGCCAGAGCAGAAGAATAACAG
A_16_P00685576

AAACCAATAGTAATAATGCTTAGGACCTCAATAAAACTTCAGTCCTGTCCGTTTTTGGTG

A_14_P105966 GCCAAGTTAAGTTGATCTGTTACAGGACTCATTATCCTAAAAGGGTCACTAATGACATTT

CUST_4582_PI426210822 ACTCCATAGCCTGAAACCATAGCACCTGATGAAATTGGACCAATCTGTTT

A_14_P136814 TTTACAAGGAAAAATCCAGGTAAGTTATGAAAAAATGGTTGTGGGGCATTTGCACC

A_14_P132043 TAGAACTGGAACATATTTCTTTTAGTCACACTGTAAACCCTTTAGCCTTCCTGGTTATCA

A_14_P103414 AAATGCAGCAGAAACTTACATTCCAACCTTAGAGACTCATAGTGAGCACAAGGAAAG

A_14_P113560 GAAGTGAGCACTAAACAAATACTCCCAGGCATGGATCATTACAAATGGAGGTG

A_14_P103837 TATGCTATGAATGGGGAAGAAGACACAGTATTATTTCACAGTGTGAAATTTCCTTCAGCA

A_14_P111757 AGACTTGATTGTGAGTCCGTGTTATATCATCTGGTCTCATTGATAGGCGGGATA

CUST_7700_PI426210822 GAGTTGGGTCAGGAGAAGGAAGTTAAGATTGAAGGAGAACATGCAGAG

A_14_P113621 CGGATACCATGTAAGACACTAACCCTATCCATGTTATGCCAGGTTTAAGGTTTAATTTCA

A_16_P01007238

AAAGTCCTTTGAGGGTAATTAGACTCATACTTAAACCTGAATAAAATGACAGCCAACACC

CUST_9703_PI426210822 CTCCTTCTCCCCAAATCCTCAAGTCCTGGTCAGTTTACTCTAAATCAAAG

A_16_P03487508

AACAACACAACATGCTACTAACATACCGACTAATAGGATATGAATTCATTACTGGGCAGA

A_14_P201621 CTCGACATATTCTTGACGAACAGCAAATATTATCTGATTTCTAACATCAGCCAATCTAAA

A_16_P02750926

CTTGAGGGATGATAACAACTAAGGGCTTACAAATGATTTCAGAATACCATGAACTGTGTA

A_14_P111183 GGCCGGAATATTCTGATGGGTGCTATAGAATATAACCTCCAGTTCTAGACTCTGAAT

A_16_P01246367

AACCAGCATCAGAATCAAACTCCTCTGTTTTACTCTAAAGGCTCTAAGTAATGTTAACCA

A_16_P20531603

AAACCACAGGATTTACATCTCTCGTAGCATCTAATGAAATACCAGCAAGAATTGTGTGTT

CUST_5949_PI426210822 TTGGAGCTTGGAAGAACCCCTTCCCACGCTGGAGAGCTGGCTTTTT

A_16_P58424528 CTTTATTGTCTCCTTTCCTTTGCTCTCTCCCTTCTCCACTCTCCAGT

A_14_P121237 TCCTATTGTGAAATAGTTTACTGAAATAAGATCTTCCCAGAAGCCCAGTATGTGAAGCAG

A_16_P18304325

AAACACGGAATTGCTTTTCTCTGTTTGTAACATCTTACTTATTAAGCCAGGTAGTCAGGA

A_16_P40327681

AAAAAGTTGGGTTACATATACACGTTCTGATTAGCATAGACATAGAATTTTACATACGTA

A_14_P117026 GTGAAAATACTAGAAGCCCAACAATCCAGATGTAGCAACCTAATACTCAGAATCACAAAA

CUST_1629_PI426210822 GACTTCTGTTGTAGATCCCGGGCACTCCTGCCCAGCTGCTCATGC

A_14_P201438 TATGAAAACTAGTCCATTTCCTAAAAAATGAAAGTTTCTTAATTATTGGGGGGAGGGAGG

A_16_P01888364

GTCCCAAGTAATAGCATATCCTATTTATTTTCACCAACTTATATAAAACTGAACCCTGAC

A_14_P108578 GGTATGGAAGATGCCTCATTCGTAATGGAACATTATATTGGACCATGTTGGGTATATTGA

A_14_P110956 CTTAGCCATGACTGAAATCCTATATTTAGCAATTTCCTTTTTAATTGTTGTGCCCACCTC

A_14_P136660 GTTGCTGACCGCAGGAAATATTTGTAAACGTAATGTGCAAAGCAATTAACTGATTATGAC

A_14_P109396 ATTATAGTTTGCCAAATACTTTCTTTTATAATCATTGTATCATTTCAATCTTAAAAGGAC

A_14_P115677 ACAGAAAAAGAGATGTATATTTAAATGCCATTTTGGGGGCTTCTCTTATACTCTCAGGAG

A_18_P12745044

TCTATCCACACGATTTCTAAAATGCCTTCCACTTTTTATACCCCCACTATAGAATTCAAC

CUST_7699_PI426210822 AGATAGGCAGGGGAGTTGGGTCAGGAGAAGGAAGTTAAGATTGAA

CUST_5050_PI426210822 CTTCTTCCACTGAACATCACATTCTTCATGAGAATCACACCATTGTCCTCTG

A_16_P19296848

GGTAATTTCCACCAGATGGTCAATAATGTTCATTCAGATCACATATTTAGATCGGGAGA

A_14_P120392 AAAATCAGATCTAATGAATCCTGGAAATGAACGGACTTAATGGGCAGCCCATACT

A_14_P109421 TTCCTTTCTCTTTGCCAGCAAAAACGATTATGTGATTTAGATATGGTAGTGATAGCCTCT

A_16_P01563156

AAAACACCCAATCATTAGTTTCTCCATTACACATTTCTAATTTCTTTTTCTTCCCTCGCC

A_14_P125744 CCACCTTTCGTTCTTGGCCCATTTTATGATGAAAATTCACATTCATTAAATTCTGCTTTT

A_14_P120898 CACAGAGTTATCACAGAGAACTGACTCCACAACACCTGCTTTTCTCATTTCAGAT

A_18_P17366233

ACCAACCCTTAATTACTTCTTGCTTTCAAATCTTCAGTAACTTAACACTCTCTACAGTCT

CUST_5293_PI426210822 TAAGGAGTTTCTCTGCTTAATTGCAGTGCTGAGCTCAGGGAAGCTG

A_16_P18978380

CACGTATCTCAGCCTATATCCCTTTTTCATGATCATCTTACCTCTGACATAACTCTTTCA

A_16_P58424345

GGGGGAGCAGAGGGAAAATCAGAGTTTTTATGCAAGTAAAGTTACATTGTGAACTTATAA

A_14_P108837 CAGGAGAGAAGTGTCTGTCCTTGACATTAATGAACATCTCTGGGAGATAGTC

A_14_P138925 TAAACAAGCATGTGTCACTGGAATGCATAAATTCATCAGTTTGGAGTAGAAACAGTCATC

A_16_P15216422

AATCTCCCCTTGATACATAAAAAAAGCAGAGGTATATGTCCAAATACTGAAGAGACCAGG

A_18_P22841609

CCTTAATTATCTGAATGTTGTTGCTAGGGAAACCACTATTTTGGCTCAGATCATCTTAGT

A_16_P01423888

CCCTCACATTAAAACTTGCTTTCAAATCATCAGATATCCTTCACTGAGAATTGAACATGC

A_14_P114617 TCCTTCCCGATACATTCCTGGCTCTTGTTTGATGTCTCCTAAATTAAAACATTTTTAAGT

A_14_P120371 TCGACCTTTAAGATCCCAGAGTTAATAAACCCCATCAGATAGTCTGAGAAAAAGTGTC

A_16_P00685252 AAAGGGACTTCGGCGGAAGCCATATGACCCACCTCTGTCATTTTA

CUST_4822_PI426210822 CTAATGTTCTGTGAGGGTGGAACACCTAACCTAGAATTTCCCTGTGG

A_14_P113715 CTGAAGAACCTATGCAGCAAGACAATCGGAGAGAAAATGAAGGTAAGAGAACTGAATAA

A_16_P02201942

GACTTTTACCTTGAAGCAATCTTCTTTTACATGACAATGGATTTAAATGCTAGGCTGGCT

A_16_P58424407 CGTTCACATCATAGCCAATGCGAGCCTCCTTTAAACACTAACGTCAAGT

A_14_P117321 GTTTCGATTGGGGTGAGTGCTTGAGTTTAAATAATAGTGGCTTATTTACATTCCATCACA
A_14_P114914 AGAACAAAATTATGAGAGAGTTATCTACATCATTGAGAAAATCCTCCTGCCACTACCAAC
A_14_P105197 TGTCCCAAGAACATAACAGTGTAAACAAATGACAAATGTTTTCATCTGTAGAAGTAGTAA
A_14_P101811 GTATAGGTGAAAGCATCACAGGTTCTGTATCTTGGATTAAAATAATAGCGAGTCACATTG
A_14_P111663 TGGTTTATTTGTAATAGATCGGGTGCAAGATTGCTGTTGTGATGTTTATTTACTTCCTGG
A_14_P123221 ACTTAAATTGTGTGCATTCCAAGCAACAAAACCAATAATCTGTAGGTTGAAGGTTGTAGT
A_14_P127275 AGGTAGTAAATAGTTTGGCAAAAAACTGAAGATAAAGTGGCAGTGCTGAGTAATCATGGG
A_18_P10303961
CCACATAAAGTTTCAAATACAAAGTTTTACATTAAAAAACACTAAATAAATCTAGTATCC
A_14_P119087 CACTCCTTGTTCCTGGCACCAAGATTTCCAGATTTTTATCTTCTAAATATTTCTCTAACC
A_14_P123499 CTCAACCTGAATTCCTGAAGCAGCCTACTAATATATATGCTCACGAATCTATGGATATTG
A_14_P136671 ATCATGTCTACAGAAAAGCACAAAGAAAATTATCGTGGGGAATTCTCAATCTAGTAGGAG
CUST_1754_PI426210822 GAACCATCAAAGTGCTGAACCTTACGGACAACACCTATTTAAAACTGAACGAC
A_14_P113442 CTAGTTACAGTTGCTTGGCTGTAAATAAAATCTCCCCTGGAATGAAGCATTTTAATTCGA
A_14_P103706 TTTTCCAGTGCAGACTCTAGGGTGAAAAAAAGTGTGTTTGTGGCTGGA
A_14_P137654 CTTCATACAGGCAACTACTTGAAGTTTTTTTTTAAGGCTTTCTAGGCTTTCAGAGGATTC
A_14_P108877 ACTTAAAGGTTGACTTGACTAGCAAACTTTTAACCTCAGTTTTTGCTTGATCCAAGTCAA
A_16_P18140575
AATTTAGAACCCATAGACGATAACTCACAGAAATTAACTCATTTTCCCAAAATACGCTAC
A_14_P109451 GAATTTCCCATAGATATGCAACCAAATCCAAACCTGAATGTTTCTAAAGAAAGTCTTTCT
A_18_P22840485 ATGTTAATTACCTGTCTGTCTAGGGAAAGACTCTGTAATGTGTGCCTCTTAGGAG
CUST_6363_PI426210822
ACATACATATAGAATGTTCTGTTTAATATTATTGGTTTATGCAGTATTTGAATATATAAA
A_18_P15885295
AGTGAGTCTAGATCGTTTTTGACAGTGAAATCAAATACCATGAATACGTTGAGGGAGTAA
A_14_P114165 AGCACTGGATTCTTTTGTCAAGGGATGAATTACTTTGTAATGGCTACTCATCTAGTGATT
A_14_P122835 CTGTTGACAATCTAGGTCTTTTTGATGAAGGGTTGCCTGAATATTCTTTTTACTCACAGA
A_16_P37434115
ACTGAGCATTTTTCCATAATAGCCGAGCAGTTTGTAATTATATCCACCACTGAAGAGAAA
A_14_P126785 GGTCAGGAAAAATGTCCATGTGTTCATTCTGATTTTCTTATTTTCTTTCCCACTTTTAGG
A_14_P121572 GCAGTGTTCTAAATAAACAGGATCTGTATTTCTTGGTCTGATGATCTCAGGGTTACAAGG
A_16_P18763267
AAAAACCAACCTATTTTACACAGGTGGATTTTGAGTTTCACTAAAGTGCTCAATGTCAGG
A_16_P30530288
TGTGTCCAAAATGTCTAAACTCTTATTGTTTCTCCCACTTATCCACAAGGATCCGAAAA
A_14_P107438 CTATAAAAATTCCTTCACCCATGGCCCTTTTCTCCTACAATGACTGTAGAAAATTAGAAC
A_18_P14132084
CTGAGGTGGTAGAATTGAGGTGAATGAAGTCAACCACAGTTTCTAATGTCTTCTTATATG

A_14_P135577 CACTAGCATCACCATTAGGATTTTCAGTTGCTAACTGTGTGTTCCTCAATTTGTTTTTAG

CUST_525_PI426210822 CCAAGTGCTGTGATGTATGTGGATCCCAGAGTAAACTTGGTGTTGTG

A_14_P113671 AGGGTTTGTCTTTCCTGAAGACTCAGGATTCACTTTTTCAGATGATGTCTTTCTACTT

CUST_3395_PI426210822 TTGCTTGGAGGGGACTGGGGGTTATTACCTCCCACAGGACTGTTTA

CUST_6063_PI426210822 TGTCTGGAATGAAGGAGGCAGTCAGGAGAACACAGTGAGAGACAG

A_14_P131093 ATATAATTTGCCATTGCTGGGCAAGCAAACATTATCTTCAAGCATTACCTCCAACTAGAA

CUST_5586_PI426210822 GTGAATAGTGGGATTGCTGCTAATGCTGAGATTGGATTGGGATTCTGG

A_14_P200721 CCATGAGGCAATCCCAATTAAATGTCCTTTGTGTATTGATCCCTTAGCATCTTTATTTAC

A_16_P00791849

ACAAAACCAATTCCCTCTATTAACACCGTTTTGTTTCAGTAAGGTTATAGCACAGTTCTG

A_14_P110658 CTTTTGTGTAGAAACATGACACACTGATTTCTCATGTTGAAGGCGAAATTAAATCTAGCC

A_18_P22840501 ACTATGTGGGAAAGCGGGACTCTTATCCTTGCAGAAGTCACTATGAAATGAG

CUST_7860_PI426210822 CTACGAGGACAGCGATTAACATCTCTCAGATATGTTCTCTCAGTGCTGAAATGT

A_14_P116973 ACAGAGGGAAAGAAATGATAGAATTTTACCTGTCTCTGCACCAGCATAGTTGGAAAA

A_14_P119693 AGCAGCCCTATGAATTGGGATTGGGTGCTCATTCACAGTCATTTACATATAATTTACATA

CUST_7156_PI426210822

AATGTAGGTTCAGAATAGTTTCTTCTTTCTTCCACGTCTCTGAAAGTCCAGCTTC

A_16_P03784874

CCCCATTCTTTCTTACTTTCAAGGTCCATATTAAGTCCCATTTACATCAGAGTTCTCATA

CUST_7763_PI426210822

CTTAAAGTAGTATCCAGGAGAATTTTGACTCCTTCAACAGTTAACTGATCTAACTCTGCC

A_14_P111372 AGAGATTATGTTCACCAGCCCAGAGATCAATCTTTTCTATTGACAACAACAAAGGGAAAA

A_18_P17382325

GCACATCTCTGTGTAATACCAAATGCTAACAATTACTGTTGTCATAAATGAAGGTCAGCA

A_18_P22841608 TAACAGGCAGCACACATGTGGCAGATCGTAGCAGCGGGCCCTTGC

A_16_P58424559

AATTACTCTCTTATCATCAGTCTTCTTCCACCAGATTAAAAACACCATGAGAGTAAAGGG

A_18_P14062998

AAAGAGTGCCTTTCTATTTTGATTTGTATTGGAACAAACTATATGGCATACGTGGCTTCG

CUST_9269_PI426210822

ACTTTAGTGCTCTTGCTAAAGAAGAAAGTCGTACTATAGACATTTACATGCTGCTGAACA

A_14_P201004 TGTGCTCCATTGAACTTTATAAGAGGAAGTGTTCCATTTACCTCTTGATATTGAGGATGA

A_14_P116711 AAAACTTTAGCAAGAGTAAGAAATGAATTCTCAGTCACCCTCTTTTCTCTCTCCCTACTA

A_14_P117535 GAGAGAGAAAAAAATAAACTGTATTTGGCTGCAAGAGTTGCTGTCCCTGTTTTCTGAG

CUST_705_PI426210822 GCAATTAGTCACTGTGCTGATGTAAATTTGGAGTTGGTGGGAATTTTTGGACT

A_14_P129533 CTCTCAGACAAGTTTCTCTTTCCTCCTCTCTTCCTGTTTACAGATAATGCTACAGAG

CUST_2340_PI426210822 CAGATAGAGATAAACTAGTTAAGGGTGGAGGGTTGGTTGGTTGTCATTCGT

A_14_P135582 CAGTCAGGCTGCATACACAGTGAGAAAAAAAAAAAGACTCATTTTCTTAGTAAACACTGC

A_16_P00685423
TCTGTCCGCCTTATCTATCTTCACTGACGTGTATTTCTTATTTTCACAAAATGAAGCCAT
CUST_9242_PI426210822
GAGATTTACGTCTCTATTGTAACTTTTGTGTTTTCTTGAGGAAGTGTCACATGACTTCTT
CUST_1854_PI426210822 GCCTTATGGTGTCCTTCAAAAGAATGGCATTTAGTGCTGTCCAACTTCAAA
A_14_P119362 AATTCTATGTGAGTCAGATTTAATAATTTGTTTTCACTTTCAATAGTGTTAGGATAGGTG
A_16_P20876994
AGAAAACACAGTCCTTAATCCAGAAATTGTGAACTCTAAATACAGGCTTACCAGAAAAGC
A_14_P117531 TGCATATGAGCAGAATTAATTAAACATTTGCCTATGACTCCAACAGTCCTCTGTTTTAGT
A_14_P123359 ACCTTCATGAGTGAAATTGAAAGTGATAAAACTAACTAGTTCCATGCCAGTAATAGACCC
A_18_P17426922
AAAGTTGGCCTCTATCCTAATGACTCAAAACTTGGTTCTTAACTACCATGATTGCTTTTG
A_14_P106153 AAAAACACAGCCTATTCTGTTTTCGTAAGGAGGAAAGACAGTAATCCACTTTTCTGATAC
A_18_P14135578 GCAGTGCATCGTGGAAGCAGGAGACCAGTGAGAAAGCAGTCTTTT
A_16_P21498744
CTTTTTCAGAATCAATGCATCATTCCTCAAGCATAGTAGCAACGTATTTGGTCAATGGTT
A_14_P103076 CTGCTTTTTTCCCTATCCTGACATACTTTATCTTTGGAACATCAAGACATATCTCAGTTG
A_14_P100331 CACTCTAGACCTTTCACAGACCTTCCATAAATCAGTGGTATTAGTAACAACTACTCCTAT
A_16_P00372286
ATCTTACTCAGGTAATGCATTAACTCCAGATCACTACAGAGAACATTTCAATTCCCACTT
A_14_P106576 TGATTCACAGGAGAAACTAGACAGACGAAAACAACAGCAAGAGAAAGGCAG
CUST_230_PI426210822 GAGAAGGGAGGACGCCGTCCGGGGCCCGCTAAGGTCGGGGTCGGC
A_16_P39303291
AACTCCAAAATTTCAGTTTCTTTCACGCTTAGGTAACAGTTCAATGCAAATCCAGATTGT
A_16_P02282307
ACCAACCAATTACAAAAATGACAAGATACAAACATATAGCAAGCATACAAATTCTCAGGA
A_14_P123738 TTGTTGCTTGTGAAAATGGCTTACCTGTCCACTTGGATCAGTCAATTTTCC
A_16_P21490873
TGGAAAGACTGATGTTAAATATCTTGACTCCTCTAAGATTAGGTAAGTATTGGGCTGAGG
A_18_P22840341 AATTCTCTAAAGGGAAAAAAAAATGAGGTGGCTGCTCTCTGGGGTTAAGCAG
A_14_P110321 TCAGTGCAATTAAAGATGCTGTTGTGAGTGCACTTCTGCTACCTGATTTAAATAAAGTCT
A_14_P114106 TCAGTAATATTTCGCCTGTTTGTTTAGAACGCACCTCTAAGTATATCCTAGGAGAGAGAT
CUST_374_PI426210822 CTGGGCAGTGAGAACTGTCCCCATGGCTAGATGGACCAGAATATC
A_14_P110344 CCTACGGTGTTTCACCACCATCAACAACTTTACTTCATTAATTCGATATCAGAACCTTAA
A_18_P22840508 ATCAAAACACACCCTCTGGGGAATGCTGCCAAGAGGTTTTGATTTAAAAC
A_14_P108597 CTACTCAAAATTCTACGTTAGAGAGACTGAAAAGACATCTAATTTCATTGCTCGGCAGGT
A_16_P20527856
AAAAAAATCGTTCATCATTGTGCAAAATTTCAAGTCCACTAAACGATCACAAGCATCCTG

A_14_P102047 CATTTGTTCCTCCTCCAACTTTTGTCATTTAGGAGAGAGACTCACCAGAC

A_16_P02162872

AATCCCAGTCTTGTGCTATTTACCCCATCTAGTAAGACTTATTAACCACCTCTGTAAATC

A_14_P111216 AAATACAGTGAACTTACTTCTGGGAAAATGGAAGCAAGGAATCTAGAGGTATGGTTTATC

CUST_7524_PI426210822

ATTATTGTGTAGAAGCTGTTTCCCATTTGAGACAAAATAAGAAACACTTGTTGAACGAGT

A_16_P16372176

CACCATCATCTTAGAACACCCTATTATGCCTTAATTGGATTACTTAATTGTGTCTCAGCC

CUST_9066_PI426210822 AATGTCCTCTTTGGGGTAAAGGGGAGGACAGATGCCAACAGAAAA

CUST_8721_PI426210822

GATGATCATAATATAAGAGTTAGAAGTCAAGAGAGATGGAAATGAAAGCAAGTCAAGATT

A_14_P127619 CTGGAGAAAGGATGCTTTCTTCCATTCTCCAAATTTTTTTGATCCAAGTAGATTAAACAC

A_16_P00685031 GGTTTCGTGTGTATTTTATCCCAGGGAAACAGGTTTTCCTTAAGGCACTAG

CUST_4466_PI426210822 GACTCTACTGGGCTCTCCCTGCTCCCCATGGAGCCTCATGCTGTC

A_14_P200838 ATTTTGCTTAAACCTGGGCAAAATTATCTCATTACTTCAACAAAAGGGCCCCATTATATC

A_14_P115334 AGTTGTGGATAAAAATGGTGGAACACAGGTCATTTTTAGTTTGTGCTTGGAATATGACTT

A_16_P15609102

TGACTTACTAGATGCAATGCCCTATTATAAGAGCCATGGATAAATAAGTGTGACATTGGA

A_14_P119366 AGGGTAACAAAATAAAGGCATTTTTCCTTTTAGGGAGGAAACAGTAAGAGCAGCAAATAA

A_14_P136912 CCCCAAATTACATAGGCACGTATGGCATTTCTAGACCTTCAAATTACTGACTAAGATATC

A_14_P139007 CTGAAGGAGTAGGACAGAAGAACTGTCAAATTCTGGAATCCTTAAAGCCATGTC

A_14_P136050 AATGATGCTTGAGGTTTCACAGAAAATATTAATTCCTGATCTGCTGCTATAAAGCATGCC

A_14_P115350 ACAGTTTTTAAAGTCTTAAGATACCACAAGAGACATTTGCATTGTCCAGAGGACACATTT

A_14_P131692 GTCAGGAGATGGAGGATAACTTTTCAGTAAAATTGACTTGGATTTTTTTGAATTGCCACA

A_18_P17265204

GTGGACACAAATGAAACAAGAATTAATTGCACAGACATAAATGAACTTATGAGGGCACAG

A_14_P202559 GGGAAAAGTCTCCATCTCAAAACATATCATCTCTAAAATGAAATACAGCCAAGATGCTCA

A_14_P124386 TTAAGTTCAGATTGATTTATTCACCAACATAATCAATTCCAAATCCCTGTCAGACCTGGA

A_16_P36141454

ACTCTACACTAAACTTCTCCAACATATAATCACAGAAACAACACAATAGTTACAACAGCA

CUST_8324_PI426210822 ATGACCCCATGAGGACCCCTTTCTGTTCTCCATCATTCCCCACCTT

A_16_P35636373

TCAATCCCATACAATTCCTAGTTTCTAAAAACACTCTCAAAAATCCACTTGAGTAGGCTT

A_16_P20978740

AGAGGAGGTATTTCAGCAAGACTTAGCATTAGACATAAAATCAAACTAATATGGACTGCA

A_14_P133386 TAAATATCAAGTCCACTAATGCCACATTCATCATATAGATGGTTACCGTGGTTCTCTTCT

A_14_P114092 GCATTTTTGTCCAGATATCCTTGTATACTCTTCCAAGTGGGATTCCTAATTTGTAGCATT

A_14_P100702 CTTACAAACTGGAGTCTGGGCTGGATAAATAGCATATCATAAAGGTAGATGATATCACAT

A_18_P14235590
AAAAACTGATGTGAAAAGAGATCCGAAAATAAGACCAAAAAATTACGTGACTCACTCACC
A_14_P129465 CTCTGAAATCTTGCTCAAATTATCTAAGTAGTGGTCTGATATTGTGTGGCACAAGTCTTC
CUST_144_PI426210822 CAACATTTAATCATTCCAATATTCCTCTCTGCAGTGAGGCAGGAGGGAG
A_16_P00977882
ACTCTGTTGAACTGACTTTACCCAATCATCATCCATTTCATAGAGATTTGCTCCGATATG
A_18_P22841726
CAGAAGGGGACACACATTGAGAAATTTCCAATCAACAAATATTTATTTATTGAACACTTA
A_14_P200195 CCACTGGACTTTCGGGAACCATCAATCTGTTTCCAGACATGGATTAG
A_14_P119789 CCCAGATTAAGACAAATGATATCCTTTTGTTGCACAGAGGACATTAACGATATCCTTTTG
A_14_P120147 TTTTACAAATGAAAGGTTTACTTTGTCTGGTAAAAGAAAACCGTCGTTACTTCCAAAGGC
CUST_2296_PI426210822 CAGGGACTCAGACATGACAACCAGAATTAGGGAAGCTGCACTTGTATTAT
A_14_P133391 TTTATATTCTAAAAGAGATCTGATTCATTAGGCATTGGAGAACACAGCAGGACAGAGGAA
A_16_P00685588 CAAGTATTGAAGGAATTTCCCACGTGGACAAGGAAGATCAGGCATTCAAGAT
A_14_P108242 TGGATTTAAGAGAGTCAATTTTTAGAGGGCTTGAATTTGAGGATACAGTTGCCTTCTAAG
A_16_P16652472
ATGTCATATTTGGGTAAGTGATAAGAATAGAAAGTGGCTATGCTAGATATTGTGCTCCCC
A_14_P108937 TTCACCTATTCAGCCAAGTGGATTTGACTGTGTTATGAAATATCTTAACATGTCAAAGCC
A_14_P112489 AAGGTCATATTTTATTTGTGAAAGTCCTTCTCTAGCCTTGCGTGAGCCTTTCTAAAAATT
A_14_P101641 CTAGATTGGCTGTATCTAGATCTCCTCCCCAACTTAGATACAAAACCTATTTTGTTTCTT
A_14_P124685
GTAAATGTAAAGACGGCAAAAGAAAATGAAATTAGGCAGATCCAAGAGCCTTTTCCAAAA
A_14_P103193 CGAAATCCATTTACGAAATACACAGAAATTTGGCTATAAAAAGGCATGCGGTCCAAGTA
A_18_P10760090
AATTATAGCCATAGACTTAAGACCCTACTGAAAGAAATTGTCCCCTACAAGTTATCCTCC
A_16_P02023238
ATTCTATAGCCATCATTAACCTCCAAGTTGCAACTAACATTAACCTCAACAGTTCTTGGT
A_14_P111684 GAATAAAGATACCTGATAAGGGCACTTCAAGAAAGGAAACAGTTTAAGAATGTCCTTCAG
CUST_8336_PI426210822 ATCATTTCCAGCAAGTCCACTTGGTGTAGACTGTGGACCTCATGA
A_18_P14137983 AGACTGATTGGCTGATTTTTGGACCCTCTTTGTCCCATTTCTAGTCAGGAATTAT
A_16_P16944398
GCTGATATGGAGAATGCAAAGATTAAAAAAGGACAAGGAGAGTTATAGACCCTAGCAGAT
A_14_P115477 ACACTGAGTATTTAAAGGAAGCAAGATTGGAATTTACAGGTAACCATAACCCTCAGTCTT
CUST_6062_PI426210822 ATGAAGATCTGCAGGTGTCTGGAATGAAGGAGGCAGTCAGGAGAA
A_14_P134681 CTTTCCAAATCTCTAGCAAGAGCATGTAGTAAACGCTCCTTAAATGCCTCCCT
CUST_8213_PI426210822
ATCTGAGAAGTTTGATGACAAGTTTAAGCCCCATAATTTCTTTGAACATTCCATATACCT
A_18_P11950419

AAGAACAAGTGACATGTCAGATACCAAATCTTCCTACCTAATAGTATGTGTCAAGGAGAC
A_16_P02740400
ACCTTTAGAGATCCGTTCTATTAATGAGGCATGATATTGTTACTCCAATTGTGCACACAA
A_18_P23445719
TGTCTACCTCGCTATTACAATGCTTTGAAACTTTAGTTCCCCTTAGATTGTAAACTTCTG
A_18_P26792747 ATGCTCTGAGATTAGACCTCTTTATCTCTTCTCCTCACCTCAACCTCAAGC
A_16_P16191696
CTTGGATTTTAAATCTTCCTAAAGCAGCACAGCATCTCTTGAAAAGAAAGCCATGAAAGA
A_18_P22840475 CTCCCCTCCTCACTTCCTTCTCTGCTTACCGTTGGATTTCTTTTTG
A_16_P01536288
TTCCCATTTTGCAGCAATTGTCTATGTCTACTCTTCAATGAACTATTAGCCGAAATAGCA
A_14_P121636 CTGGGACTGAGAAGAAAATAGCAATTGCAACTCAAAGCAACTATTTACACACATTCAATG
A_14_P109081 TAATTGGGCTGATGCCTCCCTGTCCAAATAGGAAGAAAAGATAACTCTATCTTTATTTGA
A_14_P121830 GTAGATAATGAGGCTCTTGAGCACTAACATGCACCCATTCAAAGTCATCATACAGATC
A_14_P105925 GAAAAAAATATAGTGTTATCCTATAAATACCTGCTGGCTGGGAAACTGACAGAGGACTGA
A_14_P112108 TGTCCTTTTCCTATTTAATGGAATTTCCTCATTTCCTTTCCTGCCAAAAGAATCCAATTT
A_16_P16095482
AAAGGTAAGAACCATTGAGAAACAGTTTCCATACCTTGTTGTGCAAGACTTTGAATCTGT
A_16_P20949954
ATCCATGTGGATGTAAACCATATTTCAGTAGACCAGACTTTTTGTCTAATTTTCCCAGTG
A_16_P03471164
AAAAAGCCAGGAACATAATTTTCATGACAGTTACATTTACAATATCCTCCTCATCCTGCC
A_14_P108606 CTCACAATGAATGTGTAACAGCCATTCATATTTACCAATGATCAGTCCTCAAAAAGAGGA
CUST_6841_PI426210822 AAAACAATGGCAGGGGGCTAGGTCCTGACTCTTTCAGACTGGGCA
A_14_P137329 CTTTACAGAATTTCCAGGTTTTCCCCATGGAAGAACTACAAAATCTTTTCTGTTCATGAT
CUST_4133_PI426210822 ATGCCACAGTGGTCCCTCGGCTTCAACAGTCTTGCTTGCTTACAT
A_14_P117199 TTATCTGCGTTGTGACCACAGAAGAATGAAAATTATTTGCAGTTAGAATCAGGATCCATG
CUST_1139_PI426210822 GCTGGGGCCAGTGTGGAGCACACATCTAGAGTCCTCCTGCCTGAG
CUST_4022_PI426210822 GAACTGATGATTCTCAGAAGAAGCCATGAAGGGCTCGGCCATAAC
A_14_P122303 CTTCCACTTACCTATAGTAGGGTCATGATAATCAGGGAACTGATGACTAATAAACTGCAT
A_14_P121995 AATTAAACAGAAAGCAAAAACACTATGATATAACTTTAAAATTAAGGTTTATATCACTTT
A_14_P130536 AAACTTTGTGTTTTATTTGCCTTAAGATTGTCCTAAAGCAGTTGGATGGGAAAAGAACCA
A_14_P132840 TTTTTGTGAGAAAGTAAGTTTGGAATGTGTAACTATGACTTCGGCTCCTGAGAACTAGAA
A_14_P101866 CTGACCAATAGAAATAGATCTGATGATAGACCTAGATCTGTAGGCTGGACTCCAAAATTT
A_14_P126776 AGAAATGAGTTCTTTTATATACTGTCAGAAGGCAACACCTAATTCCCTGTTGTGGTATTC
CUST_9646_PI426210822 ATAAAAAGTTATCAGGTGAGGCTGAGGGGTGAAGGCGCTTTCTCT
CUST_1852_PI426210822 ACCATGTGTGTGTGTGTAATTTATAAGACTGAGCCCTGGCCTTATGGTG
A_16_P20621134

AGTAACACACATACACCTTTGTTTTCTGTTCCACAACTACAGATATCTTTGGACTTCAGT

A_14_P115020 CATGTGTCGATGGCTTGAAACAATTAGGTTTCACTTTGTCTCTGTCAAAGTATAAATGAC

A_16_P00685445

TCCTGAACATCGGAGATAGATTCTTTATCTGGGGAGTGACTACATAATTTAACCATTTGT

CUST_1849_PI426210822 TGTGAGTATACTCACACTCTCGATTTCCTGCACACTCTCATCTGAGGTT

A_14_P107617 CTGCATGATTTCTTCTACGACATTTGTAACAGACACAACGTATATAGAATTTCAGTTTGG

A_14_P103431 CTTGCTGATCCTAAAGCCTTTCTATGCAAAAACTAGCGGAATGCTATTGAATGTAGGTTT

A_16_P00685340

CAGTGTTGAATCCATGTCAATAATTGTATTTTTTAAAAGCAAATATCACCAAGTCCAAGA

A_14_P125976 CTCAGGCCTGTAACTTCTGGAAAAGATGATTATTCAAAATAATGTTTTGGGGTAACCAGT

A_16_P01947940

ATGGCTTAGGTTTGCATTTTTCTAACTCTTCACTCAGATTTATACTTCGCCACACTATCA

A_16_P37750541

ACAGCACCTATTTTATGGAACCTTAGAAAAACCTGTAGAATTTTCAAATTGAACCCAGGA

A_14_P120074 GTGTAACCTGATCTGGCTGGCGTTAATAAAATATTAACATGTGCCTCTTGTGATCTGC

A_14_P100835 ACATCCATTTCCAAGATCAACATTATCTCGAGAGGGAAAGTAATTTTTCACATTCCATTC

A_16_P41599800

CCCATAAGCAATTCTCTAGAATTATTTTGAGTGCTCAGATTATAGATTTGCCCCCATACC

CUST_7854_PI426210822 TTGAAATTGCCAATGCCTGTTCTCTAGGTTCTAAGAGCAGCCCTCA

A_14_P134298 ACATTTGGTTCACAGTGCTCTGTTTAAGATGCCACAGCATGTAATCTACTTGAGAATATT

A_16_P15149173

AGCGCAGATAACACTAATCCTCTCCTGTTCAATCTAATAGCGTTACTGTGGTAAATAAGT

A_18_P22605007

GAATTTTTATGAGTTCTCCAACATAATTCAATGACCAGGTCAGTTTTTGATCCAGGACAG

A_18_P12991707

ACACAGACCATGTCATGGATGAACAAATGAATTTCATAGTGCATTTTGATTGGATGAGTT

A_16_P58424200 GTTCCTGGAGGAGGGCTCAGACCAGTGTCTGAGTGTCTGGTGTGT

A_14_P112796 GCCCAGAAGTAATATATTTTGAGGGGAAAAAGTGCTGCTTTAAGGAGGTATATGAACATC

CUST_3658_PI426210822

CTCAGCATCCTGAGTTCCTTTGGGTTACTAGTTCATTTTGGAACTTATTCCACTAT

A_14_P101373 AATCAAATTCACTGGGAGGAATTGGAAGTGACAAGGGAAAGATAGTGAAATAATTTCTCA

A_18_P14134862 ATCAAAGGCTGTGGGCATCTTCATCGTATGGAAAAGTAAATTCACTGGCACAT

A_16_P02595627

ACAGAAAGGTCAATTAGATGAGCATCCCTTAAACTATTCAAAGTCTCACCTACAGAATGA

A_14_P110741 GTGTGGGGGCTAGATTCATTCACGTGCTTCGTAATGAAATAATCCAAAAAATAGGAC

A_16_P18988725

TGCATTTACCAAAGAAAAAACTTGAAGTGACTGAATTTATCCAGGATTGACTAGAGTGGA

A_14_P130883 CTTTTTGCTCCCCGAAAAGGTTTTTCCATCAAGGTTTTTCCAAAGGACAAG

A_16_P20542139
GATGATGATTCACTCCATAGTTCCATCATGTATGCATCTCTAACCAGTATAAGCAAAGCA
A_14_P113145 ACAAAGATTCCTTTAGAGGCATATTCATATTGCAAGGCTCTGCTGAAGTGGTCTAAATAA
A_18_P22839594 GTTTTGCAGGCAGCAGGCACACAGGCTGGACATCCTTTCCCTCTG
A_14_P129575 ATGGTGAAAAAGTTCAAAGACTCCAAAAGTGTATTGGTAAGATTTCAGTTCCAGTATGTA
A_16_P16355236
AAACACCAATAAACATCCTCCTGACAATAGTCCATTAGCACAGTTCTGTTTAACCATGTA
A_16_P39252504
GGGCACTGAAATATTCTAGAAAGTCCATAGCACATTCTGATGTTTATACTCTAAGCCACA
A_14_P127720 CTTTTAGAGGAACTGAATCCAAACATTTTCTCCAGCCGGACATTGAATGTTGCTA
A_16_P38225338
AGTAGGGCTCTCAGAAAATACTTTCCAGTTGTCTAACCTTGAATGTTATTAACTCTTCTA
A_16_P39004576
AAGCATGTCATATGGAAATATTTGAGGAGTAGAGAAAAATGAGTGATTAAACATGTTTGA
A_18_P22841721 GACTTCTGGAGTCCCAGATACTTGAGCCTCGAGGGAGCAGATTCT
A_16_P19563001
AAACTGAAGAATGAAAGGTTACAAAGAATTCTCCTCTAACTTTGCTTCTATACCCTCACA
A_14_P200808 CTGTTCTGAACCTGGCTTTTCTAACAATGGAACAAACCTATACTTTACAAATCTGCTGAC
A_14_P105773 TCATTCAACAAATATTGAAGAGCACCCACATACCTATGGTCTTGGATCTTACATTCTAGT
A_14_P124653 CCTTTCATAGTCACAAGTTTTGTTATTTTGTTTTTTTTTGGGGGTGATGGTGTAATTGTT
A_14_P125557 TCTTTAGGTTTTCTATCAGATGTTCCACGTAATAATGCTGGTAAGTATGGAATAATTAAG
A_14_P101001 TTCAGGAATGCAAGAATAAAGAAAAGATGAGTATAACTCTTCTCCATAGCATGGAGACCT
A_16_P58424239
CTGGAACTTGAGGTTAATTCCTGCTTCAAAGCGTTGTTAGGAAGATTGAACAAAATAATG
A_14_P139358 TCCACTTGAACTCCACCTTCACATGAAAACATAAATGACATCCATAGAAACTCAGATCTA
A_14_P130563 GAAATCTCTCCTACCTACTAGAAAACGGCTCATCAAATTACATCATTAGCCCAAAAAGTT
A_14_P120876 AACACGCTGTATACCTAGATGATGACTAAATGCAAAATCCTTGGGCTTTGGTTTTTTTCT
CUST_2348_PI426210822 ACTTACGAATATGGAAACTATTTTGCCATGAGGCATACATAGTCTGAGGGGCAT
A_16_P03015543
TAACAGTCATCTTAACTCTCCTGTCTTACACATTGAAGTCTCTGTTTATTCCACTCTGAC
A_16_P58424437 ACTGAAACTCAGGCAGGGTCCCTCAGAGCGTGGCCTCAGCCTGAT
A_14_P135464 TTTCTTGTTTCTTATTAGGCCTCAGAAAGAAGTCAGTTAACGTCACCCAAAAGCACAAA
A_16_P01463892
ACCAACATCTCTGTTAGCAGAAAACATGAATTGCTATTTTAAGTTAAACTGCTCCAGTGC
A_14_P114120 ACTCTTTGTTGCTCTTGACATACATAGTAACCCTTGTAAAGTTGTAAAAGTTTGCTCCTG
A_14_P129226 AGCACTGTTGAAATAGAATTAAATGGGTCTTGAATTCATTTGTGATTGGAATCAGAAGTC
A_18_P12106782 AATGCGTTTGATGCATTCATTGAGGTAAGAGACTTTCAATCTCTATTAAGGTGGCCTC
A_18_P25712518

CCCCATTTATAGCATACTCCTACCAAAACTATCACATATTGTCGGCATAATCCTAAACCT
A_14_P124141 CATTTTTTGAAGGAAACAAAAGATGAATAAAAACTGATTCCTGCCTTCCAAGGGCATATC
A_14_P108164 TTCTCATCTGTTAATAATTTAAATCCTTTCTTGCAGCCGCAGTCAAAACTGCCCAC
CUST_7139_PI426210822
GTGTTAACAGGTATTAAGTATACCGTTATCATTTTTCCTTACTTTAGAAGATAAGCCAAA
A_16_P01752434
ATTGCTCCAAATTTACCTCTTTTCATCACCATAAAAATCTATATCCCATGATAGGCAGGC
A_16_P03387295
AACTGCTCTTTCTGAATGATTATTCTTCCTGCTACTCCATATTTTTCTACCTTCTAGGCC
A_16_P16990302
TCATGCAATCGTTAATCAAATGCAACTAGGTTGGAAGAAGATACATGCTTATTGCTTTGC
A_14_P127126 CATTCCAAACATAAGCAGCAGTTTGTTTTCTCGCACAGTAAAACATGATATTTCTATGGC
CUST_3290_PI426210822
ACGCCAACCTTAGATATGAGGTCTTCTGAAAACTTTTAAAATTTGTTTATTTTTTGAATC
A_16_P18916403
AAAACATATTCATCAAGGCAAAAATGCTCTAGCATTCAAAAGACAGAAGTGTCCCAAACT
A_14_P134543 TTCTTATCTCAATTCAGGGACAAAGAGGGATCCTTTATCTTGTGTCTGATTCTTTGTTTT
A_14_P124884 ATCAAATTTTTACTTCTGTCATCTTCCCAGAGAAGAACATTAGAGCACTTGATGTCCTTT
CUST_8197_PI426210822 CTGCACAGTGGCAACACGGAGCGCTCTGAATCCCTGCATGACAAC
CUST_3445_PI426210822
TATATTGTTAGAGAAAATCATTATTTTTAGTCAACGTATTAGTATTAATATCATGGCCTC
A_18_P14134453
CCTGAGTTCCTTTGGGTTACTAGTTCATTTTGGAACTTATTCCACTATTGAATTATCCGT
A_14_P137788 AGCGAGACCTAACAATTTATTAAATACTTCATCTCTAAACAGTGGTGGGCACTAGAGTTT
A_16_P02040793
TGCAGGTTCCACAATTCAATAAACATCAGAGCAAAGGTTATTTTAAACTCCGCAGTTAAA
A_16_P16455210
AAAGAAAACACAAAGCACAAACTCATACTTTTTTTCGGACTATAACACTACCTCTCAGCC
A_16_P16757195
CCATTCCTACTAGCATTATTTCCATAAGCCTCAAGGATTTTACATCTCATAGTCACCAGA
A_16_P16898707
ACCAGAACGTGATAAGAGTTAACATGGGATAAATTGCTTTTTTGGACATTTGACCAAGTA
A_16_P02353011
AAAGAAAAGGTAGTGAGTAATTAACACCCCTTAACTTCCAATAATGACATAAGTGACCCC
A_18_P17963397
CCTACACTCTCACCTAAACATCACATGACATATTCTGCTTCAGTTTTTATCTTTCAGTCG
A_14_P135858 GAGCCCCACCCACAAATATACCACAGTCACCATTAACGTCATATTTATTGTTATTTAACT
A_14_P133178 AAGAAAATTTGGGCTTACCTGAAAGTGCAAAATTATTGTCCAGATTAATCCCAAAGTCAA

A_16_P58424260
CCATCCTACCTTGACAAATATACCTTTATAACAACTTGGAAGCCCTAGGTTGAATTTTGA
A_16_P00777371
TCAAATGCTACATTTCCACATCTTACCTATGAGACCTACCTTAGCAATCTAAACTGAGTG
CUST_9099_PI426210822 AAAGGAAGCTTTCCCTCCACCTCTACTCCTCTGTGACTCCTGCCC
A_14_P127447 GGTTTTTTTCTCTCTCTCTCTTTCAGGTATGGAAAAATTGTATCTACAAAGGCAATTCTT
A_18_P22840284
GCAATGATGTAAGTCTTAAAGGGCTAGACAAGGATCAAAAGGAGAGGTAAGAAAAAGAAA
A_14_P102850 AGATCTTGGTGGCCTAGAATTTCTTAGTCATGGGAGTCTAGAATCATAGATAGAATGCTT
A_14_P130746 CTGATTGCAATACAACTCAAGTATTTTGGATGAGCCTAAAATTTTGAAGAGGGAAAAAGC
A_14_P109193 AGAGGGAGACAGATATGAGGGGATTTTAAAAAGGTGTGTATATCTATACAAACTCCTCTG
CUST_1268_PI426210822 CTCTGGGGCTTTCCAGGTTTCCTTGGAGCCAAGAATTTTAATCTTG
A_14_P122483 TTTGGTGAACTGGCTTTCCTCAGATGGACTGACTTATTACCTGATTTTAAAGTGTGAAAA
A_14_P105287 AATTCTTCATGGGGCAGTGCTTCCTTGCCTCTCTCATATTTTTTTTCTGTTATCATTAA
A_18_P22842094 CACACTGCTTACAAATCTGGAAACACCTCAAGGGAAAAGTGCCCATAAATGTT
A_14_P114958 CTTGAGAATGTCAAAGTCCTGTTAAGTTTCTTTCCACTGCTGCATACAGTTTGAGATAAA
A_16_P15337695
AAAAGGGGAATGTTTATTATATGGATGCAAGGGTGATATAAAACTCAGTGGCAAGAATGC
A_14_P118989 GGAGAATGGCAATGACAGAGTAAGGGAGAAAATGCTCAGTTTGTAAATGTTAACCTAATA
A_16_P17593118
ATACCATATAGTTAGCACAATGGACAAGTCCAAAGTTAAGCAGACCTGGATCTTATCTTC
A_18_P16891253
AAGACAGGTTGTTAAGATACAAAGGGCAAGTTAAACTTTAAAGTGCGCTATAAGAGAGGC
A_14_P133351 CCTCATCTATGGGGTAGTGCTTATTTGGAACAAAAACCTATTAAGTCAGCAACAGTATTT
A_14_P115061 ATTTAAGGCAGAAATCATAGTGCATATACCTGCAGAACACATTTTTTCTCTGCTGTCAGA
A_16_P21239989
AAAATTTGTTCCCAAACATTCGTAGGACTACACTGTAGCAGGAATCTGAATAGAAAACAC
CUST_4469_PI426210822 GCTGGCTTTATCCCGCTGAGCAGTGGGACAGAGGACAGGAAGATG
A_14_P106183 ACTTTTCCTTCAAAGGTGATTTTAAGATGCTCAGAAACAGATGCAACAGCAGGAG
A_16_P02053770
AGCATTCTGTAGTCTCCTTCATAGTCTTAAGCAATCATGCCATTACTTGTGTATCTCTCT
A_18_P10715448
AGGCTACTGCAACCATAATTCCAAACCTATAACAAGACCATTTTCACTTACAACTATAGA
A_16_P03124676
TTAGTCTTACTTTGAGGGCCTTATAGTTTAGGAAGTATAAATATCTAGGAAGCCCCTGGA
A_14_P139238 CCTTTATTTAGAGCAAAAATTGCAAAAATCCTGGAAGTTCAGAAGAAACACTTAGCACCT
A_14_P115604 CCAAGATCTCTTATGCAGTTCACAAAATGGAAGGAACATCTTTTGTTTTTGCCATCTAGT
A_14_P130075 ACTTTGTAGAATACAGCCTGATGTGCAAACCAAAAGTGGTGATATTGTTAAGATTCAGTG

CUST_844_PI426210822 CTGACCCTGACTGGACAAATGGGCCCCAGAGGGGCTGAACCCTTG
A_14_P127865 GAACACCCAGACCCTATCTTTGACCCCAAGGAAAAGGATCTGAAC
A_14_P127033 AACTTCAACTTCTCAAAAGTCTATGGTAAGAGTATTCAAGTCCCCTTTGCTTCTTGATTT
A_14_P128001 CTATTCAGCATCAGGCACTCTTATTGATTCATGAGGAACATTACTGCTAATCTGCTGTTA
CUST_2508_PI426210822
TCATCTTGCTTTTGAGAAATATTTCTTTCCACTAAAATTTAAGAAAACTAACGCAGTCTT
A_16_P00685624
CCTTTCCAGATTGTTAACACACTGTCTATGTCAAATCCTTACACTAGCATAGTGATGAGA
CUST_4635_PI426210822 CCAGATGGGAGTCCGAAAGCTACACAGAGCAAACAACCAAAATTGA
A_14_P139080 CTATTGTGTATGAACCATCAGTAATTTCCAGTTCATTGTGGCTAAAGATTCTGGGCTAAT
A_14_P110334 GTCTAAATGTCTCTTCTTTGACTTACTATGTGGCTTATTTGTTTCTGAGGGAGATTCAGT
A_16_P16769084
AGGCCGTTTACCACATATTCTCTAGATTCGTGCAAAATAAACACATTGACAGTAAGTTCC
A_14_P133697 TGGTGATGTATCCTAAATTAGGATATGTCATTTATTCAAGAAGTGTGTTGTGCAACTGGA
A_14_P100063 GGAAAACAGAACTGAAGAAACTACCTACTACAAAACCCAATCTAGAGTAAGTTTCCTCTT
A_14_P139036 TTTCAACAGGTTTTGCACTGATTGCAAAAATAAAGTCCTCCGAGCATACAATATCCTTAT
A_14_P119445 CTGTTAATAGTCACTCCCTCCGTGATACTTCAAGCATTTTATTTTTTTTTAGTCCCAAGC
A_14_P109983 TTCTACCTTCTTTCACTTCTGACATTTGCATTAAAACTAAGTGGTGTATTTAGGCAGCCA
A_14_P131196 AGAAGAAATGAGAGGAAGGACTTCTGTTTCAACATTCCAGGGTATAAAACCAATTCTGAA
A_14_P135608 TTTCAAACATCATAAAAGCCTACACTCACTTGATTTCACTTCTCCACCTACAACTCACTA
A_14_P112611 AAGGTTTTGTTTCTTTGAACTTACTCTGTTTTGATGCCAAATTGGAGACCATTTTCTTGT
A_18_P11706343
CTGGGCAAGAATTAGAGATGAAAAAACTTACACTACACTTTGAATCCCTACACTCAGATG
A_14_P106380 GGCTGTTTTTATAACGGCGGGGTTCACATTTGTCGTGATCAACCTCTTTTTTAT
A_14_P200821 AAACAAATTAGTCAATGGCAGCACAGAAAAAAGATAAGCCATATGCATAGTGACAGAGTC
A_14_P109827 CCAGGCACCAGAGCAAATAATTCCTCACTTCATTTCAAAAAACTAAAAGCCTTATGCATA
A_16_P40510940
ACACGTTGTCAATTAAATTCAGTCCAATCCAACTAATTCTAAAAACTCCTATCTTGTGCT
A_16_P02404578
ATCGTTTGTGAGAGTGAAAACAATGAGACGCTAAATGAACCATAGTGAGGCTGTATTTTG
A_14_P118950 CTCCAGAAGCCTAAATTTTGATGTTGTGTTACTTGTCAAGAAAGAAGCAATGTTCTTCAG
A_14_P111366 CAGAGTTGGCCACTTCTCATTTTGTCAGTGATAAAAAACAACTACCATCTATTACCTTCA
A_14_P104933 AATTTTACCTTCCTTTGTTCCAGTCCCTCGAGATCTCATTAACAAAAAGGGAAATTAGTA
A_14_P104586 CTGGGGCTATGTAGACTACATCTTTAGGTCAGAACATCTAGATTCACAGTATCTTGTATG
A_16_P02779499
CATGTTTAGTGCACAATTCGGTTAACATTGCCCTAAAAGATTCCATTAACCATAATTCTG
CUST_2982_PI426210822 TCAGTTTCTGTGAACTGTGGGTTGTTTCTCTCTTCCTTGAGTTGCAAG
A_18_P22840293

TATAGGCTGTCCTGCCATCTCAGGTCTAGACAAGCAAAATCAAGTTGTATTTTTAAGTTT

A_16_P19922112

AGAAAGTTGTCTGCTTAAACAATCTCCCAATATTTGATTTACAACCAATGCTTCTGCACC

A_16_P03434788

CACACACGCATAGTAATTACTGCCTGTTTTTTGAACTTGAAAAATGTTAGCATCAAAGGG

A_14_P105205 TCTCCAAATACATACAGAAGAGACAGTATGTTAGGCTGTGTCTACACCTGCATTGAATAT

A_14_P201465

TCACACAAATCAACTGATATACTAATCCACACCAAAAGAAAGAACTCACAAGAAAACAGT

A_16_P16370789

AGCATACACATCTCATACTGAAAAAAGCAAATGTCAACATTATTAACCAAGGGCTACAAA

A_16_P20957980

TGGTATTCCTTTTCACTCCAACTTTTTCTATCATTTACACTCACTGCTTGGACAATTTGA

A_14_P129986 AAACACAATATTAACAAACAGATGCTGGACTTTAGTGCCTTAAAGTGGCTTTAAGGGATG

A_16_P17349170

ATAGGCAGTGCTCATTGTAAATGTCCTCCAGTAATCTAAATCCTAGAGATAGTCAGTGTT

A_14_P115419

GGCAGGACTCTAAGTGAGGACAAATGACAAGAAGAGGGAGAATTTAGAAAATAAGATATC

A_14_P202430 AAAAAGTCAGTTACCTAAATCACCTGTAACCCTGTAAGATCCATTTGCAAAAATCCAGAA

A_14_P105979 CAAACTAATTACCTCACTCAGAAGAAGTGCAAGATTTAACAGTCCATGCTCTGCAG

A_14_P101538 AACTGAAATGTCTTTTCCGTATTGCAAGTTAAAACCCTGTGTTAATTCTGCTACTAAAAA

CUST_8179_PI426210822 CCTTTCCAGAATGATCTCACAAGAATCCCCGTTCTGTGCTTTCTCA

A_14_P124534 ACTGGTTGTCAGTAGAGGTTTTTGCCCCTCAGTATAAACTGTGTCCAGA

A_18_P10607720

CCCCAATGATCAAATTTGCAGTTAGTCATCAACATTTACTTGTAGTTAGAAACCTCTGCA

A_14_P120109 TCAGTTGGCTTCAACGTGGTAGAAGTTGTTGAACAAAAATTTTGGCCACTTTATTTTATT

A_14_P202122 AAATAAGACTTTTTCCACAGGAAGTTCCTCTGGTATTCACTCAGCCCCTGTAAT

A_14_P114995 GTCTTTTTGTCTATTTCCTTTTCCCTTCTTCCTTTACCTACTTCTCCAAGTTAAACCAAT

A_14_P101183 TATTTATTTATCTGTTTTTTTATAGGAAGTCCTGGCTGAGCAAGGTTGGAAGCCCTAGTT

A_14_P106895 CTGAAGTTAGGGTTTATGATGAAAAAACTTTACAATACTTCTGGCCTTAGGGTTTCTGTG

CUST_10055_PI426210822 CATCAACTGTGGAAGGGAGGAAGGGAGTTGGGAGGGAGAGAATCC

A_14_P119581 GAGAAATTATTCATTGGATTTATTTGCCATTAAGAGGCATCTTTGAATTGTGGGAGGTGT

A_14_P133144 GCTGTGCTCAGCGCAGAGAGCAGAAATCAGAAATGGAAACTGAAAC

A_14_P137841 AAGGACAAGTAAAATAGTATTTCTTGGAAAGACAGGCATTTACCAATGCACTGCATCTAG

A_14_P200179 TTTCAGATGAAGAACTGGAAGACAACCCCAACCAGAGTGACCTGATT

A_18_P16373196

AACCCTCCTTTTAATCTATTCAGTTGTAAACATCTTTATGTTCCGGTTTAAGATACACCA

A_14_P101682 AGGCAAGAGAACTAATTAGGTTATGCCAGCATGCTCAAATTTATTAAGAGGCTCACAAAG

A_16_P01282221

TTCAGATAACCCTAATCCTCTGTATGAGCCTTAGTTGATTACACAATAGTATTCCTTCCC

A_16_P01468291

ATCTCTAGTCCTTCAGATTGAGATTTCACCATCTTTAAATTCAAAACATCGGAAGGAGCA

A_14_P113509 GAATGACAAAGTGGGTTTGAAGACTGTCCTCAGTTACCTCTATGTCAATCCAAC

A_14_P104800 AATTACCTCATAAAGATGTATCTGAACACTCCCTAACAAGAAAGCACGTTTTTCTCTATT

A_14_P119262 TGTCCAATGCTTCCCTGAGGTCAAGAACAAAATAAGGTTTTATAGTCTACTTGTATTCAG

A_14_P124590 AATTTCACATTCAATTTTTCCTGTTACAGAACCAAAGCCACCAAATTCTAATGTGAGGAG

A_14_P116112 GTATACACATTTCCTTCCTTGGCCTTGCATGGCCTGTTTATTTTTAAAAGTGCAAACTT

A_14_P103075 GGTGAGGTATCTCTCTGACACCTTAAATTCACCCTGCATGGAATATAAAAAATAACAAGT

A_14_P102748 GACCCCATTGCATGCATTGGTCAAGGAATTGGTGCACATATTCTAAGTTATTTTAGTATT

A_18_P14394106

AAAACCCTGCATTTTAAACTGAACCTTTCCTATAGATTTCCCTCTGTCACTTTTGGAAAT

A_16_P58424281 TGCTAGGAATACAGTGATGAGGAAAAGCAGACATAGACCTTACCCTTCTGAAAGTT

A_16_P17941133

CCCCTGAAAGATTATGCCATACTATCATGATATAGCACACCAAGATAATAGTCAACAAGC

CUST_8249_PI426210822 GTGGAATGAGTCTCCAAGCATTCCTGCTCCTGAGCTTTTGGCATT

CUST_125_PI426210822 GTCTCTGCAAACCGGGCTCTCATAATCAGTGGGGAGTGTCTAAAC

A_14_P136934 GAAAGCAATGCATTCCTTCCTTTTCAGTCAATTGTAATCATGAGACCTTTTAGACTTAGC

A_14_P131621 ACAGAATCTTTTTACAAAGACACATAAGAAACAAGCAGTGTTGGCTGCTGGAGAGTAAG

A_14_P118338 GAGTAATCTGAACTCATCCCATTTTTAGTTTGAATGTTGCAACCTGCACTTTACCTCATA

A_16_P03542958

ATTGAGCATCCATTGATAACTCTCACCTAGATCTGTCATTCTCTTTGAAATCATTAGCCA

CUST_9627_PI426210822 GAGCATGTTAGCATGTTTCTAGGACCAGCCACATCAGCCAGACCT

A_14_P139077 ATCTAACCTCAACACATTTGTGGGTTTTTCACACAATTCTCATAAGCATTTGGTTACTTG

A_14_P104599 TAGAGAATTCACCAAAGTAATCCTCTTTAGAAGGGCATCTTGAATTATATTATCCATCCG

A_14_P118003 ACCTGTGTCAAATCCGCACAGGACTCAATATAGCAATTTATTCTTGATGTATGCAATTGC

A_14_P122017 CTCTCCATCCTGCTGAAAAACCAAGATGATCTTGATAAAGCAATTGACATTTTAGATAGA

A_16_P41454597

GTTTGACAGGTAACAAGTAGATCAAGTAACTCCATACCTTTAGAGAATTTCCACACGATG

A_16_P19774127

AATCCTTACCCAAATCTTTGTGATTCCATTTTAGGTATTTATACAGCGCAAGATGATCCC

A_14_P107417 ACTGGACATATACAGAAAAAGATAACAGCCAGGCAATGTGACATAGGCTAAACACTAAAT

A_14_P102380 GAAAAACTTGTGAAATATTTGGATCCCAACGACCTGGGGAGAATCAACTTCAAG

A_16_P20135715

GACTCCTGATTTATTGCAGTTTTGGTAATTTACAGCAGATACACAATTCTCAGAGACAGC

A_14_P111050 GCTATGTATAAAACCTGATTTTGGAAATAATATCAAAAATTACACGTGTGGTCCTTTGGG

A_14_P110068 AGTGAAAACTTTTAAAAACTCTCATTGGAGTAAGTCTTTTCAAGATGATCCTCCACAATG

A_14_P113427 ATAAGTCGCATTGTTTTGTGGTTTGCTTTTCCTCTACTAAATGATGTGCCTGCTGTT

A_16_P15007436
ATTAATACGTGCATATCTACGTAGAGATCCAACTTTTGATTTCACATGTTACCAGGGAGG
CUST_7659_PI426210822
CTACCCTAGAGTCACCTTTTTTGATATATTTGCTTTTTTATTTACTCCACCAGGCAAAGA
A_14_P133861 TCCCTTTGTGTTTATTTCTTTTGAATTCTTGGGTAGAAGTGGATTGCAGATGCTAGATTT
A_14_P107113 TCACTTTAAGTTGCTTCCGCAGTTTTTCTATTTCTTTATCCAGATGATCTTGATCTGAAA
A_16_P00685118 GTTTTTTTACCCTCAGATGTGGCCATTGGTGCACCCAAGGAGGAT
A_16_P01978019
AATGCAATTATAGTCCCTCCATCTTTATAACAAGGCAAGAGATAGTAATGAGGCACTTGC
CUST_9299_PI426210822 ATTTTCAATCTGTAGCCGTTACAGCCCTCACACACCTGAAGAGAGC
A_14_P134185 ATAAGGAAAAGACAGAGTTCCAAATTTATCACTGGGCATTTCTTCTTTTCACTATCCCAA
A_14_P137647 CATTTTTTAGCATCTCTTAGTGACCTTTGCTTTAGATGAATATGCAAGATGGGCTGATGC
A_16_P00710958
CATCCTTGGTGACTATTCAGATGCAATTCTATGTGAAACTACAGACTCCATCTAGAAAGA
A_14_P131362 CTTATAATCCTGATGAAGAAGAAGATGATGCAGAGTCCAGGGTATGTAATTTACTGAATG
A_14_P102409 TTAAGGAATCTAGTCTCGCTGGAGATTCCATCCTTCAGATGAACTGATGAGC
A_16_P00456589
GTCTCTGCAGACAGTAAGTCTTTATCGGGAAGTGTTTACTCATAAAAAAACACAGTGAAG
A_14_P114096 TGTATTAATATGTTTGACTGGGAATCAGGGAGTGAATGGCTAAAGGAATAGATGAGTGTT
A_14_P126487 GAAAGAAAAGTTACATACCTGGCTTTCTTCCGGAGTAACTTCTGAGACTCAGGATA
A_14_P131392 GAAGTATAATCTATAGGAGGCGCGATGGCAAGTTGATAAAATGTGACTTATCTCCTAATA
A_16_P21508940
GTGTGCATGTTTCACCTATTTAGAAGGGTCGTTTAGAGATTATAAAGCGCTTAAAATGCC
A_16_P39704785
AACCAAATCCAACCTGATTTTGGATCACGTCTATGACTACCTTATAACCAAACACTTAAC
A_14_P120438 GGGGACAGATTTTTTAACTACATCATTAATTTCCAAAGACACAGCCATTGTTCTTGAACT
A_18_P11292435
AAAACTCACATTGGTTTCCATTACAGTCTTCCACACGTAGAAAAATGACTCTGTCTGAAA
A_14_P125718 GACTCATGAATTCTGTCGGGTCCCAAAATACGTGGTCTAAAGTCCTTTG
A_14_P136322 TCAAATTCTCCATTACACAACAATCTTCAAGTGGATGAATGACCAAAGGTTTTTTACCAC
A_14_P134944 GTAATAAAAATTTTATGCCCACACTTCCCCAATGTCTGCCATGTTGCTGTATATGTTTTA
A_16_P19656602
ATATTGAGTTTCTGCAATTCTTACGTTGGAATCCTCAATAACCACTTTCTACCATCTCAC
A_16_P17703229
GGCATGGTGTCTTAACATAAGGTGGATGATAAACATCAAAGCAGGTCCTATTAAATGCTA
A_14_P117363 ACAAGGTATTTGTAGATTTATTTAATCTTTCAACATACCTGATTCCCCGACACTGGATAC
A_16_P00617158
AAATCACAGGTAGAATAATAGGAGAAGAACAGAATTGTCTGAGTTTAGTGCAAGGAGAGG

A_14_P102826 AAATTTAGAACTAGAAAAAATCTGGGAGTCCCATTCCCTTCATTTTTGGACTAAGGTAAC
A_14_P202238 CAGTTCTTGTGCTCACTATTTAACCCATGTCTGACTTGATGCTAAGGTTTATATCCTGTA
A_14_P131060 CTGGTCATAAAAGAAAAAACCAGAACCAAAAAACAGTGTCTTCTAATTCCAACTCGTTTC
A_18_P11802967
AAGAACAAAGACCATGATAAACATAGCCACAGTGATTTTCATACAACTTCCACTGATGAC
CUST_979_PI426210822
ACCCACAAATTAGATAACAATAAGTGATTCAAGTTTGCGAGCCATAAGAATTGAGTCAGA
A_14_P113540 ACAAAGCAGTGGTCTACAGTAACACCATCCAGTCAATTATTGCTATCATTAGGGCTATG
A_18_P22843019
GACCTACCCTAGAGTCACCTTTTTTGATATATTTGCTTTTTTATTTACTCCACCAGGCAA
A_18_P22841076 CGGATGGGTAATGAGGATTCCACCCCCCCAGAAATTAGTATTTCAAAGTATTTTT
A_14_P102336 CTATAAATTTGTAACTTATTAAGCAGCACATGGTGAGTCTGTCATTGGCAACTATGGGAA
A_16_P02234453
AATCTTACAGCCCATACTTTATCGTCTCCTATTCTACAGTGCTCTATGACTTTTGACACT
A_18_P26972590
AAAGCAACAGAGAGATAAACCTCAACCATCAAAATCTTTCCCTAGAATTCCACCTAAAAA
A_14_P139649 TCCTTCCATTGAAGAAGTAGGAAAAACAGTTACAGCACTGTTCTGAACTCATCAAAAATG
A_14_P111319 TTAAATGTCAAGGTTCGACTTTGTTGTAAATGATTCTGCCGAAGGACTTTGGAAAAGTAA
A_16_P18413656
CAGAGTGATGATTAAGTAGGGCCAACTAATCAAGAGACTGAATGTGAATGTTTATGAAAG
A_14_P120095 TCGGGAGAGGAAATTTAACATTTCTGGCGTATTGAGAAAACAGTAATACTAGGAATATTG
A_14_P113249 TCCAAGTCTCAGATCTTCTGTTTTTGAAAGAGAAAACACTACAGCATTTATACATCTACC
A_16_P03369657
AAATCCATAGATCATTTCCCATAGTAAACAAGCAATCCTATCTAACCTTTTTCCCTTGCT
A_14_P136221 ACCAAAATTTATGCACTTACTTTAAGATGTTCTTCTTGGAAAGGGTGTTCAGGAAATTCT
A_14_P122858 TTTTTGTGGTTGGTAGGCTCATAAAGAACTCTCAAAGGAGATCAAAAGGCTGAAAG
A_16_P36230760
TAGAACAGTATATTCCATTGTGAATTTTAAATTCAACACACAGCCAACTTGCCCTCCATT
A_18_P22841200
GATGGAGTTACTAATGCTTTGGAGACAGTCCCTAGGAATTTTTTTAAAATGTAGTGGTCA
A_14_P139296 TCAGGAGGAAAGTATTTCCCAGATGATTTCCAAATGATGAATCACAACTGCATCAAATAT
A_16_P19138875
AATTTGTGCATATCTCTGACATTGCTCATATCATTCATTCTCGTTACACCCATCATCTCC
A_14_P134784 CTTCCAACAGAATATGAATAACTCATAACTCATTCTACCTTCTTATTGATTTGGGACGCT
CUST_10205_PI426210822 CTGCTCTTTGGAGCTGCAGGACCAGCAGACACAAAAACCAGAAAG
A_14_P101673 GTTTATACTGTAATATTTCTGGGCTTGATGTTTCTTCTCGATAAAACAGTTGAGGTGGCT
A_16_P17483902
GAATCCTTAGACCTTGTATGACTCATTTGTCCCATCAAATTTATTTTCTGAGCCTGTCTT

A_14_P107296 AAATCCAGACAATTAAGAGATGCTCAGCAGGATGCAAGAGATAAAATGGAGGTAAGATTA

A_14_P134744 CAGATTGAGGAGTTACATCAGACCTATATGGAGGAACTTAGGAAATTGTTTGAGGAACA

A_14_P108841 ATTAATTGTTATTACTGTTATTTTTAGAACTTCAAGTCTGCAGAAGTCCAGCAGCCTGGG

A_14_P112825 AGGTCCCAACAGAACCATCTTCAATCTTTACTGGATGAACCAATGAACTGTC

CUST_5872_PI426210822 CTAAGAGACAGTACTTTGGTTAGGGGCCTGGAGCTTTGGTTTTGATG

A_14_P101643 GCCATCATTACACCGGTAACATGAGAGGCTTCAAGCACTAGGTCTCAAATA

CUST_8316_PI426210822 TAACCACACACAGAATGAATCCTTTTCGATGCCTGGTACTCTGCTGT

A_16_P19411081

TACCAGCATATCTTTTCCATATTCGAAGCCAATATTTGTCTCTCATTAATTCCCGGAGTA

A_16_P00679451

AAAAACACCACTCATATCCAGGTGCAACTTGAATTAGTTCCTTTCACCAATCTTCTCAT

A_16_P03593782

CTGACAAAGCCACCGTTTCTTTGGAATCTCATATTTTATATGAACAATCCCAGTGTGTC

A_14_P139359 TCTCAGATTTACTGAGGTTTATCTTCTGGTGGTAGATTATCCATAAGAAGAGTGATGTGC

CUST_4366_PI426210822

TAAGATTCTTTCAACTTTACCCTATGCCTGGATCATGAAAGAGGTGTTTCTAGAAAGC

A_16_P21041995

GGGCAATGTGATCCATCATTAAGCAATTCGTTCATTAATTTCATTATCCTTTCTCAAACG

A_14_P112279 TATAAATTGGGTGTTTTTGACAAAATCTAAGACCCTCAGGGAAGTCACTATGTCACTAAC

A_16_P02045182

TACCAGCAGAGAATTATTTAATCATTTGCCCCTTAGTTTTATCTTGATCACTCCTAGCGT

A_16_P00051672

GCAGGGTCAGCATTTATACTGTATGATTCAGATTTGCAGAAATAAAACTTTCCTTCCACA

A_14_P103251 GAATGGTGATCTCGCCAATATTGAAGGCATCTTGAAGAATGATCGAATAGATTGTTCTAT

A_14_P114131 GATGGACAGTTTAGGATAATAAGGGACCAAATCTGCATAACTAATTATGAGTCTGCACAG

A_14_P201735 TTTGCCAGAAGAAACAGAAAAATGCACTTGAGATTTGTATTGAAACCTTACAGTGGTTTG

A_14_P137981 CACAAAAATCGCAGTTTATTACCAAACCCAACATTTATTGAGAACAAAAGGAACCAGTTG

CUST_3858_PI426210822 AGGCAGCGTATCACACACTAGTTGTCCAAGAGATGGAGAGAGAGT

A_18_P22841353

TCATCAACATTTGGTCTAGGATTCACCCATATTTCAACTGAGTTTTGAATACTGCGAAAG

A_14_P139733 CCAAATTAATTTCACATATCTATAATTTGCTACACAAACAACCACTTTCCCAGAGGCCTC

CUST_296_PI426210822 GGCTGCTGAGAAAGGGCGGGCAGCTGAGCAGGCAGCCAAGCCAGC

A_14_P101856 CACATCTGTGGGATTTTGAAAAACTGAAAGAGATGAAGGTAGCACTGAAAGGCTA

A_14_P111394 GCACTGTGATTGGATGAACTGATGGGGATGACTGGATTGGATTTTGAC

A_16_P19079472

TTTTGCTTTTACTGCCATATTAACCATAATTCTGCACTATAAAACTCTTCAATGCCTCAC

A_16_P02450848

TCCAGATACAACAAATATCATACATAGCCCTACAATTACAAAATACTCTTGCATAGGCCT

A_18_P16034238 TGAATCTTGTCTTTATCTCTCAATCCCTTAAATCCAATTTCAAAAATAAAAAGTTGCCTT
A_14_P125716 CATTCCAGGACTCCTGCATTTTTTCCTTAAAATTCCCAAAATGGGGATGTGATT
CUST_6211_PI426210822 CTACAGATACCATATTTCAGGGACAGCCCCTTCCCTCCTGAGCAG
A_14_P111580 ACCTTACAAAAATCAAAGGCAGAGGGTTAATTCAATCCTCTTTTGTAAGGATTGTAGCAA
A_14_P114889 TCTGATTGTCAAGAACAAGGAAGAATATTTGTGTATGCCATTAGCAGCAGCAAACATGTA
A_16_P18594799 AGTCAAATTTCCATCCTAGAATAATCCAACTATCCACATCTTTCAGTCCATCATCAGGAA
A_14_P139298 TGACTAAAGAAACTCTCTTCCCTGCATTTATTCTATCTCCACTTCCTGGTTTTATTTGTG
A_14_P104694 ATTTTGTTCCTGTTCCCATTGACACAACTTCCTTTGTCACTGTGGGAAAAAAAC
A_14_P103423 GCATTTTATACTCCCGCATAGATGCTCACCAAATAATCCTGTTGTTGATGACGATAATAG
A_14_P121495 GGAAATATAAAGGGACAACACCTAGTTTGGAATCAACAAATGGAGAATCTATGACACTTC
A_14_P139988 TCTGCTTTTAGCACTTTTTTTTTTCTTTTTTCTCCCCAAAGACTTCAAGATTCAAGTGGA
A_16_P40290028 ACCTTTTCGAAACTAAAGAGCAATATTCTTCGCATTTGACAAAGAGTAAAGGGACCATTT
A_14_P116528 CTCTAGGCTAACTAAGATGTCCTCATTAGGCATTCACATAGCACTCTGTATGTTCATTTT
A_16_P17993076 GACATAGACCCTTCATAAAAGCCACTTTAAAACACTGAAACAATTACATTTGATGCCTCA
A_14_P106468 AGAACCCGTGAACATTAATTCTCGATTTACATAGTGAGTAATTGAATAATGAAGGGGCTG
A_14_P114476 GGTCGTCCTAAAACCTTCAAGTCTCTGGAGAATGCTATTGAATGGAGGTAAC
A_14_P126375 GAGTAGTCCACCAACAAAGCGTATATTTACTTCAAAGACAGAAATTTCAGCATTCTATGG
A_16_P15655960 AAAAACCTTGAGAGGCCTAATATGAGAAAAGTTTATCAACCAGTTATGGGCTCTAATTCC
A_14_P200204 CTCATACTTATGCAAGGAGTTCAGCATGATCTGAGGGAGACAGATACAGGA
CUST_1050_PI426210822 CTACCCACCTCATGCCCTAAGCCCGCCTTCCCACCTCTATGCACC
A_14_P100691 ACAGAATGAAGCCAACAAATACCAGATCTCAGTGAACAAATACAGAGGAACAGC
A_14_P117879 TCCAATGCTCTTTAGAATGTTATTACGAATAGAAAGATGGCCAGGATGACCTTTAGTGTT
A_16_P01781691 AAAGCAAATGTCTTGGGTAATGCTTCACTCATCTTCTCGTTTATCTTAGAACCTGAATGT
A_14_P121530 AAAATAAAACCATGCTCAAAAGAGGCAGGCAGAGGAGCATAAACATATTTTTAAGTGAGT
A_16_P16192045 ACGCATGGTCAACCCTCAGGGGAAAACTGTTACCTAAAGTATTTTTATAAATATAAGCCT
A_16_P18858078 ACCAGAAAAATAATGACTCAACAGTAACATTTCTTCCTCATGAATACAGATTGGCATGCC
A_14_P108808 TACATTTATAAAGATGTGAATACATTCTGCTCTGGCTGAAACTGCATTTTGGGCAGTTTC
A_16_P16647838 AATTGATGCTGAACTAATACCATTTTTGAGACCCTGAAATTACCACATGCATTGCAATGG
A_14_P138683 CACTTCTAAGAGATCTTCCTTCTAGGTCATTCCTTACCTATCTCCCATTTTTTCTGAATT

CUST_585_PI426210822 CTTGCAGATAAAATACCATAATCTACTCTTGCAAATCAGGCCATCCTGGGGT

A_14_P130988 AAACTGTATTGATTTGGGGAGCCTCTATGTTGATTTCAAAACAGATTGTCTCCTGTGTT

A_16_P15945972

AATGGAATTTCAGTCTTCTCCTCTTAATCCCCTTATATGCAGAACACTAAATAGGATGGA

A_14_P130855 ACTCCTGACTAAATTAATATTCCTTCAAAAAAAGTGCTGCTTGTTTACGGTTTCTGCAGT

A_14_P130605 TATGAAAAACAAATTAAACCATCTCCTTACCCAAATCTTGAGAGGAAACAGCCTCCC

A_16_P18585127

ACTAGAAGAGGCCCTTTAAATGTATCTCAAAGACAGTTTCACAAATATGCCAGAATTCCT

A_16_P17142141

TGAAGACTTTAGACCCAATTCTAACCCTACTTGTGATCTGATACTTTATGACTGTCTCAG

A_14_P201819 TTACTTACCTCAAGCACGATTTCTTTCAGTTGGAACTGTTTTTGCGTGACCTTATCT

A_14_P122160 CACTATCCCATTTCACCAAATTCTCCTAAGAATTGGGATCCCTAAGAAAATCCTG

CUST_2992_PI426210822 AGAGTCTGCTCAGCAGCTGCCTCCACCACATTGGTTCACCCAACT

A_14_P118161 CCAGGTGATTCTCATCTAAATGTTCAAGTTAGCAACTTTAAATCTGGAAAAGGAGATTCT

A_14_P108284 AGACCTATATGAGGTGCTTGACTTAGTGTGAGTGTTTTAGATTTAGCCACTTCACATATT

CUST_6419_PI426210822 TAGATTATTAAAGAACAGTTACCTGTGTCATGAAGGAGAGGCCCCCAGAC

CUST_6319_PI426210822 AATGGCCAAGTGTAACAGGCAGCACACATGTGGCAGATCGTAGCA

A_14_P116977 TCTAGTTTAACTAAACACTTGATCCCCTCAGAATCCTAATGAGACAGTCATGAACAGAAA

A_16_P01543793

CTCACACGTATTTTTGTCATCCTATGTGCTGTAGTCATACTCTTCCTAACGAGATCTTAA

A_14_P115775 GAACTATTTATTTATTTAAACCTGCACAGTGGGTTTAAGTCGCATCTTGAGATTGGAGCA

A_14_P137193 ACCAGTTAGTCAATGCCATTAATCAAAATCCTATAGCAGAAAACGCATGCATAAGTCTGA

A_16_P30952373 ACTCCATTTCCCTATTATCTGTTTCAGAAACTGTCTGGGCAGAAGATAAATCCAGTG

A_14_P126457 AAAGCAAAACACTGTCCTAGATTTTTATCCTAACTCTTTCACTTTTCAGCCATCTGAGGT

A_16_P41041999

AAAAAAAAGGGCCCCAAATTTGATTTATTTCCCCATCCAAGTAAATTTTAAAAGGCTGCC

CUST_4784_PI426210822

TGGGTGAAAAGAGTAGGATATAGTAAAATGCAAATGCTAGTTTAGTAAATGGCATAAAGA

CUST_6231_PI426210822 CAGACAGGGAGCCATGTGTGGTTTCCTTGCTAAGATTTTTGCAGT

A_14_P135561 TGGAACCATATCATAGGTAACAAGTGTGTTTATCCCTGCAAAATTAAAAAGAAGGGCCAT

A_14_P117287 TAAGCAAATTAGCATAGCCATGTTGTTGTTGTTTTCCGCACAGACATCCTTTTTGAAAAT

A_16_P18164389

CAAATGCAATGAGCCAATATTTTTTTCACATTCTCCAACATATAGATGCAGAGATGAGGC

A_14_P123218 CTGTGATTCCTGTAACAAGTGAATCAGCCAAATGATATTTGCCATCATTCATTGCTCTGT

A_14_P110090 CTTCCTTCAGACAAACAGATGGATACTTCATCATTTCTGTCATTATCCTCTCCTACTTCA

CUST_4453_PI426210822 GCTCACTGCCCCCATCCATGTTGCACACCCCTCTCTACCTGCAGC

A_14_P105678 GGGAGTTGTCTTAATGCCGAGGTTAAATAGCAAACCCACTATTTTTACTTAGTTGGTATG

CUST_1900_PI426210822 CTTCTTGGGGAGGGGCTGCGGGCTACCGGGCATCTCTGTAGAAAT

A_14_P101198 TAACAAAGGTGCTGTTATCAGAATAGTCTTCTGGGTATAAATTAAAAATGACTGAAGTTC

CUST_794_PI426210822 AATATGATTTTCAAAGTGAGCACTCAGAGAGCCCACCCGTGGTTG

A_14_P116940 TTTTGATCTTTGTGAAGACTATTTTAAACCTCCATTTGGACCATATCCTGAAAAGAGGTG

A_14_P115109 CCAAATCCCTTCTGTTGATTCTGCTGACAATCTATCTGAAAAATTGGAAAGGTATGTTCA

A_14_P112074 TGTCTATCAACACTGGTTAATCTGGTGGCTAATAGGCTTATGTATCACTTAGGTCTTCAA

A_16_P58424556 CTTCACTTCAGCAAGATCCCCAGGGATTCCTTTGTCTGTTCATATTTAAGAAG

A_14_P106815 TGCTAATTCCAGGCACAGCAGAGAGTTTACTCACTATAACAGAGAAGAGCAAACAA

A_16_P58424390 CAATTGTTGCCTGTCGTGTTTTCATTAATTGTTGCCTCTCTAATGGATCATTCCCATTA

A_14_P101252 ACATTTTCCCTAACCTTTTCCCATGAACCAAAAGAAAGATTAAAATTCCAACAGAAGGAA

A_16_P18194860

ATGCATTCTCAGGATACTGTGTTTGTAAACATCAAGATGTTGAATGTCCACAAAGTTGAG

A_16_P02208070

ACCTTTCAACTAGGCATCTGAAAGTGTTTTATGGCGTATAGATAAAAATAGACCCACTGC

A_14_P121523 ATAGACTCTTGTGTGGTAATTGTTACTAAATTTGCCCCTCTTCAATCTGTAGACTACCAT

A_16_P21196563

CTTCCTAGACTCAGAACATTCAATCCCAACTACTTTTTCTTGCTGCTCATCTAACAATTT

A_16_P15903519

CTCGTTTAACTGACAGTAGAATCTAAGAACTAACGGCAAATTCTGTCTTATCTGGAGGAT

A_14_P133895 CGAGTGTTCAATAATGGTTTTCTTGAAGCGCCGAATTAGTAACAAGTTCTGAACTAATGA

A_14_P106426 GATCTAGTATTTGCAGTTCCATGTGAATTTGAGAGGAACACACGTTCTTTAATTGTTTGG

CUST_5336_PI426210822 GATATCTGGAGTTCAAGAGGCGTTTTCTGGAAACAATTTGTCCAAAGGCT

A_14_P131912 CATGAGAAAGTTCAAGCAATCTCAATTTGTTAAACAGCCATCATCACAAACCTCAAGTTC

A_14_P130971 CAAGACACTTTCTGCTGGAGGTTGACATTTGTTTTAGTAGTTTATGTTGATGCAAAGATC

A_14_P125525 GTTGAGAATTTTTTAGTGACCTTGATCAAGATATGCACATTGTCCATTCTGTCAGTCATG

A_14_P131797 AGAAGTGTTTCTGAGTGGATTAGGAAAGGTAACCTCATCGTTTATATGCACTTGTCTG

A_14_P105109 AGGATTTATGTCACAGTAGAAAGTAAACAACCTAACGCAGAGTCTCACATTATTTTTCCC

A_16_P00778758

GGTCAGACACCATTAGGTTTTGTCCAACTAGAGAAGTAAAAAAATGCCAATAATCCATCT

A_16_P36230431

GGTTTAGGAAATGGTGCTATGATATAGATTAGTTTTCTATGAATAACTTGGCTTTATGCC

A_14_P115063 GAAGCAAACTCTAAAATTATAAATGACAACCTGAATTATCTATTTCATCAAACCAAAGTT

A_14_P122090 CATACAGTTGTCTAAACATAGGGAAGAGCCACTGTTACCTTAAACTTTATCATCATCGTC

CUST_4488_PI426210822 GAAGGCTCTCTTGGGTTCTTTGCCAAAGTTCAGATGTCCCCCTTT

A_16_P16263887

AAAGATGATTTTGCAGTGTCCTTTTTTGTCATAGTATTAACATTCTCACAAGCACAACCA

A_16_P32194502

CCCAGGTTAGGAACTTTAAGACATTTTGCTGACATTTCATGAATCATATTTGCACACACT

CUST_7855_PI426210822 GGTTCTAAGAGCAGCCCTCACACTTGCCATCACACAGCTATGTAAA

A_14_P136271 CTCTAGGGTAGAAAAATCATGTCAAATCTAGATTATGATCTGAGTCAGTGTCAATGCCTG
A_14_P107006 CACTACAGCACTGATCCTGTTGCATAGCAATCCTGAGAAAACCCAGAAA
A_14_P101211 ACAACCTATGTGAAATGTAAATCGAGACCTATAATTCCTATAGCACCAAGTTTGAAACCC
A_16_P16020742
GCCCATAACTGTAAACACCCCCTTTTATCTACTATGAATGCAAATTGTTAGAGTGGTAAC
A_14_P116837 CTTCCGTCATCTTGAATAAAGTAGTAATCCACTGCACTGCCTAAGCGCTTATCTTCAT
A_14_P101926 AACAGTTGTGGTGTCTGGCTCATGTTGTTTTTATTAGTAACAATACTTGTTGGTTTTGGA
A_14_P112721 ACTATTGCTTAGGATGAGGCTCGATTTAAACAGGGGATCGATTTAAAGATACATGTTTGA
A_16_P21587195
ACAACTAAGCCTCATTTAACCAAAGAAAGAAAAGTAGCATTGTCCTAGTTTTTGCTCTGC
A_16_P18747806
CCCCAAACTCAAAGATAATAATTGTGGATAGCAGACTTTTACTGACAATCCAACATCAGA
A_14_P111704 CTCTCTTAATTGAGCATTGATAAAGGAGAAAGGGAGTTGAATAGCAATTTGGTCACAAAG
A_14_P103480 ACTTCAAACGGAGTGTCCATAGGAATTATTTTTCACATTAATCAGGAAAAGTTGCCTTTC
A_14_P132466 TGTGATAGCCCAGGAAGTAGAATCATTGTTAAGAAAGTTGCAGGCACCAAATTAAAAAAA
A_16_P00685083
GAAATAAACTTTGGGAGTGAGAGAACAGGGGATGTTGATTGATTTCCATTATCGTATCAC
A_14_P121448 TTTCTCTTAACTGCTGCCTGCTTATTTTTCCTAATACTGGGACTGACTTACCTAGGAAT
A_14_P200818 TTGCACTCTATTTTTTCCTTTTGTTCAATACTCCCTCCTCCTATGAGCATACTTAACTCT
A_14_P117202 CTGTATTCATTAGACTTCTGTGTAGAAGATTCTGCCCAGGAATCACAATTGTTATGCTAG
A_16_P20204093
AGCCCTATGCTACTTCTATGCTTCAATTACCTAGAACATCCTTATGCTTTCTTTACTGCT
A_16_P18925660
AGACTCTCTAGGCTTTCAAATGTCACAATGCCTTTTATTCACAAAATCATAGCATACTCT
A_14_P122833 CTTTCTCCCCAGGATACATTTTTTGAGGTTGAGAGGATACTTGTTTATAACTGAGGATTT
A_16_P38100370
TCCCTTGATATCACATCAGTATCTAAAACCTAACATACGTCAAATACTCCTTTGAGGTCA
A_14_P121811 CTTTATTATGGCTTGTGAACTGCTGGAGTCTGGCTTTACCTTTTTGTATGTGAACAAATC
A_14_P109917 AAAACATTATTTTGACCCATGATACATTTTGAGGCAGAATGATTCAGACTTCTGATGGCA
A_14_P202310 GGGCTCATGCATTCTTTTTGAGTTACTTCTCCTTGATTAAGCAGTTTCTTATACTCCCAT
A_14_P113957 AAATTATCTATGGCTCAGAGCAAATACCAAAACCTCATGTAATAGTCAAAAGGACTGATG
A_14_P104717 TTGCCACCTTTCCTCCTCCCTATGGCGTTATATCACAGTATTGAAAATTATAAATTCACA
A_14_P127148 TTTTGAAAATGTCTAATTTACAGCAAGGGCATAGTTTAGGCACAAGAAGGATAACTGGAG
CUST_1266_PI426210822 CATGGCTGTAAGGAGGAGGCCAGAGGTCTCCAGCAGGGCCTCTCT
A_16_P01679648
CTCACAGATCCTGGTTGATGTCATATTACAGACAATTACAGACATAATTAGCCCCCATTC
A_14_P133356 TACCTAAAAGCACTGCTTGTTTTTCCATTTTCCTCTCCAGTAATTCTTTCAAACTCTCAT
A_16_P15866660

CACAATCTAACTCTCAAACAGGTTAAATCTAAACTCTCTACACCTTTATCTAGCAAGTGC

A_18_P14137149 GGCACCAATCTAATGGCTCTTCTGGATAATCACTTCACATGGGAATCCTAG

A_14_P109637 AATAAATGTGGGTTTTTCCCCTCTAGTAGATATCAATTTCAGTAATGCAGCATGTGACAG

A_16_P15911150

AATACCCAGACTTCAAAACTACCATATCAGTTTTAACTTCTGAATCTCCTTCTACATGGG

A_16_P02306232

TACAACCTTAGGTCTAATACCTTAATCTTCACACAGTTCTAGATCCACTCAGATACGCAT

A_14_P121128 AAGCGAAATAATAGCCTCACTATTGTGCTTTTCCTGAAATTGGAGATGAAGAAAGGTTAT

CUST_7526_PI426210822 GAAACACTTGTTGAACGAGTAGATGGGCTTCCTCTGGGAGCACAG

A_14_P115771 AGTCATCCAAAACTGATGACCTGGGGTCTCTTCGATTAAATATATGTTATACAGAAGACT

A_16_P00644660

AAGAAGGAAAAAGATGTCTCTAATTTACCTGTCCTAACTTCTACCTCTAGACTTACCACC

A_14_P110149 ACTTCTTTGTGATTCTTACAGACAGGGATCTTAATGCTGTAAGTGTGTTAAAGTCTGCAA

A_14_P121648 CTTCAGTTCTTCAATTTTTTTCAGGGATTCTTGAACTTCCTTCCATACTTGCTCATCAC

A_16_P15273514

AGGGCAAGAAAAATCATTGATGAGACTAGTCACTCAATACATAAGTAGGTCTGCAGTTAC

A_14_P120196 CTTTTCTTTTTCTTTTTTCTTTCTCCAGAGACCAGCACTGCGATATCCTGAACGTTT

A_14_P122052 CTGTATCGGAAGTTCTCCATTCAGAATCTAGTGTGTTTGGTCAGATAGAAAATCATCTAC

A_14_P107122 CAGTTCTGCACAGCATCCATGCAAATTGTTTTGGAATACATGAGTTTGTCAAGAATATCT

A_18_P21333374

TATTTCTGCATATGACTCTCAAAAAAAAAGCCTCAGGAATAAAGTCAGATGACATCTCGG

A_14_P111552 TCACATCCGTAAAGCCCACTGATTCTTTTACTACACTTTTTATGAGAACAAGACATTTTC

A_14_P137645 CAGAAATCTGTGGTTCAAGATGGTCATTGCAAACTTAACCAATCTCAGCATTCTATTCTG

A_14_P123272 GGCATTTTGGATGATTTTTGCTCCTAGGGTTTGAATGCTGCTTTGTTTTATGAGAATAAA

A_14_P115760 AGGTTACTCTTAGGCATACTTTCTGCTTCAGTTTCCCTATAACATGAAAGTCCTTAAGAT

A_14_P129645 CTCATGCATAAAGGGGAATGAATGTTCTAGAATTTTCCCAATCCTCTTACCAACTCTC

A_16_P41212734

AGATCTCAAGGTTTTTTCCCACTGATTCTCTAAGTCTTGGCCTTATCTAACACATGAAAC

A_14_P138011 GAAATATTTTAAAATTTCTCTTGACACAAACCTTGGTCCTGGTCCTTTACCATTTTGGAG

A_14_P100799 GAAAAGTCACTCCATATATTCCGTGAAGTCAACCAGTTCCATCAAGGAGAAAGTC

A_14_P132646 TCAAATGTGCCTTTTTTTTCCAAGTCTCTCCTCTAAGACCATGCTGAAATTGAAGAAATT

CUST_1891_PI426210822

CTAACACTGATGCTGTGGAAAGAGGTTCTTCAAAACTGAAACCAGATAAACCAG

CUST_8894_PI426210822

TAAGATGGGTCCAAGAGCTAGAACCAGAATTTAAACAAGCAGATACAATTCCAAGG

A_14_P129662 TAGGAAACAGACAGCATGAGATACTAAAGTTAGACAACAGGAAGAAATCTGGGTAGCAG

A_18_P16635042

TGCGCCTTATCCCAAATAGAAATTATCTTTTCAAACTACCACTACCTAATTGTTTCCGAA

A_14_P119738 CATGCAAGTCATTATATTCATTTTAAGGCATAGAATGTATAATATTGTGAGAAAGGAGGC
A_14_P115713 CTCAAAAGACTTCAATGCTCGATGCTTCCTCCAGCATGTTCTGAGATC
A_14_P120928 GTATGTATTACTCGGTTCTTTACCTACCAAGTCTTTTGCAAAAGACCTTTCATTCTCTCC
A_14_P135770 ATAATTATGACTATTCCTAAAAGTAATGTGCTCTCCCATTACAGCCTTTGCGTCTCACAG
A_14_P138007 AACTTTCAGATGAATGCTTACTGTGGTATTTGGAATGTATGTATCTTCTGAACCCTACAT
A_16_P17364221
AAAGAAAACGTTGATCTCTATTGACCGTGATTAGTGGAACACATCTAAAATTGACTGACC
A_14_P112455 AGATTACGTTAATGGATGCCAATTATGTCATGGATGAAACTCTAGGGACAGCAACATTTA
A_14_P125874 ACCTCATAGAGTGGCTTAACATTTTGTTATGAAGAATCTAATATTGTCACTGCCCAATGG
A_14_P101488 CCTGCAGGCTTATATATTGTTGTTTCTGAACTTGACAGGAAAATTGGCTTTGATTTTGTT
A_14_P103856 AATACTCTAGAGTTTCAGACTAACTTGATGGAATATTCAAGAGGCTTGATAAGCCCTGAA
CUST_3887_PI426210822 CGTTGAGTTTTGTCTCAGGATTTCAGCGGAATCCAAACCATGTGACAT
A_18_P14135769 TCTAAACTCCAGCCTCATATTTGACTTATACGGGAGGTCTTAATGTTTCCGGCTTATT
A_16_P00685578
TACTGTTCCATTTTGAGCACTATGGTGATATTTTCATTATCAAGAGTTCTTGGGAAGAGG
A_14_P110081 ACTCTGGAAACCCTTCCAATGATTGAACCTCTCTTGCACTTGAACTGTTTGATAATA
A_14_P109059 CAAATGGAATATTCTGCATTGCCTGGAGTCATAAAGATTCTAAAAGAATAGCAACCTGCA
A_16_P19774177
AATTCCAGACATAGTTGAAGTTACCTAGCATAGACATCTAAAGCCATATACTCCAGGGAT
A_16_P20366109
TTTCTTTTTAAAGAAGGCTACAACACAACACATACTACTCTGAAGTCGAATTCAAGAGCC
A_18_P22840481 CTTGGTCATGATAAAAGCCAGACACTCGTGGGATGGCCTGTAGGATATA
A_14_P138561 TTTTCTGTTCTTGCCGTTGGCATTTGTTCGAATCTCACCGGATTTATTTGTTTAATACTT
A_14_P135075 GTCTATTCTGCCTATATAGAGATGAATCATTTATTCTTGGGCCATGGCAACGTTATTTGG
A_14_P119972 GTTGGTGAGCATACCTTGAATTCATAGGATTCTTCAATGATCACTTCCAACTTGGTG
A_16_P16944079
AAGTTAGCAGATAAAGACTACCTTTGTAAAGAATGACAGCATTCATAACTAGCCAGGTGG
A_14_P120720 CTTTGGGGAGCAGATTCTACATCTTATTCACTCATCTGGAAGAAACCTCCATCATAAT
CUST_4634_PI426210822 GATTCCGGTGGCCAGGGACCAGATGGGAGTCCGAAAGCTACACAG
A_14_P111455 GGGAAGAAAAACTTTAATTTCAAACTGAGGGAGAATTCATTACATTGTGGTTCATCCGAA
A_14_P127697 CTGGAGTTCAGAAGTGGTATTTGGCAAAACCTCTGAGAATCTAAAGAGTTGATTATAGTT
A_16_P41624904
TCAACACAAATGCTTGATAACATTCCACAAACTAACATCAGAAGATAACTTTCATTTCCT
A_14_P102866 AAGCCCAAATATATTAATCCTGCATTCAGCAAAGTGGCATAAAAATCACCTGGTAAGTAT
A_16_P21427495
AAAAGTGTGCCTAGCATCATTAAGAGTCTGTGGTTAAACATCAGGAATGAAAACCTCTAG
A_18_P22839791 GAGCAGCCTTTTAACAGAAACACGCGTAACTTTCCTGCAGATGCACTTC
A_14_P133611 ATGATAACTTACACTGCCTCAAAGTTTCCTTGATTTCTTTCATGTTGATTTCTAATCGTC

CUST_6354_PI426210822 AAATTTTAATTGAGGTGCGTTACAGGGGTGCACTATGATCTCTCTCTGGAG

A_14_P132582 ATTGGCTTATAAGGCACAGAAAATCTACGTATTTCTTGCCTTCATTTGTTTTGGAGAGTT

CUST_7696_PI426210822 ATATGCTGTTCAAATGACAGGCCTTTTCCCCACACCTGTGTGTATTC

A_16_P01417884 GGAACTCCAAGCATAAATTTCATTACCCCATAGTAAATCATCTATGAGAAAGAACGCAGA

A_16_P19117505 AGCGTCTTCAAGTTAGAACTGGTAGTATCTCCATAGATGACTTTCACTGATGTTTCAAAC

A_14_P122486 AAAAATAAAAACCCCTCACAAGTCAAGTTTACTCAGTAGCCAATAGCCTAAAATGAAATT

A_16_P16281500 AGCACTATTGTCTTTCTGAATCAGTCTTGAATGTGATCATCTCTCTATTCTGAAACCCAT

A_14_P104428 TTTGGGCCTAGAAGAAAAAGATCAGTTGTATCAGTCTAGCAATGAATTAGAAGGTTCAAG

A_14_P102447 CTGAACCGTTCAAAACCAAGGAAGGAAAATTATAGCATCAAAAGGGATTAAAACTAGTGA

A_16_P03219454 AAGACTGCTGTTATCCCATACAAAACATGGTATTAGTCCTTTCCAAGTAGGTTCTGATTA

A_14_P201880 GTTGCTTTCTTGCCTGTCCTGCTTAGTTTTTACTTGCTGGATGATACCATAATGTATCAA

A_16_P19747927 GATATTTCCTTTATGCTTACCTGTGATGATTCTGCAAAACCTCTTTTCTAACCCTGAGAA

A_16_P36230765 CAGCAAAGCCTCACTACAGAGTGACAATTCCTGGGGTGCCCTGTG

CUST_1610_PI426210822 TCTTGCTGACCTCGCCACTTGGGCTTGCCTTTTCTGTGAGCTGTT

A_14_P126185 GTGAGCAGGATATTGACGGAAGTGGCCTTTCTTTCTTCTTGACTTTGAG

A_14_P139438 CATTGGAGCGGTGAGTTTGCATTTCCAAGGCACAAGGTTATCCTAAA

A_16_P21531964 AAAGTCCTTGGAATATCTGAACTTGCATAACTGAACTATTCCTGTCTGAATACTCATCGG

A_14_P115967 AGCATTTTGAGTAATAGAGGAAGTTTCTTCCAAATGGATGAGGGAAATAAATGATTTCCC

A_14_P101798 CTAACAAAGTTGGCAAATATGAATTTTCATCATGAGATTTTTCCAAAGCCAGAAGAAAGA

A_14_P138769 AAATCAGAAGTGTAAAACATACAAACCTAAAATCTTTGACGTCTGCATCAATCCCATTCT

A_14_P107650 ACCCCTTATCAAAGGAAAACAACCAAATATTTGTTTACATTTGATTAGGACATGCTGCTG

A_14_P135259 AGTAATGCTGTGATGTGAATAGATACATTTGGTCATATTTTGAAGGGCTTCAATGCTGAC

A_14_P124937 CTTACCATTTTGCAATCCCATCCAGAGTTGCTTGTGATCTTTTTTCTGCATTTCATTGAT

A_14_P139492 GCCATATGTTACTTCAATATTCTCCTGACTTAAAAAAGCAGTGGAGTCCATGCTATATGC

A_14_P103714 CAAAGATGCTCCCATAATACTCTCAGATAGTGAAGAAGAAGAAATGATCATTTTGGAACC

A_16_P01958066 GCCAAGGCCTAATAAGTTCATTTTTTTCATTCAAGCACATTAATGCAGTCCTCATGAGAT

A_14_P104330 TACAGGATTTTAAACCATTTTCCCTTCTGTTCTTTGAGTTCTCTGTTCTACAAACCTTCT

A_14_P102553 TTGTAATATATCTAATTGCAAATGGTGCTGTGGTTGGCACCATGCAAAGATAACTTGCAT

A_14_P129068 GTCTGTGAAGGGCACAAAAATGAATTAAATGTAATTCTTAGTCTTTAGGAGCCTGTTGAG

A_14_P131895 CCTTAAATAAGTAAGATGGTAATGGCTCTTCCTCTTCCTCTTTCACTTTGGCCAAAATCT

A_14_P138865 CTCATGACTCTGGGCTACTCTAGTGTCTGCCTCAGATTTACTGTATTTAATAAGATGTTT

A_14_P109041 TGACACGATTCTTTACCAAATTGGATGCAGACAAATCTTATCAATGCCTGAAAGAGACTT

CUST_9157_PI426210822

TAAATTTAGCAAATTTTGGTGGTCTTGTTTTATAATTCACTATTAATAGATTAGCATTGT

A_14_P120048 GATGCCTTTTAGTGTGTTCGACTCTTTGAATCTTGAAGAAATCTATAATTCAGGTAAACA

A_14_P200096 CTCACCCTGTTTCTTTCTGTGAAGCGAGAATGTCTGAAAATAAATAGGACCATGC

CUST_7098_PI426210822 CTGGCAGCAGCCTCACTCTTGGGTTTTCCTCTCTGGAATTAATTT

A_14_P104077 ATCAGTAGTATCTGTCAGGAGACAATAAGAGGTAAGAACATGTTTGCTATCATGCATGAG

A_14_P106959 GTGACACAGTTTGTCAAAATAAATCTTTTTTTTTTTTCACATGCTGATGTTGCTGCCGAT

A_14_P139983 CATAAAAACAGTGTTTCTTTTTCATTGCAGATTCCAACAATGTCTCACCCATCTTGGCT

A_16_P19725665

CATCTCTACCAATCTTTCTTGACTATCACTCAATACTGTCTAATAACCTCATGCCAAAGT

CUST_3020_PI426210822 CAGAATGCCCATTCAGGTCACCTTCCTCTCTGCCTGTTCATACCT

A_14_P120469 ATAACCTCCTCCCTAACAAAATACACATCACTGAAATCACTACAATTTGGACACCTAATC

A_18_P12433221

GGGATCAATGAAGTGACTAAATATGAAAGGAAACAGGTATAGAAGTCTGACTTGGGTTTT

A_14_P126581 CTTGGAAGAATGTGATAGGTTAACAATAATGGATGAAAGCAGTTGAACTATTTCGGACAG

A_18_P22236059

AAAAAAATTGCATTCAATTTGAGAGCGTTGAGAAAATTCTGAACCCCAGGTTAAGATCAG

CUST_7966_PI426210822

GAACTCTCAAAATTCTCAAAACATAAATACCTGTATCCCACTTCATTTTTAAGAAGAGTA

A_16_P00003777

AGGCCTCAACAAAGTAAATTGTGAAAACATTTCCGGTATGACTTACTTCAGAATTTAACT

A_16_P01363161

AAAAGCCACTTCACAGAATGAGTTGTTCATAATGTAAAGTGTTCATAACTCCCAAGAGAC

A_14_P137610 CTATGGAAAAGTTCATATGAAGAAACCAGGCTATGATCTACAAACTGGCAACAAATTTGT

A_16_P21376903

ATCAAAACAGTGAATTCTGATAAGACTAGACGCATGTTACTCTTAAATTGGGCTGTCTCC

A_14_P109845 CCTTCCAATCACTTTAACCTCTCCCTGATTCACCCTTTCCAAACTACTC

A_18_P20511409

CCTGTTTTACACTGAATTCCTTTTACCACTGATAATTTTGGATATAAAACCCCCAAACCA

A_14_P120290 GCAAACCTGATATGATGCCTTCTATAAAGGACCTGTTTCCCATAGAATCTCTTCTCTAAT

A_14_P136170 TCCTCCTCATCTTTCTCAGATTTCTGAATGTTCTATTACAAATGCATAAATATTGCTAGA

A_16_P18652704

AAAATCCAAAAATCCTACAAGATGAAATTCCTTGACCTGTTTACTCAGACATCCCTACTT

A_18_P14359682

GATTCCAAAAAAGCAATCCTACAGCAAGTTTCATCCAATTCACTTTTTTACAACTTCCGA

CUST_7943_PI426210822 ATGCAGCGTGGGATTTCCTTTCATGAGGTCAAAGTCAAAGGTAAGG

A_14_P133056 CTAACTTCTTCATGTCGTATGGGTCTACCCCCTACGTATCTGTCCCTAC
A_14_P132586 AAAACATTGCACCTCTGGGATTCTTAGATCCAGTCCTTTAATCAAGGATGAATATTTTCT
CUST_9190_PI426210822 GGTTCTTGACTCCATGACTGTTGGCCAGTGACCTAAGTTCAGTCT
A_14_P123562 TGTAGGACCTCAACTCTCTATGTTTGCCAGTGGTTTATTTTACAGGTAAGTAGTGTAATC
A_16_P35019226
ACCAAAGACATCCAGAACTTTTTAGTTTAACGTGAGAATTATTCACCTAATTTTCCCGCA
A_14_P128661 CTGTTGGCTATGATGGGTGTCATTACTGTTAGTTATCACATTCTGGGGTTATTACATGAA
A_14_P134800 GAACAGAATAGTTGAGGGCTCTGAGAGATTTTTTTTCCCCCACGGATG
A_14_P127750 TTTATACAAGGTTTATTTTCTCGTAGAAAGGTCCTAGGACTGGCACACTCAATTCCATC
A_14_P131143 ATCAAGTGAAAATTTTCACCTACCCTTGACCTTTTCAAATCAACTACCCAATACACATTT
A_14_P115883 ATGAGTATATAGGAAAATAATTTCTCGACTTCCTTCTCAAACACCTCCAATGTGACAGCA
A_16_P20628585
AAAATGAGAGATGGTTTTCCTTATAACCCAATTTCCAAAGCCTTTTATTACAGAGCGTCA
A_14_P127277 AAGGCTCAGAGGAGCCAATAAACCTGTAAATGAATAGACGCATGTTTGAGTGAGTAAAT
A_14_P133923 TGAAACCTCATACCTCTTAAAACCTATGGACCTCATTCTTCATAGCAACAATTCTGAAGA
A_14_P136888 TATCTGCTGGTAAAATGTTTGACTTTGTTGCTGTAATCAGGTGAGCGTGACTTTGTAAAG
A_18_P16350523
AAACAACCATGACATTTATCCTACGGACATTCTAATGCTGATAGTAAGGTTAAAATCCCC
A_14_P123158 CAGGAAGGAAAAGAAGAACCACAGAATTATACAGATAGTAGTTCCTCTTCTACAGAGTCC
A_14_P133601 TCAGTCGAAAAGTAAAATATCAATGATTTGGGGGAATATTTTCTCAACCAAATAAGGGGG
CUST_7425_PI426210822
GTAGGTGACAGAGCAAAGAAAACTCTTGAAAAGTTTCTCTTAGAGCCTAATAGCAATAAC
A_14_P123882 CTTTTTTTTCCACTGAGAAGTCCTTTCCATTACAAGAGGTTACAACACGGGGTTTTA
A_18_P22841878 AGAAGAACTCCAGGGAGAGCACATATCAAGAGAAGAGACCTAGGAGAG
A_16_P02308668
AAAACCATCTCTGATACTCTAATAAGCCATTCTGATTTTCAAATATTATCAGCAGTGCAG
A_16_P15548924
GAAGCAGGAAAATCTTCTAAGGATGATCGAAATCTCCACTCTATAAACCATCCCAATTAA
A_18_P27284294 GTAGTGACCAGTTCCCCCAACCCTGACTCCCCTGCAGGATGGGGC
A_16_P00685073 AGATATAAACTGCCAAGTCTGCTTTCAGACACAGGGAGTGTGCGATGTTTTTAG
A_14_P116660 ACACACCCCAACACATTGGTTCAGCAAATACTAAACTGCTTCAAAAAGCAGC
A_14_P108901 CCAGATCTCTACTCTGATGATGAAGTTGAAAACATCATAAGCAATGTGAGGAATGAAGTC
A_14_P131472 AAAAATAAAATCAGAATATGCCCGCAAATGCTGGGGAGAAGAATGTTAGGCATTTTATTG
A_18_P16319444
TCCTTTTAGAAAATCCCCATACATTTAATAGCCCAGTTATAAACATACTGCCCATGTAGG
A_16_P36234083
TCCATCTCATCAAGCAATTTACCTGAGTATTTCTTTGAAACAAATAATATGCCCTCTCCA
A_16_P16172199

AAATGAGTATCTTTTCAAGAATGAATGCTGACCTGAATATAATGTGGAATTACCCTGGGC
CUST_493_PI426210822 GCGCTCAAACATCTGTCTTGTGAAGTTCTGTTCTGCCTGCTTTCC
A_14_P132247 GACGACGCAGTTCCACAGTCTGATGGAGTTCGAGGAATTTATAAGTATTTAATAAAACAT
A_16_P00881969
GCGGAACAATGAGTGTATAGTCATTTTAGGCAGCACTAAAAAATCACATGATGGAACATT
A_14_P125590 TGGCAGAGCAATCTGCCGCACAAACTGCAAATTAAATTAACTACACAGACTAAAAACTAT
A_14_P104747 ATTATAGAGAAAACATTCATCTAAAGCAGCGATTTTGAACCCTAGTATGTTCCAGGCTCA
A_14_P122916 TTACAATCCACTGAAAAGAGATAATTAAGTCACCACAACCTGGTCAAATTACCTTGGTTT
A_18_P22839935
GACACACTCTTCATTATGTGTGGCCTCTAACATTTTTGTTATGCTCTACATAAATCTGCA
A_16_P01198581
AAACTTACAGTTATCTGAGCAGTTCTTTCCATCTAGACATATGCCGACAACATAGCATTG
A_14_P138838 CTTTTATGAATATTTAACCCACCTGGTAAAAAATGAGAATTGTATTCAGGAGGAGCATCA
A_14_P123613 TCTTGGTTCAAAACAATTGTTGGAGTAATTCAACATTCTTAAAACTATCTTCTCCAGAAA
A_14_P137376 TGTCTACTCTTCAGTAAGTGGTCAAAACCTGGCTACTGGAAGGAAATGCAAT
A_14_P120334 TGCACTCTGAAATAAGTGATTTAAAAAGAAAAACATGATACAGAGATCCCATCCTGACCT
CUST_4611_PI426210822 TCTTCATTAATGTACGTTTATACCAGGAGCAAGTGGAGCTGGGGAGG
A_14_P136532 ATGTGCCTCTTAACTTTTTTAGCCAGTATTTCTAAAGGAGACTTCAGATTGTTCCTACTT
A_14_P201539 TCCAGGAATACATACTCTCTTTTGGCCTTTCAGAAATCATTTCCATTCTTTCCTCCTATA
A_14_P109141 CGACAGTGTAAAAACTTTCAGCATTTTGCCATCTTGTGACAGTGTTTAAGTATTCAAACA
A_16_P15485301
GCAGTAGCACCAATGATCAGTATGACTACAATGCCTTATCTTAGCACAAGGACATTTTTA
A_14_P121837 CTAGTTGATAGAAACTTAGCCTGTTAGAACTTGAAGTGGTTTGAAAAATCAAGCCAATCC
A_14_P112107 CTCAACTCTCAGGAGGGACAAGAATTCAGTATAACCTGCCTAGTGGATGA
A_14_P133500 ATAAAGGATGCACCATGTTTTTGGTTGCCTCTAGGCTGGAATTTAAAACAAAAAACACTT
A_14_P129464 TATTATTTAGACATCCTCTTTCACCCTTTGAAAAAGGGACAAGAGAGATTGGCATTCTTT
CUST_6198_PI426210822 CCAGCACACAAGTTCTTCCAGGTTGGGGGATTTTATGTGTGTACTT
A_18_P15061629
CAACATTGAGACCTACTAGAAACAACAAATACCTGCTTACATATGTTAATTGTTTGCCCA
A_14_P137419
ACAGGAAAACAGGACATAAGTCTGCTTTCCTTAAAAGAAAAAAGTTAAGAGACAGGACAA
A_16_P21598965
AAAAGGCTTAATTCATGAAGTTGCTATAGGTTTCTCTTTCTCAAAAGTGCTCACATGAGG
A_14_P123059 TAAATCTTGCCACCTATTGGTAAAAAGGAAGAACTAAAACTAAAAGGTGTTTGCCGTTCT
A_16_P03638830
GCCAAAGAGAAGTTCTTTATAGCGATCAGATTGACCTAAAGAAGAGTTTTCCCACATAAA
CUST_10221_PI426210822 AACAGAGATTTGCCCAGTGTTTCTCAAAGCTCTCCCCAAGGCACC
A_14_P132574 GAGTGTCTTGATGTCATGTCTGAAGCAATGAAACCTGGAGAGAGCAAGAAAAAAAT

A_16_P20891954
AAAATATACTACCCCTATCAAGGTAAGGAATACCCCTAAGCAAAGTAGTCTTATTGTGCC
A_14_P111001 AGTTAGTTAAAAATCTCTTTCTCTCATGCTTACCCCTTAATCCTTTTAGACCTCTGATGG
A_18_P14241953
AAGACAGATCATCGTTCAGTTCTAATGCACTAATTTCATTTATGCTTCAAAATGGGCTTT
A_16_P01639919
CCCATGCTTTTATAAACCTCTGATACACTAACAAATCCAAAGAAAAATTGAAAACAAGCG
A_16_P01005510
ACAGTCAGTCTAACCAAACTACATGTATAGTATCACTTTAGCCATTTCACATTATTCCAC
A_14_P118420 TGGTGTATTTTAAGGAAATAAAGCAAAGTTGTTTTGTTGGTGGGGAAATAAATTTGGGGC
A_14_P126923 CTGTTGTATAATTAGTTCTCTTCTCATCCACACCCATAAACCAGTTCAAGAAAGGGATGA
A_14_P128524 GGCCCATGCTAAAACTTATATCTAGAAATACGGCAAAACAAAATGAAACAGATCTCTCCT
A_14_P124480 AGGATATACAAGATAAATAAATTCTTACCAAGTGTCTACCAAAGGCCAGGTTTGGGACTA
A_14_P200383 ATACCACATTACGGTGGGCACCATCATCAAAGATCACTCATTTATCAGATGCCTAATTA
CUST_1046_PI426210822 CCAACCTCCAGGCCAAAGGCAGGACGCTGATCCCTTGCTATGAAG
A_16_P16621450
ATGTTGCATTCTCAACCTTTCTTGATTGCTAAAAACTTGAAATCGTTCACCATTAGTGCT
A_16_P01266554
AGGTGTGGCTATATTTCTCAGTGGCTCATTGATATTACTTCATACCAACTTGGAAAGAAA
A_16_P01677114
AAAAAAACCAAAGTTGTAAGTGCTGATGTATAGGCACAATACTTAGGCTTCAAGGACATG
A_16_P18996053
GGCAATGATAACTTCATCCCTAGTTCATGCAAAACATACTCAGGAATTCTACATAGTGCT
A_14_P101365 TTCATTCGTTCTGGATTTTCAAGGTTCACTTTGAAGGAATGTGACAAATCCAACTCAA
A_14_P111032 TAATATCTGAGGATGACCAGAAATGGTGAGATGTATGTTTGGCTCTGCTTTTAACTTTAT
A_14_P118091 CTGCTGAAAATTTCTTAACATTCAAGCCGTTTGGAGTAACTACATCTAGAAAGGCAAAAC
A_16_P00511213
GCCAAAACACTCTTTTTATAGGTCAACTGTCTTTGATGAGTTTCCATAACCTACAGAACA
A_14_P112972 CTTAGAGCCTCTCAAGATATTCTGTATCCACTAAAAACTAAGGTTGCTATGGAAACCATG
A_16_P19789155
AAGGCTAACCCTGATCTTAACACTGTATCAATACCTTTTCTTGGAACTCAAAGACTCTAA
CUST_5624_PI426210822 TGTGTGATACCCCCCAGAGAGTTGACTCCTAATTAATTGCTAGTTGGTC
CUST_10373_PI426210822 ATTGCCATCCTGGGTCAGGATGAAACGCCCGTTCTAAGTGAGAAT
A_14_P122012 AACACATTTCATTATGTCTGATCATGTCCTGTGAATTACAGGAAGTTCTCAGAAACCTTT
A_16_P39681866
TTGTTAAAGACATTTCCAGAGATACCTATTGCTGTCAGTTATATCACCTGTTCCAATGCA
A_14_P134727 TACCTGTGATTGTTAATAAAGTAAGTGGTAGGAGTGGGGCAGTTTTTCTCTACCTTTTAT
CUST_8211_PI426210822 CCACCTACCCACCCACCTGGTAGATAATCTGAGAAGTTTGATGAC

A_18_P22840660 TCTTTTGAAAATACTGTGAGGAGCCATCTGGCTATTGACCACTTCTTCCTTC

A_14_P108313 TAAAAGTGTATCTTGAGTCCCTCGTGCATCTGACCTCCTCCCACAAAT

A_14_P112052 GTTTGGGTTATTCCAAGTGTGTCTTATTTAAAATTCCAAACTTCAATCATGCCAGTCTCT

A_16_P20844045

GCAGGCCATTAAACTATGTTAACAGTCATATCAGTTATGTTCAAAATGCTGCTCCTTTGT

A_16_P20800685

AAATCTCATTTGCCAGCATCAATTACCTACAATGAAGTATCTCTTCCACACACATACTCC

A_14_P111359 ACATAGGAGAGCGGTAACTTTCTGCTGAGCAAAAACCACATTAAACAAACATATTTCTAG

CUST_9721_PI426210822 TTCACAGAAGGAGTGACCCCCGAACAAGTATTGAAGGAATTTCCC

A_14_P108041 CTGACAAATGTAATTCTGAGAGTCCCACACATTTCACTGTCTATTTCTGACATGATGAAA

A_16_P00482788

AGCAGGCTTTTAGATCATTTAAAACCAACCTATCATACTATAGATGCTTCATTGCAGACT

A_16_P36230593 ATAAATGTTGGGGTTACCTCCACATTCTTTCCTTCTCTTTGGGACCCTATACT

A_16_P17905375

AAAAAAAAGTCTCTTCTGCAATATGACTGTCAGAAAATATGAAACCTCCTACTGTGCAAA

A_16_P03456081

ACGAGATCGAGATTATTAACCTGGACTTATGAACCATCAGAGAATGAAAGCACAATTGC

CUST_4687_PI426210822 GCAGGATAGGTGACAGATATGAAACGATAAATGTGGAGGCAGGGAGA

A_16_P17126300

ACACCCTTTCAATTAAGCATGTCCTCTGATCATTTTTCTCTTAAAAAATCAGTGTGTCCA

A_18_P11518058

AAAAATCTCAAGCCTATAGAATCGGATACAATTTAAACGATCCCCAGCATAATTTTGCCC

A_16_P16191862

TGCACTCTGTTCTTTGGGCTCTGAGAAACAAATCCTTTGGATTTTCAAAACTATATTTAG

A_14_P109940 CTGGATGTGTAGGTGAAAGAATTACCCAGATGCAGAGACTAACAAATATTATTACCACAG

A_14_P119377 ACTCTTGATGTTTTCTCATTGGAAGGATAGGCAATGAGATCAGAATCAGATTTAGAGACA

A_14_P113683 CTTCAGGTGCAGGATATCGTCAAGCCAAACACAGCACATACATTCATCATAACA

A_16_P21453370

GTAGAAAATACAGTATTCCCTACATAGTTGTCATCCAGATTCAACGTTTCAACACTCTGC

A_16_P00685422 GCCTCTGGCATTGTTGTAACTCAGGCACCTGAGCATAACTAGTGAG

A_14_P117693 GGTGTCACTTTTTAAACCACTGTATAGAAGGTTTTTGTAGCCTGAATGTCTTACTGTGAT

A_14_P113851 CTATTTTGAAGATTACCTACTTCCCTTTATCTTCATTGGAAGATAAAGTCCCATCACCAG

A_16_P01184948

AACTAAGTCAATCTTTTCCTACAACTCAGCCTAAACCAAACATCCAAGAGAACGATTTTT

A_14_P130184 CAAAGACTGACCAACTTACCACAGAAGGTTTCCTGGAATCTAGAGGAAAGTT

A_14_P136190 ACATAAGGGACCCTTGGCAGAAAGAGACTTCAAATCAGGTATGTTTTTCTCTCT

A_14_P105552 CTATAGTATTGCTTCAGTGCTACCAAAATGAATATGGTGGGCTTGGTAAGTGTTAATCTG

A_14_P131515 ATGGTAGAATCTAGCCATGAATTGCATCTTCCCCCGTTCAATTCAAGTAGATTTATTATC

A_14_P125008 GAAGTGTTTTCTTCACCTCACAGAAGAGATTACTTTCGCTTTTTTCAACTATCAGTTGAG

A_16_P20160706 TGCTGCAACCTATAGATCCAAACACAGTTCAATACATCTTTTTTTTCTTTGTCCAAACTG

A_16_P16153938 AACATGTTCTCAAACTCATGTCTACTTTATAATGCTACCATCAGAAGTGATCAAGGACCA

A_14_P104256 TCATATGTAGAGGCTCAAGCAATCTATTTCTTCCCTGGACCTAATTTCATAAGTTTGCTT

A_16_P39068704 AAACCAATTACAGTTCATCCTAACTCCAACATAGCATAACTCCTATTAATGATCACTTTT

A_16_P15822437 GTTGCAACAACCAGTCATTCAATGTGTATCTTCTCAGTCTATTTCAGCCTATTCAGTCTA

A_14_P138321 AGAACTCAGAGTTCATGCCCCCAACTTTGACCTCTTTCTACTTTATATTAGAAGCATATT

A_16_P16496247 AAGGACGATTCTTTCTAATCTTTGGTAAGCCAGTTTTTACAGAATGAAATGCTCTTGGAT

A_16_P03591623 GCAGGGAGAAAAACTTACATATTTTGAAGTTCAAATCAACCACATGACTATAGGAGGGTT

CUST_9473_PI426210822 AATGAGTGTTCCATGCAGACTTTTATGCTCTACCCACAAGATAAGTTGTCCAAG

A_16_P00685251 CTTGTTTCTTGGCTTTGCTCTTTACAGCCTCTTAGAAACTTGAGTTCTATCTGGATTCAT

A_14_P114002 GACAGGGTGGAGTATTTGTTGTCCTTATGTTAACCTTGTCACCGTTTTCAGAAA

A_16_P00685389 TGTCTAAGACTTAAGGAAAATCTAAGTGATCCAACAGAATATTATTAAGCACCATCCAAA

CUST_5559_PI426210822 AAAAAAAAAAAAAAAAGATAATCTTCCAGATGTTGGCTCAGTTTGAGGAGGAGGAAAGAT

A_14_P137677 GTTAGAAATATGATTTTTTATTGATAGGCATGGAAAATAGGTTTGGTTGAGGATGGCGTG

A_14_P127247 GATGGCCTAATTTCTGGGGTCGCTTTCTATTGGGCACTTCAAACAGT

A_14_P139903 AGCATCACTATCTGCCTTGTTCACTTATTTGCTTATTGTCTTCCTTTATATACATGGTCT

A_14_P118672 CTCGATTTATTTTTTTAAATGATCTGCTTCTAATAGTGGATCTCTCAAAGGGCCGTGCTG

A_14_P123380 CTAATTGGGTGAACAGAAAGTAGTAAAAGGAATTCCACATACACTCTGCTTTGAAGGTAA

A_14_P123466 TAATTTCAAGTAATTCTTGTATTTTAGTTTCCCTTGTAGGGCACAAATTGGTGCTAGCAA

A_16_P00685429 AAGAGATGTGAGAATTCCGTTGACACTCACTGACCACCTAGAAACCAGA

A_14_P137889 GCGGCGCGTTTTAAATTTCAATTATTCCGACCTCTAGAAATCGTCAGACACA

A_14_P106101 CTTTTGCTTTTTATGTGTGATCTGTGTAACCTTCTGAGGATCTTCTGGCCATTTGTATAA

A_16_P16586819 TAGAGAAGCCACTATTCACTACTTTTTTCCATGATTAGAATTACACAGACTAGGAGACCC

A_14_P136518 CAGAAGATGCATGTAATAGATGCCACCTGGGGACTTTAACTGAGTTCAAATTAGTTTTTT

A_18_P15470971 TCAGCGAATCTGTTATCACTCTTATACTTAACGTCTTTCAAATTTACTCCTTTGGCATCT

A_14_P125607 ACTTGAGAATTAGAAGAGATTTCTTCAGGATGCGCAATGCTACGATTCCTTAAAGTTCTA

A_14_P105029 AACAGAAATGCATTTGTAGATTCATAAATCTGGCATTTTTGTTCAGCACATCAGGAACTG
CUST_3741_PI426210822 CTTACCCTGAAGAGCTTTGGAGCCCACAGCAGAACTCCCAGCCAG
A_14_P138301 CCAAGTTCAAAGAACCCTAGCACAAATTTGCTTTGGGATTTTCTTTTCTGGAAAAAAAAA
A_14_P121588 CTGGTTTATTTACTAGTTCTGAGGATCAGTGTCCTAATAAGCAACCTCTTAAGAGGATCA
A_14_P133469 ACCTTTAAAGACTGTCTCTTAGTAACATAATTCTCAGACTGAAGCAATTTCTCATAGTCT
A_18_P14841742
CAGTTGTATTTTTACATTACCCCAAATACCTGTGAGAACCAATTAGTCTCCACAACTTGC
A_16_P15497637
ATGAAGTCGTTGCTTTTATTGTTGATCTCTATGGACCTAGCATATAGCAGAAACAACAGG
A_14_P125554 GTCCTGGATTATAAATATAACAATACTTAAATGGCAGGGTTGATCTGGTGACAGGTGTCT
A_14_P131735 AATCCAGTTCTTCAAGCCATAAATGACCAAGATCCAAGCAATCTGAATTTGTTTTTGTGA
CUST_4895_PI426210822 CTTTTGATGGGCCGGCTATTCAGAGGTCAATGAAATAGACATGAGTACTCTT
A_16_P16191651 GAATGAGTTTTGAAGGGATAGGAGTTGCTGGGTTCTCATACCAGCACAG
CUST_5906_PI426210822 TTTTTCCCAAGACTTCCATGATATTTTCCCTGATAATCAAGGAAGGGAGTGTTG
A_16_P16336438
CATTAAGAAACTCTCCTTCTTCTAAACACTCCAGATCTTCATTAGTTACTCCTAATCCCC
A_14_P138504 CGCTTGTAGAAAAAGCCTTATTTGTTTGGTGGTATTCGTTATCAAAGTGCTGTTTATCAG
A_14_P128833 GGCTTACTTTTCTTTGAATACATCTATTGCCTTGAGAATTCACTTTCACACAACACACTG
A_14_P130645 CTGTCAGGGTTACACTTATAAATTTGATTCAATCCTTGGCAGGATGTCTACAGCATTTAT
A_14_P133529 CTTTTGAAAGTCACTGTATAAACCAAGTCTTAAAACTTTGTGATGTCCCATCTACTTCCG
A_16_P15298869
ACCACTACATTTTCACCCAAGGTTGTTCTAATAATCCCATGAGGTAGTAATAGTCATGAA
A_16_P16191195 CTGTTGCAGGAACTCTTGGCACCTGCATACCCACTGACATTCCAG
A_14_P107288 TGTTCAACCCTTTCTATATACTCTGAGTTCTAACAGCATACACCCAAAAAGTTCAAGTCT
A_16_P58424372
TAAGTTATTAAGTTGTCTACCTTATTTAGTCCTCAATGTCTTTGCAAAATGGGGAGGAGG
A_16_P39161740
ATGCCATTTTAAATCCTCTAACCAAATATGTTTCACTATACACACCACTAAACCTAATGT
A_14_P111885 TTTTTGTTTTTGTTTTCTGCCAGGTGGAACACCTACATAATTTCATTTTGTTTCGGTTTT
A_16_P00759559
AGGGTGATTAGAACTGCATCTTTTTAACTCGAGGATTCTCAAGTATGCATTCAACTTACT
A_14_P135420 GAAGAAGTTATTGCTCACATTACATTGGCTGTAGATCATCTCCTTGCTACAGTATTCACA
A_14_P135690 GCCTCCAGAATTGAGGATGACAGTGAGGTAATGAGGAAATGTATTTGAAAATATTATGGA
A_16_P35867728
TTGTTACCACATCCCACTATAAGAGATTTGGCTTTCCACTAAAAAAAAAATTTTGGTCCAC
CUST_9983_PI426210822 TGGAGAACAAAGCCTGCTCAGTGTTTGGCATCTGAAAAGACAAGG
A_18_P22843576 AGGCTAGTGGGGGAGAAAGATGCAGGATGGTGGTTTAGGGGATAC
A_14_P121289 TTTTTTGCAGGATACAAGACCCTCCTGAAAGGAATTTCCGGGAAGTTCAATA

A_14_P134505 CATGAGCCGAAGAGAGTTTGTCCACATGAAACCTACAAAGAATGAAAACCACTTTTAG
CUST_6302_PI426210822 AAACACCATGCTCACCTTTCCTCCTGCTGTCCTAGGCTGAGCCTG
A_14_P136309 ACATCCTGTAAGAATCACTCCATAAATAAGGACTTTTCTTACTAGGAAGGAAGTCTTCCA
A_14_P133890 GTAGAAAAAAGGGTTATATGAAGAGTCACATAATGGTGCTTCATTGTCAACAACCAAACA
A_14_P129334 TAAATTCTTGGGAAGCCTCACTTCCTTGTTCATTTCACAGATGTATGTGGAAGAAACAT
A_14_P125305 CATGTTTAAAAGACCAGAGAGACAAGCATTTTGCCAAGATCTTCCAGGGAAGAT
A_14_P134690 ATGACTTTGCACATTTTTGCTAGCAGATGGATTTTTATCGCAAGTAGCAGTATTCACATC
A_16_P41003167
CACACTCAATACGCACCAAACTCTTAGATCTCAGATTGATTCTCACTAGTCATTTGAGAA
A_16_P15685293
AAATCTCACCTGGAAGAAACTATGAACATCTCTTAATTCAAGATCCCTTTTTCTGTAGGC
CUST_7303_PI426210822 ATCCCCCCCCCACACACATAAATAGAGAACAATAAAAGATAACGTCGGA
A_16_P18074862
GTGAATGTAGGTTATCTCAACTTTCTGAATGACTGAACTTTCAAGGCAATCATCATCACT
CUST_7382_PI426210822 GAACTGAGCTGGTTGGAGCTGGGGAGGGGGTGCGGGAGGTACTTT
A_14_P114189 AGAACTATTCAATATGTTTTTGGAACAATTCTTCATTGAATAGCCTGTTCCACACCCTGA
A_16_P00231997
AAACCAAAGGGCAGAAACATTTTTAGGAGAACATACAAGAGGGAATGGGAATTTGTATTC
A_14_P128760 AGTGGTGACTAGTGGTAAGTTAGCAATAAGAACTGTTTGCATTCAAGTCTAGACTGAGAA
A_16_P00684993 CGGTTTTGGGGAACCCCTGAGGAAGGAGTATAGCCTCTCCAGCGC
CUST_4935_PI426210822 CTATGGATCCATGAGCTCACAGACTCCCCTAACGTCCATGAAGAT
A_16_P58424101
GGGAGGCCACTGAAATCAATTTTGGGTATTACATTTTAACTAGGGAAAGATATGATCAGA
A_16_P19951141
CAACTGTCTTACCAGAGAATCATTCGTAACAGTCAATCTACCAAAGGATTCTATGTTTGG
A_14_P117292 ACAGAAATCTCCATTTTTTAACCTACCATCAGACATGCTCTTTAAGATGTAGAGGAAACA
A_14_P202120 CTGCAAATGGACTTAGATCTAGTAATCAATTCAACAGTCCCTGGTTCCATCAAATAAACA
A_14_P115072 TTACTTATATTTCTGCACTTAAATGTGCATCAAAGAGGATAGCAACATGGAAGGCCTGAT
A_14_P115725 TGTGCAATAATTTATTTTTAGGCCTTGTTACTTTCTTCTTTAACTCCTTTCCTCTGGACA
A_18_P14135911 ATAAATAGAGAACAATAAAAGATAACGTCGGAGGATGAACCCACGAGCGTGGTTTG
A_16_P15560511
ACAAATAGCATCAGTTTGTTTTTCCTCACTTAAGTCTCCATAACCAATGTAGCCAATGAC
A_14_P106687
CTAGGGAAAGAGTGAGATTCTTCAGAGAGAAGAATGTAAAGAGAATAGTGAGACTGTAGT
CUST_9447_PI426210822 CTTCTCCTCCATAAAGTGCAGTTGGCACCAAGGCCTCAGAGACAC
A_16_P21048231
AATAGATGAGTCAAATTGGTTTTCCTCTTTAGGAAACGAGATAAAGATTATCGGGAGGTG
A_16_P03698720

ACGAGCACTTTATACAACTTCAAATATTTCAGTTCTGCACTTAAGTTTTATTGCCCACCC

A_14_P102090 CTTCCCTCTCTCAGATATGCAATTTCATCAGCACCAGAAAGTTTGGGAAGTTTTT

CUST_1228_PI426210822

GTAATTATGATGCCATGGTATGTTAGACCTAAATTGTGGAATCTGGCTTTACTACTCACT

A_14_P107920 ATCTCAAACCAAAATGATCATATGCCTATATGGATATGTCCTGAGTGATTTGCCCACATA

A_14_P135917 TTTGTATGTTTAAAATCTCTTGGGCTTTACATTCCATGGAAGAAATTTGGGAGATGCTCA

CUST_5043_PI426210822 AACTCCAGATTCCTTGCACTGGAAGCTGGTGTTTTGTGTTTGGAA

A_14_P137658 TAGACAGGTTGGGAGTATTTAAAGCGCTTTACAGTTTGCAAAGTAGATTCCTGGTC

A_14_P100403 TTACTCTGATGGAGGTAAATGCTGTCTGATCTATGAAAACAATCTTTCTACACCCAAGAT

CUST_2356_PI426210822 ACAGTTTGGGGGCCACATCTGAGTCACAAGAGCTGCCCTAGGCTG

A_16_P17054199

AATTGCGTCATTATTTGGGTCATTGTCAAATCAATGCTTTAGTTCTCCATATGTTAGCCA

A_14_P201308 CTTTTGTTACCTCTCCACTCTGTAACTTTAGTTTCTGCTCAGATGGGTAAGAAGAGAG

A_14_P120702 GCACTTATTAGTATATGATTTTACCTGTGTGCATGATTCAGGGCAAGATCTGGTTTCCTC

A_18_P13866063

AGAGCCCTATCTTCAGATTCTCAACTTTACGTTAGTTGCTTAGCCATATATGAAAACCAA

A_14_P100386 AGTCATACTATTCTCTATGCACACTACTCATACCCACGTCTTGACTCTGTTTACATCTTT

A_16_P40072912

CCTGTCCTCTATCCTTATCTTAATCTTTCCAACCTACTATTATGCTCCAAGAGTGTTACC

A_16_P02042485

AAGTTGCCGAATTTCTGTCACTTATTTGTTCTGTCTTCTTTTAAGTTAGCCCAGAATCTC

A_14_P138465 GAAATATGTTACTACAGGAGTTTCATGAAAACTTACCCAGAAATTGCAGCAATGCTTTGC

A_14_P112673 TTTCACCAGTCAAGAAGTAGTGCTGATCAGTATTCAAAAGGTTTCACTTTAACCTACTCA

A_14_P131389 GTAAGTTTGTTATAGAAGTGAGCAAGAATGATCGTGTGCATTTCTTCGTAGTGTTAGCAC

CUST_10213_PI426210822 AATGGTGAAGCAAGCACTGGAAGGTTCCAGAAAAAAATGCAGAAAAACATTGA

A_16_P18842587

AAAAACACTAGTTACCACTTCACCTAGAACTCTTTGCTTATATCACCAGAGACTTAGAGG

A_16_P00685250

TCAGATGGGAAGATTTCTGAATGCTGAATAGTTTGTAATATCCTTCAAAAAACCAAACCA

A_14_P136494 AGAGAATATCTCTCTGTGTTTAGATGAAACTGGTCCATTTGCTTCTAAGTGTTCTTACCT

A_14_P202066 AGACTGTGTTGTTAGTTATCCCTCAACATCTTCTAAGGTGGCAGGAAATAATATTGGAAA

A_16_P19366241

ATTCCTCATTGCAATATTTTCTGATCCCCTTATTACCTTTCTTAGACACCCAATACCAAC

A_14_P128608 GCCACTTCAACATATACTTCAAATGTGGTCTCTGGATTCTGCCTACAAAAGAAACAGAT

A_14_P122532 CATGTAAACATCCGGGAGGTCCTCTTGAAAATAAAATTCCAAAATGTGTCAACATTTGG

A_14_P133288 ATGTATCTTCCTTGACCATCCTTATCAGATATTATTTTCCTAGAAGCAAGTCTGCTGTCC

A_14_P125167 TTGAGGAAGAAGATAAGGGCTCTGTGTTTAAGTATTTTGAGATGAAACAGAAGGCACATA

CUST_8156_PI426210822 GATCCTGCTGCTTTTGCAAACTGTGGATGCCCCTGAGGGACTGAT

A_14_P138052 TATAATTAGATCCAAAGAAGTGGATGACAACCTATAGTTAGGCACAGGTCTGGAGTAGTG

A_14_P131454 TTATCACTAACAAGAAAAATGACCAGATTAGATGTTCCATGCATCCTGATGTGATGTGGC

A_14_P128096 TTATCCTACTAAGTTAATTTTTCAAACCCTGGGACTCTATACCAGTCTTCATTAACCCCA

A_16_P17754046

ACAGGCAGTAATTCTACCATGTTTATTTTCATACCAAGACTATTGAGTGCTATTGAGGGC

CUST_4051_PI426210822

CACCTCCAACCACAATTCTTGTTTTAAAAAATGAAAATAGAGTGAAACAGAAATGGTAGA

A_14_P111314 TGGATTCAGACCCAAGAGAATAAATTCTGAAATGATGTCCAGCAACTGTTGAAGACT

A_14_P103748 AGTTCTGTTTTGAGAAACACTACAGAGGGCTTGATGAGATGTTGGCCAAATCT

A_18_P22841554

TATTATTACTTGCTGCTGAAGTAGGGAGTTGGCAAAATGGGTTGCTAAGTTCTAGGTAAA

A_14_P118221 TATACTCCAGCAGTATGTCTATTTTTTAAGAATGGTTTGACATTCAGGATAGGCAAAGCA

A_14_P115247 ACAGAATTTTGGAAGATTTTCAGTCTAGTTGCCAAATCTGGCTCCTTTACAAAAGAAATA

A_16_P18070040

AGTTTAGTAAACAAAGGCAATCAGGATTTTCCAAGCAAAAGCAATTACCATGTCTAGCAA

A_14_P125473 CTGAATCTCCCCAGATGACTTAATGAGAAAAAAACTGCTAAAACATGGTATTCATATGGG

CUST_8193_PI426210822 GATTCAACTCTAAAGTGGAACAGAGAAAGCCCACTGCTGGACAGC

A_14_P201628 ATACTTCCGGATGGTAAGGGAGATGTTAAGATGATTTGTCTTGAAGAATGGTAAACTTTT

A_14_P108918 CCAATTAAAAGATCATTATTACAAGAAATTTCTGCAGCAGGGTTCATTCTGCCAAGCATT

A_16_P21427302

GGCTCAGTTCCCTAATAACACATATCAACTCTCTATGTAGGTCACTCTTGTTCATTAGTA

A_18_P14042474

TCACCTCTTCGCATATTATCAAACAATCCACATTTGACTTCATAACATTTTGAGACAGTC

CUST_8568_PI426210822 GCTGTAGTGAAGAGGCGAGAGTCCCCAGATACTTGTTTCAGAGGC

A_16_P01020318

TCGTCATGTCAAATATTGAACCTGTGAGAGTAAAGTTATGTCCATATGCACTATGAGACG

A_14_P109207 AAGTTCAAAAACGCCGACCTGAAATAAGTAGGTGAAGATTGTGTGTATGTGTTTAAAACA

A_14_P116654 CTTAAGGAAGATGTTAACACCTGTAATCCACTAAGGAACTGAATGGCAATTTGCTCAATA

A_14_P107209 CTTGTATTCTCAACGGAGCAAGAAAGCCTGTAATAAATACCTTTGCAAATACAGAACACT

CUST_6217_PI426210822 CAGTAACTGTGGCAACCATAGAAGAGGCTGGCCACGTGCTCATAG

A_14_P122415 ACCTGAAGATATAACAACTTCCAGCACATCTAGATCTGTAGTTCTGTGCATTTGATCAAT

A_14_P131764 TCACTGAGAATTCTGTCATCAAAGGTTTGGTTTTCTGTAGGAATGAAAACACACAGTATC

A_16_P18865739

AAACCTTGGGACTAGATTTAAGAACTTGAGCATAACACCAATTTGACCTCATTCAGTAAC

CUST_3330_PI426210822 AAGCTGGGCTTTGGAGACCACGCTGGAAGTCAGCTCACTGAGTGT

A_14_P130897 CTCTCCAGTGAATAAGCAACGATGAGTGTTAGGCTGTTTATTCAGATACCCTCAC

A_18_P19542417

ACACACATATACCAAGGTCAAGTAGACAATAGTAGTGGTAACCATTTATTCACTGAACCA

A_14_P100136 ACACCCATTTCCAAAAACAATCTGATATGGCTCATTATAAAAAGCACAGGTGCAGTAAAA
CUST_8084_PI426210822
TATGCAAAGCATAGATAGGGTAGACTTCATTCCCATTGCACAAAATCCAGAAGG
A_14_P118052 AGTCCAGTGCTTTTTAGTATAGGTATTGAGGGCTATGCTTTGACTTGAAATTCCTGTTAA
CUST_5558_PI426210822
TTTTCCTGATTATGGGGAAAAAAAAAAAAAAAAAAAAGATAATCTTCCAGATGTTGGCTCA
A_14_P111558 CTGCCATCCAGTAGCAGTTAGTCTTCATCCCCACGTGAACAAAATG
CUST_7306_PI426210822 CGAGCGTGGTTTGTGTGGGAGGATGTCTTGATCCTGGGCTCGCAG
A_14_P139535 CTTATCTTTCAAGAACTAGGCTAAGTCCCATACCTTCTTAAAACTTCCTCAGACTACTCT
A_14_P109094 ATTCACTGACAATCATACTTAAAAGGGAATATTTTGTTTAATGTTCGGAAGCCCTCCACA
A_14_P107200 CTACCAAAGAATCACATTTACATAAGACCTTTATATGTATAGCGATTTGGGGAGAGAGGG
A_18_P14134377 CCCTGGGCTCACTTTTTGCTTAGAAAAGTCCTGATGGAAAGGTTGAG
A_14_P101947 ATCATACCCTAACTGTATTCATCAACACTTAGAGTAAGCAAGGCTCGCTGGAGAG
A_14_P115887 CTAATTGGATAGTGTGCATTTGAGACTCTGTCTGACTTTCGTTCATATCTTCTAAGGTCA
A_18_P10673427
AACACTAACGGGACCATTAGAATAAATGAATGAACCTAAGATCTTCAGTCCGACTTGATT
A_14_P128759 TAAGGCTGCTCCAGCAATCAGGAAAGGAAATGCTATACTTCACATAGAGAAAAATAGG
CUST_9249_PI426210822
AACGTGTCTGTGTTCAAAACTCATAAGAATGCTTTCAGAATGCAAAATTTAAGTTTGTGG
A_16_P02835850
CAGGCACATATTACAGAAATAGGAACATAGGGATCATGATGGGAATTTGAAACTTGTTGA
A_16_P31273322
AGCCTCCAAAAAGTTGTGCTAGTGTGTGTTCAGAATTCATTAAAATCTATCCAGCATTTC
A_16_P15504834
GCCACATTTCTACCTCTTATCTCATGAAACAAAACAATGATTTAAAGACTGGGCTAACAG
A_18_P10011464
AACAAACAAGACCCATTCAATTTTCTCCAATTATACCTGGAGAGTAACTTTTGCTCACTC
A_14_P132686
TAAAGGATTGCAAAAGAGAACACAGAAACATGACAGAAAAACAGTTATTGGTCTCCAGAT
A_16_P02629619
AACCAAATCCTACATGATTCTACCACAAAGAAGTTTCATTCCCATCTTTCTTATTCGTGT
A_16_P19460107
CCCTAAGAATATAAGGCTAAACTTCTGAAAAATCCTGTCCTATTTCAGTTCTCTTGGACC
A_14_P101731 ACACCAGCACAGGTATCAACACCCCCAAGTTCTTTTACATCCCAAA
CUST_9649_PI426210822 TACTGAGGCCCACAGAAGAGGAAAGGACCCCTGAAGCCAGAAACT
A_14_P200316 CTGTCCTTTAATGACCTGACTTCCATTGACCCTGTGAGTTCCTAACAGTAG
A_14_P100908 GTTTTGCATCTTACGCTACAGGTATTTCAAGCCAACAACGATGCAACTGAGG
A_16_P00932436

TAGACATGTAGCATTCTATCTTTAGCCCTATCAAATTCTCAGTAGATTTCAATCCATGCC

A_16_P37209144
AAATTAATCCTGCTACAGGATCGTATCTTATGGGGAAGAGTTTAGAATCCAGTCAAGACT

A_14_P122405 TTTGTCATTTTACTTTCATGAAAACCTTCACTGTTCTCCTTAGAGGCTCCATATAGAACC

A_14_P111441 TACCATGGAGAGATGCCTCTCTTATGTCTTCAGCCACTCACTTATAAAGATACTTATCTT

A_16_P17414202
GCTGTATAATGACTCTCAAAGTCTTGTTTGTAAGGTAGCATCTCTCAAAATCAACCAGG

A_14_P113295 ATCATCCATGTTTTCTGAAGGCTGCTGTAAAAATGCAAGATACTCTTCACTGGTGAC

A_14_P128499 TTCAGAATCCTCCTAAAAAGGCCCCACTAGTGTCCACAGTGATTTATTGTATATTAGAAT

A_16_P21525978
GCATTTGGATGCTCTGAAAAAAGTATACTTGTCAAGGAATAAACATAATGGGATGGCAGT

A_14_P121921 CCATTTTGTTAGTCAGATTCTAAAATCTGCCACACCTCATCTACTGAAAGGATGTAGAAT

A_14_P134465 GTGAGACAGCACTTTAACCACTAGATGCAAGAAACTTGGAATTGATTTGTCTTGTAAATG

A_14_P113816 TTGGGTCGTCTGCTTCCCTTGTATTTTTCTTCCTTTTTTTGTAATATTGAAAACGACGAT

A_14_P114603 TGACCCATGCTGTCTAATAGGCTAAGAATAGGGATGTCGTATGTCTATTGTGTATCAAAA

A_16_P00685514
TTCCTTGAGAATTGTCTGACTACTTATTTATTGGTATAATGGTTGTTATTGTGGTTGTCC

A_16_P00640991
CCCTTATTCCTTCAAATCTTTTTTCTACGGTCCACTTACATTTTTGATAGCAAACAGCCT

A_14_P135590 CTGTTCTTCTAACTTCATCAATCCAAGACTCATAGGTATTTTGGAACGAACTGGAAATTC

A_14_P128692 ATGACACCACCAAAAAGAAATCCAGGAAGAAATAAGACATGAATTTTCCTGACTGATCTT

A_14_P121307 GGCAGTATAGATGCTTTTACTCTTGTAAGTAGGAGACATTTTTCAAGTTGTGTAGCCATG

A_14_P124981 CTGGGGATTTTATCTGCACATTTCTGTAAATTCTTACTCTTCAGGGAGGATGCTAG

CUST_8865_PI426210822 GGTGATGGGTTGTTACCAAAGTCGATAACAGGAAATCTCACTGAGGATAAAC

A_14_P116620 ACCTGAGATTTCTTCTTTTGATTTGGATTTTGAAGGATGAATTAGTTTACTAGCCAGAGA

CUST_7640_PI426210822 TTTCTTTGTCCTGCTTCTCCAAATTCCCCAAGTGCATGGGTAATCA

CUST_3040_PI426210822
TCATATTTCTATAATGAAATACATACCTATCTATTTACTTATTGACTCTGAAATAACAGA

A_16_P20013060
AAAAACCTCACTAATGCAGTATTCCTTATTCCCAAATTTTACTTGTCTCTGCTAATTTAA

A_16_P18225623
CACAGATCACAATCATTATCATACATTCTTCTTCGCATCTCAGATAAAACCTACCCCTTC

A_14_P129415 TTAATTGGAATACGGCAAAATTCTGAATTTTTCTGCCGTCTCTATTCCAACTTCAGAGTT

A_14_P128103 TACAATCTCATTTTGCATCTCGTCTTCCAGAAAACCTGAAGAATGAAAACCCACC

CUST_5248_PI426210822
ATTTTAAAGTAGCTTATAACATTTTAACTGTGATCTTATTTATTATGAGTATAGAGATAA

A_14_P111597 TTTTTCTGAAGTTAATCCTACAAGATGGAAGGAATACACTGTTTCTTATGCCTCTACCCA

A_16_P15800463

AAGTGGGACTATTCAAGAGATAACTATCACACACTTGCAAATTATACTATGCCTTGGTGC
A_16_P00732865
GGAGCAGAACTCAAATTACTCTACCAAACTGAGCATTTATCAATTATTTTTGACCCACCT
A_14_P102082 CCCCAAACTTCTTTCCTCCAAGTTCATTTAGAGACATCTCTAAGTAAGAGATCTTTTAAG
A_14_P139367 ACAGATACCCCTTTCCTGGAACCTAGGTAATTTTTTTCATTTTTTAAGTAGGTCAAACAG
A_14_P104842 CTGTTCTTAAAGAAACCTCATCTACAGCATATCTGACTGACACACTTGCTAAACTAAGCA
A_14_P110930 TCTGACCAATACCTTTGCTTTTCTTCTCCAAGTATCCTTGCTTGATTACGTTATCAAGTT
A_14_P123980 CCAGTAATAAATTGAGTATCAGATCTAAACTGCTGCTAAGTGTTCAGTTTCTCTCCCAAG
A_18_P14133798 TGATTACAAATATGTTATACCTTGCACTGTGTCCTGTATGTCTGTTATGTTTATTTCTGA
A_16_P01006409
AAAGTGGGAGATGAAGTATACCCAGTTCTTGACAATCTAGCAGCAGATAATTGATTTTGT
A_14_P125629 GATAGAAATTTCCTTTCTATTTTGTCTCTCCCTCTCATAGCCTGAAAGAACACGTTAAGT
A_14_P106685 TATATTCCTGATATTTAACAAGGAAAAAAAATTCTGCAGGGGAGAACTTTCCTGAAAGAT
A_14_P134391 ATTTGGAGGAAATGGGGGAATTAATATAATACAAGCCTTTTGGAAGGTAAGTTGCGTTAA
CUST_2901_PI426210822 TGTGGTATTTATCTGCTGCTGTCTTCAGCTACATGACCTGCTCTCTGA
A_16_P40225498
GAATTAAAAGAAAACTCATTACCCTTATCTACCTACTACTCCTACCTTATCTCATGTTCC
A_14_P105425 CTGGGCCAATAGCACATACCTTATATATGTTAGTGACTACCATGATATGAGAAAAGACGT
A_14_P103353 CTGACAAATATCACGTGGTCTCTGGGGCTGATGATTATACAGTTAAATTATGGGATATTC
A_14_P101587 TTTTTGTGTCTGTCCCTCTTCCATATTCTCTTCCATGGCTACCTTTCTTCAGTA
A_14_P104480 TTTTCTGGTTTTGTTTTTCCTCAAAACAGGAAGGGGATGATTATGAAGTCATTCCTAACA
A_18_P14133479 AGCCTGATGTAAAGTTGAGGAAACGTGTGTGGTCTCATCGTGGATAGAATAG
A_16_P15832675
AAAACCAATCAACATATTCACCCTTTGTTACCTAACATCTTACTGGGTTTTAATCTGGCC
A_14_P136173 TTAGCATTTATTATTCTTTCTAGTTATGTGACAGGCACTGGCTGATGACAGTGACTGTGC
CUST_5266_PI426210822 GTGCCAGGGTTCCCAGGTCCAGCTTTCTTCGGGTCATAGAGTGAG
A_16_P20034311
AAAAATTCCAGAATCTACTATGTAAAGGCTTTGACTTAGTGCACTATGAATGGCAAAGGG
A_16_P21543827
AAAAAACCTCACTCATGTTACTTTTACCCCCATATCAAGTCCATTCAACCTAAAATGTGT
CUST_10189_PI426210822 GTCCAAAAAAGGAATTTGTCCCAGGTCCTCAGAATCACTGTCTAGGA
A_14_P200028 AAAAAGATCTCATGATAAGGAGGCAAACGGGAGAAGCAGAGGAACAAAAAAGAG
CUST_3193_PI426210822 ATGAGGGGCTGGGCCTGCTTTGTTTAGGCTATCTTTAGGTATTTTTTTCTT
A_14_P109712 CTTACGCCCTCTTTCTATTGGAATTAGATGATTTCCTTGGTTCTTTCAATTTCTGAGATT
A_14_P116763 GAACACTTTGGCTTTGAGTTCATTCCGCTCCTGAAGGATCTGCTC
A_14_P127805 TTTATCACTGAAGATGTTTGTGGATTCCAAGATAAGCAAGAAGCATTGGTCTTGTCTTTT
A_14_P101152 CTTCCAAACGAGACATCTTACTTCAGGTGGTAACTAGTGAAGACCGTTTTTATTTTGTAA
A_16_P20075819

CAGTTAGTCTACAGCAGATCTTGTAACGTTCGACTGAATTCTAGTTCCACTAACGATAAG

A_14_P131951 TGGTGCTGTGTGATACATCCCTTATTCGTATAAAGGCTGCATTTCTTTTACTTTGTTTTC

A_14_P125987 TGGGAAAACACATTATGTAGCACTGAAAATACCTGTTCTTTTGTGATGCTCTGCAAGTAA

A_14_P110841 GCTATGAGTTAAAGAATGCAGGATAGAGACATAGAGTGATATACCCCAAGGAGGTAAAAT

CUST_8870_PI426210822 CAAGGACTAATTGTGTATGCTTCGGAAGCGCAAAAACAGGAAAACGCTTTT

A_14_P138983 AGTAACTGACTATTGAGATCATGGTCTTCTTGGAAGGAATAAATGTAAAAATGTTGCTCA

CUST_6898_PI426210822

TAGATGTTTATAGTCATCTTGGCCTGGCCATTAAGATGATGAATGAACTTGCTGCT

CUST_7992_PI426210822 TGAGGGTGGAGATAAAGGAGGACGACCACAATCCATGGACACTAT

A_16_P01834550

AGCACACAAAAATGGTCAATTTTATGTTCTGTTTGATTACAAGTATGTCCAGATCAGAGA

A_14_P135963 AACGCCACCTTTTCTCTTCACGATGGTAAATTAAGCCAATAAAATACTTCTCAGCATCAG

CUST_3690_PI426210822 ATTCTGGATGTGCCAGCACTTTTCCTACTCATCCCCAGTTATGGAAT

A_16_P18211403

AAACCCATCTATTTCAGATTCCAGTTCCATAGTTAATCATTCAACAGGGCTATTGCTAAC

A_14_P202231 CCTGCATATGTTCAGAATGTGCAGAACAGAATTAAAGTGAAAAAAGCCAGAACAGATACT

A_14_P135732 AGACAACGTTGATGTTTGGTCTAGCAGTGTTGCTAAGTATTTTAAAAGCAGCAAAGAGTA

A_14_P124425 ACACCATATAAGCACCAGTTCGAAGAGCACCGAATATACTTCATAATGAAAAGAGAAAAG

A_14_P114624 TTGCAAATGCCAAAAGGGCTAACAATGATAAGATACTTTCACTTCCAGAATTAATCTCCT

A_14_P133887 CTAAGCATTTGCTTCACCTGCTTCAAGGCTGCCTTTCTAGACTTTAAAGACTTG

A_14_P110079 TTTTTAATTCTGGGTGCAGAGGAACACAGATTATCAAATCCAGTTGGTATTTCTTATGCC

A_16_P15753693

CCTAATGTGCCAATATACCAATGATGAAACCATTTGCATCTGATTAACCTCAAACTCTGA

A_14_P137553 AAAGCAGGCAGAAGAAATCTTGAATGCCATACTACCCCCAAGGTAAGAAAGTA

A_14_P117077 CTGCTTCTAGAAATTTCTCTCGTATTCTATTTCCTGACATAGAGATTGCCCATTTTTGGT

A_16_P01862000

AACTCCAGTGATCTTTGCTTACTTGTAACATTCCCTTATTTGCCCTTTGTGTTACCATTT

CUST_8364_PI426210822 TTAGAGCTTGTTTTTGAGACAGTAGAGTGATTGCTGTGAACAGCACCTCTG

A_16_P00685074 AGAAGAACAGAAGACCTGCTGAAATCCAGGCTTTGGGATTTAGAACCTG

A_14_P129753 CTCCTTGTTCCTGCAGAGTTTATAACTAACTACCTGGGTTACTTATTGTTCAGGTAACAA

A_14_P110864 CACTATAATATATGGGCATTACTGAGAGATACTGCCCATATGTTGTCCTCGTAAGCAAGG

A_14_P120797 GCGTGGACAAAGAAAGGATCCTCTTAACTATTCAGGATGTGATGGTAAGATCAGTT

A_14_P137898 CAACTAAAAGATACAGAGCCTTCATTACAAACATACCTTTTGATGTGAAATGGCAGTCAC

A_14_P108257 TGTTGCTTAATAGGAAGCAGGAAAACTTGGCGTTGATAAGATAGGAGATTTTAGTCGAAA

A_14_P121590 CCAGACACATGCACGGTTCTTCAAGTGTTTATTCTCCGTCACTCAG

A_14_P130342 CTCTTCCTGACAAACAGTGACAATTCTGCGTGAAATAATTCTAGATTTCAAGACACAAGT

CUST_438_PI426210822 AACACATATATGAGGCCTGAGTTCCAAGGGGATCCAAAAAGCATCTTCT

A_16_P02845549

AGTTTTAAAAAGAGTGTGTTTACTAATGTGAAGTGAGGCTTAAAGTTCAAGTGTCCTGCA
CUST_2821_PI426210822
CAAATGTTCTACCTTATTTAATATGTTCATCCTTCTTGGCTGTCTCATTGGCAATTTCTC
A_14_P102923 CTAGCAGACTACAGGACTCAGAGAAAATAAAGTAATTGACAAGGAGGTTCTGATAGTCAA
A_16_P02597768
CATGCTGAGTCCCATAGATTCTTGACTTTTTCATAACTGCAAAACATTACACTAGACCAG
A_16_P21436291
AAAAAAATGACTATCTCCCTTTGGTCAATCTGTAGTGCACTAAATTTGTTGAAGGCTGTG
A_16_P16106731
AAAGCCCCCTGATAATGATTTACCAAAGGAACAGAAGTAGTATTAGACAAAAATGGCATC
A_16_P01760990
CACCAGACACTCAATAGAAAAACCCAAAAAACTTTTAGATGTAAAACCTCCATGACTTCA
A_14_P113141 AAAGACATTGGACGTTCTTTCTAATTTCTGGATATCAACCCACGGAACCACAAG
A_16_P21429110
AAAAGTTTTCCTAATGTATCTCATGATTTGCAACAGGAACTAAAGTCAAGTGGCTCTCAC
CUST_52_PI426210822 ACCTTGAGGAAATCACCAAAGATGTTTCAGGAGTCTCCCTCTGATG
A_14_P126341 AAAATACACATTCATGCTTACCCAATCTAAATATGGAAGGACCTCATGATCACTTAAGTT
A_16_P21070976
TCAGTCTGATCAGTTTATGCCTCTTCAATCCTCCAATAGTTACCTTTAAGATGAAGTGAA
CUST_3654_PI426210822 CTATTCTCAGAGCAGAGAGCCACGAATGGGGCACTCGGGGCCGTG
A_14_P202475 CTTGACGAATACGCAGTAATTATTTTGCCAGTTGAGTTTCTTCATTGGCAGAAAATATGC
CUST_4734_PI426210822
AAATAACATTTTATGTATTTTGCATGTTTCTCATAAACTGATTGTGCATTCTAATTATTT
A_16_P02832735
TCAGCACAGGTTATGAAATGACAAAGTATCAACTATACCAAGAAACCTATATCAGGGCTG
A_16_P00815132
AAAAACTGGGGTATTGATCATAAGAACTTGGAAGACAAGGAGTATACTTCAGAATGATCC
CUST_7726_PI426210822 CATATTCGTGGTCAAGTAAGGCCAGAGTCTAGACTGACATCAAATGCTTGTATG
A_18_P22842642
ATATTGTGGAAGGTAGAAACTGGAAAACAGCAAAGAGAAAATGGTTCCAGAATAATTGGA
A_14_P138324 ATTTACACCTTCATGTGGAATATGATCGTCTCAAATGTCTTCTAGATGCACGGAGTATAT
A_16_P20812079
AGATCTTGGCTATCTTCCATAACCATTCCCTAAATGACAATAAGAGAATGTTCTACGGAA
A_16_P00685092 GGAAAGACTGAGTGAACAGAGGAATGAACTTTGGATCTGGACCTTTAGG
A_16_P02771712
GCTCAGTGTCATCTGTTAGAATCACCATAGCCTTTATAATCACACCTAAGATACAGATCC
A_14_P118601 GAGCCACGGACGTCAGAATGTAAAATCTTAGAACTCAAATTCACCTAGTTTTTAAAAGGT
A_14_P128867 CTCTGGGTTGATCCAACTCTTGCCAAAATAAAACTTTTCGTAAAGATCCATCTAAATTAG

A_14_P118391 TTGTTGTGCAAATCCCCAAATTTCTCACTAATTTTTGTTTTTTTGTGCATAACTTGGATG

A_14_P130100 TGGTGCTTCCGCTTTATTCTGATTAACTTTTGGTTACACACATTGAAAAATGACCTAAGA

A_14_P103203 AAAGAAACTACAGCAGATTCTCAAACCACAACATCTAAGTCATTACAGAATCAGTCTCTG

A_14_P136535 CAGGGAACGTCTAATAATGGAATGAACTTGTCTGTAAACATCCAGTTTAGACATGCATC

A_16_P00685086 ATCAAAGTGCTGAACCTTACGGACAACACCTATTTAAAACTGAACGACGAAGTGAT

A_18_P22842487 ATTTGTTTAGTGGTTGGGGTAACACGGGTGGTCCTCACTCGGAAG

CUST_5921_PI426210822

TACAACTAAAAGTTGTCAACAGGCTCTTCTCTCCAAATTCCACTGAAATGAGCAAA

A_14_P139010 GTTCCTGGATAAATACCTCCTTCCTGCGAGCCTGGAACAAAATTAGAGAAATGTATT

A_14_P138234 CAGATGTCTTTTTCACAAAGCAGTATATTTTCTTCATCTCCTCGGCAACGGACATTG

A_14_P122814 TTTGAGCAACATATGTCTACTACATCCTACAGAAGAAGAGGAGATGCTCTTCTTACTCAT

A_14_P104168 CTCAGAATGACATATCCCTATGTGTTTGTTTTTTAGTTGACTTCACGTAAAGGCATGGTT

A_14_P133221 CAGGATGAAGACACTCCAGTTTTTCTTCCTTTTCTGTTGCTGGAAAGCAATCT

A_14_P109491 ACATGGAAAAACAAATCAGAAAGGGTCCTTGTTTTTAGACCAAATTAAAACGTTGCTGAA

CUST_3046_PI426210822 GAGTTATTCTTTGCAGGAGAGGACCTCAGTACAGGCCCTACCGTG

A_14_P136198 TTGTTCAATGCATGTGTCTGTGTGAATGATTGAAGGCATTTCTAACCCATTTTAAAGATG

A_14_P124713 GAAGACATGAATACACTGAGAATCTAACTATGGGTGTTGTGTTTCAGGATACCATATAGG

A_14_P200425 CTTCTTGCTGAGAAATTTCCTCTTCCTCATTCTCACTCATGTTGCCTGCA

A_16_P02836315

CCAGTGGGAAGTTAATTTATAAATAACTGAACTGAGAGAATCTTATCTAATGGCTCAGCC

A_14_P116285 ATATCATTAAAGGTATCAGGAAATGTTCCACTCATTGTCTGCTTCTCCCACCATATACCA

CUST_2138_PI426210822 GGAGCATATGCACATTTTCCCAGGGAAGCAGAGGAGGACACAATG

A_14_P108208 TACTAGTGTGCAGGTAATACTTTAGCATTTTGAAAAAAAAAAATCTTGGTTTGGGATACT

A_16_P16190995 ATGTGAAGTTATGCTAAGGAGATCGGGCTCCATCCTATAGGCAGGAGTA

A_14_P105441 TCTTTAAAAAGGGCTTGGATGTTGATGCTGAGGTCTTTTACAACTTCTCTTTAGAGTTTT

A_14_P135819 ATGAAATAATTCACCCACCAAACCTATATCAAAAAAAATCTGTCAGAATCACAAGTTGAA

A_16_P03604476

ACTATAAACTGAAAACTCTGGTTTCTGTCCAAATTTGCAGAGTAAATGTTGAAATGCCCA

A_14_P140001 CAATACTTTGGGAGGATGATGATGACATAGAAACAGAATTAGATCTGGATTTGTCCAGAT

A_16_P21596684

ACCCACACAGATATTTTATGACACAAACTTCAAGTATCTTCTTTGGACAATTTCTGGCAT

A_16_P19598566

AAGCCCTATCTATTGTATGCTTAGTGTCTAGAAGAACTTTTGTCAAATCTCCAAGTCAGT

A_16_P58424139 CTGATCTGACTTCCATCAGTGTGGATTGGTTTTGCTGATTCTAGAACTTCCTATGAAT

A_14_P117844 TAGATCCCGTATTTTACTGGCTGGCTTGTATTCAGGATCCTAATGATGCCTGAATATTTA

A_14_P134521 AGAAAACTGAGTATCCCAGGGCTTAACTTTTTACAAGAGGATACCAGGGGAAT

A_14_P129623 CTAAAAGAACTGAACATAAAAATCGACAGTGCTTTGCAAGTAAGTTTTTCAGAACTGACA

A_16_P17253728

AAGTCATCTGTTCAGTCTATCTTCTCAAAACATCTTTCTGTTTTAAGCCATTGTATGCCC

A_16_P17845058

AAAAGAACAGATGCCCTTACTTTCAGTCATACCTCTCAAATCCTACAAATCCTCTTATCT

A_16_P02035970

ATCAAGTGTTTTTCTAAAAATCCCGATCCATCATGTTTTATGTCAAAAACCTTGAGTGCC

A_16_P21588540

CCTCCAAATCATTTTTTTTTTGTTTAGATGGTTGGTGTGAAAAATCTAACATGCGTGTGC

A_14_P139847 TGACTCATCACACAAAGAACAAGAACATTTTCCATGTATAAATGTTCCTCTCTCAGTCTC

A_16_P19844746 AAAACCAGACCATGATATCACTCATTCTTGTGTTGACTGACCTTTAAATGCAAGCCT

CUST_2591_PI426210822 GGCAGAATTACACTTTGGAGTGGGAGCCAATTCTTAGGTTTTGTTAATAACCTG

A_14_P106362

ACAACAAATCTAACAGACTGCAGATTACTGAAAGTAAGTCTGAGTGAAAGGAATCAGTCA

A_14_P125774 CTCGAAGAAAGTGCTGTGACTGTTATGTTTGATAAAGTGCAGTTCAGCAACAAGTCA

A_14_P109612 GATTGTTTAAGGTCTCTCTTCAAAGATTATAAGAGTAACCCGTTTACTGCACCTAGTGAG

A_16_P20206128

GCTAAGACAATCAAGGTAAGCATAAAAACTGAAACCAGATGAGATTCTCTAGAGCCCATT

CUST_909_PI426210822 GTTGCACCTCCCAGGTTTTCCCCAGAAACAAATGATACTGGATGTA

A_14_P103549 AATGAATGTGGAAAAAAGTGAAAATGAATTGCCCAAGTGTTTCCCTTTTAATTAACTGGC

A_14_P135013 TTCTGATACAAATGCAGACAGTTTCCCAGTTGTCAAAATTGAACATGATACGAGGAAAAT

A_14_P101721 ACTCTCCTATATACTGGTGAGGTATAATTTCTCAACTAGCCTCACTGTTCCTAATGAAAT

A_16_P17148643

AAAAGTCAAGAGATATTGCATGTCTTCCTACAATTTGTCCTCTCACTAAAAGAACAGGTC

A_18_P13078500

AAAACATCTTCAGATTGTGAGACTAATACCATGAACACCCTTCATATTAAAGTCAGCGTG

CUST_4815_PI426210822

ACATCAATCAAACTTGAACACCATCAGCATGGGCTTTAATGAAATTCTCACATATGTATC

A_14_P105998 ATAATGTGTGCTGGATCCTGAGGCTCTTTTTTGTTGGCTCCTCATCTATCT

A_14_P200140 TCACCTTCAGTAGGTTAAATCCCTCATGCGATTTTTTACTCTTAACACATTTTAGGTCAC

A_16_P18923959

ACTAGATCATATGCAGACCTAATTTTCATCTGAGTCACTTCTATACTCTCCTGTCACCAA

A_14_P130452 GTAGAATGGTCAAGAAATCTAAAGCATTGGTTTTGGAATCACAAGTATAAGCCATCATGG

A_14_P114201 CTAATTAAATGGCAGATATTACTTGCAGAAAGTAAAAGAGAAGACTGACAGAGCCAGGTC

A_14_P137424 TAACTGCAGAGACCAAAATGTTAAGTCTGGAAATGTTTGGTAGGCATCATTGCAATAAAA

A_14_P124269 AGGTGTTAACCAAATGCCATTATGGAAGAACCTTACTTCATGGAAGCAGTCTTAAAATTT

A_14_P101825 TTGGATATCTCTTTCTCTAACTCCTTTAGTTGTCTGGCTTTCAAACAGTAGTTTAGGTGT

CUST_817_PI426210822 TCCTGCGGAAAGACCTGCCGGGAAGACCGCGATGATGAGTGGATG

A_14_P116810 ATCCCAAGTTCATCTCCCACCAGGACATTCGAAAGCGAAACTATGTG

A_14_P114331 GGATTGAAACTTTTCTAGAACTTTTAATAGACGAGCCCTCATAAGTAAGATCCAGGCAAC

A_16_P20389533
AAGAACCTTAGAATGTTAGAATCCTTAAAGTCTGTTGCTTTTATCATGTGTTCCCATGGC
CUST_4314_PI426210822 GTAGGTGCTCAATAAGTGCATGGTTGATTAGCAGGCCTCCTTGTTTTTC
A_14_P127429 CTTGTTGATTTAAAAAGTTAGGCTGAGCCTTGCTATGTCTTCTAGCAGACATCTAGGTAA
A_14_P118429 CTACATCTGCAAGTGTCATAAAGGCTTCGATCTCATGTATATTGGAGGCAAATATCAATG
A_16_P16172286
TCCAGGGGTTTTATCATAAACACAGTATGTCTTATCTAGGTATTCTTATGCTTCCACAGC
A_14_P123596 TGTCCTTGGGTATGGATGAGGTAGAAGCCATAAGACCACTGTTAAAATTAATATTCCTTA
A_14_P105140 CTCTTTATGTGAGACTTGAGTGGCTCTGAAAAGAGCCTTTGAGTTTTAAAGCACCTAA
A_14_P101201 ACGTGGCTCCAGTTTATCAGTATGAAATCCATTAGGCACCAAATGAAAATTACTATATGG
A_14_P121679 CAGTTTCTTTACTTCAACAGTGCACCAATATTCTTTCACAGGTAAAGTTTTGCATAAGGG
A_14_P106646 CTGTGACCCTGGAGATGAGGTTTTATCTTACAACATCCTCTACAGCAAGTG
CUST_1698_PI426210822
TTATTGCAGTGTCGAAACTTTAGAAAATAAAGGTAATCCAAAAGTAGGGATTCCCTGTTG
CUST_2566_PI426210822
TATTTTCTCATTCTAATTATTCTCTATCCCAGAGTTTCTAGCTGTGGCCTTGAATGGCTT
A_16_P15502969
AAAAACTTTCCAATGTCTGATTTCAAGAAATTCTCCAAACTCATATCCAGCGACACTCTT
A_14_P126882 TTGCAGTGGTGGTCAAAATATAACCATGATGGGCAGAAATTTTGATGTAATTGACAACTT
A_14_P115877 AACGACCTTAGTGTTGACTGTCATTGAAAATTGTTGAATTAGGTGTTCCTGAATTAAAGG
CUST_6515_PI426210822
TCTCCCAGTATTCTAAAATATTCTAAATGCTAAGGAAATAAACACAATTTTATTTATAAT
A_14_P109447 GAGGAAGGAACTACTCAGGGCCCATTAATTAATGAAAAAGCGGTAGAAAAGGTAAGTATA
A_14_P135636 CACCCCCAAATGACTTAAGGGTTAAAGATTATAAGGAAACAATTTTTACAAGGCAGTCAT
A_18_P15661368
CCCCTCATTTACCTAATCTCACTTTTCCATATGTAAACCAATTTTAACCAGAAACAACGC
A_14_P124242 TCATTACAGAAATGCTGGTGTAAGGTGTTCAGAAGACAATGGAGAAAAAATGGAAATACT
A_14_P119620 GACTTTCTTCTGGTGTATGGGGATGTCATCTCAAACATCAATATCACCAGAGC
A_14_P116938 TGAATTACAATAAAGATCCCATTAATATACCAGTCTTGATGACTGGAGGGAACCAGGATA
A_14_P122923 GAAGAGGGAAATCCTCATCATTATTTTGTGGCAACACAGGTGATACTACTTTTAAAACCA
A_16_P41654165
GCATACTTCATCACATGAGTAGACACATTTGTGAAACGTATTTACTAGACTGGTGCTTCA
A_16_P19518122
AAAAAGTCACAGTTACTAATCCTGATACTGGCCGATATGTCCATAAAGATGAAACCATGA
A_14_P131803 CCACATAAAGAGAAAACCTATGGCCTCAAATTACTGGTTTAATCATGTGATTAGGCAGAA
A_14_P121875 GAGATCCTGTTAGCCCAAACAGCACTTACTTTATCTTCAGTCTAACACTACTGCAAG
A_14_P113969
TTTCTACTCCGTCGACACTAAAGAAAACAAACAACACTGAAGAAAATAAACGGAAACCAA

A_14_P104285 ATAGTCTGCTTCTCTCTTACCATGACGTGATGGACAAGGTATTCCCAAGAG

CUST_6733_PI426210822

ATCTTCTCTGTCATCACACAGTCCATTAGTTATGTATTAGCTTCTAAGACTGAATACAAA

A_16_P16204734

ATCCTTAGACTTCCAAATTGTCTCCATTTTAAGGTTAGAGCATCTAAAAGCAAACCCAAG

A_16_P15081431

CTCCCTAACCTGACATTCTAAATACTGGTAATCAGACTCTAAACTACCTCTTTCTCTGAA

A_14_P115649 ATGCAATCCTATTCTGTAACTAAGGACACAGGAATAGTGTTTCTCAAACAGGACTCTC

A_14_P200876 TTCTGGAGCACTTACTGTATGCCAGTTGTATGTGCAGCATGTTTTCATATGTAAAATTGT

A_14_P104264 CCATCATAGGGGATATGCTTTCTAGGTTCATTTAATCATATGGGGTTATGCTTTTTCTGT

A_14_P108821 TCAGTGTTCTAGGTTTGATAAATGATAATGCTGCGTGCCAAATCTTTTATCCCTATGCAT

A_16_P17440701

AGGCACCAGAACTTATGTATCCTGCTGTTTAGTATGGAACTTATCCCATTAAAAACAACA

A_16_P16602563

CAAGTAACTGAGGACAACTTCATAAAAAGAATGTGGCTTATGCTAAGAAGAATGCAACCA

A_14_P114623 GGTTAGAGATCTATCTGTAAGGTTCACTTTATGTTAATAGCATTCAGAGCAGCATGAAGA

A_14_P111615 TGGAAATGAACCCTCTACAACAGGAGACACCATTACTCAGTAAATGCACATGTAAAATAA

A_14_P127786 ACAGAGAGAAATAGCAAAGGCTGTCCATTTTTATCAACTTCTTCTGATTTGACTGTTGTT

A_16_P15637844

AAACAGTCTGACTCTTACTGTGATTATTGCTACCCTAAGTTCCTATAGTGCTTTCTGAGT

A_16_P18562998

ACTCACTGTGTCACTTAACACTGTTAAGAACAGCAATAAGGATTATAACTTTTGGGAGGA

A_14_P119754 CTAGATTCTCATGAAGGATTGTTGAGTCACTTTTACCTGGAAACTTTTGGTTTGGAAATT

A_16_P16976433

CCTCAAAAAACCCAGACATTATCACTATCTCACCTGCATTCTAATTTCTTCACCTGATTT

A_14_P115207 ACAATGTTCTGCTTTTTGTCTTTACCTATCCTAATAGAAGAGGTTCAGAAAACTCTCACG

A_18_P22840296 GGCCTGTCGTGAATTCTGTCACCTTGCTCAGTCTCTTCACATCTT

A_16_P16486376

AAGTATACTCTTACTTGTCTCCATTAGTCTCCTTGTAATCAAAACTATCACCTAGGCCAG

A_14_P135406 GTTCACATTCTTTTCTTTCTCTGCAGGTTTTGCATGCAGAAATGTGTCAGTCTG

A_16_P01099558

TTTTACCCATCTCATCTGTCTAAACTCCACCTAACCTCTAAAATCAACCATATTTTCCTG

A_16_P19060366

TTTTTCCCACTAATTCCAAAGTCTTTCACCAAATTTCTTTATAATTTGCCCCTACGCTTC

A_14_P109870 CTTTAGAGTAGAAAATTGCTATCATTAATTTTCCTTCAAACTTTCTCTGCCCCCTCTTGC

A_16_P00685012

TCCCTCCATTTACTGTAACCTAGGCTCTCCAGGATAAAAATTATTATTGCCAACCTTTTA

A_16_P00367343

ATGTTCAAAACATGTATCAGTGGTTACCTATGCTTTCGGACAGAATATTCCTACAAGAGT
CUST_8783_PI426210822 CTTTGGACAGAATACAATCGAGATGTCTCCCCAGTACACAGAACTGCT
A_18_P26979724
ACAAGTTGCATATTCACTTCTTTATATATTTGTTTTCACGTCAAGTTGTCTACAATGGCA
A_14_P106802 TAAAGAAAATATCTCTGTTATAAAGAATCAAGCATGAAAATAACCACTTCATGAAAGGCA
A_14_P110184 ACAAAGAGTAGGATAGATTTGTTGTGACTGGTGGATTATCTTCTAATGTGAGAATTGTAG
CUST_1517_PI426210822 ATACTAGCCCCAGGGTCCTTCAAATGAAACCCATCCTTCTGACTC
A_18_P14134547 ATAGGATGTCTGGGACATGGGAGGAGGCCATGTGCCTCCTCAATT
A_14_P127297 ACGTTGTTGCTTGTGCATAAATTATACACAATAGCATAAGCATGCATCTCTGCCAAGATT
A_14_P202521 GGCTGTTTTTATTGGGGGAATAATTAAGAATTGACTCAGTATAGACATGGAAGTGAGGAA
A_14_P200477 TTTTGCTACGGTTACAATTTTGAAATATTAACTGAGCCTCAAAATCACCCTTTCTGTCAA
A_14_P127032 TTAGTCACAGGTCTTTTATTATTCACAGCATAGACGCTTAACAAAGGCTGGGCAAATATT
A_14_P115199 TAAAGCCATCTCTAACAAACTCTGATGAAGAAACAAAGCAATCTCCATGTCCAAAAGAAA
A_14_P133315 AGTTGCAGCAATTGTTATTGTTAATTGTTTACCTACACTTTTTCTCCCAAAGCAGGATGT
A_14_P129242 CTTTACTACCTTCAGAATCGTGAGCCAGATCTGTGTCCAAAAACTTCCTCTTTC
A_14_P111986 CTTCATCTTCTTCCCCTTTGGCTGCAGATCCTAATTATCTCATGGCTAATGAAC
A_14_P114782 CTACGTTTCACTGGTATAATGCGCCTCCCAAGAAACTGGTTTAGAAAATTACGTCAAA
A_14_P123620 CTGAACAAAACCATAACTTCACAGATCATTATCTAAATGTGGCCTTTGACCTTTCTCAAG
A_14_P105149 CCACATCTCTCTTTGTGAATTCAACCCTTTCCCCAAAAATCCTAGGCAAG
A_14_P138127 AAAATGGCTCACAGATGTTCTGTCCTCAGAAGATATCAGAAAAGTGGAAATAAAGGTGTA
A_14_P119359 TGCAGATTGACTTTTACATGCTGTGTAGTATTCCTTAGGTGCAATAGATAGAAAGTGAGG
A_16_P18736282
ACACATTTTCTACACCAAAACAGTGCTTTAATTGCAGGTTACTTTTTCCAGTTCACTTGA
A_14_P134871 CTGGTTGCTCCTTTCCCTAGTTTTGCTCATTTAAGCCAAAAAAGTTATTTCTGAGAGAG
A_14_P118598 GGGAGGTTGTTGGGTAGCAGTAATAGAGGTCATGGTTGACATAATAGGACTTTT
A_14_P111011 GGAAGTGTTTTGCGCTCTTAAAAATAACTCTTATCTCTCAAGGTCTTTCCTTTTGTGACT
A_14_P133200 GTAAAGAGACATTGCAATTCCCACCTGTTGATTTTCTTTGAAAGTTTGGATTAATACTTC
A_14_P117632 AAAATTATGCAATGTTACAAGGTCATCCAAGACATCTCTCCTACAGAAAGAAAAAAAGGC
A_14_P129972 CTGTGGTTTGGTAAATTTTAGGCAGAGAGAAAAGAATGAGCAATGAGATAAAGTCTGTTG
A_16_P02271147
ATCACTGCTCTGCATTATAATAGGTGTGCTATAGTCAGGTAAAGGTAAGGATTGCTGTTT
A_14_P200371 TTTTTTGTGTTCTGTTACCACATTTTCTCATTCTCTTCAATCTCCCAATTCCATGTCCAT
A_14_P201795 TTTGTGAAGTGCCAAGGTCTGGAAATCACTGACCTTATAATCCCATTTTTTTAAATCTCA
A_16_P01654685
TCCCTCAACTTAATATTGTTCCCCAACTTAAGATTGATCCTTCCAACAGATTTATACCAG
A_16_P20760745
AGAGTCTGTTCATTAATTAGCATGGACAGCATAAAAGCAAAAACAATGACACTCCTGTTC
CUST_10147_PI426210822 GGAGGGATAACAACTTTGTGTGTGACTGGCACCTTTAAGAAATACCTTCAG

A_14_P109947 TCCATAACATCTTCATTGTGTGATATACTTGGGGAAAGTTCATAGTTCTGGGTCTCTAG
A_18_P10219562
GACTCAGACAAACCTATGACCTCACACAAATCTGTAACTTCATGTATCACAGTCTCTATA
A_14_P107544 ACATTGTTTCTGAAAATTTTTTCAGTGGGTCAAAACTGAACTTTTCTGCCACTGATATGT
A_16_P03809624
TTTCAAGAATCACATTTTGCCAAAATTGCCAATGTTTGCAATTACCTGTGACAGGAAATT
A_14_P109995 TCTAACTTTTGATGAAACACTTACTATTCTTAGGCAGCCCATGAAAACTACTGGAATGAG
A_14_P133874 TATGTAAGCCACAACAGCAGACGTCCTATCCTTTTGCTTTTGTTTTTAAGGGATCAAAA
A_16_P01933619
TCCCCAACTAATTTGCTTGTAACCTTGTAACTGGTTAAACTGATAATACCTCACATGTCT
A_14_P128099 TGCGTAAATTGCAAGGCTGATAACTCTTATTGTTAATTGTTCCTCGATTCAACTCAGTGA
A_16_P19743041
AAATTCTCCTATTCCTAGGTAAACATGAATACTGCTTACCCCTGTAGTTTATGATCAGCC
A_14_P102970 AAAACACTTCTTGTCTTTGACAGCAGTTCGAAAACTTGAGTAGGACTGTTTTATAAGAGT
A_14_P109469 ATCCTCGTCTTCTTCCTGGGCTTTTATTGAGACAAATTGCCCTAATAAAACAATAAGAAT
A_14_P119937 AACACCAGGTCAACTTTTCTTCTAGTAGTTTTGTGACTTTGGTTTTCTAATGCTTACATC
A_14_P122500 GTTATTACCTGTTTCCTAATGGCCCTGATCCTGTTTGCTTCTTCTCCTTC
A_14_P105431 AATCCAGCAAGTTTTAACTTGGCCAAAATCCACCAATCTTAAACTTTGTGCACC
A_14_P202034 CTTATCTTTTGAGTTTTATATTTCCTTGTCTCCCTCTGTTGCTGGGATATGAAATCTCCA
A_14_P130689 CAGTCCCAAACCTAAACCTCGGGTATGAGAAAAGCATTGAGATCTAGCAATAG
A_18_P17046274
ACTGTGTTGCTGTAATGAGTCTTAACTCTAACTACTTCTCACAAATTTTTGAGCATGGGT
A_16_P03803015
GTCCTAAACAGCATATTCCACAGATACACTATTTTGACACAGGAAAGTAATAAAGGGTCA
A_14_P134791 TGGAAATTGAGAATAGTTATTTCCAAGGGGAAAAATACATGGACACTCTTTTGAGCAACA
CUST_1076_PI426210822 CTTAGAACCTTCAAATGCCTGGGCATCGAGGACAGTCACTAATAGTGATT
A_14_P137206 TGCCTAGCCATAAAACAGGGTGTTTTAGTATCTATGCAATCTCAGCCGAACTTAAAAAAA
A_14_P201738 CTTCCAAGTAGGTAAAATTCCATGAACAGACAATGTTAATTTTCTTAGGTCAGCATCCTG
A_18_P22839213 TCTTGAAGTGAGAGCATAAAGGAATGGAGGGCATTTGGTTGCCATTTC
A_18_P13171323
AAGGACAACGAACTAATTTAAAGTTACAAGGGTATTGATCAGAAAAAGCACTGAGCATCC
A_16_P00925579
CAGGTGTCATTAATTTTCTCATTCTCTCATATGCATTGAGTAACTACCATACAGCAAGCA
A_14_P113120 TGACCTGGTTTGTGCTGTTAAGTTTTGAATTTGAATCAAAAGACTAAGAAAAGATGTGCC
A_14_P123886 GAATAATCTTCTCTCCTTTCAGGCAATGAGACCATCTGCCCACCAAATATCTACAT
A_16_P19343484
GGCTCAAACGATATGTCAAAAACATTGTCCAGATAAGTAGTATAGGAGTTTTGAGGAAGC
A_16_P18447102

AAACTCAAGTTCAACACAAAATCCAAGTGATCAAAATGTTTAACCCTTTTATCCGCAGTT

A_16_P58424348

CTTGTGTTTCTTGCAGAGTGGCTAAATTCTAAGAACAAATGCCCCAAGAAAATTAGGAG

A_14_P123237 TTCAAGCATGATGATAACAGTTGATGATGTTCAAGCAACATTTAGCCAGCATTGCAAAAT

CUST_3571_PI426210822

GGAGATTTGTGTGAAGAGGGTGACAGAGAGTTGCTTCTTTTAAAAAAAATGTATTAAGGG

A_14_P200219 TAGTGCACAGTGACTTTTCTGTTTCTTATCACTAAAGACTGAGGTGAGGTTATGAAACTT

A_14_P112055 CTAAGAGAGATGTGGAAAAGCAACTAACAAAGAAATATGAGCATGTTTTGAAGTGTCGCC

A_14_P131249 TCTGCCGAACACTTACCACAGATGAAACAAGTTGTCTTTAGAATTTCTTCTTTTTTCTGT

A_16_P19525074

GCCTCATATAGTCTATTTTCCCCAACCATTTTGTTAGTCTACACAGGTTCTAGGATAGTT

A_14_P114725 GAAGACAGAGAACACGCATGATTGATATGAAGGTGTGTAATCAATCAGCATTTTAACTTC

A_16_P18544942

AAAATACCTTCTCAGATCCAATAGTAGGCAAATTTGAGTAAATATACCCATGCTAGGAGA

A_16_P16686880

GCCTGGAAAATAAATGTCAATACTTGTGGTAGTTTCTGAAAATTTAAAAGTGCTAGCCCC

A_14_P123257 ATTCTCATACTGCTTTGTGGGAATAAATCATTTTCTCTGCTTACTTGAAACAGCATCGTC

A_14_P117246 GGTGCTTAATTGGTGAGTTCTCTAGATGCCTCTAGTTAGGCATTCATCTTTATTTATTGG

A_14_P105986 ATACCACTCAGATCAAGGCTCCTTATTAACTTGAGAACAGCCTCTCTTTCATTTTATGTT

A_14_P107574 TACTACCAGCAAGTTGATCAAATTTAACTATGTAGCAGTAACCAGACACACTAGACAGCA

A_18_P22839229

GGCAATATAGTGTCTGATCCAGAAAGCAAGAAAGAAAAGGCAGAAAGTCTGAAGTTTTAT

A_14_P112775 CAGCAAAGAGGGTAAATAGTAACCGAAATGTCAAGTTGTCTCTGGCTTAAATTAAGCAAA

A_18_P14132755 CCTGCTGTCTCCTGGCTACTTTGGACTTTGGCCTCCGCTTCAGTT

A_18_P22839978 GAGTCTACCTCACAGGTGTGCCTGATAAAATTTTTTTGGGCAGTGTTTTCCTT

A_14_P112490 CAAGAAAAAGAAGAAGAAAAGTTCCAAGAAACACAAGCGACGCAGGTATTGTCCTTT

A_16_P36510870

TCCTCCTCTCTCTTATGTAAGCGATAAGCACCATTAATAGTCCTGTCAGTCTTGATTAAT

A_14_P103479 CTTTTATACTTATTTATGATGGCCTGTGAACAACTTCAAGGATTTTCTGGTTGGAGACAG

A_16_P18449335

TCCCCATTTTACCTATACTTGCTACTACCTAATTAGACTGCTCTACATTTCTATCACACT

A_16_P16191222

TCTGGTTAGCAATCATCACATCCATTTAGGATTAGGACAATTCACACAATAGAATAGGGT

A_16_P15932387

AAGTAATCACGAAGAGTGTAAAGTCTTTTGATTGGTAAGTCTGGAAAAACCACTGTTCAG

A_14_P138868 TGTCATAGATGACTCAGGGACTTCAGGACAGGATAAAACAAAACTAAATACTCCTTTCAA

A_16_P00327646

ACAAGGAAGCAGAGTTGTAAATCGGTATTAGAATGTGATCAAAAGGAATGTCTGTATGCA

A_14_P132687 ATATCGAAAGGGCTGAGGTTCATCAAACTTTCAGAAGCCTCTTCTCCTAAAAACAAATA
A_18_P14139256
ATGTAATAACTTCAGTCAACAGTGATATCTTTTCCCTGAGTTTCTCTTGCCCCTTGATC
A_14_P121499 TATATAAGCAGTCTGGTTTAAATACTTACCTTCAATCTGGCTAGTTTCTGAGCACGGTTT
A_14_P108265 CCTTGAGGGTGCTGTGACCTTTTACATAGGGTAAAGTTTTTTAATGGCACTTGATTATTA
A_16_P15263482
AGAAGAAACTATGAGTGAAACGATGAGAAAAACCTAATGCATGATGTAGAACTGAGTGGT
A_14_P137814 ATTTCCTTACTAGATCCAAAGGCATACAAGACAAGCAGGAAATATATAAGGAACTCCATT
A_14_P115019 CATGTGAAGACCAAGAACCTGCCTTTACATCCTGCTTGACTAACAAAATCTAAATATGAA
CUST_3862_PI426210822
GAGTCTCACAAAACTACACCCATGCTGCCCATGGTTTATGTTTAATGAATGAAATG
A_16_P17063420
AAAGGGTGTCTAAGTTCAACACAAGAGTTCAACAAGAATCATAAAATGATGGCTGCATAG
A_14_P115687 CTCATTTCTCCGGTTTATATTTGGTGCTATCAAGGTGTATGCACTTGTGCTAAAAGGAA
CUST_8745_PI426210822
TACAAGAACAAATTAATCTAATCTTCATAATAATTTCTTAAGGAAATTGAGGCAAGGAAG
A_18_P25505095
CGGCCTATCTCCCTATTATAAGACATTGCATTACACTGAGTTTGTTCTTTACAAGTGCAT
CUST_10401_PI426210822 ATCGAAGCTGAGAAGAACCGGAAAGAGAATGAAGACAGTTGGGACT
A_14_P201554 AATGGAGTTTATTAAAACTCAAGCCCTTAAAGCACCATGATGAGTGACAGAAAATTACCT
A_16_P00654876
ACAATGACTGTGTTACTGTTTTACTTTTTAAGACCAGCAACCATTTTATAGTGGGGACAC
CUST_3671_PI426210822 GTTAGCCAGTTTCCTCAAATTGGTCAGTATAATCCTACCTCTTGGAGCGATAG
A_16_P02581612
TCCCAGCAATTAAACTAACTAACTCAGATCATCAGAATAGGTTGAAATTTCACTGCATCC
CUST_7365_PI426210822 AACTCAGCTGTCTGGACAACAAAAGTGGGAGCTTTTGTTGTCTAGAAGT
A_14_P134867 ACTGATGTCAGAGTTGCCAGCCAAGATAAGATTGCAAAAACAATTTTTTAAAGTGGCATA
A_14_P201487 CATCAACTCATATAAGGACAACAGCAATGATTTCATATGCCCCATCTGCTTTGATATGAT
A_16_P20289671
CATCCAAAACTTTATAGGAGACCTCTTGTTCTTACTTCAGCCTAAGACAAAAACCAGATA
A_16_P15612304
AGTGATTTGACTAGAGACCTAGATAATGATTAAGAGCAAATATCAGCAAACTACTCCCCC
A_14_P132854 AGTATGTCCTATGTGGTCTCCTCTTTTCTTGTAGTGAAAATCTCTTTTCTCTGATGCTTT
A_16_P02232668
TTCGTTGCACCTTTTTTACTTTTATGAACTCAAGCGTTACACAAATTAGGCTCCTAATAT
A_14_P114712 CTCTCGTTCTGCTTTAGAGAGGAACTTGGGCTACAAAAGAGATTGGAACTG
A_14_P135689 GTCTCCCTTTTTAAACTCATGAAGGTATCTCCACATTAGATTCCAAATCTGAGAACTTTC
A_14_P110580 CACACATGATTCAGGGATAATAATGCAAATTATTGATGTTTCCTGGACATTTACAAACCA

A_14_P136576 GATGTTTTGTATTCTTAGGTTGTAAGGGAAGCCATTTTAGAAGACTTGCAGAAAATTGGG

A_14_P139671 TTATTTTGTCTATTATCCCATATGTAGGCTATCACATTTTGCCTCCTAGCCTTTGCCTAC

CUST_8422_PI426210822 TATTGCAGAAAACCAAAGACACAGTTTTCTAGGGAAGAGCTAGCTGGAGC

A_16_P17715305

AAAGCAGGGCCAATATACACATCTCTGTAAAACTTTTTGGAAGGTATATCAGTCTTACCA

A_14_P136203 CTACTGCAGCAGAGAAAGAAGAGTCTCCTTCCTCTTCCTCATCTGTTTTGA

A_14_P111863 CTTTATGAAGTCCAAGTCAAGTTTATGATTTCCCTAATATCCTCACAAGGAATTGGGATG

A_14_P128853 CATTAGAAAATTGGAGAGACATGGTGTTTTGAGAAATGTTCACCTTGTCTATGCAAAACT

A_16_P15347299 AAGATGAATACACTCATTACTGGTTTCCCAACATGATATAAACCTAGGACTTCACCCC

CUST_5556_PI426210822 GAGAAGTTCTGGGAGAGCAGTTCTTTCTTATTGTCCTTTTCCTGATTATGGG

A_14_P129203 CTTTTTATTTAAGAATATCATAATACCTTACCTACTGAAGCACGAACTGTTGTAATGTCA

A_14_P137098 TTCATTGTCTACTATCCTCCAAAGAACATGCTGACATTTTTAATTTGATGGACTGCTGAG

A_14_P124779 CTTTGTGCAAAGCCACTTTCAAGCACAATATAGGTAAGATGGGGATGTGTTTAGAATGTA

A_16_P02393380

AAAGGTTAGCAGCAACACTTTTCCTAGTAACCTAGATCCAAATCTCTAATGATCCCATAG

A_14_P134827 TACATAACCCCATGCTGATGGGTTTTATTTAGTATAAAACATCCATCAAACACCAGTCTC

A_14_P118078 TTGGGCAATAGGTGGGCTCCAAATAAAGTTGCATAGAAACCAAGTTTTTATATGTCAAAT

A_16_P00733745

AAAACAGCAAGACTAATCCTATTTCCACTTCTTTATCCACCTTTAAGAGTAACCTGCTGT

A_16_P00685143 CTTTGCTTTTATCTCAGAGGCTGGCACATTTGTAGGCAGGACTGTGAG

A_14_P103493 ATAACTTTTCGGGGAATTATAATACTGTGCTCAGCCAAGAAAGCAAAATACCAAAAACCT

A_16_P21198866

AAAAAAGTGATCCCTACCTTATCTCTTAGTGATTGAGTCAAGAAATTATCGTTGCCCATT

A_14_P118274 TATTCAAGTCTGATTTTGGATTCCACTGGCTCTACTTTATTTGCTAATTGCACAGACGAT

CUST_9270_PI426210822

GAAGAAAGTCGTACTATAGACATTTACATGCTGCTGAACACAGAAATACTGAAAAAGGTA

A_14_P103252 ATTCCAAATCTCCATTTTTGTATGCTGGCAGATATAACAGTGCTCTCTAGTAATCCTTCT

A_14_P121733 GTTTTTTCCGGAGGAAATTAAAGCAACCAGGCATCTGTTTATAAATCCCTTTCACC

A_16_P00533794

AAGTTGGACCAATTATATGCTTACCTATGAGAATCTGATTCGGGATACAGAATATGAGGG

A_16_P01973952

AAGCCACACTCCTATCTTAATCATACACTCAATAGGCAAACTCAATGGATAAAGGGAATT

CUST_5354_PI426210822 CGCAGCCCCGAGGAACTCTGAAGAAACACAAGCTTCTGTGGCCCC

A_16_P20432194

CAGAAACTCTCAAAAACCACAAATCATTTCTTGTTCTGACAACCTGATCTACCAACTCTG

A_14_P117788 TTTCATTCTATGAGAAGAATTATGTGTGTGTTGGTAAGTCGTCCATAGGTCATACATTTC

A_14_P118257 CCCAGTGACTTTATGCATCTTATAACCAAGAAGCCTTCAGTAGAGCAAGTCTGAG

A_14_P125676 GGCATTCTCTTACAGATTTACAAATACCGTTACTGCATCTAAGAAAATGAGCAGCATTCC

A_16_P20561919
AAGGGACCTGTCATATTCAGTAAAGAAGCGAATTGAATTAAAAACACAAACCTGACAGAA
A_14_P116338 GTTCTCTAACACTGACCCACATGTAATATGTTGTCAGATGTGCACATGTTTTTAAATAGT
CUST_250_PI426210822 AGCACTTCCCAGTGACACCGCCGGTGGGGCAGGTAGTTATCTCTT
A_14_P129356 GAAGTGGTGATTATCAAAGAAAATAATGTGCATTTGAAAGTTCAGGCTACCTCTTGTGAG
A_14_P138003 CCTTTCCACATTGTTTCTATTTCCTTTATTTTGATGAACTGTTCAAATGTCATGGTAAAT
A_18_P15711528
ATAAAGTGGTTCAATTTTAGCCTTTTTTCTAACACCTAGCAATGTATGACCAGCATCTCC
A_18_P14134932
GTAGAGTTCAAGGAAAAGTTAGGACAAGGAAAATGAGTCTTTACAAAGCAACCAATTCCT
A_14_P112355 TGAGGCTCTCATAAGTGCCTACTATATTTCAGGGCACATGATTTTACCAAAGTATGTAAC
A_14_P125931 CACATATGTCTTCTTTAGTTCTAAAGCCTCTAACTGGGCTTTGATCTGTTCAAAAGTGAT
A_14_P112780
AAAAAAAACTGAATAATCATTTTGGGGAAAACAAAGGACAGCCTAGGGAGACTAGATACA
A_14_P137185 GAAGGATAACACCTATTCTCTCCTTACCTAACTCTAATCCTAATAGGAGCCAAATGAGAT
CUST_2363_PI426210822 GAAGCTTGTTTTCCTGGACCTCGGAAGCTAATCACACAGTGAGGC
A_14_P116399 ACCCTATCATTATATTAAAAGGTTAAAAAAAAGAGCCCACTTCGGCAACCTGTAAGAAGT
A_14_P118385 CATTATTGTCTGCATAATCTTTAGATCCAGCACACACTTACTGATGAGATACTCCTCAGT
A_16_P17622657
AGCCCCATGACTAGTAGAATAAATGTTAATTGCAACTTGTCTGATTCATTTCTCCTACCT
CUST_2842_PI426210822
GTGAAGGTAAATTGGCATAGCTGAAGCAGAGAAGAAGAAACCAAGATGCAAGTTA
A_14_P126209 ACATTTACATATTCTGGGACATTACTCATTTGTTTCCTGGCTTTTAACAGCACTGCAAAT
A_16_P18430148
AACTGATGAATTGGAATCAAATTATGTTACCCGTCTTTAACTAACATCAGGTGGAGTGTC
A_14_P128659 CTGCTAGGTGTGCACTGATACATCTGTATTTTTGCAACTGTTTCTTTTCCAGTCAAATAT
A_14_P138935 AACCAGGAAAGCAGATGTATCTCTGGAAAAGGAACTCCAAGGAATGAGCATATTTA
A_18_P22842086 AACACCTAACCTAGAATTTCCCTGTGGATCACTCACCCCCAATGATG
CUST_9884_PI426210822 AGTATTGTGCCCAGTCACTGCTTGGGCTTGAGCAAGGGGCTGGAC
CUST_4902_PI426210822
CTTGGGCCATATGCTTATCTGAGCTATAATATGATTCATATTGAAAGGAGGGGGACT
A_16_P36230785 ATGCTCTGCCTGTGATGTGTGGTGGCCTCTGGCATTGTTGTAACT
A_14_P201360 AATAAGTTCCAAAGTTACCCAAAGTCATTTCCAGTTCCCTCATATGTCATTTCTTTTGAG
A_14_P108511 TTCAACTGTTATTTGAACTGTTATTTTCAGGTCAGCATCAAGAGCCAAATGTGAAAGTCA
A_18_P22841250 TGGTTTAGGGCTGAGGCTGTAAACCACAGGGGATTACCTATGTTTATCT
A_14_P104179 GATGAACTTACTCGTGCTTTTTAGTCTCCCGTGTGAACTGAATTGTTCTGTATTTTATCT
CUST_5712_PI426210822
AATTTGTGGGGGGAAAATGCATTCTAGGCTTCTGAAACCCTTTTTAGAAGAAAA

CUST_4350_PI426210822 TCTGTGACTGAGGAGACGTGCCATGATGAGAGTGATGATGGTGAT
A_16_P16566768
AGGAATTCTCAGATTGATCTTTTGGCTGCGATTGTATCTAAATGTCACATTGTTCACTTG
A_14_P111192 CTCCTTTTCTCCACCAGCAAAGTCGACCAAGAAATTATCAACATTATGCAGGATC
CUST_2877_PI426210822 GATAGGAAATGAGGTGGAGGTGAAAGGTCAGGTAACTGTGGGGGC
A_16_P19250633
TTGGAGTTGTGACTAAGAAAGTTAAGTATCTATCTGAGTGAGTCATTTGAATGGCAGAAA
A_18_P14133518
TCTTACGTCTCTCTTCTCGAAGAAATTATTCTTTGTTACTTAATTCAATATTTTGCAGTC
A_16_P21619059
AACAGAAAGTGAGCCATTTAAATGGAGTCTACAGTAAAAATGATGGAATGATGATGCTCC
A_14_P116975 CTGATGAATTTACTGTCTCCCAGGAGATTCTTGGTTCTAATTCACTCCTCATTTTCTGTA
A_16_P30952623 CTTTGTATCTTCCATATTTGGAAGAAAAAAATCTTCTCCAGATTTTTCGGAGGCCCC
A_14_P116015 TATCCCCGGTTCAATTTTGTATTTCTGCCCAATTTTTAAATCCTTGCCATTCATTTCCAA
CUST_7536_PI426210822
AATCCCAGAGAAGGCATTTTTTTTCTCACATACTCTAGGTCTCAATCTCTTAACTTCAAA
CUST_4735_PI426210822
TGCATGTTTCTCATAAACTGATTGTGCATTCTAATTATTTAGTTTGGAAAATGTAGTCTC
A_14_P117075 GGACTTTGAAGTTCTCTTCTGAGAGAACCTCATGAGAATATTTGCATAAATTCCTGGAAG
A_14_P134152 AAACATAACGTTACTTACCTGAAAGTCATCGTAAGGGAAGAGCAGCATCTCCC
A_14_P119625 AGAATCTACTAATTCTGTTTTCTTAACTTGTGTCCTACTCTCCTCTATACCTCTTCTCCC
A_14_P124139 TTAACTTATTTGGAGACTTAAGTTCTTGATTTATAACATGAATCAAATGCGAGAACCAGG
A_18_P26792484 CTCTCCTCTCTCCCCTGCACCCCTTAACTCTGGTCCTTTGCAGGA
A_14_P109222 TTCTAAAAGGTTTGCTCTTTTTAGGTTGCTTATTGAGGGCATCTCCTAAATGTATGTTGC
A_14_P125156 TGATCTCCAAACCTTTTCACCCAAAATTTTAGAAAAAGAATTAGGAATTTCAGTTGCTCA
A_18_P12742892
GAATTAGAATGCCAAACAAAATCATCCTCTCCTTATGGAAGTTAAATTCCCAAAGCAACA
A_16_P15093810
AACGAATGCATGTATTGTTTACTCTTTGCTTCCTATTGAATATTTGCTCCCTCTTGCTCT
A_14_P112588 AAGCCTGGGTCAGTATATGGTTGACATTCTTACTCTCAACACCTTGGCT
CUST_1007_PI426210822 GTGGGTTTTGATGGCCTCTCAGGTAGGAATGAGAAGTTTTAAGATGGAAAA
CUST_6769_PI426210822 GGATAAGTGCTTAAGGTACATGGATGAGCCTCCAAAGGATCTGATGTCA
A_14_P109667 GAGTGTCTCTGCATAACATACATCAAGCAGCCTTTTTCTTTTTTTAAATCAGAGATGCCT
A_14_P120082 TGGAGAAATGTATGAAAGGATGATCACAAACATGTTCATATTTTCACCAGGATGGTGAAA
A_14_P112405 ACCTGATTGACAACCTCAAAATATATGGCTAAGGTTGTAGTGGGACTAAGTCCACATAT
A_16_P17683006
AGTGATATGTGCTGTCTTCCTGAATAACACAATGAATAACATCTAAAACTCCTCCCATCA
A_18_P15652474

AAATACTTTCTTCGTCCTAGTCACTTTTTACCCTCCTACTTTGTAAACATCCTAGAAGCC

A_14_P113479 GGGCTACTATTTCACATGTAATTTATTCCCCCATCACTGAAATTCTCAATGACCAATGTA

A_14_P122940 AAAATTAAGACAAATTTCAGTGTTGGCTGCCTAACGTGTTTTCTGGCACATATATTTCCA

A_16_P18487590

AGAGGCATCCTGTAACCATAAACCATGTAAAATCCAACCTCCTGAATCATTTCAAAAATT

A_16_P31225027

TACTTCTGATCTTCACTTATGCATTTCACGTGGTTTTTATAGTCATCGCCCCTTTAAAGT

A_16_P36183220

CCCCATCAAATGAAATTGAAAGGACAATCATTAGGCATACATAGTAGATAAACTAAACGA

A_16_P18096995

AATTGCCATATTAATGTGTTTTCACCTTCATCCTCAACTAATGGGACCATGGAATGAAGT

A_14_P134308 TCCATGACTTACTGTCAAAATTTGTAAGGACATGCTCAAAACCACAGAGCACATTTTTAA

A_14_P202291 CTTGGAAGTCACTAACTCAAATCATGACCTTTCTACCTCAATTCATTCTGAACATTTCTG

A_16_P20207412

TAGTGTAAAAGGATACCACTTTAGACTGTAAAGAATGCTTAAGGGTAACGTAAGAACCGC

A_16_P00015999

GTTAACACCAGAGACAATTCCTAGCAGATTTCCCCAAACATACTTCAAACATCGATAATG

A_14_P135587 ATAATTTACTTATTTCAAAAGCACTTTAACCCATAACAAACCCAACACCCACTTTCTTCA

A_14_P129031 GGTTCATTGTTTCCACCAATCCAACTTTGAGAATCCTGTTAAAGTCAAGGATAAATGTAA

A_14_P131403 CTTTCTCATATTTGATACTCCCTGCAATCTGGCCACATTTTGGAGAATCTCTCTTAAGTT

A_14_P112169 ACGGCTTTTTTTGACACAATTCAGGATTTGTGGGATTTTCAAAATAGTAACATTCTGGCT

A_14_P114123 ACAATTCTCCTCCAAGTTAGACATTGGTAAAGTCAGTGCACCAGAAAAAGCATATTTACC

A_14_P110146 ATGCTCATATGTTCTCTTGGGCCCTAAGCAGATTAAGAACAATTATTAAAAGCCCAAGTA

A_16_P03586365

TTTCCTACAACCATAAGTCAAGACCCCAATAAAAGTTTCTTACAATCTAGTTCATCCTGC

CUST_7030_PI426210822 ATGAAGATTTGCCTTCATCCAAGGGGAATGTCTGTGTGAGACAGTG

A_14_P115679 TAAGAGGGTCTCCCTTACCATCAGCCTTGAAGAAAATGATGGTCACATTCATTTAT

A_14_P110043 TAAATGGATAACACTTGAGAATAAGCCCACTATTGTTTAAAATGGCAGGAGGTGATTCAC

A_14_P129243 GATTCAGCAATGGTGGAATGGAAGACAGAACTGGAAGAGAAAGAAGGAAAAGAT

A_16_P20265397

AAAATGTTTCACTGAATAAGACCACAATTGGTAACAGCATCTAAAAAGTGTCATGTGGCC

A_14_P112123 TTGAAATTGTAAGCATCCGGTCAGTAAGAATGCCCATCCAGTTTTCTATATTTCATAGTC

A_14_P131171 TAAAATGAAAATGTTGCTGCTGTTATTCTCTTTCCCTGGGAAGGCTGGG

A_18_P22841194

GAAGAAAGTTAAAAGTCTCCTTCAAAATCTCCCCCATAGTGCCTCATCCTCTTACTAA

A_14_P127448 TGTTACATGAAAGAACTTACTGTCCCTTTTCTGCAAGTATTTGGAGTCAGAGATTCCTTA

A_14_P104702 TGGTAAAACATCTAGTTATTCACATGTAGTGTATTTGGGGTGTTGTTATGGTGTGACTGT

A_16_P02635021

ACTTGGAATGCATGTGTTCAGAAGTCTAGTCTCAAGCCAAAAACAATCTGTTCGAAATAG
A_14_P121372 CTTCCAGAACTAGAGATGATGTTCAGACAACCCAGTAGGTATACATCACATACAATGTAT
CUST_1909_PI426210822 ATGAACTTCATAGTCCCTGTCAAGTCTTAAGATGACCCCTCCGCAAA
A_16_P03741159
ATCTTCTAGCAAAGTTGCCTAGAATGTGGTTGTAAATGACTGTCATGTAGTAAACTCTGC
A_14_P110325 ACTTAAGTGATCACAAACCTACTGGAAACAATATAACCCAAACATCTCTTTCCACACATT
A_14_P112278 AATGATTTGCTTTTTTTAATCCCAGGATTTCCTTCAAATGCTACTGGAGAACATTCCAGA
A_14_P128203 GTAAAGATAATCTTCTCCCTCTCCTGCCTTAGTCTAGGTCAAGTGTTGAATTTAAGGTAT
CUST_3505_PI426210822
ATCTTGGATGTGATGTTTTCTGTTTTCAGTGGCATAGAACTAGATAAAAGGTGGCAG
CUST_1775_PI426210822 GCGTCCCTTTGGATGGAGTCCTTGCTTTCTGATCACAAAACTGGT
CUST_842_PI426210822 ATGGTGGGGGCCTCCCTCCTGGAAGCCGTTGTAGGTCCTGCACAG
A_14_P102591 CTGTTGTGCTTAGAGATGGTTTACCTTCTGGCTTCCAGGTTCCAAGT
A_14_P121806 ACTACTTCTCAATTTCACAAACGCTACATCCTTACACCCTGATCATCAATGCCTAAATAT
A_14_P104188 CATGTCTAAGGGTCTCTGGCACATGTATATTATATAGGTCCTGCTATCAGAGAGATTCAT
A_14_P122534 GTAACAATACCTAGTGAGAAGATGCATCTGAACTCTAAAAACCATTTCACAGTTTGCAGC
A_18_P22840692 GGGGAGCCCTGCCCTGAATAAGAGCATGGAAGGCCCAAGAAGAGT
A_14_P124228 AACAGATTGTTCTAGGAAAGAAGGTTGTTCTAAGAGCAGAGTTATTCTTTTAAGGTGGCT
A_14_P111280 ACCAAATCGCCCTCCAAGAACAACTAGAAAAAGCATACGTGAAAGTTTTTTTAATGTTAC
A_16_P01883348
GCATGATGCCTATATCTCAAACTACTTTACAGACAAAATTACCCTCTACAACTTTCTGGG
A_18_P16336269
TGGAGACACAACACATTTATTAGATGATCAGTTTTTTTTTTCCTGTTATGTCAATGCTCA
A_14_P106512 ACTTTACCCATCCTGGAGGAATTTTTTTTTTAATTTAGCCACAAACCTCTTTCAAACTCT
CUST_6352_PI426210822 AGAAATTCAGTCCCAGGAATGATTTCCCTGACCCACTGTAAATTTTAATTGAGG
A_14_P107210 TATTAACACAATACAACCATATAGCATAATTGTTTGGGGCAATTCCAGAACACTCCCTGA
A_14_P135023 ATTGGATCATTTCAAAATTAGCATTTACCTTTGCTTAGTCCATCTGCCTTTTCACTGGTA
A_16_P00685408 CATTCACACTGGGTTCCATGTAAACGACACCTCCACAGAATATAATGTGAGTG
A_14_P126369 CCTAAGACAGCCATCTCATTGTTGCAGATTTTTCATTTTGTGATTTTAAACTGCCTCCTA
A_16_P15611880
GTAGATGGAGGAATACACATGAATTACCATGACAGATGAAACCTACAGTTAAGAAATGCC
CUST_6347_PI426210822 GTAAATGCTTTTTTCTCTCACTTTTTGAGCATAAAACTGGCCCAGGTGGGTA
A_14_P113681 CTTAAGAAATGAAAGTAGAAAACATGAATGAGACGGTGCTTAAACAATTATTGACCTAAT
A_14_P116553 GCTCAGAAATATTTTATATTCTACTGACTGGCATGCTTGTGAAACCATTTTCCATTTGCC
A_14_P130057 GATAGTATCAGGTTTAAGAGCATTAGGTTGCATGAAAATAGGCCACTGCATTTAGACATC
A_16_P19030073
GTTCTGCAAAATGAATTTGGCATTGTTACCTAAAGGAGAAGGAATGGATGAAGTGAGTAA
A_14_P114829 TACATTTTCTTTTATTTTTCCCATTTTTGTAAGTAAAACCAGTGAGTCTCTTAAAGACGC

A_16_P19774536
CCCTTAAAGACTATAAAACCACTTTATTCCTTCAGATACCAGAAAATTAGGCTTATAGGG
A_14_P119900 CTCCAACACCAGAGGATGCATTTTCTTTTTAATTAAACACTAAATTTTCACTGCCCAAAT
A_16_P17575250
AGACTCCTCAATCTGAAGTTTCCCTACCAAAAATACGTTCTTTTCTATGCATATCTGAGA
A_16_P00766755
CAGATGTTGACCATCCAATAAATGACATCCATTCCCTATATTACCTTTCAATATAACTCC
A_14_P113436 TTTCACATTCCATGGCTACTGATGAGAAAAAAGGATTTTTAAAAATCTGCAGGGATGAGA
CUST_4800_PI426210822 TTTGCTGAACAGAACAGCTCTTAAACCAGACCTTATCTGTTCCATGGGT
A_14_P136465 CTCATTTACATTGATATTGTAGGACTTGCTCAATTCAAGAAAACTCTTGGACCATCCTGG
A_16_P36738765
CCATTCTCTGCCATTTCTTAGATTCCTAACACTAACAATAATCCTTTCTTACTCCCACAG
A_14_P102010 TTATGTTTAATTGGCAGTGGAGAAGACAAATAGAGTTTACAGGGGAAGAGGGAAATGTT
A_14_P132604 ATGATTTAATTTGGGGCAAATTATAATTGATTGGGATTCAGAGGAGATGAATGGGGTTAG
A_14_P100033 ACAGCAGTTGCTCAAGAACAAAGTTATTCTCCTTTTCATCTCGAATACTCAGAATATGTG
A_14_P111829 TCAAACAAGTAAAGAAGACCCCAGGAAGTTACAAATAACAAACCTACACATTTTTCTAAA
A_18_P14351548
ACCAGTAGGTGTTAATTGATAATTGGGCTTTATGGAACAAGATTACGTTTTTACTGTGCA
A_14_P117546 CCTACATTTTCAATCTGTTACTTATCTTGGATTAACTTTTCTGCAAACCCACCTACATGG
A_14_P118580 TCCAATGGTATCAAATACCTGTTAGAATGGCGTGTCTGCTATAATTAAACATGGGCAATT
A_14_P115087 AGAATGCAAGAGATACACAGGCCGATACAAAACAGGTTAGGAATTGGCAGT
A_16_P16700311
AATCTTTTGCCACTACTTACTCAGATTACGCATCTACTGATATTCTCATTGGTGTCTAGG
A_14_P123267 CTGGTTATAAATTCTCTTTGTAGCAACAATTGAGTCACTTCAAAAGGAGTTCATTGAAGG
A_16_P20163465
AAAGGGAATGTTTTGCATTTTTCTAGAATTGATGAAACTGGTCAACTGTTACTCACCCTC
A_14_P138107 TAGTTTTTTGTTTATTCATTCGCTTTCAGTCTTTTGCAATCTCTGGCGGCAACTTAAAGA
CUST_2213_PI426210822 AATAGGCACAGTGACGTCTGCCATCACAGCCACCCTCTGGCCAAG
A_14_P100549 GAGAAAAAGTATCCAGAAGGCTGAGCAGGTCTAAAAATGAACCAAGAAAAACAAACGT
A_16_P18227888
TCAACTCCGTTATTTACTCACATTCAAGAAGTTCAAGTATGACTCACATGAAAACGTTCA
CUST_7337_PI426210822 GTTTCCAGGTGACCACAGAGGGTCCCACCTCAGTGTGGACATTTC
A_14_P201220 TTCCTCTCATCTCCTTGTATCATACTGGTAATGAACCTTGATGAGTTTTGGATAGATGAA
A_14_P121183 GACTACATAAACCTCGATGCATTATTTATCCATCCCAGAATTAATTCCCATCCAAGCG
CUST_1445_PI426210822 GGTCTGTGGAAGGTCTCCAGCAATGTCAGCCTCACATGTGATTAAT
CUST_3125_PI426210822 AAGAATAACAGCTGCTTCTAGATGCTTCAGGGCTGTGTATAGAGAGATGCC
A_14_P136124 AATCCATATCCCAGCATTTTTCGGATTAATTTTGATCACTATTACCCGGTGCCATATATC
A_14_P130448 TGGCAGTCCCAGTTCTTTCTATGAAATGAAATGTAGTTGATGCCTTTTTTAGTGTATGTG

A_16_P15234173
GGGAGTTTACTGCTTAGTAAAAATTTCACTTCATTGCTCTCTATTCATCCCTACCATGTG
A_18_P16810920
AAATGTGACCATACTTAAGGAAGAACTTGGCTTCAATGTTTTTAGGTCAGATTGTCAAGC
A_14_P127145 GTCAAGGAATCTGAAAAAAGTATGTTGCAAATACGTAGTTGCGTTAGGGAAAACGTCTG
A_14_P130376 TTTTCTGCTCCCCCATATAAGAGAAAACTTGAAGTAGATAGCAGATTGACGTTATGCTTT
A_16_P16251595
AAATGACCTCATCATGGAGTAACTTCATCCTACGGTTCAATTACGTATTCAGGGCTATT
A_16_P40053805
AAAGAGATGCAGACTCTAATTGCTGATTAACACATTGTTTTTCTCTAAGCCCTTCACATG
A_14_P107769 CTTGTTTTAAGTAGCACCCAACATGTTATGACTCCTTGTCTCCATTAGGTTAAACTTCTT
A_16_P21364078
AAATGCAACTCTCTCCTTAACATCAATCTACTGAAACAGAGATTCTAAGATGCGGAAACA
A_14_P131663 TTTTTTTGAGGGTTTTCAAAACATTTTGAAAAGCATTTACTTTTTTGACCACGAGCCATG
A_16_P03808022
AATTGTAATTCACTGTAATACTTGAATGCCACAACTGTAGTGCTTCCATTATCGCTGACA
A_14_P120177 ACTGTGACTTTATTTCTTCACTTCCTTCTACTGCTAAATTTCTCTGATTCAGAGTTTCGG
CUST_5702_PI426210822
GTTTGAAGTATGTGTGAGAAAAACAACTAATTCACCCTTCTCACTGACCAGTGG
A_14_P129712 TGTCTTGTGTTTTGTTTTAGAAAGCCTTGTTTTTTATCTTTGCATCTGCGTGTCTCAGTT
A_16_P36230288
TGGTGACTACTTTGTCAACCGTCTATGCTAAGGCAAAGGAATATGTTTTAATATATAATC
A_14_P128444 CTATACATTTGAACTATTTCTGCGTAGTGACTGGAACCAGATTTAACTGAATTGCCCATT
A_14_P122219 CCTAAACACTTTTACATAATTTGCACATCAAATGTGCATCTATTTTTCCCCTGGAACCAT
A_14_P115792 CAACACCAATAGAGATTGCTTTGTGTATTTTGTAGGGTTCTCTGTTTTGAAGACAGAATT
A_14_P120384 TAATTCAGCAGGAGCATTTGGATAAATCTTAGAACGATCTATCCTTCCACTCCCAC
A_14_P116708 AATTTCCCTTTTAACTTACTTGTCACTTGAACATCTTCAGGCTATTTGTGAATGCCAAGA
A_14_P124322 TATTTTCCATGTAGTAACTATTAACGTTCTGGGGAATTAGTGCTCTGTGTCACACTTTGG
A_14_P139640 AAGACACTTCTCAACCATGGAAAAACATCTGGATTTCATTTGGTAGTTTAAAGGTTTTTG
CUST_3471_PI426210822
CAGTTCTTACTGCTGTCTGAGAATAAAACATAATATCTTTGGTCTTCACAGAGGTATCAG
CUST_90_PI426210822 TGAAGGCTTGGCGATAGTATTTGCCCTTCCTCTGAACTTTCTGGAT
A_16_P01537298
ATCTCCCTACATAGCCTGAAATTTGTTGCATCACTTTGTCACTATTAGATCACATCCATT
A_14_P118188 CTCAGAAGTAGGTGAGAATTATGATCTCTAAAACGCAGGTGCTGATTTCTAATCCTTCTC
A_16_P39974007
ATGCTGTAGTGTTCTCAGATCTAAGGATGTTTGCAAAAATTCATTTTCCTCTCCCTTTAG
A_16_P03596296

AGCATGACTCACAGTATCTTACACACCTTGGATGTATATACGCACTTCTTATGTCGTAAG

A_14_P100513 AAAAAAATACATACTCTGTAACTGATGTTACACTTTCCTGTCCAAAAGGTCCAACTGGAT

A_14_P113015 AGAGGAAGAGGATGAACTAAATTCCCGTTTTCTGAGGTAGTATTATAGTATTCTTTGCCT

A_14_P114518 GTGCGACTGGAAAAGGGAACACAGTGAAGAAGACTTTCACAATATTTTGTATGATTTTTT

A_14_P103449 CAGTAGAAGAGGACATATACCTACTTGAAAAATTTTGCCAAGGAGCAGATGAGATGAAAT

A_14_P110601 CTGGGTTAACTGCTCTAACATGATCGATGAAATTATAACACACTTAAAGCAGCCACCTTT

A_14_P131016 ACCTGTAGGGGTTGATCATTTCCTCTGTGACGAAATTCAAGTAATTCCTAAAATTTAGAG

A_14_P138225 CACAGTATAATGGTCACTTCTTAAAGATGCAGTCAATATCACTGTAGTGGAATCCATTCC

A_14_P124212 AAAGAATCAGTGTCCCAGAAGATGTTTATGACAAGGGTTGCGTGAAAGACGTA

A_14_P117117 TTTCATCTTCAAGTTCCTGAGGTAAGACTCTACCAATCCTTCAGTATACATGCTGAAAAT

A_14_P131939 CTTGGTCACTTGAATGTTAATTTTAATCCCCAGGCTCTGGTGCTGAAACATATTGTATAC

CUST_5720_PI426210822 GGACAGTGTCTGGCTGGGGAGGAAACGTTAGCCTCTCTTTTCTTA

A_14_P119547 ACTCCTTCAACTTTCCTATCTAGGAAACTTTCCTATACAAGTTGCTAGGCTGTAATTCAT

CUST_8120_PI426210822 ATGTACATTAACAGTGCTGCATAGCTTCAGGAAGCCCTAGGATCTGTAGC

A_18_P13338810

CCAAGGAACAAGTATTTAGTCATGATTGCCACTCGTTATAGGCTCTGTTAACAGTGTAAA

CUST_10225_PI426210822 CTACTTAACCTTGGGATGTGCTAATTTCCCAAGTGTAGGGGGACTTTG

A_14_P126738 ACCCAACGGAAGATAGAAATGACCGGTTAAGAGATTCTTATTTCTTTACCTGTGAGTG

CUST_2406_PI426210822 AGGGCTTTGCCTGTTAGTGTGAAACATTTCTTCAGTGTTTCCTACAGTGT

A_16_P15807478

AGTGTTAAAGGAGATGACTGCCCTATAACCTATGTTTTGCTGAATGTTTACTATGAGGTT

A_14_P116534 GATGACTCTTCCTTAGTGTTACAGATCACTTTTCTCTCCTCTTCATCCAAGTTTACATTT

A_16_P15711058

AGCAAATCCTCCATTTTCCTAACCTGTCATTACATCAGTAACTATCCTATCCAAGAATCA

CUST_6399_PI426210822 AGCAAGACAGAAACAGTTTCCTATCCCTGTAGGAAAGAGAAATCTCGGAG

A_14_P120793 CTGCTAACTTTCTGGGTGAATAAGGTTAATTCATAGGCATTTAGGCTATGTCTGGAGATA

A_16_P20425521

ATTTGAAACCTCCTGAATTTTGTGGACATGTAAGCATTCCAAAAATAGAGACAGATGTCA

A_16_P20902743

TCATGTATGTGAATAAGACAAGCCCATCATATGAGTTGCACAATCCCAAAAGACTATACT

A_16_P41109733

TGTGGAGGCAACAAATTTAAATAACCAAACACAATGAACCAAAATTATCGATTCTCTGAG

A_14_P108473 CAGGCCACTGATTTATGTGAAGATCCAGGGTCCCTCATGAACAAC

CUST_6831_PI426210822

TTGGCTTTTGTGACTCCTGATTTTTTTAAAATAATGCTTGTTGATTTTTTTTTTCCATTT

CUST_442_PI426210822 GAGAAGCTGGCAAGGGTGCTGTTTTCTGAGCTAGTGCTGAATGCT

A_14_P104401 CAGTTCTGCTTTAGATATGCAGTTTTGATCACAGTGTAATTTTCCTTTGAATTCCAGCCA

CUST_5045_PI426210822 CCTCTGCCCTCTGTGTCATGCTGCAGTAAAGTATGGGTCAACATC

CUST_3709_PI426210822
ATGAGACCCAGATCCCTGTCACTTGACAACATAAAGAATATTATTCTTCATACCAGCAT
A_16_P15250704
ATATTTGCATCACTGATTTTTTCCCTAACAACCCTATACCAGCAACTTAAAGGCTTATCC
A_16_P58424181 TTAAAGACATACTAGCACCGCGCTGGAACTTTCTTCCTGATGCAACCA
A_18_P15703829
AAGTCAGAGAAAGACTTCTACTGTTGCACTTATCACAAGATAAGCAATCACAACACAGAG
A_14_P106656 TAAGAAAGTGCCCTGGTCACATAATGGTGGTCGATTTTTAGAGACTGTGCAA
A_14_P126825 AGCAGAGGGACCCACAGTTTCATTTCAATGGAACCAAAGGTAAGAAGAA
A_14_P136925 TGAAAAAAAAAGTGCCTTCAGTGACCAAACTGACCTGTGATTTAATCACTAGATCTGATT
CUST_6816_PI426210822
TGAAAACTATTTGCTGGCTGGAATATTTGTACTTCACAGTGATTGCAAGAAGCAGC
CUST_8944_PI426210822
AGCCCCTCATATTAGCCTAGCTTATGTTGTTGTGATCATCAGACTGTTTCTGTTTT
A_16_P16902554
AGACCTCCCTAGACTCTAAATGTTTGACCTTTGACTTCTATTTCTAAACTCTGCTAGAAA
A_16_P00685559
CAGTTCCTCAATTTAAACATCAGAAAGAGTGCTGTTCTTAAGGAAATCATCAAGAAAGAC
A_18_P22843022 CTGACATGTATATGGTGAACACCATTTACTTAGGGCAGTGCCCCCTACT
A_14_P139050 CTACAAGGGACCTTGTATTTGTTCTGAGTATTTACTGGCTTACAACATCTTTTGGAATTA
A_16_P03679892
ACACCCAATGAATCTTTACTAAAAGCACCAAAGTAATATGAGGCAAGGAGATACTACACA
A_16_P02572303
AGCGTATGGAGAAGAATTGTTCTAAAAGTTTACCAGGAAAGATTAGGATGTCACAGATCT
A_14_P135005 CTTCCATAAAATTTTCTCTGCTAAGTGGAAAGAGGGTCCTAGAACTCAGAGGAATC
A_14_P111518 CTAGATTTGTCCATTTTTGCGTGTTAGTAGGGTTTTGGAGGACAGACATATTTCTAACAA
A_14_P139021 CTACCTTTCCAAACGTCTGCTGCTGCTGAAGATACAAGAAGCCTTGTTTC
A_14_P117315 CTTGCTAGTAACAGTGGCCTTTATTATTTTCTGGGTAAGAGAAGCAGCACTGCTTTTATG
A_14_P102825 GTTATACCTTATTGGTGTGGACGTTGTCTAAATTTCGGTAGCCATGGCACAAGAATA
A_18_P16393523
GACTCTCATAGCACTTCACAAGAATGAAACACCATACGATAGAAAGACTCATCTCAAGTC
A_18_P22839050 CTTTGAGAATGGGTAATCAGAGGGGAGGAAGATGGCCTTGGCATAT
A_16_P03449763
AAAAGTAACTGTTTCACTAAAGCCTCCAGTATTTACTGAAGACTGTCAGAATCATCCTCC
A_16_P21354411
ACTCTTAAAATTAATCCAGAATTTGAACAAGCCTATAACTTTGAACCTATGGATGTGGAA
A_16_P01414352
ACCTTTTTCGCTCATCTTCTAAATACTTGATTTCCAGACACTTTAATTCATAGGCTTGCA

A_14_P101317 AAGGACAATGAAAAAGATGCTGATTGTCATTCCTGTCTGGTATTGACAATCAGGAAATTA
A_16_P02138653
GCGAAAACTAGAACTATAATTTGACATGTTAATCATCTCTACCTTGCTTATCTCTGCATG
A_14_P125623 ACCTTCAAAGCACATGAATATGCCTTTTCTCCAACATTAATGAACTTAGGATCATCATCA
A_14_P201058 GCAACCCTAAACAAAGTTTCTGCTAGTTGGTTGAGACTCAAAATCTGTTAACAAATTCAT
CUST_1595_PI426210822 GTGCTCATGGTACAGCAAAGTGGGGACATGGTGGGTGTACAGAGT
A_16_P17100550
AACTACCAGTAACAAGTCGAATCTCTCTCAACAGTTAGTGTTCATGAATGTCTACTTTGG
CUST_5737_PI426210822
GCAACTAAAAAAGAAAAATGAAACATAGAACACAAAAAATTCCAGGGTGAAAAAGGAGAA
A_14_P135149 TAATCAGATAGGTAAATAATAGCAGTTAGAGCAGAATGAAATGGTGTAGCCAAGGGCAGA
A_16_P03425992
GTCATTGCAAAAGTAGAAAGTGACCAAATTACAATGACAAACAAAACTCGGATCTCTACC
A_18_P22839044 GTAGAAGATGCTGAGTATTTTTCTAGAAGACTGGACTGGACGGGTGGG
A_14_P128977 GCATTTGAGACACAGTGTTAGACATTTGATGCATTCATGTTCTTTTCGATTGTCTTACAG
A_16_P19046139
CCCAGAATAGCAACAGTTATCATTTAAAACCAGTGGCTTCTATATACAGCAAGTGCTTCT
A_14_P121161 TAAGAAAATCTTCTTTGGCTTTGGCTAAGAATTCTTTCACTGAAAGGGAGAGAGGG
A_14_P110705 CTAAGTCAAAAGTAAGGACTTTCAAAACTAGATGCTCCATTCTCAGAACTTGTTTCTTGG
A_14_P124705 CTGACACAACTTCTTGTTTCAGATTGTCTCCTACCTACTTGATCTTCCAGAAAAAGATGA
A_14_P131740 CACTTCCATTAGCATCTGAAGGTGCATGGTAATCGGCTCTGAAATAAGAGTCAAAAAT
A_14_P116835
AGTGAAAGAAAATGGACAAGCAAACATTAGCCTGAACAAAGGCTAAGTTCTGTTTTTAAC
A_14_P104714 TCTGATTAATTTGAACCTGAAAGTCTAAGCCCAAGTCATAGTCCCTATTTGATTTAGCCA
A_16_P37934650
AAATGAAACACTTAGTTCCTCTCACATCTCTTTAGGCATCTGTTACATCTTGAGTGAGTC
A_14_P100459 ACATTTTAAATAATGCCACATGGGTATTGTTTTCTGGTTGAGCAATATGGAGTTTCAGAG
A_16_P58424071 AAGAACTGGCCTGGAAGTCTAGGATGGATAGGATTTTGCCAGTTGAAG
A_16_P18683539
AATCGATCTTTTGAAATGGATGTCAGTTTAGGCTCAAAACGTGTGATTACCTAAGTTCTC
A_16_P19606333
AAATCAGGTTTGCAGATCAAAGACATCACTGAGTAACTTAATTACCCAAGATTCTCAACC
A_18_P22840352 AAGAATATACCCAGAACCAAGCCCTATCCTTGGGGACAAAGAGAAAACATTC
A_14_P139390 CTTTTTCCCCCTTACCTTGTTCTCTCTCATGTTTCATGATCTGTGTCATAGATATTTCTT
A_14_P123185 GGAACAGTATACCCACAATTCCACCCAAAGACTCTACAAAATAAATCTTTCTCTCCATCT
A_16_P21403305
CCAATTACAATCCCATAAGGTCACTAAACTTCCCATTTAAATTTCTTTCAGTGTAGGGCT
A_18_P22840514

AAAATGTTCACTTGCTCCAAGGTCAGGTATTAAGTTAGCAAAGTCCTAATGCTTTTGCTA

A_14_P114639 GACAAGCACCGTGTGGGTATTACTCATTCCGCTCTACTAAGGAGG

A_16_P16031579

AAGTGTCTGCCTCATTAAAATTGTCATCCCTGGTTTACTATTTCAAGTGCAGTATCAACT

A_16_P19252945

TGCATTATCAAAGAACTCCATCAATGAAGACAAAACTCTATTGCATCCCTAAACTGATCC

A_14_P201569 ACACACATCTCCATACACTGGACATAGTCTACTTGCAACAGATAGATTTCATCTCTCTAT

A_14_P103668 TGCATAGTTTCGGTCATCTAAAGGAGGTTTTAAACAGTATAACAGGTAAGTCTCCATATG

A_16_P18866203

AGCCTGTCTCCATTTTAAGATTCAAAAAGAAGCAACATTTTAATTCCTTCCAACTTTGCA

CUST_5570_PI426210822 TTGATCTCTTCACTGAGCCCATGTTGGTACCCAGGACTATACAGAAG

A_14_P101057 AATTTTATTTACGAAGTTAGACTAAAAGAAGAAAAAGAACCCGTTATGAGCCTTGAAAAA

A_14_P133350 CACTATTCAAATCTGCATCAATATAACTGGATAGAAGAAAGCAATGGGCAGACTTCATGT

A_14_P106443 GGAGTCAAGAGGATGCAAATTCCTTGTTTCTTGAAGTGGATCATTTCTCATGTTGTAATA

A_16_P18138144

AATCAAGACAAAAATCAATGAACCAGGTTATCTGTTCTCAATGACTCTTCCAAGATGTCA

CUST_391_PI426210822 TCCAGAGCGGACGTCTTTCACACCCCACCCCGTCCCCTCCTCCTT

A_16_P35945513

AACAAGCATCCAAAAAATTTGATAAAGTAACAGTGTTTTTTCATAGCATACCAATTTCTT

A_14_P129558 CTCAGTGAATATTCAGGTCATATGCATGCATATATTTAGACTGGCAGTGTTGTGCAATTG

A_14_P124032 TTGAGAGAGTTGGAGTTTTAGATAACTGAATATTTGTCCCCTATAGTTTGGGGAACAAAG

A_14_P117569 TGGACCTAGAGGGGAATGCAGATTCCTTTTGTTTGGAGAATGTTTTAAATATTTTCAGTA

A_14_P128178 TAGCAGCAAGATTCTGGTAACTGAGAAAGGTAATAGCACTTCAAAACAGGAACAGTTTAA

A_14_P120394 CCTTCTCTACTTAGTTCTCTTAGACTGATGCTCCCAAGCACTGACAAAGAAGTG

A_14_P126510 GATTTCCTCCCTTCCATCACCACCAGTTAGTTCTAACTTGTTCTATAATTTAGAACTTCT

CUST_9904_PI426210822 TCTAAGTGACACATTGAGCTGAACACTTGGAAAGAAGAGCTGCCTTACATG

A_16_P03035573

AAACACATGCCTCTATAAAGGATAAGTTCTCAGCATACTTCAAATTGGATACAGAAGGTT

CUST_7165_PI426210822 ATTAGTTTTGGCTTTGGGAAATGAGGTTTCTTTGACTTGGCTGGAAGTATACAG

A_14_P201792 ATTACTGGGATACATAACTTGTAAGTAAAAAGGTCACACTAGAATAGGCTGGAACATGGT

CUST_1348_PI426210822

TTTGAGGTGGATAAATGTTTTGTTCAGTAGTGGAGAGGGCACTAGCTATTGTAGT

CUST_6638_PI426210822 CAACGTGTCCTTCAATGTTTCCCGGGAGCTCTTCTTCATCAACAT

A_16_P21494299

AGGCCAGATGAGGAATAATAGAAGGTTTAAGATACTGGATAATAAAAAGGTGGACTGGAT

A_16_P00685328

TGTCTCATCAGAAAGGGAGGCATATTTGATAGCAGGAAAAAGACAACCTTTTTTTCATTA

A_14_P201672 GGGTATTTATCTCATTTCATCCATTAGCACTGTAATTGCATTCTAGGGAACTCAGCAATT

A_16_P21383851
AAAATGCTACTTGCTCATCTTCTGAATCCTTTCTAAGTTCTGCTGATTTTTGTGACTGTC
A_14_P118955 CTGCATAACGTCTCAGATGCAGACACTTCTGACATACAGAGGAAGAAAATAATGAAGATA
A_16_P36150419
TGTTTCCCAGACAATTCTAATCGCCATAACTTTCTAGTGAAACTGAAAATTTCTTGAGGG
A_14_P106521 GAGAGTTGCAGAAGTATCTGCATCACATTTTCCTGACTTTCTCTATTCTGAGTCATATTC
A_16_P17708573
AGGCTATGTCAACATGTGAAAGTCACATTGAAGATTTAGAAACTCGCTATTGTTGTCACT
A_14_P134052 AAGTTCCTGGAATAATATTAGTCAGAGTAATATAGGATCTGCAGGAAGTGTCTCAAGATA
A_14_P112158 CCCTGGTTGCTTTGTGTCTAGTTTTCTAGACCCTTCATCTCTTACTTGATAGACTTACTA
A_16_P03588381
GCTCATCCTCATACCAAAATTGCTAATTATGTCTCTAGTAATATGGTTGATGTCCAGGAT
CUST_1373_PI426210822
CTAAACCTGCCCTCTTGACATATATATGAAGGAATCAGGTTAAAATAATAGTAGTGCCCT
A_14_P129798 AATCCTAACTCTAACCCTAGACCAAAGTCTGTAGCAGTAGAAATCCAGAAAATAATTCCT
A_16_P16182656
AAAAATTAGTGACACCCTAAAAGGTAGATATGTCCATATCTGTTTAGGTCAAAAGAGGCC
A_16_P36231077
TATTAGATGGAATCAGAATCGGGTGGAAATGTCATCTCACTGTCACAGATGTTATTCTCT
A_14_P135313 TTCCCATTTTTGTGAAAGCAACCAAATCATGTATATATTAGTAAGTGCACGCACACATGC
CUST_7587_PI426210822 GGGGTTTGCAGGTGAGAAGGAACTCTTCACCATGAGTACATTTCTATG
CUST_7054_PI426210822 AAATGCAGCCAAGCTCTCCTCTGACAAGAGATGTGAGAATTCCGTT
A_14_P113805 GCTCCACCTCCATCTCTTATACCAACAATAGAAAGGTAAATCAGTATGGATACTGATTTT
A_14_P201181 CATTCCACGCGTCTTTGCAATAATTACTAAATTAGTCAATTGCCACCTCTGTAAACAAAC
A_14_P114691 ATCAACTGAACATGATCCGAGCAAAGAAATATTCTCTCTTTTTTCATTTCCAGTTCATGG
A_14_P131882 GTCTAAATCCCATTCATGTCATTGTTTCCCATCGCTCCCAAAAAAAGGCTG
A_14_P115343 TATTTAGAGAGAAAAACCATATCCTAAGGGGTTGCCAAATGAACGTAACTTCAGAAGGTA
A_16_P18703944
AAGGAATTACTTGGCTACAACATATGCAAAAGTTTACCTTGATTAGAAAACATCCTGGTG
A_14_P122144 TGCTCAGAACAGTAGCCAATAAAGAAAATGATCATCAGCAAAGTAACTTTTGAGCACTGA
A_14_P125011 TGTTATCCACAGACATTTTTCCTGTATTCCTGTGTTACGGTAATTTTACCATCCTGTGTA
A_14_P201024 ACAATATACATGAAGTCCAAAGGGAAATCAGAGTCTTACAATAAAGGCTTTCTGTAAGGC
A_14_P130796 ACCTGAACTGATGAAATTCTTCAGCCAAAAGTTGTTCACATGACATGTTAGATCTTTACT
A_16_P17030219
AAATTCAATCTTGTCTCATGCTCTCATTTTCTTTGAAACACAAAATCCATACTTGGCTGC
A_14_P133518 TAATGAAACGGTTTGACTTGGATATGCTGTGCACATTCAATGAACTTAAGGAATTGTTTG
A_14_P112658 TGGTGTTCTTTCTGAATCTACAAGGAATAACGATAGTGATAAATCAATGAACACCACCAG
CUST_1882_PI426210822 GATTAGCCCAGATGAGCTGAGACCACTGGTAATTGGAGCCTTCAG

A_14_P107631 TCTCTCTCAGTCACAGACATAAATTCATTCTTTTGATCAACACTTTTACTCTCAGGGTGA
A_16_P01434189
GGGCTACAGAAGTGTAAATTATGGTATCTGCTGTTAGAGATCTCTGTTTTTGAACTGACT
A_16_P16975318
AAAACAAAACCAAGACTCTGTTCTTCTCATCTCTTTGATTGAGTTATCCTTTGCTAGGTG
A_14_P108928 AAGAAAGGTCGGAATTTCTTTTGGGTCAATATTTTTAAGTGTGTGAGGAGATGCTCAGTA
CUST_6439_PI426210822 ACACCCCTCAGATTCAACAAGGGTGGTATCTTGTGTGTTTCCTTTAAGTTAT
A_16_P17269474
AAACGTCAAGGAGAGTAGTTAGAATGCATGTTGTCCCTTTAGACACATTGAAGACATTTA
A_16_P03012235
CCCACCTTATTTGGCTATTTGAATCACATACAAAGTCTTCCTATACCCTAAAACTGCTTT
A_14_P106541 TTTACAATTTGTTTACCTGTAGGGTCAACTTTCAGTGGATCCAAATGGACATTCATTTTG
A_14_P135982 GAAGATTCTCCTTAAACATGTGGAATATTCTCCAAAATTTTTAACATTTTTACTCCATTG
A_14_P127216 TTTAAATAAATTAAACTCTGGACTTCAACTCACCCAGGAATCCCTAGAGAAATCTAAGGC
A_14_P107474
CAGAATAAAAGACTATACTTAACTTTGAAATCACCAGGAAAAAGGCTAGGAAAACAGCCC
A_14_P201122 CACACTAATCTTGATGATAATTGGCTCAATTTTACAATACCTTCCTAAAAAAAATAGAGC
A_14_P138487 GAAAAAGAATTCTGTAGAATGGATGTTACAGGAAATGTGCCTGCCTGAGTAGAAAGAG
A_14_P137740 TCCTAAATAGAGTCAACAGAAGAACAAGTGATGGAAATGGCTATCACTAATGGAGTCATT
A_18_P22840507 CAAGATCACCACAGATTCAGAACCCTGTGGAAGATCAGGAAAAACTGTG
A_14_P122380 GTTTCGAATGTTCCTTTACTCTGCAAATATGTTTGTATTAACTGTCACATAAGATGATGT
A_16_P15800815
GGTCTGGCACCATATGACTTCCTGTTTTTAGAGTTTATGTCAGTTACTGTGGTAGAATTA
A_14_P119299 GCCAGATATTTCAATCTTTTTCTTTTTCTTCCCAAACATGATGCCAAAACCTGGGAAATA
CUST_3316_PI426210822 CAAGCCTCTCAGCTATAGGGTGCCCAAGGAAAATGGGAGATTTTT
A_14_P105671 AGCCAAGATGGTAAAGCCAATTTGGTTGCAATAGTGTCATAAGGATAGAATATTACCATC
CUST_92_PI426210822
CTGGATATCAAAGCCACGTGGAAGTATAGCTTGTCTTATGTATGAAAAACAGGGC
A_16_P17622468
AAACTGGCACTCTTAATTGCATACTAGACACAGGATCTTTATGATGTAAGAGCAGACAAG
A_14_P122295 ACTAGAAAGCCAAGGTCTTTTATTAAAGGTCCCGACTGGTTTTCCCACTGTATTT
A_14_P131668 GAAAACAATCCTTGCTCTTTGAGATATGGGAACAGTGACTTCTTCCTTTGGATGTTT
A_14_P109601 AAATAATCAGGAATTAAGTAGGTCAGGGTCATGTCATTAGAGAACTTTACCATCAGGCAA
A_14_P139487 TTTTTTTAGGATGTGATGTGTTCATTTGTTCCACATCAACTTGGAGTCTTCAAAGTGAAG
A_16_P36230168 AAGATGACATGTTGTGACAGGGACACATGATGGTCCCTGATGATTCCTCTATATATA
A_14_P109743 GATCATCTAGAAGAATTTGGGGTCCAAACTTTGAGGCAGAGTTCTGGGTATAAATGTTA
A_14_P115672 CCTGGATTTTCATTATAAAAATGTCATTGTAGGGAGTAGAGACTCATATCATGGCCTTTT
A_14_P134739 AAGTAATACAATCAAAGATGTTTTTCAGTCCAAACATACCTGCAGAAGGGAAAACAATCC

A_14_P122412 GTGCATTGATTTTCAGACACAAAGACCATCACCACCATGGCTGAAGACAT

A_16_P01330169

ACCTCACCTTTGTATTCTACCCACACTCATATCACTAATGGATGTAGTCAATTCTTGAAA

A_14_P125532 GCCAGTGCTTAAATTTTATACCCAGGAATTGTTTGGCTTATTTCTACTTCCTCTTCTTTA

A_14_P110500 TTTTTCTCCTCTGGGCAGCAGCAGACACATTTGTCTTTAAAAAAAGAAATTTATGTTTAC

A_16_P39698926

TTTACAGGTCATTTTGTCAGTCCTATGATATGAAACAGCATTCTACCAAGGTTTTTCGTG

A_14_P117553 CGGTTGATCATGGGGTTGAACACTGACAGTAAATTGTTTGGAAGAAATTGTTTACTTAAA

A_14_P130406 TATTTTTTCTTTTGCAGATGGTGATGCCCAGTCACTTAAGGAACACCTTATTGATGAATT

A_16_P40225684

TTTCCTCCCATCATCTTTCATTTTCTCCTGACTCATCAATATACCAGCATAACATAGGAT

A_14_P123284 TGATTACAACTCTTGATATGAAATTAGAAGTTCGCATGAATTCAGTCTAAGGAGACCACT

A_16_P00057959

ATCCATCCAAATCACATACCTAGGCTCTATACACACATGATTTCTTGTCAGAACTTTTCT

A_14_P134415 TTTAACTCTCCATAATTGATGCCTGCAGTAGTGTAAGGCATTTCATACTAGTCTCCTCTA

CUST_8704_PI426210822 CAGAGAGGCAGCAACCGCAATCAAAAGAACTAATACCAGAGGGTG

A_14_P134408 CTGTTTTCATAAGCAGGAAAAATAAACAGAAGTATAAAGGAATGTGTGCAGGGGGTTCTG

A_14_P202451 TGCAGAGACGGCAGATGGAAAATATGCTCAGAAGTTGTTTAATGACCTTTTTGAAGATT

A_16_P01947461

CGCTACATAAACACCTTTTTCACTTACAAGTTGCAGAGAATTTAAATGTTACCCCTGTGT

A_14_P109760 GTCTCTGAAAGGTGGGGTAAAGATAAAGCCTTTCAAATTTGAGGAAGACCTTC

A_14_P106624 ACTTTCAACTACCACCTTCAATGTTTTTGGACCACATATATTTTTCCCACTCTGTAAAAC

A_16_P16243867

AAATCAGAAGAATTCTTTGAAGATTAGTCCCCAAGAGAATGATTAGAAACAGGCTCAGAC

A_16_P19368759

AGCCCATTCCAAATATTAGTTCTACCATGAAAACTTTTTATCAACTGGATCTCTGCTTGG

A_14_P119416 GAGGGTAAATGGGGATCCAGATGAGAAACGGATGCGGAAAAAAAAG

A_14_P124104 TAGAAGTGCCATTTGGAAGATTGAATGGCCCATAAACATTTTCATATTTCATTAGCCGAT

CUST_6865_PI426210822 GGGTGATGATGTTCGAAGGGAAGCTCATTTTTGCTGTTTCTAAGGAGTAT

A_16_P00147089

CCTAGTCAGTCTGCAGACATTTTACTCCATCAAGGATTCCTACTGAATTTAGTGAAAGAA

A_14_P135893 ATATTCATAAAACCAAGCCAGGCACTTCTTGCTTGAAAAATGTTCCTCTCCACTTATTAG

A_18_P11807757

AAACAGTTAGTCACCACTCTTCCTATATCACGACACTGCTTTTTTTACAACTCTTCTGTT

A_14_P100972 GCTTATTTAAATGGTCAACCACATGGTAATGCTGTTAAAAGACAGCCAAAATCTTCTACC

A_14_P201076 ACGATGACTCCTAACAATGGAATATGGCCTGGGATGAGGATTAACTGTTCTTTATTTATA

A_16_P20245894

ACGTCAATCTTCCCAAAAATACTATCTCATTCACTCTCTTTACATTCCATTCATCCAACA

CUST_7672_PI426210822
CAGAGGGAAACTTTGAGTATGTTAAGACCCCAGGAAGGCTTATACTTGATCTTT
A_14_P111424
ATCAATAAGAAAGAACCAGTCGAAAAACGGGGTAAGGATTTCAGAGAAAATACAAACATC
A_14_P121605 CAGATTTGCGTCTCACAGATAAAAATCTAGTTCCCTATGGACATCCTCACGTTCAT
A_14_P117631 TCTTTAACTATTCATACACTTTTCGAGTCTACAAGAGTGGTTCAGAGGGTCCAGTC
A_14_P201218 ATAGATTAAAAACAGACCTAACAGGTCACAGAATCTGAATTTCTCCTCTCCACCTTCTAA
A_14_P102508 ATGTTTGGTGAATTGAAAGTCGAACCTGGTATTTCCTAATTCAGGGAACCATATATTTGA
A_14_P121436 GACTCAGCCAAGATTATTTTTAAATATTTCCCATTCTTATGACCTATCCACCACTTTGGC
A_14_P124194 CTCACAATCAGGCAGATGTGCCATTGAGAAATAACACTTAAAAGTCTTTCAAATGCTCAA
A_14_P113694 TTAACATAATCCCCCAATCCCATACAATTCAATTTCAGAAAAGTATTTAGGGACTGTGCT
A_16_P15533780
AATCTTATTACCACATTAGCCTCTTACAAGATGACCATTGGAAAGATTTCCCACAGAAAG
CUST_8453_PI426210822 CAGAGAAGCACACTTTGTGAGAACCAATGGGAAGGAGCCTGAGCT
CUST_1290_PI426210822 TATCTGCACAGCAGTTTGTCTGTCCATGCTTTGGTGCCAGTTATTTCA
A_14_P139653 GAATCTTGTAACAAATGTTGCTCTTCCTGTGAAATCTGAACTTCACGAAGCAGACAA
CUST_5238_PI426210822
CTGGGAAATAGGTTCTTGCTACAGGTACAGTTTATAATAAGGATTTATTTCATAATAGGG
A_16_P00432213
AAGAGCCTACTGATCCAATTTCTACGAGAAATGAAGACTATCAAGCATTCTAAATTTCCT
CUST_3683_PI426210822
ACAATTCTGTTTGTTGGTTATCATGGGTAGACTAATACCTGTCAGATCATATAACCCGAC
A_14_P122401 ACAAATTACCAAGATAATAAATATTCAAATTGCACAGACCATCTGCCCCTGAACAGATGT
A_14_P103396 ACTATAGAAACCAAAACAACCTGTTGATTGAAATGTCTTCCAACTTCCCAATGAATTTCT
CUST_1329_PI426210822 CGATTGGTGGTCTGCTTGAATTACATTCATTAATGTAGGCTCCATGCATTGAGT
CUST_5760_PI426210822
CAGCTTTAATTTCATGAACGATTTGCCTTTCATTGATTCTTGAGGATATGAATGTTTTAG
A_14_P137946 GGAAATTTTTTTTCCAGACTTCCTAGTCCTTACCCTAGCATCATTCTAAAAGGTGTTATT
A_14_P138058 CTGCCTCTCTTTTCCATTCTTGCCACTCCTTTACGAACTCCTTTTGCTTTT
A_16_P20049880
GCTAATGATTACCAGAGATAGATCTACTCAAACTGCAGATAGAAATCCTCTGATCATGCC
CUST_1525_PI426210822 GACATGGGGGGTGGAGGGAGGATGAAGGGTGCTCTCTGGCTTCCA
A_16_P15116270
AAAAGAATGCATTACTGAGATATCATCAATTGTGTTCTGTCATTACATGGCAACTCCACC
A_14_P104037 TGAATTTGGGACATAATAACAGCAGTGGAGTAAGTATTTAGAAGTGTGAATTCACCGTGA
A_14_P113069 TTACTGAATCACCCTGCAGATGCTGTCATCCTATTGTGGAAGGTGAATGAT
A_18_P26794214 CTGCCCCCATCTCCTCATCCCCACTCTCCCTCCCTGGAGTTTCTG
A_14_P130654 TTTAAGATCCATTTTCATTACTTTAACCCTCAGCGTTCTGAGTATCTTCCCCAAATCTGT

A_14_P102707 AAGAATGGGAAAAGAATGGCTCCCTGAAGAAAGAAAGGCTGGGAATACAAAATA
CUST_1866_PI426210822 CTTTCCCTAAACTATAGTATCTGCCAAAAAACCCACCCCCTGGATCTAG
A_14_P110715 TCTTGGTGACTTCAAACCCACCAAGTTGAATACAGAAAGGTAGAGATGGTTATAAGAATA
A_18_P22839943 CGGCTTGTTTCTTTTTGGATGGTAGGAGGCATCCAAAGCCTCTTTTAG
A_16_P00685602 GATCATCGCCATCAGTTTGTTGGTGGGAATCCTCATCTTCCTGCT
A_14_P201694 ACCGCCTACGTGTCCTCTAAAAGGTTTTTATCCTACATATAAACTTTTTACAAGAGTTGG
A_14_P118733 TTCAGAGTTCTCAATTTGGAAACTTTGGCAATATACCTGCTCCAATGACAGTAGGAAATA
A_14_P135647 GGGAGCAAAACTACCCACTCTACCAAGTTGGAAAAAATGAAGAATGCTTCAAAC
A_14_P123203 TTCAGAACCACAAAACACTGTATATTGTAAAAATGTCCCGTTTGCTTATGCCTGAGTGAA
A_14_P103537 GACTTACAAGTTACTCAAGAAGACTGAGTATGTATTTTGACTGCCTGCACTTCCTGAATA
A_14_P135241 CATTTCCCTCTCATCACCATGCACATTTTTGATGGACATATTACTTATGACTCTTTGTTC
A_14_P107453 AAAGATTGCAAAACACTTTATTTCCATCACATATGTGCCAAGACTTGTGTTCTGTATCCA
A_14_P109281 CAGGCTGGCAAGAAAAACTTAAACATTATTTACTCAGCCATGTGCGAGTTAAAACCAATA
A_14_P135423 ACATACAGAAACACTATGCACAGACACATTTTTCCACATTATGTTGCTGCTTCAGTAAAG
A_14_P117002 CCAGGAAATATGAAGAGATATATCCTCCTGAAGTAGACGAGTTTGTCTATATCACCGATG
A_14_P108558 AGAATAGATGCATTTATGTCTTTTCCTGGAAGATCTCTTCTACGCTTCTTGTCTCAAAAC
CUST_6778_PI426210822 ATGGAAAGAGGCTGCCCATGTTGAACAAAGGGCCAGTAACATTTC
A_16_P20008929
ATCAACAGAGGTCTATTGAGGTAATAAGAGGTCTAAGGTAAGCATATGTTCAAATCCTGG
A_16_P20171132
GCATTACTGTTCTTTAAAGTAGATGTAGAATTCAGGTCTTCGAGATTTTTTACAGAGGGA
A_14_P105723 ACAGTAGTTCTTTTGCTGCTAGTATTGCAGTTTCCTTTTTTCTGATCTAGACTTGTTGAC
A_18_P13168348
CCATCAAATGCCATTAAATCTATTGCTTTCTAAATCCCTCATCCTAAATACAGAGACCCA
A_14_P108317 ACCTCTTTGAAAAATACCCCTTTTTAATTGTATCAAGAACTAAAACCCCATTCCCTGTTT
A_14_P120312 ACAAAGTTAGCGTTACTTTTAAAAATGAAGACCGTGATATAAACTGGTAAAAAACGGGCC
A_14_P108104 GAAAACTTACTAGAAAATCTGGATAAACGGATGAACTGGAAGTGGCCACTACGATTTTAA
CUST_3054_PI426210822 GATGAGTTAAAATTATCAGACGAGATGGTTCCCTTGTGTTGGGCATAAACCATG
A_14_P100603 ATTATTCTATCTCCCTTTCTTTCTGTGAGTTTATCCAGTATCCATTTGCTGCTTAGCCAA
A_14_P107414 CAAAAGTTTTTAAAATATTGTGCAGGTTAAATATGTCACCTGTTGGCACAACCTGACTGG
A_16_P21256817
AAAATACAAAGATGAGACTCAAAACAGACAGTGGTTTCATATCAGTTAACCGTATGGTCC
A_14_P113393 TACCTGTGTCTCCACTCTGGCTTTATCAATTAAAGTCTTAGCATCAGCAATTAGCC
A_16_P36960024
AGCCCTTACATCTGACAATTTCTTTTAGTTGAATTCACTACAACAGGAATGTCATCAAGG
A_14_P135957 AAAAAGAAAAAGAAGGTGTCCTGCTTTTCCAACATCAAGATCTTCCTGGTGTC
A_14_P138990 GTGGATTACAATTATAAAATGACAAGATTTCTGTTTTAACCTGTGCAGTTGTGGGTATGT
A_18_P16224964

AAAACTGGGAAATTTTGGAAAATCACTGACATTAGCAAAAGGATTGACAAAAACCTCACA
A_14_P112361 TAATAGTTCTTCCTGCTTTCTGTTGTGCAGAGGTTACAACTCTTGGCACATACTAAGAA
A_18_P15913909
CACCATCACAACACTTATAAGGAATTCTACAGGTGTCTTTCATTAACTAACCCATCTCAG
CUST_712_PI426210822 TTTCAGCAGTTTAGACAGGGCAAACTGCTAATTCTTCTCACCTGTGAAAACA
A_14_P102154 TTGGATTCCTGGGATGATTTGCACATCAACTGCTCAACTGTGGATTGTTTTTA
CUST_7570_PI426210822 GAAGTTCAGCAGTAGCTGCCCCTGGCCTCTGTTCTCAGTCCACAG
A_16_P17845060
ACATCTCTGCCATTCTACAAAGTCTCATCAAAATGTCTCATGAAAAGAATTTCGAAGTTA
A_16_P19662665
TGACAGTCTTGTGTTTTACATTTAGCCTTATATGCTCCTTTATGAGTTCTCTGCAAAGCA
A_18_P16483275
ACAAGGCCTTTGTAAAAGATATCCCAGCATCTTCTTGATATTGAGTTCAGTAAAGGAAGT
A_14_P138628 TGGATCTTTCTATACTTTTTTTGTGTGCTGCCATAGATATCTGCCATTGGTTGCCAAAAA
A_14_P112244 GGGCAGCAGAAAACTCATTGTCCCCTTCCTCTAATTAAAAAAGATAGAAACTGTCTTTTT
CUST_9343_PI426210822 TTTATTTTCCTTTTGACCTCAAGATCTGGCCCCAGACAAGGGAATTTGA
A_14_P133314 ATGCAACCCAATAGCATAAATGTCCTTTTTCCCCCCAAATATGGTCTAGATGAGTTATTA
CUST_4416_PI426210822
CTTCTTGTTATTGTTTTTCTTCCTGAAAGACAAAGGGAGTTTTATGCCATGGTATTTTGA
A_14_P110312 GATATAATGTCACACTTAACCAGAGAAAAAGTAGGCAGACTTGAGAGATTCCTAATGGAC
A_16_P20759080
CCAGTGACATCTAGATGTGATTAGAAACTCTTACCATTTCAACACTTGTAAGTTCAGGAC
A_14_P111027 GCCACTTCAATTGGATTCAGTTCTGTGCTCGATTGGCTAATTACTTGTATTCTCTTCTT
A_16_P15984344
AATTGCGTTGCTAACTTTATCTGGGAATTCCTACTCTAAAAGCCAAGTTTCATTCTCATG
A_14_P110537 TGCCTTGAGTCACCTTTTCATACAGTGGTCTTTTAGTGCTTTTAGAGTTATGAGTTAAGT
CUST_8008_PI426210822 ATACTTTGGCTTACCTTGGATGGCCATGTATTTCTCTCTCTCACAGCC
A_14_P134519
CAGCCCAGAACCCATGTGAAAAAGACAGGAAATAAACACAAAATTACAAACAGCATAAAA
A_16_P30952397 GAATTGTGCTGTTCAGGGTGAGGGATGGAGCCATTCACCATTCAT
A_16_P31679346
CCTGAACAGTAACTGATTTGACAAGTATCGACACATAAAGTTATGGCATCAGCATTCTCT
A_14_P139769 AAAGAAGAAGAAGATAGGTCAATCCCTTAAAATGGTGTGTATATGCCCATATGCATTTCT
A_16_P36406190
AAATGATATGCAACTTATAACACAGCCATCAAACTATACACAAGTTTCTCATTCTTCTGT
A_14_P121452 CTTTATCAAAGGAGAGTCACAGAACAAATGTTTGTTTGTAAAGCGTTCCAAGTATTTTGC
A_18_P22841041 CTGTCACTACCATGCTGCAGTGAGCATCTTGGGTCACCTAACTATTAATTCT
CUST_8873_PI426210822 GACATACACCCCAAGTTTCTTCAGACCAAAAAGAACTGCCTCAATGTTGTTA

A_14_P105912 ACTTGTTTTATTTCCTTTTCTACAATGTATTGTTCCTGGGGACAGTTTTTGCCTTCTTAG

A_14_P101908 GGGTTTTTTTTTCTCTCTTTGCAGAGAGACAGCACTTCCCAAGTTCCTTTC

A_16_P00685630 CTCTCAGTAACTGGTATTCCCCAGCACACAGTCTGGAAAACACTACAC

A_14_P136295 CTCTGGCAAAGGTAAAAAGAAAACCTCGAAAATTCCAGAAAGAAAATCCCATATTTTTCT

A_18_P16813055

AAGTGGATAGTAGGTGTGTCAAGTAAACAGATGACTTCTGTTTCTGAGTTAGCCAATGT

A_14_P117893 CTTAGAATCTGATCTAGTGTGTTCAGTCTCGATCCCTTTTGCTCATAACTTTCAAAACTC

A_14_P201136 GAGGTGAGCAATTCATATTCAAAGGTTGCAAAGTGTTTGTAATATATTCATGAGGCTGGA

A_16_P21192497

AAAATTGCATACTTGTTCTGTTTTCTTTTGCTCTGTTGCATATCCTAATGACTTCACCCT

CUST_5919_PI426210822

CATGAGCAGTGACAAGGAAGAAGTTTAATAATGAGTAAATTACAACTAAAAGTTGTCAAC

A_14_P104322 CTATGCTCTATGCTCTTCTTTCTTAAAAACAAGGAAATGGAGGCATTGAGTTGTTAGGAA

A_16_P01250246

ACTTGTCCACACAGAAGTTATAGAACTCCTACTGAAGGTATTGTATGTTTTATGTCAGGG

A_14_P104088 GGAATTGCACAAGGAAAAGAAGAGAAGCCTTTACTTTGTAACTGAAATTCTCCAGAGTTG

A_16_P03714810

AACCCCTTATTCATCTGGCATATTCTTGACTTATCTAAAATTTCTTCTATTCTGTGAGCA

CUST_6237_PI426210822 TTACTGTGGGCCAAGCCAAATTCTGAAGCAGCGCAGTTGTTTGTT

A_14_P128907 GGAATTCTTTTGTAAACAATACATCTCTGAAAAGTCCTAAAATTAAGACCAGTGCCCAAG

A_14_P202514 CTAAATGTTTTTTTTTTAATGTCCTTTCAGTTTTTCTTGTGTGAAAAAATGAACTTTTGCC

A_14_P139357 AAAGGGAAAAAGGTCAGGCTTTGAAGTGGAAAGAGGGAACATCATCAC

CUST_7172_PI426210822 GCAATGATTCACCCAGCTTTCTAGTATAAAAAACTTCCTCTGGCCCACC

A_14_P113302 CTTTATACTATTGCCAAACGAATCATATTAAAGTTGTGCTGTAACATGTGTTGCATCTGG

A_16_P15929015

AAGCAAGTCAAAATTCTAGTTCCTTAGTTCCTTACAGTTTTCATCTTCTAATGCTGGCCT

A_14_P138424 GCCTTATAATGGTATTTCTTTACTTACCAGGAAGGTTGTGCCATTTGTTCACCGCAAATA

A_16_P19050838

ACACAGAGCCTATCTATTTTCAAGTTTATAGGAGAGTGATTTGATATACCCCATTCCTGC

A_16_P31433969

AATGGATTACGTTATGCCGAAACAGAAACATCAAACTGCCAACGTTTATAAAAAAAGAGG

A_14_P114423

TGGAAAAAAGGTAGATAAAAATCTAGGCTTATGAAACAAGTTGAGCAGAGGTAGGAAAGG

A_14_P134910 ATGACAAATAAAAAGACCGTCAACCCTTATACAAATGGTAAGTAGAGATTGGCCTTTTAC

A_18_P14134538 CTGGGAAGGAATGTGACATGGAAGAGATTTCCCTTGTCAAAATAGAAGACACTT

A_14_P112556 ACGGACAGCATAAATACTAGTGCTATTATCGCTGATAGTATGAGGAGGAAGAAGAAGAAT

A_16_P15568274

ACCAGCCTTACCTGATTACAAAGCCTTTAGAAGTTACATGAAAAAGAGACATTAAATGCT

A_16_P00685276
CTTCATATGATCTCCAGAAGCAACTTACATGTAGACATTTTTCTGAGAATTCCAGAAAGA
A_14_P202214 CTTTGAACTGGAGGTAAGGCCTCTCAAAATGACTTGAAAATGTTTCTCTCTTATCATTGC
A_14_P137317 CTACTACCCAAATTCTCCAGAATGATCTAGTTGATGTCTCTGACCTCAAGACATTATTGA
A_14_P101395 CTGGTTCATACAACATTGGAGTGTGTGAAAAAAAATATGCCCGTGGAAGGTAAAAATTAA
A_14_P126109
GACTTGAAGAGCAAAGAAATTAAAAGAGCAACACTGAATGAACTGGTTGAGTATGTTTCA
A_14_P116516 TCGTAATCAGCATCCAACTGTCTGCTAGCAAACCATTTCTAATAATTAATTTGGCCATCA
A_14_P131059 TCTTTCTCTTTTAAAGTCAGTTAAAGTCTTGTCTAGAGAATGCTTGAGGACTTCCCTGAA
A_14_P101067 AAATCCAACATTTGCAAGAACAAGCCAGGAAGGTAAGGAATGACTGTTAGACAG
A_16_P02367750
AAAAAATCTGAGCAGAAAATACAAACAATGCTTTGTTTCCATACAAAACTTAGCCCCTGG
CUST_6243_PI426210822 CTCCCAAGTTTGTTCTGAGCCACCTGATATAACCTCAGGTGAACAAG
CUST_241_PI426210822 CTGAACCTCGCAGGGCCTGGAGGAGTCGGGGCACTGGAGCTGCAC
A_14_P118297 CTAAGAAATGTAAGGAAAAATGATAAGCAGGAGTTTTCAAGGAGCAAATAGTCTTGTGGG
A_14_P105562 CTAAATTTTCTTGATCTTTTGCTATGTTGGTAATGGCAGCACATACACTTGCCAGAACTT
A_14_P137083 CTGATGGAGTTAAATACAGTGTTATGATGAGCAATCTATCCCTTCTCATCTGTTCTCTGA
A_16_P00081650
GCAACACATGCAATCAAAAGGTTGCTTCTTCTTAGAAGAAGATGGTGAAATCATTAGTCA
A_14_P102788 TTAAAAAAAAGCAAGTGTGCTTTTGTCCAATTAGTGACCATATAGAGTTGGAACAAAGGC
A_14_P103317 CTTTAATATTTTTATTTTGGCAGCCTTAATTATTGTCCTTGCTAATGATGACGGCCCTGG
A_14_P124266 CTGGTATGGTTCAGAGCAGCCAAGAGAAGGATCAAAAGTATTTGGAAATTAATTGTTTTT
A_16_P16445148
AAAATGAGCATCTTCGAAATGACATTGACTAGAGTGCCTATTTTTCAAACTTGGGATTCC
A_14_P109274 CTATGGGACTTGAGATGATACAATTTGTTTAAAAATCTGCTAGGCATGTGAGTCACAGTT
CUST_2331_PI426210822 AGTGGGTCAGAGACAGAAGGGGACACACATTGAGAAATTTCCAATC
A_16_P17585421
AGAAGCCTTTTGCTCTTAGATTTGCAGCAATTGTTTACACTAGAACTATAAGGAGGAGAG
CUST_569_PI426210822 TTTTCCCAGTTGGGCATGGACTCCACCAATTTCATTCTCTGACTT
CUST_1153_PI426210822 CAGGGTGGGAAATAAGGGATGGATGGGGCTGGCATGTGGAGTATT
A_18_P12019910
CACATACATGGAATCTAGAAACACTTTTAATCCACTATTTCAAATGCCTAATCCCAGTGC
A_16_P20630124
ATTGATCCTCCACAATTGAGACTTTAGCCAATATGTGCTATTTGATTCCAAGATGCTTTC
A_14_P110513 TGTTAATCTGCTACTCTTCATTTGTCCATCCTCTAACAGTAGCCATGACTCAAAACC
CUST_5555_PI426210822 GGAGCCTGAGAGAAGTTCTGGGAGAGCAGTTCTTTCTTATTGTCC
A_16_P39218605
AAACAGAAGAAGAATGCTCAGAAAGTCATCAAAAGGACATTTTTTCCTATGCCATTCACT

A_14_P110363 AAATAGATTACATTACCTTTCTAGATTCTTGCCAGAGATACTTCTTCAAAATTTTCCACT
A_16_P16022218
AATGAGGGAGGACTATAGACTAAACTCAAAACTGACTGAAATATTTTACAATTGCAGGCA
A_14_P136587 TAAACTCGGACAGAACATGAAGACCTTTATCCTATGAGTGTATAGCCCCAACTTAATTTT
CUST_7866_PI426210822 CATGTTACATACATGAGCTTGCCATCCATGAGTGTTTGGGGACAAGAC
A_14_P105836 AGTTTACTGAGTTTCACTGAACCTGGGAGAAACTGAGTGTAACTTCTTGGTAACATATAA
A_14_P121764 TTGGAAAAATTAAAGTGATATATCCTGAAAGGGACAAAGTATGGGCTTAGAACCCATGAG
A_14_P105775 CTTGGCTGCCCATTTTTGATCTGAGTTGAGGGTAACTTATTTGTCAAAATTAATTTAACA
A_14_P103160 ATTGTTTTTTCTTCCCTGTTAGGTATTAATATAGTAGGGAAATAACACTCAAAACAAGTC
A_14_P124529 GAAAGTCTCACTACCATAAAATTATGGTTCAGCATCAGATTAGCATTGCACTCAGTAGTC
A_14_P126759 GTGCAAATGCCCCTGGATTAACAGTGGATATAGATATGTTGCTAATGACTAACAATTCTT
A_16_P17915378
TCAGACTTCGCCAAATATTTGAACACATTACATCTCATTTTCCATAACTCTTCTCTCAGT
CUST_5986_PI426210822 CACAGAATGGCTTCCTTTTGAAACAGTTCCTTCTGAAAGACTGTTTTAGCCTT
A_14_P117080 GAGAAACAAATCAACCACGATTGTATGCATTTTGATGTGACAGGGAAATAAAGTAAGGTA
A_16_P55109335 GTTAAGATGCATGGTGACCCCACTTAATGGCCAAAATACAAGGAAGGCTT
A_16_P00433564
ATCGAGACAACAAAGAGAACAAATTCTTTTACAGGTCACAACTAGTTTCATCACAGGAAG
A_14_P118896 CGACTTTAACTTGTCAAACACTAATTCAATCTTCAGCCATATTCAATCTTCCACTGGTTT
A_18_P14053205
CAGAGAACTCTCCTTGTGTAGACTTTAATCTGACTCCTTTAAAATAAATCCCTGCTAACC
A_14_P106933 TAAGATGAATCTTGGCCCTGTCACAAACATTGCAACTTTAAGAAAACTATAGATCCACTG
CUST_4750_PI426210822
GAATTAAGGTATTTCTCCCATATTCAAGGACTTCAGTTTCATTACCTGTCCAGAAAACTA
A_14_P105214 TAATCTCTTCATAAAAAAGGAATATTTTCACCCTGATTCTAGCCAAGGTCTCAGAAGTGG
A_18_P19646561
AAAATCAGTCCTGGCATAATATTTCCTTTAAAGACTGATCCTGTTAAATTGTGGGGAAGG
A_16_P02228925
AGAGCACATCATTTCATTCTCATGTCATTCTAAAAGCAGAGCAAGTTATGATCAACCCTA
CUST_5234_PI426210822
TGTTTAAAATTATTTTAGAATTTTCTTCTTTGGCTTTAATTTAAATTTTTGTTTGTTTAC
A_14_P133940 AATGAGGAGTGCTTTGTAAAGAACAAAGCCCTAAAAGTGCAATCCATTATTATCCTACTA
A_14_P201227 TTCTATTTGCTATTTCAATATGTGACATGAATCTCTGAGGCCAGCATGTCACACGTATAC
A_14_P129079 TGAAACTATATATGTCCAGATAACACTCAGCAAAGTGCCCTTCTTGCATTTGTTTCAAGA
A_16_P01728413
TCTGCTCTAACTCAAGATTTCTACCTCCAAGATAACTACTTTTATCCTTCGTGGCTAAAC
A_14_P124830 ATACAGCCAATACTGTGGACTTACCTTAGTATCTTTGATAACTGTAGATGTCCAGCCTTC
A_16_P39928378

TCCACTACTAGGACAGTTACTGACAATAGATAAGAGAACAGATGAGACATCAAGATGCTT

A_16_P01070546

CTACTAGAAGACGGTATTACCACTTCCTATCCTACGAATTACACGCTACTTCTGGTTTTG

A_16_P15936530

ACTCTCTGGCAAGTAACTACAATCTATACTGGTAGGAAAATCAACTGTCATGAAAAGCAC

CUST_6431_PI426210822 GGGGAAGCTGAGAGCAGCCTCTTACAGGAGTGCATGCCCTATAGA

A_16_P17829090

CCCAGACTGTGAGGAATAAGCCAAGTAATAAGCAATAACAGATGGAATATTGAGGTTATG

A_16_P38874278

AATGCCTTAAGAAACTGATTGCTGTAGACATCAGTCAAATACTTTAGGATGAGGCTGTTT

A_16_P38065503

TTAAAAAACCCACATCTTCCATTTTAGACATACCATTCCCATTCAATCCATATGTCCAGT

A_14_P121754 AAATCATTATCATCATTGTGATCCTGGACTTGTGGTATCAGAAGTTTTCTGGTTGGATAT

A_16_P02682444

AGGTTTCTGTTTAGATGTTCCATTATTACTCCAACACATACATCATAAGTATTAGAGCCA

A_14_P110152 TTTGGTATACATTATATCACAGAACCTTGGACAACTGGACCCTGTCCTTAATTAGATCTT

A_14_P136006 GAATACTGGAATAAACCAGAAGAAACTAAGAGTGCATTCACCCTGGATGGCTG

A_14_P201502 ATAAAACTCATTGCTATATAAGCAAATACTGGTGATTTGGCGACTGTAGCAGACACTTGT

A_16_P01570049

CCAGCAAATACGTTATGTATCCTAAAGTGTTCTGCCTTATGATTACACCTTACTCCAACT

CUST_7712_PI426210822

GTGCTTCCAAAATTTATTTGAATTCTAAATTAACTAAGTGAGTTAACGTGGAAGCCGCTT

A_16_P16497051

AAACCTAATGTATCAGCGATATCACATATCCAAGAGAGTATATGTCATCTGTGTCATCTT

A_16_P36787787

AAAAAACTGGCTCATTGATTCCTACTTCATTCTATAAATTATTCTCTAGGTGGAAAGGCA

A_14_P113854 ATGAGGAAAGAAGTCTGCAAGGAGAGGAGCAAGAATGTGTCTCATAATTCTTTTATATTG

A_14_P101467 CACAATCACATACACCTAACCCTCAACGATATACAGAGAAAGTTTTGATACTAATTTTCA

A_16_P01051832

CCCTGATGGCTTAGAAAATTGATATACTAGCACACCTGATCTATTAATCACTCACAGCAA

A_14_P133924 CTTTCAGAGTTCCTGACTGCCTATACATTCACATTGTTCAACAAATGTTACTTAGGGAAA

A_14_P105534 CTCACGCACACACAAAAGGAGAAATGTTTCAAAATATGTCTCTCGTGAAATTCCACTTTT

A_16_P36499494

GATAGAGGATTTCCTGAGTTTTCGTCAGTAAAGTCTTGAGTGCTCTAGAGAGAAATAGAA

A_14_P120736 ATCTCACACAACAGAAAATCTCAAGCCAAAACAGATTTATTTCTTCCAAAGACCACTAAC

A_18_P22840875

AGTTTCCATCTAATGGTGAGGTGGTAAAGGAAATAAAACTTACCAAAATAGAAGAGGGAG

A_14_P136419 GACAGAGGTGTTAATCTTGAGGGTCTAAGATTCCCTCCTGCCTATTGTG

A_14_P128664 AAAGAGACTCGAAGTGTAATGAACACATCCAATCCAAAAAATAAGGTAGGCTAGATTGAG
A_16_P02990830
ACTCCAGGAAATCACTGTAGTCCTTTTAACTGTTACTGATTTGCTTAAAATGTGAAAGGC
A_16_P15904946
AAAAATGAAGAGAGGCATCAATGTAGTCAACCTTTGCTTATATCTGTTCTTGAGGGAATG
A_16_P20353086
AACCAAACTCGTCTAAATTTATTGATTTGACAGGTGACATCTCTTGAAATAGAGCACACG
A_14_P130036 GTTCTAAAGAGACAGAAACTCACCATTGGACTGGTCTTCTCCATAGTTTTTATGGGAT
A_16_P17793809
AGAAAATGTCCAATCAGATTCAATCACACAAAGCATTTACTATTTTCAATCACTTGCCCA
A_16_P58424623
ACCTTTGTGCAAACTATAATAGTAAATAGCACCGCTTCCTAAAGACTCTTTACTTCCATC
A_14_P110013 AAGAGAACATAAAAGACAATACCTGTAAGGTAATCCAGACATTCTAGCAGTTTCTTCTCT
A_14_P126595 GTGAAATGAGATGTGCCATCCTCCTTGCAAAATGTCATTCCCCATTCAG
A_14_P130661 CCCGGAGAGAAACTCCAAAACAATCTGACGTCTTCTTTAGACATTCCAAG
CUST_1529_PI426210822 GCAAGACTGCCTACCTTCCTTTCAGAAACTGCTGTTTAAATCCAAAGAGAAC
A_14_P112973 TGGGCAAACCTTGTTTATTCTCTACTAACTTGGTTGTAATCTGTTAATCAAAGTAGACCC
A_16_P18304488
GGATGGGAAAGTATTAGTGAAATCAGCAGATTGATCAAGACATATGGAAGGCATGAAAAT
A_14_P118125 AACAAGCAGAAGGATGATGCAATCTCTGACATAATTAAAACCGGGTAAACCACAATAAGT
CUST_578_PI426210822 GACTGTTCTCAGGGTTAGCCATGCTACCCTCCCTACCTCCACCTC
A_16_P17822265
AAAAGCACATTCCATACACTCTACTATGGCAGAATAAGGAGGTTCACAGATAATTGAGAG
A_14_P128574 AAGGCTAGAAGATGCAAAGAAGGACTTTGAGAAGAAATTTCGGGAAAAGACCAAG
CUST_953_PI426210822 AGCCGAACTCATTTGGAGAGATTGATGGAGTTACTAATGCTTTGGAGACAG
A_14_P103388 CTAATGCAGATCCCTCTGATTCACTAAACACAAACCCTAAAAATGAAAGCAAGTAGGAAA
A_14_P127926 AAAACCTGTCACCACTAAATGACACTCTACCAACAAAAGAAGTCTTCCTATCTCAAAAAT
A_14_P134564 CATGAACAGCGACTAGTTAAGTGGAATGAAAAATGCTAAAATGGGATACAAAACACTGTG
A_14_P129086 ATAGAGTGTTTACTAGACTTGGTGCCTCAGACAGAATAATGTCAGGTGAGTTTTTTGTTT
A_16_P17730145
AGCCCCTGACTTAATTTTGTTCTTTCTATTTGAAACCCCTACCATCAATTAAGATTTGCT
A_14_P135280 TCTGGCCAAATGTGACAGATGCAGATCTTAGAGGACTTACAAAGCACTATATTGGTAATT
A_16_P00684988
CTTCTGAAAAGCCAATGTTGTTTTCACCCCAACATTTAATCATTCCAATATTCCTCTCTG
A_14_P107995 CAAAGGGAGGGACAATCCCCATATTAACAATGAGCAGCATGAAAGGAAAC
A_14_P132051 GATAGTGTTCCAGATTCTGAGTCCTCTGAACTTTGACCTCCAGAGTTATTAACATCAAAA
A_14_P119223 ATGGTCATCTCTAAGGCCATTCTTTGCTCATGGTCATATAAAAACAGGTGAAATTTTGTT
A_18_P11098035

TTGCATGCTCCTTTATAAAGAGTGTAGAGTATTTTCACTCTCACTGAAGTCTTTAGCAAT

A_14_P109425 CTTCAAAATAAATATTCTATACAAAGAAAACCTGTGGCAACTTTGTGGTGGGGTGGAAAT

CUST_5857_PI426210822

TATTTTATCTTTCCATGATTGTGCTTAATTTTATCTTATCAGTACAAGCACTCATATAAC

A_14_P116352 ACAGAATTAAGGGAAAATCCACCAGTAAAACCAGACTTTTCTAAGGTAATGTTCTTACTA

A_14_P200016 GGGAGCAATTAAGGAAGGACTTTAAATCCTTGGGAGATCTTAAAACATTGAGTTCTAAGC

CUST_6664_PI426210822

TTTTAGAGTAAATTAATTTGAATGAAGTGAAGTTTATATAGCATGACTCAAAGGTACAGT

A_14_P126856 ACTATTCAAAAACGACATCTGAAAACTCATATGGTTCTAACTTTTATTATTAGGTCAGAA

CUST_9132_PI426210822 ATGTGCAGAGCAAGGAGGAACTCTGCTCCCACGCCTGATTTTAAT

A_14_P116504 CATAAGAGTTTACTTGGTAAATACTTGTGATGAGGACAAAACGAAGCACTAGAAGGAGAG

A_16_P17013167

CAAATCAGCATAGGAAGTAAAAGAAATACCCCAATCTCTCTAAGTAACAACTCTAACGGG

A_18_P14130469

GGTAGATCTACAGTTCCAGGATGGGATACTTTTTTAAAAAGTAAATGTAAATGTATTGCC

A_14_P111473 GAACTAAAATAGGTGATCTGTTTTAGGATGGCTTGGAAGAAAAACAACTTAAATTTGTCA

CUST_617_PI426210822

AGCTTTGGCTTTGGGATCAAATGCTGAAAGCAAACCTAATGGGTTTAAAGTTTTTAATC

A_14_P104597 TGAAGGGGTGAACAAAATAAAGAGGGCTTTACTGTGCTTGATCAGACTTTTGAAATTTTA

A_14_P132228 ACCTAAGCAGTCCTAAACAAACTGAACTCTGTTTACTTTATAAAAGAAACAGACTGCCAC

A_14_P136990 GGCAAACTTCCTTAAGGTCCTCCAATAGATTTTCAATGATTCACATCTGAAGTCTCTTAA

A_14_P120659 CTTGTGTGACTTCTCACTGAGCACTCCTTTACAGACTTACAAGTCTTTACTCTGTG

CUST_4134_PI426210822 CTCGGCTTCAACAGTCTTGCTTGCTTACATCTACTAACTGGGCAAGA

A_16_P02491491

GCATGCATCTAAGAAGATTCCTAAGACATCCCAATTTACTGTTCTAAAATAGCCACTTCC

A_14_P117483 TGCTTACCAATTCCTGAACATTATAACATGCACTAGGGTTACAAAGAATACAGCACACAT

A_16_P17393538

ACCGACATGCTAGATTAACCTACCTTGCTCTACAATAATTTTTTGGTTACAGCCCATTAA

A_14_P139396 CTTCAAGAGATCATTGCTTCTAACTTTCACATACTCCTCTCAAGAGATTCAACTGCATTC

A_16_P17185727

CCTTACCCAATGTTCTATTTTAACCAGATTAGCATCAACAAAAATAAAACCACTGTTTCA

A_18_P17651896

AGCCAGCACTGTTATATCCTCCCAATTTGGTTCTTACATTACTTCACTCTTCAGAACAT

A_14_P106149 ATGATGATGCATGAACAGAGACTTTGTTTCCATGCTGTCTTTATTGGAGGTCTGT

A_14_P132663 TCAACCCAGTAGTATCTTTTCATTCATCACTGATATTACGTGACTCTTTCCGTCACATAA

A_14_P113704 TATCCAAGAGAATAAACTCAGTTTTACACCAGCCTGAACAGCAGTCAAAGATATTCTCTT

CUST_906_PI426210822 GACCAGATTGTCAGGCTGGTCTGGCAGGTTGACCTTGGCTTAGTG

A_14_P114799 ATTAACTGGTAAATGTGGAACATCTCCTTCATTAATTAGTCTCAGGTCTTGTTCTCTCTG

A_14_P131627 TTTGCCATTCTAGCAAATAGCACAGAAAAACACACATGCCTATTTCTAGTTTTCTCCATC

A_14_P109267 TCCAAAAATATGGTTACTCCTCCTACCAACAAGTGTTGTTCAGTAAAATACATCAGAGCA

A_14_P103441 CTAAGTCTGCTCCTATTCCCTTCATGATACTGGGCAAACTATCTGTATTTTTATGTTACC

CUST_2503_PI426210822

TTTCAAGATGGAGTGGGGTAAGGTAAGGAAACAACAGTAAGAATTTGAAAAAATCCAG

A_14_P132146 ACATAAACATAACCGTTCATAAATGGTTATATGCTTCAGGAAATCAATGTGTATTTTGGA

A_14_P105849 TACTTATACTCTATAGGGTCGTTGAATAAATGGCTTAGAATGTGGCTGATGGCTGTGTG

A_16_P15718805

AAAACCCATTTCAGTGAGATATGGACATGGTTATTTCTTTTTACTCAGCAAACTCTAGGC

A_14_P125679 GGAAAGTTCTCATTTAGTTGCAAGACCACAGAAATCTTACTTCACGGTATAATTACAGGT

A_14_P101711 CTAATAGGCTGCCATAATTTCTTAATTCTCTTTCACTTTCAGACTCGATCTACCCATCTC

A_14_P102558 GAACTTGTTTAATAAATGGGAAACTGACCTTTGCTATACTTTTCCCACGTGCTTTCATTT

A_14_P127811 AAAGTTCACAGAGATTTGTTAACAAGAATTGAACCAAGCCACCCATTAGTTCTATGAATG

A_14_P110230 TTCTTCAGTCTCCAAGAAGAGTTTTTAGACAAACACACACTGATGAGAGTGCTTTCAA

A_14_P119991 GAAAAAGTGGTTTGAGGTTTAATTTGTTTGTTGATTAACTGGATGCCTACTCTGAGAGTA

A_16_P01213975

ACACATAAGTGATCCATGATTTTTTGCATCTCTTCCACGTATACCTTTTCTTGATTGCTC

CUST_9438_PI426210822 CACGTCAACAGAGTTGGATTTCCACTGTGGAGCCAGGACTATTTA

A_14_P121354 GGGTGTAGTCCCTTAAATGTGTGAGATATTGGAAGAGACGTCAATTTACTAATTCTGAAC

A_14_P106333 GACAAGGAGTAATTCAGAAGCGTGCTTTAATTTCTCAGAATATTAGGAATTTGGGAGGAA

A_14_P132995 GTTTATGGGATTGTCTTTTTCCCTTTTTCCCCTTAGGAACCACGTGAATGTT

A_14_P115016 TTAAACATTTATAAACTGATTCCCTTTAGGCAACTCTCACCAAGCAAGAAGCATTTAGCG

A_16_P20322932

ACAGGCACCATTATATTATGCATGCATTGTCTAAACAGATTTCCTATACAGCACCATGTG

A_14_P129272 GTGATAGTTACCCTGGTCATATTGGTTGTAGTTGACTCACGGTTAAAAAAACAGAGGT

A_14_P135835 GGAAGAGATGAAAAGAGAAAGGCAAAAACGTCCAGAGACAAATGCAGAGACAT

A_14_P126541 AAGAAAGTCCAGATATCTCAGGTCCTGTGAATAACTTAGTTTCTCTCGCATACTCTGTTT

A_18_P14132983

TCACTTTGTGTCTTTTTAGGTTTGTATTGGAACTATTTGCAAACAGGTCTTGGCTTATCT

A_18_P14830483

ATGTGCATGGAAATTTTGAAGTAAGCAGTAGAAGAAACTTGATAGTAAGAGCACAACAGG

A_16_P18931719

AGACCACAAATGAGAAATACCACTTTACATGTGCATACCTTAATGATGACCTCTTCTTGA

A_16_P18993574

TGCTAACACCTGAACTCAATTTTTACCCCTAAAAAGACATATCCACTTATCTTCATTGGT

A_14_P120588 AGAGGAAGGAACCTTTTTATTCCTAAATAGTTCATCTCAGGCCCCCCAATTCTATTTA

A_16_P35022496

ATTCTTCACATGTTTTTCCATCTGAGATATGCAATAGTTCTCCTAATCCCAAGAAATGGC

A_16_P03035400
TTCCTCTATGTCCTCTCTAAAACCTTGTGTCATATTCTTGTAATTAACTAGTCTCCCCTG
A_14_P107468 CTGTTTCCTGCTTTCAAAAAATTTCTGTTCAAACTACTCAGATTGTGATCTCCATGGAAA
A_16_P00633021
ACCTTGGCTTTAAGATATGCGATCAAGACATACGATAGGGGAAAACAATCAATGCTTTAT
A_14_P116684 CTTGTTAAGCCACAGGTTTGAAATGAAAAGTCTTTACTGTTCCCCTTAAGGAAATGTATT
A_16_P00678577
ACACAGATTAATCATCATCCTGAGTTCATAACACTCTTACACTCTAATCATGGTCCATGC
A_14_P116685 GAACATGATGGAGGCTCATTTTTAATGTCCAAAGTGGACCAAGATCTCATTATGACTC
A_16_P16703279
ACTGATGCTAACACTCTTTTCTAAATTTGCTAATCTAACCTACTTTTTATCTACCCACGG
A_14_P111930 ATAGATCATATTAATAAAGAGAGAAAGCCCAAACCAAAGGAGGTGAGGCCACTGTG
A_14_P120660 CTGCAGGGAAAATCAGAGATATTCTCCATGACCTAGAAGAAATTCAGGAAAAATTACAAG
A_14_P125784 ACTGATGTATTAAATCAGGGAGTCTTCTTACACTTTTGTAAACTCTACATTGCCTAGCAC
A_14_P136490 ATGCCCACATCGCAGGAAGCCTCAAATTTAACTACCAGAAATATTCTGAGAAAATAAAAT
A_14_P108122 CCTCATTATGAGAATTTATGTCCATCACTTTGCTTGATGTGATAACAGTGACATGCTAAA
A_16_P00736251
ATTGAAGCATTCCCAATTAAAAAAGCAATCACACCTCAAATCTTTTATACCTGACCTCGT
A_14_P111579 TCCAACATGAAAACAACTCCCTTTTAACATAAACTTTTTTTAAAGGCCCCAAACACATCT
A_14_P124518 ATTATAAATAGGGAAATACACAGCCTCCCCTACTCTGGGACTTTCTATGGCCTAA
A_16_P03713639
GGCACACAGTTTAGTAAGAGGGGAGATATCTATGAAACTAGTTCTAATTCAAGGCACAAA
A_14_P102766 GGCAGGGAACGAAGGCAAACTTTGTAATTTCTATTCAATTTGTGTAAACTTTTCCAATTC
A_14_P105413 AGAGAAACTTTAGTATGAAACCTGTAATTCTAAGAATTTTCTTCTGTCCTGAGTCTGGCC
A_14_P103062 CTATTAGATGGCTTGGTCTTCTCCCTTCTGATCTTAAAAGGTTAGATAGTATAGCAGAAC
A_16_P15933039
AATCACACTCCAGAGACAACTAACAGTGAACTCAGATTAAAAAAAATCTCTCGACAATGG
CUST_2757_PI426210822
CGTTTTATTAACAACAAACTTGCTTTTATTATTGGGACGCTAACTATTAATTTACTAGGC
A_14_P130313 ACACTCTTTGTAAATCAGACGCTAAGACTCCAATGTCCACATATTTGCCACATTTGTTAC
A_16_P19954533
GAGATACCACCCTTAATTGTGAAGTAAACCCTGTATCTTTTTAGCATTGATCCTCAAGTT
A_14_P105247 GTCATCTGGTCCAAGGTAAATGATACATCTAGTTATATTTCCTGAAGAGCAGAGTGTGAA
A_16_P02476066
TGAATTAAGACCAAGTTCTAGATGCAATAGTGTTCAATGGATAGGCTTTATTCTCACCCT
A_14_P123674 CGGATCGCTTGTAACTTGATGATACTGTTTTTTTGTTGTAAGACTGAGAAAACCCTGTAT
A_16_P18654109
GCAGTCATGTATAGCACACTTACTTTACGAAAAGAAATGAAAACATGTTAGAGGATCCAC

A_16_P18940101
GGCAAAATTATAAATGACCACCTTACTGTGAAATATAGATCTTGAGCATTTTGAAAGGCC
A_16_P02365877
AAGCACTTTCACTCCCATAATTTAACTGTATCTTCTTAACCTCAAATTCTAGACCACAAA
A_16_P32247434
TTCACTTTTTCCTTGGTTAGCATGATGTTCTCTACAACTTCTTTTAGACTCTGCTTCCTA
A_14_P130773 GAAATCCTTCCTGCTTCTTTCTGGATTCCCTACTGAATTCTGAAGGTTCTAAAAGA
A_14_P118576 ACAGCGTGTCAAGAGAGCATCGTTAAAGATAAGGTATTCTGGGGTTTTTTTAATCATTT
CUST_8864_PI426210822 CTTGCCATGGCAGGGGTGATGGGTTGTTACCAAAGTCGATAACAG
A_14_P130723
TGAATAGAAAACATGCAGACAGAAAAACTATCATGAAAATTTAAACATGTGAACAGAGGG
A_16_P18713683
GACTCTGGCTATAATTCCAATGTCTGATTCTAGAACCTACAATGTAAAATATAGGCCCTC
A_14_P121870 TTCACCAATTTCTCCTTTAGCCCCTTTATGACCCTAACAAATGCAAAATAAGTAATTGTC
A_14_P137852 GGCCCCTAAATCTCAGCCTTGGAGATCTTTTCATGCAACTGTAATTCAAATTTTAATAAC
A_16_P17465780
AACAAGTGCCAACTCGAGTTTTTAAGCCTAAAGAATGACAAACTAAGAGGTCTTCTAAAC
A_18_P22841078
TGAAGACGTTTATGAAACACCTCCCTTGTTTTTATTATGTTTGTGTTCTTCTTATATTTC
A_14_P110691 GGCAAGAGACCAGGCTAAGTCACTTCCCTAAAGGATCTTATCTCAATCTGATTT
A_14_P114696 TTTAATAGGAGACTCCAGCAAGAAAATTTGAAGTCCCACTAGAACATGATATTATTGGGG
CUST_3964_PI426210822
TTTTAACCAATAAGCTATAAAGCTGTTGAACTCCCACTGGGATGTAGTTACCTTTTCC
A_14_P100236 CCTATGCAAATTGCCTAAGAAGTGTGATCCTTTTTCTGATTCACTCATTTCTAATCACAG
A_18_P13569918
AAAAATACACTCCTCCAGTTTTATCATGCTATCTTGATTTTCTTGCCAAAACTTCAAAGG
A_14_P130433 ACAGTAGTGGTGATTAGACTTCAAATCTTTTGTGATATAGGCAAATGTTGAGAGGTCTTC
A_14_P103929 TCTCTTTATTTTCCCAAATCTGTTAATAAACCTTTTGACAGTTGAGATGGCTGTGACGTT
A_16_P00910666
AGACTTAGTTTTGCCCTGAAAAAACTTTAGATGATTCTGTTTGCCTAAATCTCCTACAGC
A_16_P17221365
AAACCATAATGTATTGTTACCTTCTCTTCAAAGTTACCAGTAAGCAACCTCACTTTTGGG
CUST_8761_PI426210822 CACAATGTGAATCACTTATTTTGCTGGCAGCAGCAGTTGACAGTGACAC
A_14_P116615 GTAAAAATTTTGTGTTTTTGTTTAGGCAACTCAGCATATCTGCCACCAAAAATGTAAAGA
A_14_P135056 ACTGGAATTAAAAGGATTTAAGAGATCATGGTGTGCATGTTCTAGGGTAAGTGAAAAGAT
A_16_P00045852
GCCTTTTACAAGATACACAACCCATTCTACACTCAATTGCGTCCTATAAATCAGGTTAAC
A_14_P111273 CCCCCTCCTTCAATTGCTTAATCTTGACAATTATTGTCCATTCTTTTCAAATTTCTCAAC

CUST_8637_PI426210822 CCTGTTTGATACTAGAGTCTAACTACATGTCATGGACATTGTCAGCAAACCACC
A_14_P121554 CACATAGTCTTCCCAGATTCACCAAGGTTAGGTTTTTACCATATTATCATAGTTGTGTGT
A_16_P16191848 AAATCAGCATCCTTTTCTTCATTGCTTACCCCATTCATGCTTGAAATGGGATTTC
A_16_P16462613
AGCAAATCCTACAGAGATCAGTTCATTCCTTCTAGTAGTCTTTTTGCTTGCAAGTCTAAG
A_14_P109638 AGGACCTAGATGGGAAATGTCTCTGCTCATCCGACCTCTCAAAAC
A_14_P100574 AATGAACATGTTTCTCTTGTTTCTAAAACTCCATCCTTAATGAGCAACACTGTCAGGTAG
A_14_P125053 GCTCTGAGAGGAGAAGAAAGTTAGGGAGTGGGAACTTTCCTACCAG
A_14_P103836 GAAATGTCTTCTAGGGTTGCTATGCGAAAGCAAGACTGTGGTTTCATTCCAATTT
A_16_P02356961
CCTGTCCTACCTGGATTACACAGGTATATTGATACCTACTATACGGCAGACATTATTCTA
A_18_P10885205
TGCCCTTCTTTTTCATACTATCTACTCAAACTGCAAATAATCTGTTTCACTTCCATACCC
A_14_P134473 TCAGTTATACCTCACCAAATGGTCAAACAACTAGGTAAGTTCTTAAAATACTGGTCGAGA
CUST_6434_PI426210822 AGGCTCATGAACTTGTGCTTGCTGACATGTATATCTTCTTCAGTTGGTCC
A_14_P112651 ATCTTTCCTTTGTTTCTCTGCTTCAGTTCTCCCATTATATTGCGAGTGCTAGATATGAAA
A_14_P136331 CCACTAGCCTCATGGCCAGAGAGAATTTCAAAATATGCAGGTCATTTAGTAATAATCTAC
CUST_5637_PI426210822 CTTCTCAGGCTTCATCAGCAGGCCCCCAAAGAGACCTGGCTACAG
CUST_3174_PI426210822 TTTATTTCTGGCAGTTACTGTCTGGGCTGTAGCAGAGGACTGGGC
A_14_P111114 CACTTCTGCTGCACTGCTTTCAGCATGCAAGTAGGTATTTCATTAAACATTCAGAAAAGT
A_14_P138129 TTAAAAACAGCCATTTTTAAAGGGAAAAAACCCCCTAAGGTTGGGAGATTGAGAAATACT
A_14_P123263 ATGATTAAGAATTTGTCATGTGGACTTAATTAGGTGGTTAGAGAGAGTGGTAGTCTGTGT
A_16_P03434375
CACTAAAAACTGAGTGTCTTGTTCCCTTTTGTGAATAAAATTCCAGCCAGAACATTTGTT
A_14_P105832 TTCCTCTCTCTAAAATAAAGACCCTTTTTCCTCTGATTGAAGCCAAGAAAAAGGATCAAG
A_14_P128625 CACAGATCACTTTGTTTACTTTCTCCACAGATAAATATGCTGGTGATTGAACTGAAATCC
A_14_P124682 TCATATTTCTTCTTAAGAAGTCTTTCAAGTTCTTCACCAAAAATGTTGACACGTCTCCTG
A_14_P113707 TTGACCTTCAGTTCTTTTCTAGGTTATACCAAAAGATTACAAGACAATGGCCGCTTTAGC
A_14_P127589
GAGAAAAAAAGGTTGGCTGGAGAAAATTAAGTTTCACAAAACTCAGAACAGATAGCAGAT
A_16_P03561976
GGCTCCTTCCATTAATAACTCTATTGCTCACAAATTTATAGATTTCCATTTTCACCCACC
A_16_P16192922
ACATCCTATTATAGCAGACTAAACCATTCCTAACAGATACCTTTTACCCGTATGTATGAA
A_14_P102282 TAGAATTACCTTTCTGGTCCTTTAAAATATCAGTGGACCAAATTCCATCTTCCTTTTTGT
A_14_P117187 CTCACATAGAGTTCGTGCTTCTTACAGGCTTGTTTTTGCTCACTTGTGTTATAATCTGAA
A_14_P129148 AGTAATGTCGTGCAAATGAAAATGTGATACAAGAACTAATGGGGACTAACTCCTCAGTAA
A_14_P136965 TTACATTTTAAGGGGACAAAAATGAAGACCGAAAACTAGGGATAGACTTTTCTGGATTAG

A_16_P00685401 CATCATTTTGATCTCTTTGCCATTGAATTCCAGCAACCTTTGAAACCTTTGTCACAAG
A_14_P120775 GACATGAAAAGATGCAACAAAAGTTGCTAAAGATTGAAACATTAGATGGCCCCTAAGGAC
A_14_P137481 AACAAAGGAGTCGCTTGTCAAGGATCTGGTTAGAGAAGACTAGGGTCTT
CUST_8854_PI426210822 TTATCAAGGAGCCCAAATGTCTTGTGCTCACAGCTGCTGTGACAT
A_16_P15212024
AAGACACAAAACCTAATGTGCTCAAATATCCTCTCTTGTTGTTTGAAAAGTTGTCTGGAG
A_14_P124358 AGGATATTGGGCATGAAATTAAGAGATGACACCCAGAAGTTCAATGTGTATTTGTTGATT
A_16_P00591573
TAAAGACAAATTCTCAGGTGTATGAAGTCTTCCCTTCAATATGACCAGTAGCAGCCATAT
A_14_P123902 GATTTAATGAAAAGTCCCAATTTCTTTGCAGAGCATTTTAAGGAACTTTACCCACTGGAT
A_14_P136820 TACCTAGATATTTGTGCCCCGTCTGGATTTCTTTAGATGTTTTATTTTCAACAGCGTCC
A_14_P106675 CTCAAAAGTCTTCTCAATGGCTGTGATCTGGCTATCCCTGTCTTTGTATATTTCTTC
A_16_P18504380
TCCCAAAATCCTGTCCATAAGTCATATATCTGTGTTTAAAACTTCCATAAATACCTGCAA
A_16_P15570126
AAAAGAGGCTCCCTAGTTAATTTCACACTTCCTCCATACATTTCAAAAAGCAAGGTGT
A_18_P22842104 TTAACAACCCACAGGCCTAACAGGACCAACATGGGCCTGTGAAAT
A_18_P14133385
GGATTGTGAACATTTGGAAACCAGTATTTTTTTCCTCTCTGAATTGCCTTCTGTTTAGA
A_14_P113212 AAGGCAATTAATTAACTTCACTCTGTCCCTAGGTCTAACAGTAGGATTTCAGATAATTTT
A_16_P03225827
AAACAGTCCAGAGTTTTAAAAGAGATTCTACCACAACTTGATCCGAAAATAGTCTGCTCT
A_14_P105801 ACCAATATTCTAATCCTGCAGTAAAAACAGCACTATTAGACAGGAAGCCAGAAGATGTAG
A_14_P103031 ACATATAGTTAGATAAAAGATGAGAAGATTCCTTCTGGCATGTTTCTGTTGGCAAAGGGA
A_14_P127552 TGAATCCACCTCTAGGAAGCCTGTTATTATCTTGGAACAAACTCATCTGCATATGTATTT
A_14_P201644 CTGTGTTTTTGATACTGATATTTTCCTATGCTGAATAGTTTTCTTACTTTCAGGGAAGGT
A_16_P17175788
AAAGCAAGCCAAGACACATATTTCATTAGAGAGTTTCCTTTTCAATATTTCTGGGCCTTT
A_14_P201011 CTGAGGAGAGAGCAATGCTCATAGTAGGTGAGAGTATCATTTGTAGCATTTGGAAAAATA
A_16_P00685495 AAGAGATGCTACTCAAGAGAGGGCTTAATTTGGAAGCCAAGTGGATGGATAG
CUST_6410_PI426210822
ATAGTTTTTATTATTTGCTTTCTAGGACATGGCCTTAGCCTTCATAATACTGTTTGTTTA
A_14_P121620 TATTGATTCTGATTATGAGAAACCTGAAACTCCTGAGCGTGTGCTTAAAACCAATTTGTC
A_14_P121299 AAGAGAATACAAACCAAGACCAAATGTTTCTCTCATCCTTTTATACCACCAAATCTCTCT
A_16_P01034422
TCCCTTCATCAACCCTAATACTACTATCACATCATTCTCAAAAACAAAAACCTTTCTGTT
A_14_P116687 ACTTACCAAATATTTCCCCTCCACTAGCATATTCTGTCACCAGATAAATCATCCGTTCT
A_18_P16126210

TTTAAAAAGTTAGTTCACTGAATCAGAGTTGTTAGATAAGATCTTGTCTTCGTGAGAACA

A_14_P133943 ATACGGAACAGCCAGATCTCTATTGCCTATGAGTCTAAATGACTTACAATTCTATCATCC

A_14_P138797 GATTGTTCAAAACTGAATTCCTCAAATTGAGGGGTATGATTTTCTTTATTGTCCTTGGTG

CUST_3075_PI426210822 CCTCATCACAGTGTGTCGGTCCTTTCTGACATTTTGACCTCTCATTTA

A_14_P111554 GAAAATAAAAATGCACTAAGGTTGGGTAGAAGTTCTGTTTGCACTCACTAATTGTGACAG

CUST_1781_PI426210822

TTAATTAAAGGCTCAGAAAAATTTCTCTTGCTTTCTTAACTCTTTTAATCTTCTGGAAAG

A_18_P15651552

AAACAAGAAAGAACCAAAGGTCAAGGAATTCTACTTCCCAAAGCGAGTAATCTTTCTTAT

A_14_P103988 AGGTGTCATCTATTTTTTTCAAACAGAAGGCAAAAAACTTAGGGAGAGGGATTTCTATTC

A_18_P26177238

GCACTTTCAACTGAAAAATGAGCATCCTAAAGCACTAAAATACTCTTGAAAACCAACCTG

A_14_P138412 AAAGCCTTCAAATCTGAAGAAAACATACACCCAGAATCACAGTTAGGATCTAGATCTTCA

A_14_P201701 GAGAAGGTGGCATTTAAATTCACGTTAGGAAGAATCCCTAATTGGTAGTTTTGCTAATTC

A_14_P139416 TCGCAGTTCAAAACATCAACATAAAATGGCTTTGGAATTTTACCATTTCAAATTACCCGT

A_14_P113385 ATTCTCACTATACTCATTTAGAAGCAATTGACCCATCTTCCTTTCCTTTCAGTTTAGCAC

A_14_P136106 AATGGTAAGTCCTCATTATCTGCAACTTCAGTGGGTTGTCAAATTCAATGTATTGTTTGT

A_14_P100511 TCTCCTTCTGTTTTAGGCAGAATATGAAGTTACTCCAGATGAAAAACTGGGAGAGAAAG

A_16_P20256494

ACACTGCAGTTTACTACCATTTATAAGCACCTTCATGTATTTTCGTCACTTGAGTCTCAG

A_16_P30952281 CACATCCTACCCCATGGCTTCTGCTACATCATCCCCTCCAACCTC

A_16_P58424187

TCGAGCATTTAGTATGTGCTGGTTACTGTGAGTCACTACATGTGAGATAGTTACTGTTTT

CUST_2862_PI426210822 CTATCCTCCATCTGCCACATGGGTTCATAGATAAAGTCAAACATCCCTTTACA

A_16_P00312521

GTGCAAATGTTCTGATTGGGATGTAACTAACACTCATCCATCTAACCCCTCTAATGTAAT

A_14_P132203 CTTGACTAAGATGATCAAGAGATAGAAGCACTAAATGGCAGACCATTGTTTGCTGAATCT

A_16_P17856979

AAAAATTGACAAACCAACTTTGCTTATTTGGAAACTATCCCTTACAATTCTACAGCCCTT

A_16_P40562185

TTCAACTGAAGTCAGGATTAACCAAGACAACATGATTACATACTTGGCAAAGAGATTCAC

A_16_P01207983

GTTGTCCAAAGAGAGTTTTCTTTTTCTCATCAAACACTACAGATCAGCAATGAAATCAGG

A_14_P119345 TTCTTGTTCTTCTATTTTAGCCTCTTTGTGAATCTGTTCGATAGTTTTAGGCCCTTGATC

A_16_P35144159

ACACTGCTGGTTTAAACCTAATTGATGACTAATGTTGTAATGCATCTGAACCTACACTGA

A_16_P00685281

GTCAGACACATTTTTCTAGTTTTTCTAGTTTTCTACCCTTCAAAATACTTTGTGTTAAAC

A_14_P114188 AACCTACTTCCGAAAGTTCTATGGAAGATTCACCTACTACAGAGCGATTGAATTTTGACA
A_14_P110107 TGGATTGTTCCAGGAAAATTTTTAGCATTTAGTGGACCACATCCTAAAAGCAAAATTGAG
A_16_P41747010
TCCCTGTATCTTTCATTTTCCTGTATATCTAAAAGCAAGATTAGTTCTAGACCACCAACC
A_14_P106809 GTTTTGAGATATCAGAACTGGATGCTAAAAAGCCTGATGTTCTCAGTGCAACCCTAAA
A_14_P202499 GTGCATGAGAACTCCTCTCCAGATGTAAATATATAACCGAGTTTGAATTCTGACACTTAG
A_16_P35668270
AAACCACTCTGTCCTAGATTTCAGCCTGAAGAAAAGTTGTTAGAATACACATCTTATCCT
A_16_P19426191
AAACTCTAACAAAAATGTGCCTTATCACTTGCTTTCTACCTTCATCTACCTACTGCACAA
A_16_P19701585
AAAACCATCTCAAAAGTCTCCTCTCTTTTATTTCTTGACATATCTCTGATCTCCAATGGC
CUST_5071_PI426210822 TAAGTAGGAATAAGCAGCCTTGTTTTGGGGAAAAAGAGGGACAGACCTA
A_14_P132981 AACCGCATCCAGAATGCTTTTAGTTTGTGCCACAAAATATGAAGAAAATGTTTTCAGTTT
A_16_P18774026
GGCTATTAACCAACATAACTCTCACTCTTGAATTGTAGTATTAAAAAGCCCACCAATCTG
A_14_P105693 AGTAAATCGCATAAATTAGGACAGTCTCAGGCTAAGTGTTCATTCTGCTTTTACTCCATT
A_14_P100312 TCATCCAGTTTATCTTTCACCCCCATTAATGCATAGAATTGAATTCTCTAATGGCACATG
CUST_2365_PI426210822 GAGACCCCAGCTTCCCCTCCTCCGGGGCCCTCCCAGTGTTTTGTT
A_14_P118948 AGACACACATTTTTCTAGTTGACATAAATCTTGGGCGGTCCTAAATTTTAGAGCCAAATT
CUST_6115_PI426210822 GTTGGGTGGTGGGGCGAAGTGCATTTCCCAAAACTGTTGGCTAGC
A_14_P109241 TAATTCCCTGCCATCCTCATTTACAGCCCAATAAAATGACAGGATTCGAAAGAAATAAAT
A_18_P12318737
AAAATCAATTTTCAGGTGTTTTCTCCATGTATTTCCCACATCTCAATATTAAGGCCCACT
A_16_P16489273
AAAAACATTCTCCTTTTTACCTTCCTATAGTTTTCCATGAGGCATAATCATACCTCTCCC
A_14_P200222 GTCTTCTTGTCTACTCTCCAGAACGGCCATGATTTCCCAATTCTTCATTCT
A_18_P17364440 CTGACCTGACTCTAGTATCTCCCCACTCCTATATATCATGCCCAGTGTATCTTT
A_16_P15920813
ATTATTTGGCTCCCTTCATTTTTATTGCTAATCTAGGACATCTCTTGTATGGACTGCTGC
A_18_P22841617 ATTGAATTCCAGCAACCTTTGAAACCTTTGTCACAAGGAACCCTAGGTTTCA
A_16_P02718700
ACCGTCACGTCTATATTGTCAAGAGTAGTGTTCATTTTATCATGCAGTGTCTTCCTTATG
A_14_P111568 TGTTTCTTGCTTCCAGTCAGGGTTTTACTATTCTCTAACCTTCTGCAATATAGAAGTTGT
A_14_P130116 CAGTGTGGACGTCGCTGCTGCTATTCAACTAAAGACAAATTTTAGTAAATTGAAAGAGAT
A_16_P18539196
ACTGAATAGGAACACAATTAGTTTGACTTATGCCAACTCGATGTCATATACAACTCTACT
A_14_P200645 ATCAAAGGTGGGAGATAAAATGATAGGGTTTTACGTGAAAGGTATCCTAGAACATTTGCT

A_14_P109336 TATCTTTTCTTCCATGTCTTTCATCCTTACAGCAAATACTTCCCTGGACAAGAAAGC

A_14_P104196 CTATAAATCAAAGTTTCCTTGACAGAGCACAGTGCAATTAACACAAATGTTCTCCTAGTG

A_16_P00768062

AAAAATTGTGAGTCTGAGATTTTAGTAGCCTTACCGAAATAAAACTTTGGATCATCTGCA

A_14_P134003 ACGGAAAATTGCAGTAGATGAAGAAAATGCTTTCTTAAGGAAAGAATTCAGTGACTTGGA

CUST_3700_PI426210822 CTGCCCTGTAACTCCATCACACCCATCTGAAGTCCTGCCTGGTAG

CUST_4155_PI426210822

TAGTGGAAGTTAAATAATGTTAAGGATAGGAGGCTTGTCCCCTCCATCATGATTTAGTAT

A_16_P19115561

GCCATGTTGACTACAACAATAGCATTGCTTATGGTTCAATCTGATATGTCTGTGTGAATG

A_14_P104823 ACGGTGAATTCAGCCTTGCAGTGATTTTTTCAGAGATGAACAGAATTTAAAGTGTTAGAA

A_16_P21467329

AGTCTTTCTACAGCCTGAAATTACATAGCCTTTTCCTTTCTCACAATTGTTCCCATTACT

CUST_8427_PI426210822 TTTGTTGGCTCTGCCCTTCTGACCTCAACTTCAGAGTTGCAGATT

A_14_P102156 CTCCCTTCAAAATTAACCTTTCCAGCGAAAATATAATTTCACACAAAATGAACCACCAAG

A_14_P113417 CTACTCATGTAAGATGGCAGGAGACGACAAACACATGTTCAAGCAGTTCTG

A_14_P128900 GAGAGACCCACACGGCACACCTTGTTGAATGTGTGACTTTTTGTTTTTAATAGAAAA

A_14_P110520 ATCAGATAGAAGCAGGGAAGGTAGATAACTTTTAGGACCTTGATGTGAGGAAAAGATAAA

A_16_P01255356

ACTGAAACAAGACACTATAGAACCTTAGAAACATTTCCTGATATGCCCAATTCAAAGTCA

A_14_P126378 GTTCGAGATCCACGCTATAGAGAGGATCCTTGCAAACAGTGCAAGAAAAAGTAT

A_16_P03282681

TTTTCAACTCTCAGAAAAGCAGATTTCATGATGTCATACAATGTCCTATTCCACTGTCAG

A_14_P100671 CAAGAGCATCATTTCGTAGAGGTCCAGCACATGTTAAAGCAAATAAAATGTAAAGCAACA

A_14_P133650 ACCTGCTCCCATGTCTGCTACAGAACTTTTATGTGTTAATGTTAGTTTCTACATCTCTTT

A_14_P115578 CTACAGACACCCAGAACATCTTCTTCATGAGCAAAGTCACCAATCCCAA

A_16_P18089917 CCTGTGTTTGCTAATATTCTTGTCATCTTCCTTAATTGTTCTTGCTATTTGCCATCACCA

A_14_P126129 TACGCTAGTATAGACGGCAATTCTATTAATCAGAATATGTGATTCTCAGAACATCCCCAC

A_18_P15447085

AGGAGCAAGTCATTCAAAATCCTCTACTGACAGATGATATCTACAATAATTTCTTCCCCT

A_16_P01830742

CCAAACCCTTCACGAATTAAAAAAAATGTAAACCCTACAAATTTCTTAACTCCATCTGCA

A_14_P118834 GCAACAAGCAAGTGACTGAACTGAGCCCAAAATAATTAAACTCTTTGCTTAAATAACTGC

CUST_743_PI426210822 TAGTGACGATGTCCTTTGTGATATCTGCCAGCCTTTGCTGGAGTC

A_14_P119500 GTAAGTCGTTTGTGATACAACTTAAGTGAAACATGCTTGCACTTCTATTTTGGGGGTTAC

A_16_P00197971

AACTCCTCTTGATCTTTCAACACATTTTTTCTACTTGGAGTATTTAATGACTGGCACTCC

A_16_P17273450

AATGGAGTTTAGAAAACTACTTCAATTAGCACAGGACTGAAAAAATTCATTGCCGAGTGA
A_16_P16179019
AATTAGCATGAGACTCAGAGATAAGATATCCCAAACACAATCTGTTAATACCCCAGGATC
A_14_P132725 TCGCAGGGAAATGCAAACTTATTCTCTTCTTTCCTCAAGGTTTGGTCTTGTATTTTAAAA
A_14_P104594 ATCAGGCTCTTTATGGTGCATGTGCTAAAGACTATAGGTAGGCCAACTTCAATTTTACAT
A_16_P19057513
AAACCTCAGTAAATACAATGATCTCCACCTCACTACAACTTACACATACGTTGGTTTTCT
A_16_P03624601
TGTGACTAGTCAACAATCTTTTGCCCACATTTTACAGAGATTAAATACCTTGTGAGAACC
A_14_P109285 GAAAACCATGTTGAAGTAGAGGGGCAATTTTATTCTCTTAGTAGTGAAGAGAGAAGTCAT
CUST_916_PI426210822 GGGGAGGGGAATCACTTCTTGAAATAACCACTGAACAAACTCTGTATAGG
A_14_P124774 ACTATAAATGTTTGCATTGAACCTTATCTAAAAGATGACTAGTGGTCGTGTTTGCATGCC
A_14_P101136 AATAAGTGTCTTCCCTACTTCAAGGTAGTAAGGATCTCCTGTAGCCTATTAAAAAGGAGA
A_14_P115194 TGCACACAGACCTGTTCCTAGGGACAAACATTAATTCTCAGAAAGTTTAAATCTACTTTC
A_14_P125715 AAAGAAACAAACACCCCCAAGAAGAAGGAGAGTAAAGTCAGCATGAGCAAAAA
A_14_P109908 AGATGTTACAATAATTTCAAAGCACAGTGTCACCCTGGAAAAGTCACATGGCAAGT
A_14_P123337 CAGGCTGATGTTGTCAAAGTTGTCATAGGCATCGATAGATGCCCTTAAGC
A_14_P123891 ACTTAGGTTGGCTTAATTTGACCTGTGTTGACAATAGTCTTACTTTAGAAAACTATGCCC
A_14_P139730
TTTTATTTATGAAAGATGGAACAAACAAGAGAATGAAAGGATGAGTGTGCAGAGGGCAAA
A_16_P00685049 TTAAAGTCTGCAGGCTGTTGAGACTATGGAAGAGAGGGATGGTGGAAATT
A_14_P202252 CTGTCTCACTTAATTTCACATTTAACAGTGTATTAACTGGGTGAGGAAGAAAGGCAAAAC
A_16_P00381376
ATGAAAACACCCTGATTGAAATACAAAAACCCCTTTAGTTTACCATTTATCCCACACTTG
CUST_8574_PI426210822 AGAATGCTCACTGGGTCAGGTGTGATTCAGAGCTCATGACGGTTT
A_16_P00174384
TGAATTAACTAAAAGGCTTCCTATAGAACAGTTCAGCAGTAATTATTCCTCTTGTCCCCA
A_14_P109361 TTAGGATGAGATGACTGTGAAGCACAGGGTTCAATATTTGGAGTCACTATAGCATAATGA
CUST_9735_PI426210822 TCAGGAGCACATCATGCTCAGACTTATTTGGTGGTCAGTTGGCTT
A_14_P201772 CATTATTTTGAGTCGATTGGGTAGATCACAGAGTAAGCAGAACCATATATCTGCCAGTTA
A_14_P128476 AATTGAGTGTTTTCTAAAGAAGTAACAAGGGCTGGCTAATTTTGGTTTTTTTTGCTTGCC
A_14_P121527 GGATGAGGTCTTGAAACATTTTTCTCTCACATCTCAACTCAAAAGAATTTTGGAAATGTA
A_14_P104522 TTTTAAACATTACTGCTTTTGTCTGAATTTTTTGCGTTTGTGTTTCTGTCCCTCTGAGTC
A_14_P113176 TCAGATTAGGGGAGGAAGGTCATTTGTGCAGGAAAAAAAGCAGTTCTAAGC
CUST_6107_PI426210822 AGAAATCAGTGGAACATTTCATTGCTGTCTGGCTAGCCAACTCCGATAAAA
A_14_P124020 GAAGGTCATGGATTTCATGGTTAAAAAAAATAATGGCACCCCAATGAGATCACTAATATG
CUST_1875_PI426210822 TCCATAGGTTGTCTGATGGGAAAGACTGAGTGAACAGAGGAATGAACTT
A_16_P00685042 CTCTATCCCTTTAGGCCTGTGCTCATCGCTGGAAGAACATCTACTATGAAG

A_16_P39449817
AGATTGGCATTTCTCTTCTAACTACCAGATATCAGCCATATTCTATAGGAACCCATTGTT
A_14_P117322 CATGTTAGGTGATAGGTCCATTTGGCTGACATATTTGGAGAAGAAATTAAAACCCTTAAG
A_18_P22843033 AGATCCCACCAGGGGCAGATGAGGTGTCACATTGCATCACATCAC
A_14_P116231 TCTACTGCAAGGAGGACTTCTTCAAGTAAGTCAGAACGGTCCGTCT
CUST_9585_PI426210822
TAACATAATTTAATATGCCAAAAATGAGAAAATAGTCAGGATGAGGGATGAGGAGTACAC
CUST_8456_PI426210822
GGAACCTGTTCCCTATGAATTCATGGCATAATAGGTGTCTAAAAAATGAAAGACCTCT
CUST_5503_PI426210822 ACCCCATTGCAATGATTATAAATGAGTAGATCGGAACAGGGAGGACAACA
A_14_P108435 TTAGAAACACAAGGATCCATTTTCAAGTAATTTCAAAAGAACTATGTTGCTTTCCCCACC
A_14_P114380 TGGTAGGTTTCTGGATCATAGTTGTAAATCTCATTCATCCATCCCTGATGAAGAAAAACT
A_14_P132911 CTAGATAGACACTTAGGGTTTTGATGAATGGAGATTAGTTGAATGGCATTTTGTGTGAAG
A_18_P10035623
AAATCCTACCTTTCGGGGATATCTTAAATCCAGTCTCTTACTGCATGTCCTCTTCTTAAA
A_16_P17938521
AAACCTGGTTGCTGATACACTTAAAACATTTCTTAGAGTTTCCCCATTTGTGTTTCTTGC
A_16_P37685151
ACAGGGCCAATAAATGATAGGAAAATCTCAAGCATGATCTTTACCATCTAAATCCCATTC
A_14_P103820 CTTCTCCACATTGGCTTTCAGTAGTGCCTTCAGGGTGTCATTCTC
A_14_P113101 CTGATCTCCAATATTTGTTTTCCTTTAAGCAAACAGCAAAATCCAAGTCTACACTGGGTC
A_14_P105991 TAGACATTTTAGCACCTGTTTCTTCCTGTAGCCTCTTCAAGGAGTTTCCTCTT
A_14_P129574 ATGCCCTATTTCTGAATGCATGTTGTTGAGGTTGGATCAAGATGCTGAATTAAAGTATTT
A_16_P00095354
ACCATTTCTAAGTATTCTGGCATCCCAAATTAGAAACTTTGACTTACTACTCCTGTGACA
A_14_P114353 TTATTTAAGAGTTCCTTCTGCTGCTGAAGTTTTGAATTTTGTTCCTGGTTAAGTTGGTTA
A_16_P02826023
CACTCAAGAGAATCAGATAAGAACACTAAAAGACTCTATTCCATCTCATTATTCGCACAT
A_14_P103439 CCATATCATTTTCCAGACAGAGGTTAATTGAAAGGACAAGTTCAACATACTTTAGCCAAG
A_14_P123666 TTCTTTCAGAGTCCTGTTCGATTTGATACCTTTGATAGCCTTTCACTAAGTATTCCAGC
A_14_P115416 CTTCTAGACAATAAGCAGCAAGTTAGAGACTAGAACAATTTCACGGATAAAAGACACCTG
A_16_P00016115
TAACAATTACCACCATAAAATAAGAAAACGGACCCAGAATAGAATTTGAGGCAAGAGACC
A_14_P118933 TATATCCTGTCTTCTCCTTGTCTTCACAGTGCTGTTTGGAGAAATCACACGTTT
A_14_P129308 ACTTCTCTACTATGAATCAGAGGATGAGGTTGTATAATTCAAAAGCATTGCCTTAGTCTA
CUST_8564_PI426210822 CTCTGTGTTCTTATAAACGATCACCACTGAGCCCAGAATGACAACAAGAGT
A_14_P133313 CCTTTAAGTGAGAATAACTAGATTTTTTTTTTTTTCCTTCAACGCTTCCCTAACCTGTGT
A_14_P119677 CAATTCAGTTTCATAAAGATGCAGATGTTGCATTCCCTGTAGATACCACTCTCCTTAAAA

A_16_P20663793
TCAATTACCTTCAGTCTTGCATAACTAATCCTATAATCTCCATAGAAGTTACTGCCCTGT
A_16_P00757570
AAAAAAAAATGGAAACTATCATTCTAATCTTTCTTCAGAGAAATAGGCACAAAGATAGCA
A_14_P201286 TTCCCTAAAGTCACCAGTTTTCAACCTGTCATGTATTCTTCAATCTGGACTTTAAAAACT
A_14_P102523 TTAAACCAGGATCTTAAGGTGCACATAAACATGTATTAGTTACAGAATGTTGGGAGGACT
CUST_4093_PI426210822 ACTTCTTCCCGTGTGATGATGGCTAAGTGCGCAGAACATGTGAAAT
A_16_P17073394
TTTGTCCCAACTTTTAACCTCTTATTCAACTCCAATTACTAACCTCAATCTTTCAGTCCA
A_16_P20643178
AGAATCATAGATCATACATATTGTTTGCAAATTGCTTTTTCTCACTTTCCAGAACCTTGA
A_14_P137208 ATATGTCCCATAAATCCCTTTTAACATAGATTATTCAGACACTGTTGTTTGCAGGGTAGG
A_18_P14136359 CTTATACCTGGAGCCTTATTTAGGGGATGTTCTTGCAGATAGATGGGGAGAA
A_14_P108037 CTTTCTTTCAATTACATGGCTAGTAGTAGGTAAAGTGGGACATGGCATTTTGAATCACAA
A_14_P109392 TCTAAGTTGGTTGTGGATTTTCCCAATTTCCCAATTTCACCTCCTGTATGAACTAATTAA
A_14_P201280 ACTAAGTTCATTTTTCTCTGTTCTTGGCTGCTCCTCTAACCTTGAACTTTATTTAGTCAG
A_14_P136404
CTTAAGAAAGAAAAATCAGAAAACTTGGGGCAATCCAGAAACTAGACAAAGACAGAGATT
A_14_P109006 AACATCAACTTTGGATGCCAAAGATGAGTGCACAGAATATTCTGATTTAGAAATCGTGAT
A_16_P15623712 CCCAATTCCTATATGCTATTCTCCATATCACACCCTACTTATTTTCCAGACCTCATACTT
A_14_P102232 GAGGTATAGAAAGCCCCTTCTTTGATTTCTGGATAAGGTGATGTTCACACATTAAGATAT
A_16_P18770899
AACTTTCTCTTGTTTCTGCTAACTTGCACTAAAAACCAATAATTCTGGCATGTAAGGTGC
A_14_P123908 TAAACAGGTGCAGTATTTTCACATCTTGCAGTAAGTCAACTCATGAGGCAGAACTTAAAA
A_14_P107184 ATCTATATGGTAAATTTGGAGCATGGAGAATTACCAACAAAGAGCCATTGACTTGGGTTA
A_14_P131365 TTCATTTATTTAAGGGACCTTCTTAAGGGACTTTTATGGAGTGCCTATTGTATACCCACA
A_14_P116988 GTCTTGATTTTCAGACCATGCATGGAATTTTGGAGAGAAGCAAATTTTGCAAAGATATGA
A_14_P127539 TTCTTTCAGGAGAGGGAAGTTGGATTATCAATTCTTTTGTAAATGTGTATGGTGATATTT
CUST_8279_PI426210822 ATTGCTGCAAACTGAGCTTGAATTCTTCTCACCTCTTATGTAGCCTCCG
A_16_P19474198
AAAGGCCACATCTTTTATTCTAGACTCTCTTTCAATCAGAATGAATCCAGTGATAGCCAT
A_16_P30952230 TGAATACTTAGCCATCTGCCTCCTATTTTCTTATCGTAGGTTGTGACAGAATGCCTTT
CUST_2920_PI426210822 TGGGCTCCTCCAGGAGCAGGTTTACAACGTATTTCATAAAGCACTTT
CUST_7347_PI426210822
TAAATAAATAATCTCATTTCCTAAAAGTTCTTGCAAAAATGTCATAGAGTCTGATTGGTC
A_14_P109159 TGAACACGTTTTTCCTTATAAATTAGTAATCATAGGGCTTTACCATGGGATGAGTGGAAT
A_14_P102916 TGGATTTTTTTTTGGAGGGAATGATCTTTCAGGAACTCTGAAATAAGCATTGAGGTATTG
CUST_10029_PI426210822 ATTGCCATGGGTCAAGGTAGGACAGGCAGGGTTTGCTGACCAGTT

A_14_P115173 ATTAAAGTCATCTACCGGCAAGTTGACAATCTTGTCCGTAGGAAAAGGAATCTTCAT

A_14_P105462 ACTGCATTAGACTATGGCCTAGGTTTTGTTTTATTTTCACTTCTTATTTTTGCCTCTGTC

A_14_P139226 GAGTTAGTCCTCTAGAATGATCACGCACTTTAAATACACCTGGACTTAAGTAATTCCAGA

A_14_P131787 TATTAATGACAAAGGTTTATGTCCTAGATCAGTTCTTCTTTAACTTGGGATCCACGGATG

A_14_P134289 CGTGTTGTTCTGTAGCAGTTCGGGTGAATATCCAATGAAACCATGTTCTGAAATAA

A_14_P102164 ACCATTCTCTCTTTCTTATATTTGCCTTTATTGCACCCCTTAATTTCCTTCAAACTCTGT

A_18_P11613762

AAAATGGAACATAGTCATTGGACCTTTTCAATTTCCTATGGTAACCAAAGACCAAACTGT

A_14_P122451 CTGAACATAGTGTGGATATAGATGTGGATATTGATTGGCCTATTGCAGAGCAAAGAGTAT

A_14_P104642 CCATCTTACATAAAAGGGAAAAAAACAAATGATTTTCCTCAGACTCCACTGAATATGGCT

A_16_P03338154

CTTGCAGAATATGACGCTAAAGTTTTATGGGACCTTTAATTTTGTATCCACCTTCTCAGT

A_14_P108575 ACTGTAGACTCAAAAACTTCAGATACAGTCACCAGTTAATATAAAACTAGTGGGAGACTC

A_14_P106099 CTTCTCGGACAAACTATTTAGTGGAAAAGGCTTACATTTTCAGCCATCAGTTTTAGATTT

A_14_P122568 TCAAAAGGCTTCATCTGTCCTATTTACAGAATAGCAACTATTTTTCTTGCCACAATGACG

A_14_P134773 ATCCATAAAGATACAGTTTAATATGTCCTGTCTTTTGGGACTGATGTAGTGGCTTGAGAA

CUST_9391_PI426210822 TCTCCTTAATTCATAAGCCAGATATTTACCCCTTCCCCATCTCTCCATCAG

A_14_P125861 GGGTGTCTCCCACAAGTCATTTCTCTCAGAAGGATCATTACCTAAAATAGCAGAA

CUST_5457_PI426210822 TAGCAGATTGTGACCCATGCCTTGTAGATTAGAGTTGTAGACTGAACCACA

A_16_P01359640

TCTATTTGATTGTATGACACCAGGAATAAACAGAAGCAAAATGATTCATCTCCCAGAAGG

CUST_4771_PI426210822 ATCTACAACTCCTCAGAGCCAGCCTTAAAGTGGTCTGTGGATCAG

A_18_P13244566

CCCCCACATATTTCAAATACTGTTCTTCTTCATTATTGTCCTCTACCATAATTAGCACGT

A_14_P130683 GAGTCAAACTGTTAAAAGGTTAGTAGAGATGGGTGTGGGATTACAAGGATATTCATGTTC

A_14_P138221 GATGGAGGGAGGACTTCCAAAATCCTACACTGCAACCTGAAATAAAATGATACATTTAAA

A_16_P17904346

ATGACAAGAGACTAGGAAAAATGATCTAGGAGATCCAGCATACATGATATAGGTAGTGCA

A_18_P11848822

ATGCACACTTTAACTAAACCTGAGGAAAAGATTCAAGTTACTGTTTTGGGAGAAGGTATG

A_14_P107759 TTACAACACGCATTTTTCTCTGACTAAAAGAGGCTAGCGGAACCATGATTATTGTTAACA

A_16_P17682306

AATAAGCCCTGTCACTTAAAATAGAACCCTCTACCTACAGATTATGTTTTCCTTTTGACC

A_14_P115451 TTACAAGAAAAACTACAACCACTTAAAAAAGCACAACCCCACAATAAGTGGGGTGATTAT

A_16_P19506829

GACATCCAGAATTGAGTTTACAGAACCTAATGTCACCTACTTATATAATTACAGCAGGCA

A_14_P106916 TTCTAGTTACCTTGACCATGAACTTATTTATTGCCAGATATAGTGCTTTGCCTGCCAAAT

A_18_P14132120

AAGTGGATTTGGCGATCAACCAAAGCAGAATAAAATGTTTTATCCCAACCTTCCTTTTT
A_18_P26792470
ATTAATAAACGTGGGAACCTTAATTTAACATGATGTCAATAAACATGATTATGGTTCTTC
CUST_1083_PI426210822 GTAGTTTCAAGTTTTCTCAGCCTAATTTTGGGTAAGCTGGAACCTGCTCC
A_14_P104643 ACAGGCACCAGGCAGAAGTTCATCATCTTTAGACTTAAGGAATTAACAAGGGT
A_16_P19245604
AAACTCAGTTGCAACATGAATTATATCTCTCTCTGAAACACTAATTCTTGAAGGGCTTGC
CUST_1483_PI426210822 ACAGCTGGCATACACTGACATGGCTGTGGTTTGCTTCTCTCTCCT
A_14_P138099 TGGGGTGGGGAAGTATTGACTTCATCATCGTTTACTTTTTTTTAGTGTTAAGTATTGCTT
A_16_P00298655
GAGAAACTCAGTCATGATATTGAGAATAATTCCGTGTGTATAAATTCCACGAGACAAGGC
A_14_P102139
CCTGCAAAACACATAAAAGGGAATGGAAAAACATTCTTTAAAATACACTGGGAACAAGAG
A_16_P16406933
GAGGCCAGTAATATCCAGTAACTTCATAGTCTGTTCTTATTTGCTTACCTCCATTCTTGA
A_18_P16049111
AAGAGGTTAGAATGTTCATCTATTACTGAGCCCAGATATGTAAGTTGATGGCATTTTGTT
A_14_P104321 TTGGTTCTGGATATCATTTGCATCTTGTGGCTCAGCAAACCCTTTTATTTTCATGTTAAA
A_16_P02574063
CAGGCACTGTGATTAAATCTTGAACTGGTCCTAGATTTTTAAAAAGATTGCTGATCCCTA
A_14_P111867 AAAAATATCCACTGCTCTACTCTTCGGCCAGTGGGAAGAGTGCATTTTTTAATCAAAATA
A_16_P17939645 TTGTCTTTTTTTTAGAGGATAATGTCTACCTACCTTCCCGTGTATCTATCTTCCCGTG
A_16_P01539300
GATCCCTACCAAAATGCTTCTAGACATTACCACAAATATGCTATCCTTTCACCTACAGAT
A_14_P115988 GTGCAATTGAGAGGAAATTGCCCAATTATTTTGTTGCTTCACTAACAAAATGCTTACTGT
A_14_P201311
AAGGAAAAGAGAGTTTGGTTTCATTAGCCAGAAAAAAGAATACAAGAAGAGGATTCCTGG
A_14_P135661 GATGTTGAATACATCTAATAGGTATGGGCTTCCTGTTCTAGGGGTGAGTAATATTCCTAT
A_14_P132419 CTTTTAGGGTTTTAGGAAGATTCCCCAAATGGCCACAAACCTAATAGGGC
A_16_P40749674
AGCATTTTCTCAACTAAGTGTTACCTGGTAGACATACAGAATTCTTGTGTAGCAATACCG
A_14_P137436 CAATTAGGGTCTCCCTGCTGAGAATACAAGTGAATCTACAATATGATGTTATCAGCATTC
A_16_P00685017 TTATTAAGAGAGGCAAACCTCTGGTTAACACCATGACAGCAGTGTCAATGCTTT
A_14_P108442 CATGGCAAACACTTCATAAGGTATAGGAGATATTCAGTGAATTATCACAAAGCCAAACCT
A_14_P105317 TTTTACTTCAAATTCTCCCAGATCCTGAAGTTATTTTTTATACCTTTTAGGGCATCATAA
A_14_P112959 AGGTCTCCCCAGCACGTGTTAATTTGGTTAATAAAACTGTGGATCAAGGAG
A_14_P139976 TTCATGGCATATTTATATCAGACCTGCAATGTAGAAGCCTTGAATGTTTAGAAGTTGCCT
A_16_P39746888

CCTGACGTAACTTCCTTTTTACTAGTCCTAATTGCTTATTTCCATTCTGAATCCACTTCA

A_14_P119689 CATTACCTTGGTCAAAGGGCTTGTTGGGATTCCAGTTTCTGAAATAATCATCCTATATAA

A_18_P13442696

AGACACTATGATCGATAAGTCAGGATACCTAATTCAGTTTTATACCTTTTGCTTGCCTCA

A_14_P118199 GAAACACCAGGTGCCTGGTTGTCTCTATGTCCTAATGCATCACTAGC

A_14_P130296

ACCAAACTTACCTTACTTAATGGAGAGGGAGCACCAGATCTAAAAGGCAACATAAAAAAA

A_14_P117615 TTCAATAATTATTTTCTGACTTGTTGGTGTGCCTCATCAGATTCCACAAAATAAAGGGGA

A_14_P139778 CTCTCTTTCATTGCAGACGAACAGGAGGCATTGAACTCAATCATGAACGATC

A_16_P16851080

AAAGAGAAAAACATGCAGAATCCTTACCATATCGATGCTAAAAAGAAGTCAATCCTCCTT

A_16_P38222998

CTGCCTAATCTAATGACATTCTAAGGCCAAAGAAAAAACTGATTTTGCTCCCAATTCTTG

A_16_P03586240

GTATTCCTAGCAGCCATAAATCTAATGACGCAGAACTATAGCATTCTTGTATCATCCACC

A_14_P111703 CTCACCTTATTTAAAAATTCTCAAAGCACACGTGAACTGCATACTTTCACTGAGAAGAGA

A_16_P00685259 CTCTTTGCTTTAGGATATGATGCCCAGAAGGGCTCGAAACTGGCAT

A_14_P106529 GAAGTCTGTATCATCTGCTTCCAAGTCTGTTATGTCCAAATATATTTTAATTATGCATTT

A_16_P40976872

ACTATTGTCAGTTTCCTTACACTGTTTGATCTCTTGTCTTACCTGCTTCACTAATTTTCT

A_16_P16845807

ACCCAGTATTAAACTTCACCTCTCTAGAAGGATTAGTAGTTGACCACCTCTTAAGATACT

A_14_P104906 TCCTGGAACGCTTTTTATTGGGTTAGTTTACACAAATTCTTTCAAAGTCTTGTTCTCCAA

A_14_P101736 AAACCAAGGAAATTCCAAGAGACGGATGCTTAAATCAGAACACTAACTTCCTAACTTATG

A_14_P117231 ATTCCAACATAAACCAAGGAATTATTTGTGAGAATGCTTTGTGATATCTCAAGCCCCTTT

A_16_P17164548

TCCAACCAATTGACCAATAACCTATTAGACTTACTAGAAATCCAGAAACACCCTCTTAGA

A_16_P17775663

GGCTGGATATAAAGTAAGTGCTGGTTTATACCTAAGTCTTAGGATCACAAGTGTAGCATG

A_14_P107490 GTAGGTGGGTTTTCTGGAGATTAGTAGATACTGTATAGAGTTGTATACATAAAATTGTGT

A_14_P111645 ACTAAGGAACAACATTCCCTTTGAAAGGACATTATTTGCCTGTTGTATTTAACTTCACAG

A_14_P108925 ATGTTGCGGCTTATTTTCACTAATCACATCTCATAAATCACTATAAAAACAGGTCACGGC

A_16_P16532205

AAAATCCATGCATCACACTGAATTCTAATCCATTCCTCACTTAAACCATACGAATGCTTC

A_14_P116379 CTAATATCTGTCTCTTTTCCTTCTAGGACACTATTGAGGACAAACTTGACACCAAACACT

A_16_P17155037

AGAAGATCTGACAGCATGTATTTGATAACAGTAACTGGAAAAAAGTAAACCACTTGTGCC

A_14_P102858 ACCAGTTAAAGTAAATAATTTGGGACTTCAAATGAATTCCAACCCTCCAAAGTTGAAATG

A_14_P132751 AGGCTAATTCTCTAAGTTCCTATTGTGTAAATCAGAAAGCAACCCTTATCTGCTAAATGA
A_16_P15252034
GCAGTGCATCTCTAATTGAACGCTAGATTTTAACTTCTAAAAAATTGCTGTATGTGCCCC
A_14_P202549 TTTCCTGAATAATTCATTTGTCCATGTGTGTATACGTCTGTGTGAGCATATCTTTACCCT
A_16_P15017772
GGTGGCACCATCTTTTTTACACCTAAATGAACAGATTTCACAACAAAGATTTCTAAGTGC
A_18_P21780115
CCCCTTCTATTAATCTTCTGTAAGTTGATCTTTATAGCCATCTAATCCATTCATCACCGC
A_16_P19655348
CCCCAATTCACAATCCTCTACTTCCTCATCTTCAATGTTCCTACATTACACTATTGTAAA
A_14_P114186 CAGACCTTTGACTTCCTGTTTTTGATGAACCTAGTGAAAGAAAAAGAAGTGCCTTTTTAA
A_14_P118502 GGAAGTCAGATCTTCATGGTAAAAACACTTTTGCATGAGATACACAGTCTTACATCTTCT
CUST_6907_PI426210822 TATAACGCCAGGTAGAAAAGCCTCACCTGGCTGGAAGCCCACTCG
A_14_P103209 ACGCCCCCTTATATTAATAGTTTTATGCTTCAAGAAGGAGTCAAAATTTCAATTTGTCAA
A_16_P17279281
AATCATCTTTATTTGTCAAGTCCTGTATAAACCATGCTCAACAATAAGTTCTCCACACCC
A_16_P02718308
CACAGAACACACATTTCACTTTTGCTCTATTAGGCTCAAACTCCAATTAGGTGTCCTAA
A_14_P130653 GAAATCTTTTTTTGGTAAGACTTACCTTTCTCTTCGGCATAATGACTATGTCGGTGAAGC
A_16_P02393766
CTACCAGAGGACTCATGTAAAAAGATAAGATGATACTCCTTTACTTGCATGTTTTGGTCC
A_16_P19858301
AAAATCACTTCTGAGAATTGCAAAACATTCCAGTCCAATGTAAGATGTGTTCCCTCTTTT
A_14_P103889 ATTTGATATTCAAAAATTTATAGGAAGCCCTTCAGAATGTAAGTTCTGTGAAGGTGCGGA
CUST_9134_PI426210822 GATTTTAATGAGGGATGGATGCATCCTGCAGGGCTCAGAGGTTCT
A_16_P00194444
TGTCGCCAACATTTATAGGAATTGCGTAACAGATATTTTAGAAGTTAATTTCTTCGGCAG
A_16_P18162311
GATGGTAAGATGCAGGGATATTATAAGACTATTACTGGGATATCAAAGCGATTGCAGGAG
A_14_P124401 TTCTTTATATAATCCAAGGAAAGCCAGGTGATTCACCAACAACCTTTCTACTTCTAACAC
A_14_P102053 AAAATGTCTTCCCTTCAAACTTCAATTTGCAGACATCTGGCCTAAGAAAAGAAAATCTCT
A_14_P118968 CTTAGTTCTTCAAGGACATGATGTGGAAGTCATGACTTGAGTAACTTCAATAGCACTAAC
A_14_P108488 CAGCATGCCACAATCTGATTGCTGACCTGGATGGAACAGAGTGAAATAAAT
CUST_4457_PI426210822 CCTCAAGTCCCAGCTGCATTACCAGCCAAGGATGTGATGCATAGT
A_16_P21624120
AAAAAAAGAATCCCAGGAAATATCTGCTTGGAACTAAGGCTTTACTAGGCAAAACTGTTT
A_14_P110772 CTCCTTTTGTGCACATGTCTATTTGCGCATTTACAAGTAGGCATGTGTGTAAGTATTTAC
A_16_P00645534

AACTAAAGGGATGTGATGAAAAATTCCCCTAGATAACCTAATAGACTTGGAGGTCAACAT
A_18_P22841213 CTTATCACACACCATATGGCTAGCAGACAGTGGGGCCAGCATGAG
A_16_P15618321
CCAGTCTTATCAGAATAAGGGAATTCTAGGATTTCAGGAACTATAGACCTTGCTACTTCA
A_14_P103877 AATTAGATCTTGCCCATTAGAAACTCAGTTAGGTTGGGCAGATAAGGCATAAATATGCAA
A_16_P18482837
ATCTGCTTTCCCATTCATTATACTTACAGATCGCTTCTCTTGTATAAGTTGACAAACCCA
A_16_P20658342
CCATCTCCATTGTTGTCAGAAAAGTCACAATAAAAAGGTCCATATTGTCTAGTTCCCATA
A_16_P02305535
AGCAAAAATCCTACAAAACTCCAGTTTAGTTCTTAAGAGTGCAATCATAGTCATTGTGTT
CUST_8047_PI426210822 GCTCAGAGGTAAGGGGGGGTTTAAACACTTTTTTCAAAGATGAGAGATTTA
A_14_P123006 AGGAATTCTTTTTGAGTGCTTTAATTTTTGGTCTTTAGAATTCTAGTGCTCAAGTTATGA
A_16_P39963689
ACAAGTTCATGCCCTATAATCAGTTGAACAAGTCCACATAGTTCTTCCATCTTGTCTATG
A_16_P18531879
ACCTACGATGCTAAAAAGTAAAGTTCCCTCGTAATGAATTTGTGACTCACTAGGTGTTTT
A_16_P37667203
TCTTCACAATATTCCATGTGACCACTATTCTTTTCTAACTTAAACTCACATGCAACCACT
A_16_P00009821
AACATGTTGCTGTTGCTATTTTGTTCAATACAGTAGGCTTTTCAAAAGAAGTTATGGGCC
A_14_P137671 GCAACAGTAACTTTCTTTGCTAATATCATGGATCCTTAAAAGTGACTCCTGAAATGAGCA
A_14_P130310
CTATGACAAGAGAGGAAACAAAAAGTTAAACAAGCAAGCCTGCCATAAGTGAGAAGGTAA
A_14_P114734 CTCAACAACCATTCTACAACTCTTTCTTAGCCTAGTTTAGTTTTTCTCATTGACCTCACT
A_16_P19126547
AAAATTACTGCATTATCCTTAACTGAAACACCACTCAAGGTGTTGTTGGTGTAATTTGCA
CUST_4931_PI426210822 GGTGTCTCAGCAGATACTGAGTTTTGCTGCCCTGTCATCCAAGAG
A_14_P113727 TAATTCAGCCTTGTTTTTCACTCTGAATATCTTGTAGATCTTGGCTTTTCTTTCCTTCAG
CUST_2755_PI426210822
AGCATCTGGTGGTTCATTTATGGCTTCAACCAGATGCGTTTTATTAACAACAAACT
A_14_P128244 GACAGGCATTAAAGTATTCAGAAATTGTTAAGTCAGAATGAGTGGGTCTGGGTATTTACT
A_14_P102148 CTGTGTTTGAGATTTTGAATGCTAATTTGGTGAGGTTTACTCATCCTCTTTGCTGATCTA
A_16_P20620281
CCTCTCTTTTCTCAACTAGTCTTGAACCATAATAACCTAGAATGAGTTCAAATGAACCGG
A_14_P110104 CTAGAATCCAAGTTATGAGTAATGGGTTAGTAGTGAGTTCACAGGCATGCAGAAGAATAA
A_14_P135154 AACATTTTTCTGAGTGCTGACATTTTTAGATTTCATCAGACATCTCTGGCATTGACTGAT
A_14_P107306 CGTTCTTGTCCTCACTAACTCTCCTGTCAGTTAGGCATCTCCACAG

A_18_P16215626
CTTTCTCTTTTCCTTAACAAATACAAGAGCCATACCATGTAATACTGTGCTATGTGCCCT
A_14_P113198 ACAATCATGAGTGGCCATCAGATTTTGAGAGATGCTTTCCACATCTAGATAATTTTGTTT
CUST_8413_PI426210822
TTTTAATTCTGTGCACTCATGTTTCACTTCAAATTAGTTATTTCAGCTATGGTCAGATAT
A_14_P101298 GATTTTAGAATAGAAGCCATGAATGTAATCCTGTCTTTACAACCTAGCGCTGAGATGATG
A_14_P116985 CATGTTAAAGGGGTCAATTTGCTCTAATGTGTCTTTAGTCTTGACTATCTTCCAGTGAAT
A_14_P101092 CAAATAACTTCTATAACTTACCCATCAGTGATCTTTCCCATTTCTTGGAGACTTTCAGGA
A_16_P21265989
AAAAAAGAACCCCACAGAGTTAAAAGTTGGAGTGTTTTTTGCTTGAAATGTTCTATTGCA
A_14_P138458 AATACAGAGTGTTGTCCCTGATTCTGGGTTTAATCTGGTAAGAATCTTTACAGAGGAC
A_16_P32481954
GATCCGAAAACTTTACCCCTTTACAATAAACTCAAGGAAAGCAAAGTAAACTCGAGGAAC
CUST_6490_PI426210822
TCTGGAAGCTTCATGTAATAACTTCAGTCAACAGTGATATCTTTTCCCTGAGTTTCTCT
A_16_P16970043
ACAGTGGTATCTCCTTAGAACAGATTAAACCTTCATGCATTTTAACGTATTCTCCTGAAA
CUST_10030_PI426210822 TCAAGGTAGGACAGGCAGGGTTTGCTGACCAGTTGGACTTGAGAG
A_14_P136678 CTCGACGGTAGAAATGCTACATGCTTACACATGGTTAGAAAACTTTAGCATCTCTATAGT
A_14_P107712 TACCACAGAAGCACCAGAAGAAATGCCAGGATTGGGTTTTTATATTTTGGTAAATTTTTG
A_18_P22842270 CTAAGTGACTAAAGCAAAGTGCAGCATGAGATGCCACCATGTGCTGC
A_14_P125848 GTTCAGTCCGCAATTATCCTACCTGAAAGAGAGCACAACACAAGAGGCTTA
CUST_3880_PI426210822 CCTTCTGCTTGCCCTCAGTCTCAGAAATTCCAAAAGACAGCTTATGAC
A_14_P118679 TGTCTTCTTATGTCTGCGCATGTAACACTGTATCTGTAAGTGTGTAGGTTCATGTAGTTT
A_14_P201175 GATCATTATGCAATCATGCCACCTTTAGAGCAATTCATGGAGATACCTAGTATGGATCG
A_14_P114236 TGAAACCAGTGCTAAATGGAGGTAAAATAAAGTTTGTAAGAGCCCAACCACCAG
A_14_P126240 CTTCCAAGATATTACTACCATCTGCCATTAATGTTCATCTGAGTTATTCCTCATCAGTGC
A_16_P01094411
AAGTGATGCCTTTGGAAGATCTCTCACTAGGTATATCCCTTCAAATAGAACTTACTCATG
A_14_P117868 CATAGGTATAATCAGAGGATATCACTCGCTATTCCCAATCTAGGAAATACTAGCCAGCAA
A_16_P18124924
TTACATTTCAGACTTTCAAGAACCTTTGTAACCACTCATTCTTAATCTCTTATCCCTGCC
CUST_4981_PI426210822
TTGGTTTTTTTGCATGGTGTAGATTTAGAATATAACTTTTACAGGGAAGAAAGGGGTTAA
A_14_P113633 CCAGATTTTCTACAATGAACATGACCTCATAAAATCAAGAGTGTTTTTCGTTTGTTCTGT
A_14_P113845 GAAGATGGCCAGTCAGTTAATTTCTCACCTAAAATCTAATTAGAAGGGTCCACATACTTT
CUST_8155_PI426210822 GAAGTCAGGCTGTGTGGGCACCTGATCCTGCTGCTTTTGCAAACT
A_16_P37850226

AGTGCAAAGGCTTTATGATTGATCATCTACATCTATCACATGATTATCTTCTGGAGGTGA

A_18_P22842307 AGTGTCTGGACCTGTCTCAAAGGCCTGTTTGCCCTCCTCAAAGGC

A_14_P107373 GGATTTCGCCTAAGTGATATTACAGCCACCTCATTCTTCTAAAAGTGGATATTTTTCTGT

A_14_P123125 CTGCTTTCCTAAATCTGCCATAATCACCATTAGTGAAAGGTGTCTTGTAACTTAATTGCT

A_14_P122091 GTTTAGGCTCCAGTGCAGCAAAGACCCTAACATCCTTAAGTAATGCTGAACT

A_14_P129770 ACAGGAATTAACCTGAAGAAACGCAGTGAACAATTTTCAGACCTTAAGCCTATTACTACA

A_18_P14899271

AGAGCACTAAGGTGAGATTAAAGTAAAAGTAGTTCCTCCTAGATTGACACTAAATGGTGC

A_18_P12132699

ATTAGGAAAACATCACAACCTGCATTTGAAGAAAAGAGTAACGTTCAACTTCTAACGAGG

A_14_P130126 ATGAATCAGGTATTTGTTTTTCAGGAATGTCACGGCACAGAGTCATTTAGATTTAAAGGT

A_16_P16086835

CACCACAAGCAGATTAAAAGGTATACCACATTAAGAAGACTCCTTATTGTTCCAAAGCAA

CUST_2259_PI426210822 TCCATCATGCTCTACCATGTTGATGGGGAAATGAGTGAGGAGAGAATT

A_16_P20918895

AACTCTGATTTCCCAAAATCTTTCCTATTCTCTTCTCAACCTAATTCAACAATGGAACCT

CUST_9854_PI426210822 CCCGCTGAGCTCAGTGTCTCAGTCTGATTCATAGTCAACAATAAGGTTTAG

A_14_P108405 CCTCAGACGAATGTGAATATTCAGAAGGCTCGATTCTCCTAGCAGCGTTTAT

A_14_P200711 CTCTACACTTTGTTGAGATTAAGAAAATTAACAAAGGCTGTTTAATGGTGCTCTCACCAG

CUST_2597_PI426210822

TTTCATGATGTCATCTTTGCTCTCTTCATTATCCTGAGCATCTTACTTTGGTTTGTCAG

A_16_P17369847

GTTGTTAGCAACTAAGTCCAAAAACTCATTCTTGTATCTGTGGTTGATCATACTCAAGGG

A_14_P131007

TAACGGTAACCTACCAAGAAGTAATACATTAGTGAAAGCAAGGTCACCAGGAAAAAAGTA

A_14_P128609 TTTAGAGTGTTGGTTTATCTTTAATATTTTTATAGCACCCCCTAAGATCATCCCAGGGGA

A_16_P03663092

ATCTGCTCCAAATACAGTAAAATTTTGTTCTAGCATAGTCATGGGTAAAATGGACTTGGG

A_16_P00072385

TGTTTCAATCAAAAACCTATGTTAGACGCTTACTAAGAATCTCATACACTTCTGTTCCCA

A_16_P58424389

CTCCCAGTCTTTCACCCTACTTCTATTTCACTACAAAATAGAAGCAATCACAGAGGAATT

A_14_P123658 CCCTGTTCTTATAGCGCAAAACTGTTAATTCCAAAATTCTGTCATTCTAATGCTGTTCTT

CUST_5124_PI426210822

TTTTGATGACCTTTTTTATTACAGGTTTGGGAGCAACTACTTTCTTCACTCTCTAAACTG

A_14_P127731 CCACATTCTTTTAATTACTTACTTTTACAGAAGACCACTGGTCCTGGTGGGGAAG

A_14_P105489 CTCATTCTTCTGAATCTAATATCACTAACTCCTAGACTTTTTCCGTTTTCTTTGGATACA

A_14_P113425 CTGCCAATTCTTGGACATTCAGGAAACGCTTGTTGACATTATCTTTTTTCTTCACAATCT

A_14_P112170 AGTGCCTCTGGCCAAATGCTCAGCACAAATATACTTATAGTGATTGCACACTTCATT

CUST_7445_PI426210822 ATTGAAGCATGAGATAGGACGAGAGATTCCCAGGACCTGGAAGAC

A_14_P118015 TGCTTCCCATGGACCCAGGAAATAAGGCATATATACAGTGACTAAAACAAAATGAAAAAA

A_14_P137823 ATTTGCATGTGCTTTACATATCATTTGCTCACACTGCTGTAAACCTGACAGCATATTCTC

A_14_P119281
ACTGCAAAGGAAGAAACAAAAGGGCTCTGAAAAAAATAATCCGATTCAACAACTTTATGC

A_14_P135233 GTTTCTTCATGTATGTCCTCTATTTCCCATATTTGAGGAGTATGAGTCTGCTTGAGTAAC

CUST_9669_PI426210822 CCTCCACAGTCCTGCCCACATGTGAGTTGCCCCTTCACACCACTC

A_18_P26795549 CTCTCTCACTTCTCAAATCTCTTTCCTTTTTCCACCTTCTAGGTGTCAAAGACAG

A_14_P106105 ATAGAAGTCTTACAAAATGGGTTGAGAGTTTTTGTTTCAATTTTTATCATCATAAATGGG

CUST_1102_PI426210822 CATGTAGGGAAATGAAACTCTGGCCCTGTTTATATCTGTATTGTCCCAAACAG

A_14_P117086 AAGGTATGCTCAGGGGTCTGTGTTCCACATTGAAAATTAGGTTTTGTCTTTTAAAGAATT

A_14_P126660 GAGGAAGTGGCAGTCAAACTCTTCATTACCAAAGTTGTTCATGATTTCTTGATGTTC

A_16_P19784491
AATCTTTCCAAAACTTTTTGACACTTCACACTGAATCAACCTATAAGGAAACTCAGGGTT

A_14_P136182 TATAGAGAACTAATAACTGGCCCACCAAAACCTGTATTTGCTTCTTTTCCTTTCTTCTCT

A_14_P126610 AAGGTCTTGGATTATGAGAAATAGTTGGCAAGAGATTGCACCTTATTACTGTATACCACT

A_14_P138302 GAATGTGAGCAAAGTTTTAAAGTTACCTCGAATCTTTGCAGACTCAGTATTTGTTCTGTC

CUST_2212_PI426210822 CTGAGCCATGCATGAATAATAGGCACAGTGACGTCTGCCATCACA

A_14_P138440 CTCAAAAGTTTGCATGATAAGCCATATGGCTCCCTACTAATAATGGTTACCGAAATGATG

A_14_P115913 TGAATCTTATTATCAGTAACTTGAAGAACTTCAATTAATGGTGGGGTCAAGGACATAGAC

A_16_P41871183
GGGCTTCATGCATATTTACATAAAAAAAGGTAGGGTTCTTATTATGGTCCTTGTTATGGG

A_18_P16168032
AAAAAGGAATTCCAAAATTACTTCACTCTGCAATCAATGAACCAGTGAAAATTCACCTGC

CUST_7290_PI426210822
ACCTTCACGCTTTGTCTTTAATTTGAGAAGCTTATGTGGAAGGAAACAGCACATT

A_16_P41087272
CTCGTTATTACCAGTCACATCAAGCATAAGAAATCCCCATGATAAAGGACTAGTTACACT

A_14_P108608 TTCTCTATTCTGAAGCCTTCTACTAAGACCTGTAGATCTTCTTCCATTTGAGGTAAACTC

A_14_P115897 CTTTTCTGTTGTGAACGGTCTCTTGGGGTAGATATTTTCAGATTATGAATCTAGTGTCAT

A_16_P17078358
AAACCCAAATACCTACATAAGACACTCGCTTTTAAACTCTTAGCACTACTTTCGGGAATT

A_14_P117998 AATCTAGTGAAATAAACCGTATGCAATGGACCATTTTAGTGGCTACAGTGATTTGTTAAT

A_14_P100043 CACACACTTACCCCACAGTAAGTTCGCTTCTAAACTTTCAAAAAGTTTCACGTC

CUST_5057_PI426210822 GCTGCCCCTCACACCTGCCCTTCTATAGAATGTTCTACCCTTTAATA

A_16_P19079570
AGGATCGGATCATACTTAGTATTGCTACCAAATTTCCAATATCACTTATCTTTCTGCCGT

A_14_P105884 AATAACCATAGTTCATTTTTTTTCATCAGGTATGAAAGTATCATCGCCACTCTGTGTGAG
CUST_9932_PI426210822 CCTTTCCAGGGAAGAAGGAGTATCCTGTTAGAGCTTCCTTCATGG
A_14_P133824 AAAAATAAATCACTTGGCTGAGGTGACAGAGATTCTTCTTTTTTAATGAGTTGGACTCAA
CUST_2543_PI426210822
CCAGGGTCTTGGTTTATAGCTTACCAAGTATTTCCATGAATTTACTGACTTAAATATCTG
A_14_P128243 TTTCTGGGCACCATTTTAATTCCACTGAGGAATAGAAGTTTAAATTAGGAATGGGACAAA
CUST_7831_PI426210822
AAAATAAGGTAGCTGAGCAGCTTTTGAATGATCAAAAGTACTGTTGTCTTAGGTCAAAGC
A_16_P38388551
CACCACACTGTTTATTTTTCTTCAATTGCTCCACAAACATTAGGTTTTATACAGCGTTCA
A_14_P100672 GGCATTAAGAAATGCAAACAAATAATTCGTGTTCATCTTCCCAAAACTTTTGATCCAGTG
CUST_2780_PI426210822 GATATTTCCGATGTTCCCTGTCGCTGTTCCTCCCAGCTGGTTTTG
A_14_P130906 AGACCTGAACTCACTGGAATCAATGCTTAGATTGTGTTTCTTATCTTTTTACATCCCTTT
A_14_P106283 ACTTTTTAACCCTTTAGGCAAGAAGCAGTGTAAGAAATATCTTGTCAAGGATTATAGCCC
A_14_P135087 CAGCCTACCAAAGAGACCATCTTCTCTAGAAAAACTAACAAAGACTGCCATTTTAATGAT
A_14_P113165 AAGATGATCTCCCACTCCAAAGAGAAAAAGGTCTAAAAGGAGTAAAGAGAGGTCTT
A_14_P123274 TGCAAAGCGTGATATGTCTGAACTATTTAAACGGTTGGGTTTACTGATTAATTTCTCCTC
A_14_P102212 CTGCTTGGCTTAATGCCAAAGAGTGTTCTTCCAAATAAAAATGGTAAAGACCTTTTTTCT
CUST_639_PI426210822 AAACCTCTGGTTAACACCATGACAGCAGTGTCAATGCTTTGGAAAAGC
A_14_P116163 CTGGTAACAGAGACCAAAATAGTTTGGAGACTAAAGAATCATTGCACATTTCACTGCTGA
A_14_P103050 AGGGTTAAAAAAATCCCAATTTAGAATTCTAACTATCAATGCTGTCTTGGGAGAGCACAG
A_16_P01683225
TTTGCCTCATTAACCTATTATTCATCCTACAAAACCTACTGTTTCTTGAGAGTCTGCTGT
CUST_1814_PI426210822
CGAAACTCAAACAACTTACAATCACTTTAGGGTTAGAGGGTGATGACAAGTTTGG
A_14_P125953 AGGGAATCAATCCAGAATTCCAGTGTTTTGCTGAAACCATATTTTTTTGTTTTTAAGCCA
A_14_P102045 ATTATGTTTTTAACAGAAGAAAAGACCTCTGCCTTGCAGCGTGTAGACAGATTTCTATTC
A_14_P119706 CTGCATCTGGGCATAAACATTAATCCCCGGGATCTTCCTAAAATATTAATAGAAACCATC
CUST_29_PI426210822 AGAATCACTATTACCTCCTCAACCCCAACTTCCTGCAGGAGCTAG
A_16_P19878548
AGAGCCATTGTTGACATTTATGGATAGCAGATATATTGTTTAGATCTCTGATTAGCCCCA
A_14_P133437 AGAGGCAATATACACCATGCCAATAAGATACCCAACTCATTATTCTTTGGTAACAGTCAA
A_14_P108246 ATTACCATTTTTACTTTTATTAGAATTGGCTCAAAAAGAACCCAAAGAGGGCAATGCTCA
A_14_P123706 TTTCTCATAAACACAGAATAGCAGCCCTCCCTCCTCTTAAATGCAAATAAAATCCTTTTT
A_18_P14131925 AGTATCTGCTTGCCAAAAATAGGGCTACCTGCAAAATGCTTTGAGCACTGT
A_14_P117446 GTGACAAGATCTCCAGTGAATATTTCTTGATTTATCCACAGACACTGTGATGGAGTTTAT
A_14_P135854 GCAAAATTCTCAACAGAAAAGGATGCTGTCCTATTTGCACTGAAAGTAAGTTTATTCCTT
A_14_P102567 CTGTCAGTCACTGATAACTTACATACCAGTGATTGATGAGAAAATGTCAGTGCAGACTTT

A_16_P03554898
ATCCATGTCCTTGAAACTAACTAAACAATTGCACCATAACTACATCAAATTCAGTCCCAG
A_14_P102575 ATCTAGATTGCAGACGTAAATTCTCATCAACCAAAAACACCAACTTTCAAAACAAACAGT
A_16_P01180131
AAAATTGCTATGCTCTACTTAGTGGTCACTAGAAATTCCAGTAGTCTGAGGATGTAGAGA
CUST_5776_PI426210822 CTCTGGAAACTTGTGTGGGGCTATAGAAGGAACACCAGTTCAGAAATG
A_14_P103461 AAGGTAGTATTTCCACCCAAATTATTGTAGTTTTTGTCATTTTTGGTGTGGCTGAAGGTA
A_14_P113749 TAAGTTCCAGAAGAAAATTGTAGGAAACTGTCTTTCTCCTTAGAGTGCGAGAGTATTCTT
A_16_P19660712
AAAAACCTCTCTAAACAAATCATACTTCCAGTCCTGATCTATCTTGCTAGTGATAAGCCC
CUST_1862_PI426210822 GTTTGAGAAGGAACATTGGGTTCCCAAGACTGGTTCAGCCCAAAG
CUST_4139_PI426210822
TTAGATGGGCTCAGGAATTTTGTTTGGAATCCTTTTGGCCTTAAACAAAATAATGATGAG
A_16_P02145976
TTCCCATCACAAAACACAAACTTATTTCCATTTCCCCCTATTTTACTCATCTTCAATCAG
CUST_183_PI426210822
TCCAAAGTTGGAAATCAGTGTGTTTTAGATCAGTGGTTTCCTGTCTTCTAAAACCTA
A_14_P110529 TATTAAGTGTCAATGAATGGGACGAAGAGAGCCCTACTACTACTACTACACGTTTCGAT
A_16_P41817619
CTCATCATTTCCTTCCCTTAAACCCATTTAAAATACTTTGCCATATGTTCAGACAGTTGG
A_16_P16178497
AAACACACAGAATGAACTCACTATAACGAATATAGTTCCACATATATCTCTCTCCACGGC
A_14_P133229 CCCTTTCCAGGGCAGTTTAATTGGTATCATTTGTAAAAGGTCTTTTCCATCACC
A_18_P16553457
AATAACCCAAATTGAAACTTGACTTCCTCCAATTCTTGAGTCTATCATAACATCCTTCCA
A_14_P123490 ACTTCATTGTCCTTTCTTTATTGCAGTCAGAGTTTTCGAAGCCTTTTTTTCATTACCCTA
A_14_P131397 CCATTTAGTTGGCAGACAAATAAACCTGATTTAAACCAGGCACACAGAGAATATAGGTAT
A_14_P135208
GTGAACAAGAGTGAGAAGATGCTGTGTTAGGAGAAAAAAGAAAATAAGTAGAAGGTAGCA
A_16_P15378776
CATCTTGCCACAATTTCTAAGAACTACTGTCTAGTCAACTGGAATTCCTTTTACAGACTG
A_16_P40941822
TTTATCTTATCAAACCAATCCCATTTGCTTTACACCTACAAATTTCTCATTTGGCCTGCT
A_16_P40974014
CCTCGCAATTACTTTCCATGCTTCGTATATAAACAGACACCAAAAATGTAAACTGTCCAA
CUST_9998_PI426210822 GTTGCAGGAGATGCTGTCATGGGTGGGGAGAACCCTGAGGTCACC
A_14_P137497 TATTTAGGTGTAGAGTTCGGTGCTCGAATGATAACTATTGATGGGAAACAGATAAAACTT
A_14_P101284 TATATAAGGAAACTGCCTTATTTTTGAAGTTGCTGGTTGGGATTTCTCTGTTCTTTCAGC

CUST_5000_PI426210822 TTTTCTACCGGGCACATCTTTGTGCGCAGCCTGGTTTGAGCTGAT

A_16_P37356341

TTCATGCCTTTGCTATAGATTTCCAGTTATGACTAAGCAGGTATTAGAGGGTAATTTGGC

A_14_P131586 AGCAACAAATTAAAATCTAAATCACAAAAGTAATTATCCAGTGTATTTCAAGTGCATTTT

A_14_P133873 GTCCTCCCTCTTGGTGGTGGACAGATTCTTGATTTATTTATATTAGTGTGGTAAATTGAA

CUST_957_PI426210822

TGTAGTGGTCAGATTGACATGTGTGTATATATAATGATAATCAAGGCTAAACACAGTCTT

A_14_P126550 TCAAATGCTCTTTTAAGGCATGAAGATTCTTTTCTGATCCCCACATATGAGCATATGGTA

A_16_P38241513

AAAAGTCAGATACAGCAATTGTTTTGGAAATATCCATCATAGTCACCTGATTGCTCCTTC

CUST_565_PI426210822 CAGCCTCTGACCTTTCACTCCCTAACACACAAGCTGGGGACCAAT

A_16_P01952742

ATGACCAATTCAGTATGCATGATATCTCTTCCCAACATAATATTTGATTAACTCGGCCCA

A_14_P137747 GTAACCGTCTGTTTATAAACAAGGGTCACTATTCCACGAGAGGCCTTACAA

A_16_P01661355

AACCATTGCTACTCTGCTATTACCTACAACTGCTATGAAATCTGAGTCCAGGTCTATTTC

A_14_P131591 TCTGGGAAGGAGAGAAGTCAGACAGACATAAATGGTGATGATGTTTTATGGTTAGTAAAA

A_16_P00685265 CCCTTGGAGGAGAGAAAAGGGTGTTCTTCCTCCAGCCCAGAGAAA

A_16_P01884533

TTCCCTATGAATAGCAGACATCATTTAATCGGTTTTCCAGTCTCATAAATACACTGCCAT

A_16_P20184030

TGCTGACATCAACAATAGTAGCATACCTTTTTCCTCACATGAAACATACTTTCAGGTCTT

A_14_P117071 GACATCTGACATTCTAGATTGGAGTTCTTGATTTCCCTTCCAGTCCTAGATTTTTTTAAT

A_14_P200767 ATTTAGAAAAGCCAAGATAAAAACCACAAAACCTTACCATGGAAGTTTAGGGGTCATCTA

A_16_P39640995

AACTGATAGATCCCCCTTAAAATTGAAATGTGACAGCCTCAAAACAAGTAACTCATTAGG

A_16_P19593937

GCCACACCAAGTATTATTCATAACCTCTCACAGTCTCAATAGTAAGATAGGAACATGCAA

A_14_P128246 GATTGCTGAAATTTACCAAAATGTAAAGAGGCAGATACAAAAGGCATGCATGCAAATTGT

A_14_P130020 GATAAATGTGACTCCTCTTCTCCTTCACTGCATTTTGATAACAGACTGTCTAATCTAGTG

A_16_P16763515

CCTGTCCCATCACTAACTATCCATCATCTAATATAATGAATTCAATGCTCATGACCTAGG

A_14_P101818 ATTCGCTCCTTATTAGGGCGTGAGTGCTACATTCTGAAAGGATTTATTTTGGGAC

A_14_P133206 CTTCCTAGCATGACCTGGAAATTTCACAAATAGAAAACAGGCAACAGTGTTATATGATCA

A_16_P15883182

AAATGTGCATTCACATCCATACAGAGCAAACAGATTAACAATAAAGAGAGTTCGAGAACC

A_14_P124687 TACATACTCTGTGGATATTGGATGTCATCATCTTTCTCACTGATTTTTCCTTTGGACTCC

A_14_P135805 AAGCACTCTGCTTCACTGAACTTGAAACATTTTAAACATCAGATTCTTTTAAGGCGCCTT

CUST_1024_PI426210822 ATGTTTAGTGTGGCAGAAGGACGATCCAGGCCTGCATCCTGGCTG

CUST_1244_PI426210822

GTTCTTAAATTTGGGATTGTTGAAGTCTGAATTTTACTGTGCTTTAAAATTGTGATGGAA

A_14_P129240 CTTACTGGCACACTAGTATTATGTGTGTTAAGGGCAAGTGACTGATTATTTTACTCTCCA

A_16_P15181266

ACACCATCATTCAACCAGAGTTACAACATTTTGCGTTGCTATGTCTATCTTAACTGTCAT

A_16_P18752216

ACCACACATTTACTTGTCTTATGTGCACATATGACTCACTAAAGATGAGCCAAAGTCTTG

A_16_P19341858

AGGGCTTTTACAAATTGAAGAGAACAATCAGTGCTATAAACCATAACTAACCCTACGTCA

A_14_P100617 TACCTTTCTGGTTTTTTTACTTCAGTCTTCGTATCTAATTGGCAGATGTAGTGACAGGAT

A_14_P124062 TGATATGGCTGTTCCATTACATCTCTCTAGAAACCTTATACTTAACTCTCACCTAGTGGT

A_14_P111584 CTCTATTTGCTCCTAAATATCACTTTGTTTCTAGGCCCTCAGACACTTGTCAATATGCTA

CUST_4154_PI426210822

TGAGATGGTTCGTAATTGATTAGTGGAAGTTAAATAATGTTAAGGATAGGAGGCTTGTCC

A_16_P17582807

ACCATGTCTTGCCTTATTCATTTTTTCATTTCTCAGCAACTTGTTAAACTCATAGATGGT

A_14_P201062 ATTAATGTTCTGGAATGTTCCCAAGGAAATGAATGTTCCATTGAACAGATGGAACATGTT

A_14_P116800 ATTTTTCTCCAATTTCTGACTTACAGGTTAAGGAATTCAATTTCCGAGCCAAATGTATCT

A_14_P104095 CTAACTAAGGAGGATTCCATTTGATTTCAGCAAACAAGTTTTTGTCAAATATGGGTTCCT

A_14_P106695 ACATGATGCAAAGATGGAAAGAAGGGCATTTACTTCAGTTACCTAATACATCTCTCATTC

A_16_P19182882

TTACCAGAATCTTTAGACACATCCTGTATGCTTAACTGTGGCTTTATGCACTTCTAGTTG

A_18_P14131216 CTCGTAACTCTCCTTTAGACACACTCCTCTATGGCAGACAACTTCTGATACATTCAAT

A_14_P124355 TTCCATTGTGCTTTGAATGGAAATGTGTGTAGGCTGAACCAAAAGTAGGGTTTAAAAC

A_14_P137597 GATTTTCAGCATGCTCATGGGTTACAAGAATTGGAATTTATTCGAGGACATTCTGATACA

A_14_P201206 AAAAACCATGAGGATTGTGCAGTAGGAATTTCCATTTCATCTTCAAGAAAGATGATTAGG

A_14_P110639 CCAAAGGTCTTTTCCCCACCATCTTTAAATCAAAGTTGTTTAAAACAACATTAATTTTAA

A_14_P136306 TTACCTACTACAATATTACCTGGCAAACCGCTCCTTATCGTTAGACATCTGATCATCATC

CUST_421_PI426210822 TTGTCCTCAGGCAGGCATGAGTTGTTCAGAGGGGCAGAAGAGAAA

CUST_5627_PI426210822 TGAAACAGAGGGTATGGAACTAAATGAAACAATCATGGGAAGTCCTGGC

A_14_P135532 TGATTTCTGAACTGCCAAAGGAAAATAACCATGATTTGCTGCTCAGAGTTTTAAAAACTG

CUST_6466_PI426210822

CAGTAACATAAGCAAGTATGTATCATTAAAGATTTATCACAAATCAACCATATTATATCG

A_14_P127371

TTCACTAGAGAGAAAAGTTTAAGCAAATCACTTGATGCCGAAATCAAACAGCAAGCAACA

CUST_8409_PI426210822 ATCAGCATCCTTTTCTTCATTGCTTACCCCATTCATGCTTGAAATGGGATTTC

A_14_P119768 CAGCCAAGGAAAGGGGCTTTCCTGATAAAGACAAGAGTTGGTTAGAGAAA

A_14_P101638 TCTCCTTCATGCAGGAAATTTACATGCCACTTATGTGATAGAAGTTTCACAGAGAAGTG

A_16_P00020739 AGAAAAGAAAAGATTACTAACTAAAAGTAAGTCCTTGATATCAGCCAGATGACACTAAAT

A_14_P108802 ACGTATGTGTTATGTGTGTTTCACTTACCGCATACTGGAACTTTTCATTATATTCACGGT

A_14_P105975 CAGTAAAAAATTTTGACCCAAAGACACGGTAATCTGAGTTTTAAGATTTGGGGATTTGAT

A_16_P35879837 TCCTTCACTATGTCACTTTCACCAAGTATCAAAGACTGATGACAAAACCAAAGAATAGAC

A_14_P130106 AAGGGTCAAGATTTTTTTCTTCTGAAAATTATGTTCTGAGTTAGGCAGAACATAGCCATG

A_14_P133257 TGGGGGTAGATTGTGAAGACATTTGCAATATTCTATCTCCCTGACATTATAATTGGGAAA

A_16_P20239735 GGCCCACATAATATGAATGAAGGAACAAATCACCATAAGAAAAAAAAGTGCCCATTTTGT

A_14_P124487 ACTGCCACTCCTTTCTTTCAAGGACAGTGTTTTTTGTAGTAAAATCACTGGTTTATACAA

A_14_P110407 CTGGATAGATCAACTTGGAAAAGGTTCATAAAGTTCATTTTCAGCGGTAACAATATATCG

A_16_P16386240 AAAAAAAGAGTGTATGCCAAGACTGGTATAATTTAACATCTTTATACGTGTTCGGGAGGG

CUST_6037_PI426210822 ATAGCTGTTCTCACAGGAACCATCAGAGAAATGTTCATTGATACTTAGATAACAAACCTG

A_16_P18128266 CATAATCCTATGATTCCTTACCCAAAAGACTGTATAGTCCCGTAACTTCTCTATCTCCAT

A_14_P116959 GAAAAGAGCCTTTGGGTTTTAAGACTGATGAAAAAGTGACTTTACATTTACGCTCTTTCT

CUST_2916_PI426210822 TTCTAGATGAGCTCCGGGCCCCTTGTGTGGCATGCACTGTGCTTA

A_14_P135898 GAAATCTGATCAGCAACAGTGATTCAGCATGGCTGCAAGTGAAGCCT

A_14_P127337 TGATTATATGGTAGTCTTCTGAATATGGTCCTCTTACCTGACACATATGCACCTCATCAA

A_14_P109769 AAATGGAAACCACTTTCAGATGCTGATCAGGTTTGCAGTCCAATTTTCTAATAAAGTCTT

A_14_P120781 CCAGGAAGCCTAGCAGCATAGAACATCTGGAAGAACTCAGCAATTTCAAT

A_14_P107462 CTGGTAAACTGATGAGAAGACAGACAACTTTGTAATGATTGGCGTTTGTTTGGCATTTT

A_14_P131266 AATGTTCCTGTAACTTACCATATATTTGGATTTAATTGCAGTTGGTGGTAGGAGTCAAAG

A_14_P136411 TTAATCATTCTGTAATTTACCAGCCAGAGTCCTGTCATAGTTCGTTGAACAAACTTTCCA

A_16_P01389350 TGCTTCACTATCATCCATGTCTTGAATGTATGCACAATCCCTTCATCAATAGAGTAATCC

A_18_P22841209 TATTCTACTTCTTGCCAGAAGAAATGAGACATTCATGTCTTTCTGACTTCTCCATTACCT

CUST_6227_PI426210822 AATGACATGGCTATTTCCCTTGTGACTGGACGCAAGGAAAGGATCTAT

CUST_6949_PI426210822 CAGCATCTGAATTGGGGAATTGACATATACAGATGCCCGTGGAATTTAACAG

A_18_P22840695 CCTCACCCTATCTGTGCCTCGTGTCTCTTCACTTCCTTTGTTCAC

A_16_P03087247 AAAAAGAGGTAGCCCTAAAATGTGTGAGACATTAAAGGTTGATTGTAGAAGATACACGCA

A_14_P126911 TCCAAATAATAAGTATTGCTTCCAGAAACATGAAAGATAAGTGTGTGTGACCTGGAATGC

CUST_5605_PI426210822 CCACTTCTGGCTGAGGAGAAGATGTTCTATGGAGAAAAGGAGAATTGG
A_14_P126314 AAAACGCGCACTAATGATTCTGTTTATTGAGTTATATATGTATATATTCCGTGTTCGCTT
A_16_P16509971
ATATGACTTACCCAATGTTGATTCTGTTTCCTCAATCATCATGTCTACTTGCACTCATGT
A_16_P18404582
AATCAAGAGCCTGAATAAGCAATACCCAGCATAATTTCATTGACCACATTCAGTCTATCA
CUST_2257_PI426210822 CCATCAACTCTCCTTGTTTCTGAGCCTTTGATGCTCCATCATGCTC
A_14_P124703 ATAAATGAGCCATCTGAGTCAACAAATAAGGTGGAGATGTGGAATTATTCCTTCTGTAAA
A_16_P58424385
CTAATATCAAGACCCTTTCTCAGTGTTGTGAGGTTTCAGAAGGATGACATATTTGACAAC
A_14_P102595 CTTAAAATCTGTTGAAATGAAACTGATCTGTTTCATCTCCAAAGAGCAGACTGCAGAAAT
A_14_P115711 CTTTTACATGAACCTAATCGCTAACCCAACTCTAAACCAAACCCAAATCTTATTTCTTGG
A_16_P15874645
AATGATTGCCACTAGTCAACAGAATCTCTTGAGTATCTTATACCCTAGCATGCATGCTC
A_14_P139257 ATTTGTTTGGTCATTGAGGGTTTGTTTGTGTTTTCATCAATGTCTTTGTAAAGCACAAAT
A_16_P01752883
TCCCTGCCCAAACTATTATGACCATTATAAATTCTCTGTCTAAACTATCATGATGCTGGA
CUST_1465_PI426210822 CCTCTTTTAGGGAAGATTGGTCTTCTTCCAGGAGACTCCACACTG
A_16_P20239471
AGAGCAGTGTTTCAAGAACTCATAAGCATCAAATCACCTCCTATATTTGGGTATGAACAT
A_14_P110951 TAAACAGTCACTGAAATTTTAATTAAGAGTGCCATACCCATGAGGCTGGAAAAGTATGTC
CUST_2105_PI426210822
TATTCCTACTTGCTTGGCCTTGTCAAATGTCTGATATAAGGAGCATTTTTATTATGGTTG
A_14_P123154 CAGGCTTACAAGTTCAAGAGGCAGAAGGTAAATTCCTGCAGCCTTTAAATGAA
A_14_P123118 GTAACATAGAAATGTTTTGTGTCTTTCAGAAAATTATGCAACACTTAGAAGGAGAAGGCC
CUST_1389_PI426210822 TGATCTAGATTTGATTATAGGAGCCAGTCAGGGGCCCCCATTGTTT
A_16_P58424074 TAGACTTATTGTGCAAGGCTGCTGAAACTTAAGCCAAAGGATTCTTGCTTGCATAG
A_18_P22842454 CTTCCTCCTGAGACACCCGGGAAGGCTGAGTCTACCTCACAGGTG
A_14_P105007 ACACATATCCTTTCTGTTCTTGCTGGAATAACTCTTCATGATCTGAGATAGAAACCATGA
A_16_P03755643
TGCTATGGTCATACGTCTACATACCTTTGCTTCCTAACATAAAACATCCTTAGAAACCTC
CUST_177_PI426210822
GGAGTTAAATTCTCTTCACCAGATGCCAAATCCTACGTTTTTCTTACCACATCATG
A_16_P00013746
AAAACTGGCACTTTCCCATTTATTTTCATCACAAACAAATAAATAAGAGTCAAAGCGCCC
A_18_P11313810
CCTGCAATACATCAACATTTACTACTCAGTCAACTTTGAAACAGATCTGAAAACCTAGCG
A_18_P13948099 GTGGGGCCTAAATTCCATCCTGCGGGATCCCAGGTCAGGAACAAG

CUST_3186_PI426210822 TCAAGAGCAGGATGCAGTCTCTACTGCAAGTGGAGCTTTCTCAGT

A_14_P133031 AAAATAATACCTTTGACTTTGTGTAAAAATAATGCTTTTGACTTGTTCTTTGATGAATGT

A_14_P125494 AGATTGCAGGAGCCATCGTGTGGGGAGAATTTAATAAAAGCCCTTTTGAAAAT

A_14_P101240 GAAGGGAAGACAAAACTGGATGTTCTTTCTTGTTGGATAGAACCTTTGTATTTGTTCTTT

CUST_9437_PI426210822 AAATGGTCTGGAAGTACACCCACGTCAACAGAGTTGGATTTCCAC

A_16_P17453166

CAGTAGTTTACTTGCCGTAAGAATCACATCAAACATTAAACCTTTCCACCTTCATTTTCC

A_16_P15791094

AGGAAAAGTGATACTAGAGACTTGAAACATACTACCCTCAATGACAATAGTGATGCATGC

A_14_P136085 AACCTTAAAATATAAAACGTTCCAGTGTGCTGAAGCCAAGTATTACAGTAGTTTAGGGTT

A_14_P113958 CCCCATGTAAATTTCATGATTGCTTTCCGTGATGTCATTTTGAAAGAGGACAGA

A_16_P00685522

TGGATTTTCAAAACTATATTTAGAATTTTCATAAAGTCTGTAACAGACTTGGCCCTAGGA

A_18_P22841432 AGGAGGCTAGGAGACAAGGCTGGGCTTGCCTCACTCCCTGTCGCC

A_16_P16711196

CAGAAATGGCACATTATAGATCATTTGTTCCAGTGAACAATAACACGTATCACCCATGTT

A_14_P126005 GACAACAACGGTGCAGTCAAGTTTCCACAACTGTGTAAATTTTGTGATGTGAGATTTT

CUST_6846_PI426210822 AGTAAGTGAGGTCTTGACTGAGAGAACAGAGAACGAAGAACAGAGGCTA

A_14_P130086 ATGTAAAAATTCATGCCTCATCACTTCAAAACAGGAAGTTAGGAGAGTATCAATTTCCCA

A_14_P114597 CTTTAAACAGGACCTTTGCCTAGGATCTTAATTTTCCTATGCTATTTTGGATGCTCTGTT

A_14_P112852 GGTTTAGGTGTAAAGTATGAAATTATCAGCATACTAGAAGGCTTTGGTGTGCTTACATGC

A_14_P109668 GACTTTTTTGGGCTGAGAAAGGTCATGGATAGTATTCAGGTTTTTAAGTTAAGAATCTGG

A_16_P21587350

AATCTGACCCATAGATCATGTTGCAACAGAAATCTACTTTAGGACATAAAAACTGGCCTT

A_16_P16404596

AGATCCTCAACTGCCTAAATTTCAGATTGGACATTCATATTCCTGCACAAATGAGAATGA

A_14_P113478 GATATCTCAATATATGAATCCTCCAGCAGTGTGTTCTCTCAGAAGTTGTAGTGCATTTTG

A_14_P128000 TTACATGTTCCTTTTCAACTCTTATTATGGTGCCCTATTCTGGATTCAGAGACAAAGTTC

A_14_P124763 TGCTGCTTGATGGTTGGATTTTTATTATCCATTACTGCTAAAACATCCTCACTGATGTTC

A_14_P116755 GACTCCTTAAATGTAGAAAAAGCCTAAAAATCCAGTATATGTCCATTCCTTAAATTGCCC

A_16_P01023838

AAAACAGTGTTAGGATCAGGGTCATATCACAGGTCAAATGATAATAAGAATTTCCTTGCT

A_14_P120758 AATACCAAAATAATGAATAAATCATGAAGAGTCTGTCCTTTTGTGGGTTTTGATGGAGCC

A_14_P122424 ATGATTTTTATTTTCAGCGAAAACTGAACCACCTAAGATTTTCCGTGTGAAACCAGTTTT

A_16_P03555808

AAAAAATCCCTCATAGACATGTCTGTTCTTACTCATTATCTGTTATATCTGGGACTGGGG

A_14_P202484 CCTAGCAACCAAGAATTTGTCTTTGTCTCAGCAACTAAAGGCCATTTATGTGGAGTA

A_14_P129583 CCTTGTTATGCTATTTTTGGAATCCAGGAAATTTGATCAAGCATCTTGTTAAAGGAACTC

A_14_P103646 ACAGAATACTCAACTAGGAATGAAAAGATTCCCAAAGAAAGATCTGGACTTTGTCCACTA

A_14_P136245 TTATCCTAGGTGACTGCTACATTGAGAAACTTGGTTGATTCATCATTAGTAAGAAGTAAG

A_14_P106708 TGCAAATTCAAACAAGGAACCTCACTGTGAAATAAATTCTACACAGTTATCTGAGAGGTG

A_18_P15885174

CCCCCTATTACCCATTATTTACCATGAACCTAATTCTACAGACATACTAAAGTTCTTTGC

CUST_221_PI426210822 CCGCGGGCGCCTACAACCTCGACCCGCAGCGCCCCGTGCACTTCC

A_14_P127721 ACAACTGTAACTGCTTTAAGATTATTTAAGAATCTACCTGTAAGAAAGCAGTTTTACTCA

CUST_3454_PI426210822 GACTTGAGCAAACTTTTGACAAATAGCGCTCTCTTTCTCTCTTGGGCG

A_16_P18446167

TGCTTTTTGTTCTATACTACAGAGACATCACACATCTTCATATTCAGGCCTTTTCACGTT

CUST_9410_PI426210822 ATGATCTGCAAAGAATCCTTAAGAGAGACCCTAGTCTGTGTTTATGTGGGTG

A_16_P19073827

AATTCCAGCATGTAGTTTCATTTTCAGTTTCCCCTAAATGTATGCAGATTTGACAGATCC

A_16_P40258222

TCAGTCATTCTCAAACCTTGTCTCATATCAAACCATCACATAACATAAACTCATAGACTC

A_16_P15682188

AAGGAGTTACTCATGTCCATTATTAACAGCACAGCCAGAATATATGTGATGCATTACCAC

A_18_P10384810

GCCCCTGTTTTACTTTCAAGCCTACACACAATAGACGTTATTCCAATTTTAAATCCTCTT

CUST_5864_PI426210822 TTTGTTGGATGGAAATTCAGCAGCACTGGAGGACTAGACAGCTAGAAAA

A_16_P16246988

ACATGCTTATCCCACATCAACTATTACCTCAGATAAATTCATTATCCATAATGCATCCAT

A_14_P201816 GGATGATGTTTCTTGCTAAACAGAGGTTTACCTGGTTGAACACCAGAAAAAGTAGATG

CUST_41_PI426210822 GAGTACACTGAGGCTGCCTGAAAGAAGACAGGGAAGAAGCTAGAA

A_14_P108658 TGTTTTTCCCAGACATTTTCCGGGAAGATCATACTGAAATTTTGGTATGAGTGTAAATTC

A_14_P124129 ATTTCTAGTAGTGAGGAGAGTTTTGCCAATTTCAAACCAAACAATGTAGGGTGTCC

CUST_583_PI426210822 TCTTCGGAGGGCATTTGTGTCTGCTCTTGGCCTTGCAGATAAAATA

A_16_P15177422

AACATTTGCCACATTTCAAAAAATGACAATGACAGACGATTCTTTTTGCCATTTACCCTT

CUST_9658_PI426210822 CAAAAATCAGAGGGATCTGGAAACCTGAGGGCTATAGGCTGCGTC

CUST_7150_PI426210822 TGTTAAAGGCCTAAAAGGTTGCATGTTACATATAGAACAAGGCCCTGGGAAA

A_14_P133709 TAGTGGTGGTCCCACTGTAAATGGGAATGTGCATCTGAAATACTTAATTAACTTGAAATG

A_18_P10888733

ATCCAGACCTTTTAAACTTATCTGACTATAGCCCATCTAGATAATTGTTCCTTGCAAGCC

A_14_P135860 TGCATTTCCATGTGTCTAAATCTGCAAAGTGGATCTAAACATTTCCTCTCTCTCCATAAT

A_14_P138106 TAGACATTGTTCATAATGCCTCCCTACATATGTAAAACACAAAAGTGGATTGGAGACACA

CUST_4524_PI426210822 CCTAGTGTACCCCACCCTTAGCCATTCCTGCAGTGGACTGAGTGC

A_14_P132482 GTCTCATTTCTTCCCTTTGTTCCTTAGTCATCCAAATAAGCCTGAAAACCATAAGAGATA

A_14_P101283 TAATGGGTGAAATGATGCAAGATTTGAGAAGTCAGTGTGAGGCAATGTATAGGTCATTTA
CUST_8127_PI426210822 TGCTCATTTCTTTTCAGGCATTGGTGAAGATATGGTCTGACTGCCC
A_16_P00388665
AAATTGGATACCTCAATCACATCTGTCATAGGAATCTTTTAAAGCACCAAGACACTTGAC
A_14_P135205 AACTCCAGTTTCATTCCTGCCTTTTGATTCTGTGAAACCCCTCAATCATTATAATGATTA
CUST_269_PI426210822 CTCATGGGGGCCCAGGTGACAGCCAGGCCGGGGTTGATTTTATTG
A_16_P17823392
AAAATTACCCCTTCATTTCATCACTAATCCCGTAACTCCCTTAATTAAGTCAATGGTAGC
A_16_P18782530
CATCCGATTAAATCAAGTGTTGTTACAGAAGTGGTTTTTAACCTGGCAAAAATGAGACAG
CUST_8526_PI426210822 AGATGTTAACATCCCTCATGATGTTCCATTCCAGTTCCATGGCATCC
A_14_P102910 CTTTTACCATATGCTTATCATCTAGTGCATAGAACCTCCCTGTTTGTCAAAGCACTCTAA
A_18_P22841604 ATGAAGATCTGCAGGTGTCTGGAATGAAGGAGGCAGTCAGGAGAA
A_14_P107787 GGAAAGCCCAGGATACTGAACCATAGGAAATGGGGTTTTATGTTTTTTATTTATTTTGGA
A_14_P113397 CTTCCTGTAACCCATCCTCAGTAACAAGATAAAGGCTTTCTATTCAAACATTTTTGACTC
A_16_P41733761
AGTCCTCTTCACTTTCATGCTTATGTTATCCTCTTATTAAATAAAGTAATGCCAAGCCCA
A_14_P134487 AAAAGGGAAGGGGGATGTGATAAGAGATAGGTAAATGAAGTGCCTAAAAATTAAACATTC
CUST_7913_PI426210822 CAGCCTAAGCTGGCTACACTTGGAAACCTCTCCTCACCCTTGTAC
A_16_P20221151
AAGTCCTCCCTTATGACTAAAAGTAAAGAGTAAACCAAAAAATACTGTCTCAACCCTTGG
A_14_P202019 CCAACAGTGTCAGAACTTTTACAGCATAAATTCATTACTCAAATTGAGGGCAAAGATGTG
A_14_P127463 GACACACCTATGAAATCCATGATTATATTGACATAGAGCCTGCTACTAAGTCAGTGACTT
A_16_P41331094
AAATGGGGGGGTTTAACAAAATAGGATAACTCATTGGATGAGATAGCATACGATCACTAA
A_16_P16582532
AAAAGAAACTTCCACTTTAGAATCCACATTTTCAAAGGCTTCCGTTATTTACACCATTGC
A_14_P117671 TAATACTTACTTTCTACTGTTGCTCCTTCGTTTGGATTTGAATATCCCTCTCCTTTCCG
CUST_10250_PI426210822
GACTTGTTCTCATGGGTTGCTTATTTTGAGTTTTTATTTTTAGAGAACTGATATTTTTTC
A_16_P16099065
AGAAATTACTAGAGGAGTCTTACCTATGCTATGGCAAATCAACCTAACAACTTTAAGGCC
A_18_P14413394
AAGGACAAACTCATCAAGAATTAGAAGACATGTCAACATCTTATCTTGGAGTTTTGTGGC
A_16_P19272934
TGTTTTAGTGTCAACTTCAGTGGTTTGATTACAAAGTAAATGACCAGATCCTAACTTGCG
CUST_1089_PI426210822 ACATTGCCTTTGGGCCTCCCTGGGTTTTCCTAGGTTTCAAATTTT
A_18_P17124219

TGCTTTCTCACTATCAAGAAACAGACATTCTTCCATCTAGACTAATATGATCTCCAGTGG
A_14_P126994 CTGCTTTCAGAAAATAATTTGACCAGTCACGTATACTGCCTATGAATGGTAACAGGAATT
A_16_P18183561
AAAATCCTTTCAACCTCACATGTATTTTTGATTCAGACATCTCTGCTCATGATACCTCAG
CUST_1931_PI426210822
AATTTTAAAAACAGGAATAAAGGAAATATTTCAAGAAACAAAATACTAGCTTTTTCTCAG
A_14_P121126 TCCAACTTCCTTCTGATTCTATCATACTTTTTCCTGGAAAAGTTTTTCCCAGTGTAAGAT
CUST_1316_PI426210822
AACTCCTTGGTGAAATTCAGAATAAAGACTGGCAAGCTATTTTTCCCCCAGTATTTTA
A_18_P11577420
TGTGCACCTTGAATTACTTGATGCAATTCTCAATACATGTATTTTTACCAGCAAATGCGA
A_14_P115997 ATCATTGAGAGCCCACAAAAGGCAATCCCACTACACTTTAAGATTATGACGAAAATATTT
A_14_P134313 GAACTTTACTTTTCTTCTCTCAACAGACTTTGCAGAACAAAATGAAAAATCAAAGGGACC
A_14_P121571 ATATCCTACTTTGGCTCTGCTAACTGGACCTGATAACTCAGAAATAACCTAGTTTATGAC
A_16_P00685425
ACTTTATGAGCACTCTTTCAATTATTGTTTTATATTACACCAGTAATTTTTAAGTAGTGT
A_14_P100518 TCTATATGAGTCTCACATGTAGAATGTCCTCAAAGACCCTCAATCCTTTACCAACTGT
A_14_P101514 AATTATGAAAACCACGTGTTATATCCTGTTGCTAGTCTGAGTCATTTGGAATTGTGGGTA
A_14_P112639 ATTTGATTTCTTTTCTTTTGGACGAATCAGTCGTTTCTGTTGTGATTTATCGTGGTGTTG
A_14_P134704 GTAAAATGAGAGTGATGGTGATATCTCTGTTCTCTGTTTCATCCCATGATTTTTTGAGAG
A_16_P38603732
AAAAAAACTGCACAACAATAGTAAAACAAGACACAATAATAGTAAAACCTAACTCAGAGA
A_16_P37501129
GCGAATTACACATCACTATAACTATGAGCCTACGTTATGAAAATTTGAGATAACCACATG
A_14_P202530 CTTTAGCCTCTTTAATTAAAAATTGTGCAGAGAAGACCTTTGAGGTTCATGAGGATATTT
A_16_P17500752
GTGGTTTGTCACAGAATTCCTTTCAATGCTGAGATTTTCAAATATATTGCAACCATCGAG
CUST_4483_PI426210822 CTTATAACTGTGGTGTAGACCTCAGTCCAGACACCCTCCAAGAGATAC
A_14_P124739 TAAAGAGAAGGGGCAGGTCACAGACTGATGGTCAAAGATCTGAAAATCAAAG
A_14_P129114 ACTGTAATCATGTGGGGCATTAGTTTTTTTAAAAAGATGATCAGTGTGGCTTTAGGATCT
A_14_P131508 TGTCGAATGAGAGAAGAACTGTAGCATTGGAAATAAGGTAATGCTCTGTTTGAATTGTTG
A_16_P16351810
ATGTGTGTTGAATCACTTTCCATCAAAAAACGTTACTTCTATCTGACATTGACTCTACCC
A_16_P00685534
GATTCAGACCAAAAAGTATGAAATCTTACTAACTTATTACAGATAGGTATAGTGCAGAGA
A_16_P17295994
ACATGAATTTTAACACCACCTATACCTTCAATTTTATTCATCTTCCACTATTGACCACCA
A_14_P128022 CACTTCCCTAGGCTACTCCCCTATGAAAAATATTTTGATTTAGTTGGTTCATTTCACATG

CUST_3877_PI426210822 TTGTCCATCGTGTCTCACCATCTGAGGAGCTGTCATCCTTTATTCA

A_14_P138812 GCTTTTCTGCCATTTTCGATTTTTAAATGATGAGGGGCCTAGAATAGCAAAGGATCG

A_14_P139426 AACTTTTCTTCAAAACTGTGACTTTCCACAAGTCTAATGATTGCTGAGGATAATGGTGAA

A_16_P16215372

CCTACCTAGAATGGCAGTATTTCAACCTAATGAAGCAGTAAAATAAGTCTCCAGGAGTTA

A_16_P00642064

AGCCATAAGAATCCTTATATCTCCAGAAATAAAATTCCCTCAAACAAAAATCTTGTCCCA

CUST_4871_PI426210822

ATCTATAGGCTATTGTGTATAACTGGAGGGTAGCAGAAAACAAGTTCCTTGTGAGATC

A_14_P119505 GATGAATGTAAGTTTCCACATTGTTGCTGTTTCTGCATTTAAACATAATTGGGAACAACT

A_14_P126916 CTTATTTATTTAGGTAGTAGTCCCCCAAACTTGAATCTAGTGAAAGGTGGCAGACATTTT

CUST_4509_PI426210822 CTCCTGCCCCTGGGCTCCAGGGCTCTCGATTTTTGGGGTAAACAG

CUST_1287_PI426210822 GATAAAATGTAGCCAGGAGACCAATCTGCTCTAGTCTGGAGTGGCTG

A_16_P58424382 CGATAAGCAAAGCCAGAGAAGGGTCCTACCCCTGATGTTTAGATTCTAGTAG

A_14_P115187 TTCCTAAAGGACAGATATTCACACATTTTTGGGATTATGAAATTCAGCAAGTTCTTGGCT

A_14_P114469 AGTATTAACACATACCTGTTTTAGGATGTATTGAAAAGAATCCTTGTGGATTTCCACTTG

A_16_P15749896

TCTGAATCTCATTTTTTGTATTATTTTCTTCTAGCAAATAATAGGTAGCAATTGTCTACC

A_16_P20634579

GTGGTCCTAGGCTCTAAAAAGGTTATTCTGTGTTTCAGCATGTAATTTCAAATACCCAAT

CUST_6316_PI426210822 CCCCGCTAGCTTTGATGAAATGCACATACACTGCTTGCATTTTCTGTG

A_16_P18413122

AGACAGAAATTTGGCCAGTCTTATGTTCTGACATCCAAAACTATATTAGGTGAACTCAGT

A_18_P15284791

AAATAGGACCATGCTTTTCAACATAGACTACCCATTAGTTCTCCGTTAAATGCACTAAAC

A_16_P20080522

CTAAGGCCAATAAGGATAACATTATCTACCCAGAGAGTTTAGAAGAAAAGTAGGAGTCCA

A_14_P108139 TTCCTCCTCATTTTCCATCTTACAAATCATCAATATTCCTGGATGTAGGATCATGTCTGT

A_14_P134553 ACCATTATCAGCAGAGTTATAGCACTGATAAAACAATAGTAATAGTGGCCTCTAGCCTGT

A_14_P114532 AGTATTTTCATTTTTTGCAGATTTTAGGTGATGGGCAAGTCAGAAAGTCAGATGGATATA

A_14_P106707 GAAGGTGAGGATTTTAATGTTTTGGAAAGAGTGGAGGGACTGAAAAAATCATGATCTG

A_14_P126996 TGTAAATGGAGATTCCCAGGAAGCGTTAATTCATTTAGTAGCGTTGACATGTTCATTTTC

A_16_P19718511

AATCTACATCTGCTGCAAAATTCACTTTTCACCAACTGTATTTTTCTGCAACGGTGTTTC

A_14_P101679 ACCTTTATTTGATACTTTATAGGTCACTATCATATGACAAAGGCTTTGCCGCAGTTCATC

A_14_P137349 CTTACGTCAACACATCTGAGGTCTCGTCATAAACACTGTTTAAAAAGTAAATTGAAACCC

A_14_P127913 GAACTTAATTTTTTAAGGAACTCAAGACGACAACTTCCAAGTTTTCTTCCCCAATATCCA

A_14_P131612 CTTTCTCTGACTAGTAAAATTTCTTGGGGATACCACAAAGGATGTTAGTTTCCTCCAATA

A_14_P100852 TAGGGTAGAGTGGCATATTTAATATGCAGGCCAAAAGTTTGGATCGTTGATTAAAAGTCA
A_14_P112196 GATTGTGTAGTTTTTTAATGCAACAGAATAGGCTACAAAATATCAGAGGGGATTTCTCTT
A_14_P119902 CTAAGAAGAACATTTTAAATATACACTTACTTTCTGACTGTCAGCAACGAGTGGTCCCAG
A_14_P114699 GTAACTAACTGGAAGGAATTTTAAATTTTTCTAGGACTTGCCTGAAACTTTCCCGAAACT
A_14_P114217 GAAATTTAATTGCTATTAAACAGGAGGAAGGGGAGAAACATGTCCATAACCTGTATGTTC
A_16_P00685357 AGAAAGGGAGAGAAACCAGATTGTGGAACACTTATGAGTCTGGACTTCATCTT
A_16_P03713286
TTCAGAAGTGCACTTTTTAGATGAACTCATTCAGAACAAACATTGACCATACATTCGTCC
A_14_P102585 ACACAGGCAAAAGGAAGGCTCCTTGAAATTCAGATACAATGATGCCTAAAATAATTAAGT
A_16_P39312597
TCATCCTAGATGAACACATTTCCCATTATCCATCCAACTAGATAGCCAGTTTAGCATTTT
A_18_P14132713 ATTTCATCCTTGCCTCCGCTGATGTCTAATTCTGGGATAACGCTGG
A_14_P115215 CTTTAGCCCCTCTTTGAAAAATTTGCTCCAAGAAAAAGAAAACACGTTTAACCAGAGATA
A_14_P117609 AAGAGACTTTCAGTCTAGAGAGTAGGCAGAATGTATTCTCATAAAGCCACTGGTATTACA
A_14_P121345 CTTATCTCAGTTCTCTTCATTCTTATGCTGAGAATTTGACTTCTCAGAGTTTTTTGGGCA
A_14_P201028 ACGGGGTGGGGGTAGATGATCATTATTGCTATATTAATGCCAATCAAAGTGATGTTATAA
A_16_P21639150
ACTGGTGATAATCTGAGAAACTATAATACTAACACATGTCATGGATTCCCTAACTGCTCA
A_14_P129982 AGGGTAATACATCTTCAGCAGTCTCCCCTGTGTATGACTAGTTACTATTCTTTCATAGAA
A_14_P131342 CTACAGTGCAAGAGTGGCTTTATACAAGATGCTCTAGGCAACTGTATTGGTAAGAG
A_14_P135785 ACTTTCGATGATTGGACTCAGCCCAGATAAAGAGGTGCTTGTGACAG
A_14_P116672 AACCAACTCTATACTAACCTGTGGGAAGAGAGAGTAAGTTGAATTGTCAATGGTGAATG
A_16_P17041349
AAAAACGAGGATAGCAGTCTTTTTACATGTTGATAAGGTTAATCAAGGCAACGTCATTGC
A_16_P03406704
TTCACCATGCGCATTTTCTAGTTACCTTTCAATCATGCTTCTCAACTTTAGTAACGCTAA
A_16_P02829321
ACATATCTCCCCTATCAGAATACGAAGATGAGTCTAATACATGTGACTGTGAAATCAGGT
A_14_P132940 GTAGTTTCAAGTTATCAACCCACATTCCCCACCCCTACATTTTAAAGTAAATTTCAGTGA
A_14_P101261
ATGAAAGGCAATAGAATTGCAAGGCTAAATGGGAATAAAAAGGGAAGGGAAAAAGAAAAA
A_14_P101693 CTTATGTTTTTCCCCTAGAAAATACCCTGTGATTCAGACAGATGATGAGCGAGAAC
A_14_P130879 CTACCCAATGATGGTCTTTCTGAAAGATTCTCCATTCATTCCATAGAACTGGATAGGTT
CUST_4481_PI426210822 CCAAATATTTAGCTACGGCCACACCAGCCCTTGTTCTGCTTATAACTGT
A_18_P13773952
TGCTACAAAATTACATAGAATGGTTAGTTTGGCAAAAAATAAATATCACCACTAGCACCA
CUST_9775_PI426210822 AGAAACACAGAAGTGAATCAAATAGTGTGGTTTAGGTTCACCCACTGCCT
A_14_P126611 ATCTTGTATGATCAAATACTTCACTTTTGAAATGAAACCCAAGTTAATGTTGACTGGCCC

A_14_P105738 GCTTGTCCGACCTGGAAGTCCTCATTGTTGTTATAGATGATGGCATTTTCTG

A_18_P15129245
TCCATTGATAATGTTTCACACTTCTATTAGCCACAGTGAATACATCTTTTCCAAGTAGGC

A_16_P18756946
AATGTCACTTCTACCATGATACCCTATCTGCTCATCCTATCATACTTGTTTCAACTTGTC

A_14_P116980 TCTTGCCAACGATGCATTTCTAAGTTTCTCTTTTAAAGAAAATTTCCTGGGTCTGAAAAG

A_14_P115732 TCAGAAAGGAAAAGAAAAAATGACACAAAATACCCTACCATTAAGTGGATTGCCAGTTTC

A_14_P108491 TTTGGAATATGATGACACTGGAGCCCTGGTTTGCAATTTATGAGTAATAATGATGATGAC

A_14_P129915 ACACTAAAAAATATTTCAACTTGATCTCAGCAGATGAGGCTGTATTGAAAGTGGGTAGAG

CUST_4140_PI426210822
GTTTGGAATCCTTTTGGCCTTAAACAAAATAATGATGAGAGAAATCTGCCTCCCAT

A_16_P19038348
ACAGATGCCTTATTTCCAATATACCATACTTCCGCTTTTTATCGTTACTTCTGCCTTTAA

A_14_P105580 TACTTAAAATCATTTTTCCCCCATACTTACCCAACACAGGTTTTAAGAAATTCTTTTCGT

A_16_P03014853
GAGCCCATAGAAGACTGCAATAAATCCTCAATGGAAATTTTGTTCTTTAGTGCCTGATAT

CUST_10366_PI426210822 TAAGCTTTGGGCTACAAACGTTGCTTCACTGTTTGGTGGTGATGACTA

A_18_P22841223
GATTCCAGATATTAGGAAAACAAGACAGAAGGGTTTCACCACTAGTCAGTATCAGTAGAT

A_14_P107694 CACCTGCTCATGGCATACTCTAGATACGTGGTTTCTAGTCCTACTGTCTG

CUST_9556_PI426210822 CACGGGACTTACCTGTTTGTTTGTCAAAGCAGAAATGCCCAACATTTTG

A_14_P135071 AAGGTATAGCACAGATGGCAGGACCTCATTTGTAACTAGGTTATATTATATCCTATTGTT

A_14_P106686 AAAATCTGATCTAATTTAGTAATCCTTCTTCCTTCTGGTGTGATTTCACTAAATGAATAG

A_18_P26461684
AAGTTGAGCATGTATGAGTTCATCATCTTCAAATTCTACTCTTCCTACATCTTCCTAGGC

A_14_P122126 GTAAAACAACTTCCGAGGGGTTAGTGTGCTTATTTGTAATAAACCACCTTCATAAAGGTA

A_14_P104273 CCTCAAAACCTCAATGTTCATTCTGCTCCCTAGATTATAAGTGAAAAACAAACAAGCAAA

A_14_P120160 AAAGAAATTTAAAATTTCACTTCTTGCAGATGGCAAGGATCTTTCATCTGGCACCCTATA

CUST_10039_PI426210822 GAGTGAGTGGTGCTTAGAGGAGGCTAGGAGACAAGGCTGGGCTTG

CUST_6273_PI426210822 TATTTAAAAACCAGACTTGTGGAGTAATGTGTGGGAGACCCCTTCAGG

A_14_P112687 CATGGATTCCTTGTTTGTGTTCTCTGCTATAGCCAAATATATGTGCACGTGTATGTGAG

A_16_P38201988
TGACACTTTGGCTATTAACTTTCTGGAATCAAGAACATCATGTCTATTGACTAATCATGG

A_18_P22842081 ATTCACAGTCATGTGTGGCTAGGACACCATGTGGTCCAGTCTGAC

CUST_6501_PI426210822
CAATTTATTTGGTATTTTTTGCTCATATCTCTCATAGAAAGACAACAGATGAGCAATTTT

A_14_P134960 TAGGACTTGGTGTTTTCTTATTCAAATCAAGTAGAACCTCAGCAGTCTGACTTCACATAT

CUST_307_PI426210822 AAAAGGTGTTCAGCCTACTTGGATTTAAAACGGGGATGAAGAAAAGCTGTG

CUST_3265_PI426210822
AGATGTTCATAAGATTGTTCTTCTATGTTCCTGAAGGATTTTTCAAGGAACTTTGAAACC
A_14_P103658 GCATGGGTCTTATTTGGCACACAAGAAACTGATGTGGAAATATAATAATGTGGTGCATAT
A_18_P25060029
CGTGCACTGTATTTATGATCCTACTGCTGTTACTATCTGTGTTACTTAAAGCCTTTGCTC
A_18_P17741044
GAAAACCACTCACATTGTTCATGTTCCTTAGATATCACACATTGAATTGTCACCACTCTA
A_14_P120583
AAGGATGATGGAAAACATTGAGGAAAAATTTCCTTCTGGAAAGAAAGGAATCAAAACTGA
A_14_P123137 GTCTGGGCTTGAGAATCAAGAGATCTCTGTAAAGATGCACCTCATACATTTACTAAGTAA
A_14_P106705 CTGCCATAGGTTTCCTCTGCTATTTTAGATTTTATTCCTGTGAAAATATATCCATGGAAT
A_16_P01811890
ATTGACATCACCTCTATACGTTTTCATCAACAACTCTGACATTTTGACATGACAACCTTG
A_14_P126264 AAACCACGTGATGTGCTTATTCTCCCACACGACCGACTTTTTGCT
A_14_P117036 CTTCAAGTATGAATAGGGTCATGAATGTCTTGGGTAGAAGTTACACACATAAAGGTGAAA
A_14_P119315 GATTCCTCTCCAAACACTTACAGCAATCTTGACGTTCATCTGAGCCATCATC
A_14_P108462 GCTACTTTTACGAACGCCTCAACATGGATTACTGGAAGATCCTGGCG
A_16_P15509372 GTGGATGAGCAGATGCTCAATCTTCAAAAGAACAGTAGCCACTTTGCTCAT
A_14_P101526 TATTGCAATTTACACTTGAAGCAAGTTACAAAAAATTCATTTCTTTGGCTTCGGACAGAA
A_14_P117598 ATACAAAGAAAATACTTAAAGGGATCAATAATGGTGTCTTCTGGTTGCAGAATGCGAAGT
CUST_2493_PI426210822 TAAAAGAAAACCAAGTCCTCTTGCGTGTGGACAAAGCAGACCTTCAC
A_14_P137479 GTGCTTGCTCAAGTCATGGTTTTGCCCTTTAGATTATTTCCTTGAAGTGCGTT
A_14_P124482 CCATTTGGCCACACTGTCATAATGCTATGCAATCCCATTGTTAATTTTTTAATAACCCAA
A_14_P122878 AGGCGGATCTGATGCTATGATATTTATAGAACACACATATCACATTAGCATCTTGCCATG
A_16_P03381857
CTGGTCCTTAATGGTTATCATTTCCAAGTTTATCCTCATCTAATTATGACAACATGCCCA
A_16_P00502105
GAGATTGTGCACTTTATATGCTTCTTGTCAAACTCATTTGGTATTTGAAATCACAACGCC
A_14_P135306 GTATAAACGTCTGGAGATGAACAGCAACCCTCTGCACTTCAGGGTATCTT
CUST_8591_PI426210822 CTCTCCACACCCTTGGGACCCTCAAATTGTTTCTCCCTCTCTAAC
A_14_P131961 CTCAGACTTAAAATTCCACATCAAGTGTTCCATTAAAAGAAGATAAGGCATTCTGAGTGC
A_16_P21425933
AAGAATTAAAATCCTGAAAGCCACATATTAGTCCTTAGCAAACCTATCATACTGCCTCCA
A_14_P103133 TAATGAATTGAATTAAGTTGGTTTTCGGATTCACTTCTTGTATATTTTGCTGCATGTAAA
A_16_P18255957
AAAACCAGAATTGTTGAATGTCAAACTCCAGTGATAACCACAAAAATGTGAGAACAAGTC
CUST_8959_PI426210822
GATCATTATTGCTGTTTCTCTGGCCTTATATTAAATAGTGTCATTGCAATTGAAAAATTA

A_16_P58424098
TATTTGATTTAATCTAGATAGGAGGGTCAAGAAAGGTCTGGGACCTTTCGACATGATTAG
A_16_P15831669
AACTTCCTGTCAATAGCATTTCAGTAGCAAAGATTGTTTTCCAGATAAACACATACCTGG
A_14_P120128 CTTTCAAGATGTTAGAAAGTTCAAGCCAGAAGTTTCTTCAGGGTTTGGTCTATCTCATTG
A_14_P100383 AGAAACTCGGGTTTCTAACTTTTAAAGTGGGATTATATGTCTTCTCCAATATATGGGCTC
A_14_P114116 AGAAAGCCACAAGTGGAAGATTTCAAACCCAACTATTCCCTTGAATTAGAGAGAAATTTA
A_14_P100775 GCTCTCTTGGTGATGGTGCTAAGGATAACTGGTGCTAGAAGGCAAC
A_16_P15820296
AACATTACGGGTTTTTCAACTACTTGCACTACTCTCTTCAAAATGTAGCCTTACTGAAGT
CUST_1251_PI426210822
CATTTCCTAGGACAGTGATCATTTCCAGGGAAGGAAATAGATTACCCATTCAAAG
A_16_P16426894
AAAAACCTGGTCACAATGTTTAACTCTACGAGTGAAGAATGTATTGAGCACCACCTTATA
A_14_P127937 CTTCTGATCAGTATTACAATTTCCTGACCACGCTACATGGGTTTGAAAAAAAAATCAGTT
CUST_7835_PI426210822
CGAGTTCCAGAGTACATTTTTGAAGACATTTATTTCACACATGCAAAAACTGAAGAAGTC
A_14_P119836 GTTTATGCAAATCATCAAAGGGATTCCTTGAGGCAAAAGGTTTGAGTCTGCAGTT
A_14_P104033 CTGCAAACAGGTTTATTAGCAGAACATGAAATAAGAGACCCCAATAAGCCCTATTTATGT
A_14_P113181 CACAGATATGTGTATCTAGATGTTTCTAGTTTGTTGTCTAAAAAGGGCGAATGCAACAGC
A_16_P17048138
AAACAATTCACGTTACTTCTACTGACAAATTTGCAATGTCACAATTTACCCAGACTCTGC
A_14_P200415 ATAAGGTTTTATTTTGAAAGTCATTTGATGAAAGTCATTTGAAAGACACTGAGGAGGGAA
A_14_P113818 TTCAGCAGATCAGATGATACTTACTCAGAGCAATTTCCACTCCTTTGCAGTAGCATATTA
A_14_P128166 TAACTTGAAATCCTCCTTTAATAAACTACAAGGCTTCAGACATCTATCCTGCTTCCCAAA
A_16_P02668585
TGTGCCATCCTTTACTACTTCACTCATCTAAGCATAAATATATGAATACCTGCCTCTGAG
A_16_P02600440
AGATTCTTGTTTTTAATTGTCACCTCAATAGAGATACCTACAGCGATAACTTCACAGGCA
A_14_P102492 ATTAATAGATCAAGTGAATAGTTTGACCAAAAACTTCAGATTTGGGGGCTTTGGATTCTG
A_16_P01353264
AGTCCATGTCAAGTGAATTTGAAAAGTGAAGAGAACTCTAGTAACATCGAATGACCAATG
A_14_P114496 ACCTAGGATAATATGATACTGCCTCTATGAAACCACCTGGTCAACCTTTGCTTAATTAAT
A_14_P202523
GTAATGAGATTCTCAAGGCCTGAAGAAAAGATAACAAAACTAGACACAGCAATGAAAGCA
A_14_P130266 TGAGATACATGGTTATTATGAACAAGAGGCTGCGTATCCAACTTGTCCTGGG
A_16_P01835269
CCACTTCCATTCACAATATTTTGACCTCGAGTCTCAAAAATCATTCTAGAATCCAGTCAT

A_14_P111749 CTGTTGAAATTGTTACTTCAACTTCTGCTGGTAAGTTGTTACTTTTAATATTCTTTCAAA

A_14_P119192 AAAGTCACAGATTGACACTTACTTTTCATTTGCTTGAAGATGGACTTGTTAGGTTCAAGC

A_14_P127264 ATAGAGTGAAGATAATATTGATAAGCAAATGAACATCACAAATGGCCCTAACATGGGCTG

CUST_7592_PI426210822

AACATTTGACTCCCGCATAACCTTTTCATCCAAACACCATTCATGAGTTGTTTTTATG

A_16_P37699099

CCATTTTTCCCATATGGGTTATTACACACGTGCTTGAAGAATAAAAAACGGTTGTCATTT

A_14_P122060 GCTTAGGGTATTTCTACAACATCCACAGTTTCGGATTCTACGTGGTAACTCAG

A_14_P105128 GTTTTAAGAGATCCCTTCCTTCCTCTTCGGTGAATGCAGGTTATTTAAACTTTGG

A_18_P20378373

AAGGCAAGTATTGAGGTCCTTCCTTATGCATTTCTATGGTGTTTATGATGACGATGAAAT

CUST_10409_PI426210822 CACTGATGCTGTTCTCTTCTTCATTCTATCAAGCCCAGGTGCCAG

A_16_P02845480

GAGCAAACTTTCATGACATATCCTAACTGCATCCTAAGATTTCCATCCTAAGCATTTTGC

A_16_P18965056

ATTCTCATTGCGGTTAACCTCATTTTGAATTAATACTGGTGTTAAGATATTCCCCACGTC

CUST_8604_PI426210822 TCTTTTGTGTTTGCCCAAAGCATTTCTTGAGGTCCAGCTGTCTGTAAG

A_16_P21520649

ACAATGACTCATTAAACTTAGGTTCTGGTAAATGGTCTTCTTCTAACATATGTGACGGGA

A_14_P138184 TGCTTCACCCAGTCCTAATAGTCATGCTTAGTATAAACCATTCATGTCATCACGATTATC

A_16_P16768295

AAAAAATCAAGATGGTGAAATACAACTCAGGAACACACATGTATTAAGGCAGGTTTGACA

CUST_2633_PI426210822 AGTTCCGGTACATGGGCTGGGGGCGAGGGGCTCTCATCTTCTGAG

A_18_P22842653 GTGTGGACATTTCCATGTGTCCTCCTGAAACCCTCTGGTGAGCAT

A_16_P00996046

ATACCTGTCCAGTCATCAAAGTCTACAAGTTAAACCTAAGGAAATTACATTGACCACTCT

CUST_7887_PI426210822 CAGTCTCTGTGTCTACTCAGAAAAGACTCATGCTGTGCAATTAGAGTGTGG

CUST_6077_PI426210822 TTGAGTTTGCTCCTCTCTCCAACCATAACAGTTCCTGCTTCCCTG

A_14_P103909 TACATGGGGGATGATTTTGGCACTTTTTCAAAGAATTTGTTTCTGTGTATCTGAATTTGC

A_16_P41330916

TTACCTTGACAATGAAAGATTCAAGTCATAAAAGTCCCTAAATTTTCTCTACCAAGGGCC

CUST_2229_PI426210822 AAATGCATCTCAGGCTATGGGAGACCATCCTGCTAAGCAGGTAAAAG

A_14_P132029 GTAATGAAATCCTCAGTGGGCGCAAACATTTTAGGGCCAAGAACATTGACTT

A_16_P17877886

CTTCCATGAGAACTTCAGTAAAATCCTGCATCTTCCAAATACTTGTCTTGTATCTCAGAG

A_14_P105817

AAAAAATCCAACACCTTGAACAAGAATACAAGAAGGAAATAAATGACAAGGAAAAGCAGG

A_14_P137200 TATCATCAACAATAAAAGGGCTATTGTTCTTCCATAACCCGTATTTTGCTTTCTTTCCCA

A_18_P16718188
ACCCAGGTTTTCAAATGTTAGCAATACTAAAGGGTGCACTTATACAGATGATATTGACCA
A_14_P125647 CCTTACTGTATGATGGTAGACATATAAAATAAATTTTTTACAAAGATATTTTGGTTGAGA
A_16_P01278783
TGGACTTGCTAAAAATGCCTGTATTTCTTACTTGCTCAACATAAATCAGTCTCATACCCT
CUST_9427_PI426210822 GACTTGGAGACCTGGAGCTGGAATTTGAGGGTCAAGACAGTCTCT
A_16_P19819680
AGCCTTTCAAACCCTATGTTTTTAAAACTGCATTGTCACAAGTTGAAGTTTACCGAATGT
CUST_1940_PI426210822 CAGGGCTGGTAAAATGACAGCTGACTTCTTCATGATCATCAGCTAGCATAAG
A_14_P137957 TGGAGCAAGCATTTTGAAGACTTCAACGTATTGGATGGAATTTTTCAAAAATTTCACCAT
A_16_P00582112
GAATATCTTTCCATGTCTGCAAATACACTCCTATCCCATTACAATTAAAGCCTGACAAGC
A_18_P16838748 CTGTAAACTATGCTTATATCGTCTTTACATCCATAATTCTCTATATCTTGTGCTGTCTGA
CUST_6083_PI426210822 ACAGTTGGTCCATGGTGTTGTGGCTGGAGGCCTCTGAATACCCAC
A_16_P03302599
GTGCACTCTAATAATCTGCAGTCCCAAATTAGTTATGGTTATTGATAGTGTTGGCCTCTT
A_14_P130722 CTAATCCATTCTAAGCATACCATCAGGATCACTTGAGTCTCATTCTCATAAAATAAGCCG
A_16_P01557195
CCTTGGTATTTCATCTTGTTCCCACCGAAATAAAGTATCAGCAAGAGAATTTCCTGATAA
A_14_P111851 ACATTGTGAAACATAAAACAACATTATAAAATGCCAGGAACATATGTCCCAGCACACTTG
A_16_P30952628 ACTGATGCTGTTCTCTTCTTCATTCTATCAAGCCCAGGTGCCAGC
A_14_P110633 CAGGCTGAGAAATTTCTTATTGATGGGTCTGCTCATTTCCTGCATAGGAAGAAATC
A_14_P111564 ATTCACATCTATCTCTTGCTCAGGAGTTGATCTAAGACAGGCTCTAATTGCACCTTAATA
A_14_P201711 ACTGATGCATCATAGTTGACTTCCACCAGTGTTTTAATTGTTTCATGGTTGCAGATATAA
A_16_P20261200
AAATGGTTGACCAGTTAGAGACATAGGAAAAAGTGGAATATTGAATTTGCATTGAAAGGG
A_14_P131206 TTGGCATTAGATGCAACAGAATCGATGAGTCAATTTAAATCGTTTTTTTTCCCAGTCTCT
A_14_P134103 ACATGAGAAGGATTTTCAAGCACACTTGCTTCTAAATTAGCGATGTCATCAGTCTCATTT
A_14_P110577 GAGGGAAATCGGATTTTCTTCACTCCTATGAGGCGCTTTTAAGTGTTTGATTTTTCT
A_14_P121518 TAGGTATACAAGTGGACAAAAAAAGTTGCTCATTTATGCAATCAACAAACATCTCTGGAT
CUST_3350_PI426210822
TACAGTATGTGAGATGCAGTTTGTAGCAATTGCTATGGGTAAAATTTTTATTTTAAGAGG
A_14_P122179 GATGGTGGCATTTTCAAGATGTGGCAAGAGGATCTTGAAGTCTGGTAACATT
A_16_P58424275
TAACTGCTGCAACAAACCCTCAGTTTTCAATTACTTGACCTACTAGATGTTTGTTCATTG
A_14_P128480 TAACTATGATCTTTGTATATCCCCTGCAGGTCTTTTCCCATGTGAATGTTTCATATGACT
A_14_P104146 TTTTTAAAAATTGCATGCTCCTGCTAAATAGGAATGCCTTTGATGTTTAAATGTGGCTCC
CUST_5950_PI426210822 CTTCCCACGCTGGAGAGCTGGCTTTTTGGAGGTGGGAGTAGAAGC

A_14_P139529 AGGTCAGTGTCTGTGTATTCATCCACTTTCTTATGCTGCAGAATATAGTCGGAGAC

CUST_7635_PI426210822

CTTTTTTAAGCTTGGAAAGAACAAACTTAGAATAGTGTAGGCCTTGGCTTCCACATG

A_16_P17027724

AGAAATCCCGATGTAAGAGCATAGACACACTAACCCTAATGTATGTTTGTTACTGGATTG

A_14_P115122 ATAATGAATTGGCTGACAAGACAATTCAAGATGGCAGAATGGAAACATGGCAAAAGAAG

A_14_P135074 CTTTAGCAAGCATTGTTATATGACCCAAAAGGATCTTTAGGCAACTGAGAAAGTTTCTGA

CUST_466_PI426210822 CACGCGTGAATCACTGTGTTGGGAGGGTGTGAGATGTGCTCCTTG

A_14_P133051 TGACATCTGACAGATGAACACTTTCAGCAAACAGCAAATAATAAACAAAGCATTGTGTGT

CUST_9046_PI426210822 CCTACAACATGGAAATGCCATTTCCTCCAAGCCGTTTATCTGACCA

A_14_P200830 TTAACAGTAATTCTGACTAAAATCACAGCAGTCCCTCTGGTAGAAAGGCAGTAAATTCTT

CUST_3440_PI426210822

TGTCACACTTATTCCTCTGTCATGTATGCTTAAGTCTGTTTTAGATGACCCCTTGTAAA

CUST_3021_PI426210822 CATTCAGGTCACCTTCCTCTCTGCCTGTTCATACCTCCCCTGGTG

A_14_P126419 ATGTGAACAAACAAGTGAGAAAGAGCACACATATAGCACTTATTAGGCATCTGGCATTTT

A_14_P125219 CGGTAGGTAATGCTGTAGCAGTCTGACGGCTCATTTCTGAAATAAATACATAAGGAG

A_16_P39431284

AGTTCCTTTGGCATGAATATAGTATTTTGGTTTCAATTTCATCCTTACCCTACGCAAGAT

A_14_P114356 GGGATTACAGATGGAGTTGGAAAATCCAATAATTTGCACGATAGCAAAAATCATCTTGAT

A_16_P58424531 TTTTCTTCACATCTGACCTGAAATAGGACTCAGGCTAGAGAATCCAGCC

A_16_P20912761

CCCTCTCTCATACAACTTTTGGAAGCACTATTACAACCAATATTCAATATCTCTCATGGC

A_14_P201689 CTGGAGTTCTTTCAATCTCTTGTTTTCTTCTGCCTGAACGAAAAATTTAACTTCGTTGTT

A_14_P135705 AATTTGGCCAGTTATCCAATTGATGAACTAGTAGATAGAGCCAAACAATCTTTTCAAGGT

CUST_8652_PI426210822 AGCCGTGTAGCATAGCCCCTGAGCACAGGTCTAAGATTGCACCAA

CUST_4798_PI426210822 CATTGGAGACTCCTATTTCTCCCTCCGTGACATCTTTGCTGAACAG

A_16_P20780109

AAATTCATTTTTCCCGCTAGAATTTCAACAAGGTATCAAGTTCTCCTACCACTAAGCAAG

A_14_P102075 ACAAATAGTTGCAGTTTTCCTTTTTGTTCTTGAGTATTAAGTAATAAAAATCAGCCAGTC

A_16_P18293661

AGTTCAAATACGTCCCTCTATGGAGAAAACAACTTTAATGGTTACAGTCTATCAAGATCA

A_14_P101606 TTCAGGGGAGATAGTTGGGATAGTTTGTAGTTTTGCTTAGGTTATATGTGTCTGTTTTCT

A_14_P118176 GTATTTGCCATACTAACCAGTTCCAGCAGAAAATGAGGTCGTAGCAAAAGCATCA

A_16_P38151201

AAATATAGCAGATGATAAATACGTGCTTCTTACTCTCATAACTTGTCATACAGAGCAGTC

A_14_P108062 ACCTAGAAGAAGGTCAATATCTACCTGCTGAAAGTAAAGAGATGAAAAACATCTCTCTGT

A_16_P01984391

AGAGTTTTGTATTCCCTTTTTGTAGAGAACCACAAAGCATATCAAAACGTCAGTTTCCAA

A_14_P124633 CTCTTCTTAACAAACAATAATACTACTCAATCCGGATACTGAGATTGGAGAATGGGGTTC

A_16_P39279421

ACTTATGTCACTACATTGCTCCATAGATTCCTTATACAGCAACATCCCTATAACAGAGCA

A_14_P119138 CGACAAAGGGCTTACTCTAGGTGGACTGAAAATTCGATGTTTCCAATTCAAAAC

A_14_P130809 ATGCCTATGGACTATTTTTTAAATGCCTACTGTGAAGACTTTTGTGCATTGCACAACTTT

A_14_P131806 AAAAATAAAATGGAATTTGATTCTGGAACATGGATCATTTGTCCTTTGACTTCCATCACC

A_16_P40431993

AACTTATGAACTTCTCAAAAAAGGTCAAACTGAATTGCAAACTTTGTATTCTCACAAACA

A_14_P125360 TTAGGAAAGTGATTTCATACAAAAACTCTTCTTTTAGTGACCCCTTTCGGTTGATCTTCT

A_16_P02558516

TCTGATGCTGCTTTATTGCATGTTAATACTTCATTACTGGACCTGATTCTGTTACACTCG

CUST_1496_PI426210822 GAGCAGATTCTGGTCCTCACACCAGAGGAGGCTATTTCTACCTGC

A_14_P123717 GTGGAACCTGAACCCTAACCTGTAGGTAGAGGATTCTAAATTTGAAACTCTTTTCATAAA

A_16_P17313238

AACTGCATCATTTGCCATTCTTGTCCTTGTCCTTAAATTAATCCCAATATCCAGAAGTTG

A_16_P02871353

ACAGCAAGGAGTTCTTATTCTTCAGTCTTCTCGAATAACTTTCAAATCACCAGTAGGATT

A_16_P00685367 AATTGTCTTACAAAGTAACTCAGGCATTGATGTGGGGTAGAGACAAGCCCA

A_14_P136275 CTAAAGCAGAGAAGAATTGAATAGTCAAGTTAAATATAAATCAGCCCTCAGTGTCTCCAG

A_14_P124527 TTTGAGGGAGTTATGGATGGATAATAATGCATTACAAGTGTTACCTGGGGTATGTAAGTT

A_14_P113600 AGTGAAGACATTTCAATAAGCATCAAGGTCATTGAACATTTTGTGGTGTTCGTTATGACA

A_16_P16711049

CTCCGCTCCTCTAATTTGTAAGTTCTACTCAATGAAATCTCTCCTTTTGCTAAACAGACT

A_14_P116739 CTGGTCAGTGTGGCAATTCTCCGCAGGTTTTCCCTGACTTTAATAAA

A_16_P17692255

AAACTCTATCAAATTTGAATGATCACACCTGCTTTTTGACATAACACACTTCCCAAACAG

A_16_P37308178

ATTTGGCAAGATAAGTTCATTCAATTCCCTACATACCTTTACATCTCCAGACAGTCGTAT

A_18_P14137349 ACTCTGTTAGTGGCATCCTCAGACACACAGTCACCCCTTGTATCT

A_14_P118872 AGTTGGATTTCATCTTCATGTTGACCAATTCACTTTTCTCTTATCCTCAGTTACAGACTG

CUST_8123_PI426210822

TATAAAAATTTCTAGTTTCTTAAATCACAAATGTTAAGATGACCATATTATCTTCTAAGC

A_14_P119291 CTGGTGCTAGACCAAAGAGAAAAAACAAGAAGTCTTATGATTTAACTCCGGTTGATAAAT

A_14_P122776 GTTGGTGGCTCATTGGATACTCAAGATAAACTCCAAATGAAAGTATAATGGTGAGGACAA

A_14_P106729 AAGATCTCTCCTCTGCTCTTATCTGCAACAAAAGACTGATAAAAATGAATCTGACACAGA

A_14_P139845 ACCAGAACAAATACTGTATCACTAACCACATCCCTAGGAATTAAGTTCCTTAAGGGTTTA

A_14_P111627 ACATGAAAGAGAGTGAGATTGGATTTTTGTTAAGATGAAAGAAAGGAATCTGTTCCCATC

A_14_P137725 GATCCGACTCACCAATACCTGCATCTCCTTGTCTACGCTTTCCAC

A_14_P133663 CTTTCTTCTCTCTACCTTAGACAGATTTTCATGAAGCAAATTGCTTATTTCCCGGGAATA

A_14_P139919 TAAAAAGATCTGGGATAAAACCAGACTGAAAGGAATACCTCAGAAGAGATGCTTCATTGA

A_16_P00247158

CCCTGGAGTGTTTGTATTTTTAAAAGTCTATGGGTTAGATTGATGCTTAGCACCAAAAGA

A_14_P103981 ATGACAGAGTTTTTCTTCCTGTATACTTTGCTCTGTAAAGCCAAACTTCACTTCTAGCAA

A_14_P109360 CTGTGGAAGAATTGCTAATTACAGCCACCATAAGGAAATATCAATGATGGACGTCAATAT

A_14_P201416 AGTCAAACTATGCAGGAATCTCTGCCTTGTGCCAATTTAAAATTCTGAGTAATTTCAGAA

A_14_P120184 ACAAAAAACTGGAAGAGGAAATCCAGACCCTTCGAGTTTACTACAGGTAAAATTCTTTTT

A_18_P22841925 TTATTAAGAACCTGTGCTCACCTCTGACACCTCAGTTGATTGGGGAATTTG

CUST_2323_PI426210822 GGAAGTGGTGAGTTCTGTTTTTTGCCGTATCAGCTTGGTGTTCAATCTT

CUST_7183_PI426210822 ATAGTCTGTCAGCTCTGTGGGGTGGGTGTGCCATTTGGGGGGAAAT

CUST_8210_PI426210822 ATACACTCCTCCCTCCTTGTTTTCTCCTCTGTCCCACCTACCCAC

A_14_P127576 AGAAGAAAGATTCCTTCCTGGGAACTTATGAATGGGACTTGTTTTGACTGAATTAACTTA

A_16_P00685334

TATTTTCATTATTATATTTAAACGTCTCTGCAGAAGGAATGGAAGGCTAAGAGGGTGGGA

A_16_P15558514 GGTAACCCATCCTCATATTTGCTATGTATCATTCCCATAAATACTACTGGTCCCTGTATT

A_14_P100277 CTCCCTAATCACTGAAATCATTTGTCTTAGTCACCCAAACTATTTTATATTACTCCCCCT

A_16_P16998034

ATCAGATCGTCATTGGAAGTAACAGATCACATAATCTCAACCATTAACTCTTGTCATCCA

A_14_P124610 CATGTCTTATCTCTTTAAACCTACTGTGCTTTGACCCTTCTTTCTGCCTCTCTC

A_14_P136292 AACAGTAAGGAAATGGTCCTTAGAAGGGATTGTTCTGATGTGCTGGATTTTAAATAGAAT

CUST_3358_PI426210822

AATGTGTTCTGTTTTATTGGTTTTGTCTTTATATTTGAGATGTGATTATAAAGATCCCTT

A_16_P21498771

AAATGGAGAGGAGGTTGAACAAAATATGTTCTAGTCTTTAATTCGGGTCCTAAATGAGAC

A_16_P16244947

ATTACTGTTACTTCTTTGCTTTTCATTGTCCACAAAATCACATATAGGAATGCACCAGGC

A_14_P108072 GGAAAATTTCATACCCTGACATCACTTCTAGATGAAGTTGTCCTGAGACAGTTAAGAAAG

A_14_P100432 TTTGGATATATGAGAAAGCCTCATATGTCTTCGATTTGGACATATCCAGAACCATTTTCC

A_16_P03030918

GGTATTTCTATGGCTACCAAGGATATCAAGTTCCATGAAATTTGGCTTACCAAATCCTAA

A_16_P58424103

TTTAGTCATATTAGCCACATTTAAGTGGCTCAATAATCACAAATGGTTACTGGCTACTCT

A_14_P119483 CTGAAAGACGCTTGCTGCACAACTGTCAACAGACTTCCAACTACTGAG

A_16_P01860694

AGGGCTACAGTCTGATTTTCTTAATAGAACACATTACACCATTAAATGGATCTCCACACA

A_14_P132557 TAAATTGAGCAATAATCTGAGCATATTTTCAGCCAGCATCTGTCTGTGTTCCACTTAGTT

A_14_P107705 ACAGTAATTCTTCGACACTGGGAAACATAACGGATTCAAAAGAATTCTTCATCTCAGATT

A_14_P107866 CAGACCAATATATAGTGGATACCTTTTGTATGTATGCACAAGGTGCAGTGTTGGAGATAA
A_14_P129140 CTAATTGACCAATCTCTAAAAGTTGTTGATTTAACAGTCGAAAAAGGGGTGAGAGTATCC
A_14_P138864 ACTGATGTGGGTGTAAGTTTTATTGGTGATAAAGGCTTATTCTTGGTCTTAGTGTTTTCT
A_14_P110205 AACTACAGGCCATTTTACCCAAACTCATACATTCATTCATTTTAAAAAGTGCATCCTTGG
CUST_9338_PI426210822 AGCCAGCTACAGCCTCCCTTGGGGAGTGGAGCCCACATGGCTCCA
A_14_P110225 CTAAATTTTGTTCAAAGCCTAGGAGAACCTGCTGTTTATGAACATTCCTGTAAAAGGAAG
A_14_P120522 TTTATTCCATTTGGCAACCCTTATGCATTGAGGTGTATTTTTCATCCATCTCTATGCTAC
A_16_P40927262
AAATGCTCATAAACCCCAAACTACAGAAATATTAGATAAGAATTGCCTTCTACCAACACT
A_16_P21137656
AAATCTTCTTGTTCCTTTTGTAACTAGATAGCATCGACATTCAGTAGAGTCAACTCAGCC
CUST_3055_PI426210822
GATGGTTCCCTTGTGTTGGGCATAAACCATGTGCTTTATAGAAGTCAGTATTACA
A_16_P18869675
AAATGACACAATTTGGCAGTAGACTAAGAACATCATCAGTGATCCTGAGATTACCTTCAT
A_14_P135327 AAAGAGGCTTGGGATAGGTAATTTATTATGACTATCTTCACCCTTTCTCTAGTGTTAGGT
A_16_P15006551
AACCTTTTTAAAGATTAGTGCTCTCTAATACCTCAGGGTTTTTTAAACACAGCAAATGCT
A_14_P106051 AAACTTTTAAATGCTGGTTTTATCTTTGCCAAATATGATGCTCCTTACTTCAGTCCAGGT
A_16_P21506653
AGCGTATAGGTAGTGAGAAATAAGGTAGCACAAATAGAGGGTTGAAAAGTATACAGAAGT
A_16_P20815066
TCCCCCTCATACCAATTTACAAAGACTTCAAATCATAATCCAAAACTTCTCTCTTTCGTT
A_18_P14137009 CAAATGCTGCTGCTGATCTCCTGGCAAGCCTCCACAGATAGGCCC
A_14_P131954 TTTGCTCAGTATCTATTTTTAAATGTCTCCGCTTGAATTATCCAGGCTCCCGTTCATTAA
A_14_P117480 CCATGAATACCGCGGAAAAAAGGAATATAAGCAGCAAAATTTGCTATGGACTTTCAATTT
A_14_P127832 CCTGCCCAGACTATTTTGGTAGCAAGCGTGGAAATCAGATCTGAGAA
A_16_P03481256
GGCTGGAGTTCTCTAAAATTGACCACTACCTAAAATTGACCACTACCCTATGATATCTTA
A_14_P201151 CTTCCTTTTTTATCCCTAGCAATCTGATTATTCAATGTTTCCCCATGTTCTTTGCTTTCT
A_16_P30952358 AGAGAGCATTGCCAGCCTGGACGATCTGGACAATGATGGGTTCCC
A_14_P127343 GTTTTTTCTAGGAACACATGTTATCTTTCCACTTACTCCATACTGCTTTGACATTACCTG
A_14_P126347 CCCATTTCCAGAAACTTCCCCTCTTTTAAACATGGTTAGAATCATGTAAAATAAGGGTAA
A_14_P120307 GAATATTTGACTACTCGCCTTTAGATCCAACACAAGATTTCAACACACAGATGTAAGTGC
A_14_P122357 AGAGATTCTCCCCAACACGAATTCTTTTGATATCAAGAAATGAGTTGTCCACAAAGC
A_18_P14134572
ATTTATATCTTTCTTAGACCTTTCCTAAGACAGACCACATCTGCCTTACCCGTCACATTA
A_16_P19213066

AACAAATGCTTGAATACACTTTCTTCTTCTGCATCTTTGATATTTGCCAGTCATCTCTCC

A_14_P107137 ATGCTGGTGCACAGTAAAATCAGTGATGCTGGCAAGAGGAATGGTTTAATTAACA

A_14_P112248 AACACCCCTATCTTCTAGGATTGACACTGGCTGTCAAACTCATCCTTCAA

A_14_P110871 TTTTGATGTATGTCAGCAATAATTCCAGAAACCAAACTTTGGAGAAGATAGGAAAAGGGT

A_14_P138232 AATGACCATGTATCTTAGATTCCCTGGGCAAGCCAATAATATTTGGTGACCATACATATT

A_14_P100054 GTTTAATACTGTGCGGTAGGAACGAAGAGGAATTTTAATTACTGACTCCTAATTGGATGG

A_18_P22841293

CCCTAGCCATGAGAGTGCATTATGTTTTAAGAAATGGTCTTCAGACTCTAAAAAAAGGAA

A_18_P22840738 AAACCGCTCAAGATGGCACCAGGTGATAAAACAGATCCACTTGCTAATCT

A_14_P116752 CATGTATAAATTCCACTCATACTCTCCTCAAAATTTTGGAATACAAGGCTTCCTGTGGAT

A_14_P115580 CTCCTGCAAAGGTCTTTAGGTATCCTAGGAAATATGAAATAACAAGAACTCAATGGATGT

A_14_P125859 AAAAACTGCTCTCTGCTTAGGGCAGTAACACCCCCAAAATATCCACTGTAA

A_16_P18025481

ACCATATTAAAATTGGATGAATCATTCCATACCCACAGATAAGATTTTGCTAGGGAAGGG

CUST_6455_PI426210822

CACATCACAGGTAATGAATGAGGCTCATTTGCACAACATACAACTTTGGACATC

A_14_P108154 TTACTTCACCTTCTATTCTCTCATCTACTTCGATGTGGCTCCTCATTTGGGC

A_16_P15654832

GTATGCAATATCCAGAAAGAGATCCTCAATGAAATCCTTTTGACTTCGAGATAACACTGC

A_14_P109446 CTTCAGCAATAATTAGATTAAACCAATCAATATCCTGCATTAGGCCTCTGACTATCTACT

A_14_P137949 ACCATAAAAAAAGAGGCATTATCACTTTGAACAGGGAATGTGATTTGTGTGGTATAACCA

A_16_P19803869

TTCTCTATGTGCTGTTTATATGAATGTAGACTGGTTCAACAATAATACTGCTGTCAGCCC

CUST_1233_PI426210822

TATTCTTAAATATTCTCAGAAGGTGTCTAACATATCCTGTTTTAGTGACTAGATAATGAA

A_16_P15712572

AAAGTGAGAGAGTAGACATGTTTCTTTAAGTTAAACAAGTGAAGTCCAGCATAATTCCCC

A_16_P02866659

AAAAAAAAGGTATTTATGAAGCAGAATCCATAGGATGGTTGATCAATTGAGCATATGAGA

A_18_P22840283

TGTTACTTAATTCAATATTTTGCAGTCTAAGATATTTGCTTCATGCTAATTAACTACCTG

A_14_P138132 TTTTTCAAAAGGCAAGCAATACATACTTACTTGGATCAGATTTAGAAAATGTGTCTCTGT

A_16_P21270751

TCCAAACCCATATAATTACTCCCCATGATCTACTCAGTTTAATCAGATTCTGCAGGTTAA

CUST_9914_PI426210822 TACAACCCTTTCAAGACCTAGGGCTTGGATTTCAAGTGCCTTTTCAG

A_16_P19671497

AGAAACCAAACCATAAGTTCCTGACTAAATTGAACAAACTAAGGCAGACTTTTACCAACA

A_14_P202075 CATAGCCTGCTCTTTGAAATGAAAACAAAGGCATTTATTGGGGCATAAAACTAAATTCAT

A_14_P128745 TTCTGTCATACATTGATGATGTTTCTGCTGTGACCCAATAAAAGGCAGTTCTAGGTATAA
A_14_P113661 AAAATCTACCTGGAAAACATTACCCTTGGGATATTTTTGTTTCTTTGTCCCTCATTTTCT
A_16_P03730958
AAGAATATGGAACAAAGTAGGCTCAATAGACTAGTTACCTTTATAGGCAACTCTACCCCA
CUST_6921_PI426210822 CTTGAGTGAAACTGCCTGGGAGACAGGCTGGGCGCTTCCTGCGGC
A_18_P14137238
TTACTGTGTTTCATGTTCTCCCCTTGGCAACAGATAAATTAGTCAAAGAGATGAGAGTTT
A_18_P12063763
GCACCAATAATGCTAGATGCTAGATTCAAACTACAGGATAACTATTTTACAGACGGCCAA
A_16_P20429611
ATGTTAGCCTTTTTACAGAGAATACAACATCCACAGTGTTTACATATGGCATTTGGAGGT
A_18_P11304931
AAAACCAAGTCTCCTAACCAAACTGTCAACTCTTAATGATGTCTTAAACATGACCCAGAA
A_16_P41768890
CGCAATACTGCTTGTTAATCTGCTACTATTGAAAATGTTTCACTACCTCTAACTTCACCC
A_18_P14137779 GTCCGCTGAACATGACAGTTATCCTGGCTTCACAGTAACCTTTCAGAG
A_16_P17214334
AATCTAGGACCAGATTTTCAACCTATAAACTCCAGGAAATTTTTCAAGAGAAGTATGGCC
A_14_P100296 ATCTGAGAGAACATTGACAGTTTTCCAAAACCTTCCTGATTAAACATTCATTTCCCTTTT
A_14_P130314 CTTCGACCATTATGTCACTTACATTTTTCTCAGGACCAGGATAAATCTTCTTCCTTACAA
A_16_P01849575
AAATGGGTGAATTAGCATAATGACGAATGCATATCGATCAGTGTTTCAGAAAACTGCATG
A_14_P202061 CTTAGTTTGATGTTTGCATACAGTCAAACAAGAAAGAAAATGAAAAATTTCTCTGTCAGC
A_14_P123532 AAAAACAGAGTTCCTGGCCTAGGAAGAAGTCTGGAAAGATATACTCCATAATACAGTATG
A_16_P03605348
CTCAATTTTCAAAGGATAATTTGGTAACTCCATAAAGAAAATCCGTAAATAAGAGAGTGA
A_14_P133058 GTTTTTTTCTATTTAAAATATGACTCCTGTTCACTGCTTTGGTTTCCCTGTCTTTGTGCT
A_14_P123233 GCTGAGAGGTAGGTTACTGGTTTAGATAAGAGAGACTATGGCTACACTTATCAATGTAGT
A_16_P17740249
ATGTGATTCACTTTATCCAAGATGAGTTCTACGATTTTGTGGTCTATGCTGTTAGTTGCC
A_14_P128299 TAAGAATAATTTCTGAAGCAGTATTTGCCTCTTCCTTCTTTCAGAGATGGAAGGATTGTG
A_14_P114542 TGATGCTCTTTTCCTTGATTTCTTGCTTAGTTAAGCAAGCAGCATTTCACTATTCCTCAA
A_14_P103306 CCTTTGAGGCATTCCCTATGGCTTAGAGTTGTAGACTTACACTCAACCCT
A_14_P123181 CTGTGACTTTAGCCTGTATTTATTGACATGCTGATGCTCAGATATTTGAAGTCTGAACTG
A_14_P116193 GCAAGCATTTCTCTGTTGACCATGGAGTGATATAGAGATACATGAGCCTTGATGTAAATA
A_14_P123389 AGAACCCAGGCTTTTTGTAACAATGTGTTTTCTTGCCTAAATTCATTTATCTGGCATCAT
A_16_P19045712
GAGCAAATGAATACATAAATGAACTACTCAGGGACCTTATGGAAAAAAAAAAACTGTTTA

A_14_P139901 ACAAAGTATTAAACGTGGACAAAGATGTAATTGGTAATGTCACAAAAAGGGGCTCCAATA

A_14_P101309 CTTGAAGGGACATACCCTTTCTAGACACTACCATTCACTAAAAGAAAAAAAAATAAACAA

A_14_P129146 ACTTGGCCAGAAAGTCAGGAGTTGTTCTTTACTCCTTTCTCTCTTTATACATATGTATGT

A_16_P41749264

AAAAAATCCATTGATCTACCATGGTGCGTTAAGTTTCCAATTCTCGGGAGTCTTCTTAAA

A_16_P17007448

CTCACTGGAGAATAATGTATCACTCGCAGATATTGAAACATATGTCAACCCTATCCACTT

A_14_P125421 ATGCAACCCCTTAATTGTGCAGATAACAAAAAAAGTAAACACAGAAAGCCAAGTTTGCTT

A_18_P15024886

ATCATGCTTATGAAACCCCTTGTTAGACTTAGTTTGTTTCTCCTTTTTCAAGATAGCACC

CUST_6596_PI426210822

AAATATTCCATGGAAACCTTGGACACTGTCTGATGTCAGGACTACTTATGAATGC

CUST_3024_PI426210822 GATGTCATTAAAAGCCGGAGCCTTCCCTAATGCCTGACTTCACTTACTTAAAT

CUST_5940_PI426210822 CTGTTAACTGCTGGCTGCCCCACCAACAAACTGGAAAGAATCTCC

A_16_P00996349

TCCAACTTTGAAATCACAGAAACTTCTTATGACACCACAAAATACATACTGACCTTCCAT

A_14_P202397 TAGAAATCAATGGGGAATCTGTGGATTTGCATATGAAATTGGGAGATAATGGAGAAGCAT

CUST_1715_PI426210822 TTAGGGTTGTTGTCAGCATTGGTGATGGTTCTGGGGCACAAACAT

A_14_P117549 CGGAATAGAATTTACATATATTCACATAAATTCATGCATCCCAGTGGAAAGCACTGACCT

A_14_P200603 ATAGTCTAGGAAGAACCCAAATAGTGAATTTGCCCTGAGTTTATACTAATGTGTTCCGTG

A_14_P130018 ACAGGTTAAGGGGATACTTATGTCTGTTTAAAATCAACGCAAAAATCAAACCCAGAGATC

A_16_P21205734

AATTCCCACTCTTTTAAAAGCAAAAATTCCTTACACAAGTCTTGGTCATTCAATGTCTGC

A_14_P116139 ATCCCTGAGACTCTAGGAGTTTTCTAATAAGTTGGAAGTTATGATTTCCACTACGTGATT

A_16_P17266388

AAAACTCAGAGTCTTCTTAATCCTGCTTAAGAGAGGTTTATGATGCTAAGGACTGTCAAA

A_16_P02189809

AAATATTGTTCTGTATAATTCAGTGGCCCGTTTTATAAACATTGTGGTTCTACCTTGGGG

A_16_P16181033

ATTAGCAGTTTCAAAAAGACCCTGAAGTGTAAATGGACTCTGAATTCTTGTCTCCATTCT

A_18_P22841404 ATGGTATGCAGCCAGTCCAGCCATCTGAAGCAGAACGAAAAAATGGTAT

A_14_P118180 CAATAGGCTGTGGCAATAAATAATGTCACCAATCTCATCAACTATTAACTGGCCACAAGA

CUST_7810_PI426210822 ATCCACACAAGGAGTGGTACTGCTTGCCAAAGCCCATGCTCTGATA

A_16_P36519424

ACACCTCAACAGTTTTAATGAACACAGAGAATTCTCACTATGCACATCTATAGTTGCTCA

A_14_P127762 CACACACCCTCCACCAAAACAAAACAAGCAAAAACTTAACAGACTTTCAAATAGTCTTTA

A_14_P134499 GAACAGGCATGGTTTAAATCTTTTTTCTTGGAGAGTAAACATTGTCACATTTCTGCCTCT

CUST_8746_PI426210822

ATCTTCATAATAATTTCTTAAGGAAATTGAGGCAAGGAAGAGTTAGAGGGAAGGCCAAG
A_14_P103752 TCTCCCCTTTGGACTTGATAAACGCAGTCCCATTCATTTAATCTCCAACTTAGAAAT
A_14_P123207 TTGGGAGAAGAGATGTTAATTTGAATTCTGGTTCCGGCTTTCAAGCAAAATATTACTCTT
A_16_P36989840
TTACAGTGAACATATTTTACGCAAGATGGACTTAGTCACAACTTAGACATAACGAGCCTG
CUST_6382_PI426210822 TAGGGCTGAGGCTGTAAACCACAGGGGATTACCTATGTTTATCTGG
A_14_P112061 ACGTTCTAAAAAAGTAGCCTTTGTTTGAATGATTTCCCATGCAGTATCAAACCGATGATT
CUST_3089_PI426210822
TGGAGCTGGTGGAAACTATTTTTATTATGTATTATGTGTGCTCTTCCAAAATTTTATTTC
CUST_6806_PI426210822 GGATCTGCATTTCTGGTAGAACTAGTCTGTCTGCAGCCTTACTGTGTTT
CUST_8969_PI426210822
GTCAATATGAAGAAATACTCAATAGTAAATCATAGCAACATGATTTAACATGAGATATAA
A_14_P139913 ACCAGGGGTGGTTTTTTTACAGAGACACTTTGAAATGAGTGATAATATTACAGACAACAT
A_14_P116386 CTCTAATTCCCCAAACCTGAAACTTCATACCCTGGGATTCTAATACCTATGTCCT
CUST_5588_PI426210822 CTCTGGGGACGGTGGAGCTTGAATGCTACAAGCCTCCGTACATAC
A_16_P41885096
TGTGAATAAGGTATGATTTCACATATTTTATTCTGTTAACATTCTTGTTGAGAGTTGCCA
A_14_P116188 CAGATAACAGACACCACACTGCTTTGTAGTCTATACATTTATTGAGTAAAAACAAAATCA
A_14_P104091 CTGTGTAGGTTTACATTGCCTGAGAGTAGGAAAAAAGTATTTTCATCCTGAAGACCTAAT
A_14_P101066 AGATTGGGTCGTGACTGAATGGAATTCAATATGGCACTCAGTTGGTCAC
A_16_P17837453
TGGGTTCTTCTGATCATTACCACAGAATATAACTGCATATACTTGATCCTAAAGCAGCAC
A_14_P118849 GATCTTACCACATTTTCCATAGATCACATCAAACTGCTGCATCAATTCTGCTTCAGTTTT
A_14_P112936 ACAAAAATTCAGGGACAAGAACTTGCCTTAGTTCACTAGAGACTCAGCACTG
A_16_P19249465
AAAGTCTTATGCTTTAAAGGTGATCCAATGTCCTTTCTATCCTGTTGCACTACAACTCTG
A_16_P21179885
AAAGGCTAAGGTTTTTCACTACCTACAATATGCGTCTCTTGATAAAAATTTCCAGCCTTT
A_18_P14959011
TGCAACTGTTTATACAGCATTTGAAATCCTAGACTTTTACTCCCATGAGACTACCATAGT
A_16_P20423658
ACACTGAGGAACAAAAGAAATGATCCCTTGTCATCTAATCTTAATAAGAATGTGGAAGGC
A_14_P128052 TTTTCCACTTGATTTTGTGGCTTTAAAACATGCCAGTAAATAGTTGTGTTTGTTTCGGTT
A_14_P114571 AAGAAGGTATAGAGGAACAAGTTTTAAAAGACTTGAAGCAGGTTTGTAGCCGATACAACT
A_14_P108903 CCACAAATCTGTTTTCATCCATTTTACCACTATGCTGTAAAGCAATTCAAAGAATCATTC
A_14_P109093 ACAAGGAGGCCAAAGGTTCTATTATTTATTGATATTCTACCTTATGTGAGGTGTGAGGAA
A_14_P103907 TCTAGGAATTCACTATGCATGATACCATTGGATGTTACCTGGATATAGATAAGGGACATG
A_16_P18440826

AAACGATAAGTCATCCAGAAATTGAACTTCAATCTTGTTTCCAAAGAAACCAAGAAGACC

A_14_P109897 TGCCTCCTGCTTTAATCCAAGCATTAAACTTCATGCTATTCTCTTGTCAAAGAAATTTGA

A_14_P132296 ATAAACACCTTCGTTTTACTCCAAGGGTAAGAATTAACTTTGACTAGGAAAATCAGTAAT

CUST_2564_PI426210822

CTTGTAGGCTGGAGGTGCTTAGTAAGTGGAAATGCTACCTTATTTTCTCATTCTAATTAT

A_14_P133368 CTACATATACGTAACAATCTCTAGATTCATGGCAGGGAGCAGGAAGTTTGTAGATTGAAA

A_14_P118721

ATACATTTTGAGACAGAAATTCACAACGGTCAGTAAAAAGGTAAGTCAGCAAAGAAAATG

A_18_P12031881

AACTGGGAATAAACAAAAGGACATTGCTTCTAGATCTCACCTAGTTCTTCAGAAAGTTTC

A_14_P117418 CGTAGCATCAATCACATGTTCATTGTTTATTCGGAACATGTAGATGCCTCGATTCTAGAA

CUST_5656_PI426210822 AGGAGCTCCCTTCCCCCTCAGAAGAAAATCCGGACACATGCTAAG

A_16_P01185392

CCCAGTAGAGGATGAGATATGAGAAAGATATTGAAAGGACTTAAATACTAGGAAGCAGCG

A_16_P39399378

ACCTCAGGAAAAACCACATCTTCAAATTCATCTAATCTAGTGTCTTCAACAGTCAATGAG

A_14_P122530 AAAAAAAAAAAGAGCACGGTAATAAAATTTCCATGAAATATTTTGTGTAAGCGCAGATAA

CUST_3870_PI426210822 GCTGGACAAGATGAACTAGAGCCCTCTTGTGGTCCCATCATTCAG

A_16_P16892395

AATCTCAATTCATTTGCTGTAGTTCACATTTCAACTCTATATCAGTTCCTGGAGAGAACC

A_16_P16559066

AATCACTTATTTGCTCACTTTTCTTGTCTATGCTCTAGAATGCACTTACTGCTTAAGCGG

CUST_3586_PI426210822 CTGCCTGTCTGTGGCCTCTGCAGTTTAAACAATGACTGTCACTTAAAC

CUST_392_PI426210822 AGCGGACGTCTTTCACACCCCACCCCGTCCCCTCCTCCTTCTCTG

CUST_6727_PI426210822 GAATCACCCCCACCCCCCATTTTTGTTTAATGAACTACTATGTCCTTAAATCT

A_14_P120543 CTTACCAAGACAGGAGTATAAGCCCTGACTTATCCAAGCCAATATGGCAAGAGTTATAA

A_16_P36613544

GACATTTTTATTGCCTATAAAAGTTAGGTGGTATTTGCCTGTATAAATAGTGCCCCTCCT

A_14_P113057 CTTAACTGCAAGCCTGTTATTTAAGAGAAAATGGCCTCTGTTTCTAACATTGTCAGTTCA

A_14_P120562 TGAATACAACTGGAGGAACTTAATTCATCTTGCTGTCACTTCCATGATGCTTTTCATTAG

A_16_P00685414

TCATTTACTCAGAAGAGATATACTGATCACCTACTTCAGACCAAGATTAAATGCTGAGGG

A_14_P119986 TGAAAAAGTCACTGAATCATTATTTTAGAACTGGAATAACACCTGAGATCTAGGCCAGCA

CUST_7574_PI426210822 CATACAAACTAATTGCACCCCAATGCTTGTCCCAAAGTCTGCTCAGAT

CUST_8611_PI426210822 CTTGTAGCTAATGTCTCTGAGCCCCTCTGCTGTGCTTGTATTTACCTTAAAAAC

A_14_P201086 CCAAGACATCACTGAAAAGCAAAAAAATCAACACTGACCTTACTTTGGGTTTGAAATATTT

A_14_P109789 ATATACAAATTGCCGATCAGGTGATAAGCCGTAATTTGAAGCATTCACACTTTTCTGAAA

A_14_P100356 GGACGCATATCATTAAGGTTCAAGGTTTTTTCTTTTCAAGGTGAAATCTACATTCAGTGA

CUST_10113_PI426210822 CTACTAGGTGGCAGGCCCTGGGCAAAGGGACAAAGATAAATAAAC

A_18_P16673229

AACACCAATCTTCCTGTAGATTCCTGAATGAAATCATCCTACTTTTCTACCCAATTCTTC

A_14_P108061 AACCTTTTAAATGATTTCAAATTCTCCACAGATCTCAAGAGTTTACTCCTCAGAGTGGAT

CUST_2337_PI426210822

TTAATTTTAAGAGGCAGCAGGTAAATGCAAGTACAACTTCTACATGAATAATTGTCCTTG

A_18_P10850315

GAAGAGAAACATTTCAAGTGGTAAGGTAGAATCAAAATGATAAAGGCCTGCTGCTTTCTT

CUST_4425_PI426210822 CCCAGGCCCACGTTAGCATCCAGAGTGGCATGCAGTGGCAGAATT

A_14_P125631 TTCAGATGTCCAAGAGTTTTGATCATCCAATACTCCACACATACTTTGGGCATATAATCA

A_14_P114081 TTATTTTGTGTCCTTTTCTATTTCTGCCCTCTCTTTCTTGTTCTCCAAAGTCTTTAACCT

A_14_P107771 GCTGTAGGAATTGGCACAGTTCACTTGCACCAGCATGAAGATATCCTC

A_14_P100468 ACATACATTTCTGTGGTGACTTTTCTTACACCCTCTGCAGATATTTTACTGTAGTGAGTT

A_14_P115515 ATTTTCCAATTCCTTTATCTGCATTTTGTAAATACCTCAAAATAGTGGAAGGCAAGGCCT

CUST_4356_PI426210822

CTGAGCTTGTTTTGTCTAATATTGCCTGTGCCAACAGTATTTTACCAGAATTGAATGAAA

A_16_P18855165

AACTGAATGTTGGCTTATCCCTAGTTAAACTCAGAAGATCTCTAATTTCCCAGAAGTCAT

A_18_P22840708 CAGCAGAGGCCCTTTTTGGGGGCAGGAGGGATTGCATAGCAATAC

A_16_P17103431

AGTATTTCTTACAACAATATGACCGTGGAATTTGATTACAAAAACACTCCTGCTATCCCC

CUST_539_PI426210822

TTTGTTGATTCATTCATTTATTCATCAGACTTTGAGTGTTGACACTCAACACTGAAATGA

A_14_P135077 ACGATTAATGTTTGGAGATACTCTGGTAGATGTTGCTAATTGTGAGAATGCCTTCATTTC

A_18_P25916560

AAAACCAAAAAACACAAAAGAATAGGCCTGATTATTCCTATTTTGTGATTGGAGTCCTGG

CUST_10402_PI426210822 AAGAGAATGAAGACAGTTGGGACTGGGTCCAGAAAAACCAGTGAG

A_16_P20769577

ACCTTCCAACGTATAAGACTTCCTAAACGTATACCTACTTCTAGCATCTCCTTTCATCTT

A_16_P17756732

AATCACTATTAATTGACCATTCCACATTAGTTTCCTCCATACCAATCTTATGCTAGGCCT

A_16_P17694956

AAAACTCTCACTTCTAGGGAATCATTAGGGTTAGAGACTGGTAATGTGATAAGAAGAGTC

A_18_P15402035

TCACACACAGTTATTGGACATACTAAATGCCATGATATTTATGAGGCCTACGGTAAATGA

A_14_P100717 TTCTTGTGGACCGAAAGTTTCAGCCCTATAAGCCTTTTTACTATATTTTGGACATCACTT

A_14_P102243 CTGCACTGAGTAAAAAGCCCTACCTATGTTTTGTTTCTATCTTTGCACATCTCTTTGAAA

A_16_P15972417

AGCCCATATGAAGAACTAGTAACAGAAAGAAGATCAGTTTGCTCTCTTAATTTCCATCCT
A_18_P12588448
AAAAATGTTCCCCATGTCAATAGATGGTAATTGTAAAACACTAAAACTGATGTCCAGGGT
A_14_P107830 GTGATAACATCTACTCAAAGAAAACGATGCTCCATTTTGATTGATTCATCTCTGACACAT
A_14_P108384 CTAATTTCTTTTCTCAAAAGAGTGTCCCTTCTTCACACCTACTCACTTTACAACTTTGCT
A_14_P129977 TATCCAATGGAAAGGAACCCAAATACAATATGAAACACCTTCTAATGTTCCCTTCTCAGA
A_16_P19394488
AAGTCCTGGATCCACAAATACAGAAGGTTCCCTGTATTCCAATTAATCTTTCTAGTAACT
CUST_9209_PI426210822 TCTTGAGAATGACTCCTCTGACAGGCCTCTTTTCATTTGCAGGTG
A_16_P18367979
AAAAAACACCATTCTGCTTAATCAACATTTACTCCATCAGAATCAATATCCTGCAGAGGG
A_16_P03530666
TAGCATTGACTCTGGAATCCTTCTCATCATGATTTTCAAATAGCCACTCAGCATCAAAAT
A_14_P127621 TCATTTGTGGATTACATCAAAACCAAGCCTATTGTATTTGAAGTCTTTGGGCATTATCAG
A_18_P22842657
AGTTAACTGATCTAACTCTGCCAGATCCAGATTTATCAGTGGTTTTGCAGTGGTTTTATC
A_18_P26795194 TATTCATGACCCCTAGCGTAACCATACAGTCCCCTACCCATGAGC
A_14_P108301 GGAGCAACTCATTCCAGCATCAACAATAAGATAACCTTTAAGTATGGCACACTTGTATTT
A_14_P123878 AGTAACATAGATGACAATTCAACGTGATGTGGTGAGTGCTGTAATGAAAGGAATAAATGC
A_16_P19914526
CCAGTGGAGCCTAGAATATTTTCTTAAAAGCAAACTTCCAATATGAATCCAAAGACATCA
A_14_P130788 TATAATATCCGTGTGAAAATGTCTTATGCATATCAACAAATGGTATGATGGCTCCCCTTT
A_14_P128740 CAACCTTTATCAAGTCCTCTACTTCATGGAAGTCCTAGATGGGGGGAAAAAAA
A_14_P128937 GAAACTGCATAGTAATGAGACTGTGCACACTGATGTTTACAAGATTGAAAGAGTCTTTCT
A_16_P17466782
ACCCAGAGTTTTACAAATATTTACAGTCATCTATGCCAAATATCTTTGTGCTGAGTCCCA
CUST_6159_PI426210822 CTCTCCTCACATCCCAGCCACGCATCAAGGATGAATGGACATGTG
A_14_P117261
GCAGGGGAATAAAAATGGTAATAATAAAAGAGGGAGATGAGGTAAAAACAGGTTGTTAGG
A_18_P22840855 CTTTATCTGTCCTTGCCTTGCAGAAGGCCAAGGCACCTTGAGCAA
A_16_P19455465
TTTCGACTACTCATATCATCCAAGGCAGTTTATCTTTTGTCCTACATTAGCACTGATAGG
A_16_P16691833
AAAGGCATAACACTGGATTAAAAAGTAGACTGTTAGGAGTCACACATCAAGATTCTCTCA
A_14_P102848 CTCTGCTATCTAATTCCTTTCCCCACTTCTAACTCTCCTGTTTGTCCCTAGTATAAATAA
CUST_2452_PI426210822
TTTAGTACATATTTGGGAAGAAAGATACAAGCTAAATATGTTCTTAGAATTTCAGCCAGC
CUST_7241_PI426210822

AATTGATGGCCATTTTTTGTCATGCATCTTCTGAATTGGTATGTTTTTCTGTGTTCAGAA
A_16_P01664464
TCATAACTGTTGCTATTATTTCAACCACTACTCATACTCTAATACTACTACATCCACCAC
A_16_P15654136
AAATCTTCCCATCCAAAACATGATTAGCCACTCTATTTTGTTAAATTTTTGCCTGAATGA
A_18_P14136533
CAGTTTGTAGCGTTATTTTTTTTAAGCAGTAAAATTTCAGCGACTATGGTCTGTGCCAGA
A_14_P114566 CTGGATAGTCCAGGTCTAGAACAATGATAGTATTTCAGCAGTGGAAATCAGATCCTAG
A_16_P37938801
TTTCTCCACATTCCAACTTACTTTACTCTTTCCAACATTTCCCTTATAACAAAGTCCTCA
A_14_P123440 AATAGGAGGAAATTCCTGGAGAAGGTGTAGATAGAGAGAATGTTGTATGTAACTCTGTTT
A_14_P100856 CGAAGAAATATCAAACAGATCACGGCAGAAATCACCAACTCAACATGATTGGACAG
A_14_P113391 GATATGGACGGATAAGAGATATTGATCTGAAAAGAGGCTTTGGTTTTGTGGTAAGTATTT
A_14_P130079 ATTCAAATTCAGGAGCAAACTTTGCTGAGTCACATTAGGGGGAGGGTAATTAATTAATTA
A_14_P104016 TTCCAGTGATATTAAAGTGACTGTTGGCATTGAAGAGTCCCAGTGAATTACACTTTATGA
A_14_P139801 ACTCTTCCCCAATATGCAGATAATTTGATGCTCTTTTTCACCATAAAAATATTGCAGGGT
A_14_P135697 CAATAGTCTACCAAGTCCAAGACAGTGTCTTCTGCTGACTCACACACCT
A_14_P131331 CTTTTGTCTGGTCAGAATGCCTCTTCGATTACAAGGAAATAAAAGATGATTCCTTATGTC
A_18_P24017881
AGTTTCAACACCCTATACTCATCCTATCAGAATAGCCTAAATTTTCCCATCTCCCAAAAG
A_14_P135736 ATTTTAAAAAGCATTTTTTTGCACTTGGTCAGATCTTTTCTATCACTTTGCAAAGGCGCC
A_14_P100313 CACTTCTGATGATGTAGATAAATGACAATGTAGATAAATTGGGAGCCAAAGGTTTTCAGG
A_18_P10245386
AATCGCCAAATAATGAAATCACCAACTGGAAATTAGAAGTAAATGGTCATAGCAAACCCT
A_14_P106078 TTATCAAGTTTGAGGTATTCACGACGTGAAGCAAGTGCAGCAAGGTCAATAACAAA
CUST_206_PI426210822 GAGGGGGCAGTTCGGGGCGCCCCGGCGGGTCCCAGGGGCTGACTC
A_16_P16509675
GGAGAGGTATAGTCTTTAGGGAAAACGTTTATGACAGACAATACTCATAATGCCCATTGG
CUST_7548_PI426210822 TGAAGGGCTTGGTGAGAGCAGCCAGGTCTTCTATGCAGTGATCAG
A_14_P129365 TCTAGTTCCTTAATAGCAGAGAAATCTGCAACAGGACTTGATCCTATGATGTTCTAAAAA
A_14_P132926 CCAGGAAATCTTGCCTCAGAGCCAGGTCTCCTAATGTAGAAAATAATATATTTGTTCTTT
A_16_P01167900
AGCGTAATATGGTATGGGTTGATTCAGAAAAATCAAGATGAAATCTCAGAACCAACTGTC
CUST_4708_PI426210822 CCAGGGGTATTAGCCCTGAGATACACTGCTGCTCTATGGGATTTT
A_14_P105327 GAATGTAAGTTTGGTTCTTCTTTTTTCCTTCCCAAGGTCGAAGTCATGTATTAGAAGTTT
CUST_1873_PI426210822 CAGATTGAGTAGGTACAGATGGATCAGGAACCCACATTTTTAAGAAGCTCCAT
A_14_P131584 AGATTCAAGTTCTGAAGAAGAGAAGAAACCCCCAACTAAGGCAGTAGTCTCTAAAG
A_14_P120627 TGACAGCATCACCTTCCTATTGCATAACTCTGGAGCACTATGTTGAAAGATGAAAATAAA

A_14_P131781 TTACCTCTCTCTTTGTTGAGCCCTTTTAATTTTATGTGGTTCTGGGGTTTTGTTTGATAA
A_14_P124956 AAGAAAACAAAAATCTTTTCAGCACAATTTTTGTGTTTTTCCCCCGTTCATCCTTTCTTG
A_14_P136359 TATTTCTCTAGGGACCTTTAACCTTAATGTCAGGGTGTTGATTTGACAAGCATCTCTAAA
A_16_P17006310
TCAGGCATAAAGTCTATAACCTCAAGAACATGAACTGTCAATTTATACTACCGTGGACTC
A_18_P14139083 ACTCTTGACTTTCCAGCATGTTGGCCTGTCACACGCAAAGGAAGA
A_14_P100219 GACATCGAAGATATCGTGTCTCAGGAAGATTCAAAACCACAAGATAGGCATTTTGTAAGA
A_14_P106934 ATTCTTTTTGATCCCAAAACTTCAGTTTCAGAATTATTTGCCATTTTGAGACAGTGGGTT
A_14_P129816 ATGCACCAGTTTAGCCTAACCAACTATTAAAATGTTGAAATCCTTCCATACTGGAAAGAA
CUST_8190_PI426210822 AAAAACAGACTGAAGGCAATGAGGTGCCATCTGCCCTTGGAGAAT
A_14_P123950 TTCCTGAGAAACCACTTACAGTCTTTTTTTCTGGGAAATCCAAAATTCTATATTGACCAA
CUST_4847_PI426210822
TAACTGCTCAAGTCTTTCTTAGGTCTTATGATGGAACTGAAAAGCAGGCTACCTATTTTA
A_14_P119550 GTTTTTATGGACCACATGGACTTTTTTCCTACCAGTCCTTGCTTTTTTAGACCAAAATAA
A_14_P113495 AAACCTGATTGTTGTAAATGGTAATTGGATAGCATAGTCATGGTTGCTGGTTTATGGATT
A_14_P107255 CGTCTTTCTCTTACCTCCTACTTCTCTTTCTTGTAATGAAACTCTTCACCTTTAGGAGAC
A_16_P03199892
CTCTCTGATGTTCCAGGATTATCATAACTCTTAAAAGGTCAGTCCGTAAAAATACCCTCT
A_16_P18907124
AACACACTTCATTGAACTAGCAGAACCCAAATTTTTGTTCACCCTATGTGTATATAAGGA
A_14_P125734 CTATTACAGAAATATCATAAAGAAGTGAGACCTGTTTACAACTGGACCAAGGCCACCA
A_14_P136507 AAAAACTAAATGGACTTCCAGTCTTTCATTTTTTCAGCCCAATGCTCAAAGGCTGAA
A_14_P129012 TCTTATGTCTAGAAGTAAACGTTTAGATCTGTTAGAACACTGCCTGTCTTTAAGGTGGAC
A_14_P115408 AAATTCTTTTTAATCTCATTTCTGTTGGTGAGGCCATAAAGTGACTGGAACATCTGAAAA
A_16_P00915729
TAACTGCCTATCTGACTACAAAAAGACTTATTTTCTCTGATATCCTACCACTTCCCTGAG
A_14_P134594 ACACTCTCACACTTTTCTAAATATGGTTTTCTGACCACCTGAATGAATCACAGATTCAAT
A_14_P124321 TCTGTGAAACGGTTTTCCTGTTTATGACGGTGTTGTTGAAATTTTGAAAAATACCACACA
A_14_P133578 TGATGGGGAGAATATTAGGGGATTTTGTTTCAATGTTTAACCCAATCATGTATCATCAGA
CUST_2079_PI426210822 TTACCAAAGCTCAGATGGGGAGATTGGCAAGGCCACTGAAATAGT
A_14_P126230 ACTAAGAGGGTTCTATTTAGATCCTTGAGTCTATCAAAACCTGCATTTCCAAACTATGCT
CUST_358_PI426210822 AAAGTCAGCACGGACTCTGCAGGCGGCAGAGTAGAGCGTTCCCAT
CUST_4651_PI426210822 TTTGTAGGCAGGATGGCCATGTATGCACAAAGAAAGCTTTGGGGC
A_14_P200266 GTCCGACCTCTGCTGTATCAGGATTCGATTCTTGGTGTTAAACAAGACAAC
A_14_P133679 GGCAGTTTCCTCAATCCTCTCCAAGTAATATATATTGAGCAGAGGTAGGATGCTTTG
CUST_7378_PI426210822 ACAGAGGGAGGAGGGAGGAGGGTCTCAAATTAAAAAGACAAGAAAC
CUST_3015_PI426210822 GATGGCTCTGCTGACTAGGAATTTGCCAGCTCGGCATGGCAAGAT
A_14_P106413 TTGTTTTTTTTTCCTAAGTTGCCTTACACTATTGATTCTACACGAGCAGATCTCAACACC

A_14_P127550 TGAGGGCAAGGATGTTCTTTCTGTTGAAGGAGAAAGTTTATATTGATGGAAAGTCATTAT

CUST_8333_PI426210822 CTGTGAGCCCCCACGGGAGGCACGCTGCTCCCTCACTGAGCCATG

A_14_P123668 TACACACTGAATGAATGAATGGATGTCTATCACATCTTTGGGAAAAAGGACATTCACAAT

CUST_5714_PI426210822

AAACCCTTTTTAGAAGAAAATCATGGTTTCCTCAAACTCTTCAAGCTTATTGAGTTTTCT

A_16_P18978567

TCTAATGCTAACCAAAGTGCTTTTCTAAACTGCTAAGAATGGACTGAAGATTCTGCTGAG

A_14_P102140 CTGTCTTATAAAGTAGCCGCCAGCCATTTGAGTTACTTTATAACTCGAATCCAAATTCAT

CUST_22_PI426210822 ACTCCCCATTGCACTCAGCAGATGTGGCCACCTTCCAGTATCACT

A_16_P03808047

TATACTTCGGGTCTTATAACTTGAAAACTTCTTCTTCTTAAGACAAGTTCGTTCAGCCGT

A_14_P118277

AGAAGGGAAGGTGATCATAAAGGAATTGCTAAGAAGAAAAAGAATGCACACCTAAAAACA

A_14_P138915 TTTTTTCTTTTAAAGAAATAGGTTTGTGTGGGCTGCCCAATACACTGCTGTGTGAAATTA

CUST_986_PI426210822

ATTTCAGTCTATTTAGAAACCTGCTATTTTAGCACAAAACAATATCAAGGAGAGGAGTGA

A_14_P123857 AAGACAGAAACATCCTCATCTGAAACTTGGGATTCCTACTCAGTTGCATTTTAGATACTA

A_14_P120426 CTCACCACAAATAATTTCCTCTTAGTCAAATGATAACACCAATGTCTTGGGCCACAAG

A_14_P108858 TCCACAGGCATGTAAGTGTAACGTTTTAATTTTCTTTTTATGCCACAGCAAAGGATGAAT

A_14_P103292 AAATGATCCAGGAGGCTAATATAAACAACAGTGCCAATAGTGACTTATAATTTATCATAC

A_14_P107638 CTGTCTTAGTGTGCCTCAGTTTATCATATCAACTGTAACCAAAGCAAGGAGCACTG

A_16_P18219501

CTGCCACTGTAACATAGAGTTAAGCATCTTACGTTGTCTCAAATTGACTGATTGCTAGAA

A_14_P123231 CTGGCAACCGTATATCTTCAAAGAATAGTTCTGCGGTATCCTAAGAGACCATACAAAAAA

A_14_P106469 GCTATTCCTCAGCCTTCTCTTAGTGACTTGACAATATGAATGACAGTTCTATTCCGATAA

A_14_P124319 CCATCTACCCTGACCTTTGACCCTACAGCCTTTATCCTTCTTATCCAC

CUST_8920_PI426210822

TGAAATCCACTGGCTTTTATCATGAGAAGTGAAATTGTTCATGAAATAATGGCAGCCT

A_14_P131323 CTTATGTTTTATGTCAAAAGTGGGAGAAAGGGGTCAAATATTATGAGGAGAACTATGTCC

A_14_P117170 TTCCATCTGATTGGAATCTTCGTTTCAGATTCTTACTCTTCCTCTCCTTAAGGAATCTTA

A_18_P14136309 CCCTGTTATTATTCTCAGGGATTTGGAGGGTGCAGAGGTAGATGAAGTAAG

A_14_P123311 TCATTCCACTAATGTCATCATCAGACCCAGCCTTGAGAATTATTATGAAGCAAGAAAATG

A_18_P16391049

TGTTATTCACTACTATTCTCCACTTTAGGCTCATAGCAAATTGATGCCGTTATAAGGGTG

A_14_P136935 ACACTCACAGTCAAGCAGATCATGAGTAACACAATCTGAACTTTCATGTAATGTGAACAA

A_14_P116753 CATGCACTGTCTACCTGGCAACTCTACTGTCTGCCCACTTCCTATT

A_18_P22842457

CATCTCAAATGCCTCCACAAATTTCAAAGAAAATTGTCAAGAGAAAAGTTAAGCACCTCA

A_16_P19457344
TGACCAACATTTTTATTCATATCTCACAAGAAATCCAAACAAAAAGAATTCCTCACCCTG
A_14_P138828 TGGAGAAATCAAGTCCTTGATCTTGAGATAGAGGTAACAATTTCACACTTTTCCTTCC
A_14_P126569 ACACTCACTCTCATTGTTATCAAATAATAATGAGGTAGTTAGGGCAGACCTAGTCCAGAA
A_14_P138254 GAAGGTAAAGGAAACACTTAAAATAATGACTGGGGTTACTGGTATTTATACGCCAAGAGT
A_16_P17036636
AAGTAGAGTTAGGTTTACCAGCAAACATAAAAATTCTCCTTTAACCCTGCATTTAGGTCC
CUST_9652_PI426210822 CTTTATGGAATCAGCTCTGTTACTGCAAGACATCCCCTCAAACTCCC
A_14_P111320 TTTGAACCAAAGAAGACCAAATAATCTTCACGATATACTTCCAAGTCCGAGTGTTTCATT
A_14_P133801 TCTGACTCATCTGAGTTATATTTAGTTTTCAAGTGGCAATAAATTTATCTACCTTCTTTA
A_18_P23594170
AAGTCATGTTCACGTTCTTTCTTAACACAATTACTGAGGAATTGTCTTGGTATGTGGATC
A_18_P22842830 CCTTTTGTTGCCTCTCCCCACCTGGGAGCAAATGCCTAGGACTTT
CUST_5275_PI426210822 CATGAATCTTCTCTACCCTATTGCTGCCTATCAACTTGACTTCCCACCTTT
A_14_P110155 CTACTCAAGAGTGTCTTCTCTTGCTTTCTGAATGAATCATTTTTAGCAGTTTCTGGGTAT
CUST_1785_PI426210822 TAGCTATCAGGCAGAATGACACTGGGACAAGGTCCATGCCAATTT
A_16_P20831198
AAAATGTCACATTACGAAGTGGATTTTGAAAGACTCTGAAGATTACTCCTGAAACTTGGC
A_14_P113087 GTTTTATTCCTTTAAATCACATCTCAAAAGGCCACCAATGGCTAGTTTGGGATCTTATTC
CUST_6512_PI426210822
GAGAAAAGTTAAGCACCTCAGTAGCATTTGTAAAGAGATGACACCTTATGTTCTAAATAT
A_14_P103601 CCGTGAAAGTGAAGACAATTTGTATCTCAGCACTCTACTTGCATTCAAGATATCTGATAC
CUST_5080_PI426210822
TTGTTTAATTAAAACTCATAGTTCATGTTAAATTTGTTCAAAGTTTCCATCTAATGGTGA
A_14_P201243 CTGCTGAGTTTAGAGAAGGGCAGAGATCGTAATGAATTATAAACAGACTTAGGAATGGTA
CUST_3707_PI426210822 GTGCTCCAGCTGATCATTTTTATGGGACAGTCCTTCCTTGATGAGAC
A_14_P113283 GATTTGCTTTTTTAGGAAGCCCCTTTAACATGCACTAGCAGAGTGCAACACATG
CUST_4185_PI426210822
TTCCAGATATTAGGAAAACAAGACAGAAGGGTTTCACCACTAGTCAGTATCAGTAGATT
CUST_894_PI426210822 AGAACTTGAGAAGTGCCTGGGGGTGTAGGCAAGAGACCAGTATCC
CUST_3834_PI426210822
AGGGAAATAATAATGGGATAGAATAACTGCAGGGAGTAGAGAGATGAGAAAACATTAGAA
CUST_1364_PI426210822 CCATAGCTTTGTGATATAGGCTGTCCTGCCATCTCAGGTCTAGACAAG
A_14_P105500 CTTAAAAATAATCTCTTCCTCTTTAGTGGCAGGAGATGCCAAAGATAAAGACATCTCCTG
A_16_P18079784
CGGAAAAAAAATTATCGAGTTAGTCAAGTGCATTTTTGTCATCTTCAGTTATTCCCACTCC
A_14_P118718 CTGCTTTGAGAGGTGAATATATACATAACTTCCCATCACTAAGCAAATGAGCAATCTGCT
A_14_P101377 ATACCACAATATACGACCAAGAGTTGTATAACATATTCTCCCTGAGCACCAATGATTTGC

CUST_9971_PI426210822
AAATAAGACAACTAATTTCTTTGGGATTACAAAATAATATGTATGTATTGACCTAACATA
A_16_P18378772
ACTCATTATGTCTGATTGCTAATTACCTCTTTGAATGATTCAGTTATGCCTGCTACTAGA
A_14_P106613 TAGTAAAACAGCATGAGGCTTTGAATACCAGCCCTGGCTATTTAAATTGTTTACCATAGT
A_14_P115563 AATCATATTAATTTGGACCCATGGGATGAGTGTTTTATTCATGCTGTTTCCAGGAAGG
A_14_P131211 TGGCTGCAGAACATTTTATTGATCAAAAAGGTGCTTATCCAAAAGTCTTTCTCTGAGAAT
A_16_P19219471
CGATCTCCAATCTGATTATCTGACTACTGAGATGTGTATAGTTCAAATTAATACGCCCCA
A_18_P12230151
ATCCAACTTCCATTTAACCTACTTCTCTATTCCATCTAAAAAACATACTGACTGGGCTCA
A_16_P17884610
ATCCACCAAATATCCACAGAACAACATGTTACTGAGAATGATAAAATGCCTGAGTCTACT
A_16_P19506245
CACCAGGACATGTGATACAGTGAAACTATTCCACTTATAGTAACAGCACTTAGTAATTGC
A_18_P17321852
TGTCTCGATTGCATGTTATCTTGATGTCCAGTATGTTCCATATAAATTATACTTTCCAGA
CUST_208_PI426210822 CAGACCAGCGCCGCCCGGCCCGGGACCCGCTCGCGCAGACTTCTC
A_16_P16191108
TGGTTTTCTTGGTGATTACTGAGTGCTGTGGCCTATCACAGAAAAAAAAGTCATATTTTT
A_14_P120141 ACTATTAAAAGTGATCTTCTTGGGTGACACATTGTGACTTTCTAAAAACCTTTTTGGTGC
CUST_9280_PI426210822
CCAACTTTTGAACCTCAGGTCACTTTGCTATTATGAGTTTATCACAGGTGGTTCATTA
A_14_P130124 CTGCCTTCAAAACCAAGCACTTTGAGGTAATAAAATGCCTAGTCAAATAAGTTATCCTCA
A_18_P22842446 ATTAGTAGTTAATGCACGTATTTGTTTATTGTTTCAGCGGAGGGTGCAGGACGTC
A_14_P102547 CTTGAAATACAAAATGAGCAATGACTTGTCTGTTCTAAGCCAAACCTAATCTTCTTGCAC
A_16_P00158316
TACTGGAATACATAGACCATGTAGTATGGAATTTGTAGACTATGAAGGGGTCATTGATGG
A_14_P102138 CAGTTTCTTTCCAACTTTGGTAGAATATATTGTTGTCCTGCTTTTATTCCTGCTGCCTTA
A_14_P129735 CAACAGACTGAAAACCAAAATTCAGTGTCTGCTTCTAAAATAGCATCCTGTGTAGAAAAA
A_14_P128831 AGAATTACAGTTATGGAGGTGTGATTCAGGATGACCATATTCCATTTTTAAGAAGAGGTA
A_14_P104962 GCTGGCTAGTAGTTACTATTTGTGAAGTTGGATTGATTGGTTTTGGAACAACCTAAAAAG
A_14_P132129 CTGCCTTGTCTAAAACCTGGGATCTTAAAAGTGTTATTTTGGCTTTTATTTTTTAAAAAA
A_16_P16114509
GACCAGACCATTTTAAGTAACAATACCTATTTTCCCTTGATGACAAAACATTTCGACCAG
A_14_P111368 TTGTATTATTGTTTAAAATACTTGGTATTCTTCTCAATGGGGATCCTCCCTAGAATGGGC
A_14_P134392 TGTGTATTTTCTTGCAGAACTCTCAGAAATTGAGCCCAATGTATTCCTTCGACCTTT
A_14_P112848 CTATCTGCCTGAAAGTGGAAAAAGGAATTAATGAACCTGTGAAGTTCAGTTCTGTAACTA

A_16_P19528663 ACTCAGCCCAAATCCTATTCTTTTCATCAATCTAGCAAATTTTACCACTTTTTACCAGAC
A_14_P107858 AAAGAAAAACATATCTGGACAAAAATACACCAAACTGTCTTCTTTGGCTGAGGAGAGGAT
CUST_1084_PI426210822 TAATTTTGGGTAAGCTGGAACCTGCTCCTTCTGTAAGTGCTGGACTATTTATTTC
A_14_P126135 CATCTGAAATTCCAAGGATACATGACAAACCTGTTTCCATTATGTGCTGGCGTTT
A_14_P200701 CACAATGTAAGCAGGAGTAAGAGTAAGAACTCAAATTCAGAAGCCACTCATAATAGCAGA
A_14_P201933 CATACACGTTATATTTCCCCATACAATAGAGTGAGACTAGAGGACTATCTTTTGAACCAG
A_14_P129368 ACATCTTAACCATTTTGGGTGTCATTTAGTCAGTCAGTCATGAAACAGTAATATGGAGAC
A_14_P100992 TGTTAACACCCCAAAGCATGGGTAAGGAAGAGGCTTTATGGTAATTTACTCCATT
CUST_1997_PI426210822 GTTAGAACTGCTGTACGCCAGCCTAGGATCCTTCCATGTTGTTCT
A_16_P17150649 AAGTTAAGGTCAAGTATCAAGCCAATAAGGTCAATAATTACATCCCCCTGATCACTATGA
A_14_P128095 ATTTCGATCTCCATGAAACTTAACATCGTCCCATTTTTCCAGTTGTGTTTGAATGTCTAG
CUST_7219_PI426210822 CTAAAAAGTTAGGACATTTAACCTATACTATACAAGAAGTTATCAGCTAAGCAAATAAAA
A_16_P19872513 AAAGCAGATCCAAGGTTACATTTAGTCTCCATATCTAACATGAGAATCTCTGGTTTTCCA
A_16_P58424510 AACGTCTCCAATCCTGCTAGCACATCTTTAACACTTCTCTATCACCAGCAAATCT
A_16_P19901122 AAATGTTGATAGAGAACCGCTTTTCTAAGAAAGATCTGAATATTGGCCTTACTCTGCTCC
A_14_P109740 GGAAAAATAACATTGAGGCAAAACTCCAGGTTAGATACCGTTGTATTATGAGATCTCCAA
A_14_P100349 CTTCACGACCAAAGGCACTAAGGCACTATCCACAAAGGCTTTTTTTGGTTAAA
A_14_P107008 TAGATTTTTAAGACTTATTTTTCAGCCAAACCTTCTTATCACTGGTCTGGGAGAACTCAC
A_16_P00685474 CGCATAAATCAAGTGTGCTTTTCATCACTGTGTTTTTAAAAATGTTAACTGATGATTTCC
A_16_P01533084 AAAGTGGAGATAGTGTTTTTAATTCTGCCCTTAACCTAGACAAAAAAGTGCCTCATCAAC
CUST_10073_PI426210822 TTTATGTCAGAGTGTGCAGTCTGTTGAGGCAGTGTTGTAATGTGATAAGGG
A_16_P37312025 GCCACCTCTTTATTTCCAAGTTTTCAGGCTTTTATGATAGAAAATTGCTCAAACTTCACA
A_16_P58424458 TTATTGATTGGCAATAGCCTTCTTCATAATTGGTTTCCGTGCTTCCACCTTTACCT
A_18_P22840498 AAAATCTCCCCTTCCTGTCCATAATTCAGACTACGACAGCAATTAGAGGCA
A_14_P111910 AAATCCTGATGGCTTGAGGATTTCATTTCTCCAGCCCAGAGCATATTAGCATAAG
CUST_5741_PI426210822 AAGTCCCTAGAAGAGAGTTTTGAGCTGAAATGAAAAGTTGTGATTATAGAGAGACATGAA
A_18_P17212657 ACCTAGATTTCAAGTCATTCACCTTCAATTCCTGAATTGCAATTAGATAGAGTATAAGCC
A_14_P130142 CACACCAAATTCCAAGCAGCCACTGATTGATTAGTTTATAGGGACCCTATAATTATGATT

A_18_P13525350
AAAAGTGACTCTTATCCAGACTCCTTTAAAAGAAACACCTTTATTGCAAGATCACACGTG
A_16_P18152977
GTCAACAAGGTCTATCAACCAGTGTTACAAGTATGTATGTCTAAGACACCTCAAGGTTAT
A_14_P110359 AGAGAAGTAGCAGCACAAAAGGAAAAGATCGATTATCATTTACATGCTGAATGTCTCAAG
A_16_P15013573
ACAGATGTCACATAAGTGGATTGACAATTGCATTATTCCAGATTAGCGTATGACAGATGG
A_16_P16724625
CTGGTCTCTCCAAAAATAACAAGAGTTACAAGGATATACTCACATGGGATTATTGCACTG
A_16_P39763466
CCCATTGTCCAGTTTTATAACCTAGACCATTAAACCCTTCTCAGACTCTAAATGTTAAGA
A_14_P201080 CCAAGATAGCATCTGAGTGCCCAAACATCTAAGGGAAAACAGACATAACAAATTACTAAT
CUST_93_PI426210822
CGTGGAAGTATAGCTTGTCTTATGTATGAAAAACAGGGCAAGGCAACTTTTGAAGAAT
A_16_P00725270
CCCGTGATAGACTTACAATCAGTTCACCATAGATACAAAATTGGGCACAATTATCACTAC
A_18_P22840489 ATTGCATTTTGCACTCTGACCCAGTGCTCCTGTGTTTCCATGGTTAG
A_14_P116041 ACTAATGTCATAGATATCTATGCACCAAAGTGAACTGGCTTTCTGACATTAGCCAAATTC
A_14_P133357 TGTAATGGAATTGTTCCAGCACATCTAATAACAGAAGGGTGGTTGTATTTATTGAACACC
A_14_P139232 TTACAGACATAAGCAGGACCTTGGCTGCATGAAGTTTTAACATGGTAAAACTCATTGTAT
A_14_P104792 CTTTTTAATTCTGATCTTCATAATCTACCCCTGTAGGGAGTTTAACTGTTTAGGCCACAA
A_16_P17830984
TGCATATACACAAACCACTATCTTCTCAGCATGAGTATTCAAGATACGTTCCTTTATCCC
A_16_P02254197
GGGAAGTTTAGCACTGGTATCTCTAGAACACATAGGTCATTGAACAATGTTAGTCTCAAA
A_14_P130499 CTCTTAACGCTGCTGAACATAACTTGAACATAATGCTCGTATGGAAACCCTTTTTTATCT
A_14_P110891 CTATAAATGAATGGAATCTTGCCAATAGGCAAGTGATACTTGCTGTCTTCCCCTTTTTAA
A_16_P15434543 AATCGCATCCATAGCAAATTCTGCAGGTAACCTCTTTTTACAGTTTCCTGATTGCTTT
A_14_P136096 AAGTGCAAAATTCATTCAAATGTTTTTTGAAAAAAGTTGAGGCCTGAAGCAGCAGTCTAC
A_16_P15114336
AACATGCCTTCTCATCGTAATTTTTTCCACACACATTTTTCCTACACCTGTTTATTGTGT
A_14_P128619 CTTATATATAAAAACTTTTCCAATTTCCTATCAACCCACTTGCAGAAGTAACCCTTCCCC
A_16_P15928050
AGGAAACATGTTATGGAGTCCATCTTAGAGAAAAATCCTACACTGATAACCAGAATACCA
A_16_P21456329
AAACCACAGCAGGGTTTATAATGTCAGATCTTACAAGGATAGAACTTGCCTCTCTTAAAA
A_14_P133746 GAAAAGTTCAAAATGTTTCAAAGCCCAGCAATTTACTGTCTTTAGCAAATCCCATTCTAC
A_14_P122627 ATCTGTTCAGTAGCAGTCATGGATCTCTAAAGAGACATAATCACTGGCTTTAGAAGTAAC

CUST_6645_PI426210822
TTCTACAAGCACTAATCCTTTCTGTGGACTATCATTTAGAATGATTAGAACTTTTTTCTT
A_18_P13325389
AAGTTTCCTAAGTGTCTACGTCTATAATACGAGCATTGAAATATCTTTCCTGCCTGATCC
A_14_P114060 CTAAGGTTAACCTCTATCTTGATTTTTCTTTCAGAATCAAGCATTATGTTTATCAAGGCA
CUST_4897_PI426210822
AAATAGACATGAGTACTCTTCACAGACCTTACCGTCTAATAGGAATGATAGCAAGTGAAT
A_16_P37671764
TTCCACTGATCTTCCTTATGTCCATAAAGTCTATACTCTTTACCTCATCTATGCCATACA
A_14_P131445 ACTGAGTCTTTCCAAAAGCAAAGGAATACATGAAACCCTTTCTTGCTTTGTGAGTAAATA
A_16_P01684350
AAACTCTAGAGGAATTTGAGAACACTGATACGCTTACTTCACAGAAAACTGAAGTCTGTT
A_14_P116211 CAGAAGACACTGCATAAAATTAATGTATTATCCAGCCAATGAGAATTTGGGGAACAGTTC
A_14_P119958 GAGTTCGAGGAATTCCAATAGATAGGCTCTAAAACTATCGATTTGGAAATCGCAGTTCT
A_16_P18798094
CAAATCCATACAGCAAATAACAAAAAGATCAGCCTCTTAGATACAGCAACATTGGGAAGA
A_16_P00175274
TGGCAATCCACATCATAATCATGATTACCATTTCATTGAAACCTTGTATAATCCTGACAC
CUST_9207_PI426210822 CTCTACAATAAGAGGCTCCAGCATCATGATTCATTCTTGAGAATGACTCCTCTG
A_14_P103391 TTTATTAATGTTACATTAACACGAACTACAAAGAGACCTTTCGTATGTCTGATACCAAAG
CUST_1216_PI426210822
ATTTTAGGACTCCGAAATGTTAAGTCAATGCATGTGATAAAATCAAGTACAGAGATTTCT
A_16_P02696332
ATGGCTACACCAAAAAAATACCCAATTTGAGTCAATACATCTAAACCTTCCTCCTTTTGT
A_16_P58424347 ATTGGAGGACATCTCTGAGGTTTTCACTGTCATGGTGACTCACGTAG
A_14_P101206 GGGAGAAAATAATAACAGAGTATTCTGTGCCACTGATGATTGCGTATCAATCAGGAATTA
A_14_P120701 AGAGAAGGGCATACAAGGTTAGACAGAAGATTTTCTTTAGACACTTTAACTCACAACATG
A_14_P117421 CTGAAAAATGAACTCTTAGAAGCAAAACGTAGAAGTGGGAAAACTCAACAAGAGGC
CUST_157_PI426210822 ACCTTCAGATTCCTTCTTGAAGTGAGAGCATAAAGGAATGGAGGGCAT
A_14_P137407 TAAAAGCATACAGATTCTTAGGCCTGATCTAGGTCCAGATCCTATTTAAATCTCTGAAGG
A_14_P114627 ACTGAATATAGTGTATTCAACATACCTTCACTGACAGCATGGGGTTTAACAATGCAACAG
A_14_P126700
CAGAAAGGGCAACAGAAAACCACATGAGGCAAAATAATTTTGACAAACTGTTATGAAATC
A_14_P109764 CCCTCACTTAGTCTAAATGTAAGGTGTCACTGTATTACGAGACCTTGAATTAATAAGCCA
A_16_P00913492
AAGTGAGGAAAATGGATTCAAAAATACATACTTCCCCAAGGATACAAAGTAAAGCCATCA
A_14_P124038 CTGCACGTATTCATCGACCATCTCCCTGTTAGAATATACATTCCCCTCTCTC
CUST_4399_PI426210822 GAGCTGTGATGCCCACATGTTTTAGTGTTGGCATGTTTCCCTCATTT

CUST_10119_PI426210822 CTACGCATGCACTGTGGTGCTACTGTTTACCTAAATCAGAAAGCCCTTATC
A_18_P22839797
CTGGAGATTAAAGCAATGGAAAGACTTTTCATGATCTAAGAGTGATCCAAGAGATAGTGG
A_14_P101949 CATTCAGGAAGTAGATGTCTTTGATGAGCAGGCTGAAGAAAGGAATGACAATCTG
A_14_P131922
GAAAGAAAGGGCTTGCTAAGAAAAAAGGGAGTAAAAATGATGATAGGGAAGTGTCTAATG
A_14_P100757 ACAGGAGTAGAGTCAGTATGTTTGATTATTTCCTGATGCTCTAAAACTGTTATTTCCCCA
A_16_P16191176
AAGTTACAGCACAGAGTTTTAAGAGATTGTCCGTAGTTCAGACTGTCATCAAAGTGACT
A_14_P101143 GATTCTCCTTTTTATGAGTTAAATTATTTATACGAGTTGGTAATTTTTGCTTTTTAATA
A_14_P130055 GTGCTTCTTCCAACTCTCTATTGCTATCAGTAAGCATACATGTTTGCTATGAATGAAGGA
CUST_7577_PI426210822
CGTGTGAATAGACAGGTCTTCAGTTCCAGAAAAGTGTATACTCACACAGGAAATA
CUST_7341_PI426210822 CCCTTTGGTGTCTGGAGCCATGGTGGCTGGTTGTCCTTCCCACTT
A_16_P19963481
AGAATCTCATAAAGCAACTGATTAAAAACACATACCAGACACTCTAACCTCTTCTTTCCA
A_16_P01249924
ACTCACCCAGAATATTACCTGTCTTATATCTTTGAAGCACATACGAAATAGCCTTTGTGA
A_18_P22839223 TGTTCTTCCCTCACAGCCGAACTCATTTGGAGAGATTGATGGAGTTACTAAT
A_16_P16114913
TTTCACCAGAGTAGAAGACTAGCAGAGCATCTATTTACAACCTCTCAATTTATCATGGAG
A_14_P104306 CATATGGTTACATTTAACCCTTTGAAAGGTCTGCATCCAAGATCTAAACGCATTTCTTCC
A_18_P14589668
ATACCGAAGTCATACTTGATTACAACTAAAAATGGTTTGTGAACCACCATACTAGTTGCC
A_16_P16191193
CTCTAACATGAGAAAACGCTTATGGAGATAATCAGTGGAAAAATGGGATGAAAACTGCAT
A_16_P16191933
GGAAGTCACATAATGTAGGAAACGTGTCTGTGTTCAAAACTCATAAGAATGCTTTCAGAA
A_18_P14134875 CTCCAATCTTTTTATTTCTGGTTGGGAAACCCTACACAGACCAGTGTCTGT
CUST_693_PI426210822 GGAATAAGCTCTCCAGGGTCCCCTCCTGCTTAGATAACCTATGAAT
A_14_P200903 ACAAGTCAATTCATCTTTCCTCTGGCTCTTCAACTTTAAAATGTTTTTCTGAGCAATCTG
A_14_P119560 AAGTAAGTGGGTATACATTTTTACCGCAAATGTTTTACCACTAAGACCTTAAGAAACTTA
A_14_P133749 AAAAAGAATCTGGCCTTCTTGAGGTCTAGACTCTATATGCTGGAGAGAAGGAAGA
A_14_P202042 CATGGTAGTGACACCTGTAGTCACTTCTGGTTTTATTGACCCAAATTTAAAAACTCCTAT
A_14_P102696 CAGTCCTTAAGTGTGTCAAACATTACGGTATGGTTCTTGGGATCAGTGACTA
CUST_1519_PI426210822 GCTTCCCAGTCCATGGAGGATTACAATGTAAAGCTCCTCATCCTG
CUST_9199_PI426210822
TGCATGTTATATGCCATCTCATCAATTATTGGGGGACCTAAGATCCACATTTTCTATTAT

A_16_P01171126

AAGATTTGAAATGGGAAAGTAGACATATAGGACACAGAATGAGAAGACTACTACCTGCAG

A_16_P16952444

ACACACACATTCTAAAAAATCTGCCAGAAACATATTGTTCACCCTAAGAGTGTAATGACA

A_16_P32316793

AACTTGCTGGACCATTTACTACCTGTCATTATGGGAGAAAAGGTATATTTTGTGATTTGC

A_16_P21626237

TTCGTTCTCATAACAACTGTAATTTTGTCAAAACATGCAATGTCCTAAATGCTACCACGG

CUST_9182_PI426210822 TTGGTAGCAAAACTCTGGCTTTAGAGTTGGACCATGAGCAGTCCT

A_16_P00816004

AAGAGAGACAGATTTACCGATCTGATTGACCATAACTCAGACTACTCATTAACCTTCAAG

A_16_P58424447 TTATTTTAGCCTGTTCAAGTCTTCTGCACTCCCTTCTTCTGGGGATACTTTTCTT

A_16_P18399697

AATACCATAGAGCCTGTAGATATCCAAATACCATTTGATGATAATGATTATGCCCTGGGC

A_16_P20709486

AAACAGGAATCCTACATTTCTCCAAAAGTGAGACATAATCTAAACTATGGCTGCAATTGG

A_14_P116412 GTTATGACTTATCTCTATCAAATAAGGGCACATTTCAGTGGCCAAGAACTAAATGTCGTT

A_14_P109078 CTTCTGACTTCCTTTTCTCTTTCCCAGTTCTTCCATTCTCTCAGGGC

CUST_10191_PI426210822 CCAAAGGCCGCGGAGTGGGTAGGATCCCCCAAGAGACCAGATGTC

CUST_964_PI426210822

CTCCCAGAATGCTTTTAGTTTAGTATGAAATGCAATATTTGGATCTTCTAGGAATTTTCC

A_18_P26795246

CCCTTTCTCTCAAGACCTATAGATGTCTGTATAAAACCAATATATACTTCCTACGTAGCC

A_14_P135066 GAGTAAAACGATCAGCAAACACATTATTTGGAAGTTCCAGTAGTTAATGCCTGTCAGTTT

A_14_P114645 CATAATTTTTCAAGATATTACTCCTGGAGCCTTATGTTTTTTTCCTCCCGAAATCCTTGC

A_18_P25044874

AAAAATCACCAAAATCCAATGAATTTAGGGAAACCTACTGCATTTCAACTGAATGGTTCC

A_16_P18080577

CCTGCCAACTTAGTTCAATTACTCAAAACCTTAGACATACTGTTAAAATCTTTGCTACCC

A_16_P16351696

AAGGAAATTTTACCCCAGACTATAGATCACTCGTTTAACCTTTCTCATGAGAGTCTTCTT

CUST_3667_PI426210822 CAAACCCTTGTTTTGCTCCTCATCAGCGACAGTGGTAGGGAAGAT

A_14_P139362 TCTAAGGATGGTGGGGTATAAAGATGGGAGTAGAACTCCGACTATGTCTAAAACATATTA

A_16_P00685358

GAGAGGAGAACATTGAGATGGTATAGGTCATTTTACCCTCTATTCCTCTCAGTGAAAG

A_16_P18412546

CCCTTTTAACCTTTACTTCAATTCCTTAAAGTCTTTAAAATCTCAGTTGGCATCAAAAGT

A_16_P19257898

CATGTTCACAACGGTCACTAATTACAGAAACTCAGATTTAGTATGTTTCCAATCCCCTTT

A_14_P113589 TGAGTTCATCAAGGCAATAGGACTCTGTATAACTATCCTCATTTGCTTAAATTCAAGGGT

A_14_P112512 GGTCCAAAGTCCCAGAAAAAGAGGGATGGGACAAAGAGAGAAAACATAC

A_14_P131594 ACATCACAAGCAGGTAGGAAAGGAAATCTTCTAATACACTGTGATCCACTGAGTATTAAT

A_16_P01578412

ATTTTCCCAATGCTTTTCCTAACTGCTTACACAACCACTTAATATATGGCTTTCCATGTC

A_14_P121362 TGATGATGACGATGACAATGATAAAATTCCAGATGACAGGGTAAAAACAGTTTTCTATCC

A_14_P136883 CTTATTCATTTTGGTGGGATTTATAGGAGAGTTCCAAAGTTTTTCAAGGTGAGGACTTTT

A_14_P129926 AAGCAATGGCAGCCTGAAGATCATCTTTGTTATCATGAGTAAGGTCTATAACTTCTGTAA

A_14_P116176 TCAAAAAACTGTGCCTCACTCTAGAATCTTGCTTAGAAAATTCTGCAAAAATAAAGGCCA

A_16_P01722923

AACTGACAGAATGACCAATTTACTATGGATCTTTTCTGCAATATCTTTCATTTCCTTCCT

A_14_P135529 CCTACTCTGGACTCTTCCTTGATCCATTTTTTTTTTTAATACGGAAGTGTCATATGTTGC

A_16_P00685499

CTTCAATAATATAAAAATTTCTAGTTTCTTAAATCACAAATGTTAAGATGACCATATTAT

A_14_P138187 AGCAGCCAACAAAATCTCTTAAAATCTCAGAAATGTTCAATAAACATTTGCAGAGCGAGA

A_16_P03269588

ACAGGTCTTCTAGTCTGATCAGTGCAGTATTCACATTTATTATATATGTCTGTTCAGGCC

A_14_P107798 CTTCAGGCATTTTCAAAAAATTCATTTTGTTATCTTCGCGAACACTCACATTAAAGATCC

A_14_P111941 CTGAAACAGATTCCTTTAGTAGACTTTCAAAGCCTCCTGATAAACCTGATGAAAAACATC

A_14_P111741 AATCCCACTATTTCGATGAAAATTACAATCCTTGAGAGATATGTTTTCCATGTGGCTTCT

A_16_P01883249

ACTCACAGGAGTATATTTGAGTAAAGGCAGTGTTTCTTCATTAGAAAGAGTGCAGGTTTT

A_16_P00685148 ATCAACATCACAGCGCCTCAGTGTCACGACGGACAGCAGCCTGTG

CUST_7170_PI426210822 AGAGCATGCATACTGAGCATCTGAGCTTTTTTAAAGCAATGATTCACCCAGC

A_16_P38831131

ACTCCGTTCATTTACATTACATCATAGGATACCCAAAGATGCATAAATAGCAACTTCCCA

A_14_P102865 CCTAAAGAGAAATTCTAGGCTTAAGTGTAAAGAAAAGGAAGTCCAACCATAGTCTCATGT

CUST_7878_PI426210822

ATTTGAGACTTTATTCTGTTTGCAGTGGGGACAGACATATTTTAAGAAGCTTCCTCTG

A_14_P117608 CTTCCTGAAGTCTATGAACAAGTGTTATGAAAACAAATCATGATCACTCTTAACCTTCCC

A_14_P116892 CTTTACTACTTCTACAGACTCCAGCAAGTCAGCAGAAGCCAATCTTAAATCTAGGTAAAT

A_14_P116700 CCACTTTGGCAAGTGGCAAGATTTTGGTCATGATTTTGTTTTATGGTCATAATAAGAATT

A_16_P00180491

AATACTGCTAGGGTCAAGTATATATCCAGCACTAAAGACCTATGTATCTTTGTCAGCCTC

A_18_P16216461

ACACACCATACCAATAAGTGACGCTCAAGAATATAGATTTCAGGTAATTATGGCAATTCC

A_16_P16246613

CCCAATCTTTGATGCATTATGTTCTACTGTCTGCTAAAACCATCATCGTAGCCTTTTAAT
A_16_P00732931
AAAAACACCACCCTACATGTTTATTAATCAGTTTTCCAAAAGTAAGTTGACAATGGGTCC
A_14_P107729 GAAAGTGTGCCAGAGTATGTGCTCACAAAATAAAAACTATTACCATTACCCAACACTTCA
A_14_P100155 CTCGATTCCTCAGTTCAAACCCGAAATCCAGGTCTATGAGAACATCCATTT
A_16_P16191774 ATTTCCATGAATGTTTTGGGAGAGGTGAGGAAGAAACCTTTTCTGCTCATTGTT
A_14_P111073 ACTCTCCAAAATACCTACTTGCTGCCATTTTGTGGCATGATAGTGAGAATAGATATTAGG
A_14_P110346 ACCATTTGGGGTCTCAGTTCACTCTTTCCTTGTTTATTAAATATCAACTTTTCCTGCCTA
CUST_4998_PI426210822 ATTTTCAGTTACAGGTGAGCATCCGATCTGCAGCCAGGGCTTTTT
A_14_P139343 CATTTCTTGTGGCTTGCACTTCAATAGGTTTTAAAGGACAACTGCAAAGAGTTTCTTGT
A_16_P16614558
AAGATAATGAGCCATTTCTTAAAAGCGTTCACCTAATCTTAAGAAAGGAGGTAGCCATCT
A_14_P109840 CCACTTCTAGGGACACTGGGGAACCTTGTTAAACGTTGACATCAG
A_14_P125282 CAAATTCTGTATGTCCAGATACCATTTAGAAGCAAACTCTGATTTCACATTGAGGCTGAA
A_14_P113117 AGGTCTCTGTGGATGATTGACTTGGCGTGTAAGTAACTGAAAAACAAAACATCATTTTAA
A_14_P201369 AAAATGACTTGAGCAATCTTCGTTTTTATAAGAATAAAATTCCATTCAAGCCAGATGGTG
A_14_P121821 CTCAAAAGGATACAGTGAAAAATATTCCCCTTACTTCTCTTCCCTAACCATCCAGATTTC
A_18_P22843574 GTCATTTACTACTTAGGGTCAGTTTTTCTGGCAGGTCAGATATTTGGAGTTGGTGTTT
A_14_P106662
AGAAAGGGTTTCAGTTCTTGATAAAAAACAAAGTTCAGGAGTGTTCCTCAAAATGGAAAA
A_14_P109014 AAACTGAAAATTTTGAAATATAGTGACTACCTTTCCCTTTCTTCGCCAGTTTCATCCAAG
A_14_P118166 TTTTATGCTCTCTCTTATCCTGGAAGAAAGCAAAAGCATGACTAGGTGAGATTATGTGTT
A_16_P02518288
AGAAATCATGATTCCTAAGACTTTCCTTAACAACCCTAGTAAACCTAGTAAACATGCCGA
A_14_P114811 TAGCACAGTCTCTTTAAGTGAAGTCTCCTATCCCTGATATTCTGAATTTCTCTAAACCAG
A_16_P00733949
AAAAAACAACACCCTTAATGTTTTTCATATGGCACATAAATTTTACAATACCTGCAGGAA
A_16_P02273356
AAACTTCTCTTTGTCTTCTATTTCTGTTCACAAATCCTTAGTTCTGAGTAAATGCTGCCC
A_18_P11458038
AAGAAGGGTTTAATTCGCTTCATAGTCGTATAATACTGTCTTCACAATTCTGCCATGCTT
A_14_P108851 CATTTATTGATTTCAGAGGTGACTTAGAAACACAGGAGGTGTCGTGATAAGTAGTAGAGA
A_16_P15674025
AAAAAAAAAATCATGTCAACGCATACAAAACCTGAAAGATATTCCTGACCACTTGAAGGG
A_14_P125138 GGCTCCCCGGAATTGACACACACTTGCCTTTTAATTGATGAATAGTATTGATCAATAAA
A_18_P16264876
ATGCTTGGCAAATACTACTACCTAAATCTCAATCTGTAACCAGGAATTTATGTAGCACCC
CUST_5575_PI426210822 GCCTAATTAAAGTGGAATCGCTGCACAGACTGTGTGTGTAACATGGAGA

A_14_P107675 TAAGAACAGAAAGGTTTCTAAATGCCAGACTTTTCCTTGAGGCATTATACTGATCCATTG

A_16_P16191472

CATATGTTATGAGGAAATACACTGTTTCAAATGCAGTTTTCTTTGTGGTGTCTCAGCAGA

A_14_P127837 GGATGCTTTACCAACTACTCTTTCTGAACAGCAATTATACCCTCCTAACTAGACTCAATA

CUST_1685_PI426210822 TGAAAAGATCTGTTTCTGAAATATATGTGGGTGACCAAGGGACGGGG

A_14_P134767 TCTTGGTCCATGAAATGTAAAGGTATAGTAACCACTGAACTAGAGAAGTAATCTTTTTTA

A_14_P138544 TCTGCTTCTCTATGGTGATAAGATCTAGGGGATCAGAGATCTTCTCATAATAATCAGCAT

A_16_P16191657 GTCGCATCACACAATAATCATCAGTCTCTCATGCATCTTCATCCTTGTCATCTTCA

A_14_P132769 AAGTATACAGGCAAAAACACTAAAAGAGAGAAGGTGATCTATTCCCTAAACACTTGCTCA

A_16_P00969297

ACAGCATCCCATTTTAGTTATCAATCTAACGTCCTATGACTTCCCAACTAAATTCTGCTT

CUST_5309_PI426210822

CCTACATGTCCAAATGCATTACTCATCCAAAATAAGATATGCCACGACTCAGGTTT

A_14_P118301 AGTAAGGTAACCCAATGACAGCCATGGGGATTAATGCAAAAGTGAAATACTCCAAAAAT

A_16_P16191442 AAAGAGGTGGTGAGTCTTTCTAAATCCTGAGTCAAGATGCTTGACCACTTATTAGG

A_14_P133533 ATTTCTCATACCATTTCGAACACATCACTTGCTCTTGATTTGATGGACTTAAGGTCTC

A_14_P115908 TTCAAACTAGGTCCTTTATTTTTATTTATGCATGACTCCCCCATAGCAAAAGCAGCAGTA

CUST_245_PI426210822 AAGGAGTATAGCCTCTCCAGCGCCGCTGCGCGGGGTGGGGCTCCC

CUST_3222_PI426210822 ATGACCTGCCCCTTTCCTCCTGGAACCTGCTCCTTACACTTCTTT

A_16_P17076625

ACCGGATTCTGTCTTTTACACAACTCACAGTTAATTTGAATAGTGTTTTCCTAGTCCATC

A_14_P114984 CTATCTAGGGACGAGATCTAGAATCAACTGAGAGAGATGCTTAAAAATCACCAGTTACTT

A_18_P13849723

GGGGATAATGTTTGGGAATGAATTAAAAACTAAAAAATGAAATGATACAGGGTGACTCCC

A_14_P200923 GAGTAAAATCAGTTTTGTAAATATGTAAATATGTCATAAATAAACAATGCTTTGACTTAT

A_18_P17313827

ATCAATTCCTATATCCCTCGGTATCATAAATTCCAAGAATTGTGTGTAACATCCTCTTCC

A_14_P117889 GACAAGCAAGAACTAAACCATCTTTATGTATTAAACCAAATTGACAAAAGCGTAAGGGTG

A_14_P138832 GTGATCAGTTCCTCAAAACTGTGAGTTTTCAGTCATGTTGTTTCTTTAAGTTGGTTTAGC

A_18_P22841290 GTTGTGAAGGCCACTGCTAACCAGGACAAAGGGGGAGCATGGGAG

A_14_P106034 AGGTTTTATAAATATTTACTGAACAAAACTCTGCCTGTTAACTGGCTGACACAGAGCAAG

CUST_4738_PI426210822 CTTCCCTGAGGCAGTAGGGGAGATCATCACCTCTGTAATGGTATAAT

A_14_P134344 TTTGCGCTCAGAAGTATATTCAATGATTTCCCCAGAAAATTCCAAGTCATCTCCTAATAC

A_14_P134843 ATAGGTGTTTCCAGATCTCATTACTGTTATGCACACATTCAAATAAAACCCAACAGAGTC

A_16_P17343832

AAATAAGGGTGAAGTGCTAGAAGTTTTTTGATCAACAAACTTTGACATGGCTTTCTCTGT

A_16_P16191725

CAGAAAGAAGTGACACAGAACATCTAAGGCATATCATAAACCATTTCTGACCCTGATCTC

A_14_P132167 TCCAAAAAACCAACTCAATTACTTGAATGTCCTCTGTTTCATATTCTCAAACCTCCATAT

A_14_P111725 ACTTAAACACACTCAGCCTCACATCTTACCCTGTTCTCTTTATTAGGGTCTGTTTATAAA

A_14_P125109 CTAAGCCTCTAAAACAACAGTTCCTAATTTCCTCATTAGGAACTCCGTTGAGAATAAGAG

CUST_5144_PI426210822 CTTTGCTCTGCCTTGGCCGAAACATCTATCCAGGACCAAATTCTTAAA

A_14_P120309 TTCTGCCAATATTATACATAATAGGATTCCCTCAAAAGACAACGAATGTTTGGATCCTGG

A_14_P119231 GAGGGAAAGAACATGCCGTCATGCACTTGTTCTATGCAAGATTCTTTAGTCATT

A_14_P117550 ATAAATATTCGCCGCCCTTTCCAGGGATACTCTTGTAGAATTAGTGTGCTTAATAATTCT

A_14_P112650 CATAACCATTTCTTTGCTGCACAATGTCAAATGCTGAGGGAGCAAACACAAGA

A_14_P105774 AACCTAAGAGATGGAAATCCCAACAAAAATATTTGGAAAGATAGACACTCTGGGATAGTT

CUST_1036_PI426210822 CTTTCTGGTCCCTCTGCTCCTCTCTTCTCCCACAGAAAGAAACAT

A_14_P126449 AGGGACATTTTCTTTTTCTAGAGAGGCCATTTCAATCGAATATCTCATGGCCTACAG

CUST_3223_PI426210822 GAACCTGCTCCTTACACTTCTTTGCCTTGGTTCTTATGCTCACAGAAG

A_14_P122513 GTCAAGTATGCTGCAGATCAGCACCAGATTCTCCAGTTTTGCACATTTATTGAGAA

A_14_P136042 AATGATCCTTGTTCCAAACACTACACTTACTTTTGTATTACCTTCTTGTCCCTTTGTCTT

A_14_P102390 TGTGGTTATTGATTCTCTAAAATTAAGTGAGGAGATGAAGCCCAATCTAGATGGTGTTGA

A_14_P122368 ATTTTGTAGAATCTGTGTCATCACTAATTGGGGATATTTGCATTTCAAAAGAGGCAGAGT

A_14_P116964 CTATGTTCAATTTTCAGCCTGGAGATTTGCTCTTCCTCTTATTTCATTTACCACTTCATG

A_14_P113870 CTGACAGAAGGTGGTGAAGTAACTAACTTGATACCAGATATAGCAACTGAACTAAGAGAT

A_14_P132761 CCAAGGGTTCAATGAAATACTCTTCATTATCACTTTTTATCATTCCAGCCTAGAGAAAGC

A_16_P00318255

AACCTACAAACTCTAAGAATACCTTGTATTTACCCCTATGATACCAATGAATGCATTGCA

A_14_P105784 TTCCCATGTTCCTGGACATATCCAATTCTAGCACATCCACATTTTTAATAACTTAGTGAT

A_14_P134060 ACGTCAAGGTTAGTTCTCTGAACTTTCTCATTCCTATGGATGCAAACACTGTATCTAATT

A_16_P17692833

AAGTCACTAAGCAGACTTCTAAAATTTGTAGCCAACTTAAATGTTGACCTCTGTGGATGA

A_16_P02939626

AAACTGCAATGCTAAAGTCTGTAAAATCTGTGCAAGTAAAAATCACCTATAGAGACGCCA

A_14_P100309 CTTTTCTGGAGATTCACACAATATATTTTCTGGTTTCAGATCACGATGAGCAATGCCT

CUST_9765_PI426210822 CTAGGTCTGGAGTTGAACCTCCTCTTGGATGGTATTGACCGAGCT

A_14_P111848 CAGGGTTACACCACTGATCGATATAGTCATTTGAGCATGTCCATAGCATGTTGTTC

A_14_P107465 CTTTGTTTTATTTTCCATGTTATTACTTGGAAATTCTTTTCCCCTGGACCCTCCTAAGTC

A_16_P17264851

AAAAAAATTATTCCAATTCAGGGGAAGTATCTCATCAGAGTCTTTCACATAGCCTGATGC

A_14_P202173 AAATATTAGCATATGAAAAATTACCTTGCAAGGGCAATGATAACGAACACCCTGCTGTTT

CUST_5115_PI426210822 CCTATTGGCTGAATGTCCACTTCTGTTCTATGCACTGTGCTAAAGGGT

A_14_P129590 ATGTCAAGTCCTCCTCAATACTACACCATCTACCCTCAAGATAACTCTGCATTTGT

A_16_P18298454

ACTGTATTTTAGGATAGCGTCATAGGACTTGATAGACAGAAATAATTTGCCCCTCATCCA

A_14_P105381 GATAGCCTAGGGTTTCTAATAGAGCATAATTTCCTCCTTTCTCCTAGTATATGATGGTAG

A_14_P135721 TGAGTGGACTATATCTCAGGTTTTCCTTTTATAGGCATACTGTAAAGCGGCATTCTAAAG

A_18_P14754637

CATTGCTTAATCTTAAATTCATGGCACTCCACTATCAATTGTATGCAAGACCCTCTCTTA

A_14_P112948 CTCTCATACTGTTAGTGATGTCTGGCTGTTTCTTCATGTAACCCACTTTAGGTTTACATT

A_16_P15718917

AAAACTGCTTTCAGTAATGTGAAAAGTCAACCTAAATGTGAAGTATTACTGCTGCTCCTG

A_14_P118519 ACCTCCTGGAAGTCTCCTTCGCACAATGCGTTAACTTTACTTATATTTACTGTTAATGTT

CUST_7370_PI426210822

ATTTAAGCACAGCATTCTTTTCCCATGTGTCATTTTTCAGCAGTTAAAAATCCATCAGCT

A_16_P00648741

AGGACCTAATATAGTTCTTCTGCAAACTGATTACGTTACAAGGGAAAATACTCATTTCGA

A_18_P14136399 GGCTTTGCCTGTTAGTGTGAAACATTTCTTCAGTGTTTCCTACAGTGTTCTGATTT

A_14_P108226 GGTGTTTCCCTTGTATTATCTGAGGTGGAAATCATTAAGGTTTAGAGTAACGATGTGTTC

A_14_P118940 TTCTACTTCTGGCACGTTTTCTTTCCTCTCTAGTATCATCATATTTCAAGCACCAATCAT

A_14_P201317 TTTCATACAGAATAATGTGATGCAAACAAATTACACAGTAAACTAAACTACCATATGGCT

A_14_P119484

CTGGCAGATGTTACCACAGGACAAAATTAGAATGTGTAACTTGTAAAACAGCAGAGAAAA

A_18_P14132066 TACCGGGCATCTCTGTAGAAATGGTAAAAATGCCTACGTCTAGTTCACAGTGAT

A_18_P11069618

GGGATTCTAACTTTTACAATGCACCAACTATGTTACCATTCCAATACTTGTTCTTCTCCT

A_14_P200170 GCTAATTGATTGCATAGAACCTTGCTCAGAATAACTGAATACCTTAAAGGATTGCAGATT

A_14_P130025 AGTTATTCCCATCATTTCAAAATGTTTGGAAGGAATGATTCTTGCAGCAAAATCAGTTGA

A_14_P106723 TTCACATCCTCATTCTTATACTGCTTTTCGTGGCCACTTTGGACAAGGTAAG

A_14_P121467 AAACCTTCTTCCAATACAACCATCTATGGGTAACCTTAAAAAACACAAGCACAGATCTAT

A_14_P129910 AACCTCCTACATATGTTTTCTTAGGAAGATTCTTCCCTATCAGTTAAAAAGGTAGTTACC

A_16_P18330333

AAAAGTCATTAGTGGCATGTTGATGATAGAGTTTCGATTACTATGGTAGCCTTTCAACCA

A_16_P38463570

TGGTGAATCACAAAGTGCATCTCTTTTTAGGTTTGTAGCAACATCAACTCCAAATGTTAA

A_16_P36307163

ATAGGTTGGCAGTTATCTGAAACAAGTAGTCTAATAAAGAAGTATGCCCTTATGGCTAGC

A_14_P108898 CTTTTACAGTGACACGTTGTTTGTTCTTTTTAAATCTTGATTTCCATTCAGCATGTGGGA

A_14_P114894 TCTTATTTTCCCCTCCATAGCAATTCCTAGACGATCCCAAATATAGCAGTGATGAG

A_18_P11711584 TATGCAGAGAGGGAAGGAATAGGGTTAGTAACAAGAGAATCTGAGACAAAGGC

A_16_P03661839

ACATTTTTCAAAGACCATGGAAATAAGACACAATCAATACACAGTTTGAAAGTCCTTCCC

A_14_P102666 TATTTGTTAAACAAGTAGTGTTGTGTGAGCCTTGGTGAAGACGCTCAGTCGTTTTAAATA

CUST_6050_PI426210822 AATGTGCCCTCCTTTCTTGTTCAAGCCAGGCAAGTGCTAGGCACT
A_16_P17834869
ATGCACTTTCCTAGATTCCTCAGTTAATTCAAGATTACACCCAAGCAGAAGATTAACACT
A_16_P41849842
TTGAACCCCATCTATAATTCTCAGTTTAAGAATGCTAGTTTTGGAGATAAGAATGTGCCC
A_16_P15850820
AAAAAGAGCACATTACGTATTCAAGACCTGGCACAAATTCATTAGTGTGATTTACCTTGC
A_16_P39482996
TCTCTGAACATCAATTTAATCTGCAATCAATCCCTAACAATTTGTCCATGAGTCAGAAGC
CUST_6147_PI426210822 GTGAATAGGAACTCATCAGAAACCTGGAAAATGTTGTCAGAACATTAGCCGTG
A_14_P119276 ATTGATTTTCTCAAACCTGCACTTTGCAGATAATGGCCACCTAGATCAAAAAGATAAGTT
A_14_P106073 AAATGATACTGATGGCAGACAGTTGGAAATTAATAATTTTCCTGATCTGCACTCAGGTTT
CUST_1544_PI426210822
TCCCTCTAGATTCTAAACTTATCTCTTAAGAAGGTTATTCCTTATGTATACTGCAAGTTG
A_14_P104976 CTTTATCAAAACTGAGGAAACTATATTTCTGCTTCTGCTGATATCTAAAACTGGGCCTGA
CUST_2957_PI426210822 CTTACCTCATGGCCGGCTGTGGGCAACTTGGACAGCCTGATCCAC
A_14_P118261 GGGAACCAGTGGTTCCCAATTTTTTTAACATCAAAAAGAAGGATCCCATATTTTGAAAAA
A_14_P129423 GGGCCAATGCTAAGTTACAATGCCAAATTTTATTTTAGTTGGTAATGACTTGGACCATTT
A_14_P117683 GTTTCTGCCTCAACCACGGACCAATAATATGAGATCTTTGTTCACCAGTTCTAC
A_14_P127735 AGCAAAACTGAACCTTAACAAAATTGCTGTTGTCATGTTCAACTATAGGTTGTTTCTGCA
A_14_P109520 ACTGTGAAGTTCCTAAACATGTCTCTTCCACTCTTTGTCTAAACTTTGTAACGTAGATGC
CUST_4301_PI426210822
TAATAAGAAAGGGGTTAAATAAATTGTATGTTGAGCACCTAGAAAACATATGCATGTGCA
A_14_P133193 TATATCCAACACTGCAAAGCATTTTTATGGACAACGACCACAGGAATCTGGAATTTTATT
A_16_P00926382
AGAGAGAATTTGCTGACATTAATGTGTGAATGGCATCTATACTGGACACTGCCTTAAGAA
A_18_P14136196
AATGATAGAAACCAAATGGAATGATGCTGGGTCATTTACTACTTAGGGTCAGTTTTTCTG
A_14_P134168 ACAAAACAATCAAAACTGCTCATAGACCATACTCTAGGACTTTGTTCCAAAGACAACAAT
CUST_7556_PI426210822 GTCTAGGTAAGAGGAGATTGGAGCTGACCTGTTGAAGCAACAAAGAGA
A_14_P126190 TGTAGACGATGGTATTTTGAAACCTCACTAAAGTGAAATTCATGTGAAAATTGCAGGCAA
A_14_P118805 GGCATTTTGCAAATGTAGTTGGTTTAGTTTTGCTAGTTAACAGTCAGATAAAGTAGGCCT
A_16_P58424571 TTGCGTAGCACTGCAGCAGTTGTTTTCCTCATTTGTCTTGCAAGTGAATATTCAT
A_18_P15124120
ATACCTTTGTCAGAAAACATTGCTAACATCCAACTTGTTGATCAACACTTTCTCTCTCAC
CUST_6378_PI426210822 GTGGTAAGCTTTGAACAGAAGAAAGCGTATCATAGGGGATGTTGAGCTTGT
A_14_P133027 TACTTTGGGATTCAAACTCCTCTTAGACTTCTCAGGATTTTGAATGACTGGACTATAGAT
A_14_P107017 CTACCTTGACATAATCATAGAAGCAGCCTTCAGAAGGCTCCAGGTCAAAC

A_14_P201499 GGGACTTTCTAACATGACCCAGTTGTCTTGGTGCAAGACTTTATTCTAACAATAAATAAC

A_14_P121457 CTTCTGTGTTATTTCTGACTTCTTAACACTATTATGTTTATGTTGCACATTACTGAAAGA

CUST_4105_PI426210822

TATTTGCTTTTTAAAAAATAATGTAGTTTTAAAGAATTCTTGCTATTTAATCTCAAGCCC

A_14_P109187 TTCAGGTTACCTGCAAGGAATATGGTATTTAGGGTAATGAGTGATACTTAACCCAAACTT

A_16_P17823253

AAATCCCAAACACTCAAACAAATTTGATATCCTTCAACTGAAACCAAGTTCTGTGACATC

A_14_P112007 ATCTCCACAGTTGTTAGAAAAACATTAAAATCCATGCGCCGGGCTCTCATTTC

A_16_P16102672

TCTCATTAAGGTTCTCTTTCTTTAATACATTGTGCAGCCTAATAGTTATCAGCAGCCCTG

A_14_P127669 ACTTACATTGGCCCTTGATACTTGAGTCTGTGGATCTATCAACCTGAAATTTTAAAAGAT

A_14_P113991 TGAAAAGAGCCTTTTGTTTTTTCTGACACAGAAGTCTTTTTACCAATGACAACCTTAAGT

A_14_P200084 TTCAGCCAAGAGGATGATAGAGAAGTAGACAAACTCTACTGCTTTCCACTTGATATTGT

A_14_P119561 TCCAGATGAAGATTTAAAGACTAACTGGTCTAAAACAAAGCCATACAACTGCATTTCCTT

A_14_P101036 CTGGAATTATTGTGCATGTCTCTCTCTAGGTATACAGCCTTGCTTTTTGTGAATTCAATT

A_14_P116197 ACAGCAAGATTTTAAAGATATGACAAACTACACATTCTACAGTTGCTGCCAGCAGAGAAA

A_14_P114783 TCTACACAAAAACGATCAAAATACTCTGGAAAACGTCTTGGTTTCTGTCTCAGAATTGTA

A_14_P124886 GAAAAGAAGTGGAAATTCTATGGCTTTAAGGCATTGAATACCTGGCTTCTTACCATTAGA

A_16_P15584710

AACATGTAACTCCTTAGTGATTAGACTGCCCATGAAAATAGACACATATCCTAGCCTCTA

A_14_P107805 AAGTTTGGTCTCTGCCTTTGCCAGATTTTGCCACATCTGGAACAAG

A_14_P109307 CTTTAGTTGGAATAGACATCCTGTAAAATGGATCTGGCCTGATCTTTAGATTGTAAATGG

A_14_P138197 CGCATGACCCATTTAGCACAGAAGTTTTTCCCCAAGGCTAAGGAT

A_14_P127983 CTAGACAGAAAGTCTGAGGTGTATTTGTGTTGGATTTAGTAAGTCATCTGTGAGATTCTG

A_18_P13981976

CTGCAACAATTCTATTACACTGATGTTTTTTGAGCAACATACCATTTTTTACTGCCTCCC

A_14_P137315 AGAAAAACCTTGTTTCTTTTCTCTTACAAAATCTTTTCTAAGGACGTAGGGAATGAACGC

CUST_9590_PI426210822

CTTTCTTGTCTTCAGGAGGGAAGATTTTGACTTCAAAAGTAACAAAATATTTAAGAAGGG

A_16_P20310423

AACTTCAAACACAGTCCATTTTGTTAAGTCTCAAGAAACAGTAGAAAAATGTCCCAGTCA

A_14_P131639 TCTAAGAAAACAATAATTACCCCTGTGGGATCGTCATGATTGCCATGAATACTAAACACT

CUST_4206_PI426210822 GAAAAATCACTGTTGGCCCCACCCTCTAGCATTGGAAGCTTAACA

A_16_P18155585

GCAGATACGGTGAATTTTAAATGCTTCATGGTAAAGAGAATAGAGTGGAAAGTGAGATGC

A_14_P121540 GAAGAGGGTGGCATATAAAGCAGGGAGTAGTTACTATAATCACACACAAAAATAGATACC

A_14_P139859 CTAACAACTCCTTATTATGCTGATCTATAAATATAAACCCTTCCTGGCTGTTTGGAGAGG

A_18_P22840490

TTAAAATTATCAGACGAGATGGTTCCCTTGTGTTGGGCATAAACCATGTGCTTTATAGAA

A_14_P118442 CTCATCAAAACAGAACATGGCAGTTTTAAGTTGATAGGGCTGATGCCGCATAAAATC

A_14_P101031 TTGATCTTCACTATTTCCAGAAGCCTTGAAACCTAGGCAACAGTTTCAATATTTTCTAGT

A_16_P38774632

AAAGCCAAACCAGACCTTAATTTCTACCTGAGTATGAACCACCTTCAAATTTTCAAGAAT

A_14_P139838 GGAGGCTAAAGGTCTTTGGTCATTACTAATAACACTCTTTCAGTATTAACCTGTATGTCC

CUST_1951_PI426210822

TCTTTCAAGCATCAGGTAAAAAGGTGAGGTTCTTGGTATAAGTGACTATTTTTTATTTGA

CUST_9969_PI426210822

GTGAAAACAGCCTCTTAGAGTATTATAGATGGATATTTTAAAATAAGACAACTAATTTCT

A_14_P134996 TACATGCTGTTTATGTTCAGAAGACAGAGAGGCTGGCATTTATCCTAGGACTAAAATAAG

CUST_8077_PI426210822 GAGGGGCGTTCAGGTGGGTGAACAGGGAGACACAAGCTTTGATTG

A_16_P01891118

TACTGTTGCTACCCTACTTAAATGAATCTCCAATGCTTCCTAATTGTTACTTCTTGGCTT

A_14_P118302 GGTAAATGTAGACCTAGAAGCAGTTAGACAACTCTAGTTATTAAGTCAGGTGTGGTTGTC

A_14_P119144 TTTTATTCTGTTCAACAGTGAGGAAAGAGAAGATCATAATCAGCCTGAGATCAAACTGAC

A_14_P110072 CTTCAGTTTTCTAGATGGGAAATATCGAGACAGTTATACAAAAGCAATGACAAGGAGGAT

A_18_P10821762

TCCTGGCTACTTCATAGACTCTTTCCACCAAAATTTACTTTTCTCACACTTGAACACAA

A_14_P117218 GGTCCTGACCACATAAAGCATATATGTTAGACAATAGTTTTACCTCTCTTCTGGCACAAA

A_14_P126403 GCCGTGGACAATTTTCCTATTCCTATTATATTATTTTCACACCAATATGGACACACCCAA

A_14_P110638 GGGGATTCAGGGGTGGGGAAGACAACATTTCTTTATAGATACACAGATAATAAATTCAA

A_14_P129974 AAGAATCCTGTCCAAGGCTTCCTCTTCCTCTGAGGAAACTACATTTCTTTTTC

CUST_9542_PI426210822

AAAAAACACACTACTAGGATATGAAAAACTTCATTCTGGGCCTGGTTAGGTCATAAACTA

A_16_P02499269

AACAAAAGTTGGAGTTGAGAATATGTCAGAGTTTGAGATCTAATGAACATCTTCACAGGG

A_16_P16852183

TGCTTCTCAAGATACAAATCTCTCTCACGTATCCTAAAAGTCATAATGCCAATAAACCCT

A_16_P16827120

ACCACTACCAATTACTGAAATGTTACAACTACCACTTTCTATTCAAGTCTTGTGTTCCAC

A_16_P16828540

AACAACACTGAGTGAAGATTTTTGCATAGAAAACATTTCATCAATCTGACCTATTGGCCA

CUST_1778_PI426210822

AGCAAATAGAAAGATAAGAACTAGAAATTATCATGACAGATGGCAACCTAATGCTGAAGG

A_14_P127005

AAAAAAGGTTGTGTAAGAATAAAGTGGCCCAGAGAATGATCATGAATGCTAAAACACAGA

A_14_P104313 TCGACGAACTAACCTATGCTTTAAATTCCTCTTTCCAGCCCCGTGAGT

A_14_P202315 CACGTTCAAATTAACCTCTCCTGTTCTTTTCCTTATGTTATAGGGTGATCGCACAAC
CUST_6447_PI426210822 CAGAATGTGGATCCCAAGGGTTGGGTAGGGTTTGTCCAGAGCCTT
A_16_P15158645
TCACTATAGACCTGGTAAACAATCAATTACAATCCCATCTGATATAGTTGTGACCCTGTC
A_16_P00369637
ATTGTCATCCAAAGCACTTCAATAAAATCTCCTTTTGTATTTACCTCCTGATAACCGCTC
A_14_P116630 CTGTGTTGTTGAAAACATACTTCTGTGTTTACACTTCAATAGCCAGATTGGGAGTGTTTT
A_14_P117976 AATTATATGCTTTAGATCTCCATTCAGGTCTTCAGGTCACCAATACATTAAGCAGAGCAG
A_14_P101710
AATCTTGAAGAAACTCTGCAACTAGAAATAGAAAACTGCAAGGAGGAAATAGGTGAAGAT
A_14_P113880 GATGTCCCATTACTGCCCTCCTTGGATTATGAGAAACTGAAGAAGGATTTGATTTT
A_16_P17260942
AAACACACAAAAAGTGGATCTTGACGAATTTCCTAAACATTTTTCTCATGCTTGGGTAAC
CUST_175_PI426210822
TGTTTTATTGATTTACTAAAATTACACAGTTGTTCGGGCCTGGGAGTTAAATTCTCTTCA
A_14_P119077 ACACAGTCGCATAGTGTATCACTTTGAGAAGTCTACAAGTATAAACTAAGGTGATGCCAA
A_16_P01690805
TCCCTTCTACCCTAATACCAAAAATAAACAAAAATCTTCTCTAAATAAGACTCACTTGGA
A_14_P129978 CTACTTCTCTGTGCTTCAATTAATCCTCTTAAAAAGATCCACGCTGAGAACTAACTATGC
A_14_P117379 CTTCAAATGTGAAATAGTTCAATAACACAATGACACTTTGCCAGTGTATTAATCTGATAT
A_14_P125118 CCAAAACTAGAAGGGAAACATGACCACCACCTGACTTGTTGCAGAG
A_14_P137750 CCACCACGAATTTAGAGATTATTACTTTGTAACAATTTCTCACAAATGTCAAGTCTTCAA
A_14_P118343 GAAATATTCTGTCTCCATCTCCACTTGCTAAAGTTGTTTCCATCCTTCAAGACTCAAATA
A_14_P202189 CTGTTTACAGAACAGTGGAAGAGACTGCAGCCTAAAGACTTTTAAAATTAACTTGGCATC
A_18_P22841932 GTCTGCTACATGAATAGGCGTCAAAAGTTGGTGGTGAGGAGTTTGCTTAC
A_14_P120269 AAAAATATACTTCACTGCCCTGAAAAACAGACACAGGGAGAGTTGGTTGTCTCTT
A_16_P40051869
AACAGCCAATTCATAATCATGTGTATACTTCGCAAGTCAACATATTCATCTCAATGGTCC
A_14_P132980 ACATCTATTTGTGAGAGACCTCTTGAAATTGTTTAAATGGTTACATCAAGAGTTTAATAT
A_18_P22839941 GGCCTAGGCTGTGGAAGGTGTGGAATGTGAAACTGTCTAATCCAATAA
CUST_9890_PI426210822 ATGGACATTCCAAAGAGATGGCAAAGCTTGGAGAAAGACCTGTGG
A_16_P18513128
AGAGCCTTTCCATGTAAATACTTGCCCTAATAACGTATCTTGTAGGTTCAATCTTTTGCA
CUST_2619_PI426210822 AAGTCCCTTACCCTCTTGGAAGAGATCAGAGGTGGTCACCCTCAG
A_16_P00685516
CACCAAGCAGCATGTCATTCCTTCCTCTCTCCTACTTATCCTTTTTAAACATTTAGTTTT
CUST_8278_PI426210822
ATATAAGCAACATTGAAAGCATTGCTGCAAACTGAGCTTGAATTCTTCTCACCTCTTATG

A_14_P133456 TTTCTTTCCTGAGCATTGCCCTTTTCTCAGGAGAATGCATTTTTGTTCAAGATTTTCTT

A_16_P18890159

AAAATAAACAGCATGGTCAATAAAACTGGTGGTGTTATTTAAGTCCGTCTGTTATCTCCC

A_14_P105185 TTTTCACTCTTCTGCAATTTAGGACATTAACCCATATGTCAAGGTCAATTGCATGCAATT

A_16_P03368551

AGCACCAAGATTTCTTTTAAATTAGTCCCTTCATTGAAACTCATACTCTTCCACAGTCTG

A_16_P18525794

CTGAAGAAGCCAACACTAGTTCTATAGAGCCAAATTTTATCTCAAACTCTACTTCCCAGT

A_14_P122734

TATAAAGCAAGCATAAGAAATCATGAGCCAAAAAACAAGAGTCAAAAAGGCATGGGAAGT

A_14_P108006 TGGATTTTCGAAGACTAATATTCCTAACCCCATTTAGAAGCCAGATCTGACAAGTAAACA

A_16_P02133219

ATACACATGAATCTATTGAGCCCTATTCTGTATGTGATGCTAAATAATCCTCCTAGCCCT

A_16_P21560685

AACTTGACAGATTCCATTTTTCTATGCGTATCCAAACCACATAACATAAGCAAACAGTGG

A_14_P125512 AATGTTGGTGGTAAATATGTAAAGTTACAAATATGGGATACAGCAGGACAAGAACGATTC

A_16_P21684384 AAACAGTGCCATCACGACCCACTTCTACAACCAAGTTACCAAAATTCACAAAAC

A_16_P20972423

GGGTGCATCTGCCACTTAAAAATACATTCCAGAGAATTCACTGTGTATAACCATCATTTA

A_18_P14666189

GGCATACACTTTGTATTTAACCAAGACAATCTTCAGACTCAATTCCTATTACCAGCCATC

A_16_P20281522

TTGCAGTTTTTCTAAGATTCATACCTATAGATTTACTTATACACCTGCTCCAAACTACCT

A_14_P114317 TAATTTGTTTTGCTTGCTTTACTCACTTTCATTTGGAAAGAAAGGGACCCTTTTGTAGAC

A_14_P200297 TCTCTTCCTATGACTTCAATGTATGTCCTCTCATTACAACAGATGTTCTTAGATGGGAAT

A_16_P15532849

ACAAGTCCTCGACATTAAAACTACAGGTAAAGATCACAGACTACTTCATGTTTGGACATT

A_14_P117239 CTAGTCGAGCATTATTCTTATAAAGCAGATGGTTTGTTAAGAGTTCTTACTGTCCCATGT

A_16_P36230676

GAATTGTGCTTTTCTTATATTAAGATGTAAAATTCAAGGCTCAGTATAGCACCTGGTGGC

CUST_2173_PI426210822 GGGATTGTTAGAGCCTTATGTTGCGTCTGAGGTTTTCAGGCTCTTC

A_16_P16191582 AGGGGCTCAGGGGAGGAATTCCCACTGATTTGCAAGAAAGGAATT

A_16_P00152842

TGCTTTATTGTTGCTTAATTCCTTATTGTCCTTACATGGAAAACTATACACTCGTGGAGG

A_16_P02445562

GAGTGATACTTGTTGAACACATAGGAAATTTCTAGCCACTTGAATATGCATATTACTCCA

A_14_P120182 ACTCATCTTTCAACCAACAAAACATCAAGATTTCCATCCTCCATACATGCCTTTTTTAAA

A_14_P107684 ACATACTGAAACCTATAGAAAATAAGACACCCACACATCTGTCACATAGATGTAGCAGAG

A_14_P116757 ACATAGCCAGATTTCATTGAAACTTCTGTGGAGAAGGTTTACTGTGTAGATTTTTTGGTT
A_14_P108266 ACACACATTCACTCATATTCTAAGAAATTGTAGAGGCTCTGGATGATTTTACCTTCCAAC
A_16_P15114793
ACAGTTACAGAGTTACCAATATTTCTCCAGCAGAATTTCTAGACATAGTGAAACTCTCCT
CUST_903_PI426210822 AATGTCTCAGTGACCAAAGGCATTATGTGGACCATGGACCCAGCA
A_14_P139033 GCTTTTTCTGTTTTAGTTGTTTGATCATGTGAAGACTGGAATTGAAGATGTTTGTGGACA
CUST_10_PI426210822 CCCCCAGGATGCTTAGTCATATCCAAAATGTCAATCCCTGTCCTTT
A_14_P101622 ATCCCTTCAGAGCCTTAAAGAATTAATGACCTTCCTCTATAGGTTCTAAGGTAAAAATTG
CUST_9993_PI426210822 CTGCCTCTCAGGGGTGTCCTGTCTCCTCTGCACAGCCCCTGCCTC
A_14_P116692 GGGATGCTTAGTCATGATGTTTTGGTCTAGTGGATAATCTACAAAGAGGGTGGTAA
A_14_P108220 ACCAGTCATTAGGGTATAATAATTGGGATACGAGAGACTAGGGAAGTCTGGAGTC
A_14_P104704 CTGTGGTATGACAAAAACTGTGCCCCAAGTTTATTCTGATATATACCTGAATGAGACTAG
A_14_P130133 GTAAGTGTAAGCAAACTCTCCTATGAACACTCGCTCAAACCAGCCTTTCAGAAT
A_14_P117176 GAAAAATCATTTATTTTTGGATTCTAAACCCTCTCTGAATGCAACAGAACAATGCAACCC
A_14_P132205 TCTATGTTTGCAACACAGTGAGTGCTCATCATTATGAGAAGCAAATTTCAGTAAGTGAGA
A_16_P01793329
GAAATGCCCTAACCATTCATCCATGTTCTATATAGTTCATGTCAACCTGATTCCCTTTTA
A_16_P01144042
GCATTGCATCTTCCAGAATATTCTTTCAGTCTTCACAATATAGCAATGACTATTTCCCCG
CUST_9568_PI426210822
AGTCTCTTTCCCTAACAAGAAAATGTCAGCCTTACGAGTCAGGAAAGCTTTTAC
A_18_P14185250
ACGATACAGACTTATATGAAAAGCAATATCTTCCCAAGTTCCTATCATTGTATTCATGGC
A_14_P101304 CGTTTGCTTTCTTTGTATGATAATCAAATTACTACAGTTGCACCAGGGGCATTTGATACT
A_16_P01567929
TAAGAACCCTACTTATAAGCCCTATTCATTCTTACCAGGATAACCTTTGTCTCCTCAAAG
A_14_P200198 CCAACATTTCACCCAAAGGATCAACCAATTTTACACCATTGTCTTCCTTAAAAGAAAAAC
A_16_P18444908
AAGCCCACTCACATTAATGTCTTTCCTATTTACCTTCATATTGAATGCTTCTACTTTCCC
A_18_P15932489
GTCTCCTTCAGTTTTAATATGATCCCAAATGACTCATACCATCTAGACAACACATGGAAC
CUST_1957_PI426210822 TGTGTTTATATGGCAGTGCTGTCTGCTTGTTGGTGTCTTTACCTTCTCAG
A_18_P12658345
CATTTCAATCCAACCTAACAAGATATTTTCCTCCTCATACTACTGATACAAACTGCAGGA
A_16_P37085224
AACCCTTACATGCATGTTACTTGGTAAAACAAACTGAGATGTTTGATGCTTTCTTTGAGA
A_14_P122283 GCAATTCTATCTCAAAACTCACCTGTAGTTCCACTTGTGAAACAAATTACTGCAAGATCT
A_14_P138289 AGTAAATATTAGATAAAAGCCAACAATGTGGAAGGAAGATGTCCAACTCAGTGACTGAAG

A_14_P128832 TTTAAGCCAATTGGTGGGGAGATTGAAGTAACCCCTTCTGCTCTAAAACTAG

A_14_P104808 GGGGACAAGGCCGTTTCTTTGTTTCTGAGCATAAAGAAGAAAATAAATCTTTTACTAAGC

A_14_P120677 GCTCCGATTCGACTCTCAGACCAAGAGCATCTTCGAGCAAGAAACTAATAATAAT

A_14_P100078 GTAGCAACCCTAGTTCATGGGTCCATCAGACATATCATCAGCCTATTTCTATATTATCAG

A_14_P135379 TTTTAAATTTTAGGGATTGAGTGTTGCTATAGTGGGACCCACGTTTCAAGATTTGGC

A_16_P01208794

TTTCAGAGGAAAATTGACAAACCAAACTAAGTAGCAGTAATTCATCCTACTACCAACAGC

A_14_P114110 GATGATAGCAGTAAAGGATGCACATCGTCATGATAGGTAAGTTGTCCCACTTGATTTATA

A_16_P02426765

AGACGATAATCTGATTGATTTTGCTGAATATCACCTATCTCATTTGTAACGTCATCCTCA

A_18_P11105413

AAAACATCTGCTGAAATAGCCATACTATGAAATTCCACCAGGATGTTTCAATAACACTGG

A_14_P118202 TTAACTCAGCATATCCCACACGTGAATCTGAGAACACCATTTATGTAGACGCTAACA

A_14_P126451 CTGGAGGTTACCTTTGCAACCAAGAGTGTTATCATTTCTTTAACAGTTTCTTCAATTAGT

A_14_P110955 CTAATGACCATAATCATAGTAAGCAGTGGCATGACACAGATATCAAAAAGTTGAGTGGCA

A_14_P124625 TTGCATGTCACTCTTTAGCAAGTTTAGCATCTTAGGCTCACAAATAGGTTAACTTGTGTT

CUST_9961_PI426210822 TATTCATCAGTAATGGTATTGGAGGCTTTAGCTGATGAAGGTCTGTCTCTTCCTT

A_14_P110782 GTTTCAGATTGGGAGACTCCATCTAAAACCAAGTGGTCACTTCTTATGGAAGATATTTTT

A_16_P37750408

TGCTCCAACTCATCAAATATCTAACTCCTGCAATGTGTATTGTCTTATGCTTGACATCAA

CUST_9353_PI426210822

CAAGATTATTATTATTATTCAAGATTGAAAATACAGCATATCCTGATACATAAAACCCCA

A_14_P100688 CTGGTCACCTTGTCTACCTGCCACAGCCCTATTGTGAATAGACTCTAATAG

A_18_P11094568

ACCAATCATTACACCATTTTAAGACATCCTCATTTTTTTTCTTATCTTCATCCCTGTTGT

CUST_3176_PI426210822 AAATGGCAGAGTGCACCCGTAGGATGAGTGCTAATGGCCTAGCTA

A_14_P121127 GTTGGTTATTATTCATTGGCTGCTAACCCAGGAATTTCCCCAGCACTTCT

A_14_P118615 TTGGATTTTTAAACTTAGAAAGGACTTTTAAAAGAAGTCTTCCCACTTCCAACCCAGAGA

A_14_P133457 CATCTGAACCTCGACGCAATCGATCAGAATCTGAATCTTTAATGGGTGTTGAT

A_16_P19328183 CTTATGCTCCTCTTAAACTCATATTAACCTTCTGGCTCTATTTCATATCTGTCCTTCCCA

A_14_P110301 ACCTTTTAAAAGCAGGAGTCAGTTGTAAAGTAACCAACCCAAAGTGGAATGAATATATGT

A_14_P104430 CTCATCATTTCTCCAAACTGACATGCTACAGAAATGTCTTCCAAAAAGCGTTAAGTTTTC

A_18_P17347909

TGTTCCTGACTCCTAACTCTAATAAATGTTGTTAACGCATTATAAATTATGAGGATAAAC

A_16_P00931357

AAAAACTGCACTTCATATTACAATAGGCTAACAATTCAACATATACCCCATTCCAAGACT

A_18_P22843230 CTTGTCAAATGGACAAGGATGGAAGGGCATTTCAGGAGGCAAGAG

A_14_P108872 CTTTTGGAATGGCTGTTGCATTGACAAGGTTTACAATTGAAAGCGTAAACTTGATCAC

A_16_P17500067
AAGCATAAGAACCACACTAACTATTGCTAGCATCCCTATTTTGGTCATCTGTTCATTCAA
A_14_P116327 CTTTCAAATGCAAAATAAATTAATTTTTTTGTTCTCAGAGGGAAACCCCCAACTCCTCAC
CUST_6342_PI426210822
GGGACATGGACGTGCTATTTTTTTTAAAGTAATACATATTTACCTTAATTATCTGAATGT
A_14_P136059
GGTGTGAGTCTAATAACCAAAAGAAATAGTGGTGGTAATAAAACAAGGGAACAGATCTGA
A_14_P118928 TTGGCACCTAACAAATTTTTTATGGCCATTCCCTTTATCAGGTGTGTTCATATTTCTTGA
A_16_P17354356
GACCGAAAAGGACTTTATCTTTACTCATCCCTACCAGATTAGATAATCACGTGGAAAACT
CUST_6308_PI426210822
GAAAAGATTCAAAATCTAGGAGATTCATAATTTCAATATATTCCATTGCTTGTGACTATT
CUST_6475_PI426210822
AAACTTGGTTAAATTCATGGGCCTAGAAGATCAACATCAAATCTTTGTCTTCAAGACCT
A_16_P16996580
TTGTGTCTTACTTCTTTTTACTTATCCCATGATTGTCACAATAAAGGTTTGCTCACATCA
A_18_P20176881
ACTTGATGTATATACTGCCCTTATGCTAAAAAAAAACCTTGAATAATCATAATCGCACCTG
A_14_P103649 ACTCTGGACAAAAGTGAGAGTTTGTAGGTCAATGTTGGGAAAATTTTTAAGCAGAATACA
A_14_P139016 TTAGGTGGTAAACAACTGTTGGCTTAAGTAACTCATCCAAGGCTTCATGGTTAATAATTC
A_16_P01292590
AAGTCACCTGAAAACAAGAGAAACAGATATCACTTTGCACAATACTATTGACCTATCCAA
A_14_P137900 TGACTCACGGTGAATTCACTGTGGAAAAGGGTCTTAAACAAATTGACGAATATTTTTCG
A_16_P15248988
AAGTTACCTTTTTTCGAAGTTCTCATCTGCACTATCATAGACCTTAAGACAACGAAGCAA
A_14_P131271 CTGGGTGAACTTCATCTTTTATTTTTTCTGGGATATCTTCTCCCATAACCTATAACAGGA
A_16_P17218125
AAACAAAACTCACGTGTATTTTATCCTTTCAAACATTCGAACAATCTCATGGTCACAGGT
A_16_P17929463
AGAATGTGGTCTTCCTCAACTTACGGTAGGAAAAGTCAAGAATAACTTAAAGGCTGATAA
A_14_P201198 TACAAAATAAGAGAATGAGTGGGTCCGAATTTTAGACAGTATTTTGAAATAATGGGTAGG
A_14_P115538 CTGCATCAAGGGCCAGTTATCCTGCAGCAGAAGAATTATTGTTATTGTTACTATTTGTTT
A_14_P122495 TTAAGGGGGAGACTATTCTGGAGAATATAGAGTTTTTGGAGGATACGGATATGAAATCTC
A_14_P121391 GGAAAAGAAAATCTCACGTGTTTGTTCCTTGTGACTTCAGTGAAGTGGAGATATAAATAT
A_16_P17024320
AAATATTTGTGTATCCCTGATCAAACAGACATCTAATGATAGCAGTCATCTCTGGATGGC
A_14_P112399 ACAGGATTCTGGGAATTAGTGACTGTAAAGAGAAAAGGAGTAGGAAAGATACTTAGGTAT
A_16_P16854071

CACACCATTTCCACTTTCATTCTGAACTCTTAACTGACACATTTACAGAACCTCTTACAA

A_16_P38265622

AAAACCTGAAATGCCATATTTATCACAACCTTAGATAGTCACTGTAAGTCAAGGTTCCTC

A_14_P120306 CTATCTTAGGGACCTTAACATTCTGATCATCTTTATCTTGCTCAACTCTTCCCCATACTA

A_14_P114975 ACTTACAATTAGATAGAGTATTTGTAATTTTGTTATTAAAAACCTTCAGCAGCCTCACTG

A_16_P03331570

AACCCCTCCATGAATTGATCAATTACTGAAACTCCACAAAATAAGATGAATCCGTTTACA

A_14_P102473 ATGTGTCCTGTTCAAATTTTTTAATGTCCTCCTTGACAAGAATCATCTGTTCTTTCAGGA

A_16_P03474400

CGACCTTTCCTTAACTGTAACAATCTACTTTCCCTTAATAACCAGAACTCATCCACTTTT

A_16_P16731227

CATAGATGCCAAATGACCTAACTGAACATTGACTCCTAAACATATATTGAGACCAAAGGC

A_14_P122780 CATGGTTAGACGTAACATCTTGTGTGTTTTTCTTTAAATCAGAGAGGAGAAAGAAAGGAC

A_14_P102656 CTATAAAAAAATTGAAGCAGATGATTGCTGAAAACCTTAGAATCTTCTGGACCAACCTGA

A_14_P106519 AAAGAACAGCAGTGTTTTCAAATAATGCTGGATAAGGCTTTGTTTTTTAAAGGTAGTGGA

A_14_P135486 TTCCTGTTGATAATTTTTGGTCCCATCATCTCTGTATTTTGGTTTGCAAGTTTAGATCAC

A_14_P102509 TTTAATCTTCCCTCTACCTCTTTCCATCTATGACTCAGTGTTCAGGAATTTGCTTGTTAA

A_14_P116509 GTAAATATTGAGAAAGTTAAAAGAATAAAGACATTTCTTTTGGAGTTTCCATATCTTGGG

A_16_P02910911

GAGGTTTTGCTGGGATATTTTGCTACACTACTATCATTGCAACAAGTATACCTAAGGGAT

A_14_P102909 CTCGAAAATCACAACCAAGAAGAAAGGCAACCACTGAATACAGGAAATACAGTATTGTTA

A_16_P01430857

GGCTACTGTGAATTTTCCGTTTCTAGGATCCATAGAACTAGCACAAGTTACTAAAGTCAT

A_14_P109772 TTATGTCACAACAACAGAAAAGACTCCCAAAATTTAGGTTTATTTGGGGGGAAGGTAATA

A_14_P122471 CAGTGTTGTTCAAATTGTGTATGTTCTGGAAAGGAGTCACTTTGTCTACTTGAACTTTAG

A_16_P03364595

AAATCATCCAAATGCAAGTGTTTCCTCGTATTCGAATCTCACATTGACTAGTGTGTGTTG

A_16_P19073414

ACCCTACACATCAGAAAAAATGGCATCCTTTCATCTACTGTTTACTTTGTCTGAAGATTG

A_14_P119365 CTAGAGGCCTTATATTACCACTAGATACTAGGGATAGATTGGTGGTAGTGAAAGACAGAA

CUST_2835_PI426210822

TGGACTCTGTTAAAAAACATTTTTAAAGAATGAAGATTAATTTTGATACACATAAGAATG

A_16_P30952527

GTATGTGATTTCAAAGAATCTTAGAATATATATTGCATGACAGGAAATGGAGGTCTGCTA

CUST_8429_PI426210822 ATTCTCTCTCTAGTGCCCAGGCCACCAAGCAGCATGTCATTCCTT

A_14_P110605 AATAATGTTTATGTTTATCACATGCCTTGCACTGTGAACACAAAGATGAACAAGACTCAG

A_16_P03665750

TTGATGCTATCCTCAGAACAAATTTTCCAACAGATGATAAGTCAATGAGAACACACTGAG

A_16_P16191167 TTATGGTGTCCTTCAAAAGAATGGCATTTAGTGCTGTCCAACTTCAAATCCCAAA

A_14_P121691 ATGAGATGACACCAGTTCTGATTCTTACATTTAATGGAAATGCCAGAGAGACTTCATTAC

A_14_P124445 GACAGAACTGTTTTACTTATGGAACCAAATGTTGAAAAAGGCTTTGGTAGTAAAAGTGGT

A_14_P111996 TCAATCCAACACTCTCTCTCAATCCATAACCTATGAGCATAGCCTTGGAGAAATAAAAAA

A_16_P00685625

TAGAGAATCCTATTTCCCTGTTACATCCTTCCACAGTTTCATGATTACATTCTCCTTCAT

A_14_P110821 TGGTCAAAGGATCTTTCAGCAACACTCTGGAACTTCATATGAATGTAACAAAATTTCAGT

A_14_P101019 TTCCAGATTAATGTGTTTTCCCCCTTCAGGTCATCACAAGACTAGTCATTTCATTAAATT

A_18_P26984465

TCAGTCCCTCATCAAATTACAGCACATTTTCAATGCTCCAATTATGTCACTGTAGAAATG

A_14_P120445 CTGATTATGTAGATAACAGAAGGACAATTTCCTTCACAAACAGAAAAACCTGCACCAATG

A_16_P20234310

CACTCAGTCAATTTTCCTTACAAAACAGACTTTGATAATGATTTGCCCATTAGACTTCCC

A_14_P118341 ACTAGCACACTACCGGAAAGACAATACCTCCTTTAGTATTCATTATTAAAACACTGCCAT

A_16_P21132554

TGCCTGTGATTACCTATACATTAACTCCTGAAGAATCAATTATAATCTCAGCAACCTTGC

A_14_P120855 CTTAAAGTCAGTGTGCATACATATCATCATTCAAGGTTAAGTAATCCCAGTTCAACAACC

A_16_P00510617

TAAAATGTCTCAAAAGTTTACATGCACTCAAAGTAGGTTAGAATAAGGAATCCCTGGAGG

A_16_P36214511

AATTTCTCTGGTAATGACAATCCTTTTTTCCACTTACCCATAGTCAAAGACTTGCATCAA

A_14_P109137 CTTCACTCCTTCTCATGGAAGACTTTTTGTCTGAGCATTGATAGACACTTGGTAAC

CUST_5149_PI426210822

ACCTGTCATAAAATGTAATGGATGATATATTTTTAACTGCTGAGACCAGAGAGTTCACAT

A_16_P15614018

ATCTGAATGACAGCAAAAACTACATTCGTTAGTGATATGTAGAGCAAGGACAGAATCAGT

A_14_P133427 GATACATCCATATGTTATTGATACATCGTTGCTTTATGTCAGAGAGCAGGGCAGAAGATT

A_14_P138870 ACAAATGTCCTATAGTATGTCCATGGGAAAAAAATGGGTTTGTGGCAAATACTTTTAGGA

A_16_P39189528

ACGTTTGAAGATATCCACTGATATTCATCTAATGGGAGTTATTTTAGTAGAAGTCGGAGT

A_14_P112411 GACACAAGGGAATATCTCTCATCTGCCACACACACATAGAGTAGTTAAACAATATTCAAG

A_16_P02711524

AAAAAAGAAAACTTCGAAGAAACTTACACAGGAATACACAAACTAGCATTTGGGACTCCT

A_16_P00685212

AAAAGAGTGCTCAACTCAAGATTAGGAATCTATTTTTCAACTACCATTAGCCCCACTTTG

A_18_P22840735

TGTTTCATATACATTGATACTATTTAGTTGCATTTTTATATGCTTTAGGAGCAATGGGAA

A_14_P106091 CTAATGAAATTACTTTCATTTCAGAGTTTTGAAGCACCATGTTGTTCCGTGTCATCTGAA

A_14_P103702 GGTAACGACCACTTGTTTTCTAATCTGCCTAATACCTAGAGCCAAATATTTCTACAGTTG

A_14_P125856 CTTTGAAGTAATTTTCAGTTTTATTTGCTCGAAAGAGAGAACACTTGAGTTTTTGCCCTG

CUST_3016_PI426210822 CTAGGAATTTGCCAGCTCGGCATGGCAAGATCCCTGAGATGGCAG

A_14_P134751 CATCATTGTTACAAATTGCAACCGCTCATATTCAGTCCTAAGCAAGTTTTTCTTTTCCTT

A_14_P124605 TTGCCACTGCAAGATGTGAGTCGTTGCTAAGTTTGAGGGTCACATTC

A_14_P138713 AACGTGGAAATCTTGTTTGGTGTATGTTTAGGGGCAAATGTTTTAAAGTTTTGCCTAAAT

A_18_P10065510

CATCCCACAACATATGAACCTTTTGCTCACTTACTTTTTCTACATCTTCTCATCTCCATT

A_16_P18867664

AATCTTCAACCATATCAGAAGACACTGTAACTCTGAACTATACCATACAAAGAAGGGTGG

A_14_P111875 GTCTATAAGAGAAAACCTATCATAAGCCCAATTTTTTTTTCCACTTAGGGTAAATGTTT

A_18_P22842280 CTAGAAGATCAACATCAAATCTTTGTCTTCAAGACCTAAGGGAATCTGGATGTGCC

A_14_P104463 CATTTCGGAGCAAGTAAACATGGCCTTTGTTTTAATATTTGTTAAGTCCCTGCCG

CUST_9598_PI426210822

TACAATGGAAATTTTGCCTTTGGGAATGCTCTTAATATTTATCATATCAAAGTGATGACC

A_16_P18079934

AAAACCCCACACTTTATGATTGGAGTATCAAGTATCAAGACACCTTAACCTAACAATCAC

A_14_P108576 ATACAAATACTTACACGCCTTTTAGGAAATTGATGTAATCCACCCAAATCTAAGAAATAA

A_16_P00850203

AACTGTCTGTATTACCTTTAAACCAACCAATTAATCCATCAACTAACCAGTTTACACCCC

A_14_P125549 TCAAGTGAGAAAATTTCACTTCTACCAATTTTCTGGGGTTTAGTGATCGATGCCAGTA

A_14_P112246 AGCATATGCCCTGACACAGTTAAATGATTAACAGATTACTCCAAATGTCTTTGGCATAGA

A_14_P130493 GACCCAATCATCTTCGCAGTTCTGATTACCTGTGATTAAGAAAATAAATAAGAACACCCA

A_14_P122206 TCTTCTGTTGATGTTTGACTGTTTGTGAATGTTGTCTGCTCTTCATTAGTATCATCTTCA

A_14_P107191 TACTTTGTTATAGGAGAAGAGTCTAATCAATGTGCCTTTATCCTAAGTGTGACTGTGGTG

A_16_P20876160

AATGAACATTCAGAGTGCTCTAAACCCTTAAAATATCGCCATTTATTTTTTCTGTCCCCA

CUST_9339_PI426210822 AGTGGAGCCCACATGGCTCCAGGCCTCCCGAAGGATGTGGAGCAG

A_14_P109573 CTCAGATACTGCAGTTTTTTCTTCCCTGTCTCAAAGCGGTTGTTAGCAAAGAAG

A_16_P03605532

GAGAACACAACTAATTTTAACAATGGCCTTAGGGAAGAGACAAATCTGTCCCTTTATTCC

A_16_P18423349

CCTTCCATTTCACTTCCTATCTCCTCATAGAATAACCTCCCTAAATACAATCTCACCATA

A_14_P115611 GAATCGATAATTTGGAAATAACATATTTTCCTACGGGTGAAAATAGCCAGAGAGCAGGAA

A_14_P108458 GGTCAATCAGTTGGCCCATTATAGTATTCTGGGTGTCTCTTTGAATATTATCAAAGATGT

A_18_P12769179

GCAAATTCAGACTCTAAACTATTCATACACTATCTCAGAAAAATTAACACTTCACAGCCC

A_18_P11814598

AAATACCACACTGAGAGTTAAAGACGATGAAACAAAGTTTAAACCGAACAGAATCTTGGC

A_14_P107868 TCAGGGACATGGATTCTCATTTTATGCGCGTGTTAAGTAGACAAAGACAGATATATATGC

A_14_P139835 GTAGTTATCGTAAGGGGCCATGTAGTTGGAGGACTCGATGTCTGCATATTT

A_14_P137446 AGGCTAAATACAAGACCCCGTTACCAATTTAATAGTAGTTTTCAAACCTGTCTCCTCATA

A_14_P127627 TTAAAGCCATATTTATCATCTTTTCTCCAAAATGGCTACCTTGCCTGAATCCCAATTTCT

A_16_P01472260

GTCGGACACTTGTTTCTTTTCCCTCTTACCATAGAATAACGTTAAACAAAAGTGGAAAAG

A_14_P137878 GTTCTGTAGCATTAAATGCCTGATCTCACAGTATTATGCTTCACTAGAATGAAGGGGTAA

A_14_P115754 AATGTTTCTGCATGTGTTAAGGTTGACACTTCTGGTTTTTTTCCCTCCCTTTATATTAGA

A_16_P17333162

AGTTTATGCAGAACCTTCCATATCTCTCATAAAACCAGAGCATTCATAGAACCAGAAACA

A_16_P20261927

ATTGAGTATCTTTACCCTTTCCTTCACAACATTATCAAGATCACTGAGTTAACCCAGGTG

A_18_P22841233 TACAACATCCTCCTCCCATCGGTATGCACCCCTTCCTAGGAGCCC

A_16_P17005980

AATGATCAAAACAAACCCCACTCTATTGTGAATCATCTTGACGAGAATGATCCATGTCTA

A_14_P136080 CAGGTGTCTTTATCCAGGGCTAATGAAGCATTTAAATCACTATTGCCAGGAATTTTTCTA

CUST_4624_PI426210822

TAAATTTTTAAAAATTAAGTCTTGTTGAAGAGTGAGTAGAAACCAGAGTGGTGATAATTT

A_16_P36449528

ACCCCCACTAAATTAAGTGCTTTGTCCTTAGTATATAAAAAAGTGTGGAAGACCATAGCA

A_16_P16094744

ACAGCACCTTAAGTATCTTCCTAGTGTTGCATAATATTCAGATAGCGTTTGTAATGGTCA

A_16_P20969632

CTTCTGAAAACAATGTCTGTTCCACTGTGTTATTCCATTACATCAATCATAACCCTGTGA

A_14_P111248

CTGAAAATCTAGTCGAGTAGGAAGATCCAGGAAACAATGCTTCAAAAAGACTGTTACAAA

CUST_1320_PI426210822

ACTACAGAAAAATAATTGTAAAATTGATGTGTTAAAAAGCTTTGAAAGACAGAACTTTCT

CUST_8416_PI426210822

TCCTTCCTTTTCTATGTCTTCAGGTTGTTTGGTGAATATTGTTCTTTTTTGAATCAGGAG

CUST_4845_PI426210822 ATTCAAGGTGTGCTGAGCCCAGTCCTAACTGCTCAAGTCTTTCTT

A_14_P133791 AATATTCTGTGCATATCTGACATACTTCTTAAAAATAAAGCATGTCGTCTGCACTGCCCT

A_16_P02131797

TGTGTAGAAGTGAAAGAGGTGAATAAGTAGACTAATCCAGAGATATGAATTTGCAGGCTG

A_14_P105435 TCCCGCATCTTGCTTTTGAATCAGTTTTATATTTCCAAAGTGCCTTCTCTGTTTTATTCT

A_16_P40323859

AAGAATCTGCTTCTAACCAGTTTGTAAGTGTTCAAGAGCAATTCAAGAGATTCTGACATT

A_14_P111055 CTTCTCTGCAATCATGTCCTTCCTTCGTCCAAAAAAGATCTAAAAACAGGAATAATTGAG

A_14_P119758 ATCCTTGGTGATGATGTAATCAACTTACCATTTGTTGATGGGCAGATAATTGCATTCAAA

A_14_P104251 AAATTTTGCTTCTCACATTAGATGTTTAACGGAAGTTTAATTTAACTCCTCCCACCAAGC

CUST_7833_PI426210822

GTTGTCTTAGGTCAAAGCTAATTAAACTGAGGTTTCCTCGAGTTCCAGAGTACATTTT

A_14_P200575 CCGAGTGGAAAAAGAAACAGAAGTTTCTAGCACTGTGCTATTTATGTAAATGAACACACA

A_14_P115833 TAAACTTACCTGTGTTTATCTTTCAGGATGCTTAAATGGCAGAGGCCAAGCC

A_14_P131138 GCATGATAAAAATTATCTGTGAGTGGAATAGCAATTCCCATATTAGATGGAGGCCTGTAG

A_16_P19728407

AGCATAGAGGGAAAATAGAATGACCAGGATTAAATTGAGAAAGTGAGTATTGCGTCTACA

CUST_3073_PI426210822 TATGAGATGTGCATGTGATCCTGTGACAAGGAAAAGAGGTGGCCT

CUST_9344_PI426210822 TCTGGCCCCAGACAAGGGAATTTGAGGTTTTACTGTTTTGACCTT

A_16_P01782981

GAAACCCCTCAAGACCTATAAAGTCTATGTTCCAAGATCATCAGAAGTAACCAGCATTAT

A_16_P58424582 AAAAGGCTTGGCCTCTTCAAGGCACTGAAAGACCAGGTAGCAGGAA

A_14_P130921 AAACAAAGACAAAAAAAAGTGAGGCCAGAATAACTTCGTCCTGTTTTACAAATACTCTGC

A_16_P20079681

AAGAAATCATAGATATGTGTTCAGTCATAGTGAAGTCTGCTAAATTCCTGCCTTCAGACA

A_16_P15853768

AAACGGCATTGCAAAGTTTTTCTAACACCATACATGAATAACTCGTAAGCAATCAAGACA

A_14_P126853 GGAAAATAAACTTCAATTCCATTTTCATAACTTAGTCAAGAATGCTTGATGAAGAGGCCT

CUST_4420_PI426210822

TTATCAGATGGGAAGATTTCTGAATGCTGAATAGTTTGTAATATCCTTCAAAAAACCAAA

A_14_P120716 GGGTGTCTTTGCTTTCTTTCCTTCAGGAAAGTTTATATGGACTATAATGCAACGACTC

CUST_6757_PI426210822 GAATAGATAACCACTGCATCACACCAGGGTCACCATTCACACTGG

A_16_P02050032

AGGCTCAGTATCATTTAGTATTACAGCACGATTAACAAAACTTGATGGATCTCATCTTCT

A_18_P14139224 CCTCCAGGGCGTCTGGAGCATGCACACATGTCCTGTGATTCTTTA

A_14_P129040 CTTCCAATTTTTAAAATCAAAATGCCAACTTTCTGTTCTTACCTTTCCTGCAGATTCCTC

A_14_P128577 AGACTTTAATCCCCTATTGCTAGACTATCAGTTTCTCATAGGAGTGCAGTATGATCTAAC

A_14_P200147 CCCATTATACTAACCCTGCCTATTGGACCTTCTGATGCAAGGAGCAGATTAA

A_16_P37530764

TGAAACATCACCTTACTTTTGAACAGAACACAAACTTACTTAGATCGATGCTTCTTCTGA

A_14_P200883 AAAAGATCTACTTCTGGCTGTATGTATGGCAAATGAGTGTTCTGAAAAATCCTCCTTCTA

A_14_P108463 TATGAACTACAAGTATTTGTGAACATTAGCCATCCAAAATCTCTTCCTTCTCCCTAACCA

A_16_P18065452

ACAAGAGCACAATTATATTCCTACCCTGTTCCAATATAGAGTTGCCTAGTCAGAATTGTT

A_14_P135740 GGTCGGAAGTCCACAATACAAACATGTTTCCAACACTTGTATTGGAAGATAATTTTTGTT

A_16_P17151488
AGCCCAGGTTTCATAAATGTCCTCATCGATGAATGGAAGTATCTGTATTCAAAAATGAAC
A_14_P102657 CTAGATTAACCCCTTTTCTCCAAAAACTATAGGGACTGAGTAGAGATCAGTTAACTGTCT
A_14_P131784 GTATTTTAGTTTCTGCACCAAAAGGAAGGGTTAGGGTGATTTCGATGCAAATTAATTCAT
A_14_P101119 TCATGTTTTTGCTTCAGGGAGTTATATGGGCTAATAATCTTAAGCCAAAATCTTGTCTGG
A_18_P22843227
AACAATCAGATTTTTAACCACATAACGTGTGCACGGTGGTAAATTTAACTGATGGAGAAA
A_14_P124147 ACACTGATTATTTACCCTGTGGTATTTTGTAGCCAGATTAGGATCTTCTCAGTTAGGTAT
A_14_P136159 AATTTTGACCTGCTTTAAGATGGTTATCAACAGAGGTTTCAAAAGTTCAGCATTTCTGAC
A_16_P18541717
AACAGTGACTCATTAGCCTTATTTTGAATACATGTAACCTTATATCTGATCCCTGGCCAC
A_14_P124084 ACACTGGCAATGTTCAGGAGGACGTTGCTGTAATAAATAAAGTTTCATTTGAAAGTTTTC
A_14_P135998 AAACCGGGAGTCTGATCCATTTTGCCTGAACGATAAGAAGAAATAAGAACTTTTTCTTAA
A_14_P200834 ACTTTTGCAGTATGAGCCTTTTAAATGTTTTTCCTGTTCTTTCTTTCTTCACTGTAGCCT
A_14_P119378 ACAGCCCCTCAGATGCCTTTCCTTCCACCTTTTTTTATTTTTTAAGAATCCCAAATA
A_14_P200632 ACATGGATACCTGACATTTTGGTAAGTTGAATATATTATTTTAATTCATTTCTTGTTTTT
A_14_P114500 CTTACTTATCTGAATATCTGTGCCTGGGAGAATGCAAAAACTTTCAGCGAAGTAACAAAA
A_14_P112683 ACCTGAGTTGGACTTTTTCTTCCAGAGCAGAATAAGAGAATCTTATCTGTAAGTAAAGGA
A_16_P58424570 AGAACTTCTTGAATCCATGTTGGTGCTGAAATTTGATTGGCACACACCAAC
A_14_P132830 AACAGCAAATATGGCAAGAGACAAGGCAAGATAAAATTAAGGGAAGTGAATAATTTACCA
A_14_P131812 TTTAACTCTCAATTGGAGGGGGAAATATCATTCTTCCATTAGGTTATTGCACAGCATTTT
A_16_P02038127
AACTCTATAGAAACTTCCCCTAAAAATTTCCTAAGTCGTCAACAAATCCACATTGCCAAA
A_14_P124998 TCATCTTTTCCTCTTTGTTTAGAAAAGCATTCATATTTCATGTCCAAAGGAAAATGCATC
A_16_P02358833
TCTCCTTGCTAATTACACACATGTTTCTAGATACTCTCTCAATTCTGGTTGTCTTCAAGC
A_16_P00299192
TGAATTCCCCTCAAAACAAACATAACCATGTTCTTTAACATTTACTGCTAATTTGCTCCA
A_14_P133463 TTCTTACTTTCCATAGACCAGTGACTGTGGACTGCTGTATCTTACTGTATATCTCTTTCT
A_14_P136130 ATGCCAAGGATCTCCAGATATTCTTCTTCTCCTTCAAATTCCCTTCCTGAAAAAC
CUST_3572_PI426210822
TGACAGAGAGTTGCTTCTTTTAAAAAAAATGTATTAAGGGCTATGTAAATAAAAAGAAGA
A_16_P37357810
CACCCAATCATTTCAAGAAATTTAAAGCATCCTTCAAGTTCTATATATGTGGTCTCGAGA
A_14_P106789 ATAAGCAGATCTATATTGGCGTTGTTGAGAATGGGCACATAACTTGCCAATCTGAAAAAA
A_16_P15714571
AAAGTTTTGCATCTGTTGACATATTCTTCCACCTGTGAATTTAAACTGGCTGTGTAACAA
A_14_P117310

TGAGAAAGAAGAGGGTATCAAAAGAAGAGAGTAGAGGAGAGCATCTGAGTAAGATTCTTA
A_18_P13863546
ACCATATTCCATTAGTTTCCCTAGACCATCCTATGACTTCTACCTTTTCCTTACATTTCA
CUST_8307_PI426210822 ACCTGGAGTGTCACTTTCAGCCCATCAATATCACCCTTCAGGTAC
A_14_P121720 CTACCCACATACATTTGAGATTTTATGGCAGGATCCAATAAATACATTCACCTTAAGGCT
A_14_P201347 GCGTAGACAATGTTTCTGTTTGTTTAAAGAATACACATGTATTTGCTTACAAAGGCCCAG
A_14_P200210 TCCCAAATGAAAGGGGAAAATAATTTGTGTTTCAAATGCATTTTGGTTTTTGTCTGGTGA
A_18_P21067100
AGCCAGCATCGTAGAACTATTCCCTATAGTAGTCATGGACATTGTAAATTTTTTGATCGC
A_16_P15557524
AAAAAAACCCTCAGATTTTCTTCAAACAGTCTCCTTACAATCAGAAAATGGAAGAGGATG
A_14_P124729 GCTTAGAGCCTCAGAAGTGGAATTTGGAATTCTGAGAGAACAATCTAGAATTCTAGGAAT
A_14_P127671 TTAAATAATAGAACTCAAAACCAAACGGGCTCTTGTATTACACTTCAGAACAGGTCTTCC
A_14_P104912 AAAATCATTACTGATTTAACAAATGAAGAATTCTATTGTAATCTTTTATCTTGATGAAAT
A_16_P18870402
TATCATAGCACAGTAAATGCCATCTAAGTCATTTAATGCCAGGAGAAATATAGGAGTGCC
A_16_P00685052
TGCCATGGTATGTTAGACCTAAATTGTGGAATCTGGCTTTACTACTCACTTATTGCTATG
A_14_P104552 AGTAGGTTTGATTTTTAATACCCCAAATGTCAAGTTTAACCACAAACCTGGTCAAATTCC
CUST_6061_PI426210822 ATGTGAGCAGAAGTATGGAGAACAGGACTCCCATGAAGATCTGCAG
A_18_P13170689
ATTTTGTCTCAACATTCAGGTATGATTTGCAGATGACTGATAACAACCATCATGTTGTGG
A_14_P136192 GAATAAGGAAGAAATATGCAGTTTCTATTTGGTCATTCAGGATTGGTAACATTCCTTCAG
A_14_P118081 TTTCCTCTATAACCATATCTAAAGATGGGTTTGGAGAATGTGTCATCCTTAAGGGCTAAA
A_14_P104096 TTATTTTCCATTGCAATGATGAAGATACCTGAAAGTTTAAAAAATGTCACCTTGCCTGGG
CUST_10391_PI426210822 AGGATGTTTTTCTTGCCAGTGTTTTCTCTAATCCGAGGGGTCCTC
A_14_P117827 TATAGGTAATTGAGGTGAAAGCCAAAAGAAGAACTGGACCACTTAACTTTGTGACTTGTA
A_18_P13912173
AAAAACTGCCACATATTGTTTTCAGGCATATTCCAAATGTCTAAGAGATTTATAGAGCCA
A_16_P20344959
GGCTGAATATTTCATTCTATGCGTATACCACTGTAAAGAAAACTCCATTTCCTCTCTACT
A_14_P119908 CAGCACCTCCCTCTTTCATCAATATCCCTGAATAGTTCTTACTAGAACACAAATGATACA
A_16_P15231305
GGCATGCAACCTAATTAGACTGATAGACCTAATGTAATCCTAGGAACTCACACTACATTA
CUST_8486_PI426210822 TATTTTCCACTGAGACTTGCTGTGTGTGTTCCACACCCACAGGTC
A_14_P201577 CGGATTTATTTTTTAACACACCCAAATCCGCAGTCATTAAGTGACAAGACTTTTGTTTTC
A_16_P39757755
CCCTCAGAGTAGCAAATAAAAACAAAAGCAGTTACCTAATTTGGTTTATTCACACACCTC

A_14_P134961 AATATATTTTCCCAATTGAGACCACACAGTTCAAAGAAACCTGAACCAAGTGGAACTTC

A_14_P116707 TGTGGATAAATGTATCGCTTCTGTCTGATGATGTGTTATTTCTCCTTCAAAACCAAGATG

A_14_P127736 ACAGACTATTTTTTGACAACATGTATGCCTTGGGTGGGAGAAAAGAATTTAGTTACATCT

A_16_P40572874 TGGGAAACAATTGTTTAGAGTTCAAACCTATAGGAACCTCATTAATTTCAATCCACACCT

A_14_P133638 GCGATCCACATGGTCAGATGCTCATCACAAAACAAATAAAACAATTTACATGAATGCTTT

A_14_P132096 CTGAATTTCTCCCTTTTATTCCAATTCAACAATTTTCATGGCTTTTTGTGTTTGTTTTGT

A_16_P17592750 TGCCTGTGTATTATAACATCACCTTAGTCCTTACTTGTAGAAGAGCACATAATAGTCCCT

A_14_P102450 AATAAATTGTGTTAAAATGGTGGGGCTTCATAGTGATTTTGCTGTTGATGTTAACGGTAG

A_16_P00295787 GATGAACTGTCCCAATAGTTAAGGTTTTCCTGATTGCTAACTCTCATCATACCTACTTTG

A_14_P133373 ATATGGTTTTTACACTTGCTTACTATAGGTGGAGGAACTGCAATGTCTGGTGGAG

CUST_8254_PI426210822 ATTTGCAAATTAGCATATTTTTGCCTCTGTTCACAGCTCTATTTTTAACTGTATCATTTA

A_14_P100431 TAGTAGCCTATGACATAATTCCAGACCAGGTGAATCTCAAGATACTAATCCTCACATCAT

A_14_P200582 CTTGCACTGTCAATTTGAGCCAAATCAGTAAGTTGCTTCTACAGAAACAACTTGAGTTTA

CUST_2950_PI426210822 ATCAGCTTTTCCATCACCATGACATCATTCGACATGGCAGGGACA

A_16_P16278589 AGGCAATGCAGTTAACCTTTAGCAATATAGAGCACTTAAGAGTTTTGCAAGCCTTGTTTT

CUST_8004_PI426210822 GTATCCGCAGCCCAGCGTGCACTCCCATGTCCTTCCAACCCTGAG

A_14_P120266 ACCAGAAGAAACAAAGGAGGTTTATTTCTATAGAGAATTGTTGGATCTCAGGTCCAAAAA

A_16_P03094834 AATTAGGGAACCTCAACTAGTTCTTTGTTTTATGTTAGGAAAAATGGGAAACTACACCGC

A_14_P125632 ATTCTTCTAACCAGTTTCTGAGTATGAGACCATCAGAGCAGTAACACTGAGTAGTAGTAA

A_16_P36484755 GTTCAGCATGCATAGTCTATTTGTATTAGTCATGTCCATATTTGCAACTCAAAACACAGA

A_14_P110530 AGTTTTGTGCTTATAGTGAATCGGATCTGTAAGTTGACCACAGTTCAGTTTACTGGTTTA

A_14_P134675 AACAGTTTGACTCATGGCTAAAAGTCACAGGCAAGTGTAAAAAATCTCTACTCTTAGTTC

CUST_7978_PI426210822 CTGGGTCATACACTCAGATCATTTTAATCTCCAAGGCTCTTTGGTCCC

A_14_P100505 GATACAAAAAAAGTTTACTCCACTCACCTGTCTGTTACGTTCATCCATAATCCTCGTAAT

A_14_P200339 AAATGTTTCAAAGTCACTCATACTGAAATTCCAAGTTGTGTTTCTTTCATATCGGTCCAG

A_16_P01635487 ACCATGATTTACAACTATCTCAAACTAATACACCTCATAAACTGTCATACTGGTCATACA

A_14_P116709 ATCTACGGAAACAGAAGACGAGAACTTTTGCTGTAAGTTCAAAAGTTACTTCTATGTCTA

A_18_P22843207 ATATTGGGATTCATCTGTGGCAGTTTGCAGAACATATCAGGGGAGAGAG

A_14_P101367 TGAAACTTTTCCTTTGATTCCGCCATAGTTAGATGGATCCATCCAGAGAAGAAATTC

A_14_P128130 CTCCTCTTTCTAGTCATACAGTTTGTCAAGAAAGAAAATAGTGCACTACACCTGTTTTGA

A_18_P13972213
TCCCTATTATAGTTAAGGCAACCATTTTTTCTGATTCCAAACTAAAGACTTGTGATGTGA
A_14_P139521 CTATCGATTGTAAAAGCAAAATCCGTAACTATGTGCCGGGGTCATCATGTGTTATAAATG
CUST_3798_PI426210822 TAGTGGGAGGTTTTAAACTATCTGAAGATCACACAACTGTGAGAGCCCAG
A_16_P16248029
AAAAAGGCTAACAGAATAGGACAATTTAATCACATGCCATTATGTTCATAGGGGTTTGGG
A_14_P122739 CTCCTCAAACTTCCTTTAAAACTGTTTAGAAAAATAAGACTTCCAAGTTGTTCCCCAATG
A_14_P104729 CAAATAAGGAGCGAGGTTGTGAAAACTGGAAAACAAAGGCTCTTTTCAGAGC
A_14_P103003 CTATTTGAAAGTCAAGTTGAGAACAGGCTTAGTCAGCAATATGAACCAAAAGTTGCATTC
A_14_P200464 TGAGAGAGATTACCTTTTGTCTGGTCATACAGAAGAAATCTTTCAAAGAGTTCATGTTGG
A_18_P14132495
GCAAGCAAAAATAAATGTTATGGATGGGATAAAGAAATGTTACAATGAAACCAGGAAATC
A_16_P03251837
ACACAAAGGGTCCTATGTAATTCACCTGATAACATATCATAAAGATCTTTGCCAATGTCC
CUST_2514_PI426210822 CCAGGAGGAAGTAAGGGAATAGAAAGAAGTCAGTGTTGAAGCTTTTGTCC
A_16_P19697994
ATACGTGTCAAGATTCAAGACTGCATCTTGCTATTCTGCTTTAGGAATTCAGTCTGCTAG
A_14_P111576 ACATCTGTGAAAAATGACTGGTCTAAGGGAACTGTATATACCACACAAAGTAGATCATCT
A_14_P115176 GACTTCAAGTTCAGAACCAAAAAGCAGAGAATCGTTTCATGTGACATGATGTTTCTATAG
A_14_P103420 CACCAGAAGAAAAGGGCAACTGAGATCCTGAATTTGCTGTTGAAAGATCTG
A_16_P18577330
CTTTGGTCACGTATAAATGCAAAATGAAAGAGGTCATTCTAAAGTCGTGAAACTGAGAGA
A_16_P20860119
ACACTACCACAATAATACGTCTTCCTACAGGTTTACAGGAATCAGTAACTGAACGTTCAG
A_16_P16191308
TCATGCCTGATACACAGATGTTTGATTATCAGAGTCTTTTTTCTTTCTTGTTTCCTGTGG
A_14_P127679 CTGTGCCAGTTTGCTCTGTCTTCTTGCTCTGTTCCTACATTCAAATTTATAGAATCAAAA
A_14_P138583 AGACAAAAAGACACATCCAGAATATTTTCCCCAACGGTAAGGAGATATGGCAAAC
A_18_P11966711
AGCAGAAATTTACAGGGGAAAAAAAGACATAACTTTGGCAATTCTAAACTATACCCAGGC
A_14_P126045 CTCAATGGACCTGTTGACAATAACAACATTCCAGAGGTAATTTTTTTCAAGGATGAGAGT
A_16_P02836140
ACGTTGAGTGAATATCAAACAATCCCGATGTATTCAGTAAGGAGAGTATTACTGCTTCTG
A_14_P119575 GTAAAAAAGGAGGCTTGCTTAACTGTCTATACCTCCAGCCGATTATAAACTGCACG
A_16_P16725646
AGCAACCTATAGGGAGATTAAAAGAACGTCATTGTAGTATACCTAAGTCCGCAGTCTAAA
A_14_P114506 ACCTCACATTTCTCTTGTTTTTACAGTAATAAAAGACTAGTGGCCTTAGTGCCCATGC
A_14_P115638 GAACACTCAACTGTCAAAGATGAACTATGCATATCTCCATCTTTCTTCCCATCATTTGAT

CUST_7199_PI426210822 TGTCATTGAGTTTGGGTGCCCTCAGAATGCTACTGAAGATGGTACAA

A_14_P139338 ACGAAACATATTCTTCAAATACTTGTATTCTTTTCCCATCTCCTCTGGATAAAAGGAAAA

CUST_5638_PI426210822 GACACACAATACAGCCCACACCTCCAGAACCCCGGGGCTTCTTTC

A_14_P107884 AGTCTTATATGTAAGCATGGCAGAGAGAAGCCGAGTTCTTGCACAGAATCTTGAA

A_14_P107338 TTAGCAGGGTGATGCTTCATAATCATGTATTATTCCTACCTCTAAGACACCAGTCTGTAT

A_14_P111556 ACTGAAGGGGATCAGAGGTGTCTATGCAAATGATATGCAAAGTCCATGCAAATAA

A_14_P122234 TTCTGCTGCAGCCTAGCATCATTAACAGAGAAGAGTTAATGAATATAAAAGCGTAAAGGC

A_14_P100528 ACTTGTAATTTTTACAGATGGCCCTATTGAATTTCTTCTTCCCTGATGAAAAGCCATACT

A_14_P135616 ACCCATAATCTACTTGTTTCTAATCTAGTGCAGGTTTAGAAGCCAGATAACTTTGCTTGT

A_16_P03739305

GAATTTGAGATGCAATGATAGTGAGATCATTCCGAGTCATTGATATTACTAGAGGGGACT

CUST_801_PI426210822 CAAATACAGCCCTTCAGTGAAGTCTCCTGGGGCTGTGTTTAAGTG

A_18_P15552806

AAAAAAACTGGAAGATTCTCTGACTTGTAGATTCTGAAAATATAGGTTCCCTGGTCCTAC

A_14_P113682

TACAGAAAAAAGAGGGAAGAAACTAAACAAAACACCAGGAATGGATCCTTATGAAGAGAT

A_14_P131530 ACATTTCCGAAACAGTCTCCATCTACCTAGGCCTCTCACAAGTAAGTAAAACAATTTTAG

A_14_P115492 CTTAATAATTGTGTATTTAATGGAATAATTTTACAGTCTAATTTCCCATTCTTTTCCTTA

CUST_9470_PI426210822 AAATGAATTCCTGAATGAGTGAGGGGTTGGGCATTCCCTACTCAG

A_14_P110219 GATTTCCATTGATTTGTAAGACTATGACCCTTACTGCAACTTTTTGTCACTGGCAATTAG

A_16_P19346165

AAAAAGACAGTTTTTCATGTTTCCCATTGAGTCATCTCTCACATGGAGTTCACTAGAGAT

A_14_P109965 TGAGAATCTAGCCGCACTTCAAAAATGTGTGCAAGTGTTTATTTGGAATCCCTTCTATTT

A_14_P128439 TTATATGTAGGTGGACAAGGCTTAGAACTGAAGCACAGGCTAGAAATTCATTCATTCAAT

A_14_P123398 ACATCGCCTCTTGCAAATAATTTATGAAGATGAAATGCTTTTGTAACTTCCTTCACCTCT

A_14_P111735 TGAACCATATGGAAAATAAATATCTCAACCATCGACATATGCTTTCTTGAACCTGCCTTC

A_16_P39228751

CCTTCCACTCAGTAGAAATAACGCCCTTATTTTGAAGTTGTTTACCACTCTCATAAATGT

A_16_P02722120

TATAATTGCAAAGAGAGACCATTACAATTTTCCATCATCATCCTATGATCAGAGAGCACC

A_16_P18729152

AACTTTCAACAATCAGTAGAGAACACTTCATTGTTTGCAGAACTTCTAAAGGTCTCAAGG

A_14_P202164 TGTAGTGCACTGTGTTTGGAGTGGGTTTCAGAAATAATTTTATTCTTGTCAAAAATTCCG

A_14_P117649 AGAAATTTGCATTTCTTTTTTTACAGGATTTTCCCTCCTGGTGAGTCAAAATGAACAAGA

A_14_P128732 AGCAGTCATGGGACCCAGGATAGGTAAGTCCGAACAAATTAAGGAAG

CUST_7213_PI426210822 GATGAAGAAGTAAAAGTTTGTGTTCTGGAGGGGTGGGGCTTCAATCTA

A_14_P133075

TAAAGTATCAAGAAGACAATAAAGAAAAGGGGAAAGGGCACTAAAATAGGAAGAGAGGAC

A_14_P136156 AAGACGAATCCATTTTACCTCCTCATATTTCACACTTAGGGATGTTTGTCCATTCTTACT

A_18_P14771424

AAATTTTGTGTCAAATGGAGATCCATACTCAGAGACATCAACATTCTACAGAACCACAAC

A_14_P109675 CTGGCTGCAAAGTATTGGGTTTTTCCTGAGAATAGTGCTTTATAGTTGATTTCCAAGTTA

A_14_P132484 GATTATGCTTCTACTTCAGCATCTGTATCAAGACATAGTTCCAGTGTGTCATTGAGTTCA

A_14_P101807 TTGCCATTAATAGAGACCTCAAACACCGCCTGCTAAAAATACCCGACTG

A_16_P35573168

AAGATCTTCATAACCTCAAAAAGTGAGTCATCAATACGAGGCTTTTAGAAACGATTCCAG

A_14_P119054 CTCTAAACCCCCAATACTTAAAAGTCTAATTGCTGTCCTGTGGTTTCATTAGTCTGATAG

A_14_P127527 CTCTCAGAACAGAGGAGAGCAAAGGGGAACCCTACTGATTTCAACAAAA

A_14_P200121 AAATCTGATAGGACTGGTATTCATCATTTCCAGTTATTTCAACTGCAGGACCACCTTAAA

A_14_P101470 ACTTCATAAAGGAAACTGCGTATGCAGATTCAGTATTGTGTATCTTTGGACAATTAGATG

A_16_P33665764

AATCACTGTTACCCTCGATAACAATAGTATGTGGAATGAGTTCTATCATCGAAGCACAGA

A_14_P104968 CTGGAGAGGATGGCTAGCCTATAGAAGGTCTCATATTTTATGCTAAGGAAGGAGTT

A_16_P36770302

TGTGTCCTCAATGAGAAAGAGATAGTTATCATCAATGTGTTCAAACAAAAGGGTAAGTGA

A_14_P106772 CCTTCTTAGGATCTAAAGGACTTTTTGGTCTAGCAGGTAAACCTACTGAAAAATAAGACC

CUST_3985_PI426210822 TCATCTGTGTACAGAGTATATTTCCTTGGAGGCAGCTTTGCTGAAGGC

A_14_P104022 TGAATGTGCTACATCTCTAAATGCCGTTATGGAAGACTTGTGATATATTTTGGTTCCCAA

CUST_1027_PI426210822 CAGTGTCTGTTAGCAGAGCTGCCCTGCCTCATGGTGTCTTGCGTG

A_14_P119675 GCAACCTACGTTAGACCTCTATTGTTTCACTGAGCACACAAAAATCTTTCCTTTGAAATA

A_14_P124130 TTCTCCTTATTGTAGGCCGCAATGTTTACCGAATGCTTTTTAAGAGCAAAGCATATGAG

A_16_P03225647 AAGTTTGTCTTATATTATTTCCACCTTCCTAATACCCACTCTTCCTTTTTCTGAAGTATT

CUST_6266_PI426210822

GCAGGCTGATTGCTTTTTAGGATTCCAAGTTTCAAAAGGAAATCAAATCCAATGTATTTA

A_14_P120903 ATGCCACCAAAAAGAAATGAAAAATACAAACTTCCTATTCCATTTCCAGAAGGCAAGGTT

A_16_P15992779

AAAGATGTATGCATGAATGGAAAGTATTAAAACCCAAGTCTTTAATGGTAGGGAGAGGTG

A_14_P117405 CTACTTTAGGTATTATTCTGTCTCCAGAACTCCACATTGACCTAGACTCACAACAATATC

A_14_P109410 GAAGACCTTCTTCGGAGGCTAAAACTAGTCAAAATGTATAGATCAAAGGTGAGAAGAATT

A_14_P136874 ACTAGAAAACACTGCAAATCCCTCACAATGAATAAATATTATTCCTGCGACTGTAACTGG

A_14_P114666 CTCTGTTTTTCTCTATTTTACGTTGTGGCATTTGGCCCACCAATTTTATTTGGAAATAAG

A_14_P135993 CTGAGTTTGACATGGAAATTGAGAGTTCAATCTTGAAATGATTGCAATGGTGATGTTTGA

A_14_P134638 TTTGTTTCTGTAGGAAAAGCAAAAGGAAAAGGATTAAGTTCCTAATTGTTGAGTAGACAC

A_14_P133225 TATATTCTATTCAGAATGTCTTACTTGCCAATTACCCTTCCTGTCATACACATAGGGACC

CUST_5963_PI426210822 CTCCTTTTTCTTGACCCCAGGTCCCAAACCTGAATGTGCAGATTT

CUST_9769_PI426210822 CAGCAGAAGCCTGGGTGAGATGTACTGCCATTGTGATCATATTCTCA

A_14_P137545 CTCTATAGTCTCCTTGTTTTAGTGAGCACTCCATAAATGTTCAATGAATGAATGATGGCC
CUST_10231_PI426210822
GGGTTTCCTGGCATGTGTATGAAGAGTTACAGATTTAGACCATGTATCTCTTAAGC
A_14_P100398 CTTTTTTCCCCTCCAATAAATGAAAAGTTAAATTGGTTGTGCCCATCTCTGTATTTTCTC
A_16_P15104881
TCCTACTAGTTGCCTGTATAAAACATCACTCACAAGACTGTTATCAGTATCCAGAAGCAT
A_14_P138240 TAAAACTGGAAAATCTTTGTATAGGGGCTCCATTCATTTGACTCAAGGTTATAGACTTCC
A_16_P16290368
ACATTTAGGGCACATTCTTAAACATGAGATATGAACTGCAATTAATCAGCACTAGTAGCC
CUST_357_PI426210822 ACAGACCCCAAAGTCAGCACGGACTCTGCAGGCGGCAGAGTAGAG
CUST_7232_PI426210822 GTTCTTTTCTGTTTAAGTGGGTTCTCAGAAGTGGTTGCACCTTCAGCA
A_14_P132884 CTTACCTGGGTTTGCTTTTCACTTTTGCTTCTCTTGACTTGTCACTTAAAGTCTTCAG
A_14_P114221 TTTTTAAAAAATAGGAGTTGAGGAGCAAGCGTTTTCTAGTGGAAAGATGAACACATCAGC
A_14_P202369 TTAATGGAGACCGAGAGAGTTTACTGCTAGATGATTTCTGAGAAAGTTTATCGTCTTCTA
A_14_P118754 TGAAAATTTTTCACCCATTTCAAAAGTAACTGGGTTTTCACCAGTTAAAGCAGAAAGTGT
A_18_P14132290
ATGCTGGAAATTTATTTTGGATCGTAGAAAAACTTCATGTGGTGGGAAAATGTTCACTTG
A_14_P126301 CTGCAACTCCCAGTCTCTCAGGAAACATGGCTAAAGTGAGAAAGACATTAGAAT
A_18_P14133800
TCAGTTTGAAAAAAACATAATCATTGTGCAATGACAATTTGAGACATTTTCAGGGTCCCA
A_14_P132954 ATTAAATGCTATCGTTGATAAACTTACGAATCAACATGCAGAACTCTCCGGCCACTTCTT
A_14_P118454 CCAAAATCTGGAAGCAAAGTCAGGACCACTTGCATATTGTCCACCTTTTACATAG
A_14_P133773 ATTACATTTGTGGACACCAGTTTGGTTGAAGTGGCCCCTACTACATTTAAAGAAGAA
A_16_P15996443
CACCACTCAATTTCCTGTTTAGGTCTGTTCAATGCATCTTACATTAAAACGTAGTGACAA
A_16_P16206334
CCATCTAACTTCATTTTCCTACAATATCCCTACCTACATTAATACCTCATCCACTCAACA
CUST_6430_PI426210822 CTTGGAAATGCAATAGCAGCTGCCTTCCTAGGTGGGGAAGCTGAG
A_14_P136557 CTGATGTCATCTTTGGACAACTTGGTGAGTAATAGATAAGTTGGGTAAAAAGAACAAGCA
A_16_P21561220
GCACTGAAACGTGAATTATCATTCTACCCCATCATTGCATTTCAACTAACAATGAACTCT
CUST_9964_PI426210822 AGCTCCAAAACAGCACCAGGGACCATAACTGCAACTTCCTGACAC
A_14_P110985 AGGCTTAATTAAATCCTTCTATGTAGTCTCCGTTAGAGACATTCCTAAGTCCTCAGAGTA
A_14_P105097 AATTTAGCCCTGTTTTACCTTGTTTCTCTCTCCTTTGCGTTGATTTCATTCTCCTTCTAA
A_14_P114395 AGAGTGTCTGCTACATTAAGCATTTCTTAAATAGAATTTTAAAAAATGGGGCATTTGCCA
CUST_4493_PI426210822 GCACAGAGATGAGGGTGGCCTCTGGGTCAGAGGGCTTCCTGTTGA
A_16_P03101404
TAGAACGATCTAGACGAAACACTGAACTTAATAGCAAAACCACTTCTTTCGAGGGAAAAC

A_14_P129644 TGGAATTATTTGGTCAAAAGAAATGAATCTCTATGGCTGTTCCCACATATGACCATTTTG
A_18_P14136778 GTTCCTGTAATTCTGCCCAGGTGTTTCCTGTCAATGAGTGTGCTCTAG
A_14_P114386 CTACAAACAACCAGTAAATTAATGTATAGCAAGCCACCATCAATCTTCCCTGTGTGTG
A_14_P138386 CGTGTTTCCATTCATGTAGATAAGGAAGATATATCTTCCCATTTGCATCAACATGCTTTC
CUST_5166_PI426210822 GGCTGATCTGAGCCTTAAGACTTATTTGTGGAGACCTTGTGCTGTTCT
CUST_2352_PI426210822
TTTTCATTAGAAGTACATAACAGGAGATTAATAAACTTCGGTGATTCAGCCCACAAGGC
A_16_P00017737
ACCCTTAAGGTCTTTTTAACTGGTAATTGGTTTCCTATCCATCAATCGACCATTCCTTAA
A_16_P15951374
AAAGCCTAGTAATGAAGATATGACTTTCATTTGCATCACACTTATCACTCTACTCATGCT
A_14_P121916
ACGTAAAGGAAAGAAGAGTTGTGAAAATGTAGTCTGTTGATCAGTAGAGAGAAACCAAAA
A_14_P105761 GTGTTAATGGCTGGCATTAATAAGGGATTCCTCTACAGGGGATAGTTTTTTCTTTTCTTA
A_14_P108342 AGAAGTGTGTTCAAACCCATCGAGGCACTCCTTCTACAATGAGGTAGA
A_16_P38436215
AAATAGACCCAAGTCAAACTTATCTTACCCCAGTATTTTACATGGCGATGGATGTCTTG
A_16_P18537315
ATGCTCAAAACATTTTTTTAGTGGAAGGTTTTAATACGTCTGTAATTTCTCCGGTGTCTT
CUST_3206_PI426210822 ATAAGGCACATCCTCCTGAGGGGAGGGCAGGTACTTTCTCACGGC
A_14_P138841 CATGTCGAGGATTCTTGGAAAGCCTTTTGCAGCGTGTTCTCTTTGC
A_14_P103047 CTTCAGCAACTCCTAGTCCATCTGCAAAGCCATCAAATAGGCTGAAAAAAAAAAAAAATA
A_16_P20032029
AATCCTTGACAGAATTTGTAACAAGAAAACGTCTATTGACTTTCTCAACTTAGAGCCCTG
A_14_P128271 GTTTTTTGTTGCTCGGGGATTCACTTGAAATTCATATTTACTTTGAGCCAACATTTTCTC
A_14_P118809 GGGTGATATCTTAATGAGGATTCTTTACATCATGTAACTGGATATAGGAGGAAAGAGGTG
A_16_P17616244
ATTGTCCTCTATAGCCTACCTCAAATCCAATTCAAATCAATAAAAGATTGTTGCCCACTG
A_16_P16191671
CATGGGCCTGTGAAATATTCTAATGGGTAAACTAAGAAAAGCATGACTAAGATACAGAGG
A_14_P124211 GGTTCCCTTCTCTTACCTTAATTGCTATGTTGTTGGATTCAGTAGCACCACTAGT
A_14_P134195 CTTTGAAGTTTATAAAAGTTTGTTTCCCCCAAAACCCAAAGCACTTTTAATCAGGTAGTT
A_14_P132957 CAATGCAATGCCAATCAGATTTTGCTTTTTGAACCTGGAAAGAAGACATATTTCTGTAGT
A_16_P17633317
AAAAAGTAGGATCTGAACATCAGCAAAAAGTAGGAGAAGTATTAATAAAGCCTGGGAGAC
A_16_P16668555
TCGTCAAAGTCAGGTTCAATACTATATGGGAAGCCATGATATAAGAAAAACTGTCATCCA
A_14_P126600 GTAACTGAACTGCACAATATCAAAAATATAACCAGACTGCCCCGAGAGACAAAGAAG

A_14_P123343 TAAGAAGAAACAGAATTATGTGTATATATGAGAGAAAGAAACAAGAATGCGTGAATGAGG
A_14_P109650 ACGTTCTAACCACAAACAAGGGAGATTTTCTATGAGTCACCGGGATTTTGC
A_14_P118088 TGTGGAGAAAATCAGACACCTTTTCAGTAAGCATTTCTTTTGTTTCTGTGATCATGTTCA
A_14_P129429 CTCAGCAAAAGGAGAACATTTTCTGAAATCCTTACCTCTCTGTCTCCAAATATAATGTCA
A_14_P127170 CTTACTCCTTTGCAGTTTGGTAAAATGTTTTATCACTGCTGCTGGCTGTGAAGAC
A_14_P111072 CACACCTTGTCAAAATTTTTCTCCCTAGAATCCTAGACTCACTGTCTGATCCG
A_16_P19306946
GGACACTTACCTATAGCCATTTTCTACAAGAAGCACATATGATATAATACGTCACCTTGG
A_14_P121727 AAATATTTAAACATGGAAAACGTGAAGACTGTTTGCCCTATGGTAAGGTTTATTTTTAAG
A_14_P120879 TTATATTGGCGCTTTTAACTCAGAGTTTTAAACCCCAGGAACAGAGACTCCTAGTTG
A_14_P110735 TAACAAAGAACTGGCTTAATCAGAAAACAGTGTGAAATTCTGCATGCTTATGCAACTCTC
A_14_P105841 ACTGAAACTCATTGACATAGGCAACAGTTGCAGGTAAATCTCATTTTGATTAAGAAAGGA
A_14_P135643 CTGCAGTGACTCAATCCTCGTTTCCTGTTTTACTGATTACAAAATATAATCGTGTTTTGG
A_14_P129804 CCTGGAACTTGATATGGATCCATATAATGCAAAAAACTTTCATGGTAGAAGGATGAATGC
A_14_P201365 ATCGTGCCATTTCACTCTTTGTGGTCCTCAATTCATTTTCTAATTTGTTGATCGTGTC
CUST_633_PI426210822 CAACATCTCTGAATGGAAAACACATGAGAGGCTGCCTGACAGGATG
A_14_P121361 AGATAAAAACGCCATCACTCACTGTGCAAAGGAAGGGGTCAAGGATAG
A_16_P02233762
TCCTAACTCAACAATTCAAATTTAGTAACAAATTGTGCGTATGATAAATCCACTGGTCCA
A_14_P201310 CTCCCACCAAACACTTTCTTGATTGTTATTTTTGTGATTTACAGTGAAGTTTTGTCTCAC
A_16_P01804418
ACACCAATGGCTGTAATTTTTATGTCTCTATAGTGCAACTTTGTAGGTTTTCTCATCGCA
A_18_P10378375
ATGTAGTTATGCAAGTGCACTTAATTTTATGGAACATCCTCATGATTCAATCTGCTGCTC
A_18_P15830087
AAAATGTGCAGATGTATACCCCATAATCTCAATTTCAGTGTCCTTTATCTCCTTATCCCT
A_16_P00085425
CAGTCACTTTCCTTTTAGTATCTCCTTACCTCAATACTCCTACATACAGACATTAGCCAG
A_14_P135787 CTAATCACACATTCCTATGGCAACCAATTCTGTATAATTGCTTGAGTCCTTTAAGGTCAT
A_14_P126964 ATACACATTGACAACATATTCAGTTTCAGGTTTCAGATCTTTCAGCACTGTGCTAGTTTC
A_14_P137575 GACTTTGGAGAGATGAAGCCGTTTGCTGATTTTCTAAAGAATAAAGAAAACCCCTCATAT
A_16_P16689142
AAAAAGACTGTGCTTTATTTTTCAGTCGACTAAGCAGTATGTTCCTGGTAAGTCGTAGAA
A_14_P107067 ACATAGAAGTATTTGCTAAATGGACCACAAAGACTGTCCCTCAAGTCTTGTGATTCAAAA
A_14_P127320 CTTATCCTTGGAAGAAACGCAGACATGTGAAGAATCAGGGACATGGAGA
A_14_P130998 CAATTGAAATCTTTGCCTTGCCCAGAGAGTTGCAGTTATTTGTAATTATAAACAGTAAGT
CUST_7414_PI426210822 AGACTTCTCAGCATCCCCTGCTCAGAAGGGGCTCTCAGAGTCCAT
CUST_8363_PI426210822 GGGAAGGATGGGGTTAGAGCTTGTTTTTGAGACAGTAGAGTGATTGC

A_16_P18414161
AAAGTGTGCTTGTTATTTGGAGTTAGGGTTAAGACATTTGGAAAACAACCATTGTAAGGA
A_14_P138339 TGTTTCAGACAGAGGTGCGTAGATGGTATCTCTGGGGTCCAGATTTTAAA
A_14_P102001 AAAAGGGAGGTTTTCAGTATTCAATTTTCCACAGTAATAAGTTGGTTTTAACGCATGCTC
CUST_2909_PI426210822 CTTTATTTCCTGCTTTAGAAATGGGAGACCACAGTGCCTACTTGGTCATG
A_14_P201135 ACTAGAGTCCAACAACTTATAAAGTATGGAGGTCTGTGTCAACTTCACTTTGTAATGCCT
A_16_P16119218
ACAACAGACCTATTTAACTCAAAGCCAGCATCTTTAGTAACAGAAAAACTCGTTTCCTCT
CUST_4825_PI426210822 ACCCCCAATGATGGCATGTCTTGCTCTGGACCCATGATATAATGAT
A_14_P108254 GTAGAAGACCAAAAGGCCAATGAGACTTCTTTCCTATTTGAGATCGGATTTGAGAATG
A_14_P130724 ACTTTAGGATAAAGTATTCAGTTATCAATGAGTCTTCCCTCAATGGTGTATGAGGCAGAA
A_14_P200027
CTTAGGAAGGAAGACTCACAGAAGTTGAACCAAATAATTGAAAATGATAGGAAGACTAGG
A_14_P139663 TTTGGGAGACCATGAAATTAGAGGCCTTTGTCTATTTTCAATTTGCAAAAGGTATCAGAA
A_14_P131660 AAAGTGCTAGTCTTTTCTGTCTATTTAAGGTGATGCCTGATATTGTCATGTTACAGAGGC
A_14_P139198 CAGATTCCTTATCTAAGGAAAGTTTTAAAAGCCATCTCCAGTATCCATTCTTTATGGCAG
A_18_P22841243 ATTTGCCCAAAGAGACTAATCCAGGACTAGAACACAGGCACTGAGTTTCAA
A_14_P105274 GTCAGTCAGTGCATTTCTACTGAACTTCATGGTTTGTTATTATCTGTCTCAAGGTGAAAA
A_14_P127101 TGGTTTATTAATAAGTGAAGTAGAATCACAGAAACGGTGCGATGTCCTACATGTTGACAT
A_14_P108406 ACATACCCCTCCTAATTCTTCTGGGTAAATTTCTATGCCCATTAACTCTAGACATACATT
A_14_P122068 TTAAGAGTAGAACAGATTGTGCTCAAACCCTGCTGTTTATAATTAACACGTTGCTTCCAA
A_14_P125566 CCAAAAGTTGACTTCTGCTTTTTCAAACAGGTAGTCTTCTTCCACCTAAATCAGATG
A_14_P104361 TGCTAAACAAGTGGTGCTGTTTGTCATTGATACATGTTAATGAGAATGAAATGGGGACTA
CUST_2486_PI426210822 GTTTTAAACTGATCTACTCTGAATGGCTTAGCACCAGGAGGGTGGATTTG
A_16_P16881775
AGGATTACCACATCTGAAGGAGAATATAAGCAGGTTTAGAATAGTCAATACTCTCAGGAA
CUST_5735_PI426210822
AACTTCCTAGCTGTTTTCTGCCTCTTCTGACTAATTAACAGCAACTAAAAAAGAAAAATG
A_14_P202059 AGATTTGGTTCTCTTCTTTCTAGAAACCCATTTCAGAAAAAAGGAACCAGATCTTAACAG
A_18_P14136754 ACTTCTCATGGATGCAGCAAAAACCCAGTAGGATGCACCTCTCAAG
A_14_P121930 GTGTCTGTGAGTCTCATGAAGACACACAGAAACATACACATTATAACTGCTAGACTAAGC
A_14_P119382 TGGGAGCCAGAAGTTACAAAAATTATAGAAGACTACCTAGGCATAAAGTCTATCTCCAAG
A_14_P108497 CATTTGACCTACATACTGTAAGCAACCTGTGCCTTTTTTCAAAAACACTGACTCAAAATT
A_14_P138913 TCTCAGGATCTCATCCCCACAAATTCTACTTCTCGAAATCTAGTATAAGTATATCACCTG
A_16_P01269131
AAATGACTCAAACCTTCCATTACTGAAAGTTCCAAATGTTTCTTTAATGGCAATCACCAG
A_16_P37025717
TTGACTCATTGAGACACATCTCTATGTAAAGCCACTTTGATGTAAATGTCCCATCTGTAA

A_16_P16191414
CTACATGACTTCTTGATAAGATTCTTTCAACTTTACCCTATGCCTGGATCATGAAAGAGG
A_14_P125656 AAGTCATGGCCTGAATCCTTACCTGTAAGGCATCAGAATGCAAATGAAAATTAACTTAAT
CUST_2827_PI426210822
AAAAAGAGCTTAGAAATTTCAACATTGAATGAAACCATACCATAAAACTTGGGAACTGGT
A_16_P20984019
AAAATCTGTGGTCAAGTTAAGAAATGTTGCTTGGTAATCCTTGATCTAGTTTCCTCACAG
A_14_P103429 CTTTCATTCCTCTTCTTATCCATTTTGGTTCCTCAAGCACAAGGGAGAAACT
A_16_P01180019
TTTCTGATGACTGCATCTACAAACATGTTTCCTAAGAATTTAAATGGTTCATGCACTGCC
A_16_P16901060
TGCACTATCTGGTAAACTCCCAAATTTCCTTGAGCATTATTCTAGAATCTAGCCTATGAG
A_14_P107075 TACACATGTTGTCTAAAGCAATATTCAGGGTATTACTCCTTTCTTTTTTTCCAGCCTGCA
A_14_P138511 AGTTACTGCAAAGAAAGTGATGCACAAACATGTGAAGTTGAGAGTAAATCTCAAGCATTT
A_14_P201422 TTTTTTTCCAGTAATTTGAACTCAGTCTTGAAGTCTTACAGTGACAGTTCAGAGCCTTAC
A_14_P116439 AAGTTTCTATCAGTGTGGATTTTGATGAGCCTTAATTCCATCATCTCCATTCAACAAACA
A_14_P134813 TTCTTTTCTACAGAAAGATGTAGCACTTAAGCCTCAGGAACGTGTGGAGAAACG
A_14_P118623 CAGCGTTTTATTGACCCTCTGGCTAAAGAAGAAGAAAATGTTGGCATAGATATTACTGAA
A_14_P136602 GTGCTTACAGGAAAATGTTTAAACAATACTGGCATTTACAGTTCTCCATGTTTTGAGAGC
A_14_P102620 CTGTGATACATCCATAGTGGATAAAATAAATGCAGGCCTTGGAAAATAGGCTTCCATTAT
A_14_P122969 CACTGTGCCAGTCAGTCTGCAGACCAAACAAATAAGACGGTAACTAAAAGTAACATAC
A_14_P107433 TATTTTTAAATAGGCTATTACCTTGTTCGATACGAGTAGCCAGATAGAGCATCTCTCCCT
A_14_P131821 ACTGAAGAAAAGAAAGGTTGAATTTACTAGGTGTGTGCTCCTTTATAAGACCCATTCTAG
A_16_P17496514
ATGAAATTTAACCTTTCTACCATGGTATTTTGCTAAACTCTGAGTATACCCCTGTCAGCA
CUST_3438_PI426210822 TAAGGTTTTCTGAGGGCCCGTCATGTCTCAGACTGTCACACTTATT
A_14_P110180 AGGCTTCCAATGTGGTGGCAATAAATGTCCCAAATAAATTTATAACAATTGATTTTCCCC
CUST_2533_PI426210822 CTCAGTGCATTTAGTGATGTACAGATTCAGACCCTCTTGGACTCAGATACTTG
A_14_P106299 CTACTCCTTGCCTGAGCCTCTAGTGAATTAAAAAAAAGGCAAAAGGAACAAGAACATTTT
A_14_P132734 CTCATGTTTTGGTTCTTTTATGCTAAGTTTTACTTCAGCATTTCTTCTGATGTATGGCCC
A_14_P127459 CGTTGACATTAGACCAAATACTTTTGATTCCCAACTACTCGTTTGTTCTTTTTCTCCTTT
A_14_P114058 ACTCATAAAACTAATCTAGGAAGATTTGTTCCTTACTTGCCCCAGAAAGGAACAAATTCT
CUST_9315_PI426210822 GTATTCTTCAAAAATGGTGCTGCAGACAGTTTTATTGCCTGTGCCATACTTGC
CUST_9480_PI426210822 CATTTAACTGCTAATTCAGACCTCCACCTGGGAAGTGAAAAAGAAAAGTGGTT
A_18_P14137183 TATTGGCTGAATGTCCACTTCTGTTCTATGCACTGTGCTAAAGGGTGGA
A_14_P110351 AAATGATGAGAGGAAAAAGTTGCTTTGACCTTTCAATCCACTTATGAAGCATGGAATTAG
CUST_342_PI426210822
ATAGAACCTACATGGGGGTGACTTAAAAGGATCACAAATACTCGTCAAAATGAGTG

A_14_P134258 GTCCTCTTGTCAAGGGTTGAGACACTCTGATTCTTTTATCCTATTTTTAAAAGAAAAGTT

CUST_8722_PI426210822 TAGAAGTCAAGAGAGATGGAAATGAAAGCAAGTCAAGATTCTTGTCTTGGGG

A_16_P17560861 AAAAAATTCCTGTAGAACATTGAAGAACTGAGTAGTCCTTTGTCATCTACATAGCAGGCA

A_14_P113626 GACAGTGTCACTATTTTACAACACAGTAAAACATGGTCCCCACTGGTTTTTTAAGAAAAT

A_14_P114948 CCTCTTCTCATGTTAACTTTATTCCAAGTATGTAAATGCTACCAGAAAGAGTAAAACATG

A_14_P106295 CTTAGTCTATTATCCTAGTGGAAAATTCTTTGCTTTTGACACTTTTAAACAGTTTTAGAC

CUST_5377_PI426210822 CGCCCCCAAACTTCACACAGAGAGTAGTATGAAGTCCTCATCTCT

A_16_P00000776 AGTGTCAACAGTTCTCAAATTCAAATGCCCAAATGACCATTTTTAGTAAGGAAGTCTCAT

A_16_P00685607 CACCTAGCAATAGACCTGCAGGCATAATGGAGGGTGGTGAGTGCT

A_14_P101310 TCCAGTGTCGCAGATTTTTGTGTAACTTGTGATATTTATGAGCACTTGGTGGTGTAAAAA

CUST_242_PI426210822 CCTGGAGGAGTCGGGGCACTGGAGCTGCACCCCTCCCCGGTTTTG

A_18_P14134010 TAAAACCAGAAAAAAATGCTCACAGAACATACTAGCCCTTCTCTCTTGGGATATTTCATG

A_16_P21439651 ATATCGTTCCTTAATCAAAAGCAATGTTACCATAACCTCTTCTTTTAACCCTTCGTAGCC

CUST_1608_PI426210822 GAAATGTGGTTGAAGGGTCCAGGCCAGCGCGACTCCCCTCCTCTG

A_14_P119216 TTTATGACTTAAAATTCACAGTGCTGTCATTCTTGCCCAAAACGAGACCAATTTCTACTT

A_14_P116170 TTCCAGAGTCTACCATGGCATATACTATATGCACTAAAATTCTTGCAAACAGGAAGACTG

A_14_P111321 AGTTCTGATGCTTCAATGAACAGTAAAGAACCATCTCTCTTTGTCCATTAGTTTTCTCAC

A_14_P124320 CTGGTTCCAATTTTATTACTTTAATTGCTGCTAATTCACCAGTGTTAACATTCCGTGCCT

A_16_P02103120 AACATCTTACAGAAGATACCAAAGGTCCTATGTTAGAATTTTTACAAACCGACTGAGGGT

A_16_P19415708 ACATGAGAAGATACAGATTCCTCAATAAACACATACCCTCTGATTAGAATCCAGTTTGGT

A_16_P02419029 AAAGAGTATGTGATTGAGAGATGTCAATATCTGAGAATGATATATCAAGACTCCCCACCC

A_16_P03034834 AGATTACTTTCACTGTCAAATCCCTACTATTTCTCTCAATACAATCTGCTGTTTCACCCA

A_14_P201749 CTATGTAATAAAAAACTATACTCTGTGTTCTGTTAATGCCTCTGAGTGTCCTACCTCCTT

A_14_P202142 GGCATACTTTCACCTTCATCTGACTTGCCATACAAGGTAATTGTCGCTTTGGATTTAT

A_14_P121001 GAGAAATACTTAGGACCCATCTACACAAAGTTGAACTAGATGCAAGATAGCAGATAGCAT

A_16_P35693648 CCCAAGTTGAATAGTCTCTTTCCAAATAGATCAGTTCTGCAATATCTATAACTGTGCCCT

A_14_P133260 TATTAGGAATTGAATGACCTTCATATAGAAGCACTTGCAGTGATAGCCAATTGCCTTGAA

A_16_P19319348 GACTTCAGTGAGAGTGAAAATATGAGAAAGGTTAATGCAGACATAAGGAGAGTAGAGTCA

A_14_P202239 ACATTGTGATTTGCAGCCACCTAGCACTAAAGCATAGAACTTAAACACGAATCCCTAAAT

A_16_P36838281

TCCATCTCCTCATTATCTCATCCTTTTCATCTGTTCTTTATAACCAAAACCCTTTGCAAA

A_14_P120096 TCCCTCATTTCCAGTTACGAATGTTCTTAGGGAATAAGTGTAAAGTTCTGGAATTGTATG

A_16_P21450321

AATGCTTTGAATTCCTTGTATATTTCACCACGTTAGATCTAGCAAACTACCATCCCTCAT

A_18_P13180411

ATCTCCCAAAATAGTCAGAACCAATTTTACCTTCTTTCATTTCAAAGATTACCCTAGGCC

CUST_3830_PI426210822

ACCATTGCAAACTTTGACATGTGAGAGTTTCCAGAAATACAGAGATTCTTATTTTAATCC

A_14_P129237 CTTAACTTTAGTTATTCTGACCTTGACCTCTGTCTTTATCCCAATTGTGTTTTTACCTGG

A_16_P01297586

AAAAAAAACTCTCTGTCTTCTAGCAACTACATCAGTTCTTGAAAGGATATTGAGGCCTTG

A_16_P36230880 ATGATGACTCTGCTTTTTAAGGTTATGCCCTGGGCATGATCTGAAACCAG

A_14_P114161 TGTGGCATAATGGAAGTATTTCTCTTAAAAGACTGTGCAGTAAGACCAGAAGTATCTCTG

A_14_P138274 TGATATCTCAGTTTGTGACACCTTGAATCAAACTTTGGCTTTCTTTTTTGGTAGGAGAAA

A_16_P01567067

TGTCATACCTTATTGTGTCAAAGTTCATCCATTCTACACTTCTACATCTACATCCCATCA

A_14_P120765 AAAAAAGAACGATCCTCCACTTGACCAAGAAAAAAGTGATTCTCCCAGAAGCA

CUST_1231_PI426210822

AAATGTAAATTTTCTAATTAATTTTCAAGCAACATAATAGTATTCTTAAATATTCTCAGA

A_14_P135053 CTCAAGTATGAAGTAAAAATTCATTGAGTTATACATATAAATCAAATTTCATGATACAAC

A_14_P101686

TAGTCCATGCAGCCACAAAAATACAGAAAATGAAAAGCCAGAGTGATCAAACAATTTCAA

A_14_P138930 ACGGTGGAACAGAGAGTCAAATGACAAATGTGAAGTGGTTATTTGAAATAGAGTCTCTAT

A_18_P17570764

ACTTTTCCATGCTGTTTAGAACCTACTATGCATTCTTAACTGATTTACATTACTCCGCAA

A_16_P38385191

CAGGCATTCACACTTAGACATCTTAACAAACTTGCTGTTAGATCGAATCCAAATTCTGTT

A_14_P131355 TATCCTCTTTATCCTGGGCAGTAAATGACAAGATTCTTGTCACTTGATTGAAAGGTTAGA

A_16_P19532549

AAAACATGGCCTTATTTTTGAAATCAAAACCTTCAGCATAAACACATGTCCCTTTTGTGC

A_14_P101103 TCCATTTCCTTGTCTCAAAAAAAAAAAAATACTCTATGGGTCTGAATGGCAATATGTCCTC

A_16_P58424122

ACCACTTGCTCTTTCTGAGCAAAAATATTCCATTTGTAAATAAATGGACAGAGTTAGAGT

A_14_P100104 ACTAATTTTTGACTCCTTTTAGTGACCCATGCTAATAATGTGGAACCATCTCCTATTAAA

A_14_P114978 GGAAGATTTTTATTTTTTGGGAACCTACCATCTTGCAATTCTGTCAAGCACATTAAGTCT

A_14_P127319 ATGCTTAGGAGACATAAAATAACTTTTCCCATAAAAGTCAAGCCAGAAATTGGCTTCAAC

A_14_P130404 ATAGTGGCAAAAGAATTGAGGATAGCACAAAAGTAATTTTCGTTTCAGGATTGGTTAGGA

A_16_P17501772

AGGATCCAGCGATAACTTGACTTAAAGTTCTATTTGCCAGAATATTCAGGGTTATTGACA

CUST_722_PI426210822 CCAAACTGCTCTTGTGTGGGTGAGGGAGGCAATGTGATCTGTTTG

A_14_P131622 TAATAGTTGGGAAATAGGATCTCAGAGAAGAAAGAGTAATAGGAAGGCTGCCTTATGGTA

A_14_P102834 TGAAAACAGATAGGCAGGCTCTAAATTGGATAGTTGAGAATTTTATTTCACTAGGGAGGA

A_16_P41580219

AACACCTTCAAATGTGAAACTCCCACTTAATTATGTTGCCATTACATTTTAGTCCATCCT

A_14_P130780 CACAAACACTGCTCTACCAAGTCTGCTATGATGTAATTCTGACTCGTGGATGTG

A_14_P123997 AATAACAAAAAAACTCCAAGAACTCTAGATAGTTTGTCCAGGGGACACCTATAGGTTATG

CUST_1061_PI426210822 GTGCTTACCAAGCATTATGAAGTCTCAGGGACCGAAATTCACCAAGC

A_14_P124539 CTTGCACTGTCTGTTTCCTTTGTGGCAACCAAACTACAAATAAGAACAAAAATATAGACT

A_14_P124112 TAAAACATTCTGTTAGAAAAGAAATGTGGATCTCGCACCCAGAAAGCCAAATGAAATTTC

A_16_P01807747

AAAACATCTCCTTGCATGTTTAAAACAAAACTCACTCTGGTAGTGTTAACTTTCCATGCA

CUST_7090_PI426210822 ATGGGCAAGGAAGCATTGATGAATTTTCTGAGACAGCTGCGATTTGTCTA

A_14_P105970 CACCCATTCTTTTTAATCCCCAGAGAGCATACAGATGAGCCCCAGT

A_16_P15650063

GTCCACCAAATATGATTTTAACCCTCTCTCTATCCAAAAATAACCACTACTTAGATTGGC

A_16_P36508721

TGCAGACTACTAATCGTTTATTTGAGATATCCGATCCAATTTTAATTGCCCACAACAACA

A_14_P133383 CGTGTCAGGTGCTTGCTCGAATATTAAGCAACTCTAAAACTGTGTCAAAGCAAA

A_14_P138088 GTGCATGTGAGCATGCACAAGTATCTTTGTGTATTTGAATATCTTAGCAACCTTAGCAAA

A_14_P120250 GAAGTCACATTGTGAAGATGGACTTGATTGTGTCTCTGTTTCTTAAGTAAAGCCTGATAT

CUST_1933_PI426210822

AAATACTAGCTTTTTCTCAGAGTTGAATTGTTTACTTGTAATTTGGAAGCTTCTTGGGTC

A_18_P22841708 TAAATGGTAGGGAGTGGGTGTGGAGCAGTAATGCTGTGCATATTCTTGTT

CUST_9349_PI426210822 GTCTGTAAGGCTTTTCTCACTGATTCTACAACCGTGGGGGCCCAT

A_14_P133038 TTAGAGCAACTTCTCTCTTTCACTTCACCAAAAACAAATCTAAGGTTTCCTACATGTCTC

A_14_P123655 AGGATCAAGGGGAGACTTGATTAATAAACTGTGGGATTAAAACTTTGTCTTAACAGAAGT

CUST_858_PI426210822 AGACAGAATTCTGTGTTACCACAGATCAGTATCACAAAGGAGTCATTTCTGGG

A_16_P00932601

AAGACAGACATATCTGAATGAGGACATACATTACCATGATTATCTAGTCAGAGCCACTGT

A_14_P111879 ATCAAGAAGGTTATCTCACTTTGGTCCTAAATTCAGTGGATGCTCCAAATCCAAAGA

CUST_5646_PI426210822 CAGGTGCTCTGCTGCCTTTCCACATCCTTTCCTCTGAGGGGGGTC

A_16_P20223175

ATGGGTGAGAACGGATCATTAGTATCCAGTTAGAACATCTTCCTAGAAATTGCTCAATAC

CUST_10050_PI426210822 AAGGACTACTAGCCACAGACGCAGAATGATCAGCCACCTCTGATT

A_18_P17316531
AGTGTCCAGCATTTATTGAACTTCATTTAGGCTATTTCTACCAGAGTTATCATTTACCCT
A_14_P108647 TGGAATGCAAACTAAAAGTTGGAGACAACGAACATTCCTCTAATGTAAACATACTCTGTG
A_14_P123617 AAAAGATGAGAAGTTAAAGACTGTGGAAGAATTACTTGAAACTGGACTTATTCAGGTGGC
A_14_P201820 TTCCTTCTCATTGGGTTGTAATTTTCCATAACGGGTTAATAGAAGACTGGATTTTAAGGT
A_14_P134912 GTCTGTGTTTCTATTGGGATTACTATAGTGACTTGTTAATTCGCACTAGTTCTTATGTTC
A_14_P134273 CTCTCGCAAAAAACTCAAATTTAGTGCGACTTAACCTTTCTGGGTGTTCTGGATT
A_16_P16320401
AGGAACCATTAAAGACAACAAAACATACCCTCTCATATTGCTATATTTCTGTGTGCTTCA
A_14_P109545 GTCAACACTTTCATATCTGAATGTCTTTGCCCTTACAGATAGATGGCTCCCCAATA
A_16_P21096862
TCCTTGCAATAGCAGTAGTCTTTCAACTTTTAGGACGATGACAACAAGATAGATCATACC
A_14_P106848 CCTCGAGTTTAGGGATTCTCCTCACTTCTAGCCCAAACTTTGGATAAACTAACATA
A_16_P37811349
CAACAGCCCTGAACAATATAGACATTAGAAGCATTTTTTTCTTTTACCTTACTACATGCG
A_14_P122081 CCTGGAAGATGATATGGACTCCCATAGTTTGAGACTCACTAAAATTTCAAAATGTTCTTC
A_14_P114697 ATTTAAAAAGACTTACATCATTAGATTGAAACAATCGCGTCATTGCAAAGAAGGCTTCTG
A_14_P132071 AAACAGGATTTTGAATATCTGAAGATATGTCCTGAGGGAATCAGAGAACAGAGTTTAGAC
A_14_P103030 CTCTTGACTTTAAATTAAAATATTGGGGCTTTTGCCTCAGCACTATTATTGGTTAGTGGG
A_14_P105566 AATTGTCCTTACCAAGAATACAGGATTGTTCCAACCACAGAGGTGAGTTTACTGTTTT
A_14_P131535 CTTAAATTATGCAACTCGCAAGATGTAAATGTCATGTGGACACAGAAAAGGGACAGAGA
A_14_P102471 GGAAAATGACAGCCTTCATTTCTCTGAACATACTGCCTTATGGGACAGATCAAGTA
A_16_P19164487
CCAACATTCAATAAATCCAGATGACAGGTTACAAAACACAATCAATGATCCACACTTCTC
CUST_3436_PI426210822 GAGAATTCCTGTTTTGAGGAAAGATTCGGAGCCAGCTAAGGTTTTCTGA
A_16_P18593339
ACTTCAGGCAGTATTTACAATCAGAACACAGAAGTTACCTAATTCTATGCAGTTGTGAGA
CUST_3669_PI426210822 GCACGGAGGTTCAGTGGTTGTTTTCATTCTTTCCTGTTAGCCAGT
A_14_P117185 AGTGAATTTTTATTTTTATTTTCCAGGTCTACAACTCGAACAAAGACAGCCAGAGTGAAG
A_14_P112616 CTGACTCTTAATTTGTAAATAGGCTCCATGAAGTTGTCCCTGGAAGCAGAATTTATTTTC
A_14_P119048 AATGTCATGTGCTCAGACACTGTGAACATTTTGCTTGGTAAGTTTCAAATTCAAACTCTA
CUST_9603_PI426210822 GCTTTTACCTGCGCGGAAGACACAAGATCTTTGGAATCAACATACACATC
A_16_P01260898
ACCCTGACAGTGAATATACTATCTTCTGACCTCATACTGAAACAGTGACAACATCTATGT
A_16_P16191471 ATCTGTAGGTGGTGCATGAGTATGATATAGTGTGCATGTGTGACATGCTGC
CUST_9220_PI426210822
CACCAGTTTTCTATTTGATATTTATTATTTCCTAAATCTGGTTTTAATCAATTTGACTTA
CUST_2805_PI426210822 TAACTGTCCCTGCCATTTTTCTGGCTGGGGAAAATCAACTTGCTAG

A_14_P131964 ATGGGACAAATGTGTAAACTTTATTGAAAGTGCCCTCCCTTATGTTGTTGGAAAGATG

A_14_P123893 ATGGGGGAGACTCTAGCATTTGGAGCATTTACCTTAGTTTTTGGTCTTTAATTAATCATT

A_14_P113659 CTTTGATCTTCACAGAGTCAGGGCAAGTCTATATACAGGAGGACGAAGTCAG

A_16_P16236532

ATTACACCGGTAATTCATGAATACAGATCCTCTTTACAGATATGGCTTGATTCTCCTTGG

A_16_P02890055

CCCAATATTCCCCCTAATTTATGACCATTGTATGCATCTTTTCCCATAAAGATCCTAAGA

CUST_417_PI426210822

CAATCTGACAGGTGAGAAAATTGTGGGGTTCTAGTTGTGTGTCTCAAATGAAATTT

A_16_P30952519 CTTGACTGTGGGTGGGGATGACAGATTTCATGCTTTCCTCTTTCAG

A_14_P137731 CTGGTGATGCACAGATCACTTTATTTGACTGATTATGGTGACAATTATGGTAGTATCACA

A_14_P200555 CTTTGGAATACAACGCTCAAAACTCATCTTGCCGCTCTTGCCTTCTG

A_14_P130069 CATCTATCTCACTTTCATATCCTCAATCTATAGGAGTCAGAACTAGGGTCAAATCTTGCT

A_14_P137150 TGAGTTAGTTGTTCCATCAGTATAGGAAAAGGAGAAAAATGATACACACCAGAACAATCC

A_14_P137832 CTGTTACTAATATGATTTTGGCATCACTTTTTAATTCTGACTTTGCCCTTAACGCTTCCC

CUST_3493_PI426210822

CCAAATGGAAATACAAATAATAGATTGAGGTCACATTGACTGTTTCACGTTGTACTTAGA

A_14_P109655 GATCTATGCTTGTTTGTTTTTGTAATCCATATCATAGTTGCTTTCTTTAATTGTTCCTTC

A_14_P136089 CTAATTATATTTCTTACTTTACTCTTCACAGCATCATATCGGATTTGAGAAAACTCACGA

A_14_P105898 AATGATAATCATTGGTTTTAATAACTCTAGTTTATGGGAGTCGAGGAACAGAACACCTGC

A_14_P131996 ATAGATAGCCTCAAAGATGTCATGCTTGGGATTGCTTTAAAATAATAGAGTGGGTGGTAA

A_16_P00685061

AAGGATTAGAACTGGATTCTACTCTACCTTGAAGGGATTAATGACAGTCACAGAAGTTTC

A_16_P02655348 TGCTCTAGCCCTATCTACTTTTATTTATTACCAGCCATTTCTAGTCTACTTTGCTTATGA

A_14_P108329 CACTATAACCTAATTATCAGCCCTGGATCAAAAATGAACATGATACTGGCTTATAGTATC

A_14_P200546 CACCATGACCTCTGACCTTGGTAATCCCGTTAGATGGTATAACTTGTGT

A_16_P02465691

GCACTTCAGGATGTGTTATTGTTATTACAAGACCAACCAAAAATATGAAAGGCTGTTTCC

CUST_8903_PI426210822

CAGATAACATATTTATGTATCGCTTTTTCACTAAAGAGAATCTGACGGCATCTGGAAATT

CUST_5511_PI426210822 AAGAGCTGGCTTCCTGCCTACCCAGGGAACAGAGAAGCTGCAGCC

A_14_P124809 CAGAGGGCATCTAATAGAATAGTTGTGGGAAAGAAACGATGGACACATTTAAATTGATTG

A_14_P112477 ATATTTCAGGATATTTCTTTTGGGTTTCTTTCAGTGCTAGTGTTCCGCAAATGTTTCTCA

A_14_P117620 ACTGTAGGTTACTGTTACTAGGGTTCTGATGATCAGAAAACTGTTCATTTTCTTTCTGTG

A_16_P02375679

ATTCTACATCTTGGCACATATAACTTACATCCATTCACTTCATGAATAGTAAGTCCACGT

A_16_P00617710

TGGTCACTCCTATAGAAGATACATTTACAAACCTTCTATCAAGAACAGTTATTAAGCCCC

A_16_P18645052
CCAGCCCCTATCATTTTAAGATGCACATGTTTAAACTTACCAGAGATAACAGCAAATCAA
CUST_7644_PI426210822 CTTGCCAGCCCATTGTCTGCATTTGGCATCTGCGCTGGAGTCACT
A_14_P105846 TTGTTAGCATCTTCTGAAATCTTCCCCTTGTTTTTGAGATGTTTAGATTTACAAACCCCA
A_14_P126778 GGCAGGAAACATAAAAATAATTAAGACACATCCTTGAGTTCCAGGGTATTATGACTTGGT
A_14_P117207 CACCAGTCCCAGCAAAATCCTCTTTGTATTTATTTTGCTAAGTTATTGGTGGTTTTGCTT
A_14_P135020
GAAGAAAAGAAACAGTTGCTGAGTGGACTAGAAACAAATGGAAAATGACAAGTAGCATTG
CUST_7590_PI426210822 ATTTCTGGCCAGGATTTTAGTAAGTACCTGAGCCCCTTCAAACATTTGACT
A_14_P103013 GAGTGTCCTTGTTAATTGATACCTAATTGGCCTTGGTTGGGAACATAGCCATG
A_14_P101869 CTTCTTTGCTGGGTAGGTTTTTCATTGGGATTTCGAGTTCCATCTTCTGTTAC
A_14_P103009 TGATGTGACTTCCATGTAGATATAAAAATGATTGTGCCCTAACCAAACTTCCTCAGATTG
A_18_P22841075 CCCCTTTCCATGTTATTTCTGCCCACCTTCATCCTCTGATCATGTTTATTTTTC
A_14_P126982 CTCTCATGGCACTTTATAATTGGAATTTCAGTTACCTTTGCAGAAATGCCTTACACTACT
CUST_2800_PI426210822 CTGGTGTTACAGAGGCTTAGGTTTCTAAAAGAGGGGTGCTGCTTTG
A_14_P114062 TTGTGCCCGTGGTTTTAAAAATATATTCTGTGTATGCTGTTTCTCCCTTTCAGATTCTAG
A_14_P137176 CTAACTGCAGTAAAGTTGCTGGTGTGTTTCTTTCCTCTGATAATGTCTGTGTTTTCTAAA
A_14_P103263 TAGTAAGAACTAGCAATTAGCCGTTATAGCATAATTACCTTTAATATGTCATGCCCCCCG
A_14_P113832 GTTGTAGAAAATACACTGCAAGAAAAGGAGCAGGTGCTTCAAAAATGTAACCCTG
A_16_P00245600
ATCTCAGACCAAATCGTTTTTCCTCTAGAATTTCACTGTAATCCAAACCAGTTGAACATT
A_14_P133509 GAAAGTGGGTTTGATGACAATGTTGTGAGGCTACAGTGGAACCCAGATAAATTTATG
A_14_P118951 CATATTTAGAGGGACTACTCTTGGGCAACACTGAATTTCCTCATTTCTTGAATTCACTAA
CUST_1725_PI426210822 TGTAATGATCGGAAAACCATGTTCTAGAACGTCCCTGAGAGGGTCTT
A_18_P14136149 GCAAACAGAAAATGTTGGTGCAGCATTTATGTCAGAGTGTGCAGTCTGTTGA
A_14_P104034 GAATTAAAAAACACCCCGAACAATAAATAGTAAACCGAATAAGGAGGAGAGAGGCGTC
A_14_P136969 TGACTTGCATAACCTCAAAAAAAGTATCTGGTCTCCATTGAATTTATTTGAAGGCAGGAT
A_14_P118354 AACTCACATGGAAGCAACTGAGGCATTGGTGGTTACAAAAGACAATTTCTTTTTATAATG
A_14_P100893 GCTGCTGTTAGTTAATTACCATCAATTTACATAGGTCAGAAACGATGGACAAACCCGAA
CUST_6418_PI426210822
GGCTTTTATATTATTTGGCGTAGATTATTAAAGAACAGTTACCTGTGTCATGAAGGAGAG
A_18_P14135409
CAGTTCTGCAGAAAGCCATGCCTAGAAAATTTATAATTACTCTGATACCAGATGGCTTTG
A_14_P127695 GGGGATGACACATTTTTATTATTTGCTTTCTGTTGTGGTATCAAGAATTACCGGCATATG
CUST_6484_PI426210822 TTTTTCTCTCTGGCTGGAATGTACTCCCTGTTTTTCTGCCCATCAATAG
A_14_P126604 AACTGCTCACATATTTTGCACATGACGCTGTGCTGCTCCTGATATTTTTCAAACTG
A_14_P110496 TATTGGATTTCCTGCTAGACTTCAACGAGGATTTTTCTTACTTTGGCAGAAATATCAAGC
A_16_P02537658

AAGTCAAGTAACTGGGTAAAATACTTGCATTCCTAAGGTAGAAGGTCAAGAAATAGGCTT

A_14_P112110 AAAATTCCCCTGTATCCTGAAGGGGCACTAGTGTTCAAATATAGAAGATGCCAGTTTATA

A_14_P130774 ACACAACTGCCCATACCAACACACAAAAGGGACAAAATACACATATTAATGGGAATTATC

A_14_P129763 AGGACAAGCAATAACTCTCTACAGGAATATATTTTTTAAACGAAGAGACAGAACTGTCCA

A_14_P139352 GAATGGACTTCCTGAACTAGAGAACTTTATACAAGGCCTTATGATTTGACACGTCTACTA

A_16_P19420675

AAACTCTGATAGATGCACAACTCTTATTCTAGACTCTATATTCCAGAATTTCCCAGATCA

A_14_P119412 ACCTTTATTTTGCTGAGTTTCATTTGGATCTTCTTAGACACCAGATCAGACCAGTATTTC

CUST_7862_PI426210822 CAGTGCTGAAATGTGGTGTGTGATACGACAACTCAGCTACCCAGT

A_16_P36231022

GGTATTGAAGATGGAATGACAATGCTTTCCAAAGCCTCCTTTTTTTTATCTTTGGTTACA

A_14_P104476 TCGCTTTCTTGGCTTCCAATTCAGCCTAGAAAGAAAAAAAAAGCATATGTCTTGATACAT

A_14_P124676 TTCTTCCCTGTGTGATTTTTGCCATCAAAATAAAAATTTGAGACTCGTTAACCGAAGTCC

A_14_P116373 AAACGCTAAAATCCTCCGAATGAAATAAAACGAAAACATACTGCTAAAGAATAGCCCCAA

A_14_P102325 GTATGTGTCTATGTATGTAAGTTTGTTTTTATACAACCGACATTTGCAGAAACTGTCCTC

A_16_P38184867

CGGATATTACAGTTTTGTTTTTACTCAAAACCTGATCATCAAGCAATAACCTGATTCCCA

CUST_951_PI426210822 GCTGTTTTCAGAGAATGCTCTCCTTCTCCTGTTCTTCCCTCACAG

CUST_5221_PI426210822

AACTAATATATAGTCATATTAAAATCTCTAATCTGATTGAGAAATTTTCAGAAACAAGGT

A_16_P16865544

AAAAAAAAATGCTGCTGTCATGCTAATATTGATCACCACTCATGGTCTAAATGTGATGCC

A_16_P19561883

CCTGAAAGTAAACCACTAACGATTACTCATCAGTCAAAAATTTACATTTACCCTGCCCAT

A_14_P201568 TCTTGGCTTGAAGAACAGTAAAATCACAGCGGAAAATAAAAAGAAAGTCGCGTTGTTATA

CUST_8701_PI426210822 GTCAATAGGACAGACAGATGAGGAGTAGACCCCCAAGAATCACCC

A_16_P18694669

GGAAGAAATCACATACCTCTAAATAGACATATCACTGACTACAAGATAATGAGTGCAGCA

A_16_P16297276

ACCGATATAGTATTCTTGGCTTTATATGCGTAAGAAATCCTGTTAGACTATGAAAGCACC

A_16_P00685065 GTTTCTTTTTGGATGGTAGGAGGCATCCAAAGCCTCTTTTAGGGAAGATTG

A_14_P137170 GAGGCACAGACATTAGTATAGTCCCTTGAGAAAATTAAAATGCTACATCATGGGAACTTT

A_18_P17317862

ACCGTAAAAATGTCTGAAAAATGCATTCACTACATATGTTACTAACTTTGCTCTTCACCT

A_14_P134051 CTACCAACCTTCAAATAAATCGGTCTCTGTGACCAGGTATTTGTAAAAGGTTAATTGAAG

A_16_P18867301

AGGAACATCAGAAACAATTACCGAAGCACAAAATTATAATCCATTGTCTCATCTATACCA

A_16_P21276445

TCATTTTGGGGTCTGACATACATAAAGCATCACAATTCTCTTTCAATGAAATCTTCCCTT
A_16_P20778926
ATGATGGATACCTGGTAAGTAGAAAACAGTGTTGAAGATCCTACTACAGATGAAGACTTG
A_14_P113122 TTTCACAGATCTGGTAATATTTTTCATCTTGTATGCCATCCTGAATGGGCACATTTACAC
A_16_P16075726 GAGCCCCTGAAAATCTTAATTAAGGAAACTTCCAATGTATCACATAAGGCCCAAAAG
A_16_P02721993
ATTGCTAGTTTCCAATCGTTACTCTAACACATCTCAGACTAACATGTTTACACTGCCTCT
A_16_P20436696
AAGTTTTCATCACAGGAGTAAGATGATCAGATTACCACATGAGGAAGATAATCTAGCACC
A_14_P201740 CAAAGGCTGTTAAGTATCTCACGCATGTGGGAATTTTATGTCGGCTTAAATGAAGATTTA
A_14_P109088 CTGCTAATGTCATTGTGTGTGTCAGTCTTGGACAGATCACTATTTACAGTGGAATGTG
A_16_P17427690
CTACAGAAGACGATGCTTTTCAAGAATAGACAATTCCATGTCACAGATAGTGTAGGTGTG
CUST_1601_PI426210822 CTGCTGTCTCCTGGCTACTTTGGACTTTGGCCTCCGCTTCAGTTC
A_14_P124722 CTACTCCCTCACCCTTGAATGGAGTAACTCATGAGCATTCCCAAATGAG
A_16_P03411972
ACACCCTCAAATAAATTAGAACCTGAAAATACCATATTTATCCCTCAGATACATCACCAT
A_14_P129664 TGGCCTGAAAAATATACTTCAGTGTTCAATGATTTGAAATCTCTGAGATATCAGGGAATG
A_14_P107258 CTTTATAGAGATTGCTCAACAATAGTATTGAAGCAGGTAAAAGAATTCTCCCTGGGGAAT
A_18_P14134204
GAGATTTGTAAATTCAAGATATTGATCATTCAAATGAAACTTCAATATAAGCCATAGAGT
A_14_P133566 GGCTCTTTTTCTCCTTCTGTTCTAAGATTTTCTGCAGCACTTTCTTCTCCTTCTTG
CUST_3090_PI426210822
TTTATTATGTATTATGTGTGCTCTTCCAAAATTTTATTTCACAGGGAGGAGCATATCCAT
A_14_P100496 CTTCTTGATCTTTAATCTTTTAGCAACCTTCTGATACTGGATTTACCTTCCTAGGTGTGG
A_18_P22843221
AAGGCTTATTTTACTCCTTAAGTCTTCCTATTTCCTAGTCACATCTCATTTGCATGTTAT
A_18_P14136738 ATCTAAGTAAGGCTGTTTGGGGCAATCTGTTGTTTCCCTAGGAGTTTTCTG
CUST_3600_PI426210822 CTCTCACCGTTCTCCCCAGGGTGGGCTGTGGGCCTTCTGGTGCTG
A_16_P00685488
AACATAGGTAAAAAGATGTGGTGATTGGAGGCAAAACTAGAATCTAAAAGACCAAGTCTG
A_14_P114550 TGAGTTCCAAGCCCATCTGAACAAAATCTAGAAACATTTCTTTCTTGTGACCTTATTTCT
A_14_P135022 CTCGCCCAGACATTCTGAAATCACAGCGCCTACTCAGCAGAAAATC
A_14_P100572 TTTTGGCCAGGCATTTCTTGAATGTGCTAGTCCCTTTTTAATTCTCTAAAAGGAGAATTA
A_14_P136756 CCACGAGTTTTTGATGGAAGTCTTCCCACAGCACATCACATTTCTAAAAGAAATAAAGAG
A_14_P200185 GAAGAACAGAGAAACGGCTATGAGAAAAAGGGCCGAAGAGTGAGAAG
A_14_P127069 GCAGGTAGCCTGTCTTGCATCATTCTTGGTTGATCTGTAATTAACCATTATAAGTCATAC
A_14_P112174 AGTAAAACTGTATGTCTCTTTCACTCTCACCATCAGTTTTGCCTACCCTTTCTAAATGTA

A_14_P200090 AGGTCATGGAAGAGTTCCCCAAGTTTGACAATGATGAGGAGGATGAAAA
CUST_4846_PI426210822 TGAGCCCAGTCCTAACTGCTCAAGTCTTTCTTAGGTCTTATGATGGAAC
A_14_P200246 GTGGCCACTGTAACTTCAATACCCAACCCTGCATTTCTGGGTTACTT
A_14_P126892 TACTTAGGAGAACTGGTGGTTTGAAAACACTAGTAGACACAGAGCACATAGTTTTGATTT
A_16_P15791540
AAGGTAACCCAGTCTAACTCAATCAGACTATCTCTTCTCAATACATGTGTTTTCAGCAAA
A_16_P20478565
TCCAAAAACCTTGAATTGTTTACCTCTTTTCACCTGAAATATTCTAACAGCGCCTTATCA
A_16_P02975478
TGGAAGTAACAATCCAGTATCAATCCAGTAATGTGAATAAGATTAAAAAGGTGGCTTGCG
A_14_P124317 TTTATTTTGGATGACAATGAATTGAATATCTAATAAGGAGATTTAAGATAAGACCCAATA
A_18_P13036127
AAAAAAAAACTTATGATTCTAAGTGTGAGACACCTTATAAATGTTAGGAATTAGCAGGGA
A_16_P16191017
GGTTTAGAGCAATGATGTAAGTCTTAAAGGGCTAGACAAGGATCAAAAGGAGAGGTAA
A_18_P16446566 ACTCCCTATCTGGCTTTATATCTTACGTGTCTACATTGCTTATGTTCGGAATCTCTTACT
A_14_P200175 CTCTCGGTAGGTTTGTAAATACTTAACTGATGGTAAAATGTCATGAACCCCTACCC
A_14_P121346 ATAATAACCTTAAATGATAAAAACAAGGGATACTCCTTCAGAGTGCAAAGCAGTCGCTTC
A_16_P20207257
GATCTCTGTTCCTAAAGTCTCAGTCCATAGTTTTCTCCAAAATATTACCTCAAAGAGTGC
A_16_P16910557
TGGTCTCTCTTGTCCATAATCTACAGCATTCATCCTCTATATTTACTGAGAACTACAACG
A_18_P13668322
TGACTGTAAAACCTCCCTATTATTTATGTTACTGAGCCATCTACAAATTTCCATCTCCCT
A_14_P109269 GGTTTCAAAAGCCATAAAGCCTTTTGTATGGCTTGGACCTAGTAAAGACATTGTTGAAAA
A_14_P131711 TACTGACACTTTAGGTTTTGCCTGTTGAGATTAGGACCTCCTATTTGTATAGGACAATTT
A_16_P41398122
TGGTCGTATCTGTAAACTTCTCTCTTACATACCATTTCGATAAACCATCTGATTTCCACT
A_16_P18157911
AAGATTTCCTACTTTCTCTATTCCCATCATTCACATCAAAGTTAATATGTTCCCGCCTTG
A_14_P201971 GGATCATCATATCGCCGGCCAATGAGACGCTTGGTAGCATAAAATGTATTGTTT
A_14_P137314 ATATAAAGGGCAAAACCACTGGAGGATGCACACATTCATGCATTCAGTTAAATAATCATT
A_14_P111217
AGCAAACAGACTAAATGAGAACTCTTGCAAATATACAAGAAGAGGATATGAAGGCCTGAA
A_16_P20434012
AAACCTAATGGCTTTTCCATCATGGAATCTATGATCTATCTTATCTTCAGTCTTCCCAGA
A_16_P41153744
TTACATCGAGTTCCAGATACATCCAAGAACGATCACATTTTCCTTAGAAATTCTAGAGCA

A_18_P14834533
AAGCCCTCATTCTTACAATACTCTTCTAATGATAGTTCCATCTTTATGAACATTCCAGGT
A_14_P103879 CATAACTAAGAAACCGCTAGCAAAATTGTCCCTCTATGTCCCTGTATATTCCCTGTAATA
A_14_P117041 GCTTGCTGATGAAGCAGTAGAGGAAGAAGTTGATGGCTGTGTTCAGAAG
A_14_P131677 AAAACATGACAGAAAATCAGTCTTTACCTATTACCCAGGATATAATGAGCATCTCCATCC
A_16_P36230966
GAACTGACCTGATTTCCAGGAGGTAAAATTGTAGGGATGAATGATAATTTTCTAAGGAGA
A_14_P107501 TTCCTTATGATAGAGTTTATAAAACAGTCCTGTGTAAACTGCTGGAAGTTCTTCCACAGT
A_16_P02867572
GAGCCCAACTATATTCCTTCTCAACAGAATACTTTTCCCAATACCTCAAAGAAATGCAAT
CUST_3343_PI426210822 CAAAGGAAGGCACAAAAATTCTAATCCAAAGGCTTTGCCCGCCTTAAG
A_16_P02573185
AAAACATGACACCCAATAATATTCGATGTCTTTTTGATTCATTCATCCAGTCAACTCTCA
A_14_P128962 CTGCATCCTAAGTTACATTTTGAAGAAGACAGAGAAAGAGGAGCAGATGAAGTTGTTAAT
CUST_6800_PI426210822 CCACAGGATTAGAAGTATCTCTAACCTGAGCACCTCACTTTCTAGACTCCTA
A_16_P19875451
AGGTTGAGAGTATCCATAGTGTAACTTGAAAATGACCCTTACTGAGAAATAGCATGACAC
A_18_P10301688
GGGCCTCTCTTCTTCTATTAAGAAAATCCTACATCTGTTATCTTTCTTGCCTACAACTCT
CUST_7562_PI426210822 CAAAGTGGATGTGCCAACCCGAGGGAAGAGATTAGTAAGCTGCTG
A_14_P117313 TTTTGTTTTTTACAGACTTTAATGTTTTTCAGAACCTAAATATGCACAGATACATTCTTC
A_14_P124929 AAAGTCATTTTACCTTGGCTTAAAACCCTCTCTCAGACTGTTTGGTTTTTGAGCATATTC
A_14_P114833 TTTGGAATAAAGGCAGAATTTTTTCTACAGAGACTTGGTAGATGAGGCTAAAAACTACCT
CUST_6396_PI426210822
TCCATGTTTCACTGAAATCTGCAGGAATCGAAAACAATAGGCAGTCACTAACTAAAATG
CUST_8314_PI426210822 CTGGTGTGGTGTGTGTGTGAGATCTGTAACCACACACAGAATGAA
A_14_P137838 GGTCTACATAGCAGGGAAATTAATTGTTGCACTGAATGGACCAGATTTAGTTATTCACTG
A_14_P104215 CAGCCTCCAACTCTTCTGCGGTAAAAATCTTGCCTATGAATCTTTTGGCATCATATATT
A_16_P15193097
AATTGCTCAGGTAAAATAAGATGACTATTAGAGGCTCCTTTAGAAGAATAGCAAGTGCCC
A_16_P16965180
AAGAAGTGGTGTAACATCTATCTCTCTGGACAAGTAGATCAACAAAGCAATCCTGAATCA
A_16_P36230426
GATGTATCCTTGCTTGTTTAATTAATGAGCCAAACAGTCCAGAAACTTAGGTTACAACCA
A_14_P200835 GAGTGCCAAGTTTTACAACCAAATCATCTAGTGTATTGCAGTCATCTAAATAACAGCAGC
CUST_7434_PI426210822 CTCATTCCCTGGAGGCTGGTTTGGACAGTGTGAGAGGCATCCTTC
A_16_P01229583
GCTTTCTCCTCTAAAGACTATTACTAATACTCCTAACTCATAGTAGACACATCTCATGCC

A_16_P19655068
TCTGACTACTCTGACATCTTCCCACAATATCAAGAAAAAGTATCCCCTGAAGATTTACAA
A_14_P139864 GTGGTTCTGGTCGGCATCTTTATTATCACTTTTTAAAATGATCTGGTCTCCTATGGATTT
A_18_P15904641
CTGATGGGAATTCATGTAATTGAAAGCGTTTATCCTCTCTTAGTAGAAAAATTGGGCACT
CUST_10299_PI426210822
ATGTCAAATCCTTACACTAGCATAGTGATGAGAAAGTGAGCCAGCTTTTGCTCTT
A_16_P00685182
GTTAAGTAACCTGACTCAGGCTATGAACTAGTGAGAGACTTCACTGCCTCTTCATAATAG
CUST_3757_PI426210822 AAAGCTAGGTTGGCAGATGCAAGCACACATGCCTCCTATAGTGTTG
A_14_P100120 AATGCCGTTGATCGTCATATTGAAAATGGTAAAGGGGATAAGATTGCTATCATCTATGAC
A_14_P109465 GAATCAGCAGTCCTGTATCTATGAAGAAACAACTGGAAATGATAGGTTCTTGAATTCCAA
CUST_4923_PI426210822
ATTGCTGTATTCTTGCACCCTGTACCACAACATTGTTAGGATAAAGTTTCATAAGTCTTA
A_16_P19563333
TGCTTTGATTCTTTAACGTTACCTGCATTAACTTCTGTAATAAATGACTACATGCCATGG
A_14_P139842 AAGTTACCTCAAGACTATGTATTCCTCCCTATTTTGGAGTCAGTTTCCATTTCTACAGTG
A_14_P110272
TGTTCTTGAGGAAGAAAAACTTATGAAGTGTAATACACAGAGTCATGAACACAGGCAAAG
A_14_P115610 CTTCAGAGTAATGTCTTTCATTCTAAAAATGTTACAGGGATTTTATTCCCAGACTGGAGG
A_14_P119903 TCCTGTTTTGTTTTTTTCATTTGCACTCTCCTTGTTTTCTCAGTATGTTTTCCACAATTT
A_16_P01596231
AAATAAATGGTCATAGTATTGTCCCAAGAGATTCATTTCTCCATCTACTGCTGTGAGAGG
CUST_7315_PI426210822 CCTCGCAGCCAGCTGAGGGAGCGCTTCTCTTTCCACTGTCTGATT
CUST_4731_PI426210822 ATGGCTGCTGCTAAGAACCTCATTCTTATAGGAAGGATTCAGTTCTTTCCC
A_16_P16770366
CATGGTTCCCTTCATTACATGTCAGTCATCAACATATCAATTTGGCTTATCTTTCAGCAA
A_16_P03794516
AAAAAATGCTGAAAGTATAGTCTTTTGGAGGTCAACTAGATTCCTCACATTTCCAAACCC
A_16_P02411952
CCAGCCTTATTTTTGAACGTTTCAGTCTTAATCTACTGGTAGATATTTCCCATGTTCCAA
CUST_1291_PI426210822 AGCTCCCTATTTCATGAGTCTTTTAGCTTTGGTCCCAAAATTGAATTCTGCTCA
A_14_P100305 GTTTTGGCCAGGAACAGTAACACATTCCATCTAATGGACTTAAATTAATTAGAATCAGGC
A_16_P02145516
GCATGCATAACCAAAGTATTCAGTAAGCAGTTTAGCAGTGATATTCTCAACAAGTGGAGT
A_16_P19165413
AACTTGTCGCGTATGCTTGCCTTTAATTCTTGGTTTCTTGAATTTATGGGATCTCAAATT
A_16_P16191652

CTTGTTTGAATCACTTGACTTTGAAATCCCAGGAATGACTTTGCTGTGTGATTAAGTGA
A_16_P18058401
TTCCTATTTTTCCATCATCCTATGTCTTCCTAAAGTCATCCTTAATTTCATCACAGCACC
CUST_3435_PI426210822 CAGTGACGCGTCGGTGCAGAGAATTCCTGTTTTGAGGAAAGATTC
CUST_3429_PI426210822 AAAGTCGTGTTTGGGTGGGAATCACAAGCAGGGGAAGTGGGGGAG
A_14_P129685 CAATAAATAAGGAATAACTGAAACCAGACCCTTTAGGAAGAGACAGAAATTCCATTACCC
A_16_P15849541
AGACTCAGACAATATCACTCAGTTGACTAGCAGTAGAGAATATTCAAGCCATCTTTCTCT
A_14_P118785 AAAAGATTTCATTTATTTCAGTCCCAGAGTTTGAGGTTATAAAAGCCCCTAAAGAATCCC
A_14_P117188 TTTCCTTAAGTATGATTAGAAATGGCCATGTATCAATGCTACTCAGACTCTCAGACTGTG
CUST_7702_PI426210822 TAGGAACTTTATGGCTCAAGCCCGAAGGATTCCCCTCTGGGAATA
A_16_P00184829
ATGACCAGGAACACTATACAATGAACACAAAATCAACTCTCTCTTAAGAATTCATCAACA
A_14_P117244 ACCTGGAAATGATGAAAAGAAAATAAAGAAGATTCCATCCCTGAATGTAGATTGTGCAGT
CUST_5316_PI426210822 ATTCCCATTCATCCTTGTGTGTGAAACATTTTTCCCCTCACCCTATATGC
CUST_9830_PI426210822 TGTAAATATGTGTCCTGGGAGCAGCTGGAAAGGCAGGAAGGAGAC
A_16_P21591919
TTAAGACACTATCACACTGATACACAGAACCTTAAGGAAGATTGACACCAGAAATTACCA
A_14_P117822
CAACATACTTACAGCCTTCTTAAAAACAGCAACTAAAAGCAAGTCACAGAAGGCGTAAAG
A_14_P106103 TTTATCTTTGTATTTCTAGTGAATCAGCATGGACGTGTCCCATGAAAGAGGAAACACATA
A_14_P133950 ATTGTCGGGTAAAGTTATAATTAGGTTATCTCCAGCCGATACCGTAAGTTCTTTCACTAA
CUST_7173_PI426210822 TAGTATAAAAAACTTCCTCTGGCCCACCCCGAGCAAACCCTCATT
A_16_P17594867
TATCAAAAGAAAAAATAGACCTCCCATAACTTTCATGAAAAAGTCAATTTCCCTGCTCCC
A_14_P133453 ACACAAATGTCATTGAGATGTGACTACTAGATTGCCAAAGGAGAACTTATTTGCATACAC
A_18_P14134530 GCTGAGTTCTGAGAGCCATCTGAGGCATGGTTCTGCTGAAAACATTTATTC
A_14_P100993 GAGAGGACTAAGAGATATGAACCTTTCCAAACTGGTATCTTCTCTGAATTCTCTTCTTTT
A_14_P201477 CTGTTTTGCATTTTCTATGTATGCAGCCATGTATTCATATGCAGCAGCCTGAGCATT
A_16_P21625667
ACAGTGATTCTCATTCTTAATATTTAAGCACACCCCTAGTTCAATACCTCTTTCATGACC
A_14_P109467 CTTTTATGCCAGATTTTGGGGAACTTAGTAAAAAACTGGCTGAGGTGTGGAAG
CUST_2326_PI426210822 CATGGGAAAAAAGGAAGTCATGCTCTCAGCAGAAGCATTGTCAGGATA
A_14_P136256 GACAGCAGACTATTGAACTGGGACATCACATCCTCAAAGCACACATTTTTAAGGTAATT
A_16_P30952494
AAATACCACTTCCTTTTTCACTAGTATGAATTCAGCGTGATCTTTGATACAAGCCACCTG
A_16_P21157689
ATGCTCACATTTGAAATTTTTTCCCACCCTACTATAGACCTCACTATTCTTGCAAAGAAG

A_16_P21198125
ACTGATCTACTTGTTATCTAGTGCTATCTATCTCCAAAAATACCTGATCCTCATGGTAGA
A_14_P112614 GCTGAATGATGGATACTATGGTGCTCCATTTGAACATAAGGTAAGAAGATCCTTCTAATG
CUST_1451_PI426210822 TCACTTTCCTGGGTATTTTTAGTCACTCACTTTGCAGAACCTTTGATGGTCTA
A_14_P120669 TCACACTCAAGTTTATGGGAACTGAGGTATAACATCCTAGTAACTTCTTTCTTGCAAACT
A_16_P01579784
AAGATAAAGAGGCAATGTTTTCTAACACAAGATCTCACCTATTGGTAAGTCTTAAAGCCC
A_14_P127889 AGTCCAGTTAGAGAGTCCAGAGTCATCATTTTTAGCCTTGTCAACAACAGACAC
A_14_P127383 CCTAGACATGGGTCTTAAGATCCAGTAGTCAGTAGGTCTGTTGCAAAGTCAG
A_14_P137175 ATGATTTATCATTTTCCCTTCACTGCAAAAACTCCATGCTCAAACAATGTGAGTTTACAG
A_14_P132571 TCTTGAGTGCAGATGGTGATGATAATAAGTTAAACTTATTCATGAACATTTCCTCGATGC
A_16_P35873535
AAGGGGTTAAGAATTTATGTCCAAATATTCACAATCCAAGTCATGATATGCTAAGTGCCA
A_14_P121561 CTCTGCTGAGAACTCTTTTCCACTCCAGGTATTCTTTTGAAATTTAATTTTTAACCTCTG
A_14_P125114 GAAACAAGAAAATCGCTCACAAGTATTTCATGGGCATCTGTATGACTAGAATTTGTTGCT
A_14_P108131 AGAACAATGAACCTAAGACCTCTGGAAATAGAGTAGATGATGATGAATTTGTTCTTGCTG
A_14_P129447 CTACAATAAACAGTAAATACTCACAAAAGGAGGGGCTTCACTAATAACTGGACGAATCAC
A_14_P112499 GAATCGGCTCAATTGGTCCAGCATTGAGCAGTCACAATTCTCAGGTTA
A_16_P36529467
TGGCACAAACAATAAGCATAACTGACTCTAATTCAAACTGCAACATCTTTTTTCTGTTGG
CUST_1419_PI426210822
TAGTCTCTAGAGTCAGTGGGATGGATATGCTTTTTGCCAGACTTTTCACATTTAATATGT
A_14_P139687 GATGTTTCCTCTATGGCCCAGACTAATTTCCCACCCAGGTGAAAATCATTTAAT
A_16_P16937260
TGAGCCACATATGAATCTAGAACAAACGTAATATGCTGTTAATGATTACTCTCCAGACTG
A_18_P14132656
TATTCAGAGACACTTTGCATTGTTTTCCTACATGAATCCTTCCTTTGACACTTTGATCTC
A_14_P101612 CAGCATTGCAGTAAGAAGAATTCCATATGTTCCTTTCTTCCTGATTTTACCAGCACAATA
A_16_P02886137
ATTCTAGGAGGTGAGATTGAAATGAGTTGACTTAAGCATAAAGCCAAAATGAAACCAGTC
A_14_P135567 GGGCAAATCTGGATGCAACACTCTTGAGAATCTTCTTTTACTAGAACATAATTATTGGGA
A_16_P19142967
CAGTTACGCCAATCATTCTCATCAATTTCTTAAGAGAGAAAACGACTATTCCTGTCACAG
A_16_P00781179
AAGCCTCAAAAATTCCTCATATTATGCCAAATGTATCCTTGCACTTGTGACATAGAGACT
A_16_P21660639
TGCTGCTCCTCAAATTTTTAGTTATCTTTCCAGACACATAAAAAGATTAACTCAGATCCC
A_14_P116311 ATAAAACATTTATAAGAACAGTGAAACGCCAGGCAGATCCTGAAACAATGAGATGTTTCT

A_16_P30952585
AAAAAGGTAAGCCCTAATGAACCGCACTTGTGGATGCATTTAGAAGCATTTGAATTGT
A_14_P202417 TGAAAAAAATGTTTGGAGCCAAATTTTGCAGTGCACACATCTTTCACACTTCCATAACTA
A_14_P119819 CTCGGTCATAGTGGAGTATACACATGATAGCGACACAGACATGTTCCAGGTAT
A_16_P18000820
AAAAATATGCAGATACAGACTTTAGAGCACAACTATTCCATCTGAATCAAGCCTTTTGCC
A_14_P119031 CATCGCCAGGCTGTAAGGTTTCTAAGTCACCCTTAAATTCACTATAGTGAAAGAATATCT
A_16_P58424534 GTTGAGAGAGTTGGGTCTGAAAGATTGATAGTGTGTGCCATTGATTAGAAGGAGAT
A_14_P117538 GCAAAGTCAGCAGGAAATGATTATCCATTGACAGTTTTCATGATAAGTCTTGGAAGCATT
A_16_P00621517
GTGGCATTTAGTTTCAAAGTTTCCTGCAAAATCTTCTACATTCCCCAAACATATCAAGAC
A_16_P16809080
ACCGACATCTGTAGAGTCATTCTAACAAATACCAGAACTGATAGCCTTTACACAAAAGAG
A_14_P101366 TTTAGTTCCATGACGCGGAATATCAATTTCTTTGGGATCCACAAAGTTCATTGGTATTTC
A_14_P201226 TTGCCTTAGGAATATAGTCTTTCCTGGCACTCACTTAGCCAAAAATCTTTCTAGAAGG
A_14_P109471 ATTAAGTTCGAGGTGGCTACCGATTTCAGCACATGCACTACTGAAATTTACACAAAAAG
CUST_987_PI426210822
GCTATTTTAGCACAAAACAATATCAAGGAGAGGAGTGAATGTGGTTTGCAGCAGA
A_14_P130591 TATCCCAGAAGAGTGGGGATTACTGTGACTATCTGAAGTTTTTATACTTGAATTTTTCTG
A_14_P122084 TTGATTGGATTTGCATCTCTTTGGCAAAGATGTGTAAATGTTGTCCTTGTTCCTTTAAAG
CUST_8875_PI426210822 CCTCAATGTTGTTAGACGCTGAGAAGTAATGATCTCCCCAGCTGTCA
A_16_P20210040
TCCTTCTTAGAGACAGAATTCATAGTAACTGCCTTAAACCCATTTTAAAGAGCGAAAAGG
A_16_P40079670
ACAACCTTTGAATATTTCTATGCAACATTTACATCATTAACAATAATAAGCACCTCCCAC
A_14_P137707 GCAATCTATACCGATTATCTGGTATAGATTTTCTGCAAAGGAAAACTGGTCTCTCAGAGA
A_14_P128188 TGACAGTGCCAACATTGGAACCTAGAACTGTTGACCAAAATGAAATAGATTATTAATGGT
A_14_P125858 ACATACCAGTGCTTTACAGGGAGGCAAAATGATTTCTTTTTGCTTAATTGTGCTTGTTTA
A_14_P101627 CTAGACACAATCTTTGTTATGTTGAAATCTCTGACAATAAATTCACCCCTTTCCAGAGTC
CUST_10019_PI426210822 GCGCAGTAACTGCTCTGGAAACTGAGAAGAAGGTGCCATGAGTCC
CUST_4372_PI426210822 CACAGATGTTCCCTCCTAAAAAAGGACATGCTGGCCTTGCTTTCTTA
A_14_P100367
TGTCCTGAAAGATAGCCCTTTAGGCATGCACAACTGTGAAAGAAAGAAAAAAAAAAATCA
A_14_P123052 CTCTTCCAGCCATTTTCTTGCTTTTGATTAATTCTACTATGACGCCACATGCCTACA
A_14_P106271 CTCCTGTGTTCATTGTGACCTCAATGTTATCATAATCTCAGGTTGCCTATAGGTATAAAC
CUST_7884_PI426210822
TATCCGGCAGAATCACTAATTTTATCACTCAATAATTTTATGTATTAAAAATGCTCATCC
A_14_P114546 AAGCCCTGAAAAGATACTACATTTTGGAAAGAAAATGAGGTCCACAATCTATCTTACCTT

A_14_P121103 CTATCCCAAATAATTAATTTTCCATCTTGAGAAGCACTGACTAGCAGCCTAGAGGAACAA
A_14_P131649 CTGAATCACTGTTATCATATTTGCAGAATTCAAACACTATGTTGATGACTGGCCTCTCTT
A_16_P01668323
GCCAAACTTCATAAACCAAAAAGCAGTCTAAATCTCCTATCCCATAATGACACTCACTTT
A_14_P115118 CTACATTTCCCAAACTGCTGTTCCTTGGAGCACTTCCAGGAAGTGTTAATAGATATATC
A_16_P17125718
AAAAATCACAACATTCAACTGAATTAGCATCCTTCTACTTCCAAATCCTGTAGTGAGGAG
A_14_P119294 TTTTGTATGTGACAAAAATTTTGTTGGTGCTCATCTGAGAGGGTTTGAGCCATCTG
A_14_P106231 TCAGTCTGTGGAGAATCAGAGAGAATGAATTTGAATAATAAAAGCCAGAACAAGGCGATA
A_14_P132328 AACTTTGAAGAGGGGAAATAAATACCAGCACTAAACAGACCTCTTAGAGTATCCTTCTAC
A_14_P112864 AAACCCATGTTTCTTTAACAACAAGAAAACACTCCTCCTCATCTTTCCTCTTTAATCTCA
A_18_P12311859
AATACCTTCATGGCATTTAAAGCACAAAATCTGTTCTTGTTAACCAAACATGTTTGTGGC
CUST_8440_PI426210822
TATAAGAAAGGTGACATTATAGACATCAAGGGAATGGGTACTGTTCAAAAAGGAAGGC
A_14_P125923 GAAGGATGGACTATAACATTGAATCAATTACAAAACGCGGTTTTTGAGCCCATTACTGTT
A_16_P17208197
AACCCACACATTTTCATTTCCATTGCATTTATTCTAGTTACCTTCACTGATAAACAGGGA
CUST_7880_PI426210822 TTTTAAGAAGCTTCCTCTGGCAGCCAGCCAAATGGACTGAAGGGG
A_14_P118814 ATGGAAAAACCACAACTGATATAGGGGAAGGTATATGCCATATGTGAACACACTAAAATG
A_16_P18981019
AATAGGAGAAGTCAAATATAGGTTTATTGACAACAAAGGCACTATTAATGTTGGACCGGG
A_16_P15792300
AATAGCACGTTTCAACTAGGAATCCATTCGTGTCTATCTTGGTAATCCTATACAGTCAGT
A_14_P129042 ACTGCTTTTTTACATGTGCATTCCCCCTTAGTTTTAAGAACCATAAAAATTCCTGGAATT
A_14_P122695 CTGTAGCACTGACTTCATTAAAGAGAGGAGGTGGATTCTGAAAATATATACAAGCAACAA
A_16_P20698958
CTCCTATAAATTTGCATACCCCAACATAATACTCCTCACCTATATGTATTCCCCAGTTGA
A_16_P19954623
AACCTATCACTCAAGAGTTCAATCACTGTCTCTTTGAGAATGACATAGAAATTCCTACCT
A_16_P37836841
AGAGCACTGACAGTTTTTCCTCTCCAAATTTTTGAAACAAAGCCTATAAATGACAGTGAA
A_16_P35841778
TGATTTTATCAGACATTCACTCTTCTTAATGTGGATCAGTAGTTCTGTCTCATACCAGCC
A_14_P107185 TGCTGCTATTAAATTCTGGTTTGCTGAAATTAAAGTAAACTAGGGGCCTTCATTCAGAAC
CUST_5344_PI426210822 CTCCCACCCCGGGAGTCATCTGTCCCCTTTGCCTGGTCTCCCATG
A_16_P16097498
CCTCTCAGCACTTAATCTAAACGACCTGAAAGTAAACAGAAAGGAAAGCGTTAGATACAT

A_18_P22841402 CAAGGTGACTTCTGGCTTCTCAGTGACTCAGCCACACTTCTCTTTTT
A_18_P15242729
ATCCTGATTGGCTTAACTCTTAGTGTCCTGTGAAATATCTTGCTAATATTTGTCAGAGGC
A_16_P16540414
AAAGTTCTCACGTGTTTTTAAGCCTTTCTACCAGACCTTGAACTATCCATTAAGTCAAAG
A_14_P101021 CTGAGTCCCTGACATGCTCTGGTTTTCTCAGAACTAATTAATGTGAGAGCAAG
A_14_P114683 CTTGAGCCGATGCCCAGCCCTTTATAATTTCAAGACTACATTAGAAGACTATAGAAATAC
A_14_P128260 TGTGACTTACTTTAGATTAAGATCACCGCAAGTGTGATCACAACTTCCCATTTTAATGCA
A_16_P01845803
TCACCTCAAGTCACTAATCTGAAGTCTCAATATCACGTCACTTTTTCTGTTAATACACCA
A_14_P128736 CAAACGAACAAAACTTGGGGAAAAGGATCTACCTCATAGCGTAATAGACAATGACAATTA
A_16_P18825277
AAAATTCGAGGTCATCTTAGAGATGCTGAAACCAAGAGATGTTAATAAATGAGTGACACG
CUST_4275_PI426210822 CTTATTGATGGCACATTTGATGACTGTTAGTCCATGGAGTGGTGGGG
A_16_P16919393
AGCAAAGGAAAAACATCGATAACAGAATCAGTCATGAGTTTTGCATAAGTTTACAGCCTG
A_18_P13632898
AAATGTGCTTGGAATAACTGTATCTGCATATGTCCTCACATGAATTCAACTTTGCCAAAC
A_14_P139858 CTTTGGGAAGAAAAAGGGGTTTAAGGATTAGAAAGGAAGAAACCTAAAATCTTGGCC
A_14_P124244 GCGTAGACTCAAGCCAAGCCTTAAATTTAGACGGGAAATCTTAAGGTTTTACTTTGAG
A_16_P00018420
TAACCAATTATGATGTGTTAGCCTGAAGTTAAAGGAATGGAGAATTCAGTAGACCTACCC
CUST_8375_PI426210822
AAAATTATTTAGAAGGGTTCAAGTAACACAGACCATGTAATGAAGAGGGTATGAGTGTTG
A_14_P102593 CTGGCCCTTCCTAAAGTCTGCTAAAACTTATCATTGACAGATTTTGCTTTGTCAATCATA
A_16_P21464716
TATCATAGTTCACCTTTTTTGTGGCTACATTTCATCTGTCTTGTCTGAACAATTCAGACC
A_14_P136489 CTGAAAGTTAACCTCCAACCCAATGATAACTACTTTTTCTATGTGAGGGCCATCAATG
A_14_P105326
AAACTTACTCAGAAAGGTGCTCAGAAGTTGGGATACAAACAGAAACTGAAATTAGAAGAA
A_16_P02166255
TGCACATGCCCATATAATCCAAAAACAGATATTTTAAGGAAGAGGTAGCATTTAACTGGG
A_14_P130014 ATTTTGTAGTCTTCAAAAATTAAAAAAGGAAGCAAAGGGGATACATCCATGTTGAAACCA
A_16_P18198939
AATACCACATTACCATTAAACCATGTCAACCATACTGTTTCTTAACTGTGCCATCTTGAC
A_14_P102730 CTGTATTTTTATGTTTTTCAGATTCAAGTCAATCTGAAAGTCCCAGCCAGCCAAGTGAC
A_18_P16735036
AAGAGACCATTTCCATATCAATTAATGTCCAGAACATTCATACAGAGAGTTGAAATCGGG

A_14_P135759 AATGAGAAGTCTCTTATGAGGAAGATGGCTCAGGATTTAAGCAGAGAAGGACC

A_16_P21144114

AAATAACCCAAACTGAGAATGTTAAATCTCCCTTATGAGGGTTAGAAGTAGATAACTGGC

A_14_P100018 TGTCCTTGCCCAGTAGAGAAGTATGGTATTTGCATAGCATTTTCAAAGTTAGTCTTTTCT

A_18_P10682425

TCACAGAATACTTAACTTACCCTATCACAAAACTCATCTAACCTGACTGCAGTTACTTGT

A_16_P01854922

TGAGTTGGCTTATAGTAACTACACTTGGAATCCTAGATAAAAGAGAACGATGCATTCTGG

A_16_P02240511

GAAACCACAAAATAAGTGATACCTCCACCACTAGTAAAACTTCGCAGTGACTTTCTAGAG

A_14_P124748 ACTCTGTGGAAATAATATATGAGGGCACAAATATTAAAGATTAAACGTGGCTGAGGCTCA

A_16_P36663657

ATTCCTTTGATCAACCCTACTGAATTCATGAACACATCAGGCTAATATTTCCCTTTTCTG

A_16_P00378939

TACTATCATTTTCCTATGTCACCATATCTACTCCTGAACCTTTAATCACTCAACTCCTCC

A_16_P21498682

CCTTTTGATGACAGCAATAATTACTCACACTTCTTCTATGTTCCCTCATAATACCCAACA

A_16_P15610405

AAAAATGACACAAAGCATGAGTCTTCGATGTCATTTCAATCATAATGCGAAGCAGTTCAA

A_14_P110904 CTCCATTTTGCCTGCATGCAACGTGCACAGTTCTGATTTATTAAAGACATAACAATTATG

A_16_P17742298

ACCCCAGTTCTGATAAATCAAACCATTTAGCATGTCTTCAAAGTCTATACATAGGTCCTT

A_14_P103769 GGATGCACTACATTTCTGCTAGATTTGCTCAGTATAGAGGAAGAAAGCCATTTGTAAATT

A_18_P15353956

AATGGCTACCTGAATTAGCATTCTAGTGTCTATCCTTTTTTCTGAAATGTGCTTTGGACA

A_14_P113355 GCAGCCCTAACAGTATGAAGTATGCTGGAATCATGGCAGAAATAATCATTGTGTTG

A_14_P102158 GGGCATGTGTTTTCTACTGTTCTGCAATCTGCGTTTCTCAAAACTATTACTTGTATTTCA

A_14_P120820 TTGAAGGATTTGTCTCTGGTAGGAAACATACTCATTAACTAATAGTGAATTGGCACCTCT

A_14_P108521 TTCTTTTTTGGTAAAATTGGGATTATGACTAGGAATCTGTTTTTTGGAGTTTCCCAGGTG

A_14_P127809 GGGAAAACGCTGTGAAGACTTCATTTTTGAAAATTTTATTACAAAATACACCCGAACCTG

A_16_P16148534

AAAATGCAGTTAATGGGAAGTCATCCATCTTCTGACATAGTTAATTCAGTTTTTGCCACA

A_14_P108563 TGATCTCTTCCTGGTTTTATTTCAGGTTTTTATTTTGATGTAATGGAGATCACCCCTGAA

A_14_P112168 CTAGGTTTCTGAATTCTCAGATGAAAACATGCCTTCTCAATCCTGTCAATCTCCTC

A_14_P131294 CTGCTAAAAATCACCTACCTTTGAAGTTAGCCGAAGAGTTGCTTGTAGCAATTTCCTT

A_14_P112407 CAAAAACAAGTGCATCCAGAAAGAAACAATTTTGGAAATACAAAAGTCTGCTCTTCCTCA

A_14_P119111 GTGTGACATGTATCTTCCGATGCAGAATTTTCAGAGCAATCTCTGGATCCATG

CUST_8299_PI426210822 TTAAATTGAGACAAATGTTTGCTTCTCAGAATGGCAGTGACCATGGGTGC

A_16_P21510334
AAAGCAATCCTTAGCAACTTTGTTAGCGGTTCTCAGATATGACTACATGGATCTACATTC
CUST_5905_PI426210822
AAGAACACAGGAATTTCCTCTTTTTTCCCAAGACTTCCATGATATTTTCCCTGATAATC
A_16_P17396719
ACCAAAACCCATCATCTTTCATTTCTATCTCCACTTTATCTCCATTTCAGCCTCATATTC
A_14_P111255 GCAACGTTTCAGTTGTTTCTTTTTTTAAGTCTGAACTTTGGAGGACAGAAAAAGTGTGTA
A_14_P113738 TTAGTTATGGGTGAAGGTCCTGAGCGAGGAAGAGTAAAAATTGGTATGTTATATCTTTGC
A_14_P113021 ATAGGTTCCGTGTCTTTCACTTCCTCTTAGTAACTCATTATTATTGCCTGATTCTCATGT
A_16_P20297520
AAAAAGTGTAAGATGTGCTCTCTTTGCTTAAGAAATGCGTAATTCATTTGTCAAGCCCTG
A_16_P00685378
AGAGCAATACCTTTCTTGGTTAGTAAAATAAATCCCTAAATATTTGCCACAATCTCTAGA
A_14_P125748 AAAAAGAATTGTTCGAAGACAAAATCAGTAGCCCATGATGAATAAAAAGTGTTAGTCACA
A_14_P138402
ATTATAGAAAGAAAAAGATGACAAGTGGAAAAGAGGGAGCGAACCAGTTCCAGAAAAAAA
A_14_P138929 ACTCTAGTTTGTTTCAAGAAGAGATTTTGGGTAGACGTAATCTTCACCCTTTCAAATTAT
A_14_P133348 AGATGGAGATTCAACTATGATACCCAATAGATGGAGACTGTTGCTGATTTTTTGGTTATC
A_16_P00685044 AAATGCCTGGGCATCGAGGACAGTCACTAATAGTGATTGAGTCAGTCACTATTTA
A_14_P137124 TTCCCCTTGCTTTTGTTATTTCTCTTACATACTTATTCTGAATCTGATGCCCTTGAACTC
A_18_P14750337
CAATTGGCCCTAAAATAGAAACTGAAAAACAGGACATAGAATTTTTTCACCAGACCACAG
A_14_P139476 TGTAATAATGGCTGAGCCATGTGCCGATTTCCAACATGATGAATCATTTGTTTACATTTT
A_16_P15889390
AAAACAGTGTTTTTATTACCGACTGGAAGAAAGTTTATGAACCCTCCATCATATTTGGCA
A_14_P201199 GCAGAGGAATTTGTCTTCTTCTTTAACACCATTAAACTAATAGGATTTTGCAGACTACCT
A_16_P40936907
GCTTTTTCACAGGACAATATATAAGCCCATAATTGAAAGGACAGAAAAATTGAGTGTGCA
A_14_P113926 TACAAGTTAAAGTGAGACATGAAGCACACACAATATGGATTCTGAGGACAAGAGATGTAG
A_14_P125975 CTTAGAACTTTAAGCAACAAGACCACCACTTCGAAACCTGGGATTCAGGAAT
A_14_P103754 GAAAAAATCCATTCTAAAATACAGAACAAACTCTTAGGACCCCCAGTGTATATTCCACAG
A_18_P22842466 CAAAATGGTCAGGGAGGTCCCTTCTTAGAGATGAGAAAGTCACTCAGAG
A_16_P17071328
CTCCAGTAAAGTCAGTTGCACTGTAGTTCATCTTACTATTTGATATTTACAACCCAACCG
A_16_P00685537
GTTCTCTTTGTTAAGCCATTCTGGTATCCCTCAAATCCCTTGTTATGACTATCCACAATT
A_14_P101808 ACTTTCCAAAGGTATTTAAACTACTATCCCTTCTCACTCCTGTAATTGACACCCTATTTG
A_14_P100566 TTACTTTTTATTCTTTTACATTTTAGGTGGCTGCCTCTTTGATGAGCCGTATAGCACATG

A_14_P108739 AGCATTACTGGGTTACTATGCACTTGCGACTGATTTCTTGGCTTTTTATCATTTTGAACT
A_14_P103687 CTACAATTATTGGACACCAAATGTATATGATCACGCTGACTGTATAACCTCATGCTGTCT
A_16_P39325915
TAACAAGCCCTATTATTTCATGACCAGTTAGCACTAAATGTCCCTAAGAAACCTCAAACT
A_16_P39638425
AAAGACTCCATAAAAGGACATCATATCATGTGCTTTAACCCCAATTAATCCTCTTCACTC
A_16_P01414464
ACCAGACACCATATTCTCTAGGTCTTTACCCAATTTCATTGAAACTACTTAGCATTCAGT
A_14_P113472 ACTCTTCACCTGGATGATAGTATAAGAGGTTTTCAGGCTTTAGATTCCTATGAGTTATCT
A_16_P38074672
ACATCTGTATGTGCTCACTATTGGTCCTTTTCTAAAGATTGGTCTGCTGATATATAGGGT
A_16_P00685320
TAAAATAAACAGTCTTAGGGGTTACTTGTGACTACTGTGGGACTTAAACACTTTCAGACA
A_16_P02937933
GGCCCGTGAAATAAATTATAACATCACTTAGTTTCCATGATATTCTTCTGCAACACTGCT
A_14_P115960
GACAACAGATCAGAGAACAAAACGAGAAAACATAAAGGAAAAGCGAAAGGCTATCTTAGA
A_16_P15580880
ACCACTTGCCCCATAATAAAATTATCCCCCTTGATTATCTTCCTAAAATACTACTGGAGA
A_14_P200858 ACAAACTTTTGTCCAACATTAAGCCATACTCAGAACAGGTCAGAATCTATCCATTTTTAA
A_14_P111242 TCTGAACTAAAGTGACATTTAAATAATTTGAAGATATGACTGCTCCACCTCCTTTGACCC
CUST_6658_PI426210822 TAGGTCAGTAGTTGTGAAAGTAAGGGCAGATCACTGCTTGTCGTGTTAGT
A_16_P35491165
CACCATGTCACAAAACACATCTTATATTTGCTGGACCTTGTATGATCAGTAATTCCTACG
A_16_P01681728
AGTTGTGCTAGGAGAAATTGATGAAGTTTGCTTTCAATATTCAGACAGGATTTCTGGAAG
A_14_P114020 GCTCTCTTCACAATGGTAAGTTTGCATTTGTTTGTGTGATTCTAGGCCTCCTCTTATTTT
A_14_P106543 GAAGCCTGTCATACATACAGTTCTGAAGAGAAAAGTTTATTCTTCAAATGAGAAAATGAG
A_14_P122671 TTTATATAGATCTGACACATTCAACTTGTGTCCAAACCTAAAAGCAAGAGAAGAGAGACG
A_16_P41418838
GACATCCTGGTAGAAATCTTAGTCCACAGTCAATCAAAAAGAAGCAAATATCACTACAGA
A_14_P123220 TTGGCCAGAAACATATTTTTTAAGTAATTCCCTCACTGACAGGTTCCTACAAGTAAAGTT
A_14_P104963 TCTTGAAGTTACTCTCTGCAGAGTATCTGATGCAACAAATTTGTCTTTGCTTGGTAATTC
A_16_P35741759
ACACTGTTTTCTTCAACATCAAAATTGCACACTTTTCTACATACTTACAAAGAGGCTTTC
A_16_P36230556 CTGTAGAAGGATGTTTTCTCTCATGGAAAACCCCAACTCTGAGCCC
A_14_P130707 TTACAGCCTAAACTGAGTAAATTCTCACCAAAATCATTCTTCCCCAGGTGAGTATAAAGA
CUST_556_PI426210822

AAACCAACAAGAAGATATCAAGGCTTTTTCTTAGATCTGCCAGAAGTCTGTAATTTGCTC
A_14_P128646 GATTGGGAAAGGGATACCTTCTTTGTTATATTTGCTGGAAAAATACAGGCTTGTTTTCCT
A_14_P112678 AATTCCTTGCACTCTAACCAGTTCTTGGATGCATCTTCTTCCTTCCCTTTC
A_14_P117050 GTGGGCAAAATAGTAACTCTTAGACATCTGGCTCTGACAACGTAAAGATGTTAAAATTGT
CUST_6161_PI426210822 AAGGATGAATGGACATGTGGGTGTCTTCCGTGGGAGTTACCTGGC
CUST_5888_PI426210822 CAGATCTGGCAGCCTGCATGTCACCAGATGGATTTAGGTCACATC
A_14_P101572 CTACGTCTCTTTATTTTATTAGAACATGGGCACAACCATTTATTCCTTTTTTGTGGCTGC
A_18_P12559831
AAATCACAGGAGAAAGTGAGTCAATGACAATAACAAGAATCCTTTTCAGCAAGTTTGTCA
A_16_P02946930
AACCTGAAATGTATAGATTATCCATCTAAACCGTGATTTATTTCATCTGGGGGTCATTGG
A_16_P00685165 ACTTACATGGAGGAGACGTGTCGTCACTATGTGGCCCATGTGAAG
A_14_P126854 TTTGTAGTCACCTGATTTCTGGCCCAATCTTTAGAAAAAAGTCACCCAAAGATACAATAA
CUST_1552_PI426210822 CTCCAACAGGCCTGTCTAGTATTGTATGACTTGAGTCAATAGGCAGTGG
CUST_3397_PI426210822 ACAGGACTGTTTAAGCTCTGGAAGTTTTCCTACCTCCTGCAACTGAATTT
A_14_P114667 CTACAGTGTTTAAAACAAACAATTCAGTCCTTGACTGTAGTAGTCATGTCCTATAGGTCC
A_14_P104220 TCTGCTAAAATGGGCACTTCATCAAGATAACAGGAAAGCAAACTTAAAGTAACGAGATTT
A_14_P128878 AATATCATGAAAGGGGGAAATGTAGTGACCAGTTACATTCTCTGCAAAGACACAAACAAA
A_14_P106374 TTCCCGGCTGTCTTGCCACTTGTGTTACCTGTGAGGAAATCAATATTTAGTAA
A_16_P15924782
ATTGGCCTAGTATTCCATATTCCCATATCTCACAGTGTTATGATCTTCAAAACTTCAGCC
A_14_P113096 AGAACTAATCATATTAGAAGGTCCACCAATAAAAAATCCTGAAGTCATAAACAGGTTCTT
A_16_P00892159
TGAAGTATGTTTTGGATGCATTATGAATCTTCAATTGACAGATTATTTCTTGCTCGGGGT
A_14_P111811 ATTCAGACAATTGAGTTTGCTGAACAGCGGATCCCTGTATTAAATGAATATTGTGTGGTT
A_14_P110539 CTAAGATTTTTTTTTCTTCTTTTGTCAAAAGCAAACTTCCATTCCTCTGTGTCACGAAGC
A_16_P16191068 CATTTTGCACATGGGCAGTCTTGCTAGGAAAGGCTTGTCCTAAGTC
A_16_P01957997
AAAAGTGATCATCCATCAGTATTATTTTCTAATTGCCATCAAATGTCTATGACCCTCAGA
A_14_P138602 CTTAGGCATGCAGGAAGAAATCCATTTCCATATACTGGTTACTGTCAAACTATTCAACAT
A_14_P132656 TGTTTGGATACTATCAGTGAATAGGTGGCAATTGTGAGAGAGAATTTTACGGAGAGAAAA
A_14_P126682 GTAACTACACTTGTATTTAAAACAACCTGCCATCTTTTCACCTTTAATTGATGGCACCTC
CUST_8294_PI426210822 AACCTAAGATGAGAAGACACTACTATTCATGCCTGGTCAGAAAGCACAGACT
A_14_P138899 AAGAAGCCTTTTTCCCAAGTTGCTAGAGATACAGCAGCCATTATTTTAATCTATAACGCA
A_16_P02872668
AATCTGCTTCCAAAAAGAAGTTTCACCTTCTGTCATCCTTTATTCAGAGTTAGACATCCT
A_16_P16497404
AAAAAAAAAGCCATGCAAACATGTTAATTTTGTCCCTCTTTGATTTGCTATGGAAGTCTC

A_16_P03032802
AACATGCTGATTCAATAATTTCGAAGTTTCACTAGCATTCAAATTCTTCGTAGACCGCAG
A_14_P130497 CTTGTATCTGTAATTTTCCATACTACAAAGAAATTTGCTGGCCCTGACACATCGATTTTC
A_16_P00717305
ACAAACCGCATAAAGTAAATGTGTTAGTGAAGAACCTTTTGTTAAACTACCTATGGCTGG
A_16_P18302820
AGGCCAAAAAATTACAGAACTGTTCTTCCATTCTACTTTGCCTATACTAAGTGATCCTCT
CUST_249_PI426210822 CTAGGATATAGGGAAGAGGAAGGTGAGGGAGCACTTCCCAGTGAC
A_18_P15000636
AGTCTTCCAGAAAAATTGGTCAAGTAAAGAATGGTTACATAGGCAAATGAAAAGTAGCGA
A_16_P16277118
AAAGCAGGTTGATACAGAGTTTCTTGGAAAGATTCCCAATACCAATTTAGTAGAGGAAAG
CUST_4003_PI426210822 TCACCTACTAGTGTTTTGTTGAGGCTGCTGTGTAACTTTGACTTTCTCTGC
A_14_P124572 GGAAATGTATTAAGGTGATCGTTCCTTTTTTGACTCAGTTTGCTCTAGAAAGGAGAAATA
CUST_9489_PI426210822
TAACAGGTTTTCTACACGTAGAATCCCTTGTGTAGATGAAAAAAAAAAATCACTGGGCA
A_16_P18703812
GTCTACTGAAGCCTTACAACTGTCACATATACCTGAACATACCAATACACAAAAGTCCTT
A_16_P20613655
ATATTTCAAGTCTGAAACATGTCTAGAGAAAGGGTCTACTCAAATACACCATCACAACCT
CUST_6895_PI426210822
TTCTGCTTTCTCTGGTGTCTCTTGTCAAGTTAATAGTAGCCTCAAGTAAAATTCAACC
A_14_P114454 GTAGCGTTCTGATTATTAAATCAGCCTCCTAGACACTTCAGAATTATAAAAGGATGTGCC
A_16_P20883640
TGGCATACCATTTCACTACACCACATGGATAATTTTCATTTAAAAATTCTGGCCAATGCA
A_18_P24736495
CATTACCCGTAAGAGGTCTAGAATACTTATTTGTGTTGTCACTTCTGACTACCTTCAACA
A_14_P118118 GGGCATAAACAAGACCAGGAGTGAATATGGCAGTGTTTTCTTATTTATTTAGTGGTTTCT
A_14_P201938 GCTGTAGTAAGGCTCAGATGGCACTTCAGGACCTTTTTTTATTCTTATTTTTGTTTTCTC
A_16_P17440469
TGTATCTTCTGAACAAACTAAACCGCATATTAATCACATTACAAATTTTTTGAGGGCCGG
A_16_P39887604
GTTCTTGCTATCAAAACCTTCGTATAACCAATCCTGTTATGTTCATCCAGTTTTTCTGCT
A_14_P126458 CTAGAGAAGGGTTAATATTAGCAAAGTTTTACTTTTTTTTTTCTGCTCATAAATGACTC
A_14_P138709 AAGAGAAGAATATTTCTCTAGTTGCTGCCAGGCATCTACTTTCTTTTCCATAATTGTTGC
A_18_P16945767
GACACCACTACAATCACATCAACTCTAAATCACATACTTACCTTTTATATGCTGCCTGGA
A_14_P102732 CATGTCTGAGTCACCTGAATTCCTTCACGTTACTTTCTGTTATCTGGAAAACTGC

A_16_P02726559
AAATAAACCACCCATAGTCACAGTTCACAGTATCACCTCCATTGGCAATTTGTATTCTTA
A_18_P22606443
TGTCAATTAGAGTGATAACACATTTCTTTATTACTCTATTCTCATTATTGATCAAAAGTT
A_14_P120429 GAACAGGAATTGGCAATTTCCAAACCTCAGGCCTGGATGATGTTCT
A_14_P122949 CTCATATGGGACTTTTTGCTACTGAACTATCTGACCTTTGACTTACTTTCTGACAGTTAC
CUST_9112_PI426210822 CATGTAACCACAGTCGGGAGCACAACTGGCCAAGATTTTCATTCA
A_16_P00685045
TGTCCCAAACAGAAGAAGAACAAACTTCATCTTAAAAATAGAGGATGATATAATCAATCA
A_14_P202492 ACTCACAAAAGCCTATTTAACCCACTGTATTTGGAAACTAACATCAACTTCACTCAGTTT
A_14_P108830 GGCAGAGAAAATATGCTGCTGCAACTTGTTTGCTTGAACAAATACTGAAATGATAAAAGA
A_14_P111802 TGGCTACATTTTATCTGGACTGTGGGGGCTTCATAAATGATAGTTATTTTGTAGTTCAGT
A_16_P21152548
AAAGGGACTGATAGGATTATGAAATGCTACTTCTGAAACTTCTATCCACTTTCTACCCTC
A_14_P116471 CAGGCAGAAGTAAAAACACCAGATAAAAAGTGAGTGTGGAGAGGTGAAGACTG
A_16_P17211513
GAACCCTGACTTCATTTCTATTCCTCTTACTGCCAAATAAATATCACTCACAACCTCAAA
A_16_P18901379
AAGGTAAAAGTTGAAAAGAGCCTCACAAAACTGATAGCAGTCATTATCTTTGTGGAAGAG
A_14_P133595 TGAAATTTCTAGAGGACACTAAGGATAGTTCCAGTATACACTGCACTTCATGTTAATCCC
A_14_P116583 AGGCAAAATATTAATTTAGGAAAGTGGACAGAATGTGTAACACTCCAGCATTAGGATCTG
A_14_P108867 ATCTGACCTAAATCTTATGGTATTGTTTAGGCTGCAGTATACCCTTAGCCCTAAGATTTT
A_14_P115893 AGGGGATGAAAAGATAACTTACTTCACCGATCATTCCATTCCACACATTGTTAACTTTCT
A_16_P36230738
TAGGTTAGCACTCTCTCATAACTCAAGCCTGAGGTTTATTCTTTTAATCTTCCAAAAGCA
A_14_P101111 CTGAAAACCCATGTCATTATTCCTAGACTATGGATATAGAGGTGGCATACGCTTGTTTAA
A_14_P112103 CATGACTAATGGATTCCTCAAATGAGTTTCTGATCTTATTTAACTTCCAACCAGGCTCTT
A_16_P00908769
ACCTGTTCAGAATCACACAAATAAGCATCAGGTATTCAATGCATCTCTTCAAGACCATAT
A_14_P114071 ATAAATTGTGTCTTCCTTACTGATCTCTGTAACCCAGTGCCAACATAAATTCCTAGTAGT
A_14_P105918 CTCTGCTCTTAAAGTTTGGCATTTGGATTCTTTAAAGATTGTGACCATCCCTTTAAAAGT
A_16_P02746696
AGCAGAAACTTAAATGGATCAGAAACTTGAGTAACACCAACCCATAGCAATTTCCTTTTT
A_18_P22843205 GTTATCCCAGAGCAAAGGAAGGAAAACCTCCTCTGCACAATGCACT
A_14_P104632 AAATAATCACAGAGTCATTTTAGGAGAGTCTGAATTAGCATTTCACATGCTCCTCAACAC
A_18_P22842472
TTTATTTGAATTCTAAATTAACTAAGTGAGTTAACGTGGAAGCCGCTTTTACCAGCCCAC
A_14_P127582 ATCTGAACACAAGCAAATGAAAACAGTTCACCTAACACCCATGCAAATTATAAATTTCCT

A_16_P03065559
AAACCACTACTGTTATCACCAAATGCCTTTCTAATTACTTGAACCATTGGCAGTTTACTG
A_14_P129202 CTATTTATGGGGAGGAAATGCAATCAGATGATAAAGACAGTTGTTAAGCATGATGTGAGT
CUST_8557_PI426210822 ATAAAGTCTGTAACAGACTTGGCCCTAGGATGGCCAAGTCTCCATC
A_14_P132886 CATACTCACTTCTGCTGTTTTGCCCATTGCTCCAAGTTGGTCATAATCTGA
A_14_P109364 GGAGGCATATAGTATTTGTCAAACAACTGAAGAACATCATTTAAAGGTCAGGCAGTTCAA
A_14_P132792 ACACAGGTTCCAGATGGATTTAAAGTAGGAAAAATATCACCCCCTGTATACTTGACAAA
A_14_P133717 CTACTTAATCCTGAAGAAGAGGATGATTCTGACTATGGACAGGTGGTCATATACTTAACA
CUST_3651_PI426210822 AACACTGGCCATCCTTCAGCTCTTCTGCTTACAGCTGTTGTGCTG
A_14_P105937 TTTTGTAGAACTCGAGATGGAATCTGTAAAATGAATCTTTGAGTCCTTGCTGGCCTTATA
A_14_P121744 TCATCCAAGGATGGCTTCTTTTTCAGTAGTTTCTTATATAAAGCCAAAGGAAAATGGAGG
A_16_P58424547
GGACACCACAAAGTTTATCAAGATATACACAATTGGAGTTGCAGAGAAAGACGATAGAGA
A_14_P126626 CCGCTCAGTTGCCATTAAATGGAATGTGAGGGTTTTAAAAATGTATTTATTAAGGACTCT
CUST_9963_PI426210822 GTCTGTCTCTTCCTTGTTTCCACTGTGTCCCCAGCTCCAAAACAG
A_16_P19887298
ACATGACTATAGTTCCCTGTTTCAGATTTAACAATGCAAAAAAATCACTCTACTGGGCTT
A_14_P116854 GCAATCTAACTTGCAGTTATGAGTTCATGTGGGCAATGTTAAGTCATTTCAAGGTAAGAA
A_14_P135573 AGGCAACATTCTTCCGATAATTCTTCTTAACACTTAAAATCCATCTGCAAAGAACCTTTA
CUST_510_PI426210822 CCACTCTGAGTTCTACTGGCTCCCACCACTTATTTAGGAATCTCTGAA
A_14_P102752 AAAATTCTGAAATCTTAGTCATAGATGTGTATGTTTGTGGCAGAGAAGTGTGATGGCGAT
A_14_P103961 CTTCTGCAGTCTGATGTTAGAACTGTATTATAAAAAGCGTCTTGCTAATGTGCTTGGTTC
A_16_P18825924
AAAGATAGCAACCTGTTCAGTTATGATAGACGATATCTGTGAGATAAAGACAAAGCCGTG
CUST_10277_PI426210822 ATGATGTTTCAGGGCTGGAAGAGGTCTGAGAATCCCATTGATCTCTTA
A_14_P107339 TCTGTCCTGGAAGCAGAAGGAGTCTTTGAAAAAGGTAAGATAAACAGCATAAAGTCTTAC
CUST_9414_PI426210822 TGATAAAATTTTTGTCTGGGCTACATGGAATCATGGAATTTCAGTTCTGCCCAG
A_16_P16827418
AGCCGAAGATTAACAGATACTTTCTCACAAAATTTAGAGCACTTTGAATAGTAGGTCCCA
A_14_P105079 CAATCTGATAAGACATGTTTGGCCTTCATTTCTTCTGTTTTCTTAAAGGGAACATTGACT
A_14_P135996 ATTCTGGGATTCCTTTCATCGTTGCTTTTTGTGTTTTCTACCTAGAGCGGAGAAAT
A_14_P129011 CCCAAAGAAGGAATAACAGAACAAAAAAGTCAACCTCTGACTACTTCTGGTTTGTAAATT
A_14_P103207 TGTGACTCAGTGACTTGTGTTTATTCTCAGAAACCAGAGTCCACAGCG
A_14_P120011 TGTTAGCCTAACAAAACCCTCCACAGGCTATCTGTCTATTTTCTAACTAAAGGTTTGAAA
A_14_P137054 GAGAACTTCCCAACTCAGATTTGGGACAGGGAGTGTTCTCAAAGAG
CUST_8706_PI426210822 AATACCAGAGGGTGCAGTAACAAGGCCAACAGAAGGCTTCTGAAT
CUST_10181_PI426210822 AATTAGAACCATGTGCCAATGACATTCAGTGGGGGCTTGTTTATGCTTG
CUST_8187_PI426210822 TGCAATACCCTAACTTCATTGGTCCATTGTTCGTGGAACAGGCAC

A_14_P137389 TGGTTGCAATAGAGCATGCTTTTGTATCATTTGAGTTTTGTATTCTGTGCATGCTCAAAA
CUST_2968_PI426210822 GTCTCTCGTTGTGACTCTTGCTTTCTTCTAGAGGAGACAGTCTGATGTAG
A_16_P15854486
AAAAAGAGGCAAGTTAGTCCTTAGAAATGAACCTACTTAGCATCCTTAGAAATGGATCCT
A_16_P15946364
ATAGGCACTACATATTGTCTTTCCATAGTCATTACTGGCCATACATGTATACTCTCCCTC
A_14_P109930 GAGGGTATACAAGATATAGGCCAAAGGATAATTTAGGTTCTTATGACTGTGTCCTTGTTC
A_14_P118523 TGGAAAACTGTGACCAGTAATTACCTAGAGAATCAGTGACAAAACTGAGTAGCCTTAATT
A_14_P109075 TTAGAGGAATGTTCTAAACCCCTGTCCAAATCCTAACCCCTCTAAAATTTCCCTTTATA
A_16_P20096126
ACCCAAATTAAAAGAACAGGAGTGTCATTAACCAAAAAGGAGAATATTGCTAAACGGAAC
A_18_P13231877
AAAAACTCTAACCAAGTCTCATTAAACTTGCATTGTCACCTGAATTCTATAGTGGAAGGG
A_14_P117522 TAAAGTTTCTGGAACATAGGTGTTCAATAAATGACAGACGCTACCATCACAGATTACCAA
A_14_P129916 CTTAGGAAGAAGACAGACTGGTTCATTGAATGCCGCCTTATTTATTTCTGGTGAG
A_14_P105117 ACCCAAACTTGAGAAAACTTGCCATAAGAATAGAATAACTGGAGGTTTTATTTCTTGGCA
A_14_P127972 CATATGTATTCGTGTCCAAAGGTGAAGAACATAATGTATTGAAGAATGTGGTGGGACTTA
A_14_P201337 GCTTAGTGTAAGAAACTCAGGGACGTCTTTATAAAAGGATTTGGGAAGAATGTAAAATGC
A_16_P16456113
TCTTTCCTGATAACATCCTTATTTCTCCATACTCTACACCTTATAAAAGCAACACTGCGG
A_16_P20524039
ACCCTGCATGTAATCTTTCACTTTTATTTACCACCATGATGTATCAACATACACCAGGAA
A_14_P131165 TTTCAAAATATTTCCTGATAACTGTTAGAGCAATGAATGAAGACCATGTCTTATAACTGT
A_16_P02951324
ATGAAACAGACTCCCTATGCTCATTATCAAAAAGCCCATATGCTATCTTCAGTTCACATT
A_16_P01809070
AAGTTCTGGTAAAACATATCAACTCTGGACAAAGACTACTTCATCAAACGCATAAACAGC
A_14_P131637 CATTTGATCAGGTCAACGATGACTATTGCGACTGCAAAGATGGCTCTGAC
CUST_460_PI426210822
TCTACAAGAAGTCAGATGCTTTCACAGGCATAATATTCTTTAGTTGAGTCTTTGTAACAT
CUST_7293_PI426210822 ATAGGTTTTGGAAAGTCCGGAGTAGACAGCATGAATCCAGGGTTTCTAAA
A_18_P15049358
AAAAAATGACCAACGTAACTACGGATGTCTCACCATAGAATTCGCATTTGGAGTCATT
A_14_P116530 TCAGTGGATCTGATCTATGCTGTCTGCTCCCTAATTTTAGAGAATGTTCAAATATCTACC
A_14_P125184 AGTAGGTGAAGGAACCTTTTGTCAGGGTCATGGTGACAGATTACAGGATAAA
CUST_9578_PI426210822
ACTTATAATAAAGGTGCAGATGTACTGATTAGACATGCTGACATGTACATATAAAATATG
A_16_P16440497

GGCTCAAGAAAATTCCAAATTTCTAATCCAATTCACACATCAACTCTATAGCATTGCACC
CUST_572_PI426210822 TGTGGTGTGGATTGTACTGCTCTCCCTCCATTTACTGTAACCTAGG
CUST_4204_PI426210822 GTAACCAGGGACTGCAACTTCTAGGGAAAGGGGAAAAATCACTGTT
A_14_P129569 AACACCCTCCTTTTCTTCAATACCAAATACTTCCAACTAAAGAATGTTACTGAGCACTTG
A_14_P119313 CAAAGGAAAACAAAGGGCCTGACAAGTATATCAAAATGAAATACTGAGTATATGAAGGGG
A_14_P113836 TTGTATCCTTTTACAGGAAGAGGATGAAATGGATTCTGGCACGATGGTTCG
A_16_P00332120
AAAAAAATTACTTTGGTCTCAAACAGGTTATGCACAGATAACATGTGTCCAATCAGGTGA
A_14_P115723 CTTTATTTTAATGTCTTATAATTTCAAAGGTCCTTCTATCCTAGAAACCAGAGATGGGTT
A_16_P03607322
ATCCCCACTTGTTTTAAATCAATGCAACTTAACAAGACACATATAATCTGTAAGCAGGGG
A_14_P126103 ATTTGCCATACTTGTCTCCTCTAGACTTCAGATCGAAGTAGACATTGCCTGTTTATAAAG
A_16_P01797017
AAGGTCTTGTAGTTTTAGGAATGTCTCTGATAAAAGCAATGCTCTTACTCTCAAGCCTAG
A_14_P128415 CTTTGTTCCAGGTCCTGATCTAGAGATCTAGAAAATTCCTCTTCAGAACTTGTCATTTAA
A_16_P17702980
AATAGCATCCCAATAGAAAACCTTACACTGAATGGAAACTATGATACCTCTTTCTGCCTT
A_14_P122338 CTTACACTTCATATGGAGTTAAACTTGGTCAGTGTTAATAAAATCAAAACGTGATTCTAC
A_18_P22839591
ACACCCAAGTGGAGAAATTTTAGGAACACTATAATTCATGGATTTGCCATTTGTTTTTCC
CUST_5368_PI426210822 CACCATGTGCTGCAGATGCTTGGACTGGATGGATACCTAAGTACC
A_14_P113046 AAGCCTGGAGACCTCTCCATTTAATAAGAAATCTTCTGCTATTTCAATCTTCATGGAATT
A_14_P103118 CCTTACTGACCTTGAGATCAAAGTGAGCAATTTTCTTTGTGTGAAGGTAGTTCACC
A_18_P15135293
GGACCCTAATATTCGACTTCTGTAACCCTTATATCCAATTGTTATGAACTCAAGTCTGTG
A_16_P15171825
AATTTGCGTCTTTTTACATTCTCAACACCTAAAACACCTAAAGCACTTAAGACACAGCAG
A_14_P126910 ATAGTTCCAGGGTTTAATTAATGGCAAAGATTAAAGAGTGATAAGAATCAGGCCCCTGAT
A_18_P13465266
AACACTTGCTTAGCCAATTAAAACAGCAAGATTTGTCTAGTTTCCTTGTCTAAGTGAGGA
A_16_P18602166
AAATAAAAATTCCCCTAAATTGTTCCCAAATACCACAAATTCCAAAGTGATAGACCCCAG
CUST_6353_PI426210822 AGGAATGATTTCCCTGACCCACTGTAAATTTTAATTGAGGTGCGTTACAGG
A_14_P116000 AGCGGAACAGAAATGTGACTAAGATCAACCCAATAGTATATCCTTCACTTCCTTTGAATA
A_14_P118230 ATATTTTTCTTCAAAGGCTTGTTAAACCAATCATGTTAAAAGGAAATTCTTGGTTTTGGT
A_14_P119969 GAGGAAGGGTCAACAAAGACAAACTTGTCTTGGGGGTTTTAGGAAC
A_14_P107360 GAGGTTATTATTAGGTCTCCACATCTCCGTCAAACTCTGTTCTGAAGAACTCTTAAAATT
A_14_P134483 CAGACAAATGTCAGTATCTAGAAAATGCAATCCTACTGCTCCTAGTATTTCAGTCTCAGA

A_18_P10556888
AAGGGCTATATAAAATCACAAAATGCATCCTCTTAATCACAAAAACTCTGATCGCTGTGT
CUST_7039_PI426210822 GTTCTGTTTGCAGTGACTCCCCTCAAAAGGCAGAATGCAATTCTCTAAA
A_16_P18252383
AATTGTTATTGGCTCTGTATATCTCACAAGAGGTTATACTGCATAATTCACCCAGTCACC
CUST_7010_PI426210822 AAGGTGACTTCTGGCTTCTCAGTGACTCAGCCACACTTCTCTTTTT
A_14_P107084 TTGTAACTACAGTGATGATGCCAAAGATGACAGGGACATAAACAGGGGAAG
A_16_P01728689
CCCCTGGTCACTAAGAATAGTAACAGTAATCCATTTTTAAAGAATAGCAACCAAAATGCC
A_14_P103617 TCAACATACTGTGTCAGGTATACTGTTTTATAATTTGGTTGTTTTAGCCTTATTGCACAC
A_14_P120273 TCAGGACAGTAAGCAGAACAATCAGAATGGAATTAAATCTCTAAAAACGTGTTACAGTGG
A_14_P100142 TGATACAGCCAGAAATACTGCATAGGTTAAGCCTGAATTCTAGGTTTTATGGCAAATTGT
A_14_P108128
GTCAAGAGACTCTTACAACAAATCTAAGACACTGTCTAAATTGAGAAGGGTCAAAAACCA
A_14_P122924 GTATACCTTCAAATTGCAAGTCATCTACTGCTGCCATTCGGCCAGTGAGT
A_14_P124270 TAATTGTGTCTCTTATTCTCTGTTTTTTGGCAGCATCTTCTCACTAAGTGGACCTTGATT
A_16_P19373840
TGAGAGTGTCCCTAATATGTCCCTACAATTATTCCAAATATCAAGATAGGTTCCAAGTCA
A_16_P58424329
GATAACTGCAGCAAGTCTAAAAAGCAGCCAAGTCTATTGAGAAGAGGAGATCAATTAACT
A_14_P124094 ACCATAGTGGACAGGAAGATGAAGAATGGCTTTCCCAGGTAAAACTCTGAAATA
A_14_P130136 ACATCATCTAAACCTGCTATATCACTCCAAGTAACCTGGCAAAAGTTAAGGTCAACATGA
A_14_P132317 ACTTACTCTGTATCCTTGGGCTATAAGGATCTTCTATTTTGAAAGCAGGATCTAAAGCAC
A_16_P00908229
AAATTCGGTGAATTTTTCTTCTAACTCTCGATTACAGGTCCAAATTAAACACGCATTGCA
A_16_P40450453
AGGAGTGACCACTAAACTACAAACACATATCATAAACCCCTTGAATAGAGTTGATGTCTT
A_14_P120695 ACCTGAATGCTGCGACAAGCAGATTTTTGTAAAATTTTATATTAGTTTTTAATGTCAGTG
A_14_P132005 TAGCCTAATTCCTGCTACTACACACAAAAGGGTTCTAGTGGATTTTTCTTCTCTTTGAAA
A_14_P109605 CAGAACACAAGCAGTCATCCTTACAAATCGTAGTTTTACCCCAGTGATAAGTTTGTCTAA
CUST_2763_PI426210822
GGATATTTCATGCTTTAATTAAATGCTGGGTTGAAGGCCTAGGATTTATCCTGTTGGT
CUST_8553_PI426210822 GGCTGCACTCTGTTCTTTGGGCTCTGAGAAACAAATCCTTTGGAT
A_16_P00339516
ACATCATGAGGCTCATCTTACAATTACCCAAAGAAAAGCATGTTTGAATTCCAGAATTCA
A_16_P18151159 TGTGTCATATCCATCTTTATATCTTGTGCATTTCCTACTTCTCATTCCAGATCTTCTCCA
A_14_P126464 GTGTAAAATTTTAGTTCTCCACCTTCATCTATGGAAGATAAATCAGATGTTGGTTATGGA
CUST_8051_PI426210822 TTAAGCTTTGATGAATCCCAGGATGCTGAGGAGGAAGAAATGGTTACTCA

A_16_P03702028
AGCCCAGTTCAACATTCTAGACATTAGAGGGTTAGAATCTAATTATCACATAGTTCCCTG
A_16_P15292271
TTTAACCTCAGGAAAACAAAGATACAGTAGGCTCACTAGTATTTACCCACCAGTTTTCAT
A_16_P16792410
CATGACCTCTCAGTCTGAATGAGTGCTATCAAAAACAACACATAATGTATCAGGGAAAAA
A_16_P02601315
AGACAGATCCCAAGAGATTTTAATGCCCATATTTCGAAAATACAAATAAGCATTATTGCA
A_18_P14135734 CCTGGCATCTCTTCAAGACAATCATAAAGGCCTGTTTAGGAGGAGAAAGTTAAA
CUST_440_PI426210822 GCATCTTCTCGGGGTTTCTGGGAAATTTCTCTGAAGATCTGGAGAA
A_16_P02333992
TGCAAATTTCTCATATGCCTCATAAAACACTTACTCATCTACATCTAAAGCCAAGAACCA
A_14_P108951 CTAAAAATGCAAATGGGCCTCAAAAAAAATTGACCTGGGCTCACAACATCAAATTTATTT
A_16_P17286610
TTATACATAAAGCACTGATCCCAACTACATGTGTTCCTATAGTTTAATTCCACCTGACCC
A_14_P120186 CCACTTATTGATCAAGGTTATGCTTAGGCACCCATGTCATTTAGGTTTTCAAACTTTATC
A_14_P108404 ACTTGGTCACCTTTCCAACCTGTGCTTTTAACCACTACACTTTTAACTACTACTATTTCT
A_14_P133266 CTGTTACTGGAAAGATTTTTACCTCATTACTTCAAGGGCGTGCTCAGTATCTACAAAGAC
A_16_P58424229 GACTTGGGTTTCCTCAGAGCATGGCAACCTGATTCCTAGTGAGATTT
A_16_P17798212
AAAAAAAAACCCTCATTCATTTCACACCACTCTGATGTTTTAGCCAACAGTTTTTTCACT
A_14_P139479 GGCAGTTTCAAAGAGTATAGCCCGAACTCATTCCTGGTAAAACAAAAGGAAAACATTT
A_14_P126012 CAACAGGATGCAAAAATTCTGAATACCCCAAAAGAAAAGTAGATGTAAAGGCATACTGTT
A_14_P115784 AATGTTTGTGATATTTTGCTTTTGCACCTTTAAAACAATAAGGCGCTTTCATTTTGCACT
A_14_P133494 AACAAAATTGCACTAACCAAAAATAACCCAAATTTAAGGACCAGAAGCATAAGCCATGAA
A_14_P136642 TCTCAATTCTATAGACTTTCAAGCCTATGTATGAATATGAAGGGGTTTTTTTTTTTGCT
A_16_P16471061
CAACATCCACAACTATTTTTGCACATTTCTTACATGTCTACTTCTGTTACATTCCATGGT
A_16_P18294402
ACCTCCACTCTATAGAGACCCTGAAATTAGTAAGGATGTTCATTAAAGCCATTGTGTTTT
A_16_P36230445
CACCTTTCACCATTGCAAACTTTGACATGTGAGAGTTTCCAGAAATACAGAGATTCTTAT
A_18_P22843017
ACATGGATGTTTTGACAAAATTTCAAGGAAGCAACTGGCTTTGTAGTGCTTTCTTTGTC
A_14_P132436 TTTAATTTTAACTCATGTTCATCCTGCAACAGAAGCATTCTCTAGGTCCCAGTTTCCA
A_16_P36989357
TTTGACCCATGAATGTTATGTCCATCACATCTTATCAGTTAATAAACCCACCAAGCAAAA
A_18_P22839423

TAATTAAACTCCTTTTGAAAATGATTTTAAAAGAATTCCCATAATATTCAGGTAATAGCG
A_14_P132016 ATCATCAAACATTGTAACAAAGTAATTTCCAGAGCGTTGCACAAATATCAGGCCAATACA
CUST_1074_PI426210822 GCCAAGGGGCACTTGGAAGGAAAATGTATCCTCTCAGACTTAGAAC
CUST_6774_PI426210822 AGGAACCTAGGTTGGAGCAGGGATGAGAGGGTAGAAGAGAGCAAA
A_16_P17298901
ACACACATCACACTAGAATTTCACTTGATGGGCTGTATTCTGAAAACTCATCATTGTAGT
A_16_P02338541
GGATAATGGGCTTTAATGATTTCATCACCAAATAACCATGAGTATGTTCCTCTCTCTGTT
A_14_P129390 AATGGCCCCTAAATTGTTAAAACTTGGAATGGCTTAATTTACACATCACTCCCTATTTTC
A_14_P125983 GAACTATGGCAAATTCACAAAAACTTATTCCGACATTCAGAGTTGTTTCCTCCATTTGCT
A_14_P127024 TAAGAACTAGTATCATTTTGTGATAGTGCCTCCCTCCTGTGTTCACAAACTTCATTATGC
A_14_P137702 CTAGTTACACATGGAGACGCTTCAACTGAGAATGATGTTTTAACCAATCCTATCAGTGAA
A_14_P129280 CTGTTCCTATATATACATGCACACACAAAATAAGCCAGACAGATGGCAATTTGATCTTCC
A_16_P02055131
CCTCTTTTCTGTCAAATTATCTCATGACCAGAAATCATTTAAATACTTTCCCCATCCCTG
CUST_8443_PI426210822 AAGTATTACCATGGCTAAACTGAAAGAGTCTACGGTGTTACCCAGCATGCT
CUST_1652_PI426210822 GTGGCGTGGTGCGGTAAAAACAAGAGCTGTTGGTTTTAAAGGTTTTTG
CUST_5553_PI426210822
TTGGTTGAAATTAAAATAAAAGAAAAAATTGTTCTTAGATTGGGAAGATTGTCCAGGAGC
A_14_P100059 CCCATTACTGGAAATGATCTATCCCCTCCAGTGTCTTTTAACTTAATGTTCAAGACTTTC
A_14_P108400 CAAAGGGGCCTCTGAACTAGTGGCAGAATTGAGCACACTTTATCAGTGTATTA
A_14_P109648 ACTATGCTTTTCTTAAATTGTATATCTGAGGAATGCATTGGTGGGAGGCTCTTGAAGATT
A_16_P40518305
TTCCTAACTTCAGAAATCATCATACAATGTGTTGACCTTTCCTGATATCCTGCTCAATCT
A_16_P03804679
AAAAATGATGTAATGATGTTTAGGGCTCCATGAAAATCTTCTTCGACCAAAATACATCTG
CUST_10324_PI426210822
TGTGGGTGTGCTGTGGGGATGATGTCTATGACTTTTTCTTAATTATCCAGAAAATT
A_16_P16787536
AGTGGAAGTAACGTATTGCAGAATGTTCCAACTTATCAATAATGTCTTATCGAAAGGGCT
A_16_P15079045
AGCAAATCCTAAACACCTTCATAGAATAAGAATCCTCAGTGATTATGCTAACTCCTTCCA
A_14_P202005 TTCCATGTGTAGTGAACTACCTATTCTGACAGCACTAACCTTTCTGAAGTATTAGCCTAT
A_14_P124238 ATTTGCTTCTCTGCTGACCTTGCCAAGTTGTTCGAGGTGGAATTAAACAC
A_16_P02597156
CAGTGCAAGGATATCTCACAAAAATTCAAGAGCATATTAAGTCTCAAGTCATCCATCTCC
A_16_P40215983
ATCCCCCAGGAATTCATTTATATCATTTGATCCATATGGAAATGTTTCACACAATCCACT

A_16_P00938270
AGGTTTGTGAAATTTAGACACCACACATTTCAGTTAAATGATACATTGATCCATGTCCCA
A_16_P00389581
AAAGCATAACTCCTCAATACTTAATTTGTAGCCTGAACATTGTATATGATTTTCCTGGGT
CUST_4823_PI426210822 ACACCTAACCTAGAATTTCCCTGTGGATCACTCACCCCCAATGAT
A_14_P202532 CCTTGTTTTTTTGTGCTCTTCCTCGCTTAATAAATTTGGAGGAAGATCTGATCTCATTAG
A_14_P106603 AAAACCCATCTCTGCTAACCATACCCATCTTAAAGATACAGGTAAGAGGTATTAGTTGTT
A_14_P121256 CTTTCTAATCCTAGAGTGAATTACTTGGGGGCCAAACAGATAGAAGATTCCCAAG
CUST_4609_PI426210822 TGGCCCCATTCCTGCCTGACTGGCAGTCTTCATTAATGTACGTTT
A_14_P128405 GGTGACTGGATAGTTTCACAAATGAGTAATTCCCAAAATGATTAAATGAACCTTCCCAAA
A_16_P02530543
ATGGTTTATACACCAAAGAGGTAGAAGGTTTTGCTAATCTGTATTAACAAAGCCTGGAGT
A_14_P134128 TATGATTATCAACCAAATTATACTTTGGAAAGGCAATCATCTCGTCCCTGCAAAGCAGTA
A_16_P00341652
AAGAAAAACACAGTGGGTAGATACACTTGAAAGTCACTTCTGAAAGAGACCTTGAAGAAG
A_14_P114738 TTTTTTAAGCACTGACAAATGGTTCTAAGTTTGAGAAGATAAACGGTAAACGTTCAGGGA
A_14_P125002 ACTTAGTGACTCATTGGACATAGAAGGATTGGACTGAGTCAATGATAAATGGGATAATGA
CUST_7255_PI426210822 TTCTACTTCTCCTTGGGGCTTTAACTAAAAAGGAAGAATTGTCTGTGGCTTCTT
A_18_P22839957 GAGGGGGTGCCCCTTATAGGAGGGTGGTGTGGGCTATAATTTCTT
A_18_P14134439
AGTTCTCTGCCTCATATCATTTCAGTCTATTTAGAAACCTGCTATTTTAGCACAAAACAA
A_18_P16381250
CTTCCCCTCCACTAAATCTACTCTCTATTCAGTATCAAGAAAGACTTAATCAGCAGTGAA
A_16_P18551474
CCACATCCTCCATTTCAATATTCTCAAAATCCAAACTTACCCATATTTCACTGTTTCTGC
A_16_P38546223
CAAACAGAGGTTATAACTTGGCTTATAGATCCCTCACTTTACTATAAAAATCAACCCCCC
A_18_P22842275 GAGCAGTATGTGAGCCCTGCTCTCCTGCTGTGAGCAGAGCAAGAG
A_14_P103468 TCCCCTCTACCATGCCATCTTGCCCCTGTATGATGTAAATTTTTATCACTAAAAATATCA
A_14_P118816 AGAATCAGAACCCCACTTTTCTCTGGTGTCATAGTGTTCAAATTAAGACTCACATAAAAG
A_16_P35635421
AGCAATGACTATTCACAATCTACTCAGCATAAAACAGGTTCATTAAGAAAGGTTCTGCAA
A_14_P115271 CAATTTTTTTGCTTGGCAAATGCCTACATGGATCTATGAAGGTTTCAAACAGACAAACAC
A_14_P202262 TCCAGGAATATATTTTGTCTAAAAATAGCAGTCAAATAACTTACCCTTTGCAATTGGCTG
A_18_P11862648
AAAAAGTTTCCACCTATCACTTACTCACCAGTAAAACCATGGTGTATACTGTAACATTCC
A_18_P14135445 AGGAAGTTTGGCTTTGCAGAGTGAGGACCCAGGCAAGTTCATTTTTTA
A_16_P38485982

TTCAACACCTGGAGATGTATGATGTCATACTGAGAAAGCCATATTTATCCACAGATAGC
A_14_P131108 AAATATGATTTTGCTGTTGCAGTTTTGATACGTATTTCCAGTGTGTAGATCCGTTCATCG
A_14_P111778 TAGATGGAGGAGCATGTTGAATCAATTATTCCTCTAAGGATCTTGAAGTTTTGAGTGAAC
A_14_P129527 AGAATGGATGATTCTAAGATCTGGTTTGGTTCAAGATGAATCTACTACGCACTGTGTTAA
CUST_8097_PI426210822 GGCGCTAAACCATGGGGGACAAAGGTCACCTAGAAAATCTGAACTATAT
A_14_P113981 CTGCAAACCAAACATTATCTTTAAAGGTCAAAAGATCTTACTAGGATGATCTGCAGGCC
A_18_P23434449
ACACTGGTGCCTTTAGAATAGAAAAGTTGAGTCACATTCTGGAATCAACCTAGTTAACAG
A_16_P02296844
TTCTATTGTGACCTGGTATTTCTATTGTGACCTATTTGACCTAGACAAATTCCTTCTGCA
A_16_P36231070
GTCCTGGTCAGTTTACTCTAAATCAAAGTTTCCAGTCGTCATGATGAGTATGATCATCAT
A_14_P136736 TTTGGTTTCAGTATACAAGAAGAGTCTTGGAGATGACATTAGTTCCGAAACATCTGGTG
CUST_8562_PI426210822
AGTAAATTCACTGGCACATCTCACTAAGCAAGTAGGAAAGCTCTCTGTGTTCTTATAAAC
A_14_P110628 CTCTTACATCCATCTAGTGGATTACTAAATTTGTATATCCATTCCCGGCTCACATGGATA
CUST_9541_PI426210822
GCTGTTTTTTGTCAAAACAGAAAAAACACACTACTAGGATATGAAAAACTTCATTCTGGG
A_16_P20766363
AAATGACACAGCCATAAGAAAGTAGCATAAACACAATTCAGAATTCACAGGGTTTATCGG
A_14_P134785 GAAGGTGTATGAACTGAGCAATGTGCAAGAAGATAGCCAACCAATGTGCTA
A_16_P01809367
AGACGTATTTCTGCTTCAAAGTCTTCAAAGTTCTCCATGTTCTTTACATTCTTCAATGCG
A_14_P122005 TTCCACTTTAAAACTTTGCTGTTTCTCCACTCTGAATGATCTGCTTTTATAAGAGGACTC
A_14_P114492 TTTTTTTTAAGATAACTATCAGGTAGGTAACCAGGTGAGGCCTCAAGGCCTGTT
A_14_P105792
CTGTCACAACCACCACAAACAAAAATAGCCTTAGGGAAAAAAAGGAAAAAAATGAATAGG
A_14_P115811 GAACACTGACATACCTGTAAAATGCAACTCATGATATCAGATGCGATTTTTGCATGAGGA
A_18_P22840716 ATGAAAGTTCTAAGGCTCATGGGTGAGGCCTAAGAAAGGCATGTCAGTT
A_18_P22842484 TTTTCTGCCTCTTCAGTGTTCAAACCCAGGCACAGGGACTGTGTT
A_14_P202167 CACTTACAAGAGAGATACAATTATATAGGATCAAGAGGAGGCCAGAGATTGTAGAAGTTA
A_14_P112485 AAGGACGCCAGAGGTCTTGCGAATATTTTAATTGGTTGACATTTGAAATTTGTTCTTAAG
A_14_P132124 CTCCTCTTTACTGCTAATCATAGCCTATGCACGTAAACATAAACAGCATGCATGTAAAGT
A_16_P21569617
GAAAAATCCAACATGTCTCTTGTAAGACTTTTACCTAAAGCCTTTCTCGATCTCTGAACC
A_14_P106842 TGTTTCCTTTAGGGAAGTAGAAATAGAAGATCAAAGTGAATCTAAAGGAACCATGGACTC
A_14_P117025 CTCAATGATTTGTTTGAATCCTATTTTCCTTTGGAATTGTTTCTTCTCAAACACCTGCTC
CUST_632_PI426210822 AGCTCAGGAAGCAGCTTTGTTGGAAAAATGCAGGGCACTTAAACTG

A_14_P108945 ATCTCTTGGAGGGGAGAGTTCAGAATATGAGAGTTAAAACTCCTTGAATACTTCATGTAA
A_16_P35288841
TTAACCATTTGTCTGTTATCACCACAATAAGTTGACTGCAGAATTTGAAGTAGCAACTCC
A_16_P19504392
AGCCTGTTATCATGCACTTCTTTACCTATGAAATTTTGGCCAAATCTAAAGACAATTGCC
A_14_P125493 CTGAGACTTTTTCTCTCCCTAAATGATGATGCACAAAGCAGATAAGCCTCTTCATAATCA
A_14_P137220 AGTCATGAAAGTCTCTGAAAGAGTTTTTTTTATCATCTGTTTGGGAGAAGTGTGGGATA
A_14_P131219 ATTCAGGAAGGAATTTGTCATAAGTCATTCACCTTCGATAAGGGTGACCAAGACTTAT
A_16_P00685135
CTTTTGGCCAAGAACCTTCCATAATGTGCCATAAAAATATTTTATAATTTCCTGCGGGTG
A_14_P105748 AAACACAGTCCCTATGCTATCATTCCCTAAATATGTCAGTGTGTGGGGGTTATC
A_14_P116546 GACAGTTTCTTGGAAGCGAACTCGTTTGGTTCTTTTCTCTCCATCGCT
A_14_P127364 TCTTTTCCTCCCTCTTAATCTTTTAAAGTTTGCCAAAATAATGGAAAATCCTGGGATACC
A_14_P121130 TTAAGGATTAATTGCCAGCAGCAATCTCTGTCTTTGTTTGTTTTCTCTAATGGAAAACCT
A_14_P105356 GAATGGTGGTTACTGTGTCTCCTTGATTACTGTTGATTAAATCCTGAAGAGAGATTTTCA
A_14_P134828 ACTCTTGTCTTTGTATGTTTGTTGTTGGTTTTTTACTAGATTCCATACCAGTTCTGGCAT
A_16_P01682671
TGCAATAATATCCATATGTCCAATTAAATCAATCATCCTTAATGCTCCATCTCTTGAACA
A_14_P103839 TGCAGAAGCAGGAGATGGCAAATTTTAAACCCTATTTTCAATCAAACCTGGAAAAAAAAT
A_14_P201457 TAAAAAATGACAAATTCAATCTGCTCTTGACTTGTGTGTCCTAAGATTTCCACTAAGTGT
A_16_P02252716
GTTCTTTGCTTAGTGCAGTCACTCTTCTTATTGGACATAAATCAAGTGCGACCTTATGC
A_16_P01154142
AAAGTTCCATACGACACTATGACATAAACTTTTTCTGCCCTAAGTTCTTCTAATCAATGT
A_14_P135273 AGTCATAGATACAGAGGTTTCTCTGTAAGGGTATTCACCACAACAACGTTTATACAGTTT
A_16_P58424388 GTTCACCTCTTTCTCCTCCAACTCCCAGTCTTTCACCCTACTTCTATTT
A_14_P101466 ACTTTTACCGACTTCATATGTAACCTTCTTGGGACTTGCTGGATAATTCCTGTAATATAC
A_16_P17112360
AGTCAACTGTCAAAGACTTACCTATTCTCAAAGTTCAGTTCATACATTATCTCCTCCTCA
A_14_P201544 ACCTCATAAGATGTTAGAAATAGTCTGGAGATATATCACAGCAAAGAAGATGGTGACCAA
A_14_P116789 TTTAATATCCCTTTTCCCGTCTCGTCTCCTGACTTGCCTTTGGAAAAAAATAAGATTATT
A_14_P201849 TTTAGTTTTGGTAGATTTTTTTTCTGACAATGTGACCAGACTGAATTTCCTCATAAAGAA
A_14_P128756 ACACTGGCTTCATTAACAGTGATGGCCCTCACTCTGTCATTCTTATTAAATAAAAATTAA
A_14_P108952 ATTCTTCAAGGAATTCCAACAGACATTTTCACACAGATTTTCAGGCATATAGATGCTCAT
A_14_P139451 CTGGATTCACACAACTGGAGACCACACAGCAATGACACAAGCATG
CUST_745_PI426210822 GGAGTCCCCTTTGAGGGCCTCAGACTTCTGTTGTATGATGTAGAG
A_14_P134197 CTCTGACTTTAGTGATCTTGACTTCTGTCTGCTGCTATAGACTGAACAATTATTGCCTG
A_14_P122897 ACACATTCACTCTACCACTAAATTGTTACTGACTCCTTCCTCTTCAGCGTTTGTTAAATA

A_14_P105170 AAAAACTGGGTTCCTGTAAGTTATGAATGGCATCCAGGGATATTTGGGTTACTTTTAAGA
A_14_P127847 AAACAAGACACTTTTCATTTGCTCAGAATGCTTGTTGTTAAAGTTAACTGAGGTTACAAA
A_14_P131689 CTTCATTCTCTATTTCCCTCCAGTGGCATCCAAGAAAAACATAAAAATTCCAAATCTCAA
A_14_P115710 TGAAAGGAAGATATTAACTAGGCAGGTAAGTCATCAGTTGGTTAATTCTTTCTCTAGAAC
CUST_7242_PI426210822 CATGCATCTTCTGAATTGGTATGTTTTTCTGTGTTCAGAATGTCATTCCTCAGCC
A_14_P108666 CTGTAGTTGCAAAGGCTACACCGAAAAGGTTTGTCACCAGAGTGGAT
A_18_P22840674 CTCAGTAATGCAAGTTCTCTGTTCCACGTTAGGGGTCACATTATGGTTAAAGACAAT
CUST_9766_PI426210822 CTGGAGTTGAACCTCCTCTTGGATGGTATTGACCGAGCTGTCCCT
A_16_P17901354
AAGAATGCATGGTTTTACGTAGTTAAGTGCCTAGTGAACTGTAGACATAGTAAGTCTCTA
CUST_2639_PI426210822 CTGGTCTATTCCAGGAGGACAAGGGAATCAGGGAGAGCAGGGAAG
A_14_P129052 CATTGTTAGAGAAGGAAATTAGCATGTGATTGGGGTGACCACATAAGTTATCATCAAAAC
A_14_P105041 CTCACAATTCCCAGACTGGTAAATAATGTTGCTTCTAGTTTTTTGCTACTTAAGCAGTGT
A_18_P14133476 CCGGGGTTGATTTTATTGTGTATTCACCAGCCTGAGCGTCCTTAG
CUST_6805_PI426210822 CTGTTTTCCGTATGTTCTAGTCCGGATCTGCATTTCTGGTAGAACTAGTCTG
A_14_P139371 ACTCAGATATTAAGAGGAAATTGAGAAGGCTATGAGGAGATACCAAGATGTCAACTTTCT
A_16_P18567997
ACAGTCATTTACCTAATGCTCTGAACATCTTTCTTGAGATAAAGTAGTGCTGTGAACTGA
A_16_P16986889
AAAGTAAAAATCTAGGCTATCTTTCCTTCTCTAGAACTGTATTTGGTCATCCCTAGGCAC
A_16_P02287133
CACACAACTCATGCTATTCTGGTATCTTTGTTCTCAGTTTCTACCTAGTGAACTCTTCAC
A_14_P123783 ACAGAGAATGGGTAATTCAAGAGGCCTTTCTTTTAAAATAGACTTTTGTGACCCACTAAT
A_16_P58424255 TTAGTTCTAATAACTTAGGGGTTCCAGTGACCACTCCCCACCTTGCT
A_16_P00685131
TACTTTTCTGTAAATGTGAGGTGAGCAAGGTGTTCACAGTTCACAATGGATCTGAATTT
A_14_P100720 CTGAACTTGTAGTCAGATAAGAGTAAACCTTTGAAGTCCTAAAACGACAGAGGAAATCTT
A_14_P118448 GGTGAATGGTTACCATGAGGATCTCTTCCAAGTCATACATTCAAAGAACATACTTCTTTA
CUST_8629_PI426210822
GATGTACAGCAGATAGATATTTTGGCAGAATGACAGAGAGTACTGAACCACAGTCATTT
A_14_P124629 GCCTTTTCTCTTGTAGTTAAATTATTTGAAGTGATTGAGACTGAGAAAACGCTCTACCTT
A_14_P103791 GGGAATCCAAACTCTAAAAGAAGGACGCATTTTAGGTAAGATCTAGTGGCTAGATCT
A_14_P134915 TTCCATGTATCAGAAAAGATCCTGCCAAAAAAGCCAGTAGTATGCTATGCATTTCCTAAA
A_14_P121966 CTTTATTTTTAAGGGTCATACAGCAATGCTTCATACTGGCTCATGGCATCCCAAAATAAA
A_14_P102872 TTTCATTTCTTTGCAGAACTGAGCAAGGATGTTGGTATTTGCTCCGTTCTCAAG
A_14_P126339 ATGCCTGTGACTCCACTTACCCGATAAGTTGCTCAAAGATTCAGCAAAGT
A_14_P138530 GATTGTTACGTCTTTCTTTAATTCTGCCAAATAAGTCTTTTTTTCCCCCAAGGCAAAATC
A_18_P15970706

TGCCATAGTTACCACGATTACAGTTAAAACAGTTCATTGCATAACTAGTTCCTTGAGGTT

A_14_P139874 GATGAATGACACTCTCAGAACCCAAGGTCAGAAATGGGGAAGATAAACAG

A_16_P15777962

AGCATACTTCATCCATGCCACATTCTAAGACCATTTTTCCATGACCTACACCTTTTTTTT

A_18_P22841716 AAAGTGCCATTACCCACCTCTAGGAGGGATGTGTTGTTTCCATACAAC

A_16_P18595637

AAAAGAATTAGAAACCTCCTATCAACAAAGACAAGTTTGCTCTATGTATCAGAAACCCCC

A_16_P16191512 ATAGAGTGAGGGTGGGAATCAAGTCACTCAGATATTTCTCCCGAATTGAAATG

CUST_6476_PI426210822 GAAGATCAACATCAAATCTTTGTCTTCAAGACCTAAGGGAATCTGGATGTGCC

A_14_P115249 TTTTCCTACATGCTTTCTGCACATTGACTACCAACATAGTAGTGTCTTTTCTGCAAATCG

CUST_3996_PI426210822 GCTTGGCAGTAGCAGATTCTAGATTGTCCTGTTTGCTGGTTTGGG

A_18_P22840908 CAATAGAATCTTTCCCCCCTTGAACCTCACATTATGCTGAGATGTAGGATGAAG

A_16_P16426344

AGTTCCATGACTCCATAAATCCCAACTCTCTAAGTTATAAATGTCTAGGTCTATCCCATC

A_14_P102275 AAAACAGGAAATCCTACCCACTATATGCAACTAATTGTTTAGGAAGAATTCCTTCTAGGC

A_16_P19935132

GCTCAATGGCCCTATAACATTATAATGTTTCCTGGAATTGGATGAAGACTACTCACCTTA

A_14_P115901

AGTTGGAACATACAAAGAAGAGAAGCCAGAAACACTCTCAAATAACTGAGGAAAAATTCT

A_14_P117205 CTAAGGTCATTTTTTTTTTTCTCACTGAAAGGGTGTGAAGGTCTAAAGTCTTTCCTTATG

A_14_P137157 CTCTCTGTTAGTTCATACCATAACTTTCACGAAGACTTTCAGAGGCATAGGAAACATATG

A_14_P133888 GATCCAAGAGATTATTTGTCACAACCATTTCAGACTACAAAGAAAACACACACATTTTTG

A_16_P16770995

AACCCTCAAGAAGAAAATCTGTAGATACATGAATGCTTATTCTTTTACCAATGTCCCCTG

CUST_1341_PI426210822 AGAGTTCCACAACCTGGTTCCAGCTCCTCTAAGTGACTTCTAAGTC

A_14_P117151 GTTTCGAGGATGTCAAGTTCGGGCATATATCAGGTATATTGCTTTTGTCATGGAAAC

A_14_P108307 CTACTTTCAGGGAGAGGAGATTATGAACATATTTAAGCAACGTTTCTGTTGCTTTGAGAT

A_14_P103664 TCTATGGCTATGTTGTTGTCTCTTATTTTTCCAAACGTGGAAACCTCTGTTTTGTAACTT

A_14_P102942 TTCAGACTTTGATTATCTATTGGATTCTAAGCACTGGGTTACAGAAACAAGACAGAGTCC

A_18_P13245966

AAGAAGAGAGGTCTGGTATCAAAGATTTGCTGAATTCACAATTGAAGAGGCTTATGTCAG

A_14_P111805 CTATTTTCTCTCTCTGTGAAAAGGAAACCAGGAAATTGACTCTGTGCCTGTTTTCAA

A_14_P134985 AGGAAGGTAATAATTAACCACTTACTGAGTTCAAAGCCATCTTGCTGTTATCAACAGCAT

CUST_3373_PI426210822

TTAAAGAATGTAGTAATTAAACTCCTTTTGAAAATGATTTTAAAAGAATTCCCATAATAT

CUST_4434_PI426210822 AAAGGGACTTCGGCGGAAGCCATATGACCCACCTCTGTCATTTTA

A_14_P125248 ATGAGGTCTGCTCCTCGTGCTTTGTAATAATCTAAATTTGGATGGAATTGATAAGTCACT

A_14_P110722 AAACATTTTTCAGTTGCCATACAACATGGTGGGCCCACTATTTGAATTATTACTAAAAGG

A_16_P00468263
ACGATTATTGCCCTACTTAAAAAAATTGGCATTTTTTTAAAATCTAAAAATCTATCTGAA
A_14_P138685 TTAAGCAAATAATTAGCCTGTGTGCAGAAGGCCACTCATGTAAAGTAAGAAAAATGCAAC
A_16_P15778076
GTTGTTCTCTTTGTAGATACGTATTTGGAACTGCACTTACCATATCGTGAATGAGGCTAC
A_16_P01880070
AATGGTGTTAGAAAGTCAAAATGCTCTCAGTCATTAAAGAACAAGATTAGGCTCACCTGT
CUST_6208_PI426210822 ACTGAGGGGAGCTTTTCAGGGCCCACCATACATGAGCAACAGATA
A_18_P10333082
ATTTGAGAACTCTAATCAGTTGGTCACAGATAATACTACCAATCTTGGTCTGAAACAGGG
CUST_8426_PI426210822 TAAGATTTCTGGCTTTTTGTTGGCTCTGCCCTTCTGACCTCAACTTC
A_16_P16817213
GAATGCCAGTTGCTTTAATTTTCACTGATAGAATCTGCTTTCTGTATCGTTAACCTCCAC
A_14_P111947 TCCATTTGCATTTAGCCAACCATGATCTAATGGTGCAGGTCTTTTACATTTCTAGAGTTA
CUST_1381_PI426210822 ACACCCACTAACTTTCCTAGACTAGAGTTGCCAGGCCCCACAACT
A_18_P22841398 GTGTCTGGGGAAGGAGAGGAGTGGAGAGGGTCAACCGGAATGTAG
CUST_9845_PI426210822 GGGAAGTGACAGGGCATTCTTGTGGAACTTGACAGACTAAACTGTAG
A_16_P01405574
TTTAGAGCATCTGTGTTTACAGTATAACACAATGTGGCAATATTAGGGAAACGTGTCAGC
A_14_P118131 CTCCTTGCTCAACGTGTTTTCATTTCATGTTTATAACTAGGTCTCTCCTAAACTGGATTT
A_14_P118378 CAAATGACCTGGGAACTTGAATGAGGAATGGGATGAAGATAGATAGTGAGCAG
A_14_P116302 TCGTTTCATTCATACATACTTAAAACACATTACCAAAACTCCCCAAAAAATCCCACCTTG
A_14_P108386 CTCACACACAACGACAACCACTTCAATTATGAGAAGACACACAACTTTAAGGTAAGCAA
A_16_P02318143
TTCAGAATTATTAACCTGACTCAATTAAGTGGAAGTTGTTCTCATTCTCATTGAGCCTCC
A_16_P17045301
ACCCCCTCTATAAGATCATATATCCAATTCATTCATAACAGAGTTGCTCAATATGTCCAC
A_16_P02216882
CGTCACATCAAAACATCCTACTATTGATCAACTTATTAGCATAAAAATGGTCCAATGGGC
A_16_P16941749
CCCCAAATGGAATCCTTTATAATGTCTAATCTCCACATACCACATTTAGCAACAAGGTAG
A_14_P121155 CAGACAGGGATTAACCGACTTGTGCTGGTATCTAGGTGCTTGGATTG
A_16_P18453468
AAACGTGAACAACAATCTTAAAGTCCGGATAATCTTCACTAGACATCCTATGGCAAATTC
A_16_P17086920
AACAGGACATGTTCTAGTTAACTTTACCCCATTTACACAGATACCATGTATACACCAACC
A_14_P127010 ACCTCAGAAAATCAACAACTGTCATTTTCAATCTTATCTCAAGACCAACCTTCAGAAATG
A_14_P104228 ACATACTATCCTGTTTCCTCCTCAATAGAATTGCCACAAACTGCATGCTAAATAAAGATG

A_16_P15237741
AAAAATGAACTGTATTGACTGATTAAACCAGAGTGACCTCTAGAAAAAAACAGTGGGCTC
A_16_P16191631
CTCCATTACAGAGATAGTTTTTATTATTTGCTTTCTAGGACATGGCCTTAGCCTTCATAA
A_14_P124396 ATGACAAGAAGCCAACCACTAAATTTGAACTAGAGCGAGAAACAGAACTTCGCTTT
A_16_P01784775
AGAACTGGGAAGATTAAATTCTGAGACGCCTAAAACCTTTGAACATTGATCGTAACTAAG
A_16_P21483195
AAAAAGGCTAACTCATTTGAGAGTGAATGATCATCAGCCGATGTAGTTTTCCTTAACATC
A_14_P104414 CTGCAGGTGGATTTGACCAAGAGATTTGGAAATCTAAAGAATGGTGTCAGTGATATAAA
A_14_P113044 TCGAAAGCATTCTAACATACGATTCATCAAAGGTTACCGACTAATTCAAGTTTGGGATTT
A_14_P105658 ATTTTCAGTGGTATATACCCTATTTCTCTCCAGATTACACATCCCACACAATTGAAGAGA
CUST_2548_PI426210822
CTTCAGAACCTAGAGCATTTTCCAAGAGTCACTGAATTCTAGGTATGTCTTTCTCTAATT
A_16_P18891087
AAAAAAGCACTTTGCAGATCATAGCAATTACCAATATCCACCTCATCAATAAGTTCCAGG
A_16_P20336143
TCACTCGGTCTATTTTTCCCAATTATCAGTGTATTTGAACTCTATTACTCTACTCGCACC
A_14_P134119 TCTTGCTAACATTCATGAACAAAAACAAGGTCTACAGCCATAAGAAATTTGACAAGGTGT
A_16_P16614343
AAAATAAAACCCATCTTGCAAGTTATGACCACCTATATTCAAATGGTAGCCTAGGACTGG
CUST_6531_PI426210822 TGGACGGATGGACAGACTTGGCTGCCCTATACTATTATCCTAGTCA
A_14_P134304 TGGGACATTTTTGGAGTGCATGAACATATGTTTATGAAATATGTTTGCCATGGATTGCAT
A_14_P115309
AACAAGAAACTGATGAGAACGGACAAGTGTTTTTTGTTGAGTAAGTGTCTGCAAAGAAAC
A_18_P22841283 TGCCTGGGAGCAGGAGAGGGCTTCATCTTGCTGATTTCCCATTCT
CUST_3802_PI426210822 GTGATTTTGCTATGCGCCAAAGCAGCTGCCTTGATCCCTCAGAGC
A_16_P16464482
ACAAGTCACTTCTCCCTACTGAAAGACATTAAAAATACATGAAAACCCTTTTCTAGATCC
A_16_P15972688
ACATCAACATAGGATTTAACCGTATTTTTGTGCTTACATCTAAATGGCCTCATATGCCTC
A_16_P15718459
ACATACAGTCAGAGTAATTATACTATCACTGGGTTAAGACAACTTCTAGTGTGGGATCCA
A_16_P15570545
CCTCAGCACTTCTATAGCATTTATCTGTGGTTCTCATCCATACAACACATTTTGTTGCTA
A_14_P130426 TTACAAATACATCTTACTCAGAGTTGAGACTTCCCTAGGAAATGAATGAGATATGCAGGC
A_16_P00378040
ACTGACTATAAGCAAACTTTAGTATCTAGCCCCTAGCATAATATGTGATTTCCCCCGAAT

A_16_P03492986
AAGCCCTACACTCCTATACAAAGTAACGTAGCAAAACTATTATTAATGGCAACTTCTGGT
A_16_P21455762
GTCCACAGACCATTCTATCAAGTGACAAATTCCTAGACTGAAATGACTGTAGATACTTTC
A_14_P103692 AGAGTAGGCGACTTGCATAATGAGCGCATTTTATTAAATAGATAGTTAACGCACTGCTTC
A_14_P137540 CTTTGATGAGTAAGGTCTTGACCCTCCTAAACAGTGTCTGGATCCTAGAG
A_14_P139758 ACTTTCTGGTGTATTTCTATAATCAGCGTGTGTGCTTAGCATCTTTAAGTGTGAGTAGAG
A_14_P123951 CCTAGGACTTATAAAATGGCTTACCGTTGACAGGTAGACTTGAAGTTTGGATTGAATAGA
A_14_P134856 CACTGCTTAGGAGTCAACATCTTCATCATTGGGCTTTCTTTAAAACGTGCACTTTGTAAT
A_14_P110470 CAAATACTAGAATGTCCCTAAAGTAGTGCCATTCGAAAGAAACCCTTAATAGGTCAGTTA
A_14_P104160 TGAATTTCTGCAGCAAAAACAGAAAACCATGACATACTGGAGTGTATTCAGGTTTGTATT
A_14_P125413 GCATCCGTGCTAATAATTACCATTCAGCCATTGAGGAAAATGAGGTAGATTTATGCATAT
CUST_2297_PI426210822
ACATGACAACCAGAATTAGGGAAGCTGCACTTGTATTATTAATGTGGCTTTTTAACCC
A_14_P105701 CTTTTTAAATGCAATTGACAGAATCCCAACAAGAATTACAAGAGGCATGTGTGAACTCAT
A_14_P110089 GGGGTTATTTTCGGGGCAAATAATTAGAGAAAACGAGGATCACACAACAAAACA
A_14_P119649 GAGACTAGATGACTTTTGTCTAGGTTCATCTCTGTGAAGTGGAGGGAATGGAAAAATT
CUST_385_PI426210822 AACATGGACAAAGCCGATTTCTGTCCCCTTCCTTTAGACATGGAC
A_16_P01418763
ACCTGATATTTGCCTTGAATTCCTGTTTTACCTTCACACAATTAAATCTTGCCTTCACAT
A_14_P101982 GAGACCAAGAAGTTGGGGAGAAAAGGAAAAAGAAAATCACCTGACATCATCTG
A_14_P103740 ACAAGAAACCACCTGTGATACTTCAGTGACAGAGATGTAAATCAATAAATGATTTTGGGT
A_14_P111087 ACTGGGTAACACTGACCATGTCGTTCTGCTTGAGACAGATATTAGATTTTTTTTGGAATT
A_16_P18646958
AGACCTACAATACATGCACATCATTTTGGGAAATTCAAGGATATCAGATTAAGTAGCCCT
A_14_P103372 GCTGTTTCTACCACAAACTACTACTAATTGTTTGGTCACCAGTATTAGGATCCTAGTTTG
CUST_1512_PI426210822
TGTGGTAGTAGCATGACTTTTGGCTGGTAGTACATTTTTAGGGACTGTTTTCTTG
CUST_7777_PI426210822
TCTCATAACCATCAGCCTTGATAAATGCTAATGTTAAGTTCTCTAAGTTAAAGCCAGTGT
A_14_P136452 CAAAGATGAAACTGCCCAACTGACATAAAATACGACCAAACATCTGGCCACTAAATATTA
A_18_P22841018
AAAAGGACTTCTGTGTTGAGTTTAAATTAGCAACAGTAGCAACAATAACACCAAAAGCAG
A_16_P16191274 GACACAGCCTTGCCTTGTAAGTCAACTGTTTGCTGGCGTTAAACAAGT
A_14_P201899 GTTTATGCTTAGACAACTCAGAACTAAAATTAATACTGGGTGGTGGTTCAGATCTCATTG
A_14_P108750 CATAAATCATTCTTACCTTGCTCTCTCTTTCTAATTCACTTCGCAGCCATTCAAAGTCAC
A_16_P03677119
AAGGCCTTTTTCGAATGTTAGATTTTTCCAGAACATAGACACCTCTCAATATGGCTCTC

A_14_P115577 AGAGGTTCCTTCAGGAGGATCAGGTAGGTGTCATCTTTCAGCAGAT

CUST_5445_PI426210822 CAGCTCTGAGGCGTGCAGCACTGAGTGCGGTGCAGGAATATGCAA

A_14_P110265 GGCCTTGAGAACATTTAAAATACAGAGCATGAATGAAAGTCATAAAAAATTTCCTAGAAT

A_14_P105924 CATCTTCTCCGAAGTTCCTCAGTGACATTTTGCCCTTTAGCCAGTTC

A_16_P17774578
ATAGGAAACCAGTAGTTATGTCAGGTTGCTAAGTATTTTCCCACTGAAGGACAAATATTC

A_16_P21080876
GATCCACTACCTAAAGAATGCCACTCTTAACAGTTTTTTCCATTTAATTACCTGTGTCCA

A_14_P112834 CTTCTAGTTGTAGTTATAGTTCTGGTTTTGGTAGGTTATGTTCCCTTCGATTTCACCATT

A_18_P22842670
GCTCTTTAAGGCTGCTAAGCAACCATGAATCATGGTTTGCATTTTTCTAGATGGATAATA

A_16_P00341470
AAAAAGTGCTTCAATTACTATTACAATTCAGGTCCCTTTACAAATCTATGGCTCTTCCCG

A_16_P15585347
ATCAAGTGCCTGACTTAAATCCTGAGATGTAAACTTCAAGAACATACTTAGTTCCATGGC

A_14_P124209 CTTCAGAAGGCTTTAGTGTTCCTTAATCTTATTAAGGATTTGCTAACCTTTGGGCCTAAT

A_16_P02152747
AAGGTTCTATATCTGACCTAACTCCAATAGACTTCCAGCAAGTTAAGTTCTGTTGATCAG

A_14_P115085 GGGAGGTGACTTTGAGTTCATGGATGATGCCAGTAAGTAGTAGATATTTGGGTATTT

A_16_P21550805
ACTCCTTCTACAGTGCATTCAGGATGTTCAAATATTTTTGCCTTATTAACTAGGCCATGA

A_16_P36230801
TTCCTTCAAATGAACCTGTTTGGATTCTCTTATCTGTGCAATACAACCTAATGTAAGTCT

A_14_P130738 TTATCTGTGCCAAGGTGAAGGGGTTATGAACCCCATATTTTTGTATCTTAAGATTATTCC

A_14_P113761 GAAATGTTCTCTACATTTTGCCCAAGGAGTGGATGACTGCAATATTTACAGTTACAATTG

A_14_P122254 GCAGACTATACAGGTTACTTACTCTGTCATGAAGGAAGATAAGAGCCAGCAATATGTCAT

A_16_P37023824
AAATATTCTCTTCAGTTCAATTCCAACCTGCAAAATTTGTAGACGTCTCCTTCTTACCTC

A_14_P100213 CAGCATTTTACACACTGAAACTATAATCAGAGAAACTGGTGATACATCCAGATAGGATCC

A_14_P127088 ATGGGATGGCTCAATTGCTTCAATTACTGACTACCATTAGTTGAGTTTCTACAATATGCA

A_14_P115322 TGCCTGGCATCCATGCAGACTTCTGGTTTTGCTATTTTTAAATAAACACTTTTTATCGAA

CUST_5054_PI426210822 CCTCTTCCATCAGGAACACTCACACAGTCATTCTCCTCACATACC

A_14_P135569 TCCCCATTATTCTAGGCCAGCCCTTCTCCCTTATCCTAGTTTTAAACATGAATTTATAAA

A_14_P118189 ACATTCAGGCTTTTGTCTTGATGCCCGTCTTTTGGTTTACGTCATCATTGTAG

A_14_P134065 TAAAAATTTCTCACACCGGGGACTTCTAGGAAAATGGAGATCTATCCTCATCTATGTTAT

A_14_P133858 ATCTTTTAAAACTCACCTGTTGAGTTAGCACTGGAGTTGTGTGGAAGCCC

A_14_P109230 GTTGTATTTTGGGATTGATGGGGGAAAAATCCAAATTTGTTTGTTTGCTTCCCTTTTT

A_14_P137947 TGACTATTGCATTTGAAATCTCTTTTCTAGATCCTGGAGTCAAAATCTATGGCTGTGCAT

CUST_6576_PI426210822
TCACATTTTCATTTCATTTCTAAAAATAACTTTGGAAGCAGAGATGTGCTGTTTTAGTCA
A_16_P30952588
CGCACTTGTGGATGCATTTAGAAGCATTTGAATTGTTGACATCTGATTTTGGGTAAAAAA
CUST_8710_PI426210822
AAGACAGTGTTTCCATGAAACAGAATAAACTAGAGATTTGTAAATTCAAGATATTGATCA
A_14_P133831
TAAGACAATGAGACTTGAACATTTTTAACTAAACAGGAAACAGAAGGGCTAACACAGCAC
A_16_P17633532
AAACAGTGTCATCTTCACAATCTCTAAGAGTCTCCCTAACTCTAAAATGTCCTCGATTAA
A_18_P22470805
TAAATGCAAGATGTGTTACTTCTTACGACTTCTCATCTCAGTAATCCTCAGACTCAGCAA
A_14_P104817 GGGAAAGCATCTCCTTGAATGAAAATCACAGACAAATCTATGCAGACCACTGAAA
A_14_P126359 TGCATTAAAATGTATAAAATGCACAAAAAGGCATAAAATGGGAAGGATGTATCCACCCTG
A_14_P107551 ACTAATTTTCAAATTGAAGATTTCAGAAACACTGAACTTGGAAAGACCCTGAGAGATGAC
CUST_5215_PI426210822 ATGTTAATTGAAGAGTAATTGGTGCTTTTTTGACCCTCCTGAGCACTGATGCTT
A_14_P105994 AAATGCTGGGACTGAGGAAATAAAGAATGAAATAAATGGAAATTGGATTTCAGCATCCTC
A_14_P112693 GCCTCAGTGGCATTGCCTACAATCATACTGAAATTCTAGAAGCGAAAATGGTTAAGTT
A_16_P35564679
TATGGACCTAGGCTTTATACATCAAGATCCACTGCTATTTAATGAGTTACTGCCTGATCA
CUST_2373_PI426210822 CTTTGTCAGCACGCAGGCTGGCTCCTTTGCTTTGACAAAGAGAAG
A_14_P103000 TTTCCTTATGATGACTTTAGATCTCTGTTCCTTGGAATTCTAGAGTGATCCAAAGGTTTC
A_18_P15417208
AATTTCTGTTTGGTCACATCTCTCAATGTGTGAATCATCTGAAAGTGAACTGATGGAAAT
A_18_P22840287 GAAGCCGTTGTAGGTCCTGCACAGGCTCCCTGAGCCCGCTGTTTG
A_16_P30952312
GAGTGATTCCCACCAGATTCACAAATTATTGCTTCATTGTTGGAAAGAGGAGTTAGTTG
A_14_P135828 ATCTGCTGCCTCCAAGATCAAATGTTTCAGTTCCTGCGCAAGAAAAAGAAATAG
A_18_P14135388
TTTGAGAGCATTACTTCTGACCTAAGGCAGAAACACTTAGAAAACAAATCTGTGAAATGG
A_14_P125780 CCTTCTGACTGCACAAGGCATCATGCAAGATGAGATAATTTGTAAATGCAGTTTCATTTA
A_14_P130324 CTCATGGCTTTAAAGAAAATACCGATTTCAATGCCATCAATGGTAACATGAAGAGTGTAG
CUST_4269_PI426210822 CCTCCAACCCCATTCCAAATTTCCTCTTAAGAAAAGGAAGGAGAGTTTG
A_16_P00690831
ACATCAAAAGCAAAATAACTGAACACACTAGTAGAAAAAAAGTGCATAAAAGTAAGGCTG
A_16_P17918444
AAAACACACCCATTTCAAAACAAACTAGATTGATGGCCTTAAAAAACTTGTTTCAGTGGG
A_14_P115484 AATACACCTTCAAGAACTGTAGGCTTTTCTTTTATTCCCATCAACAAACGAGAGGC

A_14_P117032 GCTTCTGGGAAAATCCCTTGAAGTGATCATTGCCTCCTGCTAGTCT

CUST_6322_PI426210822 GTCAAGTCATACATCGCTGCACACCGGGGCTGTCTTAGGACATTG

A_14_P101451 TTCATGATATCTGAATTTTATTTTAGACAATCCATGGACACAAGGGACCAATCACTGCAG

CUST_91_PI426210822 ATTTGCCCTTCCTCTGAACTTTCTGGATATCAAAGCCACGTGGAAGTAT

A_16_P21004926

ACACTTGGAATTGTGAATACTTGGGAATACTTAACAAGTCTCCTATAGTATATGCCTCCC

A_16_P20722563

TACTTGCATTTTCTTCTTTTCCAACTGTTAGTGAACTCTATTCCAATCAGCATTAAGCCC

A_14_P120896 TTAAAAAAACAACACCTTCTATGAGGGGAATTTAAAAAAAGAATGGGTTGGGAATCTCCT

CUST_6033_PI426210822 CCTGGCCTCTCAAAATAGCCTTTTGGCCGAATCCTCCATTAAAGA

A_18_P15191176

ACGATTCCATTAGTCTCACTTATCAAGGCCATCTAACTCTTTGTTTAGATAAGCATTCCA

A_14_P102205 CATCAATTTCGTCATCTTCAAACATTGCTAAAAGTTCTCCTGTCTGGTCAATAGAGTTGA

A_14_P122511 TCTGATGTTTTTGCATTTATCTTAGGAACCATGGACACTGAATCTAAAAAGGACCCTGAA

A_14_P100461 GGGGAAATGAACTCTATCCTAAAAAAATAATCTGCAGTGCATTTATATTGATGTGCAAGAT

A_16_P00906116

CCTTGATACTACTAGAGACATCCCCATGATAAAATGAAACAAAAAAACACATAGGACCCA

A_16_P17580711

AAAGCATTTGATAAAAAGCCTAGATCTAGACTGTTTGAATGATGTGGATTCTCAACAGCA

A_14_P130717 GGCATTTTAGAAACATCCCTACGTTCTGCAAAGAATCCCATTTTTTGTTGACTTTCTTAA

A_14_P108719 CCCATTGGGATTAAATATTTTTGAATAGGATACACTCTTGAGAAACTCGAGAATGGACTG

CUST_8452_PI426210822 GAAGCACCAGCCTGCACCACCCAGAGAAGCACACTTTGTGAGAAC

A_16_P20843923

ATAGACGTTTCATAGAGTCTTTCTCAAACAAGCCTTTGATATACTCGTTTTCTCAGCACC

A_16_P16022347

GGAATCCAAAATCTCTTATGAGGCATATTAGGTATCACATGTGACATAAGCAAAAGCCTG

A_14_P135975 CTTCTGACGTGGAATTACAACTCTGGAACTGCTTCTGTTTAAATCCTTTGCTGAATATTT

CUST_5026_PI426210822

AGATAGTACATTGGCATTTGTGAATCATTTTAGATGAGTTTGTCCTCAGTACAAATCGTG

A_18_P22839802 ACTTCATCATGGCCGTCTCCTCTCTGGGGCTGCTGCGGGAGATTT

A_14_P124931 ACATTAAACACAACAGAGGCTTACTAGCAGGAATATTTTTTCCAAGGAAAAATAGGGTGT

A_14_P125017 CATTAAATAAATGGAAGTTTCAGTTTCAGGGACTTGATGTCTGCTCAATTGTTTGAAGCA

A_16_P02826449

TGCAACATGACCATATTAAAACTTTAACCACTATTCACATTTCATATTCTCCTTCACCGC

A_14_P123368 CTCAAAAAAGAAAAAAAGGCAGTTTAGGGTATTTATCACACCAAAAATGGTTCCCCCTC

A_14_P130389 ACTTATTTTCACAAGATCCAAAAGGGCCATCCTGAATCATAAATCCTGAAGAATTAAAAA

A_14_P104929 TCCTGAAGTAACAGTAATTGAAGATCCCCAGTCAAATTTGAATGATGATGGTTTTACTGA

CUST_1237_PI426210822

CTAAAATGATTTTAAGAGCCCTTGGCAAGTTATAATTTCTCAAATACTGTTGAAGATCAT

A_14_P126336 TGGGCGTGGAAAGTGCCACTCCATGAAATGAATCTATATCATTTTAATAGCATATTCCTC

CUST_2210_PI426210822 CCCCCTCCCTCTGTTGGTTAGTATTTTCTCAGGAAATTCATACCTGAG

A_14_P130170 CTCCCATTTCAACTAAGTCCTGCTGCAGAATCATTCTGGCTTCTTCCACTATAA

A_18_P14136761 AAAGCAGACCTTCACCATTCAATGCTGGCTGGCTTGGCTTTAGAGTTAATTATT

A_14_P131221 CTAATCCTTTAATGGACATGTCTTTCTTCTTAACTGGAATGAAACATCCACTCATTGCAG

CUST_3871_PI426210822 AGTAATCTAGACCATGAAGGAGGTCTGGATCCTGGGGAGGGAATC

A_14_P100064 ACACTTCTGGAACTGTTTGGTCAAATAGAAAGAGAATTTGAAAACCTTTATATCGAAAAC

A_16_P02729116

GACTTCACTCCTAATACGTTAAACATTCTAGATTTCCAGAAAACTTACACTTTTTCACCA

A_14_P136545 CTAAGCCTCATAAGTCAAATCCTCAATTAGCAGATTTCAACCAAGTGAACAAGAACTTGA

A_14_P116478

ATTAGAGAGTCCAGAAAATTAAAACGAGAGAGTCCAGAAAATTCTCACAGCACTAAAGTC

A_16_P03618476

AAACCCCCTACTTTATTTTAAGCCACTGCATATTGAGTTTTCTGATTCTCATTGTAAGCA

A_16_P02516338

AAAGGAGCATTAAAATAGTTTTTCCTAGGCCACTATAGAACATCTCCAGTTGAGAAGTGA

A_16_P58424404 GTCCTTGACTCCTCTTTTCCTAACACCCTTCATCCAGTCCATGAGAAAAAT

A_14_P128144 CTGAAGAGTTTTCTCCGAGACTTTCTTTTCTTCAGCCTGAGACAAACCAAAATACAAA

A_16_P19403054

CCCAGTTGAGTCCCTTTATTCATACACCACTTATTGAACAATCTATGCACGTGTAAGTAA

A_14_P117223

ATTAGGAGGAAAGAGCAAAAGCAGTAACAAAACATGAATAGAGAAAAAATGGCAGGTTGA

CUST_790_PI426210822

TCCTTAAGCTTTAATATGCCTGGGAAAGATCTAACAGGTTTCACTTTAGGACCTC

CUST_1064_PI426210822 CTTCTGGCTGCACTGTGGACAGATGTCCTGAGGCTGGGGGGAGAG

A_14_P128991 TGGAAAACCAGGTAAGCACCGAAGTCCACTTGCCTTTTAATTTATTTTTTTATCACTTTA

A_14_P127054 CATAAAATGGGAAATGAGGGTTTTCTTTTCACTCTGTAGTAATAGGTAAACCCAGTATTG

A_16_P20824541

AAAACCTTTCCACTGAAAAATCCCTCTGATTTAAAAGTAACCCCTTTGAAACATAAGCAG

A_18_P14135074

CTTCAGCAGGCAAGGAAAATATTTATTTGAAGACGTTTATGAAACACCTCCCTTGTTTTT

A_14_P107837 CTTTAACTGTGTGCCCTTATCTACCCTATCTATGTAGATGTGCCCTATCTATGTAAACTG

A_16_P18861203

AAAAGTCCTTGTTGAATACTTTTCAGGACCATACTCTTAGTGCTAACCCTTTTAAACAGG

CUST_4103_PI426210822

TTGTATTGGAAAAATCTGGGATTCTTTTTCCCTTGGTCATTATTTGCTTTTTAAAAAATA

CUST_4530_PI426210822 ATAGAGAAGGAGTTGGCAGCATTTAGAATGGAGTCAGGAGGAAGATTTCTC

A_16_P36083067
TCAGATAGAAATAGACTCCCGCATCTAATTTATTCCTGCATGAATTTGTCAACTGTGCTT
A_14_P120008 ACTATACTAGGAGAGAATGTTTCAGAACTCCCTGATGAATTTCTAAGTGAGCAACTTGAT
A_16_P18123048
AAATATCCTAAACTGCTTCTAGAGTGAGAATCTCTCATCACCTACAGATAGTCTTGAGGC
A_16_P01166408
ATTACCCTATGATCACAAGACACAGATTTGTAACCCAAAGAAATCCATAAATCACAGAGG
A_16_P00042602
AACCTGAATTACATCCAAACCATCGATATTTGCAGTTAATAGCAAAGAGGCTTTACCTGT
A_16_P35495496 AGAACTGGGTGAACCCATAAATGTAGAGTTGGTTTTGACCTGAATAATCTGAGAG
A_14_P111193 CGTAAAACATATGTTGGTGATCAACTTCAAAGAACCTCAAAGGTAGGTTTCTACTAGAGA
A_14_P124056 GAGGGAAGATAAGTGATAAAGGGTCTTGAAACCAAATTATCTTCTCATTCTGAGAAGTGA
A_14_P200100 GATAGCACTTCTCTTGCACTTAAGAATGGCATCCATAAGATTCTGTATTTGGCATTTAGA
A_14_P108332 ATCAGCAACTCAAGAGCATTGTCCTTTAAGTGAATGCTATCAAGGTTTATTCATTCATCC
A_18_P17362083
TCCTTGACACACTTTTTAGAACTAACAGTTGACAGAAATACCTCAGAAGAAACAGTCAAG
A_16_P33506577
AAAAAGTGTCACTGACCTTTTCATCAATCCTTGCTGACAATGTATTAAACAGTATACAGA
A_18_P10601866
AAGAAAACTCCATCTATAGACTCCAAGGTGTTAAGTCCAAAATAACGTTCCTCTGAAATC
CUST_4811_PI426210822
GTATAAACATCTGAATTCACTGTTAAACACTCCATGAACTGTTGATAATTCCCTAGAAGC
A_16_P03366047
CCCATTTGTCTTTTCTCTTTCCTTTATACAGAAACTATCACTTACTCTTCCTTTAGGCCA
A_16_P41705966
AAATAGGCTAAAAACCTAAGCCATTAGATCTCTGGATCATTCGACTTGTTTACTTGGTCA
A_14_P109489 ATATGTCAATTACAAAGAAATGAAATGTTCAAATTATTTATAAACCTGATTTTTCAATCA
A_14_P132179 AGTCCAAAGTAGAACTTGAATCCTTCACCCTTCTATACCAGTGAATAAATGTGCTCATTA
A_18_P14139037 AAATCGAGTCCATGAGGCTGAGGAGGCCACTGGATGTCCAGTCTT
A_16_P16222875
TACCCTTATTTGCCACTTAAATCCATGTGACACTATATTTCCAACACCAACAATCACTTC
A_18_P14133174 CTAAGATGGCAGTTCCTTATCTCATGGGAAGGTTATTTACACTGGTTCTCATGAGA
A_14_P108063 GTGGGAAGGCCTGGTGCTCATCATCTTGTATGTGTTTTATATTCTGATCATGAAGTAAG
A_16_P01131663
ATGGAATGTAGAAACACTCTAATTCACAGACAGACTCAACTAGTTTCAGAGTTTAGCAGG
A_16_P16657223
AACTGAAGTATGTCTGATGTTTTTGCTACTACCTCATCTTGCTATTGAAGCCACTGTTAC
A_14_P105997 GATTATGAAAATCCTGTGCTGCAGTGTGATTTCATGGGAGAGGTTGGGAAAT

A_14_P200304 GATAAGGAGGATGATAATGTGTATGTTTATTGGAAGGGTCCTTTCTGTGAATCACCTTCT

A_16_P36192705

CTCAACTGCAACAAGTCTTACAGAAAATAAGCACATCTCTATATTGTCATCTGCCCGTTA

CUST_10107_PI426210822 GTACTGAAAGCTGAGCAGAGCAGCTGGCCATGCCGCCGCATTCAC

A_14_P120865 TAAGAATTCTTTCAACAGTTATCTTGTAGACTGGTTCATTAACTTTGCTGGCATGGGAAG

A_14_P123628 GAAAGACTCCTCCTCTTCATACCAGTAGAACGTGTCAATAAACAGATAAAAATTTATTCC

A_14_P115076 GGTTTCTTTCTTAAAGGACTGATTTTTAGAACTCCACATTTGAGGTGTGTGGCTTTTGA

A_14_P201043 GTTCATATATAACATCCTGTGTAGGTGTTTTTTTGGTGAATTCTAATGGACATTGAAGAT

A_18_P22840297 CCTTCTTTTTCAACCGTGTGCTTGGTTTCCGCCCATCCTGTTCAG

CUST_3789_PI426210822 CCAAAATGCTCGCCTTCCTGGGTTCTGCTTTCATTAGTTTGAGAGA

A_18_P22842268 AGACAAGCAGCAATTAGGTAGGCTTCTAGGTCAGGTGGCCAGTTAG

A_14_P132413 AATAGAAATGCCATTGTCATTGATCACCGCTATCTCAAGAATGCGGGCAATCTTAC

A_14_P130444 GTGGCACCCACTATAAATGAACAATTCTGTTAAGTAATTGTGAAATCGTCAGTGGTCATT

CUST_1632_PI426210822 GAGGAAATATATGGCTTCTCCCAGGCCCACCTACATACCCCGGTT

A_14_P105584 GGACAGGAACTCTTCTCTCTTTTGTTAATAAACTTCCAACTCCCTCCTCAGAC

A_14_P135278 TAATCATTGCATCCTATGATGCCAACAAGAAAGGTATGTAATTCTCTAGCCTAATCTCCT

A_14_P129005

CTTAACACAACAGAAGAGAAGGACAATGGGAATATTAACAAGCAAGAATAACTTTCCACG

CUST_3008_PI426210822

TTCTGTGTGGGAGCACTTTCCTGTATGCATTATTTTCTTCTTAATAATGGCACCTC

A_16_P02040381

AAGCGTGCTAATTACCAAATGTCCTGCATGATTAACTAGGAAATTAACAGGCCAAATACT

A_14_P102086 AGTCAATTACAGTTGTTTAACTGTATCTGCATGGACATTCCTAATTACACCAACAGGACA

CUST_9268_PI426210822

TTGCCTAACAGCACACTGTAACTTTAGTGCTCTTGCTAAAGAAGAAAGTCGTACTATAGA

A_14_P102172 CTTCCCTTCTCTCTCTCTCAAAATTATAGGAGACTTGTAGTTTAGTGTGGTTTTCTGTTT

A_14_P107932 TTGTATTTATGCCTCTGTTACCCTTGGCCTAACTTTTAAAAGCCAATTTTGTCAACTGAT

A_14_P111523 CTCCAGTATCATTTATGAGGAATGAAGGGTTAGTAGCATATGCTTTGGAATTAGGTTACC

A_14_P128604 TAACTCCTCCTGTATCAGCAACACAATAACATCTCTCTGTGTGTGGATCTGTGTATATAT

CUST_6680_PI426210822

TATAACTTTTCAGAGGGTGTGAAGTCACATTTTTGACCACCTACTATACACCAGG

A_16_P58424565

GTAGTTACTGGGTGACATAATTCATCAAACTGCACCCTTTAGATCTGTGGATTTTAATGC

A_14_P108667 TCCTTTTATGTTTCTGGGGCTTCTTGGAAAAAGATACAGGTTTCATTTTAGCCAGG

A_14_P132636 TGATATGATATGGAATGACCCATATATTTAGTCTCTGCCCCTGATTTCAGATCATAGTGG

A_14_P125867 ACTTTGCTCCTTATACCAATGTCTATGTTGCTTTCCATCCACAATCTACAAAAAGCCAA

A_16_P00906830

ATTGAATCGCATGTTGACGTCTATTACGATGATTAATGTTCTTGTCATTCCCGTCACTAG

A_16_P36729612 AAATTATTAGTCACTACAAACCAAGAAAATACATACACCAAGATTCAAATATACCTCCAG
A_14_P101763 CTTCATCTTGCTGAGCAAAAATGAAGGAGCCTGGACCCAACTTTGTTACT
A_14_P117789 TTTACAGATGGCTGCATTACAAAGTCCTTTCTATGGTGACAAAATGAATTTATACTCACT
CUST_1893_PI426210822 AAACCAGATAAACCAGGGCCCTGAGCTGGCAGGGAGGAGGGATCAA
A_14_P115093 CTACACTCTCTGATTTTGTGCTACTCCTTTGTAAAGTCACTGCCTTAAGGGG
A_14_P108885 CACTTTGAATCTATCTGCACCGTTTATTAGCCAGTTCTACAAGGAATCATTGATGAAAGT
A_16_P38478269 AGATCCATCATAAATGCTTCTTTTTCATAAAGTCATTCCATATATTTCCCACCAGACACA
A_16_P01066433 ATGTGCTCCTACCTAATGTGACAACACTATGTTTCACACCTATGAAAACAATCTCATCTT
A_18_P23688479 ACTTCTCTTAAAGGTAATAATTCCTTTCCTATTGATCCAATCTCAAACCTCATGTGCTCC
A_14_P125039 ACTTTTAAGACCCTCACTATTTCTAGAACATTTCTTACGGTCTTCACATGCTACATCCAA
A_16_P15059815 AAATCCACTTGTGTGTTATCAGTGTTCCATGCTATTAATGGTCAGTATCTTCAAAAAGGC
CUST_5717_PI426210822 TGTCCTATTGAAAACCTTGTGCAAAAGTATGTGTGGGGTGAGAGGTG
CUST_5251_PI426210822 GAAAAGGCATCCCACTTCTGAAGATTTCTCGTTTCTAGGAGTGTCTACAAGATTA
A_16_P20879222 AATTTCCATGCACTTTCAAAATGACAAGACTCAGAAGCATAATACCAACTTCCTTTGTCA
CUST_4965_PI426210822 GAACATTACTCAAGTGCAGATAGGGAAACAGTGTAAGAACAAAAAACTTTTTTTCTATCT
A_16_P38575806 ATGCTTCTTCCTTTATAGACAACCCAATCAGAATAGACATTCAAACAATTCATCTGTCCC
A_14_P131875 TATTTCAATGAAACAGCAGAGTCAATAGGAGGATTCTAGAGAGATTCATGCTGCTTCTAC
A_16_P30952370 AGTCACAGTTCTTATTTGTGACTGTGGTCTTTTCCTGTGACTCCATTTCCCTATTATC
A_14_P127432 ATTAACATTAGCATAAATGGTTTTTAGAGGAAAAAAATAGCAAAAAAAAATGGCAAAACT
A_16_P15345853 AAAGAGGCTACTGTGTAAATGATCACTCTGACCTGAAGAACAGTTCTGATAATTCCTAAG
CUST_4070_PI426210822 CCTTGAGCCTCAGTTAATTGTTTGAGTTTTCTTGAGTATAAAAGGAATCCTGGCTG
A_16_P02535617 AAAAAGCAGACAAAGACTTTTGAGAGCGTAGAATTCAACAATGGCAGTTCATAACAGTGA
A_16_P02925601 TTTCCAAAGCCTGAAAGTCATTGTATATCCACCTTAGATAAGCCAGAAGAAACTAACCTA
A_18_P14132941 CTTTGAACAACAACAGTAAGTGCCAGGGAACATGGACAAAGCCGATTTC
A_14_P201983 TGCTATACCATGCGTCTGTCGTTGTGCTTTTTTCTGTTTTTAGACCAATTTTTTACAGTT

A_16_P58424119 AAGATCTACTTAAGAGGTATGTGAGGTATAAAGTGCCTACCACAGTGCTCAGTCG
A_14_P103608 CTGTCAAGTGCTGCTCACAGACAGCCAGGGATTGTTAGAAAAGTATGGTTATATACTATT
A_14_P100005 TAGTCTGCAAACTTACCCATGAATGTAGGAGATCCATATCTGAGCAGGAAGACTT
A_16_P03772344 AATTCCACTCCCTATTACTTCCTCATTTGACTGTCTCATACTAATTTGCATGACAGATCA
A_14_P105414 CTTGGCTTCTCACTTCTTTGCAGTCAACCATTGATGAGGGATGTGTG
A_16_P00622444 CCCTAGTAAGGAAATACACTTTTACTCAGAAATACCCACAATTCCTATCAATCTGCCATC
A_14_P123078 TGGGAACTGTTTGGGAATTGAATAGGAAGCAGTTTTAATTGGAGAAAATAGAAAATTGCG
A_14_P108278 GTAAGGCTACACAACTCGGAAAATATCAGTTCTTCAGAAAAAACATGACAAGAACAAGAA
A_14_P131318 ACATTAAAGTTAACTCTGAGTTTCTGGCTTAATTTAGTTTGACCATCAAGGCCTAGCCTA
A_14_P100797 TGAAATCTGAATATATCGTCACTCACCATCTCAAACAACAGACGAACTAACTTCTCAGAT
A_14_P116986 CTAATTTCACACTCAAAGGAATTATTTTTTTTAACCTGTGAAAGCCTCAGCAGTCCTGA
A_14_P111694 CCTCTGACTCATCACTGTGATCCTTCATTATTTCATACATTTTGTCCAGATCTGTTTTTG
A_16_P16192038 TTTGCTTCACGATTTGTGTAGGACACGTCTTATATGTGCATGCACTGTTGG
A_14_P121957 GCCCTGATATAACTGGTAAGGGAGATAGACCTTAAACAGGTAATCATACAAAAGTAGAGT
A_14_P134962 AAGGATTGGCCACAACAAAAGTTACAAATCCTTGAACATATGGGGTTTCTTTATAGATCT
A_14_P105010 TAGCCAAACTTTGTGTGGGACTTCTATTGTTACTAATTGTAAGGCTGTAACTTTGGTTGA
A_16_P16157904 CTGGAACGCAGAATTATAAAGTAAAAGACAGTGTTAGACAATATTTCCAAGGCTCTCCAG
A_16_P03287877 GATCTCCCTCACAACATGTTGAACTAGATCTTAAGTCACTTATTCATTTGCCGTTTCTAC
A_14_P132824 ACCAGATCATCTAAACAACAGATTTTAAGACAAGTAGTCATCGTAGCGCCTAGTAAAGCA
A_14_P109092 ACCAGTATTTATCCTTAACACTCTTGCACCTTCTGAAAGGTATCTAGATGGGAATTTCAA
A_16_P30952414 ATCACAGTGTGTCGGTCCTTTCTGACATTTTGACCTCTCATTTATTGGCAG
A_14_P139038 ATAAAGGACCTCATCTTTGGTTATTAGTGGGTGAAAAGAATCTCCATCTGTTCCATTAAT
A_14_P138908 AGGATCAGAGCCAGATCTTTAGACGTGATGGATTCCCAAGTTTCGTTCTTAAAATAG
A_14_P104951 ACATGTCAAATTAATGCACTCTTTAAAATGATGGTGGTCACTTTTTGATCCATACTGCCA
A_16_P00340491 AAAAAAAGTTCCTTTTCTGAAACTAGGCAGGTATTTCTTGCATTTTGTTCTCGTCTCTCT
A_16_P00590323 AAAAGTCACACTGATCTCTGTATTCCAACTCCTATCTTTGGCATTTCCTATGAAGTTACA
A_14_P128638 GACAACACTGAAATTTTTAGCATAACCTTAAACCCACAGAAAACTGACTTCCTGTCTATC
A_14_P100065 CTTATAAGGAGATGCCACTAAAGTATCTGATTAGGTTGAAGCAGTGGTCAAAAGCATCTA
A_16_P19007031 AGTGTTTCAAGGTAAACGAGTAAGTCAGAGAATCAATGGGATGGAACTTATGTTAGGAAT
CUST_2435_PI426210822 ATAAAGTAAGCATAGATGCTAGTGTGTCCCATGGTGAAGTTGTGAACTCTGGTT

A_14_P129582 GAAAAAATTTCTGTTGGACTTTTAGACTGAATTCCTCTGTATTAGCAAATAGGAGGCTGG

A_14_P106884 AAAAATCAACAATGATTCTAAGCGTCATGGAGACTTAGGTATGCTCTCAAAAGTGAAACC

A_16_P03123282

GCACAGTAGTTGCATCCATAAAGCACTTTTTCCTTGAACCCCTTCTCATCTATTTATTTA

A_18_P22842261 GTCCTCAGGCCACCCTAGCCCCCTGTTCTCCTGGCTGGCTTTATC

A_14_P117502 GCTACATGTCTCAAAATTTGAAAATGTGGATTTTTTTGGTCCCAATTTTTCACTTTCCCA

A_14_P108784 ATCTTGATATTGTTTTATAATAATTTGAAGTCTAAAAGACTGCATTTTTAAACAAGTTAG

CUST_1521_PI426210822 ATCCTGGCCCCTGGACTTGGCTCCCTGAGGCCACCTCTCTCTTTT

A_14_P132784 GCAGGGAAAAATAGAGGCAGTTTTTATGACTCAAATGATACTTGCAGACAAGCATTAACA

A_14_P131236 TTAAGGCTTTCAGGGCTTTTCTTGGTCTTTCCTTAAAAAGACTTGGTAAATTTGCATGTC

A_16_P18524339

GTGCCCCAGTTAAAATTTGTCTATCAATTTCATTTAAGGGATCTTTTGTCATACAAGTCT

A_16_P40931853

AGAGAACTTCTCGTGGTGATTTGCTGTCTGATTTATAATTGCACCCTTTTTTTAAAGCAA

A_14_P105244 ATCCTTTGACTCGAGAAAAGGAGAAACTCTTTTTCAGCATTAAACACAAAGACAGTATCT

A_14_P106424 CTGGGGGTTGGCAGAAATGTGTCGCCATATGTGATAAACATATTAAATAGGTTCAC

A_14_P134941 AAAAAAATTTCCCCTAAAGGATTTCTTAAAGGCCAAATGTTAGGCTAAATGTCCCTCATT

A_14_P118419 AGTTTAGGATGTGCCTATATTTCTTGCAAATGAGTCTCCAAATACAAACCCCAAATCATT

CUST_2019_PI426210822 ATCTCCCTCTTCCCTTCAAATGTACCTGGGTTTGAAAGCCTAAAGG

A_14_P129018 CTTCTTGAAAATGGGCCCCTGCAGAATAAATCTGCCAGTTTTTATAAATGCTAAGATCTC

CUST_4709_PI426210822 AGATACACTGCTGCTCTATGGGATTTTGCCCCTCATTCACAAGCA

A_16_P17883501

CGCAAGCCAATCTTATTTTTTACCCTTTGTATCAGAATGTTGCATATCCCAAGTCTTTCT

CUST_429_PI426210822 CTCATAACCTCAGTGGTGAGCATCTTTGCAGAGTTCCTCTGTCCT

A_14_P134948 GTGGAGGAGGTCAAACCAAGAACTTTTACATTTGGGAGATTGAAATCTATTTCTAAAACT

A_14_P116533 CTTCAGGAGCCACATAAACTACTTCAAATTCAATCTCTCCTCTCTAAAACAGGAAATGAA

A_16_P19008628

ACATTTGGCTAAAGGAAACCTAGTAGAATACACGGTTTGAATAAAACATAGTCCGGAATG

A_14_P128046 AACAAGAACATGTCAGAGAATGTGAAATAGGTTCTTCCTTATTGGCAGAGACTACAGTTA

CUST_5981_PI426210822 GAACCGTACAGCGGCCTTCTGTTTCCTCTTTGCCAACCTTTTATTTTA

A_14_P127133 CTGCAGAAGGTGACATATGTAGCGAGTATTTCTAAGTAAGTTTCACTGTCCTTTCTCCT

A_14_P111672 ACGTAGGAAAATGTGAAATGTTCCTGTTCTTACATAAAAGAACTCTCAGAAAATACCCGT

A_14_P119931 AGGCTTGGGTAAAATCATTTTAAATAACATATTAAAATATGTTTGGGGGGACAACCCAGC

A_14_P129954 ACAAAACACCAATTTTATTATAAATATCAAGAACCTACAGGGTGTTTATGGGCCAGCATA

A_14_P102814 AACTAGAACATACCACTCCCTGACTGTATGCAGTAACAGAGCATTTATTGGACTACATTA

A_16_P02628560

CTCTTCCTACATTTCCTCTTCCTAAGCAAAGTAACATAAAAACTCTAGACAATTGGCAGG

CUST_4295_PI426210822 GTTTAGATTTCTTTTCTGGAGAGGTGTCCCCAGACTGCTGTGCATAG

A_14_P124817 AGGGTAGAGTAGCAGCCTCTTTCTGTATCATCATCAAATTGTATTTAAGGAGCAATTTGG

A_14_P114954 AACAATAATATACTCTTTTGAAGAAAGCCATCCTATTCAGGTATCATGTTGGCAAGCCAG

A_14_P132921 AAGAAGTTGCTTCACTTCAGGCTGTTATGAAAGGTAAAGACAGAGAAAGATCAATTTTGT

CUST_9337_PI426210822 AGAGCTTCAGTTGCCCTGAAGCCAGCTACAGCCTCCCTTGGGGAG

A_14_P113995 TGTCATTAATAAAAAACACATGGAGATAAGTCTCACTACCCCTCCAGATTCGTGTCTATT

A_14_P114510 CACTCTGAGTCATTTGTAAAGAAAGCAGAGCAATTTCAATTCTATATCTCCATGTGTGCT

A_14_P105259 CAGCCTATAGTGGACAGATTCACCTGATGGACTCTTGGTTATTCATTCTAATCAGTTC

A_14_P107626 GCATCCACTCCCCATATGTGGCTGAGATAACTTACTTTGACTGTCTATTTGGTT

A_14_P120608 CTTTAATATGGAAATAGAGTGTGTCGGGTTGACTATGCATAAATGCTTAATCGCATATGC

A_14_P201384 CTCATCTAGAACATTTCTGATGTTCTCAACACAAACTTCTCCTCTGTGATGCTTTTTTGA

CUST_3446_PI426210822

TTATTTTTAGTCAACGTATTAGTATTAATATCATGGCCTCTTTTCTTCTCATTTTGGAAG

A_16_P17619221

AACACCTTTTTCTGGATTTGCCTTCTAAACAATAATCCCTTCGAAATGACTTTGTGAATG

A_16_P19591421

ACCAATTTTCCAAATTCCTTATCCCTGGTATTCAAAGTCCTCTAAAAACTGTCTCAATCT

A_14_P133750 GGCAATGTAGATAACAGGGAGCACACATCTAAAGTATGTGTGCTTAACATATGAACAGTC

A_14_P138172 CTTGCCTTGACTTTACAGTCTGCATAAGTTTACAACCAGGAAAATGAAAGGAACAATTTT

A_14_P106224 AGCACCTACCTGGCCATACAGATGATAATCAAATTCATAGATCTTGTTGAATTTTGAAAG

A_16_P19790104

AAAACAGATGGTGTATCTACTTTTTCCCACTAAATTCTCTATGTTGCATCAAACCTCTGG

A_16_P00154395

CGTGGCCGATATTAAACATTTAATTGACTAGAGAAACCAATCATAGAACAAAGCCCCTTT

A_14_P100871 TCTTCTATCATTGATGAAAAATTATCTGAGAGTTCTAAAGAGGGTGCAATGTGGTTGGAT

A_14_P102006 ACACTTAAGAATTAATAGGCTCTGAAAAGCACCACAGGGACTTCACTGTAAGTAAAGTAT

A_14_P112119 TGTCCTAATGAAATGCTGTCTAAAAAAGCCTTAGCCTCACCAAGTTCTAAATCACATGAA

A_16_P18302508

ACAGAAATCCTGCCAATACTTAAAAACAGTCTGCATTCTACATATCCGAGTTTCAGAACT

A_14_P201257 TGGTTTTGCCATTTTGGAAAAGTAGTTCAAAAGAAATAGACTCGAAATTTGAGCAGAAGC

A_14_P117221 CTGGATTCACACAGGCAATGCACAGGTGAGTCACTATTAATTAACACCGTTTTAAAATTA

A_14_P130559 CTTACTGGCTGAGAATCCTGCTTTAGGCTAATTCTCCAGATCTGAAAATAAAGATTCTAC

CUST_3601_PI426210822 GTGGGCCTTCTGGTGCTGAGCTCCCTCACATCTGAGCTGTCTTAG

A_16_P03703359

ATACTTGTCACTATCCCTTTCCTAGGATCCTACTCTGAACTCTGTCTTTTCATATCAAAG

A_16_P19749700

AACTGTTAAGACATGCAAACTCTTCCATTCTTCTACATAGGATTGTCTGTTATCACTGGT

A_14_P119533 TCACATGCTGGAACGCACCAAATATGAAAATGGAATATCAAAAGTGGGTAAGAACATTAA

CUST_748_PI426210822 CTTTGCCTAGCTGGAGTGTTGCAGGAGGAGCAGCAGCATTCCAAG

A_14_P138539 TGATTTATTTTAGTTCCATACTTGGAGGTCAAATTTGCTATGCCTGCAGGTATGAGACAA
CUST_6075_PI426210822 TTCCTCTCAGTGAAAGTACGTTCCAGCTTGAGTTTTGAGTTTGCTCCT
A_14_P119824 CTTTTTAAAGGAATTACAAGTGGAACTTACATATATTTGATTTCTCTTATACCGCTGGAA
A_14_P103365 AAAGCCTTCAGAGACACACAATAAATTTGGACCAGGGGATTTTTTAGTTATTAATGCTCT
A_14_P130510 ATCTTAAGCATGAAGACAGACATGAACCTGAAAGAGAATAAGATATCTGTCAGCAAACCA
CUST_1674_PI426210822 ATTGCCCTCTTGCCTGCAGGCTCTATCCTCCTCTCTCTATTTTTTTT
A_16_P01492105
CGTGCTGCCAAAATTATATTCTTAAAAATATCCCAAACCATATAAGACAGAAGCCATTCA
A_14_P130280 GGGATCTTATAAGGCTCTATCTGCATGAATCAAATCGAGTTTATCGGGATAAGATGGTAG
CUST_7650_PI426210822 TTTCCATGAATGTTTTGGGAGAGGTGAGGAAGAAACCTTTTCTGCTCATT
A_16_P58424472 CTGGCCCAGGCTCCTGTAGGGTTCCAAAAGAGTGAGTAGAAGTTC
A_14_P122783 AGAATACAGACTGCTACCTCCCCGATGCCTTCAAAATTATGACTCAAAGGTAAATATTCA
A_14_P100100 ACCCATTTGTGACAACATCTCTTAAAGTTGAAGGTCTACACATGTGTATCCATATTAGCA
A_16_P19664056
ACCAAGCCCATTTTTATTAAACTGCATTTCAACTAAACACTAAGATTGTTTTACCTCCGC
A_14_P114330 ACAGGTCATATCTGAGGCATCAGCCATCGAAAAACTTGGTAAGAAAATGATAAAATTCTG
A_16_P37619552
TGGATTCTACAACCTCTATCAAAAGTCCCAATACTCTAAAATATCCTTGCTTGGTAAACA
A_18_P10526683
TCCTCCCTTAGTTCTTCATTCCTCAAATACATAGTTCAAAAGTCACAATCCTTCATTGAG
A_14_P138425 TATGGCTTTTTTGATAGTGTATTGATCACATTCCCTTTTAGGACACTTTAGGTTGTCTGG
A_14_P135923 TCAGAAGAACTTTACTTTGATGCAGACTTCCGTGTTAGGTTTTCTTCCTCTTCTTTTTCA
A_14_P100667 TATATTTCTATTTGACAGTGACGGTGTGGGCTATAGTCATTTGTATCCACCAGCAAATAG
A_14_P119079
ATTATCGGAGTGAAAGAGAAGACATTCGAGCAACTTCACAAGAAATGTCTAGAAAAGAAA
CUST_4106_PI426210822
ATGTAGTTTTAAAGAATTCTTGCTATTTAATCTCAAGCCCAAACATGGTTTTTAGTGAGG
A_18_P16000240
GGCTGCTGCTAAATATTATTATCTCTACCCACAACTTCTTAGTATAACCGTTCCACTGAG
A_14_P131361 TGCGTGGTCATAGAGTATGCAATTAAATGATCCATAGACGCCATCATTCACATAATACAT
A_16_P02136532
AGAACTTGCCGCAGTTTTTTTGAATGTAACAACCCAGTTTAAATAAGAGTCTCTGAAACT
A_14_P101173 AGAATAAGAGTGAGGCTTGGGATGGAGACATCATATTTTTCTTGGATAGATGAAAACACT
A_14_P112230 ATGTAACCACTTTAAAAATAGTTACATTGTTTTCCAGATTAAGTAAATATTTCAGTTAGG
A_18_P22841785
TGGTTTTACTTGCCTTTAAAATGCTCAATTTAAAAAATAGTAAAACGTAGAAAAGAGAAA
A_14_P101619 AATGAATGAAACATCTTACCTCCAAATGATATCCAACTCTGATGAAGGCACAAACTGGA
A_14_P110415

TAAACACAGAGGTAAAAAATTCTTCAGATACAGGGAAAGTAAAGTTGGATGAGAACTCCG
A_16_P36640387
AAATCCCATCTAAGAGAAGGTTCCTATAGGAAATTGGCCCTTTAACTCATACACAGATTA
A_16_P16996410
AAAAAAAACACATTCAGCATATTCATGTAAGGTTGCAGAAAGATTCCTGAATAGGGCAGA
CUST_8112_PI426210822 ATGTGACATGGAAGAGATTTCCCTTGTCAAAATAGAAGACACTTCCTGCCT
A_14_P111186 ATCCTTTGCGTATAAAAATCCATTTGACATAGGGTAGCATTCAACATTGCTAATATGGAA
A_18_P17340740
AGAAAAATTACTTAGGACACATTAATTGACCCTTTACAATCTACGCAGTGATCTACCAGA
CUST_2125_PI426210822 CCTCTCTGACCTGCTGGTGGGGGCCCCCATGTTTTCTGAGATCAG
A_14_P138178 CTTATCTCTTAACTAGAGACTTAAAACAGTGTCCAGGTGCCAAAAGGTAATTTCTTGCTT
A_16_P17904092
AAATCAGCCTAAGATTTGTCCAAGTAGTCTCATAAAACATTGAGCCAAAAGGATTTGACA
A_14_P109877 CTTCATTAGCATATACTGACATGCCCAGAGATGTGACAATCTTTCCCATTTTTTACATGT
A_14_P102760 TTGAGACCATCAGATAAGGGATCAGATTAAACATGTGGAAAAGAAGCCATGATAGAAATC
A_16_P17717230
AAAATTGCCCAGATTATTCTTTTCTTCTTGGAACACAGATTTGTTGATAAGTGCCAAAGG
A_14_P105506 TCACTTCAGAATAACAGGGCTATTTATTGATACAAAGGAGAGGTGTTCAGATCATCTTGT
A_16_P36963852
TGCTGATAATCTCTGAATCAAACTACACTATTAGACTCGTAGCCCTTTAGAATCTCTTGC
A_16_P18091186
ACTTGCAGTTTCCTTAAAAATCCCCTTTCAGATCATAACAATTCTTCAGTCCAGACAAAT
CUST_10430_PI426210822 AAGAAACCTCATGTAATGAATCCACCAGGCTGGCAGTGGTGCATATAAA
A_14_P122770 GGGTATAACTTTGCTTTCAAAAATGATTCATGATGTTAGTGAACTGTTGGCAAATGGCAT
A_14_P116431 TATTTTAAAACAGATAAAGTTTTAAAAGGCAGGTGTGGCACTCCATGCTAATGTGAGTGT
CUST_4012_PI426210822 ATCTGCTTGCCAAAAAATAGGGCTACCTGCAAAATGCTTTGAGCACTGT
A_16_P02034672
ATGTTTATGCAGACAGTTACGTTAAGACTTTGGATTTCTGTTTCTTAGTGAGCACTCTCG
A_14_P135559 CCCCACCTTAATGTGAATTTGACTGATGAATGAAGAGCGTTTCTAATAAAGTTTGTCATT
A_18_P14135477 AATGTGAGAAGCCCGGAAATGATGAGGCAGCACTGGATCCTGCTG
CUST_5060_PI426210822
GTAGAGTTTCTTTTTTAACACTTATATGCACATTCTTGTGACAGGCACAGTGAAAGATTT
A_14_P117104 CTTGCTATAATCCTACTTCGAGTTCTGGAAAGAATGTGTGAAACAGTATTTTGGGCATTT
A_16_P16638890
TACTCAGGTCCTAACACTTCTACTATCACTTTTGTAAAAATGACACTCATGCCTACAAGG
A_14_P102330 CTGTTCTTTGATATTGCATATTTTAGGCATGATTGAACATGGTCTCAGTGACTGTTCTCA
CUST_5323_PI426210822
CCCATCTTCACCTCCTCCCCTAGAATAACAACATCATAACTGACTTATTTCATCATTAA

A_16_P00685036
CCCCTTTCTTATGATTAGTTGAAATCTGAGTTTGAGCAAGTCTTGCTGTTTTCAGAGAAT
A_14_P107865 TTCCCAGACTCAAATTTGGAGTTAAATTTCAAAATATCTCCCTCTTCTGGAATGGTGTAA
A_16_P21522649
AGGCAGACCATTTTCTTAGTTTTTCACCATTCGAAAATATATATCCTATGTCCCTTCCAC
A_16_P16334934
CCTTTCAAGTGCTAATACCTTTTCGTAAATCCACCATTACAAAAATTGCTCCTACCGATA
A_14_P133618 TAAAGTAGAAGAGTGAATACTCCTACATACCTTTTTTGCACTAATTTTTACAGATTCGAT
A_16_P16568335
AAACGTAAAACTCTTTCAGATCCCTCCAATTGCAATGTGAACTTTATGACCTTTATTGCA
A_16_P35236215
CACAACTCAGATAAACCCATGACCATAATTATCCCTTTTCATTTCTTATCATACAGGTGG
CUST_10371_PI426210822 TAACATCAAAGGGCAGGGTCTTTTCAGAACTAAGCTAGAGGAAATTGCCAT
A_16_P20005894
AAAAACTCCATCAACTAAAAAGTCACCTGCAACAATTTTTTTCAGAAAATACTCTTTGGA
A_14_P119651 GATAGTGTTTGGTTTCTGTCTCTTGCCATGTGCATGTGTATAAATGCTGCGGATT
CUST_6390_PI426210822
ACCTTTCTTGGTTAGTAAAATAAATCCCTAAATATTTGCCACAATCTCTAGAATCTTCTA
A_14_P131730 CTGTCAATGGAGGGACACTATCAGGCAGGTCACTATCAATGTGACAG
A_14_P128231 CTAAGCGTATTTGGGGACAAATGCAAATTGTTTCCCAGGCCATAAATAACTTTTAAAAAT
A_14_P129170 CCCAAATGGTCATTTTTACCCCGATTCTCTATGGTTACTAATAGTAAAAACTCCAGGAAA
A_14_P114016 GCCCTGTATATTGCTTCATCCTACTTTCAGACAAATCCATATTTTTCACATCAAACCTGA
A_14_P101915 CTATAGCAGTAAGCAGAACAATATTACTTGGTGTAAGATACAGTTTGGCACTCCAACTGT
A_14_P104948 ACAAAAGTTTCCATAGCAAAAGTTCAATGAACAACACAAAATAAATTTACCAAATACAAA
A_14_P201096 GTGGGTAGTATAACCTTATGAGAATATTCTTTTAAGGGAGAGGTTGACAAACCACTAGTG
A_18_P15597295
TGCCTAGCATAAAGAATGTTCCACAGATTGATCATACTAAAAAACTGGCAGTCAAATTGC
CUST_7988_PI426210822 GGGAAACCTCAATAAGTGTCTGCCCTTGGAAAATGGATTACATGTGGAT
CUST_8061_PI426210822 CACTGCTGCTTTTGGACATGGTGCTGTATCCCTTGATGGAGTGGG
A_16_P21664488
GCCCTGCACTACTCTTTAAAGTTGAGTTACAAATCTGATTGCTAATATTCCCAAACCATC
A_14_P119360 ATGGTTTTTTGTCAAGATCAGATTTACAGTGTTGTTCTGAAGAAAGTCCGAGAAGAGATT
CUST_9952_PI426210822 ATCATAACTTCTCCCAACCTCATGGTGGTTCATTTGGAGGTGGCC
A_16_P20302864
AGACAGTTGGGTTAGCAAGAGTTAAAGCATTTTACTGTTCTACGTTTATACTGTCCTACA
A_14_P121484 GAAATTCTAAGGTTACTGAGGTAGTCTGACCTTGGCTAAATTACTGAGAGCATTATATGT
CUST_5507_PI426210822
AATAATAAGAGTCCAGTAATTTTCTGGTATGAGTGAAGTAAATGGAAGAGTGTTAGTGGG

A_16_P15167627
AAATTGGAATGGTCCTAATAACTAGATCAACCAACCTTAATATGACTCTGCCTTGATCCT
A_14_P132457 AGGAACATCTCGAGATTCCATTACCTTCCCCAATCTTTTCCAGTTTCTCGTAT
A_14_P108589 AAAAAAGTCAACACTTTGGCTTATGTGGGTGTCTCATGTCCAAAAAATCACTTTAAAACT
A_14_P121343 AATATGAGAGGTCCCCTGAAAGGTATAGAACTTCGATGTGACAGTGTGTTTTTATTATGA
A_14_P131421 GTGTTTTCTCCCCCACTCCTCACATATTCGAGGTATATATCAGAAGTCAAAAACATCT
A_16_P41571608
GAGCCTGATTTATTGACCTATTTCCTTGTAGAGCAAAAACTTATTGTAAACCCATCCCAT
CUST_5493_PI426210822 CCTGCAAGTCTGTCAGTGGCACAGATGTGGACTTTCTTTAGTGAAAAG
A_16_P21568899
AGATCTGTGCGGATATTTTAATTGTTCTAGACACAGTCATCAAAGAACTATCATGTGGGC
A_16_P18041282
GCACTTTGATTTTTGAACCAGTTCAATATATTACCCTAGTCAAGAGGTAGTGGTAATAAC
A_14_P127316 CCTGGAAAAGCCTGTCCTTATTATCAGGACGGATTTACTACAAAGTAAGGATATTACTGA
A_16_P15439362
CCTTTTGGAGAAAAGGCAAACACATTTATTTAACAGGAGAATCACAGAGTGAGCATC
A_14_P116372 ACGTGGCATGATGCAGGTTAACTGTAGTTATTATTAAGTCTGACTAATAGTAGTGTCGGT
A_14_P200535 GTCTAGATTTTCTTAGTTTCTCAAAGGGATGAAGGGTCCTCAAACCCAAAAAAGAGATT
A_14_P110430 AGTGACTTGATTTTAATTTTAATTTTTAGGAGAACGACACATTGGATACAGAAGGGAGGT
A_14_P124289 TCCAGGTCATGTATTGTTAACATTGCTGAGGTTTGTTTAAATGCTTTGATTGTCTTGCAA
A_14_P126973 TAAAGATAACAAGGTGCTGGTATCCCTTGTAATGGATGTGATCTGTATGACTGATAACTT
A_14_P127235 TCTTGAAATAGAAAAAGCCTTCCCTTCCCCTTTCTAGCAAAGAAAAATTAACCTTTAGCA
A_14_P120749 ACTGAATCAGATGCATTGTAAGTCCAAATATAAGTGACCTCTGAAGTTGTATATGCATCT
CUST_6259_PI426210822 TACCAGGTAGAGAACCTCTAGATTTTATGAGCTTGCTTCCCTGACTCCAT
A_14_P108654 GCTGACCATTAGAACACTCTCATATTGTTTGCCATCTTTACCTTCAATATCACAATCAGA
A_14_P105942
GAAGTGCAAGTGTCTGCCACTGTGTTAGTTCTCAGAGAACTTTAGTTAAAAAAAGAAAAC
A_14_P110928 GCTTATGACTCCTCTAGAGTGAAGTTTGCTTAATCTTTTTCTCTGATGTCTTCCTTAGTT
A_14_P111787 TGCCTTAGACCCCAGGAATACAAATTAACAAACTGCTAGACAAGACTATTATTCTGCTAG
A_16_P03204285
AAAGAAGGTCTCACTGTGATACACTTTCACTACGTTTAAAGGCAAAGAAAAGTTAACACC
A_16_P00685480
CAGTGAGGTATTGGGCATTACAAGAGAAACTGGAAACAGAGTTAGGTAAGCATGTAATAT
A_14_P138194 CTCACACCTACAAATGGCCAGCCGCTTCTGATTCTATTACTTTTATTAAATAGTGGGTT
A_18_P22841280 GTTTGTGGCAGACCCAAGACTATGCCTGCCTTGGCTTCCAGGCCC
A_14_P115237 CTGCACTCCACATGCAGGCTTCCCTGTTCACAGAAATTATTAAAAGATTTAAGATAAAGA
A_14_P129933 ATATTCACAAAAGTAGGGGTCTGTTTAGGGAGGCAGTAAGTAAGCATACTATTTCAAGTT
A_16_P17164476

AAGGAAGCAGCATGGTTATAAGTGACTAACACGATATTAGGCAATTTGTCCAAATTTCTC

A_14_P106731 TAATGATGAGACTATTGGATTTGAGGATATTCTTAATGGTAAGTGTCATTCAGCACCTTT

A_14_P135718 CAGAGAAGCAAGAATCACTGTGAGCAAGGAGTTGCTTCAAATTAATAGCCTTACAAATCT

A_14_P122520 TTTACAATCTTGGTTAATGCTCATTCCATCACACGATACCATCATCTCAGATCATGTTCA

A_14_P113066 ATTCTTTCTAAAGACTCTTTACTCCAAATACTTTTTTCAATCCTGGTTCAGATGTCCCCA

A_14_P113437 CCCTTAGCACCAAATGTCGGATTGCGAGTGTTTGTATAAAAAACAATTTAAAAAAAAGGT

A_14_P131159 ACCACATCAGAAGAAGAGGGGGGTGATGAAATTAACAAATAAAAAGTATGGGGAAAAC

A_14_P201937 TAGTTGTAGTCTGTGAGAAGAAGATGTGCTCTACTATCTTGATGAAAACAGCAAAGAGGA

A_14_P115736 ACTGGTTTAAGGCTGTTTTGTCATCCTCTCTCTTTAGAGTTTTGCTTTTTTAAAGATGCT

A_14_P102085 GGTCTGACACTACATGGTCAAATTGGGTTTATAAATACCCATTTCCAGTATACCCTACAT

A_14_P135471 AGATGTTTATTTCGGTCCTGAGAATCTCTCAATAGTTGTGCAAGATTAGCCTAGAAATAT

A_16_P15052592

CTCCGTCTAAAACCTAATCAGCAGATTCCTAAAACAAATTCATCTTTACCTTGCATGACA

A_14_P134669 AGAACAAAAGGTGATACATGTTGATCAAATATTTTTCCATTGCGTCATGAGGGTTATCCA

A_16_P20335612

TTTGCCTTCAGACAAAAAAACTCGAACTTACATGTATAACACTTTTTTATGGCCCATCCT

A_14_P119776 AAAACAAGACTTTTCCTATACCCAGAAATCCCTTTGCATTTCCGAAGATCAGATATTTTG

A_16_P00685115 GCTTGTTTGAGCCTCTTCACCTCGCTGCATACACAGCATACAGAC

A_14_P116633 TTGTTTTCACAACTAGGATAAAAATAGGTGTTGATTCTTGCAGGGTCCTTTCCATTCCTA

A_14_P104975 ATGACTCACAAGAGTCTTGTCTACCTCTGGTTCCTGTGCAGGTATTTGTTTTT

A_16_P00685460

GCATTTTTTTTCTCACATACTCTAGGTCTCAATCTCTTAACTTCAAAAATGGCAGTGGCT

A_14_P139485 TATCAGGTATTTTATTTCCAAAGCAGCATCCCCAGTAGTATTCAAGCAGATATTGCATCC

A_14_P117065 ATTATTCTTTTATTCAGGTATTTTATTGCAAACTGTTGGAAGAAGCAAACCGAGGCTCGT

A_14_P136479 AAAATAAGCACCTGCAGAAAAAGATACAGTCTGGCAACTGCTGATACTATCAATTCATCA

A_14_P121109 CTTCAAATCAGTGGAGAAAACATAACATAACCTACCAGAAAACATTCACTCAACTGACTG

A_14_P106530 TAGTGTTTTTCCTGGATTAAAGAGCGGTATGGAAAAGTTTCTTCACCCTATTTTCAGAAA

A_14_P121988 CAGAATCACACAAATAAAAAGTCTTTTTAAAAGTCTAGATCAGCCAGGCGCGGTAACAC

A_14_P135908 CTGTTTGAAGCAGAAACTTTTTTGGCTTGCATGGAGTAAATCCTAATTTTCCCATTGTAA

A_18_P14136640 GCTCCTGAGACCGTGCAAACTGGGGGATTTCAGATTGCACACTTG

A_18_P11841757

CCTCAGTTTTTCTTTTCATACATTACTACCATATCACAAAAATAGGATAGCAGTCAGCCC

A_16_P17119532

AATAGTCTCTAGAAAGTCAACGTATCCAACATTTATGGAAAATCGACCACAACCATTTGG

CUST_2084_PI426210822 TTTGAGAATGGGTAATCAGAGGGGAGGAAGATGGCCTTGGCATAT

CUST_2293_PI426210822 CGCTGAGGGATCTTGTCCCCAAATTAATGCTTGAACAATCAACAACCAATAAT

A_14_P102381 ATTGTGGCTCATTGCAGTTGAAAACAAGACAGTGTTTTGAAATAAGGACAAAATTTTGCA

A_16_P19138270

ATGTGCAACTGATTTCCAATTCCCTACTTTTACCCAACTTTGTCACTTAAATGCAGTTTA

A_16_P19278912 GCCCCAGAATATTACCTTTTTGCTTTTATGCTCGTGTTTCTACTTTATCTATCACCATCC

A_14_P103841 TGCACTTTCAAACCAATCCTTTATAACAGAGACTAGACTAGCAGGTGGAGTGATATATTG

A_16_P03800051

AAAAGCCACTCTAAAAAGAAGACGAGAACACCATACCAAAAAAAAACAGACATTTCCAAG

A_14_P127232 TGCCCTAAATGTTTAGACTTTTTCTAAATAAATAGAATTAGATATCAAGCCACCGGCGGG

A_16_P03621884

GGAGAAGAACATGAGCATTCATTAATACCAACCCATCTTTCATTAGATTCCCATCAACAT

A_14_P105607 ACAGAGCGCTGTATGATTATAAAAAGGAAAGAGAAGAAGATATTGACTTGCACTTGGGTG

A_14_P201930 GAAATAAATTTGGCATTTACCTCATGTGATTAAAAATCCACTGGACACTTTAGAAGTGCA

A_16_P01672811

GTTCCTGCTACTATAACCAGAACTTCTCTAATTGTCATGTGCAAGGTATTTCTCCAAAAC

A_16_P15846197

GATCATTCTTTCTCAAACCTTCACTGATAAAACCTCTCTCTCTTGATAACAATGGCTGAA

A_18_P22841355 CACTCATGATACATAACTTACAGGGAAAGGCCCTGACTCCCTTTTTGCATAG

A_14_P112543 CAGCCCTGCTGGGAAAAATAATCAATGGATTTTATTTCCCTTTAAAGTTAAAGTTGGACA

A_16_P37073840

AAAAATGAGTCATTCCAATGCACTCCTTTTTTAGTAATCCCAAGAGATTAATGGCTGACA

A_14_P120000 GTTAAAAATTGTGGAAAAAGTCTTTCATGAAGCCTTAGAATAAAATTTTATCTTGTGTGT

A_14_P103634 ACAAAAGCAGGTTTCAGTGAAGCCATCTGGTTGTTACCTAAAAGAGGAGAG

A_14_P135287 CACAATTCAGGAGAGCAAAATCTATGTAATTTCTTCAGCCAAAAATGTGTCACAACACTC

A_14_P120773 ACCAGAAAGATCGCTAGGTATTTCTCCAAATACCACATACTCTATCTACAAGCATAAAAC

A_16_P20096719

AATAAAAACAATAAAGTAGGCATCACTGGTATTCAACTCAGTTTTGAGATGGGGAAGGAG

A_16_P19992222

GCTGCCATTAATTACACTCTCTTATGCTCTATCATTGAAAGTATCTCAATCCAGTTCCCT

A_16_P21384737

ACTTTCTGAATCAGCCTAATACTCATTTTGACACTTCTAACTTATCTTGAAAGTCCCCGT

A_16_P19623336

AAATGATGCTCCTTGATACAAAGGTATGTTTCTGTATATCTCTTAACTCGATGGAGAGCC

A_16_P15654588

GCCTCCAATCTTACAGTTTTTGCCTTTATAATATTTTCTAATCTTTTACCCCTGAACTCC

A_18_P14179324

ACAGACAGAACGGATTCTCTTGATAACAATAATCAGCATTAAAACTAAACTCAGGCCTGT

A_14_P116237 GGGAAGGAAATTCGATCAGACAAATTTAGCAACATATTGTCTCATTTTACAAAAAGGCCT

A_16_P17695068

AAAGATGGTTAAAAGAGCACATCAGATGTCTTAAGGAAGTCCTAACACTACTTTAGTGCC

A_16_P18052387

AAAAGTTACAAAAGAGGCTATGAGTAAACTGGTAACATTATCAGACTGTTTCAGGGATCC

A_14_P200699 CTGAGGTGTTAGTGAAGACTGAAAGTTTTTTTCCTCCCAGGTGTTTGTATTTTAATGATT

A_14_P124513 AATGTGCTTCTTATATAGATCTGCAACTATGTGGGACCAGCAAAGGTTATTGTTCAGTTG

A_14_P117435 ACATCTGCACCTAAACACTCTAAGAACTCCGATCATTAAGAAACAAAGCCATATAACTTC

A_14_P101595 GATGCAACATCAATTTAGAGTCAGGATTCAGTTATCTTTATAGTTAGCACCAGATGCCAG

A_14_P104888 CTACAAAGTAAAAGTTTTTCCCTTAACTTAAAAGTTGAACCACTGTAGACAGTGATCACC

A_16_P37580880

AACAGCAACAACAAAACGATTAGCAAACCAGTTTTTCTGTTATTTTTCAACCAATAGGCA

CUST_1603_PI426210822 TTCAGTTCCCAGGCTTTTGGCCTCCTTAAACAGGAGGTCTTTGAA

A_14_P119316 TTTAGTTCTGCAACAGTTTAAACACCATCATTTTCACACCAAAAGAAACCAAAATCACCT

A_14_P127700 TCTAAGGAGTGCATTTTGAGAACTGTGGACATATGTAGGAATTTGTAATTCATAGGCCAT

CUST_9287_PI426210822

GAGAGGCCACTTCCAACAAGTTGCTGTAAAAATAATTACATGTGAAGATAAAACTAAATA

CUST_1315_PI426210822

GTGTCTGCCCAAATGCATTGTTTCCTGTATTGTACAATTATCAGTTATTTAGATTTGCCA

A_14_P137229 TAAGGGATATTATCAGATGTTACCAGGAGGAAAAAGGAAGATCTTTTGGCTAAACACTGA

A_14_P109773 CTTGGGTCACATCCGTGGCCAAAGTAGAAATTAAAATTTTGCATGAACAAGTAATAACTT

A_16_P19487043

CACCTCCTTAATCTTTCCAAAGTAATATTCAGAAACTTCGATTCAATTCAATCCCATGGC

CUST_1664_PI426210822 TCATCAACAGCTTCCTTCAAGGGGGGTAGGATCTCAAAACTTTAAAATGAAG

A_18_P19745385

TTGGCTGCACATGAATTTAAGGACATCTAAATTTCCAGAGCATTTATCACTCATAAGGAA

A_18_P14132243

CTCCTTTTCCCTGGAAGTTTTCTTGCCATATATGGTGATAAGTGACTTTATCATGGAAGA

A_14_P117109 CAATGTTTAAAAAGAGAAACAAACTGAGTTCCACTGAAATTAAGAGATTTTCTCGGTCTC

CUST_1759_PI426210822 AGGTAAGAAAGGGGAAATGGGAGTGATTCCCACCAGATTCACAAAT

A_16_P58424627 TTAGACATAGGCATTTGTGTGGAAGCAGGGCAGGGTTCTGAGAAAGTAATT

A_14_P201712 CCTTTAGAAAAACTGAAGATTACCCATAATCTCCTCTTATTACTTGAGGGCCTTGACTAT

A_16_P18200308

AAAAAACCCAGCAGATACTACTTATGAAACTCAGCACTCTAAGATAAACCAATTAGAGGA

A_14_P122180 TTTTAACTTCTTATGCTCTAGTTTTTTCAAAGAGACATCCCATGAGTCTGTGGATAGCAG

A_16_P18478440

AAAAGATCCTCCTAGATGTTTGAAATCACCAATTGTCACACTCTACATACTTTTTGTCCG

CUST_8744_PI426210822

AGATACTGTATCAAGTACTTTACAAGAACAAATTAATCTAATCTTCATAATAATTTCTTA

A_14_P118229 CATGGTAAAACTTCAAAAGCAAAGGACATGCCAATGAAGAGTAATGTTTTTCCATTTCAC

A_16_P18142066

CGACAGTAATAACTCCAGAGCAAACTTATAAAACAAATTTAGAGCCTTTATTACCCCAGG

CUST_3357_PI426210822
TCTTTGAAGTCACACATGATAATGTGTTCTGTTTTATTGGTTTTGTCTTTATATTTGAGA
A_14_P135543 CTTTCCATGCTGATTACTTTTCTTTTCCCTCAATTGTTGAAACACCATGTGTTTTTTCTT
A_16_P02137175
AATGCAAGATCAGTCTTAACATCAACCTCATATGACTTCTCTTTCCTAAGACTTCATCCA
A_16_P00685264
ACCTGCCATAAAGAAACATGTTACTATTAAAGTATAACCAGATACTCACATGTGACATTC
A_14_P111116 CAAATGATCTGATGCAGATGTTTCCCTAACATTCAGAAAAGGAAAATAAAGGGAAGACAT
A_14_P126763 TTCTAGCGTCTTTTCCAAATCTATGATGCAATTAGAATGTTTACCTCTCCCCTTCTCATC
A_16_P03099605
GGATGCGCCTTTTCATAAAGTTTCCCTTACATCTAAAGCATTACAAGATCATGGTAGAAG
A_14_P110035 GCCATTCTGTTATGTTTGTAGCCCCCATGTTTATATTTTCAAGGATTCACTATTGGATTC
A_14_P109195 AGCCATAAGCAATCTCCATAAGAAGAGAACATTTCAGCAGTCTAATTCAAAGACTGCATT
A_14_P116215 CTCTTTCTTCCCCAGAGGTTGGCAAGCCTTGTAATAAAAATTATTTTACTGAATCCATTT
A_14_P107681 TATGGCAAAACAGTGTGACTGAGAAATCAAGTCACTTTAATCTCAATTTCCAGAAGTGG
A_14_P113316 GTAGTTTTTATAGGTAAACTTGGGATCAGAAAATCACAGGTCATGGAGTCAGTTCAGTTA
A_14_P132680 CTAACAGAAGAAAAGGAGAAAATAGTCTGGGTCAGATTTGAAAATGCAGATTTAAATGGT
A_16_P21605029
AAACAATACTGCACTTAGAATGACAACATTGGATCAGATTCACCCTTTATTCAGACTGAG
A_14_P200757 CTTATACTAGTAAGAGACTTAAGCAATGCCATGCATTTATTACAGGGAGGGATCCAGAAA
A_14_P111295 CTGATCCTTGATCATTTACAGAGTGCTTTTCCATTCGTTATGTAATGTAATTAATTAGCA
CUST_3146_PI426210822 TTTAGGTTTCTTTGCAGCTTCTTGCTTTGGCTGAGAAGTTATCCTTTGTTCCTA
A_14_P118720 TTTTTTCCCTTCTTCTTTTAAATGACTAATCAAAGTCAATATCACTAGTGCAGAGAGGCC
A_16_P02536122
AATGAGTTTTGCAGCATACCTAATATTTCTTGCCAAATCACTAGTAGACTGAAGCACAGC
A_14_P130504 CGTTCTTTTAGGATGCCTCTACTTTCAGTCCAAGCACTTTGATAGGATAGTTAATTACTC
A_14_P124159 GACATGTGCACGGAAGGACTTTGTAAGGGACCAGGATTCTCAGAAT
A_14_P102719 TAATTTTTAATGTTTAACTAGAGACGGGGAGAACACTGACTCTTACTAAGTTATTCCGGG
CUST_6064_PI426210822 GAGAACACAGTGAGAGACAGGGAAGGAGAAAGGGAGAGAAACCAG
A_14_P119933 GAAAAGCATTGGTCATTGATTTTAAAACCGTAATTGGGGTCACAGGTCATATTCAAAATG
CUST_6807_PI426210822 TTTCTGGTAGAACTAGTCTGTCTGCAGCCTTACTGTGTTTCATGTTCTCC
A_18_P13536724
CTGAGTTTCCTGTCTAATTTCTGTCACAACACTTATCACAGAATATGCTATCTCTCCCAC
A_14_P202411 ACACTATTAAAAATTCTACGTGCTTTACGATATGTGGAATCTTGACCTCACTTGTCGGAA
CUST_4363_PI426210822 ACAGCCTTTAGCCCACCCTTTGAGAACCACTACATGACTTCTTGA
A_14_P139740 ATGGCCATCTCTATACACAGAAAATTAGTGAACAAAGATGGGTCAGATTGCTTTATGTCA
A_14_P202552 TTTCTCTTTTGCATAGCCCCATCTTATGTCCCTAAACCAAAGGAAAATTATCTACTCAGT
A_14_P106080 CTCTGATGGCTCTTTCAGGAATAAGGTCAAGGACATGAAGACTAAGGAAACATCAT

A_14_P124132 GGGATCCAAGATGTCTTGGTAACACATTTGATGATAGGGCTGGAGTAACA
CUST_6465_PI426210822
CTGGACGTCTACTTCACAAGCAGTAACATAAGCAAGTATGTATCATTAAAGATTTATCAC
A_14_P104000 GTATTGACAGAAATAATCTGAAACGGTCACAAAGCCATCTTCCTTACTTTACTCCTAAAC
A_14_P129552 CCTAGAGAGATCTGCGGGTGTAAATCTGTAGATCTGCACATCTCAAACTAATGCTAAA
A_16_P21568666
AAAAAAAACCAATTCCACTTTCAAGAACAAAGACATAATGCTGCTCTGATCCTTGAAGTG
A_14_P134763 ACTGGCTTTGCTTTTATTTTATGGGCACTGTAAATTTCGATATCGTCCTATTTACCTCTG
CUST_3874_PI426210822 TCTTAGGCACTAGGAAGGCAAGGAGATACTTGGGGCTGGAAGGTG
A_18_P11274087
AGACCAATTCCTCATTTTTAACTATTTATGACCACAAGACACTTTACTTTTAGGGAGCCC
A_14_P129104 TTAGGGAAGGCCTATTTTGCTTTATATATCGGACTTAGATTGTGGTAATTGCTCTAGGTG
A_14_P127465 CTTCTGCAATCAATTCATTTATTTAGCAAAAAAGAAATTATGTGTGCCGAGAGCATGCAG
A_14_P126242 CTTCCAGCAGTTTCATATGAGGTGGTTGTGGCTTTTCTTAACTATATTGTGATTATCCAT
A_14_P130118 CAACTTAGGAATTAGCCTGAGTCCTTGGCTTAAATATATTTGTAGGAATTTAGGCAGGAC
A_14_P104739 TTCTGATTAACTGATTTTGTGGGATTATTATAGGATTTTAAGGGGATTACAGGGCCTATG
A_14_P131571 CTGAGATCACTCTCTCTCTCAAAGCCAAAGTGAGTGCTGTCTTAGATTTAGTAATTAGAA
A_14_P136368 AAGGAGTTAAGGCAGTTTAATTCAAGGATGCGAAAACTACGTCTATGACATAAACATGAC
A_16_P00445407
GAGCCCCAAAGTATTTACAATAATTACGGTAAAGCCTCAATATATGCGAATTGGGTGATG
A_14_P110032 ATGCCCAGTGATTTCTTGATTGAAGTTTTCTGTTAACTGCATCCTAAGGACATTAATCAT
A_14_P137542 TTGAAACTTTGCACAGGGTAATTAGTAGATTAAGCCATAGTCCGCAAGGTTTTTTAAGTG
A_14_P127860 AAACACAAATCTTCCTCCTCCTTTACACCTTTTATAGACCCCAGATTACTACAGATTTCT
A_18_P16877726
ATGTCCTAGCATTGCCATAGTTTAAAGTCCCACATCAATATTTGTTTATTCTCCAGCCAT
A_14_P107953 TGGGGATTTCTATAGGAGCAAAACGTTCAGACCTAACGTGGGTGC
CUST_8139_PI426210822 CTGGCTTTTGCAGACCACTGGAAAGTATCCCAGCTCATTAGCTTTT
A_14_P132217 AATCCCTGAACAATATAATCTACCTGCCATCGATGAAAAACTCAAAGCCAAAGAATTCAA
CUST_5355_PI426210822 CTCTGAAGAAACACAAGCTTCTGTGGCCCCGTACACGTAGGACTA
A_16_P21022412
CTCCATAAACAGGCATCCCTCAGAATATAAACCTAATCTATTCCCAAAAAAGTAGTATAT
A_16_P58424338 TATGTGATGTGACGAGAATGTGGACAACCTTAAGGGGCTGGGAGA
A_14_P121491 CAGATTGATACACTGGCTGCAGAATACCCATCAGTAACAAACTATCTCTATGTTACCTAC
A_14_P107109 CATTCTGGAAAGGAAAGAAATGAGGAATCAGGACTATCAGGAGACAAGGTGAAAAA
CUST_9546_PI426210822
ACTGTCACTCAATTTCCTAATATTGGTACATGCCATGCTAATAAGTGTGCTAAAATGGAT
A_14_P139578 AATTAGTGTTCGAGGAGTCAGCAAAATATATCAAAGTCTGACACTATTGGGTTACTCTGT
A_14_P126142 CAACATTATGATGAATGTCATGGTAGGGGCAGTTAAGAAAGCAAACACTTTTCAAAAAGT

A_14_P102476 TAGTAATTTTGACCAGATAGGATGGTAAGGTCAGTTAATGAACTAATGAACCTCAGGGTT

A_16_P00685193

TATTAAGCAGAAAATTTTAAAGTGGTTTTCCTTCTCCTTTTCTTGGGTCTGCTTTCTTGG

A_16_P37016798

AAGTTATGTTTCTGTTCCTACCAAAACTGTGCAGTCTTTCTAAGCAATTCTGTTGACTTC

A_16_P00685151

TAGAAATTTCAACATTGAATGAAACCATACCATAAAACTTGGGAACTGGTTAGTGAGGGA

CUST_3573_PI426210822

TAAAAAAAATGTATTAAGGGCTATGTAAATAAAAAGAAGAAATTAAGTTGTAACAGTAAC

A_16_P00846177

ATGACTGGATACTGCACATTTTTATAAACGTAGGCTTAAAGGAATTCCAAGTTTGGCAAC

A_14_P120841 GACTTCAAAGTGTTTCTTTTATAGACACTGGAACAGTGTGAATCTGGTAAGCATGTTTCA

A_14_P129789 GTTTTGAGGAGTTTTGGAGCAAGTTATCTCAAATGGGAATCTTGAGAGGCTC

A_14_P120857 TAGTGGCTGTTGCAACATTATGTAAAGAACAAGGAATAACAGTTGTAGGAATTTGCTGTG

A_16_P01423744

GATACAGATTGCCAACAGTAAAGAATAGATAAACCGCATACATTAGAGTCTTAAGCCCCA

A_14_P200127 TTCTCATTTGCCCTTTGAGCACTTTTACTTAAACTTGCTTGTAGTTGCTTTTATCACTGC

A_14_P115228 CATTGGTGAGCACCTTTGCATTATGTTACGCCCTTTCCTTCATCACCTA

A_16_P17425121

GGGGAAAATCAGAGAATAGATTCCATGCATTGCTTAACATAAACAGTAATATTCGGGCTT

A_14_P102044 CATCAATATTCCTCTCACAGGTGCTCCCTTCAAAACCTGGTAAATGAAGAACAAAT

CUST_8222_PI426210822 GTGTCTCCAGTCCTGGCTCACAGGAGGACCACTTTCTGATTAGCT

A_14_P107782 TTCAAATAGGAGCAGCACTTTGAAATCCTTTTTCTTCACTGTGGATTAAAAACATCCAAG

A_14_P121410 AACCTTAAAAGTCAAACCAAAATACCCCTAACCATCAGATTCAGTATGAAACATTGACGA

A_14_P114574 CTTTTCCTAGTTTTTCTTTCATAGAAGCGTATTCAAACTTTTATTCCTCAGCCCCGTCTA

A_16_P02195831

GATGACACTTAGATTTCGAAGTTTCCTGTTAACTATTGTCTTCCTAATCAACACAGGCTC

A_14_P102938 TTTTGTATATGGTCTTCTTTGAAGAAACTTACTTCTTGCAAGCCCTGGCATCTTCCAATT

A_14_P201775 TTTCTGGTCTTCTACGTCATTCCACTGTCGATCATCTCTGTTTACTACTACTTCATTG

A_14_P139879 AAACAGGAAGAGGTAAATGAACTTTATCTTGCTAATTAGTAGTTCTCGGTCTTTTCCAGG

A_14_P101289 CAAACTTACTCACTTTGACATATTGGACTAGGATTTGACCAGATTCCAGATGATTCACAA

CUST_660_PI426210822 AAAGCAGAGAAGTAGCTTGATGCTTTGTTGATGGCAGCCTGTTGGT

A_16_P16191953 ACCACATGTCAAAATGTCAGAATGGGGTTCAAACCAAAGACTACTCTTACAAGAC

A_14_P110505 ACCCAAAAAATTTGCAATTCTCTTTGGAAAGTTGAAAGCACCTTTAAAAGCCACAATTTGC

A_14_P130249 TAGTTCCATAGTGGACTCATATAAGTGGTAGAATCAAGTGAGCACTCCAAAAATAGTGAC

A_16_P00638627

TTCTGACTATACACCATTTTCTCCTTAAACATGCACTTCAAAACCTTATTCAGCATAGGA

A_16_P19540647

GATCCAGAGCAACTAAGAAGAACAAAATCTCCTGTAACACTCATTGAACAACTGAGTTTT

A_14_P103346 GTGCACACATGTAATATCAAATTGACTGGACCTGTAATGTTTCCTTCAGAGCATTCTTAA

A_18_P16928471

AAATCCCACCTTAATTGTGTGTGCCTTATGATTACAGACCATTAGAGACCATTGGTAAAT

A_16_P18926753

TCCAAAAGAACTCTACCAACAGCAAATTTAAGCATCTCATTCAAGTTCACTTGTAAGGAA

A_14_P107194 AAATCCCTAATTGATCAGTTTTTAGCCTGCTTATGAAGGACAACTCTACATAAAGCCCTA

A_16_P58424448 TTTTCTGCTTCCTTTGTTGGGGTCTCCTTATCTTGCCAACCTTTCAACATT

A_14_P137586 TTCACCTGATATTGACAAACTGGACGTTGCCACAATGACAGAGTGTATCCTTTA

A_14_P116057 CTAATATGCAGTCAGATATAAAAGTCCAGATTCGTTTGGAGAAGAATGTAGGTGGCAGAT

A_16_P15713710

AAATTGACCTTCTCTCTTATTTCAGAGCAACTTAATTCTGCCAAAGACCATCATAGAACC

A_16_P58424150 GTTTGAGTGAGATGCCCCATGAGTGGGAGCCCAGTTACTTCTAAG

A_14_P105264 GAAGAATGTAAGTCGAACCTTATCTCCTTCCTTACATTTCATTGTGTCCTCGTTTCTAAC

A_14_P104039 TTCGTGTCTGTTTTGCTCGATTCTTTTCCTAAGTTAAATAAATGCAAGCCTCTGAACTCT

A_18_P22841068 TTATCAGGGCTATTTCAGTCTTTATTGCAATGCCGTCTCTCTGGAATTCAGACATG

A_18_P15520531

AGTTAACACTGTCCTTTTGGATTTTCAGAAAAAGAGCAAGTAACCTTTATGGAGACTGAA

A_14_P202133 CAAAGCAACCTGAGAATGAAAGTCCAATACTGGCTGCAATATTGATCATTCCTAAGTTAA

A_14_P111779 ACACGCATGGTCAACCCTCAGGGGAAAACTGTTACCTAAAGTATTTTTATAAATATAAGC

A_14_P127956 AAAGGATTTTTCCCAAAAAGAACCAGGATTTCAAGATCAATCTATGAAACCTGATCACTT

A_14_P125186 ACTGTAGCCTGCGTCATGGCTCAAGGATTCAATGCATTTTTAAGAATTTATTATTAAATC

CUST_3286_PI426210822

ACATGCTAAATCTCCGGGTCATCTTGAGAAATCAAAGAAATAGCTAAGAAAAAAACAGTT

CUST_3827_PI426210822 ATCTACTTCTGTACTTGACCTGGGTGAGGACCACTGGGAACATTT

A_16_P16191738 TTCTCTTTCCACTGTCTGATTCTGGGTCTCTGCTTCCTCATCTGGATA

A_16_P00685416

CTGAATTTGGCTAATAAGATGTTGCATTGTCTCTGATCTCCTTGAGAGACTGAGATAGAT

A_14_P108184 CTGTTCAATATTTGAAATTTTTCTCCTTTGTGAAAACTCAGGTCATCTTCTGTCCGTGCT

A_18_P22840350 TGATTTTTTAAATTGAATCCATTTTAAACATGGGATTTTATTTTTATTTAAAGAATTACC

A_14_P118663 CAAGTCACACTCATCGGCTTCACCCTAATGTTTTGGTGCGAATTCACT

A_16_P16408531

AACTCAGAAAGTGAATAATCAAGTTTTGCAAGTTCCTCTTAATGAAGACCTAACGTGTCC

CUST_3391_PI426210822 ATTTGGGATTCTGTTCGCCAGCCCCTCTGCACTCTGAAGATGCAC

A_14_P136555 CACTTCATCATTCGCTTAAATTTCAGGTGTTCCTGAAAAGCCTCAGATTAGTGGATTC

A_14_P201558 CCTTAGTGCAGCAATCTCAGAGAGTGGCAGAGATTTGCACTCACAATTTGTAATATATTA

A_16_P16191754 CTATACCAGAAAGGAGAAGAGGGGGACTAGATGTTTTTGTGTGGTAGTCTCTTT

A_16_P18959398

AATGAACAGTTTCTGAAGCATGAACAAAAGTAAAATAACCTGTAGTGATTACCTGACCGC
A_18_P26222179
CGTTGACCCTCTATTTACTAGAACATAGATTCATGAGTGCAATATAGATTCATCAACCCG
A_14_P110286 TCTTTCTTCACCAAGAAGCACAGAATTCTGCTGGGCCTTTGCTTGTTT
A_16_P58424284
TTAGCATTTGCTGAGTAACAAACCTTCCTCAAATTTAATGGCTTAACACAACAGCCATTT
A_14_P200296 CTATCTCTTTCCCAGTGGGCTGAAAGATATGCTGAAAACAGCATGGCTAC
A_14_P105728 AATATTTTCATCTAACAGTTGACACCAGAATCCCTGGTAGGGAAAATACCACTATGAGAT
A_16_P03198342
CCCCTTGTATACAAGATGGCTGTATGGTTTTTCTCCTCCTTAGTGTCATTTTTATGTTAA
A_18_P22841203
TCCTTCAGTCAGTTTAGTTTGTGTTTGTCTGATGTGAGAACATCATTACCTACCATATGT
CUST_937_PI426210822 CTCTATGGCAGGTGCGAATGTAGGAGGGTTGGCAGGGACTGATGT
A_14_P124992 TCAGAAACTGAAATAAAGCCTTTGAAAAAAAAATCTGTAAAACATCAACCCCCAATCAGA
A_14_P128131 AGAGGTGATCTTGGCTTGTCCCTTACTTAAAATATTTTAATACCTTCCTTTGGCTTTCAT
A_16_P02006832
TACCCACATATACTTCCCTAGATGACTAACACAAAATATCAGTCCCATAGTTTATCCAGG
A_14_P201110 ACCTGAATGCTCTCATTCACCATTTAAAATACAAAAGTTTGCATTTAATCACAGACCACC
CUST_9862_PI426210822 TCCTCCCTCTCCATCTTTCCTAACAGTTCCTCACAGTTCATCTGT
A_14_P102965 AAATGTGATCCTTGTGCGGCTCTCCAAGATCCAGCGAGATTTGTATACA
CUST_4730_PI426210822 GAAGCCAGCAGGAATGGCTGCTGCTAAGAACCTCATTCTTATAGGAA
A_14_P129337 GTCAGTGTCTTCTTTGCTGCAGATAGGTGTGTCCCTTTTTGCAAAATCAGATA
A_14_P136352 GAGATTTTGGCACACTCGATCACAACATCATGAGTTTCTAATCTCTTCCACCTGTAAAAT
A_14_P104075 CTACAGGTAGTTCAAACAGGAACTGATTTGTCAAGATCTGATCTTAGGAATGCACTGTTT
A_14_P107555 ACTCTTAACATCTACGATCTCAGCAATAAGAATCAAGAAGCCAAATAACCAGTTCTCACT
A_18_P15587464
TCCACATATCAAAAAGTAAACAGAAAGCAGTATTTCCGAAACCTTTTCTTTATAAGGAGA
A_16_P01848596
TCCAATTCATCCTATAGCAAATTTCCTAAACTACAACTCTATGCATGTCACTATCCTACT
CUST_2751_PI426210822
ATAGGAAATCCTCTTAAATGTATTCTTATCTCCCTAATAAAATGGAAATTGGTACTTAAT
CUST_8615_PI426210822 GAAAGACTATCAATTTTCACTGCTTCCCACCCACGGGGGTTGTGT
CUST_5726_PI426210822 GCCTTATTTAGGGGATGTTCTTGCAGATAGATGGGGAGAAGTCTTGCT
A_16_P37566951
GTTTCAGTTCTATGCCCAAGAAAATCATCGATGGAGTGAAGTAATTGAACTATCACAGAA
A_14_P116430 GGATTTCACATCACTTGTATAGAATGCTCTGTTGATACTTGAGGGGAAGACAGTTAAATT
A_14_P129072 AATGGACCCTTATATACACAACTTCATGCTTGAAAGGTTAGATTCATCACTGCAGAAAAA
A_14_P125379 TTGCAAAAGTAAAATTGGCAAGACATATCCTTACAGGCAGAGAGGTAAATACCAGTTATG

A_14_P107573 ATTGCACCATAAACTCTACTCCTTGATTTCTAATGTGCACATTTCTTGGTTATGACTCTG
A_14_P139325 TAAAGAAACCTGCTCATAATATTTACTTAAGAGATCTACATAGTTGCCTGAGGAAGCCCC
A_16_P17082903
AAACAAGTTTCCAAGCAACATAAACTATTGTTTCCCTCAGTAAGTGTGTAGTTTACGGTG
A_16_P18845727
AAATTCCTCCCTCAACTCTTAATCTCAGATTCTACTCTGTCCTAAACTTTATAGCCCAAA
A_16_P21663013
ACTCTACCCTGAATACTGAAAACTGCAATAATGTCCCTAACTTAAACTTCCTCTTCTGTT
A_16_P39181363
ATGGTCTCAAAACTAGAACACTGGAAACAATATGTTATCTATGCACTTTTTGTTCCCTCC
A_14_P123938 CAGTCCCTTGTATGAAAATACAACAGATTGTGTTTGGCCATTAATCTTTATGCCAACTTG
A_14_P202008 GCCTTGTGTCTTAGATGATATCTGTAACCAGTTTCTGAATCCCGTTGGATAAAACTGTAT
A_14_P137255
AGTAAGTTAGTAGAAGGACAAAATCAAAACACAAAAAGATGGACCTTAACAGACCAGTGC
A_14_P136117 AAGTAGTAATACAAATTCTTGAGAGTTATGTTGACATGGAATTTGTCAGCCGAAGTGCTG
A_14_P139690 ACCTGTCACACATACCATGCCCTGGCTAATAGCGTGTTGTTGTATAAATTAATTACAAAA
A_14_P137233 CCTCTTCATTACTGGAATGTAGTGTTTCCTCAGAGGCCTGAGGTAATAATTCAAGAAA
A_16_P58424078 CATCCTTTAGGAAGCAGATGCTAGGACAGAGATAGGAGTGTGAGAGAATTGTTG
A_14_P127118 CTGTTTTTGTTTTTTAAGATGAGGATTACTTACAAGCAAGCAGCCCTGATGCAGTGTTTA
A_14_P105126 GAATGAGACCAGTGATTTTTAAACTTTTTTTAGCCACAGACCTTTTGTTCAAAGGAAACC
CUST_9987_PI426210822 GTGTCCTTTATGGCTGGGTGTGTGGAACCAGACAGGGCACAGAGT
A_14_P136927 GAATTGGTGGGCAACTTGCACTGACCTTTATCTGATTAATATAAGATCTATGTCCACATG
A_14_P111211 CATTCAAGGTAGTGGCCAAATGTAACACATCAATCATGGAATGATTTCTCTGCTAACAG
CUST_6090_PI426210822 TCTGTGACCAGGATTCATGGATGTTTTTATTTTCCTCTCTGGAGAGCATGAAT
CUST_3017_PI426210822 GAGATGGCAGAGGCCCAGGGAGGCCATTAGAAGCATCAAAAATAC
A_16_P00191704 TGTTATCCACTCCTATCTTTGTCCTTGTATCCTCTTTATTGTATCGCCAATCTCTTACTC
A_14_P114038 AAAATGCACTAGTTAAATTGGAGGTAGCACAATCATTTGTCTGGATTTGTAGGCTGGC
A_14_P100776 CACTTATGTGGCTACTTCTTTTCAGGATGAATCTCACTTCCTCAAAAACAGTAGGACT
A_14_P107849 CTGGCTCCCTCAGTGTTTCCTTTAGAGAAGGAAAATCTAAAGAAGTTCATAACACATTTA
A_14_P138498 CAGCATGCTGTTTTCTCACTTTGTCTATTTGATGATGCAACCAGGAAAATCTTGATCAG
A_14_P132913 ATCATGCTAGATTTACAGAGAAGTTTCTGCATATCTGCTACTTGTTGCATTTTGGGTTCA
A_14_P103943 TTTAATAGGGGACATGTGAATAAATACAACACAGTAAGTGCTACTTCTAGAACCAGGCTC
A_14_P110070 TTATGACCGCAAGTCAAACCTCCTGTTACTTTAACATGGGCTAAGAGATCATAACTGTA
A_18_P18074160
AACTACATTCCAAGCAATGTTTTTCTAATCCATCATTCACAGGTAAATCTGGTATGTCCC
A_14_P107721 TACCAAATGTGCCATTAAGATTTGGATGGGAAGTTATGGGTAATGAGAATATAATGACAT
A_18_P22839418 GAAAGTTTAATGTGCTGGCATGCACACACCCCGCTGGAGAGGAGC
A_14_P119395 TGGGACTGAAGGGAATGCTAATACAGTTCCGAGATTTAAAAAAATATTGGAATGTGGATA

A_16_P02910086
ACAGCATATCACTCTTCATGGATCAATGCATCATTTGAATACAGTCTTATTCTGCGTTCC
A_16_P18473365
AACCCAAATTACCCCCATAAATGTTATACCAATAACTATCCTATTTTAACACCAAGTCGT
A_16_P18316684
AACAGAGACAAAAGTTAACTCAGTTCAGTCCGATTAAATAGACAGCATCTATGTGTCCTC
A_14_P200104 GACATTCAGTTTTCCTGTCTACATAATAAGTGGTATGAAATCTGCAGTCATCAGATCCTC
A_14_P200646 CTAGAATTAGACATGTATCCTGGTCTCACTGTGGAATATACTGGTATCTAGGGACTGTG
A_16_P21382884
CCCTTTCGATTACTTCATCTCACCTAATTCCTTTCATTTACATACCAGAAAGCAAGTGAA
A_14_P132276 CAAGCATGAACAAGTCCAGAAAAAACTGGGAGATGTGAATAAACAGTTGGAAGAAG
A_16_P16191715
CCAATCCAGTTCAGTTAAATCTGAAGTTTTTGATCAATTGGGTTGCTGAGATTACAACTT
A_14_P135755 CATTATATAAAACCAATTACAAAATGTGAAAGTAGCACACAGACGAATACACACATACTG
A_14_P113882 GAACAAAGTAAGGGAATGACCTAAACTATATGAAATGCATTTCTCCCACTAAAAGGGGCT
A_14_P124306 CACTCCCATCTCCCAGACTGTTAAAGGACCCAGTTCTTACAGTTCT
A_16_P01055820
CTTCATGCCTACTTGTCCTTTATCATTAGCCTACAAACTTGATCTAGACTCATTCATCCT
A_14_P122204 GAATTTTAATGGTGTTTTGGCATTTTGTCAGTTTTCCCGATTGTAAATGAGTTCTGATAC
CUST_5850_PI426210822 CCATTAATTAAGCTTCATGGTGTTCACTCAGTCACACTGTTGTCGCTCTCATT
A_14_P127408 TACCATTATTTTTGTTTTGTGTAGTGATGCTGGAAATAGTGACCGAGTAGTCATTCAGGA
A_14_P122997 ATGTGTCGGTTTCATGTTAATTATCATTTTCAAAGCCCAGGTATATCCCTCCCTAATGC
A_14_P103217 AAGTATAAGTGGGATTTTCACCAAAACCCTATCCTTCCCCAGACTCCAG
CUST_882_PI426210822 CTTGGTGGCTGATTCTAAGTGGCTGAAGGAAACACCAGGAAACATT
A_14_P138423 AACATGGCTTTGATGATAAACGACTTTACTCTAAAAGCGGCTGGAACTCAGTGAC
A_14_P118510 TGACACTCTAGGGACTGATCCAATTATTACACCAAATTGTTCTTTTTCACCCCTATATTC
CUST_9959_PI426210822 GAGACCCGGAGTCCTTTTCCTTCAGAGCCTCTCTATTCATCAGTAAT
A_14_P126353 CTCTCAAACCTCTGTATCCACAAAGAGGCTGTTTAGAAAGTTTTTTCCAAATTCTTAGTC
A_18_P17207124
GTGTGTTAGGAACGAGTTAGAAGAATGCCAGTGTATTATTCTCCTCAATGTTTGAGAAAG
A_16_P36810703
CCTTTCTCCATGAATCAGTGCTCTTCTACTTCCTCAAGATTATCACACAATGTGTCTATA
A_14_P133722 TCCCTTTCTTGGGAGTTTTTGTCCATATTTGCTCTGGAATAATTTGGCACTGATTATTAT
A_18_P14134350 TTTTGGAAATTCCTCAAGACAACAGAGCCAGGAGGAAGTAAGGGAATAGAAAG
A_14_P103920 GTCTGTAAGAAGCATGCTAAATGCAGACATCTTGTCATAGAGACTTTTCACTGCTAATGA
A_14_P114877 CTTAGACCAAAATTCAACATGTTCCAGAACTTTTGAAGGAGGAAAGATATGAAAGCACAT
A_14_P134193 CTCTGTTGAATGTCATCCTTATTTTCCGTTGTCGCTTTGATTTCATCTCTGTCTTGC
CUST_1593_PI426210822 CTTTGGGATTTAGAACCTGAGGGAAACATGGGGTGTCAGCATGTG

A_14_P133660 ATCCCTCACCTTTCACTTGCAGAAAGCAGTATCATGAAATGGTTATTAATTGCTATTCTG
A_14_P107026 TAATAACAAAAATTGAAATTCACCTTTGGGAGGATACTCAGTTCTCTCTGTCTTCTTATG
A_14_P125143 TTCCACTTTTGGCAATGCTGTAAAGAAAAGATGGCAGTGTTATAATTTAAGGACTCCTAG
A_18_P22841931
TCCAGGAGAAACTTCTAATTTTCTGATAGTAGGTGTATATTATCAGGGTTCTCAGCATAA
CUST_6673_PI426210822 CTCTAACTGCTGATAGCTGTTCAGCTGGGACTGCTTGCAGCTTTTT
A_18_P16403678
CCCGTATCAACTTAATTTCTAACCTTCTACCACTCCATTTCTAGATCATCCAAGGAATTC
A_14_P135524 AACACATGAATCATGGAATCTTAGATCTAGAGTGGACCTTGCATCTCATCAGATCTTAGT
CUST_7388_PI426210822 CTTTGCCTTTTGTAAAAAGCCTGTCGAGCTTTCTTCACAAGGGAGCT
A_14_P115917 CAGAAGGAAAAAATGCTTGGTGATAATTAAAACAACTCTTTCCACCCATCAGATTGGCAA
A_14_P115614 AATTGGCTTTAATTATTGGGATAATTATTATTTTTAAAAAGAGAAAGAAACTGGGGATGG
A_14_P132513 CTTCCAGTCACCAGGAATTCCTGGAGAGTTTGAGAAAGCAAGAAAGATT
A_14_P136412 TTACTCTTTATTGGCTGGTGGTAAATATGGGAATGCCGTCAACAAATGTATAATGCCTAA
A_18_P12234992
AAAAAAAAGGTTGGTAGAAACCCTTAATACAGTAGCCAGAGATTTTTGTTGTCAGATGCC
A_14_P127504 TTTCAAGATTTTAGACCCTTTCTAAGTAGCAGTCCACTGGACCAAGATAATAGAGCCAAT
CUST_724_PI426210822 CAATGTGATCTGTTTGGGAAAGTGCTTGCTTTGATTTCCAAAGGGCTG
A_14_P121444 GTGACAAACACACTTAGAAAAGGTCTACCCTAAAACCAAGTCATCATTATGGAAATCCTA
CUST_3695_PI426210822 ATCTTGGGTCACCTAACTATTAATTCTGGAGGACAGATTCCAGCCGTG
A_14_P127532 ACCTGAAAAATACTGCCTTAGCCGCTGAATTTTGATAGAGCAATTCTATATTCCTGGTAA
A_14_P136274 CTATTGTTTTCCAAACATGTTTTCAGAATAGTGTTCAGCAGGATGTTAAAAGGATGCCAT
CUST_3711_PI426210822
ATTATTCTTCATACCAGCATTTCCCAAAGCATTTTTTCAAAGGATAGTAACGACTTTTAT
A_14_P202383 ATGGCAACCTCTTAAATTTATAGCATATCTAAATTGGCTACAGCGTTGCTGTCCGGA
CUST_8810_PI426210822 CTCCCTGGACCATGAATGCTGCTGAAGGCTCTCACTTTGTACTTC
A_14_P132657 ATGAATTGCATCAGAGAAATCCCATTAGATCTGGGGAGATGATTAAAGACGAGGT
A_14_P104311 CTTCTCTGATTCCTCAGTTTTTCTTCTCTTCTCCATTTGGCCCTTCGATTAGAAAA
A_14_P121358 GTGACACAGAAACGGCACACTCTTCCACATGCTTTTGAAGTATTATAAAACACTTTATTA
A_16_P17609826
AAAAAAAATCCAGTCTTACATCCCATCTGAAACTCAAAACCCTAGAGGAATTCCACTTAT
A_14_P113421 GCAAACTGGTAATATGTAATACTACATACTCTGTGTCTCCATAATTTTACTGCATAAAGG
A_14_P135713 ACATCATTCGCCTAACTCATCATGATCCAGAAGTAACTTTGATTTTGTTTCATTCAACAG
A_16_P18370557
TTTCTCCTAATGATCTCTGTATCCCTTTTACATACCTTGCAAATAATCAGTGATCCCCTA
A_14_P138201 GCTTTTCTCTGCTTTTCTACACAGATGGAGTTTGATGAGAAGGAACTCCGAAG
CUST_3668_PI426210822 TAGGGAAGATGCCCACGGCACGGAGGTTCAGTGGTTGTTTTCATT
A_14_P135417 ACAATGGTAGCATCTAGGTTTGAGTAAGTCCCTAGGTAGATCTTTTATCTCTTTGTATGC

A_14_P125862 CATCAATTCACTGTTTTTATTTTGCTTCAAGCACTGCAACATGGTTTTAGAATCTGCTTC
A_14_P114560 TCTTTCATTCCGGGGCTTTTCTGTTCTTGAAACAGACCTTTGTTCTTCTTTTTTTTTAAA
A_14_P119234 CAGGTCAAAAAGTGGTAATTTCTGAAATACCAATGAAGTGTCTGGCAGTTTAAAGTAGTC
A_16_P15213171
CCTTTGGTCATATCATTCTTTGTCACAAAAATTCTTCCATGATCCTTTAGCCTGATCTTC
A_14_P114402 CGGGCATAAACTTCAGAATCTTTGATCCTGGTCCAGTTATTACAGAAATGGAAATTAATC
A_16_P17879503
CTAGAACGCTCAGATAACTACACTTACCTACACTAAGAGTCATAACCGATGTTCAAAAGA
A_14_P107060 CAAAACAGCAAAACAGGGCATCAGGAACACTCCCAGGAATTAAGTCAAC
A_14_P116570 AAGGTCTCCTTAAATTGGGCTTGTTTTTACTTGTCCTATTTAATTCCCCACTATTAGCAG
A_14_P125930 ATATGGTAGCACTTCTTTCCTCAAACTCTGTTGATTCATTCATTATGGCCTATTGAACAG
A_14_P103186 GTTTATCTTCCAAGTCACTCTCTTCCTCCTTGTCTTCCTCAACAATTATGGTATGGT
A_14_P136798 ATAATTCAAATAAAGGCTTTTATGGTTGTTGTGGTTTTGTTTTTAAGGCTTTGCCCCTTC
A_14_P111428 CTATATTCAGTCTTTGACTCAGATGCCGAAGATGAACTTTGCGAATGTATTTATTGGTGC
A_14_P124928 TTTGTTTGCTTTGGGATCTTGTTGGATGCACTTAGATATGGAAAATGGAAGCCAAATTTT
A_16_P37341109
ACCATGAAACACAAACTCACATCTTTCTGAATCATTTACAACTTTATAGCCTGTTCCCTT
A_16_P00916421
AACGAATATTTCACAGAGGGTTTTAAAACAAATCATCCCCTATAAAATCGAGTGTCACCT
CUST_9348_PI426210822
TTTGGAAGTATTCTTCTTTCAGTCTGTAAGGCTTTTCTCACTGATTCTACAACCGTG
A_14_P125449 ACCTCCCACCACAAGCATGGATCAGCGTTAGGAAATTAAAATAATGGTAATAGAAAAATT
A_14_P116400 CTTTTCATTGGTCTTCACCGTATCCATTCATTACAATACATGCCTCCTCCAAGTTTG
A_18_P14556915
AATCCTACCAAATCTCTAAGATTACACATTCAATTTTTCAACGCTATCCCTAGAATGGGC
A_18_P14920477
AAAAGAGACACCTAATTCCATGAATCTGAAGTCTTGAGAATAGCACATTAATGCATGCCT
A_16_P16257080
ATGGTATGTGAAGGAACTGTATTCTAGGACAACTAAGCATAAAGAAATAGTGGGAAGCAG
CUST_6137_PI426210822 GAGGCCTTGTTCAATAGCTTTTCCTCCTGGAGACACAAGCAATCTG
A_14_P106220 CTTCCTCATGTATTCTATCCATAACTGGGACTTTCATAATCACTCACTCACTCATTCATT
A_14_P116384 TGGAAACCAAATGAGGAAAAGACTCAATTAGAACTAATTAGCCAAGTCAGTGCTTCAATC
A_14_P109916 CTTTTGGGGACACATTTTGGATTTTTTAAAAGTTGGGTGCCTTTTATAAACCTGTTCAAA
A_16_P39431917
TGTTACCACCTATACTCATCTCCTGTCATTGATCTTCTCATATTTAATCTGCCAGTGAAG
A_18_P22841552
TTAGGACATAAACTCATGGAGCATATCCTTCAGTCAGTTTAGTTTGTGTTTGTCTGATGT
CUST_3511_PI426210822 ATTACAATATTGCCTTTTAGCGTAGAAAAGCCCTCCTTCCTGATGCTCTTGC

A_14_P107746 AGGTGAACAAGGCCGTTGATACCATCTGTTCATTTTTGAAGGAAAACTGCTT

A_14_P108965 TCACTTGATACTCAAACAACCCGGAAGAGTCTTATAAAGTCCATTTCCAGTGATTTATTC

A_14_P110502 AGAAAATAAATCATCTGCAAACTTAGTCAAAACAGGCATGTCATGTGGGCAACTTGTAAA

A_14_P127637 AGCAGATACACAAAGAAGGACGCATGCTATGTTTTAGGTAAAAACACATGCATATCTTCA

CUST_2244_PI426210822 TCGCTGACTGTCCATCTCTAGTGGCATGCTCCTGGGTCTACTGAT

A_14_P201330 CACAGAAAGCCATCATTTTTAAGCGCTTAAGCAAAGATGCTGTAAAGAAGAAGGCGT

A_16_P17441491

AGCCTAAATATTTTTAGTCACATAAATCCTTACACCAGGTATCACTTGTAAACCCCAGGT

CUST_7875_PI426210822 AACCACAACAGAACAGGATGGGCCAAAATGGAAGCTGGGATCTTA

A_16_P16162160

GTGACCATCTAAAAACACTGACCAAGATACAGATACGATTGTTCTACCCAAAGTATGAAC

A_14_P120083

GGAGAACCTATGTGGGTTTAAAAAAAAATTAAAAAGCAAAGGCAGAATGGAATTGGGAAG

A_16_P17800478

TCCATCCACTCAGTATTACTTATCGTGTCAATCACTTAATCCTGCAGCATAAGTTCTTTT

A_14_P134737 GGTTTGGTTCTCCCGAGAGGTAAAGAACGAAGACTTCAAAGACACTTTC

A_14_P130420 AAATTATGGCTAACAACGTGGGTTCATTTTGACACTTACAGATGATTCTGTAGGCATATC

A_14_P100512 ACACATAAAGGTTTTGTTTTGCTTTGCTTGTTGTGAATTAAAATGGGGAGAAAATGATGT

A_14_P116084 CACTTTTAGCCAGAGATGCAATATATCCCCACTACTCAATACTACCTCTGAATGTTACAA

A_14_P117650 TAATGGTCAATTTTTTGTCTGGGTTCTTTGTGGCTATTGGATTTGTGATTTACAAAGAGG

CUST_5046_PI426210822 CAGTAAAGTATGGGTCAACATCCCCAAAATGTGTGACTCGCCCAAAT

A_18_P15083088

ACACAGGACCTTTAATTAAAAATTCTCCCCATATTTTCTTATCCCCACATATGCCTATGC

A_18_P17014817 AACGCTAAAGACCTCATACTGTTTAGTCCTCTATACATTTAGCCCTTTATGCTTGGG

CUST_9363_PI426210822 AAAGAATGATAATTGGTGCATAACTCCCAAGTCACATATACAGACCAGGGTCC

A_16_P03385069

CCTGCCTTAACTCAAGATTGTCATTAATTTCTTGTAGTCAGTTTAACCAGTGATAGCCTC

A_14_P128744 AGGTGTTTGAGAGAAATTTAGCGCATAAATTATGCTTCCTTCCTTTCTCAAACTCTTCTT

CUST_8929_PI426210822 CATACCACTTTTCACAGGGATGACTGCCTTAAGGGATGGGGAAAG

A_14_P100921 GGTTGGTTTTTTTTTTAATTAAAAAGTTTTGCATCTTTTAAAAAAAATCACAGCACTGGT

A_16_P18256983

ATGTAGACAAGACATCTCCCTATCTAAACAATCTGTTTTGAGATCGTGCTATTGTTGGAC

A_14_P200791 CTTACAACACAAATTGTCTTTCATTCACTAATGAGACTGTGTCTGAAGATTTTAAATTCC

A_14_P102646

AATAAAAAGAAAAAAAGATGTAAAATATACACCCCCAATCAGAGAGGGGAAGTGGGAAAT

A_16_P19327291

AACCATGCTTATTTTCCAGAATGTTCCATACATTTTCCTACCTCTAAGTATCTGCAGACA

CUST_667_PI426210822

CTAGGATGTCTTTTTATAACTTAGCAGATGGCATCTTTTCAGTGGCTTGTTCTGAACTC

A_14_P117839 AGACACTGGAACTGAGAGAGAAAATGAGTTAGGGACACATGTATTTTTAAAATTAGAAAC

A_14_P201223 CATCCATCATCTCCTATCAAATCTAGTCTTTAAGAACCCAGAAAAAGGCATTTTCTGCAT

A_14_P116596 CAGTTTGATTTGGGCCCTGCCGTATTTCAAAAGGAACCACCATTTAGTTTT

A_14_P139542 AGGGATGACAACAGATTGTGGTCTAATCTCACGATTTTTGCCTGAGAGAGAATT

A_14_P109856 TTGTGCAGCACCCCTAGGAAATTAATACTCCATATACATATTAACACGCACACACTCTAA

A_18_P13743465

AAATACCCCGATTATCTCAAAGAAGAGGAAAAAGCAATTACTCCGGATTTTGAAGATTGA

A_14_P123133 CTCCCTGGGTGTTTTACACGTAAGAGTTGCAAATATCTTTCAGTCTTTTAAAACTGCTTT

A_14_P134990 GATTTCACTCAAGAAATCTTCCAAAGATGATCTTTGGGGAAGTCAGTATAGACTTAGGAA

A_14_P106826 CTGCTCAGATTCAGATGGAACATAAGCAATAATGTTTTTCCAAGGTTTCTCTTCCTTTTT

A_14_P105719 CTGACATCAGAAGGCTTGTCTCACACTCTAGCAAATATTTAAAGCAGTTGAGACATTATC

A_14_P107713 TTTCCCTTCAGGCCTACACATATCTTCATTTTTCTAAAAGAAAAAAGAGAGAAAAGGGGA

A_14_P132027 AGGCACAGACAGACAGGTCCCCACTTGATTAAATGAGTCAGTCATTGAAT

A_18_P22841051 CATGTGTGTTTTAAAGGATCCGTGGGTTTGTGGGGAGGGCTGCAC

A_14_P117388 TGGTAATTTGTATCTGAAGGAGATTTTCCTTCCTACACCCTTGGACTTGAGGATTTT

A_18_P22842838

GGTAAAAAAAAGGAAAAAGGAAAGCATTTGAATTGGTGTCCTCATGACAATCTGGCTG

A_14_P126774 GTTAGTGTCTTGGGAAACAGTCTCTGCATATATCACCACTCTGAGTGCAGATATATTTAT

CUST_8478_PI426210822

GATGCATGTTAGAAGTGTTTCTATTTTGTTGAAATTTCCCGTCATTCAGTAGTTCACTTT

CUST_2580_PI426210822 GCATTTGTACCTCCAGTCAGTCCACAACACCCTGGGGACCGGGAG

A_14_P124060 ACTCATATATAATGCTATCTTATTCTCCATTAAAAATTCAATGTTTATGTTTTGTGCCTT

A_14_P100706 CTTGGTCTTTAGATATTGCACCTCCCACGTTCCTTAGGTTAGTTTACTTAATTATCTACC

A_14_P110596 TAAAACTCAGAAACTAACAATTCACATCCTCCCACCTTCTTCTTTCCGAAGAAGG

A_14_P115386 CGCTTCTCTTCATATCCAGTGGGGACTTTTTTTGAAGGTAAATGCCTTTTCTC

CUST_1183_PI426210822 CTGTGGGTTGAGGACGGCTCTGCTGCACTTCTGCCCCTTTAGCCT

A_14_P124756 AGTCACGTTCCGAGAAAACCTGGGATGAGAAAAGGATAAATTTAGCAATAATGATTTTTG

A_16_P38231499

GGGAACTTGGTTTAAGATAAACAATTGGTCCGTTATCACTTGCATTACATAGGAACAACT

CUST_9260_PI426210822 TTCTTGTGGATTTCCTCTGCCTGGAATTGCCTTAGTCACCAAGTTC

A_14_P129562 CAGCAATTAAGAATGAATAGTATCCTAAGGCCCTGTCAATGGGCTAAGCATGTTATTTTT

A_16_P02024158

AGGATTCTGGTCTCATTTTGTTACATACTTTTTGCTGCATCAATGAAACAAACCTCTGAA

A_16_P18637831

CCCCCTATCTTTTTTCCTATAACTACTCATGAACGATGGTATCTACATGTGTCACAATGT

A_14_P125066 TCCAGACATATTCCCAGATATATTCCTCCTCTAACCAAAAATGTAGCAGAAAACGATCTT

A_16_P36589805

ACCAATCAGCCATTTTTTTCTATGCAAATCACAAATCTCATTTATTAATGCCTTCCCCTC
CUST_8536_PI426210822 TTCCCCAGCATCTAGGAGTGTTCTCTTCCTAGAGCATGCTTTCTG
A_14_P117328 ACAATTACCAAATACAGGTATAACACAGTTGAGAAACAGAACATGAAGTATCTTGCAGGG
A_14_P111279 CAATAGTAAACTGCATTATTGGGATCCAATTCTATTGCCTGTGTGTAACAATCCACTGCA
CUST_3597_PI426210822 AGCTTTTTCCCCTGGGCTGACTTTCCACCACCCTGACCTGCGATT
CUST_8133_PI426210822 ACAGTTAAAATCTCAATTGTTCCTTTCTTCCATCACCACCCTTTGAAGGTCTC
A_14_P116324 CAAAGCCAATGTGATTTATTGAATGAAAGCACTGGACAATTACCAACAACTTGTTCCTCT
A_16_P18078766
AAGAAATATTGCCACTTATCTTTGAATTTTGATACCATCCAGTTCCTAGAGTTGTGTCCC
A_14_P108160 TAGAAATTAGACAAGTTTGGTAATGAGATCTGCAATCCAAATAAAATAAATTCATTGCTA
A_16_P15527426
TCTGTTCCTGCCAAGTATCATAAACTAAATCAGCACTCAATTATCCCTTCCAAAAAAACA
A_14_P117913 AATATTGCCAGACCAAGATACCTAAACTCATGATGTCTGTGGTGCTGTAAAATTTATACT
A_14_P106383 TTATTTATTACTGTGAAGTCAACTTCAGGAAGGGGTTTTCTGAGAGTCTTATGTGCTTGA
A_14_P104545 GACAGAGGTAGGAGGCATCATTCCTTTCTATAGAAAAAGATAAGATTCTAGGCCTCTAAA
A_14_P109833 AAATCCCAGAAGAAATAACAGTATGTCAGCATTGTAGGACAGAAAATGTCCATGTCTAGA
A_14_P129088 ACATATGGTTTCAGTTCCAAATTATCCAAGTATAACTCTGAACTTCCTCAGCAAAGTCAG
A_16_P20082599
AATTCTTGAACCCAAATCTGAATATGTGGCTTTTTCCAAGTTAAACTTCACAGTGAGCAT
A_14_P106865 ACAATTCTTTTCACTTTTTGTGCTTATAATTATATTTCTGCAGGGGCTATCTGATGGGTT
CUST_8812_PI426210822 CTCACTTTGTACTTCCCTGGGCCCCCTGCCCTTGCTAGTGACTTC
A_14_P124004 TCCTTAACTAATCTGAGTCTTTTAATACCAGATGGGGACCCTGAGAATAGATTTCCTATC
CUST_1942_PI426210822 ATCAGCTAGCATAAGCACATCTGCTGTGCACCTGGCTCTTAAGCTTTTAAATAG
A_14_P100334 ACAGTCATGATACCAATCACCAACATCCTTTGATAAGACCTTGGCTACTCATTTATATTC
A_14_P132332 ATAACAAGTTACTGAGTAATTGCTGTAAGAGGAGAACATCTTTGGAGCATCTAACCAACT
A_14_P130421 AGGCCGCATTGAGATTTGGGAAAGTGTCCAATAAGACTACAACTTTGCAAAAAATATTTA
A_16_P00389529
AATTCTTCTTTCACTTAGATCAAATCCTTTTTATGAACCTAAATCAACTCCTCCTCCAAA
A_14_P201813 AAATAAGTCACAGAGATAATTTGAAAATCCCCAGCACCATTTTTGCCAATAACTGTTTCT
A_14_P116864 ACATGTGATGTGTCATATTACAGATGGGGTCACAGTGGTTATAAAGAGTTATACCCTGAA
A_16_P03562512
AAAGCGTGCTTTTTCATCAATCTGACTCAATAGAAAATGAAGTCTACCGTAAAGTTGTGC
A_14_P107699 CTGTATAAAAGCAAAACCCATCTTAATTCACATCACCACCAGTTTAACCTTCATTAGTCC
A_14_P114923 GATTGCCCTAATTTATGGTTACCTCAGGAAACCTTCTTTTGAAGAAGTGGCTG
A_16_P00685590 GTCCTTAACTAGATGCACAGATGTGCAGTCAAGGGGAGAATGAAGACTC
A_14_P116476 GCCTCGCCTCCTTAAAATCTATTTTACACTACGGTGTTGATTGGATTCTATAAGATAAGG
A_14_P118028 CTTTGGCTATAATTGATTTTAGTTTCATTTTGCCCTTACATGACTCCCTTGACTGAGATG
A_14_P110126 AAGAAACACAAGCAAATATAAAACATTCAATTCTAAGAGAAGTTCATCAGAGACATCCTT

A_16_P20776842
AAGGCACATACTGTTTTTTTCCTGGCAAATATGAACAACAAGCATAGACACTCGAAAAAT
A_16_P17131133
AAAAATCTACTTATGTGTGAGAAGTGAGCACAATTAAGTTTCTTTTATACGCCCCCTCAC
A_18_P13359685
TGCACTGATGTTCTAATAGCACACGATATTTGAAATGATGATTTATACTCCAGTCCTTCT
A_14_P110117 CCCCAACACTTTTGTTTGCTTGTTTCTTTTTCTTTTAACGTAAGTGGAATCTACCTGTTA
A_14_P118661 ATCAGTTTTATTTTCTAATAGAGGAAGTTCCTCAGGAAGAAAAGAACTTGGAGATAGGGT
A_18_P14135161 AGAAGAGGCTCCTCTTCTCTCCCCTAGATCCATAGTATCCTGGCT
A_14_P131116 GCAAAAAACAAAGCCAAGACTATCAAAACTAAATGCCACAGAAGTTCCACATTTTGTGTT
A_14_P201373 TCCAGCAGAAGAGAAATGGGCTCCTGTTTCTGAAAGATAATGAAAATAGAAGACACTTTT
A_16_P21346044
AGTTACCATCACAACTGGATACAATACAGGAAGTTACACCTTAAATCAGTTGCCAAGAAT
A_14_P105101 TAGAAACTGAGGACCGACCAAAATTAACTATGTGTAGACTTTGAACAGTTTGCAGTTTGT
A_14_P127389 CTCATGGAAAAGCAGCACAGTGAGCAACAAGCAACAGTGGTCAGTAAATGTATAT
A_14_P137125 CTTTAGAAAGTTCACGGGTTTGTAGAATTGGGACTAGATAAGATTCTTGCCTTAAAGATC
A_14_P128104 ATGGGTCATATTTCTGAGCAAGGTCTTACCAGAAAAATTGTCATATCAAGATAGAACTCC
A_16_P17586870
AATTTTTACCCAATTCTTAACTTCAGTTCAAGAGACGATTAGAGAAATTCCCCGAGACAA
A_14_P202353 ACATTGGGTTCTTACTTAATTTTGTCTTACTTGTAGATATTTCCTTCCTGAATACGTCTT
A_16_P00533986
CACACAGAATTGTATTTCACTATGACACGTCTATTCTACCCTAATTAAATTGCTATTTCA
A_16_P00685465 TTCAAGTGCTTTTGAATTTGTATTGGTTTTTTAAAATGTAAGATTATTTTTACTTATTTT
CUST_5670_PI426210822
GATATACAGCTGGTGAAAGAAAAGGTGTCGGCTTTGTTGAGAAGTCTTACTTTGT
A_14_P125469 GTCCCTGTTTCCTTCTACTTTACCTCAGGTAACTCCATTTTATGTATCATTTGGAGAAAA
A_16_P15236110
AAAAATTCCATGCACAGGAGTTATCATGATAGAAACCGGCTTGATTCTTTTGATACTCTG
CUST_3531_PI426210822 ATCAGAAGCAAGTGGGCCTCGGAGGGGCCAGATGCCTCTCTCCTC
A_14_P138918 GGCGCAATTTCTCCTCGAAGTGCCTGTGTGCAATAGTCAAAAGCAAA
A_16_P02878400
CTCTCATCTTACCCATAATCTTCATTGTTCACTTTGCAGACTAGAAACATCCATCTCAGG
A_14_P121408 GCTGTTTTTAACTGACTTTTGTTTTCTTAGATCTGAAGAAGACACTTGAATCATGGGTGA
A_14_P126563 TTTTCAAAACCTAACAAAAGCCAATATCATTCTGAAGGTTACTTTTAGTCCCTGGAACGT
A_14_P132531 CTGAATAGATTTGCTGGATTTGGTATTGGACTTGCAAGGTAATGTTTTATCTAAAGACGT
A_18_P22605812 AATCGCTTTAAAATGTGTGCCCATGAATCTGTTCCAAGACCCAAGATGAAATTTCAG
A_18_P15708999
AAAAATTGTTTCCAAGCCTTTTTTACAAATATGATACCCTCCATGTAAATCAGATGCAGT

A_18_P25691976
ACATGCTTTATCAAACATCACCAAATATCCCAAATCTAGTGAATAATTTTCTGTGCATGG
A_14_P202348 ATTAATTCAATAGAAAAATTGCTGACTCTTGGGACCTTTCTGTGTTTGGTTCTCGTCTTG
A_14_P200465 CAATACCAAGGGGTTTCAGCCCTTTTGAGGGAGAAGCAAGACTTG
A_14_P119907 AAACCAAGACAAAATAACACGGTTTATAATTGTATGCTGTGTGATCACCCCTTAACAGAT
A_16_P18088579
AAATCCAGAGTATGAAGGATAGAAAATCAGCCAGAAGGTAAAGAAAATAGAGATGCAGCA
A_14_P115153 TTGCCGCAAAGACTCACTCTTAAATTTGAAAGAATACAGATATTTGGAGGGGGTCTTATT
A_14_P112111 AGATGAGTCATCCAAACCTGGAAAAAATAAAAGAACTGCAATCACTACTCCAAGTAAGTT
A_16_P00910742
TGATGCCTCTTTTTAAAATCCCATAGCCTGAAAAACACTTCCTTAATGTAGGATCCAAAC
A_14_P125162 TGTTTTGAAGGAGGGATCAGTGTATAAAGATGTCATAAAAAATATCTCGGCCATTACTGC
A_14_P132883 CTTCTAGGTTGTGTTGATTACAAAAACACATTTCCAAACAGGTTTATAGGGTTTCCCAAA
A_14_P131056 AAATAAAGGAATGATTTTGTTAGGTAAGGGGTGACTGAGTGGTATAGACGTGAATGAGTA
A_14_P123790 CTAGCATTTTTCATTGGTAACGTGAATCTTTGTGCATCCTTAACAGACTTAGGGCTTAAA
A_14_P125598 TTCATCAGCACCATCTGCTAGGCATTTGTCAACTTCCTACGAGAAAAATTTAAGAAATTA
A_16_P20750033
TCGGTGTTGAGTTTGAAACTACGTATCCTTATGAGCCAAGATATAGACTATACTCATACC
A_14_P114837 GACGACCTCAGTGAGAGCAGTTGAGTTTATGCAGTAAGGAAATCTGCACAAAATTAAA
A_18_P22843036 CCTGAAGCCAGAAACTCCCCCAAGGCAAATCAAGACTTTATGGAAT
A_16_P36230323 GGAAGGCTTCAAAGACAGGCTGGAGCCCCTAAGAGAGGAAGCAAG
A_18_P10895419
TATGCCTGATGATCTATTTTCTGATACGCCTGACAATTTCACTAGTTATTGCCCGTAAAG
A_14_P115824 ATTTTTTGAAGATGTTGACTTCTTGTTTGGTGCTTTGCATGGTGCTACTGAAAACTCTTG
A_14_P107455 ATGTGGAACCAAAGTGCTAAGTGCATATTTCAAGCATCTGTTCTGAATATGTTTAAAGCC
A_16_P38488261
AACTCTCTTACTGCTCCTCTGAATCTTTTACAAGTTCTTCTTTCCTACATATTGGCAACA
A_14_P200496 CTAAATGTGTTAACGGATTCCAAATTACTTAAAGCCTTTATGGGAACACGGTAGATTGTA
A_14_P200239 AAAAGTGAGCATGCTCAGTCTTTTCCTCTTATCTACAATACAAAGGGTTTGTCTGAAAAG
A_14_P129403 GAAGCACTTCCTGAAAACTGATTGTGCCTTGACATTTACCTGTAAAAAGAAGTGTAATTC
A_14_P127488 TTTTGTTCTGTCTACTCTTCTATTTTAACCTAACGGAGAAGAGGCTGAAGAGAAAGTCC
CUST_4175_PI426210822
TGGATATGCAGTTATAAATGATTTCTTGTTAAGCATTTTTAAAAGGTTAACTTCTTCCCA
A_16_P19762572
AAAACTGAAAGATTCCAAACTGAACACCTACAATGTCAATGGACAGAAAAATTCCTTTCC
A_18_P11623037
AACACTAACAGATGCATCTAGATTTATAAGACTCAACTTAAAATCTCCATTATCCAGTGA
A_18_P14134949

ACTGTTTTATATTGTAAGCAGGACCAACTCTTTTATGGTGTGTTTTATACGTCTAGAGGA

A_14_P135662

TGGAAAAAGCAAAGAAAATGCAAAAGCAGTTGCATCAAGAGAAGCATTGAAGTTATTTCT

CUST_9_PI426210822 CTCCAAGGAAAGACCCCCAGGATGCTTAGTCATATCCAAAATGTCAA

A_14_P108983 GAGTGTAAAATAACAGAATTTACTTACAACCATTTCATTTAAAGACACCCAGCAATCTGG

A_14_P125414 ATGTCACCTGACTGTTATATATTCGATCCAGAACATAAGAAGGTCTGTCAGGATATGAAG

A_14_P125793 GAACAAAGGAATTATTGATTTCTTGGTTTAAACTTGCCACCTTATCCCTAGTTGCAATGT

CUST_5448_PI426210822 AATCTGCAATCCCAAGTCTGTTTCAAATCTTTTCTTGAAACCCAAGATCACCA

A_14_P131644 GTGCAAGTTTTCCTGAGTTTAAACGTAACAATTTACCAAAAACTGTGAGTGGCTTCTTTT

A_14_P136808 GTAACACGGAGGAGGATGTAAGAAATGGGAAGAAGGAATTCTGGAGTAAATAGGAAAT

A_14_P116181 TCTGTTTCTCAGAATTTTGATTTTAGTCTGAATGTTCTGATAGTTGAGGCTACAAAGGCA

A_16_P30912590

GTATTCCACACCACTGTATTTTTATTTTTCCCAACCACATGTAATACTGTGCTACGATGG

A_18_P22840872 CTGCACCAGTGACCAGTTAAGCCCAGTGGGTGATGCCTTTCCTTT

A_16_P19432538

AACCCCACTGAAGTTTCATCACTAAAAGACAATGTCGATATAGCATTTAGAATGTTTGCC

A_14_P201137 AAAATGTGACTATATTAGAACATGTCACACATAAGGTTAATACACTATCAAATACTCCAC

A_14_P107047 GAGACCAAAGTGTCAATCACTCTTCTGACAGAGGTGATAGCAGAATGAAAAACTGAT

CUST_10182_PI426210822 TGGGGGCTTGTTTATGCTTGGGAGTAAATATTGGGCTGATGTTGC

A_14_P112319 TTATCGATAAAGTCCTAGACAATCCAGACTTGACATCTAAAGAATTCCAACAATGGAAGC

A_14_P116098 AAACACAAGTTGGTGGACTTTGTGATCCACTTCATGGAGGAGATTGACAA

A_16_P18437135

AGCATAGACTATCACATGAAGCAGAAATTTGAATACACCCTCATAATCAATCACATGGCA

A_16_P16191020 AGAACCTTCCAGGGCAAGAAGACAGTCTTGTCTCAACCAGTCATTAAC

A_18_P22840523 CTAATTCCTTATCTCCCGGAAAAGGGAAACCATCAGGAATCAGAAATGGAGAAAT

A_14_P116605 CCATCAAACATGTGTTTCTCGATTGTGTTATGAAGAAATTCAGAGTAGGAGATGAAACAG

A_14_P114933 AATGAGAAGATTAGAGAACAGATTAAGAAATACAGGCGCCATTTCCTACGAGTAAGCAAA

A_14_P119904 ACTCTTTTTTGAAAAACTTATTCAATTTCAAACTAAAGCAGACAGCAGAAGGCTGGCATC

A_14_P139086 CTATGTTAATCATACCCTCAGTTCTTCACATGATATCATCATAAGCACTGACTTTGACCC

A_14_P122196 TTTGTTAATTTTCCGTTCCTTTTTCCCACCAACCCCTTTTAATTTTACCTTATCTGTGTT

A_14_P135777 GTAGACAGAAACTCAAAAACATCTATCTGCTCTCATGCTTCTGTTCTTGTTAGTTCTCAG

CUST_1596_PI426210822 ATGGTACAGCAAAGTGGGGACATGGTGGGTGTACAGAGTGTCCGG

A_14_P124924 CTTTGCCCTTGATGCACTGATGCCTTAAAAATTAACAAGGTCATTCAGAAGTAGATTACA

A_16_P58424568

GTCAGCAGTTTACCAGAATATATCATCTACAATTTGCCTAATTTAGTTGAAATGCAGAAC

A_14_P129748 TGTTATCCATTCTCCTCACAGTTTATCAGATGGTGGAAGGAAACACTGTAATTCTAGAAA

A_18_P10613176

AGATGAATAGGACTAATGTAGAAAAACAAATCAAACTGTCCTTTACTCTCAGTCAACCCC

A_14_P127237 CTCACAGTTTGGCTAATGCTGGAAAGGTATCAGTCAGTGTATTTTTTACAGTTTTCTTGA
A_14_P119126 CACATCCATGCAGACATCCGCAAAATAAAATTTTACAAAACCCTGGAGAAGGTATTATTT
A_16_P16326589
TATCTCACTCTACTCCTCTATTAAGAAGTCCTGTGTTAGTAAAGTAGATGGCTCCTTACG
A_16_P16719690
ACGTTTTGCTCTTTTTATATGTCCCAAACGCAAATTTCTAACGTGACAAAGTAGGTTTCA
A_14_P132261 GACGACATCCCAAGTAGGATTGCTGTTTTCAGCCTCTGAATTCTTAAAACTTATTTCTTA
CUST_224_PI426210822 GACTCGTTCTTCGGCTACGCAGTTCTGGAGCATTTCCACGACAAC
A_16_P19007856
AAAAAAATCAACTGAAGAATGATTTCTGATGCTGACAATGAAAGACACTTGCTTGGATGG
A_14_P130848 TGCTGTTTCTAGCCACTTACTTCAAAGGACTATGGCTATGTTGAATCAGAAGCCATTAT
A_18_P22841020 GTGTGGTGGGTGGTAGGAGGAGGAGAGGGAGACAGAAGAGAAATA
A_16_P38287416
AACACTAACTTGCTTTAATAACCCAACATCATATCTACAGAAAACATTCTTCCAACATCA
A_14_P124331 TGTTGGGACGTTTCTTTTTCATTTGTGTTGAGAAAACAATACGACAGCAACATTAAAGAG
A_14_P131148 ACTTTAGGTGTTTTACGTGGTCTAGAAATTTAGGCAATTCATAGTGTTAACTGAAGGGTC
A_14_P107896 TACTCTTGTAGCAACTTTTATATTTCATTAAGAGGTGTCAGAGGCAACCAGGTATCTCTG
A_14_P117410 ACTCCAGAAAATATGAATTTTTTTGAAGAGACCGTAGGTGTCAGTGCAGAAGATATTTGC
A_14_P138830 ACCATCATAAACTTCAGCATGTTTGTTAGCCAGTATCCTTCAGGATTTTAAACTCCAAAT
A_14_P126253 GTGAAGTTGACTGAGAAATGCCTTAATAATGTCATTGAGAGCCCAGGATTGAATGTCAT
A_14_P119782 TGGTAATAGAAATGGCTCAGGGAGGAATGATGCCCCTTGAAGAATAAAAAATAAGAAAAT
A_14_P112086
ACAAGTTTGACAAATAAGCAAAAGGAAAGGATTAGAGGGCAGATGACTAAAATGTTGAGT
CUST_2600_PI426210822 CTCTTAAAACCGGGGCCATCTGGCTGAAGGTCCATCATATAGACT
CUST_932_PI426210822 CTCTCTGTGATCCTGCTCCCCACCACACACCATTCTTAGCAGCCA
A_14_P132050 CTGCTCTGTCTTCTATTTTCTAGTGTGGGATAAACTTAATAAATAGGTTGCCCTAGGTTG
A_14_P113554
TAAAGGGCAGAAACTGTTTAAAAAGAAAACATGCTGAAAAACAATCTGATGTTGGACTGC
A_18_P22842679 TAGTCAAATGTCTACTCCCATCAACTGTGGAAGGGAGGAAGGGAG
A_18_P13858484
TCACAAGAATATGTAATTTTTAAAAAAAAAACAAATTTAGAGCGATAGAACAGCCTTGAA
A_14_P116834 ATGACCACACAAAACCTCACTGAAGTCACTCATATAGATAAATGAAGACCAGGAAGAATA
A_18_P14134759 GCATGACAAAGGCTTTGGCCATCTCTATGGCAGGTGCGAATGTAG
A_14_P129153 ACCATTCTTACATTAAACATCAGTAGTCACAAATGAAATGTGCTCCTATAGCATCTGCCT
CUST_4334_PI426210822
AAGGAAACTACAGGTGAAACGTAACAATAGTAATTGCCTTACTTTATTTGCGAAGGGG
A_16_P32381353
CTTGGCAAGTTGTAACCATTTACCTAATTTTGATTGAAGTATGTCGTGCACATGTTGACT

A_14_P138492 TTCTCTGTTTCTCAGAGCATCTTCCACTTCTTCCATCTCTTGTTTTTGCCAC
A_16_P21314522
AAAGGTCCAAAAATTGAATCCCACTACAAATACTTTTGCAACCAGTATCAAATTGTTCCT
CUST_1872_PI426210822
TTATCATGAAGGTACAGGAAATCCAGATTGAGTAGGTACAGATGGATCAGGAAC
A_14_P119035 TAGTCTGGTGTAGCAAAAGCCTTACACATAGTAAGTCCTATAATGCTCAGTCAGTTTGAT
A_14_P100886 CTCTTGAAAAGAGGATGATTTACTTGCTTCTAAAACTGACAGGCTGCACTTGTCCT
CUST_2926_PI426210822 CTTGGAGCCTCCTGGCTAGCATAGGTGAAGGTGGTGAATGCTTTG
A_14_P132294 TTGCATCCACTTCATCGGCATTTCGATCTGCTCAAGACAGTATCTGCATTTTTTATAAAA
A_14_P119225 AACAAAGAAAAGTCAGTATTATGTTGTTCTCAGACACTTTGGCTTTTGTTGGTCCTTCTC
A_14_P115564 TGTCTCCCTCCTGGAAAATAGATGGATAATTGGACAGTAGATGAGATAATGAGATGC
A_16_P19058370
ACCAGATCTCAAGATTTTCCGATGACCTAATAATCAAATAATCACCCAAGCATTGACTTC
A_18_P12558242
AAAAAGGACCACAGTTTGGAATTTGTAAAATCTGTCTAACATCAAGTGAGCCACTGAAGT
A_16_P01989862
ACCAGAACTCTAACTGTGAACCTACTAGACTTAACACTCTTGTCTATCTTCAAAGACTCT
A_14_P135867 ATCAAAATACTTGCTGGATGGTGGACTCAGAGACCAATAAAAATAAACTGCTTGAACATC
A_14_P123814 CCTGTTACATATGCAAAACAATGATCTCAAAGTTCACCAAATGGTTTCTTTCAAAAAGCC
A_16_P00685492
GGCTTCTCACCCAATTCTTTTGCCTGTCATCTCACTGTGCTCTTTAATGTTTTTAATTTA
A_18_P10745622
AAAAGGGAGCCTAACTTCAAAAAGAACTGCAACTTATACTCTGAAAATAATGGGAGCATC
A_14_P120135 CTGATTGTAGTTCAATGCTCATTCTTATACTTACTCTCCTGTAGGAAAAGACATACCCAA
CUST_207_PI426210822 GCGGGTCCCAGGGGCTGACTCCCGCCCGGGAAGTGCGCGCAGGGC
A_14_P104795 GGTAAAAGGAAAAACAAATTTACATATGTTGATAACCCATGAGGCCATCACTGTCTCTTA
A_16_P00366854
CTAGTGAACGATAAACCTTTGGTGATGCATTTTTTAACCTGTTCAGTTATTCATCCCTCT
A_18_P17411786
CATGTATTCTCAAATACAAAAAAATACAAAATAAACACAAAGAAGTAAAAGTCAAGATGC
A_18_P13207466
ACTTTCACTAGCAAATATCTTTTTTCCTTTACACTCCGAAACAATTGACTAGAAACCTGC
A_16_P16143798
ACAACCATCCTTGAAACCTAAGACCAACACATACTCTTATGTAAACACTTGGATACTCTT
A_14_P102029 GTCTTCATCAGAGAGTCCTCCTGGAGATTTGGGTTCTCTCTATAGCCATT
A_14_P120158 CACCCATGCCTCAGTCACTCATAAGTATGGATACAAAGTCTTGCATTTCTTTCTTTAAAA
A_14_P103150 TTTGCCAACTGCTTGGAGAATATTAACATTATGCCATTTCTGAACATTGCAAAGTGAACT
CUST_10163_PI426210822 CCTACTTGCTCAAGTGGCAAAGAGACAGGCTGCAGAATGTGTTTG

A_14_P201159 AATACCAACTCATTTGCAATTCTCTTCTTTGGGATGAAGTCACAAAATTGGAAATGATGC

A_16_P41843982

CTTCCCTTTTTCTCACTTTCTCTGACATCCTATTAACCTTTACACCAAAACCTTCATAGA

A_16_P58424282 CTCCATGCATATCTCACTGTCCAACAGGCCAGCCTGGGCATATTC

CUST_3284_PI426210822

AAAGAAAAACAGCAAAAGTTTCTTCTTTATCTGGAAAATAACATGCTAAATCTCCGGGTC

A_16_P02456114

AACAAAGAACTCTACAAATTTTACCGAGCCTTTATTCCTTAACACTGACCAATAACTGCC

A_14_P139026 GATGTGACATTTCAGTATAAATAAATTGTATAAATCTATGCAGGACACGGCTCTGGGAGC

A_18_P22839969 GTTCTCAGATGTTCAGAGAGGCTTCCTCTCCCCTTCCATGAAAGTT

A_14_P128579 TGTGTGCTCCTGTTATTTAAATTTCCATATACTGAGTTCTGGAGGTGACATTGCTAATGT

A_14_P100023 GCACAATTTGCATTTTTCTAGGAAAAACGTCCATATTTTCACCAGAGTCTAATCCCAAAA

A_14_P105322 TGTCTCGATGAGCCTCATTTTTGTCACCCAAATTTAATGATATTCAAAATATTCTACTTA

A_18_P15978042

TGCCGCTCACAAAAAATATATTGAAACTAACTCTCTTACTCTGATCTCAACGATATCTCT

A_14_P126366 CTCTCTTTGCTGTTTTCAGTAGAGCATGCACCATTTTGAACGTGAATTTTCGGTAAAG

A_14_P129880 TCATAATAGCAATACCTAGTCATTGTAGAAAAATTGGGGACGATTAAAATTCCACCGTGG

A_14_P132031 GACTTTTCTACTTACTTTTTATGCCTTTCTCTTCCTAATATTGGAGGCCAGAGGAAAGAT

CUST_1372_PI426210822

AGATGGATTAGGGAGAAACCTAAACCTGCCCTCTTGACATATATATGAAGGAATCA

A_14_P127387 AAATTCACTTCTCTTCCAGATGCCAGAGTATATATGCATATTTGAACAGTCACCTGGATC

A_14_P102838 TTAAGCAGAGGAAGAACATTTTAAAATTATTACTTAGTGAGAGTGCACTGCAAGGGAAGG

A_14_P108482 CTTTTGGGTCCAGAAGACCTGCATATGGGCTGTTGACTCATGCAAAT

A_18_P16844710

TTCATCCTTTTTTCCTGATGGACACATAAAAGTCAACATTTCTAACATTGCTGGACGTTA

A_14_P120115 CTTTCTCATGCCTTCCTAGAAAAAGAGTAAAACTGAAATAAAGCCTCTTTCATTGCAGTC

A_14_P132356 ACCACAAATAGGAAAGATTTCATTCTAGAATGAATAGAGCACATATTTAGTATCTACGTG

A_14_P123777 GTAGAATTTCCTGCATTCTCCACAAAGGATGGTGACAATGTGATGACTTGGAAT

A_16_P20744286

TAGGATCTGCTGCAAGAAGATAAAGTGAGATTTTAGGGCTGAAGACAACATTTCAAAATC

A_18_P26322448

AAAAAAAGAAGCAAACACTTCTAAGACTTCAAATTGTGAAGCATTACGTTTGATCCTGCA

A_18_P22842286 AAGTCTTACCCTTTGTGAGTCAGGGGGCTTGGGCTGTCTGCACAC

CUST_2180_PI426210822 ACATTAAATAAATGAGTGCTGTGGGCATTCAGTGGTGCCTGCCATTGTATT

A_16_P16226064

AACATAGTCAAAAGCACTCCAGAATGAACTAATACCTAAACACATACCATATCCTCCACG

A_14_P201060 GAATATAATAATGTCTCCCCAAAGATTTCCTGCTGCTGTAGCATACTGTATTTTATGGGC

A_16_P17835829

CCTTCTATAAATCAGTGCTAATTATACATGAGGAAACACAGATAAGAAACCCTATGCGTG
A_14_P120943 TTACACAAATGAAACTATCGAGGCAGCAGACTAAGGTCATTATTATTATTTAGAATTGAC
A_14_P134953 ATTTTAAATAGAGCATACCTGTGTTTTATCTGCTGCCTACACTAACCTCTTCTTTGCTGT
A_14_P117248 GTCTTTTAAGAGTCCTCATAGGCGATTATCTTGGGCCACTCATGATAATTAATCCATTAA
A_14_P101624 ATTCTCTTCCTCCATTCTGTTTTCAAATATTGGGCATGAATAAACAGTTTTAGGCACAGA
A_14_P122781 AACTTTCAAAAAATGCAGATCTTGCTCAGAATAAATCGTAACAATCTCAAAGTGCATTTG
A_16_P16683057
CTAGCCAATACAGAAAGTTACATCTCTTCTCATTCAAAAGTTATTTCTTTACGTTGCTCT
CUST_7615_PI426210822 GAGGACAATCACCAGGATTTAGGGGAGTGTTTTTGTCTTTATTCACTTTGGAA
A_14_P106902 AAATGAATCGAGATTGCTTTAAAGTGAAAGAAGGGAAAAGAGAGTTATCTCTTTTGCAGG
A_16_P38360542
CTCCTTTATCGTCTGTAACTCACAATACTCCTTTTAAAATTCACTCCATTGTCCATAGCC
A_14_P100733 TTCATAGTTTATCTTGACTCAGAAGGAGGACAGATAACTACTGGCATGAAATTGAAGGAT
A_14_P112063 CAGTGTGTGATGAGCATAAGGAATTCAAGGATGTCAAACTCTTCTACCGCTTTAGAAA
CUST_4148_PI426210822
GTAAGTCCTAAGATGTTTGCTTCCTATTCCTTGTAAGGTTTAGCCATGTATAGTCATGTG
A_14_P135331 AAATTCTTACTCTTAGAGACTCATGAATTAAGAAAGAGAATTCTGCTAACTCAGAGAACC
A_14_P105018 AAAATGTTCTCTTTCCTGCACAAGTTCGCTGCAAACAGAAAATTCCTGTTTAAGATTAAG
CUST_4917_PI426210822 GTGCATGTGTGACATGCTGCGAGTGTGAGGAATGCCAGAGGGAGA
CUST_10229_PI426210822 ACTGGCTATGAAGTACTATAGCAGGCTGAAGGGGCTCGTGGGTTT
A_14_P123598 GAAGATAATAGTAGTTTGAGTTTAGGACAAATTGACTCAGATATCAGTGGATCCTCCAGG
A_14_P100052 CCCCTTCTGTGCATTTAGGTATCTTATGTTATTCAGGCTGAAGGAAAAGAGCATATTATT
A_14_P125112 CTATGAACATAGGACTGATTTCGGTTGCAAAGCACATACTTCTGTAGTTTTAAAGGAAGC
A_16_P03124183
ACTTTATGTGCACGATGACATTTTCAACTACGGAGCCATATTGAAGTTTAAACACTGCTA
A_14_P115433 AAAAGTTAAATAAAAAGAAAGCGCATTGACAGATCATATTCGCAAAGAGTCCAGGACTGT
CUST_2889_PI426210822 TTACCAGCCTTCCTACTCTTTGTGCTGGCAGCTGCAGAGGAACCAT
A_14_P124125 ACACTTGCCATTTAAGGTAATTAAGAAGTGTTTGTTTTATTGCCTTACTGTGCTGTGGTT
A_14_P103742 TGAAGAATTCTAACACCTGATGCCTTTAAGTCATCTTCACACTGCATGGGACTTAATAAA
A_14_P135297 CTCTATCTAGAAGTTTGGTAAACACTTAGTGGTAGGTTGCTAGGCATGAATTTAGTCTCT
A_14_P129449 TTCAAAAGATACTAGAAAATCTCCTTTTGGATCTGTTCTGAGAAATGGCTAAGGACAGTG
CUST_2241_PI426210822 GTGCACGGAGCCCCTCCAGGTGCAGCACGCTGCTCTGTTTCAGAG
A_14_P130865 AAAAATAGAATACACTTCTGCCTGCAAATTTGGGATTTTCCGTTTCTATCCAATTCAGAC
A_14_P115048 CAGGGAAATGATGAAGACAGCACTTTAAATGGGAGGGATTTTTAAGTAAATGCTATTAGG
CUST_2715_PI426210822 ATCTCCCAGCATGGTGGCAGCAGTGGAGTCCCCTCACCCTATCTG
CUST_5647_PI426210822 CACATCCTTTCCTCTGAGGGGGGTCCCGTCTCTGCAGGCCCGTGG
A_14_P137757 ATTATGCCCCAGTTCAGAAATTTGGCATTGATCTTAAACTGGAACATGCTTTTACTTTAT
A_14_P122505 ACATTCATTCAAGCATGGACCAACACAGAGATGATTACGTTGCTAGCCTGTTATTACTT

A_14_P135000 GGGAAAAAGAAACTTGGGAAGGCTATTTTACACAATTAATTACACATTGACACCACAGTT

A_16_P03275705 AGCGGGCCCCATCCAGAGAGATGGAGAGGAAACACTTGCAGAAAG

CUST_1298_PI426210822 CATAGTAAGTTGCATGTCTATATATACATTATTATGTGCCTGTATTATTACTTGCTGCTG

A_14_P109858 ATAGATGCTTGTTAATAGAAAAAGTTGTTCGTGACACAGTGTTCAGTGGAAAACCAGATT

CUST_6412_PI426210822 TTCATAATACTGTTTGTTTACAGATTTCATGAAGGCATTTACCCTGGCATTGTACCACTA

A_14_P128030 GATCAAGAAGTTGCAAATCTTGTGTGTATTATTAAACGTCTCCTTTTTCTCCCATGACCT

CUST_737_PI426210822 CTGGCTCTTTGTACCCTCCAATTCTTCAGGTTCTAGGTGTTGAGC

A_14_P136414 GGAAGCAGGGGGCCATTACCCAGATATTTGAATAATTTCCTTCCATCCTAATAATATTTA

A_16_P17250221 TTCTTTATCGGGACTGACTTTAATTTACACAGGTTGAAAAAAATATCTACTCGTCCAGCC

A_18_P12821637 TGGCTGATATACATTCAAATGCTTCACTTCATCTGATAATTGCCCATGACTAAACATCCA

A_14_P121339 GTTTAATCCAAGGTGGCTCCTATAAATCCTTTTTGAAATGTTCAAGTATCTCTGTCACAC

CUST_2672_PI426210822 AAAGCGCTTGTGGTGCTGTCCTTGGGTGCTCCTTTGCTTTTATCT

A_16_P03650003 AAACAGAGGAAACCATCTCATCAAATCACTCCACATTTTAAATAGAAAAGCAGAGTTCCA

A_16_P38540964 ACTCTCAGTCGCATAGATTTCATTTCGCTCTTGCTATCTCTACTAGTTAAATGACAGGTT

A_14_P101055 CACTAAGTTTAAATGTCTCCCCAAAAATTGATTATTGAAACTGTCCCTTTACTGGTTCCT

A_14_P202455 AGAAAGAAAGAAGACTGGTGTTTATGATGTATTCTACTATTTCTCCAGAGCAACCTGGAA

A_18_P11955786 AGGCCCATTGTATAGAACATCTTACTAACTCTTGAAATTACCTCGAAAAATAATTGCCCA

A_14_P106376 GATCAAGCCAACAACAGTGACGGAATGCCTCCATACATGTAAGTATTCTTTTAGGTTATT

A_14_P104535 ACTCCTATGAGACCATCTTATTTCTTGCAGATGAATGATGCTATGGGATTTGAATTGCC

A_14_P106018 ATTCAAGTGGAATTTCATCAAACAATACTGGCAGTTTGCAACATCTGCTGCTGCT

A_14_P111037 ATGCCAACTCTCCTTATAGTCTCTATATTCTCCACCCTACACTTGCAGATATTTGTTTCT

A_16_P21052139 CAATGGTCTAGCCAACAATTACTGACATTTCCTGTATGCTTGGCATCAAATAAGATACAG

A_16_P18475275 AAAATCTAGACAACTTCAGTCAAATCAGCAGACATCATCCTTACCTCTTCTTTAGCCTAA

A_14_P104458 CTGGCAAGATGTGATAACATGATCAAAGAAAAATGGTCATTATACTATCTGGTCACTTAC

A_14_P100152 ATAAAGCCACAAACTCCAGAATACAGATTTGAATTTGAATCTGAATCTCGTCTGTCCAGT

A_16_P39344494 GCATTCGTTTAGGAAGTAGTGATAAGCAAAAATGACTGGGTAGTTACCAATTGAAAACCT

A_16_P19387802 AAAAAGCGATAAAATGCCAACCTATATTTATCTCTGTCTAAATTATCCCCTGGAGAGAAG

A_16_P01693442 TCCAGCATTTGGTTAATAGTGACTAGGATAATCGTTCATGTCTATTACATTTCCCACAGC
A_16_P03418134 ATTTTTGTGGGCTGTGGAATTTGTTTTGTGTTTTGCAGAATCACTGAGTCTTGC
A_16_P18540520 AACATGCCTGTCATCTATAATGTCTATGTTCCAAATGACTAATTCAACTGGTTTGTTCCT
A_16_P37795103 TTTTTGCTTCCAATTCCAACATAATCATTCTCACAAACTTTTGTCTCAGTTCACCAAGAG
A_14_P109272 ATCCTTTTAAACAAATCCAACAGGAAATTCTTGAGGCACCTTCTCATATAAAACACAAGA
A_16_P00654936 GTTGTGAGTGGATACATACCTGACCTCTTTGAATAAAATGCATGTTAAGCACAGTAATCC
A_16_P21377487 AACATTTTTTGCTTCATTCCCTAGATGGTATCAGTAACTCTACAGACTAACAGCAGTTCC
A_16_P16572616 ATGTAAATTGAGGAAAGATAGAACCTGCTTATTTGGACACTTCATGAAAATCGTGTCACC
A_14_P104974 AATGAAATAACAAAGTGTCAGGTCTGTGGAAGTCCCTAGGACAGTATTTATTAATGAAGC
A_14_P119784 AATAAAACTGAATACAATGTAATTTATACAGGTGACACCACACACCAAAAATCCTGCCCT
A_14_P200284 CTTAGGTTCAATTTTATTCTATGAGCCCTACATTCAACATTCTACCTACCTACACCCTCA
A_18_P14131027 GAAATACACCAGATAGACACATGTCTCTGAGACCTTTATGGTTCAAGAGGCAACATT
A_14_P103181 AAAGAGGGTTGTCCGTGATCCACAAGGCATTAGAGCATGGTATGTTTTAAGT
A_18_P22839049 CTGAGATAGGAAACTTCACATATAGAGATTTCTAATTTAACATTGATGAAGATGCCAAGT
CUST_8107_PI426210822 ATTTCTTGGATCCACAGAACACTCGCCATTGTTGGGCAGACCCAT
A_14_P127392 CTTTTATCTTTCCCTAGTAGACACTGACCTGTGCTTTTATTAAGCAGAAACAGGATACTG
A_14_P130824 CTGTGCTCAATGCCTTCTCTGTGGTGAACCGGAAAAACATGTTTGTTTAC
A_14_P133041 GAGTCTCTCTTAGAAAGTGATATTTTGACTGCAACAGGTAGTATAGGACAAATGCCCTAA
A_16_P02754172 TTTTAACCCTTCACCCTACTTAATGGAAGATCTGAAAGCATGGATGAAAAATAGAGAGTG
A_16_P19186660 AGAAAACCCAAACATAGTCTCCATAAGAGCATCTAAACTTTATTCTCCTTCTCTACCCTA
A_14_P112418 TTGCCAGATCCCTGATAAAATCAGTAGTGCAATTGCAACTATGATGATGCAAAATATCAC
A_14_P202204 CTGCAGCAATAATTACTCCACAACTGCAAACATTGTTTTTGTAGAGACAGCACACTTTTA
A_18_P22841558 CTACACCCTCCTGAATTATCAAAAAGCAAGCCTTTCCGGCGAGAATCTAG
CUST_3716_PI426210822 CTCTCTACTCATGTAGAAACTCTTCTTCAGCTTCATGTTTTAATAATGGAAGAACTTAGT
A_16_P18212694 GGATGGAAGACATAGACTTTTGATGCTTTTTTGTAACTTGCTTCTTGTAATGTGACTGCA
A_14_P105418 TTATTCTAAAGCAGGTGGCATATGAACGAGCATGTGGGTAATACACACCAAGTGATATAG
A_16_P03808860

CACCTTTCAAATTCCTTAAGTTCGAACCCTAAATACATACCTGAGTAACCAAACCACTAA
A_16_P30952460 GCCTCATGTTATATTAGTGTAGGCCTCAGGGCATTCATTGCTGTCTATTTTTCGTTAC
A_14_P126450 AATCTACTGCTATCACCATGATGAAGCACATAGTATACTGGAGTCCTATGAAAAGGAAGA
A_16_P16336540
ACCAAAAGAGAAGTCAAAAAAAATTTGTCCTCCCTGAAAGAATTATCACTACAGGCACTA
A_14_P107476 AACTCCCAAACACACACGGAATAAGATTTCCAGTTTTTCTTCTCTCTTTCACACAC
A_14_P119486 ATTGTGCAAATTCTTGTCTTCAAGCATTTCTTTTCAAATGTGAGGGTTTGTTTACCAGAT
A_16_P20924941
ATGCCTAAGTGGGATGTATTCTTCAGCAATCATGTTTAAGTCTGATTCACCGAAAAGTAT
A_16_P16103695
GACCTCATATCATCTTCTAGCCTCACTATAAGCAAATTGAGAACTGCAATCATTTGATGG
A_16_P15363192
AAATAGTCTTCTCCTAAAACCCTTTCATTCTATGTGATGGACAGATAAGAGATACAGGGC
A_14_P115539 ACAAATTGAACTAGAGAAGCCTTCAATGATGATGTGAGGTGTTTTATTTTGGTTTTACCC
A_16_P35197824
AAGAACAATGAACCTAAGTCTCCGTAGTGAAATAATCAGTGCCTAGACAGTATTGCTGTA
A_14_P134967 ATTATTTTCTCTTCCGCCCTACTACAATACTTAGGATTCAATGCACAGTGGATTAGTTCA
A_14_P120760 AATCTTTTTCTTCAACTCTCATTTTTCATCATTGCTATGGAAGGTCATTCTCGGAAGACA
A_14_P137888 TAGTTTTCACTAGTCTCAAGAAAGTCTGTGGCCTAAAGCATTTAAGATCCTTAGTCCTAG
A_14_P117779 ATTAACAATGAATACATGAATTACCTTTAGTGGGCCCTTTATGTGGTCATCCTGAACTTC
A_14_P117696 ACTAGGTTCTTCGCTGAATAGAATGCCTTAGTGATTCACATTTTGTAGTTAGGGTTAGAC
A_18_P13248465
CATGCAATCCTCTTATATCACACCTAAAACCATTCTACTTATTCATTGTTGACTTGGGCA
A_14_P113175 TTTTCCACACGCTGCTTCTTACTGCTCGCCGCAATTCTGAAAAAAAAATTTTAAAGTC
A_14_P131067 CCACTTTGAGGAAGAGCACTGATATACAAAGTATACAAGCCATTTTGTGTCTCATTAGAG
A_18_P11230713
AACAGATGACAACACATGAACATAACCAGTATATCCAACAGTTTTGGCTAATTGGAATGC
CUST_1441_PI426210822 GATATTGCTTTTAATGGTACCAGAGACTTGCCAGGAGCCCAGGGG
A_16_P38292337
AGTGTAAGTAGAGAAAAGTATCAATAGAAGACGACAACATGATATCAGAAATCCAGCGGG
CUST_2154_PI426210822
TTTCAGGATCTGGAAGTACAAGTGTTCAAGTACAAGTATTCAGCAAGAACAACATGG
A_14_P132629 AACCCTCTCTAAAGAGGAGGCAAGAGGTTTCAATTTCTTCTCAGGGAAAAAAA
A_14_P115294 ACTAAATTTGTCAAGTAGAGCCATTGAGGGTTTTTTTCAGGTAAAATGTCAGTAACCACA
A_14_P139498 TTAGTAAGTCACCATGTGAGGACAAAACTTGATGAACTGAAAAGGCAAGAAGTAGGAAG
A_14_P111184
GTGAATGGAGAAGGAGAAAGTAAAGGTTGTGATGATAAACATAGAGCAAGTTTTGACAAC
A_14_P100388 TCCTATAGTCACTTTGTGGTTAAGGTATTCATGGTTTCCAAAACAATTTTTTGTTCCCAC

A_14_P106338 GAAATAATCAAGCCAATGTTCTGCTTCAGAAAATTTTACTATCTCTTCATCAGACAGGCC
A_14_P132141 TATGTTGGGGGCTTGGAAGCACTTGAACTTTTTATTTTATTAAAACCTTGTTATAAGCAG
CUST_3106_PI426210822
CTACAAATTAGCTCATGGTCACAGTTAAGGCATTTTAAAACTGGTTGGACTAGATTCTTC
A_14_P106369 ACTTGGGTGGATTCCTTTTTCAGGCAGAATGGCTATCTAGGTAACTTTCTTATTTCTTTA
A_14_P136471 AGTTACTTTTTGTTCTCTTCTCTTCTCCACTCTCCCTGAAATGAGAAGTGGAAGATAAG
A_14_P105984 TCCAGTCATGGAATGATTCCTGTTAAGTAGAATTGGAATTCCTCAGTGTTTGCAAGTTT
A_14_P111471 TAGACTTTGGGTTTCAGTTTTACAGGTTTTTACATACCCTGACATCTATCAGTGCAGAAA
A_14_P125591 ACTAACACAGTTCACCTATCTCTGCAACTGACCTTTAATTTGGTTGTTTAATTTGTCCTT
A_14_P131114 ACTGGGGTAGCCAGAAATACAGAATGCTTTATTTCTGTGACTGCATTCCTAAATCTAAAT
A_14_P106077 ACTATACTAGTAGCAAGACGTCTTCCACAGAAAACTAACATTTCTCTTGTTTTCAGTTGC
A_18_P22841390 CAGATCTGGCAGCCTGCATGTCACCAGATGGATTTAGGTCACATC
A_16_P17618996
TGCTATGCCCTGTAAAAGTAACTTCTAGCAACTAAACAATCTATCGTAATTGATTGCCTT
A_16_P39885651
GAAATGTCCCATCAGCATATCTAAATCCTAACCAGTATCTTCTAGCAGAAGTTTCGGTG
A_16_P20498535
GGCACTTTCAGTCTAGGTAAAATCTCACTCTATGGAATCTCCCTATTAAGTCTTGAATCT
CUST_133_PI426210822 CTTTGTCAGGGAGAAATTAGCATTTCCCATCGGAGACCATGGGAA
A_14_P136501 CTTGCCCCTGCTTTACAACTGGCAAATATTCAATAACAATTCCACTTTCTTTTAGTCTAC
A_14_P103673 GAGAGAAAAATGGTCTCCAAATCCATCAATACAAACAAATGCATTTGTTTACTCTCACAC
A_14_P128118 CTGATCAATCTTTGTTTGGCAAATGAACAGAAAAACACCTTAGCATCAAATAACCTTCCA
A_14_P115974 AATGGGGCAAATAAATAGGGACAAGGCTATAAATATCATTAATACCAGGTTCAGGAGTTT
A_14_P120618 TATCTTTTCTTGTTCCAGAAACGGACTCTCTAATTCCCCATTGCTTGGCTATTCAATATA
A_14_P131655 GTTCTAATATTTTTCTGTAGCCAAACACCCCTTTCTTTATAAACCTCTCATCCAGAAGAG
A_16_P20077128
TAGTTCTAGAAATCACATGGCAGAAATTAATTAGTGCAGAGAAACTACATGACACCCTGG
A_14_P105808 ACAGATACTTCAATCACTGCTGATGGAAGACTTCGAGATACACTGTAAAAACTTTGAGAA
A_14_P130967 AGGAATTTTCTCTTATGACCAGAAAGACATTAACATACACAAAGTTTGCAGACAGACCTT
A_14_P119185 ATCAAAAATCAGGACATGTCGTTCTCCTTGACCTCCTTTATCTAAACCATAGGCAAT
A_18_P14133526
CCATATTCATGCAGGTCTTGCAGCCAAGGATTGTTACATTAAGATCCAATCTGAAATTAA
A_16_P15113315
AAAAGAACAATTTCTCTTTACCTTCTAAATCTTGGCAACCAAAAATAGGAGAGCAAGGGA
A_14_P121828 CTGATCTCATCCTCTGAGTTATCTTCAGACACAGAACCTATTATGAATCGAGAGTGCT
A_18_P22840724 TGTTGTGAGGAGAGGATGAGGCCGTCATGGGAGCAGGTCTGACCT
A_14_P111028
TCAGCAACTATGGAAATACTTCCTGGCAAAATAGGAAACAGAACAACAAAGGAAAGAAAA

A_14_P100942 GGGTATGTTTGCCTGGAAGTCATAGATATTTACTGGGTGCTTTACTGGGTTTG
A_16_P01337715
GGAATAAGTGTGTAACCTGAACAAGAAAAGAGCACTGATCAAACTACAAAAAAAGGGAAC
CUST_3328_PI426210822 CTGCTCATGCGATATGCTCTGCTCACACAGATGGCACTAAGGAAC
A_14_P137509
ATTTTAAAAAATAAAAAGATGAGCAAAGGACACTGCAGTCAAGGAGCGAGAATGAGAACA
A_14_P201656 AGTCTGCTTCCGAATGAAAACCCCCCTTCATATTACAGTATTTTCAACTATGGGTAAG
A_16_P00685599
GTATTGGGGAGAGTTATTTGGTAGCAGAAGACGCAATAGATGCTTTGAATTCTGCAAAA
A_14_P127205 AGACTACTCTGTTACCAGTCATGAAACATTAAAACCTACAAGCCTTACTTCTCTGTGTG
A_14_P123594 AATTCCTGTTTCTGGTCATTACCTAAAATGAGTGCTTTACTTGAAATGTGTGGGTATTCT
A_14_P127592 ATTCACAAATAGTTCATTCTGAAAAAAAAAATGATTAATCCAATTACATTTTCCCCCAAA
A_14_P118487 TAGAAGTCAGTTTTTTGGGTCTCAAGAACTATTTGCCTTTGGATGTTGTCTTTTGAAATC
A_14_P115621 AGATTTTACTCTGTTTCTCCACGACTTTATATGCTTCTCATTTTTCTTCATGAGGCTCTG
CUST_7017_PI426210822 CACACAGGCCATCTGGAAGCTTTGGCCTCCAGATGAGGACATAAC
A_16_P18938536
AATTCCCCTAAAGACCACATATTCTTCAAACAGTCATACTCAGTTTCCATAACATAGACA
A_14_P107440 CTAAGGAGATGAGCCGAATTCTAGAGAGAAAAAACTTAGATGAGAAGGAGATGGACAAC
A_14_P133690 TACTTGGTTTAGAATCTTGGTTCTGTCACTTGCAACAAAGTTTAGTTTATGCTAGGCACA
A_16_P20860485
AGTTGTGGCCAATTATATCTGATCACTTATGACTATGTCTTCCTTGCTTAAATGTTGTCA
A_16_P36469502
TTTGGAACAGAAAATGAATGCACATTCAAAATATGTCCACATACCAAATTTTTGCCCAGG
A_14_P120664 GTGGCTGGGGGTAGACTTAATAAAGAGAGAAATTCAAGAACTGCTTGCTTTATTTAGG
A_14_P139040 CATTTAGTAGGTGGACTGATTCCATTGTGCCATGTTATAGAATGGCTTTCAAAACTATCT
CUST_10266_PI426210822
ATTATTAAATTCTGTTTTATTTGTTTCTTTCAAAAATTAACAGATTTTTAAAAATTGAAA
CUST_8771_PI426210822
TTTAAACAGCCCAGCAAATAACCCAAAACAAAAATGAACAATGCACATTTTTATCTATGC
A_14_P139681 ACAGTATATCTGAGAGTCTGTAAAAGCAGCATCTCAGCACTTCACGTAATCAATATGTGA
A_16_P00009054
AAAGGTCTGTAGCATGTATGTTTCATGTGTTTAATGAATTCTGTAACCATCAGCCATTCC
A_18_P22840477 GAATGAACTTTGGATCTGGACCTTTAGGGGTAGACCTGTGATAGAGTCTG
A_14_P105573 ACCAACTCTAATTCTAGGAGATCAGATCTTCCCTCTCAAATCTGTGGTTTAAAATGAAAT
A_16_P17632359
ATCTCCACCTTTATTCTTTCAATTCTCCACATCCATACTTCCATGTGTCAGTCATACTAA
A_14_P106607 CTGTATCTGCAGGTTCGTAAGCATAGAGACGATTTGAATATCTTCCAGTGATATCGG
A_14_P119272

TAAAAGACAAAAGGGTAAAGAAAAGGGTTGCAATGTTGTTTTTGGAAGAAATGGCTTTAG
A_16_P18921801
CTATGGCCCAAGCATAAATACATTTCCAATACCTTCTGAAAGGCTTTTTAAGTTCCACAG
A_14_P139427 TTAAGGCTTGACGCTTGAATGGAGTCTTGAATGAGGAAGAAGAATTGTCCAG
A_14_P139114 GGCATCTTTCTTATACTCAGTTTTTTCTGCCTTAATTCCCATTTACCAGCAGTTAACTCA
A_16_P19988635
GGCATTGTTGTTACATACTTTAGCACCTACTTACTCTAATTATCAACCAGTCATCTGTCC
A_14_P126699 CTCTGCTCTAAAATTGTTGACATTCATGTCTCTGAGTTACAAAAGTGCTAATTCACTACA
A_14_P137324 CTTGTGTCTTGAGGATTGCTCATCTCCCATGAGGCTTTAAATCACTTTTTTGTTCT
A_14_P102972 ACTGTCTTAATTTGGGATGATGCATTTTATATTCATAAACCTTCTTTCTCCAAAGCGCCT
A_16_P19801273
TTTGCAAAATGGGTTATGTCTTACAACTGGTCTTTTCATGCATCCATACTAAGTGATGAG
A_16_P00605995
CCCCAATTCAATGTATCAATTAACACTCACAAGATAGACAATTCTGACACTAGTCAAGGA
A_18_P14132708 CAGTTGGGCATGGACTCCACCAATTTCATTCTCTGACTTAGCGTTATG
A_16_P21294782
AAAACAGAAACTACTACCTTTAAAAGTCCCTTTTTAAAAAAAAAATTCTGAGCAGTCCCT
A_16_P00532291
AAACACAAATGCTCAACTGATTTCATTCATCCAAGTTACATATAACAACAAGTGCCTTCA
A_14_P109171 TTCCCACTTTTTAGATACTGGGGAATATCAATTCTATCCTGGTGAATTAATCTTGGCATC
A_14_P129917 AAGATGGGAAGAAAATAGAACGAATGCAAAGGAGAGGAGACCAGCAAACAG
CUST_5865_PI426210822 CTGGAGGACTAGACAGCTAGAAAAGCCAGTTTCAGGGACAGGAAATA
A_16_P17873742
ACCAAAGGATCAAGGTAAAGTCTAATATGGTGCTACGTTAAAAGAAGAACCTGTCAACTA
A_16_P01880223
AGCAGCCGTCAAGATTAAATCAACCTATTTGTGATTTTAACTTTGCTGATTGGACTCAAG
A_14_P113390 TTTTCCATCCATTATGCTGGCATCACACTCAATTTCACCTAACAGATTTAGATTAGATCC
A_16_P00461630
AACATTGTTATTCATTTGGCTTTGTCAAACTCTGTAGCATCGTCAAATGTAGAGAGTTGC
A_18_P22841259 GTGGCTTTGTGCTATTGGCATGGTGGGTCTGAAACTCTTGTAGAC
A_14_P105873 TGAGAGAGTTAGCAATATTTTCCAAATGTAGGCTACTTCCACTCTGCCATTTTTAGTTTA
CUST_1770_PI426210822
AAAGCTGACAGTTGAACCTCCAGGGAAATAAACATTTTATTTTCTCATTTTATCAATTGT
CUST_3739_PI426210822 CACCTGTTGAATGCCTGTGCATGCAAACATGATGCTGGGCTGAGC
A_14_P113711
CTAAAATTCTCTCCTGCCTTCTGTAACAACAAAACAAAACAAAAGAGGAGAAAGCAGATT
A_14_P123602 GACACAGCCTTGACTCAATACATCCAAAACCCACCATCTAAAATAAACTTATTCTCACTT
A_14_P110224 ATTGCCACAGTCAGAATCCAGTATGTGAACTAAGCAAATGATCAGAAATAATAGGTTTGG

A_16_P15723963
CCAACCACGTCTCTCTAATAAGGAAATACCAAAAAATACTCTACATTCACATCCTAGAGA
CUST_1300_PI426210822 TACTTGCTGCTGAAGTAGGGAGTTGGCAAAATGGGTTGCTAAGTTCTAG
A_16_P15016856
ATAGCCATGTAAAGTTCTTTTCCAGTGTAAAATCAAAGTTTCTCTGTATCCCACTTCACC
CUST_653_PI426210822 ATGTGGGGTGGGTAGAATAGGGCACGGGTGATGTATTTGACTACT
A_16_P18254696
AAAATGGAGCCATATGTGGATAAGGTGATTGAGTAAGTATCATGATCTCTTCATACCTGC
A_14_P119876 GAGAGGTCACAGGTCAGCAAAAACAGTCAAAAAACCCCCACAGATTTT
CUST_1691_PI426210822 CATCTGTTATCCCCATGAGCCTTCATGACCAGTATCTTTGGAGTTTTGTCTATA
A_14_P106774 CTCTTGCTTTCAAATGTGGAGTCTCCTCTGTTGTAACTCGTGCTACTAGTAAATTGTTTG
A_16_P58424459 CTTAGTTTTTTATAATACCTCTAAGGCCCTAGAAGATCTGACCCCGTTGCTGC
A_16_P41629438
AAATCTATTCCCCTTCGATTTAGTGCTTGGGTTTGTATATTCCGATGAACTGATATGCAA
A_14_P123702 TAATGAGAAAATGAATGCTAAGAACAAGGAATCCTTACTTGAGGTAAGGTTAATTTTGCA
A_14_P135366 GCAAAAGGGATAATGATGGAAATGACTGAATTAGCATTGATTTGGACATCAGGATAGATG
A_14_P129137 ATGTTCACATCAGGTTTCACAAAGTGCTCTCCAGCATTCACTAAAAATGATAAGAAATCT
A_14_P127462 AAAAAAGTTGCCTTTTTGAAGTGATGTTTTTTGCTGTCTTCTTAAACACAAGGCTTTTTT
A_14_P136884 GTAACTGCTATTGATGTCACACTTTGTGAAGTAGTCTTTGTTGCTTAAAAAGGGTGACAT
A_16_P16947622
ATTTCACCAGATGTGAATTTCAAGTAGAGCAGTTGCTATTAAGAGTTGGTGCATGTTTCT
CUST_3263_PI426210822
GAGGGAATATGCACATCAGCTTTTCTGAGAAGCTGCAGATGTTCATAAGATTGTT
A_14_P127976 CTCCAGATAAAAAAATCTCAAACACGATACAACATCCAGAGATGAGAAACATCTGGAAGA
A_14_P112653 TAATGCTGATGTTGTGCATAACACAAACTCTGTTCTAGTGCCATTTTCTATAAGCACCTT
A_14_P202305 AAGACTTTACTGGAGTCATTTCTACTCCAAGAGAGAATAGATGAACATCTTCATACCAAA
A_14_P126201 CTTACAGAATGACGTGGCCGCACTTCTGAAACCCATATCGGAAAAGATT
A_14_P105137 CATTTCTGAGAGTGAAGATACAGAACACTGCAGTCAGGGGAGAAAACATATTTTTTTTTC
A_14_P123264 TGCAACTTCATCTAAGAGAGTGTTACATGAAATTTTCTTCAAGTTTTCCTTTGGGGACAT
A_14_P122689 CACAGCAGAGACTGGAGTTGAGCAGACAAATCTTTATTCCTGAGGCTAAAATATG
CUST_7525_PI426210822
CATTTGAGACAAAATAAGAAACACTTGTTGAACGAGTAGATGGGCTTCCTCTGG
CUST_1597_PI426210822 ATGGTGGGTGTACAGAGTGTCCGGGGAGGGTGGGCAGTGGTGAGT
A_16_P15036083
ATATCTGACTTCCTATCCGTATGGTAGACATCAATGTCCCAAGAAAGTTGAAAGAAAAGT
A_14_P131786 CAGAGGCATCAGACATTGGGGAGTGATTCAAAACTTTTAAATTTGACAACTGTAAAAGAA
A_14_P119616 TTGCTGCTAGGAAAAAAGAAGTTCCAGAGTGTCAACCTATATCACCTAACAGAAATTCAA
CUST_9989_PI426210822 AGTGTTGTGGCCGTGCCCCACTTCCCCTTATAGTCTGCATGCCCT

A_14_P128039 CTCCTGTGTCTCTTTTATTCTTGGCAGTCTCTCATCACTTTTGTGAACAAGCAC

A_14_P104538 CTTCTACAACCTGAAATCTGTTTTTACAGTGAATGAAATAGAAGCCACTGCAATTGTCAC

CUST_10065_PI426210822

TTTTCCCTAAAAGTCACTATTACAAACAAAAATTCGCCTAACTAGAAGTTTTGTTCTATT

A_14_P138840 ACTTTAGGATCAGATAACTTGAAACCTTTTAGAAGAAAAAGAGGTGAGAGTTTTTAGCCA

A_14_P139013 TTTGGCCGGCTGTTTTCTAGTTCAATCTCAATTGCATCCTGGTAACTGGATATTT

A_16_P21123418 AGGCTGAGACTGGATCTTGGGTCCTTCAGTAAAATTGGAGTGCATC

A_14_P125075 AATACATGCACTCCCATATATATATGTGAGTGCGTAGAAACATCCAGAAGGATAGAAACC

A_14_P137587 ATTAGACATTAACTGGTTGTCCAACTTCAACCACATCAACTACAAAATCCTGAAGTGAAA

A_14_P130942 GAAAGGGTTTGTCCAAGGTCAGCAGATAAAGACTAGGACCAAGTCTCTAC

A_14_P119385 GCCAGTTGAGCATTGCTTACTTATGGTTAAAGTGCTTCTTAAAAGAACCATAGTCCATTT

A_14_P132478 ATTTGCTGCAGGTTTAGCATTCAAGGCTGATAAACTGCTAGAAATAAGCAGGCAG

A_14_P201893 GAGACACTGATGTTTCTTCCTTGCCTTCCTAAACAGCAACTCTTGGTGTTATC

A_14_P120536 CTTACTAGGCAATTTGGCATTAAGAACTTTCCTCATAAACATTTACAAATATTCTGCTCC

A_14_P129089 AATAAGTGTAGGAAAGTGAATGATGCGCACCTCTGCGAAACTGACAACACTAAATAAATA

A_14_P107597 CATATGTTATGTGCAGACAGACGTTTTACTATAATGGTGCTTAAAATGGATCGCGAGTTC

A_14_P113140 GAGGAGTTTTCTTTCTTGACATTGTCATCTAAGTAGGTTGGTCCCAGGGTATAAA

CUST_8309_PI426210822 TCAGGTACCCACTCCTGGAGACCTCCTCTGTCCGTGGGGGCTAAC

A_16_P18172493

GATTCCTGAACATCTGCCAGAAAGAAGTTGCTCATTTCAAGTAAGTCAGTGATTTAAACT

A_14_P129297 ATTTTGTGGGAATCGTCTACTTCCTGCACTCAAGACCTTTTCAATGTCTTTATTCTGTA

A_14_P201626 AACCAGAAGGCAATAGAGGGAGAGATTCTGTATTTAAATTTCACTAGTGGCTTTACTTTG

A_14_P101212 CTACAGAACTACTCCTGGGACTTTAGCACGTTTCCTAATTGATAATCCTTCTGAAAAAAT

A_14_P139753 TAGCAAAAGGCAAAATCTTGTGTATTAGCCACGTGAAGTATCATATGGCAATGAGAAATA

A_16_P17171963

AATTAATCACCTGAACTTGGATATAAATGACCACATCTGTCAAAATCCTTGCTCCATGTC

A_16_P39146844

AACTTAATTCTCCTCTTTCGAAACCAATTTGACAACACGAAAATAACGTAAAGCAGCCTT

A_14_P107342 TGTTTGTTTGTTTACACATGGACCCTAAGTATTCCAAAGTGAATTATGATGTGGGAAACT

A_16_P16287796

AGGGATTTCACTAGATCATTCAACTCTTTAAAATTGTGGCCTAAGACAGATAGGAACTGT

A_14_P107965 GATGGGCAATGAGTTAACTTCCTTTTGATAAAATAAAATCGGCTGACTTCACTACTTCAG

A_14_P135895 CTTCTAGTGTAAAAGTATAATTCTCCAGTATTTCATGGGTCTGCAGAAGCCCCATCTATG

A_14_P110099 ACTTAGCACTTCTCTGATGCATCAGAGATTAAGAATGTTTTTTCCCTTTTTCCTACCAAG

A_14_P109055 AATTTTTCTGTGTAAGACATAGGTAATACTCCAGCCATGTGCTTCCCCTACAGTTTAAAG

A_14_P120005 TGTAAAGTTACATTTTAAAATAAACAAAAAACTGTTCTGGACTGCAAATCTGCACACACA

A_14_P103407 GTTTGTTTGTTTGTGAAAAGGAGATTACATCAGAAAGGGCCTAAAAATAAAGGCCAAATC

CUST_7195_PI426210822 CTCAGTTGGACTATCTGGACTTTTTGATTCTGCTTCTTCCCAAAGTGCTC

A_16_P58424210
AGACACACTGGTAGATGTTTAAGTGTGTATGCGTATGTATGAGTCTTCAGTTTGAGTAGT
A_16_P38361714
ACTTGGATACAAACACATCACATTGCTAACATTCCCTTACTCCTTCTACAATGATAGGAT
A_14_P102761 GTTATAATGCACTGTAGATGTGAGTGATCGGTTAACTTTAAAATGTCCCTTACTGTGAGC
A_14_P124448 GTTCTTTATAGAGCCCAAATCTTCCACCAGGTGTTCCGATCAATCCTATCAAG
A_18_P22839219
TCTCCAAAGTCTTTTTGCATAAAGATTTTCTAATTTTTTTGTTGTTTTTTGTTTGTTTGT
CUST_2235_PI426210822 CTCGAGGAGCAGCTGGCTCTGACTGGGGATGGTGCCTACAATGCG
A_14_P137538 ACAATCTGAACTACTCTTTTCTATGCAGATGGTATGGTGTATGCTCTGGGGG
A_16_P03359514
CTGCAAAAGGATTCATCTACTCACATAGGCCTACATTTTTACTTAATATAGATTTCGCCA
A_16_P00679807
GGCATAAAGTATGGCTCCAATATAACTTTTCCCTGCTATTCTAAACACTGGTGTAAGAGT
A_16_P02737952
AAAAAAAAGAAAGTTGCATCTTAGAAAGGTTTAGGAAGGACAAGAATCATGCTTCCAGGA
A_14_P115007 CGTCTAACTTATAGGACTTCCCTTTATTCACAAATTATGACCCACACGCAGTTTATTCTT
A_14_P138949 ATGGAAGCAAGAAAAAAATAAGGTAGGCCACTCCTTTCCTTTAAAGACCTTTTATAGAGT
CUST_717_PI426210822 TACAGCAAGGGGGAAAATTGATCCCTTAATGTGGCTTTCTTCTCACTG
A_16_P15817495
AATGTCACAATCAATCTAGACAGAGCATCCTAAAATCTTCTGGCCTCAATTGAAAGAATC
A_14_P126195 GCAGATATATTCTGTGGATCAAACCAGAAGAAACAGGTAAGAGTCATTCAGTTTTTTTCT
A_14_P127921
GAAAGCATCAACAAAAGGAACCAAAGAAGTCAAACGAGAAAACTCAATGCTCTGATAAAT
A_14_P102464 ATATTTGACATCTCAGAGAACTTCTGAAGAATAACTGTATGACAGACATCAGTAGTGTCA
A_14_P120234 GCACAATTAATACATAAAAGGCATTTTTATGTGGCACACAGTCCATACCATATGACCCAA
A_14_P138672 TCTGCTCCCTGGCTGGTGGTATATTGTATAAAATAAGATCATCTCTTTTCTTCTGCTTTT
A_14_P130989 AACATCGGGTTTTACACAACTGTTATAGGCAAGGAAAGAACATTTGAGACAGAAAGTATT
A_14_P127570 AGAGGCAGAATTTGATTAAACCTGAAGATCTTCTAAAAACCTAGTTCAGAGTAGATGGCT
A_16_P36019348
TGCCCACTTACTATAACACACTTATAACATGATAAAGAGCAACATAGGTAATGTCCTTGC
A_14_P121640 CAAGACACTAAAGAAGAAACAGTCGAACTTCTTCCAAAAGCCAGATTTATCTTCCTAAAG
A_16_P18776292
AATTCTCTAAGCAGCATCTATTTCAGTAATTCTCCCCTTTTTACTTACTTGAAATGGCCC
A_14_P110975 ATTACTGGGAAATTTTCAAAAAAATTACCAGAAAATCTGGCTCAGCAGATGCAGAATTCT
A_16_P16743616
CCCTGGGTATAGAAAGTGACAACTTCTGCAGTTTATCATTGGAGCACATTATCAATTACT
CUST_7153_PI426210822 AAAGTCACAAGGCCAAATGGCATACCAGGTTTTTGGTCCTTGTTTAAGAAAA

A_14_P129487 TTAAAGAGGTAACCTGAATCCACCAATTGCTGAACAATGGAAAAAATACAACTCGAACAA
A_16_P02944844
TCCCTTCTAGTTAAAGTCTACTAAACACTAACTAACTTACTCTTTACTTTCTCAAGCACT
A_14_P101189 ACAAGATTTATTATCTTATTTTAAACTACAGAATTCCAAAACACAAGGCAAACCCGGGCC
A_18_P22841938 CTCTAACTGCTTGGAAACCACAGCACACATCTTGCGTGTGTTGATTACTG
A_16_P58424334
TGTCTAGATCTGATAAATCAACGAGTAGTAGTATCAGCATGTTTTCTAGAGATATGGAAA
A_14_P133774 AATAGGGAGTCCTTTGGAGCAATAGAACATCCCATCAGTCTAAAAAATGCTCAATTAGAA
A_14_P122151 TTTCTGAAGGACTTAAAGCCCAGTAATATAGTAGTAAAATCTGATTGCACTTTGAAGATT
A_16_P01485033
GCCTACTGATCCATAAGATGTGCTAATTGTTACCATCGACTACAGTTAACTTGAATTCCA
A_14_P109310 AAGGTTTATACGTTTCAATACATACTGCATTCTGTGCTACACAAGCCTTAGCCTCAGTG
A_14_P123379 CTGATACTGGAGCAAGATGGAGATATTTCCATTTTACTCAGCAAGTCGAAATCATTCAAG
A_16_P00692018
GGGTCTTAAAAGGTTTAACGTAGTGAGAAACTGGTTCAGTGATCCATGCTTGATAAAATT
A_14_P118594 AAGTTCATGCGCTTCATGATGATGCGAGCAGAGAACTTCTTTATCCTTCGAAG
A_14_P138551 GGGAGTATTCAGGTTAGAAGTGCCATGTGTTGACATTATTTAAGATACAGATCTGGTTAC
A_14_P137113 TTCTTCCCATTCTGATAGTTTGCTCTTGCTGTAAGTAATACTCCCCAGAAAAGACAG
A_14_P135558 ATGTCTGTGCATCGTGTGTAATAGATCTGATCGAAGGGGTGTGCATACGAT
A_14_P109005 CCAAGGAAAACTCATATTGCTAATTTTGGATGGTCTTAGTGTCAGACACTTAATTGAGCA
A_16_P15337830
GTTCGAGACACTTATAAGCAACTTTCTTCTTAGTTCACAATGTTCCATGAATGTCAGTGC
A_14_P109693 CTTGTGAATCATTTATAACTGCCACATCTCAAACCCTGTGTTTGCTTGGTAACATACGA
CUST_2874_PI426210822 AATGGAGAGTTTGGGGAGGGAAAAATGGGCAGGCCTTATGGAAAT
A_14_P107553 GATTGATACCTCCTTAGTGAGAAGGGGCTAGAAAGTAATATCAGAAAGTAAACTTTCTCC
CUST_8358_PI426210822 CATGATACATAACACATGTCCCCAGCTCACAGAGGATGTGCCTTCT
A_14_P133602 CACACACACACACTTGACTTTCGGAAACTTCTTTTAAATTAATTTCACTGACACTAAAAG
A_14_P201882 CTCTGCACTATCCTACATAGGTCCCTACTATCAGTTCAAGTTTCTGTTTTCCATTCAG
A_14_P107467 AGCAGGTCCTAAAATGTTACTTGCTCTTACACCAGGAGCATGGCAGAAAAT
A_16_P38013111
AAAAAAAAACAGAATAAATGAGGCAACATATACATAATGAAGAGAAAAACGAATAATTGT
A_18_P13379011
ACCTGCATACATTTTATAGATTTATTTGATGAGCATCTCTACACTAAGACCACGAAGACC
A_14_P134430 CTTTGTTCTGGATGTCATCTCTTGAAAGTAGAAAACTCCTATGTGTTTATCACATTGCAG
CUST_5851_PI426210822 ATGGTGTTCACTCAGTCACACTGTTGTCGCTCTCATTTGCCAATGTTAA
CUST_3291_PI426210822
GTCTTCTGAAAACTTTTAAAATTTGTTTATTTTTTGAATCAGTAGTGTGTATAGTTAAAA
CUST_8990_PI426210822 AGAAGCAGGCGATAAGTCAGGGGGTGGGTCATGTTTACCCGGTTG

CUST_4146_PI426210822 AGGGAAAACTTCATCATGATGGGAGGGAAGAAAGAAGGTAAGTCCTAAGAT
A_16_P19012783
AATAAATTTGATGTTGCTCAATGGACTATTGCTAACTCCAAAGATAACAGCACTCTCCCT
A_14_P134053 GTTGTCTCATTTATTTTTAGACTCCCAGCAGGCTTTGGAAGGGATTAAGTAGACAAGA
A_16_P00337056
AAAGTCAATCACCTGATGATTGTTTTTAAATAGCATCTCATGAACTCAGACTTACCCACG
CUST_1688_PI426210822 AATTCTGAAGGCTCAGGGGAAGGTCAGGTGGGTTTCAGCAGTGAG
A_18_P15835679
AAATGCCTCATTTATTCAACTCTGGGATATCATGAAGACAACTAAGGTTTGACACAACAG
A_14_P104873 TAAAGAAATCAGTTACTTACCAGGCTCACCTTTTTTCTTTGAACCAGACTTCTTCTTGG
A_16_P20350449
AACCTTTTTCAACTTGATCATAGATGCAGACGACATATTATAATCCACGTTATCCTTGCT
A_14_P116329 AAAAGAGTGTATTTGGCCACAGATGACCCTTCTTTATTAAAGGAGGCAAAAACAAAGTAA
A_16_P00598901
TTTCATGAAAACTCACTTTAAAAAATCCAGTAGCAAGTTGTCCTAAAACTGCCTCTCTGC
CUST_3958_PI426210822
AGTCCCAAGAGTCACCTTTGCTGATTTTTTTCCTATTATTCTTTCCTGTTATTCCATATT
A_16_P18690961
TACAATGTTCTAGTCAGATTACACAGAAAGGGCACTAATCATTTCTCTTCCTAACTCACC
A_14_P104129 AGTAGAACAAGTGCTAAACTTTGGTAAGTCTCTCAGGTATACCTTTCTCAGGGTAATATT
A_14_P136553 TTCTTTCCAAGACAAACTGTTTCACAACAATCCGGAAAATATCCAAATCACAATTTGCTT
A_14_P113420 AAGCAGAGAATGAAAGATTCTGAAAATAAAGGTAAGGGGCGGACTGCAGAAAGAT
A_16_P01391816
CTTCCTAAATTCTTCCTGTAATGACTTAAGGCAACGTATTCTTTTCTCAGCACTAACCTC
A_14_P117965 AAGGGGATTTTCATGTTAAAGAAAATGATTCACAGTTGTGAAATGGTTCAAACACGGTAT
A_18_P14135034 CTTTGCTGGGTCAATGTTACAGCCATTTATGTCCCCTCAGATGTTGG
A_14_P117820 CTACACTGAACAATCTGGCTTCTTTCCACAGTGTAGCATTTTGCTTCTTTCACTTTTTTA
A_14_P122301 TTAACTGGCGCATAACAGCCTTCACACACAGCCTCATAATTAAACATAGACATACATATG
CUST_6246_PI426210822 CTCTTCACGGATGAAAAGCCAAAGGCAGCTGTGGCTTCGCTTTGT
A_16_P16370431
ATGATCATGGCAGGACATAAAAAATCAAAAGTGTCTTAGAGTCTAAAGAAAGGTGCTGTG
CUST_9352_PI426210822
CCTCGTCTGCTTGGCCTGTAACGTTGGCAATTATCATCATCATTATCGTTATTAATATC
CUST_8672_PI426210822
AATATCATACCATTCATTCCTCCTCCTGCAAAACCAATTGAAAATATTGGCCTCTCT
A_14_P132301
GAGAGAGTCCATCTAGAAGAATGTCAAACAAACCACTGAAGTAACGTGTTGACAAAATAA
A_14_P116299 GATGTATTAACAAGACAATTTTGCCACATATCCAAAGCCATGTCTTCCAATCAGCGTTAT

A_14_P134366 GAAGTGCAGTGGCAAGCCAAGACCTATTTCCAATACATTTGTCAAAACTAGATTTTTTTT
CUST_8298_PI426210822
GTGCAAGACACTGGATTAAATTGAGACAAATGTTTGCTTCTCAGAATGGCAGTG
A_14_P101766 GTGTTCTTTTGGAATGAACACTGTTTTCCATATCTGATCTAAATGTCCTCAGTCCTGATA
A_14_P201313 CTGATTTTCATCTTACTGTTCTCCATTTGCAGGTGAAAATGGAGGTTTCCTCCT
A_18_P14784465
TGTGTCTGCCTATTTCCATGATTCTAAACACACTGTAAGAAGCCATATCACCATTGAATT
A_14_P124530 CATCTATTGGAAAATAAACAGCATGGAAAAAATATCCCATTGTCTCGCACATTCTCTTAA
A_14_P110303 TCATCAATGTTGGCCTAGTTTAAGTTCCAAGAACCCAGAAACAAAGGTTTAATCATTTTG
A_16_P02019063
ATCCAAATCTACCCAGAAAGTCTAATTAAATCCTTACGGATCTACTTCTAATTGGCTGCT
A_16_P41637097
GGCTCTTGACATGCTATATTGAAATCTGTGCAAAATTAGTCAAAATACACTACACACACA
A_14_P127748 TCAATGCCTTCATGTCTCAGCAAAGTATGCTTGGCTTTAATCTGTTTCCTTAAGAACTTC
A_14_P126408 CTCGCTCTTCTGTAAGTGTAACTCTTCTTGGGTGGCTTTGTCCTATATTTTTAAGTCATT
A_14_P137780 TGATGAATAGACCAATGAGTAGAGTTTGGTCATGAGCCAAAGATGATTAAGTGAATGCTA
A_14_P115219 GAAAACCCGATAAAAATTCTTTATTGGGGGAGGGGCTCAAACAAGAAAATAATCAACA
A_18_P16801567
TGTATCTCACTAAAATCATCTCTCATTGTCTAGTCCAAGAAAACACATACTAAGCCCAGC
A_14_P202106 GACCTCATGAAATGAGTTGCAGCAGAAGTTTATTTTTTTCAGAACAAGTGATGTTTGATG
A_16_P21263512
CCCTCACACTTGGAAAAACAATTTTCTAAAATTAGGATTCCATCACCAAGGAGAAACATT
CUST_7970_PI426210822
TATTGGTATGGAGACAGATTTTCTAAGTTCAAATAGTATACTACAGCATCTTCAGACATT
A_16_P17675028
AAAAAGAACACTTCAGGTAGACTAATTCCAAGAGCCAGTTAAACTGAAATGGAAACTAGG
A_16_P00417654
AAACATAAACTTGGTGCCATATAACTGACTAGGCTGATGATAATGACAGGGAGAAGTAAC
A_16_P01230561
AATGCTCACACTTAACCTCTACAACTAACCCAAGAACTATCTATCCCAGATTTTGTGTTT
A_14_P101028 GTATCCCTGAATTTCATACTTACGGCTCGTTGATGATCCTCATAACATTTAGGCCAG
A_16_P19102846
ATCCTGGTTCTAACTAATTGCACAAAAATTTACACGATTCTTGACCTCTTCAACACAGAG
A_14_P127295 GGACTGATACTTTGACTTTTTATCTCCAAGTCTTCATCAACTGGATCATTCCAGTTCTAA
A_14_P125410 GTCATGACCAAGTTGGTCTCTGCTCAGATAATCTCACTGCCTTCCAATTATATTTTATAG
A_14_P133246 ACTGCCATGACTGAGTTTTAAAAATTTCCAACTCAACCTTACTCTACCAGACTCTGTAAG
A_18_P22841754
TAAATCTCAGTGCTTCACACACATCAGACAAAGATATTTTGTGAGTGGCTCTAAAAATGG

A_14_P101095 TTTCTGTATCCACATGTGCACTGTATGAAACTGCTGTCCATACTCTTGTCTTAATTGATT

A_14_P114150 TATTTTTCCTTATGTGATTATTTTTGCAGAAATGACCGCTCCTTCATCTGTCCTGCAGAA

A_14_P126638 TCAAATGATTCTTTGAAGGCAACAATATTAGGGTGTTTCATTTTGGCTAAAAGAACAGCC

A_14_P123495 TTATAAGCAACAACTTTCCCCCTATCTACACCTATTTTTCTTGTCATGGTGTTTAAGGAC

A_14_P107883 CAGATCAGATGTTCAAATTTCAGATCTTACCATCATCCAACTTAAACTGTTTCTCCCTCC

A_16_P19253419

CTGAGATTGTGCTAAGTATAACAAATGTCTGAGACTCTGGATTTTCTACCTCGATGAGAA

A_18_P10645499

TGTGGGCACCTTATCTTTATGAACGGCATTAGTAAATTAATGGAAATATCCTTCAATGCA

A_14_P109318 GAAAGCCCTTTCTCCTATTTTTTTTTTTGGTGCCCATTCAAATACTACTTGTCTTTCAAG

A_18_P22843234 TCTTAATGTTTCCGGCTTATTGCCATATTCCATATGTAATTTCCTCCATTCCTTTCAAGG

A_14_P108334 CTAGTGGCTTTCTGGAGAAATCTCCCACATCTGCACCTGTTCATTTTATTAAAATAGAAA

A_16_P17729248

AACAACATGCCTACCGTTAATTTCCTAGGAAACAACTTCGTGTGTTATGTTTTGAACTTT

A_14_P108904 AAGTATGAAGTGTAAACTCTTTCTTGGGCCACCAAAGTAACTAAACAAGAAATGCAACAT

A_14_P200607 GTCTCCAGCCCACTTGAAGAATTCACGGAGTTTTTCCGATGTGTATCTATATT

A_16_P58424384 ATGGGAGGATTTATTTCACCTAGGCAGGGAGATCAGGAAGGATCC

A_14_P107254 ATACCCAGTAATCTTTTCGAGTCTTTCACAATTTTTTCTGACAGCCTCAAAGTGCAATAT

CUST_6914_PI426210822 TGTTTCTCAGTTGCTTCTGCAGGCCCTTCCCTTCCTCCTTGAGAG

A_14_P133787 ACAGTAATTTCATCTTTATGATTCTGACACTCAAATTGGGAATGTTACCTGCTTGGTTGT

A_14_P130031 AGCAAACTAAAAAGAAAAATTGGCCTTTGCTTCTTCTAGCCTCCCTTGCAAATATAAAAC

A_14_P108657 CATTGATCAGAAGGTGTATCAGGCTGAGGTGGATAATCTTCCTGTAGCCTTT

A_14_P114409 TGAGATGTTCAAAGACATCTTCTGGTGCTTCTTGAAAGACTTGATTTCTTCACATAGATG

A_14_P137319

ATGTAGAGAACAAACTAAGAATGAAAGCAATACAGACTGCAGAGAAGGGTGAAAACTGTA

A_14_P137007 TTTTTTTTTCTTCCTCTAAGATCCTTTTTCTTTATGAACAGGGCTTCTCTCTTCCAAACC

A_16_P16987720

AGGAGCATTGTAATTAGTCTACTTGAGAGTCAAAGAACATAAATAACAGCACTGCAGGTG

A_14_P111418 CTGGGATAAATGTTTTGAAGAGTTCTAACTGATGCAGGCTAAATTTTAAAGGGCAGTGTA

A_16_P40360547

AGTTCTGCCTCACCATATATTTGCTCTCTGATGATTATGTTCACAAGCATCTATGCAAAG

A_18_P22841030 AAATTCATACCTGAGCCATGCATGAATAATAGGCACAGTGACGTCTGCCATC

A_16_P16191662

TTCCTAACATATTTGAGTGAATTGAGGTAGTTAAATTATTTCCACAAATTGAGTGAGTTT

A_14_P120778 AGTTGGTAACCCGAGAAGATGAGCCTTAAGAAAAAAAAAAGTATCAGGTTCCGTTTG

A_16_P00000465 GTTACTTTGACCTCATTGGCTGTGACTTCCTGATTGATGACAACTTCAAGGT

A_16_P17495150

ACCACATCTCCAATGAAATTTGATAGTGCATACCAGAGTATCTTAGTTTTTAATCTCCCT

A_14_P119690 AGTTTTAAAAATAGAACAAGTCACACAGAACTACAAATTTAGCCCCTGAGGCTGATTGTG
A_16_P39668843
GTGCATTCAGTCGTAATGTTTAAGCCCCTAAATTCCTTCTGTATGTTCTCTAATTCCAAA
A_16_P03042271
TACATTGGATGAAACACCATTTTACCATGACTTAGAAAATCAGGATACGGCTTAAACTGC
A_16_P01816703
TGGTCAAGTCTGAAAACTAAGTTGTCCAGTGTTTTGTATTCACCTTAATATCTTCATCCC
A_16_P17545841
AAACTTGAACATAGCCACTTCAGTAGAATGACAGAACCTTACATAAAAGGGATGATGAGA
A_14_P113903 TGTAGCAGATTACAGTGGACAAGATAATTTCTTACAACGAGTGGGACAAAATGGCTTAAA
A_16_P17098223
AATGAAGTGCATGGAATGATAGACAAGAGTTTACCTCCAAAGTTACAATAACCTGTTTGG
A_14_P104001 TGATTCTGAGTGGTTTAGTTTTATTCAGAGGAAAAGTAAAAAATGCCAAGAGATTGCCTC
A_16_P16931581
CTCTCAGACCTCCGTATTAACAGAAAACTAATCAAAGCCCTCCAATCTATACAGATTTCT
CUST_5987_PI426210822 TCTGAAAGACTGTTTTAGCCTTGGGGCCAGAAACCCCTCTATTAGT
A_14_P115925 TCCTCACCTGTTTTTTTCATGGATCTTCAAGATTTCATTTGTCTGTTGAAGAAGTTGCTT
CUST_3769_PI426210822
TATTTTAGTATGATAGTTCTTATAGATGATAAAGACCTCAGAAAAATCTCACTATGTCCA
A_14_P129603 TGAAATCATGTTGCAAACTATGCAGCAACAAACATATACTATTCCTGTTTGCAGAACACC
A_14_P130068 TTTGCCATCATCACTCAAATCTGACTCCACGACCTAATAAAATAAGAGCAAGAAAAAGCA
A_16_P40488602
AAGAAGAAAGCATTAGACAGGCATTCAAGTCCTTAGATACCTTTTTTCTAACTGGTCCTA
A_16_P40963736
TAGCACATTTCCAGTTTTTAGCATTGTCATTTTGAAGTATAGTGTCACGACTTGAGGAAC
A_16_P35571025
AATGCCATGAATGACAAAATCCGCAAACAGATCAACATCAATAATCCCTTTGTTTTCAAA
A_14_P139450 GTCAATGAATGGGGAGTAAATACACAGATAATCCAAAATTGATGTAAGCATTGGAAGGGG
A_16_P00369434
ATCACTGACATATTCTGCCATGAACAGATAGAGATGCCTTCTTAGGAACATTCAAAACTT
CUST_7912_PI426210822 CTGTGTGCAGAGTCCAGCCTAAGCTGGCTACACTTGGAAACCTCT
A_14_P109429 TCTGTGAAGAGAAAAACTTGCTTGTTACAGAAGCCTCAGAGACAGATTTTAATCTCAATG
CUST_5513_PI426210822 CAACAGTTACAGAGGGGGCCAGAGTAAGAAGTGGGACTGCAAGTG
A_14_P117971
ACCAAACAAAAAACACATTTTCTTTTTTGAAAAAAAGGACAGAACAAGGGCAAAAATCAG
A_14_P103906 TATTTTCCTCTTACTGACAACAAGACGTGGGCTGAGAGTGAAAGGAACTGTT
A_14_P103072 TTATATGGGGGAAAATATTGCTTTATGGGTCTCTGGCTGGGAATTATGAGTGTTATGTAT
CUST_4176_PI426210822

ATTTCTTGTTAAGCATTTTTAAAAGGTTAACTTCTTCCCAGAGAGCTTTTACATAATTAT

A_16_P00694195

CACACTCTATGCATCATACCTACCATTCTTTAATGCATTGTATTCAAATTCACCAGGTCC

A_16_P01010403

CTCAAAGGTTTTCAATCCTACTCTAACACACATAGTGAAGTAAGCCAAGTTTTCAACAGG

A_14_P103845 ATGCTTATTTTTACCTGGGCTATCAGGAACAGATGAGAGATTCTGTTGCTCGAATTTAC

A_14_P138268 TCCTCCTCCTCTTTGAGAAGACTAGAACCAGTTTTTAGCATTGAAATATCTACAACACAA

A_14_P138955 CTATTTATGCTGCTACTGTGGGATGAAGAGTCTTATCATGAGACAGGAATTTTACAGACA

A_14_P128438 CTTCATTGCACTGGGAGGTAGTTTGTGTGCTACAAATCTTAACAACACACGC

A_14_P129398 ACATAAGGTATGATTATAAAAGTGACTGTCTGCAATAAAAGAGAACTTGTTCAGGATATT

A_14_P123535 TACATACACACGGCTGATCCCTTGAAAATGTTACTGAACAAGACAGCAAGAATAAAAACA

A_16_P16895040

TGTCTAAACACTCACAGGAATCTCTACCAACACTACAAATCATTTGGAATTTTTAGAGGG

A_16_P03639380

GAACAACCACTAACCTAATAACAAACGTAGACTTTTTCACTTTTTGTTCAAAGTTGGGGA

A_14_P103255 TGGCTTTTGTGTTTGATGTGACTGGTTCTATGTATGATGATTTAGTTCAGGTGATTGAAG

A_14_P122755 GGTATATCAATTGGATTAGGCAACAATCAATATTTGTGGAACTAAGTGGCCATCTGGAAA

A_14_P137105 CTGCTTGCCAATTACTGTGACATGTTGCTAAGAAAAACACCATTAAGCAAAAAACTAACC

A_14_P110247 GTCTGGTTTCTTGGACTGCACTTTAATAGTTAGAATCCAGATGCAAAGGAAATAGATTCT

A_16_P16191443 AAGGATTGACATCAGAGAGGCCCAGGAAGTTTGACATTGGAGAGAG

A_14_P130703

AAATTAGAAAAGACAACAAAATTAAGCAGCGACCTTGAGATAGCCAACACAGAAATCTGT

A_14_P103448 GACTGGAGAACTGTTTGCTCGTGACACAAAGGCAACGAGCAATTAATTATTCATTAATAA

A_14_P120333 AGCAACTCTTTATTGGGAAACTACAGTATCTGACACATTGAAAGTCAAAAATAAGCCCAG

CUST_3882_PI426210822 TTATGACCCTGCATTTTTGGTTAAAGAAAGGAGGGAACTGAACTGTCTGC

A_14_P104639 GTCCATATTTACTGAGCAGGTTAGATACACCGAAACCTAAGACTTTGAAAAATAATCTGG

CUST_1914_PI426210822 TACTTATAATTGATAGAGCCAGACAGCGGGACTCCTGGACCCAGTTTATAAA

A_14_P127002 TCCAAACAAGTTTTTTCCATTCTGAACAAAAATGTATGAGAAAAGACTCCTCTTCTCTCC

A_14_P120125 TGACGTTTTCAAATACCCCAAACTCCAACTAAGATAACCTGGAACCTATTAAATGGATAT

CUST_8765_PI426210822

CACATCAGAAAAGTCCTGGATTTTATGACTTAGCTATGCTTAGAGGATAATTGCAACAAG

A_14_P102790 GAGGGTGTAAAGAAAGACTCAGAGGAAATTTCAGGTATTCTTCTATAGGGCTCAGATG

A_18_P17044234

GGTATCTCATCCACTTTCCTATCCTCCAATCCCATTAATGTTATACAAAAACACGAAACA

A_14_P136999 CTATCGCTGCCTGCAAATGTTTCGCCATCTTCTTGGACAATCCTAAGAG

CUST_4929_PI426210822

TGTTATGAGGAAATACACTGTTTCAAATGCAGTTTTCTTTGTGGTGTCTCAGCAGATAC

A_16_P02991500

ATAAGGCAGTCACTAAATAGTGAGAAAAGTCACCTATTTACTCATGTTCAGATGACTCCC
A_16_P35467019
AAGGGCATCTTTGAGATACATGTTGACTTCTAAAGACCAATAGTTCAATTTAATCCTCCA
A_16_P18150936
AAAAAAAAAGTGAAAGGTATACACCCTGTATCTAGTCACTTCAGGATCCACTAAACAAGC
A_14_P134418 ACTTCAGTAACTTGCCTGAATTGAGAGAGCAAAACAGTCCGGAGAGAAAACTTAAAAT
A_14_P128043 ATCTCTTTGATGTATGCAGGATACTGGACTTGATTGGTTATTGTCACTGTGTTTCCTTTG
A_14_P120094 CCACAGTGATGTGGAGATACTTTTGCAAGTTCTTCTCAAAGAATCTGTCTTATAGAACAT
A_16_P03585347
AAAATGCAACCGACTTTCTAAGAAACCATGAGATTTCTGAATAAGGAGAAAGGGCTTTTA
A_16_P30952233 CTATTTTCTTATCGTAGGTTGTGACAGAATGCCTTTTTCTCTTGCTGCAGGGTC
A_14_P113454 CTCTGAATCATCTAAGAGAATTCTCTTACCAACATCTGGTCAATAAATTCCTTCAAGACC
A_14_P108008 AGTAAATATCACAGCCAAAGAGTTCCAGGCGAAGTGCAATACTTTGATTCCATGTTTTAG
A_14_P124142 CTTAGTCTTGGAACTTTGAACAAAACTCTTGTATTCCTTGAGGTCAGATCTTCATTCTCT
CUST_8430_PI426210822 GCCACCAAGCAGCATGTCATTCCTTCCTCTCTCCTACTTATCCTT
A_14_P100696 GTTTCTCTAATAGTGAATCAGAAACTATTAAAGTGGCCAAGATGATTTTCTTAGGCTCTG
A_14_P130920 ACTACCATTACATTTTGTTTTGAGAATATCCTCTTCAATTATTTGGAAGCCTCTGGTGAA
A_14_P115704 AAACAAGTAAGAGTGACGTTGGTTCCTTCATTGACGGTCATATCATTTGAGATGTCAT
A_14_P110940 TCCCTGTCTAATGTCTATTCTCTTTGCCTTGCCCTTACTTTTGTTCAGATCAACTTTAG
A_18_P14986011
AACAGAATGTTAACCGGATACCTTCTGAGATAGGTTACAACTTCATGGCTTCAAGTATTG
CUST_5119_PI426210822 CACCAAAGAGTTCAGTCTAATTCTGGGGGTTGGGGATAAATATGTTAGCC
A_14_P200143 CTCAAAAAACTTTCACAATTTCTCTAAGGAAAAGCAGACAATCTTTGAGATGGCCTTCAA
A_14_P134361 CTTTTCTCATCTTCTCCCACTCCTGTTTTTTTGTTAACTCTTAATAATCTGACCCTACGA
A_14_P136346 GCCATGTAGAGACGTCATCACTATTTATTCTGATTAGTTGCTGGGATCTGTAGAGTG
A_14_P100456 CAGTGATAGAAAAAGACCATGAAACATCAAAGACACCCAGGGAGAAAGATGCTTAC
A_14_P123523 ATACACACATTACTATAGCCAATACACTTACCTTGAACTTTTTTGTTGGCTTGCAACTTC
A_14_P123497 TCCTTAGTATTCATTCATAACTCGACATCTCTAAGGGGGAAATTTGCAAGAGTCTTTTCA
A_16_P00618064
ACCTTTCAACTGTAGAGATGAGCATTATGTAGGCAAAACTAAATATTTTATGTGTGGGTC
CUST_6435_PI426210822
CTGACATGTATATCTTCTTCAGTTGGTCCCTAGTGAGTCTACCCAAAGTGAATTG
A_14_P126407 CTCAAGATAAATATGGGTTAATGCCTGCATGATGCCTCTGAATTCAGGAATTGCAG
A_14_P134187 CATTTTCCTAATCTTAGCCACTTATCCCCAGTGACTACCATGTCCCCTTCTAAAT
A_14_P108191 TGATCCTTGATGTGAGAATAACTGTATTCCTTGCATGTTCAGAATGTCCTTATGTTCTTC
CUST_8782_PI426210822 GGGGATTATATGATCCAACCCTCTACCTTTGGACAGAATACAATCGAGATGT
A_16_P58424403
ACTTTTCTCTTCTAGTTATTGAATCCAAAATCCTTGAAGCAGTCCTTGACTCCTCTTTTC

A_14_P129646 CTATTGCAGAACATGTTTCTGTCTTTTGATGGCTGCCTATCTAATTAGAACTTTCTTAGG
A_18_P22841107 AATCATAAAGGCCTGTTTAGGAGGAGAAAGTTAAATTGCTCAGAGGTGCAGGA
A_16_P19429793
AAACTGACATGCTCTAATTTTACCAGGATAGACATAAACACTGCTCTGTTGTTTTTTCCC
A_14_P105040 ATCGAGCATCGTTGCTTCAGAATGAATCAACCAATGAACAATTACAGATACATTATAAGG
A_14_P109731 GTAAAGTCAACAGAATGGGTTTCCTTGTTGTTTCTGGTTTAAGATGAGAAGTCCTGAG
A_16_P00685147
GACTCCACTTATCAAGTTCTTTTAGGAAATTACCCAATGAATTGGTGGAGGTAAATGATG
A_14_P119080 GAAACTTCATTTCTCACAACATACTCATAAAAATGACTGGGATCAGTGAAAGTGACTCAC
A_14_P130573 CCAGTCTTTTTCTTAGGCGATTTGTGTTTTGATTCTGATCTTTGTCTAGTTCTGTCTTTT
A_14_P107444 TTAGAACACTCCCATGATCCGATGAGTGCTAAAAATATTTAAAAACTGACATGACACAGG
A_14_P137286 ATAATCTATCTGAAGAAAATGAAGCCCTTGGAAAATGTAGAAGTAAAACCTCGGAATCTC
A_14_P111623 AGGTAAACCCATCTAATCACAGACTCCTATTTGAAGTATTTGACGAAAATAGACTGGTAA
A_16_P18157668
GATTTAGTAGCCCTGATCTCTGTTTATCATCCCTTAACCTTAGCATTCAAATGATTCACC
CUST_10279_PI426210822
TACAACTCCCTAGCCATGAGAGTGCATTATGTTTTAAGAAATGGTCTTCAGACTCTAAA
A_18_P22841067
ATATGGATTTGTTTTGTCTTGAGGCAGACTGGAAATGCTGGAAATTTATTTTGGATCGTA
A_14_P125555 AGATTGCTGGACTCAGTATTATTCTGCAGCCCATAAGAAGAAATACTGTTTTTATCTCCT
A_16_P19012290
TCCCCTTTCTCATATATCAAAACCCATTTTAACCTTAAAAGCACAACACTAAACTCAAGA
A_14_P112181 CTCCCTGGCTCTTAATTCCCTTATGATATTCCTTTGACTCTAGACTTTCTTTTATGACTT
CUST_288_PI426210822 CTTAACACTTGTCAAATTCCCCTTTTAAAGCGGAGCAGGCTGAACATTTTTCT
CUST_1834_PI426210822 TTTTTCAACCGTGTGCTTGGTTTCCGCCCATCCTGTTCAGGCTAC
A_14_P104950 TATGCAGATCAGATGTGTGAAGATTCTGCCCAATAGAGTTATGGAGGATTCTAAGTTATG
A_16_P39345255
GACACTCCTAAACCATAATTCTACACTTTACCTAACCTATACATACTTTTTAACAACTGG
A_16_P02033513
CAACATTTTACAGATGCATACTTCTCTAATCCCACTTTATACCCCTCATCTTTATGGACC
CUST_7136_PI426210822 TTGATCATGAGTGGGATCACGATGGCATTCACTCTTTCATTCAGCAAAGTTAT
A_14_P126246 TAGCCAAGCATTTTACTCACCAGTGTCCTAGAAAACAGTTCAGTCAATGTGTC
A_14_P129316 TCTCAGATGGTTGTCTTCCTTGGCAGCAACAAAAATAAAATGCTACTAAACTAGTTTGAG
A_16_P02119142
TCTGGCACTGTATAACTCTCTAATTTTCCACACATGATATCATAATACCTCTAGTAGCAC
A_14_P122167 CTAACACACTTCTTCAAACCAAGAGCATAACTTTGGCTTTCAATGAAGTCCTAGAGAAAT
A_16_P01739183
TAATAATTACCTGTATCTCAAACTAACCATCCATCCTATGTTTGTTCGTGTTGGAATCCC

A_14_P106743 AAAGGTCTCTGTAAGTTACACTAACCTGATTCCATGACCCCCCTAAACTGT

A_14_P104477 TTGTGTTTGTAACCTCTTTGTTAGAGGATTTCTGTTCATTCTCAAGTAAAAAGGCCACAT

A_16_P00184234 AAAGAGACAAATCCTTTGCTGATAACAAAAAACACCTGGAAACTTCCTAACCTTATGAGA

CUST_2082_PI426210822 CCTCTGTGATTTCAACCCTTTCCTGCTCCGTTCTCCTTTGAGAAT

A_14_P102151 CTGCAGATTATCAATGACCGGATCTATCCATATGCTGCCATCTCCTATGAAGAAT

A_14_P134467 CCATTCCCAATTCTTTTTCTGGCTCCTGCATTTTAAATATTTTCTTCCAAAAGTTCACCA

A_14_P122282 CTCGCTGGACTTGAATGACCTTTGAGTTAAAAATAATCCAAATCATTTTTTAGGTGCAGT

A_16_P00233978 TAGGGAATTGATCAGTTGGTAAAATGGTTCATCTGCATACAAAGTTCATGCATGCTGTTA

A_14_P123634 ATGCTTTAAGAACTCTTTGTTTTTTAGGAATCTCATACAGCCCATTCAGACTAAAGGGTG

A_14_P109661 GAATTTGATACAGAGACCCATAAAAGCCCATTAAAGAGAGTGGTTTCTGATTTTTTTCCT

CUST_6759_PI426210822 GTAAACGACACCTCCACAGAATATAATGTGAGTGGTGTCCCTGGAGAT

A_14_P132944 AAATGCCAAACTCAGTGCCTTTTAGTTATTTTGATATGTGGGAAGATCATGCATAAGGCT

A_14_P125605 GCACATCTTATTTATCTTGTTACCTATAGTTTACTTTGGGTGATTGGAGGGGAATGACTT

A_16_P00539976 ACTCCACCCCAATATAGAACACAGAATATTTGAAAACAGATCTGCTCTCATGTTAGGTAG

A_14_P116451 AACTTTTCTCTTTAAGGATTCAGTGGCTTGATTCCTGACATTTCCTCTTTAAGAGGAATC

A_14_P131229 TTGGGAACCAACATTTTTCAGTAAAGTCTGCAATAATAAATTCCAACTTCTGATCTCCCA

A_16_P20573912 AGATCAAGTCAAAGTGCATAAGGAAAAGAACCGTTTTAAATGCTGAAATCTCAGAGGTAG

CUST_1662_PI426210822 TTTATAAAACAGTGAGATACCCCTGGGAACATCTGGCTTATCATCAACAGCTTC

A_14_P112016 CTGACCTTGGTATTCATGTATATATTTCAGTTTGGGAAAGGAATCTACTTTGCTGACATG

A_14_P136152 TACAGATTACAAAGCACTACTCAAACTGAAAGCAGCATGGAGGATTTATATTTTCAGAAG

A_16_P03650258 CCTCAAGAACATTTAAACTCAGATCATATCCTTTCATTTCATTTAATGTAGAGCCACCCC

A_16_P18455842 AAAATTTCCCATCAGAGCCTATATGGTTAATCAAGAAACAAAATGGTATTGTCAACACGT

A_14_P111881 CTGCAGTTTTTTTGCGATTTTTGTGGATTTCATAGCATTAGTTACCCACTCTTTGCTAAT

A_16_P17494006 AAAAAAAATATTGTTCCTCTACAATCCATGACTCATCCTTCTGTTACAGTGTTCACCTGG

A_16_P38181321 ACAACCTCCACTTATTACACTATGAAGTTTGTCCTCAGATACTAAGAAAAATCACTGTGT

A_14_P115137 ATCATTAGTGCAGTTGGAATGAATGTGTATAGGTCAGAGGTCTTCGTGTTCACATTTTAA

A_14_P200567 ATTCTTAAACACTCACAGACCAGGTATCTGTATCCATCCATTAAATGGAGACGTTCTTAA

CUST_5294_PI426210822 TGCTGAGCTCAGGGAAGCTGCATATCCAAAAATGAAAAGGAAACCG

A_16_P00685434 TCTTTCATTCAGCAAAGTTATGTGTTAACAGGTATTAAGTATACCGTTATCATTTTTCCT

A_16_P17865637
CCTCATACCACATGATAACTCAGATACCAAATTGTAGAAGAATATTTTCCACCTGCACTG
A_14_P112171 TTTAGAGGAAAAACTGCTTGATATTAGAAAGAAGAGATTGCGTATGTAGAACACTTTTTT
CUST_5113_PI426210822 TTTCTTAGGAATGGTGGTCTCAAAATGCACCAGTGCCTTCCTATTGG
A_14_P104971
AAGTCAGTCCATGTTGTTCTGGGAAAATGGAAAAGTAAATGAAATAGAGAAAGAGACTGA
A_14_P127458 TCCCTTGTCACCCCCTATTAGATAGTCAATACAAAGTCCCTTGTATTAAGACATTTGAAA
A_14_P133253 GATTGTGTGGCTCCCACACACTGAGGGTTGTTCTGAACATTCATCAATAT
A_14_P113830 ACATCAAGAAATTCCCCCAAGCAGAAAATCAACACGATAAGTTTGTGGAAAAGATGAC
A_16_P18508848
AATATAGGAATTCAGAGGAGAAAGAATTAGAAGCGGAGTAACTTACAGTTCCAGGTAACC
A_14_P130173 AGAGATTTGCTATCAGTGGTGCTTTATACAAAATAGCATAATCTGATAACATAAGACATT
CUST_535_PI426210822
TCTCTTGTTACTTGATCTGATTTATGAAACCAATGGTCTTTTTCATCCTGACTTGAGATG
CUST_463_PI426210822 GTGGTTTCCAGTTTGCTATTTGGGGAACTGAGGCTTACAGAGTTACAC
A_16_P18208585
TCCACAAATGGTTAAAATTCATGGGACGTTCATAATCACTCACTAAGAAGAAACAAGACA
A_14_P139639
CTAAGACAACCATGGAAAAGTTAGGAAACAGCCAGAGAGAGATTTTAAGGAAGATTCTAG
A_16_P17587488
AATAATTGGACTGATAGTGACTGATTCAGCACTTATGTGTCTCTTTTGAACTCTCAGGAA
A_18_P22842097 GGTTTGGGATTTGAGATATCCATCCAGGTGTTCACGTAAGTGCTGAGATTTTTAC
A_16_P00720285
GATTTCACTTCATTGTTTTCGAAAATTCTGTCAAACATCAATTTCTTGTTCATCGACCCC
A_18_P16731727
CCTGCCATTATCTTAATTTTAAGGCCTCTCATCCCTACGTATTCTGTATAAAACAGATGA
CUST_6722_PI426210822 CTTTCTCCCCAGACCCCTGGCTCACCATGCCCCCACATACTCACT
A_14_P113056 TGAACTCACCCCTGACCCCTAGGTTGACACATACAACTTACAGAGAAT
A_14_P133066 TCTGGTTCCACAGACTCTTCACTGATTACAAGTATAGATTTTGAGTGTTGACATCTCTAG
A_16_P20831599
AAAAAAAGCATCCTAATCTACTCATGGGTTTCTACAGTTAGTCTTATGTATGATGCACAA
A_14_P114343 CATAATAATCGATCCAACTCCACTATGAGCCAAAAATTCAGCCAGAAGTTGCTTCTTATA
A_14_P130620 CTACCCACTGAAATCTTGTTCAAGTTATTCTTTCTTTGGTCTCTACTTTCAGAAGTCACA
A_14_P120649 GCATTTTTGATACCTCATATTCTGTTTACACATCTTGAAAGGCGCTCAGTAGTTCTCTTA
CUST_8715_PI426210822
TATTAAAGAATAAAGTAGGGGATTCGAAACTTGGATTTTCTGAGAATGTATTGCAGAAGA
A_14_P137850 TATTCAAGTCAACACACTGTTAGAAGAAAAGAATGACCATTCCTGTGCCAAAAGAACCT
A_16_P17129529

CTTCAAAATCACTGGTCATGTCTACTAAGAATCAAATGCAGCCTATAGACTTTCATTGCG
A_14_P106797 CTGTCATCTTTGCTCTGATAGCATTATAATGATGATGAAGACAATTGCTAGGTTGGTGAG
A_16_P02221843
AAAACTGTGTTGGGTAGTTGAAATTCCTGAGAATCTTGTAGTTACTATGCGCCATTATCT
A_14_P125453 AAACCATCTTTTAAAGGGACTTCTGTGATGCTGGCTGTAATGTATGTGCATTATTAAGAG
CUST_5135_PI426210822 AAGCTTCTCAGCTTTCTGGATACCCTCTCAGGGCTCATGAAGTGG
A_16_P18129627
AAATCCTCACAACCATTGTTTTAATGATTTTTACTGGACCAGACCATATGGAACTCACAT
A_18_P16323382
ATGGAACCAGAAAAAAAAATCACAGACATCATCTCCTACAATCTCCTATTTAAGAGGAGC
A_14_P201926 CTAGCACCTAATAGTTACTCAGGAAATAAATACATGTTTAACAGGCCAGGTCGTAGAGAT
A_14_P116939 TAATTCTCCAGTATTTCTAGCCTTTCTTGAGGGTGGATGGTTGGAGCAATATTTTATTTG
A_18_P16135683
AAAATACAGTTTGGGATAGATGAATGTTCAGCACCAATCATTTAAAAGGGGCTGTCTTAC
A_16_P00914554
GTCACTGGGTTTTAAGAGTTACAGAGACATTTTGCTAATCAGCATAGAAGTTTCTCCCTT
A_14_P102645 GAAATTGTGTCAGTTTATTTACAGTTTCTTGGTAAACGTGCCTCAGGAGAAGTAGAATGA
A_14_P201240 GACCAGACAAAATTTGTTACTTTGAGTTCTTTCCTAGACCATTAATAAAGGCGACCTCTA
A_14_P139514 TACTTCATGACTGCCACGCATTTACAGATTGGTGTCAAAGACATTCACTATGTTTTTATG
A_18_P14135610
CTAAAGGGAGAGTTATTAATAGTTTCAGAAAGTTGAGGGAAATCATGCAGAGGTTCTCAG
A_18_P22841066
TGCATAGTGTATCCCTTACTTAAAACTATGAGAGTTAAGTTTGGATGAAATGACATGAAT
A_14_P200560 CTTCTTTCTCCATTTTCTAAGGAGATGATCCCTACTCAAGTGCAGAGCCC
A_16_P17805740
CACAAAGGTGTATCCATTAAGAACATTCAGCATGTCTTATCTATCTACATATCTGCCCTC
A_14_P131632 CTGCTGTTGTTTTTCAGACCTCTTCTCTTCTAGCCGCTCCATTTTTGG
A_14_P128189 ACTTTACAAAGTAACACTGATTTCCCTTCAGTCCTGCTCTTTGGTGAGATGTTAATACTA
A_14_P103043 TCTTCACCAAATTTCTCTATCACAAGAGATTGCAGACAGGTGTTGATAAATCCATACTAG
A_14_P127893 CCTGAAATGGCTTATCTGATTTTCTAAAAGGGAAGAGAGTCTCTCTTTTATCTCCTCAAA
A_14_P105684 CTTCTATGGAGGAAAGGTCAAAGATGAGTATCTGCGTAGGTAGAAAACATTGATAACTTC
A_14_P117899 TGAGGAGGTTTTAACTAATAACCTAGGAGATGAGACTGGGCAAATATTAGAAACTGATGC
A_14_P201696 TTTTATGAAAATTCATATGATGGAAAAAAGGAGTGGGCCTGTGGAAAGAGAATTATGGTC
A_14_P128981 TACATTTCAGTTATAAAAGTGGTCTCAGTGGTCTGTGAGTGTTCTGTATCTTACTGAGTG
CUST_1819_PI426210822 CTAGCAAGGCTCTCAGAAAGCAGGAGACGTTGAGGCTAAGCCTAG
A_16_P30952251 AGAACATCACTCCTCAACCCAAGTCTGGCTCTTCTCCTCTTTCTC
A_14_P124205 AAACCTGAGATGATTTTTTCCTAAAATAGCCTATCAAGACTCTTCCACCTGCTACTTCTA
A_16_P17833846

AGCAAGAAATGTCAAAAAACCTGGAAGATAAACACAATTTTCATCATCTGATGCAAGTGC

A_14_P110452 ACCTTTGATGAACTTCTTAGCAAACAGATTGGTCAGTTTTTGAGGACATACTTCAGAATG

CUST_6381_PI426210822 CATCCAATGGGCTAGAGCAGGTGGTTTAGGGCTGAGGCTGTAAAC

A_14_P113475 AAAATACTTTGCTAAGCCATCCACCTACAAGGAAGAAGATGGATACAAAGACACAAAAAT

A_16_P30952318 AACCGTGTGCTTGGTTTCCGCCCATCCTGTTCAGGCTACGCAGTG

A_14_P128508 TCTGCAAGTGTCCTCAGGAAGCCACTTAATCGTCTTGTAGCAACAGTT

A_14_P200914 AAGGCCTACTTTGGATAATTCTAAATTTGGATGACATAAGCCTGCACTAGCAAAGTCTTC

A_16_P00384287

GCCTCATGACAAGTAGACTATTGTATAAAATACCACATCCCTAACACTAATCTTACCACC

A_16_P00082484

AACCACGCTAGTGTTTAATACATGACGTCATCCTAGTAAAAATAATAGTTCCCACAGGTC

A_14_P136497

ATGCAAAAGAGAAAGATGAAAAAACAACACAAGACCAATTGGAAAAGCCTCAAAATGAAG

A_14_P120532 CTGCAGCAGGAGAGATTTTGTTAAACTTAAATGTAGGTTTGCGCCTTAAATAAACTGCAG

A_16_P02567804

AAAAGAAGGACAAAAGAGACATTTTGTAGACAGCCAAATGTAATGAATCAAGCCTGTTGA

A_14_P136768 CTTGAAAGAAAGTTTAAAAAAAGGAAGACACCCACAAGATAACTGATTGCCTATTGTCGT

A_14_P101872 CTGACTGGTAGGAAATGTAAAAAGGCCATACATTTATTCTGAATGTTGGTATCTGGAAGA

A_14_P138028 TATACAGTAATTTTGCCACATTCCGACATCGGTATGTATTGTCTTCTTCTCTAAAAGATG

A_16_P20174404

CTGTATCTTTTTAAAATGAGCCTAGGATTTGTTGTAATCAGGGAGGGTAACACACAGAC

A_16_P21559008

AACCATTTAACCTAAACTAATCAATGACCCTATTGTTCAATGCCATATCTGCTCTACCCA

A_14_P115793 AATTGCCCACAAAATATACTAAAATTTATTCCGACCCCTGACTCGTTTTCCTGTTTCC

A_16_P01843472

AAACACATCTCATGCAACCATGTAATGGAAGATTTTACAACTTTCATAGGAATGAGGCAG

A_16_P16155798

CTTTGCTTCAGAACTTATTTCTCCCCTAGAACAATTTGTATGCCCTAGTCAGTATTCATG

A_14_P136019 CTTATCAATCCCAAACTCTGGGTGTAAAAGATTCTACAGGGGTAATGTTTTATTATTCTT

A_14_P135753 CAGAGGATGGCCCCAAAACTGAAAAGGAAATGAAATAATCAAATATACCAAATCCGAATT

CUST_9063_PI426210822 CAGGGTTCCTGACAGCTTCCCCAGTCACCACCATTTCATGGTTTC

CUST_6656_PI426210822 TGCTGTCTCCAGCACACACATGTTCTAGAGAATTCCAGTTTTAGGTCAG

CUST_6229_PI426210822 GATCTATGAAGAGAATGTAGCCCCGAATCCCTCCCTTCTCTCAACT

A_16_P00709707

AAAGGACCATAAAGGGCATTTGTTTCTAATTAAACACCAAAACCCTAAACCTTTCAACTC

CUST_8399_PI426210822

CAGACACATTAATTTCTCGACAATAAGAGGCTTTTTTGGCATCAAAATTTAAAGCATTTC

A_16_P02216560

GCAGTGATGGACTAATTAGAGGCACATGATAACAAAGATATTTCTTCTCTTCTCTCAACC

A_14_P106442 CTCCCAGCCACAAATTCTTCATAACAACTCTTTTTATTTTTAGATGAAAATAAAAAGGCT

A_16_P17915973

CCTTTCTCTCTTAACTACCTCCTCAGATTACAATCTCTACTCCTTAAAAAAGAAAACAGA

A_14_P120042 ATTCTCTTGATAATTAAAGTGCTGCAAACACCCCCCAAAAATACTTGCCCTAATCATTTA

A_14_P115150 TCTGTAACCAAATACTATTTCTGAGACCCTCTTGTGTTACAGTTCCCTTTAAGCAAAAGA

A_14_P138986 ATCTAGAGTTAATTAACCTTCTTATGACCTTGTTAAAGGGTCTGCTTCCATATGCTGCTT

A_14_P201231 CATTTTGCTCATTTTAGGCATCGTTAAAGAATCTGGAGTGAAGCACAAGGGATTGATT

A_16_P00443188

TTCTCAAGATTCACACCTTAGGAACACATTTTTATCACCTCAAAATTTTGATCCATCACC

CUST_5403_PI426210822

CTTTGGATTCCCTGCCCTAACACAAAATCCATATTTATCTCAATGCAAAAGAGATGTG

CUST_4806_PI426210822 AGAGGTCAGGAGGAATAAAGCTAATGAGAGAGCAGAGAGTCCTTTGTCT

A_18_P14133693 ACTCAGGCCCTTTTATGTGGTAACCATAGCAGAAAGGAACATAGTCTTGTTGAG

A_14_P124772 TCATACCTTCTGTTTGATGATGAACAATTTGTTGTAAAAGCATTTCGCCTCTTTCACAGA

A_14_P139420 ATTTTTCCCTTGGAAATACTTCCACCATCACCAACCACTCTGAAAGATAAAAAAATGAGA

A_18_P22841889

TGGGGAGAAATCTTTATGAAAAATGCCACTTTTCACTCTTTTTTTCAAGTTTGAACTGAC

A_14_P202196 GTTGATTGTAGAAGTTGAAAACTAGGACTGTAAACAGTCTTTCTCTACCTTCTCTCTGTC

A_14_P115305 ACCAGTGTGCAGATTGCCTTTACTTTCTTGCCATCTCATCAAAGGATTAAAAATATTCAT

A_14_P126988 TAAGATTGAAATTGGCTTAAGTTTTAGGTGCACAAAAATATTCAACATGCCAGAGCATGG

A_16_P20163200

GGGAGGAAGGCATGTATAGCATTGCTCAGATTGAGAAATAGTTCAGATCCAAATATTACT

A_16_P21599090

CGAATACCCAATAAACATTTCCTGCTAACACACGAATTTTACTTATTTACAAACCACTGC

A_14_P115191 CTGCATAAAGATAAGTAATTTAAAGGTGGAATATGGCTGTGTCCAAAGTTGATCAAGGGA

A_18_P14134426 AAATCTTAATGGCCAAAGCCATAGGTCCAAAACCCGGCGTTCCCT

CUST_4550_PI426210822 CTAGGTTATGAGGGCCTGGACATTTGCCTGCCTGGGGTTTTAAAA

A_14_P119513 AAGAGCAAAATGTTTTCACAGAAATTCTCTGGGTTGCATATTCTTAATTGCCTGAACTAC

CUST_5818_PI426210822

ATAAGAGATGATGGCTTCTTAGATCTGTTTACATTTGCTGGAAAAACTTCTTCCAGGG

A_16_P01650096

AAAAACAATTACGTAACACCAAGAAGTCAGTTTTCCTTACATTCACTCCAGGATAGATGC

A_14_P123225 CAGAAGATAGGTAGAAATGATTCCAGTGGCCTCTTTGTATTTTCTTCATTGTTGAGTAGA

A_14_P128818 GCAGTTTGATGTAATTCTAATCAGAAAGCAAAAAGGCTAGTTACCTGATGGTGAAGGAAA

A_16_P21302199

AAACCTTTCGCTAGAAAAACAGCATCACCAAAATTAATCTGCTCCTTGTGTAACTTGTTT

A_14_P200458 AATAACCCAGCCTGAAGGAGACACAGCACAGAAAACAGGATTGTTAAGAAC

CUST_3466_PI426210822 GCACAGGGGAGTTTCAACTCTGGGGAAGGTGATCATTTGGGTCAT

A_14_P132741 ACAACTCCCATCCACTTTATTTATCAGAAGGCATTTAAATGGAGGAGGTTCTGATATAGA

A_14_P115566 TTTTTTTTTCCAAATTCTCCACAAAGAATCTGAGGTCACCGATAAAGCAAGGTTGCATAT

A_16_P01415268

TTATTTCTGCAGTTACCACATATGTCTAGGTTTATCAGACTATTTATTCACTCCAGGGGC

A_16_P30952344 ATGGGCTCTTACTTCGGCTCCTCCTTGTGCGCAGTTGACCTGAAT

CUST_2454_PI426210822

CTTAGAATTTCAGCCAGCTACACCTGTGTAAAAACAAACAGAACCTGCATTAGGAAT

CUST_10170_PI426210822

GTTTTTATTTGAGAGCATTACTTCTGACCTAAGGCAGAAACACTTAGAAAACAAATCTGT

A_14_P113315 CAGTAGGTCATCACCCCAACGCTAGAGGAATGAGACCATATCGATC

A_14_P131643 CTACTGCACTAACTCTGGATTCTCTAACTCTATACCTCTTGATATTTTATGAAGCCAATG

A_14_P123076 CTGACATGATGCTTCTCATTTTTCTCTCTCTTCTAATGCAGGTCCAAGTTATGAAAGAAA

A_14_P103081 AGATTTGGCTCCCGTTTATACAGTGTGGTTCTTCTTAACTTATTTATTCTCTTGGCTACT

A_14_P121958

TTTAACAAAGATGACATACCGACTGAGGCAGAGTGTAGAAGAGAATGAAACAAAAATGTG

A_14_P105067 ACTTAATCACCAGAAAATACTGTTCATCCAAACAGTTCAGATTCTAGTCAAACTGGTAGC

CUST_1921_PI426210822 ACATTCCCAGGGATTGGGGCTAGTACAGACTTGATTCCCAAGGAC

A_16_P19148358

AACCACAGGATGCATTATAATCTACCAGTCAGCCTTTTTATACAAATAACTGTCAAGCGG

A_14_P116662 AAGAGTTTTCCAGTGTGTATTCTGAGGAATACTAGTGTTCTGGAGATGTTACTTAGTGTT

A_14_P108926 TTAAGAGGCTCAATAAAACTTTGAGTTTACACCTTGCAAAAAGATTTGTATGCTACCTGG

A_14_P132856 ACTAAACATTAGATACCGAGGAAATGCGGATTGGCCTTTGGATGATTCATGAG

A_16_P19570631

TTGAAATTAAGTCCTAAAAATCATACACCACACCTTACTATAAGCCTTGTCAACGGAAGG

A_16_P16722065

ATCACCTCCATCTACTTGATTACTCTGGTCTTTATTCCAAATAGTAACTGCAATGTCAGC

A_14_P122205 TTCAGTATGGAGTAGTTGGAGTGTATTGCTTAGAACAAAAGAGATGAGACACTAACACTG

A_16_P01264757

AAAACAGGAACTGAAGTGCTTTTTCATTTTTCTCAAACCTTAGGGATCGTTGATGCAATA

A_14_P132153 AAAGGTCTTTGGAAATCAAAATTTTTATTCAGTAGAGCCAGCAATAGTTCCTCACGATTT

A_14_P110593 CTTTTGTTTTACAGAAGAGGATGTGGAGAACTTTGACGTGACCAACTTGTCTCAA

A_14_P106623 TTGTTGTTACTACGGAATTGAGCCATCATTCACTCATTCATCTATTGAGTGTGAAAATGC

A_14_P137733 ATAATCTCCACTAGATGCTGGGAGGAAAATGTAATTTTATTAGGGAGAAACAAGCAAAAC

A_18_P22841644 CAAAGCCCCTTGCACAGCACATCCACGGTGGCACTCTGTTAATAG

A_14_P137956 TTATTTTCCCTTGAAGTCATGTCTCCATAACTAAAGCCTGTTTATATGGCAAATAACCCG

A_14_P121924 AGTTCAGGTTTAGGAATAGCAAATGGTATAGAACAGTGATTCTCAAACCTTAACATGACC

A_14_P102795 AAATTATTGTAAACTTTGTGGCTTTTGGTCTGTGATGCTTGGTCTCAAAGGAAAAAAATAA

A_14_P132435 AATGAGGGAGGAGATGGAGATTGCTGTTACTATTTCATTAACAAAATTACCTATCACCTG
A_14_P127131 GTCATAGTCTTAGGACATTAAGACACTGTGAAAGCCTTTTTACAGTTGTGACAGAAGTT
A_16_P18161262
AACACTCTAATTACATAAGCATACATCCCACCTTTTATTGAACTGCCATTGTTATTGCCA
A_16_P19242125
TCCCACCTTCATGTATTTTCGAATTTGCATTTCTCTTTTATAAGGGAATTATCCTGTTCA
A_14_P122802 CAGTGTTCAGTATCAAATCTAGTTTTAATCTTCTATAACAAGTCACCTTTTCTTCCCACC
A_14_P135612 GAGAGACCAGTGAGTTACTCTGATGTTTATTTTAATGCATCTTAGTCCACACAGTTGGTA
A_16_P37020421
TGACAGCAAACTCTTCAATCACCTTTTTTTCAAAGCAGTCCTTACACTTTCTTACTTGTT
A_14_P139625 TTCCATCATTATTTGCTGTCTTTACATTTGGTGTCATTCCAGAAATTTAGTCTGCTTCCT
A_14_P118407 GAGAGAAGGAAACACTTAGAGGAAGTTCTGGAGATGAAGTAAGTGTTTGTAGTCTTATTC
A_14_P136552 GCCAAGAAAGAAAAATAAGTTCACAGAGGTGGGGTCTCTAGACAGGAGT
CUST_5147_PI426210822 GGGGTGATTTTCAGCCCAGGCCCTGAACCTGTCATAAAATGTAAT
CUST_2394_PI426210822 CTATGATCCTTCTCTTGCTGGAGGGGACACTGCATAGAAACCTGG
A_14_P100914 TTTGTTCATGTTGTCTTGTCCAAAGACCTTTATAAACTTCCTAATGCCTAGAGAACAGTC
A_14_P117992 GTTTGTTATCTGCTTCTATTTGGAGGCTGGGGACCTCTCAAAGGTAATTTACATCT
A_14_P115702 ACTTGTTGAAATCTTAGGTTCTTTGCAGACATGACCACTTAACAACTGGGTTAACTTAAT
A_14_P139121 AGACCTATACAAAAGGAAGTGTTATGAGGGGATTTAAGTGCTACAACAGATGCAAACTAT
A_14_P102986 ATGATGATGGTTTTTCCTACAGAAAGAATGGACACTTGAGAAAATAAAGGTTTTGTTGGG
CUST_4058_PI426210822 CATCCTTGGGGTCGTCCAGTCCTTGGGCTCTGTTCCTCTGATGTG
A_14_P134332 TCCTCATAAACACTCCTTTGCAGCAGTGGTTTACAAATTTCACTTGATAATTTACATGGA
A_14_P201903 ACTTGATTCCTTTACAACTCCCATGCATGACTGATTAACTCAGTTCAAATGTTAAGCATT
CUST_6295_PI426210822 TAAGCCAAGAAGGAAAAAATTCTCATCTAAACCGCCAGGCTTAAAGGTGG
A_14_P108090 ATTTTTCAAAGAAAAAAACATAGTGGTTTATAGAATTGGTAAGTCCGGCGGCTTAAGGCC
A_16_P19975887
CCATCAAGTCACATCCTGTAATAGAAGTTTGACATAATTGTTACTGTCTTGGTGCTCATG
A_14_P114250 TGTTATACTCTTGAATCCCTGTGGAAATCTACATTTGAAGACAACATCACTCTGAAGTGT
A_14_P129536 CTGAAACACCCCACATCTTAAAAAGAGAGATGCATTCAGAGTAGATTCTTGAGTTTCAAA
A_16_P02372893
AATGGTGTTTGAAAAGCATTTCGATGGAAATTTCTTAGCCACAACCATAAGTGTAATTGG
A_14_P110901 CGCGTTTCCAACATCAACTCCATTCTCCAGTTCACAATATTATCAACTATTTCTTCTTTA
A_16_P18733855
TAGGCACGTTCTATTCTACTCTAATTCTCTGCAAATTGGTATTCCATTAACTGCATGTGG
CUST_9796_PI426210822 ATAATGAATCAGTGCCTTGGTTCAGGTGACAGGAATGCATGCATTCATGT
A_14_P126937 CTCTACAGTCTGAGCGGAAAGGAGAGAAGGATGAATGAACTGAAACAAGTTG
CUST_4467_PI426210822 ATGGAGCCTCATGCTGTCCTCAGGCCACCCTAGCCCCCTGTTCTC
A_16_P03757623

ATGTAGTGTGCCTTTAACAGTTTTATCGTGTTTCCTACGATTACCATCTTTCACTCTCTG

A_14_P139279 AAGTCTCCCAAGTAGCAAACAAGGGACTAATTAGGCAACTCTCTATACATATCTACATTC

CUST_8735_PI426210822

TTCTAATTTTCTGATAGTAGGTGTATATTATCAGGGTTCTCAGCATAAATGTTGACTTCT

A_14_P139267 TTTTCTAACTCTGAATTGCTGCTAAGGTAATTTTTGCATCTGCTGTGAATCAGTTTCCTC

A_16_P03190179

TCTTTTGTGATACCAGTTTGTAAATGTGTAACTTGTCTGATCTTTCTTCTTTCAGCACCC

A_14_P116127 CAGGCACCAGAAAGAAAGTCAAGTCTTGTGTGATAAAATTCATCTTCATTCTCTACATTG

A_16_P17743742

AAAAAAAAGTTACAGAAATGTGGATTTGAGGAATTTGTCGTAAGGAAACACTGACATCCC

CUST_582_PI426210822 CCCTGAAAGGAAAAGGGCTTCTTCGGAGGGCATTTGTGTCTGCTC

A_14_P119950 CTTACCAATATCGTTAATCACTGCTCTTATTTTGGAGACAAAAGGACCAACTTCACTGGA

A_14_P136805 TTCATATGCATCTTTGTTTTTAAACAATATGCTAAACTAAGGAGGAACGGGATCCAGTCT

A_16_P18295536

AAATGCAAGTCTTGTGCTATCTCAGAAACCTCAAGGAAGAGACTATAATTAGGCTTCAAC

A_14_P111637 GAACTGGGTGATGAGCCTCTTTGCAGACAGATATTGTTCTTTCATTTTGTTTTTATACAG

A_14_P113985 TAATTCCCCAGTAATTTGGTTGACATTGGGTTGCATTTTTAAACATAACTTTTTCCACAC

A_14_P107378 GTTCCTCTGCCTCAGAGGACAGTTGCACAGATGGCTAGTGTTAAATTTCTATAG

A_14_P202159 CTTCTGTGATCAGGTCTCTTAATATTGATACAGGTTTGGCATTCTTGATAAAAGGATGCT

A_16_P19993312

ATCACAGAACTTTCATCTTTAATAACCCTTTCAGCCTAATATTTCCCCTATTCCTTCACC

A_14_P110424 CTATCATACTTTTGCCTTCTGCTCATGAGGGGTGTATCAATTTCCACTCCC

A_16_P17699771

AGTTGGCTCCTAGAAAAGTTATCAATGAATCCTGTTTGCAAGTAAACTCTGCACTAGAAT

CUST_10094_PI426210822 TTTCCTCCATTCCTTTCAAGGGGAAAGCCACTGAGTGTTACCCAG

A_14_P110717 AGGTTCGGTTTATTTCTTTAGAGCCCCACTTTTATTTGTATCTTTCAGGTAATTGCATTG

A_14_P107096 TTCTCTGGATTATTGGATGCCATTTGGGAAAAATCACGAAATATGTCCTGATAAGTTTCA

A_16_P18895643

AAAAGGTAAACCAGAAACATGGAGTTATCACAAAACAAGCATTCTCTTCTAGAACAGGAG

A_16_P15662222

ATCCTGCTACACTCTAAGTTCCACATAAACTGAAGTCATGAATACTGTTGAGCATTAGTG

A_16_P17827241

AAATCAAGCATTTCATTTGGCTTAAACGCAAAATTCTCACAAAACCTCTCCTAAGAACAG

A_16_P17732373

AATCAGAAAGTCCATTTTTGCTTATGACAATCAAACTTAACTTTAGCCTGATGCCAATCT

A_14_P120687 GCCATTTAGTTTCTGATGATCATTTTCCTGCCTTTTTTTTTGGGTGCAAAAAAAATGGTT

CUST_2596_PI426210822 TGGTTGCTCTAGTTGGTGCTTTCATGATGTCATCTTTGCTCTCTTCATTATCC

CUST_9494_PI426210822 TGCTTAACATCTCGTTGTTGGATTCCAAGTCATCTGTACTCCCCCAAAATA

A_14_P120535 AACAGAGGACCATCTGATGATTCATAGGCACAAACATGAAATGACTTTGAAGTTTCCT

A_16_P19052882

AAAACTGACCTGAAGAAAGTATGAGATGAATGAAGTAGACACACCTATGAGATCAAAGCA

A_14_P117701 CCTATTAAGTTGGTTTCATAATCTGTCATTTGAAAATCCTGTATTAGGTGTTAAGACCCT

A_14_P118860 CTGCTGGATTAGAGTTTAGATGATATTTTCAGGTCTCAGGATCCTCTGAGCATATATCTT

A_14_P201945 AGAAACTTCAGAATGCTGTTTATAGTGAACTGAGTGTGTGAGTTTTCCCACCTATTTTAC

A_14_P125769 AGAATCGCTTGTATAGAAGTTTTCCAGACATCACCTCTTTCCCATTACATTTACCTGTTT

CUST_8209_PI426210822 AAGCTTTAGCTGCAGTAACTCATACACTCCTCCCTCCTTGTTTTCTCC

A_14_P105575 CTATGTGGTGTTATTTTACTGGATGAAGGAACCATAAAGCCTACTTAAAGGTCAACTTCT

A_18_P13521458

AAAACAAAAACAGCATTACCTTTTCCTGAAGACGTTAATCTTCCACTCCTATCACTAAGG

A_16_P17491948

ATGAACCAACCATTTATCTCAGGAGATAGAACTCATTCATACTCAATTACTCTGCTCAGG

A_18_P12046251

ATGCATAAACCTGGAACTTGTTTCTCTAACACGTATGAAGGATGAACTTTGAATTGAAGG

A_14_P112047 GAATTTCTACATTCTACAGAGTTACCGGCTGAAGAAGGTAATCTTAACATGCTGTTTCTG

A_14_P121245 AGAAAAACAGAGTTTATAAGAAAAATTAGATGCCTTTACCCTAAAGCAGCCACTGGAATT

A_14_P138993 CAAAGTGTATTTATTCTGTAGATCTAAAGTCATGGCCCAGGTGCAAAGAGTTTTGAATCA

CUST_4020_PI426210822

CAAGAAACAAAACCCAGAGTCAAGTAAAAGGATGCTTTTGGAACTGATGATTCTCA

CUST_601_PI426210822 CTTAAAACCCTAGAAAGCAGTCAGTATCCACTGCTTAAATACCAGGAGCCTC

A_16_P16482166

ATTTTCCCTGTCACCTATTTGTCTCCTTTAACTTTTATTTACCCAATACCAAGCCAATCT

A_16_P19011387

AATTCACCTAAGAACCTTGCATATTCCTCCTTATGACTTTGTTGAGCATATTACATGGCA

A_14_P127757 ACTTCAAAACATGCTTGCAGTGCATCTAATATAGACCAAGCGAAGACATAGAAAACTACA

A_14_P138554 AAACTGGAAATTTGGCTTTATGCATAATAAGTCATGTGGGTAAAACATCCACATTGCAGT

A_16_P00264967

TGTGCTTGTTATCTAATAATGCTACCTACCAATTTGAACACTTGATAGCCTCTAAAAGCC

A_18_P14135334 TGATGTTGAATGTATTAATTTGTATATTCATGAATCATATAATGAATCAGTGCCTTGGTT

CUST_2343_PI426210822 CTCTCTACTGGCCTCCTGCTGAGCTCAGTGCCTCACCTGCAGGAG

CUST_3524_PI426210822 CAAATGGTAAGGCAGCTCTCGGAGCTGGCACGTTCTTTCTCTCTG

A_16_P18223309

AAGAGACATTGCATTCCTTATGATGGTAAAGTGAACAGATACATCCTAAGTCTGCAGATC

A_14_P103800 GATATACTCAATTGTAAAAGAAAGCAGTGGTTTGTGAAGACCTTGGCTGGTAAGCACTTT

A_16_P15113055

GCTTCATCATATTAGCCTTTACCTCTCTAGACAAAGTTCATCCACCTATTTGGTAAGTCA

A_14_P138729 ACTTTTGAATACTCCTTTAATTTTGCTACCTATTCTGCTGATTCCAAATGATTCTCCCCT

A_14_P118474 TTACCCTTGCTTTTCTTTTCCAGTATTACCAAAATTTGAAGTGACTTTGCAGACACCATT
A_16_P01985351
AAGTTTGTAATGCCAAGCCTATTTTTCAGTAACCATCCTTCTTATGATAAACAGCATGCT
A_16_P18598202
AAAAATGCTTTACAACACATTTCCCTATCATCATTACATAGACCCAATAAGGTGAGTGAA
CUST_6297_PI426210822 CTTAAAGGTGGCTCCTTGTCAGTCCTGAGGTGGTAGAATTGAGGT
A_14_P121531 TGAATACATCTTGTTACTTCAAGAGTTTCTGGCAGTAAGGGCACAATTGACTGTGATAA
A_14_P128374 TCGTCACTAAGTTAATTCAACATTGAAAGGGATAGAAGATGTTAATTCCACACACACGTG
A_18_P22840289 CTTTGTGATCTTTTGGGGCCAACTTTCCTGCTCTCAACTGCTCTCTAT
A_16_P19964944
AAAAAATATCATACTTAAGACCTGCATTTGTTCTATACCCAGATCATCTTTTTAGAGCCA
A_16_P20363369
AGTATCAGTCTTCATCACGTATATCTTCACAACAAGAAACTAGGGACAAATTACTGGCTG
A_14_P133865 TTCTCTTAGAAAATTAACTCCCACTTTGCCCTAGTTTTTCTTAAAGCATATCCTTGGACC
A_16_P20979969
CCCCAAATTTGGACCTTATGCTCTGTAGTTTCCTGTTATAACCAATTTGCTATTTGTGTT
A_16_P02990356 TTACTTTTCTGTTCGATTAGTGACCTCATACTTCCCCCATCTAAAGGACACCTTAG
A_14_P126249 GTCTGAGCACAACTTTCTCTTTGGACATGAAAACTATAGAGTTCATACTGTGTCATCAGT
A_14_P134306 AGAGTCCTTTGCACTACTATGGGAATGCCACCATGTCTTTGATGAATAAGGTAATATACT
A_16_P01447965
AGTGGAATAGGCAATAGAGATTAAATGAAAGGTATGCAGAGATTTTAGTGACAAGGTTCA
CUST_5705_PI426210822 CCTTATCTGTCTGTCTCAGGCTTCTTCTCCAAGGTTAACCCCAAC
A_14_P105386 GAATTTGATTGAAGCAAAGAATATCATTCCTGCACTGCTCCTTATTCAGGCAACAAATTC
A_18_P22842299 CTAAAATTAATGTCACCACAGCCATTGTAGTATGTGTTCAGCCGCCTGTGC
A_16_P18507064
GAAACGAGAAGCCTTAGTAAATCACTTCTATGACAAAACATGGCATAAAAACTTTTCCCC
A_16_P21410044
AATATACTTCCTTTGTTTGACTCATCACTTGGAATTGTGGATGTTTTGACTTATTTGGGG
A_14_P137718 ACTAAAATAGACCCTAGATTATGACATACTGCAAAACATCAAACCACACAATAGCATGTT
A_14_P138743 CAGACTCACGTGAGAGGAATAGCGTTTGTTTTGGATTGTCTTCTGCTTTTCAAG
A_14_P109900 GAAAGACCAAAGAGGGAGAGGAATTATTTTGCTTAGTTATTAGAAGTAAGGGGAAGGAAT
CUST_1832_PI426210822 ACATTTTCCTCTGATGGATGTTTTTTTTTCCCTTCCCTTCTTTTTCAACCGTG
A_18_P12768173
TGCCCCAATTTTCAATATCAACTCATCATTCTCAACATTTCTCTACTACACTTTTAAAAG
A_14_P108612 CCTGCTTGGAATGGATCTTTGCTTATTTGTGGTATAATACCAAGGAATATTCATACCAGT
A_14_P121992 TTAATAGACAACTGAAATGGAACAAATATCCTGAACTCACCCTGATCTCTTACCACTGAA
A_16_P16191926
GCTGTTCTTAAGGAAATCATCAAGAAAGACTAAGCACCATTGTTTTGTTTCTGATGTTGC

A_16_P00717251
TCCCTCACATATCTAACTAGGAAACCGTCATTTCAAATACTTCCTCTTACTATCCTGAAG
CUST_2258_PI426210822 CCTTGTTTCTGAGCCTTTGATGCTCCATCATGCTCTACCATGTTGATG
CUST_1963_PI426210822 TATGGGGCCAAGTTACAGCACAGAGTTTTAAGAGATTGTCCGTAGTTCAG
A_14_P108360
AGAAAGGAAGAAATTATACCAAATAGAACATTGTAAGCATTGTGGGGAAAAGGAGGGAAG
CUST_4848_PI426210822 TGATGGAACTGAAAAGCAGGCTACCTATTTTATCCAGGAAGCAGACAGAG
A_14_P134987 CTTTGTGTGAAATCTCAAACTTTCACCATTCTCTCCACATGTTATCCTCATCTCATTTTT
A_14_P133886 CTTGTGAACGCAGAAGGAGCAGGAGGGAAATATATTTTTTTTTCTAAAAAAAGGAAGTA
A_14_P103887 TAATAATCTGCATCACAGGAATGCCTACCGCTTCTGTATCATTTACTGGAAATACATCTC
A_14_P201616 ACCATGTTATTTATGTGGTGGTTTTACTTCTAATTATGGCTCATCATGGCTGTTAGTGCT
A_18_P22840531 CTTCAACCTGCTGTTCAGGGCCTCTGCCCTCATTCCCTGGAGGCT
A_16_P40368115
CTCTCCTGTTTACACCAAATCAAAATGTCAAATTGAAGTCATGAACTGCATAGTCTGTCT
A_16_P17714540
TCCTTAAGCATTTTCCACTTACTACCCCTAGTTCTTTTAAAAGACTCGCGAATAGTAGTG
A_16_P01679152
AAATGGGAAAATAGGGTGAATGATGAAAAACACTAAATACACACGGATGACATGGATACA
A_14_P201639 TTGACAGTAAGGATGGAAACCAACATTTTATCGGAGTTCTTGGATGATAGGAATTTATCA
A_14_P116357 TTACTTTGAACTTCTTGGTGCCAGGACTGGTCATTGTGATCAGTTACTCCAAAATTTTA
A_14_P108390 GGATTTTATCTCTTTAACAACATTAGGGAGACAAGACAGGAATTTCCCCTACTTAACAAG
A_14_P121747 TGCCCAGCATATCTCATTTCTGTTTATCCATGGAAACTGTATAAAGATCACAAGAAATGG
A_16_P16737395
CCCAATCTGAACGTTTTCTAAGATAAGCCTTAGTTTCTAGTTATGACAGACAGTAATGCC
A_18_P22841541
GGGGTGACTTAAAAGGATCACAAATACTCGTCAAAATGAGTGAGGAAGGTAAAATATAGA
A_14_P139794 TTTTGTGTGCAGTGAATAATTTTGCTCTGAGACTCATCTGGCCTTAGTTACCCTATTTAT
A_16_P16350224
AAAAAGGGGATTTTCACTTACTTACACATAAGTTCTGGACATATTACATGCTTGGCACAC
A_14_P104354 ACAATTCATTTTGGCTCACACCTACTTTAGTGTAATGTAGTGCCACTCTTAAACAGAAGA
A_16_P20281330
ATTCCATACCACTTTCTTATTCTCAGATGTGATTATTCTCTACCGAGGGCTATTTATGCC
A_16_P17286723
AAGGACAAACCTGTAACAACTGAATTTTGGTCACTTCTAATTCTGCCTTTTCGTAAATGT
A_14_P109247 CTTCATATCCTCAAAGTCACCATCATTTTACTATTGCCATCACTTTCTATTTCCAAGGCA
A_14_P101542 CAATTCATTGTCTCTTTCAGTCCCTTCTGAAGGCTGCATTTGGCAATGT
A_16_P21408562
AAAGACGAAATAGGACCTTATCATGCTCTTCCCTTTTAACACAGCATAACAATAAGTCAG

A_14_P135460 GAGAATGGATCTTGTGCTAAAATTTTGAATAATAATTCCTTGTTGGCCATTGAAGAGCCT
A_14_P123194 TTTACTTACAGTTTGTGGGTTAATCTCCTCCTCTCCCATAGGTTCATCCATAATTTGC
A_16_P39716864
TTAAAATGCATAGGTTTACTCTGGGCACTAACCTATTTATTTGGTTTGTAGTCTCCGACA
A_14_P116286 AGAGAGAAGGACTTCAGGATATTGTCATTGGAATCATCAAAACAGTGGCACAACAAAT
CUST_3831_PI426210822
GTGAGAGTTTCCAGAAATACAGAGATTCTTATTTTAATCCTTTTTCTTTTTAACTGCAGA
A_18_P15517551
CCACCTATAAAAAAGACTTAAGTCATCATAAGCCAATAAGATGTGTAGAAACCTTATGGC
A_16_P15840912
ATAAATGCAGTCGAGTTGTCTCTATGACCAACTAGCCATAAGACCATTACTTTCTCTACT
A_18_P14134874
AGACAGCACAAATTCCATGGGTTTGAGAGAAAGATGTAAATGACAGCAAGGTAGAAT
A_14_P101286 TTTCAGGGTTGTGTTTCCCAACCAGTTTCCTAAAGTTTTGGAATATACTTTGCAAATTTT
A_14_P126079 CTGTATATACGTAGGAAAACATTCTTTTTTGTCAACTTGACGTTGACAAACTTGGTGGAT
A_14_P201362 TCTATTTTAATGAAAGCAGACACAGGCTATTAACTGGGGAAATGTTGGGCTATAGTGATT
A_14_P100167 ACAGGTCCACTATGTTCTGCCCTACCTATACAATTTTAAATTCTGTCTTCTACAACAGAC
A_14_P124547 AATCAAAGAGGAATACAGTATATTGATTTGTCTTCTGATAGTGAAGATGTCGTTTCCCCA
CUST_2563_PI426210822 CTTTCCTCTGTCCTGTTCCTTGTAGGCTGGAGGTGCTTAGTAAGT
A_14_P120239 TGGAATGGGATAAAGAGAAGATAAAAATCAATCAAGAAAAATGCTTCCCCTTTGCTTGAC
CUST_7836_PI426210822
TGAAGACATTTATTTCACACATGCAAAAACTGAAGAAGTCTTTGGGGCAATAAATCTTTT
A_14_P136020 TAATAATGATGAATAGTGCTCTGTTGATCAAAAAACCCAAGAGCCACAAGCACTTACTCA
CUST_2894_PI426210822 GCCTTCCAGGCACCTCTCAATGTCTGCATATCTTGGGCTGAGTTT
A_16_P01644995
CTGAGCAGACATATTTAGCACTCAGAATAATTAGTAAGACATAGACTACCGGAATGAAGA
A_16_P37751123
ATGTGGCTCATAGACACTTTATTTCTCTGAAATTGCCTCTCAAGAAAAAACTTGATCCTC
A_14_P116558 TTCCTAGATTATTCTCTGGTAAATCAAAGAAGTGGGTTTATGGAGGTCCTCTTGTGTC
A_14_P117174 CCAAATAAAAAACCAGAATTCACTCTCCAAAAAGGCTATCAGCAGGGTTCCTTCAATA
A_14_P112385 TCCCTCTTAGATTGCTACCTGTTCTGATGACAATACACTAAAAATCTGGCGCTTGAAT
A_14_P104237 ACTTCTCCAGATTCCTGTAGTTTTTCCAGTAATTCAATTGCTCTTGCAACATCTGAATGA
A_14_P124968 CTCAGGAAGAAACAAGTGAAGGTGATGCTAATGGTAAGTAGGGGAGGAAAG
A_14_P119287 GTCACTCCTGATTACAATTTCAGTTCATTTTCTATTGGAAGTTCTTGAATAGGGGGAGTA
A_14_P101886 TGCCTTGATAAATGTTCCATTTGTTCTTCACCATCCAGATTGATGGTATTTTTATTTGGG
A_14_P101916 ACAGGCATGCTAATATATTCTTTCTTGAGGCTCCTATTTCACATCTCAGAATGTTAACAC
A_14_P106614 CGAGTTGCTGACAAATTTGACATCCTGGAAGGCATCTATTGAACACAAAAAGGTAAATAA
A_14_P125563 TCATATTTTGCTCACACTTAGAATGTTGAAGTCACATTACCATCCTTCTCAGACTTCTCT

A_14_P101204 GCAAAGAAAAAAACCACCCTTTCTATTTTTCCTGGAATATAACCTGACAAGGAATTCTTC
A_18_P14135288 CTGCAATATTCCATGTATCCCTCTAACCTCCTGGGGCTGCTTTGTTACTAT
A_14_P123925 TTTAATGTTTCTTTTCTACAGCAGAATCATGAGACAGACTTTGCCTTGTATCTACTTTTG
A_14_P104347 GGTTTTACATGGTGGAAAAGAAAACATATTTTGGCTACTTGGGATTAATTGAAATGGACC
A_14_P110179 TAGCCCTAAAAGAAGCCAAGCATCAGGTAAACCAGAAGTTTAATAAAAATGAGCAAAATT
A_14_P118509 GGTGCTAGTGAGTGTATTGCTTAGACTGTTTCATATAGTGGAACATTTCAGGCTTTCTTA
CUST_4050_PI426210822 AAGACTCCATGGAGCATGACAGGCATGCTGTGCCTGCCACAGCCAA
A_14_P116279 CTGAACCTGTAATGTTTCTGGCATGATTTTATTCTTCAAGTGGAATTCAGTTAGTTCCAG
CUST_3775_PI426210822
TCTGTCTCTCTATATCTATAACTTTTAATCTTGAAATAGTTTAAGATTCATAAGAAGTTA
A_14_P133044 GATTGCCAGGTTTTTAATCTTTGGCCATTCTTGTAAATCCCCTTGAGTAATAGAAGTATC
A_16_P16585970
AAATCATTACTCTTACCCCAAGTCAAGTTCTTATAAAGGATGTTCCAATATGCTCATGGC
A_14_P100161 GCTGAATCTCTGATATCCCACACCTTAATATAAGATGTTGATACAGTGAAGACCAAACTG
CUST_3258_PI426210822 ATTTAGGGAACTTTTTTTGAACAGAACCATGTGTCAGACACTGTCAAGAAGGC
A_14_P123344 CTTATTCCCTAATCCATAGGCTACTGCTCCACAATCATATACTCCTACTACTTCAGTATC
A_14_P123595 TTCTTGAAAATAGGATTGTTTTGAGATTTTTATGCTGGCCAAATGAAACCTCCCTCAGTA
A_14_P105376 ATTACTATTTCCTAGCAAATATCGTTAGTGTTCTATCCATCCCCTCTTCACTTGTGTTGT
A_16_P00314265
AAGTCAGGAGCAATAAATCTTTTCCAAGTTTTCCAGTTAGAAATCAGTGATGCACCTACA
A_18_P17709782
AAGTATACTCTAAAGATAGCAGATTCCATATTCCATCACTGTCATAATCTGAGGTGCAAA
A_14_P122268 ATAATTACATTTGTCACCGCTTTAATTGGATGTTACATGTCAGTAATGACTCATCAGCCC
A_16_P03669507
GGTTCCTTTGACTAAATATAAGTTCCTCAGCATGACTAATAACACAGGATATGTGCATCC
A_14_P107415 CTTTGGGGAGGTTTTTGTTTATTGTGATGATAACTCACAGTTACATGGTGTTTTACCATT
A_16_P20934713
AATTGCTATGCCATGTATATTATGACCCTGTTTGTGTAAAACTACGTCCAACTAACCACG
A_16_P19518692
TGATATCTTCATCAACGACATAATGAGATTCAATTCCCATATGTGCATCTAAGGACCACA
A_16_P00685307
AGTAAACATACCTCAATATACTTATTTCTGTTTGGGATTTTCGCCCCTCAAGTCCTCATT
A_16_P36230824 GAAAAGACTCTGGCTGACTCCTTGTGATAGAGCAAACCAAGTTGAGTGTT
A_14_P124964 AATAATAATAATAATAAAACACAAAAGAATGACTGGGTGAAGTTGGGGTCTGCGTGTGCC
A_14_P125304 ACAACATCTTCCACTTTCCCGGGAACATCCAATTCAAAAACATATTCCATTTTGC
A_14_P130896 GTAACCTTTGTGTTGTCCACCTGAATAAGGGAGAATCCATGAGCAGCATTT
A_16_P20078830
CCATTGTAATCTTCAGTGACTCATCTTTCAATTCTGTAACTGTAATAAACACATGCACTG

A_18_P13345033
ATTTCCATCCTCTACTCAATACAGCACACTATTTTTTCATACAATTTTCCCACAACCTGC
A_16_P15375135
ACCTCTCCTTCTAAAGATCTACATACATATTACCTCCAGGCTAGTTTATATCCCAATCCA
A_18_P14075042
AAAATACCCCAAACAAAGGAAAGTAAAGACCATGTAAGGAATTCAGTCACTGGTGAACTA
A_14_P101188 GGAAGAAAGATTGGCAGGTAAATAAAATTATACCTGTCACCTGGACAAATTGTTTTCTCC
A_14_P120787 AATTAGAGTGACTCAGTTGTGTTATGTTTGTCCAGGACTTTCTGCTTTAATTCTGAAGAG
A_14_P117172 CTTAACAATTGAACCCTTGAATCAGAAAGGCAAGGTGAGTTTCTCAGAAAGATGTTGAAA
A_14_P125030 CAGAAACTGACAGAGATTCCTTGGCTTTATTATATCTTACACCCAAATGAGGATGAGGTA
A_14_P102769 CTGACAAAGAAACCACCTGGTCCAAACCAGTTATTGTTTTCTTTGATTTAATTAAAAAGT
A_14_P119535 AACCCCAAAACATATTTCTTCTCTAGGAACACTAGATCCTGAAGGTAATCTCTCAAAGTA
A_14_P103703 AACCCATAAAAACAAACACAATATAATGGCACAAAGCACTTGCTTTATTGCCAGAAACTG
A_14_P122962 CCTATATAGGCATACCAAATACGTTTAGAACAATACACTTTTTCAGAGCCTAAATTAAAA
A_14_P201321 CCAATACTGGTAATTGAGCCCTGTAAGATATGTTATGGACCACTGTTGAAATATTTCGTT
A_16_P15790857
GTGCAGAGAGACTTTATAAAATCAATGTGACTCCAAGAAAGTATTAGCAGGAATTCAGGC
A_14_P118688 TAAGAAAAGTTGGCATTTCAGTTTCTCCTTGAATTGAGGACAGTGTTCAGGAGTATACTA
A_16_P02484521
AAAAGGTCCTGTTCTTCAGAATAAGCAATCTGTATTTCAGTGGAATAACCTAGAAAAGCC
A_14_P118963 CTAATAAAGGAAAAAAGAAGGGTTTTTGAATTGCCTGACCTTAGTCTTGTCTTTCTGACT
A_14_P114156 ACACAGAAACAACTTTTGGAGAAATTAAATTCTGAGTGTGACTTCTGAGTTTGACCTTTC
CUST_620_PI426210822 CAGTTGCTGTCCTTGATCTGGCTATAATTTTGCCTGTGGAATCGCTG
A_14_P202438 ATCTAATAATTTTAGGAGAGACAAAACACTTGGGGATGTGAAGTCTTTGATTTCTGACCT
CUST_1165_PI426210822 GCATCTCCACCTCCCTGTTTTGTGGTTTAAACTAGACCTACTATGGCTTTTA
A_16_P00190674
ACTATTTTCACAGATCTCAACTAAAACCCCTTACTTCACAAATGATTGTGTTGTGCTGAA
A_14_P133891 TAATCCTGCAACTTTCCTACAATCATTTTTCTGCCTATACAATCCTGCTTCTTATAGGAT
A_16_P02518056
AAATGACCAATCTACTTCATTTCATACTCACTGGAGAGATGAATCTGTTGTAATCGTGGA
A_16_P58424313 CTGCTGCTGGGTCCTGGTGCAAGCCCTTACTCTCTTTCCACTAAA
A_18_P17183270
AGTTGTGGGTCTTTCATAAGGAAAAAGTGAATACTCCAGTGTCAAACTAATGGGATTTCT
CUST_10296_PI426210822 CCACATGTAAGAGTGAGTTCAGGTGCTCTAAATCCTTTCCAGATTGTTAACACA
A_14_P139249 CTTTGTCCCACACACATCTGTAGATGTAGAATCTGAATAGTCTGCAGGATTAAATGTCC
CUST_9900_PI426210822 GGAAAAGTTGGGAAAACTTGCCTCATTGGAACATGGGCTCCAATT
A_14_P120791 TAACAAACTCCACTCAAAAAAATAAATGGCTATTAAATGCACCATCCAGGGAACAAGAGC
A_14_P100250 TCTGCTTGTAGAATTCCAGATCACTCATGAATATCGGTATACTCTTTTTTAGCACGTGAG

A_14_P119635 CCCTCACTCAAGAAGTTTAAAATGAAGTGAATGGACAATTGTGAGAAGAAATGCTATTGA

A_18_P14136357 TGCGAGCCATAAGAATTGAGTCAGAACTTTAAAAATCAATTATTTAGCAGTATGGAGTGA

A_14_P127450 TCACTCCTCTTGCTTTATGACAGTGAAGTATCTTTCAAGAACAAAGTTGGGATATTTGAC

A_16_P01926320 AAAGTGAAGTCATATGACAGAAGGTAAATGGAGTTTAAGGTCTGTAAAAGTAGATGGCCA

A_14_P122563 CTCTTGTCAACCCCTCAGCATCTATACTTCTGACATATTCCATCAACCTGTGTATTAATA

A_14_P111009 AAATGTCTCCTTAATGTGTCCTTCATGTTTCTTTTTTTTCCTGACTCAGATTCTGGAAGA

A_14_P120477 CTATCAGACCTGAATTTTCTGTATACTCACATAGCAGGCAGATAGCCTCTAGTATTTCAC

A_14_P110132 CTTCCTTGTGAGGATTATGGGTCCTGCAATTCTACAGTTTCTTACTGTTTTGTATCAAAA

A_16_P19355711 AAAAAGATCTTGAGATGCTCAGAGATGTATAGAGATGACAGATTAATGGCCTTCTCACAC

A_14_P129025 ATTTACATACTTATCCTTGGACTAACTCAGTTTTTGCAGGAGTTTTTCTTCTACTTGCCC

A_14_P130790 CTTCATTGTCATGTAGGCTTCTATGTAGTTGATGAAGATGTCAAACTCACTCATGGCTTT

A_16_P17516271 AAAAAGGAGTTGTCTTTTTATGCACATGTCAACTCATCCTGGTTAGATTGGAAACTCTTT

CUST_8118_PI426210822 TGAGAAAATGACAGTAACCTACTAAATTCAAGAACACCCCCTATGTACATTAACAGTGCT

A_16_P21297156 CTTGCCCTGCTATATTGACTACCATTCAGTCTCAGAATTAATGTCATTTCCACAAAGAAG

CUST_1505_PI426210822 TGTGAGTGAGATGACATTCAGTCAGGTTGTGCCCCATCATTGGCG

A_14_P100766 AAATTATAGAGCAATTCTGAATAAACTGATCAAAAAACGAAGAAAGATGAGCGCGTGCGG

CUST_7282_PI426210822 GAGCTGAGTGGCAGCATTGCGGGAGGGCTCTTCCTCCCTTGGTTG

A_14_P134648 CTAATAAAGGGCCACTGGGATATAGACAAAACATTGGCATTTTAAAACATTTTGGACATA

A_16_P19899855 AAAGGATTCATGACTTTGTAAACCCAAGATACAAAAGTGTAGTTTCTCCTCTATTCTGCC

CUST_8481_PI426210822 TTCAAGTTGTGACCTTCCAGATCCGAACCTTAGAGTTTCTGTATGTGATTTCAAA

A_18_P17042359 GAATTCACCTGCAACATCACACTCCTCAAGGAGACGTTTAGCAGACATTATCT

A_14_P105108 AACTCCTACTTCCTAAACTTCAGTTTGTCTTTGCTTCTTGAAACCTAAAAAAGCCTAACT

A_16_P20842937 AATCCACTCATAAAATGGACTCTAGGAACATTCTAAATCCCAGTTATGAGAAGTAGGTCC

A_14_P110302 CAAAATTGCTCAAGTATGTAAGAATCAAGAAGAATCCAAGTAATGTCCTCTCCTTTGTGG

A_18_P21867124 AAAGACTTTGCACTTCTTTAGTCCTTAGCCACTGAAAATTAATTTAGGGACAAGAGAAGG

A_14_P135882 AGCGGAGTTTTGAATTGTGTAAAGGATAGTGATTGAACAGAAAGTCATCTCTCCACAAA

A_18_P13542517 ACCATCTGTCTTTAATCATCCAAGCATATGTATTCCTGTCGTGTCTTACTTTTTGCAGTA

A_18_P15983582
AACTATGCCTACCGAAAACGTAAATCTACTATTCAACTTTCTGGGGGATCAAGATTCATC
A_14_P111972 CTACTTTATATCAAGCACTGTGTAAGACTAAATGGATTAGGGACCTCAAAGTAACCTGCA
A_16_P40047974
TGCACACTTTTAGAAATACAGCAATTGAACCTTCATAAATATTCCTTCTGAACACGTTGC
A_16_P18567302
AAATCTCCAGTAAAGTGAAGCATATGCTCACATCTACCATAGGAAAGAAATAGGAGTGTG
CUST_1436_PI426210822 AGTAGACACTCAGAACGGCAGGAAGTGGCACTGTGTTGCAATCAT
CUST_319_PI426210822 TCTTGTTCTGATACAAGCTGGCTACTCTTTGTATTGGAGTGCTTGTCCC
A_16_P19113237
ATGGCTAAACCCAGTTAAACTCCCATTGATGATGCTGATTACTCTAGGATCTAATTTTCT
A_14_P114849
CAACAGGTGGACTTGTGAAAAAAATTTTGGAGACGAAGAAAGATTATGAGAAATTGCAGC
A_14_P128473 TAGCACCTCCATTTAAAAGTTGGAGACCCTCCACCTTCTTGTTAGTTAATCTAAATACAA
A_14_P122086 CTCTGATAAGGATATCCTGAGACCACATTCAAGATCCAAAACCTTTCCTCATAGATGA
A_14_P132438 TAGAAGGGGAGAGAGATTAGGATTTTAGGGAAGAGGGGCAAAATTATAATTTCCATTTAC
A_14_P131977 TCGCTTATTTCCTTATACCTGTTTTGTTCTCTAATGCTTAGTGGTTGTAATTTGGGTGCA
A_16_P02346601
GCTTCCAAATCTGAAGGTATTTGTGCTGAAAGAATCATCTGTTGTGTTCCATGAGTTTTA
A_18_P14795784
AAAGGATGCGGCTAATTTCCAAACTAGATAGTGTCAAATTCAGAGTTGTCTACAGTAACA
A_14_P132322 TTACAGTTTTTTGAGGTGCCATTTGACTCAAACATGAATAGGACAAAGAACAGACCG
A_16_P18602791
AAAAACACATATGATTCTCCCTCTATAAACTCCTTCAAAGTTAAAATGGAAAACCTACCT
A_16_P30952379
GGCACATGAGATAAATGAAGATAAATGCATGAAGTAGAATAATTTTCAGTCTGGTTAGCA
A_14_P108585 AAAAATGAAGTATAATTACCTTTCCAAAGTCACGAACATCAAACAAGTCAAATGCCTCGA
A_14_P105310 CTCTTCTCCTCCTCTTATCCTAACAGTCTCATAGGTAAACTTAGAAGGAAGACAAATACT
A_16_P16280791
CCTCAATTCTGGCCATAATTGCTGTTTTTCCTTAGAAACTGTTAAAAGGTATGCCTTGAT
A_16_P02129971
TTTTGAAGCCATCTCAGAGTAATACAGCAAGTAACTAACCAGTCTTACAGAATACCATCC
A_14_P115396 GATCTTCATCACCATCAACTCTTAAAATCCGAGACATAACCACGAAAGGAGCAG
A_14_P128241 GCTTTCTGGAAAACTTACTTTGAGATGAAAACAGGCTACGATGGGCAGTTTCTT
A_14_P202170 AGATGGCTGAATCTTCCAAGAGAAACAAGGTTGGATAAGCGCTTCCTTTTTTTTAG
A_18_P14136762 CTTCAACAGTCTTGCTTGCTTACATCTACTAACTGGGCAAGAGAGACCATTCT
A_14_P116246 CTTTCTAGGAAGGATGTGAAACACAGAAACCACACAAGGGGATATTTAACATCGTAAAAG
A_14_P112986 AAATGGCAAAACTTTTATTGAACATCATGCTGAAGCCCAGAAGAAAATGCCATCTCC

A_18_P13489256
GCAATGTCAAGTTACAGCAAGGTTAAGAACACTACTTTGAATTAAACGACTGAGATTAGG
CUST_73_PI426210822 ACAGCCCTCACTGTTACTCATCACCCTAAACTGCCTCCAACAAATA
A_14_P130117 AATCAAATTGCAATGGGCAAGGAAAGCCAAAGAAGGGTTTACACAATATTCAATAAGCAA
A_18_P25763557
GCCAGCAAAGCCATATTTTTTTGAGTTAGTAATTCCCAGATTTTTGTCAAATTCAACAGC
A_14_P131021 CTATTGTGTTTTCTTTTTCACACTTCAGAAGAGAATCCTAAGGACTAGACTCCGCTCAG
A_14_P117882 TGTGCCCAAAAGGTAGTAGGCATTAAAAGTGATAGTTCCATTTCCTATTTTCCACATAAG
A_16_P21093802
AAACGATGATGTTGATTTTCTCCCATTATTTTCACTGCTTAAAATTCTCATGGTGCCAGT
A_16_P19892164
CTCCCTCAACTATGTATGTTGTAATTTACACTTTCCCTAAGTATCAGAAGCCTTGTCAGT
A_14_P122899 ACAGAAGCAGACAAAAGACTTTCTCGTTTTGATATTGAGATGAGCATGAGAGGAGATATA
A_14_P118392 CTATTGAAACGAATCGCAAAAATAATGTCAGTGTTTCCACTTTTGACAAAAATGGGAAGG
A_14_P109799 AAGATTTCATAAGTGGAATGCACCAAGATTTACTTAAGTCCAGACTGCTGGTTATTTAGG
A_14_P131544 CTTGGATTTTTGAATTCCTTAGAAATAATTGTGGAAAGCAGGGTTTGTTGTGAGAGAGAA
A_18_P25991773
AAAGGCCATACTTAACCAAAATGTCCATGACTTCTTTCATTACTAACAAAAAATTGCCAA
A_14_P126345 GATCACTATTCTCTTTTTTCCAGGTTTCTCTAGAGGACTTTCTAAAGAAAATTCAGCGAG
A_18_P14139459
GGGCAGGGACATGGACGTGCTATTTTTTTTAAAGTAATACATATTTACCTTAATTATCTG
A_16_P02089852
CACGTCTTTTGCGAATGATTAGAAAATCATGACATCTTTAGTCCTGTAAGAACCGACTTT
A_14_P130744 TTTTGAGACAATTGGGGGCTTGGGATTATCTTTAATTCAATTCATAAAGTTGCAACACAG
CUST_6919_PI426210822 ATTACCTGTGGCCCTTGCATCAGGGAGAGCCCAGCCTTGAGTGAAA
A_16_P00812192
TAGCCATTTTCTAGCAATTTCACAACTACTACTGAAATCAGCATAACATTTGCAACCTGG
A_14_P134226 ACTCATCAATACCACATTCTTTGTTGTATTCAAGAACCGTTAAGAGTGTGCTAATTCCCT
CUST_2869_PI426210822
AGAAAATTATTCATCCCCAGCATGTTTCTGTCACTTTCTCTGGCATTCATATCTCAATC
A_14_P133013 AAACTTCACAATCACAAGAAACAGAGCAATGGCTCACAAAGCAGAAACTGCAC
A_14_P101858 TTTACAAAATACTGTGGTGTGGTATCATATTGTAGGCAGCGTTAATGGATAGAGATGGAG
A_14_P101359 TTTCCAATTACTGGGAACCGTGACAAAAACTACCTTAAGGGGACAAAATAATTTCTAACA
A_18_P22841773
GACCAATTAGATATGTTTTAATTTTTTTATGACCGGAATCTTTTCCTTCAAATGAACCTG
A_14_P101192 GGAATAAATCGGTAGCAGCATTAAAGGAAGAAAGGCGTTTTGCTGTTTCCCATAATAAAA
A_14_P132609 CTCCATGATCAGAAGGATGGCTACTGTCAGGACAGCAAATACGGCAAAAATAATAAAA
A_16_P17133992

ATCTTGAACAAATGCTCTAAGATGCATACATTTAGCAGATGGACTCACTTATCATGTGGG
A_14_P122792 GATTTCAGAACACCCCTCTTCATCAGAACGTATCCAGCCTGGGTT
A_14_P106760 AGGCCACCTTTGGTAAACTTCTACAAGTGATTCTAATTATGAAGTGCTTTGCAAAATCAG
A_16_P16773353
AAACTGCCACATTGTTAAGAAAGAGACCATTACTGAAACATAAAGTTTGGCTTGCAAGTT
A_14_P104902 CTGATGCATCTGAATGCGTTTCTTAATGTAGGTTCTGAAAAATAGACAAGGCCCCTTTAA
CUST_7351_PI426210822 GCATGCCCACCTCCTAACGGGTTGGGAGTGAGGTGGAGTGGGGAG
A_14_P111877 ACTTCCAAGGCCTTACATCAAGAACTCGCTGGAAGTCTTCCAAGGTAT
A_16_P15414056
ATTTATGCTCAACAGAGAAGACAATTCCTCACAGAGTTCTGATCTCATTTGCCATTAGAA
A_14_P115729 ATACATGGTATTAACACCATGGATCTGAAGCAAACAATGCATCTGGTTAGCATACATTGG
A_14_P115726 TATCTTCAATAATAAGACATCCTATTTTTGGGGGGTTAGGGATAGAGATAAATATGAAAA
A_14_P106732 AAGCATCTACCAGCAGGGGCTAGCCCTTAACATCTACATTTTGATAAAAATTATACCAAA
A_14_P101724 ATTGCCCTGGGTTAGGGGAGAATAAATAAAATCTGTGGCATCAGACAGGTATTAC
A_16_P00562926
CAGGTAGATTGAAAACCCTCTTATTTGTGCTTCTCTATAACCCAAGACACAATTTTCTCA
A_16_P16191741 GTGACCACAGAGGGTCCCACCTCAGTGTGGACATTTCCATGTGTC
A_16_P15153122
CTGTAACTCTCCCTTCACTAATTTCCTTCATTGTTAAACTCCTGAAATGCTAACCATCAA
CUST_8300_PI426210822 AGTGACCATGGGTGCTTGACTGTGGGTGGGGATGACAGATTTCAT
A_14_P116717 TCACATCCACAAAGTCACCCCAAGAAGATCAAACTGCTATTATAAAAGTGTAGAAAATGG
A_14_P132873 ATTGATATATGTAGATGGATTAATGAAGGGTATATACCTTCCAACCCCAGTCACCTCAAT
A_16_P16173313
AGACACTTGAGGAGAAAAAACACGTTACCATTTTTCAGATGACATTCGTAACTTTCTTGT
A_14_P109803 AGAACTCATTCAAATACTTACAGTGAATGACAGAATGGATGAAAAGAAAGTGCCTTCATC
A_16_P03221574
TCAACAGTCACTCAGGAAATATTTCAGCAACCTCTCATAACATTGTTTTCTTCCAAAACT
CUST_8796_PI426210822
CCATCCTAGGGTGTCCTCTTTGCTCTCTCCATATTCTCCAAATAAAAATTCAAAAC
CUST_838_PI426210822 ATCTGAATTCCGTCCTCAGCACAATTAAGCATCTCCCCCTTGTCG
A_14_P116995 TGGTTTTTGGTTCAGGTATTTTTTGGATTAGCATCATCAACACTAGAACTCAACATGAGA
A_16_P03678732
AAAAATGAGGTCATGACATAGATTTGAAGGCCAACAAAAATTACATAATCAGTGCTCCGT
A_14_P115073 TTTTTAAGAAATTCCAGTGAATATGAAGGGCTCCTATCTGAAAAGATGCACATTTGGCTT
A_14_P123593 AACAGAGGAAATATCTTGGGATATAAAGAAAAAAGATAAAAAATAAGAGATATGTCTCAT
A_16_P16095640
CTGCCTGATTAATCTTGTGTTTTCAACGGATTTGTCTCAGTAAAATGCACATTACAGTGA
A_14_P123519

AGAAAGAGAAGGTGGTATTAAGAAAACGATTGAAGTCTTGAAGCAACTTTTGAGTGATGT
CUST_6931_PI426210822 ATGTCAACAGAGCTGTAGTTACGACAAGGATCAATTAAGAAGGACGTTTCCC
A_14_P125456 TCTGCTCTATATAATGGGCTTCTAGGTAAGACCATAATTGAAGAAAGGGTCAAGGCTAAT
CUST_9373_PI426210822 CAGGCAAGCTGGGTAGATGTAGGTGGGGACACCACAAGACACCTT
A_14_P123792 ACACAGCATCCAGCACAGGAATAGATAAAGTTCTCTGAAGCCTATAAAAAACATACACAA
A_14_P116469 ATACACCTCAGTTCCAAAGTTTACCTCTTCTAGTAAATTACACTTTCCCAGAAACTATGG
A_14_P202372 TTTTGATGGAAACATTACATTTTTGGAGTGAACATGTGTCAACATCACTGTGGAGAAAAG
CUST_5117_PI426210822 ATGCTGAGATGAGTGTGAGTGGCCTCTGCACCAAAGAGTTCAGTC
A_14_P115511 CTTATTAGAGATCTTGAAGTATGAATGGTTCCCTCTTTGGTTCTTTTGGTTCCTGTCTAT
A_16_P18105451
TCTTCATGAACAATTTACATATCACAACTCCAACCTAACTGATTATCCAAGAAACCCCAA
A_16_P20743391
GAATGTGTCCATCATTTTCGTAAGTCTTAGTATGCAGAGAATCTCAGATAGCAAAGCAGA
A_14_P100535 TATCACACTGGAGCAAAACTGGCTATTTCTGTGAATGATATAAAACAGGGTTCTCTGTAA
A_14_P135824 CTGTCAGCAGTATTTCAGAAGCATTTGTTGAACACCTATTGCATGCAGATGGCT
A_16_P17438480
GGCAAGGATAGCATTATCACTTAGTTTATGAACTAGTGGAACAGTTTAGGTATCTGGGAA
A_14_P113717 TTTGTTTGCCCAACAACTTCATATTGCCAAATATTATAGGTTTGCAGACTCGAGATATCC
A_16_P16077004
AAAACAGAATCGCTCTGTATAATCTGTGCTCCCATAAGAATTGCTACACTTCTCTTGAAA
A_14_P131269 TTGCTATTTATTAGGGTTTATGACTAAGCCAGAAAATTATGAAGTTGGTTCAAGGGGGAG
A_14_P124603 AACATCTCGGCACCTAGTAATGGTAAATTAGTCAATATTTGTTAGTTTCGTTTGAGATCT
A_16_P02030182
TTTGAAATCAGACCCCATTAATCAGAACTCCTCACACTTATACAATATTTTACCATGGGC
A_14_P131357 GGCTAAAATAAAGGACTTCTTTAAACTTACGACCTAATATCAGATTACGCCCCGAAAGTC
A_16_P36571133
ATTTCCTTATCCATCTTCTCTGCATCATACCAATAACTTTCTTGACTTGCTAACTTTCCC
A_16_P18591314
ACCAAGTTGATGATAACTCATCTCAGATAACTCCTATGGTGGTTAATACTAGCATCCCC
A_14_P111782 CTGTTTGGATTTCTGGTTTCTCCCCAGAAATAGTGTTTAGAAGGTGGCTACTTTTT
A_14_P137135 ACCACCAGGCTTTTCCAGAAGTAATATTATGTGATGGCTAAAATACTGAAGGCAAATTTT
A_14_P127635 TTAAAACAACTATGGGTAACATACCTGTTTCAAGTCGCGTAGAATACTGATATAAGAAAT
A_16_P02577367
ACACCAGGAATGTATTTAAACTACCAGGATACTGTATCAGTTAAACTCCATTCAAACCCA
CUST_8184_PI426210822 GATGATGGGTGGTCCTTTCAGGTGAGACATAGCTGCTGAAGTCTA
A_16_P02393019
TTGTGCACTAAGTCTAGAAGATCCTCTCTTTAAATACGTGATGATTCAGAAACATCAGGC
A_14_P137128 AGTGTGAAGCAGCATGTAAATGATTCATTAATGTTTGGGAAGTTTCCTGAAAGACATTTG

A_14_P108149 CCTCATAGAACGCATGTTTATTAACCAGATGTATTTGATATGCTTCAGTATACCTAAATT

CUST_3183_PI426210822 GGAAGGGGAGTGGCAGCCGGGCCACCTCCTGGAGCAGGAACTGGG

A_14_P139349 TGCACAAAAGTATATTTTAGTTTTTGGAAGGAAGGAGATGGTGCAAATGAATTAGAGGGT

A_16_P38204146

TAAGATTCTCTTGGACTTTGAATCCTTCTACATTTGATACGACTTTTCAAACCACACTCC

A_14_P106174 CTCTCAGAAATGATTAGTGATATAAGTAATCAATGGCCAATTAGGACAAGTTGAAACAGT

A_14_P112669 ATTAAAAGGGAGAGATAGTTCCTGGACAAAAATGCTTTCCAATTTCTTTACAGATCCTGA

A_16_P03767768

TTTGCCCTAATTCCCTCTAATACCTGATACCTTCAACTATTAATAGGCATTCACTATCCC

A_14_P111847 AGAAGGGAACCAACATTTATACCTGACCAGATGGCTAAAGTGCTTTTAAAGTTTTGTTTA

A_16_P21009457 AACCCTGCTGAACTGATTTTCTGGTTGTGCCTTGTAGTTTGTAAAACATTAGTCTAGG

A_16_P20623959

TGCCCATACCTAGAGATAATGTCTGCTAATTAAATCTATCTTTCCAGCCTTTTTAACTCA

A_14_P117565 CTCAGCACTTAGGAGACACCCCATTTTATAAGTGAAAACAGGATCCCACAC

A_16_P03308804

ACACATCACTATACGAATTAGAAAACAAATGATCCATAGATGAGAAGGTAAGTTGACACA

A_16_P16192005 GAGAACAAAGCCTGCTCAGTGTTTGGCATCTGAAAAGACAAGGCTTCTT

A_14_P126259 ATGACCAACAAGCACATGAAAAAGACATCGTTATTTAAGGCAGACTCTCTCCAATTATTT

A_14_P116259 TCTCCTAACTTCAGAGACAGGGACTCTTTTTGGATCTCTATTGACAAGTAATAAAAGTCT

A_14_P135830 ACGTCCATTATGGAGAAAGATTTGACTTACAGTTCTGTGAAAAGACATCTTTTGGTCATC

A_16_P15210301

AACATCAAAAAGCCTTATCATACCCTGATGGTTTAAATCTTTAAACAGAGCGTAACAGCC

A_16_P21660830

ATGAAATCCAACCTACGAGAATCATGTTACTGTATATTCCCCTCTCATTCTATGCTTTCC

A_14_P110600 GCCTTCTTTGTAATGCTGATAGCACACCTAGTTTTCGGTTTTATAAGACACTCTATTCAG

A_16_P18384788

TAAAATTAGTATGCATGTTGAAAACCTTGGATTCCCTGTAATTTCACATCAGCCCTATGG

A_14_P128597 TTGTTTGTTTAAAACTTCCGTCATATAGGATCTGAAACCAAACAACTTGTTGCTAGATGA

A_16_P30952645 ACTGATTATGTCTTTTATATTTGTATCGATGTTTTATTATTTCTATTAAATAGTTATATA

A_14_P105583 TGAAAACCAGAAGTTTAGGATGTTTGTGGCTTTGTTCAGGTTTCTTTACAACTAAGATGA

A_16_P16069117

GTCTGTAACATAGTAATTCTCATTTCCCTGAATGTGAACTCTAATCCAAATGATCGACCA

A_16_P00429717

AAATCCCAGTTGAACTTGATATGACAGAGCAAGTAACTTAGAACAACACTGATTCTGCAG

A_16_P00684996 CTGGGTATAAGACCGAGTGTGTCTGTTCTTAACACTTGTCAAATTCCCCTTTTAAAG

A_14_P105359 ACTCTCCATCTGAATCTGCAAAAGTATATGATAAAGCGATAAACCGAAAAACAGGAGTTC

A_16_P18577776

AAAAAGGCAAGGTATGACTCATCAGTTCCTTTCTCTTAAGTAAATGACCTAAACACCATG

A_14_P125580 ACAATGTTCCATACGTTTCCACAAAAATATTGTAACTGGCATTCACTCATTATGATCCAC

A_14_P120379 CTACTCCCAGACCCAGATACATTTTTGCTTAATGCTCTGAATAAAAACTAAAAAACCAGG

A_14_P202495 TAAAACCACAAAATACCCAGAGAATTGTTACAAATTTCCCTTCTTTCTCTGACTGTTTTT

CUST_10396_PI426210822 CTTGGGGTCACAATGGCTGATTCTTCAGCACTTCTAACTCAGCTTC

A_18_P11862770

TGAAATCCTAACCACCTTCTAAAGTCTGTTTCAAATGTGTAATTGCCTTAAAATCCTCCA

CUST_6233_PI426210822 AGATTTTTGCAGTTTATTCTGTGTGGCTGTGTGTGTTGGTCCCTTGC

A_14_P131238

TCAGGTTTTAAAGACAAAAGAAAGCAGACTCAAAACACAGACAAAGCAGAGAAGAAAACA

A_14_P138941 CACACACAAATGATACCTGTCATCTCTCCACTGCCATATTACTATAATTCAGTCAAGTTG

A_14_P136063 AAATGATAATGCAGTTGCACCTGAGGCTCCGCATCAATCCACAAATTTTTAAAGTATTAT

CUST_5710_PI426210822

GTTGAATTTGCAAAACTTCTTTTTCAGTGTTATGTTTTATTTTTGCAATTTGTGGGGGGA

A_16_P18290944

AAAGGTGTGATTACTGTCAGAAGGACTGAAATGTCTAAGGGTTGAAAAGAATTCATGAAG

A_14_P139022 CATTATCTGGGACAGCAGAACCAGGTTTGGCTCAACAGATTTCTCTTTC

A_16_P20498077

GTAAAGTCCAGAATTAGACCTCAAATATATCCCATCCAAAATTATCCTCATCCCATCCAA

A_14_P109031 GAAAATGACATTTAATGGCCAAAGCCTCGACTAACATTTACTAATGGCATCTCCAATGTA

A_14_P118807 ACGTTTTATAATTTGGTTACTAGCCTTCATACTCATTTTGCCAACCAATCAACATAAAAC

A_14_P100233 AGGGCATGAATAACTTGAATATTGAAGGATAAATGGATTTATCCCTATAGAGAAGTGGGG

A_18_P16280395

AAGGCATGCTGTGTAACTTAAACACTAGATTTAGTTGGGTTAGAAGGTCAAATCTATGGA

A_16_P21371853

ATGTCATGATTGCTTACATTGGTCTCATTCCATCAAGTAAAGAACCCTCATGAGTAGAAG

A_14_P202188

AACCAGAAGAAGAAGAAAAAGAGGACTTCAAAGGTAGGACTCAAACCTTATCACATGTAG

A_14_P201090 CATTTATTTGAAGAACTTTCCCTTGGGATCTGTCCAAATCCACTTTGATACCATTAGAAT

A_16_P20013728

GCTCAAGAATATTGCTCTCCTTTTCAACTCTATTCCAGTAAGTCCTTCATCATGCTTTTT

A_18_P13782374

AAATTCTCTTGGACTCTTCCAGATTCTTCCCTCAAATATGGAATTATCAGGCAAGTTGAT

A_14_P137444 TTCTACAAGAGGAGTGCCTATCTCCAATTTGGGGTGAGGTTTCCAG

CUST_5004_PI426210822

TGGGGGCATCTGTGTACTCTTTATGGCTATATTTCCAGAATATGCTTTTAAAGACTGT

A_16_P00417471

ACATTGAGCCAAACTTTTGTTGAACTCCTAACTAGATGCATTTCCTTTGTCATCAACTTT

A_18_P22842652 CAGGCCCTGGCTTCTCTTCTTTCCGGTAGATGAACTTGAGCACAT

A_14_P123601 CAGATTTTAATTTTTCTCACTTGTAAGTTATGGATCCCTAAGGCCCCTAGGAGACAAACA
A_14_P123988 GTAGTCTTTGTGACTTGGCCTGTCTTGGTTAAACCTCTTTCAAGGTGAG
A_16_P19920733
ACCTCCTAACACATAACACTATTACTTCAGTGAGACACAATTTCTGCTACATTTCATCCA
A_14_P130161 ACTTTTTTACATTCATATCTTACCCGTTGTTTTGGTCCCTTATGGGTGAAGATCATGTAT
CUST_6425_PI426210822 AGACTTAACTGTAAAAGCTCCCAGCCCACAGAGGTGAGCAGAGGC
A_14_P137935 AACACATCTACTGCATAAAATCACATGCTGTGCACTATGACTATACTAGTGCTCCACTGA
A_18_P17312893
TTTTCTCATTAGTGACAAGACATAGAACTTGCAAAGTAAAACAGTAACCATATGCTGATG
A_14_P128788 ATCAGACAAAAACCTAGATAACAAAACAAGCATAACGGGACATTTATGAAAACACCACCA
CUST_3640_PI426210822 GAGAGGTGGCCCACAGTTCATGCACAGGCTCGAGTACAAGCCTTC
A_16_P17150377
AAAGCATGACATTCTGAAGTGAATACTTTTCTAATCAGTCCTTGCTCTAAAATTGTGCCC
CUST_9092_PI426210822 GCAGGCACTTTTGTCTCCCATGGTCTAGGCAGGAGTCATTTTTTT
A_14_P134380 CTGTCTGAGCAGATTCCAGTCTTTCTTCTTCTAGATGCTTTGTCTTTATTTCCTCCATTT
CUST_9395_PI426210822
TGGTTTTGATTCCAAAATGACTTCATTTAAGAGCAAAGAAAGAGTTAAAAGCAACTCATA
A_16_P16382430
AGGGGATGAGAGTAAATTCACCTACTTTTACACAGGAAAAAAATAGATACGTATGGCCTT
CUST_2544_PI426210822
CTAGTGGGTACTTAATGAATATTATATAATGACTAATAAATATTTTCTGATTTAAATGTA
A_14_P128882 CCCTCCCTGTAAGTCTATTTAAAAACATCGACGATACATTGAAATGTGTGAACGTTTTGA
A_16_P58424100 CTAGCAGGCCAGGGCAATGTGCTGGAATTTTATTCTGTGTGTGAGT
CUST_3529_PI426210822 AAAACGGACAAAGCCACCAGCTGGCAGTTGATGCAGGTATCAGAA
A_18_P22840339 GTTTGTAAACTTTCTTGCCAAAGCCCTTCCCGACTGAGATGGAGTTGATATTA
A_18_P25731146
AGGAGCAACATTTAGTCTCTCATCTATCACCGAACTAGAGTAGATTTCCAACAAGTATCT
A_14_P113158 AACAGATATGTCATGGAAAGCATTAGAAGTATGGTGTGTTTGTGTGCTTATGTTCATGTG
CUST_7790_PI426210822
ATTGAATTGATTTTTTAAATTGAATCCATTTTAAACATGGGATTTTATTTTTATTTAAAG
A_14_P113578 TAATCTGTCCTCAAGGAATATTGAAAGCGTCATCAATGTCTTGAAGGAAATTACTGTATC
A_14_P113757 ATAGATATGGAATCATTTTATAGATATGGCCCAAAGTCCAAAGTCACACAACTCATTCAC
A_14_P116680 GTTGTAGAAAGAGTAAGACGAAATGGTTGGGAAGGAAATTTGGTTCTTGAGATACTGG
A_16_P18294066
AAAAAACTGACCAAAAGGAAAAATATCAAACCTGTCTGATGAATGATTGCTAAGTGGAGG
A_14_P135564 CAAAATGGATATCTTGCCATTCATTATCCTTTGATTTAACAACCATATCTAGACCACCCC
A_14_P131422
CTTTTAAGGGTGAGTTAATAAACAGCAACAACAAAGTGAAAGGAAAAAACTCACCACTCT

A_14_P116330 AACCGATCACTCCAGGAGGTTGACATTTTATTTTAACTGTATTTTGTATTCTAGAAAAGA

A_14_P106669 AATTTACAGAAGGAAATATCTACATATTGCTCCTGTCAACTTCTGGGTATTTTCCCCACA

A_14_P108605 ACAAACTCCTAGAGTCTACATGCAAGACTAGTAAAGTCTTATGGAGTCTTATGATGGATT

A_16_P41033525

ACAATGTATGACCAAAAACAGGGATTACTCTTATGACTCAATAAACTTCCGAAATTGGGT

A_14_P134068 CTACTCAGAAATTCTTGTATATCTGGGGAGAGGGGTGAAGAACAGTCAGCAAT

CUST_4962_PI426210822

GATGCTTGGATTTGTGTTTAGAAATTGGGTAACTCAGTTGTTCATGGAGGAAAAAGTT

A_16_P00685021

TGTGAATTTTTTTGTGATTTTTGTAAAATAAATTACAAGTGGCAATTAGTCACTGTGCTG

A_14_P104771 AATAATCTTCTGGAAATGGCTCTTGTAGAAAAAGGCATTGAAAGTGCTTCATTGCCAATA

CUST_3892_PI426210822 GGGTCTCTGAGGATGGATGCGTGTCATTCACACACCAAGTGTGTT

A_16_P37240363

TCTATTCCCTGCATTTATGAACTCTCTAAATCCCTTCTACTTAAACCACAGCACTAAAGC

CUST_3672_PI426210822 AATTGGTCAGTATAATCCTACCTCTTGGAGCGATAGCTGGCTGGATTCT

A_14_P115084 TCATAATTAAAGTCCTCAACAACTCTAAGGGAAGACTCTTCCAGTCAGACTTTTTCATTC

A_18_P15244640

AAAATAACTTCAGCCAAGTATGTAGATCACTTATCCTTGAAATGGGAATGATAGGTTGGG

A_16_P15494527

TCTCTGTCCCTCTCAACATAATTTTTGTGAACAGGCCAAGTAGATAAACAACTCAAAAAA

A_18_P22839937

TAGGGTTTCTAATTTAAATGAGAAGTAAAAGCCAGTCAACAACCCTCACAAAGCAAGAAA

A_16_P58424131 TACAAATCTAAGCCATAGTTGTCTTGCTTCCAACCCACTGCGGAATCCTT

A_14_P119857 TGCCACATGGGACAGGTATTAGACTTGCATGTGTGATGCTGTTATATATGTATCTATCAT

A_16_P02458971

AGGTGAATGATGAGAGAATTCAATCTCCTGGAATCAAAAGAACTTTTTGGTGTGTATTCT

A_14_P200537 TGCCGTCTATAGTCTGCATTATAATCCGGTTAAAAGGAAGGTCCATGTTTTATTTTTCTG

A_14_P137496 AAGCCTTAAACTGCATCAAGTCAAAGAAACCTGGGGCAAATCCTTAACATGTTTTT

A_16_P21239529

CCAGATTCCTTGTAAGTCAAACTCCTAAAGAATTAAAAACAAGTCATCACCTAAACGTCA

A_14_P115148 GGTGCCAGAGTCCATAACATTCCTTGGAGAATCAACAAAGTCAGGAAAG

A_16_P17173701

CACCGATTGACATATGGATAAGGCAAGAATTTTCCCAAAATTACTCTGACTACCTCAA

CUST_5438_PI426210822 AGAGCACTGGGGCAAGTACAGGTGGTCCACACTCATCAGATTCTG

A_14_P124785 GGCAAAGAGATGGTGGCCTTAGTCATTTTCTTCTATTCCTCTCTTTGAGTTAAAAATACA

A_14_P134397

TCCAGCCCAAGAAAGAAGCCAATAAAGAGTTTGTAAAAGTTGTTTCCAAGAAGATAAACA

A_14_P133153 CTGCTGTCGTTTAAATTCACTGTATTCTCTCTGATACCTGTGAGCAGCCAAG

A_18_P14135690
AAGTTCTTTTAGGAAATTACCCAATGAATTGGTGGAGGTAAATGATGCAGGAGGTTTG
A_14_P117021 AACAGTTGTATTTTGCATGTCTTTGTTTGTAATCACTGAGCCCATACAAATCCTTGCACT
A_14_P200823 CAAGTGTGCACCAATAATCGTAATTATTGGGGAGTGATAAATGTGAATTGCAACTCATAT
A_16_P39070902
AGTCAGCACCTTGTTACTACTCAACATTCAGGTAATTAATATCCTCGATTAACACACACT
A_14_P120752 AGTTGAAGTTGGGAAATGAAAGACAAGTAGAAGAACTCAATTTAGCAACATCCTTTCCTT
A_14_P127960 CTATCTTCCCCATCCTGAAAACTATCTAGATTCCAGTCTAGAACTACTCCTGGG
A_14_P118163 GTAATTTTTATTTAAAAAGGAGACTAAACAGAAGTCAGGGGTAGGTGGTTCTCCATGACT
A_14_P128510 TCCTACCCTAGGGTTTTCATAGCATGTGATTACAGTTTGTCTCCATTCTATATAGGAATG
A_16_P20757229
AACTCTAGGATCCCACTTTTAGTGTAATAACCATTTCAACATTTTCTGCAGAATCAGAGG
A_14_P201862 ACCCTTGTTTATTAGGACTGTTGTGAAAATGACGTAGGATCTGATAAGATGACTCAGTGT
A_16_P00685019
CTAGGATGTCTTTTTATAACTTAGCAGATGGCATCTTTTCAGTGGCTTGTTCTGAACTCT
A_16_P03109221
TGGATAGATTCATACTACTCCATTATCACTAAGGTTCATTTTATTTGCAAGCGGTTTTGC
A_14_P126425 TTCTCAAAGTTGATGAGATTCTCCACTAGTGTGTGGTTTCCCTCATGAATGAAG
A_14_P117523 TCATCAACAACTGAGAAATCAGCCTTATAGGATGGCAGAGATGTTTTAGTGACATATACC
A_18_P10664100
AAGTCACAAAATCAAAGTCCTCAACATTTGTCCTACTTCCATATTCACTGTTTCTAGCAG
A_16_P30952241 CTGTGTTTAAGTGCCGTGTTCACACCAACCCTGACCGGAGATGCA
A_14_P129246 CTCAAGACTTTGAAGTTAGAGAGTGTAAGAATGAGTCATGCATGGAATTAGGGTGTTAGA
CUST_6688_PI426210822 GTGAGTGTTAGCTCCAGAGTGGTGATGGAGCAGGGAGGTATCTTG
A_14_P130341 GGAAGGATTGAAGAATATTGATTGTCCATTCATCTGTTAATCCTCCTGCTTTTGTTATCT
A_14_P112869
CCTAGAGAAAAGTTGTAGAAAATTGAATAAAGAAGGGACAAGGGTAGAAAATGCACAGAG
CUST_8312_PI426210822 ATGTCTCTCCTACAGAGGGCTAAGCTCTTGTCAGGAGAAACCCTT
A_16_P17193953
AACTCAAGAAAAAGACTTTCAGAGCCTAAGAAGATTAACATCTAGTTCGGACACAATTAA
A_14_P109829 TACTTTTAAAAGTGATCAGAGAGGCACTGTTTCATCAATACCCTGTAATGATTTGCGTTT
CUST_662_PI426210822 GACACTCAGGCCCTTTTATGTGGTAACCATAGCAGAAAGGAACATAGTCT
A_14_P138916 TGTTTTTTTTTCTGTCCACGTTGGTCACCCTTATCCTTATCTCTGCTGTCAC
A_14_P131641 CATTTCAGATTATTTTTTTGAGCAGATTCTAGGGTTGAATTTCAGGAAAGTGACTCCTTG
A_14_P112393 GTATAAGTTTTCAATAAATGCCTAAAGTTCAAGCATAGTATGGGGATGTCCACAGATTGT
A_16_P20440874
GTCTGCTGATTCTCTGTTTTATTTCCTAGAGTGTCTTTTATTTTATTTCCTAGAGGGTCC
A_16_P18239574

ACTAACAATAGCCTAAAGGATTATTGCCTAGAGAGTAACATATGGAGTAATCAGGCAGGG
CUST_4246_PI426210822 AAATCTCCCCTTCCTGTCCATAATTCAGACTACGACAGCAATTAGAGGC
A_14_P109408 CTTGCATGATTCAGTTCACAGGTGGATCATATCAAGAATGCTGGTTAATAAAGGCA
CUST_213_PI426210822 CAGCCCGCCGTCCGCGCCCCGGTGGCGGGCCCGACGCCCGCATTC
A_14_P138068 CTGGAATTGGTGTAAAAATACTACTAAGAATCTCATACCTCTTTCTCAGAGTCTCATGCC
A_16_P18363892
AATAGCCTGTAATCTTTTGCCTACTTCAAAGACTTCACCATTTTTCAACACTATGACCC
A_14_P201173 CTGTAGTATTCTGAAGCAGATTTTCAAGAACTATTTTCCTGTCCCTCTCTTATGTCCTAG
A_14_P115579 CATAGCCATTATTAGTGGATTGGAAATGAAAATGAGTGTGCATTTTTCTCTCAGTTGCTC
A_14_P107149 TGTTTATTGAAACATTAAGGGACAGAAGAAGAGTAGAGTGCATATACCACTTGGACTTGC
A_14_P104565 CTAAAAGGTAATATTATTAGGTAGTTGCCCTCCCCTTTGCTTGAGGACATTGATGG
A_14_P120650 TACACCCTAAAAAACTGGGGCTAGGGAGTTGAAATATTTATAACTAAAGGATGGTCTTCA
A_14_P122105 AAATGTCCAGGAACCCCAAAGACTTACTTTGTGACTTTGAAAATACGCAATAACCTG
A_16_P17707244
AAAAAATGAAGTGCAAATTCACCAGTATATTCAGACTCTCTTAGATCAACAGACTTGGCC
A_14_P138701 ATATTTATCATATAGAGCAAAAGGAAAGTGTGGAACTGAAGGACTCAATCAGGGTTTCGA
A_14_P128116 AATCAATTCTCAGGTTAACATAAGATCACAATGAAGGTGTTCATTCTGAAGTGAAGGCAT
A_16_P40076653
ATGATCCTCCATGAATAAATTATCCTGCGGCTATGATATTTTTAAAATTACCTCACACCT
A_14_P136206 ACTCAAATGTGGTCTGCAGGCTTTTGTCGTTGAAGGAGATCTTCATCTG
A_14_P138594 AGACAGTGACTTTGACACGAAGTTGAAGCACCTTAAGCCCTGTATCTCCTTTATAT
A_14_P107195 CTATCATCTTTGCAAAATGTTGTTTTCCAATTAATCAAAAATGGCCACCCAATCTCACTC
A_16_P58424383 TTAGATTCTAGTAGGGGATAGACAGTCCTCAACCAAAAAGCCCCACAAACAAAT
CUST_1717_PI426210822 CAAACATGCTCTCAGCCCTGTTGACCATTTCCCCCTGATCTAAAT
A_18_P22840325 CAGCCGAATGTTTTCCTGAACACCAGTAAAAATCATCTGCCAGTCACTC
CUST_4438_PI426210822 GGGGTCCTTAGTTTCCTAGCAAGTGCCCTGTCCTGCTCTTGCCTT
CUST_359_PI426210822 TAGAGCGTTCCCATGTTGAGGGACCTGCGGGTGTACATCTCAAAG
A_14_P124049 GAAAATTGGGCCTGAACACTTTTTTTCTGTAATCTCTGTGAGACTACTTGACTACCTAAT
CUST_4468_PI426210822 CCTAGCCCCCTGTTCTCCTGGCTGGCTTTATCCCGCTGAGCAGTG
A_14_P110135 TCTGGTGTAACGAAAATTTCCAATGTAAACTCATTTTCCCTCGGTTTCAGCAATTTTAAA
A_16_P20768217
AAGAGCCAGAGACATTTGAGATTTTCATCCATAATGCTAAATTGATGTTATCAAGGTCGG
A_14_P119983 CTTCATGGTCCCTCTGACACTATTTATATCAGCAAAAAAGATCACTGTAAAATGCTGTGT
A_14_P119573 AAAGAGGTAACCTCTGCAAAGAAATGCACTAATATGAAACAAAGTATCAACTAAAGCAGT
A_16_P15640461
ACCTTGCTATCTGCTTCTAAAAATTTCAAGAGGAACATTCAATTTGGCCATCAATCAAGT
CUST_9972_PI426210822
TTGGGATTACAAAATAATATGTATGTATTGACCTAACATAACATATCAATTACCAAAAAA

A_18_P22841273 CAGGGCTCATCGGCCATTGCCGAGAAACACATGGTTCTCATTTAA

CUST_9680_PI426210822

CTGTGACTTGTTATTTATCATTTATGTGACCAATTATAGTTACAATCATGTTCCATGTGA

A_16_P38914896

TCCCACTGAATTCCTCTAAATATAAAATGCTTTCATCCCAGAAAAACTTGAAACTCACGA

A_14_P202184 CTTCTCACTTCTCTTAGCCTTAGTGAAGAATTGAAAATGCATTATCCAGATTTCCCTGAT

CUST_8676_PI426210822

CAGTGATTCTAAAGAACTGACCTGATTTCCAGGAGGTAAAATTGTAGGGATGAATGAT

CUST_4150_PI426210822 TAGCCATGTATAGTCATGTGCCAGTCACCTAGGGCTGAGAAAGGG

A_14_P107464 AGTCTTGTCCCTATTTTAAAAACAGAATATTGTTGCTGTTGCCTGGTTTCATTTTTTTCC

A_14_P121363 GTCCCACCTAGTTTTATTTTGAAAATCCTCAATTGGCCTCAGTCTTTTAACGATTCTTTT

A_14_P132967 TTTTCCATATCAAGTGTTTCTCGGGAAACTCTAAAAAGCAGGAAGAAATCAGATTACAGT

A_14_P118572 TTAGCCCAATATTTTGGCCATTTCTTACCTCTTTCTGTAGAATTTCTTCTCGAACTTCAG

CUST_5431_PI426210822

CTAGACTGTTTTCAGAGCTTAGATTGGTGGATAGTAGGTGCTTAATGAATGTTTTATTGG

A_14_P200803 CTCTACGGTGGTAGGGAAATACCTTTTTCTTTTGTTTTGTTTTGATTGTTTTCTTCATCC

CUST_8549_PI426210822 CATTAGAAGTGGTTTCCTGAAGACCTGGACAACTGCCTGGCTATC

A_16_P00618673

AGATGCATGAGTTCTTAAAGTGAAAATGAAGTGTTTGCAAACATAGTCTGATCAGGGAAG

A_16_P20247993

AAGAATGGAAGTAAGTTGTGTAGATGTTCAGTTAGTCTAGGTAACTAACCAAATCGCACC

CUST_8356_PI426210822 AAACAATGGTCTTCCTCTCCCCAGAGTATTCTCCCAGCAACAGAC

CUST_2592_PI426210822

CAAGTTTGATGTAAATTGGGTTAAATTTGCCTCCCTAAAATTCTGCTTCTCAGTCTTGTG

A_14_P101235 CAACAGTTTAGCATGATACAACCAGGAATTAGGATTTAAAGGTTTCCAAGTTGTGGAATA

A_14_P108860 CAAAGTTGAATAAAAATAGGAAGCCATGTTCTCTGATGAAAATCTCTGATGCCTCTGTGA

A_14_P108289 GGTCTACCAAACGACAGAGTAGAATAAAATACTTTAATGTTGTGATGACACAAAGCCCCA

A_14_P112633 ACTTTTATTGTGCATGTAGTGGTTGGCATGCTCTATACTGTTGATCCAGAGATGAATAAT

A_14_P106023 TGAGGACAAATTATTTTGTGTTGACTATTGAAGGACCCAAAGAAATAATGATTCAAAGAT

CUST_6894_PI426210822

GGGACTGATTTTCATTGTGTTCTGCTTTCTCTGGTGTCTCTTGTCAAGTTAATAGTA

A_14_P104733 CATATTCTACCTTGGTGAATAACAGCGAGTGTGTTGCAAACATGAAAGGTAATGTCTCTT

A_14_P138447 ACATGCTCCAAGGTTTCATTTCACTTTAATCCTTCTCTTTAGTTTAACAACTTACGTCCT

CUST_3510_PI426210822

ATTTTAGGTTTGGGACATATTACAATATTGCCTTTTAGCGTAGAAAAGCCCTCCTTCC

A_14_P128184 GGCCAGGAAAAAATTTTATTGCTCATCTCAGAAGATTCATAAGACAGTGAGTGTGCTTTA

A_14_P136007 ATACTCCTAGCAAACCATTGAAAAGTGGTGTTTGTTTGACAGGAATTTCACATCAGG

A_18_P22841021

TTTAGAAACCTGCTATTTTAGCACAAAACAATATCAAGGAGAGGAGTGAATGTGGTTTGC

A_16_P17742922

CTGACTTAGTTCAACAGATCCAAGTGAGTTTCAAAAATGTATATTTCATGCCGTGGTTCT

A_14_P130873 ACATCACCACTATCTTCACATAGTTCAAACCATCACTATACTGATTCTCTTCATTCCTGT

A_16_P00365494

AAACTCAACTGTCACTGAATTTGCTGATTTAGAACTTCTAGGACTAGGACTCAACTGTAG

A_14_P106738 TATAAGGTAATTGTGTGTGGGGAAAAGTGCAGTATTTACCTGTTGAATTCAGCATCTTGA

A_14_P104882 TCTTTCCCCTTTTTATTAGTCATCTTGGAAATGTATTCCTGGACCTGCAGCAGATTATAT

A_18_P12343806

ACAGACCTCCTTGATACCTAAACCTGAGTCAAAAAGAGAATTTGAGAATATGAAGACAGT

A_14_P105795 CTTCAAACCCAGTATGTGCTCCACAATGCTGTCATAATCAAAACTTCGTGGAAATTTT

A_14_P118730 AACAAAGTAGGCATAGGTTCCAATGAGGTTTAGTAAAGGCTCCAAAGTGCAATCTATTTA

A_14_P119188 ATATATAACCGTTCACTGGTGAGAGCCTCTAAATTACACCTGAGAATGTAAACATCTGTG

A_16_P20307834

TTCAGTAAGGAGAGGGACTACAAATCAGACAATGATCTTCATATTAACCACAGGATGATT

A_16_P15625394

AAAATAAGAACACCAATGAAGAACTGTAACCATGGAAACCTATGACTTTGAAGCCAAAGA

A_14_P102799 GTGTTTCAAAAATGTTGATGGTAATATGAAGTTTCTACACCCAGGGAAACAATTCAAAGG

A_14_P118094 CTCAGCCCTTTAACTGAAGGCATTAACAATAACATTAACATATTTTGGCCTCGTTTTCTC

A_14_P112827 TGAATTACTGACCACCCATAGATGTCTACTGTTACCAGGTTTTACAATGCAAATTTTCAC

A_14_P134274 ATAGAACATGACTCCAAAGAAAAGCAAAGGCTCATTAGCGAATTTGTCCAGATGTTTCT

A_14_P123538 GGAAATTGATGTGAACAGTGCACCTGGAATTTTGAAAATCTTAACTTTCCTACACTCAAT

CUST_4661_PI426210822 AAAATCAAATAACTGTGAACAAATGTCCCCTTCCTGCTGTGATACCTGCCATA

A_14_P104960 TCTATGTATAATTTACCCCATCAGTTTCCCACAGTTCTGCAACTATAAGACACGGG

A_14_P109978 TTACCATTTCATATCCATTGAAGCCGAAATCTGCATAAGGAAGCCCACTTTATCTAAATG

CUST_7658_PI426210822

AATTCTTATCCTTTCAAGACCTACCCTAGAGTCACCTTTTTTGATATATTTGCTTTTTTA

A_14_P109924 TCTTCACTTGCCAGATTCCCATTTAAATAAGACATGTATGAAGAACTATGAGAGCAAGAA

A_14_P135499 TTTGCTCATTCCTCATGGCACAGTGAGTGCTTAATAAATGGCTATTGACTGAAATTAAAC

A_14_P111213 CTGCTCTCATGAGGAGAATGTATTTTAAACTTGGGAAGAGTCATAATTCTGGGATGTTTC

A_14_P115004 CATACTAGCATTCATAGATTGAGAACCGGAAAACTCCGAGAGGTCATCTTATAAATGTTC

A_14_P129033 TTTATTAACTGAAATGGTGGGTAGAAGCAGAGAAAGTTGTCAGAAAAAAATGACCTGTGT

A_18_P22841268 TAATCATAACAGTCAGAGAGGGCAGGGAACCAGTATTGCAATTGTGAAAGGTT

A_16_P18268300

AAAGACCACTTTTCTAAACTATGGGTTATCATGCTTGGTATTTTCTCCTATCTGCCTGAC

A_14_P130112 ACCTTTTACAGGTAAGTGTCATCGCAGGTAGATTTATGCTTGGTTCTTAAAACTTAAGCA

A_14_P137287 AAGTCCTCTTAGAACATTGAAGTGCCCTTCTCATGACCTCATGGTGAATAAAGTT

A_16_P19354521

AACTCTTAGACAAATTTGATCATAATCGTTCCCTATGTTTTGCAACTCATATTCCTCCCC
A_18_P16678013
ACTAGGCACTCAATCAAAAACAAGCAACTGTGTATCTCTAATCTTAACCTTTGCTAACCT
A_16_P01784884
AAAAAAACCACAAGGGTCTAATTCAGACTGCTGTCATTTAACACATAAGCCTTCTACAAC
CUST_2651_PI426210822 CCTGGCCTAACTGATGTTAGATTTTGAGTTACTTTAGGTTTTGGGTGGGAC
A_14_P139266 TTTCCCTTACTTTTTAGGTTACTCAGAGAGGGCAATGCTTTACTATGAACTGGAAGAC
A_16_P19836374
ATGGCAGAAGCACATTAAATGAAGAATTAGAAAATGCACACCAAATATCAGACTCCTCTC
A_14_P134835 CTCCTCAGTAACTGTCACCATAAGAGAACTGAGATCATATTTCATAAATATCCCAGAGAC
A_14_P108755 CTCCCCAAATGCTGTTATTACATTGATTATGTGTCTCATAAATGAACATCCCTACCTCTT
A_14_P122433 CTGTGTATGCAAATGGGTATTATAATGGTGCTAAAGCAGACCTGGAATAATTTCATGGAA
A_14_P126280 TTGGGGCCAACGGGGAAGATATAAAAACAGGAACAGATATTTAATGAAACGTCTTTTTTA
A_16_P16943925
CCTTGGTTCTGGAAATGTAAGGTTAAAAATGTAGATTAGGTCCCTATTACCTTGAAGCAA
A_16_P19127039
ATGCTAGTCCTTGATGAAATGGAATCTTGATACTTTAATACAGAGTGGAACATAGTCACT
CUST_8630_PI426210822
GCAGAATGACAGAGAGTACTGAACCACAGTCATTTCCTCTGTCAGTTTGTAAATTA
A_16_P17445975
TCCCCTCTCAAACATTTTATACACTGTCAAACACTAATCACACATAAGGAATCATTAAGG
A_14_P115234 CTATTTATTTGCCTGCTATACTGTAATAATATGGGTTTACAATCTTTTTTCTATCGTAAA
A_14_P125840 TACAAACATGAATAAATACATTCAGAATGTTGGCACCATGATGTTGGGCTTTATTTGCTT
A_14_P131398 AGACATTTTTAAAGAGGCACATAAAATCGCCTTCAAAATTCTGGACTGCAAAAGATAGGA
CUST_4006_PI426210822 AGTTCTCTTAGTCTTTGGCTGTTTTGTTGAGTGCCCAGGTCATCCT
A_14_P111858 ACAAGGATTTAAGTATTTCTCCAGTCTTCTAAAGTTCATAGACAAAGCCATAGATTGGGG
A_18_P21192902
ATTACCTGCTTCTGAAACCATAATATCTCGGCCTTTTATAGATCTTATAACAGTCTCCGC
CUST_3952_PI426210822
CATATCTTCAGAACGTCCAACTTTAAATAATGGTTGGAGAAGCTCCAGGTTAAGTAGAG
A_14_P124656 ATGTGATTTGAAATGATGCTGTATGCCAAATACATACTGAAGAAGATTTGCCAGTCTCAG
A_16_P19038478
TCTACCCTGACTCTAGATGATAATTCCAAATTTAAAACTCCTAATTCTCAGTTACAGCCA
A_16_P36230776
ATTCAGACTTTTTATCAGGTAGGCAGTAAGTGAGGTCTTGACTGAGAGAACAGAGAA
A_14_P116452 TCCAGTTTTTAATGTTTTTTCTTGTAAAACCAAAAATATAAAACTGGCAGGGGAACGGAA
A_14_P128067 GTTTCTTCACTCAAACGTCGATGGTGTTGATAGATGGTGACTTTTTCTGTGAAAGAAAAT
A_16_P03339798

TGCATTAGACGCTAATGACATACGATTTCTCACTGACAGATTTAACAGCATCAATAAGGC

A_14_P200948 TAGTTACACTTAATGGGCCAAGCAATAGGGTGTAGCGTTTTTATAGAACTGATAATCAGG

A_16_P35109115

AACAGATTACCTCCAAACCATTTTTAAAAATCGTCCCATATTTCCTCCTATCCTGATTCA

A_16_P21189857

CAACCTTATGGAGGTCGTGTATAAGAAGATTATCAATACTACTGGATAGGTTTGGTGTCA

A_14_P125974 GGCTTTCCATTTGGGCAAGTTGGGATCTATTTTTACTCATAATTATGATTTTTCTTTGGC

A_14_P115278 CAGAGTTGGATTACAGAAACTCCTATGAAATTGAATATATGGAGAAAATTGGCTCCTCCT

A_14_P138928 CCGATAAAGGCTCTAATTGAAAATATAATGACACCTTTTCTTGGGCATGTTAATGAAGCG

A_14_P111702 TATCTATTCTTTGCTTTTGTTCTCAGGGCAGGAAGATCCCAACAGTTTGCG

A_16_P16191211 AGATAGAGATAAACTAGTTAAGGGTGGAGGGTTGGTTGGTTGTCATTCGTG

A_16_P01630462

ACTTAAGAGCACATTCACCTTCTAATTTCAGGATCTTCTTTCATAGTCTTGCCATACCTT

A_14_P124828 CAGGTGCTAAACAGAGTCTTGTTTGAAGGCTGAGAGTTTTTTGTCTTAAAGATAGAAGAA

A_14_P110065 CTGAGATAAAATGGATGGGTTTGAGAGTTGTTTGGGAGATAGGATTTAATAGTGTTTGTC

A_16_P20339971

AAGCACATTATCTGAAATGACAACTTGGTATCTTCATAGTCACATCCAGTAGCATGTTGA

A_16_P20844730

AAACAATGCAACTTTCTCATCTCAAAAGACATGACTACTTAACAAGGGGAATGTATGCTG

A_18_P16722397

ACCAGGCAGAAGTTAATTTCAATGAAATCCTATAAGTGAAGTATTAATCCATCAAGTCCC

A_18_P22841383

TTTTGCAAATATGTTAATTGAAGAGTAATTGGTGCTTTTTTGACCCTCCTGAGCACTGAT

A_14_P101689 CCACGATATCTGCAAATAAAATACTGACTTCTTCGATCTGCTGCATCTTAAAAGGGCG

A_14_P108425 GTTGAGAAGAAGGTCCTTGGAGAGAAGACTGAGAATCCAAAGAAGTTCAAGATC

A_16_P18116689

AAAAGGGAAGAAGGGAATTATCTGCAACAAGAGTTATTTGGAATGTGGATTTTAGAGACA

CUST_5209_PI426210822

ACAGACTTTTCAATGCAAAATCAAACCATTCCCAAATATCCAGCTACATATTGTTTTACC

A_16_P35517964

TCCCAGATACTATATCAGGCAAGAAACCTATGAATGGATTCTACAAAATATCCCTGTCAC

A_16_P55123166

ATTAGAAGTGAAAGACAGTGAGCGGTTGTTCATATCAAAACCAATGGGATTTAGACAAAA

A_16_P15508694

AAAAACCTGCCTGATGTAATATACTCTTCTCTTGTCTGAGTAATCCAAAATGGCACTGTA

A_14_P101727 GATTACATCAAGGCTTCAAAAATACTCACATGGCTATGTTTTAGCCAGTGATGCTAAAGG

A_14_P116521 TGATCCACATTCCTCAGTTAAGGACCAAGATAGAAACCTTTATATATACTCTCACTGAGG

A_18_P22840536 CTACCGTCTGAGCACTGCTCTGAAGGACTCGATTGCCCCGTGGAG

A_14_P104475 AGATTTTTGGGACCTGAAATCTTTTTTCATCCAGAGGTAATTTTTTTTAACGGAATTGTT

A_14_P114344

ATAAAAAACCACTTAACACAGACATTTCTAGGCTGAAAAAGAGAAGAAGTTGCCAAGTGC

CUST_3428_PI426210822 TCAAGGCAAGACTGATAATGAAAGTCGTGTTTGGGTGGGAATCACAAG

CUST_5036_PI426210822

CTAGAGGATTTTTGTTGTTGTTGTTTCTTTGAGGTTTTCTCTGAAACTTCAGATTAAGGT

A_16_P03266074

AAATGTAAATATGGTCAAGAAGATTGTAGGTGAAATCGAAGATGATATGTTGCTAGGGCC

A_14_P130441 CCTTGTGTCCTAAAAATGAAGCAGTCTCTGGAAGATATTATTCTGGTTTATCTCTGTGAT

A_14_P120755 ATACAAAACTTAAAATTATGGCACACAAATATCACACCTTCCACACATTCTTCTTGGTAT

A_14_P105269 AGAATTTAAAGTGGACAGATGCCTGTTGGAGTAAAATCAACTGCAACTTTTTGATGTTAA

A_14_P123454 ATATGTTAAAAAAAAATCTGTAAAAATCCCCACTCACATCTCATGTTGCTGAGCAGGTCT

A_14_P111912 TGATCCATCACTGTAGTTGGTAGATGTGTATAATTTACCAAAGGATTGTCCTTCATCACT

A_14_P121357 AAAAACAAAAAAAACCTATATTCTTCTGTTCAAAAGGTTCTCATTTTCCCTGGGCAGTTC

A_16_P19440807

ATCCCGTAGTTTCCATATATAGCGACTATCCTATGCATATCAGTTAGCCATACCACAATG

A_14_P104674 CTCAATAAATAAGAGTAGTCACCATCGTCACTGTCATTGTCATTACTGTTGGGTTTTCAT

A_14_P137122 CTCATTCTCTGTCCTGTTGAATAGGCTTCCGAAGATCTTCTCAAGGAACACTA

A_14_P131150 AGATCTAAGTTTTCATCATAAAGTTCTTACCCAGTGGTAGAACTAAAGTGGGTGTTCAAG

CUST_609_PI426210822 TGGTCAGAGGTGGCTATCCTGGCACCAGCTCTTCCTTTACTCAAG

CUST_5838_PI426210822 GAGATGCCTTTCAGTTCCTTAGGAAACAAATTGGCCATTTGCCCAAAG

CUST_1489_PI426210822 CTTCTTCACCGAGGTGGGTGTCTGCTGTCTGGGCATTTCTGTTCT

A_16_P58424475

TTCACAACTTTTGGAGAAATTTTTGGTTTCCACAACTAGTTCAGCGCTATTAGCATCTAG

CUST_2039_PI426210822 GATACATCAGGTTCCCTGAGAAGGGACAGAAAGGCCCAGGTATTC

A_16_P40866908

TTCAGCAGCCATTAAGTCTTTACCTAAACATTTTACTCTTGGAATCCTACGTTGTTTCTG

CUST_4335_PI426210822

GTAACAATAGTAATTGCCTTACTTTATTTGCGAAGGGGTGGTGAACACTAATATGCCTG

CUST_7695_PI426210822 TGTAATTCATATGCTGTTCAAATGACAGGCCTTTTCCCCACACCTGTG

A_18_P14587468

GAGCCATTTCCTTGAAACTAGCATCTTGTTACAAAGGCATAATGTTGAAGACTGAATCTC

A_14_P121955 CATCTTCTCTGTCTCCTTTCTGTCTGGACTCCTTGAAAAAGTAATCTCCATTCATTAGT

A_14_P126463 CTTGTGGAGTCACATTTCATAGTGTCTCATCTCCAAATCAGATAAAAGTGGTATACAGGG

A_14_P125032

TTAAAACACAAGGAAGTTAAACCGGAGGACACATCTGAGACACTGAAGAATAATCATAAC

CUST_5995_PI426210822

ACACTAGTACATTTTAACTTATGATGGCACATGATCTTTCATTCATCAAAGGAAAGTAAT

A_16_P58424442
CTTCAGACCATTTGGTATATATAATTTCCAGGCCCTTCTTCACTGTTCTGTTTATCACTC
A_14_P121655 GATATGATGCAGTGTGAGTTCAATTTCAGTGGAAATGGCAATTTCCATATGGTAAGAAGT
A_14_P109203 GAAACAAAAACTCTGCAAGGAGAATACACATTGTCCCAAAGAGAAACTTGTTTCTAACAT
A_16_P15017101
AACTTTTATCACTTTGCTGTATGAATAAGGCCACTGATTTTCTAGGTTCCATGTGCTTTT
CUST_751_PI426210822 AGTGAATGACTGAGATCCCAAGATGAAGGTGGCTTTCAGGGCAGA
A_14_P136008 TTAAACCCTTAGAGCCACCAGACAATGTCACTTGACAACACAAGGTATGAAACATAAATA
A_14_P111371 ACCGAGCCTTCTGAAAATACAACTGCCTGTGTAAATAAACCTTGTCTCTACTATTGTTAA
A_14_P108619 CCGGCCTATTATTGTTTCTGAGTTAAAGATAAGAGCAGAGCAGGCATTCACAC
A_14_P114872 CTTCCTATCTCTTTGCATAAAAAGGAGATTGAATAGGGGCTTCTTTCGTGAATAAACAAC
A_14_P103941 AGCGGTCAAATATTATTCAGAAGCAATGGGAATATTACCAGAGAACTCACCAATATCACA
A_14_P101225 GGATTAATCTCTACCTTATGCCAAAGTATGGTGTTATAATTCTCCTTTGCATACCCAAAA
A_14_P100791 CTTCACTGGAATTCTTCTCTCCTCCTGGAAACTTGCTCCAGTTGTGT
A_14_P110315 AACCCAAAATGCTTATTTCTTCTTCTCTTACTTCCCTCTCACAGAAAAGTCTTACGAATG
A_14_P121967 GTTTAAAATGTTCCTTTTGTTGAAATAACTTGTCCCAACATACAAGTGAAGTCAGAGGCT
A_14_P129919 TTTGCCTTTTTTCCAGCAAAAACATATTATGGCTGAACGTAATGTGCTCTTGAAAAATGT
A_16_P58424375 CTACTGGTGTTTTATGGAACCACCTCTGGAATAAACAACTTGCTTTCAAACCTGT
A_16_P18053798
CCCAAAGTTAGAGACAATCCAAACACTAAATTCAGCAAATATTTACTTGTGTGAGGTCCT
CUST_10370_PI426210822
TGTGAAAAGTTGTAAGACCAAATGTAACATCAAAGGGCAGGGTCTTTTCAGAAC
A_14_P102601 GACTTACACTTGTGCAACATATTACAAAATGGATGGAGCCAGTAATGTAACATGCATTAA
A_14_P133670 ACCTACTTTTCTGTCCGTTTCAGAACTTCTTATAGAAAAGACAGATCCAAATATGGCCAC
A_14_P131107 CAGTCTTATTGCACCATGAAGATAAATTTGATTCATCGTAGAGGGGATTCTAAGAAGAAG
CUST_4245_PI426210822 AAAAGGAAATCAGTTTGGTGGCAAAATCTCCCCTTCCTGTCCATAATTCA
A_14_P112444 CTCCACTGGCATTTCAGTCTGACGTGGATAAAGAAGAAGATAAGAAAGAGGTATGTAATG
A_14_P103348 TACCAACATGTGCAAAGCAATAACAGTTCTGTTGAACTCATGATAGGTGCAGAAATTTCA
A_14_P115806 ACCAGGAAGAAATCAATTTGGAAAAAATGTAGGTAAGGTCCTTTGCCTCTATAGAAAGAA
A_14_P129898 GTCACAAGAACAAGAATCATGGAGACCACTCTTCTCTGTGTGTCTTATATACATGTCATA
A_14_P130268 CTTATGAAAATAAATGGTGGTGAAAGGTAAGTAACCTCTTGCATTTGTTCACCTCTGCTA
CUST_4752_PI426210822 CCTGTCCAGAAAACTATGAGAACCACCCATCACCTCTGGTTATAGATTCT
A_16_P15515066 GCTCCTTTCTAATCTTGTATTTCCTTCCCTAAATAGCCTATATGCATAGTCCTTCTACCA
A_14_P128774 GATATAGATGCTTTTAGTTTGTTCTTAAACCAGTTTTGTTAAATGTTTACAAGGACCTCA
A_14_P107867 AGAAGGCAAAAATGAAATGAGCGTCACAATTAAAATTCTGTGACAAATATGCACATGCTG
A_18_P14137082
TCTTGATATTTTCAAATAGCAGAATACATGATAAACTTGTAAGTCCACAGATGAGTCGGT
CUST_10063_PI426210822 CCAAAGGAGTTCGCTCCTGCAGGATTTCATCGTTGTTAGTGCTGC

A_16_P21660324
AAAAAACTTCTGATTCTCTACTGGCAATACAATAAAGTCCTAAAACCTCAAATTAGGCAT
CUST_3424_PI426210822 CTATGTTTTCGTGAAGGCATAAGACCATGGGCCAACTTTCTGGGC
A_14_P110926 TGATATAGAAGATCTATCTAACCCATACATAGGAAAATGGCCTTCTGACTTGAATGCCTC
A_14_P124662 CATTTCATAGAGAGAGATGCCTACCTTCAAAAATCAGAATATTGGATCCTACATCCCTTT
A_14_P126904 GACTCTCTCAACACAACCAAATCACTTTTTATGACGTGTCATTTGGACATTTTTTACAGT
A_14_P111078 ATGAACCAGAGTTAATAGGTCATTTGGCAAGACAAACAGTAGTGGAAAGAGGTAAATTAT
A_14_P122119 TTTACAGACATTTACATTCAGGTTGAGTTTGGCTGGATGAGAGTTCTGGTATGG
CUST_8779_PI426210822 TGAGAGGTTCCTTCTAACAAGGATTAGAAACCCCTCTCATGTAGGAAGAG
A_16_P16715572
GACCACAGATAGCATTAGTCCTAAAAGACTCTTTAGGTCGGTATTTATTTCTCTGCCAAT
CUST_3037_PI426210822 GGAGGGGATGACCTCTGGCACAGAAAAGCAGTAAAATAGGTGTATTG
A_16_P15719200
ACCCTGACAGTTACTTATGAATTATACATAGGCAAGAGTCATCTGATTATACTCCTCCCT
A_16_P19282070
AAAAAGTTCCAGTGTAGATTAAATGTCCCGAGATGAGTTGATTTCTATGGCCTTCCTTTT
A_14_P114078 GGGTCGTATATCTGTAAGATGAAAATAAACAATGAAGAGATCGTGTCTGATCCCATCTAC
A_14_P128949 AGTCAGGCTTTGTCTAATTGAGTTAATTTACAGAGCACCCAGTCATACTACTTATTATGC
CUST_5018_PI426210822
TAGTGGAAAATGACTCAACCAAGTAAGATTTACAAAACAAACAAACAAAACTGTTTTCAT
A_18_P22840145 CCTTTATGATTCGTGTGCTGGTCTTTCTTCCTGGAGGGCTTCAGTATTATC
A_16_P00685426
CACTTCTCTTTTTCACTCACTCTTCTTTCTGGTCCTCACTAAGACAAAATACAGAATACA
A_14_P139229 CTGACAAAGTCCTGTTTATATGAAATATTGGCCCTTTTACGTTTTTTTCATGTTGCAGGT
A_16_P16472232
GAGATTTTTATGTTGCCTTGATCTAAGTATGTATGACCCTATTATCCAGTCCAAATCCCC
A_16_P02907632
AGGAAATCACCTACTGCATATTATTTCTGGTTTAGTTCTCTGAGATTTCTTCTTCACCCT
A_16_P37676939
GGCTCTAGTTCAACTCTAAACTCTTTTTCGTCATTTAAATAAGATCTACTTTCAAGCCCT
CUST_8535_PI426210822 ATGCCACATTTCCCCAGCATCTAGGAGTGTTCTCTTCCTAGAGCAT
A_14_P135121
CAGACAAAACATGGACTCACAATCTAAACTCTGAAAAATGACAGAAAAGGAAAACCAGTG
A_16_P16562485
GGATGTCTCAGGGGTATTTTAAAGCAGCCTTTATCTTGACTACCCTTCAATACAGATATA
A_14_P129473 GTATAACTTTGCAGCCACTTTGCCTGAAGACTACCATCCTGTTTCTCTTCTG
A_14_P128065 CCAGAAAGCACCCAAGTCTTAACTGCAATCATTACTATTTTTATCAGCATCTAACACTCA
CUST_5138_PI426210822 TCAGGATTCTTCTGTAAAAGCAGAGAACACTGCATGGAGCCCAGA

A_16_P00685263 TTTTCCTCCATGTTCCAACAACTTGTTAAGTCTGCTTCTCCATTCCATTCCTTT
A_14_P131879 AGTTTAAATGCCCTCACTATCTTTGGGTTACTAATATACTAAGCCTTTCTGGACTAAGTA
A_14_P136284 GTATATAAGTGCTTGAAAAACATATGCCACATGCTTCAATGAGCAAACATCCAAAACTGG
A_16_P17996356
AAGGTAACCTTTATCCTACAATTCCACTTGAGATACATAGTGTATACCCCTTACTACAGT
A_14_P135861 AGACAGTTTCGAAGGCGAATGTTCAGTCTGGCAAATATGAGGGTAAAAATGC
A_18_P14132445
GTGAAATGCTCTACGACTGTCAGCCAGGATTAGTAGTTAATGCACGTATTTGTTTATTGT
A_14_P115518 CTCACCTCTGGAAAAACTGTAAAAATTGCAAATAAAGGAATTTGGGATAAGAGGGCATAT
A_18_P19611931
GCTTCCAAGAGTCTACTTTAAACACACACTTAAAGCCTAAGAAAGTCTAAATAAGTCCCA
A_14_P107853 TCAAGTATGATGATATAATCTGTGATGTTCAGAGCCCTGGCTGTTGTTCACATGATAATT
A_14_P114805 GAAACTAGATTCGGTGCCATTTGTATCTATTGAATCTCTGGTTCCCTTGAAAATCTAGAT
A_16_P16030402
AGTGCACTCTCCTTGATATTTTATGCTGGCAAAACAAAATAGGTTGTAGATAGGATCCAT
A_14_P119435 GCTTTCTTTTCTATGTCCTTAAACCCTTTGATAATGTTACTGTTTGAGAGTCCCTGAAGC
CUST_1501_PI426210822 CTACCCCTATTTGATGATTTCTGTAGGAGTCTGCTTGACTCTGAGACCTG
A_14_P139053 TCTTAGACATTCAGAGACTGAGATTAGGAGGCCTAAAAGTATCCTTTTCAAACTTTCACT
CUST_9097_PI426210822 CTAGTGGTTTCTTCCTCTGACCACAGTAAGAGGAAAGGAAGCTTTCC
A_18_P11549550
CATCGAAGGTCCTCTTATGAATGCCTAGAGCAACTTGATTAGAAAATCCATCTTTATACC
A_14_P124954 TCTCTTTATTCTGCATTTATTGTGATTTTTGGAAACAGGTATCAACCTTCACAGTTGGGT
A_16_P17340095
AAATATTACTCATCCCATTGTTAATGCTCAGAAGTTCCATATTTCATACCTCCGACTGCA
A_16_P02066516
ACATTGTAGACAGTTATTTTACCTGTAAACCAGATGACAAGTCTTTACCCAACATCTCCA
CUST_292_PI426210822 GTTGAAGAACACATCCCAGATCCCCACACTAGGCACCAGGCACGC
A_14_P128318 ATCAACTTTTACTATTGGCTGACTCCAGTCATACTGTGATGAAGTTACATTTGACCATTC
A_16_P00116019 GCCCCTTATATAGACCTCTATTATCTCACGTATTACCTGACATTATCATGATGGTTTGCC
A_14_P125697 CATGAAGATATTGTAGATGAAATTGAGTGCATTGAGAAGACTCTTCTGAAACGTCGCTCA
A_16_P17434715
TTTCAAGAAGCATAAATCCCAGAATGATCGAAACATCTCTTAGATCCCAGAGTGACTTAA
A_14_P103176 GACAGCCAGTTCTCTCCTCACTTATTCACTTTTTTTTTTTTTTTTGACTGTCTAACTGGC
A_14_P122447 TTAGCAAAGAAAAGAGTATGTGCTATGCCCTGAAGGCAGGAAGTATAATCCTATGTATTG
A_14_P126282 CTACATATGAGCCCTCCAACATATCACAAATTTCAAAAAGAACTTGCATCACAAGCCTTA
A_16_P58424092
CCAGAGGTAACCATTATCCTGCCTTCTAACACTATAGTTAAATTTCACCTGCTTTTGAAC
A_14_P111634 AATGGAACACATGATTCTCGTTTAAGGAGAAAATATCTTTTGGTTTTGCTTCCTCTGATG

A_14_P139930 TCTCTAAATTCACCCTGTATTTGACGGTGCATGCGAATTTGTTATTTATGCTTAAATGCC

A_14_P105001 CTTCTACTAGGTTGGCTGGGAACATGTTCCTAGGAAGAGAATCAGCAATTG

A_14_P110998 TGGAATTCTAGCAAAACTCCGATTTGAAACGTAAAAACTACTTGTTTCATGGTTGTTGCA

A_16_P20051250

TTGTCAAATTGCTGCTTCCAAAAAGATATAGGAATGAACATCCACTATTGGGAAGAGTAG

A_16_P00690480

TCCACTAAGGATCTCATGATTCCATTCAGAGATAACACTGTCATGTATATATCGAGTTCA

A_18_P22839775 GGTGGTGCAAGGAGGATGAGATTAACCTTTGAGAACCCACTTGTCTAAA

A_16_P02766126

AAAATTTGAAACGCTTGTTACCAGAAACTTCAAACATGTAACTTCCAGTAGATAAGGGCA

A_14_P135977 TAGAACAATTCTTTATTTCTACAGTCACAGCCCTTTCAGACAACATAAAACCAAAGACAA

A_14_P103371 AATGATTTCCAAGGGAACCTCATGTTCTTTCCTAAAGGTAGAAGAGAAAAGGGAAC

CUST_2695_PI426210822 AGCTCTTTTGTGAGAGGAGGATTCGGCCTCCTCCCTGACGAGGTG

CUST_4796_PI426210822 GATGGAAAAGAGGGAACAGTGGTTCCTTTCTTTCTGCCACGCCTC

A_14_P136224 ATTAACACCTTAGAGAGATACATTATCAAAAAACAAAGTGCCCTAAGGGACTAGGTCACA

CUST_6365_PI426210822

GCAGTATTTGAATATATAAATATGAACATAAAGCCACGTACAGTTATGGATTTAAAACAA

A_16_P40266776

AATTGTCTGGCAGATTACAATGAAACCTAATTCAGAGAATTATTGCAGGTATGCTTTGGG

A_14_P101427 TCAGTATTGACATGAAAAATGCCACTTGTGTTGTGCTGAAACAGGTATGCTGATTATGTA

A_14_P126055 GTTAACCCCACCTAATAAACATAACTCTAATTCTGAAATTACCTTTCACACTATATGACA

A_18_P21331942

ACCCCATGTTCTTCAGTTAATCATTTTAAAAAAGCATTGGCTAACACTATTGAAAAGATT

A_14_P120902 GCCGTCCCTCTGATGTTGTGTCCTTGCTTTGACTACAAATAAAACAAAGAACATT

A_14_P103951 ACACTCCGGAGAGCCAGCCAAGAGTAGATTCTTTATTCATTTCTCTTTTTTTTCTTAAAA

CUST_7250_PI426210822 TGATCTCACCACTTGGAGCCTAGTAAAATCCATGCTACTGTTCTGCAATTG

A_18_P22840665 TAGGGCCATTGGGACTGGCCCAGAAACAGAATCCTGAGCAATCTT

A_14_P123087 TATAGAAGCCCACAACTGAGGACACCATTAGGTAACTGCCACTGCATTAAG

A_14_P137624 ATTGACTATTGGTGCTTGTCCAATGTCTTCTGGCTAAGGAGGAGAAAAAAGAAAGATTT

CUST_5250_PI426210822

TATTATGAGTATAGAGATAAAGGAAAAGGCATCCCACTTCTGAAGATTTCTCGTTTCTAG

A_14_P100989 GTTTTCATTCCCAGGTTGAGCCCTTTTTTATCAATCTTGCCTTATTTGATGTAAAGAACA

A_14_P134348 ACCTGTCTTGCCTGCAATGTCTTGTCTTTCATTTTAATGAAAAAAATCCCAACTTCAAAA

A_16_P15535772

TCCAGCCCTGAAATAGTAAGACTCCAAAAATAACTCGCTTCTATAAAAACTGCATGAAGA

A_14_P139066 GTCAAATCCATTCCAAGTTTGAGTTGTGAAATGAGATGAAGAATTATACTACGCTGATCC

A_16_P18825468

ACAAGTGATATTCCCGTTTGTAAGACATCATCCTAAAGGTTCAGTGATCCATAAGGAATA

A_14_P116809 CTAGCAAGCACAGTGTTAATTTTAGAAATTATAGAAGAAAAAATCAGCAAGGAGTGTGGG

A_14_P105953 CTGGATTAAATGCAAGAGATGATCCCAGCCAAGTCTATCAGAGCATCTAATGTAAAATTT

CUST_677_PI426210822 AGCCACGATGTTCTGTAAACACATAGATACAAGCACATTCCCGCGTG

A_16_P18324189

AGGCCGTCATTTCTTAGAAACAGAGCACGCTATTGTTTTATGCTGATCAATGTTTTTTT

CUST_8136_PI426210822 TTGTAGATTTAGGGGGACATAACATTGAGACTGAGCAGTTTCTTGAACCTCTC

A_14_P122652 AGGTGTTTATAGTCGTGATTGCTTAGATACGTGAGTTTAAATATTGCGTGACCTGCAGAG

A_14_P138586 CTATTTAATAGATGGTTTACATGCTCATCCAGGTTTGCGTCCTAAAAATCCTACACCATT

A_14_P125618 GTTTTGTCTTTTTGTTTGCTGTCCCTATTGTTTTTATACCCATGGGTTCAGGTTCAGATT

A_14_P113387 CAAAGTTTTCCCAAGAAAGAAGTGCAAAAGCCCTTTCATTAGATCATTTTGGACATAGAA

CUST_1970_PI426210822 TGAGTACAGGTAGCAATAAATGCTTCTCACTGAGCCTGTTTCACCAGC

A_16_P20672207

CCACTCTAGCATGGCAAATAAATTTGTAAACACAGAGATTAAACCTCTGCAGTAGTGTCA

A_16_P15484322

AAAATCGATCCTTGATCCTATATTTGGATTCCATGGTCTTATAAGAAAGCAACCACTGTC

A_16_P16325309

ATCATCTCACAATGGAAAATAATAAATTCTGCCACCATTATCTGAAATATATGGGCCCCC

A_14_P133973 GGGAATTCCACGTAAAAAGATCAGATTAAGGTAAATTCTTTGTTCTGTATTGCTGCTGTG

A_14_P133568 TCTTTGAATTTGACTTTAATAGGATTTCAACAGTGATTTGGGGACAGGCAATACATTTCA

CUST_568_PI426210822 ACTCCAAGGTTGGACCCTGCCCCCGTGCCTCTGCTTTTCCCAGTT

CUST_9562_PI426210822

ATAAAATAAATCCTCAATACTTCTTATTCCCTGGGGTAGTAATTTTAATTAGATTCTATT

A_16_P36230155

AGATGTTAAAAACTGATTATTTTCAAAAGCAATATTTTAAGAAAATGCATTCACTGGCTG

A_14_P137851 GATGCTCTCCAATTCCATTTGCTTTTGTTCTGCCCACATGCTGTCTAC

A_14_P123626 CCAGATACCTGTTCTGTTTCCATCTCTGTCTGAGTATCTGTATCTGAGTCATGT

CUST_4622_PI426210822

GATAGAGTTTTTTAGGCAGGGGTCACTGTTAAGGAAGTTTGTATTTTTTTTTTTAACTTC

CUST_9131_PI426210822 TAGAAACCTGAAGCACCGATGTGCAGAGCAAGGAGGAACTCTGCT

A_14_P107333 GACACTTATTCTTTGGTAAGTAACAGAAGAATTCCCTACAGATGTCTTCCTCTGAATGTT

CUST_6151_PI426210822 TAGCTGGCTTTAATGGTTGCTGTTTCTGGAATCTCTGGCTATAGGAATGG

A_14_P137358 ACATATACCACTCCTATCACAACAGGCTCATCTCTTTAAATATGTAAGAGATTCCTGAGG

A_14_P116556 CCTGAAACGTGCATGGTGGAGAAAATGAACTTAACTTTCCATGTAAGAAAAGTTAATTGT

A_16_P30952617 CTACCCAGGGACAGACTGCCTTGGGGTCACAATGGCTGATTCTTC

CUST_3135_PI426210822 CCTGTTAAGCCATACAGCCCCGCCCCGTCAGTCTTAGATTACATT

A_14_P118177 AAGCAGCATTTTAAAGAATCTCTTTACATGTCAGGATACTGGAGAAGAAAACTCTGAGGA

A_18_P15843638

AATCAAGGCCTTCAAAAACTTCAGTTAATCCTTAGAAACTATCCCATCTGTTTACTCCAG

A_14_P129907 TAAAATTAATTTTTAAAGATCTCTTAAAAAGCAGACACCAGCCCCAATCTCAGACCCCTT
A_14_P136810 GTGCAGATGAAATGTCCTGCTCTAAATTAGGCTTACACTTCCTTTGGTCATTTTAAAATG
A_16_P40460705
ATGCCCATCACATTCAATTCAGGGTAAAAGATAAGGTTTTTACTAGTTAAACACATCTGT
A_14_P105692 ACATCAAAAGAGTATGAAAAGTGGCAATTAAACTTGGCGACTAAACACAATTTCGCTTTG
A_14_P110241 GATTAAGAAAGTCTCTCTAAAGAGGAGAATCTTCGCAATCTGACATTCATTGATGTCCTT
A_16_P20468236
TAACTCACAGATTATTCTGAAATGCCAATTCCTCCTTTATCAACAATTCCTCTAACCTCG
A_14_P124163 ACTCACCGATACAAATGCTTGAGATTTGGTTTCATTCAACTGAAGTTGCAATTTCTTTTC
A_14_P130278 GACACAAACCGGTATCTTTACTTCCATTTGCAGGTATGTTTCAGGAGAGAGTAG
A_14_P135334 ATGTCTGTTATTTTGGTGTCTCCTTTTAACACTCTTGAGGTATAGTTTCAAGAGGGTAAG
A_18_P17385399
AAAAACGCACTGTAGTGCTATTTCTTCTCTTTTTTTCTAGTCTCCTAACTTTTCCATGGC
A_14_P110938 ATTATTTTCAATGAGGGGAAAAACGGTAAAACTCTTTTTGAAGTCATCAGACCTTCCATT
A_14_P202003 AGGATCCATTTTCATCCTTGCCTACCATACCTTAAAGGAAAAGAAATTTTCCTAATTGTG
A_16_P18752031
ACTCAATCTACATAGTGCAGATTCTCTCAAATGGTGATGATTGCAATCTTTTATCCCCTA
A_14_P100113
AAACTGCAAACCAAAAACTAGTTTACAATACTGAAGTTATAGGAAACAAGCACAAAAGGC
A_14_P135103 TTCAGTCTAGTTTAGAACCATGGAATTTTGGTGGGTAATTAAACACACACAGAAAATCCT
A_14_P109366 TTTTCATGCAGTTACCATGAACTAATACTACAATAAAGGATGGTCTTGGGTGTCAATTCT
CUST_8684_PI426210822 GTGCCAGGACTCATATTTTAGTAAACTGTAAAATCTTCCCTCCCCATGCTTGTA
A_16_P02740008
TTTTGCATTCAGAAGTAAGTGTTCCTGTCTTGTGTAATCTTTTGCCAAATCATCAGCAAT
A_16_P18784949
ATGTGAGTAACTCTTCTGTGTAAAGACCTCATTACAATGAAAGTATCTCCAGGCAATCAC
A_14_P136090 AAAGTGCTGAAGGAATTAATTGCCCTATTAGATAACGAATACCTGGAGGAATGAACAGAG
A_14_P107636 ATTTCATGATTCCAATAGATGTTTAATTACTTATCCCCTTGCTCTTTCACGCTGCCTTTT
A_18_P15024606
AAAAAAATGAAATTTTTGTCCTCCATCGTTTTAGACACTACAATCAATTCACTTTACTCC
A_16_P02949484
ACCAAAACCAAAGAAATCCAACAGAAAAAAATACCAGAAAATATTACTATAGCCTGGACT
CUST_1545_PI426210822 AAGGTTATTCCTTATGTATACTGCAAGTTGGCATGAACAGCTGGGCAGG
A_14_P124381 GCCAGTTTATCAGGACCACCACAATGTTAATTTCACTTAAAGTTCACTGTGCAAGTATAA
A_14_P109309 AAGAGAACATACATTTGTGTTTGTGTGCAAGTATTCGTAAGAATCTGTGTGAACGTATGG
A_14_P128235 TACTCTACATGCACGCTTTCCAAGGCAGAAAATCAAGATGTGATCAGTGAAATTTTAAAC
A_14_P109652 TGTCTACTGGGCATGTGGAATAGAAATAATGTGTTAGGTTTAAATCAATGGAAAGGGTAT
A_14_P105221 GAGAAGGCTCGTATTAGAAAACACACATACGTTGAAATTAGAACTAGCAATAGAAGAGGT

A_16_P03606402
ATAGCACCCTCCTAAGTCATTTATCTCTTATGCATGGATTTTAGAGTAGAACCTCCAAAG
A_14_P119566 GGAGCATTGGTTTACTTTACACATTTTGTAAGTCATGCATTAGTCTGGAATGTTTTGGTG
CUST_3257_PI426210822 ATCAGTGACACCCTCTAGAGGCATTTAGGGAACTTTTTTTGAACAGAACCAT
A_14_P133846 AAATGTAAAGTCCCACAGCAGTCGGCTTTGACATTTCCTGAGTATAAAAAGAAGTATACT
A_18_P15120272
TGTGAATATGCTACCTATTCAATTTTGCTTAACCTATGTCTGTATGAATATCTACCAATG
A_18_P13003573
TGGTAGATAATTGATGAGTAACATAGATTCGTCAAACTATCCGTTTCTCTTTTTGCAGGG
A_14_P135481 TAAACTCAGGGAGTGATTTTTTTTCTCCATAATAAGGCAACCCATTTACATGCAGACCTT
A_14_P137145 CTCATTTCTCATTTCTAGACAATGCCTTTTAAAATTACCTGTTCTCTCATTGCATCCTCC
A_14_P103756 AGAAAGATAGTCAGAAACCAGGATGATGCATACATTGAGGTATGAAAACTCTGAGATTGA
A_18_P22842290 AGATGTCCTCTGCAAATTGTCTGGCTGGGCCCACTGCTGCTTTTG
A_14_P102406 CAAAAAATACTTCATTGGTTGTGAAGGCAGTCCTACTGGAGCACTAAAATGTCATTTATC
A_14_P139434
GAAAAGAGGGCAGATATGGAGACAAAAAAATGTTCTTGGAAACTCCACTATTTCAAAAGA
A_16_P16196767
TTGCTGCAACCAGTATTTTATATATCTCCTAGGAAAGCATTGATCCTTTAGGTCTGACAG
A_14_P111268
GCAATTTATAAGAGAAAAGCATAAGAATTTCCCTCAAAAAGTGAAACCAATGGGGAAAGG
CUST_3803_PI426210822 AGCTGCCTTGATCCCTCAGAGCCCCGGCATTTCTGCCCGGGGTTT
A_14_P117266 GCATTTATTTAGTGACAAAAGACTGAATACAACTCAAATTCTAATTAAGAGGTAATAGCA
A_14_P137967 AGGATCATGTAAATGCTCAAAGATGTAATGTAGTTCTTTGTTCCTGCTTTCTCTTTCAGT
A_16_P01771462
ACTACCAATTAACTGCTTCTTCTCTCTATTTCACATGTTTACACATTCTACTGCCTCAGG
A_14_P103604 ATCACAAGGACGTTTCTCTACTTATTGATTCTAGTGATGATACAAGGACTTCATGGGAAA
A_14_P109691 CTTTGTGGTTTACGCTCTCATAACCTTGTGGTTTTATAGTCCTTAAATTATTGTAGCGCA
CUST_3344_PI426210822 CAAAAATTCTAATCCAAAGGCTTTGCCCGCCTTAAGTCCTTTTTTGAAATGGAG
A_16_P02698503
AAACAAGAAAGCATCTTTTCACTGCTATGGAAATCCAGAATTCTTTATACCACAGGAGAG
A_16_P02425137
AAAAGTTGTCACTCGAGTAATGAATGCCCTATTTCCTGTTTTTTTGCCGAAAGTTAAATG
A_14_P114732 ACCTTGTAGAAATGCAATAATCTCTGCTCCCTCATCTTTTTACATGAGAAGGTCTCTTTA
A_16_P00493789
AAACAATTCCATCAAGGCATAAATGAGTTGACTCCAACTACTGAGATAGAAAATGTCACC
A_14_P126495 CTTGAAATAGAATTTCTGGCTTCTAATTGTCCATCAATAATCTCTGCAAGATTTGGCCTC
A_14_P130739 ATGTGTTGCAATATGCTTGCAACTTGTGGAAAGAGTATTCAACACACAGTTACTTTATCT
A_14_P128671 ATAACAATAAACCTTGTTACATACCACTGATGTTAAGGTCATAATCTCCTGCTGTAATCA

A_14_P106493 TTGTGTTGTCTTCCATCTAGATCTAGTATAGAAACGCAGGTCAAAAGGTAGGAAGATAGA

A_16_P03138870

GACCTGGAATCTGAACATACTGTGAACTACTCTATCAACTCAATGTTTCCACAATAACAA

CUST_4113_PI426210822 TTCAATGAGGGGAAGACACTCTACTGTGGTTCCATTTGCTCTTTGAAAAC

CUST_3541_PI426210822 TTTCTTCTGCATTCTCTGCCTATTGCTTGAAGGACAGTGTTGTCAGCA

A_14_P202033 TTACGAATGCCTTTTCTTGTTGTTGTAATGTTTAGCACATGTATGACTGTGTTCATGTGC

A_18_P22841207

TGTTGCAATCATAGTTTCATTGTTCCTTTGTATTTTGCATTTCAAAAATAATGATATTGC

A_14_P116419 GAGGGCCTATAAAAATAATTCCTTCTTGCTTACAAAGTTCAGCAAATTCCATGTTTTCTG

A_14_P120709 TAGGCTAACTGAGTTTTCATTTGGCCATTTAGATCATGGATAAGCAGATGTTTATGCTTC

A_16_P15719999

ATATTTTCCTTGACAGATGCGTATTAAGTGTCCACTACATATCACCAGGATTTTGCAAGA

A_14_P137218 GATGTTTTTCCATCTAAAAAGCCTAGGGTAAGCAAGAAGTGAAGTGCGGTATATAGTTTT

A_16_P38372237

AAGGGATATCATAAGATTCCCAAAAAGTAATCTCCAAAATCAATGCATGCCATTCAGCAA

A_14_P102411 CTACCCAAAGGAGACTAATGAACTTCAAGCAGAATGCTGCTCCATTGGTTAAATACTT

A_16_P19817046

CACAACACAAGTTAAGGATCAGTATGTTTTTCCATTACCAAATGCATTTTAGGGGCTACT

A_16_P36191720

ACCAAAGGAAGCAAAGATTACAATAAGCCTTAAACTCATTTAAATCTCTCCCCAGAACAA

CUST_8343_PI426210822 CCAACTCACCCATAGGACACAACAGTCCATATCTCCTCACTCTCT

A_16_P01693093

ATTCAGTTACTCAGGACTTTTTGCCTTACAATAGGTTTAATTTCACAACTTCTGGAGCAC

A_14_P131906 CTGGAGAGGTAACAATCCTGAAAAAGAAGGGTTAACTTAACAAGATGTGACATTTTTTGT

A_14_P135012 GGGTCTCAGGCAAGAAGAAACTTCTAGATTTTTTGTCAGTTTAAATTCTCATGAAAATAT

A_14_P131216 AACAAAGAGTAAGAGAGCGTTCTCAAGTTCCAAGGACAAAACCTTTCACTGAAAGTAA

A_14_P129371 AATAACCTTGAGATCAATGGAGTAGCAGATCGGTGCCAAATACACTTTGGAGATAAC

A_14_P138427 TGAAAGAATGAATGCCAGGAGATACAAGCACTTTAAAGTCACAACAACGTCTATTGAAAG

A_14_P100363 TATCTGCATGAACAGTATCAAGTTTAGGTTGATAATGATTTCAGTATCCCATTTGGGCCA

A_14_P200529 CTTTATAAGATGGGTTGTGTTCCTGGCTCTATTTAACGGATAGTTCCTGGATTGTTAAAA

A_16_P02476614

ATGTGGAGTCCAAAGTAGATAAAGGCTAGTGTTAACAGGAACTCTATTATTACGTGTTGG

A_16_P19430274

GGTTCTTCCCACTGTATAACTACTGCCATGTGATATGTATCTAAAGCAAATGAGGTTTCT

CUST_116_PI426210822 CCCAAGAATGAGAGAGGATGAACTGACTTTATGGGCCTTGCTCTTTT

A_14_P132724 TACTTACTGTTATCTTACGAGGACAGTCATTAGAACACAGACCACTAAGAACTGTGGAAA

A_18_P22843578

TGGTGGTGATGACTATGTGAGAAAAACTAGACACTTGTCTTACCATATGTGAAAAGTTGT

A_18_P13868193
AAAAACTTCCTTGGGTCATAAGAAAAAAGGCTTACCTGATTTTCCAAAAAGTGTTTCTCC
A_14_P200503 ACATAGGAATTTGAGAACCACTTCACAGGAAGAGGGAAACAGCCCAATATTTATTTATGT
A_14_P201339 ATTGTCATCAACCTCACCTGTTTTTAGAAAGCAAATTTGCACCCAGTTAAATCAGACATA
A_14_P202429 CTTGTAGGAAAATTGGTCTTACTGAGAATTCCTTTAGAAAATAGTCTTTTTACAAAGCAA
A_16_P18165184
CCACTGCAGGAACAATTTATACAGCCAAGAAGTAATTGAACAGATTTAGATTTGACCACA
A_14_P124734 ATGTCTCCGTAGATAGGGGGAGCATACACTTTTATATTCTTCTGTTGTGGTTTTAAAGAA
A_14_P202535 CATTTATTTGGCTTTCTTTTTAGGGTTGATTTCTTTATTCCAGGAGTCTCTGTGGTTGGT
CUST_2803_PI426210822 CTCAGAATTGAAAAGAGGCAGGCATTGTCCTGACAGCTGGTAACTG
A_14_P137368 TTCAAATGTGAATTTTTATTAGAATAATAAATCCTATTGCTGCCACTATGTGCGTCCAAC
A_16_P39295569
GCAGTCTACTATCCTACCATCATATGTGAAATAGAGGTCATTAAACTTTCATCTTCAGGT
A_14_P113228 AATTTGAGGTTACCTATTATACTCCCAACTTGAAAAACTAGCAGTATATTGAGGTGGCTA
A_16_P18199371
AATTCACCAGACACTTTGAATAGTCTCAAACAACGTTATCTTCAACAGTTCCTCTCTCAG
A_14_P137961 GACGGTGTATGTTGCTTTTGACATTAACATTATGATCATCAATATTATTGGGGTGTTCCC
A_14_P125830 TTGATTAATGACTGTGTATGTCACCTCCTCTTCCTCTACAAGACAAGGATTGCATTAGAT
A_14_P128342 TGTAACTTTTCCTCCCAGTCTTGATGAGAATAAACTTACCTCTCTCCCTATGCTG
A_14_P123957 TTCTCCAGGGATTTAGGAAGTAATAAGATTGAAAATCTTCCACCGCTTATATTCAAGGAC
A_14_P110683 GGCAAGTTTTACCATTGTATTAATTACACCACTGGAGAGATGTTTGATGTAAGCGTGGT
A_14_P121035 ACAGCAATAGTTCTGAAAACTGTTCAGAGATCCCTTGATAATATGATCGAACTTTTAGGG
A_16_P16570130
ATACTTCATGCACAAAAAGACACTTCAGTCCTAAACCTGTATTTTAGTGTTGTGAACTCC
A_14_P117578 TGCTGAACACAATGCCATCACGATTCTGACCAAGCAGAATTCTGTTAATAAAAGAAATCA
A_16_P36230212
ACCTGATCTATGTGTTGCAAATTTTTTCATCAGCCTTTTTCTTATGTTTTAAGTTTGCCT
A_16_P00250293
ACTGTTCCTATCACAATAACGTAAAGGTCACTGGTTATAAATTTCCTTTGTCAGAGCAGT
A_14_P115385 GGGAAATAATCTTAAAGTCTATCAAAAAGCCAAGAATAACTACACTGTTTGCAGGCACCA
A_16_P16191385
AGAGGAGCAATTAATAAAACCTTCCATTTCCACATCTACTGTGAAACTCTGTGACTTCTT
A_16_P00220693
AAAGACTCTTTTGTCAGGATCTTACTTTATGTCTTAACTCTGCAGAATGAATAATGTGGG
A_14_P137450 CTAACATTAAACTATTTTCAATGCAGGAAATAAATATTTCCCCAAAGAGGAATCCCCCCT
A_16_P15738429
TGTTTACCCTGATAACAAAACCACTCTGAGTTTTTGTTTGAACCATGAATAAAACTGCAC
A_16_P03511386

CCTTGTCCTCATTTTCTATAGATCACTATCACCTTCAATAAAAAGTCCTGATGCCTTGAG
A_14_P117533 ACTTGGGTTATTCATCTTCATCTCTATTCTTTGGGGTAGCGACTAATTGATTGATTAAGG
A_18_P22841412 ACTTGGTGTAGACTGTGGACCTCATGAAGGACACTGTTTCTTCTCTTAGTT
A_16_P02506454
CCTAGAGTCCCTTAATGATCTCAGTGAAAGACTACATTGTAAGTATGGAAGAACACCTTT
A_16_P36306542
TAGACCTTGTATTTTGCACAGTCTACCTCTAAACTTATTATCCCATTCAGCGTCATAAGG
A_14_P109216 TTTATTCTCTGCTAGGCGCATTTCTGAATCTGAGATTTTCTGAGAAACTATCTGGATTGT
A_14_P111076 AAAGTCAATGGAGATTTGTTTTTTTCTCACCAACAGGAAAACAATTGCTTATCGAACTCT
A_16_P15333281
AATATTGCCATCAAAACATCAGTGGAAAGGAAAGTTCTATATCTGAGGGTGAAATTAGGC
CUST_6104_PI426210822
CATATGATAAAGAAAAAGACTATGGCAAGTGAGCTTTAAAAAAATTCTTTTTTTGATGCA
A_14_P108553 CAAATGTCTTTTTTTCTTCTGAGATTCAGGTGATACAAGGTCCTCCTAATGTCTAACTTC
A_18_P22842077 AGTGATCTAAATCAGGGCTCAGATGAGGCTGTTTGTATTGCACGGTAGGAATATAT
A_14_P132804 TGACAATTGCTAAGGTCATTCCATTAATCACATACTGACATAGCATGGTAAAGCCTGTAT
A_16_P55112190 AACATTGATTAGCCCTCATAGTGGGTTGGGGATTCCAGAGTGGTT
A_16_P20756571
AGTAAGACTGCTAAAAACCTGTGAATGTATTGTGGATACTTACTTGGGAACTTGTGTTCA
CUST_2970_PI426210822
GAGGAGACAGTCTGATGTAGAAGTAATTTCCTTTGGGGTAATTTAAAATGTCAAAATTTA
A_14_P125365 ATTATCTGCTTCTTGTTCCACTTCTTCTCTATCAGCAACAACCAATTTTCTTCCATAAAA
A_14_P126623 AATGCAGTTTAGTATCTGGGGCTATGGGAGGTTTTTATGGCTAGAATCTTCTACAAATAT
A_16_P19451913
ACAGCACAATATTTAATCCCCAAACAAGTAAAGTAAGCCTGATATTAACTGACCCTGTCT
A_18_P16720968
AAAAATTGGATTCCTTCAGGCTAATCCCTTCATGGAAATTAAAACTTGGCACTCAGAAAG
A_14_P115594 ACAATAGCCTCTAAAAATATTACCCATGGGCAGCAGTAGTTCGATCTGCGC
A_14_P135485 TAGTCAATCTCTAGGTGTTTCAGGTGGAACAATCTTTTAAAGGCATACACAGGCATATTG
A_16_P21472456
GTGTGGTCTGAGTGATAAATATATTTTTATCCTAAAACAAAATGATTGGTTGCCTTAGCA
A_14_P133392 CTCCACATTCAGTAAACACCCTTGTTCACCGATGAAGACATTATTCTATTTTAGTCAATG
A_14_P115962 ACCAACCCCAGAATTCATATTTCATTATTTTCTCCCTTCTATTTTGCACAAACAAGATCC
A_14_P108207 ATAATTTTTCCACTACTTAGAGGTTTGCAAATGCATCATTTCTTCTGCTGAAGGTCTGTT
A_18_P14135764
CTCTTTGCTCTCTCCATATTCTCCAAATAAAAATTCAAAACTGAGCAGCAAATCCTGTGC
A_16_P01846574
AAGGAACGTCTTTGAAATATTTGGTAGATCTAAGGCTGAATACAGACCATTCACCAAAAC

CUST_5757_PI426210822
CATGGTTTCAAAATCTGGAAGGACAATTTGTACAGACCCTGAGATAGAAGGATTC
A_16_P16191930
CCCTTAGACTTGTTAGATGTAGTGATACCTAGTGATTTTATAGTTACGACTCTGTCTGGC
A_18_P22841749 GACCCCCCTATACCCAGTGTGCCAGACATCATCATGAGATGTAAC
A_14_P134577 CAATTACACAGTTACACAGTAAGAGCACCAATAAAACCTGAGCATTTGATCCCAATTTCA
A_16_P02293468
AGGTCTTTTCCTGTATACCACATTTCCTCATTAGTTCTCACTACACTGTATGGCTATTTC
A_16_P19973107
AAGCCGTAAGAGAGACGTATAGCAACCTATTCTGAATCAACTTGTTCAATCTAGCAATTC
CUST_3702_PI426210822 TAGGCAGCAGACACTGGGTGTGGCTTGGTTTCTCATACCTTTTTAT
A_14_P118777 AATGTGACAGTAACAGTCAATATGAGAATTTTGGCCTAAGCAACTCTATGAATGAAGGTG
A_14_P127075 GAGGAAATAGGGGAGAAATTTGTGAATGAAAATAATTCTCCCTTTTACCTTGCAAAAGTC
A_16_P02029621
AATCATTTTCCAAGAAGTTACACAAGTCAACAAACAACAATATTCAAACCTCTGGCACTG
CUST_8_PI426210822 CTTATCACCTTGAGAAACGCCTCAGGCCTTCCTCCAAGGAAAGAC
CUST_4851_PI426210822 GATGGGCAAGCACTTCTATCAGATGCTGGTTCTGTTGTAAGACATCATCAA
A_14_P105278 ACCATCTTTATGGTGCAGACTAATATCTATTTTTCTGCTGTTACAGAAGAAGTGGGAACA
A_14_P125873 CTTATTGTTGGCACGGTTGATGATATCTAAGCACTCATCTTTATACTCTCGGAGGATGA
A_16_P36569206
GAGCCCAATAATCAGATTACAAAATTCACACCTACCATTAATTCTACTTGCAAACCAACA
A_14_P100075 TCTGCTTGCTGCCAGTATAACATGCATACAATATTTTCTCAGTAGTCACGATCATCTTTG
A_14_P123672 TGTAGACTAACGCTGTTTACACCCTTGTTCTGTCAAAGCAATTAAAGATCACTTGTGTTG
A_16_P01427229
AGGACTCCACGAATTAATGATTCCATCAGAATTTGAAATCAAACTGTAAAGCAATCCCTT
A_16_P02471535
AAACCTCTTTACCCAAGTGTAATGTTTTACCCTTTAGATCCTAGAATGCACCTAGCATTA
CUST_8804_PI426210822
ATAATTTCTCTACACCAACTTCTAGTCCTAATAATTTATCCGTATAATGCTCAGAAACAC
CUST_9640_PI426210822 GTAGTTAATCATGCCCTGGAAAGCTGGCCTGCAGAAAGAAAAGGAATTTATC
A_14_P102973 AGTTCTATAAGGCATTGACTAAACGTGCTGAGGTGCAGGAACTGTTTGAAAGTTTTT
A_16_P15836311
ATTCACTGGTCTCAAGGTAAATTGCAAATTTCTAAACACAGCACGTAATGTCTTTCTCTG
A_16_P01055497
GGGAATTCAGTCTAACAGAATTATAGCATCCAGATTTAACCAAAGATGTGAAGGTAATCG
A_18_P16258121
AAAACACACAAACATTTAAGCAACATATAGCATTATGACGTTACCCTTAAAATAATCTCT
CUST_8888_PI426210822

ACCCTTTGATAAAAGGGAACTAATAAAGCAATATATGCAGGAAATGCAGAGACAACCC
A_14_P114306 CTTCCTCCATTAAAAAAAATCTTATATAACCCTGAAAGTCTGCCGCTGTATCTCTTGTCT
A_14_P123900 TTAATGTAAAAACTTTGGAGAGCCTTTCAAAATGTTTATTGAAGGCCCGTCTCGCTTC
A_14_P138844 TAGACTAATGCCCACTACGGTTCCAATTCATTTTCCTCTTAAAGAAGTCTTAGGATTTCT
CUST_5240_PI426210822
GGATTTATTTCATAATAGGGAGCTTTTTATAGATTTTAATATCATAGTTGCCATTTAAAA
CUST_1284_PI426210822 ATAGATACACTCTGTAGACAAGTCCCAGTAGGTACAGAGGGCAGACG
CUST_3608_PI426210822 TTCAACACTGTCACCCTGGGAATAGGACCCCCAAAACACTTTATTT
A_16_P00685601
ATTTATTAGTAACAACAGTCTGGATACTTCTCTGTAGTTCTCTCCAAATGTCAAGCCCAG
A_14_P202185 CTGTTACCTCCACATATAGCCTACTAAATAGGACTTGAAATCAGAAGTTCTTGTTCCACA
A_14_P106882 GTCGCTGATGTATGAAGAACTTTTTCCTTTTGAAAGAAATTTTGAGAATGTTCACCCTTC
CUST_10241_PI426210822
TAAATTTAGTTTTTCACTTCCTCATTCCTGATTGACATTCTATTAGAAAATGTTAGCTAG
A_14_P125208 GTGTCAATGATGCAATGACATCCTTTCAAGAATAGTCTCAGAATTTTGTTGTCTGTGTCA
A_16_P00370220
AGCAGCCTCTTATTGTGTAAACTCTGGATTTAGAACATAAATACAAATGCTCCTGTGAGG
A_18_P17044137 CTAGTTTCTGCCTGAGAACTCTCCCGAGGTCTCCCTGAACACTCA
A_14_P103815 TTGAAAAAAGTTATATAATGAAATAAAATTTACTTGCAAGGGAATATCATGTAACAAATG
A_16_P16027230
GGTGACTCAATTTTCAAAATCAATTCACACTAAACTTTGCTATCTTCCTTCATACCAAGC
A_14_P110898 AAATGCCCAGGTTAGAGATTAGGACTCTTTATAACGGCCCTTATCTTTTACATTTGGTAA
A_14_P115656 TAAGAGAAACTGCGTGTATTCATTTCATTTTCTCACTCAGGGCTGTCTTTAACTCAGTC
A_14_P121825 CTCTCTACATTACTCTTATACAGGCTGTCATTGGACCATGACTTGAGAAATTCTGCTTTA
A_14_P104441 TTCAAATTGACATTGGACACTGGAATTGAGGAAGAATTAGAAGCAGTTGAAGGTGAAC
A_16_P00685183
TGGATAGTGTAATTGTTTCCATTTAAAACAATGGAAAAGATTTCATTTAAATTTATTGAG
A_14_P201897 CTGCATTGGTTTGATATGTTTAAAGTTGACTTTAACAAGGGGTTAATTGAAATCCTGGGT
A_16_P15851340
GCACCTTCAACTCCTAATTACCAGCAGAAACATTTCTAATCATAGGCTTTGCATGTTTTT
A_14_P123291 GTATAACTATCCAGAATCATTATCATCCATATGCCAACTTAAAAGGCAGAGGTATGGGTC
CUST_6770_PI426210822 CCAAAGGATCTGATGTCAGCTGATTCCTACATTGCAGAGCTAGGGT
CUST_8393_PI426210822
AAAAATCGGTTATTCAACAAGCTACATAATTGTTTAAAGGCTCCACTGCCATTGTACACA
CUST_2059_PI426210822 ACCGGTTCACTTCTGCTTCCGTTTGGCTAGAATTTGGTCAGATAGTT
A_14_P102013 TTAGTGAATTAGAGCAGGAAAATGAGGACACAAAACCCAACCAAGGCG
A_14_P109040 CTGACCTTTTTCAAAATTTTCACTCCAAGATGTCTTCAAGATATGGCATACCTTTCACTT
A_14_P100099 TGAACTCCCAGGAACGTCCAAGTGCTTTTTTTGAGATTTTTTAAAAAACAGTTTAATTGA

A_14_P114065 TAACCAATGTTGAGAAAATTTCCAGGGAATTGCTTTAGAAAGACAGAGTATACAGAACCT

A_14_P202030 CACAGTTCCTTTTTCCTCCATATTTTCACTCTCATCTTACGTCTCTTTTTAACCATCCTA

A_14_P132969 GATTTATCCGCTGATTCAGATGAAGCCCTTCGAGATTCAAATCGATGAGATTGTC

A_16_P19148698

AAAATGCTTCTTATAGGCCGTATGCAAATATAGAGAAAAAAATCTTACCTTGGGATGGGG

A_14_P134482 ATTAATCTAGAGGCAAAAAGGATTCCAGAATGGGAATGGGAATAAGAAGAATGTTCACCT

A_14_P112752 AAATCCTCTAGCAGGTCTAGCATCTTGGTCATCTGCAAAAGAAGCACGGTTTAATTAT

CUST_9504_PI426210822 TCCTTTCCATGACTTTGCCTTTTCAGTCCATGAGCTACAATGAAGTTCTACAA

A_14_P127428 AGGAGGTAATCCAGAAATTTGGAACTTCACTATAAACCACACATGACATGATTTACCAAT

A_16_P37013199

AAACCTCAAACTATCTTGAAATCATGAGCAATATTGCTATATCCACACTATTACTGCCCA

CUST_8669_PI426210822

AGGTGAAAGTACTGGATTTTTGAAAGAACTCTAGTGTTAGTCCAGAAGAGATATAAAATA

A_14_P108855 TTTTCAATTTTCTTGCAGAAACATCCCTTTATTTTGATGTATGAAGAACGTGCCGTTGAG

A_14_P121300 TTCCAGGAAGACAATGCTCTAGAAATTCACACTTATGGATTCATAAGAAAAACAGAGAGG

A_16_P17436497

AAAATAACTCCAATGCAAAAAGTGAGCATATGAATCAGGGAATCTTTCCTTACAATGGGA

A_14_P130226 TGAACTGAAGCAAAACAGGCCAGATTTATGGTTTCTTTGCAGTCAAAAATAACTTTTCTG

A_14_P112505 AAAACAGAATTAAGTTATTGGTCTGAAGTGACTGAAGCATTTAGGCTCTATCAAGGCCTA

A_14_P113411 TATTTTGTGACCAGGGATGTGGAAATCACAGGCCTAGTCAATAAAATATGTGCTTTAATC

CUST_2802_PI426210822 CTTTGGCAGCTCAGAATTGAAAAGAGGCAGGCATTGTCCTGACAG

A_18_P15488017

AATTTTCACATTTCAAAGTTAACTCAAACCTGTGCTATCAAAAAATCCCATTGTCCCAGG

A_16_P15652041

CATCCTTTATCTCTACCCTGTAAATATTGTGCTTGACTCAACATTTTCTAATCTGGCCAC

A_14_P103320 CTATCCATACAGGTGACCTGAACTATATTTGCCAATATCCTTGCTTTTCATTCTGTTTGT

A_14_P201354 GTTGCTATTGTTAACCTTAAAAGTGAATTTTGTTGTCTAAGAACTGTGGAAGTGCAGGAG

CUST_1559_PI426210822 GGAAGGGCCTGAAGGGAGGCCTGGTAGGAGGCCGTGATGTGGTAG

A_14_P118734 CTTGCCAAGATAACTATTTGCACCACTCATTTATGACCTCACCAGTAACCACATTAAAAA

A_18_P14133316

TATTTCAAGAGAAAAAGGAGGCCAGAATTTATAGAATCTAAAAATAGCAGTTGAAGTGCA

A_14_P104979 AAACGTTGCTGTAAGATGAAGTAAGAGAGGGACAATGTTTTAATATGAAGTCTTGAAAGG

A_14_P138754 TGATCCCAGCACACAAGGCAGGCAAAGAATTTTCTCAACTCTTTTTATTAAGTTAGAAAA

A_14_P202385 TTTTGACTTTGTCAATTGTCACTGAGTATTTCTTTTCAAACTGCCAACTGCAGTCATTGA

A_16_P21591996

ATCAACTTACTTCCTGCATTTGGAATTGAATCTGAAAGAGAATTAGACCCCAGACATCTT

A_16_P01644921 CTTTCCCAAACTTCTGATCATTAATGGGGCTTTCAAAACAAGTAAAACTGCAACCAC

A_14_P201132 TGCTTTATCATCGCTATTAAGGGTGGCTTTATTTGTTATAAAGCCTGAGGTGGCATTATT

A_16_P00088004
AACAATTTAAGGGTGGTCAGTAAGAGTAAGTAGCAATTCAAAGATATTTGCCTCATGCGT
CUST_9128_PI426210822 TCTTCACACCTGAAGCACATTAGCAACACTGGCTGGGCTGGAGCT
A_14_P135444 GTAGTAACTACACCTTTGAATTTAGGACAGTGGCTCACTTTTTTGTTGTTGGTTTGTTTT
A_14_P118120
ACCCCAGAAGATGTGAACTGGAAAAGAATAAAAAGGGAAACTTATAAGCCATGGTATAAA
A_14_P133345 ACCTATTAACGCTAATGCAGTAGACCAGCAAAGCGTCTTATCTAATGAGCACATGAAG
A_14_P134426 CTGTTCCATATGGGTAGTTATGGGGTTTACCTGTTTTACTTTTGGGTATTTTTCAAATGC
A_18_P10447145
ACTCAGGGTATTCACTGTGATGAGTGATGTGAGGTATGGAATCAGAAAAGATTTTAAGAT
A_14_P130488
TAAGAGCAATGAAAAAGATTAACATTGACCACTGAAGTGATGTGATCAGAGTAAGGAAGG
A_14_P108722 ACCACCAAATAGACAAAGGATGGCAGACTTTTCATAAATACATATATCTAGCAATGGCCA
A_14_P113154 TTTATTTCTAGCCCAATCAAGCATCCTTTTTGGAAAGTTCTTTCTCCAGTTTCAGAAATG
A_16_P15963747
TCACCTTTAGCCTCAATTATACCTCACTAATAGAAAGAATGACCCAATTATAGAAGCAGT
A_14_P106599 AGATGAAAGCAAAGTAGCAGTTTTTTCCTACCAGGCATCATCAAGTAATGAAATGTCTAC
A_14_P102627 ATTCTTTGAAACCAGTAACCCTTCATCATCCCAAAGATGATCTAGATAAGATACCATCAG
A_14_P126175 AACCATTTTGTGGGTTTTCAAAAATAATTGGAGGATACTTTGAGGAAATGTTGTTTCCTG
A_16_P37776587
AACTCTTCATTTCAAAGCAATCTGTAGACATTACACCTTTAATTACAGGAGACCTCAAGG
A_18_P22840281 GTTTCTATAGTGGCCAAGGGGTCCAGTTGTCCCCTGAGTCCGGAG
A_14_P104784 CTATGATCCCTACCAGTGGCTTACTATTAGAATCTCTTGCACCAGTGTAGGAGTATATAA
A_14_P117326 ACATCTAAGTAATGGGGGAAAACCTGTGCAAATTAATTCACATACATGGTTCAGAAAATT
CUST_3975_PI426210822 GTTTCTTTGGGTGGGGCTGTGCTCAGCCCTTCCGGGTGGTGAAGG
A_14_P126873 ACAAGACATTTGATTTGACTTGTCTTTTATTTGACAGTGTTTATGTAGTAAAAATGTCAT
A_16_P02698149
TCCCTTAGTTCAGTAAAATGTTCAACCAAATCAATTTAATCCTTTATGTCAGAACAGCCA
CUST_4330_PI426210822
ATCTCCCAGATGTGATTTATGCCATATTACATTCCAAGAAGGGAACATGAAGAATGTTTG
A_14_P124283 ATCCTAGGGAAACACTCAAGAGGTTATCACTACATGCTCGCTAACCTGGTAA
A_16_P58424254 TCTGCCTTTTAGTTGGGTTCAGTCAGAGGGGAGCACTGGAAAAAG
A_14_P134637 TCTTAGATATCTATCATGCTTTCCGGAAGCAGTCCCACCCAGAAATTATTAGTTATTTAG
A_16_P17138797
GGACAGCCATATCTTGTAGATTTAAGGCATTTACCGTGTATATGTGTTAGGAGTCGTTTT
A_14_P131135 CCTCAAAGCATTCCAATTCTAGGTCTTTTAAGACACTATGCCAGCCAAGCAAAATATG
A_16_P21585839
ACTTTGAAAAATACACTGCTGCTGAATAAATCACCTTCCGATGTTACTATCTTTAGGGCT

A_14_P102740 CTAGATGTCTTAATTCCCAAATAGGACTCGCTGCTAACTCTTTGGGCATACCTTC
A_14_P126517 CTCGGTTTATAGGTGTAGTGCAGACTCTACTGAAATCCAGAGAATTATCCAATATGGCA
A_14_P130233 TTTTTCATTTTGTATCCTTTATATAAGTTGAAGCAGTTGGAGGAAAGGCCTTGCCATGTA
A_14_P109538 ATTCCAACCATGTTTTGTTTGAGCCATGATCTTCCCAGATACATGAGTAGATTTTAGAAT
CUST_2001_PI426210822
TTAACTATCCGAATAGGTACACTTAGTGTATAATTGTCATTATCTTTTGAATAAAAAATG
A_18_P11685338
ACTCTCTCGATAAGCCCTATTATTTTCCTGAAACCATGTTACATTATAAGAGGCTGGAAG
A_14_P138960 ACAGTTGCATTAAAAATAATCTAAACAGGACCAGCATCAAGGGTCATATAACAGTGAAGC
A_16_P17882499
GAGTCCATGAAATAACATCAGTCTATTACAGTAGTCTTAACTGGTCTCTATGATCCATCC
A_14_P200811 TCTACCCACCTCCTCAGTATAGTTTTAGATTTTCCTATTGACTTTTTCCACCAAGGAAAC
A_18_P14134605
TTTCCATCCACTTTCTATATTCTAAAAATTACCGCTGGATTAAGCCCAGTCAAGAGAGAA
A_14_P106985 ACTTACCTAAAATTGTTGTTAGGTCTTGGAAGACTTTTTTGGTTACCAGATTCTTGGTTG
A_16_P16624173
AGCCTCTGAGATGTATGACCTTGCCTCATCTATTCTGTTGATTAAGTTTACCCAATAAAT
A_16_P36085513
GCCACCTTATCTTCATTAGGCCCTCTGTAATTACATACACTTGGTCAAGAATCTCATAAT
A_14_P105907 CACCTGTCCTTTCCCATAGTTACAATTTCCTATACTTGAATCATATACTACCTGGACAAG
CUST_4605_PI426210822 CCATCTGAGCTCTGGGTCAGCAGCAGTCATTGGAGCAGGGATCTG
A_14_P200819 GACCTGTTCTAAACTTTGCCCAGGAAATTCCTAGAAAGCAGATGAGTCATTATGTTCT
A_18_P12797234
TTAATTTAACTTAAATCTGAAAATGTCAGTCTCAAATTACATATTTTAATGACCACGTAA
CUST_1118_PI426210822 TACTTTGGTTTTCCCAGAAGATGACATGTTGTGACAGGGACACATGATG
A_14_P126874 ATCACTGACCTTCAGGCATGAAGATTTTCTCTCCTATATTGATAAGGTAGGCTGGT
A_14_P103630 TTAAAGTTTGCCAGGCTTTCTATTTGACTTTTTTGGTGCAGTTTTAATATCCATGGTGGC
A_14_P101937 CCTTTTTCTTGCCCACAGATTATAGAAACCCATACAAAAAATGACTGATAATGACTCACC
A_18_P14132494
CTGCAGAAAGAAAAGGAATTTATCTCTGAGAAGGCCTTCAGAAAGAAAATGAGTAGGCT
A_18_P22840873 GGTGGCCTCTTCCCTAGCCCCACACATACTCTTTGTTTTTCCATT
A_14_P112146 ACTGGTTAATTTCACTAAAACCAGAAATTAGTGGAAGGCTTGATTAAGGGAGGCTTTATT
A_18_P16722477
AAAAACCGGGCTCTATGTTTTTATATATTGGAATTGGTTCAGATTTAAAAGGCACACGCC
A_14_P132399 TAAAATGGTAGGTGGATGCCTTCTGTAAGGCAAAAGAGAAATCTGATCCTTAATGAAGTA
A_16_P16317608
CCTGCTACGTTTCTACTAATTTACCATCGCTTCTTCAAAAAATTGTGTTCAGAGTGCTAC
A_14_P116742 GGAGTTCTAAAACTATGACTGATGCAATTCTAAAGGCCTAAAGAAATTTCTCTGGGTTTG

A_16_P20081454
AAAAAGAACAAACAGAAAGTCTAACCCCATAGTAAAAGTATACTTCCTTAGGCCATTCCT
A_18_P15309376
TCAGGAATACTTACAGACACAAAATATGCCATGAAGTGATTATACAGTGAGACCTCTTTC
A_14_P200312 CTCTTGGGATCATAACAGTGTGATTCCTTTTTGGTGGAGTCTTTTCCTCTCATTTTATTT
A_14_P127631 AAAGGAAACAACTGCTTACGGTTTCATTTCCTAGAAACCCAGATGTCTATTTTAATGCAA
A_14_P104591 ATGCTTCCCACTCAGTTTCAACACTTTCACACGAATTATTTATGCATACATGACACATT
A_14_P131756 ACAACAGGAAAGAGACATAGAGCATGCAATTTCTTCAACACATGCCATCGATTACTATAA
A_14_P104972 TGAACTAGGATGAACTTTGGGAGCCATATGTGATTTCAGAATGAAAAAGTTATAGGCTAC
A_16_P02464077
GATCACTGTGACTTTGTAAATGCCAGTATTTCTCTCCTATCCGTATCAAGAACACTGATA
A_16_P36046279
ACCTTCTTGCTGCTATAAACAATATATTGCACATGGGAAAGATAGTTACGTTATGCCTGA
CUST_10298_PI426210822
AGATTGTTAACACACTGTCTATGTCAAATCCTTACACTAGCATAGTGATGAGAAAGTGAG
A_14_P121935 GTAAAGGCAGCAGTAATGCTAACGCTAGCAAGTAAACTGAAGCGTGACGATG
A_14_P114405 ATAAGTGTATTGAGAAGCGTTCTTCCTCCCTCCTTTGCAAGCGCAATTAAAAATGT
CUST_4695_PI426210822
TGTTTTAAATAAGGTATATAGTGAGAAAAGTGAGCATTGGAGTCTATTTCAGTTATGTGT
A_14_P110550 GCCTGGTTTTTTTTTTTTATCCTAGATTCAGGTGAGTCACTGTATTTGCAGTCTACATCA
A_14_P139989 ATAATGATTTGTAAATGCCCTTGATTGTGGGCAGTTTATCATGGAAGATTCTTCTCAAGT
A_14_P123767 ACTCACGGGAGAAAAATAGACTTTATTTACAAGTAATAACATTTAGAAAAGATCCATCCC
A_16_P38893286
CCCCTACAAAAAGAGTTTTAGAAACTACCTTAGCATCTACTTATAATGCAGCCAAGAATG
CUST_4888_PI426210822 GTTGAGGGAAATCATGCAGAGGTTCTCAGATGTTCAGAGAGGCTTC
A_18_P22840856 TTGTTTGGACCTGCTATCCATGCATTCAAAAAAATGGGAGGTTTTGGTCAAGTAAT
A_14_P127959 AATGTATGGGAACTACCCATGATGATGTAAGAGGAAAGAACATTTTTTTGTGATTCACCA
A_14_P130303 TGCTTACCCACAATATCTTCATAGGCATTTTCTTCTAAAGTGCTTTTGGATCCAAACTTG
CUST_1853_PI426210822 AATTTATAAGACTGAGCCCTGGCCTTATGGTGTCCTTCAAAAGAATGGCATT
A_14_P138614 GCAACAATTGGTATGCAGGTTTCATTTTCTCCTAACTTAGATCTCAGAAGAGCACTTAAT
A_14_P107399 CTGGATCATTCCAATCAATGATTCTAAGACCTTCATCTTTCTGCTGTAAAGCACAGAAAT
A_14_P137728 CTTAAAAACCTCACTGATCCAAGAGCACTCTCTGATAATGCTAATTAGAAATACGCTTGC
A_14_P107045 CTCTGGCAGATGTCCCAATCCTGGAGGTCATCCATTAGGAATTAAATTCT
A_14_P202112 TTCTTGATTTAAGGCTAGATTTACGTAGTTCGGCTCAAAATGGCCAAAACAATCATTCTC
A_14_P114471 TGGAAATCTCTATCTGAATGATTCACCCCTGAAAAACTTCAGAATCTATAGCCTGGATAT
A_18_P10361476
CGAGCATGCTCTGTAATAACAAAAATCACAAGTGTTGATTCCTAATCCGTATGGCATTAG
A_14_P107724 CTTTTCTCCACTTCACCCTTAAATAAGCCAGGATTTTAGGTATGATCTTAAGGAGAATGA

A_14_P110153 GTATGGTTCACTTTTTTTCTTTTACTTGCATGGTTTTTTAAACTTCTTCTTTGTGGCATT
A_16_P17895186
GCCTCTTCACCTTTCTAAAAGTAAATGAACCCAAATCTTTCTTTTCAAATGGCTATGAGG
A_14_P102060 TGCAGGCTTCTAGAACATCTGATTCTACGTGTTTCTCCACAACAAACTTAATCTGTTTTA
A_14_P125944 CACTCACTCCAAATTCCGTCATCTGAGCAATAAATATTCACTTTGCTTCTTACTACAAAG
CUST_8195_PI426210822 CTAGTGTTGGCCAGCGTTCATTCATTCTCCTCTCTCTGCACAGTG
A_14_P126974 CATTATATCCTGAAATGAAGGATGAGTTCGAAATTGTTCAAAGAGATCGCGTTTGTAAAG
A_14_P118024 GGGACCTGTGTTAACTTCGGACTTCTACTCAATTTAGCCAAACTAATTAATCTATTGTTG
A_16_P41773968
AATTCAGCAATTTGTCTCACATATTCTTTCACGTTCAGTAATTTGTCTCACCACAAGTCC
CUST_6866_PI426210822
GTTCGAAGGGAAGCTCATTTTTGCTGTTTCTAAGGAGTATAATTTGAGTTCCAAATTAAA
A_14_P127498 GAACATTGTCTCAACTGGACAAACTGAAAGTTTTCGCTTTGTTTACAATGCTCTATAGTG
A_14_P122479
TGCCCATGATTTCAGGAGGATGAAGAAGAAAGAGAAACAGAATATTAAGCAGAACAGATA
CUST_2812_PI426210822
GGAGCTTTATGCATATTACTAGGTGCTTCATTTTTATTTAAAAGGCTGATTTAATAATAG
A_14_P120909 TCTTTAAATTCCTTCAAGTGAAAGTTGCTAAGTTAAAGAGAGGCCCACTTTTGTGAGAAT
A_14_P101402 AAAGTTAGGGAAACACACTACATTTGACAACCAACATTAATTTGTAGTTGGTCCTAACCC
A_14_P112274 TACCTATCTTATCTAGGTAGTATGTCAACAAGAATCTATCCCATTCTAACTGTGCCCACA
A_14_P128245 CTAACATGGGGTGATTGGGTGAGTCTGTTATTTTCTGATTTTTTTCTCCAAAGACTTTAT
CUST_5258_PI426210822
GAATCCTCTTTTAAAAACACTTAGAAGATTTTCCCTAAACTAATTATCTGGCTTAATGCT
A_16_P17623269
ACTCTTTCGACTAGCACTTTTTCAAATAGAATTCTACTGTAATACCTGAAAATGGCATGC
A_14_P133643 TTTTTTTCTTAAGCCATCTGGAAGTTCTACTGATAGTAGAACCTACATCTTCTGTCTCCC
CUST_2335_PI426210822
ACTACATGCTGACTACTTCCTACTCATATGTATTTGTATTTTAATTTTAAGAGGCAGCAG
A_14_P130712 CTCTTCCAAGTGGGGATTTATTTATTTGTCTAGGGACTTGTGGCTTATCATCTTTATGAC
A_18_P22841940 CTCAGTGTGCGTGTCCATGTCCCATTAAGATGAATGACCTCATTGTGT
A_18_P15686851
AGGCTGAAATCTAGTTTAAATCACATCTGCTTGAATTGTTGTGCTCTTAGATACGTTCCT
A_14_P118417 CTGATTTTATGATGGAAGTCTTGATCTTGCTCGCAGAATCTCCAAATTAGAGTCCAATTT
A_14_P112115 ACTTCAAATTAATTGCAAGAAAGTATGAAAATCTAGGGTTGCTGTTCTTCTTTGGTGCCA
A_14_P137602 CTGGTAGATGGCAGTGGAGAAAATGATTTTAAATGAGGCATAATCAGTAAATAGCAGGTT
A_16_P01878436
TTGATAGGTAGCAAATTTCGTTCTAAGTATACGTTGCTCTTTCAAAGAACACTCGTGGAA
A_14_P107327 AGGACCTACAAAGGGAGTGTGCTGTAAAAAGTTTTCTTATTCTATAGAATAAATGGGTGC

A_14_P113356 TTTACTTACCTCACTGAATTGGAAATGATCCAATAAGTAGTGTTGGAATAACTGGCCATC
A_16_P00042482
CTCTTTCCCTACACCATAAAGGTATCTCTATTGATTTCTTTATCTCCGTAGTGATCCACG
CUST_8994_PI426210822
GCCCAGGGATGTAACTGAGAACATAAAGTCAATGAATAACACAGAAACGTGAGTTA
A_16_P35636488
AGTGCTGCTCATAGTGTGATTCCATAAACTCAGTAAACATTTCCTAACAAAATCATGGCT
A_16_P20863429
TGCTAACTCAAGATAAGATGCTAATCCTTTATAGCCTAATAGGAATCTCATATCCTCCCC
A_16_P19140856
GAGACCTAAGGAATATGTTGTATTCATATTGCATATCTTTCTAAAAACCCAGGTGTTGCC
A_14_P105661 CTATTTGTGTCGAGTATTTATTTCTTTGCTGTCTAATTGCTCCAACTCCCTAGACGTTAG
A_14_P117964 GATTTTGTCACCTTCCTTTACCAAATAACTAGAGGAATTGCAGCAAGGAGTTATGGATTA
A_16_P00346057
AAGGTTTTCTGCACTAATGATGCTTAGATTTTTTAACCCATTTTTCTCTAACTGCATGA
CUST_1530_PI426210822
CTTCCTTTCAGAAACTGCTGTTTAAATCCAAAGAGAACATAGACAGTTGGCAATATAGTG
A_16_P01354933
AAAGTGAACATACAGAATGCCTTACAAACACCAAAATGAAGGAATAGTCACCAAATGTGA
A_14_P119978 TACTTCCTCGACCCTATTTGGCTCTCCGAATGTCTGCAGAGGATCTTTAATATTAA
A_14_P128674 CTCCTTTCCTAGTGAATCCAGTTTCTTTCTTAAAGATTTCTTTCTCTTTTGTCCATCTGT
A_14_P112510
GTCGACGATTGAGGTTTTGAAAGATATTCAGGCTCTAAAGAAAAAAAGAGAAGCGAGATG
A_18_P15262957
AAGGAAAACAGCCCTTTATACTCTTTGACCCTATGACATATGTTAAAAAGCAGGAAGAAC
A_14_P136254 CCACTCCAATCAGAGGTTCTCTAATATTACTACCAATTCTTCCTTGATTTCCCAAAATAG
A_14_P130208 CAGCCTGCTGCATTATTGTGGCCATGTTTGTTGTTAAAATAGAAATACATCCTTCTTTGT
A_14_P102178 GTATATTCATACTTCTGTTAGGTAACAGGGTAGGTATGTTAGGTAACAGGGTAGGTATGG
CUST_7469_PI426210822
AACTCCTAACATAATTGGAAGTTTAATATATTCCAACAAGTTCAGAACAAGCATACTAGA
A_14_P109767 CAAATGTTTTGCTGACGTTACTTCATTTCATCCGGCTAAATGGATAACCCCATTTTTTG
CUST_5663_PI426210822 TATAAAATTAAAAGAAATGTGTTCTGTAATCCCTCTGGGAATTCCCCACGGGC
A_14_P110420 ACTGAAAAACTGATCAAAAGCCATATGCTGCTCATGTTTCCTAATACTCTCACACCTTTA
A_14_P127128 TAGAGGGTGGTGGACTGTTTTTCTCCAAAATTAAGACCCAAAATAAAATGTCTGTCTTAT
A_16_P00685605
TTATTTAATCCACATCGCCAGATAGATTCTTAATTCTCCTTTTTCTCCAACTCCAACTCC
A_14_P104348 AGAGGCTGTGGAGATGAGGGAGTGACTGCTATAAATAGTGAAAATACTAAAAATCACTGA
A_14_P116267 CTCAAAATAACAGTCTTCAAACATACTACATCTGATCTCAGAATATCCACAGTGTTCCAA

CUST_4787_PI426210822 AAGTGTCCCTGGACATCAGTCACGTGGCTGTGGAAGGGAGACTTA
CUST_552_PI426210822
CAGTTCCAGGATGGGATACTTTTTTAAAAAGTAAATGTAAATGTATTGCCATATTTCTCT
A_14_P107662 TCTTTGACCAATGCATAGTTTGGCAGAAAAATCAGCCGTTCATTTAGAAGAATCTTAGCA
A_18_P17346169
TGATACAAAAACTCACTTAGCACTTCATCATTATAAGACTCTCTAAGGACTGATCCCACC
A_14_P120470 AAAGATAACCAAAGGCTTCTTTAGGGATGAGTCTTAACCATTCAACCACTTCCC
CUST_10235_PI426210822
TCAGAGTCACTATCTTTAGTTTAGTGTTTATCTTTAGTCTTTAGTTTTTATATTTTATCT
A_14_P201601 GGTTTAAAAACAGGAGGAATGGCATCTCATTAAATCCTGTTAAATGTCACCTCAGTCTTG
A_14_P101910 AATAATGTTAAGCAACCAAGGCAATAATGGTTTTCCTTTCATTCTGGTTTTCCCAAATTA
CUST_1265_PI426210822 CAGAGGACAGCCATGGCTGTAAGGAGGAGGCCAGAGGTCTCCAGC
A_14_P107538 ACCTACTTTCAATAAATTCTGGTTTTGTGTTTTCTTTTGTAATCACTGTTCATCCTGCCT
A_14_P200873 GATATGAAGATATAGACGAGATTGCTCCTCACATCATCTATTGTTCCATCACAGGTATTT
A_16_P00685096
AGGCACCTTAATTAAGATCTTTCAAGCATCAGGTAAAAAGGTGAGGTTCTTGGTATAAGT
A_14_P115283 GAAAGAGACTGTGAATCTATATATACCATTTCTGGGACGAATTCATCTTCTGAGGCCTC
A_16_P20875297
AAGTAGAACTCCCTCTAAAATCATTCTAGTCCAGTTTGTCTATCCTAAAATCTCCCAGAC
CUST_1294_PI426210822 TTCTCACTGAAATGTGACTGTCCCATGTTCAGGGTCACTTTTTTTTTTCTGA
A_18_P10561351
AAAATGTGCCCTCTATTGTTAATGATAGAAGTCTTTAAAAGCACTGATGTAGTAACCCCC
A_14_P122450
GCAAATCTGAGTAATGAAGAGAAGCATCCAAAAACAAAGAGGAGGATTAGAAACCTAAGA
A_14_P130650 CATTATACTCCCAGTTTACTCATACATATCGACCTGAGGTGCAAAATCCTAGAAGAATGA
A_14_P135251 TTATCTTCCTTTCCAAATCCAAAAAGATGAAAACGACTGAGATACTTTCAAAAGACAACC
A_14_P120045 CTGGTCTGGCAACAAGATTCTTGTTTCTATGAAGGTGTAAATGGTTAGTTAGACTTTGTA
A_14_P114858 ATGACTTATTTTTCCCCTTAGAAAACAAGTTTATTCGGGGATTTTTTTCTCTCCCACTTT
A_16_P36293688
CCTGTATTCTTACCAGACAAAAAATGAAACTGTTTCCAATAAACTAGCCACTACAGAGGA
A_16_P30952303 CTCATTGCATTCCCCTCCTCCCCTCCTCACTTCCTTCTCTGCTTA
A_14_P104427 CTCCAAATCTAACAAAATTAAGATCGCTGCAGTGTTTCAAAAGGACCTTGACCTTGTT
A_14_P117373 TAGATAAACCTATCACTTATCTTTTCAGCATTCAGAATTCAACCGAAGTCCTCCCATACA
A_16_P00685098
TGAAAGTTTCCATTATACTGCCCTGTTGTCTAAAATTAAGCCAATTAGAGAGTCCTGCTA
A_14_P135047 GCCCCATAGTGTCATAGTCTTCCTTCATTTCTTCTGTGGGGGAAAATTATCTTTA
A_14_P200184 ACTGAACTTGCTTTTTTTCCGGTTTCTACATGCAGAGATGAATTTATACTGCCATCTTAC
A_14_P136713 TTTCTTTTCTTGGAGGTCTTTTAAGAAAGCATTTTTCTGGACCTTTAGGTTTCACCTAGA

A_14_P124426 GGCATTATGAAGTTTAGAACTGGTCTCCAGGAGTAAATGTAGTCTCTGTATAGGTTTATG
A_14_P120920 AATTCAAAATGTTTGAAATGGGAAGCAAATTTACCACCAAGTCAACCTTTTCTCCTGTTA
A_14_P104369 TTTAAGGTGAACTTATTTTGGCTTAGGGACTACAAAAAGAGAAGGTAATTCCTAGGGAAG
CUST_1088_PI426210822 GACAGAAGTCTCACTGGCTTATGTCCACATTGCCTTTGGGCCTCC
A_18_P22841626 GTTTATTTGAGTAGCGCCCCAGAAAGTAAGAAAGAAGGAAGAAAAGGAGGGTATG
CUST_9528_PI426210822
TGAACTAAAAATAAACTAGGTAGTTTGTTCACATAACAAATCTACATTCAAAGACATGCA
A_14_P100079 CTGCTTTTAGAAGTGCTCTCTCCTTCCGTTGGAAAGAAAGGTGTTTGTTTTTG
A_14_P112873 CATTAGAACCTGTAGAAAGAACATGAGGCTGAAAGTAGAGGACATATTCATTAAGGACTG
A_16_P19727087
AAAACCTGACTTTTTTCTAGTTTCAAGTGGTATCTGAGTTCAATAGAATGTGAAGCGCTC
CUST_1676_PI426210822
CTCTATTTTTTTTCTTTAGGTACTTCTGGCTTTTGCAGACACTTGCTCCCTGAGAAT
A_14_P119305 TGTCTCATGAAGGCATTTTTTGTCAGGGATGACTGAATAAATTTTTTTTTGTGGCGAG
A_14_P115196 ATCGTTCAGGCCACAGATATGGAAGGAAATCTCAACTATGGCCTCTCAAA
A_14_P123884 GTTTGAGGAATAATACAGCATATTGGCTAACATCCCAGTCTGATCTTTGCTTAGGAATTT
CUST_8244_PI426210822 AATTTTTCGGTTTTCAAGTGCTTCTGGGTCTCAGCCTTGATTAGCAGTAGAAAT
A_16_P02255163
AGTTTCTACAGTGAAATCTCTTTAATACACAAAATCAGCAGCATTACAATTATGCCTGGA
A_14_P121729 TTCCCTTCTATATTTCTTTAAAAGATAAAATTTGCCTAAGGCTAGGAGCCAGGAAACCAG
A_14_P111943 CTCTGCATCCATGGTGGTAACTCGGACATTGATCTGAAAAACAGAATGAAACAAATTAC
A_14_P107728
TCAAAGACCAAGGTATTTCCTTCTGCTTCAAAAGAACAAAATTGGGAAAGAAAACTCACT
CUST_8960_PI426210822
TGGCCTTATATTAAATAGTGTCATTGCAATTGAAAAATTAGAGTAAATGAACAGAGCCTG
A_14_P114288 TCTCCATAACTTTCTTGGTTGGCTCAGAAATATTGCTTTTAAAATCTGATTGGGTCTCTT
A_16_P16862672
TGGGTTACAACTAACATGACATCAATGCTGATAAACTGACTGTATTTCACTATCTCACTG
A_14_P138880 CATTTTCTATCATAGCAATGTTGTGGAAGACACAGTAAGCCCAAATTGAGAAATCAGAGA
CUST_9135_PI426210822 CAGGGCTCAGAGGTTCTGTTTCTCTAAACCCAGACCAAATGTGTTT
A_14_P111188 TATTCTTCATTGATGACACGCTCCTCCACAGTATCTTCAAACACTGAAATTTGGAGAAA
A_16_P03707282
CAACTGGCTTTATTCCATCCTAATTTTAAGCATCCCATGTGATCAATAACCAACCATTTC
A_16_P41025926
ACCTCATTTTGTGGTTATGTAGCCAAAGATACTTAGTTTCATAGTGCCTGATTCTTGCAT
A_18_P22842111
TTATCTCTCCCTCACAGCCAAGCCAACTCCTAACATAATTGGAAGTTTAATATATTCCAA
CUST_3084_PI426210822 ACTTACATGGAGGAGACGTGTCGTCACTATGTGGCCCATGTGAAG

A_14_P104564 TCTGGGAGGCAAGAGAAAAACACATATTCCAAAGCAAAATATGAACTCTGTTTAGATAGG
A_14_P113610 ATTATTATTATTTAAACACTTACTTGATCACCTGGTTTTCCACCTTCTCCAGGAGGCC
A_14_P107488 GAAAATGTAAAACATTGCCCCATGTAAAACATTGCCCCTTTATAGAAGGGCAATGTTTTA
A_16_P00611541 ACTCAAGTCTTACAATTCTCCCCTATTCCTACCTAGAGTTTGTTCATTCTCATCTTCC
A_14_P110764 CTATAGAGGAATATCCCTGCTTAGAGTTTGAAATCAGTTCTGTTAGGAAATAATGTTTGT
A_14_P113404
ATCTGAAAAAAAAAAAGAAGTTTGAACAAGAGACCAACTCTGCTAAAAACTCTGTTCTGG
A_14_P120954 TTTCTGCGTAGATACAAAAGGCTTTTTACCTGGCCAAATGATTCATGCGGGAATA
A_14_P113936 GTTAATAAAATAAGCCAGGCTTGCAACTAAGTATCTAACTTTACAGGCCCACTCACATTT
A_14_P117645 ATTACTAAAAACATTTAAGGCTCAAGGGAATTAGCCTGTGGCTTACATGATTAGAGTTTC
A_14_P107057 TGGTGAGAAGCACTTACAAAAAGAAATGGACTGTGTTAGGATTGCACATTTTACTTTGTT
A_16_P41142571
AAACGTTTTGGATTGGTTTCATTTAATGGCCTCAAAAACACGTGTTAACATTCTCCAAAT
A_14_P119352 TAGAGAGTGATGAGGAACTTAGAAAAGATTGTTTGTATATCTCTACTGAGGGTCTGGAAC
A_14_P104760 ATGCCTTGTAAGGAATTGAATTCAGAGAATTCGCCATAAATGTCTATGAAACTGGGAAAA
A_18_P22003952
AAATCCCTTAACAAACTGCTCAGTTAGCATTAAGGAGACGTCCATACATAAGACTGTTAC
A_14_P126578 TGATCAGAAAAAAAGTTGTCTTATTTGCTCACTGCAAAATACACATGGACACTGGTCAAT
A_14_P104358 CAGGATCTCATCAACTGTCTCAGGGAAACTCATTCGATGCTTATCACTGACT
A_14_P129392 CTATAGTTTTCTATAAACTGTCCTCAGGGGAAGGGCACATACAACCTAGAATAAAGAAGT
A_14_P106552 CGCCATTGACAAGCAATGGGGAAGAAACAGAAAAACAAGAGCGAAGA
A_14_P125809 GAGACTGAGAGTTGGGAGGCAAAGAAACAAAATCGGATTTCCTCAGAG
A_16_P01432460
AGTTCTAGCATACTCGAATTTTAACAGATGATGATGTTTCAAACTATTAACCATGTGCCT
A_16_P00276178
GGGCCTTCTTTCTGATTTCCAGATTTTAAATAACAGGATCTAATTTCCCCTGTAAAATCA
A_16_P16120593
AAAAAACTTCTCCCCGTATCTTTTTCAAAATAGGCAAGATGCTGAAAAAGAAAATCCAGC
A_14_P200041 ATGGAAATCCTTCCTAGAGAACTAGGATCATGGCAAAGAGTGTCACATACTGAAAATAAT
CUST_5020_PI426210822
ACAAACAAAACTGTTTTCATCATCTTTGGTTGTAGTTTTATAAAGAATGCTGGTTCATGG
A_14_P121938 GGGATAATTTAACCAAACCATCAACAACTGGACAACTTATTTAACTCTTTGGTGAGTGTT
CUST_7971_PI426210822
TTCTAAGTTCAAATAGTATACTACAGCATCTTCAGACATTCATGGAACATTGTAGTATGT
A_14_P114689
AATGTATGCCTGAATAGATAGAAAGGAAAAAAGGGAAGGGTCTACAAAAACAAAGTCCAA
A_14_P103027 GAAGATTAAAGTCATACTTGAGCAATGAAGATTGGCTGAAGAGAATCCCAGAGCAG
A_16_P36897923 TTATTCTATATTAATTTCAGCCTATAGTGTGCAATTATATGGAAAATAACGTTATGTCCA

A_14_P108525 AAACAGACTTTATTGTCTAAAACCCAGAAGTAATACAGCATCACAAAAACCATTACTGGC
A_16_P02811346
ACCTCACAGATATAGAATGGTCAACCAATAGAAAAACTAAGATGGAACAAGGTATAGCAT
A_14_P113370 AACCTGGAATAAGCCTGTTTTTTAGAAATGCACCCTTTATGAGAATATCTGATTTGCTGT
A_14_P111090 AGCAAGAACTAAGTGAGATGGATGTGGTATTAAGTAGATTCCTCAAAATGATGGCAAATC
A_16_P15831347
AAACTACAGATGGGAAAACAGATCTCTTTATTTGCACATCCTTCTCTTTATCTGAATGGC
A_14_P132258 GCTACGTTTAAATCCTAAAGACAGCCTTATCATTGAGTCTCATTTCAATTGTCGACACAT
A_18_P15270057
TAATCGGCCTCATACACTACTATCCATGCAGGACTATCAATTTTATCATGTAATTCCCTC
CUST_7789_PI426210822
CCCCAAAATTTGTATTTTTAATTGAATTGATTTTTTAAATTGAATCCATTTTAAACATGG
A_14_P200784 TGAACTGCTTTAGACTCTCCGTCAAGAATAGACATTTTTGGGCAAAAATGTGGATTTTCT
CUST_6584_PI426210822
TTAATCACAGAGCCTTGTTTGAATCACTTGACTTTGAAATCCCAGGAATGACTTTG
A_14_P111888 GAAATAGAGGTAATGTGAGGTAATTGAAAGCACTCCTTCAAGGACTTTAGGAATTACACT
A_16_P58424429 TTAAACCAACTACTACAGTGGGCAATGGGGCTTATTCCTGCAGGG
A_14_P114164 GAAATGCACTTTATAATGTGTTTGAGCAAACCTCGCCTCTCCTACAACGGTAAG
A_14_P101352 TATTTTTGTTTTACATAGGGAGAACCTAAAGAAGTAGTTAAACCTGTGCCCATTACATCT
A_14_P112125 ACACACAGGGGCTTTTGAAAGCAACCACAGTAATACAAGATGAAGATTATATGTTACAAG
A_14_P136812 CAAAATACCTCCAAATAATCTGGGGAGTGCTAAAGTATCATGGATTATATCTGACTCAGC
CUST_876_PI426210822 TTCGTGAAGCCTGTTAAGGGTTTTCTGGAACAAGCTGAGCATCTTTTG
A_16_P35287192
CCTTAAATCTTCCCAAAAGTCTGTATAAGGTTTCATGGACATTACCAAACTAAACTGCCT
A_14_P202424 CAGTATTAGGCTATCAACCACTGTTTGCTGACTGAGAAAAAAATGAATGACCAATAGAAC
A_14_P115665 CTCTCCTGCTATTTTAAATCAAGGCTGGGGAAAATAAAGGGATTTTACAAAACAAGACAT
A_14_P119716 AATATAAATTTTGAACAGTTATAGAAAATAAAGATGGCCTTTGGGTCAATAACAGAACAT
A_14_P104293 TAGAACTCGGATCTCATATTCGGTATCTGGATCTAAATGCCATAATTTGTAAGTTGGAGC
A_18_P22854755
TATTCACAAACCAATATTCAAGTTCCCATCAACAAGATGTTTTAAAAACACAGTGGCTGC
A_14_P110165 AGGTGCTTGAAAGGAGCGAGGAAGGTTGACAATATGCTTACTTTATCTGGAAA
A_14_P111804 ACCTTCTCAAGGACCCAGTAGAAGACAACTCCAATTCCAAAATTATTATTTTCTTTTCAC
A_14_P116669 TAAATGTGAATGTGAAAATAAAAGCCTCACAAAAATTCCTCTGCATGAACATCTCTGACT
A_14_P103611 TGTTAATCCTAATGGTTTCACCTCCTTTCTGCAGATTTTTTCAAACTCATTCCTAGAAAA
A_14_P112430 ACTTGTCCTAAGTATAACAAGGGATGTATTTTTTGTTGGGAAGTGACCATTTCTAGGCTT
CUST_2879_PI426210822 GTGAGGTGTCCTAGAATTCAGCAAGGAAGGCAGCCTCTTGGCTCT
CUST_4072_PI426210822
ATAAAAGGAATCCTGGCTGGGCATTGAGTGAATACAATCATAAAACCCAAATGATG

CUST_825_PI426210822
ATAGGCTTTCTCTTGCCCTTGACTTTTATCAGAAGCTGTACGAAGATTTTTGTTATAAAA
A_16_P40540151
ACAACCAATTAAAAAAAACTCCTTACCTCGAAAATCTAGAAATTCAAATGCAGACGTGTC
CUST_6144_PI426210822
GCATGCCAGCAGTTTCCTAAAATGTAATATCATCACTATTATTGTTATTATATGAGGGAA
CUST_8106_PI426210822 AAAATTCTCATTCTCTTGCCATTTCTTGGATCCACAGAACACTCGCCATTG
A_18_P10776745
AAAGGACCATTAACTGTTTTCATGTCTTCCGTCACTACAAATGATGAGGAATTCATTTGA
A_16_P17568597
GATCCCTAATGTCACTATGAGAGACTGATTTATGTAAGCCAGATACAAAACTTCATCTCA
CUST_5621_PI426210822 AGTTAAGAAAGAGCTTGTTGCAGGGGCCTTTGCAGGTGAGTCCCT
A_14_P133616 TTTAGTTTTCAAAAGTTGGATGTTGGTAACAAAAAGAATGAGGTGGATCTCAGGACTTCA
A_14_P132505 CTCCAGCCAAACCTATGCCAAATGGAAACAGGTCACGTGTTTTCTTCTTATAAAAATATA
A_14_P110921 TGTCATCCTGGAAAGCATAGTGCAAGGTTGTAATCCATTTATTGTCTCCATTCACTAATA
A_18_P22842834
TGGTGAAGCAGAATATAACTTTAATAATGCTCCCTTAGGATTCTGAGAGAAAGTTGTTAG
A_14_P101160 TACCAGGAACTGCTTTTTTTTGGTATAGAAACTATCTTTTTAAAAATTTACCCTTGACAT
A_14_P108708 ATGAAAACGAAGCCAAATTTCCCACTGAATCTCATTTCAATATATCAGACTTTCCTCATG
CUST_6035_PI426210822 ATCCTCCATTAAAGAAACCTTCGTTGTACTTTTGGGCCCATAGCTGTTCT
A_14_P118435 GTCTCGTTGCTTGATAAATTTTTACCGCGTAAGACTCTGGTCTATCTAATGACATTTGAT
A_18_P14585102
TTCATCCACAAGTATCTGAAGAGTATTATGGATCCTCACTGAATAAATACCTTGCTGCTG
A_14_P133945 CTTCTCATCTGTCAGTGAAACTCTTTAGGAAATTCTCACTACTACTTGACACATAGGAGA
A_16_P03700611
TGGAATACTTCAGTGAACCGTATTTTAGTCCTGCATAGAACTGCTTTTTCCTTGTCTATT
A_14_P112769 AGAGTCCCAGGAGTTTCCAGGCTGTAAAAACCTAAATTGTATAACATTACTCACAATAAC
A_16_P16033594
ACAAACCATCTGACTTTTCTTCTCCACTCTTATATTTCAACTACCAAAATCCATGCCTTT
A_14_P119309 CATGAATGCTGAAATGTTTGGGTCCACCTATTGTGATTATACAATAGAGATATGAATCCA
A_14_P120321
TAATTGAAAAAATCAAAACAGCAAGAAGGGCTGCCACATAAGAAGAGGCTTAGAAGATTT
A_16_P58424230 TAGTGAGATTTCCGAGAGAGTGCGGTTGTCTGGCACTTCTGTGAT
A_16_P01294516
AAAGTTACACATCTGTGCTAAAGTTTTCAACCTCTTTTCTATAGAACCCCTGCCTATCTT
A_14_P107367 AGAAGAACTTCATTAAGGAAAAAGCATTTGTTCAGGACTTTAAAGGTTGATAAGCATGGG
A_16_P39533322
CTGACACAACACCTTTGAGATCTGGAGATAAAATAGTAAAGTGTAGATGATAGATTTAGT

A_16_P16644251
CCTTCATTAATGAGCACTTTACTAATACTTAGGTCCACATTTTACTACTCTTGCCTCCTG
A_14_P125254 ATGAGAAGTTCCTACCACTGTGCAATGAATAACGAAAATGCCAGATTACTTACTTTTCAG
A_14_P100593 TAGCAAAGGGATAAATATTTCCTAGCACTTGGCTAGAACCCATCTTCTCTTATACAACAG
CUST_9075_PI426210822 CTGAGAGATAAGCAGCCATGTCATGAGAAGCATTTGGACTCCGGTA
A_14_P202415 ATCTTTTCATCAGCAATTGGAAAGTCATGATAATATGACGGTGGCTTTTTCCTCTCATAT
A_14_P119765 CTGTTCCATTTTTCTTCTTTTGCTTCACTGAAGTTCTCCTTGGTCCTGATTCT
A_16_P17026673
AAGTCAACAGTCCAATCATTTATTGCCACTGAAAGTCTAATATCTTATCTGACAGGTTGT
A_16_P41237146
AATACACAGGAACTGCCTATTATCACTGCTTAAGTATCAATCCTCTAACAACCAAAGGAG
A_14_P122569 ACTCACTTTGTGATGGGAAAATAAATTATTTGGTCTGGACAACCTGACAGTGTTACTATT
A_16_P17839659
ACACGAAATCTGTTGAGAATCAAATGCTTGTAACTAATCTGTCTTTCCCATTTAGCATCC
A_14_P119431 ATCCTATTGAGTTTGAAATTTATATAACTCTCTAGTTCATGTCCTATCAGGGCTGGGGAG
A_14_P201614 CTTTGGTAATATAAGGAACTCTAATAGAGCCAGGGCTGAAATATGTTCCAAACATCTGCG
A_14_P124649 GTATCAAGGAATAAATGAGATAATTGATACGTGATTGCCAGTGGTTGTGCCTATGTCTAG
A_16_P37667490
CTCCACTACCATTACCAAATTCAGAAAATGCTTCTCCTTTCAGAATATACAACCAATCCT
CUST_6294_PI426210822
TTTCTGGTGCTAAAGAACAATTTCACCTCCTAAGCCAAGAAGGAAAAAATTCTCATCTA
A_14_P128898 GAAATGTCCTCACCTATGTTTGTGCTTCAGGTAAGTCAAAGATGTTTGTTTCTATCTACT
A_18_P14520359
ATCAGATCCGAGGAATATCAAAATACTCTTGTCCTGAAATACATCGTGAAACTCATCTCC
A_14_P202017 ACAAGCCCACGTATCTACTTTCACTTGCTCATCCTTGCTCTACCTCAAC
A_14_P103640 CTTGAACATATATCTTAATGGCTGCCAAAACCATGTCTTAAGTGACAGACTGTCTGATTT
A_14_P115549 TTAGCAAGACAAACCCAATGAAACCATTTAATTTAAAGGCTTTTTATTAGGAACCAGGGG
A_14_P116768 AAGTGCTGACAATACTTTTAACTTGTTCATATTCCTCCAATAATTCCTGCCGAACCTTAA
CUST_6255_PI426210822 CCACGTGTGAGACTTGGGTTTAACGTGACCTGTCCGTAAATCATT
A_14_P110866 TTTCATCTAAACTGACTTGGAAATTTTTCCTCTCCTACCTAAAACGAGAAGGGAAAGAG
A_14_P118334 CAGTTTGTCAAATGCTTCTTTACAGATTGCTTTGGCTAAAGTTGATTTTCCACTTCCCT
A_16_P16736043
ATAACTGAATGTTTTCCCTTCTCATATAAGCACTGTTAAAACATGAATACAGCCCCTTGG
A_16_P58424166 GATGGTGGGGCAGGTTTGGAAATAGACAATGACTGTTCACCTTAGTAAGT
A_14_P120826 CTGACAGAGTATAATTTCTGATTTGCCAAGGCTGCAAATACCTGACAGATAGTAGTTTTC
A_16_P00208025
TCACCAGTATAAGTTACTTAGTCTTTCTTGACACCTTCTCATTACACATTTGGCATCACA
A_14_P114055 CTATGAGATCTGGTCAGGACACTAACTGGACCAGATCTGTTATTTGTTTTGAGTTTATAG

A_14_P128939 CTCTTCTTTAAATGTCTGATACATTTGCACATGCAATTCCTTTCTTACCACAATCCTCAC
A_18_P14135947 CTACATGTCCAAATGCATTACTCATCCAAAATAAGATATGCCACGACTCAGGTTTCC
A_14_P104392 TATTATCCTGCATATTTCATACATCCTGGTCTGCTAACTAGCAGTTTATCAGCCATGAGT
A_14_P109571 TATGCTTTACACTTACCAGTTTGGCTTGTGCTTCTGCAATTTTTCGGTTATTCTCTTC
A_18_P22842837
GTGTGCTTTCAAAACCATGGAGTATAAGGCTTATTTACTCCTTAAGTCTTCCTATTTCC
A_14_P130681 TCAGGGAATGATCTCCCAGTTAAATCTCTTCCAGAAACAAAGAAAAGACATTAAGATTTG
A_14_P120099 CTGAAAAGCAGGATATTATGAAGGTATTGTGGGTTTTCTTCTCCACTGTGGAATCAAA
A_14_P134138 TTTATTCTGTATTCGCAATGCATTTATCTTTCACTGCTCAGTGGATGAAATGACACCTTC
A_14_P115943 AGCAGAAAATCATTAGCATTTCCCATCATTTGTTCATATTTGTGTTTTCTGACAGTTGCC
A_18_P17382835
TCTCCTCACATGAGTAAACTAGCCTGTATCTCGATTATGTTTCAAATCTTCCATCAATGA
A_14_P201638 TCAATCACCATAAAAGAGCCTGCTTTTTCTTACATCAGATAAAGGTGCGTTTAGTCTACT
A_16_P15663429
TAAACAAGAAGCAACAACCTAATCTCCTTTCAAATCCACACTGTCCTTAATTTATACGGC
A_18_P14133377
GTAGGTGTATATTATCAGGGTTCTCAGCATAAATGTTGACTTCTATTTTGGTGAAGCAGA
CUST_5210_PI426210822
TCAAACCATTCCCAAATATCCAGCTACATATTGTTTTACCTTTGAAGAGAAAAAAGCATC
A_14_P117163 GCTTGCTCTTAGTTTAGGATGCTTAAAGCCCAGCCAAACAAAATTACAGACATACTTTTA
A_18_P22842115 TTTCCCTTTTTGGCCTCCAAGACAGGAATTCTCTGGCCCTGCTGA
CUST_7164_PI426210822
AGCCAGTTGCAGTGTACAATATTAGTTTTGGCTTTGGGAAATGAGGTTTCTTTGA
A_14_P113025 TAGGCGCTTTAGCAGCAAGTTTGAGTTGAAATGAAGAAACGTTTTTCTAGAATTTCAAAG
CUST_487_PI426210822 CTCACTTGGTCCACCAATGTCTGTTCTGACCAAAAAGGACTTCTGT
A_14_P127287 GGGGTATGCTGCTTTGGAGAACAGAGAGGATTCTTTTTTGTTAATTTTTCTGTAAAACTT
CUST_7312_PI426210822 CTTAATGGCCAAAATACAAGGAAGGCTTGGTGTCTCCGCAGCTTC
A_16_P01680616
AAGACATCAGACTTTCTTCTAAGAACATACAGGTGCTCTACTAATTGGAAAAAGTCACCA
A_16_P01501107
GGCACTTGACTTTTATTTGCTAAACAGTCAGATTTCAAGAACCTAGTCTGCTAATGTTCC
A_14_P102414 TATGAAATTAAAGAAGAGACACCTGTTTTCTACAAACTCGTTCCTGATCCTGTGAAGAAT
A_18_P25916367
AAGTCTTAACCTGAGCATTAAATACGGACCATACACATGTAAATCACGGATATACAGTGC
A_18_P22660104
TTCTTCTTAAGATAAAACCAACCCATTATGCAATTCTCCATGACATCTTCTACCAGTTCC
A_14_P136181 CTTCAATCTCTAAGAGGGGGAAGGAACTTACATGACATCCTACTGGGAATTTG
A_16_P17900078

CAAATGCACCTCATATTACTTAACACCCAACTGTATTAGTCATTTTGATTGTCTTCACTG
A_16_P19939350
ACCAACTTTACCCTCATGTATTCTACTTCTCAGTTTACAAACACTCTAGAGATTGTAGCC
A_18_P22842848 AGTGTTTTCTCTAATCCGAGGGGTCCTCCAAGCCTCCCCAGCCCT
CUST_4649_PI426210822 CTTCTCCATTCCATTCCTTTCCCGGCGTGGTGTTTGGGTTTGTAG
A_16_P02354763
AGCATAAAGGACATAGAGGAATATCCCTTAACAGTTCCATAAACATCTGTTATCCCAGTC
A_14_P117391 ATAAAACGAAAAATGTCCTGAGTTAAACAACCGTTCTAATGCCCTATCAATCTGCCTTCA
A_14_P135512 CAGAAACCCTCATAAAGTAGATGCAGAGGGCAGTAAGATATAACTCAACTTTGAAAATGT
A_14_P127323 GCTTTCCACTTTCCTGTCTCTGTGCATTATGGATCTTCCACAGAATCTCAC
A_16_P20391647
AAATGACCAGTTTAAACTTCCATTCCAATGTCATGGTTTATATGCCCACTCATATACCTC
A_14_P103422 CTTTGCATGTTTTTAATGAGCATTCCGCCTACTTTCCTCTTGCTGGCTGT
A_14_P118067 ACTGTGTCTCAATCAATTCTGTATTCTTTTGGACTCGTGTCATATTAATACATTGTCTGT
A_14_P128360 TGAAATTATGTGCACAAAAGGCGCCTCCTTATCTGGGAGTGTGAACTAAATAAATAAATA
A_14_P133222 CAAATGTTACTTGTTCCACGTTTCTTCCATGTCAGGTTGGTATCTCTACATTTCATTTTT
A_16_P19035566
AAAATCAGTCCCATTTTTTTTCTGTCTCTGAATGCTTCTGTGAGAATTACTAAACCAGGA
CUST_2233_PI426210822 TTCCTGTAATTCTGCCCAGGTGTTTCCTGTCAATGAGTGTGCTCTA
A_14_P115982 TTCTGCTAATCCTACATTCCAAGAATAAAAGTCTTCCTATGCGTTCCTAGGCAGAAG
CUST_4977_PI426210822
AGCATAAGAGGGAGTAATTGACATTCTACATGGCAAAAGACCCTTGAAAGATAACAGAT
A_16_P17491505
ATCCCCACTGTAGGTATTACTGACTATTCTGATTATTGACCAAAATTGCCACAAAAATGG
A_14_P120922 CTATGCAAATATGTAAGATCTTATTCAGTCATCAGTCTTCGGTTTTCTTCCCACTTCCTT
CUST_2931_PI426210822 AAGCCTAAACTTGCTTGTCAATCTCTGGAGGCTGGGAATCAAGCT
A_14_P120089 AGTTTGTCCTCAAAGTTTGACCTGATGTTTGGGATTGTTGAGTGAATGTGAAACT
A_14_P139467 ATAAAGTTTAATGCCATTAAGTTTTCACCCAGAGCCTTCTGTAGCCAATTCTGGTATATG
A_14_P114671 CTGTCTCATTTACCTGAAGGGTGACAAGAATCTAAGGAGATAGTATACATGAAAGTGCTT
CUST_3450_PI426210822
TAATGAGAGAGTCATTTTCTCAGTAAATATTTGATTGGCCTGAAGTTAAGTGGGTAGGC
A_14_P125627 CAGTCACCATGTTGCATGTATTTCTGCTGCTTGTCCTGAGTTCTTAAGACTTCTAATATT
A_16_P36056370
ACCCTTCTGCTAAATCAATACTGAACTTGACTGATTTCTAATTGACTCATTATGTGCAGT
A_18_P11822269
AAAAAAAAAGATAGTTTTGCCCATAAATACACCATATTTACCCTATAGATGATCCACTCT
A_16_P58424412 CTTATTGTAAATTGCACCCCACCTCCCTTGTAAGTCTGGCATGTTCTATCTTC
A_14_P126493 CAGAGCCATCTTGATCTTCAGGGGGCAAATTAGTAGCCACAATTTGCT

A_14_P201498 GAGCACATTTTACTACAATTGATTGAAATACAGGCACAAAGGAAATGAGAGGTTGATCAG
A_14_P126939 CTTCTGAAAGCACAGTCATGCCCAACATGTATGATAATGTAAATAAGTTGAATGCGTCTT
A_16_P16621210
AAAAAGGCTCTGATAACTGGAGTTAAACTCTTGATGTAAACTATTTGCTTGGATCCCTTG
A_14_P104437 CCCAACCATTGATACAACTGTGGGTTCTATAGTGAAGTCAGTCTCTGGATTAAATCT
A_14_P125087 TTTCTACACCCTGTGGTTGCATACACATTTTATTAAAGCAGATGGATTACCCAATTTACA
A_14_P110865 CTTACCTCTTTGCTGGTGATGAACGTCATAGAGACATCGTCACTGACAGT
A_16_P00685091
TTTATTCATGCCCCAAATTATTCTCGTATTTATTTCCCCAGTGTTTGAGAAGGAACATTG
A_16_P03067908
GTTGAGGACATGCATTTTAGGCTTAAATTCACTAGACTAGCCCTTTAATAAATGGACCAG
A_14_P108579 CTGTGTTTCTTAATTAGTATACTAACTGCCCTGGGGCATGTAGGTAAGTGTAAATGATGT
A_16_P20391120
AAAAAATCAGACTTTTTCCATTTCTATCCGTAAGTGTCCATATTCCTGTAGACCTGGATG
A_16_P17057747
AGGCAAAGAATTACTGAGCATACTACTGAATCCTGAGTCAAAAAAGTTTACTGCAATTGT
CUST_2088_PI426210822 CCTGTGACCCTGTTAGCTGCAGGAAATAGATTGGAGTGTCCTCTA
CUST_166_PI426210822 ACGCCCTTCAGGTCAAGTCTCTGTAAAACAGCTTATTCTGGCAACTAG
A_14_P123810 GCCACCTTACCTTGGCTGTCCAGAAGTCTGTTTCTTGAATTTTATTCAATTTATAATGTT
CUST_8380_PI426210822 AAATGTTCTTATCACCCTCTGGGGAACAAAAGAAAGAAATCTGAGGGTATGC
A_16_P41136060
ATTCCTTTATCCTCTGTCAAACATGACCTGAATTATGTCACATAACCATGTAGCAACAGA
A_14_P114641 AGAAACGAGTGTCGTGATGTGAACAGCACCTTCCCTTGTTGTTCTTATTTATATTTTATT
A_14_P134614 CTCAAAAGAGAAAGTTCATCTAGAAATCTCAAAGCCATGTCTCAGGTGAGATGATGTTTC
A_14_P132901 TAAAAATAAACTTTGTTTTTGCCTGTGTTTAGTCACAGATCATTTTGGCCCAGAATGGAG
A_14_P134764 ATTTGGCTGACTGCATAAATCGATGGAATCCATCACTTTCTTTTGTTGCTACAGTTATGA
A_14_P202201 TTTCAGATACAAATTCATCTAATACCTGGGGGTGAAGAGAAAGATATGCCTTCACTTTTT
A_14_P105652 CGCATACTTGAATGTATGTGCGTATTTATTGCTCACGTCTGTGCCATGTTGTCAATG
A_14_P103619 TTATTTAGTTCCAAGCCTCTCAAATATCCCAAATTTTCCAGTTTCAAGAGTTCAACAGGA
A_14_P108922 GGACAGTCACTGTCAATAGTGTTGCCCTGTTATTTTATATTATCAATAGCCACAGAAGAC
A_16_P17472173
AACAAGTTGTCTATGGCCTTATGAACTGGATTTTCTCATGCTTCTAAACAAACTTTAGCG
A_14_P100771 AAGACTTAATTTAAGTGAAAAACAACTTACAAGTCCATCGGTTTCCACATCAAGGCTCTT
A_16_P19648925
AATTCCCCTTAGTAAAGTGAGAATTTCTACAGCATGAACATTAACATTTCTGTGTGTGGC
A_16_P17979035
CTCATGAAAAATGTTTTCAGTGACTGATATGAACCCAAATTCTATCCGCACTCTTACCAT
A_14_P136429 GGATAATAATCTGGCTTAGTTGTCTTCTTGCTGTGATTACTGACCATTTGGAAATGATTG

A_14_P123917 TTTAATCTCTCAAATGATTCACATGTAACTGGAATCTTGGCCCTGCTTTTCTCATGAATT

A_14_P133707 TCTTAGTTCAATTCCAGACCTGCTTTTAATTATCTCGCATTCTCTGAGCCAATAAGGAAA

A_14_P200353 TTATTTTCCTCTTCGTTCATGAAACTGCTCTCATCATTTTTATATTGTTCCAGGGAAGGA

A_14_P200452 GCGGTAGTAAACCAATATACTTTCGCTTATGCTGTCTCAGAACTTCTCACAAAGTTCC

A_16_P20523242

AAAACTCACATCTGAATTAAAAATCCCCACATTCCTTTATGTGTGCTAAATTGTAGGGCA

A_16_P02728728

TGCCTATGTTTCAACACCATTACTGAAGAGTTACCCATAATTTCTCTGAGATGTCAAACT

A_14_P134806 AGTATATAATGAGAGATTATTTTTCCCCCATAATCCCAAAGGCATATATCACTCCAGAAA

A_16_P20027340

AAAAAACTGCTCTTGCATTGTTAAGTGCCACTACTGAGTTTAAATGCACTCCTTTTCAAA

A_16_P17994418

AGAAGAAAATCCTGGACTAGTGAGATATTTCAAGCACATTATCATAGATCCAGAGCCTTC

A_14_P125942 CTGAGGGTATGAAGAACCTGGGCTCTATGCTCACTTGAAAGTGACAT

A_18_P16313871

AGAAGCCTCCAATTTTAGAACTAAAAGAACCATATTGTAGAGATTTCATGATCGCCAAGT

A_16_P18408393

AATTGTGACGAAAATCTCTTTTCAGTAGATTTGCAGAACATTAAATCCACAGACGGTCTC

A_14_P138120 CTAGACAGTTCAAAGAGTCTGCTCATTTTTTTTCTGTCCACACAGATTAAAACACATCTG

A_14_P133459 TCATTGCGTTGCACCATAGATACATGGGTTTACAGCAACTTAGAATAAAGAAAAGAGGAA

A_14_P200454 GTCGGTGTTAATAAATGATCGTTGGGTGAGGGAATGAATGACAGATAAAAATACAGACAG

A_14_P116730 CATTTAAAAAGTGTAAGATACAAAGCATGGTGGAAATAACTCAGGAGTCAGTCCACTGAA

A_14_P202460 CTGGCATATTAGCAGACGTGGATTACAGTAAGGAAAATCTAAAACAGAAGATGTATCTGG

A_14_P116764 ACGTGAATTGTGAATGATAAAGATCTGGGTATACATGAAGAGTAGTTCCAATTACGAGTA

A_14_P119067 AAACCTAATGCTATCTATATTCTCCATTTCCCTGAAAAATACAGGTGAATTTTTGGGCCT

A_14_P136149 TGCTCGTGGATTTAACATTAGGAGTGTTCATTGTGTGAATGAAATCAAGACTTCTACAAC

A_14_P120058 CTATTGCTCCAAGTATGTAATATGACTGCTGATTCAATCTGATCCCAGGTAGAATTCAGA

A_16_P16191801

GTTTAATTGTATTTGGGCTTTTAGGGGTTTTGTTTCATTGCTATTTTCTAAGTAAGAGTG

A_14_P132777 AATATCCAAATGCTATTCCCCCACATCTACTTACAAAGAAAACTTTACTCATGTGGAGAA

A_14_P117601 TGGTTGCAACTCTATGGTTGTTATTATTGTTGCTTTAAAGATTTGGGTTGTGAAAGGTGT

CUST_3368_PI426210822

TAATTCAAATTTTGGAAGCAGCAAGATTTCTCTGCAATCCTCTCTAGTAGATAATAGTGT

A_14_P135073 TAATACTGGGCCCTGAAATGACACCCAATTTGTTAATGGAATTATGAAGAACAGCAATAT

A_14_P200719 CTCAGTCTGTTCCTACAGTAGAGGAAATGACTTGGAATCTTTCCTATTAGGAATAGAGAT

A_14_P111918 GTTTCTCTACAAGATGGAGAGTCACTTAATAATCTACATGGAAGGGGGGAGAAAATAAAT

A_14_P116487 GGTTCTAACACATCCTACCATAAAAACTGGAGGAGATATGTGTAACCTGGTTAATTTGG

A_14_P128530 CTCTTCCCTCACCATTTCTACTCCCTCATATTTCCCTGCCTCTTTCTAAAATAAATTAAT

A_18_P14911631
ACCCTGATAGAATCCTTTGACAACTGAAATCCAAACTAGAATTAACAGGCAAAGCAATCT
A_14_P133213 CATTTTATAAATCGTCCCTTAAAGATTTCAGATATGGTGTTGAAGATTTCCGGGAAGTTG
A_14_P122285 GTAACCAGAACAACTTGATCCTATACTCCCACTGCTTATTATCCTTAATTCCAATTGGTT
A_14_P115242 ACGAGAAATTCTAACAGCACAGACTTTAAAAATAAGGGCAGAAATCCTCAGCCATTTTGT
A_16_P38107427
GAACCCAACTATTGAGAAAAACCACACCTTTAGAAGTCTTTACGAACACTATAGACACTC
CUST_2267_PI426210822 CTGCATACACAGCATACAGACGTCGGGAGGCAGAGCAACCATGTC
A_18_P22840878
AAACACTTAGAAGATTTTCCCTAAACTAATTATCTGGCTTAATGCTAACTGCTTATTTGC
A_18_P22840158 GGTGGTGGTGGGATTACATGAGGGAAATGTGAGAAGCCCGGAAAT
A_14_P104049 TCCTACAGACCAGGAATGTGTTTCTAACAATGTATCCATTTTAACCATATTCCAGGACAA
A_18_P24817366
AATTCTTAGCATCAATCCCAATTAGTAGACGTAAACGAGGTAATGTCTTCTTCCTCGTTT
A_14_P106039 GAATCAGAACTGTATGTCACCCCAAGGGAGTATAAATGGACTGGCAGTATTCT
A_14_P124688 GCATCTCTGGGAAGCCCCAGAATCTCAGGATTTGTATATCATATGTTCTTCAGAA
A_14_P134063 CTTAGCAAACTTTAGTAACATGTAGGTCCCAACTTCTTGACTGTTTTCCTGTAGATTTTC
A_14_P133431 AAAAATTTCCCCTGCGCTTAGATTCTTCTACTCAAAACAAAAGAGCCAACAGAACAG
A_16_P02066943
AAAGTGTTTTTGAGCAATTTGAAAGTTCACACTGTCTTCTCTTGCTAAGAACCGATTGTA
A_16_P39628749
AAAATCTTTATTAATGTCCAACTACCTACAGATAGACTCTTAGGCTAAAACCCTCTACAA
A_16_P00164682
ATGACTCCAACAACTGAATTCTTCCTGTTATACTGTTACCTCCCTGTTAATAACCAGAAT
A_16_P01348644
AAAATCCTATCCTTTAGTCTCCACCATTTATTATCTCCAAAACTTATTAGGTCCCTGGTG
A_18_P17379373
CACCACACACTATTCCAATCAGCAATAAGTAAGAATCACCAGATAATATTCCAATCTGCA
CUST_4832_PI426210822 CAACAGAAGTGGTTCTCCCGTCAGGTTATAATAGGGAAGGGATGTG
CUST_7680_PI426210822
TACATGAGCCTCTTTCAGAAGTTTGACTAGCAAATCAACAAAACAGTTCACCCT
A_14_P104562 TTCCTAAAAGATTGTATTGATAAATATGCTCATCCTTCCCTTAACGGGATGCATTCCAGA
A_14_P105819 TGGAGACCATCAAACTTAAAACTGTGAATTTTCAACACTATTCTTACCCAACCTTTTCAG
A_16_P03756241
AATTTTGTTAAATTCAGTCTCCTATGCATACTACACTCCTACAGATCTTTCCTTACCCCC
A_14_P133117 GGCTGAAACAGAGAAAGGAAATGGATTTTACTCAAAAACAGTGACATAAGGAGCATATAC
A_14_P112754 CAAGGCTGTGTCTGATATTCTTGAGCCTGTTCGAGTTTCCTTTTCCAAG
A_18_P11359494

AGTTTTCTCATTCCTCTTTAGCATTCTCACCTACTGTAAACATATCTCCCCATCTCAACT

A_14_P132640 CTTTTCCACCTGGAGTCCTTGGGAATACCGGACGTGATCTTCTTTTATA

A_18_P22842459 GATTAAGCCCAGTCAAGAGAGAACCAAGTATCTGTATGGTTCAAGTATCGCTG

CUST_5433_PI426210822 AATGTTTTATTGGTGGGAGAATGAGTGAGGTGAAGAAGCAAGAATGGTAGCTT

A_14_P104014 TGTATCATCTCCTTTTTAAAGTGTCTCTTCCCTATAAAAGCCTTTTCCCCAAAAAAGTCT

A_14_P129943 CCAAGAATTAAAAGTTAATTGATACCTCAAATGGATGCTGGTCACAAAGCATTTCACCAT

CUST_7124_PI426210822

CTGTGATTTTCATGGGATAAAAGCCCTTATTTTTGTTTTGTTTTATTTTAAGTCAAAAGC

A_16_P18610444

ACTTTTGTATTTGGCCTCAGAAATCTTAGAACACTCTACCAACTCTGAAACATTTCCTTC

A_14_P126039 TTTCTATCCTAGTTCAGTCAAAGAGTGCTGATCCTTTGAAGTCAAAACATCAGTTGGAC

CUST_10207_PI426210822 AAAAACCAGAAAGCACACTGTTCCTTCGGCTTCCAGCCAAGCAAA

A_14_P118291 TTATTTGGAGATTGCAGAGAAAGCCGGTAAAATACTGGTGATTTTGTCAACACTATGCTA

CUST_3281_PI426210822

AACAGATAGTTAAGAAAGGGAGATTAAGTTGGAGTTAATAAGAACTCTGAGAAAGAGGAG

A_14_P100469 TTCATTTCTTGACCTTTTCTCCACGGTCTCTGCACAATGTGAACAATGCTC

CUST_1326_PI426210822 ATCTTTCTGTGGAATGGTTTTATCCTGGTCTTTTGGAAAGGGTACAGGAAG

A_14_P128309 ACACTTCATTTCCTTATATAGAAATGTTTCATTATATAGAAGCAGCCCTCCTCTCCATCA

CUST_6536_PI426210822

ATAATGATGGTGTAATGAGAATTTTTTTAATGCAAATATAGTTGGATGCATTTGATTAAC

A_14_P136806 CTTACTGCTCTTCTTCTACTTCTGGTGAGGAAAATACATGCCAGGAGCAAG

A_14_P129038 TCAGCCAATGTTTTGTTCTTTTTGGTTAAACTGCCTTAATCTTTAAAACCGCATATCCAC

A_16_P30952355 CTACAATGCGCACTTTGGAGAGAGCATTGCCAGCCTGGACGATCT

A_14_P104670 GAACATAAAAACATCTTTTCCGGGTGCTTTCTTCATTTGACTTTTTCCCGCTAGTTC

A_18_P16373628

GCAAGAGTGCGGTATTTTTAAGACTCACTAGTGTGCTACTTCTAATAGTGCTTAGTCAGG

CUST_7507_PI426210822

GAAAGGTAAAACAATGGTAATAACAATGAGCTGTAAAATACGACCTGTGGGGTGG

A_14_P137338 GGCGTGTAATTAATGCTATCCTAGTGACATAAACAAAGTGAAACAGAGTTTCACAGTGAT

A_14_P122735 GTGGAGGTTGGTATTATCAATAATAATGGGGGATATGCCATTCCTCATTGCTTTTCT

A_18_P15585500

ATCCTTTGGTCTTCATTTTACTTCCCTTCTTGATATTTACACTTCCTAAAACACTTCCCG

CUST_9517_PI426210822 AATTGTTATGGCCTTGGAAGGAACAGGAAATGTACTTGCATTTGGTATCGAGT

A_14_P114444 ACAAGGTGGGATCTGGGAGAGAGGGACATTTCCATAATAGTTCCATAATTATTTTATGAT

A_14_P108261 ATATGACATTTCACAACAGTGACTAGTTGAATCCCTTGTAACGTAGTAGTTGTCTGCTCT

A_14_P125675 GAACTTGGTCTGGCTTTAAGAATGTCCTCTTGGAGAAAATAACAAGAGTTTTAACACTTC

A_14_P104695 TGCCACTGAGACCAACTGGATAATGCTAATATGCTTCCTTTATGTTAATATGTTGGGAAT

A_14_P116677 ATCTAATCACATAATTCAAAGATATAAAAATTGGAATGTAGAAGGTGGGGCAAGCCCCTC

A_14_P116698 TAAATATGAAAAGACACTACCTCCGACAAACACCCAAGTTACTACACACAATTCATTCTT
A_14_P134257 TGTGATTGCAATGACAACTATAACCAAGTATACACAAGGGGCAAGATTTCTTTGTTCAGA
A_14_P109754 TGTTGCACGCAGTAAGCACATTTAAGATCAAGGAAACACTTTATTATTCACTGGAAGAAC
CUST_408_PI426210822
GAAAAAGAAAAAGAAAAAAAATCAACCCATAGCATATGTGAGTACTAAAACCGAAACGGG
A_16_P00914059
AAAATCACATCTACATGCATGTTGCTATCAGGATGTTGATTCATTAGTCATGCCTGAAGA
A_16_P19882236
ACTTTCCCCAAGCATAATCTACTTCTAAATATTTTCTCCTAGACACTGAAAAAGCCTGGA
A_14_P123948 ACAAGAATCAAACTGTATTAGGGTATCTGTGGGATTGTAATGGTAACTGAATTTTTCCCT
A_18_P14226191
AAAATTCTTTCGGTATCCCTTGAAATACACAACAGTGGCTAAGAAAACATCTTTTCCGTT
A_14_P134393 ACTGCTTTCAACAACTCTGGTTTATACAATTTATCTCAACATCTGCTGTTGATACATGGC
A_16_P16191249
GAATCCCTGAGTATGCATCTATTTCTTGAATCATGGCATCTGCTATAGGAAATCCTCTTA
A_16_P02491240
CAGATGTTGTCGATGATCATAAATGAAGTCAATGCCTCTTAAAAATCTTGCCACACTCTA
A_14_P110708 GTAAGGGAGATTTTTAGCCAAAAGTGTTCTCGCATCTCGCTCTAGATTAAAAAAACAAAA
A_18_P16542014
AAAACCTGCAAAATTAAACATCCCCACATTACAAATCTCAGTGGATGGAGAATGATGATC
A_16_P16677222
AATGGTGCAGGCTCTAAAATAGGACACATCTAGTGAAATTTATGCTATAATTGACCCCAG
CUST_3970_PI426210822 TTTCACAGCCATTTCCCGTCAGAGTGGGGAATATTGACTGTGCAA
A_14_P122668 CTCCTAAAAGGGGAGTATCTGAGTTTTCTGAGAGTAGGTCAGTCATAATCTTTGTTTTTC
A_14_P116356 GTATACTGTAGGCACACTTTGCACTGATATATCATGAGTGAATAAATGTCTTGCCTACTC
A_14_P112066 GTTTTAATTCTAGGGGGCGCATGTAGATCTTTTGTGATATATCTTTTCTCTTTCTAGTTT
A_16_P01761235
AACTAGACTGAACAGAGTCCTTATGAACATACTAGAAAGAAGAATTCTACCCTGATTGGG
CUST_8172_PI426210822 GCAGGTCTAGGGGACTCTTTTCTTCCGCCCATGCAGGGTGAACTT
A_14_P107640 GCTGCCTAATCCTCTCTTTATTTGGTCTCTGCTGAAGCAGTCGTGC
A_14_P122636 GTTTTTCTGCTGTTAAAACCAAATGCAGCAACTTAGTTTAGCAGTTTAGCGTCATTCATG
CUST_10130_PI426210822 GCTCTGACACAAAATCCACAATGAGGGCCCCAAGCTAACCAGACC
CUST_7372_PI426210822 AGTTAAAAATCCATCAGCTGAGGCCACACAAAGTTGGCACTCCCT
CUST_8764_PI426210822
TTTTTAAAGCCTGCACATTTCACATCAGAAAAGTCCTGGATTTTATGACTTAGCTATGCT
A_14_P105589 AGTAACTCACACTAAATACTTACTTTACTATTGCCTCCTTCATTGCCTCTCAAGTAGACC
A_16_P40079398
GCCACAGACATCTTAAAATTCCTTTATGCCTAACAATTCTAAATGCTCAGAGTAGATCAC

A_14_P111929 ACTTTGAACCCCTTTCTTATTTTGATCTGCCATATAAAAGTCAAATGTCCATTCTGCTGA
A_16_P16342190
AGTCTCAGCCAGTCATATTTCATTATACTATAATGCTACCACCATATTTTATTGCCCCCA
A_16_P03527952 CAGTTTCTGAGATATTTGTGGGTTTGCTGTTTCTGCTTTCTGAGGTCCATC
A_16_P20024596
TCTTCCCAATAATTGCACATATTCTGGTTGATTATCTCTTCTCACAAGTTGCTTTTCTCA
A_16_P40091654
CGACTCCAGTAAATTAAATATCACTAGATGCTATTCTCCATATAATGTCCACTTACAGCC
A_14_P100225 GCATAGAAAATCAGCAAAGCAGTGCAAAGCCAGATGGTATGTATCAGTTTAATAAGTGGA
A_14_P115977 GAACATTTAGTAACACCTATTTCTTGGTTTTAACATCACTGACAAAACTGCCATGAACCT
A_16_P00647195
TGTGCGTATCTATGACTCAAAAATTAATACATTTCAAGGTAGGAAATATGGCAAATGAGG
A_14_P132584 CTATTTCCAAAATACTAAATTCAAAGCATGGAGATGTGACTCTGCTGTGATTCTCAGGGA
A_14_P101445 TCATGGAAGAAGAGTGTGATCCTCAGAAGGTTACTTAATTATGCATAGTGTTCCTATTGC
A_18_P22842136 TTGTATTCTCAGAAGGTTGCCCAGTCCATCAAAGGCTTTCACTAGATGCTAGTTATA
A_14_P123654 TAAGAGGTGTTCCCAGGACTTTTGGGACCTACTAAAACAATGATGGTTATTTTAGATGT
A_14_P126392 GAAATCTTCACTTTTCTATACGTTCAATGTGTTCTCTGAGTGTATCGCCATCATTGAAAT
A_16_P40104000
CCCCATCTACCATTTACACACTGAATTGCATTCTTTTGATCATTATTTCCATACCAAAGA
A_14_P201288 CAATATGTGCTGTTCAAGACATGTTTTGAATCGCTTTGTTGTTCATCTCTTCTCGGG
A_14_P133584 CTGTGTCCAGCAATTTAACAAAGCAAAATAAAGTGATGTCATATCACCGTCTATGCACC
A_14_P138222 ATTCAGCAGCCAGGGAAGTTGTATGCCATTTTCAAAAAGGATGAACAATAATTTTTCTTT
A_14_P130412 CTACCATATTACCATATTCAATCCTTAAAGTCACCCTATGCCATCAATATCATCCCCAAT
CUST_6724_PI426210822 CCCACATACTCACTTGAATCAGTCGCCACTTAATACCAAAGCTGCCTTTA
A_18_P14135008
AATTTTAAATAATTACCCAGCATCTGGTGGTTCATTTATGGCTTCAACCAGATGCGTTTT
A_14_P131533 CAAAATGGGCTGCTGGACTAAAGGCTGCATTACCTCAGAACAAGGAAAA
A_16_P02873502
ATGTGTGTGATGTAACAGTGAGGTTTTGATTATCCGTAAAAACAATGAAGATTCCCCAAA
A_16_P02416282
GGGACTGCAATATGATACAAATCCATCCAAATAATCACCAAGATCTCTAAATCCATTCCA
CUST_8909_PI426210822 CCTGGGAAATGGGCTTTGTCTGGGGAACTTGTCTTTCTCTGATTA
CUST_899_PI426210822 AGCTCTTTTGAAAGTGGTGTCTGGTGCTGATAGAAAACCTAAGAACAAATGGA
A_18_P14666512
AGCCATGTGTTTTTTGTGTAATCCCAGAAGTCTCGTAATCTAATATCTCATAAACTTGCT
A_14_P200894 TATATTTATGAACAGTGGAGTTAGATACTGCTGTTATAAAACCTCCCACACTTCTTCCAC
A_14_P117064 TTTTTTTCACACTTTCTCCAGTGCTAAACTAAATGAATTTTGCTCCTGACCCCTAATTGA
A_14_P108846 TTGGTTTTGGAGTAGCAGATCCTGTTCCATAAGAAAATTTTGAAATCATTGCTGTTTGTG

A_16_P18537629
TTGAAACATTAATGATTTCACCTGGCACTAATATCTCGGAAGCACAATACTCAGTCGTAA
A_16_P21534472
CACCATCTGTAACTAGTTATCAGAATCTTCTGTCATTACAATATTACCTGCCTCCTGTTC
A_14_P116082 ATTTATTATCTTCAGCACTGCTTGTGCATGGCAAGACCATGACCTGTTTAAAAGAATAAT
A_14_P200109 ATGAATTTATCACTAGCAACAAGCAGATGGTATGCACTATGTGACTTCTCCATACAAGAC
A_16_P02974779
AAAATCCAATCTTCGAACTTTTCATCCAAGGCTAACTTTTGCTGAGAACACTTCCTTTTA
A_14_P112668 CTAGTTTTTTTCTTTCTTGTTGTTCAGTAGAAATACAGGAAAATGGTGATGCCTTACATG
A_16_P00493310
AACTCCAGATCCTAACGTATCCAGGTTTCAATAAACATGCTGACTCAAAGAAATTTTCTC
CUST_4812_PI426210822
TGTTAAACACTCCATGAACTGTTGATAATTCCCTAGAAGCAGCATATTCCTGAGGTA
A_14_P106065 AGTGAAATTAAATGACTTACCTTCTGGCTCTAATGTGGTATCCTCAACTAAACTGAAGGA
CUST_1888_PI426210822 GCAGAGGTTAGGAGCTGCTGCTCACGTTTCTGGAGCATGTGCCTC
A_16_P15539993
AAAAACAAATTTCAGAGGCCAGATTATCTTAGAATGGATTTAGAACCATTCCTCCACTCC
A_14_P116902 ATACTAGTTTTTGAAATATGATTTGCAGAGGAGTTTAATAAAGAAACTGGGCAGCCCCTT
A_16_P16163300
AAAAAGCCTTAATTCCTTTCTTCAAACAATCATGACACATTCCACACATGTTAAACCTGA
A_16_P16463883
AAAGCAAAGTCCTTCATAATCCTGTCATAGGTTTTGAACAGTTTGTTAGCATACGCTTTC
A_14_P129158 GATTTCGTATGGAAATAATGTTTTATTAGTGTGCTGTTGAGGGAGGTATCCTGTTGTTCT
A_14_P111101 CTCCTCTGTTGGTGTTGACTGTGAAGAATCAAGAGATGGTATACAATCCTAAAAGTATAA
A_16_P36230515 GGAAACGGCGAGGTCAAGTCAGCAGGGTGGATTGAGAATATGTAG
A_16_P03048099
TGTCAATACAGTGCCATCAGTATTACATGAGAAAGTGAAGAGTTATTCTACATCCTGGTG
A_16_P17100902
CCTAGAGCCTACCTTATTTTCTACAATGATGCTAAGTCCTAAGAATATTCACCCCCAAAT
A_14_P138817 ATCTGTTACAACTTTTCCTACCCAGTTTAGCAAAACACCTGTTTTATGCAACAATACATC
A_18_P22841765 CACCCAAGGACGTTTTTCTCTGTTGGGCAGACTCAAAGCACTGAG
A_14_P118525 AAAAGGACAAGCAATACTGTGTGTATGTTCAGAAAACCTCTTTCTTCATTCCTATTTGCT
A_18_P16739590
AATTACCTACTCCAATCATCTTGGTGTCAAACATAACACTTCCTCTCAATATACAGGTGC
A_14_P131002 CTCTAAGTTCCTCAGGAGCCATTCCAGAAAAGAAACCTAATAATTTACACAAATCACTGA
A_14_P122411 GTAATCAGGCTGCAAAGGCCTTTAGTTCAAGATGTTCTTCTATCTCTAACTTGTCTAATC
A_14_P102580 TGCTCCTGACCTATGGAAAACAACTTTCTTTCCAAGAAAATTATTTTTAGTCCTTTGGTG
A_14_P139303 CTGTGAGGAAAAGAAAATAGACGTTAAGATGTTGAGAAAATGTGATGGCTTACGAAGGAT

A_16_P02774090
AAATCGCTCTCCGATCTTGTAATACCAAAGCACCTCGATTTTCTTTTTAATTCAAACTCC
A_16_P21301871
TCCTCTACTTTCAAACAAATTGTTTTCTGCTCCATCCTACTCAAATCACCATCTATTCTC
CUST_5806_PI426210822
TTGTTCTAAAAGAAGTAGAGTAAGGATTTAGTGAGAAGCTGGAGAAAATATTTTCAAAGA
A_16_P16335591
ACCCTAAACCATACTACTGCCAATATGTTGATCATTCAAAAATTCATTGTGTGTCACACC
CUST_9698_PI426210822 AGCACATCCACGGTGGCACTCTGTTAATAGTGGCTGTCATTGCAT
A_14_P113311 ACATAGTGAGGAAAGGCAGGGCTGTCTTCCTCTATCAAAATATGCTATTTAGAATTTTCA
A_14_P127234 GCCATAGGCCTCCTAATCTAAGAGTTAGAGACCTTTTACTCCTTATCTTTGTAAGCAAAA
A_14_P107023 AACTAGTCTGTGCTACTTTATGTCAATATAAAAATTGGTAAACTAGAAGTAACTTGTCCA
A_16_P03049067
CTTTCTTACCTAACTTCAGAACAAGACTGCGTATGCTCAGATAAGACACAGACTGTTCA
CUST_3639_PI426210822 TAACAATTACTGGGGTCATGCCTGGAGAGGTGGCCCACAGTTCAT
A_18_P16711873
AGGGCCCATAATTTAAATGAGAAAGAGAATCCAAGGATCACACAAATAAAAATGGCAACT
A_14_P130993 GCAGATCCATATTAAAAGAAATAGCCTGGGACTTCTGTCAAATAACTGGTGAGTAAATGT
A_16_P03315044
GCCCGAAGCACTGATTATAACACGGTATCCTCAATTTGTAGTAAAAAATGGCAATTATCA
CUST_9662_PI426210822 CTGGCTTCTCTCTACCTCGTGCTCTGGAGTCAGGCAGAAACCTTT
A_14_P122972 TCTATATCACAAACACTAATATGGTCACATTTTCTTCCCCTTTCCCCCCAATAAAACTTT
A_14_P104492 TATTCAACAAATGTTGGCAGATTCAATCAATCGTATGAAGGCATATGCATTTCACCAGGT
A_14_P137012 ACAAAGGATGTGTTCAACAAAGCATTTCTGTTCTGTAGAGAGTTCCTGTAAACTATCCTT
A_14_P131844
GTTTCTGTCAGGAAGGATTAGGAAAGGTAACTTAAAAAAAGTCAAACAGTCGAACAGTTT
A_14_P111085 ATACCCTTCAGCACTGCTTGTTATAAAAGGAATGAAGAGTTCTCATTGCAAAAGTCATAT
A_14_P113530 TTAAACACAAAATCTAATGATTAGAACCAGAAGTTGAAGGCCCACTGAAGGAGTCCTTAA
A_14_P117101 TCTGAAACATTTTTTTTTCTAGGGAATCTGGAAACCCAGGAAAATACCAAATCCAGATTT
A_16_P16956857
TTTCAAGAAGTTCGATGATGACATACAGCATGGTTTTCTGTGAAATATTGTATGAGTGGC
A_16_P16114319
TCCCATGTTATTCCAATACACTAATAACCTCCACAAATACAGATTAATCACGTTTCCTTC
A_16_P02920119
ACTCCAGGACTCATATCAAATCAAGTGATACAGATGAGATAGCATCCTATCATAATTGGT
A_16_P19923167
AACCCAGCAATCTAAACTACTGAGTTTTCTGTCTAATGGATATGCCTCCCATTACTTTAG
A_14_P124247 TTTCAAAGTGGAGTTTTGGGTAGTAAAACTTCTAAGTTTGCTTCATAGGGATGCACTTTT

A_18_P14138152

CGTGTCTGTGTTCAAAACTCATAAGAATGCTTTCAGAATGCAAAATTTAAGTTTGTGGGT

A_14_P117973 TTGAGCAGTTTGGAGGTATAATCACTCTAAATTTTCTGCAGCAATTCTTTTAAGCACTGG

A_14_P108960 ACAGTTTTAGATTCTTGGATGCTGAATGTTCACCAGTTCTTTCAACTAGATCTGTGTAGT

A_18_P14763539

AAATTTTCCTCCTCTAAATGTCCACAATGCTTTGTAAATCTTTCAGGGGACTACCATACT

CUST_7614_PI426210822 AATTTCATATCCTGTGGTCCTGAAGAGGACAATCACCAGGATTTAGGG

CUST_4653_PI426210822 TTTCCTGCCACCCAGTTTTTATGAACATTCTCTTTGCCCCATGTGT

A_14_P121344 TATGATTGGTTTTCAGGCATTATGCAGCCAATAGTCACAAACAAAACATTAAGACTTGGT

A_14_P130511 CTATGCTCACGGGAATGAAGGACAGGTTGTTTTATTTCAATAAGGTAAGAATGCTTTACA

A_14_P106100 GATGGTGGTAGAGGCCATTTGTTCTACATCTTTCAATGTGCATCTGAGCATTTTAAAATA

A_16_P01399127

GGCTCTAAACTTTTACTGACCCTTCCTTTATTTCCTTAAATAGTCATTGCATACTCACCC

CUST_7633_PI426210822

TGCCTCAAACATTTGTTTTGACTCTAGCTTTTGCTTGTTTCACTTTTTTAAGCTTGGAAA

A_14_P139161 AAGATGGCTAGCAATAAAATGAGAACAGTTATGCTCTTGGGAAAAAAGACATTGTTGAAA

CUST_7956_PI426210822 CAAATGTGAAAGTGAAGGACACACCACAGCCTCTGCACTTTAGCAC

A_14_P132398 ACATAAGAAAAAAAAGTGGAATTGATGCTAATGAACATTCTGTGGTCAGAAACCCCTAGA

CUST_7457_PI426210822 CAGCCAGTCCAGCCATCTGAAGCAGAACGAAAAAATGGTATGGAAA

CUST_2782_PI426210822 TTTTGCAGGCAGCAGGCACACAGGCTGGACATCCTTTCCCTCTGA

A_14_P138568 TTCTTTTCAAAACTGGAATCTCCCAGATCTGAAACTTCAGGAATAAGGGAGTGATTTTAA

A_14_P126138 CTCTTGAAGAGAGCATAATTGGAATGGAGAGGTGGGTAACATTCGCAAATTTTTTAAAGA

A_14_P126008 CAGGAAAGGTTTATTGGCTAAGCAACTGTAGAAGGTGGAGTGGGAAAG

CUST_3865_PI426210822 ATTAAGATAGGACAATTGGACAAAAAGGCTGGGCCTTCTTAGGTGGGTATT

A_16_P01690703

CATTTGTCCTCTGCAATGAGTAAATGTTCACACTACAAAAACACTCAGAAACTTCAATGG

A_14_P200571 CATTTGAATAACATAAACAATAATTTATTCCATAGAGGGATAGTGTTGGCATGATGCAGG

A_14_P126158 CTATTCTCAAGGACTTCTCGTTACAAAGGGAACCTCCAAAATTTTCTTTAAAGAGAAAGT

A_16_P15617783

AATTGGTAAATTCTCAGGCAAGTCTACAAAAGTCATTACCTATTTGAAATGCTGTCACCG

A_18_P15241182

AGGTCATCCTTATACAGCAGAGAAACACAATACCTTTCCTCATTTAGAATATTCCCAACT

A_14_P138333 GGATAGTATCCTTATCATCTTCACCACGTTATCTCATTGTTTACATTACAGAACAGGGTC

CUST_1606_PI426210822 CCTTGTTTCTGGAAGTTCAGCTGTTTGTGGCTGAGCAGGAAATGT

A_14_P139905 CTTTTTCTTATCCTGGTAGCAGGCTATGGAACTCAGTCAACTCCCCAG

CUST_8599_PI426210822 ACAGTTTTCTGACTTGGTTCAGAATTCATTTAGAAGTCTCTGGGGCTGCTT

A_16_P39063315

AATTCCCAAGAAATGTTTTCAAACACTCTTGCCATTTTAATACACCCATTGTGACCAAAA

A_18_P15777296
GTCCGGTAATCTTCATTTCTACAGCCAACATGTAACTTCTTGAACCATATCTTAATTCCC
A_14_P130400 AAGTTTTTTTAAAAACATTCCACTGTTTCTGTTAGTCCCAGGAGCCTCTTAAAAGTAAGG
A_14_P119569 CAGGGAATTGGGAGTTGAAAGTCTCTGTAGCCATTAATTCGGGATATTTATCTTTCTAAA
A_16_P58424175 GTTGATTTTCAGGAATTGGGTTGTGAGATTATGAGGTCTGGGAAGTCTGAATTCTT
CUST_2175_PI426210822 AGGTGCTTATTGGTACCTGTTGCAGGAACTCTTGGCACCTGCATA
A_16_P17041917
AAAACTTACTGAGTAAGAGAAACAGTCTTGCAATATCGAAAGCATATGAGTGACCTGCTG
A_16_P16869719
AAGACATTCAACATTGCTAAGGAAATATTTACCTTTAGAACTTGATCTAATAACCCCCCA
A_14_P127266 TTTAATGGTAATTACATGGAAGCAAAGAGTGTCCTGATTCCCGTTTATGGTGTTTCC
CUST_8290_PI426210822
TAAAAAGTCAAAGCTATGATAATCTTGTCATTTCAGAAAGGTAATCCCAGTGCATATTTC
A_16_P19217084
AAAGAAGAAGAGAATTATGCATATTTTCTGACGTTCTGAATAAGTAGTGCACCAACTGGC
CUST_5148_PI426210822
TCAGCCCAGGCCCTGAACCTGTCATAAAATGTAATGGATGATATATTTTTAACTGC
A_16_P01824663
AGTGTGAAACAAGACTTCAAATGTTAAAGACCTTGAATCCACAACTTTGAAACCTTTCTG
A_16_P02160232
AAAGGAAGCACAAATGCATGAAGAATAACTTGAACCTCATGGTTCTTTCAAAAGTCTCTT
CUST_843_PI426210822 GAAGCCGTTGTAGGTCCTGCACAGGCTCCCTGAGCCCGCTGTTTG
A_14_P116092 GAGAACCTGTAAGTATAATTTGTATGTAGTCAGACATTCTGTAGATCAGTTCCCAGCCAT
A_18_P14133933
AAACGGGTGCTTACATCTAACTAAAAATTATTGCCTGCTGAAGAGGTTGAATTGTCTTTC
A_16_P01458973
ATCTCTTTGGCCCTTTCATTATGACATTTTTACCCCTGATTGCAAAAGTATCTTGTGTTC
A_14_P122408 ATGTTATTACATACTGGACTGTAAACTTTTTGAGACTAGAGACCACATGTCACATTGCCA
A_16_P21558384
ACCCTCCACCATTTAGATACTGTTGCTATTGCCATATACCTTGAGGATACTACTTTTAGA
A_18_P22839579 CTATTGACCACTTCTTCCTTCCACATGATGGCTGGTCTACCTACCTC
A_14_P129946 ACTATTCTAGGATGTTGGTTAAGACCACGTGGAGTTATTAAATGGGACTCAGTTTAAGAA
A_14_P122701 CGCAACTTACCCTCTTTGGAATTTGTCTTTTTCGCTCCTGCTTATTAAAGAAGGAAG
A_14_P125666 TGACAACACTTTGAAATTGTAACGATAGATGGTGAATTTATGAACTGCCAAAAGGCATGT
CUST_2187_PI426210822 CACAAGAACTCCCCCAGGCCTCCACTGGGCATGATGCACCCACAC
A_14_P200814 ATTGCAAATAGAAGTTAAGAAAATATTCTCCTTGACCTTCTGTTCAAACCTGTGAAGGCT
A_14_P132232 ATTTGAGAAATAATGGGGAGAGATTTTATGTTTTCAGAATCATCTGGAGGTCTTCTTGGA
CUST_10375_PI426210822 AAGTGAGAATGTGCTGCTGACTGTGGTGCACAGACAGCACAAATT

A_14_P112461 GGAAATAGTTCAGTCCTCGGTAATTTGTCAACCTCGTGCTGGCTTTAAAATTCT

A_14_P201133 TCCTCAACATGGCTCACAAATTTCTATCCCAAATCTTTTCTGAAGATGAAGAGTTTAGTT

A_14_P126694 CTAGGACAGTATTGGAAACCCGTGACAGTGAGAGTAATTGACATTTAACATGATAGACTA

CUST_3219_PI426210822 CTGCCTGACCTTACCTTTCTAGACCCATCTTCTGCCACCACATCC

A_18_P14136951 CTGACTGCCTGTCAGGAAGTCAAGAATCCGAGTTTGGGGGCTTCA

CUST_8800_PI426210822 CCCTCCTCATCATGAATCATTATCACTCAGGAAGTAAGGACCTGGTTTACT

A_14_P119468 ATTAAGAGCAGATTGTTTCCAACTAAAAGGCCACATTTCCTACCTCTCTTTACAGATAGA

A_16_P02596038

AAACATCCTGTCCTCCTATAATATGATTTGAGGGCCAAAATAATACAATTCACATCAGGC

A_14_P129099 CTTTTAGAAGGCTTTAGTTTTAGAGTATCAGGGAAGTGGAGTATGAACAGCAAATAGCAG

A_14_P123966 CTTGCTGGTTTCCATAAACCAAACAACCAACTCAGAAATTCGTGGGAATACAC

CUST_8608_PI426210822 CTAATGAAGTGTAACCACTCCAAACTCCCAGCCTTGGCTCGTGAT

A_14_P101065 ACTTGCCTTATTATTATACAAATCACCAACAGGGAGATTGCAAAGCATTTCCTCAGGAAG

A_16_P16191927

CAAGAATTGCTTGTAAGGATGTTCTTAAGTGTGCTTTCAAAACCATGGAGTATAAGGCTT

A_14_P101775 ATCCACATAGAAACATTTTCAGCATAATGACCAATGAAAATTCCTGTTGCCATTCCAAGA

A_14_P132993 TATCTGAATGTTTTATTCCTGTTGTTTCCTAAGTTTCCTTCAAACTCTGTCTGCATCCG

A_16_P03713366

CTGGCTTTCCAATACTTTCATTTTTGTCCTTGTCTAGTCCTTGTCTACATTTCAGACTTA

A_14_P136878 ATTACTATGATTATCACCTCCTGATAGTGAACATTTTGAGATTGGGCATTCAGATGATGG

A_14_P200523 ATACTTGTAGACCTGTCCTCCACTATTCATTTTCACAGGTATTCGAAGGTTGTGTGATA

A_14_P113055 TAAATCTCTTCCTTTCTAGGGGAACACTGTGAAGAACATGTTGCTTTTTTCAAACAGATT

A_16_P02800598

AGGTCTCCATTGAAATCATCTAACATCCCAGTTAGTAACTACTTAGCAAAGTTAGGAAGG

A_14_P132189 CTAAGGTTGTCTTTTATTTTGTTTTCCACCATGCCTGAACCAGTCAAATCTGCTC

A_14_P121028 CTATGTCTATACCACACACTTATTCTACTTTAAACGGCAATGGGAGCAATAAGCACCTAC

A_14_P139726 CTGTCAGTGCAAAATCACATTAAGTATGGTTTTTCTTTTCCCTGGGACAGAAAAAAACAA

A_14_P121801 CTGTCTCATTCAGGAGGAAAACGACAATACTCATTCACTTTACATGGAAGAATGAAGACA

A_14_P111409 TGGCAAATGGAGATCCCTAGACTTAAATGAATTATATTCTCCAAAGAACCACTAGTCCAA

A_14_P101281 CTTACCTTCCAAAGTCACAGAGTTTTAATACAGCAGTATCAGGATCCAACAAGAGGTT

A_16_P15771442

ACGGTAACATTATCATGTGTGTCAATTTGTTCAGTTAATCACTAAGCCACCCTATAATCC

A_14_P138255 AGGGCTTGTGTTTTTATAATAGTTCCTCTTTTGCTCCTGTGTCAAATGTTTCCTTAATGA

A_14_P108816 TTGTTACTGCTTCAAGCCATAACACACTGCCTTGCATATATTAATGCCTTTCCATCTAAA

A_16_P21468859

AATCTTTTCCATATAACCTCCTTCAGATACAATTTCAGGTCATTTTTGTTTCCACCTCCT

A_14_P132329 ACCCACTTGAGTTCATGAGTTTCAGTTTGAAAGTGATGTAAGGTTTAATGTTTAGACTGA

A_14_P128611 CTTTAGTCCTTTTAAGTCTGTGTTTCTGACCCTGGAGGAAAAAAGTTATACATACCTTGA

A_16_P41574202
TCCCACTCTCTTCACTTGATGATAAATTAGAAGCATCTAAAAAAACCAATCATTCCCCAA
A_16_P16713761
AGCATCTTTCATTTGCTAAAGTATGTCTTCCAGACTACTAACAATTCCTTGGAGTGAACA
A_16_P00425672
CACCGGGAACAAATGATGATTCTGCATGTTATTCTTTTTATAACCATCTATTTTGACGGC
A_14_P118634 ATGTAGAATGGCACACAGACAGTTTTATAGTGGGTGTTTTTTAGTGAGTAGTTTTCTCTG
A_16_P58424205 CTGTAGGAGGCGGTAGAGCAGCAGGAACGGGGTTAGGAGACTGAG
A_14_P202281 GGTGCAATGGTCATTTTCTACCAAGGAATTTTCCTTCACAGTTATCTTTAGCAGTAGTTT
A_14_P115259 CAAATGATATCATTGGAATTAAATATGTTGGGCCCATACCCTAGGGACACTATTGAAGAA
A_14_P124392 GTCGTGGAACAGAATTCACAGCCACCAAGTGGTTTATCATCAAACCAGTTAT
A_16_P00012252
ATGCACAGGTTTAAGGATTTCTTCATCAGAATCCCAAACAAACTAAATTTTAAAGGGCTG
A_14_P119884 GAGAAACACAAGAGAGACTACATGGAAAGGGATTTTGGGAGAACAGGAATAGTA
A_16_P03201224
ACTACAATGACACTCTCTTCCAATATCACTGTCATCTTTATCCTTTTAACACCAGCAACA
CUST_5933_PI426210822 CAGGCACAGCCAGCAAGCTCTCTGGAAATATTTGGGTTTTGACAT
A_14_P138532 TTGGTGGCATTACATCAATAAGCCATAGTTTATTTCCCCTGAAGACATGTATTTGACTGA
A_14_P117514 ACTTAGGTGATTAATGAAGCAATACCTCACTGTGATTGAAAAAGGATCCATTTTACCAAC
A_14_P201707 ACCAATATCCAAAGATACAGAATGTGCCAACACCTAGTTAAAAATAGAGTCTGCATACCA
A_14_P121516 CTAAATCCCAACTACACTGTAAAATGGGACCAGGATTTATCCATAATTTTCAGGAGATGA
A_14_P108810 TGTTGTCTCTATGTTTCTTTTCCAGAGAATTAAGTATTGAAAATTGGATTATGCCTGCAG
CUST_2728_PI426210822 AGGTAAAAGGGTCTCTTTGTGAGGCCTGGAGGGGAGAAGGTTGAG
A_16_P58424302
GATAATATCAGATAACCTATAAAAGCAGGTAGCCCAGTCATGCACATTGGTGATCAATCA
A_14_P113731 GGCCTATAAAACCAGAACTTTAGAGTTTTAACCTCTCTTTAGCATAATCCGTTTATGGCC
A_14_P106182 ACTTTTTAGGAGTGTATTCAAAGCACATTCACAGTAAGGAAAAAAAAAATGCAGCCTGTG
A_14_P103910 AAATCTAGACCCTAGGATCTTACTTTTTGGATGAATTTGTATATTTTCCTGCTTGGGTCT
A_16_P18603030
CCCTTTTTTGAAGTGTGGATAAATCTAAAGCCTAGGAAATACATGTTGCAGACTAGTGAG
CUST_10307_PI426210822
CAAAGAGTCCTAAGACAAATGCAGGAGATAATAAGGGGGCAGACTGTTAAGTTT
A_16_P58424519 AAAGAGGAGCCTGGGCGCTAAACCATGGGGGACAAAGGTCACCTA
A_14_P105307 TCCCCCATTATTAGAGATGTGGGTGGGTTTTTCTCTCAGTGATAAATAATGCAAAATAAA
A_16_P20021300
AAAAAAATCCAATCTTCTGCAGCGTTTCAAAATTATCAGTTCTAATGAATTATCGCCATA
A_18_P13318250
GACTTCACTCCTTCACTATTACCAGAAGTAAACCACTACCCCTGTTAATGTCATTTTAAT

A_16_P03407800
AATCTCCCAAGTTTAGTTGAAGGTCATTTCTATAAACCCCAAATGAATAGCCCATAACAG
A_18_P13333424
AGCATCGTGATCTGTTAGTTGACTGAAAGTTTTTGCACCTTAAATGCCATTTGTTGCTAG
A_16_P17436286
AAAACCTATAACAGCAAGGGTAATGTTCACTCTATATGTCTACAAAGGCTGAACTTGGAC
A_16_P18514819
ACAGCGACATGATTCTATCAGTCAATTCCTTCATTTCCTTCTTGTCTTTTTTGTCTGTTT
A_18_P12116655
GACGTATACTCATACGCACTTCTACTGGATTATACTCTCAGACTCTAAAAATACAGCCCA
A_16_P03406934
ATGCACTTCTGCCTACTAAGAGATCGTCTAATATTCTGCTATTGAGTTCAAGAGTTTTCT
CUST_5394_PI426210822 CTCATATGTTCAGGGCATGTTTGCTCTCCTCCCTTCCTTTCCCAT
A_16_P18088007
AAAAAAACAATTACTCTTCTGGCATATTTTGAGAGGCAATCTAAACTAGCAGGAACGTGC
A_18_P22840530 TCACGGTTTAAAAGGCTGCATGCAAAACATTTCCTCCCCTGGTAGAAAT
A_18_P16727606
CCTTGCCACATTACTTATTTACAAAACTTACATACTGATTCATCATATCCCCCAAAGTGA
A_16_P17068755
GTGTTCTAGTGGCTAAGTTATTTGTGTTTATCACCCAGATCTATAAGTTTGCCCAAACAG
A_14_P126643 ATCCTTTGTAGGGTTAGGAAAGCATCGTTGCAAGTGTTAAGTTTGTTTACCATATTTTCT
A_16_P02283931
GATTCTGCTACTCTACCCTACATGTGTTCTCATAAAACTCCAATCATTCATGTTCTCCAT
A_16_P01862029
GTCCTATTACGCTACTGAGATTATGTTATGGCATTGATCTATTATTACAGCACCCAGCAT
CUST_1295_PI426210822
CTGTCCCATGTTCAGGGTCACTTTTTTTTTTCTGAGAGTAGAGCATGACATAATG
A_14_P124044 GAACATGACAGAGCGCTTCCATAGCCTGGTCACTTTACCTGCTTTATCT
A_14_P121635 TTCAGGGTTTTTTGATTAACTCAAAACCAGAGAATGCCTGTGAACCCATAGTGC
CUST_5386_PI426210822 CAGACTTGCCCTGCTCCACTTGGAAGTCCTTGGAGCAGCCCACAC
A_14_P128485 GGTATTTATAGGACTGATCAGCAGGGAAAAATAGAATTCCAACAAATGATGATGGAGTCA
A_14_P128328 CAAACTGCTAATTGCTATCTACTTGCTGAGAAACTGCTAACAAAGAAGATTGAAAGCACT
CUST_10389_PI426210822 CAGGAAGTCCATGCATTTTGAAAAATGTCTCCACAGGATGTTTTTCTTGCC
A_16_P37837473
AGACTCACTGCTCTAAAATACTCACTAAAGCATGTGACTTTTGTGTCTTATCTTGGAAGT
A_14_P137531 ATGACACATGCCTTGCATCGAAGATCCTACACCTCAGTCCCTAAAATTATACTTTC
CUST_9354_PI426210822
AAGATTGAAAATACAGCATATCCTGATACATAAAACCCCAACTTTATAAAACTGTTAAAT

A_16_P15255231
ACCGACAGAATGTTCTACATAGTGACAAGTTGAACTCTTCCTAGTAGTAATTCCACCTAT
A_18_P17197938
ACTGAGTGATGAGAACCAATAATGCCTATAACCAGTAACAAACAAAAAGTATCTGTGACC
A_14_P139916 CTGAATTTTTGGAAATGTAATTATTAGTGTTTTCATCCCAAAGACCGGATCTCCTAGTTT
A_18_P22839608 GGGGCCCCCCAATTTTAATTTACTGAATGAGTTTAAGAACCATGGTGTATTTC
A_14_P101772 CTTCTTTGGAGTTTTCCCATCAGTCAGTATGCTTTTTGGACATAGAATTTAAGGAGTCTA
CUST_8777_PI426210822 GAAATTCAATGGTTGACTGTACCAAGCCAGCTCTCTGGGTGAGAG
A_16_P21576947
AAAAAAAGTTCATACCCATTCACTCATAGAGTTATCCCATACTAGCACCACAATCACTGA
A_14_P103870 GTTGTAGAGACGTAGCAGGAGACATTTCTGGGAATATGGTAGATGGATATAGAGATG
A_16_P21580091
AAAGGCTATGGTGAATTATAGACTTAACTAAAACATGGACTTCTAAACTGGCTTGCATGA
A_16_P18300678
CACCTCCTCTTTATTTATACAACCATATCTACAGCCAAAGTAATCTCAATGTCAGTCACC
CUST_7491_PI426210822 TGATGTGTGCATGATCAGAACACACTAAGAGACAGCCTGTGTGCTC
A_18_P11662504
AAATACCTCTTTATGTTTAGTTTGCAAACAAAAGGAGTCAATTAGTGAAGGCTTGGCTGA
A_16_P01616500
AAGGTGCTGATAATTTCCTGAAATCTCTACTGCTAACTTTTGTTCTGTCTGAACTAGGAA
A_16_P19765267
AACACAGAGGCAGGAATATATGCAGACTTGTAGACCTTAGTTTCTCTTCAATTTATGGTG
A_14_P124861 TTCAACAATGTAAGTGAAAAAATGTGTTCTCAAAAGTGGCCACTATAATCTTTTGGTGGG
A_14_P134888 CGAACCAAGTAAAATATTCCAGTGCTTTTTCTATGTAGTCTACGGCTTTTCCATCAAGAT
A_16_P16191491
AACTACTTTCTTCACTCTCTAAACTGCAAATCATTCAGGGTAATTGTGCTGGTTCAGTTA
A_16_P16191602 CAGCACATTCTTTATCTATGTAGGTTGCATTTTCTCTCCTTGGAGAGCCTCAGATT
A_14_P200965 GTTCAAAAGATGCATTTTAAAGTGTAAGAATCTTAAAGTGGAAGTTGCAGCACTTTGCGG
A_14_P121681 CAGAATGGAACAGGCCGATTTTAAACATAAAAGAAAAGAAAATTACTCACCCTGTCTAGG
A_14_P138585 CTTGGAGCAGCATGTGAAAGTCAAGGAATATTTGTGGGTATTTGCAATTTTTCTTTTTTC
CUST_8781_PI426210822 TAGGAAGAGGTGGATTTGGGGGATTATATGATCCAACCCTCTACCTTT
A_16_P02344008
AAAAATAGTGGCCCAAATAGATTTCAGTGGTCGACATTTAGACAAAGACTCTAGGAAGAT
A_14_P201529 GGTGTTCCTCCTTTTATCATAAAATGATTTCTTATAGCAGTGGTGATTTAGAAACCAGGC
A_14_P117675 TTAATCTTGATTCCCAAGGCCTTCCTTATTTGTGTCTAATTTAGCAGGACAAAAAGGAAT
A_14_P114637 GGTCAAAACAATATTGACCAAATAATATCAGATTCTAGTGAAGGACACAAAGGGGAGCAT
A_14_P201737 ACACCCTTCAAGGATCTAAAAATCATAGCAATACTATTACTGGTGCTAAGCAAATTCCTT
A_16_P58424160

CTATCACCATAGATCCATCTTGCCCATTCTAGAATTTCACATTGGTGGGAATATACAGTA

A_16_P16191566

GAGGAATAAGAGATGATGGCTTCTTAGATCTGTTTACATTTGCTGGAAAAACTTCTTCCA

A_18_P13303972

ACAAAGAGAGTATTCCATGAAAAAAAAACAACAAAGATATGAGAAAAATCATGAAACAGT

A_14_P114091 CTGCAATCTTTCCCACTAGAAATGACCTTTTCTTTCTCTAATTTCCCTTCGCAGATTATA

A_14_P132461 TCTTTTGTGAAATCCTGAGTTATTTTCTTGCTACAAACATCAAGATGAAGCCTTTGGAGT

CUST_4307_PI426210822 ACTACATGTTTTCTCTTTCCCTTCTGGACACTACTGACCTCTCATTGTCATG

A_14_P117412 CACAGGCTTATCAACCACATCAGGGACCCTGTCATTCTATTCTCAAAAAATATTTTTAAC

A_14_P121095 ATTCCGTTCAGCAGAGCATCACCATGGTCAGGAGCAAGATTGATAGATTAG

A_14_P113918 AGTTTGGCAGAAAATATAATTGAACACAATTGCATGTTTCACTTGAATGCTTGGGATACC

A_14_P120207 ATGCAAAATGACTTTTCCATATTCAACTGTCTCTTCGGCACTACTCAAATTTTAACGAGT

A_14_P113139 CTGTATCTTTTCTTATCCTCTGTATTTCTGTCTACAGGTTCCTATTTCCACACCATGGTG

CUST_2790_PI426210822 AAGCCTCATTGGACCACAGGTCCTATGGGACTGGTGGAGATATAG

A_14_P103002 CTGGATGGGAGTGAAAGTTGGCAGTCATTTTAAATATTACTCAATAATGTAGCCAGGAAA

A_14_P136527 TCCCGAATCCGTTTCTCTTCACCTAGAAGAAGGTGAAGTATGAGAATAAAGTATTAGC

A_14_P125348 ACAATTAACTCCAAGGTCTCAGTTCTTTTGATATAGGAGATTTGGCTTGTATTGTGCAAG

A_14_P126262 GCCAAAAGCATTTTGGAAGTGTGGAAGTGCATCTGATGTGGAAAGAAAATTGATATTTAA

A_14_P201376 AGCATGTCCTGTTATTCCAAAGAGAATGGAGCAAATCTCTCTGTTAGTATTAACCAATTC

A_14_P121323 CCATGGATAATTTCTCTCCAAGTTCTCTAATAAGTTCTGCTAGGTCTTCTGTATTCTGAG

A_14_P125702 TTTTTCTTTGAAGACCTACATCCAAGACTTTACTGGGGAAAATGTCTTGCTTCTGATATT

A_16_P02373741

AAAAAGTGAACCTATAAACACCACATTCAAAAAGGCTTCTATTTTGTGTTAACTGCTAAT

A_14_P112364 CTGCATTCAGTTACAGTTCTCACACCCTCAAAAAATACCTCTTTTTGGTCTGATCC

A_14_P128792 ATACAATAATGAGTCCTCTTTGTTGATAAATATACCTGCCTCGGGAGACAATGTTTGACT

A_16_P00541703

GCTCTAGTCTGACTCTAGTTTTGATTCCATCAACCTAAAGTATAGTTTCTACCACCTGTC

A_14_P115057 GAGATCTGATCAAAGTTGTAAATATGGTGTTGCCTTTATGAGTTCCTTATCCTGAAAGTG

A_14_P114128 AGTGCACATCTCCATTTCTGTTGACATGATGTCTCAAAATCTCCCTTTTCCATTC

A_14_P116094 GCACTGTTCTTCAAACGATGCCCCTCGTCACAGATTAGAAGATCAAATTTTATATTCTTA

A_14_P120018 AGGGTTGAGTTTTGAGTTTAGAGAAGAATAGAACGTTAAAGATGGAATGTCACTTGGATT

A_16_P00685005 GATCATCTTTGCTCATACCTCTGTAATCAGTGTGTGGAGCGTCAGGACT

A_14_P119415 ATCCGTTCAAGTTCACATATTAATTTTCTGGGAATTAGAAGAATCATGAATGCCAAGCCA

A_14_P137853 ATTCATCCTTTCTAATGTCTACACCATATTTTGGATAACATGCCATCATCTCACATTGTG

A_14_P122545 ACAACTCACACTCTGTGTTACAAGAATAAAATGCATTATTGGTCCTCAGTTTTTTGCCAA

A_14_P103329 TTGCAACAAGGTGAAATTTTTAAAGCATATCTTGACTCTGGAAACCATATGTTGTCAGGT

A_14_P102484 GAGGATGTCAGTATGACAATCTTTTTGCCTCATTACTAGGAAATCATCTCAGCAGAGAAA

A_14_P130104 TGCATAACTATTTTACCTGACTTTAACATGCTGGGATTACAGTATGCAAAACCCCATTTC

A_16_P21692468
AATGTATGTCCTGCTTTTGATAAACTCATTGGAATCTACATATCCCCTGATATGCAAGCA
A_14_P135217 CTATTTCTTATTTGCTGGATGTCACCATGGTCATTGTTCATGAGATATGTCACCAGGTAT
CUST_6851_PI426210822 AGAGACTCAGTGTTGGGACTGCTGGAAGAAAGACAAGTGAGGAAG
A_14_P202470
GGCAGGAAAAGAAAAAAAAATACAGAGGAACCTAAACCCTAAATCAGAGACAGACTACAT
CUST_8572_PI426210822 CTGTGGCTCCGCCCCTCCCTTGCTCAGTAGGGCCTGAGAATGCTC
CUST_9492_PI426210822 TATTGCCTTCCATTATAGGACTAGACTGTGGGATCCAGGAAAATGTTGCTTAAC
A_16_P02951572
AAAGAACCCTATTGCTAATATCTTCTTTCGTCATTCCTCATCCATACTTACCCAGGAGA
A_14_P124009 AACGAAAGCATACTGAGTGTCAAAGTCATATCTGAGGAGAAGCAGTCAAGGATGTA
A_14_P119750 CTTACTTTTCAGATGAAGAAGACAAAAGATGGTCATTAGATATGCAGGTTGGATGTTTGG
A_14_P138070 TCAAATGACTCCAAGACAAAGATTCTAGTGACAATTTACTACAGTCAGTTGGACCTAATG
A_14_P114863 GATATAAGCCAGGCCAAGTGACAAGTAATTTTTCCATGGAAAGGTGATAAAGCAATAAAG
CUST_4950_PI426210822
CAAGTCCTCAGTGAGAGTATTTGTCACTTCCTACTCTTGGTGTTTAATATCATCCC
CUST_5629_PI426210822 GTGTGCTGGGAAGGGAGCAATGCTGTCCTTGGACTGGTCACCTGA
A_14_P137494 GTAAACCAGAGTGGCTATAATCCAACCAAATTTTGGCATTTTGGTTGGACTGTG
A_14_P120205 CGTGTCTTTCTAGGAAATTCAGTTCATGAAGATAAGAAGTTTCTTGACAAATACATGCCC
A_14_P122373 CCAAGACTTCTATTTGTATAATGAAACAGGATACAGTTGTCACACTTTGAATCTGTGGTT
A_16_P18810635
AAAAAAAACCAACTCCATTTACATTTCAACATGCAGTTTTTTAAGATCTTCCCCCAACAC
A_18_P14137585
TGTTCATTGATACTTAGATAACAAACCTGTGGTTCAGTTATATGGGATTTATCCAGACCT
CUST_4914_PI426210822
TTTTTTTTAAAGCTTCATGTCTCTGTTCTCCATCTGTAGGTGGTGCATGAGTATGATATA
A_14_P126612 ACTTGCTTTAAATTTTTTCACTGTTAACTGCAATCTCTGGCTTAACCTCAGTTTGTTTAA
A_14_P104433 TGAAATGAAGTTAGCATTGGCATGTTGATCAGGTAGATGTTCTCAGTGTTTTGTATTGAC
A_14_P100985 CCTAAATTCAAATACTCAGACTGTAATTAATAGGCCCTATTAAACCAGCCCTTACCTTGG
A_14_P103125 CCTACAAGGGACTTAACATTGATTATAAGGCAAACGTGTCCAGAGATTTTTAAATGAGCA
A_18_P16993864
AATTGGAAAATTTTCCTCAGTGTAGTCTACGTCTTTGGATTCATTTAGCAAACCAGTCTG
A_14_P133667 TATAACTACTCATACCACTGACAGTGATTTCTTCTCTAGGCATTTACGGTAGGGAAACAA
A_14_P111599 GGTCTGAACTTCTGGAATAGTCTCTGTGCTAAAATATTTCTTTCCACACTCAACAAACAT
A_14_P136719 GATTGTGTTTACTTCTCTTTCCAGTTTCCATCTTGCTGCTGCAAAAGGACAC
A_18_P15117890
TCACAGCATCAACATCTTGTCTCCACTTTTATCACCATTTGTAATTAACTATGAGTCACT
A_14_P128277 CACTATTACCTTTATCTGCTTGATCTCATACTGGATCCTCTTGATAGGGTTGCCAT

A_14_P106060 ACTGTGACACTACATTTTCCAGCAACAAGAACCCTAGTCACACTAAGTTAAGGAAG
A_16_P17335617
TGAGTAGAATCAGATCTGTCAATCCAATCATTGTCCACAAACTACCAGTATTTCCTAACT
A_14_P112927 ACTTGGGCCTTGGTGCAGGAAAGCAAGTTTAGTAGAATTATCTGATATTAGATGTCATTT
A_16_P16595418
AGGATCTTCATGAGTAAGCAACAGAATCTCATAAAATCCATATCCTATAGACGACACTGG
CUST_5207_PI426210822 TTAACACCCCAGCCCCCGACACACACACAGACTTTTCAATGCAAA
A_14_P108051 CACAATCTTACCGTTAGCCATTAACAGGTATTTTTGGTGTAAGATTCTAGTAGGGTAATC
A_16_P16735309
AAAAGAGTCAACTTGATCTTTTTGACAGCCATTAAGAGAACTCTATCTCTTTGTTCCGTG
A_14_P122158 ACTGAAACAGTGACAGAAATGGAACTAAACAAGTTTCCTGCCACTTCTATATAGACCTAA
A_14_P108376 GTTTTGTTCGTTGACAGGAGAGAAGTTTTGGAAGATGCTACATTACACATTTGTTTGATT
CUST_6814_PI426210822
ACCGGAATGTAGTGTCCATGGAATTTTTAGAACTCATGATGTTTTGAAAACTATTTGCTG
A_14_P117730 AGCCCAGAAGTTTGGAAGATGTTTTCACATTAACTTTGAGAGCGCACAAGTC
A_14_P105751 TTTTGATTGATTCTGTTTGCTTTTATGTTCTAGGGGAAAACATATCCATTTCGGGGCG
A_16_P19706026
AGAGATGGCAATTTAATGATCATTTGGACCATACCTATTCCCTATCAACCTAACTGAAGT
A_18_P22841093 ATGAGGTGCTGTAGTGAAGAGGCGAGAGTCCCCAGATACTTGTTT
A_14_P200288 CGAGTAAAACCTGACTTCTGGATTCTCCTTGATGTCTTTTTGACACTAGTTTAGTTGTAT
A_14_P110007 GAAAATTAACTTCTATTAATTGCATACCTGGGTTTGCCTACTGAGAATCATACCCTGGAT
A_14_P101014 CTCTCCCCTCAGAGAAGAAGAAGAAGAAGAAAAGTCCAAGCACACACTAC
A_14_P100536 CATGTATTCTCGTCTTTAAGTTAATGGTCCAAAATTTTATTTGCCTAGTAGTGTCTTCTC
A_16_P16911572
AAAATCAACGAAGGGGAAATTTCATACTGTTGGCTAAAATGCAACTATTCAATTCATCCC
CUST_8662_PI426210822 AGTTAAAAACCTGTATCTGTGAGCAGCTTTCTCACACTGGAGGAGAAGG
A_18_P10745817
AACCTTTTAACCCTTTCTGATAAGCCTTCCTATATCCTGACATCGATTTCCTTCTTTTAC
A_14_P119277 CTTCTTCATGCTAAGTCCCCCACTAGTCTAGATTCTTAATATTTCTATTCTCAGTGCTGG
CUST_4326_PI426210822 ATTTCTTAGCATGCCCGTTGGGACAATTAGGGAGGGATTCTTTCAAG
CUST_7308_PI426210822 TGTCCGCTGAACATGACAGTTATCCTGGCTTCACAGTAACCTTTCA
A_14_P138833 ACACAAAATTAAGAATCTTCCATAATTGCTTTTGCTCAGTAACTGTGTCATGAATTGCAA
A_16_P30952603 CATGGGAACCCAGAAGAGGTGACGGTGAGTCAGCCACCCCAGGAC
A_14_P101333 GCCTACACTTGACTCACTTTTTGTTTAAATGTATTTTTGTAGTTCCTCATTCTGGAGGCT
A_16_P01762679
ACAGAATATCACCCACATTTTTGTTATTTTTCAGTTTCCTCTCAATGAATGGAATCATCA
A_16_P36230137 CTCTGAGAATGAATGATCTGTTAGGAAGGCTGGATGGCATGGTGACAAG
A_14_P113195 CTGGGACTCATTCTGAATCTTGGTTTGCTTTGATATGAAAGTAGGATTTTTGGATTTGAT

A_14_P116857 ACTCTGATGTAGACTATCTAAAAGATAATTTGGATAAATACATGTCCCAGTTTCCAAAAG
A_18_P21943738
AAAAGAATGTCCCAATTGTTGAACTACCTCATGAACTACCAATGGTATACACAACTTTCC
A_16_P15654436
AGAATACACTGAAACACAAAATAGCGTCTTGGTCTTCTTATCTCAACAACCTTGTTTGTC
A_16_P01989649
GTTTATCTCAGGTATCATCAGAAAACTTCGTTAGAATTCTAGTAGATCCCAAAACCCAGG
CUST_1439_PI426210822
AGTTTCATTGTTCCTTTGTATTTTGCATTTCAAAAATAATGATATTGCTTTTAATGGTAC
A_14_P131972 AGACCATATGAACCACACAAATCCTATATAAAGGACCAAAGACAGGTGTAAAATAGAGAA
A_16_P18657542
AAGAGTCAGAGATCATTTGCAACAGTTAAATCCTTAAATGCTTGTCCTATATACCTCTGA
CUST_787_PI426210822 TAATTCCGCACTGCTGCTATTTTTTTCCTATTTGCCCCTAAATGTCCATGCT
A_14_P115372 ACATGCAGATGTCGTTATAATTATTATCAATGCTATTCTGTCACTCCACACCCTGACTCC
A_16_P41832768
AAGTGACATGAACACACTATCCATACACCAAAATCATGGGTTTGCAATAGCATTTTATGC
A_14_P115407 GACATAAGCCATGATCCTTGTTCTGTCCTCTAGGACTGTATAATCTTATAGGATTTTCCA
A_14_P139145 CTGCCACCTCTAAAGTTGCTCTGTGAAGGAAAAAAAAGGAATTAAATGCTAAAACTACAC
A_14_P138373 TTACTGTCCTTGACTTTCTTCTCTGCTTTAACATTCCATTCTATTTGTCAACTGCGTAAC
A_14_P201637 TTTGCTGTTTGCTTCAAATTGATCTCAGAGGCATGTTAACCAACTTTAAATATTGCCACT
A_18_P16352890
GGCAATCCATTTTAATTTCCCAGGTTCTGTTAAAAAAAGATGAAATTAAGCCTCTCCTCT
A_14_P126577 TTTTTATTCTCTTTCAGCACCCTTACCCTTCTGAAGAACAGAAAAAGCAGTTGGC
A_16_P00685138
CTGTGACCTTAGATTATAGCCAGAGAGCCATATGCAAGTGAATAATAATGCAACCAATGT
CUST_5997_PI426210822
ATTCATCAAAGGAAAGTAATACTAAGATTCCTTTTCTTCAGTTTGAGTTTACTGGAGGAA
A_14_P201896 GGGTTTAATATCTAAGATTCAAGTCTTTGGAGGCTGACCTAATTTCAGATGAAGAGACAT
A_14_P116261 TTATCTGCTAACTCTGTTATTCTTCAAACACAAGTGGGTAGTGTCATTTTTCCTTCCTTC
A_16_P17206983
ACTTATCCTATGTAACTTGTTTTAAACCAACTTTTCCCACTTCATGAAATCCACAGCCTT
A_18_P14137679
AAAAAGTTGAAACATTCTTGAACAACTATATTTTACTCAAGGGCATTATAGACAGGGCAA
A_14_P128112
GGTTTAAAACAAAACAGAGGGAAAGATGAGTTTGGAGTCTTCTAAAAATCAACAAAGGAC
A_14_P113902 GGGCATTTACCATTCTTATAAAATGACACTTTTCTGGTCCTTTCATTCTTTTTACCCACT
A_14_P107829 CTATGTCCACAAGCATTCTAATTTCCTAAAGAGTTTTCTTACTGTAGTTTGCATCTTCCA
CUST_9200_PI426210822 AATTATTGGGGGACCTAAGATCCACATTTTCTATTATTTGGCCCTGTTGATGCT

A_16_P00237724
TTGTTTTCTCCAATAGAATCTCAATGTGCTCATAGTAATCCCTCATAAAGGCCTACTTGT
A_16_P02618890
TTGTGATTATAGTTGCTTTTGCAAGTTTCTTCATTCATCCTATGTAGACAAGCCAGGCTA
A_14_P118007 CAATACTTTTCATATATTATTGATGGCTCCTTAATTGGGGATTCTAAAAGCCTCGCTGCA
A_16_P18806980
CCCTCTGAATTCAAAAAAGTTTAAAGAAGTCTTCCCATATGCATACCTTACTCACCAAGA
CUST_1147_PI426210822
TATCCCAAAAATACAATGGACTTAATGATGGTTAAATGGAACTGGTGAAGAAATTAGCAA
A_14_P112126 TAAACATGAGAATTCTCTGAGGTCTATTTCTTGACCCTTTGTCCTCTTTTTAACTATCCC
A_14_P115892 AATATTTCCTGAGTTTTTTTATGAAAAGTGATAGAAGGAGAAATGGGGACTGGAAACTGG
A_14_P100295 GATTTCTCAAATTCCCTGAAATCACAGGATTGCTACTTCCTTAAATATGATTGACTCCAG
A_14_P124816 TACTTTACCTCCTTATTTTTCTGAAAGTCTTGGCTAACCAACAACTTGGACTTTCATAGC
A_14_P106423 GATGAGAAGTCCCCAGAAACTTTCAAAGAGGCGGTCAAAGCAATTATTTCTT
A_14_P112904 TTGTATTTTAATGTTTCTGTTTCTTAGTCTTTCCGTCCCCTTCTACTTATCCTGGTTGAC
A_14_P111622 GGATACTTACTTCAGGAGAAATCAATACATATTGGGCCTGAAGAAAAAAATTGAGGAGAG
A_14_P103932 TCAACTTTGCTACCATTATTGGAATGTAGAGTAAAGATGCTGGTATCAGGACTTTGGTTA
CUST_7349_PI426210822 GTCATAGAGTCTGATTGGTCCAATTGAGTTAAGCCTGGGTCGCATG
A_14_P120077 ATTGTCTTTCTATGACATTAACACATTGTGATTTCCTGGCCTTTGAGGGGTAGAG
A_14_P135924 ACATTAGCAAAACTGGCAACCCTTTAAAAACAGTTCTGAAAGCAACTGTGATATTTGAAG
A_14_P201217 ATGTTTGGATTTTCAATATCGACTTTGCTTAAAACAGAGCCCACCTGCATAGTAACTGTG
CUST_7387_PI426210822 CTGCCAAAGAAAACTCGCTTTGCCTTTTGTAAAAAGCCTGTCGAGCT
A_14_P113653 TTTATCTATCACCTTGAATGTCCACACATCAGATTCACTATCTTTCTCCCCATAATGTCT
CUST_3134_PI426210822 GATGCTGGCCATTTATAAAGCTGCTCCTGTGCCTGTTAAGCCATACA
CUST_7516_PI426210822 GAACTTCTCCTTGGAGACAGCAGGAACTGTGTTACAAAGGCATATGG
A_14_P103686 AACTGCAATTGTAATGCCAGACAGAATAAGAGCAATGCATGTATCAATGACAATTGAGAG
A_16_P58424231 TTTACTGAGTGCTGACTATACCCAGGCACCATTCTAACCTCTTTACCCAAG
A_14_P100160 CAACACAGCATTGCAAATTGAAAGATAAATCATTGCACTTGACAAGGATCCTTTTTCCAA
CUST_6509_PI426210822
TGGCTAAAGACATCTAGAGTCATCTCAAATGCCTCCACAAATTTCAAAGAAAATTGTCAA
A_14_P103088 TGTAGTAAGGTGTAGATCAAACAAAACATATCTCTGGGTTCCTTCCAGACTTTCCATTAA
A_14_P116889 TTACCTATGTTGTTTTTCTAGGAAGTGTAGGGCTTTTTTATTGGGATTGCAGACCTATTG
CUST_236_PI426210822 GCCGCCCAGCTAGACTCGGCTTCACTCTCTGAATCGAAAAGTAAC
A_14_P137292 ATCCATAGATTAACACGGTAAAAACTTATCCCACTGCTTAGCACCTACAGAGCATTCAAT
A_18_P11168804
TAACGACATATGTGGTAAGTTATTCTCACCTCATGCCTATAGTTACCAGTGGAGAGACTT
A_14_P125499 AGAGGAAAATGAGAATGGTTGGGTGCTTTCCAAACATGACATTATACGTGTTAATACATA
CUST_5645_PI426210822 CTCTGAGGAAGCAGGTGCTCTGCTGCCTTTCCACATCCTTTCCTC

CUST_8038_PI426210822
AAAGCTTATCATGCAAATGAGCTTCCAGAGGTCATAAACTGCAGAAGATGGAAG
A_14_P134922 CCCAGAAATCGTGGCTATTGTTATCACTAATTATGCTTAATTATGGTTGGCTCTTTGGAG
A_14_P100159 CCTGATTCTTGTTTTGGAAGTGCCCGTTGGAGAATGTTTTCCTTTGGTT
A_16_P16706855
TTCCACTTTGACTACACATTGTAACATCTTCTTCCACTGTAATAAGACTAACCCAACACT
CUST_8145_PI426210822 TTCTGGAGCAGTCACACGATGACAGATGAGACGGTTGGGCCTGAG
A_14_P101113 GTTGTTCTGTGCAGAGAATTAATTGAGAGTCCTGTAGTCACTGGAGCCATTG
A_16_P41669218
TTCACAGAAAGGGATAAAGAAATCACTTTGCCAGAAAGAATAAACGTAACTCCTGACTAC
A_14_P127990 ACCTTGCTCTTGTTACAGCAATTTACTCTTTTCATTCCTTCAAAAGCAAACTAAAACCAC
A_16_P58424456 GATCCTACAGAGTATCTCATAATCCAGCCAGTTCTCACCATCACCATTGGTT
A_18_P16674504
AAATTCAGTCTTTGGCCTGTATCCTGAAGTCAATTCCCTTAGGAATACTTTTCTTCACTT
A_14_P111942 AAATCCTCTAAACAGTCAGACAATTCTTACTCAACAAAGGAACATAAAGGCTGTGGTGTG
A_14_P101852 CTTGGGTGTATGTTAACACCAAATAGGAGAAAAAAGAAAAAAATATCTGAGGCCGATGTG
A_18_P17041078 TCTATTAGGTCCATCTCTCTACATCCTTTTACTTTCTTATCTCTGTTCTTATCTCTCTCT
A_14_P133233 AGAACCATAAATTCTAAGGTATCTAAAAACCTATGAGTATTTAATTCATAGAAAAAATGT
A_14_P119491 ACTACTCATCCCAGTGATATTTGCATTTGGAAAGAGGTCTTCTATGGGCAGTATTATAAT
A_14_P118810 CAGACAACTGTATCATCACCCAATATATGAAATTCCAACTGGGAATTGTTCTAAAGGAAG
CUST_6007_PI426210822
TTAAAGAAGTTTCATAAATTTGCAGCTCATTAATTTTAATTTTAAATAGGCCTTCCACAA
A_14_P120803 GTGTCTCCTCTAAAAATTAAATTCTGGTAACACACACAGAAGCCATCTCCACAGTG
A_14_P115545 ACTTTTGGTGTTAAAAAAAAAAACCTGTAAGTCTGTAACCTGGCAACATTTTACAACCCT
CUST_9430_PI426210822 CAAGATGTTGGGTTTGTGGCAGACCCAAGACTATGCCTGCCTTGG
A_16_P16191287 GAATTGGGACCTAGAGTCATTTGGATACAGTGGTTGCTTGGCCATTTTCT
A_14_P124216 AGTAACAAGCACCAAGAAATCAGAAGAGAGGTCTATGTAAATTAAGGGACTAGACTTTCT
A_14_P107982 GCCAGGAATGGACTGGCATTGGCCTTTGTATTTAATTTTAACTCTTTATCACCCA
A_16_P01326068
GTGGTTTAGAAGGAGATAACTGTTCTTTAACACGGGTTTCTAATCTTGAAATTACACGCA
A_16_P41612093
ATCTTTAGTCAGTTGCTGATATTCAAGAAAGAACAAACCACAGATTTAGAGAAGGTGCTG
CUST_8046_PI426210822 AGCTTCATTGTTACTGCTCAGAGGTAAGGGGGGGGTTTAAACACT
A_16_P18094727
AGGATAAACTTTTACCTCTAACTCTGATATGTGGATGCAACACTGTATAAGAATGCCTCC
A_14_P101227 CTAGATATTTAGGTAAGGGTTATGTTTTGTCAGAAGGGAAGATCATTTGTTAAGGCCGAT
A_14_P110044 CATCTCAGGATCAATTTTTGTGAAAAATGGCACCACTACTTCATGAATTATTTTTTGCCC
A_14_P109015 GGCACATTTTATTTCCTAGTTTCACATTCATTTTCAGTCTAGGAGAGAGGCTTCTGCT

A_14_P109057 CTGGAAACAAAGCCTAGAGGATGTATACTTTTTAAGATGCAGAAAATGAAATGAAACCCC

A_14_P128187 CTTAAAATGCTACATGAGGAGCAAATAGGAAGGTCTGTTTTGCAAAGCCATGACAATTAA

A_16_P38098958

GGTAAGACTATGTCAAACCACAGTGTATAGCAGTTTTCATTGACTCTCAGAATCCTCTAA

A_16_P18123309

ACCGGATTCAGCAACATTTCACCAGTTTGATCACATTAAAGACCATTCAAGATACATTTA

A_14_P123156 CTCAGAAGTGAAAGAACGACGAATTGTTCTCATTAGGGAACGAAAAGGTGAGT

A_14_P135441 GATAGCCCCTATGTTGCAGTTAAAATTGTAAGGAGTTGGAACTTCAGAAAATATGAATGC

A_14_P101330 AGTGGTTTCTTTGTATTCTAAATCTGTGTGTATCTGTGTGCACACACTTGTGCACTC

A_16_P20125237

CAGGTGGAATCTACAATTGTTTTATTGGGCTGGATGTAAAAATAATGACCTTGATGTTGC

A_14_P103559 AAAAACTTTGAGGATTCCCCAGAATGATAGAAAGAATAATGATCATTGGAGTGAAACCTC

A_14_P122815 GACCCCAGTTAGATGTATTTACACAACCTACCAAGGAAGAGATGAATCAATTGTGATAAT

A_14_P138887 ACCCTTATGTGAATAGGACTCTGTTATTTATCATTGTCTAAGTTTATTGGGGTGGTGACA

A_14_P104577 ACCTTTGTCCTTTATTTTCTTTTTCAGTCGGATGTCATGGATCAGAATTTGTTAAATTTC

A_16_P20793133

ATCCTTTCTTCAAAAATGAGATGATGATGCACTCAGATAGTCCTATGTAAGTTGACCTCC

A_14_P114573 CTCTCTCGAACAGTGGTAAGACAAGATGGATTTGTAAAGCAGCAGAACAGAATCA

A_18_P14135641

TACCAATAACAATTTGAGAAGAAAGTTGACATTGTGACTCCTCTGCTTCACTTTCTTCAG

A_14_P117262 AAATCCTGAAGGGGAAAGAGTATTTTGCCATCGATAATTCTGGTCTGTGTGACTAA

A_14_P125880 GTATCATGATGGAGTTTACCCATGTTAGGGGAGTTAATCTATTAATTATCAGTGGGACTC

A_14_P117603 ACCGCTTGCTTAAAATTGTCAAGAAAGTGTGTGAATTAATGATGCATCTGACATATCGTA

A_14_P114554 TAGACTTACAAGACTGCCAATTCCTCTGTCTTGCTTCATCATAAAATTGACGCAATACAA

A_16_P00719588

GCCTGCACTCAATGAAAATTTGAGCATATTCACCATGAAATATAACGGTTAGCACAAAGT

A_14_P109968 TTTGGAATTCAATGGGTAAATAAATGCTGCTTTGGGGATATTATCTCTGTTTGGTCTTGA

A_14_P201783 CTCATCCTTCTTGATCTGATTCAGGGCTATTTCCATCAATTTTTCCTGACCAAGAA

A_16_P18602901

AAATTCAAGCATCTTCCCTACCATTCTGTCTTGGAAAGTTTATTGCTACACAGACAAATC

CUST_4718_PI426210822 CTCTGAGGGTCCTTCCCACCAGGAATCTCCCCACTTGAACCTAAG

CUST_8289_PI426210822

TACTTTTATAATCCAGTTTTTAAAAAGTCAAAGCTATGATAATCTTGTCATTTCAGAAAG

A_18_P11192842

ACTGTCTTTAAGTTGAACCAGTTGTATAGTTTAGACCAATATTTAGGCCCAACTTCCTTT

A_16_P17831396

TCCTTTCCAACCAACTCTTTTTCATTCTACACATGACATTTCAGATGCCACTAATATGAA

A_14_P124302 CCAGTCGTAGAAGGGATTTTTAACACTACGTTACTCAGTTCTGTCTATGCATTTAAGAAG

A_18_P16121147
TTCCTGATCATACATTAGAGGCAGACTAAACATATTCCTGATTGTCATCTAACACACCTG
A_14_P119068 TGTTCTCCATCCTCCATTAACACCATGTTAATAGAAAATTTGACCATTGTAACTTTCCCT
A_14_P113875 CTTCTTGGTGATAGAAAGACTTAACTTCCCTGGATCACAAACGATGATGCTAATAATCTT
A_16_P20819067
ACACTTCAAGTGAATCTGAAGATATCGGTTTGAGTTGAAGAAATGCTAAATCCCCTAGTG
CUST_9216_PI426210822 CAAAGTCTGGAAGAAAAGTCTTGGTAAGGATTTGTTTAGTGGTTGGGGTAACA
A_14_P139171 ATACTAGGTGTTCAAATTCCCAAGTGATTATTGAATCTTAGGATTGAGTGTCTCCTTGAG
A_14_P117885 ACACCATAGCACAAGTGAGTTCAGTGAAAAGATTTGATTATCTCTAAAGGTAGGTGTTTC
A_14_P106123 TATATTTATGTGCAGGGAAGAGTCTATTGCCAACTTGAAATGTCACATCATGAATGCTGT
A_14_P107872 TAAGTAAAGTCTGGAAGAGAGAGATGTGATTACCTGGTAAAGAGTTTTCCACTTGACATT
A_14_P105564 ACCATGCTCTTGACTTCGTATTCTTTCACAAATTCCCACCACAAAATGTTAAAAAATAAA
A_14_P104053 GGAGTAAGAAATAAACCACCTATGACTCAAGTGGTGAAAGACATTTCACGTTCCATATAT
A_16_P15705386
GTGCAGTTTTTCATTGAAAGGGAAAGAGATATGGAAATTGCTGAATACTATGGCTACACA
A_16_P19309390
AAAGACACCTACAGGTAGATTCTCTAAATTCTTTTGTCACCATTTCTGTGTATCACTACA
A_16_P00685261
AAAATTAAGTCTTGTTGAAGAGTGAGTAGAAACCAGAGTGGTGATAATTTCTGGTTCTCC
A_14_P126013 AAATACGGTAATAATAGAAGAAATACGGTAATAAATAGCCAAAGTGAAGGAGCACGGCCA
A_14_P131874 CAGTAGCAATCCTATGCTTACTTTTAAAACTCTTTTTTCTGGTTTCCTGCATAGTGTGCT
A_14_P109780 CCCTTCACCTGACTTACAGGCAAACTCTTGTGTATTTTTTGTTAGAAAATCTTCATATCG
A_16_P18095291
TAACATTTCCTGGTTATTAGCATTTCCCATTAAACTTCCTCAATAGGTCAAGTCTACGCT
A_14_P134236 CATGTATATTGACTCTGGATTCACTTGGTAGTGTCATGGCAGTCTCAGTAGCAG
A_14_P120567 GTATATTTCCGGGCATAATTTTGAGAGACGCCAGTCAAAAAAGACTGTGTGAAGTAGAA
A_14_P107056 ACAATTCTGTGTTTGATTCAGTGCCTGAAAAGGAATCAGTTAAGTCCTCAAAGTTCTTTA
A_14_P130085 ACTCCAAAAGTTTTATACTCCATTCATGTGATAGAAAAGATCCCTAGTGAGAATGTAGAG
A_14_P104688 CAATCAACACATCAATTATTTCGAAGACAGAGAGCCAAAGTTCCCTGAGGAATTTATTTT
A_14_P139382 CTGATAAAAAATATTATGGGAACTCTGCTATGTGTTATCTCTCAGGCTACTCTGGAGACT
A_14_P131518 CTTGCTCTATGGCTTCTTTTTTGCTTCATTGCAAGATGATGCACTGGTAAGTAAAAATTG
A_18_P14916333
AACTACGTCTCAGGTATAAGATTGCAGTTACTGATTTAGACACCATAGGATTAGGGATGT
A_16_P15223350
AAAGCAGGATCAGATAAACCATACGCTCAACATTAAAAAAGATATTCAGATGGGTTGCTT
A_14_P109959 CAGAAGTGAGTAGGCAGTCAGCAAGCCTTAAGGATCAATCTCTGGAATATC
A_16_P19911057
AAAAGTATGTGGAAGATATCTCTGGAGTAAGTTACTCAGGCTACCTTTTAACACATGGTG

CUST_4380_PI426210822 ATGGCTTTGCGGCCTTCAGTATCAAATGCATTGTGATTGTGCTATGAGAATTTT
A_14_P122928 TACCAATAAAAATTGAAGTCAAGTGACATTCTATGACCTGTATTTCCAATGTGTCCTCCA
CUST_1684_PI426210822
TCGTCTCTGCAGAGGGAGATCAACCTGAAAAGATCTGTTTCTGAAATATATGTG
A_16_P38492865
ACACACCACTAATCTGTATTAAACACTACTGACCATGTTAACCTTTTATATGGCTGACCA
A_18_P22842831
ACAATCCATGGACACTATTGCCTATAGTAATGTATGTGGCACATCTGCACACAAATTTAA
A_14_P120012 TGTCAGTCTGACATTTAATGTCTTTCTATGGACAACATTAAATCTCCCTCCCTTCTGTAA
A_14_P137874 GAGCCACCACCATACTAATAAGACATGACAGTCACAAGTTTCTAAGTTATAAATCCACAG
A_16_P20217245
ACTTCATCACTTGATCAATGTTTAACCCAATTTTAACTTCCCTATCTCAGCATACTTATG
A_18_P20344008
ATGATGGAACAGCACCTAAATTCAAATCTAGTCCTATGTGATTTATAAGTTTGTGTCCCC
CUST_6525_PI426210822 TTACTGAGGGCATGCTGCCCCTAGTTATGATATTGCTCCTAGGAAGA
A_14_P136348 ATTGAATGTGAGTTCCACGTGATCATAGGACATTAGGATGCTGTGATGAGAAAAGAG
A_14_P111361 GAGTAAGACGCCATATAAGATTGCTATGACTGATTTTGGTCTAATGCTTCACTCTAACAC
A_14_P121553 TGGTCGTTCACTGAGGAAGATCAAACTGGGTGTTTGTTTTAACATTTATTGAATTTTCTA
A_16_P19076708
GGGTATGTAAACAGATTTTTCACACAGCCTAGTTAGTGTTACAATCAGGTTATGAAGGAG
A_14_P200089 GACCTGGAATTTCACATGGTTACTTCTAACTTTGCCATTGGCTTTTAACATTTTCGTGTT
A_14_P125648 TCAACCTAGTATGGGTTATGTGGTTCTAATGAATCTCCACTTGATTATTGGTCCGTTTAT
A_14_P122996 GCCTTTGATGTCCTAGTGGTGAGAAATAAGGATATCATGCTTTGTTAGGTCCCTATTATC
A_14_P113807 CTGAGACAGATGATGTGCAAAGTGTTTTCTCACTGGATTTGCACAAGTTTGG
A_14_P131555 CTATTCAAAAGACAGCATCTTTCCATGTCTATGTTAAGAGGATGAGATGTTCCTTATCCT
A_16_P21541588
AGTAGAAGGTAGACTCTAGGATTATCCACACATAACCCTTCATGTATTTTCTGATCTCCT
A_16_P19583449
AAACTGACAGATGGATACTTTGCTGAGAAAGGATTTTGTAAAATAAACGTAGTTGCTCCT
A_16_P18203168
ACTGACCTTAGTCTCAAAACTTTCAAACAGTTCCTCTCTATATCCCTCACTTGCATAACT
A_14_P136706 CTACACATTATTGTTAGGTCCTGAGATAAGCAGTTGTTCTCAGACATCTCTGGACATTAA
A_18_P15901499
TACAGAGATCACAGTAACAGTTTACCTTATCTCCAGAATTTCTAACTTCTCAATGGTGGC
A_16_P18553759
ATGTAATCTAGCAAACATACCTTCCTTTTTTATTGCACCCATACAACTTATACCACTGCC
A_16_P37820942
TGGACTCCTGCTAAGATTAATTCACTAGATACTTACCAATTAAAAAATACAGCCCAAACC

A_16_P36484145
TCAGACTCTGTTTAATCTACACTCTTGACTTTTTCAACTTTATCCTCAAACTACCTTCCC
CUST_10424_PI426210822
CACTCAAGCACTGATATCTGGCCTAAAATCTTGGAAGTACATGTCCATGAATACAAATTT
A_14_P109906 TAAAGGTGTCAGAATGTGTCAGAGAATGTGGTTACTGTGACATTTAACAGAAGGCATTTA
A_14_P105052 ATATAGAAGCAAACAAGAAGTTCTGCAAACAAGACAGTTTGTTAGCACACTTGCATACGT
A_16_P00636371
AAGACACTGTAAGTAACTCTCATTCCTCCTTATGATTCCATTTACTACTGCCTAAGAGCA
A_18_P16118129
ACGTTGACTGGTGATTTATACCTTATACCCTCTCATTAAACACTCTTACCTAACTACGCA
A_14_P135850 TCTCTGCAGAATCTGTTTATGGGATCTGAAAAAGGTAAGATGAGTTTGTTGTTGTTTTGA
CUST_4765_PI426210822 CTGGATGTGGCCACCATCTGCAGGGGTTCAGCATTGAGATATGAC
A_14_P132377 CAACAGTTCAAGCCATAAAACTCAAGCATAAATTAGATTGTGTATACCCCAGGGGCATA
A_14_P130135 GCTCTTGGATCAGACTTCTGTGCCCTTTCATCTAAAAAGCAAATTATCGTGATAAACTAT
A_14_P111286 CTTTTCCTGACTCTACCCTAGTTGGGGAATAGTGAGAAGATATATTTGTGTGATATCTTC
A_14_P105522 TTAATTAAATGCAGCCTCCCTGTTTGTGGAGAAAGAAAAATTTTTCTTAACCCTATTGCC
CUST_8589_PI426210822 ACAGATTATAGGCATGGAGGGAAGGCTCATCTGTCATTCCTCTCC
A_14_P107515 TACCAGTCCCAAGATTCCAAATATGAATGATTAAGACAAAAACTTACCCCTTGGCTTC
A_14_P132511 TGCTTCCTTCTGACTAAATAACTAGCAGGAACCCTAAGACCAGTAGTTAAAGGACTATAT
A_14_P123607 ATAAAGTGGAAGGTCTCATCTCTAAATTTCTAGTAGAATCCAACTAAAACACATACCCTG
CUST_661_PI426210822 TTTGTTGATGGCAGCCTGTTGGTGGGGACACTCAGGCCCTTTTAT
A_14_P100093 CGTATTTCGTGATCAGGTATACTAATCCTTTAACACCAAGTTCGGAATTAACTCACATGC
CUST_1665_PI426210822
GGGGGTAGGATCTCAAAACTTTAAAATGAAGTGCTCATCAACAGTCATTTCTTGTC
A_14_P116962 GAAAACACAGATATAGGCAATAGTAGTGAAAGAACATAGACTCTGGGACAAGTAGAGCAT
A_16_P19658709
AAGTCAACAAAGTTTCCCTAACACTAAATCTGAGAATTACACATATCTAGTCACCGGCTG
A_14_P126026
AAAAGGAGCAAGAATAATGCAGACAATGCATTTTGCTTTAGGAAGAGAAAATCCAGAAGA
A_16_P58424441 ATTTTCCACATATATCTCATTGGCCAGCACCATGCCACAAGGTGG
A_14_P131113 GAAGTTTTGCAACTTGTTTCATGCTGAAGAAAATGAATGAAAGCCTACACCTTTGATTTC
A_14_P109457 CAGTAGAAAATGTGCATCAGATGTTTATGTTTAATTGGTTTACAGACTGTCTGTGGACTC
CUST_816_PI426210822 GCACGTCCTGCGGAAAGACCTGCCGGGAAGACCGCGATGATGAGT
A_14_P122267 CTTGTAAATACGGAAGCCTTAGGATCATTCAGGTATAGCATCTTTGATATGGTCATTGAC
A_14_P201719 GATAAGGATCTTGGGAATTGCATATGAAGCATTTTTTGTCCTCCTACAAACAAAGTTGAG
CUST_8948_PI426210822 CCTTGGAACCACTAGCTACACAGAGGGGTAAATATCAGAAAATCCAAATTTGG
A_16_P00068077
ACATGATCCCATCTTAATATCTATGCATTGGGCACTAAGTATAGTAGTCTTTCTGTGGGT

A_16_P15991335
TGATCCTGGCTCTAGATTGTCATCTGATTACACATTGTTTTACCAACTTCATGACTGTTA
A_14_P130904 CAGGAGATTTTAGGGTTAAGAAGTGTATCACTGAAATACTTCATTCTGCTTGCTACTCTG
A_14_P118778 TTGCAATGTTCTGAGGCTGAAAATAGATATTCTGTCTCCAGAAAAGGAAGAATTAGGATG
A_18_P16343148
AAACATTGCGTTTGGTATAACCTTCCAATTTTAAAGAGAAAAAAAGTGTGCCTATGTGGG
A_14_P135813 CTCCATTCTTCCAAGACTGCATGTCTGAAAATGCTCTAGATGAACTGAATATTGAATTGC
A_14_P138496 CTGTGTTCAGTTTGGGGGACAAATTTATAAATGTCATATATGTAGACAAATGGATGCTTA
A_14_P113399 TCATTGCCACTTGTAGCCAAAAGACCTTTTTTATTACACCAAACACATGTCATTACCTAG
A_16_P18299246
ACTTCAATACTTGAATACACAAACTCACAGACTGTTAGAATCTATGGCTACAGGATTGCC
A_16_P18953679
CATCTGAACTCCTTTCTCTAATCTATCGTCTTATCTCTCCTATTCTCTCCCTAAACTGAA
CUST_8776_PI426210822 CTTCACCCTCAATTTTTTTTGTGGAAATTCAATGGTTGACTGTACCAAGCCAG
A_14_P113714 CCTCTTCTAGATTTGAATAGAAACATATTGCAGGTTCAGATGGCACCATTTGGTATTTTG
A_14_P200054 CACAAACTATCACTCTGAATAACTTCTCGGTTGAAATTCGATGGAGTTAATTCGATCACA
A_16_P15870952
GAAGGCTTAGAGTTGTAAACACATTGTTAAGGCATTTCCAAGAAAGTTTAGTGCAACAGA
A_16_P00685160
CAGTGTTTTCTGTGTGGGAGCACTTTCCTGTATGCATTATTTTCTTCTTAATAATGGCAC
A_14_P129007 ATTTCCGTCTCCTGCTCAGGAATGTCAGTGCATAATAAGAGAAATAAAATGGATTTTGTT
A_14_P102800 CTGTAACTCAGTGGTGGAGAAAACCCTCAACTCCAGTCTTTTTATGTAAAATTCTTATTC
CUST_8251_PI426210822
CTCCTGAGCTTTTGGCATTTTTGGTCATGTTTTTTAAACATGAGGTACTGATGGAATA
A_16_P16030304
AATTCACTGATTCAGTTAACCTAGAGAAAACAACCATTGTGCATAGTTACTGAGGGAGTA
A_14_P122249 TTTCTACTTTTCATGCTACTTTGGTTCTGAGAATAAAGGGCAGGTAGTCAATGAGTAAGT
A_16_P01185921
CTCTCCATAAATCATGTCCTCCATATCATTGATAACCTCTAAATTACATCAGTCCCGCTT
A_14_P119682 CAATTGGCTTGTGCATTGATAATCTTTATTTACTGTTTTAAGTTGCAGAGATGTGAATGG
CUST_3748_PI426210822 TTAGGAAAGGAGTAGAAATGGGAGTTGGGATTAAAAGCAGAGGTGAGGT
A_14_P124986 CTGAAATTCAGATGAAACATTGGACCACCTAAAATAGACCAAACTGTGAAGATCTAAGTG
CUST_6826_PI426210822
ACATTTTTTAATTTAATGAGTTGCTTGTACTTAAGAAAATAAAATTATATGAAAATTAAT
A_14_P136474 TAGATCTCAGAAGATATCCAATCCTGTCTATAGGATGCAACATAAAATTGTCACCTCATT
A_16_P03254173
TTTCACATCTCTTTCGCATGTCTGTATAAATCCTCAATCTTTAAACGGTGCTTGACTACT
A_14_P110674 CTTCTGATAAATGACGCCAAACTTGAAGTTATTGCTGATGACATGCTCGTCAAAGG

A_14_P112310 CTGGACACTGGCTATTATTCTTCAACCACAAATCAATACAAATCGAACAAAATACATGGT
A_14_P107308 GACTGACCGAGTGCTTTTTTTTTTTTTAAAGCAAATATTGCCTTTGGGCTTCTCTCTAA
A_14_P122343 CTTTTCACAAATGCCGACTTCTGGTTTGTGCCTTTCCCATATTTTATTAAATTCGTGTTT
A_14_P120450 TAGAGGTCTAGATTCTTTGGAAAAAGTGAATGGTGATTTCTCTCATCCTCAAGTTATTCC
A_16_P17439576
TGTCCTTTGACTTCACTACTAGAATTAGGAATGTATCCCCTAGAAATACTCACACAGAAC
A_16_P17140377
ACAGGAAGTCAGTATATTCAGAATGACCATCAGACTATCTTCGTCCATACTAAGCATCTT
CUST_1360_PI426210822 AATGGAGGGAGGGTGTGTGACCCAGGCTTGTTGGGGATGTTGTTT
A_14_P127571 CTTAAGGAAACCTGTTTTTACTTCTCTCCCAGATACAGAACTTCAAACTGTTTATGGTTG
A_14_P121599 GTCCTGACCAAGAGGACCAGACTTTGTTAATGAAGGTAGGTTATCAGATAAAGAACTT
A_14_P100809 TTGGGTGTAATTTCTATGACAGTCCAGCAACATCAATTTAATACCTTTTACATCTCAACC
A_16_P30952351 ATGTTTTCTGAGATCAGGGATGAGGGACAGGTCACTGTCTACATCAACA
A_14_P136526 TTGGAAGAAATTATCTGAAAATGTGTTACTGTAGGAGGTGAGGGTGCTTTTAAGAAACAG
A_16_P17242409
TCCATCCACCTTTCAAAAAAATCATTCCCAACTATGCTAAGAATTTATTAATGAGTCCCA
A_14_P120427 AAAATAACCTCATTTCCCCACAGTCCTCAAGAAAAGGATTTTCCCTTGATAAGATTACTA
A_14_P135767 GCTCTGTTTTGAATGTGGATCACCACTCGGAGTTTACTAATGTTTACAAGGCTG
A_16_P16983965
CTATTGGCTATCAATGTTTTCGGAGACTCTAAGAAGTGTGAGACATTTGAGCAGTGAAC
A_14_P116679 ACTGTTGTATTTGAAGTGTTAGCAAAGAAAGAGAATTCTGGTTCAACAGAAAAGTCAGTC
A_14_P104332 CTCTTGAATGGATACTGTCAAAGTTTATGGCTGATTGTCATATTTTCTTCTGTTTCCAGG
A_16_P18683189
AAAAAATGACAGCCAGAAATGTATGAATGCCGGATAATCACTTAAAGTCTTCACACTGTG
A_14_P122523 TGAAGTCTTCAAATGTTTTGGCATTAAAAAGGGATACACCTAGTCAAAGAGTTGACAGTT
A_16_P58424464
CACCTGATTAGAAATATCCTACTACTCCAAACACCCTATCTGTCCTCTTTATCTTGCTTT
A_16_P02026386
AACGTATCTTCTTTACTCCCTCTAAGAAAAACTTGGCCAAAAAATCATCTACTGTCCTTG
A_16_P16734958
CCTGTAATCATGCTATCAAGACTGTCAGAAACATATTCCCATTCACATATCCCATACTCT
CUST_7320_PI426210822 CACGGACGGGGTTTGATGTCCAGTAAGCCTGAGCAGCCCTGCAAA
A_16_P17614343
ATGTGGCTATATTAGTCCTGGTAACTTAAGTATGACACAATTATTGGCAGTGGTCTTTCG
A_18_P22841538 AGCGTGTTAAGCAGCAGAAGGGGTCCCAAAACGTGTTTAGTGTTTAC
CUST_3656_PI426210822 TATCTGTCTGTCTGTCCCCATCCCCACTGCCTACTTCCTCCAGAC
A_14_P113706 ATGCTGAAGTCCAAATAAGCACACTGCTATTAATCTGCATAGATCTAGGTGTTGCAAAGA
A_16_P40862594

AAACAAATCCTAGCCAAAAATGTTGACTTTTATGCAGAATTACTGACTGCCTCCAAATCA
A_16_P21310040
GCCTAAGACATTTTTAACATCACCCCTAGTAGTAACATTTTGCCTATAGTCCTTATGTCT
A_14_P114885 CCTAATCTGCCTTTTTTTTGGGATTCCTAGATTAAGCCTGATCAAGATGACAACCTC
CUST_4168_PI426210822 CCATTTGGAAGCCAGGAGCTTATCCTCTCCTCTTTGTATCAAGCC
A_14_P108554 CTATACATGCATGTGTATGCATGTCACATTCCTGGCAATTATGTGTATCCTAACAAATGC
A_14_P130663 TGGCAGCCAAATAAAGAAGAATCGTGGGTAAATAGAAGAATGGCTGTGACTATTTTTCAA
A_14_P135568 TTTGTTCCAATACAGCAGATGAAATATTACCTAGATCTTGCCTTGGCAAGTAAGATGTTT
A_14_P109960 GAGTGTCCCAGACAGAAACAGGTTCTTGGAGTGCTGAGGTATAGTTGTATTTATTTTT
CUST_5370_PI426210822 ATACCTAAGTACCCCCAAACTGGCAGGCACATCTGCCTCTGGGGAA
CUST_778_PI426210822 TGCCTCTTCTGTCTTCTGGGTGGGTCTTGCCCCTTTCCTGTAGCC
A_14_P136262 GGTTAAGAATAGTTTAAGGAATAATCAATGAATTGGGCAGTGATTACCTGGCATGATGCA
A_16_P02648927
TTTGTTTCCCTTTTGATGATATCTAGAGAACTACGTAGAGAAGTTATAATGCTGGCCTGC
A_14_P134982 ATACTTGATCTGATATTTGAACTCACCTTTTCAAACACCATTCTGGCATTGAAGACCAG
A_14_P113640 AACTGAATTATGTCCTACTTTTATTTCTTCTCCCGTTTATGGGAATTCGAATTCTCCTGT
A_18_P14131440 CAAACGAAGACTGAAGCCACAGTGTGCATGTAAAGGGAGTCCAAG
A_14_P122021 AAAGAATGGAGGGGGGCAGAAACTTAAAGATATTTATTGCCTAATGACACACACTAAATT
A_14_P126193 CCAGATGCAAACAATCACAAAATTCCTTGGAATTATATCTCAAGAAACACCAACAGGAC
A_14_P121672 ACTTAACAAATACTTGATTTGTGGGGAAAACATCATCCATGTTCTCTTCCTAACAGTTGA
A_14_P125119 GAGAGTGGATTCACTGAGTTTATACAATATCAAGCAGACCGAACTGATGATATTGACAGA
CUST_2445_PI426210822 AATCTATAGATCATCTGGTTGGCTGGATGGAGAGGTAGTTTCCACATGG
A_14_P109783 ATTCCATTATGATGTGGGGAAGATGTTTTAGGGAATTCATGACCCTAATATAGCAGTTTT
A_14_P125866 ATGGAGATCCTTGGCATCATCTCCACCATTTACAAATTTCAGGGTAACTGAAATCT
A_14_P125628 AGTGGTATGTGCCCAAAATTTGTAATCGTTTCTAACGTGATCTGGATAACATCTTAGATG
A_18_P24088604
GAGACCATTAGAAACCACTTGAATTCACATCATGAAAAAAAATCCACACTGTAAAAAAGG
A_14_P139960 ATGCAACCCCTAGTCAGTCTCCCTTACTAGAAGGAGGGGTTTTTAATCT
CUST_1534_PI426210822
AGAAAAGGCAGAAAGTCTGAAGTTTTATTTTCACTCTCAAACCAATAAATAGGAGATGCT
A_14_P123288 TCCACGAGAGGCCAACTTTTCCAATAAGAGGTAGGTGTTCTCTGTTATTTTTTAAATGTA
A_14_P106244 GGAATTTGTTTTTCCACCTGTCAGTTGGCTGCTTCTAATTATAATAAGGGGCTTCTAT
A_14_P103538 GTGGCACTTCCTTGGGGACACTTTTCTGCGAAACATAATTCCTACAAAGAAATAA
A_16_P19833920
ACACCTATCATGTTCACCTTGTTCAAAAAACACTACTTACAGGTATCTAAGAGTTCAGGA
A_14_P126348 CTGAATTATGGGAGAGAAATAAAAATTAGCAAAACATATTTGCAAAGTGGCTCAGGAGGA
A_16_P18231831
TGGCATGAATACTGCAGACTAATCCTACCTTCAGAACATCGTATGTATTAAAAGGAATGC

A_14_P126035 CAGAAACAATTTAAAAGTCACATTTTCCTTAATCCTAAATCCGCGTAGGTCATAACTGGG

A_14_P130519 CTTTATCAAATCCCAACTTACACAGAGGCAGGAAGCATTGGGAGCCAT

CUST_6820_PI426210822 TGTATATGATGGGTACTACCATGGTAGTCCCGACTCTGCCACACAT

A_18_P22839227 TGGAGTAGACACTCAGAACGGCAGGAAGTGGCACTGTGTTGCAAT

A_16_P17506432

AAAAAAAAAAAACTTCTGAGGGGTGAGTTTAAACTGTTTGCCATGTATCATATGACCTCC

A_14_P136415 CTCTCACAAGTGTGTGTTCCTTTCACTGCCTTTATTCTTCATATCTCTCTCTTCTTTTCT

CUST_7325_PI426210822 GAAGTTAGGTCAGTTCTGAGGCTCCCCCAGATGTGGGACACTCCT

A_14_P137777 CTACAGTCTTATTGCCAAATTAAGTTGAGAATCTGTGATCGTTTGGCTTCAGAAAGAGAA

A_18_P26797055 GACATCTCTTGAACAATACCAGGCCTCTTCAGGATTCCCTCGCCC

A_14_P111191 AATGATGTCTTCAGGTGAGAAGTCTCTCACGTCCACCGCAAACTCATA

A_16_P17494836

TCCCTATTTTCACAGCATACCATACAACTACTTTCTACAGAGTTTCCAACATTTTGCAGT

A_14_P103675 TTTACTTGTGGGTTGTAGAAATTACTTCCCATGGGAAACTAAAGCAATTCCTTCTTCAAA

A_14_P129679 CTAGCAGTGAACTGCATTTAAAACCTCAGGAATTGAGCGAACTCTCCCAG

A_14_P200382 CTTTTTCATTGTTGAAGTTTGCTTTGTCGATGTGTGATTTAGGACTTCCTCTTGTTTCAA

A_14_P116734 GTCATCTATGAGATCTGAACAAATAGAGGAATTCTCTAGGACTGTATACTCTCTATTTTG

CUST_1998_PI426210822 CCTAGGATCCTTCCATGTTGTTCTCCCATTCAAGTAGACCTTTTTGCAATT

CUST_4838_PI426210822 CAAAAAGTAATCAGACAAAGGGCCATGTGGCAAGAGCTCTTGCTTCA

A_16_P16191375 ATCAGAATGACTTCACCCAGACTTCTGGACCTGGAACTCATTCTCAG

A_14_P130240 ATTATCATTGAGCCTGGATTCTGTATCTATGGCCTCCTCACTGGTGTGATTCATA

A_14_P115362 TGTTCTAGAGATGCTTGTATAGGCTGTTAATTGTGATGAATAAACGTTCAACCCTCGG

A_14_P100850 AGATATCCTCTGAGAGCCAAGCAAAGAACATTAAGGAAGGAAGGAGGAATGA

A_16_P00685114 CATCAATGTGCTGTGGGGAGATAGGACACGTTTAGAGGTGCTCTC

A_14_P100841 CACTCATTCTTCACTGTTCTTGGTTGTGTATTTTTCAATGAAAACTTTAGAATTCAGGAG

A_16_P03809416

TGGATGATATGCACATCACATTAAGAAAAGTTTTAGTTTGTGTCTAAGTTCACTGCATAG

A_18_P11240836

AGATCCCACCTAAACACAATTTCTTTACAAACCTGTCAAAATTATTGAGTATCCGCTGTG

CUST_4491_PI426210822 CTAGTGGGGGATGGATGAGGGCTAAGGCATGCTTTACCCCTGGCT

A_16_P02510974

TACGAACAATTTTTTGAGGTCTCCAACTAAGTGACAAAGTATAGTTCTGAAATCAAGGCG

CUST_3906_PI426210822

TTTTAAGGACTACAATCCTTAGTTTCCTACCTACTCAAGGTTATGTTTTGTAGATGCTAA

A_14_P137642 CTTTGAAACTCCACCCTTTCTCAGTTAAAAAATGTGCTGCTATTTTTAAGTGGGAGAAAA

CUST_4011_PI426210822 ATGGCTGTGCATCATCCACCCTGGAGTATCTGCTTGCCAAAAATA

A_18_P22841279 GTCTGTGTTTATGTGGGTGCCTGGGATGGAGAGGTATCTAGTTCAATGATAAAATTTT

A_14_P126216 GTAGGAAATAATTTTCTCTTAAGGATGACCTAAGAGAAAGTAAGGCCAGGGATAGGTATC

A_14_P139595 ACATTTCTATTTTTAAAATCTTCAAACTTCCACGGGGGGTGGCAAATGTTAATTCTGATT
A_16_P17228332
AGACTCCTATGACCATTAACTTCTCCAAGACAAATCCTTTGTGAACCAGATACTGAATAT
CUST_66_PI426210822
ACTGAAACATGCAGACACCTTGAGAAAAGTGTTTTGAGTTTTTCTTTTACTCCAAATAAA
A_14_P202320 CTTTCATTTGTGAAAACCTCTGTTTTTCCTAATGGCACCTGTTTATTTATCACTTGGCAA
A_14_P102352 TTTCTGCTTTTGCTTTCCGGTTACTTTTTTAAAGGCTGACCTACTTACTGAAGATTATGG
A_14_P133043 GCTGTCTTTAGTGAGTGAGCAAAACCTCAGCCCTGCATGTTAAAGCAAAG
A_14_P137670 GAATATCTCCTCTAACTCAGCAACCTCCTTATCACTAAAGTTACCTTTAGACCAAGGC
A_14_P112530 CCACCCCAGAGTTATCAACTCGTTGGAAAATGATGTTGAAATGTCACTTTTAATATTTAC
A_14_P136219 CTTGACTTTATAATGAGTTCACCGAGCAGAATGAACCACACTTGAAATCAAATACTTTGC
A_16_P20817692
ACCCTTCAAATATATGACTAAGCCTATTACATGAAGAAAATGTTGAGAATAGTGCCCTCA
A_14_P125383 CTTTTTGCTACACTACATTGCATGTATTTGTTCAATTTTGTGATGAGAGGAGGGATAAGG
A_14_P127306 CCCTGTTTTAAATAAAAACGATTCACTTTAAAGCCTATCAAAGGTCTGTCTGTAAAATGC
A_14_P101110 GTAAGCAATTGAGAAGAGTTTCTTATAGATCCATCAGGGAGTAGAAGAAAGATAGGCACA
A_14_P108012 GTGTGCATATGGATATCCATGTGCATTTTGGGTGGGAGAATAATATTATTTCAAGGAAGA
A_14_P120807 ATTTAGCCTAGTGAATCTTAGGATTTTTCATCGGCCTTCAGTCACTAACTCCATGTCTTT
A_16_P21415914
CCATCTGATTATGCCATGAGTGAATATGTTCCACCTGATAACAAAATGAGTTGAAAGTCA
A_14_P134409 CTTATTTTTGCATCATACATGTCCCTGTCTCCCTCAGTGAGAAGGATCTAGAATATTACT
A_14_P126029 TCTGTTTTTCTTTTTGGCTGTTAATGCTTGTCTATAACATTGGAGCCATCACAAGAATGT
A_14_P107834 GAGGGTGAGTTTGTGGGGGAAATTTCATAATGTATACTTCCAGAATATTTAGAACTTTCC
CUST_3432_PI426210822 GTGCTGTGTGTCACAATAAGATAGGACACAGATGCAAAAGCAGATCGCTAC
A_14_P200663 GACAGTTGTAGCAAAGAGTTGTAAATATCATGGCAATCTCTTTCTCTGGGAACAATGAAA
A_14_P124819 GCTGCATTTCTTGGATTTTTTCCTTCATTAAGTTTCCACTATATTTCATTTTTGTCACTG
A_16_P03236825
CATTGAGTAACCAGATTCCATCTCCAATAATCACATCACAAACAGCATGAAATGTGAACT
A_16_P20258298
GTTTCACAAACTGAAGACTACGATCAATGGGTAAATTATCTTACAAAGGGAAGGATCAGA
A_18_P14789527
ATTTTTGCTATCCGCATAAACCTTTAGTGAGTCTTTCAAAAGATATGACCAAGTCCAACC
A_14_P112213 CTGGGGTAAATTTGGAGTTCATTTTGCATACTGTTGTTTTTTTCAAGGATTGTAGTCTCT
A_16_P39444686
TGCAAAAGTCTCATGATACATCTTTCTACCTCCAATTAATATAGCACTCTTAATGCCAGA
CUST_7427_PI426210822
CACTTGATAAGTGGTAGAGCCAAGTCAGTTTGAAAAAAACATAATCATTGTGCAATGACA
A_14_P109212 AAACGTTTGGCACAAACAGCAAACTAATGTATTTCAAGAAGAGATTCTTGCATGACATGA

A_16_P17683538
CCTCTTTGAACCACAATACATCCCATGTCTTACTACAGTATAGTTACTCTAAGTTAGGCC
CUST_514_PI426210822
CAGTGTTTCAGAATCACTCCTGGGTTTGTAACTTTATTAAAGAAACCCACACAGAG
A_14_P122507 GATGGATTACTCCACAACCAGTGTTAGGAAAACCATTTCAAGGAATAACAGTAAGTCTTA
A_16_P19142464
GACCTCCCAAATAGTAACCACTTACACAGAAACAACACAGATTCCTACTAAAATGGATG
A_14_P201013 CTGTAACTCCATGTCCACACTATACTAGAGTATAGTGTGTTTACAGCAGGGGC
A_14_P134906 TCTACAATAAGATTCACCTTAATCTTACCTCTGGTAAACAGAATTCCGGCACAGAATCC
A_14_P133355 ACCTTATATATCTCTATGCTTTAGAGCCTATCTAGCAGGTTTGCAAGTGCTGAGTGAATT
A_14_P201715 TCACGCTGCATTAATTGTCATTTTTCTCTCCCATGTTCTTTCTCACTTTGATACGTTAAT
A_16_P01584433
AAGCCCTGAATATAAGTTACAATCAAGCCTTAACTAGATCTTTAGATGCTTAACGCCACC
A_14_P131502 CAAGAAAAATCCCGAATGTTACCTTGTAGATAACTTCTGGCAAGAAAGAACAGACCAC
A_16_P19835390
AAAAATCATCCAGTTCTGATTTGGTCTTAGTCTGAGTTTGATAACTGGTTACCATTCCTG
A_14_P119788 TATTCAGATGATCAGTGAGTTGAGTCAGGTTTTGGATTCTTTCTGACCCAAGG
A_16_P34777188
ATAATCTCAAAGCCTCTTTTTAGGTCATTAGTGTGCGTTTAGTGAGATAATGGACCCTCT
A_16_P00797791 CTTCGGCTGTTATTCTCATCAACCCCTCTCTATATTCTGCAAGACATTGAAATCTTTCT
A_14_P137277 CTGCACATGTAAACTTAAATATTTAAACTGAAATGGGATGTCTTATAGAATGATTTGGGG
A_14_P124715 AGTGGCACGAAGATATTACTCAGGATCTTCGAAATCATCTTGTTCACAAACTGTAAGTAA
A_16_P40032194
TCACTCTTGTTTTAACTCCATTATTACTGACTCACATGAACTACTCTTCCAAACTGTTCC
A_16_P19136241
TGTGATTCTACCAAAAACTAGTCAGTAAAACAAGAACTTTATAGATCATTGTCCCCCTCC
A_14_P129294 ATTCAGAAATAGAAACTAGAAAATAGTTAAATAGTTACAAGAAGTTGCAGGGGGGCACGG
A_14_P130943 CCCTCTGAGAGGGCTTAGTCCTCATGAAAATGTATTTGTATTACTCTTGAAGTTTACTTT
A_14_P106540 AATGGCAATCTTGGCAACCAAAATACTTTCCGAAGCCTTTTCCAGGAATATCTGTAAATA
A_14_P124441 GAGACTGAGAAGTTCTGTCTGATTTATCCATACATGAGAAATGGAACACTTTTTGACAGA
A_14_P139680
GAAGATGAAGGAAACATGAGATGGAATACTCAGAGAAGTCTGAAATTTGGTAACAACTTG
A_14_P108829 TTACGTCTTCAGAGTAATTCACCAGAAACCACTTTCTTTCATACAACAGGTTTCACTAAC
A_16_P17148804
TTGAGCAAGTAAAGAGAATACAATTTCCTATCCAGTTCCTAAAATACAGACACCTCCCAC
A_16_P16191449 AGTGAAGAGGTGGGAGAGTGTGCTGAAAGGTGGGCAGAAGCCTCT
A_14_P134859 GATTATACCTGTTGTTTCTCCCTTATCTGCACAAGCAATTATCCCTTAAAGAACTCAAGG
CUST_95_PI426210822

AAGGCAACTTTTGAAGAATTGCCGTTAAGAGGTGCATTTAGCTGTGTCAATCATTTA
A_16_P58424486 CTCATCTGTAAGGTGGGAAGTATAAGACCTACCTCACGGTGTCAGTGTTAAATAA
A_18_P16653083
GAGGATCAGACAGAACATCAACACAATTCCATTTTTCATCTTATTCCTATACCCAGAGAA
A_14_P139570 AAACAAAACCAAAACCTACAGGGACTCTTCATTTCTGGCCTGCAAGGAAT
A_14_P130211 GCACACCAGATCTACCAGTTCACAGATAAGGACAAGGACAACGTCG
A_14_P101456 TAGAAAGGCATGTAATTTGATCTGAAATATTGCGCAGGAACAATATCAAGTCTCAGGCAA
A_14_P110823 TTTTCATTTTCCTACACTTTCAGAGACTAGGCATTGAAAAGACTGACCCTACCACA
A_14_P138147 CAGTTTCTGAAGTTTTCCCAAAGGGAAATAACCATTTAAACATGAGGGTCCTGATAAATC
A_16_P19774375
TGGCAGACTACAAATCTAGATTATGAACATGAAGAATTGATATGAATTCCCACCACAGTC
A_14_P201044 CCTGGAGTATTTGAGTTTTCCCAAAACTCCTATCTACCAATCTATCTGCATTTTTTATGG
A_16_P19432994
ATGGCATTGGTTAGGGATACATTAGGGACATATCTAAAGCATTCCAGTTATGCAACTTTT
A_16_P15718364
AAGACGTTTTAAAGTATATTTTCACCTGAACCACTTGAAACTTTCCATTTTGGTCTGGCA
CUST_7328_PI426210822
TGTTGTATTTACTTTTCTGGATTTATTTTCACAAATATTCAGCTTAGAATGTGCCAGGGA
A_16_P18062630
ACTGTGGGCAATAAAAAAACGAATGATGAGCATTATCAAAAAGTTCCGTGTATAGTAGGG
A_16_P02588096
AAAAAAAAATGTAGATCTGGATTACTCAGTGAATCCTCTAGAAGCCTAAAACAGGGCATC
A_14_P106488 CTCTCCAGAAAAGATGTAGGAACACCTTGCTTACTAGAAAATTTTCTCTTCGTATCTGCA
A_14_P123861 TATCTACACCAGGCCCTATGCTAGATAAACACTAAAAATATGAAAATGAAGGCATAGCCC
A_14_P100542 TCTGCCCAGAAGGACTATTTTCTGAGAGAGTAGGCACAGCATCTATAAAAGAACAAATAA
A_14_P120542 TAAATAGGTGGCGTTGCACCAATTTCATTAATTGATCTGTGTCAATGTTCTAATGAGTGC
A_16_P00685461
AAGAATGTTGCTTGTTAACAACCTTTGAAGGCTTTTTAGCAAAAACAAAGTGTCATATTG
A_14_P115409 ACTCTTCTTCTCTTGCATATCTGTTGTTCAAATTCCTTGGTAATTCCAATTTGCTAGCAG
A_18_P11356424
AAAAAAAGAGTAAATGTAGCATAGAACAAGTGGATCACCAAATCACATTTTAAACAGGGT
CUST_3459_PI426210822
ATCCAACATCACTGAAGGTATGTATTTTTAACTCCTCCTTTAAAGTGGTTTTCCAAAGTT
A_14_P115371 CTCCCTCAATGATCACAATCTAGACAGGGGACAGAAAATTATGAAAATATGACAAACAAG
A_16_P17925571
AAAGGAAAATTCCCAAAGGACATAGACAGTAAAACCAGGTTAACTATAGGATTTGTCTCC
A_16_P39225861
TCACCCTCATAATTAACTCAAGTCAATTACACCATTCTGAAATATCTCCTCTTCTAGGCA

CUST_8939_PI426210822 TGGGCTGTCATGTGATTTTGTTTACCCGTTTCGGTTCAAAGACAGAA

A_14_P125088 CTACGCTGATGCATTTGATTCCTATTGTGAAGAGAGTGATGAGGAGGAAGAAGAAATAG

A_16_P19794233

AAAAAGTTAACTATGTTGTCAACAACATCACTTCCAATGTAGCCATCATCATATCTTTGA

CUST_9874_PI426210822 GTGTCCATTTGCCTCATCCCCTGGCACACGCACACACTCTCCTAC

CUST_8006_PI426210822 GTCCTCAGGCTCCAGGCCACATGCTAAGCCTCATGGCTATACTTT

CUST_4450_PI426210822 CTTGGGCCCGCAGGCTGCCTCTGCACTGTTAGTCCTCCCTGACTT

A_14_P132373 CTTAGAACCAACCAATTTCCTCATATTCCTCCATCACACACACTGGACATATAACATTAT

A_14_P139448 GTCAACCCATTAAGGAACCAGTTCCATGACTGAATAAATGATGGAGAAATTAGGCAG

A_14_P128884 GGGGAGTGTGGACAACAGAGTTCGAATGGTATTAGGAATGTGATCTATCATAACAAAT

CUST_140_PI426210822

TAATATAGCATAGTATTTGTAATGAAAAATTGTATTCTAAACCTAAAGATTACTAAGCCA

A_18_P22840702

ACATATTACAATATTGCCTTTTAGCGTAGAAAAGCCCTCCTTCCTGATGCTCTTGCTAG

A_14_P202209 GAGATTTTGCAAAATCAATAAAGGAAAATACTTATAGAAAAAATTATGCTACACCCTCTA

A_16_P01977922

AAAACTGGTTTTAGAATGTTAAATGTGTAATTCCCTCTGGATAGTCTAGTTGAGTTGCCC

A_14_P126867 ACCAATAAGAAGATATAGATATACCTATGAAAAGACACAGGCTGGTGTCACCACAACTTC

A_14_P115154 GAATGCTACTCAAGTTCAGTTCTGATCTCACCAAGAAAAATAAACGGATCACACTGTCTA

A_18_P22840356 TAGGATGGCCAAGTCTCCATCTGGATAGCCCAGCCTGTGATCAAA

A_16_P01764621

AACACTGGACACTATGAAGATCATAGATATTAATATCACATTTTGCTAACACAGTGTGCT

A_14_P116652 TTCCTCCACAAGGACCAATGTATTTCTTTCCACTCTGGAAAGTGAAGCATCTTATTTA

A_14_P133975 ACTGAGTTGTTTATTTCCCTTACAGGGATGAGAAAGATAGTCAAGGAGAAAACATGTTTC

CUST_282_PI426210822 TGTTCAGGAGATCAGCCTTCAAGAGAACCAGTTTTCAAAGCCCTC

A_16_P00290185

TAGTAACACCTGAGTGAATGAGTAGATAATCCTAAATTGCCAAAATGACTACCTACCTGC

A_16_P35881705

ACAGGTTAGAACCCCTAAAACAGATGTATCCCACCTATGTAATGGTTACTTTCAATATCT

A_16_P40973292

TTCACCCTCCACATAATATTTGATCCCATAAATCTAGCAGTTTATACCTCAAAGGAAACG

A_16_P41646474

AACAAAATTACCAAGACATGCAAATGATGTTTCATATTGCAATGGTCATTCAGACTTTCT

A_14_P138026 CTTAAGTCATACAAGTTTCCACTGCATGTAGAATAAGATCTGGGTCACGAACATTTGATT

CUST_5213_PI426210822

AGTGGGGTTATGTGATTTTCATAGATGAACAATTTTTGCAAATATGTTAATTGAAGAGTA

A_14_P122311 AAACCACTGTTCTATGAAGTCTCACGAGGCAAGTGCTCAAGGTAAAAAAAGAGT

A_14_P127086 ATCTCCGGTCAAGGATCTGTCCCTTCTGCCTATTCCATTCCTAGA

CUST_3393_PI426210822 AGCAGCCTCTCTGTACTCTGAAGACGCATGAATTGCTGACTGTTG

A_18_P12805151
CTGCACAAAGTCAGTCTTTATTGTCACCAATATCTCGCTTAAAAAAATATCCCCTTCCTT

A_14_P120245 TGACATAGGGATTAGATCATTTGAATTTTTAACCCACAGCACAAGTCCTTCAATCGTTTT

A_14_P101579 CTGTATTTATTTCCCTCCCTTTTTATGCCTCTCAAGTCTAGTTACACTGTTTTTCATGGA

A_14_P108066 AATCCATCAAAGCGATATTCTTCCAACCACCATCTTATGTTTGACAGAAGGAATCTTAAA

A_14_P139043 AGAAAAAGTAGTTAATTATTATCTTACCACTAGTTCCTGGGGGTTGTAATACATCTCCTG

A_14_P102042 AGAATGATCTCCTCATAAGTTTACAGTATTCAGTTGCTCTGGGGCATTACCTTTTGATTA

A_16_P00001417
GCGTCTTGAGTTTTGATAAATCTATCCTTTGGCTGATTAATATGACTGAGTGGTGTCTTC

A_14_P112203 TTGGGTGAAGTTTCCTTCTGCCCGTTTTTCTTGACCTAGATAAATACACTTTGAGAAAT

A_14_P112656 TTTAGGGTTCTGATTGCCAGTTATTTTCATCAATAAGTCTTGCAAAGAATGGGATTGTCA

A_14_P123621 GATAACAAAACTCTACCATCAATAAATACAAACATGTAGCCACTGACTACTGACCTGACC

A_14_P110073 CTACCCTTGGCTACCCAAGTAGTCTAAACATTTTATCTTTATCAGGCACATATACAGTCT

A_14_P109330 ATGAACAGGATTAATTTATTTGAATTCATTCTTCAGTCACTTTGCCTTTTCTGGGCACTG

A_14_P122064 AAAGTAAAACTTACAATGATCAGAAAGCATGCTTTCTTCTGGCTTTATCATTCAACTTCC

A_14_P113567 ACATAACACAGCATAATAGGTAGAATCTCCTCATCATCTACGTAATTCTAAAGCCCATGG

CUST_2302_PI426210822 AAGGAGGATGACTTCGCAGGGGCGGTCTATATCTATCATGGTGAT

A_16_P00685506
CTCTTTGATAGTGGAAAAAAGATTTACACAATTAGTTTAGATCAGGTGAGGACCCACTGA

A_14_P132563 ACAATGCTACTGAAGTAAAAGTGCTTGAAGTCTTGTGAGCATTTGGAGAATTACATTAGG

A_14_P116018 TTCTTGCCTCCTTAATAGAATTAATTCCTAGTAATGCTCCCTTGCACTTTTTCAGCACTT

A_14_P126290 CTGGGCTAAGGAACAAAAAGTGGCATATCCTTAATTACCACAAATGATACCTAATTACCT

A_14_P134042 CGGGCGACCCCTTTAAGTTTAGAAATAATTGAAAGGAAATGTTTGAGTTTTCAAAGATCC

A_16_P15310201
GTGTATAAATTGTTTTCAAGTTGGATACTTTCAGACTGGTTCTATCTACCCACACTCACC

CUST_8936_PI426210822 ATACTAGGTCATTCTTTAACTTCTTGCTCCACCTGCTCTATCCCCAGCT

CUST_9563_PI426210822
TTCTTATTCCCTGGGGTAGTAATTTTAATTAGATTCTATTGATTCATTATTTTCTCCTGT

A_16_P19563574
AAAACAAGATCCAGAAATATCATAGGCAATCTCTTGAATGTTTCACATACACAAAGCCGC

A_18_P22841924 AGAATGTTGCCAACATTCTGGATACAAAGAAAAGCACCCTCTCTGTGACTTCT

A_18_P22842284 CTCTTAAGTTAATTTTTCACATCTTGTTTGGATCCTCAGAGCCCGTGCTTCCAAA

A_14_P109687 CAAGAAACCATATTTGTTTGTAGAGGAACAGACAGAAAATTAATGTAGCCAGCCATTCAC

A_14_P136680 CATCAGTGCAATCTACTGGCCAATGACAAGGTGGTTAAAATGTCCTTTAGTAGGTAT

A_16_P15542520
AACCCTTTCTACGTGACTCTACTGAAAAACTCCTCATTGCAAATCAGAACTACTTGTTAT

A_16_P39648255

AAACAAATCAACATTGCTATTAATTTTTTCCCAATATCAGGCTTAGTTACCAAATGCAGA

CUST_3381_PI426210822

TAAGTTGATGAATAATAGGAAAGTAAATTTGTGGGGTCAGTTAAACCACCAGACGACAG

A_14_P103886 AAAAGGCTCTGGATTCAGAAATCCTCTTCAGAGCGAAGAAACATGAAAATATGAATGTAT

A_14_P122439 ATGAAGTTAAGATGTGCATAGAGTTGGTGTGACTTCAAGGTCTGTGCTCCTAAATAAAAA

A_14_P111590 TTTAGGCATGGCCATAAATACTCAACTTTAGAGGTAACAAACATCATGGATGCTTTTCAC

CUST_2186_PI426210822 AGAAGGTGGACTCTTGAAGCACAAGAACTCCCCCAGGCCTCCACT

A_14_P121626

AAGCAAACTGAATTTCGACAGAAGTAGAACGTGTATGGTAGAGAAAAGAAATGCAGAGTA

A_14_P114775 CTTGTTCGATTATGTATTTCTGTGGTATTTTTGTGAATGGAGTTGGAAGTTTGCGGTGC

A_14_P125327 CTTCAGAGAGGCTAGTGAAGAGGAGAGGAATGTAGGGTGAATTTGAAATGTGATTAATAG

A_14_P122031 GGTTAACTCCTTAACCATATGTAACTCCTTAACCTTATGTAGTTTGATAACTGGAGGACC

CUST_8946_PI426210822

ATCAGACTGTTTCTGTTTTTTGGATTTTTGTCCTTAAATAAACACCTTGGAACCACTAGC

A_16_P19431577

ACATTCCAACATTGAACTGTTCCTATAAAAACCATCCCCATATAATCTAGGTTCTCCATC

A_14_P118349 CTGCCCTAACGCACCTGCAAGATATGATACATCCCAAGTTTTATTCAACAAATATTAACT

A_14_P117133 GCAGGGGATTATTTTCTGGTAAAATATGATAAAGGAATGCACTTGACTGGATATCTCAGT

A_16_P58424118

TTATTTAAGTTCTCTGTGCCTAACCAACCTCAAGGGTCTATGAAGAGGACTGAGTTAAT

CUST_5713_PI426210822

ATGCATTCTAGGCTTCTGAAACCCTTTTTAGAAGAAAATCATGGTTTCCTCAAACTCT

A_16_P00871748

AGTTATTCATGTGGCATAATCGGTTTTAACACTTAGTTCAAGGCTCACAAAATCCAAGTG

A_18_P16132459

AGAATAGACGTAAGGATAGTCTTAAATTCACTCAGATGTCAAGGTAAGAAGAATCCGTGG

A_16_P41616529

CAGACTGGCACCAAATTACTTCACTTTTCAGGTATGTTTAGCATTTCGTCATCCTATAAT

A_14_P102534 GAAAAGTTCATCATATAGTGGGAAAGACACACTAGTAAACAATTTCCACACAGGCAAATG

A_14_P112567 TGCCTTTGACTTCGTTTGTTCTATCGAAGCATGTGCAGAATTGTTAAATTTTAATGTATT

A_14_P130844 GAAACCCATGCTGTGAAATAGAGACTTTTCTACTGATCATCATAACTCTGTATCTGAGCA

A_14_P115034 GCAGGAAAGGAGGAAGAATCCCTTTTGAAGCCTGATTTGTGTTGGA

A_14_P110455 GCCCAGGCTAGAAGATGACAATCTACAAACACTTTTATTAGCAAAAGGAGTAAGTGCAG

A_14_P122027 TGATCAAGGTAGTGTTTAACTGCCACATTCCAAAAAGTGAATTCTGATCAGCCTTTTG

A_14_P135675 TATAACTGTCTTCTATGGGAGGAATGAACAGGTGTCTTTGGGACATACATACTTTCTATG

A_14_P130237 GATCGACTTGCACTTCTGCTATTTCTACTTCTCTTGCTGTCTTTTCTTACACTTCAAAAA

A_16_P18159774

ATTCATCTTATGAACTGACCCCATAAAATTCTTCATATCAAACAGTTCTCAACCACGTGG

A_14_P118507 CTGAGCAAAAGACATCAGTTACTGGTGTCTGTAGATAGGGTTGTTTTGTTTGTTTTTAAC

CUST_5569_PI426210822 AGGGTTCCACTACTTTAAATGCCTTGATCTCTTCACTGAGCCCATGTT

A_14_P138901 GAGAAATGAACTTCAACTGGTTGATCTTCCAACAGGTTTGTTTTTAAAGATCCAAATAGG

A_14_P125262

TAAGGATAAGACAGCACCATTCAACAAAGAAAAGGAGAGAAGGAAAATTGAGATTCAAGA

A_14_P104098 ATATTGTGGGAGAAGAGAAAAGGATGGTTCACAAGAACAAGGAAATCTTCTTTTCATCTA

CUST_6521_PI426210822

CACATTGCAGGGGCTATTTACATGTATGATTTGCATCATTGATTTTATGATACATTGTTG

A_18_P16624617

AAGAATGGTAAATGGAGATGCTTAAAGTTAGTATGCCTAAAGTTAAGATTGGTCCTTGCT

CUST_8590_PI426210822 ATCTGTCATTCCTCTCCACACCCTTGGGACCCTCAAATTGTTTCT

A_14_P106547 CTACATATGGTGATTGAGAAAATCCAGGTATGCCACTGTGCACATTACTAACATCATAGA

A_14_P139233 TTGTTTCTAATCATAAGGCCGAGTTTCCCATGATTGGTGTTTTAAGTGGAATATTGATGT

A_14_P139761 CTATCACTTAAAAACAGCCAGAAACCTAACTGCATGTGTGACTGAAAATCAAGTTGTTAA

A_14_P127869 TTGTCCACATGATTTTCCTACTGTATTTGGGAAAATTTCTTCCTCGACCAAAATATGGAA

CUST_1773_PI426210822 TGAAGTTCAGATGGTCAGGTAGAAATGAGTGTGATGGAGTCTTGATTTTCCA

A_16_P38839881

ATCCCATTTACAGAACTGATCAAAATACTGTTACTATGTCCAAAACTGTTATTTGTCCCT

A_18_P14131930 GCAATCCCAAGTCTGTTTCAAATCTTTTCTTGAAACCCAAGATCACCACAGATTC

A_14_P118068 TGTCTGCTTTTCAGAAAACCAAGAAAAGACAGAAGATCAATATGAAGAAAATTCACACCT

A_14_P106013 CCTTCAAGTCTTTCATTTCCCTCTCTCGGCTCTGAATGATCCTCTTGATATCATTA

A_14_P202368 GATTAACAAGAATATTAGACAAAAGACTAAGTATCGAAAGACTATATACCAAGAGATACA

A_16_P17556305

CCTATATTTCTCAGCATTCAAACATCATTTCCTTTTAACCTTTTACAAATAACAGCCAGC

A_14_P137171 CTGATAGTTTAGACTAATAAATGTGCAGCATTCTCTTGGTGACTGTTATTTGTCCAGCAG

A_14_P136283 GCTTCCTTTGAAATGATTTCTCTCAAGAAAATTGTCAGTGTTTACTGAGAGCCTGTATTG

A_16_P02237237

AAATAGCAACAGTTCTATGCTCTGTGTAAAATCTGATGAGTTCATCTTTGGTTCTTCCTG

A_16_P20390381

ACCAGTTACCTGCATTCTAACTACTACTAGACATCAGATAGCACTTTACATATCATTACC

CUST_7155_PI426210822

GGTCCTTGTTTAAGAAAACCAAATGTAGGTTCAGAATAGTTTCTTCTTTCTTCCACGTC

A_14_P103583 ACTTTGAGAGGCTTCTACATTTTCCTTTGTGCTATAGTGATGAGAAGGAAATGCTTTAGA

A_14_P125559 AGTAGGAGTCAGTGGCCTTTTACATGTAGAGATTGTTTATTTAGCCCTTTTTAAAAGGTC

A_14_P124224 GATTGATGTTACAAACCTCCCTACTCCTCGAAAATTCCTTGATACCTCTCACTATTCTAC

CUST_4596_PI426210822 GACTCAGGAGTCTGACCAGGGGCAAGCAGGCTGCCTCTTGCTCCT

A_14_P129572 GTTCTGTGTCTCTAACAATGTGTCTCTGCCTTCACTGTTATTACCACTAGTTTACTCTTT

CUST_10390_PI426210822

ATTTTGAAAAATGTCTCCACAGGATGTTTTTCTTGCCAGTGTTTTCTCTAATCCGAG

A_16_P19337259
GCACATTTATATTTCATGGTTTCTGAATTCAAACTGCAAAAATATCCAGATGTGGTCGGC

A_14_P137499 TTCTGTATTAAGTCCCTTTCTATTTCTATTCCCCTCACAGAGTTTGCCCTTTCCTAATAA

A_14_P126807 TGAAAGTGTGTTAAATTGAATTGGCTTTAGGATGTGTCCCCAAGAAAAATGGAAATTCTC

A_18_P10460934
AAAGTGCTAGCATTTCTTCAAATAACTAGCCAGGTTTTAATTGCTGTTAACCCTAGGTTA

A_16_P01062465
TGCATGAAACATGTTAAAACATGTCAGTATTAACTGTGAACTTTTTTTGCAAGGGAGGAA

CUST_2681_PI426210822 GATAGGCACACGGTGCTCTCCCATTGAAGGTCACAGGAAGCGATC

A_14_P120924 TTCAGTGATTAGTGGTAATGGTAAACACTGGTGTGTTTTGAAGTTGAATGTGCGATAAAA

A_14_P112396 ACACACAGATCAGTCATATTTCCTCTTCCTAAAATCAGAATTAGGCAAATAAAGACTCGG

A_14_P124042 ATGGAACTTTGTTCTTTTTTTCAGATCTTTCTTACTGGCTGCAGTTTGCTTCACTTCTTA

A_14_P101955 AACAGAAAGGGAGGTCTTACAATGAAACAGGTTTATGATTAAAGATGTTACAATTAAACT

A_14_P112269 GATTTTATCTTCTTTGCTCCATTCTGTTCCTTCCAGATAATCGTGTGTTCTTCATCAGGT

A_16_P16547515
AAAAAAAAAAAAAACTCACTGTTAATTCACAAATGCCTATTTATCTCTAACCATGTGACT

A_18_P12262739
ACCATATGCTAACTTACAACTTTTGCTAGGCTCTAAAACTATGAATATGCCCCTTTCTCA

A_14_P131185 AAATTTTAGCAATGCATTGTGAGTAATTTTCTTCAGAAGAGCCAGCCAGTATTTCTCCTC

A_16_P20097442
CCAAATTACCCTCATAACATGCTACAATTTCCACACTATTTCACTGGTTAACTGATGGTT

A_18_P22840322 TTTCCTTCTTGGCTGTCTGTGGAACCTGTCTCTACATTCTAACCCATC

A_14_P115740 ACATCACATTAAAATATCAAAAATTGCAAATAAAGCAACAGTCCCTGCTGACCACCCTTA

CUST_593_PI426210822 AATTAGTGGGCGATGGAATTACCACGGTCCAGATTCCGTCAGAAG

CUST_3990_PI426210822 ATGATAAAATGGTGTCCGTTGGTGAGCATCTTGGTGGTGAGGAAGTTTATA

A_16_P20370483
CAAACACCGGTGTAACTTTTTGAAGATAACTCCCATTTTTACTCACGTCTCTTAGTCTTA

A_14_P137469 CAGGAACAGTGTATTCATAATGAATTCCATAATCTTGGTCGTGAAATAACAACACCTGGA

A_16_P15922762
TGTGAATGACAAAACCATGAAGTTCTCCATTTAGTTCTCAACAGACTACAACTGATCTTG

A_16_P02046323
ACTTTTTCAAAGCACTAAGGATTCTTTAACTTACACTCTGAACTTCCAATAGAGCAGCAA

A_14_P104508 ACATACATTCTTTTCCCTCATGAAAATGGAAACAGATTATCCATCTGGTTCTGCATCTTC

A_14_P139609 ACTCAGTCTGCAATGATCTATTTTTGGCTTATGTCTTTTAATACTACTCTCTTTCTCTGA

A_14_P111432 ACCAATCCACTCAGAAAGCATCACACTTCTGCATAACTACAAAAATATGGCATTGATATC

A_16_P21613011
ATCTTGGTATCTGCAAACCTTAACAATGTCATAGAATGCCCCTTTAATGTAAACTCAGAC

A_14_P120913 TGCTCCAGTAACTTAAAAGTCATTTCCTTAAGAAAACAGATGACAGACAACTTGAGTGAC
CUST_503_PI426210822 AATGATTCTGCCAGGGACATTTGAAGGGCCTCTTCTCACACCAAG
CUST_1859_PI426210822
TAGGTCCAGTCTCAGATATCTTTGAGGCTTTATTCATGCCCCAAATTATTCTCGTATTTA
CUST_1600_PI426210822 TGTCCTCTGACCCCGGCCTGCTGTCTCCTGGCTACTTTGGACTTT
A_14_P202121 GTTTTAACTGAAGTGTGAGAAGGAGGTTTAAGGCAAGTAGACAACATCCTGTTGTTG
A_18_P14606538
TCCCTACCTTTGCTAGTCTTTTTCCATGTAATTACGTAAATCCAAACTACATAATGCTGG
A_18_P17042647
ACCTCCTACTTAGACAATTCCTTCAAATTTCTTTCCCTTTCTCTCAAGACCTATAGATGT
CUST_3144_PI426210822 GGCCTCTTCTTGCTAGGTCTGTTCACAAAACCTTTCCTTTAGGTTTCTTT
A_14_P138991 AATATGCTATCGAACACCAAAATGCAGGTTCTGAGTAAGTAACTGGCTGTAAATCTGAAA
A_14_P100996 ACTAACTACTCTCAGATTATGGAGCGCTCTTTAAATTATGTGACTTACCAGTTTTCAGTC
A_16_P00930657
ACAACTTTGAATTGTCTTAACCTGAATCAACAAACATCATTGTTCCTCACTTCTCATTCC
CUST_2351_PI426210822
TATCTATCCAGACCATCCTCTTTTCATTAGAAGTACATAACAGGAGATTAATAAACTTCG
A_18_P22839965 ATTGAGAATATGTAGGGGGACGAAGCCAGGAGTCCAAGGACCCCTT
A_14_P115201 TGTTAACCTCTTACTTATAAAACTCTTCCCCTCTTCCTTTCCTTTTGTGAGATTTCACTT
A_16_P00685387
GCCCCTAGTTATGATATTGCTCCTAGGAAGAATAAAATCTGCTTCACAATGTCCTTTTTG
A_16_P03323884
AGCAGTTAGAAATTCATCAGTTACACTAAGCAGCAAACTTTTTCATAGATATAGAGGGGT
CUST_1246_PI426210822
GCTTTAAAATTGTGATGGAAAAACTTCCTATGGATTCCAAAGATTTCTGTACCACTCTAT
CUST_7824_PI426210822
CTCTTTTGGCCTGTCCTCACCAAGATCCAACCAGAATTTTGAAATTTTTATTTGAC
CUST_4909_PI426210822 CATCAGTGTCTCTCTAAGGGAGGGCGGCTGGTTAAAAATCACATG
A_14_P109625 ATGCTGATTTTTTCCCAAGTAAGGATTAAATATCCAAAACAAAATGTATAACTACACTTG
A_14_P107155 ATGGGGTGAGTGGAGAATAGCAATAAAGAGGAAGAGTAAGTTTGCAAAAAATAAATACTT
A_14_P124246 ATGATCCACATAAACTGTATCACAGAATTAAAATCTCAGACTCTGCATCCTGTTTAGGGA
CUST_8083_PI426210822
AACATGACTGTGATCTAATAGAAGCCTATGCAAAGCATAGATAGGGTAGACTTCATTCC
A_14_P103352 CAAAGCATGTCATCTTTTAGAATACTTACGGTAGCCACATTTTTTACAGCCTGCTCTGA
A_16_P58424109 AAATGGTCTTTCCAAACTGTCACTTGGATCATGCTTCTCAACTGTGTAAGACTCT
A_14_P134807 CTACAAACAAGAATCTGACTTGTTGCAACAACAACAGCCATATTATTCGGATTTAGCCTT
A_14_P117886 CAGTTGGTAAATAGATTATGACGTGAGCAGTGGAGGATTTTGTGTATCTTCAGAGAATAG
A_16_P40586803

TTCCTGTAAATATTCTTTTTCCCTAACAACCTTTAATATGAGAGACTGGCAGAATGAGGG
A_14_P108100 GGACTTTGTTTGTGTTCCAGGACAAGTGTTCACTCACATCTGTAAAAACAATTTTAAGAA
A_14_P127868 AGTAATACTAAAATCACTACAAACCTGAAGAGATGCTCCCCCATACTATTGCAAATCACT
CUST_9448_PI426210822 CCTCAGAGACACGGGAGGGTGTGGCAAACCACGAGGCTGAGAGTG
A_16_P01523228
GCCTGCACTTATGTAGAGATTCGTTGGACATTCTTGTTAGTATCTGAGATTACTTTGATG
A_16_P00251828
AGACCACCAAAAAGCAGATTCTAATGAAAGATGAAATTTCAGGTAAACAAAATGACTGCA
A_14_P132467 AAGCCACCAAAAAACTGTAAGCCTCAATCAACTGAGAGGAAAGCACAAGAG
A_16_P30952533 TTGCTGTGTGTGTTCCACACCCACAGGTCTACAACACTGGCCCAA
CUST_9557_PI426210822
TACCTGTTTGTTTGTCAAAGCAGAAATGCCCAACATTTTGTAAACACTAATTTTTTTATA
A_14_P118725 CATACAGAGTCTTGTAAAGGATCCAAAAGAAAGAGGGAGCAAAGGTTCTGACATC
A_18_P22840891 ATCATGAGGAAGGTGGTCTTCGTGGGGTGAATATCATGTTTCCTC
A_14_P136704
ACCAGTCTATACAAAGAAACTGTAAAGAGTTGACAGTTGACACTAGACAAAGTGCAAACT
A_14_P106260 GAGAAGTATCTTGTAAGGAAGCAACTTCCATAAAAAGGGTCAGAAAGTCTGTTGACC
A_16_P18088378
AAATTAACCTTTGGTAATTGGACGTCTAGACTCTTTCTCAAGTTGGATAGTCATGCCTAG
A_14_P130439 ATCATTTTTAAAAAATGATCCCTGACTAGAGATAGCATGAGATTAGCAGGGCAAACTGTC
A_14_P126304 GGTTTCTTCCTTTTTTGATTACTCAAAGCCATGTTTTTTAGTGTCGCACACATTTCTACT
A_16_P40100643
AATGCTAGAGTTCTAGATGACGCATTAGTTTAAATGTCAACGCTTAATGTCTTCACATGT
A_16_P19206154
CTCATGACCTACAATATTTAAGGCTCTGTTTGTATTTCTTGTAACCCATGCTATCCTGGT
A_14_P118168 GCATCTGAAATCTCAAACATTAGGGATGCAGGAAGTTTTACAGTAGTTTCATTAAGACCT
CUST_8914_PI426210822 CCTGCACTCTTGACTTTCCAGCATGTTGGCCTGTCACACGCAAAG
A_16_P03057036
CACATCTTTATGCATCTCTTACTTAAGCGGAATCACTTAAAATCTCTTATCACAAGGGGC
A_14_P121375 AGTCTATTCATTTCAAGTTACATGATGTGGGATAGAGTCAGTTCATCCTAGAAAGCCAAA
A_16_P16947961
AGGCATCTAAGGAATTGTTTTCTATCTTAGTCACCAAGTATGTCAGACACATTGCTTACA
A_16_P18850353
ATAATATGCTGACTCAATGGAATTCTCTAAACGATCTTCTGTTGTGAAGATAGTAGCCGC
A_14_P126709 TCTTTTCTAAATCTATTGTCACAGCGTTCTTAACTGGTCTCCATTCCTCTTTTGGTCATA
A_14_P135884 GAAAAGCCAGTTCTTCCACATGTAAACTACTTGAACTCCATTTCATCTTTTTTCATACCA
A_18_P11783665
AAATGAGAACAACAATAAAAGAGCCAGATTTCTATCCAGATTTCTATTGGGTCTCACTCC

A_14_P107770 ACATGGAATTGTCTTGCCAGTGGTTTATTCACTGAAAACTTATTGTTAGAACATTCTGGA

A_18_P16294807

ACACCTTCTTTCTTCAAATCTCTAATTCCTCCCTAAGTATTGTCTAAATAGCCCCTCAAT

A_14_P130565 GAAATCAGTTGTAGGTCATCCTTTATGATGGCAACACACTGATGATGAAATTGTTGAGTA

A_14_P200291 CAGGAGCAAGAGGCATTGCATATCTACTGTCTTTTAGCGAGGACACTACTC

A_16_P19849337

ACCCTTCACAATATTCTACAGCATGTCTTACAATACTCATAGATAGAAACTACATGAGCA

A_14_P128666 ACATAAGATTTAAATAATGCACCTATTTAAGACATGTTGACAAATTGCGGGTCTGGGACC

A_14_P128420 TACCCAGCATATGCAGATCTTTTATATTCAGAATGAATAACACATGTCCCCTCTATCTCC

A_16_P15117427

TCCTTGTTGCAAATGAATACACACTACCCATCTACATTCAGAAGTCAGAAACCTAGAAAT

A_16_P19014912

TACACCCAATGTTCTAGAACACCAATCAGATCCTAATGCTTCATACTTTTTAACCTGACT

A_14_P105669 TTTGAGAACGATCTTTAATGATGTCACTGCTATTGGCTATGCATATCTGGAGTAGTGAGA

CUST_1143_PI426210822

TTGTTTTTTAGGGTTTCTAATTTAAATGAGAAGTAAAAGCCAGTCAACAACCCTCACAAA

A_16_P15908198

AAACCAATGAGCATATGTCCATGAATTCCACTATTACCCTAACTAACCTGTTCAGGAATT

A_14_P123354 TAATGTGACTATGCCTCATTGCATAATCTTTCTCCACCTACATATCTTGTTACCTGTCAC

A_14_P135372 GGAAGCATTCCATCAGTGGCTGTATTGAACAAGTAATGTATCATCAAGATATAATCACCC

A_14_P130244 CCCTTGCCTGTTATCAGTCATCTTTATGCACAGTTTGGAGTCATGTTTATTTTTATTGTG

A_14_P118415 TTCTCCTGGCTGGTGCTTATGATTGCAAAACTTGGATATGCTGTTTGAAAAAGAATTAAA

A_14_P137058 GGGAAAGGGGAGAAAATCATTTATAGAATCTTGAAAAGAGGCTTAAGTTTTAGTGCTCCA

A_16_P00120622

CCCTTCCTGATTTGATTTCAGTCCTTCATTGTTATTGACCTAGAGTAGTCACCATATTAC

A_16_P01855516

AAATTCCACCTACACAGAAAAAGTCAATCATGAGTTAAAGACCCTACAATATTGCATGCT

A_14_P119671 AAGAGAAAAATGGGACATTTTCAAATGAAGTTCTTTTTTTTGCAGAACTAAACACCCTGG

A_16_P02837514

CCCCTAATCATGCTACTAAGAGAAGAAACTGTTTTATGACCCTTAGGTATTATGCAAACC

A_14_P109837 CTATTTTTCTGCCATGAGACTAGAGGTTGCTTGAGAACAGGAAATTGGATCTCTC

A_14_P105805 AGGAAGCCTTTCTTTTGAGAAGTCTTAAAGACTACCCATCCCAAATTCTAGGAAAAGA

A_14_P137920 GTTTGTTTTTCCTCTTTCCCCATCTCCTGGCCACCTGAAATTTAAAAAGAAAAATAAAG

A_16_P01908964

TGGTCTCTCAAACAAATCTTACACTCTTTCAACTAATCGAATAACATAATCCAAGCCAGG

CUST_3833_PI426210822

TTTTTCTTTTTAACTGCAGAAGGGAAATAATAATGGGATAGAATAACTGCAGGGAGTAGA

A_18_P22841720 GTTAATGGCCACTTACTGGAAAATGTTACTAGGCTCTGTGGTGAGTGTTGTTAGAAA

A_14_P113497 CATTTCTAGCCCAGTTGGGTTTTGATTTATATAAGTAGTTTAGACCTTCTCTTCATAATC
A_14_P118696 TGCTAGGAGAACTAAAAAGGACAATGTCCATATAAGCATTTCCCAGGAAAGTTACCATAT
A_18_P14137185 CTCTCCATGCCCTGCCTGACCTTACCTTTCTAGACCCATCTTCTG
A_16_P19722500
AGGAGACCACTGGAATTAATGAGAAACTTTCTGGAGTTCTATGCATGAAAGTTGCAATAT
A_14_P139637
ACATGTAATCTTGGAACAGGAAAGAGAGCACAAAGCCAGAAAATGAAATAAACAAGTCAG
A_14_P129225 CTATATCTCAAAGCAGTTGAGAGAAGAGGACCTATATGAATGGTTTTATGTCATTTTTTT
CUST_409_PI426210822
ATCAACCCATAGCATATGTGAGTACTAAAACCGAAACGGGTGCTTACATCTAACTAAAAA
A_18_P22840304 CTTTCTGATAGTTCTGGGACTCGTGTGTCCTCCACATAATTAAGCAGTCTGT
A_14_P138724 CTTTTCCTAATGGTTTAAATTCTGGAATTACACTGGGGTTCTTTTGCCTTTTTTAGCAGA
A_14_P109148 CTTACAGGTATGCTATTAAAAAACTACTTATCCTCTTCATCCTCTAGGTCCCACAAGCAA
A_14_P131650 AATCCATCATACATTAGTGCCATAGAGTTTAGTAACCGTCCAGCAAGTGTCATCACTTTT
A_14_P100056 TGTCCAGGTATCGAGGGTTCGTAATGGATTTAACACTTAATTTCCTAAGGGGATAAAAAA
A_14_P118765 CTGGTAATGGACCAAAGACTTCCAAAATATATGTGTAATGACCTCCAGATTTCTTTATAG
A_14_P200158 CTTATCTCAAAATTAAGGAATCAAGAAACAGAAGGCCAGGGAGTGTTGGTTTGGT
A_14_P107286 ATAAATAATAAGTGGATAGCCATGTTTCATGAGATTTTTTGCCATTGGATTCCCCATGTT
A_14_P109068 AATGCCAACTCACTCTACTAAAAGGCTATTTTAAAAAAACAGTTTTTCAAAGACCACCTT
A_14_P104845 AATGCTGCCTAGTCAGATATCTACATTCTGATACCGGAGAAGGATTGTCTCTAAATATTG
A_14_P105677 CAGCATCATTAAGATCATGGCTATTGTTAATGTGGTTCTCAGTATCTGGTAGAGCCTGT
CUST_1005_PI426210822 GTAAAGAGGATAAAGTCACCAGGCCTGCCCCAGGGGGTGGGTTTT
A_14_P114107
GAAATGACAAAAGAAATTTTTAAAAATTGAGTTTAGGAAGGTTTCCACCAGCAGAGCGAG
A_14_P137429 TGACTTGTGCCTCCATCAGTAGAATTTGCTTTCTCTATTTCTGGTCATAAATTGGAACTC
A_16_P00640078
ACACTAACCGTAATTTTTAGTCCATTGTGCTATTTTGTAAATCCATTACTCCCTACATGT
A_14_P100453 GTTCAATTGATGGGCCAAACTGGAAGAACATGGAGGATTCATCCTAGAAAAAG
A_14_P121379 CTGAAGGGATGGCAGAGAAATCATCCCATGACAACCCTGTATTGTTCTAATATATATTTC
A_14_P105378 AGAATATATGAGGCTCATACTCACCAGCCGAAACAAAAGATGACAAAATAGATGCCAAAA
A_14_P200019 TTGGTAGACAGATTCTTTGGCTGAAGGTTGTTTGGAGATCAAAAAGTGCAGATAGAAA
A_14_P200362 CCATTCATTCTGGCTAATTGATTACAAGAATAACTGTGGATACGATCCCTTTAGAACCTG
A_16_P17474647
AAAACAATTTACCCTGCACATTACCATTAAACTTGAAATCCTCCTAGACAATACTGTGGG
CUST_3462_PI426210822 GTTGCCATCCCCAAAAGGGAGCTTTTAGACATTGAGAGAGGTGATT
A_14_P118350 ACTGTAAATCCATTTTACCGTCTTAATAGAACTCCTCTGTTTTTCTTCCCAAACACTACT
A_16_P17087915
GGAAAAAGAGTCAGAACCTCAAATAATTATGTTCTCCTTTAGGCTGCTATCCTATACGTG

A_14_P125408 AACTTCCCCCCTAAAATATTCCTTACATGCTTTTGTAAATAAGACCGGGAAAGAATTTTG

CUST_7994_PI426210822

ACATTTATTAGATAGAGCCCACAGAAGTATGAACATTGGAATGTTGGTTGTTTTCCCAG

CUST_895_PI426210822 TAGGCAAGAGACCAGTATCCCCAAGGAGCACAATATTGGATTCCAA

A_14_P127200 TGGACTTTACCAACATAACCAGAAATGATCAGATTTTTGGCAGCAGACTCACATTACTTA

CUST_1577_PI426210822 TGCTCATGACTCTCTTAGGGGATGAGTGTGCTTAGTGTAGGGCAT

A_18_P14670776

GCAGGGAGAATTTCTTACAGTGTTATTTGATCCACATTATGAATTAGCCACCCAAAAGAG

A_14_P123710 TCCTTTGCACTTTAAACACTGAATTGATAGGCGGTGATCTTCTTCAGATAAATTTGGTTT

A_16_P16689429

AAAAGACCCAAATACACTTTTGACCAACTCTAAGAGGCTTCATCTTCTGCTAAATCTCAT

A_14_P125089 ATCAACATGGAGTTTCAGTCGAGGTGAATTGCAGAAATGAATATGCAATGATTGGAAATA

A_14_P100690 AGTTGTTCCAGAAATCTTCATTTTAAATAGAAGTGTTTACAATGCCTTTTTTCCCCCAGT

A_14_P103795 TTGAGAAAGGATAATGAGAACAGGGAAATCTACCATTGGTGACAACAGTGACTTAATTAC

A_16_P39088420

AACCCCTCCTCTAATGTAATACCCTTAGTTGCAATACTCTGATCTCATGGTTACTGATAT

A_16_P01527676

GACTTCGCTCTATTTCAATGATATTCAGGTTTCGAGAGTAAAAAAAACGTTGGAAATGGG

A_16_P16007524

AAGTGCAACTTTTCTCTTGATTATCTTGAAATGCCTTATCATCTCCACATTTGCTGTAGG

A_18_P12784997

AATCTCCCTGAAGTGAATAAATCCACATATTCAAGCATTTGGATTACAGTTAATGGGGTG

CUST_5175_PI426210822

TAGCACTGTATCCAGCTCTTCTCACCTCTTTAGGAATTTTAAGCCCACAAATTAAGTA

A_14_P123897 GTATGTGCTGAATCTTCACAGACTTGTCAATACACAGGCAGTATTCTAAAATAGCACTGA

CUST_6044_PI426210822 AGTTAAGTGCTAGTGGAAACCAGGGTCTGAATTTGGTTGGCAAAGG

A_16_P36501692

TGCCTTAGACATTTCTACTAAACAAACCTGATACACCCTAACTCAAATGTTATTGATACA

A_14_P111405 CTTGCCTAAGGCCTATTTACAAATAATGTCTTAGAGTGGGTCATTCCTTTCAACCATTAT

A_18_P12262821

AATTGAACAGATCTTTCAAAGTCACCCATAAAATGCACAGTATACATGTTTAGGCTGACC

A_18_P16129397

GGATTGGTTTGAGCAATACAATGGACTTAATTTTTTTTTTTTTAACCATACTTACAGTCA

CUST_3614_PI426210822

ATGAGTAAAGAGTTAACGTATTGAACTTGAAAGGAGAGATTTTTTTCCCTCTTGTAATAA

A_14_P132256 TCCCAAGATTTCTCATGTTTTCCAAATTTCATTTCCTTAAAAAGCCTCTGCATAGCATTC

A_14_P112267 ATCAATCAAACATAAATAGAGAGTTGAATTGATCTTAGAGGGAATGGGATGCAGCAAAGC

CUST_8354_PI426210822 TCTAGAGAATGTGGCAGTAAAACATTTGTGGGCTCTGACAAACAATGGTCTT

A_14_P109736 CTCTTTTAAGAAGTAAAAAGAGAAAACTGAAGTCACGTTCACATTCCCACTACTCACTGA

A_14_P105568 ATGTTAAGGAATAAACCTCTTTTGATCAATGGGAATTACTGTGGGTCTAAGCATCTGATT

A_14_P122173 ATGAAAAGACCTAGCAATACCAATGTGGAAATGAAGAATTTTAACCAAAAGGGGCTATTC

A_14_P126566 AGATAAGTAAGTGATCCCCAAAGTTTCCAAAGTTCTGTGCCAGAGAATGTGG

A_14_P127744 AGGCTTTGTGGGATTCAGAAAGAAAAGAAAATTTGCTCTCTCAGCAAATTATGACATCAT

A_14_P104924 CTGATAATGATTTCATCGGTTACCCATTCCCTCTCATATCGTTATGTTATCAGGCAAATT

A_16_P58424607 CTAACTCTAGTGGGGTCCACAGTCTACTGGGGAAACTGGCATTAATCAAATAA

A_14_P135597 CTGCTTTCTGTCCGGGTAATTCATCCTTCTCTTCTCTGCTTAAAAAATCTCCTTTTTAAA

A_16_P17209279

AAAAAAAAAAAAAAAACATGTTATTTTGAAACCAATGTTACAGATGCCTATCCAAAATGGA

A_18_P22177598

AGACTAGCCTATTCTAAGTTTCTCAGATCCATTTTGCTCTATTACGTTTAAACACCTGCA

CUST_6225_PI426210822 AGGTTTTATGTGGAAGCATCTTTCCAGCAGGCACAATGACATGGCTATT

A_14_P109759 GAAGAATCGTAAGAGGATGTGATCAAGGCCAGAATTTTAATCAATCTAGTCTTTACACAC

A_16_P21222671

TCAAAAGGCTTGTGATGTTATGCTCAACTCTACACAAAACTTTGACATACACCTTGCTAT

CUST_7101_PI426210822

CAAGTGAACTCAAATAAAAGGAAGAGCACCTATGAGAGCTTTGCCTTTGACTG

A_14_P139392 GTTTTTAAAATGGTTGCCTGGATACTAAACCTCTCTCTGTGACCATTATACACGCTTTAT

A_16_P21531529

AGACAAAAAGGTTATTTTGCATGGCTCATTCAATAAAAGGGAATTGAAACGTGAACTCAC

A_14_P118480 AATATTTGTAACTTCCAATATTCTTCCTCTGAGAGAAACTTTAAATCCTTTTGGCGCTCC

A_16_P16281066

AAGGGTATGCCTTTTATTATTCTCCCATAACACCTTTAATTCACTTTCATAAACTCGGGC

A_14_P129321 AACATAAGTAATTTCTTCTCCTTGTTCTCACCACAGTATCCTAAGGAATCCTTTCAAGAC

A_16_P36230813

CTGCCATTGGTGCTAAGACTTTTTTGAGAAGCATATAGAAAGTCACCTAGAAATGCTTAC

A_14_P202354 TGACAAGAGGTTGTTTTTGTCTTTGTATGGCTACAACAGTAACTTTGGGAAACCATTTAA

CUST_7371_PI426210822 CATGTGTCATTTTTCAGCAGTTAAAAATCCATCAGCTGAGGCCACACAAAGTT

CUST_7275_PI426210822

CTTGGAAGCCTTCATTTACCATCAGTTTTTCTTTCAAAACTTCTGCAAGGGCATAA

A_16_P19831336

AAAAAACACTCAGAATCTTCAATCCCCAATTTCTTAAAGAAGTAATTCTCTCAGCAGCCT

A_16_P41201955

TCAAAATTTTCTATAGATCCTTCAACTAAGATAGCCTTGAACTGAACTGAATGCTTAGGA

A_14_P109659 CTGCATTGTAAACTACAGCGGATGAAAGGCTGAAATATAGCAGACAGCAGAGTTAG

A_16_P01472816

GGTTATCAGTAAGTTGCATAATCTGACATATCTCCAAGTTCTCCAATACTACCTAGCCTG

A_14_P201155 CTTGCTGAAGTCCAAAGATTTTTATTGCTGCATTTCACACAACTGTGAACCGAATAAAT
CUST_8315_PI426210822 TGGTGTGTGTGTGAGATCTGTAACCACACACAGAATGAATCCTTTTCGAT
A_14_P200563 TATTATAAAAATGTAACTAATTGTGATGGTGGCAGAAGCCTCACAAGGTTTGATGCCTCT
A_18_P11799120
AATCCAACTCCTGATATAAAGGCATGTAGTAGTCATGATAATCGGTTCACTATGTTGAGG
A_14_P119751 GTTAAAGTATGTAATTCCATCTGATCCACAGACTGGCTCATACTCGTGTATTTTACAACC
A_14_P200073 GTTCACAAAATGGTGCCCTATATGGGGTATTGGACATAGATATTTTCACAATGTCAGTAT
A_14_P103115 GAGAAAGGGAGGTCCAACTCACCTTAGACTTCGTCATTTTGTCCTC
A_16_P02616219
ACACACTTGAAAAATATTAGTTCTTCTCCTCACTCTACAACACTTTAAACCCAAACCTCT
A_14_P105369 ATATGTTTGCTAGAAGGCAAATTTCAACAGCAAGCAGACCAATTTACCTGACCTTAATAA
CUST_706_PI426210822 GTAAATTTGGAGTTGGTGGGAATTTTTGGACTCTGCTGTGTGGCC
A_14_P101597 GTGCTCTTGGAGATTGTAGGTGCAGAAACCAATATTAACACTTTTGATAGTGAATTTTGG
A_14_P133530
CTCAGAAATGCAGAGAAGAAAATTGAAAGCGTCAAAGGGAAGGTATAAAAACATGAAACT
A_18_P22841920
TTAACAACCTTTGAAGGCTTTTTAGCAAAAACAAAGTGTCATATTGGGATTCATCTGTGG
A_16_P37364794
TCCCATCATCAAAGTTAAAGATAAAGGCCTAATATGTCTGCTCTTCCCTAAATATCCTGA
A_14_P108200 ATTTATCTCATTTTTAGGCGACTGAGTCAAAGACCAACACCAGAAGAACTAGAACAACG
CUST_1095_PI426210822 CTCTGGGAAACGTCCCCCACAGAAGAGCCAGCAAGATCTCTGCAG
CUST_2739_PI426210822 GACTGGGCAGGTTGATTTCTTCTGCCCTGAAGCACTTTGCTATTAC
CUST_5659_PI426210822 AGAACACAAGGCTAGTTCCTTTGGCAAAAATTGGGCATCATATCAGAGCT
A_14_P109124 ATTGGCTTGGATGCCTAAGTGTATGATGCTTGAAGCCTCAGAAAAGAAATAAGCATATTT
A_14_P135723 GGTGGCACATTTTCAGAGCATTTGCAATATTAGTAACAAAACTGCAACTACAACTTTCTG
A_18_P12711313
AAATCCATGGTCATTTACCACATTACCTCAATACCGAAGAGAAAGTTTACGAAGTCTTTC
A_14_P133139 ACGATTTTGATATTCCAGAAACATCTCAACCATTCGTTCTTCTTCCTTTAATTTCACAGA
A_14_P200851 TGGCTATTTTTTAACTTTAATCCGGAACCTTGGAGTGACGTGTGGATTTTTTTTAATGC
A_14_P202399 CTTGTCCTAAACTGTCCAGTGTGAAGGAAGAGTTTGGTCTGTCAAGG
A_14_P127803 CTTTGCAGATACTAGTAGGCCAAAACTCTAGGCATAGTTAATTCAGATACTAGTCCAGAC
A_14_P119406 AAAATTCCAGTCATTTCTTCTCAGGGTTTTAAGAAATTCTACTTACGGTGTCCTTTGGAT
A_14_P126215 CCACCACCATGATGTTGAACTCAAACCCCATCTTCATAGCCTTGATCTT
A_14_P125025 CGGTTTGGATACCTAATCAGGATTACTATTTTATGATTGTGCTTTATTTCTCTCGTCTCC
A_14_P106241 TTTGTATCCACTGACACAGAAGGAGATTTTACAGCACACTATGTTTTGAGTCTCAAAAAC
A_14_P137717 CAAGCACTGGAGCAAATCAGTGATTTCAGGAACACTCATATTACCACAACAACCTTTAT
A_14_P108720 CTGGTGAAGTCATTTAAGGCATGGGGTATTGGTCTGAACTCAGAATATTTAATGTTATGT
A_14_P119376 GCCATGGTAAATAAACCCCTCATCTTGCCCAAGAAGAAGGACATTTATATGGTTATAAAT

A_14_P102359 TTATTGTGCTTATATGAGTTTTGTTGGCCTGTCCTAAAATAAGTGGGTTTACTCTTGCAC
A_14_P112014 AATCTGTTTGAGATTTTCCTTAATAAATTATTGTTTGTGTATGCGAATGTATACACATAC
A_14_P132491 TTGTGTGTTTTAACAAATGATTTACACTGACTGTTGCTGTAAAAGTGAATTTGGAAATAA
A_14_P129462 TGGCGATTATTGGTGTTTTGGTCCTATCATTGACGTTGGCTAAGAATTTAAATAGCAGTC
CUST_1253_PI426210822
TTACCCATTCAAAGGTGAAGATTTTTTTCCCCTTTAATGTTAAAAGTGGTCTCCACAA
A_18_P22841783 GAATGCCTGATCCTGCGAGTCACTGCAGCCCCCATCTCATAACAT
A_14_P104386 TGGAAACCAACTGGCAATGAGAAATTTAGCAATTTGAAAAAAGCATTTCCTTCCCTTATT
A_14_P130963 TCCAGTTGCTTACAGTATTAAGAGTTTAGGACAGAGAGAGAATGGAAAGCAATACTAACC
A_14_P121888 CTAAATGAGTTAGATGATTCTTTGGAGAAAGTATCAGAGTTCCCAGTTATTGTGGAGTCA
A_16_P58424183
AATTGACTGGGAACAACTTTTCAAATATGAGAGTTGTTTTACAGAGTCATGCCTATGTGC
CUST_7657_PI426210822 ATTCCACCCAAATGAGGCAAATTCTTATCCTTTCAAGACCTACCCTAGAGT
A_14_P111382 TTCTGACCCTAAATCCTATGCTTTTCACATAAAACTTCACATTGACCACATCCTGCTATT
A_18_P10022805
TGCTTCACTAATATGCAATCCATAAAACACCATTACGAAAACTATAGTCTGCCATGTTCT
A_14_P130333 AAAATGGCAAATGAACCAAGCAGCATCCCATCTTATATTTTAAAGTCTTCTCCCAAGTTA
A_14_P125922 TGATCCCCCAGAGCCTCTCTTTCTAAGAAGAAATAAGAAAAGAATTGACAGGATAAACTA
A_14_P201229 CTCTTCATTTTGAGAGAGAAGAAAAACTGTTTGGAACCACACCAATGATATTTTTCTTTG
A_14_P138538 TTTCTGATCCTCCACTTCATTATTGGCTTCAGCGTGCCTATGTCCATCAT
A_14_P113476 TTTTAATATTGTTTCTTCCTCATCCCCTTTTCCCTGAATAAACAACAGCAGCAAAACCTA
A_18_P22840351 ATTCTGGAGCAGTCACACGATGACAGATGAGACGGTTGGGCCTGA
A_14_P128361 TCTGATGAAATATTTATGTTTGCTCTGGAGAAACAAGTATAAAACCTGGATCTCTCTGGG
A_14_P122989 ATAACGCACCTTTTCTCAATGCAGGGAGCAGAAAAGAGAAGAACTCCCTTAAATTAATAA
CUST_5816_PI426210822 GAGAGAAAGGTTTGAACCTCCTTTAACTAGGGAGGAATAAGAGATGATGGCT
A_18_P22840884
CTTACGGCTGGCATGCCAGCAGTTTCCTAAAATGTAATATCATCACTATTATTGTTATTA
A_14_P125170 TTTATTGTTACAAAAACCATGTTATCACGTTAGTGTGGAATTCTTTAGAAGCACCGGCTA
A_14_P104558 TCAAAATTCAAAAGGGATGACTTTGCTACAGGATCAAATTTCAATGCATTCCATTCACTT
A_14_P130138 GAACAGAGATGCTTTTTATGTGTTTTACATACTGCGTTGTGCACACATTGCCTTTGATTA
A_14_P106756 TGAGATTGCTTACAGTTATCTTGACTTTCTTCATCTTGTTCTTCATGAGCCTTGGAAATG
A_16_P20205735
AACAGTGATATCCTTCTACAACCTTTGAAGAGAAAACAGATTTTTAAGCCCTATGTCCAG
CUST_9922_PI426210822 TGTTTTCACTGTTGCGTTGCCTCCTAGGTGGTCTTCGAGGCCCTG
A_16_P20342071
ACAGTGGTGTTTATCTTGTATGCATTTAATTGACAGTCATGTAATGATTCACGCTCAAGA
A_14_P139541 TATTAAAGAAGGGCTGGCCTTGGCAGATACTTCAGAGAGAGAAACTATAGAAGTTATACT
A_14_P114794 AAGTATTCATCCTTCTGGCCAAAGAAACTGGCTACAATTCTGATTCTAAAGAAAACCTTC

A_16_P16981016
AAATTCTCACGTAGTGCATAGAATATTAAACATGCAATGGTGAATTGATAAGGTCTCCCG
A_14_P130714 AAGAAAATCACCATTTAATCTATACTTGAGTCTTCTTCCATCAATAAGAGGCGTTCCTTC
A_14_P133330
AGAACTTCTGATTTAAGGAACAGACAGAGAAGTATATGAGATGGGGAGAGAATTCAAGAA
A_14_P115444 TTCAAGCCAGATAGCACCTGAGGACAGAACATATCAGGTATTGGTAGTGTTTCT
A_14_P123922 GAGCGTGAAAGCCAATCCAAGCACTAGATATACACTGATTGAGAATAGTGTTAATACTGT
A_16_P03497674
ATGGAAGACAACATCAAGTATCAGTCCTAATCAGAACCTTATAGCAGTATTTCATCCTGC
A_18_P13939044
AAGGCAACCTAATTATGTTCCAAAATATTCAGAACACATTGCTATTGGAAGACCGTTCTC
A_16_P15968263
CATCACTCTAAAGTTTGTCATTTCTACACCCTCTTGTATTTAGCAGATCAGACACCTGAT
CUST_6186_PI426210822 AGATAAGGGATGTGTCCACAGCACGTCCACAGGCAGAGAGGATAA
A_14_P117009 GTTTCTGATGTGATCTTGTTTTGTCTAGATTCCAGAATTCTCTGATGGTTTCTGGACT
A_16_P20559650
AACGCTTGAGTGTCTTTGATTTCTAAAAGATAACACTGGTTTCCTGTTAGGAAAATGCTG
CUST_8062_PI426210822 ACATGGTGCTGTATCCCTTGATGGAGTGGGCAGGTCACCTGCAGC
A_16_P36230734 AAAACAATCAGGCACGGGGTTTTTATGCTGAACATTCCACGGTGCTTAGTTATTTAT
A_16_P15518845
AAAATCGACTCCATCACATATGTTCTGATCCTCAATTATCATATACATGTCTGTCACCCA
A_16_P40267979
ATGCTTACAAGACCAAATCTACTATCAACTCTTTTCATAATCCTCTGACTACATTTCTCT
A_14_P133124 CCCAGGTGAGTTAACTGTAAGTTGTCCCCATTTGGTCATCTCAGGATAAAA
A_14_P111284 CTGAGACTAAAAACATGACAGATTCAGAAATGAACATAAAGCAGGTAATAAGTATGAAAT
A_16_P17703854
AAAGATAACTATCTGTGACACTGTAAATCTTTCTTTCACGTCCTTGAAATCAAGTTGGCC
CUST_1478_PI426210822 GTCTGGTGGATTCCTGAGACTTTCCTTCCTCACCCCAGTCTGGAT
A_14_P121629 ATACAATTCTGAAAATAATATTAAACAGCACCCAGTCATTCTCAGAAAGCCGTCACACAA
CUST_3132_PI426210822 CTGCCCAGACCAGCTGCCTGAGGGCTTGGGATGCTGGCCATTTAT
A_16_P17062854
AATGCCAAAATGATTTCTTCTGTGACAATGCATATCTCCTCAACTTTTGCAATTTCCTCA
A_14_P117735 TGTGTTTCTTTATCTCTGGTTTTGATAGGGTTTTCACAACATGGTATGATTGGTGTAACT
A_14_P139571 CAAAATTTCTACAAAGAAGGTCTTCATACTTGGGATTCTTCTCTCCAGGGAGG
A_16_P40754406
GTAGTCCCTAGTTTTTATTCACTTACACAAAATCTCCCTAATATCCCTGAATCAGACCAC
A_16_P21098423
AAAACACCCCAACCTCATTATGTGACATTATCTTCTCAATAGTTTTCCTCTAAACTCTGC

A_14_P103465 CTAACACAGTAGAGTCTCATAATGCCTTGGCGAACACCAATTTGATGCTTTAAGATTTTA
A_16_P16950148
GTCAATCTCATTCTTGAATCCATATCAAAACAGACACAATTCATCTCAGTGCATTTCTCC
A_14_P100578 ACATGGGCCTCCCTTAATTTTAATATTCTTTGCTTTTCGGCTTTTGTTCAGATACTGTAT
A_16_P20241591
AAACTTCAAAGGGCAAGTTAAAAATGTTTCCTAATTCATTTCAGCCTAAACTGATCCCTG
A_14_P134358 CTCTTTTAGGGGAAAAAATAAGTGGGTTGTGTTTTTAAGTCATGGTTTAGTGGTAGGTAG
A_14_P127615 CATCTCAATCATCTCAGCCTTCATGTGATTTGCTTATTGTGTTTCTCTAGTTTTCACTGA
A_16_P00654863
ACCACATCCTCAAGGATTACATGCTAATTGGAAGATAATATACTCCTCCATCAAAAACGA
A_14_P105410 TTTCCCACTGTCTAGAGACTCGAGTTACTGTGGGAATGTGTGAAAATAATTGGATTTTTA
A_16_P00685407
CCCCTGCAATGGATTTTTCTGTCTTCTAATGCAAAACTACTTTCATAGATTTATTCTGGA
A_14_P131753 ATGCAAATCTTTTGTAATTTCAGCCTGATAGCGCATATCAAGGTGGAGTCTTCTTTCTC
A_14_P125891 GTTTAAGGCAAAGATCTTCTGTTCTTTGAATTTAGATAGAAAGGGAAATTGCACATGGGC
A_14_P127835 ACCAAACCTATAAAGCAATGTCTCCATACTCATGGATGAGTTGCATCAGAAGATAATTTG
A_14_P100101
GAGAAGAACAGTTTTTGAAAATTTCCATGAAAACAAAAAAAATAAACCGGCAAGTTGACA
A_16_P18156974
TAGGAGCATCACATAGTTATTTTGTAGCAAGGAACTAGAATCTAACCCTCAAAGAGATGG
A_14_P105195 CTTTGTCAATTATAAAAAAAATGTAAAAGGATCAGCGCCTGTGTTACTGAGGCTTTGAGG
A_14_P104052 TAACTAGAACTGCAAAACTATGCAGGAGATATCACCAGTGTCCTGTATTTTTTTCTCCTT
A_14_P107860 CCTCATACATACCTGCTAAACACATCTAGTTTCTCATCATGAGAATTGAGATGCTGTTGC
A_16_P16574140
TTCATACCATAAGCCATAATTAACATGTGTAGACTTCACTGATACTTTACCCACCAGAGG
A_16_P16755557
CCCTTGGCCACTAAATCTTAAAACCCTACCTTCCTCTTAAAACCATTTCCTTTATATACT
A_14_P127520 CCTGCATTCTCACCAACTCAAGAAGATCTTCCGAGCAAAGACGCAAT
CUST_283_PI426210822 AAGAGAACCAGTTTTCAAAGCCCTCACATAGCTCTCGCCCAGCTC
A_14_P113745 TCTGCTGGAAGAAATGTTATTTTTTGTTGGGGTAGAGTATTATAGAGGGCTTCTTAGTCT
A_16_P01861963
AAATTAGGTCTTAGACACATGAATGGGACAAAGACTAAGTGTCAAAATCCCAAACTCAGT
A_14_P106206 AAACGCATTTTAGGGAAGCAGTGAATTCTCCACTTGGAATCAATCTACTGATTGATAATT
CUST_2205_PI426210822 GGTCTTCATGTGTCAGTGATTTGGGGTGAGAGTAGGTATGACTTTTTCC
A_14_P113414 AAAGAGTAAAGGAAACCCAAGTCATCAAATACCTTCATGGCATTTAAAGCACAAAATCTG
A_14_P134565 TAGACATATTAACATTTTCTGTGCGCTGGGAAGTTTGTGTTTGGGTTCAATGCTTTTAAT
A_14_P116738 TTGGACACTATATTTTTTTCTAAATTCAACTTGAAGATGTGTATTTCCCCTGACCTTCAA
A_16_P18724122

GGCCATTGCTAAAATTATCAAGACAGTTCCAGAAAGATGAATGGTTTCAATTTGCATGAG

A_18_P15993109

AAACTTTCAGGATTGCATTTACTTCCCTGTTTTCCACTAAATCTGATGCACAAGCAAATC

A_16_P37702850

CTGTAGTTTGCCTGTAAAAACCTATTCATTTATCACTTCACCGAACATTATGACCAGAGT

A_14_P115273 CACAAATCTTTCTGTTCAAATCCATTCCTATTCCCATTCCCACATATTACCTGGATAATT

A_14_P118796 ATTTGTCTCCTTGCCGTTACAAGAAAGTAGTGCTGTTAAGTTTAAGCCATTTTTGAATGT

A_14_P119470 TTTACTTGTGGGTTCTCAACTTTTAAAATGCCATAAACTTTGAAAGCGGATGGATGTTAT

A_14_P127444 CTGTGCCTGCATTTGCCCTTCTGGCAATCTATTATAAATTAAGAAACTGAATTGAAGGTA

A_16_P38133887

TACGTGTCCTCTAAGTTATAAGTTCACAAATAAAAGCCCAAAGGAAGTTAGGTAGCAGAC

A_16_P58424236

GAGTTTGGTTAAAAGGGAAAAGTAGAGAATGGATATTGGTTAGGCATATAGTCTCTGCCA

A_14_P137599 TTCCCTTTTCTGAAAATTTTTAAATTGTTGGACTGTGTTGTTCTAATATTTTGTATACAT

A_14_P100437 CTTCTTTGGGTTAGGAGTGTTATTTCCTTTTGGTTTAAGGATCCTGCTCTGGAATAAATG

A_14_P201814 GCCACTGTAGGTATAGATGACATCTTGTCCATAGATTGGCAACTGTTCATAAGGATTAAT

A_14_P112344

AACAAGAAACAATGTCTGTAAACACAAGGAAAGTTTTTACAACGCGAGACCAAGAAAAAC

A_18_P11822633

ATAAAGAGTTCAGTCTGCATGATTAAGGGTTCATTCTAAGCAGATTATCACGTTGTAGGC

A_16_P58424201 ATCTTGGGCTTCCTAGGCCTTGGGAAGGATTTGCCTTTACTCAGA

A_14_P113751 GTGTTGTTGTGTGGCCTGTTCCTGAAAACATTGAAATCACTGTAACACTTTTTAAGGT

A_14_P103901 CCTCCCCCAGACACTACATAAGTATAAATTGCTAATGATTACTGATAAACAGGACACTGA

CUST_8726_PI426210822

CATTCACAGTCATTGCAAGAAAATTTATTGCTTTCCCCTCAAAAATCATCACACAAGTAG

A_14_P117052 GAATGGCCCTTCTAACTTAAAGTATTTTATCATGACCAAGTGGCTCTCATCTTTTGGAAT

A_14_P110679 ATAACTTTAAGACTCCACCCAGTAATAGGTCTAAAGCCCATAGATGGGAATCAAGTAAAA

A_14_P123373 GACAATTGCTTACTTTGCTGCAGATTTCTGTATCAACGGTGAAATTAGATGGAAACGTAT

CUST_3997_PI426210822 TAGCAGATTCTAGATTGTCCTGTTTGCTGGTTTGGGGGCTGCAGG

A_14_P113924 CTCATTTCACTTCTTTCTACACAGTTCAAATGGGATAATGTATGTGAAGGTCCTGTATCT

A_14_P131049 TTTACACATTATTGTCTGCCTTCCTTTTCTCAGTGGGCTCTTTATTTGTTAAAAAAGTGC

A_16_P36575473

CAACATGCCCCAAATTCTACAAATTATTTCATTCAAGACAAACATAAATATAGCCCAGTC

CUST_7636_PI426210822

ACAAACTTAGAATAGTGTAGGCCTTGGCTTCCACATGGATGTTTTGACAAAATTTCA

A_18_P13616678

AAAGATTAAAGGCCAAATCTGAATTCCTATCCCCTAAAATACTGCTCCAGATTCTGATTT

A_16_P17102762

ACAGTGCAAATAGTGACTTAATTTCTCCCTATCAAGGAATATCAACCCCAAATATACCGA
CUST_6951_PI426210822 AATTTAACAGAGAATGTGAATGTACGGCTACAGTCCGTCACCATCCAGC
CUST_4900_PI426210822
GTATAATAAATAAATAATTAGGGGTTGATTGTTACGAGTGCTATGAGGGGAATAGCCAGG
A_14_P104581 TTTGGTGAAAATGTCATCTGCTACTGCAAATTTTACTGGACTTGTGGGCTTTTATTTGAA
CUST_6304_PI426210822 CTTCCCTCTGTCCATCACAGCCTCCCCTTAGGGTCATTAACTCAG
A_14_P127877 CACATCGATTTTGTTTTCCTGCAAACAATCATAATGAACATCCACTGAAACCTTCCTAAA
A_14_P113946 TTCCGGTCCTCAATATCCTTTCATTGGCTAAAATTTAAGATTTTGTTTTAACAGCCCAGC
A_14_P113912 ACTTACAAACAGGGTGACATATTTTGTAGACTAAGACTGGTCAGATGATATTCTTTCCCA
A_18_P15901602
TCACCTATGAATCCACAGAATAATTTTCCTTTCACATGTCTATATTTGCATCTGCCTTGG
A_14_P136531 GATGAAGTTCTTTGAATGAATGTATTCGATGCGACTGATCTGTGAGCAGAGCAAG
CUST_7818_PI426210822 CTGACAGGCAGCAGAAGGGTAATCCATGACATCCCTTCCTGTAAC
A_14_P127207 ATTTTCTTACCATTTGGGGTCCAACATTCACATTTATTGGTCCTAAGGTTTTTGGTAAAA
A_16_P41569554
ACATGCTGATGACTTACATTCGAATTGGAATAGTGACCTTAGAAAAAAATTTTGTCTGCA
A_14_P103037 CTGACACTTGGGATTACGTCTCAATTCTCTTCTTCAAGGTCATACAATAAGTGCTCTTC
CUST_3523_PI426210822 ATAAGAAATGTGGGAAGAGAAGAGGGCAAATGGTAAGGCAGCTCTCG
A_16_P01602661
AAGTAACTCTCACACTCTAAAGCCTTAATCTGCAGTAAAACACAGGATTTCGTCTACATC
A_14_P121519 ACGTGTAAAGATCTGAATTCTTTGTAAATGTGTCATTGATTAGGAGGCAATTACCCCAAT
A_16_P18538576
AAATGATCTGGAGTGGTTTCTAATAGTCCTGAAGAATAGAGGCAGTATAATTACTGAGGC
A_18_P11098877
AATCTAGAGGGGTTGTTTTTTACCAATCTCCCCTATGTAATTCTGGAACACTTCATTTAG
A_14_P134217 CAATTGAGAGAGAAGGGAGAGAAGAAAACTTAGGTCAGAATAATGGAACTCTTATGTTCT
A_14_P121497 TTTTTGTCCTGGATAATGGAATGTTAAAGTATTCAAAGGCACCACTCGATGTAAGTAGCA
A_18_P19543312
AAAAAAAGTTAAGGCTGATATCATAAGTAGAAACCAAATTTGCAGCATACTCAGAGAATT
A_14_P130367 GCACTTTATCTCAATTCTTAACTGCTGTGTTGCCTCTCACTTTAAAGATAAGAAATTAAA
A_14_P139793 CTTTACATTTGGACTTGGACGCAAGAACTTCAAGAGGATGGAGTCACTTGT
A_14_P108863 GATATTCGTAAGTGCTTTTCTCTGAAGTATGCCTGACTTTGGGAGCATTTGTTAATTGAT
A_14_P120210 AAAAAACAGGTTAAAAATACGGAAGTTTATTGTAGGACACTCAGTGTGAATAAATGGAAT
A_14_P201263 GCTAAATGCAGATTCTTTTTGGCCAAACGCACCGTTTTATTTCCCAATAATCTCCAT
A_16_P36230895 ATTTCCTTTCATGAGGTCAAAGTCAAAGGTAAGGGACACAGACGGGA
A_14_P123821 CAGATGCAAATATCAAAGTCGTCCAGAAGTTCAGATGTAAAGGAGGACGAGG
CUST_8108_PI426210822 ATTGTTGGGCAGACCCATCAATGTGCTCTCTTGGTCCTTCCACAC
A_14_P109226 TTCATAGCCCCACAGCAATCACATTATTTAATCAGAAAGACATTGGGTCACAAGATTTT

A_14_P201033 ACCCTTGACTCACAGAATGTTTTGTGTGATGTCAAGTAGCAAATGCTTACATATTTCTAC
A_14_P126739 CTCCATCTCTGAATAAGTGAAAGGAACATTTTGGCCTCTTTGATTATAAAGACATGCAAG
A_14_P138673 GTGGTGTTGTGTTTTAGATGGTCCCCATCATTTCCCAATGTGGAGATATGT
A_16_P01332768
AATGCTGTAAAGACTGTTAAGGAAACCATCTTAGTCTACAGATGCTAATCAGTTGGTTGG
A_14_P131766 AGTGTTTTCCCTTGAGACCTGTGAGTTTTTTCAGTGAAAAAATATTCAAAAGGTTTGTCA
A_14_P126742 GTCAGTTACAGAAATATGACACTGACCCTTTGCATTTACAAGTTGGTTCTAAAGAGTGGT
CUST_7634_PI426210822
ACTCTAGCTTTTGCTTGTTTCACTTTTTTAAGCTTGGAAAGAACAAACTTAGAATAGTGT
A_14_P128617 CTAAGAATCAAAGAAGCAGTTTATTCCTAATTTCACAGTCTCATCTGTTCATAGTGGGGC
A_14_P126927 AATTCTTCAGCAAACTGTTCAGCAACACTAATAAAATTGCTTATGAGTTCCCCACCTTTA
A_16_P01693487
AAAAGCAAACCAGTATGAATGAATTCATAACACACACCTTATCCTTACATGTGACGTGTG
A_16_P17822851
CAAAAGGCTACAAAAATCAAAACCATCCACCTATGTAATGAACCATTTCTTATCACAGCC
CUST_2217_PI426210822
CCCTCTATCAAGATAACTCATATATGCACTTTGCAAAACAGTTTTATAAACTTGTACCAA
A_14_P117417 ACTACTGGACAAATAATGACCTGTATATCCGTGCTGTGAATGCTGAGTTCATCTATGG
A_16_P15114597
ATGTTCATGATTTCTAATTGGAACAAGGATAGGATAGCACTCTATATGCAGACAGAACCC
A_18_P14133663
ACAAAACTGTTTTCATCATCTTTGGTTGTAGTTTTATAAAGAATGCTGGTTCATGGACTC
A_14_P103540 AAGAAGGTTTCAATTGCATTCAGGTGAAATAACCACAAAATGACTGTAAGCCAAATGTAG
A_18_P22842138 GCAGGCTTCTTGGGAAAGCAGATATTGGAGCAGAGTCTCTCCTTTAAAAAAATAAA
A_14_P101290 CTAGTATCTTACTGTGATGTTGTGCTGCTTAGAATTCTCTGTTAACCAGAGTGAGAGCAC
A_14_P112400 AAGTGAATAAAATGTCCACAAGCAGTGTTGTCATGTAGTCAATGGCAGATTATTAAATTT
A_16_P16564754
TTTAATTGCGTTTTCAGTCAAAAATCTCAGTTATGTGGTTCAAATTCATGTTGCCAAGGC
A_16_P01409946
CTTCCTTACCACCAATATATGACAGATCAACATTTCAACCTAATTGCATCTGACTGACAG
A_14_P201075 GCGGTATAAACTTGTCATCTTTAATATTGATGTTTGGCGTGGGTGTTTGAAAAGGTATTC
A_14_P124012 CTTTTGTGGTGATTCTCCTCATAGTTATTTTGAAAGGAATCTATCATCAGAGCATGTCAC
CUST_3807_PI426210822 CAAAAACCCAGTAGGATGCACCTCTCAAGTGGTTGGCAGCTTTTA
A_14_P117461 GAACTCATGTTTCAGTTCGCGAACATTGACTCCTTACGAAAGTCACTTCATTCTAACTA
CUST_5839_PI426210822 GAAACAAATTGGCCATTTGCCCAAAGAGACTAATCCAGGACTAGAACACA
A_16_P00935341
CGGTCTGTATCTATGAGCAACTAGCAAACATCACTACTGTCAAATATCTTTATATCCCGT
A_16_P19000932

GCCCAAATAAGTTTATGCCACTTCTCTTTAAAATCCTTCAGTAGTTATCCTCTGTCAACA

A_18_P22839761 CTGCTTCTGCTTGGGAATTCTGCAGTTGTGCCCAGCAAACACCTG

A_18_P13082267

TGCAGTTTTGCATTGATTATGAACAGCCGTATCTATAGACATATTGATGAATCCCATGGA

A_14_P115308 CTGCTTTTAAGTTTGTCTTGCGGAGGGTTTCTTCCATGAGATTTTCATAAACTATAACTG

A_16_P00909229

TTGTTATGTAGCACCTGAAGTTGTCAATAATGAAAAGTATACGTTTAGTCCCGATTGGTG

A_14_P119219 CTGTATATTTGAGTTCACCAGCAATAACTCCCCACACTCGAAGCAGGTC

A_16_P36425146

CCCAAATCTGGCTGTATAACTACTCCATAAAGTGATCAAACACATTTATAGTCTTCAGGC

A_16_P58424497

GAGAAAGGATGGTTAATTTGCCTGACAACACATTGCTGGTATATATCTGAGCCAATAATC

A_14_P106929 GGCTGCGTTATGAAAATTGCTTCACTTTGAAACTTCTGGTCTTGGTAATATAGAATTTCT

A_14_P134013 GACACTCTGAGTCCTATTTGAAAGTAGTCGGCTTTACAAGATTAAAAACAAATTACAGGG

A_16_P37957704

AGCACACTTAATAAATACACTCTTTGTAGCACACTTGATCTTTATAATTCCAGGAGTCCA

A_14_P106075 CGTAAATGATTCATTTCCTTCAAATGTCCTATGCTGTTTTCCAGCAGAATCTCCATTATG

A_18_P10526657

GCCAAGAGATGTTATAACCACATAAGCACAATGATTCCCCTAAACATCCATTTTGTGAAT

A_14_P119946 GTCATGTTTTCTCCTGCTGAATATTTCTCTTTGCATTATAGCAACATTCAGGTAAGCACA

A_14_P100436 GCAACATGGCTTTTTGAGAGCGCTAGTGTTTTTTACTTACTGAAAAAATCATTCTTTGGT

CUST_904_PI426210822 ATTATGTGGACCATGGACCCAGCAACCAGCTCTGGCAAGGACCAG

A_18_P16739265

AAGACAGGTTCAATTAGGATCAAGAAGAATTCACTCCATTCTAGGACTTTTGGTTGTGTT

A_14_P124936 TAAAGAGATAAAATAAGACGTTCCTCACCAAGTTACACTGCTTCAAAGTCAAATGCTTGG

A_14_P129453 GAGTTTTCTCAAAATGGAGAGAATTTCTGCAAACAGGTCACATCTGTTCTTCAGCAAA

A_14_P133721 CTGCCAGAACCTGGTATCTGTATCTGTAAAACCAAATTAACTTTTGCTTAAATGGGAAGT

A_14_P106640 TATTTGTTTATTCATTATTTACGCCGTGGTAAGTGTGGAGTTGCTCAAGAGCACAAATTC

A_16_P39579783

GGCAAACCCTAAAGTGTTTAATGTGCCTCATATCTTTGCTACATCAAATTTTTGGATCAG

A_14_P103140 ACCAACAATTTCAAGATGCCAAAGCAGTTCTAGAGGAAGATGAAGAGGTCAC

A_14_P103822 CTGTGAAACTCTAAAATTGCATTACTTACAGAACTTTGAGTTCTTTCAACCCAGACAAGG

A_16_P21658932

AAGGGTAAGAACAAAGATAAGCCTTCACATCTCTTTATATTCCTTCTCACTGACTTCTGA

A_14_P131190 TATAGGTTGCTGAATAACCATCATGATGATGCCTCTAAATTCATCTGTCTTCTAGCAAAG

CUST_3515_PI426210822 GCCCCTTTTTCCTTCTGCTCTACCCTGCAAATCTTTTCCTCTTTCTAA

CUST_3553_PI426210822 AGAATACGAGAAGCCAGCTTAGAAATTGCATGGGTCCTTCCTGCATTTT

A_14_P127066 GAGGTGGAATCTGTAGGAAAGAGAAAAAGCAAAGTCGTTCACTGACTGAGTAG

A_14_P132933 ACTTTTACCTGTGCCCCATCTACAAGACACTGACTCGTGCTGGTAT

A_16_P19474494

TGGCTGCTTAACTTTACTTTCAACCTATCTAACTTACACATTTAAAATTCGACTCCCTTC

CUST_3380_PI426210822

TCTTGTTTCCTGTGGCTTTTTAAGTTGATGAATAATAGGAAAGTAAATTTGTGGGGTCAG

A_14_P100782 CTCAGATTCAATGCAGACCTTCTTGTTGGGCAGGTCAATGTCATACTTAACTC

A_18_P13720470

AAATCCCTGGTTTAACAAATGAGATTTTCTTACCATAAGACTTAAGACACGCTAATGGGC

A_16_P00739560

GTGATGACGTTGTCAGTTAAGAATAGCCATCATTTGCTTAGTTAAGTGTGGAATTGTCCT

A_16_P36959721

AGTCATCCCAAAAGTATTAGAGTCATCTTAACAATCAGCATTTTTCAAGGCATCTTCAGG

CUST_8462_PI426210822 CTTACTGGTCTCTGTGACCTCCATCTTTTTCCTCATTTCTCAGGAAATGATTG

A_16_P21132283

GTCCCCCAAGTAGATTTAAACATGCACCTAAACCTTATTTACACCCTTATGATTTTCAGA

A_14_P125279 CTAGAGCAGCACATCCCCACTCAAGCCGTTTCTAATGGTAAATATGTCTG

A_14_P126214 TTCCCTAATAATGAGGATGATGTGGAATCAGTTCTCTTGTTGCAGGACATTTCAGTTATA

A_14_P118599 AAGTAGTGTATTTTGATCTCCAATGACTTACAGTTCTTGAATTTTTTAAAGCATAACAAT

A_14_P101409 TTTCAAGAAACAGTCACATTGCAATTGCATTTGATGACACCAAAGTTAAAAACCGGTATG

A_14_P128685 TTAAAAAAAGGTCTAAATTCCCTCAACAATTAAGTCCAAATTAAAATTGGCTACATCACT

A_14_P135468 ATTACCATTACTCTAAGAAATATACTCGAAATGACTCTTAGTCTCCTTTCCCCTCCCCT

A_14_P102842 AGCACAACTAGAGATCTGGTTGAATCACTTACATGTCATTCCAGACAGTAAAACTCTAAT

CUST_8932_PI426210822

GTATTTTATTTAGAAGACAGTTTTCAGTTAGAAAGGGAGAAGACATCTAGAGTTAAAGTT

A_14_P120367 ATATCCCAGTTTCAAGATAAAATGCATGCATTTAAATGCGGTCCATCTGGTTGAAAGTTG

A_14_P115229 GGTTTGTGTAAAGGTTCAGTGTTTATAAGAGGGCTTTCCTGTCCATTTTTATACTTGATC

A_14_P202101 TAAAAGAGAATAGTTTGGGAGGTGGGTGTTTCTGTGATTTTTTTTTCTTTTACAAAAAGA

A_18_P13319480

AAGCCTCTCTGAATTGTCTTTCTACCCATCACATGTATCTAAACTTGACAAAATACAACA

A_16_P03622064

TTTTCACTTTGAAATCACACAATCCTATCTTATCCCCTAGAAACTAAGAAACAGCAGGTG

CUST_158_PI426210822 TCTTGAAGTGAGAGCATAAAGGAATGGAGGGCATTTGGTTGCCATTTC

A_14_P138350 CTCATTCCTTGGGCTTTGCGTAAGGAAAGAATGACTATGGTGAAGGTAATAGAAATTATA

CUST_6977_PI426210822 GTCACTCAGAGGCTGCCATGGCCTAAGACTGAGACAAAGAGAATA

A_18_P15389987

CCAAGCAGAATCCTTTAGAACACATTCCACATTAGTTTACTTTTACACCACCTACAACTT

A_14_P129601 TGCCATGAAACTCTATACACTGAAATGCCTGTTTCACAAGCCTATATTAATCTGGACAAT

A_16_P41027362

AGTTATGTTGTGCCAATTACATCAGTTACTGTAAACAGAAGTTCTCTTTGTGTTTCCAGC

CUST_6675_PI426210822

CCCCACTCTGTCAGTATATAGGACTTAGTCTTACTATTTTATAAACTCACTAGCCTCCTG

A_18_P14589092

TCCAGTTTCTTATTGTGACAAATTCCATCGAAAACATATTACAACTCAATGTGCTTTCCC

A_14_P122215 TTACAGGAAAACTAAAGAAACTGTCAATCTTGAAGGTGGATCAGAATAGACTCACACAGT

A_14_P104128 GAGAAAACAATAAGGTTCTTATGTCCTGTGTTCAGGCAAAGAAACACAAGACATAGACAT

CUST_8928_PI426210822 GACCTTTTCAGACTCCATACCACTTTTCACAGGGATGACTGCCTTAAG

A_14_P106127 ATGCATTTACAGTTTAATAATCTCTAAGGTCCAATCTAGAACAGACTGTCCCTCAGCACA

A_18_P14587324

ACTGAATTAGCCCCAACACTTTCATAAACAGCAGTTTTGAAATACCCCAGTTCATGAAAT

CUST_1308_PI426210822

TCCTCTTTAACCTAGCTGTTTAATTTATTTACATTTTTTCCCCCATTTATATGGTGGTCC

A_14_P138982 CATTTAAATGTCAGAGCGACGACTATTAATTAGTCTCACCTATGAAAGAAATGGCCTCAG

A_14_P104908 GGTAAAATAAAGCAGAGAAGAGATTCAGTATGGGAATTTCTGACAGGATGATCCTTTTCA

A_16_P58424172 ATATTAGCAGAGCCTCACAGCAAGCCTCTGGCAAAACAAGTGAGGTTT

A_16_P18007335

AAAGTTAGGTTAGAGTCAAGAGATACAATGACAAACATGAGTTATATTTTCCCACTCCCC

A_14_P125767

ACAATCAAATCACATGCTTAAAATCAAAGAAACAAGGTTGGCAAAAAGTGAACAAAGCCA

A_14_P124726 GTTCTGTGCCACCTTTATGCTTCTTAATTCCCCAACAGGAGATGGCATTT

CUST_5969_PI426210822

TGTATGTTACAAAATAATTATAACAAAAGCTTTGTTGGGAAGATTGTTTAGGTTTTTTTG

A_14_P126445 ACAAAACCTCATGTATTTCCAATAAAGGGTTTGTTTACTCATCTTTCACATCACACAAGG

A_18_P22841201 ACAGAAACCTGACACTCTGAACTTTCAGCATTTCATGACATTTGAGGTCTGACTTT

A_14_P109473 TGGTGTCCTGAATACAGTATTAAGAACCAGGCATTAAGAACCATTGTCAGTAATTTGTCA

CUST_199_PI426210822 CTGGATCTTACTGGTTGTGAGACAGGGAGGGAAAGAGAGACAAAG

A_14_P201858 TCCCCATCACCTTTTCTATAGATTTCAATAGGTTCAAAACACAAGTCTCTTAAAAACCAC

CUST_4233_PI426210822 AAGTGAAAGCGAACTCAGAGTGGGACTTGGGATGGGCTTTGGAAC

A_16_P02347921

GTCACCACTTACCCTTAACTGTCCATGACATCCAAAAGGAAGTAATCATATAAATAACCA

A_14_P127662 ACTCTTTAATATCTACAGGGCATATTTTTAGGCTTAGAATGCCTGAATTAGCCCCAGATT

CUST_3171_PI426210822

GCCAAGGTACACTCAAGGTGGCTGATACTTTATAGCCATACTTTATAACCATGAGAAT

A_14_P102440 CCATACTAAAAAGCATGCAATGACTGAGGTTTTCTCCTAACCTTAATCTCATCTTCTCAA

A_14_P127988 CTTTTACCCATGCATTTTTACAAAGTTGCAATTTTCACATAACATGAGAAGGACCAATAA

A_14_P135157 CCCTTGATTCAAGTTTACACAAAATGTGCCTTTGGAGACTATCCTGTTAATTTAAACAGT

A_14_P114086 GTTACAAGCCTGTCATAGTTCTAATATTTAAGTTACAGGCTGCCTTAGAACGAAGAACTG

A_14_P120651 CAGAGTTGCTGGGTTGCCTAATTACCTCCAATATTTTTCCTCTTAGAACAATAAAACTG

A_14_P124340 GCCATATTTTTTCTGTGTCATGATTCAGTTTCTTACATAGGCAGTGAACTCATTCCTTTG

CUST_3893_PI426210822 AGGATGGATGCGTGTCATTCACACACCAAGTGTGTTAGAGACAACA

A_18_P22840157

AAAATTTAAGGAAAGCAAAGACCTGCATCTGTCTCATCAGAAAGGGAGGCATATTTGATA

A_14_P124495 ACCTACAAATCTGCAGATACATTCTATTGATCTGAGACTCACACTTTCTCCTTTTAACCT

A_18_P14132521 GCTGTCCTCGGTATGAGATTATGGTGATGTTGAATGGGGGTCATTAGAAG

A_14_P137504 GCATTTAAGGAACACATTCCCTCATCCAGATATTTATGCTTCTTTGGGTTCTCTCTAGAT

CUST_1599_PI426210822 GTGAGTGGGAAGGCAGCCCCTCTGGGCGCCACTCTGTCCTCTGAC

A_18_P11097667

TTCATACTCCTTCCATTTACATACCCCAAGAAATAAACTTGATAGTTTCCATGCTCCAAA

A_16_P17147843

GCCATGTTCACTCTGTTTTTGTTATATTCCATTACACACGTATAAGGAATGGTTACCAGC

A_14_P200212 TCCCACATCCCAGAAGAGGTGGGGGTATTCCCTAACATAAAATGTTT

A_16_P01643791

TTTCATAGGATGCCTACATTTGAACATCTAGCCAAATTTTTAGCCGATTAGATTCCAAGG

A_16_P30952529

AATATATATTGCATGACAGGAAATGGAGGTCTGCTATTTTCCACTGAGACTTGCTGTGT

A_14_P121459 TGTTTTTTCCAGAAACAGCATTTAAATTTAAAGCCCTAAAGAAGGTTGCGCAGTTAGCAG

A_14_P107058 ACATCAGTTTTTCCATATTAATGCATGTATGTCCTGGTAATGGTTTTGTGGCAGAATCAT

A_16_P36230587 CACTGCTGCTATATTCCATGCTTTTGGACAAGAGCATGTTCAGTATTGTCCAG

A_14_P132711 CTGGATCTTCCAGATGACTTGATTGGATACTTTAGTCTATTCTTAGTTCGAGAAAAAGAG

A_18_P22842825

GCCAAAAAATTTGACCTTAATGAAAATAGAGGATTTTTAATGGAGATCAGAATTTTCACT

A_14_P125684 TGTGTTTGAATTACAGGTTCTCAGTATTGACCTATTTTCTTCCCTATATGATCCTGTCCT

A_14_P112404 CTTTATTCTTCACAGCACAGGGATATTAAAGAAGGAGGGAAAACACTAAGACCAGCA

A_18_P16788704

ACTCTCACCTTACCCAAATATATGTTCTCAGAAAACAATCTCATTAAGTAACTGCATCCC

A_14_P116805 CTTTTGGAATACCTATCAGAGTATATACACATGAGGCAAGTGGAATAGTGGTTTTTGATG

A_14_P113320 ATGGGACTGTGTTGAGTAAGAGCCACATTTATTTCTTAATTGGACAGAGCCTCAAG

A_14_P100405

CTTTCAGCAGGAACGACAAAATTCAAAGACTGAAAAGGAAATAATGGGGAAATAACCTTA

A_14_P136208 GGAAAGTTAGGATTTTAAGCATTCTGACAGCATACTGATTAATGTTAAATTTCTAACATT

A_14_P139804 AAAGATTCTGTTACCTACATCTCCAAAGATCCTATTAAGCAACCAGTGGGTATATTGGAA

A_16_P01861604

AAGTAAAGAAATGCTTAATCTTACCTTTTGTGAATTTCATATAGTGAACACGGTTTTTGG

A_14_P134533 GATTAGTGTTGGTCTGAGAGTCCTGCAGGAACAGTTCATACAAGATACATATGTAAGAAA

CUST_6280_PI426210822 GAAGGGGGCTCCCTTGAGAGAAATGGCTTTCATACAAAATGTCCTTA

A_16_P02379163
ACTTTCCTATCATTGAGAATCTCATGGTCTCATATTCACAAGTAAACGTGCCTCTCAATG
CUST_8801_PI426210822
ATGAATCATTATCACTCAGGAAGTAAGGACCTGGTTTACTATCTGAGAACTTTAGTCCTA
A_14_P126251 GACAGTGGTTATTTTTGCTAAAGCAGAAAGGAAATGTGATACTATTGGGCTGTGTTTTGC
CUST_579_PI426210822 TACCTCCACCTCCATCAAGGCCCTGTCCAAGGTATCAGGTTTCTA
A_18_P15592045
AAAAGAGGCCATACCTAATTTTATCAAGAACTGAAGGTCTGTTTTATCTTTTGGCTCCAG
A_16_P15449094
AAAATGACAAATGCCATTTCAAACAGACTTACAACCCATTTATATAGGCTTTCCCTGCAA
A_14_P104216 CTCCTTAAGGAAATTGACTATGAGATATTTAACTTTGACTTTCCAGTGACTGACAGCCTT
A_14_P201355 ATCTCCATCTGAGCCTAAAGTTGCCCACTGGCACCAATAGATTCTGTTT
A_16_P00685545 GTCCTAGGATTCATTAATATTATTATATTTTAGTCTTTGGTCAATATGAAGAAATACTCA
A_14_P113673 CAAGGTGAAGGAAAATTCTCAGGATGACTAAACCACTACTAATAAATACCTTTTGCCTGA
A_16_P18315506
TCCACCAACACTGTCATATAACCTGCTAGAACTCATGTTACAAGACTTTATGGAGAAAAA
A_14_P131917 GCAACTCATCAACTATCTGAAAGACAGCCTCTAGGTTTACTTCTGTCTTTGTATTTTCAT
CUST_7401_PI426210822 AAAACATTTCCTCCCCTGGTAGAAATGCTGCATTGAGCCACAGCC
CUST_1822_PI426210822
TGAAAAGAAGAAAACAGATATATAGTATTTCATACAGTACTTGTCCGAAAGAAGACACTC
CUST_4004_PI426210822
TTTGTTGAGGCTGCTGTGTAACTTTGACTTTCTCTGCTGTCTCCTATTTAAGAGTA
A_14_P131232 AATTTTACTTTATTTTTCAATGTTTTTCAATCACCAGTTTGACAATGGCATTGGCCCGTG
A_14_P110744 ACAGTAAGTTTAATCAGCAACTTGGATATGAAGTCTAAGTTAGGAACCCCCTAATGACAA
A_14_P201965 CACAAAGAGGACTTAGGTTTCAATGCTGGCTTTGTGACATATGAGTGTCTTTAATACTTT
A_16_P17518501
GCATCTCCTATTTCTCTACCATCTTAACCAGAACTACACATGATTTTAAAACTGCTCTTG
A_14_P120403 CTAAATTAAAAAGAAGATTCCCATTTCCTCTCCCCATCGCACTTTCCACCTTAATTTTAT
A_14_P130157 GTGGAAGCAGAAGAATGGGGTAAATTTCTTCACACCAAAAATAAGGTAATCACACATG
A_18_P25342639
CACTTGGCGTAATACTTTCCTTTAATAACTCACTTGTTAAGGCTAAATACCTGTTGAGTT
A_14_P130032 TTGAAATTGTCAATCTATTGCCGCCAATCTAAATTGATTTGACCCAAATGCCAATCAGTA
A_14_P128865 GGTTCAAGTTAATTTTTAAATGCATCACTAACGTGTAAATAGTGACTGTTTGTTCCAGCA
A_14_P106030 ATCTGTCCACCACTTATGACACTCTATTTCACTATTGTGTCATTTTGATGATAGAGGAGT
CUST_8483_PI426210822
CTGTATGTGATTTCAAAGAATCTTAGAATATATATTGCATGACAGGAAATGGAGGTCTGC
A_14_P115996 CTATTTGGAGAAAAATTTTAGTCTTTTTTTCATTCTAAATATTTTTCTGGAAATGGTAGA
A_16_P00366935

AAGATAAGTCAACGTTGGGCATTTTTATCACTATATTCACATAGATGACTTGGTGGCACG
A_16_P21412620
ACTGCCATTTTATGGCTCTAGAGAAATATGCTTCACAAGTTTAAACTCAACCTGGTTAGG
A_16_P03119398
ATACCTGAAAGTAGTATGTGGTATGTAACAAGCATCCAGTATTCATCATCCAGGTTCGAC
A_16_P00921367
AAATCCAGCATCTCAATTATCATCCCCAAATTTCTATCAATATATGAAGATGGCCAAGGC
A_14_P110805 TGGCTGCATTTAGTTTTATTTTTCATTAAGCAACAGCCTCTTAAGGAGAAGATCTCTTCC
CUST_4112_PI426210822 CTGCAGTTGAGAGGTACAAGAAATGTTCAATGAGGGGAAGACACTCTACT
A_14_P200761 CTAGTTAAAACTCTTTGGTTTCTGTCTCTGTAAAAAAAAGTGTCTAATTGCATTTGGGCA
A_14_P133782 TTGTGTTTGAAAAGGAAATACATGTCCCAGGTAAAAATCCAATCAACACAATTAGGAGTC
A_14_P115177 AGAACCGACTCCAGTTGAATGTTTAGATTTTTGCTAAACTGTTTTCTTTTTCCCTTTTTT
A_18_P13543989
AAACTGGGCTAAGACATACAGAAATTAAAACTCCTCACAAAAGAGAAGATAAACACTGCC
CUST_6701_PI426210822 AAGCCTTTGACCTGGAGGGGCATGGCTTCAGGTGACCAGAACTGT
A_14_P113308 AATTTATTTTATGTTAGGGATATGAGGCATCAATTTAATTTAAACCAAGTGCCAAGTGGA
A_14_P139929 GGTATAAACTGGGAGTTCTAGAAGTTGGAGATAAAGGGGACATAAAGATGAAATCAAAAC
A_14_P125179 AACTATTCAAGAAAACTCACTTGACTTTTAGCCTGTGGTCTTTCCGAAAATTAGAGACTC
A_16_P03463917
TTCTTGCCTTACAGTAAAATTGCAAAGAAGAGTAGCATAAGAGGTAGATCAGAAGACTGC
A_14_P120414 CTTGGAATTTTTATTTCTGTATATGGCCACCACACTCTACTTCAAAGAATGTTGCTCTCA
A_14_P117710 TGACAGCAGCGCTCGGTAAGTGTTTCCTTATTGGTTCAAATATGTAACTAATGGTC
A_16_P39905701
ATATTTCTGTCATGGCTCATTCAAAGACTAGGACCAATGATGGAAAAATTACATATCCGC
A_18_P22840482 TAAGCAGGTAAAAGGGACGCATTTTGCCAGCAGGAGAGAGAAGTTTG
A_14_P113201 ATTCCACCAGGAAAACAAGAAACTGAAAACCTGAGCAATGCCTAGAAAATAAACGG
A_16_P16988989
ACGCCTATAAAATATGCATCTTAGATCATCTTCCGTTTTAGAAAATTGATCCCACCTGCT
A_16_P19860346
ACTCCTTTTTGTCTCCATCAATAAACTAATTTGCCTCCATCAATAAACTAACAATTGCTT
A_14_P126436 TTCAGTGAATATTTTTATTAAACTCTACTGTGGACAAGAAGCCTGTGGAAAGGTGTTTCG
A_14_P102846 CCAGAAGGACATTTTTTCGACATGTGAAGAAAGCATCTAGGAGGAAAGCCAAA
A_14_P138382 CAATGGATATGCTATTAGACACAAGTTCCTGTCTTCATCTTCTTCAAAGCGCACAGATAC
A_14_P135052 TCATGAATGGACACAATACATTTCCAAGCAGAATCTCTGAACCTTCCAAACTCATTAAAT
CUST_10105_PI426210822 GGTTGTTCCCAAAAGTGTTGCATAAACGTGAATCGTGACATGGTACTGAAA
A_14_P107419 AATTGTTTTATTGTTGTCAGCGCTGCAGAATGGTGTGTAATCTTTGTATTCTGGAGAATT
A_18_P16214336
AATCAACCTAGGACATTAAGCACATTCGATTCATGTATACTGAGGAGATAGGATTAACGG

A_16_P16595243
TTGCTAAACCACAATACTCTAACCAGATGCTATTTACCTGTTTTTGATCATTTGCAGCAA
A_14_P138792 TTTCCGAGCCATACAAAGTTTACCGAATTGTTCCTGAGGAAGAGCAAAAATGTAACTAT
A_16_P16191587
TGATGGCACATGATCTTTCATTCATCAAAGGAAAGTAATACTAAGATTCCTTTTCTTCAG
A_16_P19856704
AAATTATAGCAACACCCTACTATACAGTTCACATCCAGAAATAAGAAAAGACCTACATCA
A_14_P124218 GAGTGAAGCCTTTTCTTTATCACTTCATCCAAATCATCTCCTATCTCCTGTGTTTGAGT
A_14_P127986 ACTTTTCCCCATTATAACCAGCATTGTCTCTGCCAAATATCAGGGCTTATTTCCTATTC
A_18_P14138233
TTTTTCCCCCTCTATTAAAATGCGTTAATTTGGAGTCTTTGCTTACTAAATGCTGTCTCC
A_14_P117190 AGCCTTGCCAATGATGAAGGAACATAAAATTCACTTACCATTAAAATCAACTATGGTAGG
A_14_P132037 CAGTTTGATTTGGTCAAATTTAGGTCAGATTATTAACCTGAGAAGCACATGCATAGGCAA
A_14_P106317 CCAACTTTCTGACTAGATCATGGCTTCATACCTTGATTGCTCAGTCTGAATAAAATGAAA
A_14_P118286 CAAGACTTGTTGATGAGTTGGATGTGGAGGAAGAAAAAGATACAAAATAAAATCCACTCT
A_14_P107876 CTTCACTACCCTTTATGCCAGGAACATCAGAAAGCCTGGGCTTTGAA
A_14_P131289 CTCAGAGATAAGCCACAGAGAAATGCTATTTCTACTCTCCTAAGATCTGGGAATTCACAA
A_14_P117589 TGAGTAGCGATTTTTCCCACCCTATTAGGGGAAATAGTTGTATCTGTATACACTGTAAAA
A_16_P40260339
ACACAAACTTTTAAATTCATTGTTTGGATATAATGCTGAAATAACCAATGAAAACCATTG
A_14_P116721 CTGCCTATCTGACAGGTCCTCTTTTCCTTTATAAAAATGAAAGCAAAAAGAAAAAGGGGT
A_14_P111758 GAGGAAGTCATTTAGGAAATGTTCAAATAACCAATATGTGGAAATGGACACAGGGATCTT
A_14_P200208 GGATGCTCAGGAGAGATAAATGCAAATGCAAAAACTGTTTTATTTTCTAGTCCTTTGGTG
A_16_P00424606
AGGAAAGAATATTGCACTTTTATGTATCTGTTTTATTAAGTGGTTACTCTTCCAAGGACA
A_14_P125483 ATGTATTTAAAGAATCAAAAGAGCAAAATTTCTATGGGTCACAGGAATCCATCATTGCTT
A_14_P122041 CTGGTAGTCTGTGGTTTATGATTGTCTCCTTGTTTGTCTTATTCAGAAACATTCTCTCAA
CUST_39_PI426210822
TTTAGTGTTTACCCTTCCCTAAAGACATTTGCTTAGAGTTTGGAGTACACTGAGG
A_14_P125730 ACAGGGTCATTCTGAATCATATTAAGTAGCCATTGCAGTTCTTCATAACTTCTGTCCACT
A_16_P17893906
GGAGGATTGTTGTCAATTAAATGAATATGGCCTACCAGTAGAAAAACTTTAGTGCCAACA
A_14_P129327 TATTTAAACCCATCATGGTCCACATCTTCCTAAGACATAACAATGCCTAACATTGACTGA
A_16_P16191909 GGACAGAGCATCCTGGTGGGGATTTTCGAGATGTGCTTCTCAGAC
A_14_P110549 ATCACCTCAGACATCTGCATTTATTACTGCAACTTTGTCCTTACCTATAAACTAATCCCT
A_14_P120179 AAAATAAGTTCCCCTGAAAGTCTTAACCAGGTTTACCTGGACAATCTGTGCAATATACTA
A_14_P100808 CTCACTAGGCTTCCACTTCTATTCCTTCTATGAAGTTTACAAAGTCTCCAGAGGTAA
CUST_10204_PI426210822 TATGTCTGTTACGAGGGTTCAGGGAGCTGCTCTTTGGAGCTGCAG

A_14_P126598 TAACGAACTGTGTTCTCAAGAGTTAAAGAGGTTGACTTTAGAACTTATAGGATGTGGAGA
A_14_P128352 GAGATTATGTTTGTTTCGGGTGAATTCCTGGACAAGACCGAGGATGACT
A_14_P139520 AAGTTCACATTCCTCTGAACAAGTAGACTTTCACAGCAATACAGAACCCTTGTGTT
A_16_P38178602
ACCGCATAACTCTTTCTTAATGAAGCATCTTCCACAATATACATTACCTAAACATCTGGC
A_16_P02120093
CACTGCCACCTCTTCATTATCATTCCTTGCTATTATATCATTCCCCAAAAAACTCTACAA
A_14_P123991 GGCAATATTGTATCTGTTTAGAAAATGGGCTTTTCCAAAAGCAAACAAAGATAGGTTCCT
A_14_P111682 TGAACTTATCCCATCTCTTCTTCCAGATTTCCATGAAAGTATTTTTCTCACTCTCCAACA
A_14_P110809 CTGAAGGGACTTAACATACTCTTAATGGCTTTCCTCTCTCTTGTTTTACCTTATGCC
A_14_P137992 CATAAAGTTGAAAAGATAATAGGGGTGGGAAGGAGAACAGTCCGAGTTTGAATG
A_14_P112228 GAAGTATTCTACAAAATCCAGGAAGATGTTTATGAGACCCTAAAGGATAGGTATTACCCT
A_16_P16578775
TAAGTGAAAAAGCCACAACCTTAGATATTTACAACCCATTTGAACTCAGGATTCATGTGC
A_14_P139069 CACAGGTTTGATGTGCTCATTTACACTCTTTTAATGTTAATGAAAACATTTGTTGCTGCT
A_14_P131736 TTATTTGCTAAATTATTCTCTTGAGCAAAGATCTTTCCTTGAGCACTAGCCATGGTTGCA
A_14_P137564
CTGAAGAAACAAGAAGAGAAATGCTCTTACAAGAGGAGGAGAAAATGATACAACAAAGAC
A_14_P136565 GGGTTACTATCTTTCTAGCCTACAATTTTTGTGTTCTCTCTGCATGCTATTCACACACTA
A_16_P19232110
AAATACTAGATGTAACCCGATGAGACTAGATGTTCCTTCTGCTGTTAATGAAGAGGAAAC
A_14_P136739
TTCGAGAACTACGATAAAAAGCAAAGAAAAAAAAGTAAGGACCCTTGGTAACAACATGCT
A_16_P18476100
TGTGGTCATTAAAGCAGAAGTCTCTACGCTATTTTAATTGTTACTGTCATATGAGCCTCC
A_14_P117394 CTTTTAAACTGTATCAATAGCAGTTCTTAAATATACCTTTCCCTGCATCTTGCCCCATGT
A_14_P110872 ATTGGAGAGGAATTTGTTGGACAAGACAAGATATGTCTTAGTAATATCCTAAGAAGATCC
A_14_P114167 CACAACACTAAAACCATCCAGGAATATTTGGGTAAAATATAAGTCTCCTCCTTGAATGCA
A_16_P18407661
ATTTCTCACCCTATAAGACTTTTCCCACATGATCAAACTTACCAGACAGTATCCAGTTTC
A_14_P128010 GAACCCAAATTGGTTTCTGTAACAAAAGGAATGTTAAATTCCTTTGCATTGAGACTAAGG
A_14_P113770 TTTTATTGATAACCTAAAGAAACCAGTTGCCACTCACCTATAGGCATGGGGCTAATTTAA
A_14_P101202 AATGTTAGTGAAGTGGCTTTTATGTGCCAATAAGTTGTGTTTAGCAGTCAGGAATAGAAC
A_18_P22839392 ACCCAAGGCCAGAGGATGCCCCAATGTCATGTGCACTCTATCTAC
CUST_1285_PI426210822 AGTAGGTACAGAGGGCAGACGTGGGTCCTCAGACTGATAAAATGTA
A_14_P124596 AAGTTTTCGCTCCGTATCTCAAATGTGCATGAAGGAATTTAACTGCACGAGTGAAT
CUST_6803_PI426210822 GGTTGGTCTGATAGAGCCTCGCTGAGGTTCTTATACAAGATCTAGTAGCATA
A_14_P120355 ATAAGGGTTCTATTAGCAGACATGCATGTATGCACTCATTAAATCACTTCATGTGTTGCC

A_18_P14132671
CTAGGGCATTTAGAAGATAACATGCTTTTTTCTGGTTCTTGCAAATTACTTTCGGTGACT
CUST_9129_PI426210822 TAGCAACACTGGCTGGGCTGGAGCTCTGCTCCCAAGCTTCCTCTC
A_16_P30952462 GTAGGCCTCAGGGCATTCATTGCTGTCTATTTTTCGTTACAGTATGGATGAGAAAA
A_14_P137880 TGAAGAGGCCTTCTAGCATCACCTAAAGAATACCTGCAAAATAATTTAAACACACCAAT
A_14_P123763 GCTATATTCATGCAATCAAGTTAGCCCAACAAGGAAACCTCTTAGAAAAAAGAGTAGGTA
A_16_P03635188
AAACAATTTTTTAAGCAGTGATAGAATTTTCCCTCCAGATTTAGAAGGAATCGTCCCTGA
A_14_P137876 GTGGTAGTTAACTAGGAATGTAAATTACTAAAAAGGATTGCTTATAATTAATAGTAATTG
CUST_1211_PI426210822
AGAAAACGAGAGCTTTGGTGGGACGATGTATAGGGAAAACTACTTGTAAATTAAAGG
A_14_P114034 CTTAAGTCTGGACTTAGTATTTGAATTTTCTCCATCGATTGCCATCTTATCTCTGCCTTT
A_14_P115106 CTGAATGTGAATGCCCATGCTATTGTTTATAAAATCACCCCTCCTCCTTTTCTAACTC
A_16_P16191392 CCTCATGTGTGAACCTTAATATATTCTGCCAAATATTCTGTTTATAATATCTAACCCTCC
A_16_P15249152
TTGAACTGCAATTTGAGCATATTTTTACCCTTCAGACATTACAAACATGCCATAACACCC
A_16_P37954201
GTCTTCTATTCCCAGATTTGCATTAGATACTTCACCTGATAATTTAATGAGCACACCTCA
A_14_P117439 TTATAAGGTGAAGTGGAACTAAAACCAGCAGCCACTAGCATCAATATCTATACTCTCAGT
A_14_P114209 CTCTTGCTTTTTTTAATGAAGGGAAGACTGGAAAAGGGAAAGTGATGGCAATAAAAGTAA
A_14_P200848 ATAGGGGATTGCTGAAAAGGAATGGCCTTCAAGACTCAAATAATATATGCTATATTGGTG
A_14_P112795 AACTTTTTTTTTTCATCTCTTGTTTAACCACTGCTCAAGTCTTGTCATCTCTTTCTCCTC
A_14_P135795 CCCAAAGAAATACAACAAACAGTAGAGGTCTAGTCCAAGGCAGAAGATATACAAAATTCT
A_14_P122611 AATATGTCAAGATCAAAGGTTTCAGTGGCTGTTTACTGGACACAGGAAGAATTCTG
A_16_P38133330
ATTGGACAAAAAGAAGCCTAATTCTGGGTCTTTCAATACTTCTCCCCCAAGACTATTATT
A_16_P02893723
AAAACTTAACACACTTCTAGACAATATACCTCAAAAGAGATTCTTCTACTTGCTTCACTA
A_14_P130232 CTGCCACTGGCATCCCATTAGAAAGTGATGAAGATAGCAATGATAATGACAATGATATAG
CUST_734_PI426210822 CTTCCTCATGGAATCAGATACAGACCCTCCAAGACTGACCTGGAA
A_14_P129060 CTTTAATTACAACTCCTTGGAGCACGCTCACCTGTTTTGCTGTTTCAGTGAG
A_14_P102864 CTTCTAGGCCATTATCAAGAGTCAGGATCTGTCATTATATTTGTGGATAAGCAGGAACAT
CUST_989_PI426210822
ATGTGGTTTGCAGCAGAAAAAATAACAATAGATTGTTTTCTGTAGTTTAATGTCATACGA
A_14_P200966 ACTCTCACTTCCCTTCTGAATAGAGGAGAGGCACTCAGAATAGTATTTTAGGGATATTAT
CUST_5984_PI426210822 ATGAAGGTGCTGGTCCTGATCATTTAGCCTCATTTCACAGAATGGCTT
A_16_P15113777
AAACCTCCCATCATATAGCAATGTCCAGAATCCCTAAATAAATAGAATATTTGCTGGCCT

A_14_P137311 TAGCATATGCCAGAGAAGTAGAAAGAGCCATTGTATGTCTGGGTATAATCATATAACCAG
A_14_P108993 ACAAATGCTTATCGTCCTACACCTCTGTAAAAGAGAACTTTGACAAAAAAGGTAACATCA
A_16_P17876837
AGGACCATTGCATAAGTTACTTGTTTCAGTGTCTTTCTTTAATGCTTTAGTGTGCTTACA
A_18_P22841710
TGATTCATTCATTTATTCATCAGACTTTGAGTGTTGACACTCAACACTGAAATGAATGAG
A_14_P119511 CTCTTGAAAAAAACAGTTGAGGTATTTCACATAAAAAACTGAACAATCAGGTTAGCCACG
A_16_P15798379
AACGTTACAAGTGCCTAAATTACAAACATATTCCACTGTCTTTTTAATAAAGGGCCAGCC
A_16_P16407861
AAAAAAACTTCTACACTAAGACTTTACCTTGATTGCACATGAAATCTAAGCCCACCTTGG
A_16_P17143727
AAAATTGACAACTGTTTGCATCCATAATTTCTGAAGTGTCCACTATGAATGACTCTTCCC
A_14_P106503 TGCCCATGATTTGGAAAAGAGGCTTCTCTATAACCTTGAAGAAAACAATTATCTGCAAAT
A_14_P115134 GTTCAGTAGTCACAGATGGAGTTCAGGACGAAGATCAAGACTAGAGTAAAAGTCATAAAT
A_14_P139181 TGGGCACCTCTCTGGTTTTTAGGCTTTAAAGCAGATCCAAATGTTATATTATAGTCTTGT
CUST_1128_PI426210822
GTTGATCTGAGGTCTTTACACATTCTCTCATCTAATCTTCCCAGAAGCTGTGAAAAA
CUST_2307_PI426210822
TTATTAAATACTAAAAAAAGCATTCTTAGAATCCCAAGTTCGCTAACACTCAGGGATGCA
A_14_P128777
GAAAGTGAACAGAAGAAACTGGAAAGGAAATAAGAACACTGGTTTTAGAAACTGCTTTCT
A_14_P123269 CTGTGGAGACACCAGGTAAGATTAAATAAATGCTTTAACCAAGTCATTAGCAGAATGCTT
A_16_P17621048
TTTGTATCCTCAATCTGCTCATTAGTGAAATCCTAGGAACACTACGAAATACTACCTTCC
A_16_P35680701
TCATTCAGCCCTATACAACTCAGATCTTACGGAAAAATCTACTTTTAACATTTGACCCTC
A_18_P19550117
ACTGTTTTAAGTGATATTTGAAAAAGGAAGATACATCAACCCAATTAAAACCACATTTTC
A_14_P116364 CTTCAAACCACCAACTTAAAGGTGTAGAAATAGTAAGCATAATACCGCCATAACCAGTTT
A_16_P21340489
AGAGGCTTTCATTAGAACACTTGGAACGTTATTCAGATGAAATGTCAAAATGAGCAGCG
A_16_P00828375
AAAGGATTTTCAGGTCTCTGTAGATAATTGTTGCTCAATTAAGTCAAAGGATGGATCACC
A_14_P116136 TAGGGTCTAATATATTCACAAAGAGGTCACAGGAGACTTCTAATGATACCTTTTATGGGC
CUST_1972_PI426210822 CAACATTACCCCCTTCCCTAATCCTCAGCCCTCAACTGTCATTGC
A_14_P132660 GTTGCAGGTGATTTTCAAAGCCAAGTCAAAATATTCTCCAGAATTACTCAAATACCGGT
A_16_P38853476

CAATCTCAGTCGACATTCTAGGGAAACAATTCATCGATTCTAAAATAGTAGATCTTGGGC

A_14_P121680 CTGGGAAACTGGGTTTCTCATTAAAGTTGTTTCCCACTCTGTATTCTCTGTGAAATT

CUST_1025_PI426210822 AGGCCTGCATCCTGGCTGCTGGCACTGCTCCAGTGTCTGTTAGCA

A_14_P202386 AGTGACACTAATAGCCAACTGGACCAAATCATTTGCCTTATCATTCATAGTGTCCTC

A_16_P16056232

AGATTTCCAGTTAAAATGTAGGCTTGTTCATGTATTCTTCCATTGTCTGAAAGCGAACAA

A_16_P16488187

AGGTTCCCTATATGCACTACATCCTCCATTTTTAAAAAGTTTAATGATGCTTACGCGACT

CUST_1003_PI426210822 GTTATTTCACATGTCCTGGGACATGTGGCAGGGGGGCTGTAAAGA

A_16_P00993182

AAAACTTCCTAGTATATTCACTAGCACTCTGATACCTATGATAGTGACATAAGGGCAGCC

A_14_P119539 ACAAGAAACATCAAAGCGATCATACTTATGAAATAATGGTAATGATGAAGTTGCTGGGAA

A_18_P13561106

TACACATGCACATAATCAAATTTCTTCGCATTAGTTCATTTCAAATCTGTCCACATCAGC

A_16_P16191670 AAACCTTTGTCACAAGGAACCCTAGGTTTCAGTTCGACAGAAACTTGACAAA

A_18_P15209880

CTGTCAGTAATATCTGCAGTTACCAATAGAAGGTGTGCTATCTATTTTCACAGCAGTCTG

A_16_P00584299

TGCTTTTGTTAGCCATTCTTAGCATATCCTTAGTAGCAAGAACTATTTAACACCGCACTG

A_16_P17213900

ACTCATGGAAAATTGCTATTCTCAGAATACCTTCACTACTTCCAAGTTGACTACATTCCT

A_14_P124877 GTTCCTAGTCTAGGCTTGGTATCTGATAAGTTACCTGTAAATTATTGCAAACTTCCAGTC

A_14_P114912 CTGGACTCTCTGGCTCTGCATCACCTTTCTCTTAAAAAGGAAGCAGAAAAAAAT

CUST_4492_PI426210822 CTAAGGCATGCTTTACCCCTGGCTCCCAGGCACAGAGATGAGGGT

A_14_P119290 ATATGAAGGATTACATACCATCTACTGCAAAACGCAGCCTGGGATTTCAATAACTACAAA

A_14_P107183 TTTCTCCTCTTCAGAGAGATTTTTTTTATAGCACAGATTCCTTTGCCCCTTTTATCTCC

A_16_P17897717

AACAAATCCTCTCCAGCATAATCAACTCTTCCTTTACTGTCAATATGGAATTCCTTGTTC

A_14_P132517 ACTGTGGGCATTTAATATTAAAATCAAGAAATATTCCCAAGGAAATAAACCCTCCCTCAG

A_14_P103322 GTGAACTCGTCATGAGGGCGCAAGGCAATGTGTATCATCTTAAGGTAGTATTT

CUST_10228_PI426210822 TAATGGAATAGGTAGGACTGGCTATGAAGTACTATAGCAGGCTGAAGGGG

A_14_P134326 ATTAGTTACTGTATAGGTCTCAACCTATGCATTCTTTCATTGTTGCTGTGGAAACAAGCA

A_16_P17833232

AGCCATACACCCATCTCATTAGGATTAGTTTTGTTAATCTATAGATCCACTTATTCGGCC

A_14_P124815 CAAGCAACTTACCTTAAAAGTCCCCACCACCTCTTTTGATCCTCCTTC

CUST_6790_PI426210822 CTGGGGTTATGTGTACATCTTCATGGTATTGACTGTTATGGCATGCATGGA

A_18_P17015194

AAGATAGAAGAACAAAATTTCACCCTAATGTAGTGTCTTTAGACCGTGCAATGTTGTGCT

A_14_P133881 CATGTTTTTCTTGAAGTAACGTGTCCTCACTCTTGAGCCTGAAATTTCTGCCTTAAC

A_14_P131971 TTTGGATTTTGATTGCCAACTGGTCTCCATAAGCATGATTAACATTTAGAAGGGTTTTTG

A_14_P139699 TAGCACTGAGACAAATCAGGATATCTGAAAGGAGGTCTTTGTTGAAAAACAGAAATATGC

A_14_P126148 GATTGTTGGTCTTCCTCTTCACTTTCGTCTTCTTTGCTTCTGATCTGTTATCAAGT

A_14_P112191 GGCAAAGATCCTTTTAGGCAAAAGACATTTTCACATCTAACAGTAACATCTCTGATGGTC

A_14_P128033 AAAAATATCTCATGGCAAAAGACATTATAGACAAACATAGCCAGTGCAACCTGTTCTACC

A_14_P133192 AATTGTCCAAATGAGGCTCTCATGTCTAGTGATTATTACCATTACTGCAGAACTCTGTGT

CUST_8144_PI426210822 CAACTCTTCAACTTAGCTCATTCTGGAGCAGTCACACGATGACAGATGA

A_16_P16863773

TCCTATGTATCTTGTCTACTTCATCCACCTATCAACCTCTTTCATAAATGCAATTTCTCA

CUST_1008_PI426210822

GAAGAGGTCAGAGGTAGGCCATAAGAACGCTAAATGTTATCACTTACGAAAACAATG

CUST_8446_PI426210822

ATTGTTATAAACACACAAGTTAAGGGCAAGATTCTTGCCAAGAAAATTAATGTACGTATT

A_14_P138257 ATGAAGCCCGGCTAGGTATAAAATTGATAAGATGCTTGTCACATTAATGGTGGTCTAATA

A_18_P14133996 GTCTTGCCAGATCCCCTAGAGTGAACAGGGGATTCTTTATCCTCTC

A_16_P03807008

TCTCTACCTTACTTCTGCAATTGCCAGTATACTGTTAAACCATTTATTAGCCTTGACCTC

A_14_P202338 ACCTGTAAGATTACTTAAGGGTAACTTAAGTGTTTCGCTCAAGTGTTGTGTTCTGATTTC

A_14_P202503 ACAAAAACCAGTCATTTAAGTTCTTGGCAGTAACAACATATTTATACCTAGTGCTTCATT

A_16_P36847181

AACGTGACTTCCTATTTAGTGTTACCCTCTGCTACTCTTGTATTACCTTTCTAAATCACA

A_18_P20393293

AAAAAAAATTAACCTCATCTTCTACTGGGAATCCTAGTTCCTTGACTCTTTCCATTCAGA

A_14_P113901 TCTTTAAGAATCTCTCCTTACTGGTCACAGCAGTCTTGAAATAGCCAGGTTCAATCATA

CUST_2393_PI426210822 GTGAGAGCCCTCTGGGCATATGGCGGCCCCTATGATCCTTCTCTT

A_14_P129516

TTTTTAGAAATATAAAGAGAAGGACAAACACAAACAGAAACACAAGAAGCAGCCAGAACC

A_16_P01643673

AAAAATCACACTTTTTGGGAGATGTTGTTCAAGAGAGAGACTGGAAAAGCATGACTTAGT

A_14_P137393 CTCACCCTCATCATCTTAGAAGCATCAGGATAAAAAGGCTTGATTTCTTTGGTATAATCT

A_16_P18310969

GGGAAGATGGAATAAATCAATGGCAAATAGTGAAGAAGGAATACAGATATACCATCCACG

CUST_7248_PI426210822 GCTTCTCTTCTTTCCGGTAGATGAACTTGAGCACATGATCTCACCAC

A_14_P110390 TTGCTTTTAATTTTCCCCATCTGATTTTATCTCTGCGTTTCAGTGACCTACCTTAAAACA

A_16_P00685372

AGAAATGCTGTTCTTTCAGTTAACAGTTGTGTTTTTTCCAAGAAAAAAAAGAAATACAAA

A_16_P38524965

AGAATTGCTTGTCTAAACATCCAATCACAGAATCTTTCCCTTTCACTGAAATTGATCACA

A_14_P130692 TTCTTCCTTTACGAGTCTAAATGGCTATCAGGAATCAAAGACACATGGACTTTTTGTTAG

A_18_P22839940

TCAAAGGTGAAGATTTTTTTCCCCTTTAATGTTAAAAGTGGTCTCCACAAGGGATAAAAA

A_14_P109683 AACACATACATGGCCAAATGCAATACTGAAGAGACAATCATCCTGTATCTTTTATGGTTT

A_14_P113179 TCCTCCAGTTACTGACAAATTCTCCTTGAGCAGGAAATGGTATCTTAAAAGTCTTTTTAC

A_14_P107334 CTCCCAAGGTCGAGAAAAGTGCATCCTTTTGTTCTAATGAATCTGAAATTCCATTTTAAA

A_14_P112708 CCTTTTTGTAAACAGAGGCTTTCATTGACCAGTTTTCCCTCCTTGTTGACATCTAT

A_16_P00148321

GTGCAGTTCAGAAACAATTATGCATACCATTAAGGATGTTATCAGGGTAACTTTGCAGTG

A_14_P122322 GGCATATGTTATTGTCAGGCTATGTTAAACGGTTAGAGAGGAAGAAACTTAGAAGGCAC

A_14_P126606 ATGCTTAAGGAAATCTAAACATCACCAAACTAAGGCTTGAGGCTTGTAAATGCTTTTTCT

A_14_P132411 GAAGACAGATGCTGATACATATAAAAATGTGTAGGATGTATATGGGTGTGTGTGCTGCAA

A_14_P133420 TCAAATTCATTCTTCTGCCCAAACCTACCATTTTCAGTCCTTAAAACTAAACAGGCTAAA

A_14_P133829 AGAAATATGTCAAGTGTGAACGTGAAGATAAGATTCCCAGTTCTTGAAGGAAACTCCATA

A_14_P102880 TATATGTGTCATGTTATCGCACGTGAACCACTGCCTATGTTAAATAGGTTTTTCTTTCCT

A_14_P131680

AAAAATTGCAATTGTCCAGCAAATGCAAATGTTTAAAAAGGAAACTTGAGGAACCATGGA

A_14_P121176 GTGAGTTCGGAATAATCACAGCCCGAAGAAAGCACCTTGTGGAAGAT

A_18_P14789142

GGTCTCATTTGCCAATTATTCATCATTAAGCCATCCTTAATTATTGCCATCACAGACCTA

A_14_P201977 CTGCCTGGTCCATTTTCTTCTATTTGCAAAGTAGTGGTAACCATTTCAAGATAGTAACAA

A_14_P111149 TTGTTGTGCAATCAGGGTTAAATAGGGTTAGTGTCTATAAAGGAGCACATTGTTCCATAA

A_14_P100918 CATGTCTGAACAATTAGCCTGCTTCCTCTATACCCATATCTCACAATCCAGATTTATTTT

A_16_P00629762

ACTAAGTTCTTCACTGTCCAGGTTGAATATTACAGGTTAAGACACAGACATTTCAGAGTA

CUST_8303_PI426210822 CTCTTTCAGAATCATGTCTCCAACCTCCTTTGTATATGGCGAGTCCGT

A_14_P125261 AAGTTACTGGCATGGTTTGAGAGATAGAAGGAATTGATTAAGAAACCTCTCAAAGTTTCT

A_14_P113792 TTTTAAAATTAGTGTTTTTCCTAGTTTGCACTGATGCGTGTATGGATGTGTGTGAGTCAG

A_18_P17392570

TGAAACCTCTGTCTCTAGTAATTTACCTCCTAAATTGACTTATTTCCACTGTGGCACTTA

A_14_P119823 ACACTGCAGACACAAATAGCAATTGCAAAGTCATCATGATAATTTGCATGAGTGCTTATC

A_14_P201545 TCCTGAAATGAATCCTTTGTCAGCAGTTAACATGTTTCAGAAACAAAATTCAAAACCCAG

A_14_P111665 CAATCATGTAGCCTGGTGTTTCAGGAAAAGATCATAAAGCATCTCTAAGATTTCTTTCAG

A_14_P117580 AGGCGCTAAAACCAAGCAGAAACGCAATGTTATTTACCATTATGACTTTATTCAGTGTTA

A_16_P19006520

CATCCAATCCCAGAGTTTTGATGATAATTCGCTCCAACTAACATATGTGCAACATCTATA

A_14_P136492 CCCCATGACAGACATTTTTGATGATTATCAACAGAAACTTTATTTCTCATCGGTTCAGGA

A_16_P41115072 AAAAGTAAAGAACATTTTACAGTTTAACAATCTCGCAATACCACTAATGATAACAACAGT
CUST_4937_PI426210822 ATGAAGATCATCACCAGGATTTGAGGGAATGCTGTGGTGCATACTTTGT
A_16_P16783837 ACACATCCAGGTTATAAACTTGCTGCATACATAGAACCAAACCCTAATTGTCTGATGATA
A_14_P107974 CTCCTTAAAGAGAGAGACTTGGTAAATTATCTTTACCAGGTGTTACCCAAGCCTGT
A_14_P133084 CTCTATCGTTGGCTACTATCTTCAGGAAATAATCAGTCCAGATTGAAGAGATGGAAAATA
A_14_P135987 CATTTTTAGCGTCGATGACAAATGAAAAAGAAGGGGAAAAGGCTCAGAGAATTAAAAATG
A_14_P100385 CGCTAGAGAAGCAATTTCTGACCCCTCTTTCTTTCTCTGGTCACTCAATTTC
CUST_1609_PI426210822 GACTCCCCTCCTCTGCCCTCTTGCTGACCTCGCCACTTGGGCTTG
CUST_2295_PI426210822 AATCAACAACCAATAATTTCTGGTATCCAGGGACTCAGACATGACAACCAGAATT
CUST_3650_PI426210822 TATAGAATGCCTGGGAAATGCAACACTGGCCATCCTTCAGCTCTT
A_14_P101269 CCACAACAGAATGATCTTGAGATTCCTGAAAGTCCTACCTATGAAAATTTCACCTGAAAG
A_18_P13086925 TAAGGCAATGATCAAGTCATCTTCAACTTGATCACAGACAATTTTGAATTTGTCTGTCGC
A_16_P19080559 AAAGCCTAATTTGATCGTCAAAGTGAACAACGAATTTATTATTGTAGCAGAATCCACCGC
A_14_P121962 TTTAGAGCAGTGTCCTTTTATTCCATGGAATATTATCCCAACAGAATTTTAACAGGAGGC
CUST_2750_PI426210822 CTTGAATCATGGCATCTGCTATAGGAAATCCTCTTAAATGTATTCTTATCTCCCTAATAA
A_16_P01697615 TCACTGCAGAACATAAAGACAATTTCAGTCCAACATGTCAGAAATCTCACTTTCAGAAAA
A_14_P106641 GAGTATTTTCTTAGGCATTTTGGACAATCTTTTTGCAGTAGGTTCCATAGGTTTTCCC
A_16_P16191903 CCTTGGTTCTCAGAGTAGGCAATGGGTAATTGAATTAGCAAAGTAGACATTCAAAACATT
A_16_P03712428 ACAGTAAGGAAACCACAAAGATAAACCCAAATAATAGAGCCAATTTTCTTTTTTTCCGGG
A_14_P120022 AATTTTATGAATCTGACAGGCAAGTAAAACATAAGACCCTCCTATATTGGGAGACTCAAG
A_14_P124802 GTCAGTGCTCAAGAAATGTCTACCAAATTGAAATTTAAATAGATACAGCCTGTGGGTTTG
CUST_9083_PI426210822 TTGGGTTGTCTGCAACACATGAAAAAAGCATCATGTCCCCTTGACTTT
A_14_P133468 GTTCTCTTTTATACTCCCAAGGAGAGTTCGTGCATTCTAGTAAATGCATCATCTTTCTTT
CUST_9529_PI426210822 TAGTTTGTTCACATAACAAATCTACATTCAAAGACATGCAAACAAATTTTCCACTTAGTC
A_14_P120369 CAGTGAAACTGTGAGCATTTCAGGATAGGGAGAAGTGCTGAACAATTTCAAAAATACA
A_14_P113193 GGAATAAAACCCTAATTAGTTAGATTATTTCTCAAATTGGAATATGTGGCTTCCTGGGGC
A_14_P128051 CACTCACTATGCAAAGAAGTCATTCCCTCTAGTTAGTGTTAAAACCAGTTATGGGTCTTC

A_16_P20887921
AACAAGTCATTTGCCTTTTTATTCGGTGAAGTAATAGACTATGAAGAAATCTGGCTTGCC
A_14_P100165
GAAAAAAGAACCCAAATTCCATCCAAGTTAACCAGCAATGACTTAAAAAGCAAGCAAAAC
A_16_P18161761
AGTGGAGGCAGATATATTCTTAGAAGACCTTACTCCTGAAATGGTTATACCCCTTACATA
CUST_3304_PI426210822
TTCCTGTTCTTTTTCAGTAGTTCTTTTTCACTATTCCTTGATTTATTTAACCTGTCCAGG
A_16_P19116643
AATACTGTCCCCTAGTTTCCATATCTAATTGCTAAGGATTTATGAATTGACATAGCCGGG
A_14_P122667 ACCTCCCCAGTTACGATTAAGATAGTAATCGTATCTGGATTTGGCCTATTTCATACAATT
A_14_P104829 AGAGATACAATACTAGAATAATTCACCCTAATGTGTGGCCCAAAGAATCTAGGTTTTCCA
A_16_P17927185
ACATCCCTGGAATGTAATATCCTAACACCTTCTTTTCATTCTCTTCCATATTGCACATTG
A_16_P18226629
TCAATGCATGTTTATCAAGCATCATTAGGTTTTGACTCCACATAATGCTAGTCTCTGACT
A_14_P129061 AACAAATTCCTCCCAGAGGTTGAAAATATGACTGGTGACCCAAACATTTCTGTATAATTA
A_18_P22736957 AGAAACCTTCACTTTCCTTCATACCCTTAACAGACTTGTGTATATGCCATTTGTCCAC
A_14_P136302 GACCAAATATTCCTTTCCCAATCTGAGGTATTCTTGGGGCAATAAAATGAAGTTTCTAAT
A_16_P19617978
AACACTTTAAACACAAAAAGAAACCTTTCCTCAAACACTGTATAGTCGATTGCTGAGTGG
CUST_9754_PI426210822 TGGTTCTGCTGAAAACATTTATTCCAGCCCCAACCACCCTGTCTC
CUST_2420_PI426210822
AAGAAAGTCAAAGTGAGCCCCTAGATAGCTGTAAACCTGGTTCTATTGTACTTTGA
A_18_P22841028 CTCAGAAAGCCCTCTTTCCTGCCCTGATATCCTTGTGAACATGACTTTTT
A_14_P139166 TACCTTTTAAAACACTGTTAGTGTTCCCTGTAGATAAGGTAGCAGGTATTTTTGCCAATG
A_14_P133677 GCATGGAGGGAGAGGAATAAAGAGAAACAGAGTCCAGGAAACACTGT
A_14_P136583 GTGTATTCATTTAACTGTTCATTTGTATAACCCCGTTTAAGAATACTGTCCTCCAAGTGC
A_16_P17130574
ACTTTACCCTTTTATCACATTTGTCTTGATTTCCTCTAGAAAAAATCTAGTCGCCTGAGC
A_14_P123995 TTAGGTTTCACATAACAATTAGACTTCCACACAGAAAACAAAGCCTTTGAAAGACTTGCT
A_16_P18312630
AAAGCCAATTCTGTGTTACATCCTTGTTAGAACATTGTGCAATTCAAATAAGCGAGTCTG
A_18_P22842479 TAAGATTTCTGGCTTTTTGTTGGCTCTGCCCTTCTGACCTCAACTTCAG
CUST_3191_PI426210822 CTTATCACACACCATATGGCTAGCAGACAGTGGGGCCAGCATGAG
A_14_P113558 TTTCTCCTTTCACAAGTTGTTGGAGAAATTGTCCTTTTCATGATTTCAACTCTAATCCCA
A_18_P22841073 AGTCATAAGCCTTTCTCACCAGGAGCACTGGACTCTCCTTTAAGAAAGTTTT
CUST_6726_PI426210822 TTACCCAGGAATCACCCCCACCCCCCATTTTTGTTTAATGAACTA

CUST_3105_PI426210822
ATGCCTTTCTCAGTGGTCTACTACAAATTAGCTCATGGTCACAGTTAAGGCATTTTAA
A_14_P132892 TGCTCCTTAAGGAGTCTCTTTCTTTAGTAGAATCATACCCTTTGCCTTAGTTTTGGTAAT
A_14_P139741 AGTCTCTTGAAGTTTCACATCACATGGTTTTCAAATATCCTTTGTGGTTTCTTTTCCAGA
A_16_P17829145
ATAAGATCCCATTTCAGTTGATGTGTTTGGAATACTTGCCTTACTGGTCATAATAGCACA
A_14_P126316 TTGAGTGATTCTCACTGAGCAACAGTTTACAGTTGCTAAATTGCAAAAACACAAGAAAGT
CUST_3110_PI426210822 ATTTTAGGATGCATTTTTTGGCAACCTTTCCCCTCGCACTCTAAATAAACTTGC
A_14_P121160 GATAATAGTAAGTGGACTGCTTAAGTCTGTTTCCCTTCCTAAGGCACATGTTCTATCAAT
A_14_P135350 ACTGTTACCACCATACAGGCGATTAAGAAGACCCCTATGCAGTAAATGGCTAT
A_14_P113368 CTCCCATGTCTTCTAAGTTTGTATTTTTCACTGCATTTAGTGCAGAACTTGTCATCTGAA
A_16_P19971106
GCCTGGTATCAGCATTATGATCAAAGATTACTTAGAACTGCTATAGATACTCCACATGCA
A_14_P129900 CTAAATACAACTATCTCCCAAATTTTGGGCCATACTCATTGGCAACAAACAACAGAATTT
A_18_P13181484
AAGTCCAACTCTGGTTTATGCTCTCTTGGTAAAAATAAACTCTAACATTTGCAGAAAGCA
A_14_P108600 GTGAGTAGCATTTCCTGGGTTGAGTGCACAGGCATTTTTATTATTCGAAATAGTTTAACA
A_14_P202069 GAGATTTCGGTGATCATATCCATATTTGACTGTTTCCTATTCCATAATTTGTCGCTGTCT
CUST_7129_PI426210822
CTTGTTTGCATGGTTCAGAACTAGGCATTGCAAAGATAACCATTTACTCTGATTTCAGA
A_16_P30952619 GTCACATGACCTGATCACTAGCCTGTCATCCTTGGTCTTTGTATCTTCCATATTT
A_18_P22841774 CTCCTAACGGGTTGGGAGTGAGGTGGAGTGGGGAGGGGATGGCTC
CUST_1980_PI426210822 GAGATTACTCTGCCTGGGTCCTCCCCACCCTGTCCCTAATCCTCT
A_14_P100139
TCTTACAAAAAGAAAAAGGAACAAAGAATAAATAGTGACCGTGAAGAAAGGTGGTCTTGA
A_14_P106702 GATGACCAATGACTCAGAAGGGAAAATGGGATTTAAACACCCAAAGGTTAGTTTG
CUST_523_PI426210822 ATGGCCTTTCCTACCCCTTCCTATCTAGTGTCCAAGTGCTGTGAT
A_14_P136542 TCACTTTTAAGAAAAAAACCCCTGCAGCACATGCTAAAATAACACTAAGGAAGGGTTTAA
A_14_P132752 TAGCACTAATCACAGAAGGTGCTCTGACGGAGTTACTTTGCCTCAAAAAAAAGTATTTTT
A_14_P116608 ATCTTTCAATCTCTTTCCCTCTTCCTAAAATAGGCTCTTCTTAAAGTCTCTTTGATCCAG
CUST_812_PI426210822 ACCCTTTGAAAGCAGCCAGCAGAACATCACTCCTCAACCCAAGTC
CUST_3694_PI426210822 GCTGCAGTGAGCATCTTGGGTCACCTAACTATTAATTCTGGAGGACA
A_16_P40363209
GTGTGGCTGCTTAATAAGAATAATCTAGGATAGCACAAGTTGATCATTCTTCGTATAGCC
CUST_327_PI426210822 TGTTCTCCCCTTGATTTCTGTTCTCATGCTGTTGTTTTCAAGTATTTTCCTCC
A_14_P110425 GGTGCTGACAGACATTTAGATATCTTTAATTCAAATTTCCTCACTGAGGAAGAATTGAGG
A_14_P134907 AGACTTCTCTTTGAACACAAATGCATTAGCCTTGTGGCTAGAACACCCTCTT
A_14_P107477 CTAGATTGACAGTAAGTTTAAAAATGCCAGTTAGGCTGATTCTAATCCCCAGTGGCT

A_14_P118632 TAAGCAGTTTTCTACCAATTCTGGAAAAAATCAATCTCACCTAGTGTCACTGATCTAGGA
A_14_P132225 ACACTAGTTGAATAGGAAAGATAGGATCTTGGTAGAAAGAGACTCTATCGAGTGAGATTG
A_18_P13726005
AGTATCAGTTTATGCCAAAATCCAGTCAAATACATGAGTCCTTTGCATACAATGGTGATC
A_14_P138977 CTTATATTCCTCCACCTCTCATGGTGTGACTTTATGTAGATCCTTAGTGAACACATTCTC
A_14_P120047 AAGATACTCACTTAAGACTATCTTCCTAAGAATACAATTGAGGATTGCTCAATGGGCTCC
A_14_P112836 TTTCTAAAGGAATTGGTGTTTCCTGATCTGGTATAGTTTCTGTTTGTTTTGGATTGTCCA
A_18_P13300603
ACCCAATTTTTCTGAGATTCTCAAAAAAAATATTTTCTGATAAGGGCAAAGATTGTTCCA
A_14_P136844 CTGATGACAAATCCTCGTAGAGTCCCATGTTTTCTATAGAAACCTTCAGGGAAGA
A_14_P105417 ATTGACCTCACAACCGAGCAAATTGAAGAGACGTTAAAATACTTCCGTAAGTAGTTGTC
A_16_P00007806
AAATCTGGCTAAATCATCTGTGCAATTGTATAAGAAGTTGTTAATCAAGTCTCGATGGGC
CUST_3704_PI426210822 GTTTCTCATACCTTTTTATTTCAGTCCCTGGTGTTTTTGTCATCTTGTGTGGCA
A_14_P131092 AAAAGAAAACCCCGTGAAAGAATACTCCAGATAAATGGAAAAGTTACAATTGCTCTAGGA
A_16_P18253804
AAAACCAAATATACATGTTTCCCCATTTTCTCTTACATCAAGCCAAATCTCTCTAGACCC
A_16_P16888909
AAACTCTTTTGACACAAGATGTCTCATTTGACTTTCACAAATGTCTCATTTGACTAGCCC
CUST_9702_PI426210822 GAACCACATGAAAAATGAGCCTCCTTCTCCCCAAATCCTCAAGTC
A_16_P18381931
AAGAGCCCTTAGAGTCTATCTATTCTAGACGTTTAAGAACTCATCATGCCAGTAAGAAGC
A_16_P15958760
TGTTGAAAGTTATTTCTGCTTCCATACAGTAATCCTCTCTATTAGACTTGCAGTAGTGCC
A_14_P107425 ACATTGTAATGTTCAAGATACAATACGTCACTACCTGTGCTAACTGCGCTAATTGGATGA
A_14_P129724 TTTTTGAGATACCTTTGGGCTTAGATTGGCATTGTTTTATTCTAAAAACCCAACTCAGTG
A_14_P121267 AGAGTTTTCCTAGCACCATTGTGTAATTTATCCTTTTCCCCTGTTTTAGAATGACATTTC
A_18_P11070171
AAAGCAAAATCATCATCAAAATCTAGTGCCACCTAAAATATTCCCACAGTTATTCCTGGC
A_14_P110492 ATGTTTTCCCACTGGAATGTTGCCTTTTTGCTTACTGATATGTAAGCCTTCTTTTTAAGA
A_14_P127653 GTTGCCATCTCCAGTTATTATCTAATCACACTGTTATTACCAGGTCATTAGAGAGGGAG
A_14_P107668 TTAGAGTTTTAACCCTTCTGTAAACTCATCACCAACTGTTTTAAAGCATGACATCACCTG
CUST_9296_PI426210822 AAATAGGCACAGAGGTAACACACTGACCTGAGAGGATGCAGGCAT
A_14_P120422 TCTATCCACAATCATCAAAGGATCCCCAATTTACACCATGAGTTAATTCCTAGGTAATTC
CUST_6470_PI426210822
GGAACCACTTATAGCTGACTACATTTTTTAATATCTTCACTTTGGTGTTTTATTATTGTC
A_14_P115914 ATATAACACCAATTCTTGCCCCAAAAACAGAGTTTCTTGGTTATATCTTTGGTTGGCAAT
A_18_P27216045 CTCTCAGTGCCTTCTCTTCTTCCATCCATCCCTCCTAAAAGGATCT

CUST_2470_PI426210822
TTATTTGTGACTGTGGTCTTTTCCTGTGACTCCATTTCCCTATTATCTGTTTCAGAAA
A_14_P130424 TTTTCAGATGTGGACTTTATTTGGTTCTACAAGACTTCTCTTAACAGTCTTAGCAACCAC
A_14_P120616 TGACCTGTTCAAGGTAGGGAAGTGATTCATTAAAGACAAGAATCATATGCAACGAAGAAA
A_14_P130903 ACTCTTTGAAGATTTCCTTTCATTTCCTTTTCTCTTCTCTCTTATTTCTCTAAGCCCCAA
A_14_P126519
GCAAGATGAAAAAAAGCAAATAAAAGATACTTGGGAAGAAGAAGACTTGGAGGCAATTTT
A_16_P01297143
AAGACTCTGGTCTTTACTACACTACTGTTACCAAGGCTAAGATCATACTGCTGATTCAAT
A_14_P116389 ACTTACTGCGGAGACATTTTCAAGTCCTTTTAGGTCCTCTTTAAATTTCTTTTTGGAACC
A_16_P41874212
TAGAACTTTACTGCTACATCAATGAATGACAAGTCTCTCTATTCAGCATGAAATCCCTGC
CUST_4789_PI426210822 GAGACTTAGGGTGTGGCTTTCCCCATGGACCATGGTGGGTCTTTG
A_16_P37751597
TGAAGACACAAGAACTACATACTGATCACTAACCTCACAAGTATAATTAGACTCAGATGG
A_14_P105115 ATCCAGGGCAACAGTAAGCATTATAACGTGATCCACAGACATTGGGTCTAAAA
CUST_9999_PI426210822 CTGAGGTCACCCAGCTGTGTGGGATCCAAAGAGCCAGGACCTGAG
A_16_P20880232
ACATCAAGCATTAGATCAGAACCAGAAATATCTTTACCCTGAATGAGATCTTGAAGACCT
A_14_P102199 CTGAAGGTGAACTTTCCAGTGTTGATAAAGTTGAATTCACACTGGACACATTCATTTAAC
A_14_P123708 CGGTTGGCCTTCTGGTCAGTTGGATAATCTTATATAAAAACCTGTTATTAAAGTTGATGA
A_14_P109646 AATTAAGAACTCTGCCCATGGCACATTTTGTAAATACGTGTGTATTTTTCCCCCC
CUST_6923_PI426210822 AGTGAGGAAGTGGTGGAGAAACGCAGATGTCCATTTCAAATCACAAG
A_14_P137807 TGATGAATTGCTTGACTTAGCAAAGTTTCATTTCGGTGACTCTTTATGCACACACAAAG
A_16_P58424134
CAAAGGCACTCAGATTTGAATTTTGATACATGTTGGCAAATTGCACTTCAGACATGTTTA
A_14_P136696 TTAAGAGTTGGACTTCACAGGCTCAATATCCACTGCTTGGTCCCTTCT
CUST_6970_PI426210822 TCAATGTGTTTGGATTTCTGTCCTTCAACAGAGGTGCCACAGCTC
A_14_P124553 AAGAACTGAAAGTGGCTTGTTCAAGTGAGCAAATAAAAAACTGTTTGCTTATCATCTTCC
A_14_P103102 CAGTCAACCACATTGGATAATTCAATTGCAATAAATTGCTTATTCTGTGCCATCTCCTCC
A_14_P137347 CTTTCTAAAGCCATCTATTGTTGGCTGTTAAAATTATAGCATGTTCTGCCGTCTCTTCAC
A_16_P40410498
CTCCACTTAAACCCAAGAATCACAGAACTTTTCTTATCACAAAAGGCAAAACAATTCGAA
A_14_P121893 GGAAATAACTTTGTATTATTTCCTTCAGCCATCATTTTTGGGGTAAGGGGTCCATAAAAA
A_14_P132575 GATATATTCACTGTGGTTTTATGATGATTTGACATTACAGAAAAGAGCAGAGAAGGGGCT
A_14_P202439 CTGAATAAAGACGATAACTATTTTCAAAACTGCTTTTCTTCAGGGACACAGAAGCACTTG
A_18_P24944641
TTGTTTAGCATTACCATTTCTATATCAACCTACCTACAATACAAACCTACCTACACATAC

CUST_5129_PI426210822 TGAGAAGACAGGCCCACTGTGGTTTGAGTAACTTGGAAATCTTGTG
A_14_P137396 GATTTTATTTTAAAAGATTTTCTCTGCTTTTCTTAAGCCACATGGGTGATCACGTGCCTT
A_16_P03747620
GCCTTGATTTCTATCAGTTCTCCATACTTGCCTAAATATTCTTTGAGACTGCTTTTACCC
A_16_P16191146
CATTTTTCAACTTTTATCCTGGGTTAGTCTAAATCATCAGAAAGCAACATATCCCATCCC
A_14_P132778 TTTAAAGGAAACTTCCTGTGCAACCCAGATTATCACCTTTGAAAGTTTCAAAGAGAACC
A_14_P124759 TAAATTTGTATTTAATCTTTGACCTGCAATCCCACAGAGTAGAATGGTGTCCCTAGGTAT
A_14_P108097 GAGTTTGCTTACCACGGTGCATTCTTTATGCACACTACTTAGATTTCTGTAGGAAAGAG
A_14_P104551 CATCCTTAACATTTTTCATTACTTTTTAGGTTCAAAATGAGCCTGTTTGGAACAACCTCA
A_14_P109728 CTTGGGTCTGAAAGAAATCGGTCAACAGGACTGAAAGAAATGGACACAAAG
A_14_P135394 CAAACTTTGTGCTCGCTGTTACCCAGAGAATCAAGATCACATGTGAGACATTTAATAATG
A_16_P15967828
CTCCTTTCAGTTTTACATAAGACATTCCAACTCTACATATAAGACAAGGCTCACAATGGG
A_16_P19654965
AGCCTATGACCAAGACAGTTTTACATGCTCAACCTACATTTCACATATGCAGTTACAAAA
A_18_P14354039
AGGGAATAGTAGACAATCAAGGCATCATTGTATAGCAGAAAAGTCAGTAGGTAAAACAGG
A_14_P117304 TGATATACTGAGTTATTCTGAGTGTCTCTCCTGACCTGTTCTAGAAAATGCATAACTGCA
A_16_P02401232
TCTCTTTGGTCTACCAATATTTCTCCTCATTTGGCTATCTAGTGACTGATTAACTCACAG
A_14_P121882 ACCAAAGAATCTCTCCTTCTACAATGGGAACACTAAATGCTCTCAAGTTATATTTCAGTG
A_14_P103775 TCCTAGGCAAGGAATTAACATTAGGTTAGGAGGTGACACTTTGGATACTTGTTTTTTCTA
A_14_P128353
GACAAGGAAATGGCTGATTTCCTGAGAATCAAGTTAAAACCTCTAGACAAAGTAACCAAA
A_18_P16974506
AGATGTGATCAATCAAAGTCACCCATCTTACCATATCCACCTCATTGTTCCTCTAAAAAG
A_14_P122040 AATCTGGAAACCTTTTCCTTTACATTCTACAAGTTTTATTCTGCTCCCACAAGGCAATAA
CUST_7113_PI426210822 AAAAACCCAGATCCCCTACAGAAATACAACACCGGTGACTTGCTG
A_16_P19516288
TAGGTTATAACATACCTGTGACATCACATGAAATGCTGTAGTCAATTTGACATCATGGGG
A_16_P00685335
AAAACCATCTAGTTTGAAGTATGTGTGAGAAAAACAACTAATTCACCCTTCTCACTGACC
A_14_P121007 TAAAGGAGGATCTAAAATTCATCTACACTTCAGGGGAAAACTTAGAAGACAATCTTGACC
A_16_P21199276
AAAAAATGGCAGGGCTAAATGATTACATTCTTGGGTTATATCAGTGGGTTCCATTAAACA
A_14_P104786 CTTATTTACTAGTGCTTATACTTCATCTAGTTAACCCTCAAAGGGAAAGAGCCCTGTGTT
A_16_P39650945

AGTCCCGCTCCTAATAACTATTAACATGCCTAGTTTCTTCAACTTTTTCTACCTCAAGAG
CUST_977_PI426210822
TAAGTCCAAAAACTTAGCAAAACCAAAACATAACTGATTTACCCACAAATTAGATAACAA
A_14_P134363 ATAGATCAAGAAGTAAAGGAGAAAGCGGGGGGAAAAAACTTCAAAATTTCCTTCTACATA
A_18_P14779933
AAGGTCCAGAAAAAACTAACACAATTTCTTAGAAGTCCAACAAATCACTTCAGACAGAGC
A_14_P114461 TAAAACCATGTTCACCAGAAGGAAAAGACAGACATAAACTACCTTTGATTTATTTCCCCT
A_14_P104148 AGGTTGAGTTTTTTTTTCTTTACCAAGCCCAGAAGTTTAATTGACATGTAGTTTGTAGGC
A_16_P03435323
CCATTTGCAGATGCCTAAAAGATACAAAAAAGGATAAACAGTGGCTATCACCCAAATTTG
A_14_P121625 CTTATGTTGCCTTCTTTGCTAACTAAAGTTTCAACGGAGTGTTTACTCATGGCTCAATAT
A_16_P00685542
GCTTCCAAAATATTTCATTATCTGTGGAAGGAAAACATTTTTGGTATGCATGTCAAGTGC
A_16_P21244434
CCATCTCCCATTGATCACTATAATTCACAAATTCTCCTAAAAAACAGAAACCACAGTTGA
A_14_P139601 CTGAAGAAACTGGTGTATGGAATCAGGAGGCCAGCATATTTATTTGTTTATTTACCTGAA
A_14_P136871 AGTATTTTGAGTCTGGCAGTTTTGTTCTCATTAACAGATCAAGACAAAGGAAAGGCTAAG
A_14_P124853 GAGGCTGTGGAAGTCTTTAACATATACCTAATCTGTCAAGGGTTTTTACTTTCCTGGTAT
CUST_3602_PI426210822 CTCCCTCACATCTGAGCTGTCTTAGCACCTTCTTTCCACCTGTAT
A_14_P121612 AACAAAATTATTATGTAAATAGTTGCGCACAGTGGTCTTATGCCTGAGACTGTGACAGAA
A_18_P13190114
CCCCTCAATTAAAATCTGTCATATTGTGAGCAAATTCTCCATATGTGTCATCTTTTCCGT
A_18_P15064372
TCCTCTTGTTACCTTTTGTCCCAACAAAAATCTCTACAATGAATTTGAATTCATCCCATC
A_16_P19859512
GGATAAACAGGATCACAAAGTTCATGACAAAAGCATAATAGAAGTCTCATAACCCTCTGG
A_14_P119608 TTGCCGATTGAATCTGATTTAGTAAATGCAGTTGCTATGCATTAAACTTTCAGGAGATGC
A_16_P36230114 CTCCAGAGAATTTACCTGCAGATTACCCTCAAAACATGAGACAGGTTCAGAG
A_14_P118942 TGAATTCACCCTCTGTTTACAGATAACTCTCTTCACTATTCCTAGGAGGAAAAAGAAATG
CUST_10339_PI426210822 TCATTCAAACTGCACCACCTTATCGACTTCCCCTCACGGTGAATG
CUST_1793_PI426210822
CCTAATGATGACACACAATGCTCTTTCGCACTCATGATACATAACTTACAGGGAAAG
A_16_P31951964
AAAAAAAAAGATTGTAGTGGTAGTCTAACTCCATAACGCTGAGGAAATACATCATTTTCA
A_14_P134802 CTGGGCAAATTCACAGCCTTTACTCTTCTAGTGTTTACTTCTTCTATGCATTTTTTGTGA
A_14_P112001 ACATGCATGAAGTTTTCCATGATAAAAATAATGGGGGAAGTGATGTGAAAAGTTTAGAGC
A_14_P103177 CAAGTATTTAATTGCTTCTGTCATCTCTCCTTGGCTAGAATGAATGAAATAGGAAGTCAA
A_16_P20595862

AATCGACAAACGAGAGATACACGCATTCAATTTGAAGTTTTGAAGACATTAAGAACCGAA
A_14_P101730 GACCCCAATTGGCAGAAACACTTTTATCATTTTGAGGTAAAGAGACTTTAATTTTCAGTT
A_16_P03439392
TGTTTACCACTTTAATCATCAATCCACATCTAGTCACAACAATAAGTCAGAAGGAGCAGT
A_14_P109046 GAATACTTTACATATGCTTAAGAAGGAACATAGCCTGTTTGCAAACAGTGAAAACCCACA
A_14_P120428 GCTTTAAAACCTTGAGGTGATTCATCTTCCAGGCTCTCCTTCCATCAAGT
A_14_P129800 TGGAAGAAATGTTACTGAAGCAAATCTTCTCTGCTTAGATTCATCTGGCCTAACTACAAT
A_14_P120102 CGTGGGGTTAATAAATGGCTTATCCTTCTCTCCACCCAAGTTTCCAC
CUST_7852_PI426210822
TTCTTGAGACAGCATTTGAGAAATTATTCTCTGGTTTTCCTTATTGAAATTGCCAATGCC
A_16_P17841866
TCCATCAGATTATGTTGATAACTCCGAATTCCACATTCTTTTGCTACAAAGATGCTTCTC
A_14_P103936 ATTCTGAGGATGTCTATGCAAAGTTGGATTCTTGTTACAGTGTATCCAATCTGAAGTATT
A_14_P106397 CTACCTGAAAAATAGGCACTTAGAGTCACATGTGAATCTTAGACCACATTAAGTGGTCTA
A_16_P18593587
AAGTCCTAAGTCATGCTACCTTCTAATCTCATAGAATCAGAGTAACTGAATGGATTTCCC
A_14_P104111 TTCAAGTATGAGTCAGAGTTAACCATGTAGAGGAAAGGAGAGGGTGAATGTTCTAGAAA
A_14_P114111 TTCTGCTGAGACATACATTTGCAATGCCAACTAATGCAGGTTTATTGAGAAATACATGGT
A_16_P02482205
GCAAAGATGCCTTAGTCTACCTGTTATCTGTTAGTATGGTGTTCAAGAGGAATAGAAAGT
A_14_P120060 AATAAAGAAAATCCACGTGATAAAGGAATATCTAAAGATCTAAGGGTCTGCATCTTTCTC
A_14_P110232 CTTCAACACAATGGCTGATTCATCTTTTCTGTGCCAGTGCATTTTTATTACTTTGAAGTT
A_14_P103145 CTCATGTGTCTCTTGTTTTTTTTTTTTCAGGGTCAACCAGGAACTAGAGGTTTCC
A_16_P01911927
GTGTCCCCTTCTCATAGTATATCTCCTAGATGAAACACTAACTATATACGAAGAGGCCAT
A_14_P118134 CTCATAGAAAATAGAGGAAGGATGAAAGGGTCTGCGGACAATTTGGAGAAAGCATAT
CUST_7775_PI426210822 GTTTGCTCACTGGTCCGCGTTCCCAGTCAGCTAAGAGTCTCATAA
A_14_P133626 GGCCATTATTCCCTGATTCATATCACCTTTGATGACAGTAGTAATAATGACACTAATGGC
A_16_P03750072
AGCACCTAACAAAAATGCAAGCAATTTCTATCAGTATATCTCCTAATCTCAGGTTTCCTT
A_14_P100471 CTTTCACTCTAGCACTTGCCATACCAAAGGGCAATTATTTATCTACCTATCTGTATCTCC
A_16_P18577384
AAAATTGTCATTAGAACAGCAAATAGGCCATGTGAATTCAAGTCACCCATTTCATCACAT
CUST_6329_PI426210822
AGATGTCCTAAGATTTAGTGATTCCGGAAACTACTCCTAAAGTCAAGGCTGATGA
A_14_P120861 TATGACTAGTAGAGATATGTCATTTTGTGTCATTGTTCCCTGGTGGTCACGTGATAGATA
A_16_P18746405
AAAAGTATGTGAGCACTTTGGATTATCACTACAGTTGAAACCAAGAACAAGCAATGTGAG

A_14_P124864 CTGTGGCCAAGGTTAACTGTATACAGACTCATTCAGTATTTATGATTTTGCATAGTGGAG
A_16_P00685483
GAGCACAGAGAGATGTTAGAAAACTAATCATCATTGAGATCAGTTATATCCGAGACTGGA
A_14_P113658 CTAAAAGCCATGTTTTAACTTTCCTAAAAATTTCTCATCATAAAATTTGTGACCATTAAT
A_14_P124822 ACTGCTGTGAACTGGGTCTCTGTGAAGATGAAGGACTTGAAGTTCTC
A_16_P19879775
TACTTTCACCTGATCAATCATAGTTACCTCACAGATAAACCCTACATTTCTATCTGCCTG
CUST_395_PI426210822 AAAGTGGTTGGGTGCACTGGCTTCCTTTCTGGAGACGTGCTTCCT
CUST_6279_PI426210822 CTTTCCAGACCCAAGGGAATGGAAGGGGGCTCCCTTGAGAGAAAT
CUST_10042_PI426210822 AGAAGGAAGGTCAACAGAAGGAAGCCCCTTCTCTCTACTTGGCTA
A_16_P21382021
AACTAGCATTTTAAACCCATGAGTAAATGACACTTTTCTGAGTTTAAGTCTCCCTTTCGC
A_16_P15291453
AGCCAAAATTTCCTCCAAATGAACATATCTCTCATTTTATTCTGAAAGTCCATGAAAGGA
A_14_P138556 CTAGTGGTATGCTTCACTTGGGACCCAGAGATACATGGGCATTATAGTTCAAATTATAAT
A_14_P105194 ATGTGCACTTAGCCAGAAATAGAGAATTTAAATAGTGGTGAGTCTACCAGTCATTCCATT
A_18_P23553273
AAAGGACAGATAGACTGGAACAAACCATCAAAGATGCATTTTACGAAAAACACAAGTTTG
CUST_5236_PI426210822
AGGATTGTAAATGAAACTATACTTGGCCTCAGTTTATTCTCTGGGAAATAGGTTCTTGCT
A_16_P16032051
TCCAGTTACAGATGAAGCATAGTATTCGTATGACATCCAATACTTTTTCACTGGTTAGCA
A_16_P17139679
TCTTACGATGTCAAGTTACTCTACCACATTTATAGACTACACATAGATACAGCCCACTCC
A_14_P127747 GAATATGACAGAGGTCACTTTTCTGAATTATAGTTGTATAGGTTTGGCTTTCCTGGTGTT
A_14_P119099 AGCCCTGTTATATTTGTTCTGTCATTTAAAATTAGGAACTCACCAAAAACTGTGTTGGTG
A_18_P12434241
AAATGTCCCAATATGCCTCAATTCTGCGGAATGATTTTACTAACGACTACCTAAAAGTCA
A_14_P103166 AATTTTCTGATGAACGTGCTGCATATTTCAGGTGTTATCATAAGGTAAGACTCAATTGCT
A_14_P123171 ATATACAAGCATGGAGGGGCACCACAGATTAAAGTAAGATCAATGTTACCAGAACAATAA
A_18_P11719819
AGTGAAACTTGTAATGCCAATTGTCAATACTACTTTCCAGTCATATCATTCTCCCAACTG
A_14_P119885 ACAGTAAGAATGGCATGAGGAGTCAATTCTTCTAATATGGAAGAATTATCTGCAGTGTAG
A_16_P03218766
GAGTCAATGCCTGTCTAGATTTACCTATAGACTTGACAACTATATTGTTTGCCTGTGAGC
CUST_4872_PI426210822
GAGGGTAGCAGAAAACAAGTTCCTTGTGAGATCAGACACATGAGCTAAACATTTA
CUST_7942_PI426210822 TTCCTCAAATGCAGCGTGGGATTTCCTTTCATGAGGTCAAAGTCAAA

CUST_7520_PI426210822
CTACTCAGTAATTATTAAATGAGAGCATGAACAAAGGAATGTAAGATGTAACAGAAAACA
A_18_P17306238
TGACTTTCTAAATCCCACTAACTTAAATGACCAGTTCAATTATACATTCCTGCAACCCTT
CUST_10337_PI426210822 TCTTGCCAGTTGTTCTAAGAAAGCTTCTGCTCTTTCCCAGTCATTCAAACT
A_14_P101803 AAGGTGGTCTTATGTTTACTTTCCTCACTTTGTTTGTGACGTTTTGTTCTTATTCACTGA
A_14_P133827 ACATTGTTTCCTCAAGGCAGAATTTTCCTGGTTCTGTTTTCTCAGCCAGTT
A_14_P125568 GAGACTCTGTGCTGCAAGAGGATGGATTGAGAATAACTTTACTTGTTTTTTGGGAG
A_16_P16928934
ACAGCATTCTCCTAAATTGATGATGTATGGTCATGACATGTTTTAGTTTATGCCTCCACA
A_14_P101837 CAATGGTCACCTTTTCATAATTAGCCACATCTTTAAGAAAACGGGCATTAAGATTCCTAG
A_14_P101415 GTGGTCTTTGAATCCACAACCTCTAGTCTGAACACAGAGAACACAAATTTGTCTA
A_14_P106368 CAATCTTTGCTTTATCCTCCTCATTCAATGTTTCTGTGAGAGCAGAAAGAAAAATGAAAC
A_16_P20231485
AAAAAACCAATGAACAGGATACAATTCGCCTTTTTTTCTTCATATACCACCACTCTAGGG
A_16_P16842660
TTGACTTCCATGGAAAGTTTTGTGTTAGTATGTGGTCCCATTTTTAACCTATTTGCAGAC
A_14_P130140 TTGGAACAGGAGGAATTGTTTCTACAGCATTTTGCCTGTTATACAAATTATTTACCCTGA
A_16_P03603060
AGTTTAGTCCTCACATACCTATGTTTTACATCAATCCCCAAATAGTTCTTTCCCACAAAG
A_16_P03662124
CTGCAACAAAGGTAGATACTAAGTTCAGCAACATTTGAAGAATCTACTAAAAGATGGGGG
A_16_P16191892 ATCTCTACTTGAGATCGCCCAGTAGGAATATTTTGAACAAATTCCTTTAACCCATGG
CUST_2517_PI426210822 ACCCTTGTGGGCCTTTTTAAGTTTGAGAAAGCAGTTCTTGTTCTGGAAAATTT
CUST_6836_PI426210822
ATATTGACTTTTTAAAACCAAGATATATAATTGTTCAAATATGTGATGCACGTAATACAT
A_14_P101149 CTGCCAGACTTTCATTTTCGATGGTAAGTGAATAGAAAACATCCAGAATCCTGTGAG
A_14_P109641 CGCTCAGAGATGCTGCTTCATTTACCCAGGAGGTCATATTCTTTATATATATTTTTTGTT
CUST_10108_PI426210822 CATGCCGCCGCATTCACAGCACCACACAAGCCTGAAGGTCTGCAT
A_14_P122525 ACAGATAATCCACATGTCAACAGGGACTACTTACGATGATGGAAATTAAAATCGGTATTC
CUST_7540_PI426210822 GACCAGTGTTTCAAAGGCTAGTGTAGAACACTTGCCTCAGATGATTCAAGAT
A_16_P36230986
ATAGATGGTGATGTAGGAATCATGGCATCCAGAAAATGAGTGTGATTTACCCTTTGATAA
A_14_P121603 TGTCCTGTAAACATCTGTCCTGTAATGGCCTTTAAAAATGGCCTTTATACAAGTCAGTTA
A_16_P18998520
ACATTACAAAGCATTAAGTTGGATATTTAGAACTCGGCACACTAAATTTCATCTGGGGCT
CUST_7739_PI426210822
GTGCTTTTCATCACTGTGTTTTTAAAAATGTTAACTGATGATTTCCTGATCCTGAGAGTA

A_14_P139881 AAATACATGGATGAAGGAAATGACCCAAAAATATTTGGAAGAACTTGACAGTGTTGGAT
CUST_3108_PI426210822
CTGGTTGGACTAGATTCTTCCCCCTTGACTACTTTCTATTTATTTTAGGATGCATTTTTT
CUST_1681_PI426210822 GATCTAGCCTCCAGGGACTTGTCAGAGCCAGCAGCTTCTGCCAGT
A_14_P132673 TGTTGCTCTCTTGCCTAAATCTTCCTCTGAAGTAAAAGATCTCAAAAGGACAGATCATTA
A_14_P118101 ATTTGATTACTCTTGAAAGGACATTCCATTGCTAAAGATTCAAGTTTCAGTTCCTCAGCC
A_14_P123656 CTGATTCTGTTCTGAGGAGACTTCGCAATTTCACTTTATATGTTTTAGGTATGGGTAAGG
A_14_P111204 TAAACTTCAACTGGCTGGTCTTCACAGTAAATCCACCTATAAATTGGAAAACAGAAAGAT
A_16_P16767922
AAGTCTTATAACTCAGAGCAGTAATCCAGAAGTTACCAACAATTTCCAACTCCTATCCTC
A_14_P132800 TCACACCTTTCCTAGGAAGTAAGCAAGACAAGCATCAGACGGTGG
A_16_P19025001
AAAATTAGTTCCTGCATTGTATTGAGAGACATATCAACTGAACTCTAAGGACTGGTGAGG
A_16_P40730036
ACACTTGGACATATAACCTTTTTTTCCTACAGCATCATGATCATATTAGCCACACTTTCA
A_14_P120190 AGGTATTCAAGTGGAAAAAAAATCCAGTGAACTCTAATAGATGGTTTATTGGAAACGCTCA
A_14_P129620 ATTGTCCAGGCCTCTCTGACTCAAAATGCCTACACCTGTTGAAGGATAA
A_14_P122600 ACAGCAGTATTAATCTGATTCACAAATCTAGAGTATTCAGCATGAACCTGAAAGAGAGGG
A_16_P00349900
TGAGGATAGAGTTGATAGCATCTTATGACCAGTTGAAGAATATAACCAGGGCTTATGCAT
A_14_P137180 GTTGACCACAAAACCTTCTTTGTCAAAGATACACCTGTTTATAACACTTTGGGAAAGAAA
A_14_P133597 CAGGCTGAACAGAGAGGCTTAACAATCTAGAATGCTATAAATCAAATAAACAAGACTGCC
A_16_P00416914
AGATCCCAGAGGAACTATACTATTGTCTCAATTAGTCCAAACTGCATTTTCTACTGGATC
A_14_P110339 AGCCCCACCATTTAATCCTATACTTATAATTGGTGTGCAGCAGATGAATTTTTAGCCTTA
A_14_P120001 TAGACCCTGGAATATTCTGAGTGATCAGTTCTCTTTTCCCAGTTATCTTTTTCTTACTCT
A_14_P131614 TGTATTGAAAAAATTCCTAGTATTTCAGTAAAAATGCCTGTTGTGAGATGAACCTCCTGT
CUST_9763_PI426210822 GATGTTGACTGCAGAGAGCATCTTGCCCTTGTCATTGACCTTCAG
A_14_P135431 AAGAATAACTGCAAAGCAGATTAAGACCCATCAACAAAACGGTCCATTAACTCAAAAGTA
A_14_P128029 ACATTGCAAAATGAGACGATTGCCTACTTTCTCTGTTGAAATTAAGCAAGGTTTAGAGTT
A_18_P14132440 CAAGAGGTTTTGATTTAAAACACGAACCTTCTTTCACAACACAAGTGCCCCAG
A_16_P36230379 GGCTTCAATCACATTACATGCTGTGGGTTTAGAGTGTTGGATGCTGGTTTT
A_16_P16151727
CTTTTGTAGACCATTGTAAGCCTAGGATCATACCTCTTTCGTATTTCTTCTGCAAAATGC
A_16_P40309822
ACTCCTAAATGCTTAATGTTCACAGATGTGTTCTTGTTATAGTCAGTTTTGGATGCTGGT
A_16_P19762271
AAAAAAAGTGGCCATTAATAGACGGAACTATACACTGGACTAGTTGTTGTTTTAAAAACA

A_14_P137482 GACCTCTGGGTTTTTCTACACAGGTGTATGATTTAATTTATGCAGGTCTATTCTCACTT
A_16_P16191334
CATAACCATTTTGAGGTCATTTAATAAGGGATTGCTTTCTGGATAATCTCGCTCCTTGG
A_14_P107450 TCAAAAGGTTCTGACTGAAGGATGAAAAATGTGCCAGGTGGAATCTTTTCTTTTTTAAAC
A_16_P01164569
GCTCTCTGTTATTGTTCAACTTCTTGCTAGATACCACCTTTCTTAGAAAGAACTATGCCT
A_16_P18764439
AGACCTCAGGATTGCTAGATATAACCAAAGGCTTACTCTTTACCTTCTAAAATTGCAGTC
A_14_P129638 TGAGAATTAATGAACTAATAAAAGACAAAGGAGCGCCCTTTCCTTTTAGGCTTCTTATGC
A_14_P107737 ATCCAGAGTTTCATTTTTCTGCAAGTCCGTCAGAGAAAGAAATGTAACAGAAATGTTATC
A_14_P201485 CTCGTAATATTCCAGAAGATAATGCTGACATGGCCAGACTTGATTTCAATCTTATAAGGT
A_16_P16162916
GCGGTAAACTTCAAGTTAGACAAGTCTATAATGTGACTCATGGAACTTTCTCATGCTTTT
CUST_7731_PI426210822 CATTATTCGATTCTTCCAACCACTGCACGTTACAAACAGGGCTGTAGC
A_14_P123191 GAACAAATACACCTCTTACCTGATGATTTCATATGGGCAACAGGATTTATGTATTGCCAG
CUST_9808_PI426210822 TTCTAGCTGCCCACCTAAGAAGCTCCATTGTATACACTGGGATGCTA
A_14_P119355 ATTGTCTTAGACTGTCAGTAAAGTAAAGCCTCATTAATTTGAGTGGGCCAAGATAACTCA
A_14_P106110 ACCAAACACAGAATTCTTATGGAAATTGGTAATTATTTGGCACAGCAGTCACTCAGAAAA
A_16_P03042865
TCTCATGCTATTGATCATGCAACAACTGAAATAACCTAAACTACCACTCTGTTCCATTCT
A_14_P101920 GCACAATTTTGGATTCTTAAGTTTTTTGGGAGACTTCACTTTATCAGCAATTGCATTTGA
CUST_10342_PI426210822 GTGAAGATACCTGCTGGCTCTCTTGACAGTTCTCGTCTTTGGCTT
A_16_P15982344
CAGAAAAACCTCGAGAAAATGAATAAAAGACCCCTCTAAATCCTTACCAAGATTAGGCTA
A_14_P122790 ATTTTGGCAAAACAGCAGAGATATGCTTTTCTTACCTCAAGGCTGAAGGATTTTCTTTTA
A_14_P137291 TTTTACTTACATTGAGTGAGAATCCCAGTTAAGGCATTTTTAAAATTCTCCTTGGCTTCC
A_14_P128700 GATTTTGCTTTGTAACATCAGGGAGGTGTTTATTCGACTGCAACATGAAAATAAGATGCT
A_14_P111341 TATCTTTTCAGAACTTAAGGCTTCTTTCCGTCTTAATTCAGTCCTGCTGTTACAATGTGA
A_14_P115639 GAGTTTTCTACTCTGCCCATTTGCCCATTAAGCCTCACCTCGCTT
A_14_P111099 TAGTAAACATTTACCGAACATGACCAACCACCCAAAAGTGTGTTAAATACAATTTGGGAA
CUST_6756_PI426210822 TAAAAAGGAGCATAAGGAAGAATAGATAACCACTGCATCACACCAGGGTCAC
CUST_8554_PI426210822
TCTGTTCTTTGGGCTCTGAGAAACAAATCCTTTGGATTTTCAAAACTATATTTAGAATTT
A_14_P136551 CATATGCCAGGACACATGTCTGAGTCTGGATATGTGTTTATAACATAAACACTAGACTGA
A_14_P104541 TTTAACTGTAGATGACAACAGTGGAATTAGACTGTGCTGCAATATCTTAGGGAATGCTAC
A_14_P114367 AAATACCAGGAAGCCATTGCTCATTTCTGGTGATGAAATATGCTCTTATTTATTTTCCCT
A_14_P137923 ATATAAATGCAATTCTTACATTTTTCATCAGAATCACTGGGGGACTGAATGGTTGGTAGA
A_14_P106407 CTAAACTTTTATTTCATTGGCTTTTAGGTGGTGCTGGCTAGAGTGTATACGTTTGGAC

A_14_P112508 TGCTTTGAAGGCTTCTCCAAACGTGTCTGGAAGACTTATTTATCCAAGATCACAATATTT
CUST_4501_PI426210822 CATGTCCTTAGCGCAGCCCTGGGGGTTATCAGGGTGGCTCAGATG
A_14_P125760 CTTGGGAATTATTCCCTCTTTTTCTAGTCTGTGGAGACGGAGGGTTTAAGATCAATAT
A_14_P108681 CTGGATTGAAAATATTCCCAGTTCAATGGACAACAATTCTTGACTGAACCTAGGATGAG
CUST_3444_PI426210822
GTATAGATTTTATTCATGCCTATATTGTTAGAGAAAATCATTATTTTTAGTCAACGTATT
A_18_P22840469
TAAAATGTTCCTTGGAAATGGCAGGCAAAAACAAGATACAGTGAGTGTCCCTATTTACAT
A_14_P132700 CATCATTAGACTCTAAGAAGTCCTGGTTGGAAGAGATGATCTGCCAGAGAG
A_14_P139717 GCCACTCACTAAATCCTAGCACACTGGTGCAATCAACAGAATTTTTGAAATTCTCATTTC
A_14_P130198 GCAATGGAAAAAGTGGAGAGATGAAATTATTCTCAGGACAAGGTTAGTATCAAATGCATG
A_14_P201666 TTTCCACATCAGATTGAAATTAAATACTCACCACACAGAATTCAGTAAAACCCAACTACT
A_14_P108751 AAATGTTTCAACCGATTTTACCCTATGTTTTCAAGGGTATTATAGAAGGGGAGGTAAGAT
A_14_P125844 AATTTACCTGTTTCATCTGTAGCGGGATCATTCACCAGATCTGCAAAGACACATAAC
A_14_P107352 TATTATAACATTACAACAGTGCACAAGGAAAGACAATCCATAGCGAAAGGAACAGAAACC
CUST_1689_PI426210822 GTGGGTTTCAGCAGTGAGAGACTTGACCTGCACAGCATCTGTTAT
A_14_P125471 ACAGAATACAGTATTGTCATGGAACCAAGACTGACATATCATTCTGTGGTCAAATGCTAA
A_16_P00685199
TAAAATATGTCAGAAAGAATGAGGGAATGACTCAAACAATTTCAGGAAATGGTTCAAGGC
A_18_P12095068
AAAACCACACACTTACCTCTTACCATTTTGTCTGCTAAAATTAGATTAAAGTGTCTCTGA
A_14_P107742 AGTAAGTCAAAATCTTAGTAAACTCTATGATCACTTTGGCATTCCTCCAACAAACTCTGC
CUST_9592_PI426210822
AACAAAATATTTAAGAAGGGAATTCACATCTTTCTGTTCTAATTGGTATTCTTGCGCTAT
A_14_P129586 ATCCTTAATACATCACTCTGCATCTTGCTGATCTTTCACAAAACCAAGGTCATACCC
A_14_P133589 TAGAAACATTGGAATTACCAGAACAACTAACCATGGTCTCCATCTCTGAATTCTAGACAA
A_14_P108213 ATTAGTAATTTCTTGGTGGCTTTTTGGGATCAGTCTCACTACTTCTTAGTCTCACCTTAA
A_14_P139128 TTCCCACCATATATGCAAATAATTTTGCTTTTTCCATGAATGGAGACCATTGCCAGATAT
A_14_P110611 CTTCTATTAAAGAATCATGCTGAGCACAAGATCAGAGAGGTTGTGACATTGCAAACTC
A_14_P133188 GTCACAAATGGCTTCCATTTTGAGCAGCAAATTTTGCAAATTAGTTTGTTTGAATATCGC
A_14_P113383
TACAAAACAACAAAATAAAGAGTTTAAAAGTCTGGAAGGAAAGCAACATTTACAAGGGCC
A_16_P41845806
ACGAATGAAACCCCAAAACTTGACAATTCACCTATAAAATATAAGAAAAGCCCTTGTCGT
A_14_P137344 CAATCTCAATACAGCCTGAATAAACCAAGACAAGACCTCTGGCTTGTGAGC
A_14_P124833 TCAGGTGTTTTTTTTTTTTTAAGGCTGCTTTTGTGGAAATTAATGTCAAAAGCCCATGCA
CUST_4982_PI426210822
AGATTTAGAATATAACTTTTACAGGGAAGAAAGGGGTTAAAATGTCTGGTGCCATCTGTC

A_18_P22841195 CTAGGATATAGGGAAGAGGAAGGTGAGGGAGCACTTCCCAGTGAC
A_16_P00348612
CTCTTCATGACTTGCACTCTAACAAGTTCATTCCTCACTCAATGTATTAGACCCAAGTAG
A_14_P137214 CTTTTCTTCATGGAGGTGTCTCATTTCATTATCTGTGAAGAACTCTAGCAATTAAGGCAT
A_14_P130431 ATTCTCATAAGAGCACAGGTTTTTGTTGTTTATATCTTGACCCAAGACAATTTCCCTGAA
A_14_P133718 AAAATGATAATTACAGGTAATTACTGTAGCATAGACCTCAGTCTGTTCACCTTCCAACCT
A_14_P107279 ACTTTGTTTCATATCATATGTCTTTTTGCTCACTAACAAGGCTTATACTGGCTTAACCCT
A_14_P127049 ATTCCCTTCTACTACCTCCAATTCTTGAGAATGTTTACATCTTGGGGATCAATAGATACA
CUST_7919_PI426210822 CCTCTCTTTTCTTGGTGTGATTTTCTTAGAGTTTGTCTTTCCTGAACCACCAA
A_14_P125837 TTTCTCAGCCTAAACTATACTGTGTCCTTACAAACTAGATCCATTACCCAAATTCCAAGT
A_14_P135671 TATGACTTTTCTTCTCCTTTCTCCTCTTCTTCTTTGAAAATAGAGGGAGTTCGTAATATT
A_16_P02097074
GGGGCTTGTTATCCATCTAATATACAAATTCTTCCTTCTATACCAAGTCATGTCTTGCCA
A_14_P121023 ATGTCTCTCAAACATTACTGGCAAACACAATGACTGCTTATGAGAAAATCTGCATATGTC
A_16_P36811041
TCGTAACATGCAATACTCTGAAACTTTTGAACTACCTACTTTCCACTCTATCTGTATCCC
A_14_P113003 GCAGTCAGTGTAATGATTTCTGTTTAGCATCTGACCCTCTTGACTTCTCACTTAAAAATT
A_16_P36369406
AGATACACCTTTTTTTAGAGCATTTATCCCCTACTTTCATATGTAATTGCCACTTGACCA
A_16_P21298776
ATTGAACCCTTTTCTTTGCAATGCTTCATCTTTTCACAGACAGAATTTCTTATGCCAAAC
A_14_P116052 CAATCTGTGCTTCGGTCCCGTAGTAAATTCTCCCAGACTCATTAAGAACATACT
A_14_P139594
GAGGAAATAGGAAGAGAAAGAGTATCTGTGACAAACAAGAAAAAAGGAACAAGAGACGTA
A_16_P37493451
TATGCTTCTCAACAAGTTCAGTCACTTTAGTCTACCTTAATGTTAACCTCACACCCAAAT
A_14_P116470 CAGCATGTCTTGAGTGATTTTATTTCTGGCATCCATACTTTGCACATCTTCTTCACTG
A_14_P117758 CCTTGCTCGCTAGAATAAACTGCTTTGCGCTCTCTTCTCTGTCTGAGAT
A_14_P136509 CTGTGATGCTACCACTGCTCTGTTCTTTCTAAAACATTGTCTTTTATGCTTGGTTTTCAT
A_14_P127754 CTTATATTCAGACACATGGCCTTGAACTCGTAATAACTCACACATGTAAGCCACAATAAC
A_14_P114297 AGGACTTCGTATCTTCTAAAATTGCTTGACTTGAGCAAGTTAAAATCTTCTGGAGTTTGC
A_14_P132755 TAGGGATTCTGTGAGAACTCCATTCCTAATTTGTAACCAAATCTCTATCTTCCTCCTAAT
A_14_P125370 TATAAGCAAATGTCAAATAATGTCACACGTCCTTCTCTGTATAATTCTCTGCCTCCCTAG
A_16_P58424182 CAACCAGAAGGACTGCAGGGGAATTGACTGGGAACAACTTTTCAAATAT
A_16_P02440107
AAGAGGGACATTTTATTGTTACATATTGACTATGAACTTAGGCCTTATGAAGTCCAGCCC
A_14_P115311 AATGCAATTTAGCCCTTTTAGTAATGACTATGCAAATAATCAGCCATGGGAAAGCCATTT
A_14_P118380 GAGGCTATGGATGAGTTTTCCTTCTCTGGGAACTTGCTCTCCTTATTGG

CUST_7710_PI426210822 TGACACCTCAGTTGATTGGGGAATTTGGGATGAAGTGCTTCCAAAATTTATTT
A_18_P11432354
ATCGATTTCAACAAACCTTTCCTTAAACCTTTAACGTTACTCTTACAAGCAAATACCGTT
A_16_P15841512
AAGATCATGCTTTGTTCGGTAACTAATACATACTAAGGGATGATGCTGAAATAACTTGGG
CUST_1927_PI426210822 CCACCTATCCCTTTATCTTCTGTTGTAATGCCTGGTAAAGTCTTACTGGGAA
A_16_P03609470
CATCTGCAAATACCTTTGGAAAAATACACAAACATGCTGATAATCTCATTTGCTTGTGGG
A_14_P202272 ATGAATTGGTGTCCCTAGAAAATGATGTGATTGAGACAAAGAAGAAAAGGAGTTTCTCTG
CUST_2826_PI426210822
TATTTATTTTATCATTTTTTAAAAAGAGCTTAGAAATTTCAACATTGAATGAAACCATAC
A_14_P124077 ATGAAGTGATGTTATTTCTCGACGATATCCTTCAACATTATCTTGCAGCATTTCATAACT
A_14_P118042 TGCCAAAAGATACCTTAAAATATCCATGCGCATTATTTCTCTGTCCTAGCCAAAATGTTG
A_16_P16341248
ATTCCCATCAATAAGCCTAAAAAAAAGAGAAAAGTCACACTCTAAATAGCAGATCCTGGG
A_14_P106549 TGTGCTTTTCTCTTGTGCTGCTGAAACTCACTATTTGCAAAGTATTTATCAGGTAGATGT
A_14_P135656 TTTTCCTTTCCAGATACTGAGGAGAGCAGACAAAAATGGTAAGACCAAAAATCTACAGTA
A_14_P121913 TCCAGAACATGTTGATTTCTGGACGAGATATTAAAGCAAAACTGCCTTAAAAAAAAAAAA
A_16_P36230338
CACCTCAAATGTTCTACCTTATTTAATATGTTCATCCTTCTTGGCTGTCTCATTGGCAAT
A_14_P124585 GGGAAATCTTTGCCTGACTAATAGACTTGGAGTAATGATGGTTATAACCTGTTGAGATTT
CUST_1683_PI426210822 AGTGCGTGGGCTCGTCTCTGCAGAGGGAGATCAACCTGAAAAGAT
A_16_P15663921
AAACTGCTTTCAAACTCATTTGTCATTTTATTGCACGATACAGTCTTATCAGTTAGGGGG
A_18_P22841618 AATTAAACTGGTCTTCCAAGTTGGGGTTTGGAGGCAGTAAAGCACCA
A_16_P17971654
GGTCATGGTGCTGTATAAATTCAAGGTAATGATGGTATTTGAACCCCATAGAAGGATATT
CUST_9901_PI426210822 GAACATGGGCTCCAATTGCTGTGGCTCCCATGGGAAACTACAGTA
CUST_4290_PI426210822
TCTTAAAACTTGTCAACCAGTAGGCCTTCAAACCTCCTGCCCTTTAATATTTATTGAAA
A_16_P16582731
AAAGAATTTTGGTCCTTGCTTAGGGTTTAATCAAACAGGAATTAACTTGCCTTTGCATAG
A_14_P126585 GATCCCTTGTGAAGCATTTGAAATTACATTGAGAGCATTCTGAATTTCTTCACAAACTGT
A_14_P109989 ACACATTTTACTCTTCCAAGATACACAGAGTAGAGAAAATTAACATTCCTGGCACTTACT
CUST_4604_PI426210822 GGATCGGAGTTCCAGGGCCATCTGAGCTCTGGGTCAGCAGCAGTC
A_14_P102312 GTGCTTTTTTCTCTCTGCAATTACCTGTCATAGCATTTTGTGCTCACCACGAAG
A_14_P139062 CTCATCCAGGCTGCAATGCAACAATGATATAATAGGATCAAAACCTTGCATTTAACAAAG
A_18_P14399244

AAAAAGCCATTCTGAGTCTAATCTGAAGAGGATAAAATCAGATATGAGGAGAGAAGGGAT

A_16_P58424344
AGCACAAGAGAGCATAATAAAGAAGGAAACTCGTTGGTAAAGGTAAGTATATGGACAAAA

A_16_P01472089
GGAGTGATATGACTGTATACAACAACGATAATTGCGACTATTACACATATTAAGCCCCCA

A_14_P202266 GAATTTGCAATGAGCCTGAATTTTTGGGAATAGTTACTGAATACATGCCAAATGGATCAT

A_14_P131651 CTATCCCAGAAAAACATCAATTTGATCTTTGCAGTGACTGAAAATGTAGTCAATCTCTAT

A_14_P107674 ATTTATTTTGCAGTCACTTACTGTAAGTGCTGTCCTCATCTTTGGTGCCATCAATGG

CUST_6550_PI426210822
GGAACTTTGAATGGTAATCCTAATGTACAGTGATGAATGAGGACAGAAAACAAGTTCTAC

A_14_P119167 GGGATGGAATAGGAAGGAGATGTATGCTCTTTACTCTACATGATTTTTTTACTATGTGC

A_14_P139039 CTCAACAGGGTCTTGAAATAAGGAAATGATCTTATTATCTTTAAAGATAGCCGCTGGCTG

A_14_P127596
ACACCTGAAAATCAGATGCAACACTAACTTGCAAAGATTCCCACAACATAAAAAAGAAGT

A_14_P116422 GTGAATAAATTCTATTTTTAGGTGACTGTCGTTTTGTTTCTGCAACATTTCGCCTCTTCA

A_14_P201931 CTAACCTGAAGAATTTTTGGCAAAATGGTATTCTTCCTGAGTGAATTAATACGTAGCCTC

A_16_P18032311
AAAAAGGTAAATTGAAAAGCCCGTTATATAGCAAACTCCATAGAACTCCACTTCTCTTCC

A_14_P136787 AAATCATATCAATATATTACTCTCATGGAACTTGCACATTCTAAGAAGGGTCATTTTTTC

A_14_P134523 TGATGGCTAAACCAATTCTTGAGGGATCAAGGAAAACTAAGATGAATTTTGCAGATTTCA

A_16_P16767578
ACCACTATTTTTGCCTGTAACCAAATAACTCAGATTATATTATCTTGAACACTGTGCTGC

A_16_P17623521
ACTCAAGAGTGATTCTTTGCATGTTCTTTCACTAGATCCTAACTATACACAACTGACTCC

CUST_6775_PI426210822 GATGAGAGGGTAGAAGAGAGCAAAGGAGGAGGGGCAGGAGAGAAC

A_16_P19966564
GGCAAATTTGGAGGAAATACTAACTTTGACACAGAAAGTTTAAACTGAGCACAGTGAGAT

A_18_P15002585
AACCTAAGAAAGTCATGAAGTAAACTCCACAGTATCGTGTATGAAATTCTATGCTAGGCA

A_14_P114148 CTACAAGGTAAAAATTTTCCATCTATGAAGTTTTCCAGCATCAAAAGTGCGTTTGTTCCA

A_14_P101927 GTGCACCATGAGAACTTCTGACCATGAGAACTTTGACTTCCGGATTTG

A_18_P22839041 CTTCTCCAGTTGCCTCCCCTCAGTGTGTCTGCCAAGACCTGAGCC

A_18_P22842126 CCAACAGAAGGCTTCTGAATAAGCAAGACTGATGTCTTCAGAGCAATGCAAA

A_16_P00685554 GTTTCCAGGCTAGTGGTTTCTTCCTCTGACCACAGTAAGAGGAAAG

CUST_3645_PI426210822 CAGAGGTAGATGAAGTAAGCAAGCACTATTGTGCTTCCTGTGTTGAAGTCAG

A_16_P16191224
GGGTAAGGTAAGGAAACAACAGTAAGAATTTGAAAAAATCCAGAATGGTCATGGAATTCA

CUST_4794_PI426210822 GATTCTAGACTCCTTTCCCTCGTACCTCCTAGCCTTATTGGATGGAAAA

CUST_9841_PI426210822 CTCTGCCAAAGCATGGAGCACGCATCCAAGGGGATGAACAGCATC
A_14_P105468 CTGCAAAAATAGTGTCTTTGGGAGAAAATAGAGTCTCTACATCGATACAAGAAAAATAGG
A_16_P16026421
ATAATGGTTATTCAGTCTCATGCTTACCATCCATTACGAATGTGTTTCTAGTGAAGACCA
A_14_P121298 GGTTCAGGTCTGTCCCAAACTTCAGTTTATGATATTTGGCCCTAAAAGAGCAAAT
A_14_P115485 TAGAATTTTCTAACGTGGTCATTACTTACATAAAGCCATAGACTGCGGGGATGAAATCC
A_14_P125147 GCACTCACAACCTGAGAGTTCAGATACACTTGATCTTTCTTATCCATCTCTTCAAAAAAA
CUST_1867_PI426210822 CCAAAAAAACCCACCCCCTGGATCTAGGGCTGCCTGAATTCCTTTG
A_14_P127975 TGTTAAGTGATCACTTCTAGTATCCTTTGCCAATTCCTTTCTTCTTGCATCTGCAATCTT
A_18_P12456157
AATTGCTGAAGTGCTAAATTGAGTGTGACAAATCTAACAGGTAATTGCCAAGTAGGTTTC
A_18_P10235237
ATAGGATGAATAGGCATGTAGACATTCACTAGTCAGTTTTACTGTGTCAATAATGGCAGG
A_14_P124251 AGGTTATTTTATCTGATGCTAGTGCTCCTGGTGAAGGAGAACATAAAATCATGGATTACA
A_14_P131699 CTTTTTGTGCTCTGTAGAAAAGTGCTGAATGCATCACTAGACACAACTCAAACTCTATTT
A_14_P130750 CAGGAAATGTGATAGAATCAGTGGCAATCTCTGTGTTTCTGTTATTAACATAGCCAAACT
A_16_P15114300
GGAGTTAATTTTCTCATAACCCTTACCCAGTCTTAACAATTAAATTCACTGAAGCCCACA
A_14_P115459 AATCTTATAAGTCAACAAAGTTTTAATGCACATGCTTAGGCTGTTTATGTTTTTCTTAGC
A_16_P36329255
CCATTCATTATTTTTGAAACCAGCCTATACCTTTCAATCCAGATAAATCGAATGTGGCAG
A_14_P122469 TAAACTTAGGCTGTGATAAATTGGTAGGCTTATTTACACTCTGTGGAAGAGAGAGAGAGA
A_16_P36820358
ATTTACTTCATCTCCATATACTGAAGACCGAGTGTTAGTTTCTATCATACCTATGCCCGT
A_14_P133800 TCCAGAAAAATCTTGCTGTAAGCCATCATGGGGAGAGTCATTAAAGGTAAGTAAAATCTA
A_16_P19171528
GTCTTGCAAGGTATCTAAGTGCTGAATGAATCATCAACAAGAGACAGTCTTTTTTAGTCC
A_16_P18190182
AATGAAACTGGATGTTGAAATTTGAGGTGAATAAATCATTGCTAACAGGACATCTCAGGG
A_18_P22842453 TAGAGACCTTGGGTAAAAAGTCTTCCTCTCTTTGAAAGCCCCAATCCAG
CUST_9724_PI426210822 AAAAGGATCTGGGCAGAGAACCGAGTCTTGGCCACAGATGGAAAT
CUST_5105_PI426210822
GAAGATCTGAAAAGAAAAACAGTAAAGAGATGTGAGATGTAAACACTGAAAGGAAGTAAG
CUST_5464_PI426210822 GGGAATGCTGCCAAGAGGTTTTGATTTAAAACACGAACCTTCTTTCACAAC
A_16_P16688728
GCCTTCTTCACATCAATTCACACAAGTTGTTAAATCTCAGAATATACGATTTCCCTGCTT
A_14_P100656 TGAACCTAAATTACTATTAGCAGTAGTAGCAGCAATGACCCTGAACACTATTTTCCAAGC
A_16_P19190289

AAGTCATGAAATCATGACAATGATTGCTTACTGTGGTTAGTCTAAAAAACTGGTCAGGAC
A_14_P133901 GAGTAGGTCTTTTGGGAAGATTCCTTTGCTTTCATGGAGATGAGATATAATGTTGATTTC
A_14_P105525 CATAAAATTAATGAATTCCAACCTCACTTGGGAACACAAAACTGCTCCTTTCACTTATCT
A_16_P20051449
AAGCCATTACTCCGAGTTTCTACAAAACCTACAACTGAATTTCATTTGATTTCTGACCAA
A_16_P02237091
ACAAATGACCACTTTTATTCCAGACATAGATCATCTGTTAGTTTTGATTTTAGCACATCC
A_14_P122066 CTTCTCAACCTGGACCATATAGAAACTTGGCTTTCCAGCCTAAAGATGGATAAA
A_14_P106841 CTGGGAAATGCACGTAACATCTCATGAATTTTGTATATGGTGGCCTGTGTTCATATTTAA
A_18_P13260611
AAGGTTCGACGAGTTCTAAAAAGAGGTCTGAATTAAAGTCTTTTCTCCAGGTCTAAGAC
A_14_P124190 CTGCGTGCCAAGATCTGGTTTGTGCCAGGTTTCAATTTTAATCAGAAAGTCTT
A_16_P58424147 TATGGCCACAGAATCTTTGCCAGTCTGCTGCCTGAAACTTGTTATCGTAA
A_14_P136362 AGGAAACTTTGCACTCTGTTATCTTCAACCTCTTACTCTACCTTCCTCCCTAG
A_16_P17379017
AAAGAGGATTTGTAGGCTACTTTCTGAATGAGCACGTTACTATTCCATAGACTGAAAACA
A_14_P129476 ATAATTCTTTTTTTTTCTTTAGTGGCTTACAGAAGAGACGAAATGTGGTCTGAGGGACG
A_14_P115510 TGTCTAATTTTCAAGTTTGTTTCACCATCTAAGTTGGATGTTTCAATGTAGCACATGGCT
A_14_P201268 GAAGATACCCATTTATATTTTCTGCTTCTGATACCCCAGTTATCTTTTCCATTTCTGCAG
CUST_4171_PI426210822 GCCCACATGTTGGAGTTATAGCCCATCTTTGTTCAATTGATTCAGATTCAGC
A_14_P132446 ACTATTATTAGTATCTGGAATCTAGCATCTCACGGAGAGTCCTTTGTAGGACATGGAATG
CUST_10011_PI426210822 TCTCATTTCGGCTCACATTTCTCGAACGCTTACTTTGCCCCAGTC
A_14_P119598 TCCACAAGTAGTCCCTGGATTTCATTTCAGCCAACAGTTTTAACAATAGTTTCTACATTC
A_16_P40232118
AAACAGAATTATCTTCCCTCAAAGAACCTTTGTGGATTTTCCTCTCATTGCAGGATAGTT
A_16_P19655406
TTCCAAACCTTAATCACCAAGATAAGCCCTAATTTCAATATCACTCCTCACTCTCAGAG
A_16_P00413489
ACCCATAAAAGCATCTTTGTTTCTAGGTTTCTCCATTCAAACTATCCTACCCACAACTAT
CUST_8531_PI426210822 TATGATGTACTATGGTTGGAACTTAGCATGAGGTGAACAAACCACACAGGC
A_14_P113143 GATAATTCATTTCTTTGTCTTCAGGCTTACAATGGCAACACTGCCCTCCATGTT
CUST_1218_PI426210822 CAGAGATTTCTTTCATCCTTTGCCCTTGAGACCACTGAGGCAAGAA
A_14_P131819 AGCATTAACCTGCTACTCATGAAAGTTGGAATAATTCTCTCTTTGAAGCACTTTAGAAGG
A_16_P19968351
AAATAGGTATGATACACCATCTGGCATAACATTGCATTGAAGGATACTGGGTGATAGACT
CUST_5680_PI426210822 TCATGCTATGGAAATCCACTGTTTGCACAGCCTGGACATTAGGTAATAGTTTC
CUST_6074_PI426210822 TATAGGTCATTTTACCCTCTATTCCTCTCAGTGAAAGTACGTTCCAGCTTGAGTT
A_16_P15329028

AGATCTTTATCTTCAGCCTAACAACGTATGTATGCTAGAGCAACTAACACTAAGGGTCTC
A_14_P136703 CTTTAGGAAGTGACAAGGGTCTGTTGCTGCCATTGTTTCATTTCTCACTGAA
A_14_P125723 AAGTAAGATGCTGTCTGTCTTTGCAGATTCGAAATGCCATATAACATCTGGTGCGAT
A_18_P22150855
AAACCATTACTTGTCTTACATCACCAAAATACTTGACCTATCTTTTCAAAGTCCATGTGT
A_16_P36230874 AAGGTATGTCTGGATGAGAGAAGGTCTTTGCCAAGACAAGCATCTCTTGT
CUST_2453_PI426210822
AAAGATACAAGCTAAATATGTTCTTAGAATTTCAGCCAGCTACACCTGTGTAAAAACAAA
A_14_P123244 ACAATATTTTTGTTTTTACAGATATGCAATATGATGAGGATGATGATGAAATCACCCCGG
A_18_P26374791 GACTGCTAACATGCGTTCACACTGATGCACTTCGGAACATGCGTG
A_16_P19078205
ATGGTCAACTCCAGTAAATGTTCTTGAAAATGGTCTACTCCAGTAAATGTTCTTAACCTG
A_16_P03794328
ACTGCTGATGATGCAAACAGTATCTACCCACATTTAAAGTGACTCACTAAAGAACACTTA
A_14_P137345 GAAATACCCTTACATTCATCTTGTCTGCAGATTTTCCAACTTTGTGAATAGTGGGTTTTT
A_14_P130558 TGTTTTGCTTCTTTCAGTGTCCTATAGGCATACTGGCACTCATTAAATGCTTCCTAAAAA
CUST_739_PI426210822 AGGTTCTAGGTGTTGAGCTCAGGGCAGTGGGAATTAAGATGGGAC
CUST_2671_PI426210822 AAGAAAAAAAAAGCACTGAAAGCGCTTGTGGTGCTGTCCTTGGGT
A_14_P129354 TGATATGCCTAATTAGAGAGGTAGAAGAACTAATAGACAGAGAGGATGGAGACCTTCTTT
A_16_P39584431
ACAGAGTAGTGTTGACATTGGTGACAGTAGAGAACAATCATGAACACAACACAAAGAAAG
A_14_P125141 GATGATACACATGTTATCATGCTGTATTTCCTTCACTAATTATATGGGCTCGGTAAGGGA
A_14_P102541 AACTAGACGAATTGGAAGGAGAGAAAGACTGATGGATGTTTTCTTTGTAAATGATGGATT
A_14_P201987 CTGGATTTTGCAAAGCCCTTGCATTGTATGACATGAGGTTATTTAAGGTATAGTTTTGTC
A_16_P02114848
TGCTACCCTTACCCCATAATCTTCCTTTATATGTAATTGACTCTAACTAACAACAGTGCC
A_18_P14136528 CCCACTGGTGCCTACCGTCTGAGCACTGCTCTGAAGGACTCGATT
A_16_P16450433
AAATGCACCCTTTACTTTTACCAAATTGCTACATTTTGCTTGAACATCAAAGGTCTTTGG
A_14_P201021 CCACTGCTGAGACAGCACTATAGATTCTGTAATAGACAACTCCATCTACCTGAGTAG
A_14_P122406 TTCTACTGTTGTTCTCAGGGTAGCCAGTCACTGTATCGATTTAATATTAAGTCTCATTCC
CUST_4073_PI426210822 GCATTGAGTGAATACAATCATAAAACCCAAATGATGCTGTTGGCCTCATCTGAG
A_14_P122169 TTTAAGCGCCACAATTTTTATTTTACCAAGGAGCAAAACTGAGTAAGTTTCCTGACGAAA
A_14_P201248 CTAAACCAGTTTTGTCCTTTAATGCATGTCAAATATTTCTCCCATGCTTCTCTTAGCAGA
A_14_P120734 GTCTTCCTCAGGGTCAGCATGATGTCTGGCACTCAAGTATATATTTTATAGTGATAAACA
CUST_4341_PI426210822
AATTTACTGAATGAGTTTAAGAACCATGGTGTATTTCCCAAGCTTCGCTAGTGAAGATG
A_14_P127728 AAACCTATGGAGAAGATAAGCCTCAGCAAATCACCCAGTAAGTAAGAAAACATAATTGCT

A_14_P121142 CTGAGGCATTCTGCTATGAAAAGAAATTACAGATGAAATCCATTAGTCAGACATTTCCAG
A_16_P02839310
ACTGAACTTTTCAACACATTCTCAATTCCCTACGAAACTCAACAAAACTAAGCATTGTGT
A_16_P36230044
GGCAGTCCATTCTTAACATACTTATGTAGTCACTTTTCTGTCTTGAGGCACACAAAAAAA
A_14_P134846 CTCAGGCAATTTGTTCACATTTTCTGTTGATCTCTGAAGATGCAAACTCTGCATTATTTT
A_14_P133934 ACCTTTTAATTAGTCTAGGAAAATTCTGAGCCATCATCTATGGCCCACTCTTTCATTTAG
A_18_P13314935
CCCTGAATAATGTATCTTCACCCTTGAATAAAAAATGCCCCTGAATAATTTACCTTCACC
A_14_P102308 AGTCTGCCAGAGTTCCACAGCCTGAGTTTTTCCTATTAAAAAAGTTTGATAACTCATTA
A_14_P105396 CTTCCTCTTAAGTTGAGAGTTCTGTTTTCTCACAAGAGAAATGTAGATTTCTGGAGAGAT
A_16_P30952468 AGAAGGTAAGGAGGGCATCCCTGTAAAAAGAGCAGTTGTTCCATCTG
A_14_P100229 CTTGTAAATCTGTCACTGAGACCTTAGGATGGTTAATAGGAAAACTGTTATCACTGCAGA
A_16_P16009408
AAAAGGAGAAAAAATTCTGTCATCAGAACGAGAACAAGAAACTTAGACTCTTGCTCCATC
A_16_P16789011
AGCCTGTTAAAATCATACCCAGAATATCAAGCCATAAGAAAACACAGAAATTTCAGGAGG
A_18_P13623721
AAGATTCATAACCTATCACCTAAAATCGTTACTACTTCCAATCAATTTTTCCCAATCCCA
A_18_P14136764
AAATACAATAAAACATGGTCAACCCTTGAAGCCAAGGGCTTTGCTAAGTCCAAAAAC
A_14_P200432 CACAATACCATCTTGAACGCCCACAAAAAGGTTTGGTTTTGTTGCCAAAG
A_18_P14134021 TCTTTCTTTCTCTGTTGTGTGAAGGTCAAGTTGTCTCCTCATTTGGAGATCAGATA
A_14_P115635 GAAGAGAAAAAAGAAAGTCAGTTGAATGTAGCAACCTCTCCCTGTTGTGGAG
A_16_P18617144
AACTAGTCCCCAGACATTTCTAAATTACACGTAGTTACTTGAACAACATGTCCAGATTCT
A_14_P113549 GTGCACACAGGACTCTCATTACTTTTTTATGGAATCTGCAAGGTGTTTTTAGGCTTTTTA
A_16_P58424301
TTCATGTTGCAGAAGAGCATGTGGAGTCGGAGATACTTTACAAGCATCTATGGAAAATA
A_18_P10735373
AAAAACAAAGCCTACTTTTTCAATGAGGACAATTATGCAGTATTCCCATAATGGCTTCGC
A_16_P02394071
AAAAGCCTTTCTAACGTTATTGCTCTATCCTATGCACTATATCTACTCATGGAAGCCTGA
A_14_P107368
CAAGCAAAAAGGAAGATACAACATGTTACTTGGCCCTGAAGAAAAGTTTCCAAAAATGTA
A_14_P129047 TTGCAGCATCAATTTGATACTGTCTGTCTTGAAATACTCTTTCTGTAGTGCTTGCTTGTT
A_14_P200396 AATAATTTAATAGAGAAACAATGAATGATACTCTTTACACTCAGCCTCCTGCAGGGAGGA
A_14_P127378 CAGTTGAAGTTGGTTTTTTGTAAATGCTTATTGGGAATTTCTAAAGCACTGACTTGGAGA

A_16_P35897447
ACCAGAAAAATATTTACCCTCAAATTGCTTCAATACTCCGAATTCATCAACAAAGTGTCA
A_16_P18855428
ATATCTTTCCCTGTCTCTGTAATTCAAAAACCTAAACCTTTCTTATAATCCCACCACTCC
A_14_P114918 CTTCTAGGAAGTAGATGTTCCATATCTTCAAAATGCCTCCTCCAATTTTGTAAGAATGCT
A_14_P132848 ATGCAAGAAAAACTTGGTGATATAGGAAGGAAAATGAAATGAACTTGCTGAATGACTGCA
A_14_P103022 CAGTTTTCTTATTTTCTAGGAACTTCCTGTAGAAAAGCCCCCAAAACAAAACAAACCTTA
CUST_5311_PI426210822 GTTTCCCTCCTCAGCCACCATGGCTTGAATGGCTGCATTTAGAAA
A_14_P128570 CTATTAATCCGATTAGCGTTTCGCATAAAAATAGAGTCGCGGATGATGATGATGGTGATA
A_14_P113528 GAACTCTGCTACTGCAATTAAGCCGATCTTTACAATTCCAGGGAAATCTATAAAAACAGA
A_14_P102915 TTAGAACACCAATTCCTTTTCTATTAAATTTCCTTAGACTTGACCCATCCCATTTCTCCT
A_16_P17748608
ACTGATATGTGGCCCTTTACTATGTAGCAATTCTGTTTTTCACTACCAATAAGCCTATGC
A_14_P111652 AGCATATATTGTGAACAATAGTCTCAGAAGAGCAGATAGGTATAAATGTGGGAATGCAGC
A_14_P113456 CGAGATAACAGCACCTCCCTCTGGAAACTTCTACAGAAGTCAGTTTTGTTTTCTATTTT
A_14_P104668 GTCTGGAAACAACCCTACCATAATAAACCAGTTTGTAATTCATGGGGACAGGT
A_14_P113765 CTAAACTAGGATTATACCATGAAACATCTTAAATACCAGGCTGAAGAGGGGTTCTAAGTC
CUST_8326_PI426210822 GCTGCCTCAGAGGATTTGCTGTGTCCCTTCCTCCTGAGATTTTCT
A_14_P109435 CTCCCTATCTGACTCACCATCCACATTTTATTCATAACTCACACACGGCTTACTA
CUST_2036_PI426210822 ACAGGTTCAGGCCTGAATGTCTGCTTGGATGTCCTTATCATACCAA
A_16_P18468395
ACAAAAAGAACAGAATGGAAGGTGATATGTTTGCATGTAGTAGAATAGGGGCTTCATAGA
A_14_P200386 AGCCACTTGGTCTTTTGCCTTACATTTATTCACTTCATTCTTTCATCCCACATATTTACT
A_14_P115040 ACTCTTTCTTTTTAGGCTTTGGCATAGGATCAGAAATTCTGAAGAATCTAAGCCAGGTAT
A_16_P41897024 TTGAAAACGAAACAGAACCAAACTCCGCTTATTGCTGCGAGTTTATCCAGG
A_14_P124960 GAAGTAATTGGATTTTCTCACTTGTAACTACTAATCCCCTGCCTCTTTCTAATAATTCTC
CUST_8665_PI426210822
CTCTTGTTAATATGCAAGAAGGGGAAAAGTCAGAAATGATAGCTAACAGGGAGTGA
A_16_P16191228 GGTGACAATTCCATGTTCTTCAGAACCTAGAGCATTTTCCAAGAGTCACTGAATT
CUST_7471_PI426210822 GCATACTAGATCTTAAGGCAATCTCATGGCCCTGCAGCTCATCCTTTTTT
A_14_P200009 AAAAGTTATAAAAGAACTCTGTTACTACTGAATTTGCAGGTTTGACATGTTTATTGTATT
A_14_P111692 CTCCCCTCCACCTTTGTAGATGAGTTTGTGCATCTGAGAACTTCTTGTAAATG
A_14_P126415 CTTTTCATTGTAAATAACTTTCATGAAAAAGGTTAAATTGCCCCACGGGGCAGGAG
A_14_P129985 CACTAGTAATAAACTGGCTAGGTTGATAAACAATTTCCTGCCCATTTATAATGAAGGGCT
A_16_P20001959
AAACTCTTCAGTGACTCAATTCTAGTGGCACCAATTTTAACCAATTTGTCAGACAATTGA
A_14_P130040 AGCAAAAAAATGAAAAAGAAAAATGATTTGGAGTGTTTATGTCCTGCCTAGAGTGAGTGTG
A_14_P123100 TACTCACTTTGATGAATCTATGAAGTATATTACAAGTGCACCGATGCTGAGAGCAAAGAC

A_14_P137212 CACTCCTTTCCCAAAACTATAAAGGGAAGTGAAAAAAATTCAAAAGCACCTAGTGTTTAT
A_14_P101802 AGCACAAATTTTACCAATCTGACCTCTTTGAAGTTGCAGAATGCTTTGAAATTCTAATGG
A_14_P123129 ACCTTTTGCATGTTCTTTGAGATCTAGATATGGGTTAATCACTCAGATCTGTAAGAACCA
CUST_3842_PI426210822 ATGAGGCTGTTTGTATTGCACGGTAGGAATATATCCAGAAACAGTGGGC
A_14_P112598 TGGGCTTTGATATTTTCCAAAAAGATAATAACTCATTGTAGAGTAAGATCAGGACTGGGG
CUST_1771_PI426210822
CAGGGAAATAAACATTTTATTTTCTCATTTTATCAATTGTTGAAGTTCAGATGGTCAGGT
A_14_P137862 TACAAATTCCAGTTATGTTACTCCCAGGAACATCATCAGCGGAAATGAAACAAACAAAC
A_14_P109814 ACCAAAGACCAGTTGTAAAGAGGGTCTGTTAACATTTTAATCAGTTTAAAATTGGCGGTA
A_14_P132644 CTGTGCTGAGTTCAGCATTTGCTTATTTAACCACTACATAATGACAGACCAGTTATTAGG
CUST_3922_PI426210822
AATAGCTTCAAGGAAGGAAATATATTTTTACATTGAAAGAAGAGAATATGAGCCATGTGT
CUST_2462_PI426210822 TGACGTTTGGCCAAGACAAGTAATAATAAAGGAGTGTGTGGCCTTCAACT
CUST_2107_PI426210822
GAGCATTTTTATTATGGTTGTAACATAACTTTGTGTCCTCAGCTTGCATTTTTAAAAACA
A_16_P19655995
GGTTCATCACTTCGCTATATACTTTCCTATCCTTCATAATGCTCAAGTATTCCTAAACAC
A_14_P118862 GTTTAGTTCTATCATGTTTTCTGCCAATTACCATGCATAGACCCTCAGTAGAATTGGATT
A_16_P30952285 GCAGGACGCTGATCCCTTGCTATGAAGGTGAGCATGGATTGATTTTT
A_18_P22841768 GAGTGAAGGATGGACGGATGGACAGACTTGGCTGCCCTATACTATTAT
A_16_P15658171
TGCCATAAAAAAAATATGTAGCCTTGCCTACTCATGAATGCCTAACACAATACAGTGCAT
A_14_P107287 ATCTTTTGACCCTTTCTAGAAAAAAGACAAAGACTTATTCCAGGCAACCAAGAAAGAATC
A_16_P17824290
ATTTGGTAGGATTGTAATTTTTCCGACAAATTGTATGATCGCTACACACGTTATCCTTCC
A_14_P100763 ACCTGAATTTATTACAAAGACCTCAGTTATTTAAGTAAGGGATGAAGGTAGTTGTGGGAG
CUST_10335_PI426210822 CCTTGAGGATTGAAGCTCAGAACACAAGACTCTTGCCAGTTGTTCTAAG
CUST_2314_PI426210822
TTTGGTGACTACTTTGTCAACCGTCTATGCTAAGGCAAAGGAATATGTTTTAATATATAA
A_14_P117085 CTGATTGTTGTGTTTTATAAATCTTCTGTTAAGGCAGAAGGGTGATTATGAGTGGTTCAC
A_14_P120612 AAAATCCTTTCAACAAAGACTTCAGGCACCACTGCATTCCGAAAGCCTATTTTTAC
A_16_P18300244
AAAGGTAAATGAGGGTAAAATCAATACAAGATGGCTATCTATTTCCCCATAGAGACACCA
A_14_P122764 ACCCTTACATTGTGTGGGTTAAAGATCAAACATCTTAACAGATGCAGGTTGTGAATACTA
A_14_P120283 GTGTTACTGCAGTAATGTATGAAAATATGAAAGCAGCAGATGTCCAAATTACAAGGGCTG
A_14_P122703 TGGAAATCCTAGGGTTTTATTCTTTTAGCAGGTTTTGTGATGAAAATGTCACCATCAAAC
A_14_P122244 GTTGAACAACTACTTAAGATTCCTTAAATATTATTATTTTGAGGGGCTCCCATTCTGGGG
A_14_P201669 CACTTTCATTGAGGCAACTTCAACCCACTTTTCCTGGTTAAGAGAGAAAAAAAAAAATATG

A_14_P128500 AAGACAAGAGAACAAACCATTGTTGAACAATGTAATTAAGGTTTGGTGATGATGCATCTG
A_16_P16901642
AATAGAACCGTAAGAATTGGCTACATAATGGAGATCCAGATACCTACTGAGATCCAAACA
A_14_P102839 GGTCAAATCATAAATATGAATTTACTACAAGTGCTACCTGAGAAAACAAAGTTGTCAAGC
A_14_P139327 TTATGGATGGTGAAGGGAATGGTATAGAATTGGAGAGATTATCTTACTGAACACCTGTAG
A_16_P02158760
AAGCCATCAACACGATTTTATTTTATTTGCATAGCCTTCAAATAGGAAAGTCTAGGTTCA
A_14_P107374 CTGAGAGGACTGCCACATTTCATTAAGGAAGAGGGGTGCCTAATCTATTC
A_14_P139228 CCATCTCAAATGTTCCCAAGATAAATTCATCCTTCAGGAAATGGAAATGAACTTGCTT
A_14_P116026 GAGGAAATAACAATGAGGAATGCTGGACAACACATAATTAAGTGCCAAGTGGTATGATAT
CUST_6394_PI426210822
TTTCACAGCAGTGAAAATTGATATTCATATTCTAAATAATTCCATGTTTCACTGAAATCT
A_14_P136395 CTCTCCCTCGCCTGTTTTCCATTTTTTAGAATATATTCACATTCATCCTTCTACCTTAGT
CUST_5775_PI426210822 AACATGAATTACATGATTAGGGCCTCTGGAAACTTGTGTGGGGCTATAGAAG
A_14_P126440 TTCTAACTGCTCAAAGTCTTGAGAAGGGAAAGATTTTAGCAAGGCTAGGAAAATAAAAAT
A_14_P102059 CCAGTAGCCTGGGTTCTTATTCTTTAAATCAATTTTTTTTAAGTGAATGGGGTGGTCTTT
A_16_P18087620
AAAAGCAATCCTCATCAACTTCAGCCATTACAGATCATTTTTAAGAGTGGAGTATTAGGT
CUST_1062_PI426210822 ACCGAAATTCACCAAGCCTCAGCCAAGTGACCTAACAGCTGCCTT
A_16_P58424250 AAGTCATGAGGCCCAATCCATCAGTAGAGTGGGGAAATAGAGTAGAAATAGATC
A_16_P00096921
TGCAGTGATTATGCTTCCTTAACCTACATTTCTTTAAAGAGCCTATGACATAGCATCCAA
A_14_P111106 CGTTGTGATTTCAAACGATTCCATACCATCTCCATCAACAATATTGTATGTGACTAAGCC
A_14_P131304 AAGTGTAGATATTTGAATACAAACATGGGGTTAGAGTCAGGGGACCTTATTAACAGTCAT
A_16_P01841987
AAGTAACCAAAACATCTAGTTTCCCCAAACAGAGATTCATCCAAAATTTTGGTCAGTTGT
A_14_P109377 CTCTATGAAGAAAATCCATTTCCATGACTGAAGCATTGGATATAAATATGGTGTCCTGCT
A_14_P128923 CATCCCATATCTTTGATTCAGGGAATTCTTTGGATGATCACTTACTCACAAAGACTAAAC
A_14_P129850 CTGATAGACATTCCCAGTGTCATTATAATCCCCTGTGATCTAAAATACACCCTCAGTC
A_14_P138345 CTATGCTCTCTCTCCATTCTGAGTTTATTTGAGGTTGTCCTCTAAAGCAAAAGCATACTA
A_14_P138609 AACAAGTTTTAAGATGGTATTGCTCACATCACATGAACAGGTTGGTAAAAGGTCCACT
A_14_P103821 CTATCTATTAATGTCACGGGAAAGTCAGTATTCAAGTATCTGACAACATGCTTCTGCAAC
A_16_P21695090
ATGAGAATGCCGATTTTATTGTTTACTGACTCACTTCCTTAGACTACAGAGAACAAACCT
A_16_P18658568
ACACTAGACATTTCCTCCTGAATTACTATTTACACTCTGTCCCAAATATTTTCCCCTAGA
A_14_P117197 TAGATACTTCTTCCCTTAATTGTATATGAGTTGGATCATTTTCCCGTTCATTTTGCAACT
A_14_P113347 CTGCAGCAAACCGTTCTTCAAAATCCTCAAACGAATAGCCATTCTGAAAGTG

A_16_P15118935
TTCACATTTGATATTTCCATAGTTCACACTCAAGACTTCTAATCAGAAAAAACAGCCCCA
A_18_P22840349 GAGTTGGGTCAGGAGAAGGAAGTTAAGATTGAAGGAGAACATGCAGAG
A_18_P14132762 TTCTTGGCATCGGGCTTTTCTTTGATGTGTGCATGATCAGAACACACTAAG
A_14_P122289 CCAAGGGCAACATGCAGTCTTGGACTTCATTATCTTATTTCTATTACATAGTGCACTAAG
A_14_P119703 GACGTAGCATCTGTCTATCCATTCAGGAATTTCGCTTGCCTCTCTCTTTCTTT
A_14_P101246 TTTGTCCTGGTCTTTGCTTTTACTGTAACAGTTACATTTTGCTGGCCTTATCCTGTT
A_14_P126957 CAGGCAATTATGTAATAAATACTGTCTGGTAGGCAGAAATATTCCAAATACAGAGCCCTC
A_14_P126309 CGTATATCAAGAGACCACAGCATGGAAACGTCAACAATTCATTATAAACACAAAAGGTGA
CUST_9292_PI426210822
AAGACTAAATGTGTTAATCTGTACAAACCTATCCACGTGTGAATGAATAACCTTAGAAAA
CUST_5698_PI426210822 CTCTTATGGGTATCAGCTTCTGGGTAAATACTCTTTTCAAAGAGGGCACCAT
A_14_P101884 AGGAGGAAATTGTTCCTCGTCTGATAAGACAACAGTGGAGAGTATGCATTTATTTATTTA
CUST_8824_PI426210822
ATTCCTTTAACCCATGGCAGAGGTTGCCTTAATTTCAATGTGAGAATAAGAAGGT
A_14_P128106 AGAACTGAGTATTTTCATGGAATTTGTGGAATTTTACATACAATCCATGCAGGCTTCTCT
A_14_P135969 GTGAACTGCTTGACTTTACCACCTGAAGGAGTATTTACTGCAAGAATTAACAAAGCAG
CUST_4248_PI426210822 GCAGCCTCCCCTCCAAGGAGAAAGAAACCCTCCACGTGTTTTATG
A_16_P17021746
AGCGGGGATAGAGATTAATCATTAATCTGACTAGGAGTTTTAAATTAGCCGGAAGATCTG
A_16_P20928818
ACACCCTGGAAATCTAGAGTCTTTGAAGTAAAATGATGATGACACCTTCTAAATTTACCC
A_16_P15035496
ATGTGGCAATTAAAGGCAAATGTATACTTTTTCAGTCATGACTAGGGATTACTCTGCTGA
A_16_P17576649
AAAGGGGACACAACTTTGTATTAAAGCATCCATACTTTATTCATTTCCATGAGTCCACTC
A_14_P200142 AATACCATCTGGGAGCATAAAGGTGACCTGAAGGTAGGGTGATATGTCTTAAAG
A_14_P134422 GTGTATACTTATACTCACCTGCAAAACGTTCGGATATAATCCTGGTTGATTCTGATGAAG
A_14_P115648 TGTTCTTGGAGCCACAGAAAAACAGATCTTACATTGTATGCCCATTGTATGATGAATAAG
A_16_P03529994
ATTTCATTGTAAGTGCACAGAAGTAAATATGGAAGGATGCTAAACTCATTACATGACCCC
A_16_P18430285
AATGTAGGGAGCAAACCATTACTTTACAATACAGAGGTTAGAAGTTCAGCATGTAGTGAG
A_14_P129792 TATACTTTCTTAATAAAAAGGAGAAAGAAAATCAACAAATGTGAGTCATAAAGAAGGGTT
A_14_P101585 GTCTCTTTGTGTGATAATGACCTACACATGGACAGGGTCCAAACCATCTG
CUST_7886_PI426210822 GTATTAAAAATGCTCATCCCAGACCCTGCAGTCTCTGTGTCTACTCAGAAAA
A_14_P128348 ATCTCATCACTGTGCTGTAGGTCTCGTCACGTTGAAATCAGTCTCTGAGTATTTA
A_16_P15580153

TGAACAGTAGCAAAAGAACCAAGATTAATAGCACTAGAAGGACATATTAACACCGAACAT

A_16_P17435884

ATTTAAACAAGGAGTCCTTTACTGACCTTAACTTGGCTAACTAACTGCTTAAACAGTCCA

CUST_3086_PI426210822 TCAGTCCTCTCCTCCTTTTTATCCTCAAACTTTGATCTCTGCAGATGTTCA

A_14_P136318 CTTTAGATGTGTCCCATGTGGCAAGAAAGGAGACAGTGAATTTTGTCAAACAATAAAAAT

CUST_6076_PI426210822 TACGTTCCAGCTTGAGTTTTGAGTTTGCTCCTCTCTCCAACCATAACA

A_16_P18256691

ATCGTCCAGTGTCAAGATTTAACAGTTGCTTTTACGAATATTGAGTCTCAGTTATGTGCA

A_18_P12131306

CCCCTCTTTTTGTCTCATAAACACCTGTTAAAAACCTGTAAATACTTATGTTCCGAGGAT

CUST_1932_PI426210822

AGGAAATATTTCAAGAAACAAAATACTAGCTTTTTCTCAGAGTTGAATTGTTTACTTGTA

A_16_P17030818

AAAGAGTTCTTAAACCCCTTTGGTAACAGATTTAATGGTCCTGTCTTCAATACATGATGC

A_18_P17402956

AGTTGATAATATTTATCACTTTTTTGTTTATTTTCAAAGCACAGTTGATATAATCACGTA

A_16_P00729240

AAGGATCCTTCCACGTAGATACATGAATTTCCATTGTCCTAGTTAGCATAGTTTCATCTT

CUST_5975_PI426210822 AACATTTCTGTGGCAGGAGAGGGCTGAAGGAGAGGGGCAGGCTGC

A_18_P15060639

GTGCTTCGGATCTACACAGTAGTTTTGCCAATTACCCTTCTAAGAAAAGCAATTTCATAG

A_18_P14674409

AAACTGCTAGTGAGTTCAATGGTTAATTAAATGATCCAAAGAGAATCCATCGTCAGCAAC

A_14_P117931 CTGGTGTGTTTAAGACAGAGGACACTGACTTAGGCAGGCTACATACTATTAGATTTTTTA

A_16_P21697128

GTAAGCAAGTAGAATCAATCTTCACACTGATCCTATCTTTGAAGTCAATCATACGCGTCT

A_14_P126087 TGTGACGAGTGTGGATGCCAGTGACAAGATGCTGAAGTATGCACTTAA

A_14_P117894 CTCAGGCACAGTGGTTTTCCTGCCTAAAAGTAGTATATGAAGATGACTGTTAATAAGTCA

A_16_P03793414

CGAAGATCATCAGAAAAAAAGCATAAAAGTGTCTCTTTCTACTGATAGCATCTCTCCCAG

A_14_P114756 ATGTGTTATGTTAAAGTAACAGTAAGGTATTTGAAAGCACAGGCTACTGAACAGGTCCTA

A_14_P138471 CTCTTAACATTCAAGTTGATACCTATAGTTACAACAACCGCAGCAACAAGCCTGTGC

A_14_P101935 GAAACACTCAGGTGCCAAATGAGAGGTAAAGAGACTCCATGCATTACTCAAC

A_14_P102268 ATAGGGTTTGGTAAACATGACAGGGTTTGGAAATGTGTCACTAGAATAAAAAGACAAAGA

A_18_P14132341 GTCTAGTATTGTATGACTTGAGTCAATAGGCAGTGGGAACCACTGTAAGTTCACAAG

A_14_P112046 GGAGGCCAAACTTTTTTTTCTTAAAACATTTTTACTTCTTTCAACCCAGAAACATCACCA

A_18_P10511072

ACGCTTCTGAATCATGATCCACATCTTACGATTTTCAAACCATTAATTTTTTTAACCCCC

CUST_9168_PI426210822 TCACATCCTGTCCAGCCTTCAGCTACAACCAGTGTGATATGGTGATATAT
A_14_P200685 CTACTCGAAAAAGGGAAAGCATTTCCTTGAGTTTGCGTTTTAAGGGAAACTTACAG
A_14_P103956 AGCAGATCTGACAAGATCCATAAATTTTCGAGTTAAATAAACCAGCGATACATCAAAACG
A_16_P15587925
AAATCATCCACAGTTCTACCCATTTAAAGCAACCTTCTATCTATGACATCAATTACCGCT
A_14_P132739 TAGGTTAGACCAAAGCCATTGCTTTTGAAGTTATTAAAGTCTGTTGGTGCACAAAAAGAC
A_16_P02361491
GTTCTCATTCTTCTAAGACTCTCTAAAGAGCAAGCACTTTAATTTCTGGTATTCAAGGGG
A_18_P14347279
AAAACAGTGGAGAAGATTTTCACATGTCTAGAAAGGTAACATGTTCAACTTAGGTTTGGC
A_18_P22841615 CTACTTCAGATGATCCCTGGTGTCTCTGTGCAACCCTCAAAATTTGTAGC
A_16_P39410115
AATAGCACAGTCAAATCCTATTTCCTTTCCATTTAGTGAAGTGGATCGAAATAATGCAGG
A_16_P00184281
GGTAGGCTGAAATAAAACAATCCATTCACTGTGACATTATAATGGCCCAGAGTTAACAAA
A_14_P124949 CTAGAGACAGCAATCAATATTTGAGTGTAAGGAAACTGTCTGGGGTGATTCCTAGAAG
A_14_P127423 CATATGGCACAAACAAATCGTTATTTTCTCTAGGATACCAAGTTTCCTCCTTCCATTAGA
CUST_7230_PI426210822
GGATTTAGTAATTTAGTAATGTTTAGTAATTTTTTCAACAAGTTCTTTTCTGTTTAAGTG
A_16_P18923839
AATGAAAGGAATATGACCATTACTCCATCTGTCATTTTCACTCATGCCTTTAAATGAGCC
A_16_P00685493
GTAGAATTCAGTGTTGCAAATATTAGACCATTGCCTAGAATTCACCATAATGGAACCCAA
A_14_P110092 TAAAATACATGCAGGAATGAATGATTCAGTTACATAAATTGTGCATTTGAGCAGGGCCAC
A_16_P16673177
AAGAGACCCCCAATTAAATGTCTTCTACTCTTGATATATCTGGCTCCCCATTTATTAACA
A_14_P131656 GCAGCAAAGACAATAAAAGGTGTCTAGAGAGGTCATATGTGTGTGGGTCTGT
CUST_4415_PI426210822
CTCCAGTAATGAATTTCAAGCTTCTTGTTATTGTTTTTCTTCCTGAAAGACAAAGGGAGT
A_14_P101140 GTCCAAGAAACGGCATAATCTCCAGATGAATACACTATAAAGTTCACAATTAAGGTGGAT
A_14_P112389 AAATCTTTTTCCAAAGAGCAGCAGACCATGGATAACTTAGAGAAGCAACTCATCTGTC
A_16_P18937180
AGACAGAAGCCCAAATATTATGACCTTCATCTCTTCATACATGTTCTGGTAGGAGTTATG
A_16_P16588135
TCATATCCCCTTAAGTCTTCTACACATTGAGTATCTGGATTTACATCACTTCAGTCAAGG
A_16_P41246040
ACAACAACACATATCAGATACCATCCTGAATATAACAGTCTAAACCTGACCTAACATGGT
A_14_P129126 CTCCTGAAAGTCTCTCTTCAACTTTGTGAATAAAGCAAACACTTTGGCCATCTAAAAATT

A_14_P102418 TTTTCCTTGTTCGCAACGAGATATTTTTGATCCCCTCAGTAAGTAATATTCATTGCATCT
A_14_P101660 CTGAAATCTCTACCTTATAAAGCCTCTTCTTTCATTTCAAAGCATTATGCCAGAAGACCT
A_16_P15249974
TTCAGGCTTAAAATCACCAGTTCCTTAAAAAACTCCATATTTTCCCTTGGCTACCATACT
A_14_P118945 GGGAGTTTTTAACAATTTTCTTAAACTGTGTTACAACACTACCCGTTCTTCCAGTTTTGT
A_16_P02848567
TCTTTTCCAGTCAAAGGATAAACCACTTCTCGAATCTAAATTACAACTGCAAACACAACA
A_14_P200400 ACAATTTTGGTGCCTCTCCTTTATCAAGATAACTTCCAGCATACTGATCTCACTTTGG
A_16_P18818145
CCCACCAGTTATTTTCATGTGCAACACTAATGACCAAAATTTGTTTGATCTATTTCCCTC
A_16_P00003452 ACAACGTCACACTCATTACATGAAGTTTCATGGTAAGTGGCGCATCCAAAAC
A_16_P16798514
GGAACATACATTCTACCTTATTTGACTAAACACTTATAGCAAGAAAGCACACTAATGGGC
A_14_P113967 CTTTTGAAATCTCACAGAGTTCTGAAGTGGAAGGCCGATAAATCTCTTTCTTATAACTCA
A_16_P21645113
AAAAAGCCTGATTCAAACTAACCTTTTTTTTTCCTGTTTTTTCCCAATATAAGCCCCTCA
A_14_P132925
CACATGTAAATAAAATAGGAAACAAATTCCAAAGCACTCAAGAGAACAGAGTTAGGGGAG
A_14_P119890 TACCACTTGAACTTAGGGCAGCAGAGGATTAAAGCAATTATTATTGAAAGGGGTTTTATG
CUST_1707_PI426210822
ATTAGCATGTTTTGTGTTTTTATGTGATGACTTCCTTGTTAGTGTTTAATCACTGTTCTG
A_16_P20391867
AATTCTGCAATAACTCAACTTTGAGTCTGGTAATGTCTCTATCAAATGCATACCTGGAGG
A_14_P123253 GTCAGATTTTATTGCTAAAGGACACGAGGAAACTGTTTAACAATGTATGTGGTATGCCAT
A_16_P19758981
ACTACAAGTTATCCAAAGCAATCTTACTCATTTATCTCAGTTGAACAGAAAAACCAGTCA
A_14_P138822 GAAAACAAGAAAATGTTTGGGAATTTGTATATTGGGAATGGGCAGGTGTCTACATTCATT
A_16_P16992677
AACATTTGTAACTCAGGCTAGATGAATAACTGCTGATTGACATCAAAGTCTCTCCAATGG
A_14_P138275
GAGGAACAAGAAGCAAAAATGGAACTTAAACGCAGACTCAGCAGAAAGGTAATGAAATC
A_16_P01206069
TCAGACTCCATTATAAGTATAGCCTTCTTTCCGAATCTAATCAAAATAGCCAAAGCACCA
A_14_P108498 GCTGCTAGTTATCTTATCTCAATCTTAGAATGTTTAAACCGGAAGGGGACTTAAGAGATG
A_14_P114474 TTGCCTAAGCCTTTATATTTTACACACCAAGATTATTGTGATTATTCCCACTTAGCAAGG
A_14_P133965 CCCATATACTTTTGCTGAGATTGATGACAGTGAATATGACATGATATTGGCCAATCTGAG
A_14_P128723 CTGACTAAGTAGAAGTCAGGAAACATATCTGAAGCCTTGCTTAAAAACTTTCCACAAAGT
A_14_P136918 AATAAAAATTATTACTTTTTAAACCACTAGGGAAGCAGAGAGCCGTTTCCCACCATGTGA

A_14_P112648 CCATAGACCATCCGCTGTCTTCCTCTGTAGAGTTAACTATTTTCTCTTTTATGAGGAAA

A_16_P17142778

CCTCATTTTATAAAGCCCTTCACAGATTCTTTTTTCGTTCCAATCATCATTTCACTTCCA

A_14_P104526 AAATGTTTGGAATTTTCATTTCTGAGCATCCATCCCTCTCCTATGTGCTTTTGCATTTTA

A_14_P121219 TGAATCTGGGAATAACTGGAGAGATACTCCCAATTCATCTAGAATGACTACTAACAATGG

A_14_P137926 CTTTTAAATACAAAGAAGTGAGGATTTTGAGATTCGAGGAGCATAAGAACACAAGAAGCC

A_14_P110246 ATTCTTCAGGACCCAAAAACCGTAAGAGAGTATTCTCTTTTCAGAATTTTCACCAATACA

A_14_P116371 TCAATTTTTTAATTTGTAAAACAAAGGGTTCATATCTGAGGCCTTTTCTGGTCTGAAGCG

A_16_P17579704

ACATACTTTCTTCTGTCTTTAGTTTTCAAAGGTCCCAAAGAAATCAAACAAGCATCCACT

A_14_P116019 CTATTTGGGATTCTAAGAGGACACATGAGTCACATAAGTGCATTTAACTCAGACAACTTT

A_14_P107977 ATTTCTGAATCATCCATATAAAGTCATGGGTAGCAAGAACAGATAAAGATGTGGTCTGTC

A_14_P137360 TCACTTACACATTCTCTACTAAAGGAATTCTAAACCATTTTTGCATCACAGAGCAAAGGG

A_16_P01486976

AAAAATATAGTCAAGCAGGCAAATAAAGATACCTCCACTTTACTAGCATCACAAAGTAGA

A_18_P13433079

ATGGTTTGCCTTAAAAGGAAAAACATAAACTGCAATCTACAGTGTTCTATTCATCGTCCC

A_18_P24734938

AAAACCTCTTCCTGAACTTAATAGTAACAGAATTGCATCTCATTCTTGTGCTTTTTGCCC

A_14_P134555 AGTGTATTATTTTAGTTGGTTAGTTGGTTTGTTTATTCCCCCAATGCAAAAAAGTAGAGT

A_14_P136506 TCTCTAGCCCCAAATACTAACCTTCTAAATCTGAGATCATCACTTCTTGCTTATTCCTGA

A_16_P02989865

AATAATAAATAACGTCTCTGCTTATCTTAGGACCATGTTCCAGTAAATGAGGACTGCAGC

A_14_P124733 TAAGGGATAGGAATTTCTTTTGCATTTGCTGAGTTTTGGAAAGGTTGAGAAATTGAGAGA

A_14_P105593 GGAAATTTCACCGTATAATTATGGAAGATGAACGTATTTTCCCGAAGTCCAAAGTAACTG

A_14_P106453 AGAATTTTCAGAACTACCTGGGATCCTAAAAGAGTCTTTGTATAATAGTCTCGAGGCATG

A_14_P103970 CTGTTTTCTAAAAGATTCTGTTTTCTGCCAAAGTCATTTTGTGAATGGCCACTTTAGGAA

A_16_P19464679 CCACCTGTATCCTATCCTAAGTATGTTTACCTGTTATCTCATTCCTTTATCCTTGTTGCT

A_16_P16192026 AAGGTTCCAGAAAAAAATGCAGAAAAACATTGACTCCATTGGCTTGGAAGTGAGT

A_14_P132626 CACATCCCCTCCAAGCAGAGAAGAGAAGAAGGAGATGGAGAAATTGT

A_16_P35869282

AAATGGAAAATGTCTATCAGCACACATTAACTCAACTTCACACAAGTGATCAATCTGGAC

A_14_P120212 GCACTCATTTTTGTAAATCTAATTTTGTCCTTAGAAACAGCCTGGTTGTTTTCTTGTCGT

A_14_P106952 TGACACCTGGGCTACAGTTTCAAAAGAAGTTCCATCTATTTAATCTGTAGAATTCAAGAC

A_18_P12604631

TTCTTTTTTTTCCCTTTCCTTAAGCCTGAAATTATAACAGTTCTCAGAAAGTAAGAACCA

A_16_P01117570

AATTGCTGACTATTGCTACACTCTATTGACATCCTTGAATCTAACTCAGACCGGTAGATA

A_14_P125537 GAAAACGCCAAGATACTTTGTGTGAAGCAGAAACACCAAGGTCTTCACTC

A_14_P126207 ATCAGAAAATTCATGCTCGGAAGAATGCCTACAGGAATGAAAACTTAATAATTGTGGGAA

A_14_P126081 CTGGAAACAACTGAAAAACTGGGCAAATATAGAAAGTCCTTTGAAATAGGTTTCTGAGTT

A_14_P109165 GTGGGTAAAATTACCTTCCCATTTATCACCATCAGAAACATTCTTCAGATCTAAATGTGG

A_14_P201255 AACTGGCAAAATGGCAAGTTAGTAGCAGGTTTATAACAAAACTTTTAAATGTTTAAAGGG

CUST_5516_PI426210822 TATTGAGGGAGGTCAGTGAAATGTGAAGGTTGGTATGAAGAAGGGTTAGA

A_14_P125217 TTTGATGATTTGAACGTGAAAATGAATCCTACTTTCTGCTTTGAGAAAGATGTAGGGGAT

A_14_P202044 ACTGCACAAATGTATTCCAAATGTTGAAAGTATGTGCTCGACTGAAAAATAGGTTGGAGA

A_14_P124872 TCCGTTGAATCGAAAAATGTCGAATATTACTTTTCTCAACTTTGATCCTCCAATTGAGGA

CUST_56_PI426210822 AGATGTGGATTTGACAATTTACGAAAGCTAAGGTAGAGAGGGCCCTGAAAAA

CUST_6692_PI426210822 CAGAAACGCAGATGGGCAAGACGACCCTCAGGGTGGCAGGAAAAG

A_16_P41098443

ACAAGACTTTAAGGGCTTAATTCTGTATGCTTGTTTGCATCTTCTTAACTGCCTACTTGA

CUST_9986_PI426210822 CTCTTAAAATGAGAGGAGCTGTGTCCTTTATGGCTGGGTGTGTGG

A_16_P17208453

AATCAGTTAGCAGTGTTTTCTCTAATTCCCTTCAGTTAGTCTGATTATTCGGTCTCTTCA

A_14_P105346 CTTCCAATGGAATCTGATTACTGTTTGGTATAAACTGGAACTTTGCAACAACTCCTCTAT

CUST_2350_PI426210822

TACATAGTCTGAGGGGCATTTATCTATCCAGACCATCCTCTTTTCATTAGAAGTACATAA

A_18_P15511328

AAATGTTCATCCACAACTTTCAGTTCTTCAAACCCAATCTTCATTCTTCTCTTCACTGAT

A_16_P15541388

AAAACTCTACGCATTTAGAGATCCAGTTACCTTCTTAATCACATGATATGCCTCCATAGG

A_14_P126642 GTAATCACATAGCCTTGCCTAATTAGTCAGAAAACAAAGGATTAAGTGAGACAGTCACAG

CUST_25_PI426210822 CCAGCCCTTGCCCAGGGTGCAAGCCTGCCTTCAGGAAGGATGACT

A_14_P118873 GATTCTGTAGTGTATTGATTCAAAAGAACAAAACCAAAGATCTCATGAAGCAGGTAAGCC

A_16_P15895700

AAAAACCAGACTATCAGAGCATTCTCAGGTAATATTCGAGGTAAAAGAGTATTCTTGGGG

A_16_P00684991

GGTTTCCTGTCTTCTAAAACCTACCTTACCTTTAAAGAATCTGTTGAAATCTGTGGATCT

A_14_P139328 AAGGAGTGGCGTTGCTACTTACTGCTGCATCTGTGCTAAGAAAACCAAATATAATAAAAT

A_18_P11869650

TTCCCTTTGATGACTCAATTCAATACTCCACAAAGATTTTCATCCTAGTTCATGAAACGT

A_16_P30952497 CTAGTATGAATTCAGCGTGATCTTTGATACAAGCCACCTGTCTGGGGAAGA

A_14_P107318 AGAGAATTACTTCTTTCTGTAGTTAATAACACAAAGCAATCCTGTTCAGGTTCTTTGGAT

CUST_2359_PI426210822 TCCCAGGGTGACAAGCTAAAAGTCACCCAAGGAGCCCCGTGCTCG

A_14_P109806 CATTCTAAATCTTTCGGGCGCGGAGGATGAATTTTTCTATCTTTTTGTTGGAATTAAGAT

A_18_P22840127

AGTGTGGAATAGTTTGGGAGCAGCATGGCAAATTAACAAAGCCTGTTCAAGATTAAA
A_16_P16491766
ATTATCAGAACCGGCTTTGTTAGTATCATGCACTGGTAACCATCTATATTGATCACATGG
A_18_P22841237 CAGTTCTCCGCTGGAAAAAGGGACAAAAGATTGCCCAAAAGAATCAG
A_16_P37116839
CCTTCTACACACTGAACAAGATCTTACATGAATTCCTATAACTTCCTTACTTCTTACTCC
A_14_P136263 AAGTGAGTGGCTACAGAACTGGATGTTAGGGGAGAATTAATTTTCACATTTTTGGTAATT
A_14_P104419 CTTACCTTTATGATGGTTTGCACTGGATACAACTTTCCCCCACAAGGTTACAATAAATAT
A_16_P03112975
TCTTATTGACATGTGTATGCTGATTTCATCCCAAAAGAAATGATATGGACATTCCCAGAG
A_14_P101474 CACTTCCTTTTCTTCATTGCAGAAAGAGACATCGGGAAGATTCTGATGTGGAAAT
A_14_P122435 AAATAACTCACAAACACCTGTAAAAACTGTTACAGTGACTGGTGTGTGTGATAGAGGG
A_14_P115171 GGATAATCCATTCTGGCATGATATCAAAGACAGAGTGAGAAAATGAAAAATTTCTGTCCG
A_16_P02040123
AGTCTCTGTCTTTTGTGTAAAGCAGAGTCGATAATCTGTTTTCTCAGCATCTGTTCTGT
A_14_P104959 CTACAGGCAAATATTCTTGTTTATTACAGGTGATCGGAGGATAGTATTGAGAACCAAGAG
CUST_718_PI426210822
GAAAATTGATCCCTTAATGTGGCTTTCTTCTCACTGGAGTTTTGTTTTGTACGTCATT
A_14_P108890 TTTGTTCAGGGCATCTTTGCATTAATCGCCACTGCAGGACCAACATT
A_14_P105025 TACCGTTTTCCTTTGGGAACTTCCTTTCCCAAACATTCATGTAAATAATATTTCTGCACA
A_16_P37791856
AATGGCATATGCAACACATTTACAAGTTGAACATCATTACCTTCAGAACAATCTCAAACT
CUST_1414_PI426210822 GTGGTGTGTCTTGCACTATAGTTTCATTCAGCTCATGGGGTCTGAATTTC
A_18_P16412944
ACTTTAAAGAAACCCGGGATTTAAAGAAAACCAGAGTTCTTAGATAATGAATCACAGGCA
A_14_P202379 GGCACTTGCAGAAATGCAACTTAATTTCTAGTTCTATCATATCTGTCTCCAAAATGGACA
A_18_P11628322
AACCATCTATCTCCATTTTCACCATATCATTCATCTGCTGTATTCCTGAGTATTTGCACA
A_14_P122951 TGCAGGTAAAACAGTCAAGAAGAAAACGGCATTTTGAGTGTTAGACTGGAAACTTTC
A_14_P119589 TGTTCACCTGTGAAGACCTTCAAGGAATTGAATAGGAAAATACCTAGAGTTTTGTTTCTT
A_14_P132815 TTACCTACTTACTTCTTTTCTTACCCGTCCTGTTATTGGGTGTCACTTCATTTTTCAACT
A_16_P16104536
ACTGCTAGCCTGAAGATTGTCTAATGAACCAAGAATAATTACTTGTTTGAGAAGTCTGGA
A_14_P200682 CAGAATTGGTCTTGTAATACAGACTTCAGGGATTGGAGGATTTTCAAAAACAGGAATGTT
A_14_P128787 CTGGGGAACATACTTACACATTTATTTGCAGATAAACACAGAATTGCACACGTGGATTCA
A_14_P113199 TTGGTTTTATTATCAGACTTGTTGTGATCGATGTCAGAGTCTACAGAGGAAAATGTAGGT
A_16_P40976177
AGTTGCCTCAATGAAATAATGCAGTTTCTGCCTTTTAATAGGACATCTTTCATGCATACC

CUST_7364_PI426210822 TGTCAGAAGCCCTTTGAAGAACTCAGCTGTCTGGACAACAAAAGTG

A_14_P102357 GCCATGCTCATTATCGTCTGCTTCATCTTCATTAGCATGATCCTCTTCATTCGAA

A_16_P00202930

AAATGTGGGGAGAACAAAATGACAATCAATCAGTGAACATTTGTAGACACATTTGGATTC

A_14_P135147 CTGGCAAAAGTATCTTCCTTCATCAGAAATTGAGACGTTTGTCAATGATACTTTGAGTTC

A_18_P27181550

ATAGTGGTTCAAAAGTTTGAAGTAATCGTAGACATTGTTCGATTTATGTCTCCTTGGACC

A_16_P19702765

AAAGGTCAACCCTTCATTAAACCCAAGAAAGAGCCTAATAATGACTATCTGCGCTATTTT

A_14_P100840 CTCTCCTTCATTTTCAACAGTCTGACCCTTTTTAATATTCAATATGGGTAGAGGGTGTAA

A_16_P15534308

AGAAAACTTAAGTAACCCGAGTAGATTAAACCTAGAAACAGATTTTCAGATGAACAGCCC

A_16_P02020811

AGAACTGCTGCTCTAATATATACCTAAGAGAAACCATTTTGTTCTAGACCTCTACTCCTT

A_14_P128613 ACCTCCCCAAGATATCCTATAATTTGCTGGTCAGAACATATTTTTCCTCTTTGGAATTAA

A_16_P02554461

ACACAATAATACACACTCACAACTGATGATGATGACTATGATTGGCAGTAATAGCAGTGC

A_14_P138050 CAGCACAAAGTTCAGAGAAATGCCTCCATCATAGCATGTCAATATGTTCATACTCTTAGG

A_16_P21590652

CGGATTCCAACTTTTTTTTAGCAGCCAACCCTTTATTCAAAAGACAACTTACAGGAATAT

A_18_P22842822

CAGCAATTTAATAATTTCCTCTTTTAAGGGGAAAGGGGGGAGTCTTGGATTTTAAATCTT

A_14_P133633 CAGGACTTTTCAGGCAAGCCATAAATGGTTGATGGATGAAAGTTATTTAAATAATCCCTT

A_14_P129734 TTACAATGGCATGGCCTTTGAGAAAACAGAAGTCATTTTACAAATTCACAATGTTCCTCT

A_14_P139694 TCACTTTTCTTAAATACTTAAGAAAATGTATGGTGATGCTCACACCTTTCCCCTCCTACT

A_14_P130954 ACAGCAGATGATGACAATGGCTTCAGTATTGAAAACTTGACCACACTAGGCTAACAATAT

A_14_P107875 ATCTCCTATCTCTCTTGTGACAACATAGTTATTCTAGTTATTCTGCACCCACAATTATAA

A_14_P107358 CAGATAACTATTTAATTTTTTTTAAGAAAGTTGGAAAGGTCTCTCCTCCCAAGGACAGTG

A_14_P125945 CTTTTCTCAGACACCACAACTTCCAAGAAGGATCACTGGTCCTGAAC

A_16_P17473420

GAACAGCCATCTTCTGATTAAACATCTTCTCTCCTTGTTAACTCCTAATTCCCCATAAAA

A_18_P14042125

GCATCTTGCCTCATATGTCTCTCTGAATGTAAAGATTTTGCCTACATCTGATGAAAACTG

A_18_P16358423

GACAGATGCATGATACTACTCGCTTTTCAACTAGAACACAGGAAAATTTATGGACATTGG

A_14_P125539 TATCTCTCCCTGCACTGATACTACATCTTACCGATTAAAGTTTTATAACACAGGGCTTTT

A_16_P18076247

AGGGGCTAGATGAAATGCTAAGTATTCAGGAACATTTAGAATAATTTCCAGAAGGTCCAT

A_14_P113888 AGCCCTGCTCTAGATTTCGTCCTGGATTGTTATGGTTGTTTTTTGTCTTATTTTAAAAGA
A_14_P123420 CAAGTTCTCTATATCCTTACATCCCATCAATATCTGTCAGGCATACTGTCAGTAACTACC
A_16_P15106090
GCAACCTAATCTTTCAGTGCATGACAAGAGTAAAAACTTCAAATCTCAAAACCAGGTCTA
A_14_P125091 CTCTGGATTGAGAGATCACGATGTAATTGTCAACATAAATGGGAAACCTATTACTACTAC
A_14_P135184 GTGATAATGGAAATGCTTAAAATACAACTTTTTCTGGCATGCTTTGCAGTGAAAGGTTCT
A_14_P113965 ACTGGCAATTATTACTATGTTTGTGAAAGGATCCTGAATTCCTTTGAAGGAAAGTTCACA
A_14_P131370 TAAAAAACATTTGGGTTCATCTGAGTTATCCCAAAATTGCACTCACTACCTTCCTTGTAA
A_14_P137944 GTTTGTGTTGCAAAATGCATCTTCAGAGCATCTCAAGATAAAGTGATCTCCAATAAACAG
A_16_P31437845 ATCTGCTCATCTGATATTCAAACACACAGTATTCGTGAATAAGTTGATTCTGTCCCCC
A_14_P108826 ACCCCTGGAAAATTAAGGTCCAGTGAGGTAAATTGTCTTGATTAATTATATGACATGCAC
CUST_6467_PI426210822
TATCATTAAAGATTTATCACAAATCAACCATATTATATCGTAAGTAAAGTAAAACTGGTG
A_14_P110822 ACTCTTACGTGGAGGATATAGAGTAATAAAACACGTAATTTATTTGAAAGCATTTATAGA
CUST_8072_PI426210822 GCTTCTGGGGAGTTACAAGATGCCTTCTCCAGGGCAGATCCAGCA
A_16_P20330015
AGTTTCCAGCAAACAAAAACTCTATATGGCCTAATTTTCAAGCAGTTGAGATTGAAGCAG
A_14_P132431 TAAGCCAATTCAAAATGTGTTTGACCCACAGAAATCATCCAAAAAAGGAAGGTTTACTTT
A_16_P16191478
ATTCAAATTGAATAGGAACCATTTTTAATTTGGGGGTAACTCCGTCTTCACCATTAATGG
A_14_P131505 CTGGTTTTTCAGAACCTGAAGAATGAGAGGTGTCCCTTTAAAGGTGCC
CUST_6226_PI426210822 AAGCATCTTTCCAGCAGGCACAATGACATGGCTATTTCCCTTGTG
A_14_P136333 CTTTCTCTTCTAGATTTTATAGGAAGACGAAACTTGTCGGCTTTCAAGACATGGAGTG
A_14_P139550 CTAATGCTTTGGTTTATCTTAGATCCCCTCTTTCACCTCTATCTTTTCACTTTCCTTGAA
A_14_P124871 ATTTTTTCCAATCTTGCACTTGTATTAACTAGAACCCTAATTTGTCCATGAGGCTGAAGT
A_14_P120452 CTGAAAAAGTAGTCTTTGATGAGGTGACAATGATGAAGTTCTCTCTGGCACTGCTA
A_18_P22841898 CTGAGATGAGTGTGAGTGGCCTCTGCACCAAAGAGTTCAGTCTAATTC
A_14_P120347 TGACCACTGGGAAAGGGAATTTCATCTCTGCAACCTAGAAATTACTACTCTTAAATACAA
A_14_P124161 GAGGAGTGTGCTTTGACACAAATTTCTTCTCAAGAAGGTATAAACATTGATGGGATGTA
CUST_5455_PI426210822 TTCACAGTGCTTCAGACCACTGTAAGTGTAGGGGAAAACTAGCAGATTG
A_14_P201449 TGCTATCTCAAATGTCATCTACCAGACTGTTGAGGGCAAGAGTAATTAGAAAAGAAAACT
A_14_P104730 AACCCAGAAACACCATTCTCTCCTATTAACTCCTATTTATTTTTCAAGACCCAGATGGAA
A_14_P125668 ATTGTCTATCAGTGTAGAATTCTTTCCCCCATTATCTACTCTGTGTTTTACATCGGTTGA
A_14_P133079 TTCATTCACAGCATTAGGCTAAACCATACTCAGTATCCAAACAGTGGCCTTTGACTATAT
A_14_P201396 ACTTATCAAAAGAGGACATCCAAGACATTATCTACAGAATGAAGCACCAGTTTGGAAATG
A_16_P21470392
ACCCCTTTGATGTTTCATTCTTGTCATTCATATAGACCTCGATATTGTTAAATTGCCTCA
A_16_P20796471

CAGTTACTAGTTCACAAGAGATCTGAATATCTACCTCCTATTCTAGTATCTCCCACAGCA
A_16_P19093533
ACCCTTTTCTGATCACAGATTAAGCCAATCAACTTCAAACTTATGAATAGAATCCTGTCC
CUST_4137_PI426210822 CCTGGACCACCACTCTGATTTGGATTTAGATGGGCTCAGGAATTT
CUST_9755_PI426210822 ACCCTGTCTCCCTCCTCCCTCCCCCACACCTGCACTGGCTATTAG
A_14_P100335 GTTCGCTTCAAGCGAATATCTGGGACATCTATTGCCTTTAAGAACATTGCATCAAAAATA
A_18_P14136585 CATGGCTAGATGGACCAGAATATCCTGGACAATGTTCCAGATGACGTTTGTAT
A_16_P16191769
AAGGAAAAACTTCCTGAGTGAGCATCAAATACTTTAATTACTTCTTAGGCTTCAGAAGGG
A_16_P01400694
TTATACTCCACTTCTCCTATTGCTTAGAGTAAAACACCAAATTCTTATCCCTGTCTCCAA
A_14_P125168 CTAGTCTGCAGATTGAGTTGGTAGGAGGTGTGATTTGTTTACATGCACCTTTGTT
A_16_P02546865
CACGATTCCTCTCATAGACTGACTCTCTAGGAAAAGAATCAAGTAAGAAATACCCTCCTA
A_14_P137084 CATCCCAAACTTTCTATGATGCTTACATTTCTTATGACACCAAAGATGCCTCTGTTACTG
A_18_P18521102
GACAGTTTCTGTGAAATTGGTGTTGGATAAAATAAGAATATTTTTGGGGGGGCCAATAAT
A_14_P117485 TCTTTTAAATCATGTGTCTCACAATTCAGTTGTTGAGGAGTATCTGGTGGATCTAACAGA
A_16_P00935274
AAAGGAAAAGCACTTTCAAAGACTTTTCAAACACAAAACACTATGCATTAGCCAAACCAG
A_18_P14036993
TGCAGTAATAGATAACCCCAAAACCTCTATGTAAACATAAAGTTATTCCCCGATAAAGCA
A_16_P18805251
CACCATCATCTACTCTTATCCCTCATCTTAGAGACAGAAAAGAATAATACGTGATCTGCG
A_14_P109252 AATTAAACATAAAACTTCAAACTTACCCTTTAACTTGGCTCGAATTTTATTCGCAGTTTT
A_16_P18819905
AAAAAGACGTTTTCCTACATTGCTACAATCTTACGATTTCATTGCTGATGTTTGAGAGGC
A_14_P115835 GCAGACACAACCCAATAGGAGTCTTAATTTATTTCTTAACCTTTGCTATGTAAGGGTCTT
A_14_P200220 AGGGATAATCCTTCGGAAAAGTCTCTGGATTATATATTAAGTCCTGAGAATAACACGGAA
A_14_P200489 GGACTTTTCTTTGATAATCTAACAACCAGACTCCAAACTGAACAAATATGCAATGTCAGC
A_14_P101923 TTGGTTGTGAGGAGGTAGAAATTGCTTTTCATCATAATGGTAAGAGTTCAGGAATAATGC
A_14_P201222 ATTTCTGTTTATTGGCCAATATGAAATGTTTGCAGAGCATCATAAACAAACATAGCCCTG
A_18_P11898002
AAAATATTACCAGTTACCCTAAGCATGGATTCCTTTATCTTCCTTAACATTGACAAGGGC
A_14_P129757 CGGTAAATGAATATATTATTTTCACGGTTTCTGAGCAGAAGTTAGAAACACCGGATGCAA
A_14_P200292 CTTACACAGTTAACTTGATTTTAAAATCCAAGGCCAGGAGAGAAGAATCCAAAAAGCAAT
A_14_P130105 CTGGCTTCTCTGCAGTATCTTCTTCCTCTTCCTTGATAATCACTGTCTTCTG
A_14_P120497 GATTCCTCTGCTTTTCCTCCTTTTTACCATCTACTTATGTCTGAATCTTACCTCTGATCT

A_16_P20587540
AGAAGTTCATTTAGTCCGTTAATCCATTGGCAACTATCAACAGATATATCCTATGTGCCC
A_14_P138852 TTTGACCTGAATTGAGGTCATGCTAGCCCTAGTTTTCTTTCTGCATATCCAGAAAATTAA
A_14_P116808 CATTATGACTCCATGGCAACAGATGATTCCCTGATTCTCCAGAGAGATTACAC
A_16_P00956477
TTGGTGGCATTTTCTCATATACAGTTGAATGACTCAGATAACATAATCGCTAGCCTAGTG
A_14_P120145 AGGCTTAAAACATCAAAAGCAATTTTACTTGTGAAAATTTTCATCAGTTTCCTCCAGATG
A_14_P118885 GCTTTCACAGGCAAGTATAGAGTCAAAAAGAAATGAGAAACGGCCTAGATCTCAATAAAT
A_14_P104324 TCACAATTCTTTACCTGATTATTACAATTACCAATAATGAGTGCGTCAGCCAGAGACAAC
A_16_P15882102
GCAAATTTAAAGTTCTTACTATCTCCCACTGTTTGTCATTTCTGAGTCTACCATGCAACA
A_16_P36230054
GATTGTGCATTGTTAAGAACCAAGTGGCTAAGTTCTTTAGGGCTAACTCCAAAGAAAAC
A_14_P104634 ACAAGGAACAAGTGTTGGTTGTTAAGCATGGAAAATAAACCAGTAAGTGAATGACATCAT
A_14_P109263 GATGAGAGAAAAGACTAACCTGGAAGTTTTCTTACAGAAATGATGTATCTGCCATTCTCT
A_14_P127209
AAGAAACAGAACAGACACAAGAAAAAGACAAATCTGACACAGGATTTTGAAAAGCAGGTC
A_14_P200742 CAAATTGGCCGAGATCCTTCTTCTTTGGATTAAATATAACTTGGAAGCGTCTTTTTTAAA
CUST_6277_PI426210822 TCTGGACACCTCTTCCACTGAGGCTTTGTTTTTAAAGAGGACTTTCC
A_14_P102857 AGAAGACATGTTTTCTGAGAAACAGGACATACCTTTCGTAGAGGTCTGTATTTTTTTTCT
A_18_P10575488
AAAATGTGTCTCTGACATCAAATGAACTCTACTGAGATTCCCTGTATTATGCCAAAAACC
A_14_P101257 GCAGGTCCCTATTATCATAAATGAACATAAAAGTGCTCTAAAAACACTCCACAGATGTGA
A_14_P120842 ATAAATGTTTTATTAGGAGTGTAAAATTAAGAATCTTTATAGATCTTTTGGAAACAGTAT
A_16_P01970402
ACCCATTCCTTTTCAAGATTCTCCTTTCATATATCGTTTAAGAAAATTGCTCAACCATCT
A_14_P114267 GGGTTTAAGGAAATAACGTGCAATTGTTTGTAAGTAGAATACTAGTGGCTAGGCTTTGTG
A_14_P114336 TGCAAAGATTCTGTGAAGATATAGAGATGATGATTGGATTCCAGCCTAACATCTTCTGGA
CUST_6709_PI426210822
AATAAAAAGTGTGCCACAGCCAAATAAGCATAGAATGAAGCAAAACAATTTTTATAAATA
A_16_P00404352
GAATCTTCATAATTTGCCTGATGTGATTACTGAGTATGACGAGATCTCATAGTCCATGCA
A_14_P138069 CTCTTTGGGACCTTCGTGGACTTTGAATTTTTACTATTGTTCATTGTGTCTAAAATCTTC
A_14_P129267 TGAAACTGGTTGTTGGGATTATTGCAAGGACTTGTTGCAGATATTGTTCATTACATCATT
A_16_P03034098
GAGGATGTATTGTTCCCATTTTAACCATAGAAAGAGTGCTATTGTTCCTTTTCTCCACAT
CUST_7673_PI426210822 ATGTTAAGACCCCAGGAAGGCTTATACTTGATCTTTGTATCCAGAATGTTGCC
A_14_P118763 TCATGTGAAACTGAAATTTCCTTCTACAGAACAAAACTTTTTGGGGGCTACTTACCATAT

A_14_P118096 ATTTGAGATGATGGAACTATCAAAGCCAGGTTCTTTTGATCACGAAGGTCAAAACATC

A_16_P17643683

CCAGTAAAGTCTCAGTAAATACAACCATTACAACTTCAGAATATTTTCTCCTACACGTGC

CUST_2839_PI426210822

TTTCTACTATTTTGGCCTTGAAGCAGTGTTAGACAACGTTCACTTATCGCTGTTG

A_16_P16191148

CCTATCCTTTGTTAAGGCTCTTCCCCAAATTAACCATAACACGTATTGTTTAAGGCATTG

A_14_P115731

AGAAAGACAAGATAGAAGCCACAGAAAACTCAAATCATTCTCAATAGTCCCTAGAAGAGA

A_14_P121890 CACATATAAGTTAAAGTAGGAACAGACCACAGATACTGCCAAAGAGAAGGGCATTATCAG

CUST_459_PI426210822

ATGAATTTCGTCAGATAGTATCTACAAGAAGTCAGATGCTTTCACAGGCATAATATTCTT

A_14_P109376 CTGGTCCACCCAGGGTAAATTATAACATAAGTCTTCCAGCAACAAAAGCAGTTC

A_14_P122758 CTAGTGTGGTGAATAAGACATTCATAATAACCCTTGGCTCATAAATGAGAATAGAGGGAG

A_16_P19688449

ACAATGCAGTAATACAATAATTCCTTCCGGTCATTTTAGATACTCTCATAAGATCCCCGT

A_16_P19063005

ATTGGCATGGTAAGTCTTTTTTGCATGGTTAGTGAAATAGTCAAATACAGGTTAGCATCC

A_14_P127507 CACATGGTCTGACACGTATTCTTGGTAAGAGGTCTTAGTCATACAAGGCATAGCATA

A_16_P37541182

TCGCAATGGAAATATAGTAATCCCCCTGATTTATAAGGATGTGTTGGTAAATAGTCTGCA

A_16_P15725577

AAAGTTCATTCGGGTAGAAAGATCAAATCATATGCAGCCATACTGGTTTAGAGTCTACTT

A_14_P111143 CCGGACAGTGTTTTTTTTTTTAACTCCTCTTTCTTCCTCCTGCAAAAATTGCAAG

A_14_P124074 TCATTAAGCCTTTGAGTAACGGCAGAATATATGGCTGTAGATCCATTTTTAATGGTTCAT

A_16_P35872291

ACAACACTGTATGTGGTAAATGCTTCACTCTGATAACTCAGTCACAGATTTAAAGGTGAA

A_16_P39137239

ATCCATAGGGCCATTATTTATTGAACTTTGCAACTCAGATTCAAAAGTTAAAGCAACCCT

A_18_P22842486 AAAACTCTGGCTTTAGAGTTGGACCATGAGCAGTCCTGCTTGCTG

A_18_P11798495

AAACAGAGAAATGCCCATCATATCCAAAGGAAGTAGCAAGAAAATTTCGACTTTTCCAAT

A_16_P18870939

AAGTGTTATTCCAGAGATCAGATCTACGTCTATGTGGGAGACATACTCATTGCTCTTAAC

CUST_7553_PI426210822 GCAGAACATATCAGGGGAGAGAGGTGTGTGCAAGGCATCCACCAG

A_14_P111818

AAGTTTCTCAAACAAAAACTATGGAGAAAAAAAATATAGGATGAAGGGCAGTGGGGTATG

A_16_P16866828

GTTCCTGCAAATACTAAGTAATGCCATTCCTCTCATTTAAAAATAACCCTGAACTCTCCA

A_14_P133922 TTGTGATTATCTCTCTAGGACCGCCTGTATTTTCATCCTACAACAACACGCTTGATTAAA

A_14_P126234 ACATCAGAAACAACAGATTATGGAGTCCAGGAGAAGATGTTTATATGTGACCAGAAGTAA

A_16_P30952289 ATGGATTGATTTTTCCTCATCCCCCTACCCACCTCATGCCCTAAG

A_16_P00684985 TGACTTTATGGGCCTTGCTCTTTTGGAGGGATTAGAACTACAGAGATCACTTTT

CUST_5595_PI426210822

TTTGATAGCAGGAAAAAGACAACCTTTTTTTCATTAACCAGAGGAGTATTTTAAAAGACT

A_18_P19607908

AGTATATCCCCGTATCACTGAATATGAGTAAAACAATGGATAGCAGCAAGAATTATGGGG

A_14_P117935 AATTTAAAAATGCATACCTGTTTTTTCCTTGAATTTGCTTTGGGAAATAGGTCTGCTGAC

CUST_3933_PI426210822

CTAATGGCTCTTCTGGATAATCACTTCACATGGGAATCCTAGGACTCCATATTATATTCT

A_14_P101537 TGCAGCATATGTTTCACATACCTTTTTGAGATGTCCTAGTCTAAGTTTGAAAATTTCTTC

A_14_P113481 GACTTTGAAATAGACTTTGACTAGCCCAGAGCACCCAGATTCCAGTATTCATGT

A_14_P113392 AATAGAGTGTGGTGTCATGTTTGTGAGAGAGAAAACACTTTCGAGTGCCAGAA

A_14_P128802 TCTTCTCTTCTCTGTCATCTTGTTACTTACTGATTTGATAGCAGGATCTATATGCTTTGA

A_16_P15189905

ACTCTCATCCATTCTTGAAGACCAGTGAAAAGTCTCATATCTTCCATAAAACTAAGACCA

A_14_P128765 GATCTTTGTGTTTCATTCTTTGTAGACTATATATGTGTCTATCTGCAGGTGCTCACAGTT

A_14_P128534 CTTACTCAGGGAAGAATATTGAAGTGCAGCATTAAAAATACCCATTTATGTGTGAGGGAA

CUST_6139_PI426210822 AAGCAATCTGTCCTGCTGGGTCAGTGGCAGTCCTTGCATTGAGTC

A_14_P136005 CTGATTTTTATCCAGAGTGCTGTTATAAAAGCATTAGGTTGGGAGTCTGAATTCTTGGAA

CUST_8555_PI426210822

AAACAAATCCTTTGGATTTTCAAAACTATATTTAGAATTTTCATAAAGTCTGTAACAGAC

A_14_P127204 CTGGCTTAGCCATGATTAGAACTCCAATAGGTTCTTAAATGATGATAGAAAAGGTGTAGG

A_14_P130368 TACTGCCAAGCAAGATCTTTAAAATGTTAGCATCCCTTAAGGTTTGGTTGAGGACAT

A_14_P127548 CTGCCAACAGTGCTGACTTTCCTTTAACTCTGCTTTTACTGTCTGATTGTTTTTGTTTAG

A_14_P118369 GCATAATGTAATGATGATGACCTTTGACCTCTATTAACATCTGCACCTCTCTCACAAAGA

CUST_3956_PI426210822 CTTTGGGTTAGGATCAAGTGTGGTGTAATAAAATATCAGTCCCAAGAGTCACCT

A_14_P135733 GCAAATAACTTTTACCGCTTGTTCACTACTGTATCAGATTCCTGGTCATGTTATGCTTTG

A_16_P17945900

AAAAAGGGGTCTCTTGAATGATGAACACAAACCATAAGGTGAAATTGATGAACAAAAGTC

A_14_P111369 AGAACTTACACTTAATGATCTTCTTAAACGCGTTTTGAAAGTCCTTGTTGAAGTATGCGT

CUST_145_PI426210822 AATATTCCTCTCTGCAGTGAGGCAGGAGGGAGCAGAGCAGTATAA

A_14_P100698 GTAAGGTGGGGGCTCTTTATGAATTCTGGAGAAATAAACTTCATTTGTTCTTCTCTTTGC

A_14_P108971 ACTTGGAAGTAACAGGACACCCTCGGATATGTTTTTTATTTTTATGGTCTAGAGAGATGA

A_14_P107981 GAGGTGCTTTCAATAGAGAGGATTATGTAACTCATGTTTTAGTTAGGGGAGGTAACAGTA

A_14_P113993 GCCATTTTAAATGGGTCACCCCATTTAGGATTAGTGGATCTCAAATTATTAACCAAACAT

A_16_P21242333
AGCGCTTAGTTTATATGGAGAGGAAAAGGTTTTAGAGTAAGATCAATCAGTAAGACCCTG
A_16_P16865083
AGTGCTACTCTTTCATACACTGATACTCTAAGTCAATCCCAACTTTATTGTGAAACCAGG
A_14_P138516 ACGTAGCCTTAGAGGCTGAAACAATGTAAGTGCACAGGAAACATTTATTGTAAGAAAATG
A_18_P14431274
GCATTCTCAGTATCCATCCATATTCCCAACTTTGAAATTCACATTCATATCCACAAGACA
A_14_P138608 AACAGGTTGACCTAAATTCAGTGTATTTTGTGTTTTCACTAAGTAGCCCATTGTTACCTT
A_14_P115116 CAATACCTGAACAATTTGCTACTCTCTTCAGTGTTTCTTGGAGATTCAGATTCTGGTG
A_14_P106681 TATCATTATCTGTCAAACTTTAACACACAAACAGAACAACATTGCAGAGGGCATAGTTCC
A_14_P128169 AGCAATATCTGTTTCCTATGGTGCCTAATATATGCACCCTATACCACTCTCTTGACAATA
A_14_P105545 CTTTATGCCATCATCTTGGAGCCGATTAAATCCCAGGCACAATCGAGTTAC
A_14_P120692 CACCACAAAATACCACACCTATAAATATTTGGAAAACGTAAATGAACATTGAGGGGCTTT
A_14_P121644 AGGTGAGTCCTAGGTCCTAGATTTGGAGAGAACTATTATTAGAAAGGAAGGTGAGAAATT
CUST_10263_PI426210822
AATAAGTGAACATAACTGCATGTGTTTTGTGAATTCAAAGACCTCATAATCTTTTAAAAT
A_16_P21198767
AAAGGCATTCATGATACCCAGTTACTTCCTCTAATGATTGACTACTGTTGTGAAACCTAA
A_14_P117135 TTTTCTGTGCTCCCAGTTGATGGAAAAGTGAAATATACAGTCTTTAACTCCAACTCATAC
A_14_P120069 TCCCCCCTATACATACCCACTAGTTGGAATTAGTTGCCTTTTACTATATGCTGTATTGAA
A_14_P114909 ACTTTTCTCTACAGAATTGGTTTCCTCCTTGACAAAAAATGTGGCTAGATATAGGTCCG
A_14_P111237 CTTTACATTATGGGGTCTGTAAGTAAATACATGGAAACCTCATCGAATACAAGCACGCA
A_14_P113131 AAATGTAGGTGTGTAAACTTGTGAGTTTCCATCTCTCTGTGACTTGTAACGCAAGATC
A_14_P200499 ATTATTTAAGAGAACGAAAAACCACCACCAGTTCCTGTAAAGAATAAGTCACAGGTGCA
A_16_P19436249
CACTTCTAGGCTACTTTAGTCCTAACTTTTCTCAGTTACCATTATATGCAGTTCTCCCAG
CUST_4533_PI426210822 ATTCATAGATCTGCTTTGGGGCTGTCTCCTCCTCCTCAGCTGTAG
A_18_P14135953
GTCTTTGGTCAATATGAAGAAATACTCAATAGTAAATCATAGCAACATGATTTAACATGA
A_14_P103840 AAAATTTCACAGTGTTACAGAAGAAGGCAAATAGCCCATCACATAAGGAATATTTGCAGT
A_14_P115885 CCAAACTATGGACCAAGATTTATCAATAGAAGGGTTGAGGGAAAGTCACAGCATAT
A_16_P19287543
ATGGTTCTTTGAAAGTTCTCCCTACTACAAAGTTTACTCTGCAAATGGTGAACATCAGAG
A_14_P138788 TCCTTTTTCTAAAAAACAAGAGATTCCAACCTCATGAAGTAAGAGAAAATGCCACATACC
A_16_P20301055
TCAGGATGACCTAGTCGCTATTTTTTGAGGCATATATCTATAATTGGATCTAATTTCGGT
A_18_P10864572
GTTGACTGAAAAAACCTGAAGAAACATCTATGCTCTTCCCAAACATCTAAACTTAAACCC

CUST_7911_PI426210822 TACAGCCTTTCTCTTCTGTTTCTCTCAAGTCGCTGTGTGCAGAGTC
A_16_P19849904
AACTAAGAGGTCTTGCTACTCTTTATCCAGAGCGTTATTACTGATTGCGATTACAGAAGA
A_14_P116283 CAGAGTTGATGAGCAAGAAAGAACGAGAGAAGATATCCATTCCCAGAGACAG
A_16_P17988626
AGTTGAAAGCCGTGATAAGAATCTATTGTGTGCTTCATTTATCACTACAGAGGATACCTC
A_14_P126668 GTTCACCAACATTTTTATTACTGTTGAAAAGAGGAAAAATGGTAATTGGAGAGACCACCA
A_18_P22843224 AAACTCCATCATTTCCCACCATCCACCATGATCTGCAAAGAATCCTTAAGA
A_16_P01988427
AGTTCTGTCAATGGAATCGATCTCTAATTTAAAACCCAGGCTAGTATATTTCCTACGTCA
A_14_P125286 TTAAATAGAATTTCTTAAATTGCCCTAGCACAGCCACATGCTTTCTCACATTTCAGTGTC
CUST_10224_PI426210822 GGTGTGTATGTCTGAAGGAGGCCTACTTAACCTTGGGATGTGCTAATT
A_14_P127969 TTTGCATGGTTTTTATTCTTTCCTAATGGATCCTGTTTTATAATACTTCCAAGCCTGTCC
A_16_P16609276
TCAGGCAAAACAGTTCAATACCTGTAATTAGTGACCTTGTTTTATTCAAGTCTCCATCAC
A_14_P110158 CAATTTCTGCTAGGTAATTAACCATTTCGACTCGAGCAGTGCCACTTTAAAAATCTTTTG
A_14_P104104 CAAAGCAAACATAGGCCTGTTAGGTTTCTGTGGCTACTGAATATTTGTTTCTCAAAACAC
CUST_7642_PI426210822 GTAATCACCTCTGGTTCCCTCGGAGTGTTCGTGGCCAGCTCCTTC
A_14_P113575 GCCTCATCATCCATTTTTTAAGATACTAGCATTTTCTTGTTTGAAAACCTGGATTGCCTT
CUST_1800_PI426210822 TAACTACAAGTTTTTTGCCTGGTAGCATAGACCCAACAATCCTTCTACTCTGG
A_14_P116549 ACCCAATTAAAATCGCAAGTGTCTTTATTTGTCCATGAATACATTCTCTTCAGTTGGTGA
A_14_P102503 GGGATTACGATAGATGTTTTGAATCAAGATGGTCTGCGGGGAAAAAAAAATAAAAAGGG
A_16_P00741489
TAAACCACTCTTTATAGCATTGATGTCCTGATCTACCTGTTACAAATGAGGCATGAACGT
CUST_4383_PI426210822
GGAAAATTCCAAACTTTATTAATTATTGTATTGGGGAAAACATCATGTTATTGAATTTTA
A_14_P115571 TTAAAAACGTTCCTTCACTAACTATGTGGTTACCCTGGTAAAGTCAGAATACATGCTTGA
A_14_P101174 AAAATCTTGTTTTCCTTCTCAATTTTAATCACCTAACCGCGGATCCATGGGATACAG
A_14_P134484 ACCTTCTGTATTCCAAATATGATATGATCAACCTTCATGGACTTGCTCTACTGAAGGAAA
A_14_P111825 AAAGTGCTGGTATCTACAACTCTGGAGAACAGAAAGGAAAATGCTTACAATACGAGC
A_14_P200232 GTGAACTTCTGTCTATATTTCAGTGGAGATGAGAAACATTAGCGTTGGTTCTAGGATGTA
A_18_P14134815 ATATACTTGCCCCTTTAATTCAACAGGCACCTGTTGAATGCCTGTGCATG
A_14_P100348 TAACCATATATTGCATCTTTTTCCAGGATGGGTAAAAAGATAAAGAAGGAAGTAGAGCCT
A_14_P112113 TTCAGTTTCCCCATCATCTTATGGGCCTTTGGACTAGGAAATTTTAATGAGAGAAAGTA
A_16_P38143556
ACCTTTTTGAAAAGCCAATTCAATCTCACACATTTGTCTCTTGTATAACAGAGCCCTTAA
A_16_P16197410
AGCAGCAGAAGAACCATTTTAGAAACATACTTTTTCCTGATGTGATTCAAACCTTTGCTG

A_14_P110305 ATTGGTTTGTGCTGACAGATTCAAGTCTCAAATATTACAGAGACTCCACTGCTGAG
A_14_P201129 TCAGAGAGTCGTTACCTGTCAAGTATCACCTACAGAATAAGACCGACTTAGTTCAAG
A_16_P36090883
TACATTTTCACGGCTAACTATGATGAACATACACAACCATCTATGAAGTTCAGTCTTGCA
A_14_P103660 CTGGTAAACTTGGAGCACATATAAGACAATCAAAGGAAGTCTCCATATAAACTTCTCCAT
A_18_P13154013
AACATAGACATTTGCCTACATCGAATAGACATTGAGTATGCCTTTAAAGTAACCCCTTCC
A_14_P104863 TTCATTGCTTTCACAGGGAAAGAATTACTCGTCCCACTGACGAGTTCTGTAT
CUST_827_PI426210822
AAGATTTTTGTTATAAAAGCGTAAGCTCAAGAGGGGAAAGTAATACTAGCTGCCGCTT
A_14_P108842 GGAAATAGGAAGTCAACATAATGATGCTTATTCTTCTCTAACAGTAGAAAGTGGGGCATC
CUST_9347_PI426210822
TCACCGTAGGATTCGGATTTTTTTGGAAGTATTCTTCTTTCAGTCTGTAAGGCTTTT
A_16_P01606427
TAAGTGTTCTTCTCTGCATTTTCTATCATTGCCTCATAACAGTTCTAGGTCTAACGACAG
A_14_P121903 TTATAGCAAACTTGCAGAGATAGAATCAACAGACTTGGCAAATTGAGGGGATTTAAACTG
A_14_P114327 CATCTGTAAAATGGGAACAAAATTAATTCCCCACTTTCCTCACAGAGCCTGCATTTTA
A_14_P102248 TAAGAATGGCAGGCACTTTCTTCCTGAAGCAGACGTGGAGAAGTATTATACC
A_14_P119921 TGCATCCCCATTATATCTTCTGTTGACTGCTTTTTCTCTTATGTGAATCTGCTATTTGCT
A_14_P201101 AGTGATTTTGAATTTTGTGCTATTTTGACCATGAGTAATCTCTGTTTGGGGTTAAGGTTC
A_14_P126903 CAATATGCCCAAGGCAACCATTTTCAGGTTTTCTACTTAGATTCATGATCTATCTGACTT
A_14_P138682 CTATTACTCAAGAAATAGTGGGCTGTGCCCATTTGGAGAATTATGCTTTGAAAATGTTTT
A_14_P139955 GCACTTCCTTTAGGAGGAAGAGGGTGAGTTGTGATTGAAATGAGACGTTTATTTTTTTAA
A_14_P129152 CTTGGACACCAGTTATTAGAGGATGAGAAAAGTAAATGAAATGTTCTCTTTTTCACTTCA
A_14_P103175 CTCCTCATTGAAAATATGATGCTTTGTAAGAGATCCAAGTGCAGCCACCTTCTTAGA
CUST_777_PI426210822 CAAACCTGCCTCTTCTGTCTTCTGGGTGGGTCTTGCCCCTTTCCT
A_14_P133809 CTAAAACCCTGCCACCTCAAAGCAATAATAAGAGGTAAGTCTTGTGGAACTACAGTATG
A_18_P16264733
AGTCCACGTCATGAACTAGAGATCACATAATAGAAAACCATATGAGACAGTAAAACCACG
A_14_P100526 TCTCCCACTTTAAAACTGGCATAGCCCAAGAAACTTCGCTGAAAATAACAGAAAGAAT
A_18_P14132273 GTGAACTAGAGGAAGGAATCAGAAACAACTCTGGGCAATGGAATCACTTTG
CUST_3902_PI426210822 TGAACTGAATGGGAGTTCACATTTTATCTTCAGGAAAATGACCGCAAGAAGG
A_18_P12377942
ATCACACAATGGTTACACATGTAGAGTTGGGTAAATGAAAGATTTTTTAAGTAGGAGGCG
A_14_P139484 GTTTGGAAAAGCATGTTATAATATAATGTTTTCACTATATGGCCTCTGTTTTGGTGTGTT
A_14_P107460 CTAGTTTTGCTGGCCCTAATAGCCAGTTCCATGGTGAACTTAATTATTTTTTAGATGTCT
A_16_P00237818
AAAACTGAAAACCTCTAGTCTAACATTCCAAATGTTCTGTTTTTTCCAGTTCCTCTCCTA

A_14_P135725 TAAGTATAAAAGTAAAAACTGAACATTGCATATAACCCTGGTCCATCCCATTTCCTGCAG
A_16_P20497106
AAAACCCTACTGTTGGATAAATGTGGAGAAGGAGATTTATGTAAATCAACTGCATTAGGT
A_18_P16989170
AAAGGTCTGGTATACATTGGCATCAATAGACATTCAGGACAAAATAACTTTTGATTTGCT
CUST_3568_PI426210822 TTGGAACAATGGTGGGTCCAATGGTGGATGAATAGTTTATTCACATGCATC
A_14_P121701 CTTAGCGCATTCATTACAAGGGAACAAGGCAACATACATACTACAGCCTTTCACATC
A_16_P17499794
CATTTGTTACGGTTCATGAAATATTCTTCCCAGTAAAGATACAAAATGCCAACCAGAAGC
CUST_3635_PI426210822 AGACACTTGATTTAGGTGAAGGGGGGCCCCAAGGCAGTGGTGCCT
A_18_P14134779 AGACACGAGAAAGCCAAGCAGGGATTTTCAGTTACAGGTGAGCATC
A_16_P02745168
AAAAACAAAAGAAAATTGTGACCTTCCATTAGTTTCTTCTCTCATTAGTCCATGCACACG
A_14_P113351 CTTTGAAAGCAACAATTTTATTTACTTCTTGACGGTCCAAAGGGAAACTCTAGATGCTCA
A_16_P20793525
ACGTATTCCCACATATTACACTGCTGACAATTTCCTAAGTTTTCCTCTTTCACTTTGAAC
A_14_P102598 CTGGTGTCAATCGTGCTGATTTCTTTATGTGGTCAGAGCAAAATTCACATTTTTTTAAGT
A_14_P123780 GCTTTCTTCAGTGTATGTTTGGAGAGAGTTTGAACATTTTTTGACTCTTTTTCATTGAGT
A_16_P19525599
AAAAGAGCATAAATCAATCGACACAAATATCCCCTTAAACCTTGTCTTTCATGTCCAGTA
A_16_P58424258 AGTGAATTCTTTTTGTCTCCCTGGTTGAAGGAAACATCTGCTTCTGGC
A_14_P123947 ATGCAAGAAACGTCATCCAGCCAAATAGGTCCTGATGCTTCTTTAAAATAGGTCATAC
A_14_P114315 GTAAATACAGAGAGATACAGATGAGCAAGCCACAAAGCCAATTACATACCAAAGTTATGC
A_18_P11916125
TGGCTGCCCTTTAAATGATTAAAAATGATTGCTCTAAAAGGTATGGATTGTGAACACAGT
A_16_P00933518
ACACTGACATGAGTAATTGGTCCATATGATAATTCTCCTTGTTGTAATATGCCGTTCTCC
A_14_P109038 CATATGATTTACAGCATATTGTAGGTGGAAGTTCTGGTGAGTTGGACATTTTAGTATGGC
A_14_P115120 AGGATATTACCTACATGGTGGATAGGGAGAAGGTCAGTTTTGGATGAGC
CUST_9847_PI426210822 TAAACTGTAGCTGCTACTTTAACCTCTGTGTCCACAAAACCAGACCTCC
A_14_P201948 ATTGCTGTATAAAATTGTATCATGTTTTTACATGCTAGTTTGCTTATTTTTCTTTTCAGG
CUST_6502_PI426210822
GCTCATATCTCTCATAGAAAGACAACAGATGAGCAATTTTGGTTTTATGGTTTACTCATG
A_14_P134993 TAAAGAAAGCAGAGTCTCTGCTTTGCCCATTTAATACTGAACAAAATGTAGTGCAACTGA
A_16_P16628656
CTATCCATCAAACTTACATATGCCATTAAGTGCCTCTATCATCTTCTTATCCCGTCTTCA
A_14_P107495 ACCAAAAGTTTCTGATTCAAGGTAAAAAAGCAGTTACCCACTCATGTCAATATCTGCTTT
A_14_P128386 AAATCTGCCATCAGAAGCACAGGAGCACCTTTGTAACTCCTACATTAAAAAAAGAATCAG

A_14_P128586 GAGTTAAACCTTTTCAGTTTTAGTCCTGTTGCATTTTGGGTTTCTTGTTCTCCAACAATA
A_16_P15601723
AGCCTTTCTGTAGCCCTAAAATTAGTGATCATTCTATGCTCCATCATCCTGAAATTGAAT
A_14_P121838 TTTATAGCACAGTGGGATAAACCAAGGACAAATTCTTACTGAATAAGACCTCTGGTAGTT
A_14_P132407 CTAACGTTCAATCAAGAATGTCGAATCGTCATAAAAAGTGAGAAAACTCCAGGCTTTAGT
A_16_P00480899
AACCTACGACCTCTTACCTAGAATCATAAAGGATAGACATTGATGTGACAGTTTTTCTCG
A_16_P37358532
TACACCCACTGTTAACTTTCAGTTTAAGATTTCACCTCAGTCCTTATTTCTTTTGAGAGG
A_14_P120513 CTGCTGAATAGCGTCTTCCTCTTCCTCACCTTCTACATCAAGAAGGT
A_14_P110085 TGGTTTAGGTTGAGGGATCAAACATGAGATAACTGAGTTCGTTTAAAATGTTCCTATAGT
A_14_P120730 CACAGTCAAACCAATAGGTAAAATGCATGGAAGTTGGGAGCACCAAAGATTATATTTATG
CUST_6685_PI426210822 AACCTTTGTCACAAGGAACCCTAGGTTTCAGTTCGACAGAAACTTGAC
CUST_10151_PI426210822 CTGTGGAAAGAATGAAGATGATGGGGTGTTTCTCCTTTTGAAGAAAGACAC
A_16_P20304950
GAATAACGTGACCCAGTTCATTTTTAACAAGAGCACTTTTGCCATCATGTGTCAAATTG
A_14_P103998 GGAAAGTGTTGTTACACTGCAGAAAGTTTTGGTTGATAGCACAGTTGTTTTCTGTTGATA
A_16_P36613837
AGATATTACTTGTGAACTCACTTTAGTAGATGCCTTCGACTGTTATTTACAATGCCTAGA
A_14_P124080 CTGACTTATCCTATTAAACCTCCTCTGCTATGTTCACAGATTCTGCATAGTTTTTTTTCA
A_16_P21084134
AATGAAACATATCCACCAAGTATGTCCAATTTCTCTGATGACAGTGCTAACTTACATGCG
CUST_8923_PI426210822 CTGTGCATTGGATTATACTCAACCTTTCCTCTGAAACGTTGGTCTGGAG
A_16_P01722875
TTACCACAATCTATGGGAAAAGTAATGAAACATGCTGTTATCTCAGTAGATAGGACGAGG
A_16_P18198824
GGGGTTTACTTACATTATGAACACTCTCTCATTCACGAACTATATCAGAACCAGAAGACA
CUST_10340_PI426210822 CACGGTGAATGCTCAGGATCTCCTTGGCAACGTGGGTGAAGATAC
A_14_P114299 AGTAGCAATAACTATGGCAATATATTGACAGATATCGCTGCTAAAGAAACAGCCAGACAG
A_14_P133545 ATACTCGTATCTCTGATGCATTTAGTGAAAACACCATTAACAAGCCATTCAACGACGTTA
A_16_P17218695
TCCATGAACTATAAAAACTTCCAACTGAAAGCCTATCTATTCCCGTATCTTACCCTTCAA
CUST_8496_PI426210822 TTCAAATTTTGTATTCCACCCTACCCTGACATCCACCTTGAGGGTTT
A_14_P114509 TTTGAGTTCTGATGATGACAAAAATATTTTGGCAGAGACTCCATCTCAAAAAAAAACAAC
A_14_P103488 AGTATTTGCACAAGGTGATGCAGTCCATCTCTAACACTTGACTTTTCTCTCACTTCTA
A_14_P128443 GACAGCATTTGAAGGACACATATTGCAGTTGCTTGGTTGAAAGTGCCTGT
A_14_P135191 GAATATATTATCCTATTGGCTTATGCCTTCTAGACTGGAGGCGCTCTCTCCTCTT
A_18_P13654658

AAAACTGGTTCAAAAGTATCCATTCAATATCTTTCTTCCATGCACTCTATATACCTGGGC

A_14_P129884 ACCCTCATGGAGGATACATCAACTTCTAATTCATGTAGAATTTCCAGCAGAGAATACATC

A_14_P116587 CCTGTTCTTGGAGAACATTTAAACTATAAAAAGGGCCTTTGATAAAAACAGATCAATTTC

A_16_P02719124

ATTACTAGTTGCCTGTATCTCTAGTCCAACTTTCAGCCTTACTTATCCTAAAAGAACAAT

A_18_P22843225 CTTCTGTGGTTCGTTCTTGTGATAGCATTGTCCTTTAGGATGCCATCATTATGTGAAT

A_16_P00812743

ACCCCCAGTCTCTGAATTTAACTATTAAATAAACTGAAGTAATATTCTTTCACGACCTCC

A_16_P15606197

CCCCTTCTCTTCCTTATTGAACTTTAGCATGCTAGACTGTCTTCATTATTCATGTATGGA

A_14_P126673 CAACCTCCATGACAAAGGTTTTGATGAATCATCAGCACATTAATTTGTTTCCTCTACATT

A_14_P118249 ACTCAACCATCATTGTATTAGATGATACATAAGGAAAGTGACTTAGGGAAGTAAGTTCAG

A_14_P138610 ATAACAAGTGAGGATGCACTATAGTAATGGCTTGCTTTTATCAACTACTCAGCAGATGTC

A_18_P10594146

TGTGAACATTTCAATAAGTAAAGTTGAAGGAAAATATATGTGGCAGAATGATCAGTGTGT

A_14_P118845 TGCTCTAATGCTTTACTTCCCTTACAATTCTAAGTAGCAGTCTAATGAGATCACATAGGC

CUST_4124_PI426210822 AGAAGGGTCTTCATTCCTAGAGGGATATGGAATGACCCACTGCTTTT

A_16_P19746155

AAAAACAACCGGGTAGTCATAATTTCCTCATGTTTTAAATCACTGAAATCTAGAGTTGCA

A_14_P118959 GGCCACTTACCACATGATCATGATCACTAACTTCATTACAATCTCATTCAAAATGTTGAA

A_14_P103581 ATTTTCTTGAGAACAGAATAGCAAGCCTTTACATCTTCCTGCAGGAGGAAATGG

A_14_P124318 AAATTTATCCCAGCAATCCAAAATAACGTATCTGGATTTCATCCTTTGTCTCTTGAAGGA

A_14_P128716 GTATGGCTGATGGAAATTAGGCCAAATTATAAACAAATTATAAACCTACAGTAGCAACCA

A_14_P115251 CTGATTTTGATAGAAAAATCCCCTTTTCCTCTTTGAAGTTAAAGGAAGATACCAGAACCT

A_18_P17407446

AGTCACTGCTGTAAAGTTAGTCTATTAAGGTTGATTTGATGTGTCTTACGGTTTTTATCA

A_18_P22842141 TTTAGGACTGGCTTTGGGCTCATGGTTTTTATTTGAGAGCATTACTTCTGACCTAA

A_14_P124975 GAAAGAGGTTACTTTAAAAGTGGTGAAAGTGATGAGACCTATACGCCCATTTAACATGAA

CUST_6708_PI426210822

CAAGAATACAAAAGTAGTTGAATAAAAAGTGTGCCACAGCCAAATAAGCATAGAATGAAG

A_14_P107461 CTCTATTTTGCATTAAACTGAGGTTTTCCAGTATGGTTAATCGGTTCCATAGATCCCAAT

CUST_8412_PI426210822

AGACATGCCAGGATGTTTCTTTTTAATTCTGTGCACTCATGTTTCACTTCAAATTAGTTA

A_14_P103788 GTTCTTTTTTTAGGGTGAATCTCCTAATGGCTCAAGTGATAGAGGAATGAAATCACTAGT

A_16_P15996277

CCCCCTGTTAGTAGAATTTCCAAAGTTGATCATTCTTGCTATAAATGAACTCATTGCCTA

A_16_P40459488

CGCTCTAACTCAAATTTTGGAGAAATGTGACATGAGACAAGGTATCACATGATGTTAAGA

A_16_P20932354
ACAGGATTAAATTAGACACATGTGAACATTTTTGAGTTGCTGTATAGGAAGAACTGCGCT
A_14_P117861 AGGTGAATGGAAAGAGATTTTCTAAGTGTAAAAGCCATAGAAGGAGTCATAAAGCAGATG
A_14_P104684
AGAAAAGAAAAAGTTTTAAAAGAGGAAAACAGAAAAGAACTATGTTAGGATTCCAGCCAC
CUST_9566_PI426210822
GTGCCTTTATTTGGGAAATACTGTTAGTTTACCTTGCTATCGAGTCTCTTTCCCTAAC
A_14_P135279 TTCTCTTTCACTCTTTAATCATCTTGGAAATGGGCTGAATTCAAAACTTGGTGAGAAATC
A_14_P121443 TGCAGGACGCTTTTAAGACTAAATTATCTTCTTAAGGTTTCACAGGCAGTGTGAATTAAA
A_14_P109734 TGATGATTGTTTTATTTCATTGTTTTGAGAAAAGGTGTTTGGATGGACTGGTGAACTAGG
A_14_P132530 CTGCACTGGCTTCTAGGACATTTACCATTAGGAAGATTTGATCTAAATATTACCCTATGG
A_18_P11402760
ATCAATAGCAAGTCATTTCTAGTGTCTCAGCATAGAATCATCCTAATAATTCCTCCTGGC
A_14_P123081 ACACCATGTAAATCATACTAAGTGAGACAGAATATGGAAAGACATACGGAGTTCAGGATT
CUST_3371_PI426210822
GTTTGGGGAAAAGTGAGGTATAATAATACAGGAATTAGTATTAAAGAATGTAGTAATTAA
A_14_P111195 TAGCAACTAGGAAACAGGCTTTTGTGTAGAATTTTCAAAGCAAGACACATAATCTCATCT
A_16_P19880401
AGGGCATAGAAGTAACCTATCATGGCATGATTTTATTGAGTCTCAACATTTGTCAATCAA
A_14_P112886 TATAGCATGTCAGTAATTTGGTTTTTGCATATTTCCGTGGAGATCAGATATCCCAGACTT
A_14_P106536 TCCAGGATTTTTGTGTGTGTCTTTTTGTTAAATCCATTTCAGATCATGCTTGCATAGTCT
A_14_P108401 AGATTTAGTAAGTGGTATTTCCATGAGATACCATCTCGTGGAAACCAAAAACCACTGTTT
CUST_4519_PI426210822
GGAATTTATTTGCTGGAGCATTTTCCATTTTCATATTCAGTTTAGACCTTGGCTGAAGTA
A_14_P200927 GAGAGAGTAACTAGAATTAAACGTGCCATCATCCTGTTGAGTTTCTTGATTTCTTCTTTC
A_14_P124905 TTTTTTCATAAGTCTATCTTGAGCATTTCCTTTTATGCCGCAGAATACAAACCTGGGAGA
A_14_P100707 CATCACAGAGTGTTTTGAATTCTTGGAAGAAACAACTGTTGTCTATAACTTAACGCATGA
CUST_10248_PI426210822
ATAACATGCTTTTTTTCTGGTTCTTGCAAATTACTTTCGGTGACTTGTTCTCATGGG
A_14_P124698 TCCTCCAGTATAAATCTGGCTCCTTATAGGAATGCACATTATTTTACAAACAGACCTGTT
A_16_P20127389
TGTTGTGCTCTCCTAATATTACTGCTTACTCATGATACATACAAGACCGTCAACATACAA
A_14_P126086 AAAGGAAAGATCTTAAAGTCTAGGTTTTAGATTTTGCAGCGGTGACATTAAATGCGTGTT
A_16_P30952456 TCGGTGTTTCCTTCAGAGCATTGTTCTCAGGCTCCTTTTTCTTGAC
CUST_5317_PI426210822
TGTGTGAAACATTTTTCCCCTCACCCTATATGCTGTTATGTAAACAAGTAATTTTTTAGG
A_14_P201800 TGAACTACACATTCCACAGCATCAATGAGATGAAAATAAAATGGATATTCAGAAGCAGCC
A_14_P128728 GAAACTATTAATCTTCCAAATGAAACCTTTTAGTGCATGGGTGTGCTGGCCTACATATTT

A_14_P118201 CAATTTCTGAGTTACTCTATAGATCACGTCTGCTATGATTTGTTACCCCAAATTGTCTAA

A_14_P114261 AATTTGGGACTTTCAGTGTATGCCATTACTTATGAGAAGTGCTTTAGTTATGAGTTGTGG

A_14_P107888 GAAATGAACTGGTGAAGGGGTTTTGTAAAGTTTTTGCTTCCTGCTATAAGTTCTTTTCTT

A_16_P03241470

ACAAAACCCTAACATTTAGACTTCTGATTCAAGATGGTTACCCTTAACACGTATGTTTGC

A_18_P14136593 CTACAGTTAAAATCTCAATTGTTCCTTTCTTCCATCACCACCCTTTGAAGGTCTCTTT

CUST_200_PI426210822 TACTGGTTGTGAGACAGGGAGGGAAAGAGAGACAAAGGCAGGGAG

CUST_9628_PI426210822 CACATCAGCCAGACCTGGCTCTGTACAGACCCAAACCTGAGAATG

A_16_P36094905

ACCCAGTGACCAATATTAAGGATGCAGATAATCTACCAAATGAATCCATATTCTTTCCAC

A_14_P134253 AGGGGTATATTGTGGATGCTCTACTTTTCTTGGAAATGCAAAAGATTATGCATATTTCTG

A_14_P133372 CTAGTAAGTCTGATATATCAGCAGCACGAATCAGTGCATACAAACCTACCTCAAGCC

CUST_5031_PI426210822

AAAGGCTTTTACTAGATGTTTCAGTGACATAAAGTTGAAGACATAAATACTGTTTTATAT

A_14_P133699 TGAACTTAGCACATCTACTAATATGAAACCAAACCAGGCTAATCAGAGTGTTGGGTATTT

A_14_P100957 GAGGTCTCCATTGGGTAGATGGAAGTGATATGCAGCAGATACAATATTATACAATTTTGC

A_18_P16276293

GATGACAAAGGGAATTTAGCACATTTAATGAGACAATTGCTTATGTTCGTGATGAGGTTG

A_14_P202357 CTGCATTTAGGTAGCATCTGGAAAGAATCCAAACTAACATTCCATCATAAATTTGCCTTC

CUST_517_PI426210822

CAAACTTCTGCACCAGTGACCAACAATATGTGAGAGGATGATTTGTATCCTCAAATATTT

A_14_P109702 ATTCTGCTTCAGTTTATCAAAAATGGCTTAACAAGAGAAAACAGTGATGGACCATGAAGA

A_14_P109152 ACAATTTGGTCAGTAGGCCTTGAGGTAACTATTGCAAAATATACAGTGTAAGTTCAGTCT

CUST_9389_PI426210822 GTCTTAGCTGTGCTCCCTTATACCCCTGCCCCTTTCTCCTTAATT

CUST_8165_PI426210822 GTGCTGTTGCCATTCAGCACTTGCTGGTTGAGAGAGGAAGGCAGT

A_16_P37027145

GACATTTACTTATCAATCCCTCTTCACAATACTTCCTCTTATTGCATTCCTGTGTTCTCC

A_16_P16895609

AATAGATGCTGATCACAGTTCATTAATCCCATTTACTTCGTTACTAGATGGTTGCAGTGG

A_14_P122165 TTTTCCAGTAATTTCTGGGGGAAATCTGGGATAGTCAGGGGGTAAATCTAG

A_14_P119107 CTCTTGCAGAAAGTGGTTTCTGGAGAAGTTAAAGCCTGAAGGTATTATCTGCTTTGTTT

CUST_2670_PI426210822 TTATTTACCTTGAAGTGAAGTGACAGGATTTGAACTGTCAGCCCTCAGG

A_14_P101263 CTCCTCAATTATAAAGGGCACACAGCAGTTTCTCTACCCTAACATTAACTAGAGTAAACA

A_16_P19833442

AAGGCTCCATTTTTCCAAGAGAAGATTAATACCGTTCAAAAACTGTTCTGATCACTAACA

A_14_P126589 TGCAAGGTAGGAATGTTATCAGAAAGAAACACACATCCAAATACAACAAATGTGATTTTA

CUST_4294_PI426210822

GTTGCTCTAATATATATTCTTGGTTTAGATTTCTTTTCTGGAGAGGTGTCCCCAGACTG

A_16_P16583304
GAGACTATCTGTCTGTCAAATCAAGGAATGGAAATGTGCTTAGTGTGAAATGCTGACTTT
A_18_P22840123 ATAAGGAACAACGCCTGTTCTCACTAAGAAGTGGCTTGGCATTTTCTACTCTTT
A_14_P137614 CAACTGTAATAGACCAAAATATATAAGTTAAGCCTACGACATCCTCCCCTATGATGCAGT
A_14_P125924 AATCTGAGATTGGAATTCAATCATGCATCTTGTCTTTTACTGGCATCTCTGTTTTCAAGG
A_16_P19200138
AAAAAAAATAAACATATGCTACCAGTTTTCGTAAGGTAAACGATAATACGGCCTTCTCTC
A_14_P109412 AAGTCACAGCAGACCAGCACTCAAACATATTTCTCTTCTATTTCCTACTTTTGAATAAAA
A_14_P116819 ACACACAAAATATTCTCTTAGAAATAAAAATATAACGATGCCTTGGCAAGACACCAACCA
A_16_P16742997
AATTCACCCCTCTTAAAACTGGTTCTGAGATAATGACCTTATTTTGCTAGAGCATGGTAT
A_14_P128750 AGAAGTTTCCATTTCTGACAGTCCAGCAACTCCCTGCTTGACTGAC
A_14_P123074 CTGGATTAAACTAATACCCTTGGCTGTTTTTCACACTTCTTATATCCCTCAAAAGAGAGA
A_14_P136113 GACAGTATAATGAGACCATCATATCCTTGTCTGATTGAAGGTAAACTGTAACCTCCATGA
A_16_P01966937
ACTGCCTAAAAATTTAGTTCCCTCATTCTTACATTACTTCAATCTTAACACACTTCAACC
A_14_P116650 AAAAATAAGGTAGAAAGCACCCACCTTCCACATTGTTGTCTTCTGAAAGCACATG
A_14_P200738 CTCCTTAAAGCGCCAGGTGCTATACTGCTATGAGTTTCATATATAGTCTTTTTTAATCCT
A_14_P125810 TTTTTACTTTGTAGGAGATAAAATGAAGTGTCATGCACAGATGCTGGCATGCCTTTTTTT
A_16_P03710407
CCTCTTTCCTTAAATCCTTACAAGTCTTCCTTAATGTTCATCAATCTAAAAGCCCTTGCT
CUST_8613_PI426210822 TTAAAAACAGAGCCACGGTGGTGGCCTGAGGAAAGACTATCAATTTTC
A_14_P129207 ACCTGCTTTATAAATATTTCTTAAACTACCAAAGGATTTTAGTTTCCACATTTTGCGCTT
A_14_P103349 AGATATTTGGTCAATGGTTGCTTATTTTTTGGCGTCACCATAGGGAGAAATATGGTTAAA
A_14_P135952 ACTGCTTAATACCCTCCTTTCCTTCAAAGTAATGCTTTAACCCTACTTGGTTAAATTTGC
A_14_P124414 GTGCTGGAATCTTCTACGGGCCACCCCTTTATTAGCATAGGAAACG
A_14_P137143 ATGGTTCTGATTGGGATTTTTATCTACACTGCCTCTAAATACTTGCTTCTAAGACCCAAA
A_16_P20272396
AATACTTTGGGTTAATCTACTGTTAGTCAGAAAAACCAACTCATCAGTAATGTCCTGGCA
A_14_P139906 ACTCTGACCAGCAATTTTTTTTCTCTTTAGTCCACCACAAATTAAAGATGAACCTGAAGA
A_16_P19115806
AGTCTACAAATATAGCGTCACCTCAAGTTATGTCGAGAGTTCAGAAAAGTAGGGAAGTTA
CUST_3786_PI426210822
AATTGTTTTGTTAGAGTTTGTGGCTGACATTGATTACACTATGAGTCATGTGACATTCCA
CUST_4046_PI426210822 GAAACATTCCCCCTGGGCTCACTTTTTGCTTAGAAAAGTCCTGATG
A_14_P125507 TTCCCTTCTTTCCTAGGGAATATCATTGTCTATGGGAATACAATTGATACTTACACCACC
A_16_P37551776
CAATGTTCACAAAACAAAGACTGACACTAAACTGCTAAAAACCTGATTGTGTCCCTATGT

A_16_P16459919
AAAACTTTCCATCCTGAGAAAACATTTTTGTTATCAAAGACTGAAACAATAGACCCAGGC
A_14_P100987 GAACTTACCTCAAGTTTGGAAGCAATGAATAATGAGGTAATTCCAATGAGTTGAAGCATA
A_14_P126544 TTGACAGGCTGCATAATAAATCCATAGGGCTCTATCTGAATTTCAACAAGGATTATTCAG
A_16_P16191046 GATGTTCTGTAAACACATAGATACAAGCACATTCCCGCGTGCCACCTC
A_18_P15665674
AGCAGTTACATTTTTGCTCATAAACACCATTCAAAAGAAGAGAAGGTTATGATCCCTGAC
A_16_P16577358
TACTTTTGTGCTTCAACTCTTTAATTAATGTGAACCCAAGACATACTATCCAGCCTTTCC
A_14_P100564 CCAAATATTCCTTCAATTCCTAGTGGTTTCTTGGTCTCCAGAATAGTTAAAAACAGAGAG
A_14_P129303 ACAGAGATGTATGTAACAAGGTGTTGATTGATAAAGTCCTCCCAGTTGTATGGTTCTTAA
CUST_3647_PI426210822 CCTGTGTTGAAGTCAGGGAATAAGAATTTCCTCTGTGCCACAAATAATGCTC
A_14_P122013 AATTTAGAAAGAAGAGGAGAATCGCTGGGTATAGATGTAAAACAATGCTTTGTAGGAAGG
A_14_P118526 AGTGATAAGGAAGGGCCAGCAAGAATGTCAGACATATGTCAATATGTGGTAGATATATTG
A_16_P16759922
AACCCCAATAATGAATAATTCAGACACTTTAATTCATCCCTTTACTAGAGTCCTTAGGTT
A_14_P136966 ATTCTAAGGTCCCTGTGTCTGTGACATAGACATAAACAGGTGTTATCTTGTATCTCATTT
CUST_9054_PI426210822 GATTCACACTTCAAACCCACTACTTCATGAAGCAAGGTCTTGCCAGATC
A_14_P128162 TTACTGTATTTACTTTTATTTTTCCTGCGGTTCTAAGTGGGAGGTCATTATTGGGATGTG
CUST_7108_PI426210822 ATGTGAAGTCTTACCCTTTGTGAGTCAGGGGGCTTGGGCTGTCTG
CUST_1583_PI426210822 GAGCCGAGGAGAAAGTGTTCCCAGGAGGGAAGTGTCATTATGTTT
A_14_P119270 ACTCATTAGCACTGTGAGGCTTGTTCATATAAGATGCTACCAAAAAAGAGAAAAGAGTCT
A_14_P101130 TACAATTCAATCCCCACTTGGTATTTACCTTTAAATTTGGCCCATGGAGTATTTTGATGA
A_14_P108439 ATTTCTGATAAATCCCTGATAGAAGCATTGCTGCACCTCGGGTTTCTGAGT
A_16_P00025232
GCTGCTGAGGAATGGAATAATTAAAAAATACTGGATTTAACACCAACTCTCCTTATTGCA
A_14_P112859 TAAGTGGGTTTCTTAAAAAATGTTATTAACACCAAAAAAAGTTGAGGTCATCTGGAGGGC
A_14_P123423 ATAGTCATCATTATTGCTTCCAAAGAGACCTCTGATAGAGAAGAGGAAAGAAAAGGCTGT
A_14_P202276 ACTTGCCACGTCATGGAGGAACTGGAAATGAGACTTAGGAAAATGAAGTTTTATCATAAA
A_16_P02332105
CAACTCTGGGCATTTTGATTCTTTTAGTGTTGAATATTCTATTGCTGCTCTCAAGGTGAG
A_14_P131619 CTATATGTCTCCAATAACTCTACAATGTGTGGATGTTTCAGCATATGACAGATACTGGCT
A_18_P22841034 GATAAAGGCAGAAGGAGTAGGTCAGACCCCAGAAGATGGGACTTT
A_14_P106005 CTATCTTAGACTCTTGGATTCAGGATTGGGATGTGGTGAATGCTGCCTTTTTTC
A_14_P202055 ACAGATCACAAAACATGTATATACATTATTTAGAAAAGATCTCAAGTCTTTAATTAGAAT
CUST_2147_PI426210822 TTCTGCAGAACCATGAAAGGGACGTGACATGGACAGAAAGATGCC
A_14_P104922 GTGCGTTGAACATCAATCTGGCCACTCATCTTTTAAAAATTTATCTCACTACAGTTCTTG
A_16_P00685295

GAATCCAAATATTACGAGTGCAGATGCTGAAAGGAACAAAAGGAAATCTCACTCAGTCAT

A_18_P14134992 TTTCCCCGTGGTGGACACACAGTGAAGAATTTGACTCATGTAACCAAAA

A_18_P11096132

GTGCAAAAAATAAACATAAATATTTTAAAAAGGGAGGGAAAAAAACAGAATGAAAAGGGT

A_14_P112523 GGAATAAAATTTTTTAAAACCCTAGTGCCATATGATGAAGGGGCCCCTGCTTTT

CUST_473_PI426210822 CAGAAACAGAATCCTGAGCAATCTTGCCACCAGCTGACTCACAAG

A_14_P136694 ATGTTGGTTGAATTGAGGATTCCAGAAGTAGCATCAGATTTGGAAGCCTTTGAAAG

A_16_P15544384

TCCTTCCAGTTCTTTTAGCAACACTCTTTTGAAAACCATGATAATATGACGATACAAGGC

A_14_P137806 GGATTTTGACGATACCTCTGATAAGTTGGGTTTTTAATTTGCTGGTTTATCTTAGAGTGG

A_16_P01821049

CCATTGCATCATGTTTGTTACTGTAACCCTCAAATCTTTTTCTTGACCTAACTCTTAGCA

A_16_P03738469

TACCCTTAAATGAGAGAAGTGAAAAATCTACCCAGTAACTAGCCTTATTCACCTGACAAA

A_14_P103830 AGTTCCAGTTAATGTCACCAGCACTGCCTTGTTAAGGTATGAGACCAAAACTAC

A_14_P112623 CATGCCAGTCACCTACTGCGTAAGTGGACAAAGAGAAAGCAGAAGGTAAT

A_14_P124879 GAATCTCTGGGTAAGGAACTTAAAGAACAAAAATCATCTGGTAATTCTTTCCTAGAAGGA

A_14_P118099 CTTCGTCAAAACAGAACTATTGTTAATTCTTGAACTCTCCATGGTCTTTAATGTCCTCAC

A_14_P101315 CACTTTAATTTATTTATGCTGTAAGTTATTTTTGCTTCCTTCTCCTGGACCCACTTCCAG

CUST_4670_PI426210822 CACAAAGATGTGTGTTGATGTACAGCTTAAAGGGGAGTTAATGGAGCCC

CUST_4892_PI426210822

GGTGAGGCCTAAGAAAGGCATGTCAGTTGTCTATCAAGTTCATTAGTTTATTCAGC

A_14_P109589 ATTGATTCTCATTCTGAGGCAGACCTATTAACACCATCCTGATATTGCTCTATAAGGATT

A_14_P201559 CTCGATACTTTGTCCTTTAGTTTCAATTCAACCTATCAGATTTATCGAGCGCCTATTCTG

CUST_1286_PI426210822 GTCCTCAGACTGATAAAATGTAGCCAGGAGACCAATCTGCTCTAGTCTG

A_14_P130436 AAAAAATGGATAACGGGCCAATTTCTGGGCTTTACCCCCAAATCTAATTATGTATTCATA

CUST_5764_PI426210822 ATTTTAAGAGGCAATGAGCCTTTTCCTTCCTTCGTTTAGGCATGGGG

A_14_P126456 CTAGAAGATCCAAAGTCTTTCAACAGAGAATGCTAATACCTTGCTTCTTTTGGTGTTCTA

A_14_P130598 GGAAAAAAATGCAATTTGCACTTCACTTTGTTGGAATATCCCATAGCACTCAATAAACTC

A_14_P113209 ACATGAGAAGATTAAGGTAGAGAGTGAAATATGGTCAGGACAAATGTGAATTTAGAGCCT

A_18_P11624347

ACCCTTCATACTTAGGTATTTCTATTTGTCTGACTGCTGATTTGATTCCAATCCAGTGTT

A_14_P101953 GATATGCAGACGATGAAATTGGCTTGAAGAAGAAAAAGCACCTCCCAGACTATTTC

A_14_P114961 CATAATCTATTTAGAAGATGCTCCAGTGTTATGAAAACAGCCTTAGGAGGCTGTATGACA

A_16_P00991620

CAAAAGAAGACGTGTATATGCATTAGATTCCACTGTGGTTATATGAGCATCCTAGGACAT

A_16_P01844789

GTAAACTCCCGGTTGTTTAAAGTCATTAACGTTGTGTCGTTATGTCTAGTGTCAGGTTAT

A_14_P133029 TCAATGTAAAAAAAGGCATATAGAAACAAATCAAACCAAAAGAATCCCACAGTCTCCTCC

A_14_P124250 CAGGACTTATGTGACTTATATTTTGGGGAGATGAGGGTTGGGTTTTTTTTTTAATGCTAC

A_14_P108120 AGCCTGTCCTGTGTAGCCTATGACCCACTTAGTTGTTTTTTTACCCTCTT

A_18_P13899628 ACTGACAAACAACGTTTAGTTATTTCCCTTCAGCCTTATGTAATCAATAGTCCCGTTAGA

A_14_P104372 AACAGATATTCCTGAATTCCTTCCGCAGGATGTATTTGGGGGTCAATTTCATG

A_18_P12785663 TCCTAATTTATCCTGTAGATCCGAAAACTCTGAAACACTCCATAAAAAAATCCTTACCCT

A_14_P202111 TTCTGATGGATCCTAAGAGGCAGATCTTTCTAGATCAAAACATGCTGGCAGTTATTGAT

A_14_P106327 TTCAAGATAACCCATGAGTATCAAATGCTACCCATTATCCCTTTGTCACAATTTATAAAC

A_14_P131811 AGGAGGCTCCAGCGAGTGCAAAACGAAAGGCTTTTATTGAAAAATATCAAGTG

A_14_P106758 ATTCTTCAATGCTATGTAATTTTGGACTAAAAAGTCTGTTTAAGGCACATGTCTCAGTGT

A_14_P108665 AAGTTGAGAATTTTAATGTGGGAAATGTTGGAATGTAGAAAATAAGCACATCTGCGAGGA

A_14_P104881 CTTACCGAAGTTAGGTGACAAAAGATACTTTTAGAGAGTTTCTCTTAAAGAGAGCACTGC

A_14_P120155 CTGATTCAGAGACAGATGATCAATGGAGGAACTGTCTTCTTCATTTTCACTGTCAAAAT

A_14_P104722 ACAGACATCTGGTCCCTAAATCCCAGGATGAGTGCTATTTATTCAGGCTAAG

CUST_9805_PI426210822 CATGGGTTCCCTTGAGTTTGTTCAGTGCAGCTGAGGTCAGGTTAG

A_14_P101636 GACAAGGTCTATTTTCTTCCCATAACACCTCATTATGTAACCCAGGTATGACTGG

A_14_P107218 GCCTTCTATTCCAGAATTTTTATATTGCAATGTTAAGAAAACTAAAGTCTGGGCCAGGCA

A_14_P111220 TTTTTACAATGATTACAACATTTCCTCACTGCGGGATATTTCTGACCCGCTTTAGAACTT

CUST_4878_PI426210822 GCTTTTGGGAATGGTGTAGAAATCACGTGCTTAGTCTTGAATTATGATTCACCTTTACT

A_14_P106124 CTTCTTTCAACATGGAGGTGGTATTTGGTGTTGATTTAAAGAAAGTTGATTCTACCAAGG

CUST_4321_PI426210822 CAACCACCATCCCTCTCCCAAGAACCTGGCACAGTAAATTCATTA

CUST_6573_PI426210822 GAAAGATGAAAACAATCAGGCACGGGGTTTTTATGCTGAACATTCCACG

A_14_P114396 CCTATGAAACAAGTCAGATACGTATTTCCTTTCTGTAGGATACAGACACCAATAGGTAAA

A_14_P108876 ATAATAATTGACTCAAATAAAACAATTTACTCAGATCCAGCATCAGCATCTTGTTCTTGC

A_14_P122018 GGCATTTTCAATTGCTAAAAACTCCCAGAAACAACTTCATGTTAACTGAGGCTCATAAAG

A_14_P111677 TTACCTTAGAAAATTGTGCATTTACCCATTTTGTGAATGTTTTCTTTTGAACATCTTCTC

A_14_P112730 TGAGTGATTTTTTCCTAGCACTTGTTTCCGCTTTCACAAAACACTTTTGGATTTGAAAAT

A_14_P112867 ACCTAATATCCATACTTCCATGATGATGCTGTTCAGAACAGACCAACATTTTTTTAACCA

A_16_P36230182 TTTTGGAGAGAACCATAAAAGGCTAAGTTGATCCATTTAAAAATTCAAGAACAAGCCTCA

CUST_4780_PI426210822 ACATATACACATTCCCACATGTGTGGGGAATGAGAGGCTACATAGCC

A_14_P117095 TTTCTCACCAGCCTTTTCCTATGCTGAACAAAAATGTATGTGTTTTTTTACCCATGAAAG

CUST_121_PI426210822 CATCATATGGTCAGTCCTCATTTCACATGGTTCTATGGTAACTGAGATAAAATCTCAGAC

A_14_P120969 GTGCTTGTTTTAGTGCTTTTAAAATGGAGTTTAAACATTTGTGCCATAAAATGGCGTGTT

CUST_6448_PI426210822 GTAGGGTTTGTCCAGAGCCTTGGGTCCTTTCCAGAAGGAGCTTGG

A_14_P126849 TGGTGATTTTATCATTGCCTTCATTAGTGTTACTGTTATTGGCTATGAGGTGTTCCTTCA

A_14_P122936 CTTTGGAGACTATAAAATTGAAGTAGATACCATAAACTTCATCCAGGTTTTCAGGCTGTA

CUST_944_PI426210822

GTTTATTTTCTTTCTCTTTTTCCTCTTTTGGGAGGAAGAGCAATCCTTATACCTTGAAAT

A_16_P18327923

CTGGAGACTAGCACTACATTTTAACCACTCTTGTAAATTATGTCACCAACATGCTATCCT

A_14_P201374 CTCTAGGTAGTTCCTTAGACTTACACTTTTGAAATGGAGCAGAATGGTTTTCAGGG

A_14_P101683 ATAAAGGCAAATAATCGTTTCTGGAGAGGTGATGTCTCTTTATCAGTTTTCCAGTAACAG

A_14_P109166 GAAGTGCAGAGGGCAGAGTTCTATGACTTACCATCATGGACTACCCTTTAATTAAAG

A_14_P106364 TCAGCAATACATGGATGTCTTTCAGGGTCCAGGTTTATAGATCTACATTGTCCTTTTTAA

A_14_P109534 CTCCTGACTGGAATATACTTTACCACTGCAACATCTGAAATGAAATCAGAAATGCCTATT

A_16_P20614990

GTCGTGGTTTACTCTAATGATGGCATTTAGTCTCCTGAATTTAACACAATAGAGAATGGG

A_14_P106097 CTGAATCGCTTTAGTAAATAAAGGGTCTCCAAGAATAAATTCATCCGAACATGCATCCTT

A_16_P02435064

GAACCCAAGTTAATATGGCACATACTATTTCCACTACTGATACTTCCACTAGACCTTTGT

CUST_3981_PI426210822 CAGACTTCTGGACCTGGAACTCATTCTCAGAACCTTCCAGAAGAC

A_14_P200130 TATCCTGAGTAAATATTTTCCTATCCCACTCTCTATCCCTTCACCACTGAAATAAAGGAA

A_14_P123355 TCACTTAGACAACTTTTGACAAATCCATGTATTGCTCCTGGCCTTTGTTTTTGCATTTAA

A_16_P18775238

ACTATCACGGCATAGTAATGACAATGAATTTTGATTTCCCGGAACTGATATTAGACTCCT

A_18_P16450187

ACTGCTAGAAAAATCTTATATCCCTTCTCATATAAAGACCTCTTGAAGTATTGCAAGCCA

A_14_P115902 CTATGTTCATTATCCTACTATCAGCACCGACATGCTCTCTGTAGTGAAGAATCAAAATAT

A_16_P20226214

AAGGAATCCTTTAGACATCCAACTACTCCTATCTATTAAGGTCACTAACAGATCAAGTCC

CUST_9644_PI426210822 CGGAGACGTTCATCTGTCTTCGTGCAGCCATAAAAAGTTATCAGGTG

A_16_P41685915

CTGTAACTGGAAAATTCTGGACATGCTTCAAAATTGTCACTATGCTTTTATCCAGATGCT

A_16_P01454867

CTCTTACAGACACTCCAAAATAGAATGGCTTAAACCAGTAAGAACTTGAGTTCTTCCACA

A_14_P120530 CAGGTAGAATCTGTGGGGAAAATGTGACTTTTAAGGGCTCTGTCTGTTTTTG

A_14_P117436 ACAAAAGCATATTGTTATCAAAGGAATCTGTAGTCACTAGCGTGAGCCAATGTGGATTAA

CUST_7169_PI426210822

AATTAATAGGGCAGTGAGAGGAAAGAGCATGCATACTGAGCATCTGAGCTTTTTTAA

A_16_P16191144 GGCACTTGGGTGGGAGTTTGTTCAGCACGGACTTCTGTTGTAGAT

A_14_P112304 TCTTCAGCCTTACCCGGAAATACTTGTGGTTTCTAGATCACCATCTTTAAATTTACTTCA

A_14_P108144 GAAGAAGGTCCATGACTTTGGCTTGTTGCTTCTTGCCATCTTCACT
A_14_P122461 GTTTCCAATCTGGAAAATGGTCTTAACATATGAGTTGTTTTGCCTAGTTTGAAGAACATC
A_14_P129631 CTCTGAAAAACTCTTCAACACTGTTATTGTAAACAAAGGTAAGACCCATTCATTCTCCTG
A_14_P129264 ATAGGAGAGTCACTCTTTATACAAATCCTTCTGATGGTCTGATATATGTGTGTGTCTTTA
A_14_P123189 ATGTGAATTTTAAAGAGAAAGTGTATGCATGCTTTGGCTCAGCAGTTCTCTTCTTTTTCT
A_14_P110060 TCGAACTGGTATGACTTAACATGGAGTAGCACAACAATTGTTACAAGGTCTCATTTTCAA
A_14_P111231 CCTACCAACTCTGGTATTGTTAAGTATGTGTATGTGTGTTTAATGTTTGCCATTAAGGTG
A_14_P129654 AAGAAGCATTTGCCTCCCTAATTTGCATTATTTTCATCTATGAAGCAATAGAAAAACTGA
A_16_P00129664
AAAGTGGTCATTCTAGCAAGTAGCATTTTGCAATCATAAGTGCTTATGTGAACCCAATCT
A_18_P14040480
AACTACTGCTTGATACCAGATGACTTAATCTCTCTAACCATGATGTAATGAACCAGAGGT
A_14_P138251 TGTTTTGATTGACAATACAAAATTAAATATTAGTCCAGTCCCTCACTGGGCTATCACAGG
A_16_P30952625 AAAAAATCTTCTCCAGATTTTTCGGAGGCCCCACTGATGCTGTTCTCTT
A_14_P107585 TTTTACAGATCATGTGAACCCAAAAGCATTTTTCAGTGTTCTTCGCATATATTTGTCTGG
CUST_2807_PI426210822 CAGCTCTAGAGACTGGATTAAACCCCAGAACAAGATAGAACCAGTGTTGAT
A_14_P131646 ACCATATGTTTGGAAGAATGATTTGTCATAACCCACTTTCCCAGAAGTTGTTATTGTTCT
A_16_P36230877
TCAGTGGCACTCAAAACATTGTAATTATAATTGTCAAAGATATCTTAGACCAAAGGCAAA
A_14_P107015 CTCCGTTTACAAGAATGCTGTGTGGGCTACTGCACCAATTTAACTGACTATTATGATTTT
CUST_5777_PI426210822 GCTATAGAAGGAACACCAGTTCAGAAATGTCACTCCTGTCATCCAGACA
A_14_P106226 CTTAAAACAGAACAGCGAAAGTATTGTAAAGTAGACATTGGATAATTGTTGAGCAAACGG
A_16_P16569055
ACACTTAAGTGAAGAATGATTGACAGTGCATCATAAACCATTTCCCATGATTCAGAAACA
A_14_P127934 TCTAATGCCTGGGTTTATTCATCTTGATTCTCAACCAATATCTAGAGAGCAAAGGAATGG
A_16_P02697560
ATCTCTGTTCCTTAACCGAAAACAACCTCCATTTTTAAGTTACCTAAAGGCAAGAAATCA
A_16_P19419925
AAATGCTTTGCTAACAAAATATCCAGGGAGATTTTATCCACTTCTAAAGTCAAGCCACTG
A_16_P15831531
AAACTTCCTGTCTTTAACCTGCAAATCCCAATGAATTCTGGAGTTAGACATACTTGTTTT
A_14_P118045 AATCATTGCCGTTGACAGATTGGAGATAAAAAAATTTAAAACATGGACATCGGACCATTT
A_14_P201953 GGATACACACAGTTCTGGAGTCCATTGCAGACCAAAGTATTATTAATACACATGTTACTA
A_14_P127188 AACACAGTAAGTATGAGAAATGACATAGGATAAGGTCAGTCTACACTTCCTTGCAGTATG
A_14_P111258 CAGAGGGTGTTTTGGTGCTAATTGTGCAAATACTATGGTTAACCGTAACTTAGTTATTGA
A_16_P21418907
AGTCTACTACAGCATCCAAAGTAATCTCATATTGCCATACCAAGACAATTTTTCAATGCT
A_14_P119191 CTTTCAATTCTACTTACGCTCCATTCAAAGGATCTAGAGGCCAAATGTCTGCTG

A_16_P16349438
TTTACCCTTTCAGACAAACTATTGAAACTTTACAATCCATAACTACTTTTACCTCCCCCA
A_16_P20628454
ACAGACAAGTGGGTTTATTACAGGCAAAATGCTACTTTCTCTATTTAATACCTGTCCTGG
A_14_P102686 ACTGCAATTCGGGAAATTTCTCTATTAAAGGAACTTCGTCATCCAAATATAGTCAGGTAT
A_14_P135094 TGTCTGTGCAGGAGATGCAGTTTTTGTGTAGAGAATGTAAATTTTCTGTAACCTTTTGAA
A_14_P119624 CCCTAGTTGTGTCATCATCTTTGTCATCCAGATTTTCATTGTCTTCTGGTTTTTTAATGT
A_14_P108725 TTTGCCTATAAAAGAGAAGAGAATATTCCAGTGGTTACAGTAGCCTGACATAGCATGAAG
A_16_P16799250
AAATCTTGAAATAGCCCATAACTGATACAAGGAACCCATGATATCATCAAGAATTCTGGC
A_16_P00019703
TCATGCTTTCTAGCCTTGTATTTACGTTTTCAGCCTGTAGAATATCTGACTGTATTGCAC
A_14_P135193 TCAAAGATATAGAACCCAATGGTTTCACTGGACAAAAATAAGCACTTCAATTTTCCAACC
A_14_P110797 AAGAACACAAATCTGGTTTTCAGGCTTTGGAATTATTGGCTTTGTGTGATTTAAATCTGT
CUST_5653_PI426210822 TTAGTCTGTCGCAGAGGGAGAAGATCTGGGGAGGACCCATATAAC
A_14_P101303 TTCTAAGATGGCATAGTGTCTTAAGACTTAGAATTTCTCTGGTCTTGGAGGATCACTCTA
A_18_P13647644
CTGCCACTATTGTCTAACCATTGACCAACTTTTCAGTATTATCTATTGTCTCTGCTACGT
A_16_P15416146
TCTCCTCTCAAATGAGTTCAAATTTAGTCTTCCTAACTTCTTTCCTATAGCCTCTTCCTT
A_16_P15623256
CTAGTCTTTTGCCTTTTACTTTCCAATAACCTTCTAGTCATAGTAAATCAGCCTGCTGCT
CUST_1907_PI426210822
GAAAACATGATCCAGTTTTATAGTTTTTCCTTCAGATGATTGTGATGAACTTCATAGTCC
A_14_P106527 AATATTTTTACATGTCATATATTAATATTTAATGAGAAACAAAATGTCACATAAACAGCC
A_14_P126070 AAAAATAATAGACTCGCTAGTCAGTAAAGATATTTGAATATGTATCGTGCCCCCTCTGGT
A_16_P58424297 CTAATTCTGCCTCTGCTACTTCATTCCTATGCAACTTTGGGCAGGTTTCCTAAT
A_14_P124208 CTATGATCTTCTCCCAAAGGAGTTACAGTTACCTCCATCTAGAGAAACATCTGTAGCAT
A_14_P115462 CTGCATTCATTTTTCTACATCTACTAATAAAGCAATATGCTATTGCCTGGCACATGGCTG
A_16_P18082315
ATGACTGTCCTAATCATATCTCAGGCTCAATTTTTCCTTTCTTTTGCAATTTCAGATGGC
A_14_P137708 GATCACCCAAGCACATGCAAAAGGAATATAGTTATCTTTAGTGGGCTTGCTAAAGTATTG
A_14_P115045 TGAAACTACATGCTAGAAGTTGTATTCTAAGAATATGCTTGTGGAACACATCATGGGGAA
A_14_P100115 TTTCCATCTGACACGAATGTCATGTGGCTGAATGTGACTTTATTTGTTTTTCTTTTGTTC
A_16_P00685150
GGTGCTTCATTTTTATTTAAAAGGCTGATTTAATAATAGAAAGAAAAACTCCAGGCAAAC
A_16_P58424174 GGGTCAAGGATCTGAGTGCATGTGGTTGATACACAAATGATCCCAAGA
CUST_9013_PI426210822

AAGCTGCAAATTATTTCTTTAATTTTGAGCGACGTTAATCCACATGATCTAAATCATACA

A_14_P128911 ACTCTGGGAGGAGTTACTTGAAGTTCCAGTTGTTGTTTCACTGGAATAAATCTG

CUST_5421_PI426210822 CTCATACTCAGCACCCAAGGTGGCAGCTGCACAGAGCAGAAATAA

A_14_P104420 AAACTTCAATAATAGGCAGTTTATGTCAGCACATGTAACGGATGACTTTTTGTTGTGGAA

A_14_P201771 AATTTTTGTCATTTCTGAAATAGTGACTCTGCCAGATTCTCAAAATTGCTCACGAGTTCA

A_16_P16190988 GAGTTAAATTCTCTTCACCAGATGCCAAATCCTACGTTTTTCTTACCACATCATGGTG

A_16_P01415087

TTTTTTTCCATCCTAACCATTGACTCTCTTTCCAAAATACCAAAAACATTTCATAGCCCC

A_16_P40094792

GCCAGGCTTTATCTAAATTGATTTCATCAGACTTGTCAAGAATTCAAATCATTTCCCTGC

A_14_P109134 AATGTGATGGCAATGAATGTTCAACTCCATGATCTCTTTACATACTGTCTTAAGCAGAAG

A_14_P112026 CAGGGCTTCTATGTCAAACTATTTGTGCCTCAATCAGGCAAAGAATATAAAGGAAGTATT

CUST_88_PI426210822

TTCAGTGGGAAAGTATTTGCTCAAATCTCTGCCTGCTATTATAGTTGAAGGCTTG

A_14_P119143 ATGGATTCAGGAAAATACATACCTGATTGCACTTGCAGATAAGTGACCATAGGAGC

A_14_P100545 CAGTAACAGCAACAATGAAAAATAAGATTGCAGGTGCATACCTTAACCTCCTGATTGGTA

A_14_P117648 GTAAAAAATTAAAAACAAAAACAACCTACCCACAACGTGTTCCATTTGTCATGTTCTGGT

A_14_P123718 AGCACTGTCACATACACACTCTTCTGAAATTCACAATAACCTTTTAAGATAGACAGGGTA

A_14_P121646 AATGTGTTCTTCCTGTAACATTAACAATAACTCCAGTTCCTATCCTAAAAAGGGCACTGC

A_16_P01397650

ACTATTGCTTGTATCTTGTAGAAGGTTGTATTTTTGTTCTAACTGTGTAATGCTCAGGCC

A_14_P114292 ACATTCCTTTTATTTTAAAATGCTGACCACTAGATGATGAGGATAAAATGCACGGGAACT

A_14_P121377 CTCGTGTTCGCCTCTGTCTACATCTACAGATACTTCTTCCTTGCGCAG

A_14_P107290 AGCACAGTGGCTTATAAGGAACCAGCAGTGTTTTTCCCTGAGTAATTATGAACTTAGT

A_14_P100674 ACTGGTTTTATCAAAAGTTGAGAGAAAACTTCAACAGGTGAATATGCATAGGTCTATTTA

A_16_P00864684

ACTTCTTCATGTTTACAATTCTTTTCTGCATTACGATTGGAAGATATCACTGCTCAGGCT

A_14_P202431 TAACGTAAAATAAAAAATGGTTTCTCTCCCCTCTATGTGCCAGACAATGAGGAAAAGAGA

A_14_P121918 ACTTCCCCAGGATATGACTTCTCTATTTCTTTGTCCAGGAGGAATATATTTATTTTCCCT

A_16_P01190558

AAACAGTAATGTGCACATCTATTTGTCTTCCATTCCTTCTATTATGCCCATGTTAGCCTT

A_14_P112422 ATGACACCAACTTGCAAAATAACTTGGCGATTGACTTCATAGTTCAATGGCTGTTAAATA

A_16_P58424346

TCAGAGCACCTAAGTATATACAACACTGACAGATCTGCAAGGAGAAATTGATAGCACTAT

CUST_3322_PI426210822 CCTGAATGTTTGGTAAGGGGTGCTGGAAACTAGGGCAAGAAAAGA

A_16_P20881876

AAGTAATTCAGTTGAAGTCATTCCTCTAATCATTAAAACCCTGGTCATGGTATCATCTGG

A_16_P58424376

TGCAGTATATCCCAAAAGTAATTATTTGAGCATGTAATCAATATTAAAACAACTGTGGAG
A_14_P118027 GACCAAATCCAAATTACCTTCTTTTAAAGTCCATTTGATTGAGCCTGTCCTCTTGCT
A_16_P21507536
AAAACTGATGTTTGACCAAGGATTCTGTAATATAAGTTTGAAACCTGCTAGAGTTTTGCA
A_14_P133187 CTAGACTCAATGGAGTCAGGGTTAGAGAAAATAGTTTCCTTCTCATCTACTGTGTCATAA
A_14_P117329 CCTCATCCCCACACACCAAACATATTTTTGAAGGTCTTGAAGATCCAGTAGAA
A_14_P125472 ATGTGGAACTTAGAAAAGTAGGAACTTGATTGGTCTCAGATTTGTCTTTGAAGATCAGTT
CUST_44_PI426210822 AAAATTTCATCTCCTGCCCCACTCCCCTACTCGTAACTCTCCTTTA
A_14_P200842 GGCTTGGATTATATTGTGGATAGTGAAAGGTGTGTTTACCCCTTAAGTCATCATAGTATG
A_14_P105679 CTATATATTAAGAGAATAGAGTCACCAGGTAGGTAAGGGGCACTGTAGAATTCTCTGTCT
A_14_P128123 GAATTTCCTCTCTCCTGTTTACAGAGACCACAGATCAAGTATCTAAAAACATCCAATTTC
A_14_P138731 ACTCCTGACCCAAAAATCAGGCATGGCATTAAAACGTTGCAAATTCCTTTACT
A_14_P119724 GGAAAGGAAGCAAGCAAGCAGGGAAATGAGACTAATATATTCCAGAAGTGTATTATGCAA
A_16_P20032564
ACCAACCCTAATTCTCCATTTCACCAAACAACTCTACTCTCCAGTCTTTTTGAATTTTAT
A_14_P136728 CTTGGTTGAACCCAGTCGCATATAGTTAAAGATACCTGAACCATAGAATATGGCAACC
A_14_P122166 CTGACCATTGTCTGCATGGAAGAGTTTGAGGACATGGAGAGAAGTCT
CUST_4682_PI426210822 CTCCAGGGAACCTTCAGTGTGCCTGAAAAAAAGTCAACCTGGAAT
A_14_P128909 CTTCTTTTACATTTTCATCTGACACCCACTGGGCAAAGAAAAGGTCAAATACGACTATAA
A_14_P125813 TCACCACTCTGGAAGCATCCACACTCAATTTCTAACCAAAAATAGCCTGTCAT
A_14_P122575 CTTGATTATCCATTCTGTTAGCATTTCCCTCCTTTGACTATCAGGAAAAGCAACAGTTTA
A_14_P125875 CTTGGCATTGAAAGGAGGTGTTCTGCAATGATTTTTTTTCTTGTTTAGAGAAGTTTACTT
A_16_P02030224
GCATGGATATTCTGAAAGCAAAACTGAAATGTAAACTTCAACTCTTTCTCACAGACACCT
A_14_P201448 ATCAGTTATTTGTGAACAGCAAAAGAACATAAGTGGTCGAAGTAGTTCAGAAGAGGGTTA
A_14_P126880 CTCCTGTTCCTCCCTATTATTAAACTAAGTGAATTAAGGTTTAAGCACCCTTTGTTTGCA
A_14_P133825 GAAATAGGCATTTGAGGATTACATGTGGGGAAGTTTTGGTTAAGTATAAGTATGAGGTCT
A_16_P02900030
GTCTACAACCTTCTCTAACTACTTCTAAACATAATCACGACTAATATATGAGCCAGTGCA
A_14_P124614 CTGCAGTAACATTCTGTTTTCCCCTCCTTAATCTTCTTTATCAAAGTCTTAATCCTTTGC
A_16_P18998623
AAAACTTGCTGTAGTGTAACCGATAGAATTTGACCATCATTTCTTTTACTAGGCGTTTGG
A_18_P14138043 TTTGCCTGGGCCTGCAGTCGTGTTTATTTTCCTTTTGACCTCAAGAT
A_16_P21241988
TGTATAGTGCAATAACAGCCTAGGAGAATTAACAAGCATTTTGCCATCAATACTTATCCT
A_14_P112093 GCCATATTTCACTTTCTATGTCTTTCTTCTCTGAAATCAGGTGAACATGCAAAATCAGAC
A_18_P14218829
CAAGATACTCCTTGATTAACTCATGTTCCAAACCATTTTATCTGCAGAAATCACCATCCT

A_18_P13499954
AAGCCACATTGTGTCAATTATCCAGATCCTAAACTATGACAATGTCATTAGAGGCATCAT
A_14_P115949 CTAATTCTATTCCTGTGAATACTTCTGACTGGTGAGAGAAAAGACAAGAGTAGAATGGAG
A_18_P14136417
GCATAGTATTTGTAATGAAAAATTGTATTCTAAACCTAAAGATTACTAAGCCAATTGGCT
CUST_4346_PI426210822 CTGGGAATCTGTTTTCAACGTGTATCCTGAGTCATATTTGGGAAGCACTG
A_14_P110988 GAAGTCACAGAGCAACAAGAATTATACATCTTTGATGTCACAGATCAGAAAAGCAGAGTT
A_14_P137507 CTATAGAATCCCAGACAAATTCAAGGGACCAGGATTATCATTACTGATTCCAGTTATGTG
A_16_P16798925
AAAACTAGGGGAATGACAAAAATTAGCAAAGGACACTAATCACAGTATGATAGGCCATTG
CUST_10242_PI426210822
CTCATTCCTGATTGACATTCTATTAGAAAATGTTAGCTAGGAAGACCTGTTCTTTATTTA
A_16_P20515337
ACACTACCAGTATAGTTGAAACATTCTATGAACACTTCCTTAATAACATTCCCCCTCTCT
A_16_P35813965
AGCATGGATTCTGCCTGAGTTACTTCCCTTTTGAGAAATCATATCTCAAATACATAACCT
A_18_P17372593
AATGGGCAGAGTGTATGTATACATAAGTCAGTTTCAAAATTTCTGGGTTCCTCATCTACA
A_14_P127040 TTCAAAGATAACTTGTTTCTTTTTAAGCCCACAATTTCAAACTCTCTTCAAAGTGGAGCC
CUST_5443_PI426210822 TCACTCGTATGAAGTAGGAGTGAGCCTGTGCCTGATTTTGTGCAG
A_16_P03302414
AAATGGCTTGTTATTATATCCTAGAGGAATTCCATGACTCATTGAAAATGGCTTCTGTGC
A_16_P20362276
ACAACACATCAGACATTTCCAATTCTACCTCCAAAAAGAGATATGTGAACTGTTCAGATC
A_16_P16191154 GTTTTGTTCTTCCATGTTTGCAGTGGACTGTAATGATCGGAAAACCATGTTCTAGAA
A_14_P135985 AGTGTATTCAGTTCTCTTCAACCCGCATTGGCATTTTAATTACTGTTGGGTAAACTAATT
A_18_P14138205
TCTAGGAGTGTCTACAAGATTATCATAGGTGAAAGTAGGGTTGGATGCAGAGAAATAGTA
CUST_6026_PI426210822 TATCCTCTGTGGGCCCAGTAATCAATAATGCTTGAACAACTAGTGTGGTG
A_16_P17832615 CTACTACTGGCATCTTACCCTATTTGTCCTGTCATGTATCTATCCCTTGATACATTCATC
A_14_P115024 TTCCAATTTCATATGGTTTGGGCTTCCACTAAAGTGTCTAGTAGATTAGAAGATCTCCTT
A_18_P14133119 CAGCCAGAGCACAGGGTATTATGGCCAGCAACTGGGTTTCCTCAG
A_18_P11936056
AATCAAAGCATTAGAGAAGGGTTATTCAAAGTCCCATCATACTGATTTATTTCCCAAGCC
CUST_4017_PI426210822
TAGGTCAAGGTTTTCATAATTGAGTTTTAGGGGGGCTAATTTTAGACTTTCTGAAAATCA
A_14_P118282 TTCTTAAGATGCTGAGGTATTATGGTGATAGGCATACATAGTCACCTGTATTGTCACAGT
A_14_P139791 AACTCTTCCTCTTTCTTCTTCTTGAGAGAGACAAGATGAAATTTGAGTTTGTTTTGGAAG

A_14_P131842 TTCTCGGATTAACCTTTAATTCACCCAACTAAGAAATTTCTCCAAGCCATAAGCATATGA
A_14_P109911 AAAAAACATACCAGGCTGTAAGTCAATGATCTTTGAACTTTGTCTTCTTCAACTTTGTCC
A_16_P20739086
TTATTTCTTTAAATGTCTTTAGAAAGAACGCGTTTACTGGTTTACCACAATCCCCATCCC
A_14_P109824 ATTAAAACATCATACAATTAAAGGCAAGCAACAAACAAGGGAAAGGTTTTCAGCGTATGC
A_16_P02220400
TCCTGCATCTAAGCCTAATATATGGCATCAAAGTTCCTAATATATGGCACTCACTGAACT
A_14_P127643 GTATACTGAAAGGCAGGGAACCATACAATTCAGTTCTGGATTTCAAGATCTTCAAGTG
A_14_P118946 ACATCCAAGTCCTATATCATGTTATCTCAGAAATCTAACTCTTACACAGGCCTAAGATGC
CUST_2626_PI426210822 TGCTCCAGAGATGAGGGATGGGGGGATGAGGTTGGTGAGAGTGAT
A_14_P129233 ATTTAAATAACAACTTTCCACAGTCTCCATGTCCTGAATTGTTTTATTCCTAAAAGAATT
A_16_P01847105
CAGAGTAGGTGATGTTTGTCTTGCTGGTGAAACAAAGGTTATAAAAAGAGAACATGTTTG
A_14_P200579 TGTTGATCACATACTGTTCTGTGTCAAGCACCACACCAGGCTTTTTCC
A_16_P15843444
AAGTCTTTTGAATCTTTCCTTGAACGATCTACACCTGCATTTTATGTTGACCTCTTAACC
A_14_P137017 AACTGTAATAGTCAGTGAGAGAGGTGAATTTCTGCTCCATTCAATAGTCCAGGTCTT
A_16_P00685000
TAGGATACAAACATGCAAGGGACTCAATTTTCTTTTTTTCTAATAACACCCAGAGTGTCT
A_16_P02750844
CTTTAGAATGTTTTTGACTCGACTATCACCAGAACTAACAGTCTACTTCTCAAAAGGAGC
A_16_P18152520
AATGTTTTTCTTGCTCTCCATACATATTTGAAGTGGGCAATGATATTCAACACCATGCAG
A_14_P108291 CTTCACTCATAACGTTTAATAGTATCCTACCTCACTTCATTCACTTCCCAACTTCCTTTT
A_16_P36864904
AGGGGACAAAGATATACTAAAAAGTAACCTGGGTTATTAATCACACTTAAGTGAGCATCA
A_14_P132069 AATTTCCCACTACTTACCTGCCAGAATCATTCAGCAGTGGAAGGATATTGTGATAAC
A_14_P129924 AGTGCACTGAGTGTGATGTTTGCTAAAGGAACAGATAATTGCATCAAAACCTATTTCTAG
A_18_P16527536
AAAAACCCAGGTGTTAATTTTTGTAATTGACCTAACTGCCTCATTAGTGTTATTTTCCCC
A_14_P137937 TCAATTGGAAAAGTCAGAAATTGATTGATGGTGACTGTTATCCCTAACCAGAAAATGTCT
A_16_P58424312
CATTTCTCTAATCTTCACAACAAGCATGCAAGGTTGATTCTGTCATCCCATTTTACATCT
A_16_P01769709
TTCATTGCAGACAACATAGAATTCTATTGGGCAGATATTATCCACTGTATCAACTCAGCA
A_14_P103801 TTATTCTGCATTCCCTATATGTGAGTGTCTCAACCTAAGTAAAACTCACTTCTGCTGTGC
A_16_P17336047
AAATTTAGCACATGACTTATGAAGATCAAGCAACCCAAAATTTATCTACTGTGGCCTTGC

A_14_P129436 TGAGGAGCCAAAATCATCTGAATTAAGTAGAGATCTCATTTGTCCCTGACTCATGATAAT

A_16_P17844796

GGATAACCCCATATATCAAAAAATGCCACTAGTTTCAACATAGACATAGCAGTGTATCCC

A_14_P200533 TGGCATAACAATTTAACCAAAGCCCTAAAAAGTCACCCAACTAGTTTTAAAAATTGTGTC

A_14_P126111 CTTATTATTCCATTGGACTGTGAAATAACGTTTCCGTCTAAACTTAAACCAGTGCCTTTC

A_16_P19639144

AACTGAAATTCCCATAAAGAAGTTCATGATTTATGCCCAAAAACCAAGATTTTCCTCGTG

A_14_P132864 GGATTACAGTGGGAAATTCTAGGTGATGGGTTATATTATCCTTTAGAACTTTTCTGCAAC

A_14_P138617 CTTCACAAAACTATGTGTGTAGTGTGCAAACTAGAACCAGTTATACTTTCATCACTTCAC

A_16_P15220285

CAGACTCTAATCCCAATATCTCCCAGATATTCCACAGATACCACATTCTAACCAGAATAA

A_16_P20515179

CCATGTCAATAAGCATTACAGTAGTAGGTAACAAAAGCAAATATGCATACATGTGTCACC

A_14_P127630 ACTAAAAAAGAGAGGGATATACAGTAAAGCAGTGATTGGTATGAAAGGAGCAGGGTTTG

CUST_9570_PI426210822

TCAGGAAAGCTTTTACTTTCACTATCACATATGAAGAATGAACTAAACACCTAACTTGGA

A_16_P19457770

TGCGTGTGTATCATTTTTTTTAGGAAGAGACTCCAGAATATTAGCACTGCTTACATCCAA

A_14_P120655 ATGTATTGAAATCCATATTAATTTGGAAAGACGTGGAGAGAGGATGAGGTTTTGACATGT

A_14_P125415 ATACACTTTTCCAGATTTAGATAAAGGTGAAGGTTGTTTGGGGTTTGAAATGTTTTCCTT

A_14_P123944 AGAACTTGATCTCTTAGCAATCTAAAGCAGTGGAATTGGTGTTAAAAAGCAAGGAAATGT

A_14_P115417 CATGAATAAGTCTCAATGACGGATAGGCAGTTCGCACTTTCAGTTTATTCTTAAGGATTA

CUST_581_PI426210822 TTTCTAAGGAAAAAGGAGAAGGGCCCTGAAAGGAAAAGGGCTTCT

A_18_P14122332

ACACTCTTCTTAATGTCCAACTTTCCAAAATATCGTATCTGATATCTACTACCTCTTCCA

A_16_P18537595

AAAAATGTTGAAGTTGAATCCTAGAACAGTGAGCATACCATTATTCCTTAAGAAGGCACC

CUST_5134_PI426210822 CTAAGGTCTAGGGGAAGCTTCTCAGCTTTCTGGATACCCTCTCAG

A_16_P16372780

AAATGCAGTTTCCTAGCAATTCTATTTGTGCAGGTATCCCTAATAATCAGTGACCAGATT

A_16_P01246654

ACATCTCATGTTTCTATAGTCTCAACACAAAACAGTAAGAGATATTACTCCTCCCTGTCC

A_14_P119562 GTAAAAGTTAAGGTGCAATCAGAAAACTAAGTGTCACAGAAATGAACTTATTGGCCACTC

A_14_P117406 CTCAAATGTGTTTAAAAGGAGAAGCAATCCAGTGAGCAGAGGAATAACATATGAATCACA

A_18_P22605645

TCACTACATCATGAAATCATTTGTAATGGTTGCCTAAGATAATTTAGTTTTTAGATCCCA

A_14_P102346 CCTGAAATTGACAACTGATTTCATTCCTAACCCTGCAGTGTCCCTAAAATACTCATTC

A_14_P124138 CATAGATTTGAATATAACCTGCCGCTTTGCAGGTGTATTCCACGTGGAGAAAAATG

A_14_P134383 ATCTATTCCATTTTTTTGCAAAGTGGAATGCCAGTTTAGTTTTCCAAACCTGTTGAGAAT

A_14_P201761 TGTTTTCATCTTTATAAAGTCAGAGTTGGTGTCTATAGGCTCTCTACTGCCACATTCAAG

CUST_8016_PI426210822 ATTTAATTCAGATCTCCTCCCAGTGCTGACTCTACACCAGTCAAGACC

A_16_P19850177
TCGTTCTACACTTTTATACATAACCGAATACATTCTCCACTTCAGATGAAAACTGCCACA

A_14_P118488 AGACATACAAGGAAACCATTCCCCAACATGGCAAAAAGTGCCAAATAGCAAATATATTAA

A_14_P131807 TTTGCACAATGTCGCATGTCTTAAACATTCAATCAAACATCGATTACTTCCAATTTTGCA

CUST_8656_PI426210822 TGGCTTATGAAGGGAAGAGGGTAGTGGTCTTTCCAAAGTTAAGTTTTACTCA

A_14_P120917 CTTACTCATCTTTCCTTCTGATTCATTTCATTCTCCTACTAAATACCTTCCTTGCAGGAT

A_14_P128463 TATTCATATCCCTGTCAAAATTATTTAGGAACTTGTAGCGAAGAAGGTCGTCGTCAGTTA

A_14_P106881 AACTCTATAGATTTGGAAGAGATTAACCTTCCTTGGCTCATATCTTTTGCATCTCACACA

A_16_P02992222
CTACAACTTATACATTACCACTACAACATTGAACAAATATTACTAGCATCAATATCACTT

A_14_P135360 AATACACACACTGTTTTATTTGCTAGATGGTATATTCACACATCAGGAGGTCAGTGTCAT

A_18_P14393790
AAAAGAAATGCAGCAGTTTGCTTTGAAATTCCTTGGTTAGATGAAGAAATTGCAATGTGC

A_16_P20015841
AAATCCTGTGAGTAAAAATAAACGTCTAGGTAAGAAGTGTATATCCCAGAGCCAACACAG

A_18_P16640655
CATCAGTTCACAGTATTCTTCATCAAAAATCATCACCAAGAATCTTACTAACAGACCACA

A_14_P130272 TAATAATTATTGCGGTGATTTTCTGGCTTAAGCAAACATCTGCTGACCCCTAACAAGTTA

A_14_P101754 AATTTCTGCTCCTGCCTCAAAAATGTCCAGAACGATGTATGAATTTTAAAACTGTTAGAA

A_16_P20882487
TCCTAAATTCTGACACCTTTCAAATACTTTGTATCAGTCGTTCCTTAACTATTTGCAGCA

CUST_7920_PI426210822
GATTTTCTTAGAGTTTGTCTTTCCTGAACCACCAAGATCTGAAAAGGCAGGATTAG

A_14_P127588 GTCATGACATTTCCAACACTGTGTGAATTATCTAAATGCGTCTACCATTTTGCACTAGG

A_14_P124962 CTTTAACATTTGAGTTTTATACAGTTTCACTGAGGAGGCGTTAGCCAAAGGATGCTTTTA

A_14_P137306 TGTTTGGTTCAAGTAAAGCGAGTGATTTCCAAAGGTCTCAAGTCTAATTTGGTATTCCTA

A_14_P139004 TGCTAGAAACACTAACTTGCAATAAAGTGCAGTTTTCATGCAAACTTAGCCATCAGTTTT

A_14_P117912 TTAGTCACTGTATATAAACAAAAGAAGTCACAGTGTGCCTGGACATTATTTGTCCCAGAT

A_14_P108967 TGGACTTGCGCTTTATATTTTTTTAACAAAATGTATTCATCTTTCTTGGAACTGAAAAA

A_14_P201662 ACAAAACGGGACACTGATCAAAATCAACTTGGACAAGTGACAAGAGTGAGTCTTAT

A_16_P00788766
ATCATTAGGACAAGGTCAAAATAATCCTCTAAGCAGAATTTCCTTACATTCCCTCAACAC

A_14_P108256 TGATGTATTTGTCTTTCCATTCCCATGATCTCTAGAATATAAGGGCATTCCAACACATTG

A_16_P20889331
AAATGGAATTGTGAAATGGATGAGTCTGATATCGAGTGTTTTCAATCTCCCTATCTGTGT

A_16_P21085212
AACTCTCTCTATTCTTTCACCATATAGTCCTTCACTATCATGGAATCCTCATGTTTCAGG
CUST_949_PI426210822
TCCCCTTTCTTATGATTAGTTGAAATCTGAGTTTGAGCAAGTCTTGCTGTTTTCAGA
CUST_10244_PI426210822
GAAGACCTGTTCTTTATTTATTGACTTGCATTTATTGAGAACTGTTGGGAATAATATTGT
A_16_P35261835
TCAGACTTGTGTTTATTAATGCAGAAATTATGAGGTGGTCTCTTCATTTGACAAATGCCT
A_18_P14870503
CACTACCAAAGTAACAGACGTAATACAGCAATTCTCCTTTACCACAAAATCCTTCAATGA
A_14_P100123 TAAGAATGATATTCTCAGCACCAGCACATTAAGCACTGCAACAAGTTTCACAGAACTATT
A_14_P139624 AACAAAACCTACAGTCAATTAATTTCTATCTTACTCTACTTCCAACCTCCAGGCAAGAAG
A_16_P16074053
CAGTGACTAAATCTGGTAGCACATTAGCAGTATCACTACTCAAGGGCCTATTAAACATCA
A_14_P113062 TGATGCTGTTAAATGTTTGTGTCATATATCTGACTCCCTGTGATTTGATTTCATGACCAC
A_16_P03108441
GCTACCTGGTTATGTAATTCCAAATTCATTCCTCTTCTAAGACATACAATGGCACTCAAG
A_14_P138409 AGTGCATGGGCAGCATGCTTATTTAAACTGTCAACCATAACTCAAAGCATTTCTAGTTTT
CUST_7438_PI426210822
GGAATGGTAATTAAATGAGTCCATGTTTAAGGGGCACTTAAGTGGATTAAAATCAATATG
A_16_P40174807
CCCATGATCAACAGAATAAGTCATCACATGATCCAACTAGAATGTTACCTGAATCACTTC
CUST_2062_PI426210822
ACTTAAGAAATCACCAACATGTGTGCAGTTCAGATGTTAATACAGACACACATCTCCA
A_16_P36230611
GTCTTTGTAAACTGTGAAATGCTCTACGACTGTCAGCCAGGATTAGTAGTTAATGCAC
A_16_P00728906
TCTACTGTGACTAATTTAACACTTCTGAACATCTCGATGTAGGCTAGTATCTAATGGGCC
A_14_P118049 ACTGGATTTTGCTTAGGCTCGTGTTGAAACTATAAATCTGCTCTACTTTAAGAAGCCTGT
A_18_P22839215 TCTGGGCAGTGAGAACTGTCCCCATGGCTAGATGGACCAGAATAT
A_16_P15193656
AAATGCTGAATGCAGTTCCTCTAGAAAAAAATGCTTAACTTTCTGTCAACCTTCTGGTTT
A_18_P22841588 ATGTTCAAAGGCTGTGTGAGTGAAGAGGTGGGAGAGTGTGCTGAA
A_16_P38540333
GACAAAACCTCAGCCATATAACTTGTTTCTCAATCTTCAGTCTTATTTAATAGGCATGGC
A_14_P124260 ACTTTTTGCATTGATCGTTATGAATACCAGACCATTTGTAAGTGTGGTTGAAGAGCAAAA
A_14_P124563 TTGACATGTCATTCATGAGAGGGACAATCAATACGATGATGTTGTTGTGAAAGTTAAAAT
A_14_P131652 AAGGTGGTTAGTAATTCCATCTATGCATATCCAAATGTATTCCCATCTGTTTGCAAATGT

A_16_P20165883
CAGAAATGCTCAGTCATTTATAGCCACACGTTTTCTATTTTTCACAAAGTGCCCTAGAAT
A_18_P22841567
TGTGAAAGATACAATTAATATTGCTTATTAAATACTAAAAAAAGCATTCTTAGAATCCCA
A_14_P126354 CTACGGCATTGCTCAGTAAGTTGAGGTCAAAAATAAAGGAATCATACATCTCAAAATTTT
A_14_P116247 CACTCAAACTCCTCCTTATTTGCTCTTGTCATTTTTGGCTTCTAAATATCAAGAGTGTTC
CUST_7742_PI426210822 CAGCAAGAAACCCTGAGAATATTGAACGTCTTTGCCAGACAAGGAAAAGT
A_14_P133754 TGACTCTTTTCATGAGTTATTCATTGCCCCCCTTTAATATAGCAGAAGGATTTGGTTGTA
A_14_P109271 CAGAAGACGAGGATATGGCTTGTTATTAATATTCTCTGCTTGATTGTTCCTTTCTGTTCT
A_18_P14588981
GGGGAAAATCTCATGATTTCAATGCATAAGCCTGTATAATACCTTATTCATTCCGAATGC
A_14_P113871 AGCATATTGCTGTAAAGCCTTTGATTAAGAGATAATTCTTAATAAATGTCCCAGGTCCCC
A_14_P122131 CCTTTCTTAGCGGTTCTTACATTTCTCCAATTCAAACGCCATATAAATTACCTCCCTTAA
A_14_P129673 ATCCACTTTGGCAGCAAAACTTCTTTAAAAAATCTATCCCCAAACAGTAAGTTGCTCTAA
CUST_3460_PI426210822 CTCCTCCTTTAAAGTGGTTTTCCAAAGTTAGTTGCCATCCCCAAAAGG
A_14_P112671 CACATTGGGAAACCTGTCAGAACATTGTTGGGAAAATAAAGTGAATCAATAGAATGTCAA
A_16_P02951303
AAATTCTGCTGATTGACACTAATACCGGTATTTACCAATCTTCTGTAACGAGGTAGACTC
A_16_P40127409
AAACATGACAAAATGAACGATAAACTCAGAAAACTTCCCTAAAATGAATTAAGCCCCTCC
A_14_P125060 AGGCTTTTATGTCTGTCGAGTTAATAACAATTTCACCTTTGAATTCAGCCAGTGGTCA
A_14_P113048 CTCATTGTTGGCTTCCATAGAAAATTATCTTGACATTGCATCTGTCCAGGCTAAATACAA
CUST_1881_PI426210822 GAATCTGGATTCTGGATTAGCCCAGATGAGCTGAGACCACTGGTAAT
CUST_1469_PI426210822 TGGGCCTGCAAGGGATCTCTGTCCCTTCTGGAGTAAATGAAATAAT
A_16_P15132723
CAGCCCATCACCTTTATATTACCCATAGTCCTTTACATAGTATTCTACTCATAGTTGTGG
CUST_9059_PI426210822 CTTCTGACCATGACAGCTCCTTTCACTGAGGCTGACCCAGACTCT
A_18_P15474245
ACCTGGTAATATCTTCAAGGTTCCTATTTAATACGTCATCCACTGGTTATTGTATGGAGC
A_16_P00444380
AGCCTAGTCTCTAAACCATTATCTTTGCTTAATACAACTCTCCTAACTCTGAAATGCTAA
A_14_P102402 GAGGGGGAAAATGTGATCCTTTCATTCTTCTCATTTGTGGTGATACTAATTACATGTTTA
A_18_P16603972
ATTTGCTCAGTGCTAGAAATATTTGTTCCCAAAAATAGAGTATCATAACACACTTGGCCT
A_14_P109863 ATGGGATTTGCCATTTACATCTACCCCACCCTCACTCCATTTATATTTAGTATTTATCAC
A_18_P22841649 TCAAGAAAGCCCATCTGCTGGCATCTCAGGCATCTTCTCATGCTT
A_18_P14133439 CATCCTTTCTCAACGAATCTCCACCTCCAGAGAGAGCAGATCTTTCT
A_16_P41352255

TCACCTGCACTGAATATAACTTACACCTTTTAATACAGGACTTATTTGACTTTCAAGTCA

A_14_P100384 CTGGCTAGCCAAGATTGTTTTTCTTTCTTTTATATGGGCTATGAGAAGGATCTGGTTTCA

A_14_P120965 CACCACCATGTCTTTACATCTCTCATCAACTTGCTTATAGTATTACAGAGTCATCCTCAA

A_14_P123090 GTCATCAATGACATCAACAAAGAGGAAAGAAAGCACGCGCACATCTCTGT

A_14_P202086 TCTGTTTCAAATGTTTGACCTTGATGGCCTGCAGTGCTCTATCTCTTTTATGTATTTTAC

CUST_847_PI426210822 GCCATTCTGGGGAAAGAAAGGGAAGAAGCTGACTCATCTGTAAATCAAA

A_14_P200917 ATGGCCATGTCTGTAAATCAAAAAGAACGTGAGATATTAGAGAGAAACGATTGTCTCAAA

CUST_1136_PI426210822 AAGATGTACTCCTCAGCACGTTACCTGGGCGAGTGGGTGCAGTCT

A_14_P133171 ATGAATTGGGTAGAGTCTCTGGAAGGTTTTAAGCCCATTTTCAGTTCTAACTTACTTTCA

A_14_P111696 GGACTTTACTTAACAAAACACTTGAATCTGCGGTCATCAAATGAGGGTGGAGAA

A_14_P100638 GCAGGCTGGATTTTTTGCCTTGATGAAAAGGTAGTTAAATTTCACACTTATTATAGCACC

A_14_P125791 AAAATGGACTGAATTGAGAATGACAGACTCTGGGTCTCAAAAATGTTTTTTTTCCCATAA

CUST_8218_PI426210822 ATGGTAACAAGCAGCATGGCTCCTGGGATTAAAATCTCAGCTGGA

CUST_3864_PI426210822

TATGTTTAATGAATGAAATGATGATTAAGATAGGACAATTGGACAAAAAGGCTGGGCCTT

A_16_P16854543

GGGACAGATTCAGAAATATACAAACATGCCTAGTAACCAATTTCCTGAAATTGCAATGCT

A_14_P106554 GACTTTTGTGCCTGCATGGTATCGATGGTATTGCTAGTTTTCATTTAATTACTGTTCACT

A_14_P119590 TGCTTCTGCAAACCATATTTATGTAAGCCACACAACATAAAAACCAACCTGTAAGTTTTC

CUST_2621_PI426210822 CTCAGCTTCACCCTCATCTATGTGAACCCTGATGCCCTAACCCTG

A_14_P107942 GACAAGACATTTATAACACAGTCGGGAAGAAACATTTCCCTAATTCATCTATGACCAAGA

A_14_P201624 GAGACGTCTGATCTGGTAAGATGTTATCTCCCTCTTTTATAGGTGCATCTAAATTAAGCA

A_14_P105820 ATCAATAAACTGCTCTCAATCTCCGGGTCTGTGAGTAACATTATCTGCAGCAAAG

A_16_P41112722

ATATCACACCTAATCTCACCACTCTACCTCATAATCAGCATTTGGTGTTCTTATTTCTGC

A_16_P19856988

ACACATCATCCCCTATTAACAAATGCACAGAGTATCCATCTTTCATCTACAAAGTCTGTT

A_14_P126583 GTGATCGTTGTGCTTTTTTTTCTGTGTTGGTTGCCAGTTTATAGTGCCAACAC

A_16_P16705975

AAAACATGCTATACCCTCATTCTTGAATTTTGCACAATTTACACATATGGGCTCTCCTTC

A_16_P19746691

ACATCCTTAAGAGCCTGAAGATACATGCCAATCTCATTTGCTTATGTAACACTACAAAAA

A_16_P17973305

GCCAGGACATATACACTATCTTTAAAAATGAATCTAACCCTTAAATGAAATCCAGCAGCC

A_16_P18339312

TCTCTGCTTTAATCCTTATTGTTTTATCCCCAAATACAGACATATCAATCACACACTCTC

A_14_P111781 CTCTAGGTTGATGTAATTAACTTCTCTGGCCTCCAAGGTAGTTTGATGTTAGTTCAATTT

A_14_P128677 ACCTCCGAAGATCTGTCCTTATAAACAATTTGATGAAAAGGATCCATGGAGAAATTATCA

A_16_P41272794
TGGTCAACTAACAATTTTTATCTTTGTGGCTACTCACTATTATTCAGACCTCACTTTCCA
CUST_3646_PI426210822
GTAAGCAAGCACTATTGTGCTTCCTGTGTTGAAGTCAGGGAATAAGAATTTCCT
CUST_2520_PI426210822 CCAGTGGTTCAGCTACTTTTCTGTAAATGTGAGGTGAGCAAGGTGTTC
A_16_P00685275 CTTATCTGTTCCATGGGTGCAGAAGGGTGCAATGTGAACAGAAACAG
A_18_P16029848
CAGCGAGGTATCTTTACCATAATACCATTTGACTCACATTAGTAAGAACTGCAGGATTCA
A_16_P00467932
AAACCCTAGCAGTGATAACTTGAACCCCATCTTAAATATGACCACACTGAATTTTTTAGC
CUST_840_PI426210822 CCTTGGGAGAGGAGGAAGCAGCTGGATGCTGTCGCCAGCATGGTG
A_14_P137221 GTAAAATACTTCTGCTGCTATCCTAAACAACACTGGAGACAGTGAAAAATACTTCGGTTT
CUST_5475_PI426210822 CAGGAAGCTGATGGCTTCTGCCTTGACTGGGAAGTACAGACCTAG
A_16_P21615994
TTTATGTGGAATACCAGTATCTAATGAGACAAAAGTCCTTGGTTAAAACTGTGGTGGAAG
A_14_P201617 TTTTGACCTAGAACCCAGAAAAGTATCACTCTTTAGCAGAGAAGTAAGCCTTAAAGTGTT
A_16_P35618008
AAAACATTTTTCAGAGAAATTATCTACATCCTGCTGAGAATTTTATGCCAAACTGCAGCC
A_14_P134793 CTTCTTCCTCTTCTCGGTTCCCTACTGTGAAATCGCAGCGACATTTA
A_14_P124462
ACCCAAAAGTCGATATTAAGAGGGAAAAAAATCAGAAGTCACTGTTTAAGGACCAGAAAT
A_16_P36965263
ATTGTTAAGCATAACGGTCAACATAATTGCAACAGTTGAACTTTAATCATGACTCCGAGC
A_16_P16887035
TTACTGCCCTTTAAAGAATTACTACAGAAAGTCCATTGCTTTACTTACTGGTCACTGGTT
A_16_P18766082
ACAGACTCACCTCATAATATATCTTAATTCCAGAAAATCACATTCCCTTTACTCTTTGCA
A_16_P03472279
CAGAGTCATGATGCATAACTACCATCCTTTAGATACTTCATTAGTTGGATGACCACTGTT
A_18_P15117149
TGTAGCCCCTAAAAAATCTCTCTTATCCCTAAAAAATCCTCTTTCTGCAGTAATTTTCCA
A_14_P116794 GAAATCAGACATTACACGTGATTGGACTTGTTGATGTCTAAAATTCAGTAAGCGCTGAAA
A_16_P41781820
ACTTAGGCCATTCAGATGTCATATACACCATAACCAGACAAAATCTCTTCTTTAGGCATT
A_14_P107508 ATTTCCATGGCACTATATTAAATACTGTCTTACAGCACAGGAAATGGCAGCACAGC
A_14_P114377 CCACAGAATGGGATGTCAGTGCTTGGTTGTTTTTAAACACTGTATTTTTAATTTTTCAAA
A_16_P20243358
GCCTCACAAAAATCATATCCCCCAAAGTTGTCATTAACTCTAGAAATAATCACCTTTTGC

A_14_P100085 CAGAGAGGCACACGTCACATCTTTTGCTTTGGACTGAATTTTAACTAAAATATCATCTTT

CUST_8055_PI426210822 AATTGGATTTCTTGGGGATGGTGGTTGCCTAGTGCTGCTTGCATA

A_16_P41845121

GATTGCTTTTAAGGTGTTCAAAAACTGAGATCAAGCATTGGATCATAAAGAGCAACAGAG

CUST_7806_PI426210822 CTTTTGTTGCCTCTCCCCACCTGGGAGCAAATGCCTAGGACTTTC

A_16_P58424221

ATTGACTGTCTCATGAAATAGTGAGTTTCTTGTCACCAGAGAGATTTAAGCAAGAGCCAT

A_14_P106396 ATGTTTTCAAATGTATACTTGAAGATGTTTCTCCTTTCTCCATTCTGCTGTCAAGTTGCT

A_14_P122134 CTTTAAATTCATTTGCTAAACCTCTGTATAGAATTTGTTTTTGTCTCTTCCCAACCAGTT

A_14_P120223 CTGAAGCGACTTCAACTTTAATAGGATAATAACTGAGCAGAGTGTTCTGTGAACAGGATA

A_16_P02078513

AAAAAAAAATCCATAGGAGTTACCATCTTCTATTCACATTCAGCCAGATACAGATTTCTT

CUST_4768_PI426210822 CATCTCAAGAAGCCCTTACGGATGCCTCCCTTCCCTTCATGACTC

CUST_9753_PI426210822 CTGAGAGCCATCTGAGGCATGGTTCTGCTGAAAACATTTATTCCAGC

A_14_P120503 AAATCCCTAAACTGAAGGGAAAAAAAAAGTTTTTCTTCGAAATTTACTTCCAGGACGTCA

CUST_9852_PI426210822 TTAGCACAGGGGGCTGGGTTTTACAGCTAACCCCGCTGAGCTCAG

A_14_P105460 ATAGGTCTTAGTGAAGTTTCAATCTGTGAGATGAGATTGTGTTGTTCATTCTGTCCTACA

A_14_P200053 TGGGAAAATAAAGTGTTCTAACACATTTTTCGTATTTTATGACGTTCTTTCCCAGGACGG

A_14_P201406 AGCCTCTCTATTCAGTGGGATTAAAAGGCTAAATATCAGAATTTAAGCAGGTGAGTCAAA

A_16_P15942074

AAAAAGAACAACACATTTCAGTTTCTCTATGCAGTGACAACTTTCTATTCTAGGCCTAGC

A_16_P19953889

ACTTTAAGCAACTCAGATGTGAGATTATGTCCTCAATCATTTACAATCCCAAGCAGAGTT

A_14_P134566 ACTACTTTCTCTTCGTCTCCCCTCATCCTTCTCTTTCCAAATATTCCTATCATTAAAAAA

A_16_P01396459

ACATGGAACACACTGATTATCTTCCATTTCCTAACAACACTGTATGTAATCAGGATTGCA

A_14_P101596 GTTTTCTTCAGTATCGGAAAATACAGTTTCTAGTTTTAGTCCAGTAAGTGTCCAGGGATC

A_14_P102374 CTTAGAATATGCATCTAACAGCAACCAGTTCATGCTTTCAACATTTGGAGTTTTCTTTGC

A_14_P103424 CTTTAGGGGTAAAGTGGGGAGTGATGATACTTAGTAAAGTATCAAGAGATAAAGGAGTGA

CUST_4258_PI426210822

GCCAGAATTTATAGAATCTAAAAATAGCAGTTGAAGTGCAGCTGTGTTTAGCTTGTCAGA

A_14_P123077 TGAAAACTGAAAAACCAGCATTTCTATACCACTTTGGGCTTTGGTTATAAGTGCCATCTT

A_14_P112241 CCACATCACCAACACTTCTTTGAATCTTTTTCATGATGATATTCACCATGTGCGCAAAA

A_14_P138659 CATCCTGGTAGCAGAATTCAGATTATTTAGTGAGAAAGTCTTGACTTACACTGGAAATCC

A_14_P201228 CTGAGAAATATTCTTTTGAGACATTAATCTCAAAATAATCCCTTAAACGCATGGGCCAGG

CUST_8845_PI426210822 AGAGGGAGAAAGCGTGTGTGTTTCTGTGCACAAGTTACACTCAACA

A_16_P39927904

AAACCCAGTATCTTTCTCCTGTTAAGTTCATACAGACAGACTTCATTGAAATTTAAGGGC

A_14_P106257 GGCTAACGAAGGGTTTATAACAACAAAAAAATGTGACCCTCCAGTCTGAATTCTGAA

CUST_7322_PI426210822 GAGCAGCCCTGCAAATACAGATGAGCCCAAGTCACCTGTACAGTT

A_16_P19221833

ACTCCACAGCCTCAATTTTTCTTTATATCCCATTCTATCAAATAACACTCATCTTCCCAA

A_14_P111052 ACACACTGGTAAAATATGTCTCAAATTCAAGAGTAATGGTAACAGATGAGTTCCCTTCTC

A_14_P133909 CCCAATACTGGTAAAGATGTGTGTCTAATCCATATGGCCATATTGAACTTCAAAGTGTTT

A_14_P200433 TTCTTTGAAGTATCTCCTGTGTCCTTTCACCAGGTAAAGACTGTAAGCCACAAATAG

A_16_P17189281

GCAGTAATATTAGCATATATGATAGACATGAGACACGTTTTAAATCAGCAAATTACCCCC

A_16_P18149188

GAAAATCTAAACGTAGGACAATGTCATAGGAAAGGTGAATGAGAGTATGATGCCCTGATT

A_14_P115990 CTCAAAACATTTCAAAAAATAGGAGGAGGAAGATAGACTAGGCTATTGTAGAGCATCAGG

A_14_P122016 CAGTCTAGATGTTTTGAAAAGGAGAAAACCATCAAATGGTCACATAAATTGAAGTCTTGG

A_14_P105152 GTTTCCACTTCTCTACTGTTTCTGAAAATCTTGAGTAGTTTCCAGTAAAAAGTAGGAGGA

A_14_P138481 CTTCCAAAACAGTATCACCTGGGTTGATATCCATCATTGAGAGAATCATATTAATATCCT

A_16_P58424248 AGAAACATGTGAGACCTCTTAAGGGGTAGGCTCAGAACTGGCACTATC

A_14_P126315 ACAGGTGTCTTCTATTTGTGGCAAAGGATAATGGCTTTTCACTTAGGTTGTGACATAA

A_16_P40344830

AAGAAGCATCCACACTAAACTATACCTAAGGGCTTATAAGAGACGAAAAAAAATCCCTCA

CUST_5765_PI426210822 CTTTTCCTTCCTTCGTTTAGGCATGGGGCTGAATGGAGCTGAAAT

A_14_P113860 CTGTCCTGTCTAGAGCCCAAAGGTGGATTAATCTGCTTCATTTTTTACTTTTCAAATTTT

A_16_P19251654

GAAGCCATGAGAAATGATCACAAGTCCTGTAGATTCATAGGTGCCTGTTAAATGTCATT

CUST_2903_PI426210822 TCTCTGAGGATGGTTTTGAGAGCCATAAGCAGCTGATGTTAGTCTTGTG

A_16_P20686258

CATTTTTGTTTCCGTTTAAAACATTGCTATCAACCAGTTGGAATATAGGCCCGGAGTATC

CUST_5067_PI426210822 GAGAAAATGTCATGAGGGAGGAAATGCTGGGATTTGCCTTGACCT

CUST_8636_PI426210822 ATGGAGAAGGCCAAGCTCCCTGTTTGATACTAGAGTCTAACTACATGTCAT

A_14_P138174 GAATTCTGCCATTCCTAAAGGGCACATAGGCCATTTCTAATTTCCTTTAACTCATAATTC

A_14_P108526 CTGCATGTCTTTGCTACCAAAAATAGGAACCGTATTTCTACTAAAACATAGTTGGTTCCT

A_16_P58424561 GCCCAGTTCTGTGGCCTGCTACCTGGGGAGCCTCTGGTTGTAATT

A_14_P121539 GACAGTCATAGGATGCTGATATGTTTTTCACATATCCTGGCTTAATAAGAAGATGTGCAC

A_14_P135318 TGTCTCAACCTCCCTATTTTTCTTCATTTATCAATGAACTGGTTTCAGTTCATTCTGCAG

A_16_P01026320 CCCAATATCCTAATATTACTAATTGTGCTATATTCTCTTCGTCACAAACTATATCCTGGG

CUST_9607_PI426210822

TTATGAGCAATCTCTCCCTTAATTTCTGGATAGAGACTAATCTGCTCTCACAGGAG

CUST_897_PI426210822 AAGGTTGGGTTTGAAGAGGGAGAGGTGGGCTAGCTCTTTTGAAAG

A_14_P107043 CTTCTTGACACGTAAAGGTTTACTTCCTTTCTTTGCCTCTTCATCTGCAATCTTAAACAT

A_14_P124568 TAGAACAAGATTGAGGAGCCAGTGTGAATATTTTCATCTGGCATTTCTTTTCTCTACTGA

A_14_P117724 CACAGATGTGATGCTTTATAAGGTAGATTTGCATTTGTTAAAGGTCATTCTCCACTGAGT

A_14_P106388 AAGTAAACCGTAGTTGCAAGAATATACCATGAAGATTAAAGTAGGCTGGGTTTCATTTCC

A_14_P111124 CTTATTTGTGGTGCTGACCCTAGATAAGTTATTAAATTTTGTAATGCCTAGGCCAAAGGT

CUST_410_PI426210822

ACTAAAACCGAAACGGGTGCTTACATCTAACTAAAAATTATTGCCTGCTGAAGAGG

A_14_P115276 TTCCCTTCCTTAAAGGTAAAAATAGTCAGTAGTAATCCACAGACACAGGCATAAAGATGT

A_14_P116083 CCATAAACTATTCACAGCCTCAGACTCCTCGTGGGTTGTTGTTTTTTTTTTAAATGTATG

A_16_P20234452

AAAAGTCTTACAAATCATGCTTCTAGTTGCCATCTTAAAAATTATGTGATGCAGTGTGCT

CUST_9859_PI426210822 CATTTCACAGAGGAAGAGAAAGTTGGTTGCCTACGGTTGCACCAG

A_16_P02480614

TATCCTATCAAACCTACTTGTCTTTGACTCACCTATTTCAATAGTGCTTTCAATCTCCCA

CUST_2999_PI426210822

TGTAATCTTTCTTACTTCAGGGCAGTTGTTCAGGGAAGTTGTGATCAGTAGAAAG

A_14_P123894 GCATAATAATAATGATAAATTAGGAACATCACAGACGTGTTCACACCATGCCCAGATGTG

A_16_P17712767

ATCACTGTCCAATGTCATCATAATTTCACTGCCACTTAAAACTACACTTCAGCCATAGAA

CUST_1708_PI426210822

TTTTGAAAAATGCAAAGGAAATAAACTTTGGGAGTGAGAGAACAGGGGATGTTGATT

A_16_P02168228

ATGGAAAGCATTCACAATCTCTACTCTAAACCAAGACTTCTTCATCTACCTCAGTAAGAG

A_14_P102908 GTCAGTTGAGACCAACTGAAAAGTGATTGCTTTCAGTAAGTAACCTTATGATAACACGAC

A_14_P131968 GACAATTCTCAGGTTCATGCAGTTGATCAACAAATGCACATTATAGCCTGAGGAACAT

A_16_P17273781

AGGTGGTCCTCAATTTACTATCAGTGTAGTCAGTAACCAACAATTACTATTACCCATTGC

A_16_P35538657

GACCGTCTTATCCAACTATACATGCTTCTTGACTCTAGAAATTGTTGAATGGAAGTGCTA

A_14_P123463 ATGCCACGCTGTCTGTCTAAATACTTTTCTTTTATCAGTGCTTACTAGATATGTCTGGAG

CUST_3464_PI426210822 GTGATTATGAAAGTACGATAGCTTGCTCACCACCTCTCTCCTCTGCTC

A_14_P118126 TCTATAATTGCGCCTAATTTTATTCACCGGAGGGACGTCCTTTACTTTCAAGATTCTAAA

A_14_P114432 TGGAATCTCCATTTGGGTGTATCTTGTTAATAAATTCTGCAGGAACTGTTTCTGTTTTCT

A_16_P19948346

GCATGAAAACTCTCAATCCAATTATTCCTATACGCATTTTGAAACAAATCATTTTGGCCC

A_16_P00685612 TCATATTTGACTTATACGGGAGGTCTTAATGTTTCCGGCTTATTGCCATATTCCATATGT

A_16_P00685315

CGGAAACAACAAACAAAACAGAATAAAATCCTCTTTAACTTTGTTTAACTTGGTGTCCCA

CUST_1961_PI426210822 ACTCGCTTTAGGAGACATTCTGTAGCAAGATGAGTTATATGGGGCCAAGT

A_16_P19252044
AGTGCTGCATAAACCACATTCAAAACTAAGATTTTATTTCTAGACAAACCTGACCTGTGA
A_14_P126052 ATGGCTCCAGGTTGCAACAGGAATAAAAAATGCTGGATTTAGTATATGTGGCTATTACAT
A_16_P00608642
AAAGGATCCATATCTCCAAGGTAACACCAAAATTCCTATTGGCTGAGAACAATTTGTCTT
A_14_P136579 TTATCTCTACTCTCTAACCTCTCATACAGAGATTTCCTCTGGGGACGTCTG
A_14_P126516
CTAAAAGTAAAAAATCATAGAGCCGATTTGGTGGTCTAAAGAAGAGAGTAACAAGGCAGT
A_14_P130028 TGGCAACTATGGTAAAACCACATGATCTAGTCATGATACTGAAGAATCCAACCAAATGAT
A_18_P23937642
AAATGAGCCAACTGATTTCTACCAATTCTACATGCTTCCTACTTTTGTAAAATAACTCGA
A_14_P107951 ACAATAATAGCGAAAAGCATGGGCTTTGATATTAGACATGGCTATGCATCCGAATATCTT
A_14_P125394 TTCTGATTAATTGCCAAAAAAATGATCATTGTTTCCTGAAAAGCACCCTGAAAGAGGGAA
A_14_P133209 AAGTTACTCTTTTTCTTGTTCACAGGCTAAAAGGTCAGATTCCTGAAATTAAACAGACTT
A_16_P16728147
ACACAATGCATCTTCCTCTATTGAGACTTGACAAGTAACACTTAGTTTCCCACATAGTTT
CUST_5322_PI426210822 CCCTACCCCCATCTTCACCTCCTCCCCTAGAATAACAACATCATA
CUST_7977_PI426210822 TTCATCCTTTTCTCCTGCCTGGGTCATACACTCAGATCATTTTAATCTCCAA
CUST_7532_PI426210822 GAGGCTCCAGACAACTGACTCAAAGACAGAGAGGACCATAATACCA
A_14_P112210 TGAATTGAGAAAAACGTTGCGTCTTTTTTTCTAAATTCATGCATTCCAGTAAGTTGGGAC
A_14_P138757 TGTTACAATTAGTGACATCAATGTGTTTACGATCCTTCATCATTTCAACTCCCACTTTGG
A_16_P03028146
AAAATTACCAGGAAGGCTTTAAGAAAAGACAACAACCAAGTAGATTTCCATACTCTACCC
A_14_P119361 AGAAAATATCTGACTCTGCATGAGACTAGGCTTATTCTCATTTATTTGGCAACTTACCCT
A_14_P124115 AAAGATGCAGCATGTCGATATGTGAGGTATAGGTCTACTTGATTTAAATGGAGACACTAG
A_16_P18423896
CTTAAAGAGCACAGGGTATTTTCATGGATAAAGTCTCCAGATTGCTACGTATTGTCAGAG
A_16_P30952284 CAAAGGCAGGACGCTGATCCCTTGCTATGAAGGTGAGCATGGATT
A_14_P112527 TGGTTTCTCGGTATATCAGCCATGTTTTTCCATGGCAAATAAGTAAGGCAGAGTATTAAA
A_16_P03016372
AAATCAGAAACACGCTTGACTTTTACATGCCTCTTAAACTCATCTACCAAAGTTCCTCTT
CUST_4264_PI426210822 GGGGCAGCAAACATTTCCAAATAGGGATTGACTTTTAAGGGGAGG
A_18_P12078230
ATAGCAATCCCAGAGAAAACTTAACATGATGAAGACCCATATTAATCTCCAGCAAGTTCA
A_14_P127633 CTTGAGAGACCATCAAGAATTGGATTTGGGGAAGAGGTGAGTATTTGCTATACTATATTT
A_14_P124182 TAGGTGTTTTCTTCTTTATCTTGTCTGGCTCACTGATAAATTTTCTTCAGTGTGGTAACC
A_14_P126110 TCTTTCTTCATCCTTCTTGCATCCATCTTCTTTTTAATATTTACTGATTTCTTAACATCA
A_16_P15070795

AATGAGAGCAAAGATTATTCATGTCAGTCTAACAGCAATTATATTAGTGGGAGAACAGTT
A_16_P18659822
AAAAAGGAAGTTGAGTAACACATGTAGAAGGAAGGTAAATAATTATCACAGCCACCTGAA
A_14_P119714 CTCTTGTTCTCCATCTAAAGTTGGCTCATGCAAATGTAGACTGGTTAGCCTTTAATATAC
A_14_P133476 ACAAAGGTTTCTAAATCCCTTCTTTTCTATGCACTTTTTTATTTAAGAGGTGGGGTCCC
A_14_P121551 CTGGCAAGGAAATTACCACAGTGACAACAATACACTTTCATGTTTTAAATCATCTTCAGA
A_14_P103492 ACATTCCCAACTGTAAATCCAGTTTTGAAGTTGCTTTTTGATATTCTGTAATGACCCATG
A_14_P105069 AGAAAAGAATGGAGGATGTCAATACAAACCTTGCTACTTTCAGCATTCTTTTCAATCTCT
A_14_P201847 CTCTCCCCTAAGATTAACACTTTGGCCAAAATTTGGCAGCATATTATTCTTTAAAGTCTG
A_14_P200322 TTCATTTTCTCAACCATTATGAAATACTGTTTCTGGATATGTATAAAATCTTTGTGAGCA
CUST_10372_PI426210822 CTTTTCAGAACTAAGCTAGAGGAAATTGCCATCCTGGGTCAGGATGAAAC
A_14_P117011 CTGACATGATATCCATGAAGGCTTTGGAGGTATCAGAGAACTTCTTAATAAGCACAGG
CUST_8188_PI426210822
GAACACAACCAAGACAATAGAGTGCCCTTGATAGATATGTACAGGGACCAAAA
A_14_P103158 CTATTATTTCCTGTTTTTTATTTTCAGCATGTTGGAACAGGATTCCAGGAGAATGAACCC
A_14_P115633 CTTCCTTTCTCGAGCACATTCAGGATTTCTCCATTCTTGGGCTCATGT
A_14_P100055 AACTTACCTGATGTGTGCATTACAGTATCTTTTTGCATAGTCGGTCCATCTTCCAAATTT
CUST_4381_PI426210822
GTATCAAATGCATTGTGATTGTGCTATGAGAATTTTGGGGGGAAAATTCCAAACTTTATT
A_14_P116199 AGGAAAATACTACCTTTGCAGAGGCATGATTTCACAAGCCATTCTCGTATGTAAATTTGC
A_14_P129681
ACAGATAGAGACAAGGGTTGAGGAAAGAGTAAAGGTGCATAGAAATCTGTGCTGTTAAAT
A_14_P200909 TTTTCATTCCATCTGAATTTTACAAAGCCTCAGGTTCATGCGTCAAAGATAGGGATAAAT
A_14_P137768 TGATGATGTCTTTGACCAGAGAGTAATGTGGTAAAAGAGATAGTGAACATGAGTTGTTGT
A_14_P115121 CTACCTAGAAGATGAGGCAGAGGAAAGAATCATTTTTGTAAATTCAAATTCCACAAGGAC
CUST_2286_PI426210822
TACACAATAGCCATTGTCTATGGAATTCTCCCTGATGGAAATTATGGCACGTAGTTTA
A_16_P21081399
AACTGGATTTAAACTAGGGAGGATCAAAATGTTTAGGTAACAGAAACTAGCAGTCCAATG
A_14_P118930 ATTGTCTGCAATAAAAGTGGCTCGATATGGCACGATATTGGGATGGTTGAAGA
A_14_P136043 TCCAGACTATGTTTCCTACCTTGCTGGAAATATTAGATGTCCTTGTAGTTTTTCTCATGT
A_14_P127859 GCCCAGGCAAACTTAAAAACAAAGGAAGAGGTAACACTTAAAATTTATAGCACATACTGG
A_14_P133859 TGCAATGCAAAACTCTGGCTCATATTCAATTTCTCAGTTTCAATCCAGAATGATCAGGTA
A_14_P200182 CTCCGTTACTTATATACATGGAATGACACACGGTCTTATCCGTTGAAACTGTGATATGTA
A_14_P127183 GCATTTGGCTCCAAGACTGGGGACCAGAGATATTACATAGATTTTTTTCGATTTTAGTAA
CUST_424_PI426210822 GAAGGGAAAACATTTAGAAGGGGAGCCCTGGGATCATAGGACCTG
CUST_6553_PI426210822
TGTTGACTACTAATAATGATTTTCATATAAAAGCACAGCAAAGAGATCCATGTACAAAGT

A_14_P201836 ACATTGCTAGAATGAGTCCTGATAAGTTAACAGGTGAAATTTACATACACGTGACAGCAT

A_14_P138655 TCTACACTGTTCTATGCCCAAGTGTTCTTCTCATTATGAACTACAGAGAAATCCTAGTTC

A_16_P02320942

AAACCTCCTTTTACAGTTTAACTAGACCTTGATAGTGGTAGAAACATACTTTTCATCCCC

A_16_P16761048

CACATATCTTTTCACCACAAACAGTTTCAGTCTTAAGATCACATGTTGCAATCCATGAGA

A_14_P119692 TGTCCCTCATCTCTGTTTTGTGTTATTATTACCCTTATTATCTATGTTACAAACCCAACC

A_16_P21425077

ATACAGAGAGTAGCCCTGTATACAACAATCATTCCTAACTTGATAACTGAACATAGGCCA

A_14_P115078 ACAAGATTGTGTATTCCAGGGTGCTCTACAAAAATTATAATTTCCATTATGTGCTACGGC

A_14_P202366 TTCATTTTTCAGGATGTAAACAGTCTTCGACTTTCTCTTACGGATAATCAGATTGTCAGT

A_14_P102334 ATTTACCTTCAGCCAGTGCTGGTGATTCTGTCCTTGTCCTTCTATCTGTTTAAG

CUST_8708_PI426210822

TGAATAAGCAAGACTGATGTCTTCAGAGCAATGCAAAAAGACAGTGTTTCCATGAAA

A_16_P38204683

ACTAAGCCTGATCGTATTGTTGATGGCCTAATTGTGAGAGACTCTAAAACTGACTTTTTT

CUST_7716_PI426210822 CGCTTGTTGTGTCTGGAAGAAGGAAAGGGTCTTAGAGGAACACAAATT

A_14_P139278 TCGTCTGATGTGAACCATGTCTTGTTCATCCTTAATTTTGTCCTTACGAATGGATTTTAT

A_16_P19404502

AGATCAACTACATGTCCGTATACTGCAAGTTAGAATTTAGTTAAGAAGGCCCTGAGTAGA

A_18_P16536129

AATGAAAGATCACTCCTGTATACTTCCATGCTCAACAATAACAAAATAAAATGCACCCCC

A_14_P101844 GGCATTATCTTTCCATATTTATGTTTATACTTGGGCCTCTTTCTGGTCTTTCCAAGTAGT

A_16_P01194110

CCAGGATGTTACCATGTATTGTTAAAGCCCAAAACTAAGTATAACTGAACCCATCCAATA

A_14_P109539

ATAATAAGAATGTGCGAGAAGAAGAGTCCAGAGTTCACAAAAATACAGAGGTGAATTCCA

CUST_8421_PI426210822

TTCTTTGAAGTGGAACCAACTATTGCAGAAAACCAAAGACACAGTTTTCTAGGG

A_14_P126842 ACAGAGTCCAGGGAAAGACTTGCTTGTAACTTTATGAATTCTGGATTTTTTTTTTCCTT

A_14_P116208 CCATACAGTAGGTCTGATTTCCCAATCTTGCCATATTTTTTTAGGCATCTGGTTCTATTT

A_16_P00685597

TTATCACCAGGGAAGAGCCAGTAAGTAATTCATTCAGTTGACAGGTAGCCTATAAAGAA

A_16_P18224427

ACTTCCATGTATGTGTATTCCATTAATTTTCGCTTCTTCAATAAAATTTCAGGCCCATGA

A_16_P21381773

TTGAATACCAGCAATAAGCCAAAAAATGTCACTCCTCCTAACACAGATATTATGGGTTC

A_16_P19847722

AGCGGTTTTTTAACAAACTAGTAGGCATTCTGGAAATATTCATGAGAAACAGAACGAGG
A_18_P14134978 CTGGGTCATCTGCCTTCTGTTGCTTAGCCATCTTGCCTTGTCATT
CUST_148_PI426210822
TTTGCATTGTTACAGTAGCAGTAACAATTCAACAAAGTATGTATGTAACTGTTTAATTGT
A_14_P202153 TGTTATTTCCATTCCCAAGATGTTAGTAGAGACGAAGATTTTTCAATTTGTCTCCTTGAT
A_18_P22841352 GTTTGAGCCAGGCTTCTGGAGAATGGTGGTGCCTCTGGTGAAAAG
A_16_P01363454
CTAGAGATGAGTATCCCCTTTTATGCTAGGAATAAATTGTTTGCTAATCTGTGACTGGAG
A_16_P36230358
ACATACCTATCTATTTACTTATTGACTCTGAAATAACAGAGGTAGATCCTTCCAGGTTTA
A_14_P119243 CATTTCATTTTATGGGACTAGTCTGCTTCCCAGAATAATCATCAAATGCAAAGGAATTTA
CUST_5165_PI426210822 GACCCTGAGGAGGCCTGGGGCTGATCTGAGCCTTAAGACTTATTT
A_14_P124658 GTCTTCCCAAAATTGGCATTAAAAAAGTGGCTTTTCTCTGTAAAGAATCTAGCATTTGCT
A_14_P120545 ATGTAATGATCAGAAATTCTTGCTAATCTGCTCCTACTTATTGGATGTTGGCTCTTTGGT
A_14_P114001 CTTTGCTATCAGGGCTCTCACTATTTGAACTAAGACTGAATAGAGTCACAAGAGATACTT
A_14_P118882 TCTTGCTTCTTTTTTTTCTCCTGTTCTCTAAAATTCTGAGGCCCAGTATTCTTTGACAAA
A_16_P00238841
AAAAAAGGTCAAAAGGAAAATACCAGAAGATGCAATATGCACCTCTATTCCTTAAGCCTC
A_14_P139963 AGAAAGGTGAAGGTAGCATTTAATAGTGGTCTTGTTTGGATTGTGAGAGGTTGAGAG
CUST_4104_PI426210822
ATTCTTTTTCCCTTGGTCATTATTTGCTTTTTAAAAAATAATGTAGTTTTAAAGAATTCT
A_18_P11858251
CTAGTATCCCTTTCATGAAACACTAACCAAATCTATATCAGTCCTTTAAACCCAGTGACC
A_14_P122847 ACTTCCTCATCCTAAAAGTAAACAATGAAAGACGCTTTAGAATTCAGATCTTGGATTATC
A_14_P116656 ACTAAGAACTCTTGCAGTTATTGACATAAAGAATTATTCTGCTCTGGTTAATTCCCCATA
CUST_6364_PI426210822
GTTTAATATTATTGGTTTATGCAGTATTTGAATATATAAATATGAACATAAAGCCACGTA
CUST_1420_PI426210822
ATGGATATGCTTTTTGCCAGACTTTTCACATTTAATATGTAACTGTTTGTTTAGGTTGAG
A_14_P104713 GTTGAAAGTCGCTTTGATACCCAGATTTGAAATGTATAGGCTAATTTGTAAACGCCATGA
A_16_P58424176 AACCCTGGCAGTCAGTGTAGACCGTGCCCCAGAATTACCCCACAT
A_14_P110799 AGAAGGGACAAATGGTTAAGACTTTCCTTGGAAATGGAACTTGGAGAGTTGTTT
A_14_P137824 AACATTTCAAGGGAAAAGTTTTGGTTCTTTGTTTTTTCTCCTATCGACGAATCTCAGAAA
CUST_6046_PI426210822 AGGCATAGTGGCTCCAAGCAGAGGTAAACTGCTTTGGTTATCATTGAG
A_14_P134489 ATCCCTTCTGAGAGATGGATAGTAAATTTTGACAAAGACCTTTCAGATGGTCTTGTTTTT
A_14_P111690 TGGTCCAGTTTCATCTCACTAAAATCTCAGTAAAATTGCTTAAAAGAACATTTTGCCCCT
CUST_382_PI426210822 TATAGCTCCCACTCCCTGAGTCTTCTGGCGTCCTTTGAACAACAA
A_14_P129775 CTTTTTCAGATTAAAGTGGGAATCATGTCATGGTAGTGACATTTTGGAGAGTTCAAGTTT

A_16_P00685618
GCTATGTAAATAGTCAAGTAACTGAAGAGAACTATAAACATGAAGTCTTGAGTTAGTCCA
A_14_P105726 GAAATTATTGCTCAAAACATCTCCACATTAATATAGTTTATTCAACGTATTTTCATTGAC
A_14_P105457 ACCATACTTGATGGTAAATTATAAGCAAAGTGCCGGAACAGCCAGTAATAGGTTGATAAA
A_14_P105771 ACCAGTTGTTTCGTTCACTCAATGGTAGTCCATCTCTTACTGTTATTCAGTTGTTTTTAT
A_14_P200038 CTTTGCGTTTTCAGCGCTACTCCTTGATTATGACAATGTATGCATTAGTAATAGCAGCAA
CUST_6657_PI426210822
ATGTTCTAGAGAATTCCAGTTTTAGGTCAGTAGTTGTGAAAGTAAGGGCAGATCACT
A_16_P16484917
GCCATACCAATCCTTGATGCAACATTTCATAATTGGTTTTCATCTCTTTTTTAGGATGCC
A_16_P17287265
ACTGAGTTTACAGATACTGCCAGAATTTTACTGCTACCATTTCTATTCGCTTTTGCTGTT
A_14_P139003 GGCTAAGCAATGTATGAGATACAACATACAAAAGGAGGTATTTTCTTCCCATACGATAGG
A_14_P200515 TTAACAGCATTATCTGTTTGCACCCCTCATGGGTGGTTAACAACAGCAATAAAAC
CUST_5810_PI426210822 GAGATGAAGACCAAGGTGAGGAAGGAGCCAGCAGAGAAATCGAGT
CUST_9774_PI426210822
TTTGCATTGATCAGAACATAAAAGTTTCCAGAAACACAGAAGTGAATCAAATAGTGTGGT
A_14_P132182 GCTGAAAAGGGTCCAATTAAAACAAATGGAGCCAAATTTTCTGCCTGAGAACTTG
A_14_P121859 ACACATATATGCACGTGGGTAAGTGTAACTAGAAAAGGAGAAGAAGAGGAAAGTTATTGT
A_16_P35795741
TCATTTTCTAAAAGCCAAGTAACTTTTTCACCTCAACCTTTACGAATAACACTTTGCCCT
A_16_P15312310
AGTTCAGCATTTTCCATGTCTAAGTGCATCAGTATTTAAGTTCAGTTCTTACTCTTGCCA
A_14_P130767 AGTAAAAAAAGAAATGCGCAGGGGCAGAATTAAGGAATTTAGATTTTCACCTATGAGCAA
A_14_P133655 CTGATGTCAGTGCCATTTAGACAATGTGTGTGTATGCACATGCACAGGG
A_14_P104209 ATGCATTTCCTGGGAATCTGATCAATTCTACCGGCAATTCTGTTTTCTTTGTAAGATTAA
A_14_P132042 AAGAGAAAAATAGACTAAAAGCCTTGCAGCGCAATAGATCTACATACATCACAAGCACAT
A_16_P20345990
GAGGCAAAGAATCTAATCTCAAATTAGTCAGGGTATCCACAGATGACAATACAAGTGAAC
A_16_P16733464
AAGAAACGAGCCAAGTCTTATCTTTCCATCTGTCTATTAAGTATAAGAAGTTCCTCCAGC
A_14_P114520 CTCAGGATTATATTTGTAATCAAAAAAAAAAAATCACTTGGCGCAGGAGGGAGAACTTTG
A_16_P16191189 ACTGAATGCTTTGTTGCAACTCAACTTGGTTGCAGATGGGCTCTTACTTC
A_18_P11284038
GAGTGGTCTCTATTTACCAACATGAAACAGAAAAATACCATCTCAGGCTCCATATTCCAT
A_14_P114822 AGGAAAAATCTCCATTTTTCTAAATATTTAGCCACCAACTCCTGAAGAGTCCTTTCAGAA
A_16_P19433681
TGAAGTCTGTTGCATTTCATGATTCTCAAGTCTAATATGACATCTTATTGGCTCACTCAC

A_14_P139324 TTGTTACCTCCCCAGCCCTTAGTCTGTTGCTTAATTCTGTTTAACATTTCTCC

A_14_P133546

AGTTCCAAGAAAGAAAGAAGGTAGTGGTATGTTATCTAGTAAAGGAGAAGGAAATGACGA

A_16_P30952272 CTTTTGGGGCCAACTTTCCTGCTCTCAACTGCTCTCTATCCCTTTA

A_16_P00154158

CAACTGATCACATAACCACTCATTGACAAAACAGAGATTAAACCTATATTTTTGACCCCC

A_16_P35872087

CATTCACCTTCTCTTAGACCTAGATGGCAAATCATAGACATAAACTTGTGCATCCTAGAT

A_18_P22839415

CCAGAATGGTCATGGAATTCAGGAAACATTTCTCTAAATTTTATAGTCATCTTGCTTTTG

A_14_P200762 TGAGATGGGATTCATAGTCAAGAGTCAGGATACACTTTATTTCTCACAGGTGTTAGTTCT

A_14_P119743 TTCTGTTTGCAGGAAAAAAGAGAAGGCATAGATCTAAAGTAAATGGTCTCCAAAGAGGTA

A_16_P18969217

CAATAAAATGAATAATCCTGCCCATAATATGATGCCGATAACTGAACATGTATCCTGCGC

A_14_P109062 TATTAGCATTCAGATCTAAACCACTCACTAGTTCTGGGGAAATCACCTAATGATTCTGCT

A_16_P00236775

TACCTGCTGATCCAAATACTCTAGGTTTCTTTGCAACTGAAGATCTAAAAGTTCATCCTA

A_14_P126414 ACTTTGTATCCTATAAGCGAGGGACTATAGGGGTTTCTTTGTTCAAATTTTTCCCACATC

A_14_P201986 ACACAAAAGAAAGCACAGATTGTTTACCTGTTGTGGATTTTAGATGTAACAAATGTTTAT

A_14_P126468 TTTCTTTTTAGGCAGTTTTATACAATGACAGATCTGTTCTGGAAAATCATCATGCTGCGT

A_14_P106456 CTTCTGATTGGCATCACTAATTAATCTTGCCTGTTCTTTAGCGATAAGATATATTCTGTA

A_18_P12099807

AAACTGGCTTACAATGTCTTTCATTGAACCATATCTGATTGATTTTCATCTCCAACAGCA

A_14_P132321 ACACATGTCAAAACTGTCAGATTGACTACAGATTCAAGATAATGAACAACCATGGTAAAC

A_16_P00965968

AGCAGTTTCATTCTTTTCTACTCAAAGCACATTGTTCACATGTTTCACATTGCAAAGCAT

A_14_P129296 GCAACGTTTATTATTTTGCTTCATGACTAACATATGCGACTCTATTCCTTGGATGGCTTT

A_14_P124025 GAGAGAAAACTGTCCCTGAAAAAGGTATCATCTTCTAACGCAAGTGTGGG

A_14_P102061

AAGGAGTCAACACAAGTGAGAGGAAATAAAACATGACTGGATAGAAAGTAATGCTGAGAT

A_16_P16784311

GGCTTGTTGCTAAGATATTACCTTTGAACTGCCTAATCTACACCTAAAACATGACAAAGG

A_14_P115095 ACTCTGATTGCCTTATAAACTTCAAAGAATTCTCCTCTGCAATTGGTAAGATGATTTTTT

A_14_P138846 TTTTCTTTTGAGATGGGTGAATGGTGATATTCTTTCAGCAAATAGTATGTTAATGAAACA

A_14_P100122 ACGCTGAGATAGGCTGATTAGAGTCATCACTTTCTTCCATTTCTAAGAGTAGTGATTTCA

A_14_P200746 CTACTCTTTAGAGAGCCCCAGCATCTTTGATGTGGATTGGAGACAATTGC

A_16_P20524774

AAATGATAACTTGGAAGTTGAGATTTTGTCATAAGCACGTATAGTCATAGGCAGGTGGTC

CUST_490_PI426210822 GCCTTTGCCCACCCAGGCTGACTGCCTGTCAGGAAGTCAAGAATC

CUST_4387_PI426210822 CTTTTAACTTACGGGTCACCTGGTTATTGCCTGTGAGGCTCACTG

A_16_P31222296

AGCATAACAGACGTCACTATGGAAAATAACCTTTCAAGAGTTATTGCAGCAGTTTCTCAT

A_14_P129506 CTTGAGCCTGACGTAGACTGTGAGGAACTAGGATTCCAAACCAGTTC

A_16_P21248742

AGCAGTTCATCTATCTTTCACACACACCAAATTTAATCTTCATGTTAGACAATGATGGGG

A_14_P117869 GTCCTAACTCTAGATCTTGCATTAGTTCTACTTCTATGGACTCTTTTTGGGTTCAATCTC

A_14_P137527 CTTTATCATGCTAAGGGTCAACATATCCCCAGACAGAATCGTATTTTAATTCAACAGCAT

A_14_P111107 TTTTCTAGATGATTCTTTGGGATTGGGACTTAAAACAATGGTATAAGCCTCATTATCAAG

A_16_P35647754

AAGCAACACTAAAAATCATCTACTTACCTCCCTCAATAATTTCAATTCTTAACTGTCGCT

A_16_P33810993

AAAATAGCACAAGAAACACTTACCAAATATAAGGTTATATCTTCCGCATATACAGGAGAA

A_14_P107261 TGTGTGCTTACGGAATGCGGAAGTATCCAGTCTACGTATCTCTATAAAAATTGCTTTCTA

A_14_P109210 GTTCAAGTAATTATGGCTCTCTGCTGTTTAGCCCATATCATATACAGCATAAAGCACACT

A_14_P202070 CGTCATGATTGCAACTGGGTGGGTCAAAGGTAAATAGGATGATGACAATGAAAAAT

A_14_P136588 AGAAAAGATGCTGCTAAAATTACTATGAAGTCTTTCCCCAAAAGGCATTTGGTTACATCT

A_18_P12434214

ACTCAAAACAATCCACCTGTTTGGAAATGTCTGTTGCTGCTATTGTAGAAGTTTCTTTAC

A_16_P21144840

TGAAATCTTTACTTGTTCACAAGGTTTGTCCAGAAATGAGTTGAACTTCTAACCCGATTA

A_16_P37086379

ACTAAAACATTCCTACATAGCACTTACATAACTTATCATTATCAACCTACACTCAGAAGA

A_16_P19839723

AACAAGAGTAAAATTCTCTCGATAAAGTCACATTCTCTGCAATTAGGACTAGGAGAAGCC

A_16_P18610182

AAAGAGATCATATCTGTGTTAAAAGAATCTACCCTCTCAGAATCCAACAGTCCATTCACT

A_16_P03610011

AAATTCGACAGTGAAATCTCATCCTACAGCATTGGAAAGACCTCTAATTTTGATCACAAG

A_18_P13802856

CCGACACAAATTTTCCTAGTGGATATAGACCTATTTTTTCACAAACGTAAGTGCAACAGT

CUST_6925_PI426210822 ATTTCAAATCACAAGGCGGCTTCAGTATTCCGCCGCGGGGCTCAG

A_14_P104606 AACATAATCATTGCGTAGATTTTCTTTGAATGAATGTGAAAACTCATGAGGTAGTGGTCT

CUST_1860_PI426210822

TGAGGCTTTATTCATGCCCCAAATTATTCTCGTATTTATTTCCCCAGTGTTTGAGA

A_16_P00766676

TGTTTCTTTGTTACTCTCCAGTGGATACATCATTAACTACACCTTTGACTACCACAAAGA

CUST_9719_PI426210822 CAGAGTATCTGTGCTGAAGAGGTCAAGACAGGTTTCACAGAAGGAGT
A_18_P22842091 ATAAGCAATGCATGTAGCGGCTAGGGCACTGAGGTCCCGTGACCT
A_14_P122253 ATTTCCACATAGAGTTGGACACCATGATTGCTCAAGGACAGGCAGTG
A_14_P132497 CTATTCCAAGGATTTTCTGCTTATCCTTCTCTTGAAATGATATGAATACTGTCCTTGGCT
A_14_P102640 CTTTCTTGTAAATTCAGAAACAACAACAAAAATCAGGGGGTCGTCAAAATACTCATTGAC
CUST_1966_PI426210822 ATCAAAGTGACTGCATGCCCTGTCTCTCTGCCTCCATCCTTTCTC
CUST_7117_PI426210822
TCTGAAACTCTTGTAGACACCAAAGAAATTGGTAGGTTATGGAATGATTGATTATATCCT
A_14_P103285 CTCTGCCCCACTTTAATCCCCATTGCTTACCTTACAAATACGAAATAAAATATAAACTCC
A_14_P102775 CTCAAATGAATAATGTATACGAAGATATATCAAGGTAACATTTATATAAGTTTAATGTAA
A_14_P132518 TAAATGCTTAGGGCTTAAACAAAGGCATTTGCAAAAATGGATCAGGTTTTGTGGATCATA
A_14_P134321 CTTGAGGACCAAACACTAAAAGCCACATGATAAAGGAGAATGCTGATTTCTATTTATGCA
A_14_P117838 GACTTCAAAGACTGAGATTTAAAAATCATTATTTGCCACACTCTTTAAGGAGATGGGGG
A_18_P16231620
CCCTACAATGGCTTTTCTATGATTTCCTCTACTCCATTCTAAACACTCTTCCTTGTGTAT
A_18_P15902960
GGTGCCTTACATGTAACTACTCACAAACTTTTTTGATCCAAAATTTAGAAACTTCACACG
A_16_P20268645
ATCGTCCCTAAATGTCCAGATCCACAATACACAAAATAAACTTCTTCAATAAAGTTCGCC
A_14_P129579 AGGATGAACAGGAATTAGTGAAGCAATTCTCTATTGAGAAGGAGCAATTCTCTATTGAGA
A_14_P119207 ACTATTTTGTGTCTTTGAGTGCTTTGGCTTCATGCTATAAATTGTGCTCTGTTTATTTCC
A_14_P108186 CAACTCCTTTATCTGAATTCACACCAAGGTATAAAGGACAATGGAAAGAAGAGTGTCTAA
CUST_4293_PI426210822 GGAAATGAATTCTGTTTTTGGTGGAGTGCGTGAAGTTATTTCCCTCCATTAGTT
A_14_P112011 TTTGAAATAAAGCCACGTGCCCAGTGAATCCCAAAGGAACCTCAACTAAAATAAAA
A_14_P116074 GTCCCAAACACATTACACTGAAGAGATTTTGGTGAGGAAACTTGCTGGAGTTTT
A_14_P120801 CAGAAGATTGTGTGTTTCTAGTATCTTTTAAGTATCTGATACTCCCTCTTCTCCAGTTAG
CUST_4779_PI426210822 CATGTGTGCACATATACACATTCCCACATGTGTGGGGAATGAGAGG
A_16_P03671123
CCTGCAACTAACAACATTTCATAGTAGAAGGTAAGAATTGCAATACGTCCTTTATTCCCC
A_14_P111497 CTCCATGTATGAGAACAGCCTAATCCTGGAGAACATTACTAGTTTTGTTTCATCTTCCTA
A_16_P00702951
TTATGACCTGTGGCAGTTATATCACAACATTCATTTCCAACATTGTCTAAAAATGAGGGC
A_14_P129787 CTTTAATGCAAATAGTTTAGAAAAGAAAGGACCAGTCTTCACTTTCTGCATTATCCCCAC
A_16_P00524466
AAGTCTAGCCCTCCATGATACTTACATTCCTTCATAAATGACTTTGTCGCCTACATTTTA
A_14_P123068 AGGGAATTAGAGGGCTCTGGACACCTTACCAAATATTTAGAAAATGAAGAGGTAAATTTT
A_16_P31210167
AACTGTGAATAAACTGTAGCCTACATTACTCATCCATTTTTGGATAGTTACCACTGGGAG

A_18_P22840468 ATTTCTGGGGACAGTGAACAGCAATGAGGACCACAGGGAAGATTTA

A_16_P19840736

AAAGTTATCCTACTCCCCTCATATGAAAATGTCCTTTCCTTTTAATACCTCACCTACACT

A_16_P02053157

AAAACATCCCTTACAGTTGGTAAAACTTACTGCTGAATCAGTTAAACAATAAGGCCTTGC

A_14_P131301 TCTTAAAAAAAGATTACCACAACAGATAAGGCACTGGTATGATGGCTTAATTTTGGTAGA

A_16_P17892655

AGGCAATAGCAGACCTAGAGTAACATACCAAGTGTAGATAGTAGCCTCAATCAGTTCAAT

A_16_P39911812

ACCTCACTTTTAATAGAGTCTCTAAGGAATGCTCTTAAATATCCATGACAGTGGTAGCAA

A_14_P131750 TTCATAGGAATTGTGAACCAAATAAGAAGGAACCAAACACATAACAAGAGTCCTTCATGT

A_14_P128484

AAAAATTCTATGAAAATTTTGGAAAGCAAAACAGGAAACCCAGTTATAGGCAAGGGCAAA

A_14_P132962 TTTTCAATTCCATCCCCAACTTTTTCTAAGTCAAGTTCAGCAATTTCAGAGGTAACACAG

A_14_P103327 AATCATTTCACTACCACCAAAGTTATGCCAGTTTCCTTGCATTCTGCATATACACATTTT

A_14_P107430 TTGCCCGGGCATTTTGGTAAGTTTGAAAACCACTTATATTTATATTGGAAATTTAGGGCA

A_16_P18941169

TGGATCTGTCTTACAAAGAAAAGCGATAACTTTCTAGTCTCTTATGCTGTCCAAAACTCA

A_16_P16677944

ACCTCATCAGGTTTTTTTCACCATCACTACTAGTTACATAAATCCTACGTGAAATGCAGT

A_14_P103647 ATTTGTGTGGAACCAATGTCACATCATCAAAATAAATTCTAAGAGTCCTGGCTTTGATTC

A_14_P101835 AAAATAAAGGAAATCAGCACAGCACACATTACTCAAACGTTTCTGGACATATTATATGTT

A_14_P118554 TTCCATATGGTTATACAGTCGAATATCCCCTTCATTTATCAGTAATTATCTTTCCCTGTC

A_16_P40308998

AGTGTGCAAAATCAACATGAAACTCCATTTTAGGAAAACAAAGTAAGTAGCATTTCCGGT

A_14_P101041

ACATTCAAACAAAGAAACGGCAAAGAAGATTAAAAGGCCCAAGTTCAGTAAGTAAAATCA

CUST_1536_PI426210822 AATAAATAGGAGATGCTACCCTCCAACCCCCATTCCGCACCCTCA

A_14_P107556

ACAAAATGAGCAAAGAGTGAAAGTCAGAGGAAAACACTGAGATAAAAGTGCATTCATCAA

A_16_P03547069

CGAGGATAATGGAATTGGAGGATTGTTAACTAAGTCGCATTGACTTAGAGATGGATAATG

A_16_P01006729

CCCACTACTGATTTTTCAATTGGCACAATTCAAAACCATTTAGTTACCTTTGCTTCTTGA

A_16_P02323380

CTTGTATGAAAAATCCCCATATTAGCCTCACTTAATCTCAATAGAAGCCTGCTGTTTACC

A_14_P124018 TGCAAAACAGAACAAATAAAAATAGAAACTTTGACACCCCTCAGCAGTGACTAGTTGCAT

A_14_P120582 AAGATCAGTGAGTTTGAAGAGGAATGCCATGAATGCTAAAATTAATAGGCAGGAAACAAG

A_14_P132932 TCATGCATGCTCTACTTGAAGGTCTATTTCTATCTTTTCAATGCTACCCTTACCCACTAG

CUST_3853_PI426210822 CTTTTTGGGGGCAGGAGGGATTGCATAGCAATACTGCCTACCCAG

CUST_5947_PI426210822 ACTGACAAAATCAAAACCACAGTTGACTTAGGCTTCCTTGGAGCTTGG

A_18_P22839426 AAAAATCTCACTATGTCCATGCCCAAGAGTTCCACCATGAGCACATGC

CUST_4913_PI426210822

ACTTGAAATGATTAAACCCTTTTTTTTTAAAGCTTCATGTCTCTGTTCTCCATCTGTAGG

A_14_P107927 AGCCAGTCCACTATAAAGAGTGATGATCACTATGATTATCTCGACATTGACTTGAACTAC

A_16_P21598330

AATCATCCTTGCTAATCTTCAAACGAATACTCCTGTATTTCTGAGAAGTTATTGGTGCCT

A_14_P115145 ACACAGCAGGCTGTAGATTGCACTATAGTGATCATAGGCATCTGATCTTAATGACTAC

A_14_P109888 CTCTGCTTCCAGATGGACACAGGTGTGAGCCATTTGTTTAGCAAAG

CUST_3670_PI426210822

TTCAGTGGTTGTTTTCATTCTTTCCTGTTAGCCAGTTTCCTCAAATTGGTCAGTATAAT

A_14_P108308

GAAACTCAGAAGAGGAGAGAAAAAGAGACAGGGCTAGAATAAGATTAGTATGATGTCACA

A_16_P19035804

AAACTAGTTTTCATCAGATCTGTGTCAACTAAGCATTGGATATCTGAATTACAGCCAGAA

A_14_P118326 GATCCCCAAAATGTTTGAGTATAGATACGTCTGCTGATTCCCCTCATTCTTTTAGTGT

A_16_P36573960

TCATCTTCACTAGTTCCCAAGTTTTTGAACACATTCCTTTAACCTAAAACTTTCCAGTCT

A_16_P16923218

AACCTTTCCCGGATGATAATTCTTCACTTCTCTACCTATTGGAATAAAAAAGGCATTACC

A_14_P112828 TGTGAGGCTGGATGAAGAAAATTATAAACCAAATATACATTGGAGCCAAGGTAAGCCAAT

A_16_P37298650

CCCTCCATTCTATACCAACTTCCTCCTAAAAATAAAAGAAACTAGTAACACAAAGACCCA

A_16_P02013639

ACGTTAGCCCTGATTTTCTTACTCTTGATTTCATACTGTTCAGTCAACATAGTTCTGGTT

A_14_P102496 GACACTCCTGAAATATGCACATTTTCAGAATGTTCTGCAAGAGCACCATTTCCTAAAATT

CUST_4673_PI426210822 AGGACATATGAGTGCATCTCAGGGTGAAAGCTGGCTAGGAGCTAT

CUST_9228_PI426210822

CTTTGCAACCCACCAAGCACAAAGTAAAATGTTTCTTCCACTAGAATGAGTCTAAAC

A_18_P14136578 GATTTACATCCCGCCCATACTCTGCTGTGAGAGAGATGGTATGCAG

A_14_P200164 AATATGGTGAAACCCAATTTCTTACATCATATCTGTGCTACCCTTTCCAAACAGCCTAAT

A_14_P137312 CTTTAACAATGAGGTTAAATTTTTCCCCCAGGCTTATGTGTTATGGACTTTTGTTTTTCC

A_14_P125225 TTTTGTTTGTTTTCTCTTTAAAGAATGGTTATGCTCCTGTTGAATTTTTCCTCGAAGGGA

A_16_P15369478

AAAGTTGCATCTCTACCCTAATTAGGCAAACCATTTTACCATTTAACTTTCTGAGGCTTC

A_16_P19921152

ACATACTGCATACAACAGGTTCATACTTCAGCATACTCAGTTAATGTGAGGGTCATTTTA

A_14_P115924 TGACTGTGTATGGTCCAGATAATCTCTGGTGTAGCATAAGATTATGTTAGAATTGAGGGA

A_14_P114358 TCATTAATTTCTTCTCTCTTAAAGTGACAACTTGGAGATGAAGGAAAACCCATCCATCTT

A_16_P30952640 AAAACTGTTACCTAAAGTATTTTTATAAATATAAGCCTTTTATACTGATTATGTCTTTTA

A_14_P139457 CTGTCTTTCAAAATGTGTGCACTGACCCCCTTGTTTGATCAAATTTGTGTAAAATTTTTT

A_14_P102347 ATTTTAATTTTCTGCAAACTCTATTTCACATTCTGCATTTGAGGATGCCTGTGACTTGGA

A_16_P00685242

CTCTAATATATATTCTTGGTTTAGATTTCTTTTCTGGAGAGGTGTCCCCAGACTGCTGTG

A_14_P202248 TTTTATAGATAAATAGAACATTCCAAAGAGATGGCCTCATCAGCAGTGAGACATCTTAGG

A_16_P16471370

AATAAATCTCTACTTGCAACTTGAACTACTGAGTCTCCATAGATGTTTCAGACCTGTGTC

A_14_P114717 CTGAAAGGGCTTTCCCCAGAGGCTAAATTTATAATCTAATAACATCATTTTTCATCTCCC

A_14_P126124 TATTGATAAATATGTCCAGAGGACAACATGGGTTCTTAATGGATGGCTGAGTCCAAATAT

A_14_P114072 TATGGACCACTGAAAACTAGGCTGTCTAAGACACTTCAGAATGCAAAAACATGCAATTAA

A_18_P17383398

TCCAAATATCAGTATTAGATAAACATCAGACTTTGTCATCAGACTTTGGATTTATGGGTT

A_14_P120733 TCCCTAATTCAGACCCAGCCTCAAGAGGAAAGGGAGTAAAATAAAACTAACTTGTTT

A_16_P41912704

ACAGCCATCTCTGTAATTACAATGATAATTGCTTCCAATGTAAAATACCACTTATCTGGC

A_14_P114790 CTTTCCGAAGAGACAAACAGTATCTTACAAGAGTTGTATTTGACAAATAACAGCCTCACA

A_14_P120987 GTAAATCTTCACTTTTCTTGAAATTGGTGAGAGATAAACTACAGGACAGAATGTCCCCTT

A_14_P201537 TAAAGCCTACAGTTTTGAATACTACTGAAATGACAAGTTGTAAGTTGGGTCTCCAAAGCA

A_14_P110746 TGTCATGGGTTAGCCATGGCACTGCGATTGATATTTAGTCCATATCTGTTGTTATATAAG

A_14_P123467 CAGCCTTAAATTTTCAGAGTCACTACACCGGAGGTGAGTTTACTAATCACAAATGCATTT

A_14_P131528 CTAGGGTTAAACAACAGAATGAGTTACTTAGGGAAGGTTAACCTATTTCCCATTTCTCAA

A_14_P107898 GCTGTATTGTAATATTACCAGGGTTGCAAAAGAAAAGCCTAAAATGGAGACCTAAATGCA

A_16_P01943010

GATGGGCTATTTACAGTTCCTTCTCTTTACCTCTAAGAAACATACCAACAGACACTAAGC

A_14_P133983 CTCTTTTATGCAGTGTGATGAAAAATGCACAAAAGCATATTTTCACATGGGAAAAGCCAA

A_14_P130065 ATCTGATGTTTTATAGGTCGAAGGTAGATCAGATTCAAGAGATTGTGACAGGAAATCCTA

CUST_255_PI426210822 TTCACCAGAAGCCTTAAGAGAGAGACGTGTTTCTATAGTGGCCAAGG

A_14_P114354 TGCTGCGGACAACTTCAACTGTCTACAATTATGATTACATTTGGGACTTTATCTTCTACC

A_14_P119130 CTTTAAATTGACCTGAGAACTAATATCACAGCAGTGGCAATTCCTGTTTCTTGGATTACA

A_14_P108990 TTTACAACACAACCTAGGCATTAACAAGCAAGGAAAACTAGAAATTGAAATGAAGCGGTT

A_16_P40104915

ACAGACACAAACTAGACCATGACCTTTAATTAAAAACCCACATGATCAACTTTCTGTGAT

A_16_P58424593 CTCAGGGAACTCTGTTCACTCCCATGCCTCTATCATGAGGATCTTTTATCAT

CUST_1382_PI426210822 ACTAGAGTTGCCAGGCCCCACAACTGCCCCCAAAGTGTGTGTGGC

A_16_P00915394
GGCCATTAGTGAAATGAATCTGTCAAGCATTTCTCTTTACCATGTTTTAGAATGTCGTCT
A_14_P112737 CAGGCTAGATATTCCAAACAGCACCTACCATATGACTCAGCCTGGACTTT
A_16_P18199176
AAATGGAATCAGAACATCACCAATCTGTTTATCCTCTTTCCAAACTATGTCTCCTACTTG
A_16_P18447685
ATTCCCATTGATAAGAGACTTCACAAATTCGAAAATAGGTTGAGATCAGTAATCCAGGTC
A_14_P129227 GGTCTAAGAAGTTCATTGACTTCATTGACACATGTCTCATCAAGACTTACCTGAGC
A_14_P133485 GTGAATGGGTATCTTTCCCGGAATAATCCTAATTTTTCTAAGGGTGAAGTTTGCAAC
A_14_P102043 TTGCTGCCTCCATCACCAGACTTACTCTGAATCTCCTTATTATCCAGATTTTCTATATCA
A_16_P15940463
ATCTGATACCTTAGTAACCCAAGTAGAATGTTCTTTCCTGACTAACAAAGACTCCATACC
A_14_P103483 AACACCCGATTTTAAAATGGCAATGCTGCTCTATTTCTTTAAGTAGTTAGGGAGTCTCTA
A_14_P106055 ATTTAGTGAGTGAATATGGATGGTAGCATCCTGAATCTAAGTCCACTTAGGATAATCCTG
CUST_8327_PI426210822 GATTTGCTGTGTCCCTTCCTCCTGAGATTTTCTTGCCAGCTCATC
A_18_P13903519
ATCCCCTTTATTTGAGAGCAAATCTTCTGAATATCCATTCAATGAAACTCAGATACCCTC
A_14_P120671 AGAAATACAAAAATTGCGAGGAATGGCTGGACTAGTGAAAGTTGTGATAAACTATGGTTT
A_16_P02356134
ATTATTTTAAAAGTCCACACCCAAAATGAAAAGACCATAGACTATACCAGGTTCTCACGG
A_16_P36998301
ACTCCAAATTTTTGTATTCTCACAACCTTGTAACTGTCTCATTGAACATACCACACTTCA
A_14_P129841 AATGTCCCCATAGAAGAGAATGATAGCAGTTAGATGTGAAGAAAAAGCAAGAAAACCTTT
A_14_P126267
CTAGAAAAGAAGATGAAAGCAAGGGCTTGCAAGAGGGAAATGAACATATTTTACTGATGG
A_14_P131859 TGAAAACCTTTAAAGCAGCACTAACAATACCTTCCTGGTCCTAAATATAACTATATCCAT
A_14_P131935 ATCAGTAAAGCAAGAGTTATGCCAAATGCTCATAGTAGGAGTTTACTTCGACCTCTTTTT
A_18_P13362508
AATTGTGGAGTAAGAACCATCAAAATCATGGGATTGTAGATTTTCTAACTGTTCACCTGG
A_18_P17123465
TACGAGCATTGCTACACTTATTGGTTCTCATCCATAGTTTTCTTAGCATAGACTCCACTT
A_16_P01159923
AAACAGAACTTATTTCTGCAAATTCTAAGGTCCTCTCTGTTTGAATTAGAAGTCCTGCAG
A_16_P16125584
AGTCCTTGGTAGTAATAGGTCTGTTAAGAAGAAAAACATTCTGAAACTTCTGGAACCTTC
A_18_P22840326 TTAGCAGAGTGAGCACAGATGTTCTCCCATTTGCCAACTGGTTTCTAAGAATATTTG
A_16_P01656082
AAGAGACACCAGTAATAGGAAATCAAAAATGGAACAATCAGAAAGATAGGAGAAAGTCGC

A_14_P125121 ACCTTTTAAGAATTTTGTAAACCGTTTGTCTGTCTTTTGTTACTGTTTTATGGTGCCAAG

CUST_6019_PI426210822
TAGTGGATGATCAAATCCATCTCTATTGAATTGTGCTTTTCTTATATTAAGATGTAAAAT

A_14_P129136 ATCTGAGCACTTACTGCATTCAGTTCAGTAGCAACAGACTGATCATTTGCTACAAGAG

A_14_P122631 CTTTTTTGGTCTTTGTATACATGCTGTCACAATGCAGTTCTTCCTTTGTCTCTTTATAGC

A_14_P120127 AGCATTTATCTGTTCAGCACGATTAACTGAATACCTGGTCGCTACTAGGAGGAAAA

CUST_9254_PI426210822
AATTCTTGGTAGGATAAATTAGTGAGCTGTTTTCTCCCAGTTGCTTGACTTAAAGGC

A_14_P108729 CATGGGAAATCACTGAGACAATTGGCAAAGGAACTTATGGGAAAGTTTTTAAAGTATTGA

A_14_P126206 ATTTTTAAACAGAAGATTCTATTTCATTACAAGGACTGCAGATCCAAGCCTTGTCACCTA

A_16_P18773910
AAATTATTGCTCTTTCCAGGATTTAAATACGGACACCATAGAAAGATTGCTGTTGTCACC

CUST_2134_PI426210822 CACTGGGGGCAATGTTACCCAGAGGAGATGAAAATATGTGATATCTGTCT

A_16_P41239586
GAAAAAAGTATCATGTCAATGAATTTTTCCCGACTGAAGTAAGATCACATCGAAGCCAAC

A_14_P106389 TAGAGAATAGAGAACCATGTCAGAGCAAAATTTCTATCCATAAGGTCCATTCCTTCTGTT

CUST_6768_PI426210822 ATGTTGGACAGAATCCTTGGATAAGTGCTTAAGGTACATGGATGAGCCTC

A_14_P114036 GAATGTAGGAAGACTTTGAGTTGATGTTTTGATGGTCCAAGTATCTTAATGTGGGAACTA

CUST_2997_PI426210822
TGACTTTGCTTAAGCGAACTTGGAAATTCACTTCATTTAAATGTAATCTTTCTTACTTCA

A_14_P135399 TGTCTGTATCCTTTTCTCCTTTTTCTTGTGATTCCCTCTTTTTTCTTCTGAGTGTCATAT

A_14_P119204 GGTTTTTAAAAAGGAATGAATTATCACTTACCCATTGGCTGAGTTGGATCCTGTAACAAT

A_14_P130088 TATAATCTATTGAATGGTGCAGTTAGGTTGCTCCTCAGTTTTTCTGCCCACTTCAAAATA

A_14_P201454 TAGAGGCCAGAACAAGGTTCTACTCCCTGTGGTAATAAATGAAAAATACAAGAATAAAGC

A_14_P139243 GTAGTGCATGCTTAAGAGAGTTGATTCTAGGAGATTCTGATATGCTTGGGCTGAGT

A_14_P139105 ATTAAGTTTGAAGGACTGTCACGCAAACAAATTGGTCTTTCATTTGGAGTAATAGAGGAT

A_16_P17261231
CATCCTGTTGCCTAATCTTAGACTTGTCCTATTTGTGAAGCATTACCATTTTTGGAGTTT

A_16_P15019659
AAAACTTTGTCATTTTTCACCCAATGAAGTAGCCTAGTGAAAAAGATATATGGGTCTGGG

A_16_P17429819
TCAAAGTCAACCTTCCATACTTCTATCTATTCTTGCAACGGATGTTACATTTACTGTGGT

A_18_P14133694 ACTAAATAGGGACCAAATAAGTGAGTCCATGCTTTGGAAATGATGACCTGAGAGT

CUST_316_PI426210822 TATTTCAGGGGTCCATGGGAACCCATGTGTCTGGGCTGCTGAGCT

A_14_P102314 TAGGAACGAGTGGAATATCTCTTTCTCATAATTTTTACGGTGGAAGCGTTTTTAAAAGTA

A_14_P200488
GAAAACATGAAATAGGGAACTCCAGACTGACACAGCAGTTGTTTTTGAAAAGGAAATAAA

CUST_7339_PI426210822 GTGTGGACATTTCCATGTGTCCTCCTGAAACCCTCTGGTGAGCAT

A_16_P03524252
AAAAGAGAAAGGAAGAAATCCCTTTTTAATTCCTCACTCTGATGATTCTACCCTTCATGA
A_14_P131928 ATTCACAGTGATGTCTGAGGAGATATTCATGATCTGAGGAGGAACTGTAAGGAAAC
CUST_8803_PI426210822
ATCTGAGAACTTTAGTCCTAATAATTTCTCTACACCAACTTCTAGTCCTAATAATTTATC
CUST_1049_PI426210822 ATGGATTGATTTTTCCTCATCCCCCTACCCACCTCATGCCCTAAG
A_14_P201279 CTCCAAAGGTGGAATAGATAGTGTAGCCATGATCCTTGCTAATGGTGCATG
A_14_P107480 CTCGATGAAGCCCAATAAACCTCTTTTCTCTGAAATGCTGTCTGCTTGTGTG
A_14_P126795 CTAATACATTAAAATGAGGAGTAGGAGGAGGTCCCATTGCCATGGGTCTACTTT
A_14_P137875 ATCTAGTCTCCTAGACCAACTTCCTCAAGTTAAACTAGTTTTGTGTATTTTCTTTCCCAC
A_14_P105324 GGTTGTTCACCGTAAACTGTAATACAATCCTGTTTATGGATTTGTTTGCATATTTTTCCC
CUST_8977_PI426210822
CACCTTAGGTCAGGCTAATCAACCAGCTGGTATCATTTCTCAGTTATTGTATGACTA
A_16_P20223783
TACTCCCATACACCTCTTAAATTCTAACCAAACATTGCTATTTTTATGCAGTTCTGCCAC
A_14_P113945 TATGCCCACGAATATACCTGAGACAGAAGTTTAAGGCATTAGAGATACTTTCTATGGGC
A_14_P110328 TCAGAAGGTCAGGCACAAAGTCACTCCTTGACCCTATTTATTGAAATATCCGTTTTTTT
CUST_7821_PI426210822 CATGAGTCATGAACCATAAGCTCAGCTCTCTTGTTCCTAGAGATGATGTCTTTG
A_16_P16191457
AAAATGCAAATGCTAGTTTAGTAAATGGCATAAAGACATTGAAGTGTCCCTGGACATCAG
A_18_P12802140
CTCAACTAACTTCCCTATGCTCTTTCCTTATTACATGAATCACATTAGGTTAGGCTGTGT
A_14_P112997
TGTAGTTAATACTGGAAAGAGAATTGGGAACTCTGAACAGCAGTCTTAGACACAAAAACA
A_14_P114927 CAGACTTTGTTCTGAATTTCTCTTTGCTACTTGATAAACCTCTTTTCAGACCATGATGCT
A_14_P107832 CTCTAATTTTATTCTGGGATTCTGTCAATCTTGCAGTCTTCCACATCACAGAAAGAAGTT
A_14_P122643 CAGACAAAGATATGCAGATCTGCCTGGAGAACTGCACATTATTGAACTTGAAAAAGATAA
A_14_P107840 ACCATATGCCATTCTGTTTCAGCATGAAAACAGCCTTTTTTTCCTCATCATAAACGTAAT
CUST_4178_PI426210822
GAGAGCTTTTACATAATTATCCATTTAATGTGTTTTTAATGATAAAGAATCTAATAGTTA
A_14_P132910 CGTTCTAAAATGCACTTGTAGTTAATATCTTTGGAGTCTTGCTTCCAGGTTTTTGAGTTG
CUST_5008_PI426210822
GGAAGATAGTGACTTATAAAAACCTCAAACATTTATGTTTCCCTTTCCAATATAAAAAGT
CUST_7697_PI426210822 ACACCTGTGTGTATTCAAAGCACCATGTCATGTTCTGTAGGGAGATAGG
A_16_P03023606
GTTCAGAGAATGTTCTCAACCAGAGTAGATACCAAACTGAGCAATACTATGTCACAAAGG
A_16_P16458349
AAAAAACGTCTTTAAAAAAAATAATCACTCCCCTTAATGATGAGTTTAATGCAAGGTCCT

A_14_P119426 CTGATACATTTAAAGAGTCTTAAAGAAGGAAAACGGGCTCAGCAAGATTGAGTCTATTCT

A_14_P102481 TTGTTCATCTCATGATTCCCTACATCTGACATAAGGAAAGTAAGTGCTCAGAAAAATGTG

A_14_P126852 AAATCTCTTTCTAGGACTCATCTCTGCTTCTACAATCAACCCAGAATGTGACTGTAAATG

A_14_P112098 GATTATTGGCTCTGTGAAGTTTTGTTTTTAACTGGCACTGTATGGGATCCTTTATCTTTT

A_16_P17177864

ATAACTCATGGAATACTGAAACAAAATGTCAAGCCGTTAACAGTTTTACTCTGTGTGCAC

A_16_P17001526

AGTCTCTTCCATTTTCAATATGTAACTGAGGCATCCTTCTACTGATAACAGCCAAAACTT

A_14_P121080 CCTGCCTTTCATTGCAACACGGTGTGTTCATTTGTTTAAAACAGTTTAATGAGTAAGTTT

A_14_P117760 ATAACAATCCTCTTTTGACCTAATATTCCCTCTTTTCTTCAGGATCTGACTTCTTGCATT

A_18_P16253036

AAAAACCTCTTTCGCCTATTTAATGATGCCTCCATTCTAAACTCTTTGTTCCAAAATTGG

A_14_P109239 ACTTTAAAAAACACTGGACCCTTTATCTAAGTCTCTCTGGAGATGACAATGGTAAGAGG

A_16_P21200296

AATTTTATAATGATAACAGAACCTGGTCCGACTTCTGTGGAAAAGGATTGATCAACAAGC

A_14_P201194 AGGCTTGATGAAGAATAGCATCAATTTAGAATTGGACTTCTCAAGAATTCATCTGTGTGG

A_18_P15908009

AACCCAGCAATGCCTTATAAAATATCTTCACAATGATTTGTCCAAGATTAAGTGGTAGCG

A_16_P01892213

CCTCATAGTCTGCATCTTCAAACTTTTTCCATACTGTAAAACTGTCTTCGCAAAATCTCA

CUST_2775_PI426210822 CTTCAGCTTCCATGGCAAACACGTTCCAGGAGAGATTGGTAATGA

A_14_P106035 GACTTCTTGGTTCAGATCCTGAAACTCTTTATACTGCTCTTTATGTGGTTCTAATGCAAG

CUST_6575_PI426210822 AGAGGAATGAGTTTTGAAGGGATAGGAGTTGCTGGGTTCTCATACCA

A_16_P20896905

CATCAACACCAAAAATGTCACAGTATCAGTGTTCTCAATAGTTCCAGTCACATGTTTAGT

A_16_P20107037

TACTATTCCCCCTTTTATTAAAAAATGGAGCAGACACATGATTAAAATGAGGACCAGGTC

A_16_P19870716

AAAACCCAGAGCAAATGTCTTTAGATTGTATGCATCATCTGTTACTAGCACTCTCGTTTT

CUST_6187_PI426210822 ATGTGTCCACAGCACGTCCACAGGCAGAGAGGATAAGATCCCTGA

CUST_8855_PI426210822 CCAAATGTCTTGTGCTCACAGCTGCTGTGACATCTGGAAGCATTAAC

A_14_P120231 AAGGATTTACTGTCCCATGAGTTTCAGGATCAAGAAACAAATAGTTAAATGGCATGTGAA

A_14_P105862 CTCATTACTGACATCTATTTTTTGTCTGCTCTTTGCTTTTATTGATTTTTTTCCCCCACC

A_16_P39623705

GAATCACAATCCCTTCTTGAATCATTCCACACTTTGTTGAAAACATTTTCTTCAAACCAC

A_14_P104465 CTTCTATTTCTTGGAGACACATTGAGAGAAAAATGCAGAAATAATTTTTACCCCCTGGAT

CUST_3088_PI426210822

GATCTCTGCAGATGTTCACTTGGAGCTGGTGGAAACTATTTTTATTATGTATTATGTGTG

A_18_P14137093
ATAGTCAGCCTTAGGGAAACACTGAAGAATTTATTTTTAAACACATAAAGATTGCATTCA
A_14_P134478 CTTTCAGCACACCACAAGGCACGATGTAAGAAATGAACAAATCAATAAACCTATTACTGA
A_14_P101285 TGTAGATGTTACATGCTCTTGCTGTTAAATACGATATATAGCCCACTTCTTTCTACCCCT
CUST_1033_PI426210822 ATGCACCCCAAGACCCTTGGCCATGAACCTTCCTTCAGCTTTCTC
A_16_P01869401
AATGAGGATCCCTGTTAAAACATCACTGAACATTAGAATGTAATTTAATGGCTGAGTGGC
A_16_P00408867
AATGCTAGATAGGACCAATCATCATCTACTAACTTACACAACTGACATCAGTATTCCCCT
A_14_P115536 TGATATCTTTCCACTTTGCCTTCGGAGGAGAAATTACACACTAAGTTTATGTCCTTAGAT
A_16_P17470631
CTCAAGGTCTTTCCAAGTAGTCATTAGACAAACCATACCATTGATTCTCCAGTTTTTCAG
A_14_P106384 TGACTATTTTGTAACCTACCCTTTCAAAATGAAACCTTTAGCATGCTTTCATGCTTTTGC
A_14_P100397 TTCTATCATGTCCTTGTTCAGCATTCCAGACAAAATCCAATCCTTATGGTCTCTACAATA
A_16_P40310121
ACTCATGGACTCATGTTGATTGGTGTCAGAATAAAGACTATAATCAGTCCAATTCTGTGT
CUST_103_PI426210822 ACATGATGGCTGGTCTACCTACCTCAGTTGGCAAAGTCCCCTCAA
A_14_P125452 TATTTATAACAACATTTTCAGCGTGGCAACTGCAGTTTCAGAATGGTGGAATTATACCAG
A_14_P118875 AGAAATCCGTTCTTAAGTGAAATATTGATAGGTTTATTCTGCCATCTTAAGCATGCCTCC
A_16_P00866590
ACAAGCATATCTTTACACTGGGCCTTTCTTAGTTGAAGTTCATTATGGAAACAGCAATTC
A_14_P100522 CGGTATATTCGGAGAGAAGACATGGTTTCTCTGTTTTATCATAAATCTTGGTAGGGAGAT
A_14_P129774 ATTTCTATGTGCGTTTGTAGACAGCATCAGTGTTAACAATTTTGGCAGCCGAACTC
A_14_P134402
GATTTTGAAAGGAAAAAAACCCTTGGAACAGGTTCATTTGGAAGAGTCATGTTGGTAAAA
A_16_P16191628 TAAACCACAGGGGATTACCTATGTTTATCTGGGGCAAGTTGCAGGATAAC
A_14_P136791 GTAAATTTTTTTTCCCAAGTCCAGACAGAGAAACAGCCAGTAACTTTTTACTTTTGGATG
A_14_P137883 CTGCTGAACCCATCTGGATCATGCTGGCTTAAACTCTTCGTTTGTATCCTATT
A_14_P119567 TCAATTCCTCATCTAGAGATTCTGTCCCCTAGGATCATGTAAGTCATTTATGCTAGGTAA
A_14_P103949 AATATCCTTCTTTTTTCAAAGGATATTTCCAGGCTACCATCCTCAATCCAGATCATTCAA
A_14_P108305 ACTGATTATAAATAGTATAAAACATTGTTGAGGACTCTTTCTTGGAGTTGCCATTTGGAA
CUST_10273_PI426210822 GTACCCAGCTGTGTGGCCCACAGTCTAGAGCCTTGAATTCACTTT
A_14_P135706 GATTAGATAGGAGGGTCCTTTAGCCAGGGGCAACGTTCACAATATTTATCAG
A_18_P22841742
CACTTCACATGGGAATCCTAGGACTCCATATTATATTCTTATCCTGTTGAAAATTTGACC
A_18_P15772563
AACAGAACTTTCTATCTCTGGTCTCATTTTTTCTAACCAACTTTAACATGTCCTAGCAGC
A_16_P00506763

TGTTTCCTGATAACATTTATTCCTGATACTCTGCAACTCTCAACTCTTATGACAATCCCT
A_14_P103261 ACATTTAACTTGATACTTACTCATCTGGATTCTTTCCTGCATTGGCATAAGGATTCTCTC
A_16_P19874361
AAATCCGCATTTCTCCAGATAGTAGTGATTTCAAACCAAGCAAAACTTATTCCCGTTTAA
A_14_P108095 CTTGGCGGATTTCATCATTGCTGTAAATGTGATAATTTCAGTTCTATCTAGTCGGCTACA
CUST_9331_PI426210822 TGAAGCCTTCAAGGCTCATTCTCCATGTGCCCTTTCAGCAGAAAC
A_16_P20608202
TTGGTATCTGTGTTGGATAAGAATCCTTAATTCTATAACCTGAATAGTCAATCTGCCCCA
A_16_P31224758
TGCCAAATTCAACATATAAAGCAATTACCCACAATTTTTACCTTTCCAGTGTTTTGAAAG
A_14_P131217 TTTTTACTTGTAATTCCTTTCCAGCCCCATTATAATGGCAAAACACTGCAATCTGAATGT
A_16_P18531598
ACGTAATCTTGGTGAATCACAAAAATTCAAACTGTATACTTGTATCCTACCTCAAACCCC
A_14_P120328 ACATTTAAGAACAAAAACACTTCAGACACATGCACAAAAACCGGTTATATGACCTTCTTG
A_14_P108939 TCTAAATAAACAGAGACCTTTTTCTCTTGGTTGAGCCTTTCCTCTTCTTTTCTGTAACAA
A_16_P38560876
GTTTTCCTCAACAACCATATAATCTACAACCTTCTATCCATAATATGGTCAACTCTGCCC
A_14_P110260 TACAGAAACGTGAGAGGACAGGAACAGTTAGTCTGTGTAAATGTCTGAAATATATGTGAG
A_14_P134001 CTACGCAGTAAAGTAATTGCAGGCTGTGTCTAACTACAATTTGCAAATGGGATTTTGAAA
A_14_P124225 AACACATTCCCTACTGCTAGGATTCCAGCAGCAACTATCATGTTTATAAATGTAGCAAAT
A_14_P112897 TCCTCATCCCCATTCCTATCACCACAATTATTGTTATTATTGTCATCCTTATGGATGTTG
A_14_P111400 TTCTTCAAATAGGTTATGTGGGTATGCAATTTATGTCGAAAGCAACAAGAAATCTTAACC
A_14_P133328 GACAAACAATGTTTTCCCCCTAAGATAAATTAATTACATTATCCTGATTGGAGGGCAGGA
A_14_P129199 CACTGGCAAGCCAAGAATCAATCTGCATATGGATTTCATTCTACCCAGTGTTTTAAC
A_16_P00000136
ATAGTGCAGAGATCAAGGAGTATGGACAGGACTTACAAGTTCTTACAAAGGGAAATAGAG
A_16_P00685253 CTTGCTTCATTTCTGAAAAAGCAGGTTGTGGCCACCCTTTCATGAGC
A_14_P134514 ACAAGATCAGTGGTAATTTGCTTTTTGGTAACCATGAGAACATTAAGCCATTTGTTTCTG
A_16_P00470495
AAAACTTGGACATCACTGAAATTTTGCTGATCACTATACTTAACCTCTCTATGCTCTCCA
A_16_P03424197
CTCTGATGTTCAATTCCCCTACTTTGTATATTGAGTCAGTATGAGATGCGATTTGTTTGG
A_14_P136633 ACAATTTAAAAAAAGAGTAATACTAACATGCTGTGTTCTTACCCCTATTGTTCCAAAACT
A_16_P20805954
TATTGCCTATGCCTATGACATAGTAATACCCTATGACTCAAACTACATTTCCCATTCCAG
A_16_P16577672
AGGCTCTGTTAGTTTTTCTATTTTCTGACAATGCTACTAGTTTGCAACATACCCTCGAAA
A_14_P139180 CAGCACTTGTGTGAGTAAATATGAAGGATCCAGGGTAGATAACAGCAAATATACTGTTTG

A_16_P30952579 TTCTCAGGACTGTGAAAAACCAGGAATTTCTTGCCTAACAGCACACTGTAACTTTA

A_18_P13084043

CCAAAGCCATCCTAAATGATACAATGCTATTCAGTTACTCAACAAAGACCAAAATGCTCT

CUST_5313_PI426210822

TGAATGGCTGCATTTAGAAACAGCCCTTACTTCAAGCCCCTATTTCTTAATTATTTGTC

A_14_P112148 TCCTTTCACTCATTTTAGTCTGTCAGGAGAAATCAGAAGAAGACTCTATGGATTATGGAT

A_14_P109542 TCTTTGGATGCTACTTGAGTTAGCATCCTGCCAAGCAATATGAATTCTCCTTTTTAAATC

CUST_6798_PI426210822 AGAGGAGGCCATGCTAGGAGAGAAGGGAGAACAAGCAGGGCAGGC

A_14_P133771 TATCTTTACATAGCCCAGTTAATAATCAGGCTCCATGCTCTGCTATGAAATTGACTGTTG

A_16_P02175724

TGTGAATTGTAAAGCACATTCAAAAATCTAGTTGTTACCATCAATAGCCCTTGACTCTCC

A_14_P127623 GGAGGCATACAAAAATATTTGTCATAATTACTTATGGTAGTGAGACTTGGGTGGGGAAAG

A_16_P36230905 TATATGGTGAACACCATTTACTTAGGGCAGTGCCCCCTACTCACAG

A_16_P03123240

ATTCTACCAAATGGACTTCTTTTCACCAAATCCTCTAACAAAGCAAATAGCAATAGAGCG

A_16_P03315414

AGCGTCCTGATTAAATCAAAGATACTTAGTCCTCTAACCTGAAAGTTGAGAGAAAAGGAA

CUST_8874_PI426210822

TCAGACCAAAAAGAACTGCCTCAATGTTGTTAGACGCTGAGAAGTAATGATCTC

A_16_P19299667

GTTACCGGAGGATAATCATATGAACTTAGCAGGTAAATGACTTCACATCCATCTCAAGTT

A_14_P139142 GCTATGTTTCTTATGGATTTCATTTCAGTGCTAGTGATGCCATAAAAGGAGAAGGCTTG

A_18_P16201648

TGATCTTGGAGCCGCATTTTAAACAGTTTATTCCAAATTTACTCTCTTTGTTTGAAGTCA

A_14_P100991 ACGATAAATTATTTTTGAGGACTGTATATAGTGATGTGCTAGAACTTTCTAGGCTGAGTC

A_14_P115654 AACTGACACAGAGAAGAACATTTTCTCAGGCTCGTAGAGAGGTGGGATAG

CUST_9714_PI426210822 AAAGTTGTATTCTCAGAAGGTTGCCCAGTCCATCAAAGGCTTTCACTAGAT

A_16_P16663802

AAAACAAACTTCCAATTATGGCCTCTCTAATCTATGAAGACACAGTCTAACCTTTGTTGG

A_16_P21542139

GCTTACAGACATCACTAGCAGTAATTATCAAATTCCAGACTAAACCTACCTTTTGCTTCC

A_16_P16531804

GGCAGGATATTGGAGATAAAGAACGTAACTAGCAAAAGAGCAATACTACTTAGGGGTTTT

CUST_2260_PI426210822 ATGTTGATGGGGAAATGAGTGAGGAGAGAATTAGAAACCCAGACATCTTCT

A_14_P131747 CTAATCACAGTCCAGAATTAGAAGACGACAGAGAGATTGAAGAAGAGCCTTTATCTGAAG

A_14_P114795 AGACTAGGAAAAGCAAACAATAAACGAATACTCAAATGAATGCATCATACGATGTCAGGG

A_14_P104462 GGCCTTAAATCTTCTATGACAAGTTTAAGTGCAGGATGCACAATGACATTTTAAAGAGGA

A_18_P16006384

GGCCTTACCCAAAATTTTTACCCTAGACATTAGTATTGTATACATTTTACACCCAGTTCC
A_14_P103278 ACTGGGCCAACACTGATGATCGTAATAGCCATATGCACATGTAATAGAAATTAAAGAGTT
A_14_P107948 ATCATGTGTGGTATATTCACAAATGCAGACAAAGAAAATTCTAGAAAGCCAGGGGTGAAA
A_18_P12812354
TTAATGCAAGAGAAAAAGATTGAAAACCTTGACCCTTTAGATTACTCCGAACCTCAATGA
CUST_299_PI426210822 AGGCTGAGGCTGGCTCAACTGCCTTGGAGCTCTTTGACAGCTAGA
A_14_P127562 AACCAACTAGAGAGGAATGAAACATTTAGTTGGCAATAAACTGTCCACATACATGTGTAG
A_14_P124357 GCTCTGCTTTCTGGACAGATAGAATTACTCATGTGTATGTGTCAACCAACCAAATTAAAT
A_14_P109791 CTCCTAGAACTCGTGCCAGCGAGAACTTGGTAAATGTTAGTTGATTAAATATTAGTCAAG
A_16_P03645713
CACTTCCTTTCTTCAAATATGACTTAAAAGCAGACTTGAAAATGAATCAGATGACCCTGG
A_18_P22839399 GACTGATGTGCCTTTGTGGCCGAAGCACCCGGAAAGTGCCATTAC
A_14_P126469 TGGACTGGAAATCAATTTCCAACGACACCCATACTCAGCATTTTTAAAAGTAATTTTGTC
A_14_P129107 TAATTAAAATTTCCTTAAGAGGAGTCACTTTTAAAACTATGTAATAACCGCCGGGCGTGG
A_14_P130952 TCACAGGCTACACATTTTATTGCTTCAGGTTTATTTTGCACTAAACAGGTATCACAATCC
CUST_1311_PI426210822
CTTGTAAACTGAGGGTAGTTTTATTCTCTCTTCATTTTTCTGTATAATTGCCCTTTTTTT
CUST_1239_PI426210822
AAATACTGTTGAAGATCATATTGGCACAATTTGGTTGGTAAGTGTTCCTTCCCTCTT
A_14_P137138 CTGGGTGATGCGGTTTTCATAATAATCAACTGGGTGAGAGTGATGATAATAAAATTTGAC
A_14_P120424 GTATTTTTGTTTTCCCCACTAAACCCACTAACTCCATTATGCTATGCATAAATTCCATGC
A_14_P103085 ATACACTTTTGGTTCCAGAAGTAACACTTGACACTCTTCTTGCAGAGAGGAAGAA
A_16_P16191078
AAAACATAACTGATTTACCCACAAATTAGATAACAATAAGTGATTCAAGTTTGCGAGCCA
CUST_2378_PI426210822
TCCCAAGCTGCTAATATGCATATGATAAGGGCAGAGATTTAAAAGAAAAAAAATTCTTTA
CUST_3282_PI426210822
AGATTAAGTTGGAGTTAATAAGAACTCTGAGAAAGAGGAGAAAGAAAAACAGCAAAAGTT
A_16_P00685111
CTCACCTTGACTGTTTTCAAGGTGTAAATTGTGTTTATAGTGAAAAAGAAGACTTGGTCA
A_14_P102493 AAAGAGAAACGTAAAAACAGATAGAGATTCTGCCTGTGCTTTTGTTTGACTGTCTCTAGA
A_16_P36230499
AGTAGCATATCTATTTTCTCATTTTGCATTACAATAGCATCTAGTAACATTCAAGCCTCA
A_14_P117020 TAAATCAGTGGTTGAAGTGATTGAATACATGGGAATGGGAATATCAGTGAAGTATTTTGG
A_14_P125645 TGATTAGGGGAAAACAGGTCCATCAGTTTCTCAAAAAAAGTAAAAGTGGGTTATAATTGG
A_14_P125019 CTCACCTTTTCATGATGATACCAAAGGTCTAATTCCCTCACTTGTCTCTCTATTGCTAG
A_14_P115525 CAAGATAGTCTGAGATTAGAGAGTGCTTTGACTAGACAAAACTTAGAACCATTTCTGTGC
A_16_P21508332

AAATCAAGCAAGGGCATTAATTTTGAACATTAAACTCATGGTAACTGAATGCACAACAGT

A_14_P107814 AATTTAGAGGGGACATACATGGCAGAAATTTAAGCAATGAGGAGATGATACAGGCAG

A_16_P01331861

AAAACATCGAGTAAACTTTCATCCATTCAGGGATCAACTAATCCAATCCATGAGCATCTT

A_14_P103747 TGTATGAAACCAGTGCATGATATAAAACTATAAGGATTGCACTGAGGATCAGGAGACTTT

A_14_P128180 ATGAAGTCCTAATACTTACAGTTGATTTCCATTCAGTATTCGGTATTTTGCTCACCTGAG

CUST_4210_PI426210822 TCCTGTATGGCCATTCTAAATCAACTCAGGTCTGACCTTTGGAAATACGTAC

A_14_P116861 ATTGAGTTTAGAAGTGAGTGTAATCCAGCAATACAGTTTACTGGTTTAGTTGGTGGGTTA

CUST_4877_PI426210822 GGCCTTGTGTTGCTTTTGGGAATGGTGTAGAAATCACGTGCTTAGT

CUST_2742_PI426210822

GGACTCCACTTATCAAGTTCTTTTAGGAAATTACCCAATGAATTGGTGGAGGTAAAT

A_16_P16191053

ATTTTCTACTCTTTGAACATCAGTGTGGATTTTGTTTGAAGTCTCATTGTCCAAGACCTT

CUST_2769_PI426210822 CTGCAGAGCAAGGCCTGTCATTACGGTGGATGTCTCCATCTTCCT

A_16_P01810120

ATCTAGCAATTTCACTATCAACTCCAACAAATTCACTCTTAAAGCCAGAAAAGCATTCCA

A_14_P129353 CATCTCAGCCATGAAACATTCATACTAGTGCAAGCATGTATATCCTTGTGTTAACACTTC

A_14_P124778 CATGGGAGAAGAGGCATTGATGAGGAAAAATCTACAAAGAAAGCAAATATCAATTTGTCA

A_14_P104716 ACAAAAAGAGATCAAAAAGTCCTGTAACCTGAAACTTCCCTCGCATCCTAACC

A_14_P116228 CTGCTTTGAGATATGTATTTCAGTTCACAAAACCAATTACTGAGGCTCTATCTGCTAGGT

A_14_P112821 AAGTAATTTCAGATCTTGAATAGCAAGTCTCTTTACTGCCTTCCTGGGATCATTCTTCAA

CUST_6376_PI426210822 ACCTGAGAAGGAGGACAGGGTCAGATAAGGTGGTAAGCTTTGAAC

CUST_775_PI426210822 CTTACTGCCATTTCCCCCGTGTTCTGCACTCACACAGGCCCAAAC

A_18_P22842495 GAAGAGGTTTCAGGAGAGAAAAGTATATGTGTGGGGGCGTCAGAACAT

A_14_P133532 TTTAAAAAACTGAGATTTGAGTTCTCTGTGTCAAAACCACCATCTGTATTTGGGGAATTC

A_16_P20481740

AAGTCTAAATCACAGAGCCTCATGATTAAAAACCCACGTATTTTAAAGTCTCACTTCGTG

A_14_P118197 AAAAGTTCCACCAGAGACATTTATTCTTCTTGCCTGAGGAAGGTGAGAACAG

A_16_P00685511 ATCATTTCCAGCAAGTCCACTTGGTGTAGACTGTGGACCTCATGAAG

A_14_P131259

GGCCCAACACTCTGTGTTTAGAAAGAGAATTTTAAAACACAGTCAAAAGAACGGAAATCA

CUST_4711_PI426210822 ATTCACAAGCAGAGGGCCTACCCTTAGGCCCCAAGTGTGTCAAGC

CUST_9205_PI426210822 AGGAAAGGTGAATTGGGGTTCTACCACTCCTGCCTCTACAATAAGA

A_18_P13977749

AATTCTTTCGTGTTGACTTCATTAAGTTTCCGTTGAAGAATTCCTCTAGGTATTACCGTG

A_14_P116500 GAGAAGAAATTGACCCCAAACATCATCTGCACCGATGACATCTTATAGGCAAA

A_16_P18492045

AAAACTACTCCATCTGAATGTTTTAGGGGCATCTTATATACGGAGTCTGTTTGAAAAACG

CUST_10067_PI426210822
ACTAGAAGTTTTGTTCTATTTTAAAGTAGATGAAACCTCATCTCTCTCCCATAGTGTCAT
A_14_P126590 CTTTAATCATGTCCATAATTCACTCTACTCTGGATGAAAATAAATTGTGCCCAGAGGATC
A_14_P100580 TCCAGATTACTCAGAAATACTTCTTTTTTGTAGAACATTTTTGTGGGCTCATGCTCTGTT
A_14_P127853 CTTGAAAAGTTTTTGAAGGGAATATTGGGAGGCGTTTTTTTTCCATGTTTAGATAGTCAC
A_14_P109678 AAAGTATTGTTTCTGATCTGGGATCAAGAACTTATTTTGGAAATTAGCACCCAACACTGA
A_14_P108982 TTTGCCTTTTCTGTTTCCTATATTCACATAATCAGAAGAGCCTGTTTTTTTAAAGGCTGG
A_16_P01709038
CCCAAGTCTGCTGTTATTAATCCAGATACCGTTGACATAGAAGTATTGTGATCAAGTGTA
A_14_P137114 GACCAGTCACCTACTTTCTGATCAATAAAAATGTCGACATGCATTTCTTACAGGGCT
CUST_2066_PI426210822 CATCGGACACACACCCCAAAGCATGTCATTGCCATATTGAAGTTTTATTGAA
A_14_P127823 TCTCTTGCATTGCAAGGGTAAATAAAAGTTACATGCCTATCAGGAAATCAAGAGTTTCAG
A_14_P130258 CGAGCGTCCAGCATATATCAAGTCCCAGGCTAAGGAAATACGAAAAAATCTAATACATAA
A_14_P116217 TTACTTTTAATTGCCCTGAAAAGCAAATACTTCCTAACGGCAGTAATGTGACTATGACCA
A_14_P123135 GTCAAGCCAGCAGACATCAGGACTTACTTTCTAATTAAGCCCAGAGTCACTTTAAAAA
CUST_1094_PI426210822 CTTCTGGGTACTGAGGTGACACAGGTAGGGTGCTCTGGGAAACGT
CUST_2005_PI426210822 AATGGCTCTGCCTTCCCGAGAACTCTGCTGGCTTGTGTTATTTTA
CUST_2459_PI426210822
ACATTATCATGGTAGTCTAGGTGGATTTTTTTCATTAACACAGAACTTAGAAAAAATTTT
A_16_P16876684
ACATTGATCAGCCTTATTCTCATAATCCTCAAATTACAATTCTGATCTTGAACCCCAAGC
A_16_P16144786
ACTGATCATATACAGTTTCGAGATATTTCCTATCCTCCTAATCCTTACACCTGTCACTCT
A_18_P17401494
CAACCTGACAGTCTTAATTGTTGCCTAGAGATGTTACAAAATTGGCTTTCTAAAGTCTGA
A_18_P14136561 GAAGTTCTCAAATCTCCCATTTTTTGGAGACCTTGTCATAAAGAGACCTTGAGACT
A_14_P104130 TATAGAAGAAGAATCTTTTTTCCCCCTTTCTTTTTTGAAGGGAGATGAGGCAGTCATATT
A_16_P02446221
ATGCTATGTAGAGGAAAGTAATGAAGTCTTCCCAAGTATTCCATACCTACCATTTCTCAG
A_18_P22842082
AAATATTCTGTTTATAATATCTAACCCTCCTATCAGATCCTAGGCCATTTGGAAGCCAGG
A_14_P121458 GATCTCTCATCAACAATAACACTGAACCCTCCTCTCTTTCCACAAATTGACACAGATATA
A_14_P102483 ACTGGTTTTTCTTCACTTGTCAACAAAATGGAAATGGTATTTAAAATGGAAAATGGCAGG
A_14_P120009 AACTACTACCATGGGCAAAGTTCTTGTGAAATTACTTGGGAGTTCTCACATGGTAATTAA
CUST_6251_PI426210822 AGTTTGCTGCAATTGCAGTGAGAGGGAAGGTACCTTGTAGAAAACCT
A_14_P104569 CTTCTATGTAAGAGATTTAATGTCCCTGACCCTTATCCAGAGTAAGTTGGATAAACCTTT
A_16_P18894964
ACCAGTTCCACTTTAATCAAAGCATTTTACTTGACTTGTAACCAATCACCTATGAAACCA

A_14_P124746 CTTGCGTTATCACTTGACATCCCTGAAATCCATTTCTAAGGTAAAATGGAAGTCTAATTG

A_14_P130742 AACTTTATTCAGCCATATAATCATTATGCTGGGTCCTGCCCTCTTTGCCAC

A_14_P128886 ATTTCAAAGGCAGTTCTTTTGCACTTAGATCATTTTTGCTAAATCATTTTGGATGGGGAG

CUST_2599_PI426210822

ATCTTACTTTGGTTTGTCAGGATTCTTCTCAAATAAAACACACTCCTCAACTTTTACAGT

A_14_P102528 TGGAACATAAATATTTGTGCAGTGAGTGGGCATTTCTCTAGAAGTGTTTCAACAGATACA

CUST_1166_PI426210822

CTGTTTTGTGGTTTAAACTAGACCTACTATGGCTTTTAAGGCCACCATTATTTCCTCAAT

A_16_P39232723

ACAGCCATAATTTTAATCAAATCCTTTTTATCAACGTGACTTCTTAGACATTCACCTCCA

A_14_P133086 ATTTAGATCTTCACAGACTGGATGTTAAGCAAGTGGAAAGGCAGAACAGCCT

A_14_P128250 GGAACTATACACTGTATTTTAAATTTATCAGGGGAACCCAATGTTCTGCAAGGATCATTC

CUST_3738_PI426210822 TTTAATTCAACAGGCACCTGTTGAATGCCTGTGCATGCAAACATGATGC

A_14_P117305 AGTTGGTGATGATTTATTAGCCAGACAAGAATCAACAAACAAGTTCTAACATCTGTTCTC

A_16_P02380498

TTTTAGATCAGTTAGGGATGTGATGTAAATGGTAAATCCTTTAGGGAGGAAAAAGGCAAA

A_14_P122019 GTCCAGATTCCTGCATGGAAGAAATACCCTGTCGCTTCAGATATTCTTCAATTTTC

A_18_P22841425

TCGTCTGCTTGGCCTGTAACGTTGGCAATTATCATCATCATTATCGTTATTAATATCCAA

A_14_P105734 AGTATTATGACCTGAAGGCCTCAACATTTATCTGGACTGGAAATGATTCGAGATTTGTG

A_14_P100347 CCAGAAGTTATCTCAACATGCATTTGTCTTTTCATAAAAAAATTACGTAAAAATGCCCCC

A_14_P109557 GTATACGAGATATTCAGTCTTCTTCCACTGCTAGACGAAAATGGAGAGAATTGATCTAGA

A_14_P103521 GAACTTACCTGGGGTTTGGGTTGTCATGGTTGTTTCTAAAAGAAGAAAAAGTCTG

A_14_P105513 CTCCACTTTATTAGAATGTGATAAAAATGAGACCTAAGTGGAAACAATCCCTGTCCATTG

A_14_P100887 AGATACCTCAGTTGTCTGACTTTGCCAATTGCTTAATTTCAGAATTTAAAAAGGGGAAAG

A_14_P200068 CATTTCCTGGCCAGTAAGCACAGAACAGAGGGGCTAAATATTTTATGGTTTTATTCAT

A_14_P115382 AAGTATGGCGAAATCCACTAAATTTATTTAGGGGTGCTGAATACAATCGGTAAGCATTTT

A_16_P00513145

GCACTGTTTCATAAAACCCAAAACTTATCTTGATTCATGCCTATATGTTGACTGTCACCA

A_14_P126439 CCAAATCACCTGTTAAAAGATAAACAGCAGATCTAGGGCTGAACATAGAGAGAAACTATC

A_14_P201943 CTAGTATGAGTTACTGTTCTGCTTTACTTCACTGCAAAATATCTTTTGGGTTTCAACAAC

A_16_P02036085

AAAGCCAGATGTAGTCCATTATTTACATAAACTTCCAAGAATCTATCCTATGCGTAATGG

A_14_P107292 TGACAAAAAAACAAAAGGTAACCTAGTCATTTCCATGGAAAACCAATTGAATTCGGTGAT

A_14_P137972 TCATTTTTTCCTCCCCTCTCTGCTATACAGGGAAATGATAAAACATTTTATATCTCCCTG

A_16_P18464626

GTTGCTCTGATGCCATTATATATGCAGCAGTAATCAGTTACTGGTCTTTTTCAACTCTGT

A_14_P102878 CAGTCTTTCCCCTTGCTCATTGTATGTTGATGAGTTGATTAAGTCTAACAGATTCATCAA

A_14_P115059 TGTCCATTCTAATTGGTTAGTTTCTGTTATTACACTGATTGCTGAATAGTTGACACCCCA

CUST_8338_PI426210822 CCAAGCGAGAAAGGATATCCTGAAGGTCCCAGAGCTGGGAGAAGC

A_16_P02948988 AGTCCATGTGATTAGATGTTTCCAAGAGTCTGGAATAACATACCCCAAACTGTTAATAGT

CUST_5312_PI426210822 CCACCATGGCTTGAATGGCTGCATTTAGAAACAGCCCTTACTTCAA

A_16_P58424399 ATTTCCTAGTGATCGTTTCTAGTCTGTCCCAAATGTATCCTAGCCCAGACC

A_14_P106876 CTTCAGAGGAGGAGACATCAGATTCTACAAAGTGGACTATCTACAAGAAAAATAGTCTTG

A_14_P113725 GTCCTCAGCAAATCTTAAATTTGCATTTTTCTCACCCTTTAATTAGAGCCAGTTAGAGGG

A_16_P16234231 AACATTCCTTGTCCTCTGTAGACTAACAAATACTTGAGTATTTCTCTTCGTCTGAAACCA

A_14_P111226 GATAACGTTATTTCTACCCATCACTGGCACTTGCCCTTAATCCGAGTCATTTTG

A_16_P02037984 GCAGTTACTGATTTGAAGCAACTAAACATTGGAGTGAAATCTCTTATGATAGCCCACTTC

CUST_8747_PI426210822 ATTGAGGCAAGGAAGAGTTAGAGGGAAGGCCAAGGTAAATTGGTAAGT

A_16_P01340311 TAAAACTGCACAAACCTTTTTCTTTTCCCTCTATTTGTCCTATCAAGTTATCTCAACCGG

A_14_P112584 TGAGTATATTCTTGATCAGCCAGTGATTATTCCACTCTCTACTGCCTTTAATTGGAGATC

A_14_P111387 AATATTTCACTTTTAACTAACAGACCCCAAAAAACTAAATCACACTCCTGTATCAACCAT

CUST_5488_PI426210822 TCTGTGCTTATTTGTTTCTGAATTGACCCAGTGAGGTAGTCATCTACCCTG

A_16_P21635961 AAATCCAACATCTCCATTTGATTCATTTCACATTGCTGTCATTATACCATGGGCTCAATT

CUST_1358_PI426210822 CTTCCCAGGTCCCTCCACCCTGCACTTCCTGGACTTGTCAATGGA

A_14_P138590 GTTACTCTTAGACCTAGTCTTAGAACCTCTGAAGACACACTTTGTAATTTGTTGTTTGGC

A_16_P36352387 AACATAACAGATGATACCATTTTATCATGCAAGGTATGGATCTAGGGAATGACCAATGGT

A_14_P136953 TTTAGAATGCCATGAAATATCTAGTTGGGGCATTATCTGCCATTTTGCAAGGTAATCTTC

A_14_P118585 TCACAGGGATCATCTACCTGCCTTCTGAGAACAATCTTTTGGCTTCATGTAAAA

A_14_P134120 GAATTTTCCTACTTGCTTCAGCGCGGTTATGCATTTCCCTAGAAATGCTGT

A_14_P109246 ATAGACTTCTCTAACAAAACCATGGATTCTAGGAGGGACTGGGAAAGAGAAAAAC

A_16_P02181866 ACAGTCATTCTGTGATAGACGATCATTCTGAAATCACATATAAGACTCTTTCCCTCTCTG

A_14_P116154 AGGTCATCCACGATGATGAGAAGAACGTTCAGAGCATCTACACAGGAG

A_16_P01132506 TGCAAATTCCTACTTCATACTAACAACCACACAATCTTTACATTTCCTTCATCACTCTCA

A_14_P129355 CTTGAGGTCTCTGACATCTCAAAGATTCCTGACATGTAGAGAAATCTGTCATCTC

A_14_P133585 GAAACAAGTTTTTTATAGATTCACTTACTCATTTCTACACACCAAGCCTGTTCAAGGCCT

A_18_P10835288 GGACAATTCAAGAGTTTTAGTCATTTTACATCCTTAACGGTTATTCCACAAAAAGCAGCA

A_16_P15049024
ATTAGTTACACTTCTCAATTCCTGGCTTAAAAAAAAGACATTAAACTCCTACGGGCACAC
A_14_P130762 GCATGACTTGGATTAATAAATTTGCAAATACAACTGGGTATGCTCACTCAGTTTGACCAT
CUST_5035_PI426210822
TTATGGTGTGTTTTATACGTCTAGAGGATTTTTGTTGTTGTTGTTTCTTTGAGGTTTTCT
A_14_P117619 GGGGAGAGATTGTTGTGAAAATGCCCACATGTGTTATTTCACCTATTCTCCTATTAC
A_14_P120401 TCAGAAGTCTGGTTACGTTACTATTATAGGAAAGCAAGATCAAAGAACCTAACTCTCTGG
CUST_1140_PI426210822 AGTGTGGAGCACACATCTAGAGTCCTCCTGCCTGAGGGCTGAGGG
A_14_P132608 CTCTGAAAAGATTTTCCATTTTAACCTTACCTTTCCTTCTGCAGGTAAAGATGGAAGCAG
A_14_P120937 ATAATATTTTTTCATTGTCGTCTCTCAGAGGATATGCCACAGGACTTCAAGGCTTCC
A_14_P202566 GACCACTTTAGAGAACAACAAAGGTTTTCAAAGTGAAGAGTGCACATTTCTTTAATGAAT
A_14_P108074 CTTAAGTAACACACAAATCGGGAACAGGAGGACAGAACCGTTGGCATTA
A_16_P19133504
CAACGTGTCACTAAAGTAACTAGCCTATACTTAACGCATAAGTTTCATCCATTCCATTGG
A_14_P113042 TCCACTGACTTCAAGACTCATCTTAGATGTATCTAAGGACCTCAGAGAAGGTCT
A_16_P01956205
CATTTTGTCCATTGTCCATTTCTCTTCATAGCCACTACACCTACTTTATTCAGACCATTG
A_14_P134040
AATAAATAAGAGTTCCCTTGAAAGTAAAAAGGTGGGAGACTCCAAAAAGTGTCCATAACA
A_18_P12309290
ACACTCTTAGATGTTGCCTTCTTCTGAAGTAGATTTACAACAAGAAACCTAGGAAACACA
CUST_1827_PI426210822 GAGATGAGAGCCATTTCAGCATGAGAAGGTTGACAGCTGCCATTTTTTT
A_16_P58424439 CTTCTGTCTTTCCTCTCAGCCTGTCTTCATAGGGGTTTTCATCTCCATAC
A_14_P116877 ATTTCAATAGGTTTTGGTGAGTGAGTGTTTAGAATGGTGAGTAAGTGTTTAGAAAGTGGT
CUST_1274_PI426210822 TTGACGGCTTTTAAGTGTGTAGTAGGTTTCTTTGTAGCTACCTGTTCTGTTGG
CUST_5185_PI426210822 CTAGTGTTTATTAGACCTCCATAGTATCTTCACCAACTGAGAAGGTGCCCC
A_16_P37911472
GGTGAGAAGCATTTAGGCATTAGAGTCATTTTAATCCAAATTTTCACAGAGAAGAATCCC
A_14_P130358 TGAACTAGCATTTTTAGTGACCACTTAGTGCGGGTCCTAAACATACTGTCTCATTAAATC
A_14_P134155 AATGTGTATTTTGATACCCATTGGCATACTATGTATATTATTGTATTATACTATGACATT
A_14_P115780 GAATCAAAGGGTCTTTGTTGGGACACATACTGACCGAAGTGGATCTGTTTTAATTAA
A_14_P121011 AAATAACATTTGAGCCAAGCATCCTGCTTTATAGACTTGTGATTCACTGTCTTACAACAG
CUST_4645_PI426210822 TAACAGTGGAGTTTGACTCAGATCCCCTTGACCTCACTTGGCTTT
CUST_8785_PI426210822 CAGTACACAGAACTGCTTTTCCAGCAAGTTCTCTTTGTTAAGCCATTCTGGTAT
A_16_P01482628
ACTCACCACAATTTTTCTAATCCCAATTCATATGATTCTATAACACACATAAAACAGAGA
A_14_P130315 CTGAATTCAGGCCCAGATAATTGTCTTCTTTTTCCCCAAGAAACATTTGAATTGTATTTC
CUST_1924_PI426210822 ATTTTGCTGGATATTCTAGTGTCAGGGCACAAAAGCACTCACACTAAGATGG

A_14_P201215 AATTCTGCTGGGTTAATGTTTCAGCATATTGCCTAAAGGAAACTTACACATATCACTGTC

CUST_4725_PI426210822

CTTTGTTACTTTTCATGCAGACATCTGGAAAGAATTTGTCAAGAATTATAATAAAGAATA

A_16_P15713439

ATACCCAATAATTTATCACGAAAAGGTTAATGAGCATCCAGATGAGATAAAACCCAAGGG

A_14_P107341 TCTGGCCATTCATTCAACAAGAAGAACTTGGAAATTTAGATGTCTGCCATAACTGATTTA

A_14_P125509 AAAAAAGAAAAAAACAGAGAACTGTCCCATGCTTCTCAAAGCCATTAAACTGCATTGCTA

A_14_P113304 ACCTGCTACATACCAGATCTATTACATGATAGGATGGAAATATCATGAGTCACAGGTAAC

A_16_P00685419 CTTATCAGATGGAAACTCGGCATTCCTCAGAGACCCTTCAGTGTTTCTC

A_16_P36463925

AATGCTTACATGCGACTAAAAATTTGTTCCTACAAGGATCACTATTATTTCTCACGCCTG

A_14_P133582 ATTACTGAGTGTGTTTCAGGAGAAAAGTGACACTTCCACAATCATTAGTAGTTCATAACC

A_16_P03102671

ATCACTCTTCTAAAACATTAGTGCTTCATCTCGATAACGAAACAATGTCAGTGCATCCTG

A_16_P17218016

ACCTACCATGTAACTAAAATACTTGAGGAACAATGAAAAAAAAACCTTCACCCCTTATGC

CUST_2837_PI426210822

TTTTGATACACATAAGAATGAGAACTATTCTAGAACTCTCAAAAAGGGCAAACGAGTAAT

A_18_P10777906

AAATGATCAAAGAACTCTATACCTGAAAACCTGAACCTAAAATACTTGTGCCCTGTAAGC

A_16_P15022785

AGCCAGTCACCAAGTAGTTTGTATTCATCTGCTATAATAACTTCTTTGCAGTTCACTGTG

A_14_P113611 TTTTATAAGGCATTTACCTACCTTTAACGCAGCCTTTTAGTTGGGACTGCACTTCATG

A_14_P119548 ACCATGGCTTTTTATTTTCTCATGTTTTGCCAATCAGCATTTTCTACAGTGACCAAATAG

A_16_P16702175

AAGAATGATTTGTGTGAAAATTCACTGCATCATGAGTTCATTTTCTTTCTGCCTTTTCCC

A_16_P38293383

GGCTTTCCTAAATTCCACTTCAACTTTCAAATGCTTCATTGAAAAGTTCTGGGTTCTAAT

A_14_P101725 CTGAAGAGATACAACACATCATGCCAGGATTTCTAGTTGTTGGTGAGGCC

A_16_P03519072

ATTTTCCACTCTATGCAAATCACTCACTTTACGGAAATCATCTATCATATGTTCCCAAGC

A_16_P03061440

AAACCAAGATCTAGAGGGATTACAGGAACTATAGTTAACCCTAACATCACATCTTGTTGC

A_14_P135006 ACTTTCTTTTCCCATGACCAAGTCTCTTTGCAAAAGTCTTCTTAGTTATAGAAGCAAACA

A_14_P100773 GCTGTGAAACCACCAAAGTCACCAAATGAGGTTTATGTTTTGTTTTCCAAGTTAGATATT

A_14_P119095 ATCATAGACTGTTAGTTTTATTTTGGTCTCCTCATAGATGGGATACTCAGATGGGAATGT

A_18_P14480225

AAAGTCAATTGTAGTTCATCACTTATGCCCATACCTGACAGAATTATTACACCGTCACTA

A_16_P15323587
ATTCTCACTCTACCTCAAATTAGCAGCATAGCAGATAGTAGCAAAATGTAATCTTTGGGG
CUST_3727_PI426210822
CCTACACTATGGAAGACTTTCTGAAAGCCCATATCTCATGCTAAATTTAGCTGGATTTTT
A_16_P19776955
AACTGCTGCTACATAACAGTTAGTGGAAGAGTTAAGGAGAAAGAGTATTAGCACATACCA
A_14_P105567 TCTGGAGTTCAGAGGAAGAGGTAGGACCAGTGCTTTTTTGTTTCTTTTGTTATTTTTG
A_16_P15676725
CTGTTTCCTCCATTTAAGTGCACTTAAGATTTGTCTTTGTAACTATTCCTTGACACCCTT
A_14_P126786 CTTCATATGCTTGTAGGTGGAGATTATCTGAAAATTAACAACACTTGGAATATTCAGTCC
A_14_P139580 CTTTTCTGCCAGTTGGGCAACTCTTAGATAAATAATTATTTCTCCAATCCTTTCCTGATC
CUST_2582_PI426210822 TGCCCGTTAGACAGCACGTCATTCCCTGTTGGCTGCAGAGGTGAG
A_14_P130453 CTGCCTCAGTTTGGCAAAAAGTAAACTTTCCCTTTCCCAATTTATAAATAATCTGTCTGC
A_14_P108333 TTTCACCAGATTCTGGAACAAGTAATAAAAATAGTGCCACTACTAACCACACAACTGTGA
A_14_P134501 CTTGGCCAAAAAGTTTGTTTAGGTGATTTGCAGTGTATTTGGAGGGATTTTGATAGTAAT
A_14_P110267 CTGACATCTGCTCTCGAATTCGCATCCACTTATTGTCAGATGTGGGAAATCTATATAATT
A_14_P130165 ACTAAAGCAACACCAATTGATTTCCCTATCAACAGACTCCTTTTATAATCACAGTTGCTC
A_14_P136299 AATAGAGAGGTTTTCTTAATTAACAAAAGGACGTATGCCATGGAGAAACACACCCG
A_14_P139547 AGTTGGGTGAGGACGATGATGAACAGGAAGATGATGACATCGAGTATTTCT
A_16_P00715832
CAGAGACAATCAAGTCCTATCCTACCAGATCAACTGAGAGATTTCTATTCCCTAAAGTTC
A_14_P114950 CTGGCTGTAGGAGTTTTTGTGGTTGATCTAGACAGATGCTAGATAATGAATAAAAACTGG
CUST_1934_PI426210822
AGTTGAATTGTTTACTTGTAATTTGGAAGCTTCTTGGGTCTTATTGTGTGTGGCTTTTAT
A_18_P15690522
GTCTCTAGTATTTCAGTAATCACCAAGGCTAATTACTCCATTTCAATTCCACATTCTCCC
A_14_P106242 TTGTTTCACTAAAATCAGTCTTTCTCTTGAAAGGCAAGATGAGGGCAAAATGAATTGTTG
A_18_P13493109
CCCCAATAAGTAGACTGCAATTTTTAAACACCAGCAACTCTTAATAAGCACACTTAGGTC
A_14_P108217 CCTGGCTTTACTGGTCTTCCTTATTTTTATTGTTTCTTTTACTGTGTGCTATTGCCAATC
A_14_P113448 CGAATTTTTTCAATGATTTCTCCCACATCTTCTAACTTTGGAAAGGAGATAAGGCACTGA
A_14_P202073 CTGTGAATATACCAATTCCCCTTTACAACTGATGCAGATCATTATGAAATACTGGAAGGC
A_14_P134377
CAACACCACATAGATATAACAGTGCAGCAACAGCAACCAAAAAGTCATTAAAACAAGAGA
A_16_P16158020
AAATGGTTGAGTTGCTACTGCATAATTCTGTGTTGGTCAAAGATGAGAACAACTTTATCC
A_14_P106445 TTAAAGACTCTTTTTCTGCTCCAGCACACTGGCTAAATGAAATAACACAATCCTCTAAAG
A_14_P127338 AAATATTGTTTTCCTGTCTTTCAGTTACAGATGAGGACACAGTAAAGAGGTATTTTGCCA

A_14_P114862 TTCATTTAGGTTCTTATCCATTGGTGCTGGAAATTTAGCATTATGTGGCTCTTTACTGTC

A_16_P03575913

CCTGACACAAAATAAACCTAAATTCTTGGCCTTTAAAAAAAAGTCACAACACAAATGACC

A_14_P138954 ATTTAAGGAGAGGATTATTGTTGATTCCATTTACTCATGCTTGCAAAACTAGAGACCCCT

CUST_8204_PI426210822 AGTGGAACACTGTGCTTCTCATATTTGCATGTGTAGCCAGCCAGG

A_16_P17013314

AAATAGCAATAAACTCAACCTCCCAATAAAAGTTCTGCCAAATCGATTTGACTTTCCCAT

A_14_P123561 CTTATTGTTTGCCAGGTCACAAAAGAAGATAAGTCCTTCCAGAACATTATGCGTTGTTAT

A_16_P36231083

ATTCATAATATCAAAAAAACACCTGTTTACTTTAAAGGGTTCCTACTGTATTGCTGCAAA

A_14_P133201 GGGAATTCACTGTATTTGAAATCATACATTCATCAAATATTTAGTTGGCCTGTTCCCGGA

CUST_9198_PI426210822

TTTCCTAGTCACATCTCATTTGCATGTTATATGCCATCTCATCAATTATTGGGGGAC

A_18_P16318629

ATTAGCAGTGGGATACTAGATACGTGAAGCCAAATGATAAGTGTGCTTAACATCATTTGC

A_16_P58424257

CTTTTAAACTGACCCCCCTTACTGTTGTAAATAGTCCCTTCAGTGAATTCTTTTTGTCTC

CUST_8952_PI426210822 CACAAAGCAAAAGACCATCATTCTTCAGAAGGATCTCCCAAAGAATGCCAT

A_14_P119140 ATTTCTTCCAAATGCACAACCAAATATTCACTGTCAAAGAAAACTGTCACTCTGAGCATA

A_14_P130822 GAATTGGACTTGGTCACGAAGAGTTTGTTCGATGAGTTCGGGAGAATTC

A_16_P19827829

ACTGTGTAGAGAGAGACTGTCAATTTGTAACACTTATAGGGCAATGAACTACTAACATCT

A_14_P113620 ACATCCATTCACAGTGACTGTTGAGTCTAATGACAGACACCCAAGTGCT

CUST_528_PI426210822 CAGTAGGCAGCATTGCAGAGGGTCTTGGCACCTCTTTTGCACCAG

CUST_5548_PI426210822

GATTGCAAAACTTGATGAAACAGGAAATCACTGTTGGCTTAAAATTGCAGAGGTCAT

A_14_P139524 CTATGAAAATACATCCTTACACGAAAACGTGATACAGTTTTTCCTCCTACAGACAGGGTT

CUST_8146_PI426210822 GACAGATGAGACGGTTGGGCCTGAGCCGGCCAAACAAACGGCAGG

A_16_P41416022

TGTTAAAACAGGTGATATCCTCTCTTAGACAAACCATTCTTCAATGCCTTAACCTAAAGG

A_16_P39336723

TAATCCAAATTCTCCCATCGCTAATTTTGAGTCACACAAATTCATGCTGTTCCACCATAT

A_14_P122774 AGGAAAAAAAATCGATATGTAAACATCTTGCCTTGTGAGTGTCTTTAGTGTTTCTTGGGAT

A_14_P129346 CTTACCCACTAAGAACATGAATGCGCTCTGTATTGTATTTCAGCGGCGTCAATT

A_14_P122170 CTAAGAGTGACCTTTAAAAATGGTTTTAACAACCCAAATTCCTCTTCCTGGTTGATATGT

CUST_8872_PI426210822

AAAAACAGGAAAACGCTTTTCTGACATACACCCCAAGTTTCTTCAGACCAAAA

A_16_P38426974

TCTGTCAAATCCCTTATCTTTACCCTAAAACTAGACTAAAATCCTCTTCATTAACCCAGA
A_14_P131102 AATGGTATCTTGACCAAGTATAGGAGCAAGGGTTCAATTTCAGTTGTATGAACATGTTAC
CUST_2397_PI426210822 CCTTCGCTCTGCCCCTCGTCCCAGCCACAGCATACCTTAGAAATG
CUST_6717_PI426210822 AAGAAAGTTGACATTGTGACTCCTCTGCTTCACTTTCTTCAGGAAGGAGT
A_14_P116723 GTGTTCTAGATAAGTTAGAGAATTTGTCCCTAATTGGCTTACTGATAGATAGACCTGGGG
A_16_P15651717
AAAAATGGTGAGAGCATTTCAATATGAAACATCCTGTTTGATAGTGTGTATGTCTGTCGC
A_16_P19643948
ACCACCATCTACTTCTTTCACCATCACTTCATAAAGGAATTTAACATTAAAAGTAGGGCA
A_14_P118808 AAAAACAGTGCCTTTTTCCAGCATCTTACTTAGGTGCTGTTCAAGTATCTTTCAGCATAC
A_14_P136230 TGCAAACATTTATAATAGACCAATAAAAAGTTTGCCCTGGGCTAGAGTTTGCCATTTGTT
A_14_P131401 GTTGTGCTTCTTTCAAGTTCAAAGTGGGTAAAAGAGAGGGTGATATGATTTACTGGAAG
A_16_P35419581
AAACACACAAGAACTCTGTTACTTTAATGTCTAGGAGAAGTAGAGATTTCTGTCTGTGCG
A_14_P124747 GCAGAATAGGAAGTGATATGTATACTACGACTGAATGGAAAACAGTAAGTGTGTGGACAA
A_16_P19318592
AACCAGCCTATGGATCAATTTTTTAGTCTGATATGTGAAGTTTGTAAAGCACAAAGAGGC
A_14_P115497 AAAACAAGAGCAACAATAAGCATTGAATGAGACATTTGTGTTGCCCACATACTGTCTTAA
A_14_P130013 GAAGGAGATAAGTTACTCAGATATTAACTGGTTGTAGGCAAAGGGAATAAACATGGTGAA
A_16_P16632543
CTGTTGCCTCAACTAGTTTACTCAGTTCAAATATCCGTTTAGTGATTTTTGCAGCCATTT
CUST_2122_PI426210822 CAACTCAACTTGGTTGCAGATGGGCTCTTACTTCGGCTCCTCCTT
A_14_P102245 GCATATAAGGCTGGAACCTATCAGATTGAGGGAACAGCACTTCTTCAAGG
A_18_P13086300
AACAGTTGTGATACCAACGATGAAAACTGATGTCCACTTTTGAGTAGGATGTAAACTGTC
A_14_P111227 CCTATGGTCCATTTGAATCATGTGACGATACTCTGTATCCTGTGTATTGTTGCATAAAAT
A_16_P58424190 ATTTAAACCCGCAAGTCTGGCTCCTAAGACTTGGGTTTAAACCACTACATTGTAA
CUST_5458_PI426210822
CTTGTAGATTAGAGTTGTAGACTGAACCACAGAGAAGGGTTTGGGATTTGAGATATC
A_14_P104772 GAAATAAAATATGACCTCCTCGTGATTTTCAAGTGGAGTTATTGGCTTACACAGAACAAC
A_14_P123150 AAAGAATCAGAATAATTCACAAGAGTTGCGATTAGCAAAGGACTCAGAGACTACAGTTTT
A_14_P106913 TCCAGAGAGAATATTTTATGAAATAGTTTCCATGCAACTGTTTCAACCAAGTGCTGTAGG
A_14_P202371 ACACAGCATGGATACTGTAAAACTCTTAAGTGGGATCACATTATATGCAGATTGCAGATT
A_16_P18940437
AAAAAAAAATCCCAGTCTGTATCTTATCTTCCAGTTATCAGATCTTTCTACCCATTGGCC
A_16_P15801446
ACACAACAAGGTTTCAAAGTTAATTGAGGATCAAGATTTTCTGCTGTTGTCTGGTAAGTT
A_16_P18001382

ATAAAGGTCTTCAAGTGCCATAGAGTAAACTTTCCTGCAATTCTCTTCTTAGCATCCATT

CUST_7154_PI426210822

CAAATGGCATACCAGGTTTTTGGTCCTTGTTTAAGAAAACCAAATGTAGGTTCAGAATA

A_14_P124973 CTGATTTTCCATGGTAGTTTTATCTTAAGTGAGCAGTCATTGAGATAGTTTTATTTTTTC

A_14_P201290 CCTAGGGATGCTATAGATAGACTGGGTTTTCTCAAGAATTTTCAGAGAATTTATCGTGTT

A_14_P102768 GTTTATGCTTTCAGTTTTGTTTTGTTCTTTTTTAAACACACATACACAAAATTCTCCTGC

CUST_3934_PI426210822

TCACTTCACATGGGAATCCTAGGACTCCATATTATATTCTTATCCTGTTGAAAATTTGAC

A_14_P123165 GCTGTGGAAATGTAGAAGAGACACCATTAACCTGTCCCTGTTTTTCTATTATTCTTAAGT

A_16_P00685305 TGATGATATCCATTACTATATGTGCAATTGCTTATAAACTAATATATAGTCATATTAAAA

A_14_P127995 TTAGATCAAATTACTCATTAAACAAGATGATGACCTTGAAGTTCCCGCCTATGAAGACAT

A_14_P109510 CCCCTTTGGAGAAATATTAGGAGTTAGAAAACTGCAATACAGTGATGCTTAGACTCATAG

A_16_P18931106

ACGAACAGAACATTCTATACAACCTCTAGACATTATGATCACTTACGTTATTACCCCAAA

A_14_P107134 TCGCAGTGATATAAACTCAGTGGAAGTAACTCGGAAAACTTCAGATTCTTGAATTTTTCC

A_14_P117515 AATAGTGAGATCACACCTGGATCAGAAAAGTTATGAAATGCTGAGATGGCATTTTTCACT

A_14_P112689 TGGAACTAGGGTATAACTTCACTGTCCTTGGAGAATGGTGGATGAAATCTCAATTT

A_18_P22839237 CTCTGTTTCAGAGGGGCAGCCTGCTCGCTGACTGTCCATCTCTAG

A_16_P19941840

AAGGTTTACAAGTTGCATCATATCTCCACCTTCAGTTTATTACACTCCTTTAGTCTTCCG

CUST_6903_PI426210822 AATAATAAGTCTTTTCCGCCCCCTTCCTGTCTTTAATCCAGTGCCC

A_14_P200364 CTTAATGATGACTGTTTTTTTTGATTGCTTGAAGCAATGTGAAAAACACATTTCACCGGC

A_16_P15670043

ACTCTCCACATCTACAATGAGTTATGTAAATCCTGCCTACCTAGTATTATCTTGTGGTTC

A_16_P16696267

AAGCGGTTTATAGAAGACAAGAAAAATCTTAGTTTCAGTTTCATCAGAATACCAGCCTCA

A_14_P111171 GATATGGTTAGTTTGCTACTTACGGGTGAAAATACATCAGTCCCTACCTCTAAACTTAAG

A_14_P121937 TTAATGAACATCCTCCTTAGGTAGTAAGACTGAATCCTAAGTTGACGGGAAAAAAAACTG

A_14_P100445 CCTATCAAGACATTAGTTTTTGTTTCTAATGTGGGTAAAATGTTTAGGCCAGAGGTTCTG

CUST_8081_PI426210822

TTGACACTATTTTCAAGATCACATAATTTACATCACATAAAACATGACTGTGATCTAATA

A_16_P16191936

TTGTTGACATCTGATTTTGGGTAAAAAAAAGGAAAAAGGAAAGCATTTGAATTGGTGTCC

A_16_P01083336

AAAGCCACATATCTGCTAATAAGCCTTATAAAAATTCTGGACTTTTGCAATCTGATGACA

CUST_8067_PI426210822 GCTTAATTTGGAAGCCAAGTGGATGGATAGGAAAGGGGATCGTGG

A_14_P122912 ATGTGGAGGTAATGAATTTGAAAGATGTTCCAGTCAGGATTCAATCCTTGATACACAG

A_16_P19648094

AAAACTCCAAAACTACTAGGGTGTTCACATGATTTAGGGATATGATATGGTAGCATGGGA
A_14_P108245 TTAGTAGAGGAAAAGTTCTGATGTGATTTTAAAAACAGAATCCCTTCTCGCCTTACTTAC
CUST_10089_PI426210822
CAAAGACCTGTTAATTGATGGTCTAAACTCCAGCCTCATATTTGACTTATACGGGAG
CUST_7868_PI426210822 ACAAGACAGAGGAAGAAAACCTTGGGGGAAGGGCTAAGGAAAGGT
A_14_P125820 GGTCCCCATAGGTGTATGGGGGTCTCTATAGATAATATATGTGCTGTGTGTAATTTTAAA
A_16_P20984727
ACATAGAGACCCAGAATACGTTATATGATGATCAGCCAACTTAATCAAGGATGTTCTGTC
CUST_4955_PI426210822 CTTCCTTTATGTTCAGCAAGGAACTCTCAGTGTTTGTCCATTTCCTTAGGAA
A_16_P17164891
AACCAGAGATGTGCCTATACAATTTGTGTTCATTAATGTGCCTCACTTTTTTTCTCAGTC
A_16_P41450744
AAATAATCACTTCCCCTATACGTGATTGTTTCAGCCGTATAGATTCTGACTGTGACTCAT
A_16_P19914041
GGGGCAGGTCATAAAATAAAGGATTACAGAGCAAAGATATTAGTGAAAGAGAAGCATCAG
A_14_P117469 GAGCCAGATCTCTGTTAATGAATTTTCTTTTCCTGACATTTGTTGGTTTCTCGTTACAAT
A_14_P103474 GAGAAACAAGGTGGTCACAGAGAGGCTAGCAGAAGATATAGGAACCC
A_14_P129399 ACATTCTCTTGTCAGGTGATCATAACTTGGGTTGACTTATACATTTCTCGAATCCATCTA
CUST_8501_PI426210822
CTGTGATACCTGAATTTCTACATTGAGACCATTTGGTTAATGCTGTAAGAAGACCTCTT
CUST_2357_PI426210822 CCCTAGGCTGCGGCAGGGATGCTCTTCCCAGGGTGACAAGCTAAA
CUST_757_PI426210822
CTAAACACCTTTTCCACAAATACATAATATGTAATGCACTCTATTCCAAGGCATGTGGGA
A_14_P111708 ATCAGAATCATAAATGAAAGGAGGTATTTCACTGGTAGATGTGTCTATGCCTATGGAGAC
A_14_P113134 TAGGTGAGGAAATTTCCTAATAGTTACACTGTTACATTTGTGTTTGCAGGCAGGTAACAA
A_14_P111964 AAAGCCTGTTTCCTATTGCAGATTTGTATCTGTGTTGAAAAGGCCAATGTTCACTTAATG
CUST_7077_PI426210822
TTTTTAGTCTTCTCTTTTTTGACCTATAAAAAAGGTCAATTATATCTTAAAAGTTTCACC
CUST_3116_PI426210822 TTTGATTAGCTACAGAGTGGCCCTTGCAGCAGGCGCTGGCAGGGT
A_14_P200487 TACAACATGCAGAAACAAATAAGCATGCTAGGTTTTCAGGAAACTTTCACAACCTTCATT
A_14_P135186 CTCCAGTTCGGCATAGGTTAAGTTTTCCTCAGCAATCTTTGGCTATAAAAGAAAAAACAA
A_14_P120356 CTTATTGTTGGTAGAAAAACAGGCTAACACTTTGAATAAACATCTCACAAACTGCTGCTC
A_14_P106130 TAATACATTACCAGAGGTTTCACAATTGTATTTCTAATGGGCCTGTTTCTTTTCAGCAGG
A_14_P105106 AAACTACTTTATTTAAATAAAACCAGGAAAGACCCTTTCCCCCTTACACACCAGCACAT
A_14_P125884 CTGAGCCTTTGCATTCATTAAAGTATCTGTTAACAAGTGACTGAAAACTGCTTTCAAATG
A_14_P130698 GCCATTTCAACAGTGAGCGAGTATTGGTTTTGAAAGTAAAATAACATTAATGAGTGGCTG
A_14_P103697 CTTATCCTAATAATTACGAAACTATGAAAATTCTCGGCTCTCTCTATGCTGCCTCAGAAG
A_14_P120566 TTCTCTGAGAGAAGTTAACGGATCCTTTGGGGATAAGCCTATGATTTTGATTTCTCATTA

A_14_P121290 AGGCTACACTTTGCTTTCAGAAACATTTGAAAGAGGGACATTTTTGCCAATTAATAGATG

A_14_P125832 TAAATTACTTGATTTTACGTAAATAAGATTTCCTTATGGTGGTCCCTAAACTCCCCTGGG

A_14_P115240 CAGAAATACTTACTTTTTGGTTGAAAAGGTTTGTCGTCATCCATGAAGTTGCAAGGTC

A_16_P03634946

ACCCAGTGTAGGTCACAATAATTTAAAGACACTATAGACATTTCGACGCTATATAACAAC

A_14_P120988 TCTTCTGTTACTTCAAATTAGTAGGAACTTTGTTGTTGGATGTTAAGTGGCATGTGTGCA

A_14_P114029 CTCCAGATTCACTCAGATAAGGAATCAGTTCTCATTGGACACGGAATAAACTGATAAG

A_16_P35165983

AACTATTTCCCTACTGAGCCTTTATATGCTTGGACTTTTCTGTAATCACAGCATACTGTG

A_16_P36421438

ACCACAGTGACTACATAATTTTTATCCACATCCTAAAGCACTTTATTATATCACCCTGCA

A_14_P116317 TGCTTTCTCCTATTCCTTTGCCACAGAACATTTTTAGAAACTCAGAGAGAGGACTATTAA

CUST_5016_PI426210822

AAGTGAGTTTATGGGATACCAATGCAAACAAACATAGATTTAGTGGAAAATGACTCAACC

A_14_P123213 AGTAGTAACATACCTATAGATGGAGATTTGAAAGGGTATTTATCAGGTAGGTCCACTCTA

A_18_P22842300

TCCTGGAATGTATTCAGATCAAAGATAAAATAGAGATAAATTTAGCAAATTTTGGTGGTC

A_14_P138898 CTTGCCATATCTAGTAAGAGACGCTAGTAAGAGACCCATTAAACTAGAAGGTAATGTCAG

A_14_P116149 ACTTGATTTATTAGGAAAGGGCTAACACAATATCACATTTGGCTAACCCAGATATAACCC

A_14_P107835 TTTCATCTTTCAGTGGGAGACACTAAACTCAAACCAGATATTCTGGATCCTGTC

A_16_P37090582

CCCAAATGGAAGATCTAGACCCAATACAACCATTAAAGTAAGGGAAAAAGCATATGATCA

A_14_P105901 TATCCTAAATACTATGAGAAGAAGGAGCAACTAATGATTTCTCTCAGCAGCGGGTAAACT

A_16_P00685579

ACTGATGGAGAAATCAAGCAATTTTCCTGTGATCTTTTTTAATAGGAGTTTTAAAATGCA

CUST_4968_PI426210822 GGAAAAAAAATATTGACAGTGTGCCCAAGCTTCATGGTAGGATGGGTGTTAA

A_14_P138689 TTGTATTTGGGATCAAATAATGCCCTACTGATTATACAATGATACCCAGTGCATCTCGTT

A_14_P121414 CTTACCCTGAAACATGCAACGTCAGCAGCATGTAGAAGACAAAGAAGAGGTTAT

A_16_P02207780

CCCCAAACACTGTAAAAATCATCATTCAGTTAAACGTCACAACATAATCACATGGCTAAG

A_18_P22841904 TTTAAACACACCCGGTGGACAGTGTCTGGCTGGGGAGGAAACGTTA

A_16_P01330521

AAACTACTGCTTGTTTCATTGAACTTCCTGTTTGAAGTCATCTCTAGAAACTGTGGTCAG

A_14_P108687 CTTCCAGGACTCAAATCGTGGAAAAAGAAAAGTTCTTGTTCCTAGATTGAAACAAAAATT

A_16_P00685177

TGTTTATTTTTTGAATCAGTAGTGTGTATAGTTAAAATTTTTAAATGGTTATAAAAGTTG

A_16_P15850920

AGATTCACAGCATTGACACAATATGGGCATTACGATCTCATTCAAATTTTCTCCTTCTTC

A_18_P22842131
TGTGGGTTTAAAAAAAAAAACAATTATTTCTCTTTGTGAATTCATCCAATTCTTGGTAGG
A_14_P130799 AGGGCTCTGCTTCTTCATGATTGCAATCTGTATCTTGTTCACTCATTTGAATAAACATTT
A_14_P102452 TATGGGACAAAAATGCTATAAATCAAATCCCTTGTTAGGGTTTTCCAGTGCGTTAAATGT
CUST_2333_PI426210822
AGAAATTTCCAATCAACAAATATTTATTTATTGAACACTTACTACATGCTGACTACTTCC
A_16_P02543757
CATTTGCACACACATTTCCTTAGTTTTGATGTCAGTTGTCTCAAAAATTGTCCCCATTTG
A_16_P02229934
GAGAGAAAGTTATCCCACCTTATATTAAGCCTTACACCTTATCACAGTGCATTGGTTTTG
CUST_7773_PI426210822 TAATCAGATGTCTGCACTAGCAGTCAGGGACATTAGATAGACAGTTTGCTCAC
A_18_P22080216
GCAGGAAAAATAAGTAATTCCCCTAGATATGCGATATCACAGGCTTATTCTTATGTCGAC
A_14_P112903 CTTTTCTTTGTTAGTTGACAAATACATTTTCCGCATTCTCTCTATACCCTCTGAAGTGGA
A_14_P105794 ATTTCTGATCTTTTCTCCCAGTGAGTTTTTAGCAGAAGACAGTCAAGAGAAATTTTGGAA
A_14_P116061 CTCAGAACAGTGAGTGATGTAGGTAGTCAGTGTATCTTAAGTGAATAGTCAGTGTTTTGT
A_14_P103618 ACTTAGAGTTATATAAACCACTTCCCAGAGCAGGTGAGTTATTGATATACTAATTCCATA
A_16_P01872639
TTCATTCCTCATTCAGTTACCTAATTCCTAATCTGCTTAATTTCCCAAACAGTGAAACCG
CUST_7287_PI426210822 CATGCTTTCAGAAGAAGGCGTCAGACCTCCTTGTATGGGAACATC
A_16_P36230187
GGTCCCTTGTAAACTGAGGGTAGTTTTATTCTCTCTTCATTTTTCTGTATAATTGCCCTT
A_14_P111561 CTGTTTTCCAGGTATAAAATGTCCTTTCCACAACTCAGTGGCCTGCTG
A_14_P112745 GAAGAAATTTTAGAAATTAACAGAGCAGGTAGGAAACCTCTTAGAGGCATGTCGATCTGA
A_16_P20000027
AATCAAATGTGATGTTGTGTCTGATCTTTGTCTCCTTTGAACTGTATAACACATTCAGCC
CUST_492_PI426210822 AAGTCAAGAATCCGAGTTTGGGGGCTTCAGGGCGCTCAAACATCT
A_16_P17057882
AAATGTGAAGTATGGTCAAGATTATGAAGACAGGCTATAACAGACATTTTGGGTCAAGAG
A_14_P119775 ACATTTCCATGTGAATAAGCCATCTTTTGAAAACCAAAACGAAAGAATATGCACAATAAA
CUST_2828_PI426210822
AACATTGAATGAAACCATACCATAAAACTTGGGAACTGGTTAGTGAGGGAGGATTA
A_14_P139569 AGCAGCAGAGGTCTAATGGGGAACATCATTATTGAATTGTATGAGGTATGTAGCCAAA
A_14_P128016 ATCTGTGATATTTACCTACTGCAGAATTTTGGCTTTTGAAAATGGAATTTGGCTCCTTTC
A_14_P110053 TGCTCCGATCCATAGTCTAAATAATCACTTTTTTCTTCTTTTAAGAAGGAACTCCACACA
A_14_P111468 GGCATCATGTTTTTAATGCTTTACGTAAGTTTGGCATCTGAAGTCTTCAATTCCTTTGAA
A_14_P121996 TGCAAAATCAATTTTTGATTCACGAATAGAGAGTTGAAGTGATAAGGGCTTTGGGTTACT
A_16_P36519112

ATGCCCTTGGATTATTTACTTCCTCTGTTTTCATCAACATATGCCAAATTCCACTTTCAT

A_14_P105358 CAGGACAGAAACGCCAGTATATATACAGGATATTTGAATAAGGAGGCATTTAACCATCGA

A_16_P20026437

ACCCCCAATTACTGATACTCAGATAGAACTTGCATATTCATACCTTATAGACATCAGGGT

A_14_P128775 TGGGTATACATAGCAAGTTCACATGAAACCGCCTGGGAGATTAAATAAGGTATAATTCAA

CUST_2102_PI426210822

AATAACCTATTTCCCCATGTAACATTTATTTTCATGCCTTTCTTGTGTTGTGAGATTTCA

A_14_P128518 TACATTTTTCACTTTCTTTAAATAATTGCTTCTTGACTCTTAGACGTTCCGGTTCACGGG

A_14_P139565 AACAAAGTGAAAACTTTTCTCTAGGTGCTGTCCGATTTCTACATCAATTAAATCCGTTTC

A_14_P115762 GACTAGTTAAGTCTCCGAAAGATCTACTGAATGAGGAGTTCGTATTTAGACTTTTTTCCC

A_14_P139787 TGGCCCTAATTATAATTGGCTCAATGGCTAATCATGCATAAAACTTGGGATCCAAAATTT

CUST_4641_PI426210822

GCCGGAACAGCATAAACACTGCAAAACAAATCCAGCTATGTAATCCCTTTATTTTAGTA

A_14_P124288 AAAATGAAAGACACTTTGTGCTGTTGTGAACCAGAAAAATACAACCCATGTATAGACCTT

CUST_8788_PI426210822 CTTGTTATGACTATCCACAATTCTCCATGCCCCATGACCCTTTCTTAGCTA

A_18_P12389824

AAAAAAAATCATGTTTGTGTAACCCTTTAAGAAGAACTCCTCCTTAGCAACTCAGTAGGG

A_16_P15117490

AGACCAACAGCATCATTAAGAAACTTGAATCTCCTCTTCTTTCTTTAGCATCTAAATGGT

A_16_P01896918

ACTAGGCTGCTTTCAAGTAGATATAGGCATTTTTCAGAGAATATAAAATGGCAGGGCAAA

A_14_P114993 GATTGGCTGGTTAGTCAGTCCTAAAAAAGGTTAATGCCAGGTCAGTTATTAAATCTCCAT

CUST_4240_PI426210822 GTTCACCTGGAAATCTGGGTCTTAAACCACTTTGAGAAACACTGTCCT

A_16_P01626880

AAGGACAGACCAATCAAAAGAATTACGTGCTCTTCTAGTAAGTGTTTAGAGACTGTTTCA

CUST_3965_PI426210822 AAAGCTGTTGAACTCCCACTGGGATGTAGTTACCTTTTCCTAAACTTCCT

A_18_P17380607

ACTGGCAGAAGGTGTGTTTTCTTAATGACCTGACACAACATAACCACTCTTTGTATATTA

A_14_P114819 AGGACTATAATACCTTGGGATGTGGACACAATTTGTAAGGTATGAATATAATGGTGATAG

CUST_5930_PI426210822 TAGAAGAAAGCCCTAACCCCTCTCCAAATGCTGCTGCTGATCTCC

A_16_P30952442 CCGCCTCTGACACCAGTTCTCCGCTGGAAAAAGGGACAAAAGATT

A_16_P17993501

AAAAGCAACATTAACAAAAACCACATGTGAGAGTATTTTTAGTATGGAGACCCTGATCCC

A_14_P120315 ATCATGTTCCTTAAGAAAGGTGCAGAGAAACAAACTGAGATTTTGAATGAAGTTCTGTTC

A_14_P103503 TATTTACGGAACTCAAATACGTGGGCATTCAAATGTATTACAGTGGGGAATGAAGATACT

A_14_P120819 ATATCCTAAAATTTCCTGAAGAACTGGAACACGTAGAAAGTGCAAGCAAAGGTAATTGAT

CUST_2587_PI426210822 ATCTCTCCTGCAAAGGCCAGTGGGCCGCCGCCACCACCCGTGAAC

A_16_P02773221

AACCTGTTCCAATTATTTACCTCCTTCTAACAAAATTTTGATACATCAATTGCATGTGGA
A_14_P115495 ATCCGAGCAAAACCAAAACAAAACAAAAACCAAATAAAATGGTGGTTTAGCAGAGACG
CUST_10374_PI426210822 GTCAGGATGAAACGCCCGTTCTAAGTGAGAATGTGCTGCTGACTG
A_14_P132344 AAACTAAACAAATGGACTCACTTCTTGCCGTTTGAAGTTCTGGACATATTCTTCGAACT
A_14_P111339 CTGGAACAGTGATTTTAAATGTCCCTTTTTCTGGATCCCTTGTAAACATGAAATCATTCC
A_16_P01124950
TCACCTTAAGATCAAACCACTATCAGTAACAGGTTCCCTCATATCAAACTACAGTTTCTT
CUST_703_PI426210822
GTGAATTTTTTTGTGATTTTTGTAAAATAAATTACAAGTGGCAATTAGTCACTGTGCTGA
CUST_561_PI426210822 GAAGGAAAACCTCAGTCCTTTTGCCTTGCCTCCTTCTCCAGTTGC
A_16_P21424705
ATGAAACATTGTAGGCCTTCATTAGAAAGATTACCTCAGACTAACTTACTGCTAGCCATG
A_14_P110008 CTCATCTAATGGTTTCATCCTTTCAGAATCTTGCATGAGTCGAGTATTTCATTAGGGTTG
A_16_P38300133
AGTTTAAGGCTTATCAGAAAGGAACAAAGACATACCAAAATAGACCATCCTTGCACAAAC
CUST_8370_PI426210822
AATAGATAAAGACCTGATGGGTTTATTTCATCTTAGTTGTTTCTACTCTTTCCTACTCTT
A_14_P200274 CTATGGCCTCACAGCAGTAGCAATAACTTATTGGGATAAAAGGGAGAAGCAGAG
A_18_P13058381
TCCATAAAGTATATGCACTATGTTGTTAACTACGTGTAAGACCAAGAAATATCTCCACCA
A_18_P22842845 TTGTGATGTGAGAGAACAGGCTCTGCCAAAGCATGGAGCACGCAT
A_16_P17445442
CTCAACCTCAATATGTCATAACTCATCTCTTCACATGCTACTCAGATGTTCAACTACGAA
A_16_P17348369
AAAGATGTAGTCCCCAAAATCATCAAATCCATTGGTTGTTGCACTATCTATTAAGGCTTG
A_14_P102551 AACTTGTCAGAGAAAGCCCCTCCTTCTGGTTTTCATATTCGTTGTAATTTTCTTTACATT
A_14_P133036 CTGCCTTTCGCTATGGATTTATGCTACAAAATCCATATCATTTTCTCTTTCTTGGAAAGG
A_16_P02075223
CCTCATTCAATATGTTGATAGTAACCTAGGCCCACTTAATACCAATTAAAGACCCCCTAT
A_14_P123649 CTTAGTGAGTGAAGATACAAAGACCAAGTTTTTGAACAAAATGGGCCAGTTGACAACAT
A_16_P01171615
TTTTCATTATCCAGATCCATACATTATCCAGATCCAAACCTTCTAGAACTCATTTGTGGC
A_14_P105865 GAAAAGTTCAATCACTTCTCCAAATTCTCCGAATAAACATAAGAAAATATCTCTCCCCAG
A_16_P18997625
ACAAACACCTGAAGTGAATGATACCAGTAAAGTTACCATCCTTTTTTCTGCAATGCTTTT
A_14_P109103 CACCTTATTTTACAAATAGAGGTCGTCTACTTGACCTTGGCCACTATTTTCTTTTCTTCT
A_14_P100241 AACAGCACATTCTTGAAGATCTTCCAGTCATGAGCAGTGAAACTAAGAGTTATCTAATTG
A_16_P16681574

AGAACCTAACAATTCCCATGTCTAAAACTGTGAAACCCATCTAGATAATGAAAATGGTGC
A_16_P19315089
ACCATGGAATTGTAAATGTGTGCATAATCCCTCTTTGATTTTTGCTTGAGACCTAACTTA
A_14_P123663 CCTTTTTGAAGTTAGGTAGATTCTGTGGTCCACATTTCCCATATTACTCACCTGAAAGTA
A_16_P38365592
ACCTGATTACCTCCACATATGTATAATGCTCTGAAGATGAACAGATAGGCAATGAACTTG
A_14_P128431 TTGAAATCTCAATTCACTTCCAGAGTGTTTGCTATTTTGGTTTGAGTCTGTGCTATGTAG
A_16_P18959036
AAAAAAAGAAAGTGTTCATTGCTTTAACCCTGTTGCTACTATGTATTTCCACCCTAGAGC
A_16_P40144497
CATCCCTCTCCTACTACTTAGTGCAAGTCCAATACTGTATCTACATCACGCTTTAAGTC
A_16_P02073591
AAATGTTAGTCCCTCGAGAATATTACCATTAAATCTCCCATTACCACTAGTAACAGAGCC
A_16_P02334338 CATTATCTGCCATTATCCTGTTGTTCTGTAAGATATCCCTATATCCCACCTATATCCCAC
A_14_P114296 AATTACTCTCTTGGAACTGGCAAAGGCATAATTAAATATGGAGATTCCAAGGACTGTTTT
CUST_3049_PI426210822 CACTACAAAATGTTTGATCACCACACTCGGCTACCTGGGAATAAAGGC
A_14_P100700 AATTTTATATGTAAGAAATGTCGATCCTGATTTTCCCAAGGCTAGTGTTAGAGATGGGAC
CUST_9558_PI426210822
CAGAAATGCCCAACATTTTGTAAACACTAATTTTTTTATAACTGTAAAGATGATTATTCC
A_14_P120214 TAAAATAAAGCAAACTAACTGTTTTATTTTCCTTGGACCATAGTGCTTCCATTTTTTCTT
A_14_P112121 CTTGGAGCCAGGAGAACATGAGTATGAAATAGTATGAGTGCAGTGTGTGTGT
A_14_P117434 TATTCAATGACCCAATGGTATCAAAGTGGAAGAGGAAAGTGACAACTAGAGATTGATAAC
A_14_P201690 AGTTATTTGCAATAAAATGCTTTCCTTCTACAGTCCCAAGTTCAATGATGTCCTTTACCC
A_14_P121407 GTATTATAGTGGATCTTGACTATGCTGAAAACATCTTTAAGTGATGATGGCTATCTTTAA
A_16_P20849548
CCCTTCTTATGCCAAACAATTCAAAATTTGTCTAGCCCTTGTAATACCTTCAGAGTTTTG
A_14_P104977 CTTGAGAATTGCCCTCAGTGAACATCTTAAGCAGGTAAATCCCATAACATCATGAAAATC
A_16_P16803837
ACCAATTGCCCTCATTCCTAATAGATTCAGTTTTCCTAGTGTTATCTCCATTCCTTAGAT
A_14_P117018 CTGAAAAGGGCCTTTGGTTGAAAATGAAAATAAGAGTGCAGCAAGCAGGAG
CUST_5136_PI426210822 CATGAAGTGGCAGGCCTGCCCTGGGAAGGTCAGGATTCTTCTGTA
A_18_P14134804
TATTGCAGTGTCGAAACTTTAGAAAATAAAGGTAATCCAAAAGTAGGGATTCCCTGTTGG
A_14_P122229 TAACTTCAGTGCTTTCTGTCTTTTTACAGGATGATGCAAATAAAAATGGTGGCAAGTGGA
A_14_P118549 ATTAAGATGAGGTCTCAGTCTGCAAACAGTGACAATCTTGGTGGCTAATAGTAAGAATAA
A_16_P37821973
GCTGACTCTTACATCCAATTAATTCTGAAGCCTTTGAAAACTCTGTTAGATGTTCTTCCT
A_14_P116582 AGTGACTTGAGCCCAAAGGCAAGCGTATATCAAACGATCTTCTCCACATAAC

A_14_P132879 AATCAGTAAATATTTACAGGATGAACTGACGTCCACAAGTTTTTCAAATCCTAACTCCTG
CUST_5620_PI426210822 AGATTGGTGTGCCTGGGTATGGGGAGGCCAGTTAAGAAAGAGCTT
A_16_P02468801
GGGGAAGTATAATAGCACAAGAGAATATTGTCAGATGTAATAATTCAGAGCCAAACAGGG
A_14_P110086 AGTCACCAACCTATTCCCTAGGTTTTTTAAACTTTAACTTTATTTTGGCTTGCTGATCCA
A_14_P132024 GCATCTTTTTCACTGATGGCTGCAAGTAGTGCTTCCTGTCTGGTAAAATAAAGATTATTG
A_14_P138975 TAATTTTTAAAAAGCCAGCGTTACCTCAGGATGATGTCCAATTTCAATGTAGGTGCAAAT
A_14_P106177 GCCTCCCAATTCATTACAGTGCAAGAATAAACACACATGAATAAGTCAGAGACCAATTAA
CUST_1405_PI426210822 AACCCGAGGTCCTGCTCCCAACATAGACCACACCAAGTACAGTTT
A_14_P116503 TTAAGATTTTGTTCATCTATTGAGTTCTTTTGGGTGATTTGTTTAGGAGGAAGTATCTGC
A_16_P16880854 GGGTCCATGCTATCTTATATATCCTCTCAATGAATATGACTTCATATACTCATCCCTGCC
A_16_P16191615 GACAGTGAAGGCACTGAGGCAGTAACTGTGGCAACCATAGAAGAG
A_14_P125619 CCAGGATTAAATAACATTAATATTATTTTTTGTAGGCCAGCAGGATTGTCCAGAGGACCA
A_14_P138193 TTGGGAACGAAAAGGACGCTGTATTGATTTTTAAGTCGCATATTCTTTAACTTCAATATT
CUST_9638_PI426210822
TTTGAGGCCCACAGAAACTATAAAGATGAAGAACAGGAGAAACGTAGTTAATCATGC
CUST_8831_PI426210822 GGGATTTGAACACATATTTCCTTCCCCCTCAGTTTCTCTCACACAGA
A_16_P01723690
CACAAAGACTAGGAGTAACAAACTACAATACGTATATCGCCTTGATACTGTAAAAGTCGT
A_14_P114383 ATCTGATGCATCTTGACCTAAAAGGGAGTGATATAGGGGATAATGGAGTAAAGTCATTGT
A_16_P15133387
AAAAAACAGAGATATCCAGATCACCCTGATTAATCCCTGTCTTCATTAAAAGCATTCCTC
CUST_384_PI426210822 ACAACAACAGTAAGTGCCAGGGAACATGGACAAAGCCGATTTCTG
A_14_P133575 AGACAGGGGCAAAGATAAATAAAACAAATGCTATTTTAAGCCCAAGACAAGGATTTCAGA
A_14_P121250 AGAAAGGTTTCAACATGCAGTCTTGACTTTTATGCTTCCACTAACATCTGAATGATTGAA
A_16_P02044911
TCAAAACCACCTTCTTATCATCAAGTTGCACACTCTAATTTCTTAGAACCCCCTATTATC
A_16_P19294834
AAAAAAACTTCTCAAAATTAGTTCCAGACTTCAGGAAAATGATTTCCACATGGTAAGGCC
A_14_P109864
GTGGAAAACAATGCAAAAGAAAAGAGGCGTATTTTTAAAGGCAGTGTTGGCAAGTTTAAA
A_16_P18562879
AAAAATGCCCTAGATGTTAATGGTCAACTTCTAAAAATGTCAAGAATTTGCACAGAAGGG
A_16_P19905414
ACCTGGTCAGTGCATAAATACACAACCTAACACAGTTTTCTTTGTTCTTTTTCATTGCAA
A_14_P135994 CTGCAAGAGTTATGGAAAACTCCCCAAAATCAAACAATCCACCTCTCTAAATCAATGAT
A_14_P128239 ACTCAAGGACTATTAGACTCTAGTCTTTGGCTCACAAGGCATAGTTCTACTAACAATTCT
A_14_P133931 GCGATAATCCCGTGATTCTCTATCCGCTGAGCGCTGTTGGATATATTCTCATTATATTTA

A_14_P112931 CTTTGTGAATAAAAGTTAGTAAAATTTCCCTCCGAGATGGCCTATTATTTAATTTGGGCG

A_14_P122681 ACAATGGACCGACTTAATCCTTTTCGACTACCTGACGGCCAACTTC

A_14_P134821 ACTGCCAGTCAGGTGGAGAATGGGATTATTGTGAATAGCAGATTTCAAAATTTGATAAAA

A_14_P115255 CTGAGAAACATACTTCTTGTCCCTGTCTACTCCTTCTTAAGGATACCAAGCAACAA

CUST_1743_PI426210822

GTCTGTATAATAGATTCCATAATGCTGATGAATCTGGTGAAATCTGTTTTTGTTGCTTAG

A_14_P115303 GCCAATCTTACCGGTAGAGTAGTCAGTTTATTACAAGACAGATCCAGGATGGTG

A_14_P114612

GAGATCATACATGGGAGAACGTGGACAGAAAAAAAAATTAACTAACTACTGCAGGAAAGA

CUST_7018_PI426210822 CTTTGGCCTCCAGATGAGGACATAACGAGGTCATCTCTGCTTTCA

A_16_P21433193

AAGTAACTCATCCGAAAAGATTTTCTTTAGTGGTTCAACCTATACAAACATACCACCCAG

CUST_2360_PI426210822 AAGGAGCCCCGTGCTCGGGCAGCGGTCAGGGTCATTCTCCACGCAT

A_16_P18142452

ATCTCTGGAGTCTTTTAACAAATGTAGAAATCACAGCAACATTTACCAGTGTGAATCCAC

A_14_P100117 TTTTTAAAAAGAAACCTCTCTCTAAAACATGATTTATTTCCCAGTCCCCTGACCTGCAG

A_18_P13339368

AAACCCTTTTAAAGTGCGTTACTACATCATTTAGTATGTGTTTCGTAGAAAGCAACCTGG

A_14_P137773 CACAACAAAGGATTAATTGCTGCAAGGACATTCTTGTAAGGCTTTGATTAGTTTTCTTGT

A_16_P16716163 CTTGCCATGTCTTCTAGGAAAGAAGTGATGCTGAGTCTGAATACACAGTAAAAGC

A_16_P39598831

CTCTTCTGAATTCAACCATCCTTTAATCATTCAACAGTCCTTTAATATGCGCTGAGACTA

A_16_P31097891

GCGTTTGGTCCAAGAATATGAAATCCTATCACCCGATCCTTTAATACAGAAACAAAACAA

A_14_P123375 AAAAATCACAAATGGGATAGTCTAAGTGTTAAGCATTCTGCTTCCTATGGCAGTAAGTCA

A_18_P14135305

TCTTAAAAATACAAAAGGGACACATAACTTCATCATTTAATTATATTAGGAGGTCCGTCA

A_14_P102145 AGCCTGCTTTTAAAGTGATTTGAAGAGAGTGGCTTTGAAGGTTTCCATCCTTACT

A_14_P122686 CCTCCATAGATAACTGTCATTCTAGCCACAGAGTCAAAGAATCAGACTATTACGTAAGAT

A_14_P120806 ATACAGAGTCTTTTAGGAAAAAAATGAAGTTAACATTGTTAGATGGTGGGGAAAGTGGCA

A_18_P22842106

TACTTCAGACCAAGATTAAATGCTGAGGGGGAAGAGGAAAATAAGGCAAGAATATAAAAC

A_16_P21589723

AAAGCCTTACAACAATTTTTCCTACAAATGTATGAGGGTAAAAACCTATAGCCAGTGGTG

A_16_P21671977

ACAGCACGAGAGACAAAAAAAATATAGTTAGTTCAGAACTAGAATCAGAAAGCAAGACAA

A_16_P18144417

ACAGAAACCATTCTCCAACATGACATGAAATCCCAAGTAAGATAACCTTGAATAAGTGCA

CUST_6337_PI426210822 GACTGGCTTATTTGCCTTGTTAGAAAGAGGATGGCCTTTGAAGCAAATATCG

A_14_P134653 AGCAAAATGTTAGTTTCTCATTGTGAGTGATTCAAGAAAACAACGGTAACAGCCCTG

A_16_P58424438 CAGCCTGATGCTGCAAGGGGGATCAGAGGTGTGAGTTACGCCAAG

A_14_P101925 ACTGAGGTTGAAGTTGTGCTATAATAATCACCACACAAAATTATCTCTATTTCACCCCAG

A_14_P112581 ACTTCCTAAACATCTGTCTGGGGTTCCTTTAGTCTTGAATGTCTTATGCTCAATTATTTG

CUST_10152_PI426210822 GGGTGTTTCTCCTTTTGAAGAAAGACACCCCCCTTCCCTTATTTAG

A_14_P116974 CTACTGGGAAAATCTACCTCCGACAAGAGGAGATCATTTTAGATATTACCGAAATGAAGA

A_14_P107125 TGGATTCAGGAAGATTGTGGAAAAACTGCTGACTTGTGAGTTATTTAGAGAGATAGTTTC

A_14_P114115 ATATAATCCTGAGGTGTTTGACAAAACATACCAATTCTTGAATGACATCCGAGCGAAAGA

A_14_P109777 AAGTCAAATCAAAATATGCCACTTCCCACATCACAAATGTGACTCAGACAATGAACATAA

A_14_P124601 GTCAAAATGGCTTTGATCCTCTATGTTGAATCTTTTGCCTTGAAAGTTTAGTTTGTCCAA

A_14_P139464 CTATGAGGAGGAAGATCAACGAAGTCACAGGTGAATAGGAGTTTCTCTTCTAAAGT

A_14_P101161 AGGTCTTCTCTGTTCAAGGTTCCGTCATCATCAAAGTCTAGAGAGCAGAC

A_14_P100979 CCCATCTCTGTAAACCACAGCCATGTCCAGAATAATTCTACACTCCCAT

A_16_P00365958

AACACCTTTAAGGAAACCTAAAGAACTTTTACCAGTGACCTCAATATTGTAGATCAAGGC

A_14_P123195 TATTGATTCCTAAGAAGATAGACCAACTTTTTATGTCGGTTTGGGTGTTTGTGAGTTTGG

CUST_2089_PI426210822 CTGCAGGAAATAGATTGGAGTGTCCTCTACCTCTCTCCCTCAAACT

A_16_P03232883

AAATCAGGGGGAAGAAAAAGTTAATAGTCTGCTCAAGATCTTTTTCAACACCATGATTCT

A_16_P15823557

TTTGAAATCAGCCTCTAACTTCTACAATCTTTTCACCCCCTTTAAGAAACTTTGAGCAAA

CUST_8988_PI426210822 TTTTCAAACTCAAGGAGCGTGTGACTTCAGAGAAGCAGGCGATAAGT

A_14_P100191 GCAATGGCATAAAATCAGGTGAATGATGAATCCAGAAAAGACCATAGGAAAAATAAAGGT

A_14_P111062 GAATGTGGCCACCAATAACACATGCAATAAGATTTAAAGAAAATAGATTGTGCCCCAATT

A_16_P21246250

ACCCAAAAAAGCAAAATATAGGAACATTAACTACATTCTCTTACTGGTAATCTGATGGCA

A_18_P11571934

AATAATCATGCTACATTAAACATCTTAACAGTCAAAACACGTCTCTAACTTCTTTTCCAA

A_14_P201848 CGCATCCATTGCTATCACCACGAAAATCCATGATTTTGTAAAATTAAAGAGCGTAGTTTT

A_16_P00973016

AGTCAGAAGACCTAGACAAATTATATTTGCAACTCACATTTCAGAAGGCTTATTTCCTCA

A_14_P107233 TCCTTTCATATGATGCTGCTAACTCAATAGAGAGACACCCGATTACAAATGCTATTGATG

CUST_1500_PI426210822 TGGCTCATCTCGAGGCTACCCCTATTTGATGATTTCTGTAGGAGTC

A_14_P133265 CGCATAGTTTGTAGGAAAAGTTTATTTAATGGGGAGACTAAGACGATGCAAGATGGTTAC

A_14_P117369 ACCAAGATCTACCTAGTTGACCAAGCCATATATCTGACAGTCAAGTCCTATGTATTTTTT

A_16_P15543755

AGATCTGTCTCATCTCATTACTATGAAATATCCCTTGTGAGCAATTAATCCCTGTGTCTC

CUST_8131_PI426210822
CTATGACGGAAAGATCCTTAAAGCTCCTTATCTTGAAAACTACAGTTAAAATCTCAATTG
A_14_P126465 TATTCATGATGTTCTGTGTGCTTTTCAGAAACTGTAGTAGAACAATTGAAAACCGAGTGC
A_14_P128778 ACACACTAATTTGCATTTTATGTGGTATTGATCTGTAATGAAGAGAGGGGAAAGCCTAAG
A_14_P106474 GTTTATTCTCAGATATAAAGCGAAGGCCACATGTAGTGGAATTTGCCAATCCATGTAAAA
A_16_P01621153
TCAGATGACCACTGAACTTCTCAATGTTGGCTTTACTTGAAGATAATTATGCTGCCTTAT
A_14_P138244 AGTAAAGAAAACTTAGGATTCTAAGTGACAGCAGGTATCTTAGTAACTGGTTGGTGATCA
A_18_P17042802 AAAATGCATACCTTGGTCATCAGTGTTTCTCAACAATTCTCCATCCCACTTAACAAG
A_14_P103506 ATGGTTGAACATGACTTAGCATGTTTGCTTTCTGCTTTAAACACTAAAAGTCCTTGTCTT
CUST_1172_PI426210822 GCAAGTGTTTGTTAATCATCATAGGGTGACTGACTTGTGTACCGGGAATAGAAT
A_16_P19509441
TTGATCATAGGACCAAGGAACAAGATTACCTCTACCTTACTATATTTCTTAAGTGGCCAG
A_14_P133325 AAGAATCGCCGTGGATGCACACAAATAAGCGGCCTACAAAATGGAG
CUST_9595_PI426210822
TTTCTCAGCAGTCCATGTTTCTGGATAAAGTTTATGATTGTTAAGAAGTGTCAGTGCTTC
A_16_P18189582
AAACCAAATTTTGCAATGTTTTTCATTCCCTTTCAGTCTAAAGTTTTCATTCCCATCTCC
CUST_5931_PI426210822 TCTCCAAATGCTGCTGCTGATCTCCTGGCAAGCCTCCACAGATAG
CUST_4544_PI426210822 AGCAGCACTGGCCTTGAGGGCCATGTTTCTACCCCTGATTCCCAG
A_14_P103722 GTCCATCTGGGAAATAGATACAAATTTCATCCAGGCTATAAAGACCGGCTGTCTTA
A_16_P16191438 CTCAAGAATTTCCCAGTTACTTCTCCTGGAAGATCCGCTTCCTTGTTTT
A_16_P21386285
GAAACCAGAAAAGATTTGCAATACTTGGGCATTTTTTAAGATTGTAATTGGCTACCCACA
A_14_P139156 TGAAAAATATTGGGGATGTTTATGAGACGATTGGTATGTCAGCAGATATGCTCTTTTGGA
A_14_P138401 AAAGAAAGATGTTTCACCTTCAACCTCTTCCCAATCATTTTCACTTTCTTCCTCTTCTTC
A_14_P115333 CTGGGTGAGCAATTATGCTGTTACTATGTATGAGTAATATCTACTTTCCCTGGGCAAC
CUST_7317_PI426210822 CTCTTTCCACTGTCTGATTCTGGGTCTCTGCTTCCTCATCTGGATA
A_16_P21433769
AAACCCCTAAGATACTCAATCATGTGAGCACAAAGTTACTGTGTAATCTTCCATAGATGT
CUST_2603_PI426210822
TCTCATCATTCTTGTATTTTTTAATCTTCCTGCTGTGAGCTTAATCCAAAGGGTATTTTC
A_16_P18941083
ACCAAGTAAGATGGAAACAGTTAGTGTCAACATGATGAGTTTGTGAGATTCTATGTGACT
A_16_P18364671
AAGTCAAACTTTCCTCATGTCATATTCATAATTCCCTTGCTATAACAATGCATCTGCTCC
A_14_P104085 TCTTCTGTTTTCATATATCAACAGGAATTGGCAGCAATGAAACAGATTCTCGTTAAGATG
CUST_10036_PI426210822 AAGGCAAGATGCGAATTAGTTTGGCAAGAGGAGGAGCTTGGTGTT

A_16_P01222283
AAAAATGTGCCATAGAGAATACATTCATTTGGGACTTTTAAACTCTCCTCTTCCCTCAAA
A_14_P127409 TACATGTTACATAGGAATAATCTAGCGTCCTACCTGTGTAAAAACCAACTCTGGAATACT
A_18_P26792316 GGCACCTGTTCCCCTCCTATCTCAGCCACTCTGCCCACGTCTGTC
A_14_P118748 AGTTCGTATCATAATAAAGGAGCATGGATTATTGCAAGGGTCAAATGGCAAATAATGGTT
A_18_P22841229 TAGAAACCGTCATGTTATAAACATGAAAGCCCCAAGCAAACAGCCTTTAGCC
A_14_P107908 CATACTCACAGTTTTATGATGTCTGATCCAAACTTGTCAATGACTAAATAGCAGGTGGTT
A_16_P00725395
ACCTTTTACTGAATTCCATTTCCAATGAACTGTCCTAAGACTCATATCTGATTTCCACAG
A_14_P201488 TGAAAAAGTATTGGTTGTTTGGAGTGAAAATGATGAGCACCGGAAACACATTATCCTAAA
A_14_P118279 ATCCCGGAAGAACAATGTTGCCATCCACATCCATTGTCTGCTCTAC
A_16_P18222879
TCTCAAAATACATGCATCAGCCTTTAATCGACTACACACAAAAGTGGTATCCTAGACTTT
CUST_10087_PI426210822 CGGTATCACACTGACAGTACGGTGCAGTAACCCAAAGACCTGTTAATT
A_14_P121711 ACTAGTTGAGAGTTTGACTGTGAATTAATTTTATGCCATAAAAGACCAACCCAGTTCTGT
A_14_P138650 AATTAGATGGTTCCCTAAACAAATATCTAGTTGCACATGCATCTTTCCATTTTCCCTCAT
A_14_P200361 TAAAGGTTCATGTATTCAAACATGACATTATTCTCCTTCATGCCTTTCCTTACAGGAAGA
CUST_1425_PI426210822
ATTTATTATTAATTTGTATTATTTCTTGCTATTATGAAATTTGATGCAGGATTTGTTCAT
A_14_P121751 CTTATAAATATATGGGATGCTGCTTTCTCACTCCATGGGCCATATGTTCACATATGGATA
A_14_P112750 TCTTGTTATATTCCATGATTCCTACTTGCTCCACATGTCAGTTATGTATGCCTGCATTTT
A_14_P201324 TTAGTAGTAGCAGCAACAGAGTATGATATGACCCAAAAGCCATTGTAAAGTGCCACATTA
A_14_P113576 CTAATTCAAACCTGGATGTTCACCTGTAACTAGGAATGTTGTTCTGGTCAAAAGTTAAGA
A_14_P122122 CTAGACAGGTAGCCTCTTAAACGTGTTGTTCTGAACTTTGTTTTTTTCCACTACAGTCTT
A_16_P02721126
ATCACTTTCTGCCTCGTAGATAATAAATGATCCGAGTCTAAAAATAGCCATCAAGTTGGG
CUST_3026_PI426210822 ACTTACTTAAATGCCAGTGGGCTCGGGGCTACTTGTTACTGATGC
A_16_P17129305
CACCTTCTGCATGTATCTACAAAACATCTTCCTCTAATTGATTAATGGCAAGTCACTACA
A_14_P115847 AAAGGATGACAAAAGACAAAAAGCACACATCGGTTAACATTTTTCAAGGAAGACCTTG
CUST_7949_PI426210822 AGGTGAGGAGTCCTCCCACCCCCTGCCTCCCATAGCAGCTGGGGC
A_16_P19614414
GCCACACACAATATGATCTTCTTTCATCCATATTGCCGTAGATCTATTTTCTCAACACAG
CUST_4390_PI426210822 AAAGCTGAGTTCGCTCAGAATGTGGACTGTTCCATTTTTCTGTTGTCC
A_18_P17040226 AAACTCATTCCATGCCCAAGATCAAAACCAGTAGGCTTACCACTTTACCATTG
A_14_P106849 CAGTTCTGACACTGCAACTGCTATAAAACCAATAAAAAATACAATAGAAAGAGAGCGGGT
A_14_P123802 AGAGTTTGTGAAATGCCAAACTAGAAGTGACTATCATTGCACTTCGGATCCTATGTC
CUST_10320_PI426210822

CCACATCTTTATCGCAAAAGAATGACATTTCAAGCAGTAGCTTCTTAGATTTCCATTCAG

A_14_P114575 GGAAATTTGTATTGCTGTAACATACCTCTAAAAGCCAGTCTAGAAGTATGGACCTCATCT

A_14_P119091 CAGGTAACGATTCTCTTGTTTGTCTTCAAATAAGTCATCTTCATTGCAATCTGATGGTTC

A_14_P124397 TAATGAACAGAAAGACCACGGATATTAGTGCGGATCGTTTGCAGATTCTTAATTGTATCT

CUST_5203_PI426210822 ATTCCATGCTTTTGGACAAGAGCATGTTCAGTATTGTCCAGGAGACC

CUST_1275_PI426210822 GTGTGTAGTAGGTTTCTTTGTAGCTACCTGTTCTGTTGGGATCCTGGTTT

A_14_P111104 CAGCAAAGGATGGGTAATTTGCAAATGAACATGGATAGAAGAGCAGCAAAGCAC

CUST_4401_PI426210822 CATGTTTCCCTCATTTTTGGGGATGCCTTGGGTTGAAGCTGATTTTT

A_14_P110771 GATGTTTTTCCCCTTTTAAGTTCTTTCCTGTGTAACTTTACAATGGATATCTCCTACCTG

A_14_P106087 AGCAAATTTGAAATATTCTCACTAAAGTCCCATAACACTCACAAATGGTCCTCAACTTCT

A_14_P115695 CATTTCACATAGACATGTTTTTACAGGTGATTTTCAATGTTGGGACTCAAAGGTGAAGAC

A_18_P10185462

ATAGAGTCAACTCAAAAATAGTGTATCTGTGAATGCTTGATGTTAAAAGAGGCTATCCCC

CUST_1362_PI426210822 GCAGCCCCTTGCCTCTCTCTGCGCCTACCTCACTTCCCATAGCTT

CUST_1742_PI426210822

TCTTTAGTCCTGATAATCCCGTCTGTATAATAGATTCCATAATGCTGATGAATCTGGTGA

A_14_P124648 GTGAATGCTCTTACCTCTCTAAGATGTATGTTTTCCTTCTCTTTCTGATCTAAAATCACT

CUST_8718_PI426210822

ACAAAAAAGCCAGTTAAACTTATTCCTTTTTTTAAATAGCAATCTGGAATGAAAATATCA

A_16_P21563641

AAAATTCTACATGTGTTCCTAAATTTTAAAAAGCAAGAAACCAAAGTCTTAACTAGGAAT

A_14_P101739 GCTTTCTTCCAGCACACAGATGAAGAAGGTTATGTTAACAGTATTTAAACACAATCATGG

A_14_P105241 AACTTTGATTTTTCTAGGAAAGGTTTGTTAGTGTTCACAGATGGTTCCATCACTAATGGA

A_14_P117493 TAATAGAAAGTATTAATAAATGTTGGGTCCCGAGCACTGCACCTCATTTAAACGTTTCCT

A_14_P119577 AGTAAAATCAAGACCTAACTATCTGTGAGACCTCACTGACGATTTGCTACCAGATAAATG

A_16_P36145029

GGAAAAACACACACCAATTAAGTTGACAGTGATCCCATATATTTCTTCCTCTAATTTCCC

A_14_P114676 GCATTTGTCGTAAAACAGACTCAATTCTGCTTTTCATTGATGCTCCATTGTTATTTCTAG

CUST_3317_PI426210822 CTATAGGGTGCCCAAGGAAAATGGGAGATTTTTCTTGATTCCTGCAGTT

A_14_P112065 GAAGTAGTTTGTCCTATTAGTTTGTCCTATGGCAAGAACCTGAGGCTTCAGAG

A_14_P132590 CACAAAAGCCAGTGTCATTATTACAGGATGGTCCATCTTGCATCTACTCTAATGTATGTA

A_14_P136930 GTCTTCCAAGTTGACATGATGAAAAGAGAAACAGGATGGATTGTGTAATTCTTACTGTCT

A_16_P01345188

GTGCACTCTCAATACACACTTTTTTTCAGTAAATTTTACCTTACCCATAGACATAACCCC

A_14_P123981 AAACTCTGCAAAGACAATGTCTAGCACTTATATCTCCAGTAACTCCTGTCAAGGTTTATT

A_14_P113625 ACAGCAGGATATTTCAGGCTATAGTAAATGGCCCACTCTCCTTTCTTTGGTTATATTAAT

A_14_P110993 AAATGACTGTCCACTGGACTGAAGATGTTATAGCATCATATTTAAAATCTCCATGGGATG

A_14_P132395 TGTCATATTGCCCTAATCTCTATTTTTGATAAAATTGGATAAGGAGTGAAAGAGTATGCT

A_14_P131852 CCTCCTATACCTACACCTGTTGTAAAGAAACAAATACAAAACAGGATTTCAGCAAAACAC
A_16_P03544322
AAACTCACCACAGACAGTTAATGCTCAACCAGATTTCATAAACCCTGTTGTTTTCATACT
A_16_P03454624
TGGCAACATTGATTAGAATCACACTTGAAAAGTTTTAACAGGCTTATGAGTAAGGGACTG
A_14_P107993 TTCTTAATGGCTGGATTTTAAAACATACCTGAGGGTGCTTGGACAATGAAAATTCTTCTC
A_14_P200659 TATTCAGTATTGATCTGCCTCTCGTCTTGAGTGGACATGACTGTATTCCATTAATATCTC
A_14_P127523 GCTTGGTGTAACAAGCATGTGAGGAAAATATTTATTTATGCCTTTTCTGCAGGTGAAGAA
A_14_P201475 CTGATGCTTCAAAAACGAGAAGGTAAATATACCCCAAATGGATAAGGGATGCATTCTTAA
A_14_P113649 TAAGTAACAAGTAATCTCCATCTTCTCCAGGCTTCTAATCAGTTTCAGAACAGGAGTTTT
A_14_P134695 CTGCTCTTTGCTTCTCTTAAGGAACATATGGTCACTCTTATTTCTCTTGCTGATGTATTC
A_14_P137994 AAAAAGGATTTTGTTTAAAATACTTTAAAAATGTTATTTCCTGCATCCCTTGGCTGTGAT
A_14_P136582 CTGCATAAGATACCAGTTATGCTGGCTTCCACTTTCACAGTATTCTCAAATAGTATCTCT
CUST_3041_PI426210822
ACATACCTATCTATTTACTTATTGACTCTGAAATAACAGAGGTAGATCCTTCCAGGTTTA
CUST_4733_PI426210822 CCCATGGGCTTTGGAGTCCCATGGGGCTCTCAAGCCATGATTCTT
A_16_P03809793 CTCTCAGGCTCATTGTTATAATCCTCTCAAGGATCAGTCTCATTAGCAGACAGC
A_18_P14133801 GTGCATAGTCCCAGGGAAGCCGTCTCATGAGCCCCTCCTTCTCTT
A_14_P109531 CTTTGTGTCCATCCCTTGATCAAAAACTTTCATTTTCTACCTGCACTATACCTTATCAAG
CUST_210_PI426210822 ACTGAGGACGCCGCCGCTCGGGGCCGCCCCTGTGCTCGCCTTCAG
A_14_P102903
TTCACTGTTTTTCATGGAGAAAACTACCAATCAACAGAGAAAAAAAAAGGAGACACTGAG
A_14_P114435 ACTTAACACAATTTCCAACTAATTCAGCAGTGATCTCCTAGTAGCAAGAGAATGATGACT
A_14_P108180 TCGAAGGATAGTGCCATTTTTTATCTCATTGCGGTTCTGGAAAAGAAGAAAAAATCTTTG
A_14_P123877 ACCTTTCTTTTATGATGGGTTGTAGAGGCATCCATCTCTTCCTCTCCTATATTATCAATT
A_16_P00685571
CCTCTTTCAATGTTTAATGGTTTTGATTCCAAAATGACTTCATTTAAGAGCAAAGAAAGA
A_14_P114714 ACCAAGGGAATAAAAACCAATGTTCTTTGAAACAGCAGGTAATTTGGTTTTTCCTACAAA
A_16_P18196734
GACACCAGAATATTTGCATGATCAACGTTTTCATAACAAATTACCATTCCTGTTCTCCTC
A_14_P117046 TTTGCTTTCCTTTAGAACTCTATGAACAAGAATGCAACGGAGAGACTGTGGTCTAC
A_14_P110815 ACATCAGTCTTACTTAGAATGACATCTCTGAGTCATTTAGGACGTTCACTGTTTTCTGAT
A_16_P00685201 TATTGTGCTTCCTGTGTTGAAGTCAGGGAATAAGAATTTCCTCTGTGCCACAAATAAT
A_16_P39773474
ACTGAGGCTCTGAATCTATAAGTCCCTAAAACTCTTTCTTATGAAATTCATGCTTGTGCT
A_16_P03810157
CATCTGCCATCTTGAATTATCCATTCCACTTCTGTTTTCCATTAGCATCAATAATCACGA
A_16_P03288778

AGGACTTGATCACAAAAGATGGAAATCACGTGTTAATTGTTGAGTAAGCACATTTTGGTT

CUST_1365_PI426210822

TGATATAGGCTGTCCTGCCATCTCAGGTCTAGACAAGCAAAATCAAGTTGTATTTTTA

CUST_606_PI426210822 GGCAAGCATTGGTCCCTAGTGAGTCCCCTCCCTCTGGAAATGAAG

A_16_P19832903

AGGATGACTCTACTTACATGCCAATAATGAAACTTCAGCAGATATACACCATTCACATTT

A_18_P14246653

AAAAAAAAAAAATGTTCAATTAAACCCATAAACATTCTAACCCACTAACTGACATTTTCT

A_16_P19398194

CATTCTTTAAATTCATGCTTGATAAACTGATGCTGGCTATAGTAATCTTCTGTCACCCCC

A_16_P37726834

TGTTTTGCGTCTTCATTCTACAACACCTCAATAAGTAAAATGACAAAAGATTCTCCTTGA

A_14_P200411 CTAAAGTTAAATGAAAACTTGGTTTCTTTGAAAATCAGGCAATAGAGACAGGCTCCAGAT

A_16_P21499121

CCTGCAAATATCCTAAGACTATGCTCAGAAGATACAATCCCAATGTGTATAATACTGCCA

CUST_4060_PI426210822 CTGATGTGGCACAGGTGGTCAGTGGGGCAGGGAAGACAGAAAGAT

CUST_10114_PI426210822 ACAGAGCCCTCACCTAGTCTTGAAGACCTTTAGGAAAATCAGCTTCAG

CUST_5801_PI426210822 ACCATGGCACTGTAAGAGAAGCACAGCCAGCCCCAGCGTCCTTAC

A_14_P138702 AAGGACTAGGTGAAGTTTTAGGCTTGGGTTTAAGGGTTGTGTTATATTGTATGCAAATTT

A_14_P103213 ACATGTTGGAAAAATCCTTCTACCAGTTTCAGCATTATAGAGCAATTCCAGGAGTAGTAG

A_14_P122250 TCTTTACCACGAAATCATGGTGCAACTAATGAAATTATATTTGTCCTCACATCTCGTTGT

A_14_P115596 TTTAGATAAGGTTTCATTTTTACTTCTAGGCTCTCCAGGCAGCAAGACAACTTCTTTTAC

A_14_P106392 CTTCAGCGAATCTTATATCCTAAAACTTAGCAAGCCTGAGGATTTGCAAATGGTTGC

A_18_P22841623

GTGTTTGTATGATATTCTGTGTCAGTTGCACATGGGATGTTTCTACCAGCATCTGAATT

A_14_P115102 TAGGTTGCAGATGCTGCTAAAAACACAGAAGTCTGTGATGAACTAATGAGCAGACATAA

A_16_P15406713

AAGGCAATAGACAGAAGAAAAGTTCACAGAGTAGGTTCAGAAATAAAACATAAATGGGGC

A_14_P127957 GTGGCATTACAATTTACCATTTAAAACCATAAAGATCACGGAATTGAAACTGTTGGGCTT

A_14_P136915 CTAATAATGGGTAATCCTACACCATTTTTGCATCAGAATATAAGATGGGGCTGTTTGTCT

A_16_P15652898

ATGAAACATTGAGGCAAAAAAAAGGTTTGTAGTCCTTGAGATTCAATATCCGCACATTTC

A_16_P20798707

TTCTTCCTATCGACTTCTACCAATTTTGTCATTCTCTTTTGGCTCTTAAAAACAGACTGC

CUST_4169_PI426210822 GAGCTTATCCTCTCCTCTTTGTATCAAGCCAGGGGCCCACATGTT

CUST_7954_PI426210822 GGCTGCATCATATGGTCTTTCTCTTCGATTCACATGATGTACCCCAAATG

CUST_2778_PI426210822 GATACCCGTTTAGCTGGAGTGGGCTAGTGGGATATTTCCGATGTT

A_14_P119363 GCCTGAATAGGTCTAAAGAAAAGAGCGGCAGATAGGAACAGGTTAGGAAGTTT

A_16_P17617455

AAAAGTATTTTTGTCTGTATTTATGCTCTGTGATCTCATTTGTAGGGAATTGCTAAGCCA

A_14_P110634 ATGATTCTCCTTAGTATTGTAGTCATTGGTCAGCCTTAAATGTTCATACTTGTGCAGAGT

A_16_P15122320

AGACAGTTCTGACATACTTAAACCTAGACACTTTTAAACTTCCGTTAACCACTGAAGAGT

A_18_P11597138

ATGCCTCCTAGTGTATACTGGAGTTACAAAAGAACCCTATTTTCAGAGTTGCAAATAGAC

A_14_P200916 CTGATGATTGCTAATTAGCCATAAGTTGGGACTGTGGAATGGTCTTTAATTTTGGAAGTA

A_14_P200886 CCTAATAGGTGAAATCTACACCAATGACACAGTTCCTAAGAACGCCAACAGGAAATATTT

A_14_P133410 CAGGCAAACAACATGGGTGTTGGAGTGGTTGGAATTATAGAGTGTAATTTCCTTAAG

A_14_P122868 CATTCGAAACATGAGTATACAGAAGTCTTTGGTAATTCAGGATCACTGATGAGTAAAACA

CUST_10253_PI426210822

TGTTCTTGATGAGGTAATCAGGCAGTCTCTATGTTACATGAGTTAAGAGTCCTAGACTTA

A_16_P02202972

AAATGCACGACATCAAGTAATTGTTACTCAAACTGATGCAACTATGACCTGTAATGACGT

A_16_P16191880

GCTTTCCCCTCAAAAATCATCACACAAGTAGGAGTAAAATAATGAGTATTTGAATAATGC

A_14_P122766 CTCCTTTTTCCTTCCCAATGATTGTGTGGTCACCTTGAGATGAAACCAG

A_16_P15763483

ATTCCACGTTCCTTTATTAGTCATATGCTGCAATCCCTAATCAGATCTCTTTACACAGAG

A_16_P37311670

CCCATTCATCCTAGAAAATCAACAACTTCTAACCAGTATATTTAAATCTCTCAGTTCCCA

CUST_9156_PI426210822

GATCAAAGATAAAATAGAGATAAATTTAGCAAATTTTGGTGGTCTTGTTTTATAATTCAC

A_14_P134738 AGTTTTCTTGATTCAGTGCTCTTCCATCGACTTGTATACAAATTACAAAGGCATACTGTG

A_16_P18539449

GTCACAGAAACTTCGCAATAGTCTTAGTCTTAAGTGGTAGTTCCAAATTCCTAGCAGATA

A_14_P121698 AGAACACCAGTAGATGTTAACATCAAAAGTCCTGTGAAAAATGACAGTCGTTATACACAG

A_14_P200755 TTGAAAAGTATACCGGACAGAATTCTTAGGGAGCGGTAACGTTATACCTATGAAATGTTT

A_14_P123776 CAGATAAGTGTTCTTCAGTGTAATGTGACTTCATGCTATATATCTTTTGTAAGACATTTC

A_16_P16191871

AATTTTTCAGAACAGACCTGAAAAATTGAACCACTTCACTTTATTGCCATTCTTCTGCAG

A_14_P132102 TTAGTAAAATGAGTTGTGCAACATCACCGATAAATATCAGTCGAATGATTCTGGGTGTCA

A_16_P00685463

CTCACACAGGAAATATCTCTCAAATAACATGGGCAGTGTATATGTTATTTTTCTGGAGGG

A_16_P17301621

AAAAGCCACTTTTCTATTCATATTATTTGCCTTCCCATCTAATGGTTTCCCACATAGCAC

A_16_P37489672

ACCCCAATTTTCCAGTTAAAAGCCTCTCATGTGCTAGAATTACCTACATTTAGATTAGCA

A_16_P00685127

AGGAATGGAAGTTAAACTTTATCCATTTTGAATTTCAACATGTCAAAACATTATCATGGT

A_14_P131679 GAGAAGTTGGTTTTTAAAAGTATGGACTTCTGGATAATTGGCAACAAAGAAGATGACAGG

A_14_P106370

CTCAAAGTCCAAGAAAAAGAAAATGATCTCTACTACCTCAAAGGAAACTAAGAAGGACAC

A_14_P108587 CTCTCATAAGCACAGACATTCAATTAAAGCAACAAAGGTTTTACGGCTTTCAGTTGGAAG

A_14_P127454 CTTCAAAGGGAAGAGTCCAGATGAAGTCCTGGAGAACATTTATGGACTCAT

A_14_P126504 CAGTAAGGAAACTGGATAATCCATAACCAGGAGATTTACTGTTGATAGACGTGTATTTTA

A_16_P30952667 TAGCAAGAAGTCTGCTCTGTATCACCTTTATATAGCAGACATGTCACCCTGCTTCTA

CUST_6713_PI426210822

TAAAAAAAAGTATTGCCATGGTAAATGCATAATTTAAAAGATTAATAAAAATAATCCCAT

A_14_P123401 CCTGGGAGGCTTTAAATGATGTAGCCTATAGTTTCTAATGGTAAGGCTAGATTCTAGATT

A_16_P02298191

AAAAATACTCTCAGGCAAGTGATACATGAAGTCATGAACACTCTAGTATGAATCCGTCAC

A_14_P101109 GGTCATCAGATACTGAAGAAGAAAGCAGAAGCAAGAGAAAAAAGAAACACCGTAGAC

A_16_P38628714

AGCCTGAGTAGCCTTAAATATAATGTCATATCCTGTAAAACCAATAACTCAAGTTCTGGA

A_14_P123652 CACTAGTTTACTGATGCAAACATTAGGAGAGATCTGAGTTCTATTAGTATGGCAGGCATT

A_14_P138611 CTTGTGGGGGTTAGAGGAAATGTATTTTATTTGTTAAGCAGAAAATCAGGGTATGAGATG

A_14_P118377 CTTACCAAACTGCATGCCCCAATCGCCAAGGTAATTTATTCTTATTACTTGATGTCCTA

A_16_P20899881

ACCCACCAATACTTTTCATTTACTGCAACATTTTAGGAAGATTATCAATCTGAACTGCCC

A_18_P11914440

AAACCTTGTCTTTCGTGATCATAATCCTATAAACAACAAAAAAAATTAACACCTGGGTCA

A_16_P17537852

AAATACCAGAGAGAATACACATCAAACACCTAGAGACTATAGATTCTGCAAAGTCATGCC

CUST_4037_PI426210822 TTTAACAGAAACACGCGTAACTTTCCTGCAGATGCACTTCCTGTGAATGA

A_14_P106767 TTCTTTATTATTATGAATTCATCCAAGTAATACAAAGATGCCCTTCCCCTGCCATATGGG

A_14_P136998 AACCTCTTGCACAAAACATTGACTATCCCTACGATTTATTCTAACTGTCATCCTTAGATC

A_14_P201906

AGGGACAGAAGGAGAAGAAAAGGATTGAATAAAGAAAATAAATTGGTTCATTGTTTGGGT

CUST_8464_PI426210822

AGGAAATGATTGACTTCTGAAGACAGAAAATGGAGAAATGCTAGGGAAAACAAGAGA

A_16_P19790022

GTCTTCAAATACGGGTAAGAGTTAAAGGGTTGAGATAATATAACCGATCAAGGATTGTGC

A_14_P126597 CCTTAACATGTTTTCAGTTTGATCCTGGGAAGAAAAAATATGCCCTGAATGAAGGATATC

CUST_3777_PI426210822

TTAAGATTCATAAGAAGTTATAACTCATAAGAAGTTTTAACTCATAAAAAGTCAAAATAG

A_18_P11427421

AGCCCAAACTTACAAATAATACCCAATACGATAAAAGATAAAAACATGTGCCATAAGGAA

A_14_P102564 CATATATATTATTCCTTATAGGAATCGCAGCATCTGACCCCAGGATTCACCTTACAAAGT

CUST_3829_PI426210822

GAACATTTGCACCTTTCACCATTGCAAACTTTGACATGTGAGAGTTTCCAGAAATA

CUST_6987_PI426210822

TTTAAGTAGTGTTAAAAACTAGTAAACAATGTAGGGGTTTGGAGAAAAATGCTAATTCCT

A_14_P114690 AGATTTCTGAAGCCTGGACAACTAACGTCTACTTTTTCCCACAACTTCTCTCT

A_14_P101718 GAGACGGAACATTTGCCTAATTTTGTAATAAAACACTGTCTTTTCAGGATTGCTTCATGG

A_14_P105156 CTACTTCTTTTAAAAAGGTGATCTACACAGTAGAAATTAGACAAGTTTGGTAATGAGATC

A_14_P121494 ATCTTACATAAGAAGCATCATTAACAGAATAGATAGAGGATTATGGGGCTTTAGCCACCC

A_16_P02101684

AAAAAGCATTTCTTTTCAAGATGCCTCTTTCTATACATCGTTTAAAATCCAGCAGAGTGT

CUST_3374_PI426210822 TTCACTGCCTCTTCATAATAGAGTCCTGGGACCTTCTCTGTGTTAAGCATAT

A_14_P105159 GTCCCTACCTGACAGTTACTGTGCTCCTGGATCTTCATCTTTGCTC

A_14_P136627 AATATGTGTTATCACATGTTAAATAGGGCATATGTTTGAACTTTTTAAAAAAACTTTTCC

A_16_P40327887

CCAACTATTGAGAATTAGTTCCTGAGTAAATCAAAGAACATTATTTTATCCAGCCCCGGT

A_14_P136823 TCATATCAACATGAGGAACATCAAACTCTATTTTAACCCTGTGAAAGAGAAATTTATAAT

A_16_P37315088

GGCAATCCAGGACTTATTTACCAGGTTACAAAAGTAAGGATTCTATGATCAAAAGCACTT

A_14_P127641 CTCTTAATGCCTTGTTTTTAAATCATCCCTCTAGAATCTCATCTCTATGCTCCCTAGGTA

A_14_P200633 ATCAAGACTCTTGTTGCTCAAATCTGCATTTAGTTATTGGAAATAATCTCAGGCCAAGAT

A_14_P125043 TGAGCCCTATTGTAGCCTCCTTAAATACATGAATTTTGACCTTTGGCCTTCCTTTAATAA

A_14_P116229 GGCAAAAAGCACTAACCTGTATTGTTTTAATCTGCTGAAAACAACCAACTTCTCGAGT

CUST_2785_PI426210822 CCTGCTTATCAACATGTTTCTAATGTCACACAACCTCCAGCTCTAGGACAAA

A_14_P123134 CAAATTCGTGGATTTTATTTTTTGCCTCAGCCTCAAAAAGCCTTAGGGCTGATTC

A_14_P106880 CATTACTAACACAATCTTTAATGGGCAGGGATTGTGTTTTTTTATTTAGTCCCTGCAAGG

A_14_P119685

ATAGGAAAAAATATAGAAAAGTCAGAAGAGTTCAGATTCAGACAGAAAGAGACACGTGCC

A_16_P19540246

AAAGTGAGCAAATCCTACATATTCCCACCTATTAAGGTATGAAACTGAAAGGAGTCACAT

A_18_P14138393 CTCTGAAGGCAATGGAGTTAACAGGAAGATGGACGATGCTGAGAG

A_14_P121334 CTCTGTCAAGTTAGAGAGAGACAATGTGTAGGAAATGTTCTTTTTTAAAAAAAAATAACA

A_16_P00890019

CCTGACACAGTTAATTGAGCAAAATTTTTCACTTTCTCTCGAACTACTTGTATTCCACGT

CUST_7130_PI426210822

CTAGGCATTGCAAAGATAACCATTTACTCTGATTTCAGAGGTCTAAAGGTGTTAGTTCC

A_14_P126106 TACATTTCAGTTTCTTCATCGGTTGGATTGGATGCTTACAGGGTTTTTCTTGTAACATTT

A_14_P111503 AAAAAGGGAGAGATAGTGTGTGTATGGGTCTAAAGATAAAACAAATTTTGAGGTCCTCTT

A_18_P22841371

GATTCTTCCCCCTTGACTACTTTCTATTTATTTTAGGATGCATTTTTTGGCAACCTTTCC

CUST_8567_PI426210822 ATGAGGTGCTGTAGTGAAGAGGCGAGAGTCCCCAGATACTTGTTT

A_18_P22843398 CATGATGTTTCAGGGCTGGAAGAGGTCTGAGAATCCCATTGATCTCTTAG

A_14_P136115 ATTTTACTTTTGTTTCTCACATTTTCAGAAGGGTTTTACTCCCCTGCATGTAGCAGC

A_14_P108517 AGTTCCATATTGACTTTGTGTAAATTTCACTCTAATGAACACAAGGTCTTTGGCTCATTC

A_14_P200534 AAACCAGAACAGGTTTCTAGAACTGGTGGGAGAGAACAGGAGATGTTG

A_16_P18405395

ATCAGAAAAGAAGAGTTCATTCAATCTAAGGCGTTGTTCAAAAAATATTTTCAGGGCTCC

A_14_P125620 GTTTTAATGATGACAAAAATTTGCATCTTGTTTACTCAGCACTCTTACATACTGGGCCTC

A_14_P132257 GCGTTCTTACCCATCAGCATTACTTTTTTTCATGAGGTCAGACAAGATTGTCAGATATAT

A_16_P35552051

AGACAACATCTACTCCAGTGAATGATCTTCAAACTTAACTACCTGATGTAGTCACTTGTG

A_14_P112608 GCGTCTCTGAAAATTTTAATTTGTTCTGACAACGAAACTAACACAGCATAAGGACTTAAG

A_14_P121716 GGGGCTGAAATGGCTTCTTCTTTTTGCATTTTATGGTTTTACAATTGTAGCAGTAAAACA

A_14_P105361 AAAAAAGCACAAGGGGATATATGCATGCCTTATTTTTACCAATGAGGAAGATGGTAATCT

A_16_P15680092

AAAAATACCTAGGGTTTAATCAGTATCCACTTATTCTGACTTTTCTCCACTCACTCACGC

A_14_P105209 GGCAGTATGGTAGGAAATATGGGTTGGAAGTTCTGTATCTATTTTAGAATCTTGCTTTTT

A_14_P200859 GTAAGTGCGCCTCTCTCCAATAAAGTGATAGAGATCACTTTCTTATAGAAGAAAAACTGG

A_16_P00201823

CAACAATGGCTTTTGAAGATAGGCAATGAAATTAACAACGGTAACATTGAACTTCAGCAC

A_14_P135576 CTCCAAATATAGAAGAGGGGGGAAAATATGATGAGCCTCTCTGACATTTGTCTTTAAAAT

CUST_3721_PI426210822 TCGATAAGACCTGTTTCAAGATATGGCAAGCCCTAGTTCTGATCTCTGG

A_16_P20785996

ACCCATGACTTTTCTACTGACAACAAAAATCCAGATTAATCACCACCTTACTTCATTAGC

A_16_P17469311

TGACAGTCCAAACATTTTAACACCGTCTTAAACTTAGCATCACCTTCTTTTATGTGTCTC

A_14_P137861 CTTCCAGCAAAGGAATCTGAATTCTGGAGTGTTTATTTCTGTTTTCTTTTTTGCTTTTCC

A_16_P18376817

AGAATTTGCCCTGAAGAAAATACAAGACACAGTTGCATTTCAAATTCTTATCTGTCCCTT

A_18_P22839039

TGATCTGATTTATGAAACCAATGGTCTTTTTCATCCTGACTTGAGATGCTGAGTTTTCTT

A_14_P112347 CCCTGTGCCAGCATTTTACAGATTTAGTGTCTGAACTTCTAGATCTTATCCACTGAATAC

A_14_P126935 GTTGACTTTTGGTTTGAGAGATATGTTTCTGTGCTGTTTAAATGCTAGAAAGGAATTAAC

A_14_P102690 CTGCTCCATCTCATTGCAAGATTTTTGTTACAGTAAAGTTGGACTGAGTTTTCTAAAGAC

A_14_P108407 TTATGTTGCCCATTCAAATGAATTGCGTAGTCCTTACTACAGAATATAAGCCCAGTCAAT

A_14_P129702 GAGAGAGAGACTTAGCAGTATTACCTAGCAGTTTCTTTCCATTCATATTCTTCTCCATCT

A_14_P132758 CTCAAGTGTTGTGTTGTTATGCTGATGGAAGATACCTTGTTTCAGATTTCAATGAGAATG

CUST_3942_PI426210822 TTTAAGTTGGATAGGAAGAAAAAAATCCACACCAGGAGAAGATTGGTTGTAAAAGTTTAT

A_14_P132525 AATCCTTTGCCGATCTATCTTTATTACTTTGTAGGTCCAATAAAACTCGCCTTCTAAAGG

A_16_P16318853 AAAAAACACTAAAGGCATTCTTAGACCATTTGGAAGCATGAATTTATGCTTGGCACACAA

A_16_P18695158 ACTGCTCTGTCATTAACCATCTGAAGATCTATCAAAAAATTCCTCAAACATCAAGAACCA

A_16_P20980807 ATGTTCAATGGCTTAGGTAATGTCTTATACTGCATATCCTACATGCTAATCACTGTGACT

A_14_P102429 ACCGAAAACAAATGGAAGAAATGCAAAAGGCTTTCAATAAAACAATCGTGAAACTTCAGA

A_14_P109843 GAGTTATTTTTCCTCATCGTTCCCTTTCTTTGGTGTTCCCAGGCTGCTATT

A_16_P01891855 AAATCCTCACCAATAAATCCTATTATACAAGGCAAAACCTGTATATTATCCCTATCCCCT

A_14_P116445 AACCTAAAATAACTCGTCCTCCCATAAATGTGAAAATAATAGAGGGATTAAAAGCAGTCC

A_14_P127291 GTATGGTCTATGAAAGTTGGCTATTATTAACCATGCCTAGGGAATTCAGACTTCATATGG

A_14_P128032 CAGGATCATTGTGGCTTGGACTTCCTGATTGTCCAGAATCTGTAAAGAAATC

A_16_P15114647 GTCCCTATCTTCTGGAAAAATTTGCAGGTATCCCATAAAAACTCCAAGTATTTGACACTA

A_14_P120240 CTAAACGAGAACTTAGGACAAGTCAACATAATAATACTTTGGTCTGAAGTGACCAAAGAC

A_14_P111312 CTAGAAGCAAAATATCCCAAAATATTTCATAGCAAGTTAGGATCATGGGAGGGAAAGGAA

A_18_P22745477 TTAAATCTTTCAGTCACAGACTAAATAAAACAGTCACCTACAAGTTCTACAAGTTCAGCC

A_16_P35981870 AACGAACAACAAAGAATGTTTAAAACACCATACATATGCCTTTCTAAAGTCAGACAGTGA

A_14_P138781 TAATTCTCCCCTAGAGATCACTCACCATGAGGTTTAGTGTCCTAGAAACCAG

A_14_P120006 ACAATTCCCCGTGAGTGCGTAATTTTATGATTCAGAGGATACTGCTTCCAAATAACG

A_16_P41818075 CATTTAACTACCAGAAAGGACTCTTTACAAACTGGATCAAAATGAAAACTACCCTCCCTA

A_14_P133598 CAGGAGGAATTTTGTTGTCAGAGAATAAAAGGAGGTTGTCCATAATTGACTTTAAGCAGC

CUST_8450_PI426210822 AGCTTCCTGAAACACGTGAAGGAAAATGATCAGAAAAAGAAGGAAGCTGG

CUST_1411_PI426210822 GACTGCTTTAAAAGATGAAAGAGTCCCACACAATTCCTATTCATGTCAGAAATTTATGAG

CUST_4690_PI426210822 GGTCTAATGGTGGAGGTGAAGAGAAGAGGGCAGGCTGCAGAAAAG

A_16_P39937758
AAATTGTGACCTCCAAGAGATGAAATAAATGGCTCTTTTCATGAAAAAGAATTTGGGGAA
A_14_P118107 TATCACAGATTATTAGAATTTTGTCAGTGATCCAATGTCAGTTGAACTAAACTTGGGGGG
A_14_P202508 ACATGGAATGGTTAACAGTTGTTAAAATATCAGTTTCACCAAATTGGGAAAATATCCAGT
A_14_P131079 AAATAGCCTTAAAGGGCTGATCCTCACCTGAACATGTTTTCTTGTCCTCATTTAAG
CUST_8828_PI426210822
GAATTACTTATCAGAAACGAAAGCTTCTTAGAGCAAATTTAAACTCTACAAACTTTGGTC
A_14_P128321 TTTCGTTTCATATCTGTAACATCAAGGTTTTTCTCTTACAAGAGGGTAGATTACACCGTC
A_16_P16715252
ACTTCAATCACCAATACTAACATCCACTTTATCAGAACACTACCATAATGTTGTCTAGCC
A_14_P110182 AGATCAAGTGGCCAGAAAGAAAGTAAGAAAATAAAATTATTTTGTCCTCTTGCAACTGGG
A_16_P03738926
AAATTTGGGTTGAGGGTAAATTATAGTGCACATCAGGATGATAACAAGAAATAGGATCCC
A_14_P111685 AACAACGGCAAAAACGAGTTAACTGCCCTGCTCAGTAATAATAGTATTGTATGAGTTTCA
A_14_P126273 CACAGCCACCTTTGACTACAGACTATTTTTTATCCAAGATGTATTTCCATGTAGCAAAGT
A_14_P121473 GGAGTAATTTGACCGACAATGTCATGATCACAGTAGATATTATTCCTTCAGGTTGGAATT
A_14_P112991 GGAATTCCACATTTTAAATGACACAGCAAGATTTCCTAGGATCCTATTTTGACATCTCTC
CUST_7413_PI426210822 AGTCCACAGGTCAGGGGCAACGGGCCCTGCAGACTTCTCAGCATC
CUST_9379_PI426210822 CTTCTGCTCCACAATCAACCTGTACCACTGGCTTGAGGTTCTGGC
A_16_P36231066 AAAAATCAGAGGGATCTGGAAACCTGAGGGCTATAGGCTGCGTCC
A_14_P110326 AATTTTTTGCTTTTTCTGTCATCTTTATAGTCTATTGCGTCTGCAGACATGGATTTCAAC
A_14_P111715 GTTACAGATCACAATGGCTGTAAGAGGCAAAGGGCATGAGTGTTCTGTT
A_14_P112787 TTTTTTCACAAACATCATTTCATTGGGTAGACCATGGATGTGAACTCTGGAAACTTTTTG
A_18_P13856766
AAAACTACCTGACAATTAATGGAGGAAAAAAATAAAACAACATTTGCATTAATATTACCA
A_14_P121915 TAGAAGCAAAATCAAAATCTCCCACTCTTTGGTGTATTCATTAATGTTACTATCCAGCCC
CUST_6613_PI426210822 CACATGTTATTCTTGTTGATGTGCCAGACATGCAGGAGAGTGAGGTC
A_14_P201270 ATTCAATCCCTCTGATTTAAACCTCTCTTCCTACTGGGTCTCGCTAGTGACTAATT
A_16_P20270933
GGATTTCAGAATTGCATTTACCTGTCACATAACCCATTAAGATCTCTCCGTTAAGTTCAA
A_16_P18179266
ACATATTTCTTGGCTACCTGATCAAATTCTACAATGGTCCTAATCTGTCTTATCAGCAGC
A_14_P130281 CTGTTTTATAAGCCAGGAATTGTCTTGCTTCTGGCATTGGCAAAGTATTTGAAAATGAAT
CUST_5478_PI426210822 AAGTGGGGAGAAGAGGTTGGCCTGGGGACACAGTAAAGCATGGTA
A_14_P130917 CCATATTATTATTTCAATTACCATCTGAATTAAAAGTTTGAGCAGCGATCAGCCCACGTA
A_14_P133555 GTCTTTATAATTCCCAGACTGTCTGATATTTGTATCTAAAGAGGGAGACCTCATCACTTG
A_16_P36599493
CCCTCAACCTTACATTCTCTTCAAGCAATCCATTGTTGACTTTAAATCCATTAAAAACCA

A_14_P102256 AGCAAATTTGGGGGTGGTAATATTTTCTCCATTTTCAAGAAATATCTTCAGCATATGTCC
A_16_P30952328
AGAGTGCTGATAAATATCTTTGATCATTGATTTCATCCTAACTAGATTTTTTTTTTTCA
A_14_P103283 CTACACAGGGATTTTGACCAGAAGCCATATTACAAAAATAGCCTAAAAATACCTCTATTC
A_16_P00692385
TGCAAATATCTGAATTCCATACCACTAAGGTCTCACTTTTTAGCCATTGTGTTGGATACT
A_14_P138893 GACTGACATAACTCTGTTTAATTGTGAGGAATTTTAGGTCTAGTCATCAGCAAACCTTGT
A_14_P133971 TCACTTTTGAAAGATTGCCAATACATACCATGTCTGCTTTTAATGAAGGACCACTCTTAG
A_14_P136590 GAGGTATTTGGAAATGAAAAGGCACTGATTTTTATACTGTAAAACCAGAAACAAGAAGTT
A_14_P117772 CTGCAGTATCCAGGAAGGTCTTTATGTAGGAGGAAAAACTGCTCTTTGAATAATATGTAG
A_14_P106144 CCACATAAAAATTTTCATCTGAAAGTGACTTCTGGAATCATTAAGCCTCTTCCCATTTCT
A_14_P116586
CTGTCTCACAGACACTAAAGATAAAACAGAAAACAAGCAGATGAGTGACAATGACAAACA
A_16_P03142070
CCCCCAGAAAAACAAAGAATTTCAGAGTCAATAGCACGTTATGTTTTGACTACATCACAA
A_16_P18269070
AATCAGACATCCAAATTTTCAAGAACCACACTACATTTCAAGCCTTCTGTCACATATTCA
A_16_P20976463
TTTGTGTGTCCCAAAAACAATGAATAATAAGCCAGTGCAGTCTTTTACATGCCATTAAGA
A_14_P118651 CAGTTGCCACGTTACTGCCTTTTTTAAAAATATATTTGACAGAAACCAGGTGCCTTCAG
A_18_P11167500
GCTCAAGCCAGTATTTAACTCAATTCAATACACATGTTTTTATATCTGCTTTGTGCCAGC
A_16_P18873974
TGTGTAAAGTCGACGTCCAGAGTATTTGAAAAAGCATTTGACTCAACTCATGTTTTCTTT
A_14_P114649 CTGCAATTGGTGGCCGCATTATTATTGTGAATATTCTTATGAAACCAACAGTAACTAGGT
A_14_P121737 CCTTGAATTGTCTAACCATGGACATAAACAGTTGTCTCCCTTCTACTGTGTAGAATACTT
A_14_P134106 CCATTCAGTTAACCAAACAGAGAAAACTGAAGACTGATTAGTTGAAGTCTCACAGAGTAT
A_14_P103995 AAATACCACCCTCCGCCAGAAAAGTGCAGAATAAAAATTTTCCACTAGCAAAAGAAG
A_14_P108293 AAAAAAAAAGGCCACTGAAATGTATAAAATGGTCTGAACATGATGGTGGTATCAAAGTCG
CUST_2984_PI426210822 AGTTGCAAGAGATCAGTACTTCCGGATCAGTACTTCTGCAGTGAGATGA
A_14_P123646 TTTAGGCAAAACAAACGTTGGCATATCCTTTCTTAGATATACTGAAAAGTCTTGGGGTCT
A_16_P18105009
ATTTTTCTACAGCGACTTCCAGTTTATCCTAAGCCTCTTTATGAGTTGACCATAACAGAT
A_16_P38976991
CCACAAAAGAGATATAGGCAATTGATCCATCATCTTACTAGTCAGCCAATTAGACGACTT
A_14_P111619 ACCTGATAAAATGAGACTTTTGGCAATCAACTGTAATGTTCTAAAGGATCCACCAAAAAC
CUST_8288_PI426210822
ATGACAAAACAGAAAATGTATACTTTTATAATCCAGTTTTTAAAAAGTCAAAGCTATGAT

CUST_8520_PI426210822 CATTGTGTGATGGTGACAGTAATTTCTGGCATTGCTGCCCTCTGG
A_16_P18993775
CCTAACTAACAAAAAGCAAAAACGACTGAACTAAGAGAACAATTTTCCATCACGTTGAGG
A_18_P16727456 GCCTCCAGTCATATTAATTCTACCTCCTTAATAGTTCTCATCATTATCCCTTTTCTCCAC
A_16_P38507738
AGCAAACAAGTCAAACTTAAACTCAGAGCCTAGTATAAATCAATTAGATCCAGTCTCCTT
A_14_P135926 GATATTGTTGGTTCTGCAAAACTAAAACTCTTTTCCCTGGACAAGCAAACACCAC
A_14_P103129 TTTGTTCCCTCAAAGGTTCTTCAGAAGACCTGAAATTCTGCGTGCTCTATCTA
A_16_P00176893
GTAGGTGAAGAAATGGGAAATAAATGTCACCTAGATATCTATCTTGAACCCTGACTTTCC
A_14_P132346 TTATTATCCAATGCTGCTTTATTATTTGGCTATTGGGGGCTTCAGCCCATTTTCCTTA
A_14_P131659 AAAGCCAATTTGAATAAACAACTCTTCCCTCTCAAATGGGCAAGAGTAACTCTTCAG
A_14_P111008 GTGAAGTTGTTTTAAATTACCCTTTAGAAACAGGAGATAAAGTGAGGGAGTAGAAAGGCA
A_14_P112839 ACTTAGTTGAAGACGTTCTAAGTCACTTCGTAGTTTCTCAGTCTCAGAGTTGTATATTCC
A_14_P100207 TTTTGTTTCTGTTTGTGTTCTCAAGACAAAATGTCTTTACCGAACATTTGCTTTGGAACC
A_14_P136584 GCACCTACCTTCTTCCGTTAGTGCATCAGTAAATGTGTTATTTTGTCATTTTTCCAAAGA
CUST_8545_PI426210822 TCTTTACTGATCATCGAGCCAAGGAAAAGCCACTGGAAGGGACTT
A_16_P16191948
TATCATAGAAAAATCTTCAAGTAAGTTGTGTTGTTTCTTCCACAGGACAGTTCGTCTGTC
A_16_P01482961
AAAAATGGATAAATTGGCTACGTAGAGTTATGTCAGGCTTACTAGCACAAGATCCATGAG
A_14_P106752 CTTTGCTGCCCTGCATTCCACATCCAGACGCAGTGAAGTATTTAAATACTTGTTTA
A_16_P15024708
AACACCTAACTCATACCTCTTATTTGAAATTAACCTGCAAATCTTCTTCTTCACCAGGAG
A_14_P121275 TAAACCTTATCTTCTGCCTTCCCATTCTTCCTTTTTCCAGAAATAGTGTTTTAAGGAACT
A_16_P17136719
AAGCACCTTTATAGACACGCTAACTTATATACCTGACCTTGGAGTAATTCCTATTGGAGA
A_14_P111740 CAGCATCAACCCCTTTAATAATGATCCTTTTCTCATACTTTTCCTTTAAATCGTTGCAGA
A_16_P32060000
CCTTGCTTGTGTTTTTTACTCTCATAATTTCCATAGACTATTTGCAAAAGCAGACTTGCC
A_14_P136364 ATTTGAAGTTTTCTTGCATAACTGACTGGGTTGTCAGGGAGACTCACATACAAATGAAAT
A_14_P106213 GTTCAAATTATATTGTTAATTCCAGTTTCCCTTTTCCTCATATTTACCCCGAAGAGGTGT
A_14_P118461 CTTCTTTTCCATGAGTGGAAATAGCCCAGATTCATTACTTTTACTTTGGCCTATTCAGAT
CUST_9015_PI426210822
CACATGATCTAAATCATACATTCTCACACATTCTCCTACTTCAAAGCTTTCCCTGGTT
A_14_P113279
CAGAAGGAAGAAATTTCAGTGACACTTAGAACAGAGAATTCTCAAAAGTGAGATTTCAGC
A_14_P134496 TTTGGTTTTTTTCTCTTCTAGGCTTTGAATTGTATTCCATGGTGCCATCTATTTGTCCTC

A_14_P123004 CTGGTTCTCTATAACAAAAACAATGGACAGTGGAAAGAAACTAAGAGGAATATTGGCTTC

A_14_P107284 ATTAGGGAAGTCAGAGAGATACCTGTGACCAAAATTTTGACCTTTTCTAGTTAATTTGGT

CUST_2935_PI426210822 TGAAACGAAAACCAGGAAGGGGCGCTCTCTGAGTGATTGCATTTT

A_16_P37894295

AGCCCCATCATGTGATACTTACTCTATGCAGATTTCTTAAAGTCATGCTGAAAAACTTGT

A_14_P137988 GTGCTTGACCAAGGATGGGCCATAAACAATGAGTAAACAGTAAAGTGTGGATC

A_14_P105780 AAAAAATTTCTGCTTGCCACCTCCCACTTCACACTTTTAGACTAAATAATCAAGTATTTA

A_16_P16191077

TTCCAAGATCACCCCTTAAATAGTTTATGGCACTTAAAAAATGAGACAATACACCAGACA

A_14_P131706 AATGAGACTGAAATGGCCTACACATAGGCTGACGACCTATAATAACATAAGAAAGTGATG

A_14_P101741 AAGCCAAGTCTGCCCAAGTAACTTTATTCGTGTCTTCTCAAACATTTCAGATCAAATAAT

A_14_P136174 ATAAAACTACTGGCTAAAAGTCAACACCAAACACCTCTAAAACCTTAGGAGGTTTAAAGG

A_14_P123833 CTGATAGAATTCTTTTCTGCATCGAAGTAGCACTATTCCAGTATAGGCATTGAGATATCG

A_18_P22841740

CAAAACACTTTATTTCTCAAATAAATGATGGAGTAGGTTCCAGCGTTGTTTCTTCCTTGC

A_14_P135649 TCCGCTAACTTTTTTAAAAATTCTGTTTCCTATGCTCCCTTCAGAAGTCAGTTATCAGAG

CUST_5655_PI426210822 AGGTCACATCCCTGCATAACCTCCTCCCGCTGAGGAGCTCCCTTC

A_16_P36230433

TGCATACGTGGGTGTATTTTTAAAAATTGTGACACATCATCAAGATGGATATACTTGCCC

A_14_P102613 AGATCTGAATACTTCAGATTAAAGAGTAAATAACTGGAGGTGAGGTTGAGAATGGGAAGA

A_18_P22840148 GAGTCTGCCTCCACTGGGCAGTGCTGGCCTGGAGGAAACTTCCAT

A_14_P109937 GTCCTCCTTTCTTCCCTCGTTATTGATCTATAGACATTAGGAAGGGAGTGAGA

A_14_P112946 ATAAACTCTAAGGCCAGATTGAATGAGCATCCAAAAGACGTATCTGGAGGGAG

A_14_P110478 TTCCTCTTCTTCATTTAGATCATCTGCCCCACACTCATGGTAGAGTGGAT

CUST_9559_PI426210822

TAAACACTAATTTTTTTATAACTGTAAAGATGATTATTCCATACTTAAAACCAATTCAAA

A_14_P110062 TAAATAAATTTTTTTTTCCCACCAAACACAACAGTATTGTTTTGGTAGAAACCATGTCAC

A_14_P200235 CAGGAGATAAAAATGTCTGTAACGATCTCTGCTGCCATGAAGAGTGCAAAAACAATC

CUST_3878_PI426210822 CATCTGAGGAGCTGTCATCCTTTATTCATCCTGCCCTTCTGCTTG

CUST_962_PI426210822 TACATGAATCCTTCCTTTGACACTTTGATCTCTGACCTCCCAGAATGCTT

CUST_263_PI426210822 GTTGGTGCCCTCCTTGGCCACATCCTCTGGACCAGAGGGTATAAA

A_18_P13086403

ACAGTCTACCTGGGAGTATTCTAATTTATTTCTGAAAGATGGAAAAATGAGCAGAATCCC

CUST_7326_PI426210822 CAGATGTGGGACACTCCTCACGGACAAGTTCAGTGTTGTATTTACTTTTC

A_16_P15628390

CTTTACACCACACAACAAACACTTTCCAATCTTTTTAAGTCTATCATGGTTTGCCTCCAA

A_16_P35744410

AATCATGTTCGTATGCCTTAAGATTACACCTAGCATCATTTAGTAAGCCAAGTCATCTTT

CUST_60_PI426210822 GTCATGGGTGCTTGGGTTGTGACTCACCTCTCCAGCCCTTCACAG
A_16_P18292168
TCCCTGTGCTATTGACAAAATTCTCTAAAAACCTTTTGAATAAATGTGACAAAGACTACC
A_16_P15325198
TCAGCGGAATAGAGTTATGAGGTCTCAATGACAAATAATCTTGAAGGTATATTGGGCATT
A_14_P111935 TTCCAACAGAACATAACCAGATATACAAAGAGATAACGTGGATGATCCCAAGAAATTATA
CUST_1649_PI426210822 CTTGGCCCTGTTTTATGTAGTATGGGCTGAAGGGAGAGGGGTGTG
A_14_P200131 CTACTTCCCTTTTGTTTTTGTTAGAAATCTTTGTCTCCTTAAAACATCTGAGAGTGGATT
A_14_P105662 TTATTAAGCCTTTATACCGTTCTTCAAATTTATCACCAATTCCTGCAGATCTAGGCGCC
A_14_P136044 AAAACTTTCAGACATAAAGGTCTATTTGACTCTATGTTAAGTGGCCCCAGATTGCAAATT
A_16_P41638130
TCAGCATAACAGCAAATTTGACAACAATCAAAAACTCATCCTTTCTGAATATGTCCCTGA
A_16_P17219472
TTGGCCCTTCATCAATTAGATCTTCTTCAGAAAACTATTGTCATATACTCCTCAGACCAT
CUST_3422_PI426210822 GAATGAAGACGGATGAGCCGTGTGGACACAGCCACACTATGTTTT
A_16_P00685283
CTGCCCCTATATAAGGAATATGTTATTACAGTCCGAGACTAAAAAGATGCTTTTGTTTCT
A_14_P123830 GTCTATTTTAAGTTGCACCTTCCAGAACTTTATTTTTGTCTCTGAAAGAAAGAGGCAGAG
A_14_P201853 CGGGAAGTTTTCAAGGACTTAAAAATAAATGCGCAGGCTTAAGTTGCGAAACTCA
A_14_P137977 GAATGTGGCTCTTTAACAAGCCATCGCTTTATGAAGCAAGGTTAACAATTTCACTTGATT
CUST_2973_PI426210822
AATCAAGGAAAAGTACTAATTAGGCAGTTCTAGAGATGAAATGCTTCCAGAATGATTGCA
A_14_P117345 AAGAAAATGCATAAAAATGCTGACATGGTTATCTTCACACTACCCCACAAAAAGCACCTA
A_14_P119787 GATGGATTCCTTAGGGGGTTATATGCTTGGGAGGCTGATACAATTTCCTAGAATT
A_14_P131869 CTATTTTTTTTTTTCTGGTGGGTGGGGGTTAATATCAACAATAAGAGTAAGACCAGAGAT
A_14_P104658 CAAGACTGTGAGTTCAACTTGCACTGCACAATTAAATGTTGTGTTTGATTGTCAGTAGTA
A_14_P201093 CTGTTAGAGGAGATAAATCATCATTGTAAGGCCAATTAGTAGACGGGGCAATAACTCATA
A_14_P104549 CAGAATTATTTTCATATGAAAACATATGTGGGAACAATGTCTAGGTAAGTGCTCAGGGCT
CUST_9573_PI426210822 AAGTAAAACTTAAATGAGGGATTCAGGACATTTCCCAAGGGGTCATCTGAC
A_14_P108793 CTTCGTTTTCTATTACCGAAATAGATCACACTTAGAACCTCAAACCCCTAGACCAC
A_14_P120073 GATTGCTTGGGGTTCTAGAGAGGTCAGAGTTATACAATAGATGATAAGCCATGCT
CUST_5300_PI426210822 CTTTTGGGGGTGTTGGCCTGTTTCTAATTGCTGCACCCTCCTCTG
A_14_P125698 TAATCATATGTGGAAGAACAAGCCTGGGATTCAAAGGTCACAAAGAAAATATCACTTGAA
A_16_P40206630
AACTTACACTGTGACCTTACAAAACACTCTTATTCAAAAACCCTCCAAAAGTCTTGAATT
A_14_P128153 TGGGGTTTCAGATCTTTGTGACGAAATAGAATACTGTTTCATATTTGAATCAGAGGGCTT
A_14_P108516 CAGCACAGTCTCATCTGATTCTCAAAGCCACTCTGTGGAGTAAACTGAG
A_14_P105627 AGTGTCTTTTTAAAGGAAACACTTCAAGTTGAAGAAGATGATCGACCTGAGTTACCATG

CUST_8284_PI426210822
ACTTCTAGTCATCTTGGCAGTCTGATAAATTTAAGTTTTTTAAGTGAATTTGTCATAGAG
A_14_P202144 AAGATGAGGAATATATGTTTCACCTTTGAAGTCTCAACACCCTGAACAATACAACATGTT
A_14_P110103 TGGTGTGGGAGGATATAATGACTAAGAGTTGAAGAAAAACTTTTGAAGAGTTCCTAAGTG
A_14_P114589 ATTCTCTAATGCTGATGTTTTAGTCATTCTCATGAGATGGGGAAGATACACCCTATTTCA
A_14_P105345 CTGATGAGACCTCTATTAATACACGAAGTTTCTCAAAGTTTGTAGGAATGAGTGCAGATG
A_14_P128822 ATTTTGTAGGAAAATTTTGAATGACTTATCTTCTGATGCACCAGGAGTGCCAAGGATTGA
CUST_4874_PI426210822
CACATGAGCTAAACATTTAGGAAAACCTTACAGAAGCAAATCTTTAACGGAAGGGTTTC
A_14_P122149 TTAAGCCAGCAATATTTGGAGGATCCGACAATGAACCTGAAAGAGAAAACACGTATATAA
A_18_P14137507 CACCTGGCTCTGCCTCTCAGGGGTGTCCTGTCTCCTCTGCACAGC
A_14_P132133 CACATCTCAATCAGGATGCTTCTCTCCATTGGTAGCACTTTAGAGTCCATGAAAT
CUST_5021_PI426210822
CATCTTTGGTTGTAGTTTTATAAAGAATGCTGGTTCATGGACTCCAGACACTTCACT
A_14_P128726 ACTCCTGAATGCTTTTTTTTTTTTTTTCAAGAAAGAAGTGGCTGTGGACTCAACTAGATT
A_16_P16191297
ACAGCAAAAGTTTCTTCTTTATCTGGAAAATAACATGCTAAATCTCCGGGTCATCTTGAG
A_14_P114028 GTGGCTGAAGGAAATTAGAAATTAACTTGAAAGGCAGAAAAGAGAGGGCACGAAAATTT
CUST_5667_PI426210822
AGTGACATATCTATACCACGGTATGTTTGTAGTGACTACTTTTCAAAAGACAAGATGGAG
A_14_P137139 CTTAGTTTTGTGGGTTATTCATTTAGCCTTTCCTTGAACACCCAGATAGCATATGGTTTA
A_16_P17230620
AAAAACATCTATAGTGATTTGTTGATTGTTTACAGCCCTTTAAATTTTGAACACCCCCCC
A_14_P126116 TACTAGATTACCTTTAGGACTCCATGGAGCATCATCTGAATTCTTCTTTTTCTTGGGTC
A_16_P00388789
AAAAATCACTGCCTAAGGAAACAAATTCTGAAGAGATTTGACTCTGCAAAGCATAGACAC
A_14_P110980 CTGAATTACTACAGGAGATATCAGAGATCGAAAAAAGGTATGACATTTGCAGCAAGGTTT
A_14_P117499 ACAAATATGACTTTACGTCCAGTCGGCATACAGTGGTGCAGTAAGTGGG
A_14_P132210 AATCCAATGTTGAATATCACAGTTCTTCTTTAATGGAAGCAGAAGATTCAGAGTCCTTGT
A_16_P58424471 ACTTTTTATTAACGTGTTTAACCCTCACAGCAACCCAGTGAGGTATGTGCAGTTAC
CUST_7921_PI426210822 CAAGATCTGAAAAGGCAGGATTAGGGTGTGGGCTTAGGAGAAAGG
A_16_P18949351
AAGGCTTGAGCAAATTTAATCCAACTTCCTGCATAATATTTGATTAATCTCAGACTCGGG
A_14_P105886 TCACTTACAATACTATTGGGGAAGATACCGATGTCTTCTTTTCCTTATATGACATGAGGG
A_18_P17037952 AGTTAACAATGTAACATTGACCCCACCTTTCTCAGAGTAGCAGACCAGTCTC
A_14_P134108 GGGAAAACTTCTTTTCTAAGTTGAGTTATTCATCAAAGCAATCAGCCATTATCTCCTCAG
A_16_P01779981
AACACAGCCAACTCTTTAAATTTTCCTATGTAAACTACCCATTGTCCAAAAGATCAGTAA

A_14_P136822 AAATATTAAATGATTTAGCAGTCTCCACGTGTGTTAATGTTTCAAACGTGTATCATAATG
CUST_7102_PI426210822 CTATGAGAGCTTTGCCTTTGACTGGGGCTGGGAAGGTGGAAATAG
CUST_8117_PI426210822
CAGTATTGCAATTGTGAAAGGTTCATTTGTTAGAATAACATGTCCACACAGCAGCAGT
A_14_P100058 ACAAAACATTATTATACCCCGTGCAAAATGAATTGAGGCATCAAAAGTTTGCTTTATTGA
A_14_P139165 ACAGATGACAACCTATTAATGCTGTGAGAATGTTTCTAGATCAGTGCATGGATGGCT
CUST_4549_PI426210822 CTTTGACAAAGGATGGGATGTTTTTCTAGGTTATGAGGGCCTGGACATTT
A_16_P02835317
TACCCCTGTAATCATATAGTCCAAACTTTTCTATCCTCCTTACAACTCTTTTACCCCATT
A_16_P01426183
GTGCCTATTACCTAAACCTATTCCAATGTCTTAACTATGAAGAAACTCGGTATGTGGTGA
CUST_8431_PI426210822
CAAGCAGCATGTCATTCCTTCCTCTCTCCTACTTATCCTTTTTAAACATTTAGTTTTAAA
A_14_P131772 AGACCTTGAGGCCTTCAGTGGTCTCATCAGCCCTATTTTTAAATAATTTGTTTTCTTTTT
A_16_P15606097
ACTCTGTTAGGAATTCACTAGATGTCTAAGAGCACAAAGCATTTTATTCCCTGAAACCCT
CUST_1314_PI426210822
TTTTTTTTTTTACTAGAGCTGTGTCTGCCCAAATGCATTGTTTCCTGTATTGTACAATTA
A_18_P17363787
GAGTCATCAATTCTTATAGATTTTACTTCCTCAACAACTCATATCTATCACCAGTGCTCA
A_14_P138024 CACGGAGGATATCTCTGGGATCTTTTTCCCTAATACACAGTTATAGTTATATCCATATTG
A_16_P16191951
AAAGAGATTACATCCAAATTGCCAAGACCTAGTAACTGCTCTTTAACTATTTACCACCCC
CUST_5925_PI426210822 CATCTGGAACTTGGGGAAGCTACTGTCCATAGATCTGAAATGTCAGAGA
A_18_P16668991
TGACCATCACAGAAAAAATGGAATTGGAGTCACTCAATCAGATTAAAATTAGACTAGCAC
A_14_P118533 TGATATTCCAGACTGGATTTTTTGGGAGATGAACTAGATTTCTATGTTAGGATCCCTTTC
A_14_P121939 CAACCAAAGTAACACAACACTTTTAAGGGAAGTGAAAATAGAGGGTAAACCTGAGGC
CUST_4667_PI426210822 CAGGTCTAGCTCCTGAGAGCTAGCCTGACTTTCTTCCAGAGTCTT
CUST_4886_PI426210822
CTTTAAGACCCTAAAGGGAGAGTTATTAATAGTTTCAGAAAGTTGAGGGAAATCATGCAG
A_14_P111739 CAAGAAAAGGGGTTTTTATATGTTGATGGCCAATGCACAGGTAAGTATTTACTGGATAAA
A_14_P107030 ACTTGTTGACAGTTACAATTCTTCCACAATGCCCTGCTAGAGCGATATATTATGTTTTTG
CUST_785_PI426210822 TTCTATTTCTGTTTTGCAGTGGGAGAGAGGATACGGTTTATAATTCCGCACTG
A_16_P02732362
AGTTGGAATACAGACAAGATGATAGGAATTGAAGCAACTAGACAACAGAAAAATGGAAGG
CUST_7962_PI426210822 CCTAGACAACACAGAAGAAGATGTGGTCTGTCACCTATAGTCCTGTCATTC
A_14_P107245 CATCTGAGATTAGAAGCACTCTGATCTTTGTGACTAAAACCTACTCACTGCATTCACATA

A_14_P134599 GAATAGTCTTCAGGAGCAACTATCCCTGAATATTAAGATCAGCAGAGGGAAAAATATAGG
A_16_P16937272
AAAGATAGAGTCCCATGTGCTAAATCACTAGAGTCAAAACCAAGAAGTCCTATAAAGTCC
A_18_P15197382
CACCCAGCAAATACCAAATAACCTGAACTTTGAGATTAAACCTCTTCTATGGAACACTAT
A_14_P119235 TGATTGCACTGGATGATCCATGTCTGATTTGAGAAATTACTCAGCAATCTGATATTTTGC
A_14_P119699 ATTTTTCATCTTCTACGATCAGCACTTTTACCACCATCAGGTTGATGGAATTCTTGATG
CUST_8880_PI426210822 GCCTCCCATGAAGTCCTTGAAACTTGAGACTTAATGAAGGACACTGAG
A_16_P16191459
AAAATGGAGATGGGGTATAAACATCTGAATTCACTGTTAAACACTCCATGAACTGTTGAT
A_14_P115789 CTGTTCCATGTTCAATCGTGCTGTTAGTTGGTATTTTTCTTCCATCTTTCTTGAGCAG
A_14_P122873 CTACAAAGTGAGATACAGATAAGTCACTCTTGGGAAGAAGGCTTGAAACTGGTATGATAT
A_14_P129080 CGGCGACTTTCTATTAAGTTAACACACTGTGGCCACCTTTAATGAAAATCAAATGTTTTT
CUST_9584_PI426210822
AGCATTAAACATTTGGTAATTAACATAATTTAATATGCCAAAAATGAGAAAATAGTCAGG
A_14_P114723 GGCTTTTCTATCAAGCAGTTGGACCTTTTTTCTCCCTCCAAGTTATATTACATTTATCAC
A_16_P02938148
AAGGATGAGTGAACTGTAGAATTCAGTGCAAGAAAGAAGTGTTTATCTCCTGACGTTTG
A_14_P118450 TTTAAAATATAGCATCTGTTATTCACCCAAATCATGCATGTTATGGGGAAGAAGTCTGCC
A_16_P19847489
ACTTCACCTCTCCATTAAAATCACCACATTCATCTTGATCATTCCATTGCATAGTTCAAA
A_16_P01501363
CCCATTATTTTCTCAAGAATTATACCAGCCTGCTTCTAAGTATCCATCACCTCATTCTTG
A_14_P119592 AAACGCTGAAAGAAATGTCTGCAAACAGAATGTCTCAAAAATAGGTAGGAGATACGATTC
A_16_P02057082
AACACAAAACGTCTTTCATGGTTATTCAGAACTTGATCTTACTAACAGTTCCAACGAGTG
A_14_P113891 ACTTTCTTAGTGGCCAAAGAGCAGTTGTTGACATTGATGTCTAATTATTGAACACGACC
A_14_P137510 TTCAGGACAATTTAGGAAATACTGGGAAAAAAACCAACAGTAAGTATTGGGACGATAACG
A_18_P27173174
AACCTGAGTCCAAAATGAGTTTCTTAACCAAAATCCACAAGTCAGAAGTAAAGGAAAGTT
CUST_8579_PI426210822 GGATTTGGGTGGCTTGGTGCAAGTGAAATTCAGTCCTATTAGGAGAAA
A_14_P200006 CAATCTCAGGGCCAGATAACATCACAGATACTTTAAACCTGCAATTAATTTCTGGTTGTT
CUST_9467_PI426210822 CTCAATAAAACTTCAGTCCTGTCCGTTTTTGGTGAGCAAGAGATGAGAACATC
A_14_P137445 TCTCAGTCTTCTCAGGCTTTTACTTTAATGCTAGGATAGTCTAAATCTTCAAGGTGGCTA
A_14_P103781 TTTTTCTTGTCCTTTAAGGCCTTTCCAACTCTACTTGCAAAAAAACTTTGTGTTTCACAA
A_16_P00685359 GAAAAGTCGAAGCAAAGGGGTCACAGAGAAAGATTTCTCTGATGACTGTGTAG
A_14_P118051 TTTCTCCGTAGCCCTGCCAAAATGAGGTTTTATGATTTTAATCGCATCATAGGTAACATA
CUST_7761_PI426210822

TCTTGATCTTTTTTATTTAAATCTTTGTCTTCTGAAGCATCTTAAAGTAGTATCCAGGAG

A_14_P132620 CCTAAGGAAATTGAGACGTCAGATGGACATCATTGTGTCTGAAGTAAGAATCTTTAGTTA

A_14_P202263 CTTTGAGACTTCCTCATAAGGTTTTCTGGGCATGTTATTCATAATTTCCTCTGCCTAATT

A_14_P119917 TTGAGATACAGAGTGAAAATGGGGAGAACTGTAATCAAGACATGTTTGAGAATGAATCAC

A_14_P138151 GAATAAGAACTAGTGTCTACCGCAAGAAATAGTGACTATCTGCAATGGTTTTGTCAGATG

A_14_P104628 AGCCATTAATTTGATTAAACAAATTTTTGGCCCTAAAATTCAAATTTCCAAAGGACCTGC

A_14_P106350 CTGTTAATGGTTTTCACTCTTCACTTACTGATTCAAAGGTGTGATGTTAGTTTTACCCTG

A_14_P122494 TTTATGGCATATCTAGCCAGACATAATTGCCCAGGATTGCCACCGATGATAATTAGTT

A_14_P136024 AAAAGTCTACCGCCTTCCTGATATATTGCTTCAATTTTGTCATCAATTAAATTCAGAATT

CUST_3362_PI426210822

AGATATTCATTATAAGCATGATTCCCTTCAAAGTAATCACCTTCTAGTCATTCCTGTTTT

CUST_8937_PI426210822 TTTAACTTCTTGCTCCACCTGCTCTATCCCCAGCTCTGCAGGTTT

A_16_P15206940

CCTCCTACCTGCCTTATAATTTTATGTATGACCAGAATTTAAACTTTCCCTAAGACAGCA

A_14_P109710 AGGCTTCAAGGGGTGATATTTTAAATCAAGGTTTTAAATACAAAAGGTGTCTTGTGTTGC

A_16_P16957415 ATGTTTATGCTTGGTCTATCCTCCATACCTACGATATCCTTATACACCCCTAATCTTGAT

CUST_6880_PI426210822

AGGTGATAAAACAGATCCACTTGCTAATCTGGATAGAGCAGGAAGGACTTAAACAT

A_14_P101492 CTGAGCAAACCAACGTATACATAATATATGCTTCTGTTCTTAACTAAAAGCAGGCCTCCA

A_16_P01410304

CGTCACCATAAACCGTTTAAAGCATCCAGTAATTTACTAATAATGCGTTTCCTGATGAGG

A_14_P138644 GAGCCAGAATTTTTGGTTTCCATTAAACTCTCATATTCCTTAGCCCTGAAGAGAAAAAAA

A_14_P116573 TCATAAACTTTAACCCACTCTCGTTTATCCCATTCAAGATCATCAAATTCCACATACACC

A_14_P108130 CTGAAAATAAGAAAAATGAAAAGATCTCTTTTAGCAGGTTGGTGTTTTTGCCTTGGTGTT

A_14_P201492 GTGAGATGGAGAGAATTTGACATGTCTTGGTTTGAAAATAAGTCAAAAGAAGTCTAATAT

A_14_P113080 GATCAGTAATGCTAAAATCCAAAACTAGGATTCTGTCTTTGAGGCAGACCACATAATCAG

A_14_P138923 CAAAACTTGGTCTTTTCTGTATCTATTTTTCCTCCTATCCTGGATCTTGTATTACTCAGG

CUST_4833_PI426210822 GTGGTTCTCCCGTCAGGTTATAATAGGGAAGGGATGTGTGATCTTTC

A_14_P113005 CTTCCTGGTTATCAGGCCAACTATAGGTGCAATCACACATTTCCTAATATAAATTTCCAT

A_14_P122584 TCTCTATCTTTTTTTTAAAGTTAGTCACAACCTACAGTGTGAAAACATGGTCCTGGCCAT

A_14_P115727 GTTCGGGCATGGAGATCTAAAGTGGATTGTTACGAGAACAATTTTTTTTTGTTAACGAAA

A_14_P139754 GTAATATGCCTTCAGTGACACTATCTTCTGTTAAGCAGTCAAATAAGAAGGAGATGGAAC

A_16_P00698907

GTGTCAGACTCTTTAGGTATGTTGAAGCAGAAAACAATTCACTAGATCAAGTCACTCAAC

A_18_P16117951

CATCTTCCCATCCATTACTACTTTGTCATTGTTCTTATGTCTCATAGTTGAACTCCTGAG

A_14_P106175 GATAAACTTTTTCTCTTTGTCCTTATCCTTTTTCTTGCTTGCTTTAGGATCAGTGCTAGC

CUST_2808_PI426210822 ATTAAACCCCAGAACAAGATAGAACCAGTGTTGATTGGAATTCAGAGCACGT

A_14_P201745 CTCATCAGAGAGCAATTTCTTTTGACACCCAACACAACGGGTAAAATGTTTAATTTGAAA

A_14_P128502 ACTGATCACCAAGTAGCCACAAAATATAATAAACCCTCAGCACTTGCTCAGTAGTTTTGT

A_14_P200964 CTGACTAAATTACACAAAATAGACCATAATAGGATAGCGCTGTGAATACATCCTTCCCGA

A_14_P121075 CTTGTTTGTTTTGCCTGCTAGAATTTGTCACATGCATCTACTTTACGGATGTATAGTTTC

A_14_P126192 CTAAATGGATTTAAAAGTTCTTTGGGCCTTTAAGGGGTTTAAAGGAAGAGAGTTTTTTCC

A_16_P16991562

AAAAGTCACAGAAAAATCCAACCTTCAATTGTCTTCACACAAAGGAATGACTCTAATTGC

A_16_P16191638

TTTACTTTCATTTTTACCCAAATTTATTTCCCCTGGACGTCTACTTCACAAGCAGTAACA

A_16_P01831436

GTCAGTGTGAAGAGAACATTTGTTGACAAGATGAGGATACATATTCTGATGAGAGCAAAC

CUST_6495_PI426210822 ACTTCATGGTTTATTTCCTGGGTAATTCATTTACCAGCATGCATTCAGGAGGT

A_14_P123176 TTCAAAGCCAACTTCACATATGTTTTGAGGCTTGCAAAATACAGAATGAATACCTAGGAG

A_14_P110609 ACAATTCTCCTTAACAAGGAGCAGTTAGTGTCTCAAACATTTGGGTGTAAAGAAAATTGT

A_16_P00684979 ATTGCACTCAGCAGATGTGGCCACCTTCCAGTATCACTGACTCAG

A_14_P100351 ACCAATAAACACAGATCTTAATCAGCAGGAGGCAATTTTCTAGTATAACAGGATTCGTCA

A_14_P104198 CACACTCAGCGATGCTTAAAGATGCTTGTGAACATTGCTAACTATTGCGATATAAACGTA

A_16_P20314896

AAACATTTCTGGGTTTAAGCAGTTCTTTTCACTTTACTCTTCTAATGGCCAAAATCAGGT

A_14_P125004 TTACTACTCTCTATAGCATGATGGCTCAACTCTTCACTTTTATCTGTTTGAACTGCTGCA

A_18_P15327820

AAAATCCCTCATCCTCTTTCAAGTTTGCTACTTTCCAATATCATGTGCATTAAAACCAAG

CUST_2330_PI426210822 CTGGTCTTTCTCCCAGTGGGTCAGAGACAGAAGGGGACACACATT

A_14_P112226 TCCCTAAGTGACCTGATTTAAAAAGAAGGCATGTTTATTTCCACTTAGTTCTCTAAGTGG

A_14_P100323 ATTGTGGAACTTTTCTTTGTTCACTGTGTCATTTGTCTTCTATTTCACAGATGGAATCAC

A_16_P16997472

ACTAACACAACTTTATAGGTTCATCCATACATGCCATGTCTTTAATTGCACCCCAGTAAT

A_14_P110269 GTAATCCATGCTTTCATTTGTGTAAGGCTTTGGGATCTTTGGATATATTTGGTAATGAGG

CUST_6445_PI426210822 ATGTCTCCTTGTGACAGTTACGCAAGAAGGAAAGTGAACAGAATGTGGAT

A_16_P58424130 AAAACCTCTAACAATGACATCCATTGCCCTCCACATCCTCTGCCA

A_14_P118066 CTGATTTTTCTTCTTCCATTATCCTGAATCTTTGCTTATTTAGGATGAGTGCCTTTACCC

A_16_P16791270

AGGAACATAGAGTATGCTTGGGATATACAGACTTACACATATGAACAGAAAACAGATGGG

A_18_P14686228

CCTCATGTCTCCATTGAAACATACCCTAAAAATTCTACTCACAAACCTGTCATTTCACTT

CUST_9221_PI426210822

TTTATTATTTCCTAAATCTGGTTTTAATCAATTTGACTTACAAAGGCACAGACTGTGGTC

CUST_5235_PI426210822

TTGTTTACTTTTTAATTTATAGGATTGTAAATGAAACTATACTTGGCCTCAGTTTATTCT

CUST_3415_PI426210822 CTCGCCAGACCTCCCCACCTCCCGGACATACAGACTCCAGATGTG

A_14_P125846 GCTTTTTACTCTGTTTTCTTGCTAGTAGTAAATCACTCCTCTCCATGCTCTGTCAAGAC

A_14_P202229 ATTCTATATGGTTAATAAGGCTACTCTACCCACAGAAAGGTTACTGCGAGAAAAGTTACC

A_14_P137177 CCAGGCATGTGGATTAAAAATCCACAAGGGATTTTTTGAAGCACTATTCAACTTCATTAA

A_18_P22841922 ACATAATTGCTTCTGATGCCAGCCAGTCTGCAACTGCCAGTGATTG

A_14_P136909 ATGGCTTGCTTCAATCTTTTGATATCTTAGAGTAATTGCTTCCAATTTTTGGGCATCGC

A_16_P16464648

AGACTGGAATTGCTTCACATTTTAAGTGCCTAGTGACTATTTTCCTCTTTTAGACAGTGT

A_18_P15331595

TGAACACATGCTTATCTCTTACAAGAATTAGGTTTTTCCATTAACTACCCCTTTCACAGT

A_14_P113075 GAATCTCAGAATTGGCGCTATGCTACTGGTGGATGGGAGACTGTATTTAG

A_14_P138905 GCAGATGAGAAGAGTAATGCCAATTTTATGAATTGTTTCCAAGTTTGGGCATTTGTTTGT

A_14_P115457 ACTAATAACCCCTGGATGGTCAATCTGATAATAGGATCAGATTTACGTCTACCCTAATTC

CUST_225_PI426210822 CTACGCAGTTCTGGAGCATTTCCACGACAACACGCGCTGGTGAGT

A_14_P125429 AGTTTTTCCACATTACCTTGGAGTCTGCCACTTTATCCAGACCTAACTCTTGAAT

A_14_P101902 CTTTTTCTTTTCTCTTTTCAACCTCCAGGTGAATTCCTGATGCGGAGATGAG

CUST_1032_PI426210822 GTCCATCTTCTTCGCCAGTATGCGGAGCTCTCATGCACCCCAAGA

CUST_1690_PI426210822 AGAGACTTGACCTGCACAGCATCTGTTATCCCCATGAGCCTTCAT

A_14_P101274 CTTGTTTGGATACATCTTTCATGAGGACTCTGCCACATCCATACTTTGTCCTTG

A_14_P105673 TGAATGTGTGAGCACAATAGGGCCCATTCGCTTGAAGGTATTTTACTCAAAACTT

A_14_P133652 AGTTTCCTTTCTCTTTATTCTTAGGAATTCATCTCGGTTTATAAACAGGCACAACATGCC

A_16_P30952515 GATTAAATTGAGACAAATGTTTGCTTCTCAGAATGGCAGTGACCATGGGTGCTT

A_16_P21569285

CCTTAGTTTTACTCTCAACTGCTTGACATACAATTAACTGCTTGACATACCATTAACTGC

A_14_P107228 CCTAATTACTAATCTCCTACCTGAGTCAATTTACTGAAGTTTCCTCGCTAACTGAAGGAA

A_14_P122806 TACCTTATTGCGAAGATAACCAAAGTCCAGTGAAAAGGATGTAGCACTGTCACG

A_14_P201259 TTCTTCATCATGTTAGCACTGTCCAAAGTATTTGGCTCCTATGCCAAAGACATTAGATTA

A_14_P120915 AACACAGCCTAGATTCCTTTTTTTTTCTTTTTTAATGAAGCCACAGCATTCTGCTTCTGT

A_14_P124282 TCTTTTTCAATCATCGAGGACAATTCTCTCGCTTATAACTCTGCCTGGAGTTGTC

CUST_7099_PI426210822 CTCACTCTTGGGTTTTCCTCTCTGGAATTAATTTGTCCCCAAGTGAACT

A_14_P118818 ACTCTCTTTAATGGGAAGGGGATCGTCATTTCTTACAGCAATAGGTAAAAACATAAATAC

CUST_285_PI426210822 ATCTCCCTCTCTCACTTACTCCAGTTGCTGGGTATAAGACCGAGT

A_14_P128229 TGTGAAAATAGAAATGAAAGCACCGTTCTGGAAATAATAACCCAGTTAATGTCTGGCTAG

A_14_P104274 GTCCTTAGCCTCTGCCGGGAACAGAGTGTTCATATGCTGTTAGAATTTTTTATTGTAA

A_14_P118465 ACAGAAAATTTTGATGAATCAAACTGTGCCCAACGTTAGTTTGCACATACTTTGTGTCTA

A_16_P02142511

ACTGACACAACTTCACTTTCTATGGACACTATTCTCTTAATTACCAATCATGTATGAGCA

A_16_P03606956
AACAACTCATTCTTACTGCTTTTAGATTGTCTTGAATGCATATTTGGAGTCTAAAGCCGG
A_14_P118497 AAAGCATCACTACAAGATGAATGGATGACTCCGATACATATGAACTTCAGTTGACAAAAC
A_16_P02936646
GAACCTTATCCCTTAAGCATCTATATCCCAGTCATACTTATGCATGGAAAGGCTAAGTAA
CUST_326_PI426210822
CTTAATTTTTTCTTAGATTGTGTTCTCCCCTTGATTTCTGTTCTCATGCTGTTGTTTTCA
A_18_P10896589
ATGCCCATCCTTTTAGAATATCGGATATATGACTCAGTTGACCTGCTTAATAACCAAACA
A_14_P112923 TGGCAATTGGCAGGCATCTAATATTTGAATAAAATTGAAAAACCTTCACAAGAAGGGTCA
A_16_P18034150
TGGAAGTCCTTAAAGCATCTAATACAAGTTAATGAACTCTGCAACCATTTATACTAGCCC
CUST_8125_PI426210822
GACCATATTATCTTCTAAGCACACACCAGAATATTTCAGATGCTCATTTCTTTTCAGGCA
A_16_P41871116
AAACCTTTATGTGCATCAACAAACTATCAACCACTTTGTTTATCTCATTGAAAGGATGGT
A_18_P14131636 AGCACTCACCCTTTAGAGCCTGTTTCCTCACCTGTCACTGGGGGT
A_14_P117802 TTCGTGGATTCCTGAAAACATCAAGAAGAAAGAATGCGTGTATTTTGTGGAAAGTTC
CUST_7345_PI426210822 AGGCCTACTCTGTGGGCTGTCCTTTTTTCTAGGGCCTTGATTAAAT
A_14_P137653 ATTATTACCAGCGCTGAGAAACCAATACTTTCTTTTTCCATCTCGCTTGTGTAATTTCTT
A_14_P130524 TTTCTGAAAATTCCTTTGGTTATATATTCTCATATTCCCTCCACTCCTCATTCTGTCTCC
CUST_3801_PI426210822 GAAATGCGTAGGGTTGGTCAGTGATTTTGCTATGCGCCAAAGCAG
A_18_P16251338
ACGCATTTTAAGATAAACTCTAACCAATACCACTACTTGAAGATAATAATAATTGGCCCT
A_14_P202132 CTTCCCATCTTTTCGATACGTGAATATCCAGAAATTCTCATCTCAAGCCTTGAAATCATA
A_14_P113691 CCCACATTAGGAATTGATTTAAGCCTGAGAGTTTGAGGGCTTTAATCCTTTAAAACTTGG
CUST_2301_PI426210822 GCACCCAAGGAGGATGACTTCGCAGGGGCGGTCTATATCTATCAT
A_14_P134156 CTACTGAGAACATAAAATCTTGGCGAGTCATTAAACTTATGAAAATCACCGCTCTTGGAT
A_14_P108365 CTAGAAAAAAGTTTACGTAAATTTCTACCTCTGATAAACCTAATGACTTACTATTTTGTG
A_14_P102160 CTGATGACAGTTATTCATATTCATATTCACTGCAGCAGAAGGGATTCTTTCTGGATGGAA
CUST_5768_PI426210822 ATGAAGGGCAGTTTTGGATTCTGGGAAAACAGCCCATTGAAACAGAAT
A_14_P137403 TAGTTCTCTTGTTCTCAGAGAACAGCAGGTTGATAGTAGATTATGTCTCTAGGTAGAGAC
A_18_P12879225
AGATGCATCATGGCTTTAACTCTCTAGAAAATGGCTACAAACCCTAACTAGGTGAAAAAG
A_16_P18419782
ACCCAACTATGCACAAAAAATCTTAACTCTTTATTCACATATAAATCGAATGCTCTCCCA
A_14_P138087 GAACATGTCTCTCTTCCATTGACACACACAAAAAAACTTTACTCACAAAAGAAGCAGAAT
A_14_P129554 ACCATTGCTTTTATAGACATCCAAGGTTGAATTATAACTGCCTAAGAACAACCTCTTCTC

A_16_P15841947
AAAAAGGTGAGGAATGATATCATAGTCTGAATTTGTCCAAGTTTTCCTTCTAGCACACAC
A_14_P111336 CTGGGATAGTTGATGATTAGAAACAATTGCACAAATGTCTCATTCTGACTTCGGACATTA
A_14_P200802 CTAACAGAAAGGCACATATGGAGGCATGTCTTTTCATACAATATTCAGTTAAGCCAACAT
A_18_P17040875
TGTAAACCTCCAATTACTGTCAAGATATCTTTTTAAGGCAAACTTCTCCAATCTCCTCAC
A_14_P115975 GGCACTATAATTTCTGTAACACACTAACAAAAGCATGAAAAATAGGAACCACACCAATGC
CUST_135_PI426210822 CACTCTAGAAAAAAGAGCACAGGAAAAAGTATGGCTCAATACAGGGTCCAGA
A_18_P11191015
AGGAAGCCCTCTGAAATAATACAAATACATCATCACTCCAGATAGATTTTAAACATCCCA
A_14_P130831 TTGGAGACTGATTCTTATTTTTAGGTATGTTGAGTCTGCAATTCTAGAGGCAATCACCAA
A_14_P119825 AAGTAAATCTCCTGCCCTTTTCCAAAGAAAGCCCGTTAGATATACTGAAATACATACTGA
A_14_P117554 CTGCAAATTCATAAATTTCTTCCATTTCTGCTTCATTCACGTTTTCTTGATCTTCTTCGT
A_14_P101555 CTCACCATCTCTTAAAAGTTTTATGGAGAAACATCAACTGTCTGGATTCAGTTCCTTTTT
A_16_P21296091
GACCACAATGCAGAAAATACATAGGGGATCCACATAATTTCAGGATAAGTGCTACAAAAA
A_14_P123693 CTTGGTATCACTAGGAGGCCCAGATGACGGAAGGTAATGAACTAAACATAGTTACTTAT
A_14_P136942 CTTGAAATTTTCCCACTTGTAGATCATCTCATTCTCAGGATAGGAAACTGGAAAGGAATG
A_14_P114370 ACTTTCTGGTTTAGATTATGACTTGATTGCCACATTTGTTACCACCAAATCTTTAGGGAA
A_16_P41102903
ATCCCTGATAATCGTTTAACTTCTTAGATCGACCTGCAAGATTTTCTTACTGTGGAACTT
A_14_P114251 GATAGAGTTCTAGGCCTGTTAGGAGATACACAGACACTTTTCGGAACAAACTATAATCTT
A_14_P125231 CTCATTCAAAATGCCTTTTAAAGCATTTGATACCTTCAAAGAAAAAATTCTGAAACCTGG
CUST_80_PI426210822
TGGCATTATGTTGTACAGCTGTATTCTAACACTGATGGTTGTTGCTTAAAGCCGT
A_14_P100974 GCAAAAAGTTCCAAATTTGAAATTCTTAGACCTCAACAAAAACCCCATTCACAAAATCCA
A_16_P21558109
AAGTCCAATGTAAGAACCAATAAAGCAGAGCCAAGACAATTATCTCCTGACCAATTTGTA
A_18_P24091661
TCACCCATTTCCTACCTAAAGATGCAGTTTAGCGATAATTTACATTACAAACGGTTTTCC
A_14_P132605
TTTTTCAAAGAAAAAGGACAACCAAAAAAATTAGGGAAAGAGAATCCCTCTGTCCCATTC
A_14_P200773 CTGATACCTTCGGTTATTGCTGTAGTTTTCATCTTACTTCTCAGTGTCTGTTTTATTGCA
A_14_P121066 ATCTTGATTAATTATTACGCTGTAGAAATAGATCTGAAAGCATAGGGACCAAGGTTGGTT
A_14_P108828 AGATAATGTTACCTTCGTCTTGACATTCTCTTCTTTCAGGTGTATAAAACCTCTGAAGGG
A_16_P01292634
AAAAATGGTCTCATAGATAAAGCCACTTTATATTAAGCCCTAAATATCCATGAACCTCAA
A_14_P103983 CTGAAATAGGTGCTTAAGCCACTCATATATAACAGTAGATTCCACAGAATCTACCCACAT

A_14_P131261 AAGATTTTTGTTTTTAATATCCTGGTTAAGTTCTCCTCCCTACATGTATTTAAATTTGTT

A_14_P114528 GGGCTTTGAATATTAGGCTGGGAGGTCCTATAGATAACTCGATATTAAATGAGTCATCAA

A_16_P17031267

AGTAAGTCATGTGGTTTAAGAACTATGCCTTCCTATCTACTGTCCACTAAATCTGAGAGT

A_14_P122291 TCACCCAAGAGATTGTTGTTTGCCATACTTTGTCTGAAAAATTCCTTTGTGTTTCTATTG

A_14_P138877 TTTTCCTCTGCCTTCGGAGGGAAATTGTAAATCCTGTGTTTCATTACTTGAATGTAGTTA

A_14_P134541 ACTTTCTTTTTAATCTACTCTGTTGCATGGATATAGTGAGGGGACAGTTGACCCTTTATG

A_18_P16291168

AAATACTTGCTGCTTCATTTACTCACTTAGGTGATAGAACCTCTGGATCTGAATACTGTG

CUST_2519_PI426210822 GGGATCCATGCTTCCCAGTGGTTCAGCTACTTTTCTGTAAATGTGAG

A_14_P102424 ATCAGCCCATATCATTTTCAAAAGATTATTCCACCAACATTTACCTTGAACCCTGACATT

A_14_P100891 CCTCTGAGAGTTTCAGCGATTTCCCCACAGAATTTACGGCAATATTAGAGAAAATAATAT

A_16_P01875891

AGTCTTACCCTCTGAATATTAGTTAACTTCAAAGTCTGCAACATAAATGCTCAGTCAGCC

A_16_P02392977

TGAGAGTTGTAAATTGGGTTAAAATAGGGACACATAGTAGACAAAGCAATGGGATGTAGT

A_14_P136477 TATACTTACAGGGTGTATGGCTGGAGAAAAACACAAAAATGAGGTCTCGTCTAAGTTTC

A_14_P106751 GGGAGTTCAGGATGAGTTGCTATTATTTTCAAAGTGATGACATTTCCACTTGAAATCATG

A_14_P108429 ACTAGAGCAGTGTCTGAGAATAGAAGAATTTAAGAAAGTGAGATTAAGTCAAGTGGTTGG

CUST_6628_PI426210822 GTCGCATCACACAATAATCATCAGTCTCTCATGCATCTTCATCCTTGTCATCTT

A_14_P122037 CTAATTAAATGCTTGCCACTGGCTTATTTTGGAAACTTCATGGACTGTGTGAAAGCC

A_14_P114102 CATTTGCTGTGTATGTGCAGAAGTATTATAGAAAAATTATGCAGGAGATGGCTAGGATTA

A_16_P16418493

AAAGAACAATGAATCATACTGCTTTTTCTCCTCACTACAATCCAAATAGGCCATGTTCAG

A_14_P124990 ATCTGCATACCTGTTAATTTGATATGTCCTATTTCATCAAGCAAAATGCTGTAAATCAAA

A_16_P16706006

AAAAATACACAAAGTCTTCCCTTATTCACTCTCATAACTCCCTATGTTTGTCTTCCTTGC

A_14_P100487 GGGGATATTATTAGCGTGGGACAGAGATTGGTATGCTTGTTTACTTATTTGGTCATATTT

A_16_P40243707

AATCCCTGATACTGCCTATATACACAAAGCAATTTTTTACAAGACAGTTAATCACCACTT

A_16_P36477108

CTCATCTCAGTCGCTATGTTTTACTTATGGTCAGATGTCTTTTCCTTAACTAGGGACATT

A_14_P137810 TGGTGAAATGTTTTTTGTTGTTTTTTATGTTATCCTCCTTCCTGTTTTTCAAAGGGATCC

A_16_P03771353

TAACTATGGCTACATCTGCAAATTTACCCTTTCAATGGAATTCTTGAATCAGTTCATCCC

A_14_P101135 TCTGCATTTATCATAGCACCTGCCTGACACATAGTTTGAAATTCCATGAAGAGTATGAAT

A_14_P122677 CTGCTTGGGATGTCCAAACTAAAGAGCAATTGAGATATGTATGTCAGTTAACTCAACTTT

A_14_P202192 TGGAGAAATGGTCAAAACAACCGTGGCTCCATATATACTTCTTTCTCAATTTTGAAATCT

A_14_P124447 GGGAGGGATACTCAGGAAATGTATTGGTTTTCTTAAAACCTGTCTTGTGATCCTAAAC
A_16_P17049595
AGCCAGCATACATAAATTTCCCAAATCTTACTCCAATCAGAATTACCCATTGCCTAAATG
A_14_P131025 AGATTTATAGGAAATGCACAGATACGGCATGGTTATTCCTGTTCTTTCTCTTTTGGACTG
A_16_P20332084
TCAACATCTCTCTAACAATAAGGTTATGTCGGTTTAGAATTTTCAATTGCTTACCCCGTC
A_16_P17282881
AAATTGATCATTCCAGTGTGGTATAGACAGATTTCAGCAATGATCATTGACAACAAGCAC
A_14_P117727 TTTCTTTTTTAGTCCCGGACGATCCCCAGTATCCTTTGACAGTGAAATAATAATGATGAA
A_14_P102344 TGACCATCTTCTTCCTGTGAACTGCTAAATGCAAATAATAATGAAGCCATATGGAGTGAA
A_14_P113247 AACAGCCAATGAGATTTTTCACAAATAAAAATAGGTCTGACCAAATGTTTCTAATATATT
A_14_P131647 GTTCCTTGTCTTAGCAAATCCTACTCCATTGCCTAACAATTTTCTTTCTGTTCCCATC
A_14_P131577
GAACCTTTCCAAGGGATCTGTGACAAGAATGGTTAAATACTGAAGTGTTGAAGAAAGAAA
A_14_P108568 TTCTAAATCATGCATATTCCTGTGTTCCAGGATTTCTCTCTGATCAAACGGACAGTT
A_14_P102143 CTTTCAAACCGCAATTTCTGAGAACAGAATAATCGGAGCCATGGCCATATTTTTTCAA
A_14_P105618 GATGAGAGGACAGGGGTGATGAATAAATGCAGTGTGAATCTATAATTAAAAAAACCCCAT
A_14_P132311 CTAAATTCCACATGCTGAGTATGTATTCTCTCTGGCTGCTTCTTTGTTTATCTTATGTCC
A_14_P133651 AGACTTCAGGTAGTTATTTCGCATGTCCTTTTTGTCCGTCTGAAAGAAAGGGAAT
A_14_P105570 TGATTTAAGACCCTCAGCAATTAAATTGAGTTCATAACACCAGGTTTCATAGCGATAGGC
A_14_P130016 TAACTTCAAAATAGTTACCTTGCATAAAAATCTCCTGAACCTTTTGTTCCTTTACCCAGA
A_16_P30952380
GATAAATGAAGATAAATGCATGAAGTAGAATAATTTTCAGTCTGGTTAGCAATCATCACA
A_16_P00685084 TTTGTGTTTTTCCTTAGTTGACCTTGCTGGTCTCTTGGTTTTGTTCTTCCATGTTT
A_16_P16306808
ACATCCCTGATAACAGTATTCTAATGCAATCACATTCTGTTTTCTGTTCAACTTCTTCGA
A_16_P19638230
AAAAACCTTATAACCCCCAATATATTCCTGGACACACATAAAAACCTGTTTGCTTTCACA
A_16_P20210331
CTAAGTCTCCATCTTAGTTGCAACAGTATGAAGTCATCCCAGATCAAATTCAACCTACAT
A_16_P01220379
CAGATAACACTTTTGGAATCACACATATGGAGCAAAACCAACAATTATCTTTTCATCACC
A_16_P00685060
GACAAGCAAAATCAAGTTGTATTTTTAAGTTTTTTAGATACGAGTATGGTAGAATAACCC
CUST_5807_PI426210822
TAAGGATTTAGTGAGAAGCTGGAGAAAATATTTTCAAAGACAACTGTATTCTCCAGGGAA
A_14_P110236 CTTGGTTTTTGGATTGTTTTTTCTAACTCTGAACTGGACAAAGATGGAAGGTTTTTAATG
CUST_7965_PI426210822

TGCTCACAGTTTTCTGAAGAGAACTCTCAAAATTCTCAAAACATAAATACCTGTATCCCA

A_14_P135836 CTGTGATCCTAAAAAGTCATCAAATCCATTTGGATAAACGGTCAAAAGCAAAAAAGAGGT

CUST_6530_PI426210822 TAAGAGAAAGAAGGAAGGAGTGAAGGATGGACGGATGGACAGACTT

CUST_7138_PI426210822

CTTTCATTCAGCAAAGTTATGTGTTAACAGGTATTAAGTATACCGTTATCATTTTTCCTT

A_16_P02989210

GAACATCTCCTCTCCATCACGACTACTGTCTTTTTCACCAGCATTTCTATCCTAAAAT

A_16_P15685585

AATGCAAGTCATACCCTAGGAGAATAAAAGATTACTGAGTCATTGTCCATGCAAAGTTGA

A_16_P02678550

ATCAGTCTATTGCCAGAAACCTAATATGGTGAGTCATCAGTTTGAATCCTGAGGTTTATG

A_14_P200577 GGAAAGGCCTACACATCGAGACTCATCCATGATTGATATGAATTTAAAAATTACAAGCAA

A_14_P109027 CTACTAAATAGGGATTGAAATTCTCACCCTGCTCACCTCTCTGCAGACATTGAT

A_14_P104957 TCTCCTGTCTTGTGATTCACAATATCAATTTCGCCAGACTGTAAAATGAAAAAATTCAGT

CUST_9302_PI426210822 GTTTCTCTGGTTTGCTCTGGAGGGAAACTGGTTTGGGGCAAGTTG

A_14_P105048 AGCCAAAGAGAGAGCCCTGGTAGGTCGTTATTTTCAACAACTTTAAATTATCAAAAAACA

A_14_P126062 TGTTTCCACACAAGAGGAAAAACCTGTAACATTTTAGACCTCAAAGTCTATATACCACAA

A_14_P122799 CTTTTATACTTTCTCTCTGGGTTCAGATTGGATGTGGTTGCCATATATCTGAATTCTCTT

CUST_7423_PI426210822 GTGGCTTTGGACAGCTTGAGAATCTACAACTGTAGGTGACAGAGCA

A_14_P111614 ACCATATTTTCATGTAAGTTATCCTCATACCATGGCATAGATTTTAGCAAATTCGTGGCC

A_16_P02720928

CGCAATGTAATGCCATGTAATTATCTCTTTTCCTTTCGTATGTGTTATTTCGCCTTGGAA

A_14_P139104 CTAATGAAGTGAATGAATGAATATCATAAAACTAAATGGCTGGAAGGAGAGATGGTCGCA

A_14_P123294 GAATAATCTCTTTCTCAGTGTGATTACAAATTAATATTTGGAGGAGGGGGAAGAGGAAGA

A_14_P120700 AGAAGTGGAAATAGTTGCTCTTATGGGTAATGGCATATATGGGACCATTTGTCTATTTTG

A_16_P16191514 GTTGCACTGCGTTGTCATCTCTTGTCTGTTTCCCCTGAGACTCATTATTC

A_14_P111849 TGAAGACTCTTTCTCCTCCTAATCCTTAAATAGTTTTAAACCTCATACCACTACCCTAGC

A_14_P136126 GAGTAGAAACATGTTCCCCCCCTTTTTTTTACATCTCTCCCACAGCACTAAATATTATTA

A_14_P133957 CTATTGACTATAAACAGGGGCGTTTGCCAGTTTTCCAGAATAGTAGGAATGCTTTTTTTA

A_14_P135220 AGAGTTCCAGTTGTTATATTTCCCTTTTCTTTGGGGAAATGGAAATACAGGAAATCTAGA

A_14_P139743 ACTTGCACTCCAAATAGGTTACTATGTGGACTCTGACGACAAAATCTTTCACGTAAAATT

A_16_P00601337

ACCCTGTATTAACCACATTCATATGAGTTTTGTATTTACTCCAATAAACCAGTAGTGCCA

A_14_P111225 CCTAATAAGCACCTAAGAATTCATTGAGTGAAAAGTTAAGGTTAGCAATCAACCTTGGCA

A_14_P133691 CTAATAAAAAAGGTAAGATAGTAACCTATCTTACCTGGCCCTAAAAACTGGCATCCTCGA

A_14_P132352 TGTTTTATGTTGTGAATCAAAGTAGAAAGGAGGTTTGAGAGCCAATGCTACCCATAATTC

A_14_P105077 GTAAAAGAGTTGGTTATATTTTCACCTTTGGATGCTAGGTTACCCATGAATGCAAAAAGG

A_14_P139125 CAAAGTCTGTAATTTTTTTCAGTAAAGATTGATGGACTTTCATTACGGCCTGGTCTAGTG

CUST_7163_PI426210822 GTATCGGCTTTCTAGCCAGTTGCAGTGTACAATATTAGTTTTGGCTTTGGGAA

A_14_P200539 CAATCAGATCCTGAGGAAAGTTGACCGCGATAGAGTCCTTCGTCG

A_14_P138944 TAACTGTTAAGATTAATGCTTTGAGCACTGGAACTGCTCCTCTAAATACCCAATTTGCAA

CUST_6997_PI426210822 CTTCAGGGGTGACAGACAGTGGACCAAGGCCATGCCTGCATATTC

A_14_P109291 TTCCATTTGAGGGAGAACTAGATGCTATATATAAAACTTGCAACCACATTGGTTTGCTTG

CUST_5915_PI426210822 TGCCTGGACTTATTCTCTGGGCAGGGAAGAAATAACCTGGAACTA

A_18_P26794093 CTCCGAAATTTCTTATAATCCACCATCATAAGAGTGGGCATTGTATGGAAACTGAGG

A_14_P121382 TGACATAGAATTTTCATTGTGCAACTTTCTAGCCAGTGTTACTCCTCTGGAATATTAGGA

A_14_P134677 TTTGAGAAAAGTCTCGACAGAGAATAGGGGAGAGTTTTAAGAAAGAAATGCTGTTTGTTT

A_14_P110320

GGAAACTGTTGACTTCAAAGATAAATTGGAAGAAACGAAAGGTCAGATCAACAACTCAAT

A_14_P137449 TTCCCAATTCATCAGATTATAAGTCCTCACTCATAGCACTGACTGCTCATAATTGGCTAC

A_14_P112643

ATTTCAGGTGTTGAGAACTAAAGGGTAGAAAAGAAGAGAATAGAAAAACAAAAGATGGGG

A_16_P02684099

AAGAATGTCGTGGAAGGATAAAATCTTGTTAATTCGTGATGTCACAATTCAAGTGTGCTT

A_14_P107310 TGGAAGGTGGTGATTCTTTTATTTTACCCTCAAAAAACCAACTAAGGGATGTCTTTTAAA

A_14_P131145 AAAACAGTGGTTGAGTTGTATTTTCTGTCCAGTATTCATGAGAGGTGCTTTTCACTTTAT

A_14_P136426 ATTGACATTCTTAATTTTGTCTGTAAGTCCTTGGTATATATGCCTTTATTTGAAGCAAAC

A_16_P19187696

AAATGGAATGACACTACAAATCAATGCATCAACGTCTACTTAAATGAGAATGTCCTTGGG

A_14_P101258 TAAAGATGGGTCTTTGGAGATGTTTCTTCCTGATTGTGACATGGTCATCCATTTAAAG

A_14_P200989 AAAGTTTTATCTCTGCTCCAATTAAAGAAAAAGAGCCTGATGCTAGTCCTCCATTAGATG

A_14_P137371 GAGTTTAAATAATCATAACTCAACTCCCTAAAACTGAAGATTCTGTGTAAGGTTGGGCCA

A_14_P123441 AAGTCAGTCATTAATGATGTGGCCAGTTATTTGCAAGTGGTAAGAGCCTATTTACCATAA

CUST_3143_PI426210822 ATCTTCTCTCGGGCCTCTTCTTGCTAGGTCTGTTCACAAAACCTT

A_14_P122978 TTTGTTTTGTTTTTATGTATCAGGAGCCAACTTTCATCTTGACCACAAAGATCTCAGAGT

A_14_P131343 ACATGACACAGTAGATTGCAACTGATAGTTGGTAAAGTTTTTCTCCCTCACAGAAAAAAT

A_18_P10615408

ACGAAAAAACATTATGCTCCACACAAAGTTTTTAACCCTGGTTTTTATTGTCACTTAGCA

A_16_P00685455 CTAAAGTATAGGTCACATCTCACTGTCCCAATCAGGGACAAAAGAGCATGG

A_14_P107394 TGTGTCTTGTAAAGGCCATAGTTTGTCTCTCTGATGCAAAGTAAATATAATGCATGTTGG

CUST_7038_PI426210822 CCTTTGAATAGAGAGCTCTGTTCTGTTTGCAGTGACTCCCCTCAAAAG

A_14_P103011 CTCCCTATACAACATGGAACAGAAAGACTTGTATTTAGTCGGACCCATGTCTACTTTG

A_14_P112136 CATATTTCTTTTAAATCAAATTCAGTATGCTTTGGACATCCATCCCACCTGTCTGCTAGA

CUST_6679_PI426210822

AATGATCATATCCTTAGCTCTATAACTTTTCAGAGGGTGTGAAGTCACATTTTTGACCAC

A_18_P18074774

AAAAAAATTCAGGTTAACGCATCATTCAGTATAGCATATAAGCCTTTAGGATGCAAGATG
A_14_P112271 GTCAGTTTGCTCCCAGGAAAGCCTTTTTGTGGAAAACAGGTAAAATAAAATTTACTTTTC
CUST_7857_PI426210822 CTATGTAAACACCTATGTCTCTGCTGGGAGGTCCCCACCTCTTCT
CUST_7514_PI426210822 AGATGATAAGTGTCTTTCCTTGAACCAGTTCTTCTTCTTCCTCCTAGGCTG
A_14_P124966 TACAGTTTAAATTACTTGGTAATAAAACAGACGTGGAAACTGGTGAAAGTATAATGATGC
A_18_P18607449
TCAGATTTTGCAGGACAAGTTCTAGGTATCTACATTCTTTTCTTTCAAAAAAATTTTACA
A_18_P22839609
TTTATAATTACTCTGATACCAGATGGCTTTGCGGCCTTCAGTATCAAATGCATTGTGATT
A_14_P122828 ACATCCAGAACTCTGTCATAAATTACTAAGGTAGTTCTGGCTCTAGGAATTTTTGGTTTG
A_14_P201104 TGAAAAGTTCTAAAACGACCCCTCAAAAATGCCCACCACAATTATTTTTATATGGCTAGT
A_14_P120423 ATACAAAAGAAATATAAAGCACCTGACTTTTGCTTCAGTTTGAGAGACTACATGGAGGTG
A_16_P00925417
GGCACTTCTTAGATCACTAACATAGCATACTACTAATCCTTAAATTCCTGTTTCACACCA
A_16_P02953582
CTTTCATCTCAACTGCCTTTTAATAGACTAACCCATTTCTCTTTACTTTTGGCTCCACAT
A_16_P20865633
ACTACTCACCTTACTGCTCAAATTCTATCTAGTTTCCATGTTCCAAAACACAAAGGAAAT
CUST_9936_PI426210822 AGTGGAATCTTGGCTCCCCTTGAGGCCATTCACAACTGAGGATTT
CUST_1514_PI426210822
TAGGGACTGTTTTCTTGTGTCATTCTTACTTACTGTAAGTTTGCCCTTCGTATCAGT
A_14_P201358 TACCTTATTGAGGCGTTTTATTTCACATTCGTAATGTTCCTTTTCCTTGTTCGCAAGAGT
A_18_P13374499
ACCTTTGATTTATACGTTAATTTTCAGGGAGAAAACTTGCTATCCAATACACTCACGTGG
A_16_P17427325
ATTGTCCTTGAACCACAACTAAAAATACAACGATGCTCAGAAAAAACATATCAGGCCTTT
CUST_184_PI426210822
CAAAAGAAAACCTTGTGTTTTGTGTTCTACAACGAGAATTGATCATTTTTCACTCTCACA
CUST_6194_PI426210822 CATGACTCTGGGATGCCCCTCAATTGTCTTACAAAGTAACTCAGGC
A_14_P128798 AAAAAGGAGACCAGAACAACTTAACAACGTGTCTCCTGGATTTTACTTTGAAGCCTATT
A_14_P107609 CTTGATTCTAGGATTACTTCCTTTCAATGTCTATTTAGAAAAAAGTCCCCCTTTCTCCAG
A_14_P138873 GTTAAAAATTTCAAAGTGAGTGACACAACTTGTTTGGGGCTTTGTTTTTGTCCCAAATTT
A_14_P119180
AAGGACACCAAGAAATCAACAAGAAAATTTGAGTGTATCTGACAGAAGAAATTTGGCTTT
A_14_P128344 CTGAAGTAAACACTTTGGGAGACCGTTGCAACATAAAGCGGCCTCT
A_16_P18463730
AACACAACTTGGTTAAGTTACCTCTTGAAACTTCTGAACAAAGACATTTGGCAAATTCAC
A_14_P137795 AAGGGGGCACAAATCACATCTGATTATAAAAGGACTTCATGAAAAAATAAACTTATGGGC

A_14_P114705 ACCTCAACTGTATAAAAAATCCTCTCTCTATTCTATCCACCCTGTAACGAAAAGAAATCC
A_14_P136619 CTCATACTAACTCCATCCCTTTTCCTTTCTGAATGCAGACTCATAGTTGTGAGTAATATG
A_14_P112935 CTTTCAGAATTATCTTCTTGCTAATTTTGAGGTTGACTGGTAGAAACAACAGAAACGCTG
A_14_P115355 TCTTTTCGCTTATCATTTCAGATAAGACCAGAAATTCCCATTCCTGAAGAGTTAGATGAC
A_14_P110055 GTTACAAAAGGAAAAAAATACAATGCAGTATAAAAGATTGACAGCGTCATCAAGTTTATT
A_14_P109084 CTTGCCCTTTGTTTGGCTAACTCACACTCCAAACAAATGTTTCTTATTCCATAATTTGAA
A_14_P126244 ACAAGATATTAACAAAAGAAAGAACCAAACACCCAAGGTATTGTGCTATCAGTAGGGTTT
A_14_P132643 TATGGCCAAAAGCACTTTAATGCTGCTGACATTGTTGTTTTTATGTTCATTTGCTGGAG
A_14_P112414 CATCAATAGGTCTGAGAAAGGTGATAGACTCTGCTTTTCCAATGAGCAAAAAGATGTAAG
A_16_P00778545
CCAAATTAGAGCAAGTTCATGATTCATCCAGGACAGTATTCAGACTCTAAAAGCGTTAAC
CUST_6839_PI426210822 TAGATAATGAGGAGAATAATGCCCTCCAAAAACAATGGCAGGGGGCTA
A_14_P105681
AAAAACAAAAAACAGAAAACACGAATGCCTTTTCTATCGATTCAAGGTCTCAAGATGGAA
A_14_P121124 TTTGAGCGAATATATGAGCCTCTGGATGTCAAAAGTAAAAAGATTCATGTAGTGGACAGT
A_14_P121508 GTTCTCTTACTGTGACAACTGCTGTTGAGGTTTAATTATTTGCTACGACTTCATAGCATC
A_16_P15472947
AGCATAACCACTACCTTTAATGTAAACAAGGATTGACAGAATAGTTAACCAAAGGCTCCT
A_14_P115077 CACTAATCTCTGTTCTAGTTCAATCATATCAGTTACCTCCACTTTCTGAATGTAAGCCTG
A_14_P121113 TGGTAAATATCCCAACATCTTGCTCTTCTTTGAACAACTTGATCAGTGTGACCTTAAATT
CUST_7376_PI426210822 GGACTGATGCTGTCTCCAGATGCAGACTGGGGCCATGGATTGAGC
A_14_P130074 AATCCTTTGCTGCCTTTGGAAGTTTTCTTGAACAGCAGTTGACCAACAGGTAAATATATT
A_16_P03417008
AAATCTGGACCTCATCGTATCATTGACAAATATCTATTGGGGCTCAATATTTTCAGGACA
A_14_P127424 ACTTAGCAAAATGGTTTCAGTCCAACAGGGTAATGGGTGTTAATAGTTACTTAATACTCC
A_16_P02882536
AAGTCCCTCTCATCAAATACTGAATCTGAACAAGTCTTACAGAAAATAGGTTAGAAGCCA
A_16_P41226222
ATCTGCATTAGAAATTGACTGGATGACCTGTGTGTTCTTTGCAATTTTTCCCTGTATAAA
A_14_P106577 GCATAATCTCTCTCTTCTACTAGGGAGCAAGGAAAACTTCTTAGGAGTCTGATAAATACC
A_14_P103350 GTAAGTGCTCAAATAGGTTAAGGGAAAAAGGAATTTAATTAGAAGACATATTCTGATATA
A_14_P102807 TTTCCAACCACAATACTTATTACCTGAACCAAACCCTAAGACAAGAACAGATCTGAGATA
CUST_1760_PI426210822 AAAGGGGAAATGGGAGTGATTCCCACCAGATTCACAAATTATTGCTTCATT
A_14_P137687 TTCCTTTTATTTGTGTAGTCTTTTGGGGAAACTGAAGTCAAATTGACCTTCTATCTGAGT
A_14_P135414 AGGCAAAATTTATACATCTGGAGGTTCAGAAGTAGGTAAGGACTTCTTAAGTATTTTGGT
A_18_P12440683
CCATGATGTATAAAACACTGTTGGCCAAAAATGCACATTTGGTAATGTAAAAATGAATGG
A_14_P100086 ACTTCTGGAAGCCTCTCTACATGCCCTGACTCATTCATTCATCTAAGTATCTC

A_18_P17379639
AAAAAGATTTCACACATAGGTCTAGCCTTTCTCAGTCTGGTATGTATTCTTGAGAAAGCA
A_18_P13503158
TCATGCACTTACCTATAGGAAAACCAGAGAAAAATACATTGAAACATTGAGAACATCCCC
A_14_P138897 CTGCTGGTCTTCAGGATGACAGAATTCTACAATATTCTTTCCTAAGAGTTCCTAGAATAC
A_14_P110637 GATACCTCTAAACTGAGTAGCCTTTGCTTCTACAACCATAATTTCTCCTAAAATTTCAAG
A_14_P133423 TTAGAAAAAAAAGGAGCAGAAAATAATATGCCCACGATAATCGATACTTCAGCGTTGTGG
A_14_P114971 ACAGATATGTTGATAAAAATGGACAACGTCCATAGAATCCTGATACTTGCAGACATTCAG
A_14_P105271 ACCATTTCCCATTCCAGCATCTAGGAGTAATTAAAAAGCCATTTCCTTATTTACTTGTGT
A_16_P02599803
CAGAAAACGATTTCCTCTTGACATCTATCAGCAAATACACTGAAGAAAGCAGACTTTGTT
A_16_P01345753
AAAATAGAAGACAGTTTTGTAAACCTGATGGAACAAAACCTAAAATGCTTCATGCATCCC
A_14_P121059 CACCAGAAAAGTCAGAGTTGGTTTTTTGTTTTGTCTTTTTTTCCCCCCAGAAAATTAAAA
A_14_P128861 TTGAACTGGGGAGAACATTGGGAAGCAAACCCTCATAACACAATAATTAGTTCTTTAAAA
A_14_P139573 AGTTTATCTTTGTGTTAGACTCACTCCTTTTGTATTTTGAGCCCTTTTCCTTCTGTTTTG
A_14_P135462 TTGTATACTGATGGCTCCAGGACACAACAGGATATTGAAGGTCCCTG
A_14_P121384 ACTTCTCCTGTTATGTTTTAGGACTATGATGATGGATATGGCACTGCTTATGATGAACAG
CUST_486_PI426210822 AAATCTACAGTCTTTGAAACCACGGGCTCACTTGGTCCACCAATG
A_16_P15372876
CCCCAACCTTTAATAATTCCTACTTGCAGGATTTGACTATACAATCGCACTGCTAAAAAG
A_14_P117960 GACAAAGATTAATTAGATGCAAAAGTTAAAAGAGAGAAACAGATGAATCCTCGGCATGGC
A_14_P136014 AGACTTAGTCATTTTTTTCCTTCAATTTGACATGACTGCTAAAGACTTTTGGCATGTTCC
CUST_1631_PI426210822 CAGTACACAGAGGAAATATATGGCTTCTCCCAGGCCCACCTACAT
CUST_8230_PI426210822 TCAAGTCCACGGTCATTCAGTGCCGCTGGCATTGTTGGAAAGCAG
CUST_6813_PI426210822 GAGAGGGTCAACCGGAATGTAGTGTCCATGGAATTTTTAGAACTCATGAT
A_16_P18884086
TACTCCCCTTTTAATTCCTAAATCCAGAAGTAAGATCAGAACACATGAGAGATACTGCTG
A_16_P00685277
TGTAAATAACAGTTTTTTGTTTAATAATGGCATTTTGTATTACCAAGACCTTGCTTCACA
A_14_P109314 ACTGAAGATATATAACTCTGAGCCAGTTAGAATCCCTTAGACTTATGTGATCCTTCCAAG
CUST_1633_PI426210822 CCACCTACATACCCCGGTTCCTCTAGGTGTTCTGCTAATAAATGCAT
A_14_P126295 TCCTGAGAAAGTTAAGAATCATAGATACTGGTTGACGCTAATATCCTTGACCTTTTCCTT
CUST_8205_PI426210822 TCTCATATTTGCATGTGTAGCCAGCCAGGAGCCAAGGGCTGTGCC
A_16_P17051311
ACCTATTGTGAACTCTTCAAAGGTAGAGCATTGCATTATTTATTGTCACACTTCCTCAGT
A_14_P138694 AAAATGAACCGTTATGATAAGAAGATTGATAGTCTAATGAATGCGGTTGGTTGTCTGAAG
A_14_P110210 TTTGTCCTTTTCATACCTTTGTCTCCTAATTGTGCTACAATTGGAAGACCTTCTTTCTTT

CUST_1346_PI426210822 GTCAGGCAAAGCCTGCTTTTCCCCTGCTTCTTTGAGGTGGATAAA

A_16_P30952490 AAGATGGAAGAGAACAGTTTAAGTTTGGAATGCCTGCTGATGCCAAATACCACTT

A_14_P139749 GGATTTCAGTGCTACATCTAAAAAGAGAAGCCCTGTTGCCGATTTAATAATTTACAGTCT

CUST_8813_PI426210822 TTGCTAGTGACTTCTCCTCCCCTCTGGCTGTGGAAAGCTACTCAT

A_14_P133958 ACAATCATTCAATCAACATTTATTAGCCAATCAACTCAAGTCTACTGATCCAGCACTTTT

A_16_P17483151

AAAGTTCATAGTCTCTTGTCCTTTCACTTACACAAACTAACAGTGTTTATACCCAAACCT

A_16_P16773751

AAAACCACAAATGGAACCTTTAAATATTCACTCACTCTAGCAATCAATGTTCCAACCTCA

A_14_P131415 TTTTCCAGCAAAACCACTGAAGATGCTTAACATGGTCAAGACAATTTAACCTACAAAAAC

A_14_P127168 ACCTGCTTAGAACATGGGGTTTTGACTTTAAACTCATTCACATGAGTATTCATTATCTCT

A_14_P106316 CTGTGTTTGAAGATTCCTGGAGATAAGAAAACATTTTCAGAGAAAGAGTGAGTAATCTGG

A_18_P26111260

GGTTCTTTACATACAGAAACCTTTTACCCTTGACAGAATTAACGAACTGAGAGACACATT

A_14_P132338 TTTCCTCCTTTCCTAACATTTCTTTCCCCATATACTTGAAAATTGCTTCCATAGACACAA

A_16_P17925103

ACTCCTTCACTCTTTCTTAGATCCTATCCAGACAATAGTAATCATGAAGAACGATCATGT

A_14_P127782 AAATTAAGTTGGGTAGAGATTAGTTTGCCTGTTTTTCTCCTTGCCTTTTCACAGTTGTAA

A_14_P107291 CATTGAACGTGTCTTTAAGGGGAAATACAAAGAGTTGAACAAAGAAACTTCACGCTG

A_14_P115856 TTTCAAACATTAACTTGACTCCTACTAACTATCCTTTACTGTGGCCCATGTAAAACCACA

A_14_P112578 GCAATAGTTTCACTCAATGACTGTGAATAGCACAGTCATTATTTAGTGTCCTCGTGCTTA

A_14_P117716 TGTCAGATGAGGCAGAGTGATGCATGTGTAGTGAAACAAACTGCCATCAT

CUST_3121_PI426210822

TATTGTTTATTAGCCACTGGATTTCTGACTGATAGTTTGAAAAGTCTGCAGATTAGAGAT

A_16_P01105427

GTGCTTGCTTATACATGTTCACAAAAGTGTATCTAACCCTGCTTTTTAGTGTGATTGAGG

A_14_P122195 AACACTGCTGTATTAGAAAAAGTCTCTTTCTGGTCTTTCTGGTTTTGTTTATGAATTTCC

A_14_P130600 GAATGGATGAGATTATTTCATCCAAAGTCTGAAAATGAAGGGGTCTTCTACTCAGATATT

A_14_P102116 AGACTCAGTTTAGGCAGATTTGATTAATGGTAATTTTCAGAAAGAGAGTGAGGTCTTCTG

CUST_6160_PI426210822 GCATCAAGGATGAATGGACATGTGGGTGTCTTCCGTGGGAGTTAC

A_16_P02241326

ACCAAAGTGAATTAATCCAAAATAAGCACAAACTTTTATGTGGTCTATAGATCTTTGCCT

A_14_P129258 CTCACTCCCCCCATATATTCCCCCTTTACATGTTTTCTTATAAGACATACAGTTTAATCA

A_14_P119734 GACTTCTCCCTGTTTGTATGTATCTTCACATGGCCATCTTTTTTTTTAACTAGGAGATGA

A_16_P16191691 GTAAAGCACCAGGTCAACTTTCAAGGCTTTCAGTGGCTAATTTTTTATGACAGAAGG

CUST_4237_PI426210822 ACTATTACAGGTGGGGCCAGGACCCCACTTCCACAAAATTCAATT

A_16_P30952279 GAACATCTACTATGAAGCCGACCACATCCTACCCCATGGCTTCTG

A_16_P16994169

TGTTATTACCAACGATCTCTCTTTTCTTAATAGACCAAAACATAAACAACTCAGGCTTCC

A_16_P37384491

ACGTGTGCCTAGTTTTTATGTCTACTCCTACTAACTATGCAAAACTGAAACTAGTTCCTT

A_14_P100252 GAGCAACTCTCTTGTTCCTGAAAGTTACAAAAGAAAACCTTTGTTCCAAGTAGAAAATCT

A_16_P03062758

CTAACCCCGTGTAATAATCTCTGTATTAATCCTTCCTCGAATTAAACTGTTTCCTGCTGG

A_14_P126520 ACCAAGCACCCAACAAGAATCAGTATCCGGTTTTAGTTGGAAATTAATATGAAAGAGAAA

A_16_P37269155

TACACCTACACCCTTATGACTTATTTCCTCTAGACAAATACCATGACCTGTTTCAAAAGA

A_14_P103430 AAAAGGTGCCAAACAACCAGAAGACTTAGTTACACACAGAGTGATTTGGATTTTTATGAA

A_14_P116315 TTCTTTTGAGAGCAGATTCTCTGTCATAAATCTTTGTATCTGGTATGTTGTAGAAGCCCA

A_16_P18288150

AACTTCGGAGACAAACACTAGTAGACATTTAATTCAAAACAGGATCAGATCATGAAACGG

A_14_P133123 CTACGGACCATAGTTAAAAACTGTTATAAACAGCATGGGCGGGAAGACCTTTTGTA

A_14_P121670 ATACATCTTCAAGAAGCCCACTTTCTGTGATGTCTGCAACCACATGATAGTG

CUST_10359_PI426210822 ATGACTTGGGAATTGCAAGCTGATGATACCATGGTACCACAACCTG

A_14_P112338 CTTAAAACATCTTAAAATACACTGATGGGAAACATGCTCTTTCTTCTGCCTCTGTATTTC

A_16_P16191704

CTGAGACAAAGAGAATATGTCTGTATACAACTAAAGAAAAAGAGAAAAGGTTCGCTGGGC

A_14_P123140 GAGCAAAATATATTTCAGTGGCTTGTAGCATCAGAGAACCTGCAGTATTTCAGACAGATA

A_14_P138297 TGGCTACTGCATCTTATGATACTCGAGTATATATCTGGGATCCACATAATGGAGACATTC

A_16_P16191194 CCTTATGTTGCGTCTGAGGTTTTCAGGCTCTTCAGGAAGTCAGATCTAAA

A_14_P115033 GATTACAAAATCTAATAAAGCCGAAGTGTGAATTAAACTTAACCGCAAGAGGAGTCACCT

CUST_3378_PI426210822

CCTGATACACAGATGTTTGATTATCAGAGTCTTTTTTCTTTCTTGTTTCCTGTGGCTTTT

A_14_P128626 ACCTAAATTAACCTCTTCTTAAATAAGTGTAAGGAAAGAAGACTCGGACCTGAATACGCA

CUST_468_PI426210822 GGCCTGCAGTGTGGCTGGGGATCATCTTTGCTCATACCTCTGTAA

CUST_2703_PI426210822 GTTTTATAGGGTGTCCATTGGCATCTGCACAATCACACTGGCCAG

A_16_P30952591 ACATGGAATCATGGAATTTCAGTTCTGCCCAGTCCCTCTCAAGGC

A_14_P120613 CACATTTGCACAAAGGACCATCACCTGTCCTTCCTGGAATCTCTAAAGTTT

A_14_P109026 CTTGGCGTGAGAAGAGACATTGAGGTCTTCTCTGTAAAATTTACTTAGATACTTGTGAAT

CUST_2098_PI426210822

TTAAAAGTTGTACCTTTTTCTGTCACTTCAGCTCATGCCTTCCTAACCAATCTTTCA

A_16_P17840268

TTTCCTTGAAGCAACTGATTAACTTACTTTCCTTTTCCTCTATAACTCTGAATTCAGCCC

A_14_P139100 TAATTGATAACAGTGAGGAGAACAGATTTGGGAAAAGAAAATGAACTCAGTTGTGGATGT

A_14_P136097 TTTTTGTTTCTTATCTTAGCCTAAAGCATAGTCTTTACCTCCACCACTAGTTCTGCCTTT

CUST_5613_PI426210822 ATCTGGACTGGAGATTCAGTCATGCAGTCTGAAGCCACTTGTTCA

A_16_P00511991
AAAAGACTGAATAGAGTGAGATCACTGACAGAAACATTTTTGCCTAGAAACTCCAACTTC
A_16_P19756272
TTACTGTGGCATTAGCACAATCAGTATCTTGATGACCTACTCCAGTTCTCTACTTTCTAG
A_16_P15563564
GGTTTGGGATAACTGATAGGAACTTTTAACTGTAAGCAAGACAACATATCATCTTACTCC
A_14_P112057 GTTAATGCAATGCATCATACCTGGTTTATTTCCTGAGTGGTTGTTATTTTGTCCTGAAAT
CUST_6796_PI426210822 CAAACAGGATGCAACAGCTTGCCACAGAGTGATAGAGGAGGCCAT
A_14_P124717 AAAAGTTGACAGGAAATTTCGGAATGGATTTGAATGGTTTTTCCTTGGCATATGAATCAT
A_16_P35336138
TTCCCTTCTTACCTTTACTTTGCCTTAACACTAGACAGTTTCTCTATGTAGAATGCTAGG
A_16_P16641832
AACATAACTCAACACTTTACAAATCACAAAGCACTTTTCATATTATGTCACCCCCTCACC
A_14_P135246 TCCTGGAGTGTATGAAAAATCAGGAACTCAGGGCAAGCAACTATTTAAATATCCTTAAAC
A_14_P135342 CTCTTTGGTCTTCTTAAGATGGCTACACCTCAATTTAAGATGGGGTATTCTTTCACTAGT
A_14_P104079 TGAAGTTGAGATAATAATATCCCAGTTATGCAGAACTTTGAGTCCTAGTTTGGGTTCAGT
A_14_P130587 AGAGGACACGTAATAAAGATTCAGTTTTCAGTAATTTTCAATCTTCAAGACTAACAGCCT
A_16_P00694002
ACATGGGACTTTCAAATCTATACATGCACACCATCATCTAAAAACCACTCATAGGTCTTA
A_16_P16067411
AAACCACACATCTAAACACTAGAAGTTCATTACAGTGAATACCAAACCAATCACAACTGG
A_14_P128795 CTAGGAATAACTGGTGAGTAGTTTCTTGACCTTTTTTCCAGACCTTTTTTGGTGAATTAT
A_16_P17475144
CTCCCCCACAAGATGTTTATTGAGTGAAAATGCCTCTAAGATTTTATTCCATTACTGTCA
A_14_P125833 ACGCAGACATTTTATTAAGCAGGTTAGAAAAACTGTGCTGCTCTGCTACACTTTTAGAAA
A_14_P111671 TGGAGGAGAGAGTAGAAGATAATGACCATGCTAAGATACCTTTTCTCTCATATGTTGAGA
A_14_P110908 GGAATAGGTTGGTTCTATTTTTCTCCACTGAGGACACATTGAAGCGGAAAAC
A_14_P137572 TGAAATAAAATACGGTGACTCAGTATGCTATATACAACATGTAGACACAGGCCTATGGCT
A_14_P110367 CAAAAGGGCTTCTTCCAGTGGCTCAGCATCCAAATCCGATAAAGATTTAGAAA
A_16_P32455963
AAAGGATCTAGCACCATTTATGATTTTAAATTCCCTTGGACTTACTATTTCTGTTTCGCC
A_14_P117263 TAAATATTACTGTTTATTACCAGGCATACCCCAGTAAAATAAAGAGGCAACCAGGCGATA
A_14_P104247 TTCAAACAGAAAACGATTTTTTTAAGTGCCATTCAATGTCAAAAAATGCTTAGTTAAAAT
A_14_P102321 CTTTGGTTATATCACTGATCTTCAATATTTCCCACTATGTGGAAAAGCAACGACAAGGTA
A_14_P115998 CTGCATGTTTTATCTTTTTGTTAACAGTGTAAAAATGAAGTCTTCTGTTGTCTCTCTCTG
A_16_P38284526
CCCAAAATGAGATTATGTTCCACACCCATAACTTTAGAGTTGATGGCCTACTTCATAAAT
A_14_P124847 ATTTGCATTTATTGAGGTCAAATAAAATGCTGGAAATTGGTGCCTGGTGACACTGTC

A_14_P119793 CATCAGTCTCACAAGTCTTAGGTTTTCTTGTCTTTCTCTTACTACTTCCTGGTAGATACA

A_14_P101457 CTCTTTAGTATCTACATGAAAATGGGATTGTCCATCGTGATCTCAAACCAGAGAATCTTC

A_14_P100402 CTGTATAAGTTTTCCATTCTCTTCCTGATTGACTTTCCCTTACTAAATCCTGGGCAG

A_14_P111298 TGAGATCATCCCAGTTAATTAAGGAGAAGAAGACATGACTCTTAATCTCCATCTGTTGAT

A_18_P14136784

CCTTCAAACCTCCTGCCCTTTAATATTTATTGAAACCTTGGTGGAAATGAATTCTGTTTT

A_14_P102046 AGAACAAGACATTCAACCTACTTTAATGGTAACTTTCACTGATACCATGACAAGGTGAGT

A_14_P101551 TTTCAAATCATTCCTCTGCACTCACTTTCTATAATGGTAGAGAAATGCATATCCTGGAGT

A_14_P117243 CTGTGAGGACTTTACTGATGACTGTCAAAACACAAATGACTTAAAGGACATCATAGTTGT

A_14_P202464 CCCTGCCTACAGGATTAAATTGCTACATAGTCTGAACAAGCAACTTTCTAATAATCAGAC

CUST_9493_PI426210822 CTAGACTGTGGGATCCAGGAAAATGTTGCTTAACATCTCGTTGTTGGATTC

A_16_P20419315

TCAATGAATCCTTTGCTATTGCACAATTAGCCCTCTTTTTTACTTTCTATCAACTGCTCC

A_16_P15572397

ATTATGAGCATCCCATTATGTTTGTATCATAGGGTCCATGATAAGATACAGCAGGAAACC

A_16_P19282237

AGCCATATCCAAATTGTGTCTTTTGGTTGCATTGTAAGGAAAAAAAGTTGATTCACATGG

A_16_P18165255

GACATTTCCAGCACATAGGATACAGTAGTTAACTAGCATTTCATTAACGTCACCAGGTAT

A_14_P132260 TAATAACCTGTCTAAACATCAAGGTTAAACCTTCTTGCTAACACAGCAGTATTGCTTGGT

A_16_P03708258

TCTAGTTAACCCCATCTATAACCAAGGTTCTTCTATCCTTTATTCAAAGGCTTCTGTTCA

A_14_P115005 ACAGGGCTGGGAGAAGAAATAGACTTGCACCTTATGTCATGTAAATAATTGATTTTCTAG

A_16_P02009058

GCCACACACACTTAATGATCTCTAGTCCTAACATGAAAATTCAGCAAAGTTAAGCAGTTC

A_16_P35589538

ACACTAGTTTGCCACATTATTAACTATAGAATGACCTGACATTTTGACATTTATCCCCCA

A_16_P17711854

CTTTGCTTGTCTCACATATCATAGTCTCATTGTCATTCCAATGCTTTTTAACTCTGACCA

CUST_5772_PI426210822

TCAGGTCATGGTATTTATATGAATACAAAAAAAAATTTAAATACTGTGCTAGGTGCTGGG

A_14_P120866 TGTTTTGACGGACTGTCATTCTATCTGAGCAGAATTTCCATATAAGTATCCATTTCATGC

CUST_9676_PI426210822

CAAAAAAATGCCACTTACCATTCCTTTGGTGTACTGCTTCTAGAGGCAAAGTTTAC

A_14_P128972 ACTCTCCATGCTAAGGAGTCATTTCCTAACCCTGCAGAATAATATTCCAAAATATTTCAC

A_14_P105866 TGAATAAATTTTCTCGTTCTCTTTATAGAAATGGACCAATTTTGACAAGATATAGTGCTG

A_16_P19900424

AAAGTTGGAATTCTACTATGCCTTTTTCTCCCTAACTTGTCAACTAAGAGGAACATTGTC

A_16_P16191765

AACAAAGAGAAAGGGAGGAAGGATCATCCTAGAAGTGGATACCAGTTCTTTAAGAAG

A_16_P00007064

ATTGGACTCCTACTAAGCATTTATAATTTCTTACCACAGTTACTTTCTAGCCAGCATGGG

A_14_P120194 TGTTTTAAAGAAAGCATCACGAGGTTCATCTGATCTTTTTGCTTACTGCTCTGTCAAAC

CUST_3517_PI426210822 TTTCTAACTGCCTCTGCATTTGGAAGGGGTCAAGACTCGTTTCATAGT

A_14_P111602

GAACAAATAAATGAATACCCAGAAACAAGGAAATTGAAGACATAAGAGGAAAAGCCCTCT

A_16_P36230896 CCCTAGACAACACAGAAGAAGATGTGGTCTGTCACCTATAGTCCTGTCATT

A_18_P22839043

TTTGCATGCAAACTGTTCTATTTCTGTTTTGCAGTGGGAGAGAGGATACGGTTTATAATT

A_14_P108481 CTGAAGAGCAGCACTTAATTTAAATGCAGCAAAACTAGTTAAAACGAGAGAATACTGTTA

A_14_P102088 TATTAAGAAGGTGGGGTCAGAACAGTTAATTTGTTTGGGCTGAGAACCTTTAAGATTACT

A_14_P133789 TATTGTTGGTAGCATAGTTTCTTAGTCATTTTACCAGTTGGCACACTTACGTGTCAGTAG

CUST_2209_PI426210822 GTACACCATTAACCCCCTCCCTCTGTTGGTTAGTATTTTCTCAGGAAATTC

A_14_P128557 CTAATCTCTGCATTGCAAGGGAATTTTTGAAACCTTACCCTATTCAAGTTACTGGTAGAA

A_14_P111290 AGGTTTTTGAATGCCATTGAAACTGCTTTGGCCTTTGGTGATGTCATCTTAATTGAAAAT

A_16_P00685169 CTCGTATGCAAACACTGTCAGGCCTTTGAATGCCTGTGATTGGTGTTTTTATAC

A_14_P122508 TTCAGAGTAAAACTCAAAGGATTCAGTTTGAGCCTAGAATGATGGTTAGACTTGTAGTCA

A_14_P109234 GAGGGAGCCTAGGCGCATCTTTTTAAGCAATGAAATTACCTAGCCCCATAT

CUST_5834_PI426210822

AAGTCATTTCTACTTGGTTTTTGAAACTGCATTGTCATCTAGTATTCCAAGGCTTTAGTC

A_18_P22841055

ACTTACCAAAATAGAAGAGGGAGAAATGAAAAGGAAATATGATCAATGATTTCTGTTCTT

A_14_P201038 ACATTTTTCCCCCCTAAAGGCATAGACAACAAAAGAAATTTTATTGAGAGGAAAACACAA

A_16_P30952668 CTGCTCTGTATCACCTTTATATAGCAGACATGTCACCCTGCTTCTACACAGATGAT

A_14_P115600 GTTGGAGGAAAGGCAACTTTAAGAATGCCTGAAAGCGTTATTTTTTGCTTACATTTCAAA

A_14_P110097 TTTTAAAGATAGTGTTTTTAGAAGAAGCATCTCAACAGGAAAAACTGGCCAAAGAATGGT

A_14_P123619 TGCCAATGGGCAGGAATGTAAAGAGAGAAAGTAGAGAGAATATTTTCAACAGTAGTTGAT

A_14_P114999 TTGACCACAACGGTGAATGTGTAGTTATAAACAAAGGTCAATTTTCCAATGGAAAAGAAC

A_16_P00638898

TGCCTTTCATCATCAACTTCAATGAACTAAGTCTGCAATCAATAACTACGACACTTATCA

A_14_P105280 AATGTGTAGAATCAAGGATAATTAAGGCTTCAGCACATGTTTGTTCACAACAGTGTTCTG

CUST_6401_PI426210822 GAAATCTCGGAGCTTTCATTGCATGAAAACCGACTCCATTTTAGTACCTCG

A_14_P113541 ACTACTCAAAAATTTACCAGACCTTACAACTGTGATAAAACTTGAATGTGTATGATGAAT

A_14_P102720

CTTTTGACAAAACAGACAAAGCAAGAATGAGAAAAGGAAAATAATTGTTTATGGCGCCAC

CUST_7268_PI426210822 CTGGCTGGGAGGAAAGAGATTTTTCTCTCCCAGCAAAGTAGTAAACAG

A_14_P113729 TGGAAACTGATTTTGATCTGAAAATCCTACCAAGATAATCCCTATGCAAACTAGTTCCTC

A_14_P124479 AGTCTAGGAGCACATACCTTTCATTATTTCCATTTTATAGGTGGAAACAAGAAGACTTGC

A_14_P123350 ACATTTAACATCCATTCCATGGGCATCAAAAATTACAATTATGATGCAACCACTGTGATC

A_14_P125079 CTTAGAACAACTGACTTTAGAAAGTAATCTCAAAAGAAACCATCTGGGCCAATGGTATTC

CUST_1766_PI426210822 CTCCACAAACACAAGGTAGAGAGGAAAACCAAGGAGGGAACTGAATTA

A_14_P116829 AATGTTGCTCTTAACTGGAGAACCATGATTCTTTGGAAAAAATTTCCATGGTTAAGTGTA

A_14_P126922 CTTTTTACCTTCTCTCTACGCTAGGAGGATACTTGGTGACCTCATTCTTGTTG

A_16_P15430229

AAAAGCACAAATGCATATGATGAGGTCTTCCTAGCAATTTCCTTGATGTTCTTTTGGAAT

A_14_P132977 GTCGTGGAAATTAAGTTTATGAATCTCCTTGATCTCTGAATTATTCCAAACAGCACCATC

A_16_P16191185

AACATTGATGAAGATGCCAAGTTAAATAGTCCTTTGGAATGAAACAAATAGTTCCCCTGG

A_14_P122242 GAAAATTCATGCTTTCCACCCACCGCTGTGATTGGTTTAACAAAGGCTTAG

A_16_P18932068

ACCTATTGGTTATTTCTTACTCTAACAGGCACTTACGATACTATTTAGGCCACCTGCTAA

A_14_P131164 CAGTTAATGACAGAGATGGCGGTTCTGATCACAAATTAGATAGTTATCCTCTTGAGTAGA

A_14_P118531 AAAGACAAACAGTTTGGACTCACCTCAATGCACATCTGATCCCAGGAG

A_14_P108171 TTTACAGATTTTTAAAAGCATTGGTGCTGATGAGACCGTCCAAGGACAAGGAAG

CUST_6794_PI426210822 TAAGCTTCAAGCCAGGTGCATGAGGGCTCCCAGCTGACTTTTGGG

CUST_4531_PI426210822 ATTTAGAATGGAGTCAGGAGGAAGATTTCTCTGGGGGGTTGGATTC

A_14_P108369 GTTTTTCAGATACCTACCTCAGTAGACCCTGTGAATGCAATCTTGTCTATGCCAAT

CUST_4149_PI426210822 CCTATTCCTTGTAAGGTTTAGCCATGTATAGTCATGTGCCAGTCACCTAGG

A_16_P16191578

GAATATGAATTTTCATAGGCGAAATTACTCTGTCTGGTCGAGTCTAGACTGTTCGTTGG

A_14_P114929 CTGGCCTGTAGACTTATCATTTTATTTATATTGTTTTTTCTCAGCGGCGGGATTTTTCAG

A_14_P133753 AATTCTTTTTGAGGATCCTGAGAACAGATAGCAGATATAATGGCATCTGTTTTCAGGCTA

A_16_P03490319

ATCACAATTCATTTGTTTAAAGCCCTCATTCTTCATAAACCATACTAGAGTTTAGGCCCC

A_16_P01163677

TGCCGGTAGTGTTTTATGTTCCAATGAGCAATAAAAAGTAAAAGAAGTAGAGTAAGTGTC

A_14_P100435 CTCATGAAAATGGTCAGAGAAACCTTTATCTGTATCAAAGAATGGTCCTGCACCAGTAA

A_16_P18131799

GTCCCATTAATCACATGCAAAGTTTGTTCATTTTCGTAATTAAAATGCCTGGTCCAGTTT

A_14_P111625 CTCCAGCCCAGCCTTTTGAAAATACACTTAATGATGGTTTTGACTTTTTTTCTAAAAGCA

CUST_8619_PI426210822 CTGTGGACCTAGTGTCTGTCCATCAGGAAGCCAGGTTGTAATGAT

A_18_P11170332

AAAAACAGCATCAACTCCACTTAGTTGGATTTCACTTCAAAGTTTAAGAGTATGTCCTGG

CUST_2655_PI426210822 GTGCAAGCACACATAGATATATATTGCCCATCGTTCCTAACCCTCCTAAGG

A_16_P00323274
CTTTGCTCAACATGTTTTACAGAATGAAGACTTAGAAGACTTTATATGTGCAGCCATGGA

A_14_P111135 AGGGATACCTCTTTGTTTCTTTTCACCCCGAAATACAACAGCCCATAACAG

A_14_P119525 GCAAATAGGTTACTGCAAATATATTTACTCACCTATCTGGCTATGGATGGTAGTTGCTCT

A_14_P134574 AAAATGACTTATCCTTTTAAGGGATAATGTCTTACAACTAATCAGAGGCCACACACCTTC

A_16_P02007438
CTACCATTCACCCATAAAGTTCACCTTTTAGATTCACTTCTATAAAACCCAACACAGGTG

A_18_P15054500
AAGTAGAGAATGTCAATGGAAGATCAAGAGTTACCTCAAAAAGAAAGTGAAACGGGAAAA

A_14_P138719 GTTCCATTTTTCACTGTAAGTTCTTGAAGAACATTAAAGCATTTCCCACACATAATCCCT

A_16_P03250279
CTTCTACATTCTTTGTGTTTCAGAACAATCCAATTCATTCCAAACCTATTCGATGCACAC

CUST_5315_PI426210822
TATTTCTTAATTATTTGTCACTTATTCCCATTCATCCTTGTGTGTGAAACATTTTTCCCC

A_14_P139410 AACCAGAGTCAACTCTAGGATACCACCGCATAAAATGTTCATTTGCTTTTAATGTGAAAT

A_14_P139056 TATGTAAGGTTCAAGACACAGGAAAGAGGAAGGGAGAAAATAAGGCCTAATCTAGTCAT

A_14_P116467 ACTTTTTCTATGGCTATTGTGTCAGGTCACTGTGGATAAAGGCAAAGACAGATATTTATT

A_14_P126099 TGTCCAATTCCAGATTTTCAAGAACCATATGCTGTGGTTGTTCTTCTAGAAAAGGATTTA

A_14_P110417 AGGCCTTGAAGAAACAAAAATATTTACAAAGAATTGTCCCCTAAAATTGGTAACCACAGG

A_14_P131006 TTCAGTTGAACAAGAAAACAGTGATAATTAAATGCCAACTTGCAGAAGTAACCAGTGAAC

A_14_P105782 TAGAAAGCCAGAAGATAAACCCAATAAACTCAAGTCCTCTATTGATTTTTCCTCCTCTTC

A_18_P16263091
ACAAGCAGCATTACTCATGTTAAGATACAAGATATCACGGTTTATATTACCTAGGTAGCA

A_16_P15686134
AAAATGACTGTCAACTTGTAACCTTACTGTTTACACCACAATGTGCAATAAAGATGGCAG

A_18_P22843206
AACTATCCTGTAGAGGAGAGAGACATTGAAAATGACATATACAGACACAGAAAAGGTTGC

CUST_5794_PI426210822 ATTTTCTGGTACAGATTTGGGTGGGAGAAATGAGCTACCCTTCCC

A_14_P110778 AGAATAATAAATATATTGCTTGTAACTTCCTGCTTGTGTGAATCCCTCAGAATCCCTTCC

A_14_P200010 CACATCCAGAAAAATTAGATTGAGAGTATTAAATCAGGGAAGTTTGAACACAGATGAGGT

A_14_P102743 TGTCTACTTCCCTAACAATATTCCCCTTACTTTAAAAAACGTAAACTGACAGATGGCCAT

CUST_4954_PI426210822 GTTTCTTGACACATCTTGGCTTGTGTGAGGGGCCATAACAATAGCAG

A_14_P135630 GTCTTGAATATAACACACCTTGTTTCACCTTGTTCCTTCCCTCGATGTGATAATTACATT

A_16_P16133698
TTTCCCAAAGGACACAAATATGCCGAAACAAAAAAACTCTTATTGATGGATACCTTTTGG

A_14_P100647 CCCTATGACAACAAGGTCCTGAGATTCTACGAGATTAAGACTACTATGTTTATTAGTGGT

A_14_P121373 GTCATGTAGCATTTACCACAGTTGATACACATTTCTTCATCAATCATAGCCACAACTTGC

A_14_P201154 ACCAATCAGTCAATAACTGCCCAAACCAGAGACCTTACAGGAACAGAAGAATAT

A_16_P37168256
ATTCTTAAGAAATGGTAGCGTCTTAATGTCAGTGAACGTAGATTGTTGTCAACCTCATGC
CUST_8998_PI426210822
TAAAATGAACCTAGGTCCCTTTTAACCCAAGCCAATTCAACAAACATAGCTTTTAGATCA
A_18_P22839597 GTGTTGATTTCAGCCTTTAAGGGGGGTCGTGGTGGTCTAGGAGAC
A_14_P104213 AGATCTGCTAGACCAGGTGGATATCTATCAAGTTCTCTTTCTTTCCTGCAGAGTTAAC
A_14_P132119 AGTCAGGTAAGACTGGAAGATTATTTTATGCAACAGAAAATAAGCACTGTAGACTCTGGT
A_16_P16937040
GCCCAAATCAAACCTAATAAACTGTGATCAAATCTAATCAACTCTAAATACCTTGCCTTG
A_16_P03799907
ATGTGAGGCACAACCATAAGTCATTTAAAACAGGACTATGAATAGCCAGGCAAATTCATA
A_14_P113364 GGGAACATTGATTCATAGGGAATAAAAGTATGGGTGAAGGTAAAAAATTAATTTGGGACC
A_16_P02766512
CTCTCCCATACCTTACTACCAAATTTGTCATACGTTCAAATGTCTATTAGCAAGCAGACA
A_16_P18049769
AAACTTCTGGGAAAGTCTACTTCAAATTGACAGAGATAATTTAAGGTTTGCCATGCTCTG
A_14_P200007 CTTTTGATTCACGTGGGAGACTAAAGATGAGAATAAACCATTTATTCTTTGATTCCAGGG
CUST_4852_PI426210822 AGATGCTGGTTCTGTTGTAAGACATCATCAAGGTTCCTCCAACCC
A_16_P16267730
AATCTTAATTCAGGTGAAACTCAATCAGTCAAAAGACGAAGAACACAAAATCTGTCCTCA
A_14_P102980 ATGCACATCCAGGGGTATACTTACACTATATGTGCAAGGTCAAGTGGTTTCTCT
A_14_P138933 CAAGATCATCTTTTTCCCACAGTAAACAAAGGGCTCTTTCCTTCTGTTACATTTTTAGTT
A_16_P17342989
CCACTAAACCCTAGAACTCTGGATCATATTAAGTAAGAATGTAAACTCAATACAAGCCTG
CUST_1434_PI426210822 TTAGAAACTGAATTACTCTGTATATTTCCCCAGGGCCCTTTTTCCCCTTG
A_14_P131960 TTCCAAATATTGAGATGTTTTCTCTGTATTGGCCCCAATCATTGGAATGTTTTGTGCATA
A_16_P00090185
CATTGCCAGCATTGTATAATCTCTTGCATATCTGCTCATGAGTATTCCCAAAATACAACC
A_14_P105470 TACCAGTGGTCTTTTCAAATGTAAATCTGATCCACCTCCACTTAAAAATTCATCATTGCT
CUST_7069_PI426210822
TAATGTAAGTCTACTTTGTAAGTATTTGTTCTGAGATTATTTGTTCTGAAGGAGGCCCAA
A_16_P02351256
AAGGAATAAGCAAAGAACGAAAATCTTCAGTGACATAACCCATTCCAAAGAGAAAGAAGA
A_16_P18594683
AAAAACTGTTGGCATTAGGATGAACTATACATTCCAGAAAAGTGATTCTCAACTCTCTCC
CUST_9231_PI426210822
TCCTTTTTTTTATCTTTGGTTACATTTCTCTAAGCTCCTTATTTCAGCCCTGGCACA
A_14_P123102 GGGAAGACTAAGATAATCTATACCTCTACTACTCTACCCTTAGTCACCCCAATAATCATG

A_16_P16292721
AAAGACAAGAACGTGTATTCAGAAGGCATTACGGATCTATAAGAAGTGAAACTCAATGCA
A_14_P106508 GAGGAAGAACAGTCAATCTTTTTATCGTGGATATCAGAAAAATTAGTGTGTGGCTCAGAA
A_14_P114084 AACATCAAGTAATTCGATTTGTCTAGATGACTGAACAGAGAGAATTCTTAGCCACTCCTT
A_14_P132230 ACACTGTTAATTTTCACATGAATCTGTGTTTGAGATAGGTGGGATGTATGCAGTAACATC
A_14_P122870 GGACTGTAAATGCCTCCCTAGTAATACAGAAATATCCATTGGAAATAAAAATAGCACCAG
A_14_P120472 ACATCAAAATGTCAATACCATGGGTGACTTTTTCTTCTGTTTCCTAATTTGGGATTCTTT
A_14_P118806 TGAAAATATATCTGAATGCCTCAGATGTGACAGAATTGGACTGGAAATCCATTTAAGCAC
A_14_P124388 TTAAGTGTTCTTTCAAGGTCTGACTCTCAGGATCTAAAAAAGGAAAAGAGCACATAAGTC
A_14_P100670 CTTTCATAACATATGCAATAGCACAATTTCCAACAGGGGACTGATGCAGCCTAAATTTTA
A_16_P17833653
ATTCTGAAGCCAAAAATCAGTTTCCAACCTAAAAGTCTAAAATACCTTTCCTTACCAGGT
A_16_P36230234 GATTCCCACCAGATTCACAAATTATTGCTTCATTGTTGGAAAGAGGAGTTAGTTGG
A_16_P01193991
CTCCCTCTCCTTTCATACTATTTTGTATTACTTCCCAGATAAAAACTCTTCCTTACCCAC
A_16_P18123894
TTCACAGTGTGAGTTATGATAGACGCAATCTAAGAGTGAATTATAAAAGTGATAGGGCCG
A_16_P36230910
TCACATAAAACATGACTGTGATCTAATAGAAGCCTATGCAAAGCATAGATAGGGTAGACT
A_16_P15719908
CTGAACTTACACTAACCTGCTTTATCCATACTTCTTCCTCATCTACCCAAATGTAAAACC
A_16_P18865126
AAAAATGGGAAAATGCTACATTACAGCATCCTAGAATCAGTCTTAAGGTCAGTTTGTGTG
A_16_P00543254
AATCACAACAGTTTAATTTCAGCCTAAATCAGAAAACCAACTCTCAATTAACAGCCTGCA
A_16_P41714657
TCAGCCAACTACTAAAGGAGACAAATCATAACAAACTTTTTCTAACCTTGACATTGCTGA
A_14_P133504 GTCATGGTGACTCGGGACTTTTTAAAATTGCCTCCTGGTTGATCCTG
CUST_5607_PI426210822 AAAAGGAGAATTGGCCAGAGCCTTAAAGGTGGAGTAGGATTTGGG
A_14_P108419 GACAAAAGGCTTTCTTATTCTGGAATCCCACTGAACCTCACCCTAGACAAT
CUST_9275_PI426210822
TTGAATTGTTGACATCTGATTTTGGGTAAAAAAAAGGAAAAAGGAAAGCATTTGAATTGG
A_14_P105114 GAAAGGGTTCTTTTTTTCACCAGTAGTTTGTCTTATTAATGAGTCCTTGAAAACCTGTCT
A_14_P123638 ACACCTGGGAAGATAATAATTGCCTTTCAGTATGACTACTAGACAATAGCAAAGAAGGTC
A_18_P25341996
AAGTTACACAGGTCAAGTTGGTTTGTTTTATGGAAGGTTCTAGAACTACGAGTTCCAATA
A_14_P201211 CGTGAAGACAAGAAGTATAGGGTGCATAAGTAACTTCTGTGTGTAGGCAGATTATTCACT
A_14_P131129 TTTTCTGGCGTTTATGTAATGCTTCTCAACTCAAGGGGCTCCTTTATTACTTTTGAATGT

A_14_P111894 ATCTCAGTCGTATTTTGAGGAAAAAGATGCAAAATCACATTTTGGTATTGTCACCTCTGA

A_14_P105432 ACAAGAGCAATGAGGGTAGGGAACTTATACCCAAGTTTCATTCACAAATTATTTACATAT

A_14_P109807 GAATGCTAATTGGAATGTCTAGAAAATCAGCCTCTGTTACCATGGATATCTATGGGATTG

CUST_9234_PI426210822 TTTATCATTTCCTTTCTCATTCTTTTCTCCTGTTGGAGAAGGGTGACTGTTTGC

A_14_P127927 TCCTTTTTAGGAAATGACCACAAGTGAACATATCCAAGATGAACCAGGTAACTGTTATCA

A_14_P125232 TCAGAATTAAATCTCAACTGGCAAATGTTGCATGATATGATTTGCTTTCTTCGGTGGG

A_16_P03033078

TCATGAGCCCTAAATTTTATAATTTCCATGAATCAGCCATCCTTTTTCACATCCTTGCAT

A_14_P138394 TTCACATAGGTTGAGTGCTCATTGTGGATACCCTTTCTGGGTATGCATAAAATATAAACT

A_16_P00026136

GCTTAAATGTTAGTGGTATTTAGGTGTCAGAACTATGAGTAACTGTTCCCTACCATTCCC

A_14_P117024 ATGACAGATAAACAGAGTCTAATTCCCACCCCAAATTTTGCTGAGGTTTTCTTAATGTTG

A_14_P118313 GTGACAGTTCCACTGCACACAGAAATAGCAATGGAATCATCAACCATATACTTGTAG

A_14_P124021 TATATAGTTTAGAGTAGTGATGGATTTGGGTGTGCCCAAGGAAAAGAAAATTGCCTACAA

A_14_P115508 TTTTTTAAACCCCTTTTATTTAAAGGTCCATGCAGAATCATTACAGCAGGTGGTTCAATC

A_14_P114009 TTCACTCAGACACTGGGATCTTCCTGTTCAATTCACAAAACAAGTGTGATTACTGTT

CUST_686_PI426210822 CAGGGTTTGAATCAGCTATTGTTGTTAGAACCATTGAGTACCTTGGGCTTG

A_16_P41087420

GGTTTACTCACAAGTCTTAAGTGACGACATATTTCCATCACTGATTCATACAGTGTCTGT

A_14_P124701 AAATTGATTTTATGCTTGAGAATCTTCCTGAATGGGTTACTGCTAAACCAGTTAAGAAGA

A_14_P102774 CTTTGTGAATTCTGGTGGAAGGAATTTGCAAGAAACAGAGTTGCAAAAGAAACCATTATA

A_16_P18981304

ATTCCCAAATAGTCCAAATTTATCAAGTTGAAATCCCATGAAAAGGTAGTCTTCCCATCT

A_18_P25293549

GTTCAGAAAACCAAAATTCCTTGAATGACTACAGGATTGCATTAAACATGTTACCTACCC

CUST_8714_PI426210822

AAGCCATAGAGTAGAATAAATATTAAAGAATAAAGTAGGGGATTCGAAACTTGGATTTTC

CUST_8468_PI426210822 GAGACAGAAGAGGGGTACAGTTGTGCAAGTGATGAGCTGCAGATT

CUST_4489_PI426210822 AAAGTTCAGATGTCCCCCTTTCCCACTCTGTGGGTGGGCCCTAGT

A_14_P132992 CCTGATAATAGAAACCAGAATGAACCGATGTAGTATTTAGCACATAGAAATGTCTCCCAC

A_16_P18793704

TTCAAGCCGTTATATTTTAACAGCATTCATAGTTGCAGTAACCAATCCTGTGTCTTGAGT

A_14_P131737 TGTGCAGTAGACACCACAGTGCTCTGGTTATAAATTGTATTTGTAAGAATAACATCGGAC

CUST_7803_PI426210822

AGGACTGTTTTCTCTTCCTTAGTATGGATTTAAACAAGAATCATAAACACGTGAGCTTTC

A_14_P130005 AAACTGAGGCAGAAAGTGAATTTGACCCAGAAATAGAAATGGAAGCAGAGAGAGT

CUST_721_PI426210822 CATTTGAGTTACAGCAGACAAGGGCCAGCCAAACTGCTCTTGTGT

A_14_P138793 GTCAGCAAAAGCCCCAAATATGATTAGGAATATGTTTGCTACAGCAAAAAGTTAATGTCC

A_14_P113793 ATCAGTAACTCTGTCACTATATAGTGGGGTTATCAGGTAGAATTGCTTAAAAGACTGGTC

A_16_P32386788

AAAATAGCCTCTTTTCTCATAGTGCAGTTGTAGTTTAGAAACAAAACTTGGTCCTCTCTC

A_14_P108029 CCTCGGAGGATGTATACAATTATGTCTTCTGAGGAAGCAGCAAATGGAAAGAAATC

A_14_P121797 TCTCTGCCTTTCTGTAAAGTCCCTAGTAAATGACACTGTATATACATGAGAGTGAGACTT

A_14_P130060 AGAGAAGATGGTATTTTCAGTTCTGCCTACTTCTAGAACAGGCAAATTCAGAGAAGAATT

A_14_P201812 TTTTTCTTTGCAAATAACACAGCGATTCCCAGCCTACAAAGGTGAAAGGAGC

A_14_P106734 AGTCGCAACTCTAATCTCACTAACTTTATCTGTGTTCCTCAAATGCCTTAGACTATTTGC

A_16_P00548914

AAAAGCCAATCTTGCATACATAAACCAAACCAAGATAAGTGTTGATAACATGTTGATGAA

A_14_P125496 CTTCAGTGAAGATGTGTTCCAGTCTGTAAAGTCTTTATTGCAGAGTCAGAAGGAACTATG

A_16_P00814453

ACCGTTTCTAACTGAGCATATAACATAAAGAATCTCCCATACTTTTTTGACGTAGGACTC

A_14_P114061 CTCAATCACAGGCACGCTGAAGACCTCATCGTAACACCATTTGCT

A_16_P40301063

CCAAACTGACCATTTAGTATACTGTGCTACCCCTACATACTACCAGGTTTTCAATATGAA

A_14_P130465 ATTTGACCTTTCAGCATTACAGTTCATGAAAGGTATGGCACTGTGGTATCATATGTTCTA

A_14_P117293 AAAATATCACCAAAGAAAGGCTTTAAACTGAATTTTCTTGGTGAGATCAGTCAAATGCAG

A_16_P17042494

CTCACTGTCTATCTGATTGCTGCTAAAATCTTCTTCTGAGATGTAACCTAGAAAACGTGT

A_14_P126098 ATTCTACTGAAAAAGAGCCTGGAGCAGCAATTTCCGAATCACCTACTCTTTGTAAG

A_14_P138872 CTGCATCTCTGTGGTTTTTTTATAGGTGGATCTTTAACTCAAGACTAGCATGAAGAGTTG

A_16_P18526283

AATGAGCCCCATGTTAATTCATGTTCAACTACAGAAAACAACAAGTGAAACTTTGTTTGC

A_18_P22841228 TTTTCAACGTGTATCCTGAGTCATATTTGGGAAGCACTGGGCTCTGCTTAT

A_14_P118990 CTACAAGGCAGTCACACATAATTGAGTGACATGGATAATGATTACCATATTTAGAGGGTG

A_14_P128667 CTCACTTTGCTATAGGTGCATGGCATTTTACTCACCCATTTGCTTACCAAAAAACC

A_14_P127746 ATCATACAAGGAGCAAAGTGTTTAAGCAAGAAAAAGGAAAACAGCCTCCTTGTCCTAAA

A_16_P58424529 GCATTTCCTGGGGCCACCTCTCGAATAAACTACTTACACTCAAATCTTTG

A_16_P01892306

AAATGTGAAGACCATCTGTAAGTGTGATTTTTACCAGGATCAGACTCTAATTTGCAATGG

A_14_P104320 TGAAGAGAAAGAAACATACTATTGACATGCCCAACATAAATCGAAAACCATTTCCCATCT

A_14_P201676 TCTCTTCATTCTGGAAAAAGAAGGTTATGTGAAGATACTTACCCCTGAAGGAGAAATTTT

A_16_P18211913

AAAACCTGACTCATGAGTGTTCTGATCAAAGGTGACTTAAAACTCATATAAACAGTGTGG

A_14_P122521 CAGTCACTAAGGCATGAAACTGACAGATTTTACTCTGAACGTCCTTTTCCTTCTG

A_14_P201045 GATGGAGAAAGGCAGATAGATATAGGGGAGTGTTTTCTCTTCAATTACAGTCTGATTTTG

A_16_P19984439

AAACTCAATTTACCTTACAAGGCTTATTTCTGTTTGGCATCCTAATGAATTCTCTGCTCC
A_14_P134842 AGTGATGGATGGTCCATTAGAAAAAGATTAACAGGTGTGCTGAATGGAGGAACAT
A_14_P122894 AATGGGAGGGTTTAGAATTAAGCAGCAAAAATAAGCAGATTCCCTAAAATAAACCTGCAG
A_14_P105371 AGATGGTGCTCAAAAAGATCAGTCTATTCCTTTCAACATGCCTGTAAATGTATTTCTAGG
A_16_P30952501 CTAGTGTTGGCCAGCGTTCATTCATTCTCCTCTCTCTGCACAGTG
A_14_P122822 GAAAACCCAGCGTTAGTAGAAAAGTTTCTTTTCTTTGAATGACAGAACTACAGCATAATG
A_16_P01456870
CAGTGATCTTTCTAGAACAGAAAAGAGCAGTTCTCTTGATAATTGGCGAGTAGTCCAAAC
A_14_P120189 ATTTACTCTTCTTTCATCCCGAAACCCCGTTGACTTCTACAGTTCTAGAATTGTTTATTT
A_14_P118622 GGTCTGTTTCTATGTCCAAAACATTTATTTCAGGAAATATTTGTGCCTGCACAACAGG
A_14_P118271 ACAGGATTCATCTTCACTATCGGAGAAGGATGCTCCATGGACACAAGT
A_14_P128839 TTTTCAGGCTATTTTCCAAAAAAGAACCTGGACTGGGTAACTAAATGAAATCACCACTTA
A_14_P126532 ACCATTGTTAAGTCATGTGTTATATCATGTCCCGATATTTCACTTGAAAAATATGATGAC
A_14_P128146 TTCGTGTCATTTTCACAAACAACCTTTTGAGGAAAAAAAGTCTTAAGGACAAACACATGC
A_16_P20913860
CTCATGCCTTTACCACAAATATCCTTGAACTGACTGAACATATATTACTGAATACTGTGA
A_16_P16160076
TGAAATACTATGTCCAAAAATGTCCCTGTAACTATGATACCAACTCAAATTCCTCACACA
A_14_P115551 CCTGCCTATGTCAGTAAATGAAAGAATATTGGGTCAAACCATATGAAAAGCAAATCAGAC
A_14_P114884 GAAATTCAGCAAGTTGATCTGCAGAAGGTTGATCCATAGGCTGGTCTGTAA
A_14_P201428 CAGTTTAGAATGGTTTGGGGTAGCATCAAGTCATCAAATAATTCACATTTGTTGTCCATT
A_14_P135724 ATAAGGAATCCTATCAAAATGGGTGCCATTTAGCAATCAAGAAATTGGCAATTGCTGTTA
CUST_2900_PI426210822 GTCAGAAGTGGTATCTTCTGTGGTATTTATCTGCTGCTGTCTTCAGCTACATGA
A_14_P106901 TATATTATGACATCTGCAAAACTCATTGCTCCTGTAATTGAAACATCTTTTGCTGCAGGT
A_16_P19239039
ATCTGAAACTTTAGAATTCACTTGGAGAACTGTCATGTTTAGAATTCAGGGCATTTTGCA
A_14_P137364 TCTTCTCCCTGCAGAAATATTGGCTTTTTGTCTAGCAGCACTCATTATGCTCATTTAAAA
A_14_P106119 CTACCGCATCAGCCTAAAGAAGTTCTGCAAGAAGGACTATGGTAGGTG
A_14_P133441 ACCAACTAGAAGTGGGTCATAGATAGATTACCAGGGATGCAGTGGTGTTTAG
A_14_P103188 CTACTGCATTTTATTGCCTAGGGTTTAGAGTTGTCACTTTGAAACCAAGATATCACTTGT
CUST_9081_PI426210822 GAAATGACATTGTTGGTCATTGGCCGGGGATGCTTGGGTTGTCTG
A_14_P201508 CAAACTCTTCTTGGGTGATCTGTTTTAAATAGGAGTAAAACATACTTCTCCGACTTCTCA
CUST_9278_PI426210822
TTTTGAAACTTTTCCAGCAAGTGCCATTGGTAATTTTGCTGGTAGATAATGCCAG
CUST_3001_PI426210822
TGATCAGTAGAAAGTAAATTGGCTAGAGTTCAGTTGTAGATCTCTGCACTTGTTTGGA
A_14_P100249 GCCCCACAATGCCAATCTTCTCTTTAGGTTTGATCGTGAAGGATACTTTCTTTA
A_14_P134125 AGTCTTTTTTCGTTTTAAGATGTGAGAAGAGATGGGATTCTTCAATCAAGGCCACAAG

A_14_P200863 ATTCTAAGTGTGTGGGGAAAGGGAGTTTAATTGACTCTTATCTTCCATATTTTTTTTCCC
A_18_P22841393 AGTGGAACATTTCATTGCTGTCTGGCTAGCCAACTCCGATAAAATAAGGACTTTTAA
A_16_P19995490
AAACCCAGATGTCAATTAAAAGATGCCAGAAGAAATATCTGCAGGGATCAACATTTTACA
A_16_P01056612
AAATCTAGATTGAGAAAGTCCATTAGCAGTTATTGCAGATACAGTTACCTTCTCCTCACC
A_14_P105286 CAGACTCAGAAGAATACTATTCAATACCTTATTTCTGCAGGGCACTAAACATTAGCAGGA
CUST_4657_PI426210822 CTTGTGTGTTTGTCTCCCTCTGTGCCTAGGCCCGAGAGGCCATTA
A_14_P134140 AATTCCTTGGATATTCAGAGTCACTCTCAGTTATGACTTTTGCAAATAATAAGGCGCTTC
A_16_P36230384 GGAAGGTTGGGATGCATAGAACTGATGTGTGGGAAACTACCTTCATCAT
A_14_P110218 GCTTCTAACGCTCTGGCTTTGTGGCTTCAGACTGGTCATTATATTTCTCTTCTTTTTT
A_16_P36230138 GTAGGTGCTTTGAGGGAGATAGAAAGAAAAGTCTTTGCATTTGAGACTCCTGAGAT
CUST_7116_PI426210822 CTATTGGCATGGTGGGTCTGAAACTCTTGTAGACACCAAAGAAATTGGTAG
A_16_P15166957
AACAGTATACCTTTATTAGCCACACTAGAATTCCTAAAGAGTTAACGTCCAATTCCCTCC
A_16_P19424870
ACAACAAGTTTCTCACTCTCTTTCAGGAATCTCAGATACCACTAAAAATGCTGTCTTTTG
CUST_9239_PI426210822
TCTGTCTGGCTGAAAATTTTGCCGTATAGATGAAACATCATCAAGGTATGTATACGCAT
CUST_7473_PI426210822
CCTGCAGCTCATCCTTTTTTTCAAAGAAATTAATTTTCAAAAAATGACTCTATTGAGATT
A_14_P134447 TTGTAGAAGATGTTTCGCTTTACACTCCAAACTACACACATCTTCATCTGTCTTATCACA
A_16_P03317530
ATTTTTCTATTCCATCTGGATAAACAATCTCAGAAGTGCATTGAATTGGTTGTGACACCA
A_16_P16427636
AAACAGGTCAAATTTCAGTATGTTGTCCATCAAAAATGTCATGCAAGTTCACATTTGAGG
A_16_P19958245
GTCTACCTAGAAGGCTATGAATGATAAGAAACTCAACCTAATTACTTCGCTTTGCCAACT
A_14_P135991 ATGGGTATGAATATCTCTATATATTGCCAAGCAGCAATTAAGAACTGCCAACCAAGGAAA
A_14_P104970 AAGATTGTCAATCTGCTCTTTGGTTAAACCACGTATTCGATCATCATCATCACTTTCATT
A_14_P134809 CAAAACCCGTATTTCTAAAGTTATGGATCTTCTGTTCCCCAAAATGAATGGCTTGATTTA
A_14_P100311 TGAATGGTGTTTGTGTAGCAACACATTGTGTGCTATGTTTATTAAAATGCAGCGACAACT
A_14_P201919 TTCATGGGTTATAATAAGAGAAACACAGATGAGATGTAGATGGTAAGGAGTCTTACTGTT
A_14_P119114 TAACTATTAACCATCCCTGGGACCACAGGTTGGGATATAAGACCTCTTATTATATACTGA
A_14_P201275 GCTGTGACCTTCTGAATATAATAACTTAGTGTCCTGAGAATTGAAAGGCCTCTTGATTTC
A_14_P108992 ACACTAAAGGGAAAGCATTGATGTTTTAACAGGCCTAACTAGTTAAAAGGATTTGTGGAT
A_16_P19042973
AATACATCCATATTCGAAATCATTCCGTGAATTACAGAACAAATAAGTGACTGTCCTCCC

CUST_194_PI426210822 TTTACTGAGCACTGCTCCTACAGCTTATCTCACAACTGGGTGAAGG
CUST_8441_PI426210822 GGAATGGGTACTGTTCAAAAAGGAAGGCCCCGCAAGTATTACCAT
A_14_P130417 AAATTACAAGGAGATATGCACTTGCCCTTTGGTAATCCTGCTCCTTCTGAATCT
A_14_P129938 AAATATAGTTCTGCACCAAATGCAGTTGCCTTCACAAGAAGATTCAATCATGTGAGTTTA
A_14_P135621 TCTACTTAGTTAGGAGAAGGGAAAAACCTGCCACCTATTAAGGTTAGTCTTGACTTGAAA
A_14_P105700 ATTTGAGTTAATTAGCATTGCAGAACATGAAGTAATTGCTGTTGTTCCTCAAAATTGGGG
A_16_P02232775
AATAAGGAGTAGAGTCTATTGCACCTAAGCCTTACTACTAGCCTAAGCCTTCATACTAGA
A_14_P100832 CTGGCTTCTTCAAGTTGCTTTGTCCTGAGGTCTTTGCCCTTTAAAGTTACA
A_16_P35681799
AAACTGCACAGTGCTACAATATAGACCATTTCCCAAAAAAACTACCATTTGTTTGAAGAA
A_14_P116944 AGAAATACTGATCCTGACTGAATGAATAGTTCCTACAAGTGATTCGATCTTTGACACAAG
A_14_P202346 TAGGAATTTATAAAATCCCAGAGTAGATCATATGACTATTGGCTGGGATTATCTCCTGAA
A_16_P00060286
ACCTATGCTAAAAGAGATTTATTACTCCCACAAACTCTTTTTATCCCACTCCTTCTCAGA
A_16_P18171178
AATCAACAGGCAGTTTTTATTCTTATCTTCAGCAATGTCAGTTTCAAGATGATCTCAGGC
A_14_P106971 AGGTCAGAGAAAGGAGATTACTAATGAGACTAGTTGCGTCTCACTCTTCTAGCAC
A_14_P103873 ACCAGATAAATAAAAGGTAGTTATTTCCTAGGGTGACTCAGCAAGATATTTCTCGAGGAT
A_14_P105574 AATTACAAAATCCTGAACTGAAGGATTGTAGCCATGGTTCCTGTGAGTTTTAAAGGTAAA
A_18_P17189316
GAGTGCAGTAATTATGATCTGAATTTTTCCATGCTTTCGACCTTTAGAGAATCTATCGCC
CUST_9948_PI426210822
TTCCTCTAAGGAATGTGAGGAAGATTTCTGACCAGAAAACTTGAGTAACTGAGATG
A_16_P01481078
AAACTATCCTCTAGAAAGTGTGGAAGTTTACATCAACTCTGCTGTATATCACAGTTTGGC
A_14_P134055 CTTATAATGTTGACTTTATTTCACCGTGGAAATTTTGTGCTCAAAGGTAAGAAACCATCT
A_14_P103971 AGCAAATGAACGATATAAACAAAACTCAGGAAATTTTGGGTGAATTACGGAGAGGCACT
A_14_P108593 CTATTCCAGAAATTCCAAGATCTTACACATTACGCAGAAAAGGCGCTTATCAACATTCTT
A_14_P126878 CTTTGTGGATTAGAGCCTGACAGTTACAACAGACTTTGGAGTTTAAACTTTGTATCACAA
A_16_P17440596
AAAAATCCTAATGCAGGCTAATAATTTCCTCAACCTATCAGTCATACATCTGAGTCCACA
A_14_P124210 CGACCATGAAACTTCCAACAAAACTGGAAAGGCCAACATGTCAAAAAGTTACTC
A_14_P201658 GTGCATAGCCATGTCACAGGAAGTTTGGAAAATAAAAGAAAATTAATACCATCCCTAATC
A_18_P11374451
AGGTCTATTCACAAATATCTTGATTTCTTGGAGCACTGAATCTATCTACAAAAACCCACG
A_14_P122124 CTGAGGAGAGTAATACTCACGCAGAGACAATTTCCTCACCACTCACAAACTT
A_16_P16107357

ATCACCAGCAGATTGTAATTTTGAGCCTAGATCATCAATGTCTAAATTCTTGCTCCTCTT

A_14_P119932 GGGATCAGTTTCATTCTACATCTTCCTGTGTCATTTGAATCATTTACAAGGAGAGTGTAT

A_14_P109169 CTAGGCTACGAGTGCAGATATAATTTACATGAGTAAAGTATCCACAGGGTATAACAGGTG

A_14_P123414 AAGTCACACCAAGGTCACAGAAATGGCTACTGATTAAATTGTTTCCATCATCAAAGATAT

A_14_P103505 CTTGAATTATGTTAAAATTCATGTGGCCGCTCATCCACCAGAATTAGTTTCAAGTTATGT

A_14_P103442 AAACATCACATTCAAAGGTTGCATTGAGATGATGTATGAGGTTTGCTTCGAAATAATGGA

A_14_P105206 CTTTTTTATTTTTGTAGACACCAACACCCATGTATTCTACATTACATACACATTGGGGGC

A_14_P127810 TGATGAAGAGAGTTACATTGTATTCACCTATAGGCCTTGTGGGTAAGTAGAACTAGGTAG

CUST_7396_PI426210822 CAGGTGGCTGAGATTCCCTCCAATCTTTTTATTTCTGGTTGGGAAAC

A_14_P121228 TCGTGTTTCAGGGAACTTTTGTTAATACAACACAGTTGGTCACACAAAATACAAATGCTT

A_14_P105495 ACAAAAAAATCAAAGAGGCATTTGAAGTCTTTGACCATGAGTCGAATAATACAGTGGATG

A_16_P18360039

CCCGTTTCTTAACTATTCAAGACTCTACACATTGATTCTTACACTTCAACATTTCCCACT

CUST_2573_PI426210822

GTAGCCTAACTCTTAAGCCTAACTCTTAAGCTATTTGCTTTTGGCCAAGAACCTTC

A_18_P22841565 CTTGGCATATCACTGGGGAAGGATTTCTGAGCGGCCACTACAAAG

A_14_P132986 TCTTTTCATTGAACTTCAAGATTAGGAGGGGAAAGAGACAGACAAAGGAGACTGATAA

A_16_P20102252

AGGTATGGGGGAAAATGTAACTATCCGTTTAAGCACATGAGTTCATGAATTTTTGCATAG

A_14_P126734 AAGCAAACATATTCCATGCCTTCCCAACAATCATATCAAGTGGTTTTACTAAGAACTGG

A_14_P136857 TTATTCCCCTTCATCCTCTGGATGAATGTTAGATCTCTATATCTTCAATGTCCCTTAACT

A_18_P22841080 CTGTCCTTTTTTCTAGGGCCTTGATTAAATTTGCTTCTCTGAACACTGTGAGGTATCT

A_14_P119215 AAGAGCAGTGAAAAAAAAAATCTTCAGACCAAATTGGCTCAGAAATGTATTGCACTAACC

A_16_P19490387

GCAGTTCCTATCAAGACAAATCTACAATGGAAAACTGAAATTCTTACAAGTGGAAAGACA

A_16_P37015064

AAGATTCCACTTCAATGAATACTAGACTATACCTGTCAGAGATTCCAATAAAACCCTCAT

A_14_P105207 TCACAGAGAAAATCTCTGTGAGAGATTTTCAGTCTTCTTCCTGGAGATGTTTGTGAATAG

A_16_P20799789

AAATTCCCCAAATCTCTACATTGCATTCACTTGTCAGTTTTGTCTATCACGGAGTTAAAA

A_14_P135335 TTGAGCAGAGAATGAGAGATAAGGCAACAAGGAAAGTGACTTTGAAAATCTTTTATCATC

A_14_P100283 ATATAAATACCACAGACTTTTAGAAGATACCACAGAAAGCCATATCCTAGGAGTAAGGGC

A_14_P135040 CTAAGATATGGAGCATCTCTAAGAACAAATACTGACTTTTGCAGAGTTGGATTGGACAAG

A_14_P106730 CTCCCCAAATGCTGGCTAGTATTATTGTGTGCCTAGAAGTGTTCTAAATTACCTATGTAT

A_18_P15531852

GCAGAGGAAATAAAAAGTTGAAGTGAAATCCATGACTATTTTTGTGCATCATTAAAGGCC

A_18_P22842833 ACATATCTGGAGAGGCCAGGGGACCTGTGATACCTGAATTTCTACATT

A_14_P201781 AACAAATACACAGGATTTGGTCATTTTCTGCCATGAATCTAGGGCACGTGG

A_14_P110163 CCTTAGAATAAAACTACCTTTGACTCATAGACTCATTCATCCAATAGGAATAGAGTGCCC
A_14_P119457 TTAAGTTATAAATGGTAGATTATGAAATGGCTTCGTTTTACTTGGTGGAGGAGGGACACA
A_14_P108432 CACTTTAGCATAACTAAAGTGACAGTAACATCAGTGTCCTATGAGGAAAGAACAAGGAAG
A_16_P02475426
AAATTGGGAGTAATAAGGTTGAAAATACCAAATCGTGAGTTTAAATCCAACCGATGGCTA
CUST_3760_PI426210822 TGGTCAGGCAAATAATTGATCACTCTGTGTTCTCAAGACAAGCAATGGGTAA
A_14_P135820 CTCTAGGAAAAACTACAATATGAACAAAGGCCAACAGCAAGAATATTACCAGATGTCTCC
A_16_P17776220
AAATTAAGGGAAACAAAATTTTTAGGGGGCAACAAGATAAGATGTCGAAATTCTGGTTCT
A_14_P114634 CTAAATCTTACCAGCATAGATTTGTAAAAAATTCTGTCAGCATGGCCCTGTGACTTTTTC
A_14_P107571 GTCAGGGATATTTATAACAAACCCCCTTTCAAGCAAGTGATGCTGAAGGGATAAC
A_14_P137154 GAAGAGAGAACTGACCTTTCTAACAAAGTTCAGAGACCTAAATTACTGAACTTTTCCCTA
A_16_P35355644
AGAAACCATTATTCCACATGCCTTGAATATCCTAGAAACAAGAAATTTCCATCAGACACT
A_16_P17146741
AGTGGTCACTGAAGATTGCTACTCTACTGGTGTTTTAGACTAAACTTACTCTGTCTCTAA
A_14_P110896 GTTTCCTTGACTTCCACTTAATGTTTCTTTCATGGGCTTTCCTCTTAAAAAGTAGTGCAG
A_16_P16026886
AAACAATCTCTCATCTCAAGAAAATAGTTTGCTTTAAACCCATCAAGATCATTAGCCCAT
A_14_P139599 AATTTCCAACTGATTCCTAATAGGAGATAACCACAGCAGCAGATGATTTAACTGCTCTTC
A_14_P114253 ATGTTTTGGCTTTTTTTCCCACTTCTACCATGTTCAAGGTTTACTTTTTAAATTGAGGGG
A_14_P126406 ACTCACCAATCCTCTAATACACAGTGTATTGCTTTTCCTAGATACTCACATTGCTTTCTG
A_14_P128004 TTATATCCCCAATTAGACAGAAGGATCTTCAAGGATCAGAATTCTACTTCAGATGTCCTT
A_18_P23444933
CATGGAATTTAGGTGGGAAATCAACTCATAATTACCAGAGGATAACTTCTTGGCTCAGT
A_18_P13470333
AAACATATCTGAAGATAAGTTCCTAGCCATTGAAGTTTCAAGGTTCATAGAATCCCTGTG
A_14_P111228 CTGGTTTCTGGAGGTGTTTTGCGAGACAACTTGAATTTTGAGAAACAAAAAGTAATGAC
A_16_P00685190
AAAGGGAAGGCATTTTAGGTTTGGGACATATTACAATATTGCCTTTTAGCGTAGAAAAGC
A_14_P110745 CTAGGTGATGCATGTCTGTAATAACATATGTCAAGACACTAACATGGGAGAGAACATTTG
A_16_P00685153
AAGTTAGAGCAAGAGTGATTCAGAGCATGAGACAAGTGGTAGAGCAAACTCTGATTT
A_16_P02240028
CTACAGACATCCAAATCTCTACCTGTAAGAATATGTCTCACTCTAGATTCCCTTCATTCG
A_14_P101125 TATGAGGGAAGTTGAAGAAATCAAAGTTGATCTAAGACAAATGGCATTTTGGCTTTTTCC
A_16_P02950958
GGCTAATGCCTCTTGCTTATTAAACCTAGACTTATTAAACCTATTTGCGTATTGACTGGG

A_16_P18909754
ACAAGTATTTTTTATTCCAGTGTTTCATTTGTCTCCTGTTTATCAGATCCTGGCTCCAA
A_18_P22840294 GGCAGGGATCGGATAGGTGGTAGAAGGATTAGAACTGGATTCTACTCTA
A_14_P107816 CTTGGTGGCTTCTGAGTTCGAATATACAGCACATCCACTATTAAAATGAGCAATTGTGTG
A_16_P18091592
GACAAAGGCTACATCATCTTATCTAAGACAGGAACACTAACTACCTTTATACATGCAACA
A_14_P125316 CAGGACTTCAGAAGATGTTCTATTTGAAAATTGAGCGCTGTGTTCTTTTTCGTTTGAAAA
A_18_P22841551 CTCAGGCCTGTCACTTGGCCTCTCAGTGGGAGTCGATGCTGTGAT
A_14_P124073 CTAAATATGGCCTGTTATTTTTGACATGTGCATATTTTATATGCAGGGCTTGTGTGGTCT
A_14_P131109 AGAGTTATGGTGCCGAATTGTCTTTGGTGCTTTTCACTTGTGTTTTAAAATAAGGATTTT
A_14_P139789 GACCACATGACTGCTAGAAGAAGATGGGCAATAAGAAGAAAGTGTATCTACTTCACATAA
A_14_P101642 CAATCCTCAGTTAAATCTGGAAGGGGTGAGTGAATATAAGATAGAAGAGATTACGAGAAG
A_14_P200772 AGAAAGAGTTGTCCTGATGTTTTTCTGTGTTACTTATATTAGAGTCAGAGATCTTGGTAT
A_14_P139893 CATGCTAAGTGTTTATGGAATATGATCTTTCTGTTCTCCTCTGTCATCTAACCTCATCAG
A_14_P103770 TTGACACTGTTGAAATCATAGCAGGTGTTAAGTTTTTTAACCCAGACAATGCATTTCTGA
A_14_P108421 AAATCCTGCAGCATAAATCCGTGCTGTAAAGGGAGGATTCCGTTCACATAT
A_14_P102576 TACTTCTAATTAGTAATGAAAGTCCACAATACCCTGAGATAGCCTTAAAAGAAGCGTCGG
A_16_P01613889
CCCTATAGTCACAATCCAAGCCTTATTTCCTACTATTACCATAAAACTAATGTGGCCAGA
A_14_P131440 CTATGAATAGAAACAGTATGCATCTGAATTAGTCACCAGCCACAAGGAAACTTGATGTTT
A_18_P22839589 GACAGGGTGTTTTAAAAGTGGGAGGGCCGTGTGGAGAGAGAATTT
A_14_P128317 AGAATTTACTGACTGGTTGGTTGGTTAGTGATGCTGAAACTTTTAAAAATACTTGACAGT
CUST_3546_PI426210822 TGTTTTTGGACACAGCTATGTCAGAAATCTATTTCCCCTTCTCAGAGACCA
A_14_P131207 CACTTTAGGAACATCTCCTTTAAAGGGTATCAGATTCAAACCAGGAAATAATGCTCATTC
CUST_267_PI426210822 CTTGATAGGAGAGAGAGTTAGGCTGCGGGAAGTTCCCCCGTTCAA
A_16_P21433108
ACACTCAATTTAACAAAGCACACTTCCAATGATACATTTTCACGATTATCCCCTTTTGAA
A_16_P01402878
TTGACATGCATATTTGAACACTGGAGACAAAATTTCTGTATCGTTCTTGGTCAGCAAAAT
A_14_P137818 ATGCTCCAGAGAATGGGAAGTAAACTTTTGAATTCTCTTAGATGAGGAATGGCTTTTAAT
A_14_P126416 TAGAGGCTGACATTCTCTTGTAATCCTGAAAAGCCTTTCTGTGGACTATTTACACTAATT
A_16_P17191330
TGTTTTTCTCTGCAATCAATTCCACTAAACCAATGAAACTCTGTAACAAAATTAGCCTCA
CUST_1240_PI426210822
GCACAATTTGGTTGGTAAGTGTTCCTTCCCTCTTTTTGTAGATGACCTAAAACTTT
A_14_P131300 AGAATTGGATAGCAGAGTAATGTTTGACCTCTGGAAACAGTAAGTCAAAATGAAATTGCA
A_16_P39704126
ACCCTTGCACCGAGTATTTACTTTTAGTCACACTTTGCTATAAACTTTATGACTTGTGCA

A_14_P102828 CCTTTAAGAAGAAATGCAACAATTGCTCTCACTTTCAATACTATTTATTGCAACTGGAT
A_18_P20789855
ATCTCCTTTTCTTGCAAATATTTCACAGGACTTAGGTAGCAATAGACCATTCACGATGCT
A_14_P120034 CTGCTGGACCCACATGTCAATTTTGTCTTTAAGTGTGGGCTTCAGAC
CUST_9916_PI426210822 TTCAGGTGCCAGTGAAAAATCCATCAGTTTGCAACCAGAAAATCGTTCA
A_14_P129335 ATGAAGTGAAATTAGCAAGCCTGCCTTATTAACAAATGTGTGTAGGGAACATCATTGTTT
CUST_4272_PI426210822 TCAGTAGAGTAAGTAGTGACTGCCTAGGTGGCAGGATTTTGGATGGTA
A_14_P134474 TTTAAGGAGGACAAAATTTGCAAACTGCTCTATTCTCTGTGACAGAAACTCAGAGATTAG
A_16_P00063718
ATTACCTCCCACTATTTTTGTATACAGGTTTCTTTGCTCAATGATCCAAAAATGTGCCTT
A_18_P14135226 GATGGCAGAGAAGCCCAGGCAGAGTGCAGGGGCAGTGATGTCATT
A_14_P139408 TATACCTTGAATTTTCTCTGGCCCATAAGAAAATACAAACTCCACTTTGCCATACTCTGG
A_14_P200658 TATGGCAGAAAAGAAAGAGTTTACTGTTCCAAATTTTTATTGATGTGTTACTTGGGAAAT
A_18_P14574817
ACAGTTTTATCTTCCAACATAAGAAAACCTAGAATGAATGACTCAAAGGTTGTAGAGGGT
A_18_P22841894 CTTACGGATGCCTCCCTTCCCTTCATGACTCACTGTGATCTACAAC
A_14_P101429 TAGTCAAAACCACACAGTCATGAATACATAAACACCACTTTCGGAGCCATCTAATAAAGA
A_16_P18458116
GAATGCAATCAGTCCATCTAAGAGTTTAGCAATTTTCCCTAACAACATACTTCTCTCCAC
CUST_3261_PI426210822
ACGGTATTATTGTTAAAAACAAAACAAAACAATCTACACTAGACGAGGGAATATGCACAT
A_16_P20886664
TACAGTCTTCTAGTGCTTTGAAATCAACAATGTCTACCCAATTAAACTCAATGCTTGTCC
A_14_P116724 TAGAAAAAAAAGGACTAGAAGGCAGTGTATTCTGGAATTTTCCTAATGTGATGGAGGCTA
CUST_5927_PI426210822 GAAATGTCAGAGAGGTTGAGACAGATTGAGGGGAAGACTAACAAGCC
A_14_P111019 GTGATTGGATTAGATGATTTACTCTTACCCTATTTGCTTTCCAGTAGACAGGATGTATCC
A_18_P23417744
AGAGTTTTACTTCTTTTGCACACAAGTTGTCACAGTTAGGTGTAATGTGATTCTACGTCT
A_14_P102326 AACAAAACCCTAGCAGCCTGAAGAACTCTAAGCCAGGTTTAATTGGTTTCTTTTTC
CUST_4508_PI426210822 GAAACTTCCATGCCACCCCTCCTGCCCCTGGGCTCCAGGGCTCTC
A_14_P110426 AAATTAGGAATTTGGGGAATTTTTTTAAGAGCAACCTCTGATTTTTCCATTGATTTGGCC
CUST_2730_PI426210822 TTAGGTGAATGCTGCCTGAGCAGATGTCTGCAGTGGGAGCAGAAT
CUST_7335_PI426210822 CTCTGAGCCTCCCCCAGATGTGGGACACTCCTCAAGGGTTTCCAG
A_16_P30952454 TAAACTGCTGCTCTCCAGGTATGTCGGTGTTTCCTTCAGAGCATTGTT
A_14_P117162
AATACATTCCAAATCAAGGAAAAAAAGGGGAAAGAAGACTTTAAAAACCAGTCATTCCGA
A_14_P139330 CCCACACCTTTTGTTCCAAATGTAATTATGACAACTACAACAAAAGCAGTAAAAAGCAAG
A_14_P122442 AGATTTTTTAAAATGACGGACCTTAAAGCAAATATATGACACGCAGGGGGATTTCAAATC

A_14_P135010 AATAAAGTAGATTCAATTCCTACCTGCTTGGTTGGTGGTCACAGTGACAGAAACT
A_14_P117736 TCTGATTTACTGAAATGGTATCTGCTCCACTTTCAGATCCTGATTCCAGATCCTATTAAA
A_14_P101960 TAAACTATAGGACAAATTGCTTCAGAAATTCAGACTTGTCATCCCACTTTGAATAGGTGG
A_14_P136371 CTGCTGGGGGTTTAGAAGGATATCTTAAGAATCTAGAAAATTTGCAAATCCATTTAAATC
A_14_P116639 CCTTGACTTGATCACATTTATTCAATATGGTTGGAAATTAGCAAGAATCAGAAGAAGCAC
A_14_P103597 TATTACTTAGCAGAAAAATCATGGGCCTGCCTGAGTTGTCGTTTACTGAAAAATTTTAAA
A_14_P115591 TACCTGTTTTTAATTATCCAGTGCTTGTTTCCCTTCTGGATTCCATATCCCACTG
A_14_P101564 TATTCATTGCAGTTCTCAGTAATAATTCCACTTTACTATCCAGTCCGTTCATGCAGAGTT
A_16_P00859011
CCAGCCAGATGATGAAATTACTTGACCAAAACTGAGATTTATTCCTGAAGCAGATTCTTT
CUST_415_PI426210822 ATATTTAGGGCTGGGGGTTCAACAATGAAAACTTTTTGCTCACAATCTGACAG
A_14_P131801 AAATGAAGTTTGAGGTGAGAAGCATTCACCATGGTGCCCATCGTGTTAC
A_14_P202319 CTTGATGAAGTAAAGGTGAGTGTAGCCTTCATGCTCTTACAGACACTTGATAATGTTAAC
CUST_9377_PI426210822 TGAATGCACTAGTTCTCTGCTATCTGTTTCCACGACAGAAGCCAGG
A_16_P01621453
TAGCATTGATGGAGGTATAAACACTGTTCCCTTTAAAAAGACTAACAGCATAGAAGACCC
A_16_P21574904
AAGAACCACCATTTCTTATGCAATCTGTTTAACTGAGGTTTTCCTGTATAATGCCACTTC
A_18_P14135556 CAGAACATCACTCCTCAACCCAAGTCTGGCTCTTCTCCTCTTTCT
A_14_P110261 CTCTCATGTTCCTCCATGATAAAGGAATCATCTATAGGTGAGTTTTGGTTGCTGC
A_16_P01829914
AACCCCATCTTTGAATATCACTTGTAGACATAGTCACCTAAAGAAGCAACTAAACGTTGA
A_14_P125604 TTTCCAGAATCCACATTCATCTCAATGGAAGGATCCTGCCTTAAGTCAACTTATTTGTT
A_14_P120973 TATTTACCTTCAAAGTATATACTCCCATTCTACCTGGAACCTTATAGAAGATGCCCTCTT
A_14_P134118 TCATTGACAGAAGAGAAAACCATGAAGTCATTCAGCAAAGTAAGTGGGAAACGTCTATT
A_16_P03235960
TGCCTAAAAGATTCATCTTCATAAAAGTTTCCAACATCCTTTACCTAACCTCCATCTAGG
A_14_P136484 TTTCAAAATGTAACTTTTGGATGTGTGGATGATTCAGTGTGACATCTGAAATGATGGTGA
A_14_P138170 GATTTTTATCTGATAGGCTGGTTTCATCTCTACTGGTCCTCGACTTCAAATTTTTCAATG
A_14_P139189 ACAGTAATTCCAGTAGGCTAATTTTCTGATCTTAATATTTCCACCCATATGGAGGGTGTC
A_14_P130610 CTTGCTTGAGTTGACATTTGTGCATAGGGTCACTGACTAAAATTTGTTCTACTCAAATTC
A_16_P19762738
ACTCTTGGCACAATAGTCTAACATTTCCCAATTTCCTTATATCTCTGCCCTTTCATTTTC
A_14_P106518 CAATCAGTAGATCACAAAGAATTTGACTGAGAACCTTCAGATTAAGCCACGTTAATAAAT
A_16_P19157688
GGCAACCTCATCTTCTAAATGTAATTTCACACATGTTACTAAATCACTTTGCTTCAGCCT
A_16_P03639537
CACTTAAAATAGTTCCTCTGTATGAAGTTAAACTACCAGAATCAGGCATATTGTGATCCC

A_14_P123629 TTCTTATGTGAATAAACTATTATATGAATGAGTGCCAAGCAAACTGAGGCTCAGACACAC
CUST_8978_PI426210822
CAACCAGCTGGTATCATTTCTCAGTTATTGTATGACTACTATCTTTATTTCAAACCCTGA
A_16_P00685621
GTAATCAGGCAGTCTCTATGTTACATGAGTTAAGAGTCCTAGACTTACTTAGCAAGTCCT
A_16_P18097938
AACTAAGTATGATGGATACTTGGATGCAATGGATCATAAGTTCTGAGTTTACAGATGCGC
A_14_P110963 ACAGGAATGGGCACATCACTCACAAACTTTGAAAAGTTAAAAATACTCTCCTCAAGTAAA
A_14_P121832 AAACAGGTAAGCAGATTCTTACCACAGAAATGGAATTAGAAAGCAGTGTTCCATCCT
A_14_P128159 GGCATTTTATCCCCTCAATGATGAGGCAAGATAATGTATGTATAACTTTGGTGCTTTCAA
A_16_P01843009
AAAAAGGCAAACCATTAAAGGCATTAAAATTTCAACAAGAACGTTATCTCCACAGTCAGC
A_16_P02955432
ATGTAGGCACAAATTCCATATTCTAAAGACATGTCTCAACTGATTTGGATGCATTTGGTC
A_14_P113511 AGAGTTGCATGTATATAAGTAATTGCTCATATCATCCTGGGAATTCCTGCATGGGTTAAT
A_14_P127443 GAAAAAACTCATTACAACCCCAGTCTCATCAAATTAAACTGTTTTCACATTTCCATGTCC
A_16_P16824825
GCAGCATCTCATGTATCAAATTGATCATCGAATCAGAACTTAATCCTTAACCTTCAGTCC
A_16_P15821267
CCTGATACTATGGTATTTCTCTGCAGGGTAAACCTTGATTAGTGGCATATTCCTAATTGT
A_18_P14299318
GTATCTCTGAAATATTTTAGCCAGTTGTCTTGAATACCACTAATTCCACCAATGAAGGCC
A_14_P125268 CCTGCTGTGTCAGGGGGACTAAATAAATGACTTTGTCTTATTTAGTGATAAATCCTGAAA
A_16_P02278442
AAAAGTCTGTTAACAGAAGTTATCGATACCAGTTTTGCGTTCTCATACACTCTGTGTCAA
A_16_P01983303
GCTGCTTAGGTTATTGATAGGTTTATGTCCAAATGCACTCTAATTGAGGAATCTCTGATG
A_14_P118757 ACTACTTAGTGGTTAATAATTAATTAGCAAGCGTGACTAATTAATGGCTTCCCCTCCCAC
A_16_P19227761
AGGGCTGTCATACTAGTTTTGAAAATGTATAGTGCACTGAGATCAAAAAGATGTCCTTGA
A_16_P41747805
ATACTTTTCCACATTGAGATTCATCTTACACTTTCTTGACCACTCATTGCTTGTTTCTGC
A_14_P110879 CTGCTCTGTGGCCACTAAAAGAAATAAGTTGTAGTGGGTATAGACCAGTAATGTTTATTG
A_16_P01755696
TCATTTCAAGGAAAGACTACATAGATGCATTACAGAAACGAGTTAAATTCCACAACACGT
A_14_P112953 ACTGACACTTCAGACATTGCTGAGTATAGAATCAGAATGCTGAGTATAGAAAATAATTTA
CUST_5306_PI426210822 CAGACTTCTTAATTAAAATAGGTTCCGGCAGCCATTGCCAGGAAGCAG
A_14_P123740 CTTGGAAGTTTACAGAGAAAAAATAAAATCAAAATCAAATTTTCAAATAACCACTGACTC

A_14_P107511 TCTTGAAACATTTTGAAGGGTAGGAGTCTCTGTGGACACAGATAATGGTTTACAGTATAT
A_18_P14133389
AAATTTAGGAAGGCCTAATGATGACACACAATGCTCTTTCGCACTCATGATACATAACTT
A_16_P15987529
TATCAGAGCATGCATCATACAGAAACAGAGAACATTCCATGTCATTATACAGGTGTAAGG
A_16_P20489046
GGGTTTAACTTCAGGAAAATAGCACAATTCACATAGAGTAGATAGATTTCAAGGGAAGGG
A_14_P121562 GCATCAAGTTCCTCTCCTCTAGTTTTGTCTACCTAAAGTCAACTTTTAATGTTATGGTGT
CUST_6029_PI426210822
CATAATGCAATTGACTATACTATATAAACAGCAAGGTAGCCCTGTCTCTTATCCTGATGC
A_18_P22841252
TTCCCCTGGACGTCTACTTCACAAGCAGTAACATAAGCAAGTATGTATCATTAAAGATTT
CUST_127_PI426210822 TGTCTAAACCTCCATTTAGGGTCCTCCTACGGTACACTCACACAG
A_14_P202536 TTTTTGTTTTACGTATGCACCATTTGCTGCTGTTAGGACATCGTTTATCAGAATAAAAGC
A_14_P113778 GGACTAATAGGATGGGATAACAAGAAAAACTTAAGATTTTTATGGACAGTTCTGGCAGG
A_14_P110848 AAAATATGCTCAGGAAAGAAAACATATGGGGTTTTAACTTTCACACCCAGTAAGTCTTCA
A_16_P18155017
CAGTCATTCCAGAGATTACTCCTCATACATGAAAATCCTCCAGATTCCTACTTTCTGATA
A_16_P00008785
CCGACAGATGACAGTCATAGAATGAAACTCCCAATGAATAGAAAACCCCTAACATTTATT
A_14_P112764 AATACTTACTTTAATGGGACAGTCAAAAGTCTCGTGAAATTCTTCTCCAGAATACAGTCC
CUST_1726_PI426210822 CATGTTCTAGAACGTCCCTGAGAGGGTCTTGAGTGTGGTCTGGAG
A_16_P03644192
TACAAATCCATCCAATCAACACATACTCATCTAGTAAACACCTTTAGATAGAGCCTGTGC
A_16_P16471902
CTCTGAGTTTCTTTAGTCACCAACCTTCTATTGTGAGTAAAAATGAGTGTAGCCAGAATG
A_14_P114400 CTCTTTTTTTGTGCTTGCCTCCTTTGAAGAATCCCTGTAACCATTCATCCAG
A_14_P113446 AGAGGCATCACAATTACAGATTACAGACATTTGCAAGTAAATAATATGCAGGGTTAGAGC
A_14_P102475 AAAACCAGTGTTTTTCTATCCATCCTTTGAAATGCCTCTTCTGTAATTTCTGTTCTCATG
A_14_P128459
TAAACACAATCTCTGGAATAAATGAGACATGAAAAGTGAAGGTGACAGACTAGCAAATGC
A_18_P22842494
TTTCTGGTTGCTTTTGGATACATGGTTATATGAGAAAATTGTGTATGTCTGTTACGAGGG
A_14_P133094 GATGATAATGACAGCCAGAAGGAAGAGGAGATCCGCAAATACAGCATCATCTATG
A_14_P132176 CTATTTATTGATCACAGAATATTCCAACTACCTTTCTGTGCTGTAGGCTCCTAGTGGTAT
CUST_4521_PI426210822 TTTAGACCTTGGCTGAAGTAGGGAGGTTATGTAACTGGGATGGAACAAAT
A_14_P128323 CTGCCATTTTTTCTTACTCCATCATCTGAATGCTCAGGATAGATGAGGACTTAAACAAAA
CUST_3548_PI426210822

CTCAGAGACCAGGAAGCTCTTGAGGTACAAGCTCTTATTAAGCAGAAAATTTTAAAGTG

A_16_P18139472

ACCCCAATGAGCACATAAAATTAATTTGCAATCCCTATTTAGTGTCACCTGTCGTTATTC

A_14_P108389 ACCCATTGTAAAGGAAGTAAAACACATACATAAAGGCTGCTTCAAAGATGATTCGGAAAA

A_14_P122033 AAGTAGAAGCAAACTGTAGTTTGATGCTAGAACACTATTCTTTGAGAGGGTATGTGTGAT

CUST_7618_PI426210822 TGTAAAGATTAGTCCTAAGCAGGGTTGATTCACCGCACTACAGAGGG

A_14_P129580 AAGAAATATTTCAGTATACCTGTATGCTGCCGAACTCAACATTCTCATAATGGAAATGCG

CUST_2474_PI426210822 TGTTTGGTCAGTCCATATCGGGAGGCATTGATATGGATGGAAATGG

A_14_P114160 TCTGTGTGTTATGTTTGACACGAGACATATGAGGTGTCATGACTAACTAAGGATACATTC

A_14_P105672 GTGTATATTGTTACGAGAATTCAAGTTTTTCTTCTTTTTTATTCACACGGGGCCCATGAC

A_14_P133817 AAGAACTCATGGGTTCTAAGGGAGTAAGTGAGAATAAGAAATCATCATAGACTTCAGGAA

A_18_P17177212 TCCCCCGCTAATATTAGTTTAATGTTGTGGCCATTATATCTCTACTATCTTGGGCGATAT

A_18_P11529012

AACCCACTCCATATCTTTTACCCTCAAATATCGTTTAAAATCCCATGTTTCTTATGCTCA

A_14_P134745 ATACAAGGGCCTGAATTCACTCCTTTGGAAAAGATGTATTCGGCTGAAAGTTC

CUST_9694_PI426210822 GTGCCACTCACACGTAAGGAATGCTTATCCTGGAGGGCTGGGAGT

A_16_P18156135

TTTTATCCCATGATCTCTGACATCTAGCCACATTACATAATTATAACGACATCCCCCTAC

A_16_P21645301

CCTTTATACATGTCCCTACCTGATTTTTACTGTCCTTCATGATCCTGTTCTTGAAAACTT

A_16_P03082671

TCCATTTTCCTCAATTTCGTCTAACTAAATCTAACTACGTCTAACTACCTCTGCTAACCA

A_14_P133693 CCACACCTTGAAAATCAGCAACTGTAAGTATGGCACAATCAAACATTTCTTCATCTGT

A_18_P22839779

GGGATGCATTTTGCCTAAAGAAGATAATTTTCAACTCTTACTCAGAGTTGGAGCCATTTA

A_16_P03768876

GAAAAATATAACAATGTGTATAAAGGTGCAGTATGAGTCACTTCACGGAATGTCTAGGCC

A_16_P03506943

CGACCATGATCCTCTTTGTAGCACTCTCTTCAACATTAGTTATTCTCATATTAAAACGGG

A_14_P122759 AATTGACTTGCAGATGTTCTATTGCTAATTCTCTTAACAGTTGTATTTGAGAGTGGTGGG

A_14_P117142 CTATTTAGAAATAAATAAGCAAGCAACAAAAGCCTAACAACCACAAAACGGCCAACCTTT

A_14_P135720 GACCGATATAGCCTACACAACACAGTTTTGTGACTCCTTAATTTTCCACCTCCATATTTT

A_16_P36342436

CATCATTTGCTATGCCATAAACACAAAAAGGAAACCTTCTTAGGAAATCCACATCGAATT

A_14_P129891 CTTTTTAAAGTTGTATATCTCCAGATAGATGGCTTGGGTGAGTTGAACACTGCCG

A_14_P133903 CTGTTATTTTTCTCTATCCCTCTGCTGAACTACAGTGCAGTTTCTGTATGCATAATGTGA

CUST_2123_PI426210822 ATGGGCTCTTACTTCGGCTCCTCCTTGTGCGCAGTTGACCTGAAT

A_14_P111281 CATCGTATTGATAATCTGTTCTTGCTTTTGAGTTGTGGCAGTGAGTTTCTGATTTCTCTC

A_14_P118321 AAAAATATGGGCCACACATGATATAGGGTCTGTGGCAAATAATGCTATAGGATAATGACT
A_16_P21522159
AGTCAGTTAAATGAAGCACTTGAGCAAGATAAAGAATGATTTCCCCTTAGTAGTGACCTT
CUST_4173_PI426210822 AGCCCTTGCCTCCCATATCCCTCACCATGGATATGCAGTTATAAATG
A_14_P133929 CTAGAAAGAATAAGCATCAAAATGCCTACCTTGAAATTGAGTAAATTTAATGGTCCCCAT
A_14_P104787 GATTCTTGGGGCTATATTTGCCTCTCAAGACAAACTCCAATTATTCAAGGAAGACTTTC
A_14_P130482 CCATTTTGTAGGTCTATGGTAAGGATCACCTGGTTTAATTATTAGGACACACCTACTATC
A_14_P114883 CCCTGCCCTTCATACTTACCTTGGTGGAGTGAAGGTAATAAGTCTTATTTTTACCAATT
A_14_P201734 AACTCCTACAGTTAAACATTTACCATAATTGGTTGATGTTTAAAGGCCCCAAATCTAGGT
A_14_P200484 ACTTATCAATGGGCCTATATATAGCAGACATATGTGGGACAGCACAAACATAGAGACAC
A_14_P135727
AATAAAAAGAGAAAGGAAAAATGAAAATGCATCTCAGACCAGAATACCATAACAAAACTT
A_16_P15845294
ACTCAAAACTGGTGGTATTCTTCATACTCATCTGACCTTTATCTTCAATCACTACTCGAG
CUST_8019_PI426210822 TCCTTCTTGTCCTCTTCCCGCCCTGTCTCCATGCCTGCCCCCCAC
A_18_P13378613
AAATATGGTGACAATTGAAGTGCAAAGATCTGTTAGAAATTTTATGGAGGAATTGGTGGG
A_16_P58424595
AAAGCAAAGACTGCCTGTTTTATTCATCAATGAATTTCCACAATGTCTAGCACATAGGAG
A_14_P124888 CTTAGTGTAAGTCCAGGATTTTAAAACGGATGTGGAGTAGAAGAGACATGAGATGCTATA
A_16_P01158898
ATTTCTAGAGACTCTTACGGAATTGTCAAGATGTAGACAACTTTATGTCCAATCTGCTCC
A_14_P131231 TTAGAGAAATCATTCTCTTTCAGGCAAATGCCATACTTTCAGATTTCCTACTCCTTTCTG
A_14_P118956 CAGGCAGCCCATTGAGTAACTTCTTTGACGTAATTAAACAACTTTTTTCAGACGAGA
A_18_P22841722 GTTGTCAGCATTGGTGATGGTTCTGGGGCACAAACATGCTCTCAG
CUST_10310_PI426210822 TGATAGTGCCATGGTAGGGAAATGTGACTTCTCTGGAATTACAACTGAGTG
A_14_P114638 CAACCGTATGCAGATCAGTTTGAAAATGATGAAAAATTTTTATCCCTGTCTTCTTCCTCT
CUST_10223_PI426210822 TCACATGAGCGGGTGTGTATGTCTGAAGGAGGCCTACTTAACCTT
A_14_P130589 ACCCAACACCACATCAGGACATGTAATTCTTATTTATTTTTCACCCTCAACAAGGAAG
A_16_P03136433
AGGAAATCTAGATTGGGAGATTTGGAAGATTTGGAAGTTATTAGAAGCATGTTGAGAAGG
A_14_P107566 GTTCATAAACCCTGGGGACTAATAGATGAAAAGTCAAAAAAGTTTCTTTCTTGAGGAAGT
A_16_P41433928
AAAGCCACGTAAAAGCAAATCAGCAGTTTATTATTTTCTGAGGATACTGTGGATTTCCAG
A_16_P17538573
AAAACACATAGACAGCATTTTTCAGAACCACAGTGTTAATCTCAAGGGCAAACATTCTTA
A_14_P102415 CTCCTACTGGCTCTTTCAATTTAAAATACTTACTGCTGGAGGAGTAGAGATAGAGAATTG
A_14_P107321 CTCTTTGCTCCATTTCTCTAGTGAAAAGAACTGCCTTTATTCTTTTTAATCTCCACTTGC

CUST_3118_PI426210822 AAGACCTGGCAGTGCCTGATGCTGCAGGAATGGCCAGACACTGAC
A_16_P19427904
GTGCAAAAGGAAGATCTTGAAAAAGTCAGATGTCTGTCAATCTATGGAAGAAGCCTTATT
A_14_P105037 TTTAAGGTAAGGAATGAGGTGGCCATTTGTAGAAATTGCCCAGGAGAGTATCTTTTATAT
A_14_P125402 TGTTCTGATGATAAAAGGTATCCTGTGAAAATAAATGTGAGAAATATTCTGCTGGGCAGG
CUST_8437_PI426210822
GTTCTCTAGGCTTTTTAGAAAACATGGAGTTGTTTTTTTGGCCACGTGTATGCAAA
A_14_P123518 AAGAAAGTAACGAAATCTACATACGTAATCAAGATTGACTGGTTTTCACGATCTGTAAAA
A_14_P115700 TAATAACACTCTCATTCCTCCAGGAAATCGTGAAAAACCCAGAATTCATTCTTGGAG
A_16_P02174695
TGTCATTTAACATGCTCTGATTTCATAACACCAATCATGAGAACCTAGCAAGTCTTTGTT
A_14_P124798 TTTGAAAGCATTTTGTGCTTTCTTATTTTGTCACTAATCCAGGGATGCATTGTAACGGAA
A_14_P104579 TAAATGGGTGTGGCGCTAAGTATATCTTGTTTACCTTATCTTTCTGTGGAAAAGTTTTGA
A_16_P21385106
ATAGACAAGCCAATATATTTCCAATTCACTGTATCCCTTTTGTAATGAAATCTCCCACGC
A_16_P17693617
AAACCTTAACTGACAGTCTACATATCCATTGTTTCATTCAACAAAATTGAACACCCAGGA
A_14_P131938 ACAGCCTGTAAGATTCCATCTTTCTCAAATTACTCTGATGAACGTCTGATAAAACTTGAG
A_14_P123370 ACAAACAGACCAAAATTGCAGGTTTTCTAAAAATCAGTGACAAACACCCGTATGTTATCA
A_14_P106846 ATCAACCAGTGTTGAACAGCCTAGTTTGTAGATGTCCACATTTCTATCCATGTTTATGTA
CUST_4565_PI426210822 TATCAGTGGTAGGAGATGGAGAGGAGGGGGTGTACTTGGGAAACG
A_16_P19529634
TAATTCCCTTTTATTGCACTGTCTTTGTTATCTGCATTGGTAAATAGAACTGCTCGTCCT
A_14_P111934 GACAAGGAAGCACTACTTCTTCAAGCAAAAATATTGCCTATAATTGTTGTTGGGACCAG
CUST_956_PI426210822
CCCTAGGAATTTTTTTAAAATGTAGTGGTCAGATTGACATGTGTGTATATATAATGATAA
A_14_P107924 CTCATGAAGTATTTAAACTCTTCATGAGTTAGGGAGTTCTCACTCAAAGGTGTCCTACTA
A_14_P114155 TTTTTAAAGTAGAATTTGAGGTGCGGGAATGATTTAAATACTGACACCTTCAGGTCCGTT
A_14_P122580 ACCTACACTCTCAGAATAGCACTAAAACACTCATATTTACTGACAGAAGCCGAGAGTAAT
A_14_P126571 TTTAATTAGTTCACCCCTTGTGCATCTTGGCTTCAAGGATTCAATTAAACACAAAACTTG
A_16_P00178612
AGTAGGTGGCCCTATTAAAATATGTATAGCCTGTGCTAAAGTTAATTCTAGGGGTGAGAA
A_16_P15504298
CTTGAGACAGACAAAAGTTTAGCATTTTTCTATCATGGTCCTAACAGTATCTCCTATGCC
A_14_P117004 CTTTAGAAAAATTTCGTGAAACCTCATACACTAGTAGTTGTGCTTGCAGTATCCATTGGT
A_14_P127314 TGAGCACTAATGCATCAAAAGCAAGATTTATTTGAGGTGTAGGCAACCTTCACTAACTAT
CUST_1077_PI426210822 CCTGGGCATCGAGGACAGTCACTAATAGTGATTGAGTCAGTCACTATTTAG
CUST_6404_PI426210822 TTGTCCAGAGTTCTCACACATGACAGTGAGCTCCAACTACAACCTATTC

A_14_P123451 TACCATGGAATAAGATAAAGCCCTTAATGACACAGGCTTCTAATCTATTCGGTGACATAG
A_14_P126437 CTTTGTCAATTTTGTGAGAACATCTGAGTATTGACCACTGTTTGATCTGATACATCCAAC
A_14_P110454 GGATGTATTACAGAGACATAAACATATAAATGAGTAAGACCCGAGACCAGAATGAGGGTA
A_14_P127103 AATAAAACCATCCTCTGGATTAATAGTGAAAAATCTGTCGAGGTCAGTGTGACGATCG
A_16_P16946471
ATCTCTCCTTAGACTGAACTTACAGGACATTTTGTTTGAAACTTTATGACAGCACAGTTC
A_14_P105828 AGACCTACAGATACACAGGTTTAGGCTAACTAGTCAGAAGTCTATTAAAAACCTTAGGCA
A_16_P02039655
AGCAGTTTTGAAATTGAGCATTCTAACCTTTCTGAAGACTCCATTGATTTGACCTCAGT
A_14_P116773 TTGAAGTATTTCTTATTCACCAGGCGTTATGCCAAGTATTTAAGGAAACACTTGATCCAG
A_18_P14465133
ACCTCCTCTTTGACTAAACCAAGATTTTTCATTGTGAAGGTTTGACTCATGATAACCTAG
A_18_P15060159
TGGAACAAAAAATGTCCTTGAGATAAAACCCTTCACTCCAGTATTATCAAACGAATCTGT
A_14_P136758 ACTATGTAGGCGGTGGTGTTTAATAGATTCTGTTTATCAATAAGTGGATGAAGTGGAGAT
A_14_P120595 TGGTCCTCAGAATCCAGATTTCCTTTTTTGTCTTTTTCCTTCTTCCACATGTTCT
A_14_P105408 GACAGAATAGAAGATGTGTGGTTCTTACCTCTGACCTGCTATTCTGATTAAGTTTCTTCT
A_16_P15538508
AAATCAATCTCCATGTGCTATCAAAATAGACTGAATTCCAAGAAGAGTATCATCTCCCCA
A_14_P125218 GAAATACTCAGAGAACAAATTGGCCTCGGTTGAAATCCTCATCTCTTGTATAGTGACA
A_14_P104300 TCATACATACCTACGTTGAGTTCCTGAATCTCGGCTTGATAGATGTTTCAATTGAACTTG
CUST_774_PI426210822 AATTATTTTTGTAACATCTGGAGGGGAGCTTACTGCCATTTCCCCCG
A_16_P18877445
TCATTTCTTCTAGATCTCATTCTTGCATGAAAATCTCCTTTAATACTGGACCAGCAGAGA
A_14_P135396 TTGCACTAAGAAAAGAGGCATGTTTTCTTTTAGCCACTAAACTACGAGGTGGCATATATT
CUST_2567_PI426210822 GAGTTTCTAGCTGTGGCCTTGAATGGCTTGCAGGTATGAGATGATAACA
CUST_7267_PI426210822 GACCTGGCATGTGAAGCTGGCTGGGAGGAAAGAGATTTTTCTCTC
A_14_P137873 TCAATTTTTAGGAGTATGTGCAAAACTATCACCTTCAGACTCTAAAATATGGGGCTTTGG
CUST_6423_PI426210822
CAGGGGACTTTGGAATAACTCTCAGATATGGCAGTAGACTTAACTGTAAAAGCTC
CUST_3945_PI426210822
TTTACATTTATGTGAAGTTTATTTTTGGTTCTGATATTCTTCATAGGGTAGATATTTCTT
A_14_P122895 ATTATTCAGAAGCATAAGAAGATTGCTGTTTTCATGTTTTCCATTTCTCCCTCCAGTGG
A_14_P132325 GAAGCCTCTAACTTCATTTTCTTGCTCTTGCTCCAGATCCTAATGCTTCTGACTT
A_14_P134734 CTGCTAAACGTTAGCATCTACAAAATGAATTTGCATAGGGATAGAAGAGCAGTTACATGG
A_14_P100318 GAACGAAACTCAACCCGAAAGTAAATAGGTAAATGCCGGTAGTAATTGGCAGTTATAAAA
CUST_4419_PI426210822
CGACAATGTTGAATCAATTGTTATCAGATGGGAAGATTTCTGAATGCTGAATAGTTTGTA

CUST_6815_PI426210822
GAATTTTTAGAACTCATGATGTTTTGAAAACTATTTGCTGGCTGGAATATTTGTACTTCA
CUST_5231_PI426210822
TGGTTGAAGTTCTGTCTTAGAAGACTGTTGTAGTCAGCTGTGCTTGTCATAAAGT
A_14_P119570 ACTGGAATTGTTTGTTGAAAAGTAGTTCTGGGTCAAATTTCAGTTTGAAGTGATAGCCAC
A_16_P18548522
AAAAAAGTATGTCTAATTTGTTTGCCACTATTCAACTAGGGTCTTGCAAATAGCACTAGG
A_14_P103459 CGTTTGTAACAGATCTCACTTTGTGTCATTTTTTTTGCACAGTATGAAACCTAATGATGC
A_16_P20856199
TGAACAGTCTTCTCTAGATCTAACCATTCCCATTATCTACAATCCTAAGTAAAAGACGGC
A_14_P101026 GAAATGGGGTCACGTTCATGTAAGTCAGTTAAAAATAAACCCCAATCTCTCCAG
A_14_P120019 GCGATAATTTCCCCACCATATGCAATTTTTCATTTGTCTTATTCATATGTCTTTTCCCCT
A_16_P01165311
GCGCAGTTTACAATTAGACAATTTATAGGGGTTATTGGTCTCTAAAGGAACACAGTGAAG
A_14_P123046 TAAGAAATGCTGAAGAAATAAAGGTCATCGATGAACTGTCATTTCAAAGGTCAGGCTTTC
A_14_P124914 CTATAGCAGCAGTAGCCTTGCTCTCCTTCATGAAATATGAAAGCCTGTTTTTACTTAGAA
A_14_P132635 GATAAAAATTGGCCAGTGACTTCCTCTTTAAAGTTACCTTTGACCCTTCAGACC
A_14_P115298 CTTCTTCATAAACTGTTCTTGCTTGATTTTTAGCATCTCCTTCTGTTGAAGACAGAGACA
A_14_P118708 TTCTTACACCAGGTGGCTGTTAGATTATGCATAGTAATGTAACTAGCAAAAATGAGCACG
A_14_P108204 AGAAGTTTTATTTCCAAACCCCAGGAAGGCATTACAAATAAGAGATAGAAACCCAAATTA
CUST_7356_PI426210822 AGTCCTTGGTCCTGAGTGCTCTGTGGCCATCTTCCACTTAAAGCT
A_14_P113384 ACGCACATGTGTGTGTATGCATACTTGTCTCTAAACATATGGGATTATACTCTGCTGCT
A_18_P14137224
ATTTCTCAGGAAATGATTGACTTCTGAAGACAGAAAATGGAGAAATGCTAGGGAAAACAA
A_14_P122292 AAATTTTTTGTTTTTACCTGTTTGAGCAAACACACAGCAAACTGAACTGATCAGTCCAAT
A_14_P128733 GTCCTGGTGTGTTTGGGAATGGGACAGACAATTTTGTTTTTCACAATGGTATT
A_14_P132939 GGACTTAAAAGTTTAAAAAAAAAATGTGTAAAGTTGTAAAAGGCTCCACCTCGATGCTGG
CUST_7027_PI426210822 CAACATGCATGAGCCACTTAGAAGGGCTTAGGGTGGTGAGGGTAC
A_14_P118351 GTTTCCTCACTAACTACAGAATAAGGTGAAAATATCTTGGCCTGGTAGAAAAGGATTTCT
A_14_P136539 CTTTGCTCTCTCTGTATATACTTGTCCTGGGCTATCACTTCTATACCCTGGCTTT
A_16_P19485040
AAAAAACCAAAATGCATGTTACCTTTTCTTGACCCTTAGTAACTCCTTGTGACATACTCA
A_16_P39787199
ACCACAAAAATGCCATATTCAACACTTTTTCCTATTACAGTATAAAACACCATTTGACCT
A_14_P102165 ACCATTAAATCTCATCAAGTTGAGCAGGTCATTGTTGTTCCTTTCATCAACTCAGTCAAA
A_14_P111700 TGAGGAAGAAGCGATATTATTCTCCAAGAGTCGACAGATATCTATGACACACCCATT
A_16_P16280967
AAATCACTCAGTCGGAATTTTAAATTGGGTCAACACCTATAAAACTGATCACAACCAAGA

A_14_P123630 GGAATTAGAGAGTCCAGTGTTCCAAAAATTTGATTCAACTCTTGTTCTGTCATTATGGAG
A_14_P202311 AGATCTCCAAGAGAACTGAGTATCCTTTCCCAATTGCCATTATATTTCTTTCAGGTAATG
A_14_P102607 CATATTGCCCAGAGCAGAACTTGTTTCTGTAACAATTTATAGGAAAACATCATGAATCCC
A_16_P02504007
GCCATTCTATGACCATTTTTCTATGCTTCCATTTTTACACTTATACACAGACACTCATCA
A_14_P124655 CTAGGAAGGTCGTAAAATCGGATATTAAATAATGTTATTATATTTTTTCCATTTGTTAAT
A_14_P128918 TCTATATTTTCTCCAAGTCTTACATAGTTTCTCCCAAATGCCTTTCAGGTTACAAGTGGA
A_14_P110827 AGTAGGTATTTTCTATGCAGGTGACCACTACCTCATTTTTTAAAATCTCTGACCCAGTAA
CUST_3968_PI426210822 TAGCCCCACAGTTGATTTCTTATTGAGCCTTTGGAAAGTTCCTTGCC
A_14_P139315 ATAGGTTTTGCTGTTCTGTGTTGAGATAGCCCCACTTGAAGAACTGCATTCA
A_14_P119797
AAACGGCGAGAAATGAATTTTAAAAGTTTGTGATGGGAATTGAAACAGAAAGTGGTAACT
A_14_P200716 AATAGGGTCAACGACCCACTTATTTTTATAAGAGATATTAAGCCCGGACTGAAAAACTTA
A_16_P03101047
TAAAACCATGTCATTTCTCATCCTACTAAAAACCAGTCTACCAAAAAATGCCACCATGAA
A_16_P37584436
AGTGAAGATGGTTGAATTGAGGTTACAATGTTAGATAGTTGCTCCTCTCATGTTTCAAAT
A_18_P22840121 GGATAGGAGGAGAGAGCCTTGCTTCTCAAGGGCAATCTCTTCTACT
A_16_P30952441 AGGAGAGGGAACTGCCGCCTCTGACACCAGTTCTCCGCTGGAAAA
A_14_P139806 AATTGTGGCAATTCTAGTGTATCGAGGTGGTTTTCTGAAGCAGTAATCAGTGCATTATTA
A_14_P130955 AAGCCCCACATATTTTTATAGTGCCAAAGTTGTTTTCCTTGGCAAATGGAATGATGATAT
CUST_110_PI426210822
GTGTAAGGTGCTGATTCAACCAAATAGTGGATAGTATAGGCTGGATTGTGCATTG
A_16_P15045119
AAATCAACTAGTCAATTGAACAATCAATCATGAAAGAGGGTTTTAATGTTTCGAAGAGGC
A_16_P15943889
AAAGCATGTTAAATGAGAGACCTACTAAAGCAAGACAGTCAAACATAAAAACCAACTGCT
A_14_P126840 AGGAAGCCTCTTAAGAGTTAGCCACTCACCTAAACCCTTAAACATTTTCAAGAAAAAATG
A_14_P135338 TCCCATTGTTTTTCTTCTTTCAGGAAATCACACTGCTGTTTTCAGGTGTTATGTTTATTG
A_14_P102365 ATTTTTTTAAAGACTCTGCCAAAGCCGTCAAAGGACACTGGTGTGATATAACATTATCTT
A_18_P22843020 CTCACTGCTTCAGGCTTTAGGAAGAAACCCTCTCTTTTCTTGGTGTGATTT
A_14_P131725 CTCTGTCATTTTTCCTTGTGCTTCATCTTTTTCCTCTGTGCCATTTAACTACTTTTCTTT
CUST_9172_PI426210822
CCTCAATTTAAACATCAGAAAGAGTGCTGTTCTTAAGGAAATCATCAAGAAAGACTAAGC
A_14_P104588 CTTTTTTGTAACCAGATGTGGTGTGTCTCAATGATGTTTCCTGCTACTTTTCCCTT
A_14_P102221 ATTTGCAGCAGTTACTGAATGGCGTCTTAGGTGTTCTGTTTTGGTTTAAAGTTTATTTCT
A_14_P132593 CTTTTGTGTCTAACAGCAAAACAAAATCCATCTTTGTTTCCTTCCTCTTCTCCATCATAT
A_14_P101440 ATCTCTGTTTTTTCAAAGATTTGTCCCATCAGATGAAAAGAAGAAACAAGGTTGTCAACG

A_14_P117401 GATGCATGTGTCATAATTTAGCATACCTTGGGTGGAGGATTAGGCTATCAAAAATTCAAA

A_18_P17040090 CAGTGCCCCACTGACTTCTGAGGTCCACCCATCCTGATGGTTCAT

A_16_P03647922

CCTCCTATGGAATGTAAAGTGCAATCATTTCTTCAGATATAAGACTTTCCCCAACAATGT

A_14_P135880 TTTTTCCTGTCAGGTTAACTTATTTGTAGGTTCTGCATTATTAGAACTTTCTAGATATAC

A_14_P125230 GGAATAACAGGTGTTATGTCATTTCCTCCCAACAAATCCAATAAATCATTGGCCTAAACA

A_14_P125738 AATCACTATTTTCATTCATGTTTTCACCACTTTGCTTACAACTGGTCAAATTGCACTTGA

A_16_P15587246

AATCAGGTATGTTGACAGTTACTCATCAGAAATAGCCACACTTCTTTATTCTTTCCCACT

A_14_P120952 TCTACTTCAAAATTTTTTACACGTTTGCAGAGGTGCAATCAAATGGAATTAAGTCTCTCG

A_16_P02508728

CTCCAATACCACACATCAATAATCACAACTCTACAACTTCATAGCAGTGTTTCAAAATGT

A_16_P21346665

ACCTCCCTCATTAAACACAGTTCCTTGTAATACTGTCCCCATTACGAATAAATTAGGTAT

A_14_P126824 TCCTTGGATTACAGGAGATGAGAGTATTGTAGGCCTTATGAAGGACATTGTAGGTAA

A_16_P30952329 GCTGATAAATATCTTTGATCATTGATTTCATCCTAACTAGATTTTTTTTTTTTCATTCCA

CUST_10155_PI426210822 ACTCAGTGAAGGAGAGAGACCATATCCTCCCTCACTGCTGTCAAC

A_18_P15548796

CTTACACTCAGCATATCACCATGGACTTTTTCTATCCTTCAATTCAAATCTCATGACAGG

A_16_P02735838

ACCTTCTAAGTATCTGACACCTCTCTTTTACTTAATATGCTTTCAGTTGACCAACATCCA

A_16_P35610156

CATGGATATTGTGCTTCCATGTTCATATCCATGTATTCATCCTACTACAGACTATCCCAA

CUST_1746_PI426210822

AAAAAAAAGTTCTTGGTGTGAGGAATTTCTCCTTTGTTGTGACTGTGGTTCTCA

A_14_P123201 CTGATGTCGTTAGCATAGCATCTTACCAGATTCACATTCCAATTTAGTTCGATTTAAGTC

A_18_P25008127

ACAATCTGACCCATTCTCCTTAACTCCTATAATCACTTGATATCTTTGAAACATGTCCAG

A_16_P30952239 AAATACAGCCCTTCAGTGAAGTCTCCTGGGGCTGTGTTTAAGTGC

A_14_P202444 AAGCCAGAAATAAATTGTGTAAGTAGGAAATGGGGCACACAGATTATTTGGGCATGT

A_14_P116407 AAGTATGTGTTCCAAACTTTCCACGGTTCCAAACTGAGCGCTTCCATTAAG

A_14_P127657 ATATTTTTTGTAATTTCCACAGTCACCTTCAATGCTATACCATGTGTCTGTGACCTACCT

A_16_P00497600

AATCCCATCCTCCCTAAATTTATGTAGAACCCTACCACCTATTAACTTAAATAACTGCCA

A_16_P03800439

TGAAGAAGCATACCATCAAAGAATACTGCTTGCTAAGAACTTTCAGAAAAATTAAAGGGA

A_14_P139251 CAATTAATTCCCGAATGACATTTCTACACTATGTTGACCTCAAAATGCTTTACGACATCC

A_16_P18430412

AACCAAAATCTCAACATCAGTAAGCATCTCATTGTTACAGTATATACGAGACTCATGGCC
CUST_10428_PI426210822
GTACTTTGGAACTTGCTGTTTAAAAAGACAGATGAAATAAGTTGAAGAAACCTCATGTAA
CUST_5725_PI426210822 GTGTGCACTTATACCTGGAGCCTTATTTAGGGGATGTTCTTGCAGATAGATG
A_14_P125958 TTTCCTTCTGTTCCAAGTAACTCTTGAAATCCCGTCTTCCCTAAAAGTCTTTTTGAATTA
A_18_P21509659
ACCTGTAGAGCAAGTAAAGTAAGCAATGACTACCTATACGTAATACTATCCCTAAACAAA
A_14_P113908 CCATATCAGATAATTTAGATCTAAGAGACGACTCTCGCCTGAGAAGCCTTGATATAAGGT
A_14_P105333 CTATTCTGGGCATTTTACTGACTTATGTTTTACTTTCAGTTCAGTTCTTCCAATGACCTG
A_16_P00685528
TGGATTTTTGAAAGAACTCTAGTGTTAGTCCAGAAGAGATATAAAATAAATATCATACCA
A_14_P110670 AGATGGTTAATGGAGTATTGATGATCCACAATGTGAATCAATCAGATGCTGGAATGTATC
A_16_P02936880
AAAAGTAGACACATTGCTAAGAAATACACCAGGCATTTCTATGTTCCTATTCCCACAGAT
A_16_P39857072
ACAGCAACCCTTCAAATATAACATCTCAAAAACGCAATTTTCTTACAATATGTCCTCAGT
A_14_P135870 AGGAGACTCTGAGTGACTGGGAGTCTCAGTTCACTTGTAAGCATTTTTAAAATTGTG
A_14_P131074 ACTCCTTGCTGATGCTGTGTTATGTGTCATCTAACAGAAATGACTCCTTTGAAATAAGTA
A_14_P135945 TCTGTTAAATGGAAACCTAATAGTATCTGTAGCGAGAAGTATGGCCACCTTTATGTGAGA
CUST_855_PI426210822 GAAAATATTTCCTCTCTGCAAGGAAGGATAACCTCTCTGTGGCTGAGG
A_16_P16115181
AACCCTCTACTGAACACTTCTTGATTTAAAAATCACACTTGCTTCTGATACATGTTCTCC
A_16_P00685232
AATGCCTTGAGCCTCAGTTAATTGTTTGAGTTTTCTTGAGTATAAAAGGAATCCTGGCT
A_14_P137382 TCTACTGAGTTCATTTCTAAAGAGATGCACCAACCAATGAGGTAGAATTCCATTCATTCA
A_16_P18432137
AAAAATCTCCATGCTGATGAACTTTAAGATGCATGTTTTACATCCTTTCCCTGAAAAGCA
A_14_P138645 CAGAACACAATCTCAAAGTATCATGCTGAGTTTTGTTTTGGAAGAGAGCAGTAAGAATGG
A_14_P134360 GGCACAGAATATTCTGTAGTTAATAGATGGTTTCTAAGAGGAGAAGGGAGATTTTCTGCA
A_14_P137164 ACAAAGGCATGTTTCGTGTTGATTGTTCCCATGTAAGATATTTTTAAAGCCACTGCTTAT
A_18_P14135904
GCACAATTCTAAATTATGAATTACTAATTATTAATTTTCTGGTTTAACCTCACATCCTGT
CUST_8841_PI426210822
CACTTAAAAGAAGGGAGATGGCTGATTGAATTCTGGAACAAAAGTTACATTCCTAGG
A_16_P20034093
ATTACCTTTTGATTAGATAACTGCCCCGTTAAATATAGCATACTGACAAAAATGAAGACT
A_14_P118979 GAGAATCTAAGAAAGGATAGCCACATAAGAGAAAGCCATTTCACTCAAGAGTTTTTCCAA
A_14_P139317 CAACAAGTCCAAGTAATCGAGATGTTACAAAAAGTCACCCTTGATATTAAATCGCTCTCT

A_14_P123588 TTTTTTCCACACAATGGTTTTTTTTGGAAAATGTCCTTAAAAGAGGTCTGTATGGTCTCA
A_14_P133968 CTCTTTAAATTAACTTTCAGGAGGATTGTAACAATGTGCTCTCCACCCAGTATTTTACCA
A_16_P03418959 CCAACCCAGATCTATCCATTATGCTGCTACCTAAATCCTTTGTATTGCTGGTCTAC
A_18_P10077302
ACCCATGACTATACTTTTCTTTCCAAACATCTTCAAATCCATTATCTTACCTGAGTCTCA
CUST_2841_PI426210822
CTTATCGCTGTTGTGAAAGTGAAGGTAAATTGGCATAGCTGAAGCAGAGAAGAA
A_18_P16724933
TCAGCCACATTAAAAAAATCTCTCACCAAGTATTAACACGTCTTTCATCAGTTTATTCCT
A_16_P00808557
ACCTTTTCACACTGTTAAACTGCACATATGCTATGATGCATTATTACACCAGTGATTCCA
A_16_P17161709
ATGTCTTCGATGATTCTGAAGTTCTTTAGTTTACAGCCTCTTATTTCCAATGACACAACT
CUST_4462_PI426210822 GACACAGGCAGCCTGGGGGTGCTTGCTTCATTTCTGAAAAAGCAG
CUST_2369_PI426210822 CCACAGTATAACTCTTGTGGTTAGCGTGGAGCGTCTTTAGTGGGAA
A_16_P00130430
CTCGACTACGCGTATTCTTAAGCAATAACAACGTAATCCGTATTATCCACCCAAGAATAC
A_14_P102066 CTTCATCCTAAGGACAGACTGAAGAGCAGAAATATAGACTGTCGACGTTAGACAATGAAA
CUST_261_PI426210822 GAGTGGTACAGAGCTTGTGCCCAACCCCAGCAGAGAAGCACTAAG
A_14_P103402 AGATGTCAGAGGAGAAGAGTAGCAGATGAGAACCATAAACAATGATTTGAAAATTTTGGC
CUST_9238_PI426210822
TAGTGATTTTATAGTTACGACTCTGTCTGGCTGAAAATTTTGCCGTATAGATGAAACATC
A_14_P113533 AAGGACCTCTGATCTCTTAAGATTTTTCAGAAATTGTCTATTCTAGACTGCTCCCTCTTC
A_14_P100253 ACCTCTGATTTGAGTTACATTGAAGTAGGTGCAGGAAAACCTGCTACATAATCTCTTATT
A_14_P104357 GCTTTTTCTCCATTCTTTGATTATCGCTGGTAGTCAGTGATGATCATCTCTATGAGTCTA
A_14_P130102 ACCCCTGGAGAATAAACAATGAAGATTAAATGACTCAGTATTTGTAATATTGCCAGAAGG
A_18_P16774111
AAAAAATTCTCTCAAGGCTTATCAGGAAACCACATTAAGCAGAACAAGTCATGTATCTTT
A_14_P104328 ATGATGTGCAATAGTGCAATTTTCCTTTGCTAGTCCAGCAATGCAAGTAAGTCTTAATAG
A_14_P104103 TTGAAATTAGTCATTAGGAATGAATTTCCTTTTCCTCTGCTCCTCAGTATTCAGTTCTTA
A_14_P138961 AAGGATTTTTATTTGCATGATTATAGTTAAGCAAATTTCATTTCACATTTTTGTAATGCT
A_14_P138555 CCCCAGATTTTAGGTTTTACTCTGAGTTTTCTTTCTCCGGTTTTATCTTCTATTGTCTCT
A_16_P18175391
GAAGCATATACCACTTCCAAATATGACACTGAGCATTAATATTTTTAGACTTCCCCCAGG
A_16_P02092341
AAGAATGGTCACATGGTATAAATCAGACAGAAACAAAACAAATCAAAGTCTCTGAGTGGG
A_16_P58424266 GAGACTGACCTTGGTCTCACAAACTCATTGCCCCTTGCAAAGAAATAAC
A_16_P19260113

ACAGGTAAATTGATGTCCCTTCCTATCATCCAATATCACCTACGACAATTAAAAGCATCT

A_16_P19505498

CTCTGACTTCTTCATACTCTCTTTTGACGTATTTTCTCCCTTACTCCACAATTTTACCAG

CUST_6892_PI426210822 CTTCACTGCCGCTTTCATTCAGCGCAGGGACTGATTTTCATTGTG

CUST_7809_PI426210822 GTTCTGAGTGGAGCACCACCAGCATCCACACAAGGAGTGGTACTG

CUST_10054_PI426210822 AGCTCTAGTCAAATGTCTACTCCCATCAACTGTGGAAGGGAGGAAG

A_14_P120770 ATTTTTCTCCATTGGCTCTTCTTTAATTTCTTCCCTCTGACAACTTGTTACTAACAGAGT

A_14_P104655 GGCATGCTAAATGATAACCGAAAGAGACTTCTTTTGAATCTTCATTTGGATCAATCATTC

A_16_P19324551

AAATCTGACCTCCCTAGAGAGATAAACCTAGAGAAAAGATGTATAAGGCAATGTGAGAAC

A_16_P21455963

ACCATCATTTATACAACCACTTCTTACCCATAAATCCATGACCACACTTTTGATGAATCT

A_14_P131564 GCTTCTTATCTGTCCTGTGACTAGGAATAATTTTTCATTCCCTCCTACTATACAACTTGC

A_14_P105102 GAAGTTACATTATGCATCTTATGAACCGACTATAAGGGTGTTACATGAGAAACACCACAC

A_16_P17182669

AAAGATCCTTTTTTGACTTCTCTTACCTATGCTTCAAACAAGTTTTTGTCTGCCTATCCG

A_16_P19362734

AAAAGACAGAAACAGTGTCATATTCCTCTTTGTTTATCCAGGATATGAATCAATACGGGC

CUST_4182_PI426210822

ATTCTAGGCATGAAAATTAGCAGTATGGTACATATGAAGTACATACCAAGTACTACCTGA

A_14_P106753 TACATCATCAACTTCAAGCCGTATTTACAGATATCATCTCAGGAAAGACTACTCAACACC

A_16_P03600388 CTCTCAGAGTGTTTGTTGAGTGACTAAATGAATGAGGAAGTGAGCAGATGAAGG

A_14_P121987 TCCAAACCTCCAGTGGTGGTTTGAAAATTATATTTTACAATTAAGGTGGCTGCATTTTCT

A_14_P104276 AACAAGCCCTTTCACTGTCATGTCTGTGGCAAAAGTTTCCCCTTC

A_16_P16157171 ACCTGTTCATCACTTCATAGTCATCCTTTCCTTTATATAATCTACTCTGCTCATCTGAAT

A_18_P13641642

AGTGACTTTTACCGAAAAACCCTATGTAATAGCAATTCACCTTTATTCTGATCCTTGTGC

A_14_P139889 GTTCCAGTTATGTGAGATGTTCTGAATCTTTTTCATTTTGATCCTCTGATCCAGCATCTT

A_14_P128550 TGGGAAGTGAACATAAGATAAGGCTCATAGAGGAACTGAACTTCTAAGGACAACTATCTA

A_16_P36230165 CTCAGACTTAGAACCTTCAAATGCCTGGGCATCGAGGACAGTCACTAATA

A_14_P128695 CACTGACTATGGAAGACTTTGAAACCAGATAGGGCGGAAAGAAACTATTTCAAAATAATC

CUST_3496_PI426210822 ATGCTGATTAAAACCTGAGTTGCAAGTGTACCATGGAACATAATCTCTCCCTC

A_14_P127163 GCCTAAGAAACTTGAGATTTTGAAATCCAGCATCTTAAAGTCTCTGATTTGAAAGGTGGT

A_14_P133669 ATGACAAGAAAGAAAATTTTTTTTTAGGTCAGAAAGGATTCTGCAGGTCAGAATGGTTCA

CUST_1756_PI426210822 TATTTAAAACTGAACGACGAAGTGATCATGAACAGGCGGTACACCTACCTG

A_14_P105650 AAACATTGGGGACCATAAGATTATATATAGACTTTTTCCTTTCTCAACCAACAGGATGGA

A_14_P129382 AATATATAATCCTGAAGTGTGGGGTATTTCTGTGCTAAAGATAATCCTCAACCAACAGGA

A_14_P111105 TAAAACTACTGAACAATATGCATGAGAAAACCAGGGATTCAAGTGACATAGAAACAGCAG

CUST_7145_PI426210822
GTACCATTTACACATTTCGTCACTGCTGACAGTCATTAGCTAGTGGATAGATCCA
A_18_P17043796
TTCAACTTGCAACTTAAACACAACTTTGCCTTCTCCCTGAGTCTTTTAATTCAAACATTC
A_14_P113914 CGAACAATCACTGCTGACACTTTCCGCAAACTCTTCCGAGTCTACTC
CUST_4371_PI426210822 CTCTGACTAACTGATCTCTGGAGAGGAACACAGATGTTCCCTCCTAAAAA
A_18_P24983760
AAAACATGCTGAGAAAGTAACTGATACTCACTGGACAAGTTAATGACACTGAACAGAAGA
A_14_P119943 CTATTTATTGCCTTATCTACTTTAGCACCCAACATCTAGTAGGTGCTTGATGCCCATATG
A_14_P138675 ATGACATCTATTTTCTCTCAAAACTTCTTCACATTTTGGCCCTAACAAATTTACCCCATC
A_16_P15841686
CACTCCTGCTTAACCAAAAAATCAAGTCTGTCATTTAAAATGTCCAAGAAGTCTTCACTG
CUST_8578_PI426210822 CTTTCATTCTGGGGATTTGGGTGGCTTGGTGCAAGTGAAATTCAG
A_16_P02552154
ATCAAAATTCTTCTGGATGCTACATTAACCCTTAGAACCTAAGTATACACTTGAGCCTGC
A_16_P19608552
AATGGGCTGCTTATAACAAGTTGGTTAAACTAAAAAATGTGGGGATATTGTAGACCTTGT
A_16_P20311087
TCTGATAGAACAGTTTACGTCACTAAACACAACTACTTCTTATGTAAGTCCACTACCTGA
A_18_P14137635 ACAGAAAAAAAAGGAAACAGGTCACTGTGGACCTAGTGTCTGTCCATCAG
A_16_P17632683
CCTCTATATCCATGTGTTATCTTATCCTAACCCAATGATTTAATTACAACCCACGTGTGG
A_14_P127418 ACATGAAGTGGTTTGCCATCATTGAAAAAAACTTGTAACCCTTACAGATTAATGCAAGAG
A_14_P127768 CTTAAACAGTGGGTTTTTCAATGTCTCTCTTTAGGATTTTTGCTGGGTAAAAGCCTGTTT
A_16_P18854868
ACCCCCGAACTACATTATAATGTGCACATTATGTCACATTTAATGTTCAACATACCCTCA
A_14_P121810 ATTTTTAAAAATCTGGTTTCATCTCCACGTGCATTTTCCCATCGTTCTTCAGATTTAGGA
A_14_P100565 TGATTGGTTTATCCTCCCCACAAATAGAAAAATGACTTGAAAATGCATTATGACCATGCA
A_14_P138988 TTTTTTTTTGATCAAAACTTTTGGCCAGGTTTTTGGGTTGCTACGTTTGGTTATAATGCA
A_14_P129959 CTTTCATCAAAGTCTCAAAAGTCCACAAAAGGCTAGGAGTCACTACCATGATCTTTATTT
A_16_P17911250
AAAACATGAACAGGAGTATGCTTAGAAGATCTGCAATTAAGTTTCTGGCCTTGAAACAAA
A_16_P19793145
AAAGACAGGAACTGTAATTGTGGGTCACTAAATCTCTGTCATTCAAAAAAGATTGTCCAA
A_14_P128207 CTTTTTTTGTGTGTGACCCAGAAGTTGCTAAAATTTGGAAATCATTTACCCATAGAAGCA
CUST_2981_PI426210822 CCTTGTCCTCAGCTCAGTTTCTGTGAACTGTGGGTTGTTTCTCTC
A_14_P109942 ACTAAAAGATGGAACAAAACAAACTCCTTCAGAATCCTCCAAACTTTTGGCCATTTTTTC
A_14_P131629 TTGCATTGTCAAATCTTGATTAAGTCTGTGAGTGTCTTTGGACTTTAATTTGTCCAGGAT

A_14_P102056 CCTGAAATAGATGCTAGGTAAAGATTGGTCAAATGAAGAATGGATTCTAGTTCCAACTGT
A_16_P36490774
TCTCATACCCCAATATATCAACTTGTATGTCCTATATTCTCCATATAAAGAACACAGCCT
A_14_P134578 CATCATTTGTGTTCTGTGACATTTGACCATTGACTAAAGCATTTCTGACTCATATCGTTC
A_16_P17761856
AATATCCCAATGTAGTCATTCGTATGGAAAAGAAATCTGTAATTACCCCAGGATAGACGT
A_14_P128269 TGACGCAGACATGTCTGAAGATGCCTTCCTCAGGACATCCTTACT
A_14_P107775 GAGTTGCTGGATTATATACCCACAGTTCCCCTCTTTAAAAATTCTGTTTGTTTCTTACAC
A_14_P111351 TAGGAACCTCATTGGTTTTCATAGGCAATATACATTGTGTGCCACTATAATCCAAATCCA
A_14_P127902 ACATTGCAGATTTTTCAAGATGACTCTAAAGATGTTGGCGTTTTGAAACATTCTGTCAGA
A_16_P18210232
GAGGCTTTAAACCAGATCTGCTTTTCTACTTGCAAGTGAATGTTCTCAAGGTAAATTTCA
A_18_P22839037
TATTTTTTGCTGTATGTATGCTTGAATGTGTTTTTTACCCAGGCTTGGTAAACTATGGCC
CUST_8490_PI426210822 CAGCATCTCTTTCCCTAATCGACTCTCATCTGGTGGTGCAGAGAT
A_18_P13434828
AAAGCAATTCCTGAAAAGTGTCACCTAGACCGTTAATATAGTATGCCCTGTAGAGTCAAG
A_14_P109051 CATCTTTGTGTATTACTTCGGTGATCAAAGTTTATTATTCATCCACCAGCACTTGAAGGA
A_14_P133338 CTGAGATGTCTGTCTAATATAAATAGAACCAGGAGACCCATAGAGATTGCTTGGAATTTG
A_14_P108748 CAAGCACTTTGAAAAAAGGAGTTGAAGCATTTGCTGCTTAGATATTCTTCACAGTAAGCA
CUST_8533_PI426210822 AAACCACACAGGCTTCTGGCTACCCTCTTAAATATTATTAACCATGCTGACC
A_16_P16191700 CTGAGAGAGGTAGTTTGTGAGTGTGTATCCCTGAGATACGTAGCAGAACTG
A_14_P104181 ATCATAGTTTGCTGCTTCTCTTTGCTATCCATAAACATTTCCTCGTTCAAATAGGTCTTC
A_16_P58424479 ATGCAGTTGTCTGAAGGCTCCACTGGAGGATCTGCTTCCCTTGTG
A_14_P112700 TGAAAGCAAGGTATTTAATCCACATTTTATAGTGCAGAGCCAATAAGATCCCTTGAGGAA
A_16_P16191218
AAAAACATATAAAGTAAGCATAGATGCTAGTGTGTCCCATGGTGAAGTTGTGAACTCTGG
A_18_P22840491 AGGTATTTTTTTCTTTTTTTCTTTAAGCCTATTCCAATCTTTTTTTTTCTCCCTCTCCTT
A_18_P11716805
CCCTACCAGCATATCTAATTAACCTTCTTCTCATCTTTGTCCATAAACCTTATTCTCCAC
CUST_6864_PI426210822 AGGGTATTGTGGGAGGGGTGATGATGTTCGAAGGGAAGCTCATTT
A_16_P20189853
CTCCTTCCCATGCTAAATCACTTGATTTCAAACTTGCATACACTTAGTCCTAGAATCAGA
A_18_P22842070 TACTTGTTACTGATGCAAAGCCCCCAGATTCTAAGAGGCAGGTGTGT
A_18_P14138849 ATCACCCAGCACCGGGTAGGATTCTGACAGGACCCAGTGGCTAGT
A_14_P131186 TTGGAAGCAACAAAACTGCCAAAGCAGTTTACCCAAAGCCACCATC
A_14_P201051 CTAGTGGTCTTGCATGATTGCATGAGATGTTTAATCACAAATTAAACTTGTTCTGAGTCC
A_14_P135264 CTGCCTTCTTATAATGGTTGGACTTCTACTAGGTGGGATTATTTTTGGTGTTGATGAGAA

A_16_P02930845
AAAAAGTCACTTTAAGTGGAGACAGAAATTGCAATTCTGATCAGATGTGTAGACTGCTCT
A_16_P00379114
AAAATTCCACATCAGGTATCAAATGATGGTAATGTGAGCCTTTTACAAATGCTTGTGGAT
A_14_P136827 CTAAACTACTTCTCAGTCTCTGTGATGTTTGTTTCCTTTACTTTCTTCCACTTGGAATGA
A_14_P100885 CCAAGTAGATTTCATAGAAAACGCTGTAGCCAGGTTATCAAGTCCTTACAACAAAGTAAA
A_18_P17559917
ACAACCTCCTTTTTCAAAAAACTTTACCTAAAGCCCTATAACCTTAAGCAACTGAAAGCA
A_14_P108115 TGGTCTATAATTGTGTAGTTTTCTCTCAATTCTCAAGATTTTGAGGTGCCAGAAACTGTC
A_16_P58424280 ATCTGAACTCTTAGGCAGGTCCCTGAACTGCTCAGCCCTCAGTTT
A_16_P01487901
CAACCAGCCTCCTAAATATTTTCCTACCTCCAATAATACTGTTACTTCTCCACTCTAAGA
A_14_P135383 TGGTTATAACAGAGAAAGTAGAATCAACAAAGATGTGGGGAGTAAGGAAGTATGCTGATT
A_16_P02502857
AGAACACGAAACTTTTTACACCTGCTACTATACATACAGATTTGTTCATAGTCCTTAGGG
A_16_P00685154 AATGTCTGCATATCTTGGGCTGAGTTTGTTAGCAGGACGGCTGCATTT
A_14_P107822 TTGCGCATTAATCCACCGCTGCTCTGGTTTCTGTTAAACATTGACCTG
A_18_P13289594
TTTTTTTCCCAGTATCAGTTATGACAAACCGATTTAAAATCATTTATTGCCTACCCCTCC
CUST_5692_PI426210822 GATTTCAGATTGCACACTTGGAGTCAGGGCAGCAGTTTGGCTCAG
A_14_P116925 TGTGGCAGATGACTACATCAGTCATGACTGATAAAACATAACTTCTATCCTTGTAAGTGC
A_16_P18546859
ACATCAACCCACATTAAAGAATTTTAAGAGCACCTTTGTTAGTCTATACCACCTACCTGT
CUST_7483_PI426210822
AGCCTGTCTCCCTATATTGTTCTTAAAAATACAAAAGGGACACATAACTTCATCATTTAA
A_16_P00908168
TGACGTGAATTTTATACGTTTGTAGAAACTCTGATGTAACTTCTTCTACCTCTGAAGCGC
A_16_P18037928
AAAAAAAACACGCTGTAATTACTTTCAATTTCCTCATAAATGTTTTTCCACTAGCAGAGA
A_16_P16707223
AAAAGTTCATCAGAATCCAATGAAACAAGGAACTCAAATGAAATTGAAGCATGCTTCTGG
A_14_P128290 GAATCAGAACACGCTACTTATGACCGATCTCGTCTCATTAATGACTTTGTTATCAAAGAT
A_14_P139175 ATCTTCTTTAGGATCTGTCGAAATCGAAGAGTTAAGGCATCAGATTTTTTCTTCAGGAGG
A_14_P116411 TTTTTCCCCTCACTGAGAAAAGTAATTTTCAACTTTCACAAAACTCCATGCTGACATTAC
A_16_P00195924
AACTTGACCTTTTCCTATTTCCACTCTTTATCCCTTTTAATCTGCCTCATCAAGACTCAA
A_14_P106873 TTCCCTGGACTCTTGCTCTTGGAATAGATTAGATTTAGAATCTGAAAATAGCAATGCCAA
CUST_7412_PI426210822 GTGAGGAGTCCACAGGTCAGGGGCAACGGGCCCTGCAGACTTCTC

A_14_P104175 GTTAGAAAAGCGAAGTGCTGTAAAAACCCGGGCCTTCACGAAAAGC

CUST_9741_PI426210822 CATTACCAGTAACAGGAATTAGAAAGCCCAGCTCATTCCTCTGCAAACTCT

A_14_P112141 GGTCATGCTTGAGATGATGCATCACTTAGAATTGGATGAACATAATTAAAACAAGCAGGA

A_16_P00443133 AACTCAATTCCCACATCAAGTCTAGATTCTGTTCTGCAAAATTCTACTGCTGTGACTG

A_16_P17394396

AAGCCCTTCAACTTATACTGGTGTCTAGTTTTATCTCCCTCTACCTATTGTTCTTGTTTT

CUST_2759_PI426210822

TAACTATTAATTTACTAGGCAATATGCCCCTTCTAAAACCAGAAAAAAATGCTCACAGAA

A_16_P21372139

GGAATGTTCAACTTGTTACCTCTTACATACCTCCTTAAGTGTTATATATCGTCACCACCT

A_14_P132874 GGAGCAGAAAATACATACACTACTGCCAAAAGACCAATAGTATTGTCTACCAGAGTAATG

A_14_P121873 CTGCAAAGAATGAAGTGAGACAAACAGTTGTAAAGATGAGTTTCCACCTACTTATACCAG

CUST_7235_PI426210822

TATTTATTTGAAGACGTTTATGAAACACCTCCCTTGTTTTTATTATGTTTGTGTTCTTCT

A_14_P112965 TAGGACAGTAAATTTTGGGAACATGCTCATGAAGTGTCCAGCACATGAGATCATTTATTA

A_16_P15653805

AAAAAAATCCCACCTAATCCTTAGCAGTAGCATTCCTGAACTCCATAAATGTGGGATATA

A_14_P117298 CCATTACAGAGACATATGACAATGTTCAGCAGGTCATCTTTAATGCAGAGGAGGAG

A_16_P19056823

ATAGACCGGATTGATAAGCAAAATTTATGCATGCTGCTATTTTGAATCACTTGCGAGTTG

CUST_1968_PI426210822 AATCTCCACCTCCAGAGAGAGCAGATCTTTCTGAGTACAGGTAGCAATAA

CUST_5084_PI426210822

TTACCAAAATAGAAGAGGGAGAAATGAAAAGGAAATATGATCAATGATTTCTGTTCTTTT

A_16_P18199550

TCTTCAACACCATTTTTCCTAATTAGCATTATCAGCATCCAGTTCTAGAAAGTCCTAGCA

A_16_P00637361

AACAACCTTTGAAGTTGGTTTTGACACTTTACACGTAAAGAAATAGATTCCTGAAGCAGG

CUST_2796_PI426210822 GCTTGTAGCATCAGCCAGATGTCAGGGGACTTATACCATTTACTATGGAATTG

CUST_3296_PI426210822 CTATCCTATTTTTCTCCCAAAGGCAACCACAGTTCCATTTTCTTAACGTGTCTTT

A_14_P132451 TCTTTTTTTCCAAACATAATTCCTTGGAGTTTCTTACATGTCCCAGTAATAAACTGGGAG

A_16_P19876369

ATGTCCCAGAGCCTATTTTAAAGTCAAACTCCCCAAATTTACAAAATTTTGCTGCTATGT

A_14_P123478 GCCCTGATGCCAAAATATGTCCATTCTAGTCAAGCAGTGGTGGTTGAA

A_16_P15523883

AAACATAGGTTACTGTTGAACTGTTCCTGAATACTGTCAAACTCTTCATTTAGCACACTT

A_18_P21809891

ATGGTCATCTTAAGTAGAGACTTATGAGTGCGTATCATTGTATCACCAATCTGAGACTCA

A_14_P101738 CAGAGGAAGAGAGAATAGCCTCCACACTTGTTCCAAAGACAAATTAATAAATAACACCAT

A_14_P107316 CTAGCATTTCTCAAGCACCAAAGACAGAAACTATATCCAGCCTCCTTGATATGTCTAATA
A_18_P21946920
AAGCCTAAGGACTCTGTTAAGCCTTTTAAGTTCCTCTAACATACAGTTTTCAAGGTCTAG
CUST_5272_PI426210822 CTCTCCCTAGCTTTTAAGATCACATAACCTGCAAAGTGCCCTGAGATCT
A_16_P18298680
AGCAGTAAGCCTTGTTTTTTCCTGAAATCACCATAATTACCTCTTTACCATTTACGCATG
A_16_P17009985
AGTCAGACACAATTTGCTCTAACTTGCATTTTATCTGGATACGATAATGTCAGGCAATGT
A_14_P102629 TTGCAGTAACTACTGGCTCAGTCGGGAAGGCAGCAATCTACATATATTTATTGATTTCTA
A_14_P111238
AAAACAAACAGATAATTTGACAAACAGACTGCAAACAAGCATGAAAAGGGAATAGAGCAG
CUST_7435_PI426210822 CAGTGTGAGAGGCATCCTTCCCAAAGCAGACAAAGAGAAAACACAT
A_14_P116913 ATTCTCTCTTTTTCTGAACAATCAGGGATGTTCTACCATTTACCTTGAAGTTGCCTTATG
A_16_P02691485
AAACCCAATGTATCGTTTCTCTTCTCAAAAGCAACCTAGACAATGAACACTAATGTATGG
A_14_P136945 CTTATTGAAGATTCCTGGTATTTAACTGAAGAGTCCAAAACATATGTGGTGGGAATGATG
A_14_P202453 TGGGAAGGTAATCATTGTGGATGCTGAAAATGTTTTGGTTGCTGACAAAAGATTAATTAG
A_16_P00818644
CCACAATAACCACATATCCCAGTTTTTGTTTCTTTGTCTATGTTTACACACACTTTTGGG
A_16_P19362972
ACCTGCAGAGTAAACAATCTCAGAAGTATCGACTAGGACGTAAATAGCAGTTCCTTATAT
A_16_P18182866
ATATGCTTTGCACATTAGGATTTTTCAGATCTTCCCTATTCTTGGTTTACCCAAAGCAAA
CUST_4085_PI426210822
ATTAAGTTCTTTCTGGTGGCTTCTTTAATTTTGGAATAACCAGCCTTCCTTCAACC
A_16_P21564557
CCCCATTCCTCTTGATAGATCTTGCCTATTTATCCATTTCTCACATACAATAGAACAGTG
A_16_P36230816
GTTTGGGGACAGTAAATAAAATTGATGGCCATTTTTTGTCATGCATCTTCTGAATTGGTA
A_14_P137789 GTGGGTAACCATTGCCAATAACCTTCTTTTTAAGTGTCATATACATCTGAGAATACATGA
A_14_P103375 AGAGTGCTAGTTTGCATTCAACACTCATTTCAAGATAGCCCTTCTTCTCCATCTC
CUST_879_PI426210822
AATAATACAGGGACTGAGGGTCAATGTTAAAACAATTAGAAACCAAATTAGTTGCCATCT
A_14_P128870 CATATTTCTCTTTTTTTTTCTCAGCCTGTAGGTTTTGTCAGTTTTGACAGTCGCTCAGAA
A_14_P134300 CCATGCCTTATCCTTCTGTGTGTCCTTCAAAATAGCCAGATTGTGTTTAATGCATTATTA
A_14_P109463 GGTTTGTTTCTTTAATTAATTTAACAAGTGCTGCAGTTTGCCCTCCCATTCCCATCTAT
A_14_P109597 TTAACTTGTTTCATTTCCAGAATATTGCCCATGTTTGTTAAGAACTTCAGACACACCACA
A_16_P37247739

ACTCTGCCTTTATTCTTTAGTGCAGTATGAAGCAATAACCTAGTATTACCATCACTGCCT
A_16_P20975515
AAAGAAACCATTCTGATTGGTCATTCATTTGAAAACCCTAGGTCTAAATAAGATGAGCCT
CUST_1947_PI426210822
ATCCTAACTAGATTTTTTTTTTTCATTCCATAGGTTTATATTTTCAGAGCTGACCGAAG
A_14_P122049 ATGATGAAAACGAATCCTTATTCAGAACCTAGCAATACTACTAGAACCACCTCTCCTACA
A_16_P16552625
GCTGGGTGAATACTCTCTTGAATCATTGACAAGGACTATTGAGAGCCAACTTTTTAAATT
A_14_P122184 ACAATTGAGAATGTATTTTATGATCTTATAGTTCTGAAAAGAGGGGGCACAGGAGTAAGG
A_14_P102953 ACAAGTTGTAGGTCTTCAGATGCAAGAGAAGTTCATTTCTTAGGGGGAAAAATAGAAGAG
A_14_P108621 CTTTGGAAGAGGAATCAGGACAGCCCTTCTCTTGATACTCACCATC
CUST_10326_PI426210822 TTAATTATCCAGAAAATTCTGGCCAAGCACCTTTTCTTGCAGAGGGGAG
A_16_P36230261 ATTTCAACCCTTTCCTGCTCCGTTCTCCTTTGAGAATGGGTAATCAGAG
A_14_P107509 CGTTACAGTGCAGAAATGTTAGTTCATGTTAAACTTTTCCTCCTCACATATGATGTGACT
A_14_P137355 ACTTTACATAGGACGCAGCAATATAGTATCTTCCATAGTTTTTCATGACTTTATTACGGC
A_14_P108141 CATTGAGGTTGACAAATGATTTAATGAAAACAGAGTCAGGATTTGAGGGATTATCTTCCT
CUST_3935_PI426210822
AGGACTCCATATTATATTCTTATCCTGTTGAAAATTTGACCTTTTAAAGATTTATTTATA
A_14_P202545 CTCATAGAACAAAATTGCAAAACAATTTCAGGAACCTCACAAATTCCTAAAAATCTACTT
A_14_P201363 ACTATGTGGTATATGCAAGGTTTTATTCTCCTAAAAGCAGTAATATCCTCCTAGACTCCC
A_14_P133285 CGATGGCAGTTCTTTGACTTCTAAATGTTTTTACCTGTCTTACGTGAAACATCCTCTTAT
CUST_354_PI426210822 TACAGGTGAAAAGATGTGTTGTCTCTAGGGAAAGGGAGGGAGAAGAAT
CUST_9416_PI426210822 CCAGTCCCTCTCAAGGCCCAGCCATTATCATAGAAAAATCTTCAAGTAAG
CUST_5154_PI426210822
GTTTAAGATATGCTTTTGGTTGACAGTAGTAAAACAAGCCAAAAGAAGTTGACCATTTAT
A_14_P139497 TATTATTCAACTTTTTAAAGCAGTATGCTCTGGAAAGTGCTGTATTTTGAATGGCAGGCC
A_14_P120986 AATCGCTATAAAGCAACGTGTTGATGATGCACTCACTTCAAAACTTCCAATGTTTTACTT
CUST_5544_PI426210822
CATTAAACTTGATTATATAAAGGTAAAAGTAGAATAAGATAAGTTTTCTTAATATCTTAA
A_14_P104003 CTGTTATCTCTCCAGTCTTGTTTCTATTGATGTAAACCGAGAAGCCATTATTTTGGAAGC
A_14_P119674 GTTCAAATCCAGAGCCCTCCTTTTTCCTTTGCTCCACTTTAGAGAAATCGT
A_14_P135521 ACTCAGTTGCTTCACTGGGTTTAAGGCATGCTTTGTGTATTTAGTTTGCAAAAATAAAAC
A_14_P114994 AGGTGTTGGTGTCACAGAGGAAGAGATCATTTTCTCTGTCATCATAACCTCAAATT
A_16_P19624622
ACTAAACCCACATGTGTATTACCTTTCCCCATAGACATATTTTACACTTTGGACACCTAA
A_16_P21048114
AAAATCTCCTTCTCATCCCTAAATGAACCATAGCAAATGAACAAGATCTCATCATCATCC
A_16_P02515678

ATGTAAGTGCAATACATTGAGTGAAATTCTCTGGATGAGTTTGTGATCCTAACCCTAACA

A_14_P113799 ATATAAGACTCCCAAGTATTACTGTCATAGAAGGAAGGGATTTTTCCTTTTACAAATGAC

A_16_P20423482

GAACTCAGTCTACGTTGTTTTTCCTTATTGTCTCGTAAAGTCATTGGTATTCACATAGGC

A_18_P16971431

AAAATGGCTTTGCATGTGATTAAAGCAGTTCTCTAGCATTGTTAAGTGACTGACTTGTTC

A_14_P114736 CATCTTAGGAAGACTTAGAACCCTCTTTTTATTTGTGCACTTGCTTGAAGCATGTAACAA

A_14_P124109 ACCTTGAGTTAAGTATACAGTGAGTTATAAATAGGATAGTAGGGCCAGGAATCTGATGCT

A_14_P130960 AACCTCATAAGAAGAGAGTTTTATTCAACATTATGGCATGGCCAGTGTAATTGTTCCAAC

A_16_P39048906

TTAGGGTGATTGCGAAAGTTATACCTCCTAGGTTGAGTTTAAAATGCTAGTCTCTTACTG

CUST_1831_PI426210822 AGCATGCTTGAGAAACATTTTCCTCTGATGGATGTTTTTTTTTCCCTTCCCTT

A_16_P19038585

AGTAAAAACAGTCTATGACTCTTGAGGTATGTTAGAACTCATCCCAAAATTCAACAGTGG

A_16_P15398380

TACCATAGGTGAAGACCCTATCTACTAACACATTGTGTCTTTCATCCCTATATAATGCCT

A_14_P111628 CCTTCCCTTGACAGTATATATGGTCATGTGCACAACGACATGAATTAAAAGTGACTAAAT

A_14_P121689 CTGGAAATACAAGAATCATAAGGCAGACGCAGCAGACTTTTAAAAATCGCCATTATTTTG

A_16_P17574129

AAATCCTTCTATTTGACTTCCAGACTGCTCTATCTAAAGAACTGACGTGATCAACTTTGT

CUST_4013_PI426210822 AAAAATAGGGCTACCTGCAAAATGCTTTGAGCACTGTGGAAACCAGC

A_16_P21211442

AATGCTGATTTTTGGTGCTAAACACATCACATACAGATTCACAGTTTGGAATGTAGCAAG

A_14_P135211 CCTGGCTAATTCCCCTTCCATTCCCAACTCTCTCTCTCTTTTTAAAGAAAA

CUST_701_PI426210822 TTTTCCCTTCCCTCGGACGTGATGAATTCTCACTCTTGTGAATTTTTTTGT

A_14_P202517 TATAGAAACCAAGATCAAACCAAGTCACCCTATTTTCCTAACACAATTTCAGGAAAATCG

A_14_P112152 CTTCTAATTTGGTGTGTAGTGGCCAAGATGTATAAAATGAACCCCAAATTCTAGGCTAAA

A_14_P139711 ATCGACATTGTTGAGCACGTAGTTTCGTCATTATTCTTGTTGACTCTGTATTCAAGGTTA

CUST_9641_PI426210822 GGCCTGCAGAAAGAAAAGGAATTTATCTCTGAGAAGGCCTTCAGAAAGAAAA

A_16_P37433113

AAATGAATGAGACATTTCGGAACCAATCATACATACAAAAAAAGCATCCTTTTTCCAGGC

A_14_P101011 ATTCTGATGATCTCCTTCACCACTTACTGTGGATAAAATTGAACAGAAATTTCTCCACTA

A_14_P115478 AAGTAAACTAGTTACCTGGGTAAAAGCGCTAACGACCCAAAGGCTCTTCTAAG

A_14_P131950 CTGGGTCCAAAACTTTGGTCGAATTTTAGAACACTTTGAGAATAAAAGAATAATGGACCA

A_14_P100598 AAAGCAGTAACTACTATGCAGAAATGAAGTTCTATGTGAGTGGGAGTGAATGTGGGTAAA

A_14_P137798 TTTGAAAATGGCACAGATCTTTTCATTGCTATTCAGGCTGTTGATAAGGTCGATCTGAAA

A_16_P41573528

GTCTAGTAACACAGAACCTTACCATAAACTCTGTTTGCCTTCTTTAGTTTTTCAACCCAA

A_14_P135508 CTACTTTATTGAAAAAGAGATTGGTATTAGATGGGAAGGGGGAGAAACATAACATGGAGA

A_16_P02885804

GAAAAGCACCACATCAGATAGAAAGCACTAAATTTATCATAGTCAAGTAAAACTCGGTCG

A_14_P124851 ACCTTCACTTAATAAATGATTCATGGTCTTAGGTTATCATGTGGTTCTGCTCTCTAGTGT

A_14_P128615 CTGCAATCGACATGCAGATGCATCCAGGTCATTGATTCACTGTTCTTC

A_14_P125650 ACTTATTTTATGTTTACCATTACGTTTTCCGGGCATAGTATGAGAACAACCACAAAACTC

A_18_P22841570 TATAGCCCAGACAGTGGTCGTCTGAGGGCTCTCCGGCCTCAAGGC

A_14_P130203 TAATAGTAAAGTGGCTCTATATTTTCTCTACGCCATCACTGGGTCCTCTTCTTATTCTTC

A_16_P18648988

AAAATGACAAGTGATCATTTCTCAACATACAAGAAAGTCTATCTCAGTGTATCCATGGGC

A_16_P16607012

AAAACCATATTCTCTCATATGGCCTATTTGCTACTCAGTGACACATTGTTTCAAGATGCT

A_14_P128135 ACAAGTTGGTTGATTTTCTGCTTTTACAATTAAGTGTGGTTGGCATAAATCCTAATGGCT

A_16_P35017844

AGGCAAAAACTTTCATTCCAAACATAAAGCAAATACCCAGTTAAAACTTTCTTTCCATCA

A_14_P130347 ACTGCATTGCAGGCTGTATGATAAAACACACATAATTTAAAGAGAGAAGGCTCTTGATTC

A_14_P125665 TGTAAGCATGAGACCCCTCTGAATAATACACAAATGATGAAATGTGATGCTCTTAGCAAT

A_16_P37733292

ACCCAACTTTTAAGATAGTTCTCACTTGCAAATAGTTCCAATTAGATTCAATCCCTGCTT

A_14_P110576 ACACTTGGCATGCTTTAACAATTTCAATGGAAACGTATAAGGAAATTGGGATCAAAACCT

CUST_8695_PI426210822 AGGAATGAATTGTTTTCCCCAAGGATGACCTGGAGGAGCTTCTTAAG

A_14_P120111 AAACATGTTCAGAAGTCAAGTAACTGGAAAAATAAACCTCCTTGGCCTGATAGAAATGAT

CUST_1271_PI426210822

CTGTCTCCTATACTTCAAACGTGTTTCCATGTATTACGTGGAATTATAGACTACCTAAAC

A_18_P26792317 GGACATAATTCTCAACCAAATTGCCTCTGTCTCTCCTTCTCACTCTCTCT

A_16_P01858901

AAAAAAATCTTTGAAGCCTTCATAAATCAGCCCTTTGATTTAAATCTCCATCTCACTCCG

A_16_P21505793

AACTCAAAGTTTAAGTGCAGTGACATTTCAAAGTCTTTTTCTGGATTAGGTGACATCAGC

A_18_P12895657

AGTCTTCATAACTCCTTAAGTCTCCAAATCTCTTCCAAGTATACTGCCAATCCTTCAGT

A_16_P18485704

TCAGTTCTTAATCATCCACCAATTAGAAAGGTTACCCAATAATAGAATCCTTGGCATTGC

A_14_P201112 AGGAAATTGAAAAATATGTGTATGTTACAACCATGAAAGTGAACGAGATGGATGGTCTGA

CUST_7805_PI426210822 CATAAACACGTGAGCTTTCTCCTCATCCTCCTTTTGTTGCCTCTCC

A_14_P126621 CCAAAATATCTCTGAATAGGAACAGTGAAAAGAGTTCAAGCCCTTTCACACAAAAATTCT

A_14_P101865 GACTTCGGGCTTGATTTTCGCTTCGGAGATTTGCTAGACTTTCTTAGAGAAG

A_14_P123952 ATTTATTTAGTGATCAGTGTGGTCATTTGCTTTGTTCTAAAAACTGGGACTGCAGAGAAT

CUST_7310_PI426210822 GTAACCTTTCAGAGGTGCTCTGAGTCCTGAACATCGGAGATAGATTCTTTATC
A_14_P135407 GCTGGCTAGTTTCACCCAAAATAAAAGCGGTAATAATAGTAACATCCTAATTGATGTGCG
A_16_P02180308 TCCTTACACTGGATGAAGTAGACAGATGAACTAGACTCAATTGCTGAGGAGC
A_16_P15049579
AAGCAACCTAAGGAAATGAGATGGTTTGTAGATTTCTTTAGAGGAAGTCATGTCATGCTG
A_14_P109290 TTGAGAACCCACCACTCACTGTTATATTTAGTTGTATTGTCTCTGGACAAAATGATGGTA
A_14_P108927 TATATGTGGGTGTGGGATAATGGAAAAATATACTAGATTTTTTTCAAGGGCAGAAGCAGG
A_16_P00784860
TCACAAATAGCCACATGAAACCATATGAAGATTTTCCAATCCATTTCTTGTTATTGCGCA
A_14_P128599 TTCTTTTTGTGCATATTTCTGAACATGCATTCTTCTACCTTCTGCTGTGTTGTTGTGTTT
A_14_P127220 AACACTGCATTAGAAGTTCAAGCCTGCCATTTGTCTCCAATGCAATCATAAATAGATACG
A_14_P101654 ATGTAAAAGAATGCTTATTTCCTGGGTGTTGTTGCCAAAATTGGGTGTAATCTCTTTTTT
A_14_P118310 ATTGCAGATTTATCAACGGCTTTTATCTTGAAAATAGTGCCACGCAGTCTACAAATTCTG
A_14_P132539 CTGTAACAATGATGTTTTTCTCCAATTTTCCATTCTCATGGATTCGCCATTTTTTGATCA
CUST_5550_PI426210822 ACTCAGTATGGCGAGGTATGTGGCAGGGGGTTATTATTTTTCTAACCATG
A_14_P116073 CTTAACTCTTACCTTTGGATCTCTCAGAGGAACTCAGAGTATCTTCAGACTCAGAACT
A_14_P125687
AAGGCAAAGTGGAAGTTACAAGTCATGGAGAAGGTCATTCTAGCAAAGAAAACTTTTTAA
A_14_P137637 ATGTGGACACTGTTTTTGAAGTCGAAGATGAGAATATGGTTTTGGCATCTTATAAACAAG
A_14_P115359 ACAGTGGACTAAATAAAACGGACACATGTCACTGTATATCAGTCCTTACTTCTTCTCAAT
A_14_P137326 GGTCCCCTTCAAGTGTTGTTTCTCCAAGTAGTCATAAATGTAGCAAGTATAAATCAGAAT
A_16_P21498274
ATTTTGTGCCTATACATACTCCAATCAGAGGAATAGAGTTCTGACGATACACCTTGAGG
A_14_P123342 ACTGCAGATGAAGAATGTGAAGTCTGTTTGAAGGTATGAGAAAGAAGAAAATGGATTTTC
A_14_P135352 TGAAAGAGGGTAATGAATGAAAGAGGGTATTTTAAGAAGGGTTTCCGAATAAGTTTCAGT
A_14_P111472 TTCCAGCAAAGTAGGCTAATCATATCAGTTTGAGATGGCAATTAAGACATCTATAGTGGC
A_14_P100537 TGTGAACCTGAACATGAGATGAAAAATGAGGAGGCTGTTTGTAAAGAATCCTGAAAATAT
A_16_P19036789
AATACTTGGAAACCATTTTTTAACCCATCCTCAACTATCAAATTGTCTGCCTGCATTTTG
A_14_P138529 AACTGAAAAAGGGTGCTAGTCTCATTTCTGTTTATCTTCTTTTTCCTAGAATCTTGGTGT
A_14_P132095 GAGTGGATTGATGGATGACAGGTAAATAATCAAAGACAGAATCCCCTATAACCAATTTCT
CUST_9495_PI426210822 TTGTTGGATTCCAAGTCATCTGTACTCCCCCAAAATACCACTTCCATTAATTACT
A_14_P116770 ATAGAATGTTGGCTCAATTAAGAAACATCAGGGAGATAAATTCAACCCAGTGGTAAGTAA
A_14_P133473 CACATTTAAGAGTTATGTTTCAGCCTTGTGATTTGTCCTATATTCTGACTCATCCCTCAA
A_14_P132600 TCTGATCAGGATTGGTTTGGGGGTTGCTTATTTTAAGGTATATTGAGTATTCAAGCATGA
A_14_P108923 GATCCTTATTATCAAACAAAAATGAAAAGAAAGTTTGTTTGCCCCTCGTGACCTATTCCA
A_14_P108262 CTATAAGATTACACTGGGACTTAGATCAAATAGCCTAGACATTTAAAGGTGCAAACAGCC
A_14_P128650 AGCGGTGCTTTAATGAATGGGGAAGGCTAATACTGAGTTATAGAAGATCTGAACAATATT

A_14_P114118 AGATACAACTTTAAGAGAAATTAATTAATTTTTCCCCACTCTCCCTTGTGTGTCCTCAGT

A_16_P03541365

TGGGATACAATGCTTTAACCTGATTCAATTACCCTTTTACCTTTGTTGTCATCAAGAACA

A_16_P18536918

AAAAGATTTTGTGCAGCAGAAATACCTTGTTCAACCATTTATGTCCCCCTATGTCATTTA

A_14_P101698

AATCAACTCCAACTGGAAGCAGAGAAAAAGCAGTAAAACCAAATGTCCTTAGAAATTCCT

A_14_P112590 AAGCACTTCATATAGGTAGCAAAAGGCAACTACGTCATAATCCTAAGGTCCTTTTCATAA

CUST_1017_PI426210822 AAGTGGAACGGGCTGGGCAGGGTAGAGTGTGTGCCTCTGAGTAAG

A_16_P20775386

AAACTGCCTGTTTCTTTGATCAGATTTTAATGCTGACTGCTCTATCAATGTCGAACATGT

A_14_P114175 TTTTTATTCTCTCATCTTTGCATTGAAGATTTTGGTGCCTCTGGTTTTGCTACGACAAAT

A_14_P108532 AGCATCATTGTATATCTCTTCTGGCATTTATCTCTTGTAGTTTTAGAGTCGCTGATCCTG

A_14_P117371 AAAACTCCTCCTTACCTTTCCAGGACAATTATCACCTCCTGAGGAGAAAAG

CUST_3307_PI426210822

TGGTCTTCTATATGTCAGTTCCAGACTAACCTGATCTGATGCTTGTAGACTAGTTATCTT

A_14_P101557 CACATGGGTGCTTTAATCTCTAAGTCATGGATGAAGAACCAGCAAAATTATTAACAATCC

A_14_P100971 AACCTGGAGCAGTGATGACTGTGCTTTGGAAGAATACAATTTTTTTAACTTTTATTTGCG

A_14_P134591 AAATGGTTCTTGAATAAAGACAGTGCTGATTTTGGAGCAAAACTTCAATTTTCGCTTTGT

A_14_P133037 TCTCTTTTTGTCTTTTCCTTTTCATATCAGTCACTATGAACAAGACCGTAGTGCACTTAA

A_14_P133813 TATTGTATTGTGTCTGGTTCCTGTCTGCTTTGTGAGGCAGTCTTGTTCTTAGTATAGATG

A_16_P20124173

TATGAACCATCAACTACCACATTCTTCTCCATTTGATAACACTTGAGTCCTTTTGTTTCG

A_16_P17571518

AACTGGCTCATTGTTACAAATCACTTCCTACTGTGGATTTGAAGTCTTCTTATTTTCTGC

A_16_P01583620

CCATGGACTCATTCAAATTCATAGCAGAGATTCCTTATTCCATCTACCTCATTTACCATC

A_16_P15622599

GCAGACTTTCACTTAATTAAACTACCGTGATTGCTTTATTCATATATTTTGCTTGCCCCC

A_14_P125946 ACCACATCCTAACTCTATTAAAACCAGAATGTAGAGACTTTGATTAAAGGGTAGACACTG

A_14_P118626 TTTACCTTTGATGTTTCAAAAGCAGTGTCACATACATGAAGAAGTTCAGCAAGAATTAGC

CUST_5176_PI426210822

TTCTCACCTCTTTAGGAATTTTAAGCCCACAAATTAAGTATGGAATTTATTACATGGTAT

A_16_P18879369

GAAACACGACAGTAAAACATCTACGTTTAGACACTACAAATTGTTATTTTCCCAGTCCCA

A_14_P134006 GTAAGCCCTTTTGAAAATCTCAAGTGTCATTTAAGCACTTTTCTTTGGCATATTTCCACT

A_14_P126363

CTCAGAAAGAAAATATAAGACAAAAGTTTGTAGCAGATCTACGGAAAGAGTTTGCTGGAA

A_14_P126650 GTTTGATCTTGCCCTAACAGACCTGGGTCTATGTCAGAATCATTTGGATCAG

A_18_P14133043 ATGCTTTCAGAAGAAGGCGTCAGACCTCCTTGTATGGGAACATCATC

A_14_P121896 ACCTTTGCTCTTAAATGGAGTAGTCAGATCATTTTGTCAGTCAAGTAGTCAAAAGAAAGC

A_14_P200437 GGTTTTGCTACCATATGATGCCTAACAGAGTAGGTTCACTAAATGATTGAAAAATGGGTG

A_16_P36230178 AGTTGTCTTTTTATTGTGACCGAAAAACCTGATGAGTGTGGAATAGTTTGGGAGC

A_14_P103256 CCAAGGCGTGGGGAAATACCTCATGAATTCATGAATACACTAAATTAACTTGTCAACTG

A_14_P106165 CTGTTCTTATATATTGTTTCATTTTTAAAACTAAAGATGCATTTAAGAAGCAATACAAGT

A_18_P13122018

AAACCTGCAAGGTAAAATCGTCTGAAGGTTATCAAGATTGATACTGCAGGCTTTGTTTT

A_14_P137476 CACAGATGTAAATTGCACTGTCATTTGAAGTCACTCTGTTTACAGTGAGGGAAACTT

A_18_P17134079

AAAACTGGCTATGACTCACCTACTTACCTAAGAGTTGAATAGATTTGCGTAAGGACCATA

A_14_P109575 ATCATTATTTACACTGAAGATTAATTCTTGCTGTGAAGGAAACGATCAGGTTATGGGAGC

A_14_P124564 CTCTGGAAATTGATGAAAAAGTTCGATCTGCATGATCAAATTTGCCTCCTTGCAAATTTA

A_14_P133278 GTTCTGCTTTTTGTGTATATTTGCAGGGAAGATGTTAACACTTCCTAACAAGTCTTTGTG

CUST_9281_PI426210822

TTTGCTATTATGAGTTTATCACAGGTGGTTCATTAAAAAACCACCTCAACCAGTGCA

A_16_P19949821

AAAAACAGCCCTTATTAACTCTTCCTTCCACTGATTCTACTGTAAACTCATAAAAGCCTT

CUST_7182_PI426210822 CAAAAAGTGGAAAGACTGGTAGATAGTCTGTCAGCTCTGTGGGGTG

A_18_P22840319 GCTGTGCTCAGCCCTTCCGGGTGGTGAAGGTCTGAGGTCACACTG

A_14_P105042 ATTTGAAACCGCAACCCAGGGAATGAAATTAACCACCCGACATAACAGA

A_16_P03774196

TACATTACCAACAAAAGCATGTTCTCTAGCCTTAACAATCTAATTACCATGGCACTTCCA

A_14_P200333 CCAAGAGAACTTGAGAAAGTTGCTTTCTCAAGCCTGTTTCACCTCTATTGCT

A_14_P104333 GAAGGATATTGAGAGAGTTGAGGAATAGGAAAGATAACCTTTCCTGAGTGATGTGAAAAA

A_14_P108018 TCAGGCATTTTGGAATTAATACGCTCCAATAAGGCAAAGGGCACATTTATAGATTATTCC

CUST_1226_PI426210822

GCCTGTTCAAGATTAAACTCTGAGAAAGAAATGCAAGAGGTAATTATGATGCCATGGT

A_16_P17422034

AACGCTCTAGTCTACAGTTGTCATTGTCTACAAATTTTCCTCACAAATATTCCAATTGCC

A_14_P135832 AGCACTTTTCCCTCCTCCTTTTGTTGAAAAATTTCAACTCTTTAGAAACTTCTCCGTT

A_16_P19036350

AAAAAGTTACCCTAAAAGTGCTCAGAACACTCATTATCAAGTTCCAAGAGATGCAAAACA

A_16_P03414672

AATTAGGTGAGTTTGCTTGAACTATACGTTGAATTAGGTGTTGAATTAACACATAGGGGG

CUST_3003_PI426210822 ACTTGTTTGGACCTGCTATCCATGCATTCAAAAAAATGGGAGGTTTTGGT

A_14_P104316 GGATCTGGGTTTAAATGCTGCATCTTCAGAAAGGAAAGAATATTCTGAACTTCTAAGTTG

A_14_P100439 ACTTCAGTCTTCAAGGTTATAGAAAATCAGGTCTGTTCCAACAAATAGTGTCATTTTTGG

A_14_P139768 AAAGTGAATAAAGTTCCAAGAAGCAGAAAACGGACACTTTTGATCATGCAGCATGACT

A_14_P200641 ACTAGGAACAGTGTATTGCATAAAATAACATTTTAAAAATAGTGTGGGCACTACCTTTCT

A_14_P112518 ACTGTGGCTATTCTTCAACAGTGTTCTTAGAAGAATGGCTTCTTACACCACAATATACAT

A_14_P123940 GTCTAAATTGGCTATTCTCCTAACAAGAAATCTTTAGGAAAGTCCTCCCTAAAACCAGAA

A_14_P104058 AGTTGGTAGTGTAAAGGAAGTAGAACTCATACCTACTTTGGAATGGCTATCAGAAAGAAG

A_16_P16366058

ATTTCTTTCTCTTCCTTGAATATCCCTAAGCATTCCATTTTACCATCTATCACCAAACCC

CUST_7759_PI426210822

AGGATCATTTTAACAATGGTCACTTTTTTCTAAGGGCTTCTCTTGATCTTTTTTATTTAA

A_14_P133592 TCTTAAAGATGCATACTGGAATGATAAGTTTGAAGATGTAACTATCAACAATTCTTTTCA

CUST_5522_PI426210822 GCCTTCTCTAAAGTGTTAAATAAACTGTTTAAGATAGTGTGGGCGGCCTCGTT

A_16_P20865243

AGCCATCAAGTATGATTGGAGCCATTTTAATGAGTTGCCTAGTATCCTACAATGGTAAAA

A_14_P127775 CAGAGTTACATTCTGTTCCAAGCCCCAGCATTCACTTTAAACATTAATAAGAGATATGGT

A_14_P128216 AGTTATTACTAAGCAGTTTATCCAATTGCATCAGCATTGATTGACCTGCTGCTTATTCAG

A_14_P108409 CTCTAGCCTCAAAAAGCAGGGAGATTTTGCAGTGAGAGGTTCCATC

A_16_P20596392

GTATCTCCTATTTGCCTAACATTCTGCTAAATACACAACTATTGCTGTCACTTCTGAACA

A_18_P10278479

GGATCGCTTTCTTTATATATCACCTAAAGACCAAGACATTTCTCTCTAAAAGCCTTCCAG

A_16_P18763664

TGGATTCTTTGGGGTTAGAACATTCCTTTTATGCAATACATAAATGTCTAGTATGCACCA

A_14_P134820 CCAATTTGAGTAATCGGAGTTTTTTTAAACACCACACTGAACGCAAGGTCATAATTCAAA

A_14_P104812 ATGTCCAAGAAACCCAGAGACTTCAGAGTAACATGAGAAAAAGACAATTAAGAATTCTCT

A_16_P16674324

AAAGGTAAAGTTGTTCTTTGTAAACACTGGATTTTAATGCATCACTATAAAAGCGGACGT

A_14_P112612 ATGGGGAATTCACAAATTAAACCCTCATGCATTACACATTATGAATGGAAAAGACATCAG

A_18_P13583240

AAAACCACAGCATATTTTAGCATCTGTCATTACTCTCCTGAGTTCTTTCTATCACACCAA

A_14_P201874 CTGTAGTCTATTATATGACTACCACCATCATTGCTGTGGTGATTGGCATAATCATTGTCA

A_14_P131602 TTGAAAGGGCTAATCATGATTTTTTGGAATCAGTAGAGAGCATTGAATTTGTCAACTCCT

A_16_P16473361

ACACACCATATTTTCCAGCCATACATGAATATAATTCTAACTCCTTGGTGACTAATAGGT

A_16_P40940855

AAGAACATGGTAATGAGTAATTTTTCTACACATGCAAGTTAACCTACTTTCCCAAGCACC

A_14_P105444 TAATAGGCCCTGTCTCCTACTTCATACATTAATTCCTTAACAACATCCATAGGAAGAGAC

A_16_P15962422

AATCCCATGATATTTGTTTAGATCCACCTATCCTACATATGCAAGTAGACATAACGAGGG

A_14_P127797 TCACACCAGAAAAACCTGATCATATATATGCTCGTTTTCTGAATAAAATTCTTCTGGGGG

A_14_P112447 CAACATCCAGCATCCCACACTCAAAACTGCTAAATGTAGCAACCATTTAATTAGCAAATA

A_14_P201909 TGCAGTTAGGAGTTTGTTAACTGCCTTTTGGAAGTCAGTGTAAGAATAAGTAGTGTTGAT

A_14_P101428 ATCTTGTCTATGAATTTGACAAGTTTTGGTTTGAAGAAGAACCAGAAAGCATTATGTATT

A_14_P122297 GCATGGGAAATCGACCGTTTTCATTTCTATATCCTTCTACTATAGTGATTGCAATCCTTT

A_14_P123297 ATTGATCGAGGCTTCTTTTTCCCTTCTATGATGACTTCTGGAGACTTTCCAG

A_18_P14879561

AGTGCCCTAAATACCAATATTGCAAAAACATCCATTTATCATTTTTCACGTCTATGCCTT

A_14_P116952 CCACTCCATAACAGTGTTCTTAACCCATAGTTCTTTAGGCTCTACTAACTTTTTCCTTTT

A_14_P125155 GACTCTTAAGTTTTCAACACTTACCCATTTCATCATGTCTGAAGTTTTCTGGCTTTCAAA

A_18_P22841641

TCTGTCTGGCTGAAAATTTTGCCGTATAGATGAAACATCATCAAGGTATGTATACGCATT

A_14_P102360 ACTTCTGGACACTGAACAAAGGAAATTTTTAGCCAGCGAGTATGAATTGTATTTGCTTTT

A_16_P01183532

CATCCTGTTCTCTAAGAAAACCTGGTAAAATCTTCCTCTTCACTGTTATCTCTGTATTGC

A_16_P15821850

GAAGATCCACTTCACCAATTCTTTAATGCGTGATCTCTGACTTAATGGCAAAGAAAAGTA

A_16_P15150800

AAAAAAGTAATCAAGCCAATCAGACAAAATATGTTTCTCCATACAAACCATGAAACACCA

A_14_P116820 ATCAGAGGATCTCACATTTAGCAAGCGTGGTTTCATCAAACTTTTGTTACAGTTAGTATG

A_18_P14131315

TGCCAGTTTGTGAGAGTAATGTTATTTCTTGTGTGTGAAGTGAATGCCCTTCTGTATATT

A_14_P128311 GAAATAAGAAATCATTTGGGTAATTGCCTCTGGAAGAACTCACTAAGAGAACAAAGTCAG

A_14_P100082 CTACCTCTTGTTGCCTAGTAACAAAATGAAGAACTGTAGGTTCCCAGTGAAGAAACA

A_14_P136848 GTATGGTGAGAAAACATGAAGATCTCCCTGATGTGTTTTTAAGATACTGGGTTTCAGTTA

A_14_P116704 TTACAAGCCATTCACTGAAATACATACTTAGACAATATCACAGGATCTTCCAAGGAAGTC

A_14_P139850 CCATTGAAACTAATATTTTCTATTTTCAAGAAAAAAGCAGCAAGCCTCGGCAGCAGTCAA

CUST_7863_PI426210822 ATACGACAACTCAGCTACCCAGTGCATGCTCAGGGCGCGTTCTGAAT

A_18_P22605647

TGTTGCTATTGTTATCTCTGTAAGGCTATCCTCTCCTTTGCTTTATACTTTTACTCAGAA

A_14_P121005 CTACCGAGATTGTGTGATGATTTAAAAAACAAATCTGCTCAAGTATCCTGAATTCTGTGG

A_16_P03463884

CTTTTTCAGACTCCAGTAAGCATCACTGAGAACACTTAATTAATATACTGAGGCCTCTCA

A_14_P104592 TAGAGCACGTTTGACCTCAGCATAAGGCATATAGGCGACTGATTTTCCTATGAAA

A_14_P103291 AAATGAACACTAGACAGACGTTCAAATTTGATCTGATTACAGAAAAGCCACTGAAGGTTT

CUST_6345_PI426210822

TGTTGCTAGGGAAACCACTATTTTGGCTCAGATCATCTTAGTAAATGCTTTTTTCTCTC

A_14_P133308 CTATTAAACATAAGTATGGCTCCATTTTTTTAGTATTACCAGAAGCCCTCCCAATACGGC
CUST_2885_PI426210822 CTTCCATCCAAGCTCAGCAGAGGGTGCTGGCCAACTCTGTCATCT
A_14_P131409 TTCAGTCACAGCCTTCAAGCCAACATGTTCCTTTCCTATCTGAAAAACATAAAGCATAAA
A_14_P131558 AGATTTGTGATAACGTGACCATGTATTGGATCAATCCCACTCTAATATCAGGTATGACAT
A_14_P102573 GGAAGCCATATGTTAAGTTTTCCTTTTAACCAGTATGCATACATCACCATACACCATCTT
CUST_8405_PI426210822
ATCCAGTATAAAGACATTTGTTAAAAATAAAGATGGTAATACAAGCTCTCCTGTCCTCCT
A_14_P122749 CTTTATTAATCCCTTACACGAGCAAATGCGATCTCTGACCTACTAGACTTGTTTTCCTAC
A_14_P113194 AAAGACAGGACCACCGAAGACTCAGAACCTATAAAATTTTAGTCACCTATCAGATAAAGA
A_14_P108749 TCTTGTCTTATGTTCATTTTAGCCATAGACTTGACTGGAGTTTAGAGGGAATTGATTGTT
A_14_P138236 ACTGCCTGTTACCACGGCTTCTTAACAAAGATGTTAAATCATTAGGAGTCATATTTCATG
A_14_P108938 CGTTTGGACTTATCCAAGAGATCTATACACATATTGCCATCATCAATCAAAGAGTTGACT
A_14_P100466 TTTTCCCTAAATTCTGCTGGTTTCTGATTGACCATTTTTACTTTTTCACTCTAAATCGGG
CUST_1506_PI426210822 ATCATTGGCGGCTGTGCAGATACATGAAGCGTGTGAGCATCCCCT
A_14_P110091 GTTGTGTCTGTAATCGCCCTACTATTCAGTGGCGAGAAATAAAGTTTGCTTAGAAAAGAA
CUST_9891_PI426210822 AAAGACCTGTGGTTGGCCAGCCTGTCTGGAGCCAAAGGAGAGAAT
A_18_P22842306 CACCAAAGATGCAAGCCTGGTATCTCAACCAGAGAGGCATCTTTCAA
A_18_P22840687
TCAGACTTTCTCAGTTACACAATAGCCATTGTCTATGGAATTCTCCCTGATGGAAATTAT
A_14_P115287 TGTTTCTGTTTTTCCTCAGACAAATAAGAAGACCGGAAAACCAATGATAAATCTTTATAC
CUST_5122_PI426210822 TTTATTGCTTTAGATGTCATCTCAGAAGGATAAGGAGCAGTGGGGACAGG
A_14_P115870 TGTAACAAATAGCCCTCTTATGAGTCTCATTTATGCCCTTTTCTTTTTCAGATCTAAGCC
A_14_P104705 CAGATACATAGGTAGGTTGACCCTATTGTTGTCTGTGACATTATCTTCTTGATGTCTTTG
CUST_3954_PI426210822
CTCCAGGTTAAGTAGAGAGTAAAATCCTATTGCTTTCTGACCTTTGGGTTAGGATC
A_14_P137327 GGAACAGCATTTGATCTGTGCAAATAAACATCAGAAGGTTATATTTTCCAGACAGTTCTG
CUST_2702_PI426210822 CTTCTGTCCATCTGTGAGGCTTTGGACACTCGTGGTTTTCAGTCT
A_14_P139517 AAGGTTTGAATGTTTCAAGATCAAGAACTCAGTAAACTACCATCTGCTTTTTACCTTAGT
A_18_P16486606
TCCGGTAACATATATTTAACATATTGGGGCATATTGTGATATTTTCCTCGGAAGTCTGCT
A_16_P16085870
ATTACCAGTTTTAAGCCAAGTAAGCAAACTCAAAGAGATCTTCTGTCAGTGTGATTCTTG
A_14_P133451 CAAACATATTGAATGCAAAACATATATAGAGCAAAGGGATCACACATTTAAGGATCCACG
A_16_P15951967
AACATGATTACACATCTTGCTTTTTCTTCCACTTTAGCCAAATGTCTTAGAGACTGTTCC
A_14_P110191 TACCTTCTAAACCTGCTTCAATCAGCCCAAACTGTTCCAATAATTTAACAAAAAGCACAA
A_14_P133128 CTATTATTATACACCACATTGCAGCAATTATGTTGTTCACTGGGTGTTACTCTGTATGCC
A_18_P22840850 AAAGCATTTCTCTTGTCCTCCACAGTGAGGACATCAGGAAGTGAGC

A_14_P121157 AAAAATCTAAAAGAATACTCAACCAAATACTATTGGTGGGACTGGGTAGTGGGACCTATA

A_14_P117492 TCTTACGACTTCGAGAGAACCAACTATCTGAATCTGTCTCCCAGAATAAAATACTACTTC

A_14_P129904 CCAACTCTATGGGCCTGTTTTTGCAGAAAACAAACATCATCCGTGACTATC

A_16_P16831904

AAATCGGTTATTAATTGCAAATGGGAAAATTAAGAGAGCATAAGTGATCCACTCAGTGGC

A_16_P03713986

AGTAATCTCAATCTATACTGCTATATCTTGTTTCACTCATTCAACCTATTCATCTGTCCA

A_14_P137771 AACCTTATTTCTTTGTTTGAAGTATATTCAAAGAATTGATTGCCTCCTTGACCATGGCAT

A_14_P123411 CATCATCTAATATTTTATGCAAAGATGACCTTTCTCCCCTTCTGCACATGCTTTATCAGT

A_14_P130611 CTGAAGAATGTGATACAATTACTTTGAACTGCCCACGAAATTCAGATATGAAAAATCAGG

CUST_6918_PI426210822 CTGTGTTTGTATTTCCTCACCTGGATTACCTGTGGCCCTTGCATC

A_14_P132099 CCAACCAATAATAGCAAAACCATCCAGCAATAGGAAAAGAGAAGTTTTTGCCTAGTGATA

A_16_P01826484

ATTGTGCTACAACTTGCTAAATAGGTCTGCTTCTAGGAATAATATAGCCTCCTCAATCCT

A_14_P122750 GGTTTTAGACACAGTGGTATCTGAGGTTATTATTCTTAACTGCTAGGTCACTTGATGTTG

A_14_P123455 ACATAGAGGCTTCAGTTTGCCACCTCAGAACCTTAGTTAAGAGACTAGAACTACATTATA

A_18_P14133957 ACATGTATATCTTCTTCAGTTGGTCCCTAGTGAGTCTACCCAAAGTGAATTGCACT

A_14_P123851 CAAAGTGAGAGTAAGCAAGGAAAATCAAAGGACCTTATTTGAGTAATACCCTTCTGAAAA

A_14_P117204

CAATGAGTGACAATGATGGAATGTGAAAAAAAGAGAAATCAAGAAACAGTGACACACACA

A_14_P107323 TATTTAAGACTTTGGTGTCTGGAACATCAGTCTTCATAAACCATGACCTCCTAGATTGAA

A_14_P137038 CTCAAGACCAATTATTTTGTTATTTGTAAAATAATGGGCAGTTTCAACCCAGACAGGTGT

A_16_P16246440

GCCATGTTTAATTACCTTTACAGGCATTAACAACAGTTTACTAGAAATCGATGGAGGAGC

A_14_P122490 TGAATCTGCCTATAATTAATTATAAATAGGTGACAGTGGGCCTCTAACCTAGGATCTCGA

A_16_P16906075

GCCTCACTACACGATATCATTCATGAAGAAGGGAAATATTATGTTAACTGCCTCTGACAT

A_14_P121437 GGCACAGTCTGTCCATGAATAAAACCATATTTTAAGGCACCAGGACACTTATTATGTAAA

A_14_P132538 TTTATATTTCTAACCCTGTCTCTTGCTTGTTAGTTTCTTCTAACCAGGTGGGTTCTAAGT

A_16_P18540897

CCCAGTATAGTGAGTGTAGATGTCGGTTATTCTGTAGTCAATCTAAAATGTCCAGTATGC

A_14_P104416 CAATACTCACTGGATTTTGTGGACTTGAGAGCAGAAGTCAAATATATCCATAGCACATTC

A_14_P201665 CTCTCTGAAGTCTCATATTCCTCCTGTCATCCATTTTCCTTTATTTTTACTTCTTCCACT

CUST_7533_PI426210822 ACTGACTCAAAGACAGAGAGGACCATAATACCAGGGTTCCTCAGTC

A_16_P19820704

ATAATTACGATTTTAAGTCTGTGGTTCCTCTAAGATTCTCTATGATGACCGAAAGCCTGG

A_18_P16521547

GCCAGAGCAACGTTATATATTTACTGATAGGACCTCATAAAGTTTTCCACAGCATAAGCA

CUST_6871_PI426210822 AGGCTTTCAGTGGCTAATTTTTTATGACAGAAGGCATCGAGGGCTTGT

A_14_P130791 ACATAAAAAGACATCTTTTGGCGTCTGTTTTAACAATTTGAACAAAACCTCGAAAGCCAT

CUST_5167_PI426210822

GTTATTTTTAAGAATAAATTTTAAAAGGACAAAGTAAAACTCCAAGTTGAACTTAATGGC

A_16_P16236131

CATCTTAAGAGGTTGTTGCACAGATTAAGCGTAATGTAAAATGACTGGAACACTGTTAGG

A_14_P103126 ATTTGATGTGGACTATGATTTTTTGAAGAGACTGTCCAATGAAAACCATGGAATTGCACA

A_14_P129468 CTAGGTCTTTATTCAGTTCTTCAATTTGAATCTCCAAATCTGTTTTATGTAGGGTTAGGT

CUST_6326_PI426210822

GCCCCCTAAAGTATTCTTTGTCTTTAATCCAGTTGTTACAGTGAGTGAACCTGAT

A_14_P131017 CTGAAAGTTCTACTCTTTCTAGTGGAATATCAATAGGTGAGAAATACTTCTCTGAGAGGG

A_14_P129420 AAAAGCAAATATGCTGTGACCCTGCTATGTAGAACAGTCAAAAAATTCTGGGTATTATCT

A_16_P02258428

ACACATCCTAACCTTACAGTCTTTAATGTCTTATTTCTACAGATCCTAATCTTACAGCCC

A_16_P02684266

AGGAAACACTCATTCTGCTCATTTTAACTTTATTGTCTCATTCTGATTCTAATCCCCAAA

A_16_P30952443 TGACACCAGTTCTCCGCTGGAAAAAGGGACAAAAGATTGCCCAAA

A_16_P21422220

ATCAATCTGCCTGTCAGTAATCTCTCTCTTATCTGAACTCGGAATCTATAATCTGCAACA

A_16_P01805630

AAAAGAGATTTCAACTTAGGAATTGTCTGTGGTCATTTTCTACCGTGAAGACTTTTGCAG

A_14_P110433 CAGGCTCTCCTTCCTTCTCAGACAAGATGTCTTATCAGAAAAAGCAGCC

A_14_P113858 GGTCAAGCAGGAAAAGGACGGCTTCTTCTCTTATGATATTTGTAAAATCTACGTTGTTTA

A_16_P18892106

AGACGTTTGCTTTTCATGACAACTGCAGAAAATGAAGTAAAATCTAAATCCCAGACCTTT

A_14_P104295 ATTTTGGATTACATTCAGCCCTCTACACAAGATAGAGAACATTCTGGTATGAATGTGAAC

A_14_P137626 GAATGACCCGAGACTGGTTGTTTTTTCTAAATATAGGCAGAGAGGGTTCTTTTTATGATA

A_14_P127172 CTATCATCAACAGTTGTTTTAACATGCTGATTTCTCCAACACTGTTTAAAATTGAGAACA

A_14_P104137

CTGTTAAAAAACAAAACCAATTACAGGCTTAAGGAGCACATAAACCACACCTGAGAAAGA

A_14_P109881 TTTCATGAACCTTGTAAGGCTAGTGTTGAGTGGCTTACAAATGTCATATAATGGACTGTA

A_14_P108441 GTTGGTAACTACACGTCTAGGTCCTCACTATATTGCCTTGCACTGTGAGATG

CUST_10118_PI426210822 AGCCCCTGGCCCCACCTACGCATGCACTGTGGTGCTACTGTTTAC

A_14_P102574 CCATAGATTCAAAAGCAGTCATTCAAGTAAATTAGGTGTTGCCTATAGGAACCCATTTCA

A_16_P15527643

CCCAGCCTACTTATCTTTATCTCCCCAAATCATATATCGAAAACCCCAGTTTATTTCTAT

A_18_P22842310

AAGACAACTAATTTCTTTGGGATTACAAAATAATATGTATGTATTGACCTAACATAACAT

A_14_P124398 TGTGAAGAGGAAGCAGGTTGTGCTTACCTTAAACATTCTCTGTTCCCG
A_16_P36052369
TTCCCTTACCCTCTCATACTCTAATAGCACTATGGTCTAAAGATCGGAATACAAACAAAC
CUST_9146_PI426210822
CAACTACACACATTCGGATTCTCAAACGCCCAATAAACATAAATATTTTCCATCCACAAT
A_14_P115662
ACAGAATTGCTACAGAAAACGGGCAGAAATATAAAAGCACAATTCAAGAACAAAAACACG
A_14_P200574 GCAAGCGGGTAGTTATTGATTGGCAAGGATGTTTGGAATGGCTTTTCTTCTAAAA
A_14_P110271 CTGAGTAAGCCCATAATCCCTACATCGGACACAAGTAAGCAGGTGAAG
A_16_P37677342
AAACCATTTCAAATGTGTTCCTACTTACCCACACCTACTTTTTGATCTTTGGTCCAAAAA
A_16_P58424630 AGGAAAAACTGTTGGAGAGCAGGGAAGGAAGCCAATAAAAGATTTGTCATCAA
A_14_P131299 AACTGTAAATTGTAATGTCTGTGGAAACACATCACTATGGTTTACAAATCCATTCACCCA
A_14_P103810 CTTCTCTTATTGCTCAATTTCTGTTTTCCTCAATGGCTGTAATCCTCTCCTATAATGCTT
A_18_P11675915
AGTCCCCCAAGTAGTATAAAATACTTTTGATAGAGCATGACTTTTTACCTACGCTTGACC
A_14_P138606 CACGGGACGTTTCAATAATGAAAGCGCTAGGTGCTAGTGTCTCAAAAATCAG
A_16_P18513932
CCCATCTGCCTTACAATCTCAAGATTTGCTTTAATTCAGAAGGAAAGTTTTATCCTATGC
A_14_P124160 TTGGGGAAAAAAATGGGGAAGAAGTTTCTTTGCATCCAAAAAAGTAAAATGTGATATGGA
A_14_P202491 TTTATTATACAGTGAGAACAGAGATATGTTTTTCTTTTCTCCTCTGCTTTGAACTGGCCC
A_18_P17924028
AAATGGTGAACTATTTCAGAAATCGATTCATGTCTACTACTCTTCAAGAAATCCTGCCAC
A_14_P106855 TTATCGTGATTATTGCCACTGTCAGGATGAATGATTATGACTGGGCCAGGTT
CUST_5255_PI426210822
GCAGAGAAATAGTAAGAAAGGATGGAGGATGGAGGGGATAAAAAGATCACATGT
A_14_P115125 ACAGGAAAGGGAATTTGATTAATATACAGTCTAAAATTACCTAAGGCTCCCACGAATCAG
A_14_P114879 TCCCAGACATACAGTGAACAATAAGACATATAATAACTCTGGTCAAAACATGTGCCAACT
A_14_P125719 CTGTTTCCAGTTTGTAATATGAAGTCACCACTGTCACACCTAAGAACACTTTGTTAAAGA
A_14_P130019 GGATTCCAAAAGTTGTGTAAAGAAAAACATAAAATGAGGCATTGTTGGGGAATGGAAATT
A_14_P103827 GGATCTGCCTCACGTTATATAATTAAGATGTTGAAAATGTCCGCATAAACCAACTCACAT
A_14_P125211 TGGAGATTTTTAACCCTACTTTTGGAATAAATATTAGGAGACTATGCACTGTGCTTCCCA
CUST_2589_PI426210822 TGAACCCTCTCCAAGTATTTCTAGCTGTATGAGTCAGAGGAGGCAGAATTA
A_14_P111024 AGTTAAAGGCAGGACCTCTGAGTGCATTGATAAGATAGGGGTAGGGATTAC
A_18_P12064293
CCTGTTGATCCTGGTAATTGTCATCAAGTCTAATCAATCACAAAAATGCATCACAGACTA
A_14_P111984 GGGGCAACTATATCTTTGTTTTAGAGCCTCTTTGATGATGAAATTACTGAGGACAAAAGA
CUST_6888_PI426210822 GGGAAACCAAGCATTGAGCAACAGAATGGTTCTTAGGCAGAAGTGA

A_14_P122164 ATTCATTTTTATCACTTGCTATTTCATGCATACGTGATACAGGAGGTAGTAAGTGCCCAT

CUST_2439_PI426210822 CAGGGCATCTTCTTCAGCTGAGTGCTTTTGATTTCCTACAGTTTAAGAAGTGT

CUST_8116_PI426210822

AGAGAGGGCAGGGAACCAGTATTGCAATTGTGAAAGGTTCATTTGTTAGAATAA

CUST_4927_PI426210822

GTCTATTTTTCATCATAGTATGGGTTATTTAGAATTCATATGTTATGAGGAAATACACTG

A_14_P104467 CTAATTTGTCTTGTTCTTTCCTTTCCATATCCCAGTATAATCTCTGTTAATCAACAGGAC

A_14_P118937 CTGCAAGTGTTTTAATAGCCCATTATCAAAAATTATCCCTTGGCTATAAAATCCCCAGAC

A_18_P22841572

TCCCCGATAATCAGGCTTAAAGGGGATGGTAACTGGAGAATTTTGATATCTCAATATAAT

A_14_P126533 AACTTCTAGATCTCATGATAATTAGCCAATAGCCACGTGCTTCAGGGGAAAATAAACAAT

A_14_P111679 CTAGTTACTCATCAGGGAATTTTGGGCTGAATGTTCAGATGTAAACTGGAATTAAAATGC

A_14_P116200

TTGCCAGTTAAAGGAAAATTGTTGGTCAAAGAAGTAAAAGAAGGAAGAGCAGTGAATACT

A_14_P114451 ATTGGATTGCAGCATTAAAGACTGTGCAGAACAGGGAGCACTTTGAGGTTAA

A_14_P129174 TAGTTGCAAAGTAACAAAATGATAAGAATCCTCTTTGCCTCCAACGCTTATCAAGTTTCT

A_14_P117708 TTTCCAAATAAATTATCTATAGACCAGGAGTTTTGGACACTGTCCTGTGCTACTCTCTAG

A_14_P100932 TCTTATTCTCAGATTTTCTCCTCCGTCCTAAGAGTATCTGCCAGAGGCC

A_14_P118852 TCTAAACCTCTATACTTAGAACCAGAGCAGAGAGCCAACTTTTCAAGTATGTGGTTTATC

A_14_P127212 AATGGAACCAAGTCTATTAGGATCCAATTTGTCCATTAGAATTTACCCAAGTTCTTGCAA

CUST_6143_PI426210822 TGAGCTTACGGCTGGCATGCCAGCAGTTTCCTAAAATGTAATATCATCACTATT

A_14_P129598 GGGATGAACAATGAAACATTTTTCTGTTCCCAATTCCAAGAGTAATTGGTTATCCATTCA

A_14_P125775 TTTCTTAATCACAATTTTGAAAGTGGGCTATTGAGTCGAAATGGAAGAAATGCTGAAGGA

A_14_P125990 CTTCCTTCTAAATAGTTTTTAGATTCTCAATGACCCAAAGAGAATGATCACCACCTTGAG

A_18_P13376809

TTTGTTCAAGAACCCTTCAATATTGTTAACGACGTAACCTAGATTAGACCAATGCCAATC

A_16_P16324484

GGACTACCGATCACTTCTTAAATAACCAGCATACATACAGTGAACCATTCTAAACACCTT

A_16_P20593725

ATATAAAGGCTTATGTGTCCTTTAATTTCCATGTTCTACCTACATAATCCCTCAGCAGGC

A_14_P109279 AAAATGAGAAGAACTGAGTGCTGCCCATTAAGAAAAAGCATCCCAGATGATTAATTATGT

CUST_6785_PI426210822 TCCCCACCTCATTCTGTTTTGTTTTTAGCCCATTCTGGATTTCTGCA

A_14_P136938 ACAACAGGGTTTAGGTTAATTCTAGTGAACTTGCATGGTTTTAAATCTCTTTTACAGTTC

CUST_1791_PI426210822

GCTATAATTCTGGATTGAAAGCGAAGCTAAATTTAGGAAGGCCTAATGATGACACACAAT

A_16_P30952521 GTGGGGATGACAGATTTCATGCTTTCCTCTTTCAGAATCATGTCTCCAA

A_14_P136568 CGCCTGAGTCTTGGAAACAAAATGTTGAAGTTGGAGTCTTTGATAAAATCTTGAACTG

A_16_P21162718

ACACTTCCTTCACATGCATTTACGAGTTTTCTGAACATATAATCCTGTTGAGATCCATGT
A_16_P15260920
TGAGTCTTCTTCAAATCCTACATTACCTTAAAACATCTATGCAATACCACTCATGTTTGC
A_14_P115928 GAACATCCTGATCTTCATAAGAACTCAATCCTAAACAGATAAACCAGATTCAGAAGCACT
A_14_P110371 TACTCACAGTTGTTCACATGGACACAAAACTGCCTGATTCCATTAGAATCCATGAAAA
A_14_P139094 TGTATCAACCAAAAAAATACCTGTCAATTTGCCCTTTAAAATCCACACCCATTGTATGAG
A_14_P132105 CAGGGAAAAAAATTAACATTACTGTAATACCTGACAAAATGTTGTGAGCCACTTTTTCAC
A_18_P16877404
CATGCACATACAGGATCCATATGACAAACAACACACGAAAATAGAAGTCACACCTTTTTA
A_14_P116691 CATAAAGCATGATTCATGAATTCTGTGTATAAAACGAATGAAGAGAATGGGAATGGGAGG
A_14_P116063 TCACTTCATTCATAAACTTTGGAAGCAGCAGTTGATTCTTCATATCAGCACTCTTTTCTT
A_14_P134387 CAAATCCATCTTAATGGGTCATTAGAGGTGCCTGGAAACTGTAATTATGGTATAGCATTT
A_14_P102863 CACTGAAATGTTCCAAGAACCTGGGTCTATTTGAGGATATCAGAGTTTATTTAGGAATGA
A_14_P117062 CTGTAGACTGACATTAAGCAGGAATTTAAAAAGTCTGAAGCAAGTTTATCCAGTAGATGG
A_14_P125142 AAAATACTTACTTCGTGCTCATGTTCGATTCTCATACTGGGATTTGCATTTACTTCAAGT
A_16_P19642291
AACACAACCAATGCATAGTGTATGATGTGACTCACCAGATTTTAATGCTATCAACCTACA
A_14_P134754 CAGTCCAGCCTTCTCTTTCCCATTATAGTTGAAGTTTCACAGAAAAGATCAGGAA
A_14_P117297 TTTCTCTCGAGTCATAAACATCTTTAACTGTCTAATAAGCATAGTTCCATGACTGTCGCC
A_14_P107808 AGTTTAAATTAGCACTCTTCCCCTCTCCCATTAGTGACTAAACCATATCTCTTAACTCTT
A_14_P123726 CTATTTCTGTTGAAATATGGACCTCAGTAAGGCTGCCATTACTGCTTTCTTAACTGTATC
A_16_P18401867
GAATCAATCAAAAGGTGTTGCTTACTAACTCATGAGTTTCTGGTGAAATTACCTAGCAGG
A_16_P02471790
CCAGGGAATACTAATGCTATTATGCGGGTATATAGCACTTACATAACGTTGGTCCTTTTC
A_14_P201401 TCTCTAATGTGAATGTGGAAAGCCTCGTTGTTTATAGAACACATTTAAACTTACCATGGG
A_14_P121722 ATTTCTTATTGAGTAACTGCCAAACTGAAACAGTGAACGAAATGTCCATCTTTCATGTGA
CUST_5365_PI426210822 GTTCATAGGACCAATCTGGGTGAATGCTGCTACAGTTTGTGGATGG
CUST_8898_PI426210822
CTATTCACTCATTTATTACCCAAATCAACAGACCATTCTGCTTCCAAAATATTTCATTAT
CUST_5606_PI426210822 TGAGGAGAAGATGTTCTATGGAGAAAAGGAGAATTGGCCAGAGCCTTAAA
A_14_P111328 CTCCAAATATACTTACAAAACCACTTCATGCAACATAAGAATGTTTGGGTGCGGATTCAG
A_16_P15166732
TGTTGGTTTAGTCATCTCATAGTTTACACAAAGCATCCACATACTACAGTATTCAGTCCG
A_14_P139190 TTGACAATGAGGTTCTTCCAGGATTATCTTCGAAGCCCTGATGATCTGCTAAATAAATAA
A_14_P133025 TTTTACAGAGTTACACTGTTAGACCTTATGATAGCCAAGATAACGAGTGATGAGCCACTC
A_14_P127874 ACTGTTAATCATTGTAAGGTTTTTCAGGAAATTTGTTTAAACACTCTGAAGTCACTGGGC
A_14_P200524 GTCATCAGGTCAAAGTTGTGCTGGCATAAGAGTTGTCATCAGGGGTTAAG

A_18_P14137642 CTTTCTCTGATCCCTGAATTGGTTACTGAGGCCCACAGAAGAGGAAA
A_14_P130405 AAATTAAGAGATTTCTTAGGAGTTTCAAACAAAAATTCCAGGTCTACAGAGCCCCAATTC
A_14_P201550 GAAATGTTTCAGATTTCTAAAGAAGATTTTGTGCAGTTTACAAAGGCGGCCAAACAGAGT
A_14_P122324 CCTGTAAATTGAAATGCAGATGGAAAGAGAACATGTATTCCAGCACTATGAAAGTCAAAC
A_14_P116146 CTACGTGTGACTACATTTGGAGATAAGTAGCCTTTTTCAGACCTTCTTGATTTCAAAACA
CUST_1250_PI426210822 TAGTGTGTGTTTGCATTTCCTAGGACAGTGATCATTTCCAGGGAAGGAAATA
A_14_P119026 GTTGCTTTTATTTTTTTTCCAATTGCGCATAAGGAAAGAAACTCGTTCAGCGGTTTTAC
A_14_P121181 ACTTTAATACAAAAGTGTTGGCATTTTTATGCATGTTCCCTTCAGTCTTTGCTCATGTGT
A_16_P01472349
AGAGCCCTGTAATTGAGATTTGACCATTTACAACCTCTGAGAACTAGATACCATATCATG
CUST_3412_PI426210822 GCTCAAGGAGCAACAGGCCTTGGAATCACTCTTTGAGTAGTAGCT
A_16_P58424351
TTACTCAACTGGATTATCATTCATCTAAGAGCATCTGAAAGACAGTCTCACACAAGCAAA
A_16_P16192039 AAGATGCAGAGAGGAATGTCTGACCTCATAAACAGAGCCCACCAATGTTA
CUST_8974_PI426210822
TGGTTTGCATTTTTCTAGATGGATAATATGAAGCTATCTTATTTATCTTTCTAGAACTAG
A_14_P126448 GTCTATTTTGTATTTCCTTGAAGTTGCGTGTTTAGTTCTTTGTGACTAGTTCTTTCACTA
A_14_P108449 CTGAAGACAGTTCAATAGCCACAATGTGCAAAATAGCACAATTACATTTACCTGTCAACA
A_16_P17123141
GCAACTATAACACTTCTGCTACAATTCTATTTGACAGTCCAAAATTTCAAACTCCTCTGC
A_16_P01872077
AAAATCACATCTCCTTAAATCTGTCCATTTTGAACAGTGAAATAGAAATGCCAAGTGGCT
A_14_P120644 CTTTCTGGAACCAAAAAAATCCACTTCTGTTTATCCCCATTTTTCTACTACAAACCTCAT
A_16_P20582916
ACTGACTCTTAGGTTCATTTCTTCACAGAAGCAACTTAACATTTCATCTACCTTCCTGTA
A_14_P125090 CTATAAAAGGGGATGAAAGAATGCCCTATTAAGTAGGCATTTTGCCTTCCAAATAAATGC
A_14_P111447 ATGCTTATTCCATTTTCAAGTTGTCTGTTCAACTGCAAGGTTTTTTAATTCTGCCCAAAT
A_14_P138550 CTGCACTTCCTGATTTAACTAAAGTGATTCAATTGAAGATGGACTTCACTGCAGTAAGTA
A_16_P02279773
GAAGCCACAATTAATCTTGAGTAGCACATTAACCTTGTTCATATCAAAGTAACCCTGCAG
A_14_P200392 TGTTGGCTTTAGGCTTTAAAACTTCGTCATTTCGTGAGATTAATCTGAACAAACTGAGTC
A_14_P116161 CTAACTGCAGGCTTGTTGATGAGGATTTGAAATGTGTTTCTAGGAAGGAAGGAG
A_16_P00347316
AGAGGGCATTAATCAAGACATAGGCATCCATTTATAATCTCTTTGCACTGTTGCTAACTC
A_16_P03190282
TGGACTAATATCCTCGACACAATTTAAATCCCATCTCTTCAAGACTATTCAATGAGAGCA
A_14_P138479 GTATTGTTCTCTTGCGGGAGGTTATGAATTTGTCTTACTGCATCTTGCAAATTTAAGTTG
A_18_P15154029

CAAGAGCAAAATTTATGAACACAAGAAATTAAATTATTCTTTCGTATAATATTGTTGGCA

A_14_P121025 CCTTTTTTTTTTTTCCTAAAGCCAGAAAGTGAGCAAGGTCGTAGAACTTCCCTAG

A_14_P109174 GAGCAATAAAGAAACGAGAGTCAGGAAATGCTGTGGGAAGTCTGGATTTATGAG

CUST_9295_PI426210822

TCATTCTGTGGTTATTTTTAAATAGGCACAGAGGTAACACACTGACCTGAGAGG

A_16_P01729591 GACGCTAGCACTATTCAGGATGATCATTTTTACTTAACTCCCCTATTTCCATTGCAGTG

A_14_P132606 TTGAACCTCTAGGATGTGAAGGATTTATTTTACTTACTATGTGGCTTGCTGAAGCAATGT

A_18_P12773926

ACCAACACTGCTCTTTTTTCATATTCTGTGATTTGTCTTCTGGTAAAACACCTGTATCAG

CUST_5961_PI426210822 AGGTATGTCGGTGTTTCCTTCAGAGCATTGTTCTCAGGCTCCTTT

A_14_P118486 ACATAAAGTTTCCGGAAATCTTAGATTTGGCTCCTTTTTGCACCCTTAAATGTAAGGTAA

A_14_P101059 CTTCCGTAAGTGTTGAAGGCTGCTCCCAATTTTTCATTCTAAGAAAAATTCTTAGAATTC

CUST_6712_PI426210822

TTTACAATAGTAAGCAAATGTAAAAAAAAGTATTGCCATGGTAAATGCATAATTTAAAAG

A_14_P127270 ACTGTGGAGGGTATTTTCAGGACCATCTTACAAATTATTGTGAAGTGTTGGAAACAAAG

CUST_8322_PI426210822 CCTCTGTGTGGTGGAGGCCTCAGCCAATGTCCCAGCCCAGATGAC

A_16_P21406259

TTAGTCCCTCATATTTCACATTAAGCACCAAAAATACCCTAGTTTCTTGTTGATCCATGC

CUST_6087_PI426210822 GTGATTGTGTCACCCAGAGTGGCCAGAGATCAAGCCATAGGAAAT

A_16_P19607063

AAAACACTAGGCAATCCCTTAGTCTTTGTCAGTATATTAGATCCTCAATTACCCACAACA

A_14_P139970 CTAAGTTGAATGTCAGTTTCATACATGACTTCACATTGCAGATACCTAAGCCACTCTAGA

A_16_P18293762

AATACAGACTCCTCAATAAAATCTCCAACCTTTTCTTTTTACCATAAGAACACACTGGAA

A_14_P201210 TTCTATCCTGCTTCTCACACCCATCAAATCTTTATTTAGTCTCTCCTGTTTTACTCATGT

A_14_P201412 AATACCTCTCATACTGCCTTGCTTTAAAATGAACTTGGAATAAAATTTCAAATGCCCGGT

A_14_P106233 GCATCTTATTTTGCAAACTCATGTGCGTGAGGATTTTACTGGATTTGACTAATCCAGTAA

A_14_P136987 AATCAGTCAAATAGGACACATACTACCACTTCATTCCTAAAAGTTGAGCAAGAATCATCC

A_16_P21491965

GCCCGTGAACTATATTGTATATAGCCAGAATACTTAGCACTAAGATTAGGGTCATTAGGC

CUST_8129_PI426210822 CCCACACACAGCGCTTGACTCCCTGCTATGACGGAAAGATCCTTA

A_14_P127661 GCCTTTTCCACCTGTGCTCTTGGTATATGGGGAAGATACTAAAACCAAAAACA

A_14_P111713 CTCTTCTCTCTATACTATCTTTAACTCCACTTCTCGACTCGTCTACTCTACTTCTCTCAT

A_14_P128880 ATTCTCCATCCTCCTATCTTTCTTCTCATTGGAGTCTCCCTGTAATTTATTTATTTATTA

A_18_P22841106 AGAGTGTGCAGTCTGTTGAGGCAGTGTTGTAATGTGATAAGGGGCTT

CUST_2414_PI426210822 GTCAGGGGGCATCTCTCGTGCCTCCATCTGTAGATCCTGTGAAAA

A_16_P00046414

TTCCCCCAATTATAAGACAAATGTTTGTTGATCCTCTTAAGGTTTATCACATAGCAGTGC

CUST_8104_PI426210822	TTGATGAATTTGATAAGGGGCGGCAGATGCCCATTCAAACCTGAAAATT
CUST_5197_PI426210822	CAATTGAGGTTCCTCCAGAGAATTAACAAAATGTCCATGCACTCTTTCGTTGTT
A_18_P10845512
	AGCCCATTTCTTTTGAAAATACAAGTAATTCCTCATATCAGAATCCAGATAGTAGCAGCA
A_14_P133803	CCTTCCTGCATCACCACCTTGGTTTTGTTTTATTTTGCTTCTTGGTCAAGAA
A_14_P202235
	ATCAAAGATTTCTTTGAAGTTGCAAAGATCAAGATGACTGGCACAACTAAAGAAAGCAAG
A_14_P126889	TGTATTGTCTAAGGAACACCTACAAAATGAAGTCAAATGAAGGACTCAGATCTCAGACAA
A_14_P112240	CTTCCAATTTTCTGATTCACTTTTCTCACAACACCTTCTACATCCACAATGCTCTCTTTG
A_14_P133293	CTGCTCCTTATTCATTCTGTTGGTATTGTCTTCAGGTCTTTGTAAAAATGCAGTGACTTA
A_14_P126160	TAGTGGGAGGTCAGGGAATAATCATGGGATCTTAGTAGAAACCTAACATTTTTTGTGTAA
A_18_P22841199	ATTCTAATATTTGGAGTGGGACAAGGCCCTCCTTCCCCTTTCTTATGATTAGTT
A_14_P125515	AGTTGAACTCTTATTTTTCAGACCCTAGTGCTTACATTTTGGAAGTGTCTACTGCTTTGG
A_14_P106352	GACCTTGCTTTTTTTTTTAATTTCTAGGCAATCAGGGTCCTTTCTGGTTGGTGCTAA
CUST_86_PI426210822
	CACCCAATAATAACAGTTTAACTTTTGGCTTATGAAATACTTCAGTGGGAAAGTATTTGC
A_14_P137316	TCTTTCATTCACTTAGGTATTGAAGAAGCACCTGGTATCCACATACTACCATACTAGAAC
A_18_P15959636	ACACACACTTAGGCATGGCAGAAAATAGAACCCAGTTGGGAAAATCTAAACATC
A_14_P200069	CCCTGCTTTGGACAATATTGTCCAACAACTGTCTTCCAACTCCAGGATTAAATATATAC
A_16_P02936342
	ATGGCTTTTGCATTTCTATCTTTCCATCTGAGACTTACAACTTCAAACTCCATTTTCCAT
A_16_P02598746
	AAGAGGAGAACTGTAAGTCCAATCTAAGGAAGATATCCTAGATATGCGAAGATTGAAGGC
A_14_P128332	ACCCTGGGAGGTGAGGGAAAATAGCAAATGTTAATCATATTAATCATCTTTAGGCACTG
A_14_P105155	AATACTTAAGCGGAATTGGTGAAAACCTTTAATTTACAGAAAGTGTCACAAACCCACTTC
A_14_P129610	GCTTCCAAGATGGTGTGGACTATTTTTTAAACCATTTGACTTCAGGACACTTTGC
A_14_P103413	ACCTTTGAGAGCAATGTGAACGGCACGTTATTTTTATTAAGGAACACAAAGCAAACATTA
CUST_6391_PI426210822
	TAAATCCCTAAATATTTGCCACAATCTCTAGAATCTTCTAAATCAGGAAATTTTAATGAT
A_14_P127945	CCCCAAAGTCACAAAACATGGCTCTATAATATTACGGTAGCATTGGGGATTATAAAGAAT
CUST_4644_PI426210822	ATTAGAGGTGGCACTGGGAATGCTGGTAACAGTGGAGTTTGACTC
A_14_P117155	ACACAAATAAAACCACCCCACTAGTGTATGAATGATGCATGTTTTTATGATCTTAATTAC
A_14_P100174	AGTGGTTACTTATGAACAATACGTTTGAAAAGACCTATGAAAAAACACGTGAAATTTGGA
A_16_P58424070
	CAAAATGATGAACATGTTCTAAGTGTTGAGATAGAGGCATGTGAACACTCTTTGGCAG
A_14_P130733	TTTACCTGGGAATATCTTCTTCAATTGTTGCACATCCATAAAGAAACACAATCACTCCAA
A_16_P30952321	TGACCGCTGGCCACTTCTCTCACCCGTCCACCATTGATGTGGTAG
A_14_P131042	TTTGGGAAAACGTGACAAATAAGAAACATTTGATAAAAAAGACTCTGTTGTCACCACACC

A_14_P125361 CTAGTATGAGACCTGGCCCGCAAATCTATCCTTTCCTTTTTCTCCATAAAAATAAGTAAT
CUST_3976_PI426210822 GCTGTGCTCAGCCCTTCCGGGTGGTGAAGGTCTGAGGTCACACTG
A_14_P201811 CAAGCCTCAGACACAATCAGATTCATTAAAACCACAAGTGTCATAATCAAATTAAGAGGC
A_14_P113484 TTGTTATGCATAGCATAGCAATATGAAAGTCAAAACAACCTAGAAGATCCTGGGTCCATT
A_14_P132492 TCTAATCCCAACCTTTTCCAAATCTTTTCCTATTTTTACACAGGCTTTCAGAAATGCTGA
A_14_P117909 GTCTCCAAGGAGATAAGGGGATTTTTATTTAAAGAATTTGTGAAAGTTGGTCGATTTCAA
A_14_P200899 TTTTCACTAAAAATGCCTACTCTTCCTGTTGATGTTCCTTTTCTGTTTTTACCTTGTCCA
A_14_P137026 TCCTAAAAACACACTTGGAAATACCATTTGTATGGACCAACTTCAAATCCATGTTCAAGA
A_14_P127905 CTGACATTTGTGGGGACAACAGGAATAGGCTTTTATTTATTGGTAAATGAGTAAAGCATC
CUST_3390_PI426210822 GTGCACATTTGGGATTCTGTTCGCCAGCCCCTCTGCACTCTGAAG
A_16_P21628209
ATGCCATGGTATATTGTTATAAGTCACAAAGCAATATGGAAGGTTTCCCAGTATGTCTCA
CUST_5137_PI426210822 CCTGGGAAGGTCAGGATTCTTCTGTAAAAGCAGAGAACACTGCATG
A_18_P14457402
TTCTCATTTACCTACATGCAACGTTAAATAACTCACTCCTACTCAATTAAAAGCCCCAAG
A_16_P17567951
AAGTCTAGGATAATCACCACATCATAATCCATTCAGTCTTTTCCTTATGCCAACTGCTTA
A_16_P18132497
AGATGACAATAAGTTCGTAAGTTTATACTGTCAAACCTGTTTTTACCCTAACACCCCATC
A_18_P22842460
GGTGTTTTTGAGAGGCTCTGAGAATTCAGAGAAAAAGAACAAAGCAATAAAAAAGGATGA
A_16_P36922243
CTCTTCACTCTCACTACTCAATGAACAGACAAATAAGTGCACTTACTCACTATGTTCTCC
CUST_8040_PI426210822 CAGAAGATGGAAGAGAACAGTTTAAGTTTGGAATGCCTGCTGATGCCAAATAC
CUST_6671_PI426210822 CAAGTGGACAAAAGTCGACTCTGCTCTACCCAGTGTTGCTCTAACT
A_16_P58424502 AGATGGACTCTCATGAAGGGGATTTATTGGGGCAAGAGGAGCGAA
A_14_P114765 AGAAAAGCCTTGTTTATTTTGGAGAAGGAATTGTTTTGTTATTTTAAGCCTTAGGGCCTT
A_16_P01567374
CCTTGGGCTAGAAACATATTCAGTGAGTAATACATGAATCAACAAGATGATAGGTGTGTC
A_16_P17193155
AATCACAGAATGTCAAAAGATTAGCCTAGACAGACAAAAAATCTTTAAAGAGCCTTGGGG
A_14_P201212 CATTTCCTCCGATTTTCACTACTATAAGGTGAGTATTTTGGCTCCCTATAGGATACAATG
A_14_P117332 AATGCGGTATTTCCTTACAAAATAAACACTCTCATCAAGTTGATTTAGCAGTGCTGAGAG
A_14_P109633 AAGGGAGAAATGTATATCATTTTCAGGACAAGTTCTTCCCCTTTAATTCCTTACAGTCAT
A_14_P102699 TCACATTTTCATTTTCTCTCTTTGAAAAGTTGAATTGCAAGAGGAAAGGTAAGGTTCTGC
A_14_P122995 ACCATAAGTGTTTACAACAAAGGAGTCTGATAGTGAGGATGAGCCAGTTAGATGATTTAT
A_14_P200060 TTTTTTCCATAGGAAAGCACAAGGAGAAGACAGAAATATCAAGAGTCTCAAGAACCTTTG
A_18_P15841429

ACCAGCAATATGGAATACACCTCCAATCTTCAATACATCTCTAATCTATGTCTGCAACAA
A_16_P03660657
CCTGTTTCTGATCTGCTATCTGCTAAAACCTAAATGGAAACTAGATAACCATGGAGTTCA
CUST_7930_PI426210822 GTTTTGTCCTCTTACCTGTCTTCCCAGGGAACCCATGAAAAAGTG
A_18_P22840317 CACACATGCCTCCTATAGTGTTGTCCTAGACTGGGGTGGTGTTAGT
A_14_P130269 CTGTCTTTTCCTATTTCCCTTTGTGTTGAATTGTCTGTGATTTTCAAGACCCAATTTAGA
A_14_P100336
AGAATTGGTTAGGGAGGCAAAAGAAATATTTTGTGAGTAAACAAAAGATGAAGAGAACCC
A_16_P20207862
AAGGCTAGCCACCTCATTAAATAATAAAATGAAGAAACTAGTTATAAAACCGGTGTTCCA
A_14_P104903 TCTCATTTGGGAAGTCATTTGGCGTATAAAAAGTGCAAACACCTTTTATTTCCAAATCTG
A_16_P16871075
TCCATCAACCACTTCTACTAGTATTGCTCAGTAATTCAATTGTTCAAAATCCCATGCTTG
A_14_P104762 TTACTAAATATACCTTCAGTTCCATCTTCAGCATTTTCTTGCAGGTCAGAGAAGATTGTA
A_14_P133452 CTACAGAAATAGGTAGAAGCCCATCTAAATATCAGGTAAGTATCAGGGGGGTTATGGAAA
A_16_P40362597
TCTTCCAATTTCCTTCAATGTGTTCACGATGTATCAAGTAACTCGTTCATTTAGTCAAGC
A_14_P131553 TAATCTCTCCCTTTTATGTCTTTATAGGACTCATGGTTTTTCCAAAACCAGTGACCG
A_16_P21592660
ATTTTGACCTATGTTCACAAAGATGAAATCTGGGCAAAAGAGAAATTATCCATGGAGTGT
A_14_P117793 CTTTTTGCAGTGGGTTCTAGGTGAGAAAAAATTGGCTCGGATCTGGATG
A_14_P114761 TTTACCAGTGTTAAACAGGGGAGTTTCTAATCAAAGGCTTTTAGGAATCAATTTTGACCA
A_14_P104858 CAGTCTTTGAGGTAGCCAATTTTTTTTTTAATCCTACTTTCGAGGTGTGTTTGGAGTTG
A_14_P118113 GGTGGACAGCGTCAATGTCTAACAGGTGTTTGTTTCATATGGGTCTAAAACTA
A_14_P104833 CTACGAGACTGCTCGGAAGGAAAGAGAGGAATAACCAACTTAACCCTG
A_14_P105188 AGAGATCCCATTTCTTCATCTTTTCTTTGGGTCTCTTCCAGTAGATCTGTAATAAGAAAG
A_16_P36328276
TCCACATGTTCATCTTCTACTTGTTTTTCCTCTATCCACACTCTTAATGTCAAAAGCAAC
CUST_310_PI426210822 ATTTGGCCAGTGTGGGAAAAGACAGTCTGGCCTTCAGCTTGGTAC
A_16_P35485535
CAGAAGTCTAACCCTAAAGAAAATCGTATCACCTTCTGTAAGAAATCTGTAAGATTGCCA
A_14_P129244 ATGGTCATTGTTTATTTTCCTTCTTCTCCCTTAACATGATGAGAACGGAGAGATTAGTTG
A_14_P105090 CTGGTAGACAAAGCAAATAGTAGGTGCTTGGCAACCATATAGGAACTTGACATCAATG
A_14_P123242 CTAGTGATTTCCATAGAAGGATTAAATTCTAAGCACCAGGCAAATGCTCTTCATTGTGTA
CUST_5523_PI426210822
TAAATAAACTGTTTAAGATAGTGTGGGCGGCCTCGTTCTCAGAGAAAAGAAAGC
A_16_P21149287
TGATTCTTTCAGATGTTTTCACAACAACTTTTACAGCAACTATCTTTACACCTGCCTTCC

A_14_P102386 CTACGATGATATGACCCTTGTCATCGGTTTGAATCCCCTAAAATTACAAAGAGATATCAT

A_14_P138443 CTTGTGAAAATGCCATTGTGTGCTGGAAACCTGGCAAGATGGAAGATGATATA

A_14_P139030 AGTAAGCAGAATTTTTAGTTTTTCTGAAGGTAGGGCTTTTGGCCAGATATGCAGCAATAA

A_16_P15243388

AAACCCAAGAACCTATCTAAAATCCGAATGCTCAATCTGTTTCTCATGAAGTAAACTAGG

CUST_9340_PI426210822 CAGGCCTCCCGAAGGATGTGGAGCAGCGCCAGCCTTGTGGTTTGC

CUST_1718_PI426210822 CCCCTGATCTAAATGCTGAAGACAGCTGTGCCTCTTATGTTCTTGAGT

A_16_P41530974

GCCATCAGCACTTTTTATTTAGACCCGAATTCATGAATTTTAACCAAATAAACACAGGCA

A_16_P37071069

AACTCCCAAAATCATCCACTCTAAAAAAAATTCCTGCACTCATATTTGGTGTGTATGTCA

A_14_P119599 GTTGACCATTTGTTTCTCTTTTTTTAGGGTCTTTAAAAGTGTTATTCTTTGTCGCCCTTT

A_16_P17760226

ACCTCACTGAGTCCTATTATGTCCACTTATGTTTATGCTTAAGAAGTTCACTCCTTCATG

A_14_P107463 ATACTTTCTTGCTACCACCTTTGCAGGCAAAGAGCAGATCTTGCTGCA

A_16_P17328710

CCAGAGTTTCTAACAAACCCGTAGATCAGTTTTCTCTTCCATAGTTACTAGTTACACAGA

A_16_P17109875

TTCCAGTATGACCTGCTTTTTCATCATGAATATTGCCTATGACATCTTCAGGAAATCAAC

A_18_P14473461

AAAGGAAAGAACAGTATGAGCAGAGTCATCACAAAACCTAAGGAAAATACGGGATTTAAC

A_16_P19528418

AAATGGAAACAGAGGTAAAATCAGGAAGCCATTGATATGTGAACATTCCCTTGCATACAA

A_16_P19910481

CAGAGACTCTCATGTTGACTATTCGCAATCCTATTCTAGACAAATTTCCTGTGGTAGTAA

A_18_P14136186

AATGTTCAAAATAGTTAAGAACCTGCACAGCATGAGCACACCTACCTTTAAGAACAGTTG

A_14_P125690 ACATTCGCAGCACATCATAGAGATAGTCCTTCTCTGCATCATTTTCAATCAGAAAATCC

CUST_8415_PI426210822

TTTCAGCTATGGTCAGATATTCCTTCCTTTTCTATGTCTTCAGGTTGTTTGGTGAATATT

A_14_P125411 AAAGAGGTATGGGGTAGGGCAAGGGTATTTTAGGTTAAGGAGATTTAAAGGACAT

CUST_6157_PI426210822 CTCATCCTTGCCCTCCTCAGAACAAGTGTTTGCCGTTTCTCTGAT

A_14_P108188 ATCTTTTGTTAGTGGTAGGAGGGTGCTCCTTTAATCAGCCAACTCTTTGTTTCAAAT

A_18_P14137158

TAACATTGATGAAGATGCCAAGTTAAATAGTCCTTTGGAATGAAACAAATAGTTCCCCTG

A_14_P125269

GTGTCTAAAAGAAGACGTTCAGCGACAACAAGAAAGAGAAAAGGAACTTCAACATAGATA

A_14_P136134 GTGACTTCTTGTTAAAAAATCTGCCACATTCCATGCATAAATAGACTCCTGTGTGTGTTA

A_14_P200624 GATTTTGGAGAGTCCAAAGTCAGAGACCATGATCTTCGAGTCCTCAAAG

CUST_7020_PI426210822 ATCTCTGCTTTCAGGGAGCTCTCAGAGTCTCAGGAACAGATGTACAT

A_16_P15127242

AAATGCTGTCCTTGGAGTATATACAATGCTGTAAAAACTCAAAGGTATGAGTGTCTGAGG

A_16_P00149958

AATCCGCAAGGTTAGATCGTAGAATAAGTCTGTAATTCACCAACCTTTAGATTGCAAGAT

CUST_6944_PI426210822

TTTCCTTCTTGTTCTGAAGGATATATTACAAGCATATTGACAAAAGTCTTTGAACTCTTG

A_14_P121789 CTTTATAGAGTTAACAAAACAGAGTCAGAGACCACTGTCTTAACAGTGGAAGATGCAAAT

A_16_P03646373

AAAATTACTGAGTCAGTCAAACATGTGTCACAATCACCAATTTTGCAAAATTATCCACCC

A_18_P19482269

TATGTTTCTTGAACCCATAATTGGAAACTCACACCCTAATTTAGACACCTACCTACTGAA

A_16_P00685623

GTGCATTATGTTTTAAGAAATGGTCTTCAGACTCTAAAAAAAGGAAGACATACATCAGTG

A_14_P118190 TGGGATCTTTATTCCTTTCTCATCCCATGACTTGAAAATGGTAAGCATTTTTACAAGGAA

A_16_P58424073 ATTTAGCAGGGCTGAGTGGGACCTGAGGATTTGCATTTTCTGACAAG

A_16_P20161534

AACAGCAAAACCCAAAGTTACCATCTAGAAACATTGTAGAGAAATTTTTGAGTAAGGAGA

CUST_1910_PI426210822 AAGTCTTAAGATGACCCCTCCGCAAACCCCTCTCACTGGCTATCT

CUST_8024_PI426210822 ATGTTCACTGTAGATGATGGTCCATTGTAATGGACCAATGTCCGTTGCTTT

A_14_P124566 CTCCTTGAAGGAGAATTTGCGGGAAATGATTGAAGAGTCTGCAAATAAATTTGGGTAAA

A_14_P129103 CCAAAGAGAACTCGACAACCCTGGCAATCATACTACAGATGATTCTCTTATAAAAAGAAT

A_14_P131283 GTGCATACTTGATGCAGTATGTAGTATGTAAACTCTGTGATCTTCACAATTATCCCACAA

A_16_P02996562

ATTCACGCTGATTTATTCTATCTCCAGAATACAGGAAATTGAAAATGAATGATGTGGGGC

A_14_P200864 CCCCTTCTTATTCCTTTGAAGCCATTGCTGTGAATTACGTGAGGGAAAGATA

A_14_P135133 TTTCAGTTTTGTCTCTAATGGGCTTTCATTAATTTTCTCATGTCTCATTTCTGTGCCTCA

A_16_P17830461

AGAAAGCAAAACATCTTTTCGTCTGACTCACTTTACATCCAGAGACCTTGAATCAATGTT

A_16_P17721155

AGCAAGAGATTCCCTTAATATCACATTACTCACCTGGATGTAATTTCCCACTCAGATTTT

A_14_P101096 TCCAGAATAAAAATCTCATCTTGGGAAATCTCCATCACAAAGTGCAAATGTTCCTAAAGA

A_14_P108214 ATAACCAAACACTCTTCCTTTTCACTTTTCTCCTTTACAACATCCGCCCACAG

A_16_P01573448

AAATGGTCAAGCGATATTTCTCAGGTGTTATTCTATTCATCTCTTACTTGTTGGTTTGGG

A_16_P21607169

TCTCCATGACATGATTGCATCCAATATTTATATGAAGACAGTATACAGGCAATATGGGGC

A_14_P111699 GGAAATCTGCAATTGCACTATTTTTAGGCAGAACAGAGGAGTTTCTGTTTATTTTTGGTT

A_14_P136564 TTCTAAAGAACTGAGTGATACAATGACTAAAACCGAGAGGCACCATCTTTTCTCCAATAT

CUST_6822_PI426210822

TATGTCAGGGGGTGATAACTCTTCCTTTTAAGCCAAAAATTATCAGTCATTGTCTTAATG

A_14_P110231 AAAGGAAAAGTTATCCTGGGGATTTAGACCTTCTGTAGATTAAGATGCTCAGATATGCAA

A_14_P108253 GAGAAAAAGTTTTGTTTGTCCTTCAAGGGCATAAAGAAGAAATAAGAAATCCTCTTCCCT

A_16_P15934332

AAAGGCCGTATGTGTATTACTGTTGTAATCAGACTTTTTCTCTGGACACTGACTATCTAG

CUST_972_PI426210822 CATGATAAACATCCGCCTCTTTTCACAGCCCCTCCAACTCAGAAAT

A_14_P112786 GGTTAGGATTTCTTGCTAGGTAGTCTGAAGTGCATATGATTAAGAAATGGTAACATTGGA

A_14_P119239 GGATGAAGTCAAAGAACTGGCTGAATTCCACTTTTTGATTTTCCTATTTTCCATCTCTTT

A_14_P139828 TGAAATAAAAAATCCTTGCACAGAAATACCATTGGTCTCTTTCCTTTCAGTTTGCTCAAC

A_14_P113273 TTTATTCTTCATACAGGAGTCACCTCACGCTTCATTTCCCAGTCTTCTAAGTTTGAAATA

A_16_P00685525

CAGATACATAAAATGCCATTGTAATACCGTATAACAAATGCAATAATGGAGGCATGCCCA

CUST_3841_PI426210822 GATCTAAATCAGGGCTCAGATGAGGCTGTTTGTATTGCACGGTAGGAAT

A_14_P201739 AATACACGTCCTTCCATATGCAATGGAAATTTCTGGAAGAGTATGGAAACAATAGTGATG

A_14_P131273 CTTTCATTCATTCTGGAGTCTACTAATTTACCTGAATGGTGTTTGCATTCTGTGAAATGC

A_14_P117583

CTTCACTGTATGAAGATAGAATAATAACAAGAGAGAAGGACATGAGGAGAGTGAGAGATG

A_16_P55119454

AAAGTTGAAGGGATGAACAAATTAGAAGTGAAAGACAGTGAGCGGTTGTTCATATCAAAA

A_14_P127214 AAAAGAGCCTTTGCTGTTAGGCTGATTTTGTCTGCTGACAGAAAAACAGCAGT

A_14_P120284 TCTCAATAAACTGGCAGAACTCACAACTTTGGGAAAGGCATATTTGCAACAATTAACAAA

A_16_P15823706

ACCTGAATCTCTCTCTATTCCATTGTTACCTACATGCTAAATTTACCAACACAGAATGTC

A_14_P200247 TTCATTGCTATCGAATGAGTGAACAAGGTAAAAAGTATGAAACCACTTTTGAGAACTGTC

A_16_P41585213

CATGTTTGGTGCTTTCTAATGATTAATCCCAGCAACATAAGTTAAATTGGCCCAGTAAGT

A_18_P15461113

GTCTAAGGTCTCCAAAGTCTAAGTTCAGAACACTCTTAACAGCACTCTACAGCATTTTAT

CUST_3187_PI426210822 TACTGCAAGTGGAGCTTTCTCAGTGGAGCTGGCTCTCGGCTTTAT

A_18_P18913335

GACCCCAGGATATTAAAAAAATACGGTCTCTCATTAAAACAGTTCAATCTACAGACAGGA

CUST_3751_PI426210822 TTTGAACAAAATGGGCCTGATCTTGGGCTTCTGAAGTCCTGTATAGTGTAA

A_14_P118914 TGATCTGGTTGGACCCTACCATTGAAATATGATTTTGTGCTTCTTTAACATGTCTCATAG

A_14_P128141 TGGCCTCAGAGTAGAAAGAGTTTGTATACAAAGGTTTCTGGTTTTAGTTCATTTCAGGTA

A_14_P101522 TGTTTCCACTTGACACAGTGAAGGAAAAATATATATATAAATCCGCACTGAGGGAGATGT

A_14_P139179 TACCCTGCAAAATGAATCTTCAAACAGATGGTATGCATGGAAACGAATCTGATACAATAC
A_14_P118489 TCTTGTCTTTAAGATTGTTGCTTCTTTCCTACAAAAATAGAAGTGGGGAAAAGGCAAACA
A_14_P112943 ACCTGAAGGAGAGCAGGCCTAACCCCAACAATATTTTTAAAAACTCTCTCTATATATGTA
A_14_P138717 GGAAGGTGTATTTTAAGAATAATGATAAGTTGCATAACCCACTTCCCTGGCTTCTTAATG
A_14_P125614 TGATCCCTTGCCATTTGTAAATGAGAGAGCAGTTTTTTATGTGTATGAAGTAACTGAGCA
CUST_865_PI426210822
TGGTGCCATGGATAGTCCTGTCTAATTAATTGCATGCAGGACTATTTTTTTAATGTTTTA
A_18_P11234847
AAGGGCCACTAATAACTTCAAAACAAATTCACTGATCTCTTAAAGAATCTGTTCTCAGGC
A_14_P128691 CTCTTACAATTCAACAAATCTGGAGTTTTAGATAAGTAAATTCAGAGACAAGAGGGGGCA
A_14_P117437 CTCATCAGTTAGGAGTTTTGATTTGAGAGTCACTCGGGAAGTTGGTGTTG
A_16_P16191362
AAGTTATGAGAACCTATTGATGAGAGGCATGCAGAAATAAAAAGCCCAAATTCAGTGATC
A_14_P113716 AGGTAGAATAAAGCATCATTATTTTTTAGTCAACTGCTTTGTCATGTGCTTGGTAGGCAC
A_14_P201081 AAGCCATTATAACCATGGATATGGTGAACCTCTTGGACGGAAAACTCATATTGATGATTA
A_16_P21333631
TGACAGACCCTATGAACTATGTCTCATAACATCTGAATCCTATTGCTACAGATTTCTTGT
A_14_P122777 ACCCGAGAAGAACAGGAACTACGTATATGTAGTAAACCCACTCATTGTCAAAAATTCTTA
A_16_P16191316
ATATATTTAAAAACATATACATCCAACATCACTGAAGGTATGTATTTTTAACTCCTCCTT
A_14_P103586 AAGTTAAGATTTAGGGTAGAAGAAGGGAAGATAAAACCAAAATTCCCATGAAGTCAAAAT
A_14_P120893 TTTGGGTTGGATGCTAATAATACTCACCTCTATTACTGTGCTTAAAAGTCATGCTAATGG
A_18_P17148166
AAGAGGAAGCAGGTAAACTATAAGTGAACCAGAACTCATAAATATCCCTGGGTGTTCATT
A_14_P103878 GGCAGGACTTTCTGTGCCACAGTAAATTAATCTTCCTTCTGTTTTGTATTATGATGAACG
A_14_P101793 TAAGTCCGAAAAGTAGGCCATAGTAGTTCTACTTTTTACATGCTGGGATCTGTGATATTG
A_14_P111410 TATCTCCTGTAACATAACTGTCTTTTAAGGAGTGAATGAGTGGATTTTTTGATGGCACCA
CUST_7151_PI426210822 CATGTTACATATAGAACAAGGCCCTGGGAAAGGAAATGGTCTGTTGAAAGTC
CUST_3442_PI426210822 TTAGATGACCCCTTGTAAAGAAAGGAAGCCTGGCCCCTGAAGCCT
CUST_178_PI426210822 CCAAATCCTACGTTTTTCTTACCACATCATGGTGCCACAGGTTAAGGAATAC
CUST_7772_PI426210822
TGCATTGTATTGTAGTCAGATAATCAGATGTCTGCACTAGCAGTCAGGGACATTAGATA
A_14_P123199 AGCAAATGCAAACATTTTATATGAATCTACTAGCATGGAAATTCTCCCATGCTTACCGTC
A_14_P200939 CATTAAAATCAAATTTAGCCTCTTGCATCATTGTGCCCCTGGTTGTTGGAACAAAAG
A_14_P120836 CTTTACGGGTGTAAAATGTAACAAAACAGCACCCTGCAATAAATAAGATTTCATAAAATC
A_16_P15766700
AAAAGAGAGTTTGGAATTGAGAGAAAACAGGTCTGATTAGTAATTGATACAAACCCTGCC
A_16_P19343208

TGACAACTCACTCTCTAATTTAGTCTCCAAATAGTCTTTGCATACACATTATATCAGGGG
A_14_P116468 GACAACGAGCAAGGAAAGCCAGTTTCTAGAACATGCCAATAACGTGACTTT
A_14_P201309 TCCACAGAAATGCAGTGATAGTTTCCTTGCCAGTATTTGTGTAAAACATTTTCCAAACAA
A_16_P17514073
TCTTAAGAGAACAGTCAACATTTCCTCTTACATCATGACAACTTCATACAAACAGAGCCT
A_16_P16191134 CTCTCTGGCTTCCAAAGTGAGGGCCCCTCCTGAGTCAGGAGCAAG
A_14_P101849 GTGATGTCAAAATGCCTACTCACTCTCTAGAGAATTGGTCATACTAACATGTGAATGACA
A_14_P130084 TTCCCTTATCCTCAAAATAATAATTCTGGGCAATTCAGTCGCATGATGAAGCATGTATAT
A_14_P111127 TACACTTTGTGGTTGAATAGTGTATCAGATTTGAACAGTGGCTAGTTTCTACACGACTAG
A_14_P112458 TGAGTTGAGCATGTTCCCTTAGAATGCCAGAAATTTAACTAGCAAACACACCTGAATTTT
A_14_P109417 AGCCTGTTGTCCTATTTCCCCTGATATCATGTATCAATTGGTGAAAACAAATTCTGTGT
A_14_P135701 ATAAAAGTGCATTGGCTACCTCTATTTTTCCCCAAGCAAAATACCTCATGTTTTTCTTTC
A_14_P131540 CTGTATTTTTACGTTTTAATAAATCCCCGTATTTTTGTTAAAGGCTGGGTAACCTGACTC
CUST_4835_PI426210822
GATCTTTCCTGAGAACTGAGGCATGCTATTAAGCTTTAAATGGAGCATCTCTAAGTTTC
A_18_P17038569
CCTAGAAAAACAAAATTCATACGTTGAGGAATCACCTAAACATCTCCTGATCTTCACACT
A_14_P121904 CTTGCAGCAATGCATCTTTTCTTATTATTCATGGGTATTACAGTTGGAGAGTCATTTCTT
A_14_P109120 TTAAATTTGAAGTGAGTCGTCATAAAGTGGATAGTGCTTTACCTTGGTGCAAATCCTCTA
A_18_P15053919
AAAACATTTCACCTATACCTGGTTCTGTTAATACCTTGCCACTACCATATTATGGATGCA
A_14_P130996 CATGACCCTGATCCAACGTAAAATTAAGTTTCTCAGTTTCTTCAGATTTCTCCATGTTTT
A_14_P107697 TTTAATACTAGGGCTTAAGATGGCACTTCAAGGTTAGTAGAAGGTCATTTCCCATCAAAA
A_16_P03224753
CAACTGAGAATACTGATAGATACCCACATCCTATTGATCTCCAAGGCATTTTAACTCCTA
A_14_P133627 TCCTTATATTACTTTGTCACAATTTCTAGACCTCCATCCTAACTTTCGTTGTCTGGAGAT
A_14_P126723 CTAGAATAAACTTACCTTGTGCAATTGTAAATGGCTCTCTCTGTAAGGAAGAGACTTGCA
A_14_P121865 CTTTGACTATTAAAGTTTTTGTGATCTAATGATACAGTTTTGATTCTATAGTAATTTGTG
CUST_2768_PI426210822 TAAGTCACCTTCCTCTCTTGCCCTGCAGAGCAAGGCCTGTCATTA
CUST_5978_PI426210822 GATAGTCTCCTTATTGTTTCAGGGTTTCCTCCCGCACTGCATGAATC
A_14_P201127 ACTTTCTTTGGCTTCCCGTTCACTATCCGAATTTCAGGCTCAAGGT
A_18_P12971137
AAACCAAGGAACACTTGAATTTCTCAGGTTTTGGACATTCAGAGAGATGAATTGTGAG
A_16_P18210156
TGGGCAAGACTTAAGACAATTATGATTGGTTACTAAGTGTTCACTTAACTTCCAAACTGG
A_14_P120467 CTGGAGGATTTGTAGCCAAACTGTAAGATGTAAATAACAGACCCTGACATGTAGG
A_16_P16290457
CCAGTTTTTCCGTAAGTATATAGCCTTAAGTAGCCAATTAACTGAAATCACCACTACTGG

A_14_P134477 TTTGTTCTCCTAAGGTTTTAGTGAGTCGTAAGAGGGAGACTTTGATTCCACTTCTTAG
A_16_P20752379
TTTTTGGTTCCAGTAAACATTGTATCTCCACAAACCATTTTAAGTAAAACCCATGAAGCC
A_16_P36230991
GAAATTAGCCATGTTCATATTAAGGAGCAATCTAAGATCATTATTGCTGTTTCTCTGGCC
A_14_P131144 CTCATTAAAACCTTCACAGATCCAGGTATGTTGAGCATATAGCCCTTGCATAATAATCAC
A_16_P00807007
ACTTCAGGCCATTTTTGCATCAAAAGTCTTTGCCCTAATGCTTTTTCTCTAACATCAAAT
A_14_P121238 CTTTTGCACAGAAAGGATTGGTTCCAGTTACTTGCATTTTCAAGAAACATCAATTTAGGA
A_16_P00818204
GTCTCCTAACCTCCTTTTTAGCATCTCTGTCTATCAACCTTAAAATTTACACACACAGTG
A_18_P22840837
TATTATAGTTGAAGGCTTGGCGATAGTATTTGCCCTTCCTCTGAACTTTCTGGATATCAA
A_14_P115817 CACTTGATCACAGGCAGATCTTGATTTGTAATAGTCCCAAATATCATTGCAGAAAAAACA
A_14_P110892 AGACACCATCTCCTTGGCTGAGTCAAGATGAGTGAGTTGATTTGACACTTATT
A_16_P03000613
AAAAAGAATGTTTCCAAGAGTCAATCCTAATATTCCACAATATGGCATTTCTTCCATTCA
A_18_P11157935
CCACCCCTTTCCAAATATTCACCTCTAATAGTCATTTTTATTATCCAATCTGACAGCACA
A_16_P01311005
ATCTGGCCATTTTTAAACAAGATTCCTCTACCATTATGCTAAAATGCTCCAAACAACCTT
A_16_P00977815
GGCCATTTAACAGGTTAAACACAATCTACCAGTCAATCCTGAAAATTTAAATGCCACTTT
A_14_P132379 CTTTATTAAAATTGTTTTCAGCACTATAATTTTGTGTGAATTGTTATACTTAGACAAAGG
CUST_5649_PI426210822 CTAAGGGTTCTGGCAGGCTCTCAGATGAACCTCTCTGGATCTTTA
A_14_P114669 CTTATGACTGTGCTCCGTAAACGAAGTTCTGCTGCTCGAAAGAAAAGTCTGAAAAT
CUST_9090_PI426210822 CTTCCAGCCCAAGAGACTGTGCAGAATAGGCCTCTTCTCAGTCAG
A_14_P123642 GTGTGCTTCATGTTTTCTGTCTTTTTTTAAAACTGATGGCTGTGGTTTTTGAAGAGAATG
A_14_P113285 GCTTTAGAGGAAGGAAACATGCTTGGAAATAAATTTAAGGGAAGAAGTATTCTCCTGAAG
CUST_7309_PI426210822 ACATGACAGTTATCCTGGCTTCACAGTAACCTTTCAGAGGTGCTCTG
A_16_P00476825
GGAATCCACCTTAAAAATTAGATCTCTTTTCCTCTCAAATTCTCAAACACCAAGCATCAG
A_16_P20767197
AGGTTGTTTGGTAACATTGCTCTATGATAATGTCATCCTAGGAGACTTAGCATTGTAAGG
CUST_8099_PI426210822
TAGAAAATCTGAACTATATCCTGATGGCACTGAGGAGCCACTGAAAGTTTTTGAAAAG
A_16_P19198381
AACTCCAGTGAAATAAAAAGTCCTGAAACATCTACAATGCATGGTTGACATATCTCAGAG

A_14_P112337 AGGATTAAGTAATGATGAGTCTTCCCTTTCAACTGTTGTTAAATCTGAGACATCCTGACT
A_14_P134932 CATATGTGATGGCCTAAATACCAGGGCACGGACAATAGTCTGCTGT
A_14_P111158 GACTCTATAAAAAAGACAGTCCCTGTTTCTTCCCTTCTTATTTGTAAGACATCGTCATCT
A_14_P132902 CCACGATGCTGGATACACTGCTGTAGACATAAAAAAAAATCTTTATGTTTGGACAATGTG
A_14_P118764 TATGATGAGGAAGATGCTGCTTTCTGCCTAGAGATGCTGCTAAGGATTGTGTT
CUST_351_PI426210822
TACTTAATTCAATATTTTGCAGTCTAAGATATTTGCTTCATGCTAATTAACTACCTGTTT
A_14_P120191 CTTTCATGGAGAAAAATTCTCATGTCTCAGCAGTTACTCAAACTCAATGTGCAGTTATTC
A_14_P110794 GTTTACTGTATAAACACAGTGACTGCAAATATCTCTGAAAAGAACAGATCACCTGTTCTC
A_16_P20864856
AAAAATTCAGGATCATTTTTCCCAACTCAATGTTACTTCAACATCACTTGAACCTATGGC
A_14_P135050 CGAAGTAGTCCTCCAGTTTTCTAAGCAAGTAAATTTTGGAGGTAGAAAGAAAATGTTCTC
CUST_9801_PI426210822
GAAGATTTATCATTACCTTAAAGAGTGAGAAGGACCTAGAACTGGGACTCTGAAGTATAC
A_14_P135581
TAAATTAAACAAAAAGAGGAAAAATGAGAAAAGGTTAGCATGGGTTTGGAGCACTGAAGC
CUST_4618_PI426210822 CTCTTCCTTTCCTTCTGGTAAAGCATAAGGAGAGGACTCAACAGTAAGGA
CUST_4595_PI426210822 AAGGGCTCGAAACTGGCATGACTCAGGAGTCTGACCAGGGGCAAG
A_14_P127533 AGCACCCTGGAGTCTGCAAAGTGTTAATCCAGGCCTAAAGACAAGTA
A_14_P122391 CGCTTGGCTAAATCTTTCTTATAGAAGGCCTCAAAAACATCCTTGCCTATGTAAAAAGAA
A_14_P137560 GTTCATTTTTAGAGTAAATTTTAATTATCTGGGCATCTCTGCCACCTTCAACCTCTTCAG
A_14_P127980 GAGCCTGGCTTAGGAAAAATGAATGGACAAGTTCACTTCAAGGACTGAAGATT
A_14_P132473 CTACTAACTTTATTTCTTACATTTGGCCCAAACTGGCTTCTATTTGCCCACTCTTGTTAA
A_18_P14134797 GTATTCAACTTCTTGGCTTCGTAGCCATTTGACTTTTTCTTTTCTTTAGGGCTCTTCT
A_14_P137260 CCATTAGATTGAAGGTTCTTGAAGACGGGCAGTTTCTCTTTTGTCTGAGC
A_16_P19138717
CACAGCCTAACCATGATATAACTTACCTTAGTCTCAAAATCTCTACAAAACATCCCTTGG
A_16_P40545533
CACTCAGAGATCAATTTTCACCACTTAAAGAGCATTTAGCCATGATTCCAGGAATAGAAA
A_14_P136430 ATCATGAGAAGGCCAGGAATTAGAGAGTTTTGCCAGTAATTATTTTTCACAGCAATGAT
CUST_4437_PI426210822 TAAATCTCAGTGATGGCTGGAGCCCTGGGGGTCCTTAGTTTCCTA
CUST_5040_PI426210822 TTAAGACCTAGTGTTATCTGTCTCAGGGAGGTGGGGGACTAGGTG
A_14_P126757 CTTACTTGAGTGGAATGTGTAGTGAAAAGAGAAAATGTATTATTGCTGAAGACAATGGCT
A_16_P15613231
ATATATTTCTCTGGGTGCAATGGATATGGAAGAACACACGTTTAACTAAGGGTTTTACCA
A_16_P18925417
GAGTTATCCAGAGATCTCTTCTATCCAATGGCCTATTAATTTTACACCAAAGTCCCTTTC
A_16_P19486896

AAAAAAAGTTTCTGCACATACGAAATCATCTTCACATTTTATTTCCCTACCTTCACAGCA

A_14_P127171

AGGAAAAAAGAAAAGCAAACAAATAAAACACCTTTAAGAGGCTTGAAAGAGAAGGTTCTG

A_14_P115882 GAGTCCTAAGGCGACAGAACATGGGTCAACACCTTGATGTAAAACTTGTT

A_18_P12913246

TGTCAGTCCTCCTATCAATATGACTAGGCTCTTTTATGTAAGAACACATTCTACCCTTTT

A_18_P16373176

TCTTTGCCTTTCCTTACGTCTTTAACTCTTCACACTTTTAAAAAAGTTATTGCCTTCCAA

A_14_P134194 GCTTGTATTCCCATCTACTCTGGTCCTATTCACAATTTGAACTCCTCACTGCTATTG

A_14_P105509 GACCAGTGCAGTTTCACACCATTTTTCCTCTCCATTACTGAAAATAATTGAAGGTTAAAT

A_16_P00264485

CTTTGTCTGCCAGAAAGAATAATCACAACTGAAACAAATGTGTAACGATACCTGGATAGA

A_18_P25781334

AAAAGATAACGAGGAAGAATTACATAATTACAGTTCTCCTAGATCTCAGGCACTTCAGGG

A_14_P106022 AAAAATCTTTTCCTTGTGCTCAAATGCCTATTTAGTTCCATTTGGGCCATTTTCTTTGAA

A_14_P133757 CTTTATCTCTGCCCACTTGTTTCCACAGGGACTTTAAAAGTAAGAATGTATTGCTGAAGA

A_16_P15228258

AAAAAAAGCCAATTCAATAGGGTAACTGTTATAAGGTAAATAGGCCTCACATCCATTGCC

A_14_P119221 TGACTGCTGTGGCTCGCCCTTGTCAATACAGAATTAACAATAACAATGTTATTAATGTCT

A_16_P15529243

AAATAGATTCCTTCAAGAGGCTACTTAAAAGCACATCCTAGAGACTATCTGAAACAAGGC

A_14_P120248 ACCTATGAAATTTGCAGAGAAAGTGCCTCATTCTTAAAAAACCACATCCATTCTCAAGAG

A_14_P101653 TTCCTTTTCAAATCATATATTTATTATCATCAACTTCATCTTCCCTGCAAGAGTTTGTTC

A_14_P131329 CAGGCCACCACAACACACAGTAGATAAAACACAGTGGTTACAAACGTCTTTTAAATTTAT

A_14_P126730 GATCAAAGGTATCTAGTGTGCCATTCTACTTAATCACTCGTCTCCTCCTTAACAAATTAC

CUST_8469_PI426210822 CAGTTGTGCAAGTGATGAGCTGCAGATTGCCTGGAAACCAAATCA

A_14_P105892 TTTCAGGCAAAATTCCACTAGTGAGTTTGCCTTATAGGAAATCAATAAGTTCCTTCCTAC

A_16_P00685230

ATGTCAATGTTGGGATCTTCATTTTGTTATGAGAGATTTCAGTTACAACAGTGTGAGGTG

A_18_P14131884 GCTTTGATCTGGATATTTCCTATTGAGAGAATACACTGAGGCCCAGTGTGATTTCATT

CUST_1527_PI426210822 CTTCCAAAGTGAGGGCCCCTCCTGAGTCAGGAGCAAGACTGCCTA

A_14_P135292 AAAACCCATCTGACAATGGCTTTGACAGTGATAGCAGGATTGGTAGTGATTTTCAT

A_16_P16041599

CCTAGGACTTGTTAAAACTATGCACTACAGATATATTACTTCTTAGATGACACAATTGCA

A_16_P16403839

ATAATGTGATTGGATTCAGGTTAGGGAAAGAATGGTTGAATTTCAAGAACTTCTCGGATT

CUST_10072_PI426210822 CAGAAAATGTTGGTGCAGCATTTATGTCAGAGTGTGCAGTCTGTTGAGG

A_14_P117150 TTCCCTTATTAGTCTAATGTTTTGCTGGTCTTCAAGGACTTGAGTAGGGAAGAAAAAAAT

A_14_P103612 ACCTACCTGCATAAGATCTTGAAGGCTGTCAACAAAAGAAATCTCTGCTTCTATCATATA
A_14_P126898 AGGCTCTATTCAAGACAGCAAATCAAATGCCTGTTGCTAAGAGAATCTGAGAATATTTGT
A_14_P200887 TTCCCCATTGATATAACCATCTGTTGTCATGGGAAAGATTGTCTGATATTTGGGTTAAAT
A_14_P120255 CGAGAAGACACTACAGCCTCAAGGAGGTGTGCTTCTAGTGTTTAATTGGATAC
A_14_P121713 CAGAAAATGTAAGTTAGTGTTTCAAAAAATGTGCTCATCAGGACATTGGTGGAGAACATT
CUST_9308_PI426210822 CCTGACTTAGAGCACCACTGGCCATCCTCTGATTCCATAAGGGAG
CUST_7517_PI426210822 CAGGAACTGTGTTACAAAGGCATATGGCCCAACAAAGGACACTGA
A_18_P22840137
GGCTGGGCCTGCTTTGTTTAGGCTATCTTTAGGTATTTTTTTCTTTTTTTCTTTAAGCCT
A_16_P15188779
ATTACATTGCCCTTCTATCATTATGCTAATTCCTCCACAAGAAATGAGAAAATAAGGCCC
A_16_P01133223
ACTGCTGCTTGCAAATTATTACATGTGAAATTATGAACTCTAACTTAAGCGTTCCTTGGA
A_14_P128929 CTCTGAGTCTCTACAACTGTATTCCCGTGTTGAAGTTATGATCGATGAACTACTTACTCA
A_14_P108211 CTCTTCAAATTCCACTCACTACCAGAAATAAATACAAAGTGAGTCCCATGTCAAGGG
A_18_P11859059
AAACAAAACTATGAATTTGCATTCCCATTTAATCCAACTGTCAATCCTCTAAATGACCCA
CUST_9650_PI426210822 AGCCAGAAACTCCCCCAAGGCAAATCAAGACTTTATGGAATCAGC
A_14_P118296 GGGGATTTAGTAAATGCTTATCAAATGGGGGTGCTTATTGAATTTGAGATTTCTGTTTGT
CUST_464_PI426210822 TTGCTATTTGGGGAACTGAGGCTTACAGAGTTACACGCCTTGTTCAA
A_16_P20353479
AACTTCTACCTTGGATCATCTTAAAACAGGCAGTGTAATTTTAAAAAACAATGACCCCGA
A_16_P30952282 CTTCTGCTACATCATCCCCTCCAACCTCCAGGCCAAAGGCAGGAC
A_14_P115112 GTAAAATCATTAAGATTTTTTTTAAAAAGAGGTCTCAGTGCCTGGACGGAGTTAGGGTTC
A_14_P102833 CTCTGGAACCAGGCTCTATTTTGAAAATTGTGGAAAATCAAAGTTTCTCTGTAATGATGT
A_18_P11701718
AGTGTGGATGATTAATACGTTTTGTGCAAAAGAGAGTTCTAAAGCCAAACAGATTTGGAA
A_14_P108852 ATTCTCTTGTTTTTTAATAGAATTTTGTGACTTTGGGGAGTGCTTAGCAGATGCTCATGT
A_16_P03037000
GTAGCCAACTAAAAACATCTTTATCCCTCTTACCTTCTTTTTATACACACAGAAGCATGC
A_14_P131877 GACCTTTACTTGGGATTAAAGAGGAAGTAAATGGGACTGTAAATTGAAGAATGGAGAAAA
CUST_8056_PI426210822 TAGTGCTGCTTGCATAGCCGCCACTGCAGATTGAACTGAGAGGAA
A_14_P139582 AGCCATTTCTCCGAGATTTAAAACCTTGATATTGCCTCTCTATGAAGGAAAAATATAACA
A_16_P15863752
AAAGCAAATATGATTACACTCTTTGTCCTCCTAATAGCCTATTCTGACTCTCAAATGCCC
A_14_P105161 CATTCTACAAATGATAGCATCCAATAATGTCCCAATACTTTCTTCTTGTGAAGAGAGTTT
A_16_P16191390
CTTGTCCCCTCCATCATGATTTAGTATATCATCCTTCCCAAGAGAAGTTTTGAGAACTT

A_14_P107935 CTGCCCCTTGATGCTCAACTAAGCATCTGTTCCCTAGAAATACATGTGTC
A_14_P128204 TGACAGAGCAGCAAGATACTATTATTACTGGCACCTGAGAAAACAAGTTCTTCATTCTCA
A_14_P107065 ACTTCAAGCACTATGCTGGCCTTCAGTAATGTCAGTATTAAATGTGCTATTACATCCTAT
A_14_P121775 TATCTCTTCTCAAAACCCAATCTGGCTTAAGTCTGTTAAAAAAAAAAAAAAAGTCTGGCA
A_14_P200505 GCTGCCTATAAAAACAAATAGGATCCTTAGGGTTTAATTGGAGGACTTATAATTGCACCT
A_14_P113992 GCGAGGTGGTCTTCAATGTTTAATTAAAATGGCCTACTTATCTGTGCACTCTGACAA
A_14_P106490 ATCACAAAGTAATGAGACTCAGATCACACCCTTCCTATGCTGGGATTTAATAGAGTATTT
A_16_P19470036
AAGAAGTGTTCTTACTTGACCATATCAGAGACCTAGTTTTGAATAAGGAGACTCTCTCAG
A_16_P19910602
AAAACCCTGCATGATAAAACCATAGAAAAATTCACAAATAAACACTTGCAAATCCAGTTT
A_16_P58424142 AAAGGGCATGAGGGAACCCTCTGGGGTTATGGTTCTGTGGTTATA
CUST_3901_PI426210822
CCCAACAATTAGATAGGAAATGAACTGAATGGGAGTTCACATTTTATCTTCAGGAAAATG
A_14_P126163 AGTGCTTTTTTTCCTTTCCCTTTAGATGATTTTAAACCAGCATCTATAGACACTTCCTGT
CUST_7563_PI426210822 GAAGAGATTAGTAAGCTGCTGCCCCCTGAGACAGGGCTCCATGAG
A_16_P17489238
AAAAATGGGAAAAAGGAAGAATAATCCAATTGGCTTAGGACAGAAATACATCTCACGTGA
A_16_P35629001
TCCTCCAGATTTTTCTTTTAAGGCCTTTTCTAAGTTATACTGCATATCAAACTTCCCTGT
A_14_P110902 AGTGCAGGTAAGAAGTGTGTATTCACCTGAATTTCTTCTTTCATTAAGTATGAGAGGCC
CUST_7555_PI426210822 TTTTGTGCTGGTCTAGGTAAGAGGAGATTGGAGCTGACCTGTTGAA
A_16_P19860171
ACCCATGACAGTCTCAATAGATCATTCTATACAGGACGACAATATGGAAAACTAAGAGAC
A_14_P118320 ACTTCATCCCTGCAAGACGAATGAATTCATGACTCCTGTATTAAAGAATTCTCAGAAAAT
A_14_P130318 TTTAAATCTTTTCAAACAAGGCTTAGATAAGTTTCCAGGAGAAGTAACCCTGCTCTGTG
A_14_P113465 GCAGGTGTTTTCAAATGATTGCAAGGCTTAACATTCTGGTGCATACAAGATACTTTTTTT
A_14_P200987 ATCATAGAATTCATCATGTAAATCTCTAGCAGATGATGCCATTGGAAAACAGAAACTGCC
A_16_P21506960
TACTATAGCACACAGCATACTCATACTAAGGTTAAGATCCAAGAACAATCCCTTGTCTGT
A_14_P127311 AGTTAAACTTTCTTGCACTCTCTGCACTCTGCAAAGGTAAATGTGTTCTAAGTTTACATG
A_16_P21041528
AAGTTACGATCAAACCTTTCACTCTTAACTGGTTGAATCAATGCACACATCTAGGCTTTA
A_18_P14987503
ACACGCCAAATCTTAAAGTTGTCTTATAGTTACGCTGAATCTTTCATATCTGCCAGATGG
CUST_6253_PI426210822 TTGTAGAAAACCTCTAGATTTTATGAGCTTGTTTCCCTGACTGCATTCCAGAGC
A_18_P13864281
AAACCATAATTTCCCCTTATCCTTGTATTTGCTGTTTGAAAAATCGTATCCTCAACGCTC

CUST_38_PI426210822 AGAAGGGGTCCCAAAACGTGTTTAGTGTTTACCCTTCCCTAAAGAC

A_14_P127080 GTTTTAAGTAAATGATGGATGTCGTTATGGCCGAAGCCATTTGCTAATTGGCTCTATATT

A_14_P126104 ATGAATGAGAATGGAAATGTTTTTCATCCCCAGCCACAGTTTTCAATATTTCACTTGTTT

CUST_9594_PI426210822

AATTGGTATTCTTGCGCTATTTTCTCAGCAGTCCATGTTTCTGGATAAAGTTTATGATTG

A_16_P19572728

AAAAGCCAAGGATACTGATTCTGTTTGATCCCAGTATTATCCTCTTACATAAGGCTCTAG

A_14_P127174 TTTTGGTGGCAAAGAGACTAGATGAAGTTCCTTGCAAATAATTAGATTTCTTTCCCTGTT

A_14_P122182 CTAGCAGATTGCCCTGACATCTTGTTCATTTCAGATAAAATGTGATGGGAAACTTCTC

A_18_P16334578

ACCCCATGCGTAGTTAATAATCCCATTTTAGAAAAAAAGCCACTTTGACATTGAATTGCT

A_14_P121232 ACTTCAGTCGTTTGAATTGATAATCTCTCAATTTTTCTCTAGACGTCCTAAAGTGGGAAA

CUST_1570_PI426210822 TGCCTGAGAAAGAACCCATTGGGAGATATAAACTGCCAAGTCTGCT

A_14_P117718 AGGCATCTCTTGTGCAGATTTACTTATTCACTAAAGTGATTTGGAAAATAGCCATGTGTG

A_14_P202286 ACACATTGGCAATAAACACCCTGGTAAGAAAATAAGCAGTTAGTGTTATGTGCACAATAG

CUST_8982_PI426210822 ATTGTAGTATGTGTTCAGCCGCCTGTGCCGGAATAGGGGCTTACT

A_14_P135422 ACAAAAGCCTTGCTGTGATTCGTTGAAACTTCCTATAGAAAACCCACCAATTTCTTC

A_16_P02635242

GTGCAGTAGGTTAAACATATCTTATAGATCATGGTCAAAATTGCAATCTTATAGTGGGCC

A_14_P125726 AGCCCAATTTTAGGAGCATTGCATTTGACTTGTAATTCATTTCTAAACTTTTGAAGAGCC

A_14_P135472 ACATAACCTTTATTGCACACGGCGCTGGGTCTTTTTTCATAGAAAAAGGTATTAACACAT

A_14_P107783 ATAATTCATCTGGTGCCTTCTCTCCAATATTGTTCCATACCACAATCACTTTGTGCAG

CUST_3201_PI426210822 TAACACCTGTTTTCCAGGCTAACTGTCTTGTACCAGACTTTGTTCCATTCA

A_18_P22790290

ATCTCAGATGTTAGGATCAGGTAAGAAGAAAGATTGAACTATGTCATGACCCAGTAAGTG

A_14_P112511 ATTAATCACTGTCTGCTCCCTTCATTTGCGTAAGAAATGTGATTCACTCCAATAAAACTG

A_18_P14230861

ATAAGAATACAGAGTTTTTACATAGGCAAGAATGTCTCTGGATTCTGATGGAATAGGCCC

CUST_5911_PI426210822 TTTTCATAGGCGAAATTACTCTGTCTGGTCGAGTCTAGACTGTTCGTTGG

A_16_P16191281

ATTTTATTTCACAGGGAGGAGCATATCCATTTCTTCTTTTCTTTTAAAGTTGGCCTCTGA

A_16_P00685507

CTGTATCATTTATTTACTTGTCTCATCAAAGGCACAGCAAGAATATACCCAGAACCAAGC

A_14_P110885 GGACGCAATAATTTTTTTTGCCTTATTTTTGTTAAAACCTTACCGCACTTAAGGAAACGG

CUST_8216_PI426210822 TTATGTGCATATATTCTCATCCGCCCCTAGTCCCTCCAGAATGCTGTAT

CUST_1954_PI426210822 GATGTGATCATGTGGGAGTCAGTACTTTTTAGAAAATTGGTTTGAAGGCCCTAC

CUST_1982_PI426210822

AATCCTCTCTCTGCTTTCTGAAAGTTTCCATTATACTGCCCTGTTGTCTAAAATTAAGC

A_16_P21560890
GCCAACTGATAACTGTTAAAACTATCTGCCACACATATTTTTCACTCAATAGTCTGTGCC
A_14_P105179 TTATCAAAAACGACTTTTCCTAGTTCCACCAACTAATGAGTGAGTGCTAATGAGGGATTA
A_14_P115284 GTTCTTTTTCTGCTTTTTCCTGAACAGAACTTTTCTATCTTTCTCATAAACTGGTGGATT
A_14_P200165 CTGGTTTTGGTGATTTTTGTCTGATTGCAATGTCTGGACGTGGTAAGCAAG
A_16_P15556223
AGCAGCCATTACAAGCATATTCTGAATCTGACATCTAAGCAACATTTTGACATCTACGAC
A_16_P00361567
CCAGGGCCATATTGAACTTAGTGCATCCTGTAGTATTTTAAGAGGAGATAACAAACAAAC
A_16_P19146296
AAAAAAAAACCCACTCATGTAAGTTCAGGTATCTTGAAACTTTGACACTGGAACAAACTC
CUST_5364_PI426210822 GAACTGAGGTTCATAGGACCAATCTGGGTGAATGCTGCTACAGTTTG
A_14_P129051 CTGAGCATTATGAAGAAATTGAAGACTATGATTACAACAACCCTAACGACACCTCCAACT
A_16_P58424268 ATTTTTGTTCAACTGGAGCCTTGGCTTCCTCGTCTAGAAATTGGAGATAAAATGGTAT
A_16_P16578240
TGAACCTCTGAGTTCTAATTGGTCATCTCACATGATAATGTTTTGTGCCCCGATAATAAT
A_14_P100380 CTTACATCTTTTTTGAAATTATCATTTTAGGGCTTGTATCCAGAGTCACATGGCATCATT
A_16_P15245256
ACAAGTTACCAGAAAAGTAAGGCCAATGACATAGAGTTCTTAAGAAGTATGAAAAAGCCC
A_16_P00242363
CCCACCTTTACAAAGAAATCATAAGGGCAGATTATGTTTCTCACATTAAACACAACACAG
A_18_P14474258
CCAACAGGTATCTAGGTAATGTAATCTAATCAGGCAACTAAGATATACCCTCCAGCAAGT
A_14_P138278 TGTTGTTGTGAGATTAAACTGGCCATATGAGCATTAAAACCATCTGCTTCTGAAATAGCA
A_14_P130309 ACCATGTTGTTGGGGAGAATTCTGAGATATTTGATGGCATTATAGAGAATATTTGCTGTG
CUST_2384_PI426210822 GGAAGGAATCAGAAACAACTCTGGGCAATGGAATCACTTTGCTTTGG
A_14_P126506 AATAACTTAGATAGGTATAACGCTTTGCCCATGGTTAGGATGGGATTTGGATTCAAGAAA
A_16_P02728242
CAACCATGACTGATACATGCTATTCCTGATCTGTTTGATATTTCAGCATTGCACATATGC
A_14_P104083 CTTATCATTTCTAGGAGAAACATTGAAATACAGAAACCCTTGAGGCTGAAGTCTTTCAGA
A_14_P120993 GTTATTTGACACCAAATATAAAAATATCTACCCAGATCACATCCATGACTCTTATCATCC
A_16_P01800912
ACATGACACATGTTACATGAAACATTTCAAACCTCTCTTTATCTGCTTAAGGCCTATTCT
A_14_P112154 ATAGTCCATATAGAATACTGTGTCTTGGCTTCTTTCTAAGAAGTAGACACAGGCTTGAGA
A_14_P124544 CTTGAGATTTCAGTTGGTCACTATGATTACTAAAGCAAATTGGCAGGAATTGAGCATAGT
A_16_P03807244
AGGTCCTTATCTGCCACTAAGTCTTTACTGTTTTCAGATAATATGCATAGCAGGTGTTTG
A_14_P132458 GACAATCAAACAGTCTACAAAATGTCCCAAAATATGATTTGATCATGGAAGGTCAGTGAT

A_16_P41104740
GAGACAAACTGACACCGATACTGATTTATTCTCCTTTTCAAGCCTTTTTAAAAGATCGCT
A_14_P125260 AGTGAAAAACTTTATCTTCAGCCTATTAGTTCACCTGCCTGACAGTTAACTCATCAACC
A_14_P101697 CTCAGATTTACACTTTTCTGCACGTCTGTGTTTTGTCTGTCATAATCAAATTGATCATCC
A_14_P137063 CTGTCATCAAAATGGAACAGATTTATTAGGATGCTCACAAGAATGGAGAACACATGAACT
A_14_P131835 TTACCCAAGGGCTTCCACAACTTGTAACACTGTATAGTTGCCAGATTTCACAT
A_16_P02165252
AAAAATGAGAGTCAAACTTGGGAAGTCTAAACAAATCCATCTGGAATGTAAAGCAAGAGG
CUST_1607_PI426210822 TCAGCTGTTTGTGGCTGAGCAGGAAATGTGGTTGAAGGGTCCAGG
A_16_P40253448
AAAAACCACTTCCCTAAAATAATCCTTGAGGAAACATATTTGTCAGAAATGTTCACATCA
CUST_7120_PI426210822 GTCAGCTGGAATCCTGCGACATGTTCTAGTTTCCTAGTGTGCAGA
A_14_P110100 CAAACCTTGTGGCTTTTGATGTTTGCTTACTCTTTGCTTAATTTAGCCTGGTTTTCTAGT
A_16_P16901794
AACCCTTGATACGCTAGTATTTCAGCCTCTACTCTTTCAAATACCTGTGTCTGTTTAAGA
A_14_P139393 TCATAGGTAGGAATGTCTAGAGTCTTTTGTGTTTTAAATGTGAGGTGCTGATCTGAAGTT
A_14_P126877 CAACATTTCGACTCTTGAATCTGGGAAACAACTCAGTGGAAATTTATATACAGTGCGTAA
A_14_P132617 AGGCTAAGTGGACCAAAAAATTTAACCTGGCAGAAGCAATGTAAAGACATTTATTGTGAC
A_14_P133358 TTTTCTTAAAGGCTCAAGATTATTATATTCCCTCCAATTCCTGATCCTGGCAAGATTTTT
A_14_P104408 TTGCATTTTAGGACCTCTAGCCTGCTTTCTTGTCTTATACTTCTTGGAAACTAATGTTCT
A_16_P03663488
CCAGTGACCATATCCATCCTTCCAATACATTACTATGAATCAAGATCTTGACAATTCCAT
CUST_10435_PI426210822
CGATCAAGTGTGGAGTCACATGCTAATGTGTGCTAAAGAACTGTAAGTGTTTTTTCATAT
A_14_P128227 TGGGTTAATTAATCTTTTTAATGACCCTGATTGGAAAAACATCCCCTTAAGTCTTGGATG
A_14_P127417 ATAACAAGTTATTGTGCTGTAAGAATTCACATACAGCCGGGTAACTGCCAGAGACAAA
A_14_P108583 CCAGCCAAATTCCTTTAAAATAAAAAACTGTGAGCACGGCTATGTGAAGTTTCCTC
A_16_P17805262
AACTGATTCATTTCCCTCTCATAAAAATAGAAAGACCAGTGAATAATGACCACACCAAGG
A_14_P110740 GCACACACATAATTTAACCAGAGTCCCACATTGGGAAATCTAGCCTAAGAAATAAAAATG
CUST_1905_PI426210822
CTATGCTAATGATAGCAGCTTTCTTTTCTCCCTAATTGCAGAAAACATGATCCAGTTTTA
A_14_P128720 CTCCAGTGAAGAATACTCAACAATGACTCAAAACATTCCTAATCCAGAATATGAAAAAAC
A_14_P105350 GTCTAACCAAAGTAATCTTTATGTCTAATTTTATTAGGTTGGGAAAGGAAGGACCATCCG
CUST_42_PI426210822 GAAGACAGGGAAGAAGCTAGAACTGCCCTTCCATTTCCTGCAAAATTT
A_14_P104076
GGGTTTATTCAAAATTGGAGAAAACTATGGAATGGTGAAAAGTAACAAGCCAAAGACTCA
A_14_P130500 GAGGTGAAGAAGATGATTAAAATAAAGTTTTTACCTTAAAGAAGGGGGTGCTGTTCCAAG

A_14_P102150 AGGTAGATGCTAATGGGGATTTGTAGAATTGGTGGCTTAGTAAAAATGCTTCTGAATATA
A_14_P136457 TTTAAACAAAGATTCACATACCTGTAAGAAATGGCAACAATTCACTTCGGGTCCTTTCTA
A_16_P02238557
AAAGAACAGAGACTAAGGTATTTTGGGAAAAGAAGGACTTAATGAAAGGCCACTGATCA
A_14_P102300 CTAGTCTTCGGATTGAAATGAATTACCAATAAAGGGTTGGGAAATGGATCAAGGAGAG
A_14_P112576 CTGATGGAAGACAAGATGTGGAGTGAATGTGAAGGCAAGATACAAAAATAAGAATTTTTT
A_16_P17749700
ATTACACTGGCTATATTACATTGTCTACCCAAGTTTGTATGGTTCATACCCCCATAAACC
A_14_P103739 AATTGGTTCGAACTCTATGATCCCTTTTGGCTCTCGTAATTAATGATGCTTTATAAGCAC
A_14_P102936 CAAATCCCAAGAATCTTAAACCCCATAACCTAAACAGCCTATAATCTTCCTTTTCCTAAC
A_14_P101254 ATGGCTCCAGTGATTAATAGATGGGTTTTTAGTAATTGACAAATTCATGAGGGAAAGCAT
A_14_P110175 TTGAAGTCCAGAGACGATCAAATATAGATATCCTCATGAACACTCATACATCTTGCTGTG
A_16_P01857191
TTCTTGCCATTTTACAGACCATATTTCCAACTACTTCTAGAATGTTTTGTTCCTTCAGCC
CUST_9867_PI426210822 TTTTTAAGGGCAGGAACCCAAAGACATGAGTCTGTCTGCCTTTAAAGATG
CUST_3570_PI426210822 TATTCACATGCATCCCAGGAGATTTGTGTGAAGAGGGTGACAGAGA
A_14_P120144 CTTTCAGTTTTCTACATTAGTTTGGAGTGATATGAAAGAATGATCTGTTTCACCTGCGCA
A_14_P113010 TTTGTTTTTCAGGAGAAAGACCATTTATCTGCGAATTATGTGGAAATTCTTACACAGATA
A_14_P135498 ACTGGATTTTAAGACAGGAGGAAAAACCTTTTTTTCTGTGATGTCCTAAGTAAAGAGACA
A_14_P108959 GGACTCGTCTTTGAAGGAAAAAAAATAGCGAGTAAGAAATCCAGCACCATTCTTCAC
A_14_P106189 ACCTGTCTGAGTAGTGTATGTTATAAACGTATTTGCAAGTATTTTTCCCTGTTTTCCTTC
A_14_P135374 CTCTTTCCTCGCACAGGAGAATATGAGGCTACAGGAATTGACAGATCTTCTT
A_14_P107402 ATTATCTGAAAGCACTGCAAGAGCCTACATTAATCAGAGAGAGACAGATTGAATCACATT
A_14_P135874 CTGGAAGGACTTTTGTCTTCTTAAATATTGAAGCATTCACTGTAAACTTCCATTTCCCAG
A_14_P125877 TTAATGGAATGTAATTTTACAATGGTGGGTTCGGAAGTTTTCTTTTTCCTCTCTTTCTCT
A_14_P105654 CTGAATTCTCTTTTCCTCTCCCTCTAGAATCCACAGTATGAATTTAAGGAGGCTG
CUST_10272_PI426210822 CCTTAGTGTGAGTCCTGAGTACCCAGCTGTGTGGCCCACAGTCTA
A_16_P19603970
TGTTGACAAGTAAAAATACCTCAAATCCTACATGAAACTTCCCCTGTTTACTCACATACA
A_14_P119951 GCTGCTATAGCCTTCCTTGATAAGATTTTAGAGGTAAGTTTCTTAGATACTGCAGTTGAC
A_14_P122444 AGTATCAGCCTGTAATACGAAGCCTTTTCTGTTTCTTCATTAGCGATCAGAGCAC
A_18_P15634209
ATGGACAATAAAATTAGCCAATTTGGAACTTGATCAAGATACCTCAACTCCCATACATCC
A_14_P136308 CTGGGTTGGATGTAGGACTTGAAGTAACGTTATTCGGAACATTTCTGTTAAGACAAATTA
A_14_P133382 TATTGTTTCCTATATTCAAAACTTTAATTTGGACTTCGTTTCCTTTGGGGAAGACTGGAA
A_14_P134285 AGGGGGACTAATATTAATCAATTTCTAAAGGAAAATGTTGGTGAGAAGACACTGGAGAAC
A_14_P134825 TGAGAGTTTGTCCATAACCAGTATATTTCTCTGGTTACACAATCAAGTTCTTCAAGCCAC
A_14_P135308

AAGAAGATAGAGGTAAGAAGGGAATATAAAGTGTAACTGAAACCAAGTAGACGGAGAGTG
A_16_P21054031
AAATGCTGGAGAAACCTAAAAACACAAAGACAATGCTATTATGTTACCATCCTAGCCTGT
CUST_7506_PI426210822
TAGTAGACTTTTTAGTTAGTTGAAAGGTAAAACAATGGTAATAACAATGAGCTGTAAAAT
A_16_P00685209 TTAGTGGTCAGGCAAATAATTGATCACTCTGTGTTCTCAAGACAAGCAATGGGTAAG
CUST_3369_PI426210822
CAAGATTTCTCTGCAATCCTCTCTAGTAGATAATAGTGTGTTTGGGGAAAAGTGAGG
A_14_P110376 ACTCTTGCTGCTAGGAATCCACTATAGGTATCCATCAAAATCCCCATTACAAAAAATAAC
A_16_P15104944
CCATAAAGGTAAAACATCGCTATTTTGTTCCTTCCTACAGTCCATTTTTTGATGCAAAGC
A_16_P20320469
AAAAATCCAAATGAGTTAGGAGAGACTGTGACTTTCTAAAGCAAACTCTTTTGCAATGCT
A_16_P17586193
AACCACTTCCTCAGTATTCACATATTCAGCATGTCTCTAGTTTTGGCTTCCATACAAATT
A_14_P201970 AATACCCAGATGTCTGATTACAGACTTTTCCTTTTGTTCTTTCTCTTTGTCAGTGATTCT
CUST_1200_PI426210822 CTTCTCAGGTGCTTCCTGTTTCTGGTTGGATGGGAAAAGCTACCA
A_14_P201476 CGTGTAATTGCTGGAGATAGTCATAGAGCCTTATAATACCCAGAAAGTTTGGAAACCTAT
A_18_P17038306 ATCTTTGTAGACCCAGTGACCCCTGTTTCTGTGATAACACCTGCC
CUST_9071_PI426210822 TCCTTTCCTTGTTTGGGTGCCTGGTGAAGTCCTTTTGTTCAAATTCTAAT
A_18_P22841945 TTTATCTTTAGTCTTTAGTTTTTATATTTTATCTTTAGCCATTATCTTTAGTTGAAGTCA
A_14_P125952 TTTCTTAGAAAAATTCCAACAGTATTTTTGGTTGGTCAGCCAGTTAGTTGGTCAGTTAGT
A_18_P16331674
CCAGAAGATTTCTATTCACACTTTTCTAAAAAAAGCAAGATACAATACAGTAACACTTCC
A_14_P201094 TCTAGAACAAAACTACATTAACATTATCGACCTGGTTCTTCCCCATTCCAAACTAAGACT
A_14_P109550 GATACAAATGTGTATACTGAGAAACACTCAGACAGTCTGTTTAAACGGACAGAAGTCCTA
A_14_P112662 TTGTATTACTGAACTCATGGTCTTATGGAACACACCATATAGGTTATATAGCCCAATCCC
A_14_P124807 AACTGAAGGTATGGCTGAACAAAATAGGCTTATTGTCACTACAGTGGCATAAACACCT
A_16_P02877744
AACCTTGCCCATAGAGTAATTTAACCTGTTTTCATACTCACTTCCTAAGATTTGAGCACT
A_14_P102539 AAATTTCTTGCCACTCACTGAATCATGCCATTCATTGTTTATGAAGATCTGTAGAGATGA
A_18_P22839984 ATCTGGTGCGCTTGCTGACTTTCATTATTGCCTGGAATAGGCAATGATTCTAC
A_14_P113775 GTGCTTACAGTGCAAATGAACTTGCAAATTGATGTGTTTCTCCATGAAACGTTTAAGATT
A_16_P18379862
AACTTGGCTCTAGTGAATAATGTGACTGTTTTTGTATCAACATTCTTTCCAACCATCTGC
A_14_P125908 GCAACCCAGTAGAAAAAACCACTTTATGTTTCCATTTCTCAGGTTGTTTTTATTGAGTCA
A_14_P106056 AATTTCAATTTGTTTATATTTACCTCTGGGCTCAATAAGGGCATCTGTGCAGAAATTTGG
CUST_8543_PI426210822 AAGGTACCCCAAAACCAGGGATACCCCTTTTCCTCTTTACTGATC

A_14_P201724 GTTCTGTTGATTGCTTCAGGGGATCAGTGAAAATTAGGGAATTTTGTGTGTTGCTATATA
A_14_P200945 TACTATTTTTATACGTGATTTGAGTTCCTTTAAATGCTCAGGGGTCACCACCACAGTC
CUST_6182_PI426210822 GGGCATTTGGCTTTACAGCAAACCTATGAACCATGTATAGGGCTCC
A_14_P127519 CTAACTTTCATATTATTGACAACGTGGACTTGGTGGTTTCTGCCTACTGCTTAAAATACT
A_16_P01445619
ACACCTCCCAATTATGATCAAAGTATTAAATCTACTGAAAACGTTTTAATGAGGTCGGGA
A_14_P103826 ACTCCCAGAAGCAGATTTATGGCTTCAGAGATCATATAGCATTTAAAAAAATGCTCTGAA
A_14_P134459 CTGTATTATGATGACAAATTATACATCTATTTGGCAATGCTTTGCCCCCATTGGCAATTC
A_14_P106133 GATCTGGAAGGAAAGAGAAAAAAGAAGGGCTCTTAGATTAGATGTGCCATGAAGAGT
CUST_2684_PI426210822 GCCTCTGAAAGATTTCTGGGCTTTTCTTCATCCAAAAACAATACTTGGAGTTGA
A_16_P19689763
CGTCAGGCTAATTCGGATATAAATCTGTATGAACATGTGATCTTAAATCTCTATGGCTCC
A_16_P36239486
AAGAGGTTAACAGAAGTCCTTTATATCTGCATAACACATCATACTTCCAAGGAACTTCCA
A_14_P135571 CTATTGTTTGAGGTCTGCAGATGAAGTGAAAGCAAGAAGATAACCACAAGTTATCACAAA
A_14_P131578 TGCAAGAAAAAATGTGATACTGAATTTCCACATATGTTTTCAGAGAACCCAGCCAACTAT
A_16_P01399815
ATCTTGGCTAACAATACTACTAAGACCCAAGGCTATATTCTGTAGGGGATACTGGATACT
A_14_P125165 CTGACTCAGATTCGCTTATTCTCATTTTTCACCAATCACAGATCATGTGCACTTTTAGAA
CUST_2705_PI426210822 GGCCAGGGGTAAGAGATCCACTTTCCGATTTATTTCTAGTATCCTATAAGGATG
A_14_P100950 TTAGCGGTTTCAAATGTCATTCAGATGAGATGACAATGAATCATAGAAGGAAGACAGAGA
A_14_P104413 GAGGTTTGAATTTCAGTTTCTTTCTGCATATTGGATAATGAGCAGAGGTCATATTTGACC
A_16_P30952630 TATCAAGCCCAGGTGCCAGCCTGAGGCAGCCACTTCGGCCAGGTC
A_14_P107443 CTTGTTGTTGTTCCACACCATCTCCTTCTGTTTCATGATCTTCTTTCGTGTCAT
A_14_P138656
ACAGATTTCCTCTTCATTCTGAAAGTTCATCAAAGAAAGCACCAAGAAGCAAAAACAAAA
A_16_P21561142
TGACTGCCAGTTTACTCATTAACATCAAAAGTTGCTACCACAGATATTAGCCTATTGCAT
A_14_P103294 ACGAAGACAGAGTTATTAAACCATTCAAATCTCTGTGGTCAGTGGCATCCATTTGAT
A_16_P00250456
GTCCTGACGATGAAAAAGATATGAGGAGATAATGATGCAATCATAACACGTCTGATGGAG
A_14_P200163 CTGTTGTTTATACACAGATTTTAAGTTTACTCCTACTGCTGACCCAAGTGAAATTCCTTC
CUST_5873_PI426210822 CCTGGAGCTTTGGTTTTGATGAACAGTGTGCTGGTATTGGCTCTAAG
A_14_P125845 GCAAAGTAGATGTGTGGTCTCTTGGAATAACATGTATTGAACTAGGTAAGCATTGTTCTT
A_14_P123239 GATGTCTGCCATTTTGAACTACAAGATCCACGGACATTTTAAACTGACTAGTTTTGCATA
A_18_P22841891 CTGGGGAAGAAGAAAATCTAGGAAGAAAAGGCCACACAAGGAAGAAGT
A_16_P01892058
CTGAGTCTTCAGACAGTTTTACCATCTTCCCTATTTTAACCCCAATTTAAACATCCACAG

A_14_P113471 CCAGAAGACCGGTTATTGTTATGATGGATGAGAAAATACAGTGTTTATCAGCACATTTG

A_18_P13703429

ACTTGTCCATCTAGTAGAGCAAAATTCAACAACTGAAAGTTTTCAAAAGATCAAATCTCC

A_16_P19876917

TTAATTCTCCCTTATCCTACCAATTTGACCATCCCTTAGTTACCAGAACCTTTCATCTTT

A_14_P105683 AGCAAATAATTTAAAAGAATAAACATTGTGAACATCAATTCCCATCACAGCAAGCCCTGG

CUST_1874_PI426210822 GATGGATCAGGAACCCACATTTTTAAGAAGCTCCATAGGTTGTCTGATGG

A_16_P18445808

ACTGTTTGCTGACATATGTGACATGGAAAATAATGAATCCGCTAGAAGAATCAAGTTTGA

CUST_8020_PI426210822 TGCCCCCCACCCCACAGTAAGCTGACCAGTCCCACAGCAACAGAA

A_16_P37903544

GCATCTCAATGTATTGTCAGTCTTTCTGTCACATAGACACTCAAAAGTTATATTCTCTGC

A_14_P124339 TTCCATGAGGCAGTCTATTTTATCATTAAACTTTTTCTCTGCCAAGAAGATCACATCTGG

A_16_P58424278 TAAACCGAATTATATCCTGAAAGGCCCATCACAGTGGGAGTTAGGGCTTAAAAAT

CUST_5027_PI426210822

TGAATCATTTTAGATGAGTTTGTCCTCAGTACAAATCGTGCTGTGTTTAAAGTTCTTGGA

A_14_P125416 AGTCAGAGCCACATGATTCCCATCACTCAGACATTCTGAACCCCTATT

CUST_6829_PI426210822

GTGAAGCAGTAAATATAGGTCAGTTCAATAAATGTCTGTGTTGGCTTTTGTGACTCCTG

A_14_P129885 CTGGGTATTTAAACAACAACAAAAGGAGTTACTGTTAAATTTACAAATCCAGCAGCAGGC

A_14_P121053 AGAGTTTCTATTCATCATTTTAACCGCATTTCATCGATCTTGAGACACGGCTTTTGATAT

A_14_P121524 TAAAGAAAAGTGTCAAAGACTCTCAGAGTGTCCTGAGGCTTGAATTCTAATCTACTTTCT

CUST_2630_PI426210822 CATCTTCTGAGCCAGGCCCCAAGATAGGAATAAGGTCAGAGCAAG

A_14_P114882 TCCCACAATGGTATCAGAGGAGGTTCTTGTAACTAATTCAGATACAGAAACTGTGATTC

A_16_P18817072

AAAGAACTGTCCATGTTAAGAATCCATTTGTTCCTAAGATCATCTCTCAAGAAGGCTAGA

A_14_P117367

TGAAGTTACTTTTGAAGCAAAACGTGGATGTCGAAGCAGAGGTAAGTAAACTTGAAAAAT

A_14_P137215 TGCAATACATAGGTCATCTTCACATCAAATGCTGCAGTCTTTGTCTATTTACCGTTTTGA

A_16_P00685240

TGATTTCATACTCATGAAATTATTGTCATGGAGTTAGAGCCTAGATAAAAGTGTTTGGCA

CUST_4740_PI426210822

ACCTCTGTAATGGTATAATATGAACTCAGGAGTTGAGATTCGTAAAGCAGTAAGGAATGC

A_14_P104447 ATATTTTAGGAAAAAATGGTAATCCAGTGGCAGAGAATTTCCGAATGGAAGAAGTCTATT

A_16_P01081600

CCAGAGACTCACAATACTATAGGATGAAACTTAAGCAATGATTACAAGCATGAACTCACC

A_16_P15731053

AAAAATAGAATGTCAGGATCTCCATCTTCAACCAACTTCCAAATCGATCATGGAAAATGT

A_18_P26827501
ACCATTTATGAACTGTCTTATGTTAAGCACAAATTCCCTAGAAGATTGCATTCATCCTCA
A_14_P101407 AAAATGAATGCATCTGAAATGCATCCTATGTAAATGATTCAACCACATCTTGCCAGAGTT
A_16_P15996925
AAAAGGAAGGAAAAGATGATACCAACTAGACTTGCTTTAAATCCATGTAGGCTTCAACTG
CUST_7379_PI426210822
GAGGAGGGTCTCAAATTAAAAAGACAAGAAACAAACAATCTCCCCAAATAAGCAGAA
A_16_P19916578
AAACCAATTTGGAAAATGGATAAGGATTACAGTGACATATGATTTCCCCGAGTAAGGTTT
A_16_P00685057
AGAGCATGACATAATGCATAGTAAGTTGCATGTCTATATATACATTATTATGTGCCTGTA
A_14_P122323 ATAACTGAATGATTAGGGCAGGAGAAGGAATAATTACCAAATCCTTAATTTCTAGGCTGG
A_14_P114807 CATACTATGTGGATGTGACAGTGGCGTTTGTAATGAGAGCACTTTCTTTTTTTTCTATTT
A_18_P15970673
AGCCAACTAATCTCACATCCCCAAACACAAAAGTGTTTTAAGAAAGAAGAAATTCTTTCC
A_16_P21373659
AAACATCTATGGTCCACATTTTCTGTTGAAGTCATAAACTACTAATTTGAACCCCACTGC
A_16_P30952301 AATTTCTCCTTTGTTGTGACTGTGGTTCTCATTGCATTCCCCTCCTC
A_14_P121880 ACCCTAATAAAGGCAAGCCCATCTCATCTTCCTGAGAAAGCATGAGATCATATCTA
A_16_P41885477
TCCTATGGAAAAGGAAACAAAATAAAAAAAAAACTCCCTTTTGTGCAGACAGTTTCTTCT
A_16_P20863990
ACCTAACTCCAAACATAGACACTTCTCTACATCATCACCTATCTTTACTTCCTTTGCTTC
A_16_P36712025
ACCACACTCTAAGGCAAATTGATTCACAACAGTGTCTGTATGTTTATAGCACAAGAATGT
CUST_7776_PI426210822 CGTTCCCAGTCAGCTAAGAGTCTCATAACCATCAGCCTTGATAAATGCTAATG
A_16_P37854599
ATGCGATTGTTACTACTCTTACTTTCACACTTTCCATTGTAGACATTTCATCAGACAAGT
A_14_P122020 TCCCAGAACTTCCTCAATTCTTTGTGACAAAATAAAATATTTTCAGCAGGCATTCATGCA
CUST_9300_PI426210822 CACACACCTGAAGAGAGCTGTGCATAGTTCTGGTTTCTCTGGTTTG
A_14_P103409
AGTGGTTGGTAATATGAAGCCTCCAAAGCCAACAAAAATTAAAAAGAAAGGTAAAACGAG
A_16_P21255647
CCCAAACCCCAAATAAAAAACTCTAAGGCCATACAAATTCATTCCTAACCCCTATAAGTA
A_14_P107072 CTCTAAAGCACAAGACCTGGATGTGTGACACACAGTTTTGGAAAAAGGTCT
A_16_P19794939
ACAGCAAACATACCTACTAATGTGGTGTCTAATTCTTACTTTGTTCTATATCCCCCACAG
A_18_P17361955

ACGGGATAGAAGTTAAGTTCCAACTGTTTCTCTCATCTTATGATATGATATTTTAAGTCT

A_14_P137901 TCGGTGCCACTATTCTTTTTATCAAGACCTGAAATTATTCTATAATTTCCTTCCAGCACC

CUST_27_PI426210822 GATGACTGCACCCAGGCTGGCTGTCTCAGCTCCTGGACTCAGAAT

A_16_P01164731

GGAAGGTTTGAATGACACTAACTTACATTCAGCAAAAATGCTTTAACTCAACATCAATCT

A_14_P105686 CACTTTGCCTGACTTCTAGAGTAGGACTTCTGGTTTTAAAATCTTGGAAATCCACTG

A_16_P15860049

ACCTCAAGCAATAGAGAACCTAGGTTGTAACTATAGAAGTAATCAATAGGGGGAAAAAGT

A_14_P103070 CACCTAGTTAATTCCTCACATTCAAGTTCTAGTTATGTGCACAAGCAGTGATAATCTCAG

A_16_P41836732

AAGCACGATACTAAAAAAGAACCTATGTTGAATCACAACTCATAAAAACCATCAGACCAA

A_14_P103753 TGATCCAGGTTCCTCCAGAAATGAGATATACAGATTACTTTGTGATTGTTAGTGGAACTT

A_14_P137618 ACCTAATTTGAACCTCATTTTGTAAGAAAATGTGATTGCATCTGCAAAGGAGAGTTTCAG

A_14_P138138 ATATTTGATCGAAAGATCAACTTTGATGCGCTTTTAAAATTTTCTCATATGTAAGTGTTT

A_14_P120383 TCAGCAAATCCTATTAAATACTTTGTGTTTCCAAGCAAATAAGTGTGGCTTCTTTCTTTC

A_18_P12515330

GGCATTCAATAAATATGCAAAGTTTCCTAATCTATTACTTTGGTTCCCAAATACTTACTG

CUST_9009_PI426210822 ACACTTTGGTGACCCTCCCTATCCTTGGAACCCCAGCCAGCTGAT

A_14_P129795 AATCTGATCAGCAAAACCAATCTTCCCTGGATCACCCATAAAGGCACTAAC

A_18_P14135073

ATTCTTAGAATCCCAAGTTCGCTAACACTCAGGGATGCATTTTGCCTAAAGAAGATAATT

CUST_6748_PI426210822 GTATTATGGCCAGCAACTGGGTTTCCTCAGGGGCAGGCCCAGTGC

A_18_P14133666 CTGGATTTTTGAGGTCTGGTGGATTCCTGAGACTTTCCTTCCTCAC

A_14_P104814 GTCTATGCCTCTGGCACAGTTATATATTCAATGCATTTTTGAGGGATGAATGTAAGTAGC

A_14_P125474 GTAGAAGTCCTGTGGAGTCACTGACTGACTTTTTGGTTTGAAAACTCAGTCATAATCT

A_16_P36230864

TTCTCAAAATGTAAAATTCTTCCTTGACTCACTAAGCAACCTTCTGATTGCAACTGCTTT

A_16_P01062492

ATTCATTCTCCAAATGAATTTAGAACATGAAGCATGTTCCGTAAGTGAGAAAAGGAGGAA

A_16_P21226160

GAAAAAAGATACCACTAGACTCAAATAATTCACATTTCACTCATCCACTGTCAAACTTCA

A_14_P122620 CTATCATTTTCATGTCCCTTTGAAGGAGAAAGCCTCAGAATTATTTCACATATAGCCTCT

A_14_P129411 CCCAAATATCAAATAACTGAAAGAGTTTTTCTTTCGACCATCTGAGTCAAGATGTGGTTT

A_14_P137213 TGGATTGTGTTTCAGGGCCATGATAAAGGCAATGTATGATCCTTCAAGTATACTATGATT

A_14_P104737 TCCAAGTTCTGTTGCAAGTTTTCTCTTGCTGTCTTGCTCCTTGATTTTTCTAAAAATACT

A_18_P22841388 ATCTGGACTGGAGATTCAGTCATGCAGTCTGAAGCCACTTGTTCAC

A_16_P17598232

AGAACTGGAGATAGCACAAAAATAAAGGGGACTTCAATAATGTAGAACAAGTAAGTGCGT

A_14_P131244 GTTTTCTTGAAAACAGAACATCTGAGTCAACAGGTTTTCAGATTGGAAATTCTGAGTGTT
A_16_P21203810
GCAACATTGGTATACGAATATTGACATCCACTAACTTACCAAGAGAACTTGAACCTCTAC
A_14_P128899 CAAGCGTGACAACAATTTTTATAGTGTAGAGATTGGAGATTCTACATTCACAGTCCTGAA
A_18_P22841767
TGGTGAGAAAGAAAAGCACACATATGTTACGCTTTTTTTTTCAGCATTTCACAATATTCA
A_16_P01907956
CCCAGAACTCTAATAAAAATTAGCCTAAGTAGAAATCGAGTCATTTATTCTGACCCCTGC
A_18_P19444245
ACTGACATGTGTGACTTTGAGTTTAACAATTGCTAAACTGTGATCAAAATTATGGGAGGC
A_16_P19618566
ATGCATTTATGGATATCTCAGCATCTCGTAACTACTGTCAAATGTGTTTTAGAGACCTGC
A_16_P16947867
ACCCCTGAAAGACTTATTACAAATCACAGAAAGCCAAAATCATTGTATTCTTGCCTGAAT
A_16_P30952653 CTCATGTAATGAATCCACCAGGCTGGCAGTGGTGCATATAAACTGTGG
A_18_P13357613
AAACCTTGAACAATCTAGTGAATGAACGAATGTGTCTTCAGAGTTATGACATAACCAGCA
A_14_P116879 CCGTTTTTACAATGATTTTTTTAAAAAAAAACACCAAGGTGCATTCTGGACCAGATTCTA
A_14_P108501 GAAAAGATGGGCACTGGAGAGGTAAAAATTCTATTTAGTGATCACTCACACTTGAACAAT
CUST_2432_PI426210822
TGACAAAGGCTGTTTGTACAATAAATATTGTTGAATCAGTGCTGCCTGTGTGGATATG
A_14_P120731 GCATTCCTCCAGAAAACTGATGTGGATTTTTTAAGTGTGCATCTTAGATTTTATTAATGC
A_14_P101549 ACTAATTCTACTAACTAAGAGGCAAGACAGGCATTTCAAATACAAAACTATGACCAGCCT
A_14_P102273 ATGTCGTCTAAGATTTAACTTACCTGCCTCAGTCTCTATGTTTAAATTAGTTGTTACAAA
A_14_P117746 TAAAAAGAACAAGTGCCTTAGTTTGTCCTGACATCCAATAAATGCTCTAATATCGGGATT
A_14_P200507 TCAAGGCATGGATGCTACTTTCAGGTCAGTTTTCTGAATACCTAAAAGTCCTTTGATTTA
A_16_P58424128 CTAACCATTGAGGCCGAATTTAAAGATTGTTCTCTCACCTCACTCTCCTACTTCTAA
A_16_P20352864
AACTTTCTGGTCTTTCTAAGTTTAGCAATTACCTTGTTTGCATCTAATATGAGCGCAGGA
CUST_7934_PI426210822 GCATCTCGTCCCACTGGTGCCTACCGTCTGAGCACTGCTCTGAAG
A_14_P124156 CGGGGAAAAATGCCAGTATTAATACGAGCCAGACAAAGATTAACTTCTCAGGATTAGAA
A_14_P134660 CTTTGGTCTTTTTTGTAACTCCCAGTTGGCTGCTAAATCCAGACTTATGGTTTATTTCTC
A_16_P00300828
TCCAGGGGCTTAGTATAAACTATAAATTTTCACCACATATTACGAAAAGAACTTGCTGCT
A_14_P110887 GGAGATAATTCTCTCATTTCTGATAAGCAAAATAAGCAAAGCCTTCCTCAGGCATATTCT
CUST_2647_PI426210822 TTGATAAAGTCCCAGGATTCAGCTGAGCAGCAGTGAAGGCCTGGA
A_18_P14135552 AGAGGACAGCCATGGCTGTAAGGAGGAGGCCAGAGGTCTCCAGCA
A_16_P19747130

ACCCATGTCAATTGCAATTCCATTTATCTCTGAAGCCATATGTATGAAGAGCACATACTT

A_18_P25260998

AAAAAAAAATCCATAGCCAGATTTTGTGCTTTAATGTGGACCTAGGTCAATAGTGACTGC

A_16_P37447078

CAGTGTTGCTTACCTATCTTATAATTTCAAATTCTACCTCTACGAATCATTACCTCAGCC

CUST_4259_PI426210822 AAAATAGCAGTTGAAGTGCAGCTGTGTTTAGCTTGTCAGAGAACCTGTTGAG

A_16_P01173894

AAGGGTGTTGAATGTAAGTTGTGCAAAATTTGGACACAATAGACATGATGAAGTGAGAAT

A_14_P113344 GCTTGGATTTTTTAAAAAAGTGTTTTCAGTCTTCCCAGTCCTGGATATTTTAAGCAGAAG

A_14_P136933 TGACTGGCATCTATTTGTATAAATCCAAATGGTTTACTCTGAAGACCCTCTCTGTTTTTC

A_14_P119584 CGTGGAGTCTGTGTTTGGAAGCAAATAACAAAGTCGCTGCAACTCCTAC

A_14_P119479 GAATAGTTTGGAGTCTTACCAATTTGTAGGGGCTGTTCTTATGTTATACACCTGAAACTT

A_14_P119755 AAATCATGGCAATTAGTGTAACGGAAATGTTTACAGCAATCTCTGATGGCAGTTCTACTA

A_16_P15517416

AACGATAGGGGAAAAGATAAGACACTAACAAGTCTTCAGTTTACCAATGATGAAATCAGG

A_14_P114886 AGAGGTAGAATTGTGTTTTTTCCATACTCTTTAGTTCTTGATGAACCCCATATGGTTCTG

A_14_P137715 GTTTTGGGCTTTTCCTATTTATGAAGTTCTTAGTTGTCTGTTGTGCACACTGACTTTTTT

A_14_P139834 TACATTAAAGCACCGGTCAAACCAGATAGTTATTTTCATATAAAGGTGTGCTTTCAACCC

CUST_6349_PI426210822 GTACCTATGAGGGAGGTGGAGGTATTTCTGGCTTCCTATAAGTGAGTTTTT

CUST_338_PI426210822

TTTAGAAAAATACATTAATTTATTCAGACACTGGAGGCTTGGGAGTAGTTTTGCAAAGGG

A_14_P106348 TCAAAATCATGGTCATATGGGGGAGAAGTAAATATTCCTATTTTATGGTTATCCCACTCC

A_16_P18217712

AAAGACACAATGTTGACCCAAATTAAACCCTTTTCTGAATTTAACACTGAGGAACACTTC

A_16_P36626646

TTATTTCTCCTATCTTTCATTCTCAACTTCACTGAAGAGAAAAATACCAGCAATCAGGCC

A_14_P107606 TGGCAAAGTTTCCGAGAACAAATAAAACCATTATTAGAATTGAAAAAAAAATGTATAGAA

CUST_7407_PI426210822

CTCACCACAGCACCATTCTTAAGTTTTCTAGCCTCATTTATTTTAGAAGGGGAACT

CUST_1856_PI426210822 TGTCCAACTTCAAATCCCAAACCCTTCCCAAGTTTGGCTTTGATTTAATG

A_18_P14132631 TGGTGTTTGGGTTTGTAGGCAGGATGGCCATGTATGCACAAAGAA

A_14_P123334 TTCTCTTACTAGTCAAAAGAGAAGATGTGTGAGAATACAGAACCAGTGGAAACTTCTTCT

A_14_P121973 TGCAAAATGAAAAGAATTGTGCTGAGTGTTTCAGGAATACTTTGATATTTTGGCTGATTG

A_16_P58424514

AGGTGATGTAATTTGCAGATGTGGCAGAAATTGAAAAGATGGTATGTGAGTGAAACTTAG

A_14_P100248 TTTAGACAAATATCTATCCTGTATTCTCTTTGCCATCTAGATTGGAGCCATGGCTTTGGA

A_14_P133172 CTTGGCATTTCTACCAAATATACAGATAGAAGAAGCACAATTGCTACATCAGTTTCAGGA

A_14_P124215 ACATAGACGTTACACTCCTGAAAATAAGTTGGCAAGTTATCCTGTAGCAAATGTTCTGAA

A_18_P22842476 ATGAATTTGATAAGGGGCGGCAGATGCCCATTCAAACCTGAAAATTCTCATT

A_16_P03462711

CATCCATCCAAACCTTCTAGATATTCTTACCCCACCTAAATTCAACACCAATTTGGATTC

A_14_P115345 GTCTCCTCCTCTTCAATATGTGGGGACTGCAGTGTTATTGGAGAATCTG

A_14_P138167 CTACTGAAACTCCTCCACCTGCTTCTAAAATGCCCAGAAGACAACTG

A_14_P117472 CGGGGTTAGGAAATGAAAAACAGGGTTCTTGTCCAAGATCATTTATTGTTATTATTATTT

A_14_P109287 ACATAGTTCCAGACACGATCTTCTCACCAGATTTATCAAATCCCACACTTATACACTATC

A_14_P133607 ACTTATACTAATGTTTACAAGAATGCAATATACTGTGATGCCTTCCTACTCAAGCCTCCT

A_14_P139439 TTTTCATACCCCTTGATGTCATTTATATGCTCTCCCTTGAAGTCAGTAAGGAGAAAGAG

A_14_P130479 CGTCCCACAATTTAACAATTGCACCTGATTTCACATATCCATTAATCAACAAAGGAGATG

A_14_P136052 AGATAACTAATTGGTCATAAGAATCAAAAGACCCAGAAGGGAAAATAGAGAGTCTGGTCT

A_16_P18397523

CAGCCCCTAAAATTAGTTACCTTGAATTCACACTTACGTATCTTTTACCTTAACTCCCAA

A_18_P15712741

ACACTGACCACATACCTACTCATTCACTTATACTTTCAAGCACAAGAATTCAGGAAGAAA

A_16_P17110942

ACCGAGATAACATTTCTGAAAATATAGTATAGCCCTCATAGTCTTATCCAAGTGCACTGT

A_14_P139342 TTGTTTTTATTAAAAAAAGATTTCGGCCTTTTGTGCGTCCCACCTCGTCACTGTAATTTA

A_16_P17799068

AAAATCCTGTCCATAAGTGTATGAAATATTTGGCATTCAAGTTACCCTAGGAGAAGAAGA

A_16_P01171307

AACCAATTTCTGAACCACCATTTAATGAACGTTAGACAAAAAGGGTTACAATAGAGAGCA

A_14_P106652 ATCAGGAAGAAAATGATGTTGTATAATCTATGACTTTGAGGACAGCCTGTAAGACAGGAA

A_14_P131179 AAACATATGGGACAGTGCTTTGGGATTATGTTGACCCTACTTTTGGTCTAAATATTGAAT

A_14_P124297 GCATCTTGAGGTTTGCTTTGTTGACTCCGTAATTCTAGACCTAAAAAAGAAACAAGAAAA

A_14_P106509 ACTCACATAGTCCACAGGCTTTTTCTTTCTTTCTTTAACTTTAGGAAGCGAACTTTTGCT

A_14_P132689 TGTTTTCAAAGATCAAGTAAATCCTTCATTCCAGTGTGATACAGAACAACCTGTTCAGTT

A_14_P100587 TGGACTCTGGCTAGAAAAATATGGTCTATACATGGGCCAAGGTAGACTGTGATTA

A_14_P139819 CTCATTCTCCAATTCCTGAACTTATCTTCTCCTTCTCCCATACAATCCCTATTTTTTTTT

A_16_P18533376

ACAACCAAGTATCCCATCAAGTATCAACCAAGTCCTAGTATATGTTCTGTTTATTCCCAA

CUST_1450_PI426210822 ATGATTTCCAACATCCAGTCACTTTCCTGGGTATTTTTAGTCACTCACTTTGCA

CUST_7403_PI426210822 TCTGCAGCCTGTGCAGTGTCAAGGGGACAGTGGCTGCATGATGAT

A_14_P104406 AGATATACAGTCACTCAAGGCGATAAGATATGCAATTTTATTCAAGGGAATTCTGCCTCT

A_14_P126632 TGCCTTACCTCTTCTAAATAGACTGATGATGAGTATATTCCTCTCATTAAACTCCAACGG

A_14_P101532

AAAGGGAGTGTTTCATGGAACTAAAGGAAGAAATAGAACTAGGAACTAGAACCATAGAGA

A_16_P16756831

GGTTCAACAGATACTACCAATTCAGGTTTCACTAACAATCACAAAAAACATTTACACAGG

A_16_P38283517

ATTCCTTATGCTATGTTTGTTCTAGTCAGCATTATCCTGAGTAAATTTACACAGCCTGGC

A_14_P134527 ACTGCTTGAAGGTGACATTTGTCATCAAACTCAGAGAAATCGCTTCATACTATCAATACA

A_14_P129041 GATGTTATACTTTCTGTGGTTGGAGAAGTAAAATACCATGGAGAGAGGAAGAGTATTAGG

A_14_P111648 ATGGAAAACATCCACGTTTTCTTTTCATTTTCTTGCCGCTTTCTAACAGTATCACTCATA

A_16_P16895388

AAACCGCATATTAAATCTTCACATTCTAACAAAGCCAACTGAATTTATATTGCAAGCACA

CUST_1404_PI426210822 GCTGTCACCACCTCGGAAACCCGAGGTCCTGCTCCCAACATAGAC

A_14_P105387 CCAAGGAGACGCTGACACTTGTGTTTGAATATGTGGTAAGTAAAATAAGACTTTTAAATT

A_14_P103623 ATGAAATGTTTTAGCATATTATTGTATGCATTTCCTGGTTGGAAGTTTACTGGGAGACTG

A_14_P125323 CCTTAGAAATCTTCAAACTTAGCCTCTCAGAAAATATAACCAATCCAGGGCTATGAACTT

A_14_P139197 TAAAAAACACTCAAGACACAGACCCAAGCCGGGTTTTATTGAAATGCCAG

A_18_P22842675 TTTCAGTCTGTAAGGCTTTTCTCACTGATTCTACAACCGTGGGGGC

A_14_P132569 TGAGCAATTTAAAAAGTTCTTCAAAGTATCTCTCTCTCTCTTCAGCATTTCTCTGAACCC

A_14_P130582 ACTTCTTCTGTTTTACTTTAGAAAAAGGAACAAGTTTTTCCATTATGTTGTAAAGTCTAT

A_14_P200676 GAGTTAACTAAGCCAAATGTGTATTACAGTAACATTTTCCTAATCCTGGAGCAATGGAAT

A_14_P138119 TGCTTGATGTAAAGTCCAGGAAAGCCATTCATTATATAAACCACTGGGATTAGTCAAAAG

CUST_7073_PI426210822

AGTTTTTGATCAATTGGGTTGCTGAGATTACAACTTTCTTTTTCATTAACTATTAGTACT

A_14_P134857 GATGCTTGGTTCATTTCCTTGCCCAATTTTAGCCTCAAACTTGGTCAGAC

A_14_P138643 TGTTTAGTGAGCCCTTATTACATGCACAATGTATTAGACGATCCCGTGAATAAACAAAGT

CUST_9929_PI426210822 GAGTCTGGTGATTGCAGGTCCCCCTGGGGTCCCCACTCATAGATG

A_16_P01220348

AAAAAATAGAAGCACCAAGTTTAACTTAGGGGAGAATCAACCATCATTATGGTTGCTTGA

A_14_P128735 GTTGTTTTCTTAAAAAACAAGTTAAAACCTGACGATTTCTGCAGGCTGTGTAAGCATGTT

A_14_P111857 CTCCAAAGATAAGGTAGAGGTGGAACTAATGTGATTGCAGTTATATTGAAGAGTTAGGTG

A_14_P129785 AAAAGAAGTCTATCCTTCTCTGGAATACTCCTTGATAATGACTGTGATTCCACTTAGCAA

A_14_P135368 AATTTGTCTAATGCTCAATTTGTTCAGATGACGATACTATCCACCACAGGGTAAAAAACT

A_14_P129706 CTTAAGAAAGGACACAAAGCCTGAGAAAGTTGTATTATGCCATTTATAAATGTCCGGTGA

A_16_P02331079

AAAGGGCACATATTATACCTTTCATGTTCATTTGTTCATTTGGTATTCCTGAGGATAGGC

CUST_2477_PI426210822

TATCCTGGTAAGCTGTTTTTCTTTAAAGGCACATGAGATAAATGAAGATAAATGCATGAA

A_18_P17273297

AAGTTTACAGACAAGGCATGAATGAGATTGACCTTTTTGACAGAAGTGAGTAATTGTGAG

CUST_9367_PI426210822 CTTTGGCAGAGATGAGCAAAGGGTACCCTTGGAACTGTTTTCTGT

A_16_P58424507

CATTGACCCTAACAGTGACCCTTTTCACCTTAGTCAGTTCAGTCATCTATAACAAATTAC
A_14_P124308 TTTACTTTTCACAAAAATTATTTACACAGCAGAGGAACCTGCTCCAAAATGAAGGAAGCA
A_16_P02532875
GTTTTACTTCTCCAGACTATCATTACCTGTTATCCGGTCTATCCTAACACATGTGTAACA
A_16_P17617733
AATCATTGTGAATGTCTAAAGGCTATCAACCTTTTCCTAACCCTTGAAACTTCTTCCTTT
A_16_P00581832
CCCCTTCCATTCTGAATAAAAAACAAAACATCACTATTCTGTCCTGATTTTTAAGCACCA
A_14_P109781 CTCTGCCTGGAATTTCCACCATCTATGTCATTTTGGACAGATAACAAATATTAAGCAGAA
CUST_3221_PI426210822 CCCCAGCTCTTGTCATGACCTGCCCCTTTCCTCCTGGAACCTGCT
A_16_P16637263
AACAGAGTAAGCATTGACCTTTAGATTTTCCTTTCAACTGAAATGTTTCAAAACCAAGCA
A_14_P126283 AGTTTTAAAATAAATAATAATTCCCCCAAAGATACCAGACGATCCATTGGAGACCTTGGA
A_16_P16191484 AAGTGAATGCCCAAATGAGTGGCACCAACACTGATTATTACTGGGTGAC
A_14_P101990 CTTCCAGGAGCCAGTAATTTCAGGCAACATGATAAATGATGAAGTTGTTTGATACTTACG
A_16_P03474027
CTTGTTATTGTAAGTGCCTTCAATACTTTGCATTTTGGGATAATAAAAAAGGCTCCCTCC
A_14_P136444 CTCTGTTTCTGAAAGTGTTAATAGCATACATTTGGTTCTTGACCCTTTCTGTTACTGCAA
CUST_8510_PI426210822 GTGCCTGCAGTCCATGTTGAGAGGATTGTGAAAGCATTGCTCTCTATA
A_16_P15322855
AAAAATATGTGGCCAGTAAATAACTCTGTGTGTCATGAAATTGGGTCTGAGATTTTGCTT
A_16_P41695853
ATATTCAGACTAAATGCCAAACATCCTTTTCTATGTTTCCCAGATTTGGAACTACCTCAG
A_14_P111871 CTATTCTAGAACAGGAAGGCACATTTACCGCTATGATATTAATGAAGTCATTCTCCCCC
CUST_893_PI426210822 TTGTTCAGGTGCATTTTGAGAACTTGAGAAGTGCCTGGGGGTGTA
A_14_P200673 CAATCATTCACTGGTATGCGAAACACAGTGGTAGGCATTTAGTTGAGAATTAAACAAAAG
CUST_7973_PI426210822
CATGGAACATTGTAGTATGTGCCATCATTATAAGGAGATACAAACATAGGTAAAAAGATG
A_14_P120941 ACGTATTTCCATTATCACATTTGGTCTGCAATTTGATTAAACTGCTTCCAAGTGACAACA
A_16_P18103647
AATCTCACCTTCAATCTACAAACATGTCCTCTAAACATACAACCATGTTTCCTTGTAAGC
A_16_P03662877
TCCCTCCAATATGATTAGTTAACCTTCATCCCATATGCAGTTAAATCACCTTCAAAAAGA
A_16_P19245437
TGAAGCCCACGTTATCTTATACCAGAAATCCCATATCAACCTCTAAACTCATTTTTTGTG
CUST_3004_PI426210822
CTGCTATCCATGCATTCAAAAAAATGGGAGGTTTTGGTCAAGTAATGTACAACATGC
A_18_P22840470 ATCTCTATGGCAGGTGCGAATGTAGGAGGGTTGGCAGGGACTGAT

A_14_P121136 GCTCTATTACAAAATCAAAGGGGAGAAAATGAAGAATAGACTGCTTCCTTGGTTATTGTG
A_18_P14589316
AATTCTGACTTTGAGTTCTCATTCTACTGCAAGAAGCAAAAATTTCGCTTAAACCTTCCT
A_14_P132023 CTTAGTCCATTCTCCTAAGTAAAGCCTGAAGGAAAACTCTTAACACCTAATTCTTTGTGG
CUST_631_PI426210822 AGCTTGACCACACACAGGGAGCTCAGGAAGCAGCTTTGTTGGAAA
A_18_P11196441
AAAAAACCTGCAGGCTGTAAATAGTGTGTTCAAAATTCAACCTTCAAAAAAGGAGCAGAG
A_14_P112763 GCCTTGGGAAATTATAACTGCCTTACTAGGAAGTAATTACTTTGGCATTTTCTTCATGGA
A_18_P14130265 AAACGTTGTTTGAAGCACAGATATGACAAGGTGAAGCAAGTCATTACCACAGTG
CUST_4979_PI426210822 AAAGATAACAGATGAATGTCTCATGGCCAGTAGCTTGGTTTTTTTGCATGGTGT
A_16_P38605828
GAGACTCTTGAACTTGTCATCTCATAATCTCACAATTCGAAGTCTATTGATCTTGATGCC
A_16_P01131211
ATGGCCCATATCATTCATTACACATCCAACTTGAAATGTAGTTCCTAGTCCTGAAAAAAG
A_14_P132463 GCAATCTAACAGAATGGCAACATTTTACATAGCATTCTAAACGGTCCAATGAAGCAAAAC
A_18_P14134212 CACAGGTAGAATGTTGAATGCACTAGTTCTCTGCTATCTGTTTCCACGACAGAAG
A_14_P139631 ATTTACTTGTTATCATTAGAGAGGGAACATCAAATGCTGGGACATCATTACTAACCAATA
A_14_P120134 ATGATTTCCTTATTTATAGTGGGAAAGTAAAACTGTCCTGCGCAATGAGCCTTTGGTTTT
CUST_9942_PI426210822 ATAGGATGGTGAGAACCTAACTCCAAAACAGAAGTCTGGGGAGCA
A_18_P16621387
CGGCTTTGAAAATACCTATGCAACAAGTATTAGATGTGGATGTTGATTGATGTGTTCTTG
A_16_P18271480 ATTTGTGCCTATATTGCCTTCTACATGCTTCTATCAGAAATCTTTATTCCCCCAGTCTTT
A_14_P135018 ATTTGCATCAATATATCTCTCGCATCAAGTGGAGTCAATGAACTGCAAAAGAAATACAAA
A_16_P00886995
TGTTTAACCTTACACCATGTTGGATATGAATTCCTCACTAAAAATAACGGTCACCCATTT
A_14_P100627 ATATCTTCTCATCCCCACTCCACTATCATAAAATGGGTTTATATTCTTCCATCCTTCTTG
A_14_P138571 TCAAAACAATTTAAGTTGCAAGGCCAACTTCTCAGTTATTGGTGTCATCTTTGAATGTGA
A_18_P12143830
ATGTTAAATTTAGGCTGACATTGAGACACATTTTCCCGTTGTCTTTTTCATATGCTTGCA
A_14_P115437 ACAGCATCTGTTGGAAAAATGGCTGCATGGACATTTCTTATTTTATGCCCACTTATAAAT
A_14_P123007 AATTCTTTAGAATTTATCAAAACAGTATGTTGAGAGTCTCTTTAATTTTTAGAGTAAATA
A_14_P104201 CTAAACTTTTTCCTTTTCTTGCAGATGCATATGAAACAGCCAAGATGCTGTGTGAACAG
A_16_P17254626
TCTATTCCACAGTTCACTCTAAATACTATTCCCTGGATTTTGCTATCATCTAGAACTGCC
CUST_7482_PI426210822
AAGATAATCTTGGAAGGCAAAGCCTGTCTCCCTATATTGTTCTTAAAAATACAAAAGGGA
A_14_P134368 CAAAATAAAAGCAAGTCTCATTACTTTTCAAGGGGAGTGTAGAAGCGATTAGTCTCTGAT
A_14_P134863 AAGCAAGACTCCTTCTAAGAGCAAAAATGTGAACTTCTTTAACTATCCAAATCCCCAACA

A_14_P139475 AAATTCATTGCCAGTTTAGAGTCTTCCTTGTTCAAGCCTATTTCTTTCAATGATTCCATC

A_14_P200174 CTGGTTGTGGTGTTTTCCAGCGAGGGAAGAAAAGAGTAATTTTTTCAAAGCATTTATAGA

A_14_P115830 TTATCTCCAATCACTAGATTACAGAACTGAGTTAAAGACTAAGAACTTGGAGACCCCTG

CUST_10045_PI426210822 ATCATTCTCTTCCCACCGCAGACCAGCTTTCTCTATGTTTCCCTA

CUST_5091_PI426210822

ATCCAAATATTACGAGTGCAGATGCTGAAAGGAACAAAAGGAAATCTCACTCAGTC

A_14_P102519 ACACTGAGAAAAAGACAAATGAAATTACTAATAATTTTTAACAACCTGGTTTATTTTGAA

A_14_P123550 TTTCAGATTCCATTGTGATTGGATTTTGGGTTGGTCTTGCAGTCTTCGTGATTTTTA

A_14_P121294 TTTTCCTTTGCAGTCTCGAAGATATGACTCCAGGACCACTATATTTCTCCAGAAG

A_14_P124921 CTTAAAAGAAAGCAGAAAATATACTGAGACTCATCAAAGACCATACGTATGTCCGCACTC

A_14_P119025 ATATTCTTATCCAGGTCAACATACACATAGGAAATGACTGACAAGATCTGGTAAGTTTGC

A_14_P100130 CCCATAGTTCATCATTAAATGTGGATGTCAAAATTTCTGAACATGCTGTGAGGAAATTGT

A_14_P127026 ACTCCAGATCTTGCCAGTGTTTAGTTTTACCCATGAACTTTAGTGTATGGTTGATGATAT

CUST_7162_PI426210822 AATGCTCTACCTGCTTTCTGTATCGGCTTTCTAGCCAGTTGCAGTGTA

A_16_P00685391

TCACATTTTCATTTCATTTCTAAAAATAACTTTGGAAGCAGAGATGTGCTGTTTTAGTCA

A_14_P107962 GACATTTCTGAGGAGTGACAGGAATATTGTCACTTTTGCTGTGTGAGTTACACTGA

A_14_P121669 GCCCAACTTCCTTCTTACTACAGTCATATTAAACAGATCACATCAATGACAAATGTCACT

A_18_P16691447

AAAAAACCAATGCATCAAGTCATACACAATTCCACAATGTGTTTTCAAGTTATGGCTTGC

A_16_P02048033

AAGGTGAGTCACTAAAACTCAGAATCCTTTCTTTAGAATTTCTAGCAACCCACAGTATCT

A_14_P128816 GTCCCTTCTGCCAGTAATCCTTGTAGTAATTTCCTTAGACCTATATTGGTCATAATCACT

A_14_P113123 TTTCATTCAAGCAGTTCAGACTCACGATTTTAACTAGAGTAATCTGGGGTTTGTTTGCTT

A_14_P136715 CTGGGGAGTAGAAGCAATAATGTATTTCTAATTTGTGGGTCCCACTTCGGCTAT

A_14_P123321 ATCAAAACGGTAAAACATCCAGAATTTCTCAGTCTCTGCGTTTGGTAGTTTCTCCT

A_14_P110597 GACTCTCAGGCATGGTCTCACTCACTTTCTCTCTACTTGATGTCTCACT

A_14_P133543 CTTGAATGTGATTTTAATTTGGTTCGTTCTTGATTTTATCCTTGGAGGAACTTCCTTGTG

A_16_P17197826

CACCCCTCTCAGAATTACTCATTGAATTACATATCTCAACATCTATTTTGACCACTCCTG

A_16_P15013792

AGCGATGATTAAACACTTCTGAAATCTGGAATTACTGACTTTGACCTTTTCTTTATCGGC

A_16_P00405428

TAGTCCAGAAGTAAAGCGATATGATGTGGTTCTTTGATAAAAACCCCTTTTTTACACCAG

A_16_P00368152

AGCCCCTTCTAAAGTAACTAATCAGCATCTAACACATCTAAAGCCAACTTTCAGTCATTT

CUST_7243_PI426210822 GTTCAGAATGTCATTCCTCAGCCCCCAGGGGACATAAGTACAAAG

A_14_P133719 ACAATAGAATGTGACTAACTTAAAATTGTGGCTGTCAGACTAGACAGAAAATGGGCTTCT

A_18_P13384499
AACGGCCTTTTATCTTCTATCCTACTCACTTCACATCCTATGCTCTATAGAACTGGTTAT
A_14_P103893 AGATTATGGGATGTTCGAGACAATAACACTGTGTATTATTGTTTATCTCCTGGTAACGCT
A_14_P128226 AACTTCAGAAACATCAGAAATTACCGACATCATTGGGGAAAGCCTTAGAAAAATCTATAA
A_14_P200532 CTCTTAAAGGCAGATCTTCACGGGGCTATTATTTCAGGTAATTTACCTAAGAGCGTGTA
A_14_P102685
TGTAAGCAGAAGCCAAGAATGTCATTAGAAAGTAAAACTTTAACAAGAGGGACTGAATCC
A_16_P18003274
AGGCCAAAAAAAAGTTAATCTCTCCAGCAATTCTATACCCATAAATATCCCATACAGAAA
A_14_P106832 GAAAGGTAAGAAACAAAATCGGAATTAGAAAATGACTGTTGCCCCTCTCTTTTCATTCTT
A_14_P101662 GACTTGGTAATCCACAGGCTCTGCATATCCCTCTTTAAACAGCATGAATACCAA
A_14_P133440 CGATCAGAATGCCAAATGTGTTTGCATGCTGGGTAAGAAGGCTTCTCATTTTAAATATA
A_16_P18089474
GCACTCCACTTGTCAGAATAACCAGTTAAATTTCTCAAGTCATAACTACCATTTATCCTC
A_14_P106671 GTTCCTGAGTTAAGATTAAACAGTGTAAACTAGTTTAGTTTTGGTGGGGTTTTTTTCTCC
CUST_4993_PI426210822 TAATTTGGGGGTAACTCCGTCTTCACCATTAATGGCCATTCTTCAAAGC
A_14_P112551 CTTGAGATGAGATCCAGTGCTGGCTTCCTCATCTTCTTGTTCTAATGTAAAATTAGTTTT
A_14_P137211 AGAATGCTTTCCTAAAAAAATGTCCACTCTTACAATGCTGTCTGGGCTATTATTACACAG
A_14_P134142 GATATTGATGAGTCTCATCCAGAAATGGGTTAGGCATTTCAAGGAAATATCTACCATTCT
A_14_P132120 TGATTATGTGAATGATTTTACATGGCACATAAGGGAGAGAAAGACAAGATCAATGTCCAG
A_16_P15544604
AAATGTTGTTATCCCTATGTTAACCACTTCCAAAGAAACCTTTATTGTGATGACTCTGGC
A_14_P109418 TTACTGATAACTTGCTTTCAGGCTGTTTGTGTTCACCATAATGGTGGTTTGCATTTATTT
A_18_P22840342 ATGTCTTTTGGGGTATGCTGGCTGCTCACGTGGCTTTGTAGACATTA
A_16_P02391032
AAAAACCCAGTGTAATTACTTCCCATTATTGAACATGATGTCAATACTCTTCAGTGGCTC
A_16_P02311567
GCCTCCCCATAAAATCAATTACATCTATTCTTTGCTTTAAATATCGATTTCATCGTCCCA
CUST_1343_PI426210822 AGTGACTTCTAAGTCCCCTTGTTCAACCCTATCTTTTGGGTCTCAGATT
A_14_P131933 AAAGAGAAGGGTCATCCAGAAAGAGGCATCTCCCAGAAACAGGAAAT
A_16_P16215274
TATCTAGTGCCTTTCCAATTCCATAATCAACTACCTTTTTATTACTGATTAGGCAGCCCA
CUST_9632_PI426210822 GAATCAAAGTGCTGGTTGCGAGGTGCCAATTACATGTTGAATTTCATCTTCAA
A_14_P101965 CAGGGATATACACAGTCCTATCCAGTGTTTCAATCAAATCATTCTAATGTGTCATTCCAT
A_14_P134520 TATTGCAACCCTAAAATTTTATAATTCAACTAAACTTTCCGCAAAAGCAAGGGAGTGCCT
A_14_P110428 CTCTCTCACTTACCAATAGGAATATTGGGGGTAACTTTTTTTTTCCAGAGTTGTTGTTAG
A_16_P16878076
GACTCATTCTGCTTTATGCTCCATCTTACTTTTCAGGACACTCTATTCTACAACACTCTT

A_14_P114850 ACCCAGGGCTGACATCCTAATAAAATAACTGTTGGATTAGAAACTCCATAAATGAGTGG
A_16_P18397997
AAAAACATTTCCTTATTGTAAAACTGCCTTCCATAAGAAAAACCTATGCTTGGAACCCAG
A_14_P130022 CTGCAAACAAATATTTGAAAATTGCAGTTTGGATTGCGTTTTCCCCACATATTGGTAAAT
A_14_P114203 CTACTACACCACTTCTGACGCTGTCATTTCCACTGAGACCGTCTTCATT
CUST_4873_PI426210822
GTTCCTTGTGAGATCAGACACATGAGCTAAACATTTAGGAAAACCTTACAGAAGCAA
A_14_P110739 TTTGGAAACATATCCTTCCTGTTTGATGTTCAAAGGTCCTTTTCTGAAGTTATGATGGTA
A_14_P120603 AAATTTTTGTTTTGGCGCCCACATTCTGAATCTTTTTGTATTTGTCACTATGCTGTCAAA
A_16_P21639639
TTAAACATGTTTCCCGCTATGAGTTCTCAGAAATTTAAGAAATACACAAAGGAACCAGGT
A_16_P18446952
AGAGCCTTTTATGTTGACCAAAAGTTTTAGAAATCCTATCTGTCCTTGAGATCTATGCAA
A_16_P40937468
AAATGATAGATTCATTTCTTTGTAACCTGAAATTCCACCATAAAGGAAACCTAAACGGCT
CUST_1592_PI426210822 CCTGCTGAAATCCAGGCTTTGGGATTTAGAACCTGAGGGAAACAT
A_14_P131908 TATATGTGATATTTTAACAGAGACGTGATTGGCAGCAGATGGCTGATTAAGGAGGAACTA
A_14_P104027 CTCTGGAGATGCCACATGGAAAGAAACATTCTGGTTGGCAAGTATCTTCT
A_14_P101815 ATCAGTGTATCTTACTTCTTGAGTTCTAACTGAATGTCTGAGAACCTGTGTTCCTACTAG
A_14_P104362 CGACATGTTTCTCCAGTGACACCTCCCAGAGAAGTTGTAAGTATAAAATGAAAAATTATT
A_14_P132847 TTTACCACATCATTTTCAACAACAAGGCACATTTTCTAGCATTAACACACACAGAAGGAT
A_18_P22840877 GTCTTTTCTGCTGTGTTCAATCTCCAGTAAAATATGTCAGCACCTTAACCACTGTC
A_14_P132125 TCAAGTCAAAACATGATTCTCTGCATTGCAATTTGTTTTACTTCGTTGCTTCTAGCCATT
A_14_P114806 AAGTCAAAGTCAATTTCATTGCCAGTTTATTTTGCCAGTGTTGAAGATACCTATGAGTAG
A_16_P00685589 ATTAAGATGAATGACCTCATTGTGTGAGGTGTGCTCGAATCTGCTGAATTCCT
A_14_P103313 TTTAACAGTGGTGGTCTCTGCGTCAGAGTCAAAAGATATGATTTTATTCATTTTGCTCTG
A_14_P125422 ATGCATAAGCAAGAGTGTCATCTTCAGATTCCAAAGCCACATACAGCATAAAACTGGTAT
A_16_P15683473
AAGGAAAGGCAAATTAGACACTGTATCTCTACTCAAATTCACTTAGAATACCCGACATCC
A_14_P129740 CTAGAATGCTTGCTGGATTGAAAGGGAGTGCCTATCTATATTTAGCAAGAGACACTATTA
CUST_9396_PI426210822
CTTCATTTAAGAGCAAAGAAAGAGTTAAAAGCAACTCATATGAAGGGTTTGAGGGTTGTG
A_14_P135357 CTTATTAATATTTTAATCTGCATCCTGTGACCCATGAAAATCTCTAACCCGTGCTTTCCA
A_16_P18486587
AAGTCATCAAAGTCATCATCAAAAACATGCTAAAAGTCACTACTCAAAGATTGCTCCCTC
CUST_9506_PI426210822
AATGAAGTTCTACAATCTGTTGTTCTCAATTTTCCTGGCAGGAAAAAAAAAATCTTTTCT
A_16_P01412483

ACACATCACTTTCATCACTACGTAAATCAAAGCCATCATTCATGTTACATTGCTAAGGCT

A_14_P130301 TACGGATGTTGCAGATGTTTTACTTCAAGCAGGGGCTGATTTAGTAAGATATTTTTAATT

A_16_P15969210

GACAATCGTCCAAATTAGTCTTAAACTCTTTCCACCTATCTAACTTGCAAGGATTCCATT

A_16_P01388843

AAAATCGAACATGAGGATTAAACCCCTCTCTTACTACATAAGAATTTTTACACGCTCACA

A_14_P133244

AAGGGGCTTTTCCATAAGAACCAAAGAGCAAAAAGAAAGAGCATCTAGGTAAAATTCAAT

A_14_P124193 AATGTGCTCTCATTGTCCGCAGGGAGGTTATTTTGCATATTCTTGTTTCTTATATAACAA

CUST_9715_PI426210822 AGAAGGTTGCCCAGTCCATCAAAGGCTTTCACTAGATGCTAGTTATACC

A_14_P126497 AAGAGGGTTGCAGGAATTTTTTTTTTTAACAACAAATCAATGGTATGTGTCCCAATCTC

CUST_1366_PI426210822

CATCTCAGGTCTAGACAAGCAAAATCAAGTTGTATTTTTAAGTTTTTTAGATACGAGTAT

A_18_P22839417 GTTGGAGCCTTCATGTCCGACAGCGTGGTTCTTCTCAGGTGAGAG

A_16_P35549209

ACTAAGCCATTCATACCACAATCATTGGGATAAATACATGACATCCAGGAGAATGAAATG

A_14_P109985 ATAGACAGTGCCACTAAAATATCGATTAAACTGCTGATTCTAGAGTGAGCAATAGGGAGA

A_14_P118751 ACAACAGGGTAACTTCTTGACATACTGAACATGAGCCTATTCATGTTAAATATGCTTGGA

CUST_919_PI426210822 CTGCTGCTCAGACTTTGGAGGACAACTCAAAATGTTCAAGTCTAATGTCG

A_14_P134656 ACAGTCCTAACATACCTTTGAAGAGGCACAGTTTCTTCTCTCTGCTTTGATCATG

A_14_P122635

GAGGATAGGAAACTTCAGGAGGAAAATAAAGAGAGAGCAGAAGAAGTAAGATTATTTGGT

A_16_P19221069

CACATGGATTGCTAAAAAGAAAATATGGTCCTAGATCTCTTCATCAAGACAGCAAACTGA

A_14_P117908 TTTTTCAATCAAGGGAAGTGGCATGTATGGTTAATATAAAAGTGTAGTCGGACTGACTTG

CUST_9825_PI426210822 AATGGACAAGGATGGAAGGGCATTTCAGGAGGCAAGAGCAGCCTG

A_14_P116505 CATAAACTAATGGAATTGAAAGCCACTATTTGGAAGCAGAGTTTACAGACCACTTACCAA

CUST_1788_PI426210822

CTGTCTTTGCTTGTGTAGATGACAATTACAAGACTCTTTTCTTTAATTTATGCCCTAAAT

A_14_P130637 CAGAACTTACCACATAAAATGAGTTACCCTGGGGCTTAAAACTTAACCATATAAACCTGT

A_14_P136559 CACTATATCCTCATTTCTGTGTTTCTGTGTCACACATTCTCTGGCTCATTTATTTACTTA

CUST_6133_PI426210822 GAGAGGAGGTAGATGGGCAAAAAATCCACCAGCAAAGATAGCTGC

A_14_P132839

CATGGAACGCTGAAGTCTCTGAAAGTGAAGTTGTAAAAAGAAAAGGAATGAAATAACCGA

A_16_P41137901

TACCATTTGTATGCCGTTTGATGTTTTGTTAAGCCAAAAAAAAATGTTGTTGAGGTGCTG

CUST_5369_PI426210822 AGATGCTTGGACTGGATGGATACCTAAGTACCCCCAAACTGGCAG

A_14_P134292 CATGCAGATCCAGAGTTTCTGCAACCTGCAGTGGTTCAAGAGTAAGAAT

A_14_P107173 GTCTGGGAATCCCAGAGAAATTAGTCCTGAATTGAGTAAGTTTGAAGTCAAAAATAGACT
CUST_9966_PI426210822
AACTGCAACTTCCTGACACCAAAGTCCATACTTCTCTAGAATTACAAATTTCAGATGTAG
A_14_P124971 AGAATGAGTCATTTCCAGTTATCACCATGATTACCTTTTAATTTATCTTTAATGTTTTCT
CUST_5917_PI426210822
AAATAACCTGGAACTACATTTTGGGGAAAAAACTCACGAACATGAGCAGTGAC
A_16_P21431115
AAAAAGAAAGTGAGCATCTCCTAGAATATTACAGTGGAAGTATCCAACATTGAAGGCAGA
A_14_P123266 AAAAACTGTTCCTATTGTTGGAAAAAATTAAAGGAGAAAAGACCTCAGGTTAACCGCTTT
A_14_P131223 CATATGTCTCAGGGCACAGCAAATCAGAAGGTCTATACTGACCCTGAAGAAAA
A_18_P16274532
CTGCAAGTAATATGAAAACCAATCCCTAAAGTATGCTAAATAATATACCCATGCCTGCCA
A_14_P137827 CTCTTTGTGAAGTCTATAGGTCATGACAGGTTCAGAAAGATTCCTGAAATGAATGAAAAT
A_14_P124720 CTGGGATAAGAAAGAATAGCAGCCCTATAAGTAAAGCAACAGTTGAATCTGTAGCAAAAC
A_16_P01455604
CTTTCCCAGTAGAGCATTACAGATCAGTTAACAGTCTTCAAACTAGGGTTACATCAATGA
A_14_P105617 CTATGAAAACTAATACCAATCTCTTAAATTCAATAAACAAAAATTAAATACCATTAGGTA
A_16_P03601932
TGCTAGTGTATCAACATTTGGAAAATGCAGAAAAATTAGAGAAAGGAAAGTAGGGACTCC
A_14_P131372 GGAAACACAATAAAGGTGGCATACGAAGGAAAGGCTGGTAGCAGAGTTTTT
A_14_P132030 CCTTTTGTGAATCAACCATTCCTGACTTGTAAAACTGCAATCGTTCTGCTTTTTAAAAAT
A_18_P12877138
ACACTTTTCATACGCTTATTCAAACTGATGGCTATTTTTGTCCACTATTCCCATGTTCTT
A_14_P109135 CTATACCTTTTTTTGAATCATTTCCATCTCTTACTAATTTAGCAGGGTTGACAGCAATCT
A_14_P104874 GGTATGAGTTAATGAAATATTGATGCAGAAATTTCAATGAAGCAGGCCTGGAACACACTA
CUST_7207_PI426210822
AACCTGTAATTGCTTTCTGACATTTGAGCAAAAGCATCAGTAGCTCTTCTCATGAC
A_14_P136260 AGAACTTGATATACAGAGATGTAAAACCTGAGAACTTCTTAATAGGACGACCAGGAAACA
A_14_P123448 CTTAGTTTTCGCGAGATAACGGTCGAAAACGCGCTCTTGTCGATTTC
CUST_6177_PI426210822
ACACCCAACATTCTCCAGTACTATGATAACTATTCTCTATTGCATTCGTCTGGACC
A_14_P114953 CTCCTTGCATATCAGTTGTTGCACTTCCACGTAAGTCACTACGTATTAAGAGCCATA
A_16_P33138531
ACTGGCATTCCTCTCTTTAAAATTTCTACCGAACTTAATGGAATGGAAAAAGCAAACTCA
CUST_4311_PI426210822
TGTGCTCATGCTTTCCCTTGAATCTAGATAACCTGTAACTTCTGTTACAGAATGAGAT
A_14_P113773 TTTGAAGGGGATCTTGTCTCTTGAAAGGCTTATGTTACTAAGTCTAAAATCCTGTTTCCA
A_14_P137914 GTTGTATCTCCTTCATGCAGTTTTTTTTAATGGAAAGTAAAGAACTGGTGTTTGTGGAGT

A_16_P17131976
GGTGGTGGCTTTTTAAATACAGACAAACACATGATTATGGTATCTCCTGAAACTGACATT
A_14_P125823 CAACTTAAATGTCGCATTAGGTATCCAAAGAGCACAGGTTATAATTTCATGTTTTACATG
CUST_1474_PI426210822 TTTCTCGCACACGAGGGGAGGAAGTCTGCTGGGGCACTGGGCAGC
CUST_4701_PI426210822 CCTGGGGGAGGGGATGGTGCCAGGGAGCTCTAATTGTATTTTGCT
A_18_P22839782 GTTAATGGGCATATTGCTGGCTCAGTTGATACCTCATGATGGCATGAGGTTTAAAT
A_14_P138956 GTCTCTCCTTTTGTCCTTTCTCCATCTTCTCCTCTCTTACCTCTCAAAAATTATCAATAA
A_14_P107230 TAGAGACTTTTGATGATTAGTGTGCTAAATGAGGCAAGAGTTCTAATTCAGTGCTACCAG
A_16_P01155836
TGTGATCTTCTACTCCCAATTTTTTAAGAGTTGCATCTCTATTGAGAACACCTATTCCGT
A_18_P22841895 TCTAATGTTCTGTGAGGGTGGAACACCTAACCTAGAATTTCCCTGTGGAT
A_16_P16302497
AAAAACAAAAATCTGTGATCATCAGTAGCATACTGCAGCATTAAAAAGGACAGATCCCTG
A_16_P00416472
ATGTTACAACTCTGATTTACTTCCATTACCTCCATGAAACTTTTAGCAACACCTTGATGC
A_14_P115909
ACTTATTAAAATTCCAAAACAAAACATGAAAGAGAAAAAAAAAGAGGACTGAGGACCGGC
CUST_9538_PI426210822 AAGCACACTGATAACCCCAATATACCCACAATTCACACTTTTTTCCTTCTCTGA
A_14_P112571 TACAACATAATTTCTATAACCTGAGGGGACTCGCACATCATTACCTGATTGCTTGCT
A_14_P117241 GCTGCAGAGCCTGGTCATTTTAGTCCTCTGTCCACTTGTGAATCTG
A_14_P125757 ACTCCAGACTGATTAAAAATAAAGAGCATTATATTGAGTGCGCTCTCTTGAGCAGGCATT
A_14_P121404 GCCAGGCTGTGAAAGCATTTGTGAAGATTTTGGACAGTTTTTAAAGTAGGTTTTTTACTT
CUST_2843_PI426210822
TGAAGCAGAGAAGAAGAAACCAAGATGCAAGTTAGAGCAAGAGTGATTCAGAG
CUST_2846_PI426210822 TAGAGCAAACTCTGATTTGAAGGTTCATGAGCTGAGCGCAGAGGAG
A_14_P126130 GTCATTCTAAGAAAGTGATGTCTGCTTTTGTGTTAGCACAGATCGAACAATGGAAAATCA
A_14_P111239 AGAAAGTTGTTATTCAAAGGCTCTGAAGTTTTCCTCTTGAGAGTCCTTGGGTTTTATTAA
A_14_P127820 TTCTACTAAACTATTATTCATGCTTTTCACCTGCTAATTTTACAATTCCAGCAATCCAGA
A_14_P126460 CTGCTTACAATCCAGAGGGAACTTGTTCACCATCCATTACCACCC
A_14_P132558 CTAGAAATTCCGTCAAGAAAGGAATTACTCATTTGTTTCCTGTTTGTTCACAACATATGA
A_16_P02722916
ATTTCGAGTCCTTCCTATTAAAAGAATATTTCCTAAACCATATTCATATGCCACCACCAT
CUST_976_PI426210822
GAAGCCAAGGGCTTTGCTAAGTCCAAAAACTTAGCAAAACCAAAACATAACTGATTT
CUST_7074_PI426210822
GCTGAGATTACAACTTTCTTTTTCATTAACTATTAGTACTTAAAATATTGACATGCTAAG
A_16_P00828290
GATAGCCCCAACTCATAAAGATATGCCAAGATATGCCTAATGAGAAGACTATGTAATGCA

A_14_P117179 TGTCCCTACTTGGTCTCTAGTTGGAGAAATGATAGAAAAATGCAAAACAACTTATAGGGA

A_14_P102080 TAACCATCACAGGAGACATGTATGTATGTGTGTGGAACTCTGACGTGTATTTTAAACTTT

A_18_P12832262

ACAAGGTATGCCACTTTAAACAATGGATCTTGTATCTGGAACTGAAGTAAGTAAACAGAC

A_14_P127351 AAATTCTGTATACTTTCATTGCTTCCACAATTTTCTCTCCTCCCCATTATAGGCTAAGAG

A_14_P127276 TTTAAGGAAAGATCGGTGAGACTATTAGCCTGCATCTTTCAAGAAAGACTGCTTAGATTT

A_14_P136213 CAGCATTTATGGACACATGTCTGTCAAATTCCATCCCAAAGACTTTAAATTCAGTAGTGT

A_14_P113149 AACTACAGGAACATCTTCGTTCATCTCTGTCTCCCCACTTGTGAGATGTT

CUST_5376_PI426210822 CTTCTTTGAACACAGTGCTACTCTGCTCCAAACGCCCCCAAACTT

A_14_P109034 TTGCCAGGTTTTTGGATATATCATCTCAGTTCTAAAAAGTGTTTCTATGGCATGTCTTGC

A_16_P02028444 GTAAATCTGGTTTTACGGTTCTTAACCCCTTACTGAGACTACCTCTTTCGCAACTTTT

A_14_P114830 CTCAAATCACACAGCACCTTGTTAGTAGAATCTTTTTTATTCAGAAAAAAAAAACCCCAA

A_14_P134943 AAGCAAATGGGCTTTGAATAATTGCCAATAAGAAAAGGGCTTGCTATATTCCAGAAATTG

A_14_P118980 AGCAATGAGAAATCTAGGTTGGACAGATCATTTCACCTCGAGAGCCAATAATATGAAATA

CUST_9491_PI426210822 AAAATCACTGGGCATATTGCCTTCCATTATAGGACTAGACTGTGGGATCC

A_14_P111611 AGGGAAGATGGAAAAGAGGCAGGATTATAGGCTGAAGGGCTTTCATTTTAGAAAATAA

A_14_P101072 AGAATTGAATGAGAAACTGAAGCAAGCCACAGTCATAATTCAAGGGAAATGTATAAACCT

A_14_P113628 GATGAGGTAGCACATTAATTGCTTCTGGAAATGTGATGCTTTTAATGTAGTGGTTTTCGA

CUST_952_PI426210822 GTTCTTCCCTCACAGCCGAACTCATTTGGAGAGATTGATGGAGTT

A_14_P139042 TCTCTATGTGTTCCAATTCATTCTTCCAACTGTTTCATATGGCCCATGTATGTAGAAATG

A_16_P16191786

TGTGCTTTTCATCACTGTGTTTTTAAAAATGTTAACTGATGATTTCCTGATCCTGAGAGT

CUST_3491_PI426210822 AAGGCACTAGTCACACAGATCAAGCCACACAGCCAAATGGAAATACAAAT

A_18_P15338150

AAATGGTTCCAAGGGCATAGTAGTATGTGATAAACTTATTGATCACCAGATGTCACTCTT

CUST_7056_PI426210822 GACACTCACTGACCACCTAGAAACCAGAGACCCAGAAACCCAAAG

CUST_7683_PI426210822 CTCTGCTCAGGGATCAACAGGAGTGCTTGCTTATGTGGCTTCATT

A_14_P200172 TTGCATCCTTTTATTTTCTCTTGACTGGATTTCTTGAATCTCCTCTCTCTCTTTTCTTAG

A_14_P110832 CTTGTTAAGGGCAGAGTTGCAAGTTATACATAACAACCATACATACAAACATGCAGGATG

A_14_P134464 GTAGAGTTCAGGATGGGTGACATTCAGGTCCTATTGTTTTTGAGTCAAGGTTT

A_16_P19533020

TGGTTGTGGTGAATAAAGTAGATGTATCAACAAAAAGCACACTATATAAAGGACTGGGCT

A_14_P120577 TGCAAGGTCTAACATGTAATGCTGGTTTAGAGATCCTGGTTTAGAGACAATTCTAGTATG

A_14_P120203

CATTACAAAAAAACAAAACAAAGAAGACAAGGCCACAGGAATCAATTCACATTCATCAGG

A_14_P201260 AGTGTATTATAGAGGCTAAGAATGATAACTCTATGAAGGTTTATTATGAGATTCCCATAT

CUST_5648_PI426210822 AGGCCCGTGGGTGGGCTCCCCGGTGGGACGCAATGGCACCATCTT

A_16_P16458703

ACCCTCTACAGGTGCTATATGACATAGACATACACTTACTGTTTTGATCAATACTGGGTA
A_14_P111771 AATGTCCATAGTATTTGCTCCTTCACTTACCACGAAACAGTCTCTAGGTAGTTGTTCTT
A_14_P132289 AATGATGCTCACCGATACTGATATGTCCTCATTTCTTCTCTTAACACTGGAGTCAAACAA
A_18_P14806828
AAATGACATTGTTTCCCAATCAGATGAGTAACACTGCAAAATAACATTGTCCATATGGGC
A_16_P18616308 ACATTAGTCCTGGAGTTAGGAAGACCCTCAAGGGAAAGCAAGAAGTC
A_14_P111322 ATTTTGCTTAGGTCAAGGCTACTTTTTAAAAAGGGAGTGACTTTCATGAAGTCAGTTTGA
A_14_P133711 CTAAGTTCTTCGCATAGTCTTCTTCAATCTTTATCCTGCAAAGAGAGAAAACCCAACACA
A_16_P58424215 GGACCTTGTTCAATGCAGATCCTGATACAGCAGATCTGAGGTGAATTTCTA
CUST_2242_PI426210822 AGCACGCTGCTCTGTTTCAGAGGGGCAGCCTGCTCGCTGACTGTC
A_14_P130327 TTTTCATAATGGGAAATTCTCTTGTGTTTTTGTGGTGTGTCACTAACTAACCAGATCCAC
A_14_P135432 ACCATTTTGCTTTTCTGGCTTTCTTGCATTTGCATGGCTTTTCTGCTATTAATACATAGT
A_16_P16677154
TGGTAAAAAAAAAACCCTACTACGTATGACTCTAACCTGATACTTGCTCTCTAATGGCTC
A_14_P137247 CTCAGCCCTGGATTTATATGTCTCTTTGCTATGCCATCATAGCAGTATAAATGGTTAGTG
A_16_P18217803
AAAACTATGGTCTGAGGTCTAATTTTATCCATGACAACATGACACTTCAGGACACTAACT
A_14_P106974 AAGTATTATGTAATAACAGAAAGTTGTTTTTTTCTTTTTCCCCACATCACTGCCCCACTT
A_14_P108730 TGTCTAAAAGCACATTTTTCCTTGCTTCTGAAATCTAGAAGCATCCTATAGCCTTTGTCT
A_14_P128555 ATACTTGAACAATGTAAATCTGCGATGGATTACCTTCTTAGTTCTGCTCTTGTGATGAGA
A_14_P201001 ACATATAGCATATGAGTAACACAAATTCCACTTTGGGAGTTAAAGCATAGGAAGTTGCCA
A_14_P120635 AAATATGTAAGGCATGTTTTTTTAGGGTGATGGTTTTCTGATCCAGGCTTTCTGTTAGAA
A_14_P137829 ACAAATCTTTCTTCCTATCCCCCCAAAAAACTAGGGGAAATATTTTAAATTGTGAGTGTG
A_14_P127628 CTGTATTTGAACTAGAAATAAAACAGTCCTCATCACCTAAGCAATAGTTGTTCACTGGGT
A_14_P201414 CTAATGAAGATGTAAAGGAGTGGCTACTCTATGGCTACTACCTATCTATGCATAAAACCC
A_14_P117926 TATATTTTCATTACCTGTGAAACTAGTCAATCTTTCCCGTTTATAGGATGGTTCTTCCAG
A_14_P202563 GTCATTAGTTCTAGCCATAATTCTCTCAGTGTGTAGTGCTGGTGATTTGACAACAAAAAG
A_14_P101223 AACATCAGGAGAAGGAACTATGAGTTTCATGTAAGTTTCCTTTTTGGCTTGATTTACCAA
A_14_P118974 ACATAGAGACAGTAGGAACGCAGCAAAACAGGACCTACAAGGAAACAACTC
A_14_P115326 TCGACCTAGTGGTTTCTACTCTCACCGACTTATTCTGATTTCAGAAATAAAATGAAATGT
A_16_P01671421
AAGTCAGTAAGCATCCAGTCATCAAGTAGAAAATAGGTCACTTTTCATGGTAAGCATTCA
A_14_P113852 CTGGGTCATTACCCTTATCTATCATGTTTCCATGTTAACTTCAATGGGTTATTTGAATCA
A_14_P111904 ATGTAATACAGTCGGACTTTGCTTGCATGTTTTCGACTCTGGGAAGGAAAAG
A_14_P106858 CTTTGTGGTTCAGTGAGATGAGAAATCAGTCTCCCTCATTAAGATTTAATTCAGAAATTC
A_16_P16601986
AAAACCCTGCCCAGTAATTAAATGCATCTCACAAATAAAGTGTCACGAATAAACTTAGCA
A_14_P110324 GCTAATGGTGGAACAAACTGATGTTACAACATCTCCTATGATGTATTTCCCACAAGTATT

A_14_P101301 CTCTACTTGGTGAAAAAGTAGGTGGCTCTGAAAAGAACCTTTTTGGTTTGGAC
A_16_P36668815
AAATACCAGGATATCAAACCAAGTAGCAATTACCAAAAACATTTTTCTGATACCTCCCCA
A_18_P22606618
TTTATTCTGGATTGTGTATTTTCCTGTAGGATCATAAAGACTTTCCTTACCCTGATTCCC
A_14_P137655 CAACATTAATCCTTCAGACACCCCAATATCCTGCGCCTGTATAACTATTTCCATGAT
A_16_P20589443
ACTTTGTTGCATAACTCCCAACATTATTAGTAAATGCTCATGGTTAAATTGCCCTGTCTC
A_14_P104941 ATGTTCTGCTCCCTCAAAGCATCTATATGACTCCGGAGTTTTCCATGAACAT
A_14_P103463 GGAAATAATCCAGGGGAAGGCAGCAATACAGGCATTATTAACATGTGAGTTATTTACAAA
A_14_P117325 ACCTAAATACTGAAGGCATTTTTATGCAGATTGACTGAAACCTGAATCAAATTGGAAGGA
A_14_P139160 GAAAGAAGGTAGACAATTTAGCAATAAGCCAATGCAGATTTGTCCAGAACATGAATGAAG
A_16_P00007114
CACCCCCATTCTAAATCTTGAATCTTAAAAAGAAAAAGCCTCTGATTAAATCCTTTCCCA
A_14_P112806 TGGCGAGTATCAATGAAAACCCAGGGAAAATAATATTTCAATCTGTTGTCTTTCCTCAAA
A_16_P00233016
ATTAACCCATGAGATGTGCATAGAAGGTGTCTTCCATTTTATAAACTGACAGGTGACATT
CUST_10129_PI426210822 ATGCTTGGCGGCAGCAGGCCCTGGCTCTGACACAAAATCCACAAT
CUST_8583_PI426210822 CTGGTGGCATTCCCTCCCCACCCCCACTCTCAGCCACCACCATTC
A_16_P00713190
TGGCGTATAAACCTGATTAGAACCATAACATCATACCCATTTTAAGTTGTATCTGCACAC
CUST_963_PI426210822
TTGACACTTTGATCTCTGACCTCCCAGAATGCTTTTAGTTTAGTATGAAATGCAATATTT
A_16_P21244968
TGAACACAGTATACATCACGCTAATCCATTACTATCAATCATAGGTTTCAAGGTAGGCAA
A_14_P133524 ATGAAATCTCAACTTACCTCTACAATAAACGCTTTCCTCTCAGATTCACTTTCTATTTGT
A_16_P17192352
AAAAATCTGAGATGTAGAATCGATATGATCTAGTGGCTAACAGGTCCTCTTAATTGCTGG
A_18_P22842461
ACAATAGTAAGCAAATGTAAAAAAAAGTATTGCCATGGTAAATGCATAATTTAAAAGATT
A_14_P200583 GTTTGGATCTTTGCTGAGCAGTCCCATCGGATTTTTCTTTATTAAATAGGATTGTGTTTT
A_14_P200473 AAAAACAAGGAAAAGGGTGAGGAAATTCTGAAATGATAAAACCTCCATGAGTTTCACTAT
A_18_P14169858
GGATCCAGTTTCTTGAACGATTTATTACAGGCAACAAACTAAATGTTTTTTTCACAGTCT
A_16_P19419715
GGAATTGGAATGAGACAACTGCTAGTGATAAACTCATGCTATCCTCAGAATCACTTTTTT
A_14_P129275 CAGACTACTGTGCTTAAAAACATGGGATCCTTCAGGCCCATGATCAAGTT
A_14_P104892 GCCAGGAAAGAAGAACTTTCTGCATTTGACAGATAAAGATGGTGAACAACCTCAAAT

A_14_P127285 ACAGAGGAGATCTTAGAATTTTGAGGAAAATCTGGGACTTGAGACCTGGATTCTAA
A_16_P00940363
CTCCTTTAACCTCCTTTACATGACAAAATTTCCACTTACAAGTTAATCAAAACCCTGACC
A_14_P202556 ACCTGACCCCAATACCTTATCTATATTGAGATTGTTCATTAGATCCAAAACATCTTGTGC
A_16_P39528417
CACACAACCTCAATCTTAACTCAATGTCAGTCACATTCTACAAACTTGACACAATTCTCT
A_16_P18834566
AGGGTCAAGTTATCTTCCATCTAGTCAAACAGCATTGTGTTTTTACATCAAGAATGTTGG
A_18_P22841636
TTTACTTCAAGTTGTGACCTTCCAGATCCGAACCTTAGAGTTTCTGTATGTGATTTCAAA
CUST_6687_PI426210822 AAACTTGACAAAGCATCAGGCTCTTCGGGACGTGGTGAGTGTTAG
A_14_P202026 TTTAAATTACATACCTGCCTTCATTTAGGGTTTGAGATAAATGTCCGGTAGCAATCTGCA
A_14_P137562 CAAGGACATATCTTGTATTTCAACATCAGGCGAGAACCACTGTCTCTGATCAAC
A_16_P19282615
TTTAACCCAAAAAAATTGCTTCAGGAAAATACATCTGTCAGAATATGTCTCTTGCTCGCC
A_14_P127076 TCAAGATGGTTCATATTGGGAAGGAGACAGTATTTTAAGCCATTTTCCTGTTTCAAGAAT
A_18_P13105171
GCTCAGTGGTATGATATGATAAGTTGTGCAGAGAATTGACTTTTGATTAATTCAGCCACA
A_14_P138233 GTTGCTGAACAAAAGTATCAGTCTACCTTGTGGTAAGTTTCCTTATCATAGGAGAGGAG
A_16_P17840605
ACTCTCAATCTTTTTGTCACCGAAGTAATTAGATTACATTACCAAATAGTTGCCCAAAGA
A_14_P137941 GAAAAATCTCTAACCTTAGTGAGATGAATTTTGTAAAGTTCAGTGAATTCCTTCCATGCA
A_14_P102637 CTGTTTCTATGACACCTTTAGCACATTTCTCCTTTCTATTAAGTCTCTTCCTTCCTTCTG
A_14_P138640 AAATAGTGGTGGTTCATCTACATCCCCAACAATGATCTTCAGCATAGTAGCATCTTTAAA
CUST_193_PI426210822 TATTGTTATGAACAACAACAGCCGGCGTTTACTGAGCACTGCTCCTAC
A_14_P123222 ACTTTGGGGACAGTTACTGTAAATTCCATACATGTTTTGACACATCCTTCTGTTTTGCTT
A_18_P15082822
CCCTTCACCTCAAAAACTATCCATATCAAATCAAGAAAAATATCCATTGAACGTCTCTCA
CUST_4131_PI426210822 TTAGGGAGTTCCTTCCTTTCACCCATCTTTTTCTCCCCATGGCTT
A_14_P132723 CACATGGCCTGTATTCCCTTGTGCTAAAATTAATTTGTAAGATTGTCCCACATTTTTCTT
A_16_P03719723
ACTTGGCTAAGGAGTAAAGTTTGAAAGAATCATGTCTCAGGAATTTTAAAGCACTCACTG
A_16_P39073069
TATTGCCCATCACAAACTATCTTTACATGTTCCATTCTAATCAGGTTTCCAATGCAACTG
A_14_P106096 TTTACAATTTCATTTACAGCAAGCCCTTCTTGGTAGTTTCTTTGTGTGATTGATGCTGAA
A_14_P118073 GTGTGTGTGTGCTAATAATCCACACGCACTGCAGTAGAATATGGATCAGCAAAAAA
A_18_P15535921
AAAAAGATGCCATTGGTTAGAAATTCTCTCAATTTTCTGCTACCAATCTATGAACCTGCC

A_16_P21200626 AGTTCTCAGCCCAAGTTAAATGACTATCCTATTGGTTCTCTTACAGATTATCCATTCTGG
A_14_P136648 GTAATGCTAAGACCAGTGATGATGGCTCTAGCCCTGATGAGAAATATCCTGATC
A_16_P15842535 CTCTTGTATTCCTATGAAGTCATTCTCCCTTATTGACCAACATCATAACCTTACCACATC
A_14_P102951 CTAAGCCTGATCTCCTGTAAAAACATCATTTCCCTGATGGACGTATCCTTCCAAATATTT
A_14_P139372 GGATAACATTTATTTTGAATACAGCCATGCTTTCCAGGCAGTTACAGAGTTTTATGCAAA
A_18_P10923027 AGCAAGGAAAGATCTGATAATGTGGCAATAACAATACACTACATTTTACAGTCAGTGGCA
A_14_P110849 ACCCTCAGTCATTGTTTTCGGATTATTTTAGCAACAAATGAACAGATAAGTGACTTTCCA
A_14_P110447 AGTCTGTTCCCAGTATTCTAGAGGAGTATTTGCCATTTTTGGACTCTATGATAAGAGGT
A_18_P22842448 CAAGATAGCAGAACTTGTCATTTCTAGTTTTTCCACAGGACCATAAAGCAAAATTGGTGC
A_18_P22839776 ATGTTTCTAAAGCCAATGGGGTGGAGGAAAGCGAGGGCATCCTTG
A_14_P122042 AGACACTAGACATTTCTACCAATCGTTTGCTAACTTTACCCGAGAGGCTTCACAT
A_16_P19505641 TGAAAGCATTCTCAACATACCAAACTACAAACATATCAGCCATAAATTTCTGCAATCTCA
A_16_P01808431 CTTTCCCTTTGTAAGTTTTCTCTTTCCACAGAAAAACCTAATTTCTTAACCTCTACCGTG
CUST_1566_PI426210822 CAGGGAGCTGTGGGGTAGAGGAAGGAGGGTTTGAGCCAGGCTTCT
A_14_P134346 ACATTTTATAGTATCAGCATGGTCTGGATTTTGAGTCACCTTGTCTTATACTCTGAGGTT
A_14_P108270 ACATATTCCTAATGTTCACTTTCACGGCTGTGTTACACATTTTGGAAGAGAAGTTATGAG
A_18_P15354957 CACCTGATCACTTGCTACTAGAAAATACTCCAGACAAAATATCCTTATCAGTTGCCTAGA
A_18_P14131934 AAGATTTAGCAAAGGGAGCCAAGAAGGCAGCACTAGAGCATTTGTGGATAAT
A_14_P200039 GCTCCAACTATCCCTCTGAGAGTAATGCTTCACCTTAGTTATGTCTTTGCTATTTTCAAA
CUST_683_PI426210822 AGGATTTGGGAAAAACCTTTCATCTGACCAGCTGATCAGAAGTTTTGAACTG
A_16_P19790207 AAAGTTTCAAATGTAAAGAACTACGAAAGTCTCTTCCTTATCCTACTCCAGACTCCAACT
A_14_P133472 TTTTAAAATTGTTGCTATTTGCTGTAGTTCCCCTGTTATTCCTAAACTCTCAGGAAGCAC
A_14_P126180 GTTAAACCAAAACCCCAGGAGAAAACTTTCTGGTGACAGAAGGGCATT
CUST_5520_PI426210822 AGGCATGCAGAATAGCAAGCAGCCAGGCATCGCCTTCTCTAAAGT
A_14_P132033 AGGGCCTACTTTAATAGTTAATGGAGTAGATGCTAGCGTTCGATGAACATTAGGAATTTT
A_14_P111617 GATGACATTAAGTTTTATAGTCCCTACCATGCTAACTCTAGGCCCAAATTTGTTGACTTT
A_16_P19917455 TGCATTCACCAAATAATTCCCTTCATAATATAGCATTCCAATTGCCCATATCAGTTGTCC
A_16_P36248829 AGTCCAGTTTGTCAGTTATCAAAGTGAGTGCTTTAAAACTCTATGTTAACTTAGTGCCCC

A_16_P40649515
CTTCCTTCCAAATTACATCAGTTTCAAGAATATTCTCCGATCACTATCCCTGTTTTGTCT
A_18_P22843222
ATTCACCTACCTAATAAATAGGCATGAAAGTATATTTGCCACTACACAGGTGGAAGTCAC
A_14_P115836
AAAAATAGGACTGAAATCAGGAAAATAAAGAGAGCAGATCCCCAACAACTTCGACAAGAA
A_18_P14134273 GAAGATCATCACCAGGATTTGAGGGAATGCTGTGGTGCATACTTTGTGTT
A_14_P134044 CTTCTGGCTCCAGAACAGATCCCCTGGATCAGAAAGAAAGAAAAAACA
A_14_P102540 ACAATACTTTTCCTTTTACAAAGGGCTTTGGTAATCCTCACCTCATATGGACTTAAAGTT
A_14_P106898 AGATCTTTGGCTTCACCGTTACCAATGGTGTCTGCATCTCACTCATATGT
A_14_P138637 TATAATGAACTACACTTCTCATTTAGGTCTTCACAGGGATCTTCACAGCAGAAATGTTTC
A_16_P00924534
GCACCTCTTTTAACTAACCCGTCAAACCTAATAATTGGCCAAACTATGACAATTACATCA
A_14_P202531 CTTGTCCTTCTCAAATAGTGATCGGCATATATTACAATATAAGTTGTATGTGAAGTGGTC
CUST_795_PI426210822 CCCACCCGTGGTTGGGTGAATACTTAGCCATCTGCCTCCTATTTT
A_14_P100948 GAGAATTTCAGGGTGCAGTATACAGAAACAGAGCCGTATCATAATTACCGAGTAAGTATG
A_18_P23776966
GGATTTTCAGATGCCCTAAACATAGTCCAAACCTCATTATCAGTGCTTTTATAGATGAGG
A_14_P139568 GTAATTACAAAGGATTCTTATCTTGTCCTTTGGATGTTTCTCAGGGACTGGTTTTGGAAA
A_14_P129517 TACCTCTTCTTCTTCGAGTAGAATGAGCCGAACCACAACAATGTGAATTGCATT
A_16_P00685413 TATGTTCTAGTCCGGATCTGCATTTCTGGTAGAACTAGTCTGTCTGCAGC
A_14_P131662 TATAAAAGCAACTTCTTACCTGTCTGTCCATATGCAAAAATACACGCATTATACCCCTGA
A_16_P19757734
TCTACTGCTTTCAGTTATATACCCAATGTGTCCGATAATGTTTCTACCTCAGATTATGCC
A_14_P138813 CTCTGTTTTCTTCTTTCCAAAACTGTGCACCCCTGGATGAAAGTAAGTTATGTG
A_16_P30952548 ATAAGAGGCTCCAGCATCATGATTCATTCTTGAGAATGACTCCTCTGACAGG
CUST_10106_PI426210822 TTGCATAAACGTGAATCGTGACATGGTACTGAAAGCTGAGCAGAGCAG
A_18_P22839803
TTTGTTTAACTTGGTGTCCCATGCAGTCTATTTAATTTCAGACTCTTATTTACCCCAGTC
CUST_6118_PI426210822 GATGAGAGCTCCATCCATGAAAACCAGTCTTCCAGAGGAGGATCC
A_14_P135305 AACCTCCAAAATTAAACTAAGGGTAAGGCCAATGCATAGCGTAACCTATATGTTTGCTAA
A_14_P119364 ACCAGAAAATGCTAGTTACCGATTTGGAATACGCTTTAGACAAGAAAGTAGAACAGGATC
A_14_P117951 GAATTTTTTTAAAAAAAGAAACCATTTGGTTTTCTGTGTGGATATGAGACAAAGTAGTAT
A_14_P139533 TATAGCACTTCAATGAATCACAGACATCATCAAAGAACTCTAGCCTGTAGAATGGATCTG
A_16_P18445140
AACTCCTGCCAGAAAAAGTAATGAAACCTGATATCCACCAGAATACAAGAGAGAGTTTAA
A_14_P115911 TTGCCTACGTAACAGAATCACTTCCGCATTTGAAATGAGTATTACTGCCACATAATTGAA
A_14_P200194 AATTTAAACTCATACAGGAATATGGGAATAGGTGGGATGAAGGTAGTCTATCTTTGACAA

A_14_P122367 CTCAATGACTTAGGATGGGCATGTGCTTGTAATGTAATGAGAAGAACCCTAATTTATTGT
A_16_P16191794
AGATCCAACCAGAATTTTGAAATTTTTATTTGACTGAGTAAGGTGGAAGAGAGACCAGAA
A_14_P115921 TCCTCTTTATTTTGACTTGACAAAATTTGTCTGAGATGAATGAGAAGAGGGTGAGAGTTT
CUST_595_PI426210822 ATTACTGGTGGTGAAAGCATCCTGGCTGTCCCTGAATTACTGTGA
A_14_P202471 AACTGGTGCATTATTTTTTGCAATTCTCTTGGAAAGAGTTCGTTTTTAACTTCTGCTCAG
A_16_P19327473
AAAGACAGTAGTTTTTAACCATTTCACCAATAAGAACCTCCTTATCATTCCCTCTTCCAG
CUST_1930_PI426210822
TCCAATCCATACCACCAACTAATTTTAAAAACAGGAATAAAGGAAATATTTCAAGAAACA
A_14_P137244 GGTAACAGCACTACTCTTATTTAACTCTTGGTGCAATTCTTCATTTTCTTTCACCACTTC
A_16_P00685473
GTTTAACAAACACATGTCAGAACTAGAAAGTTGGAAGTGAGACTTCAAACTCTACCTAGC
A_18_P14136772 AAAATGTGAGCAGAAGTATGGAGAACAGGACTCCCATGAAGATCTGCAG
A_14_P131166 GAGTGGGTTCTCCAGTGCTTACATATTGTTTTCTCTCTTCATTAGTCTGAGAGTCAG
A_16_P16628494
AAGGACAAAATGACAATTCTATCATGAAGTTTGCTCTCAATAGGTTTGTAGTCTGGAAGG
A_14_P112799 CTGCTACATTCATCTTTTACAATAACTGTTTCCATTAGCAGGAAAAACAGTGTTCAACGC
A_14_P110604 CGCAAACTCATATCTTGCTTGAAACTTGTCGATGAACTCCTTTAGTATGACAAGAACAAA
A_14_P113922 ATTTGGTTTTTGAAAATGATATTGAACTTGGAGATAAGTGAGAATACATGACCCCCACAC
A_16_P00685587
GTATGATCATCATCATTATTATTTTTAGGCAACAGAACCCATTTCTAAATTGAAATATGC
CUST_6979_PI426210822
GACTGAGACAAAGAGAATATGTCTGTATACAACTAAAGAAAAAGAGAAAAGGTTCGCTGG
A_14_P115735 AGAAAAAAAATGTGTCTGTCTCATCCACAATATGTGATTGTTGAGTCCCTGAGCCT
CUST_4660_PI426210822 ATTTGTGTGTCTCAGCATGGCAGGAAGGTCAAACCGCTGCTTCCT
A_14_P136386 AAAAGGGCCATTAACGATATTGCAAGGAAAGGCCTGGCCTCACTTAAC
A_16_P15883553
TTTTCACCCTATGTTAATGCCTAATTTAAGTCGGATCCTCCTAATATACTCACATACGCC
A_16_P21582579
GGACAGCAAAGATTCAAAAAACAATTACATTGCAGCAAACTATTTTGACTAGGCTTCTCC
CUST_9727_PI426210822 CTGTGCTCAGTGTGCGTGTCCATGTCCCATTAAGATGAATGACCT
A_16_P16024979
CATCAAATACCTCATTTTCACCAAAGTCAACATGTTTCTCATCTCATTACATTTCCCCAG
A_16_P01856516
TGATCTGTATGCTATCAAAACCCTCTACACCATCATTAAAAGAACCCCAGGAAAAAAAAT
A_16_P21209773
AGCAGGATAATGTTTAGGTGATGTTCAGAATGATGATCTTCTAATGATCAGAAGTAGGCC

A_14_P134370 TTCTGCTTTGCTATTATCCAGAAGGTGTGTAGACATTAGAGGTATCTGCATAATATGGCT
A_14_P201985 CTGCTCAAACTCCAGTATTGCATTTGAGTTCCAGATGTAGAGAAAAGTAAGAAGTAAGAG
A_16_P15374716
AAAATATGACTTAAGTGTCCATGGTAAGTAGAAAGAGGCTCAATATGTCCATTCACTGGC
A_14_P123017 CTTCACTGCAGATATCTGGAGAATTCCTGCAAAACTGATTACTTCTTTTTGGATTTGCTA
A_14_P116281 CTTTTAGGTGATAAAATGATTTCATCTTGGATCTCTACCATGACTTGGTCTCCATTCTGA
A_14_P128171 CTTGCATGTCATTTATTTCAGATACTGGAATTCCAGATATTGAAATAGAAAACAATGGGA
A_14_P116992 GCTGGAAAATATAATTTCTGCCAAGAACGACACAGATATTGTGAATGATCTATCACTCTG
A_16_P37072639
AAAAGAATTTGCTCTTTAACAGTTCCTAGCATCTTTCCAACACATTTCCCATTACGTGTT
A_14_P137609 GACTGATTTCCTTCTTTACTCTCTCTTTGGGCTGTTTCCATTTCCTTTGCATTGATT
A_14_P134572 TTCAGGTGAGTTTTTTACTACAGGCTGCTGAACTATACTGCAGATAAGTAGGCTTATGAA
A_14_P200683 TTACAATCCAAGGCTCAAACATTTGGAATAAAGCCTCCATGTTAACGATTTCAATGCTTT
A_18_P22840518
TGAGATTTGAACTGGAAGAAATGCTGTTCTTTCAGTTAACAGTTGTGTTTTTTCCAAGAA
A_16_P18010898
AAAAGTCAGGTAAGTAGAGTTTGACCCTTTGATTCAAGAACCTAGCCTTTTTTATGACAA
CUST_220_PI426210822 GCGCTGGTGGTCGCGGGGATCCCCGCGGGCGCCTACAACCTCGAC
A_14_P127469 AAACAGACATAATAGCCTAGATGAACTCCCAAGAGATCTATTAAATCTTGTGGGCTGAAT
CUST_171_PI426210822
TAATACATGTTTAGCGTTTTATGGTTAGGAAATGCTGTCCTGAGAAGCTCATCAATAGGT
A_14_P107411
CTGTTGCACAAAAAGGGGAACTTGGTGTAGGAAAAAATAACTAGAAATGATAACTACGAG
A_16_P17211111 ACCTTTTTCCCTCTCAAAAATACTGATTCCATTAAACTAACACTAAGATCCACTGGTTCA
A_14_P133591 AAGACAGCACAAGGTTTTGGCAGTAAGGGATAAGGAGAAAAGAAATCCCAAATAG
A_18_P10390241
CTCCAGATGAAACGAAAGATCTTACAGACTAGTGTTAATACCCCATACTCTTCATGATGA
A_16_P18204787
AAAGAACGGCTTTTTGCAATGTAGAATTACGGCTCAATGTTACCATGAATTAAGTGAGAG
A_14_P104727 TTTGGCAATGTCACCTTCCACTGAGGTTAAGTTTTTGTTTAGTTAAGATAAGAAACAGGT
A_14_P100096 CTCTACTGTTTCTCATTCAAAGGCTGATGTAACCAAGATTTTTATCACGACTTAGGTCAT
A_14_P105749 GGTTGTGATTACAGTCATTCTTGTCTTTGGGTAGTGAACATTAAGTCAAGTTGAAACATT
A_14_P116106 ATCTCCATATCCCAAAAGAAAGCAAATATGTGAACTGTTGCTAAGAAAAGGAGCAAACAT
A_14_P116806 GCAATTCTTAATATTATTCTGCGCAGCAGAAGGGGTAAGTGGTAACATCTCACTAATCAA
A_14_P101941 CTGGTTTTTTGGACAGTCCCTTTGCAAAGTGATTCCTTATCTACAGGTAATTGTTTTTAA
A_14_P127644 AAATATCCTGTGCTACTGTGAGATCACATCAAAACTATGTGTCCTGATTTTTGACTCAGA
A_16_P21483941
ACACAACACTAAGTTAAAAATTTCTATACGCCCTAGGTTTAATATGAAAGTCTACAGCCC

A_14_P114228 TCCTGTTTTCTATTCAGAATGACATTTTGTTCAGAAAACACAGATGACCTACTACTTCGC
A_14_P101197 AACCATTTCTGCTGTGAAAATCCCAGTAAAGACCTAAAAAATAGAGATCAGCACTACTTC
A_14_P112161 TTTTTTCCCGTGTCTTCAAAACAGGAGAGTGATGGTTGTATGTTAGCAGATTTTTTCTAT
A_16_P15744496
TCTCCTTACAGTAGAAACTCCTTGTATAAAACTCCTTCAGTAAGGCTTCACTAAACAAAA
A_14_P131900 GGGAAAACAGATACTTACTGAAAGTTATGATTTGGGCTGTGCGTTGGACCTAAAA
A_14_P106935 TTTAAAAGCAAGTAAATCTGTGTGCCTCTCTGAAGAATAGAGGCATTCTCCCTTATAACC
A_14_P113441 TGCTCAAGGGCACGTTTCAACCCATAATATAAATGGCAGTCAGTGTGATAATATACATTT
A_16_P01211838
AAAAGTTGGCTGATACTTAACTCATTCTGTTAGACACTTCTCTTTGGAATAGACTGGAGC
CUST_3347_PI426210822
TTTAAATATGTAAGTATGGTCACAGCATTGACACTTCAGTGGTTATCGTAAGCTTATTTG
A_14_P104982 AACACATTCGCAAAAGAGTGAGGACTGGAATATCTTTGATGTAAAACAAGCCAATGCTAT
A_14_P118612 GAATTTGCATGAGCCCAAGTTGTTTTTCAGAAAAAGGGTGTGACTCTGAAAATGAAC
A_16_P19687608
ACCTTTCTGTTCTGCTTTAAATTAATCAAAGCCAGTCCGATATTAATACTGTAGGGGGAA
A_18_P22841253 AAATATTCCATGGAAACCTTGGACACTGTCTGATGTCAGGACTACTTATGAATGC
A_14_P115712 AAAGTGGGAACTGTAGTTTTGCTTCAGACTGGTTTAATGATAATTGTGAATGGATTTTGA
A_14_P108618 ACTGACTTTTTAGAAGGATTTCCATTTGTTGAATCTCCGTTAAAATAAAGTCCTGGATCC
A_14_P122258 GTTTGGAGTGTTTTTCCAGAATATTTAGAAAGAACAGCCTGTAAGAGCCAAGATTTAGAC
A_14_P138035 CTCTATTCTAGACTAATGAATCAACAGCCCAGGCATTCTTTTTTTTTGGTAGATGAGTGT
A_14_P130680 GATTTGGACACAAACGCATGGTTCACCTTCCTGTGGAAAAATCCG
A_14_P104728 ACTAGCCAATTTCACACAGCACTGTTACATAGATTTGATTACCACAAAAATGTGATTCCC
A_14_P113152 TCTCAGGCCATGGACATTTTATAATTTTTTGAGGTTTGGGGCTCCTAGAATCTC
A_16_P36230378
AGGCCAATAACGGTATTATTGTTAAAAACAAAACAAAACAATCTACACTAGACGAGGGAA
A_16_P16382267
CAAAAACCATCCCTAAACTATTTTTTCCCAAAGAAAAACCTTTAATGGCCATAACAGTGC
A_16_P18063253
AGAGTCCTCAGAGTTTCAATTAGCAAGACTATTTAAACTGCAACTGAAAACTACTCAGAA
CUST_8231_PI426210822 CACGGTCATTCAGTGCCGCTGGCATTGTTGGAAAGCAGGACCCTG
A_16_P00527064
CTCGATACTACCACAAGTCTCAAGAGTATATCACCTTGAATCTTTACTGCATTTGAGTGG
A_14_P130509 TAAATGCATTCCTTTTGCAACTTGCATCCTTCTCATTCACCAGAAAATCATGTATCAGTT
A_14_P124761
GACAAGAAACTCCTAATTGGGTTTTGAAAAAGTCTAAAGAAGTAAGTAAACTGACCACTG
A_14_P122629 GTGAGTAAGCAGAGAGGAACTCATATTAAAGCATATTTCGATGCCAGCATTTAATGTGTC
A_14_P137871 AAAACATTAAATCCATTCAAATGGCATGAAATGGTCCCTTGCTTTCTGATGGAAAAAGAA

A_16_P18233041 CCATCCTTCTGTAGTTACCTAAATCTCACAATTCCTAAGTATAACCCATCCATCTTCACT
A_14_P202432 GCTGCATTTCTCCTTATTTCAGAATCTGAAGACCACATGATTTGTTTTCCCGTAAAACTT
A_18_P12308351 TGTGTTACTTATAATAGACACTTCCCAAAACTAAGAGTATGAATTGATTACCACCTCACT
A_16_P17503364 TTAAAATCACTCTCTCCCACGAAATTGTATTTAAAATGTTCAGTAGGAAAAGGGTTTGCC
CUST_6615_PI426210822 CTTCACGGATGGGTAATGAGGATTCCACCCCCCCAGAAATTAGTA
CUST_2353_PI426210822 AGATTAATAAACTTCGGTGATTCAGCCCACAAGGCAGGAGGAAGAGA
A_14_P129270 TTTTTTCCCTTTGACTTTAGTTATTTCAAAAACTTTCGATACTGCTCCTATGCTCCCCAT
A_14_P124775 TTCAAGTAAACATAAACCACCAAATACTTAGAAAAGGCTTGTAAACGAGTGATCCGAAAG
CUST_2120_PI426210822 CATCCTTGTGTGAGAGCAGACACTGAATGCTTTGTTGCAACTCAACTTG
A_14_P125943 CTAGTATTCTTTTCAGAGGAAAATTCTGGTATGTTAGGGAAAAATCACATGCTTCAGTGC
A_14_P122760 GTGTCTCTCATGGAGGCGTCTCTAACCAGGGTAGGAAAACAATAGTTCTAA
A_18_P11339161 GACTCTCCACCCTATTCTTATCATTACATTCCACTTTCTTCATTTAACTGCCTACTTGAC
A_14_P118322 GGTTTACCATTCAGCATAGGCTTTATCCGTAAGAGGGCAACACATTATTTGTTGAATTTA
A_16_P37948583 GCATCCAGATCAAACTATTTCAACTCATTATACCATAATTTCCCTAACTATCTCAACCCA
A_16_P21483824 AAAAAAAACCTTTTTCTGATTTCGTATGGTTCAACTTGATACAATCCTTACTTGGCAGCC
A_14_P130434 CCAGCAAAAGCAGGTCTTATTTAGTTGTAAGTGGAAAGGAATACTCTTCTCTGCAGAA
A_14_P201649 CTACTGCAAGTTCAGGTTGAAAAATATCTTCTTTTCAGAGCAGTTGTTACTGAAAGAAAA
A_14_P119222 AATATTTTTGTTTTGCTTCAGGACAACGGGAAAACCAAACGATTATGTTTTGTTTGCAGA
A_14_P101997 CTCCACTTCTAATGCTAGAGAATGATAACTAAGACTTCTGTGCATTTGAAGGTTGTTGGA
A_18_P22842103 TACTATACACCAGGGACTTTTTGCATCTCGTCCGCATCCCCTCTT
CUST_3914_PI426210822 ATCACCTCTCACTCTCATTTTCTAATAAGCCTGAAAAGCATGATGGAGTCATAAATCATG
A_18_P14133912 AGTTCCACTGCCTCTGTTATCACTACTCTCTGTTGACCCCATCTC
A_16_P30952635 GGCCAGCACCACTTCCTTTAAAGATGAACTCTGAACTTTGGAGAGTG
A_14_P115843 TCTCAACAATCCAAAGCAGTAAAACTATTACTTCTGTTTGTGAGTGTGGAAACCGAATTT
CUST_736_PI426210822 AAGGAGACACTCCAGTGAGGGGTGGGTCTCCCTGGCTCTTTGTAC
A_14_P118788 CTCATTTATGTCCAGGAAATGTGTTCGTGAGTCAGGCCCTATAAGCATAATTTCATTATT
A_18_P17289811 CCTCTACGTGTGTTCATAGAAATATTTTATGTCCTGAGAATAACTTCAGAGGTTGAGAGC
CUST_6403_PI426210822 GTTGCACTTTAGGCTTTCCTTGTCCAGAGTTCTCACACATGACAGTG
A_14_P100067 ACTTATGTTGAAGCAGGAACTCTCAGTATACTTCACCTAGTATAGAAATGGTCCCAGATA
A_14_P108357 GGAATACTTTCAGTAAAATTCTTCTAGAGGATTAGTTCACAGGTCTGTGAGAAAACCAGT

A_18_P14139288 ACAGCACTGGTGGCTGTAAGCCACTCTGATGTTTCCTTTATCCTCAA
A_14_P117368 AAAACTATTTGGGCAGCAAACTCTTACTACTTTTAAAATGGTATGGCATCACCTAGAAAT
A_16_P03259037
AAAACCATGTTGGATGTAGGTAAGGATATAACATGACGTGCTAAATGAACAAGGTGCTTA
A_14_P134525 CTTTGATCTCTAGGTTCTAGACTGACCTGTTTATGGCATAGATATGGCTTATAGGCATTT
A_14_P110150 TATTTTGCAGAGCATCCCCCAAAGACAGATTGAAGAAATGCAGAGACAGGTAA
A_14_P134076 CTTTGTAAACATAATATTTATAAAATCAAAGCGGGAAAGCCTTGTTCCTTGCTAGAAATG
A_14_P132326 TCAGAATTACTTGATCAGAAGGACAAAACTGGGACATCTGATAAGAAGTAACCAGAACAT
CUST_3479_PI426210822 GCTCCAGCCATTGTATGTGTTTTTCATGCAGGAAGAAGTGGGACAT
A_16_P39243515
GTTCACATAAAACAGTTCACAGTCATAAAACGGTTCAGATAAACGTATGCACTCCAAGAA
A_14_P123661 ATGTAAACCTCAAACTATACTGAACCTGGACTTTTAATTTTGTTTCCCAGAGTGCAAACA
CUST_10285_PI426210822 TTAGGAACAGGACTGGAGACCCTGCAGTGTTAGAGTGGGGCATAG
A_14_P103811 CTTGAACTCAAACATGTTCCTCTGCACGAAAGTCTTGCGGATCTTCTG
A_14_P108834 CTTTACCACACGTAAAATGGTTACAACATAAAACCACGCAGCACGTTAGTGGG
A_16_P30952432 AGGATTAGTAGTTAATGCACGTATTTGTTTATTGTTTCAGCGGAGGGTGCAGGAC
A_14_P201790 CTGCTGCTGCAGTTTTTGTTTCACATATCCTCAGAGAGATTCAAAAAATTTCCAACTTCT
A_16_P21050682
TTGAAGGCACAATTCTATTCCAGATTATAGACAAATCAAAATCTTACAGACCTCACCTGG
A_14_P129531 CTCATCGAATAACTATGATGCAAGGTCATAGACAGTAGATGTCCTAGGAATGGCC
CUST_6496_PI426210822 TAATTCATTTACCAGCATGCATTCAGGAGGTGCCTGACAGTCCTCA
A_14_P122554 ACTTATCACAAGGAGACTTATTTTGAGACTATGTTCTAACTTCACAGCCCACATCTTGAA
A_14_P133647 AAAAAAAAAAAACTGTAAAAACCCTCTTATGGAGGACAGATTGCTAATCAGATCTGCAGG
A_14_P202308 AATCAAAAACGGGAATCTCATTTAGACTTTAATTTTTTTGAGATTATCGGCGGCACAATC
A_14_P120007 AAACTCTATTTGCCAAAGGAAACTTGCCTTATCACCTTCTTGCTTGCAGGTAAGT
A_16_P21209576
ACCTCAGTGAAATATCCTTGATTTTATCCGAGTATACAGGAATTTTCAAAATGCAGAGGG
A_16_P20761061
AACTGAAATGTCTTAAGGGTCCAGAACATATTGAAAATGGGTGAATCAACTAACAGTAGG
A_16_P01786132
AATCTGCAACTCAGAACATATTTTGAAACTGGTTTCAGATTTATATTGGTTAGCCCCCTG
A_14_P116262 ACACTATTTCAGGCTTGGCATATTATCTGATACAATGAAAATGTCAATAAACATTATTGA
A_14_P129130 ACAATCTAAAAACTTCAGTATCACCCTATCAGTTATTAAGGCACACTACAAAGCCTGAGT
A_14_P101964 TATGGAATTCAGTTGGGATTGAATATTCTGTGTGAATTGTGAACCTGTATGCAGTAATCC
A_14_P108223 CTCTCCACACATATACTTTATTTCCACAGTCCCATTTTCCCATGCTGAACCTG
A_14_P115393 CATTATAACCACCTAGTATATTCACTGAAGGTGATACCTGAAATTCAAGTGATCCTCTGG
A_14_P135865 GACAATGCAAAATGTAACTTGTGTGGCTTTCCATTGCTTTTCTTAGGATCAAAAATACGT
A_14_P202195 ACAATGTAGACATCACTAAAGTCTAGATTTAAAAGTCCAGTGAAAATGGCACACAGTTGG

A_16_P21659318
CCCTTGTCATTGAGTATAAATGCATTAGAAGGTGGAGAATTGCTAGAAATCAGAAGGAAA
A_14_P112336 CCCTTCAGCCCAGCAAGGTTTAATCAAAATGCAATGCTTTGCAAGTCTTTA
A_14_P132181 GGAGGGGGGATTGTTTTCAAAATCTAAAAAGGAAGATTTGGGTTATTTGTTTTTACAGTT
A_16_P18288634
GATTGGACTTTCTTATCCAGTTCAGAGCACTAGATAAAGAAGCCTTTATCCAAAGGACTT
A_14_P122798 GGCTTGTTCTGTTTTGTTTTTGTTTGGGAGGAAAGAACATGCTTATGATGTTAATCATTG
A_14_P104894 CAGTAAATAGTCCTCAAACAGTGATTTGTCTCTACCATTGAACTTTTAATGGCACCAGAA
A_14_P117775 TGCTGCAAATGATTAAGAATCTTCACAACTATGTTTTCTTTTCAATATACTCATGATTCT
A_16_P33435330
CAACAAAGCCATCCTAAGAAAAATAAAACATCCACAGCATTACATTACATAAAGAGGTCG
A_16_P39660032
AGCCTAACAACTTTAATGATCCTCTATTGTCACTGAATTAAATACTAACCCTTAGCCTGT
A_14_P126855 CTAATTAATGAAAGAATAATATTGGTGAAAGGGTCCCAGACTGAAGAAACAGCAATGAGG
A_14_P132749 CTAAGATTTGCTTTACATTGTGGCAAATATTTACATTTTAGAAAACAAACTTTGTATTTA
A_14_P126803 CGGTAGACCCTGAAGGCATTATTTCCTTTCTTTATGCTTTTATCCTTGACTTCTTCAATA
A_16_P17316203
AGTCCAGAAATGTCTTGCTTTAAGAATACCCAGCATAAGAAAATCAGATCCTACCAACTT
A_14_P116249 TGTCTTTCCTCTTTTATAAAATGCCTGCTGATGTCTATGAGTTGGTTCCTCTGTTTAGTT
A_14_P121654 AATGACATGCCTATAGTTACATACACTTGCAAGCAGGACTATAACCTGAATTCCTAGAAG
CUST_4643_PI426210822 CCTTTATTTTAGTAATTAGAGGTGGCACTGGGAATGCTGGTAACAGTGGAGTTT
A_14_P117131 TATAGTGATTCACTATTTAGGTCATTGTTCAGAAGGAACAAGGGTGTTTGCTGAGTTTTC
CUST_6220_PI426210822 ACACCCGGGAAGGCTGAGTCTACCTCACAGGTGTGCCTGATAAAA
A_16_P19841485
AAAAATCTCAGAGAGTGAATGCATATTCAGCCTTAGATTCTAAGTGACACATCCACACTA
A_18_P14917607
AAAACATCCACAAAATAGTGCATACATTTCCTGATGTCACAGAATATGAGATAGGCGTGA
A_14_P105416 CCTTTTATTTCTGGGCATATCGTGTTCGATAAAACCCTTTGCTTGTAACACTGCTG
A_16_P16751190
AAGGGAAATATAGAGCCATCAGATGATAATAACAAGGCAAAAGTTAGTATGAGACTGGGC
A_16_P00468295
GAGGTTCATTACTCTCTCTTCTTCTGTTCTTCTGTGTGTGTCTGAAATTGTCCATTTAAA
A_14_P137022 TTCATTTTAGACAAGCATTTCGTGGTGGCTCTGTATGACTACACCGCTATGAATGAT
A_14_P118405 CTTTTAAAAATATACTCCCTACAGGAATTCTGATAGTTGCCCTTCTCCCAATTTCATCCA
A_14_P115369 ATCTTCATCGTCGTAAATGTCCTCTGGTTCTTGATCATCATTCACCTACACATCTC
A_14_P136399 GCTCTCTCTTCACATTTTCTCTGGCTTCACGTTCATCCTTAAAGAAAAAGATTCAAATAT
A_16_P17878746
CTCTTCTGAACACATGCTAACAAACTCTCTTTGACTCTATTTTCCCGAACTCTGTTAATC

A_14_P109846 AATCCAATTTTGCAAAGGCTTTGTGTTTATAAAAATCTTAAGATGGAAACCTTGCTTTGA

CUST_7844_PI426210822 CTGCCTCTGATGTCCACAGGCCAATTAGCCAGGATTTAGGGTAGT

A_16_P16322132

GTTTTTCAGTCATGCAAAAAAATCAGGAAACATTCAATGTAAGTGTATACGCAAGGGTCC

A_14_P117302 CATATCCGCAGTTTCATGAAGTGTATTTTCCTGGTGGGTGTAAATACGTAACTGTTTC

A_14_P139589 CATTACTTTTAAGAATATAAGTCTCCATGAGTTGGTTCTAATTATCACAGCCTGCACTGG

CUST_1984_PI426210822

CTGTTGTCTAAAATTAAGCCAATTAGAGAGTCCTGCTATTAATAAGCAACAGTTTTCATG

A_16_P36230957 CACTTTGGCCTTATGGGACTTTGAATGGAGCAGGGCTTTCATTCTG

A_16_P03031877

CATTTCATTGACAAATCTGAAACACAGATCAAATTGGCCTTATTCCAAGACTAAACAGCC

A_14_P128894 CTTCCTGCCATTCTCTAAATCAAATATGTAAGCAAAACTCACTATTCACAAAGTGTGGGA

A_14_P131123 CTGAATGTGACAGTAATGATACTTATATCAGGTCAAGGATGCCTAAGGACCATAGTTACT

A_16_P58424279 GGCACAGACTTGGGAGCCCCATGGCCAGGATTTGAATCTGAACTC

A_18_P14264151

AAGGCACTAAAACTCCAAAACAGACTTTTCCTTCATGAAACATTTAATGTAATCCCTCAA

A_14_P113376 TGAATTATGAAAACTTTCTCCCCAAAATCTTTATACCATGCATTCTTCTACCTCCATGCC

A_16_P00592470

AAAAATGTGACTATGTGAAACAGAAGGATGAGAACTCAGAAGTCATAGCACTTTTTCCTG

A_14_P100990 TAATCTAACAAGAGAGGGTATTGTAGTGCAAAGTATAGTGTCCCGGGTTTGGATTTTATT

A_16_P40473789

TGGTGTATACATAAGAAGGGCTGAATAAACATATAGGTTGAATGGCATAGAACAGACACT

A_14_P118799 CTAGGGAATGCTGCCGAATTTGCATATTTTACCAATTATTGTTGGCTTGGATGTTATGTA

A_14_P124665 AGCATTGAGAGAGGCTAATTCTTACTGGGAGATTGGGAAAATATTTGAAGAATGGATGTA

A_14_P111726 AATCCTTAAAAGTTTATCAGAAGGCAAACTTTCGGACAAACCTGCTTTGACTTACCAATA

CUST_3348_PI426210822

CACAGCATTGACACTTCAGTGGTTATCGTAAGCTTATTTGTACAGTATGTGAGATGCA

A_18_P20230161

TGTGAACATATATACAAGAATGTTAATTACAGACTAAAACATAGCAGACTTGCCACCATG

A_14_P100411 CAGCCCACCAAAACGTCAATTTAGAATGAGACATGTGTTGAAAATGGGACG

CUST_7968_PI426210822

CTTCATTTTTAAGAAGAGTAAAGAAATTAAGTCACTTTGGTATTGGTATGGAGACAGATT

CUST_841_PI426210822 GAAGCAGCTGGATGCTGTCGCCAGCATGGTGGGGGCCTCCCTCCT

A_14_P133300 ACTATATACATCAAACCGTAATTACTTACCTACAGAAGATGTGCTCACATCCTCCTAAAC

A_14_P103403 ACTCCTTATGCTAGCAGATTCAAAGATATGCCTAACTTTATTGCCCTTGAGAAGTCATCA

A_14_P136769 ATGAATGTGTGACTGACCGAATGTTATTTTTACTATAGAAAGGGCCCTTTAGAAAACCAC

A_16_P16406189

GTAGCACCATTTTTAAAGTCCTCTTCTTAATGAGTGTCTCTAGCCAGTAAATTTCACACC

A_14_P136176 GTCCGTTTTACACGGATTTTGGAAAATCGAAATTCTAGGAAACACAGGACACCAATTA

CUST_5573_PI426210822 ATCAAATCATGATGACCTTTTAGAGTTGGGGTCTGCCCCCACTGAT

A_14_P101590 TCTAAAGTTTTATTATGTAACTTGCAGATTTTCAGGACGAGTGAAGGAGGAAAATGGCAG

A_14_P115933 GAGCACACCAACACAGACTCACACCTTTCCCTAATACTTTATTGGTTACCTCTA

A_14_P100299 ACTAGACTTGCTCTTCTACCTTCTGTGTAAAGTCATCATTTTCAAGAATGCTAAGCATGT

A_14_P118422 CTGAGAACTTTTCTCATTAATTTCTTTCCCCTCATCTGTTATTCCATTGGCATCTTCATC

A_14_P131916 CTACAGAGGTAAGGAGTTTCTATGAGTTCATTGTGAAAGCATAAATATTCACAGGGTAGG

CUST_7314_PI426210822 CTTCCTGAAGAGGGAATGAGGGGCAAGGCTCTGGCCTCGCAGCCA

A_14_P138523 CTTCTTCTACCCCAGAAGGTTGTTCCTTAACCAACATGCAAGTTCTTTCCTT

A_16_P18534051

TCACATGACCCATTCTTAATAATTCAGCCTAACCATCTGTTTGTTCAATTCCATTTAAGA

A_14_P201702 ACATTCCTAACATTCCACTAGGTGGGACTAAGAAATTACTATGAACATTCTACCCAATCA

A_14_P138693

GAGCAATTATACCGAGGAAGAGCAAAACAAACAAAAAAGAATCATGGAACCATGTTTTGT

CUST_2529_PI426210822

CAACACCCAAGTGGAGAAATTTTAGGAACACTATAATTCATGGATTTGCCATTTGTTTTT

A_16_P18927806

TGCAACTTAAGAAACCATTTTGTTACAGTATACCCAAGCCAAAGTATTAATCTCAGGACA

A_16_P02969864

ATTCAGAACCCACTAGCAAGAAAAGACATCACCTAACCAGCATTTATTACCTAATAACGT

A_14_P107312 GTGAGGAACATTGAACGATTCACTGCTTTGAAATCTTTACTATATCTCCCCATGATGTAT

A_16_P00685200

ATGCAGTTCCTGAATCAACTAATAAGTAAAAGTATACCTTTTATCACCTGAGGTTCTGGT

A_14_P103996 CTTGCTTTCGTCTTAAATTGTGACCTTTTTAACTGAGTAATGCAGAACAGACATCTAGGT

A_16_P19648258

AAAACTTAACTGTTCCTAGGTAGTGATCACATTGCTTTCAGATTAGCATCTTCAGTCAGG

A_14_P106629 GGAGGCACAGATTCCCCTTTCCAATTAGTTTTTAAGTCAAATCAATCAAATTTCAAAGAG

A_16_P20673416

AAATAGTAACTTGACCAGAATATGCAGGCTAATATCCATTCACCCTCAAAAATCCAGAGT

A_14_P123835 AGAAATTGAAACACTAAGTCAGAGTCCTGCTCAATGCCTCTCGGTTATCATTAATATCAA

A_14_P131551 TCTCGTTGACCCAAAGGATATCGACCTCAGCCCTATACATATTGGGTTT

A_16_P19916128

AAGAGAAGACCTCTCATTAGCACAAAAATGAAACCTATCCTCATATTTCACTCCTACCTT

A_14_P120895 GCATGCTTCTGATTCACTAAAATGGTTAACAGGATAAAAAAGGAATGTTCCAAGTAAGTT

A_16_P20886808

AGCCCTCTAATAATCCTCTCACCTTTGTATAACTCACTTTTCTTAAAATTCACCACCTCA

A_14_P129945 GAGGTGAGTGACTCTGTGATGGTGCTTTACATAGTTGTGACTAAAAATCAAAGAAAAAGA

A_14_P102210 CCAAGACTATCTACACAAGTATGTAACATCTGGGCATTGTCTCAGAGAATGTATGATCAA

A_14_P200569 GTTTTCTATCATTAACTCCGTTGTTGTGGTCTTCTTCCTGTCAGGTGAGAGATCT
A_14_P130402 TCTTCCATCTGCAATAATATTAGTTAGTTTGTTAAGTGTTCTCACCAGTGGTTTACGCCA
A_18_P22840896 GTGGCGGAACAGGAGCAGCAGGTCTCTGGCCAGAGTATGTGTCAG
A_16_P16191124 CTTATCTGTTAGTTTTCTTTCCACCTGGAGAGATCTTCCCCCTTTCCTTC
A_14_P109741 GAATATTCTTCCTCTAGTGGTTAGTGGTCTTGCATATGTCATTTGATGCTGGTATTCCTA
A_16_P19943641
AAAGACTGATCTTCATTGTGACTCTTATACGGCATTTGTATGTTGAAATGTAGCCTCAAA
A_16_P18696387
GGCTAACTAGGTGCAAAAAATGAAGTTAGATAGACCATACCTACAGTAAATTCCAGATCA
A_14_P129936 CTTTTCTAATAGTGGTCCATCTCTAGCCCTTGTTTTATTGAGAGACAATGGCTTGCAATA
A_16_P00685126
GTTTTTTGTTTTTAAAAAACATATAAAGTAAGCATAGATGCTAGTGTGTCCCATGGTGAA
A_16_P00000987
CCACTCCAAAAACAACACTTAGTGGCACATAAGAATATTTTCTTGCTTAACCGTCACTTA
A_14_P102912 CTTCTCTGCTCCTTTTGTTCTTTCCTTCCCTTTTTTAAAAAAAGAGGGGGGAAAT
A_16_P19884938
AGATCAATTCAACAAACTACACATTTCACACTTGCTTTGAGATAAATTGACCTTCACCGA
CUST_7628_PI426210822 CATAATTGCTTCTGATGCCAGCCAGTCTGCAACTGCCAGTGATTG
A_14_P138078 GGTGTGATAGTGCTTAGATACCTTAAGGTATTTGGGAAATTTTAGAACCATGTTCAGCAT
A_14_P136060 AAATCCAAGGTAAAAAACACGGCATGGGTATTAGTTTGAATAGGGAAAATGAGAACTCTC
A_16_P03810575 GTCTCTTCACCTGTCTGAGACTACCTACTTAATACTTACTCCTTGAACAGTGACACTT
A_16_P16191549 CTCTGAGGAAGCAGGTGCTCTGCTGCCTTTCCACATCCTTTCCTC
A_16_P00685271
TTGCATGTTTCTCATAAACTGATTGTGCATTCTAATTATTTAGTTTGGAAAATGTAGTCT
A_16_P18390193
TGTGTATTTCAATCACCAAGTAGAGTCAAAACGTGTTTCAAGAGATCACTAAAATGCCTG
CUST_2990_PI426210822 ATTCACCTGGGAAGGAGGCTGGTCAGGGGACCCCGAGGAGAGTCT
A_14_P133984 AGACATATGGAAAAAGGCTGGAGTGATTAACATGTCCTCAATGTTCTGTATGTCCTATAA
A_14_P201119 CAAAATGCACAGGCTCGAGATTTTGGAGTCAATACATGTTTAAATTGGCATATCATGCTA
A_14_P117572 TTTATTTGAACAGTTTGCTCCTGCTAAGATCATCTGTCACTACTGAAAATTATGCCTCTC
A_14_P102194 ATTCGTAAATCATAACAGAGTGTTTTTCTGTGTTGTTGCCCAAAGACATCAGAAGTTGAA
A_14_P135317 CTGCACCTTTTAAATGTTCACTATCAGATGTGTCGTCGCCATTGTTGTGATATTTACAAA
A_14_P120640 GTTTGTTCCAAATCATTTTTCAATGCTGTGATTTCCCTTCTCTTTGCTTTAAACACTCAG
A_14_P131325 TAGAGTAACAGATGAAGTTGCCACATATAGAGATTGGATATCAAAGCAGTGGGGCTT
A_16_P16669962
CTCTTGAACATTGATTTTTCCTTTATTCCCCTTACTCCTAGAACATTTCATTCCCACCTC
A_14_P139461 CATTATATCATTTTATTTTTTTCCTCAACCCAATTGCCAGGGTCTCTGAGAATCACAAGT
A_16_P15434946

CCGTGAAGATCCTACATCAACCAGTAAACAAAAATGTATTAATCACATGCTACTTTCTGG
A_14_P128208 GTATGTTGTCCCTATCTTTATGTTCTTTCCTGTTTTTAAGGGATTCTGAAGCAAAGTCAC
A_14_P100408 TGTATAGGGATTAAGACAATTACCTGTAGTGATGTAATAGATGCACTATGGCCCTGAAGA
CUST_3915_PI426210822
TCTAATAAGCCTGAAAAGCATGATGGAGTCATAAATCATGTCTGTAAAATGAAGATGTGT
A_14_P103796 ACCTGACTAGGCTAAAGGAGAGATACGCCAGGACTAAGAGAGACATCTT
A_14_P101970 AAGTGTCACTATTAGGGTTTTTTTTAATTGCAAAGCCAAATAAGGCTGTCAATTGCATCA
A_14_P134493 ACTGCTGTTATTGCTAAAAGAAAGAAGTTAACTCAGAGGAACTATGGTGTTTGAACTGAC
A_14_P131966 GAAAATGATATTTTCTGCCAGGTTTTCTATGAATTAGCATTAAATTATCCATAGTCACCT
CUST_6072_PI426210822 GGTGGGGAAGGGAGAGGAGAACATTGAGATGGTATAGGTCATTTTAC
A_14_P122561 ATACTGAAGTTTCGTCTTTCTTCTGGTATCCCATTGGCAACTCTTTCATCTGTATACAAT
A_16_P16191728
ACCACTTGGAGCCTAGTAAAATCCATGCTACTGTTCTGCAATTGTTCTTTTATTCTTTCT
A_14_P121709 GTCATTTAGCATCCTAAGTATGTAAACATGTCTCTCTTCACGATTCACAAAGTGGCTTTG
A_14_P108355 AGAGGCTATGAAGTTTCAGAACAAGGTGGTATTTTCTGCTCTTGCTTTAATTTCCTTTAT
A_16_P18404066
AAATGGTCTTTTTGACTCTTGACACTGGTAATTGATCACCTGCTAGAATGAATCATGAAG
A_14_P130702 CCTAAAAAACAAGTATTTGCTGTTAATTTTCACCATTCAGGGTAAGCACTTCTTTGCCAT
CUST_7059_PI426210822 CTGGGGAATGCACAGGAAAAGAGCTCTGCCTCCCGGAAGGTTCCA
A_14_P128785 CGTCATTTTTGAGGAAGTAGACAAACCTAAATATGTGTCTGAACTCCAAGGAATATGAAC
CUST_10315_PI426210822
AGAATCCTATTTCCCTGTTACATCCTTCCACAGTTTCATGATTACATTCTCCTTCATG
CUST_9665_PI426210822 TACTTTCTGGAGTTGCTGAGAAGGTTCAGTGATACCAGCATGAAGCTC
A_14_P109130 ATATGAGTGAACTATTAATGATCAACTAGTCAAGGGGTCACCATTAAAAGGAGCCCTAAA
A_14_P200228
ATTTTCAACAGGGCAGAAGAAGAAAGCAAGAAAGAAAAAGACAATTTCTCTAACCACACA
A_14_P107131 CACGGTTTATTCTAGAAGCCTATAGAAGAGCCACATTGAGTATTTCCAAAATCACAAAAT
A_14_P131427 GCAGTTCTCTTCATCTCCAGGAGAAGGCAGGAGAAAAAGACTCAAGAAT
A_14_P101507 TCAGGTTTTTAGGAACCATTTTTGCAATTGGAGATGTTTGTGAGGAAAAAAATCTCTGTT
A_14_P124063 CTACCTAAGCCAATAAGAAGAGTGATTGATAGTGACTGTGACCACATACAGCACTGTAT
A_18_P27285590 CACTGCAGCCAAGTCCCCATGTAGCACCACAGCCCCTATTCCAAG
A_16_P58424541
TTCATATCAAAACCAATGGGATTTAGACAAAACCAACACAGAGGAAAATGCCACTACTTA
A_16_P18306494
TCTATAAAATCTTCGTGGATTCTAACTTTGGTAACCAACTTAGCAAAACCTATTGTGGCC
A_16_P18367273
AATGTGAATCCCCTGTCAATAAATAAGATGGAAGATACAAAATACAGAAAGCATGCCAGG
A_16_P15153711

AATAGTGGAAATGTCTTGACATAATGACTGTGGAGAATCAGTAGAGAGATGTAAACCCAG

A_14_P126771 TTTTATTTTTCCTGGTGGCAGGAACATCCCTACATATTAGACAATAACTTTAACTCTGGG

A_14_P119301 ACAATAACGAATCCATAAATAACCCTGTTGATGCAATTAGTGCATCACAAGAAAATGGCT

CUST_1750_PI426210822 CTCACTTCCTTCTCTGCTTACCGTTGGATTTCTTTTTGGTAGGAGCTG

A_14_P202279 CTACACTAGAATTAACTCAGACCTGTTAGCAATGAGACTATCATGTTCTTCCATTCTCCT

A_14_P116807 TTGGGGCTGATCTTCGGAGTCGCTACGTGTTCTCTATACAAAATAAAATAATCTGTAAAG

CUST_3744_PI426210822 CTACAGCCACAACCATCCCTACCTGGAGAAAGGGGCTAAAAGAAG

A_14_P102602 AACCTTGAGACTTTTCTTAGTCTTTGAGTTCTGTCTTTCACTGTGTTATGTCTAGCAAAG

A_14_P122992 TTTCCGGATTAGCATCGTGTGTCTTCCTTTATCGAAAGAAAATGCAGACACGATAATTAA

A_16_P01908081

GCATGAACACATACAGAAAGGATATCTTGAATTCAACGTTTTTTTAATCCTGACTCACCC

A_18_P14135751 CATCCTATGCCATCAGCAGTGGGTGGTGAGTAGTTCTTTCTCTTCAC

CUST_3153_PI426210822 TTACTGGGAAGGATCAGGGTTGCGCAAGGGGTTGGTCCTGCTACTTT

A_14_P103284 CTGGAAGTTCTATGAGAAAGCGTGCTAAGTTGATATCAACTGTTTCCAAGAAAGATTTCA

A_16_P18213978

AAACATTATCCAGTCAATTGTGATTAGTTAGGCTACAGGATTATCATGTGTGTCCCAAGA

A_18_P10432299

AGCATACCTCCTAATAAATGAACAGCGTTTTCATATTCTCAATCAATCTCTAGTGGCCAA

A_14_P130686 TACCTGTTTGCAAGACCACTGTTCTCTCTTCATCCAGAGAAATAATCCTGAAGTTATAGA

A_16_P00685604

TCTTTGGGATTACAAAATAATATGTATGTATTGACCTAACATAACATATCAATTACCAAA

A_14_P115493 AATTGATTAGCATCATTGAAACCCCGCTTGGCAATAACTCTTCAAATTCCCTAAAACATA

CUST_891_PI426210822

GGCAAGTATTTACTTTTAAATTAAAAATGGGAAGGATATGTGTTGTTCAGGTGCATTTTG

A_14_P118032 ATGTGATTTGATATGTTTTCATTCTGAACCCCTTGCATAAGTTCAGGCGAATTAGAGAAT

A_14_P112727 CTTTGGGATAAGAGTTTCATTTTCATACCAATGAATTAATGTTTAGATCCCTTCCCCATC

A_14_P139481 CCTTGACAAGTGAAGAAACAGGAAGAGATGATGAGAAAGTTACCTGTGCATGAATTTG

A_14_P105605 CATGTCTACTGCATCATCCTTACAAACCAACAGATTCTAGTGTCTCCTATCACTAAGAAA

A_14_P112462

CAATGGTGTGAGTCTGACTTTGGAAAGAAAAAGGGACTAAAGAGAAATGTAACAAACTTA

A_14_P123053 AACTTTTTGTGACTTTGTGGGATTATTGGAGTTGACCTTAGTCTATTACTATACCACCGT

A_14_P121581 CTACTCCTGCCAATTCAGATTGAAGTTCTCCAAGTCTTTGCAGATCCCTATAGTAAATAA

A_14_P202156 CCTTACTGAAATAATGACGTTTTCAGTTGCTTAGTTTTTGTTGAACCTCTTGGGAATTCT

A_14_P136183 ACATATTTTCTTCTGTTGTAGGACAGTTCGTGTTTGGTTAAAGAGAGACAGTGGACAGTA

A_14_P112045 CACTGTTATCAGGTTTAGAATATTGGATAGAGTCTTCAGGAATCCTTTATGGGCTGTAAG

CUST_9748_PI426210822

GTGCCAGTCTTGCCAAATGTACAGTTTATTTTCTGTTCATTTATGTCGTCTCCATAAGT

A_14_P106126 CAATCAAGCAGAAATGGCATTCCAGATTTGGACTTGTCATATGAGACACACAAATAAGTA

A_16_P17912492
AAGCCTGAAATTATTTTGCGTGAACAACTACAAATGAGATGTGTTACTTTTACTGCGGAC
A_14_P118555 AGATTTATCAGTCAATGTAAAATGGTTAAACAATTGCACGTGCTCTTCTTTTGTGAGTTC
A_14_P135622 GTGTTGGGAGGAAAAATGTCCTTCTTCAATTTCCGTAAGATCTTCAAGTTGGG
A_14_P131431 ATACAAATAACCACTCTACTATGCTTACAGGTGCCTTTCATGCATGTCATCGTTGAAGAA
A_14_P124680 TCATATTTTTTGCTTCACAGTCTTCCTGTAAGGACTTAAAAAGACTTTTCTCCGGAACAC
A_14_P129254 CTCATTGTTTACTTACATGAGGCTGAACATCCAACAAACAAACTTTGTTTTTAGCAAGGA
A_14_P105930 GGGTTATTTATTTTGGCTGCTAAGAGGATGTTTTATGCTTTTATGGTAGATGAGATGGCA
A_16_P20107820
AAAAAATGTCTGTTAACACCTAGTGTTCTTCTCCTGGATAATGAGGACCAAAGAATAACC
A_14_P132345 GCCTTCCTGCTCTGTGGGATGTATATGACTTTTGAAATCCCAAAATTATTGATTTTTCAT
A_16_P19159338
CCCTTTGATTTGCCACTTTTGACTAGACTTACACAACATAACACAAATATCAAAGCCTCA
A_14_P100941 TGAAGCCAGCAAGACTTATACATTGGAATTTTAAATGTATGGGATTGTAAGGAGGGTAGA
A_14_P121013 CTGAACCCTTTTCATCTGAAAAATTTTGTATGCACTGTCTGTCTTATACAAATACTGGCC
A_18_P15508690
TGGATGCAAAAACTTTATATGAAAATTTCCCTTCGCTAAGAGATAGTATCATCCTTAAGA
A_14_P130325 GGAGGTCATCCACTGTCTACTTTGTGTAATATCTAGGATTCTCAACATTTTTCCAACATT
A_18_P22841711 CTTGATCTGGCTATAATTTTGCCTGTGGAATCGCTGCTGTGGTGTTTGT
A_16_P15433198
AAAACTACCTCTTAAAATAGTCTTTGGCCATGTATTCCTTATAATTGGACTCTGCCCAGA
A_14_P138421
GAAAAACAGTGGAAAAAGCCTAAGATGGTAAACTAGAAGGAGAATTTGGTTAGGGATTTT
A_14_P125440 TATTTGTTCCTATAAAATGCAACCACTTGGCAGAATGTAGTAGGTCAGTAATGGAGTGTT
A_14_P113319 ATCCTATTAAGTAGCCTAGATGATGCTGAGATAATAATGAATCATTGATGATGTCGGCGG
A_14_P120718 GTGGATTGCTCCAAATATTGAAGTTACAGGGAAATTATCTGAGTGAGGTAATTGCTTTGA
A_16_P36610291
AAAAAAAAAATACTAATCATTGATTTTGCAGGACTCAGATATCATAGTAAAAAAATCAGA
CUST_2158_PI426210822
CCGCCTACCTTTTCTCTAACATGAGAAAACGCTTATGGAGATAATCAGTGGAAAAAT
A_14_P100373 TAAAAACTTTTATCTGTGAATTTTCTATTTCTTCCTCCCACTGGGTTTTCATCTCTAGCC
A_14_P119664 AGGAAAAATACTTTCACAGACCTTTACTCAAAGTGAACCTGCTCCAGTTGAAAAATAAAG
A_14_P112261 GATGAATACATACCCTGCTCTGATTGGTAACTTTCACCATTAAACCTGCAAATTAAAAGG
A_14_P109709 CAAAGCATGACTTTTTAATAACATGAGAAGACCTTGTTTAAGGTCACATGGCCTTGTTAG
A_14_P110049 CTTTAATAAAAAAGACTAAAGGTTCTTTTAAGATATCAGTGTCCAGCCTTGGCCAAGACC
A_16_P03147514
CCTTCACAAAATACCATTCCATACGTATATTTGCTACATATAGGAACTACAAGAAAGGGA
A_18_P25384941

AAAAAACAGACTGGCTTTTTCAAATCTATATCACCTGAGAAAGATGTATGTGTCCACCTG
A_16_P18536631
AAGGCAGATCATGTTTAATGTCCCTCTAAAACTTCTGTATGGTCATGGATTAGAATGTGA
CUST_3885_PI426210822 AATTCTGGGCTAGTGGGGATCATGGGGATTTTGTAAATTATTTTGGTAGCC
A_14_P131657 CATGATCATCCTTTTACTTCTACCCCCACCTCCCTTTTAAAACAAAGAGGTT
CUST_7685_PI426210822
TTCATTCCAGTGGGAGGTTCATATTTTCTTAGTTATGTATCATGTAGTCTTCCTTGATTC
A_14_P121899 CTTCAAAGTAAATATTATCATTATGACCCTTTGTGCTCTTCACAGCCATGACTGCTACTG
A_16_P17197574
AAACAGCCCATCTAACAACTATCAGTCCATCTTTAAGAACTGGAAAAAACTGTTTAGCAG
A_16_P21040707
AAACTAGTAGACCAAAATCTCAGCCTTTAATAGTCATCCTGATGAGTTAACCAACCTTCG
A_14_P100556 TACACTTTTAGGCAAAATAGCATAAAAACATGGAGATACAAGCCAAGTGTGCTCATGTGT
A_14_P115203 AAAATGGCAGTTTTGTCTAAGGAAGAATGTTGAGGGACATGTTTATTACATTTGGTAGCA
A_14_P126053 AGGGTTTTGGGTTTCAGAGGTTTTTGACGACTGTAATTATTTTGTTTTCTTAACTGAATG
A_16_P18303874
ACCCATAAATAGTGCAGATTCGATTTCTCTGAAAAATATCAACTCTTCCAAGACAAGACT
A_14_P102714 AGAAAAGGAAAAGAGGCACAGAGATTTTTATTTTTTACAAACAGTGTAAGGGGATTATTT
A_14_P102749 GAACACACAAGTTCTTTCAACAGTCAAAGTTCTAGGAGTCTTTAGGAGATTGCAAGAGT
A_14_P138533 CTGAGTCATGTGTTCCAGTGGCTTGGGTTCACAGTGCATATACACAATA
A_14_P109097 CATACTCTGAAATCGATCAATTTTACCTTGAGGACATCTCCTTCGGAGAGTTCGAAT
A_14_P113202 CTGGAATGCACTCAATTTACATTTCACCAGAATTTTCATGAGAGAACCTGAGATTATCAG
A_14_P202216 CTCGGGAGATGCTGGTTTTCATTTACAACCTTCTGTTAATCAAACCTAGAGCAG
A_14_P102514 TGACATCAGCATCAGAACTTAATGAATTTTCTAAACTCAGTTGTAGGTGAAGGGGATTCT
CUST_4773_PI426210822 ATCTCCCCACTGGAGCTCCATGGGCAGAAAGCACTCTTTCTCTTC
A_16_P18503828
ACGTTAGTGCTAATAACCCAAAACCTTATCATCACTATCATAAGAGTAACAGTAATCGTT
A_14_P136162 CATTTTACCCTCATCATGCACCACTTTAGCCAAATTCTGTCTCCTGCATACAC
A_16_P35747774
AGAGTTTTTTTCAGCATGAATTATAAACAGGGTCCAAGATAGTAAGAACAGAGCACTCCT
CUST_4465_PI426210822 CCTTTCATGAGCCTTGACTCTACTGGGCTCTCCCTGCTCCCCATG
A_14_P104563 ACTTCATTCAATCCTTTATCATTATGACCATTTAAGGTGAGGACTACCCTAAGGATTCAG
A_14_P117726 CATTTGAATGTCATCAATTGCACACAAGATGTTTGTATCCTGTAAATAAAGATGCCCCAC
A_16_P19637303
AAAATGAACGCAAGAATTAGTTGATACTCCTCTGTGTCTTTAACTGAGTAGCCTCTGTTC
A_14_P112034 TTCATCTACTCCCTGTCCATATAGACCTAGTGAAATGGAAGATGTCACATACAGTGAAG
A_16_P58424335
TAATCATCAAAAGAAACATAAACGGACGTGGGTAATAATGAGTAATAATGGTTGCCCCTG

CUST_2823_PI426210822
TGTCTCATTGGCAATTTCTCTTGATCTTCAGGTATATTGAATAAGCTCAACTATTAAACA
CUST_7974_PI426210822
GCCATCATTATAAGGAGATACAAACATAGGTAAAAAGATGTGGTGATTGGAGGCAAAACT
A_14_P116295 CTGAGACGATGTTTTGGGAAAAAAAAAATTGGAAAAAAAATGTTACCCCAACACGATGG
A_18_P10606340
ACTCAACATTCCCCAATATTTGAAACATTTCAAAACGGATATCTTAACTGGTCAAGACAA
A_14_P104822 ATGAGAACCAAGTAATTAAACATCATTACTAGCCTAGATCCTAATTGAGGACATGAGATT
A_18_P14136601 CTTAATCACAGAGCCTTGTTTGAATCACTTGACTTTGAAATCCCAGGAATGACTTTG
A_16_P00685054
GTTGATCCATTTAAAAATTCAAGAACAAGCCTCATAAATTTTTATTAAATAAAGACCTCT
A_14_P111475 GAACTATTATTGTTTTCATTTTACTTATTACGTAGATGCAAGTAAGAATTATGGCAAAAA
A_14_P108098 TTATCAGGAGAACATTTAAATGACGATAAAATGGGTTGTTCGGTTTACTCTTTTGCGTGT
CUST_9265_PI426210822
TAATTCCATTGGAAATTTTTCTGACTTTTCTTCTCAGGACTGTGAAAAACCAGGAATTTC
A_14_P118792 TGTGTGGTGACCATGGTAGTGTTAGATTGTTTAGTTAGTGCTGTAAGTATTACTAAGGGA
A_14_P117000 ACACTTTTTTGTCTTCTGTTTTTTTCTTCCCTAGAATTGCAGTCTGAATTTCCCTAGATC
A_14_P132654 GCTGACTGCAAATGAGGAACTGAAAATAATCATTTGTTCGTGTTTTCATTTTTCAACTGG
A_18_P13860917
TGACCGTAACTACTATTCAAGAAGAAAAAAAAACTACTCATTAGAACTTAAAAACATAAA
CUST_3298_PI426210822
CATTTTCTTAACGTGTCTTTCAAAAGCTTGTCAATAAATATAGAAATCTGTGTGTGTACC
A_14_P139456 GAGGAAATGGCTTTTGTTTATTTGACATCTGCAGAAAAAGCAGTTCCCAGGC
A_14_P129621
ACATGTGCTTATAAGAGAACACACAAAGAGACAATGCAACACAAGGAGATAAAGAGATCA
A_14_P129794 CTTTCTTGGTCCTAATTTATTTGTCACTAGCCACAGTCTACACTCTACAAAAATACCCTC
A_16_P41085968
CTTTCAAATCACCTGATACAATTTGCCTCTACTCTCAATGATGAATTCTGTTTTGCCCTA
CUST_5948_PI426210822 AACCACAGTTGACTTAGGCTTCCTTGGAGCTTGGAAGAACCCCTT
CUST_7646_PI426210822 GCTGGAGTCACTGTGTGCTCCAGTTCCTTAAGGATACTTTTAGTTTATCTGATC
A_18_P16848731
TTACAGATTTAACATCCTACTTCACTAGATGCTCATAGCCTTGATCCATTACAGTAGCCA
A_18_P14874515
AAAAAAACACCCTACCAAACTTTTAAACACCAATAGAACAGATCCTATATAACCCTTGCA
CUST_5208_PI426210822 CCCGACACACACACAGACTTTTCAATGCAAAATCAAACCATTCCCAAATATC
A_14_P124710 CTCCAGTGTATTTTGGAGTTTTGATTGATACTTCATGCCTCAAATGGGGATTTAAAAGAT
CUST_2650_PI426210822 GTTAAGTAGGGGCTTGGCTCAGCCTGGCCTAACTGATGTTAGATT
A_14_P107071 TATAACCGCCAGCGGAAGATCAAGAAATACAGACTACAACAGGCCCAAAAA

A_14_P103853 ACATCATCCGAGTCTTTTACTCGCTTAATCTGCTCCCTAAAAACGGGAATATATTATCAG
A_14_P136027 TTATGTGATGGACTGAAGTGCTCCCTTTAAAAAAATGTTTTAAAGTAATCTGGGGCAGAT
A_16_P00685418
TTAATAGTAGCCTCAAGTAAAATTCAACCCTTAGATGTTTATAGTCATCTTGGCCTGGCC
A_14_P133307 TTCTGGGGAAGAATTTCTGGTTCCTACAGTATCCACTCCATCCTCAAG
A_14_P133759 GTTTCTGCTTGCATCCCACTAATGTCTAGCAAATAGGTTTTTCATGTATCTACCTAAGTT
A_14_P102288 CTTATCTGTTTTCTTACCTCTGGATTTCCGCTTGTTAAACACAGACTGCCAGAC
A_14_P116621 ACAGATTTAAAGGATCTGATGGAAGCACGTCATCTGATACTACCTCAAATAGTTTTGTTC
CUST_1826_PI426210822
CTGATATCCTTGTGAACATGACTTTTTGTGAGATGAGAGCCATTTCAGCATGAGA
A_14_P105976 CCAGAAACAACCTTCCTTATTCTCCTGGTTTACCCTTTTGTTATTCTTGTTGTTGGATA
A_14_P109012 AATAATCACTCATGTTAAAGTCACTGGGCCTGGGTAGTATATTAACCTTTTCTTTGCATG
A_16_P30952598 AAAAATCTTCAAGTAAGTTGTGTTGTTTCTTCCACAGGACAGTTCGTCTGTCATCC
CUST_4369_PI426210822 CTTTCTCTGACAGACGGCTCTAGGCAGGGGGAAGCTCTGACTAAC
CUST_4517_PI426210822
TATTGTTTATGTAAAAGGACCATTTGCCTAATGTCCCTGGAATTTATTTGCTGGAGCA
CUST_2523_PI426210822
TCACAATGGATCTGAATTTCTGATCTTGTGTTTTTCATAAGGGAAATTGACAAATTCATC
A_14_P139885 GAACACAGATCGGCAAAGAAAAAAATCCAAGATGACAGATGAGGAGATCTTAGAGA
A_14_P129584 TGGACAATTTATAAGAACTATGGGAGCAATATGAAGGTGCTTGAGAAAAGAGATGATGTT
A_18_P22840140 CACATTTGGGATTCTGTTCGCCAGCCCCTCTGCACTCTGAAGATG
A_16_P01615906
GAGGAAGTTATGTTATCTTGATCTGATGGATACTCAACGTGAACATTATTTCAACGTGCC
A_18_P14134621
TCAATCTAGACCTTGCCTCTTTCTGATCACTGTTCTTGACACTGGATCTAAATTAAGAAA
CUST_4402_PI426210822 TGCCTTGGGTTGAAGCTGATTTTTCAGCATCCCTGGTTTAGCATATG
A_14_P127445 GACATCACAATCACCTGCATAAGAGAACGAAGAGGAGGACCAGAAACAAATAGT
A_18_P22840677 GTAATTTATAAGACTGAGCCCTGGCCTTATGGTGTCCTTCAAAAGAATGGCATTTAGT
A_18_P16501633
ACCTTGCAAAGTAACTATCTGAGTATATCTGAAATTTGCCATCATTTATCAACTGCCCAA
A_18_P14679376
TCTCTCACACACAATCAGATTGAAACTATAGAGCATCATTTTCAAATCAAACTCCACCAG
CUST_370_PI426210822 ATATCACACTGAGCACAGAGCCCCAGACCCAGCTTCTCTCAGAGTA
A_16_P36231138
TAGACACTTGTCTTACCATATGTGAAAAGTTGTAAGACCAAATGTAACATCAAAGGGCAG
CUST_10035_PI426210822 CTTAATCAGGGTGGGGAAGGCAAGATGCGAATTAGTTTGGCAAGA
A_14_P107528 CAGAGACAGAAATATTTTGCCACTGTTGATTACTATACTTTAAAGTTCTATATTATGAAA
A_14_P134232 CTTCTCAGGGATACACACCGTTTAAAGGAATTGCTCATTTGCTAGTTGTATGTTTGATAT

CUST_7055_PI426210822 TGACAAGAGATGTGAGAATTCCGTTGACACTCACTGACCACCTAGAAA

A_14_P119652 TTCTTACTTTGAAAACCTGAATTTCAGGGAAAATCTGCCCAGTCATTTCAAGTTCAAG

A_14_P130916 TGCAACAAATTTAGAGACTCAAATGGCAGGTCTCTTCTCATTCTAATCTATTTTCAATGT

A_14_P138727 CTACTTCTTAAGCAGGCTTTGAAAAATGTTCATTCTGTCTATTTGTCATGGTGCCAGGAT

A_14_P110812 TAGTTATTTCAGTGCAGAATCGATTCACGTGTAAGCCTCTACCTCCTGCACTTA

CUST_10031_PI426210822 GACCAGTTGGACTTGAGAGGTCAGGGAGAAAAAAAGAATAGCTGTCTC

A_14_P116790 GTTAATATAGGTGGGCACACTAATATAAGGAGAACTAATTTTATGGGTAGGGGAGAGTGT

A_14_P104844 TTTCAGTAATCATTCAACCAACGCTTCCATGTCTCTACTCTGTCGTAACAAAGGC

A_14_P139914 TAACCTTCTGTTCGGTGATGAAATTATCACTAATGGTTTTCATTCCTGTGAAAGTGATGA

A_16_P18508257
TCTTGGTTCCTTCCAGAAATTATAATTCCTTCAGCAATAAAATCAACCTGTCAGCATTCA

CUST_7289_PI426210822 TCCTTGTATGGGAACATCATCACCTTCACGCTTTGTCTTTAATTTGAGAAGCTT

A_16_P02445254
TCAGATTGCCAAGGTATCACTAGAATTCTTCTCCTTAACCATTTTCTTAACCATTACTCA

A_16_P18549409
CAGTTCCCTGCTAGTTATAGGATAACTCAACTACTTAGCATTTGATACAAGCCTTTGACA

A_14_P135408 ACAGATAGAACACTGTTTACATCAGGGTATAGCACAGCACAATATACTGCCTTTACTACT

A_14_P118192 CAACCTGATAATAGTATTTATTCCCAACAGTGCAAAGAAAAAGAAGATGGCTGACAAAAT

A_14_P136632 CTGACAATTACCAGTGGCACACTGCACTACAATTTTCAAGTTTAGTAAGTGCAAATTAGT

CUST_5525_PI426210822
CAGAGAAAAGAAAGCCTTACTTCTGGCATTTGAAGTCACCAACGTAAGAGCTA

A_18_P24515496
AAAAAGTCACTGGAAATGAGAGCATTAACTAGGATACAAAGTATTGGATGGGTCCAATTC

CUST_9217_PI426210822 AAGTCTTGGTAAGGATTTGTTTAGTGGTTGGGGTAACACGGGTGG

CUST_2896_PI426210822 CTGAGTTTGTTAGCAGGACGGCTGCATTTGGGCAGAGCAATACAC

A_14_P138044 CTACGAATTGCTTCCTTCTCACAGAACTGTGACATTTATCCAGGTAAGGAATAACACATG

A_14_P114000 GCATATTTTCTGATGTTGAATAAAGTCTGAGTATTAACTAAAGTTTTTGCTTCTTAAATA

A_14_P114426 TTTCAGCAGCATAAAGAAAAACATCTCATTCCTTTTATAGAAATGCAATCCCCTGAGGTT

A_14_P116034 TTGTATTGCCAGAGGTGTGGCTTTTGGCATTAACTTTTATTTAATTTGGATGGCTACTTT

A_16_P02606498
GCCTCCGTCTATAGACAATGATCTCTTAAATGACCATTGGACTATAAGAATGCATCTCTT

A_14_P124491
AGAAAACAGAACAGGTGAAGGGACTAAAAACAAGTCAGGCAGTCATTTGGTAAATATTAT

A_14_P101664 TGCCTTTTCCTTCACGTGTAAAAGTCATATATGTTCTTTGGAGCCAATTTGAAAGAGAAT

A_14_P119861 CTGAGGACTTAAGTATTCACTCAACACTCAAATGATTGCTGCTGGTATTCTGTAAAAAAT

CUST_5047_PI426210822 TCAACATCCCCAAAATGTGTGACTCGCCCAAATTAAGTGGTGTCATAC

A_14_P107975 AAGGCATAAGAAGTCTTTTTGGCTTAATGGGGAAATTGAAATGTTCTCTCATGCAATAAC

A_14_P115533 CAATTTTATTTGCAGAGAACTCATGTTCTGGTTTTTGATTTGAGGTTTTGGTTTGGGTAT

A_14_P131063 ACTACTTACAGTTAATAGTGGCTTTCATATGTTATTTACTGCTATTAAATAGGCTGTCAA

CUST_4532_PI426210822 GAAGATTTCTCTGGGGGGTTGGATTCATAGATCTGCTTTGGGGCT

A_14_P107747 ACATGGTCATGAATACACAGAGAAGTTTTCATAACACCACTTTTTCATCATGATCCATCT

A_14_P138894 TGATTAGTAGGGGGCTCTTGAGCAGTTCAAATCAATTTATTCATTCACATCATTGGTTAG

A_16_P16240764 AAAAATGGAATGTTTGTTGAAGGTGTAAATAAAAGCCCATGATCTTAAGTGTCACTGCCT

A_14_P104236 AACCAATTCCTGACTACTCTATATGCTGCATAGAACCTGGAGAGGATTTTTCAAAGTAAA

A_14_P127856 ACACGAATGTCTTGTCTGTTGACGAAAGGAAATCCAGTATAGGAAATAACAAAGTTAGCA

CUST_3356_PI426210822 TGCCCTTTTAACACTGGTATTCTTTGAAGTCACACATGATAATGTGTTCTGTTTTATTGG

A_14_P200306 CTGGGGGCAACAGGAGAATATATCAAGAGGTGAGAAGTGAGGATCAAG

CUST_5505_PI426210822 GGAGGACAACACTGTGAAAGAAAGGGCATCAGCTAATAATAAGAGTCCAGTAAT

A_14_P129817 TTTCTTTGACTAACTCCTACTTACCCTTTAAAACTTCAACTCAGGTTTCACTTTCCCATT

A_16_P39364378 CGCTTCAAAAGAATCTTTCATCAAGCACCTTACTCTTAAACACTTTCCCATAATACCAGA

A_16_P19598990 AAACCCAATAACTTTTCATTCTGCCTAACAAAAATAGACCCTCCTTTAAAAAGTGGTTGC

A_14_P136251 ACTTGTGCTTCAGAATAAGTTGTTTCACAGACAAGGTTGCATATGTTAGGTGACAATATC

A_14_P116828 TACCATGAATTAAAACCATCAGTTTCTGTGGATTTGTCAAAGCATCCTCACTCATAAAGA

CUST_5598_PI426210822 ATAAAGCTGGAAGCAATTCAATTGTGTGTCTACTTTATGTAAGACCTCGATGAAAAAATA

CUST_7395_PI426210822 GAGGATGACCCTCCAGGTGGCTGAGATTCCCTCCAATCTTTTTAT

A_16_P03450213 TCATGCTCCTCAACCTAAATTCACTAAATACAGCATTCCCACTAAATATAGCATTGCTCA

A_18_P13789303 GACTTCAGGGTAGTAAGACAGTGAGTTGTAGACTGAAGACTAATGCATCTAGGTTCATTT

A_14_P119184 ATAGTCTTATAATTAGCAAATCTTAATAAGCAACAGCACTGCTTCTCTCAGCATGCACAT

A_14_P111880 TTGGAAAACAGAAATTACAGCACTGGACATTTCAAAGGTATTGAATGCAGGGATTGAAAG

A_18_P22842483 TAATTTTCTTTCCCATCCTAGGGTGTCCTCTTTGCTCTCTCCATATTCTCCAAATAAAAA

A_18_P17391833 TGGGATAGCAGACTTTGTCTAATCATCTATCTCAGAGTTTAGGATACCACACTCGTTTAA

CUST_6068_PI426210822 CATCTTGAAGCCAGTGGGAGTACAGCCAAGTTGTAGTGTAAGTGCTTTTAA

A_18_P11869943 GGGTAGAAAACGATCAAATGATCATGAGCAAGATAGTGACATAATTCTTGAGGAAAGCAC

A_16_P16233158 TTTCCCCATTCTAAATTCACAAACATAAGAACGCAATCTAAACTAGTCATCGAGTTGGTG

CUST_2822_PI426210822
TATGTTCATCCTTCTTGGCTGTCTCATTGGCAATTTCTCTTGATCTTCAGGTATATTG
A_16_P16191397 GTGATTGGTGAGGACTGTTTGACAGTGTAGCCCAGGACTGTGCAT
CUST_4944_PI426210822 GTCATTAGAAGGCTGTCCACAGAGGGGATCAGCCCCCCTCAATGA
A_14_P137019 AAAATAACCTTTTCAATAGCCAGTTGCTTGTTCCAAGGACTCTTCTTCGATGGACTAT
A_16_P19008438
AGTCCCTGATGATTTCACTGTATTGTAGCATAACTCTGGTATTCATAATGTCAATTCTGA
A_14_P200735 CACATAACTTGATTCAATTACCATTTAGTCATTTCTCCAAAGGATAAAGGCCCTTACCGC
A_16_P18396113
TGCCAAACACTGAAATTTATCTTATGTCCAAACTACTCGAAATGAAATACAACGGTGCAG
A_14_P131321 GGAGAGTTGAGTTGGACTTGAAATAGAGCACTGTTGGATTTGAAGAATATGTTACGTTAT
A_14_P108711 GAAAATGGAGTTATCCAGAATCAGCGTTGCTCTCTACGTGGTCATAACATGTGTATTATA
A_14_P139977 AAGGAGGGTTTAGAAAAAGAGAAGCCACTATAAATAGTCACCTGTCCAGTCTATGCTATT
A_14_P120185 CTGTGGTTTCCCTTGGATCTAAGAATGGGACAAAATACCTATTCATTTTTATATGCACTG
A_14_P202502 GGAACATAGGAACACAAGAATAGATAGGAAAATACCAGTCATGCCTTAGCATCACAAATG
CUST_2550_PI426210822 ATTGTATAGCCCAGACAGTGGTCGTCTGAGGGCTCTCCGGCCTCAA
A_16_P02754738
TTCACCATAAGTCCAAATCAAACGTAGAACATGACAAATAGAATTAGTTCCTCTCTTCCC
A_14_P129818 CTTCGGCCGTCTTCAAATATTGTTTAAATTGCAACTCCGGAGGAAAAGTATTTTTTGTAA
A_16_P17614982
AGAAAAAGCATTCTACACATGTCATTCCATGTTGAACTCTTAGAGCACAAAAAACAACCT
A_16_P16855081
ATTCAAAAGACTCCAGTAAACCTTATCACCAAAAAGAAAGATACTACTAAACCCAGGCTG
A_16_P21360315
AAATAAAGATGGCTTCTTGATGTTTAATATGCCCTGCAGATATCTTACAAAGCCCCAGTA
A_18_P14138717
CTAGTTCAATGATAAAATTTTTGTCTGGGCTACATGGAATCATGGAATTTCAGTTCTGCC
A_14_P127035 ATACCATTTTGTATCTTTAAGACTCTCACTGTGTGAACATCATGTGCATCAATTATGCCT
A_14_P127221 GAAACAGGCAGTTAATATCTGTCCTCAACATCTACATTCAGGAATTGCAGATATTTTGCT
A_14_P127903 TAAGCAAAGGCCATCTTTTTATCTAGGAATTGTCAAAGAGAAGATTCCAAATTGGAAGGA
CUST_5774_PI426210822 ATACTGTGCTAGGTGCTGGGGGTTATAAACATGAATTACATGATTAGGGCCT
A_14_P120453 ACTCACAATTCTTCTTTCAAGTTCAAGGCATCTTCTACTTTATCATGTCGACAGCATAAA
A_14_P128672 GAACAATGCCACGAATTAGACATGGCTACTCTAAAAATTCTCTGTGGAAACAATATTTGG
A_16_P17216796
ATGCAGGGAATAATAGTCAAGGCCACTTAATAGACACAATACATGGGGATCTTATTATGC
A_14_P102169 AGTGGAAAAATACTCACCTATTTATCTGACCATTTTCTACTTCGGTGAAATCATTGGAGT
A_16_P02426392
GGACTATTGGCAAGGTTTTTATAATCTGCACTAAGAAAGAGTTTCATCTACCAGATCAGC

A_14_P121844 ATCTTTCTCCCTATGAATTTTAAGGATAACCATGGAATAGCCCTGATGGCATATATTTCC

A_16_P01649618 CCATTATCCTGTCTCAATAAAGAATTGGAAGTGTCTGGTATCCTGTTGGAGATTTTTACA

CUST_1479_PI426210822 ACTTTCCTTCCTCACCCCAGTCTGGATGGCATCCCTGGGCCCAGC

A_14_P124797 CTCGAATGTTCACTGCTGCTATTACCAGGCAAAATTTGGAAATACAGTGTTGCTTAAATT

CUST_5007_PI426210822 GTCACATTTACCTTGGTTAGGGAAGATAGTGACTTATAAAAACCTCAAACATTTATGTTT

A_14_P117525 GTAGGACTCATCCGTGAAGATAGGTTTGATGAATTTCTACAAAGTATTAGAGAACATTCA

A_14_P123144 AAGGGTTTTAAAGAGGAAGAGTGATCCTGAGACTTGGCAGAGTTTTCTGAAAAG

CUST_7188_PI426210822 TTTACACTCTGGTTCCATCTCCTTCCCCCTGCACACATGAGTCAG

CUST_8079_PI426210822 CGCTTCTTTTTACTGGTCCACAAGACTTGGTTATTTTCTATTGACACTATTTTCAAGATC

A_14_P114892 CTTTTTTGATATAACCTGCAGTTTACGAGGGGAAGCAGTGATGCGTATCTATACAAATTT

A_16_P17592216 AATACATGACACATTACTAATCTGAATTTACCACCTCTCATACTTTTTCTCTCCCCAGGA

A_14_P104305 TTGAGGAAAGGTTGATGAAAGGGGAAAATTTGGGACTTTGTGTAGGTATCAATTATGTTA

A_14_P117780 GGAGGAACAAATCCAAAAAGAATAACAGCAGTAGCAGGTAATTTGGTAAATACTTCTTTC

A_16_P03039825 ACTCAGAACACTACTACTCATAACAGACAATGTTCTTAAGTCTAAATTATCCCCTCTGGT

A_16_P18140657 CTAATAGTCCCCAAAGAAGAAAGTAGTTGCCCTTTTATTTGACACAAGACATAAACCACA

A_14_P118352 AGCATGACCTTAGATGACTAGTCTAATTGGTTCAGCATCTTCCTTTGTTAATGTGATGAT

CUST_8109_PI426210822 ATCAATGTGCTCTCTTGGTCCTTCCACACCTGAGGCAGCCTGTAT

CUST_4061_PI426210822 CAGGGAAGACAGAAAGATGAGGGCCACATCCACAGAAGTCATTTC

A_14_P126515 CTGAAGGCTAAAGTAAACCTTGCTCGTATCAAAAAGTAAAGATTGTGCAGACCTGTTGTA

CUST_1768_PI426210822 CCAAGGAGGGAACTGAATTAAAACTGTATGCTTTAATTAAAAAGCTGACAGTTGAACCTC

A_14_P202246 CACGGACACTTAAAGTCAATCTGCGAAATAATTCTTTTATTACACACTCAGTAAGTTGGA

A_14_P128211 TCATTAATAAATAGGTTTTCTTCATTAAACACAGAACATATAACAGATTGAAACACTCCC

A_14_P131648 GAGTGAACCAAAATTCCTCCTAAGTCACATAGTAAAGTTGGAAAACAGACCCATG

A_14_P120169 GAATGACTTTAGAAGATGGTTTTAAATTTCGCCTTGGCACTTTGCCACCTAGTC

CUST_2809_PI426210822 ATAGAACCAGTGTTGATTGGAATTCAGAGCACGTGATCACCTGAAGGAAAT

A_18_P16324992 AAATGACATTCAGGACCAAAAAATAAGAGGCATGAAACTACTTTCATTGCTACTGTCAGC

A_14_P116667 TTTGCTAGGTAGAAAATATGATAAAAATGGAAACCTGGATCCTTGGTGGTCTACTGAATC

A_16_P01648559 TAAGAATCCTTTCTGGGTTTTAATGTTTCGCGTTATTCTAACAAAGCCGAATGTGTATCG

A_14_P120198 AGGTCAGTAAACCATTTGAGTTAGCATGTGGTAGCCTTAAAACTTAAAAGGTAGTAGAGT

A_14_P136082 CATGACATGGCACATAGAGAAAATATTTTTTTCTAGCACACAAGAGCAACCTGAAAGGCT

A_14_P100909 CAGTTCATTTTTGCTTTAGACGATTCCTAATTAACACCTAACGTGCCATGACACCAA

A_14_P138513 ACCTATTTCACATGGGCGTTTTGTATACAACTATTTTGATCTACACTTGATGTCTGAGCA

A_16_P18211513

AAAGTTCTGATGCCCTCATTCTGATAATACTCATCAACCGTAGAAACCATTTTGACATTC
[truncated: 614,305 more chars]
